# Exploring Novel Applications: Repositioning Clinically Approved Therapies for Medulloblastoma Treatment

**DOI:** 10.3390/cancers17223659

**Published:** 2025-11-14

**Authors:** Arthur Karaulic, Clémence Fournier, Gilles Pagès

**Affiliations:** University Côte d’Azur, Institute for Research on Cancer and Ageing of Nice (IRCAN, UMR CNRS 7284/U INSERM 1081), 06100 Nice, France; arthur.karaulic@univ-cotedazur.fr (A.K.); clemence.fournier@univ-cotedazur.fr (C.F.)

**Keywords:** Medulloblastoma, kinase inhibitors, immune check point inhibitors, mutations, non-metastatic/metastatic tumors, BCL2, Venetoclax

## Abstract

Background/Objectives: The advent of tyrosine kinase inhibitors (TKI), therapeutic antibodies and inducers of apoptosis has revolutionized cancer treatment, yet their application in pediatric tumors, particularly medulloblastoma, remains understudied. Understanding the expression of these targets in specific genetic subgroups could unveil potential repositioning opportunities for already approved drugs. Methods: We analyzed RNA-sequencing data from the R2 Genomics Analysis and Visualization Platform (N = 763 patients, multiple cohorts) and the TCGA database (six individual cohorts 828 patients) to assess the expression of 73 potential targets of TKIs and antibodies targeting immune checkpoint inhibitors (ICI) or membrane receptors and inducers of apoptosis. These treatments, FDA-approved or in phase II clinical trials for solid or hematologic cancers, and their targets were evaluated in both non-metastatic and metastatic patients when data was available. Additionally, we examined treatments tailored to mutated targets crucial for tumorigenesis or resistance to conventional therapies. Results: Overexpression of certain targets beyond predefined cutoff values in Kaplan–Meier analyses correlated with either prolonged or shortened overall survival. Targets associated with shorter survival suggested potentially relevant treatments, thereby highlighting the importance of defining specific treatments for distinct genetic subgroups. Notably, certain immune checkpoint inhibitors showed relevance for specific subgroups but detriment for others. As a positive control, our analysis confirmed the use of axitinib, an anti-angiogenic treatment, as demonstrated by our recent publication. Surprisingly, a treatment developed for hematological tumors, venetoclax, demonstrated potential efficacy in medulloblastoma. Conclusions: Medulloblastoma displays subtype-specific expressions of FDA-approved TKI, ICI and pro-apoptotic drug targets, impacting overall survival. Clinical trials investigating these approved treatments in medulloblastoma are therefore warranted.

## Simple Summary

Medulloblastoma patients who relapse after standard therapies face a poor prognosis, underscoring the need for novel treatment strategies. To accelerate therapeutic discovery, we explored a drug repositioning approach by analyzing genes targeted by approved cancer drugs in relation to patient survival. Using the R2 Genomics Analysis and Visualization Platform and TCGA datasets, we identified genes associated with poor prognosis across medulloblastoma subgroups in both metastatic and non-metastatic contexts. Among the candidate therapies, the BCL-2 inhibitor Venetoclax, typically used for hematologic malignancies, emerged as a promising option. Experimental validation in medulloblastoma cell models confirmed the efficacy of Venetoclax alone and in combination with Etoposide, a standard chemotherapeutic agent. This in silico and experimental study supports the potential of drug repositioning to expand therapeutic options for medulloblastoma patients, particularly those with relapsed disease or in need of less aggressive treatments.

## 1. Introduction

Medulloblastoma is a highly malignant, embryonal brain tumor that predominantly affects children between the ages of 0 and 14 years [1]. It is one of the most common and aggressive pediatric brain cancers, often leading to significant morbidity and mortality in affected children. Medulloblastoma belongs to the group of primary neurectodermal tumors (PNET): it is an embryonal lesion that arises from progenitor cell populations present during early brain development [2,3,4]. The tumor typically develops in the cerebellum, where it disrupts normal developmental processes, leading to impairment in motor coordination, balance, and cognitive function.

Medulloblastoma is divided into four molecularly distinct diseases (subgroups), with further subdifferentiations within each subgroup, named subtypes [5,6,7,8]. Each subgroup/subtype possesses unique transcriptional and epigenetic profiles. Sonic Hedgehog (SHH), Group 4 and Group 3 medulloblastoma (from the least to the most aggressive) are thought to originate in the cerebellum. Wingless/Int (WNT) medulloblastoma (the least aggressive form of all) arises from the lower rhombic lip of the developing brain stem [4]. Each subgroup is defined by a specific set of genetic alterations that shapes its molecular profile, growth dynamics, and response to therapy:

- WNT medulloblastoma, characterized by mutations in the WNT signaling pathway, are associated with the best prognosis.
- SHH (Sonic Hedgehog) medulloblastoma, associated with mutations in the SHH signaling pathway, display various subtypes that can vary in severity and prognosis.
- Group 3 medulloblastoma often involve MYC amplification and are highly aggressive, with poor prognosis.
- Group 4 medulloblastoma, the least understood group, is typically characterized by a mixture of genetic alterations, and is also aggressive in nature.

Despite this apparent heterogeneity, all the different forms of medulloblastoma share a common primitive embryonal phenotype, i.e., a progenitor neural cell of the primitive cerebellum and the presence of specific gene drivers for each MB subgroup has been firmly established [9,10,11,12]. Over-proliferation or early mutational state of this progenitor cell is enough to lead to the failure of a normal cell differentiation and to the shift toward a malignant phenotype [13,14].

Patients undergo rigorous treatments comprising surgery, radiotherapy (administered to patients above 3 years old), and chemotherapy regimens incorporating agents like etoposide, carboplatin, and vincristine. While these intensive interventions yield a 70% long-term remission rate, most patients suffer from the severe side effects of these treatments [15,16] and approximately 30% of cases experience relapse, with fatal outcomes being commonplace. Therefore, physicians face two paramount challenges in managing medulloblastoma: (1) **Reducing Treatment Intensity**: The primary objective is to mitigate the high detrimental side effects associated with intensive therapies, which can encompass mobility issues, cognitive impairments, language deficits, and motor function limitations. This reduction must be achieved without compromising treatment efficacy. (2) **Identifying Relevant Therapies for Relapse**: Another critical goal is to propose effective treatments in instances of relapse, with the aim of extending survival and potentially achieving a second long-term remission. Addressing these challenges demands a delicate balance between treatment effectiveness and the minimization of adverse effects, underscoring the importance of tailored therapeutic strategies in the management of medulloblastoma.

Over the past 15 years, considerable advancements have been made in cancer treatments across various types. Yet, the challenge persists in developing innovative therapies, often constrained by logistical hurdles and financial constraints. Despite advancements in treatment strategies, the development of new therapies for pediatric cancers remains challenging, largely due to historical underinvestment by the pharmaceutical industry. Treatment repositioning represents a promising alternative, leveraging the efficacy of several targeted therapies that have been successfully utilized for decades in adult cancers sharing similar mechanisms of tumor aggressiveness with medulloblastomas. For instance, reverse genetic analysis conducted by Coy et al. [17] demonstrated the potential application of antibody-drug conjugates targeting HER2 for pediatric brain tumors.

This presents an opportunity to reposition existing treatments through a molecular pathology approach, leveraging the analysis of specific mutation expressions in newly diagnosed patients or those experiencing relapses. By harnessing available data from public databases, we can tailor treatments to specific genetic subgroups of medulloblastoma, thus creating a customized treatment landscape.

Given the hyper vascularized nature of medulloblastomas and considering that increased angiogenesis is associated with the most aggressive form of the disease [18], Axitinib, a tyrosine kinase inhibitor used in the treatment of kidney cancers [19], has shown promise as a potential treatment for medulloblastomas [20,21]. This approach has led to the initiation of the clinical trial Mependax (NCT06485908), which is currently enrolling patients with relapsed medulloblastoma and ependymoma, building upon encouraging findings in six children treated with Axitinib and metronomic etoposide [22].

To identify additional therapeutic candidates, aimed at improving outcome both in the first line and at relapses while reducing toxic side effects, we implemented a systematic in silico approach. First, we cataloged available targeted therapies, including older and next-generation agents, alongside their molecular targets. Utilizing publicly accessible datasets via the R2 platform https://hgserver1.amc.nl/cgi-bin/r2/main.cgi?open_page=login (accessed on 10 November 2025) and the TCGA database through cBioportal https://www.cbioportal.org/ (accessed on 10 November 2025), we analyzed the expression of target genes and their correlation with survival outcomes. Specific activating mutations in target genes were also evaluated, given that many therapies are indicated only for tumors harboring such mutations.

This strategy identified several unexpected candidates for repositioning in medulloblastoma, including therapies traditionally used for hematological malignancies. The validity of our approach was supported by the identification of Axitinib [20] and HER2 inhibitors [17,23], as relevant treatment options consistent with previously published findings. Furthermore, we demonstrated the potential of Venetoclax, typically used in hematological tumors, as a promising therapeutic candidate for medulloblastoma.

## 2. Materials and Methods

### 2.1. Expression and Mutation Profiling

Gene expression levels and survival correlations using the Kaplan–Meier method were analyzed using the R2 Genomics Analysis and Visualization Platform (https://hgserver1.amc.nl/cgi-bin/r2/main.cgi?open_page=login accessed on 10 November 2025) and the cBioportal platform (https://www.cbioportal.org accessed on 10 November 2025). For the R2 platform, medulloblastoma datasets included the following:

- **GSE85217** (https://www.ncbi.nlm.nih.gov/geo/query/acc.cgi?acc=GSE85217 accessed on 10 November 2025, Cavalli, [8])
- **GSE67851** (https://www.ncbi.nlm.nih.gov/geo/query/acc.cgi?acc=GSE67851 accessed on 10 November 2025, Hsieh, TH [24])
- **GSE37418** (https://www.ncbi.nlm.nih.gov/geo/query/acc.cgi?acc=GSE37418 accessed on 10 November 2025, Gilbertson, RJ [25])
- **GSE74195** (https://www.ncbi.nlm.nih.gov/geo/query/acc.cgi?acc=GSE74195 accessed on 10 November 2025, den Boer, M [26])
- **GSE10327** (https://www.ncbi.nlm.nih.gov/geo/query/acc.cgi?acc=GSE10327 accessed on 10 November 2025, Kool, M [27])
- **GSE49243** (https://www.ncbi.nlm.nih.gov/geo/query/acc.cgi?acc=GSE49243 accessed on 10 November 2025, Pfister 2, [28])
- **GSE12992** (Delattre, [29])
- **GSE3526** (https://www.ncbi.nlm.nih.gov/geo/query/acc.cgi?acc=GSE3526 accessed on 10 November 2025, Roth, [30])
- **Cohort Pfister 1** [7]

For the cBioportal platform, the following dataset were analyzed:

- Medulloblastoma (PCGP, Nature 2012, [25])
- Medulloblastoma (Broad, Nature 2012, [31])
- Medulloblastoma (DKFZ, Nature 2017, [7])
- Medulloblastoma (ICGC, Nature 2012, [32])
- Medulloblastoma (Sickkids, Nature 2016, [33])

These datasets provided robust platforms for exploring gene expression profiles and their association with clinical outcomes.

### 2.2. Establishment of the List of Targeted Therapies

The compilation of targeted therapies, including kinase inhibitors, immune system modulators, and specific monoclonal antibodies, was carried out using the resources provided by the National College of Medical Pharmacology (https://pharmacomedicale.org accessed on 10 November 2025) (Table 1). This approach ensured a comprehensive and up-to-date selection of therapeutic agents relevant to the study’s focus.

### 2.3. Cell Lines

The human medulloblastoma cell lines (ONS76, DAOY, HD-MB03) and the normal microglial cells (HMC3) were purchased from American Type Culture Collection (ATCC). DAOY, ONS76 and HMC3 cells were maintained in MEM alpha (Gibco, Life Technologies Corporation, Loughborough, UK) supplemented with 10% fetal bovine serum (FBS, SIGMA, Burlington, MA, USA). HD-MB03 cells were maintained with RPMI 10% fetal bovine serum (FBS, SIGMA, Burlington, MA, USA). Cells were monitored routinely, and the absence of mycoplasma was verified monthly using the PlasmoTest kit (Invivogen, San Diego, CA, USA).

### 2.4. Cell Transfection and Cell Viability Assay

Cells were transfected with a SmartPool siRNA (four independent validated siRNA) targeting BCL2 (Dharmacon, Lafayette, CO, USA) using the RNAiMAX transfection reagent (Thermo Fisher Scientific, Dardilly, France). Cell counts were assessed using the propidium iodide (PI) exclusion assay. Following treatment, cells were harvested and incubated with PI (10 μg/mL) for 5 min. The percentage of PI-positive cells was subsequently analyzed by flow cytometry using a MACSQuant Analyzer (Miltenyi Biotec, Bergisch Gladbach, North Rhine-Westphalia, Germany, catalog number 130-092).

### 2.5. Immunoblot

Cells were lysed with Laemmli buffer and protein amounts were determined by the Pierce TM BCA Protein Assay Kit (Thermo Fisher). Then, 20 μg of protein were resolved by SDS-PAGE. The proteins were transferred onto PVDF membranes in Tris–glycine buffer. Membranes were blocked with 5% milk at room temperature and then immunoblotted overnight in 3% milk with the anti-BCL2 antibody (Cell signaling Technology, Danvers, MA, United States, Ref #2872). Membranes were washed with PBS–Tween 0.1% and incubated with HRP-conjugated secondary antibodies at room temperature for 1 h. The Advansta Western Bright Quantum HRP substrate was used as a detection reagent.

## 3. Results

### 3.1. Identifying Medulloblastoma Patients Eligible for Targeted Therapies

Table 1 lists kinase and apoptosis inhibitors as well as immunomodulators that are already clinically validated in hematological and solid tumors. Each therapy corresponds to a specific molecular target, and the associated gene was analyzed in our study to evaluate its relevance in pediatric medulloblastoma.

To assess their potential, we examined the relationship between expression of these drug-target genes and overall survival (OS) in the Cavalli et al. cohort, the only dataset with survival information [8]. For each target gene, Kaplan–Meier survival curves were generated using the R2 platform with optimal cutoffs. In total, ~300 curves were produced (Supplementary Figure S1), and both raw and Bonferroni-corrected *p*-values are summarized in Table S1 for the four molecular subgroups (WNT, SHH, Group 3, and Group 4). This analysis revealed that several drug-target genes are associated with shorter OS, suggesting that therapies against these targets may have clinical relevance in specific medulloblastoma subgroups. Genes with statistically significant associations (raw and Bonferroni-corrected *p* < 0.05) were prioritized as potential therapeutic candidates. The classification of significant targets by subgroup, together with the corresponding therapies, is presented in Table 2.

### 3.2. Stratification of Patients by Metastatic Status

Our previous work on kidney cancer and medulloblastoma showed that the impact of gene expression on OS can reverse depending on metastatic status (non-metastatic (M0) vs. metastatic (M1)) [34,35]. Therefore, we reiterate our analysis considering this important parameter. To account for this, we repeated our analysis considering metastatic status. About 700 Kaplan–Meier curves were generated (Supplementary Figure S2), and the results are summarized in Table S2 across subgroups and M0/M1 status.

From Table S2, several genes displayed opposite prognostic significance depending on stage. For example, BCL2L1 predicted poor outcome in M0 Group 3 but favorable outcome in M1 Group 3. Similar shifts were observed for CDK4, HER1, SMO (SHH subgroup), HER1, MET, ITPKC (Group 3), and FLT3, PD2 (Group 4). Conversely, genes such as BCL2L2, HER4, LAG3, JAK2, MAPK3 (Group 4) and CTLA4 (Group 3) were favorable in M0 but unfavorable in M1 patients.

Despite these differences, most genes showed consistent prognostic patterns across stages. Overall, metastatic status strongly influenced whether genes were associated with good or poor prognosis, highlighting its importance for therapeutic prioritization.

These findings underscore the critical role of metastatic status in shaping the prognostic significance of target genes. Importantly, genes consistently associated with poor prognosis can be prioritized for therapeutic intervention, as they point to specific vulnerabilities that may be addressed with existing targeted drugs. Table 3 summarizes these gene–therapy associations, providing a framework for tailoring treatment strategies in medulloblastoma according to both molecular subgroup and metastatic status.

### 3.3. Analysis of Targetable Mutations in Medulloblastoma Cohorts

Table 1 highlights treatments applicable when specific mutations are present, such as BRAFV600E. To explore actionable targets, we analyzed medulloblastoma cohorts from the TCGA database, focusing on mutations in targetable genes. Across six available cohorts comprising 828 patients, we identified specific mutation frequencies as follows: 54 patients (6.5%) carried mutations in CTNNB1, 42 patients (5.1%) in PTCH1, 21 patients (2.5%) in SMO, 14 patients (1.7%) in SUFU, 13 patients (1.6%) in PTEN, 5 patients (0.6%) in PI3KCA, 3 patients (0.36%) in PIK3R1, and 2 patients (0.24%) in FGFR1. Additionally, mutations were identified in each single patient (0.12%) for ATM, CDKN2A, ERBB4, FGFR2, IDH1, and NRAS.

Given these mutation profiles, conventional treatments listed in Table 1 may not be effective. The specific mutations identified in these cohorts, and their clinical implications are detailed in Table 4. Details corresponding to this analysis are given in Table S3.

To address this, we propose therapies tailored to specific mutations. For the CTNNB1 pathway, GSK3 inhibitors such as Elraglusib and Tideglusib [36,37] may help block CTNNB1 activation. In the PI3K/AKT pathway, options include Capivasertib (used in metastatic breast cancer with PIK3CA/AKT1/PTEN alterations [38]), RLY-2608 (PIK3CA inhibitor [39]), and PI3Kβ inhibitors like GSK2636771 and AZD8186 [40,41]. For the FGFR pathway, inhibitors like Erdafitinib and Fexagratinib may be effective [42,43]. In cases with EGFR mutations that heterodimerize with ERBB4, the inhibitor Dacomitinib could be considered [44]. We also identified mutations linked to resistance against SHH inhibitors (Sonidegib and Vismodegib), highlighting their value as markers of likely treatment failure.

This analysis provides valuable insights into potential repurposing of drugs approved for other tumors to address specific subsets of medulloblastoma, supporting more precise and personalized therapies.

### 3.4. Experimental Validation of In Silico-Identified Therapies in Medulloblastoma Cell Lines

**Figure 1:**
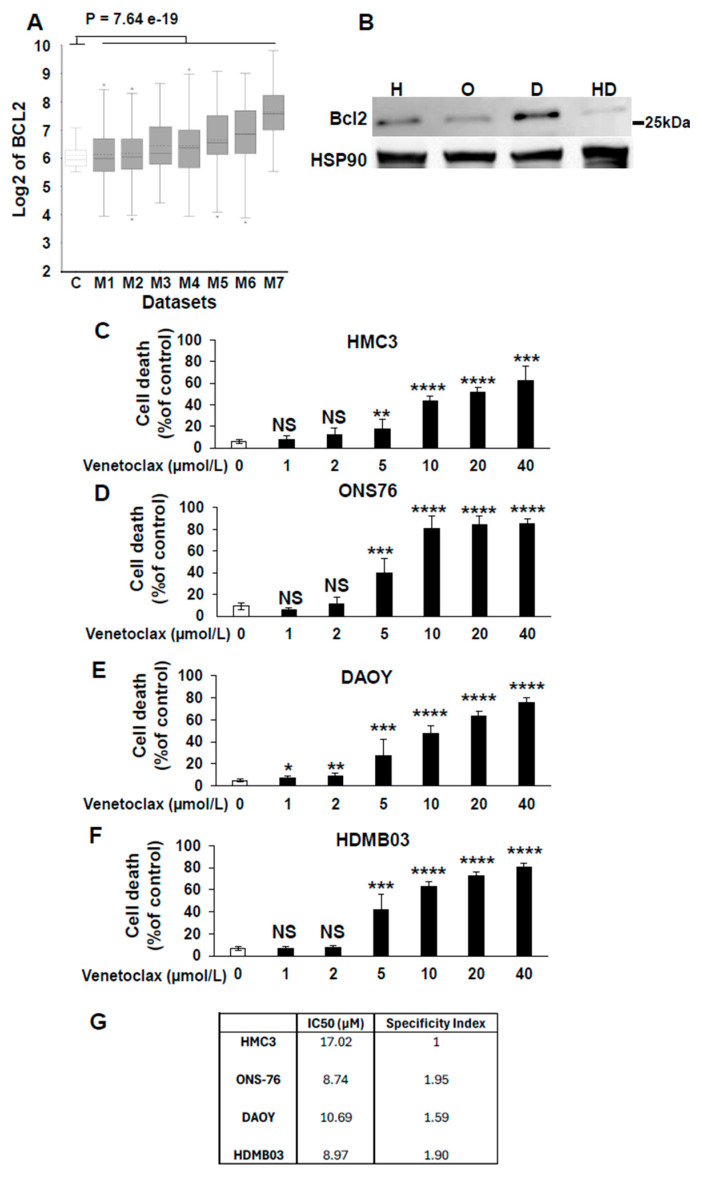
Relevance of targeting BCL2 for the treatment of medulloblastoma. (**A**) Box-dot plot showing mRNA expression level of BCL2 (R2: Genomics Analysis and Visualization Platform (http://r2.amc.nl accessed on 10 November 2025) in normal cerebellum (**C**) (*n* = 9 retrieved from Roth database), and medulloblastoma patients (M1–M7); M1: (*n* = 76) from Gilbertson database, M2: (*n* = 57) from Delattre database, M3: (*n* = 62) from Kool database, M4: (*n* = 223) from Pfister1 database, M5: (*n* = 51) from den Boer database, M6: (*n* = 31) from Hsieh database and M7: (*n* = 73) from Pfister2 database. Statistical analysis (One-way analysis of variance (ANOVA)) is shown, *p* = 7.64 × 10^−19^. (**B**) Immunoblot analysis of BCL2 expression in HMC3 (H), ONS76 (O), DAOY (D), and HDMB03 (HD) cells. HSP90 was used as a loading control. Densitometric quantification of BCL2 expression is shown, normalized to HSP90 levels (BCL2/HSP90). Original western blots are presented in File S1. (**C**–**F**) Dose-dependent inhibition of viability of the four cells lines by increasing concentrations of venetoclax. *: *p* < 0.05; **: *p* < 0.01; ***: *p* < 0.001: **** *p* < 0.0001; NS: Non Significant. (**G**) IC_50_ and specificity index of Venetoclax for the different cell lines.

Based on Table 3, several unexpected treatment options emerged for medulloblastoma, including Venetoclax, a BCL2-targeted therapy commonly used in hematologic cancers but rarely applied to solid tumors [45]. Our analysis indicated that this therapy showed the greatest efficacy in Group 3 and Group 4 tumors, with a lesser effect on SHH tumors. To validate this, we compared BCL2 expression in normal cerebellum and different datasets of medulloblastoma in the R2 database. This analysis reveals a global upregulation of BCL2 across several datasets of medulloblastoma compared to healthy cerebellum (Figure 1A). Then, we assessed BCL2 protein levels in SV40-immoralized microglial cells (HMC3) and in the medulloblastoma cell lines ONS76 (SHH), DAOY (SHH with a P53 mutation mimicking Group 3 tumor outcomes), and HDMB03 (Group 3 tumor). BCL2 expression, as assessed by immunoblot analysis, was highest in DAOY cells, intermediate in ONS76 and HMC3 cells, and low in HDMB03 cells (Figure 1B). Then, we compared the effect of Venetoclax in normal cells based on their neural origin and tumor cells. The IC50 of Venetoclax was determined on these normal cells and medulloblastoma cell lines (Figure 1C–F). Except in normal cells, dose–response experiments demonstrated a direct correlation between BCL2 expression levels and Venetoclax efficacy (Figure 1C–G). subsequently calculated the specificity index for Venetoclax (Figure 1G) [46]. The specificity index was defined as the ratio of the IC50 in HMC3 (normal) cells and the IC50 in medulloblastoma cell lines, with values greater than 1 indicating preferential activity in tumor cells. Although a specificity index above 5 is generally considered indicative of therapeutic relevance with low toxicity [46], Venetoclax has already demonstrated both efficacy and manageable toxicity in a pediatric cohort of patients with newly diagnosed acute myeloid leukemia [47]. These clinical findings support Venetoclax as a viable treatment option in children. Taken together with our data, these results suggest that Venetoclax warrants further preclinical evaluation as a potential therapy for medulloblastoma.

**Figure 2:**
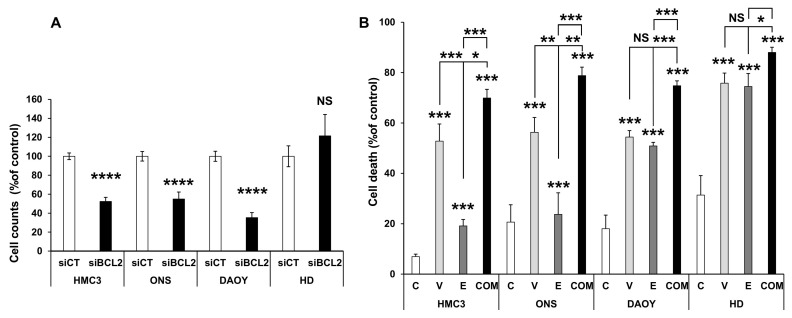
Genetic inhibition of BCL2 and combination with standard-of-care treatment decrease cell numbers and increase cell death. (**A**) Reduction in cell number following transient transfection with a SmartPool siRNA targeting BCL2. The indicated cell lines (HMC3, ONS76 (ONS), DAOY, and HDMB03 (HD)) were transiently transfected with either a scramble siRNA (siCT) or a pool of four independent siRNAs directed against BCL2. Cell counts were determined 48 h post-transfection. (**B**) Increased cell death induced by Venetoclax (V, 10 µM), Etoposide (E, 0.5 µM), or their combination (C). Data are presented as mean ± SEM. Statistical significance: * *p* < 0.05; ** *p* < 0.01; *** *p* < 0.001; **** *p* < 0.0001; NS, not significant.

To further support the role of BCL2 in mediating medulloblastoma aggressiveness and its potential as a therapeutic target, we employed a genetic approach to downregulate BCL2 using siRNA. A pool of four independent siRNAs reduced BCL2 protein expression by approximately 25% across the different cell lines. HDMB03 cells, which exhibited the lowest baseline levels of BCL2, were an exception and may require a greater degree of inhibition to achieve effects comparable to those observed with Venetoclax. In all other cell lines, both genetic inhibition of BCL2 and its pharmacological inhibition (see Figure 1) resulted in a significant reduction in cell numbers (Figure 2A).

Since BCL2 inhibition would likely be combined with standard-of-care therapy—particularly Etoposide—in a clinical setting, we next evaluated combination treatments. Given that conventional chemotherapies are often highly toxic, combining agents at lower doses may help reduce side effects while improving the therapeutic index. To explore this possibility, we tested IC50-equivalent doses of each compound (for etoposide see [20], either alone or in combination.

Depending on the cell line, etoposide alone induced between 20% and 80% cell death. However, the combination of Venetoclax and Etoposide was markedly more effective across all tested cell lines, suggesting a beneficial interaction (Figure 2B).

## 4. Discussion

The treatment of medulloblastoma remains a significant challenge, whether in the first line setting—aiming to limit the side effects of intensive therapies—or following relapse, which often represents a therapeutic dead end. The development of new drugs is particularly complex and time-intensive, often taking years to reach clinical application. In this context, drug repositioning offers a promising alternative, avoiding extensive preclinical toxicology studies in animals, though still requiring clinical trials to assess toxic effects in children, who are no longer viewed as “small adults.”

Through comprehensive analysis of available patient cohorts, we identified potential opportunities for therapy repositioning using drugs with established safety profiles in adults. Table 5 and Table S4 highlight several targeted therapies previously tested in early-phase clinical trials for pediatric brain tumors. Some demonstrated promising effects, though confirmation in phase III trials is still required. Notably, therapies relevant to specific genetic subgroups of patients in our analysis were often found ineffective when metastatic status was considered, underscoring a potential source of failure in some clinical trials. Additionally, the presence of mutations in specific genes may act as confounding factors in therapeutic outcomes.

Crossing the blood–brain barrier (BBB) remains a major obstacle in the treatment of central nervous system (CNS) tumors. Several invasive (intra-arterial delivery, intrathecal/intraventricular injections, convection-enhanced delivery, polymer-based implants) and non-invasive (osmotic disruption, focused ultrasound, nanoparticle carriers) methods have been developed to improve CNS drug delivery [48]. Importantly, BBB integrity is not uniform across medulloblastoma subgroups: WNT tumors typically exhibit BBB permeabilization, SHH and Group 3 tumors show heterogeneity, and Group 4 tumors often retain an intact BBB [49]. This variability suggests that drug delivery strategies should be tailored to the tumor’s molecular context.

These considerations indicate that drugs with apparently unfavorable BBB properties should not be categorically excluded from CNS applications. A combination of tumor-specific BBB permeability, advanced delivery methods, and potential microenvironmental effects may allow agents such as Venetoclax to achieve clinically relevant activity in medulloblastoma.

Among the repositioned therapies, treatments for hematological cancers such as Imatinib and Venetoclax emerged as particularly intriguing. Venetoclax showed significant specificity in medulloblastoma model cell lines, further supporting its potential relevance considering that BCL2 expression correlated to the most aggressive form of medulloblastoma [50]. Moreover, using a completely different approach, Garancher, A. et al. demonstrated the efficacy of the pan-inhibitor of BCL family members, TW37 [51]. However, based on our study, the use of a pan-BCL family inhibitor is not recommended, as the overexpression of certain BCL family members appears to have a beneficial effect for reasons that remain unclear. Instead, Venetoclax is strongly recommended due to its selective targeting. Although its physicochemical properties (MW ≈ 868 Da) predict poor BBB penetration, clinical studies have consistently detected the drug in cerebrospinal fluid (CSF), with plasma/CSF ratios ranging from 44 to 1559 and occasional clinical responses in CNS disease [52,53]. Standard dosing (400 mg/day) yields nanomolar CSF concentrations—below the ~10 µM IC_50_ measured in our study—but higher-dose regimens can approach micromolar levels. Such exposure could be further enhanced by the delivery strategies described above. Similar observations have been made with Axitinib [20], where significant antitumor activity was achieved despite poor predicted BBB permeability, likely reflecting additional effects on the tumor microenvironment such as angiogenesis. Venetoclax has likewise been reported to normalize tumor vasculature [54], suggesting a possible indirect mechanism of benefit in medulloblastoma.

Venetoclax has long been used in leukemia, including more recently in pediatric patients. After oral administration under fed conditions, Venetoclax reaches maximum plasma concentrations within 5–8 h, is highly bound to plasma proteins, and has an apparent volume of distribution of 256–321 L. Its terminal elimination half-life is approximately 26 h, and excretion occurs almost exclusively via feces (>99.9%, with 21% unchanged). Venetoclax is primarily metabolized by CYP3A, with minor contributions from CYP1A2, CYP2B6, CYP2C8, CYP2C9, CYP2C19, and CYP2D6. Thus, CYP3A ontogeny is a critical factor when determining doses in pediatric patients, particularly those under 2 years of age. Age- and weight-based dosing schemes have been proposed to achieve drug exposures in pediatric subgroups comparable to those observed in adults receiving 400–600 mg Venetoclax [52]. While Venetoclax would primarily be administered at relapse, resistance to this treatment may also emerge. Resistance mechanisms mainly involve the upregulation of other BCL2 family members, such as MCL1 and BCL-XL [55]. Favorably, our analysis showed that these markers may correlate with favorable prognosis in medulloblastoma, suggesting a distinct biology compared to hematological tumors. We also note that resistance mutations in the Venetoclax binding site, as well as alterations in OXPHOS, nicotinamide and fatty acid metabolism, epigenetic changes, *TP53* mutations, and activation of ERK and PI3K pathways have been described. To address these issues, combination strategies with pan-BCL inhibitors, DNA methylation inhibitors, histone deacetylase inhibitors, or cell cycle inhibitors may be beneficial.

## 5. Conclusions

Although our findings represent an initial step in treatment repositioning for pediatric brain tumors, they provide a foundation for exploring therapies previously overlooked for medulloblastoma, with the goal of improving outcomes in this challenging context. The demonstration that Venetoclax can be combined with the standard-of-care therapy Etoposide paves the way for clinical trials evaluating combination treatments that maintain efficacy while reducing drug concentrations to limit side effects.

## Figures and Tables

**Table 1:** Overview of approved targeted therapies for solid and hematologic tumors. This table provides a detailed summary of targeted therapies currently approved for the treatment of solid and hematologic tumors. It includes the associated pathologies, the generic names of the treatments, their corresponding brand names, and the specific genes they target.

	Name of “INIB” and “UMAB”	Brand Name	Target Gene(s)
**Immunomodulation**	Baricitinib	OLUMIANT	JAK1, JAK2
	Tofacitinib	XELJANZ	JAK1, JAK3
**Hematology**	Ruxolitinib	JAKAVI	JAK (+JAKV617F)
	Acalabrutinib	IMBRUVICA	BTK (Bruton)
	Idelalisib	ZYDELIG	PI3K
	Venetoclax	VENCLYXTO	BCL2
	Midostaurine	RYDAPT	FLT3, KIT, VEGFR, PKC
	Ivosidenib	TIBSOVO	IDH1
	Gilteritinib	XOSPATA	FLT3, AXL
	Asciminib	SCEMBLIX	ABL1, BCR-ABL1
	Bosutinib	BOSULIF	BCR-ABL, SRC, LYN, HCK, PDGFR
	Dasatinib	SPRYCEL	BCR-ABL, SRC-KINASES
	Ponatinib	ICLUSIG	KIT, FLT3, RET, PDGFR, VEGFR
	Nilotinib	TASIGNA	BCR-ABL
	Imatinib	GLIVEC	BCR-ABL, FILP1, PDGFR, KIT
**Solid Tumors**	Avapritinib	AYVAKYT	PDGFR, KIT
	Sunitinib	SUTENT	VEGFR, PDGFR, KIT, FLT3
	Pazopanib	VOTRIENT	VEGFR, PDGFR, KIT, FLT3
	Axitinib	INLYTA	VEGFR, PDGFR, KIT, FLT3
	Cabozantinib	CABOMETYX	VEGFR, MET, RET
	Sorafenib	NEXAVAR	VEGFR, PDGFR, KIT, FLT3, RAF-KINASES
	Lenvatinib	LENVIMA	VEGFR, FGFR, RET, PDGFR, KIT
	Vandetanib	CAPRELSA	VEGFR, EGFR, RET
	Pemigatinib	PEMAZYRE	FGFR
	Regorafenib	STIVARGA	KIT, RET, RAF-KINASES
	Sonidegib	ERIVEDGE	SMO (Hedgehog pathway)
	Lorlatinib	LORVIQUA	ALK, ROS1
	Brigatinib	ALUNBRIG	ALK, ROS1
	Ceritinib	ZYKADIA	ALK
	Crizotinib	XALKORI	HGFR, c-MET
	Alectinib	ALECENSA	ALK, RET
	Afatinib	GIOTRIF	HER1, 2, 3, 4
	Dacomitinib	VIZIMPRO	HER1, 2, 4, DDR2
	Erlotinib	TARCEVA	HER1
	Gefitinib	IRESSA	HER1
	Osimertinib	TAGRISSO	EGFR T790M
	Dabrafenib	TAFINLAR	BRAF V600E
	Encorafenib	BRAFTOVI	BRAF V600E
	Vemurafenib	ZELBORAF	BRAF V600E
	Cobimetinib	COTELLIC	MEK
	Trametinib	MEKINIST	MEK
	Selumetinib	KOSELUGO	MEK
	Capmatinib	TRABECTA	RET fusion
	Selpercatinib	RETSEVMO	RET fusion
	Praseltinib	GAVRETO	RET fusion
	Larotrectinib	VITRAKVI	TRK
	Alpelisib	PIQRAY	PI3Ka
	Lapatinib	TYVERB	HER2
	Tucatinib	TUKYSA	HER2
	Abemaciclib	VERZENIOS	CDK4/6
	Palbociclib	IBRANCE	CDK4/6
	Ribociclib	KISQALI	CDK4/6
	Olaparib	LYNPARZA	PARP
	Rucaparib	RUBRACA	PARP
	Talazoparib	TALZENNA	PARP
**Immune cell targeting**	Blinatumomab	BLINCYTO	CD19
	Basiliximab	SIMULECT	CD25
	Gemtuzumab ozogamicin	MYLOTARG	CD33
	Alemtuzumab	CAMPATH	CD52
	Sabatolimab		TIM3
	Ipilimumab	YERVOY	CTLA4
	Adecatumumab		EPCAM
	Atezolizumab, avelumab, durvalumab	TECENTRIQ, BAVENCIO, IMFINZI	PDL1

**Table 2:** Alignment of targeted therapies with gene-survival associations across medulloblastoma subgroups. This table outlines the positioning of targeted therapies based on the survival impact of their corresponding genes across medulloblastoma subgroups. It highlights the most suitable treatments for each subgroup, determined by the lowest *p*-value: Small characters (Grey background): Indicate a trend toward significance. Medium characters: Denote a statistically significant raw *p*-value. Large characters: Represent both raw and Bonferroni-corrected *p*-values as statistically significant.

TARGET	WNT	SHH	GR4	GR3
				
**ABL1**	Asciminib	Asciminib	Asciminib	
				
**BCR-ABL, FILP1,**		**Imatinib**	Imatinib	
**PDGFR, KIT**				
				
**BCL2**		Venetoclax	Venetoclax	**Venetoclax**
				
**BTK**		Acalabrutinib		Acalabrutinib
				
**CD19/CD3**		Blinatumomab	**Blinatumomab**	
				
**CD25**		Basiliximab		
				
**CD33**		Gemtuzumab	Gemtuzumab	
				
**CD52**		**Alemtuzumab**	Alemtuzumab	Alemtuzumab
				
**CDK4/6**	Abemaciclib, Palbociclib,	Abemaciclib, Palbociclib,	Abemaciclib, Palbociclib,	
	Ribociclib	Ribociclib	Ribociclib	
				
**CTLA4**		Ipilimumab	Ipilimumab	Ipilimumab
				
**EPCAM**				Adecatumumab
				
**FGFR**	Pemigatinib	**Pemigatinib**		
				
**FLT3**				Gilteritinib
				
**HER2**	Trastuzumab, Pertuzumab,			Trastuzumab, Pertuzumab,
	TDM1, TDXd,			TDM1, TDXd,
	Lapatinib, Tucatinib			Lapatinib, Tucatinib
				
**JAK1, 3**			Tofacitinib	
				
**Kit RET RAF**		**Regorafenib**		
				
**MEK**		Cobimetinib,	Cobimetinib,	**Cobimetinib,**
		Trametinib	Trametinib	**Trametinib,**
		Selumetinib	Selumetinib	**Selumetinib**
				
**MET**				Crizotinib
				
**PARP**		Olaparib,	Olaparib,	Olaparib,
		Rucaparib,	Rucaparib,	Rucaparib,
		Talazoparib	Talazoparib	Talazoparib
				
**PDL1**			Atezolizumab,	
			Avelumab	
			Durvalumab	
				
**PDGFR, KIT**			Avapritinib	
				
**PIK3CA**		Idelalisib		Idelalisib
				
**PRKCD**			**Midostaurine**	
				
**SMO**	Sonidegib	Sonidegib		
				
**TIM3**		**Sabatolimab**	Sabatolimab	Sabatolimab
				
**VEGFR, EGFR, RET**		Vandetanib		
				
**VEGFR, FGFR, RET,**		**Axitinib,**		
**PDGFR, KIT**		**Lenvatinib**		

**Table 3:** Alignment of targeted therapies with gene-survival associations across medulloblastoma subgroups considering M0 and M1 status. This table aligns targeted therapies with the survival impact of their corresponding genes across medulloblastoma subgroups, considering the metastatic status (M0; M1). The therapies are categorized based on the statistical significance of the association between the targeted gene and OS, as determined by the lowest *p*-value. The significance levels are represented using text size: Small characters (Grey background): Indicate a trend toward significance (suggestive but not statistically confirmed); Medium characters: Denote a statistically significant association based on raw *p*-values; Large characters: Represent statistically significant associations confirmed by both raw and Bonferroni-corrected *p*-values.

**TARGET**	**WNT M0**	**WNT M1**	SHH M0	SHH M1	GR4 M0	GR4 M1	GR3 M0	GR3 M1
								
**ABL1, SRC, FYN**			Asciminib, Dasatinib	Dasatinib				**Dasatinib**
								
**BCR-ABL, FILP1,**					Imatinib	Imatinib		
**PDGFR, KIT**								
								
**BCL2**					Venetoclax	Venetoclax	Venetoclax	Venetoclax
								
**BTK**			Acalabrutinib					Acalabrutinib
								
**CD19/CD3**			Blinatumomab	Blinatumomab		**Blinatumomab**	Blinatumomab	Blinatumomab
								
**CD25**			Basiliximab					
								
**CD33**			Gemtuzumab	Gemtuzumab			Gemtuzumab	
								
**CD52**			**Alemtuzumab**	Alemtuzumab	Alemtuzumab	Alemtuzumab	Alemtuzumab	
								
**CDK4/6**	Abemaciclib, Ribociclib,		Abemaciclib, Ribociclib,		Abemaciclib, Ribociclib,	Abemaciclib, Ribociclib,	Abemaciclib, Ribociclib,	**Abemaciclib, Ribociclib,**
	Palbociclib		Palbociclib		Palbociclib	Palbociclib	Palbociclib	**Palbociclib**
								
**CTLA4**								Ipilimumab
								
**DDR2**						Dacomitinib	Dacomitinib	
								
**EPCAM**							Adecatumumab	
								
**FGFR**	Pemigatinib		**Pemigatinib**	Pemigatinib	**Pemigatinib**	Pemigatinib		
								
**FIP1L1**	Avapritinib							
								
**FLT3**				Gilteritinib	Gilteritinib			Gilteritinib
								
**HER1**			Gefitinib, Erlotinib, Afatinib,		Gefitinib, Erlotinib, Afatinib,	Gefitinib, Erlotinib, Afatinib,	Gefitinib, Erlotinib, Afatinib	
			Dacomitinib, Cetuximab		Dacomitinib, Cetuximab	Dacomitinib, Cetuximab	Dacomitinib, Cetuximab	
								
**HER2**							Trastuzumab, Pertuzumab,	
							Afatinib, Lapatinib	
**HER3**			Afatinib	Afatinib	Afatinib	Afatinib		
								
**HER4**				Afatinib		Afatinib		
								
**IDH1**							Ivosidenib	
								
**JAK1, 2, 3**					Tofacitinib	Tofacitinib, Baricitinib		
								
**Kit, RET, RAF**			Regorafenib	Regorafenib	Regorafenib	Regorafenib	Regorafenib	Regorafenib
								
**MEK**			Cobimetinib, Trametinib,				Cobimetinib, Trametinib,	
			Selumetinib				Selumetinib	
								
**MET**							Cabozantinib, Crizotinib	
								
**NTRK1**			**Larotrectinib**			Larotrectinib		
								
**NTRK2**				**Larotrectinib**				
								
**PARP**			Olaparib, Rucaparib,		Olaparib, Rucaparib,		Olaparib, Rucaparib,	Olaparib, Rucaparib,
			Talazoparib		Talazoparib		Talazoparib	Talazoparib
								
**ROS**						Lorlatinib, Brigatinib		
								
**ITPKC, PRKCA,**			Midostaurine	Midostaurine	**Midostaurine**	Midostaurine	Midostaurine	
**PRKCD**								
								
**PD1**					Nivolumab, Pembrolizumab			
								
**PDL1**					Atezolizumab, Avelumab,	Atezolizumab, Avelumab,	Avapritinib	
					Durvalumab	Durvalumab		
								
**PDGFR, KIT**					Avapritinib	Avapritinib	Avapritinib	Avapritinib
								
**PIK3CA**							Idelalisib	
								
**PI3KG**			Idelalisib	Idelalisib	Idelalisib	Idelalisib	Idelalisib	Idelalisib
								
**PIK3C2A**							Idelalisib	
								
**PIK3C2B**	Idelalisib		Idelalisib		Idelalisib			
								
**PI3KC2G**			Idelalisib	Idelalisib		Idelalisib	Idelalisib	Idelalisib
								
**SMO**	**Sonidegib**		Sonidegib		Sonidegib			
								
**TIM3**			Sabatolimab	Sabatolimab				
								
**VEGFR, EGFR, RET**			Vandetanib					
								
**VEGFR, FGFR,**			Lenvatinib		Lenvatinib			
**RET, PDGFR, KIT**								
								
**VEGFR, PDGFR,**			Sunitinib, Pazopanib,	Sunitinib, Pazopanib,	Sunitinib, Pazopanib,			Sunitinib, Pazopanib,
**KIT, FLT3**			Axitinib	Axitinib	Axitinib			Axitinib

**Table 4:** Alignment of targeted therapies with specific gene mutations not addressable by conventional treatments. The table lists the identified genes and mutations, their frequency across TCGA patient cohorts (see Section 2), the associated phenotypes, and the therapies tailored to these mutations.

Gene	Mutation Type	Frequency Across Cohorts	Phenotype	Potential Treatments
**CTNNB1**	D32, S33, G34, S37 mutations	~5–12%	Likely oncogenic	Elraglusib, Tideglusib
**PTCH1**	Truncating/splice mutations (e.g., X406, E374*, I1055Sfs*3)	~5–9%	Likely oncogenic truncating mutations	Sonidegib, Vismodegib
**SUFU**	Splice/truncating mutations (T261Gfs*8, X386_splice)	~2–5%	Likely oncogenic	Resistance to Vismodegib
**SMO**	L412F, W535L	~1–6%	Likely oncogenic	Resistance to Vismodegib
**PIK3CA**	Q546K, H1047L, C420R	~1–3%	Oncogenic	Alpelisib, Capivasertib, RLY-2608
**PIK3R1**	Splice/frameshift mutations	<1%	Likely oncogenic	Capivasertib
**PTEN**	G165E, H93Y, G132V, deletions	~1–4%	Oncogenic	Capivasertib, GSK2636771, AZD8186
**FGFR1/2**	N577K, K687E, K659E	~1–2%	Likely oncogenic	Erdafitinib, Fexagratinib, RLY-4008, AZD4547
**NRAS**	G13V	<1%	Oncogenic	Binimetinib, Cobimetinib, Trametinib
**CDKN2A**	D84N	<1%	Truncating mutation	Palbociclib, Ribociclib, Abemaciclib
**ERBB4**	Fusion ERBB4-LCLAT1	<1%	Likely oncogenic	Lapatinib, Dacomitinib
**IDH1**	R132C	<1%	Oncogenic	Ivosidenib, Vorasidenib
**ATM**	Y1915*	<1%	Likely oncogenic	Olaparib, Talazoparib + Enzalutamide

**Table 5:** Overview of treatments that have been approved for clinical use or previously described in the literature. Potentially repositionable treatments were extensively reviewed to evaluate their relevance for medulloblastoma or other brain tumors. The table also indicates whether each treatment can cross the blood–brain barrier (BBB).

Treatment	Used in MB	Other Clinical Brain Tumor Use	Research in MB	Research in Other Brain Tumors	Crosses BBB
**Abemaciclib**	No	–	No	Glioblastoma	Yes (low)
**Acalabrutinib**	No	Leptomeninges/CNS	No	Glioblastoma, Neuroblastoma	–
**Afatinib**	No	–	No	–	–
**Alemtuzumab**	No	CD4+/T-cells in brain, CNS	No	–	Yes
**Alpelisib**	No	–	Yes (in vivo)	Neuroblastoma, Glioblastoma	–
**Asciminib**	No	–	Yes (in vivo)	–	–
**Atezolizumab**	No	Glioblastoma	No	–	–
**Avapritinib**	No	–	No	Myxoid glioneuronal tumors	Yes
**Avelumab**	No	–	No	–	–
**Axitinib**	Yes	–	Yes	Glioblastoma	Yes
**Baricitinib**	No	–	No	–	–
**Basiliximab**	No	–	No	–	–
**Blinatumomab**	No	–	No	–	–
**Cetuximab**	Yes	–	–	–	–
**Cobimetinib**	No	–	No	–	–
**Dacomitinib**	No	–	Yes (in vivo)	Glioblastoma, Pineoblastoma	–
**Durvalumab**	No	–	No	–	–
**Erlotinib**	Yes	Glioblastoma	–	–	–
**Gefitinib**	No	–	Yes (+in vivo)	Glioblastoma, Glioma	–
**Gemtuzumab**	No	–	No	–	–
**Gilteritinib**	No	–	No	–	–
**Idelalisib**	No	–	No	–	–
**Imatinib**	No	–	Yes	Glioblastoma, Myxoid tumors	–
**Ipilimumab**	Yes	Glioblastoma	–	–	–
**Nilotinib**	No	–	Yes	–	–
**Nivolumab**	Yes	Glioblastoma	–	–	–
**Olaparib**	No	–	Yes (+in vivo)	Neuroblastoma, Glioblastoma, High-grade glioma, Ependymoma	–
**Palbociclib**	No	–	Yes (+in vivo)	–	–
**Pembrolizumab**	No	–	No	–	–
**Pemigatinib**	No	–	No	–	–
**Pertuzumab**	No	–	No	–	–
**Ribociclib**	No	–	Yes (+in vivo, bioinformatics)	–	–
**Rucaparib**	No	–	Yes	–	–
**Sabatolimab**	No	–	No	–	–
**Selumetinib**	No	–	Yes (+in vivo)	–	–
**Sonidegib**	Yes	–	Yes (+in vivo)	–	–
**Sunitinib**	No	–	Yes (+in vivo)	Glioblastoma	Yes
**Talazoparib**	No	–	Yes (+in vivo)	–	No
**Tofacitinib**	No	–	Yes	–	–
**Trametinib**	No	–	Yes	–	–
**Trastuzumab**	No	–	No	–	–
**Venetoclax**	No	–	Yes (+in vivo)	–	–

## Data Availability

The data were from publicly available databases including the R2 Genomics Analysis and Visualization Platform https://hgserver1.amc.nl/cgi-bin/r2/main.cgi?open_page=login accessed on 10 November 2025 and the TCGA databases from the cbioportal visualization platform https://www.cbioportal.org/ accessed on 10 November 2025.

## Supplementary Materials

The following supporting information can be downloaded at: https://www.mdpi.com/article/10.3390/cancers17223659/s1, Figure S1: Kaplan–Meier analysis of the relationship between targetable genes and survival in different medulloblastoma subgroups; Figure S2: Kaplan–Meier analysis of the relationship between targetable genes and survival in different medulloblastoma subgroups considering the non-metastatic (M0) and metastatic (M1) patients. Table S1: Association Between Targeted Genes and Patient Survival Across Medulloblastoma Subgroups. Table S2: Association Between Targeted Genes and Patient Survival Across Medulloblastoma Subgroups Considering Metastatic Status (M0, M1). Table S3: Alignment of Targeted Therapies with Specific Gene Mutations. Table S4: Overview of treatments that have been approved for clinical use or previously described in the literature. File S1: Original western blots.

## Abbreviations

The following abbreviations are used in this manuscript:

ABL	Abelson Tyrosine-Protein Kinase 1
ALK	Anaplastic Lymphoma Kinase
AXL	Tyrosine-protein kinase receptor UFO
BBB	Blood–brain barrier
BCL2	B Cell Lymphoma 2
BCR	Breakpoint Cluster Region protein
BRAF	B-Raf protein, serine/threonine kinase
BTK	Bruton Kinase
CDK	Cyclin-Dependent Kinase
CNS	Central nervous system
CTLA4	Cytotoxic T-Lymphocyte Associated Protein 4
DDR2	Discoidin Domain Receptor Tyrosine Kinase 2
DFS	Disease Free Survival
EGFR/HER1	Epithelial Growth Factor Receptor
EPCAM	Epithelial Cell Adhesion Molecule
FDA	Food and Drug Administration
FGF	Fibroblast growth factors
FILP1	Filamin A Interacting Protein 1
FLT3	Fms Related Receptor Tyrosine Kinase 3
HGFR/MET	Hepatocyte Growth Factor Receptor
HRP	Horse Radish Peroxidase
KIT	KIT proto-oncogene, receptor tyrosine kinase
IDH1	Isocitrate dehydrogenase
JAK	Janus Kinase
LAG 3	Lymphocyte Activating 3
LYN	Protein tyrosine kinase Src family
MAPK	Mitogen-activated protein kinase
MEK	Mitogen Activated Protein Kinase Kinase
mTOR	Mammalian target of rapamycin
OS	Overall Survival
PARP	Poly ADP Ribose Polymerase
PD1/PDL1	Programmed Cell Death Protein 1/PD1 Ligand
PDGFR	Platelet Derived Growth Factor Receptor
PFS	Progression Free Survival
PKC	Protein Kinase C
PI	Propidium Iodide
PI3K	Phosphoinositide 3-kinase
PVDF	Polyvinylidene Fluoride
RAF	Rapidly Accelerated Fibrosarcoma
RET	Rearranged during transfection tyrosine kinase receptor
ROS	ROS Proto-Oncogene 1, Receptor Tyrosine Kinase
SMO	Smoothened protein
TIM3	T-cell immunoglobulin and mucin-domain containing-3
TRK	Tropomyosin Receptor Kinase
VEGFR	Vascular Endothelial Growth Factor Receptor
