# Supplementary material for: Exploring Novel Applications: Repositioning Clinically Approved Therapies for Medulloblastoma Treatment
Source: Cancers (Basel). 2025 Nov 14;17(22):3659. doi: 10.3390/cancers17223659 (PMC12650705; doi:10.3390/cancers17223659)
Supplement: Supplementary file 1 [file cancers-17-03659-s001.zip › Supplementary Figure S1.pdf]

ALK

WNT

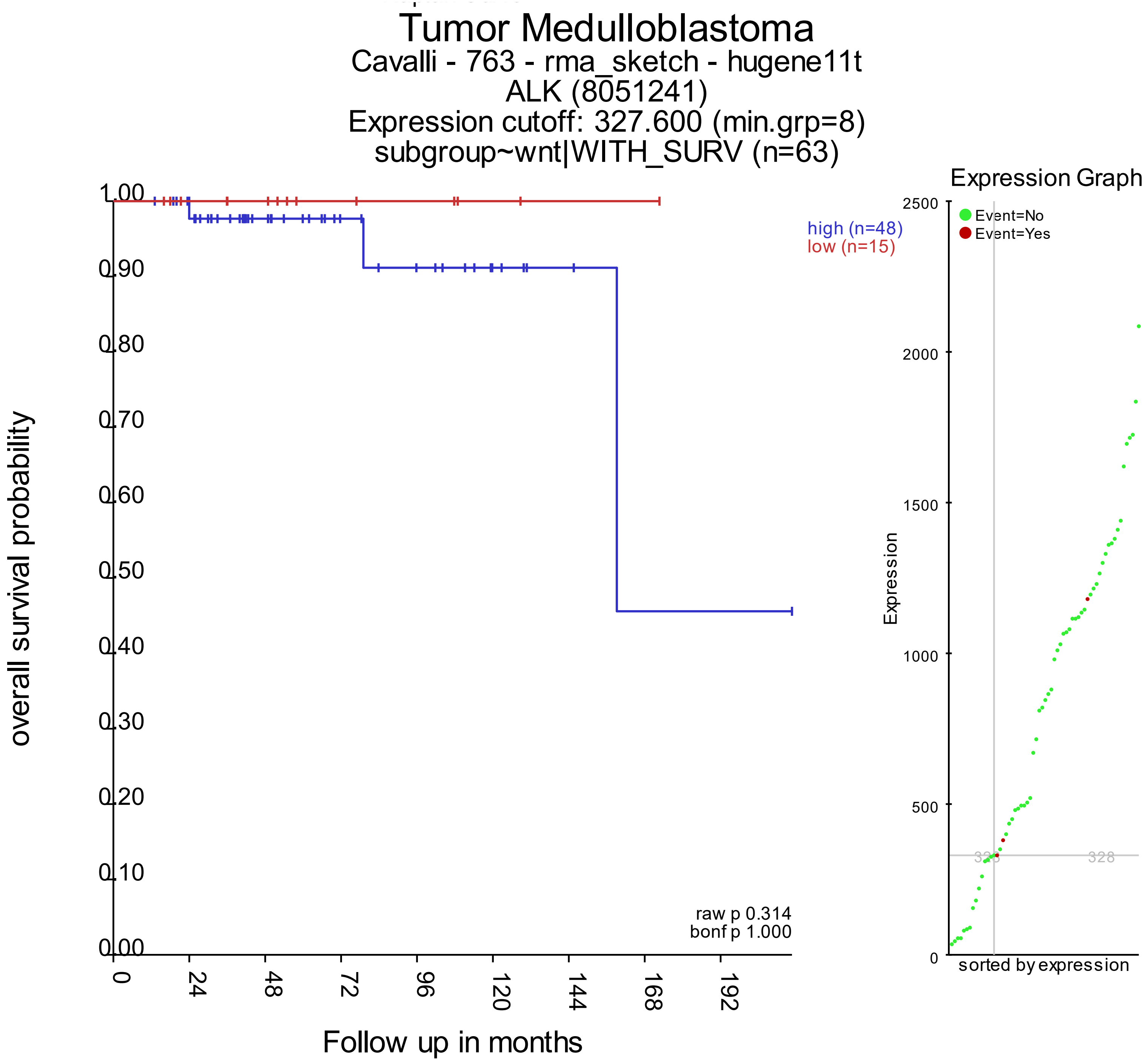

Tumor Medulloblastoma
Cavalli - 763 - rma_sketch - hugene11t
ALK (8051241)
Expression cutoff: 327.600 (min.grp=8)
subgroup~wnt|WITH_SURV (n=63)
overall survival probability
1.00
0.90
0.80
0.70
0.60
0.50
0.40
0.30
0.20
0.10
0.00
high (n=48)
low (n=15)
raw p 0.314
bonf p 1.000
0
24
48
72
96
120
144
168
192
Follow up in months
Expression Graph
Event=No
Event=Yes
Expression
2500
2000
1500
1000
500
0
328
sorted by expression

SHH

Tumor Medulloblastoma
Cavalli - 763 - rma_sketch - hugene11t
ALK (8051241)
Expression cutoff: 130.200 (min.grp=8)
subgroup~shh|WITH_SURV (n=172)

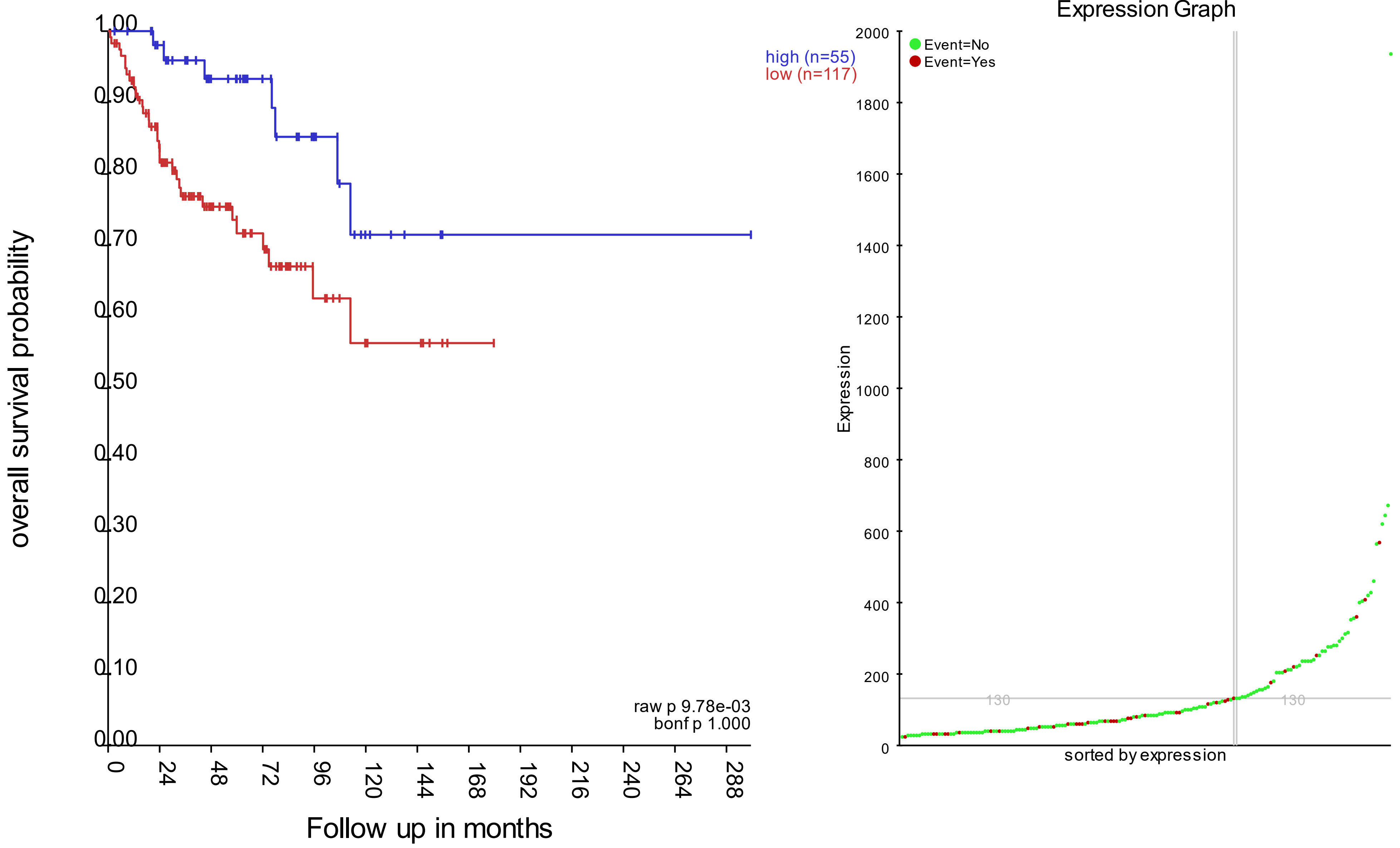

GR4

Tumor Medulloblastoma
Cavalli - 763 - rma_sketch - hugene11t
ALK (8051241)
Expression cutoff: 42.500 (min.grp=8)
subgroup~group4|WITH_SURV (n=264)

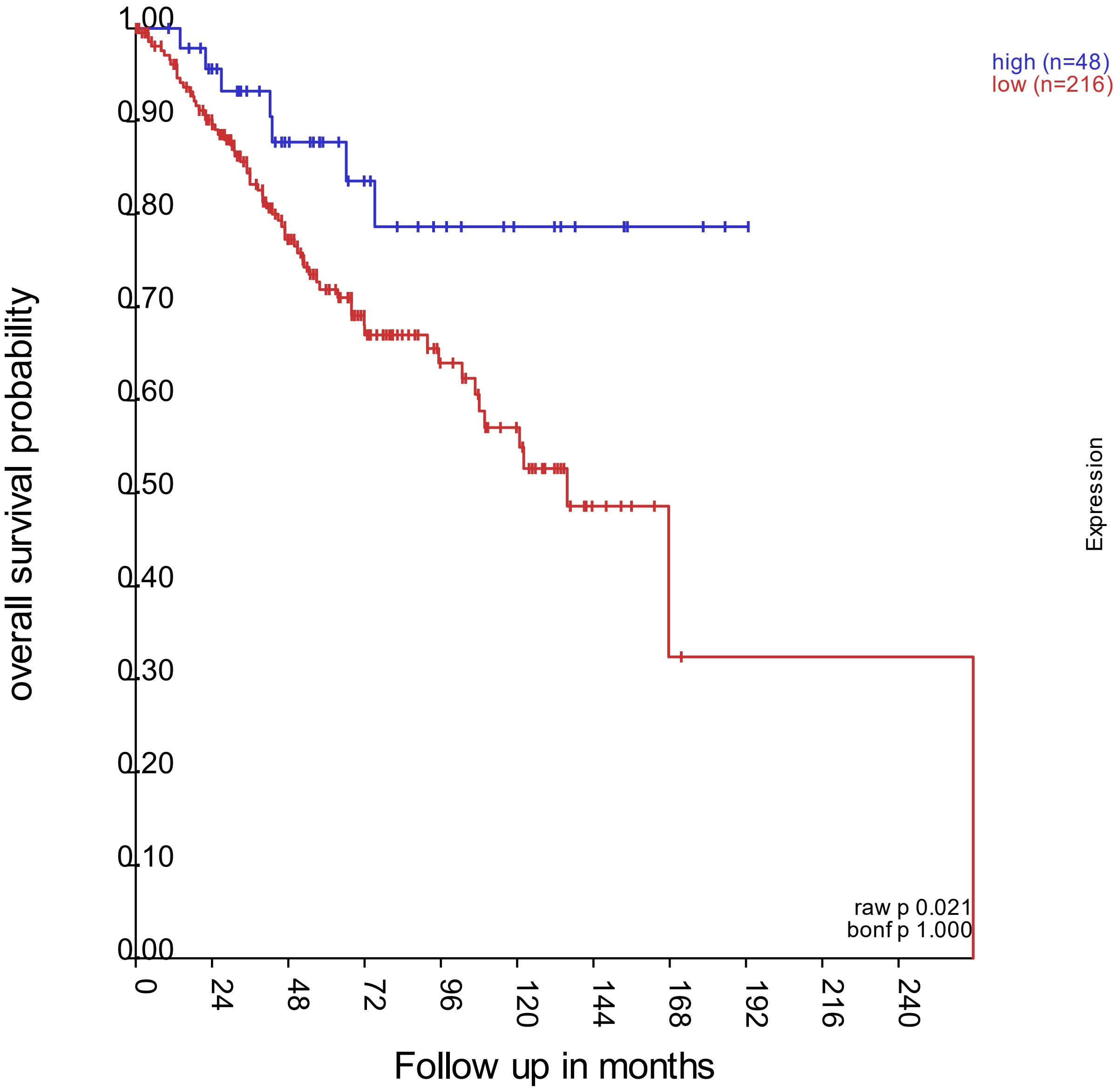

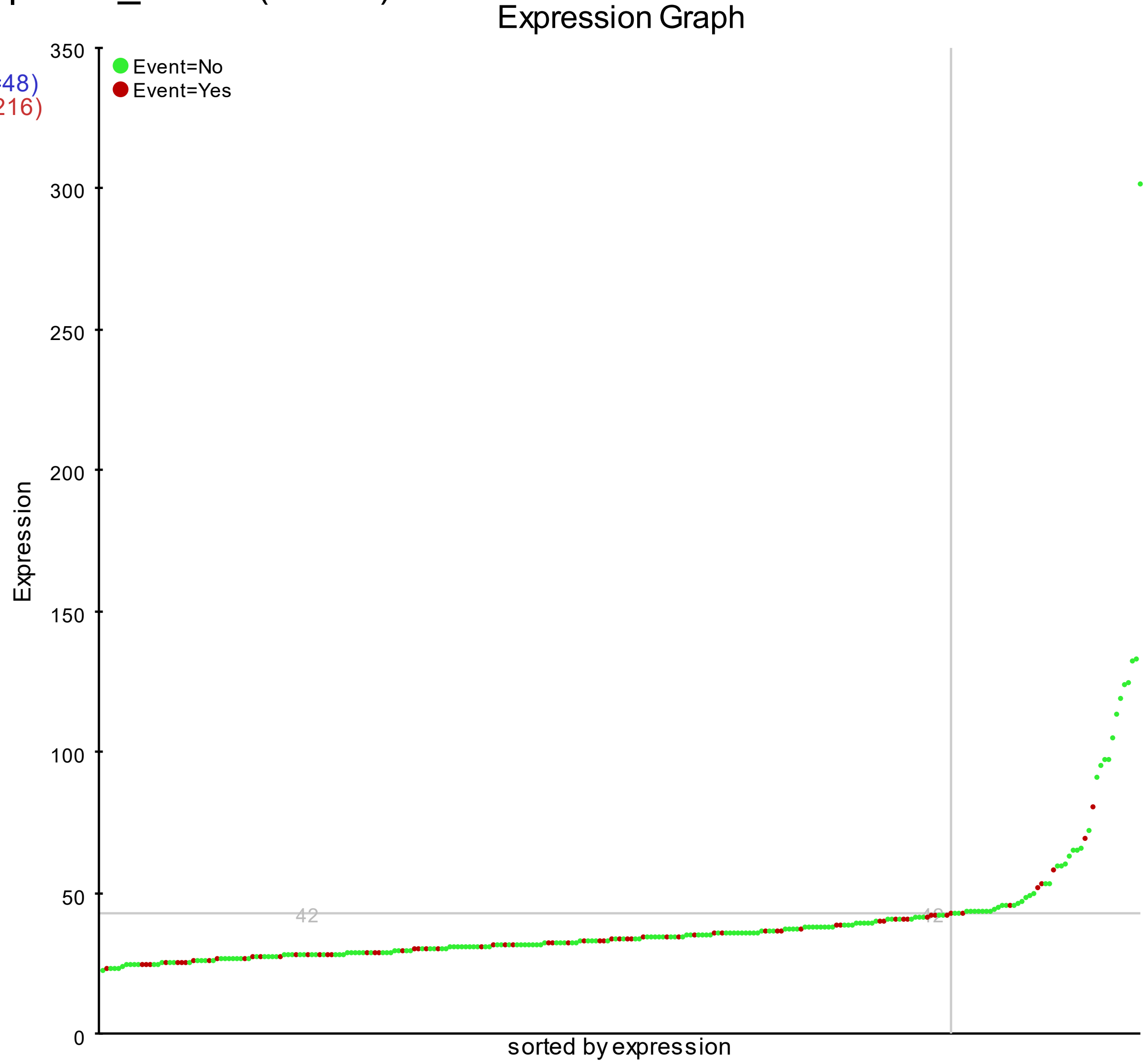

GR3

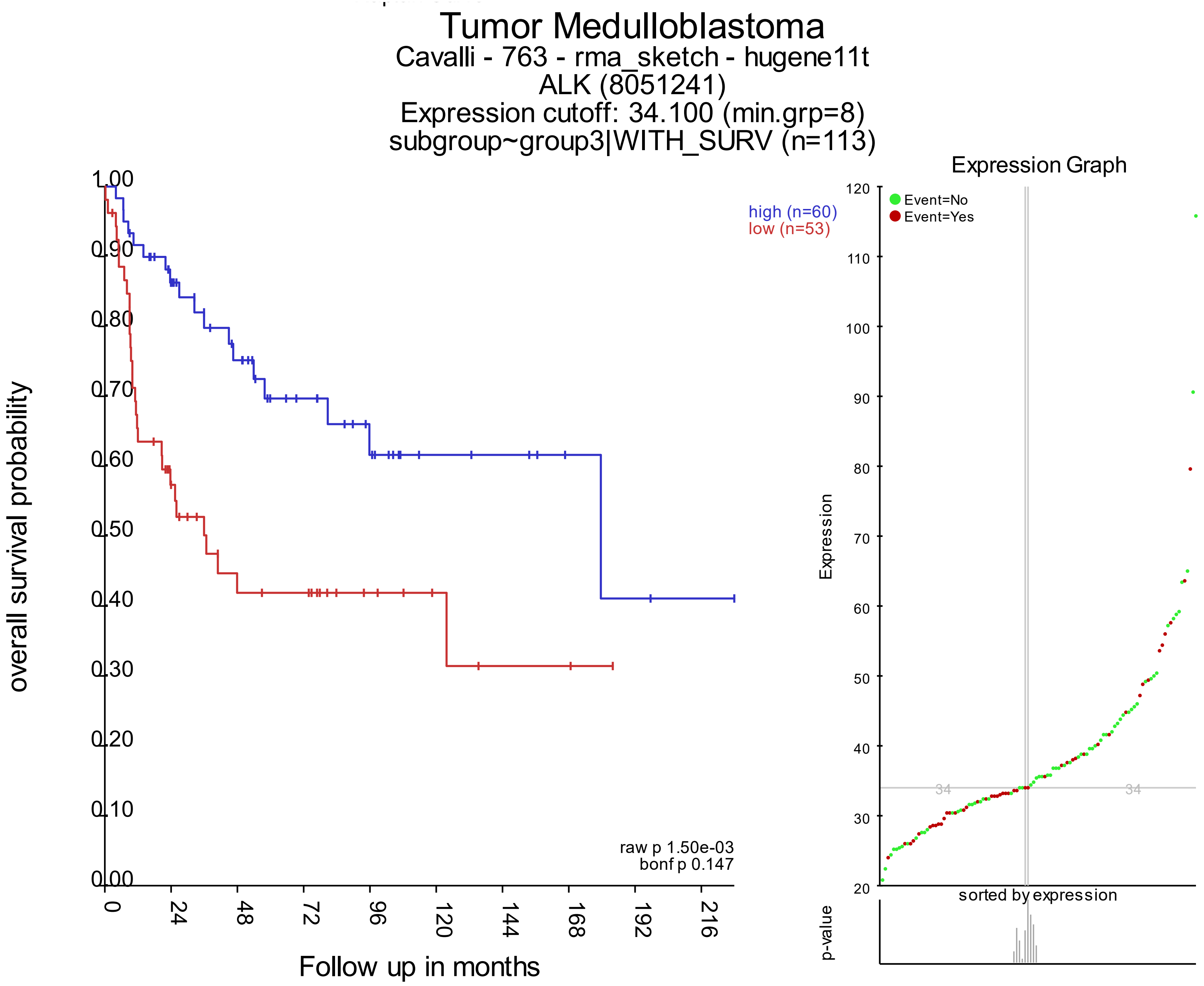

Tumor Medulloblastoma
Cavalli - 763 - rma_sketch - hugene11t
ALK (8051241)
Expression cutoff: 34.100 (min.grp=8)
subgroup~group3|WITH_SURV (n=113)
high (n=60)
low (n=53)
overall survival probability
1.00
0.90
0.80
0.70
0.60
0.50
0.40
0.30
0.20
0.10
0.00
0
24
48
72
96
120
144
168
192
216
Follow up in months
raw p 1.50e-03
bonf p 0.147
Expression Graph
Event=No
Event=Yes
Expression
120
110
100
90
80
70
60
50
40
30
20
34
34
sorted by expression
p-value

BCL2

WNT

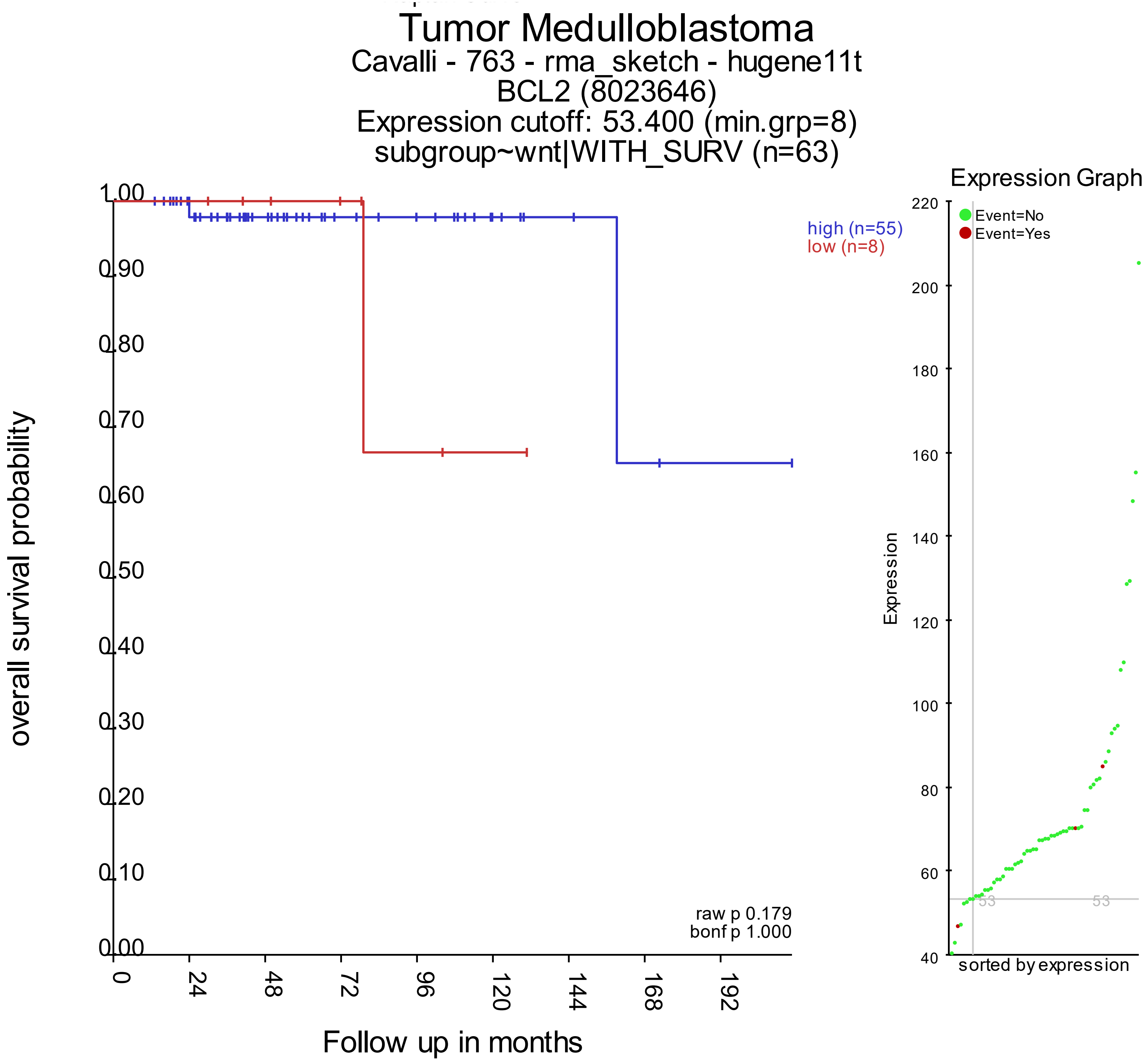
Tumor Medulloblastoma
Cavalli - 763 - rma_sketch - hugene11t
BCL2 (8023646)
Expression cutoff: 53.400 (min.grp=8)
subgroup~wnt|WITH_SURV (n=63)
overall survival probability
1.00
0.90
0.80
0.70
0.60
0.50
0.40
0.30
0.20
0.10
0.00
high (n=55)
low (n=8)
raw p 0.179
bonf p 1.000
0
24
48
72
96
120
144
168
192
Follow up in months
Expression Graph
Event=No
Event=Yes
Expression
220
200
180
160
140
120
100
80
60
40
53
53
sorted by expression

SHH

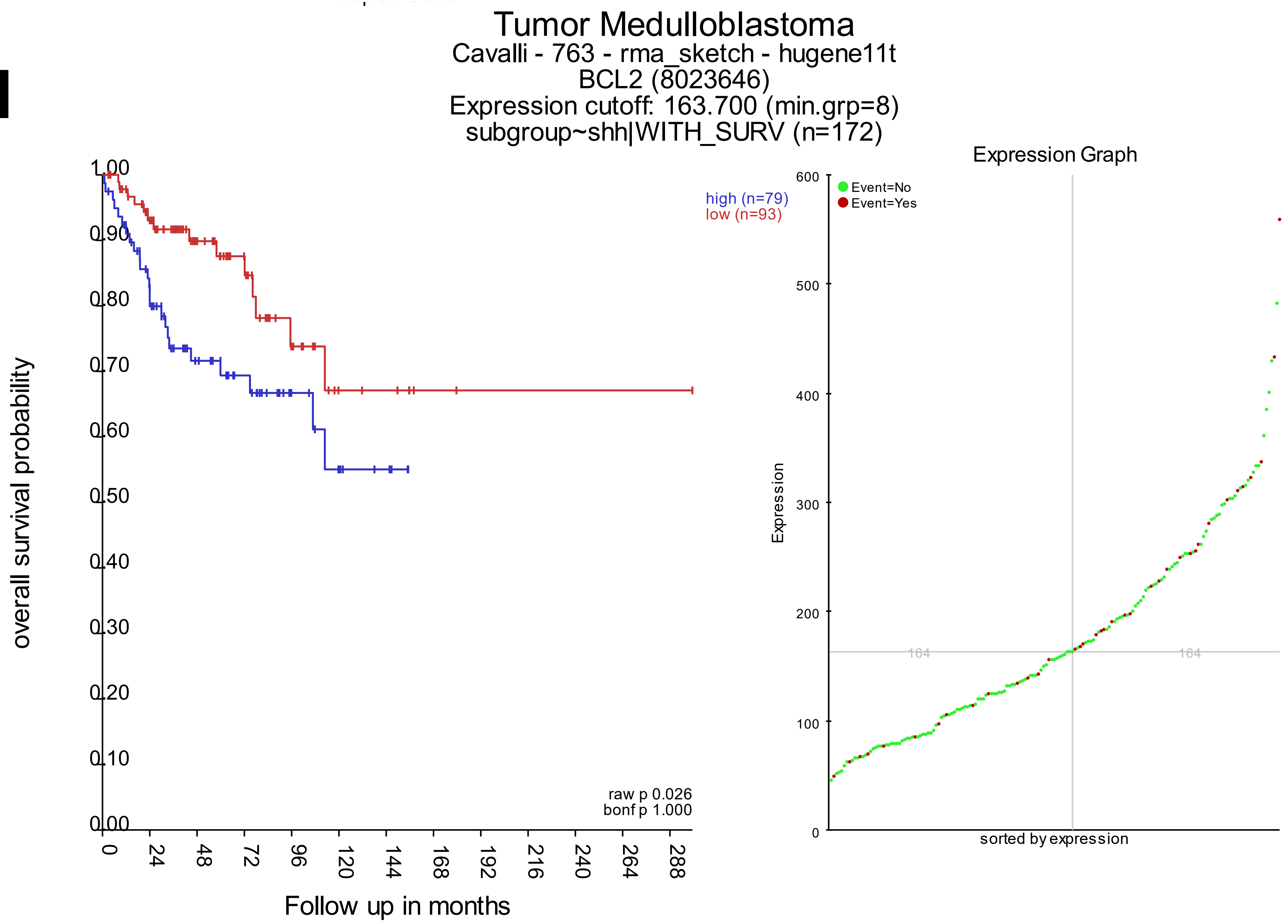
Tumor Medulloblastoma
Cavalli - 763 - rma_sketch - hugene11t
BCL2 (8023646)
Expression cutoff: 163.700 (min.grp=8)
subgroup~shh|WITH_SURV (n=172)
overall survival probability
1.00
0.90
0.80
0.70
0.60
0.50
0.40
0.30
0.20
0.10
0.00
0
24
48
72
96
120
144
168
192
216
240
264
288
Follow up in months
high (n=79)
low (n=93)
raw p 0.026
bonf p 1.000
Expression Graph
Event=No
Event=Yes
Expression
600
500
400
300
200
100
0
164
164
sorted by expression

GR4

Tumor Medulloblastoma
Cavalli - 763 - rma_sketch - hugene11t
BCL2 (8023646)
Expression cutoff: 95.300 (min.grp=8)
subgroup~group4|WITH_SURV (n=264)

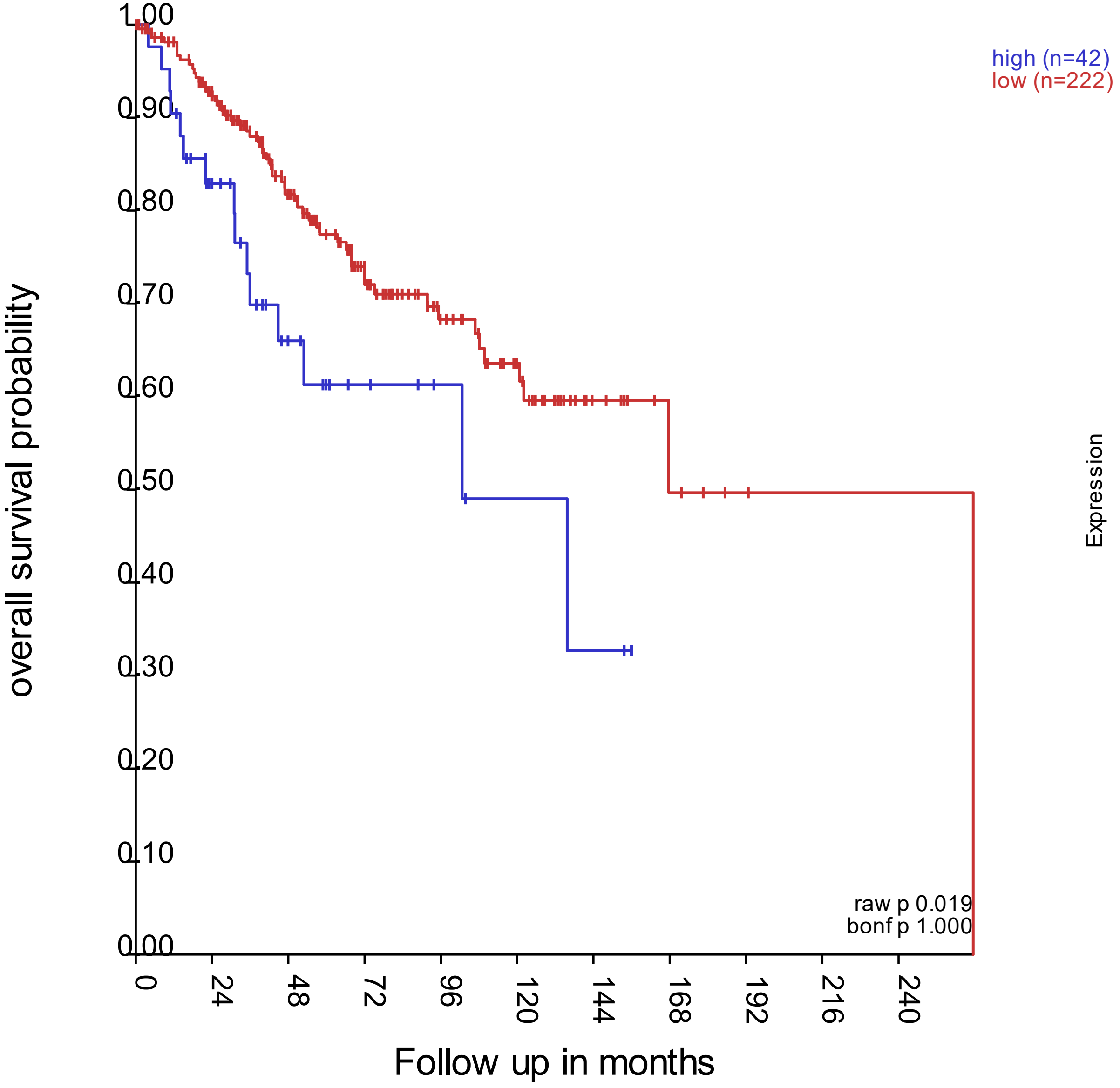

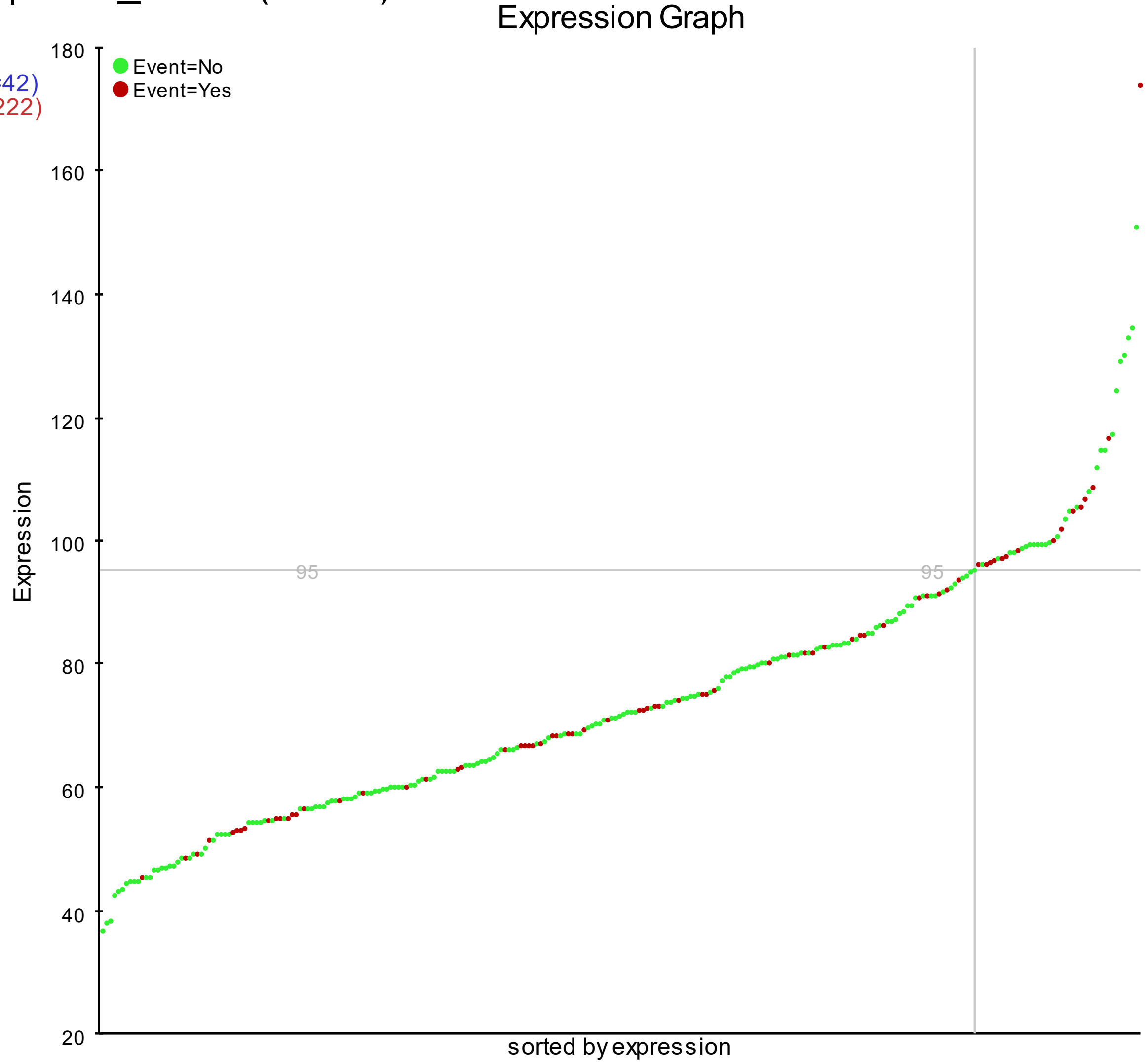

GR3

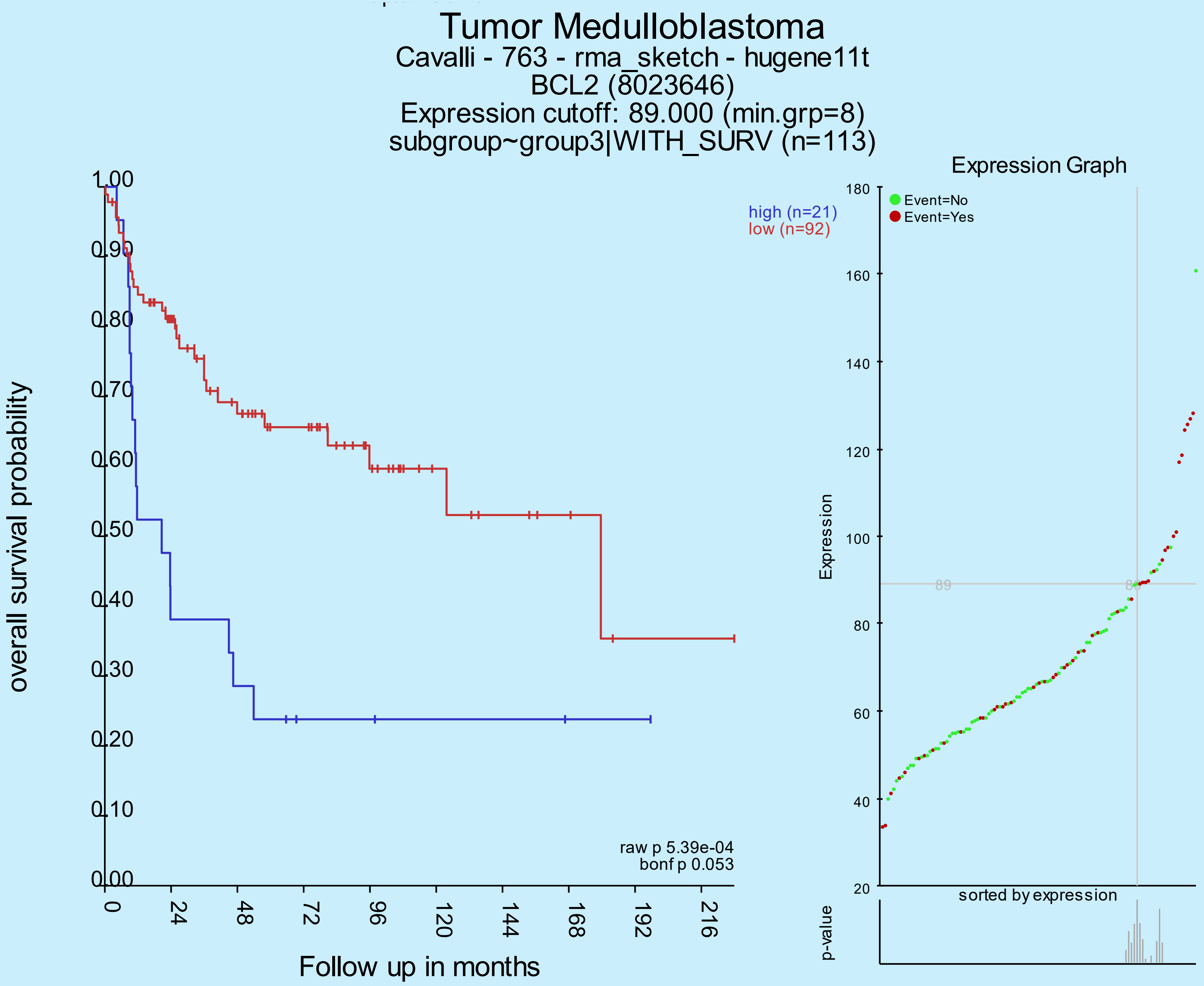

Tumor Medulloblastoma
Cavalli - 763 - rma_sketch - hugene11t
BCL2 (8023646)
Expression cutoff: 89.000 (min.grp=8)
subgroup~group3|WITH_SURV (n=113)
high (n=21)
low (n=92)
overall survival probability
1.00
0.90
0.80
0.70
0.60
0.50
0.40
0.30
0.20
0.10
0.00
0
24
48
72
96
120
144
168
192
216
Follow up in months
raw p 5.39e-04
bonf p 0.053
Expression Graph
Event=No
Event=Yes
Expression
180
160
140
120
100
80
60
40
20
89
sorted by expression
p-value

BCL2L1

WNT

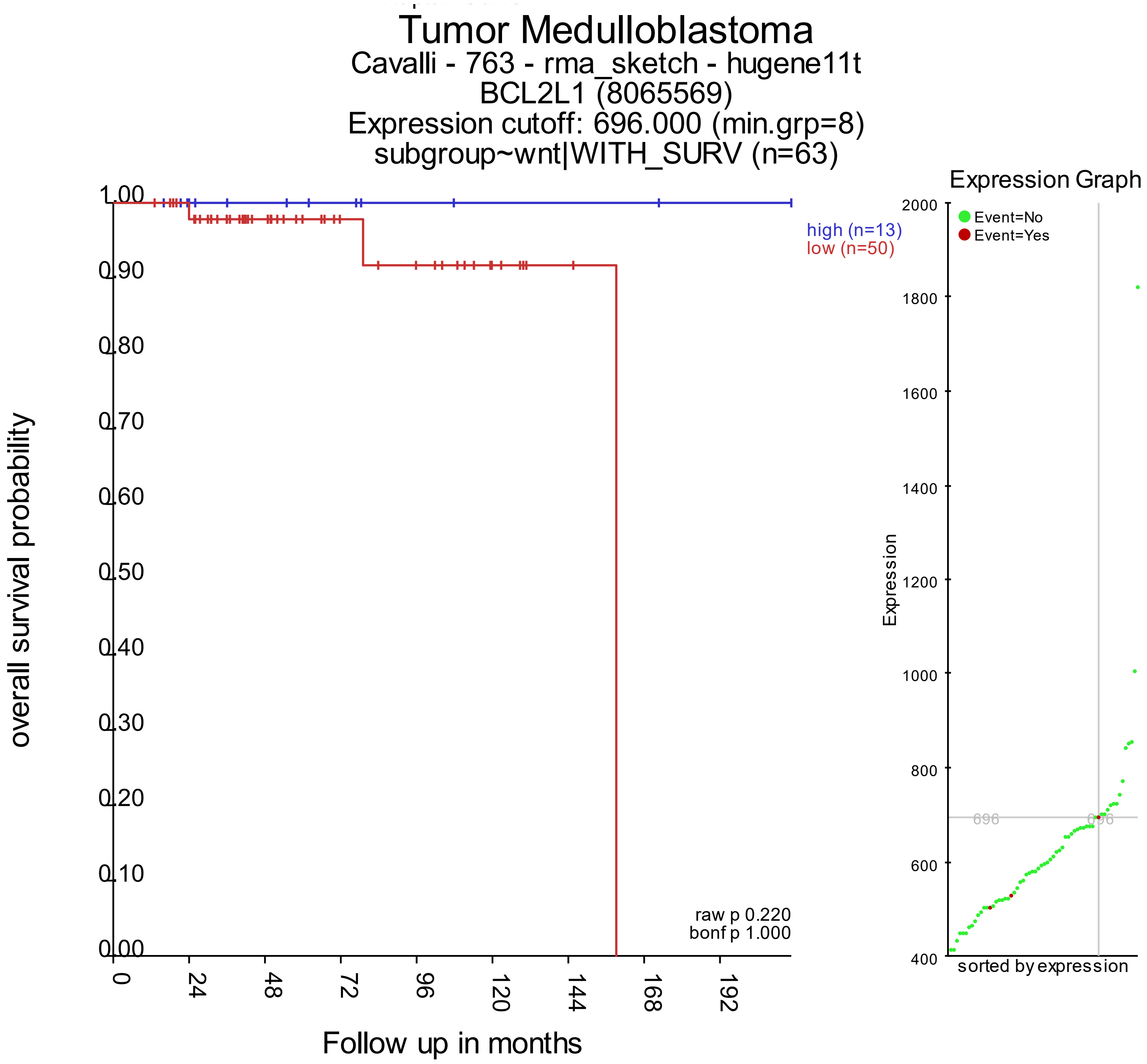
Tumor Medulloblastoma
Cavalli - 763 - rma_sketch - hugene11t
BCL2L1 (8065569)
Expression cutoff: 696.000 (min.grp=8)
subgroup~wnt|WITH_SURV (n=63)
overall survival probability
1.00
0.90
0.80
0.70
0.60
0.50
0.40
0.30
0.20
0.10
0.00
0
24
48
72
96
120
144
168
192
Follow up in months
high (n=13)
low (n=50)
raw p 0.220
bonf p 1.000
Expression Graph
Event=No
Event=Yes
2000
1800
1600
1400
1200
1000
800
600
400
Expression
696
sorted by expression

SHH

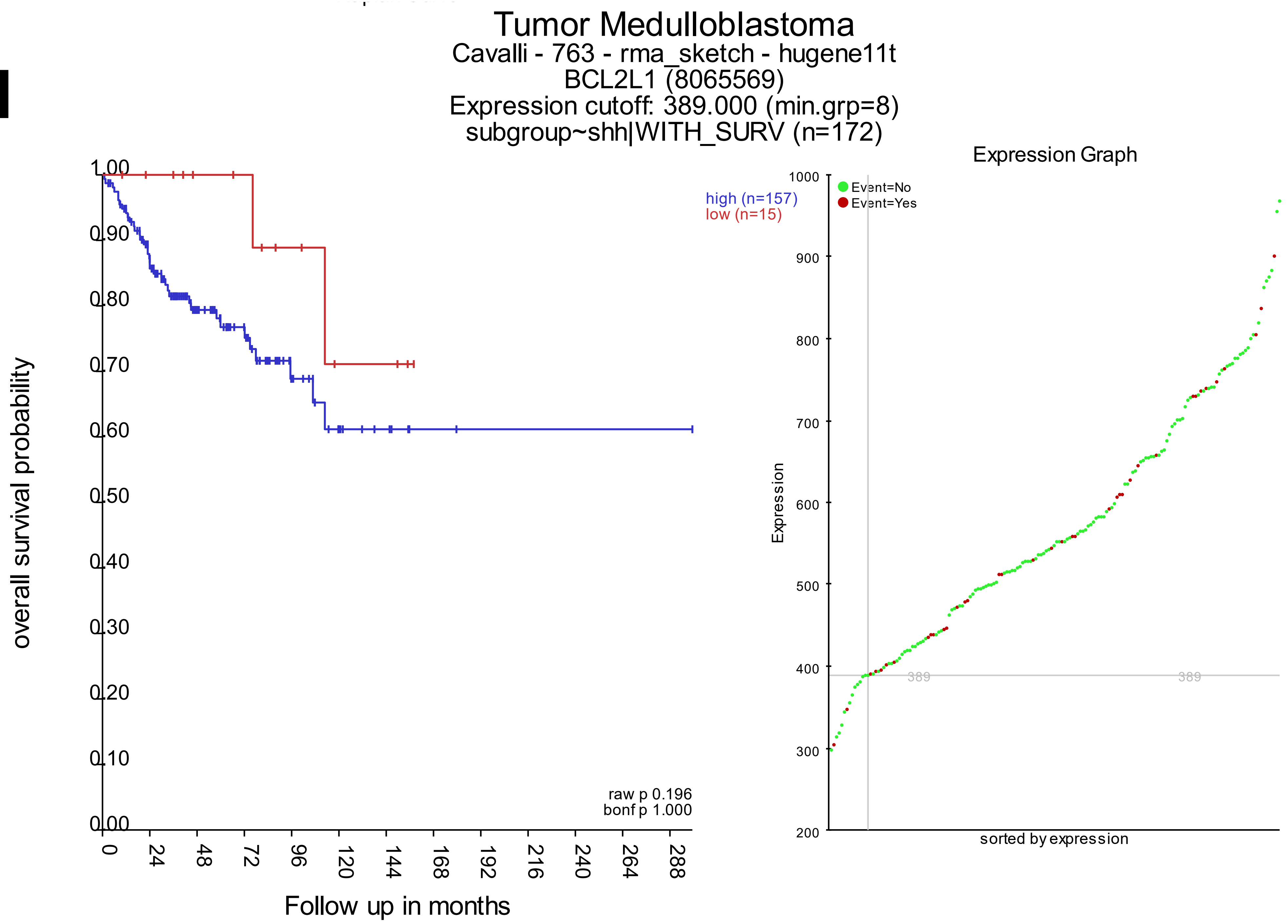

Tumor Medulloblastoma
Cavalli - 763 - rma_sketch - hugene11t
BCL2L1 (8065569)
Expression cutoff: 389.000 (min.grp=8)
subgroup~shh|WITH_SURV (n=172)
overall survival probability
1.00
0.90
0.80
0.70
0.60
0.50
0.40
0.30
0.20
0.10
0.00
0
24
48
72
96
120
144
168
192
216
240
264
288
Follow up in months
high (n=157)
low (n=15)
raw p 0.196
bonf p 1.000
Expression Graph
Event=No
Event=Yes
1000
900
800
700
600
500
400
300
200
Expression
389
389
sorted by expression

GR4

Tumor Medulloblastoma
Cavalli - 763 - rma_sketch - hugene11t
BCL2L1 (8065569)
Expression cutoff: 633.300 (min.grp=8)
subgroup~group4|WITH_SURV (n=264)

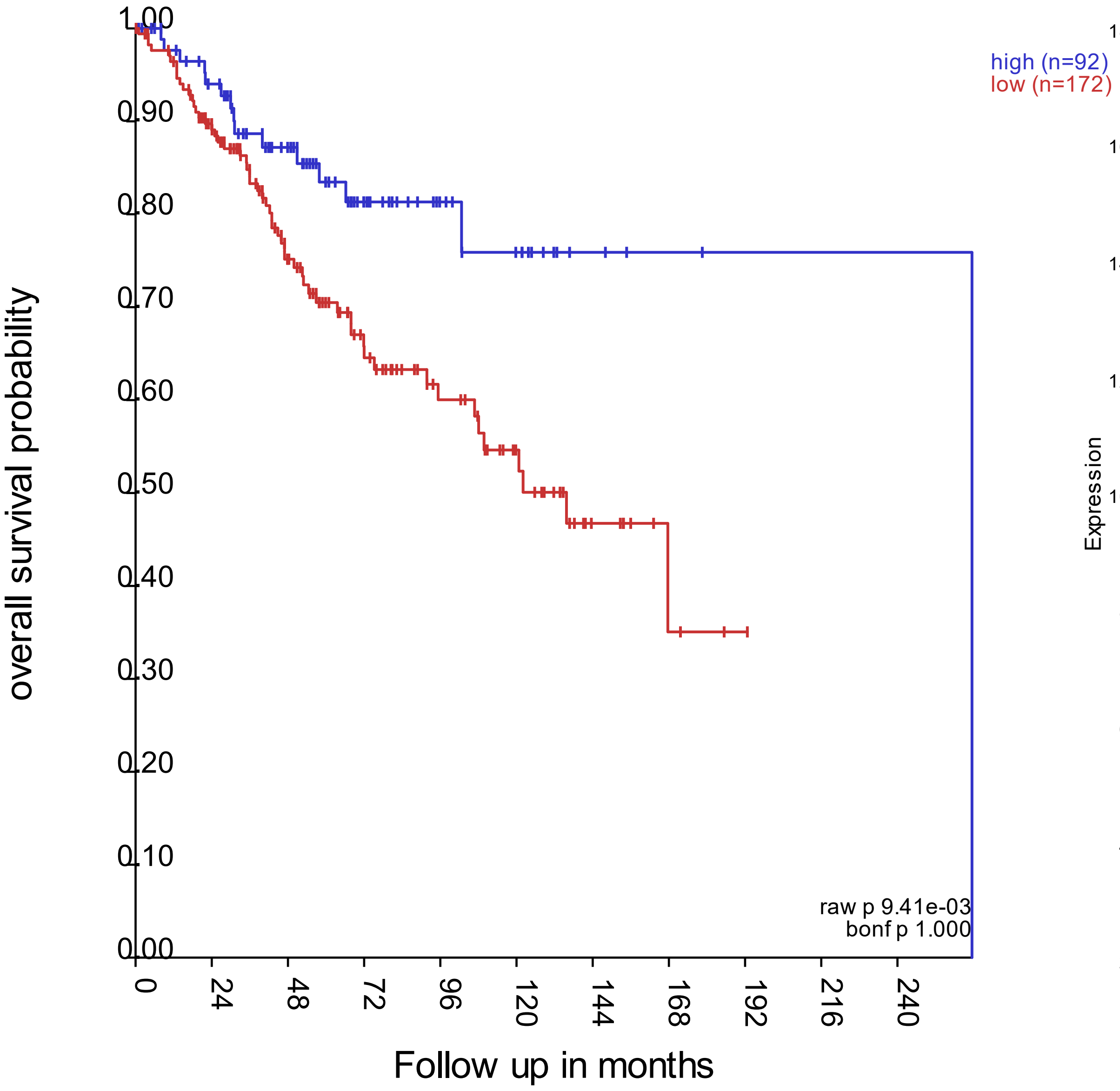

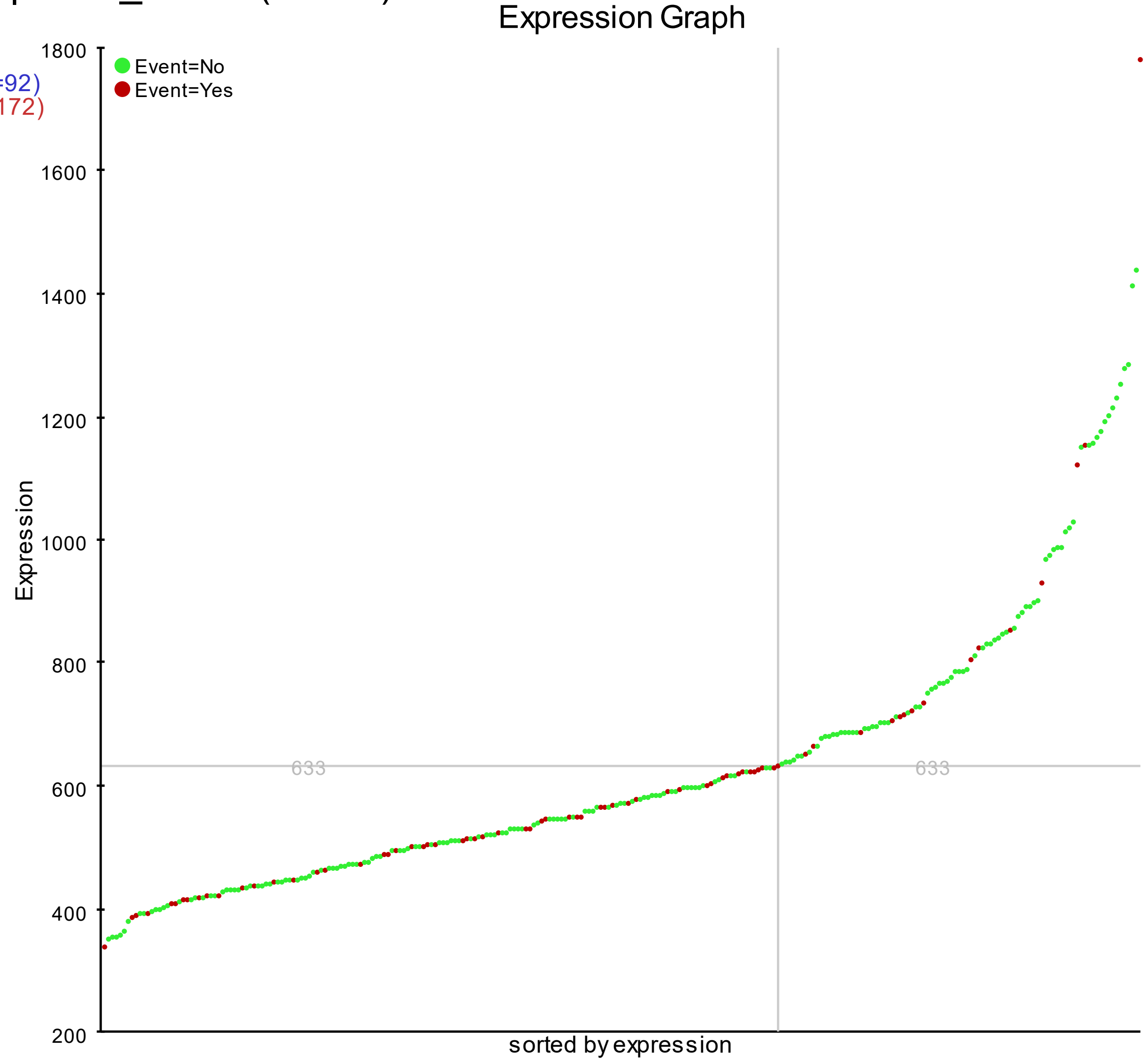

GR3

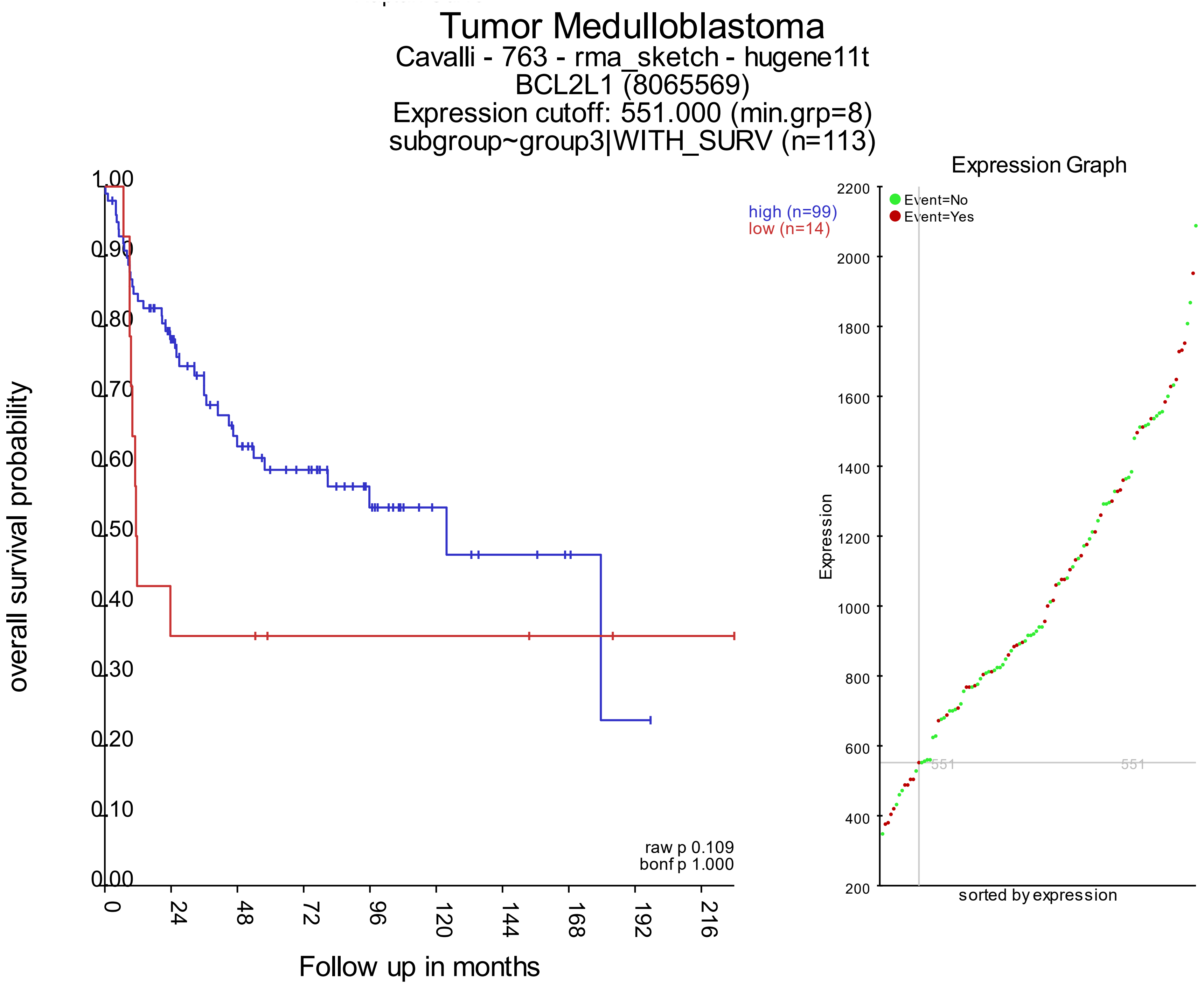
Tumor Medulloblastoma
Cavalli - 763 - rma_sketch - hugene11t
BCL2L1 (8065569)
Expression cutoff: 551.000 (min.grp=8)
subgroup~group3|WITH_SURV (n=113)
overall survival probability
Follow up in months
high (n=99)
low (n=14)
raw p 0.109
bonf p 1.000
Expression Graph
Event=No
Event=Yes
Expression
sorted by expression
551

BCL2L2

WNT

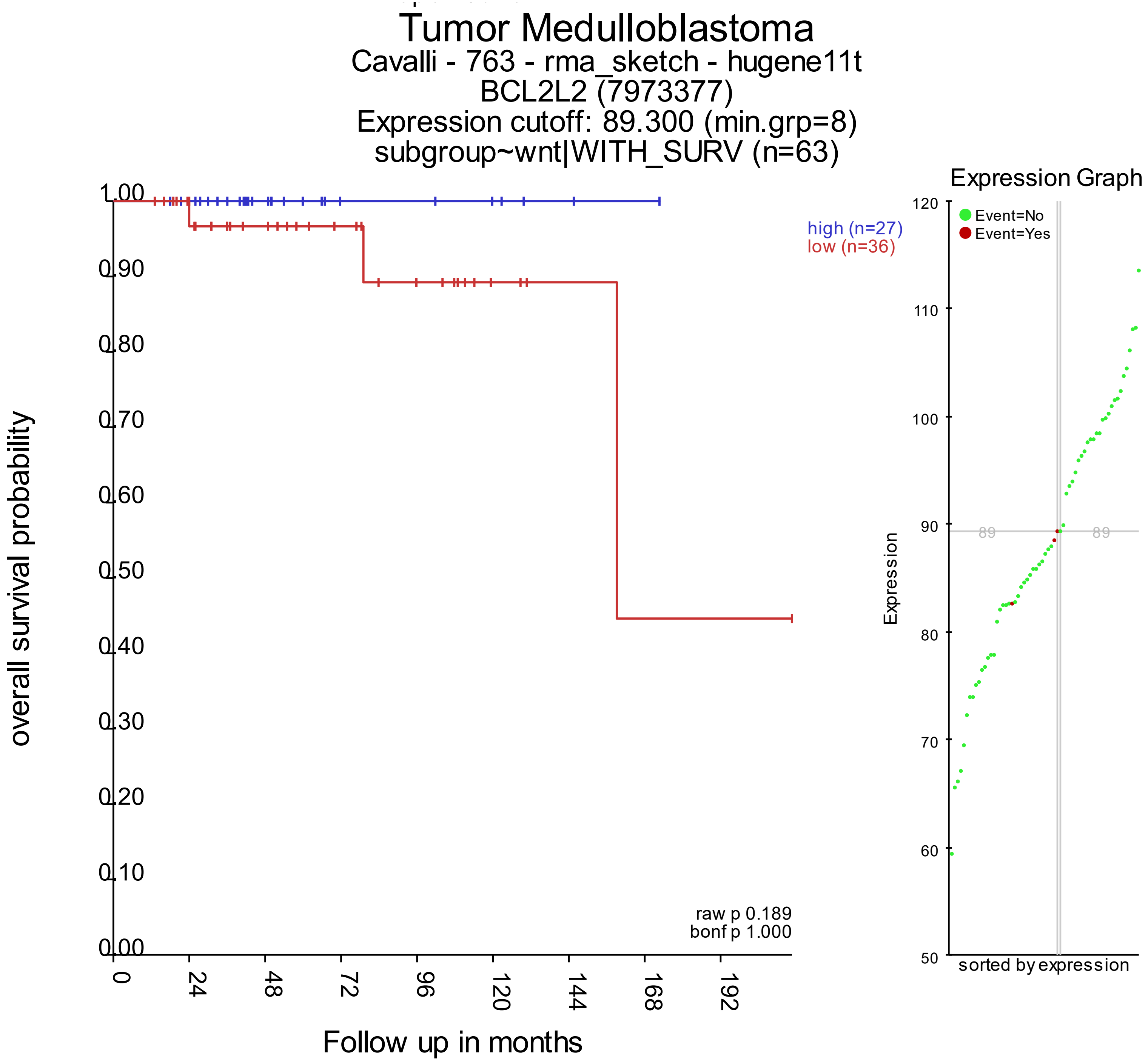

Tumor Medulloblastoma
Cavalli - 763 - rma_sketch - hugene11t
BCL2L2 (7973377)
Expression cutoff: 89.300 (min.grp=8)
subgroup~wnt|WITH_SURV (n=63)
overall survival probability
1.00
0.90
0.80
0.70
0.60
0.50
0.40
0.30
0.20
0.10
0.00
0
24
48
72
96
120
144
168
192
Follow up in months
high (n=27)
low (n=36)
raw p 0.189
bonf p 1.000
Expression Graph
Event=No
Event=Yes
Expression
120
110
100
90
80
70
60
50
89
89
sorted by expression

SHH

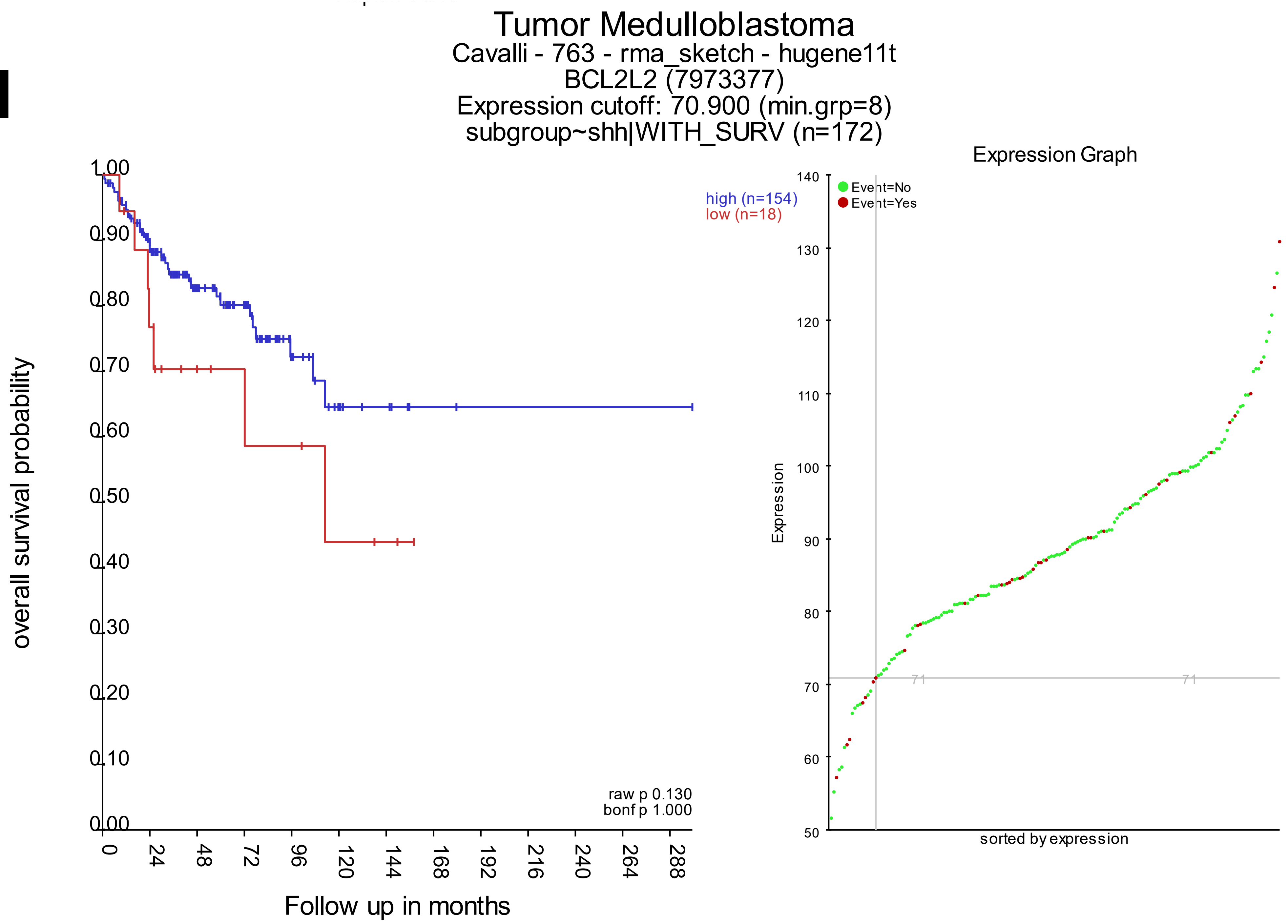

Tumor Medulloblastoma
Cavalli - 763 - rma_sketch - hugene11t
BCL2L2 (7973377)
Expression cutoff: 70.900 (min.grp=8)
subgroup~shh|WITH_SURV (n=172)
overall survival probability
1.00
0.90
0.80
0.70
0.60
0.50
0.40
0.30
0.20
0.10
0.00
high (n=154)
low (n=18)
raw p 0.130
bonf p 1.000
0
24
48
72
96
120
144
168
192
216
240
264
288
Follow up in months
Expression Graph
Event=No
Event=Yes
Expression
140
130
120
110
100
90
80
70
60
50
71
71
sorted by expression

GR4

Tumor Medulloblastoma
Cavalli - 763 - rma_sketch - hugene11t
BCL2L2 (7973377)
Expression cutoff: 73.000 (min.grp=8)
subgroup~group4|WITH_SURV (n=264)

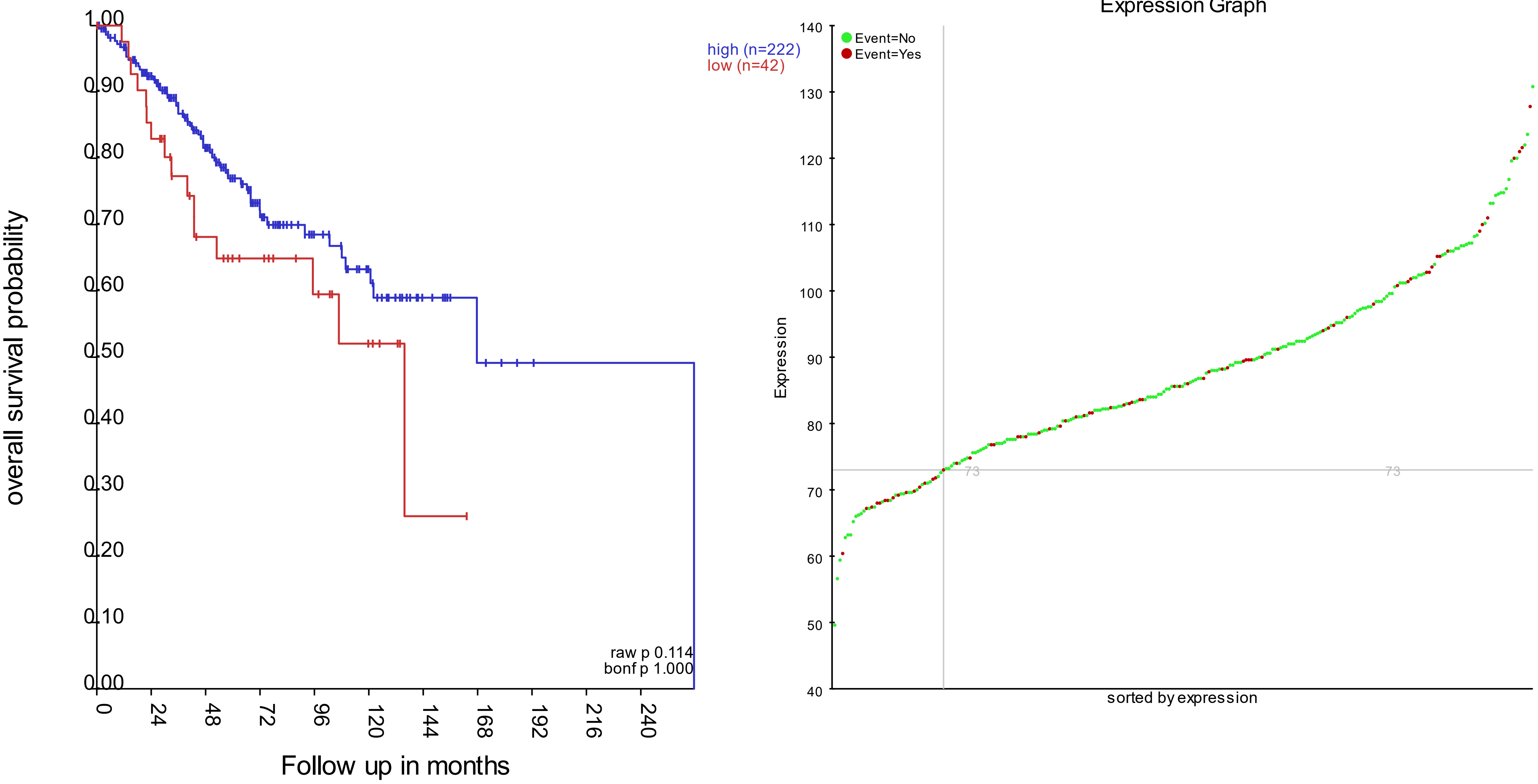

GR3

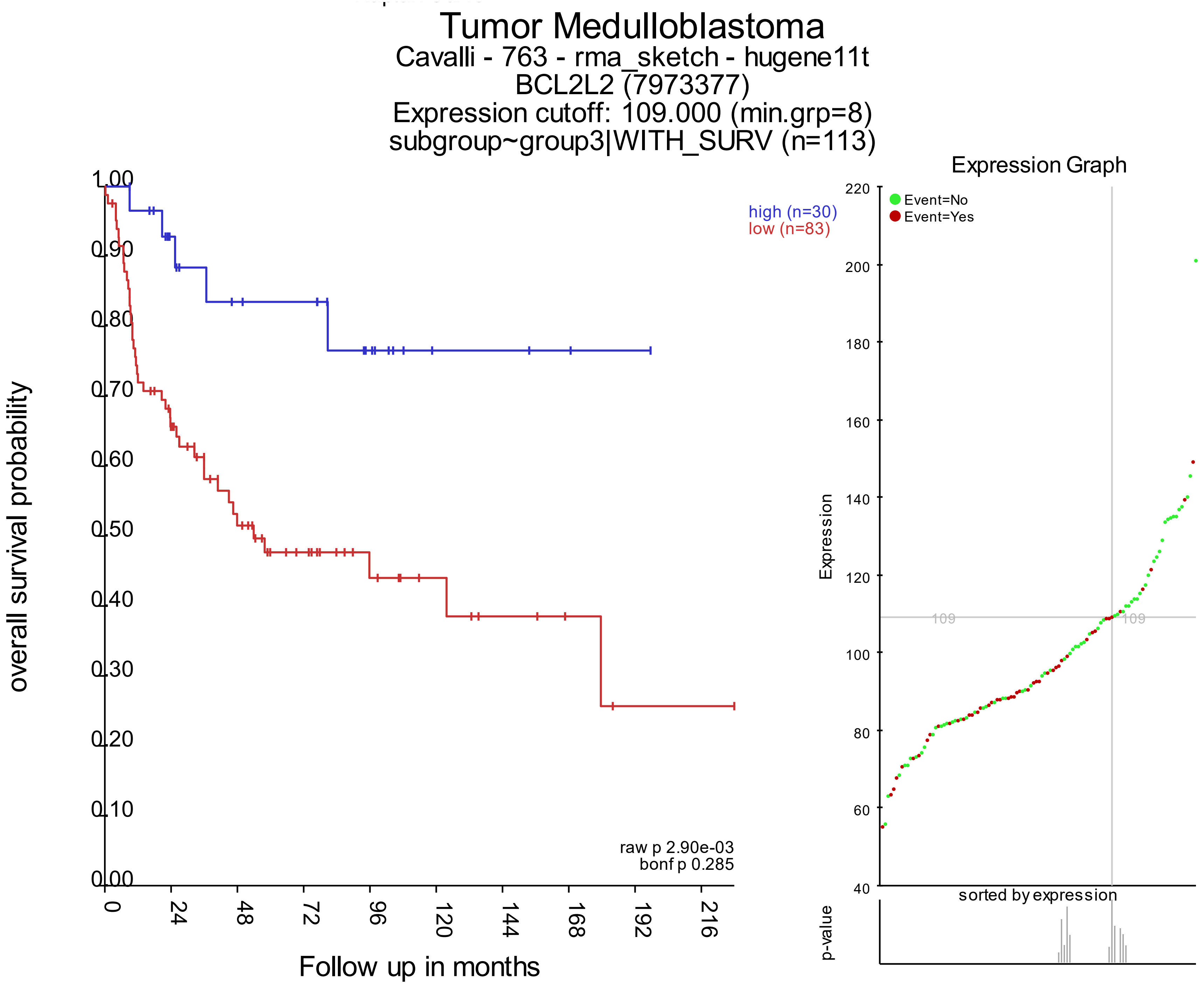
Tumor Medulloblastoma
Cavalli - 763 - rma_sketch - hugene11t
BCL2L2 (7973377)
Expression cutoff: 109.000 (min.grp=8)
subgroup~group3|WITH_SURV (n=113)
overall survival probability
1.00
0.90
0.80
0.70
0.60
0.50
0.40
0.30
0.20
0.10
0.00
high (n=30)
low (n=83)
raw p 2.90e-03
bonf p 0.285
0
24
48
72
96
120
144
168
192
216
Follow up in months
Expression Graph
Event=No
Event=Yes
Expression
220
200
180
160
140
120
100
80
60
40
109
109
sorted by expression
p-value

BTK

WNT

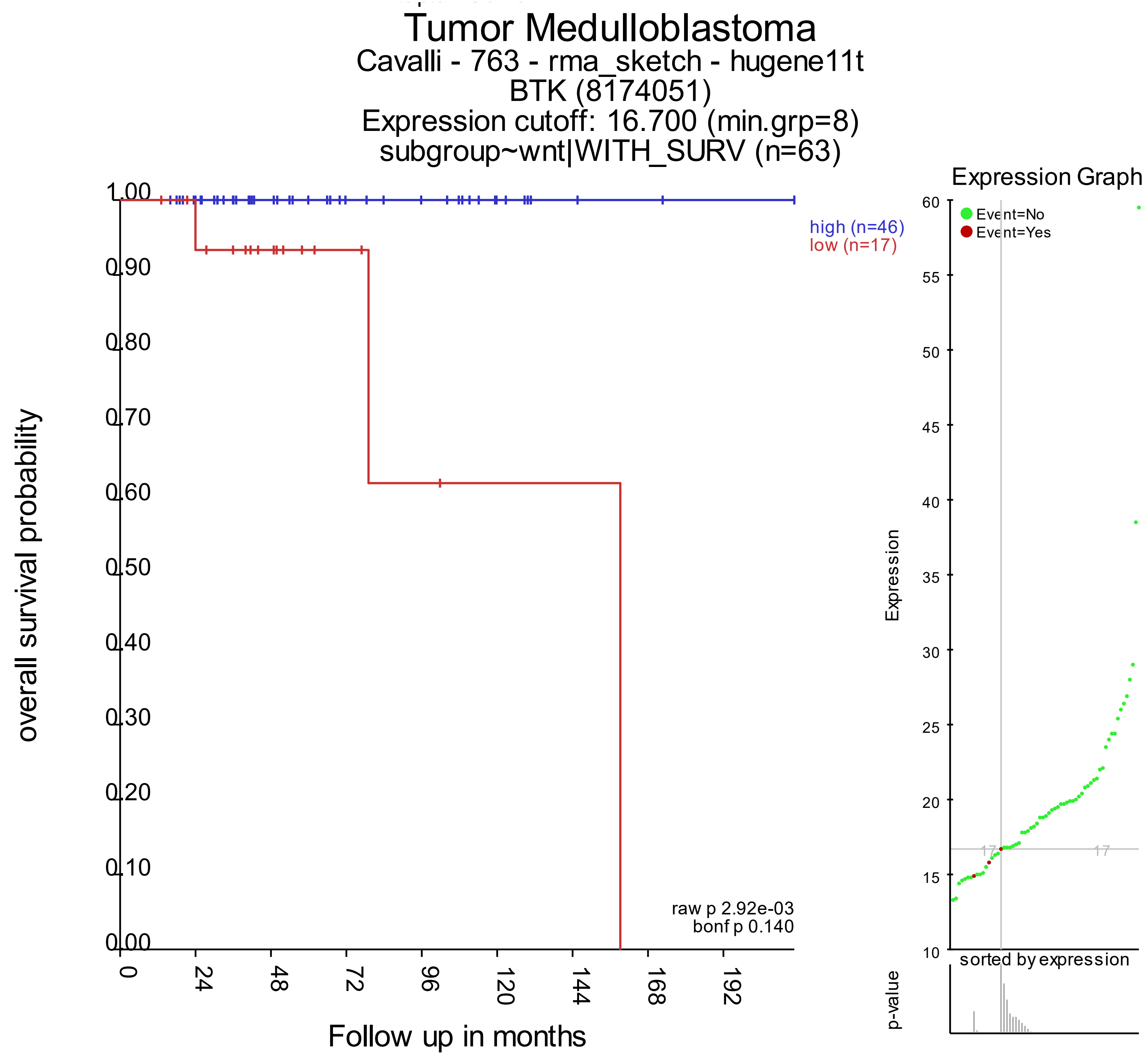

Tumor Medulloblastoma
Cavalli - 763 - rma_sketch - hugene11t
BTK (8174051)
Expression cutoff: 16.700 (min.grp=8)
subgroup~wnt|WITH_SURV (n=63)
overall survival probability
1.00
0.90
0.80
0.70
0.60
0.50
0.40
0.30
0.20
0.10
0.00
high (n=46)
low (n=17)
raw p 2.92e-03
bonf p 0.140
0
24
48
72
96
120
144
168
192
Follow up in months
Expression Graph
Event=No
Event=Yes
60
55
50
45
40
35
30
25
20
15
10
Expression
17
17
sorted by expression
p-value

SHH

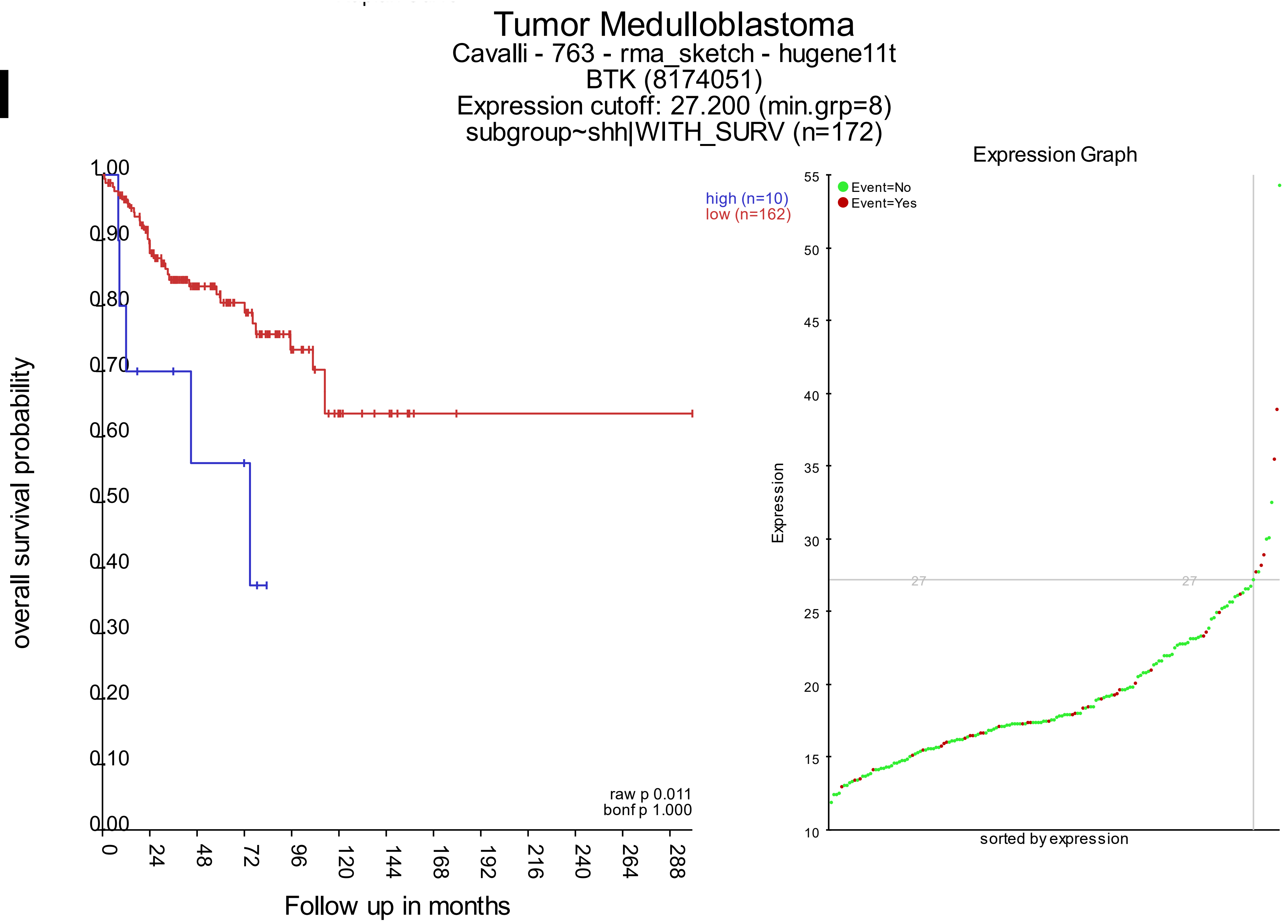

Tumor Medulloblastoma
Cavalli - 763 - rma_sketch - hugene11t
BTK (8174051)
Expression cutoff: 27.200 (min.grp=8)
subgroup~shh|WITH_SURV (n=172)
overall survival probability
1.00
0.90
0.80
0.70
0.60
0.50
0.40
0.30
0.20
0.10
0.00
high (n=10)
low (n=162)
raw p 0.011
bonf p 1.000
0
24
48
72
96
120
144
168
192
216
240
264
288
Follow up in months
Expression Graph
Event=No
Event=Yes
Expression
55
50
45
40
35
30
25
20
15
10
27
27
sorted by expression

GR4

Tumor Medulloblastoma
Cavalli - 763 - rma_sketch - hugene11t
BTK (8174051)
Expression cutoff: 13.000 (min.grp=8)
subgroup~group4|WITH_SURV (n=264)

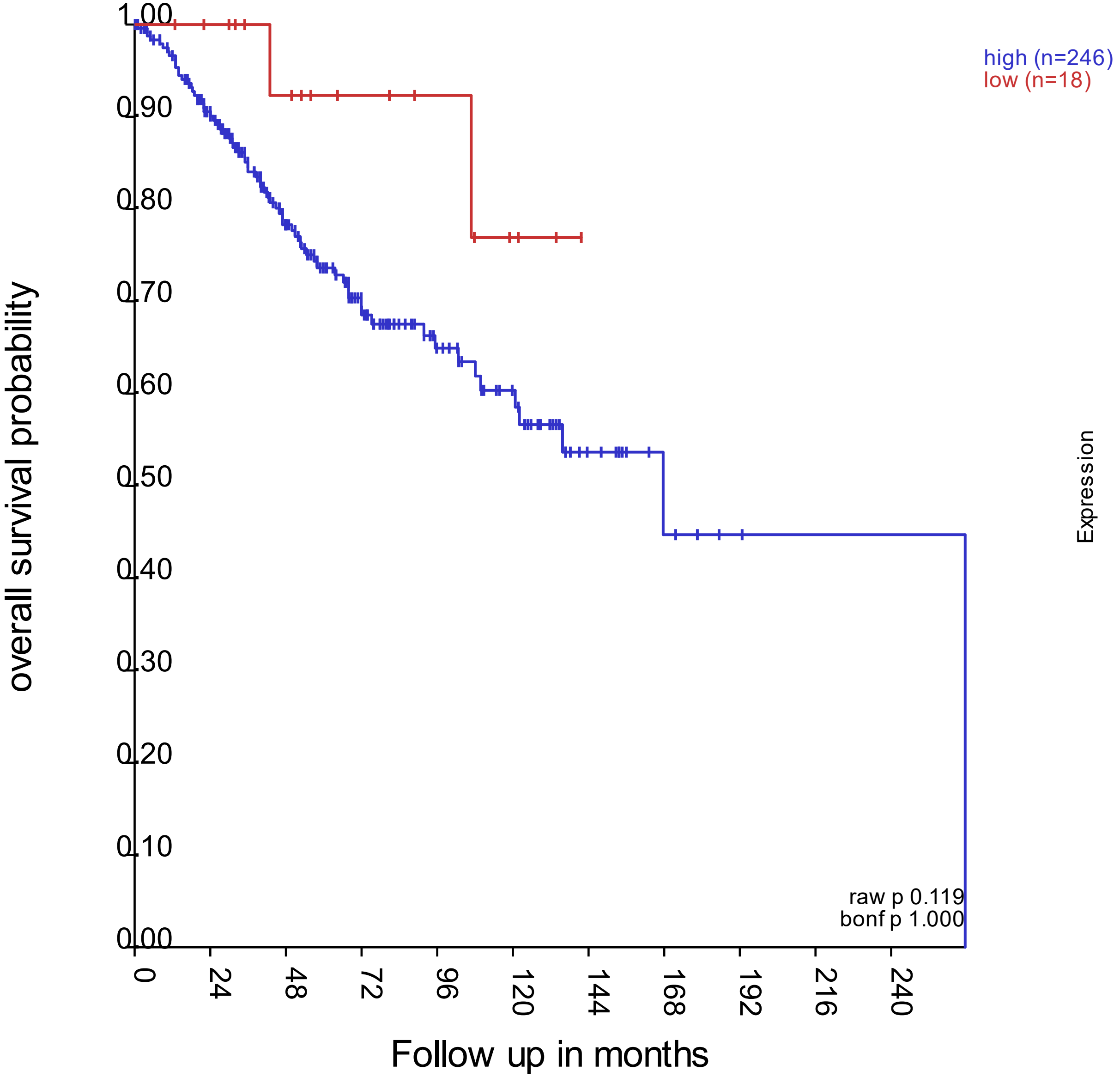

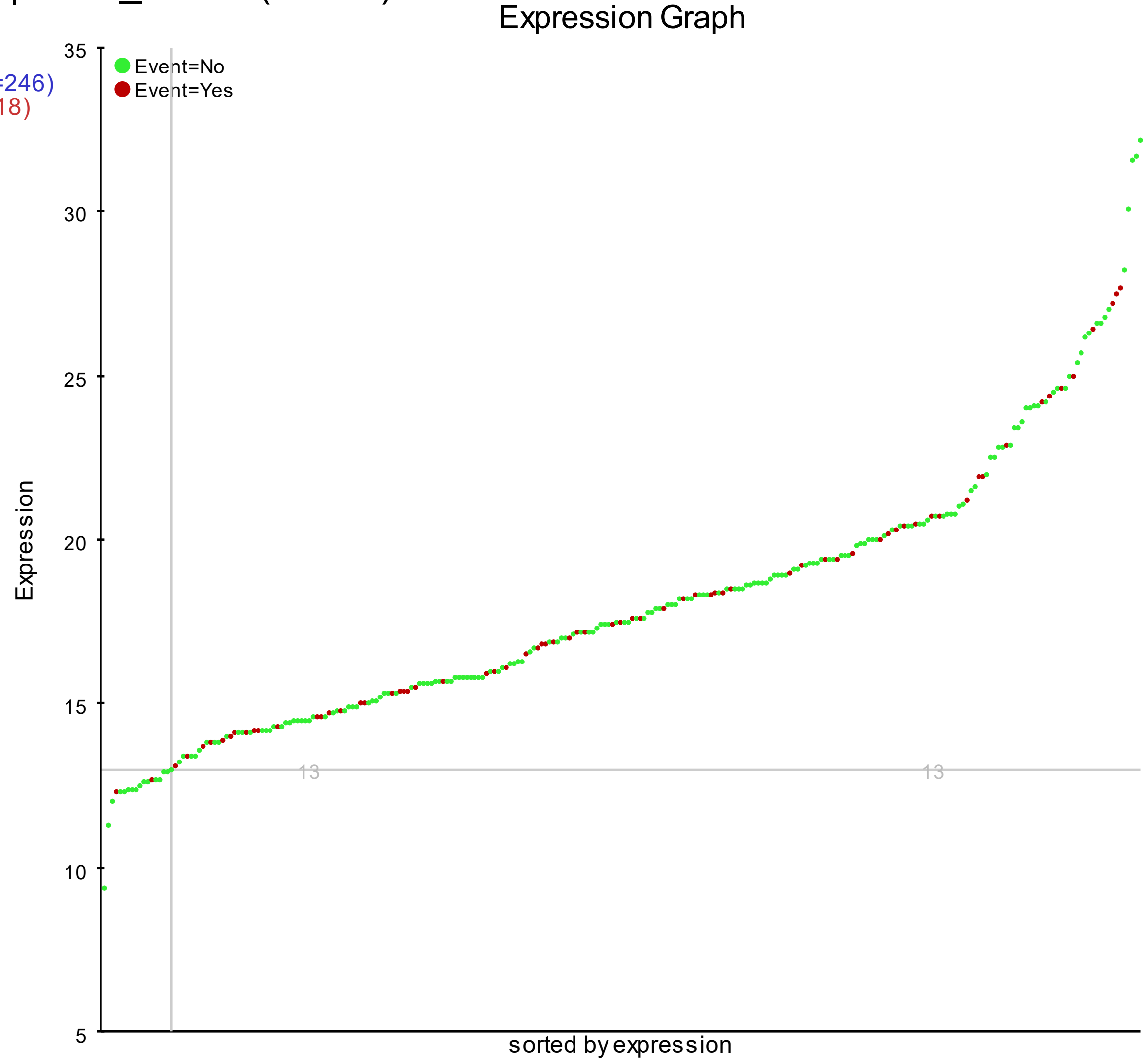

GR3

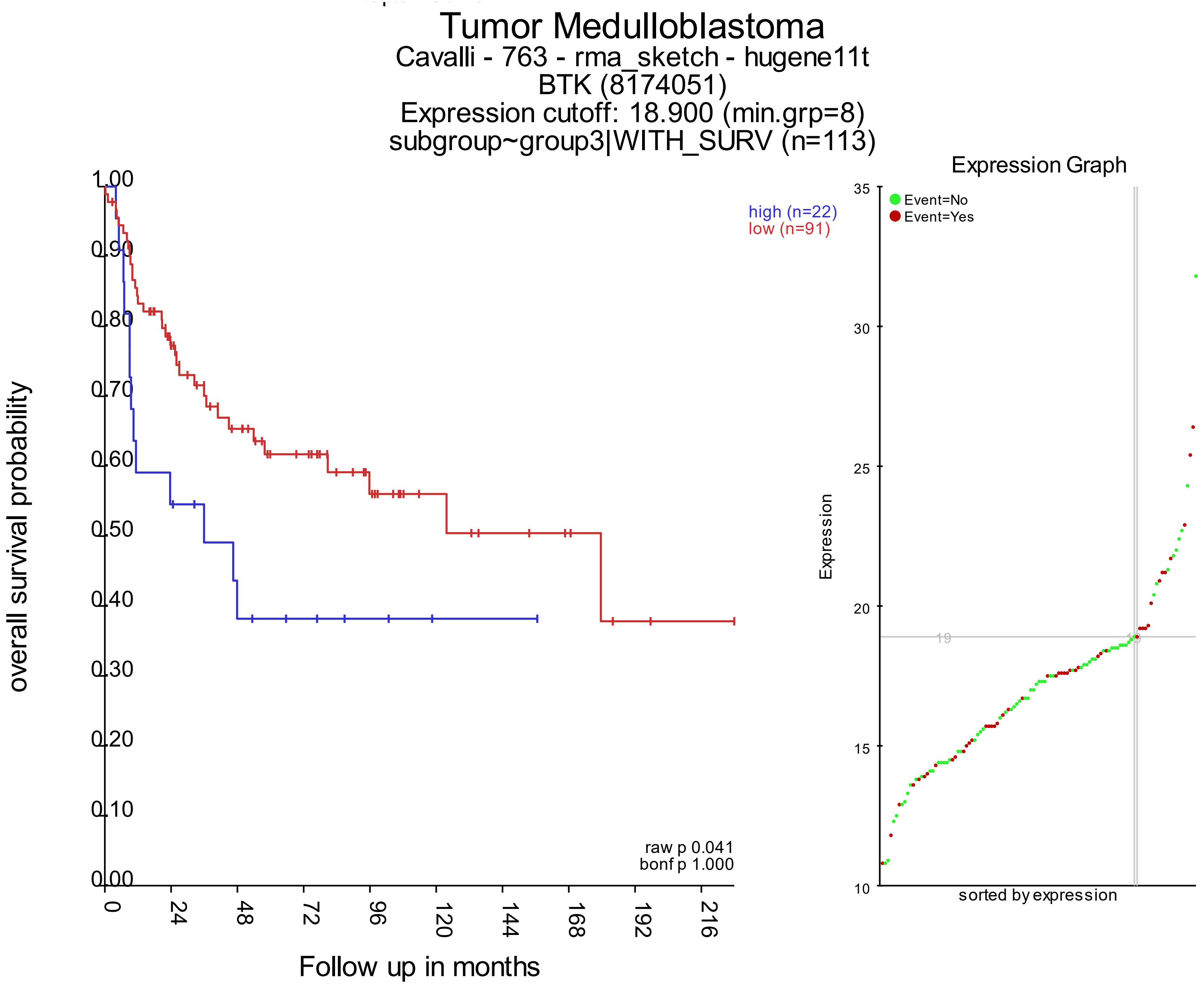

Tumor Medulloblastoma
Cavalli - 763 - rma_sketch - hugene11t
BTK (8174051)
Expression cutoff: 18.900 (min.grp=8)
subgroup~group3|WITH_SURV (n=113)
high (n=22)
low (n=91)
overall survival probability
1.00
0.90
0.80
0.70
0.60
0.50
0.40
0.30
0.20
0.10
0.00
0
24
48
72
96
120
144
168
192
216
Follow up in months
raw p 0.041
bonf p 1.000
Expression Graph
Event=No
Event=Yes
Expression
35
30
25
20
15
10
sorted by expression

CD3D

WNT

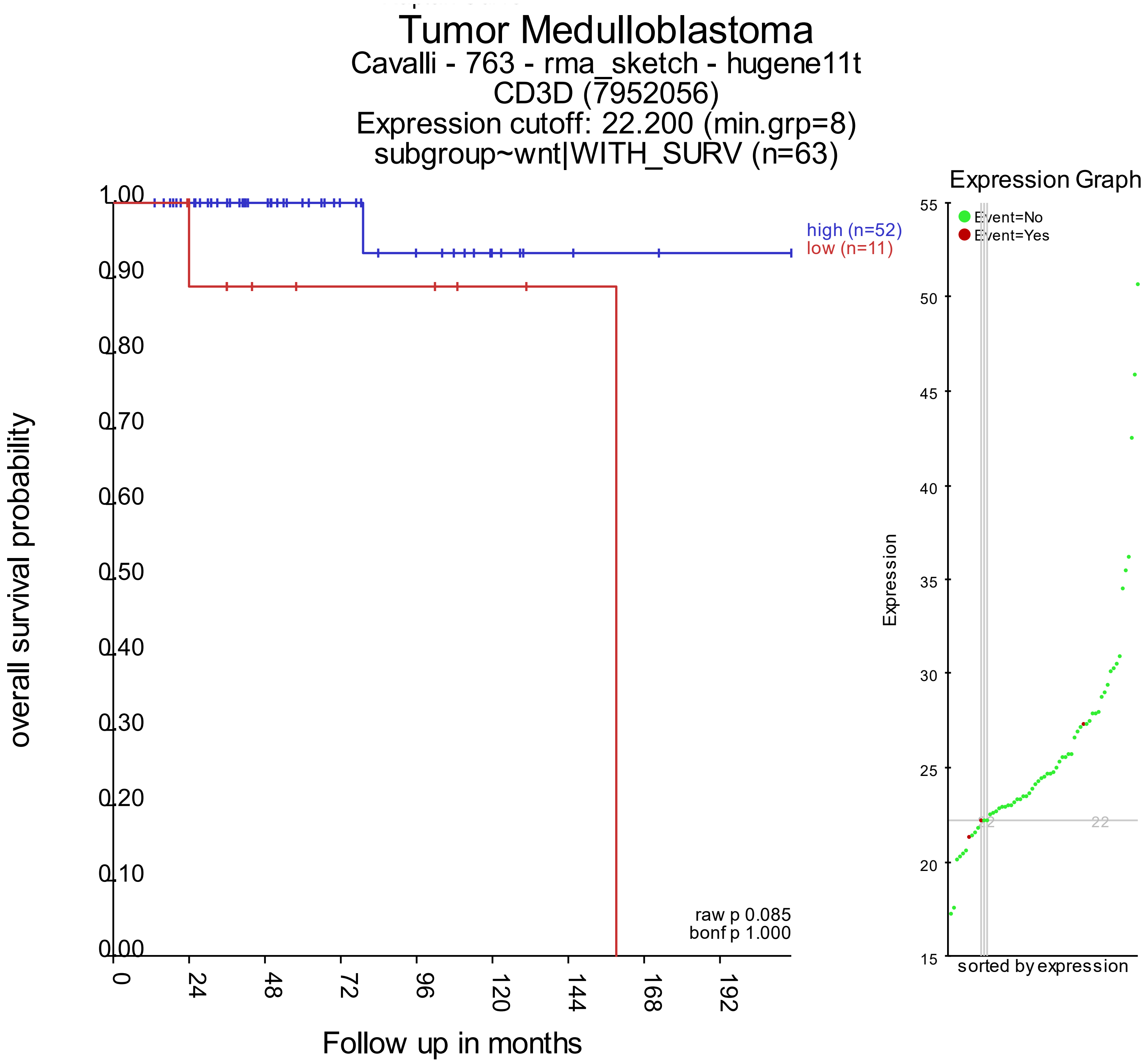

Tumor Medulloblastoma
Cavalli - 763 - rma_sketch - hugene11t
CD3D (7952056)
Expression cutoff: 22.200 (min.grp=8)
subgroup~wnt|WITH_SURV (n=63)
overall survival probability
1.00
0.90
0.80
0.70
0.60
0.50
0.40
0.30
0.20
0.10
0.00
0
24
48
72
96
120
144
168
192
Follow up in months
high (n=52)
low (n=11)
raw p 0.085
bonf p 1.000
Expression Graph
Event=No
Event=Yes
Expression
55
50
45
40
35
30
25
20
15
22
sorted by expression

SHH

Tumor Medulloblastoma
Cavalli - 763 - rma_sketch - hugene11t
CD3D (7952056)
Expression cutoff: 36.500 (min.grp=8)
subgroup~shh|WITH_SURV (n=172)

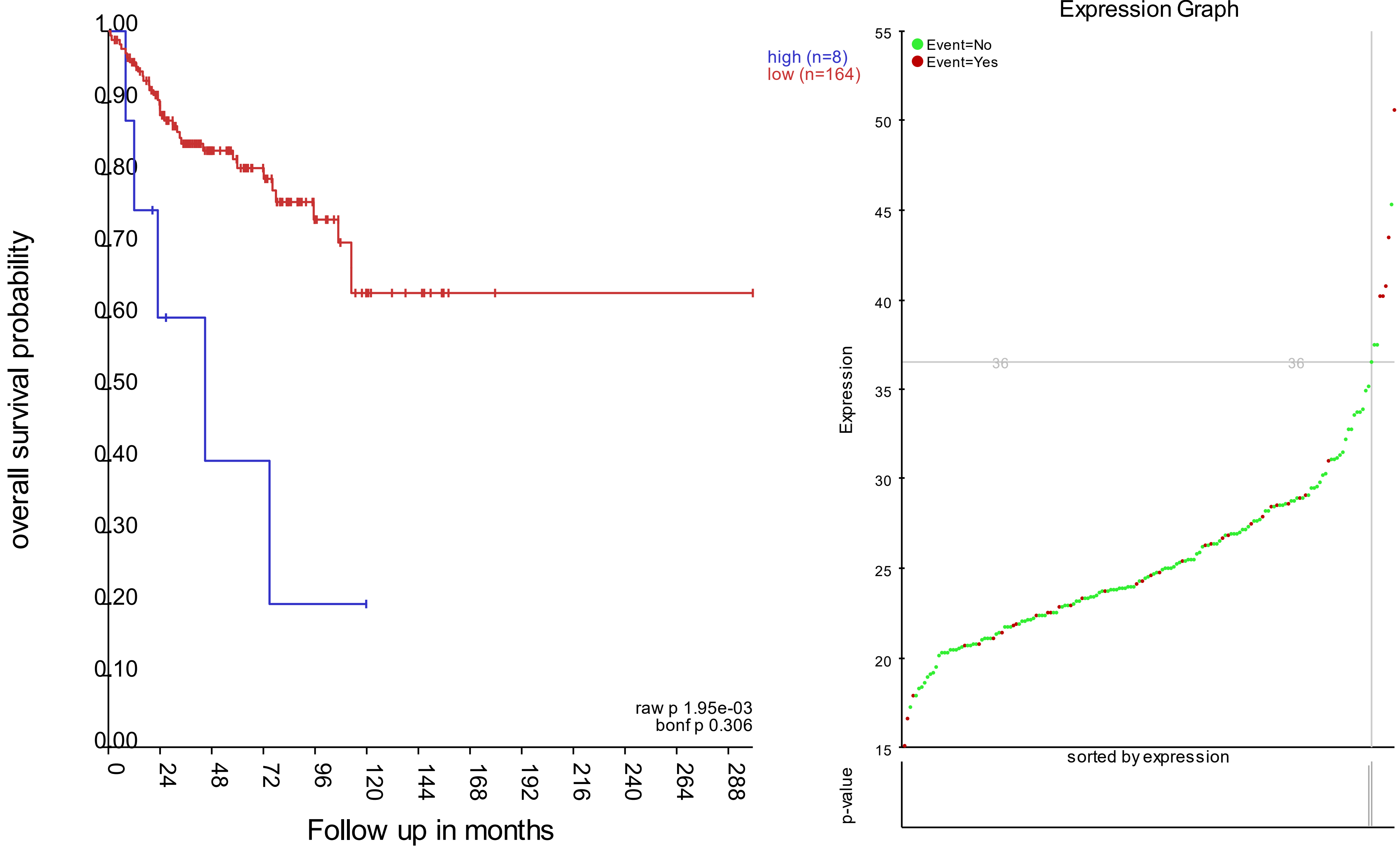

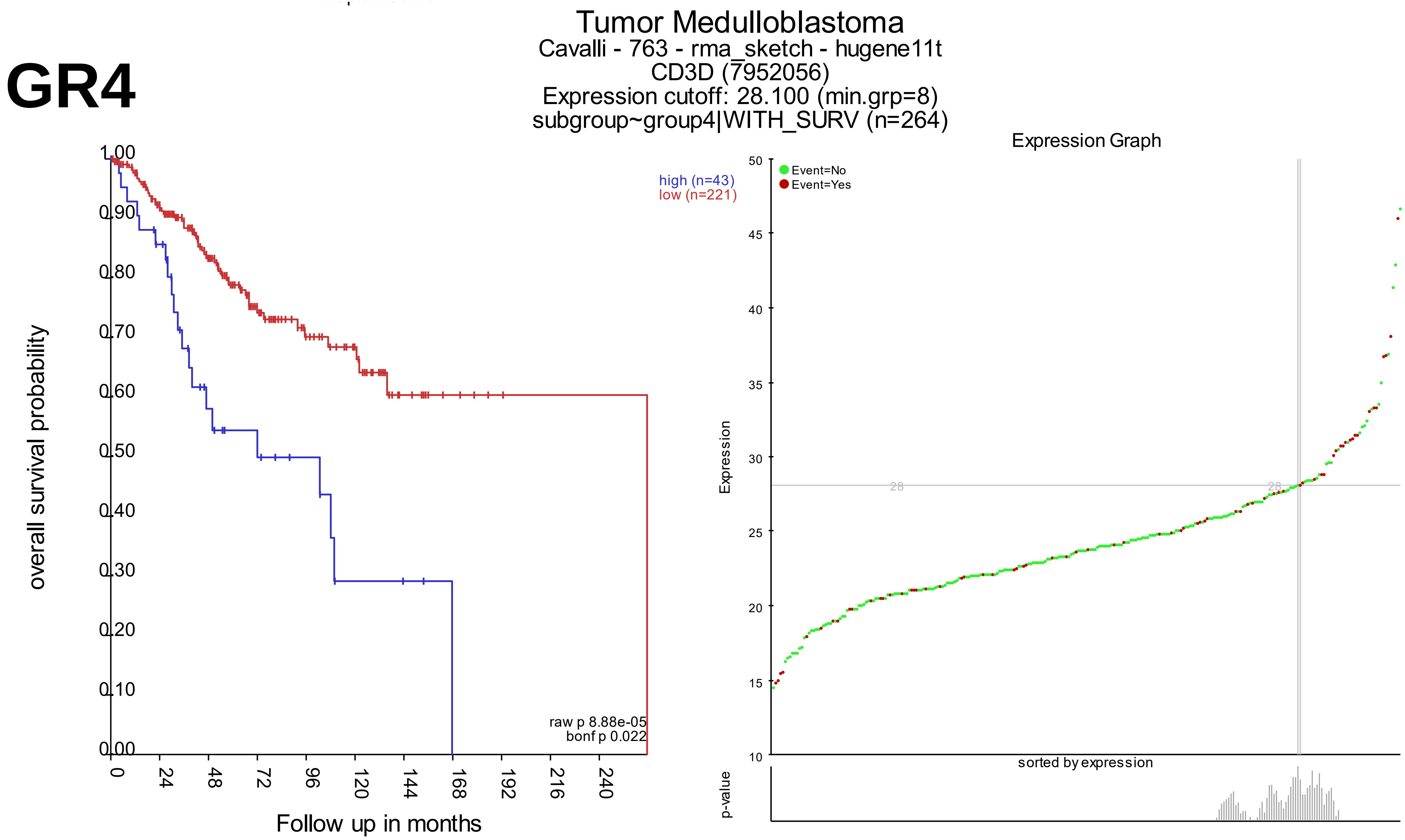

GR4
Tumor Medulloblastoma
Cavalli - 763 - rma_sketch - hugene11t
CD3D (7952056)
Expression cutoff: 28.100 (min.grp=8)
subgroup~group4|WITH_SURV (n=264)
overall survival probability
Follow up in months
high (n=43)
low (n=221)
raw p 8.88e-05
bonf p 0.022
Expression Graph
Event=No
Event=Yes
Expression
sorted by expression
p-value

GR3

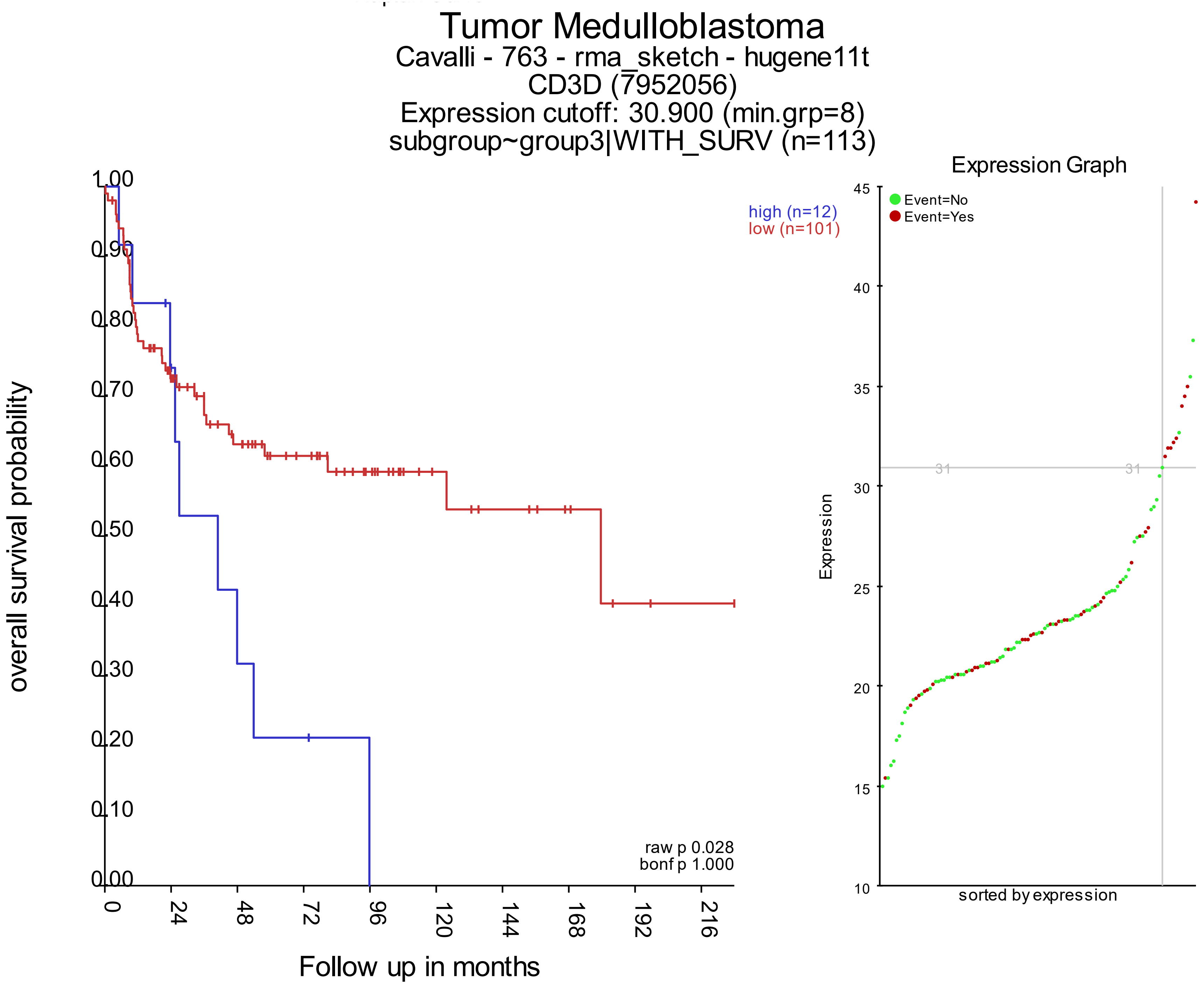

Tumor Medulloblastoma
Cavalli - 763 - rma_sketch - hugene11t
CD3D (7952056)
Expression cutoff: 30.900 (min.grp=8)
subgroup~group3|WITH_SURV (n=113)
overall survival probability
1.00
0.90
0.80
0.70
0.60
0.50
0.40
0.30
0.20
0.10
0.00
0
24
48
72
96
120
144
168
192
216
Follow up in months
high (n=12)
low (n=101)
raw p 0.028
bonf p 1.000
Expression Graph
Event=No
Event=Yes
Expression
45
40
35
30
25
20
15
10
31
31
sorted by expression

CD19

WNT

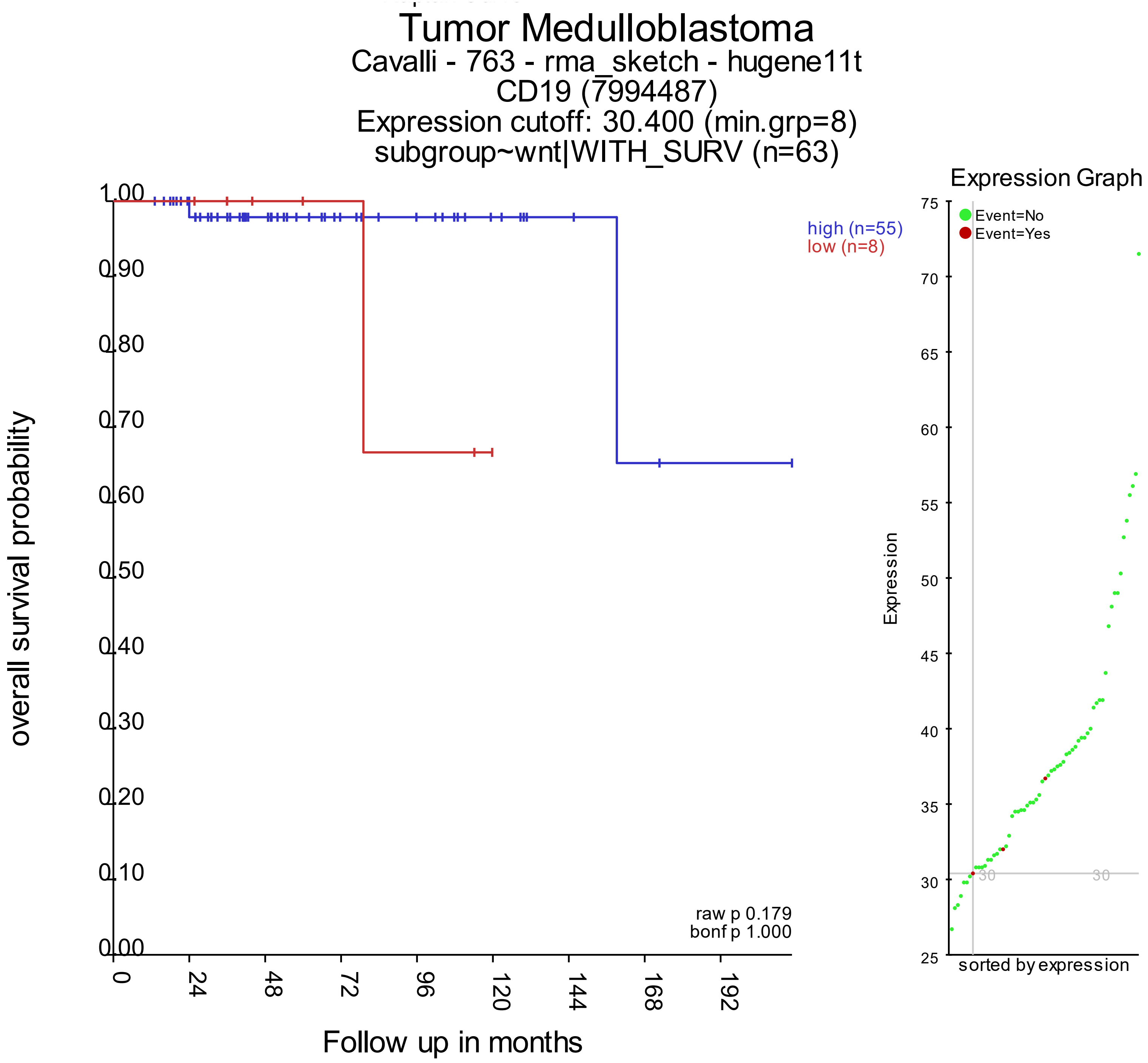

Tumor Medulloblastoma
Cavalli - 763 - rma_sketch - hugene11t
CD19 (7994487)
Expression cutoff: 30.400 (min.grp=8)
subgroup~wnt|WITH_SURV (n=63)
overall survival probability
1.00
0.90
0.80
0.70
0.60
0.50
0.40
0.30
0.20
0.10
0.00
0
24
48
72
96
120
144
168
192
Follow up in months
high (n=55)
low (n=8)
raw p 0.179
bonf p 1.000
Expression Graph
Event=No
Event=Yes
Expression
75
70
65
60
55
50
45
40
35
30
25
sorted by expression

SHH

Tumor Medulloblastoma
Cavalli - 763 - rma_sketch - hugene11t
CD19 (7994487)
Expression cutoff: 42.300 (min.grp=8)
subgroup~shh|WITH_SURV (n=172)

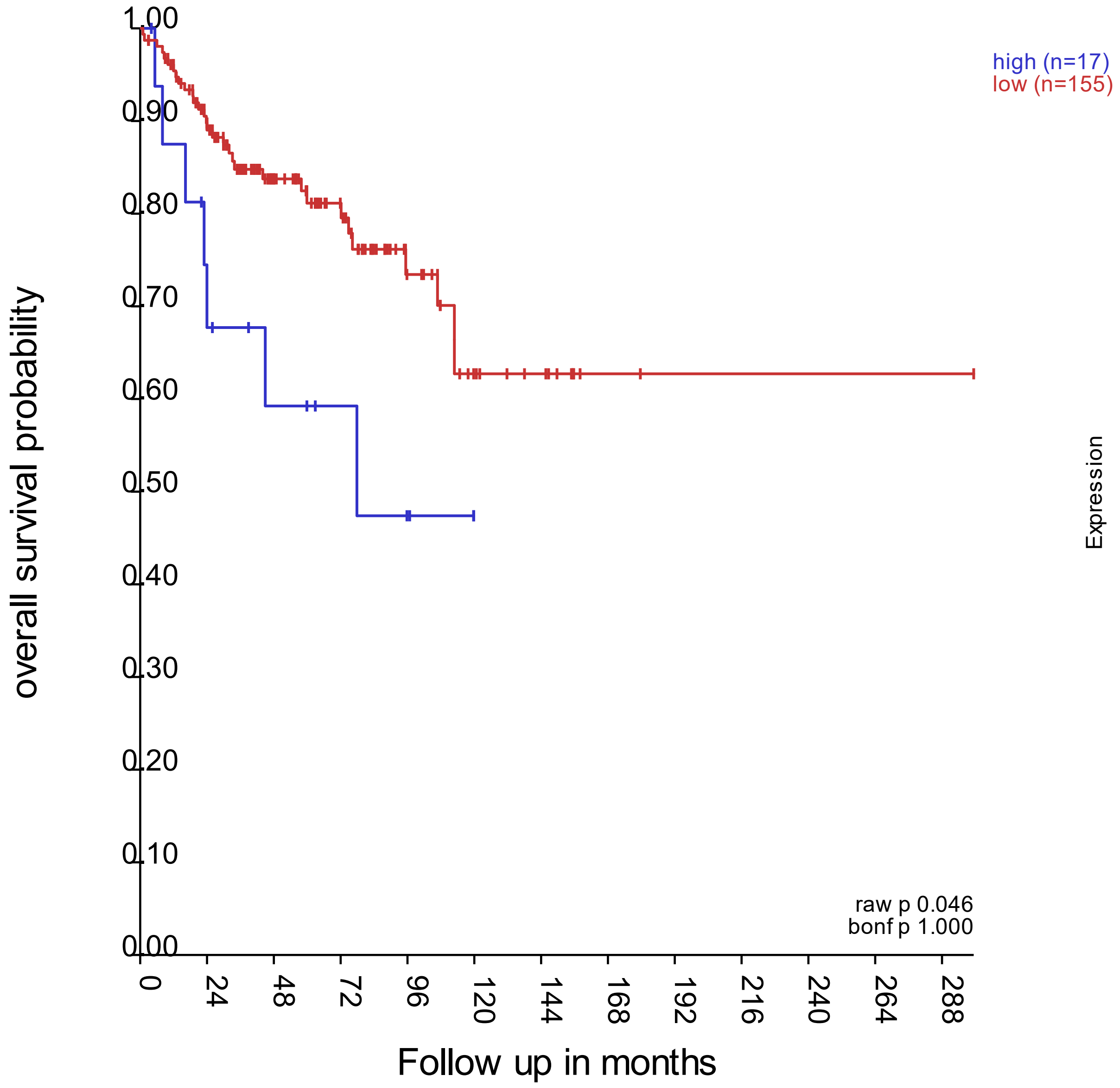

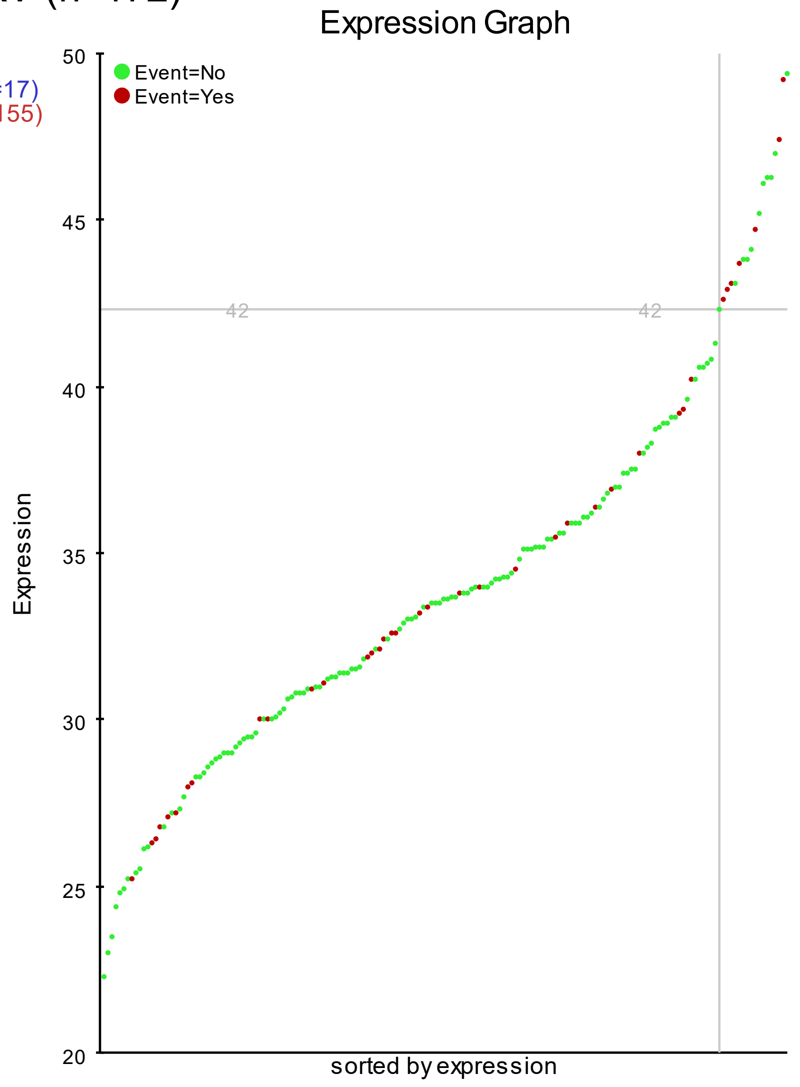

GR4

Tumor Medulloblastoma
Cavalli - 763 - rma_sketch - hugene11t
CD19 (7994487)
Expression cutoff: 28.000 (min.grp=8)
subgroup~group4|WITH_SURV (n=264)

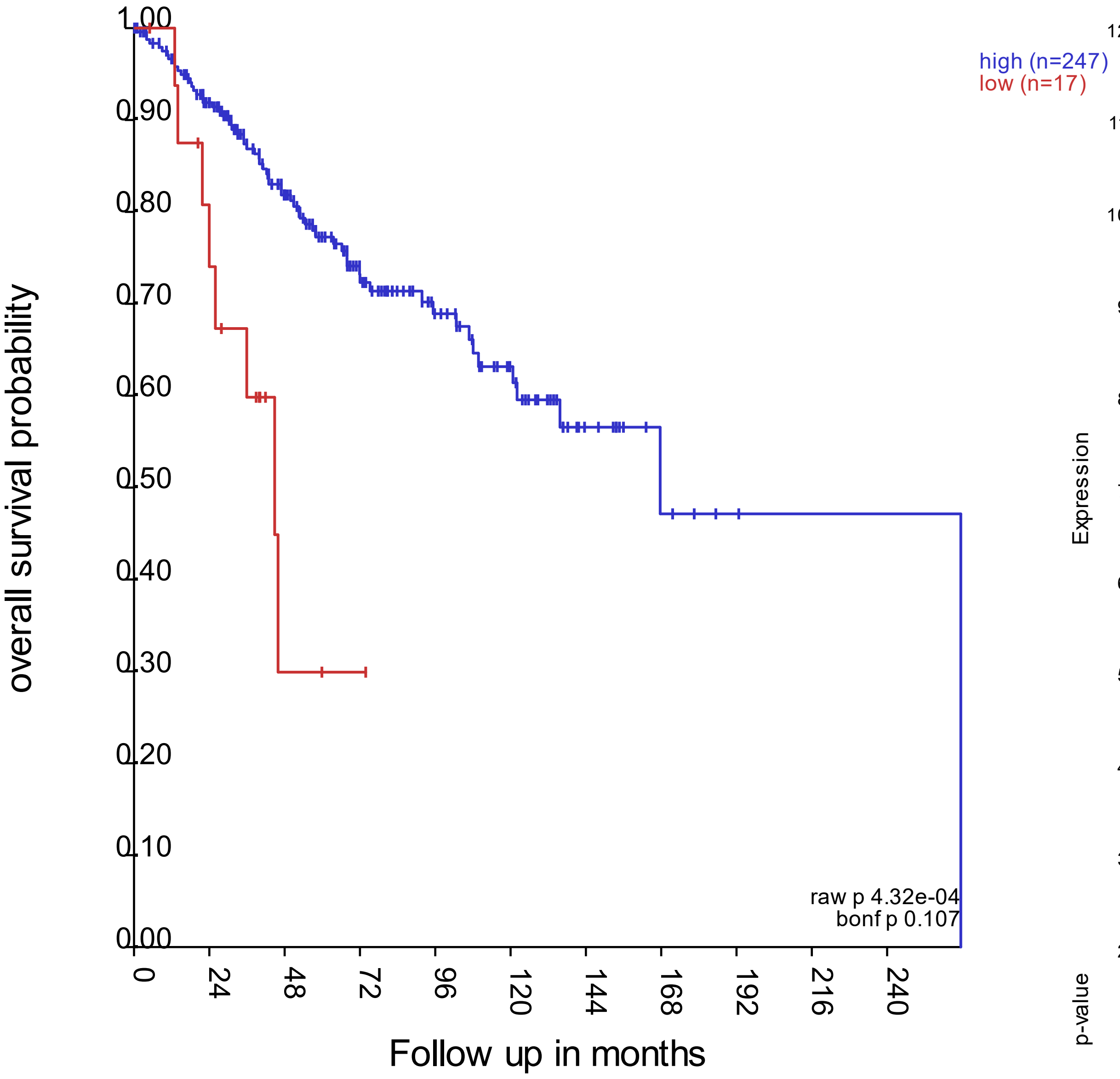

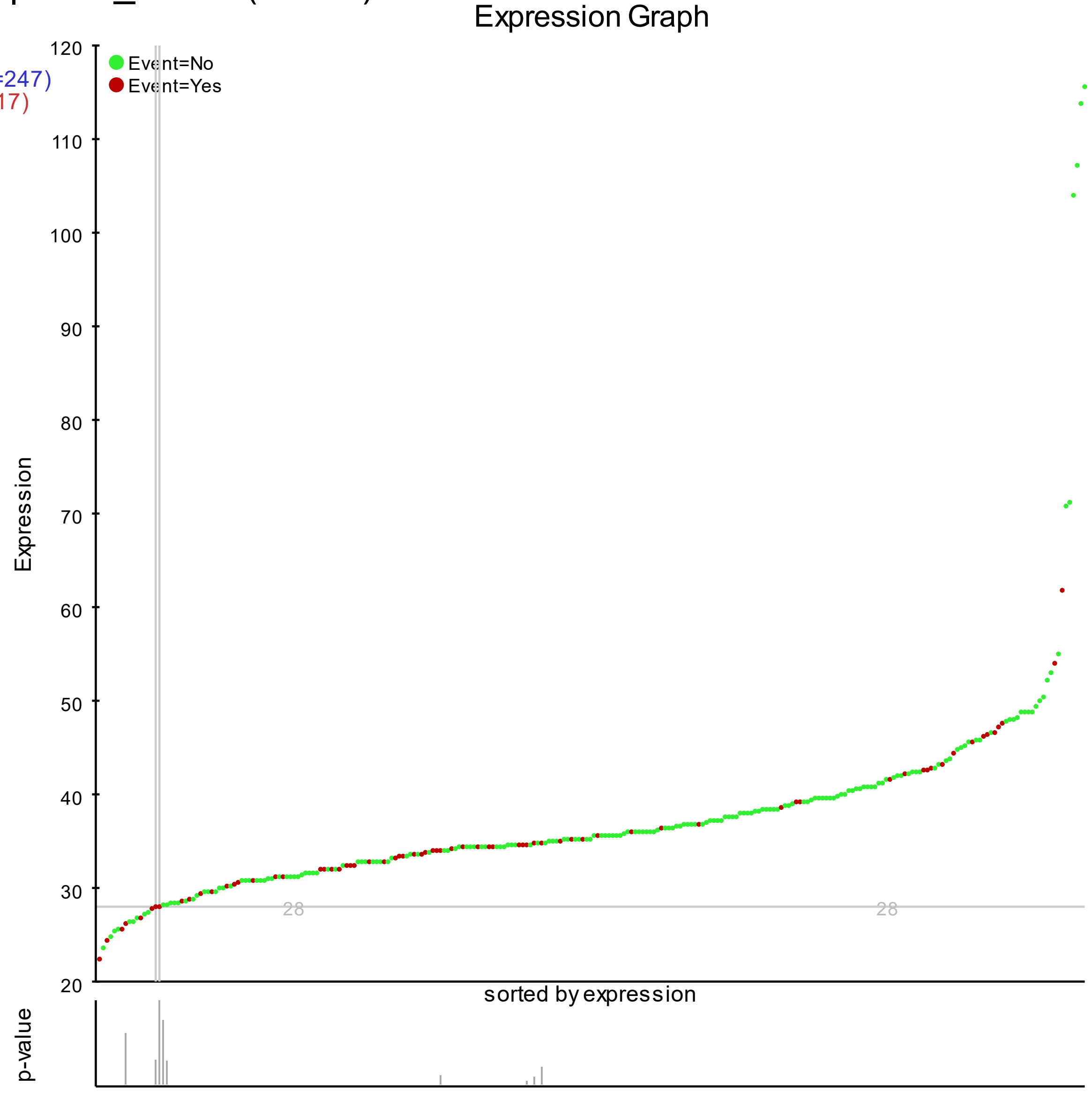

GR3

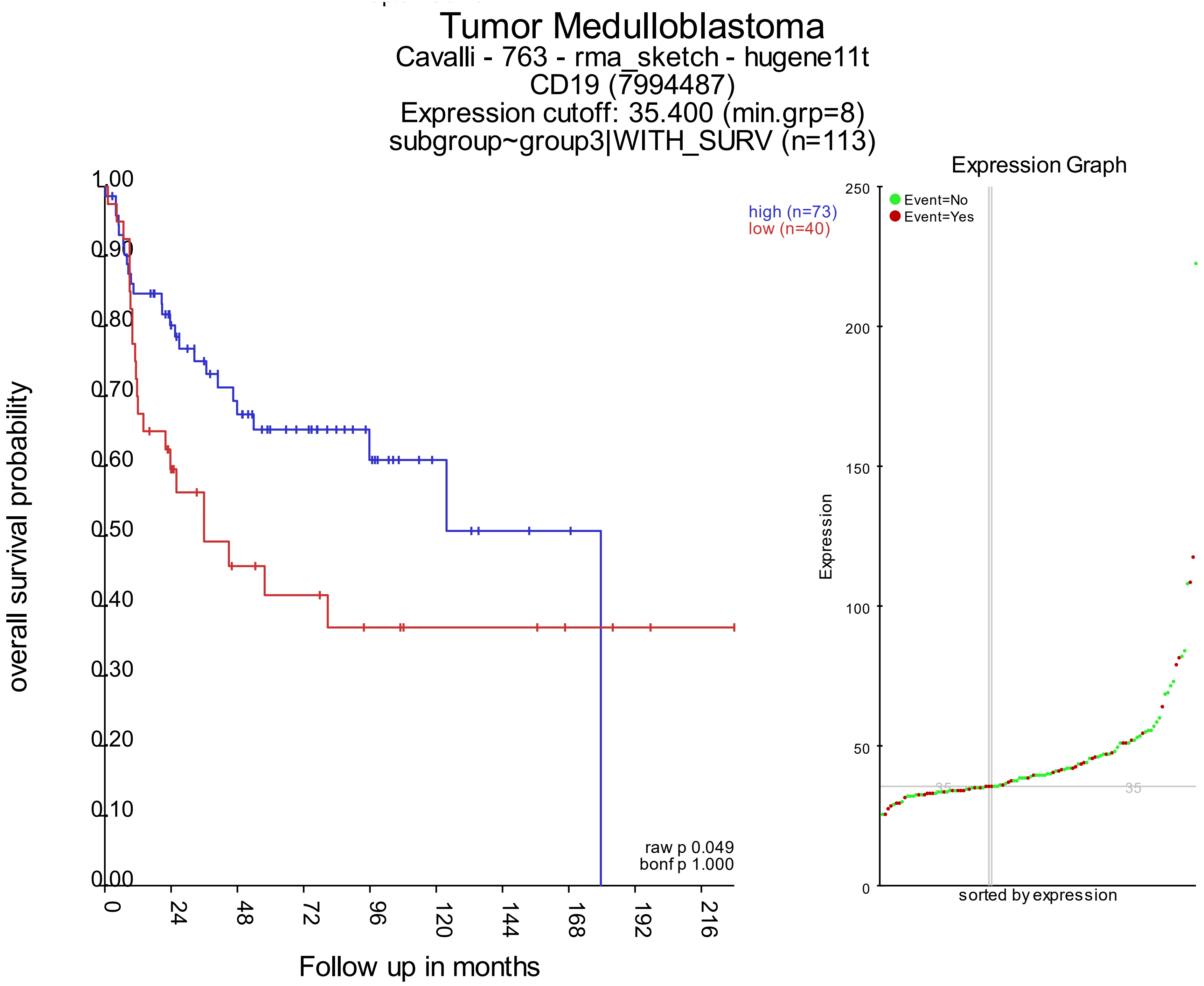

Tumor Medulloblastoma
Cavalli - 763 - rma_sketch - hugene11t
CD19 (7994487)
Expression cutoff: 35.400 (min.grp=8)
subgroup~group3|WITH_SURV (n=113)
high (n=73)
low (n=40)
overall survival probability
1.00
0.90
0.80
0.70
0.60
0.50
0.40
0.30
0.20
0.10
0.00
0
24
48
72
96
120
144
168
192
216
Follow up in months
raw p 0.049
bonf p 1.000
Expression Graph
Event=No
Event=Yes
Expression
250
200
150
100
50
0
35
sorted by expression

CD20

WNT

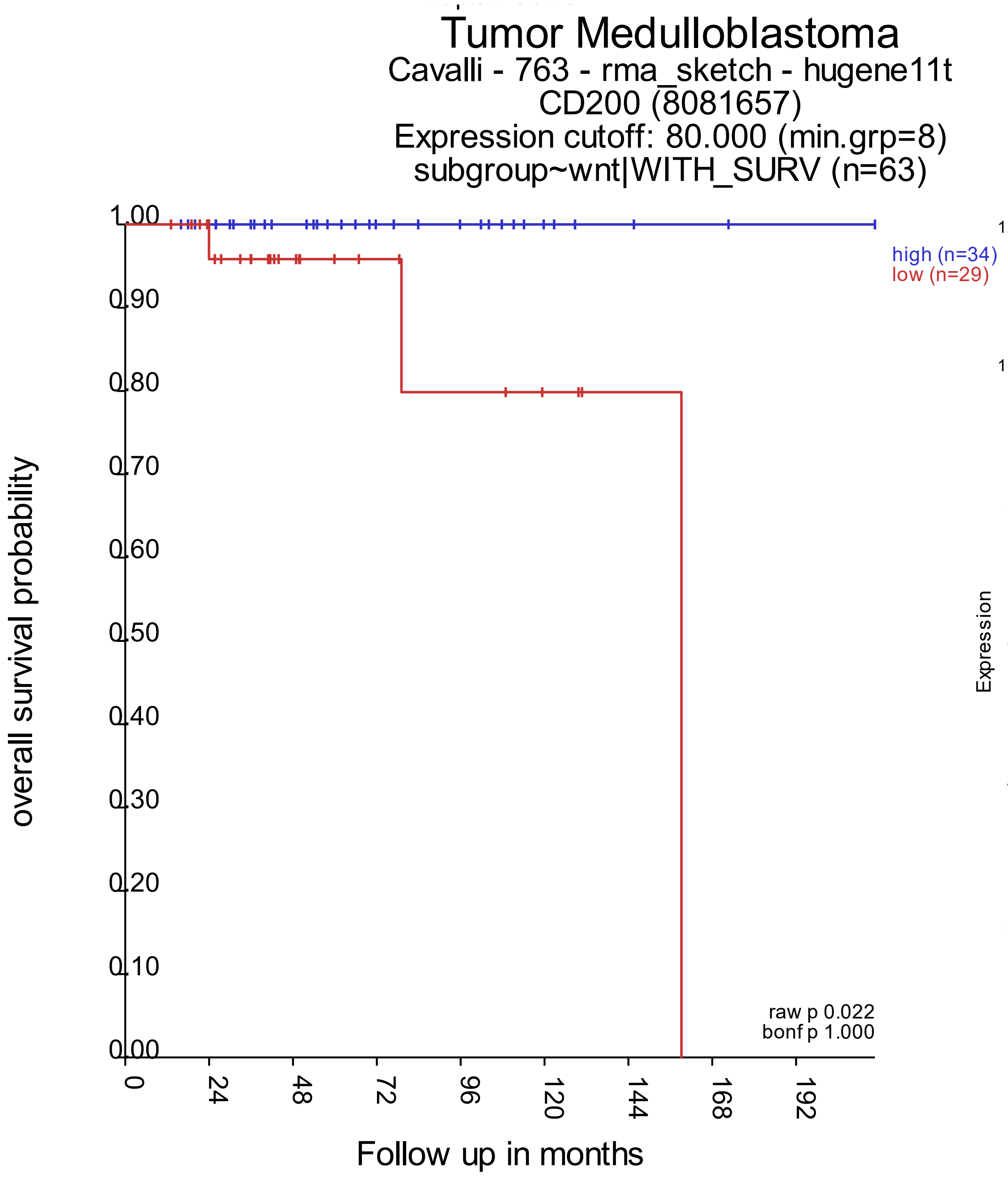

Tumor Medulloblastoma
Cavalli - 763 - rma_sketch - hugene11t
CD200 (8081657)
Expression cutoff: 80.000 (min.grp=8)
subgroup~wnt|WITH_SURV (n=63)
1.00
0.90
0.80
0.70
0.60
0.50
0.40
0.30
0.20
0.10
0.00
overall survival probability
0
24
48
72
96
120
144
168
192
Follow up in months
high (n=34)
low (n=29)
raw p 0.022
bonf p 1.000

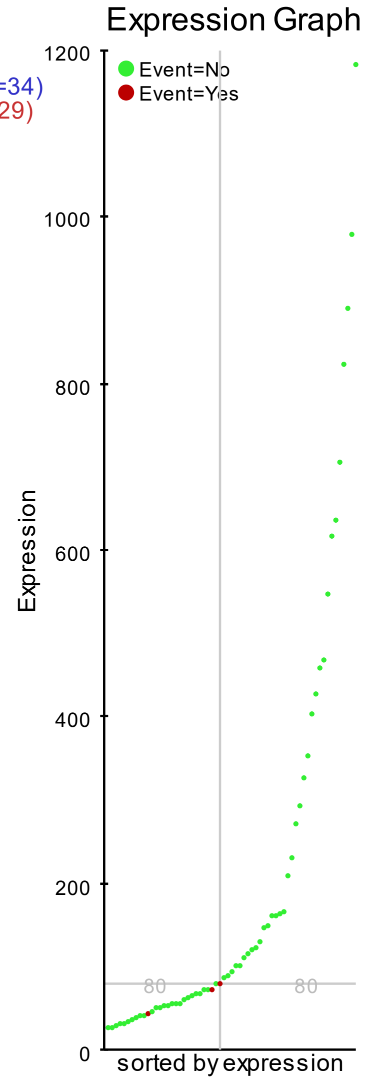

Expression Graph
Event=No
Event=Yes
1200
1000
800
600
400
200
0
Expression
80
80
sorted by expression

SHH

Tumor Medulloblastoma
Cavalli - 763 - rma_sketch - hugene11t
CD200 (8081657)
Expression cutoff: 575.500 (min.grp=8)
subgroup~shh|WITH_SURV (n=172)

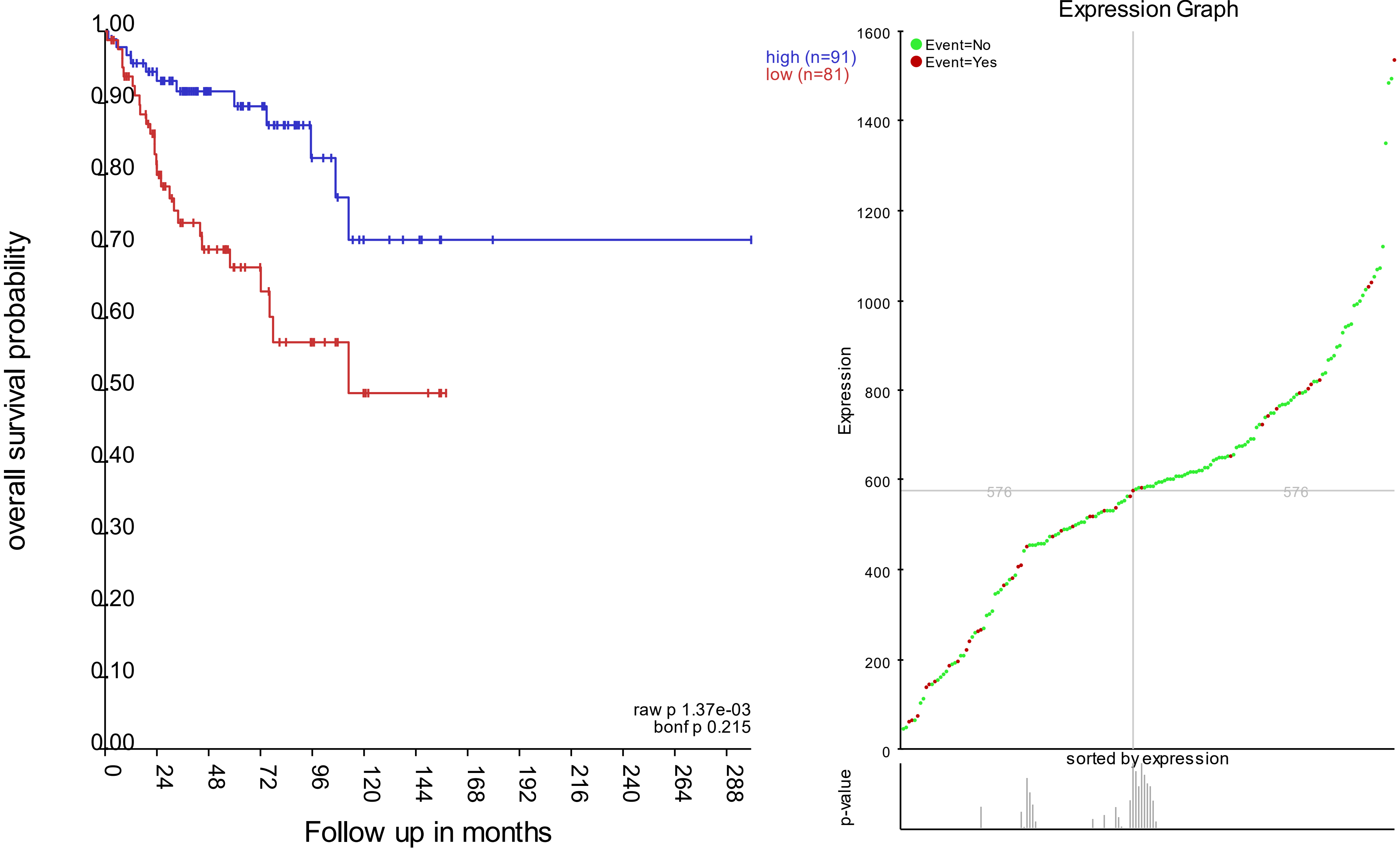

GR4

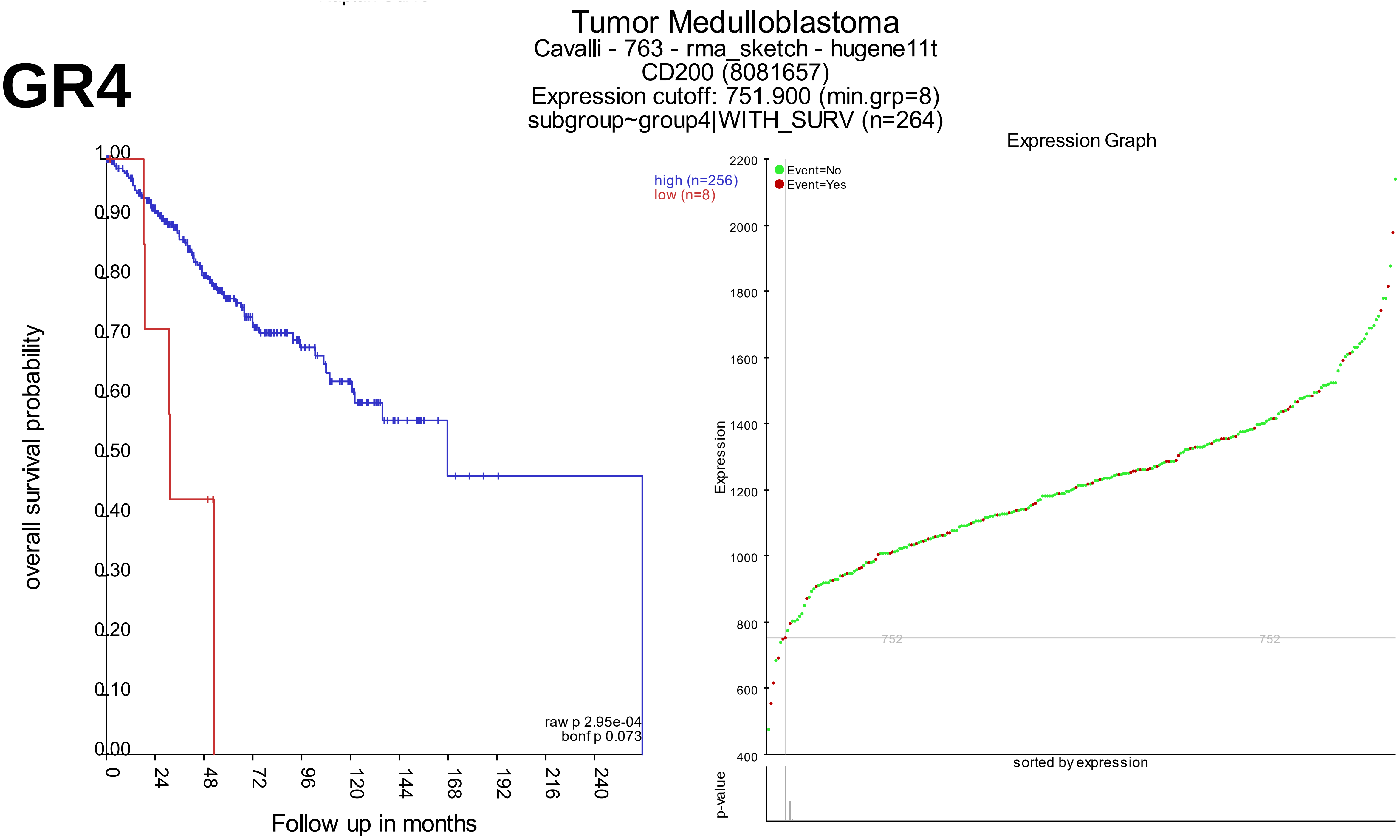

Tumor Medulloblastoma
Cavalli - 763 - rma_sketch - hugene11t
CD200 (8081657)
Expression cutoff: 751.900 (min.grp=8)
subgroup~group4|WITH_SURV (n=264)
high (n=256)
low (n=8)
overall survival probability
1.00
0.90
0.80
0.70
0.60
0.50
0.40
0.30
0.20
0.10
0.00
0
24
48
72
96
120
144
168
192
216
240
Follow up in months
raw p 2.95e-04
bonf p 0.073
Expression Graph
Event=No
Event=Yes
Expression
2200
2000
1800
1600
1400
1200
1000
800
600
400
752
752
sorted by expression
p-value

GR3

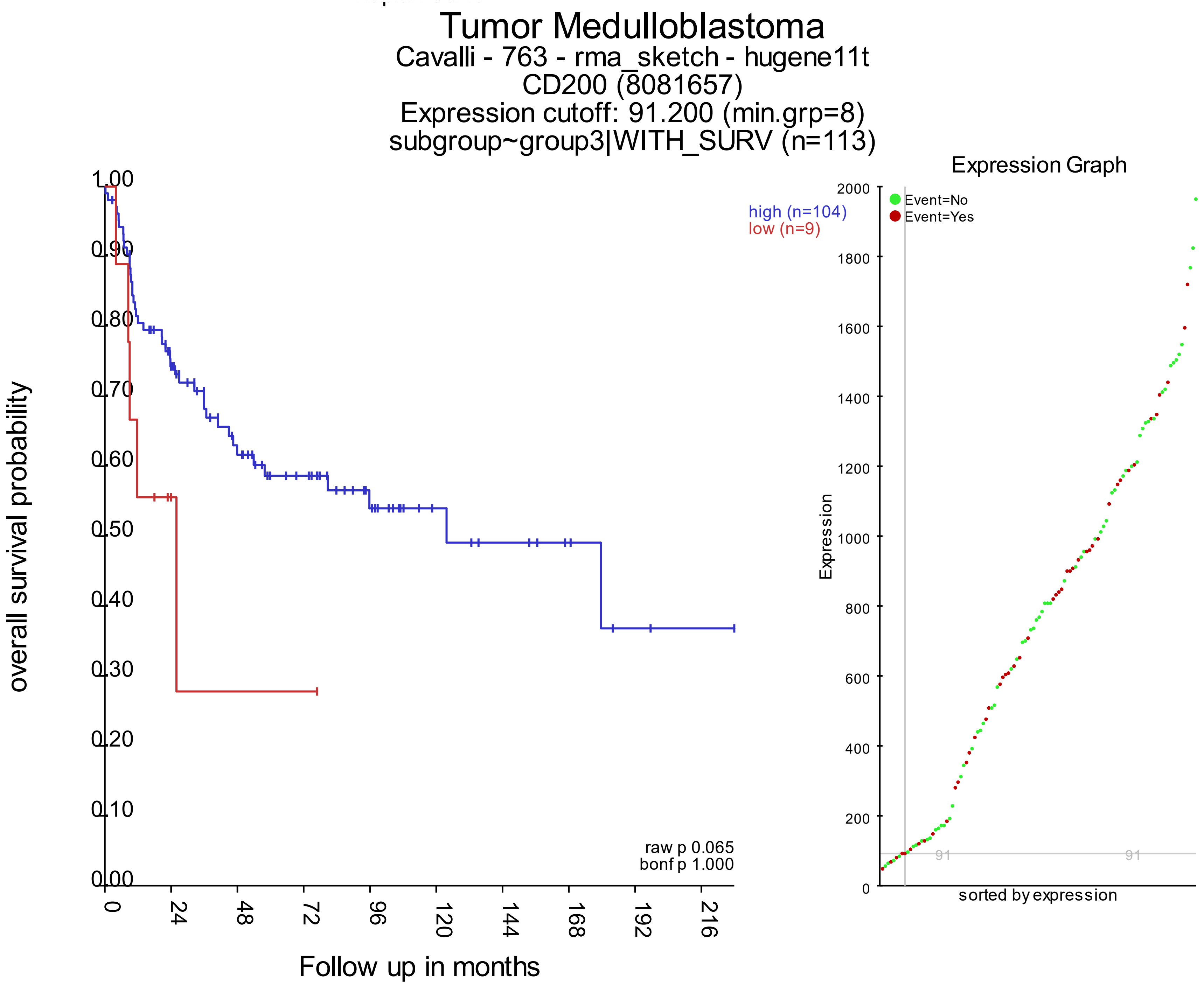

Tumor Medulloblastoma
Cavalli - 763 - rma_sketch - hugene11t
CD200 (8081657)
Expression cutoff: 91.200 (min.grp=8)
subgroup~group3|WITH_SURV (n=113)
high (n=104)
low (n=9)
overall survival probability
1.00
0.90
0.80
0.70
0.60
0.50
0.40
0.30
0.20
0.10
0.00
0
24
48
72
96
120
144
168
192
216
Follow up in months
raw p 0.065
bonf p 1.000
Expression Graph
Event=No
Event=Yes
Expression
2000
1800
1600
1400
1200
1000
800
600
400
200
0
91
91
sorted by expression

CD25/ISG20

WNT

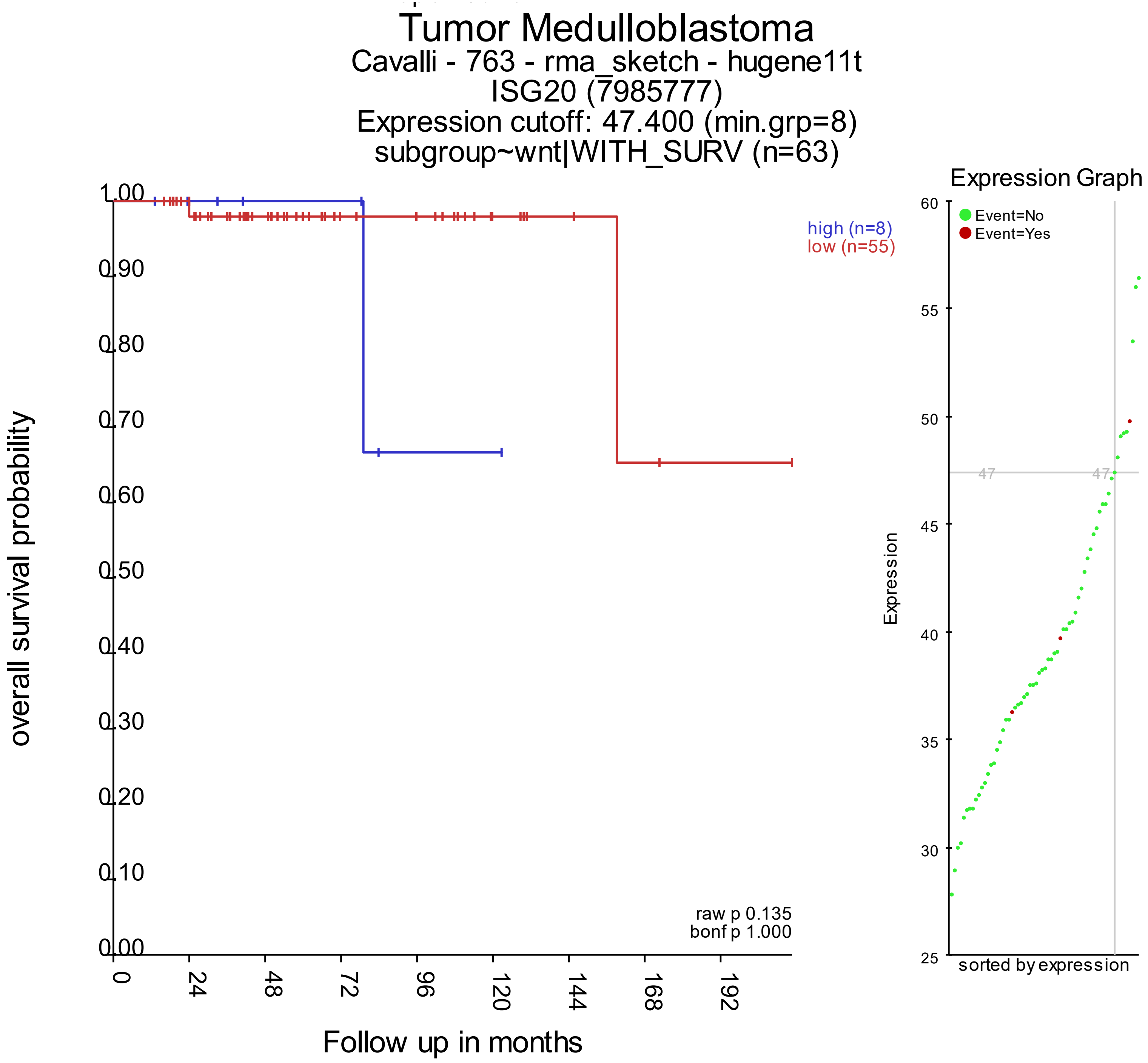
Tumor Medulloblastoma
Cavalli - 763 - rma_sketch - hugene11t
ISG20 (7985777)
Expression cutoff: 47.400 (min.grp=8)
subgroup~wnt|WITH_SURV (n=63)
overall survival probability
1.00
0.90
0.80
0.70
0.60
0.50
0.40
0.30
0.20
0.10
0.00
0
24
48
72
96
120
144
168
192
Follow up in months
high (n=8)
low (n=55)
raw p 0.135
bonf p 1.000
Expression Graph
Event=No
Event=Yes
Expression
60
55
50
45
40
35
30
25
47
47
sorted by expression

SHH

Tumor Medulloblastoma
Cavalli - 763 - rma_sketch - hugene11t
ISG20 (7985777)
Expression cutoff: 46.200 (min.grp=8)
subgroup~shh|WITH_SURV (n=172)

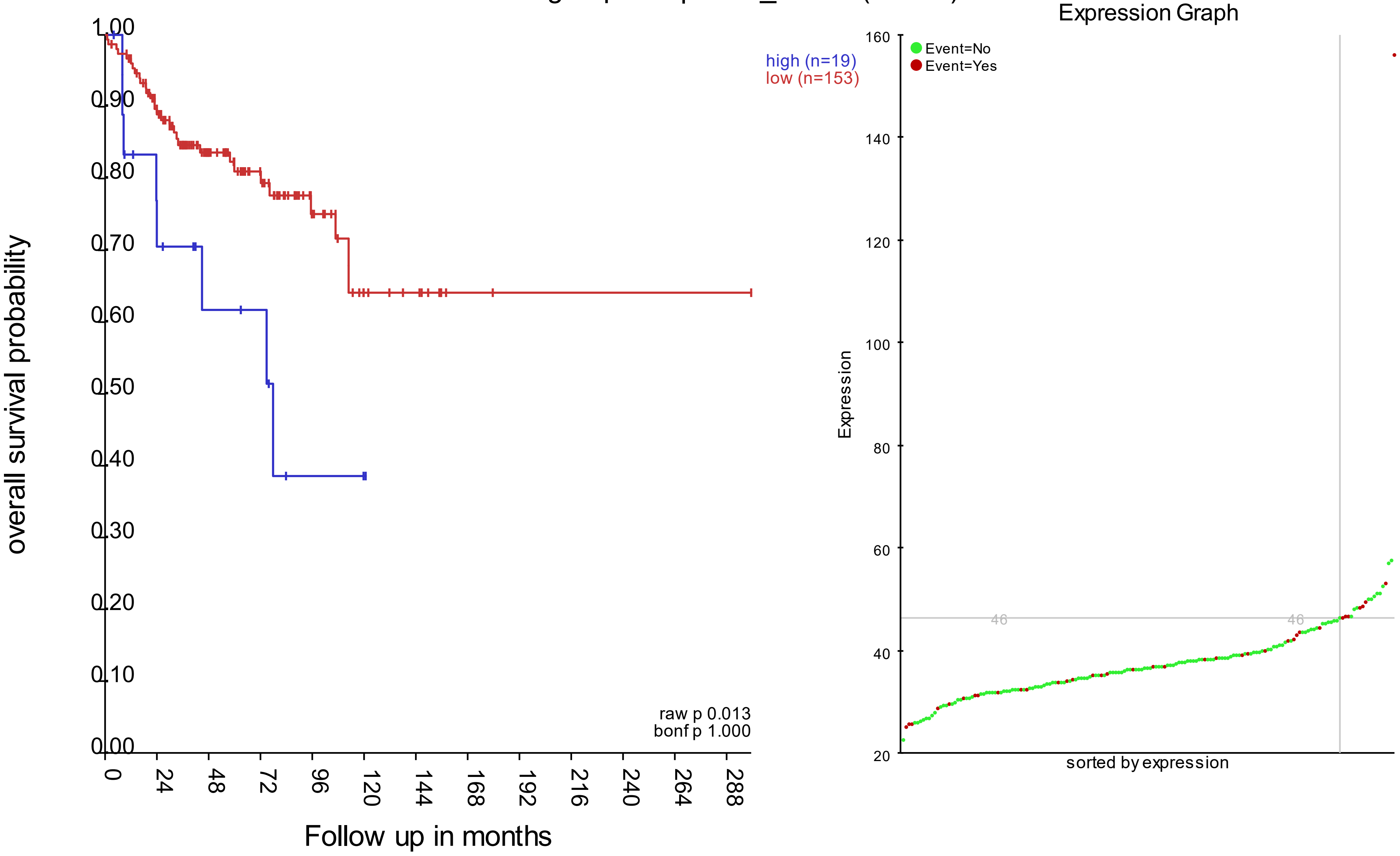

GR4

Tumor Medulloblastoma

Cavalli - 763 - rma_sketch - hugene11t
ISG20 (7985777)
Expression cutoff: 44.000 (min.grp=8)
subgroup~group4|WITH_SURV (n=264)

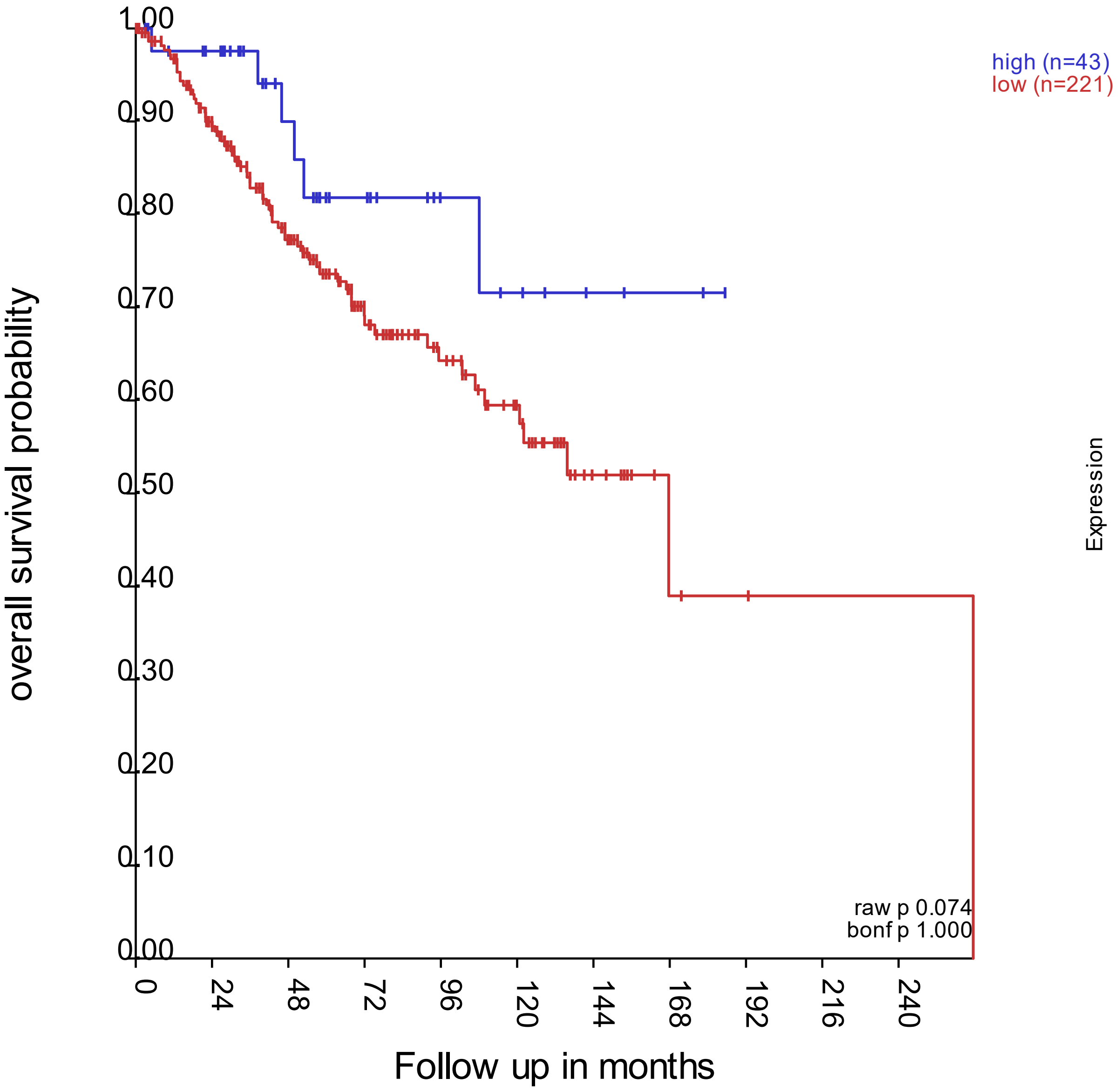

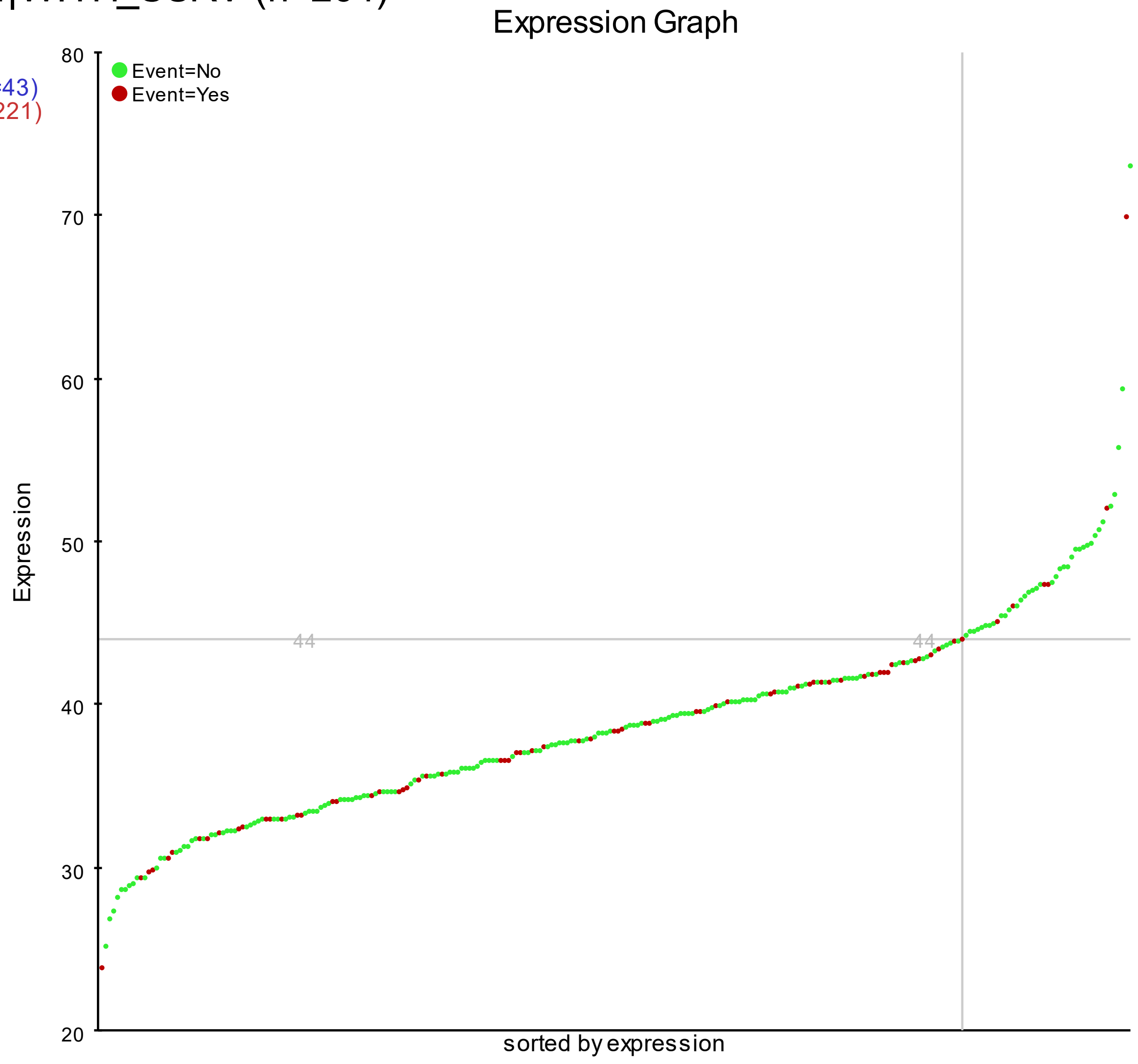

GR3

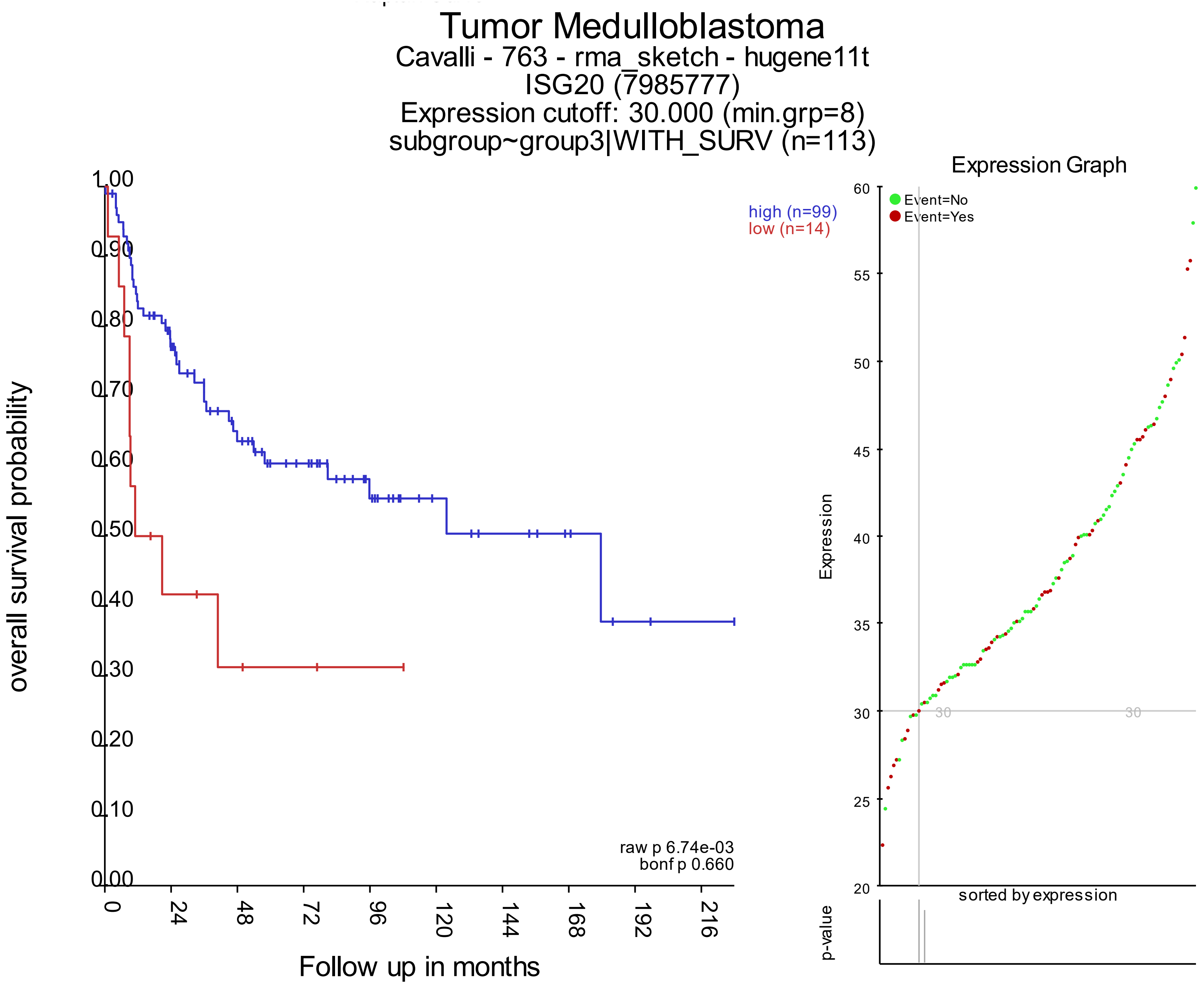

Tumor Medulloblastoma
Cavalli - 763 - rma_sketch - hugene11t
ISG20 (7985777)
Expression cutoff: 30.000 (min.grp=8)
subgroup~group3|WITH_SURV (n=113)
overall survival probability
1.00
0.90
0.80
0.70
0.60
0.50
0.40
0.30
0.20
0.10
0.00
high (n=99)
low (n=14)
raw p 6.74e-03
bonf p 0.660
0
24
48
72
96
120
144
168
192
216
Follow up in months
Expression Graph
Event=No
Event=Yes
Expression
60
55
50
45
40
35
30
25
20
30
30
sorted by expression
p-value

CD33

WNT

Tumor Medulloblastoma
Cavalli - 763 - rma_sketch - hugene11t
CD33 (8030804)
Expression cutoff: 54.100 (min.grp=8)
subgroup~wnt|WITH_SURV (n=63)

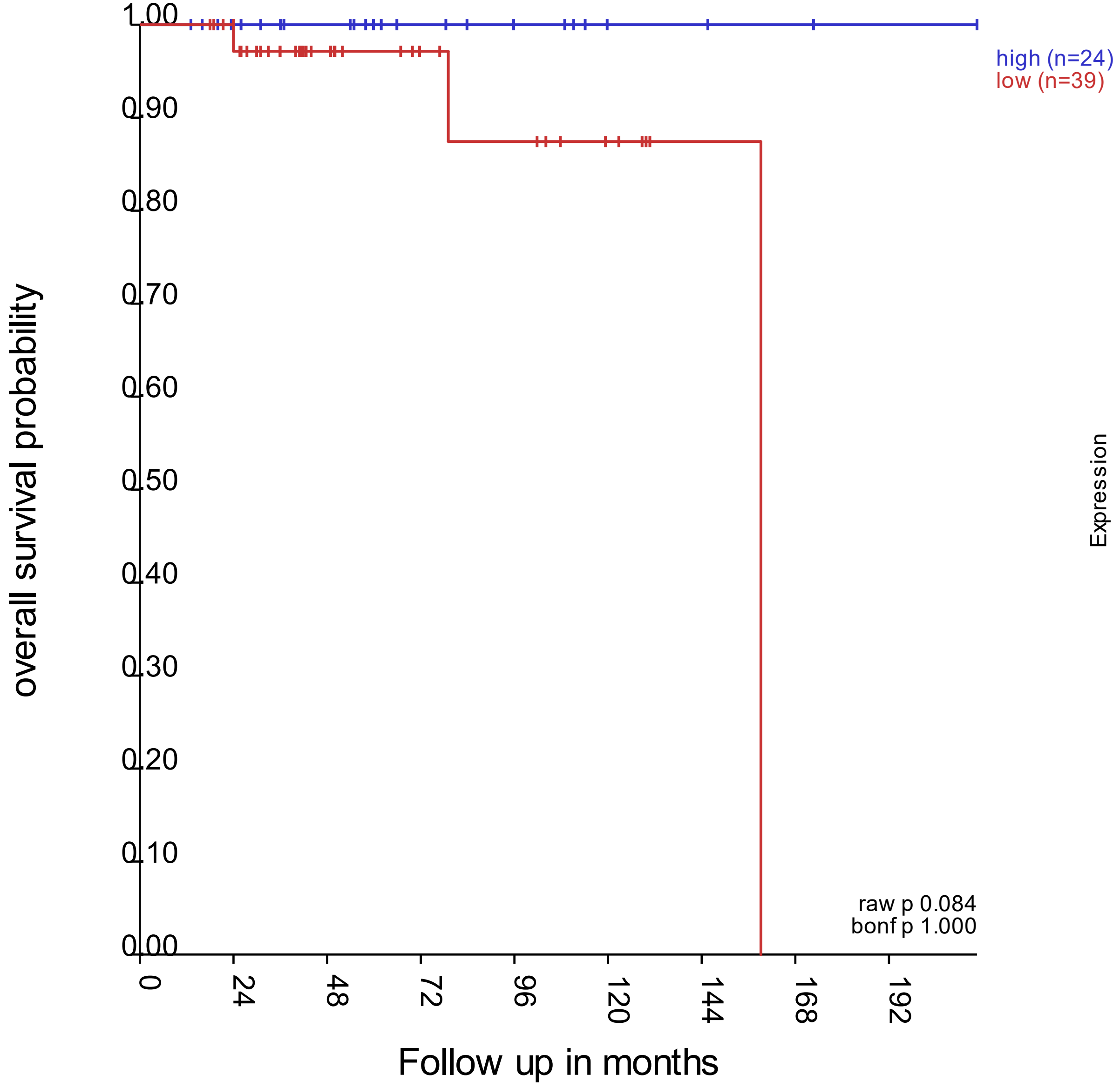

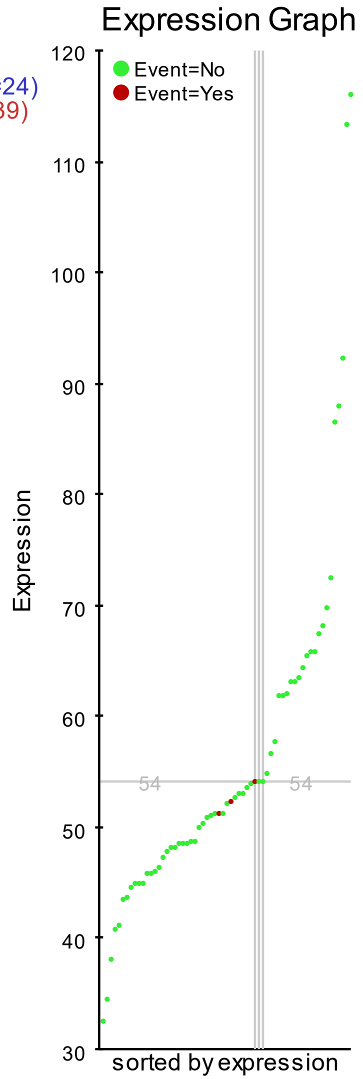

SHS

Tumor Medulloblastoma
Cavalli - 763 - rma_sketch - hugene11t
CD33 (8030804)
Expression cutoff: 88.600 (min.grp=8)
subgroup~shh|WITH_SURV (n=172)

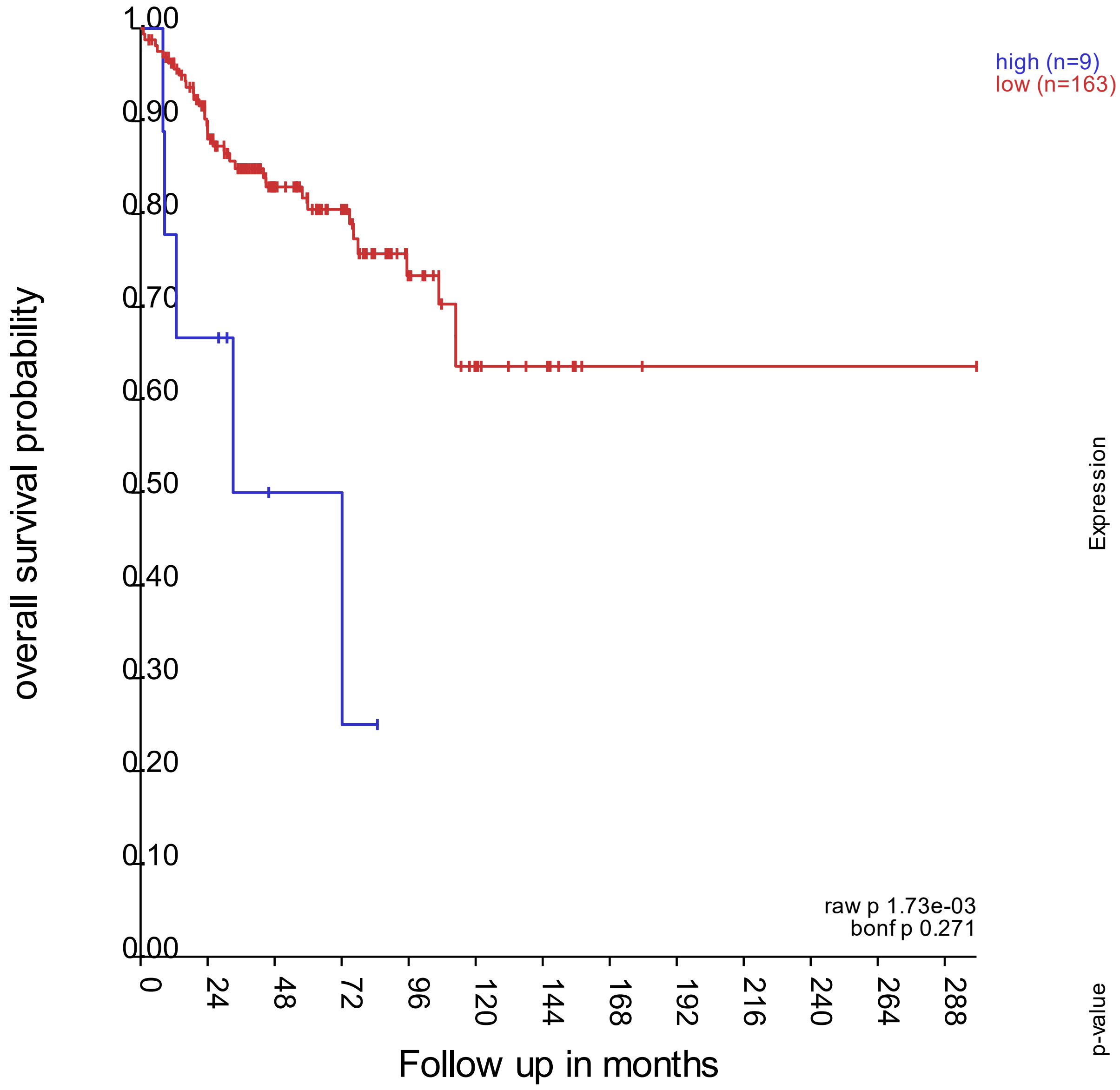

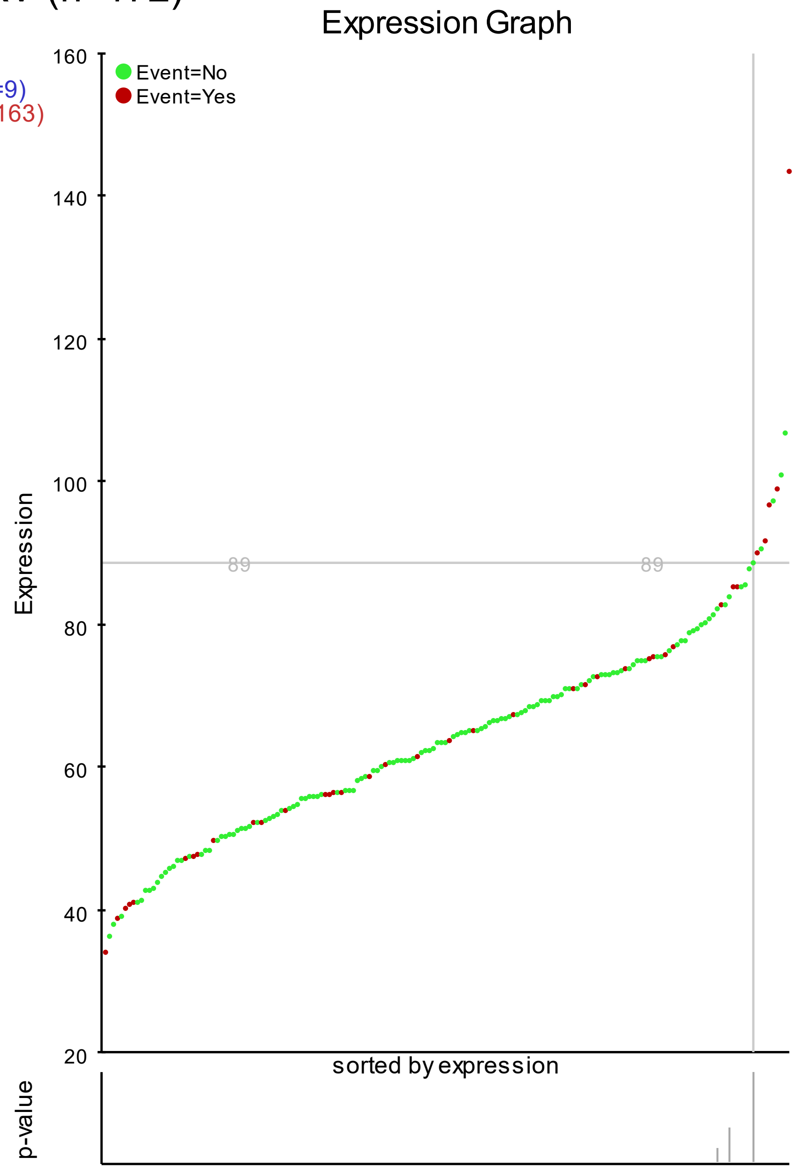

GR4

Tumor Medulloblastoma
Cavalli - 763 - rma_sketch - hugene11t
CD33 (8030804)
Expression cutoff: 73.800 (min.grp=8)
subgroup~group4|WITH_SURV (n=264)

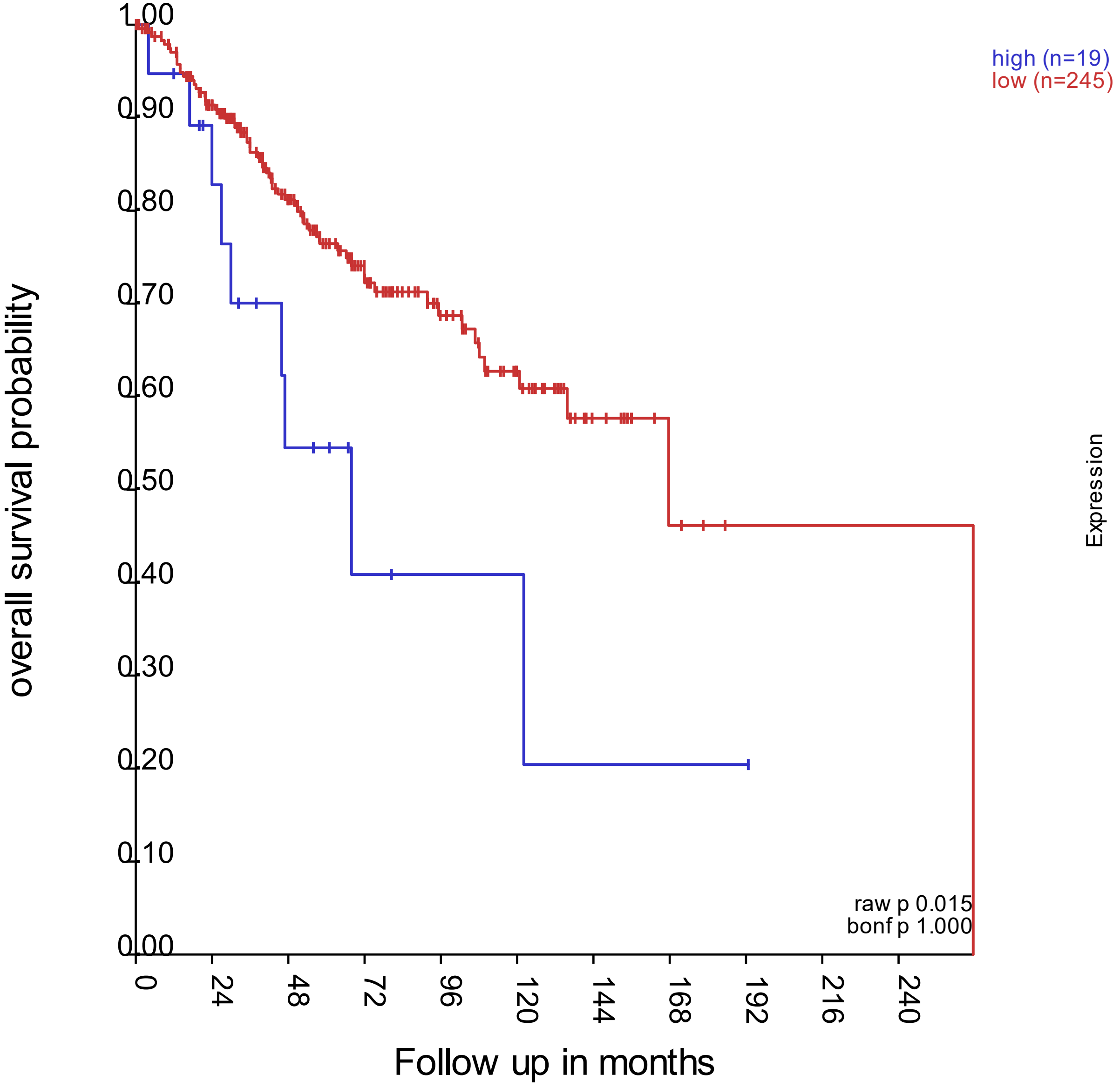

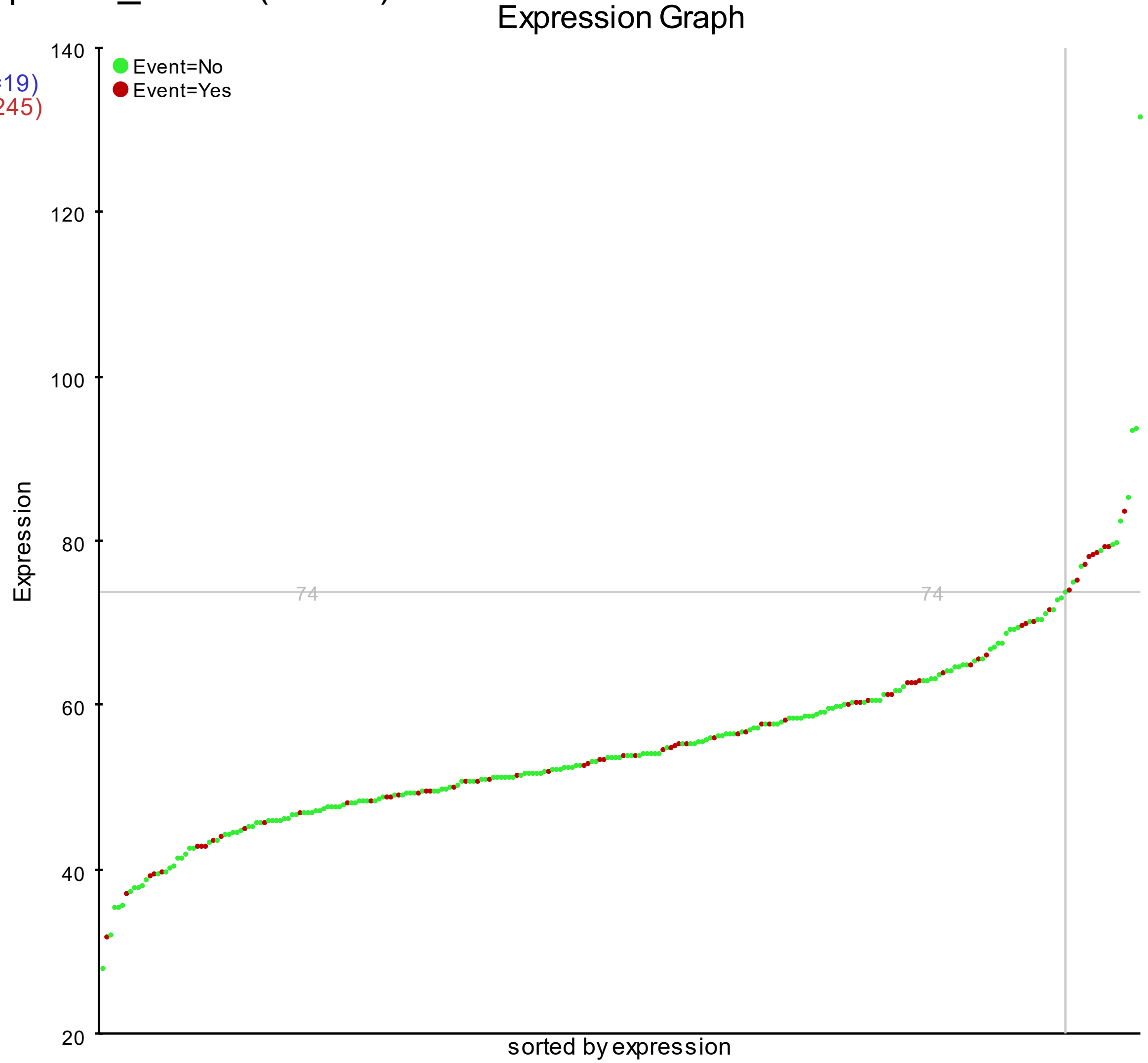

GR3

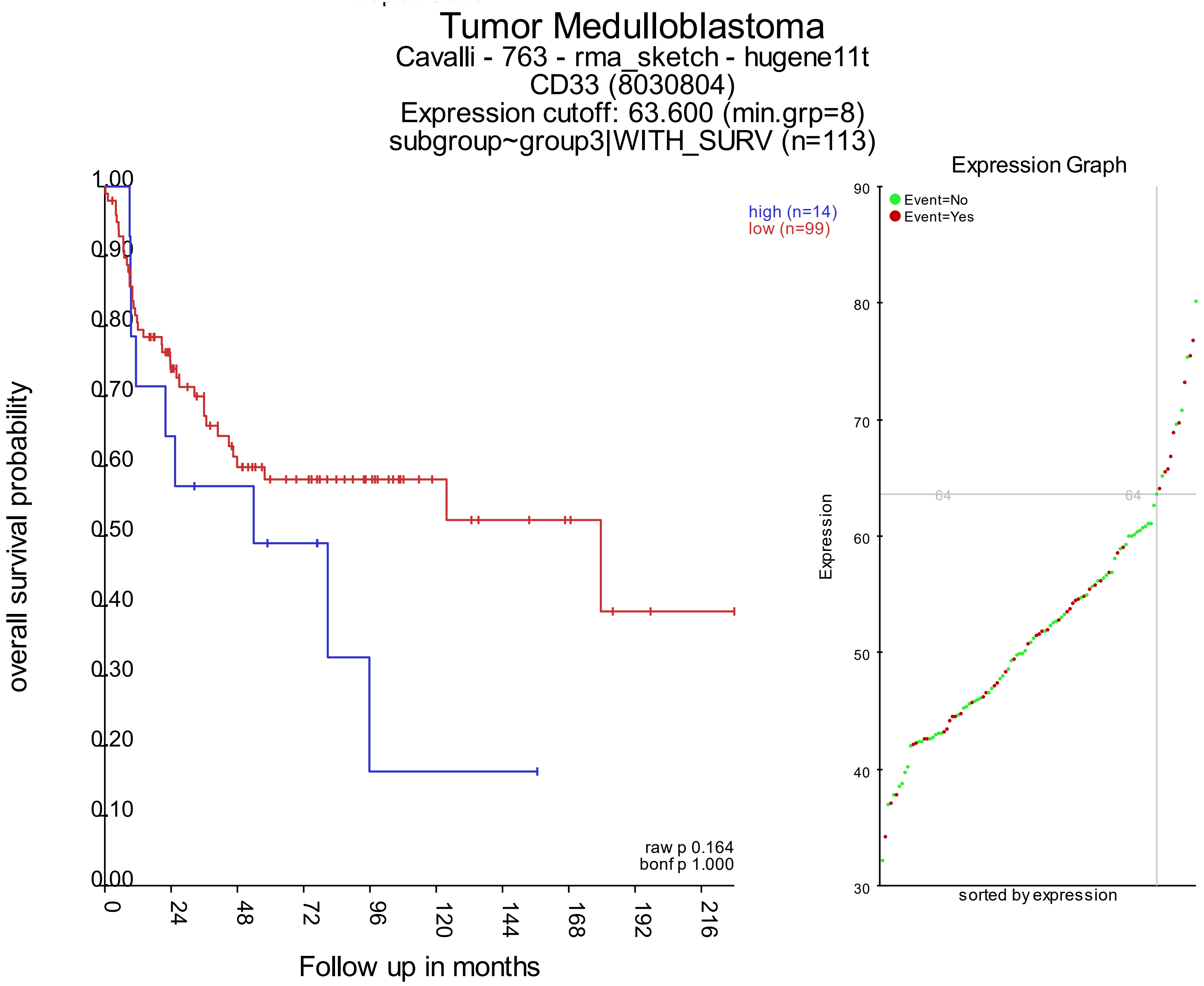

Tumor Medulloblastoma
Cavalli - 763 - rma_sketch - hugene11t
CD33 (8030804)
Expression cutoff: 63.600 (min.grp=8)
subgroup~group3|WITH_SURV (n=113)
overall survival probability
1.00
0.90
0.80
0.70
0.60
0.50
0.40
0.30
0.20
0.10
0.00
0
24
48
72
96
120
144
168
192
216
Follow up in months
high (n=14)
low (n=99)
raw p 0.164
bonf p 1.000
Expression Graph
Event=No
Event=Yes
Expression
90
80
70
60
50
40
30
64
64
sorted by expression

CD52

WNT

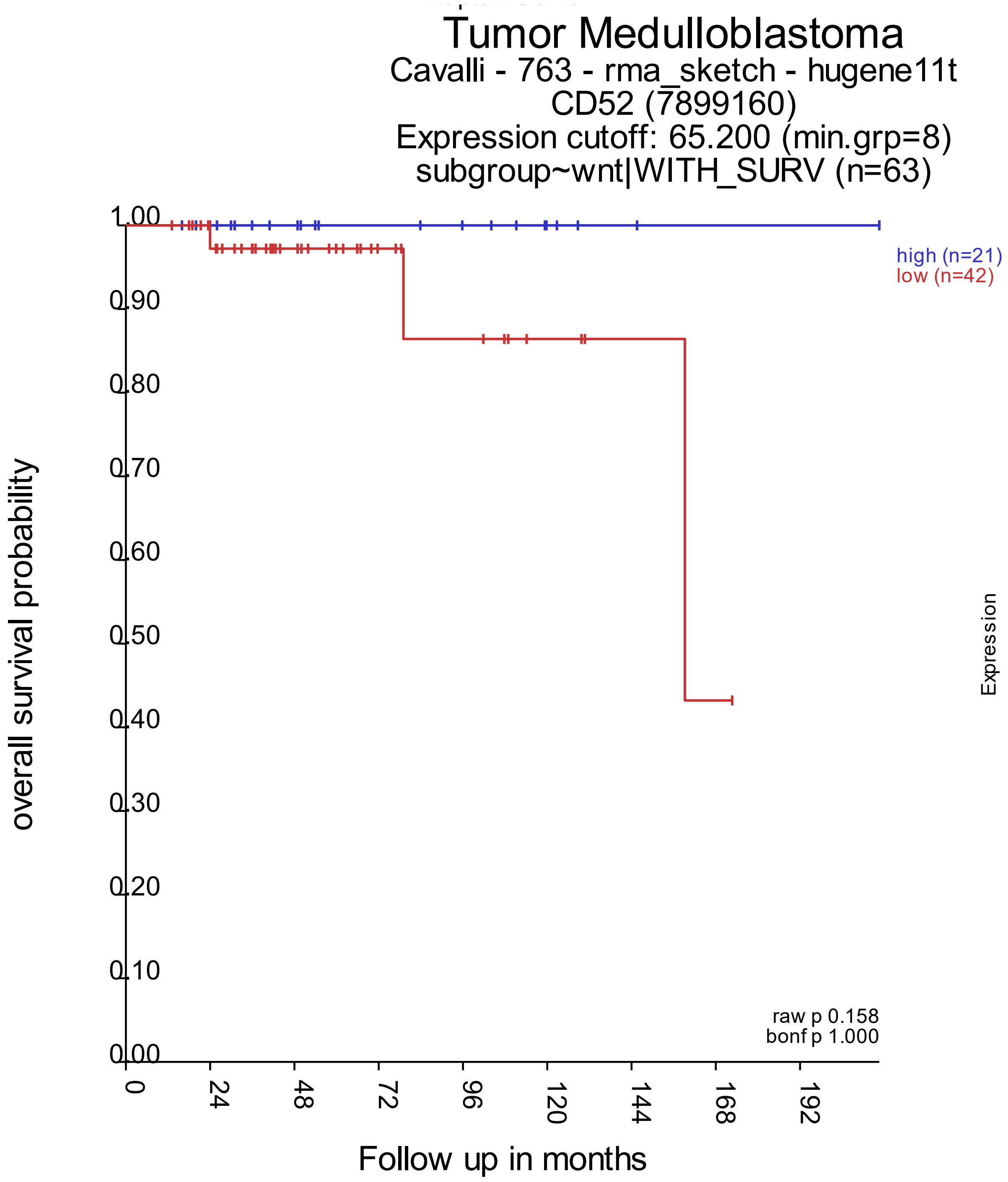

Tumor Medulloblastoma
Cavalli - 763 - rma_sketch - hugene11t
CD52 (7899160)
Expression cutoff: 65.200 (min.grp=8)
subgroup~wnt|WITH_SURV (n=63)
overall survival probability
1.00
0.90
0.80
0.70
0.60
0.50
0.40
0.30
0.20
0.10
0.00
0
24
48
72
96
120
144
168
192
Follow up in months
high (n=21)
low (n=42)
raw p 0.158
bonf p 1.000

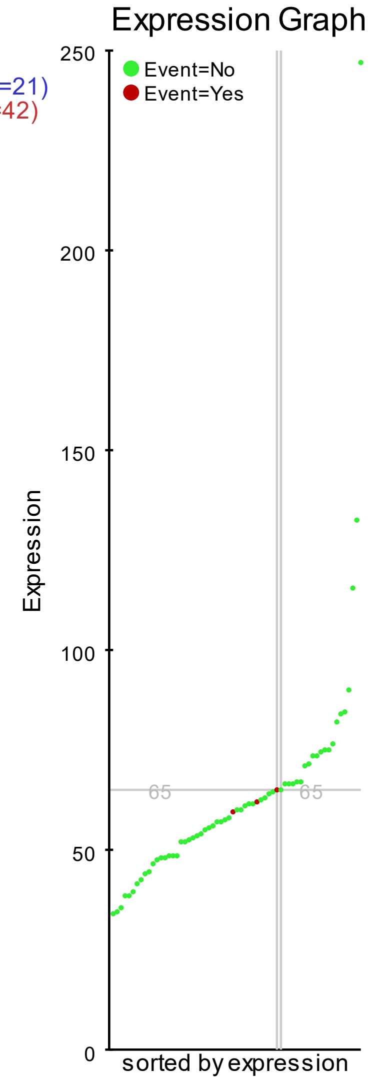

Expression Graph
Event=No
Event=Yes
Expression
250
200
150
100
50
0
65
65
sorted by expression

SHH

Tumor Medulloblastoma
Cavalli - 763 - rma_sketch - hugene11t
CD52 (7899160)
Expression cutoff: 78.000 (min.grp=8)
subgroup~shh|WITH_SURV (n=172)

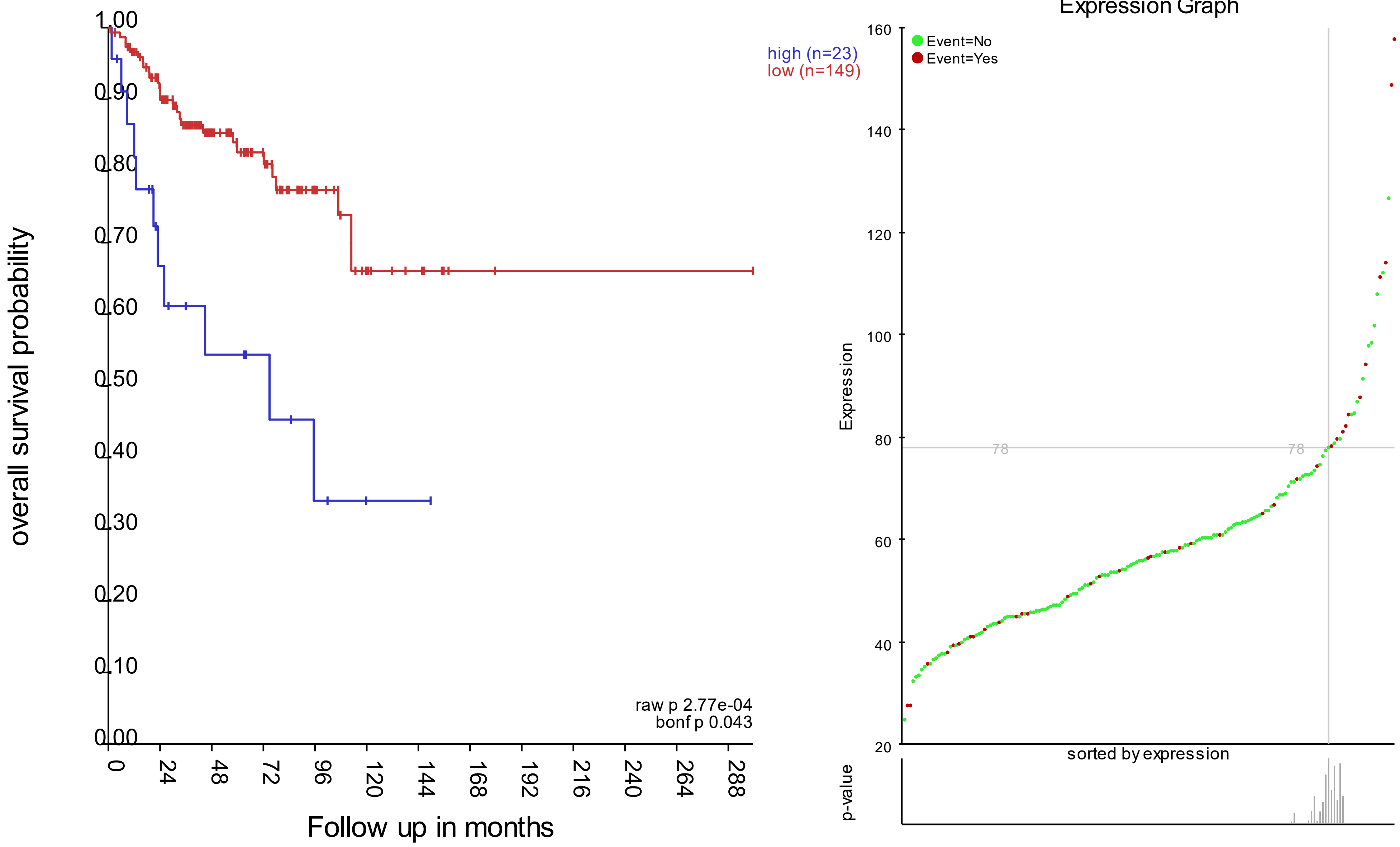

GR4

Tumor Medulloblastoma
Cavalli - 763 - rma_sketch - hugene11t
CD52 (7899160)
Expression cutoff: 67.400 (min.grp=8)
subgroup~group4|WITH_SURV (n=264)

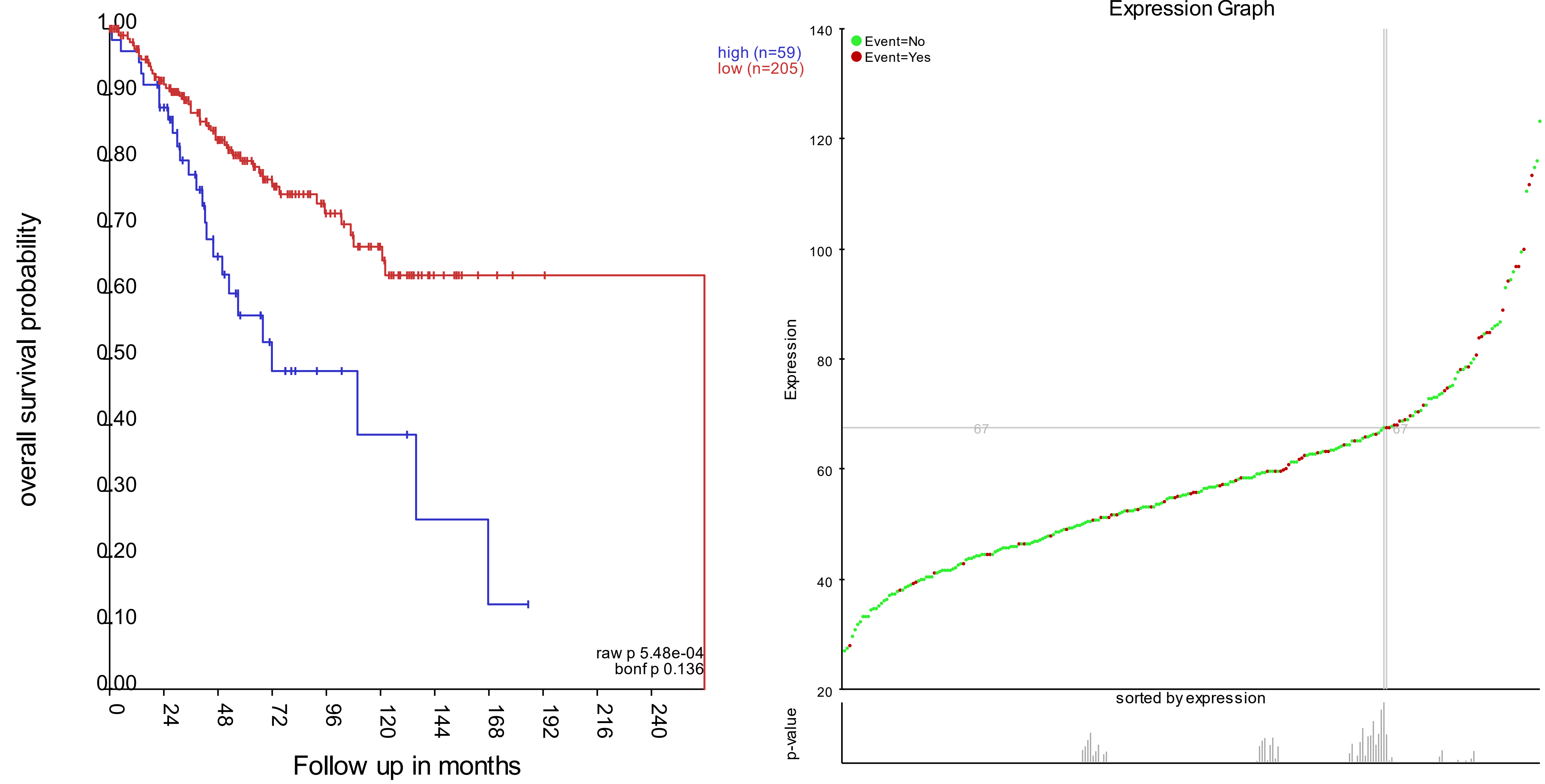

GR3

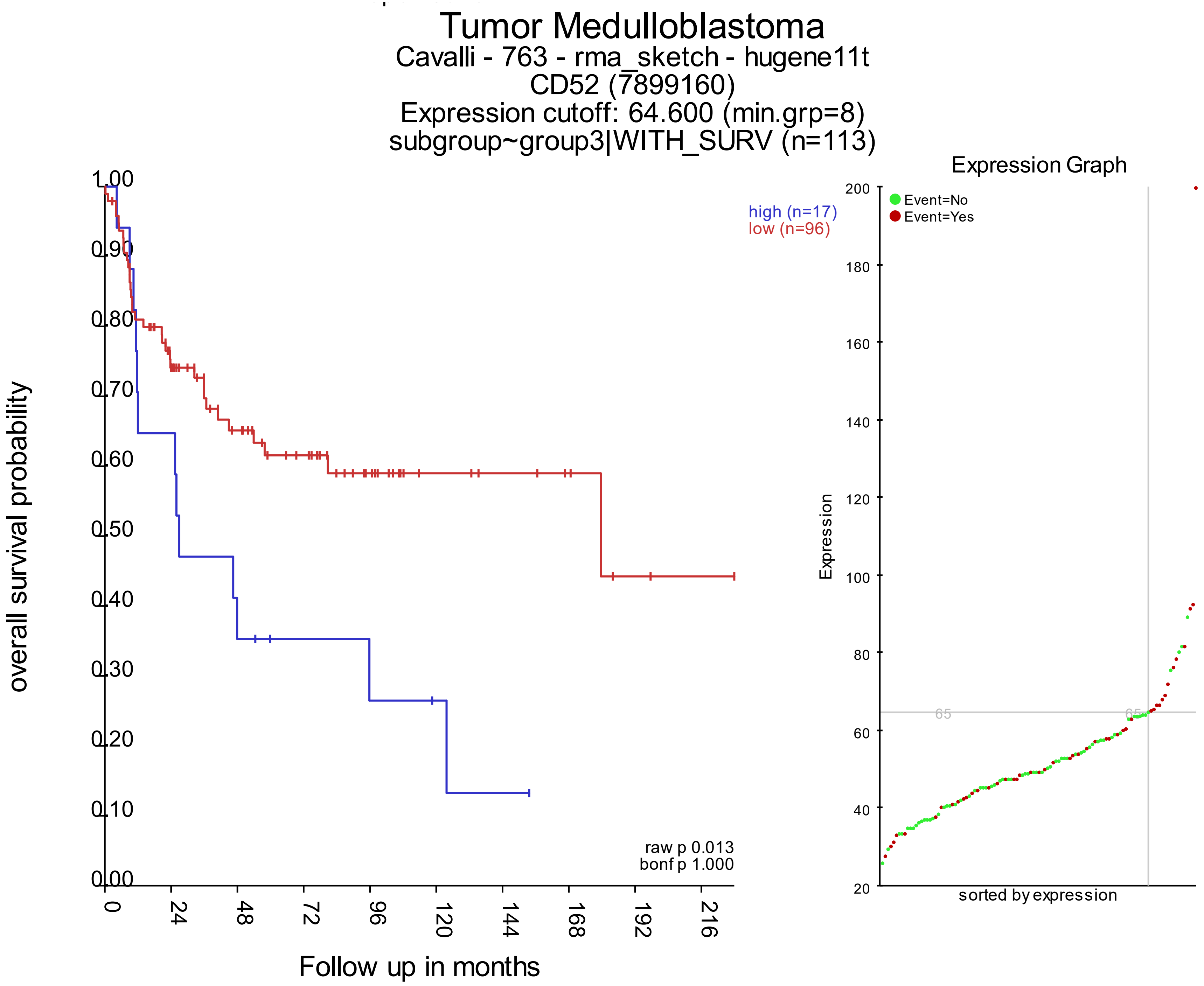
Tumor Medulloblastoma
Cavalli - 763 - rma_sketch - hugene11t
CD52 (7899160)
Expression cutoff: 64.600 (min.grp=8)
subgroup~group3|WITH_SURV (n=113)
overall survival probability
1.00
0.90
0.80
0.70
0.60
0.50
0.40
0.30
0.20
0.10
0.00
0
24
48
72
96
120
144
168
192
216
Follow up in months
high (n=17)
low (n=96)
raw p 0.013
bonf p 1.000
Expression Graph
Event=No
Event=Yes
Expression
200
180
160
140
120
100
80
60
40
20
65
sorted by expression

CDK4

WNT

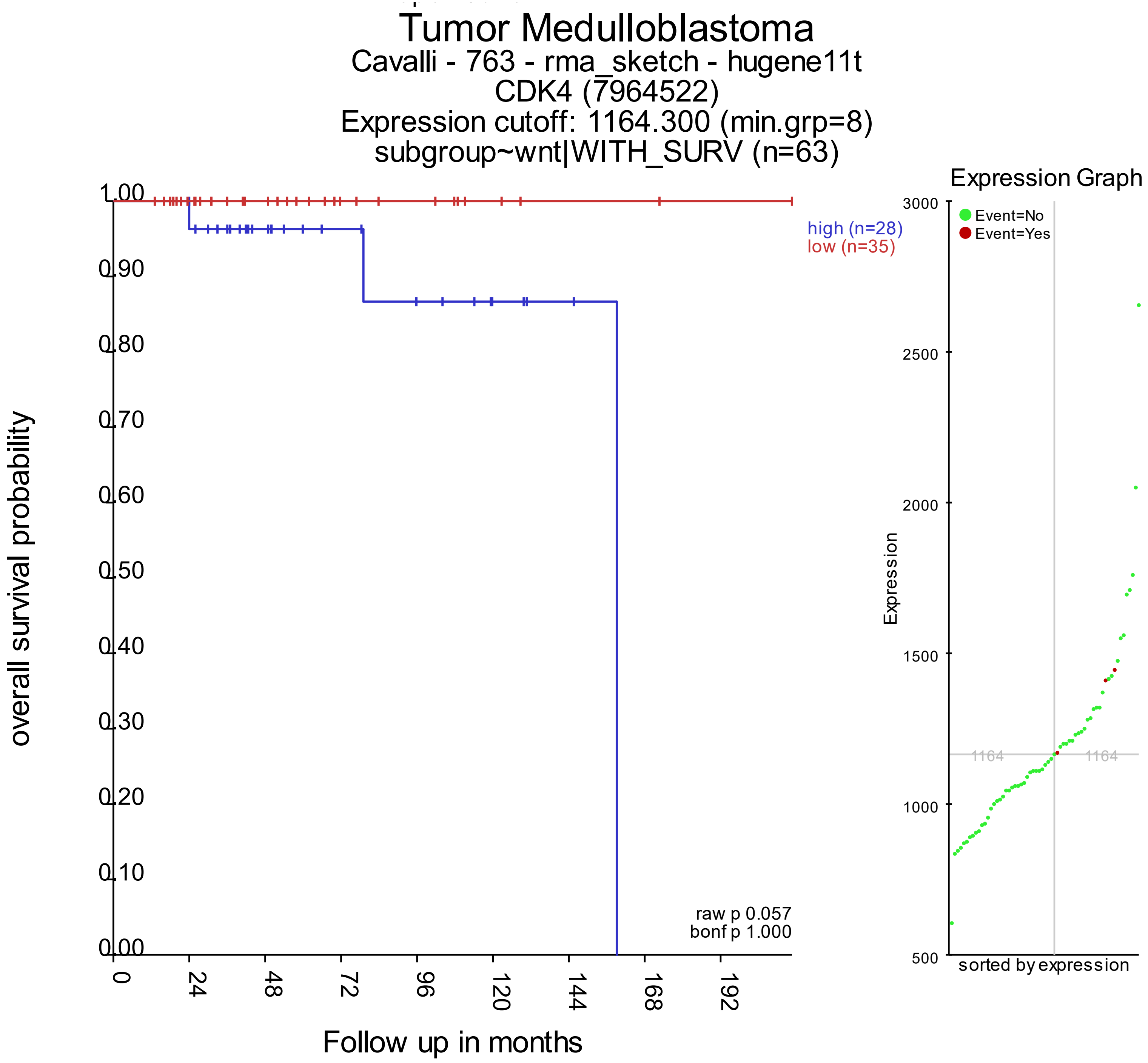

Tumor Medulloblastoma
Cavalli - 763 - rma_sketch - hugene11t
CDK4 (7964522)
Expression cutoff: 1164.300 (min.grp=8)
subgroup~wnt|WITH_SURV (n=63)
overall survival probability
1.00
0.90
0.80
0.70
0.60
0.50
0.40
0.30
0.20
0.10
0.00
high (n=28)
low (n=35)
raw p 0.057
bonf p 1.000
0
24
48
72
96
120
144
168
192
Follow up in months
Expression Graph
Event=No
Event=Yes
3000
2500
2000
1500
1000
500
Expression
1164
1164
sorted by expression

SHH

Tumor Medulloblastoma
Cavalli - 763 - rma_sketch - hugene11t
CDK4 (7964522)
Expression cutoff: 1633.400 (min.grp=8)
subgroup~shh|WITH_SURV (n=172)

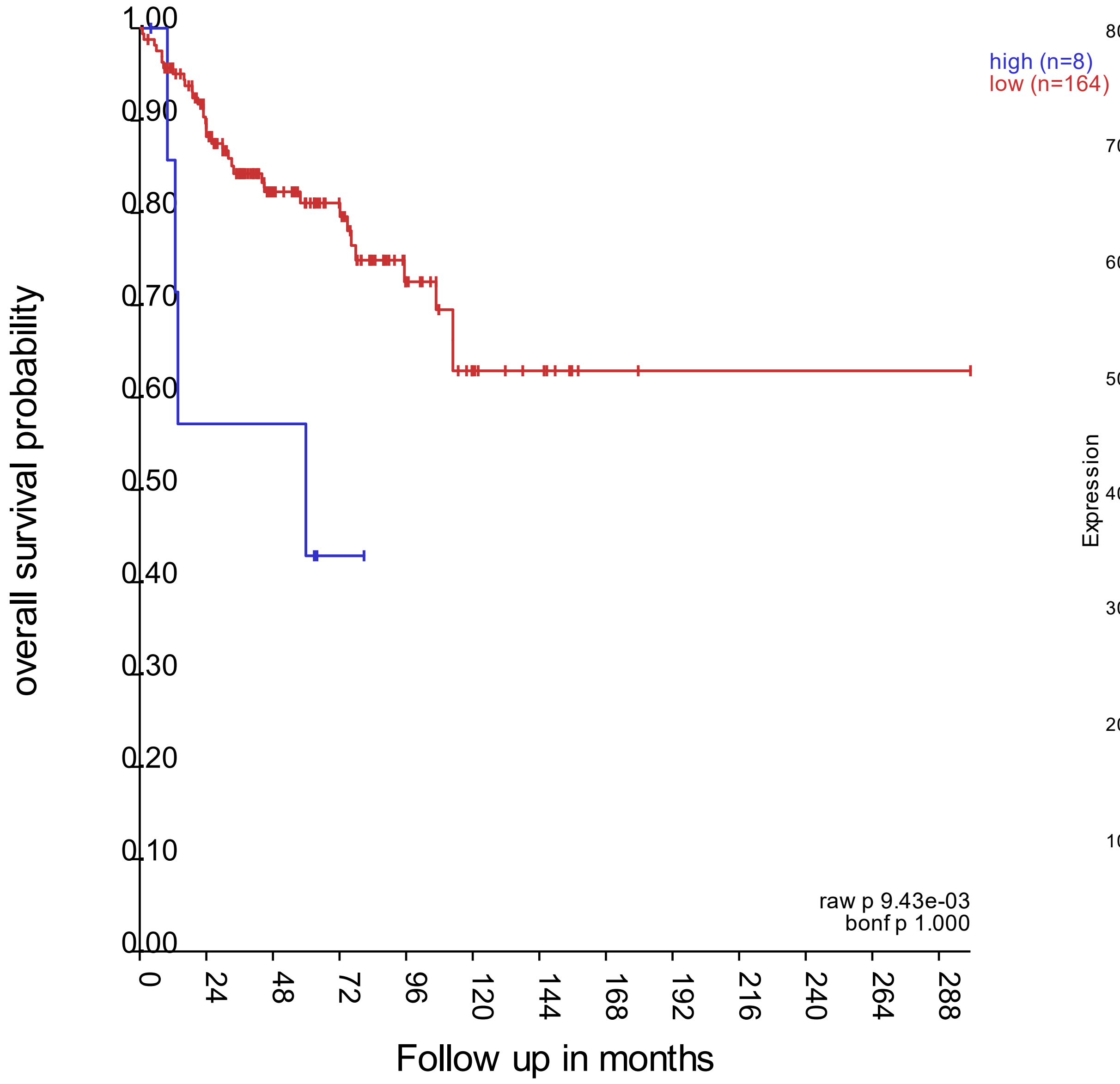

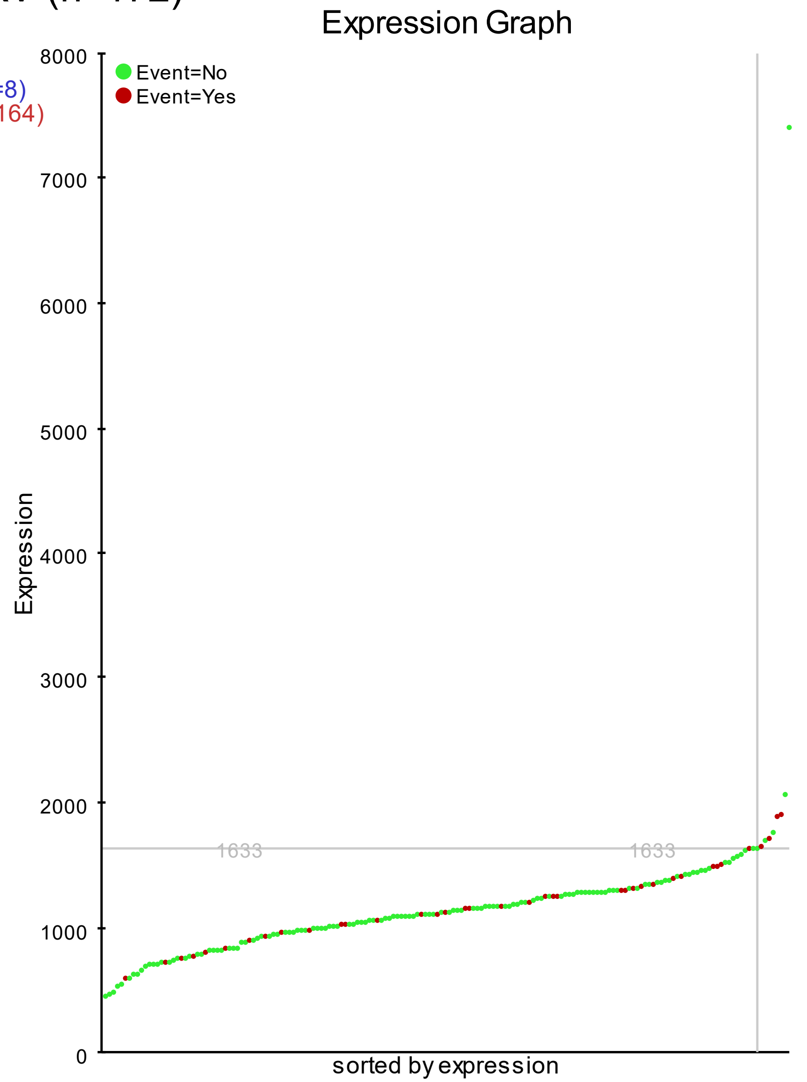

GR4

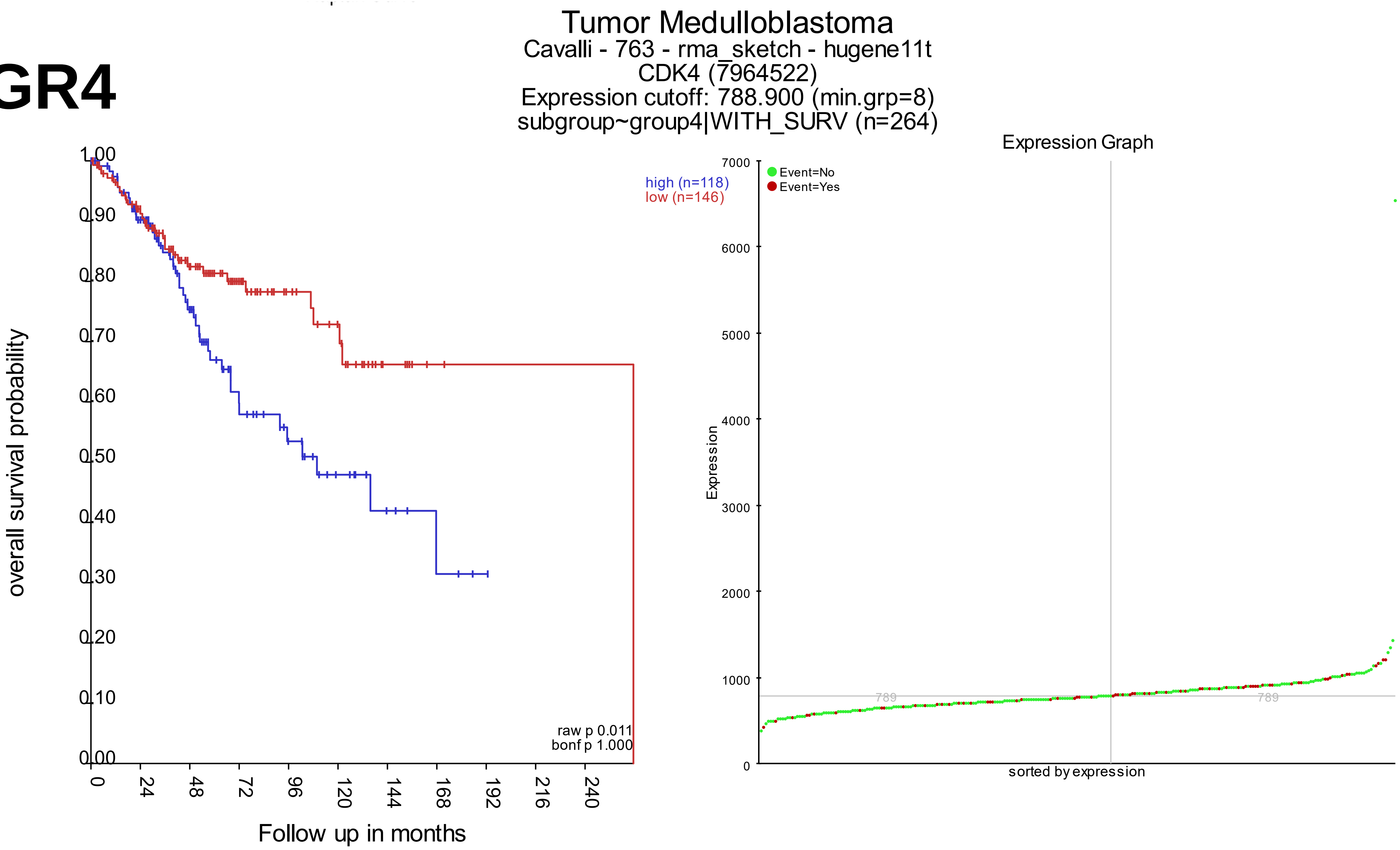

Tumor Medulloblastoma
Cavalli - 763 - rma_sketch - hugene11t
CDK4 (7964522)
Expression cutoff: 788.900 (min.grp=8)
subgroup~group4|WITH_SURV (n=264)
overall survival probability
1.00
0.90
0.80
0.70
0.60
0.50
0.40
0.30
0.20
0.10
0.00
high (n=118)
low (n=146)
raw p 0.011
bonf p 1.000
0
24
48
72
96
120
144
168
192
216
240
Follow up in months
Expression Graph
Event=No
Event=Yes
Expression
7000
6000
5000
4000
3000
2000
1000
0
789
789
sorted by expression

GR3

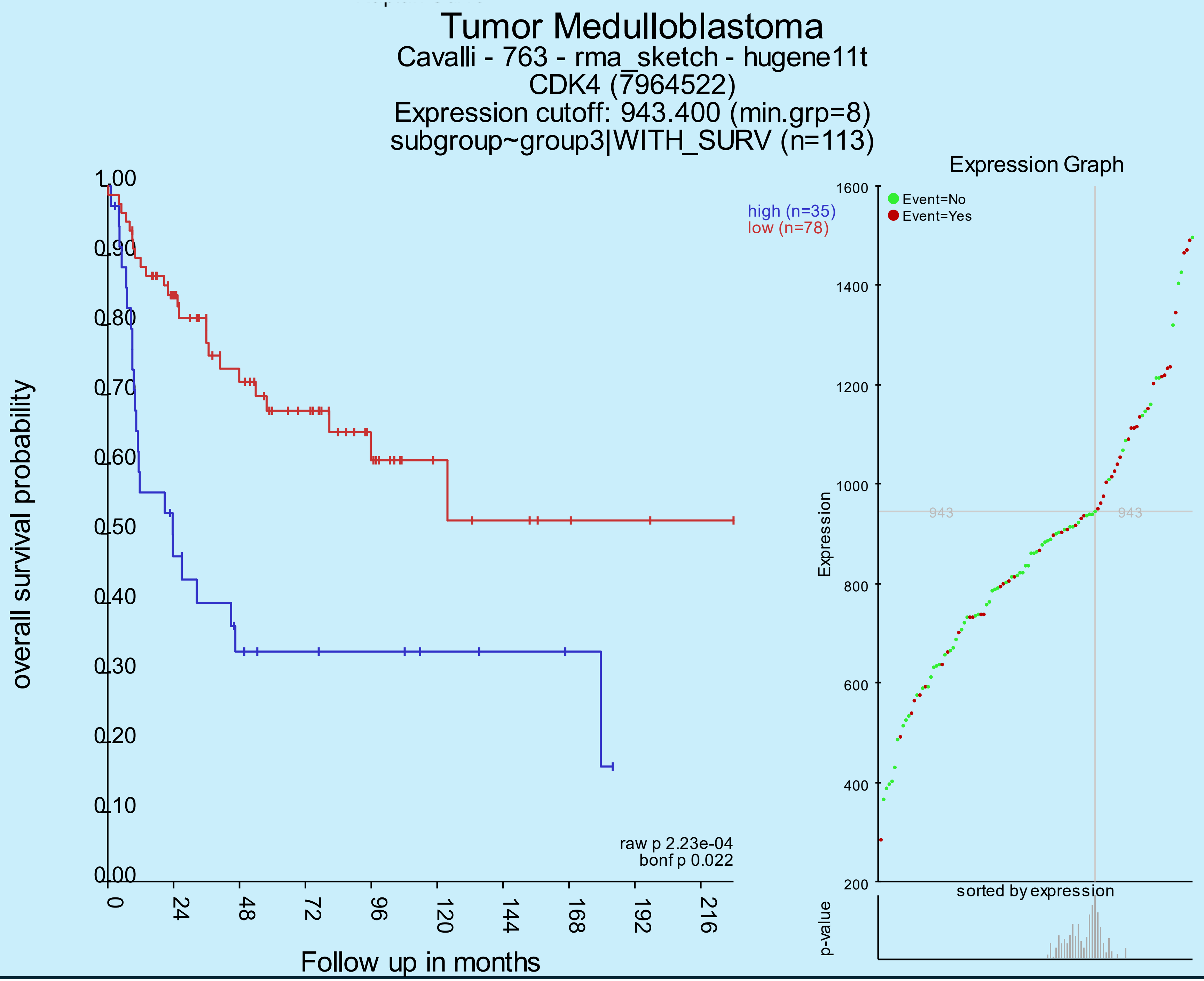

Tumor Medulloblastoma
Cavalli - 763 - rma_sketch - hugene11t
CDK4 (7964522)
Expression cutoff: 943.400 (min.grp=8)
subgroup~group3|WITH_SURV (n=113)
high (n=35)
low (n=78)
overall survival probability
1.00
0.90
0.80
0.70
0.60
0.50
0.40
0.30
0.20
0.10
0.00
0
24
48
72
96
120
144
168
192
216
Follow up in months
raw p 2.23e-04
bonf p 0.022
Expression Graph
Event=No
Event=Yes
Expression
1600
1400
1200
1000
800
600
400
200
943
943
sorted by expression
p-value

CDK6

WNT

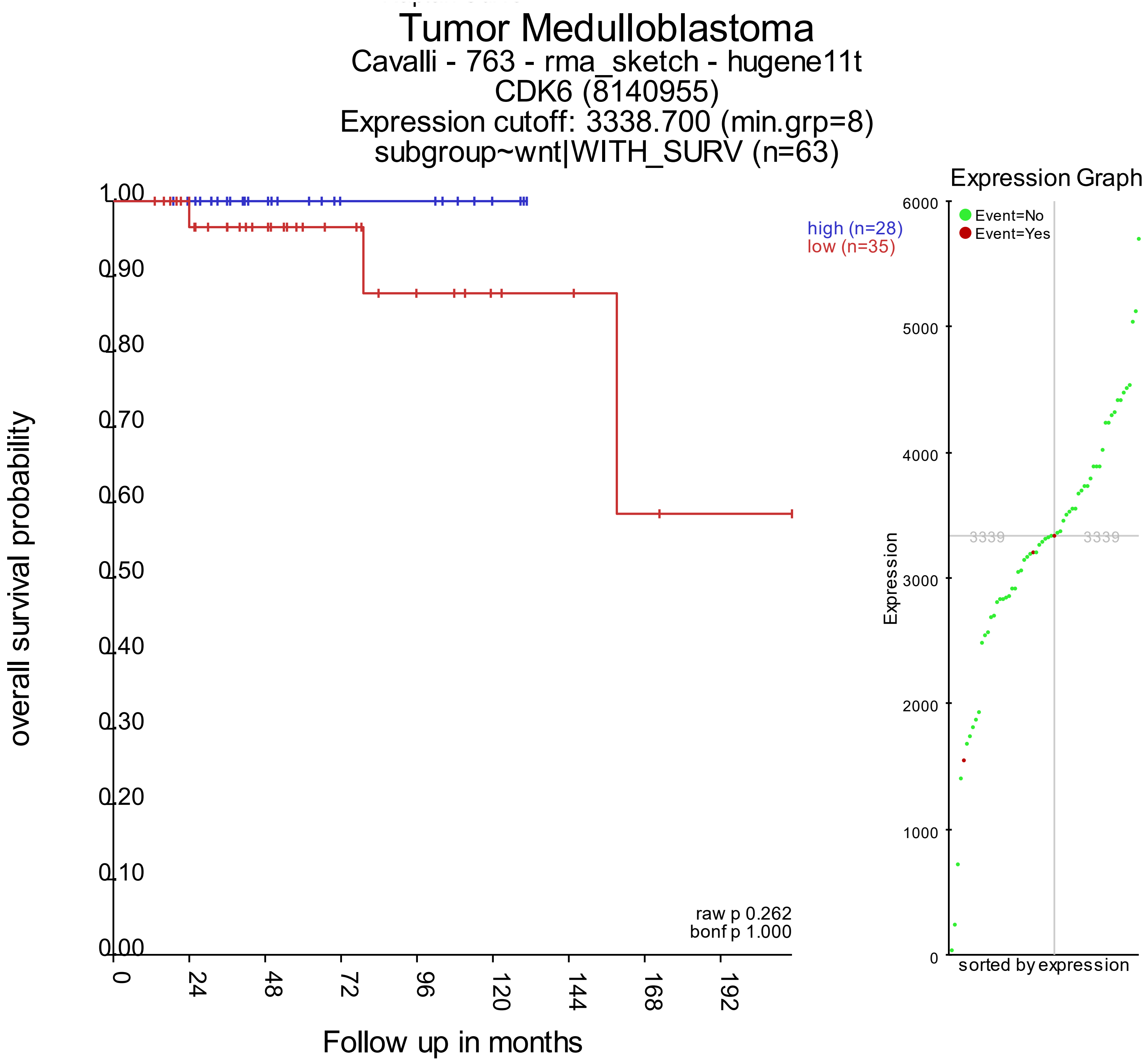

Tumor Medulloblastoma
Cavalli - 763 - rma_sketch - hugene11t
CDK6 (8140955)
Expression cutoff: 3338.700 (min.grp=8)
subgroup~wnt|WITH_SURV (n=63)
overall survival probability
1.00
0.90
0.80
0.70
0.60
0.50
0.40
0.30
0.20
0.10
0.00
0
24
48
72
96
120
144
168
192
Follow up in months
high (n=28)
low (n=35)
raw p 0.262
bonf p 1.000
Expression Graph
Event=No
Event=Yes
Expression
6000
5000
4000
3000
2000
1000
0
3339
3339
sorted by expression

SHH

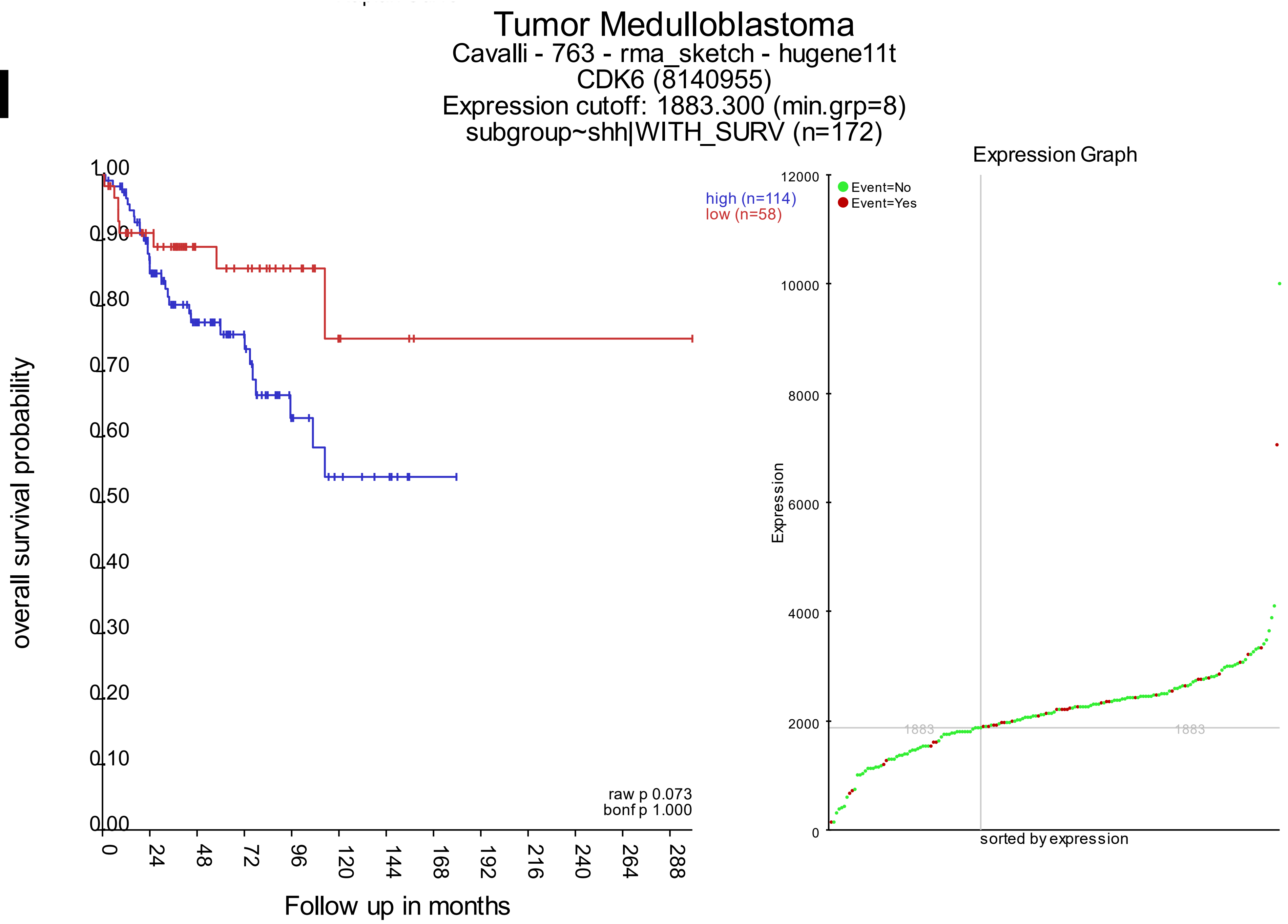

Tumor Medulloblastoma
Cavalli - 763 - rma_sketch - hugene11t
CDK6 (8140955)
Expression cutoff: 1883.300 (min.grp=8)
subgroup~shh|WITH_SURV (n=172)
overall survival probability
1.00
0.90
0.80
0.70
0.60
0.50
0.40
0.30
0.20
0.10
0.00
0
24
48
72
96
120
144
168
192
216
240
264
288
Follow up in months
high (n=114)
low (n=58)
raw p 0.073
bonf p 1.000
Expression Graph
Event=No
Event=Yes
Expression
12000
10000
8000
6000
4000
2000
0
1883
1883
sorted by expression

GR4

Tumor Medulloblastoma
Cavalli - 763 - rma_sketch - hugene11t
CDK6 (8140955)
Expression cutoff: 3307.800 (min.grp=8)
subgroup~group4|WITH_SURV (n=264)

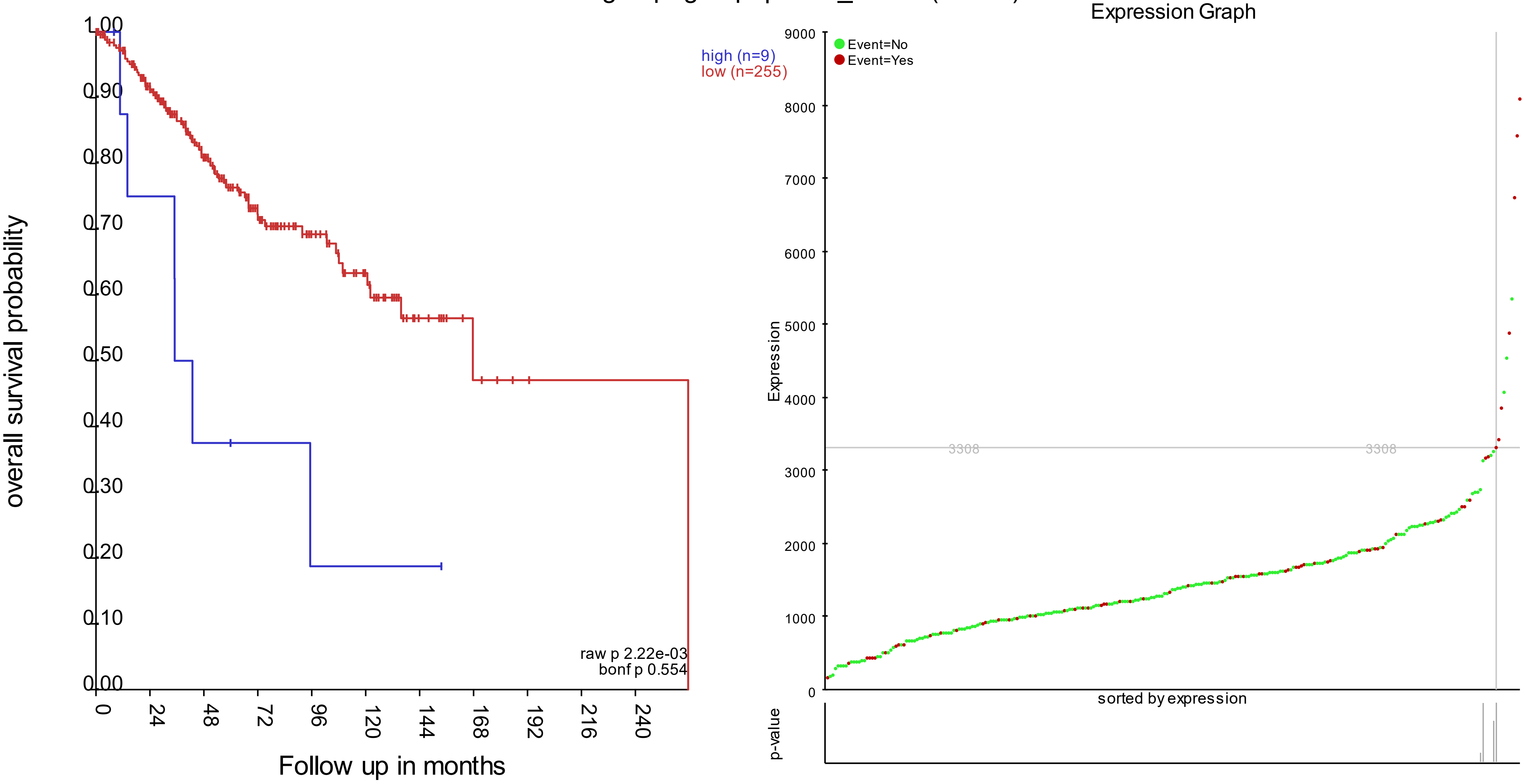

GR3

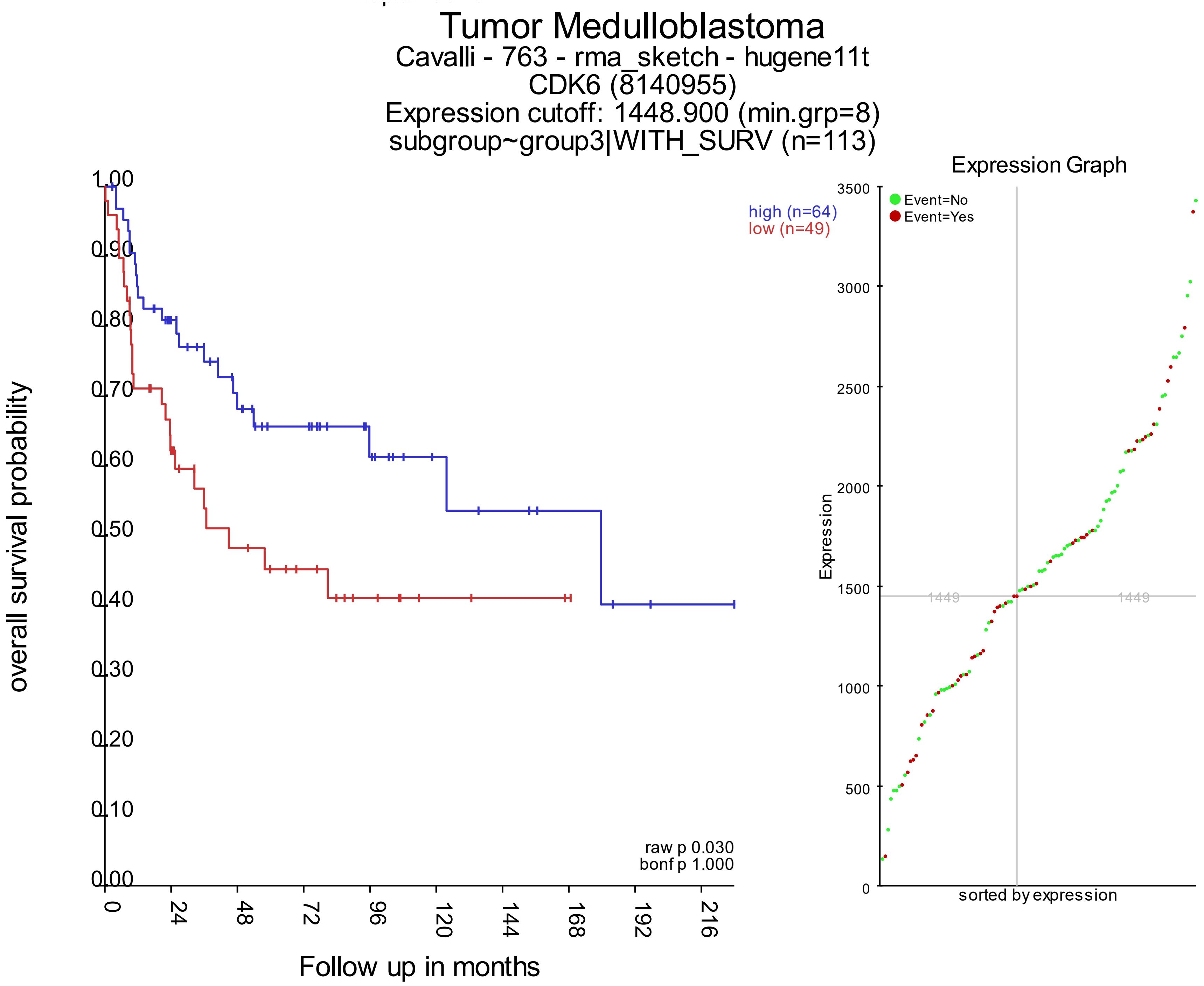
Tumor Medulloblastoma
Cavalli - 763 - rma_sketch - hugene11t
CDK6 (8140955)
Expression cutoff: 1448.900 (min.grp=8)
subgroup~group3|WITH_SURV (n=113)
high (n=64)
low (n=49)
overall survival probability
1.00
0.90
0.80
0.70
0.60
0.50
0.40
0.30
0.20
0.10
0.00
0
24
48
72
96
120
144
168
192
216
Follow up in months
raw p 0.030
bonf p 1.000
Expression Graph
Event=No
Event=Yes
Expression
3500
3000
2500
2000
1500
1000
500
0
1449
1449
sorted by expression

DDR2

WNT

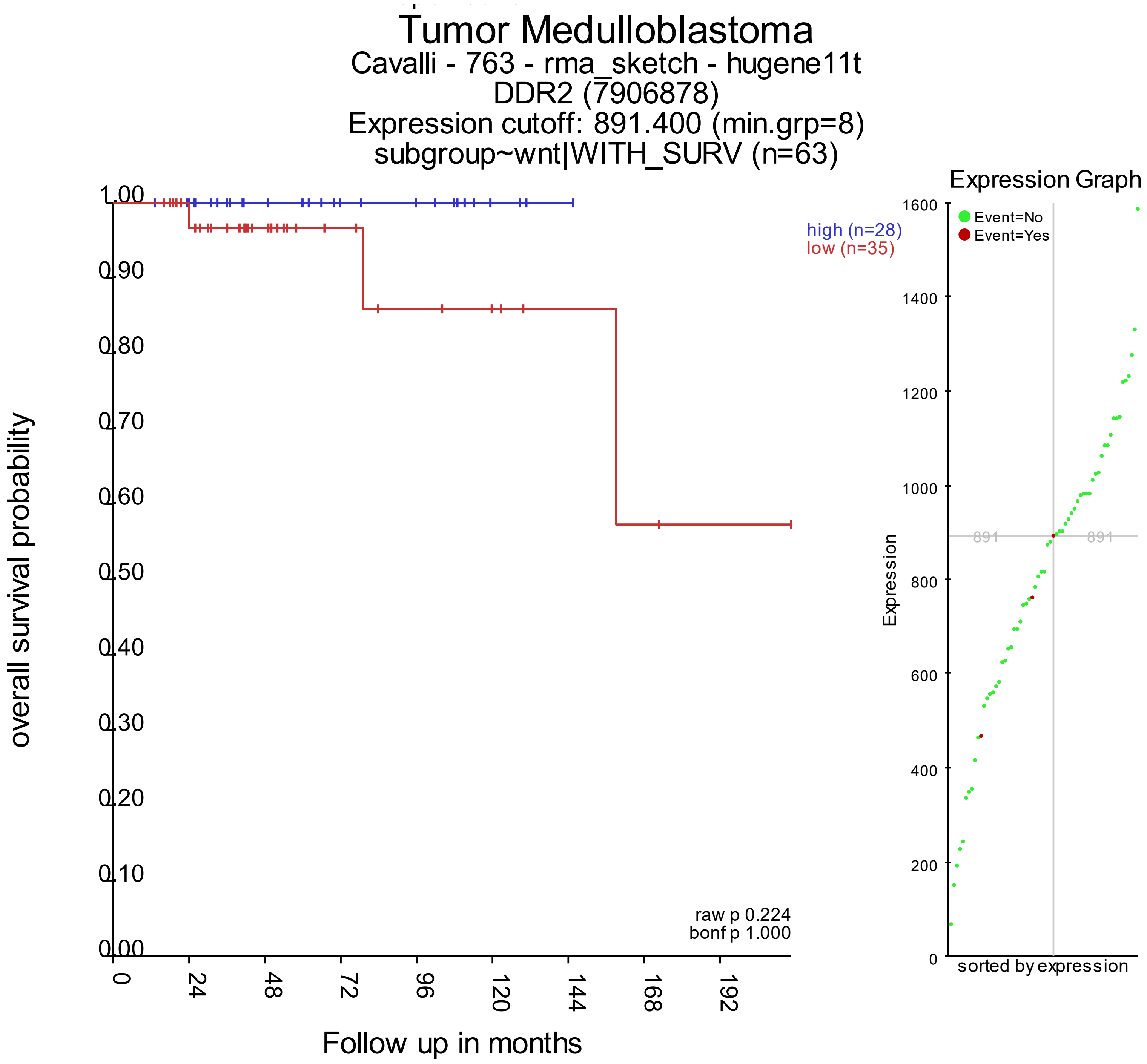
Tumor Medulloblastoma
Cavalli - 763 - rma_sketch - hugene11t
DDR2 (7906878)
Expression cutoff: 891.400 (min.grp=8)
subgroup~wnt|WITH_SURV (n=63)
overall survival probability
1.00
0.90
0.80
0.70
0.60
0.50
0.40
0.30
0.20
0.10
0.00
0
24
48
72
96
120
144
168
192
Follow up in months
raw p 0.224
bonf p 1.000
high (n=28)
low (n=35)
Expression Graph
Event=No
Event=Yes
1600
1400
1200
1000
800
600
400
200
0
891
891
Expression
sorted by expression

SHH

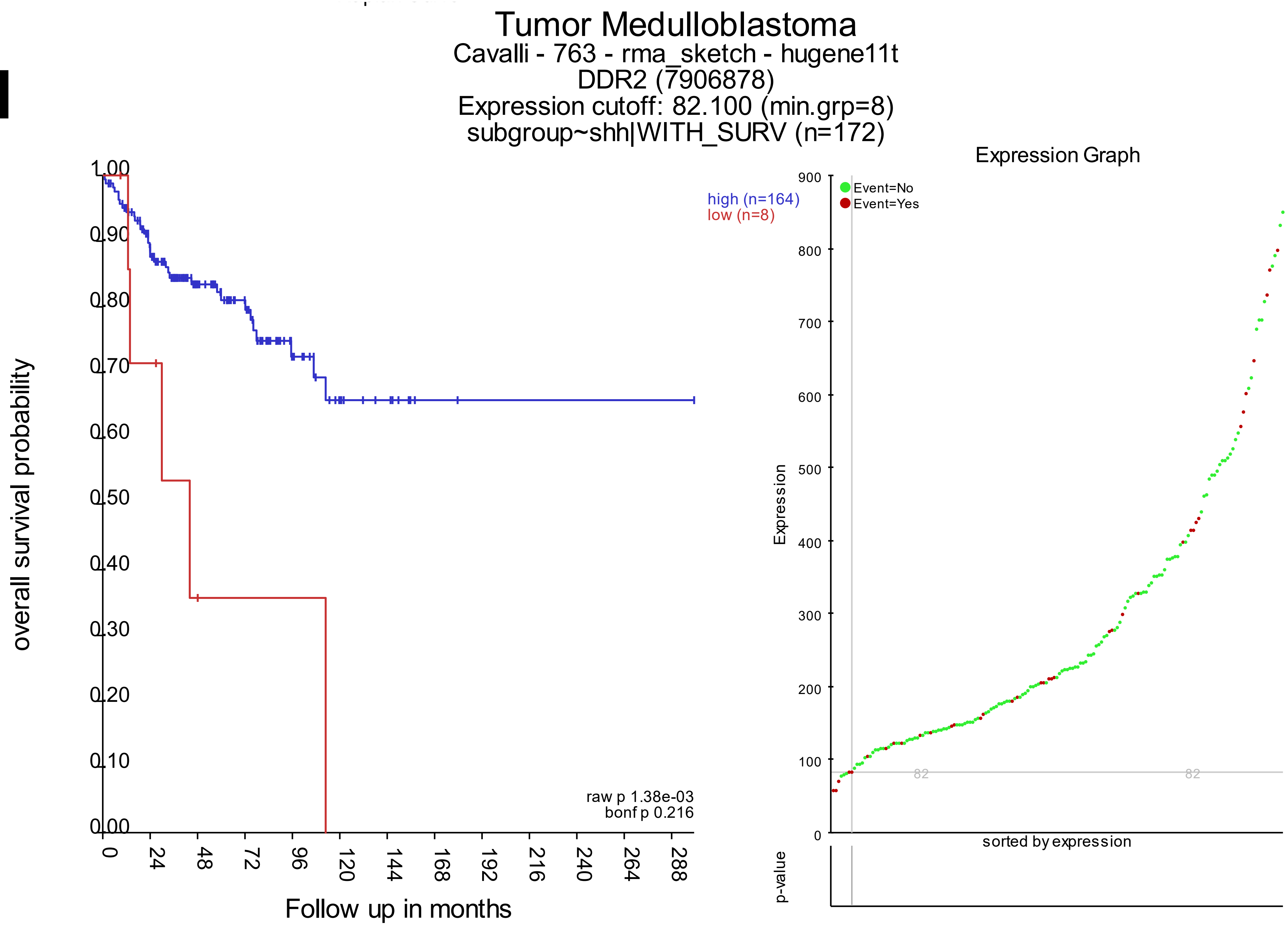

Tumor Medulloblastoma
Cavalli - 763 - rma_sketch - hugene11t
DDR2 (7906878)
Expression cutoff: 82.100 (min.grp=8)
subgroup~shh|WITH_SURV (n=172)
overall survival probability
1.00
0.90
0.80
0.70
0.60
0.50
0.40
0.30
0.20
0.10
0.00
0
24
48
72
96
120
144
168
192
216
240
264
288
Follow up in months
high (n=164)
low (n=8)
raw p 1.38e-03
bonf p 0.216
Expression Graph
Event=No
Event=Yes
Expression
900
800
700
600
500
400
300
200
100
0
82
82
sorted by expression
p-value

GR4

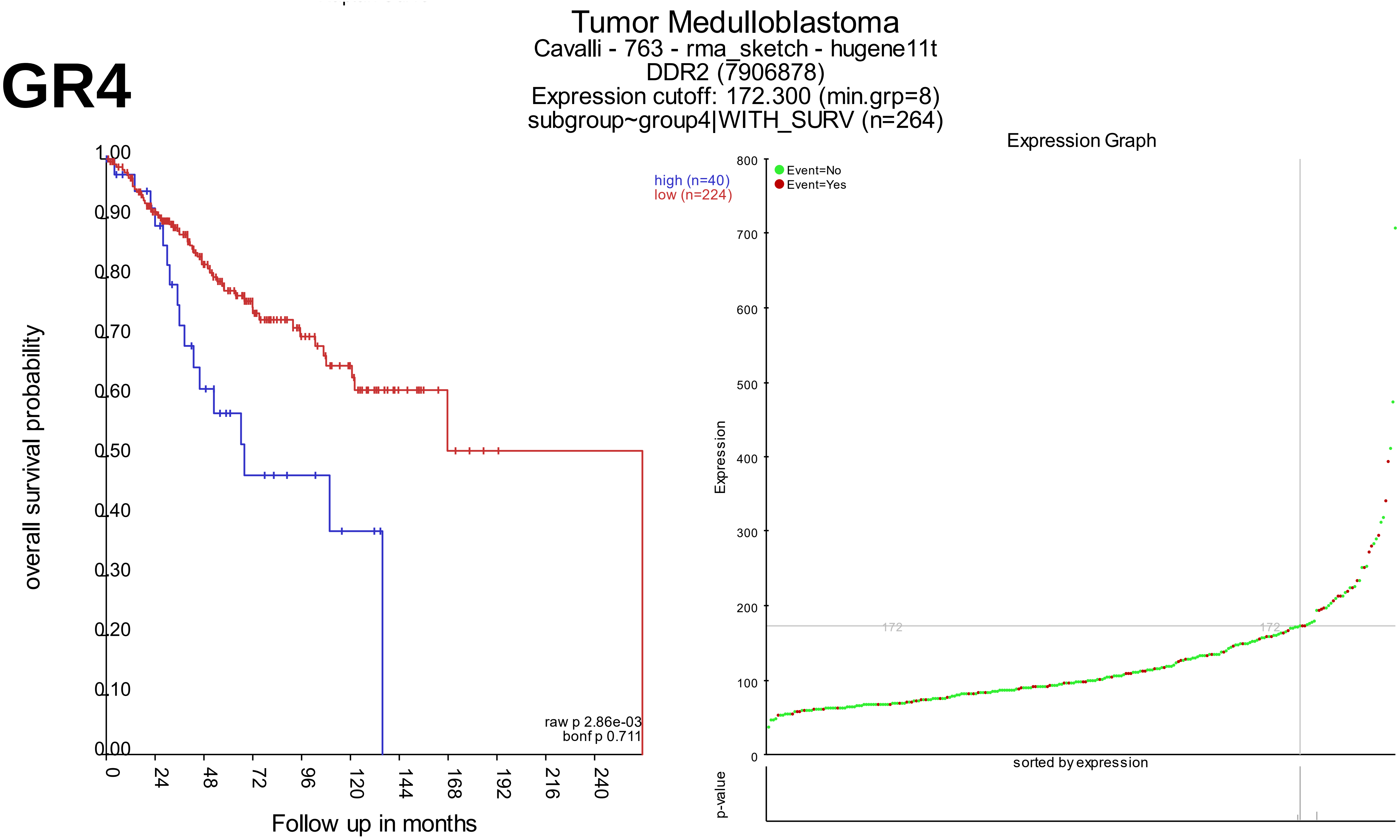

Tumor Medulloblastoma
Cavalli - 763 - rma_sketch - hugene11t
DDR2 (7906878)
Expression cutoff: 172.300 (min.grp=8)
subgroup~group4|WITH_SURV (n=264)
high (n=40)
low (n=224)
overall survival probability
Follow up in months
raw p 2.86e-03
bonf p 0.711
Expression Graph
Event=No
Event=Yes
Expression
sorted by expression
p-value
172

GR3

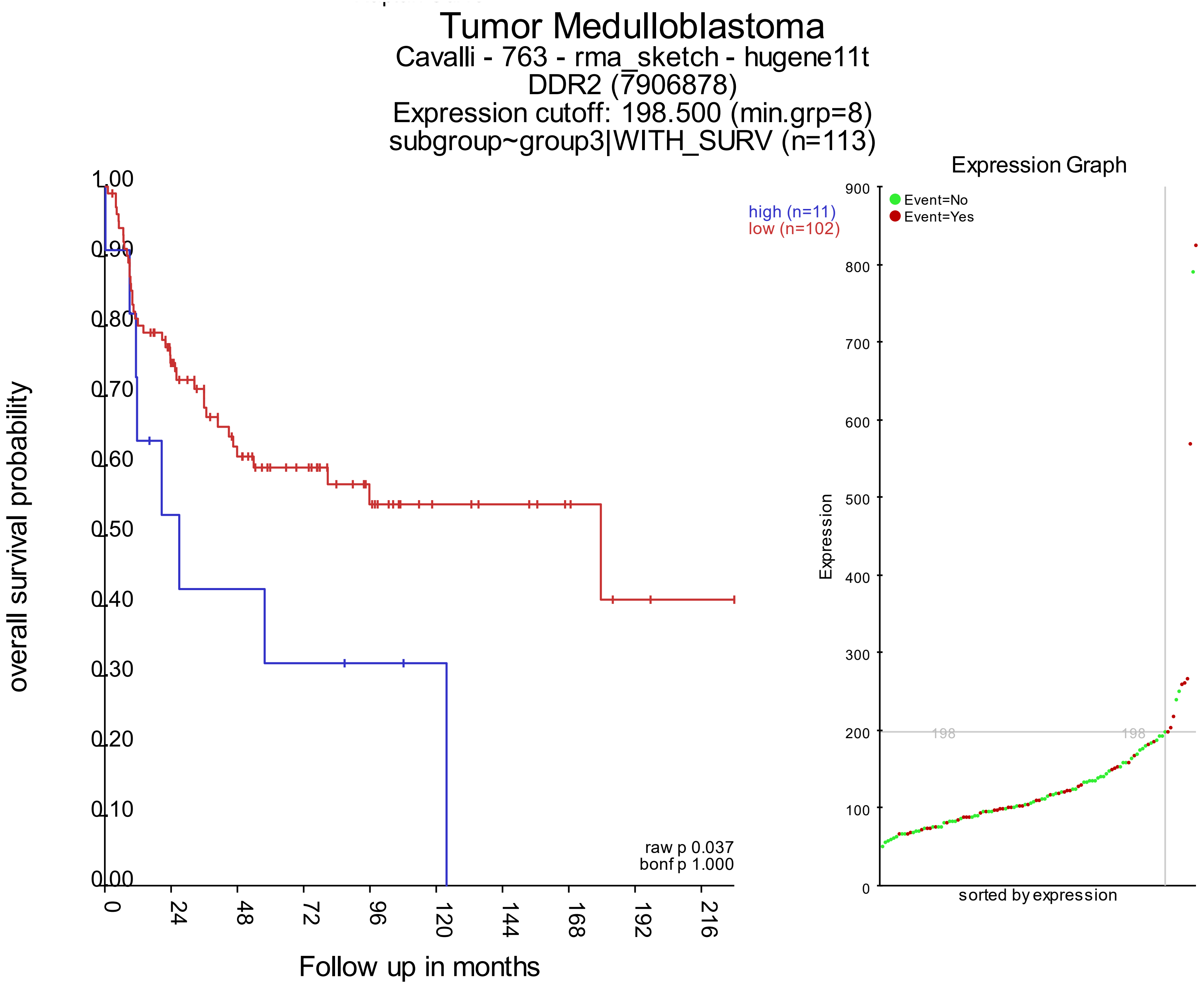

Tumor Medulloblastoma
Cavalli - 763 - rma_sketch - hugene11t
DDR2 (7906878)
Expression cutoff: 198.500 (min.grp=8)
subgroup~group3|WITH_SURV (n=113)
overall survival probability
1.00
0.90
0.80
0.70
0.60
0.50
0.40
0.30
0.20
0.10
0.00
0
24
48
72
96
120
144
168
192
216
Follow up in months
high (n=11)
low (n=102)
raw p 0.037
bonf p 1.000
Expression Graph
Event=No
Event=Yes
Expression
900
800
700
600
500
400
300
200
100
0
198
198
sorted by expression

EPCAM

WNT

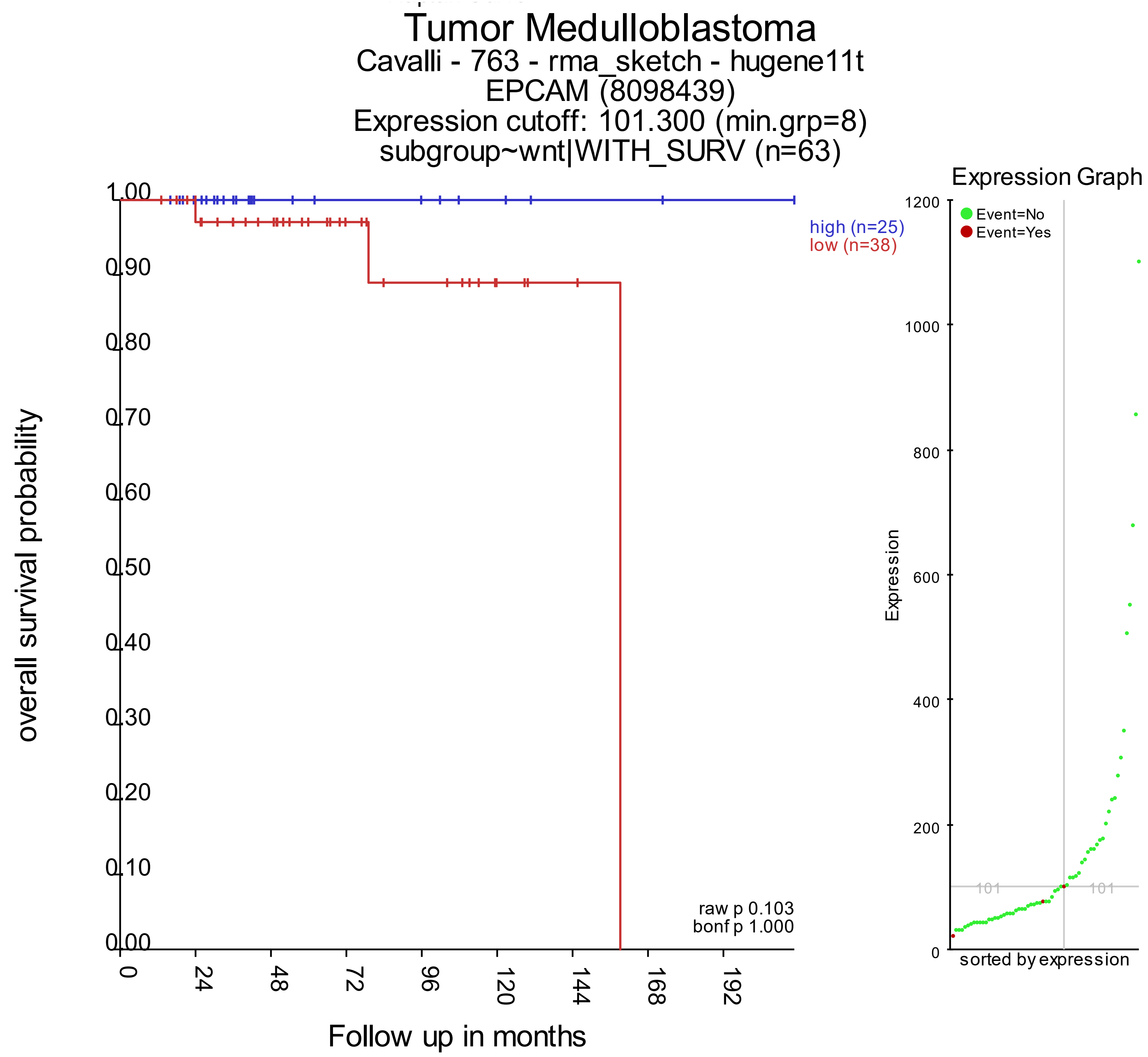

Tumor Medulloblastoma
Cavalli - 763 - rma_sketch - hugene11t
EPCAM (8098439)
Expression cutoff: 101.300 (min.grp=8)
subgroup~wnt|WITH_SURV (n=63)
overall survival probability
1.00
0.90
0.80
0.70
0.60
0.50
0.40
0.30
0.20
0.10
0.00
0
24
48
72
96
120
144
168
192
Follow up in months
high (n=25)
low (n=38)
raw p 0.103
bonf p 1.000
Expression Graph
Event=No
Event=Yes
Expression
1200
1000
800
600
400
200
0
101
101
sorted by expression

SHH

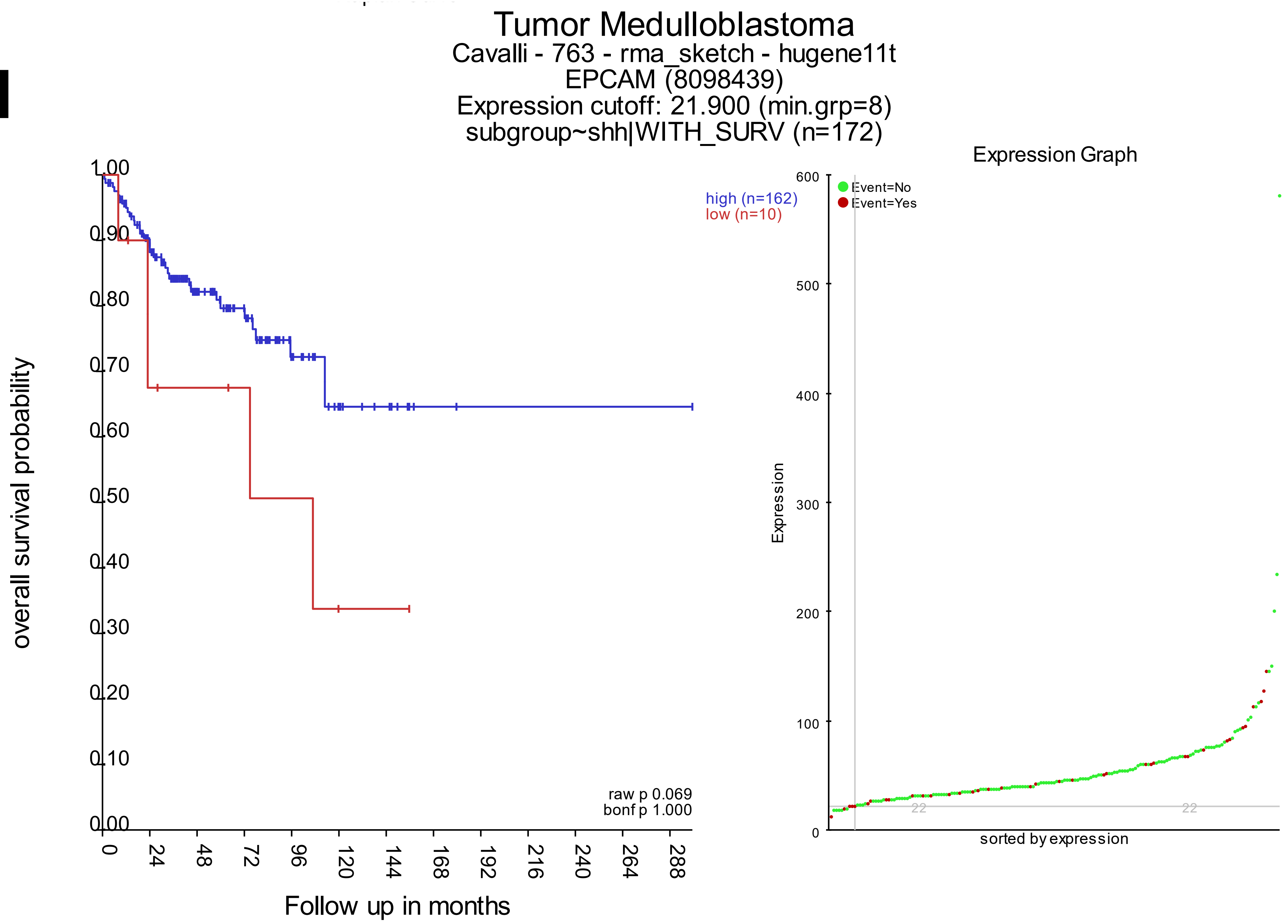

Tumor Medulloblastoma
Cavalli - 763 - rma_sketch - hugene11t
EPCAM (8098439)
Expression cutoff: 21.900 (min.grp=8)
subgroup~shh|WITH_SURV (n=172)
high (n=162)
low (n=10)
overall survival probability
1.00
0.90
0.80
0.70
0.60
0.50
0.40
0.30
0.20
0.10
0.00
0
24
48
72
96
120
144
168
192
216
240
264
288
Follow up in months
raw p 0.069
bonf p 1.000
Expression Graph
Event=No
Event=Yes
Expression
600
500
400
300
200
100
0
22
22
sorted by expression

GR4

Tumor Medulloblastoma
Cavalli - 763 - rma_sketch - hugene11t
EPCAM (8098439)
Expression cutoff: 216.100 (min.grp=8)
subgroup~group4|WITH_SURV (n=264)

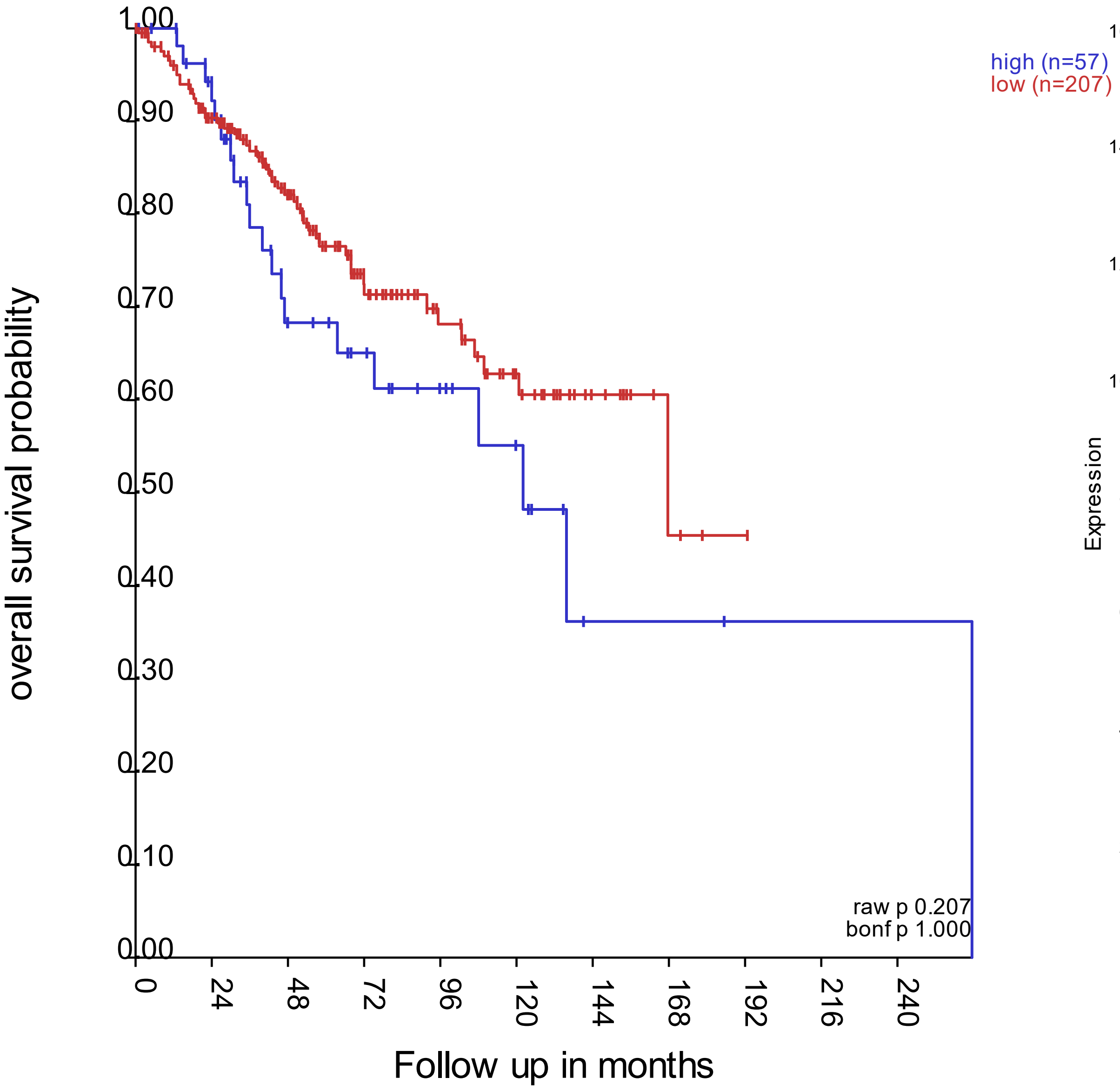

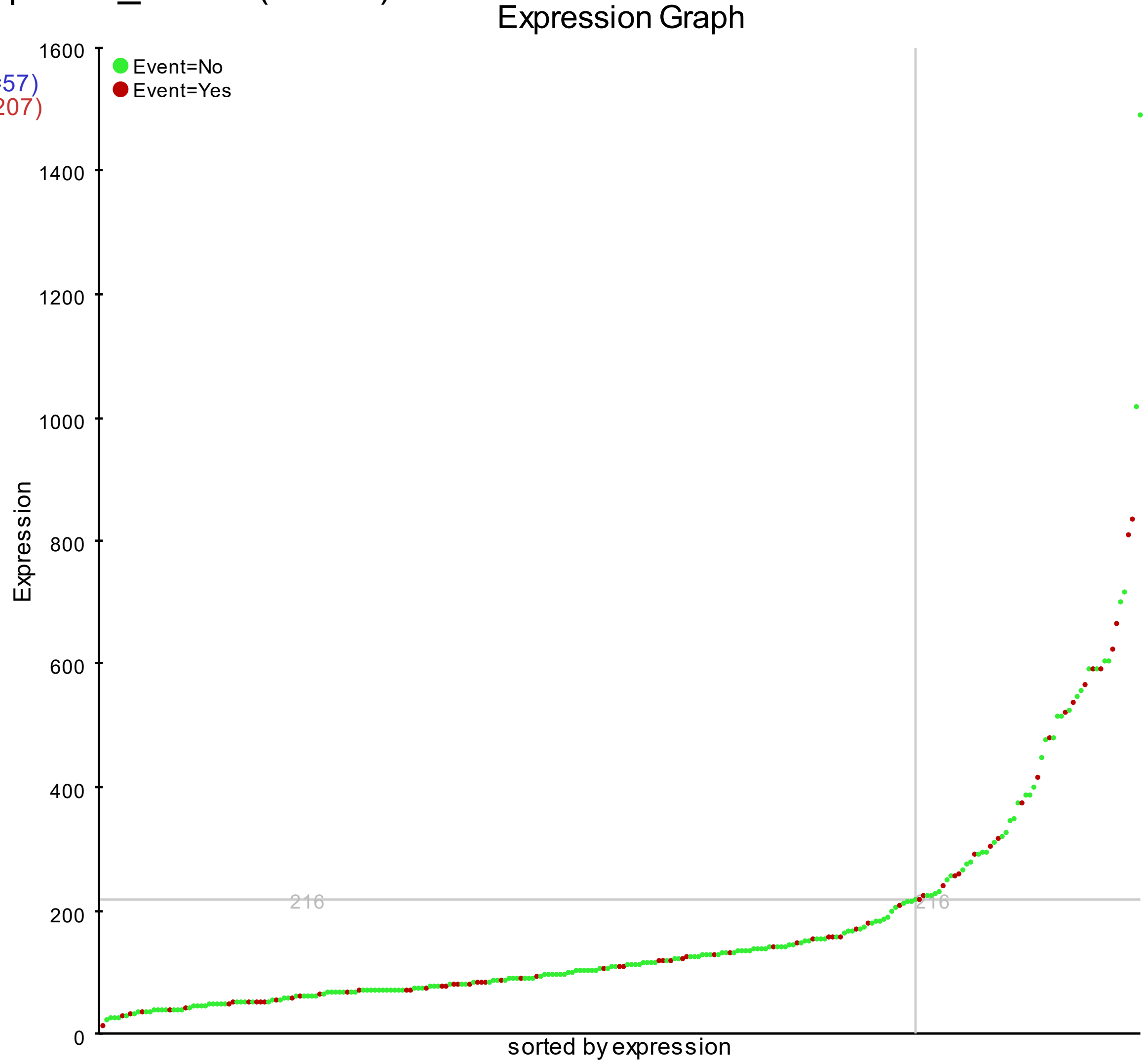

GR3

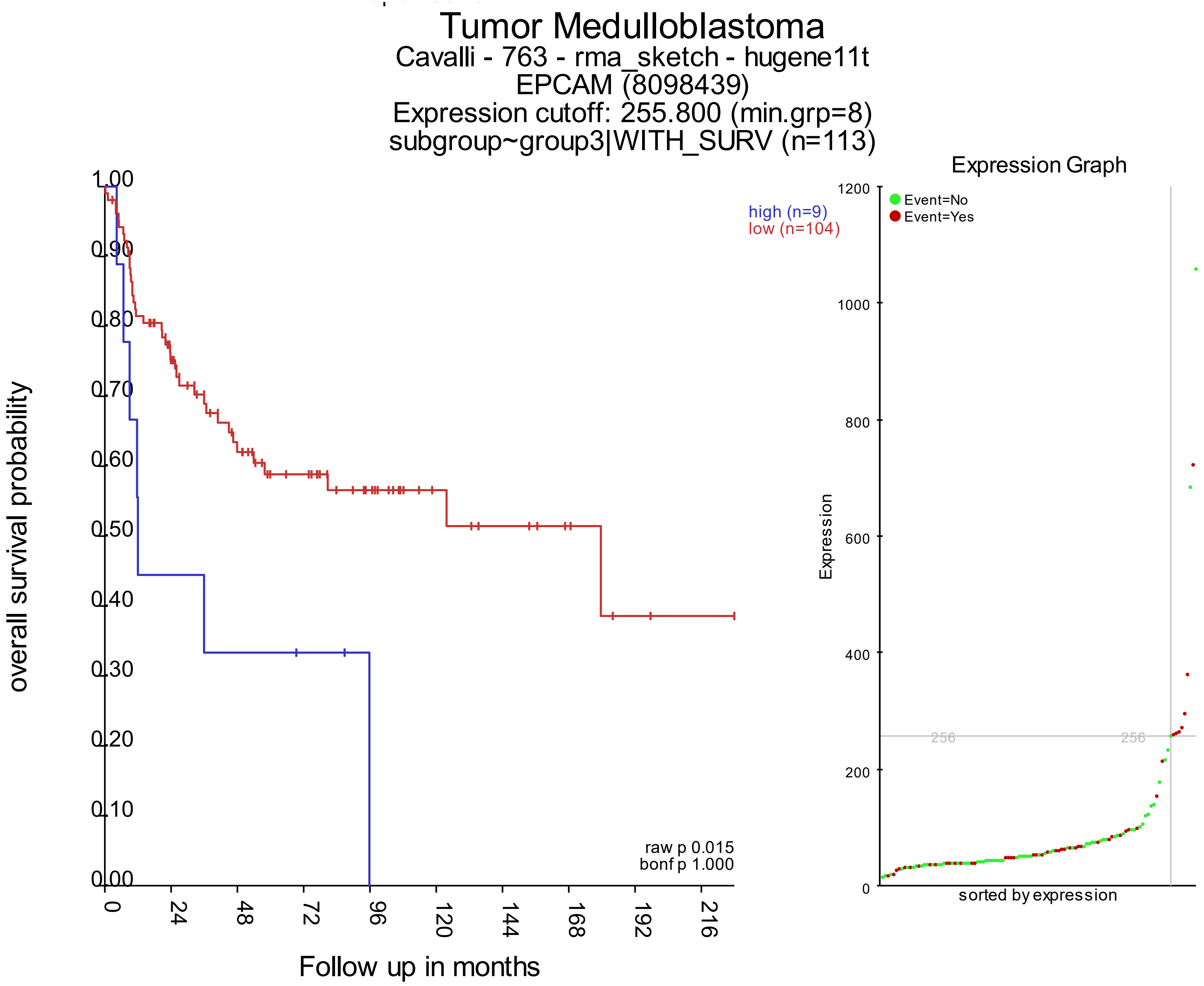
Tumor Medulloblastoma
Cavalli - 763 - rma_sketch - hugene11t
EPCAM (8098439)
Expression cutoff: 255.800 (min.grp=8)
subgroup~group3|WITH_SURV (n=113)
overall survival probability
1.00
0.90
0.80
0.70
0.60
0.50
0.40
0.30
0.20
0.10
0.00
high (n=9)
low (n=104)
raw p 0.015
bonf p 1.000
0
24
48
72
96
120
144
168
192
216
Follow up in months
Expression Graph
Event=No
Event=Yes
1200
1000
800
600
400
200
0
Expression
256
256
sorted by expression

FGFR1

WNT

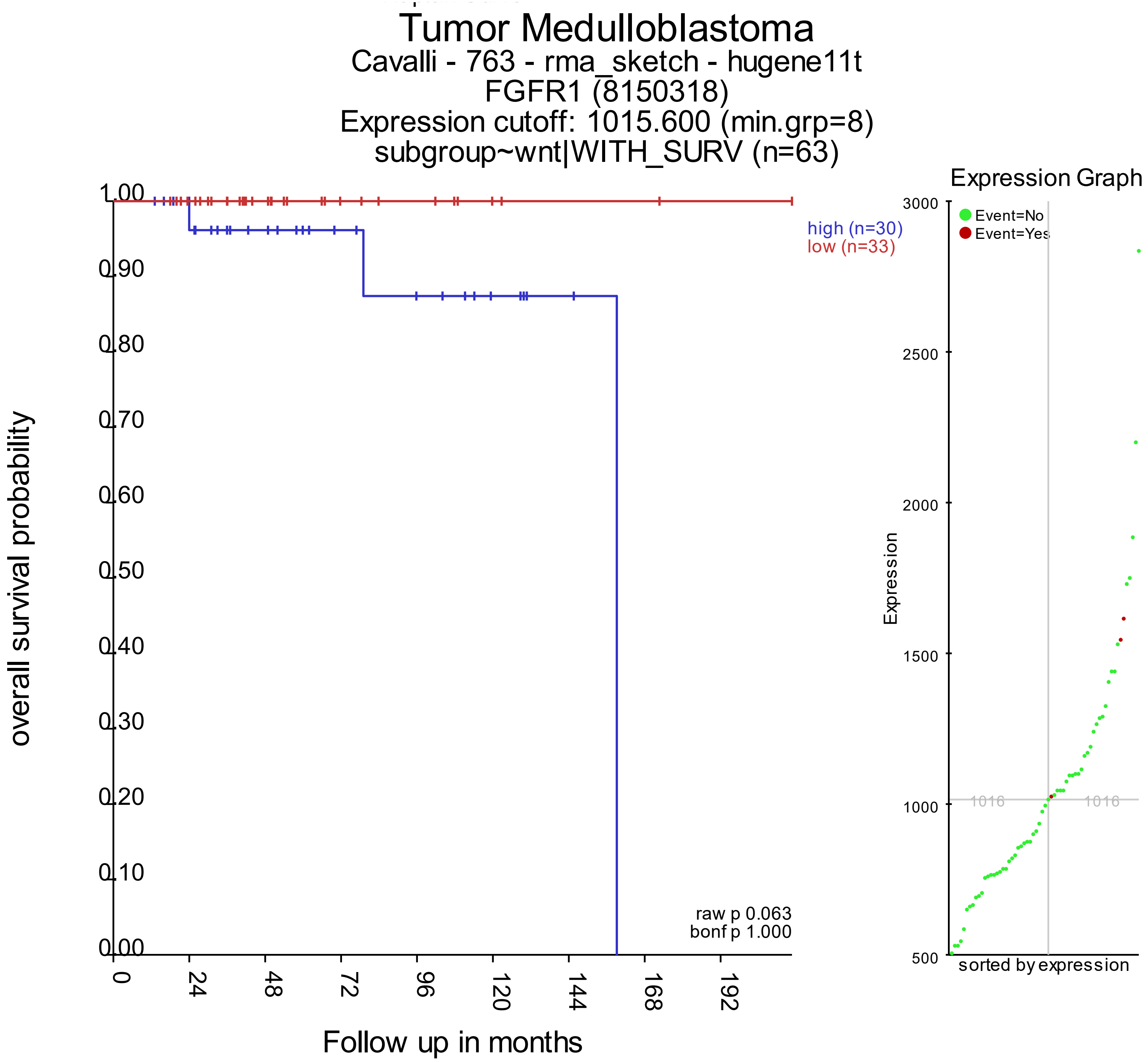

Tumor Medulloblastoma
Cavalli - 763 - rma_sketch - hugene11t
FGFR1 (8150318)
Expression cutoff: 1015.600 (min.grp=8)
subgroup~wnt|WITH_SURV (n=63)
overall survival probability
1.00
0.90
0.80
0.70
0.60
0.50
0.40
0.30
0.20
0.10
0.00
0
24
48
72
96
120
144
168
192
Follow up in months
high (n=30)
low (n=33)
raw p 0.063
bonf p 1.000
Expression Graph
Event=No
Event=Yes
Expression
3000
2500
2000
1500
1000
500
1016
1016
sorted by expression

SHH

Tumor Medulloblastoma
Cavalli - 763 - rma_sketch - hugene11t
FGFR1 (8150318)
Expression cutoff: 197.300 (min.grp=8)
subgroup~shh|WITH_SURV (n=172)

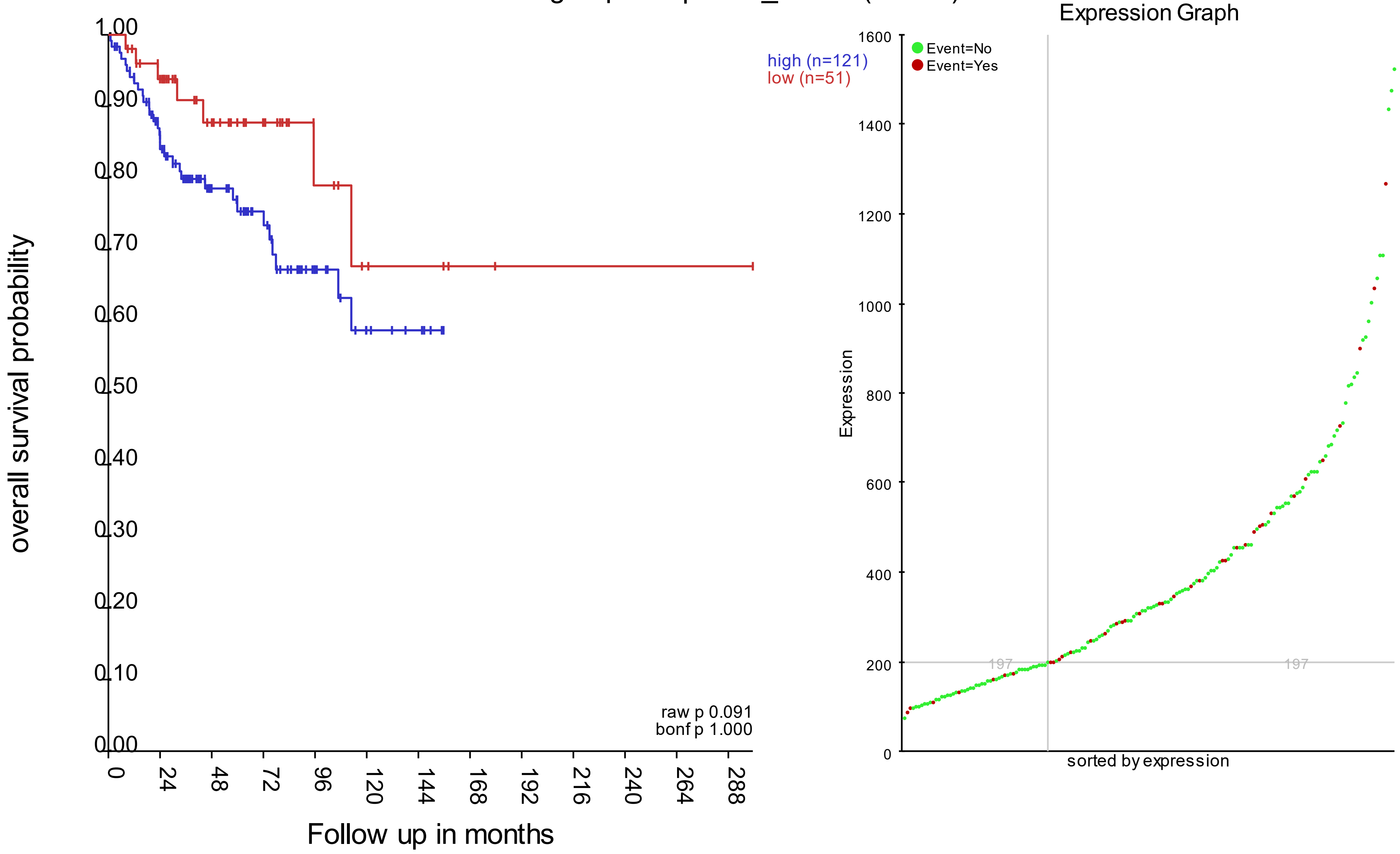

GR4

Tumor Medulloblastoma
Cavalli - 763 - rma_sketch - hugene11t
FGFR1 (8150318)
Expression cutoff: 165.500 (min.grp=8)
subgroup~group4|WITH_SURV (n=264)

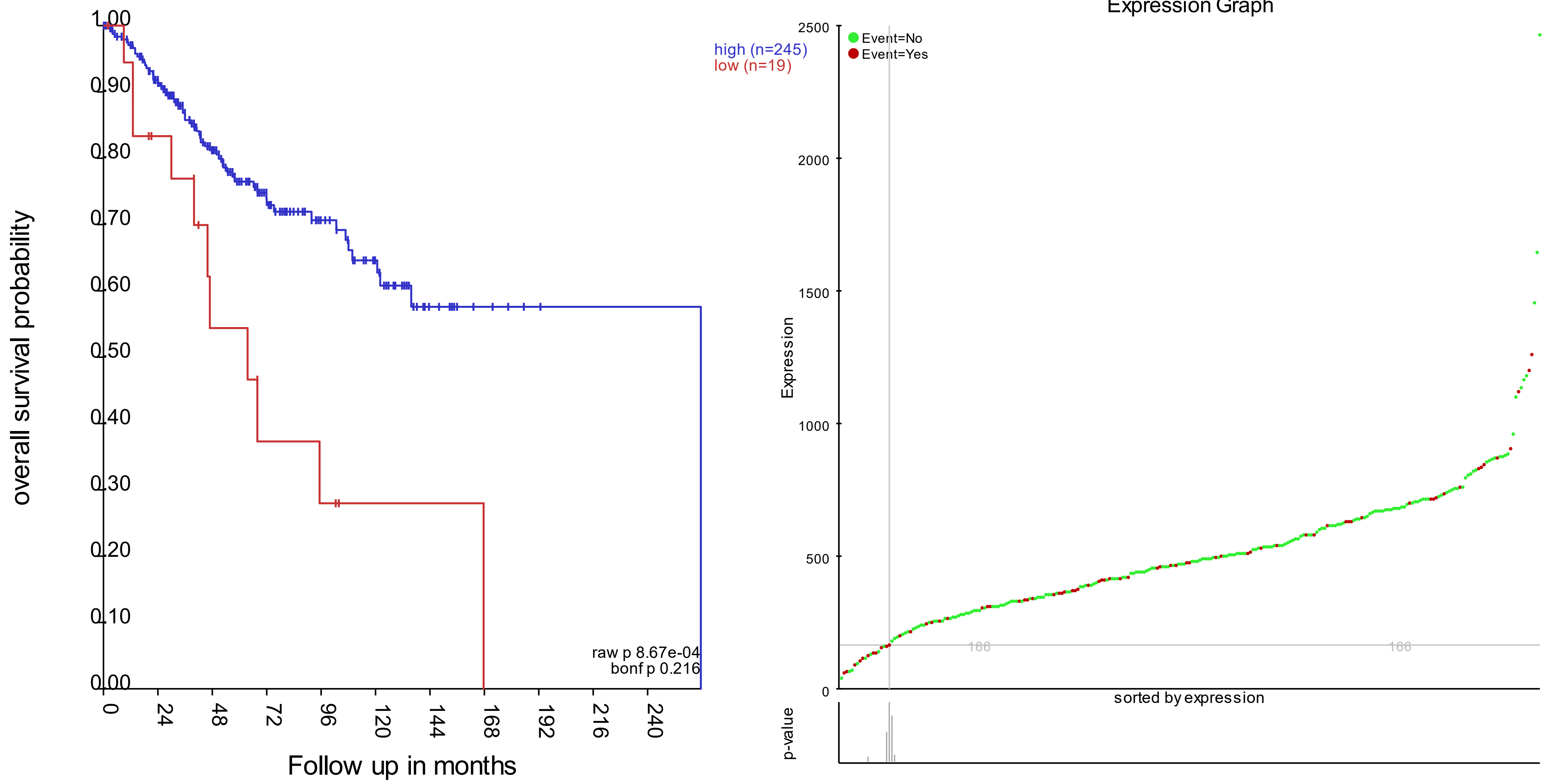

GR3

Tumor Medulloblastoma
Cavalli - 763 - rma_sketch - hugene11t
FGFR1 (8150318)
Expression cutoff: 381.700 (min.grp=8)
subgroup~group3|WITH_SURV (n=113)

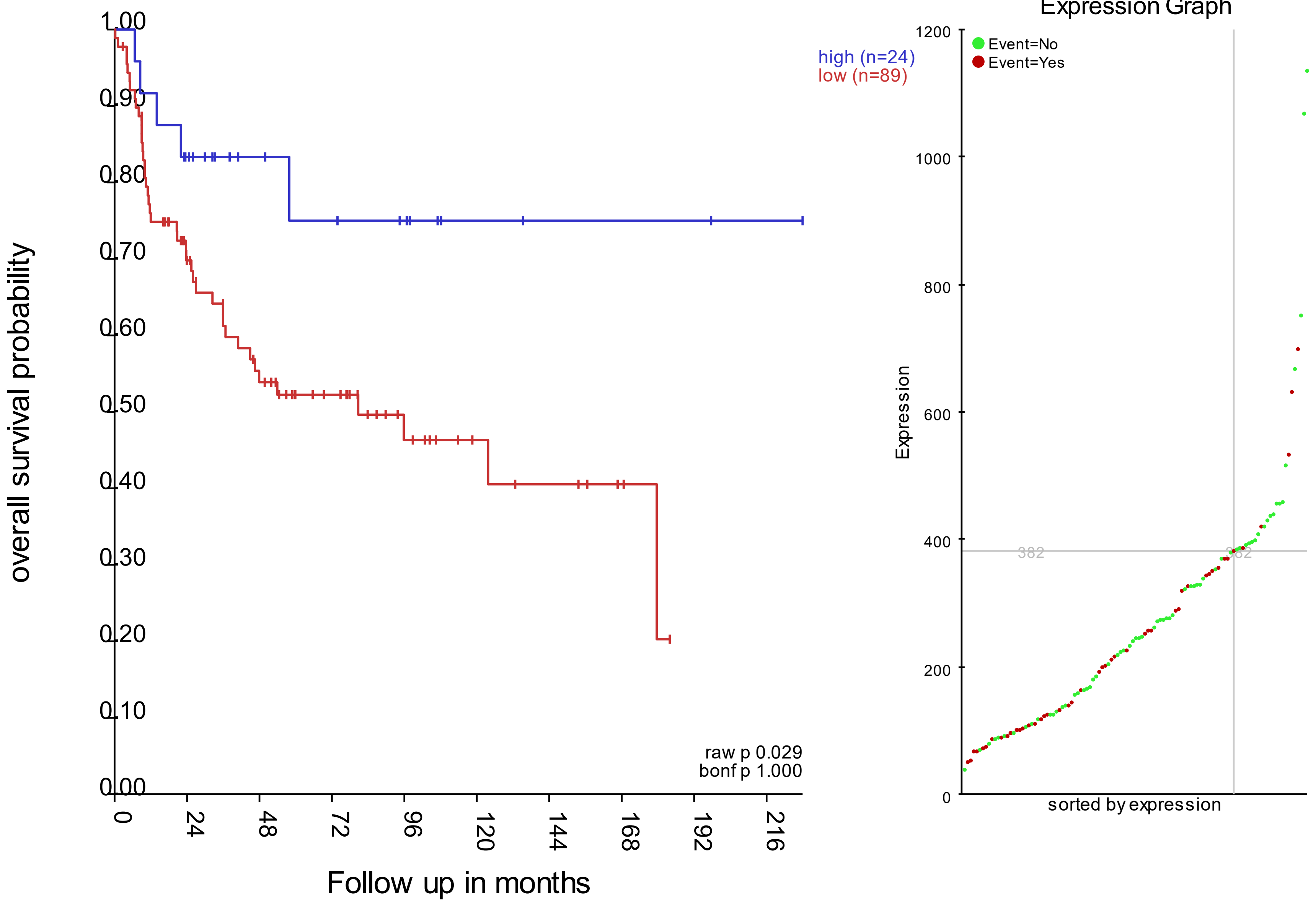

FGFR2

WNT

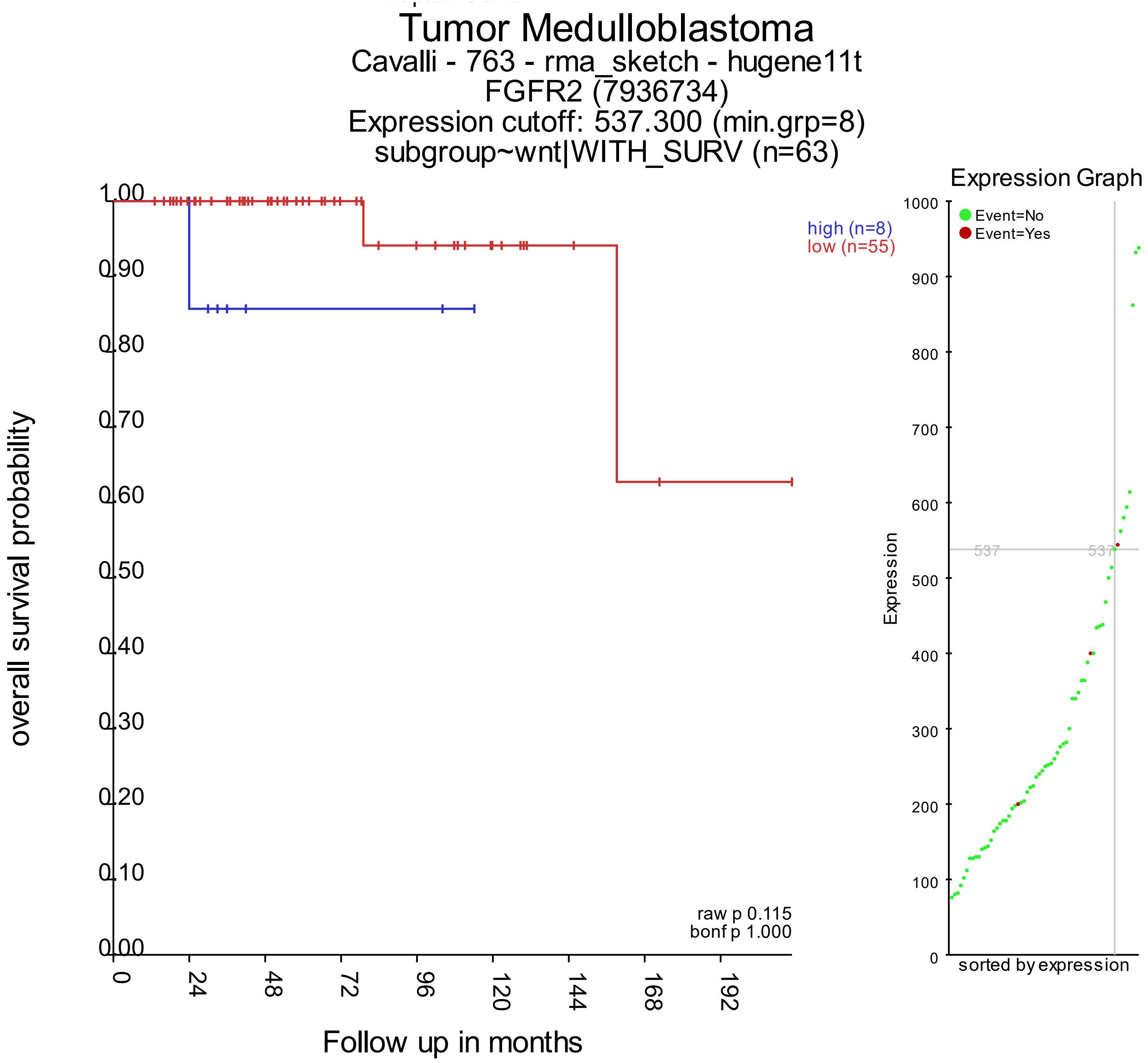

Tumor Medulloblastoma
Cavalli - 763 - rma_sketch - hugene11t
FGFR2 (7936734)
Expression cutoff: 537.300 (min.grp=8)
subgroup~wnt|WITH_SURV (n=63)
overall survival probability
1.00
0.90
0.80
0.70
0.60
0.50
0.40
0.30
0.20
0.10
0.00
0
24
48
72
96
120
144
168
192
Follow up in months
high (n=8)
low (n=55)
raw p 0.115
bonf p 1.000
Expression Graph
Event=No
Event=Yes
Expression
1000
900
800
700
600
500
400
300
200
100
0
537
537
sorted by expression

SHH

Tumor Medulloblastoma
Cavalli - 763 - rma_sketch - hugene11t
FGFR2 (7936734)
Expression cutoff: 561.100 (min.grp=8)
subgroup~shh|WITH_SURV (n=172)

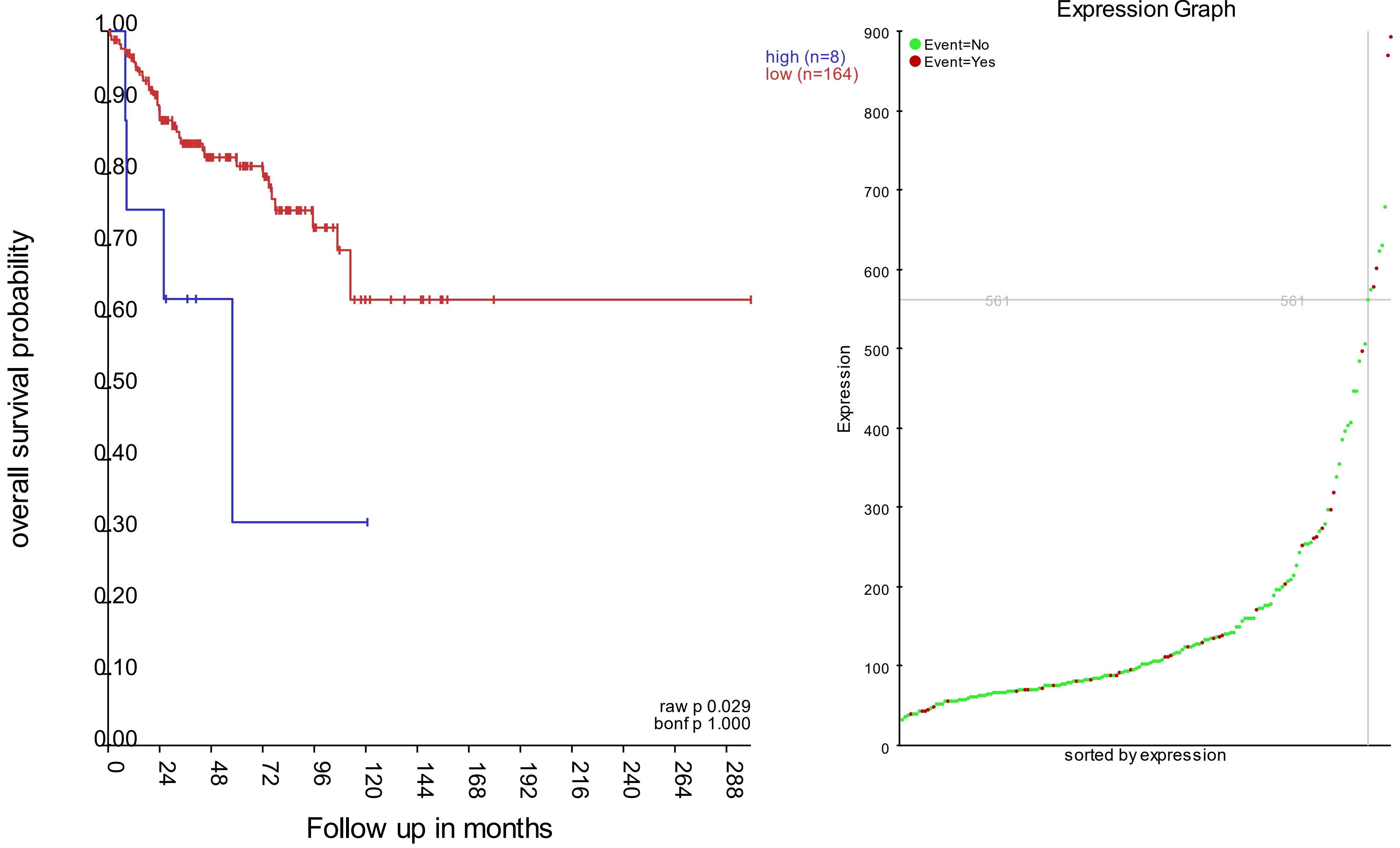

GR4

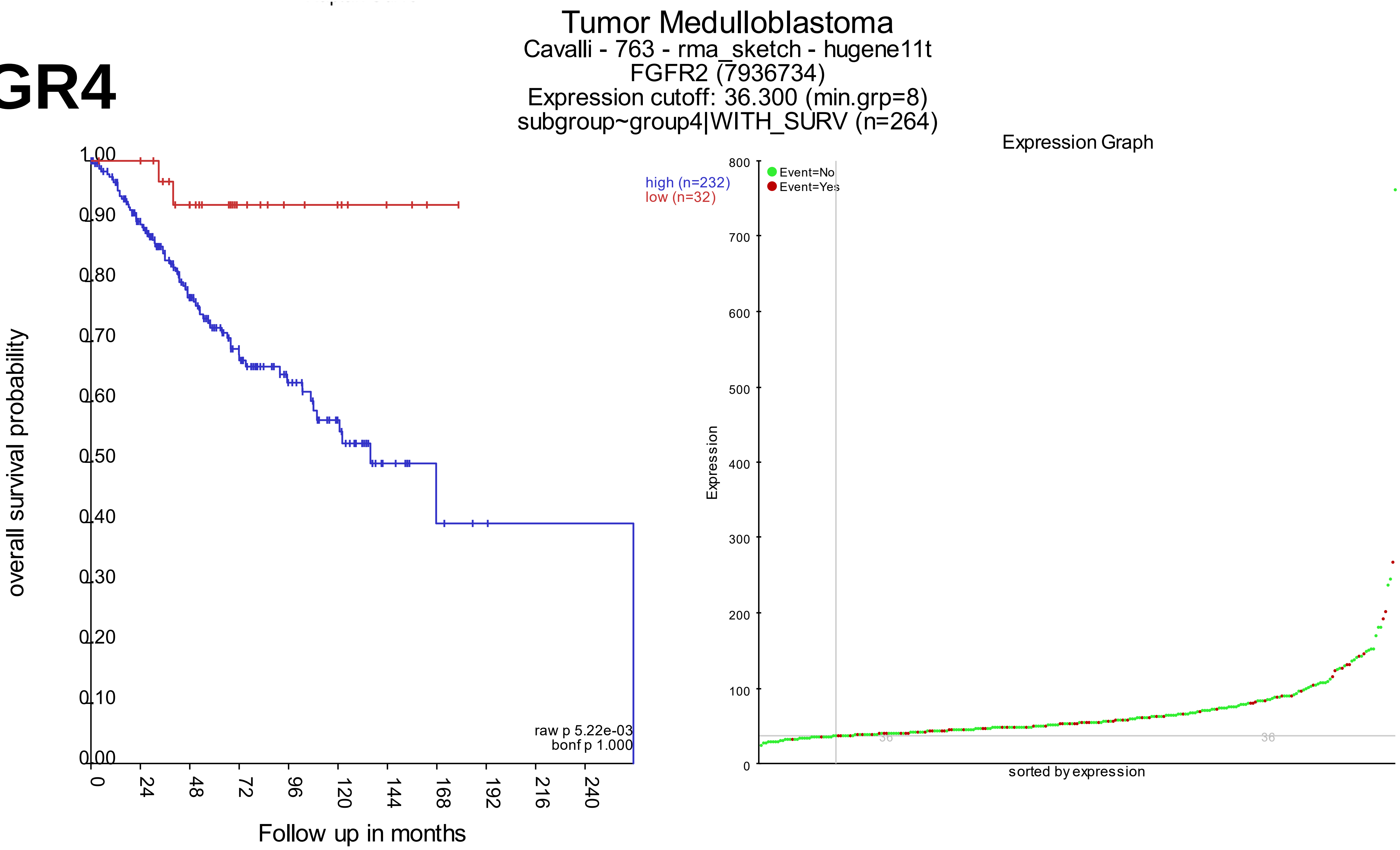

Tumor Medulloblastoma
Cavalli - 763 - rma_sketch - hugene11t
FGFR2 (7936734)
Expression cutoff: 36.300 (min.grp=8)
subgroup~group4|WITH_SURV (n=264)
high (n=232)
low (n=32)
overall survival probability
1.00
0.90
0.80
0.70
0.60
0.50
0.40
0.30
0.20
0.10
0.00
0
24
48
72
96
120
144
168
192
216
240
Follow up in months
raw p 5.22e-03
bonf p 1.000
Expression Graph
Event=No
Event=Yes
Expression
800
700
600
500
400
300
200
100
0
36
sorted by expression

GR3

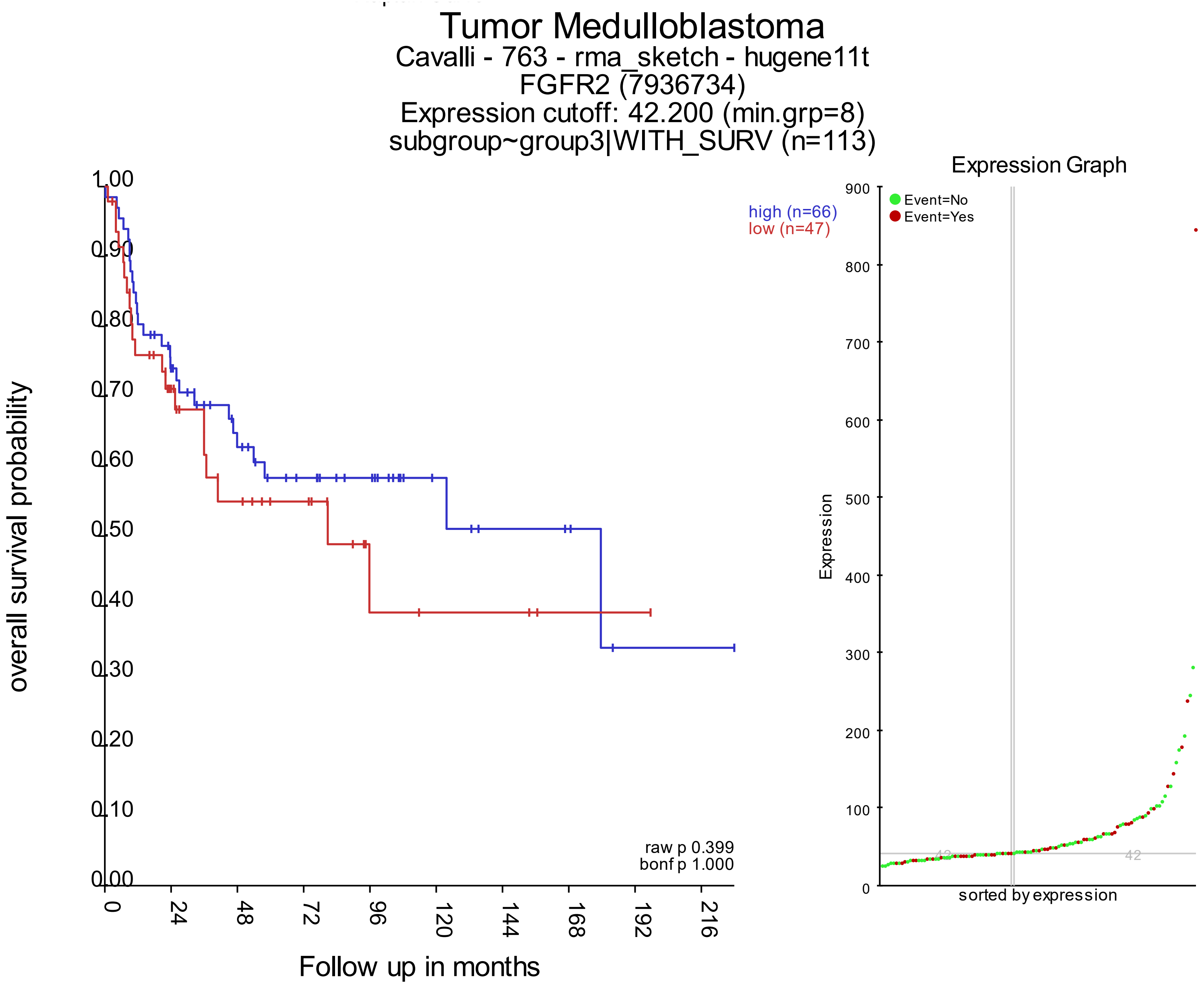

Tumor Medulloblastoma
Cavalli - 763 - rma_sketch - hugene11t
FGFR2 (7936734)
Expression cutoff: 42.200 (min.grp=8)
subgroup~group3|WITH_SURV (n=113)
high (n=66)
low (n=47)
overall survival probability
1.00
0.90
0.80
0.70
0.60
0.50
0.40
0.30
0.20
0.10
0.00
0
24
48
72
96
120
144
168
192
216
Follow up in months
raw p 0.399
bonf p 1.000
Expression Graph
Event=No
Event=Yes
Expression
900
800
700
600
500
400
300
200
100
0
42
sorted by expression

FGFR3

WNT

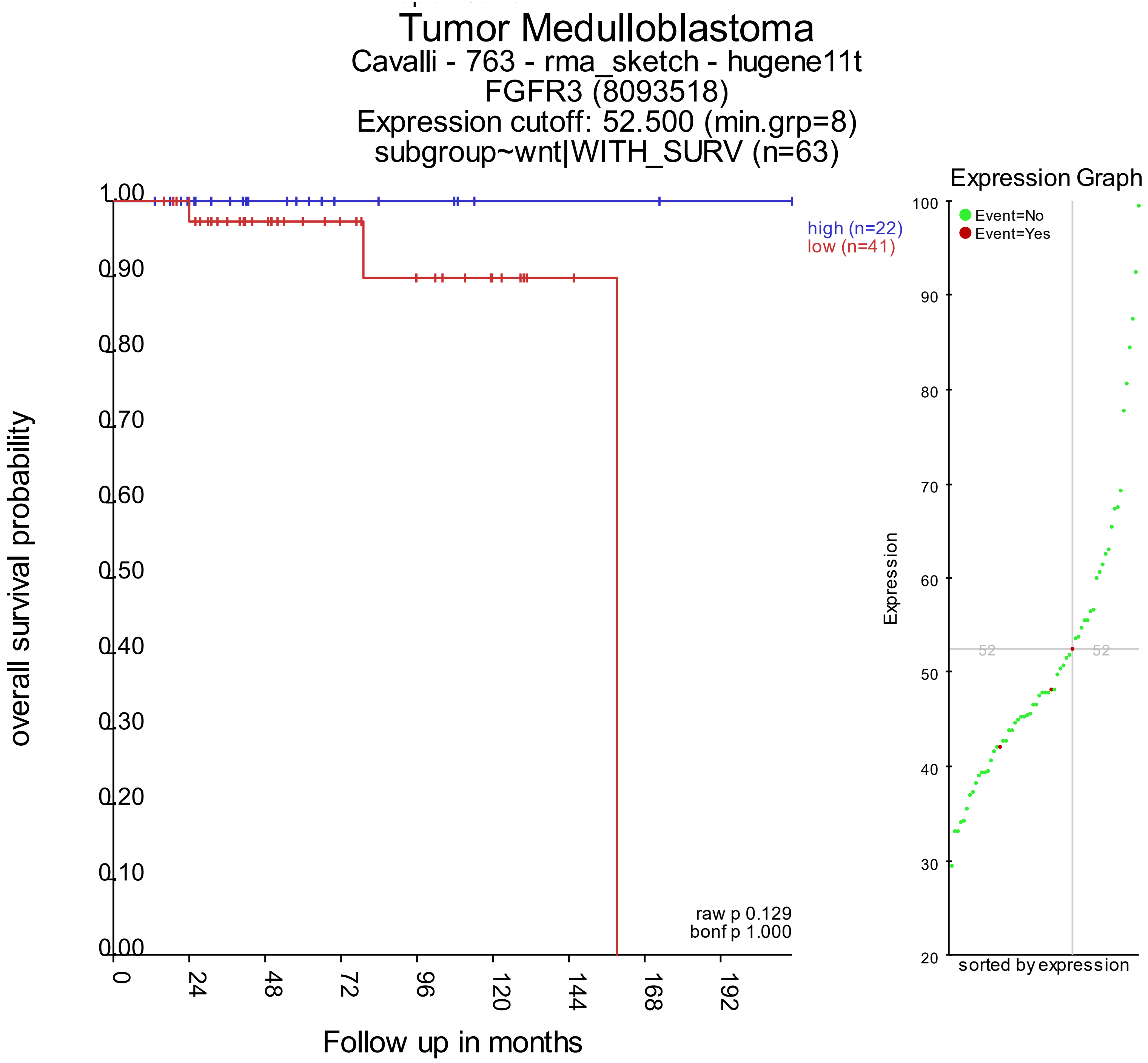
Tumor Medulloblastoma
Cavalli - 763 - rma_sketch - hugene11t
FGFR3 (8093518)
Expression cutoff: 52.500 (min.grp=8)
subgroup~wnt|WITH_SURV (n=63)
overall survival probability
1.00
0.90
0.80
0.70
0.60
0.50
0.40
0.30
0.20
0.10
0.00
0
24
48
72
96
120
144
168
192
Follow up in months
high (n=22)
low (n=41)
raw p 0.129
bonf p 1.000
Expression Graph
Event=No
Event=Yes
Expression
100
90
80
70
60
50
40
30
20
52
52
sorted by expression

SHH

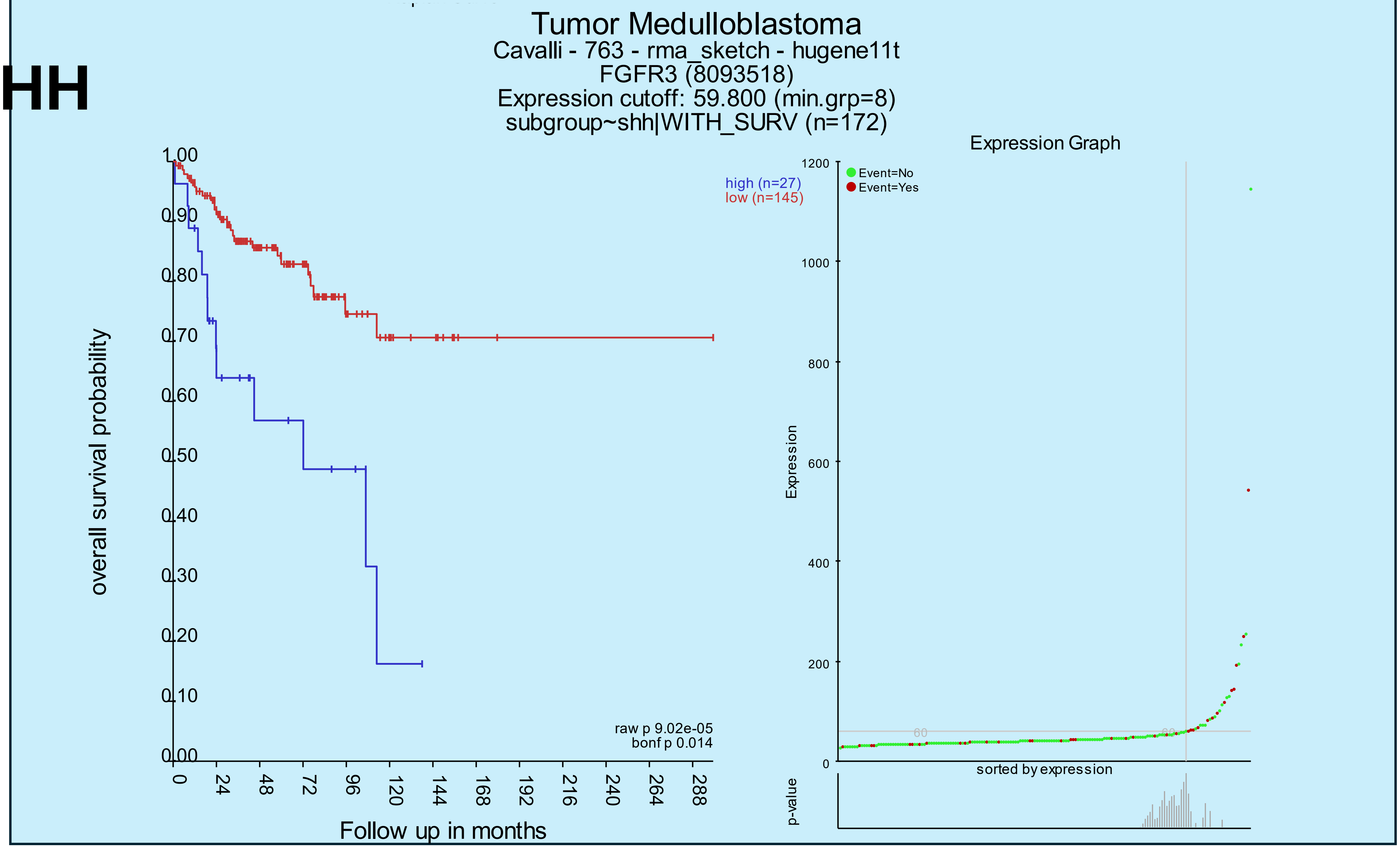
Tumor Medulloblastoma
Cavalli - 763 - rma_sketch - hugene11t
FGFR3 (8093518)
Expression cutoff: 59.800 (min.grp=8)
subgroup~shh|WITH_SURV (n=172)
overall survival probability
1.00
0.90
0.80
0.70
0.60
0.50
0.40
0.30
0.20
0.10
0.00
0
24
48
72
96
120
144
168
192
216
240
264
288
Follow up in months
high (n=27)
low (n=145)
raw p 9.02e-05
bonf p 0.014
Expression Graph
Event=No
Event=Yes
Expression
1200
1000
800
600
400
200
0
60
sorted by expression
p-value

GR4

Tumor Medulloblastoma
Cavalli - 763 - rma_sketch - hugene11t
FGFR3 (8093518)
Expression cutoff: 81.900 (min.grp=8)
subgroup~group4|WITH_SURV (n=264)

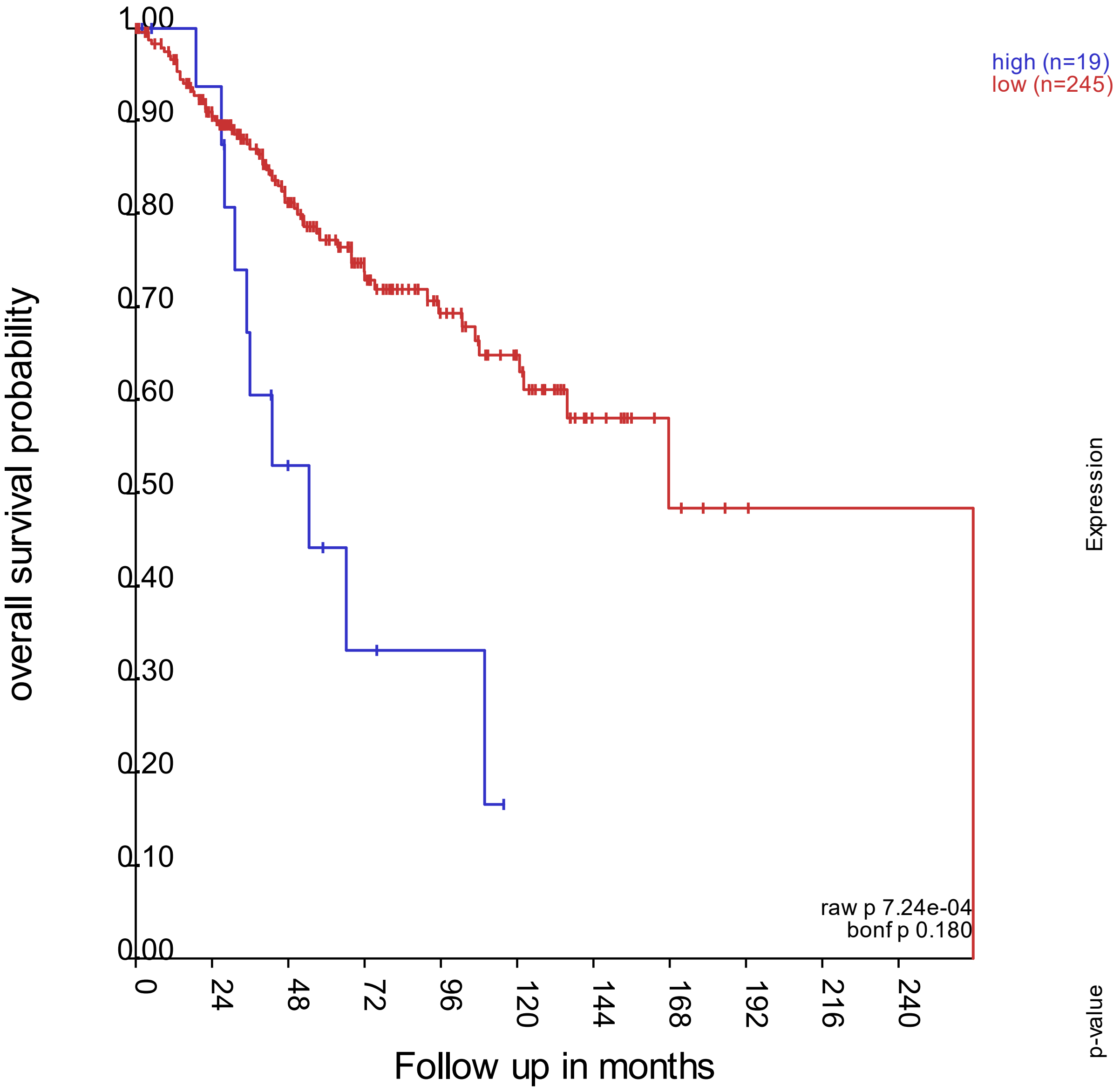

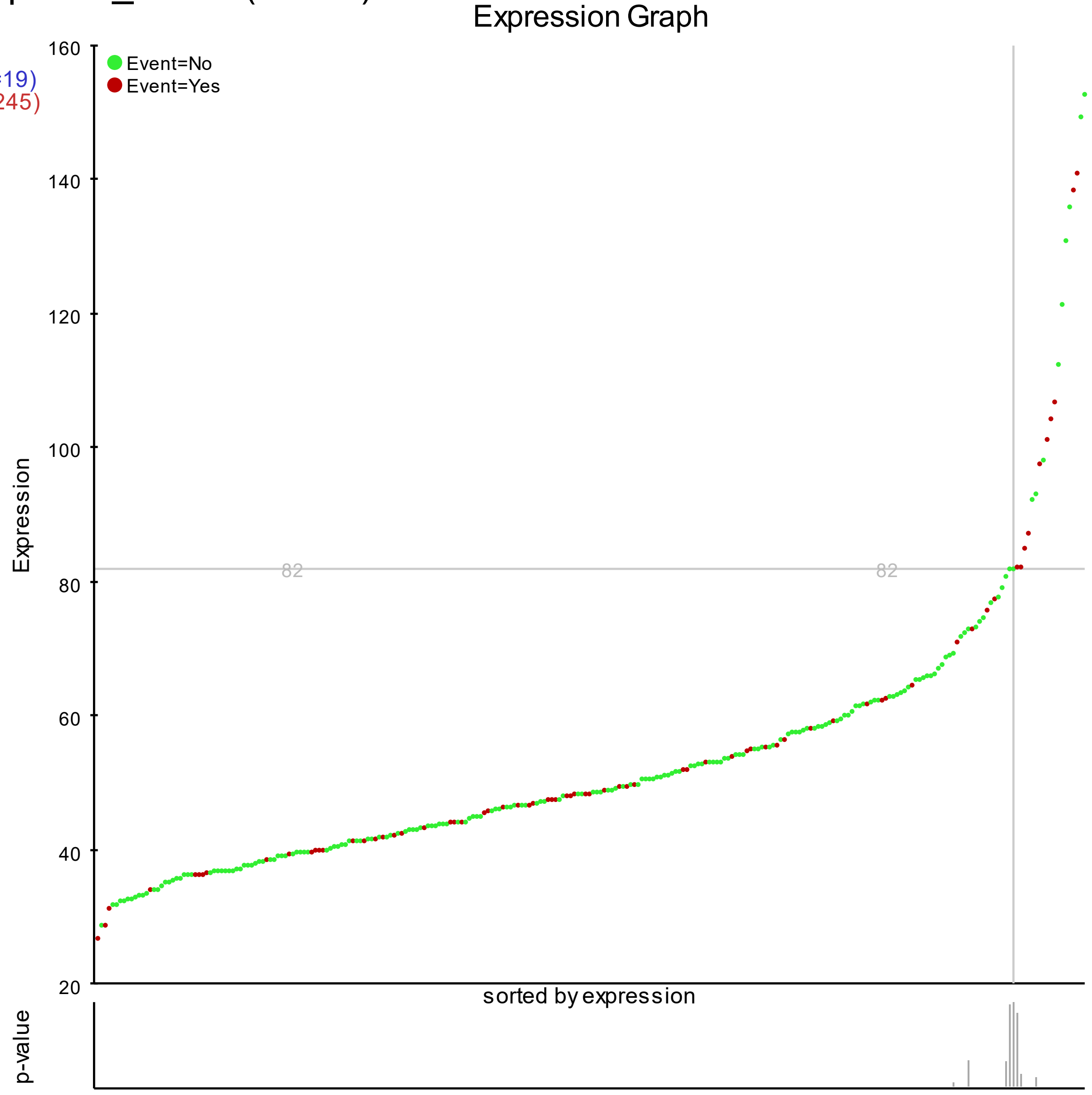

GR3

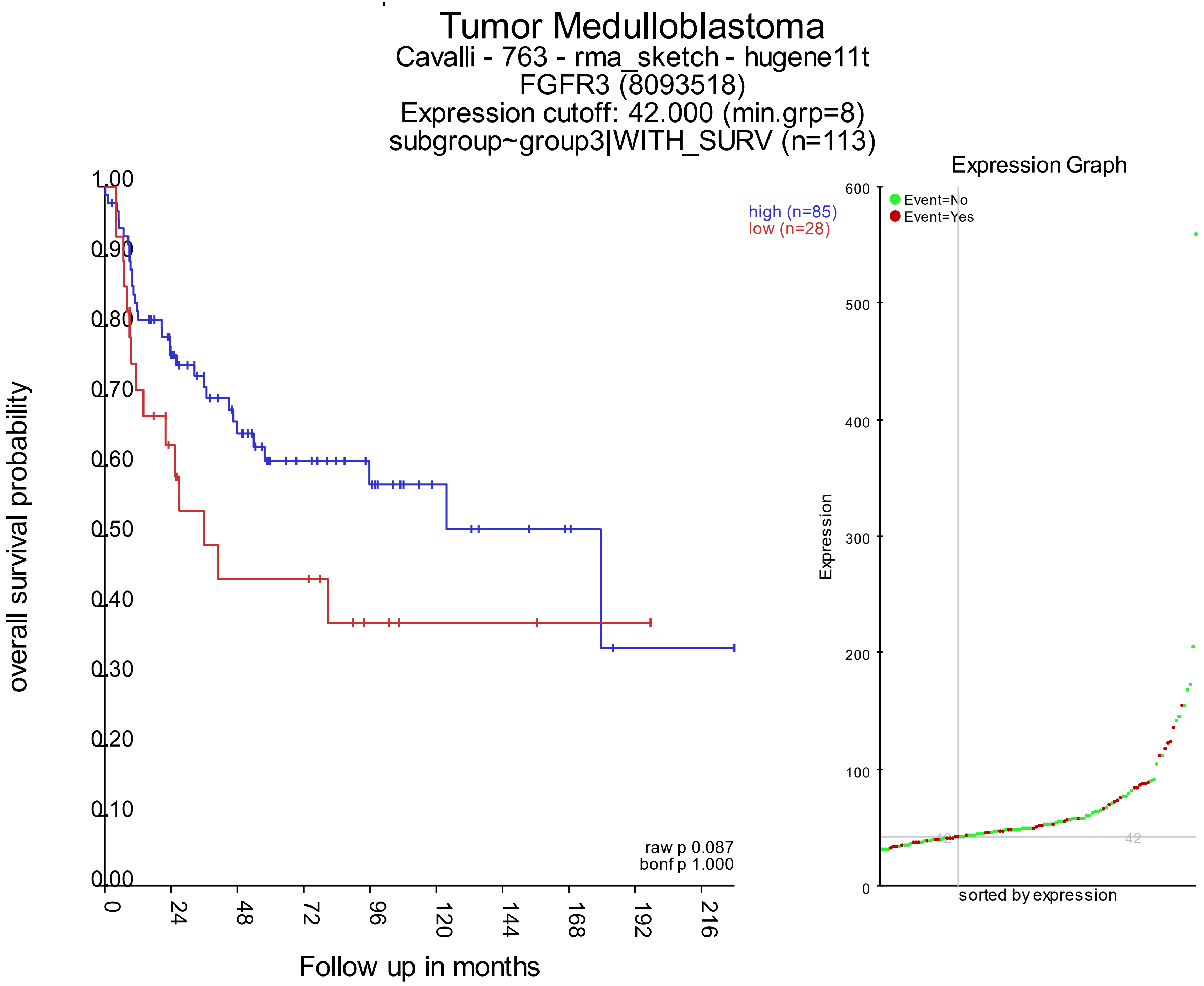
Tumor Medulloblastoma
Cavalli - 763 - rma_sketch - hugene11t
FGFR3 (8093518)
Expression cutoff: 42.000 (min.grp=8)
subgroup~group3|WITH_SURV (n=113)
high (n=85)
low (n=28)
overall survival probability
1.00
0.90
0.80
0.70
0.60
0.50
0.40
0.30
0.20
0.10
0.00
0
24
48
72
96
120
144
168
192
216
Follow up in months
raw p 0.087
bonf p 1.000
Expression Graph
Event=No
Event=Yes
Expression
600
500
400
300
200
100
0
42
sorted by expression

FLT3

WNT

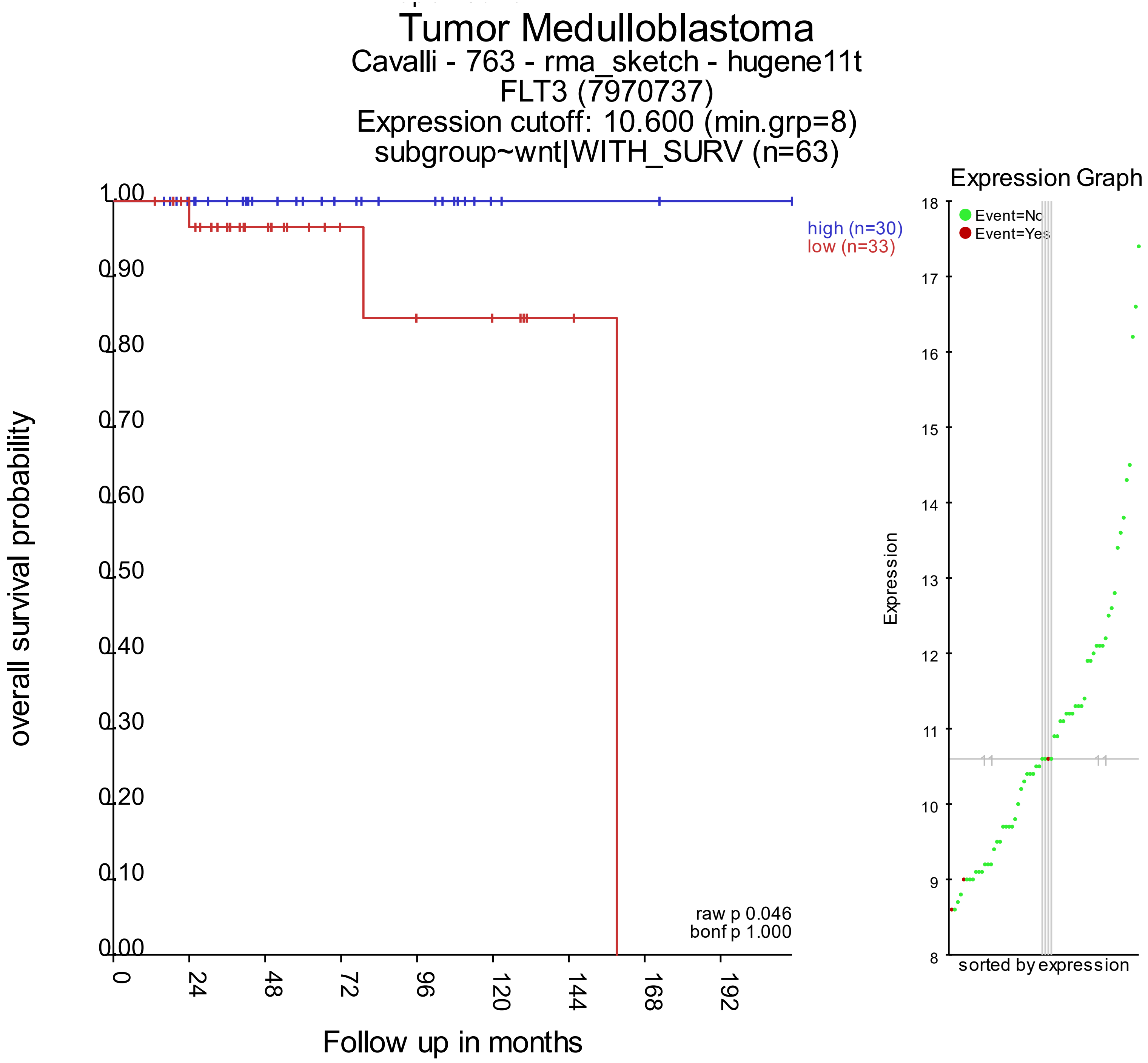

Tumor Medulloblastoma
Cavalli - 763 - rma_sketch - hugene11t
FLT3 (7970737)
Expression cutoff: 10.600 (min.grp=8)
subgroup~wnt|WITH_SURV (n=63)
overall survival probability
1.00
0.90
0.80
0.70
0.60
0.50
0.40
0.30
0.20
0.10
0.00
0
24
48
72
96
120
144
168
192
Follow up in months
high (n=30)
low (n=33)
raw p 0.046
bonf p 1.000
Expression Graph
Event=No
Event=Yes
Expression
18
17
16
15
14
13
12
11
10
9
8
sorted by expression

SHH

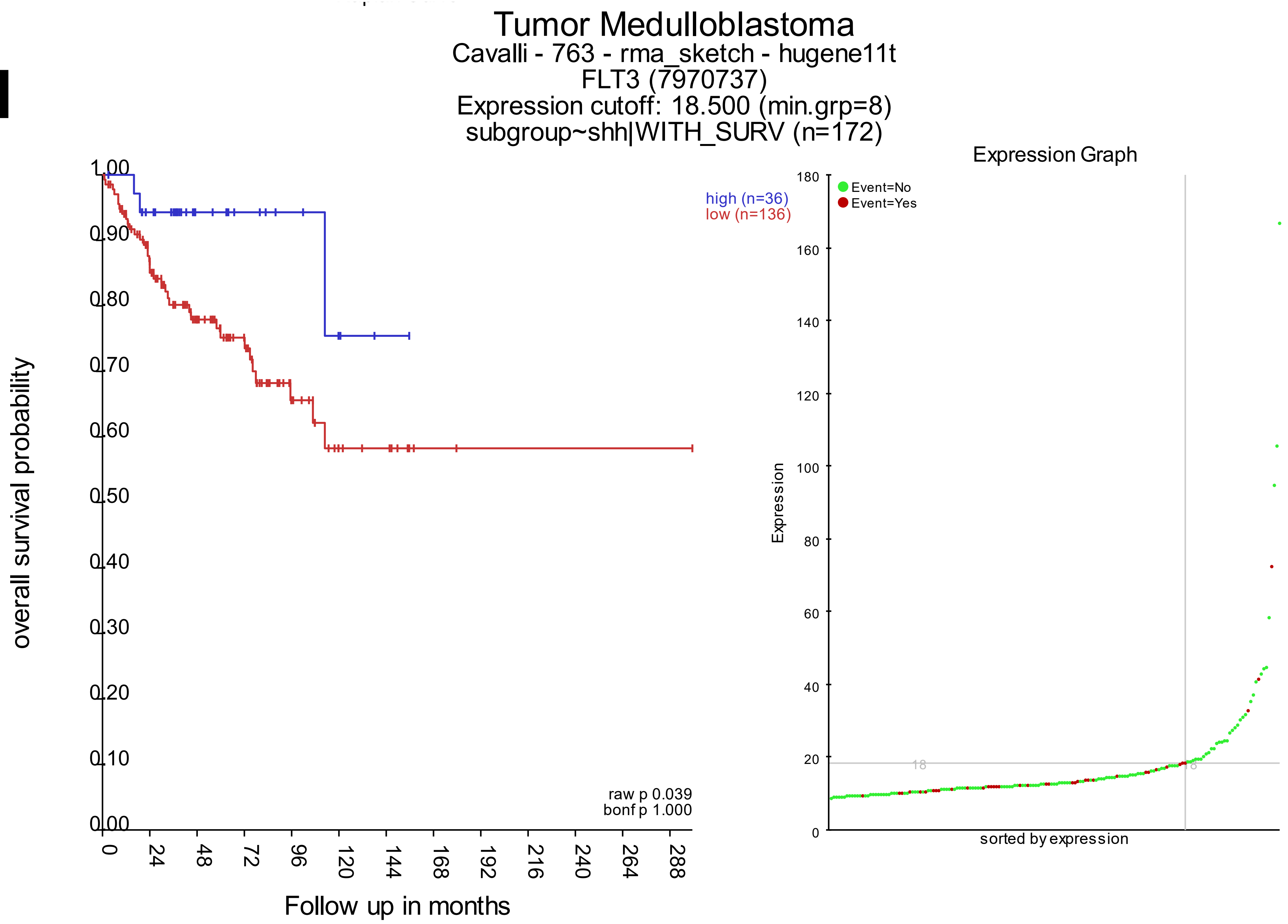
Tumor Medulloblastoma
Cavalli - 763 - rma_sketch - hugene11t
FLT3 (7970737)
Expression cutoff: 18.500 (min.grp=8)
subgroup~shh|WITH_SURV (n=172)
overall survival probability
1.00
0.90
0.80
0.70
0.60
0.50
0.40
0.30
0.20
0.10
0.00
0
24
48
72
96
120
144
168
192
216
240
264
288
Follow up in months
high (n=36)
low (n=136)
raw p 0.039
bonf p 1.000
Expression Graph
Event=No
Event=Yes
Expression
180
160
140
120
100
80
60
40
20
0
18
sorted by expression

GR4

Tumor Medulloblastoma
Cavalli - 763 - rma_sketch - hugene11t
FLT3 (7970737)
Expression cutoff: 12.200 (min.grp=8)
subgroup~group4|WITH_SURV (n=264)

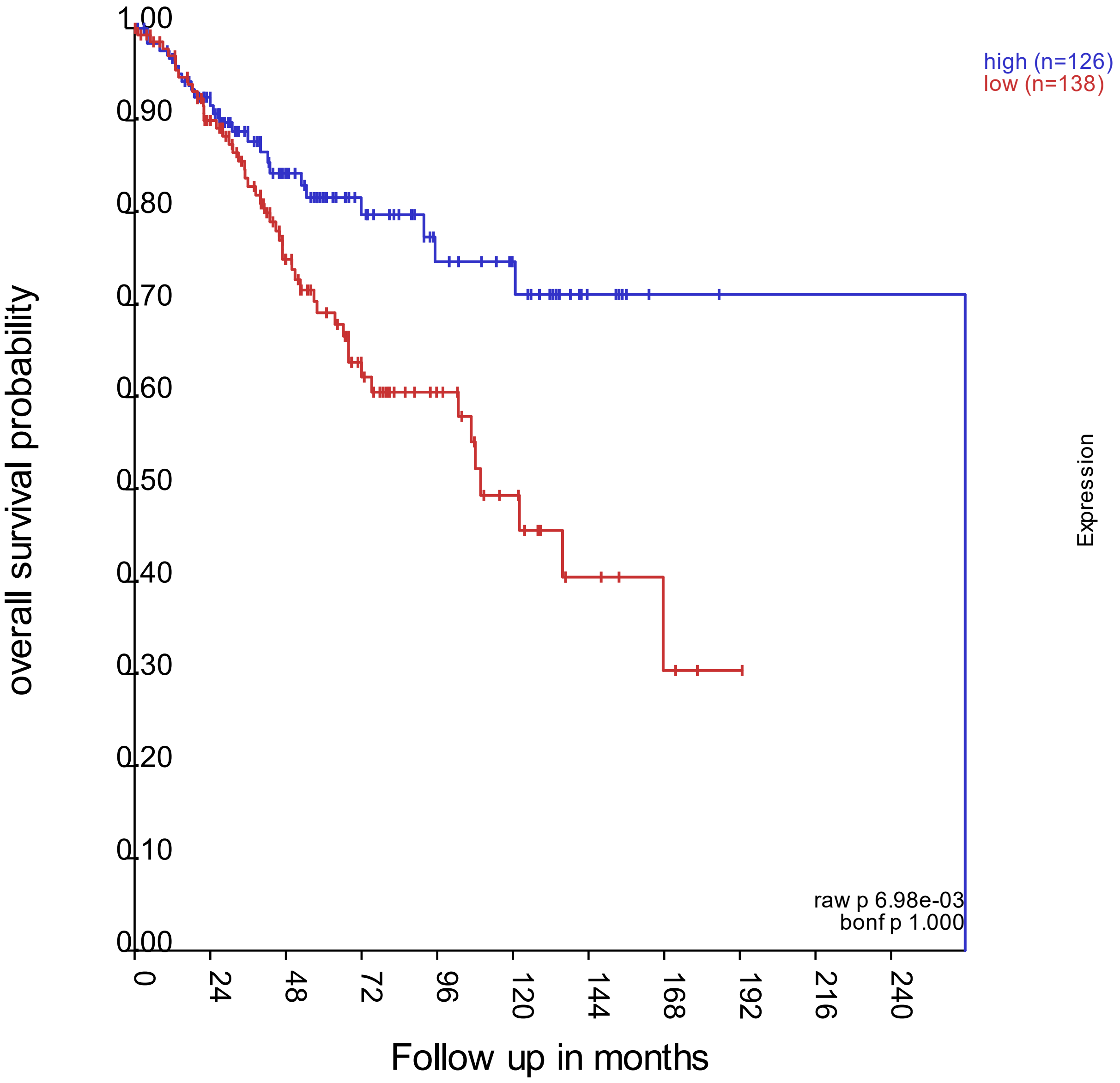

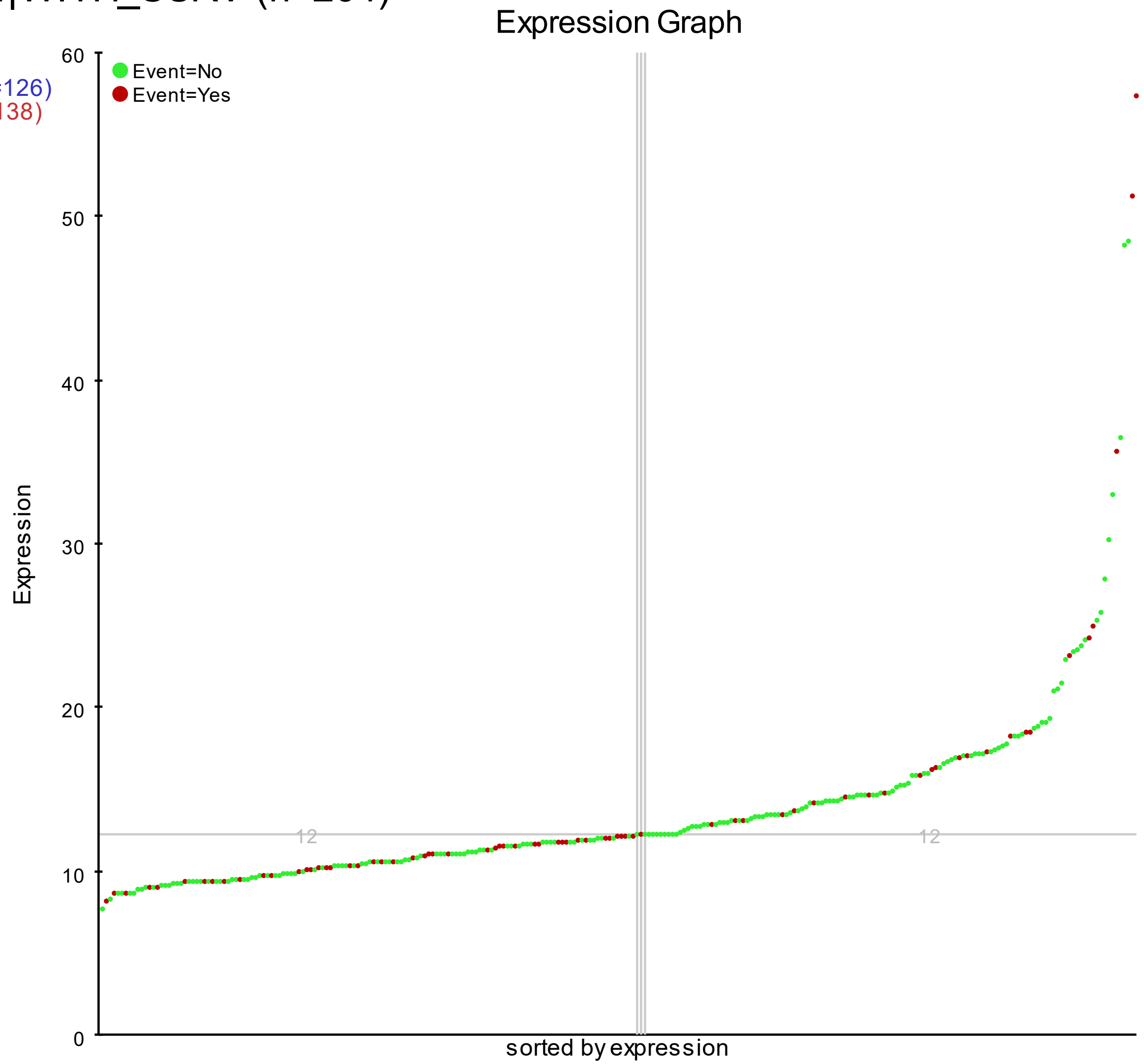

GR3

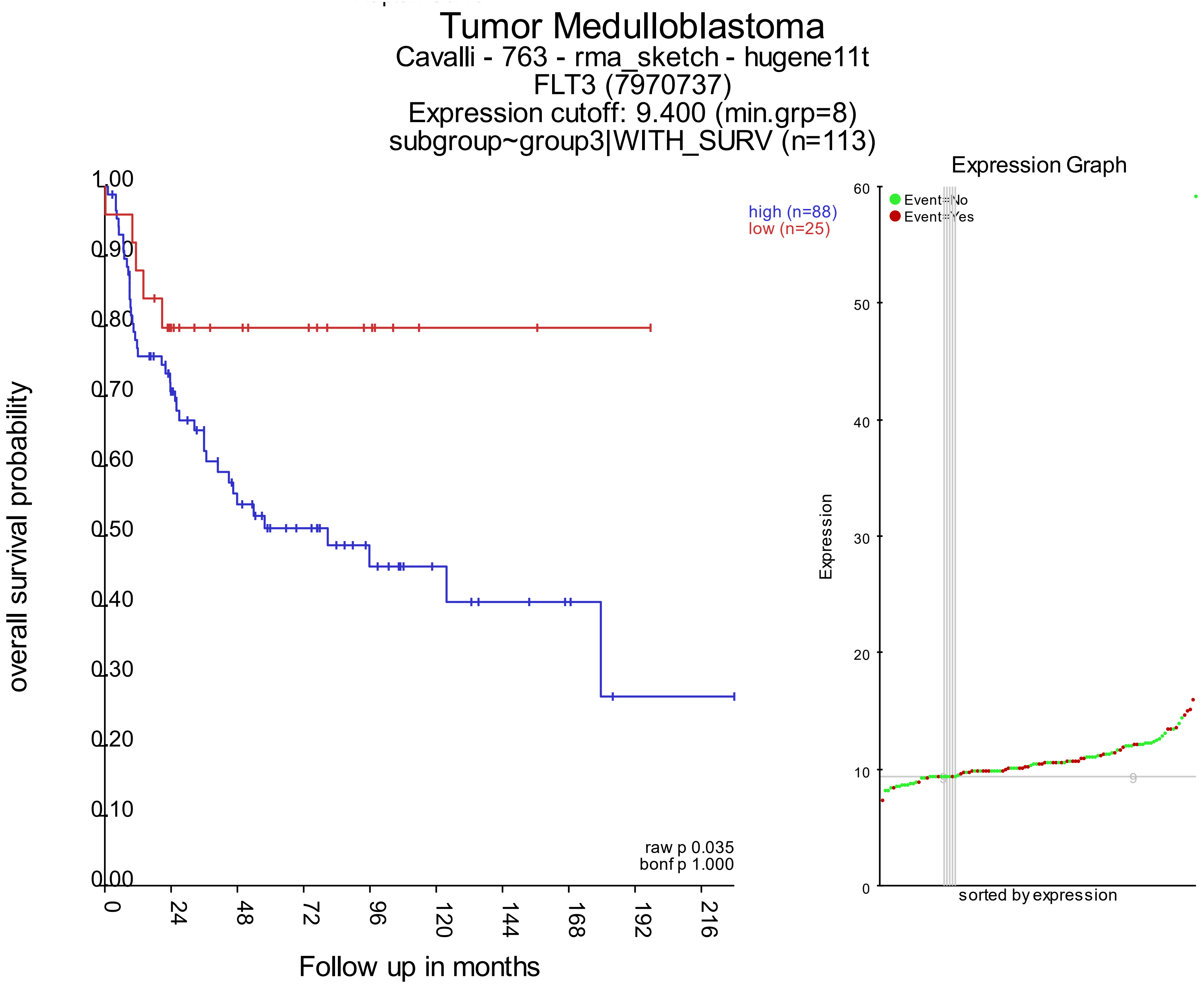

Tumor Medulloblastoma
Cavalli - 763 - rma_sketch - hugene11t
FLT3 (7970737)
Expression cutoff: 9.400 (min.grp=8)
subgroup~group3|WITH_SURV (n=113)
overall survival probability
1.00
0.90
0.80
0.70
0.60
0.50
0.40
0.30
0.20
0.10
0.00
high (n=88)
low (n=25)
raw p 0.035
bonf p 1.000
0
24
48
72
96
120
144
168
192
216
Follow up in months
Expression Graph
Event=No
Event=Yes
Expression
60
50
40
30
20
10
0
sorted by expression

EGFR/HER1

WNT

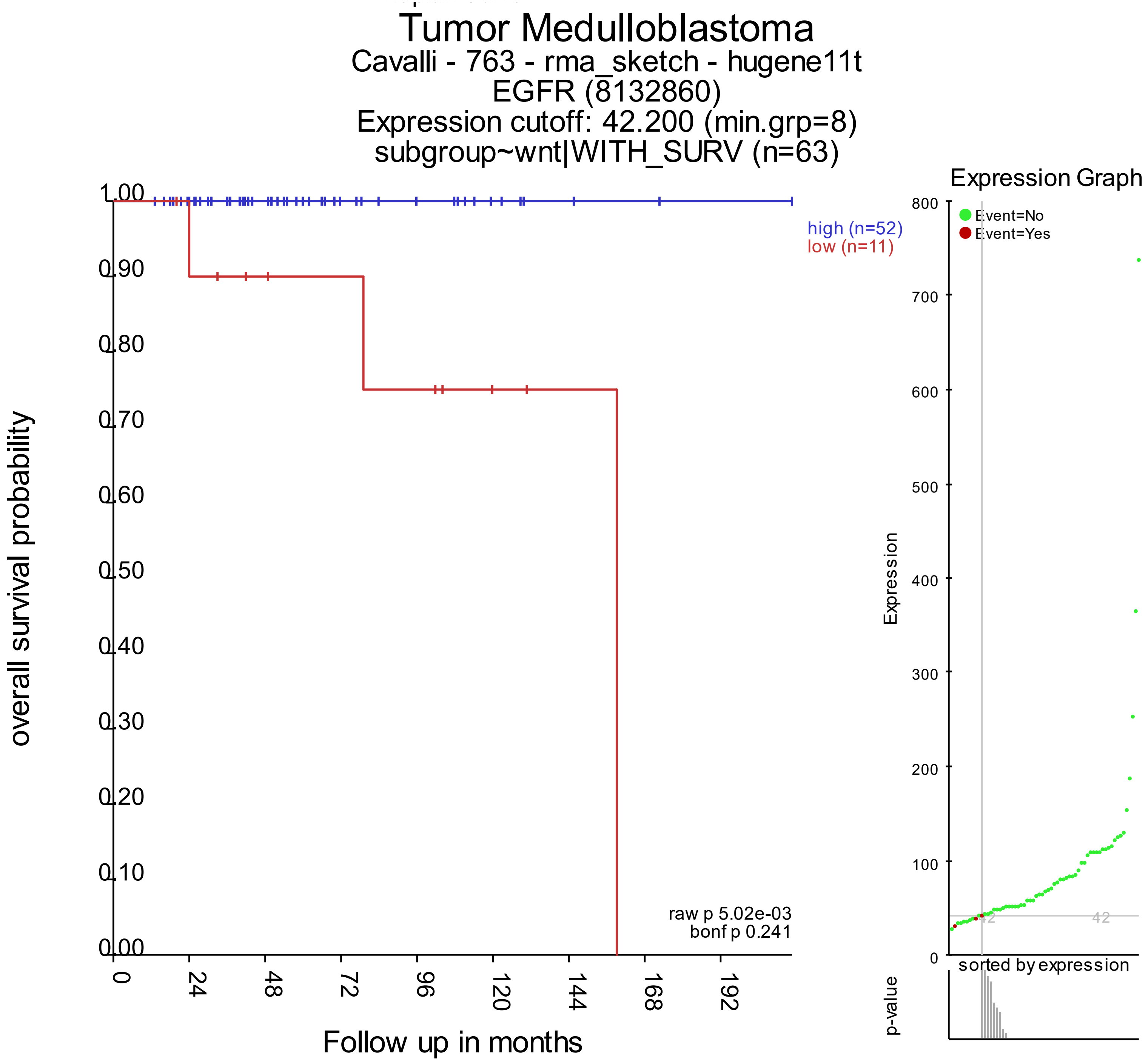
Tumor Medulloblastoma
Cavalli - 763 - rma_sketch - hugene11t
EGFR (8132860)
Expression cutoff: 42.200 (min.grp=8)
subgroup~wnt|WITH_SURV (n=63)
overall survival probability
1.00
0.90
0.80
0.70
0.60
0.50
0.40
0.30
0.20
0.10
0.00
0
24
48
72
96
120
144
168
192
Follow up in months
high (n=52)
low (n=11)
raw p 5.02e-03
bonf p 0.241
Expression Graph
Event=No
Event=Yes
Expression
800
700
600
500
400
300
200
100
0
42
sorted by expression
p-value

SHH

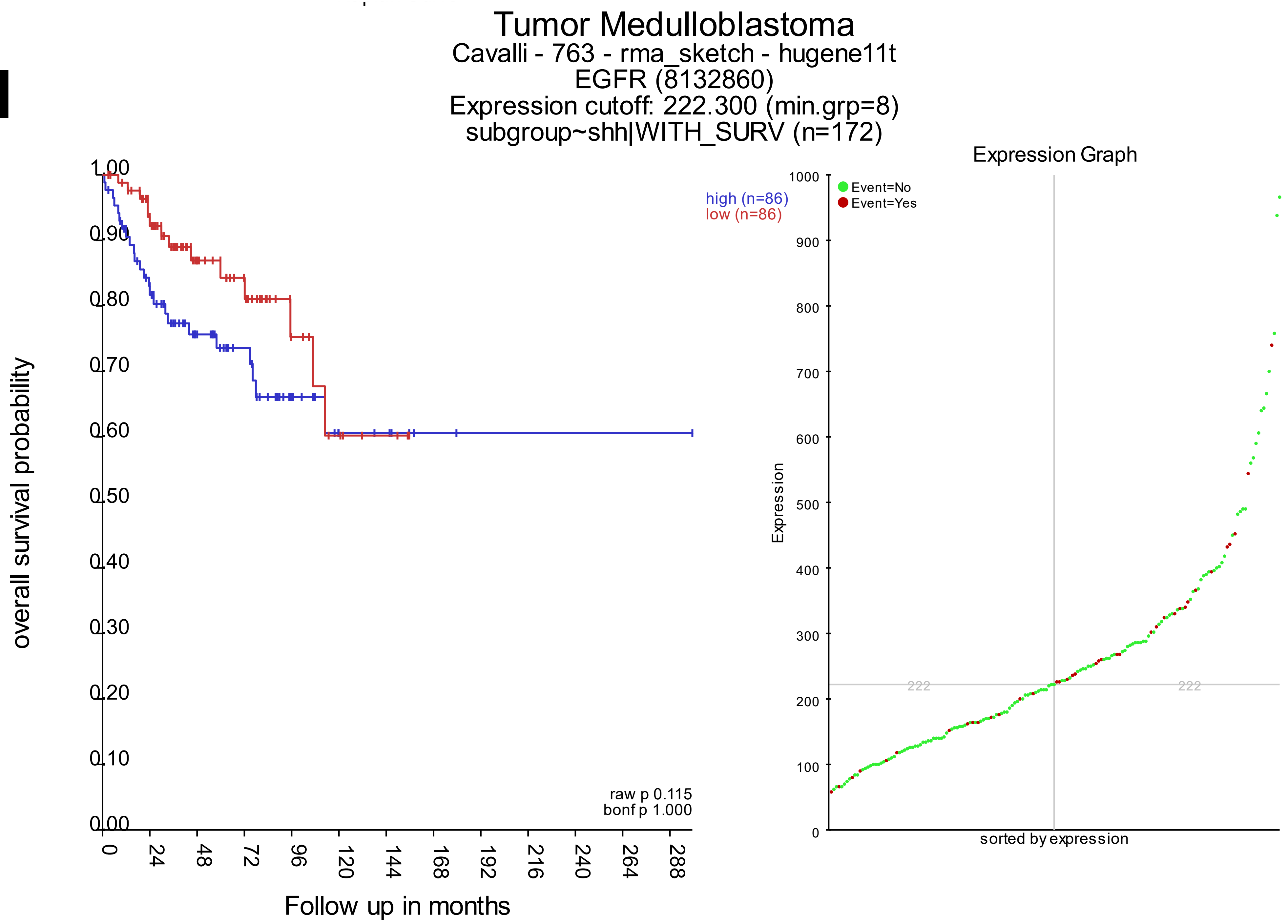

Tumor Medulloblastoma
Cavalli - 763 - rma_sketch - hugene11t
EGFR (8132860)
Expression cutoff: 222.300 (min.grp=8)
subgroup~shh|WITH_SURV (n=172)
overall survival probability
1.00
0.90
0.80
0.70
0.60
0.50
0.40
0.30
0.20
0.10
0.00
0
24
48
72
96
120
144
168
192
216
240
264
288
Follow up in months
high (n=86)
low (n=86)
raw p 0.115
bonf p 1.000
Expression Graph
Event=No
Event=Yes
Expression
1000
900
800
700
600
500
400
300
200
100
0
222
222
sorted by expression

GR4

Tumor Medulloblastoma
Cavalli - 763 - rma_sketch - hugene11t
EGFR (8132860)
Expression cutoff: 214.200 (min.grp=8)
subgroup~group4|WITH_SURV (n=264)

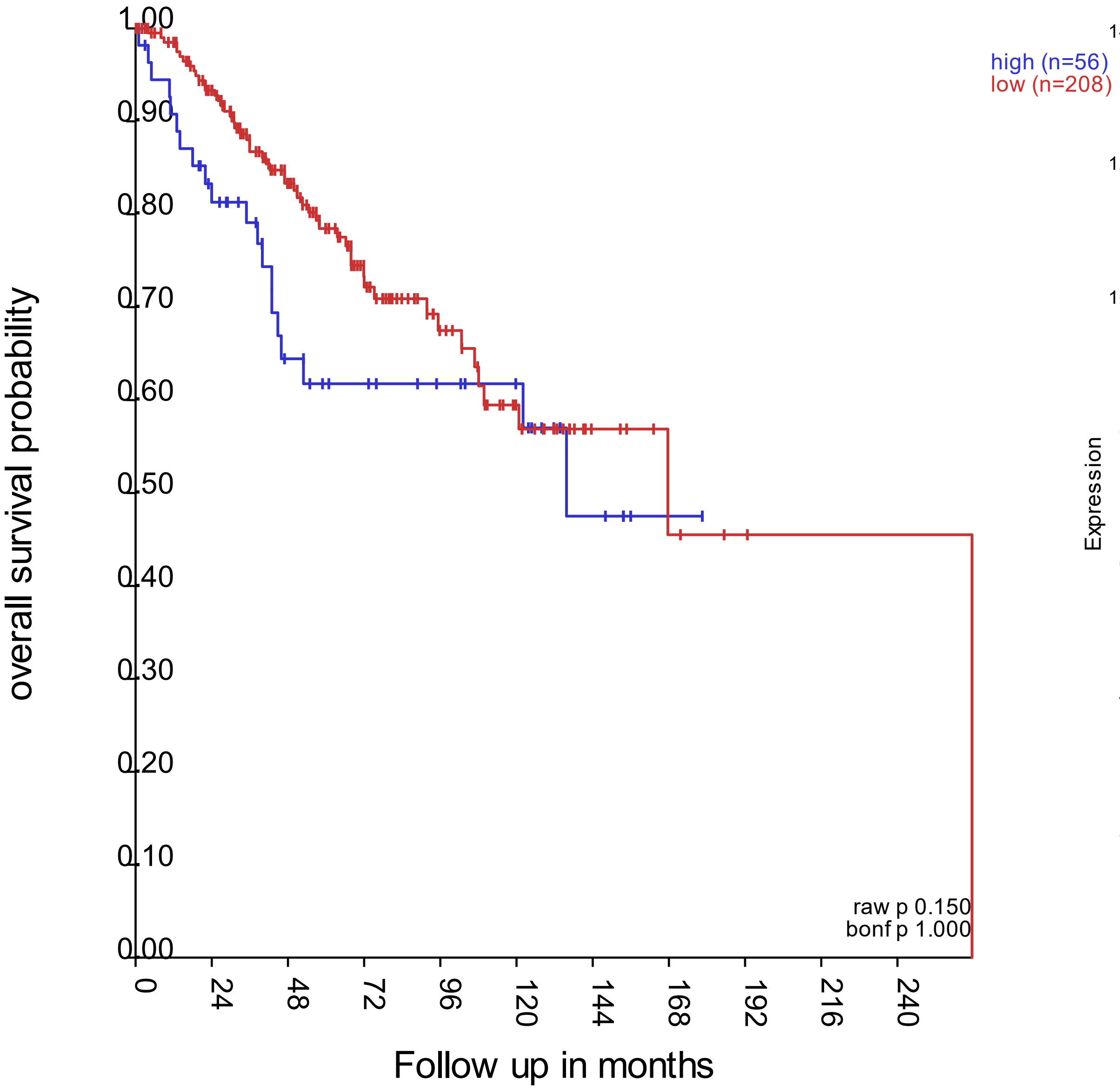

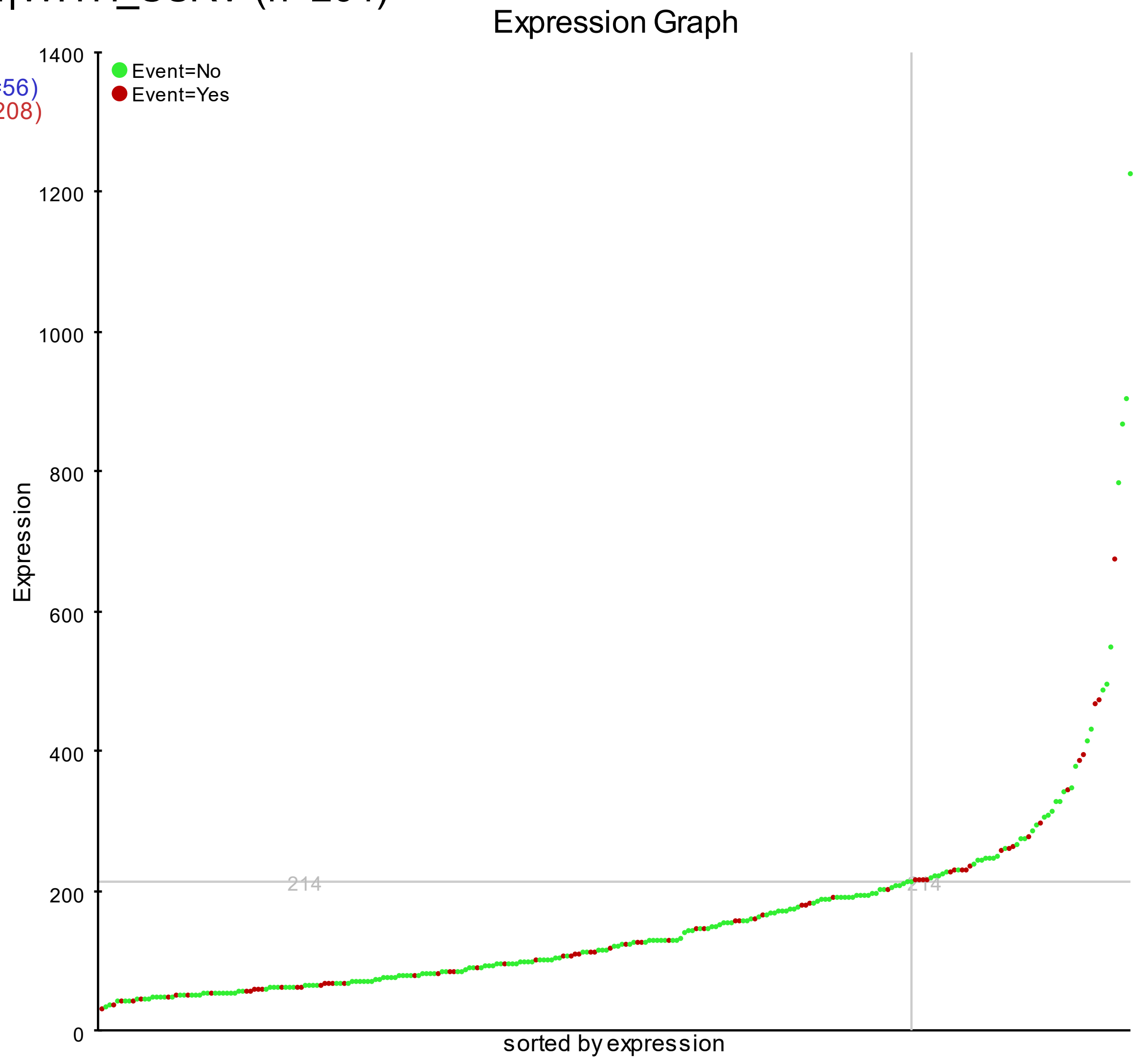

GR3

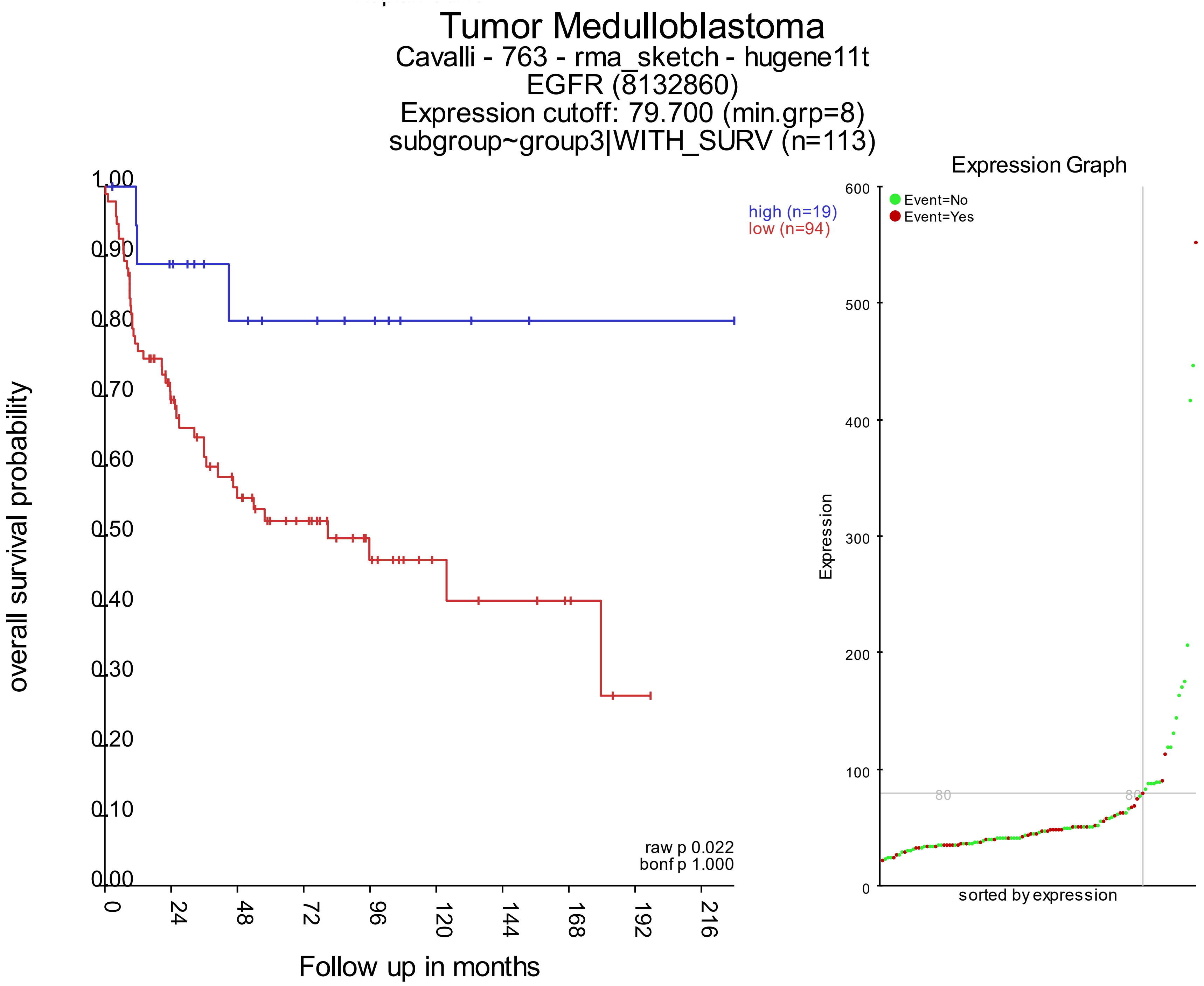
Tumor Medulloblastoma
Cavalli - 763 - rma_sketch - hugene11t
EGFR (8132860)
Expression cutoff: 79.700 (min.grp=8)
subgroup~group3|WITH_SURV (n=113)
overall survival probability
1.00
0.90
0.80
0.70
0.60
0.50
0.40
0.30
0.20
0.10
0.00
0
24
48
72
96
120
144
168
192
216
Follow up in months
high (n=19)
low (n=94)
raw p 0.022
bonf p 1.000
Expression Graph
Event=No
Event=Yes
Expression
600
500
400
300
200
100
0
80
80
sorted by expression

HER2/ERBB2

WNT

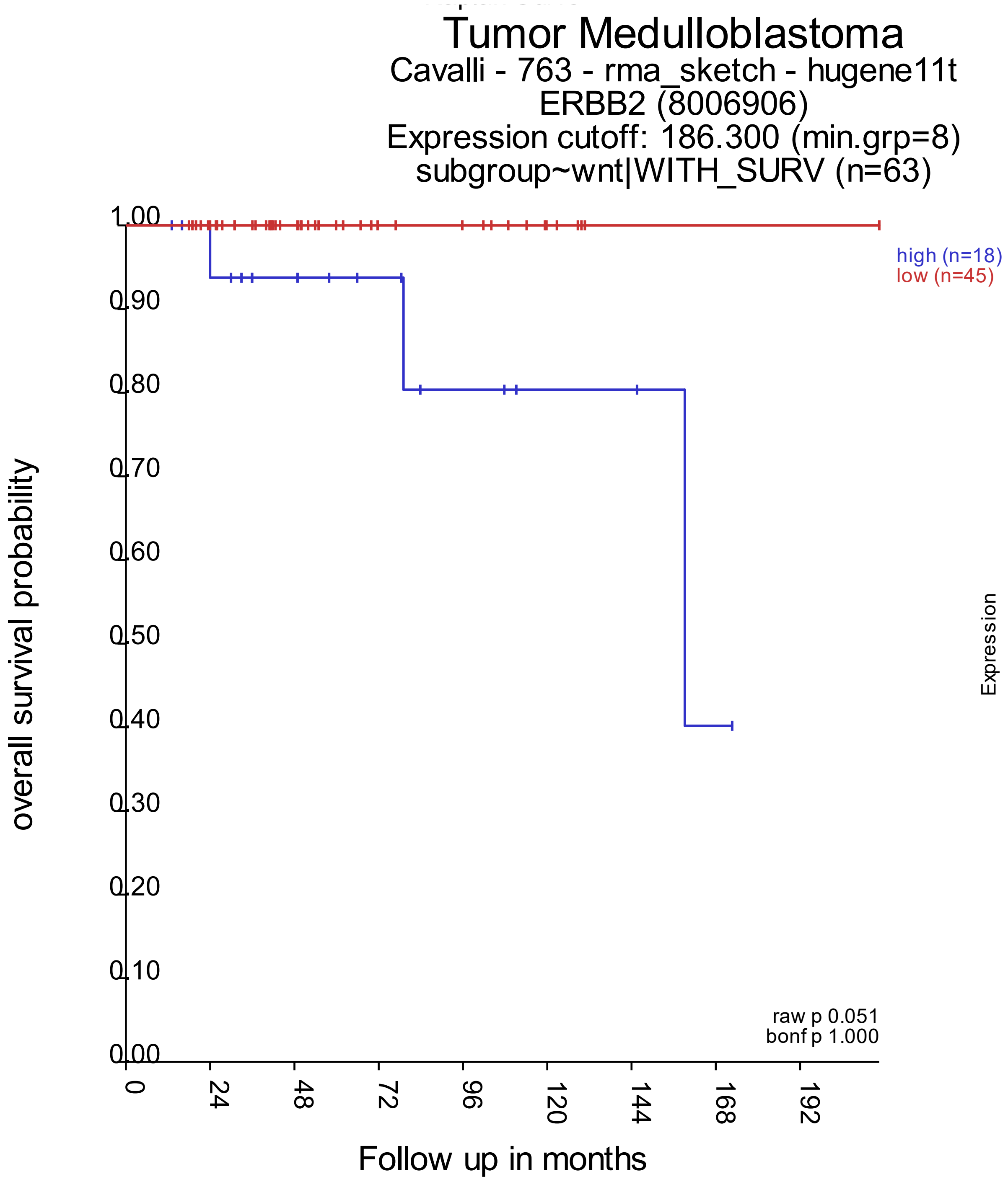
Tumor Medulloblastoma
Cavalli - 763 - rma_sketch - hugene11t
ERBB2 (8006906)
Expression cutoff: 186.300 (min.grp=8)
subgroup~wnt|WITH_SURV (n=63)
overall survival probability
1.00
0.90
0.80
0.70
0.60
0.50
0.40
0.30
0.20
0.10
0.00
0
24
48
72
96
120
144
168
192
Follow up in months
high (n=18)
low (n=45)
raw p 0.051
bonf p 1.000

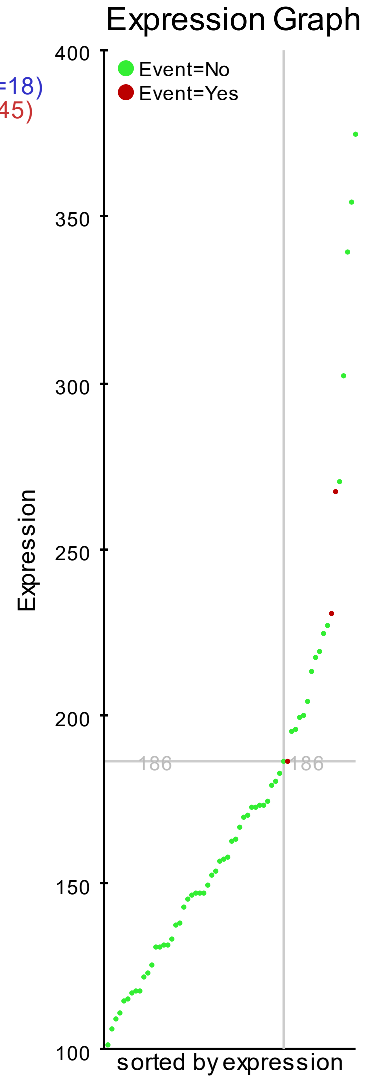
Expression Graph
Event=No
Event=Yes
Expression
400
350
300
250
200
150
100
186
186
sorted by expression

SHH

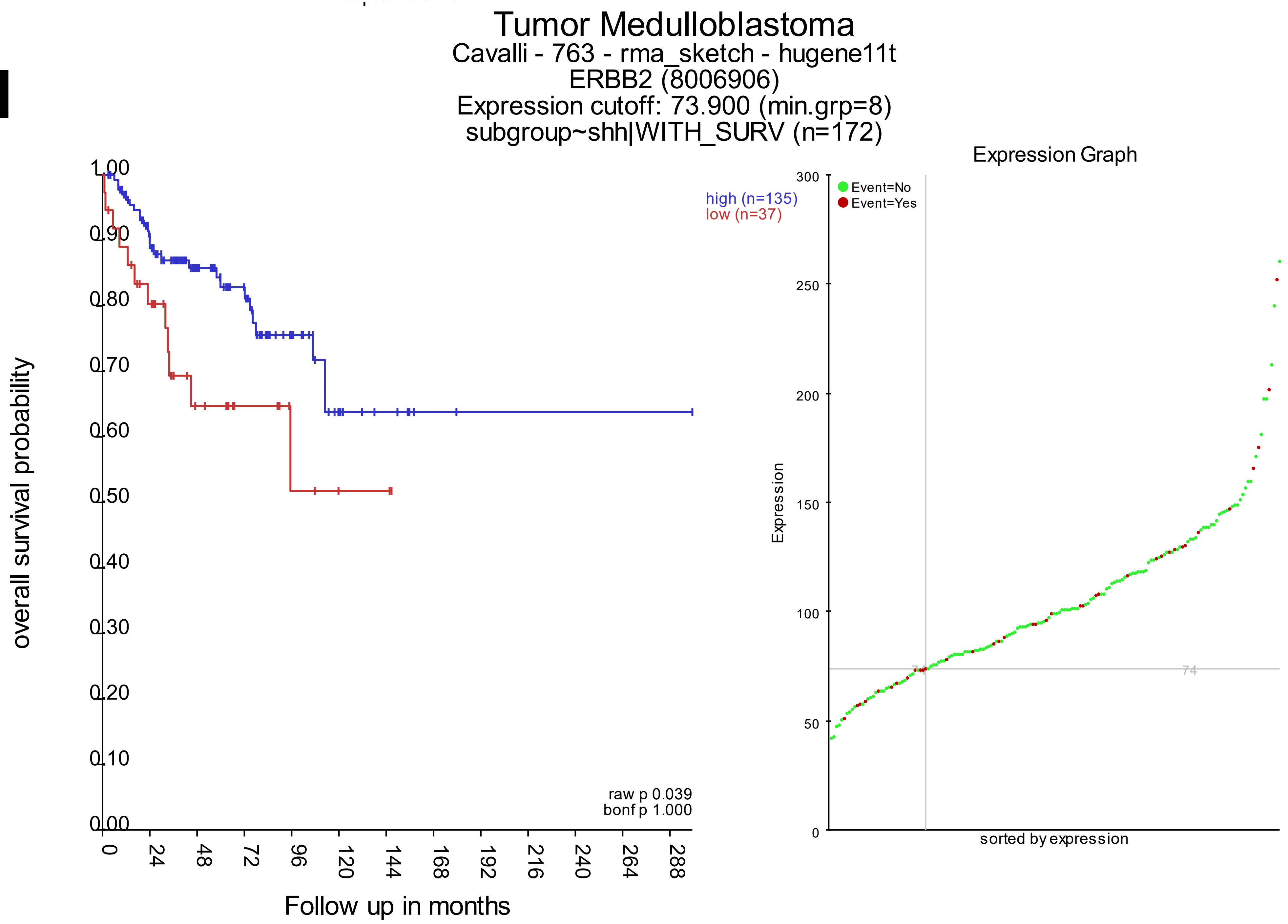
Tumor Medulloblastoma
Cavalli - 763 - rma_sketch - hugene11t
ERBB2 (8006906)
Expression cutoff: 73.900 (min.grp=8)
subgroup~shh|WITH_SURV (n=172)
overall survival probability
1.00
0.90
0.80
0.70
0.60
0.50
0.40
0.30
0.20
0.10
0.00
high (n=135)
low (n=37)
raw p 0.039
bonf p 1.000
0
24
48
72
96
120
144
168
192
216
240
264
288
Follow up in months
Expression Graph
Event=No
Event=Yes
Expression
300
250
200
150
100
50
0
74
sorted by expression

GR4

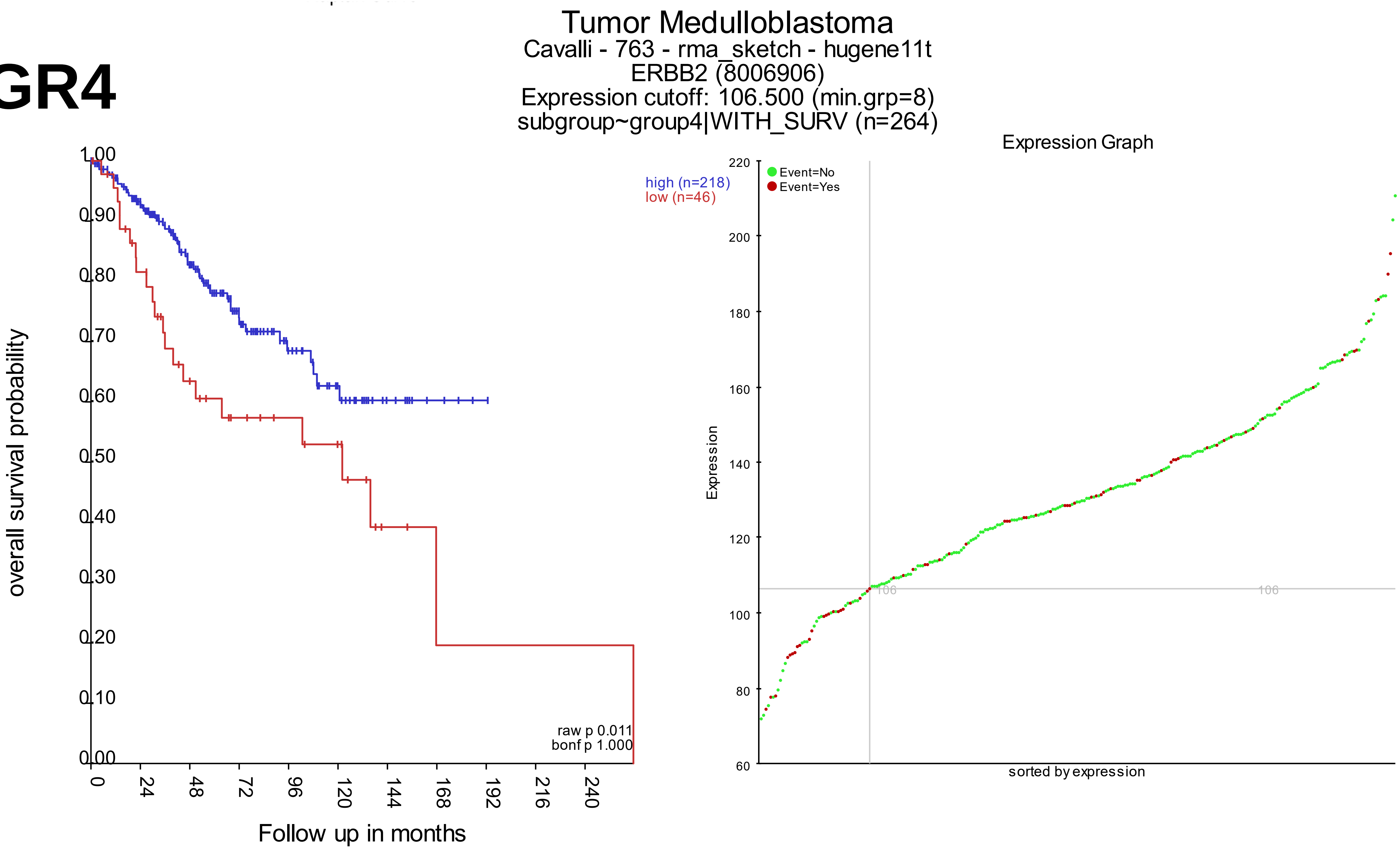
Tumor Medulloblastoma
Cavalli - 763 - rma_sketch - hugene11t
ERBB2 (8006906)
Expression cutoff: 106.500 (min.grp=8)
subgroup~group4|WITH_SURV (n=264)
overall survival probability
1.00
0.90
0.80
0.70
0.60
0.50
0.40
0.30
0.20
0.10
0.00
high (n=218)
low (n=46)
raw p 0.011
bonf p 1.000
0
24
48
72
96
120
144
168
192
216
240
Follow up in months
Expression Graph
Event=No
Event=Yes
Expression
220
200
180
160
140
120
100
80
60
106
106
sorted by expression

GR3

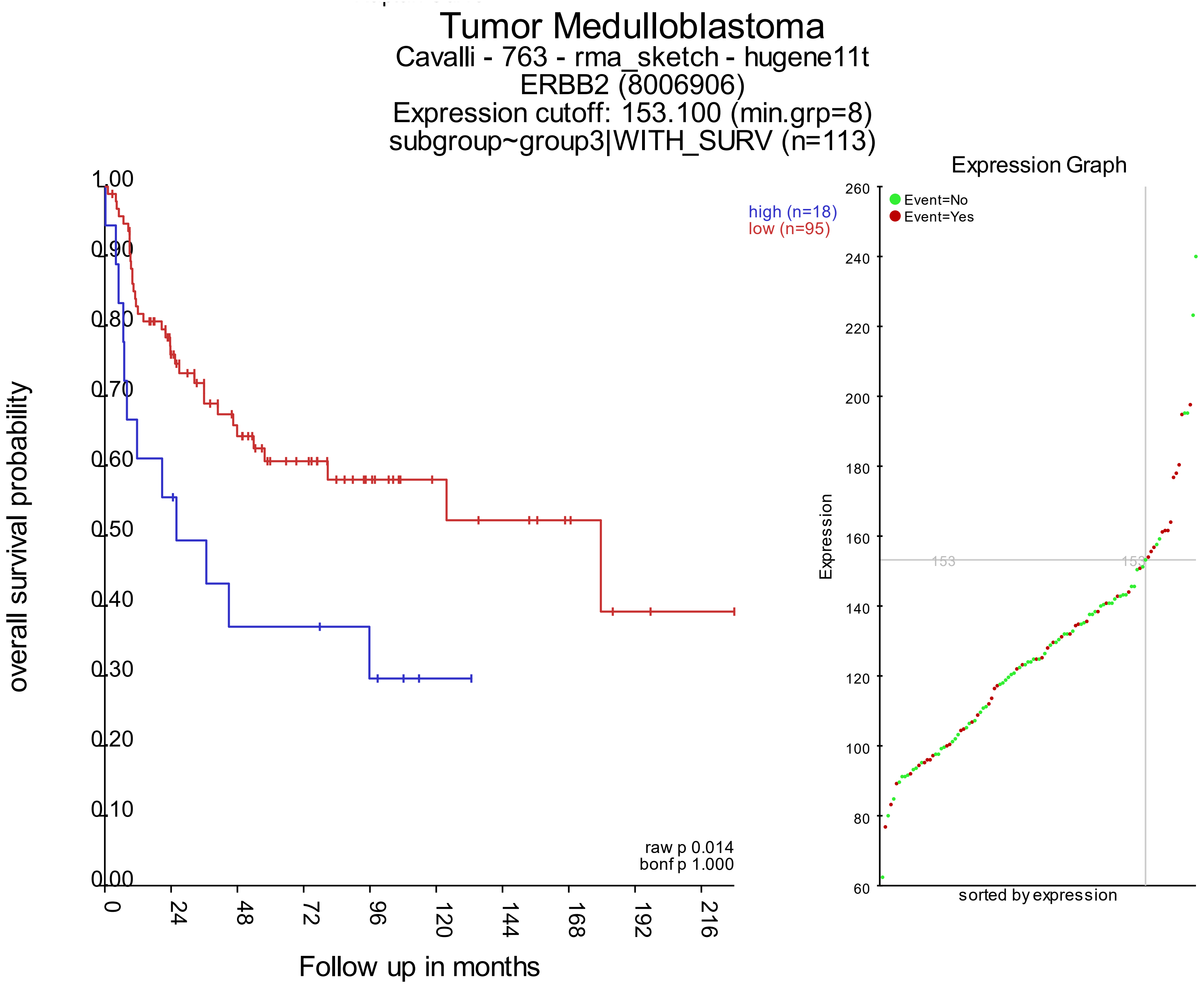

Tumor Medulloblastoma
Cavalli - 763 - rma_sketch - hugene11t
ERBB2 (8006906)
Expression cutoff: 153.100 (min.grp=8)
subgroup~group3|WITH_SURV (n=113)
overall survival probability
1.00
0.90
0.80
0.70
0.60
0.50
0.40
0.30
0.20
0.10
0.00
high (n=18)
low (n=95)
raw p 0.014
bonf p 1.000
0
24
48
72
96
120
144
168
192
216
Follow up in months
Expression Graph
Event=No
Event=Yes
Expression
260
240
220
200
180
160
140
120
100
80
60
153
153
sorted by expression

HER3/ERBB3

WNT

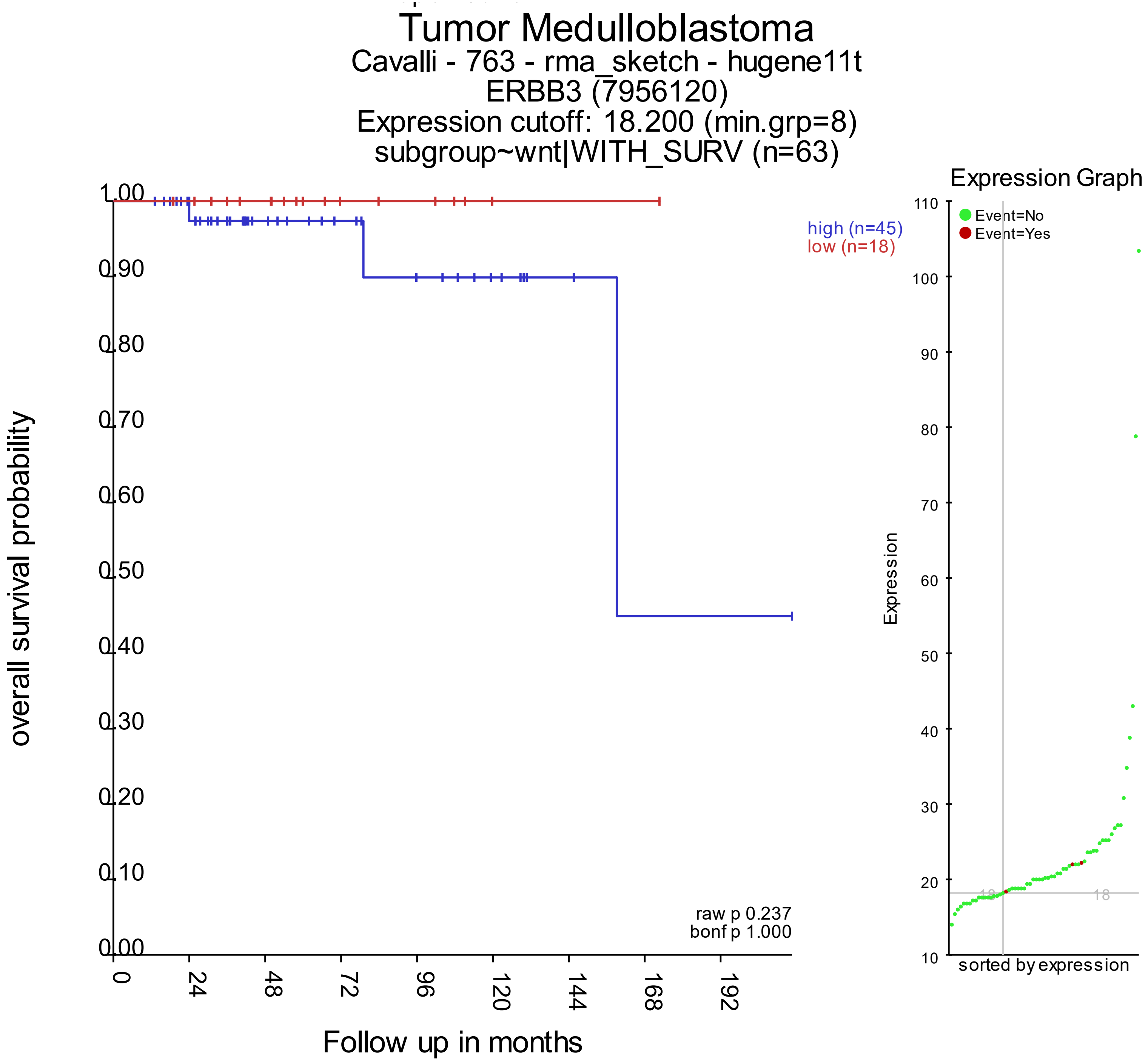
Tumor Medulloblastoma
Cavalli - 763 - rma_sketch - hugene11t
ERBB3 (7956120)
Expression cutoff: 18.200 (min.grp=8)
subgroup~wnt|WITH_SURV (n=63)
overall survival probability
1.00
0.90
0.80
0.70
0.60
0.50
0.40
0.30
0.20
0.10
0.00
0
24
48
72
96
120
144
168
192
Follow up in months
high (n=45)
low (n=18)
raw p 0.237
bonf p 1.000
Expression Graph
Event=No
Event=Yes
110
100
90
80
70
60
50
40
30
20
10
Expression
sorted by expression
18

SHH

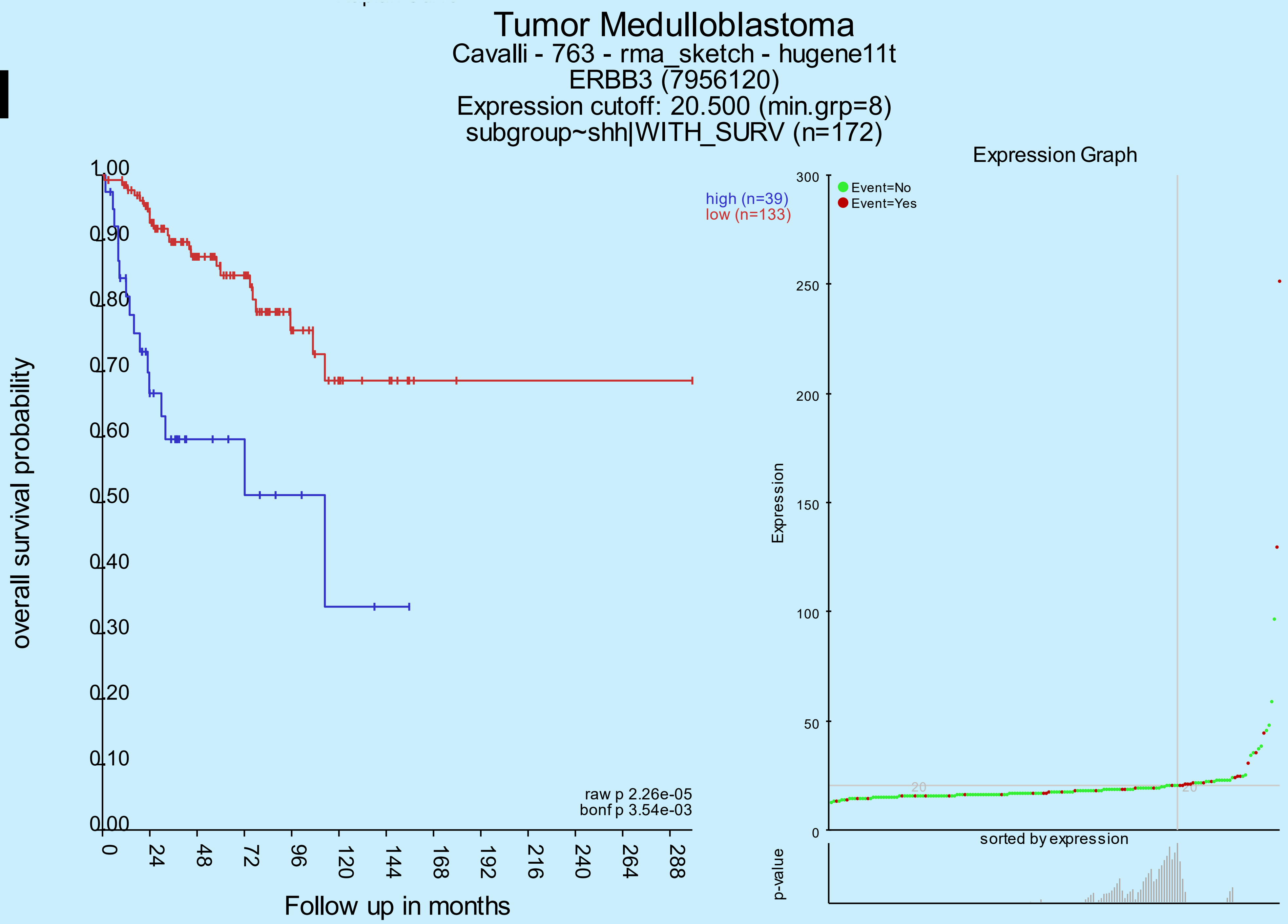

Tumor Medulloblastoma
Cavalli - 763 - rma_sketch - hugene11t
ERBB3 (7956120)
Expression cutoff: 20.500 (min.grp=8)
subgroup~shh|WITH_SURV (n=172)
overall survival probability
1.00
0.90
0.80
0.70
0.60
0.50
0.40
0.30
0.20
0.10
0.00
high (n=39)
low (n=133)
raw p 2.26e-05
bonf p 3.54e-03
0
24
48
72
96
120
144
168
192
216
240
264
288
Follow up in months
Expression Graph
Event=No
Event=Yes
Expression
300
250
200
150
100
50
0
sorted by expression
p-value

GR4

Tumor Medulloblastoma
Cavalli - 763 - rma_sketch - hugene11t
ERBB3 (7956120)
Expression cutoff: 26.700 (min.grp=8)
subgroup~group4|WITH_SURV (n=264)

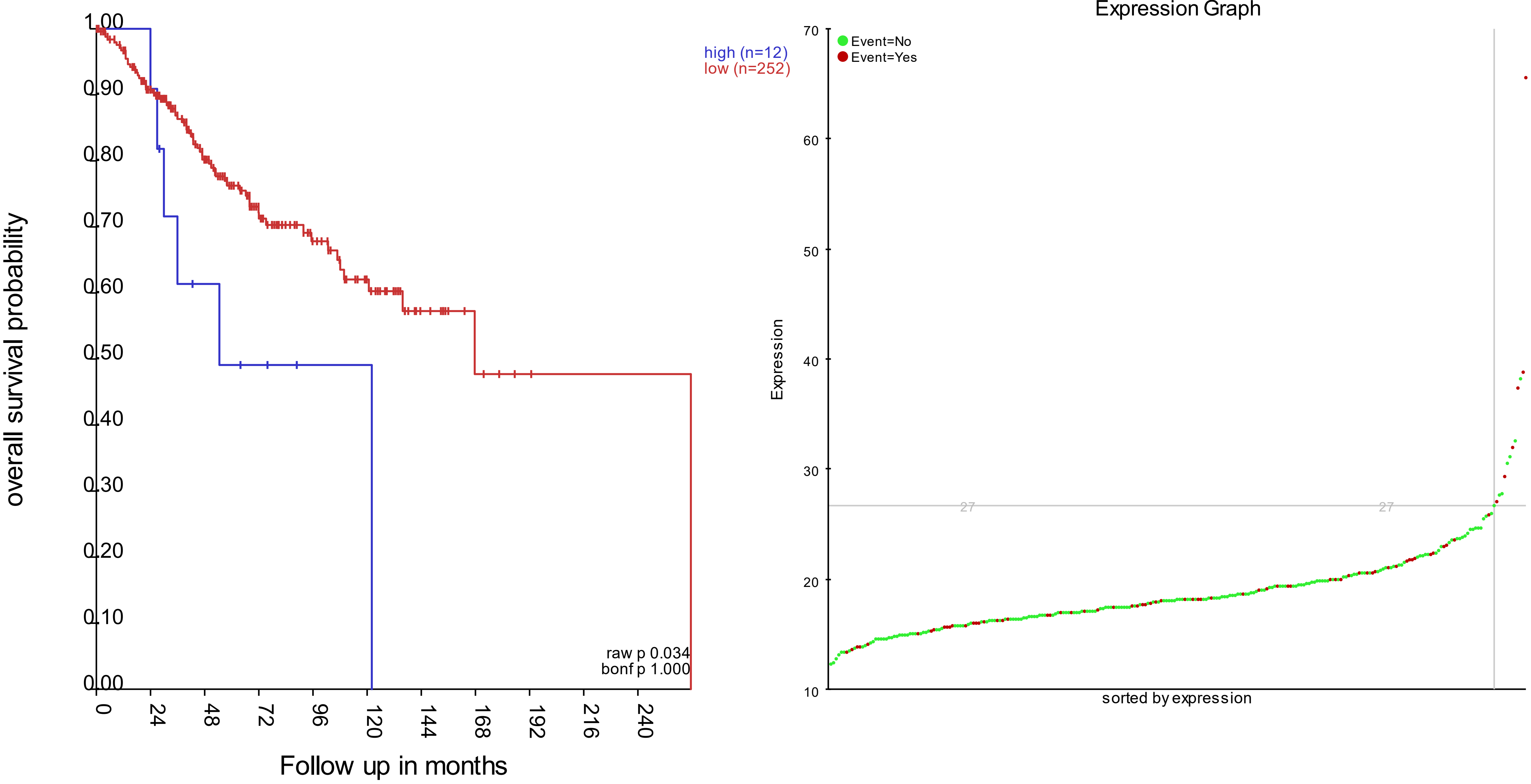

GR3

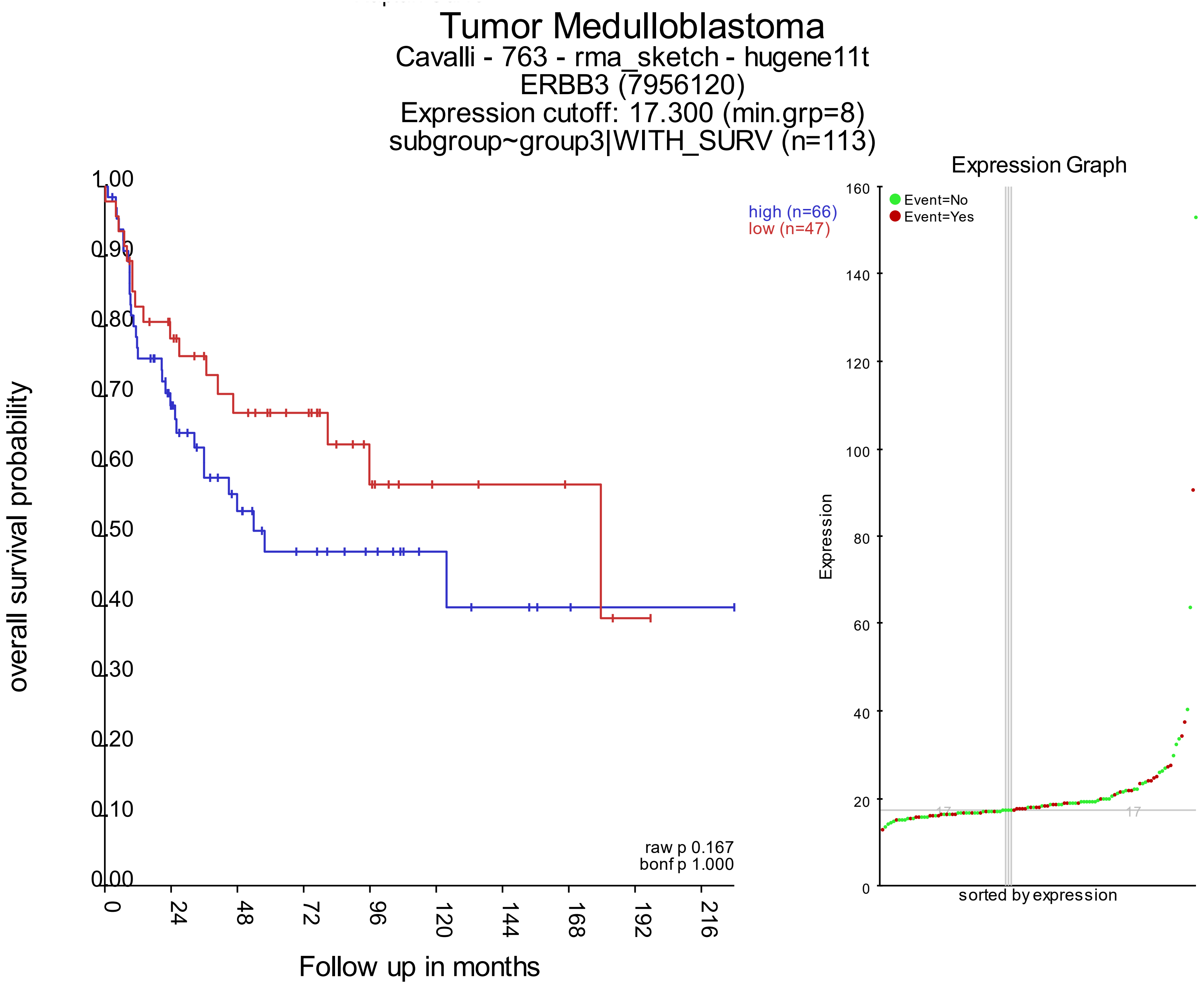
Tumor Medulloblastoma
Cavalli - 763 - rma_sketch - hugene11t
ERBB3 (7956120)
Expression cutoff: 17.300 (min.grp=8)
subgroup~group3|WITH_SURV (n=113)
high (n=66)
low (n=47)
overall survival probability
1.00
0.90
0.80
0.70
0.60
0.50
0.40
0.30
0.20
0.10
0.00
0
24
48
72
96
120
144
168
192
216
Follow up in months
raw p 0.167
bonf p 1.000
Expression Graph
Event=No
Event=Yes
Expression
160
140
120
100
80
60
40
20
0
sorted by expression

HER4/ERBB4

WNT

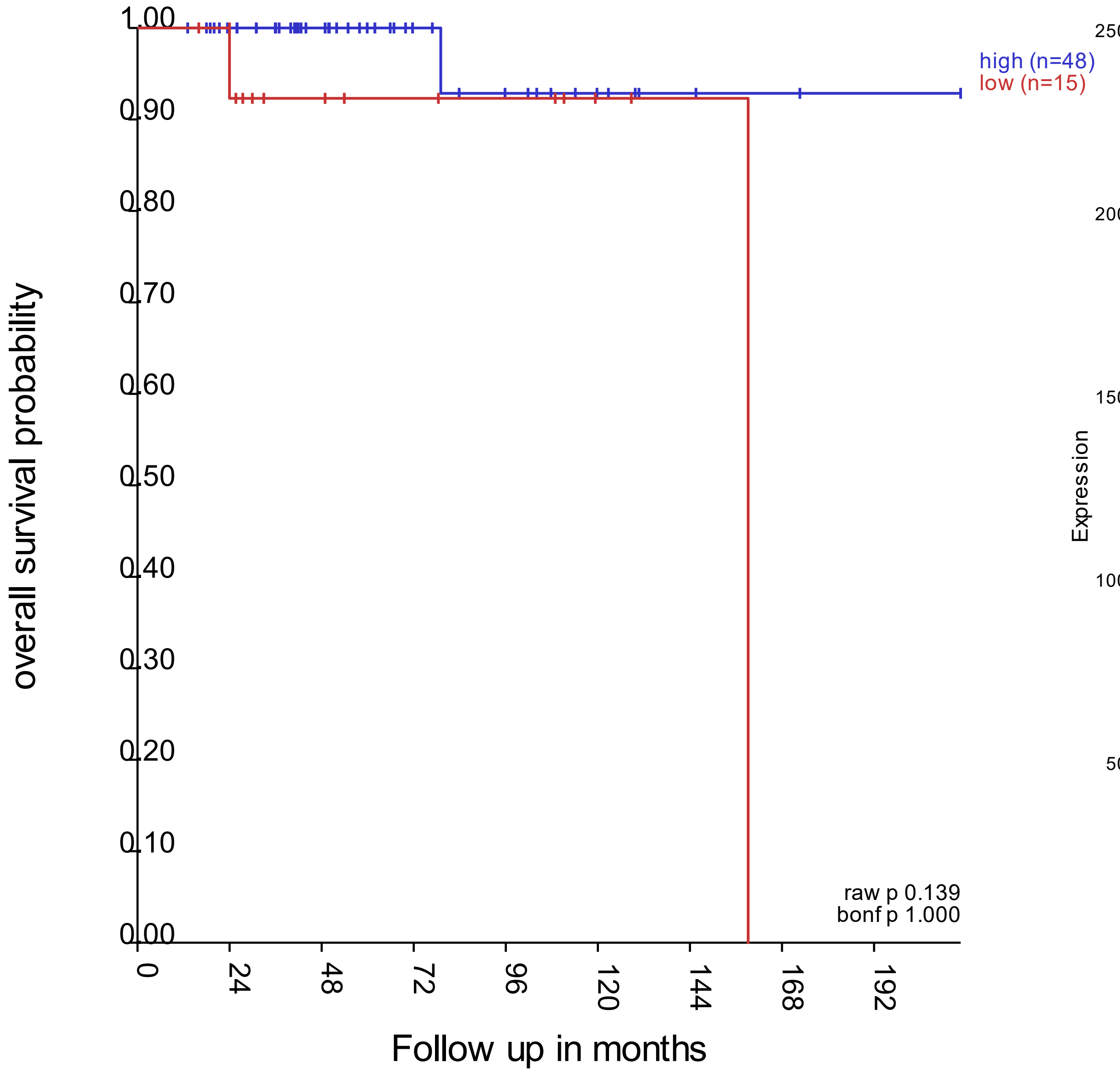
Tumor Medulloblastoma
Cavalli - 763 - rma_sketch - hugene11t
ERBB4 (8058627)
Expression cutoff: 65.200 (min.grp=8)
subgroup~wnt|WITH_SURV (n=63)
overall survival probability
1.00
0.90
0.80
0.70
0.60
0.50
0.40
0.30
0.20
0.10
0.00
0
24
48
72
96
120
144
168
192
Follow up in months
high (n=48)
low (n=15)
raw p 0.139
bonf p 1.000

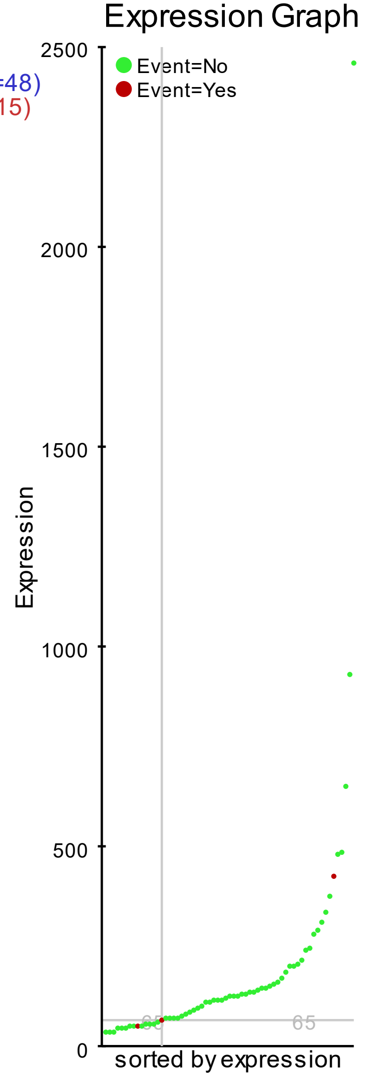
Expression Graph
Event=No
Event=Yes
Expression
2500
2000
1500
1000
500
0
65
sorted by expression

SHH

Tumor Medulloblastoma
Cavalli - 763 - rma_sketch - hugene11t
ERBB4 (8058627)
Expression cutoff: 1517.900 (min.grp=8)
subgroup~shh|WITH_SURV (n=172)

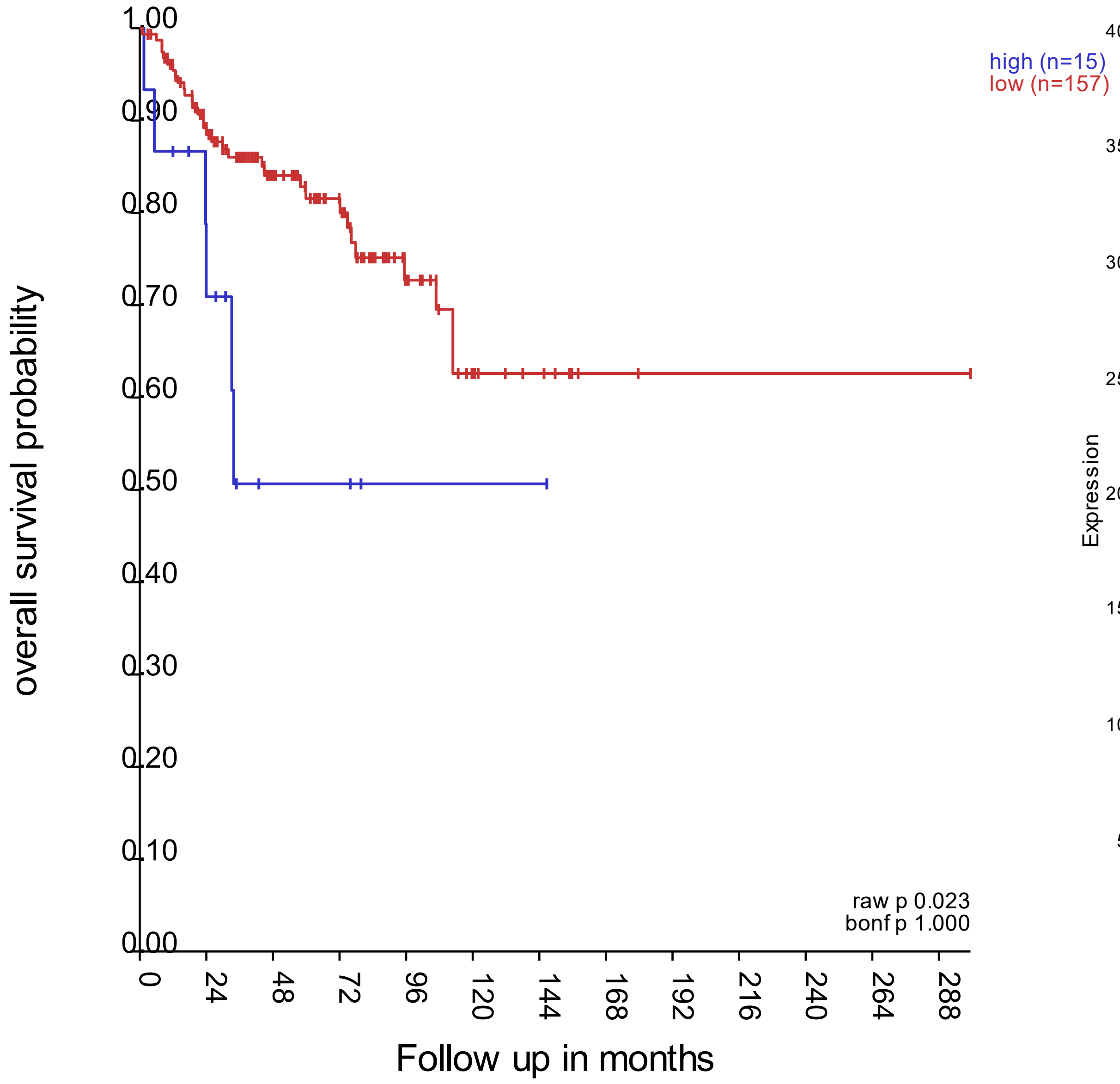

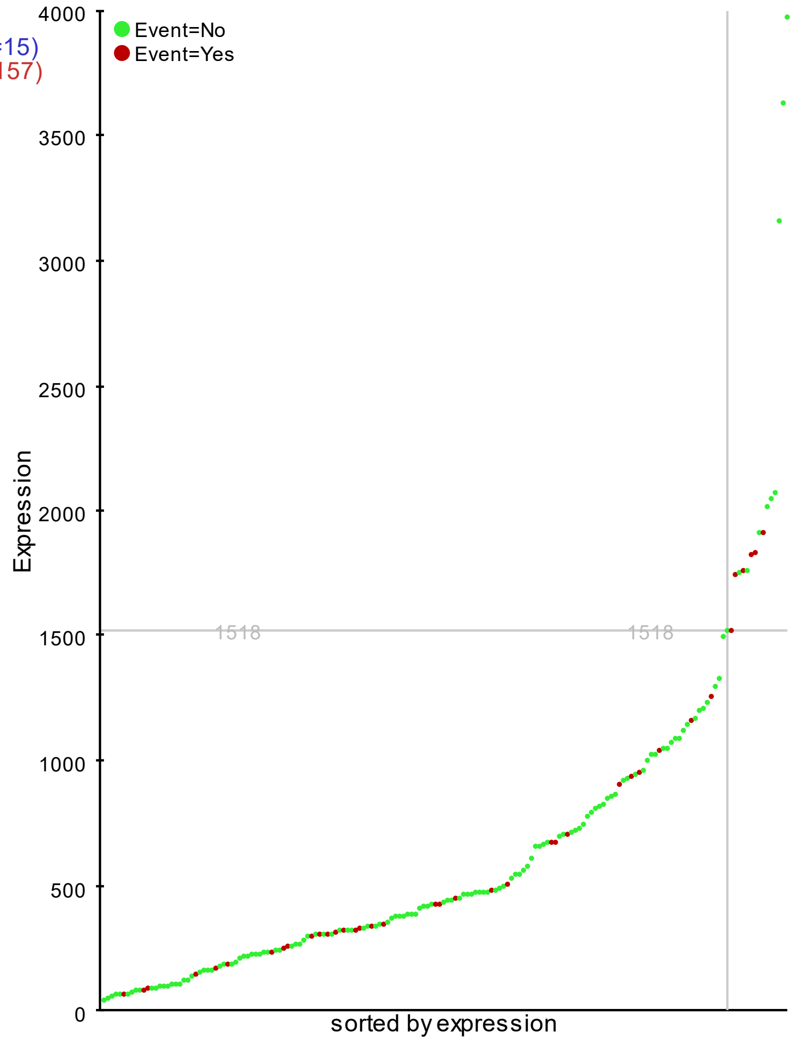

GR4

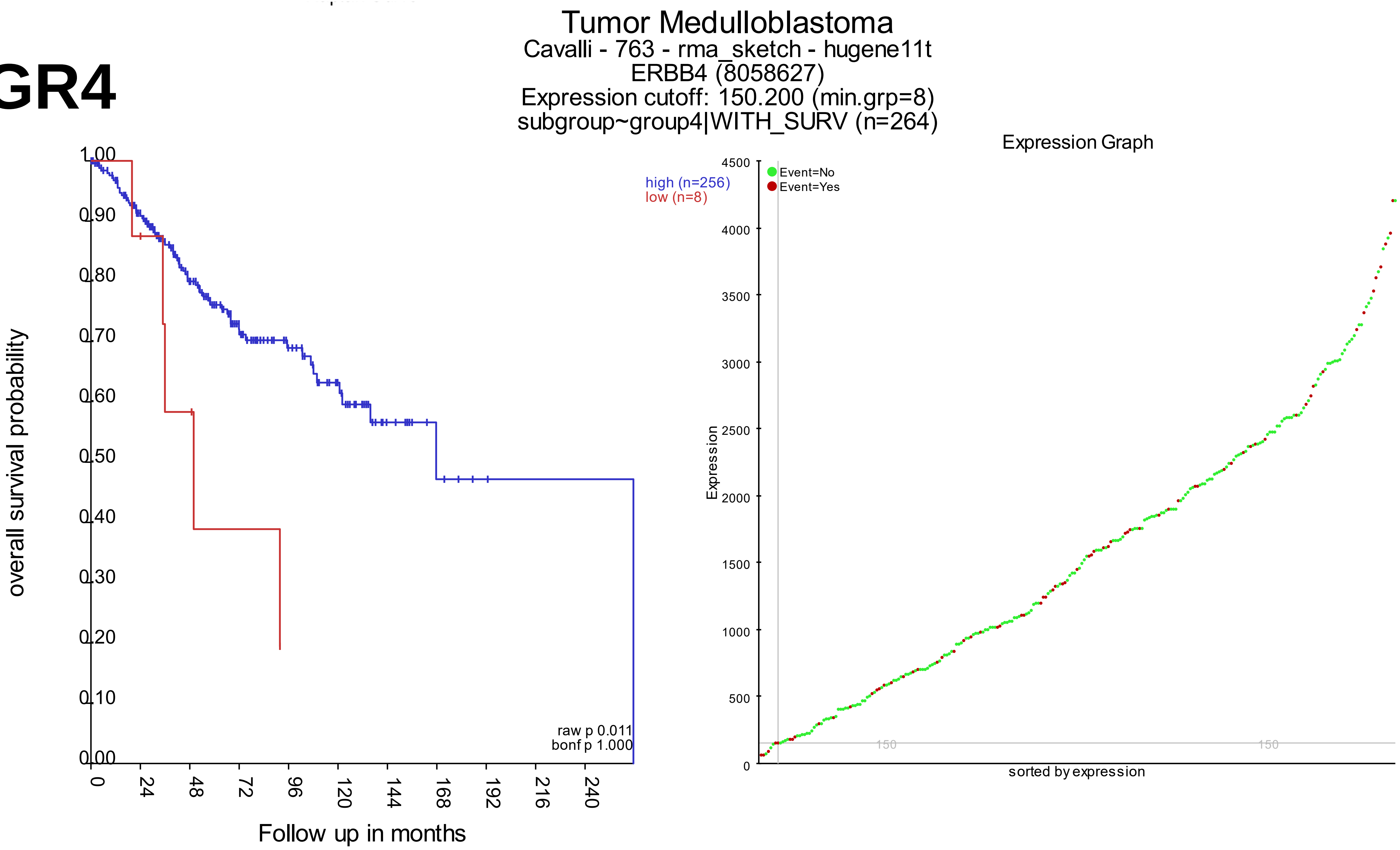

Tumor Medulloblastoma
Cavalli - 763 - rma_sketch - hugene11t
ERBB4 (8058627)
Expression cutoff: 150.200 (min.grp=8)
subgroup~group4|WITH_SURV (n=264)
overall survival probability
1.00
0.90
0.80
0.70
0.60
0.50
0.40
0.30
0.20
0.10
0.00
0
24
48
72
96
120
144
168
192
216
240
Follow up in months
high (n=256)
low (n=8)
raw p 0.011
bonf p 1.000
Expression Graph
Event=No
Event=Yes
Expression
4500
4000
3500
3000
2500
2000
1500
1000
500
0
150
150
sorted by expression

GR3

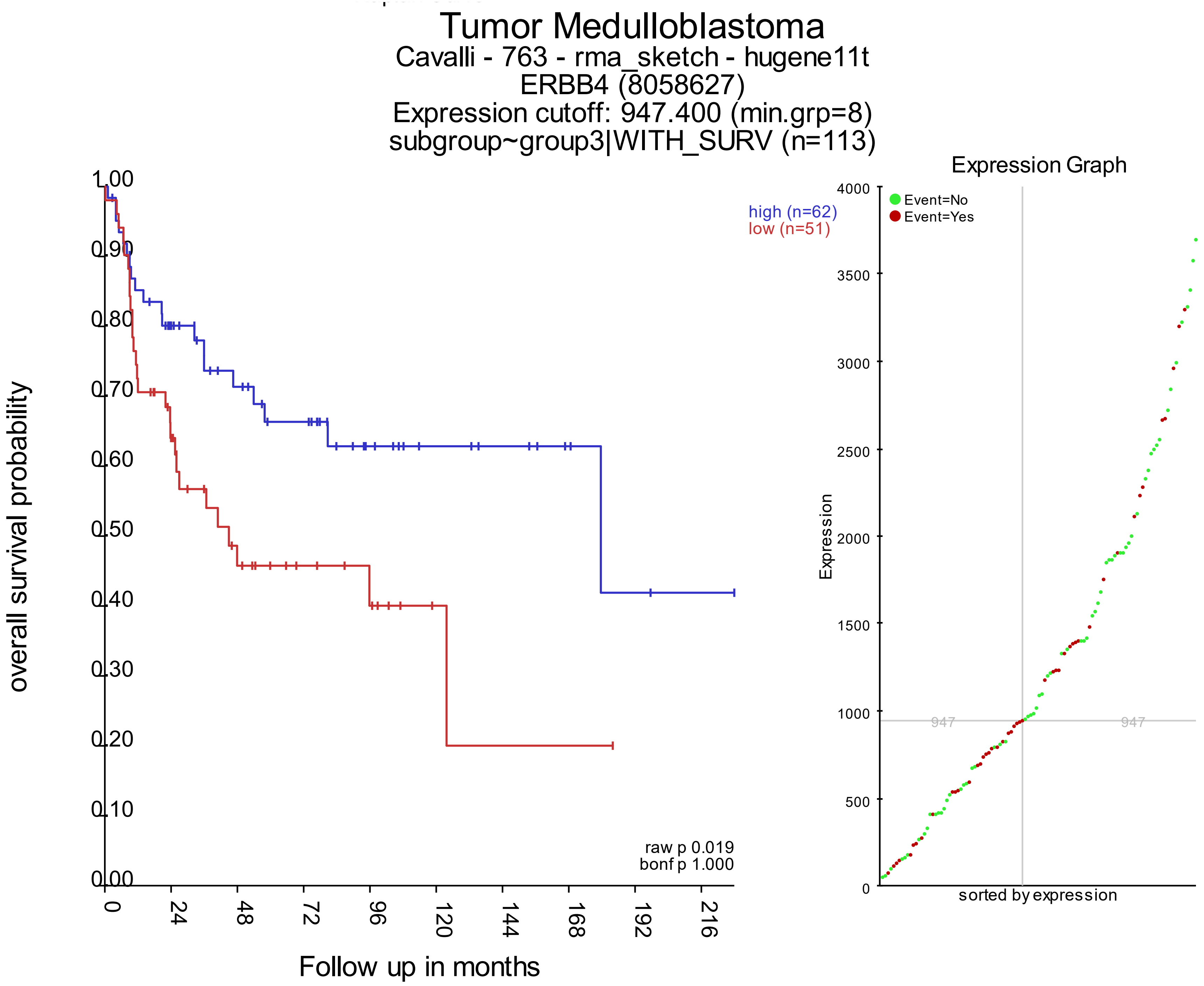

Tumor Medulloblastoma
Cavalli - 763 - rma_sketch - hugene11t
ERBB4 (8058627)
Expression cutoff: 947.400 (min.grp=8)
subgroup~group3|WITH_SURV (n=113)
overall survival probability
Follow up in months
high (n=62)
low (n=51)
raw p 0.019
bonf p 1.000
Expression Graph
Event=No
Event=Yes
Expression
sorted by expression
947

PDCD1/PD1

WNT

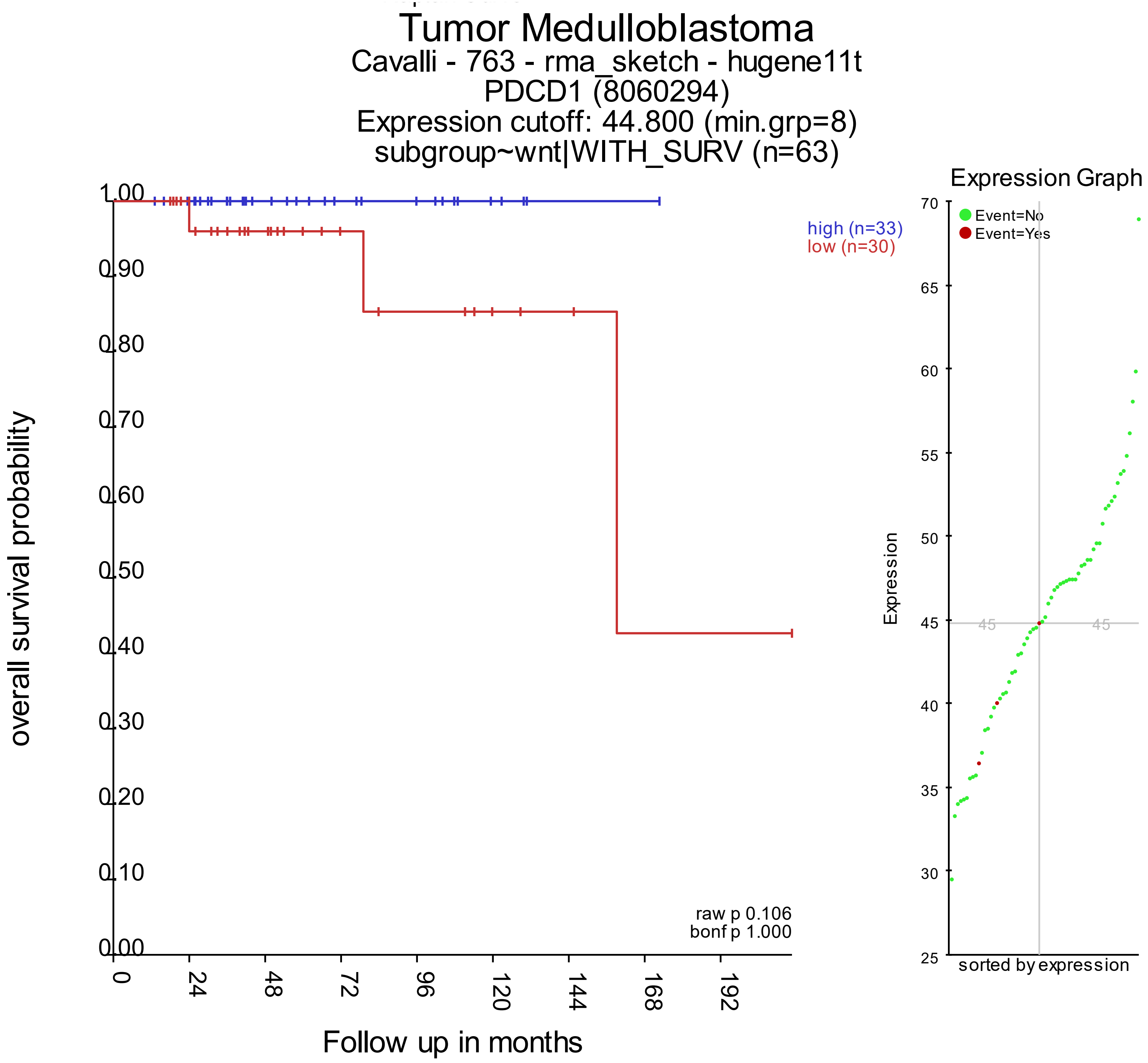

Tumor Medulloblastoma
Cavalli - 763 - rma_sketch - hugene11t
PDCD1 (8060294)
Expression cutoff: 44.800 (min.grp=8)
subgroup~wnt|WITH_SURV (n=63)
overall survival probability
1.00
0.90
0.80
0.70
0.60
0.50
0.40
0.30
0.20
0.10
0.00
0
24
48
72
96
120
144
168
192
Follow up in months
high (n=33)
low (n=30)
raw p 0.106
bonf p 1.000
Expression Graph
Event=No
Event=Yes
Expression
70
65
60
55
50
45
40
35
30
25
45
45
sorted by expression

SHH

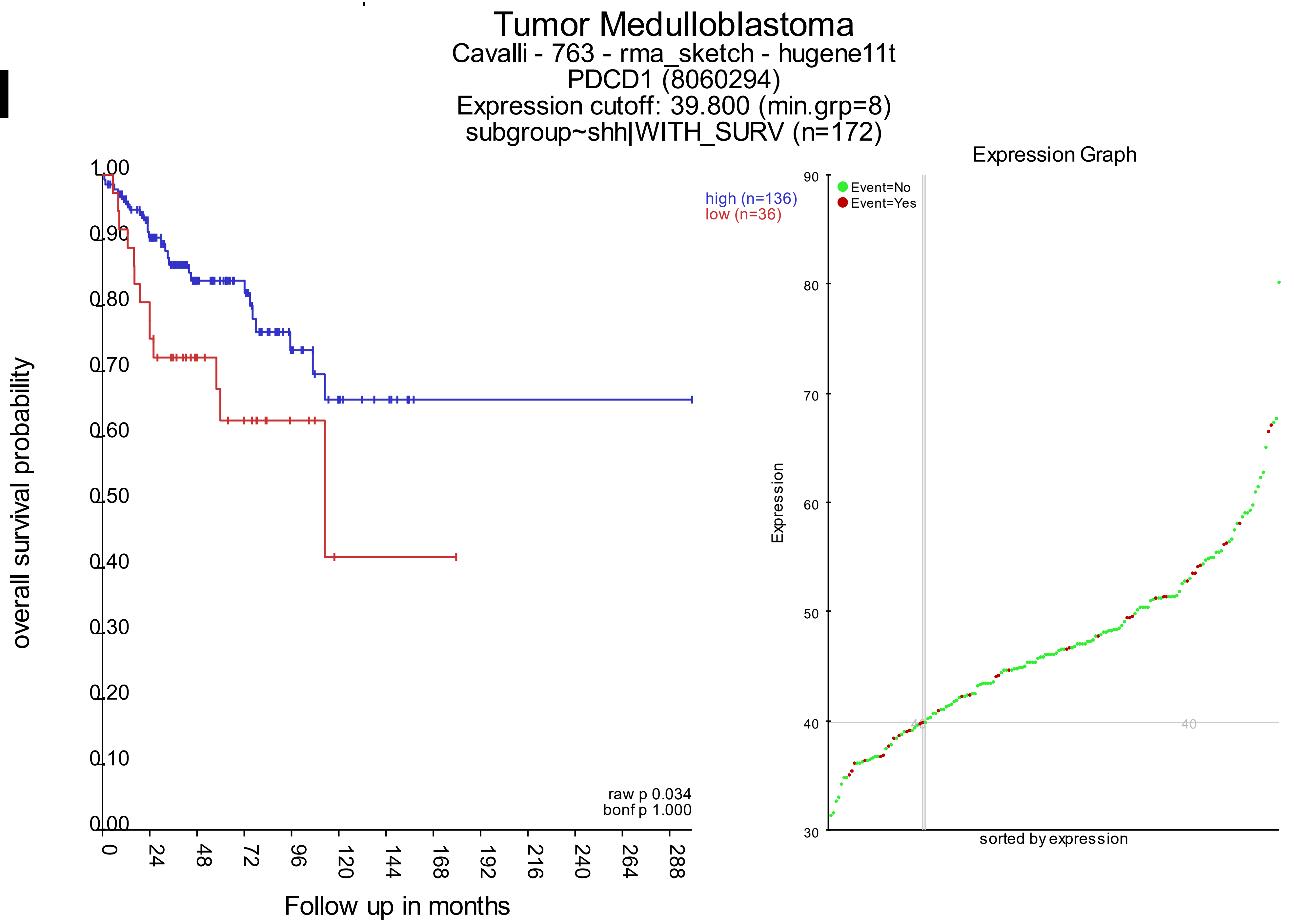
Tumor Medulloblastoma
Cavalli - 763 - rma_sketch - hugene11t
PDCD1 (8060294)
Expression cutoff: 39.800 (min.grp=8)
subgroup~shh|WITH_SURV (n=172)
overall survival probability
1.00
0.90
0.80
0.70
0.60
0.50
0.40
0.30
0.20
0.10
0.00
0
24
48
72
96
120
144
168
192
216
240
264
288
Follow up in months
high (n=136)
low (n=36)
raw p 0.034
bonf p 1.000
Expression Graph
Event=No
Event=Yes
Expression
90
80
70
60
50
40
30
sorted by expression

GR4

Tumor Medulloblastoma
Cavalli - 763 - rma_sketch - hugene11t
PDCD1 (8060294)
Expression cutoff: 44.100 (min.grp=8)
subgroup~group4|WITH_SURV (n=264)

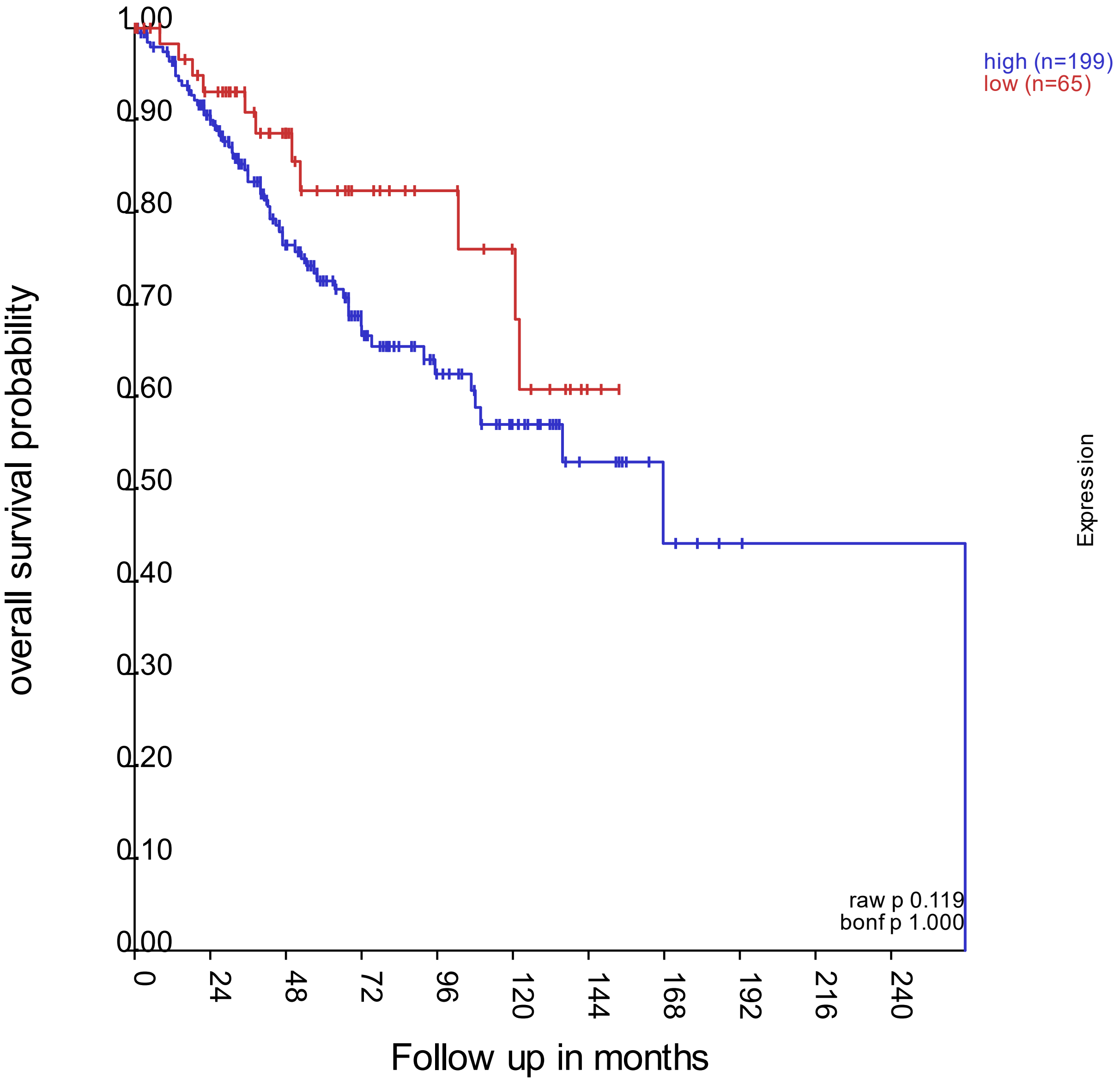

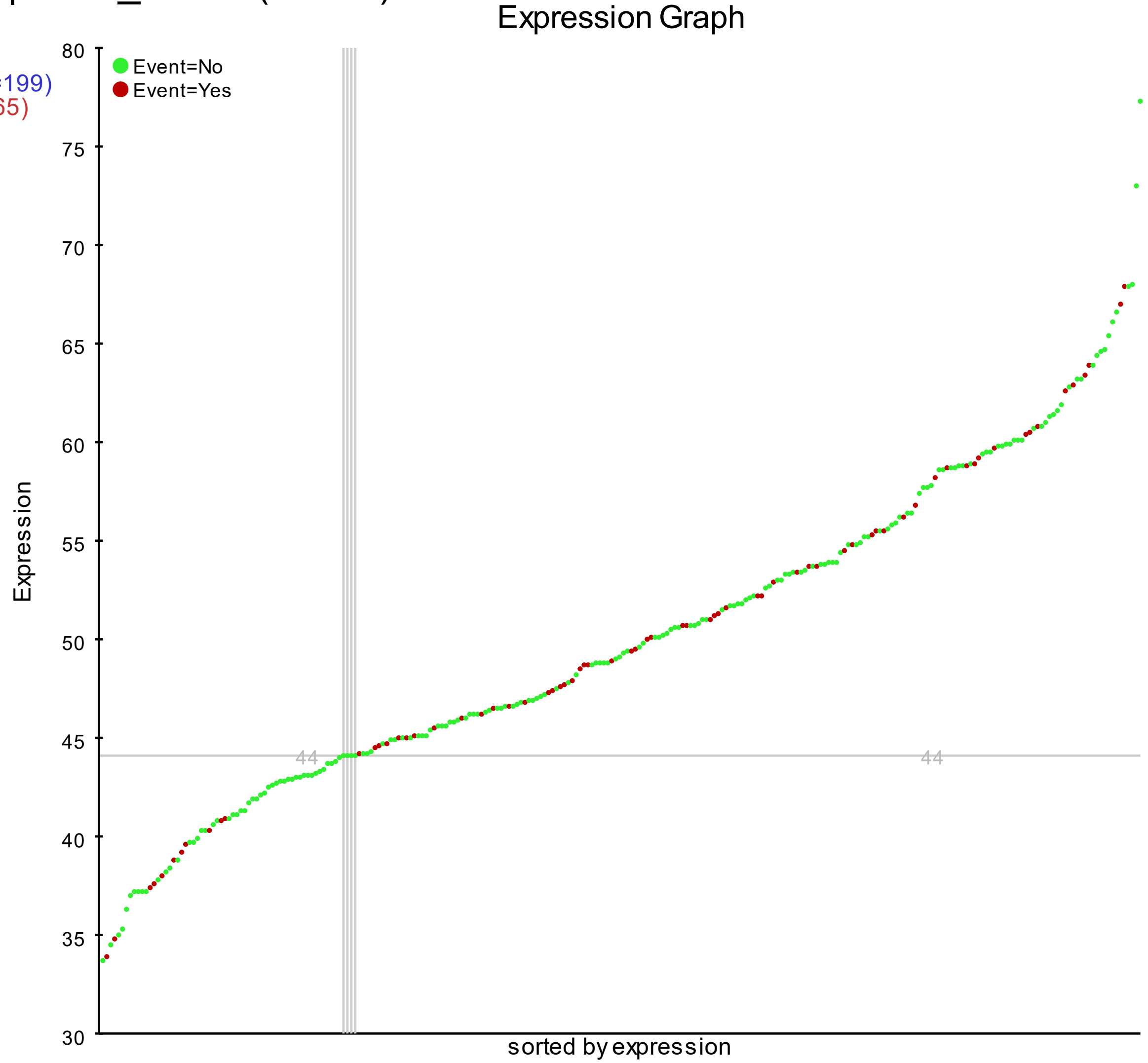

GR3

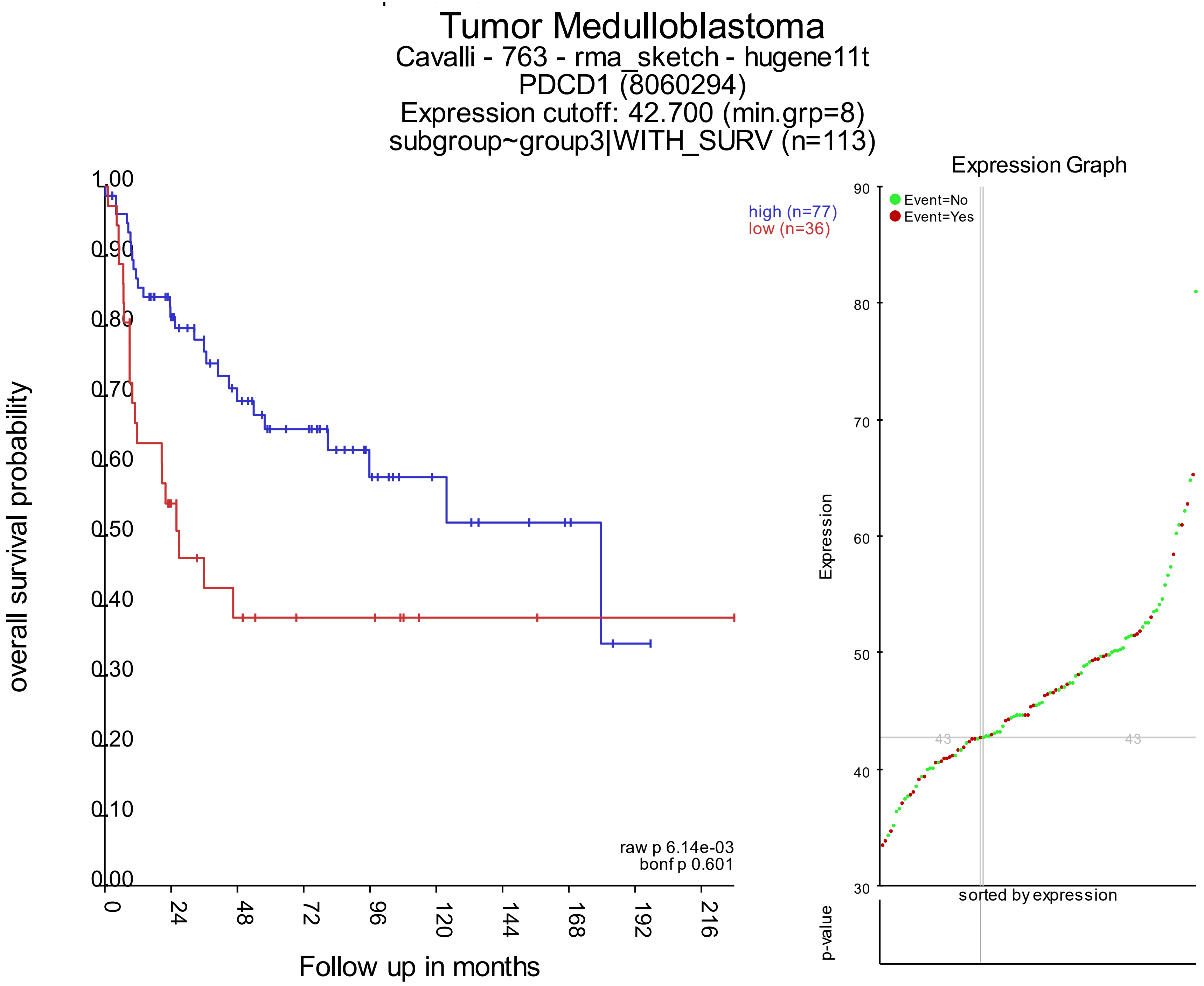
Tumor Medulloblastoma
Cavalli - 763 - rma_sketch - hugene11t
PDCD1 (8060294)
Expression cutoff: 42.700 (min.grp=8)
subgroup~group3|WITH_SURV (n=113)
overall survival probability
Follow up in months
high (n=77)
low (n=36)
raw p 6.14e-03
bonf p 0.601
Expression Graph
Event=No
Event=Yes
Expression
sorted by expression
p-value

PDCD2/PD2

WNT

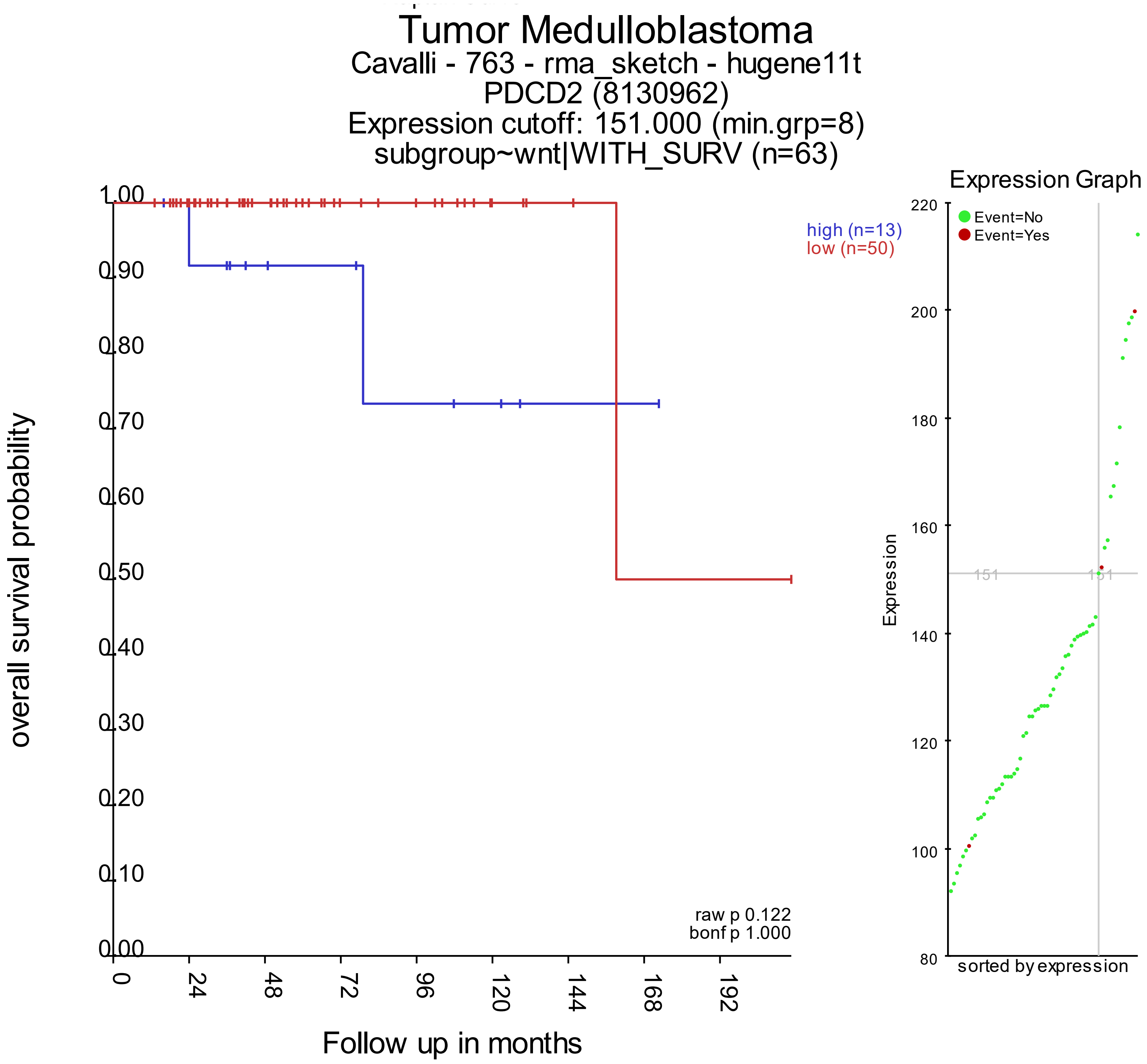

Tumor Medulloblastoma
Cavalli - 763 - rma_sketch - hugene11t
PDCD2 (8130962)
Expression cutoff: 151.000 (min.grp=8)
subgroup~wnt|WITH_SURV (n=63)
overall survival probability
1.00
0.90
0.80
0.70
0.60
0.50
0.40
0.30
0.20
0.10
0.00
0
24
48
72
96
120
144
168
192
Follow up in months
high (n=13)
low (n=50)
raw p 0.122
bonf p 1.000
Expression Graph
Event=No
Event=Yes
Expression
220
200
180
160
140
120
100
80
151
151
sorted by expression

SHH

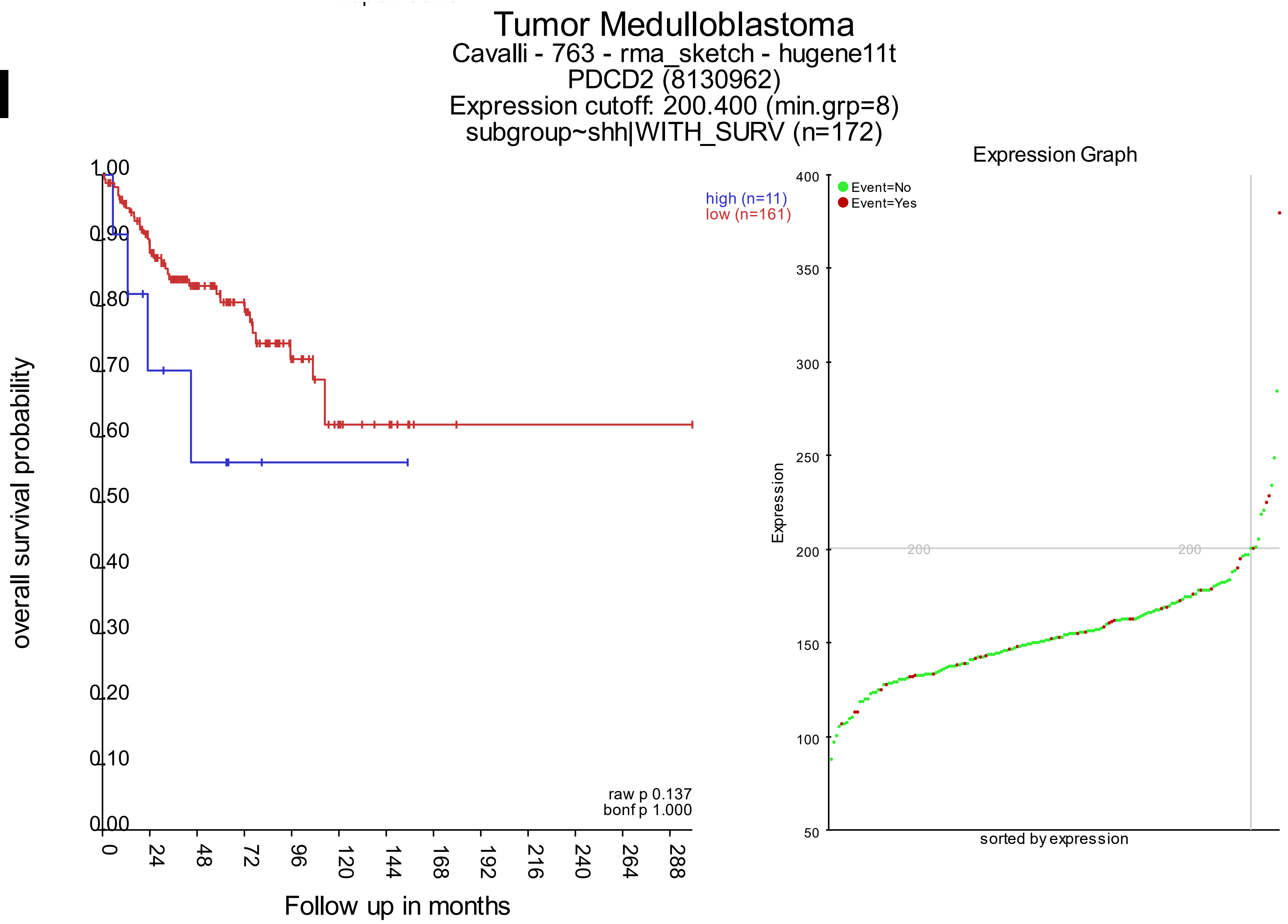
Tumor Medulloblastoma
Cavalli - 763 - rma_sketch - hugene11t
PDCD2 (8130962)
Expression cutoff: 200.400 (min.grp=8)
subgroup~shh|WITH_SURV (n=172)
overall survival probability
1.00
0.90
0.80
0.70
0.60
0.50
0.40
0.30
0.20
0.10
0.00
0
24
48
72
96
120
144
168
192
216
240
264
288
Follow up in months
high (n=11)
low (n=161)
raw p 0.137
bonf p 1.000
Expression Graph
Event=No
Event=Yes
Expression
400
350
300
250
200
150
100
50
200
200
sorted by expression

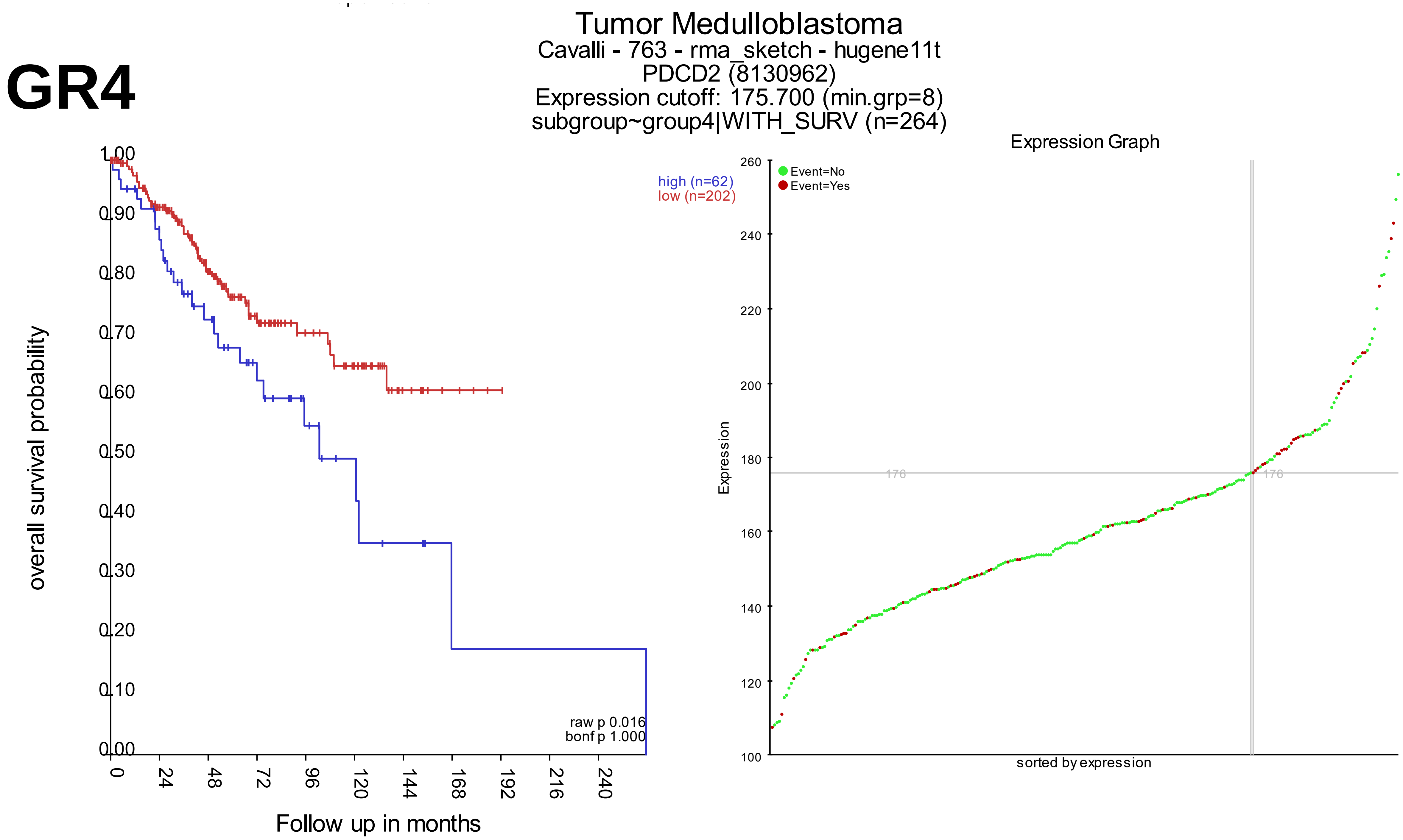

GR4
Tumor Medulloblastoma
Cavalli - 763 - rma_sketch - hugene11t
PDCD2 (8130962)
Expression cutoff: 175.700 (min.grp=8)
subgroup~group4|WITH_SURV (n=264)
overall survival probability
1.00
0.90
0.80
0.70
0.60
0.50
0.40
0.30
0.20
0.10
0.00
0
24
48
72
96
120
144
168
192
216
240
Follow up in months
high (n=62)
low (n=202)
raw p 0.016
bonf p 1.000
Expression Graph
Event=No
Event=Yes
Expression
260
240
220
200
180
160
140
120
100
176
176
sorted by expression

GR3

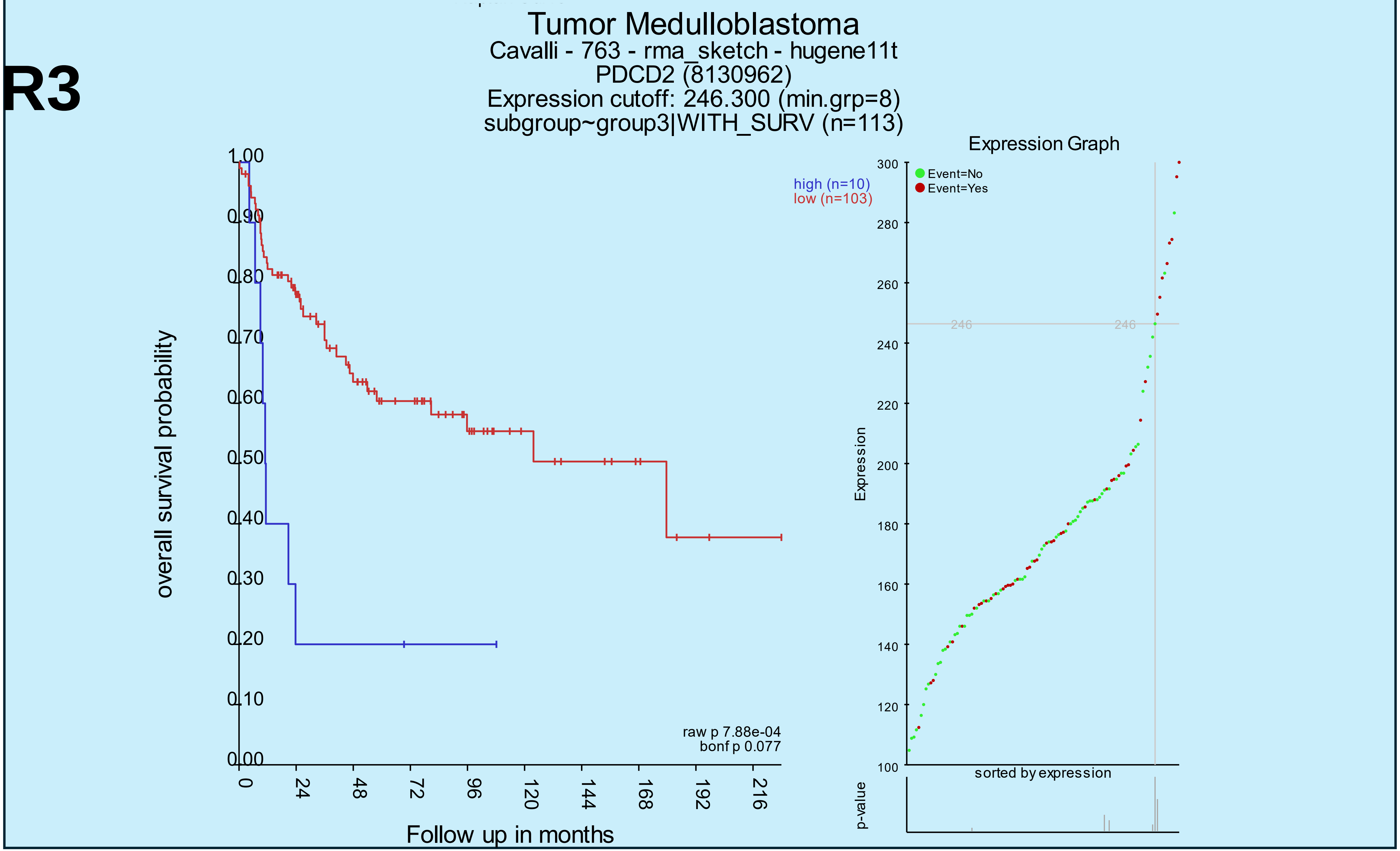

Tumor Medulloblastoma
Cavalli - 763 - rma_sketch - hugene11t
PDCD2 (8130962)
Expression cutoff: 246.300 (min.grp=8)
subgroup~group3|WITH_SURV (n=113)
overall survival probability
Follow up in months
high (n=10)
low (n=103)
raw p 7.88e-04
bonf p 0.077
Expression Graph
Event=No
Event=Yes
Expression
sorted by expression
p-value
246

PDL1

WNT

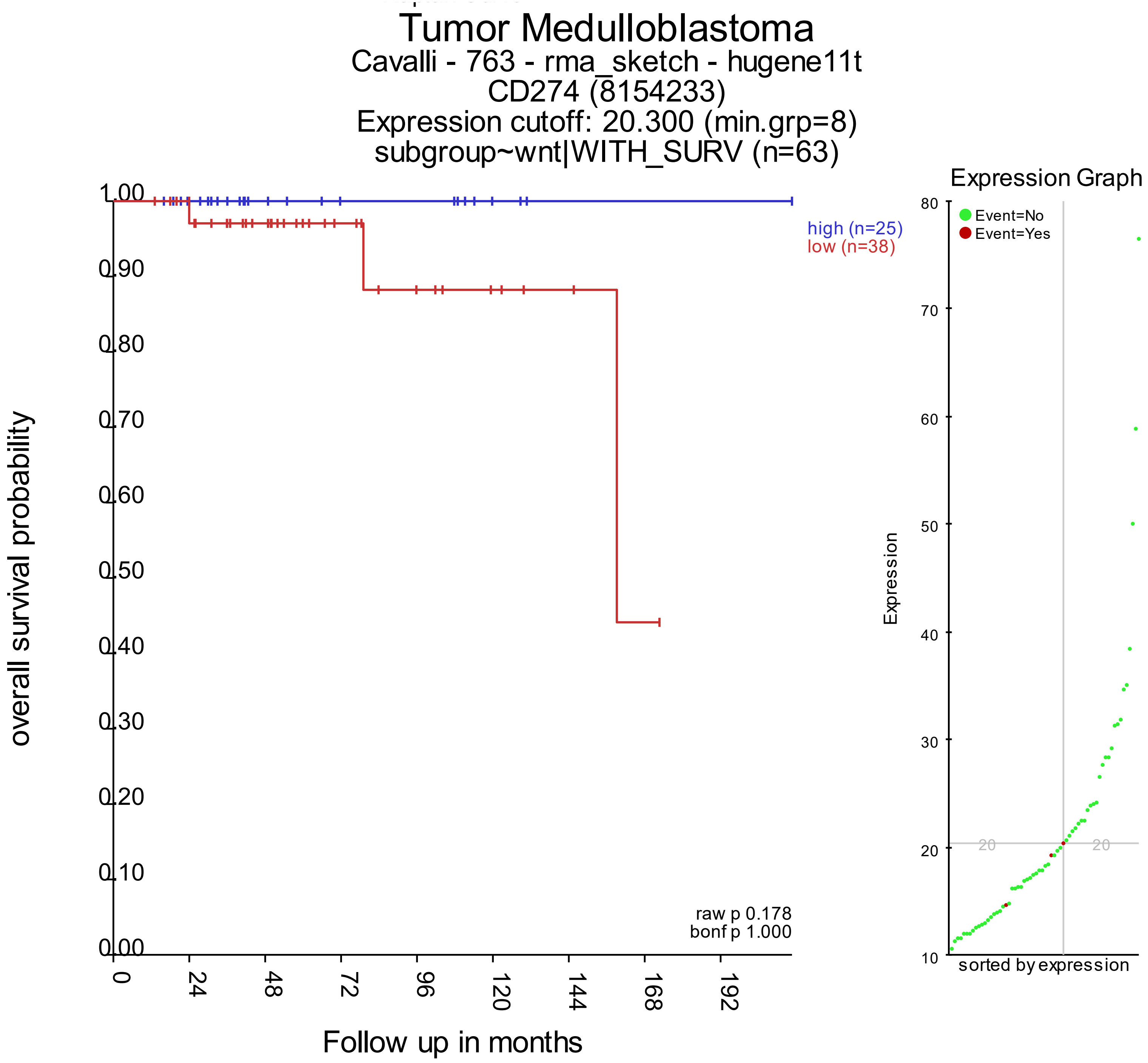
Tumor Medulloblastoma
Cavalli - 763 - rma_sketch - hugene11t
CD274 (8154233)
Expression cutoff: 20.300 (min.grp=8)
subgroup~wnt|WITH_SURV (n=63)
overall survival probability
1.00
0.90
0.80
0.70
0.60
0.50
0.40
0.30
0.20
0.10
0.00
0
24
48
72
96
120
144
168
192
Follow up in months
high (n=25)
low (n=38)
raw p 0.178
bonf p 1.000
Expression Graph
Event=No
Event=Yes
Expression
80
70
60
50
40
30
20
10
20
20
sorted by expression

SHH

Tumor Medulloblastoma
Cavalli - 763 - rma_sketch - hugene11t
CD274 (8154233)
Expression cutoff: 16.000 (min.grp=8)
subgroup~shh|WITH_SURV (n=172)

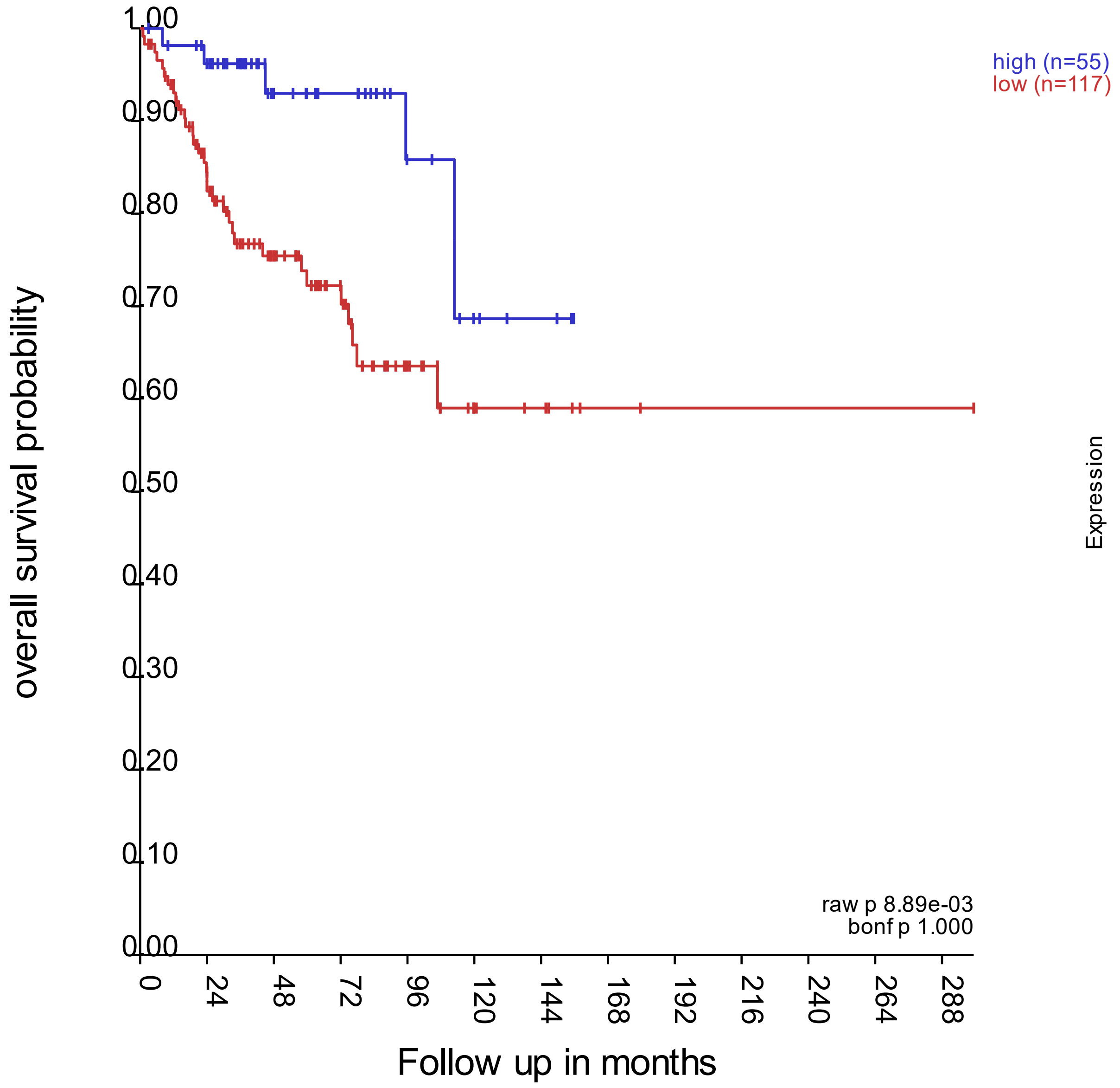

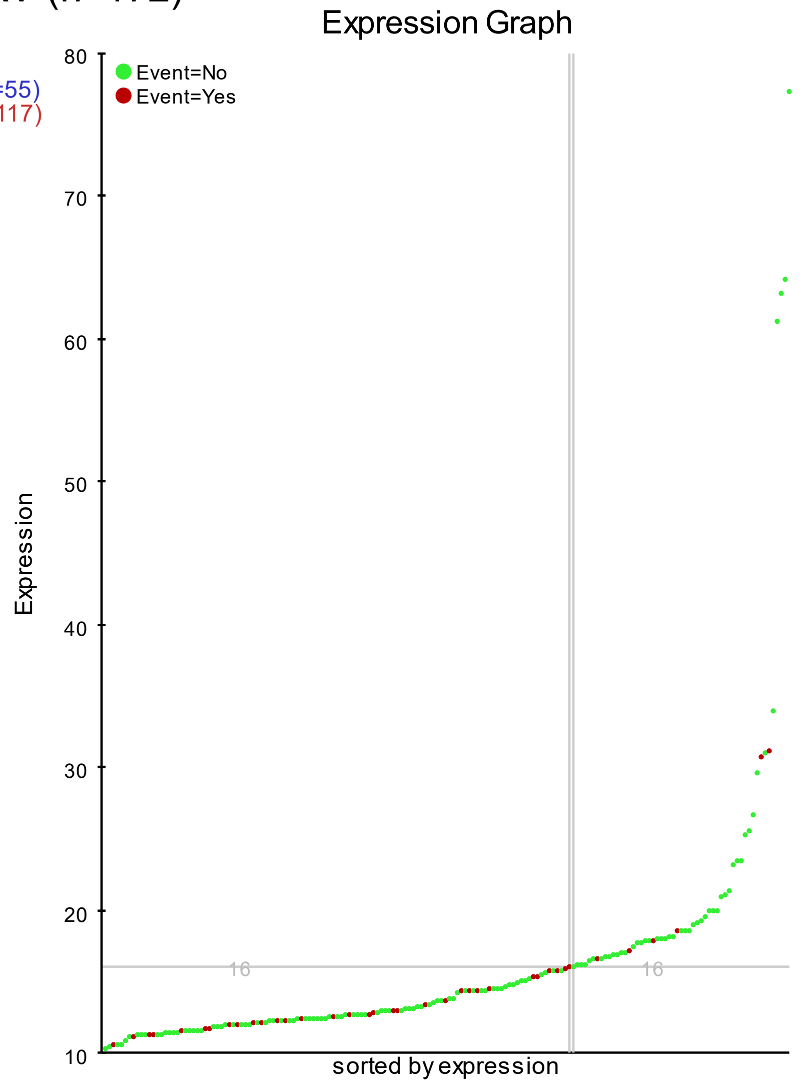

GR4

Tumor Medulloblastoma
Cavalli - 763 - rma_sketch - hugene11t
CD274 (8154233)
Expression cutoff: 16.300 (min.grp=8)
subgroup~group4|WITH_SURV (n=264)

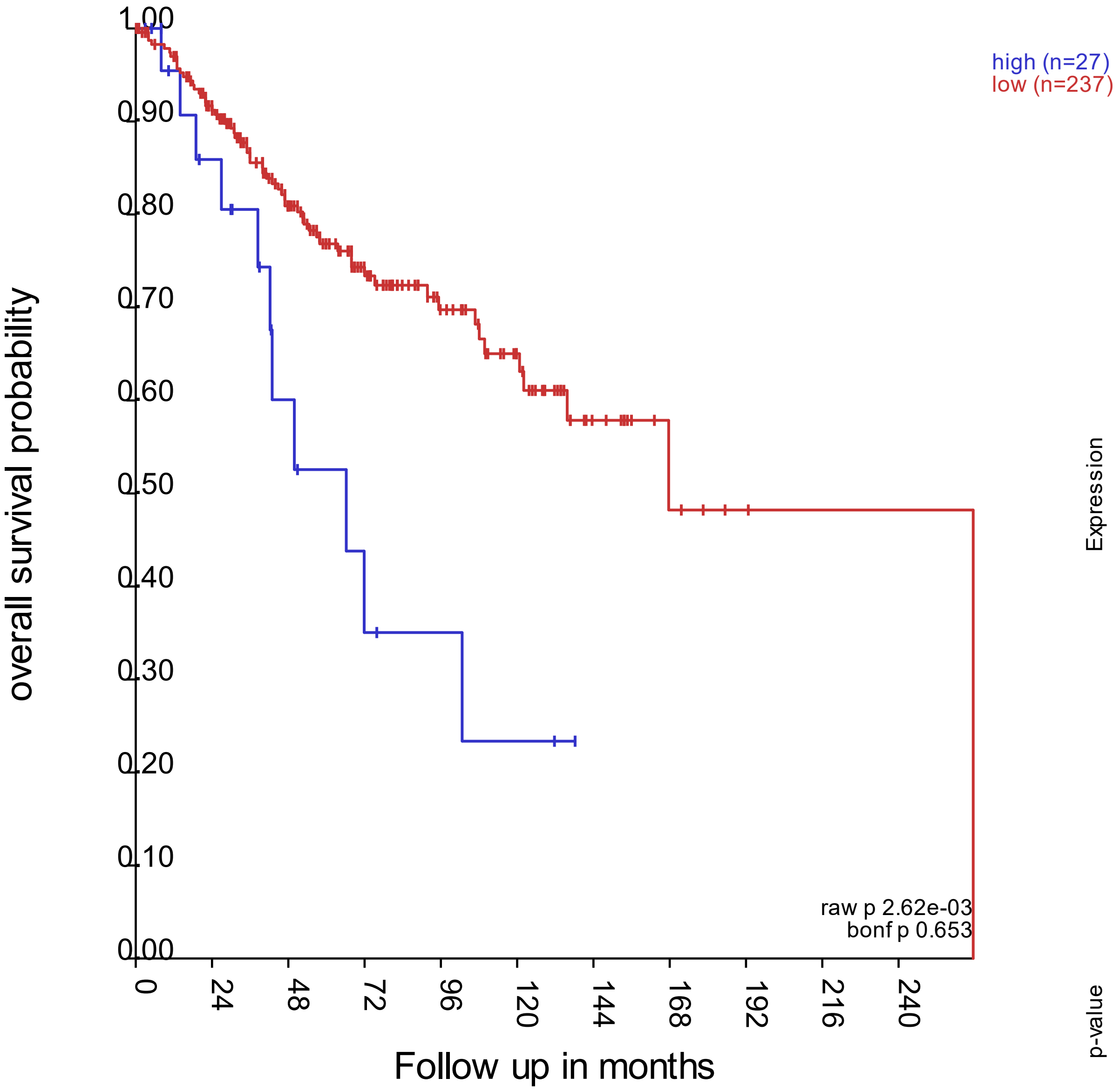

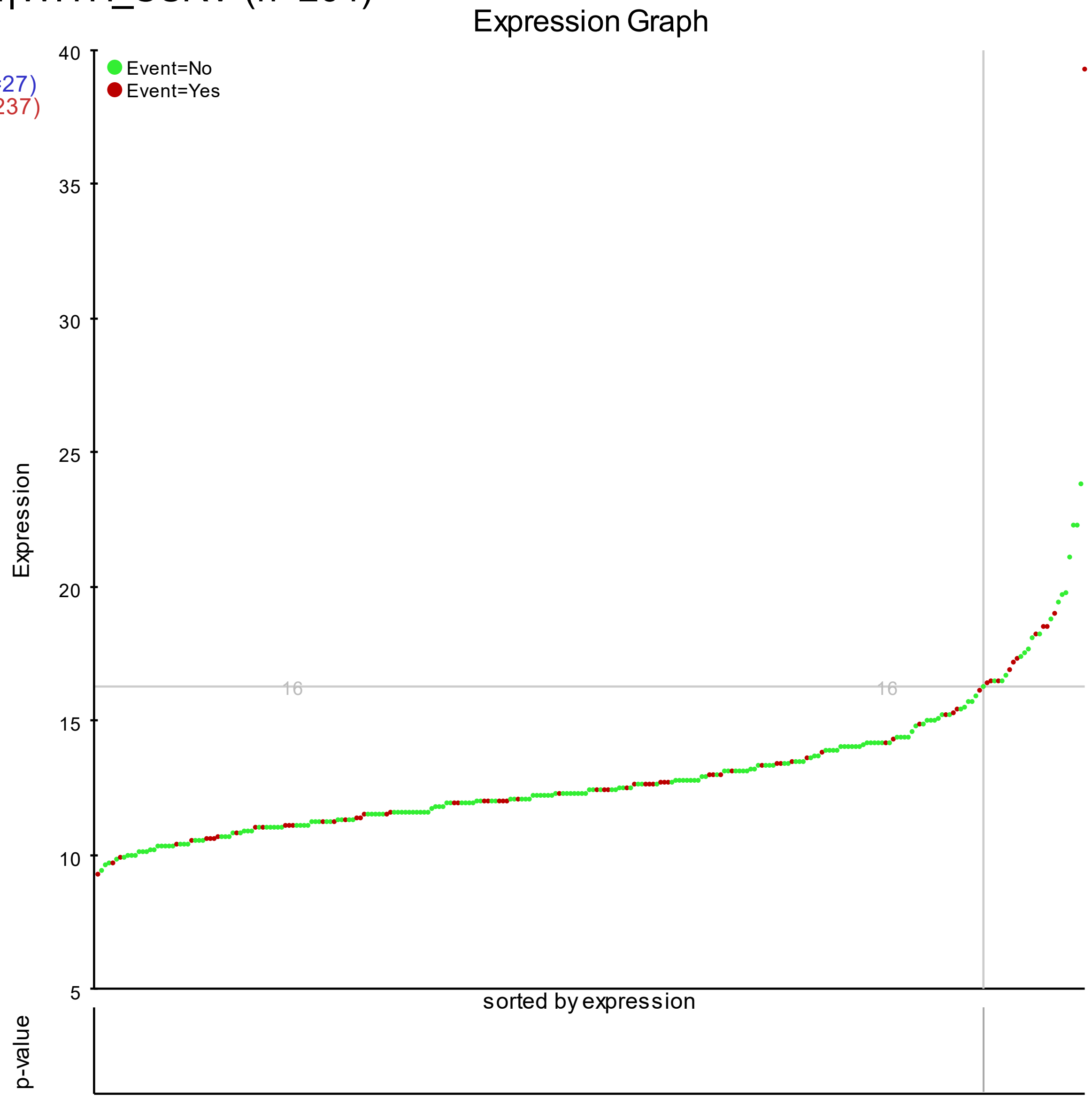

GR3

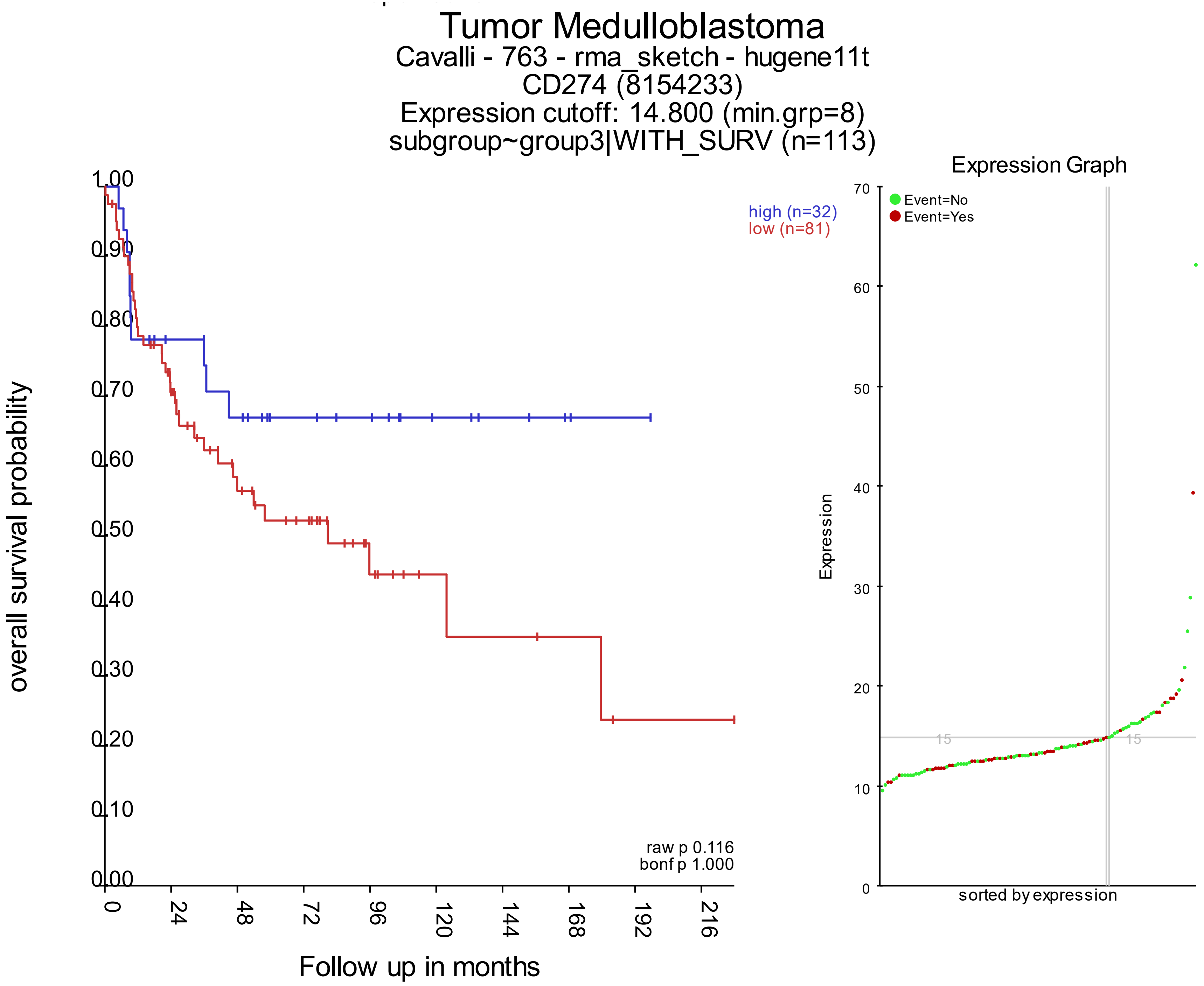

Tumor Medulloblastoma
Cavalli - 763 - rma_sketch - hugene11t
CD274 (8154233)
Expression cutoff: 14.800 (min.grp=8)
subgroup~group3|WITH_SURV (n=113)
1.00
0.90
0.80
0.70
0.60
0.50
0.40
0.30
0.20
0.10
0.00
overall survival probability
high (n=32)
low (n=81)
raw p 0.116
bonf p 1.000
0
24
48
72
96
120
144
168
192
216
Follow up in months
Expression Graph
Event=No
Event=Yes
70
60
50
40
30
20
10
0
Expression
15
15
sorted by expression

PDL2

WNT

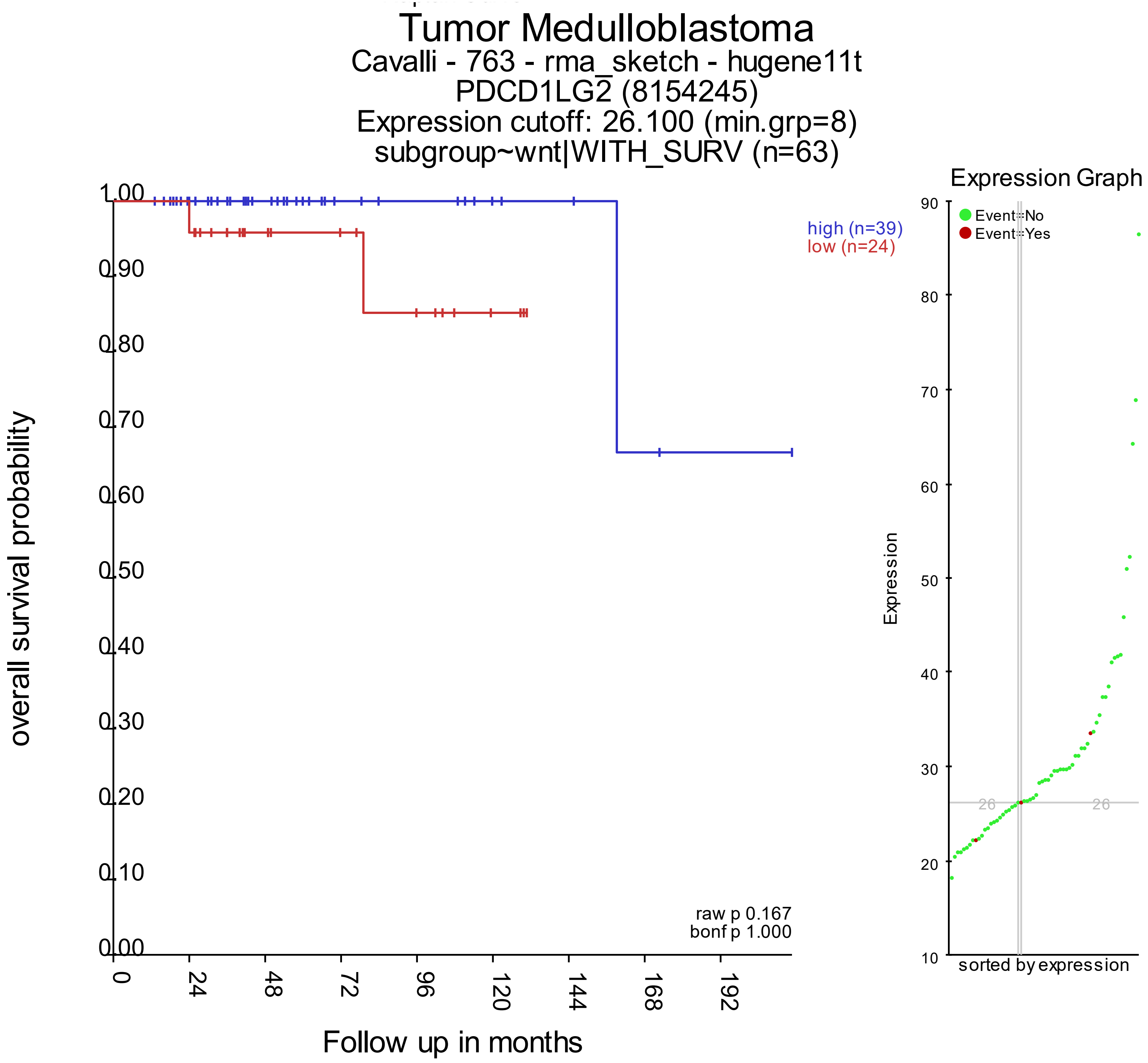

Tumor Medulloblastoma
Cavalli - 763 - rma_sketch - hugene11t
PDCD1LG2 (8154245)
Expression cutoff: 26.100 (min.grp=8)
subgroup~wnt|WITH_SURV (n=63)
overall survival probability
1.00
0.90
0.80
0.70
0.60
0.50
0.40
0.30
0.20
0.10
0.00
0
24
48
72
96
120
144
168
192
Follow up in months
high (n=39)
low (n=24)
raw p 0.167
bonf p 1.000
Expression Graph
Event=No
Event=Yes
Expression
90
80
70
60
50
40
30
20
10
26
26
sorted by expression

SHH

Tumor Medulloblastoma
Cavalli - 763 - rma_sketch - hugene11t
PDCD1LG2 (8154245)
Expression cutoff: 19.400 (min.grp=8)
subgroup~shh|WITH_SURV (n=172)

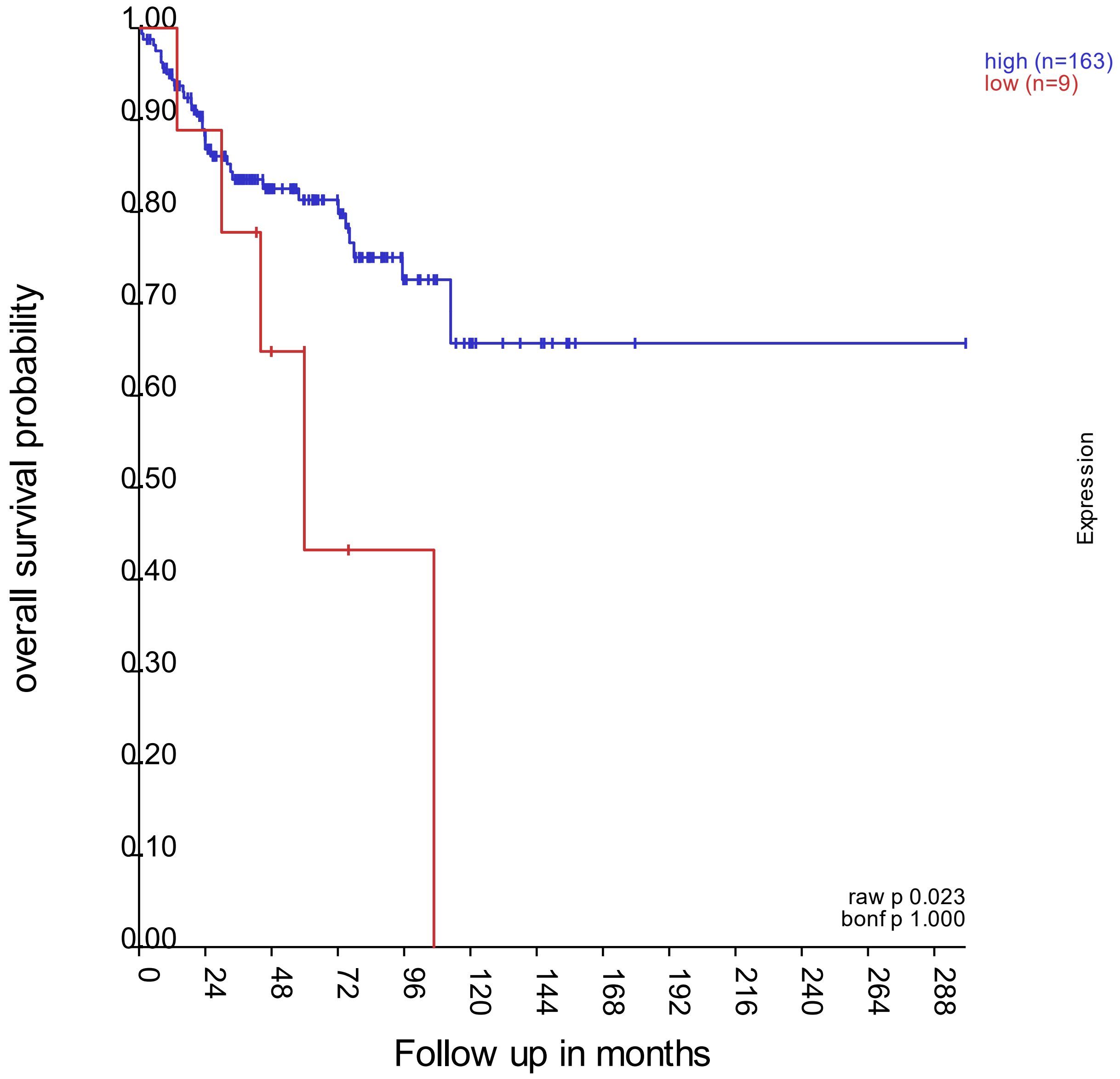

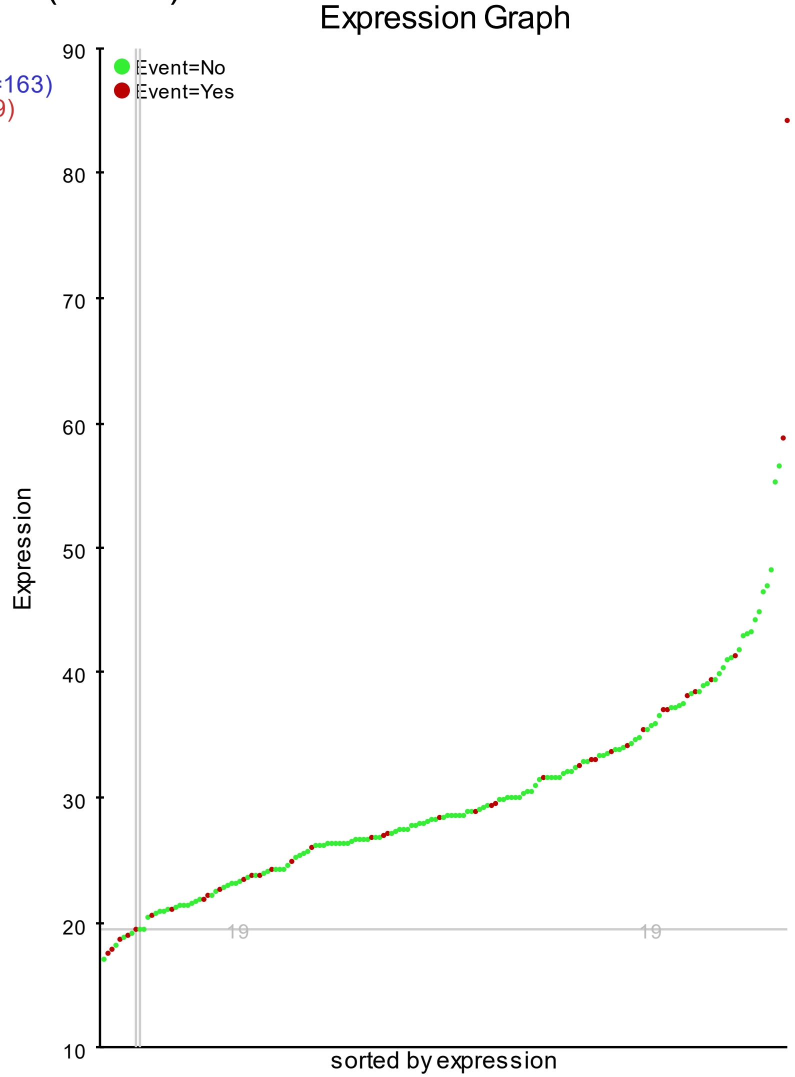

GR4

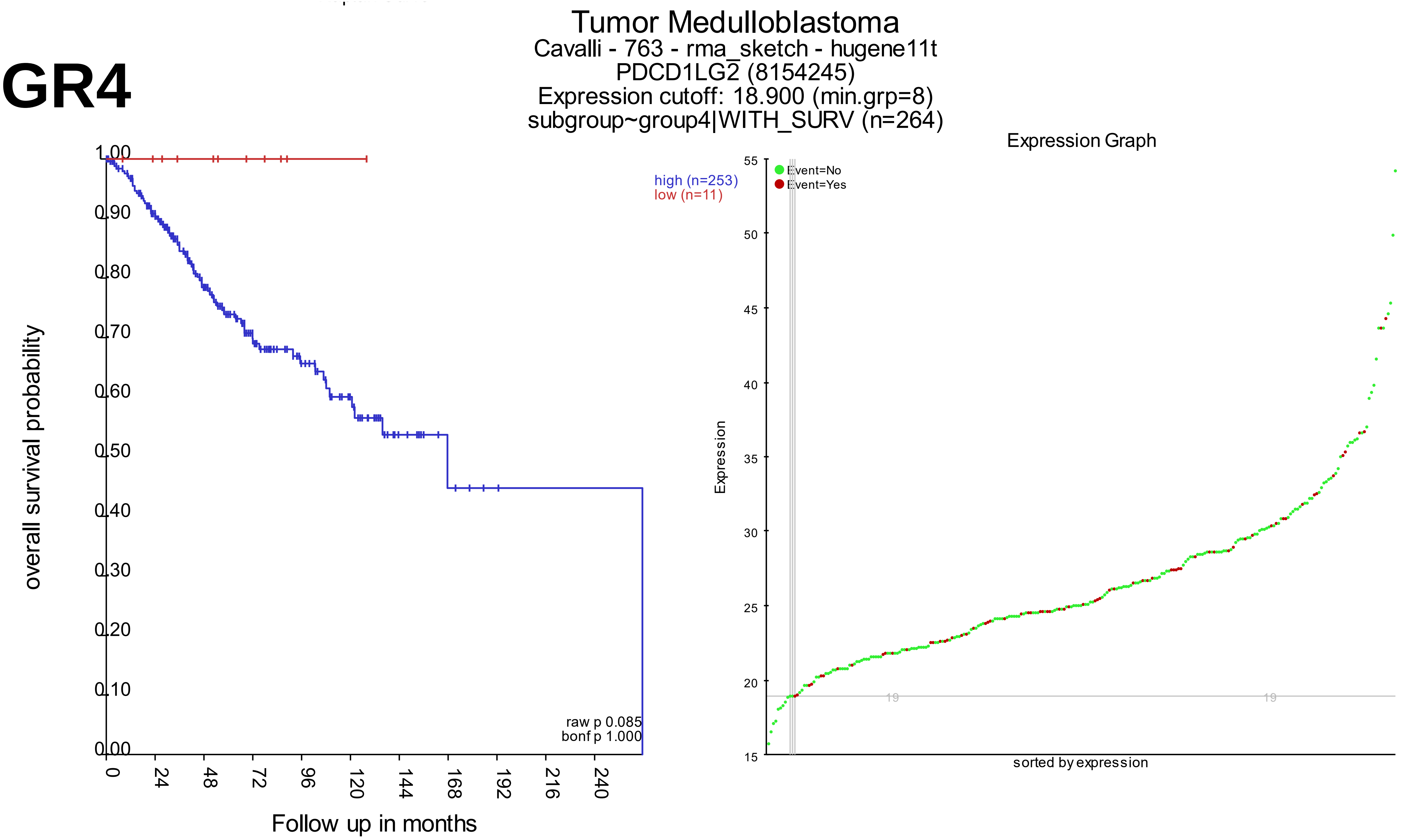

Tumor Medulloblastoma
Cavalli - 763 - rma_sketch - hugene11t
PDCD1LG2 (8154245)
Expression cutoff: 18.900 (min.grp=8)
subgroup~group4|WITH_SURV (n=264)
overall survival probability
1.00
0.90
0.80
0.70
0.60
0.50
0.40
0.30
0.20
0.10
0.00
high (n=253)
low (n=11)
raw p 0.085
bonf p 1.000
0
24
48
72
96
120
144
168
192
216
240
Follow up in months
Expression Graph
Event=No
Event=Yes
Expression
55
50
45
40
35
30
25
20
15
19
19
sorted by expression

GR3

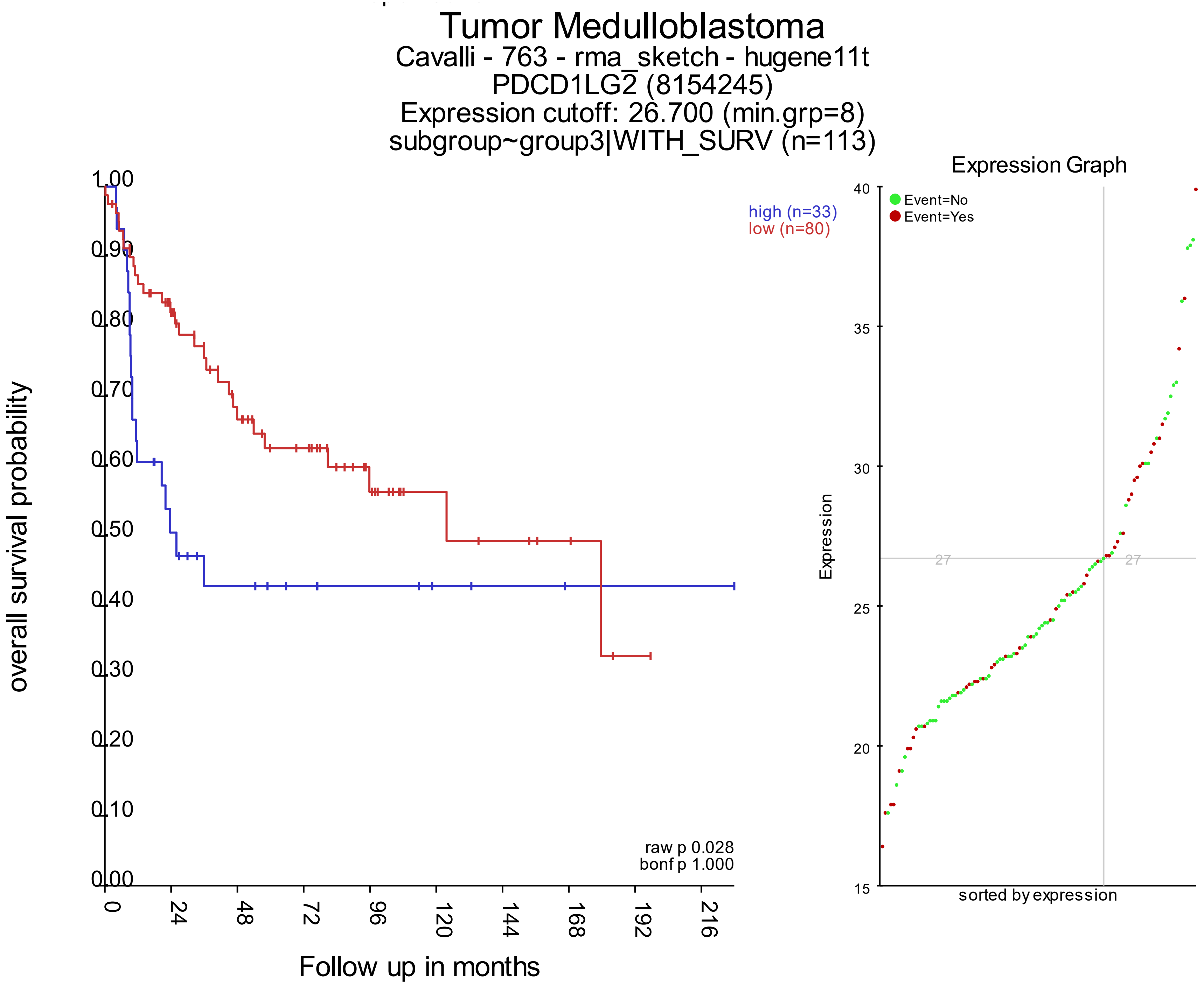

Tumor Medulloblastoma
Cavalli - 763 - rma_sketch - hugene11t
PDCD1LG2 (8154245)
Expression cutoff: 26.700 (min.grp=8)
subgroup~group3|WITH_SURV (n=113)
high (n=33)
low (n=80)
overall survival probability
1.00
0.90
0.80
0.70
0.60
0.50
0.40
0.30
0.20
0.10
0.00
raw p 0.028
bonf p 1.000
0
24
48
72
96
120
144
168
192
216
Follow up in months
Expression Graph
Event=No
Event=Yes
Expression
40
35
30
25
20
15
27
27
sorted by expression

CTLA4

WNT

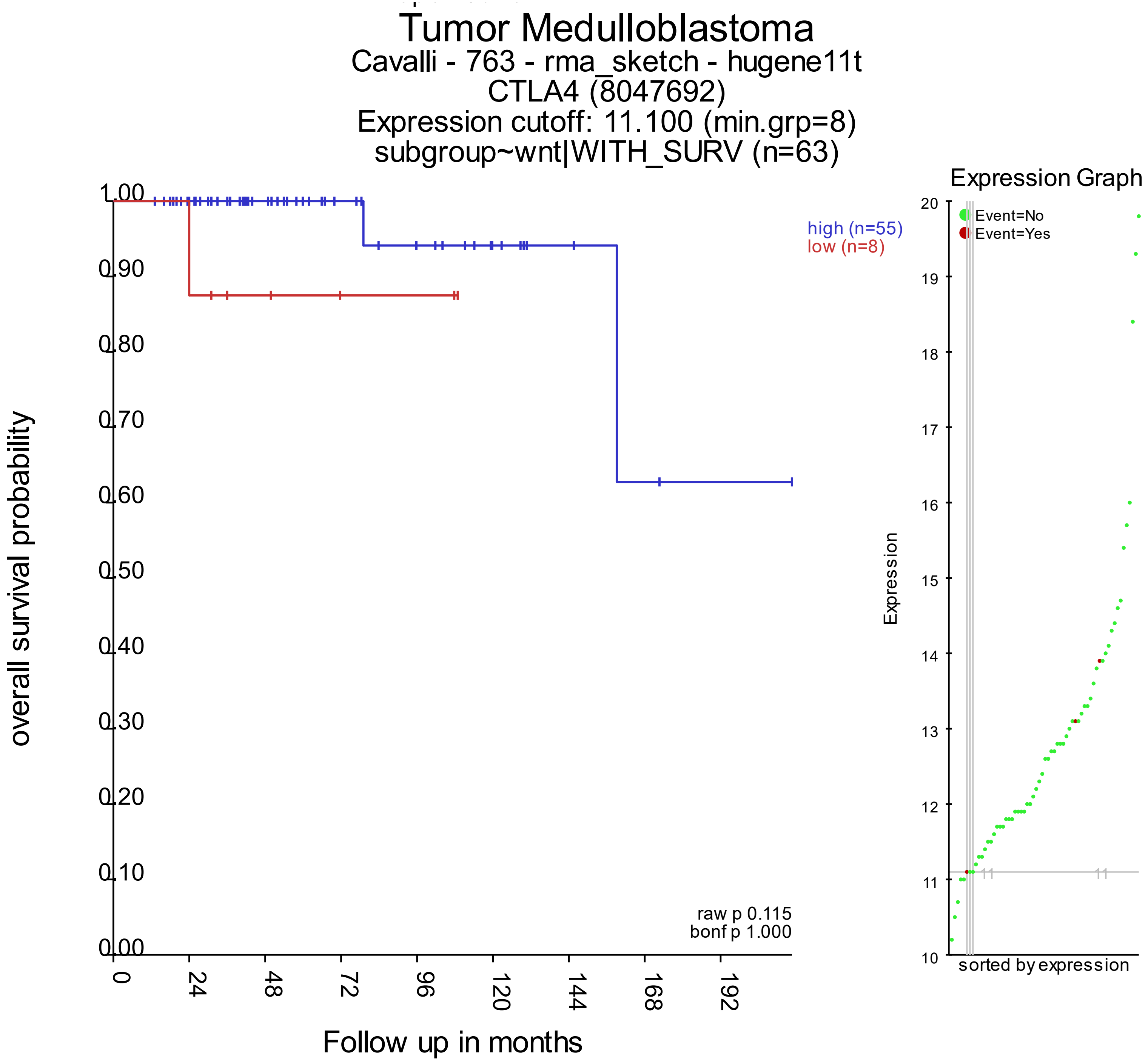

Tumor Medulloblastoma
Cavalli - 763 - rma_sketch - hugene11t
CTLA4 (8047692)
Expression cutoff: 11.100 (min.grp=8)
subgroup~wnt|WITH_SURV (n=63)
high (n=55)
low (n=8)
overall survival probability
1.00
0.90
0.80
0.70
0.60
0.50
0.40
0.30
0.20
0.10
0.00
0
24
48
72
96
120
144
168
192
Follow up in months
raw p 0.115
bonf p 1.000
Expression Graph
Event=No
Event=Yes
Expression
sorted by expression

SHH

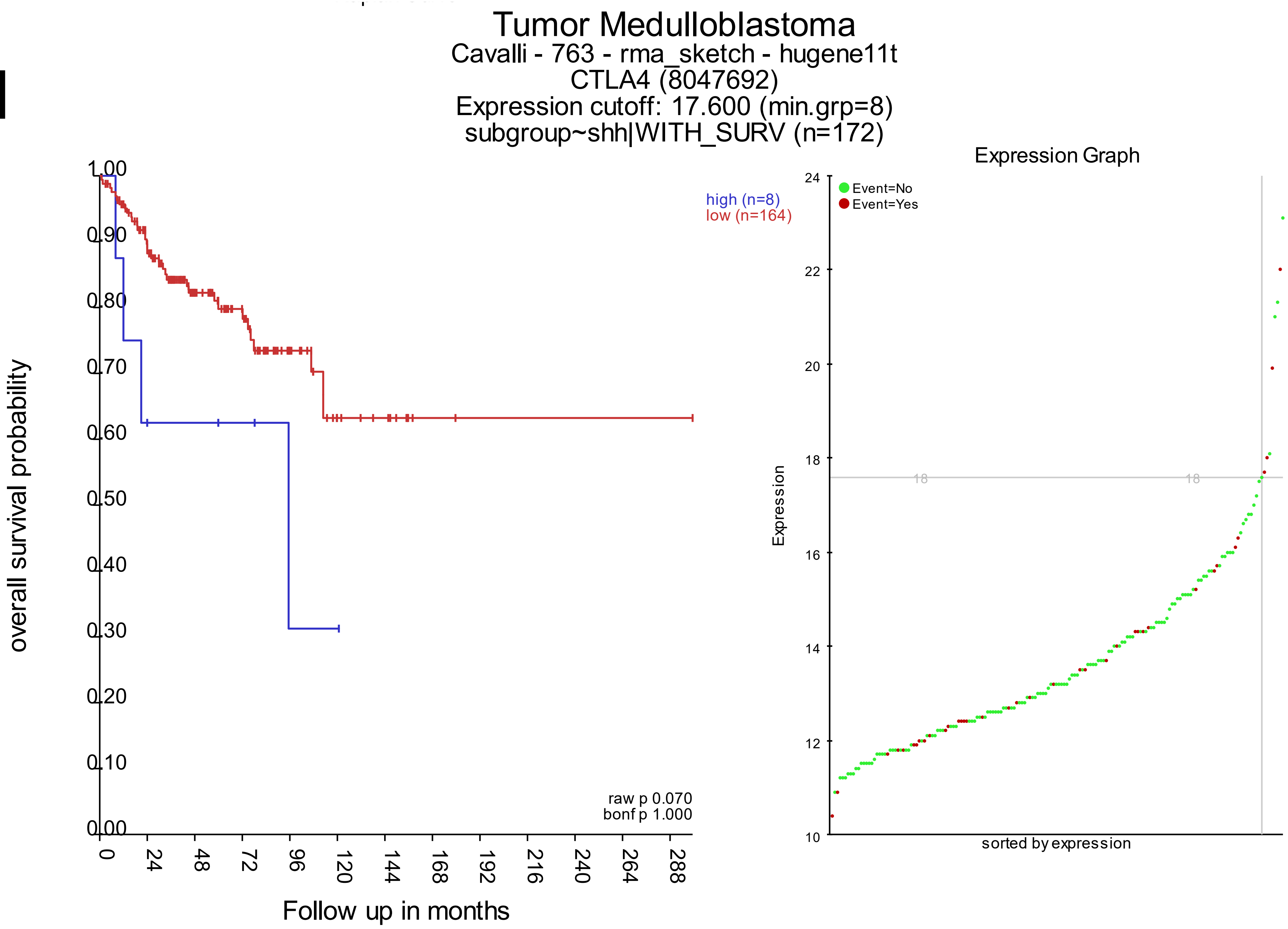

Tumor Medulloblastoma
Cavalli - 763 - rma_sketch - hugene11t
CTLA4 (8047692)
Expression cutoff: 17.600 (min.grp=8)
subgroup~shh|WITH_SURV (n=172)
high (n=8)
low (n=164)
overall survival probability
1.00
0.90
0.80
0.70
0.60
0.50
0.40
0.30
0.20
0.10
0.00
0
24
48
72
96
120
144
168
192
216
240
264
288
Follow up in months
raw p 0.070
bonf p 1.000
Expression Graph
Event=No
Event=Yes
Expression
24
22
20
18
16
14
12
10
sorted by expression

GR4

Tumor Medulloblastoma
Cavalli - 763 - rma_sketch - hugene11t
CTLA4 (8047692)
Expression cutoff: 10.800 (min.grp=8)
subgroup~group4|WITH_SURV (n=264)

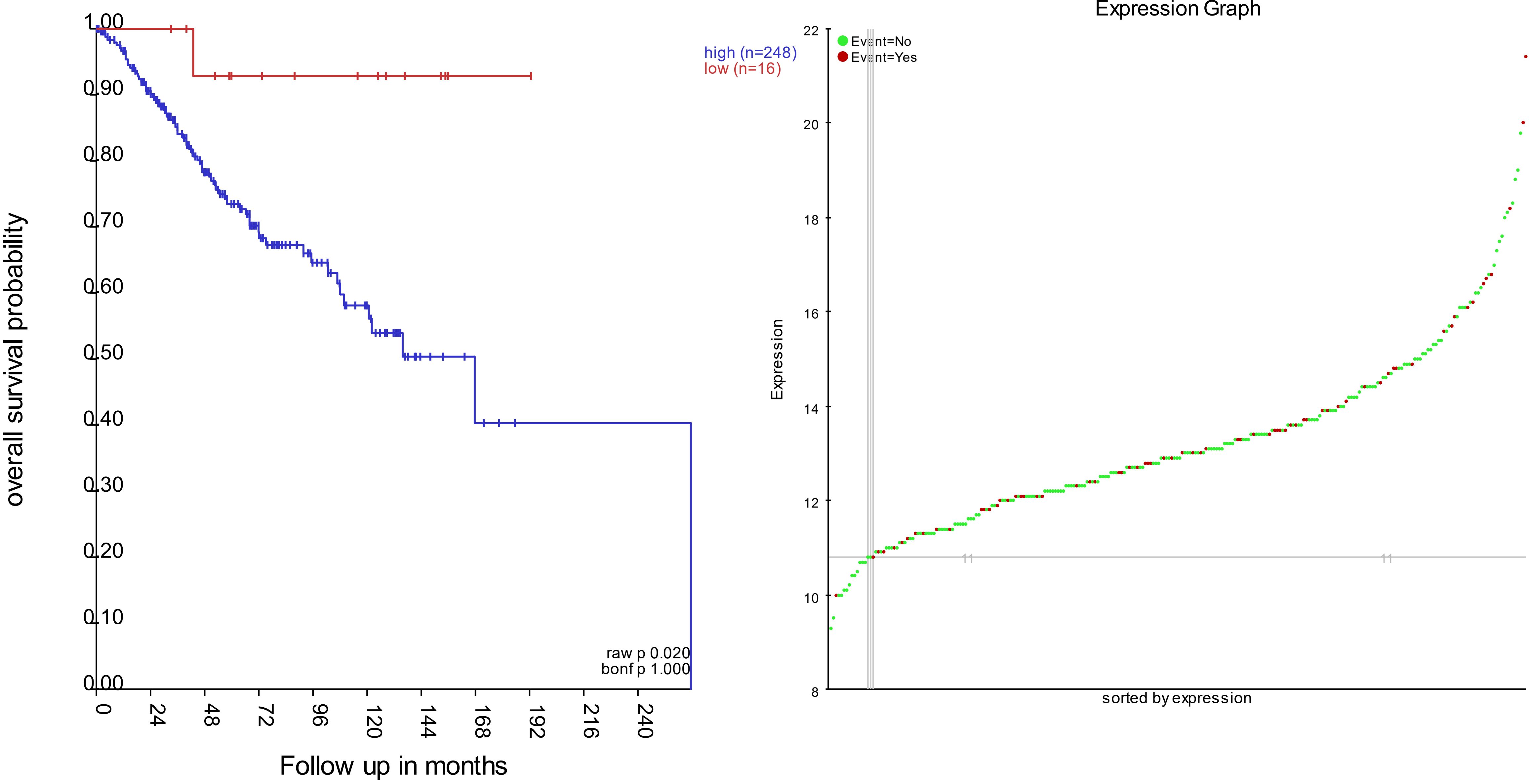

GR3

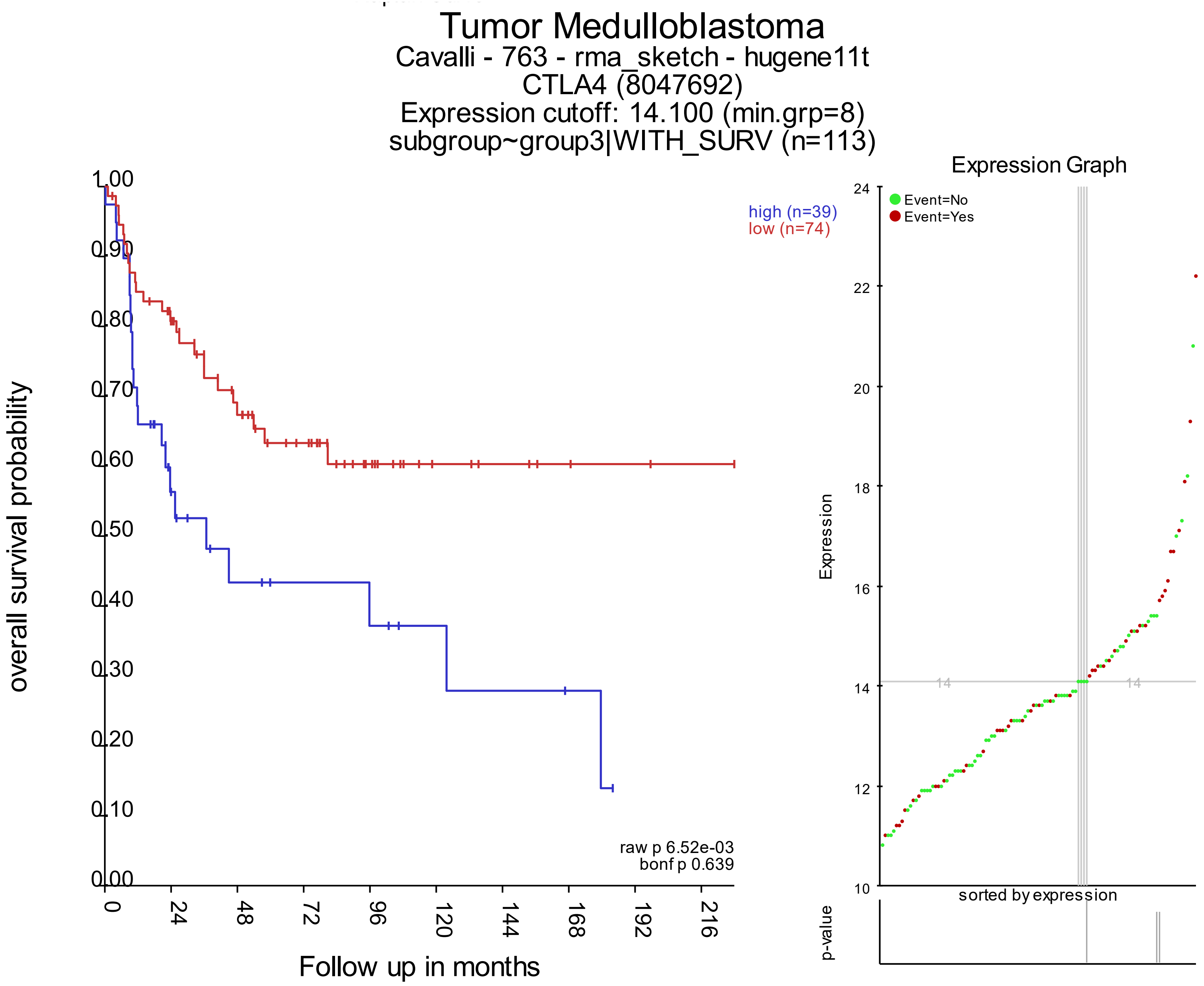
Tumor Medulloblastoma
Cavalli - 763 - rma_sketch - hugene11t
CTLA4 (8047692)
Expression cutoff: 14.100 (min.grp=8)
subgroup~group3|WITH_SURV (n=113)
high (n=39)
low (n=74)
overall survival probability
1.00
0.90
0.80
0.70
0.60
0.50
0.40
0.30
0.20
0.10
0.00
0
24
48
72
96
120
144
168
192
216
Follow up in months
raw p 6.52e-03
bonf p 0.639
Expression Graph
Event=No
Event=Yes
Expression
24
22
20
18
16
14
12
10
14
14
sorted by expression
p-value

HAVCR2/TIM3

WNT

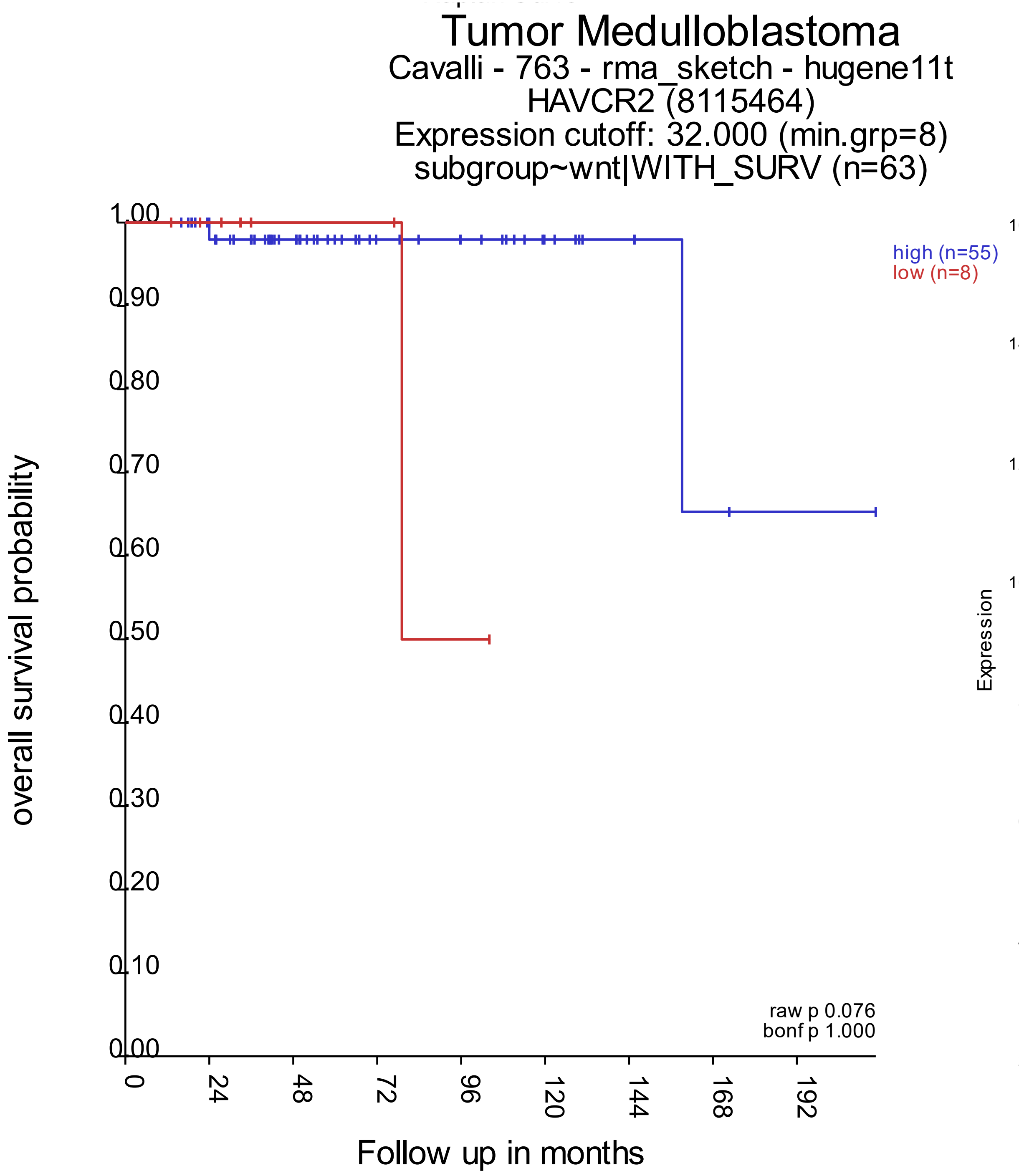

Tumor Medulloblastoma
Cavalli - 763 - rma_sketch - hugene11t
HAVCR2 (8115464)
Expression cutoff: 32.000 (min.grp=8)
subgroup~wnt|WITH_SURV (n=63)
overall survival probability
1.00
0.90
0.80
0.70
0.60
0.50
0.40
0.30
0.20
0.10
0.00
0
24
48
72
96
120
144
168
192
Follow up in months
high (n=55)
low (n=8)
raw p 0.076
bonf p 1.000

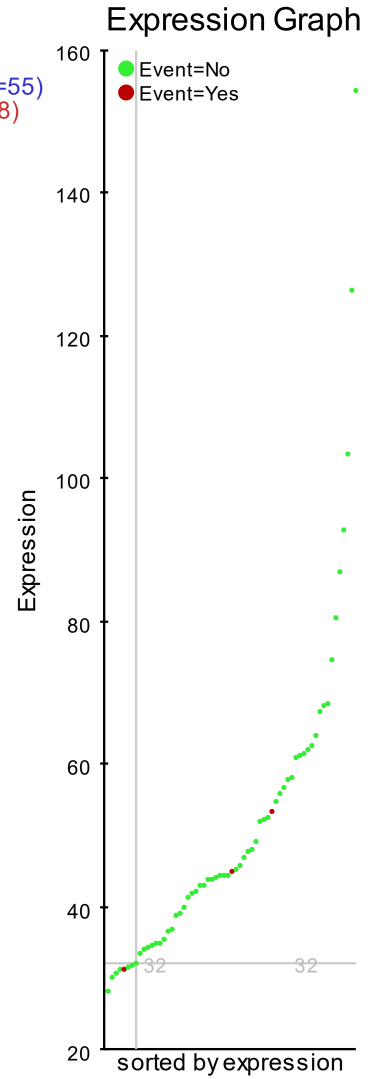

Expression Graph
Event=No
Event=Yes
Expression
160
140
120
100
80
60
40
20
32
32
sorted by expression

SHH

Tumor Medulloblastoma
Cavalli - 763 - rma_sketch - hugene11t
HAVCR2 (8115464)
Expression cutoff: 97.100 (min.grp=8)
subgroup~shh|WITH_SURV (n=172)

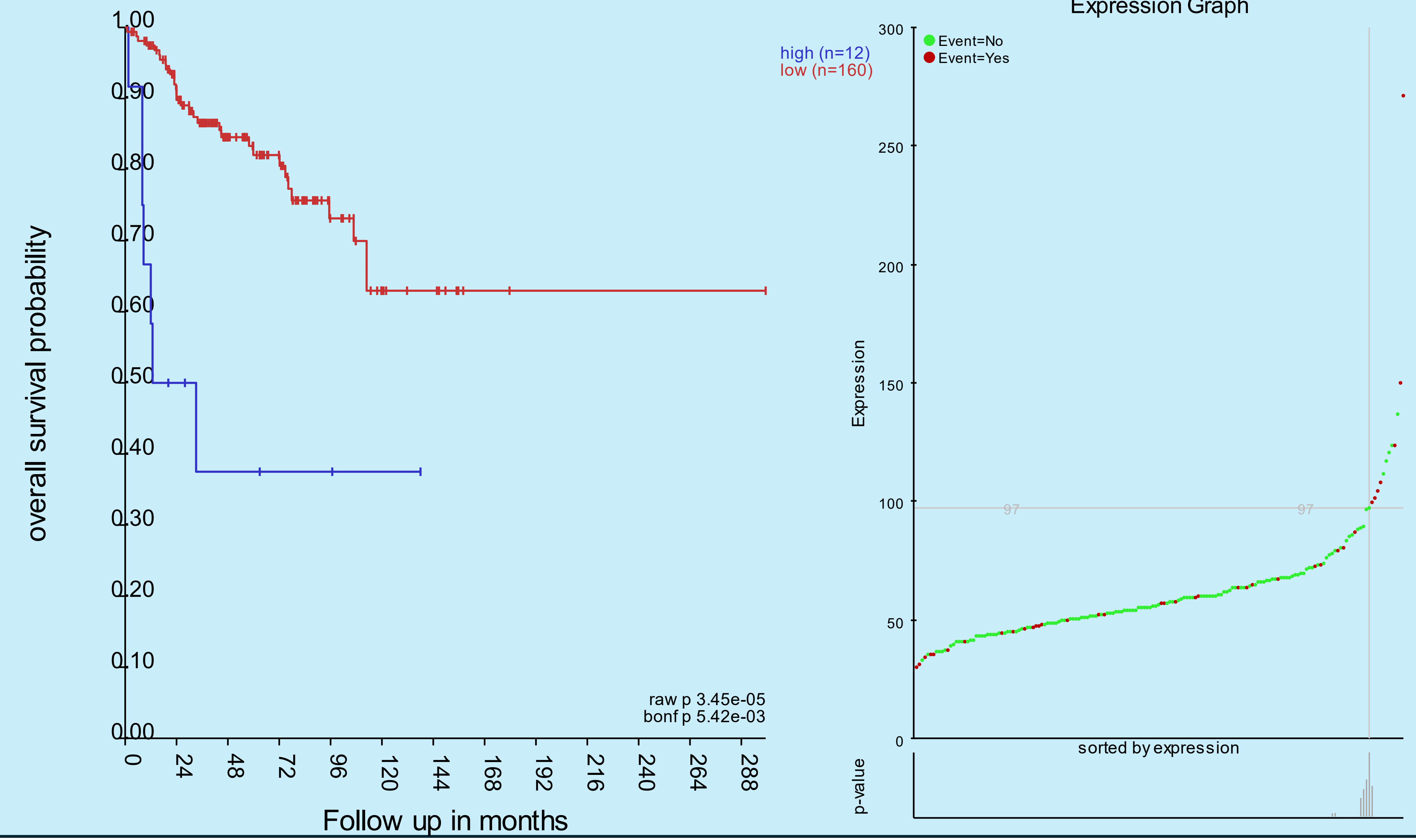

GR4

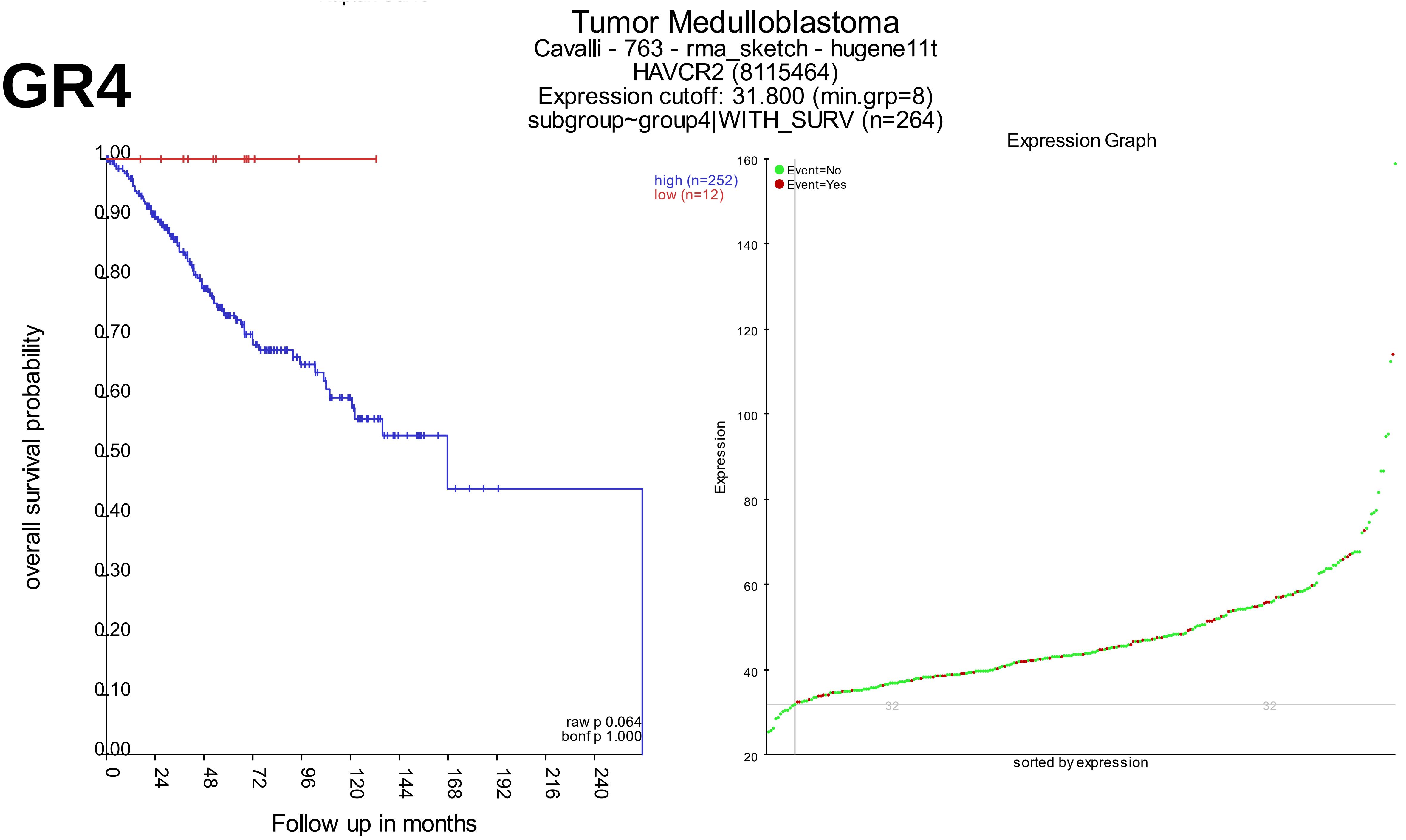

Tumor Medulloblastoma
Cavalli - 763 - rma_sketch - hugene11t
HAVCR2 (8115464)
Expression cutoff: 31.800 (min.grp=8)
subgroup~group4|WITH_SURV (n=264)
high (n=252)
low (n=12)
overall survival probability
1.00
0.90
0.80
0.70
0.60
0.50
0.40
0.30
0.20
0.10
0.00
0
24
48
72
96
120
144
168
192
216
240
Follow up in months
raw p 0.064
bonf p 1.000
Expression Graph
Event=No
Event=Yes
Expression
160
140
120
100
80
60
40
20
32
32
sorted by expression

GR3

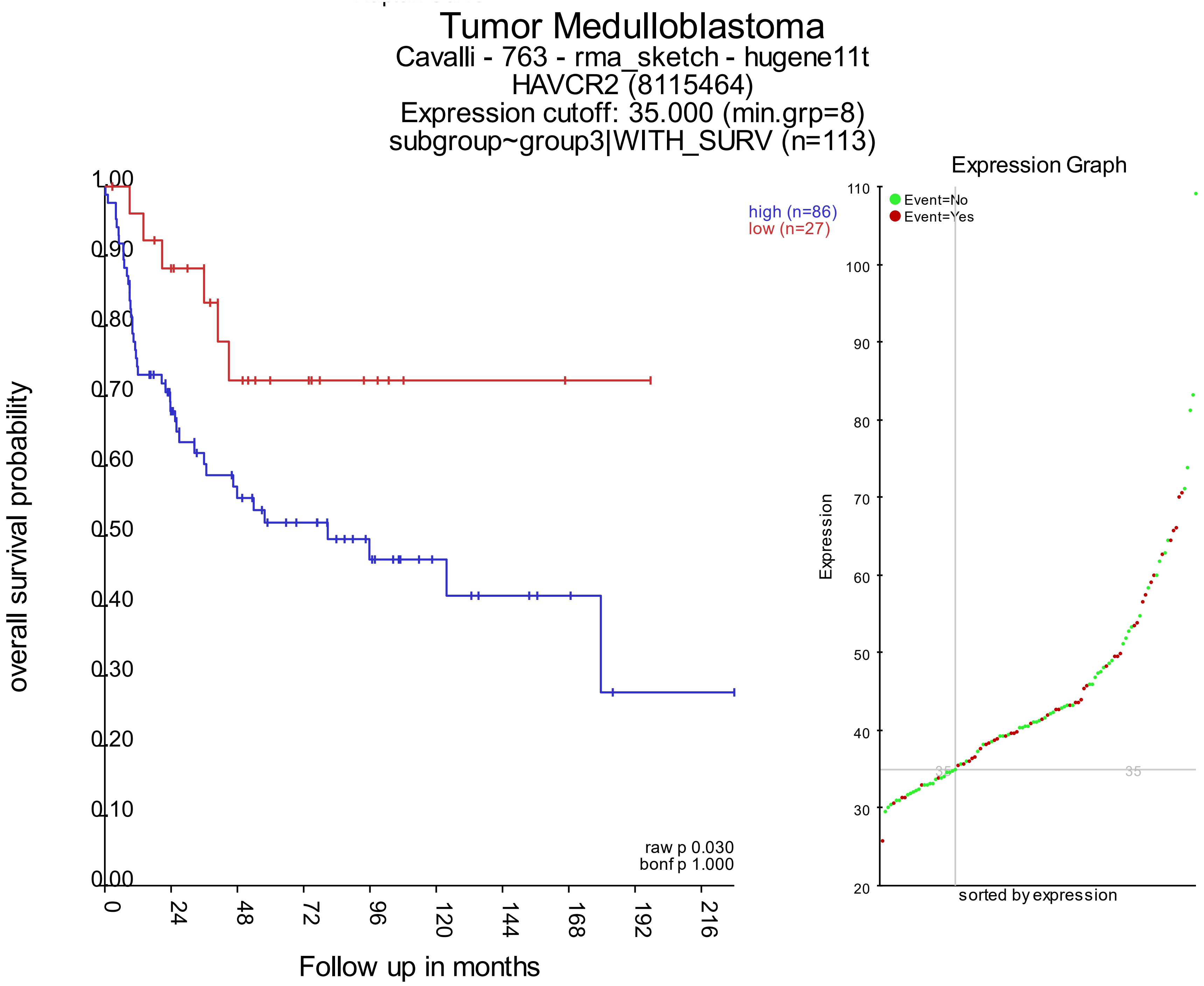

Tumor Medulloblastoma
Cavalli - 763 - rma_sketch - hugene11t
HAVCR2 (8115464)
Expression cutoff: 35.000 (min.grp=8)
subgroup~group3|WITH_SURV (n=113)
overall survival probability
1.00
0.90
0.80
0.70
0.60
0.50
0.40
0.30
0.20
0.10
0.00
0
24
48
72
96
120
144
168
192
216
Follow up in months
high (n=86)
low (n=27)
raw p 0.030
bonf p 1.000
Expression Graph
Event=No
Event=Yes
Expression
110
100
90
80
70
60
50
40
30
20
35
35
sorted by expression

LAG3

WNT

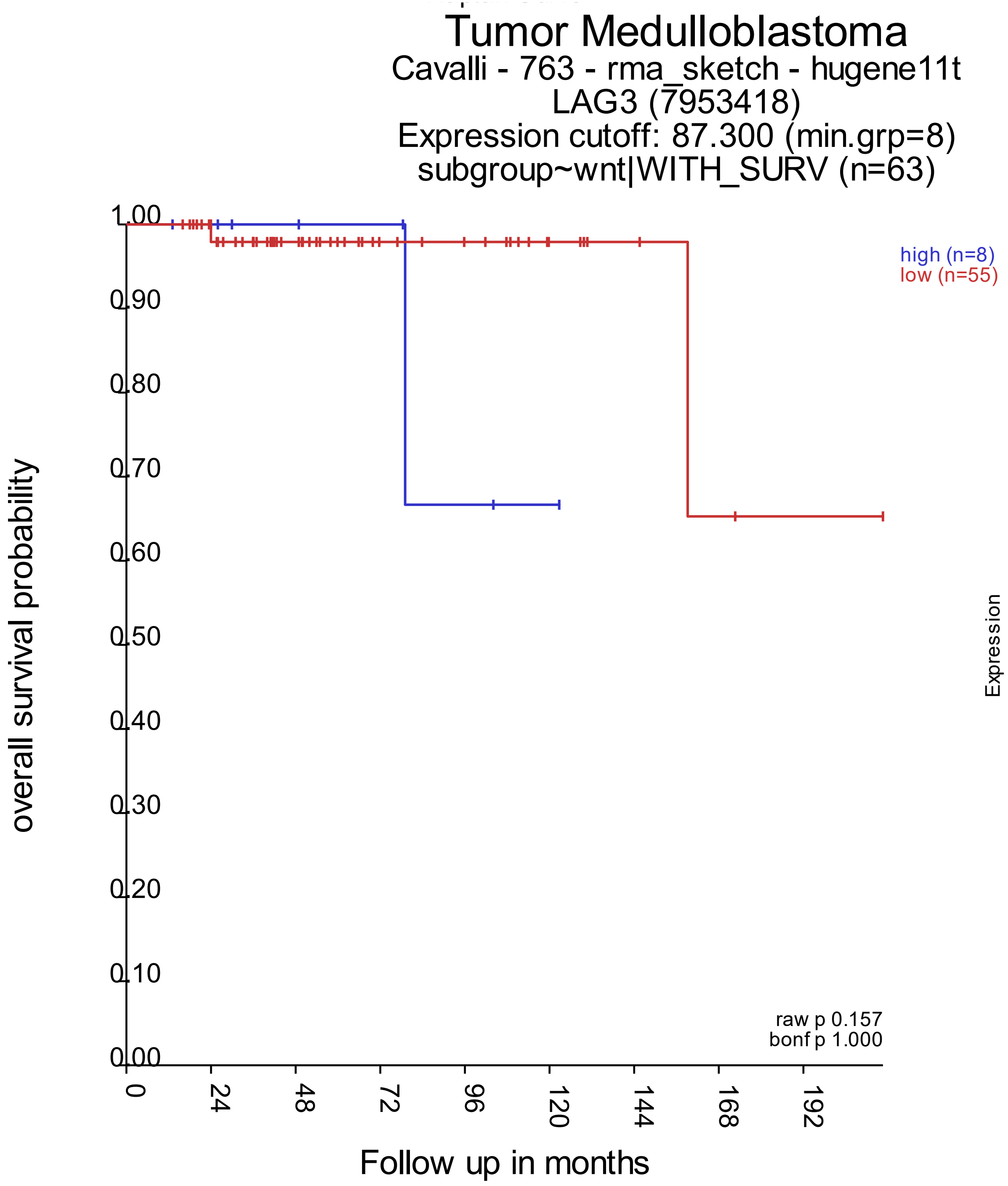

Tumor Medulloblastoma
Cavalli - 763 - rma_sketch - hugene11t
LAG3 (7953418)
Expression cutoff: 87.300 (min.grp=8)
subgroup~wnt|WITH_SURV (n=63)
overall survival probability
1.00
0.90
0.80
0.70
0.60
0.50
0.40
0.30
0.20
0.10
0.00
0
24
48
72
96
120
144
168
192
Follow up in months
high (n=8)
low (n=55)
raw p 0.157
bonf p 1.000

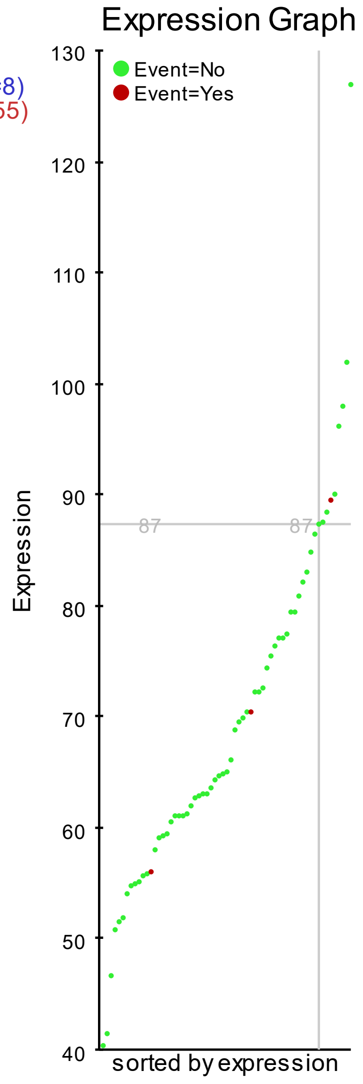

Expression Graph
Event=No
Event=Yes
130
120
110
100
90
80
70
60
50
40
87
87
Expression
sorted by expression

SHH

Tumor Medulloblastoma
Cavalli - 763 - rma_sketch - hugene11t
LAG3 (7953418)
Expression cutoff: 36.100 (min.grp=8)
subgroup~shh|WITH_SURV (n=172)

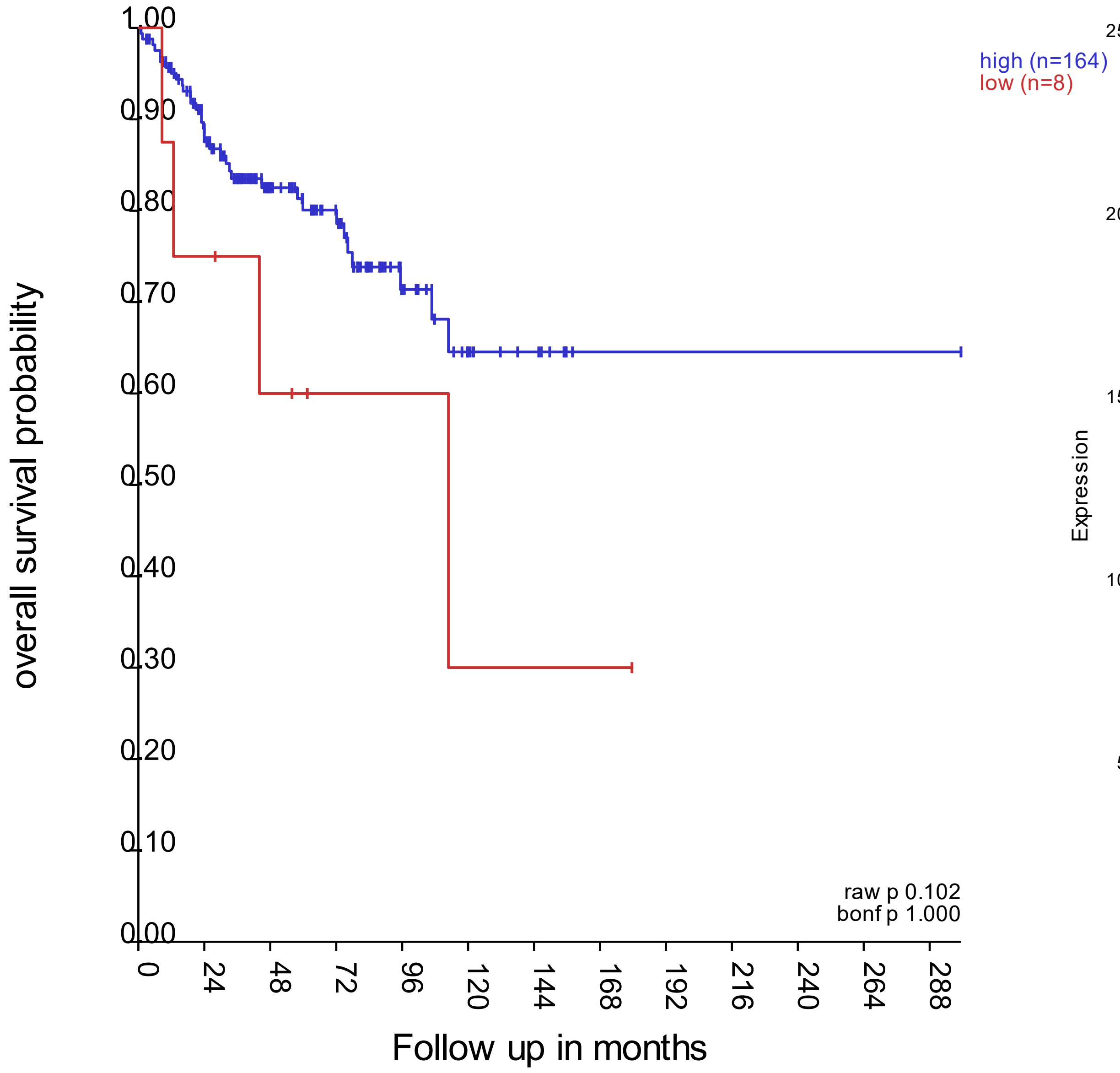

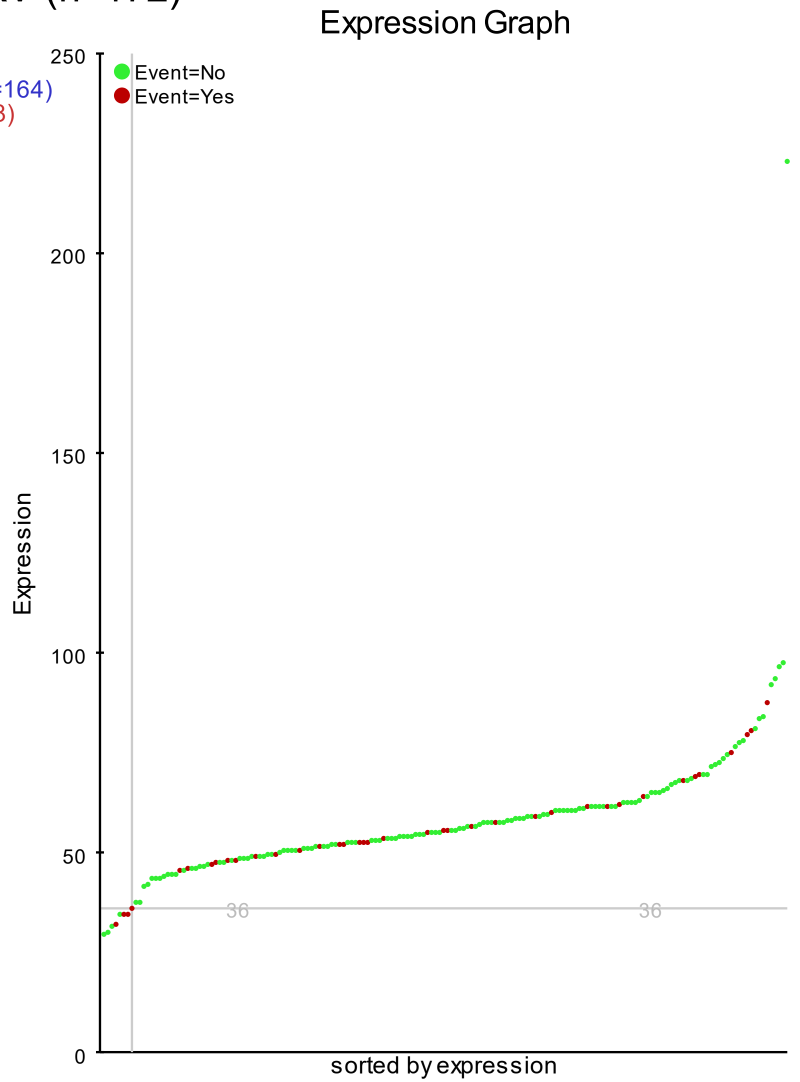

GR4

Tumor Medulloblastoma
Cavalli - 763 - rma_sketch - hugene11t
LAG3 (7953418)
Expression cutoff: 46.800 (min.grp=8)
subgroup~group4|WITH_SURV (n=264)

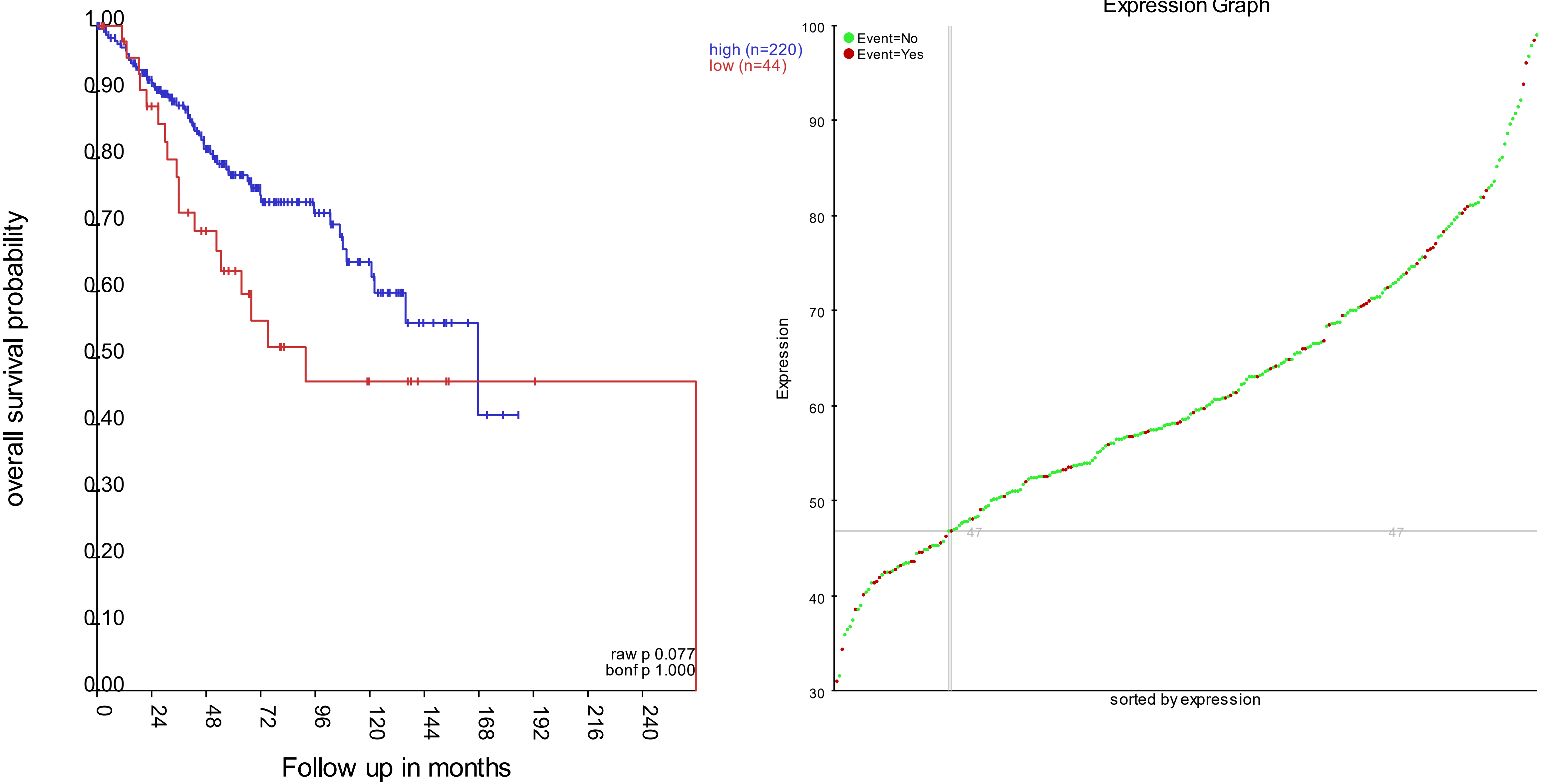

GR3

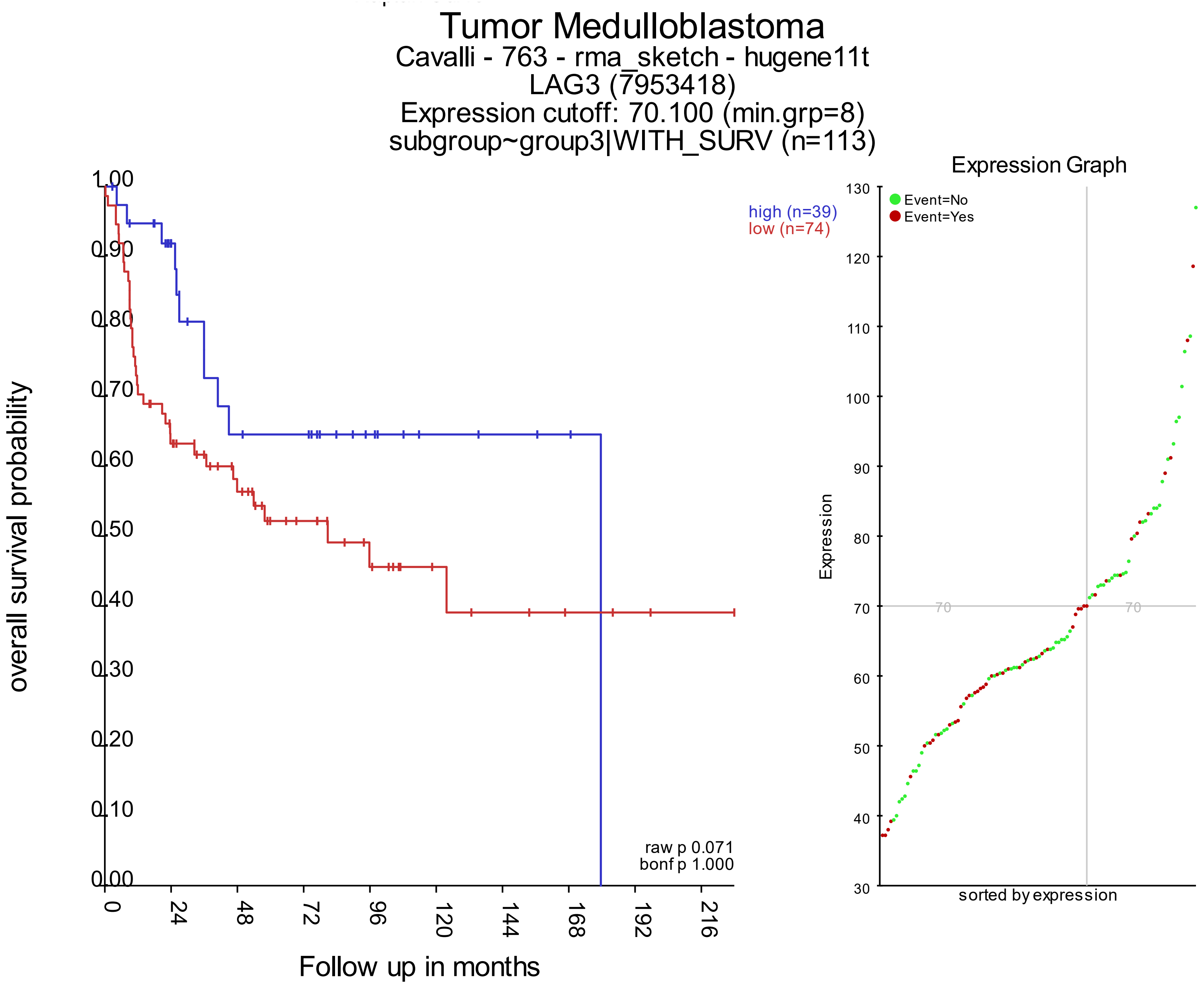
Tumor Medulloblastoma
Cavalli - 763 - rma_sketch - hugene11t
LAG3 (7953418)
Expression cutoff: 70.100 (min.grp=8)
subgroup~group3|WITH_SURV (n=113)
overall survival probability
1.00
0.90
0.80
0.70
0.60
0.50
0.40
0.30
0.20
0.10
0.00
0
24
48
72
96
120
144
168
192
216
Follow up in months
high (n=39)
low (n=74)
raw p 0.071
bonf p 1.000
Expression Graph
Event=No
Event=Yes
Expression
130
120
110
100
90
80
70
60
50
40
30
70
70
sorted by expression

IDH1

WNT

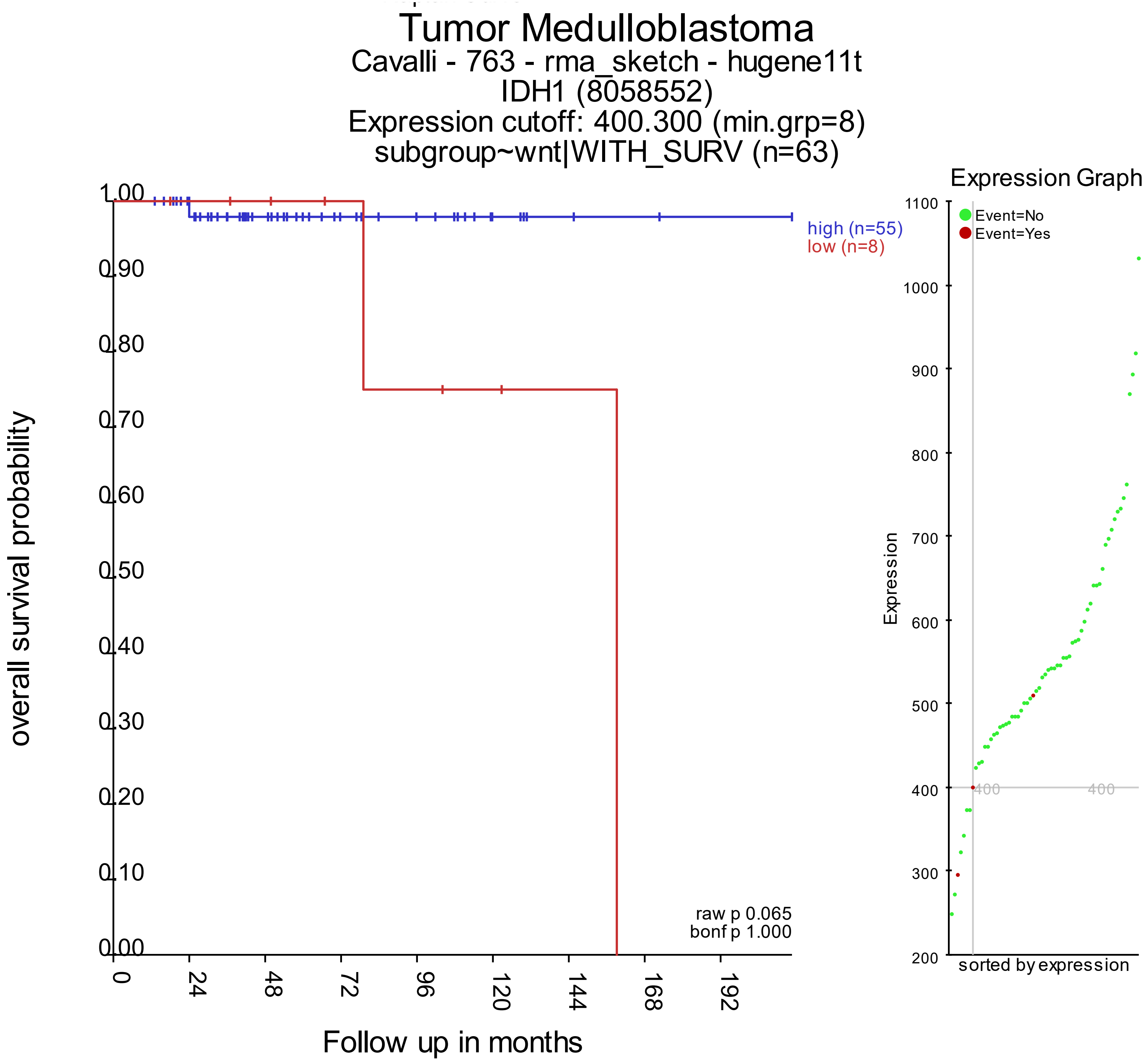

Tumor Medulloblastoma
Cavalli - 763 - rma_sketch - hugene11t
IDH1 (8058552)
Expression cutoff: 400.300 (min.grp=8)
subgroup~wnt|WITH_SURV (n=63)
overall survival probability
1.00
0.90
0.80
0.70
0.60
0.50
0.40
0.30
0.20
0.10
0.00
0
24
48
72
96
120
144
168
192
Follow up in months
high (n=55)
low (n=8)
raw p 0.065
bonf p 1.000
Expression Graph
Event=No
Event=Yes
Expression
1100
1000
900
800
700
600
500
400
300
200
400
400
sorted by expression

SHH

Tumor Medulloblastoma
Cavalli - 763 - rma_sketch - hugene11t
IDH1 (8058552)
Expression cutoff: 354.900 (min.grp=8)
subgroup~shh|WITH_SURV (n=172)

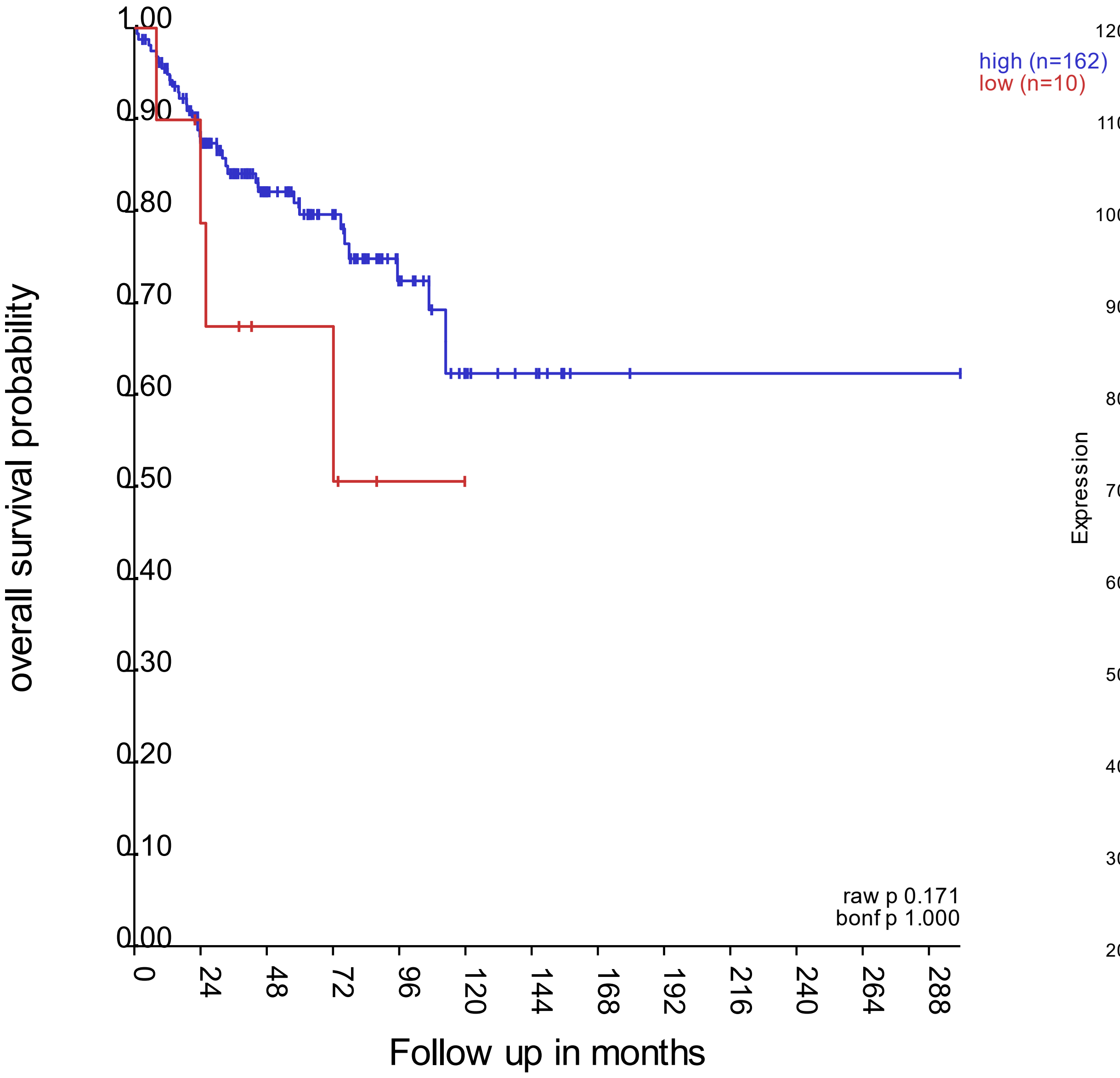

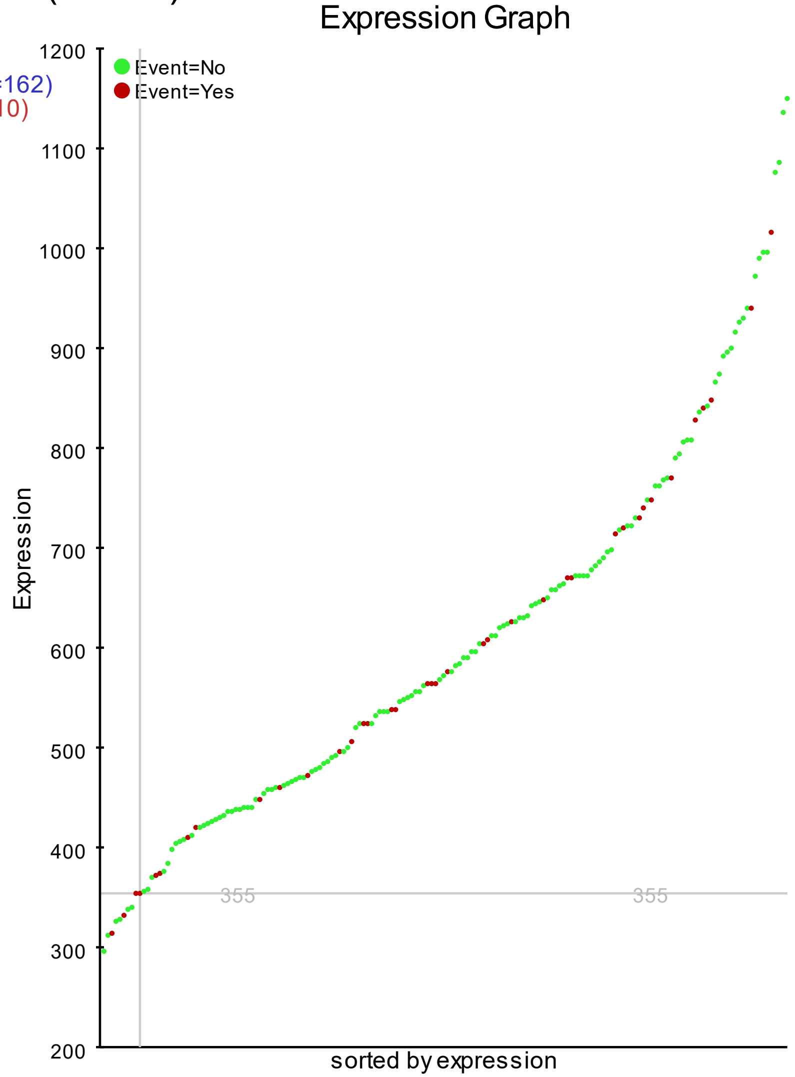

GR4

Tumor Medulloblastoma
Cavalli - 763 - rma_sketch - hugene11t
IDH1 (8058552)
Expression cutoff: 325.300 (min.grp=8)
subgroup~group4|WITH_SURV (n=264)

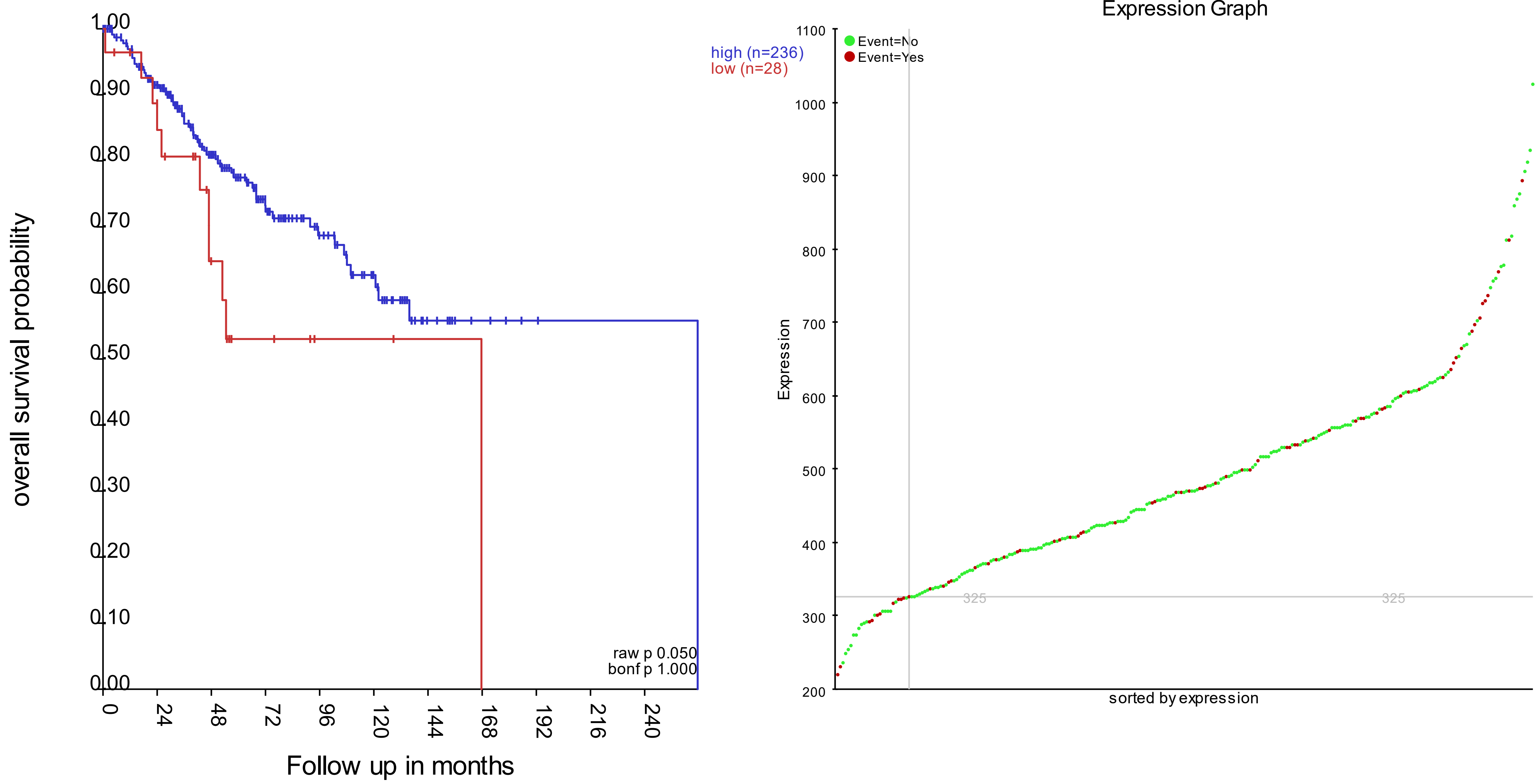

GR3

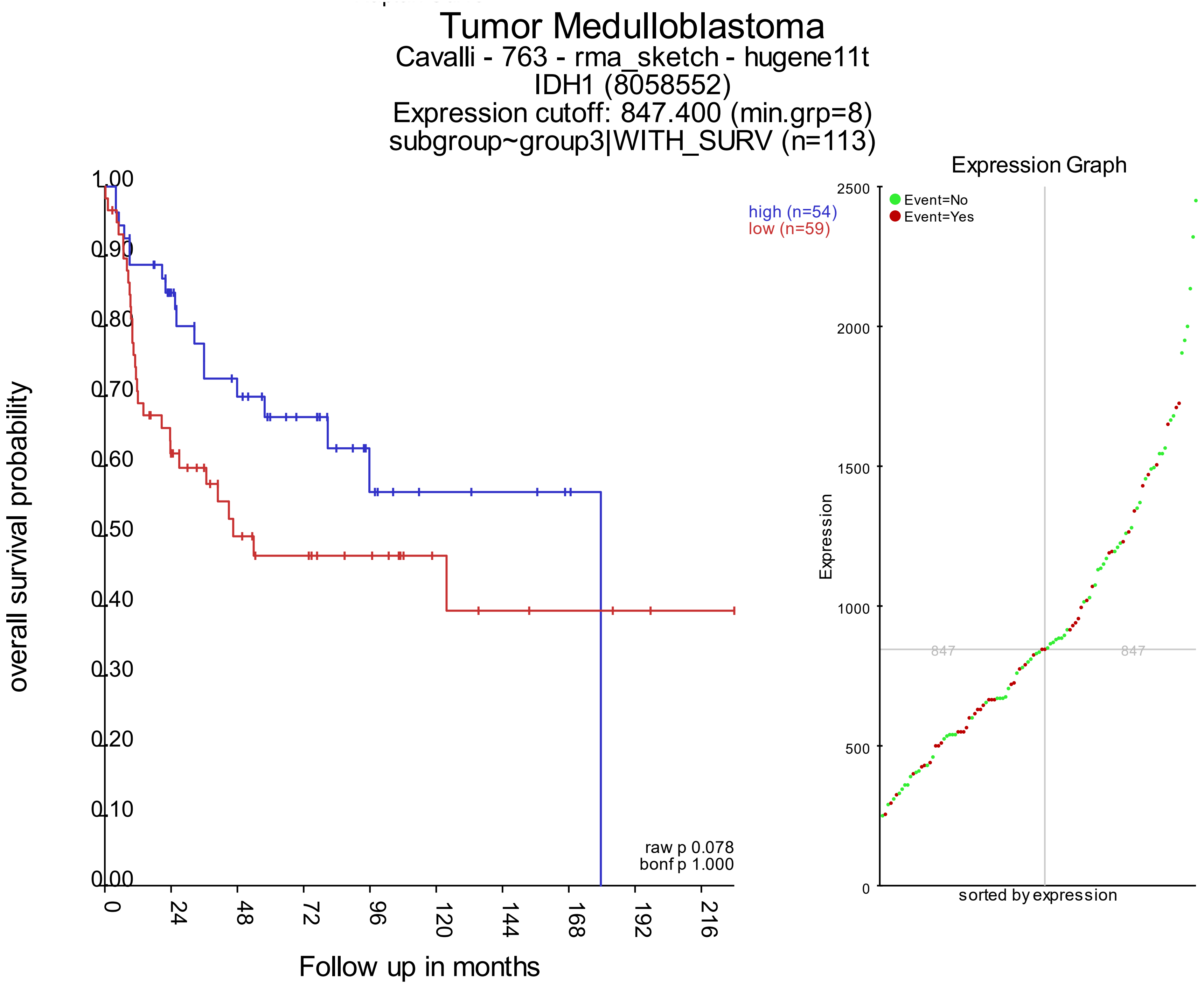

Tumor Medulloblastoma
Cavalli - 763 - rma_sketch - hugene11t
IDH1 (8058552)
Expression cutoff: 847.400 (min.grp=8)
subgroup~group3|WITH_SURV (n=113)
high (n=54)
low (n=59)
overall survival probability
1.00
0.90
0.80
0.70
0.60
0.50
0.40
0.30
0.20
0.10
0.00
0
24
48
72
96
120
144
168
192
216
Follow up in months
raw p 0.078
bonf p 1.000
Expression Graph
Event=No
Event=Yes
Expression
2500
2000
1500
1000
500
0
847
847
sorted by expression

JAK1

WNT

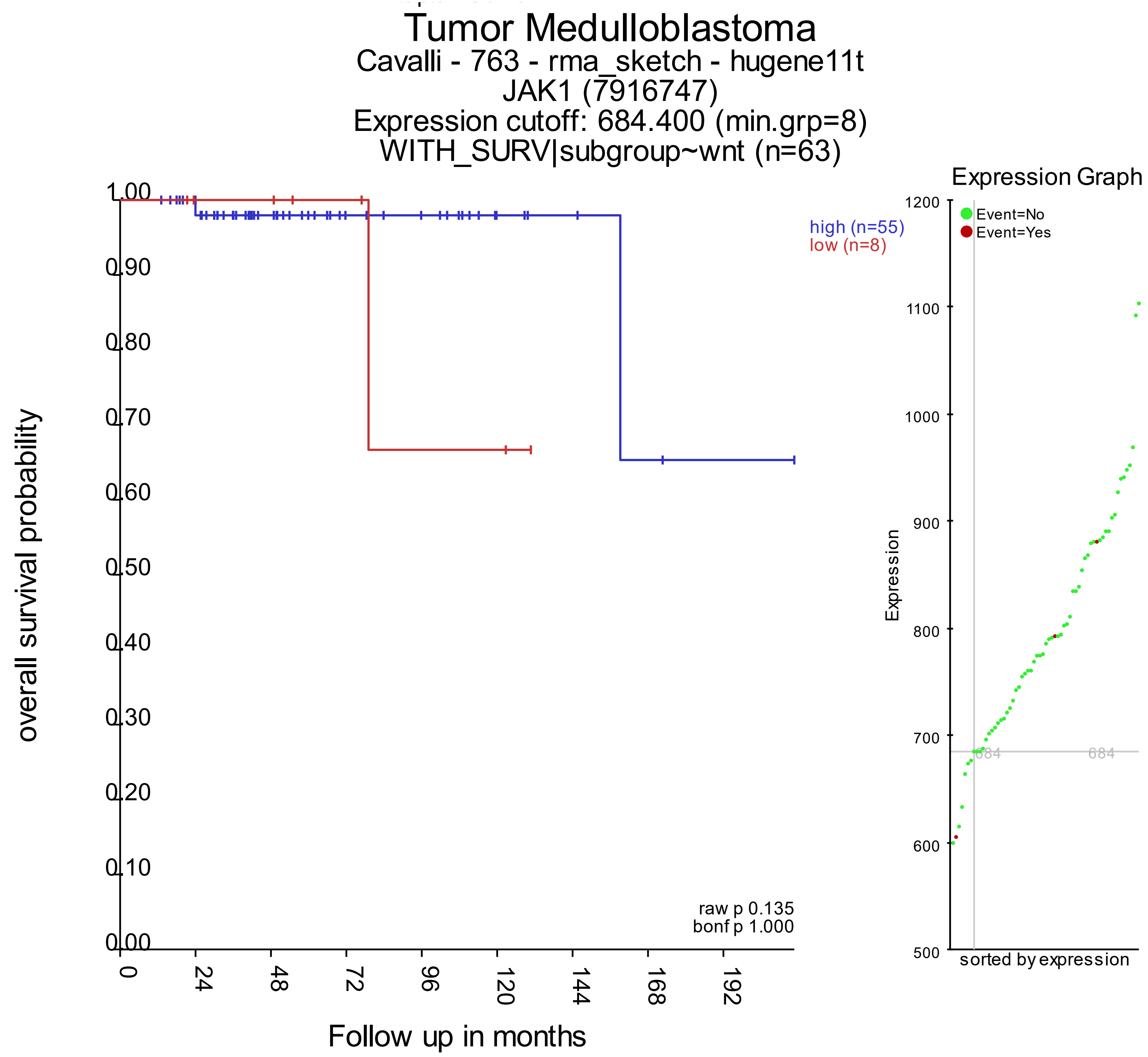
Tumor Medulloblastoma
Cavalli - 763 - rma_sketch - hugene11t
JAK1 (7916747)
Expression cutoff: 684.400 (min.grp=8)
WITH_SURV|subgroup~wnt (n=63)
overall survival probability
1.00
0.90
0.80
0.70
0.60
0.50
0.40
0.30
0.20
0.10
0.00
0
24
48
72
96
120
144
168
192
Follow up in months
high (n=55)
low (n=8)
raw p 0.135
bonf p 1.000
Expression Graph
Event=No
Event=Yes
Expression
1200
1100
1000
900
800
700
600
500
684
684
sorted by expression

SHH

Tumor Medulloblastoma
Cavalli - 763 - rma_sketch - hugene11t
JAK1 (7916747)
Expression cutoff: 597.900 (min.grp=8)
subgroup~shh|WITH_SURV (n=172)

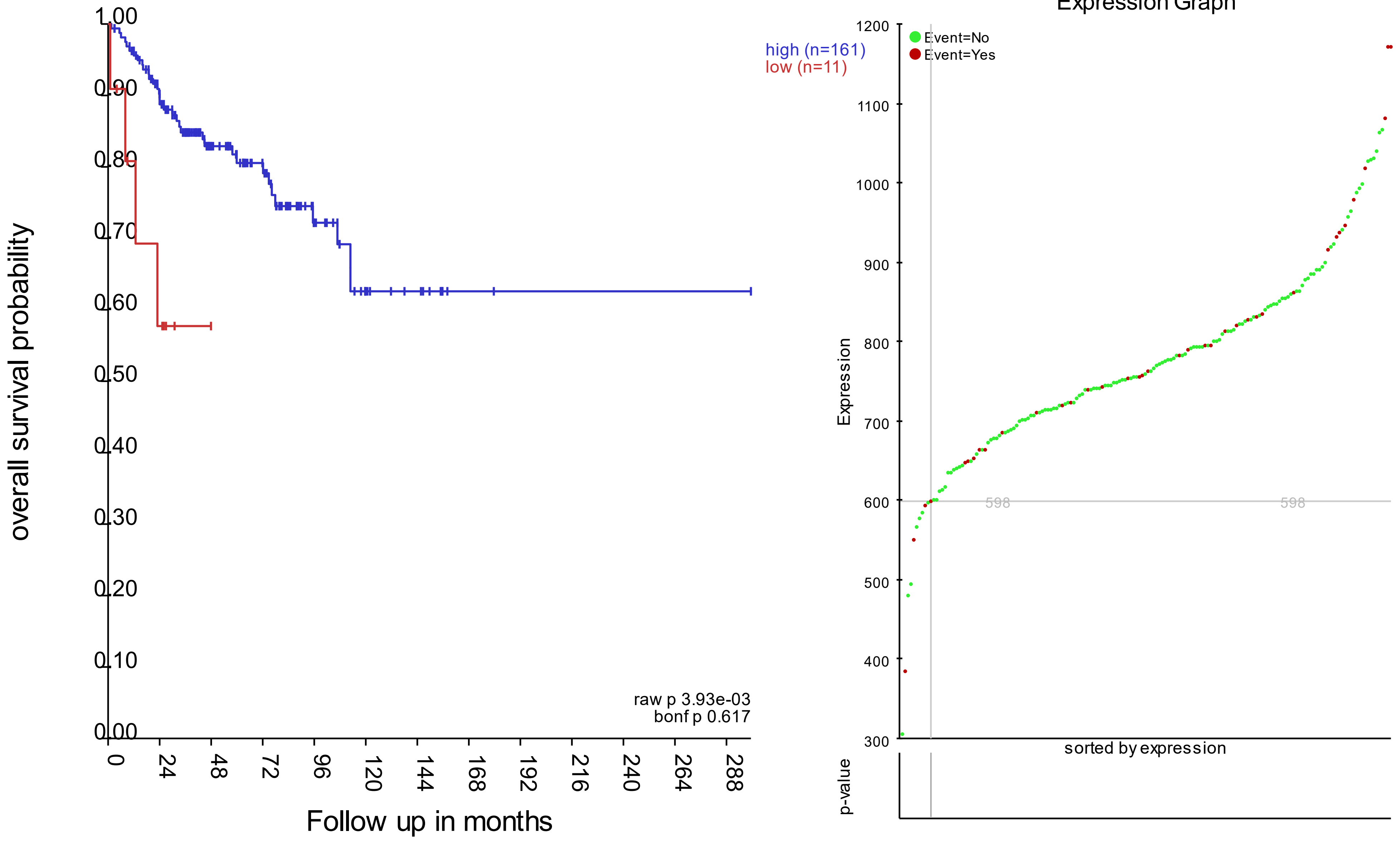

GR4

Tumor Medulloblastoma
Cavalli - 763 - rma_sketch - hugene11t
JAK1 (7916747)
Expression cutoff: 706.600 (min.grp=8)
subgroup~group4|WITH_SURV (n=264)

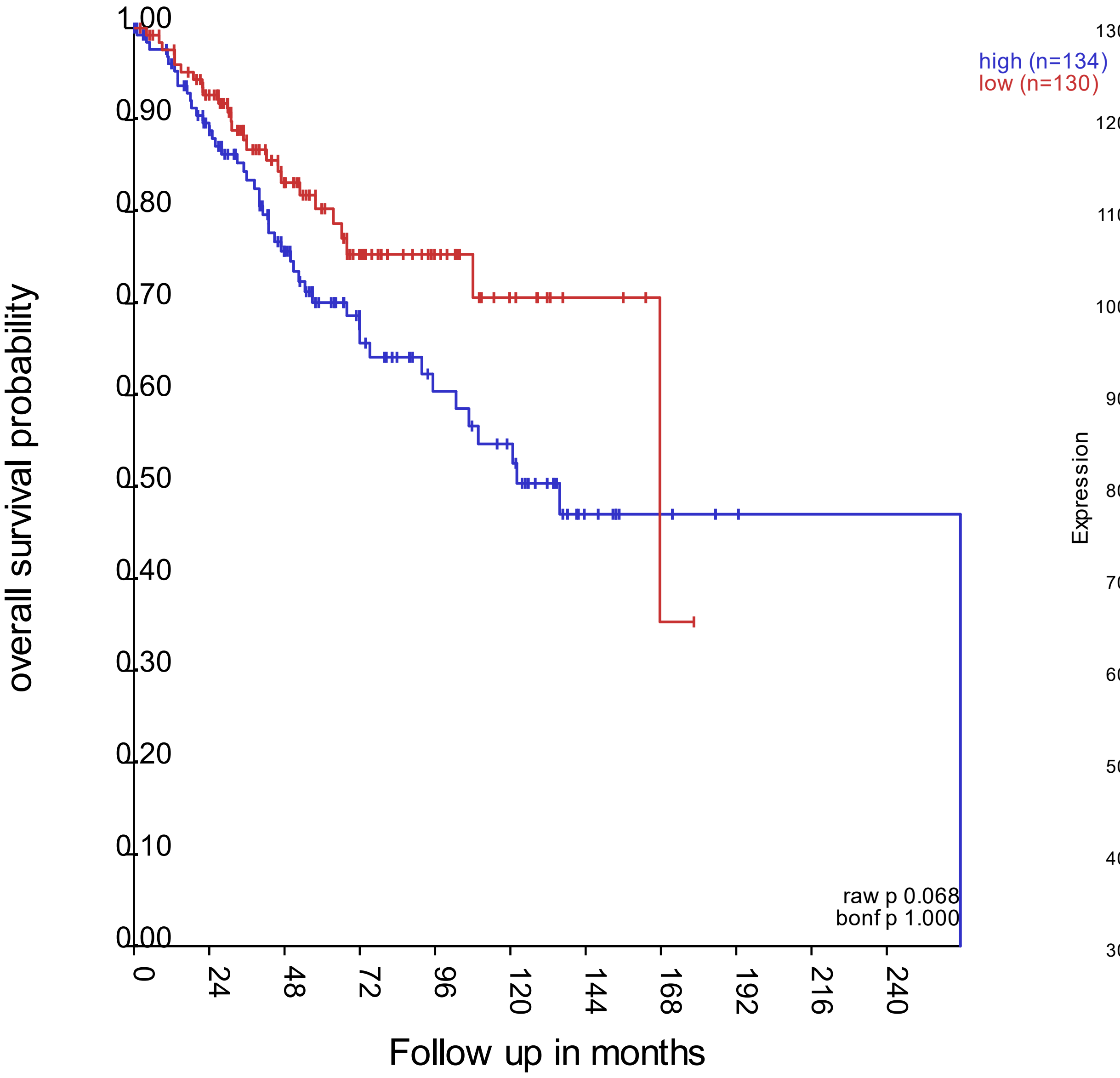

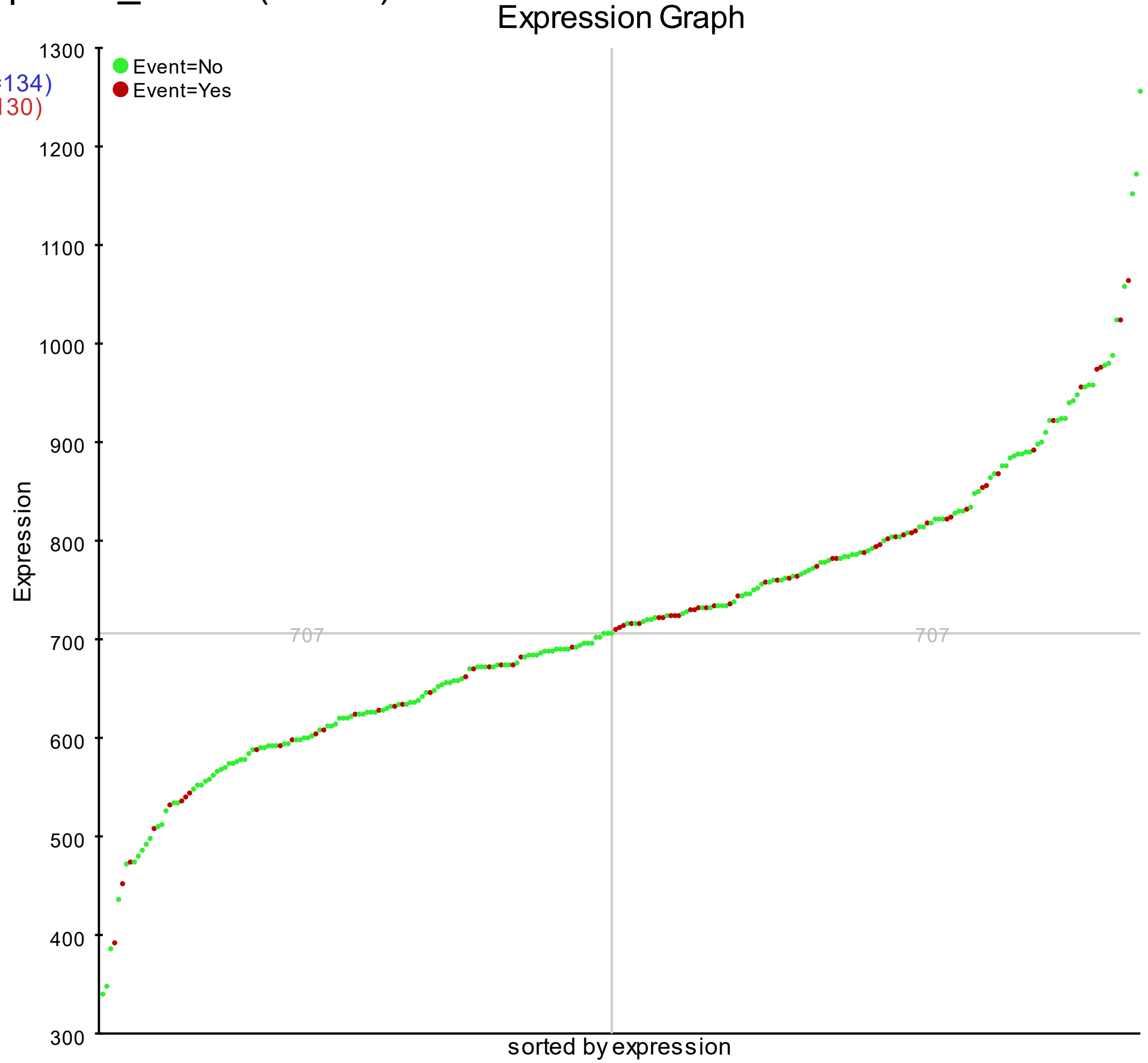

GR3

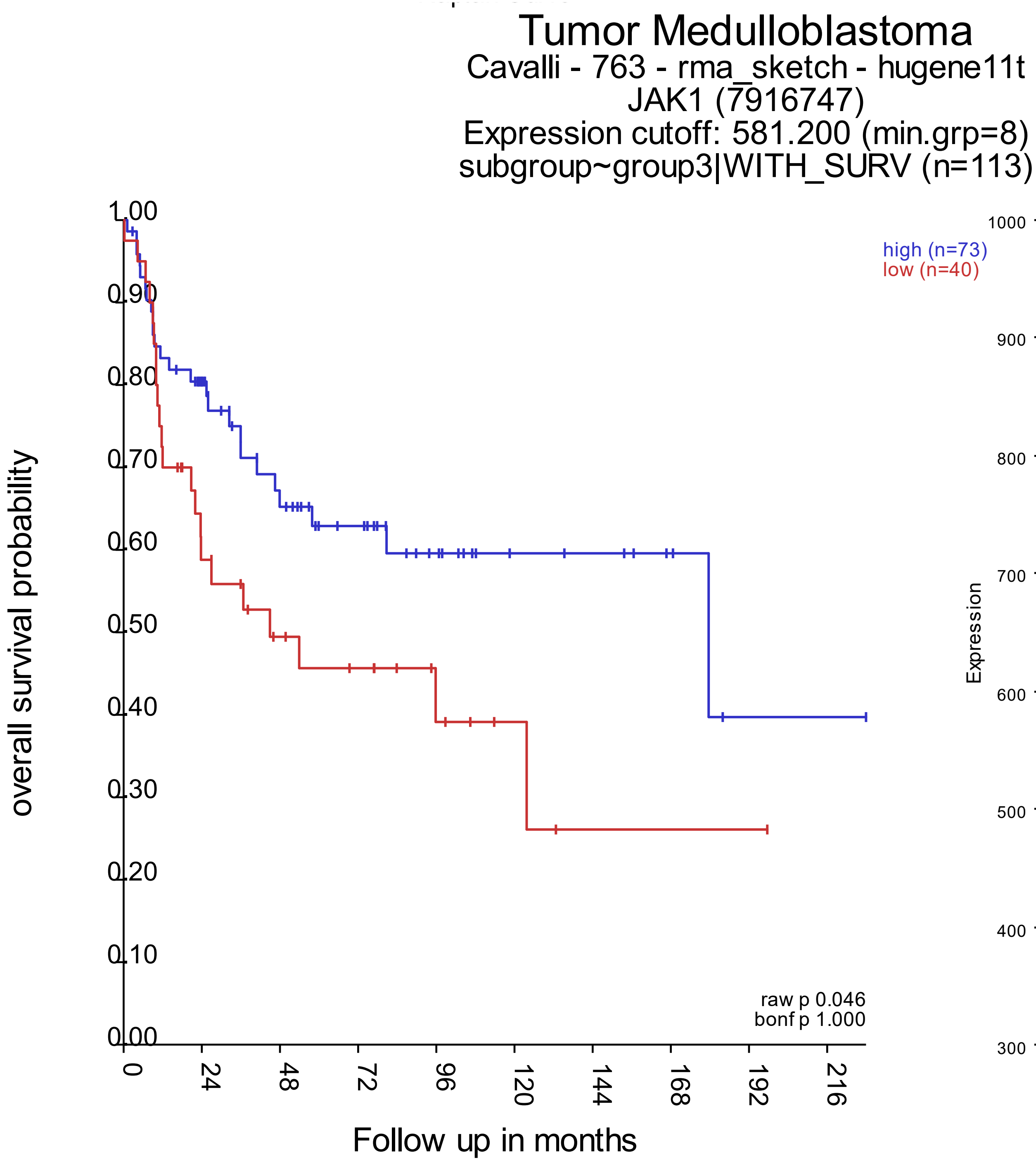
Tumor Medulloblastoma
Cavalli - 763 - rma_sketch - hugene11t
JAK1 (7916747)
Expression cutoff: 581.200 (min.grp=8)
subgroup~group3|WITH_SURV (n=113)
high (n=73)
low (n=40)
overall survival probability
1.00
0.90
0.80
0.70
0.60
0.50
0.40
0.30
0.20
0.10
0.00
0
24
48
72
96
120
144
168
192
216
Follow up in months
raw p 0.046
bonf p 1.000

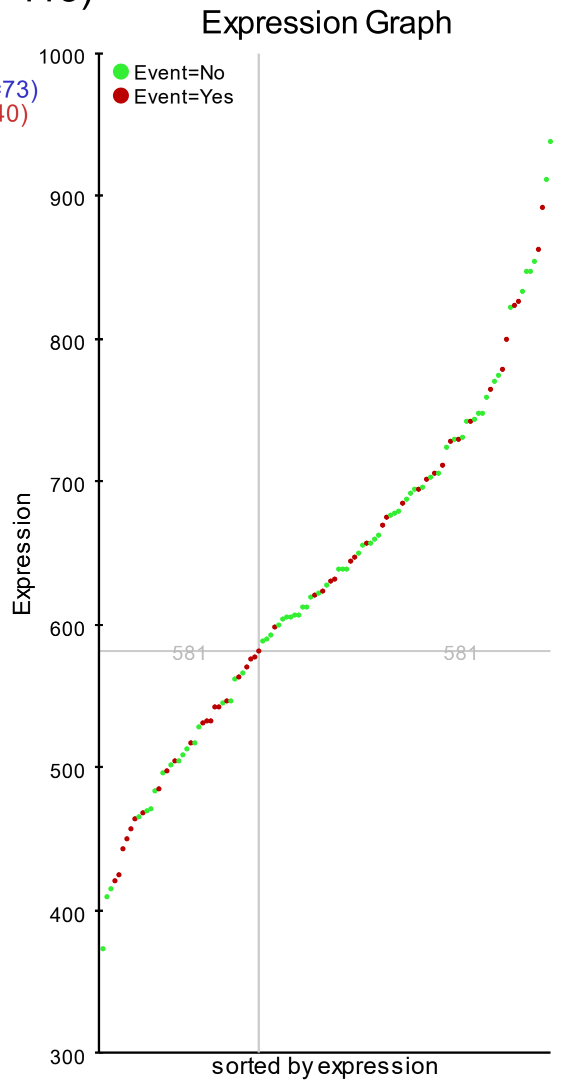
Expression Graph
Event=No
Event=Yes
1000
900
800
700
600
500
400
300
Expression
581
581
sorted by expression

JAK2

WNT

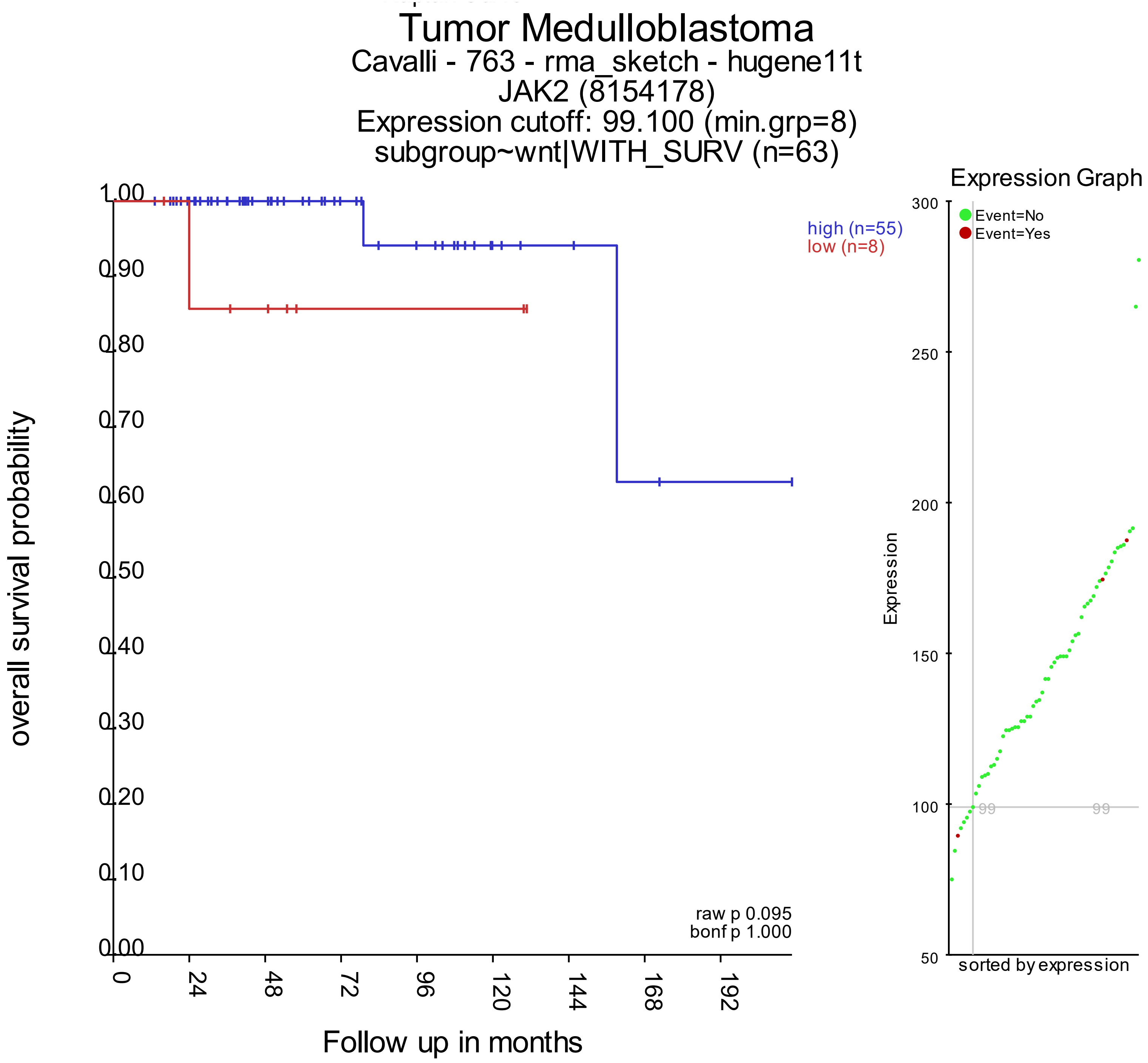

Tumor Medulloblastoma
Cavalli - 763 - rma_sketch - hugene11t
JAK2 (8154178)
Expression cutoff: 99.100 (min.grp=8)
subgroup~wnt|WITH_SURV (n=63)
overall survival probability
1.00
0.90
0.80
0.70
0.60
0.50
0.40
0.30
0.20
0.10
0.00
0
24
48
72
96
120
144
168
192
Follow up in months
high (n=55)
low (n=8)
raw p 0.095
bonf p 1.000
Expression Graph
Event=No
Event=Yes
Expression
300
250
200
150
100
50
99
99
sorted by expression

SHH

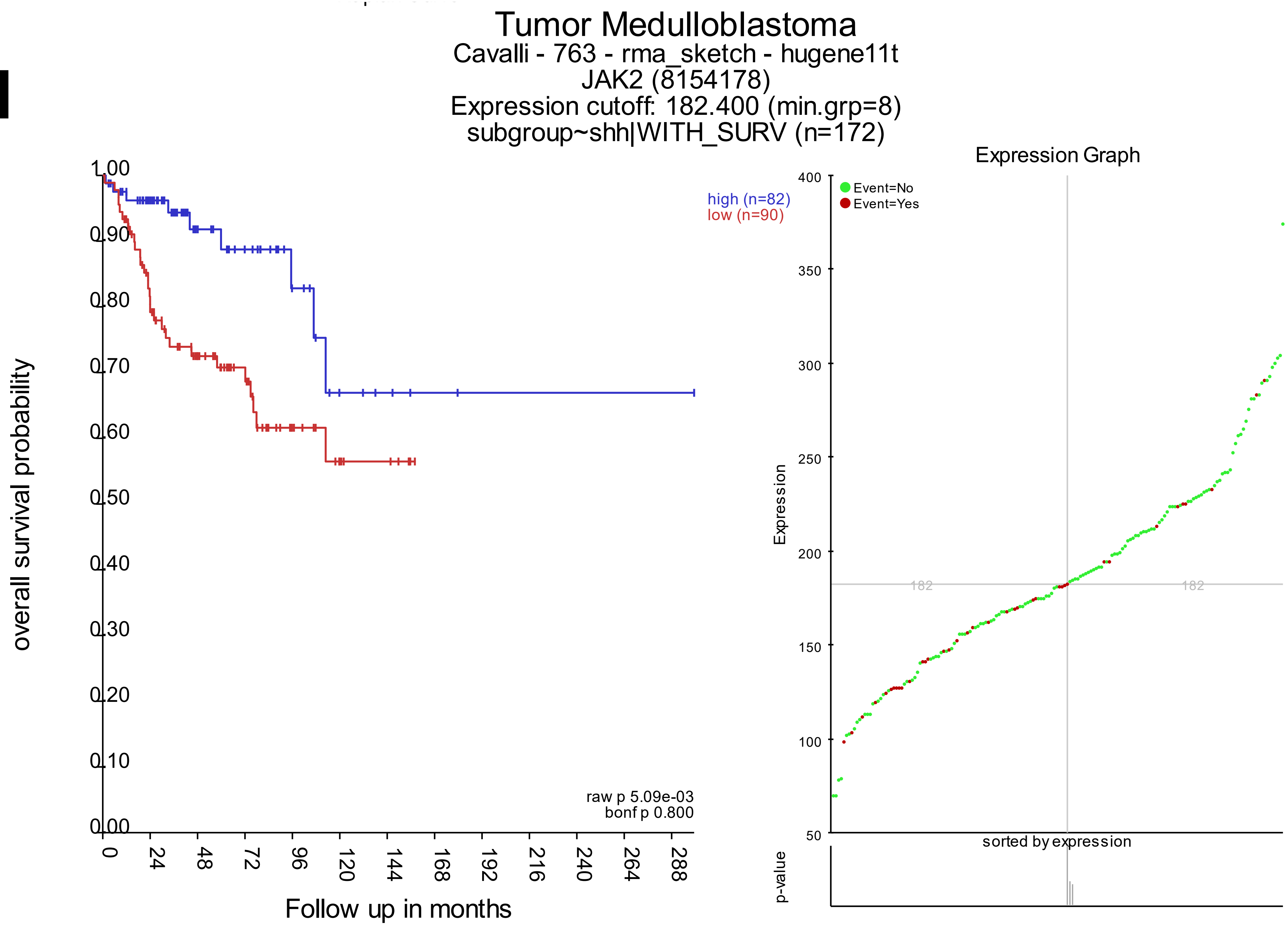

Tumor Medulloblastoma
Cavalli - 763 - rma_sketch - hugene11t
JAK2 (8154178)
Expression cutoff: 182.400 (min.grp=8)
subgroup~shh|WITH_SURV (n=172)
overall survival probability
1.00
0.90
0.80
0.70
0.60
0.50
0.40
0.30
0.20
0.10
0.00
high (n=82)
low (n=90)
raw p 5.09e-03
bonf p 0.800
0
24
48
72
96
120
144
168
192
216
240
264
288
Follow up in months
Expression Graph
Event=No
Event=Yes
Expression
400
350
300
250
200
150
100
50
182
182
sorted by expression
p-value

GR4

Tumor Medulloblastoma
Cavalli - 763 - rma_sketch - hugene11t
JAK2 (8154178)
Expression cutoff: 296.400 (min.grp=8)
subgroup~group4|WITH_SURV (n=264)

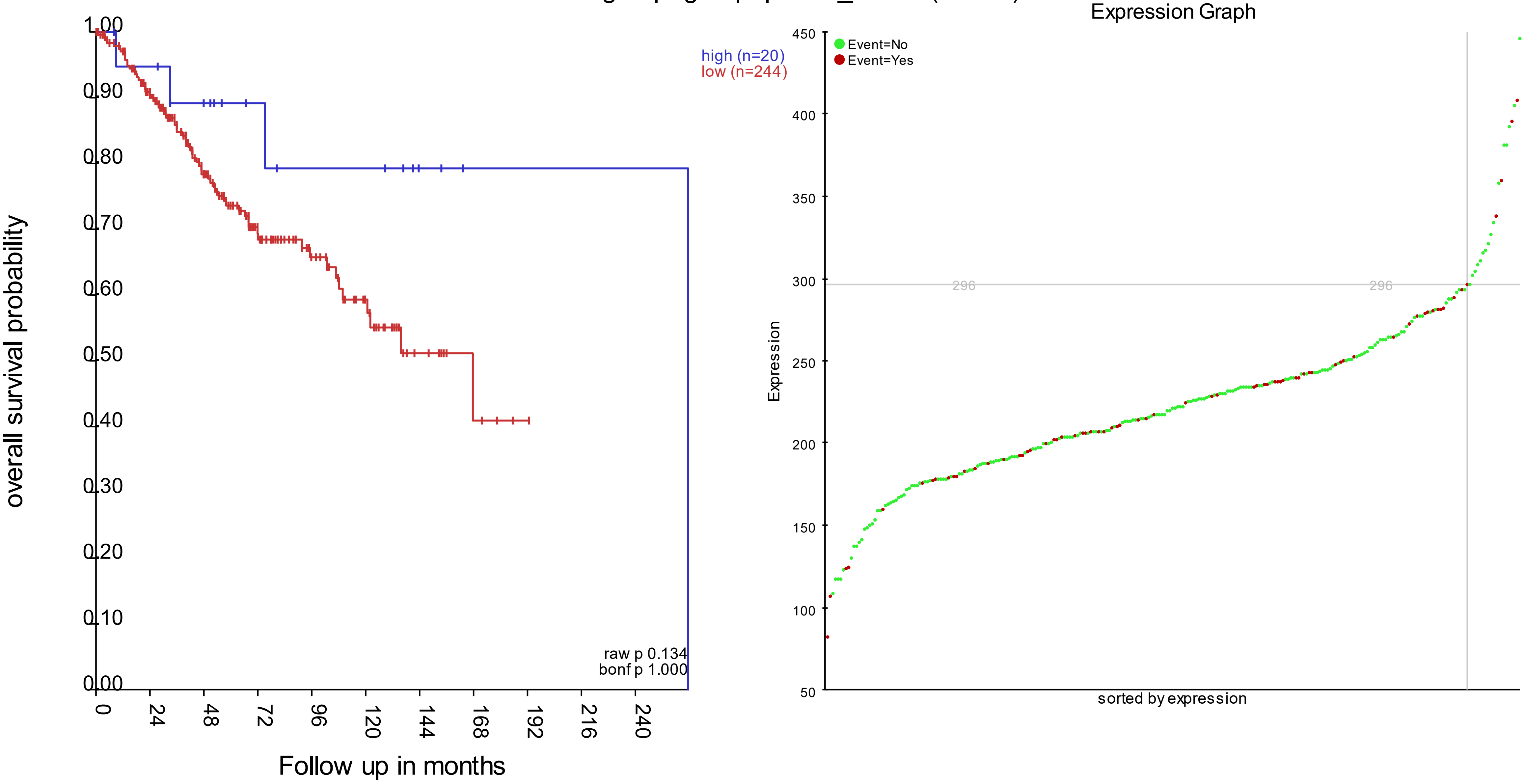

GR3

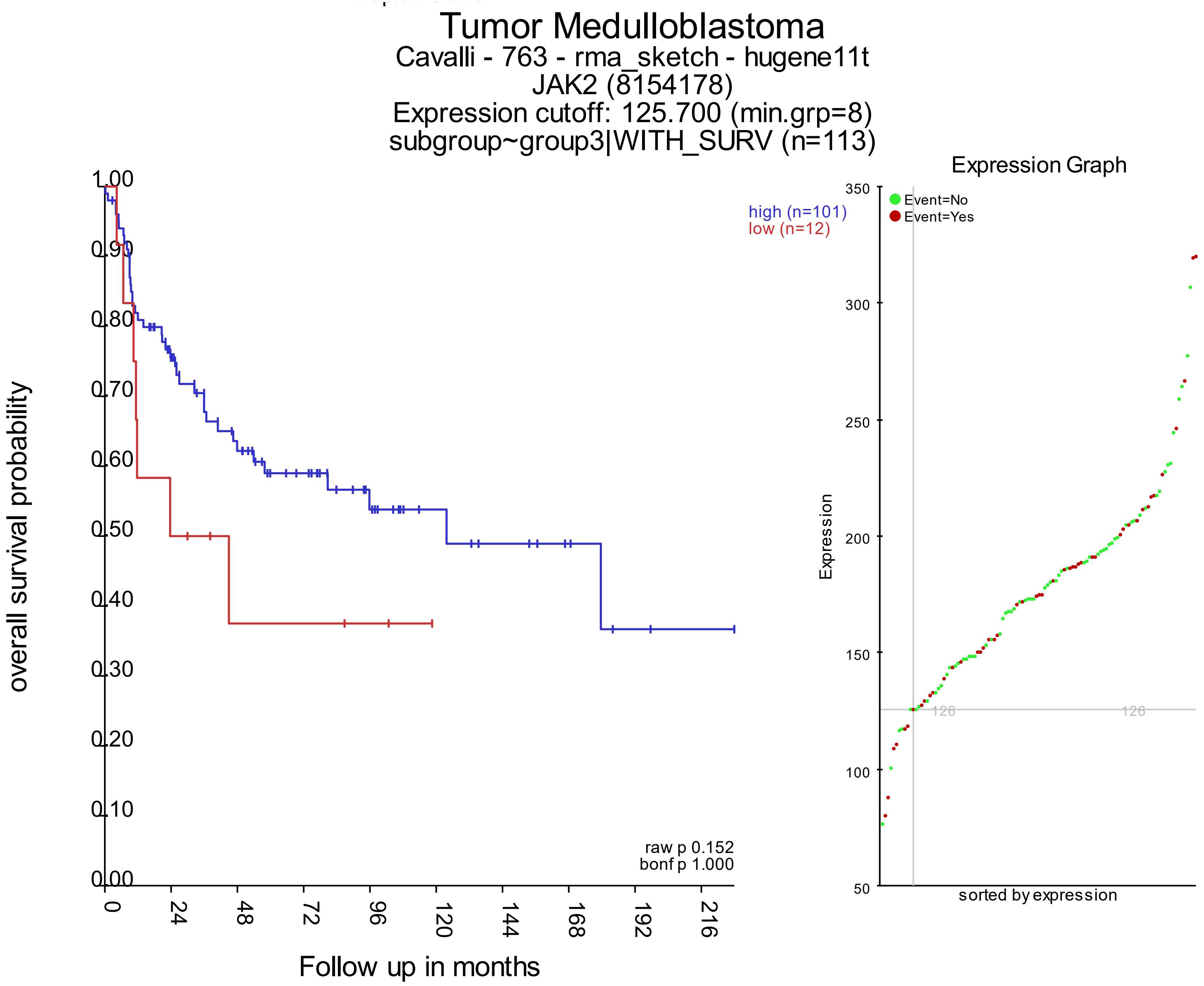

Tumor Medulloblastoma
Cavalli - 763 - rma_sketch - hugene11t
JAK2 (8154178)
Expression cutoff: 125.700 (min.grp=8)
subgroup~group3|WITH_SURV (n=113)
overall survival probability
1.00
0.90
0.80
0.70
0.60
0.50
0.40
0.30
0.20
0.10
0.00
high (n=101)
low (n=12)
raw p 0.152
bonf p 1.000
0
24
48
72
96
120
144
168
192
216
Follow up in months
Expression Graph
Event=No
Event=Yes
Expression
350
300
250
200
150
100
50
126
126
sorted by expression

JAK3

WNT

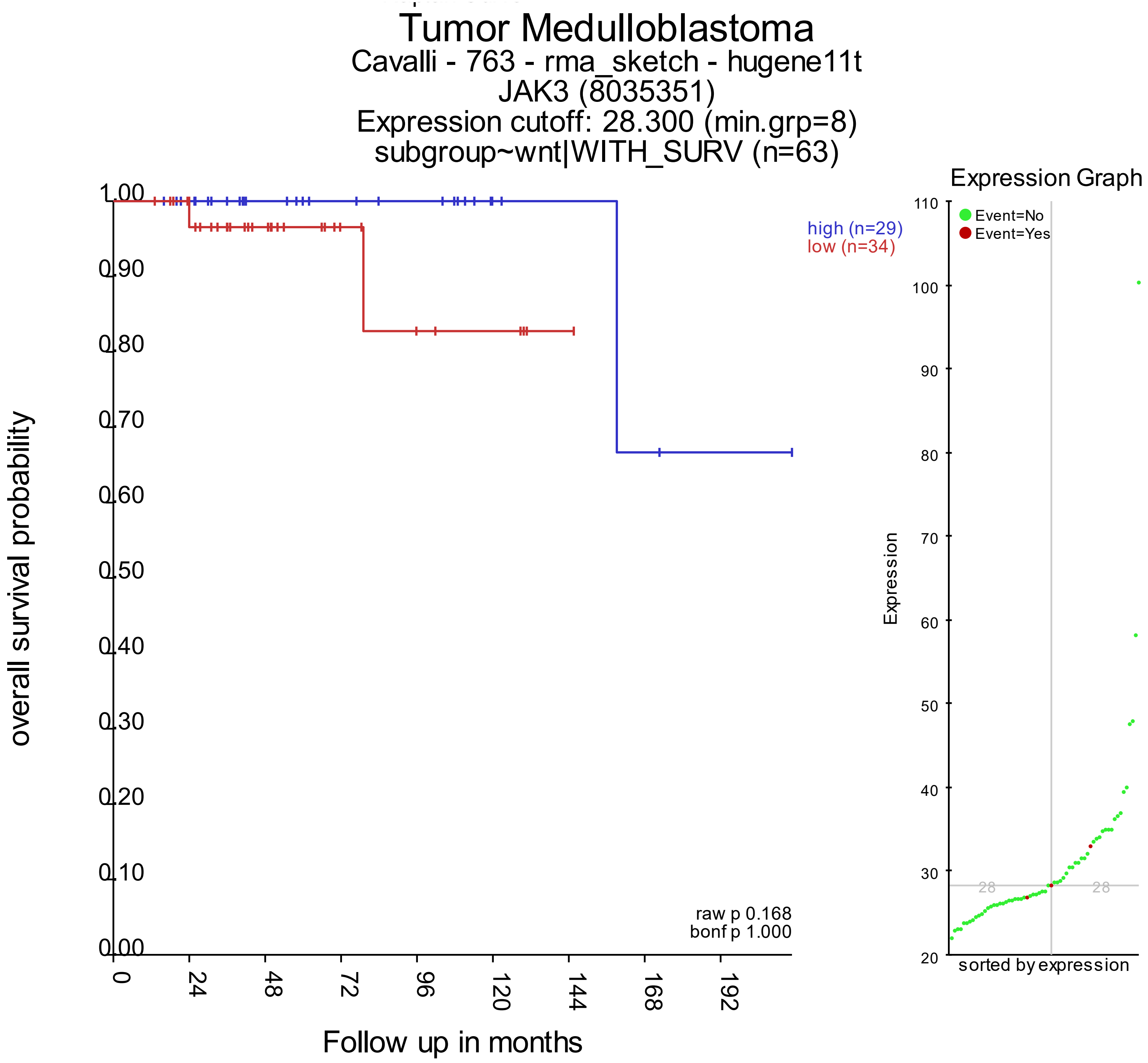
Tumor Medulloblastoma
Cavalli - 763 - rma_sketch - hugene11t
JAK3 (8035351)
Expression cutoff: 28.300 (min.grp=8)
subgroup~wnt|WITH_SURV (n=63)
overall survival probability
1.00
0.90
0.80
0.70
0.60
0.50
0.40
0.30
0.20
0.10
0.00
0
24
48
72
96
120
144
168
192
Follow up in months
high (n=29)
low (n=34)
raw p 0.168
bonf p 1.000
Expression Graph
Event=No
Event=Yes
Expression
110
100
90
80
70
60
50
40
30
20
28
28
sorted by expression

SHH

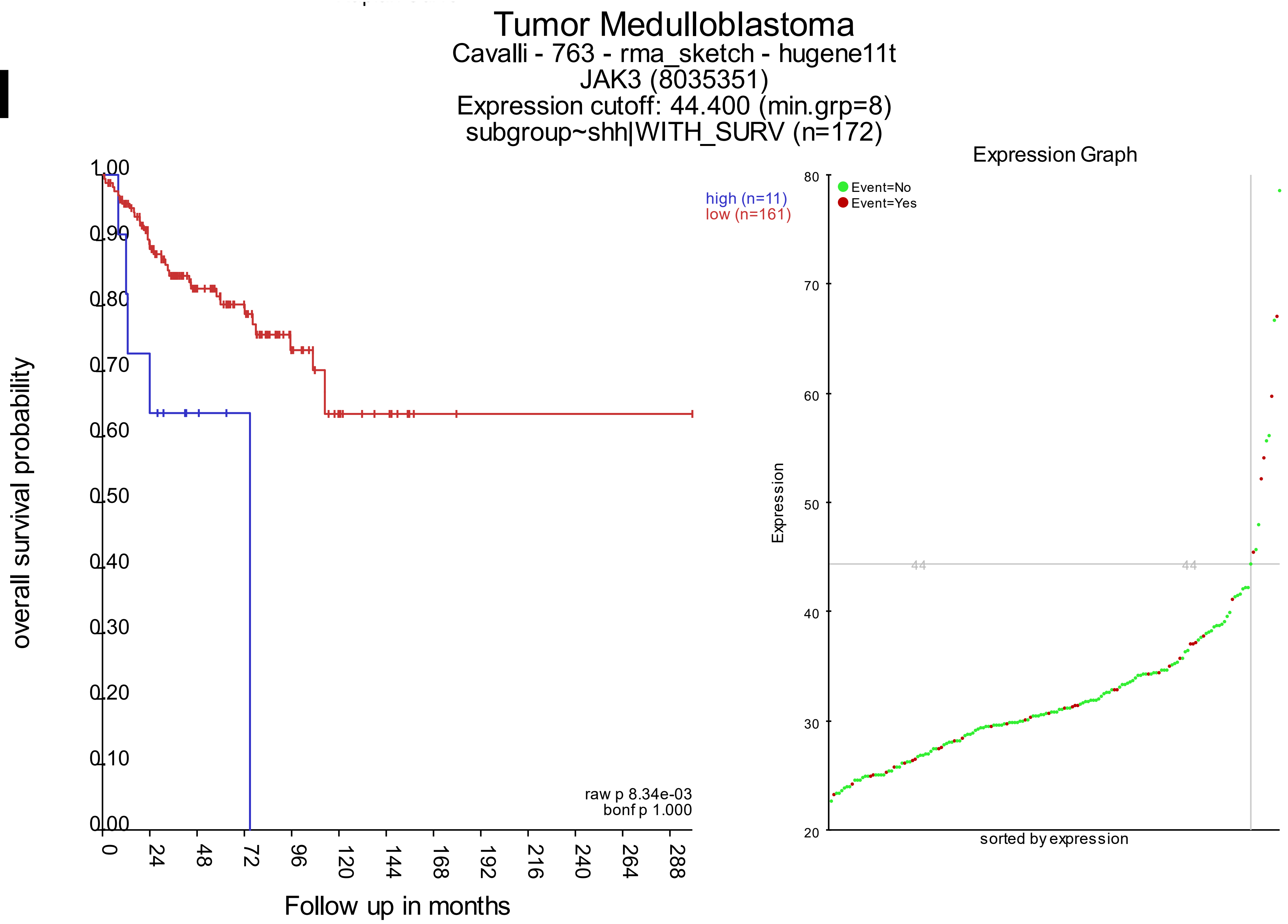
Tumor Medulloblastoma
Cavalli - 763 - rma_sketch - hugene11t
JAK3 (8035351)
Expression cutoff: 44.400 (min.grp=8)
subgroup~shh|WITH_SURV (n=172)
overall survival probability
1.00
0.90
0.80
0.70
0.60
0.50
0.40
0.30
0.20
0.10
0.00
0 24 48 72 96 120 144 168 192 216 240 264 288
Follow up in months
high (n=11)
low (n=161)
raw p 8.34e-03
bonf p 1.000
Expression Graph
Event=No
Event=Yes
Expression
80 70 60 50 40 30 20
44
44
sorted by expression

GR4

Tumor Medulloblastoma
Cavalli - 763 - rma_sketch - hugene11t
JAK3 (8035351)
Expression cutoff: 40.200 (min.grp=8)
subgroup~group4|WITH_SURV (n=264)

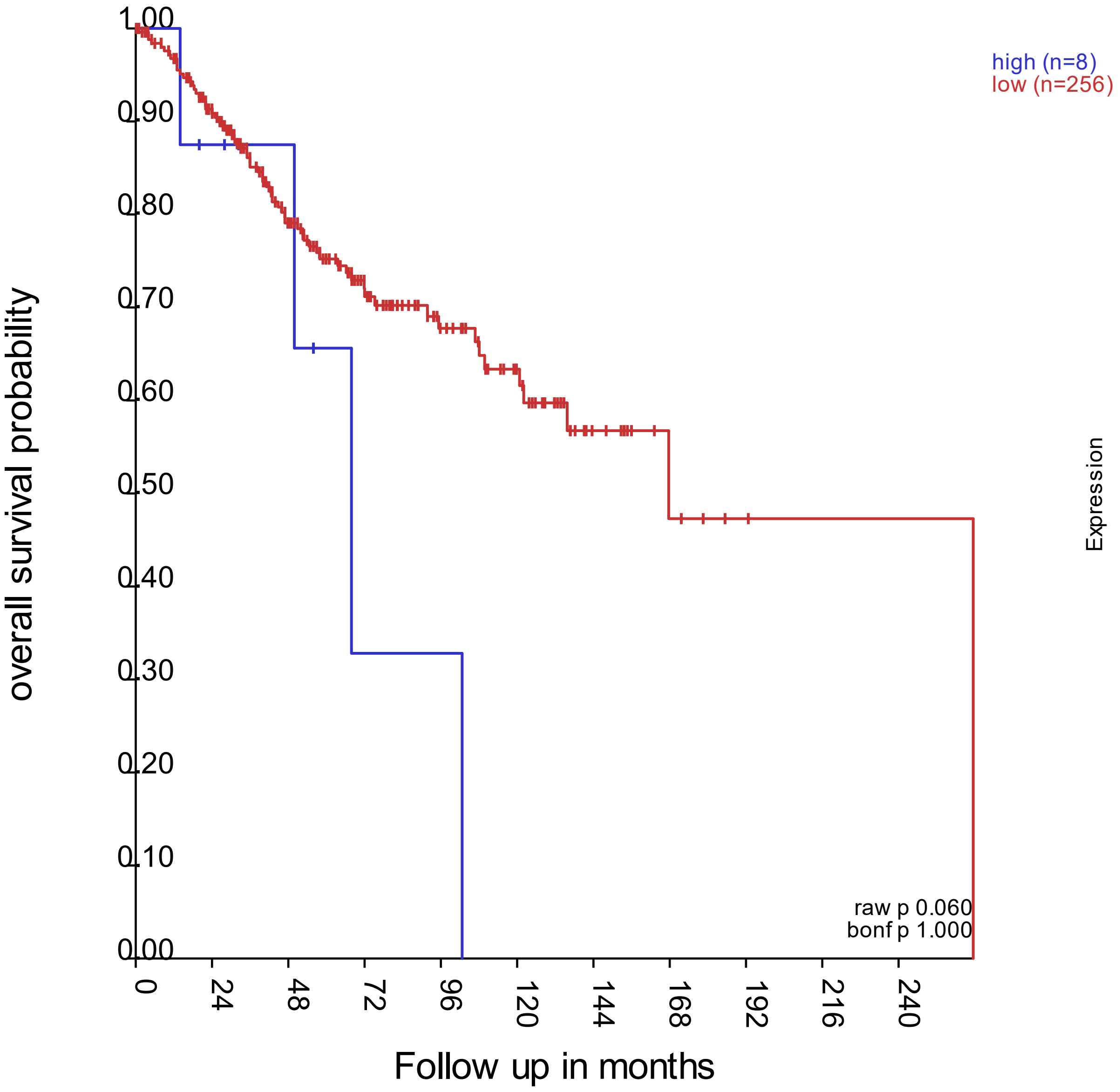

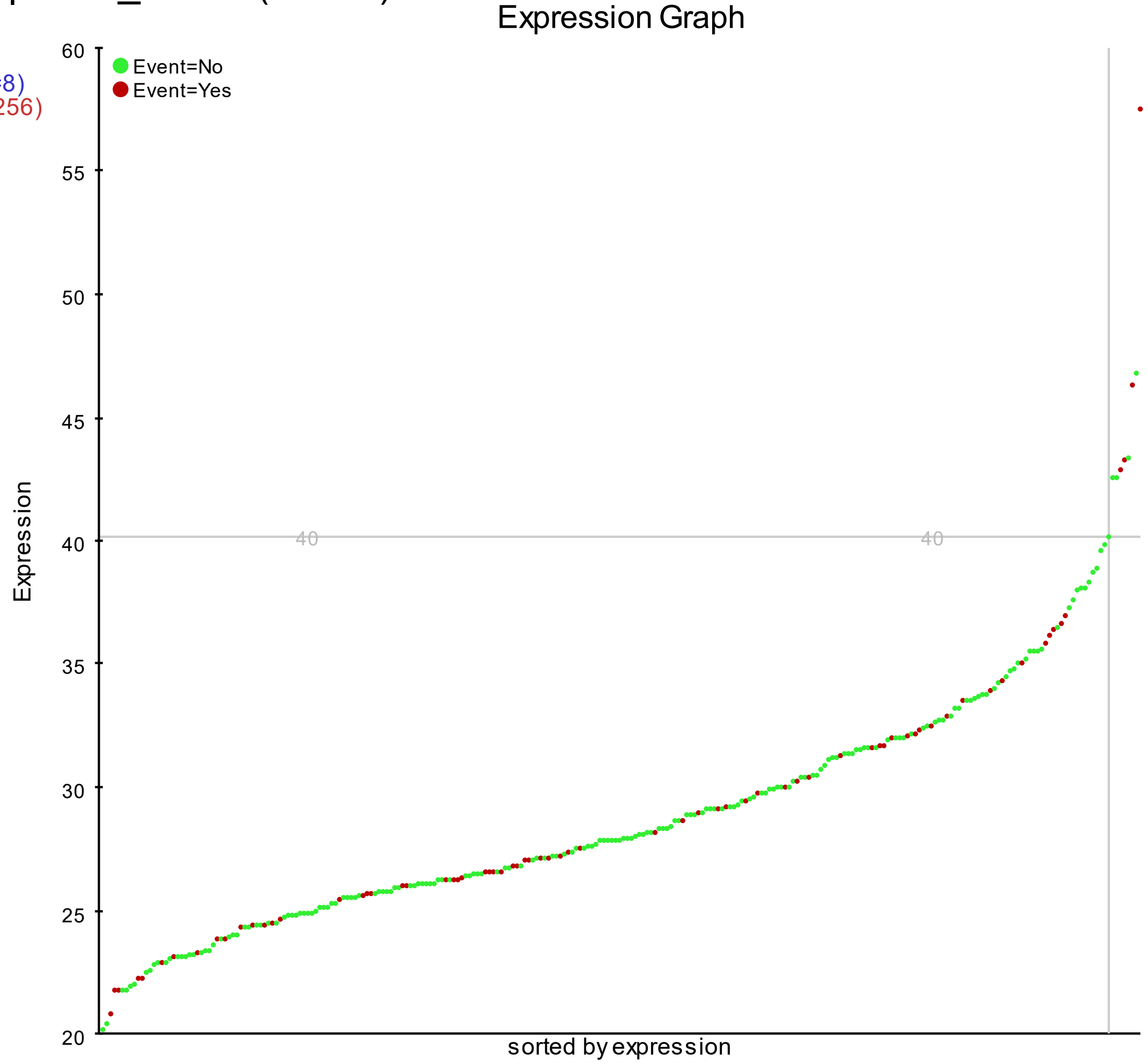

GR3

Tumor Medulloblastoma
Cavalli - 763 - rma_sketch - hugene11t
JAK3 (8035351)
Expression cutoff: 37.800 (min.grp=8)
subgroup~group3|WITH_SURV (n=113)

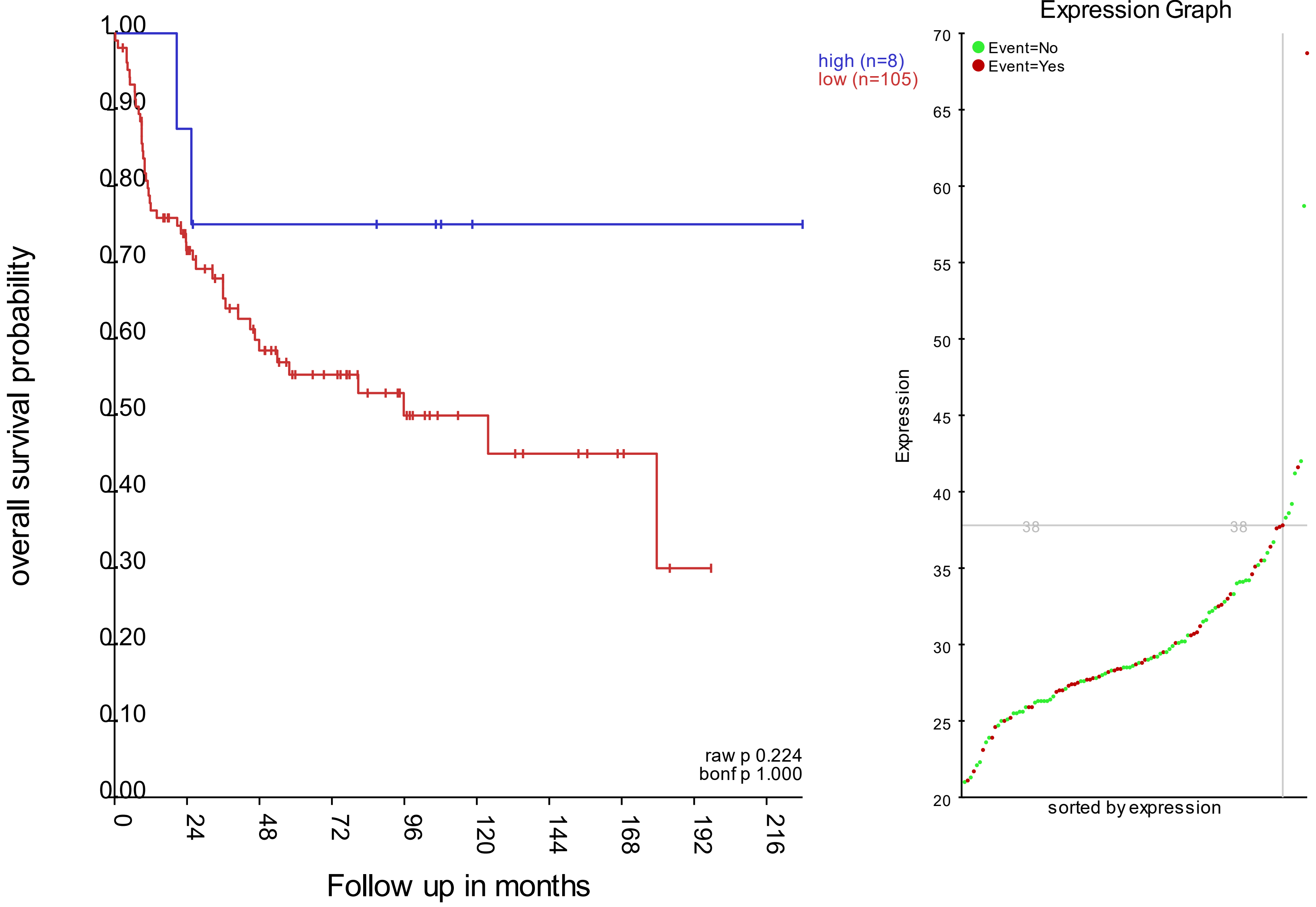

MAP2K2

WNT

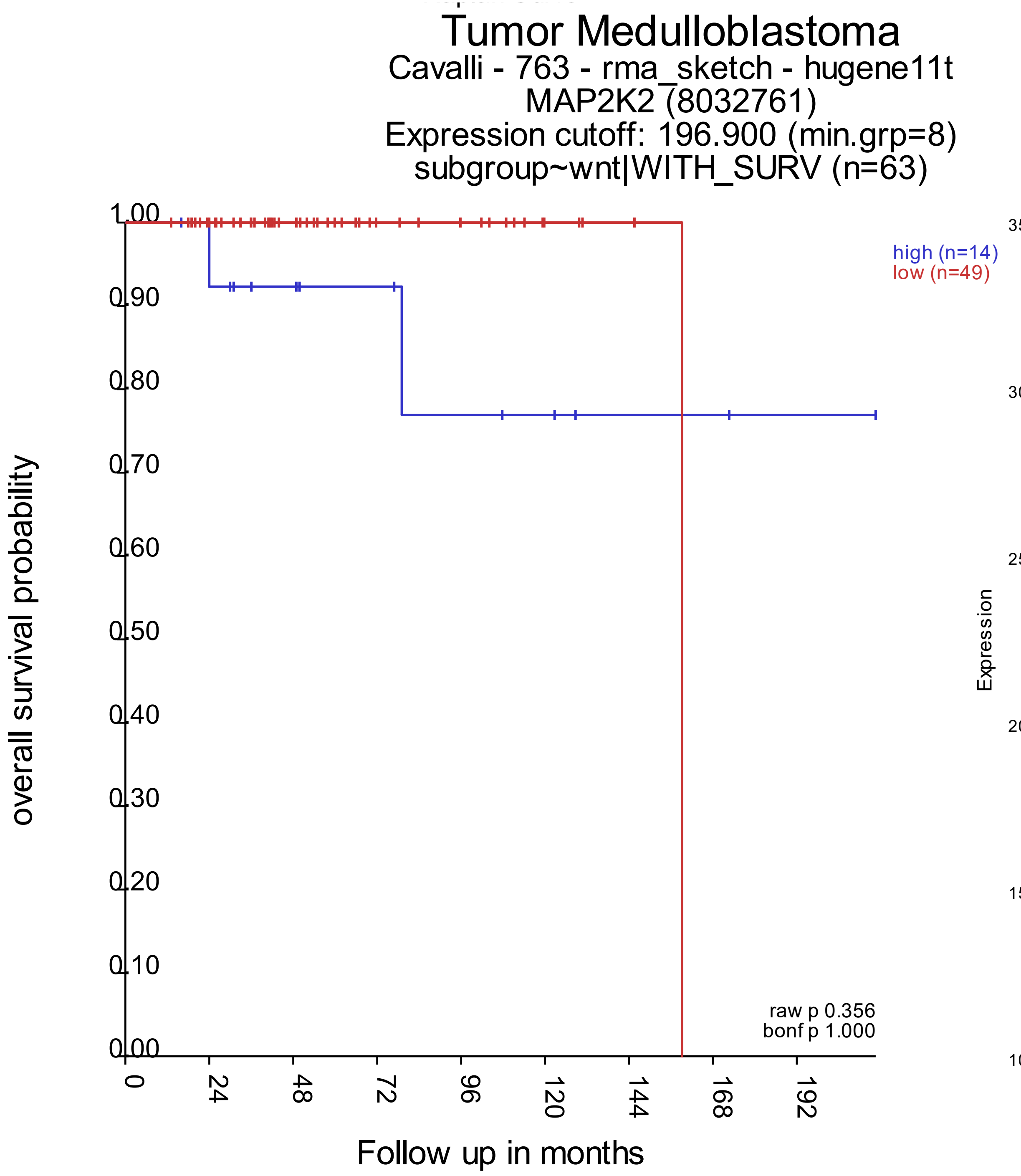
Tumor Medulloblastoma
Cavalli - 763 - rma_sketch - hugene11t
MAP2K2 (8032761)
Expression cutoff: 196.900 (min.grp=8)
subgroup~wnt|WITH_SURV (n=63)
overall survival probability
1.00
0.90
0.80
0.70
0.60
0.50
0.40
0.30
0.20
0.10
0.00
high (n=14)
low (n=49)
raw p 0.356
bonf p 1.000
0
24
48
72
96
120
144
168
192
Follow up in months

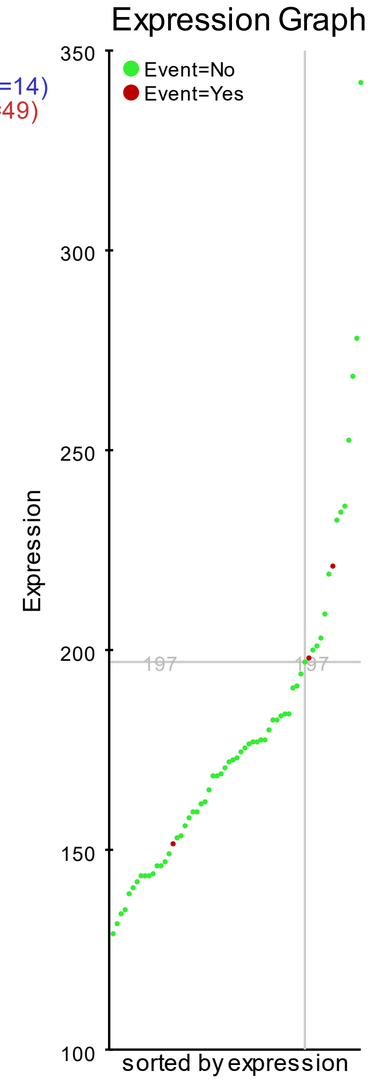
Expression Graph
Event=No
Event=Yes
Expression
350
300
250
200
150
100
197
197
sorted by expression

SHH

Tumor Medulloblastoma
Cavalli - 763 - rma_sketch - hugene11t
MAP2K2 (8032761)
Expression cutoff: 145.100 (min.grp=8)
subgroup~shh|WITH_SURV (n=172)

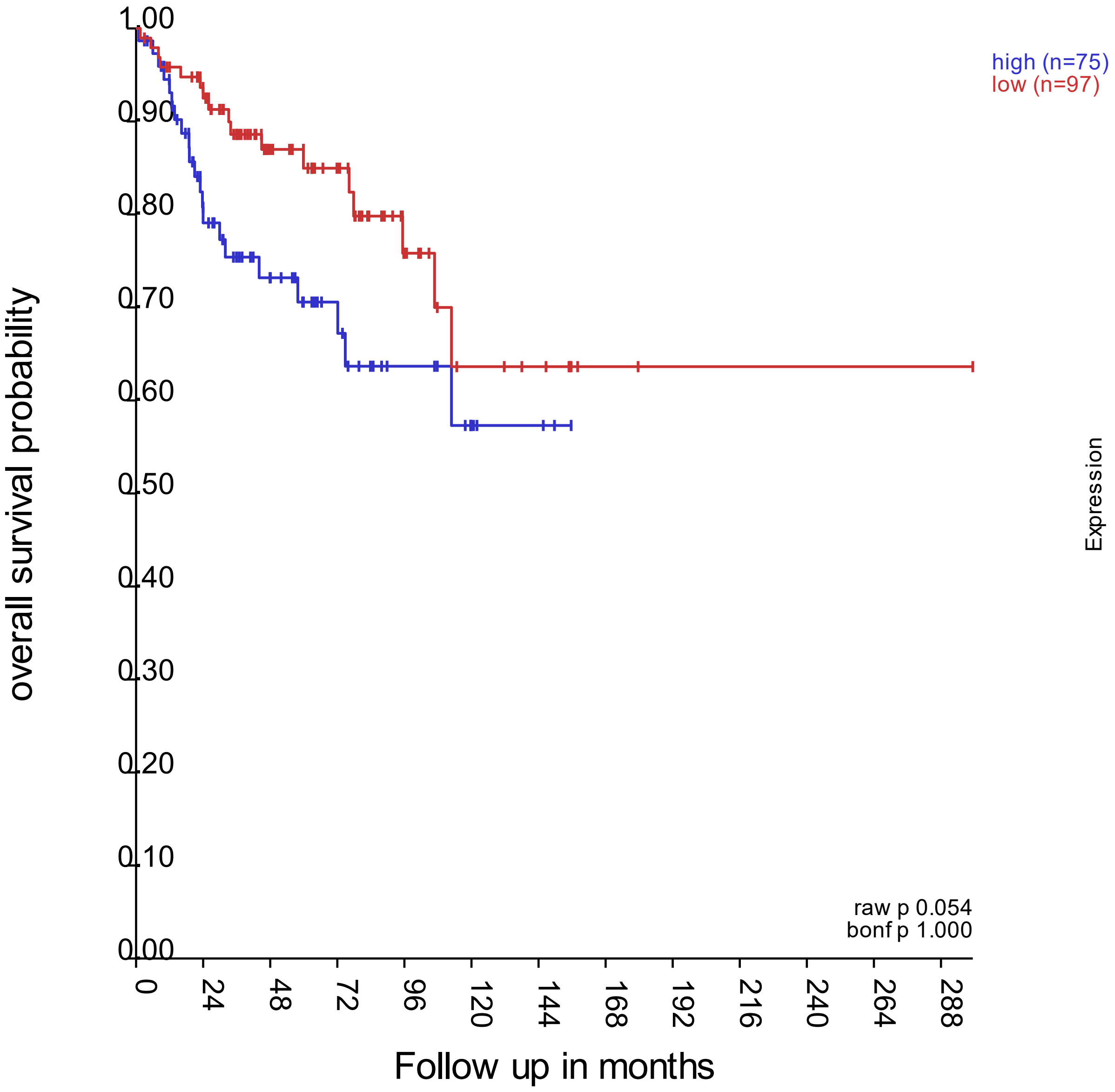

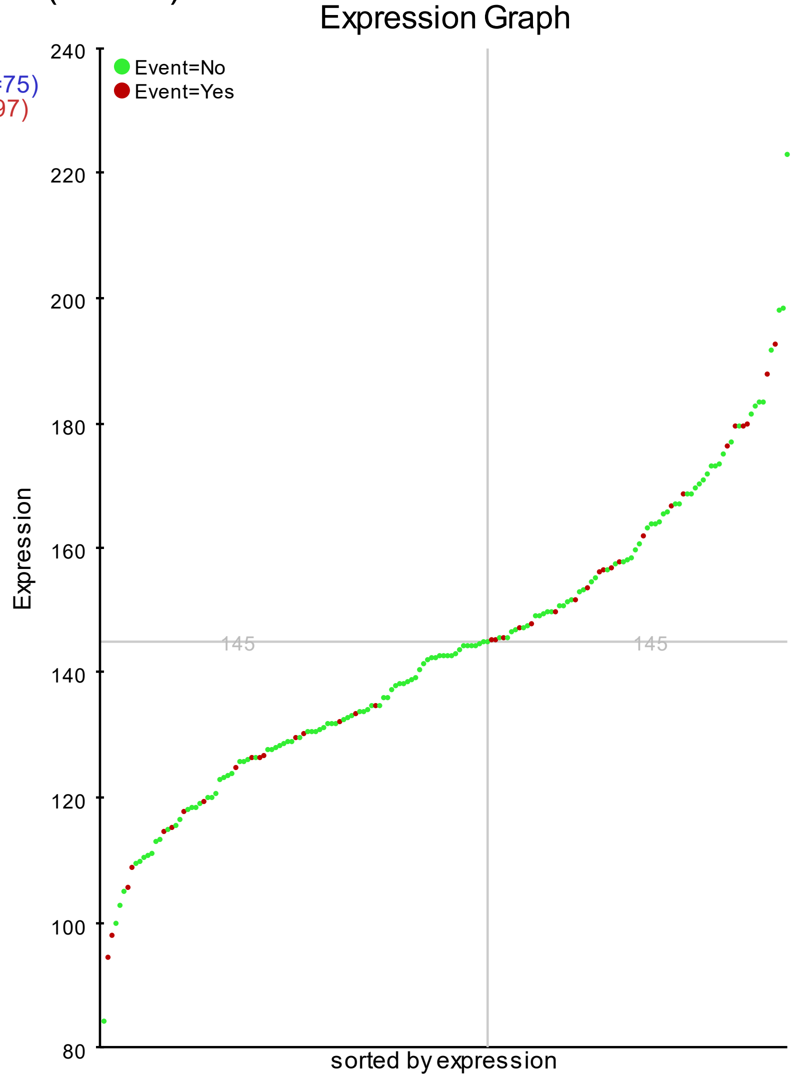

GR4

Tumor Medulloblastoma
Cavalli - 763 - rma_sketch - hugene11t
MAP2K2 (8032761)
Expression cutoff: 147.900 (min.grp=8)
subgroup~group4|WITH_SURV (n=264)

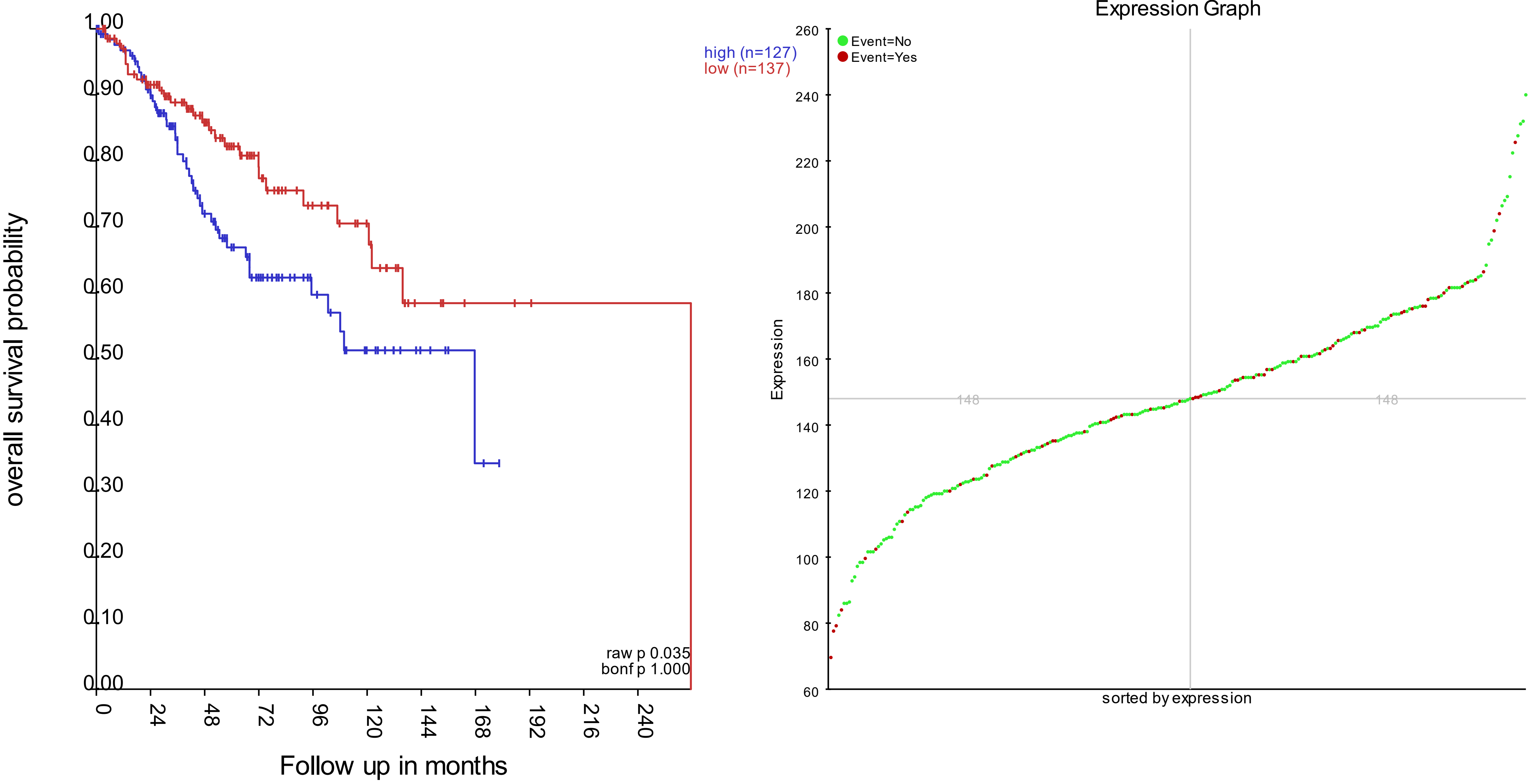

GR3

Tumor Medulloblastoma
Cavalli - 763 - rma_sketch - hugene11t
MAP2K2 (8032761)
Expression cutoff: 233.800 (min.grp=8)
subgroup~group3|WITH_SURV (n=113)

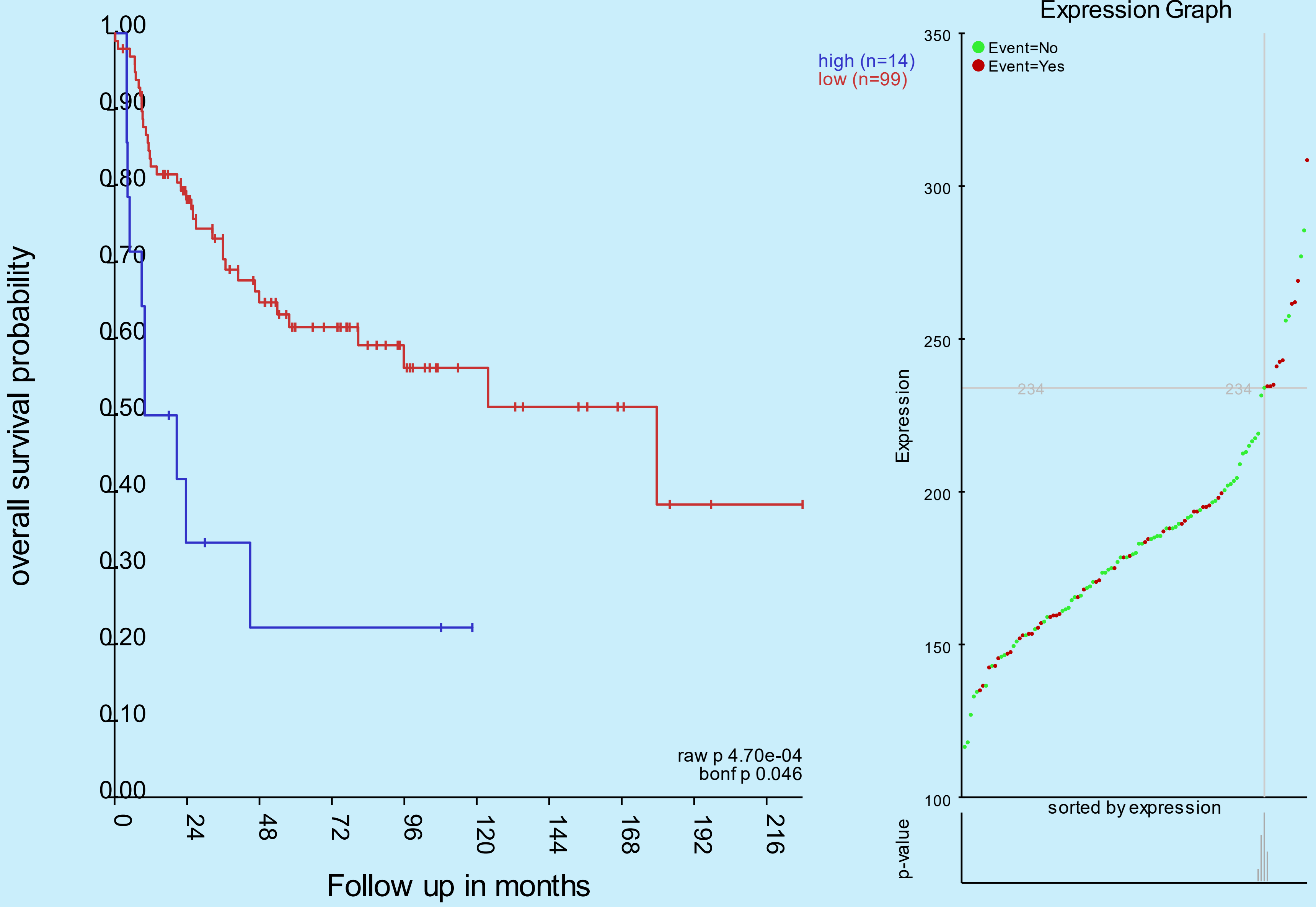

MAPK1

WNT

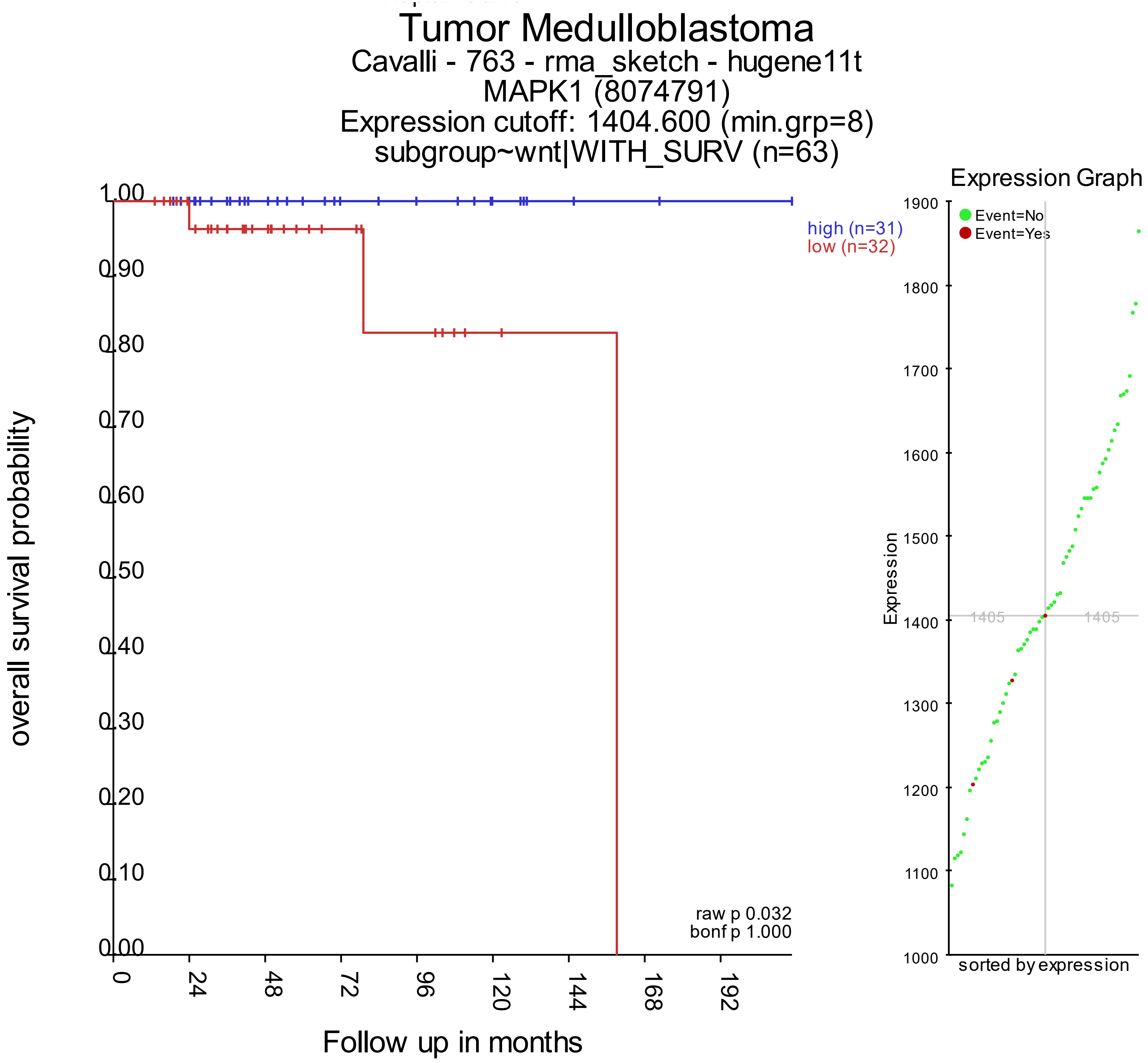

Tumor Medulloblastoma
Cavalli - 763 - rma_sketch - hugene11t
MAPK1 (8074791)
Expression cutoff: 1404.600 (min.grp=8)
subgroup~wnt|WITH_SURV (n=63)
overall survival probability
1.00
0.90
0.80
0.70
0.60
0.50
0.40
0.30
0.20
0.10
0.00
0
24
48
72
96
120
144
168
192
Follow up in months
high (n=31)
low (n=32)
raw p 0.032
bonf p 1.000
Expression Graph
Event=No
Event=Yes
Expression
1900
1800
1700
1600
1500
1400
1300
1200
1100
1000
1405
1405
sorted by expression

SHH

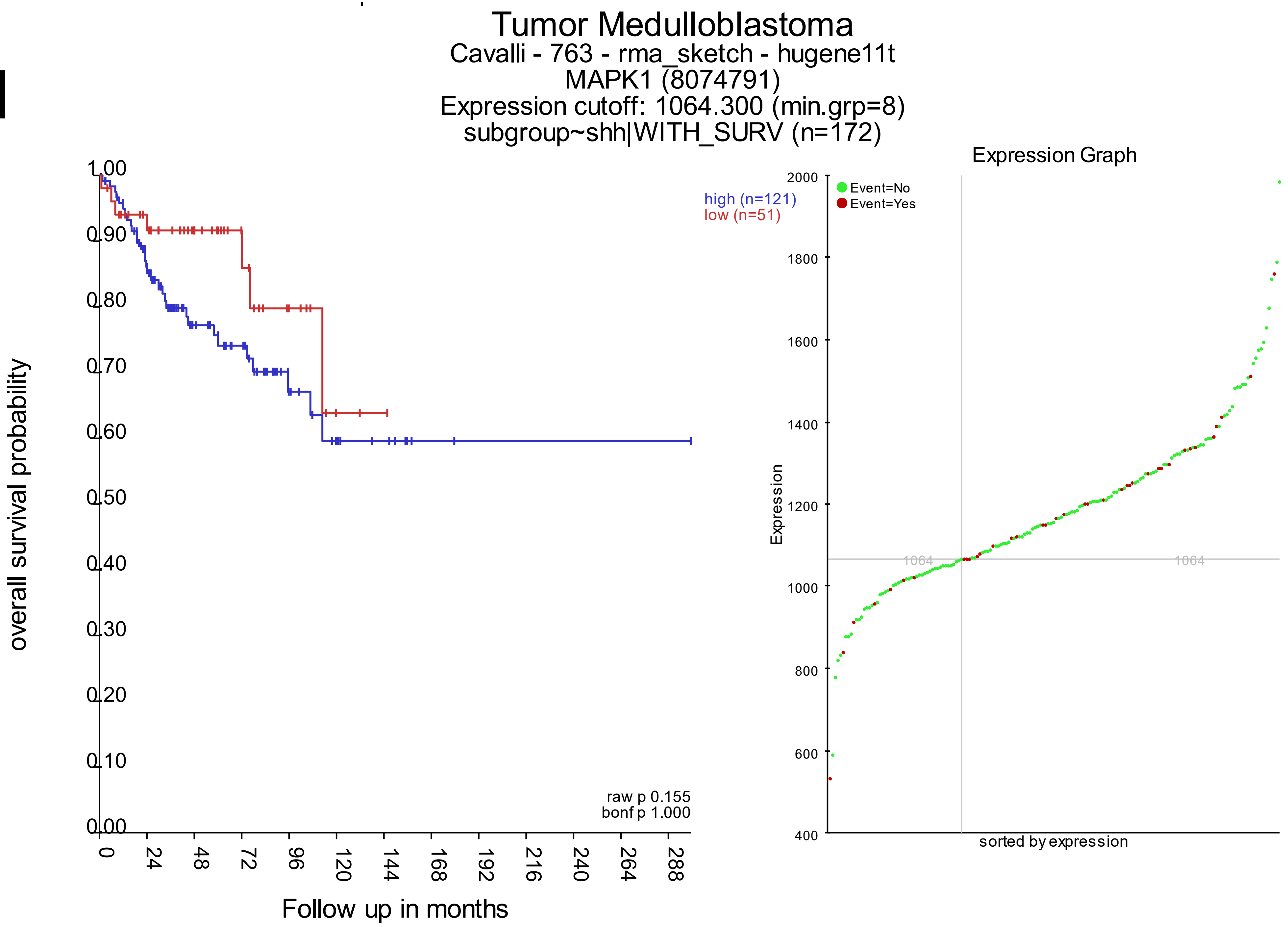
Tumor Medulloblastoma
Cavalli - 763 - rma_sketch - hugene11t
MAPK1 (8074791)
Expression cutoff: 1064.300 (min.grp=8)
subgroup~shh|WITH_SURV (n=172)
overall survival probability
1.00
0.90
0.80
0.70
0.60
0.50
0.40
0.30
0.20
0.10
0.00
0
24
48
72
96
120
144
168
192
216
240
264
288
Follow up in months
high (n=121)
low (n=51)
raw p 0.155
bonf p 1.000
Expression Graph
Event=No
Event=Yes
Expression
2000
1800
1600
1400
1200
1000
800
600
400
1064
1064
sorted by expression

GR4

Tumor Medulloblastoma
Cavalli - 763 - rma_sketch - hugene11t
MAPK1 (8074791)
Expression cutoff: 1329.500 (min.grp=8)
subgroup~group4|WITH_SURV (n=264)

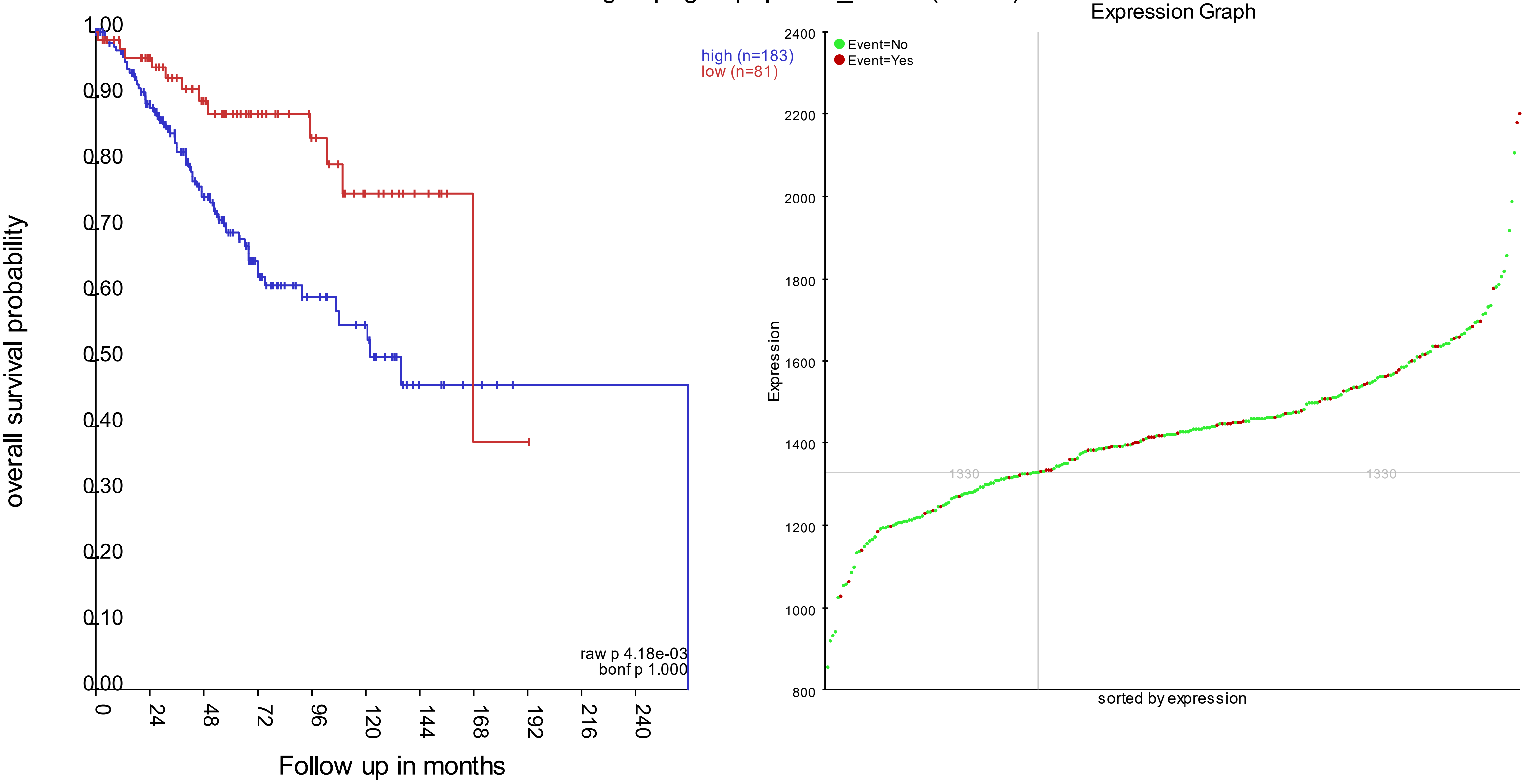

GR3

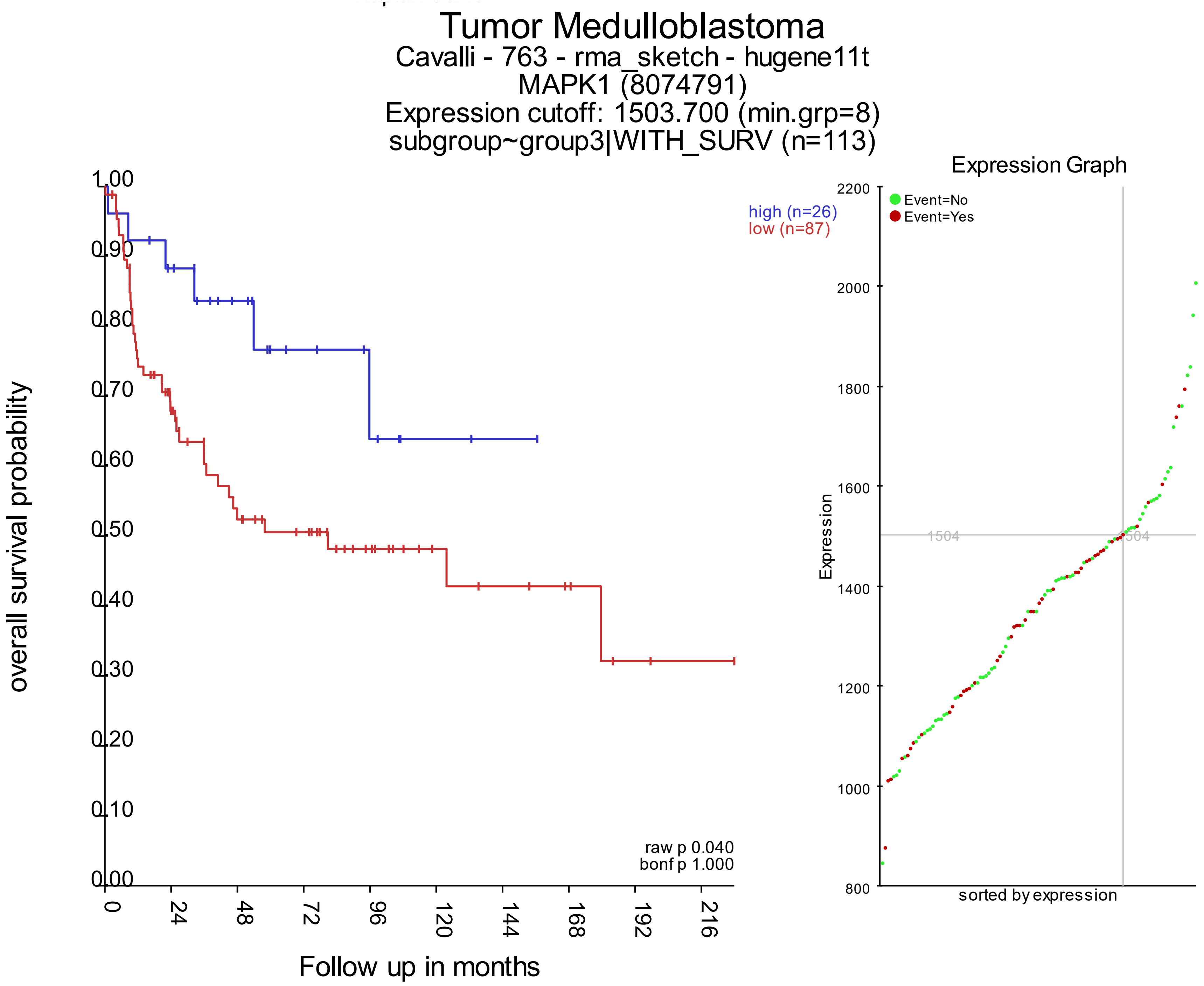

Tumor Medulloblastoma
Cavalli - 763 - rma_sketch - hugene11t
MAPK1 (8074791)
Expression cutoff: 1503.700 (min.grp=8)
subgroup~group3|WITH_SURV (n=113)
overall survival probability
1.00
0.90
0.80
0.70
0.60
0.50
0.40
0.30
0.20
0.10
0.00
0
24
48
72
96
120
144
168
192
216
Follow up in months
high (n=26)
low (n=87)
raw p 0.040
bonf p 1.000
Expression Graph
Event=No
Event=Yes
Expression
2200
2000
1800
1600
1400
1200
1000
800
1504
sorted by expression

MAPK3

WNT

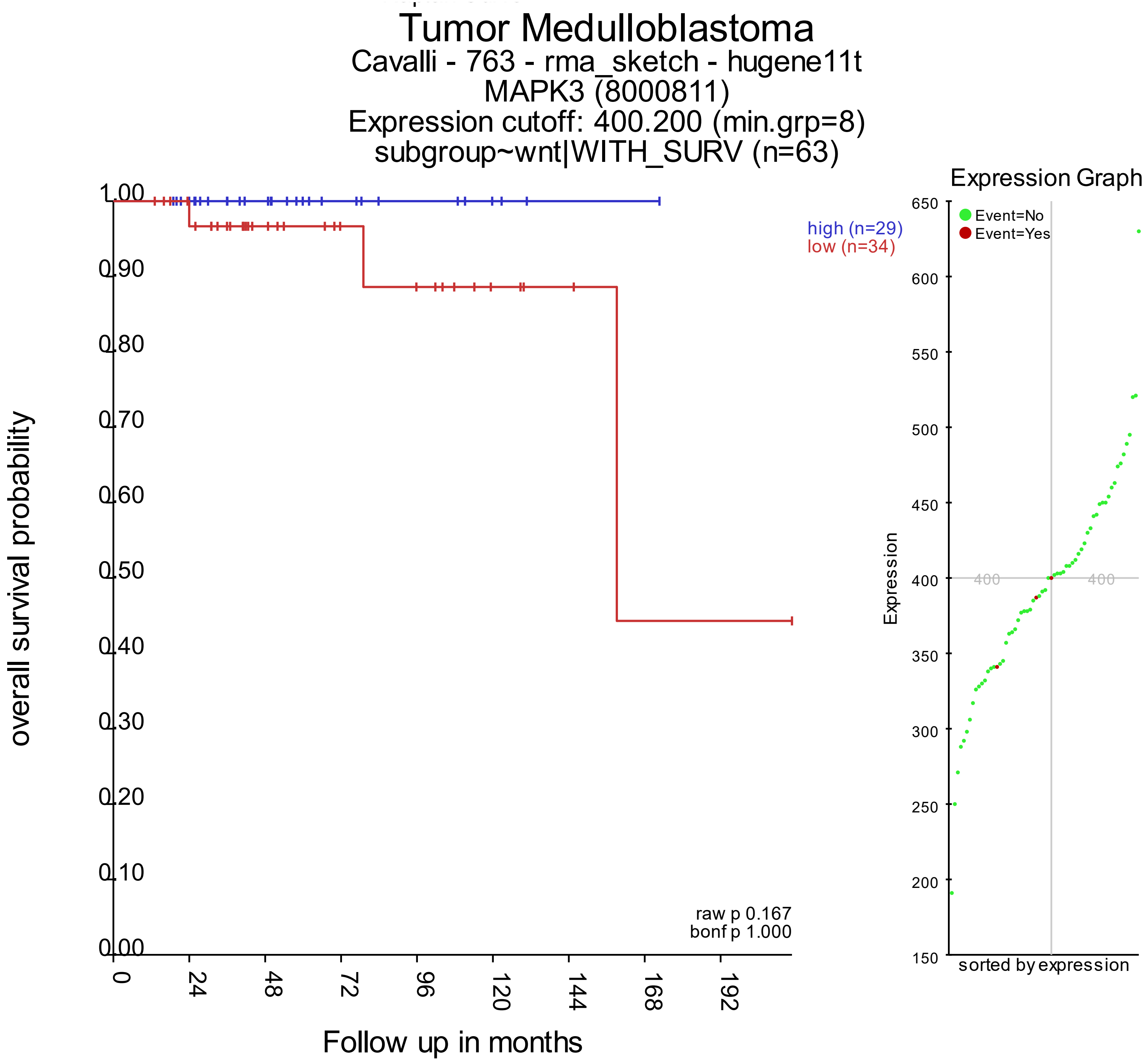

Tumor Medulloblastoma
Cavalli - 763 - rma_sketch - hugene11t
MAPK3 (8000811)
Expression cutoff: 400.200 (min.grp=8)
subgroup~wnt|WITH_SURV (n=63)
overall survival probability
1.00
0.90
0.80
0.70
0.60
0.50
0.40
0.30
0.20
0.10
0.00
high (n=29)
low (n=34)
raw p 0.167
bonf p 1.000
0
24
48
72
96
120
144
168
192
Follow up in months
Expression Graph
Event=No
Event=Yes
Expression
650
600
550
500
450
400
350
300
250
200
150
400
400
sorted by expression

SHH

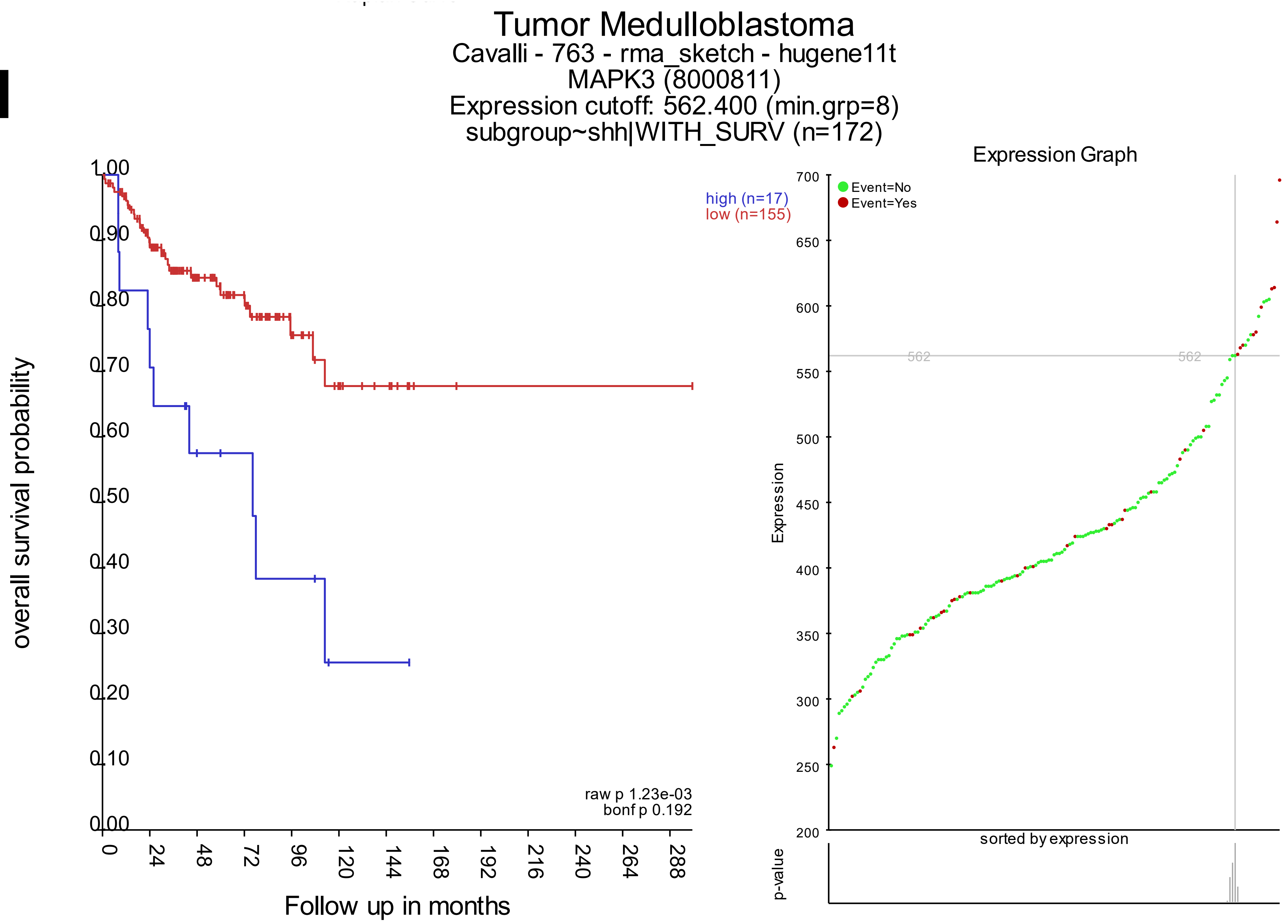
Tumor Medulloblastoma
Cavalli - 763 - rma_sketch - hugene11t
MAPK3 (8000811)
Expression cutoff: 562.400 (min.grp=8)
subgroup~shh|WITH_SURV (n=172)
overall survival probability
1.00
0.90
0.80
0.70
0.60
0.50
0.40
0.30
0.20
0.10
0.00
high (n=17)
low (n=155)
raw p 1.23e-03
bonf p 0.192
0
24
48
72
96
120
144
168
192
216
240
264
288
Follow up in months
Expression Graph
Event=No
Event=Yes
Expression
700
650
600
550
500
450
400
350
300
250
200
562
562
sorted by expression
p-value

GR4

Tumor Medulloblastoma
Cavalli - 763 - rma_sketch - hugene11t
MAPK3 (8000811)
Expression cutoff: 441.400 (min.grp=8)
subgroup~group4|WITH_SURV (n=264)

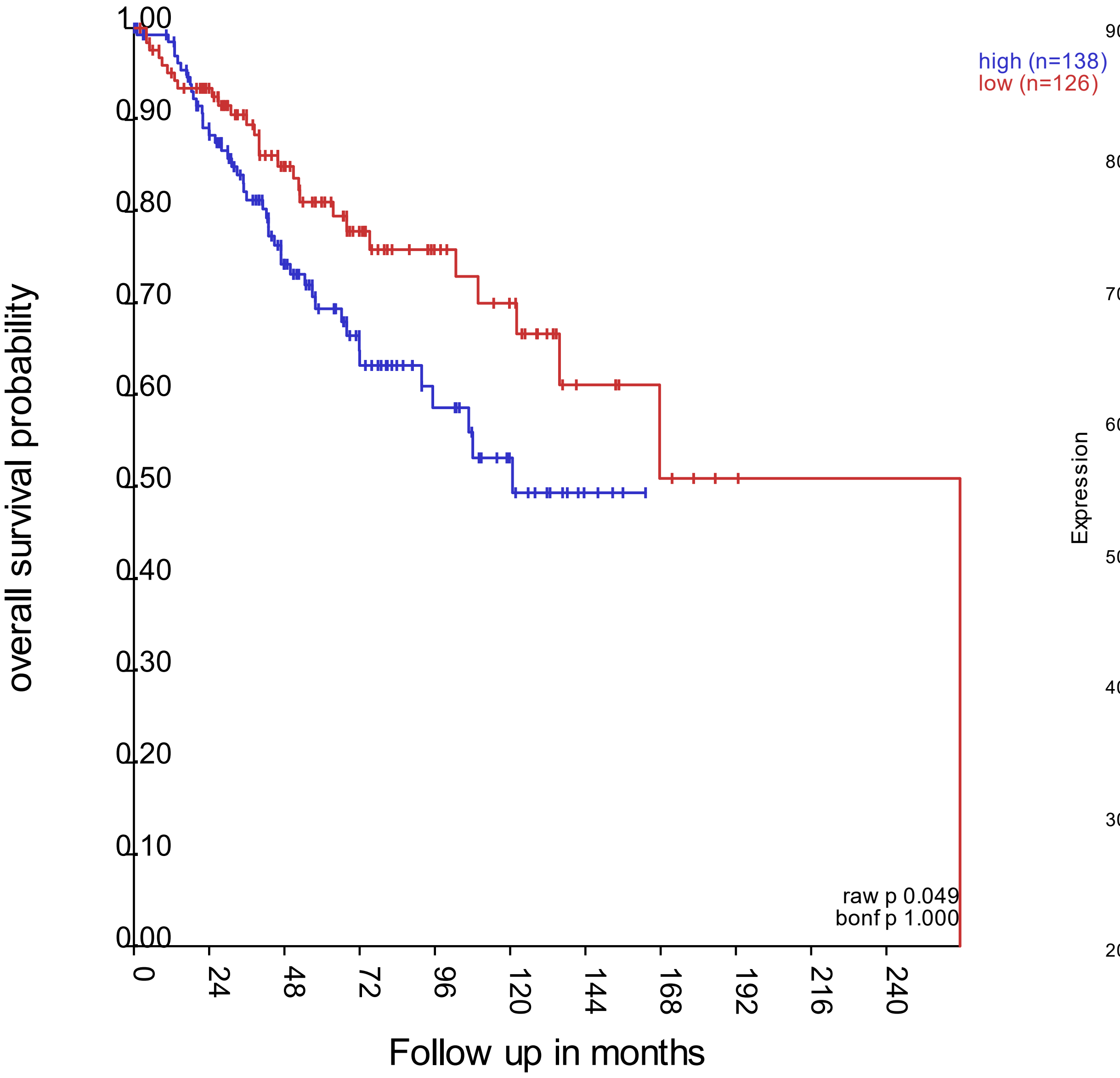

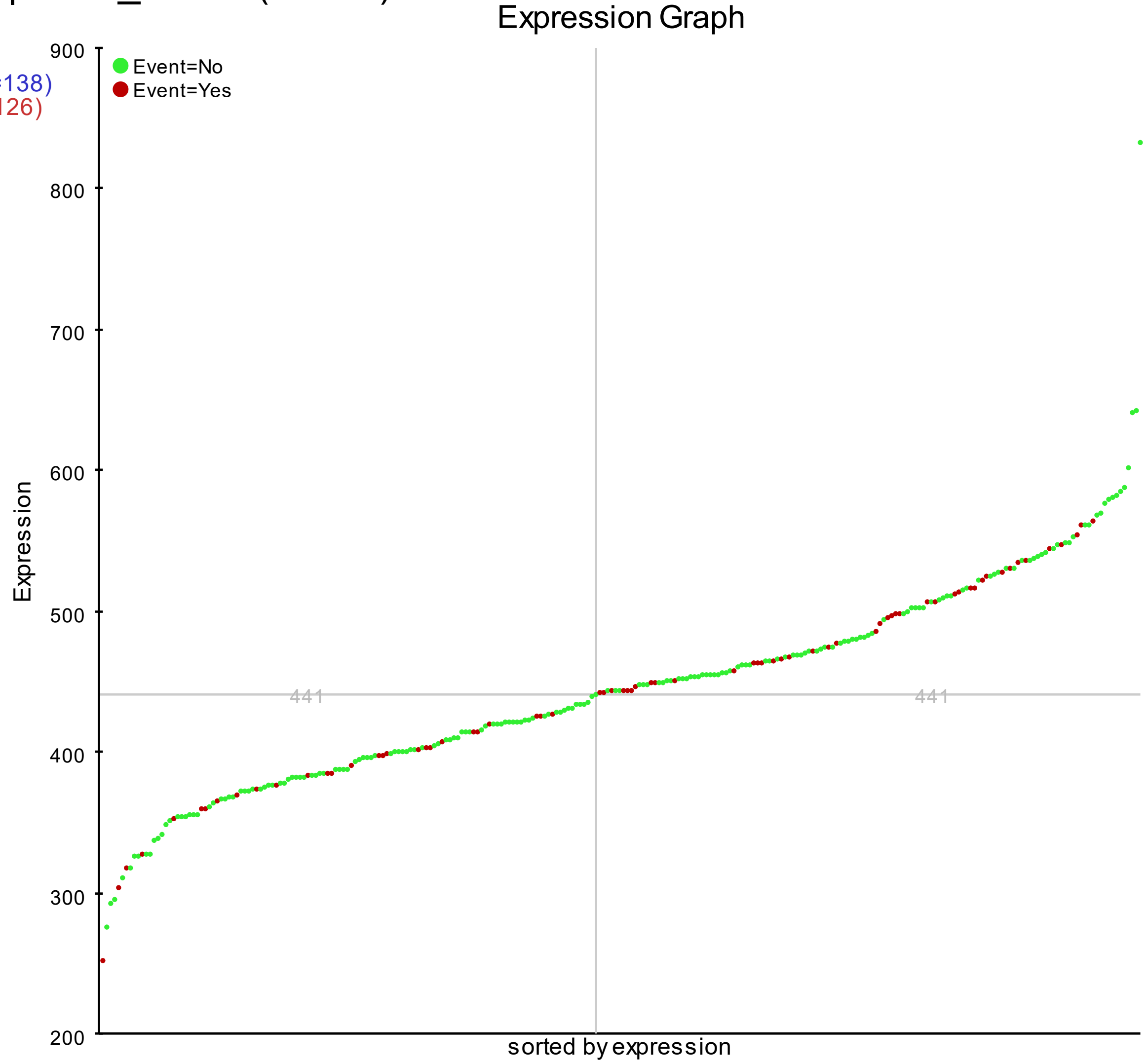

GR3

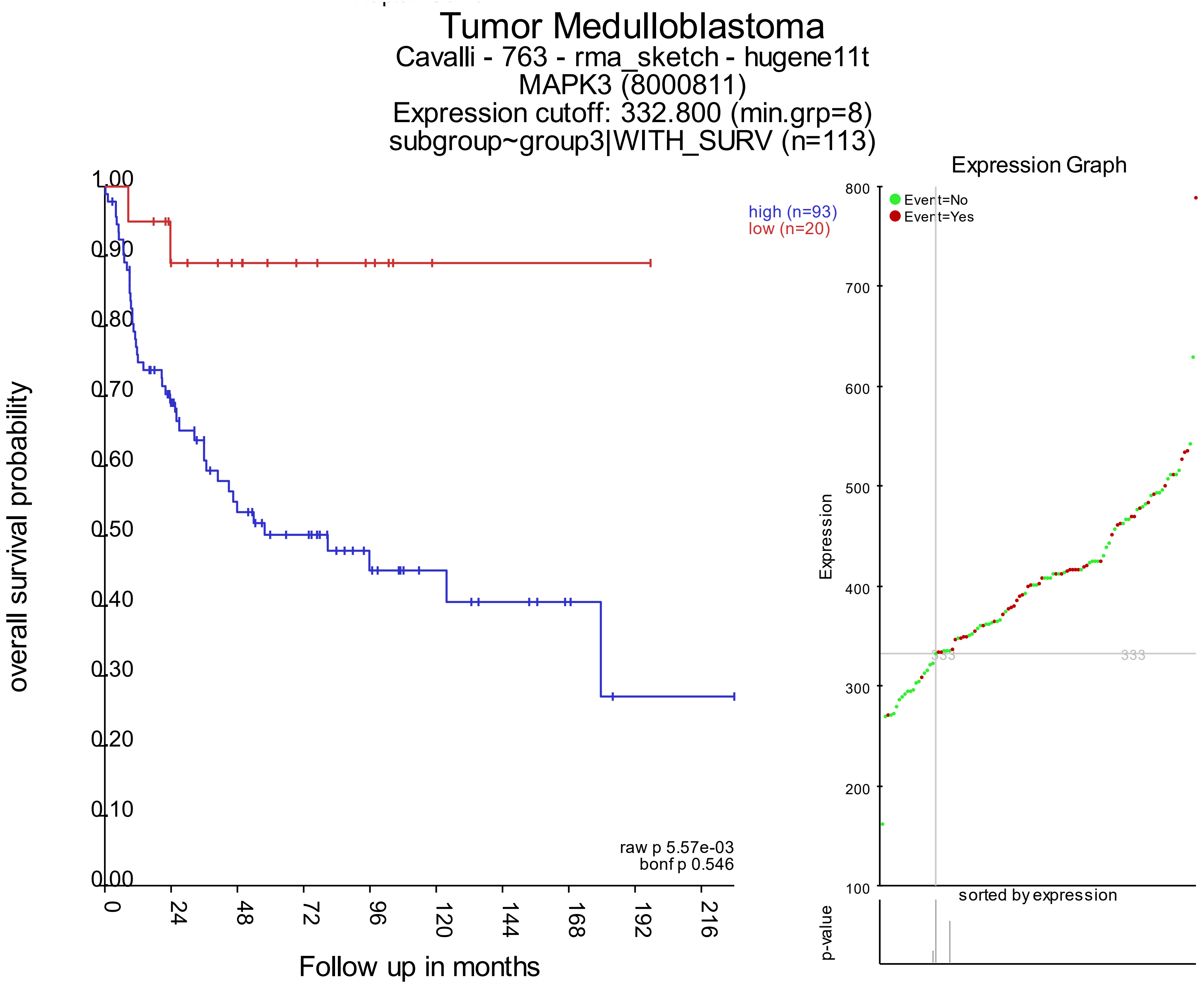
Tumor Medulloblastoma
Cavalli - 763 - rma_sketch - hugene11t
MAPK3 (8000811)
Expression cutoff: 332.800 (min.grp=8)
subgroup~group3|WITH_SURV (n=113)
overall survival probability
1.00
0.90
0.80
0.70
0.60
0.50
0.40
0.30
0.20
0.10
0.00
0
24
48
72
96
120
144
168
192
216
Follow up in months
high (n=93)
low (n=20)
raw p 5.57e-03
bonf p 0.546
Expression Graph
Event=No
Event=Yes
800
700
600
500
400
300
200
100
Expression
333
333
sorted by expression
p-value

MET

WNT

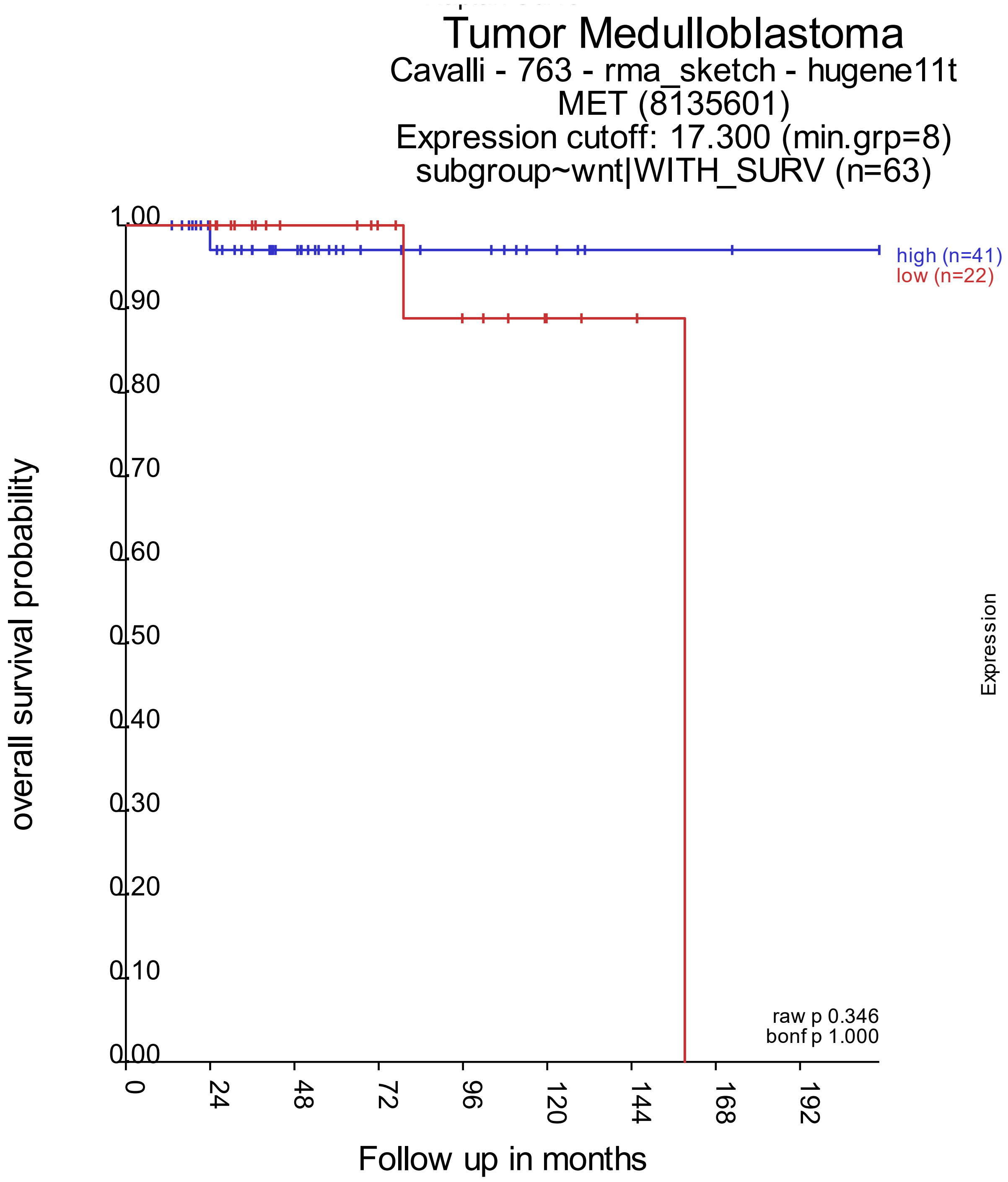

Tumor Medulloblastoma
Cavalli - 763 - rma_sketch - hugene11t
MET (8135601)
Expression cutoff: 17.300 (min.grp=8)
subgroup~wnt|WITH_SURV (n=63)
overall survival probability
1.00
0.90
0.80
0.70
0.60
0.50
0.40
0.30
0.20
0.10
0.00
0
24
48
72
96
120
144
168
192
Follow up in months
high (n=41)
low (n=22)
raw p 0.346
bonf p 1.000

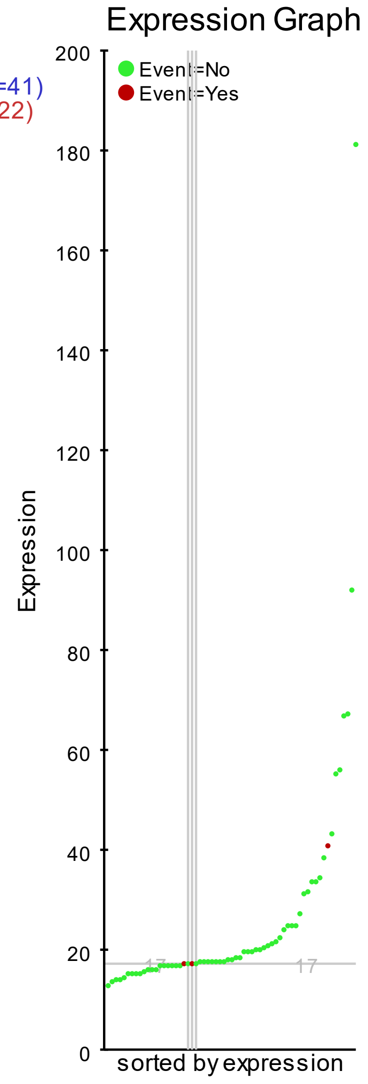

Expression Graph
Event=No
Event=Yes
Expression
200
180
160
140
120
100
80
60
40
20
0
17
sorted by expression

SHH

Tumor Medulloblastoma
Cavalli - 763 - rma_sketch - hugene11t
MET (8135601)
Expression cutoff: 358.800 (min.grp=8)
subgroup~shh|WITH_SURV (n=172)

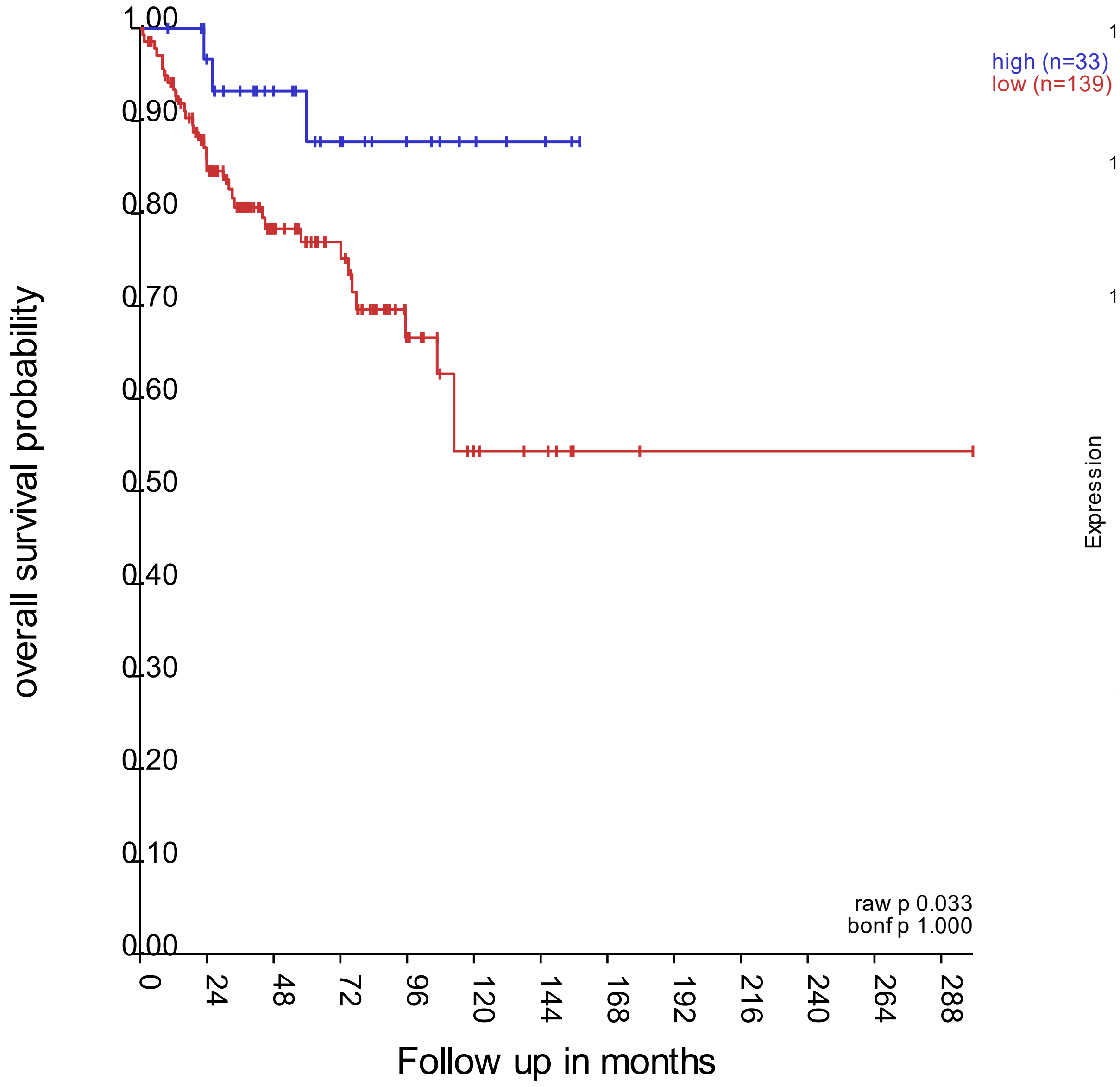

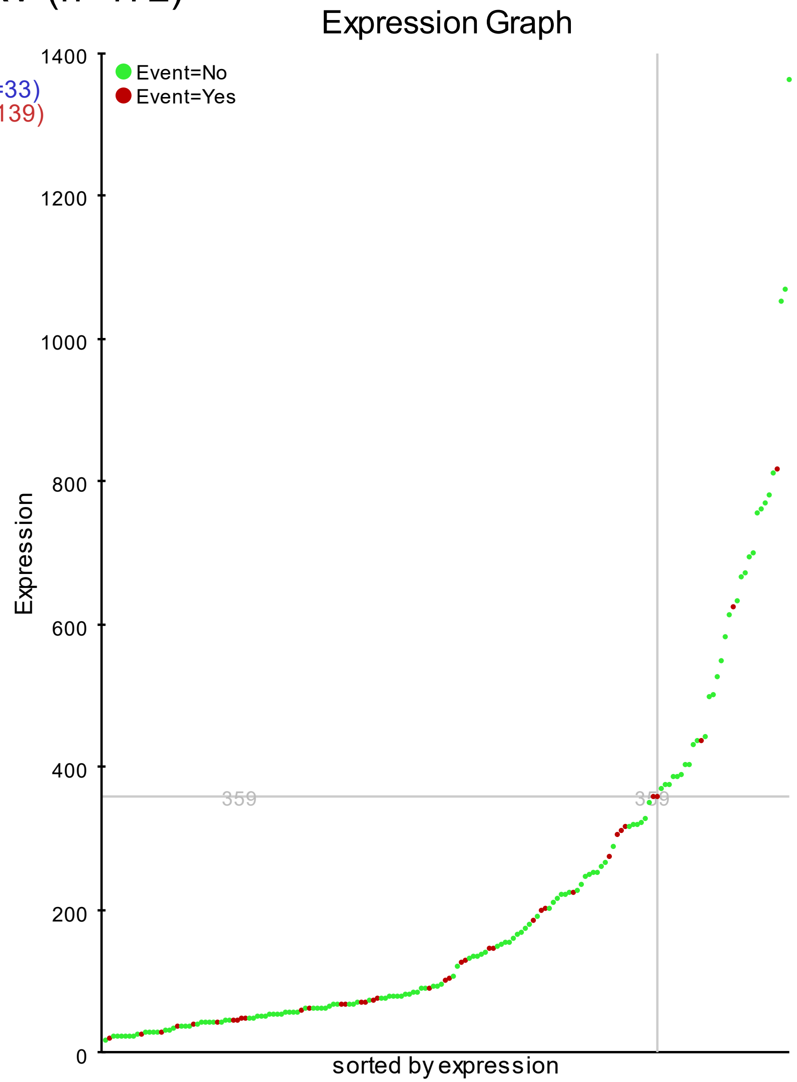

GR4

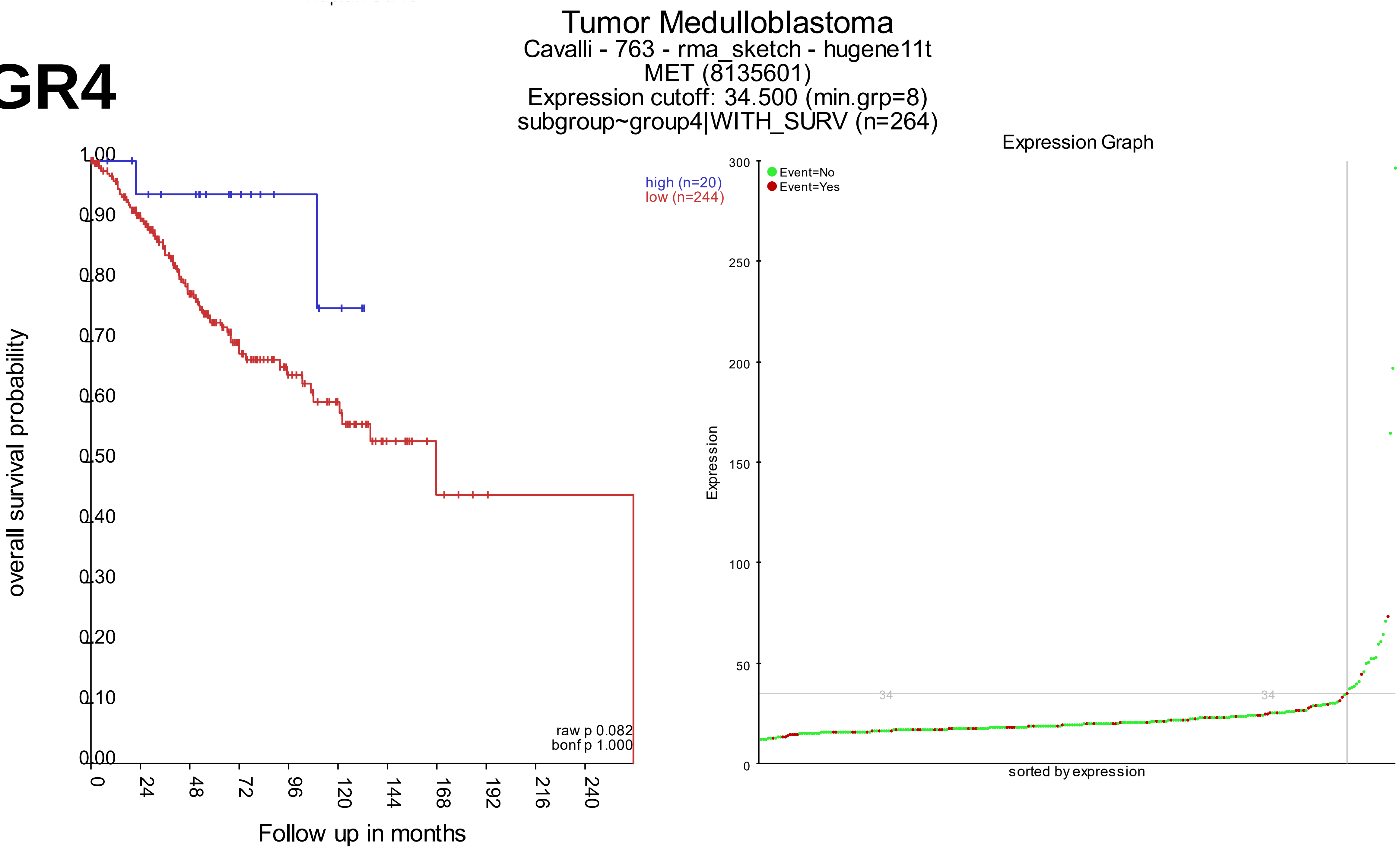

Tumor Medulloblastoma
Cavalli - 763 - rma_sketch - hugene11t
MET (8135601)
Expression cutoff: 34.500 (min.grp=8)
subgroup~group4|WITH_SURV (n=264)
overall survival probability
1.00
0.90
0.80
0.70
0.60
0.50
0.40
0.30
0.20
0.10
0.00
high (n=20)
low (n=244)
raw p 0.082
bonf p 1.000
0
24
48
72
96
120
144
168
192
216
240
Follow up in months
Expression Graph
Event=No
Event=Yes
Expression
300
250
200
150
100
50
0
34
34
sorted by expression

GR3

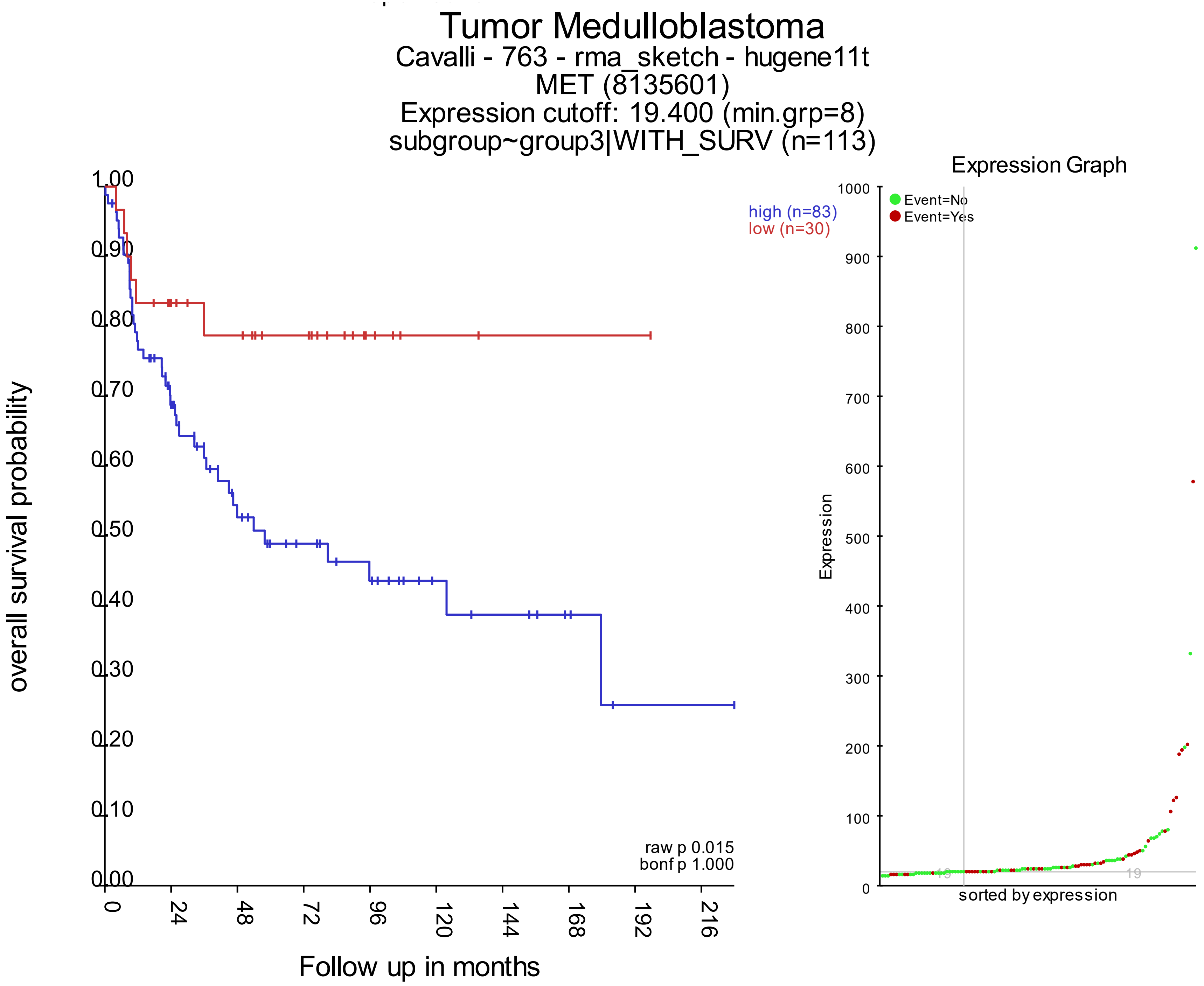
Tumor Medulloblastoma
Cavalli - 763 - rma_sketch - hugene11t
MET (8135601)
Expression cutoff: 19.400 (min.grp=8)
subgroup~group3|WITH_SURV (n=113)
high (n=83)
low (n=30)
overall survival probability
1.00
0.90
0.80
0.70
0.60
0.50
0.40
0.30
0.20
0.10
0.00
0
24
48
72
96
120
144
168
192
216
Follow up in months
raw p 0.015
bonf p 1.000
Expression Graph
Event=No
Event=Yes
Expression
1000
900
800
700
600
500
400
300
200
100
0
sorted by expression

MTOR

WNT

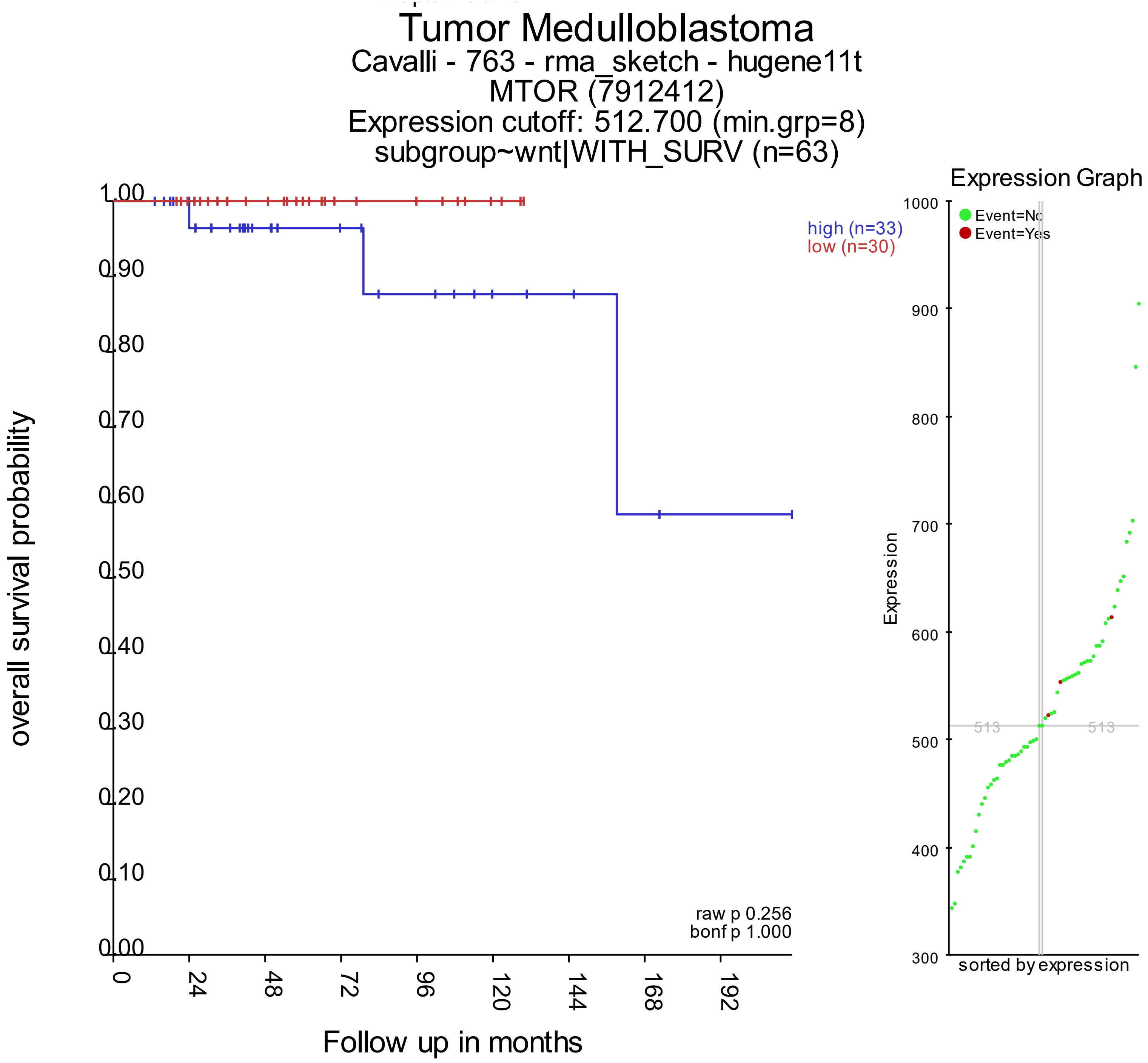
Tumor Medulloblastoma
Cavalli - 763 - rma_sketch - hugene11t
MTOR (7912412)
Expression cutoff: 512.700 (min.grp=8)
subgroup~wnt|WITH_SURV (n=63)
overall survival probability
1.00
0.90
0.80
0.70
0.60
0.50
0.40
0.30
0.20
0.10
0.00
0
24
48
72
96
120
144
168
192
Follow up in months
high (n=33)
low (n=30)
raw p 0.256
bonf p 1.000
Expression Graph
Event=No
Event=Yes
Expression
1000
900
800
700
600
500
400
300
513
513
sorted by expression

SHH

Tumor Medulloblastoma
Cavalli - 763 - rma_sketch - hugene11t
MTOR (7912412)
Expression cutoff: 406.000 (min.grp=8)
subgroup~shh|WITH_SURV (n=172)

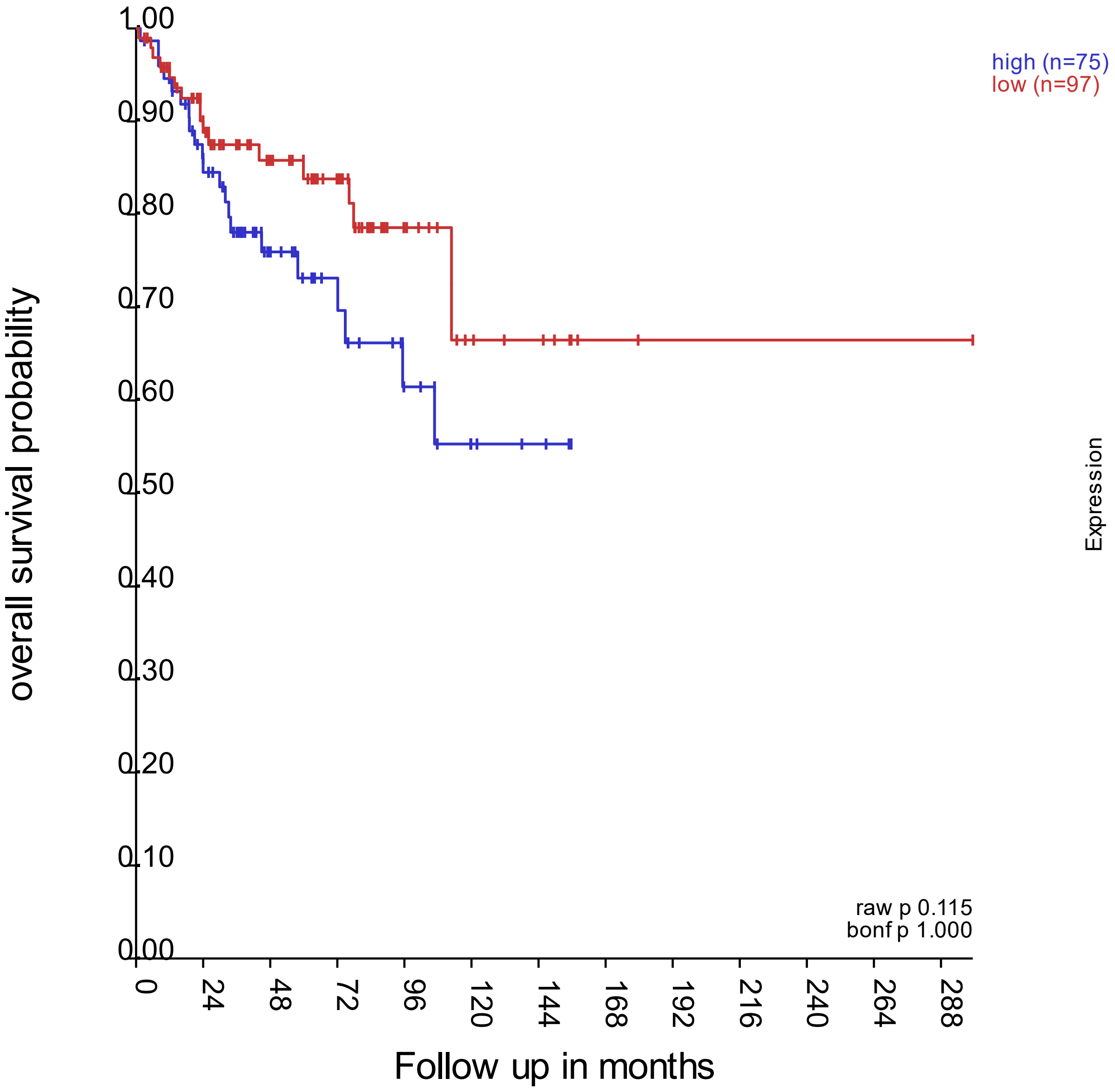

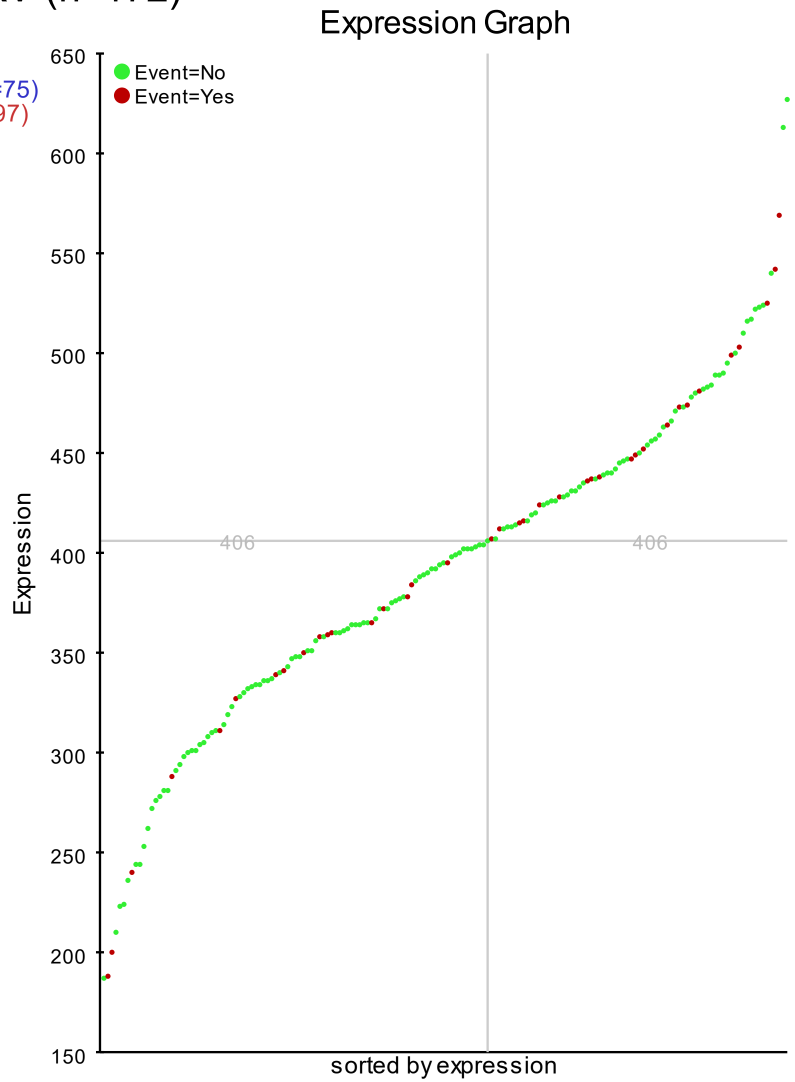

GR4

Tumor Medulloblastoma
Cavalli - 763 - rma_sketch - hugene11t
MTOR (7912412)
Expression cutoff: 640.900 (min.grp=8)
subgroup~group4|WITH_SURV (n=264)

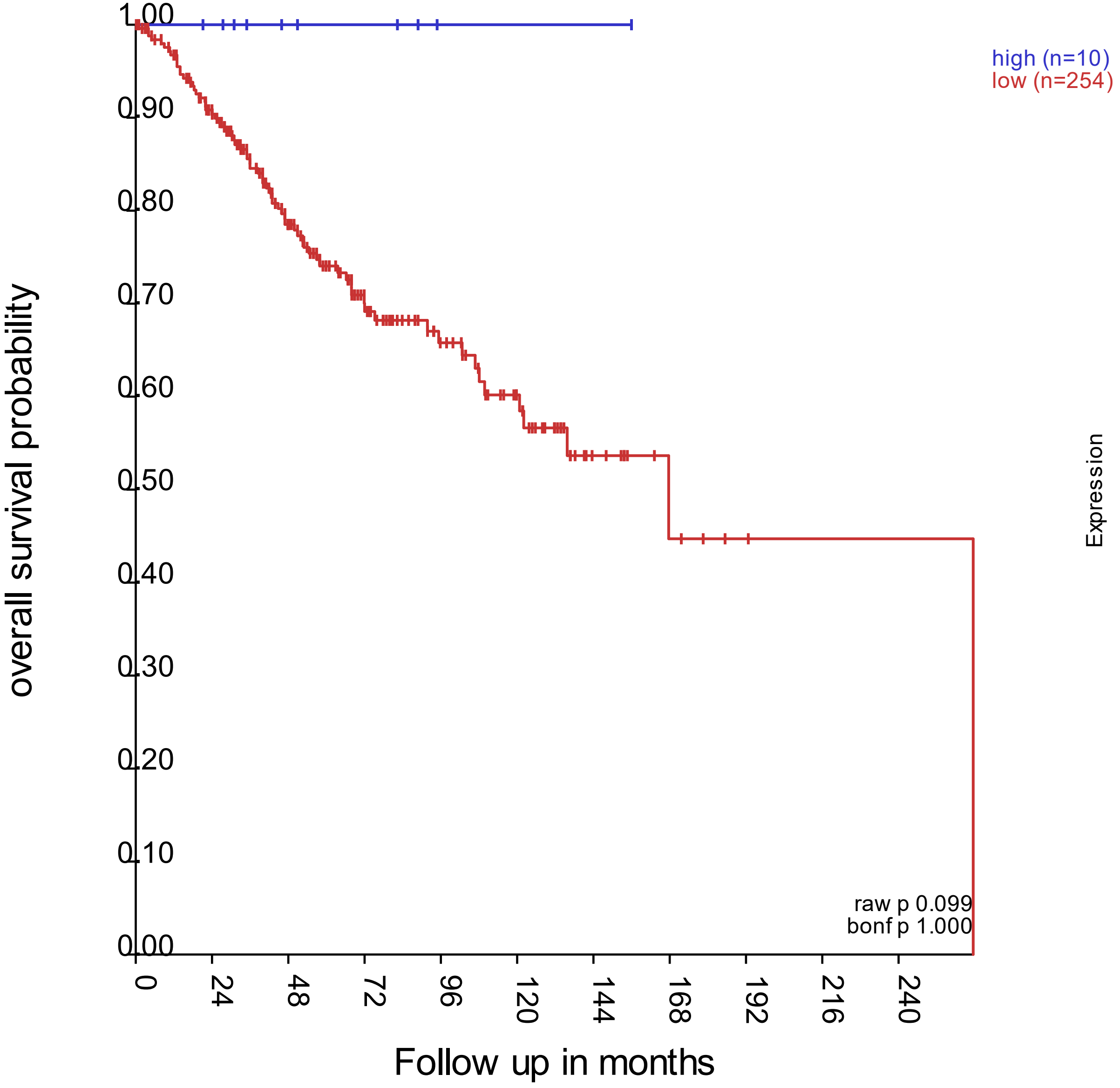

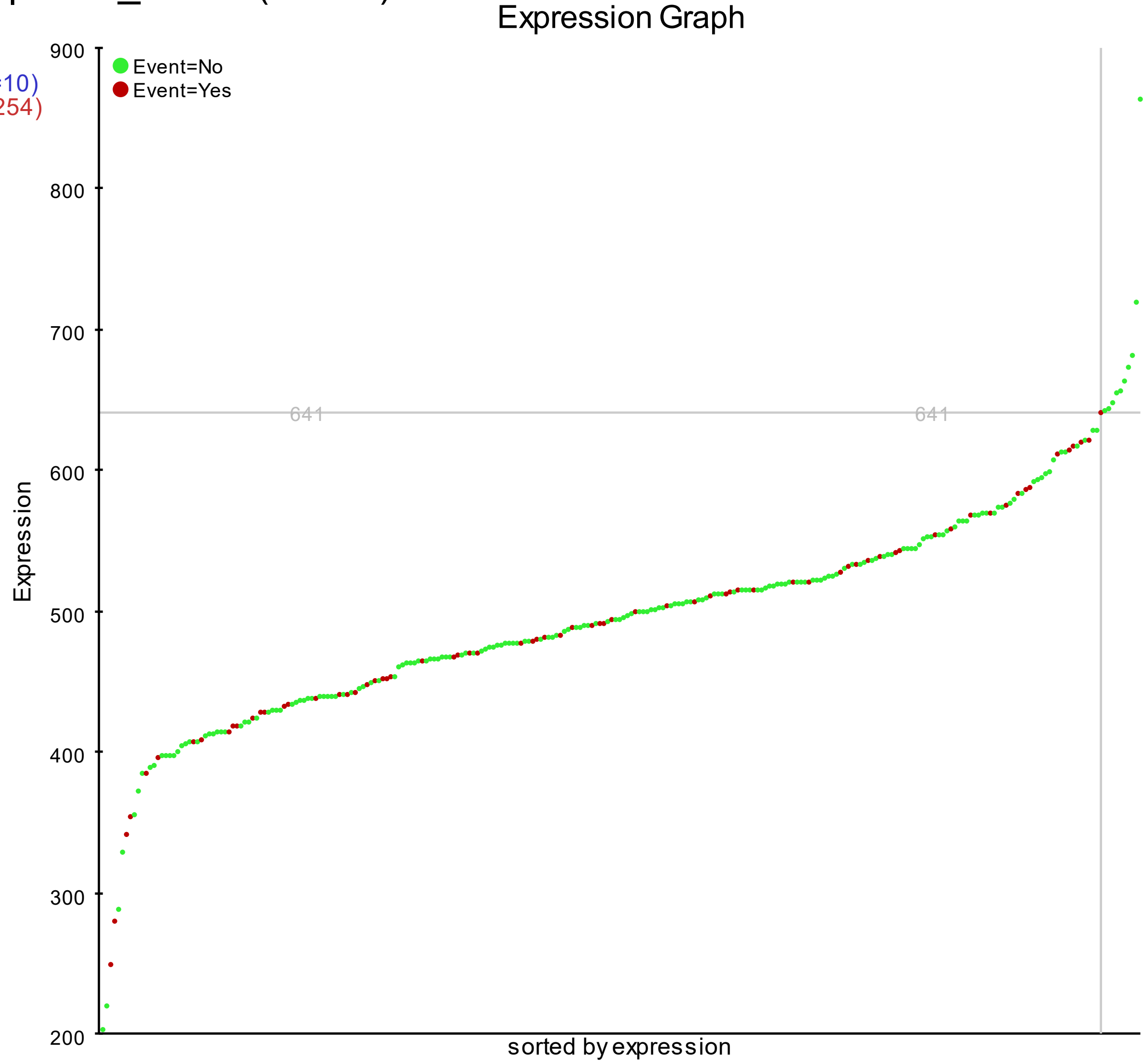

GR3

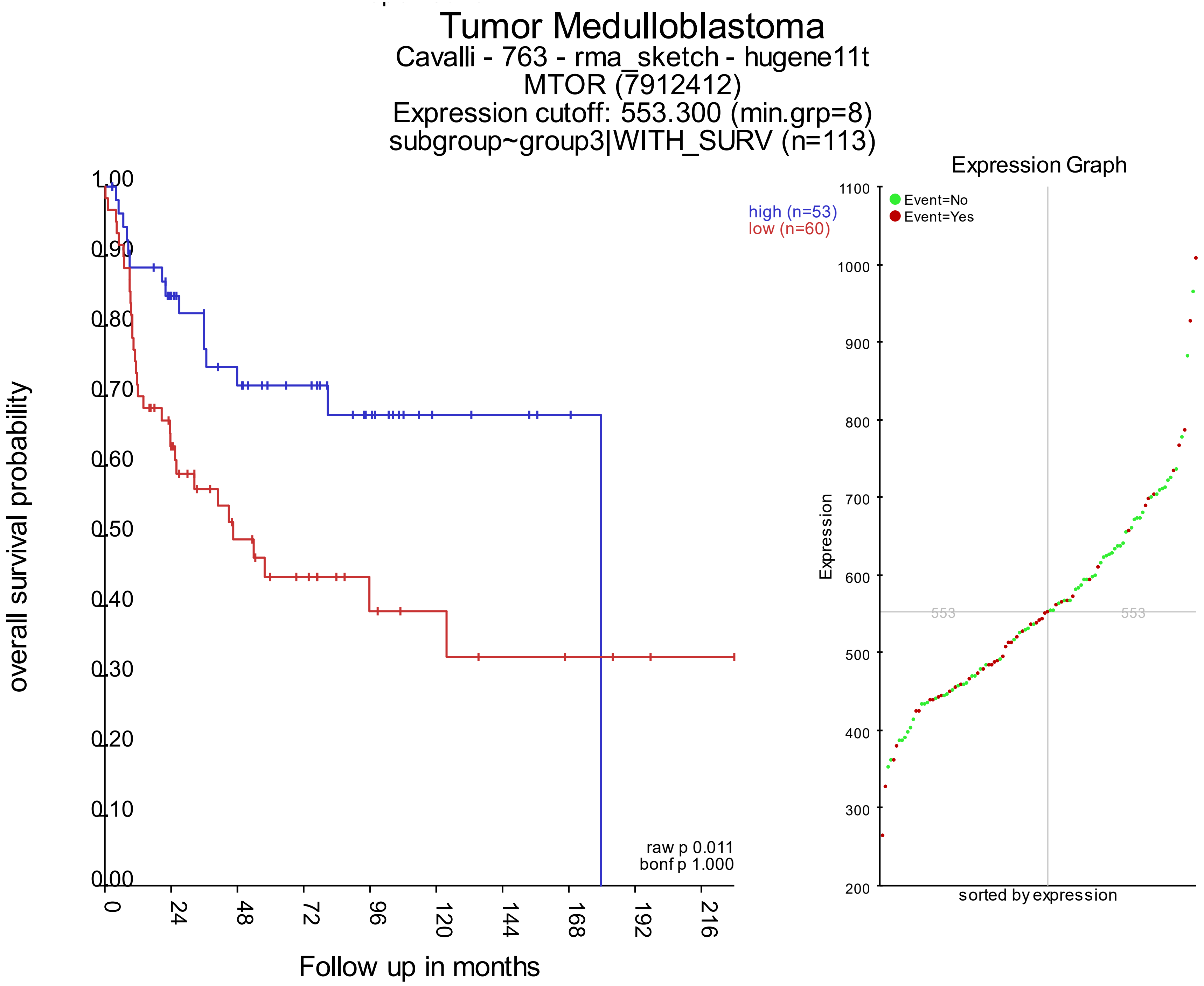

Tumor Medulloblastoma
Cavalli - 763 - rma_sketch - hugene11t
MTOR (7912412)
Expression cutoff: 553.300 (min.grp=8)
subgroup~group3|WITH_SURV (n=113)
overall survival probability
1.00
0.90
0.80
0.70
0.60
0.50
0.40
0.30
0.20
0.10
0.00
0
24
48
72
96
120
144
168
192
216
Follow up in months
high (n=53)
low (n=60)
raw p 0.011
bonf p 1.000
Expression Graph
Event=No
Event=Yes
Expression
1100
1000
900
800
700
600
500
400
300
200
553
553
sorted by expression

NTRK1

WNT

Tumor Medulloblastoma
Cavalli - 763 - rma_sketch - hugene11t
NTRK1 (7906244)
Expression cutoff: 187.700 (min.grp=8)
subgroup~wnt|WITH_SURV (n=63)

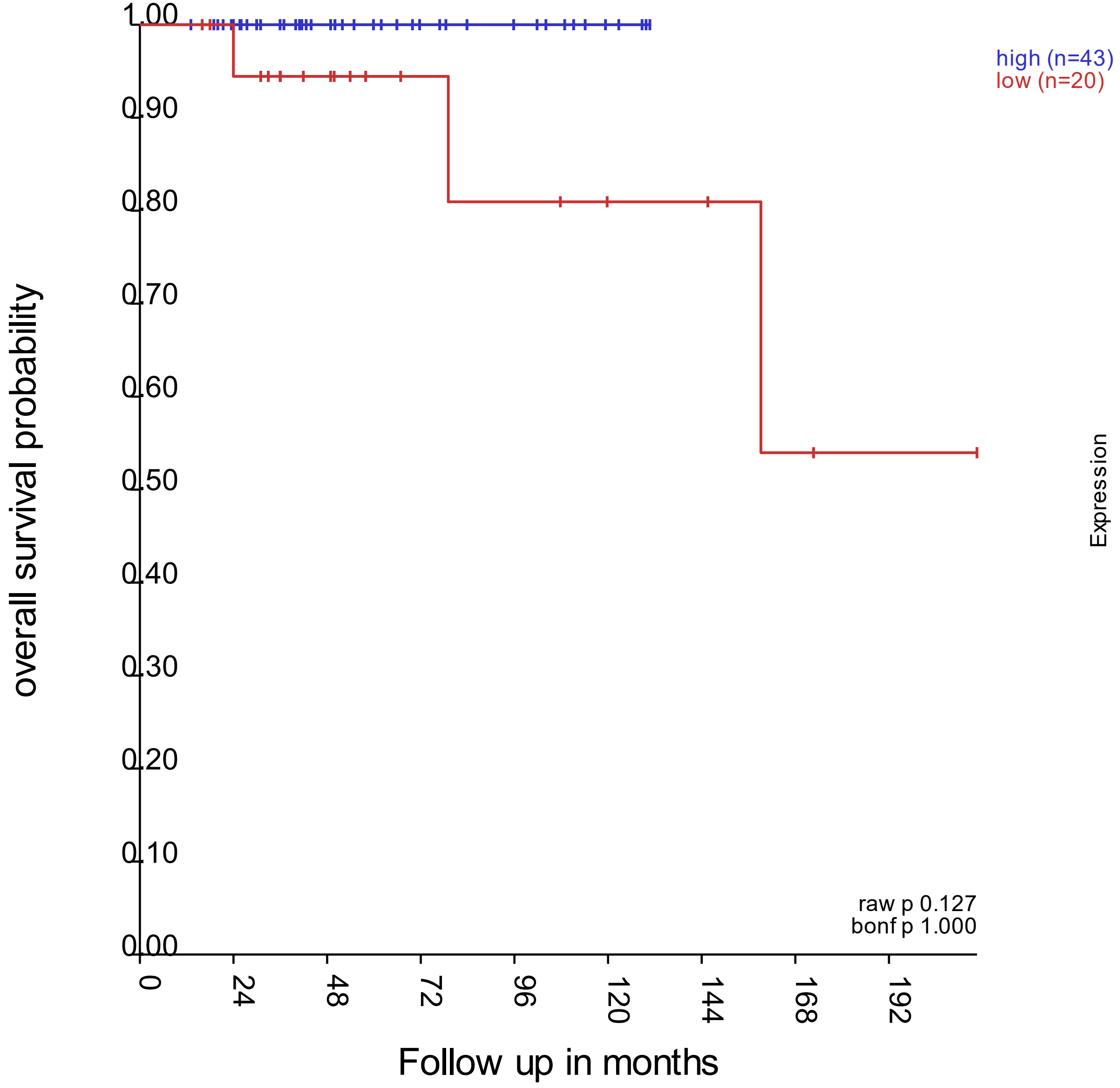

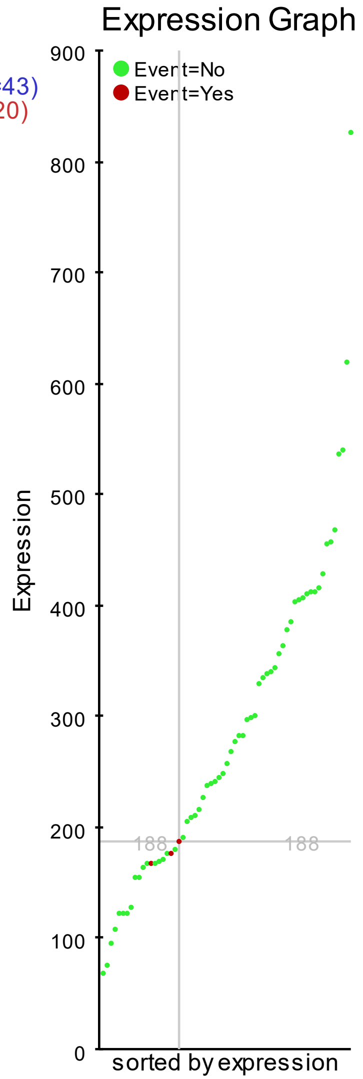

SHH

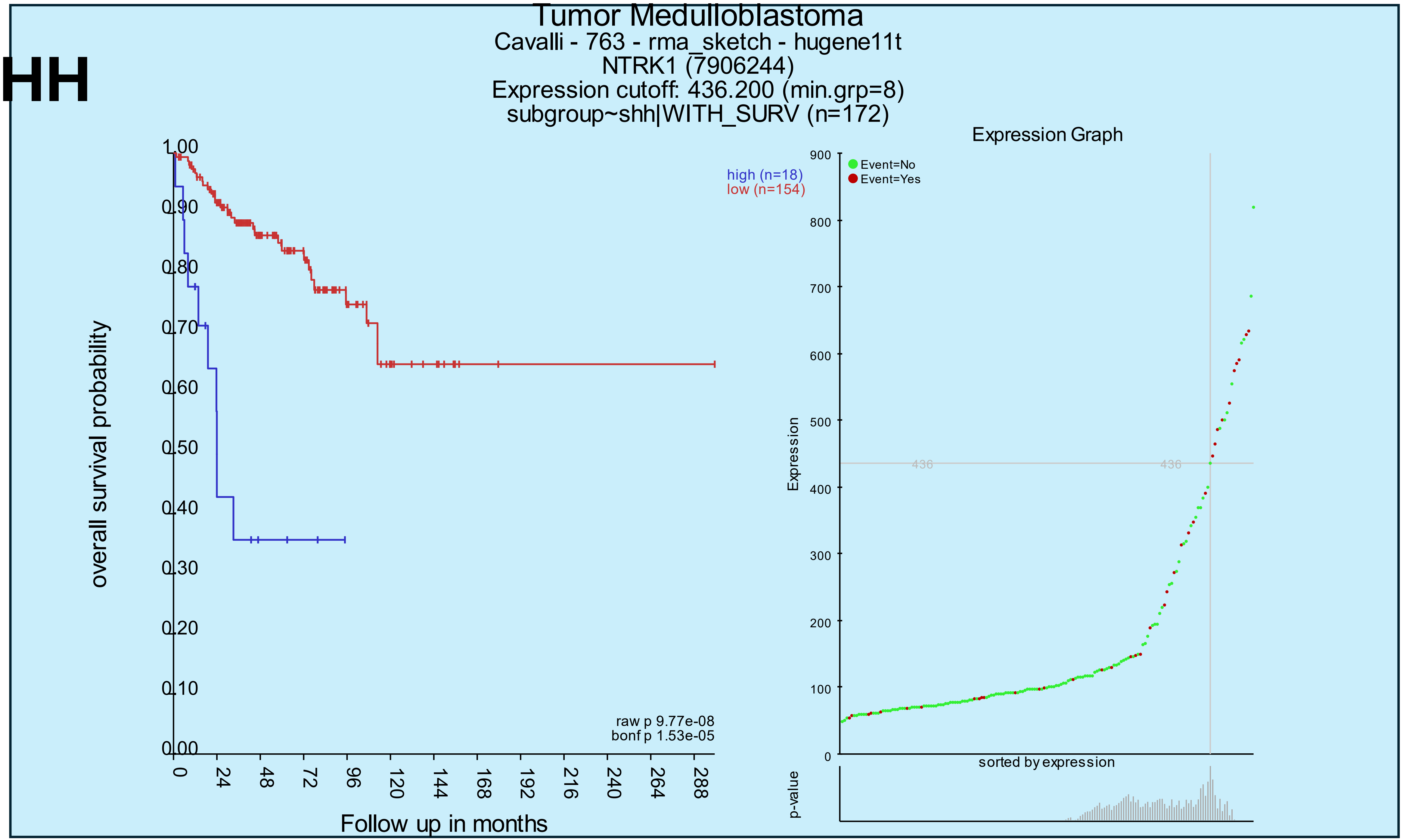

Tumor Medulloblastoma
Cavalli - 763 - rma_sketch - hugene11t
NTRK1 (7906244)
Expression cutoff: 436.200 (min.grp=8)
subgroup~shh|WITH_SURV (n=172)
overall survival probability
1.00
0.90
0.80
0.70
0.60
0.50
0.40
0.30
0.20
0.10
0.00
high (n=18)
low (n=154)
raw p 9.77e-08
bonf p 1.53e-05
0
24
48
72
96
120
144
168
192
216
240
264
288
Follow up in months
Expression Graph
Event=No
Event=Yes
900
800
700
600
500
400
300
200
100
0
Expression
436
436
sorted by expression
p-value

GR4

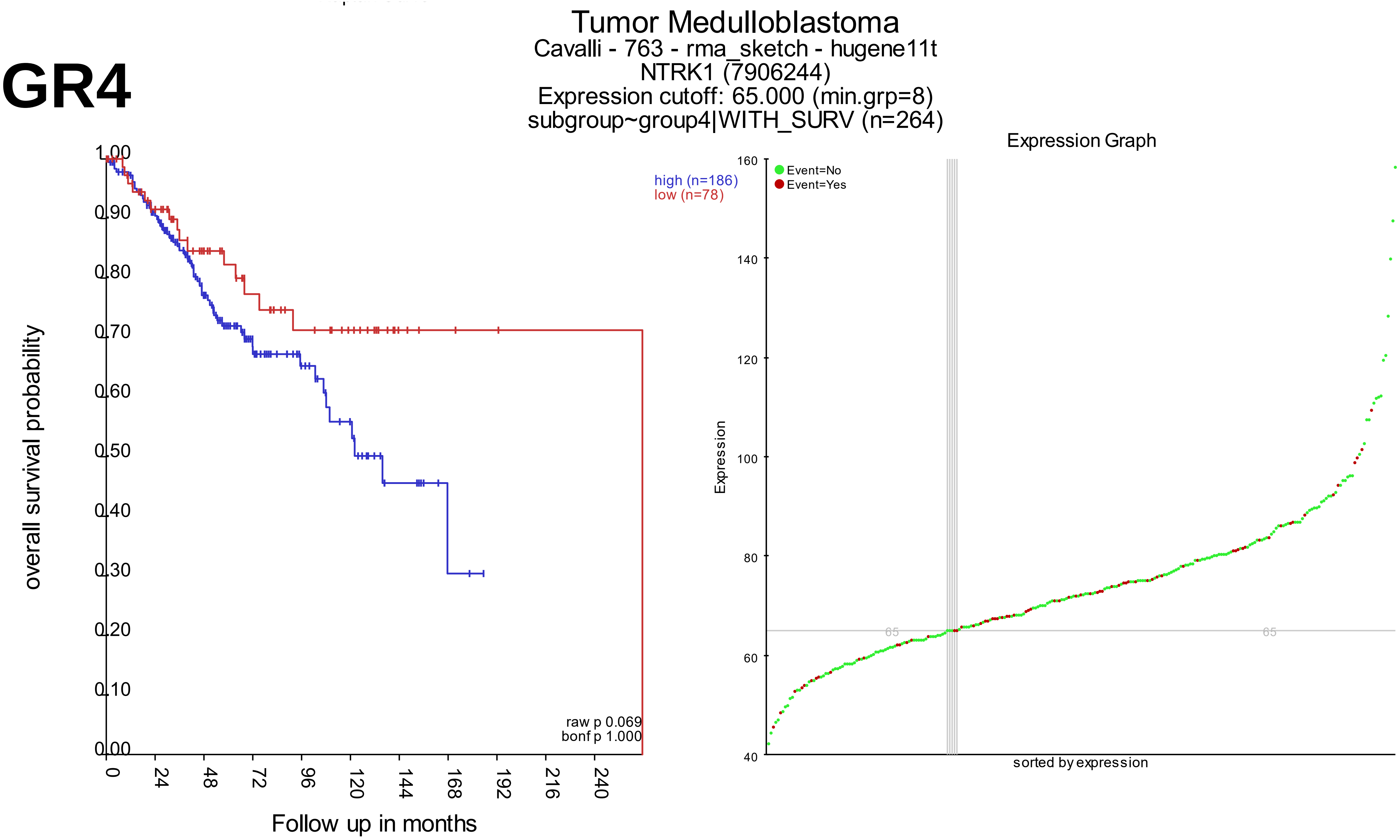

Tumor Medulloblastoma
Cavalli - 763 - rma_sketch - hugene11t
NTRK1 (7906244)
Expression cutoff: 65.000 (min.grp=8)
subgroup~group4|WITH_SURV (n=264)
high (n=186)
low (n=78)
overall survival probability
1.00
0.90
0.80
0.70
0.60
0.50
0.40
0.30
0.20
0.10
0.00
raw p 0.069
bonf p 1.000
0
24
48
72
96
120
144
168
192
216
240
Follow up in months
Expression Graph
Event=No
Event=Yes
Expression
160
140
120
100
80
60
40
65
sorted by expression

GR3

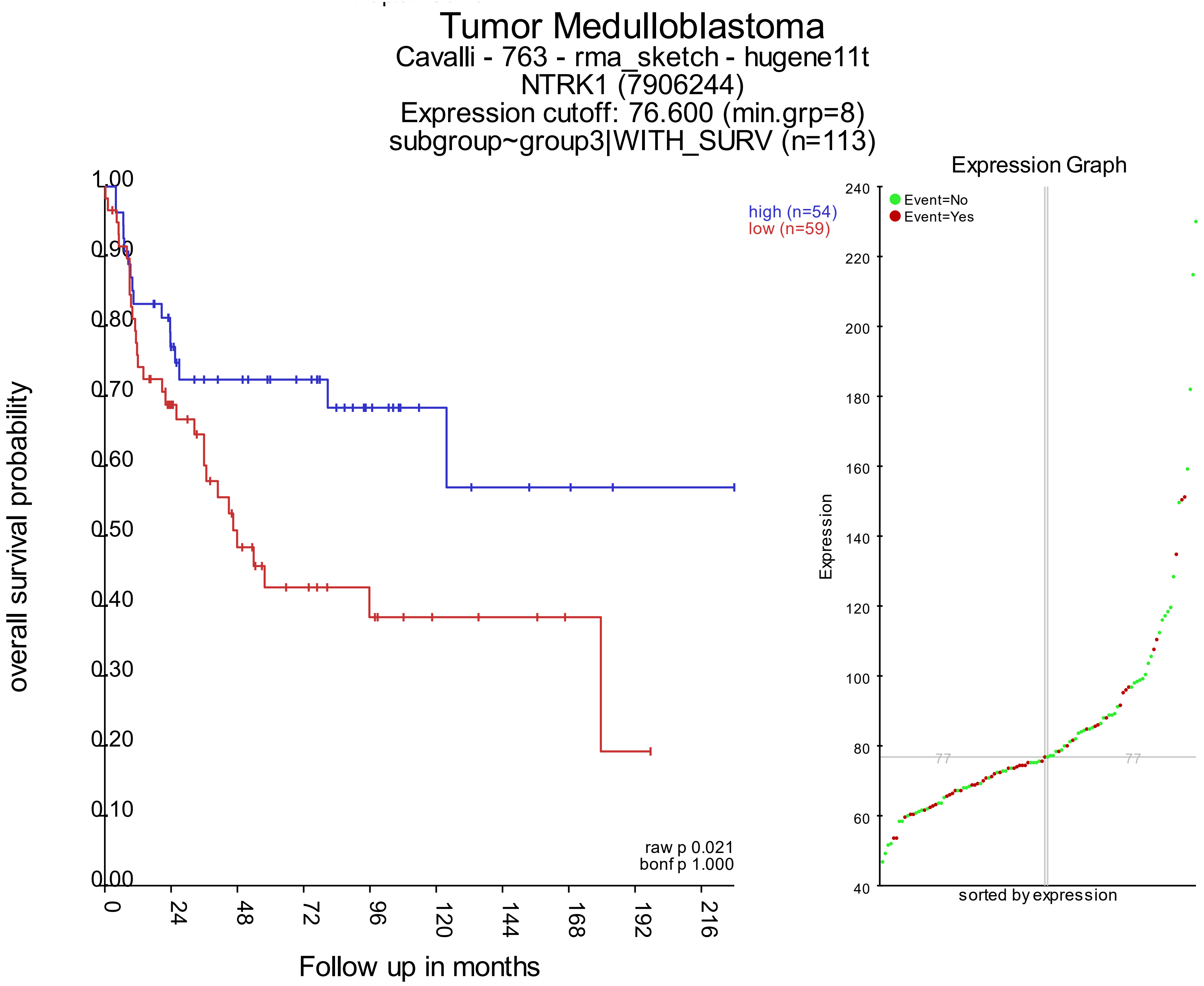

Tumor Medulloblastoma
Cavalli - 763 - rma_sketch - hugene11t
NTRK1 (7906244)
Expression cutoff: 76.600 (min.grp=8)
subgroup~group3|WITH_SURV (n=113)
overall survival probability
1.00
0.90
0.80
0.70
0.60
0.50
0.40
0.30
0.20
0.10
0.00
0
24
48
72
96
120
144
168
192
216
Follow up in months
high (n=54)
low (n=59)
raw p 0.021
bonf p 1.000
Expression Graph
Event=No
Event=Yes
Expression
240
220
200
180
160
140
120
100
80
60
40
77
77
sorted by expression

NTRK2

WNT

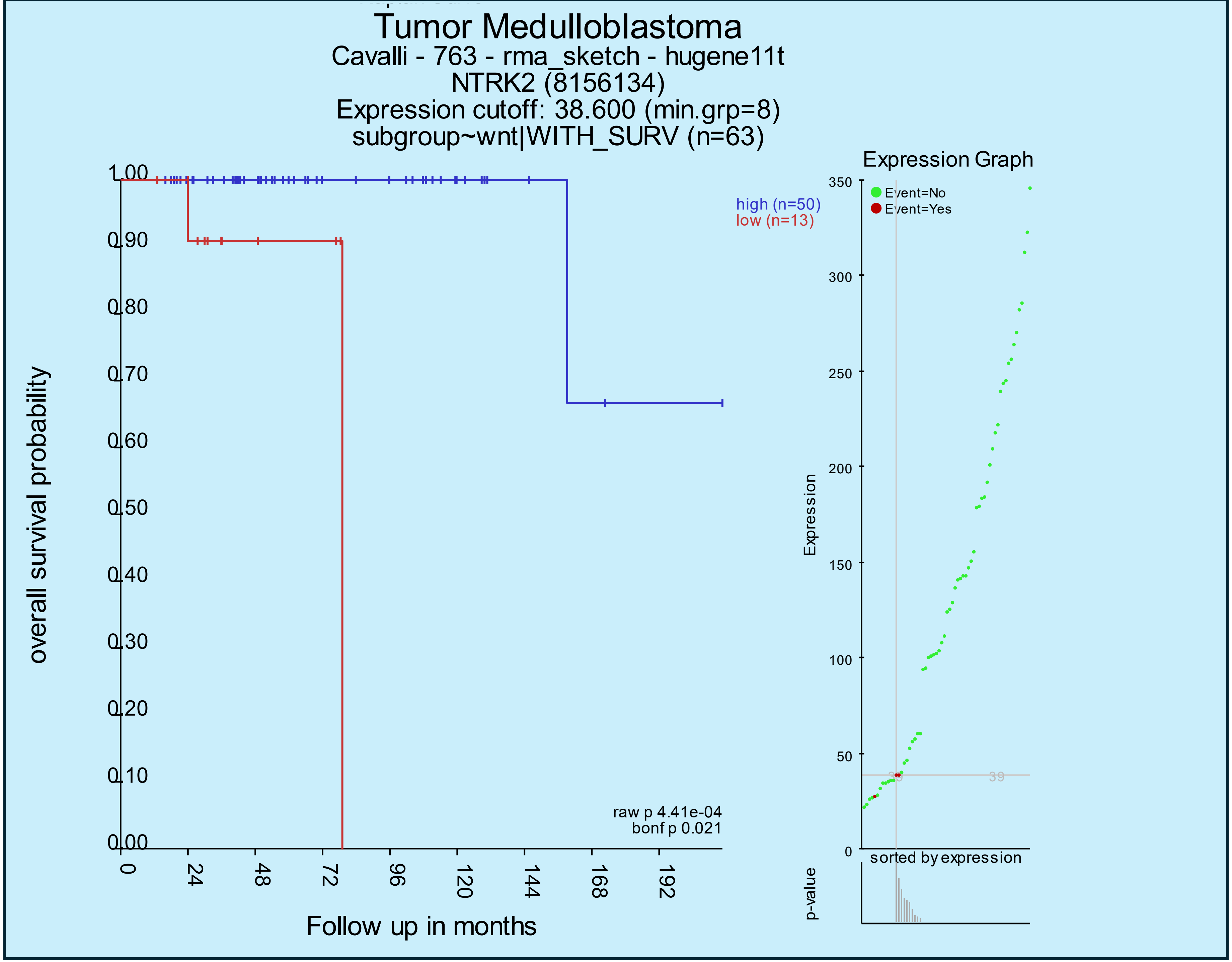

Tumor Medulloblastoma
Cavalli - 763 - rma_sketch - hugene11t
NTRK2 (8156134)
Expression cutoff: 38.600 (min.grp=8)
subgroup~wnt|WITH_SURV (n=63)
overall survival probability
1.00
0.90
0.80
0.70
0.60
0.50
0.40
0.30
0.20
0.10
0.00
0
24
48
72
96
120
144
168
192
Follow up in months
high (n=50)
low (n=13)
raw p 4.41e-04
bonf p 0.021
Expression Graph
Event=No
Event=Yes
Expression
350
300
250
200
150
100
50
0
39
sorted by expression
p-value

SHH

Tumor Medulloblastoma
Cavalli - 763 - rma_sketch - hugene11t
NTRK2 (8156134)
Expression cutoff: 53.900 (min.grp=8)
subgroup~shh|WITH_SURV (n=172)

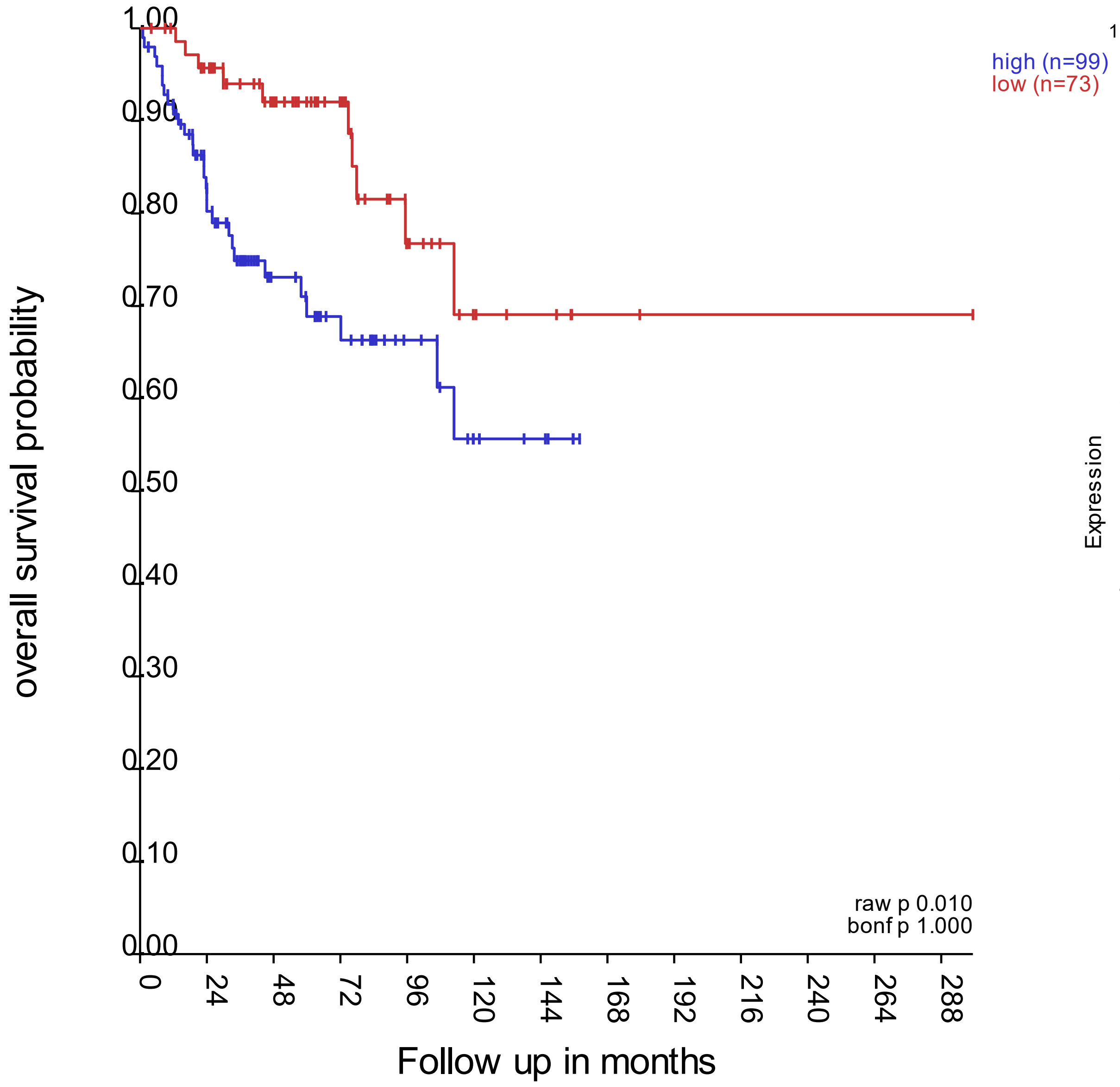

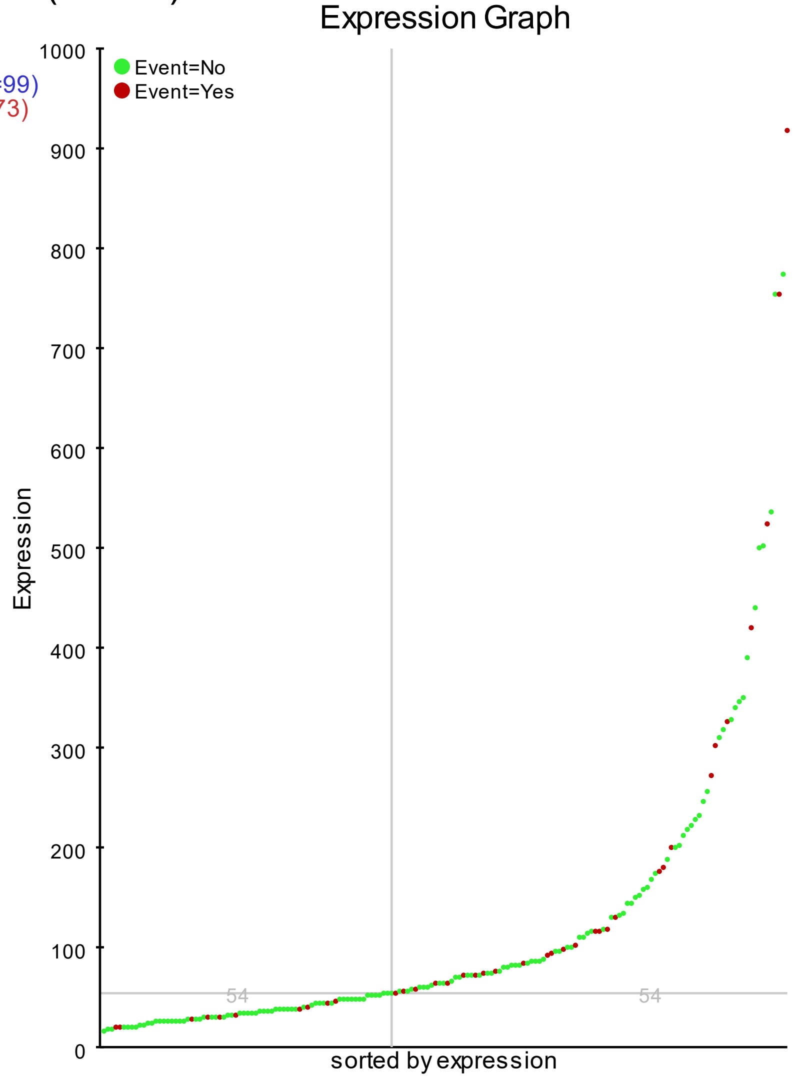

GR4

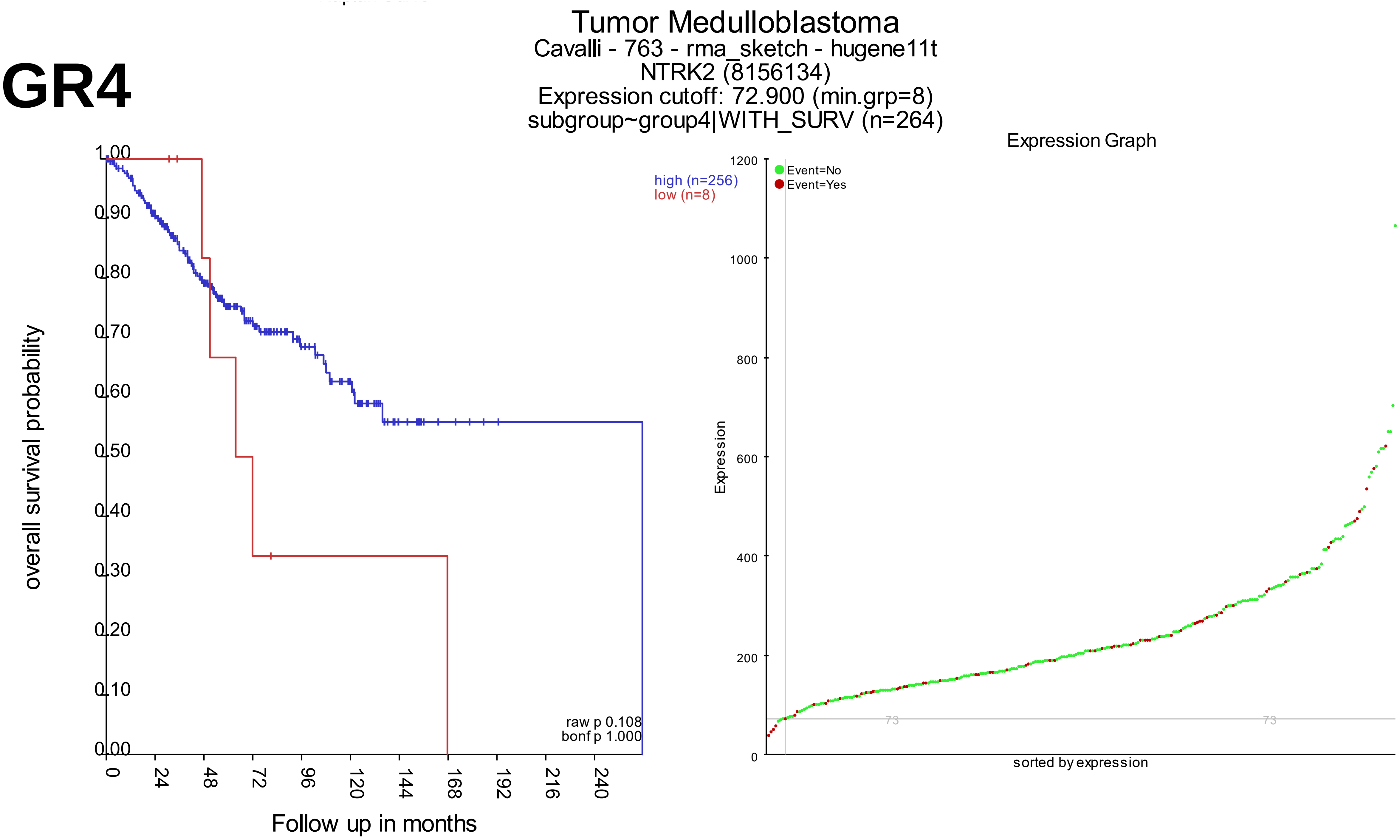

Tumor Medulloblastoma
Cavalli - 763 - rma_sketch - hugene11t
NTRK2 (8156134)
Expression cutoff: 72.900 (min.grp=8)
subgroup~group4|WITH_SURV (n=264)
overall survival probability
1.00
0.90
0.80
0.70
0.60
0.50
0.40
0.30
0.20
0.10
0.00
high (n=256)
low (n=8)
raw p 0.108
bonf p 1.000
0
24
48
72
96
120
144
168
192
216
240
Follow up in months
Expression Graph
Event=No
Event=Yes
Expression
1200
1000
800
600
400
200
0
73
73
sorted by expression

GR3

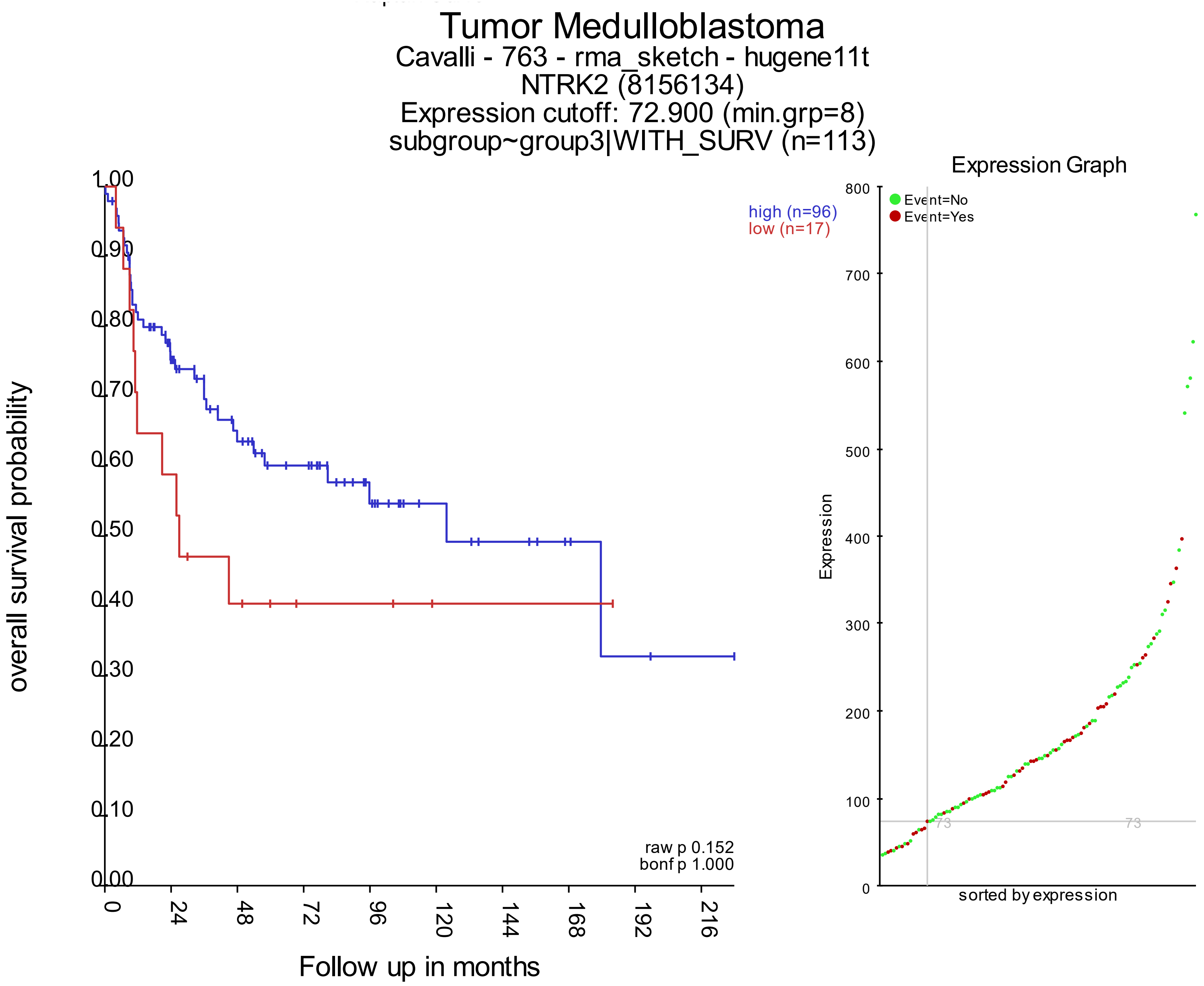

Tumor Medulloblastoma
Cavalli - 763 - rma_sketch - hugene11t
NTRK2 (8156134)
Expression cutoff: 72.900 (min.grp=8)
subgroup~group3|WITH_SURV (n=113)
overall survival probability
1.00
0.90
0.80
0.70
0.60
0.50
0.40
0.30
0.20
0.10
0.00
0
24
48
72
96
120
144
168
192
216
Follow up in months
high (n=96)
low (n=17)
raw p 0.152
bonf p 1.000
Expression Graph
Event=No
Event=Yes
Expression
800
700
600
500
400
300
200
100
0
73
73
sorted by expression

NTRK3

WNT

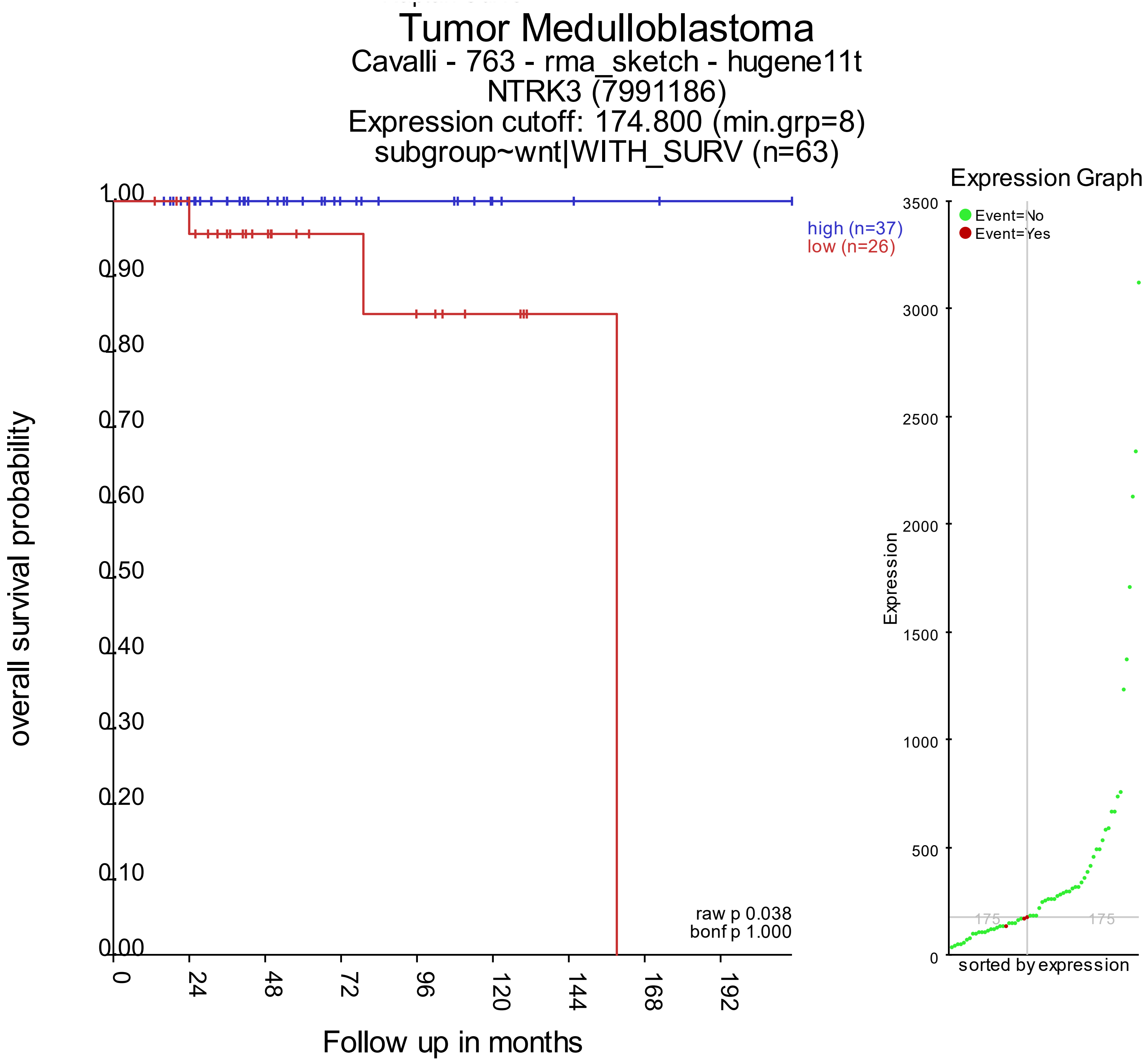

Tumor Medulloblastoma
Cavalli - 763 - rma_sketch - hugene11t
NTRK3 (7991186)
Expression cutoff: 174.800 (min.grp=8)
subgroup~wnt|WITH_SURV (n=63)
overall survival probability
1.00
0.90
0.80
0.70
0.60
0.50
0.40
0.30
0.20
0.10
0.00
high (n=37)
low (n=26)
raw p 0.038
bonf p 1.000
0
24
48
72
96
120
144
168
192
Follow up in months
Expression Graph
Event=No
Event=Yes
Expression
3500
3000
2500
2000
1500
1000
500
0
175
175
sorted by expression

SHH

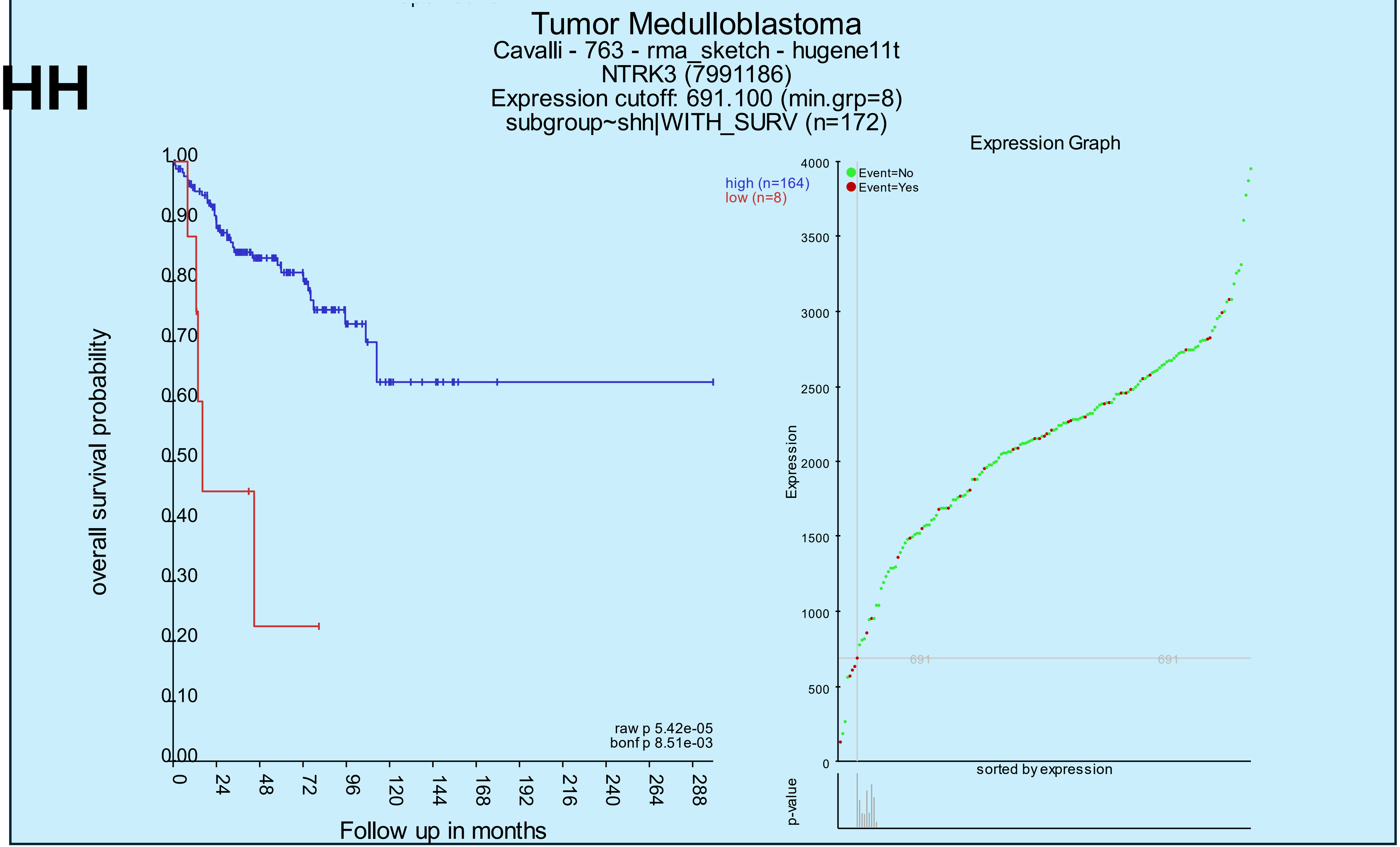
Tumor Medulloblastoma
Cavalli - 763 - rma_sketch - hugene11t
NTRK3 (7991186)
Expression cutoff: 691.100 (min.grp=8)
subgroup~shh|WITH_SURV (n=172)
overall survival probability
1.00
0.90
0.80
0.70
0.60
0.50
0.40
0.30
0.20
0.10
0.00
0
24
48
72
96
120
144
168
192
216
240
264
288
Follow up in months
high (n=164)
low (n=8)
raw p 5.42e-05
bonf p 8.51e-03
Expression Graph
Event=No
Event=Yes
Expression
4000
3500
3000
2500
2000
1500
1000
500
0
691
691
sorted by expression
p-value

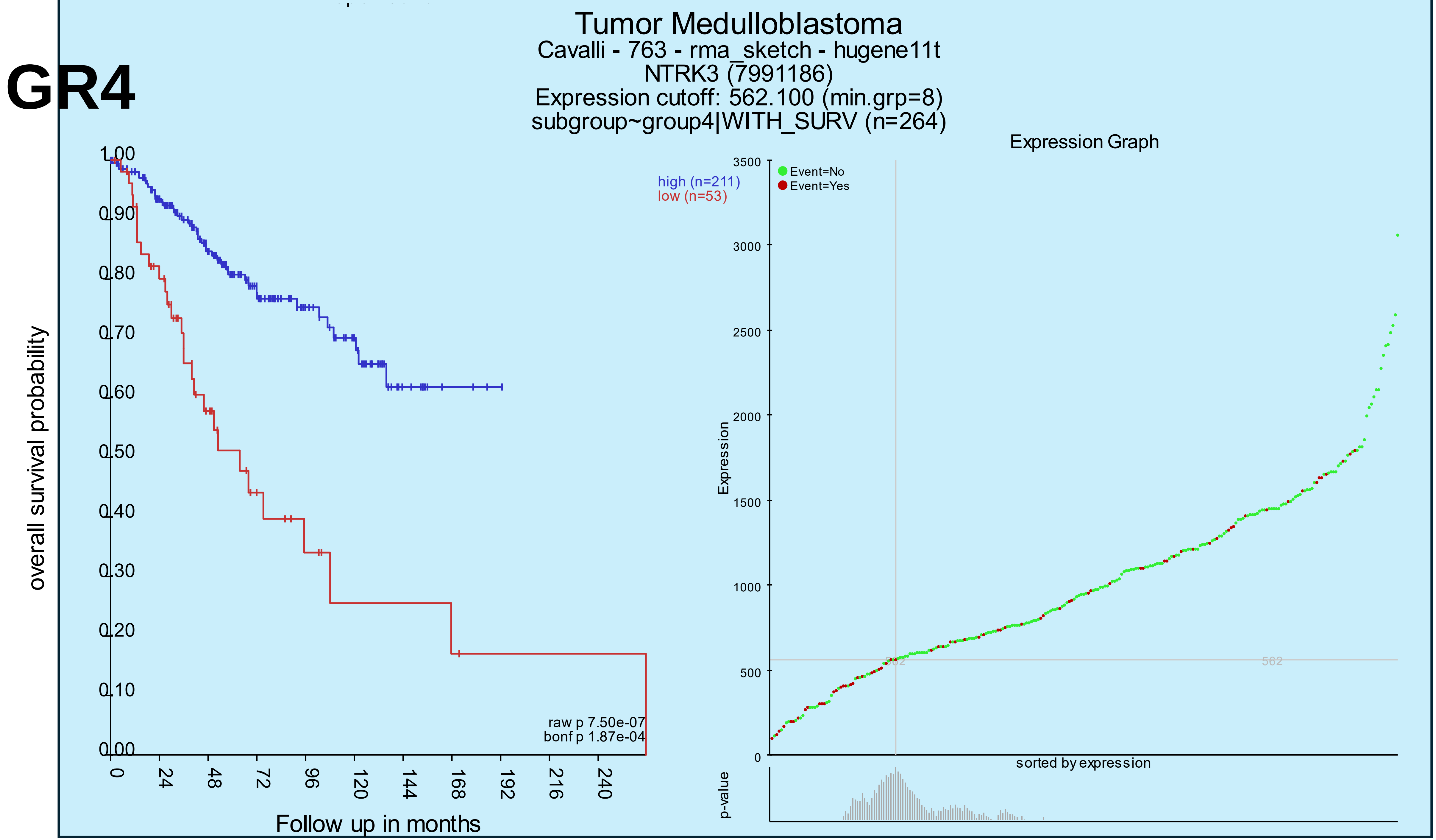
GR4
Tumor Medulloblastoma
Cavalli - 763 - rma_sketch - hugene11t
NTRK3 (7991186)
Expression cutoff: 562.100 (min.grp=8)
subgroup~group4|WITH_SURV (n=264)
high (n=211)
low (n=53)
overall survival probability
1.00
0.90
0.80
0.70
0.60
0.50
0.40
0.30
0.20
0.10
0.00
0
24
48
72
96
120
144
168
192
216
240
Follow up in months
raw p 7.50e-07
bonf p 1.87e-04
Expression Graph
Event=No
Event=Yes
Expression
3500
3000
2500
2000
1500
1000
500
0
562
sorted by expression
p-value

GR3

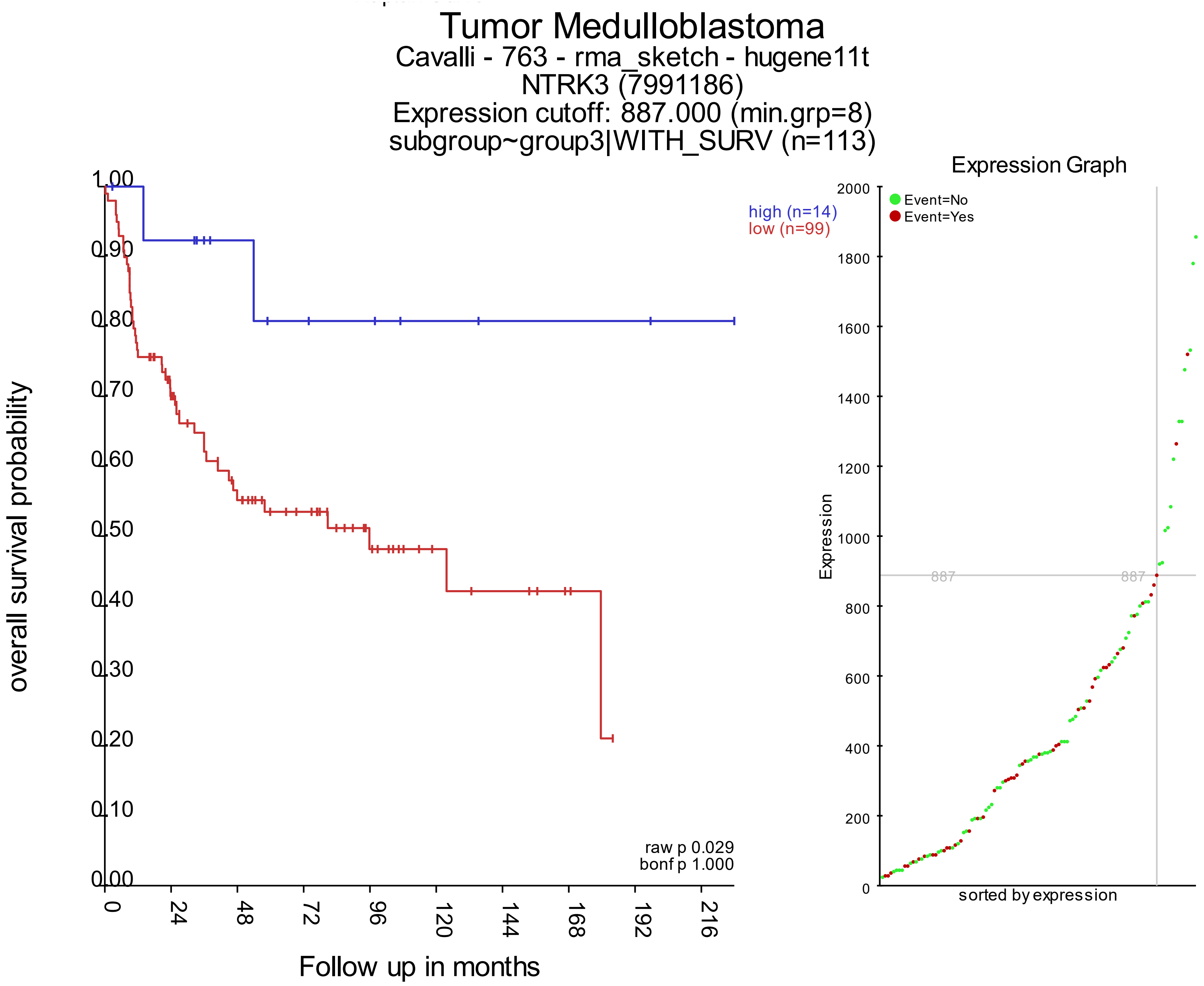

Tumor Medulloblastoma
Cavalli - 763 - rma_sketch - hugene11t
NTRK3 (7991186)
Expression cutoff: 887.000 (min.grp=8)
subgroup~group3|WITH_SURV (n=113)
overall survival probability
1.00
0.90
0.80
0.70
0.60
0.50
0.40
0.30
0.20
0.10
0.00
high (n=14)
low (n=99)
raw p 0.029
bonf p 1.000
0
24
48
72
96
120
144
168
192
216
Follow up in months
Expression Graph
Event=No
Event=Yes
Expression
2000
1800
1600
1400
1200
1000
800
600
400
200
0
887
887
sorted by expression

PARP1

WNT

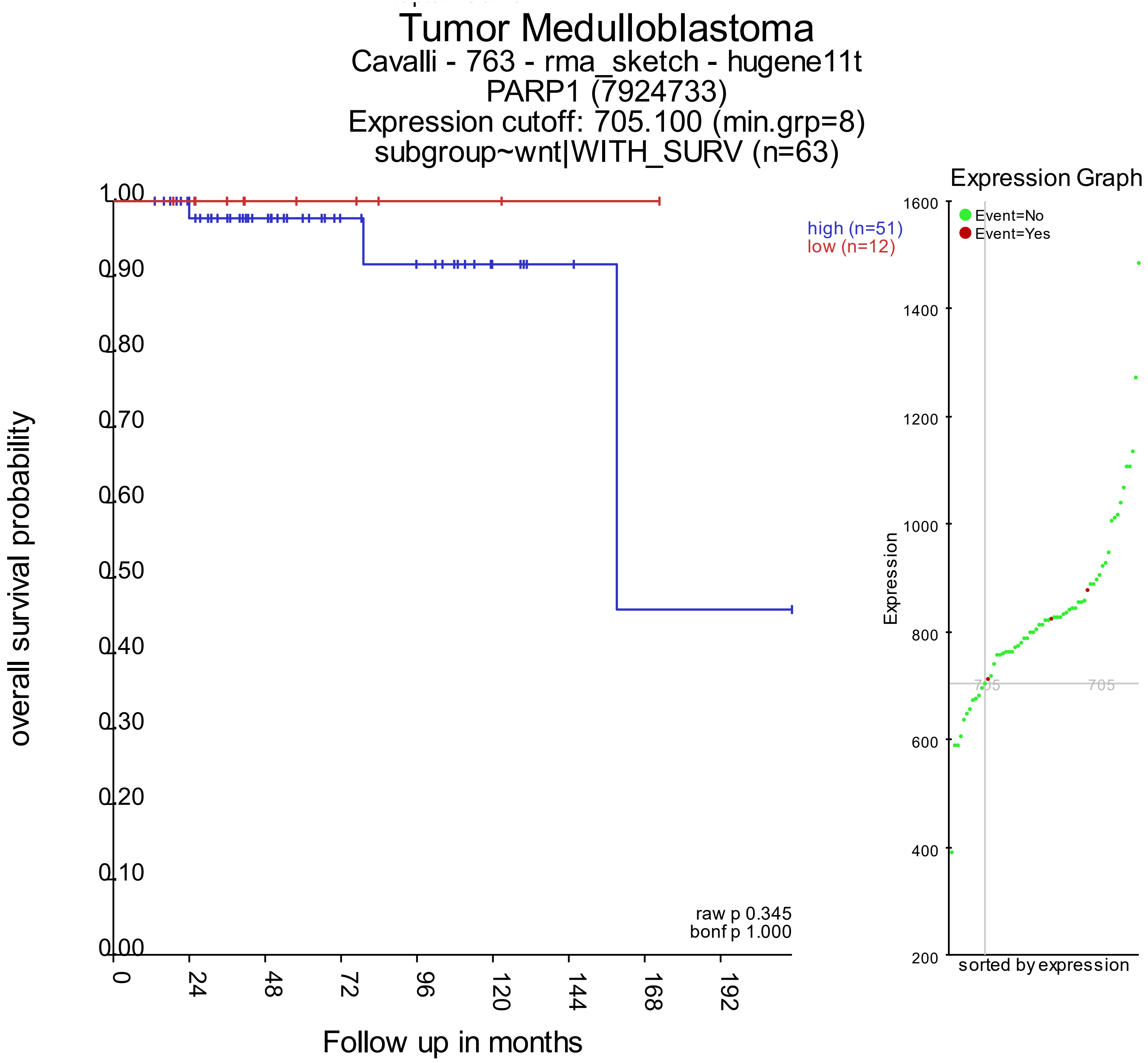

Tumor Medulloblastoma
Cavalli - 763 - rma_sketch - hugene11t
PARP1 (7924733)
Expression cutoff: 705.100 (min.grp=8)
subgroup~wnt|WITH_SURV (n=63)
overall survival probability
1.00
0.90
0.80
0.70
0.60
0.50
0.40
0.30
0.20
0.10
0.00
0
24
48
72
96
120
144
168
192
Follow up in months
high (n=51)
low (n=12)
raw p 0.345
bonf p 1.000
Expression Graph
Event=No
Event=Yes
Expression
1600
1400
1200
1000
800
600
400
200
705
sorted by expression

SHH

Tumor Medulloblastoma
Cavalli - 763 - rma_sketch - hugene11t
PARP1 (7924733)
Expression cutoff: 942.600 (min.grp=8)
subgroup~shh|WITH_SURV (n=172)

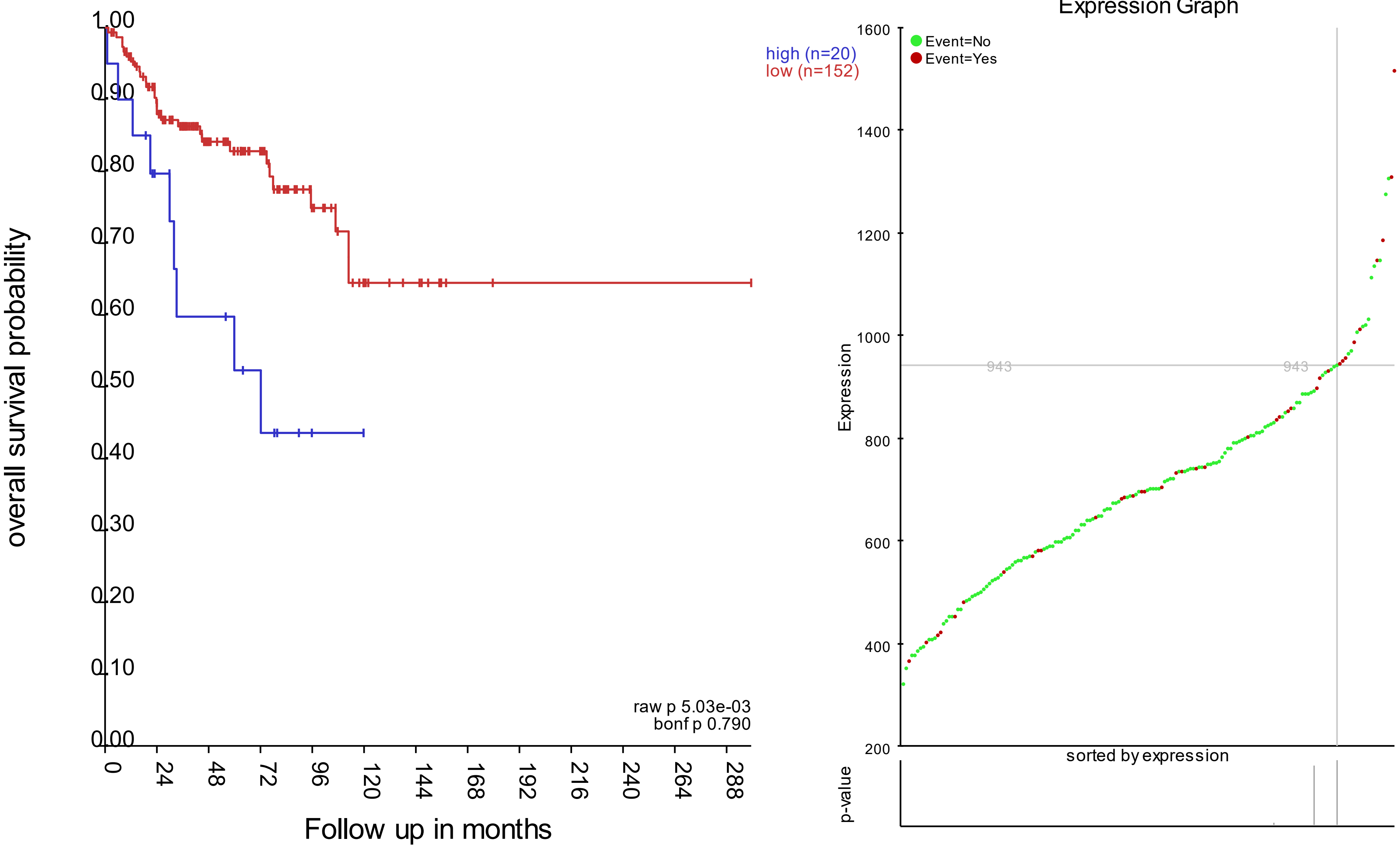

GR4

Tumor Medulloblastoma
Cavalli - 763 - rma_sketch - hugene11t
PARP1 (7924733)
Expression cutoff: 407.500 (min.grp=8)
subgroup~group4|WITH_SURV (n=264)

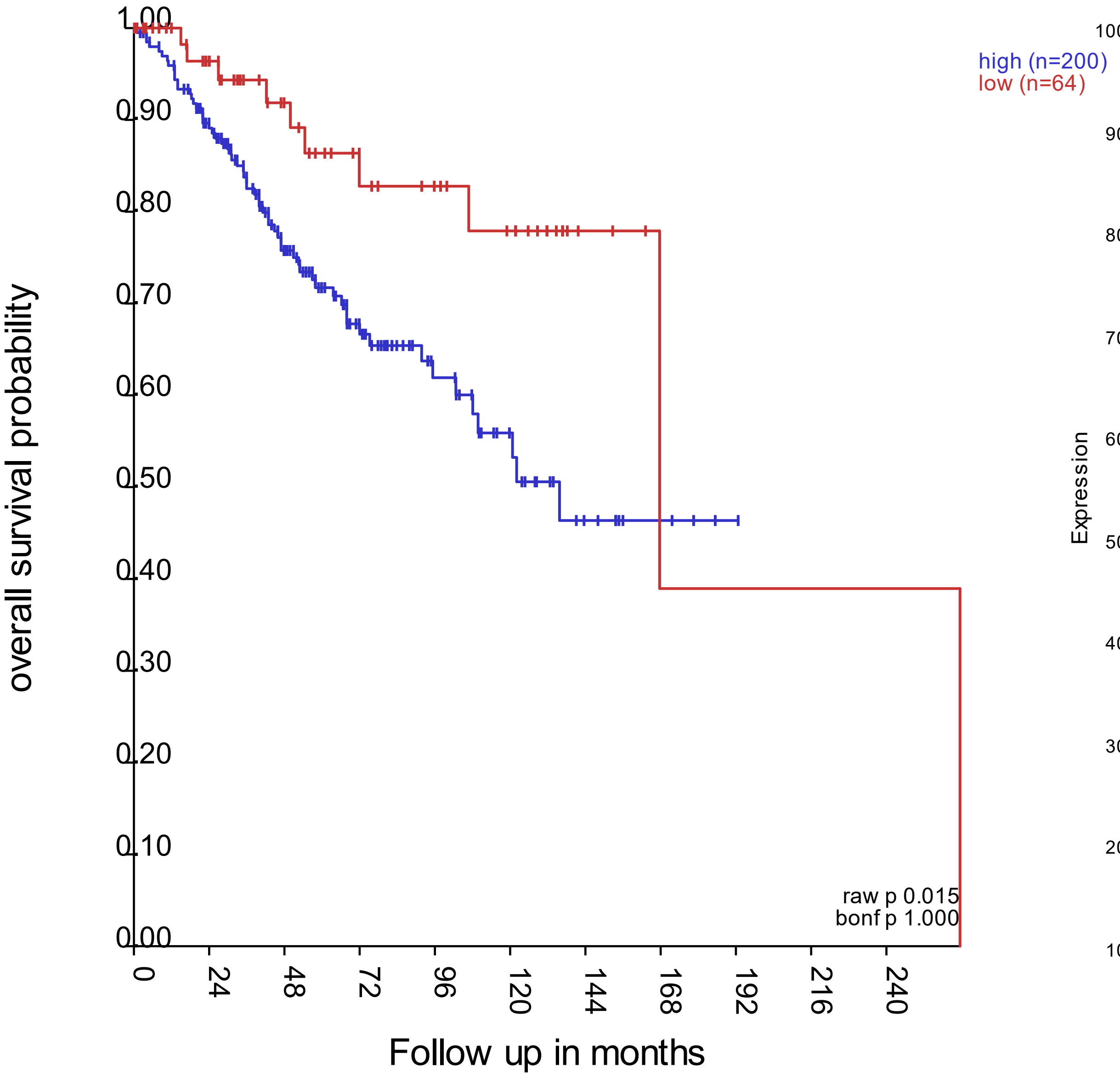

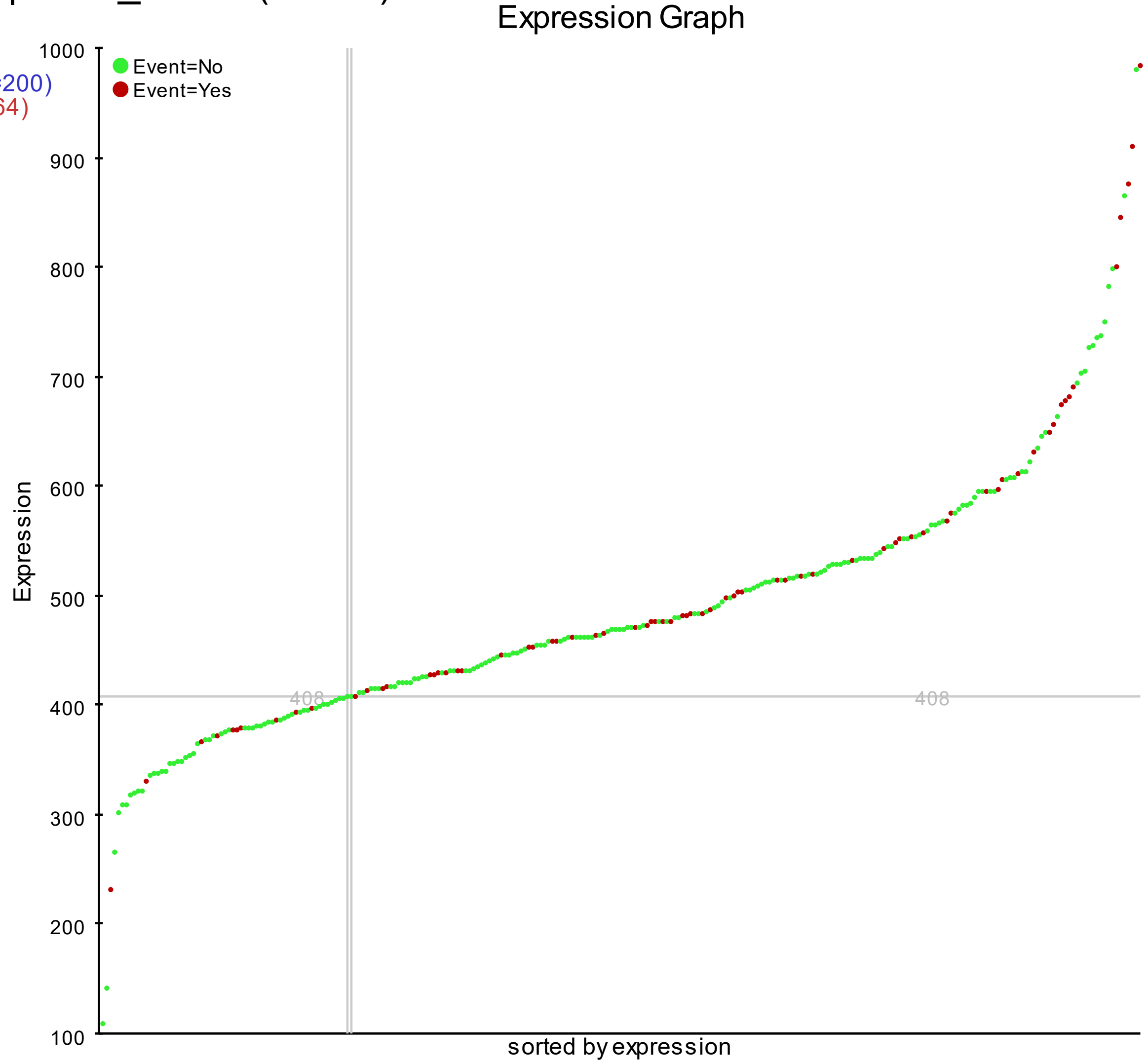

GR3

Tumor Medulloblastoma
Cavalli - 763 - rma_sketch - hugene11t
PARP1 (7924733)
Expression cutoff: 522.900 (min.grp=8)
subgroup~group3|WITH_SURV (n=113)

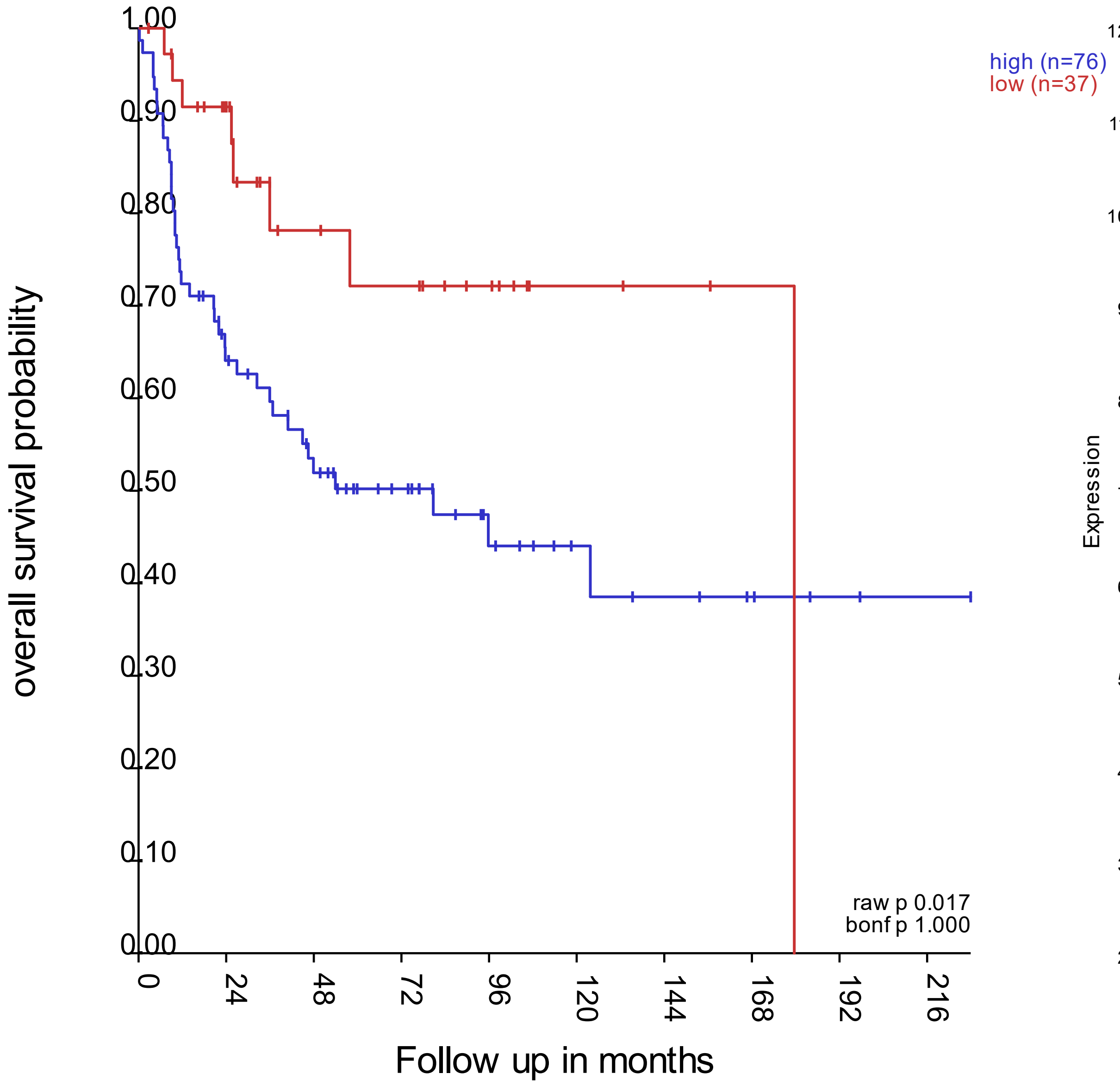

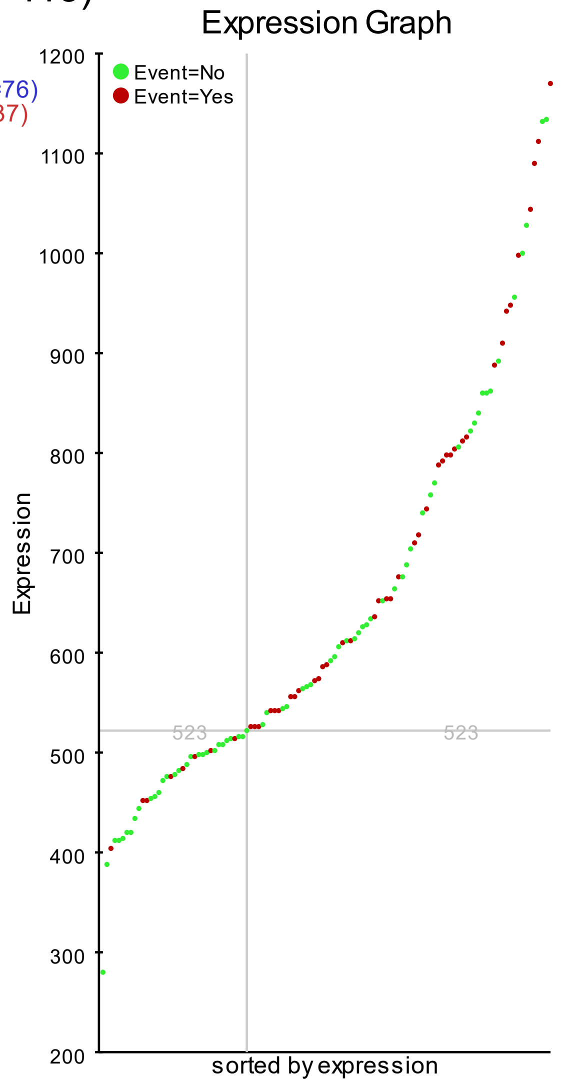

PDGFRA

WNT

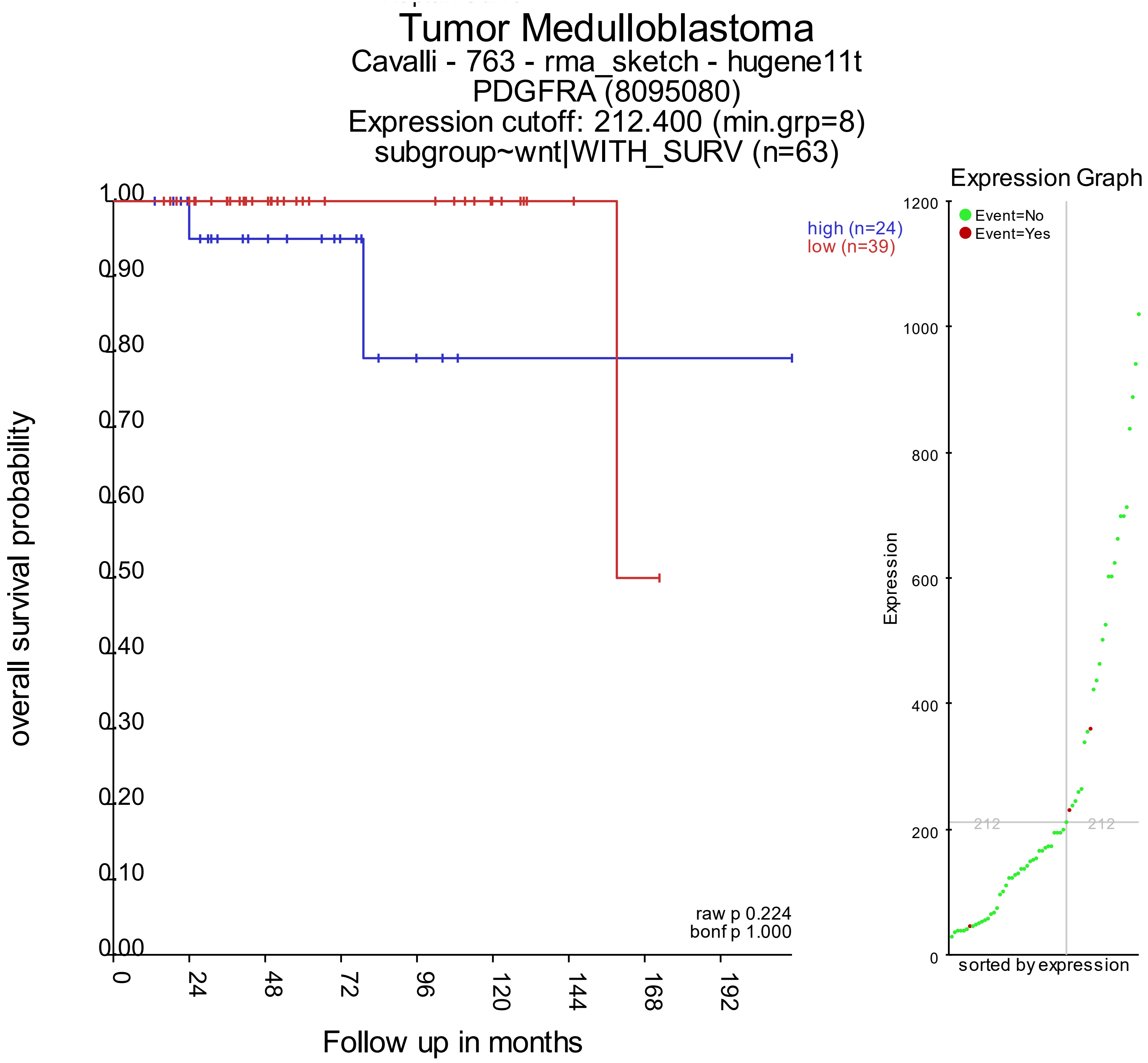

Tumor Medulloblastoma
Cavalli - 763 - rma_sketch - hugene11t
PDGFRA (8095080)
Expression cutoff: 212.400 (min.grp=8)
subgroup~wnt|WITH_SURV (n=63)
overall survival probability
1.00
0.90
0.80
0.70
0.60
0.50
0.40
0.30
0.20
0.10
0.00
0
24
48
72
96
120
144
168
192
Follow up in months
high (n=24)
low (n=39)
raw p 0.224
bonf p 1.000
Expression Graph
Event=No
Event=Yes
Expression
1200
1000
800
600
400
200
0
212
212
sorted by expression

SHH

Tumor Medulloblastoma
Cavalli - 763 - rma_sketch - hugene11t
PDGFRA (8095080)
Expression cutoff: 490.000 (min.grp=8)
subgroup~shh|WITH_SURV (n=172)

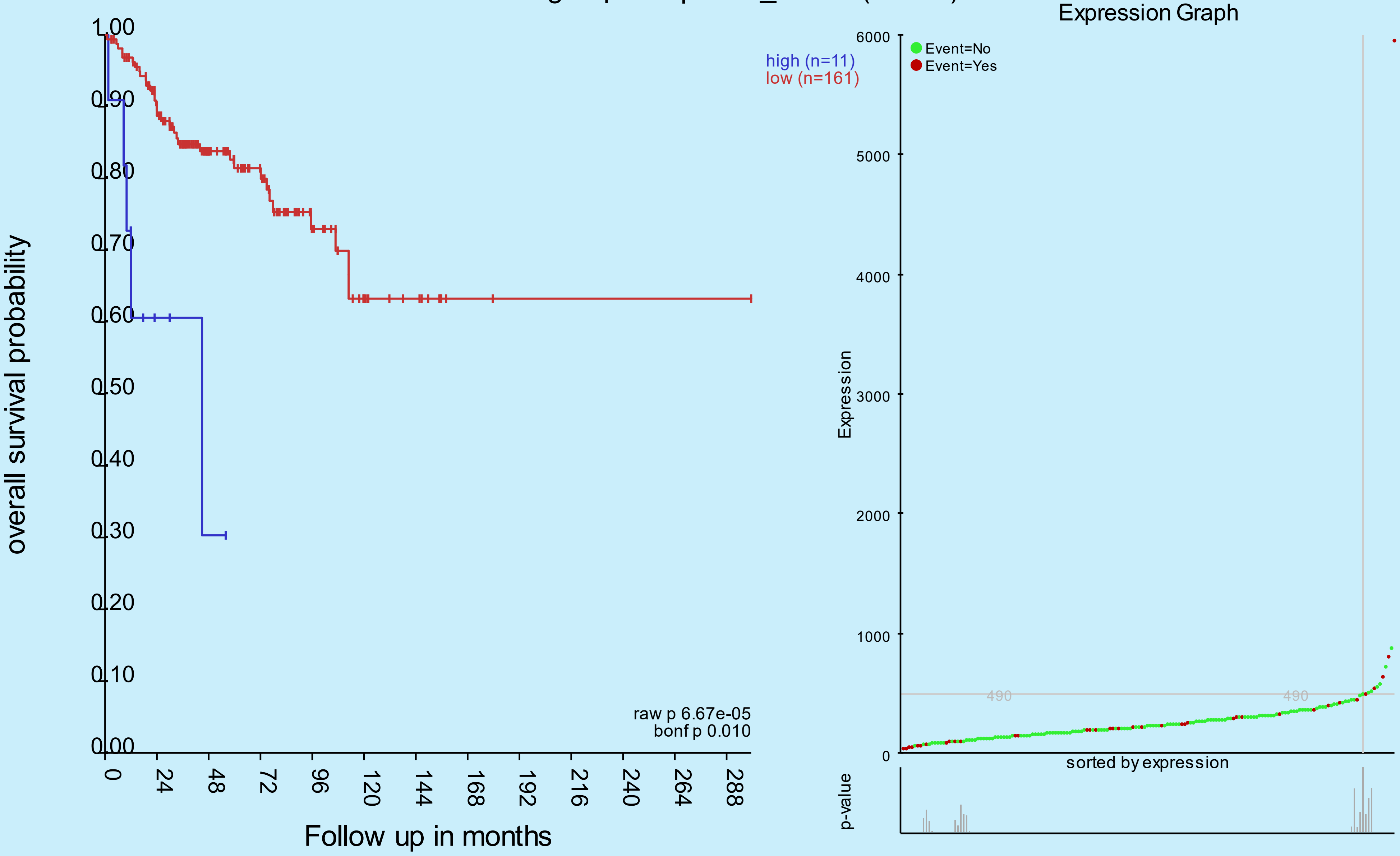

GR4

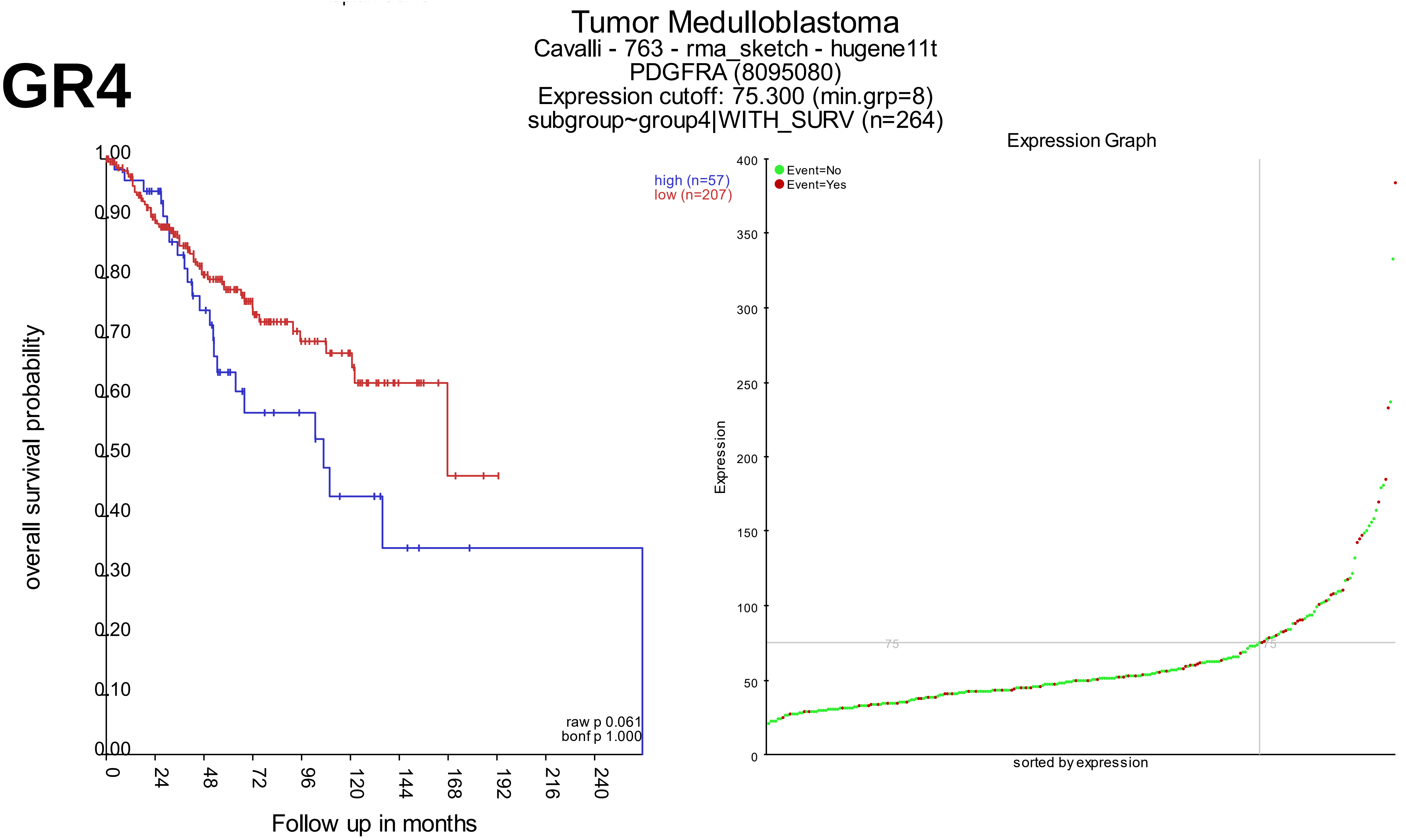

Tumor Medulloblastoma
Cavalli - 763 - rma_sketch - hugene11t
PDGFRA (8095080)
Expression cutoff: 75.300 (min.grp=8)
subgroup~group4|WITH_SURV (n=264)
overall survival probability
1.00
0.90
0.80
0.70
0.60
0.50
0.40
0.30
0.20
0.10
0.00
high (n=57)
low (n=207)
raw p 0.061
bonf p 1.000
0
24
48
72
96
120
144
168
192
216
240
Follow up in months
Expression Graph
Event=No
Event=Yes
Expression
400
350
300
250
200
150
100
50
0
75
75
sorted by expression

GR3

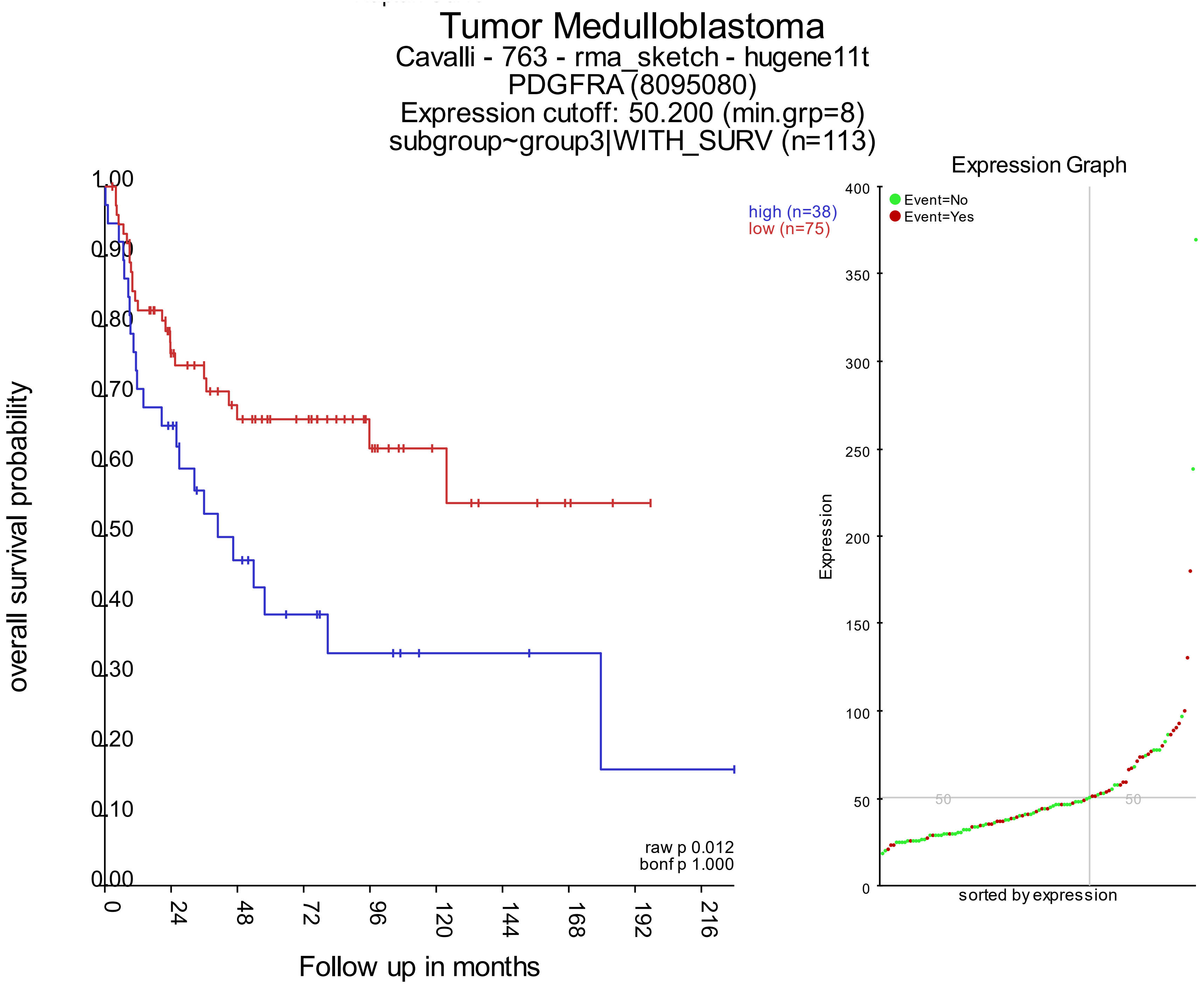
Tumor Medulloblastoma
Cavalli - 763 - rma_sketch - hugene11t
PDGFRA (8095080)
Expression cutoff: 50.200 (min.grp=8)
subgroup~group3|WITH_SURV (n=113)
high (n=38)
low (n=75)
overall survival probability
1.00
0.90
0.80
0.70
0.60
0.50
0.40
0.30
0.20
0.10
0.00
0
24
48
72
96
120
144
168
192
216
Follow up in months
raw p 0.012
bonf p 1.000
Expression Graph
Event=No
Event=Yes
Expression
400
350
300
250
200
150
100
50
0
sorted by expression

PDGFRB

WNT

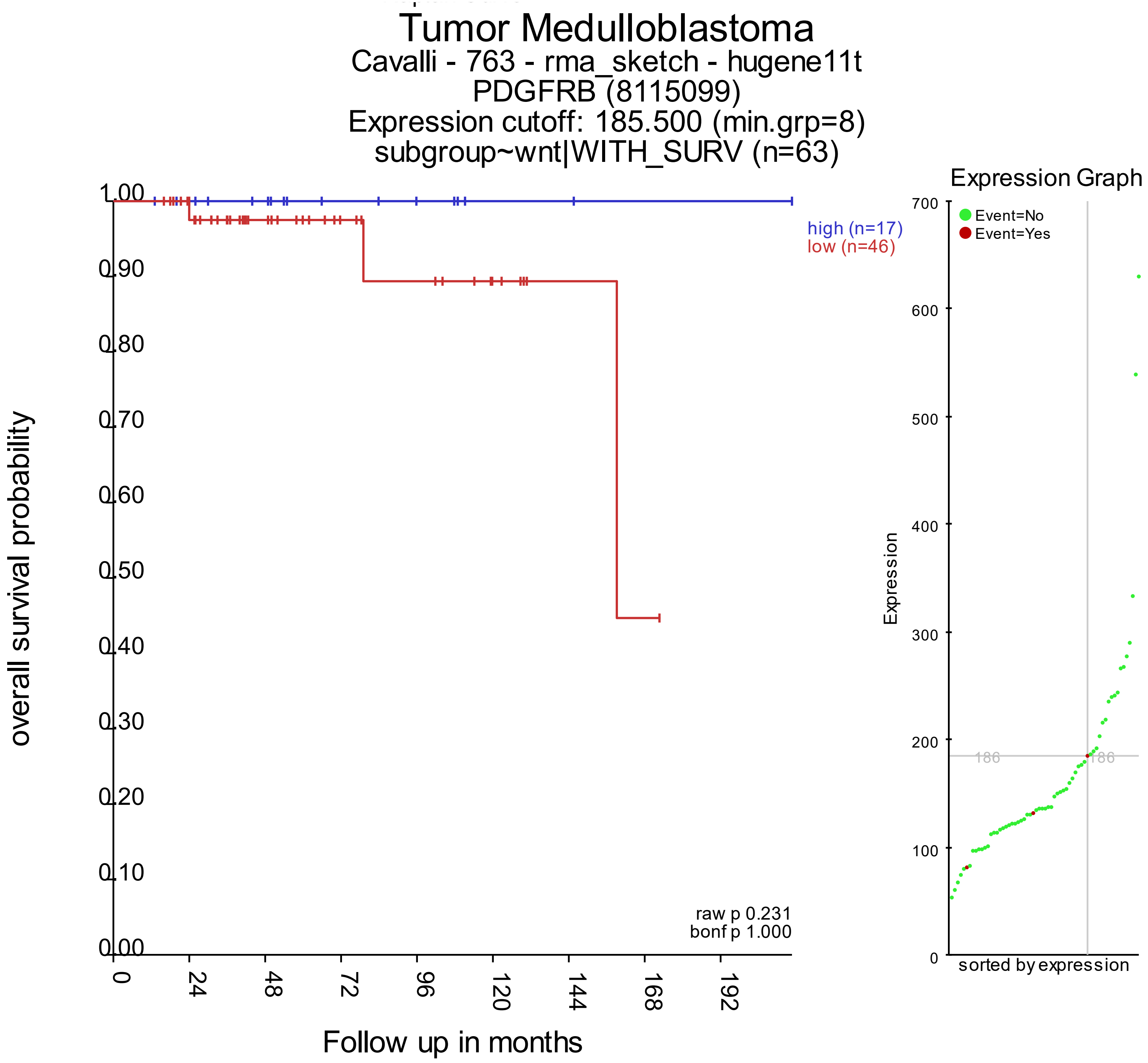

Tumor Medulloblastoma
Cavalli - 763 - rma_sketch - hugene11t
PDGFRB (8115099)
Expression cutoff: 185.500 (min.grp=8)
subgroup~wnt|WITH_SURV (n=63)
overall survival probability
1.00
0.90
0.80
0.70
0.60
0.50
0.40
0.30
0.20
0.10
0.00
high (n=17)
low (n=46)
raw p 0.231
bonf p 1.000
0
24
48
72
96
120
144
168
192
Follow up in months
Expression Graph
Event=No
Event=Yes
Expression
700
600
500
400
300
200
100
0
186
186
sorted by expression

SHH

Tumor Medulloblastoma

Cavalli - 763 - rma_sketch - hugene11t
PDGFRB (8115099)
Expression cutoff: 229.400 (min.grp=8)
subgroup~shh|WITH_SURV (n=172)

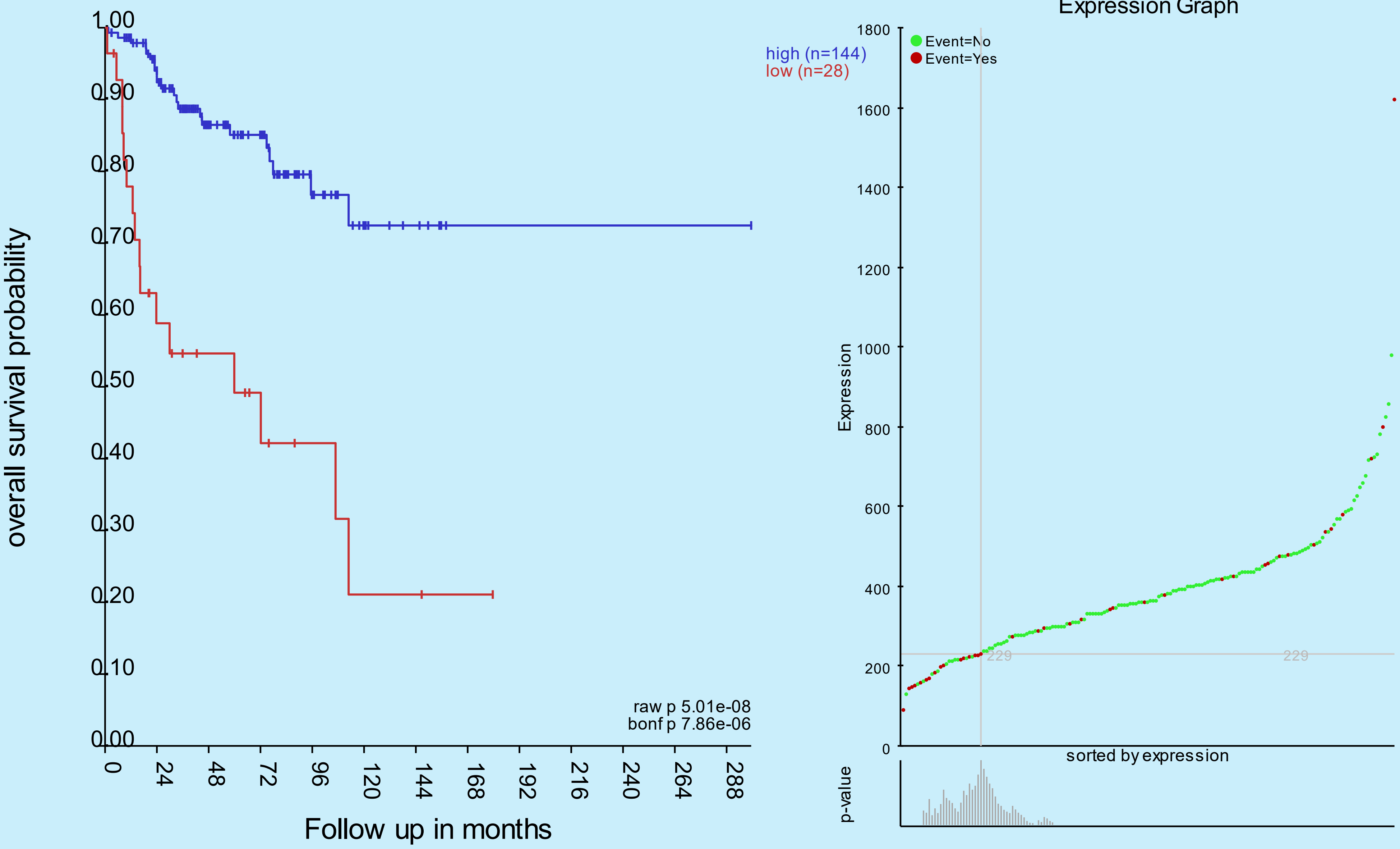

GR4

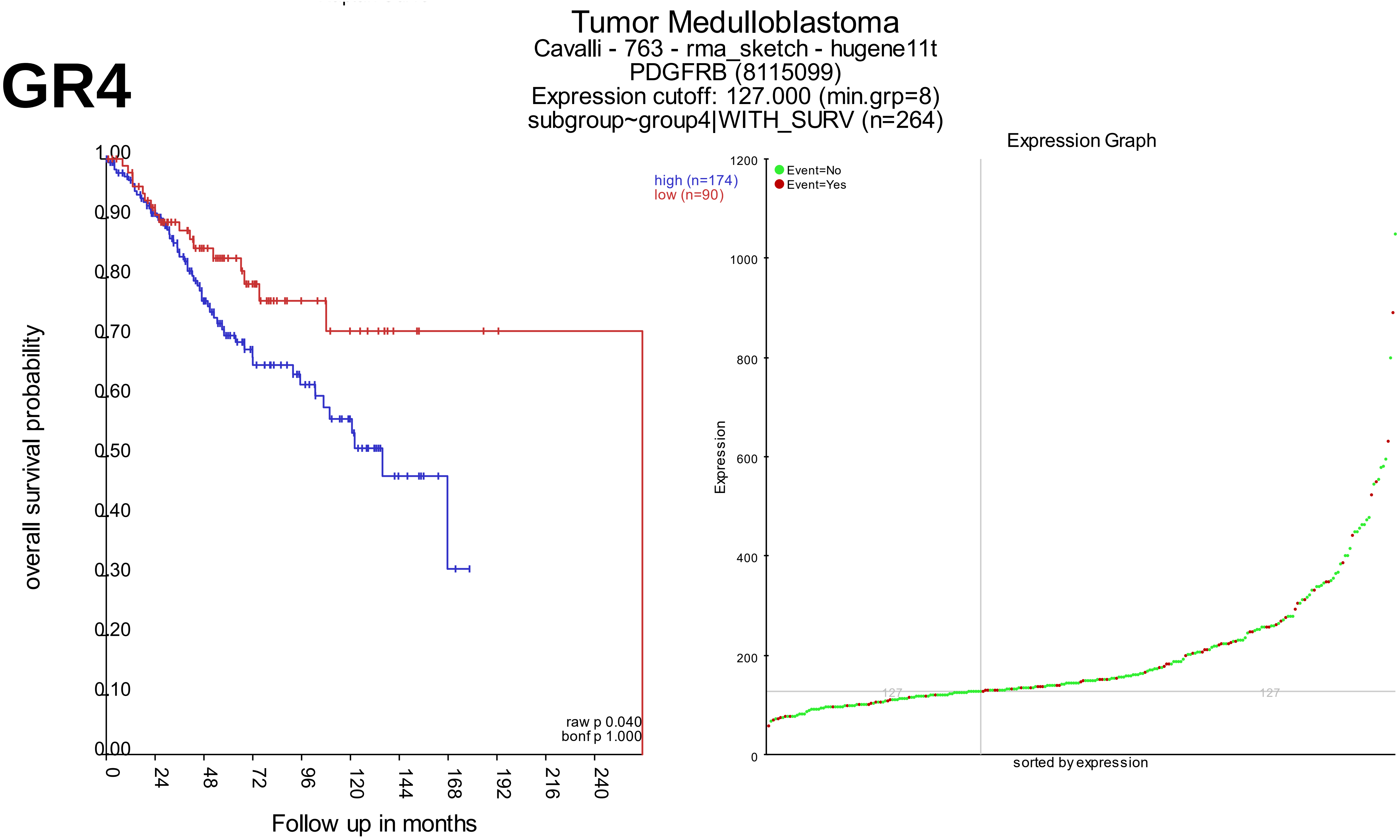

Tumor Medulloblastoma
Cavalli - 763 - rma_sketch - hugene11t
PDGFRB (8115099)
Expression cutoff: 127.000 (min.grp=8)
subgroup~group4|WITH_SURV (n=264)
overall survival probability
1.00
0.90
0.80
0.70
0.60
0.50
0.40
0.30
0.20
0.10
0.00
0
24
48
72
96
120
144
168
192
216
240
Follow up in months
high (n=174)
low (n=90)
raw p 0.040
bonf p 1.000
Expression Graph
Event=No
Event=Yes
1200
1000
800
600
400
200
0
Expression
127
127
sorted by expression

GR3

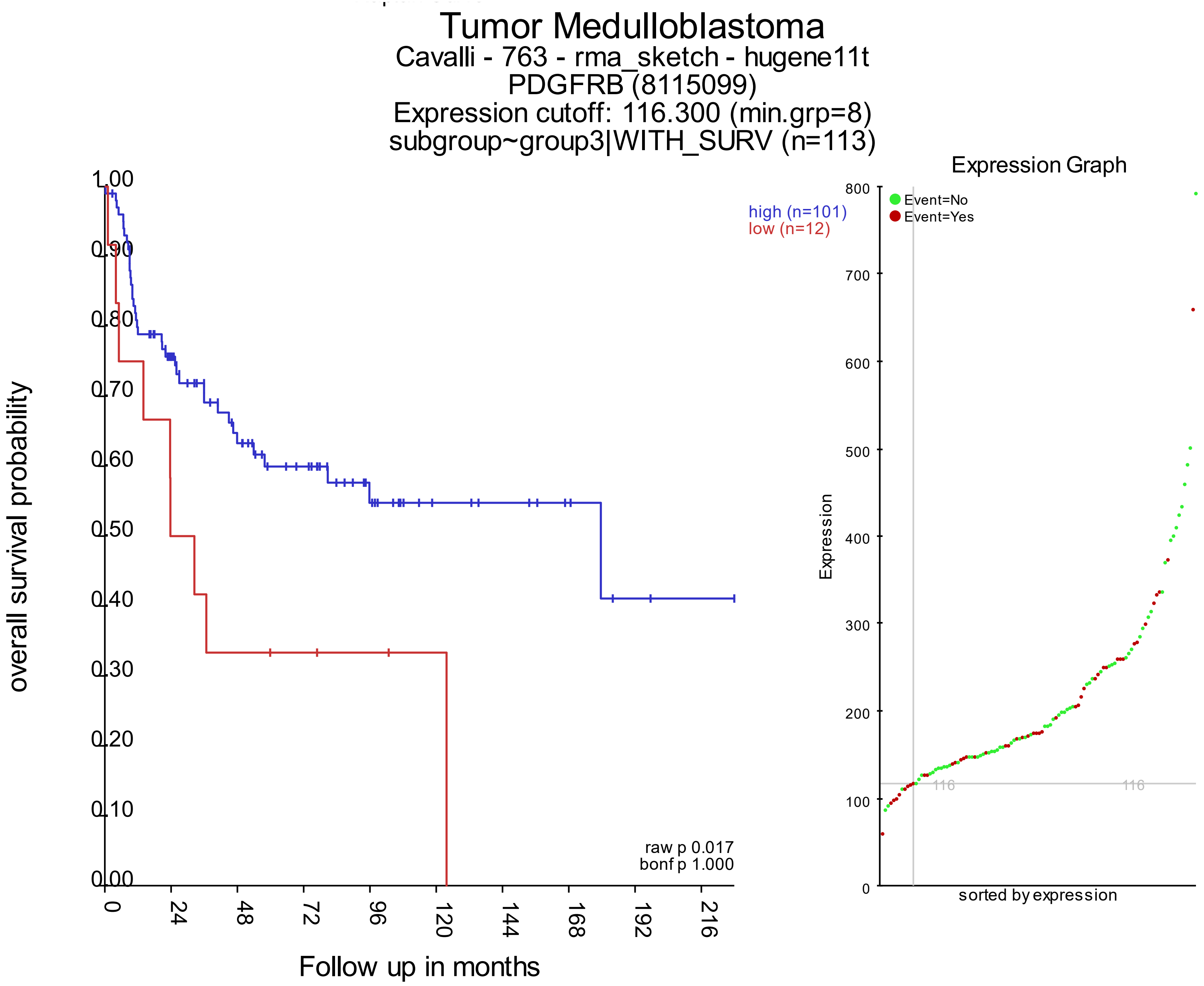
Tumor Medulloblastoma
Cavalli - 763 - rma_sketch - hugene11t
PDGFRB (8115099)
Expression cutoff: 116.300 (min.grp=8)
subgroup~group3|WITH_SURV (n=113)
overall survival probability
Follow up in months
high (n=101)
low (n=12)
raw p 0.017
bonf p 1.000
Expression Graph
Event=No
Event=Yes
Expression
sorted by expression
116

FIP1L1

WNT

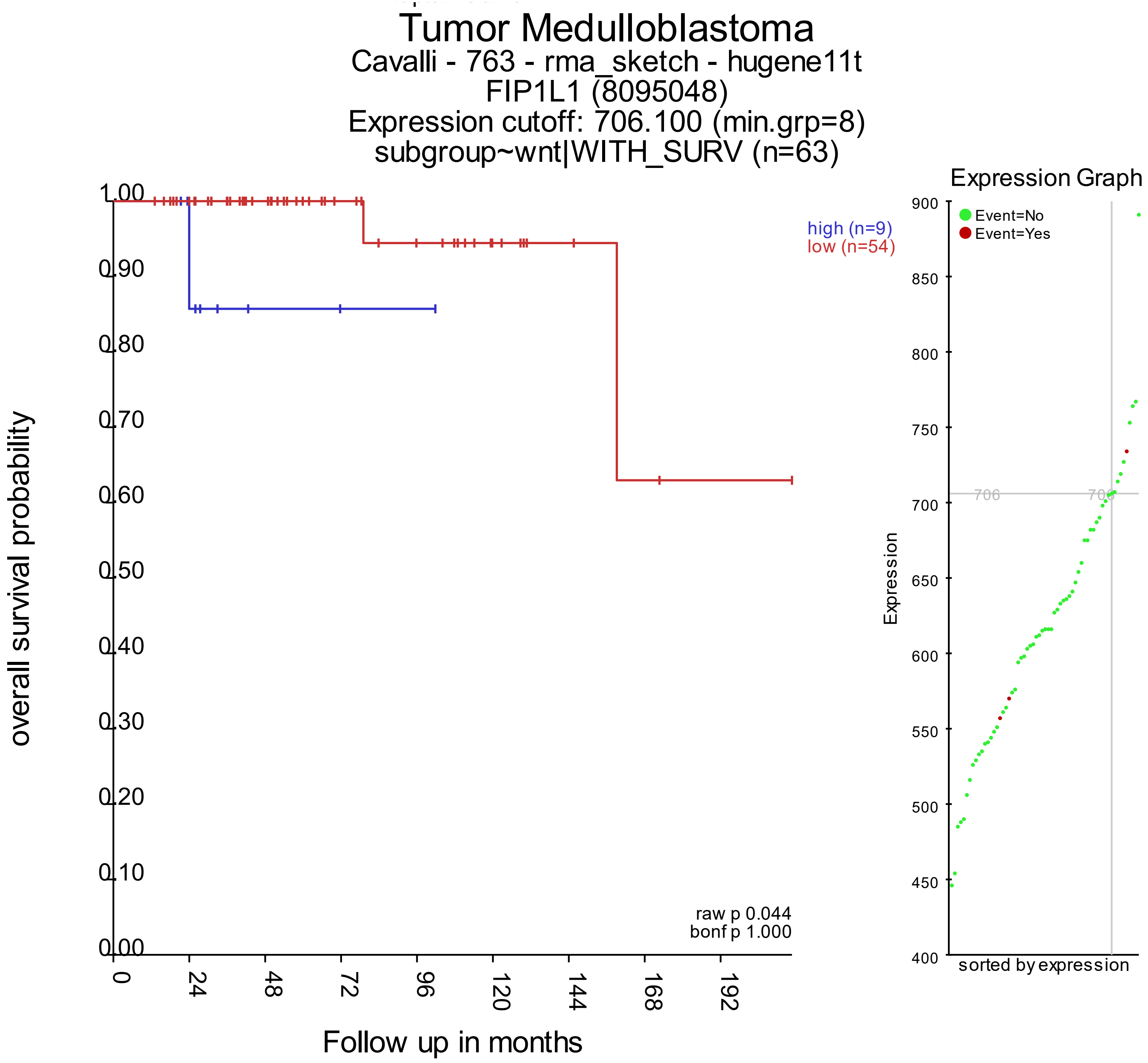
Tumor Medulloblastoma
Cavalli - 763 - rma_sketch - hugene11t
FIP1L1 (8095048)
Expression cutoff: 706.100 (min.grp=8)
subgroup~wnt|WITH_SURV (n=63)
overall survival probability
1.00
0.90
0.80
0.70
0.60
0.50
0.40
0.30
0.20
0.10
0.00
0
24
48
72
96
120
144
168
192
Follow up in months
high (n=9)
low (n=54)
raw p 0.044
bonf p 1.000
Expression Graph
Event=No
Event=Yes
Expression
900
850
800
750
700
650
600
550
500
450
400
706
sorted by expression

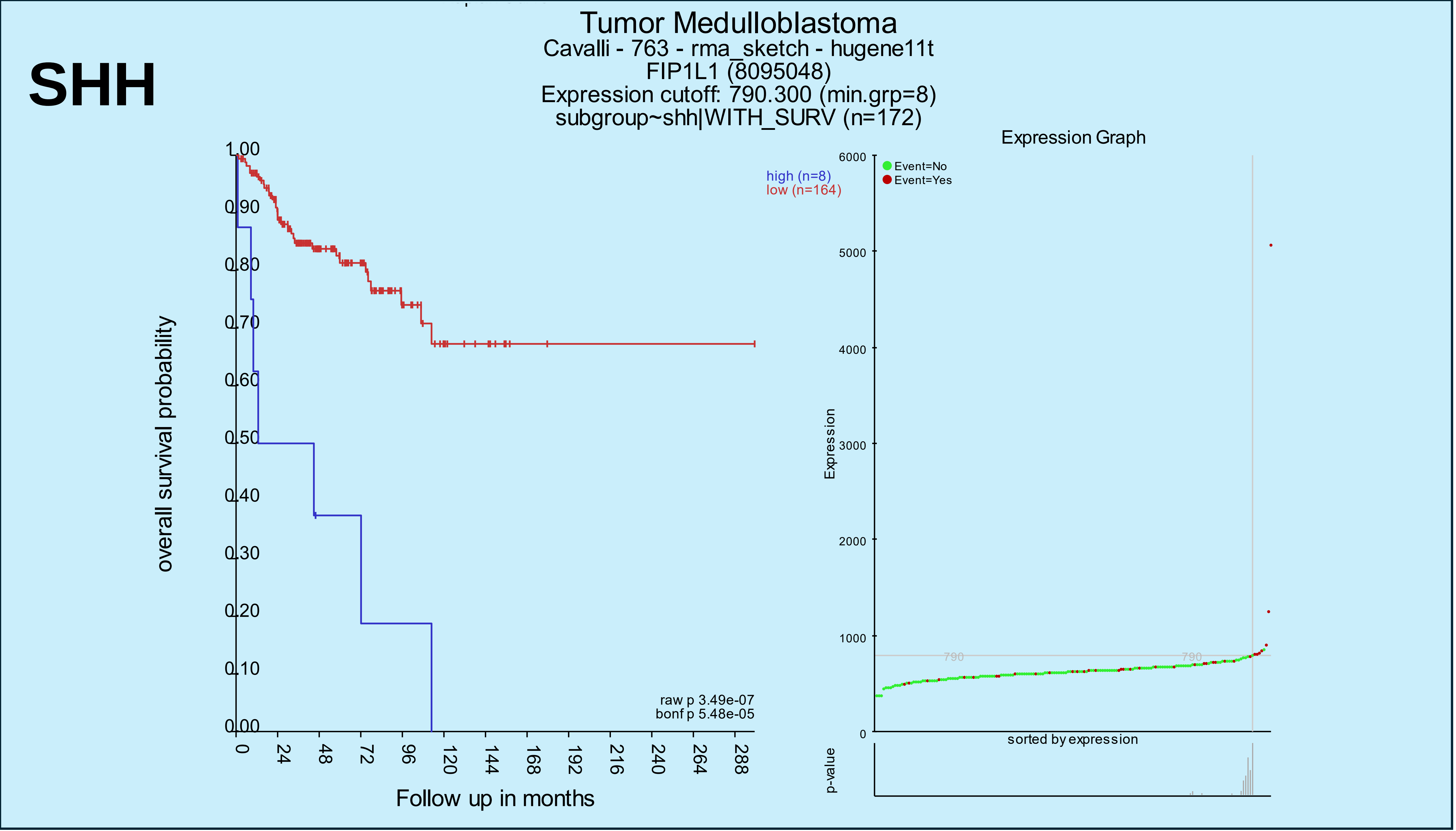
SHH
Tumor Medulloblastoma
Cavalli - 763 - rma_sketch - hugene11t
FIP1L1 (8095048)
Expression cutoff: 790.300 (min.grp=8)
subgroup~shh|WITH_SURV (n=172)
overall survival probability
high (n=8)
low (n=164)
raw p 3.49e-07
bonf p 5.48e-05
Follow up in months
Expression Graph
Event=No
Event=Yes
Expression
sorted by expression
p-value
790

GR4

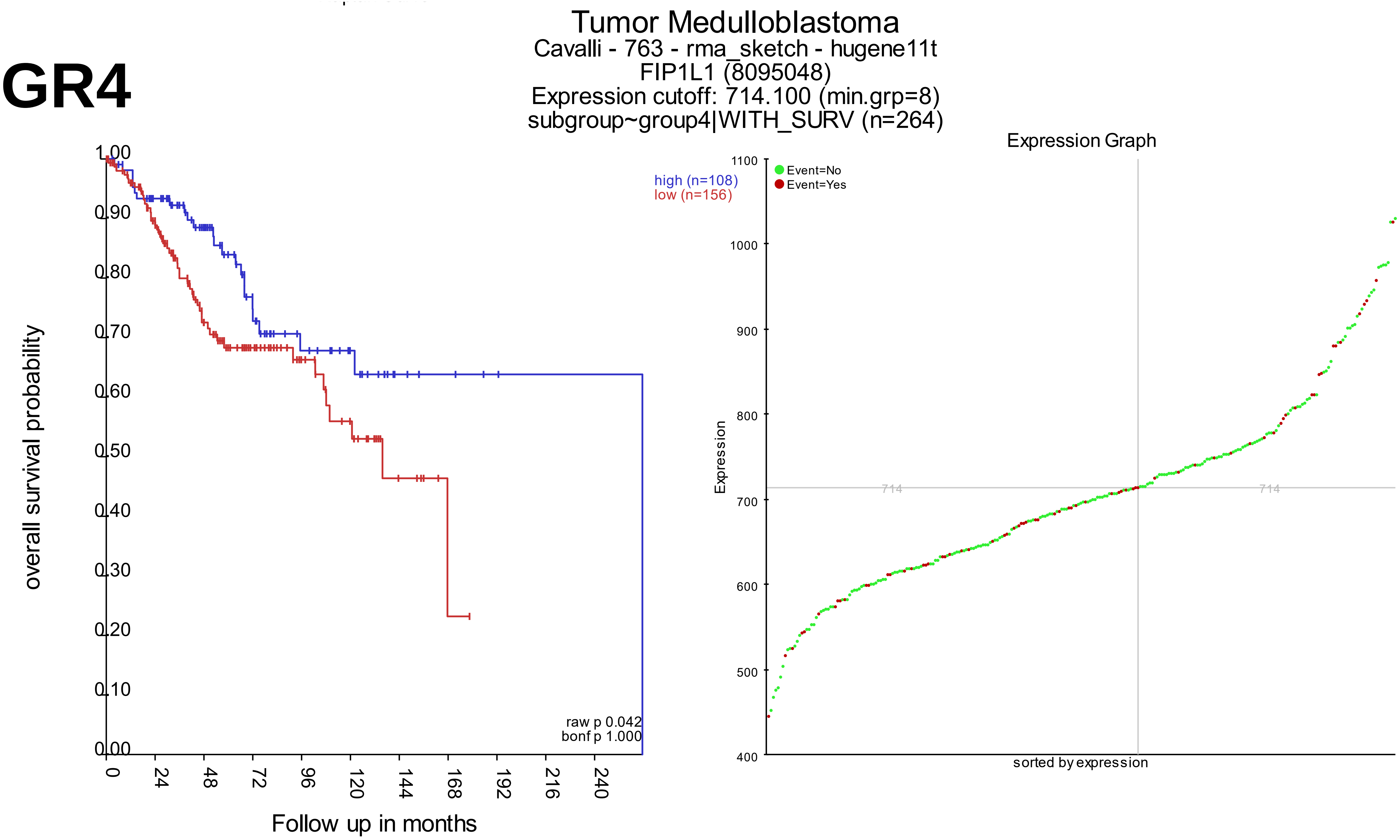

Tumor Medulloblastoma
Cavalli - 763 - rma_sketch - hugene11t
FIP1L1 (8095048)
Expression cutoff: 714.100 (min.grp=8)
subgroup~group4|WITH_SURV (n=264)
overall survival probability
1.00
0.90
0.80
0.70
0.60
0.50
0.40
0.30
0.20
0.10
0.00
high (n=108)
low (n=156)
raw p 0.042
bonf p 1.000
0
24
48
72
96
120
144
168
192
216
240
Follow up in months
Expression Graph
Event=No
Event=Yes
Expression
1100
1000
900
800
700
600
500
400
714
714
sorted by expression

GR3

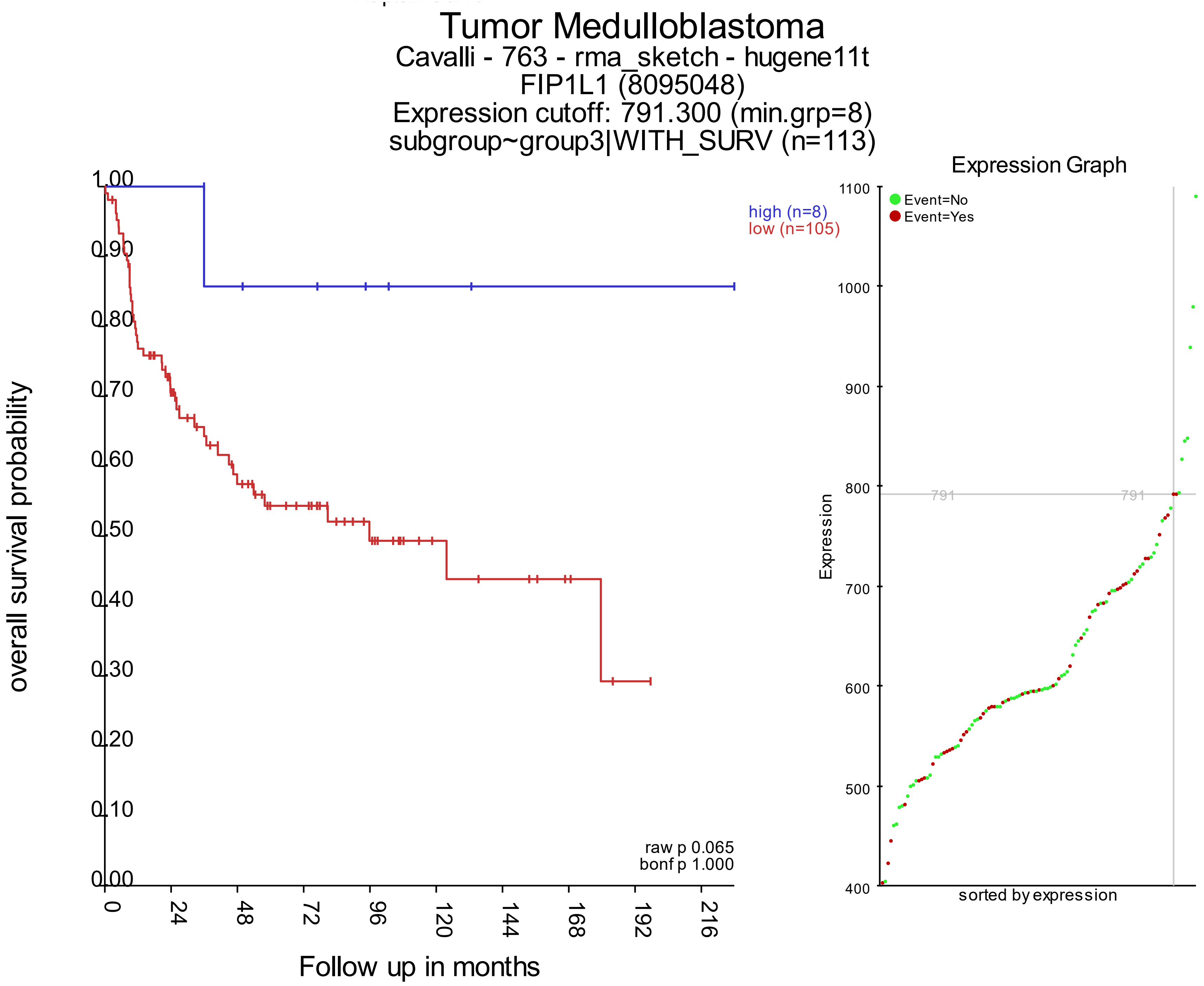

Tumor Medulloblastoma
Cavalli - 763 - rma_sketch - hugene11t
FIP1L1 (8095048)
Expression cutoff: 791.300 (min.grp=8)
subgroup~group3|WITH_SURV (n=113)
overall survival probability
1.00
0.90
0.80
0.70
0.60
0.50
0.40
0.30
0.20
0.10
0.00
0
24
48
72
96
120
144
168
192
216
Follow up in months
high (n=8)
low (n=105)
raw p 0.065
bonf p 1.000
Expression Graph
Event=No
Event=Yes
1100
1000
900
800
700
600
500
400
791
791
Expression
sorted by expression

KIT

WNT

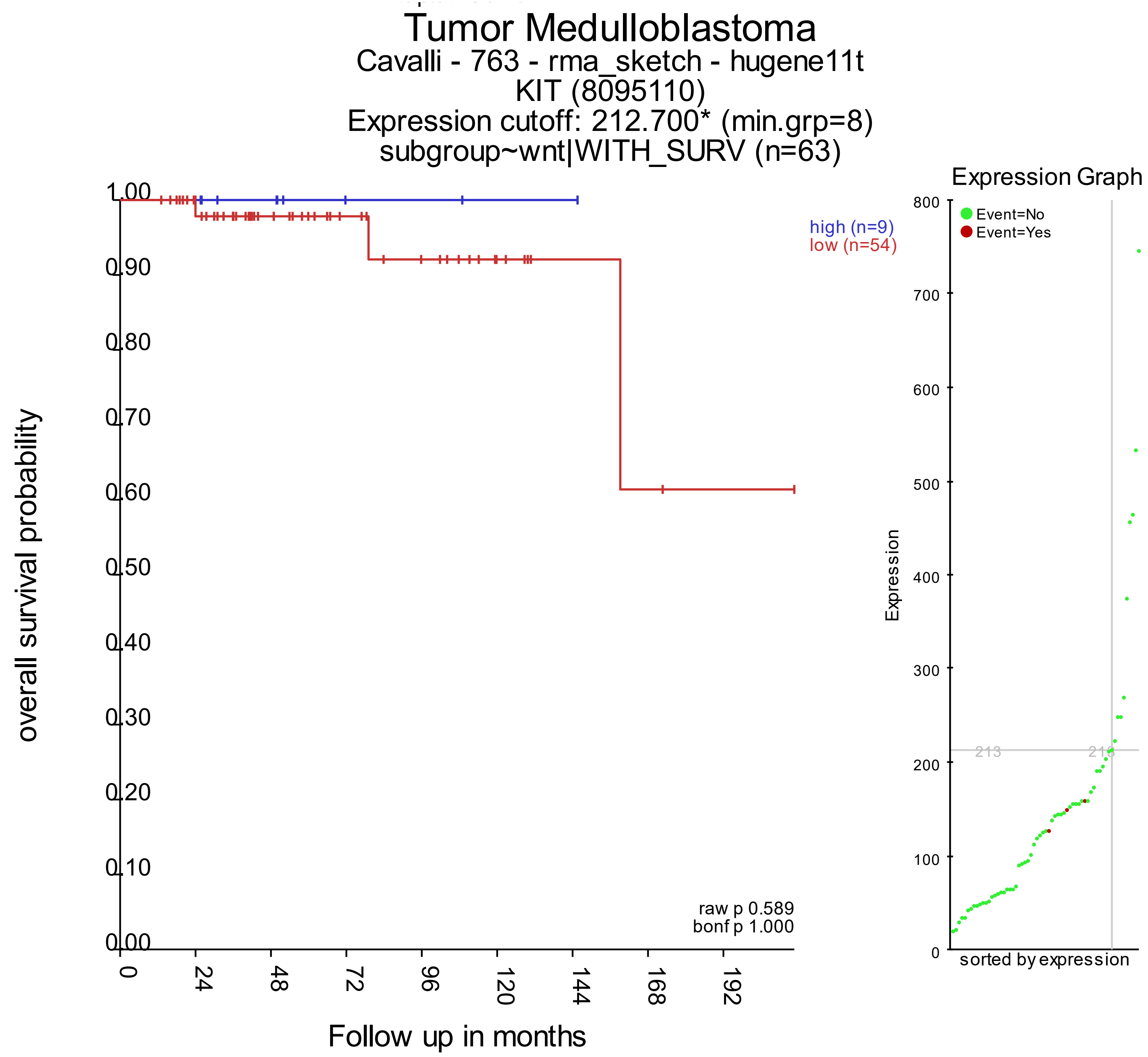

Tumor Medulloblastoma
Cavalli - 763 - rma_sketch - hugene11t
KIT (8095110)
Expression cutoff: 212.700* (min.grp=8)
subgroup~wnt|WITH_SURV (n=63)
overall survival probability
1.00
0.90
0.80
0.70
0.60
0.50
0.40
0.30
0.20
0.10
0.00
0
24
48
72
96
120
144
168
192
Follow up in months
high (n=9)
low (n=54)
raw p 0.589
bonf p 1.000
Expression Graph
Event=No
Event=Yes
Expression
800
700
600
500
400
300
200
100
0
213
sorted by expression

SHH

Tumor Medulloblastoma

Cavalli - 763 - rma_sketch - hugene11t

KIT (8095110)

Expression cutoff: 303.100 (min.grp=8)

subgroup~shh|WITH_SURV (n=172)

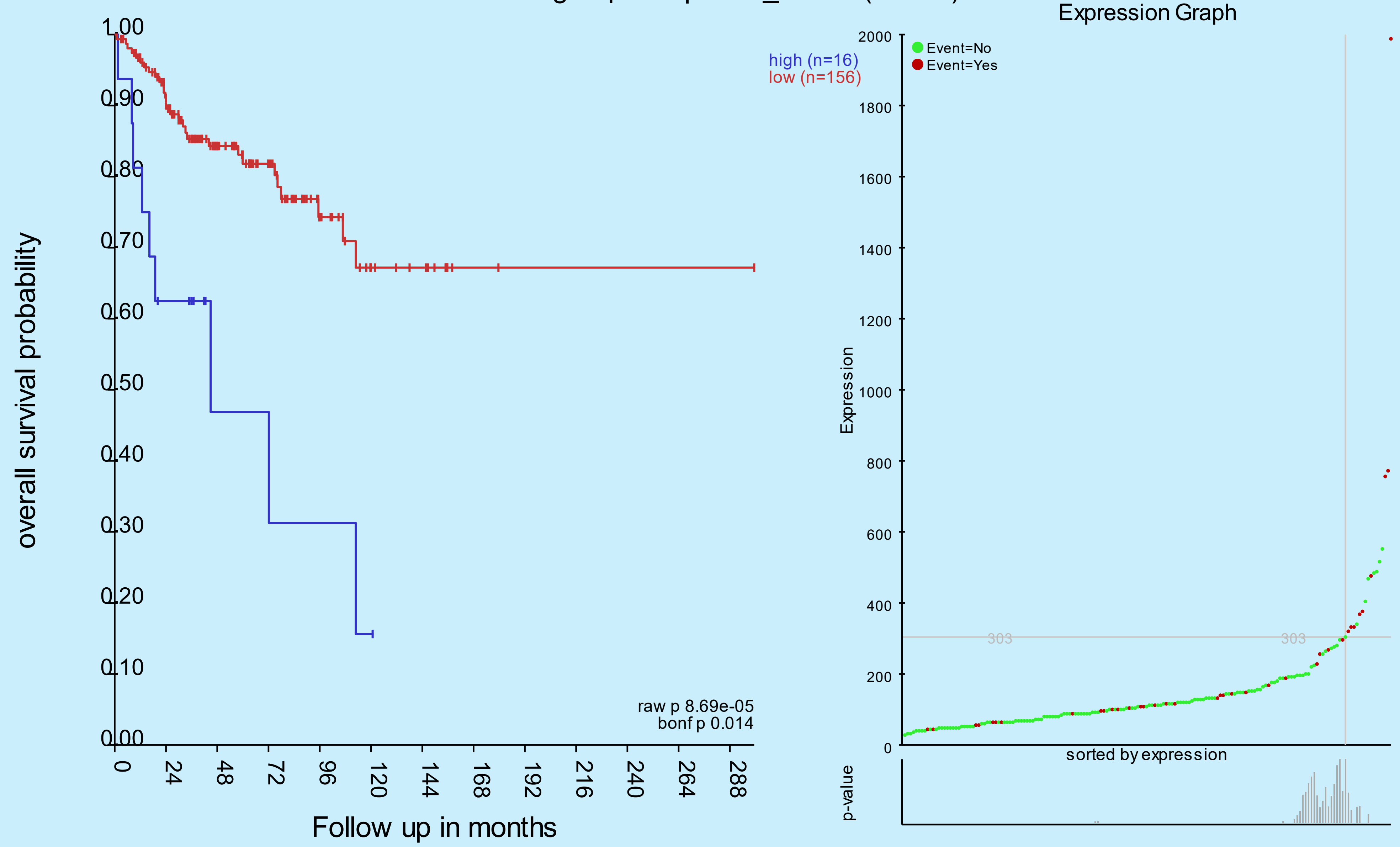

GR4

Tumor Medulloblastoma

Cavalli - 763 - rma_sketch - hugene11t
KIT (8095110)
Expression cutoff: 141.900 (min.grp=8)
subgroup~group4|WITH_SURV (n=264)

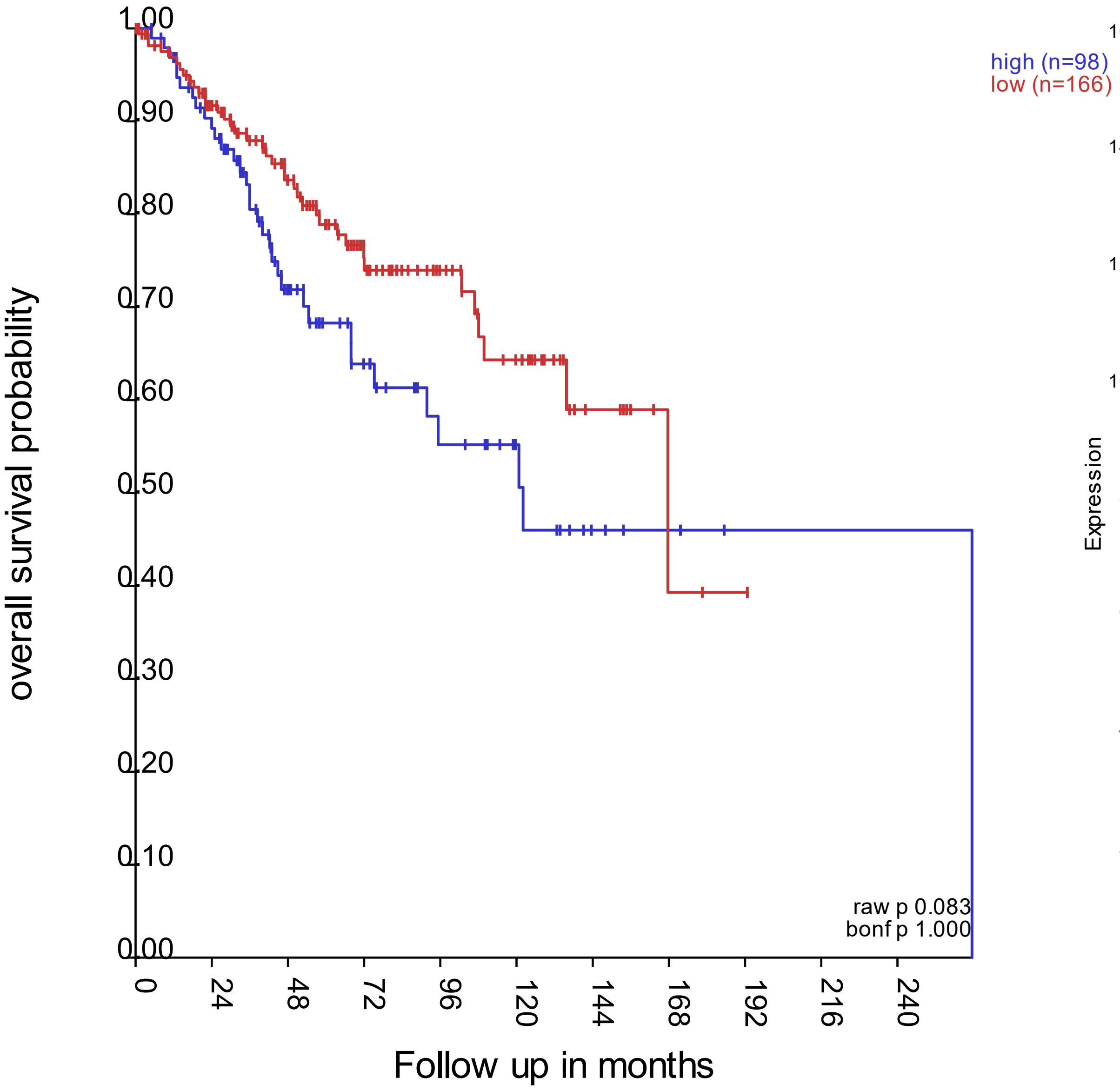

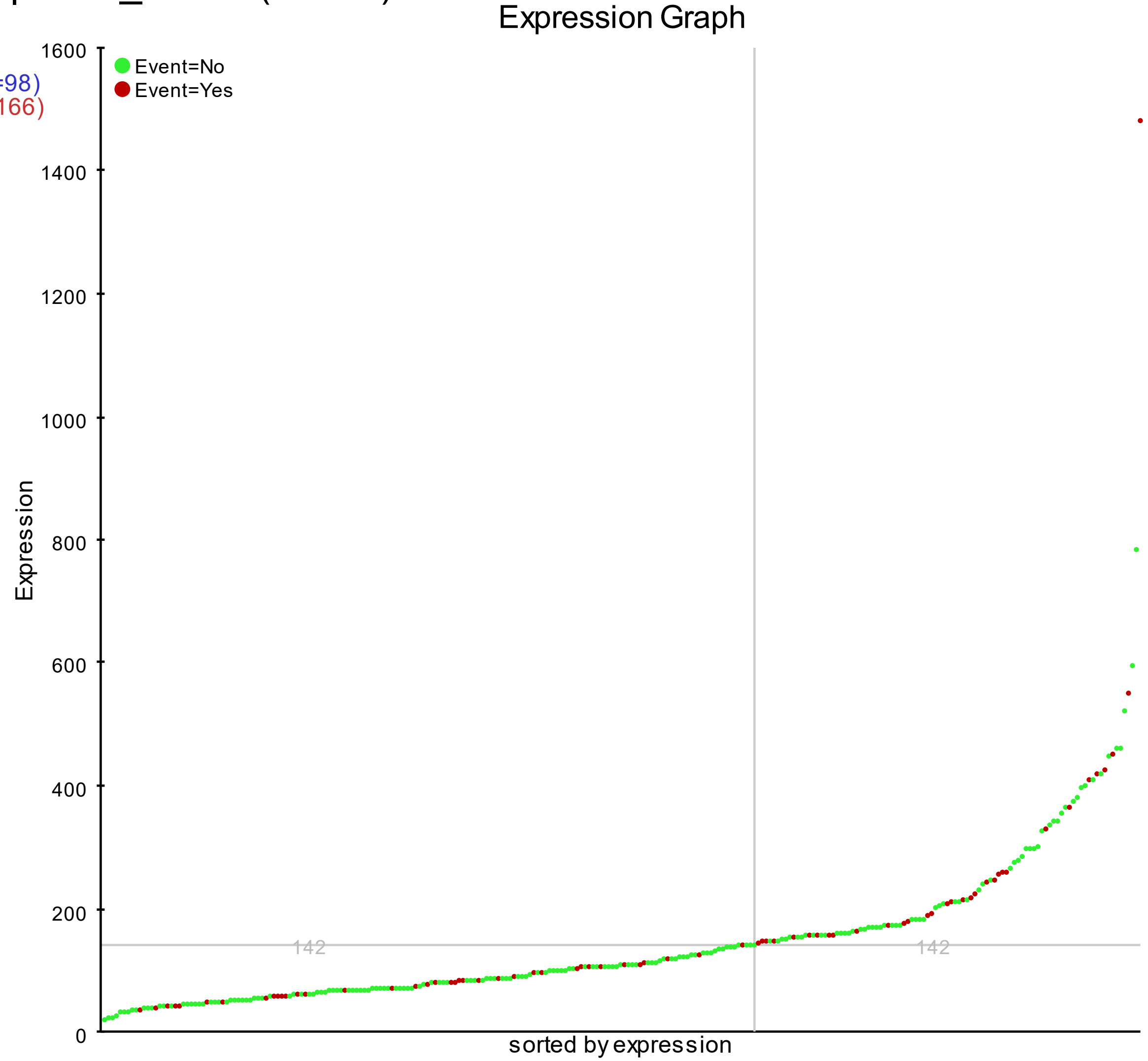

GR3

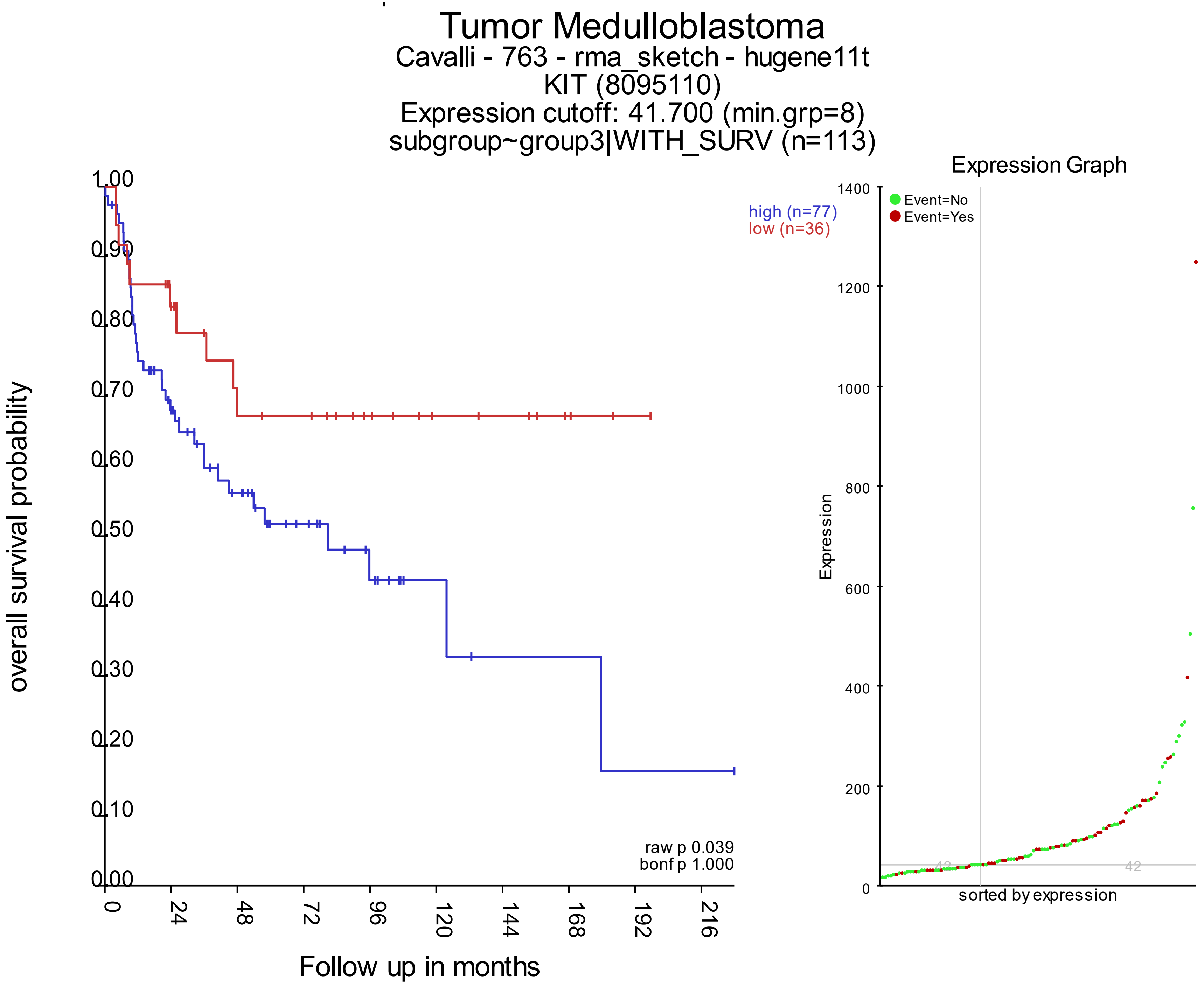
Tumor Medulloblastoma
Cavalli - 763 - rma_sketch - hugene11t
KIT (8095110)
Expression cutoff: 41.700 (min.grp=8)
subgroup~group3|WITH_SURV (n=113)
overall survival probability
1.00
0.90
0.80
0.70
0.60
0.50
0.40
0.30
0.20
0.10
0.00
high (n=77)
low (n=36)
raw p 0.039
bonf p 1.000
0
24
48
72
96
120
144
168
192
216
Follow up in months
Expression Graph
Event=No
Event=Yes
Expression
1400
1200
1000
800
600
400
200
0
42
42
sorted by expression

PIK3CA

WNT

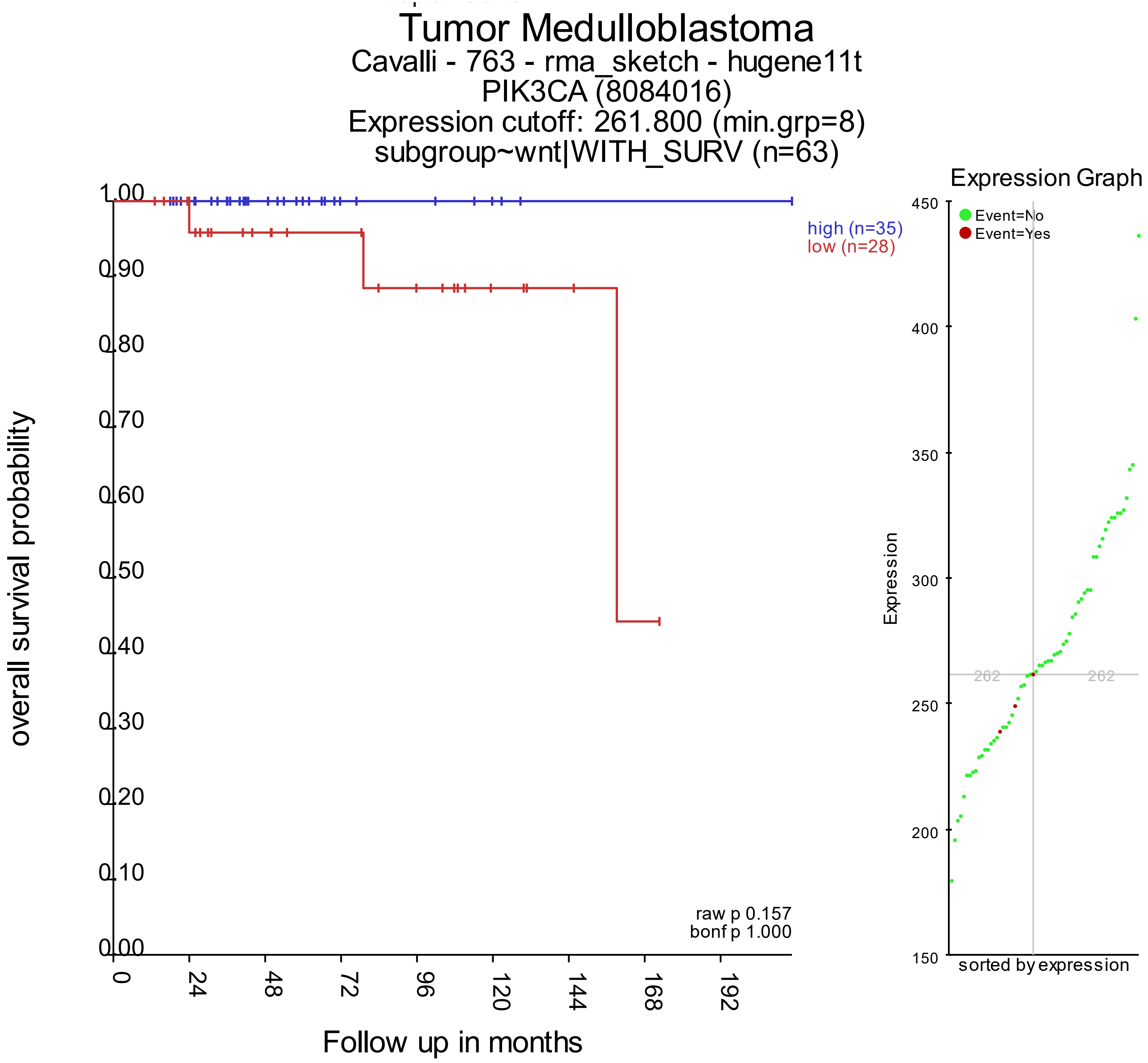

Tumor Medulloblastoma
Cavalli - 763 - rma_sketch - hugene11t
PIK3CA (8084016)
Expression cutoff: 261.800 (min.grp=8)
subgroup~wnt|WITH_SURV (n=63)
overall survival probability
1.00
0.90
0.80
0.70
0.60
0.50
0.40
0.30
0.20
0.10
0.00
0
24
48
72
96
120
144
168
192
Follow up in months
high (n=35)
low (n=28)
raw p 0.157
bonf p 1.000
Expression Graph
Event=No
Event=Yes
Expression
450
400
350
300
250
200
150
262
262
sorted by expression

SHH

Tumor Medulloblastoma
Cavalli - 763 - rma_sketch - hugene11t
PIK3CA (8084016)
Expression cutoff: 564.100 (min.grp=8)
subgroup~shh|WITH_SURV (n=172)

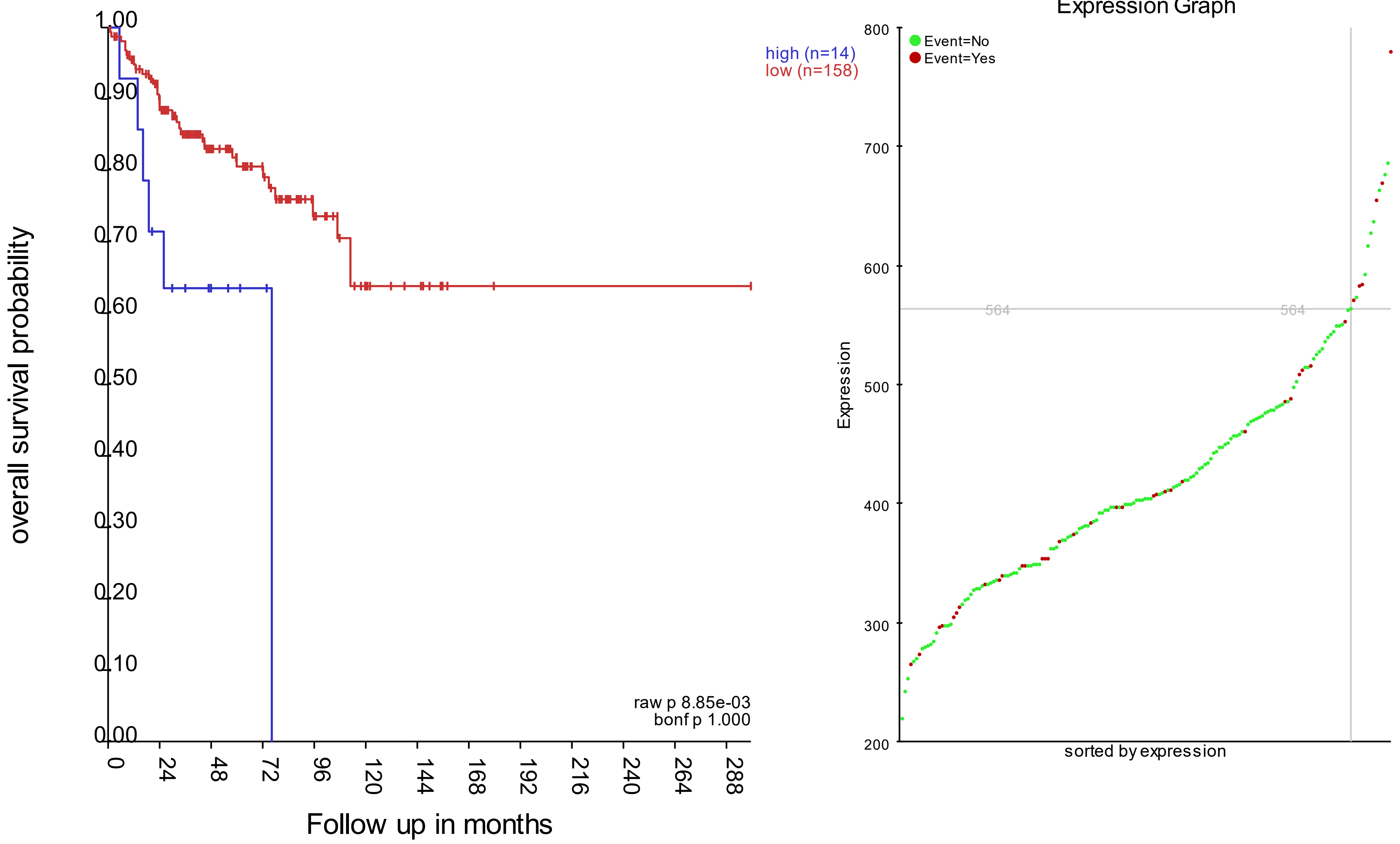

GR4

Tumor Medulloblastoma
Cavalli - 763 - rma_sketch - hugene11t
PIK3CA (8084016)
Expression cutoff: 495.000 (min.grp=8)
subgroup~group4|WITH_SURV (n=264)

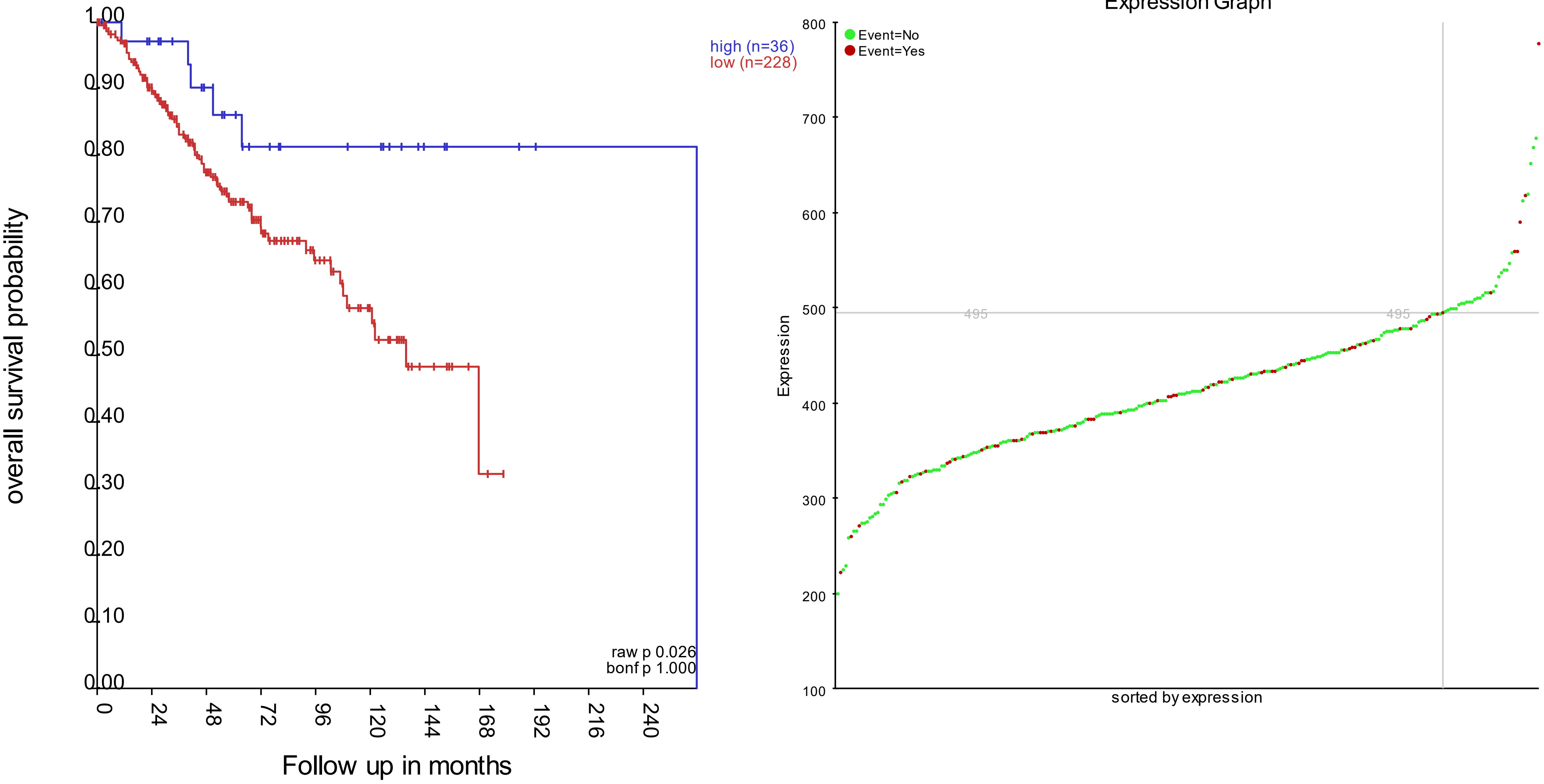

GR3

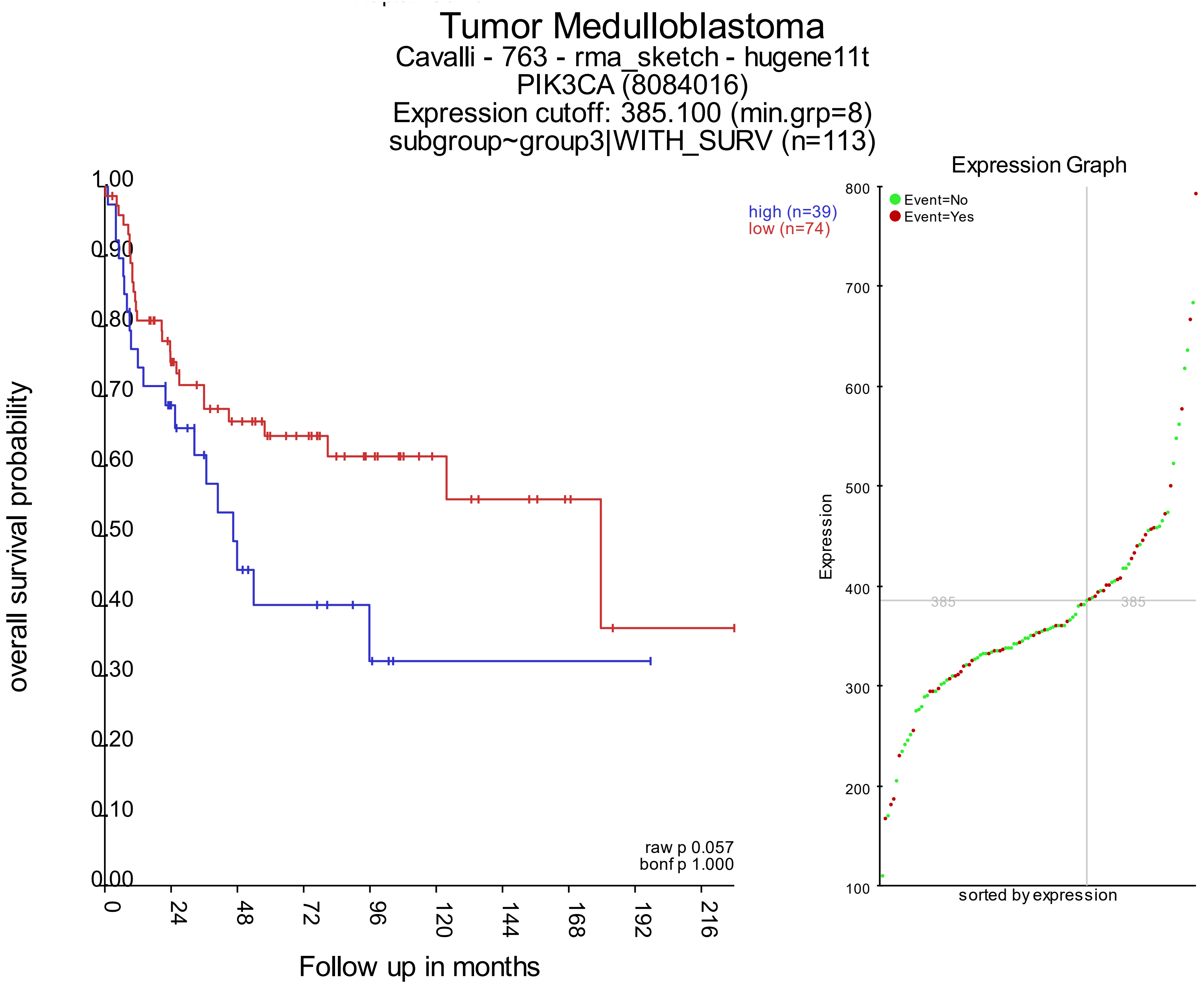
Tumor Medulloblastoma
Cavalli - 763 - rma_sketch - hugene11t
PIK3CA (8084016)
Expression cutoff: 385.100 (min.grp=8)
subgroup~group3|WITH_SURV (n=113)
overall survival probability
1.00
0.90
0.80
0.70
0.60
0.50
0.40
0.30
0.20
0.10
0.00
0
24
48
72
96
120
144
168
192
216
Follow up in months
high (n=39)
low (n=74)
raw p 0.057
bonf p 1.000
Expression Graph
Event=No
Event=Yes
Expression
800
700
600
500
400
300
200
100
385
385
sorted by expression

PIK3CB

WNT

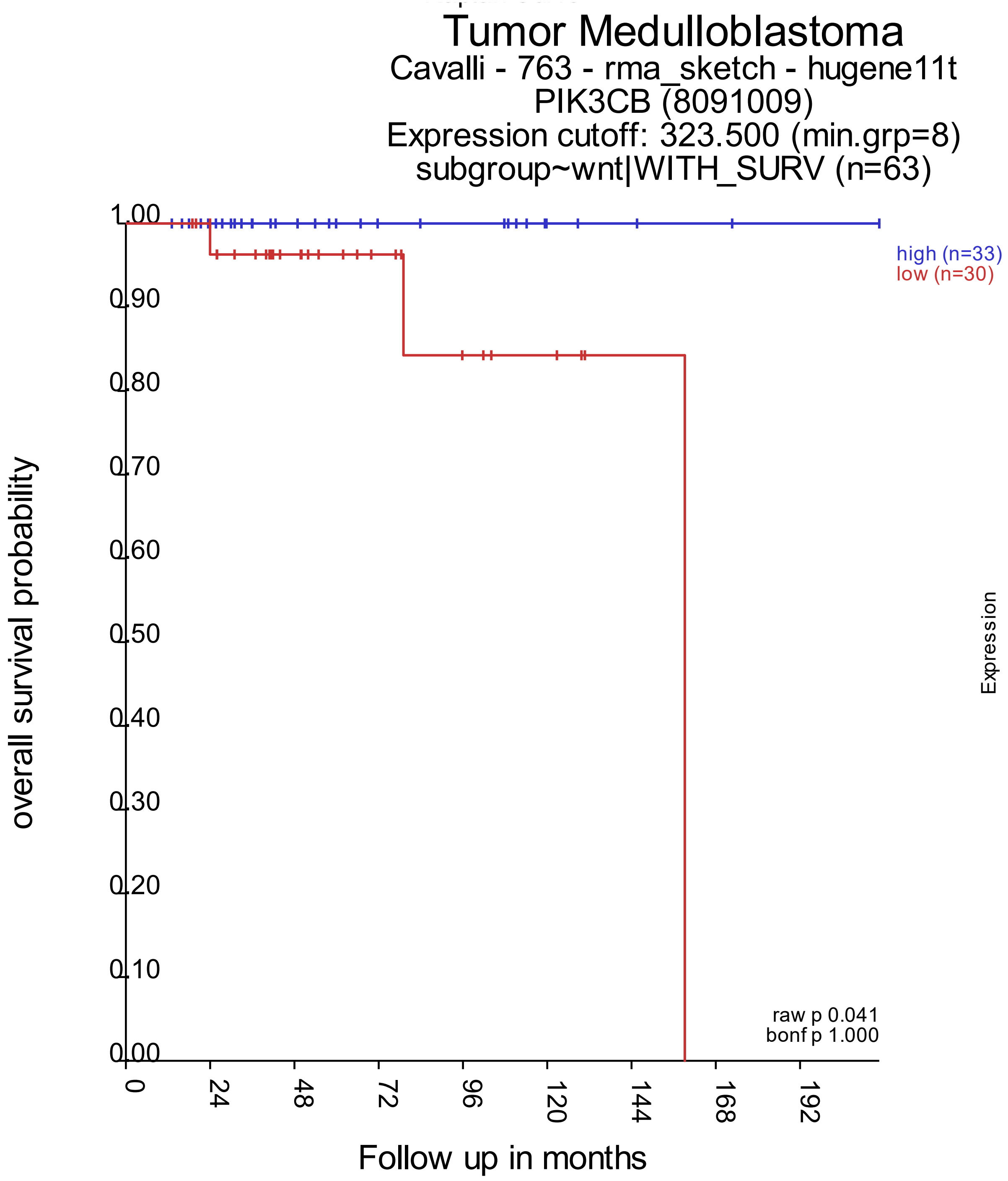
Tumor Medulloblastoma
Cavalli - 763 - rma_sketch - hugene11t
PIK3CB (8091009)
Expression cutoff: 323.500 (min.grp=8)
subgroup~wnt|WITH_SURV (n=63)
overall survival probability
1.00
0.90
0.80
0.70
0.60
0.50
0.40
0.30
0.20
0.10
0.00
0
24
48
72
96
120
144
168
192
Follow up in months
high (n=33)
low (n=30)
raw p 0.041
bonf p 1.000

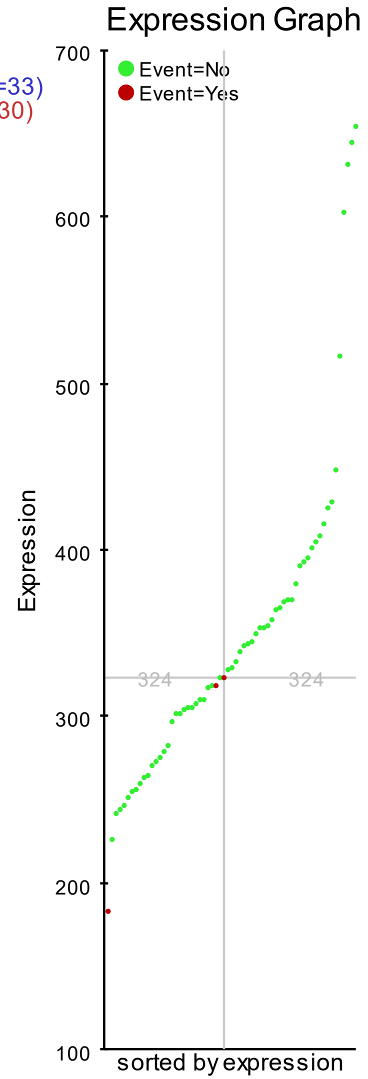
Expression Graph
Event=No
Event=Yes
Expression
700
600
500
400
300
200
100
324
324
sorted by expression

SHH

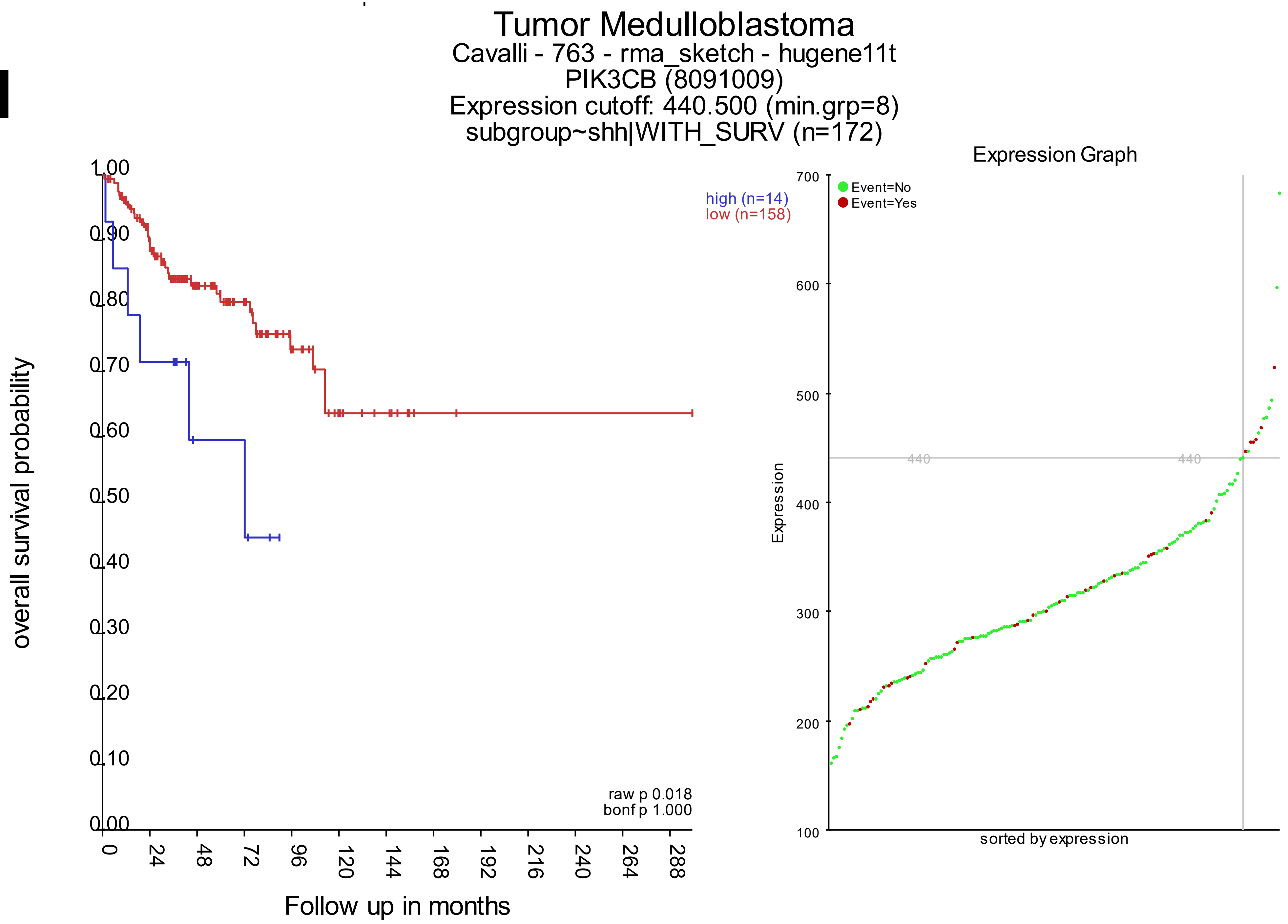
Tumor Medulloblastoma
Cavalli - 763 - rma_sketch - hugene11t
PIK3CB (8091009)
Expression cutoff: 440.500 (min.grp=8)
subgroup~shh|WITH_SURV (n=172)
overall survival probability
1.00
0.90
0.80
0.70
0.60
0.50
0.40
0.30
0.20
0.10
0.00
0
24
48
72
96
120
144
168
192
216
240
264
288
Follow up in months
high (n=14)
low (n=158)
raw p 0.018
bonf p 1.000
Expression Graph
Event=No
Event=Yes
Expression
700
600
500
400
300
200
100
440
440
sorted by expression

GR4

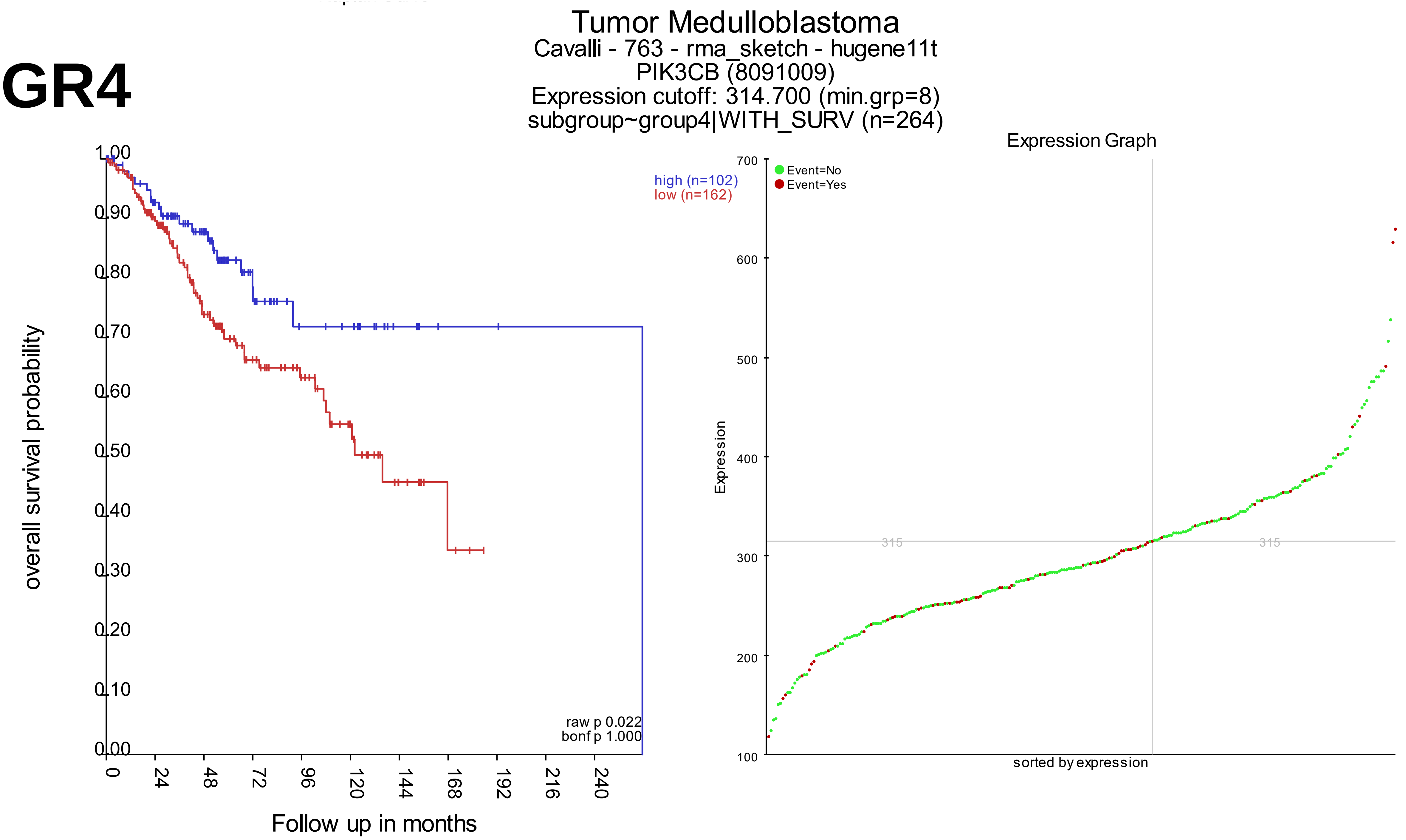

Tumor Medulloblastoma
Cavalli - 763 - rma_sketch - hugene11t
PIK3CB (8091009)
Expression cutoff: 314.700 (min.grp=8)
subgroup~group4|WITH_SURV (n=264)
high (n=102)
low (n=162)
overall survival probability
Follow up in months
raw p 0.022
bonf p 1.000
Expression Graph
Event=No
Event=Yes
Expression
sorted by expression
315

GR3

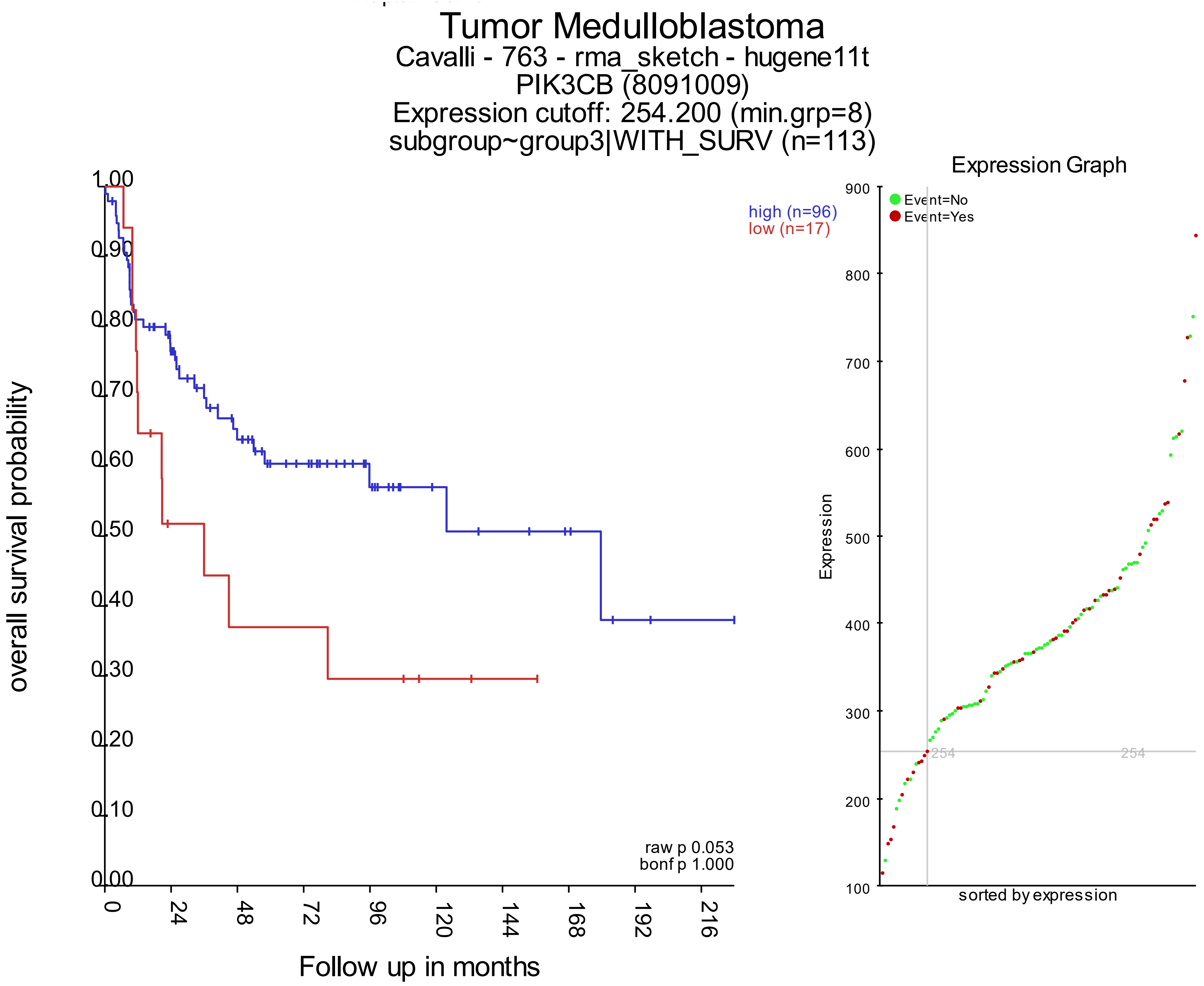

Tumor Medulloblastoma
Cavalli - 763 - rma_sketch - hugene11t
PIK3CB (8091009)
Expression cutoff: 254.200 (min.grp=8)
subgroup~group3|WITH_SURV (n=113)
high (n=96)
low (n=17)
overall survival probability
1.00
0.90
0.80
0.70
0.60
0.50
0.40
0.30
0.20
0.10
0.00
raw p 0.053
bonf p 1.000
0
24
48
72
96
120
144
168
192
216
Follow up in months
Expression Graph
Event=No
Event=Yes
Expression
900
800
700
600
500
400
300
200
100
254
254
sorted by expression

PIK3CD

WNT

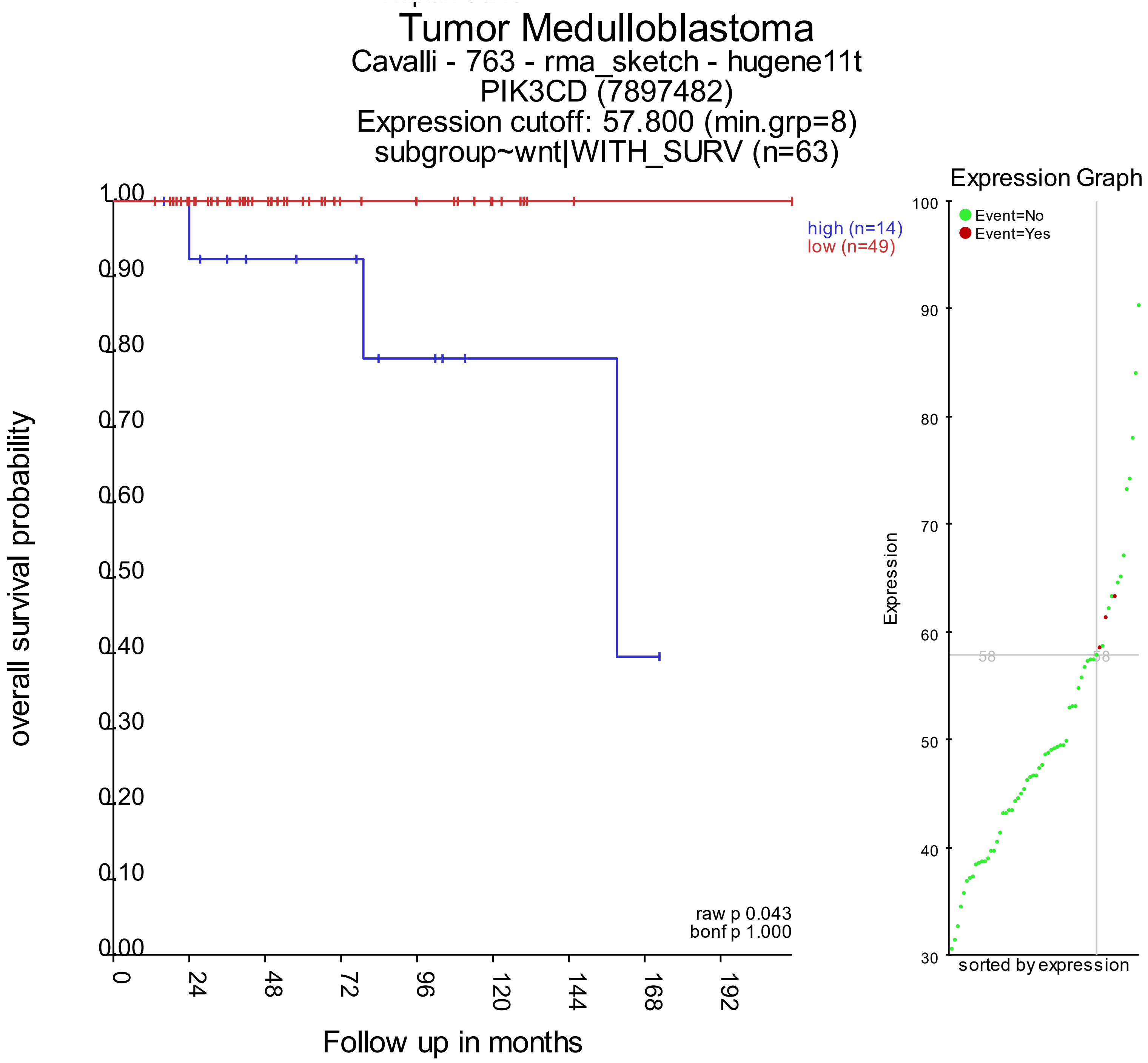

Tumor Medulloblastoma
Cavalli - 763 - rma_sketch - hugene11t
PIK3CD (7897482)
Expression cutoff: 57.800 (min.grp=8)
subgroup~wnt|WITH_SURV (n=63)
overall survival probability
1.00
0.90
0.80
0.70
0.60
0.50
0.40
0.30
0.20
0.10
0.00
0
24
48
72
96
120
144
168
192
Follow up in months
high (n=14)
low (n=49)
raw p 0.043
bonf p 1.000
Expression Graph
Event=No
Event=Yes
Expression
100
90
80
70
60
50
40
30
58
58
sorted by expression

SHH

Tumor Medulloblastoma
Cavalli - 763 - rma_sketch - hugene11t
PIK3CD (7897482)
Expression cutoff: 62.100 (min.grp=8)
subgroup~shh|WITH_SURV (n=172)

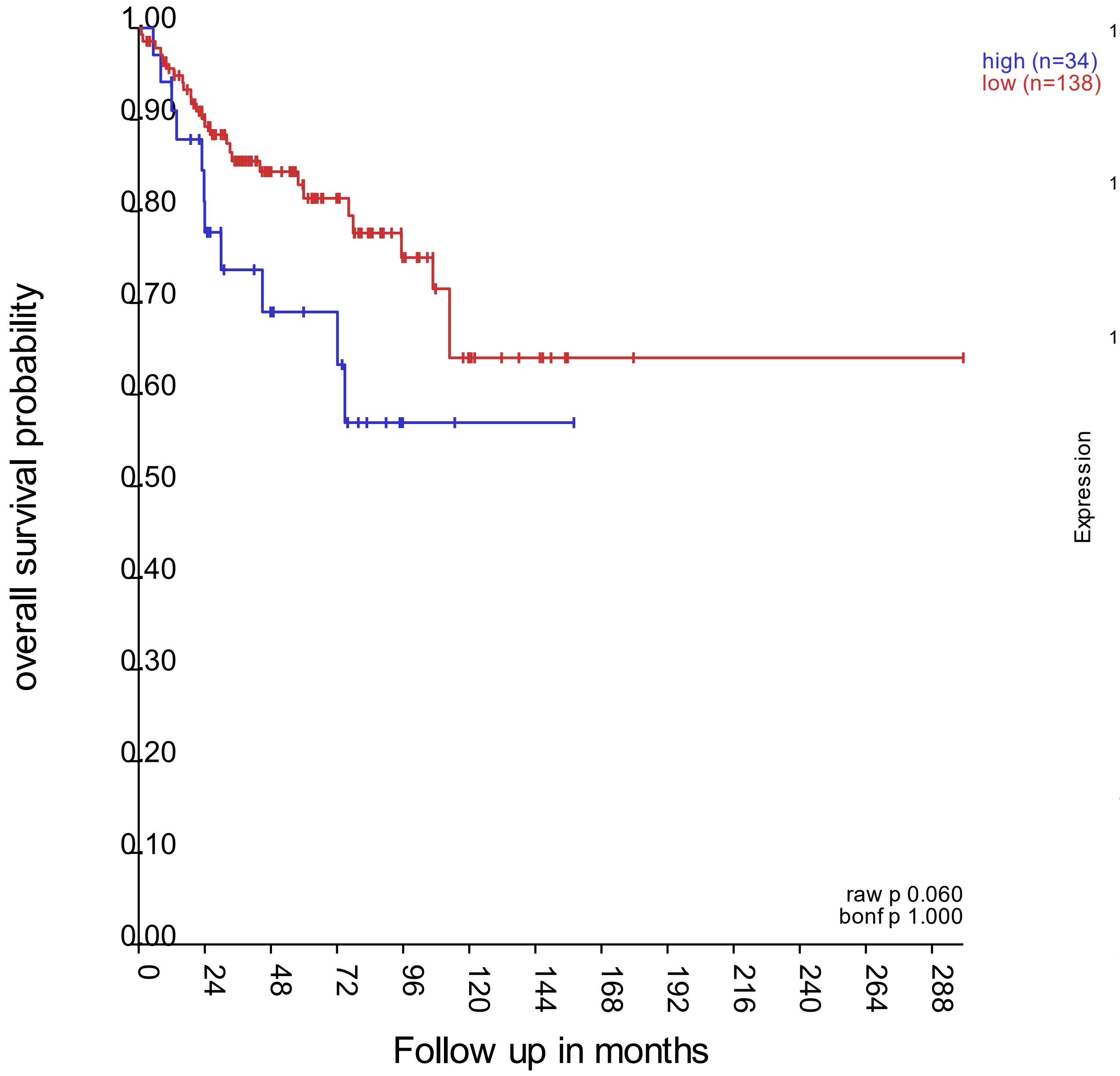

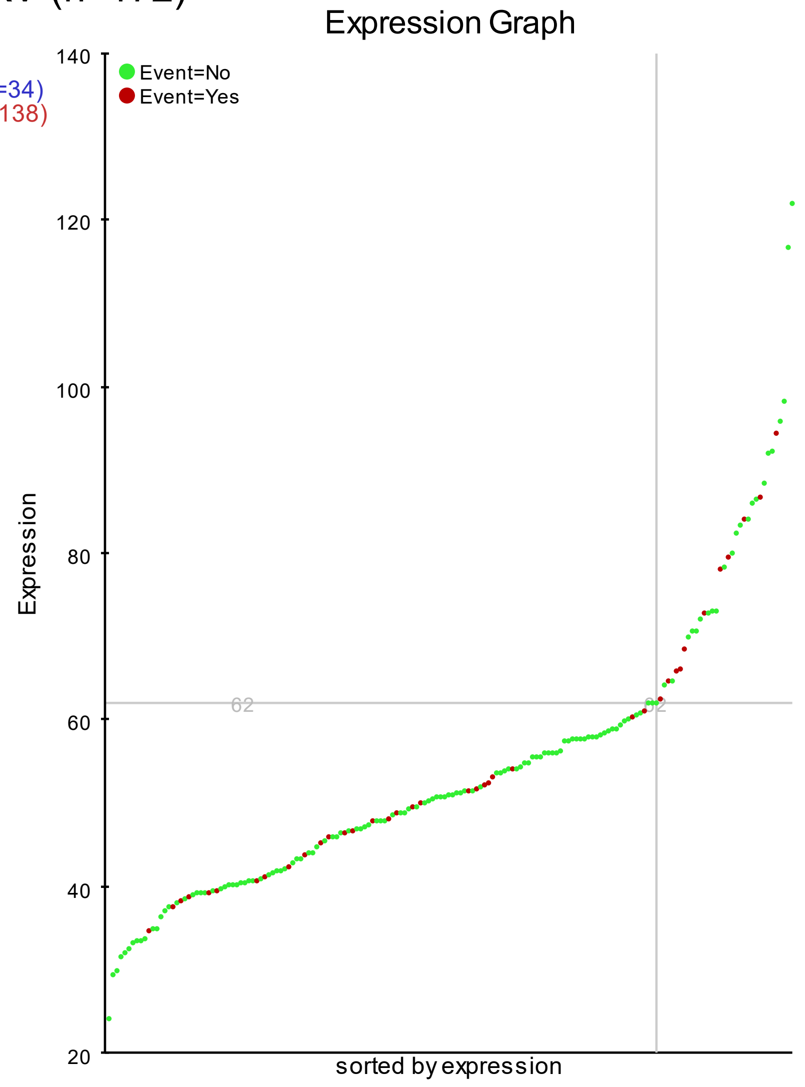

GR4

Tumor Medulloblastoma
Cavalli - 763 - rma_sketch - hugene11t
PIK3CD (7897482)
Expression cutoff: 48.600 (min.grp=8)
subgroup~group4|WITH_SURV (n=264)

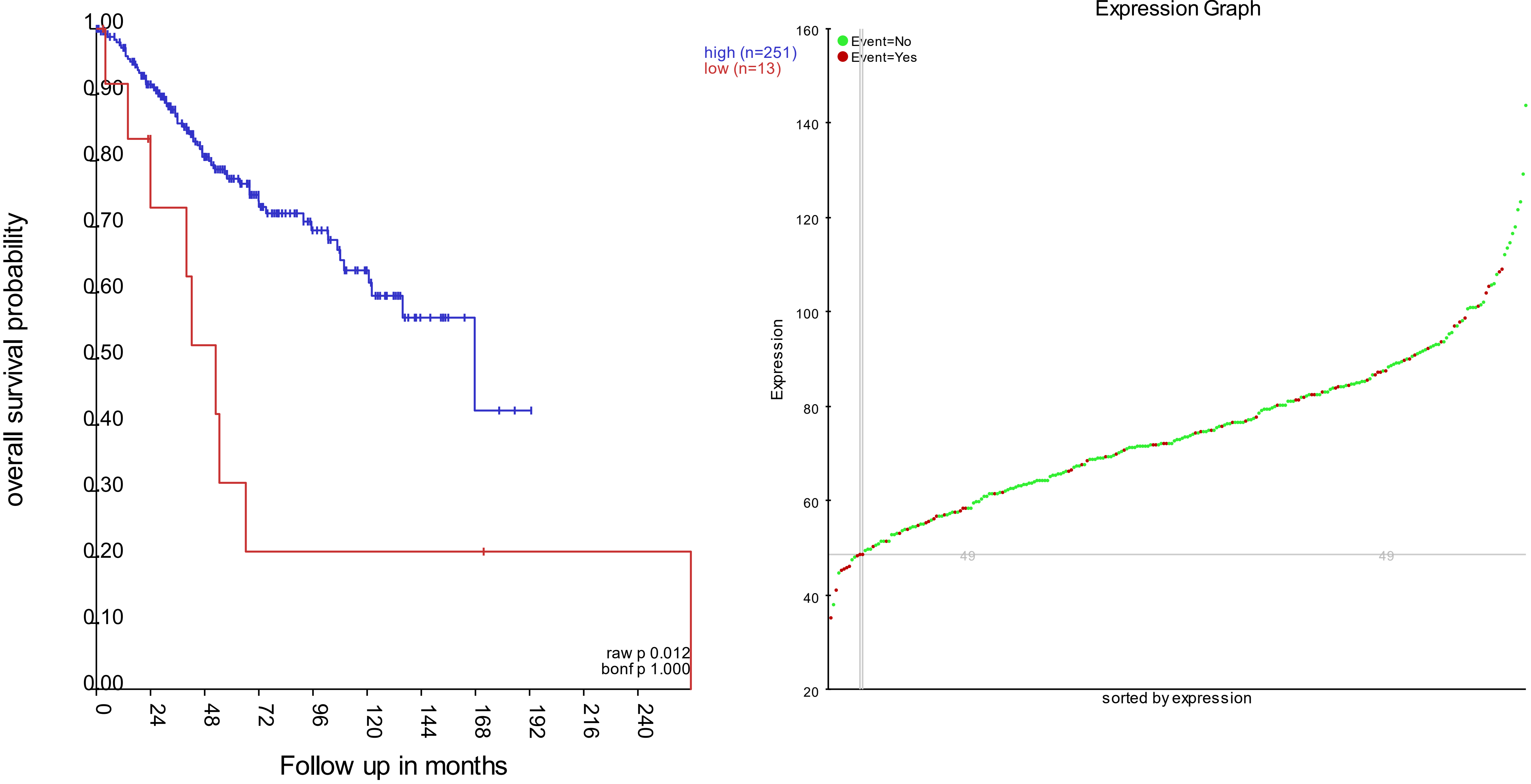

GR3

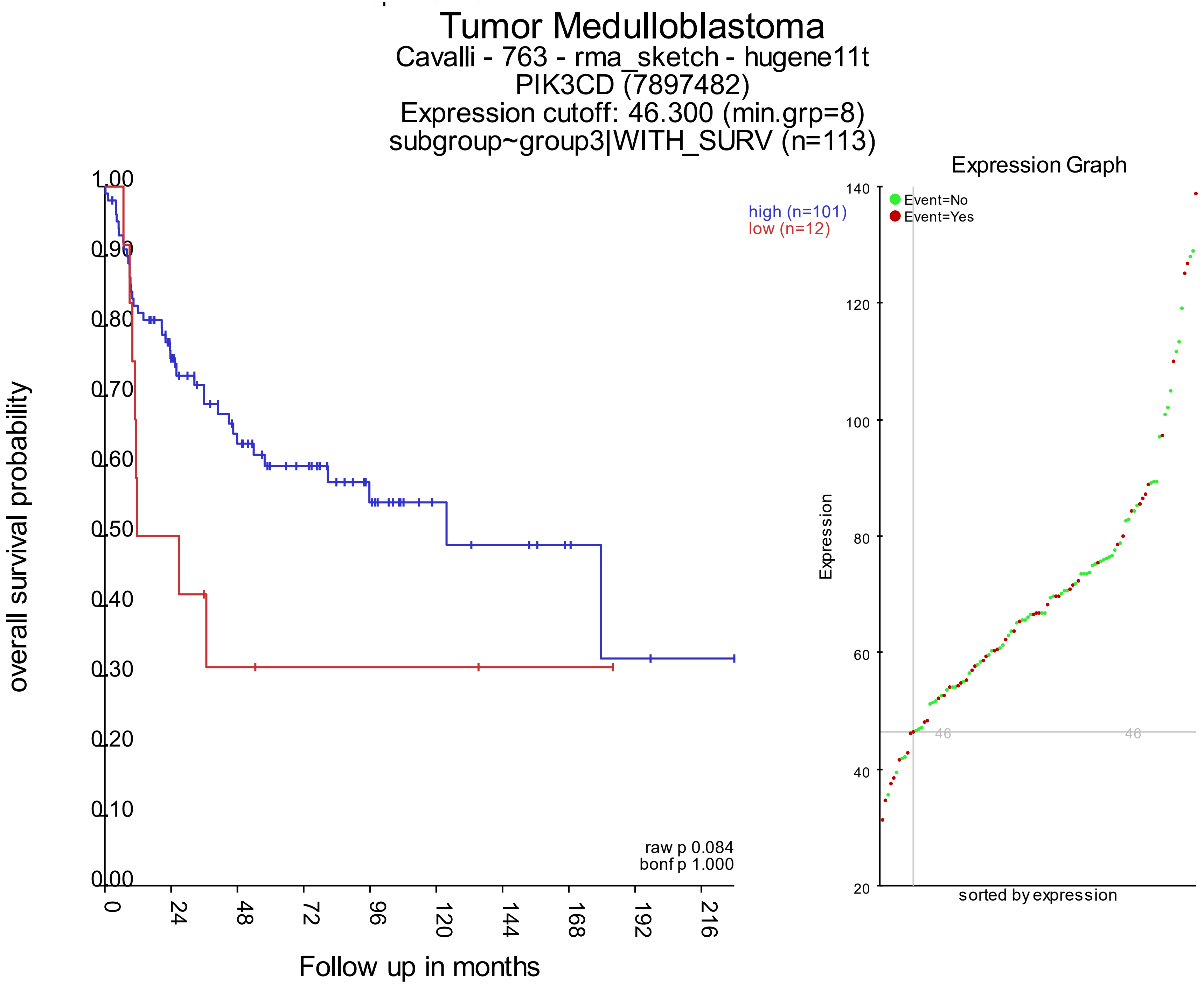

Tumor Medulloblastoma
Cavalli - 763 - rma_sketch - hugene11t
PIK3CD (7897482)
Expression cutoff: 46.300 (min.grp=8)
subgroup~group3|WITH_SURV (n=113)
overall survival probability
1.00
0.90
0.80
0.70
0.60
0.50
0.40
0.30
0.20
0.10
0.00
0
24
48
72
96
120
144
168
192
216
Follow up in months
high (n=101)
low (n=12)
raw p 0.084
bonf p 1.000
Expression Graph
Event=No
Event=Yes
Expression
140
120
100
80
60
40
20
46
46
sorted by expression

PIK3CG

WNT

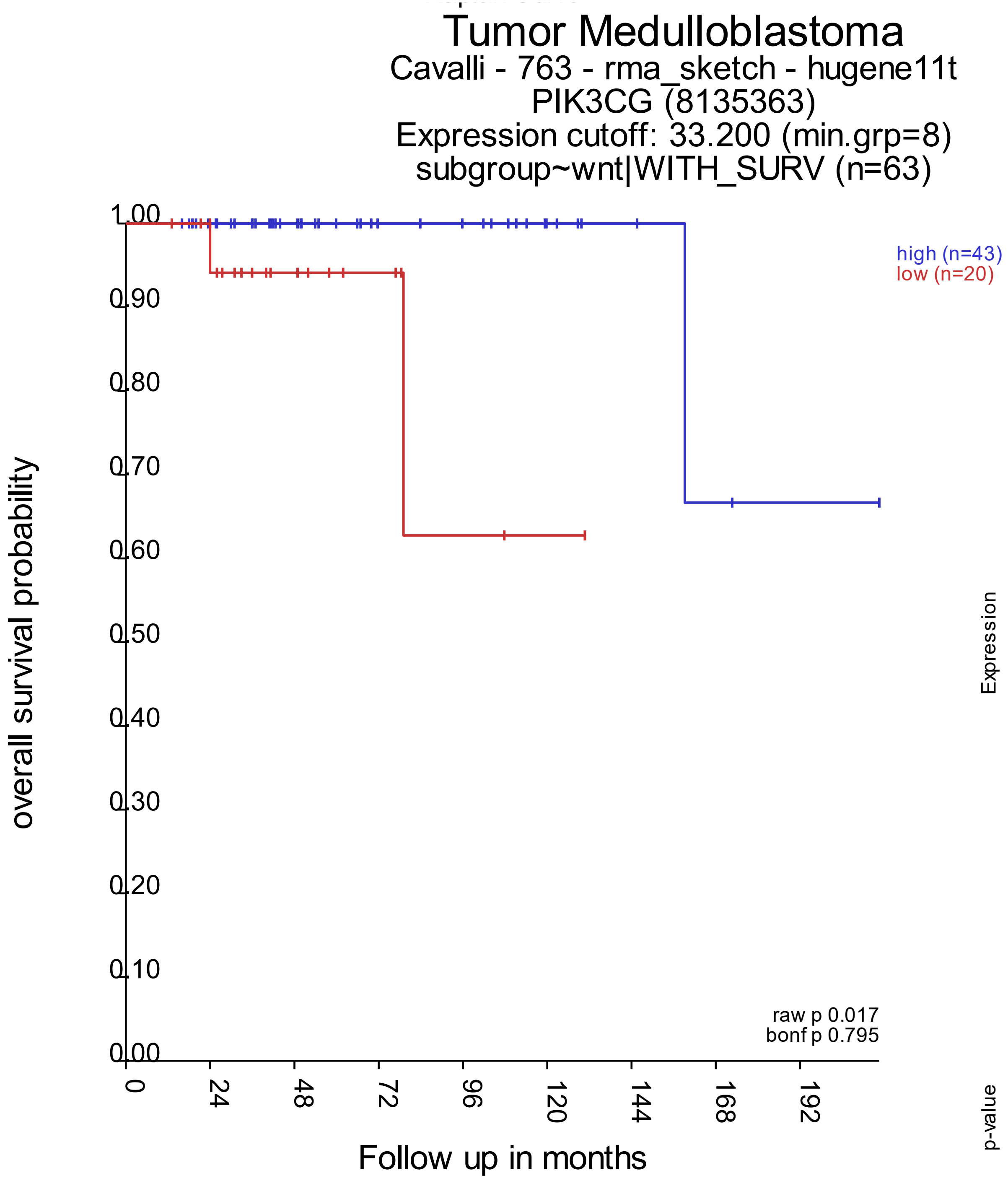

Tumor Medulloblastoma
Cavalli - 763 - rma_sketch - hugene11t
PIK3CG (8135363)
Expression cutoff: 33.200 (min.grp=8)
subgroup~wnt|WITH_SURV (n=63)
1.00
0.90
0.80
0.70
0.60
0.50
0.40
0.30
0.20
0.10
0.00
overall survival probability
high (n=43)
low (n=20)
raw p 0.017
bonf p 0.795
0
24
48
72
96
120
144
168
192
Follow up in months

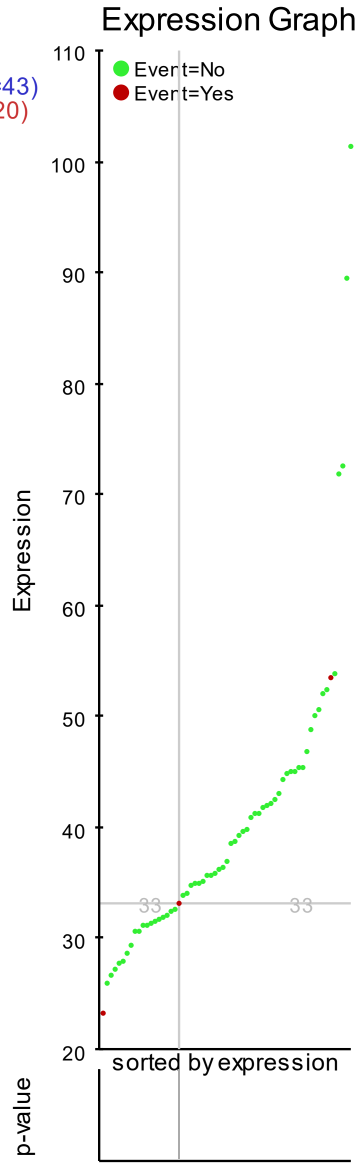

Expression Graph
Event=No
Event=Yes
110
100
90
80
70
60
50
40
30
20
Expression
33
33
sorted by expression
p-value

SHH

Tumor Medulloblastoma
Cavalli - 763 - rma_sketch - hugene11t
PIK3CG (8135363)
Expression cutoff: 81.300 (min.grp=8)
subgroup~shh|WITH_SURV (n=172)

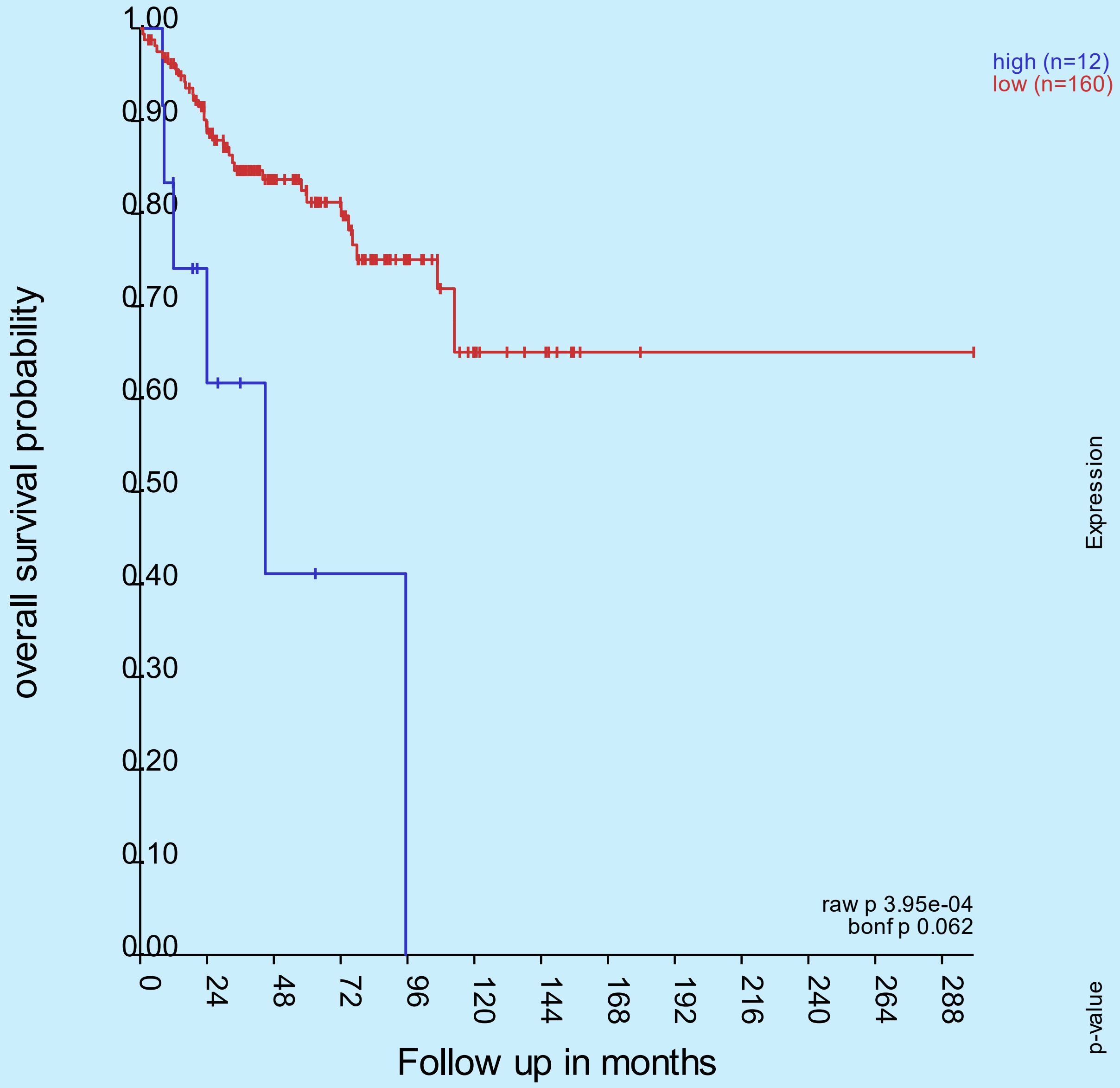

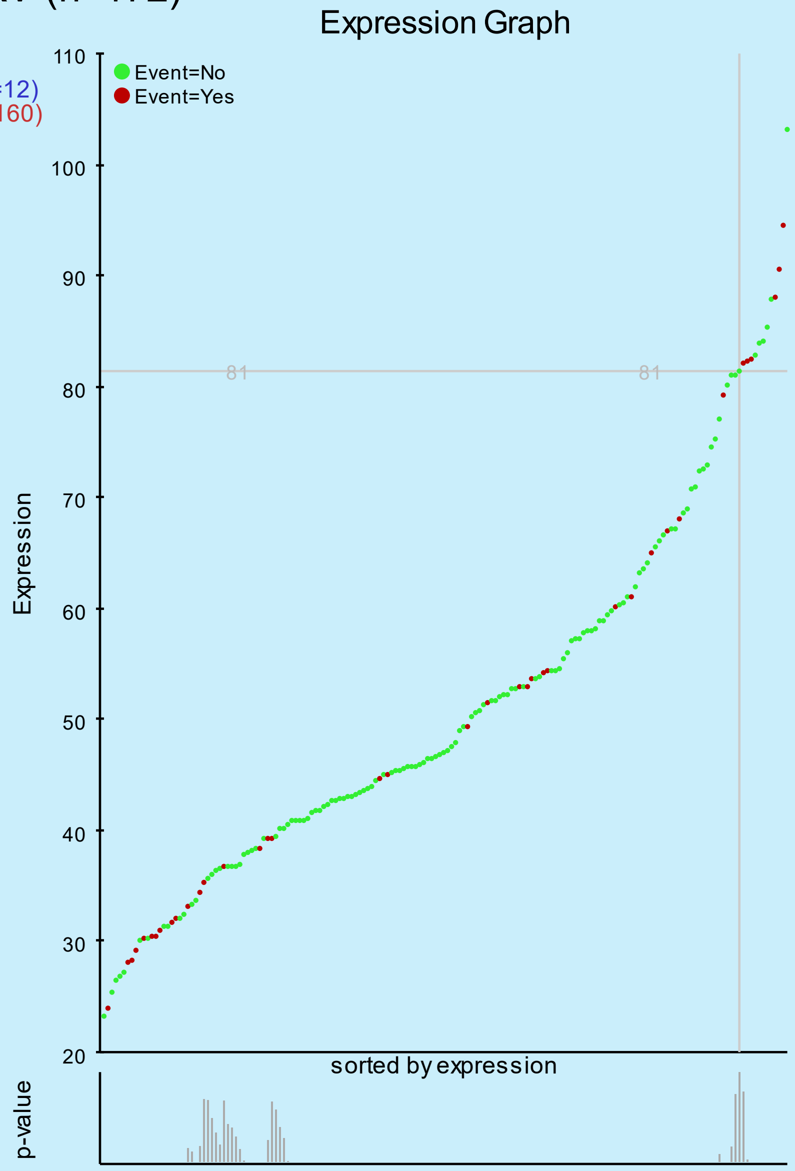

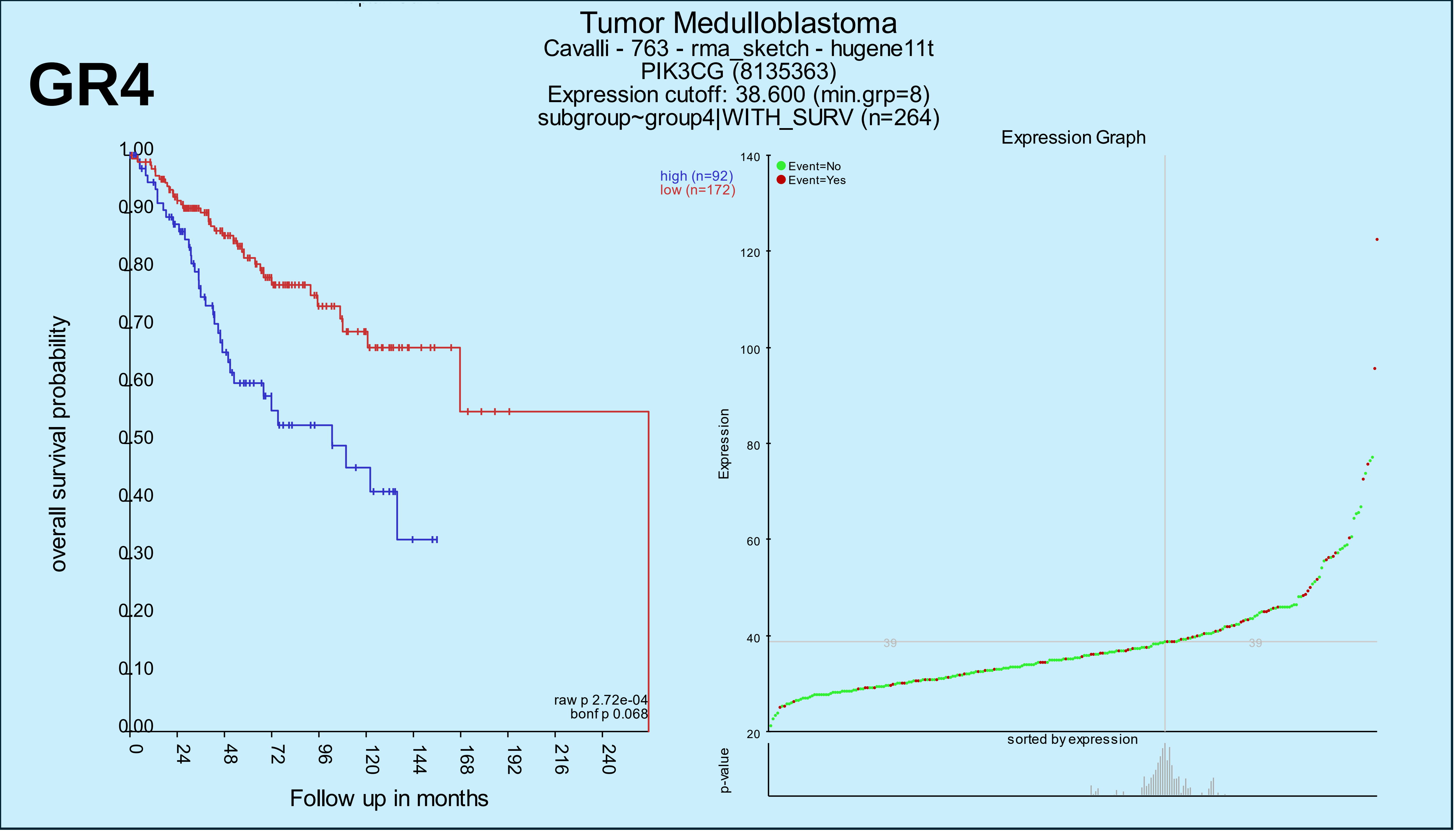

GR4
Tumor Medulloblastoma
Cavalli - 763 - rma_sketch - hugene11t
PIK3CG (8135363)
Expression cutoff: 38.600 (min.grp=8)
subgroup~group4|WITH_SURV (n=264)
high (n=92)
low (n=172)
overall survival probability
1.00
0.90
0.80
0.70
0.60
0.50
0.40
0.30
0.20
0.10
0.00
0
24
48
72
96
120
144
168
192
216
240
Follow up in months
raw p 2.72e-04
bonf p 0.068
Expression Graph
Event=No
Event=Yes
Expression
140
120
100
80
60
40
20
39
39
sorted by expression
p-value

GR3

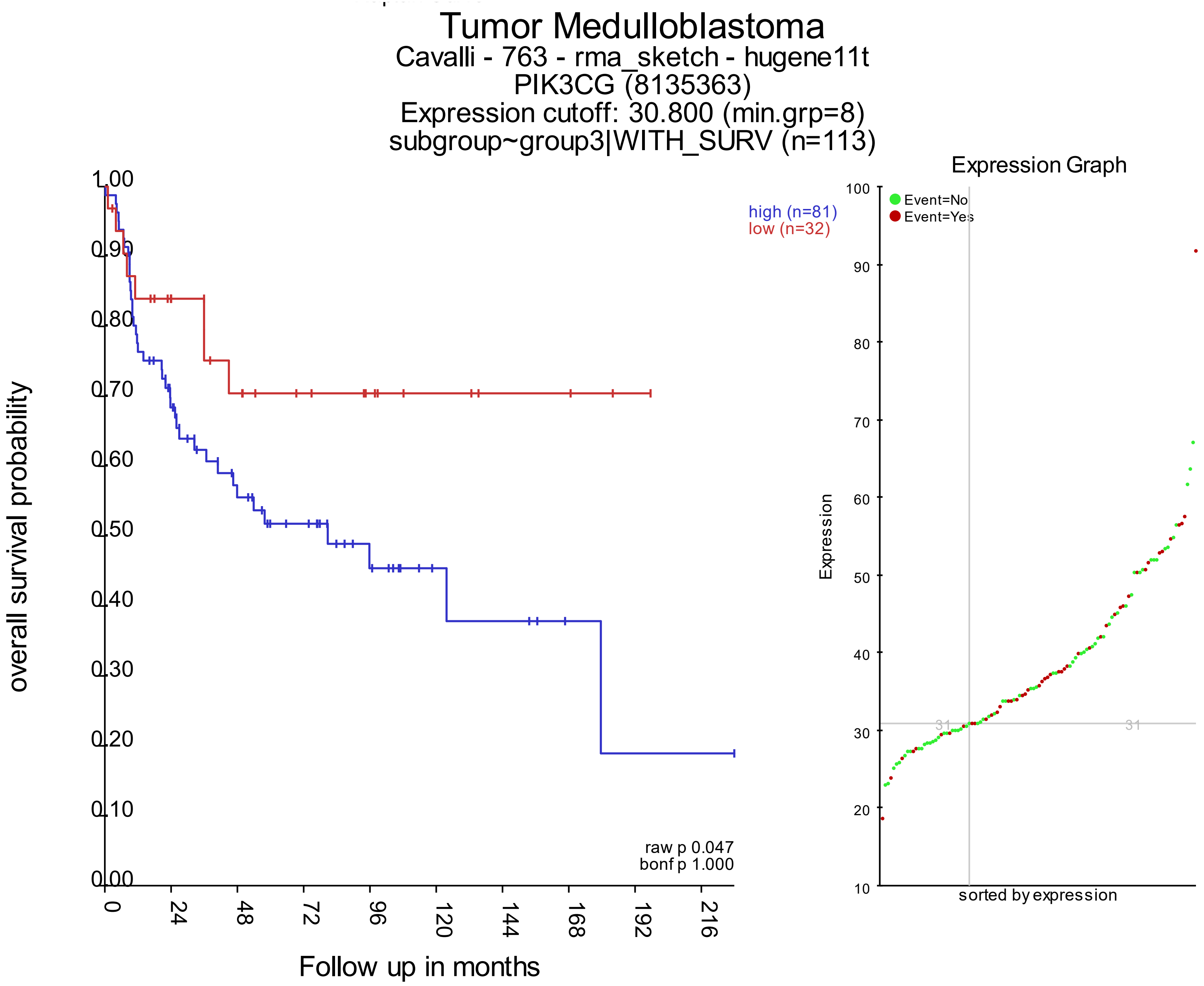
Tumor Medulloblastoma
Cavalli - 763 - rma_sketch - hugene11t
PIK3CG (8135363)
Expression cutoff: 30.800 (min.grp=8)
subgroup~group3|WITH_SURV (n=113)
high (n=81)
low (n=32)
overall survival probability
1.00
0.90
0.80
0.70
0.60
0.50
0.40
0.30
0.20
0.10
0.00
0
24
48
72
96
120
144
168
192
216
Follow up in months
raw p 0.047
bonf p 1.000
Expression Graph
Event=No
Event=Yes
Expression
100
90
80
70
60
50
40
30
20
10
31
31
sorted by expression

PIK3C2A

WNT

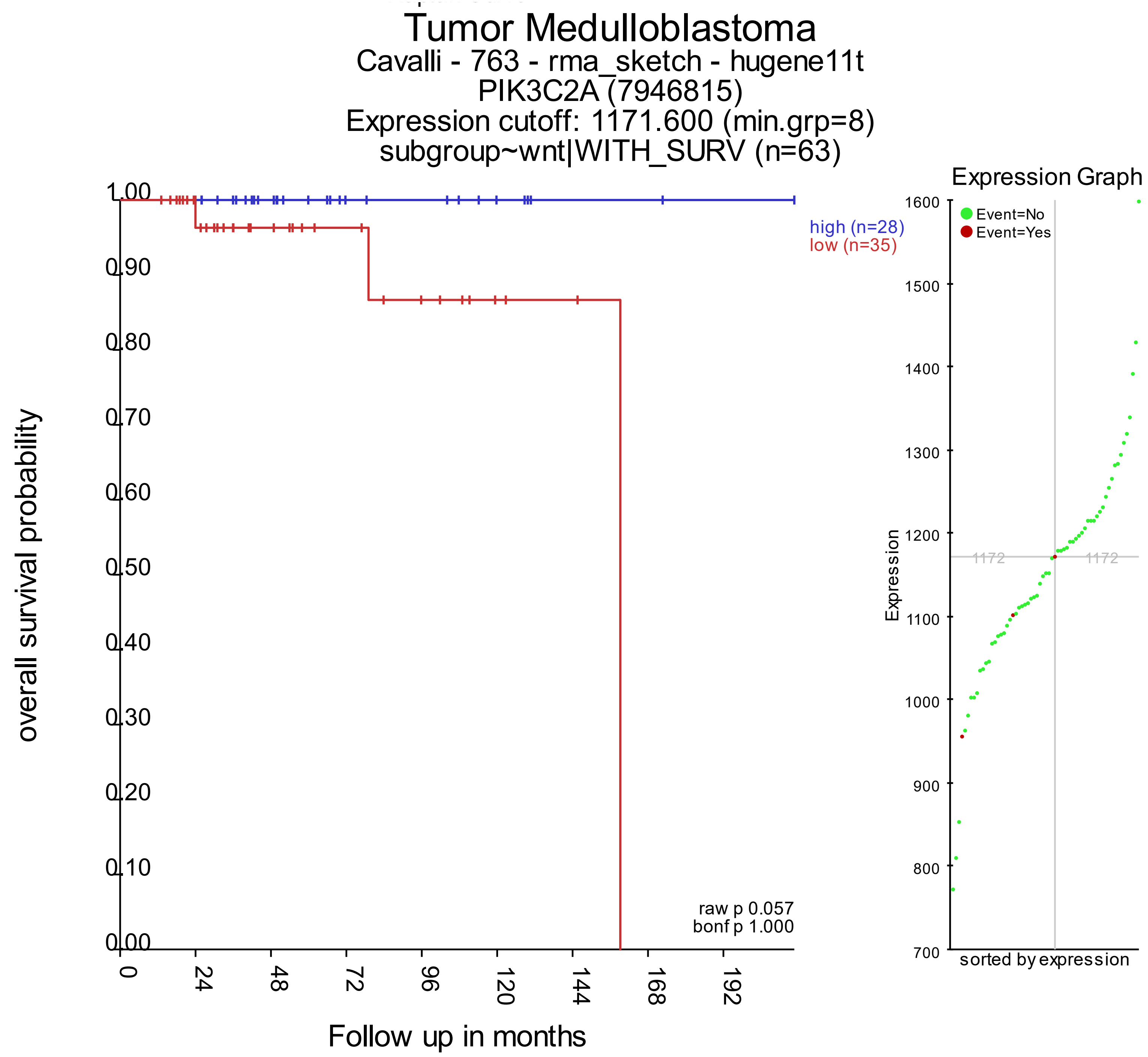

Tumor Medulloblastoma
Cavalli - 763 - rma_sketch - hugene11t
PIK3C2A (7946815)
Expression cutoff: 1171.600 (min.grp=8)
subgroup~wnt|WITH_SURV (n=63)
overall survival probability
1.00
0.90
0.80
0.70
0.60
0.50
0.40
0.30
0.20
0.10
0.00
high (n=28)
low (n=35)
raw p 0.057
bonf p 1.000
0
24
48
72
96
120
144
168
192
Follow up in months
Expression Graph
Event=No
Event=Yes
Expression
1600
1500
1400
1300
1200
1100
1000
900
800
700
1172
1172
sorted by expression

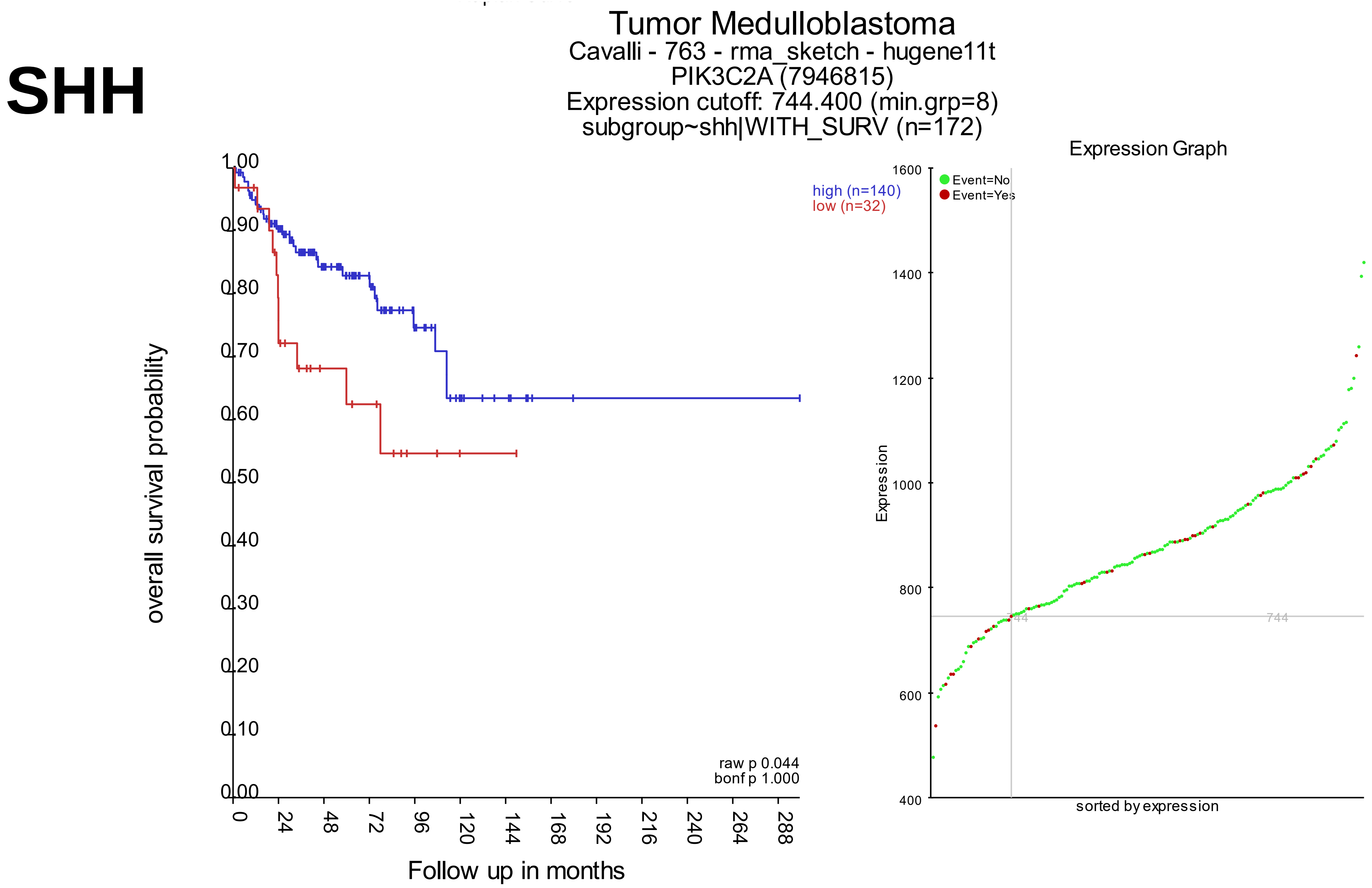

SHH
Tumor Medulloblastoma
Cavalli - 763 - rma_sketch - hugene11t
PIK3C2A (7946815)
Expression cutoff: 744.400 (min.grp=8)
subgroup~shh|WITH_SURV (n=172)
high (n=140)
low (n=32)
overall survival probability
1.00
0.90
0.80
0.70
0.60
0.50
0.40
0.30
0.20
0.10
0.00
0
24
48
72
96
120
144
168
192
216
240
264
288
Follow up in months
raw p 0.044
bonf p 1.000
Expression Graph
Event=No
Event=Yes
Expression
1600
1400
1200
1000
800
600
400
744
sorted by expression

GR4

Tumor Medulloblastoma
Cavalli - 763 - rma_sketch - hugene11t
PIK3C2A (7946815)
Expression cutoff: 1083.200 (min.grp=8)
subgroup~group4|WITH_SURV (n=264)

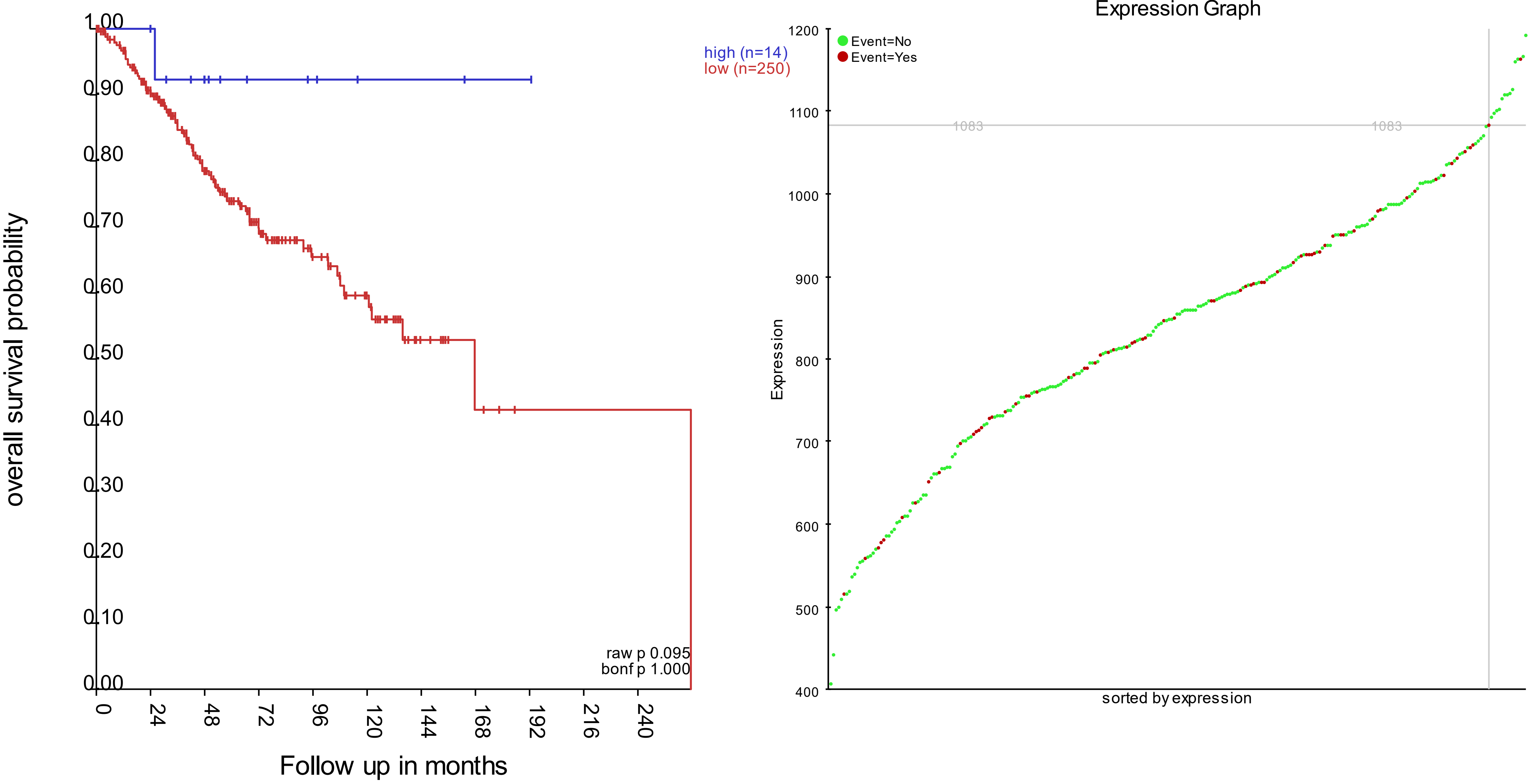

GR3

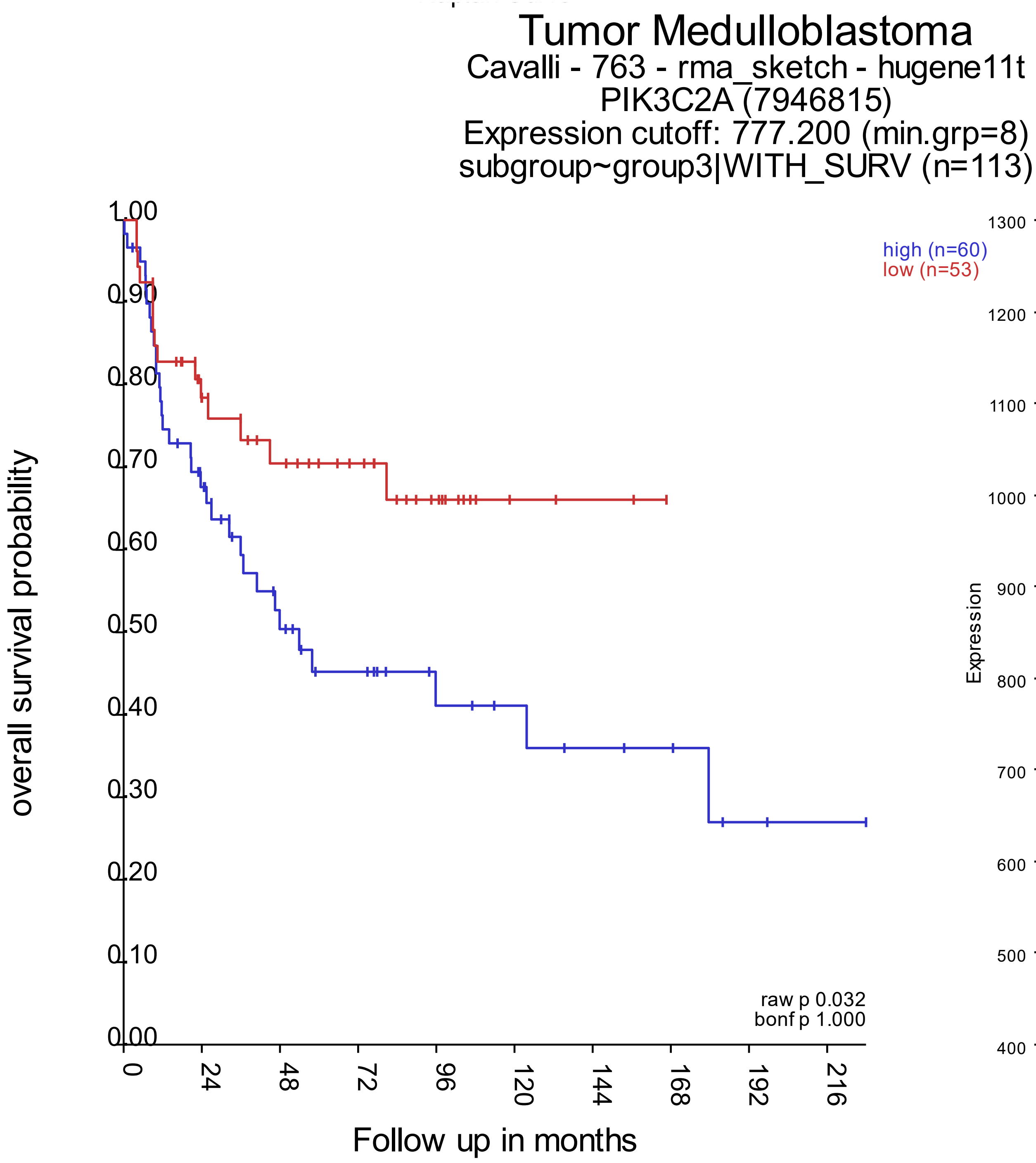

Tumor Medulloblastoma
Cavalli - 763 - rma_sketch - hugene11t
PIK3C2A (7946815)
Expression cutoff: 777.200 (min.grp=8)
subgroup~group3|WITH_SURV (n=113)
overall survival probability
1.00
0.90
0.80
0.70
0.60
0.50
0.40
0.30
0.20
0.10
0.00
0
24
48
72
96
120
144
168
192
216
Follow up in months
high (n=60)
low (n=53)
raw p 0.032
bonf p 1.000

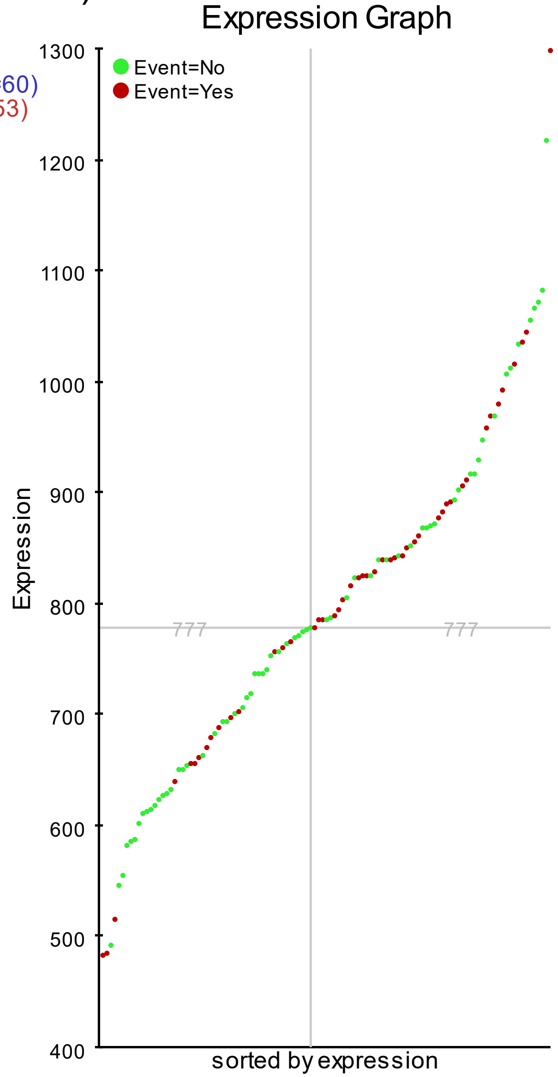

Expression Graph
Event=No
Event=Yes
1300
1200
1100
1000
900
800
700
600
500
400
Expression
777
777
sorted by expression

PIK3C2B

WNT

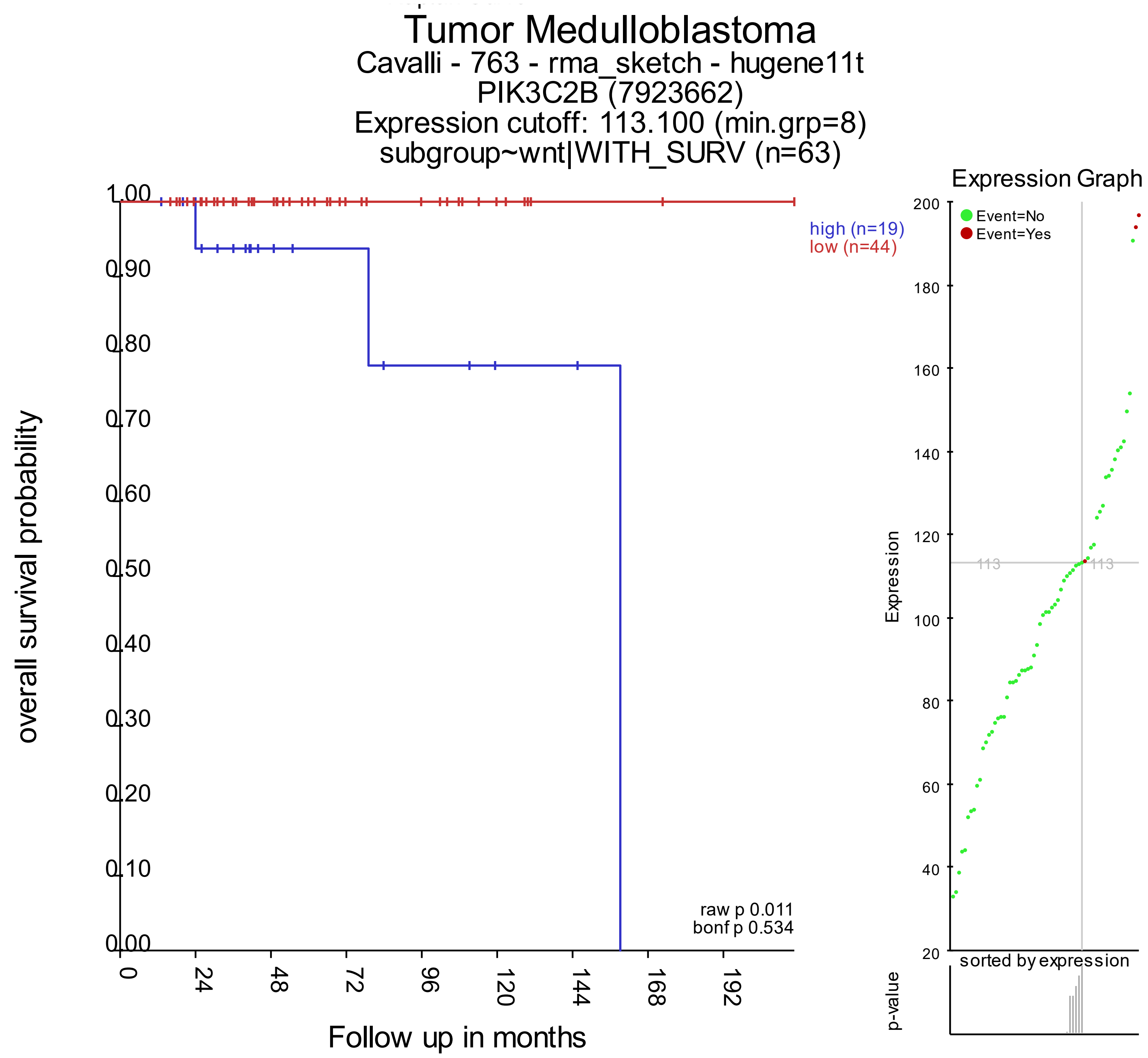

Tumor Medulloblastoma
Cavalli - 763 - rma_sketch - hugene11t
PIK3C2B (7923662)
Expression cutoff: 113.100 (min.grp=8)
subgroup~wnt|WITH_SURV (n=63)
overall survival probability
1.00
0.90
0.80
0.70
0.60
0.50
0.40
0.30
0.20
0.10
0.00
0
24
48
72
96
120
144
168
192
Follow up in months
high (n=19)
low (n=44)
raw p 0.011
bonf p 0.534
Expression Graph
Event=No
Event=Yes
Expression
200
180
160
140
120
100
80
60
40
20
113
113
sorted by expression
p-value

SHH

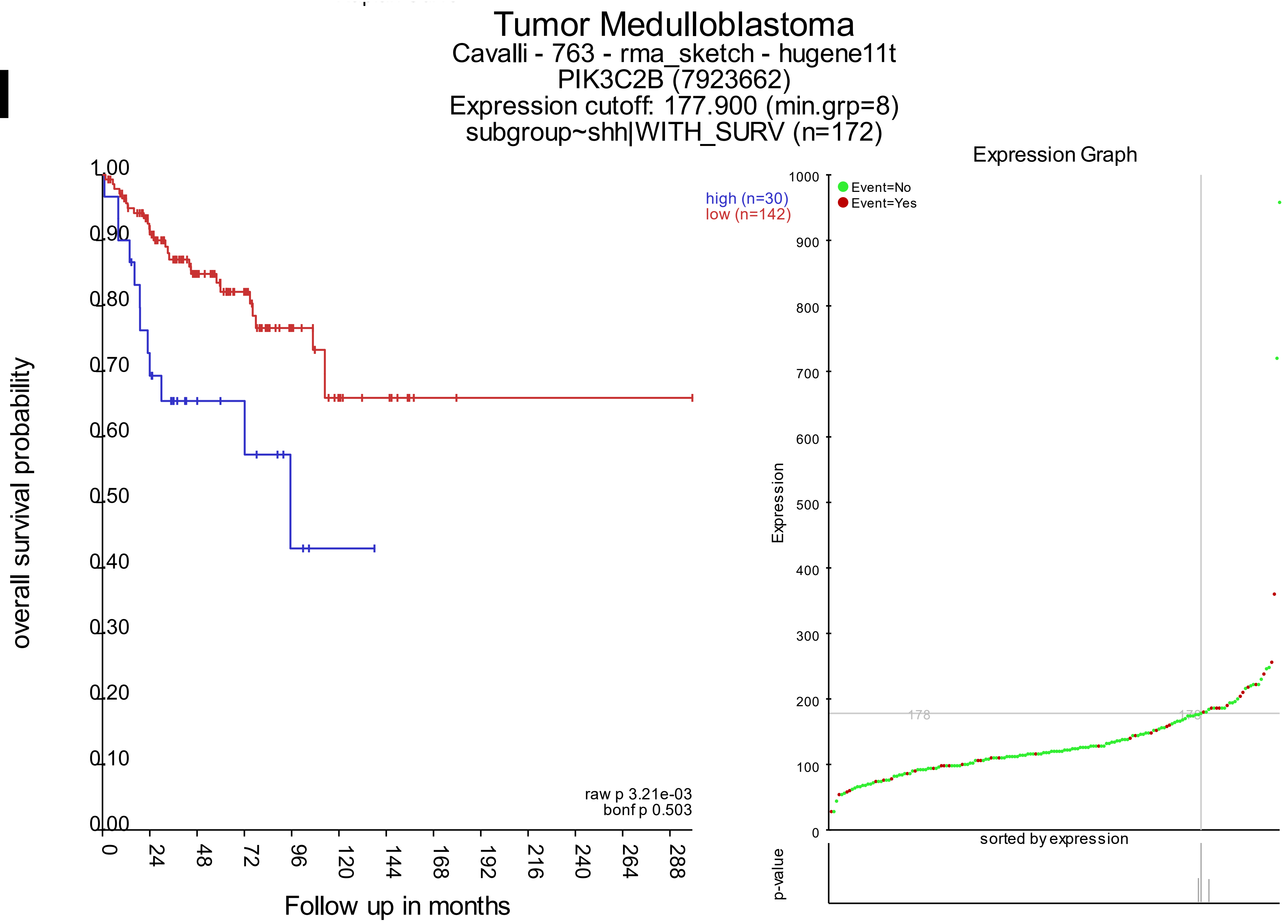

Tumor Medulloblastoma
Cavalli - 763 - rma_sketch - hugene11t
PIK3C2B (7923662)
Expression cutoff: 177.900 (min.grp=8)
subgroup~shh|WITH_SURV (n=172)
overall survival probability
1.00
0.90
0.80
0.70
0.60
0.50
0.40
0.30
0.20
0.10
0.00
0
24
48
72
96
120
144
168
192
216
240
264
288
Follow up in months
high (n=30)
low (n=142)
raw p 3.21e-03
bonf p 0.503
Expression Graph
Event=No
Event=Yes
Expression
1000
900
800
700
600
500
400
300
200
100
0
178
sorted by expression
p-value

GR4

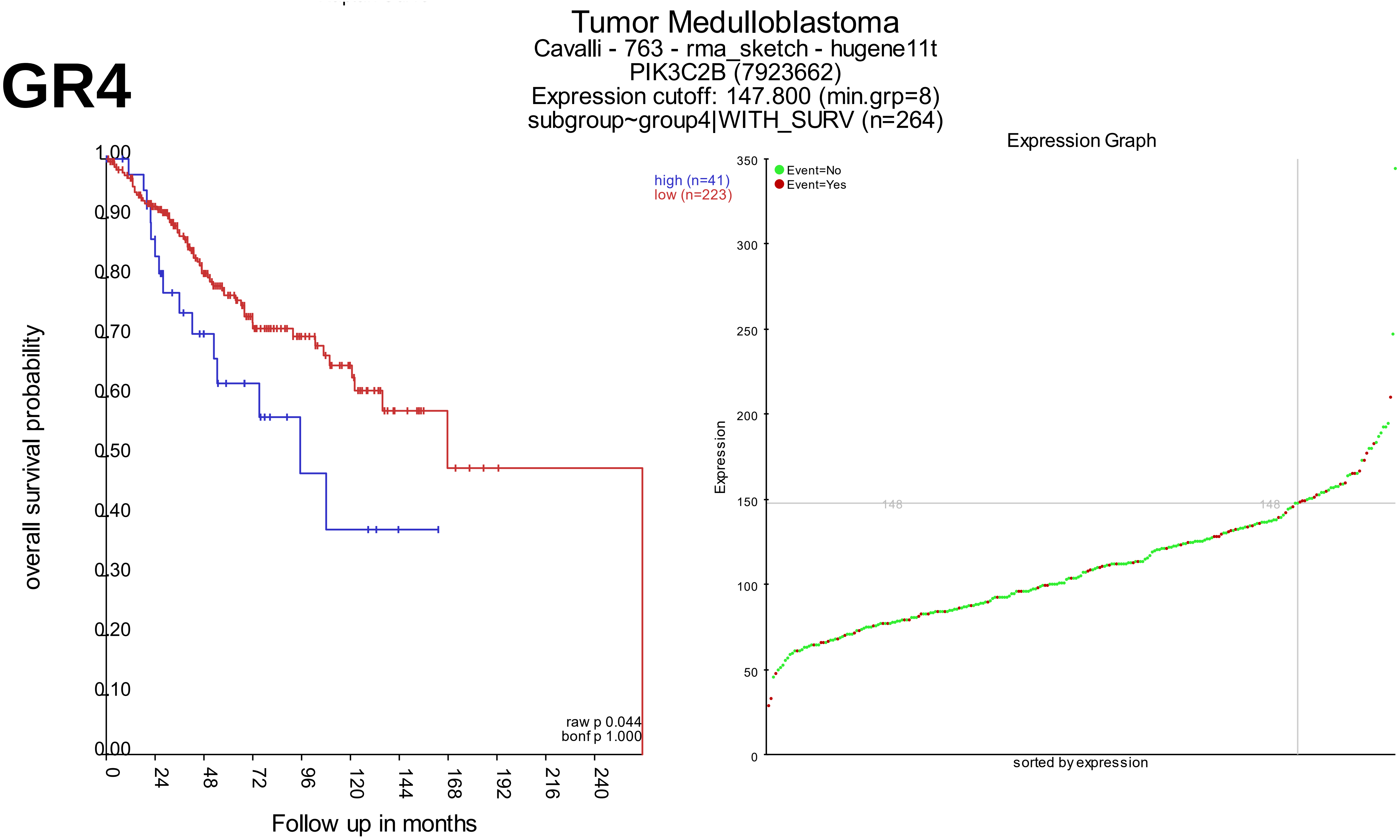

Tumor Medulloblastoma
Cavalli - 763 - rma_sketch - hugene11t
PIK3C2B (7923662)
Expression cutoff: 147.800 (min.grp=8)
subgroup~group4|WITH_SURV (n=264)
overall survival probability
1.00
0.90
0.80
0.70
0.60
0.50
0.40
0.30
0.20
0.10
0.00
0
24
48
72
96
120
144
168
192
216
240
Follow up in months
high (n=41)
low (n=223)
raw p 0.044
bonf p 1.000
Expression Graph
Event=No
Event=Yes
Expression
350
300
250
200
150
100
50
0
148
148
sorted by expression

GR3

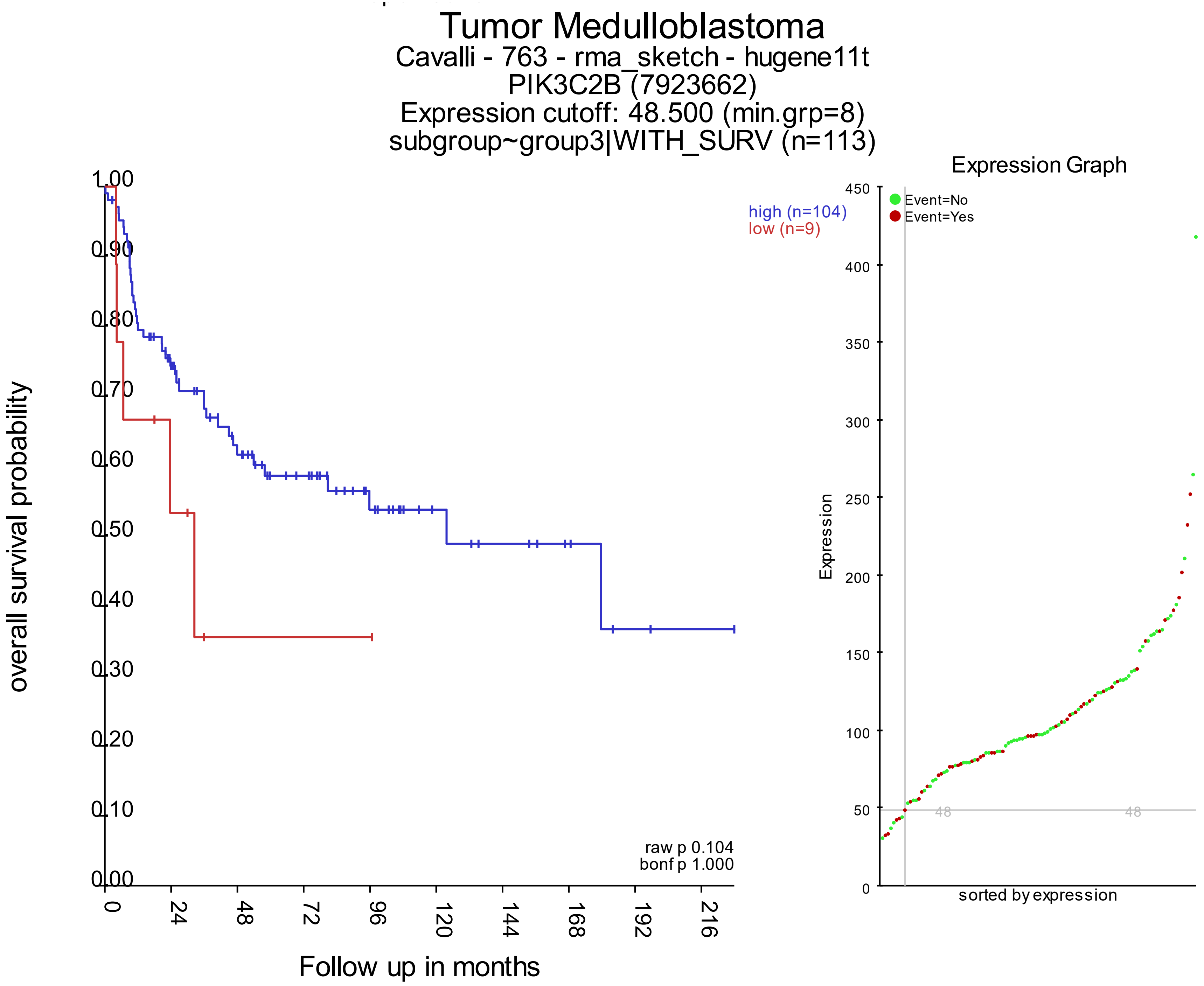
Tumor Medulloblastoma
Cavalli - 763 - rma_sketch - hugene11t
PIK3C2B (7923662)
Expression cutoff: 48.500 (min.grp=8)
subgroup~group3|WITH_SURV (n=113)
overall survival probability
1.00
0.90
0.80
0.70
0.60
0.50
0.40
0.30
0.20
0.10
0.00
0
24
48
72
96
120
144
168
192
216
Follow up in months
high (n=104)
low (n=9)
raw p 0.104
bonf p 1.000
Expression Graph
Event=No
Event=Yes
Expression
450
400
350
300
250
200
150
100
50
0
48
48
sorted by expression

PIK3C2G

WNT

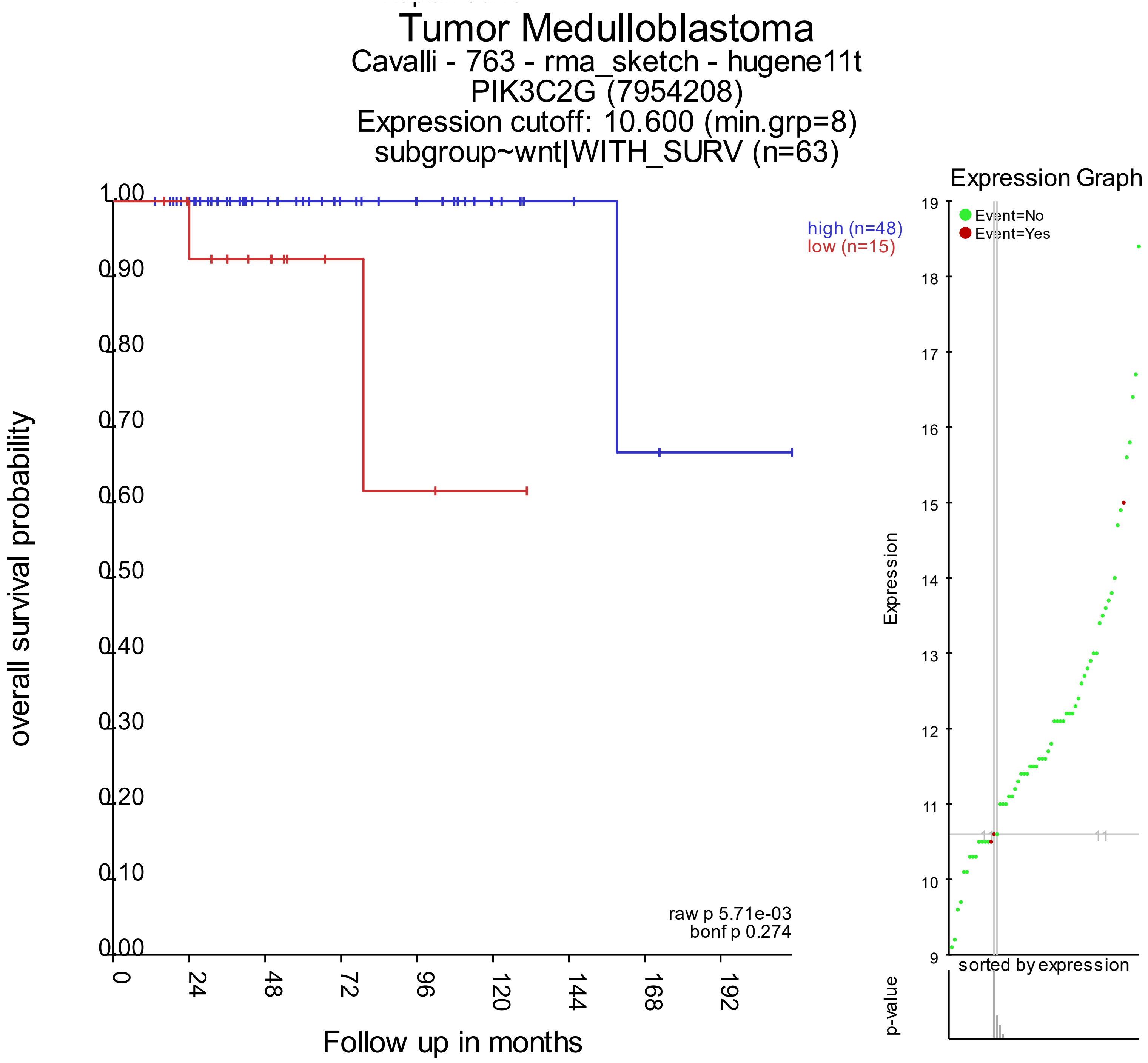
Tumor Medulloblastoma
Cavalli - 763 - rma_sketch - hugene11t
PIK3C2G (7954208)
Expression cutoff: 10.600 (min.grp=8)
subgroup~wnt|WITH_SURV (n=63)
overall survival probability
1.00
0.90
0.80
0.70
0.60
0.50
0.40
0.30
0.20
0.10
0.00
0
24
48
72
96
120
144
168
192
Follow up in months
high (n=48)
low (n=15)
raw p 5.71e-03
bonf p 0.274
Expression Graph
Event=No
Event=Yes
Expression
19
18
17
16
15
14
13
12
11
10
9
sorted by expression
p-value

SHH

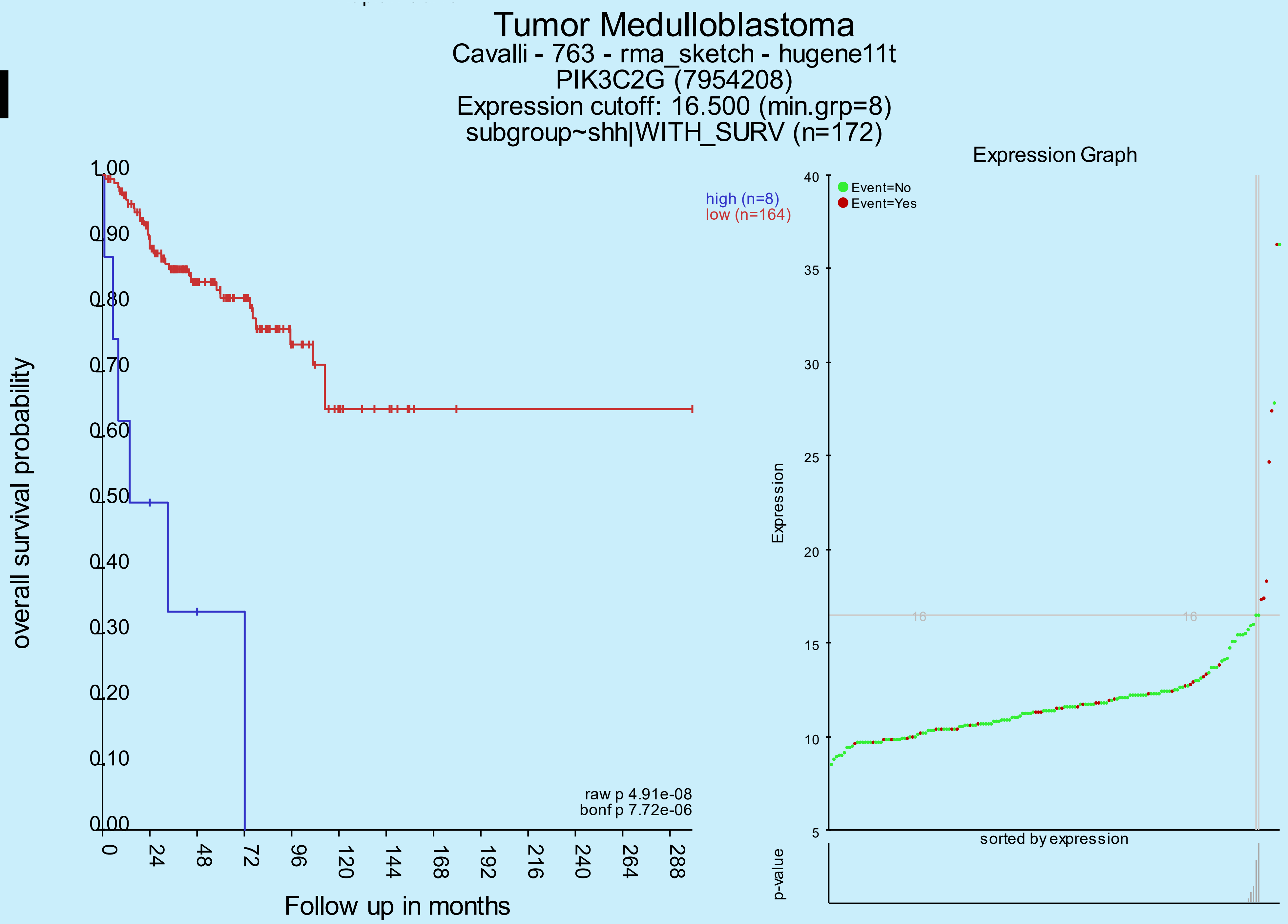

Tumor Medulloblastoma
Cavalli - 763 - rma_sketch - hugene11t
PIK3C2G (7954208)
Expression cutoff: 16.500 (min.grp=8)
subgroup~shh|WITH_SURV (n=172)
overall survival probability
1.00
0.90
0.80
0.70
0.60
0.50
0.40
0.30
0.20
0.10
0.00
high (n=8)
low (n=164)
raw p 4.91e-08
bonf p 7.72e-06
0
24
48
72
96
120
144
168
192
216
240
264
288
Follow up in months
Expression Graph
Event=No
Event=Yes
Expression
40
35
30
25
20
15
10
5
16
16
sorted by expression
p-value

GR4

Tumor Medulloblastoma
Cavalli - 763 - rma_sketch - hugene11t
PIK3C2G (7954208)
Expression cutoff: 13.900 (min.grp=8)
subgroup~group4|WITH_SURV (n=264)

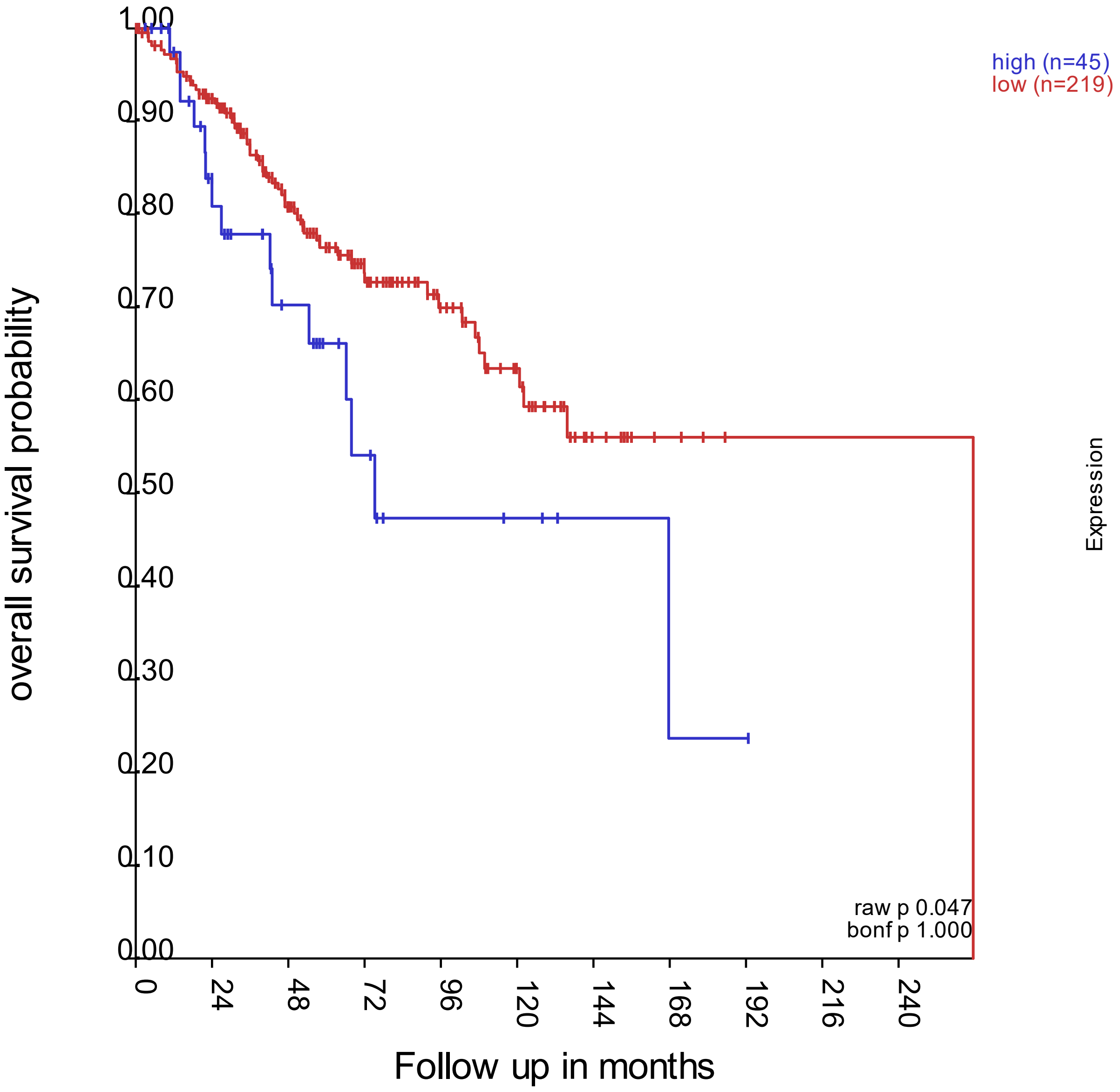

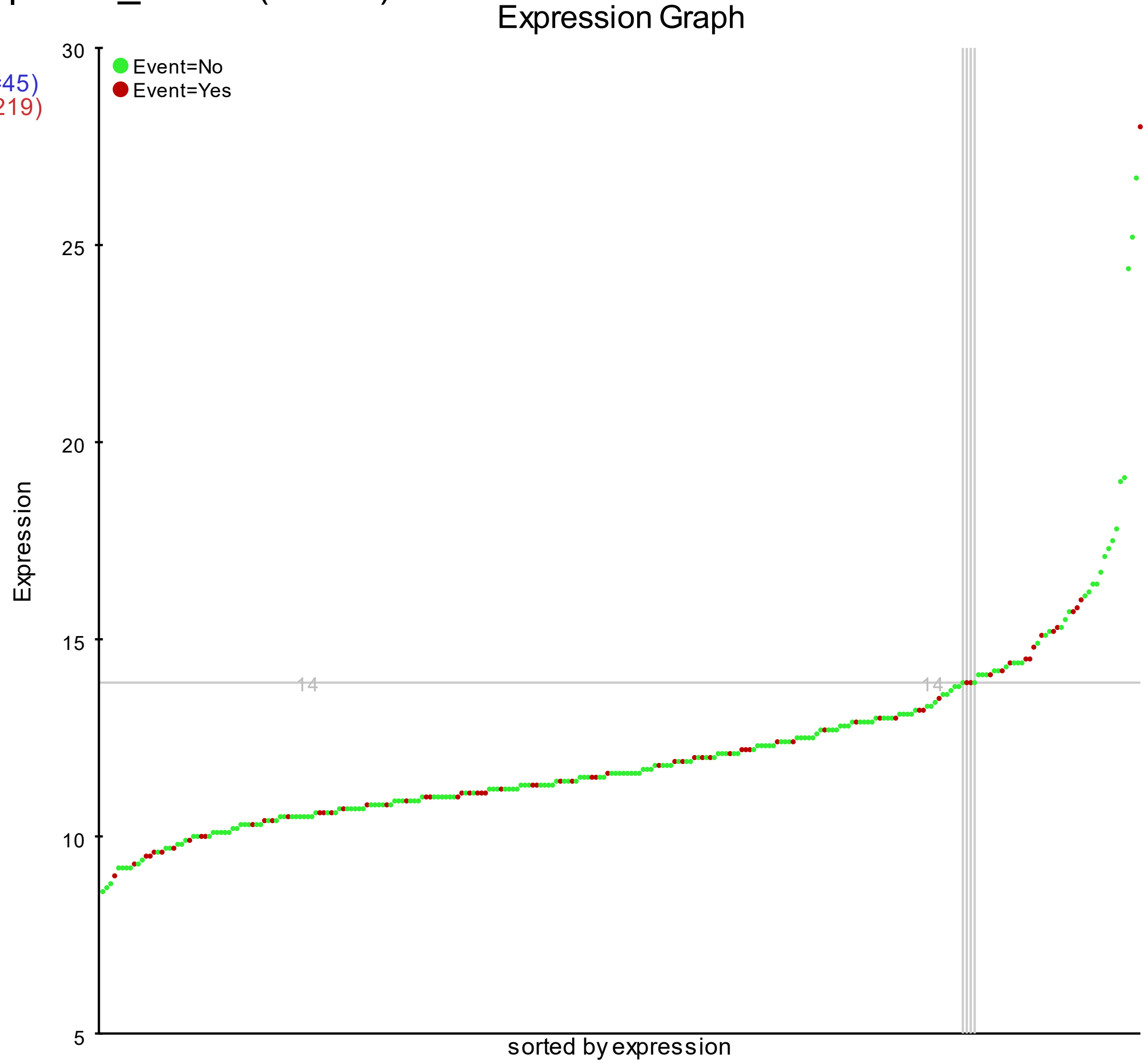

GR3

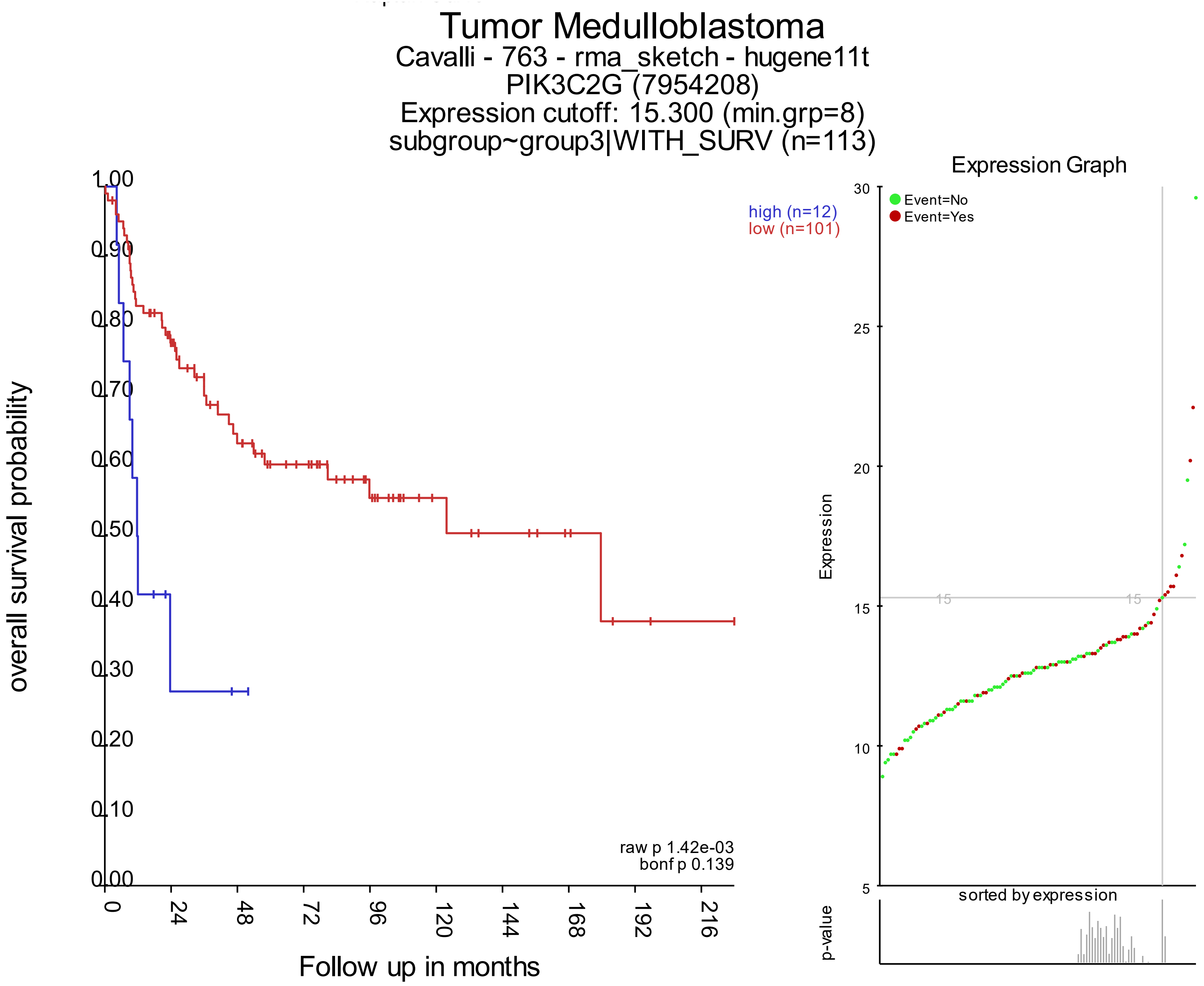

Tumor Medulloblastoma
Cavalli - 763 - rma_sketch - hugene11t
PIK3C2G (7954208)
Expression cutoff: 15.300 (min.grp=8)
subgroup~group3|WITH_SURV (n=113)
high (n=12)
low (n=101)
overall survival probability
1.00
0.90
0.80
0.70
0.60
0.50
0.40
0.30
0.20
0.10
0.00
0
24
48
72
96
120
144
168
192
216
Follow up in months
raw p 1.42e-03
bonf p 0.139
Expression Graph
Event=No
Event=Yes
30
25
20
15
10
5
Expression
sorted by expression
p-value

PIK3C3

WNT

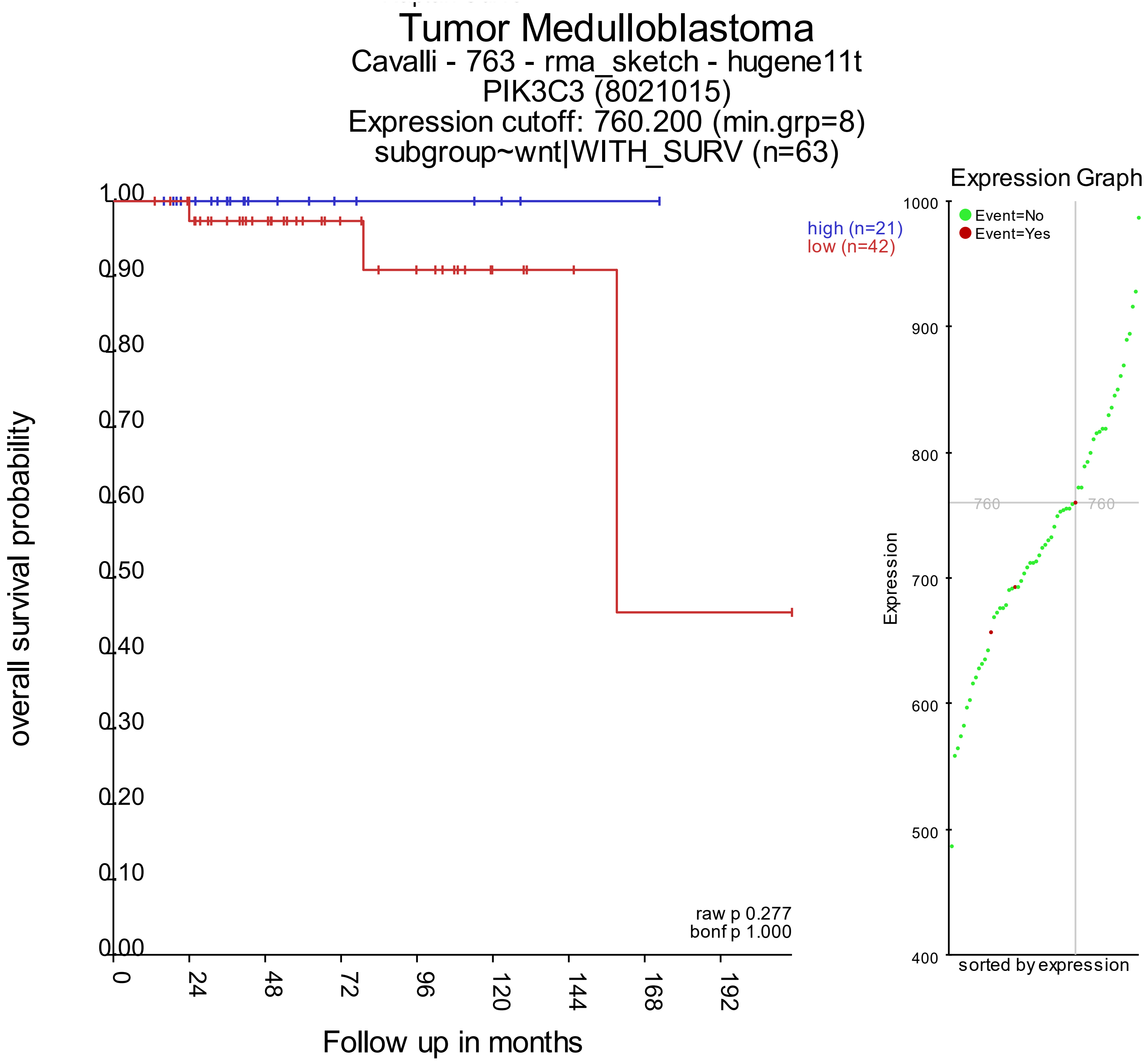

Tumor Medulloblastoma
Cavalli - 763 - rma_sketch - hugene11t
PIK3C3 (8021015)
Expression cutoff: 760.200 (min.grp=8)
subgroup~wnt|WITH_SURV (n=63)
overall survival probability
1.00
0.90
0.80
0.70
0.60
0.50
0.40
0.30
0.20
0.10
0.00
0
24
48
72
96
120
144
168
192
Follow up in months
raw p 0.277
bonf p 1.000
high (n=21)
low (n=42)
Expression Graph
Event=No
Event=Yes
1000
900
800
700
600
500
400
760
760
Expression
sorted by expression

SHH

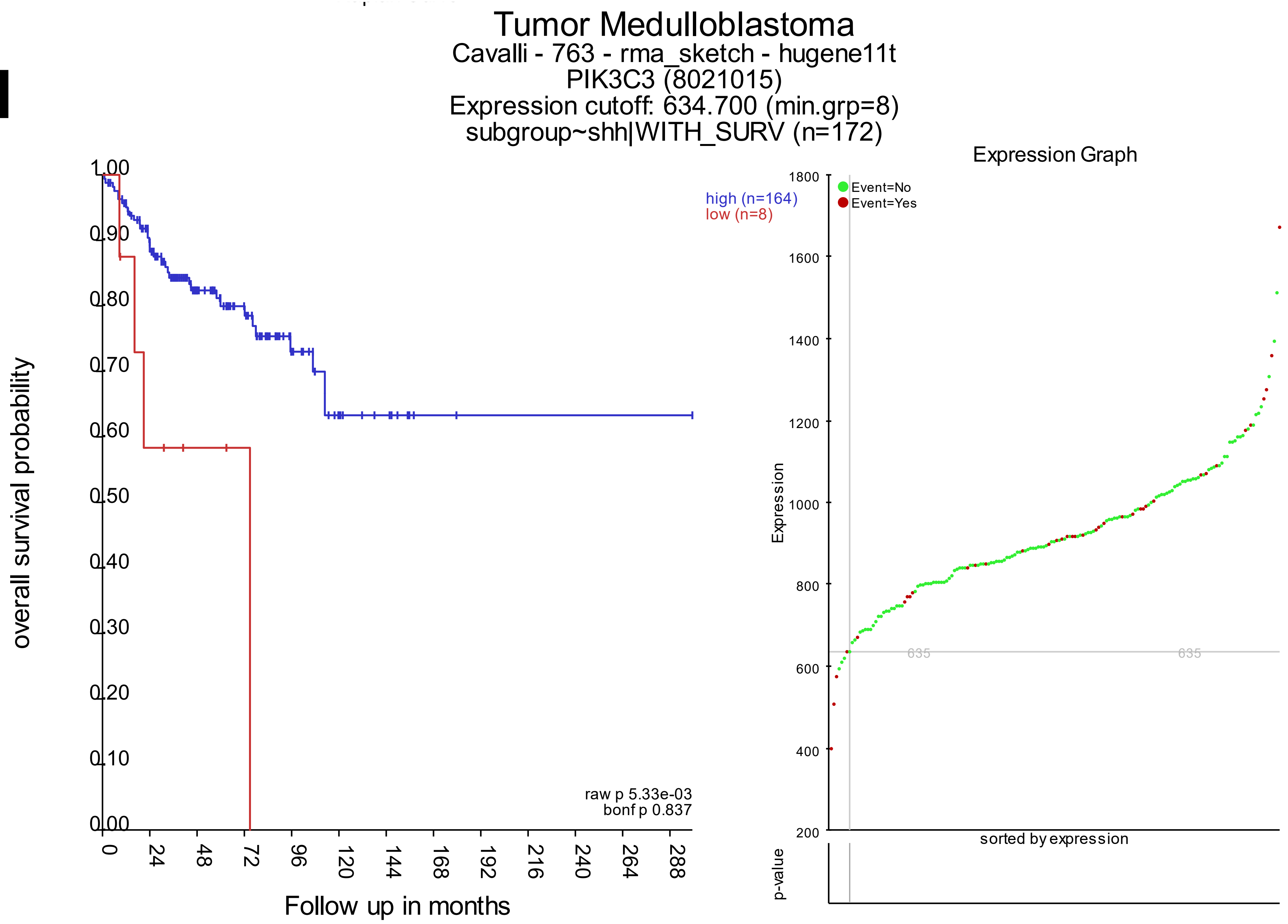

Tumor Medulloblastoma
Cavalli - 763 - rma_sketch - hugene11t
PIK3C3 (8021015)
Expression cutoff: 634.700 (min.grp=8)
subgroup~shh|WITH_SURV (n=172)
1.00
0.90
0.80
0.70
0.60
0.50
0.40
0.30
0.20
0.10
0.00
overall survival probability
high (n=164)
low (n=8)
raw p 5.33e-03
bonf p 0.837
0
24
48
72
96
120
144
168
192
216
240
264
288
Follow up in months
Expression Graph
Event=No
Event=Yes
1800
1600
1400
1200
1000
800
600
400
200
Expression
635
635
sorted by expression
p-value

GR4

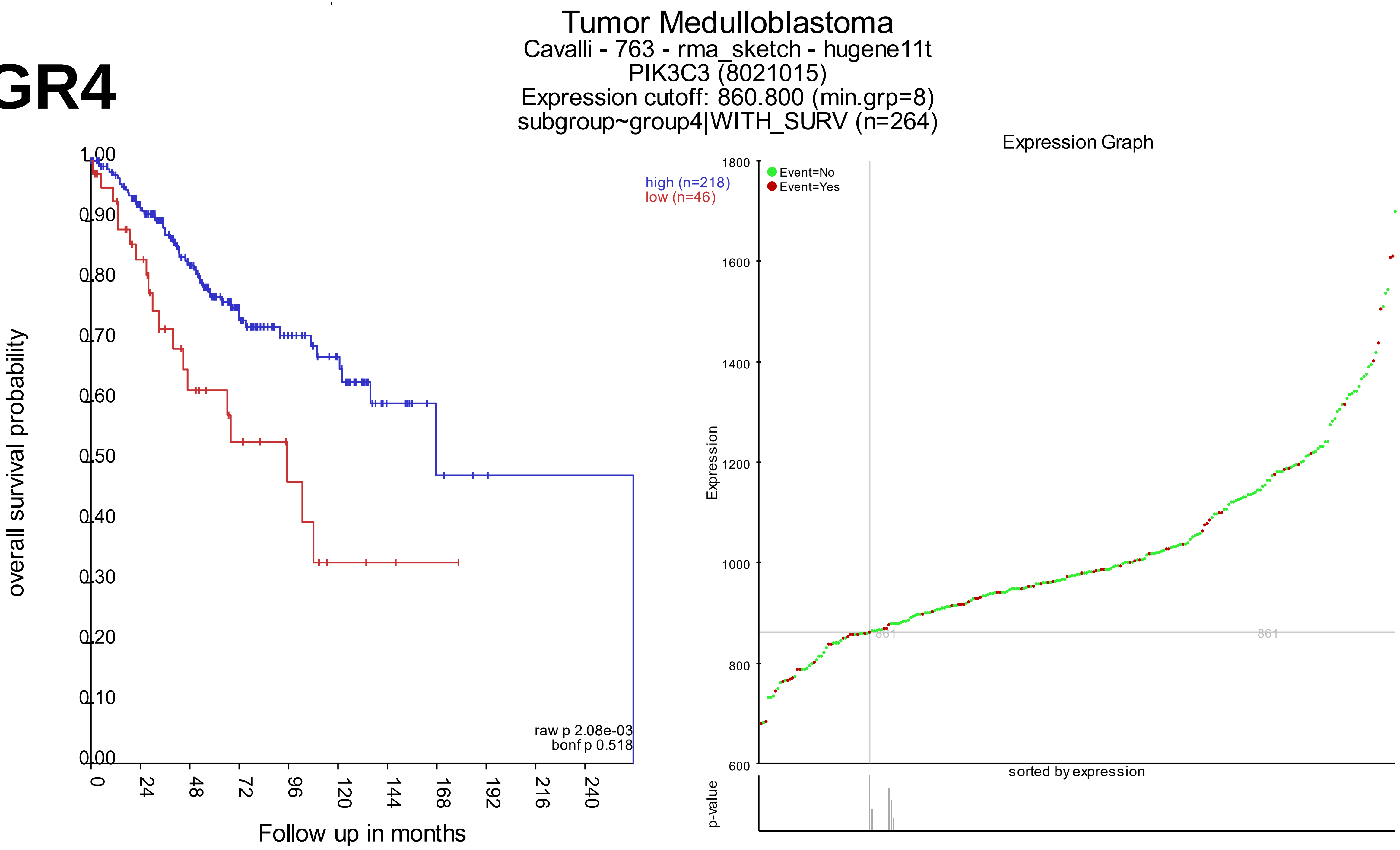

Tumor Medulloblastoma
Cavalli - 763 - rma_sketch - hugene11t
PIK3C3 (8021015)
Expression cutoff: 860.800 (min.grp=8)
subgroup~group4|WITH_SURV (n=264)
overall survival probability
1.00
0.90
0.80
0.70
0.60
0.50
0.40
0.30
0.20
0.10
0.00
0
24
48
72
96
120
144
168
192
216
240
Follow up in months
high (n=218)
low (n=46)
raw p 2.08e-03
bonf p 0.518
Expression Graph
Event=No
Event=Yes
Expression
1800
1600
1400
1200
1000
800
600
861
861
sorted by expression
p-value

GR3

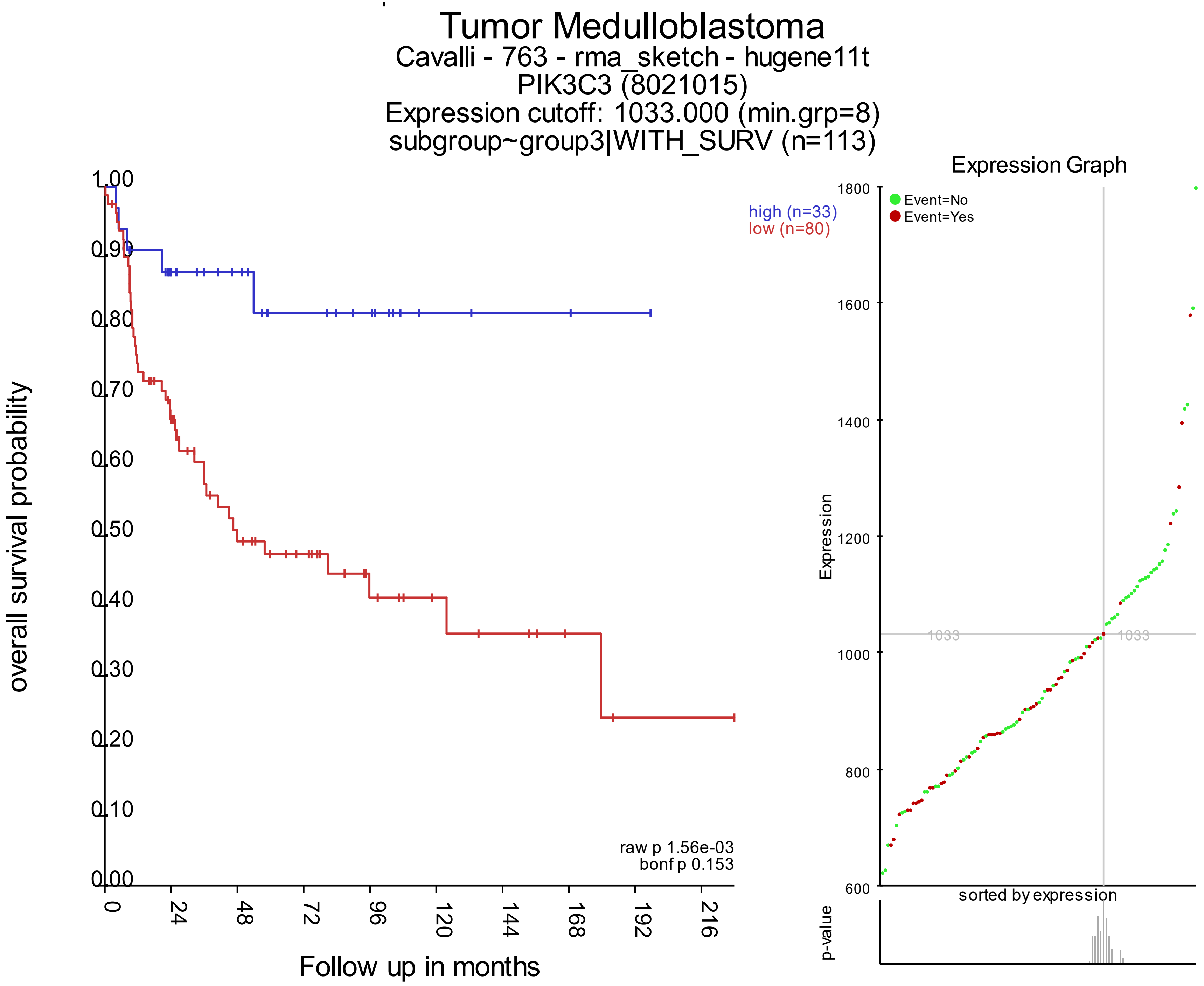

Tumor Medulloblastoma
Cavalli - 763 - rma_sketch - hugene11t
PIK3C3 (8021015)
Expression cutoff: 1033.000 (min.grp=8)
subgroup~group3|WITH_SURV (n=113)
overall survival probability
Follow up in months
high (n=33)
low (n=80)
raw p 1.56e-03
bonf p 0.153
Expression Graph
Event=No
Event=Yes
Expression
sorted by expression
p-value
1033

ITPKC

WNT

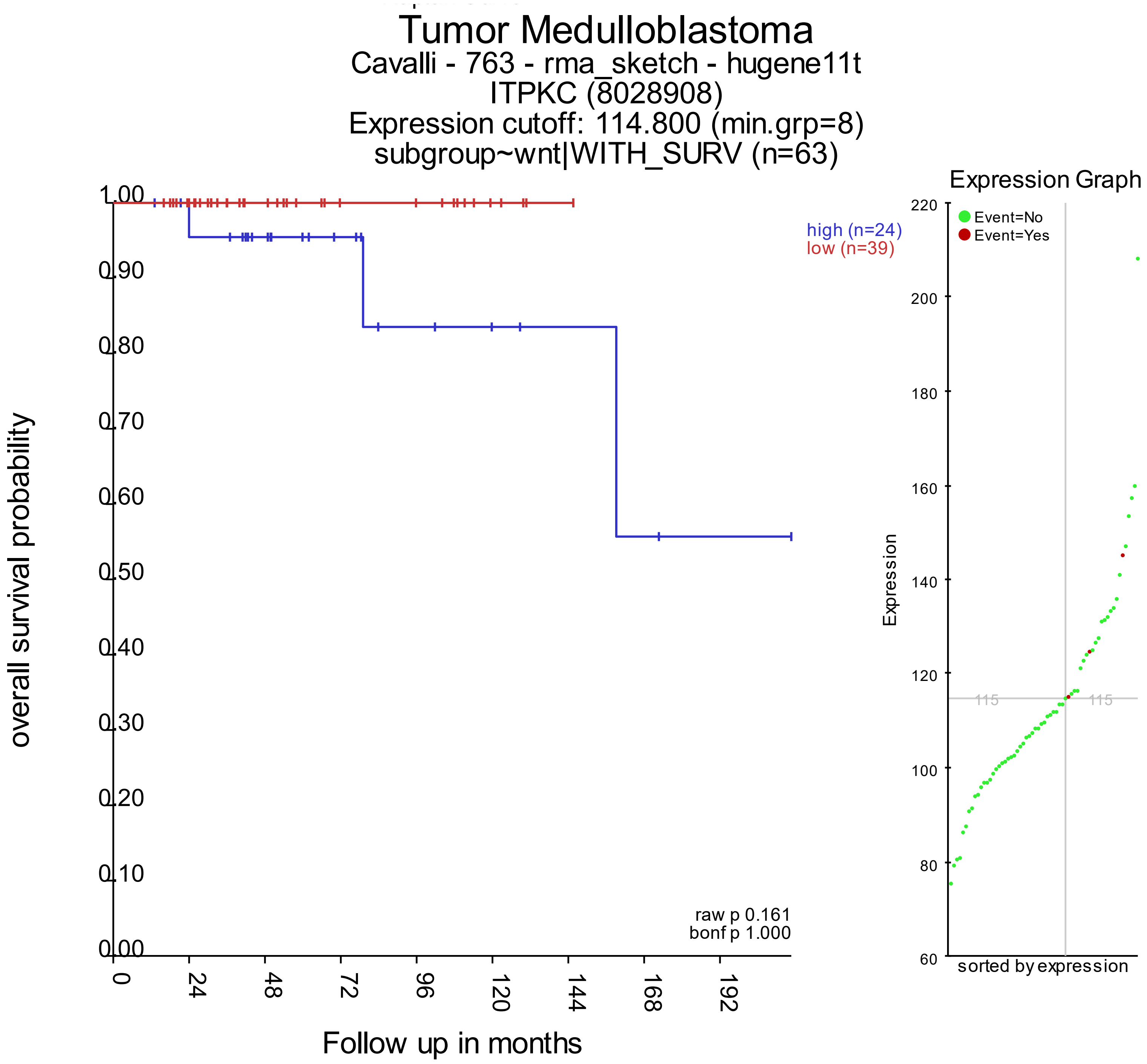

Tumor Medulloblastoma
Cavalli - 763 - rma_sketch - hugene11t
ITPKC (8028908)
Expression cutoff: 114.800 (min.grp=8)
subgroup~wnt|WITH_SURV (n=63)
overall survival probability
1.00
0.90
0.80
0.70
0.60
0.50
0.40
0.30
0.20
0.10
0.00
0
24
48
72
96
120
144
168
192
Follow up in months
high (n=24)
low (n=39)
raw p 0.161
bonf p 1.000
Expression Graph
Event=No
Event=Yes
220
200
180
160
140
120
100
80
60
Expression
115
115
sorted by expression

SHH

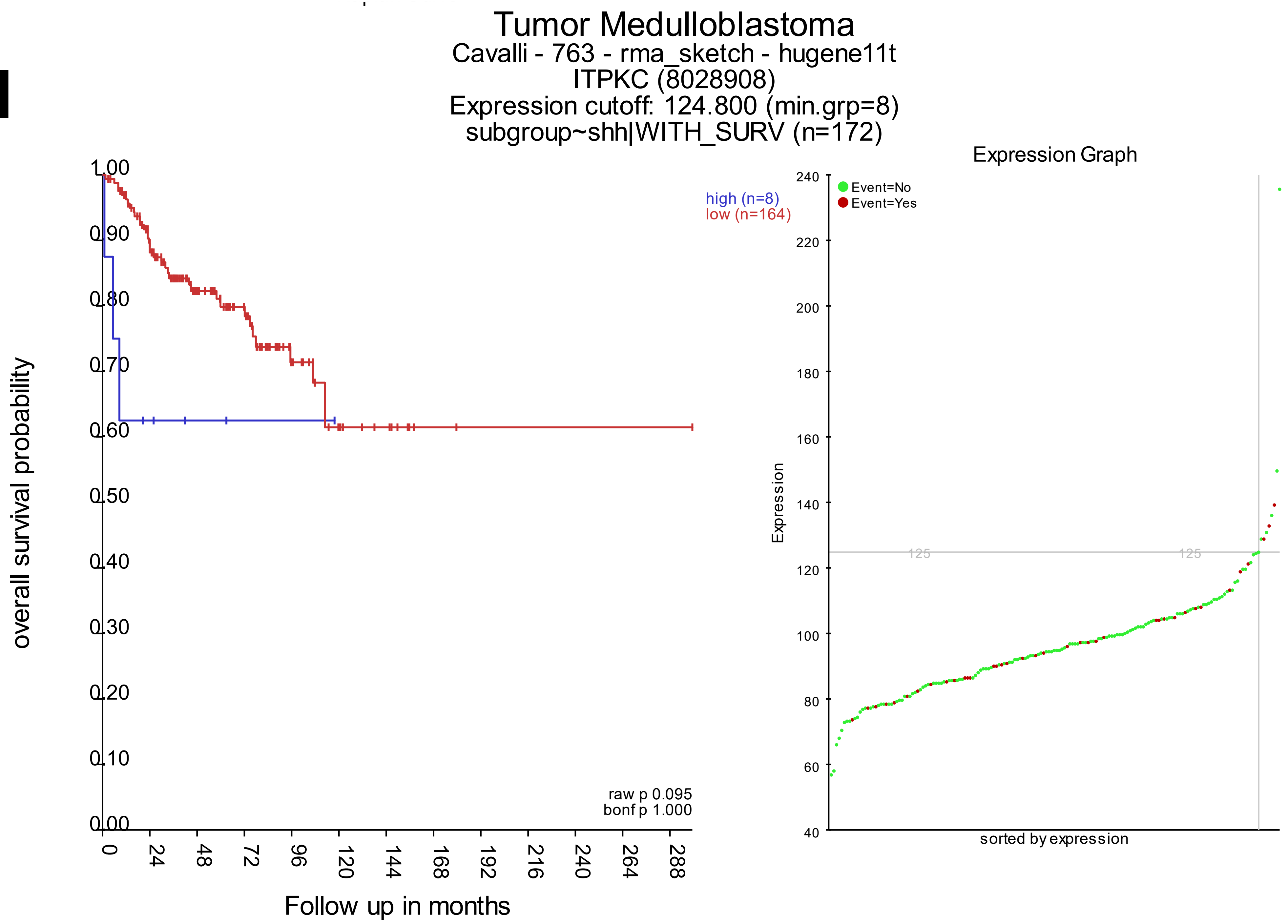

Tumor Medulloblastoma
Cavalli - 763 - rma_sketch - hugene11t
ITPKC (8028908)
Expression cutoff: 124.800 (min.grp=8)
subgroup~shh|WITH_SURV (n=172)
overall survival probability
1.00
0.90
0.80
0.70
0.60
0.50
0.40
0.30
0.20
0.10
0.00
0
24
48
72
96
120
144
168
192
216
240
264
288
Follow up in months
high (n=8)
low (n=164)
raw p 0.095
bonf p 1.000
Expression Graph
Event=No
Event=Yes
Expression
240
220
200
180
160
140
120
100
80
60
40
125
125
sorted by expression

GR4

Tumor Medulloblastoma
Cavalli - 763 - rma_sketch - hugene11t
ITPKC (8028908)
Expression cutoff: 89.900 (min.grp=8)
subgroup~group4|WITH_SURV (n=264)

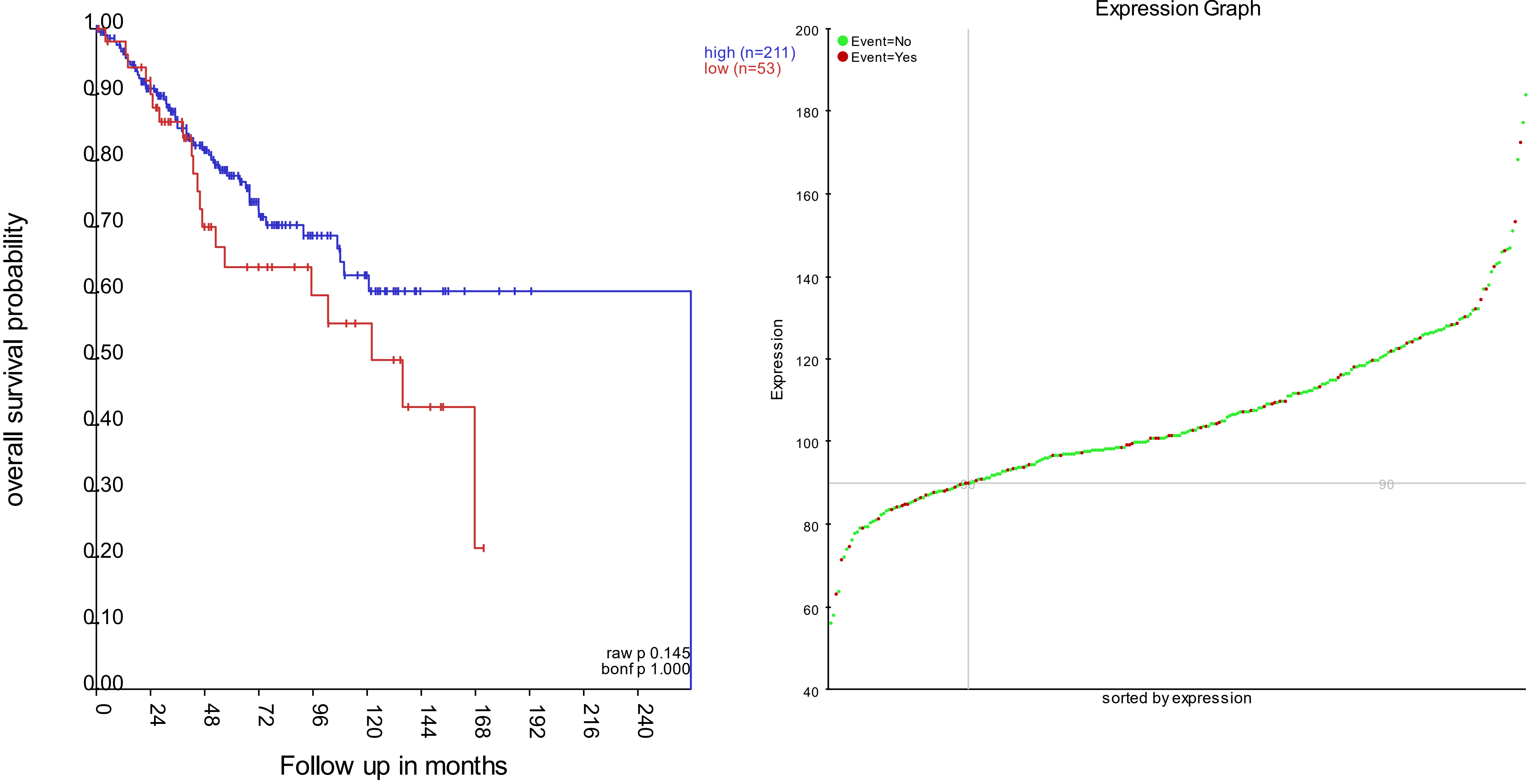

GR3

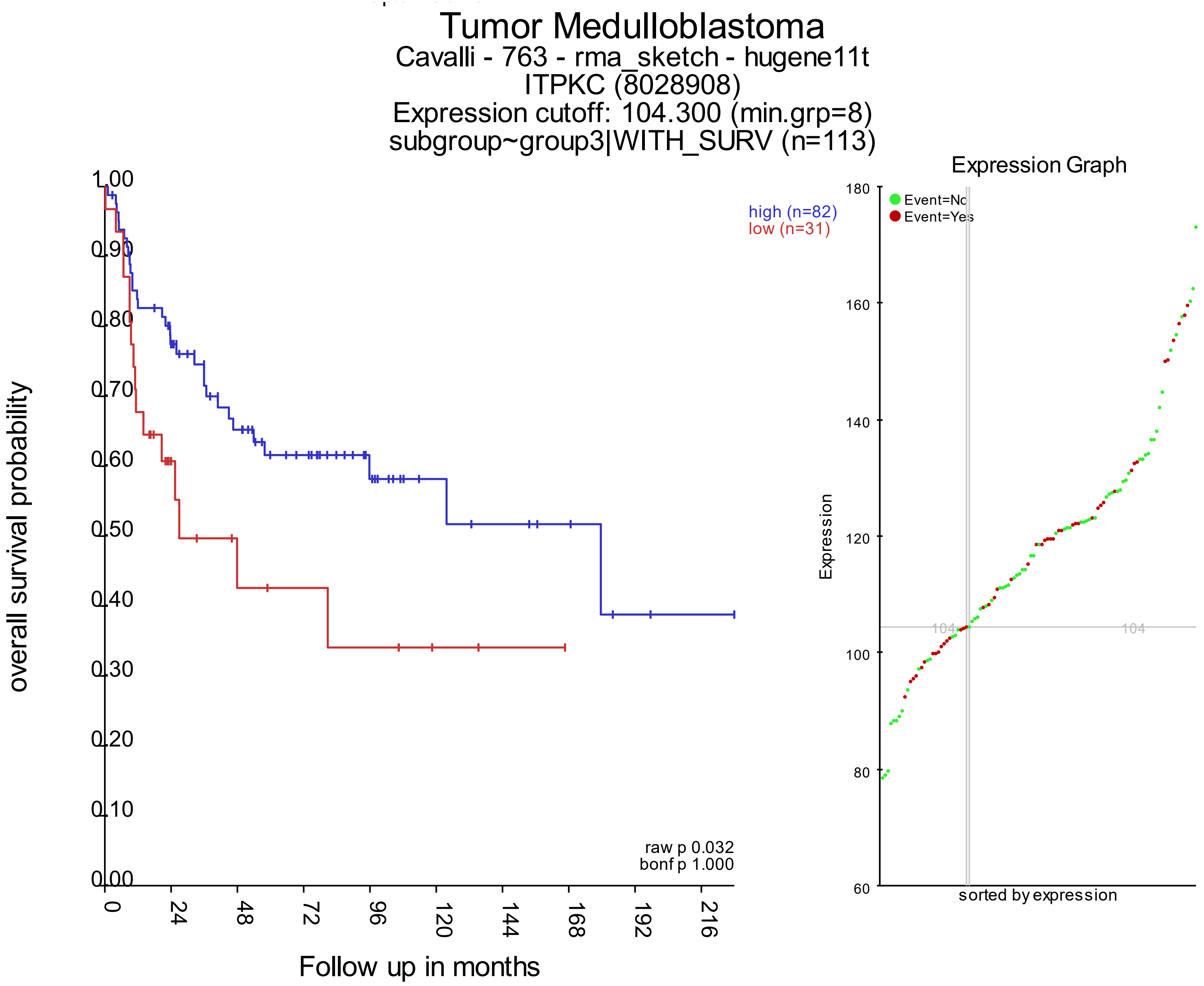

Tumor Medulloblastoma
Cavalli - 763 - rma_sketch - hugene11t
ITPKC (8028908)
Expression cutoff: 104.300 (min.grp=8)
subgroup~group3|WITH_SURV (n=113)
overall survival probability
1.00
0.90
0.80
0.70
0.60
0.50
0.40
0.30
0.20
0.10
0.00
0
24
48
72
96
120
144
168
192
216
Follow up in months
high (n=82)
low (n=31)
raw p 0.032
bonf p 1.000
Expression Graph
Event=No
Event=Yes
180
160
140
120
100
80
60
Expression
104
104
sorted by expression

PRKCA

WNT

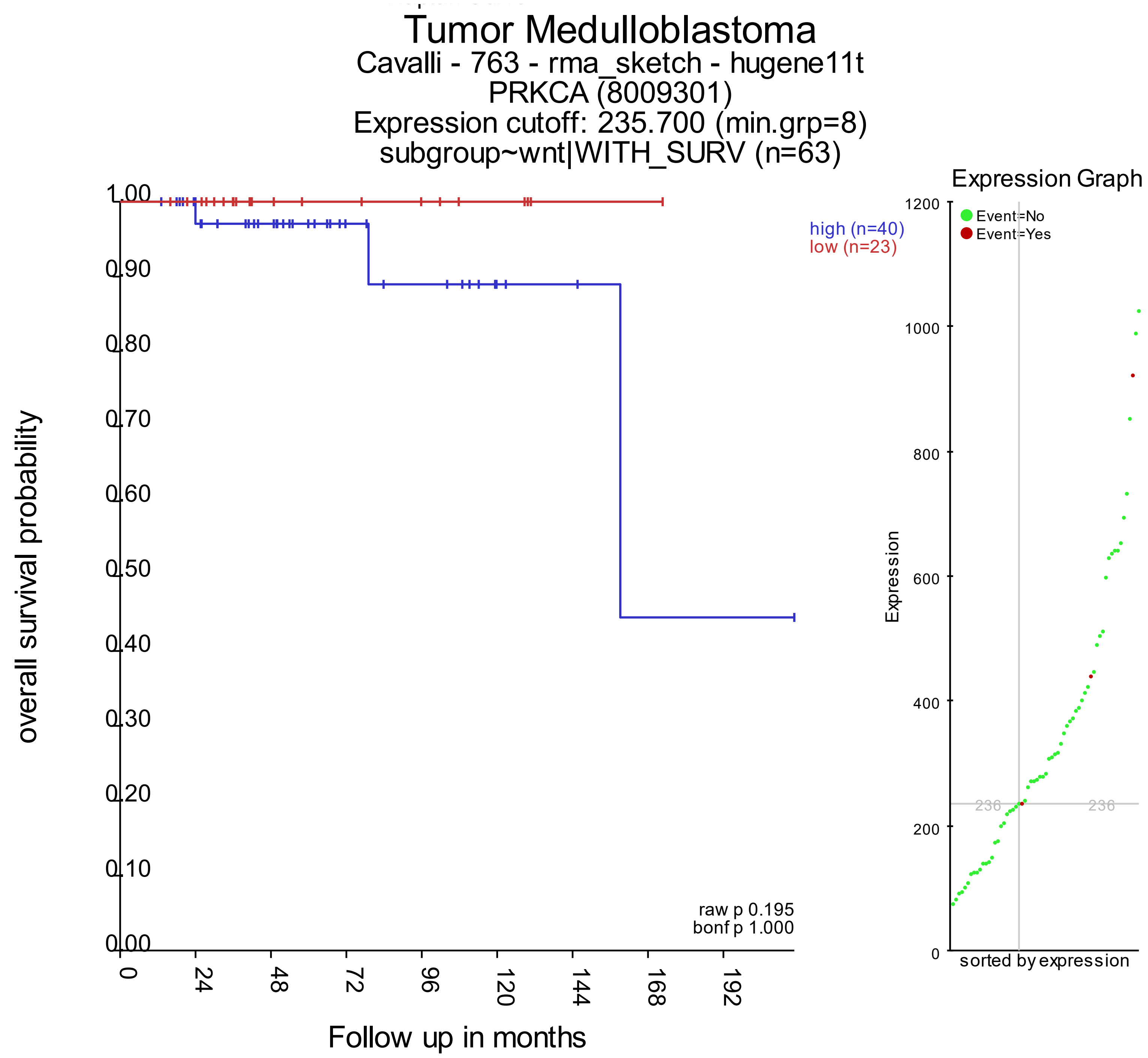

Tumor Medulloblastoma
Cavalli - 763 - rma_sketch - hugene11t
PRKCA (8009301)
Expression cutoff: 235.700 (min.grp=8)
subgroup~wnt|WITH_SURV (n=63)
overall survival probability
1.00
0.90
0.80
0.70
0.60
0.50
0.40
0.30
0.20
0.10
0.00
0
24
48
72
96
120
144
168
192
Follow up in months
high (n=40)
low (n=23)
raw p 0.195
bonf p 1.000
Expression Graph
Event=No
Event=Yes
Expression
1200
1000
800
600
400
200
0
236
236
sorted by expression

SHH

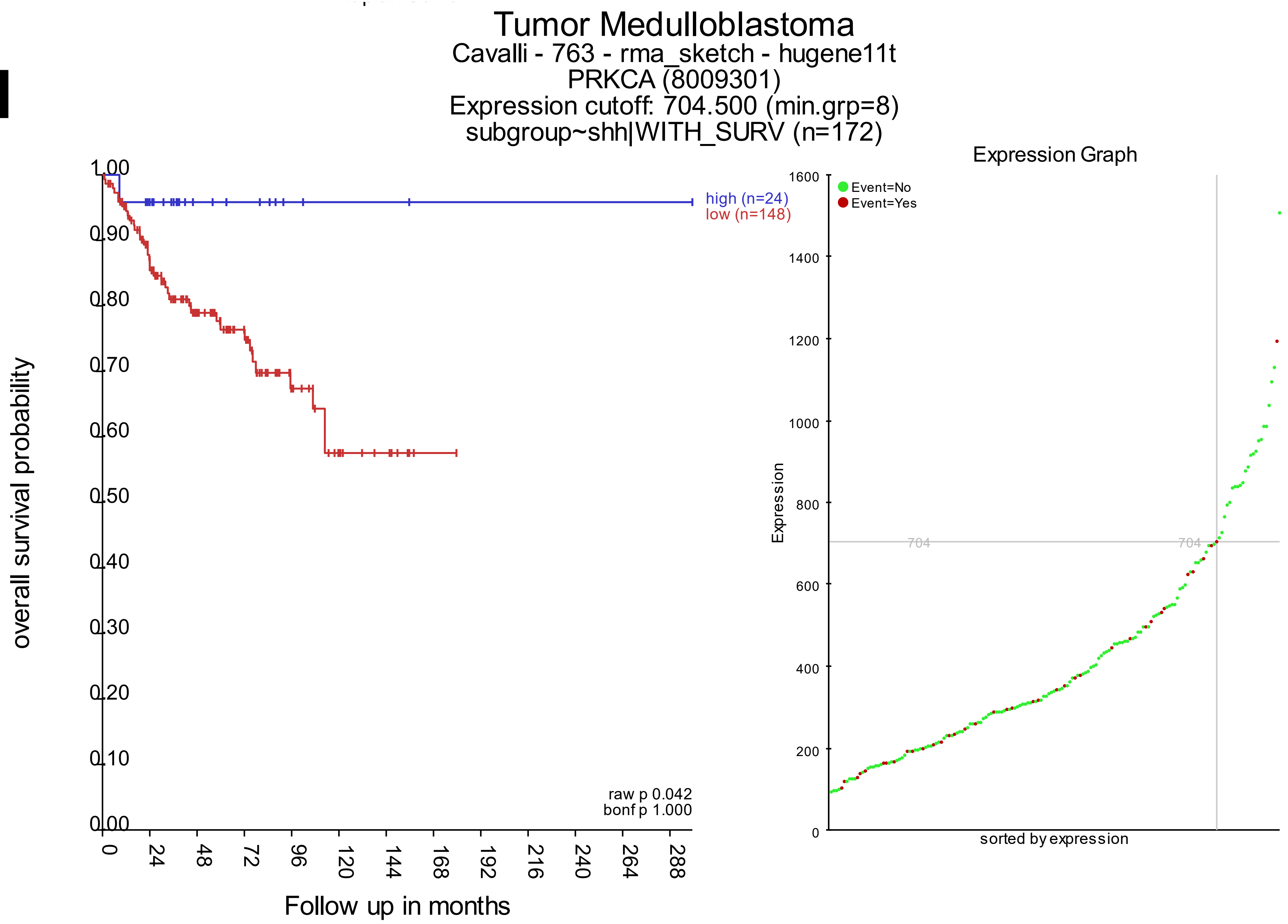

Tumor Medulloblastoma
Cavalli - 763 - rma_sketch - hugene11t
PRKCA (8009301)
Expression cutoff: 704.500 (min.grp=8)
subgroup~shh|WITH_SURV (n=172)
overall survival probability
1.00
0.90
0.80
0.70
0.60
0.50
0.40
0.30
0.20
0.10
0.00
high (n=24)
low (n=148)
raw p 0.042
bonf p 1.000
0
24
48
72
96
120
144
168
192
216
240
264
288
Follow up in months
Expression Graph
Event=No
Event=Yes
Expression
1600
1400
1200
1000
800
600
400
200
0
704
704
sorted by expression

GR4

Tumor Medulloblastoma
Cavalli - 763 - rma_sketch - hugene11t
PRKCA (8009301)
Expression cutoff: 164.800 (min.grp=8)
subgroup~group4|WITH_SURV (n=264)

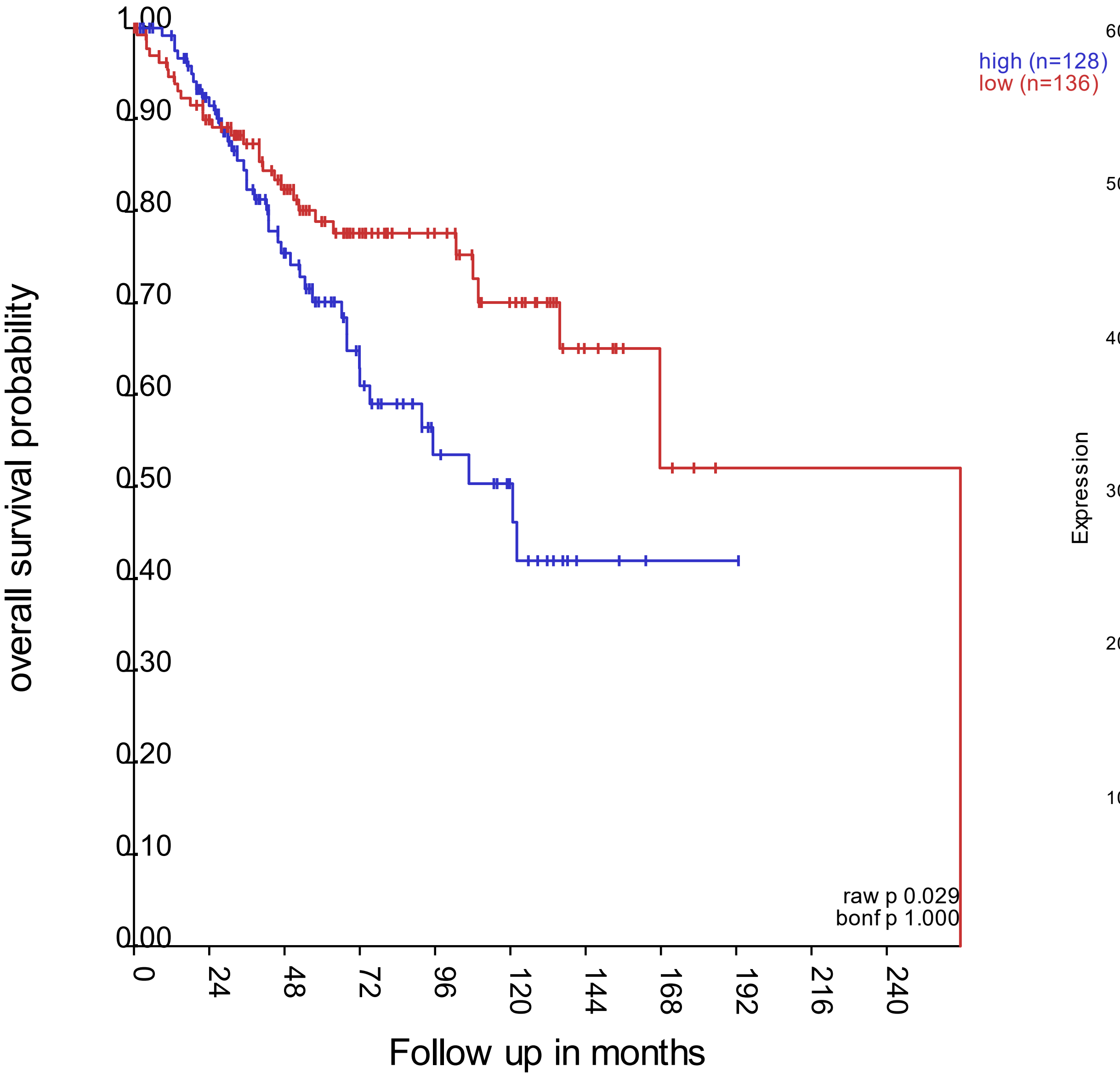

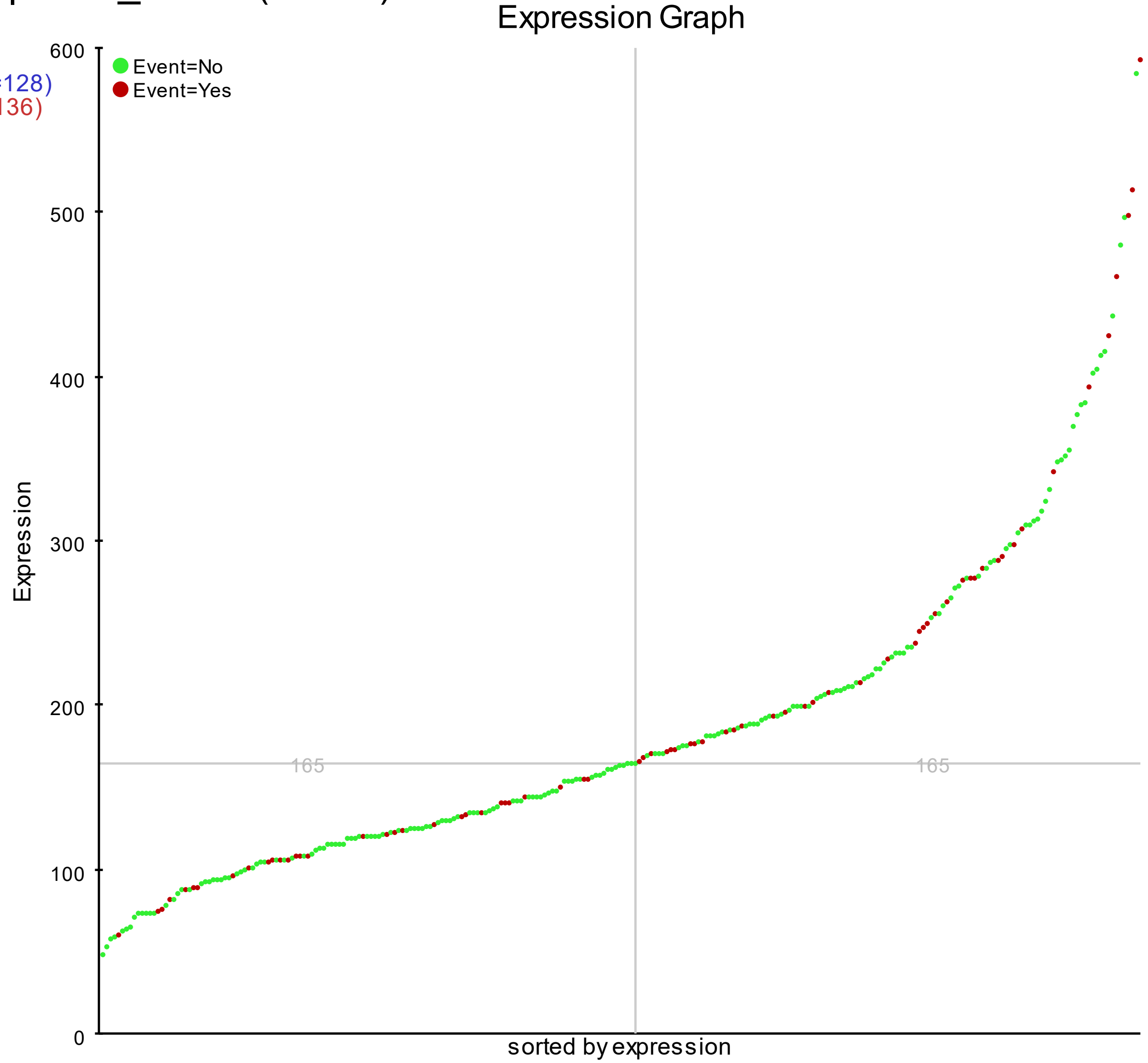

GR3

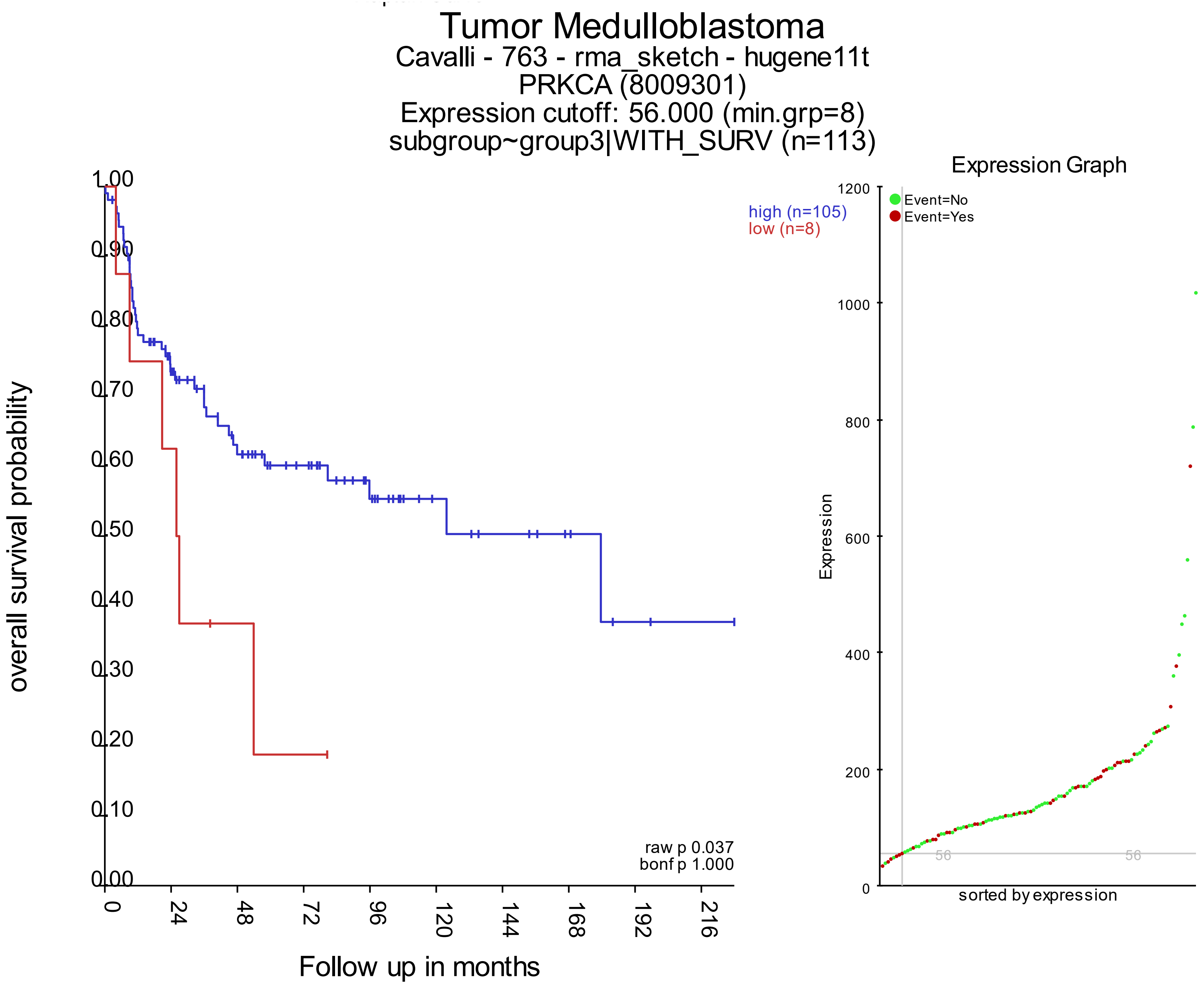

Tumor Medulloblastoma
Cavalli - 763 - rma_sketch - hugene11t
PRKCA (8009301)
Expression cutoff: 56.000 (min.grp=8)
subgroup~group3|WITH_SURV (n=113)
overall survival probability
1.00
0.90
0.80
0.70
0.60
0.50
0.40
0.30
0.20
0.10
0.00
high (n=105)
low (n=8)
raw p 0.037
bonf p 1.000
0
24
48
72
96
120
144
168
192
216
Follow up in months
Expression Graph
Event=No
Event=Yes
Expression
1200
1000
800
600
400
200
0
56
56
sorted by expression

PRKCB

WNT

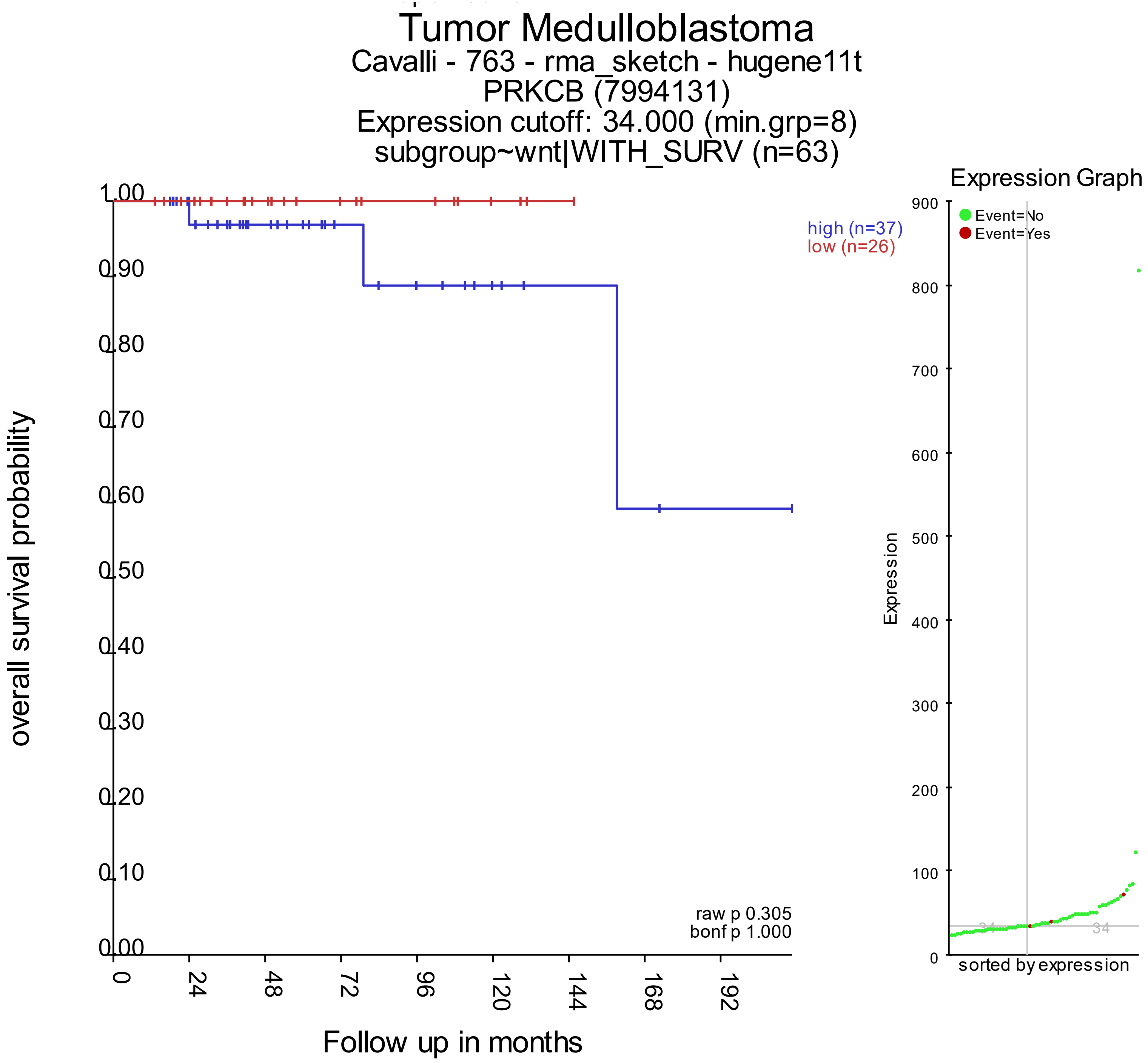

Tumor Medulloblastoma
Cavalli - 763 - rma_sketch - hugene11t
PRKCB (7994131)
Expression cutoff: 34.000 (min.grp=8)
subgroup~wnt|WITH_SURV (n=63)
high (n=37)
low (n=26)
overall survival probability
1.00
0.90
0.80
0.70
0.60
0.50
0.40
0.30
0.20
0.10
0.00
0
24
48
72
96
120
144
168
192
Follow up in months
raw p 0.305
bonf p 1.000
Expression Graph
Event=No
Event=Yes
Expression
900
800
700
600
500
400
300
200
100
0
34
sorted by expression

SHH

Tumor Medulloblastoma
Cavalli - 763 - rma_sketch - hugene11t
PRKCB (7994131)
Expression cutoff: 158.200 (min.grp=8)
subgroup~shh|WITH_SURV (n=172)

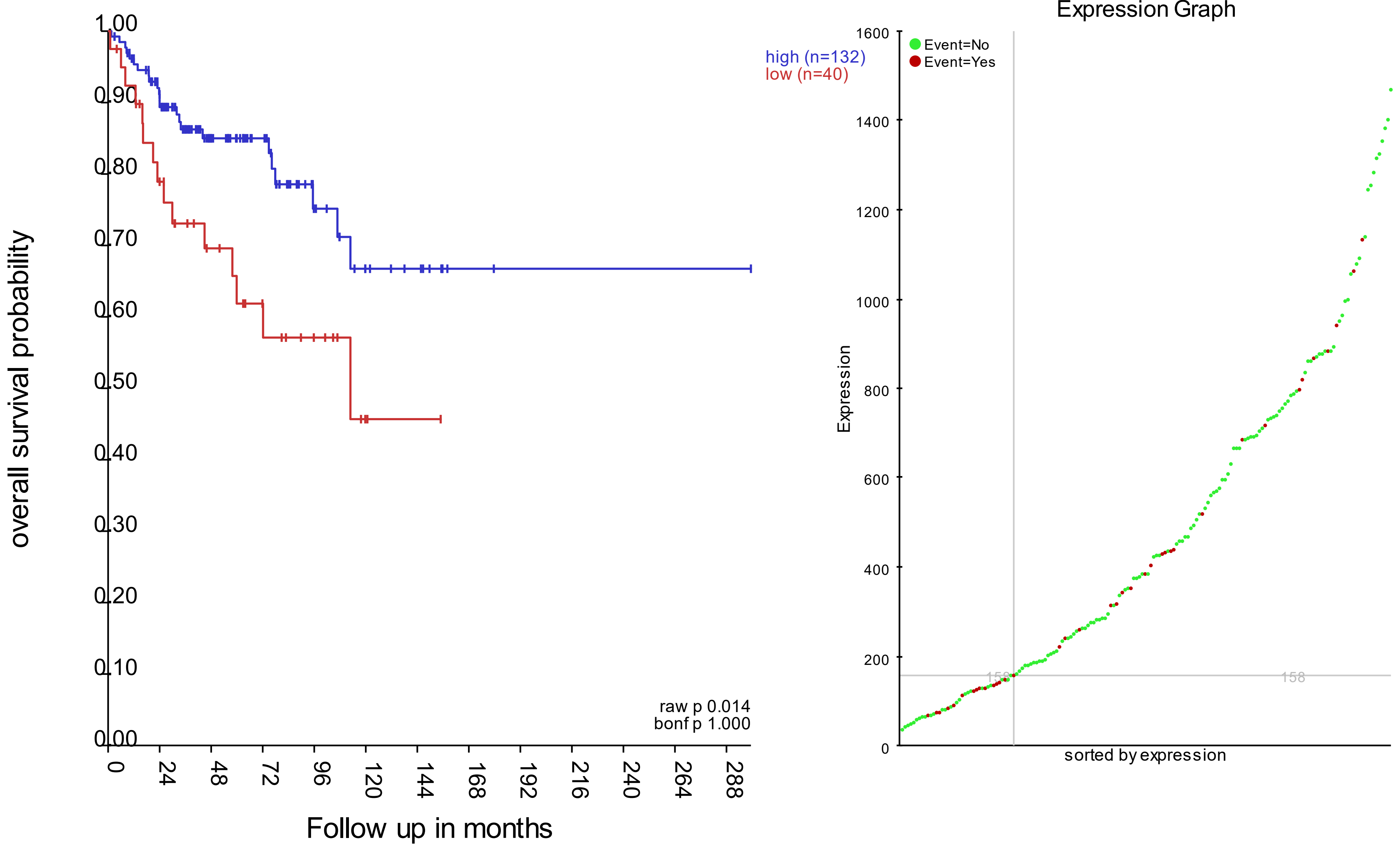

GR4

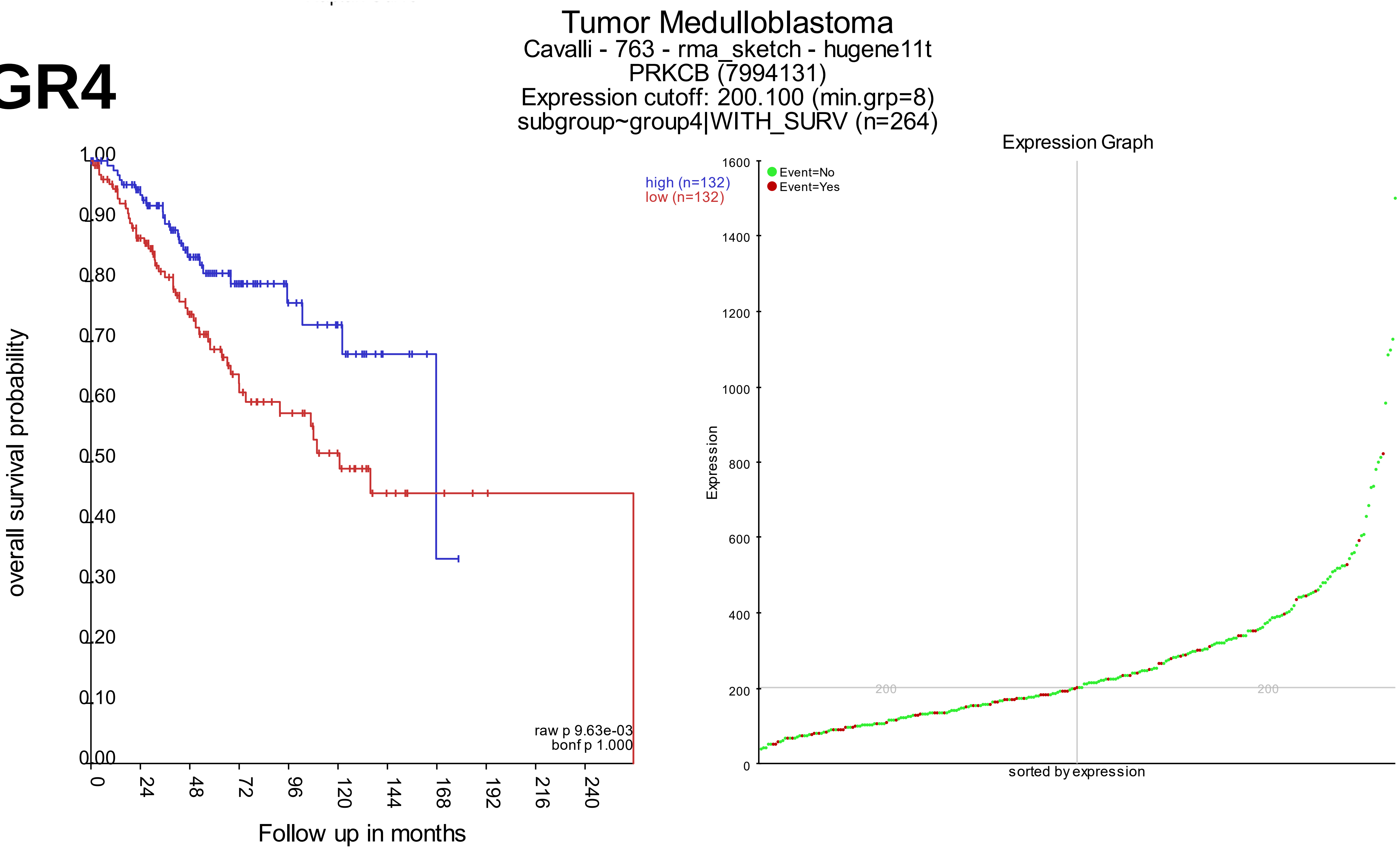

Tumor Medulloblastoma
Cavalli - 763 - rma_sketch - hugene11t
PRKCB (7994131)
Expression cutoff: 200.100 (min.grp=8)
subgroup~group4|WITH_SURV (n=264)
high (n=132)
low (n=132)
overall survival probability
1.00
0.90
0.80
0.70
0.60
0.50
0.40
0.30
0.20
0.10
0.00
raw p 9.63e-03
bonf p 1.000
0
24
48
72
96
120
144
168
192
216
240
Follow up in months
Expression Graph
Event=No
Event=Yes
Expression
1600
1400
1200
1000
800
600
400
200
0
200
200
sorted by expression

GR3

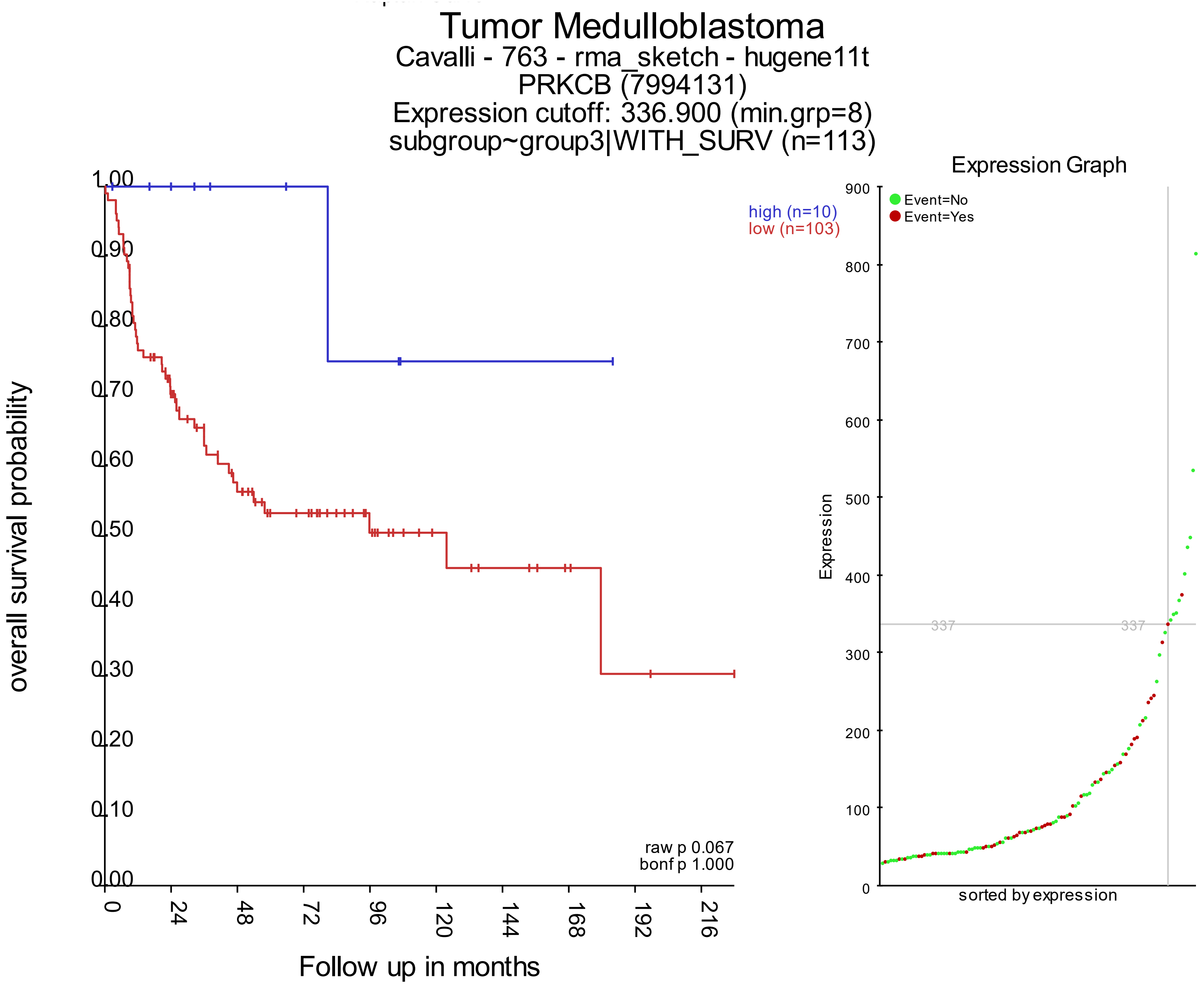

Tumor Medulloblastoma
Cavalli - 763 - rma_sketch - hugene11t
PRKCB (7994131)
Expression cutoff: 336.900 (min.grp=8)
subgroup~group3|WITH_SURV (n=113)
high (n=10)
low (n=103)
overall survival probability
1.00
0.90
0.80
0.70
0.60
0.50
0.40
0.30
0.20
0.10
0.00
raw p 0.067
bonf p 1.000
0
24
48
72
96
120
144
168
192
216
Follow up in months
Expression Graph
Event=No
Event=Yes
Expression
900
800
700
600
500
400
300
200
100
0
337
337
sorted by expression

PRKCD

WNT

Tumor Medulloblastoma
Cavalli - 763 - rma_sketch - hugene11t
PRKCD (8080487)
Expression cutoff: 87.300 (min.grp=8)
subgroup~wnt|WITH_SURV (n=63)

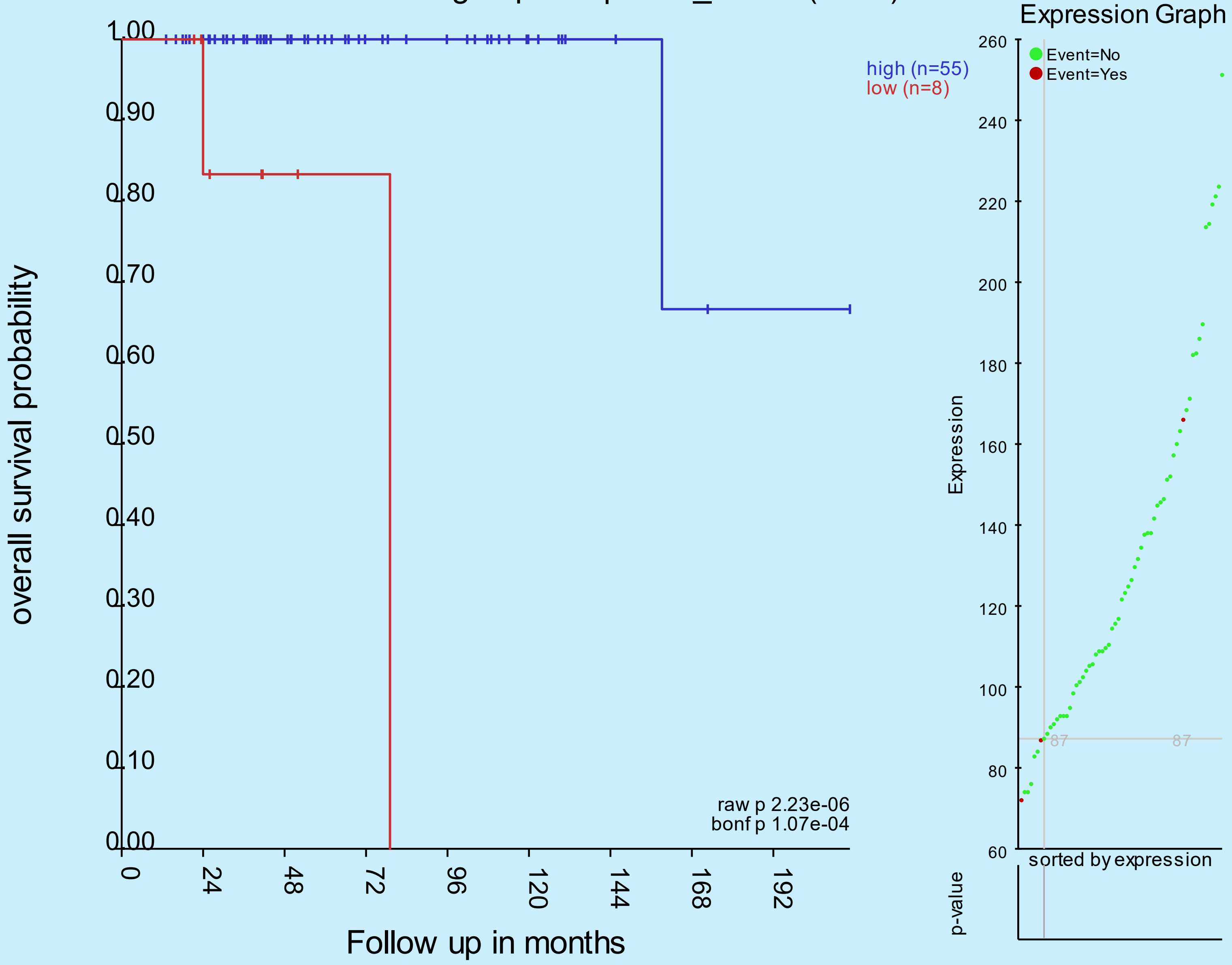

SHH

Tumor Medulloblastoma
Cavalli - 763 - rma_sketch - hugene11t
PRKCD (8080487)
Expression cutoff: 69.800 (min.grp=8)
subgroup~shh|WITH_SURV (n=172)

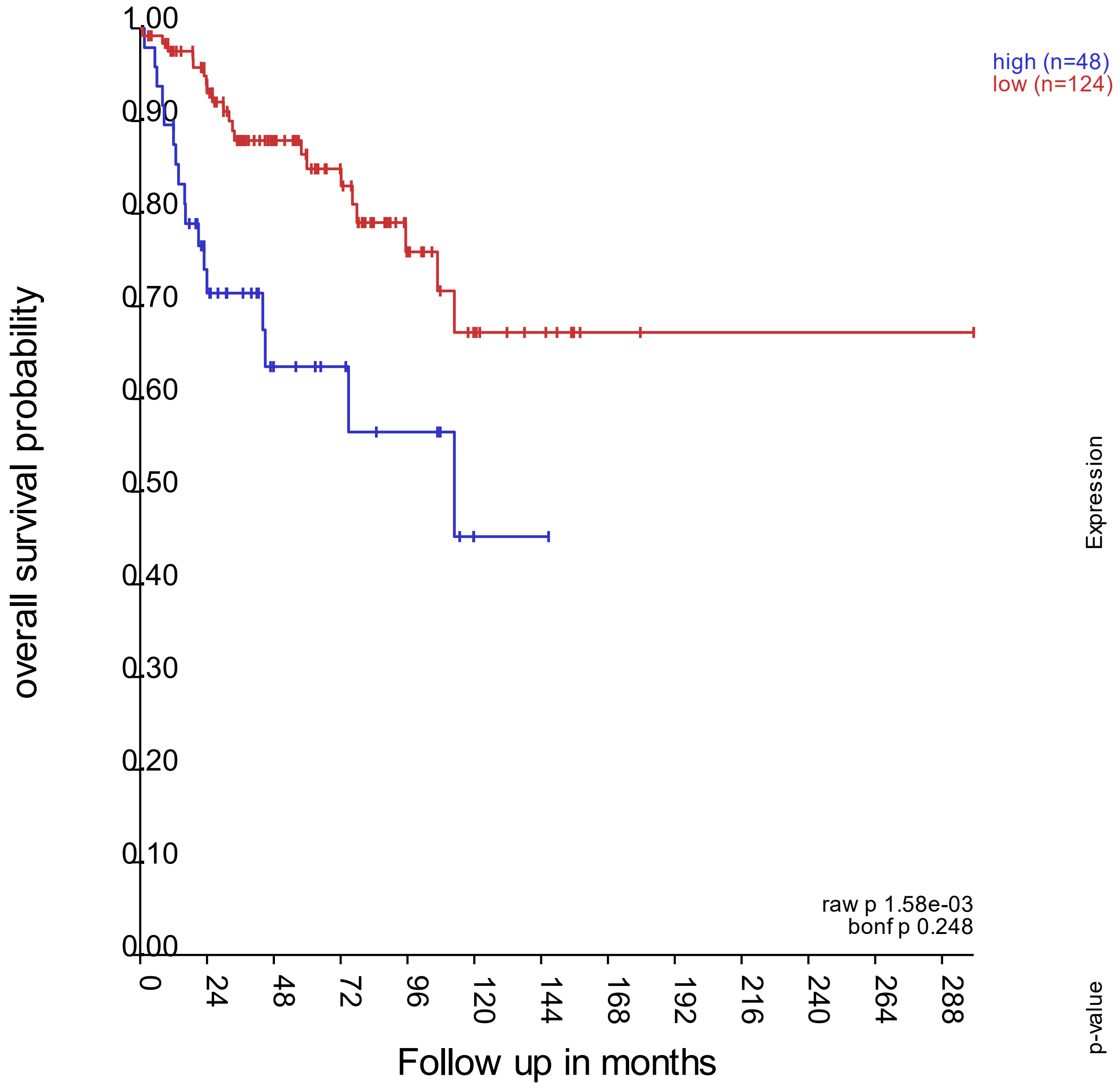

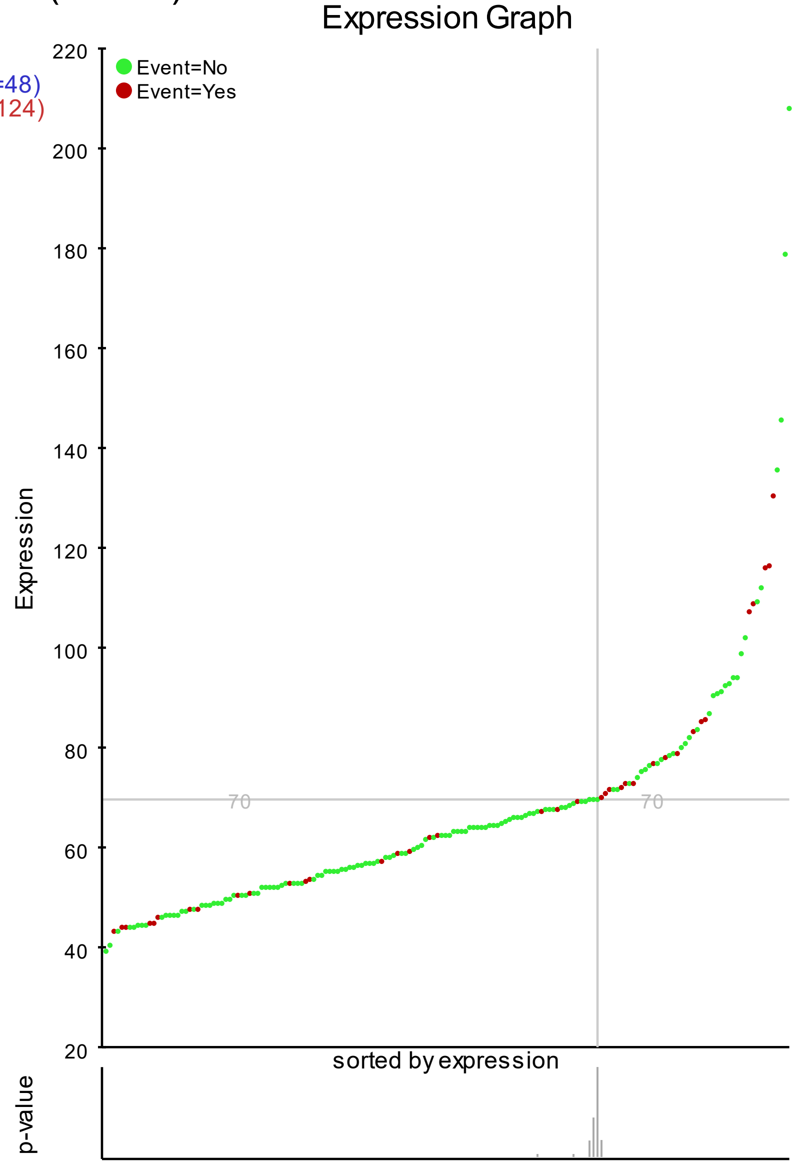

GR4

Tumor Medulloblastoma
Cavalli - 763 - rma_sketch - hugene11t
PRKCD (8080487)
Expression cutoff: 95.000 (min.grp=8)
subgroup~group4|WITH_SURV (n=264)

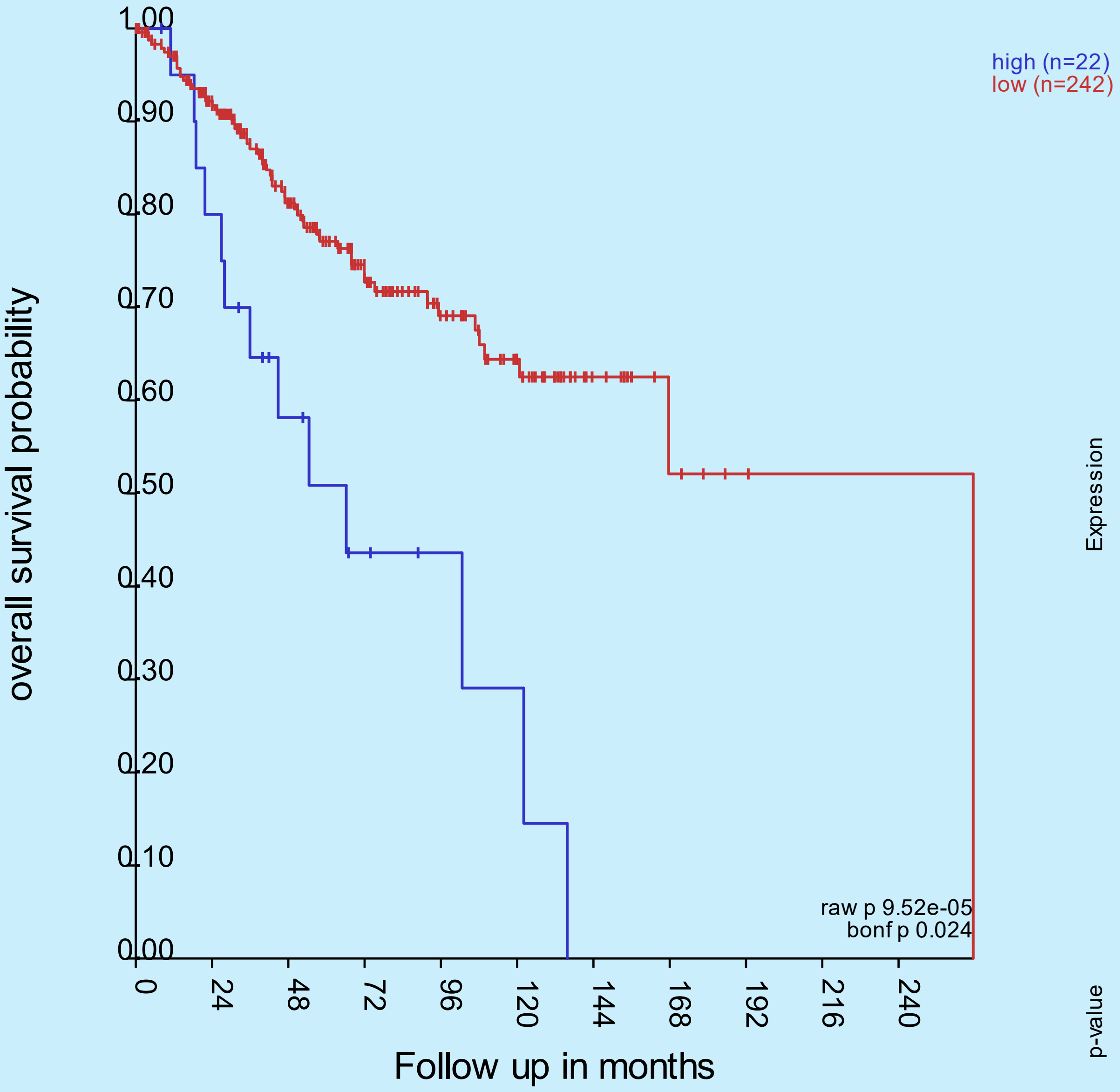

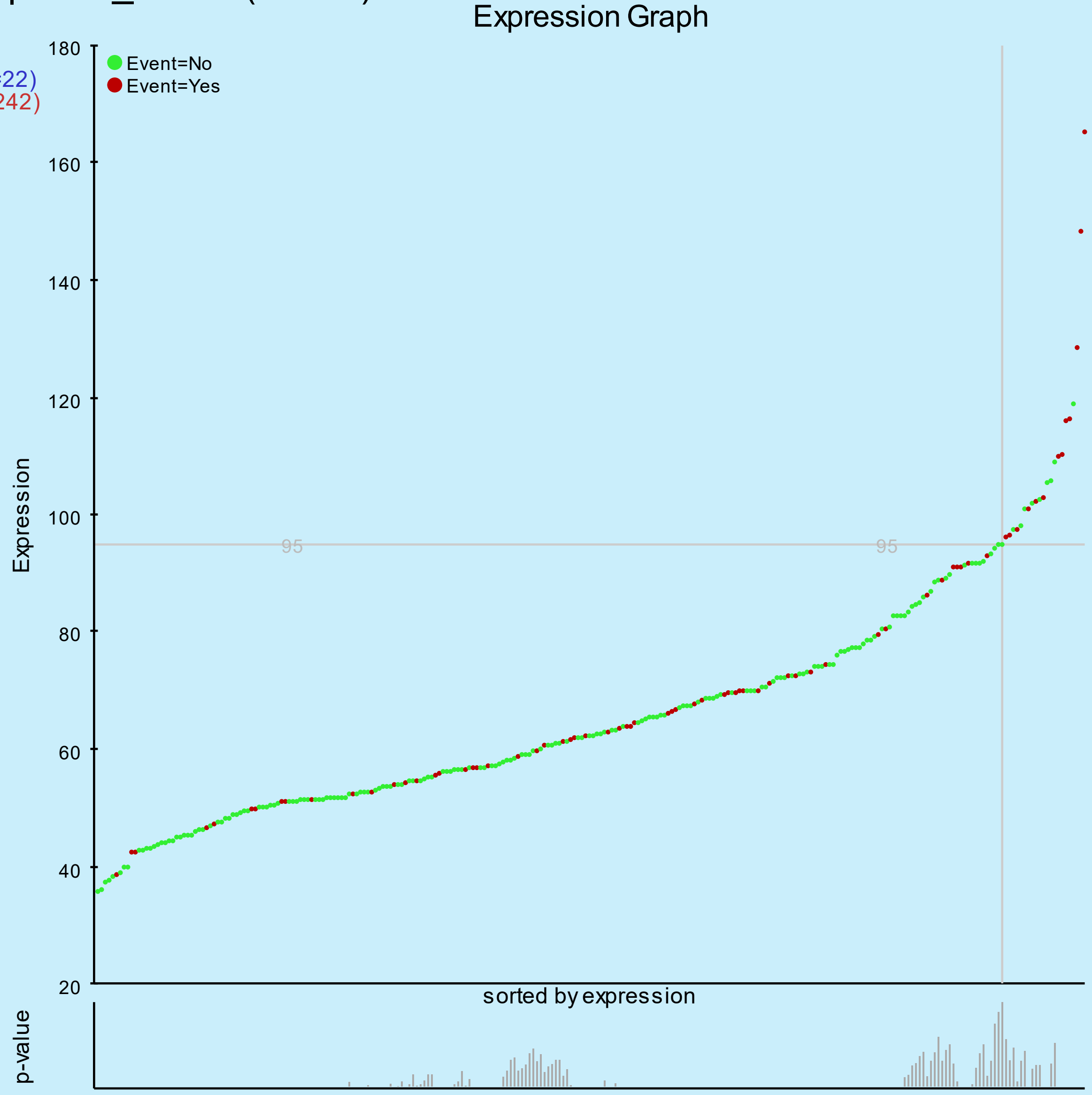

GR3

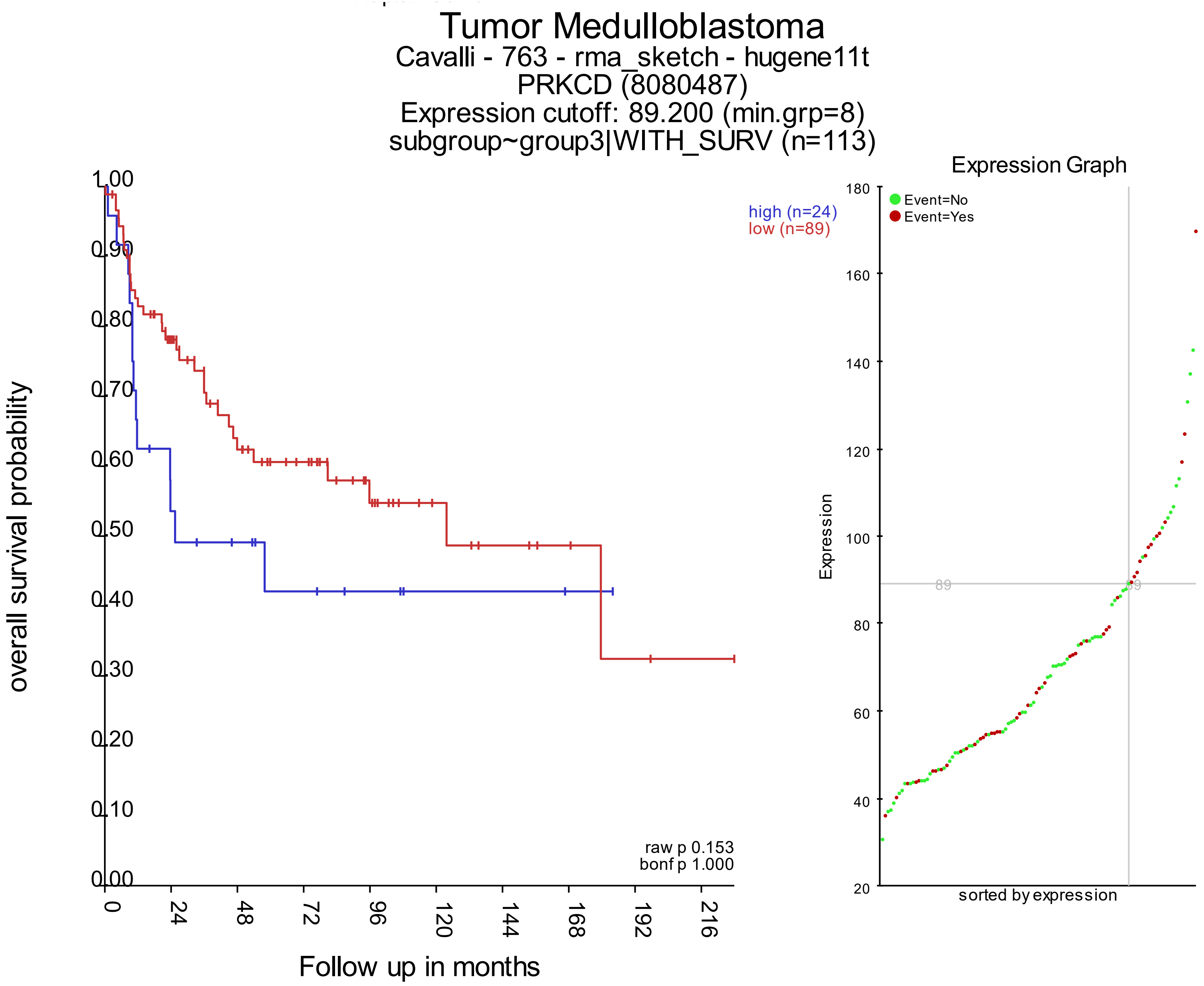

Tumor Medulloblastoma
Cavalli - 763 - rma_sketch - hugene11t
PRKCD (8080487)
Expression cutoff: 89.200 (min.grp=8)
subgroup~group3|WITH_SURV (n=113)
high (n=24)
low (n=89)
overall survival probability
1.00
0.90
0.80
0.70
0.60
0.50
0.40
0.30
0.20
0.10
0.00
0
24
48
72
96
120
144
168
192
216
Follow up in months
raw p 0.153
bonf p 1.000
Expression Graph
Event=No
Event=Yes
Expression
180
160
140
120
100
80
60
40
20
89
sorted by expression

ARAF

WNT

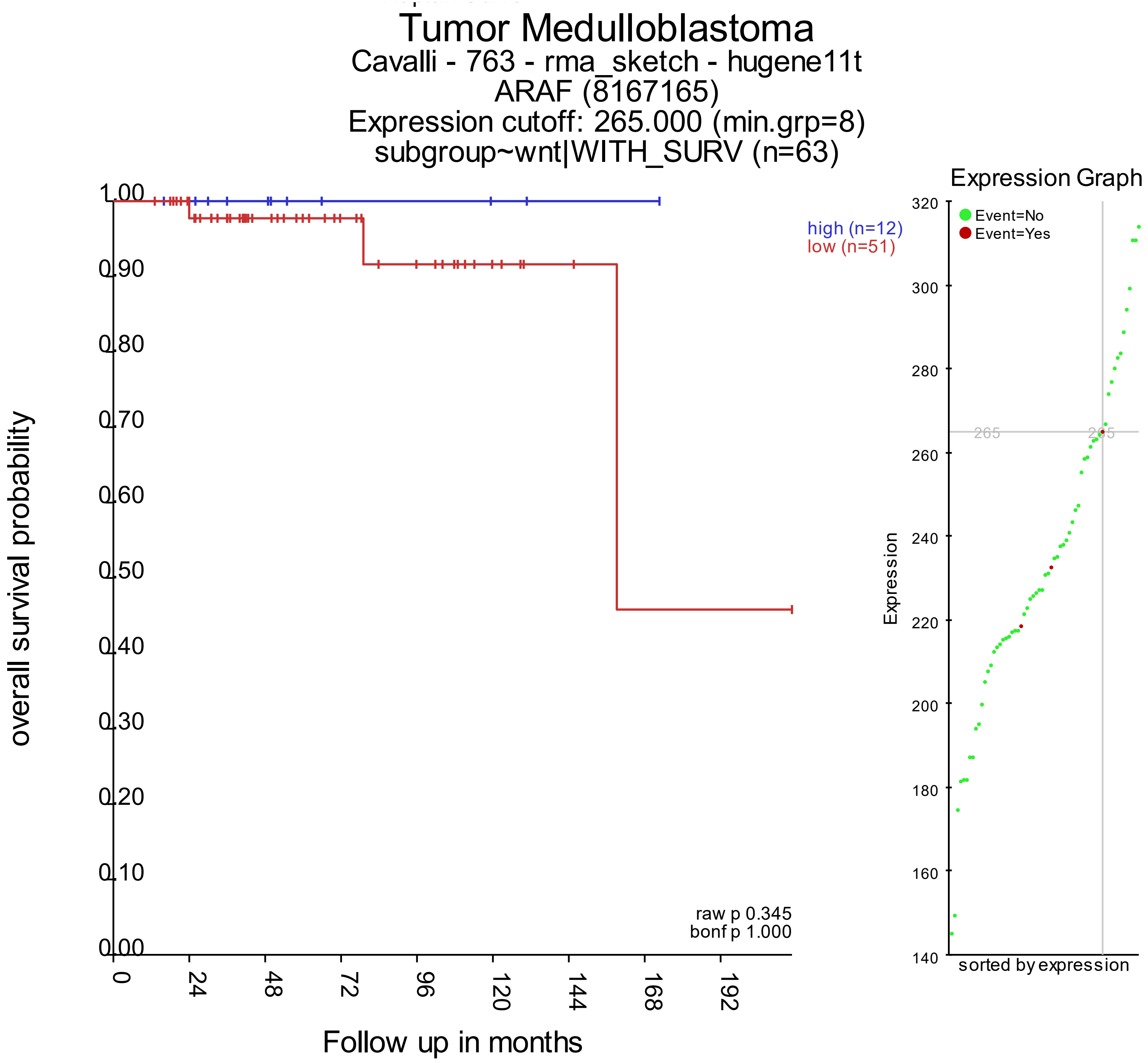

Tumor Medulloblastoma
Cavalli - 763 - rma_sketch - hugene11t
ARAF (8167165)
Expression cutoff: 265.000 (min.grp=8)
subgroup~wnt|WITH_SURV (n=63)
overall survival probability
1.00
0.90
0.80
0.70
0.60
0.50
0.40
0.30
0.20
0.10
0.00
0
24
48
72
96
120
144
168
192
Follow up in months
high (n=12)
low (n=51)
raw p 0.345
bonf p 1.000
Expression Graph
Event=No
Event=Yes
Expression
320
300
280
265
260
240
220
200
180
160
140
sorted by expression

SHH

Tumor Medulloblastoma

Cavalli - 763 - rma_sketch - hugene11t
ARAF (8167165)
Expression cutoff: 235.400 (min.grp=8)
subgroup~shh|WITH_SURV (n=172)

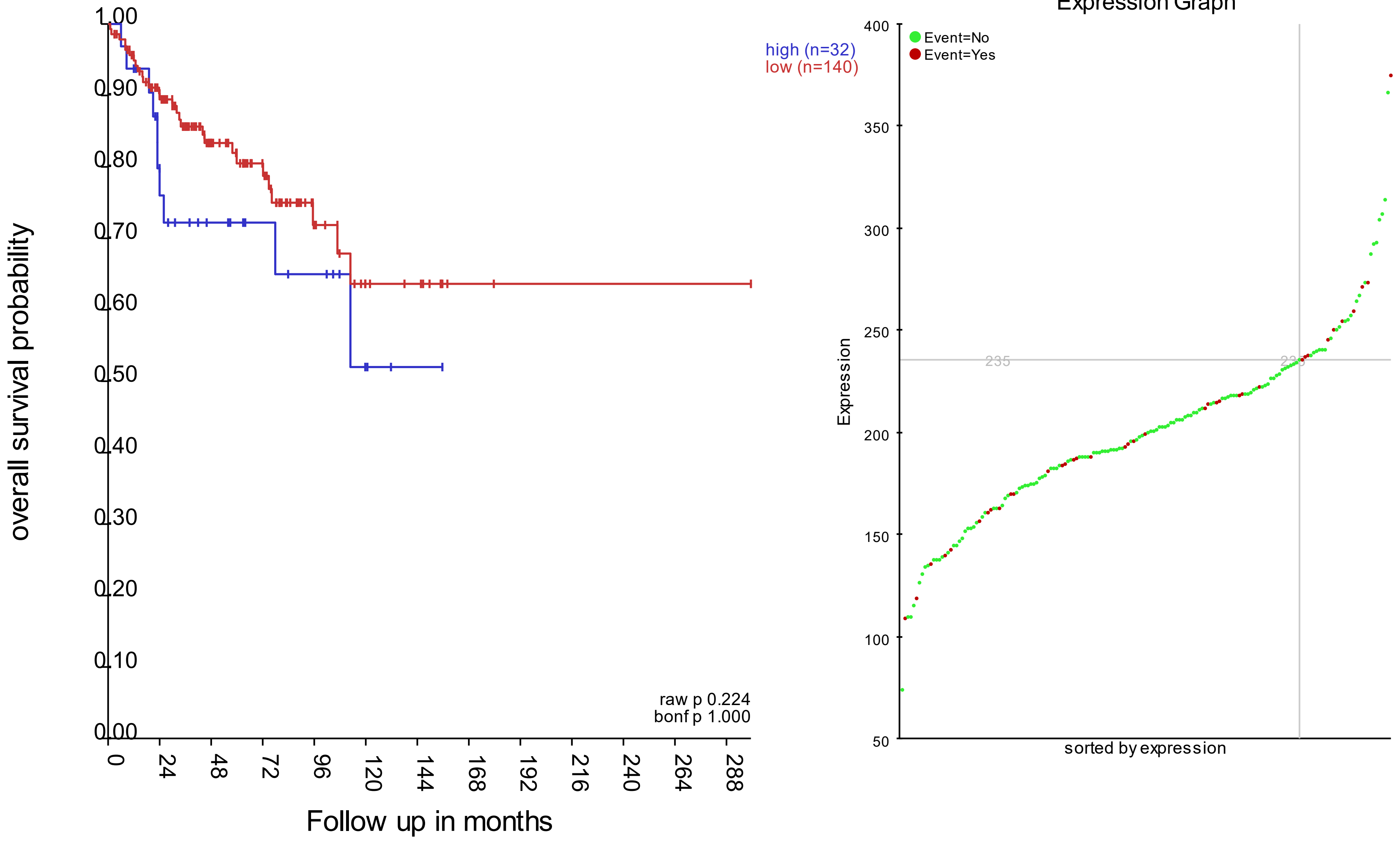

GR4

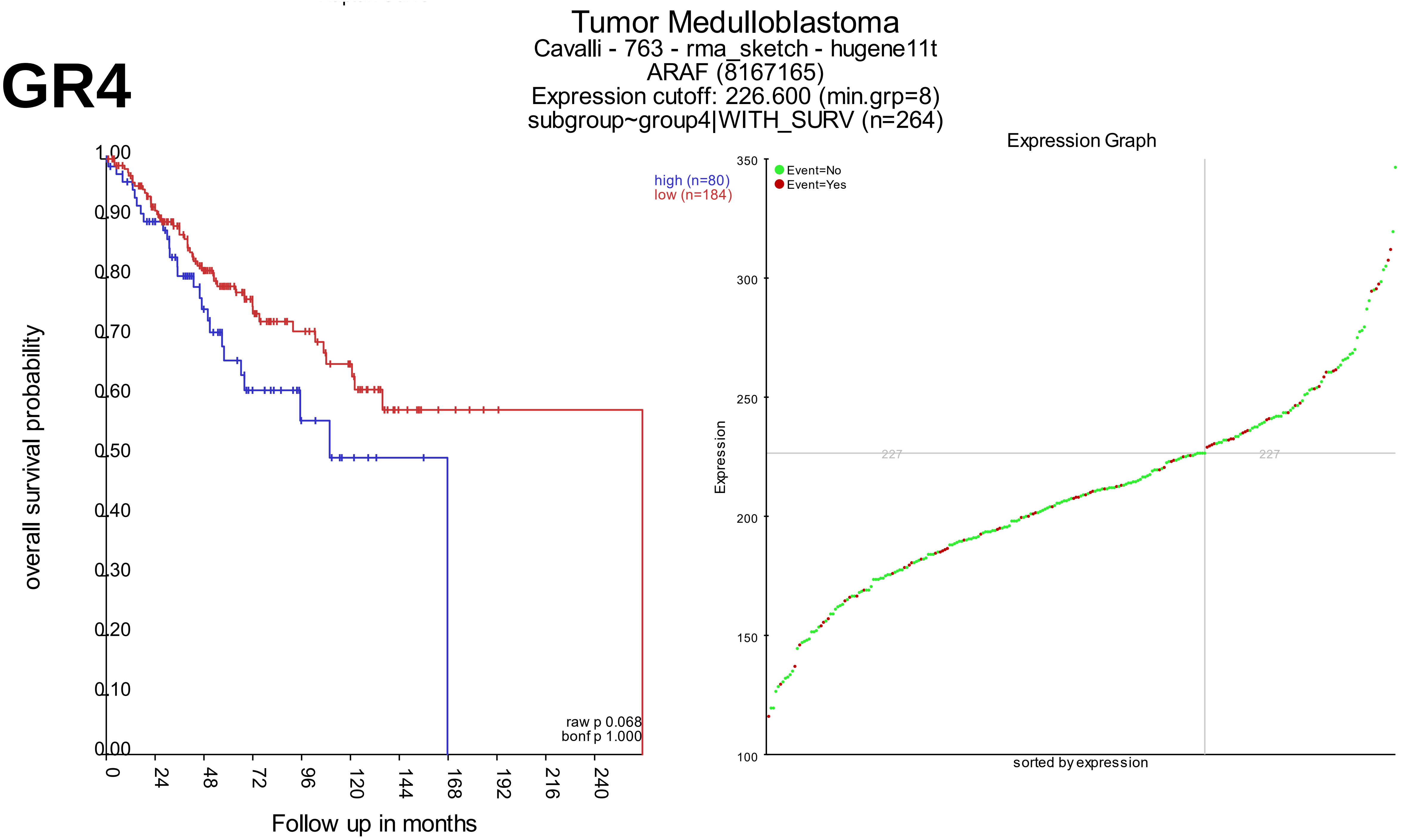

Tumor Medulloblastoma
Cavalli - 763 - rma_sketch - hugene11t
ARAF (8167165)
Expression cutoff: 226.600 (min.grp=8)
subgroup~group4|WITH_SURV (n=264)
overall survival probability
1.00
0.90
0.80
0.70
0.60
0.50
0.40
0.30
0.20
0.10
0.00
0
24
48
72
96
120
144
168
192
216
240
Follow up in months
high (n=80)
low (n=184)
raw p 0.068
bonf p 1.000
Expression Graph
Event=No
Event=Yes
Expression
350
300
250
200
150
100
227
227
sorted by expression

GR3

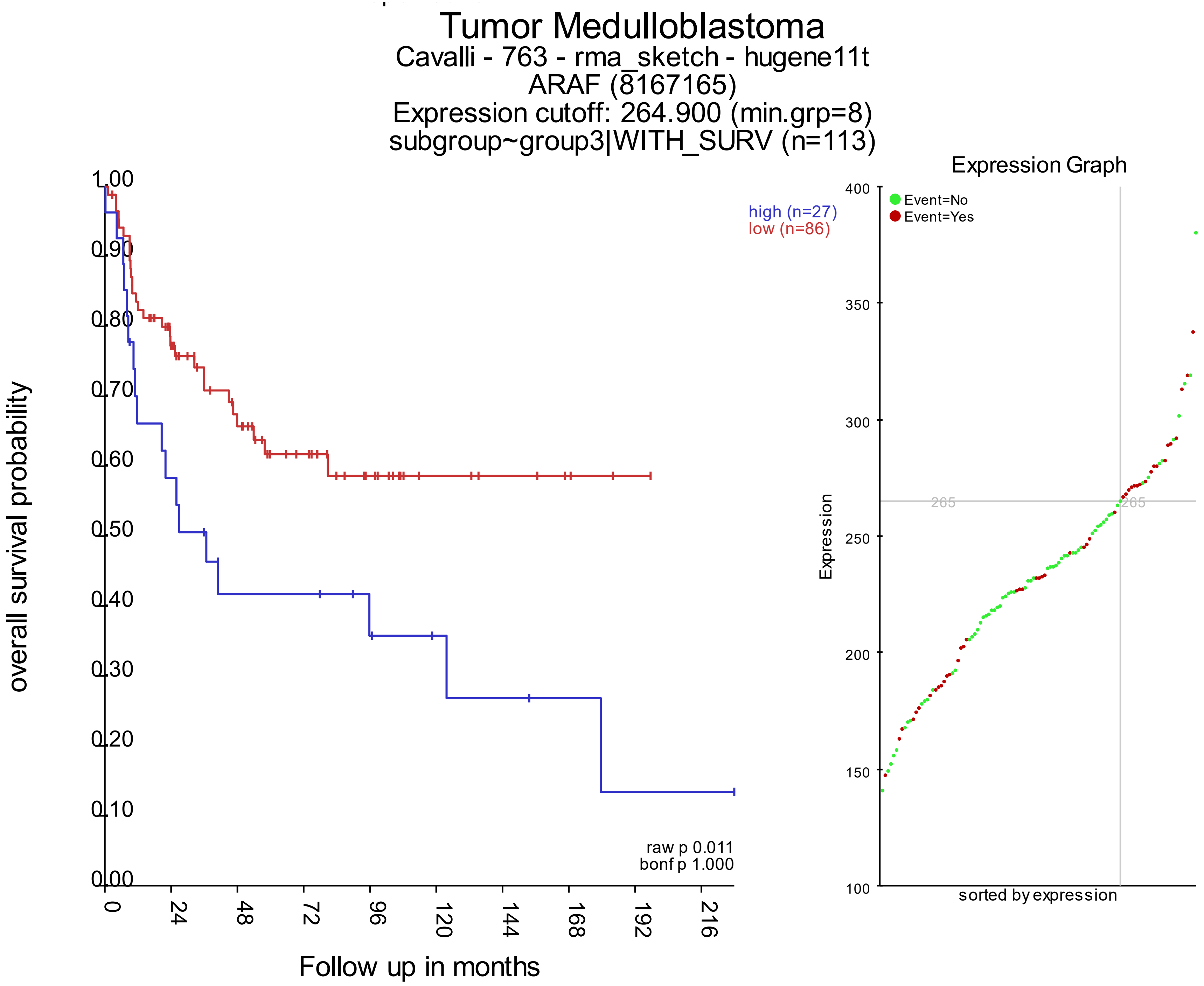
Tumor Medulloblastoma
Cavalli - 763 - rma_sketch - hugene11t
ARAF (8167165)
Expression cutoff: 264.900 (min.grp=8)
subgroup~group3|WITH_SURV (n=113)
overall survival probability
1.00
0.90
0.80
0.70
0.60
0.50
0.40
0.30
0.20
0.10
0.00
0
24
48
72
96
120
144
168
192
216
Follow up in months
high (n=27)
low (n=86)
raw p 0.011
bonf p 1.000
Expression Graph
Event=No
Event=Yes
Expression
400
350
300
250
200
150
100
265
265
sorted by expression

BRAF

WNT

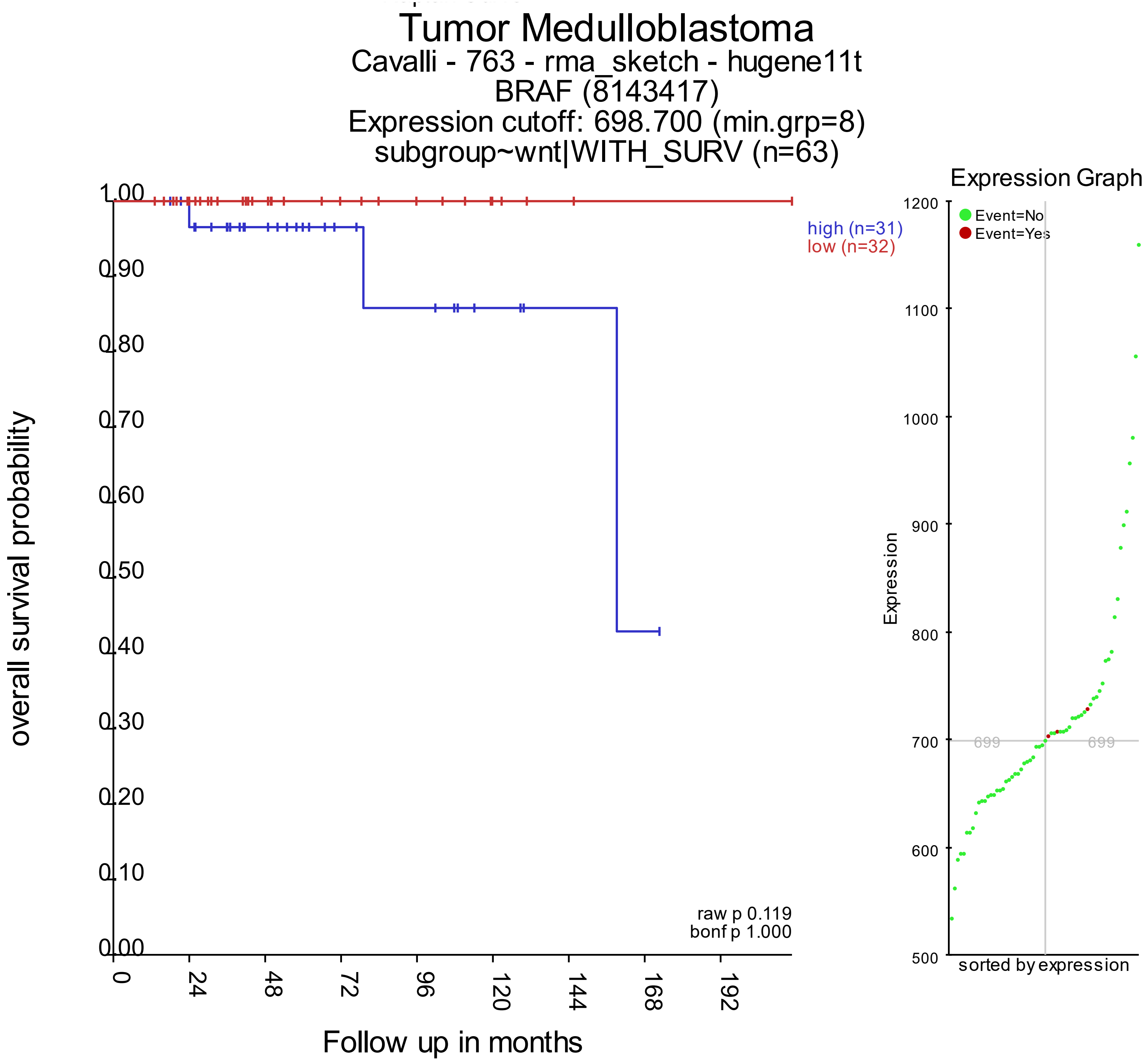
Tumor Medulloblastoma
Cavalli - 763 - rma_sketch - hugene11t
BRAF (8143417)
Expression cutoff: 698.700 (min.grp=8)
subgroup~wnt|WITH_SURV (n=63)
high (n=31)
low (n=32)
overall survival probability
1.00
0.90
0.80
0.70
0.60
0.50
0.40
0.30
0.20
0.10
0.00
0
24
48
72
96
120
144
168
192
Follow up in months
raw p 0.119
bonf p 1.000
Expression Graph
Event=No
Event=Yes
Expression
1200
1100
1000
900
800
700
600
500
699
699
sorted by expression

SHH

Tumor Medulloblastoma
Cavalli - 763 - rma_sketch - hugene11t
BRAF (8143417)
Expression cutoff: 1204.200 (min.grp=8)
subgroup~shh|WITH_SURV (n=172)

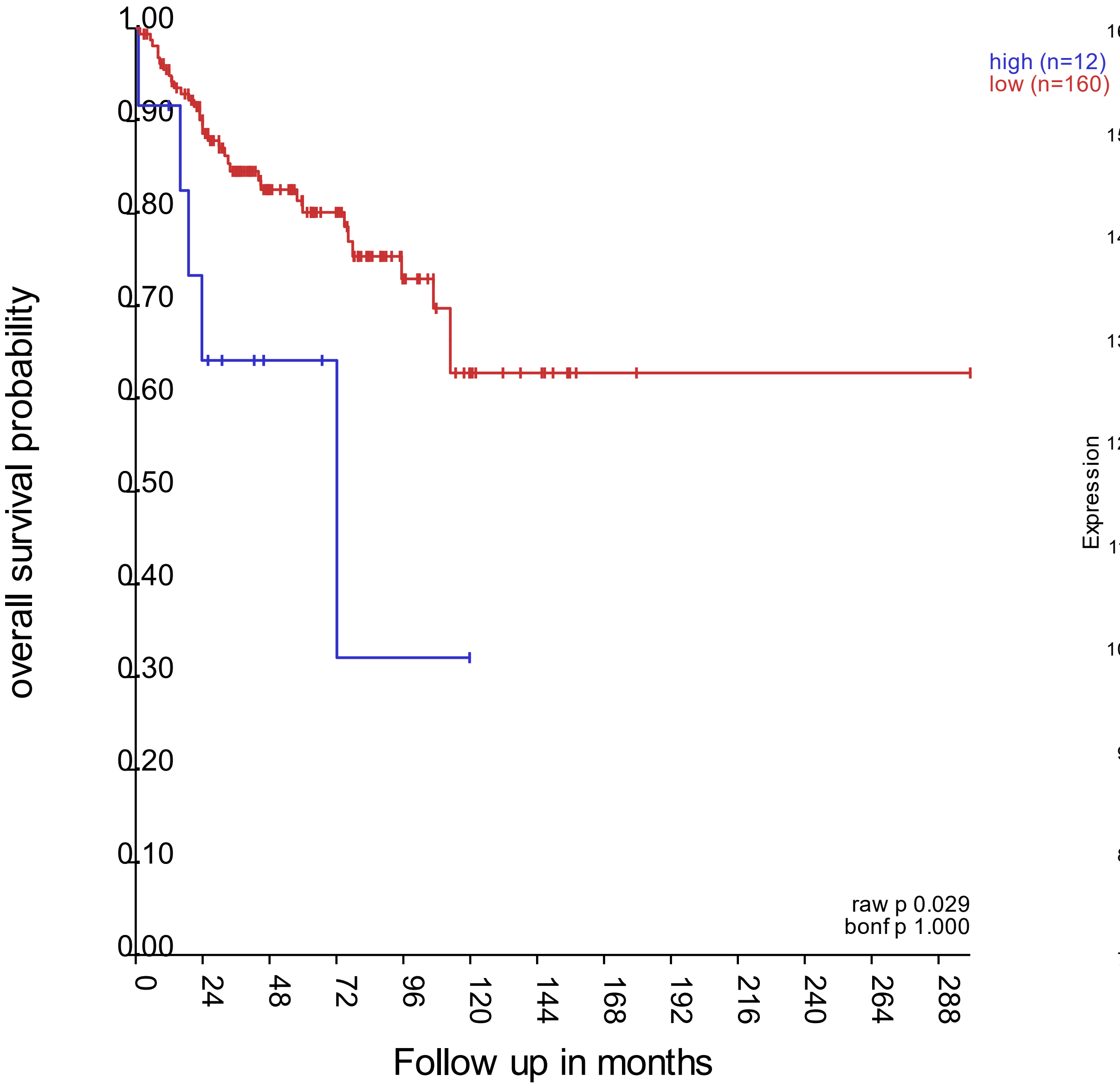

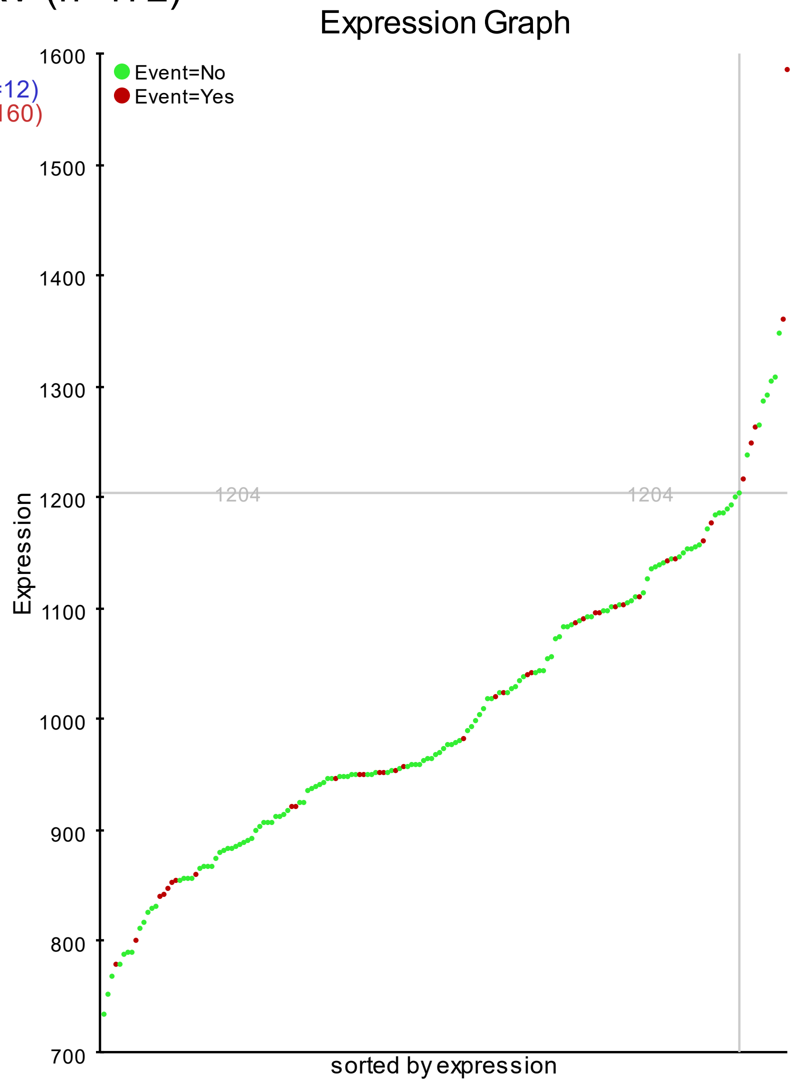

GR4

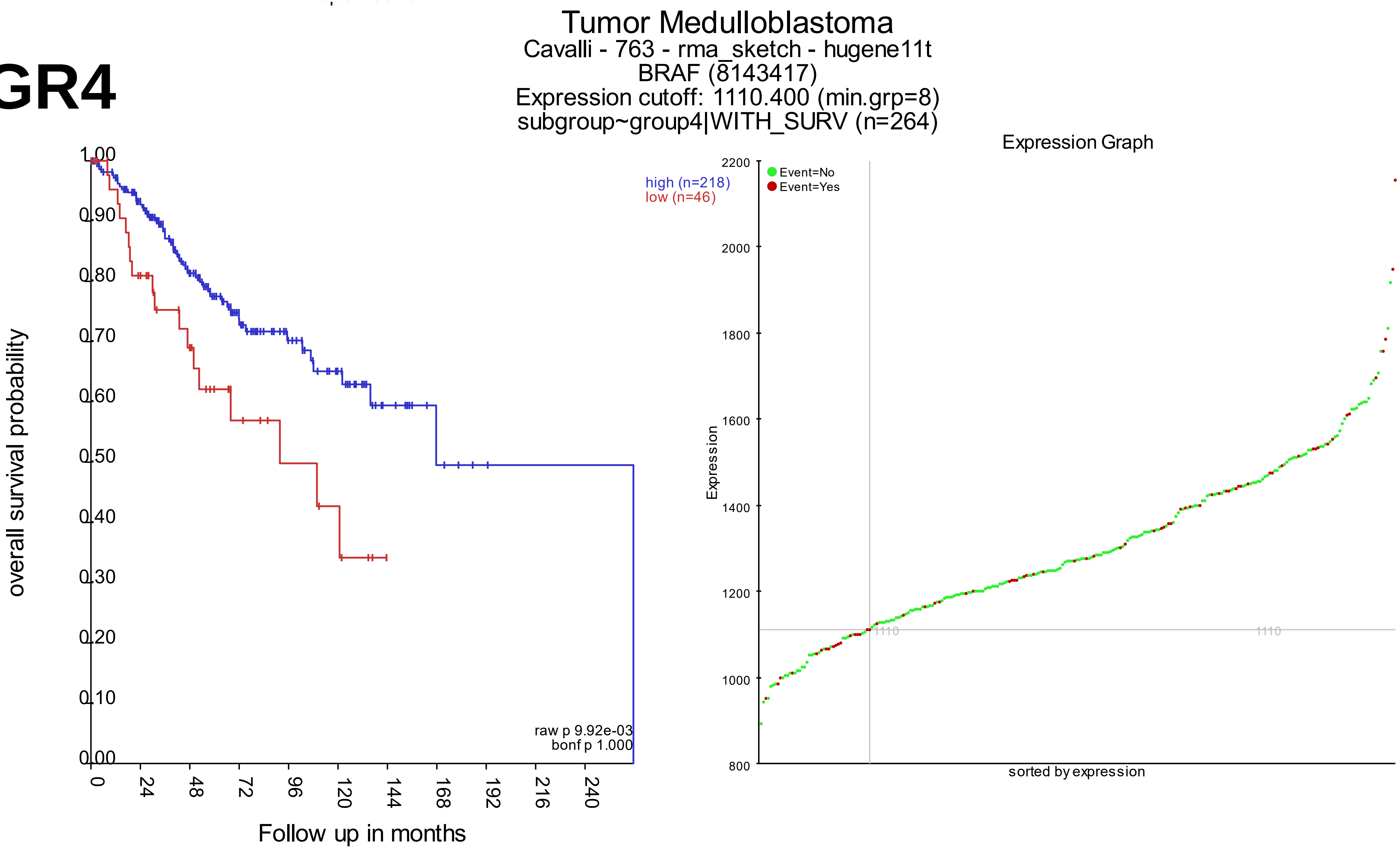

Tumor Medulloblastoma
Cavalli - 763 - rma_sketch - hugene11t
BRAF (8143417)
Expression cutoff: 1110.400 (min.grp=8)
subgroup~group4|WITH_SURV (n=264)
overall survival probability
1.00
0.90
0.80
0.70
0.60
0.50
0.40
0.30
0.20
0.10
0.00
0
24
48
72
96
120
144
168
192
216
240
Follow up in months
high (n=218)
low (n=46)
raw p 9.92e-03
bonf p 1.000
Expression Graph
Event=No
Event=Yes
Expression
2200
2000
1800
1600
1400
1200
1000
800
1110
1110
sorted by expression

GR3

Tumor Medulloblastoma
Cavalli - 763 - rma_sketch - hugene11t
BRAF (8143417)
Expression cutoff: 1041.200 (min.grp=8)
subgroup~group3|WITH_SURV (n=113)

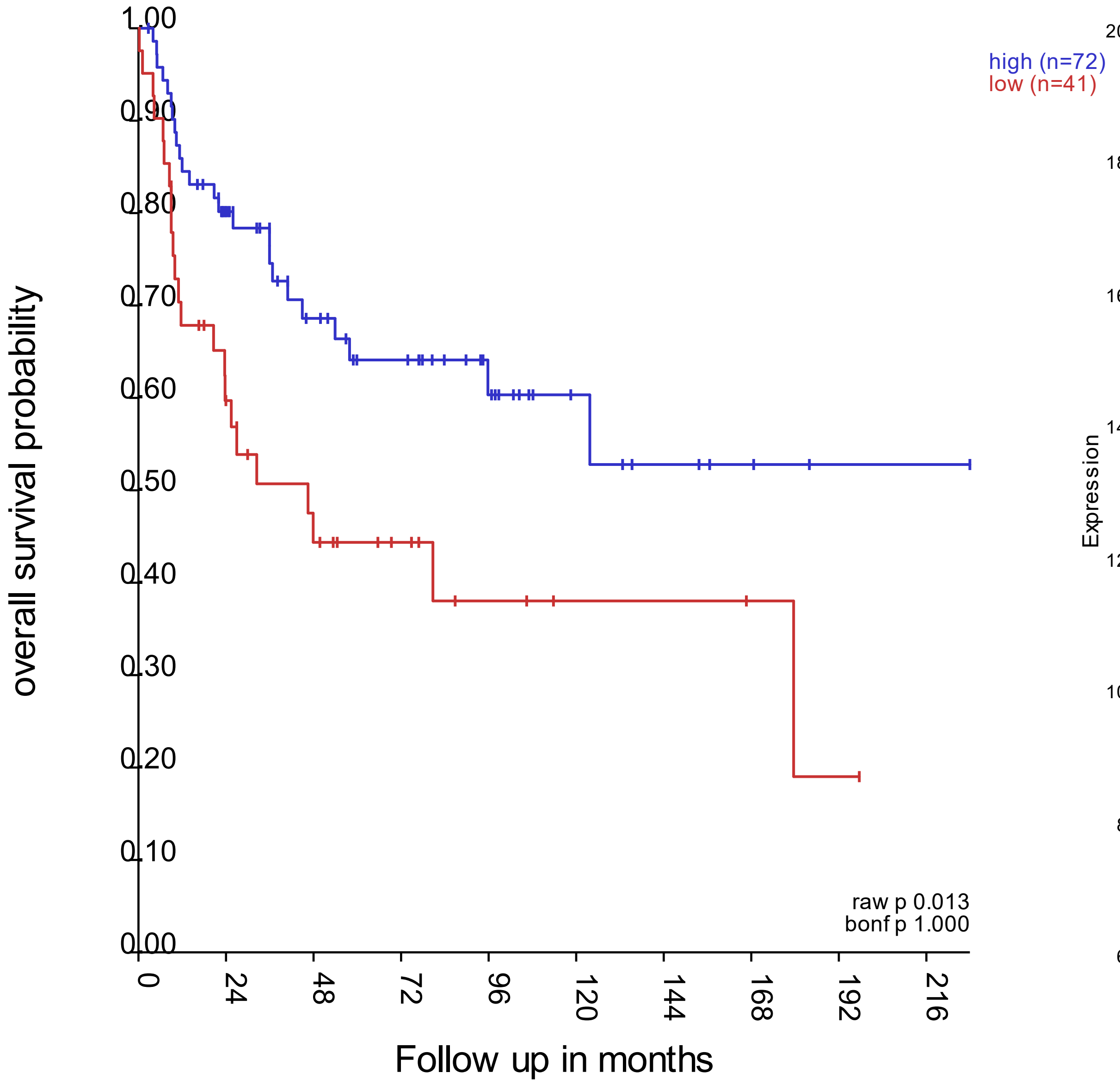

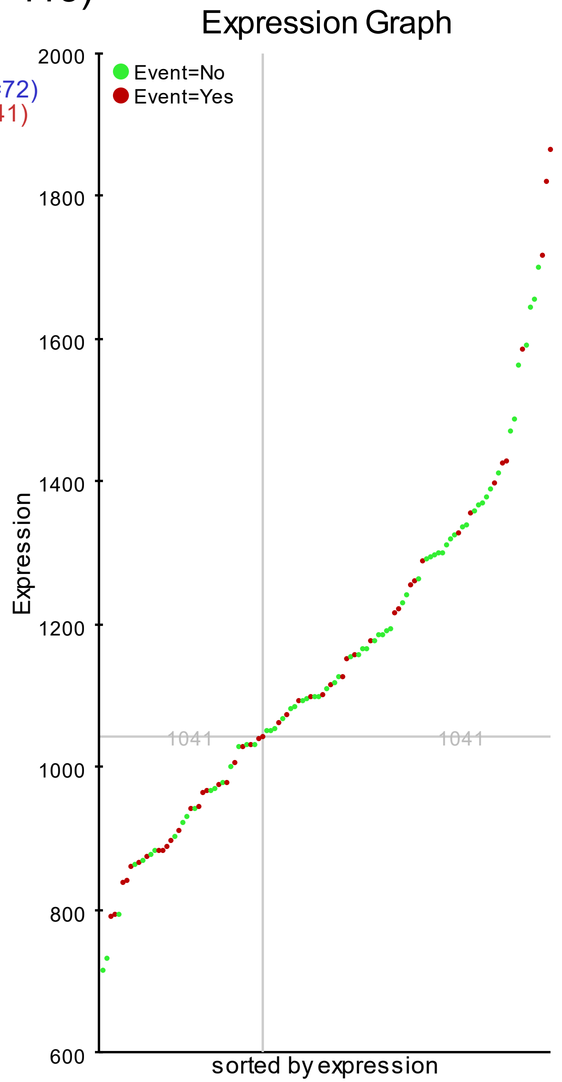

RAF1

WNT

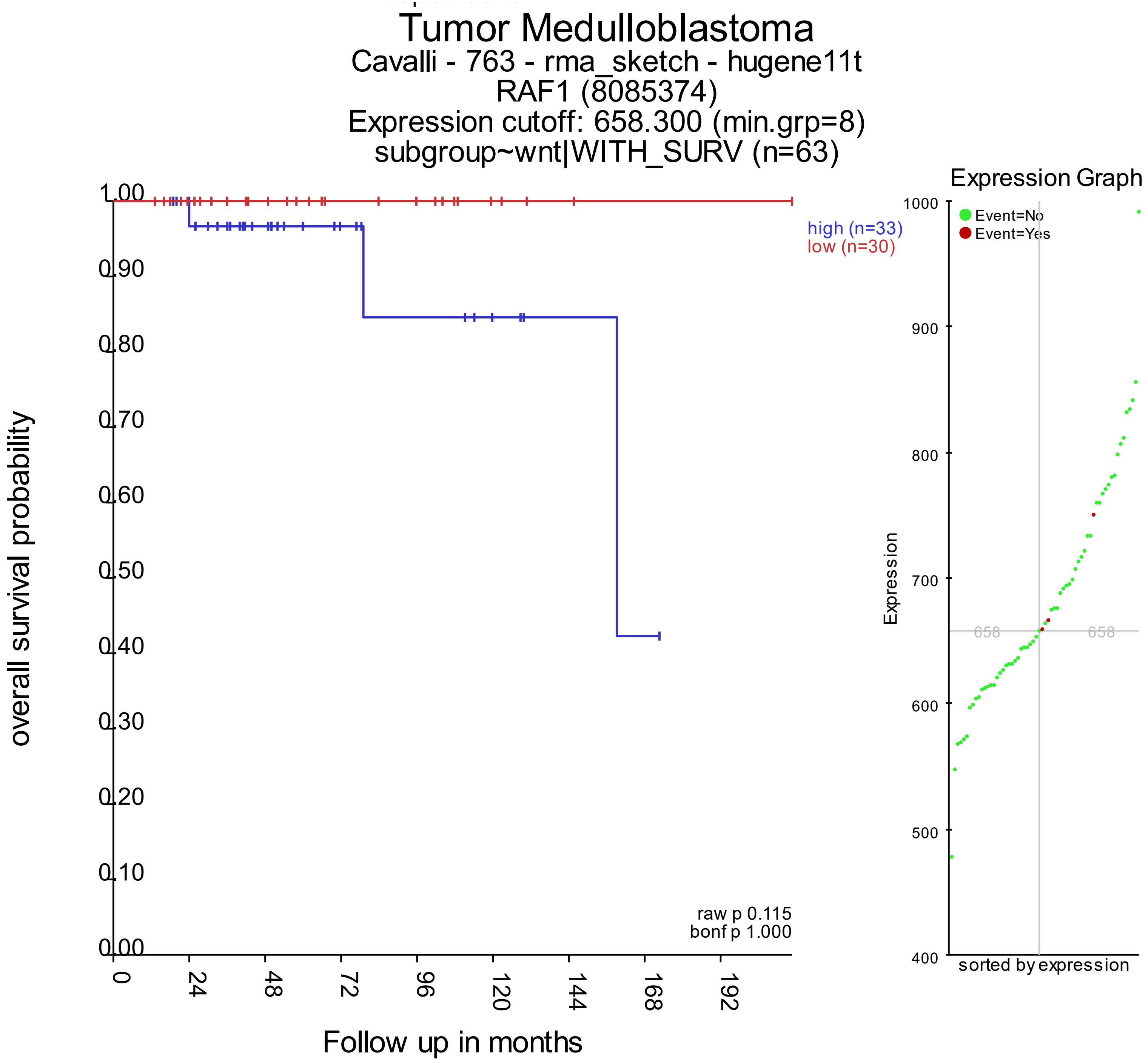

Tumor Medulloblastoma
Cavalli - 763 - rma_sketch - hugene11t
RAF1 (8085374)
Expression cutoff: 658.300 (min.grp=8)
subgroup~wnt|WITH_SURV (n=63)
overall survival probability
1.00
0.90
0.80
0.70
0.60
0.50
0.40
0.30
0.20
0.10
0.00
high (n=33)
low (n=30)
raw p 0.115
bonf p 1.000
0
24
48
72
96
120
144
168
192
Follow up in months
Expression Graph
Event=No
Event=Yes
Expression
1000
900
800
700
600
500
400
658
658
sorted by expression

SHH

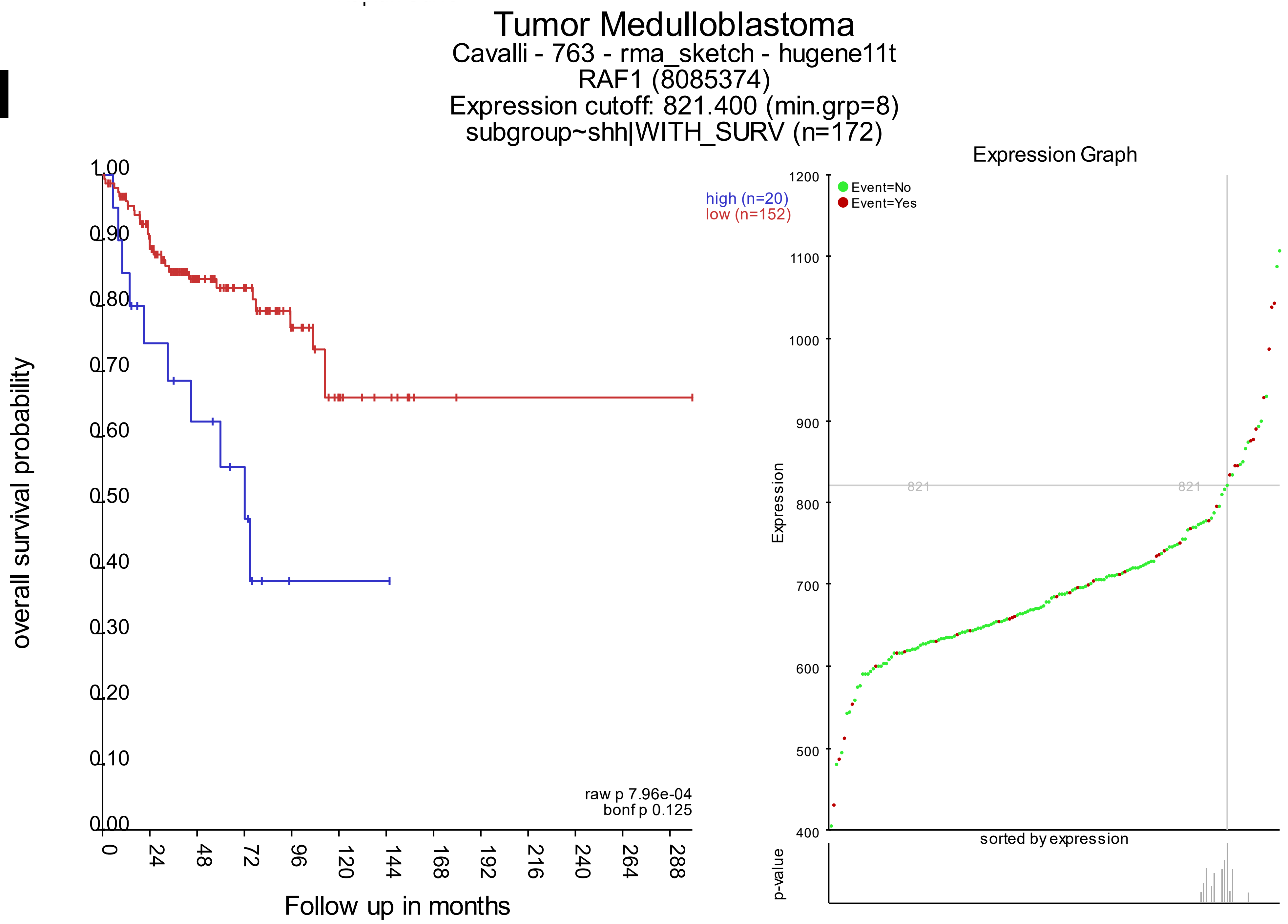
Tumor Medulloblastoma
Cavalli - 763 - rma_sketch - hugene11t
RAF1 (8085374)
Expression cutoff: 821.400 (min.grp=8)
subgroup~shh|WITH_SURV (n=172)
high (n=20)
low (n=152)
overall survival probability
Follow up in months
raw p 7.96e-04
bonf p 0.125
Expression Graph
Event=No
Event=Yes
Expression
sorted by expression
p-value

GR4

Tumor Medulloblastoma
Cavalli - 763 - rma_sketch - hugene11t
RAF1 (8085374)
Expression cutoff: 757.600 (min.grp=8)
subgroup~group4|WITH_SURV (n=264)

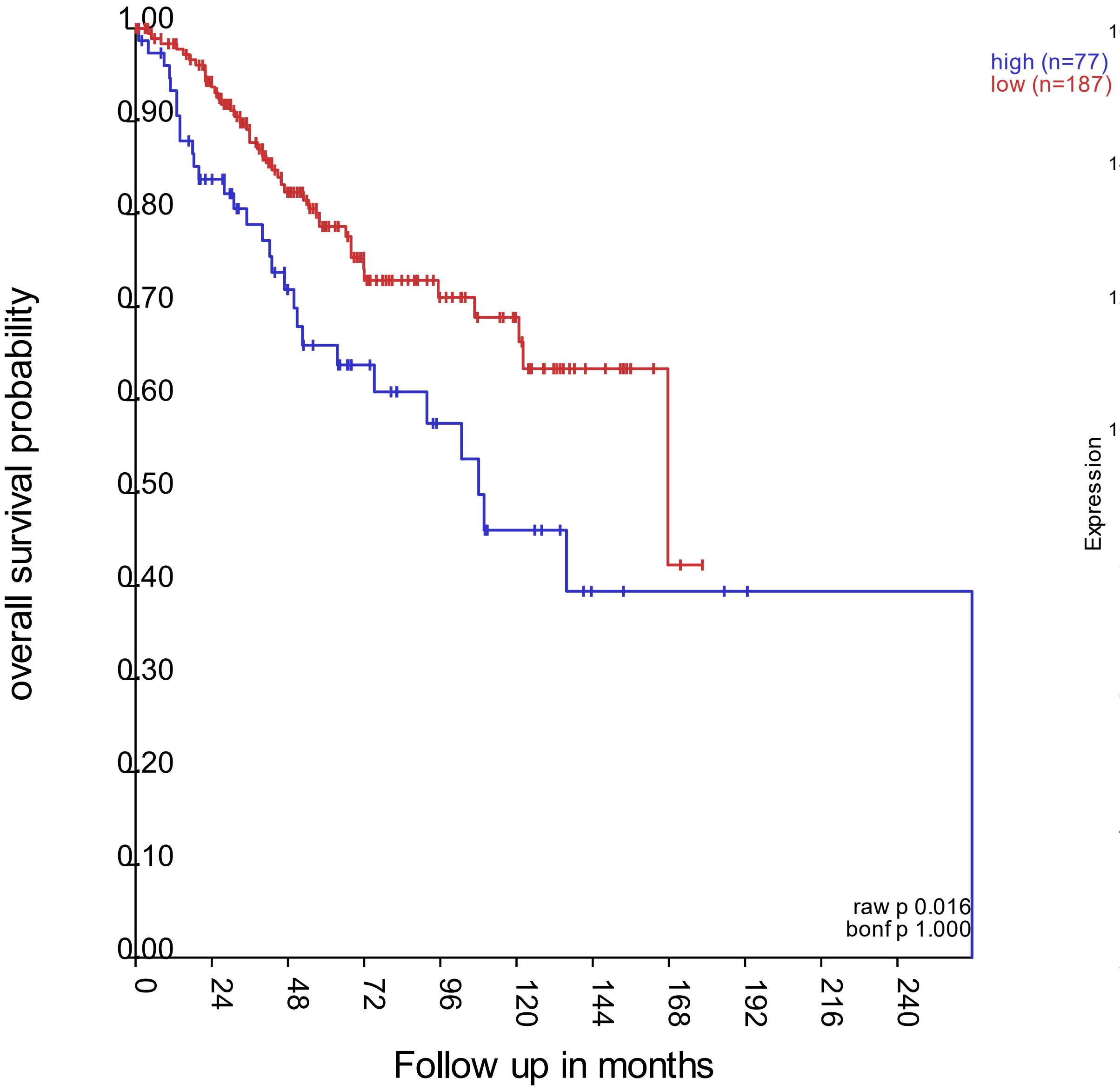

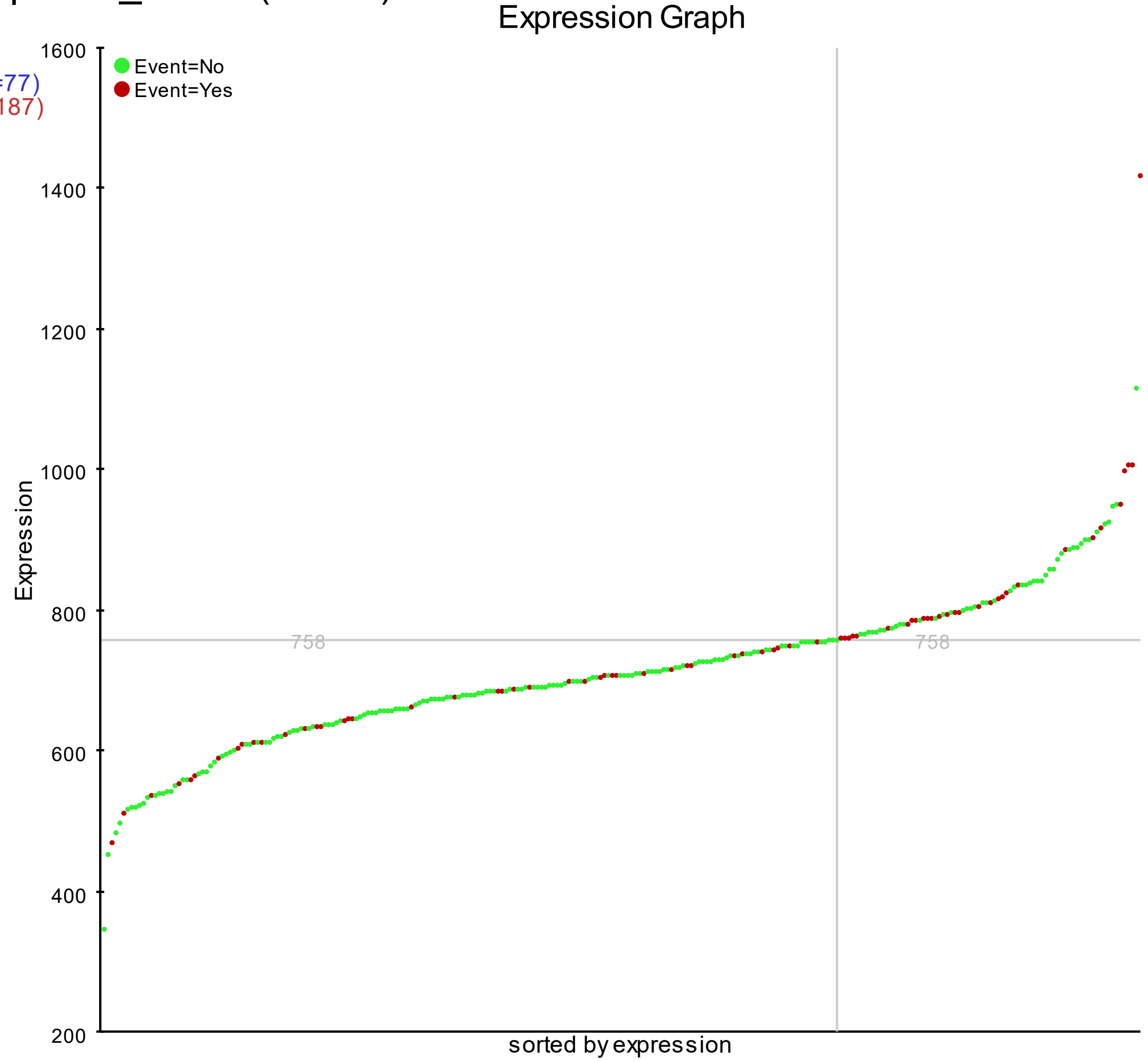

GR3

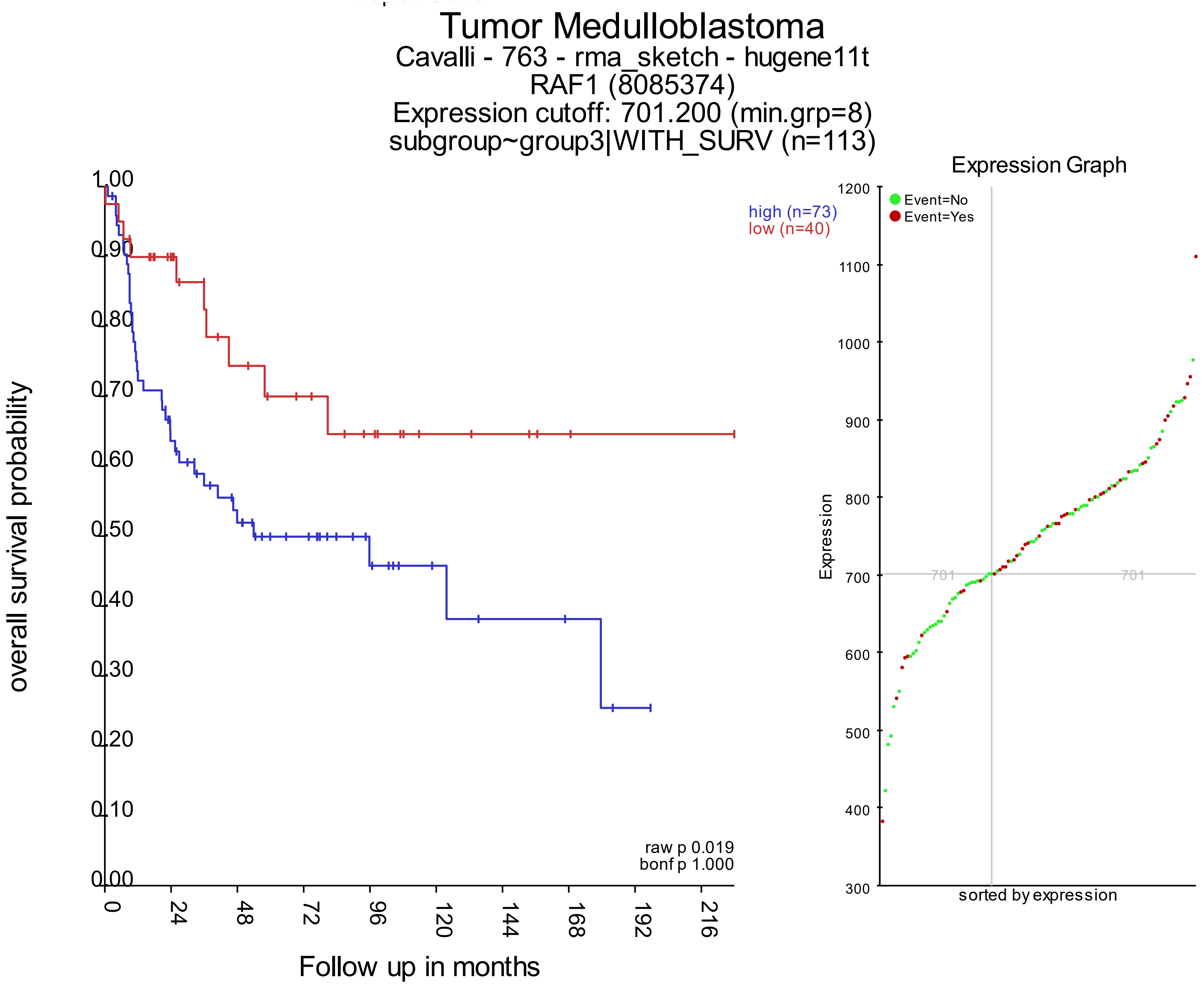
Tumor Medulloblastoma
Cavalli - 763 - rma_sketch - hugene11t
RAF1 (8085374)
Expression cutoff: 701.200 (min.grp=8)
subgroup~group3|WITH_SURV (n=113)
overall survival probability
1.00
0.90
0.80
0.70
0.60
0.50
0.40
0.30
0.20
0.10
0.00
0
24
48
72
96
120
144
168
192
216
Follow up in months
high (n=73)
low (n=40)
raw p 0.019
bonf p 1.000
Expression Graph
Event=No
Event=Yes
Expression
1200
1100
1000
900
800
700
600
500
400
300
701
701
sorted by expression

RET

WNT

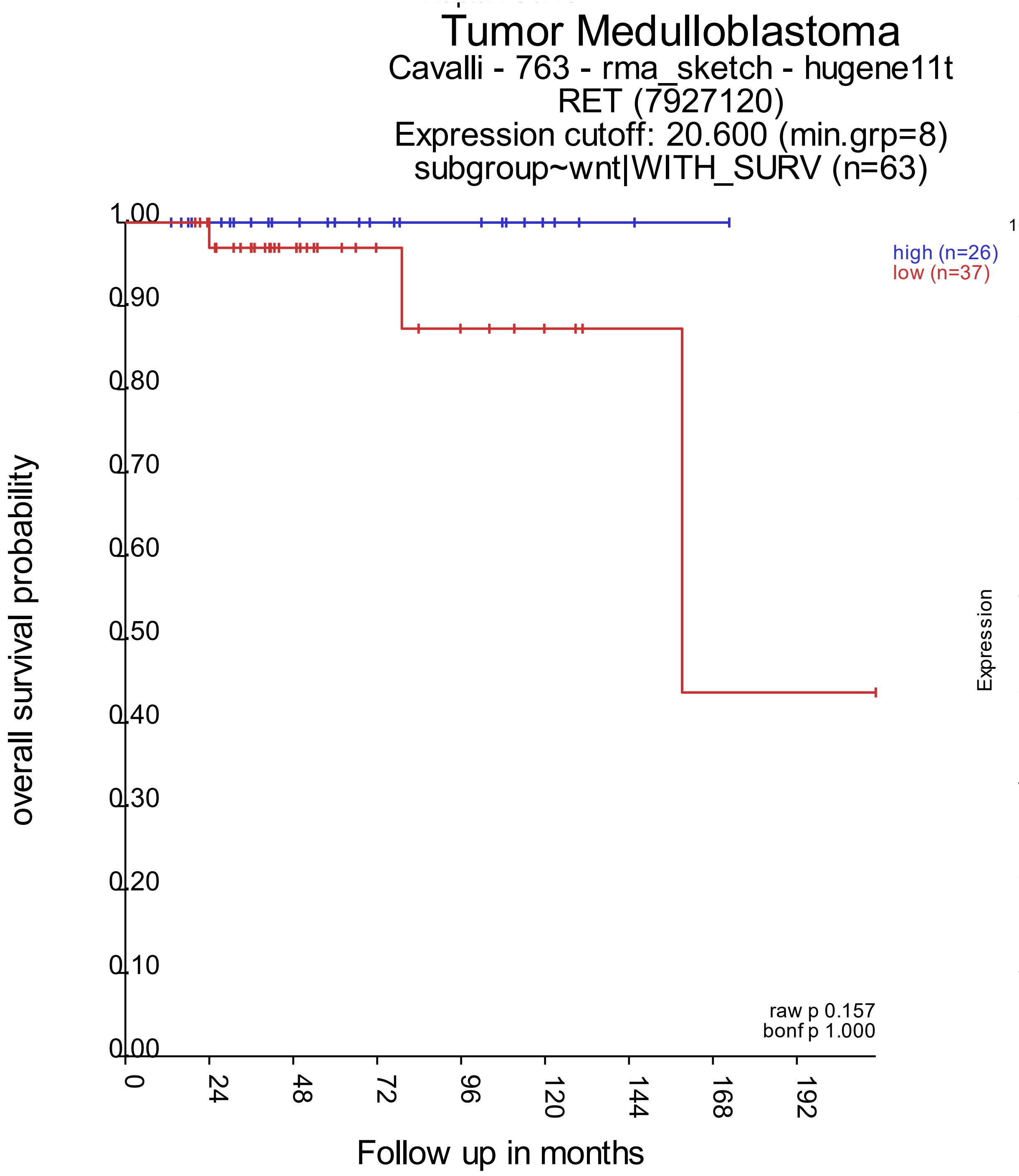

Tumor Medulloblastoma
Cavalli - 763 - rma_sketch - hugene11t
RET (7927120)
Expression cutoff: 20.600 (min.grp=8)
subgroup~wnt|WITH_SURV (n=63)
overall survival probability
1.00
0.90
0.80
0.70
0.60
0.50
0.40
0.30
0.20
0.10
0.00
0
24
48
72
96
120
144
168
192
Follow up in months
high (n=26)
low (n=37)
raw p 0.157
bonf p 1.000

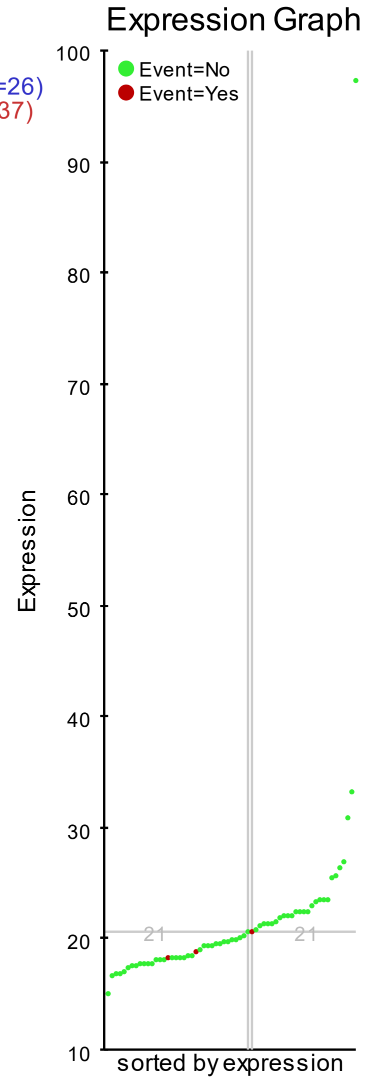

Expression Graph
Event=No
Event=Yes
Expression
100
90
80
70
60
50
40
30
20
10
21
21
sorted by expression

SHH

Tumor Medulloblastoma
Cavalli - 763 - rma_sketch - hugene11t
RET (7927120)
Expression cutoff: 22.700 (min.grp=8)
subgroup~shh|WITH_SURV (n=172)

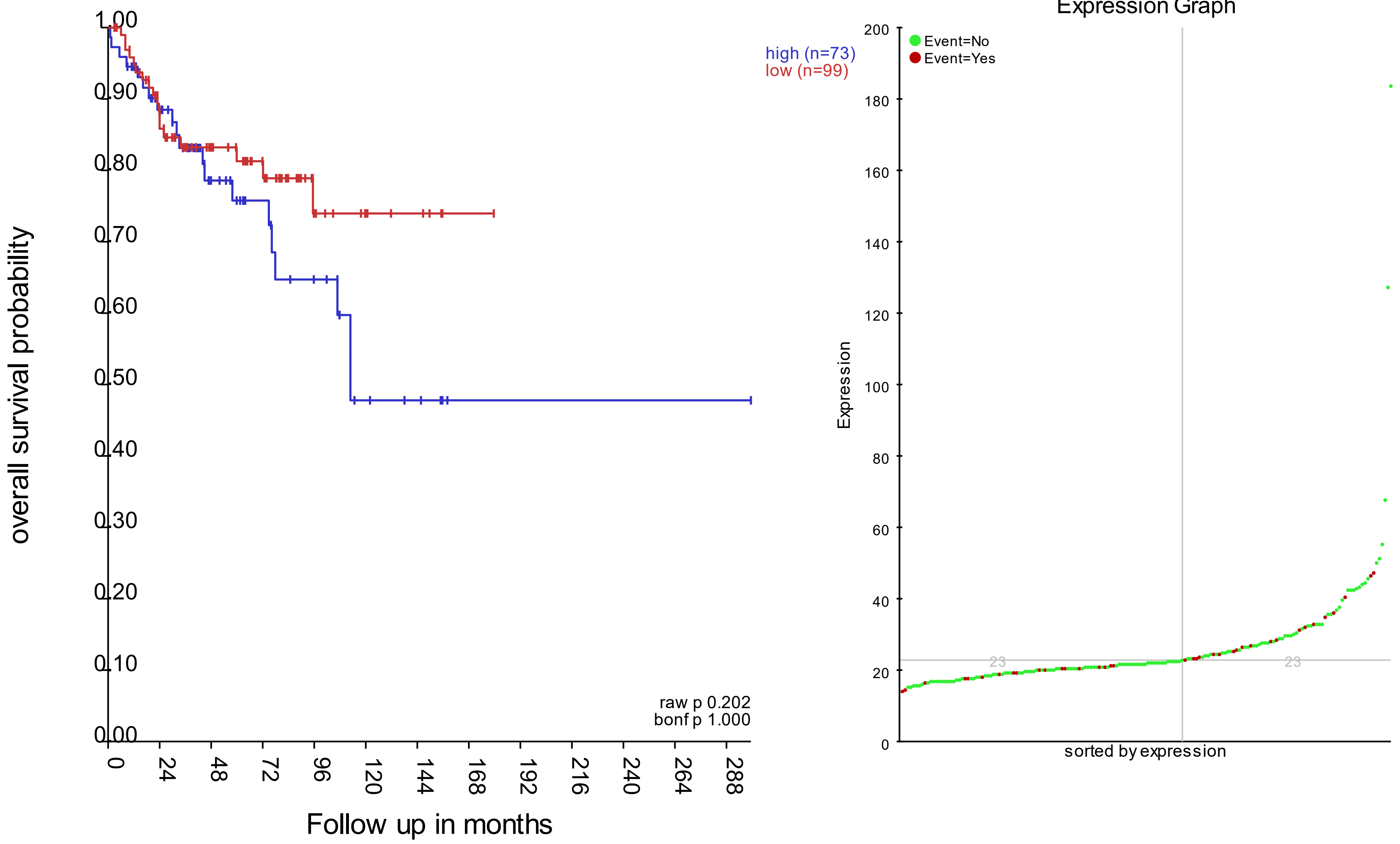

GR4

Tumor Medulloblastoma
Cavalli - 763 - rma_sketch - hugene11t
RET (7927120)
Expression cutoff: 16.400 (min.grp=8)
subgroup~group4|WITH_SURV (n=264)

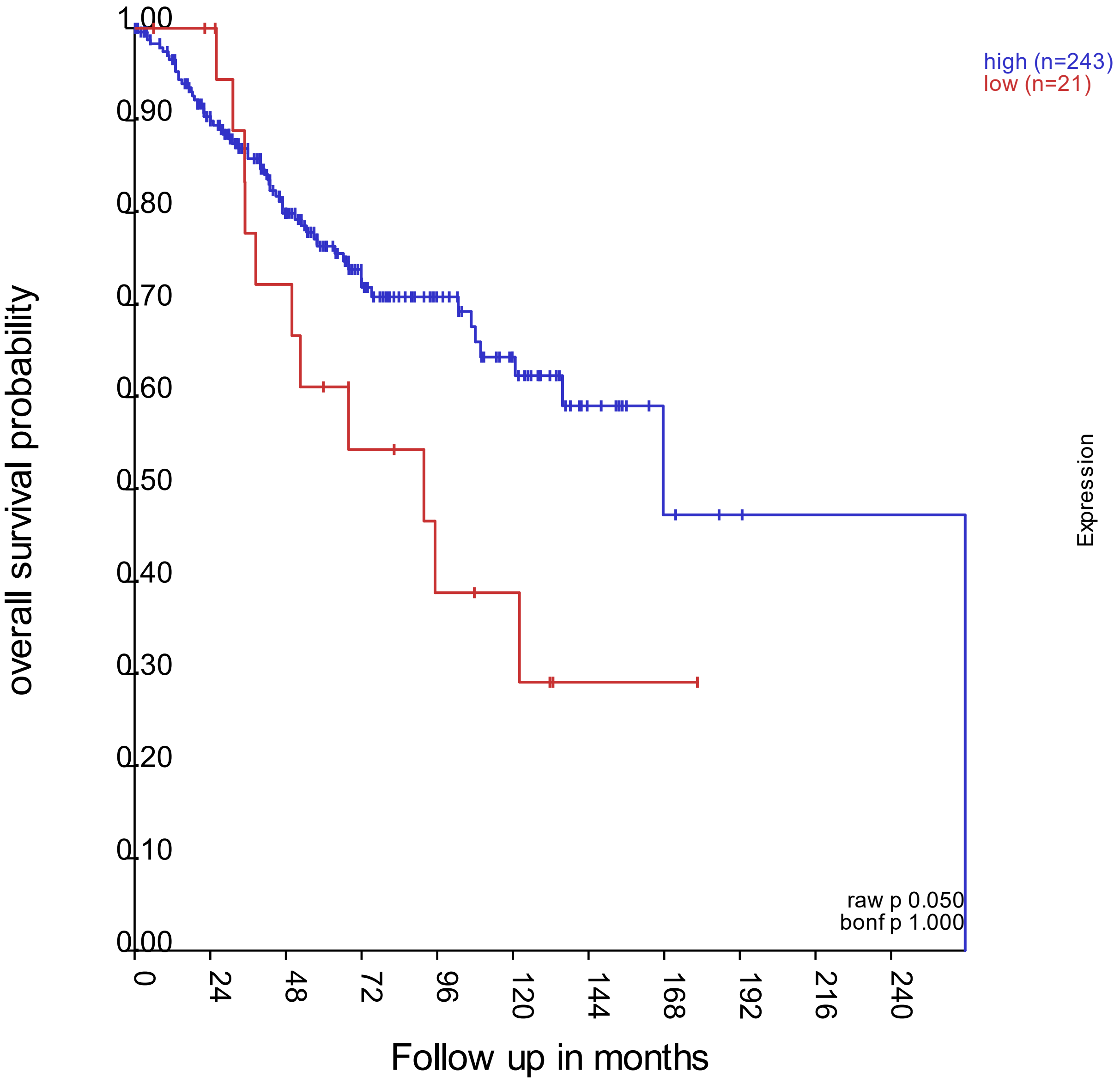

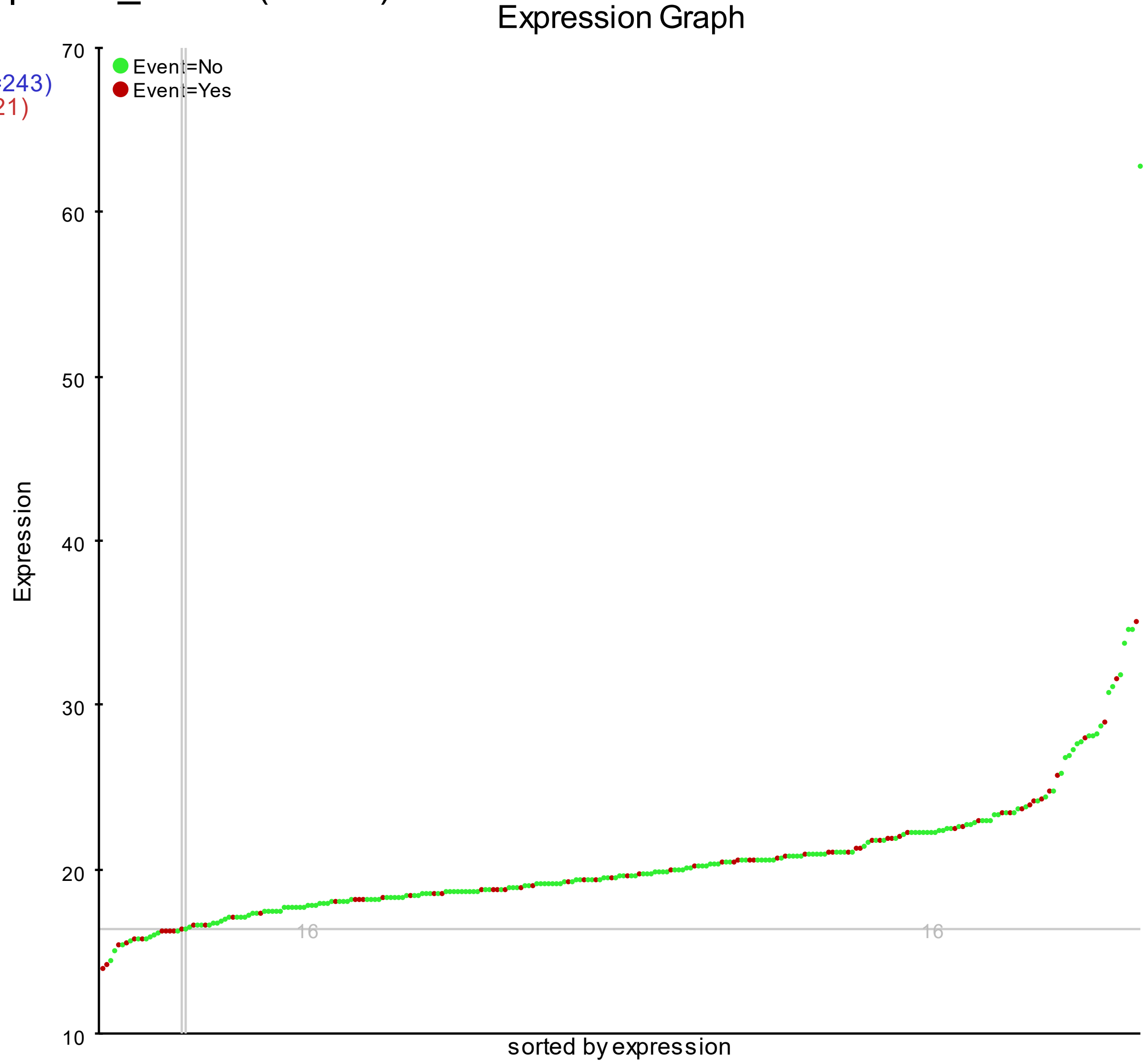

GR3

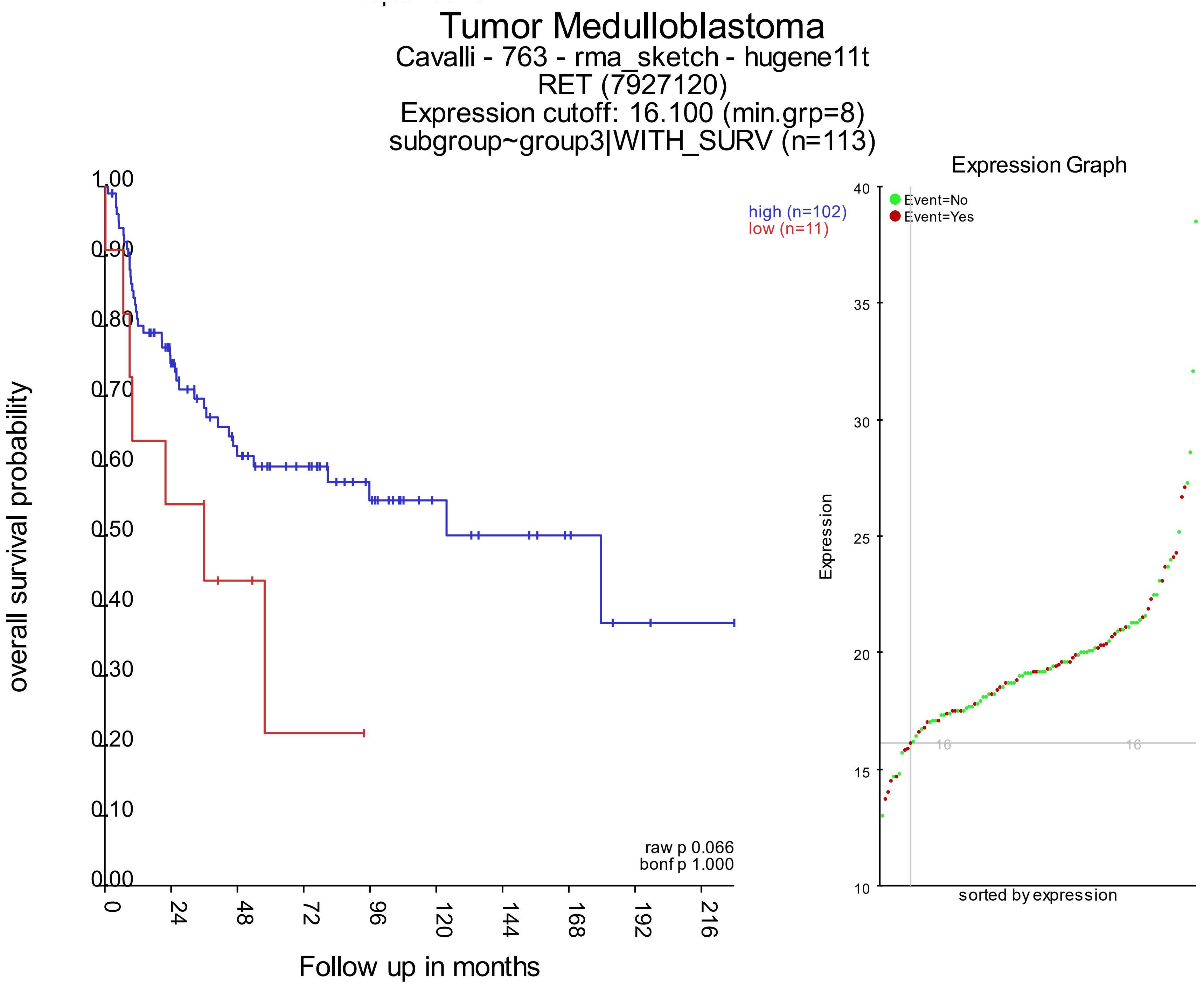
Tumor Medulloblastoma
Cavalli - 763 - rma_sketch - hugene11t
RET (7927120)
Expression cutoff: 16.100 (min.grp=8)
subgroup~group3|WITH_SURV (n=113)
overall survival probability
1.00
0.90
0.80
0.70
0.60
0.50
0.40
0.30
0.20
0.10
0.00
high (n=102)
low (n=11)
raw p 0.066
bonf p 1.000
0
24
48
72
96
120
144
168
192
216
Follow up in months
Expression Graph
Event=No
Event=Yes
Expression
40
35
30
25
20
15
10
16
16
sorted by expression

ROS1

WNT

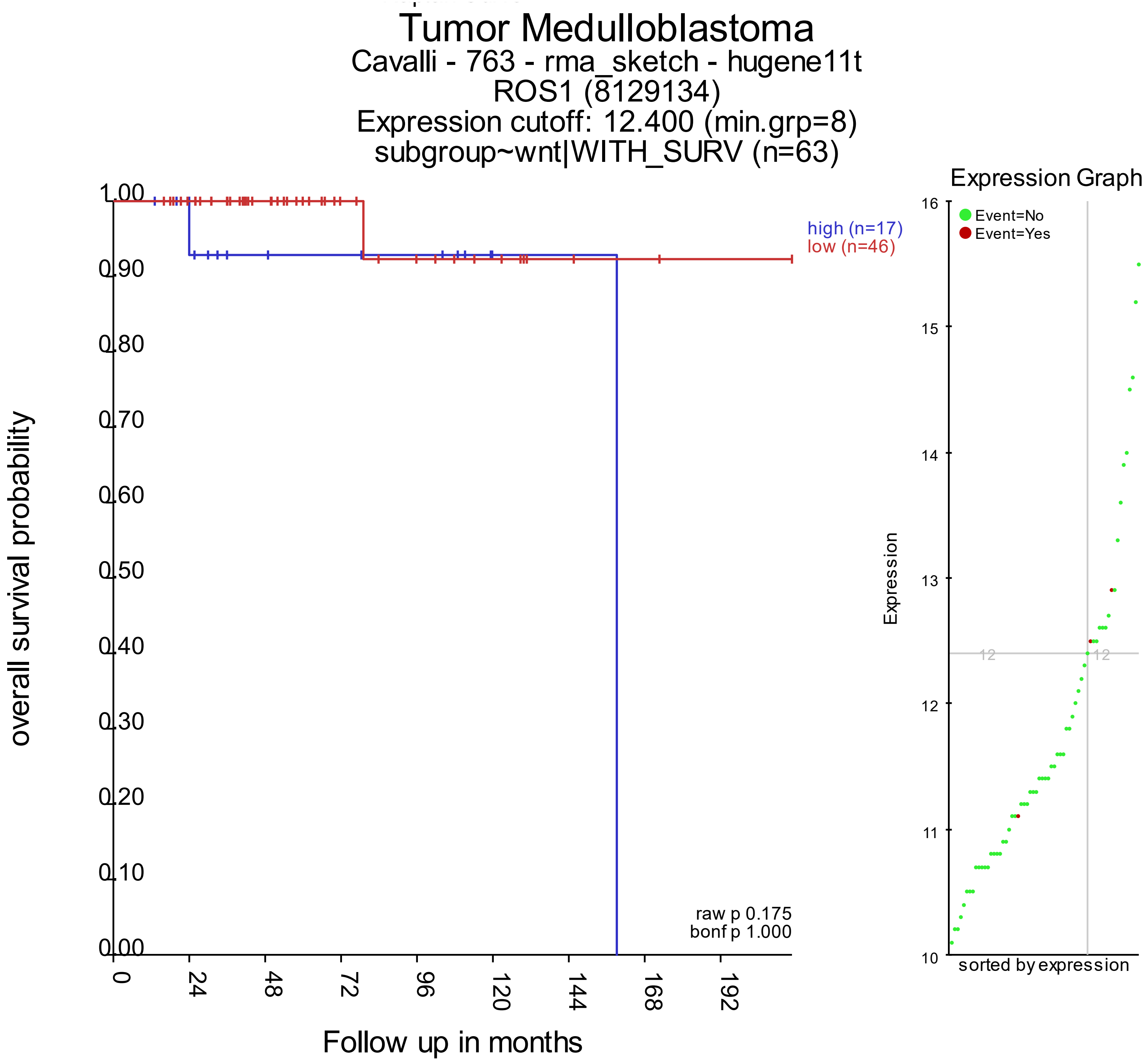

Tumor Medulloblastoma
Cavalli - 763 - rma_sketch - hugene11t
ROS1 (8129134)
Expression cutoff: 12.400 (min.grp=8)
subgroup~wnt|WITH_SURV (n=63)
overall survival probability
1.00
0.90
0.80
0.70
0.60
0.50
0.40
0.30
0.20
0.10
0.00
high (n=17)
low (n=46)
raw p 0.175
bonf p 1.000
0
24
48
72
96
120
144
168
192
Follow up in months
Expression Graph
Event=No
Event=Yes
Expression
16
15
14
13
12
11
10
sorted by expression

SHH

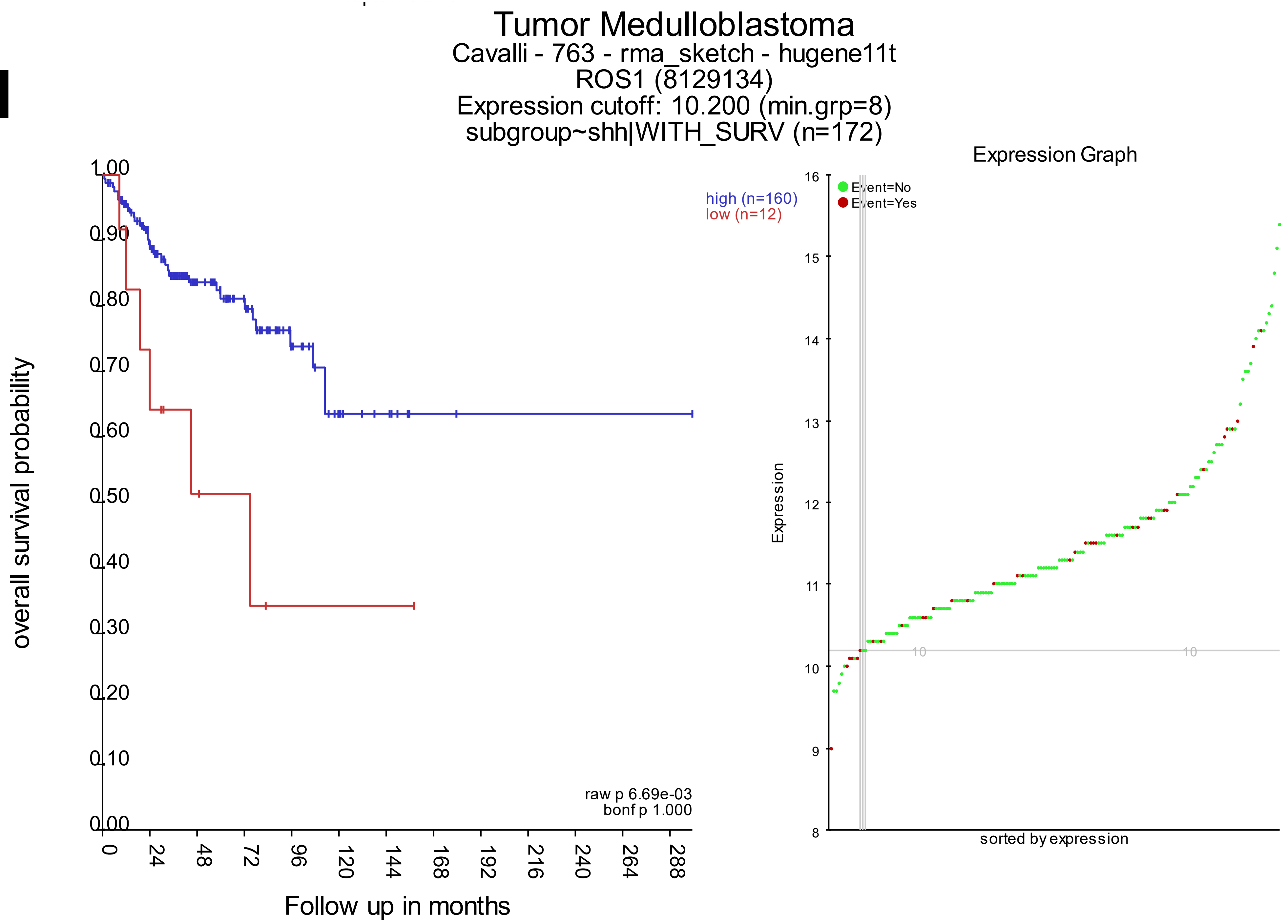

Tumor Medulloblastoma
Cavalli - 763 - rma_sketch - hugene11t
ROS1 (8129134)
Expression cutoff: 10.200 (min.grp=8)
subgroup~shh|WITH_SURV (n=172)
overall survival probability
1.00
0.90
0.80
0.70
0.60
0.50
0.40
0.30
0.20
0.10
0.00
0
24
48
72
96
120
144
168
192
216
240
264
288
Follow up in months
high (n=160)
low (n=12)
raw p 6.69e-03
bonf p 1.000
Expression Graph
Event=No
Event=Yes
Expression
16
15
14
13
12
11
10
9
8
sorted by expression

GR4

Tumor Medulloblastoma

Cavalli - 763 - rma_sketch - hugene11t
ROS1 (8129134)
Expression cutoff: 11.200 (min.grp=8)
subgroup~group4|WITH_SURV (n=264)

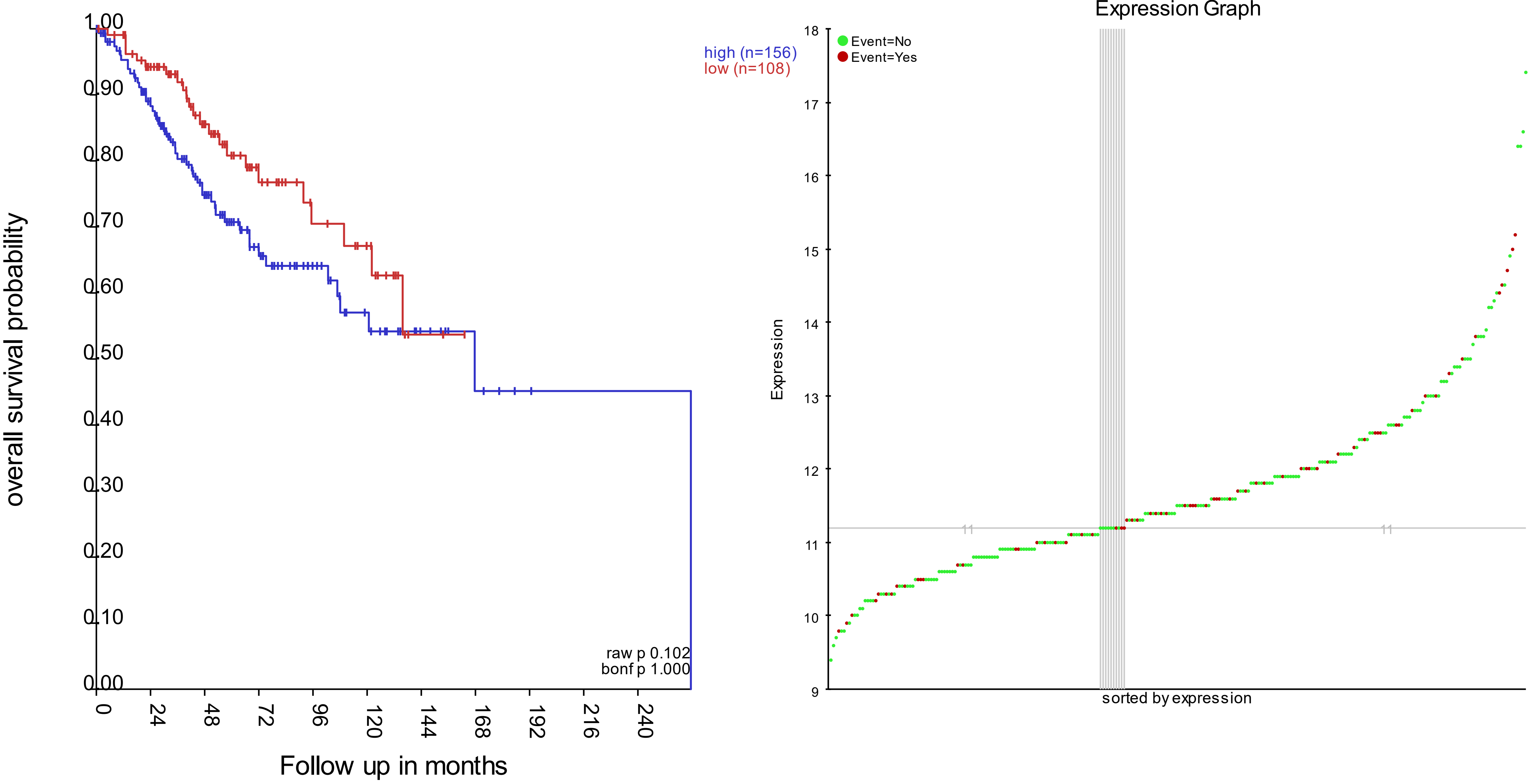

GR3

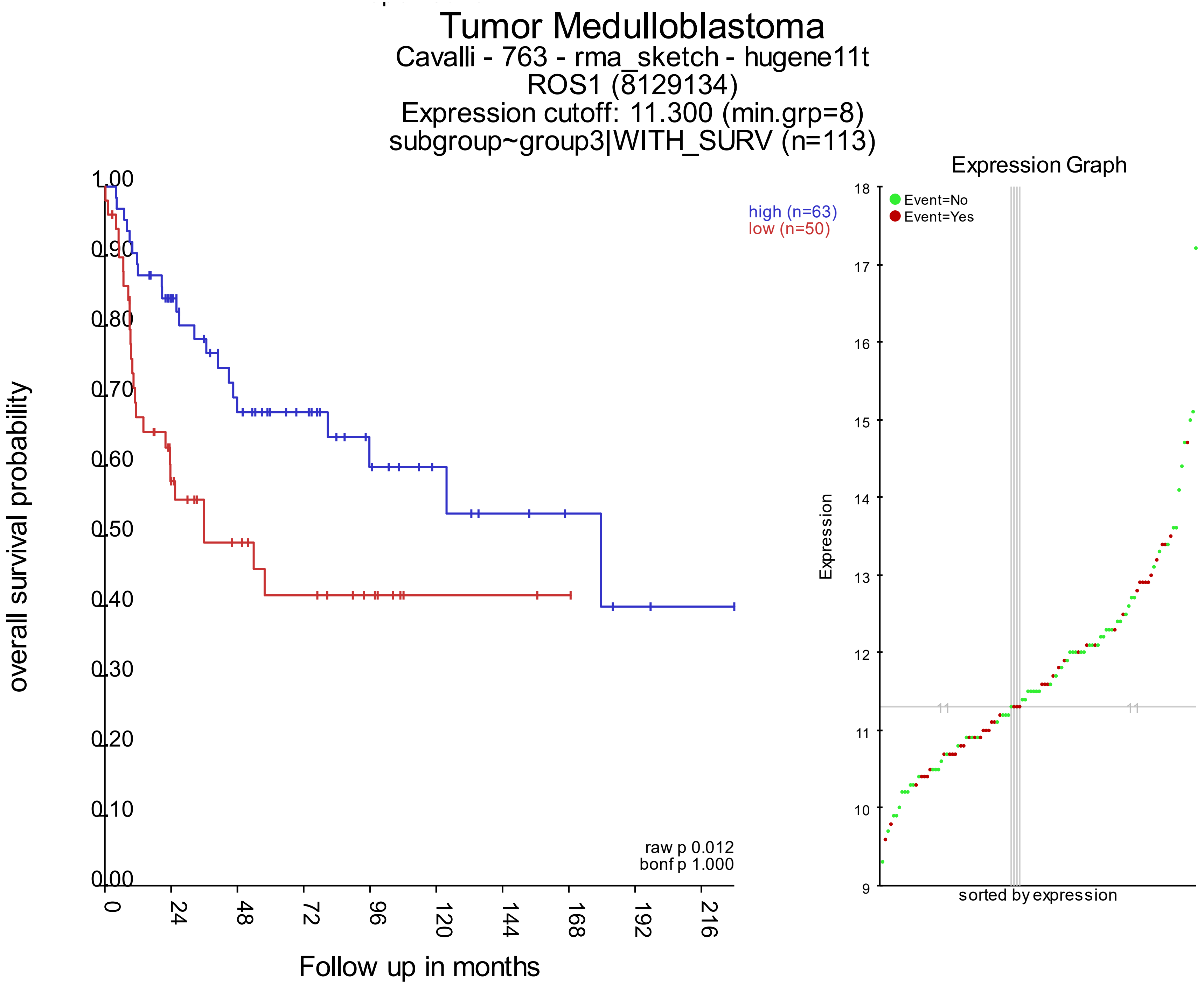
Tumor Medulloblastoma
Cavalli - 763 - rma_sketch - hugene11t
ROS1 (8129134)
Expression cutoff: 11.300 (min.grp=8)
subgroup~group3|WITH_SURV (n=113)
overall survival probability
1.00
0.90
0.80
0.70
0.60
0.50
0.40
0.30
0.20
0.10
0.00
high (n=63)
low (n=50)
raw p 0.012
bonf p 1.000
0
24
48
72
96
120
144
168
192
216
Follow up in months
Expression Graph
Event=No
Event=Yes
Expression
18
17
16
15
14
13
12
11
10
9
sorted by expression

SMO

WNT

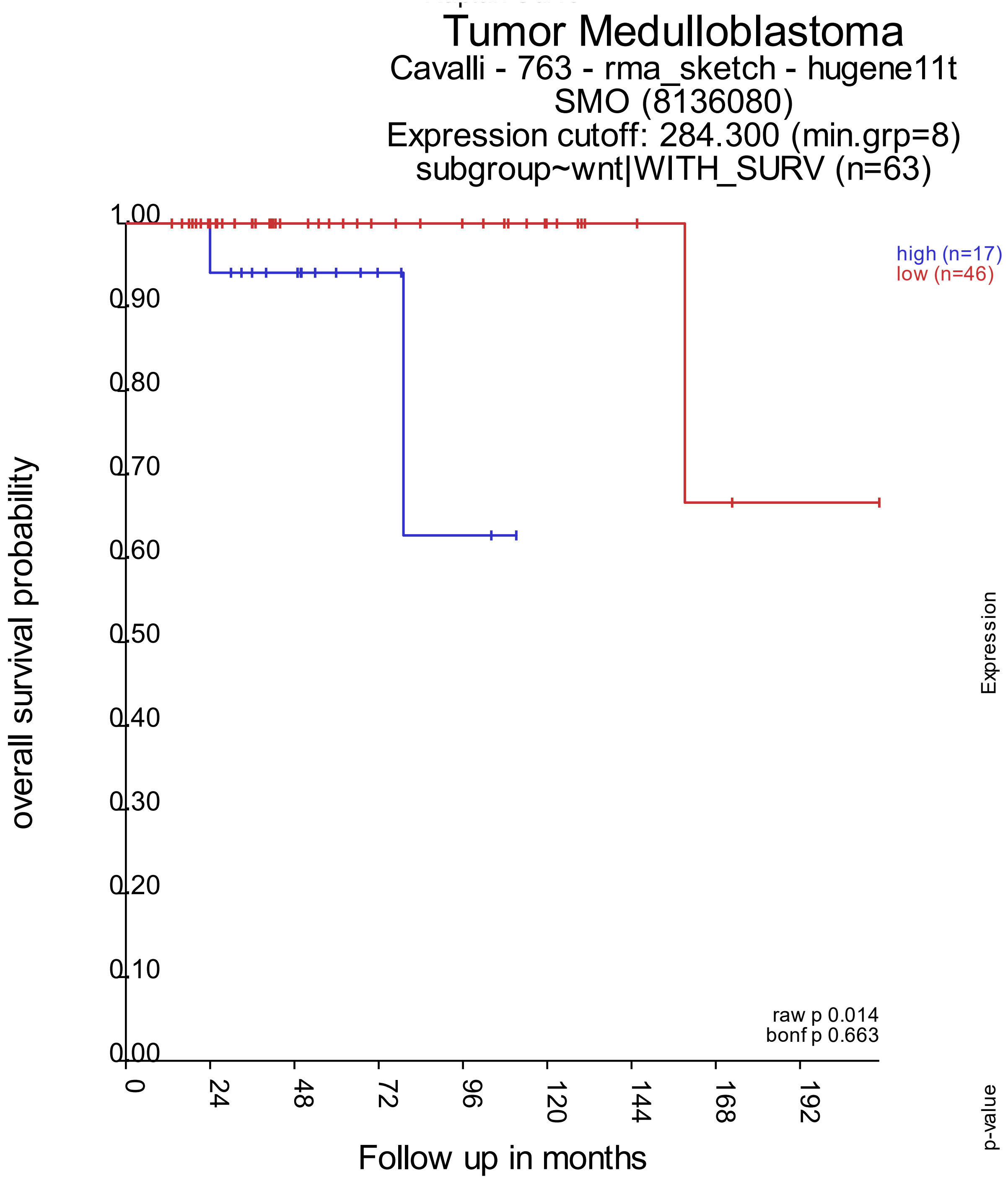
Tumor Medulloblastoma
Cavalli - 763 - rma_sketch - hugene11t
SMO (8136080)
Expression cutoff: 284.300 (min.grp=8)
subgroup~wnt|WITH_SURV (n=63)
overall survival probability
1.00
0.90
0.80
0.70
0.60
0.50
0.40
0.30
0.20
0.10
0.00
0
24
48
72
96
120
144
168
192
Follow up in months
high (n=17)
low (n=46)
raw p 0.014
bonf p 0.663

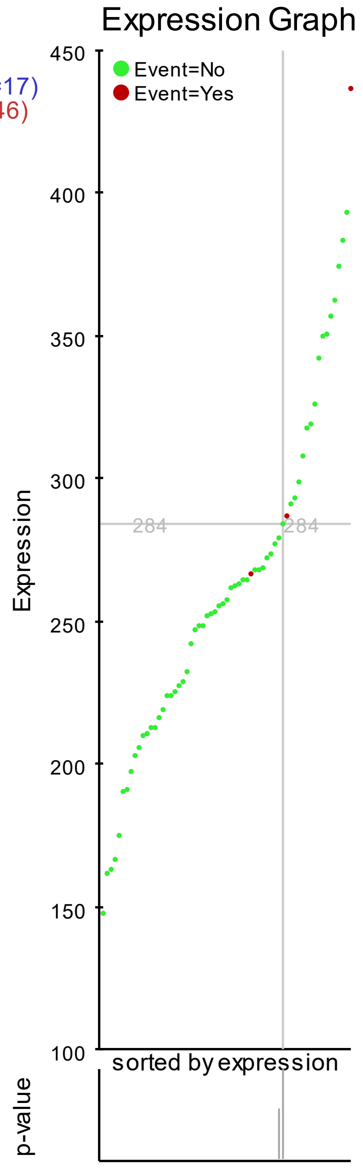
Expression Graph
Event=No
Event=Yes
Expression
450
400
350
300
250
200
150
100
284
284
sorted by expression
p-value

SHH

Tumor Medulloblastoma
Cavalli - 763 - rma_sketch - hugene11t
SMO (8136080)
Expression cutoff: 272.500 (min.grp=8)
subgroup~shh|WITH_SURV (n=172)

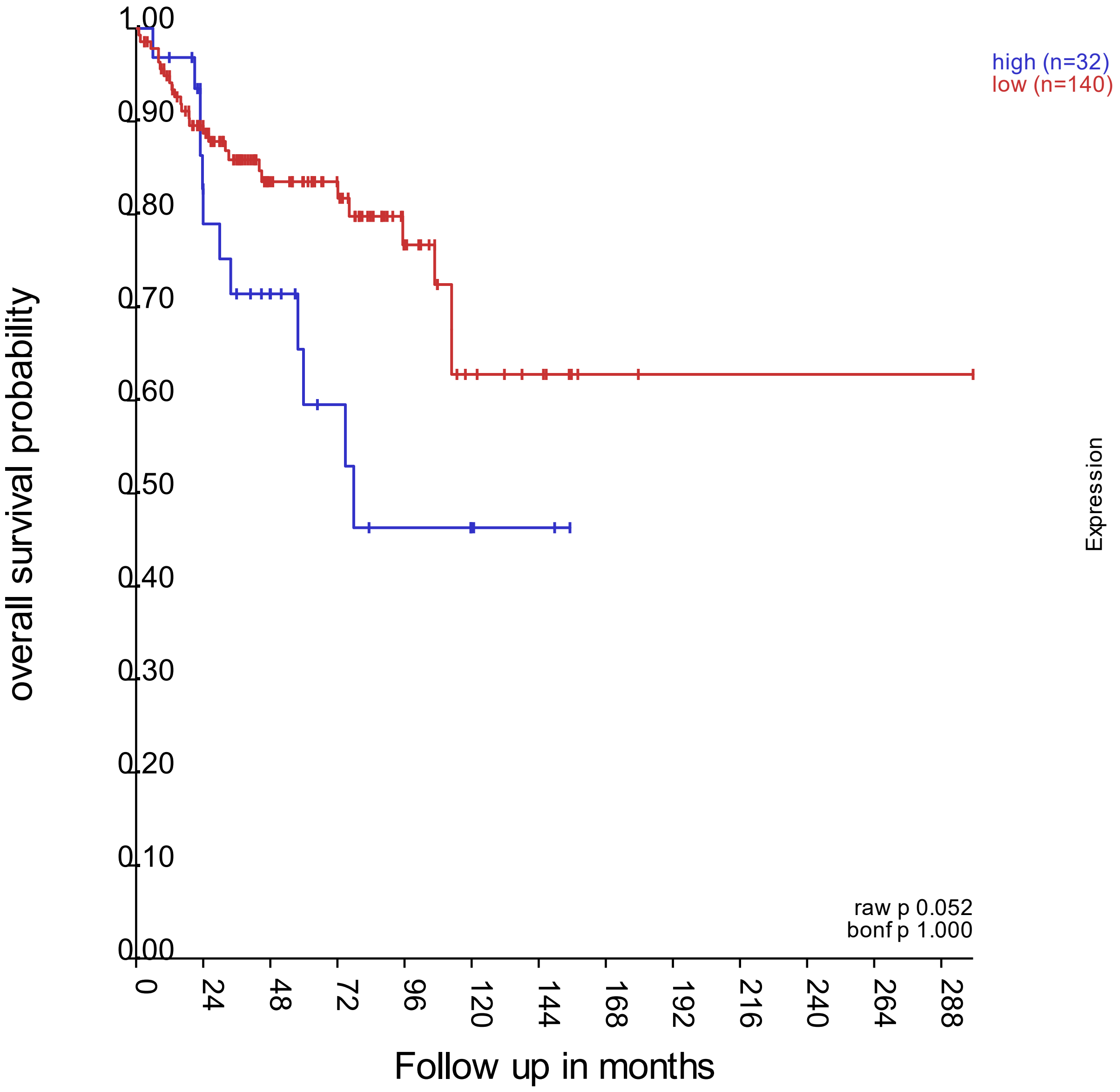

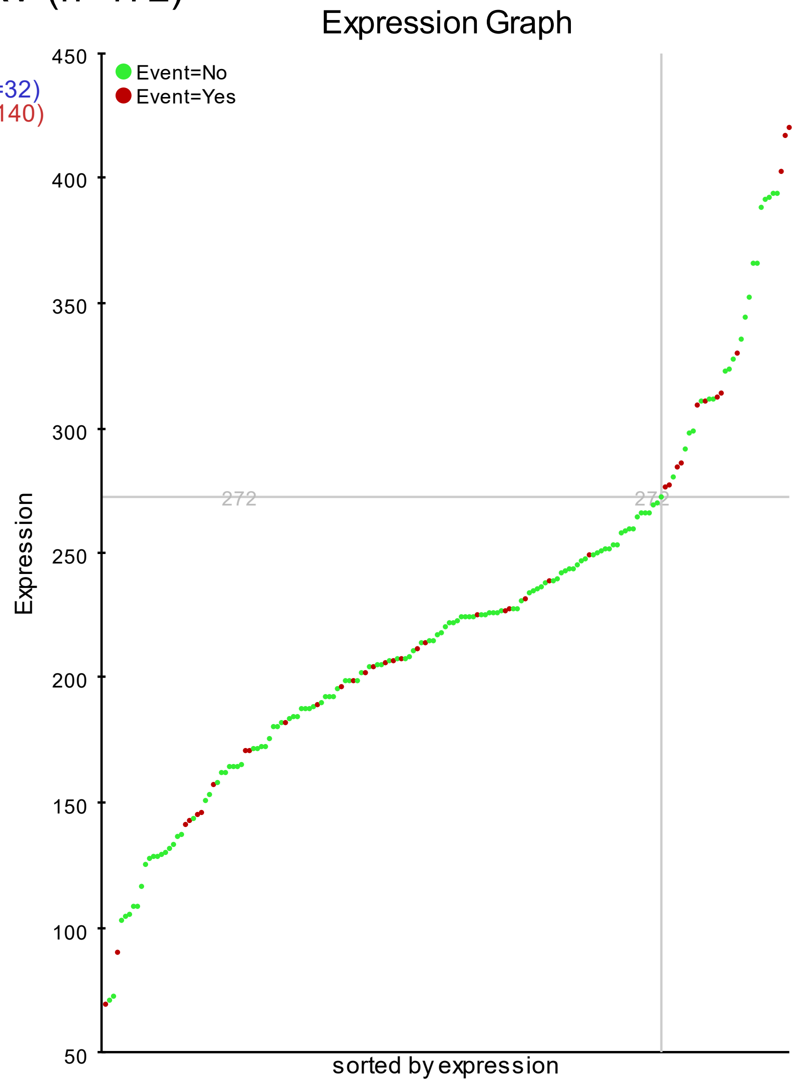

GR4

Tumor Medulloblastoma
Cavalli - 763 - rma_sketch - hugene11t
SMO (8136080)
Expression cutoff: 118.300 (min.grp=8)
subgroup~group4|WITH_SURV (n=264)

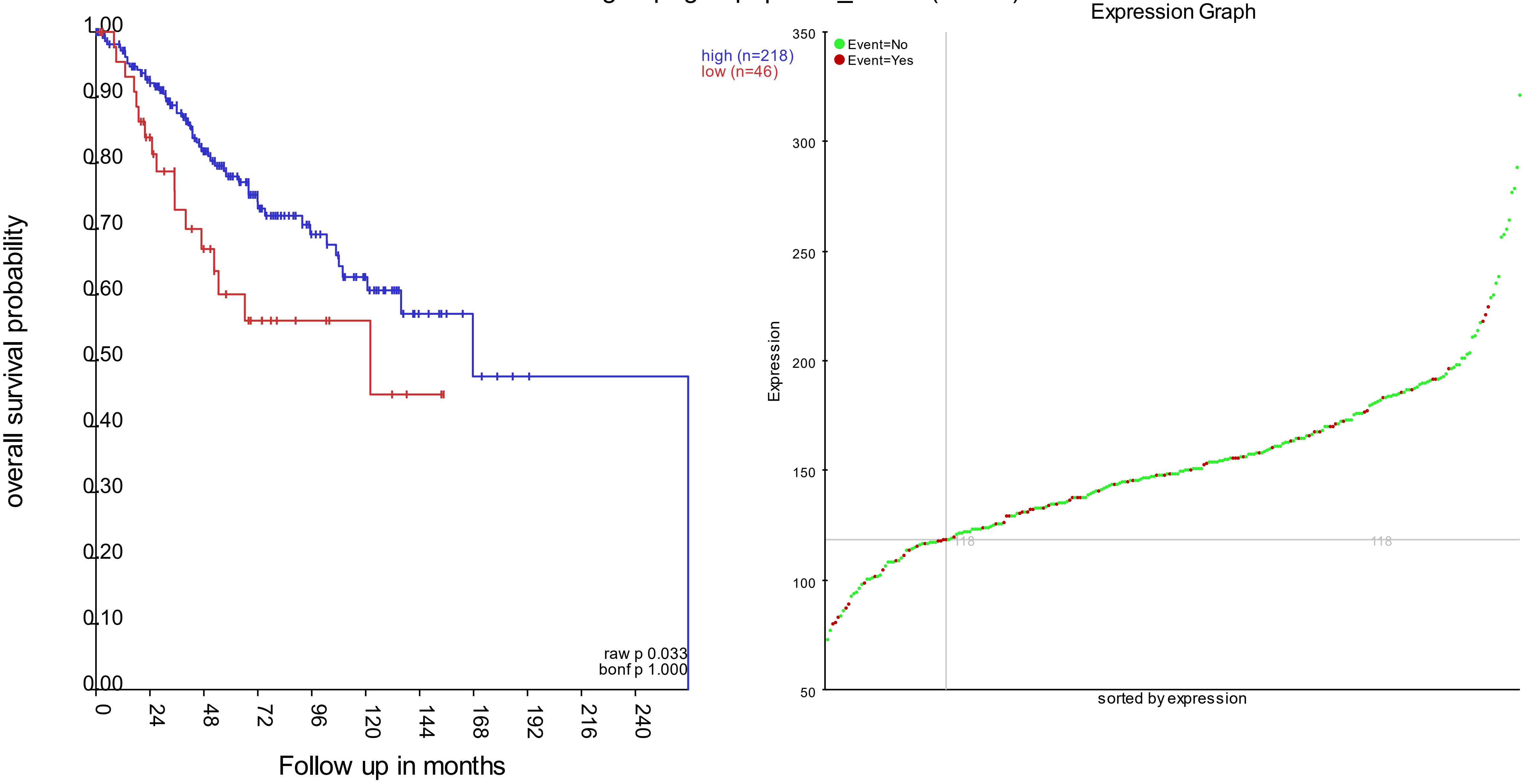

GR3

Tumor Medulloblastoma
Cavalli - 763 - rma_sketch - hugene11t
SMO (8136080)
Expression cutoff: 91.000 (min.grp=8)
subgroup~group3|WITH_SURV (n=113)

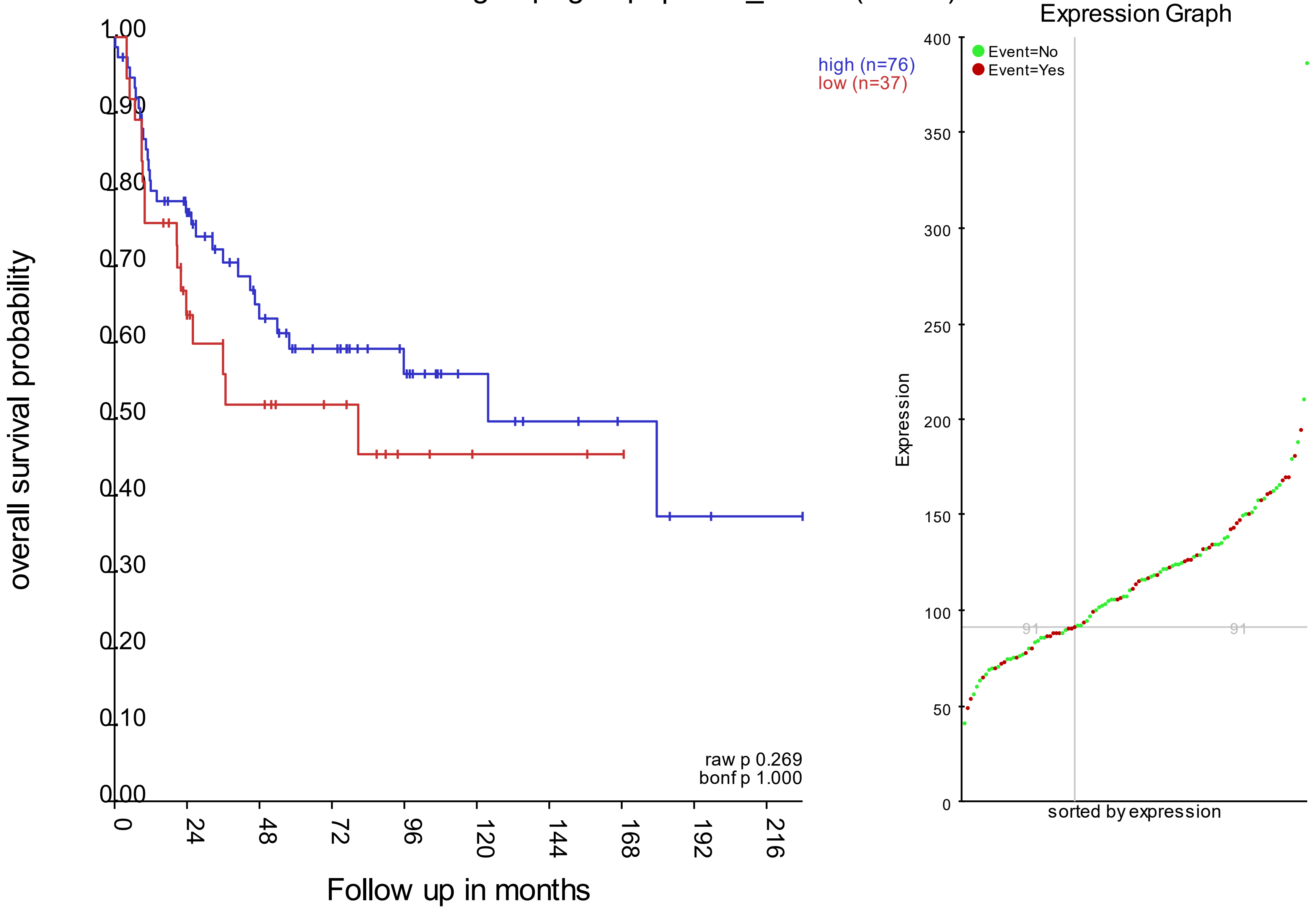

ABL1

WNT

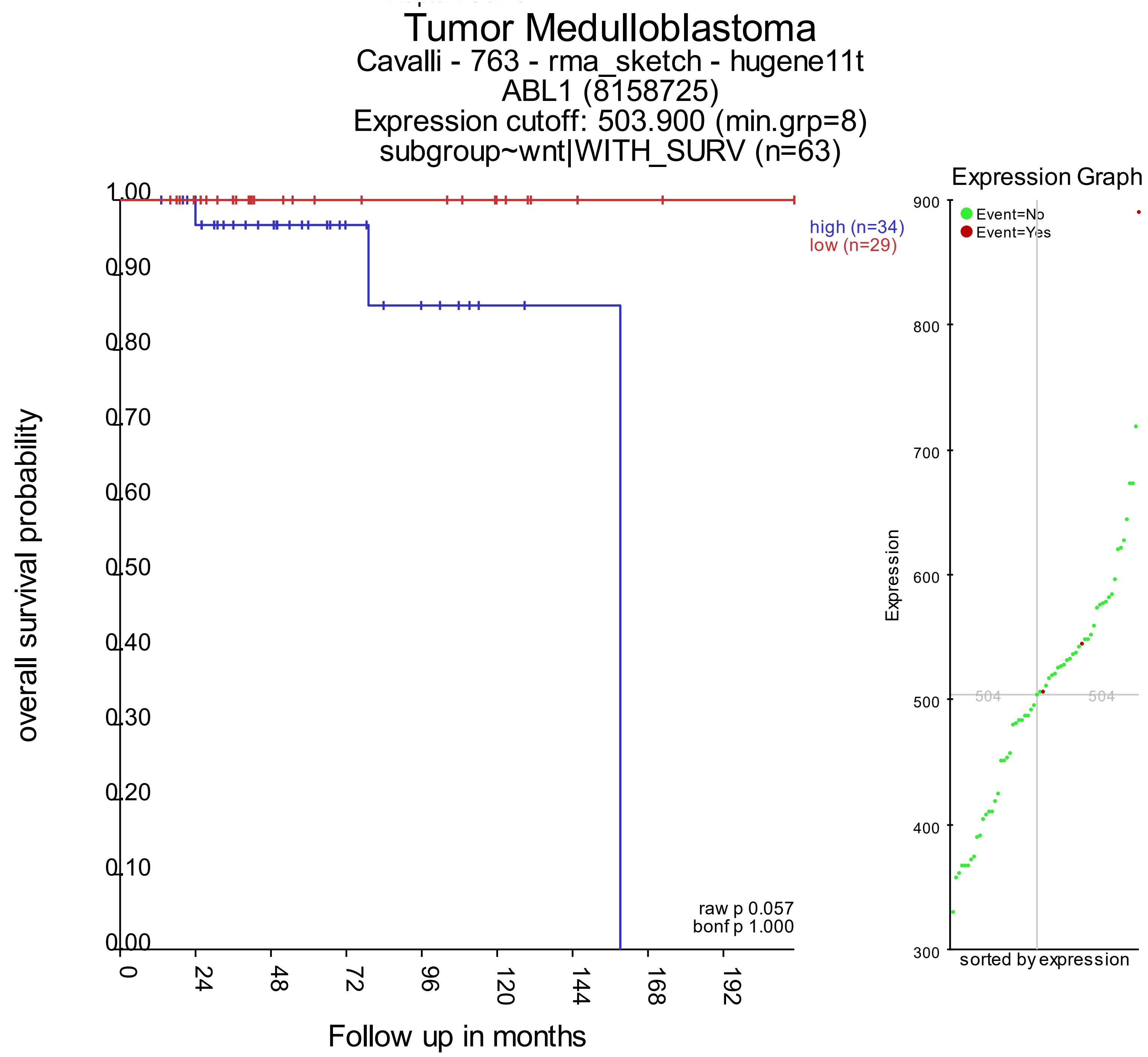

Tumor Medulloblastoma
Cavalli - 763 - rma_sketch - hugene11t
ABL1 (8158725)
Expression cutoff: 503.900 (min.grp=8)
subgroup~wnt|WITH_SURV (n=63)
overall survival probability
1.00
0.90
0.80
0.70
0.60
0.50
0.40
0.30
0.20
0.10
0.00
0
24
48
72
96
120
144
168
192
Follow up in months
high (n=34)
low (n=29)
raw p 0.057
bonf p 1.000
Expression Graph
Event=No
Event=Yes
Expression
900
800
700
600
500
400
300
504
504
sorted by expression

SHH

Tumor Medulloblastoma
Cavalli - 763 - rma_sketch - hugene11t
ABL1 (8158725)
Expression cutoff: 321.300 (min.grp=8)
subgroup~shh|WITH_SURV (n=172)

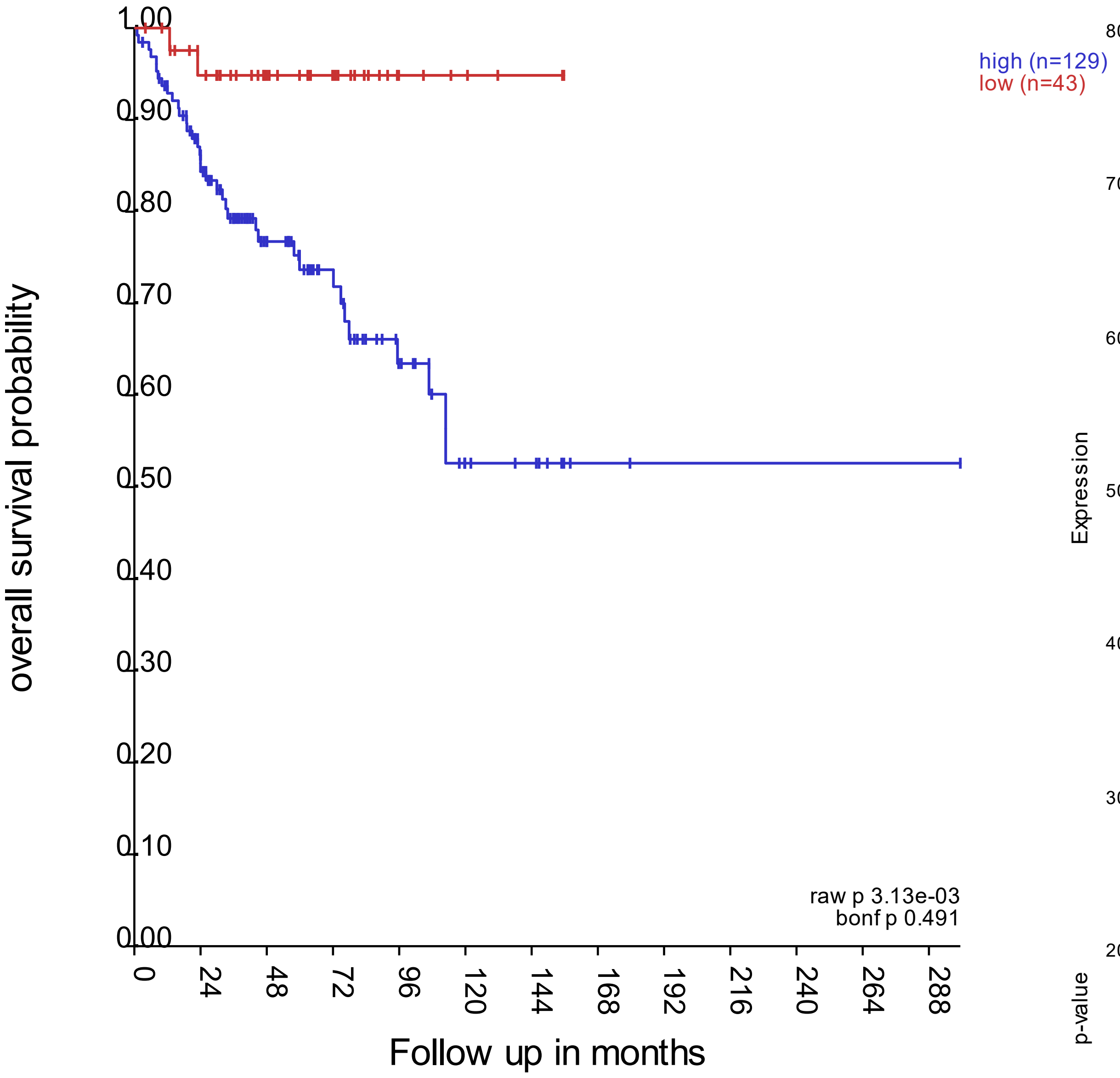

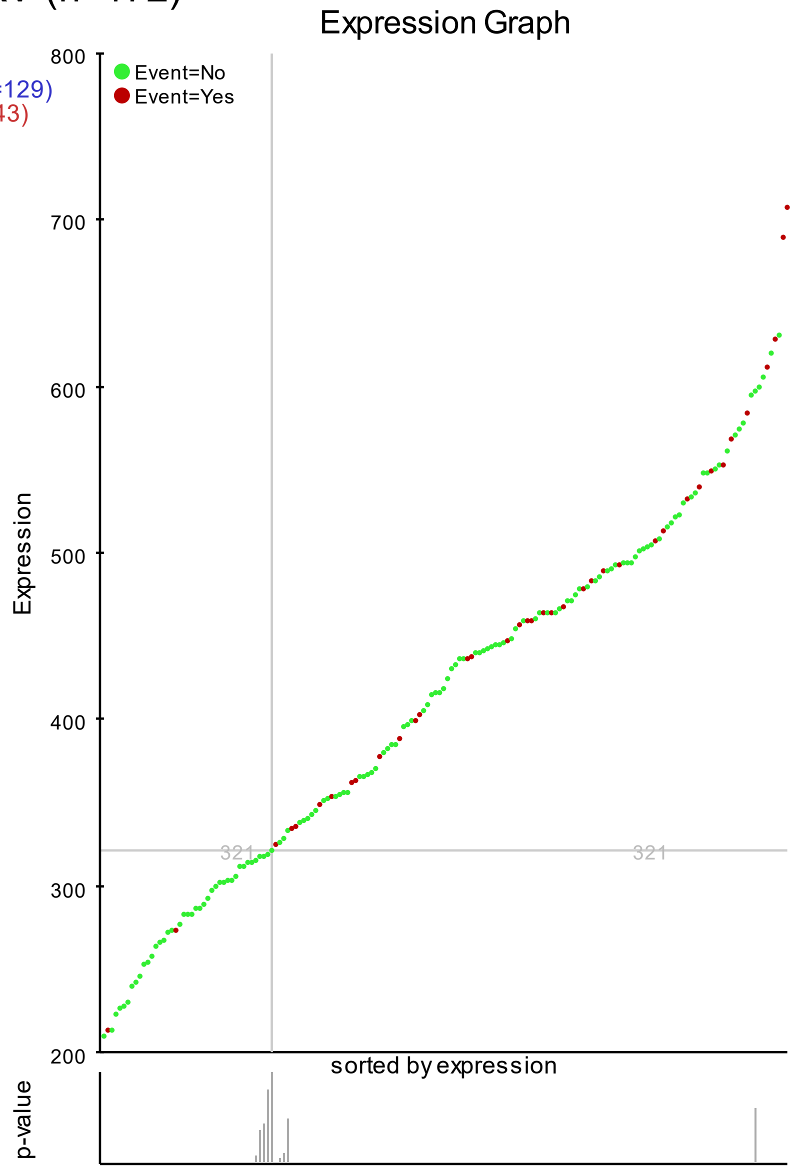

GR4

Tumor Medulloblastoma
Cavalli - 763 - rma_sketch - hugene11t
ABL1 (8158725)
Expression cutoff: 586.400 (min.grp=8)
subgroup~group4|WITH_SURV (n=264)

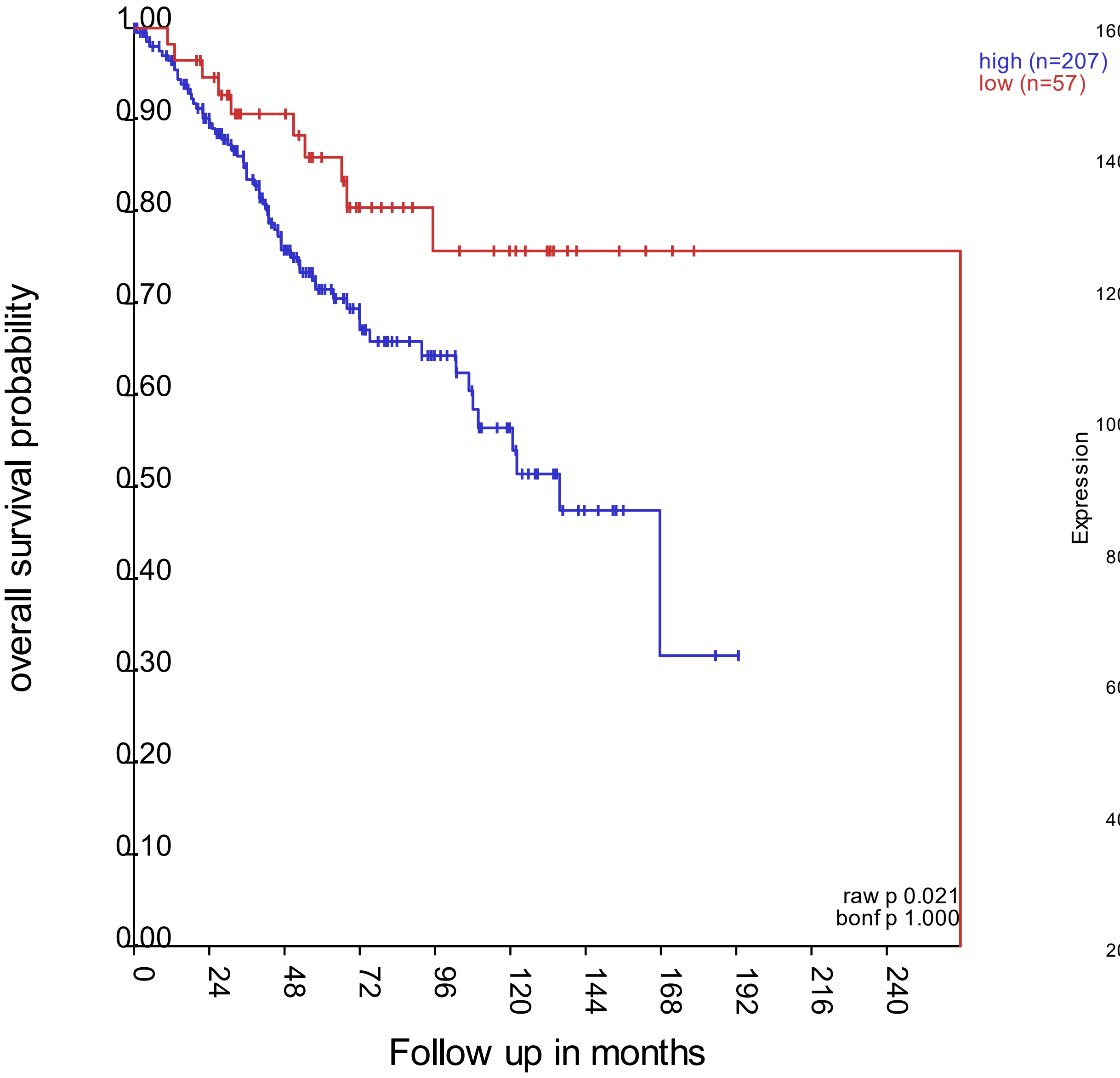

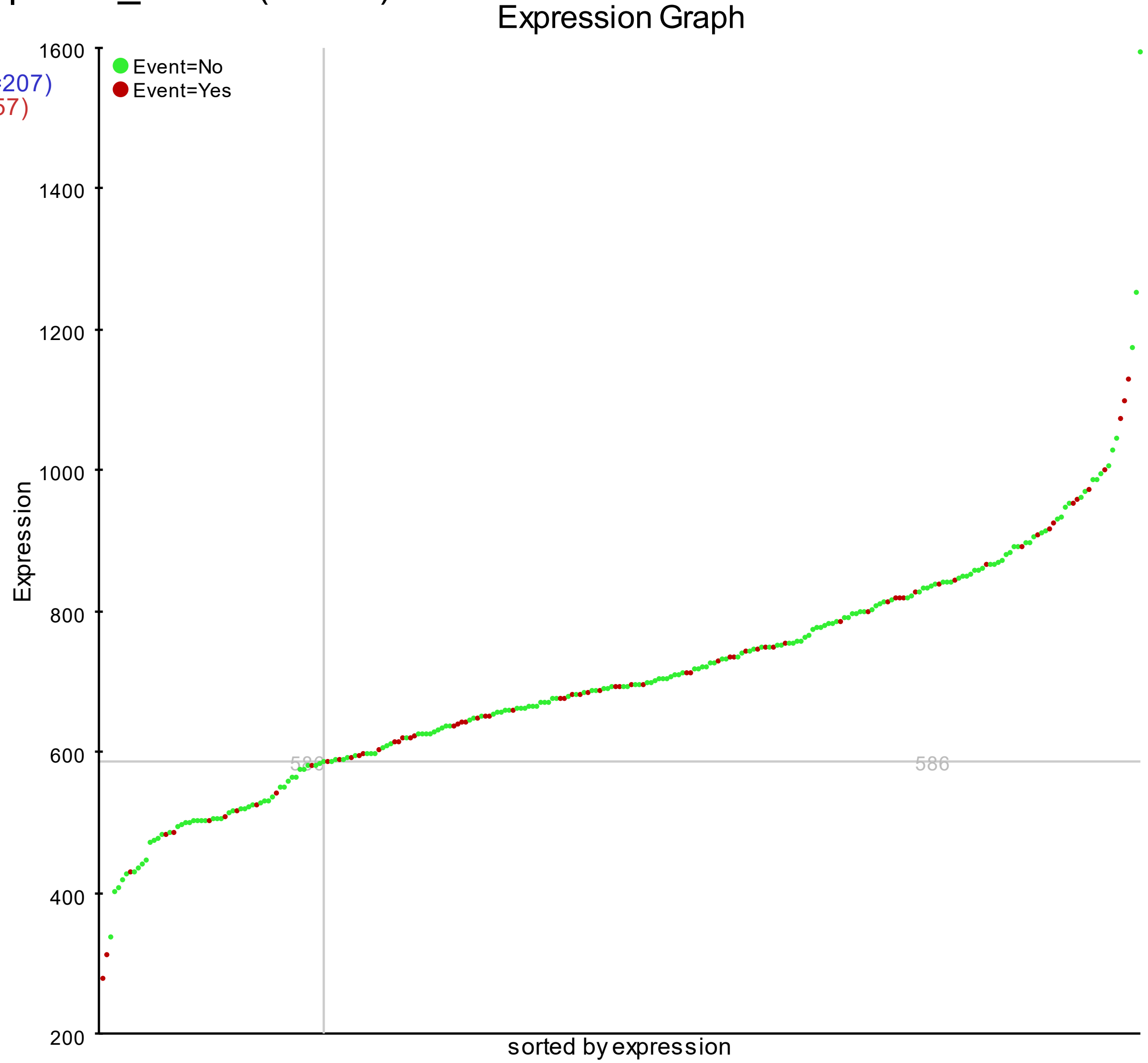

GR3

Tumor Medulloblastoma
Cavalli - 763 - rma_sketch - hugene11t
ABL1 (8158725)
Expression cutoff: 500.800 (min.grp=8)
subgroup~group3|WITH_SURV (n=113)

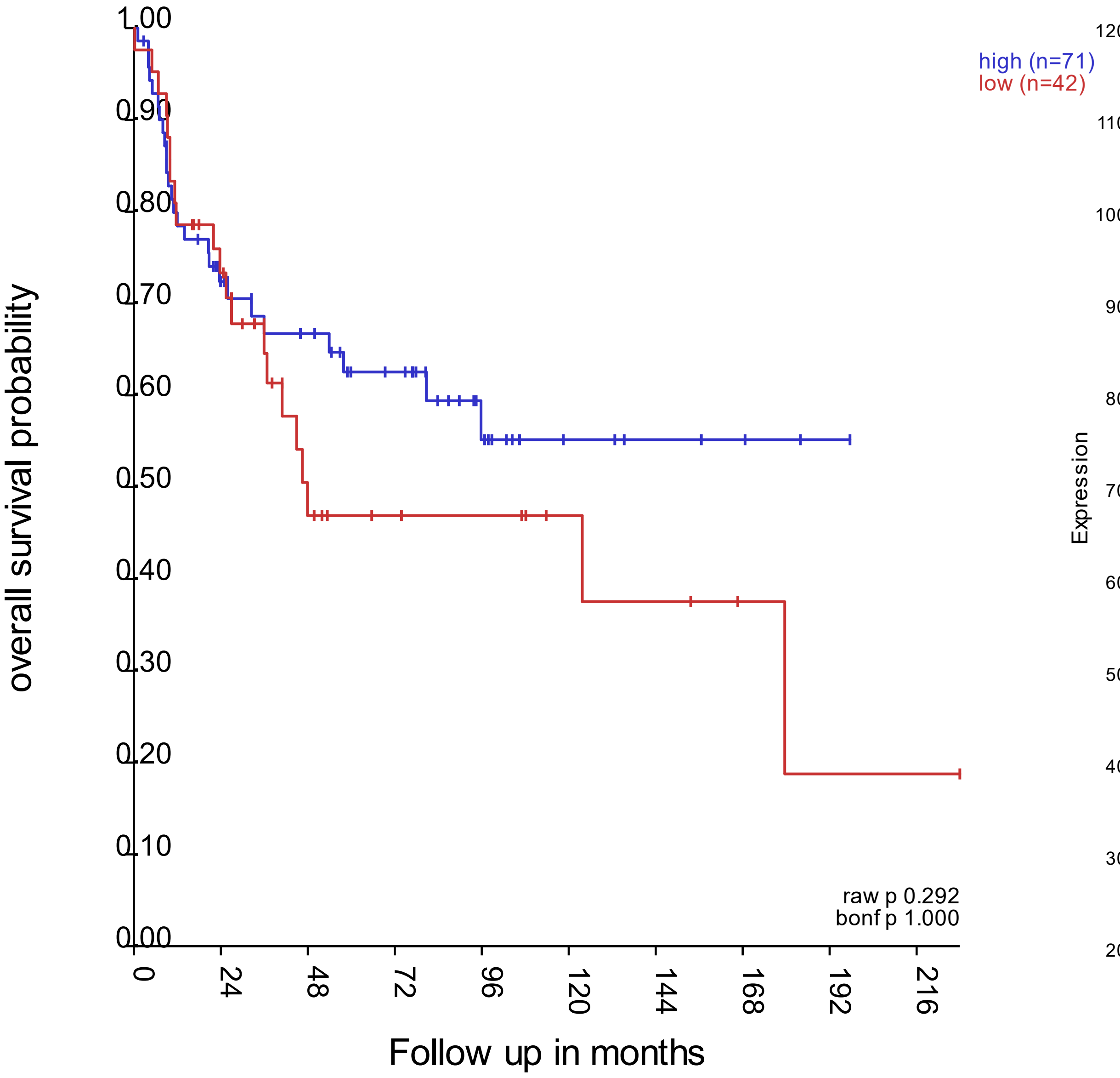

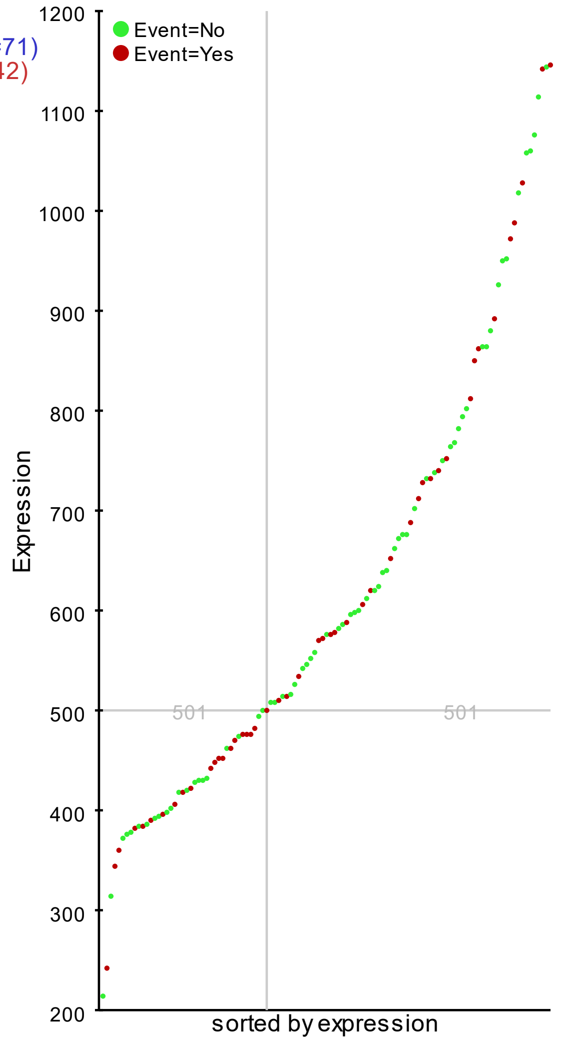

SRC

WNT

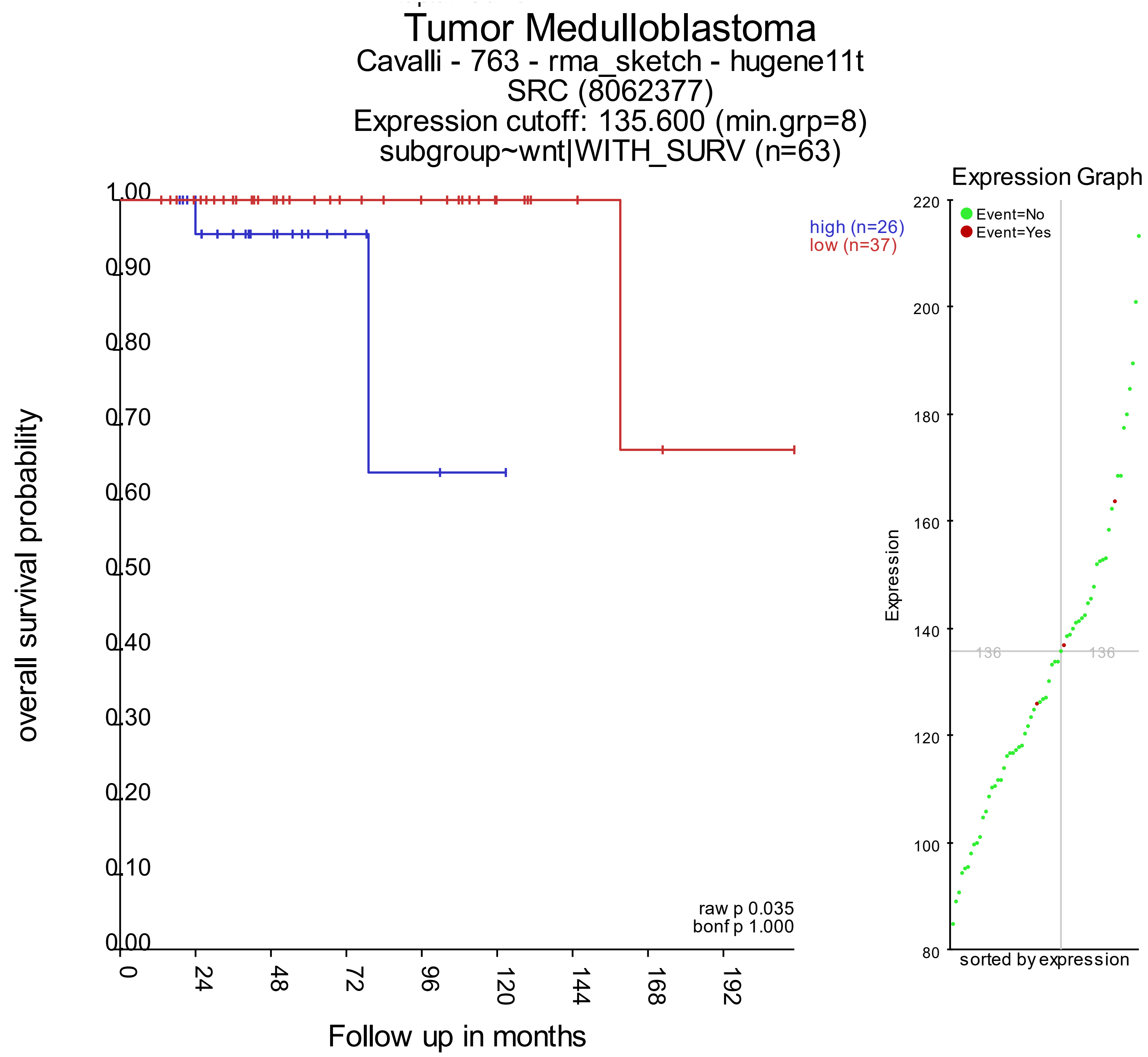
Tumor Medulloblastoma
Cavalli - 763 - rma_sketch - hugene11t
SRC (8062377)
Expression cutoff: 135.600 (min.grp=8)
subgroup~wnt|WITH_SURV (n=63)
overall survival probability
1.00
0.90
0.80
0.70
0.60
0.50
0.40
0.30
0.20
0.10
0.00
0
24
48
72
96
120
144
168
192
Follow up in months
high (n=26)
low (n=37)
raw p 0.035
bonf p 1.000
Expression Graph
Event=No
Event=Yes
Expression
220
200
180
160
140
120
100
80
136
136
sorted by expression

SHH

Tumor Medulloblastoma
Cavalli - 763 - rma_sketch - hugene11t
SRC (8062377)
Expression cutoff: 81.200 (min.grp=8)
subgroup~shh|WITH_SURV (n=172)

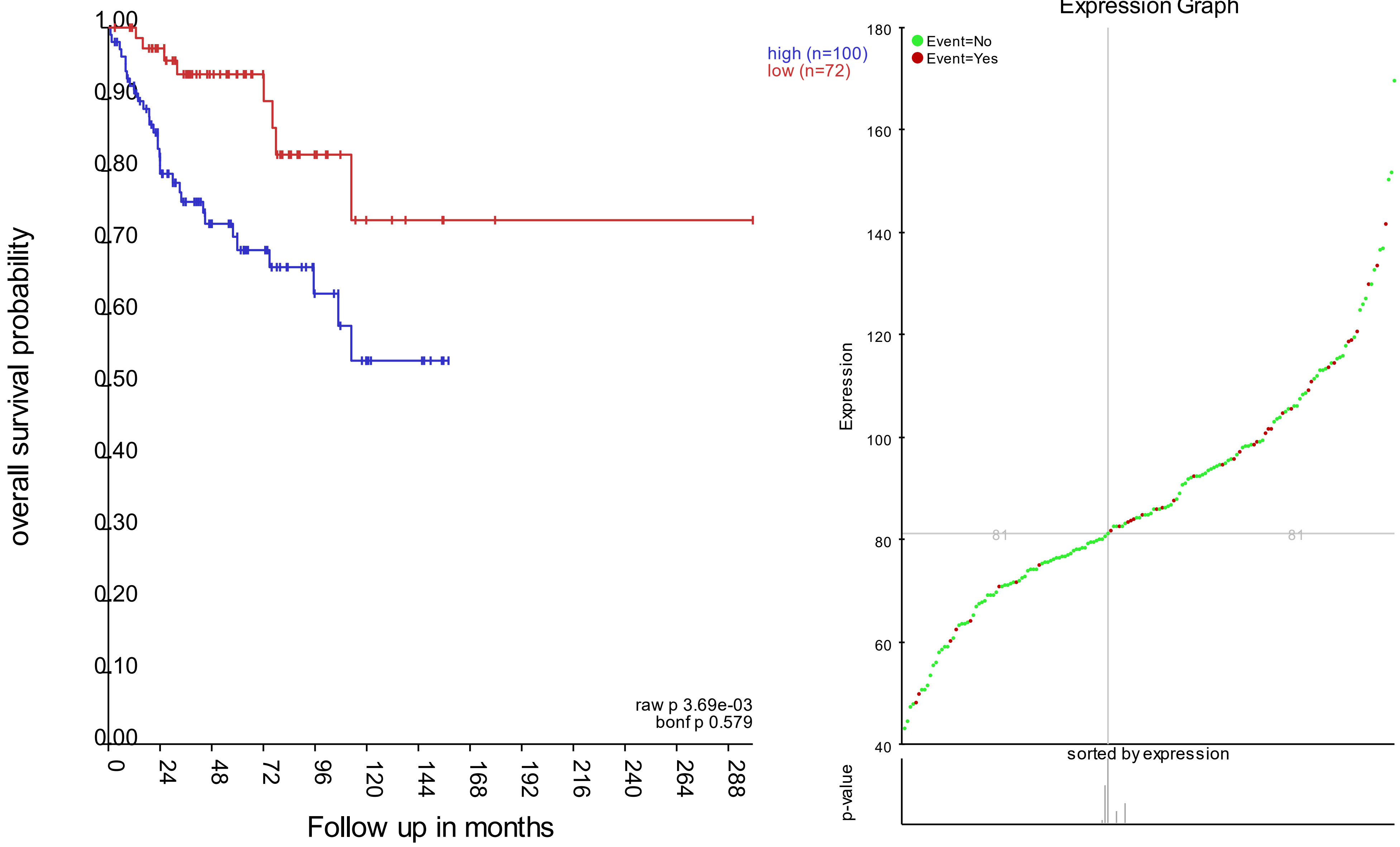

GR4

Tumor Medulloblastoma

Cavalli - 763 - rma_sketch - hugene11t
SRC (8062377)
Expression cutoff: 240.500 (min.grp=8)
subgroup~group4|WITH_SURV (n=264)

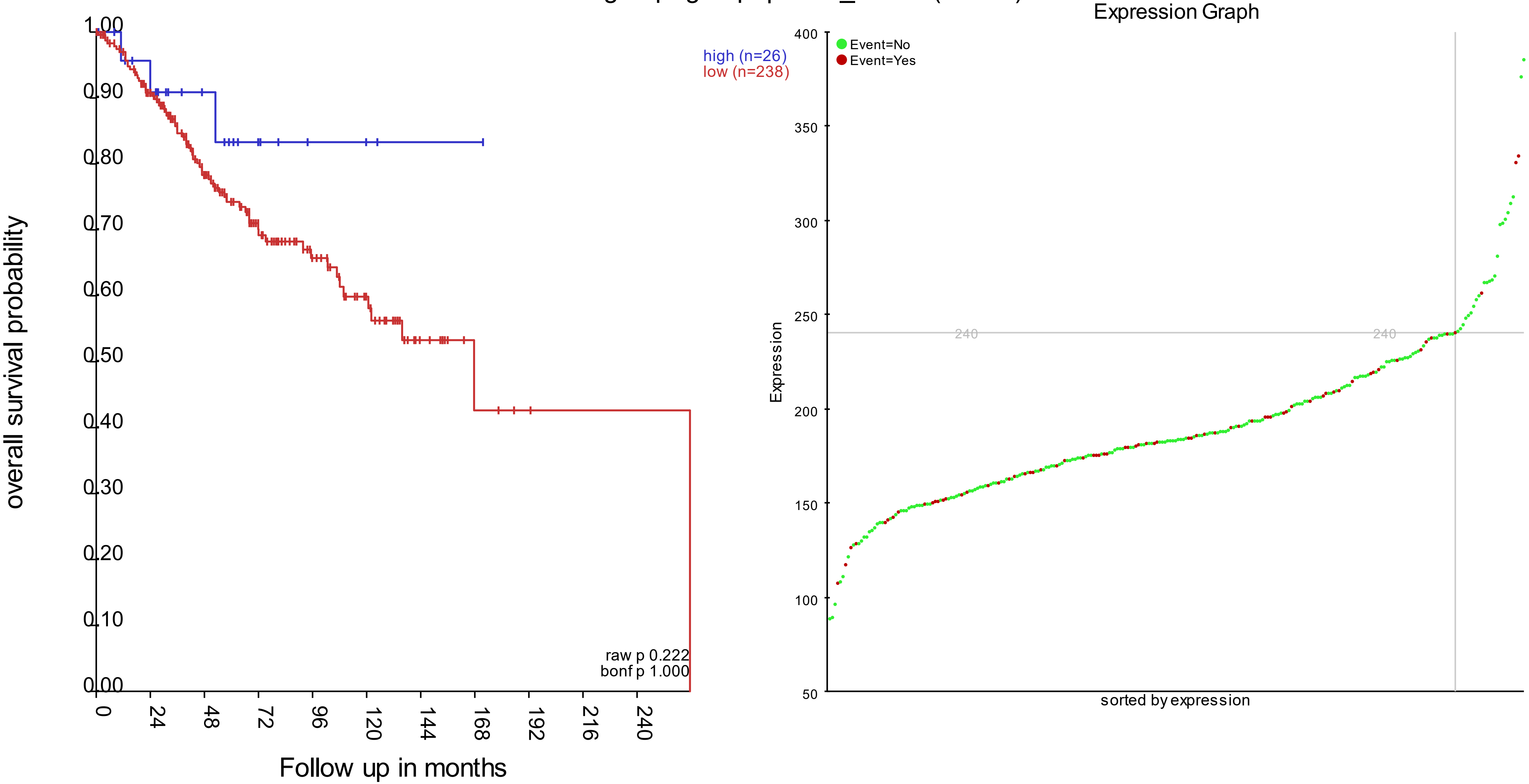

GR3

Tumor Medulloblastoma
Cavalli - 763 - rma_sketch - hugene11t
SRC (8062377)
Expression cutoff: 126.100 (min.grp=8)
subgroup~group3|WITH_SURV (n=113)

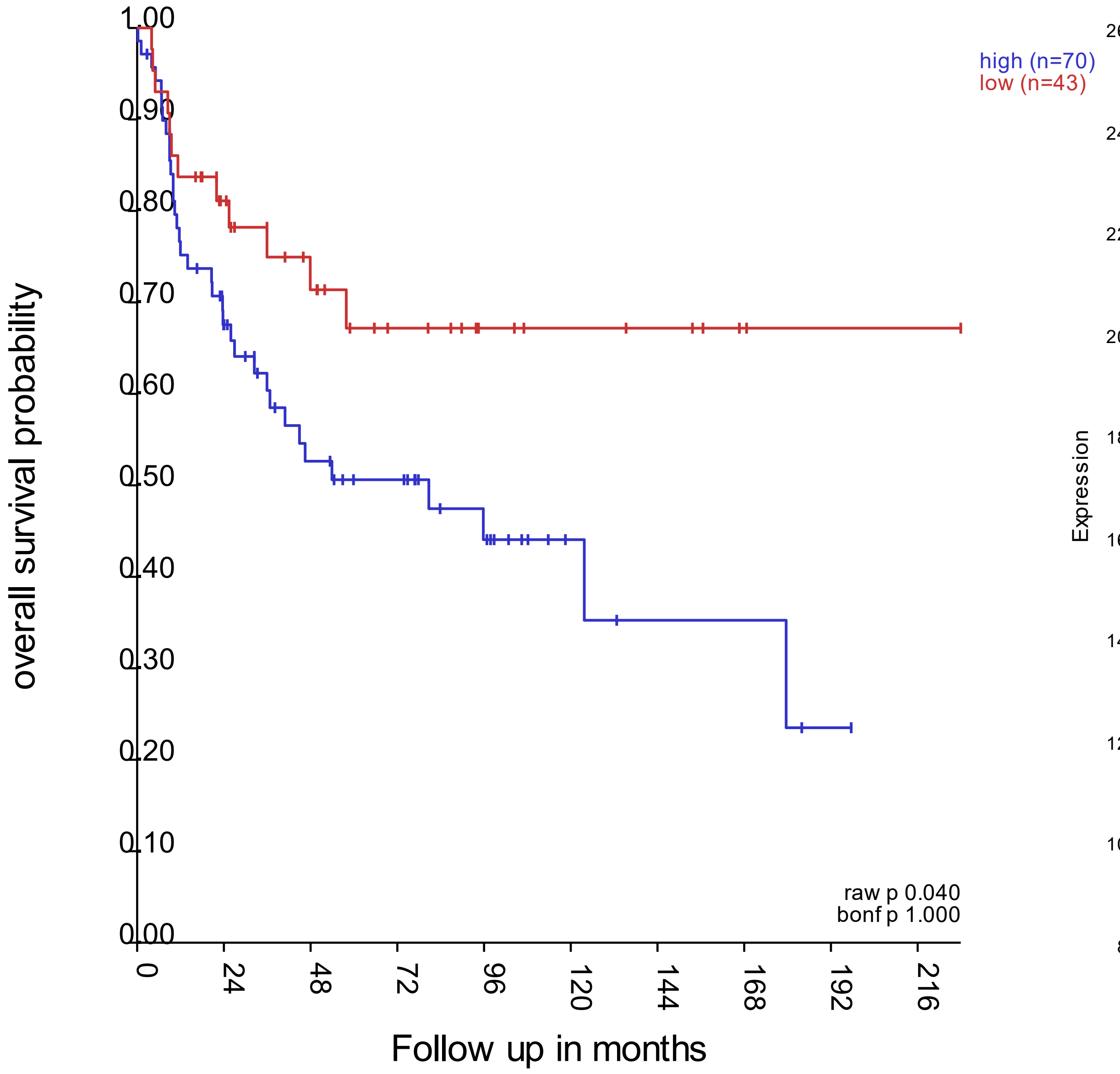

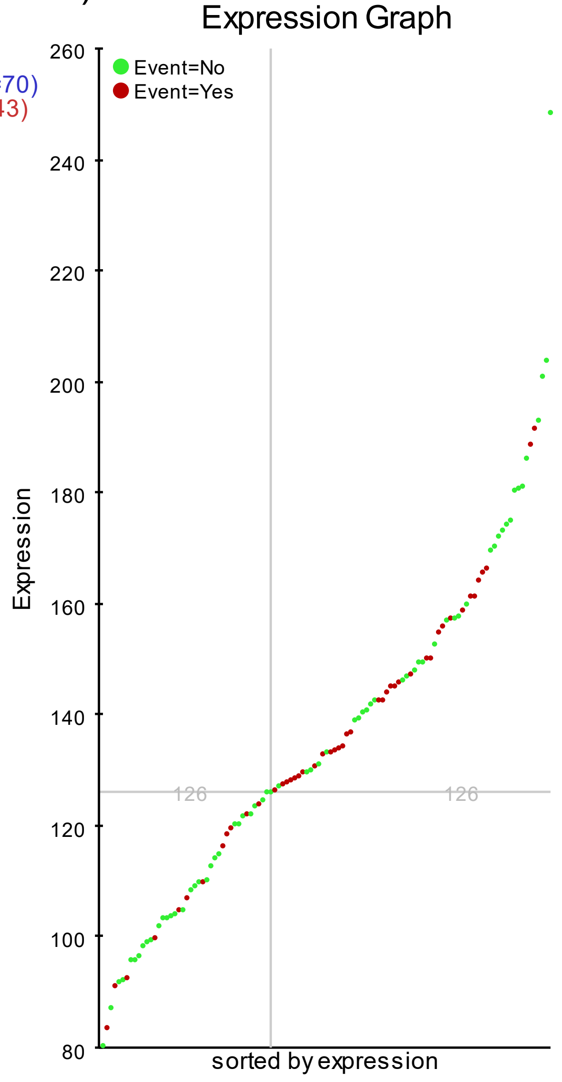

FYN

WNT

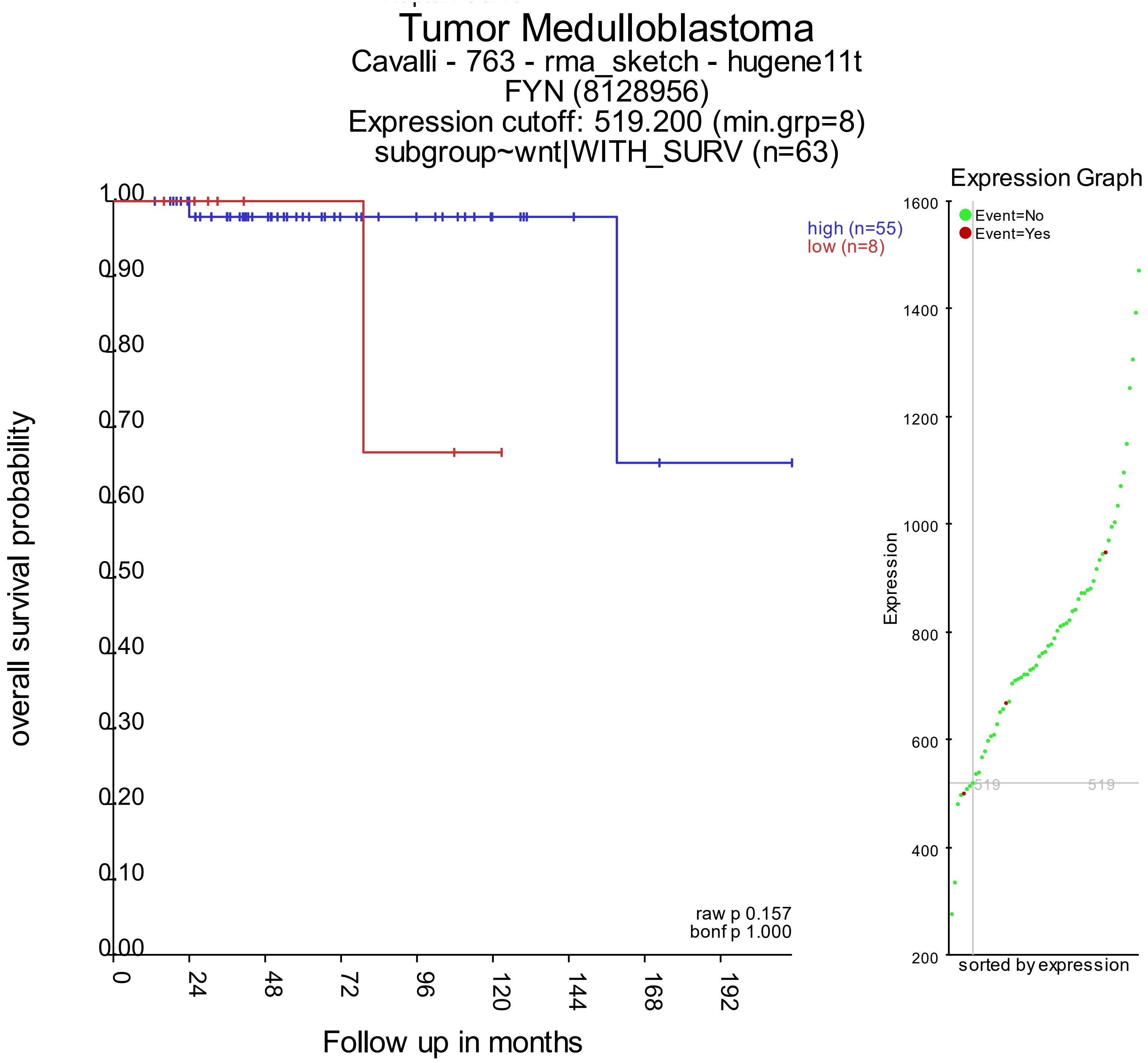

Tumor Medulloblastoma
Cavalli - 763 - rma_sketch - hugene11t
FYN (8128956)
Expression cutoff: 519.200 (min.grp=8)
subgroup~wnt|WITH_SURV (n=63)
overall survival probability
1.00
0.90
0.80
0.70
0.60
0.50
0.40
0.30
0.20
0.10
0.00
0
24
48
72
96
120
144
168
192
Follow up in months
raw p 0.157
bonf p 1.000
high (n=55)
low (n=8)
Expression Graph
Event=No
Event=Yes
Expression
1600
1400
1200
1000
800
600
400
200
519
519
sorted by expression

SHH

Tumor Medulloblastoma
Cavalli - 763 - rma_sketch - hugene11t
FYN (8128956)
Expression cutoff: 885.200 (min.grp=8)
subgroup~shh|WITH_SURV (n=172)

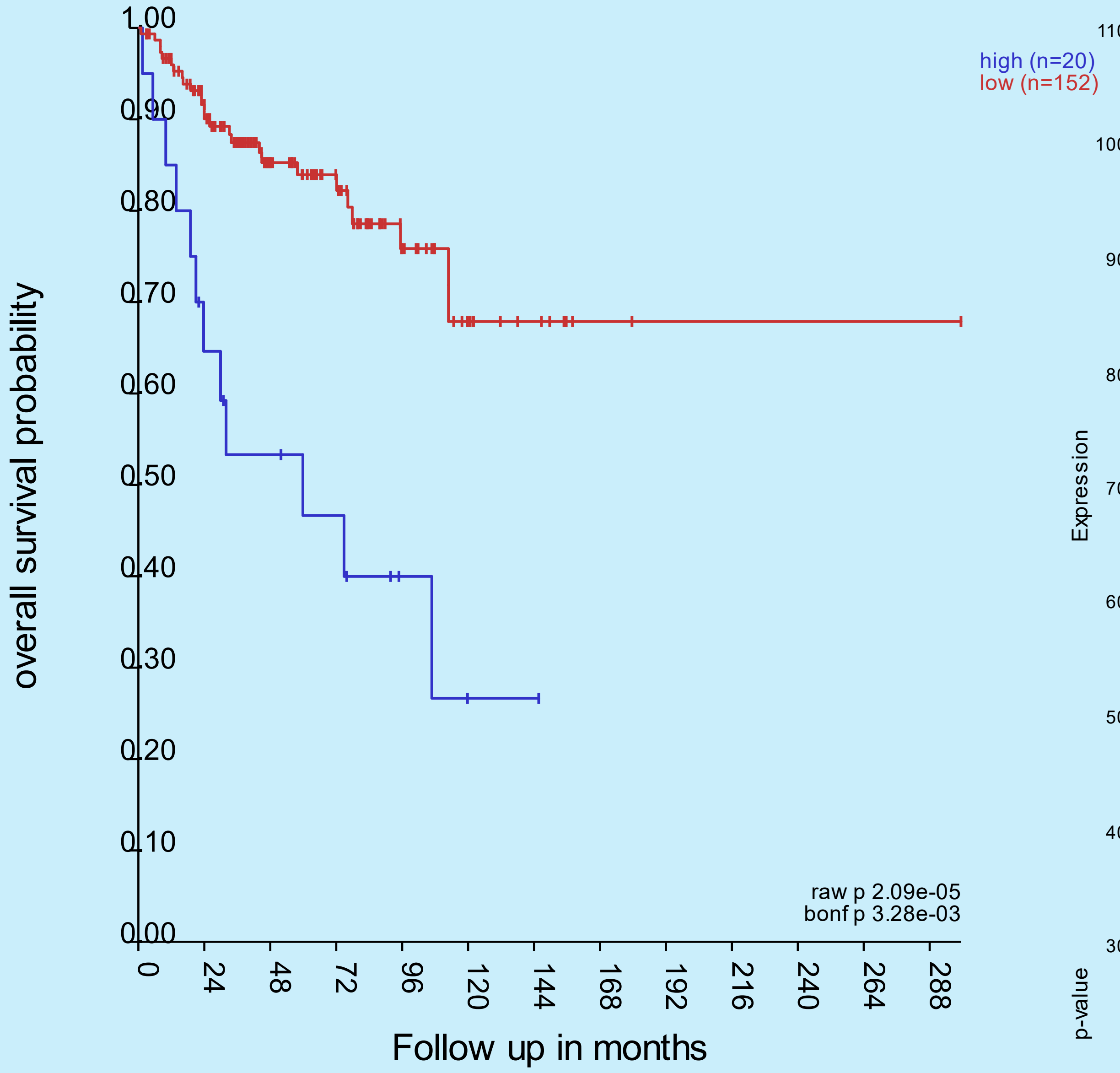

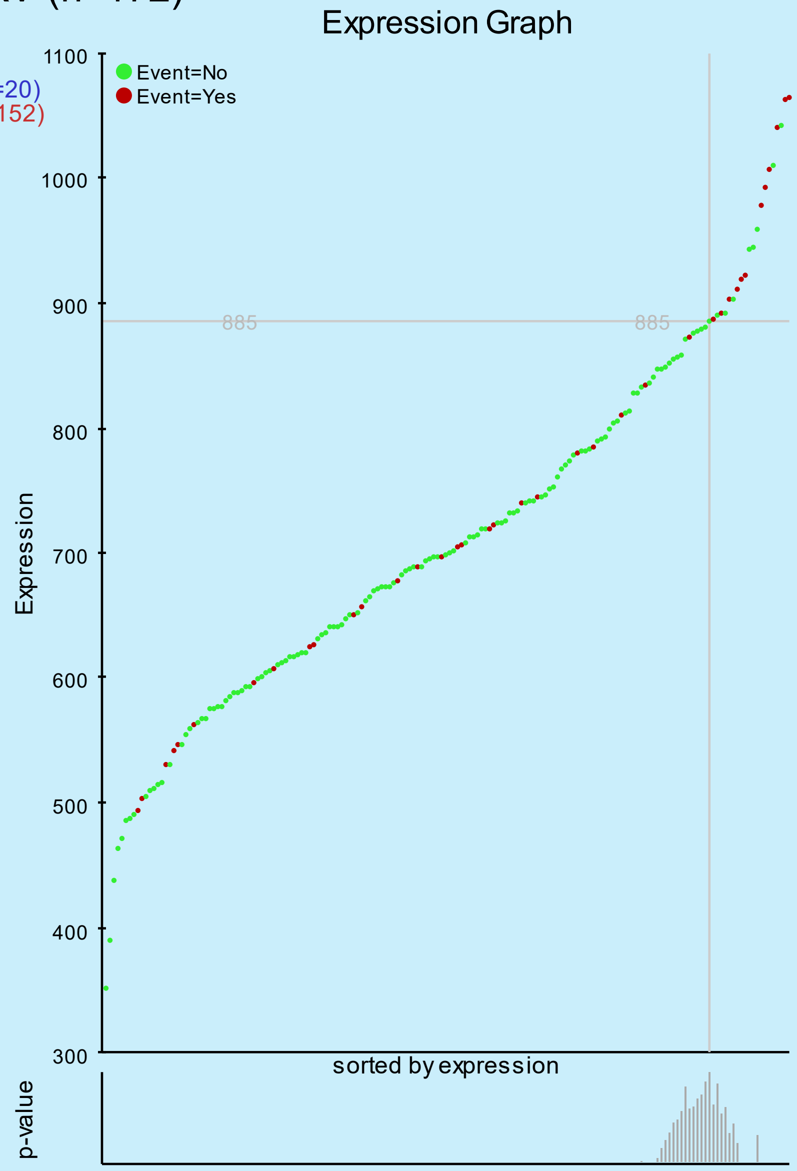

GR4

Tumor Medulloblastoma
Cavalli - 763 - rma_sketch - hugene11t
FYN (8128956)
Expression cutoff: 1304.700 (min.grp=8)
subgroup~group4|WITH_SURV (n=264)

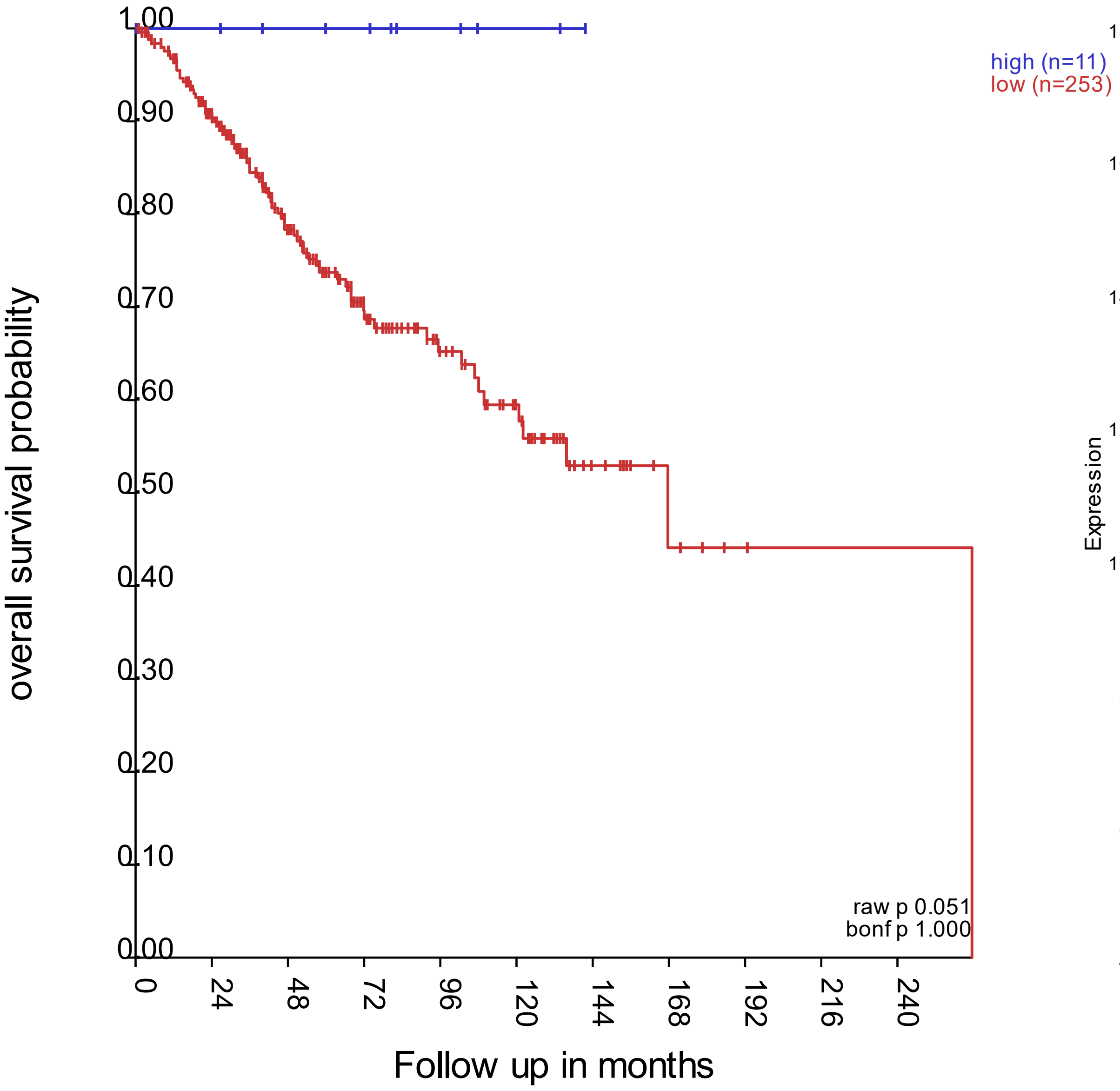

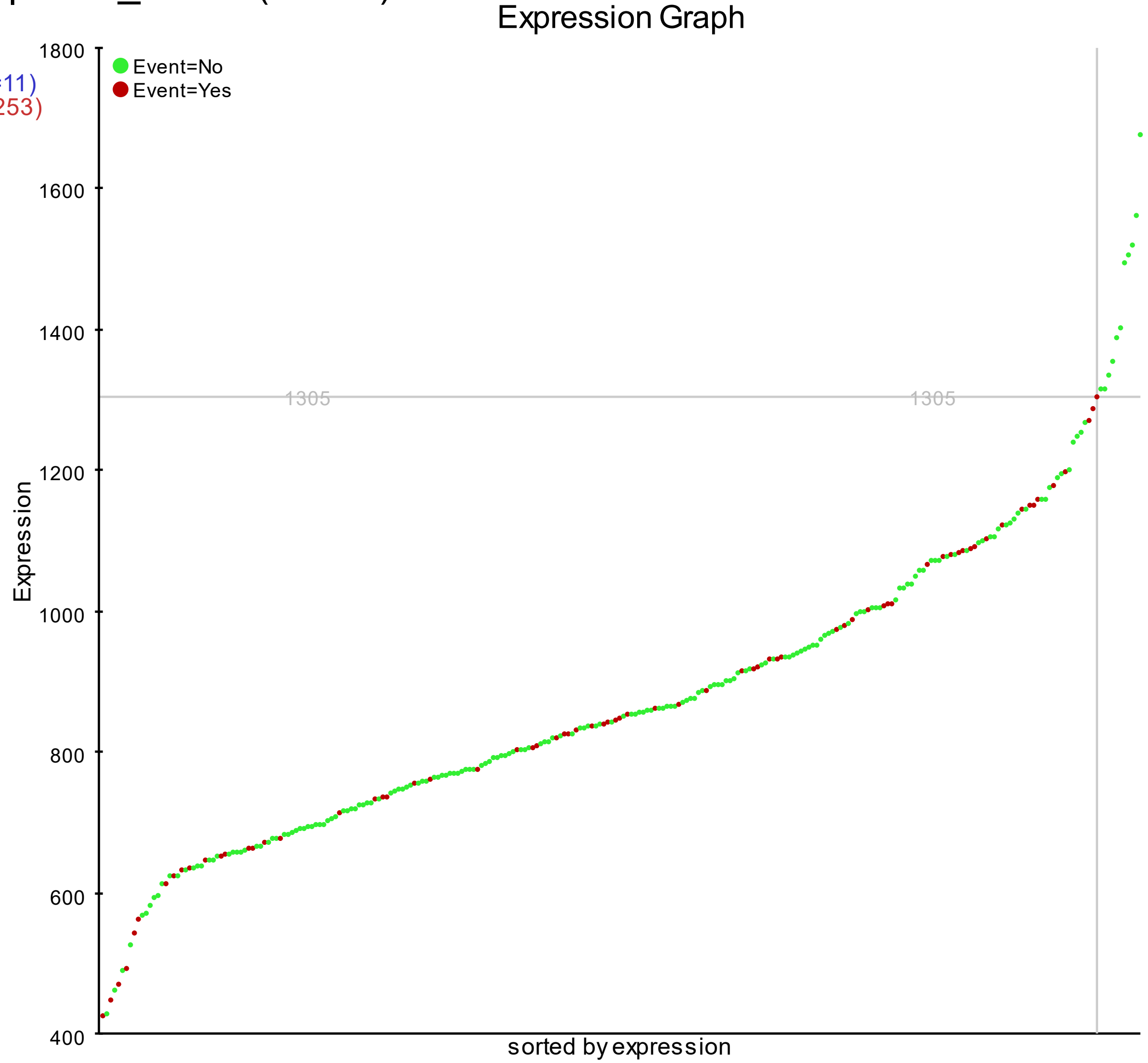

GR3

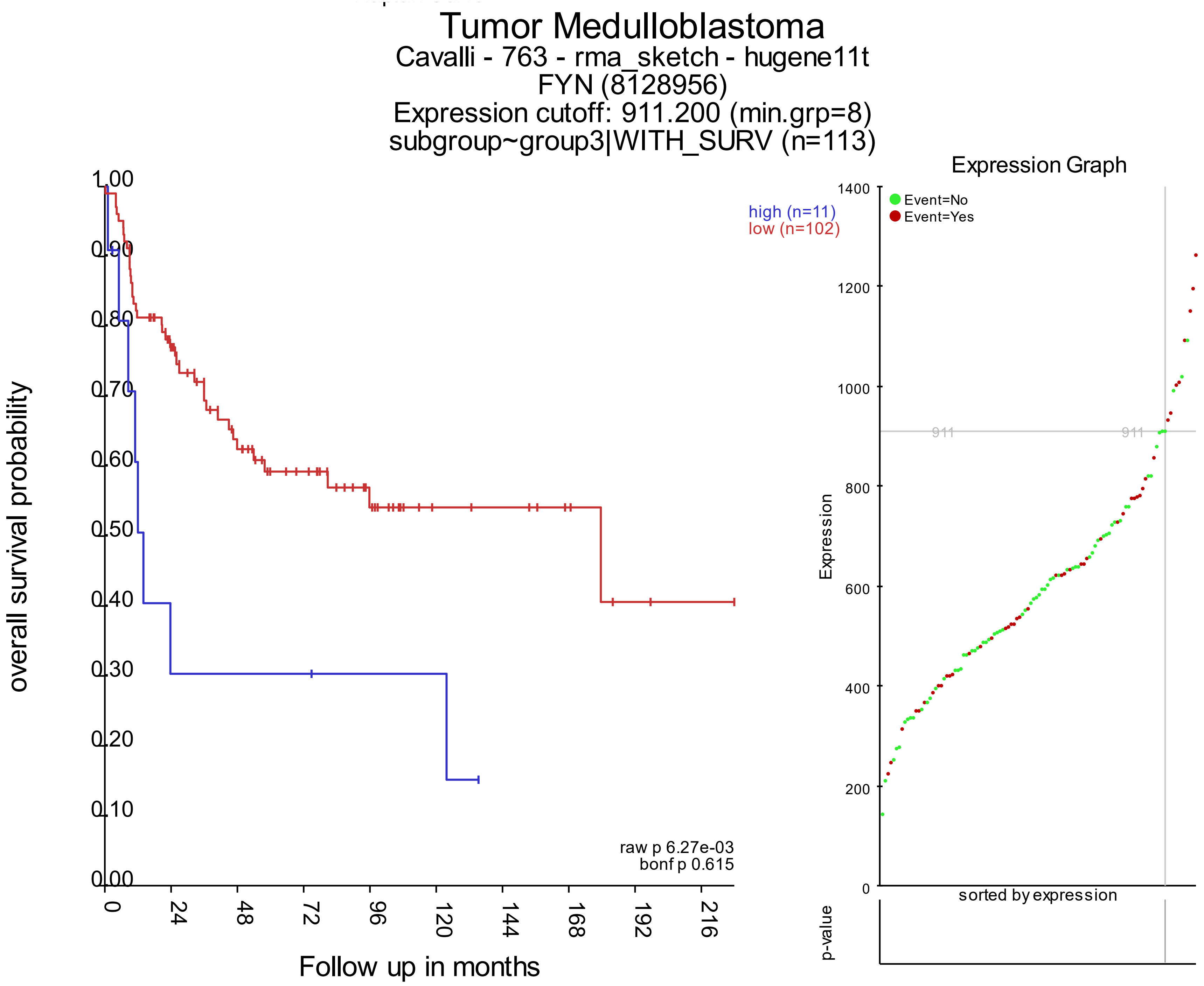

Tumor Medulloblastoma
Cavalli - 763 - rma_sketch - hugene11t
FYN (8128956)
Expression cutoff: 911.200 (min.grp=8)
subgroup~group3|WITH_SURV (n=113)
overall survival probability
1.00
0.90
0.80
0.70
0.60
0.50
0.40
0.30
0.20
0.10
0.00
high (n=11)
low (n=102)
raw p 6.27e-03
bonf p 0.615
0
24
48
72
96
120
144
168
192
216
Follow up in months
Expression Graph
Event=No
Event=Yes
1400
1200
1000
800
600
400
200
0
Expression
911
911
sorted by expression
p-value

YES

WNT

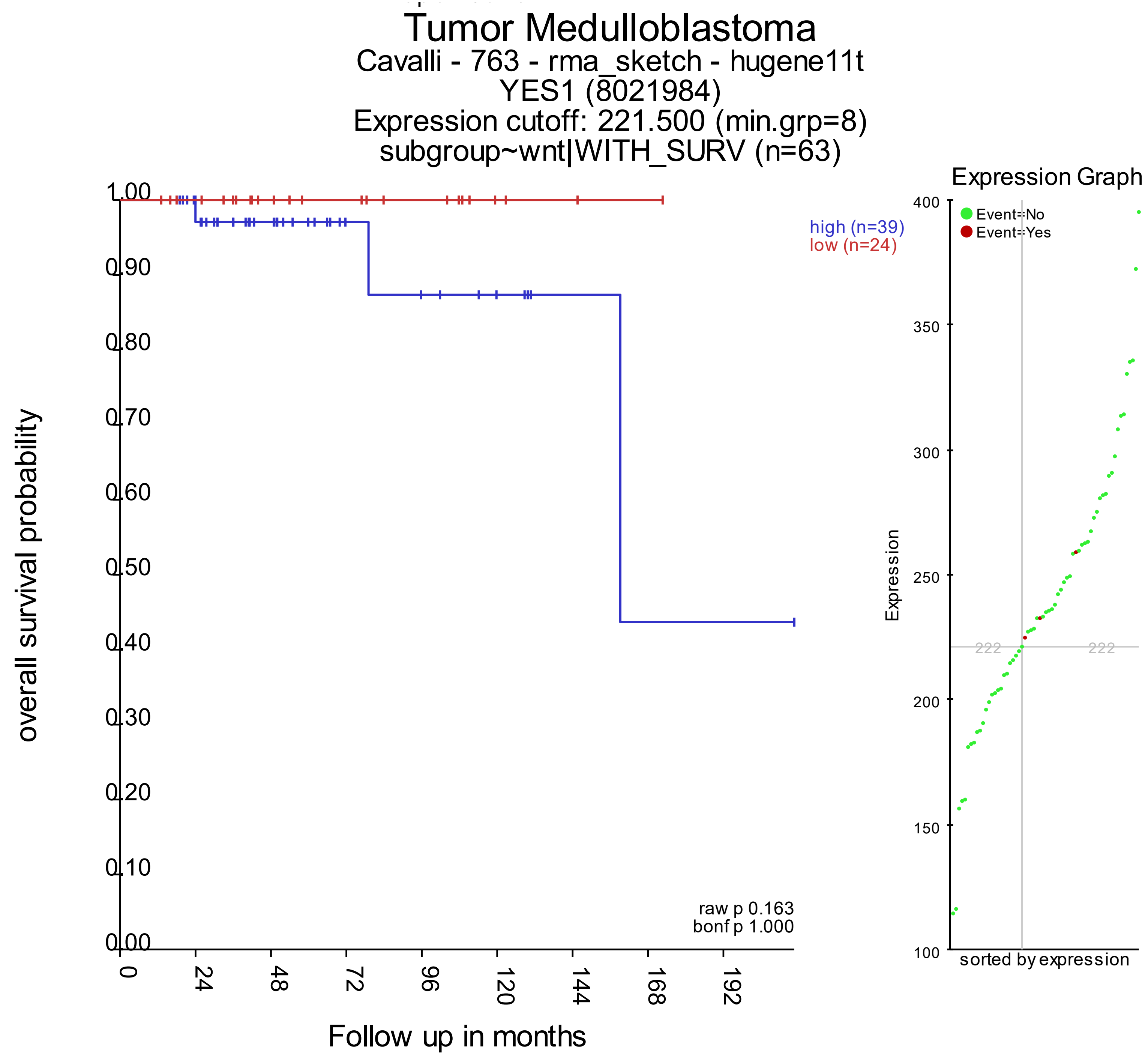
Tumor Medulloblastoma
Cavalli - 763 - rma_sketch - hugene11t
YES1 (8021984)
Expression cutoff: 221.500 (min.grp=8)
subgroup~wnt|WITH_SURV (n=63)
high (n=39)
low (n=24)
overall survival probability
1.00
0.90
0.80
0.70
0.60
0.50
0.40
0.30
0.20
0.10
0.00
raw p 0.163
bonf p 1.000
0
24
48
72
96
120
144
168
192
Follow up in months
Expression Graph
Event=No
Event=Yes
400
350
300
250
200
150
100
Expression
222
222
sorted by expression

SHH

Tumor Medulloblastoma
Cavalli - 763 - rma_sketch - hugene11t
YES1 (8021984)
Expression cutoff: 197.400 (min.grp=8)
subgroup~shh|WITH_SURV (n=172)

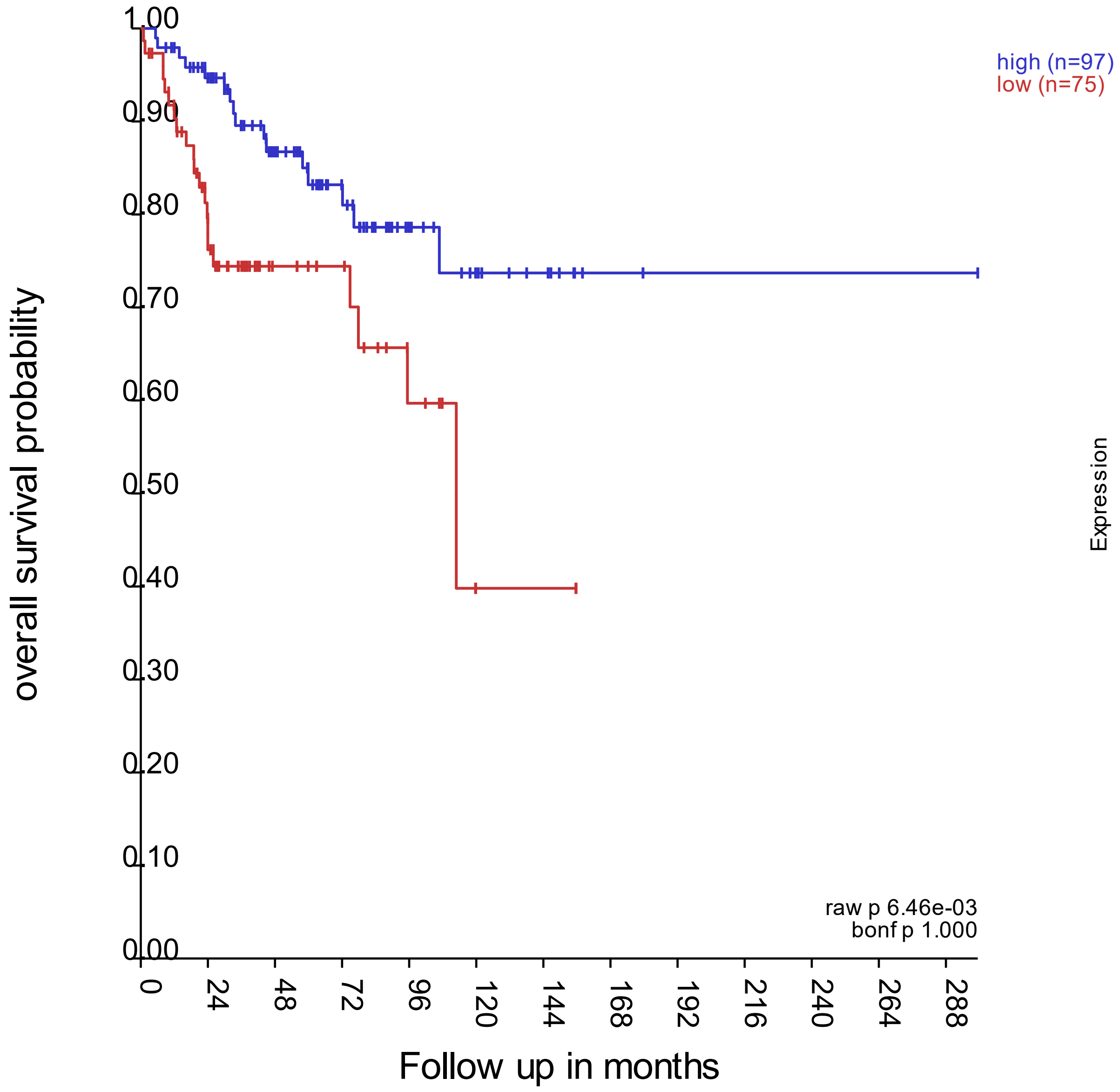

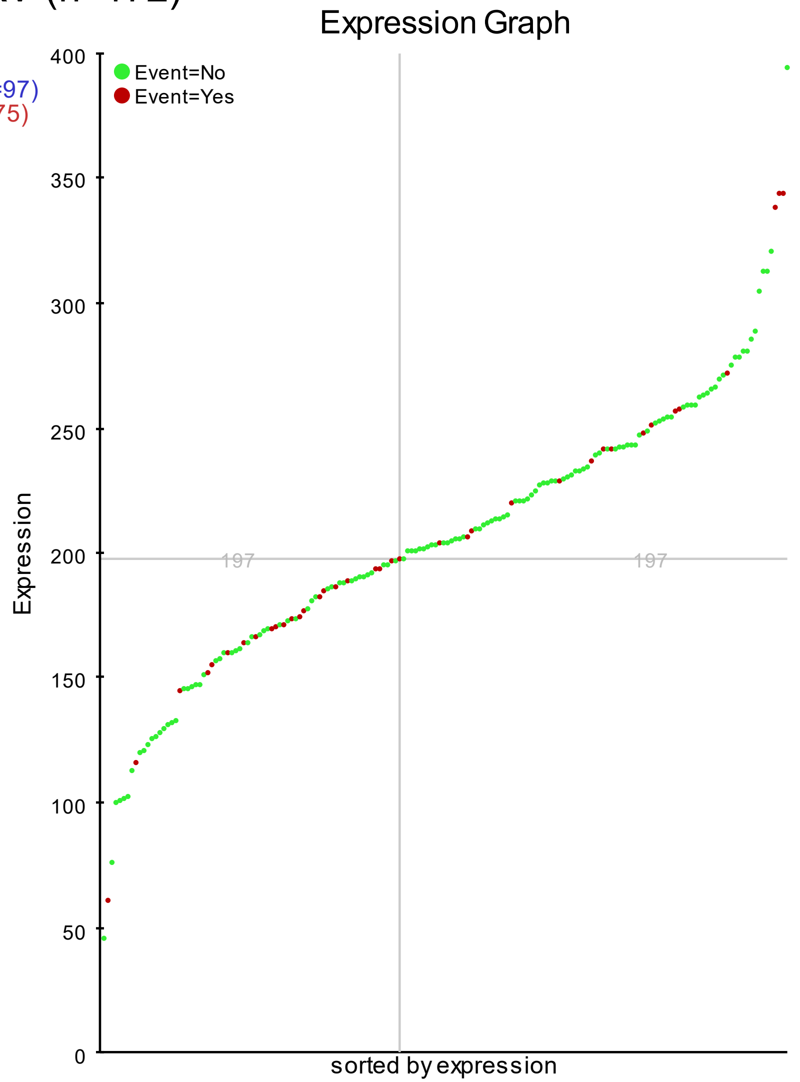

GR4

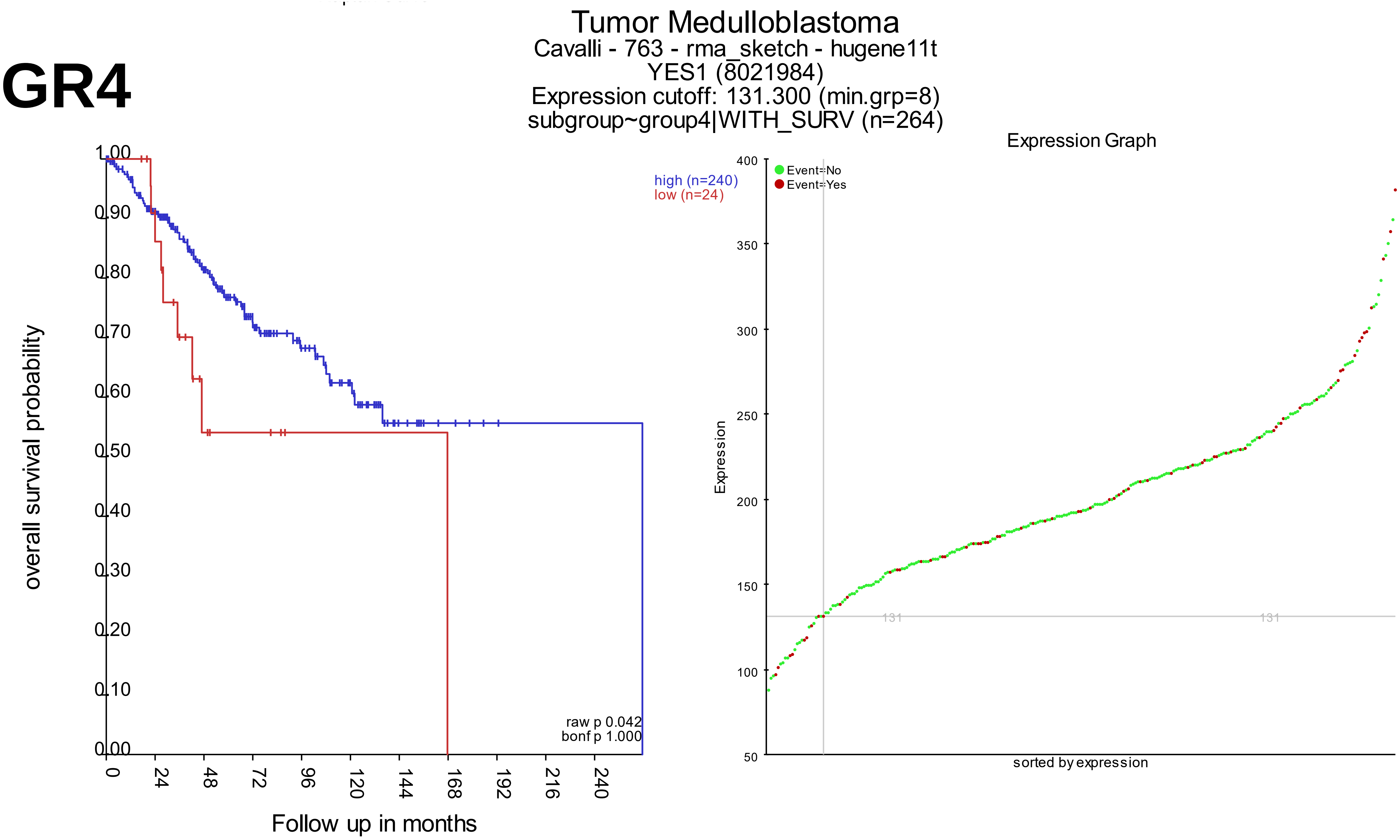

Tumor Medulloblastoma
Cavalli - 763 - rma_sketch - hugene11t
YES1 (8021984)
Expression cutoff: 131.300 (min.grp=8)
subgroup~group4|WITH_SURV (n=264)
high (n=240)
low (n=24)
overall survival probability
1.00
0.90
0.80
0.70
0.60
0.50
0.40
0.30
0.20
0.10
0.00
0
24
48
72
96
120
144
168
192
216
240
Follow up in months
raw p 0.042
bonf p 1.000
Expression Graph
Event=No
Event=Yes
Expression
400
350
300
250
200
150
100
50
131
131
sorted by expression

GR3

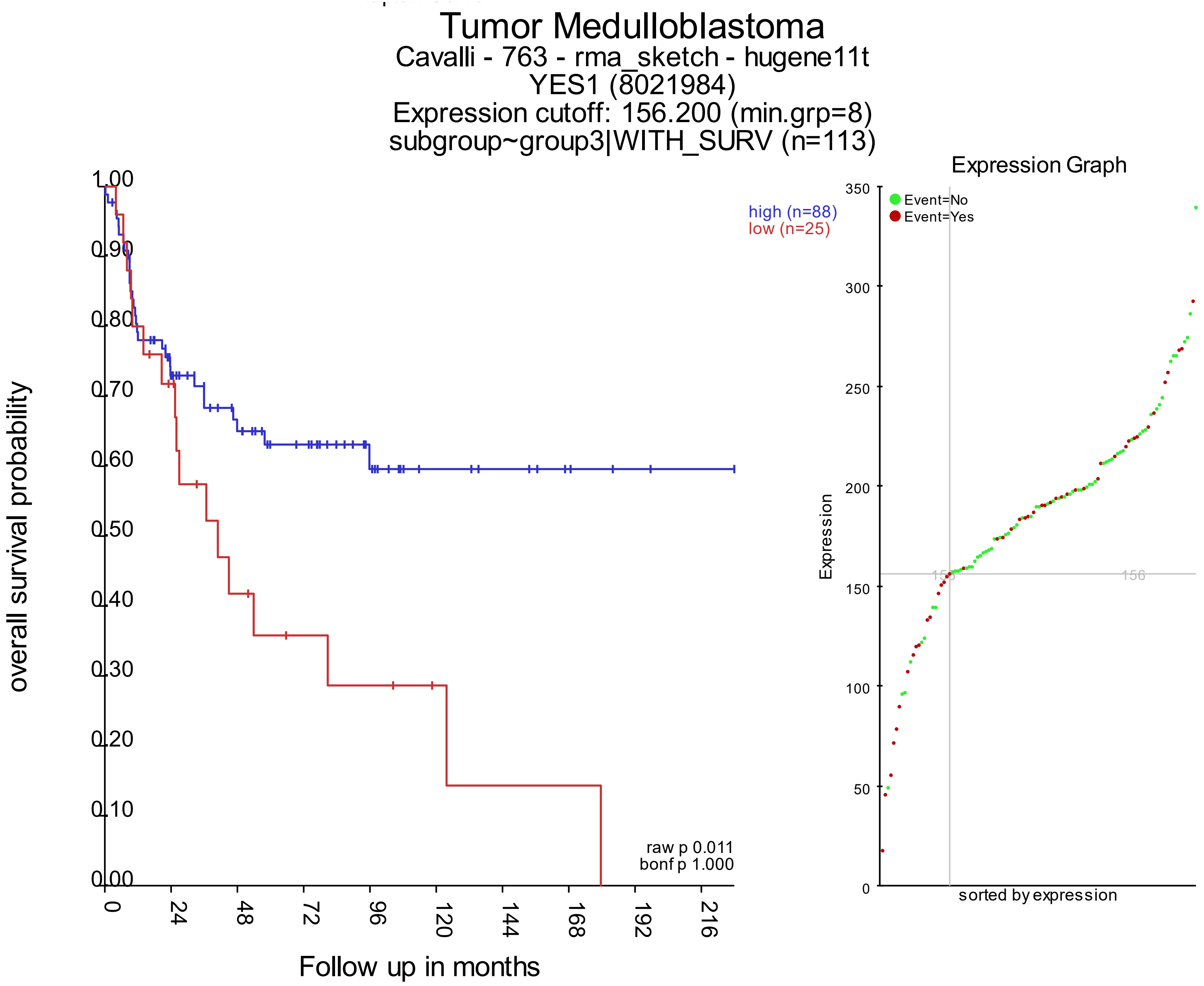

Tumor Medulloblastoma
Cavalli - 763 - rma_sketch - hugene11t
YES1 (8021984)
Expression cutoff: 156.200 (min.grp=8)
subgroup~group3|WITH_SURV (n=113)
overall survival probability
1.00
0.90
0.80
0.70
0.60
0.50
0.40
0.30
0.20
0.10
0.00
high (n=88)
low (n=25)
raw p 0.011
bonf p 1.000
0
24
48
72
96
120
144
168
192
216
Follow up in months
Expression Graph
Event=No
Event=Yes
Expression
350
300
250
200
150
100
50
0
156
sorted by expression

LYN

WNT

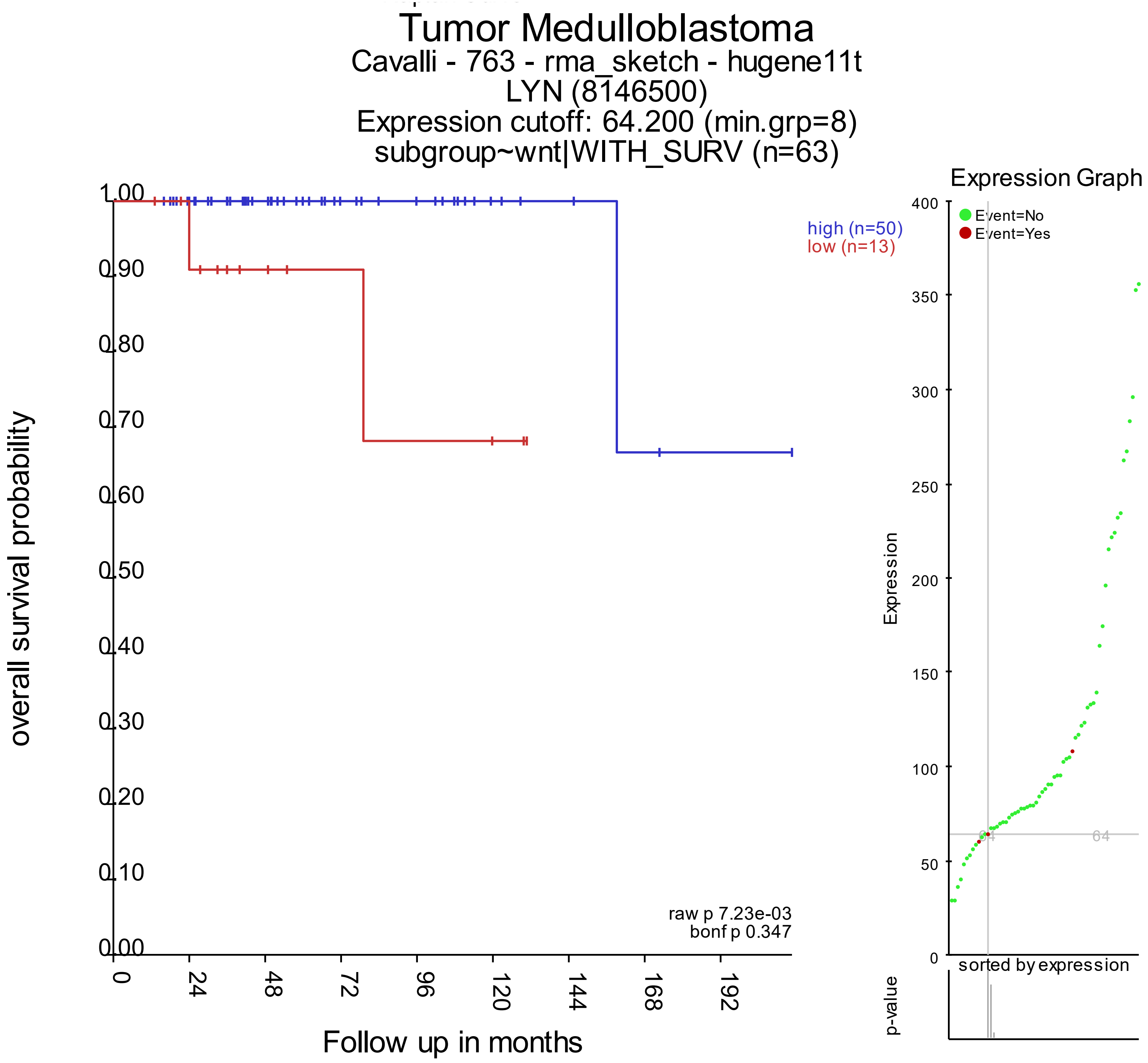

Tumor Medulloblastoma
Cavalli - 763 - rma_sketch - hugene11t
LYN (8146500)
Expression cutoff: 64.200 (min.grp=8)
subgroup~wnt|WITH_SURV (n=63)
overall survival probability
1.00
0.90
0.80
0.70
0.60
0.50
0.40
0.30
0.20
0.10
0.00
0
24
48
72
96
120
144
168
192
Follow up in months
high (n=50)
low (n=13)
raw p 7.23e-03
bonf p 0.347
Expression Graph
Event=No
Event=Yes
Expression
400
350
300
250
200
150
100
50
0
64
64
sorted by expression
p-value

SHH

Tumor Medulloblastoma
Cavalli - 763 - rma_sketch - hugene11t
LYN (8146500)
Expression cutoff: 168.900 (min.grp=8)
subgroup~shh|WITH_SURV (n=172)

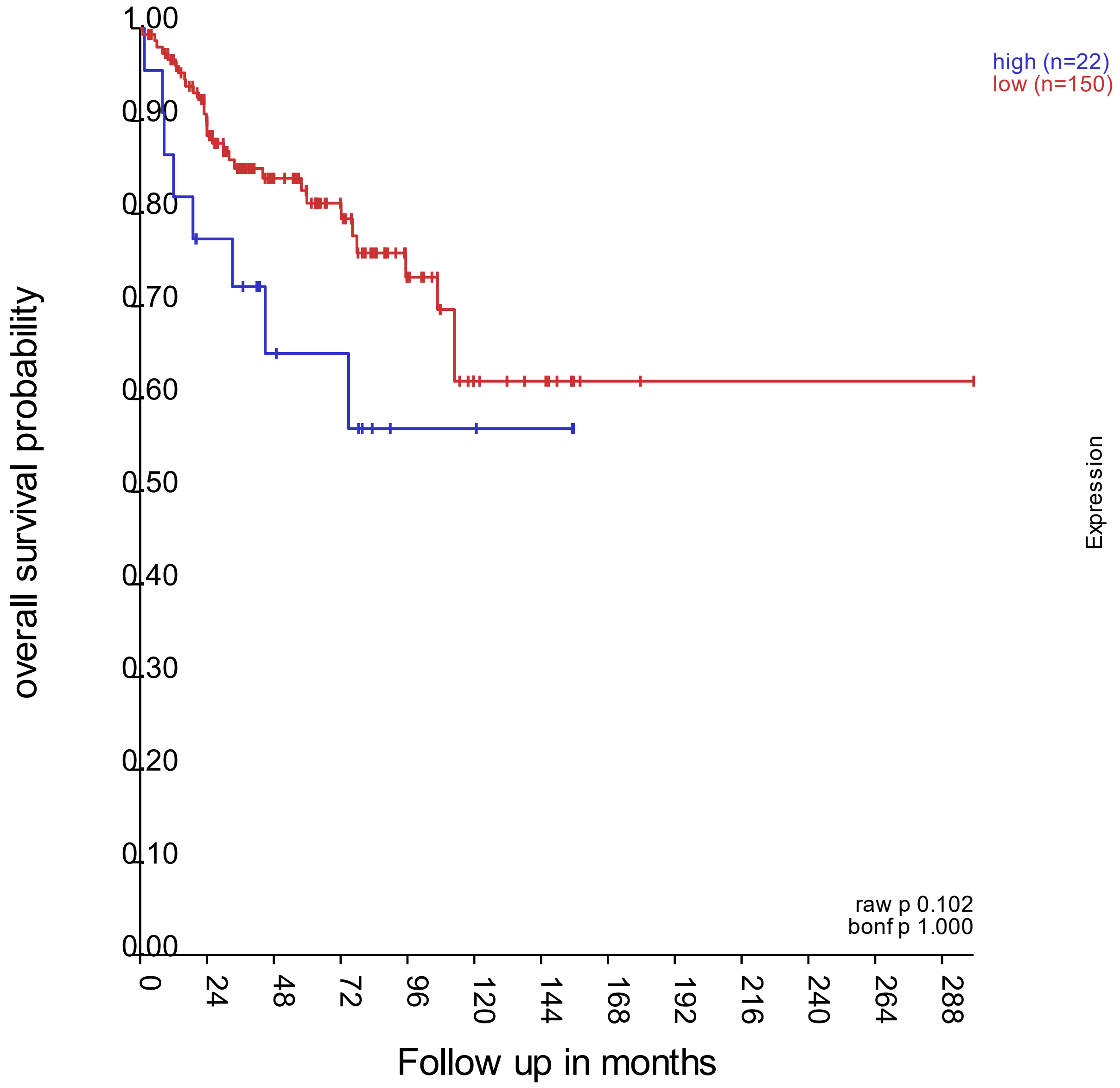

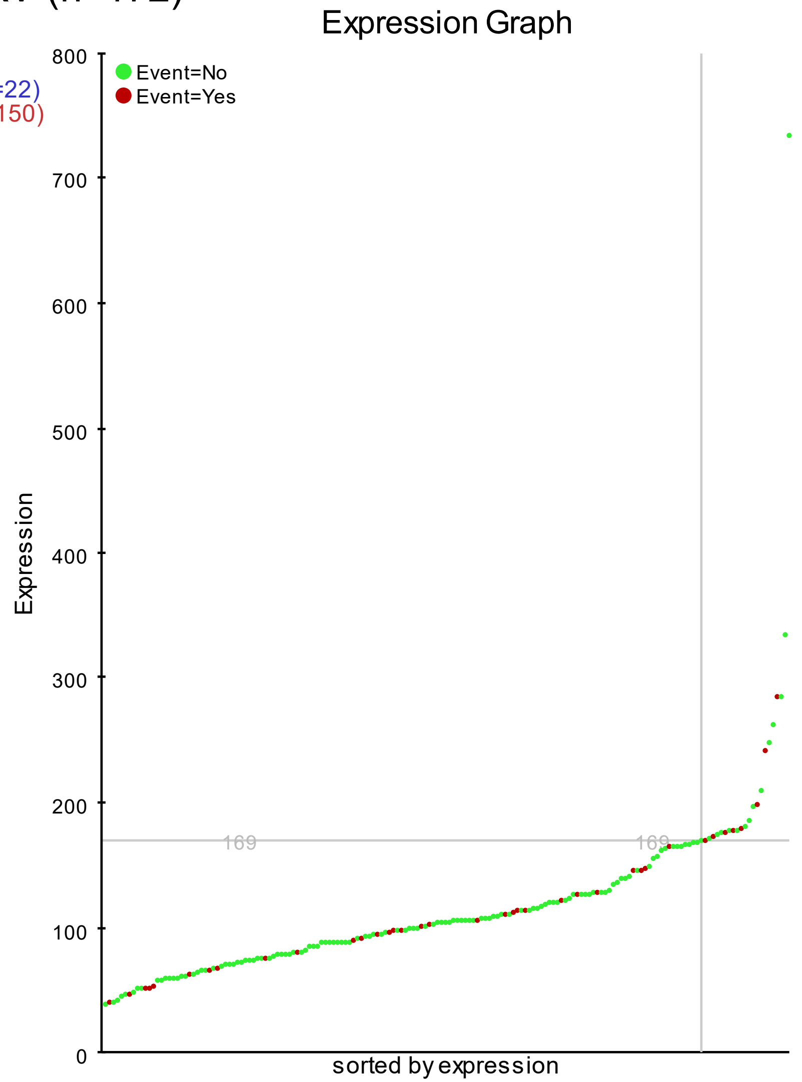

GR4

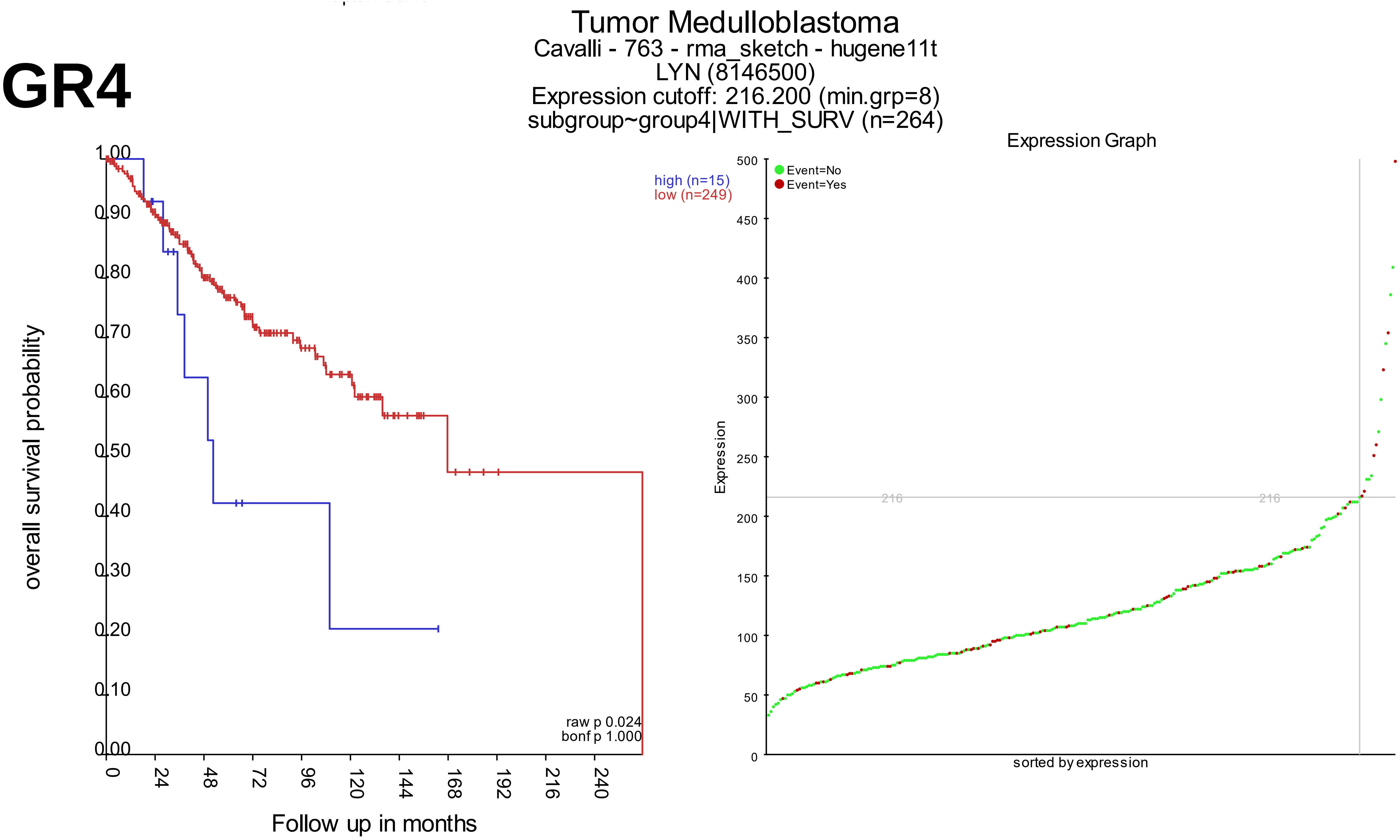

Tumor Medulloblastoma
Cavalli - 763 - rma_sketch - hugene11t
LYN (8146500)
Expression cutoff: 216.200 (min.grp=8)
subgroup~group4|WITH_SURV (n=264)
overall survival probability
1.00
0.90
0.80
0.70
0.60
0.50
0.40
0.30
0.20
0.10
0.00
high (n=15)
low (n=249)
raw p 0.024
bonf p 1.000
0
24
48
72
96
120
144
168
192
216
240
Follow up in months
Expression Graph
Event=No
Event=Yes
Expression
500
450
400
350
300
250
200
150
100
50
0
216
216
sorted by expression

GR3

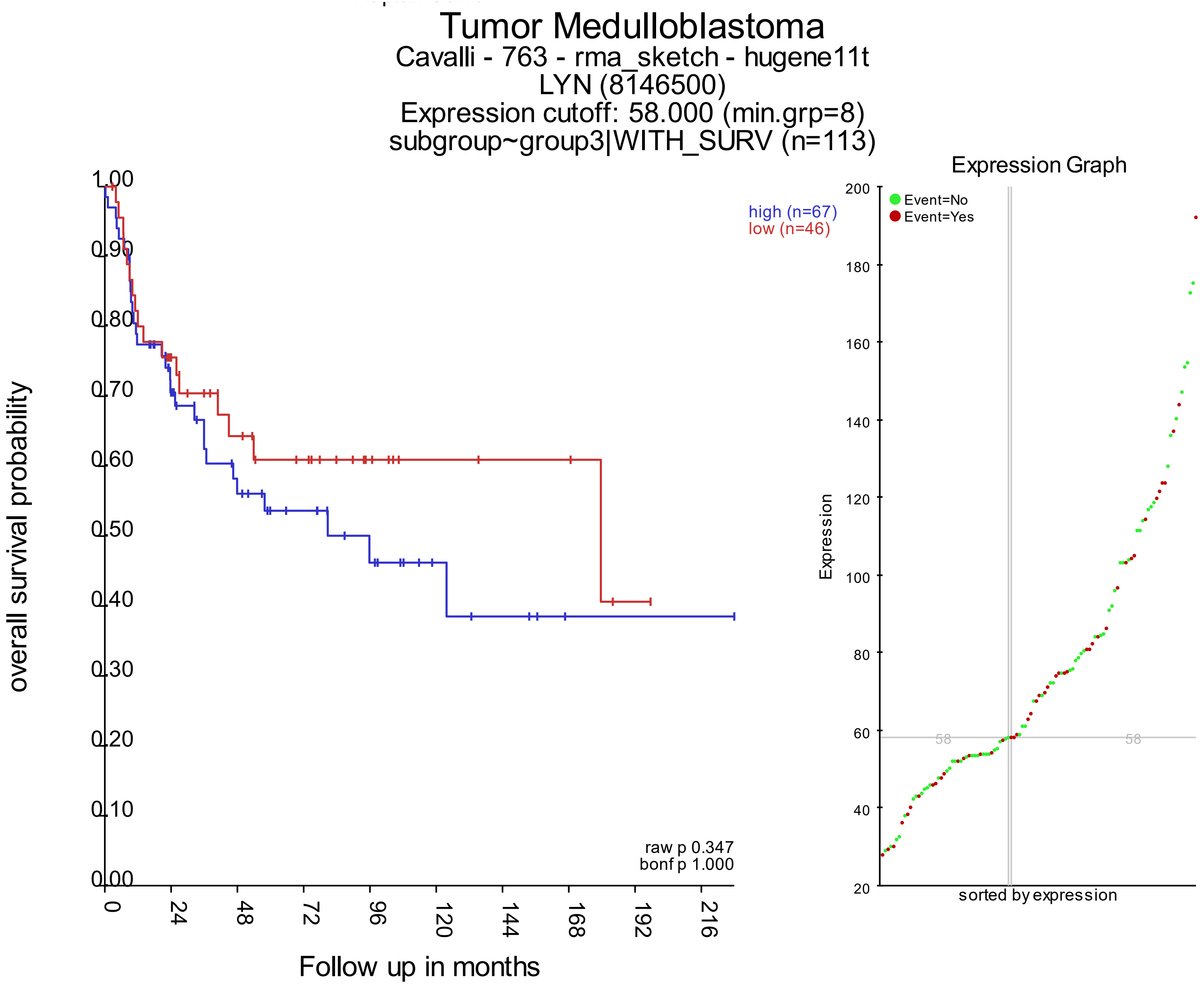

Tumor Medulloblastoma
Cavalli - 763 - rma_sketch - hugene11t
LYN (8146500)
Expression cutoff: 58.000 (min.grp=8)
subgroup~group3|WITH_SURV (n=113)
overall survival probability
1.00
0.90
0.80
0.70
0.60
0.50
0.40
0.30
0.20
0.10
0.00
high (n=67)
low (n=46)
raw p 0.347
bonf p 1.000
0
24
48
72
96
120
144
168
192
216
Follow up in months
Expression Graph
Event=No
Event=Yes
Expression
200
180
160
140
120
100
80
60
40
20
58
58
sorted by expression

FLT1

WNT

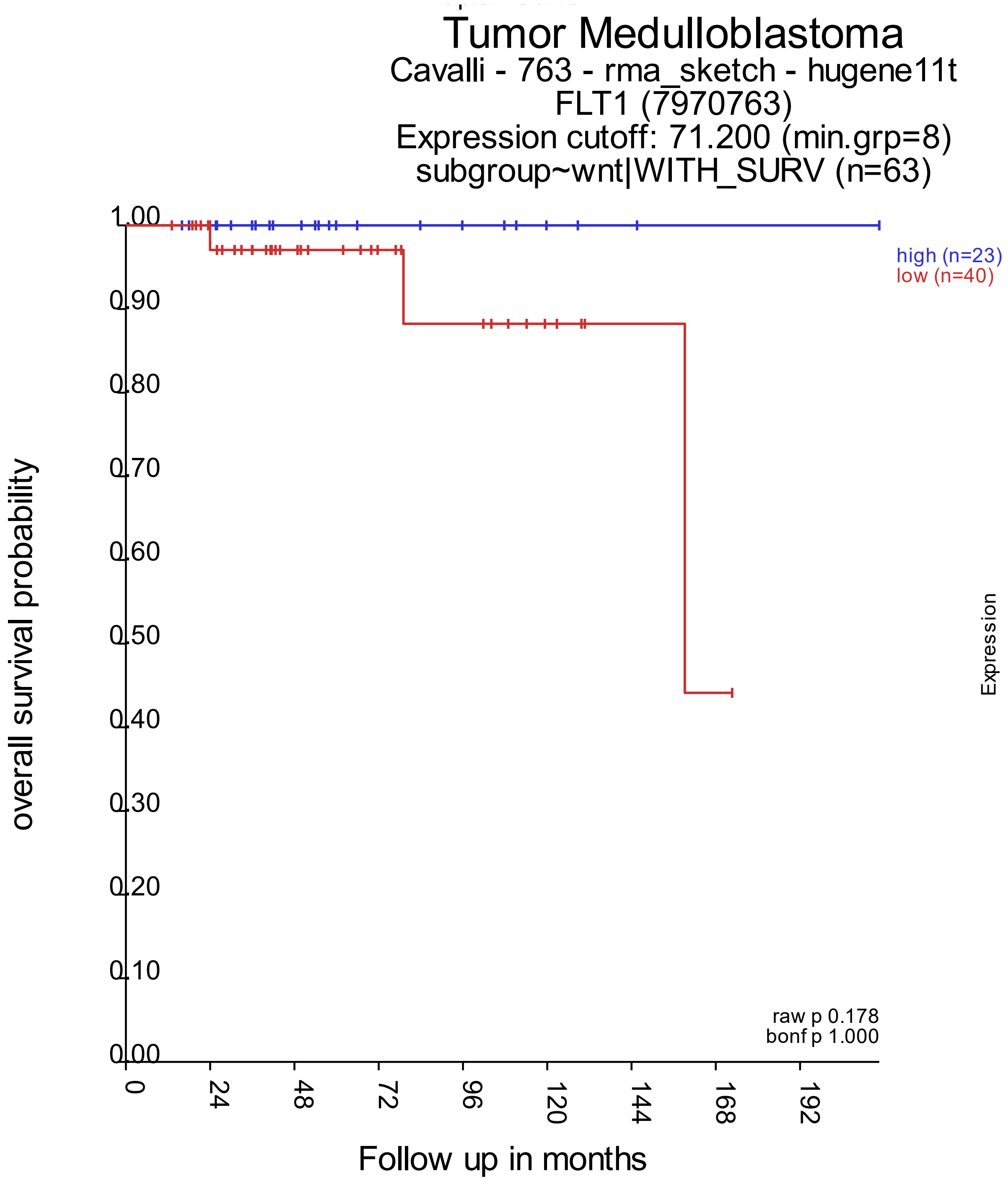
Tumor Medulloblastoma
Cavalli - 763 - rma_sketch - hugene11t
FLT1 (7970763)
Expression cutoff: 71.200 (min.grp=8)
subgroup~wnt|WITH_SURV (n=63)
overall survival probability
1.00
0.90
0.80
0.70
0.60
0.50
0.40
0.30
0.20
0.10
0.00
0
24
48
72
96
120
144
168
192
Follow up in months
high (n=23)
low (n=40)
raw p 0.178
bonf p 1.000

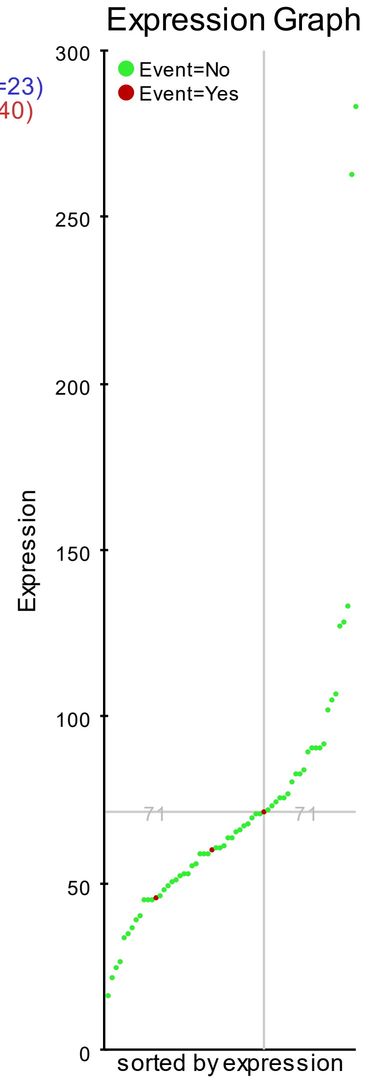
Expression Graph
Event=No
Event=Yes
Expression
300
250
200
150
100
50
0
71
71
sorted by expression

SHH

Tumor Medulloblastoma
Cavalli - 763 - rma_sketch - hugene11t
FLT1 (7970763)
Expression cutoff: 68.300 (min.grp=8)
subgroup~shh|WITH_SURV (n=172)

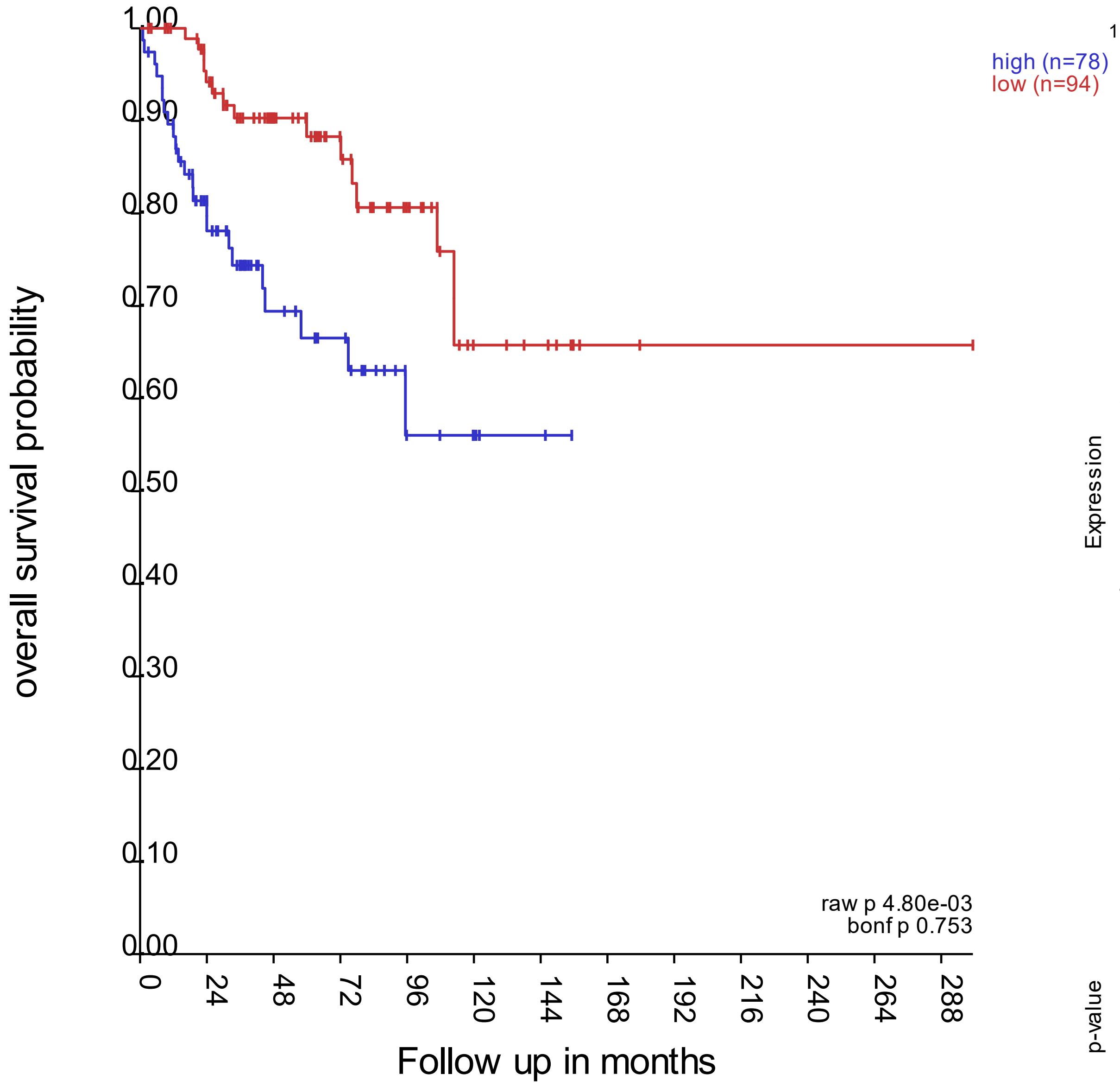

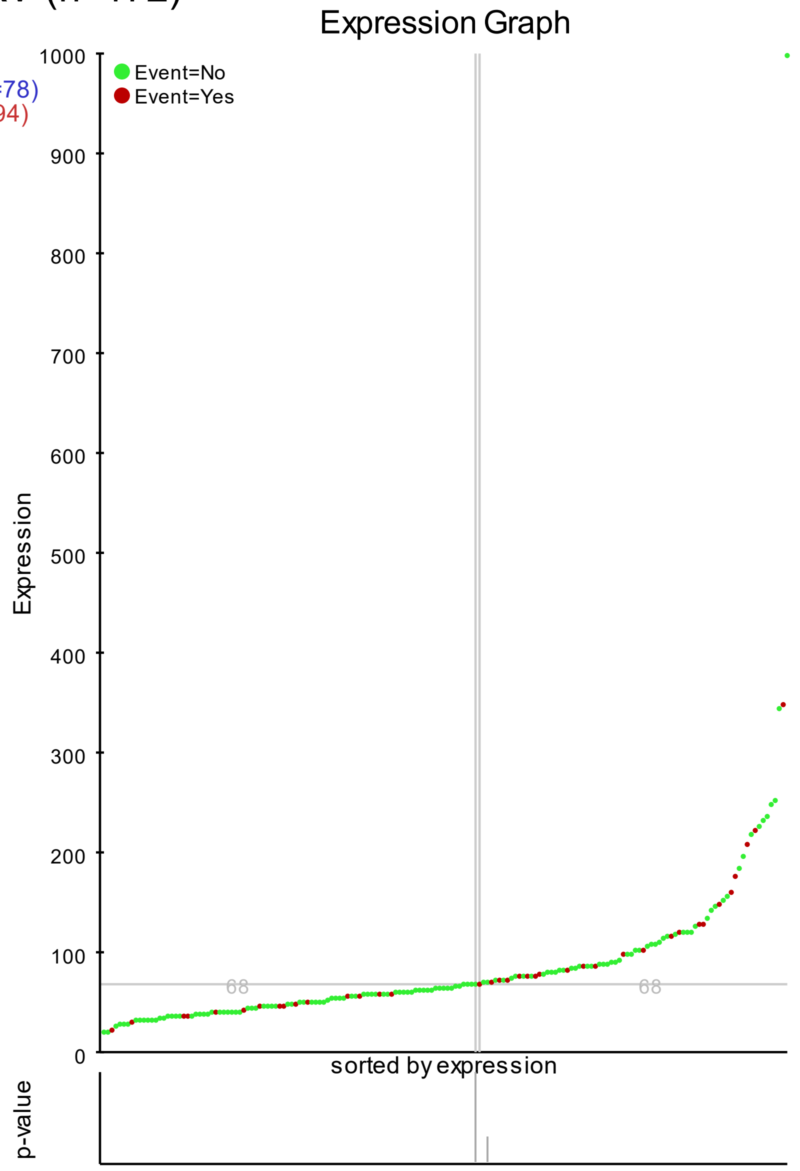

GR4

Tumor Medulloblastoma
Cavalli - 763 - rma_sketch - hugene11t
FLT1 (7970763)
Expression cutoff: 65.200 (min.grp=8)
subgroup~group4|WITH_SURV (n=264)

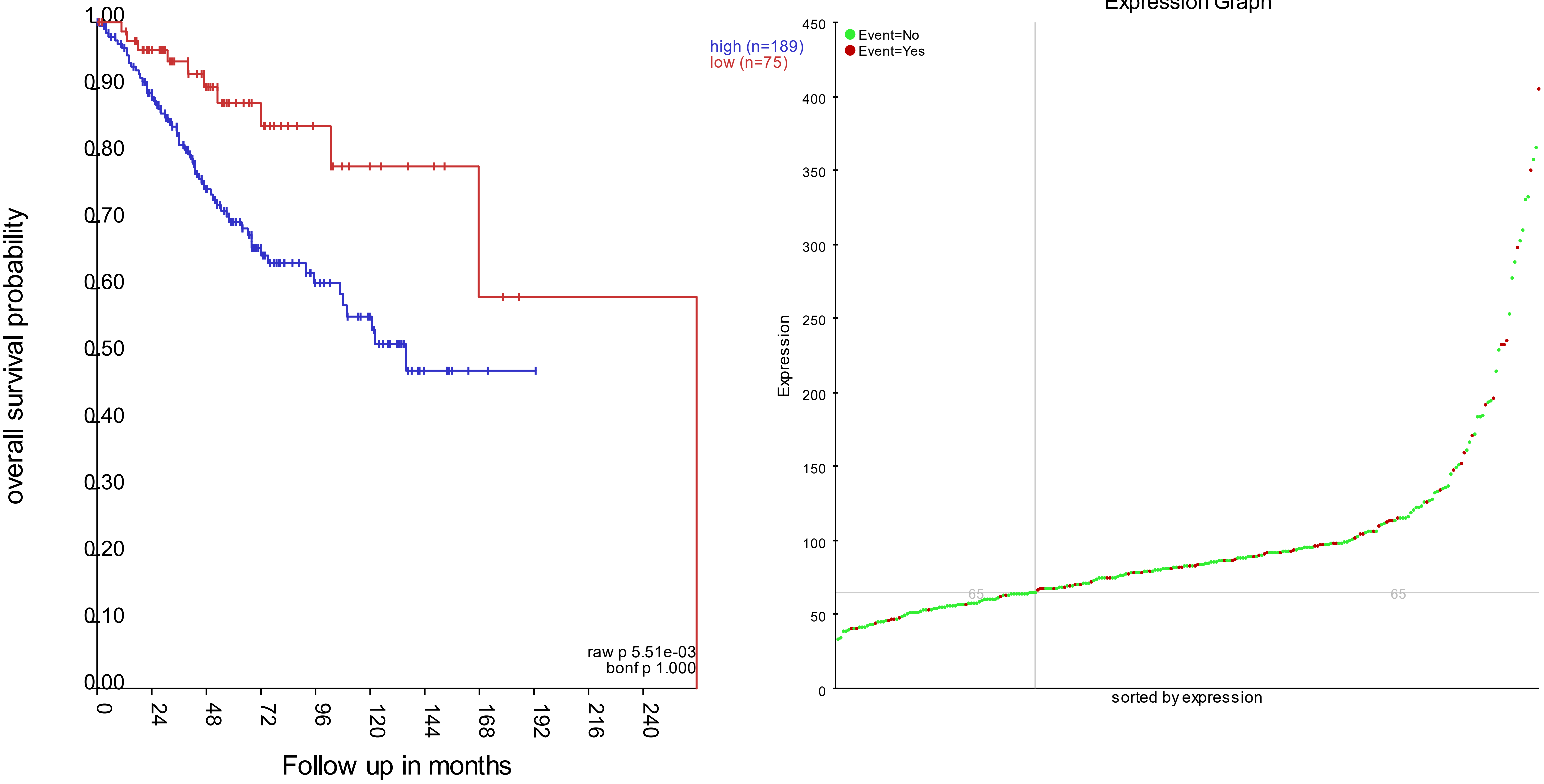

GR3

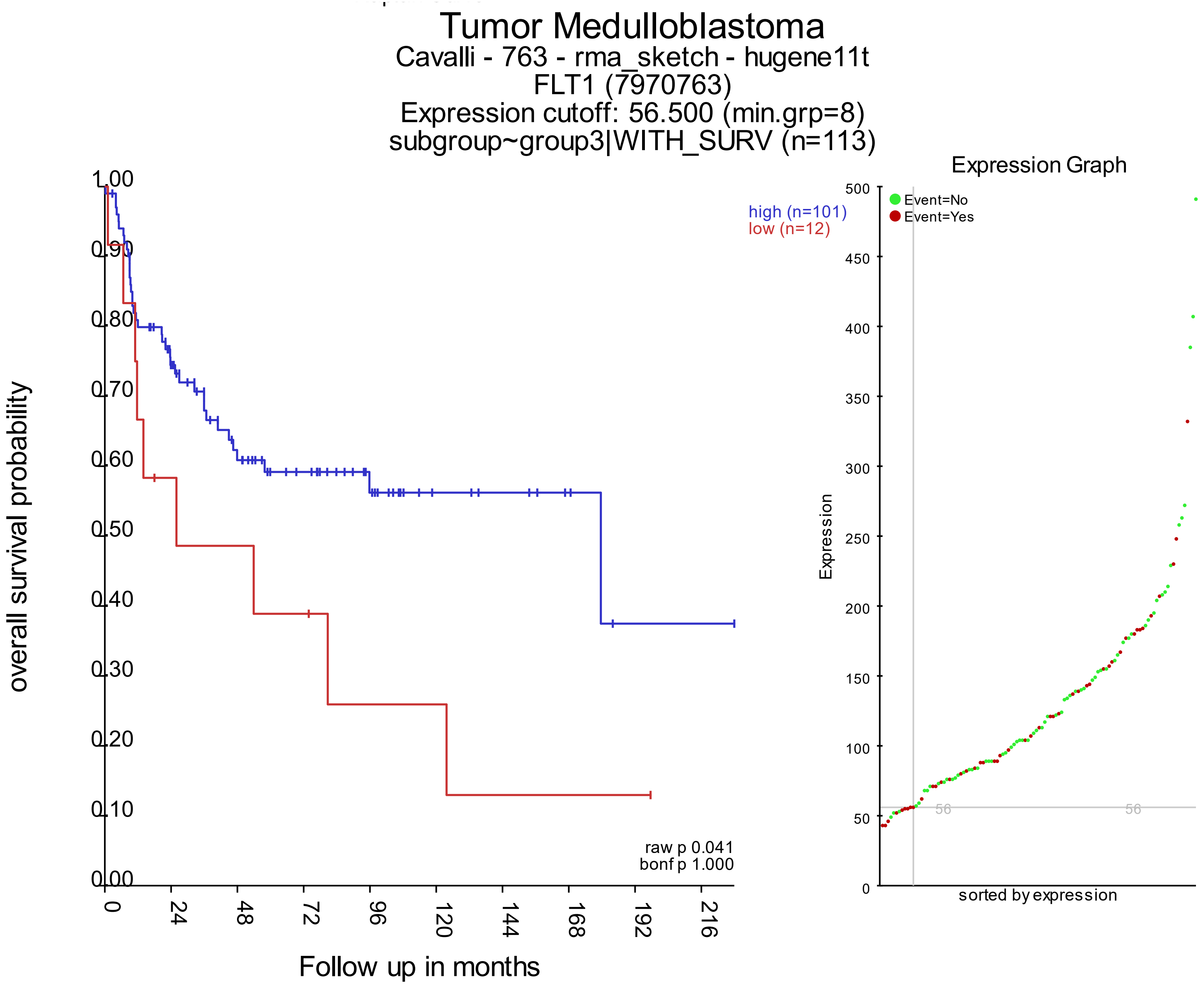
Tumor Medulloblastoma
Cavalli - 763 - rma_sketch - hugene11t
FLT1 (7970763)
Expression cutoff: 56.500 (min.grp=8)
subgroup~group3|WITH_SURV (n=113)
overall survival probability
1.00
0.90
0.80
0.70
0.60
0.50
0.40
0.30
0.20
0.10
0.00
0
24
48
72
96
120
144
168
192
216
Follow up in months
high (n=101)
low (n=12)
raw p 0.041
bonf p 1.000
Expression Graph
Event=No
Event=Yes
Expression
500
450
400
350
300
250
200
150
100
50
0
56
56
sorted by expression

KDR

WNT

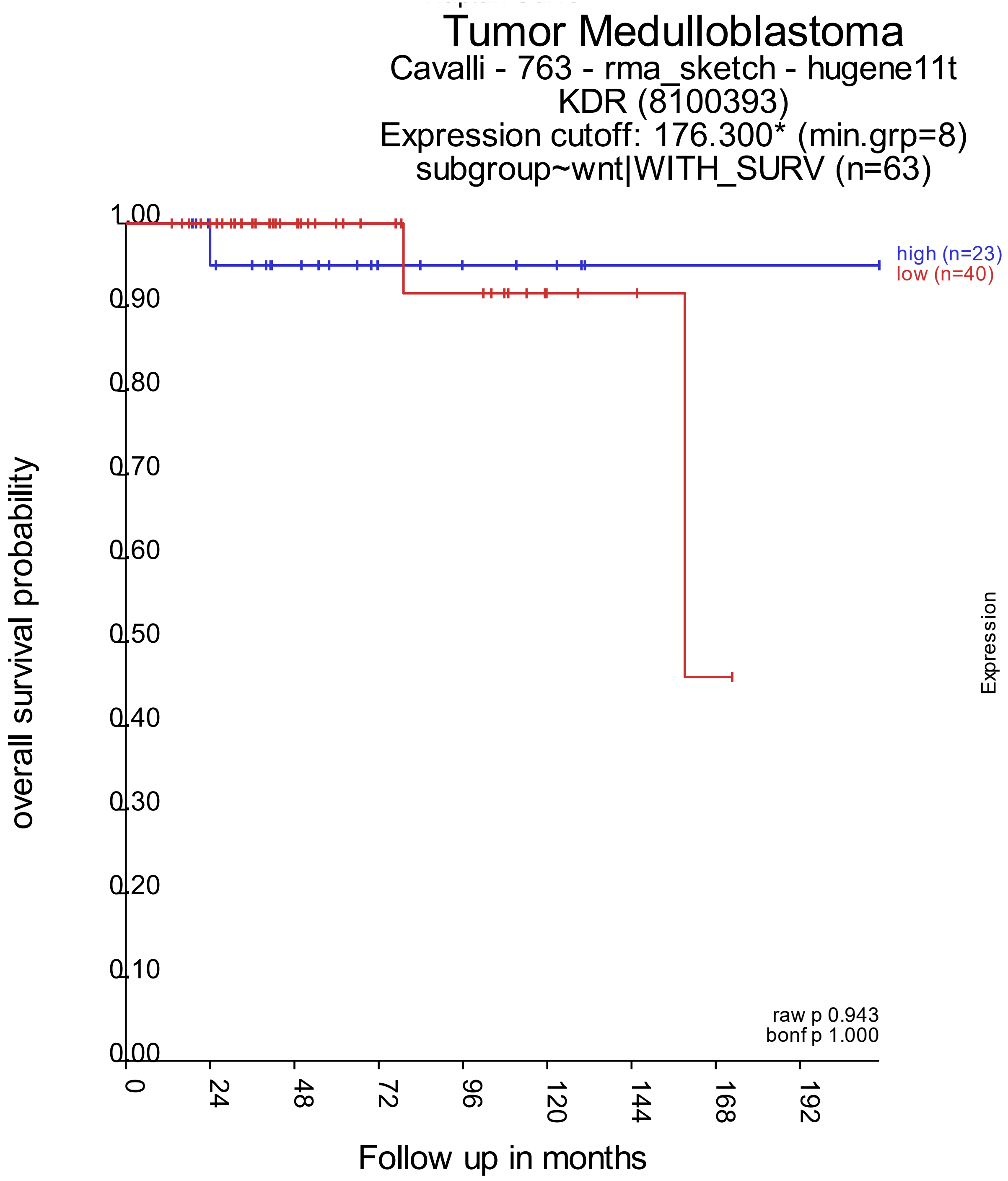
Tumor Medulloblastoma
Cavalli - 763 - rma_sketch - hugene11t
KDR (8100393)
Expression cutoff: 176.300* (min.grp=8)
subgroup~wnt|WITH_SURV (n=63)
overall survival probability
1.00
0.90
0.80
0.70
0.60
0.50
0.40
0.30
0.20
0.10
0.00
high (n=23)
low (n=40)
raw p 0.943
bonf p 1.000
0
24
48
72
96
120
144
168
192
Follow up in months

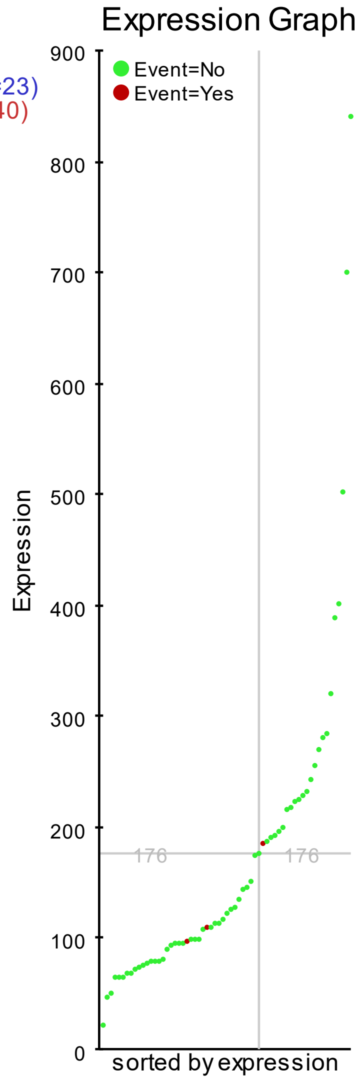
Expression Graph
Event=No
Event=Yes
Expression
900
800
700
600
500
400
300
200
100
0
176
176
sorted by expression

SHH

Tumor Medulloblastoma
Cavalli - 763 - rma_sketch - hugene11t
KDR (8100393)
Expression cutoff: 130.200 (min.grp=8)
subgroup~shh|WITH_SURV (n=172)

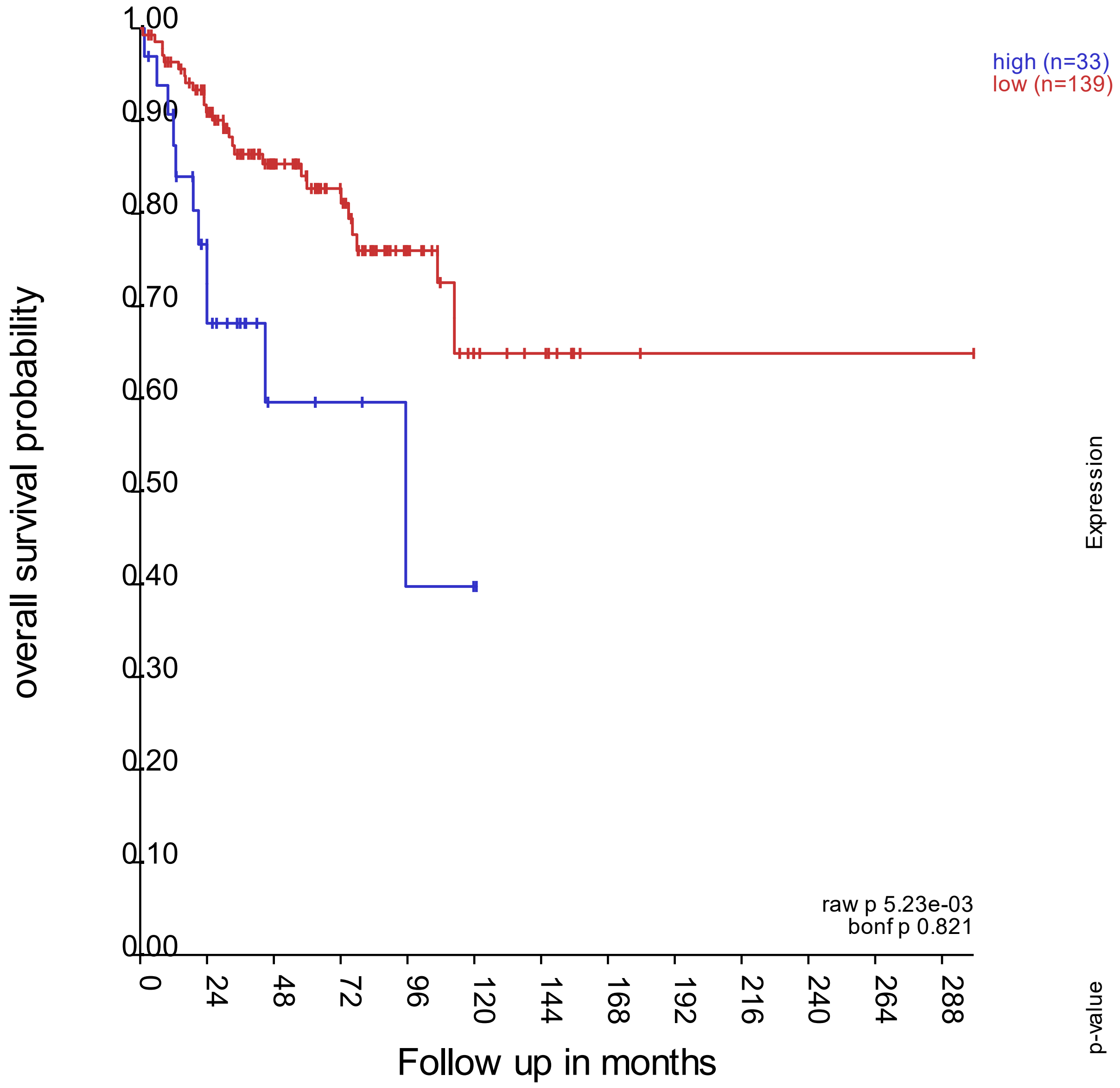

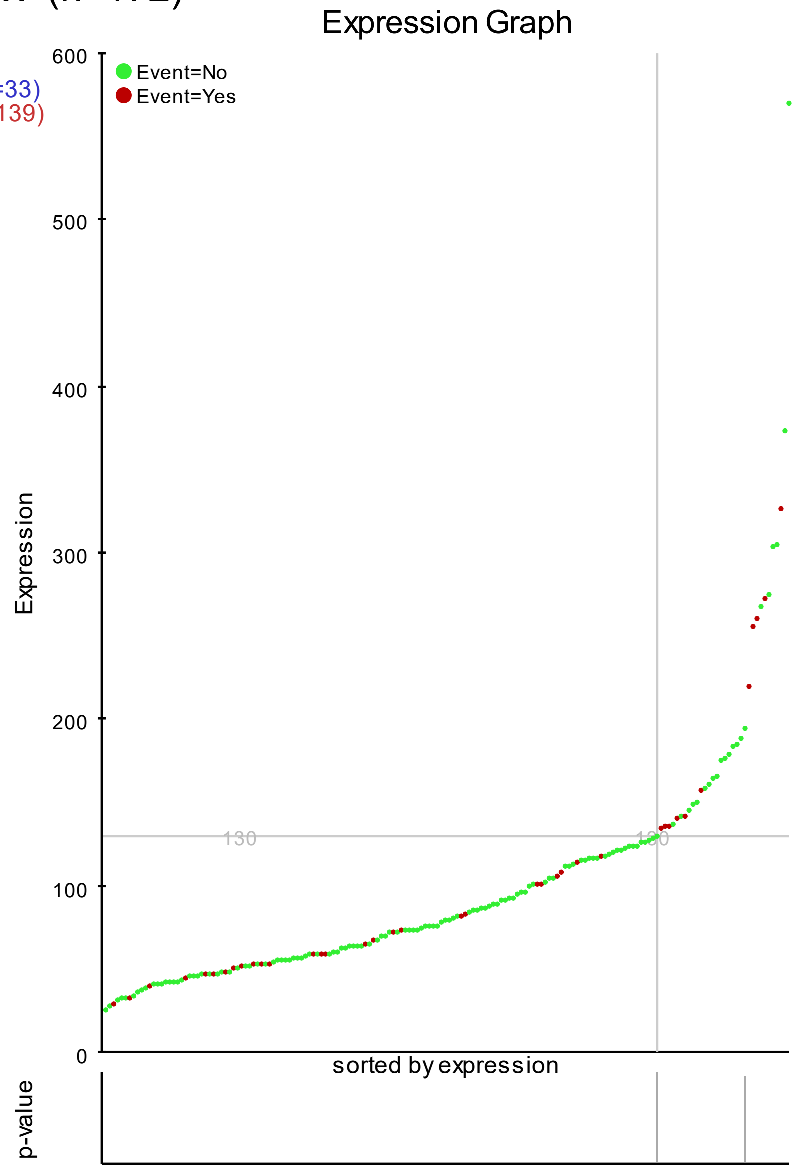

GR4

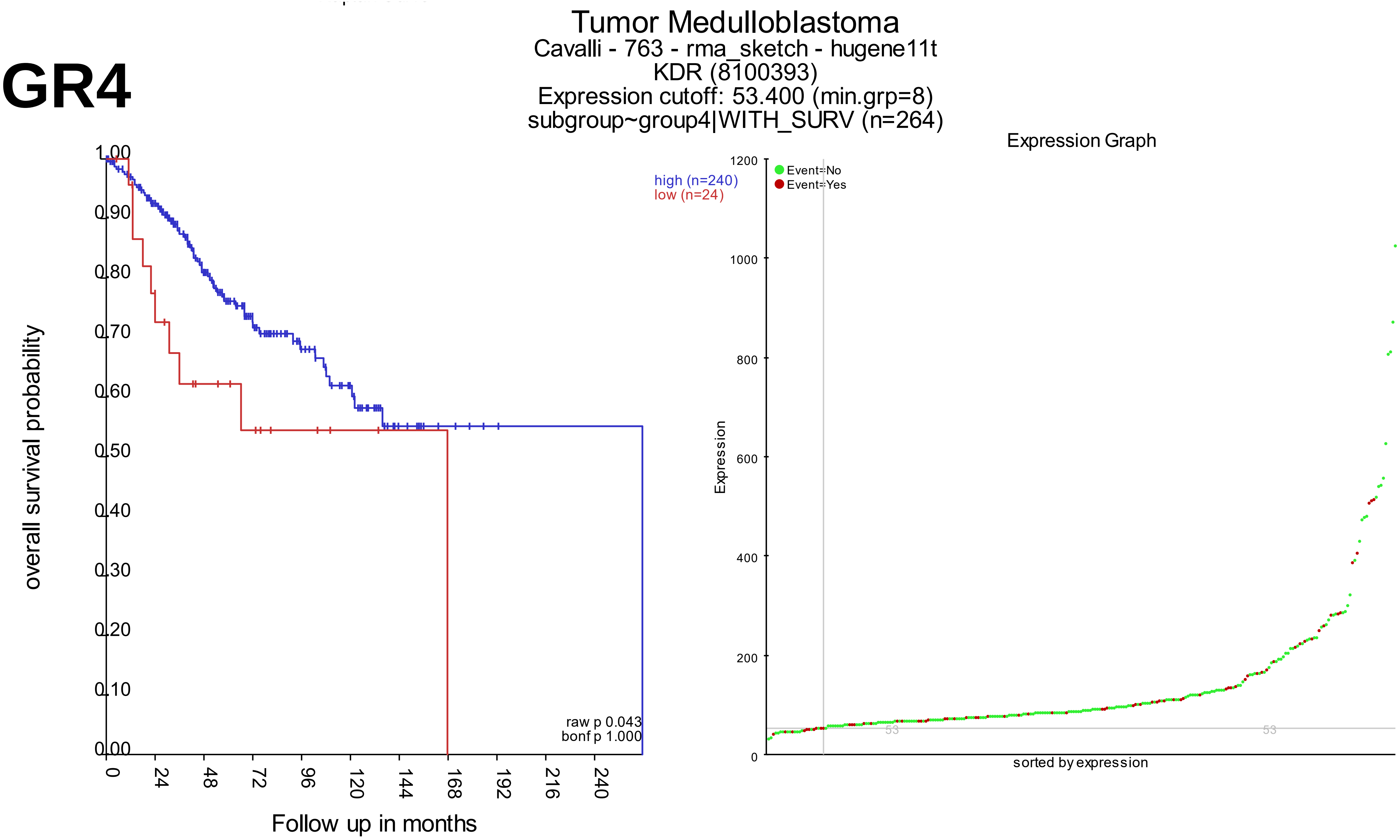

Tumor Medulloblastoma
Cavalli - 763 - rma_sketch - hugene11t
KDR (8100393)
Expression cutoff: 53.400 (min.grp=8)
subgroup~group4|WITH_SURV (n=264)
overall survival probability
1.00
0.90
0.80
0.70
0.60
0.50
0.40
0.30
0.20
0.10
0.00
0
24
48
72
96
120
144
168
192
216
240
Follow up in months
high (n=240)
low (n=24)
raw p 0.043
bonf p 1.000
Expression Graph
Event=No
Event=Yes
Expression
1200
1000
800
600
400
200
0
53
53
sorted by expression

GR3

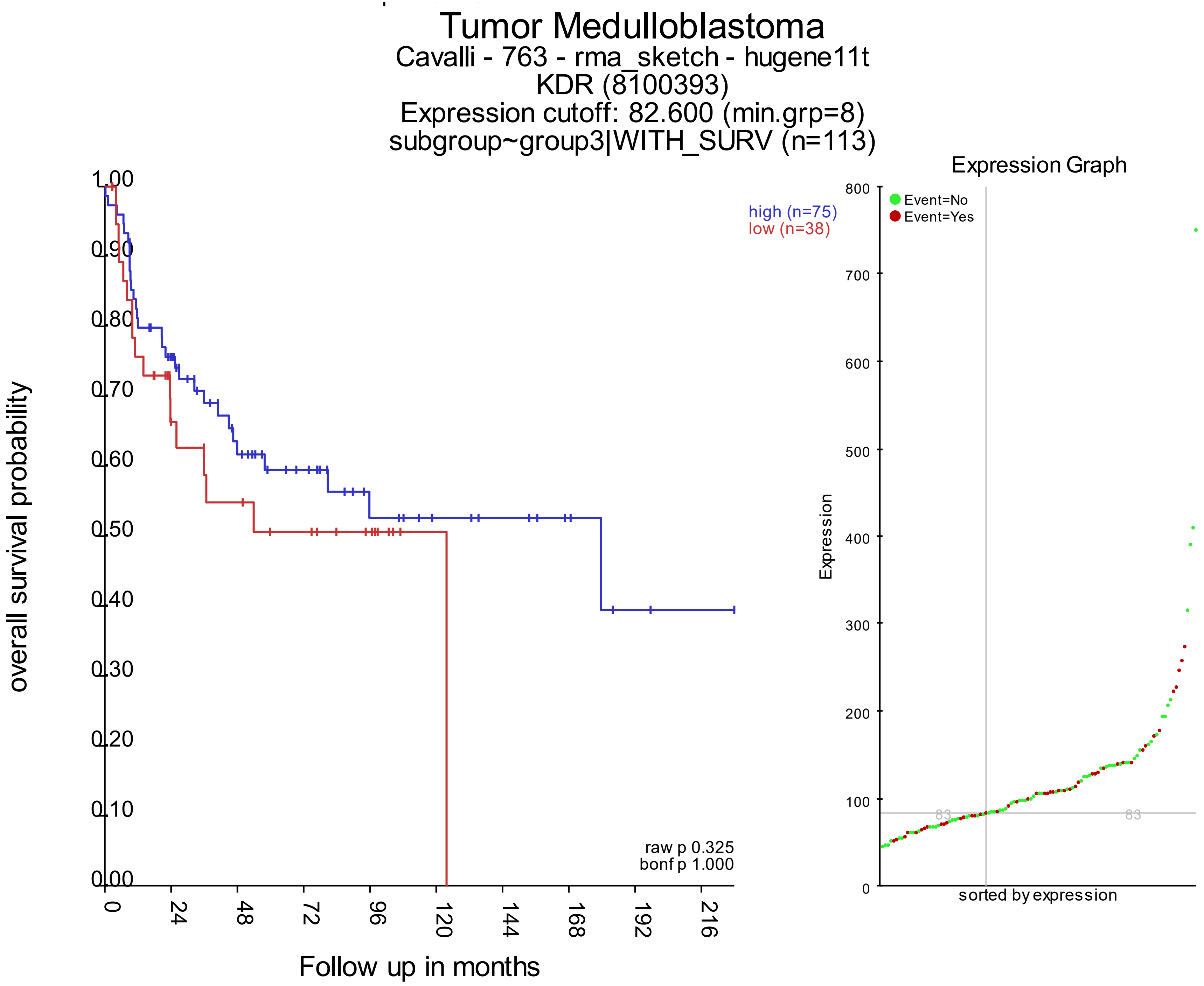

Tumor Medulloblastoma
Cavalli - 763 - rma_sketch - hugene11t
KDR (8100393)
Expression cutoff: 82.600 (min.grp=8)
subgroup~group3|WITH_SURV (n=113)
overall survival probability
1.00
0.90
0.80
0.70
0.60
0.50
0.40
0.30
0.20
0.10
0.00
high (n=75)
low (n=38)
raw p 0.325
bonf p 1.000
0
24
48
72
96
120
144
168
192
216
Follow up in months
Expression Graph
Event=No
Event=Yes
Expression
800
700
600
500
400
300
200
100
0
83
83
sorted by expression

FLT4

WNT

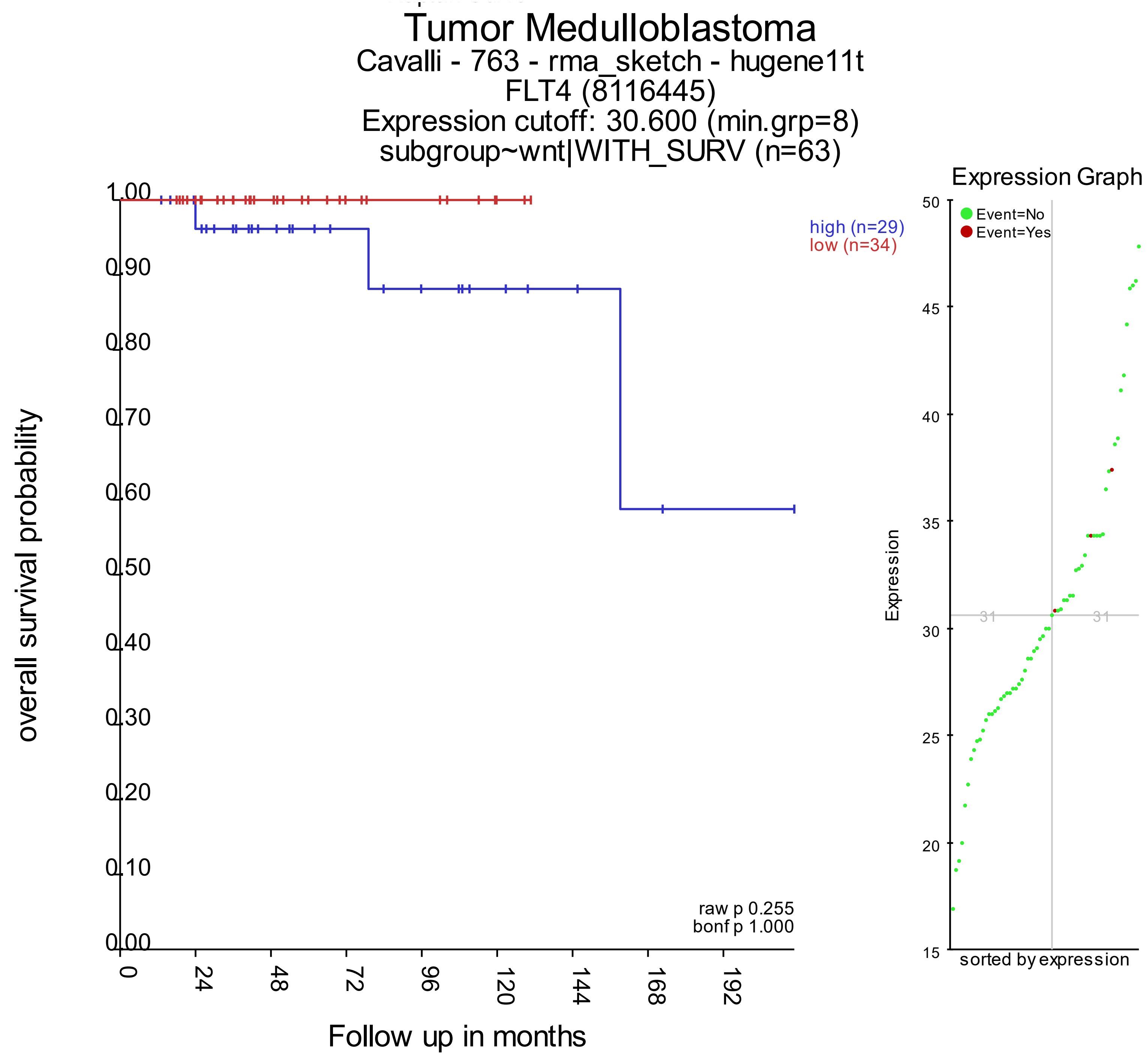

Tumor Medulloblastoma
Cavalli - 763 - rma_sketch - hugene11t
FLT4 (8116445)
Expression cutoff: 30.600 (min.grp=8)
subgroup~wnt|WITH_SURV (n=63)
overall survival probability
1.00
0.90
0.80
0.70
0.60
0.50
0.40
0.30
0.20
0.10
0.00
0
24
48
72
96
120
144
168
192
Follow up in months
high (n=29)
low (n=34)
raw p 0.255
bonf p 1.000
Expression Graph
Event=No
Event=Yes
Expression
50
45
40
35
30
25
20
15
31
31
sorted by expression

SHH

Tumor Medulloblastoma
Cavalli - 763 - rma_sketch - hugene11t
FLT4 (8116445)
Expression cutoff: 39.500 (min.grp=8)
subgroup~shh|WITH_SURV (n=172)

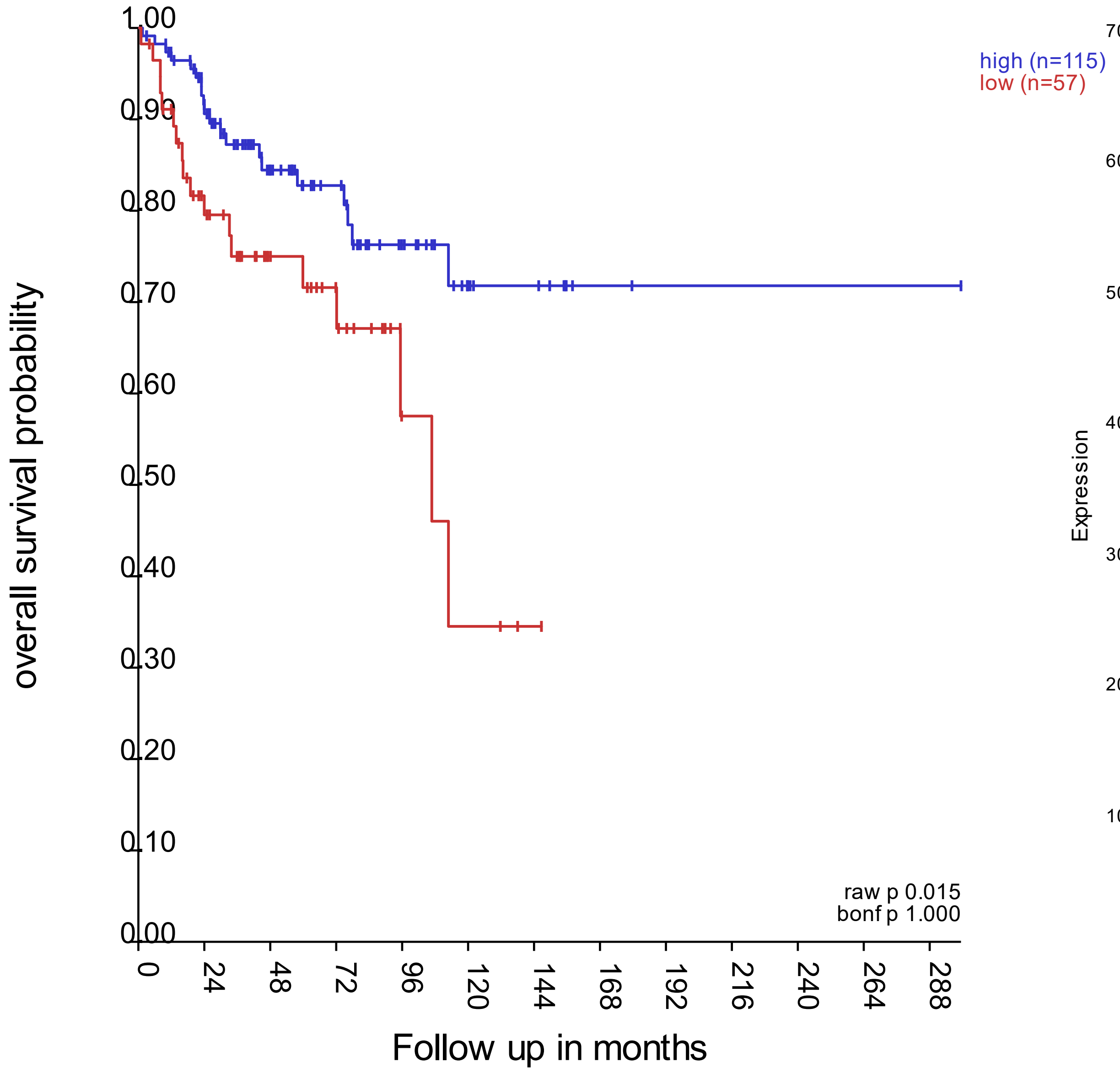

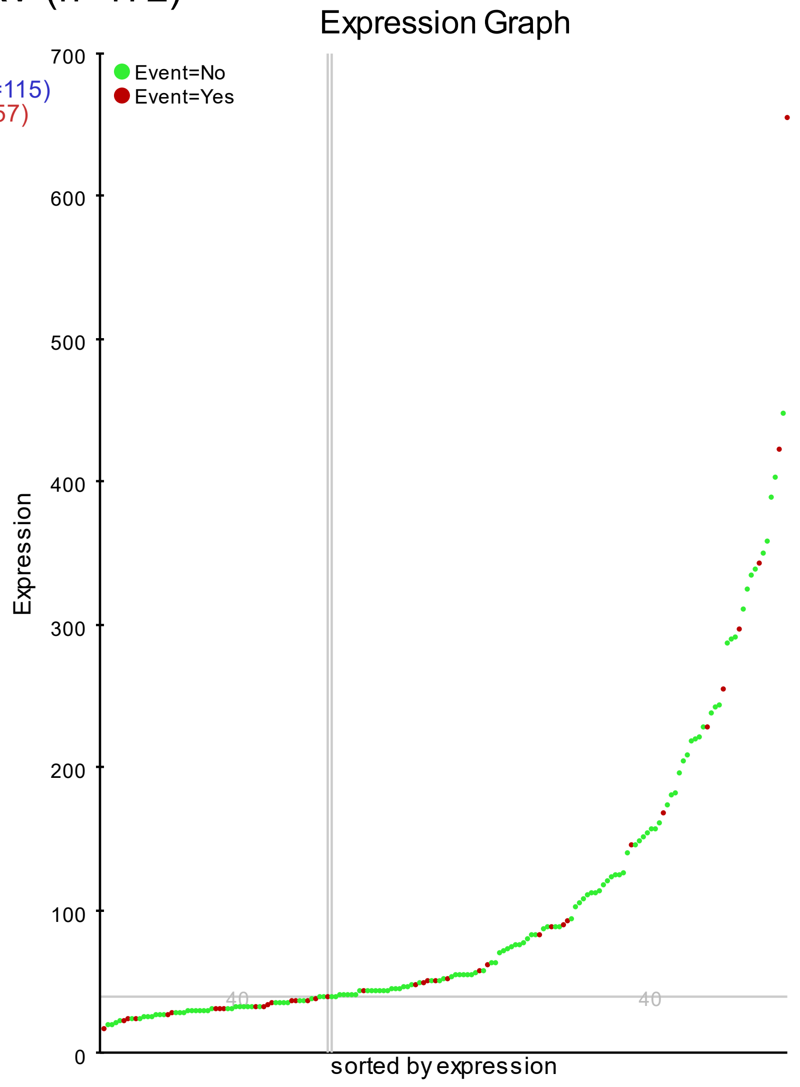

GR4

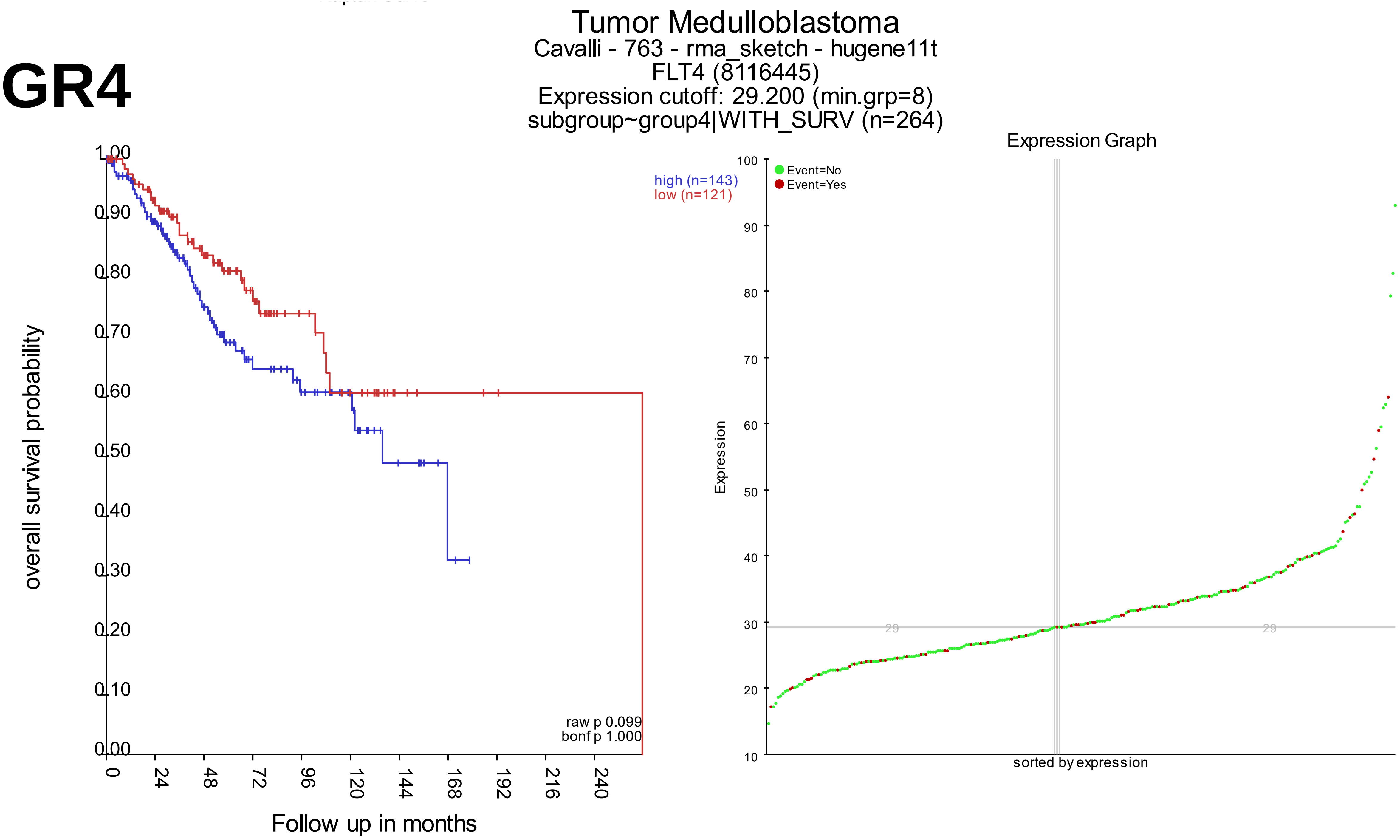

Tumor Medulloblastoma
Cavalli - 763 - rma_sketch - hugene11t
FLT4 (8116445)
Expression cutoff: 29.200 (min.grp=8)
subgroup~group4|WITH_SURV (n=264)
high (n=143)
low (n=121)
overall survival probability
1.00
0.90
0.80
0.70
0.60
0.50
0.40
0.30
0.20
0.10
0.00
0
24
48
72
96
120
144
168
192
216
240
Follow up in months
raw p 0.099
bonf p 1.000
Expression Graph
Event=No
Event=Yes
Expression
100
90
80
70
60
50
40
30
20
10
29
29
sorted by expression

GR3

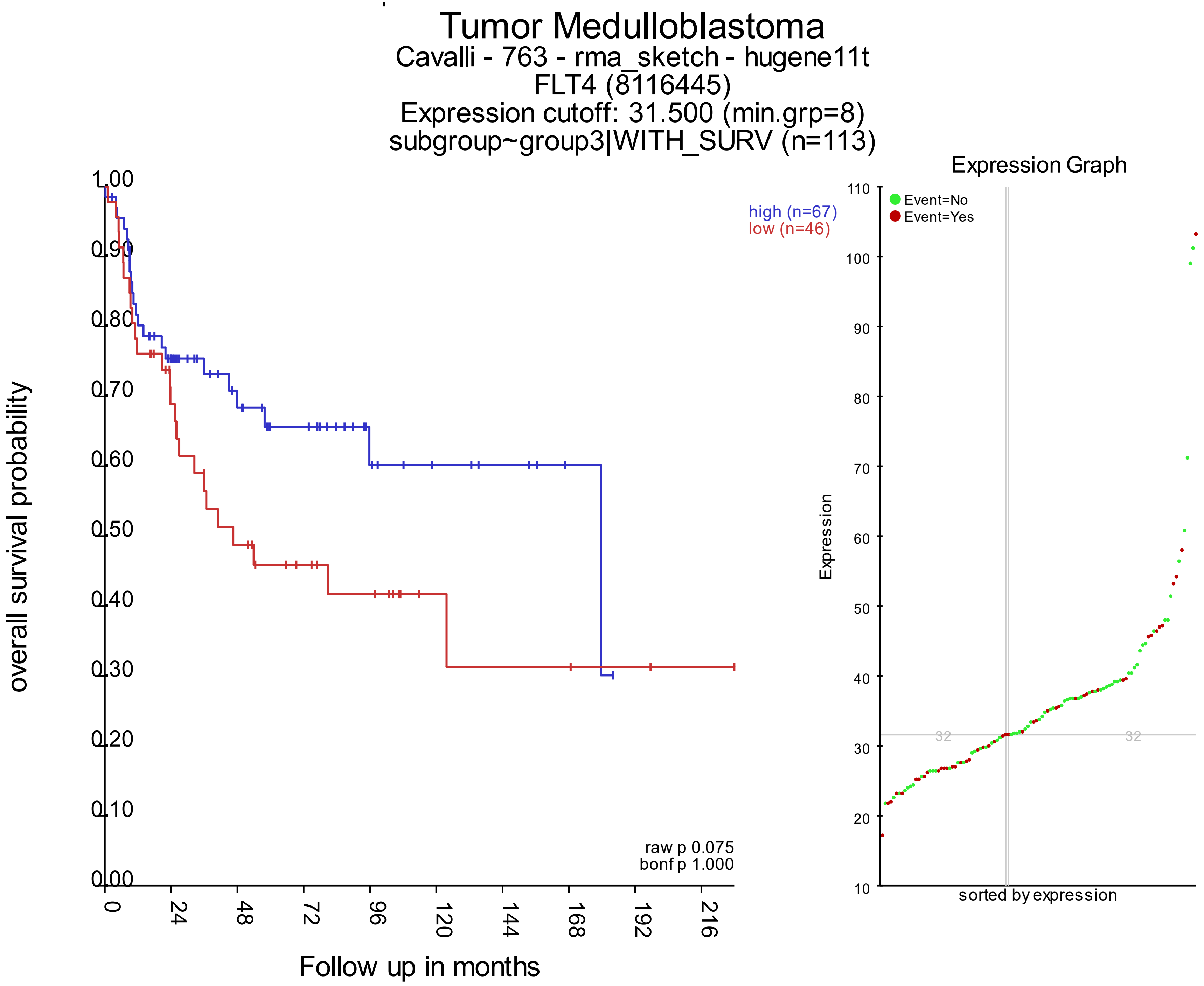
Tumor Medulloblastoma
Cavalli - 763 - rma_sketch - hugene11t
FLT4 (8116445)
Expression cutoff: 31.500 (min.grp=8)
subgroup~group3|WITH_SURV (n=113)
overall survival probability
1.00
0.90
0.80
0.70
0.60
0.50
0.40
0.30
0.20
0.10
0.00
0
24
48
72
96
120
144
168
192
216
Follow up in months
high (n=67)
low (n=46)
raw p 0.075
bonf p 1.000
Expression Graph
Event=No
Event=Yes
Expression
110
100
90
80
70
60
50
40
30
20
10
32
32
sorted by expression
