# Supplementary material for: Exploring Novel Applications: Repositioning Clinically Approved Therapies for Medulloblastoma Treatment
Source: Cancers (Basel). 2025 Nov 14;17(22):3659. doi: 10.3390/cancers17223659 (PMC12650705; doi:10.3390/cancers17223659)

ALK

WNT M0

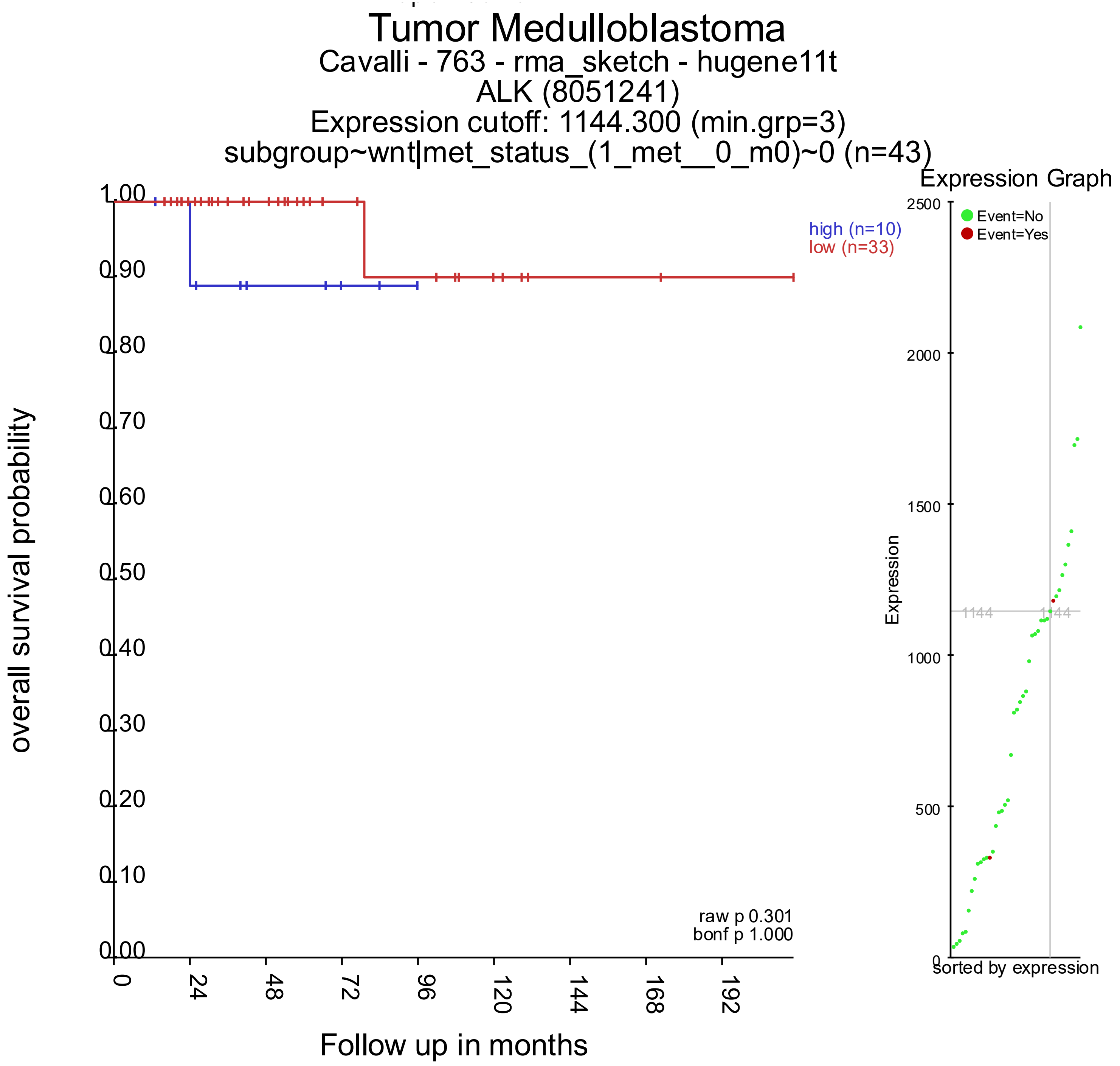
Tumor Medulloblastoma
Cavalli - 763 - rma_sketch - hugene11t
ALK (8051241)
Expression cutoff: 1144.300 (min.grp=3)
subgroup~wnt|met_status_(1_met__0_m0)~0 (n=43)
overall survival probability
1.00
0.90
0.80
0.70
0.60
0.50
0.40
0.30
0.20
0.10
0.00
high (n=10)
low (n=33)
raw p 0.301
bonf p 1.000
0
24
48
72
96
120
144
168
192
Follow up in months
Expression Graph
Event=No
Event=Yes
2500
2000
1500
1000
500
0
Expression
1144
sorted by expression

WNT M1

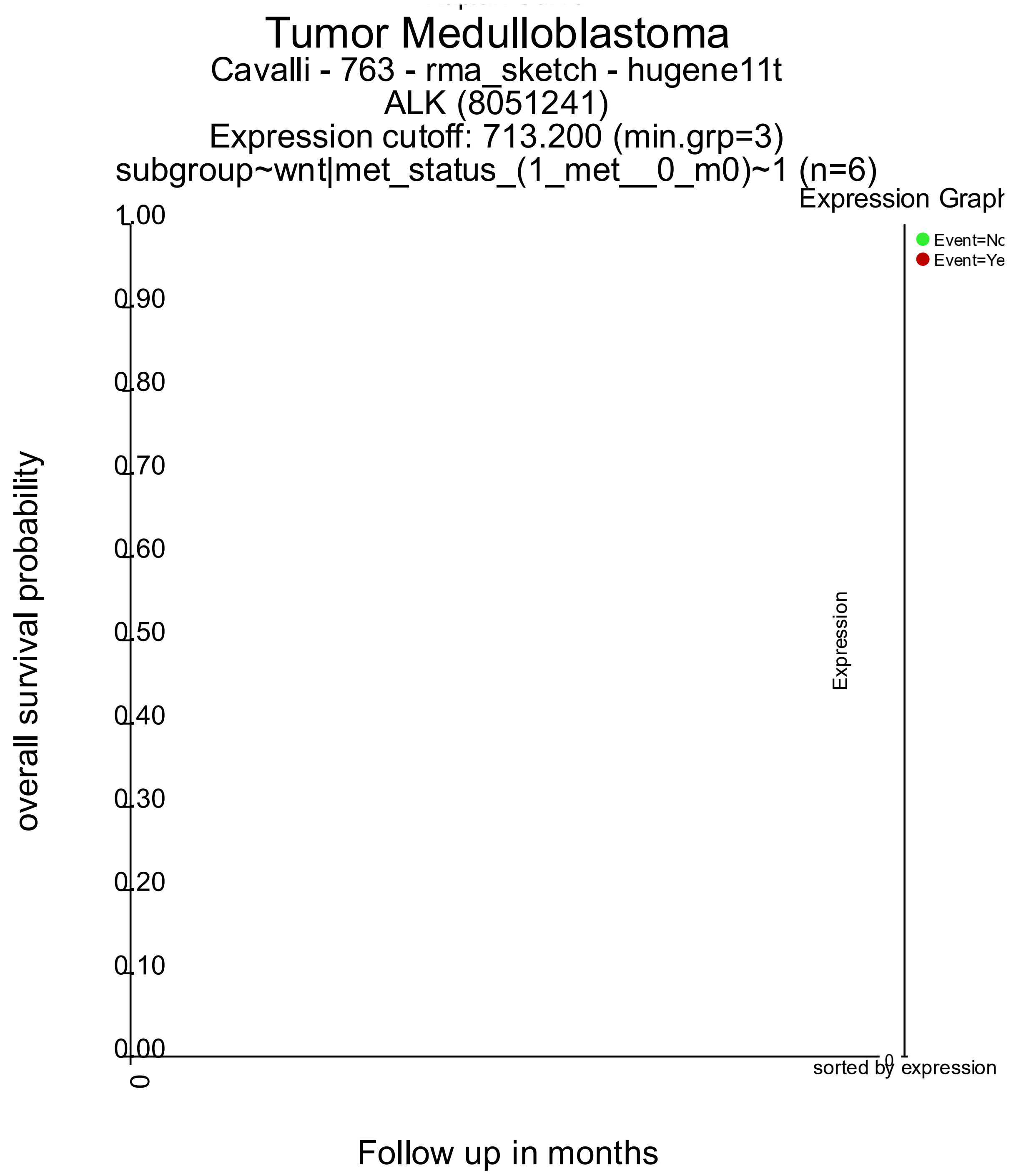

Tumor Medulloblastoma
Cavalli - 763 - rma_sketch - hugene11t
ALK (8051241)
Expression cutoff: 713.200 (min.grp=3)
subgroup~wnt|met_status_(1_met__0_m0)~1 (n=6)
Expression Graph
Event=No
Event=Yes
1.00
0.90
0.80
0.70
0.60
0.50
0.40
0.30
0.20
0.10
0.00
0
overall survival probability
Follow up in months
Expression
sorted by expression
0

SHH M0

Tumor Medulloblastoma
Cavalli - 763 - rma_sketch - hugene11t
ALK (8051241)
Expression cutoff: 125.000 (min.grp=3)
subgroup~shh|met_status_(1_met__0_m0)~0|WITH_SURV (n=124)

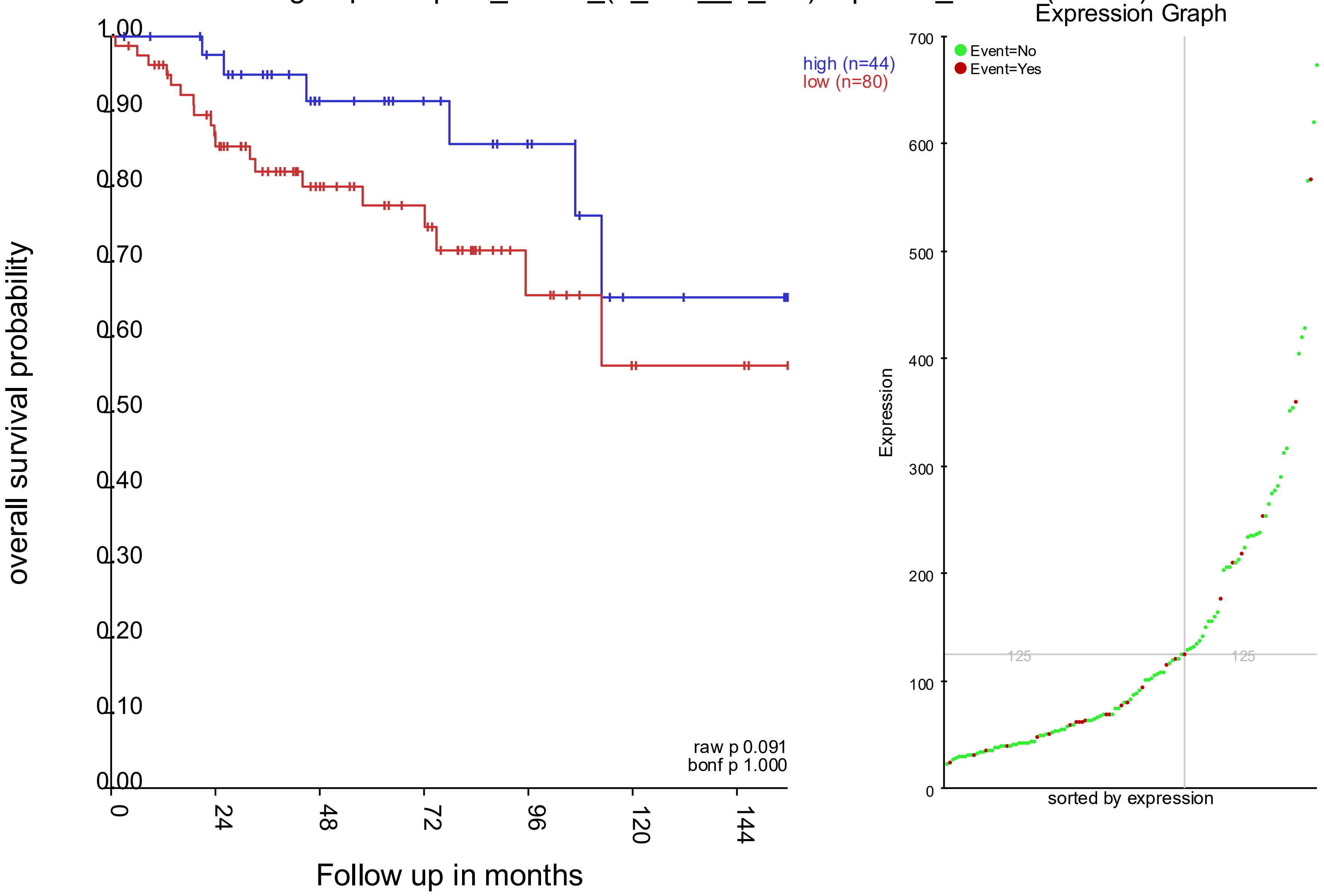

SHH M1

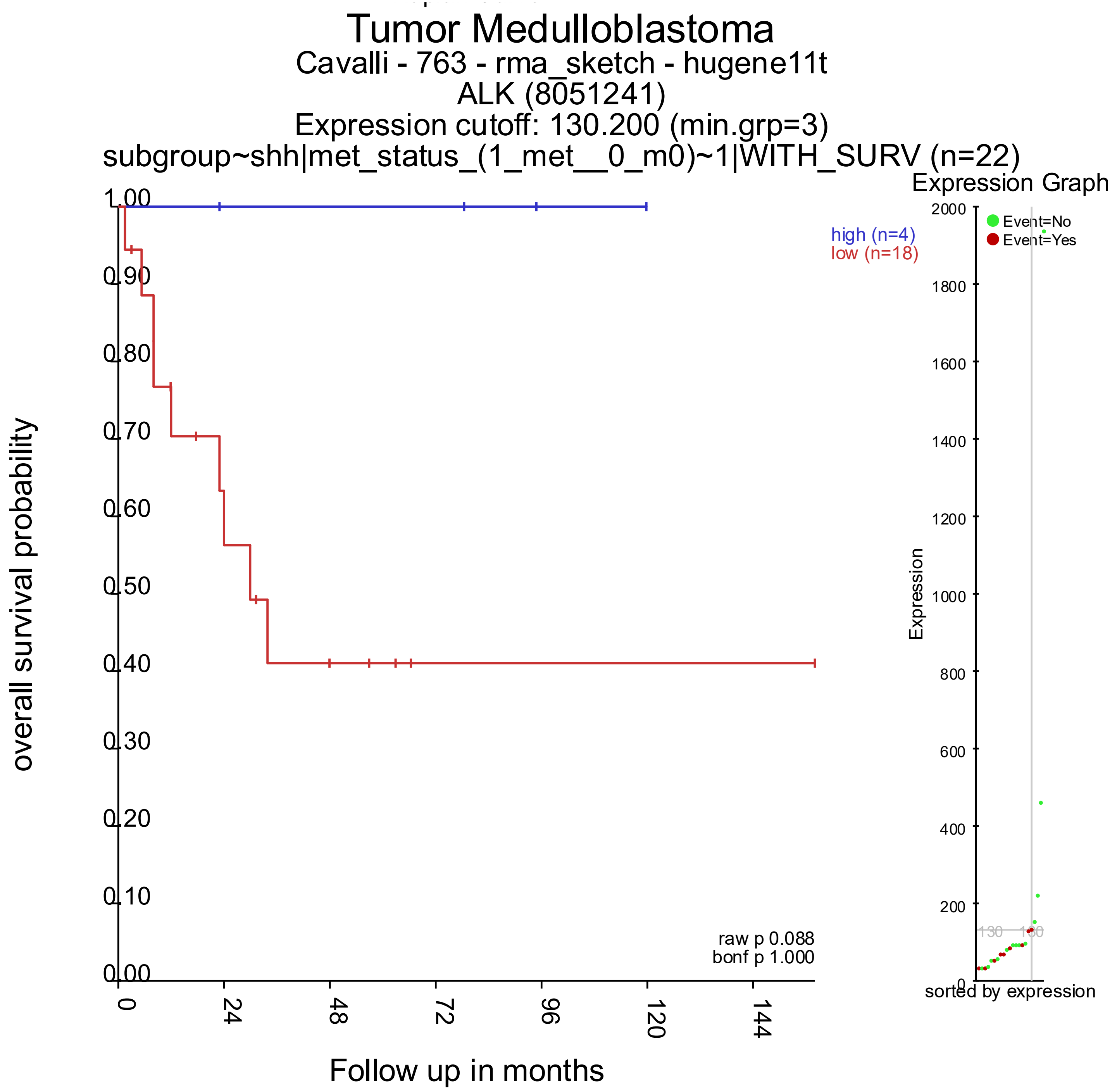

Tumor Medulloblastoma
Cavalli - 763 - rma_sketch - hugene11t
ALK (8051241)
Expression cutoff: 130.200 (min.grp=3)
subgroup~shh|met_status_(1_met__0_m0)~1|WITH_SURV (n=22)
overall survival probability
1.00
0.90
0.80
0.70
0.60
0.50
0.40
0.30
0.20
0.10
0.00
high (n=4)
low (n=18)
raw p 0.088
bonf p 1.000
0
24
48
72
96
120
144
Follow up in months
Expression Graph
Event=No
Event=Yes
2000
1800
1600
1400
1200
1000
800
600
400
200
0
Expression
sorted by expression

GROUP4 M0

Tumor Medulloblastoma
Cavalli - 763 - rma_sketch - hugene11t
ALK (8051241)
Expression cutoff: 42.500 (min.grp=3)
subgroup~group4|met_status_(1_met__0_m0)~0|WITH_SURV (n=145)

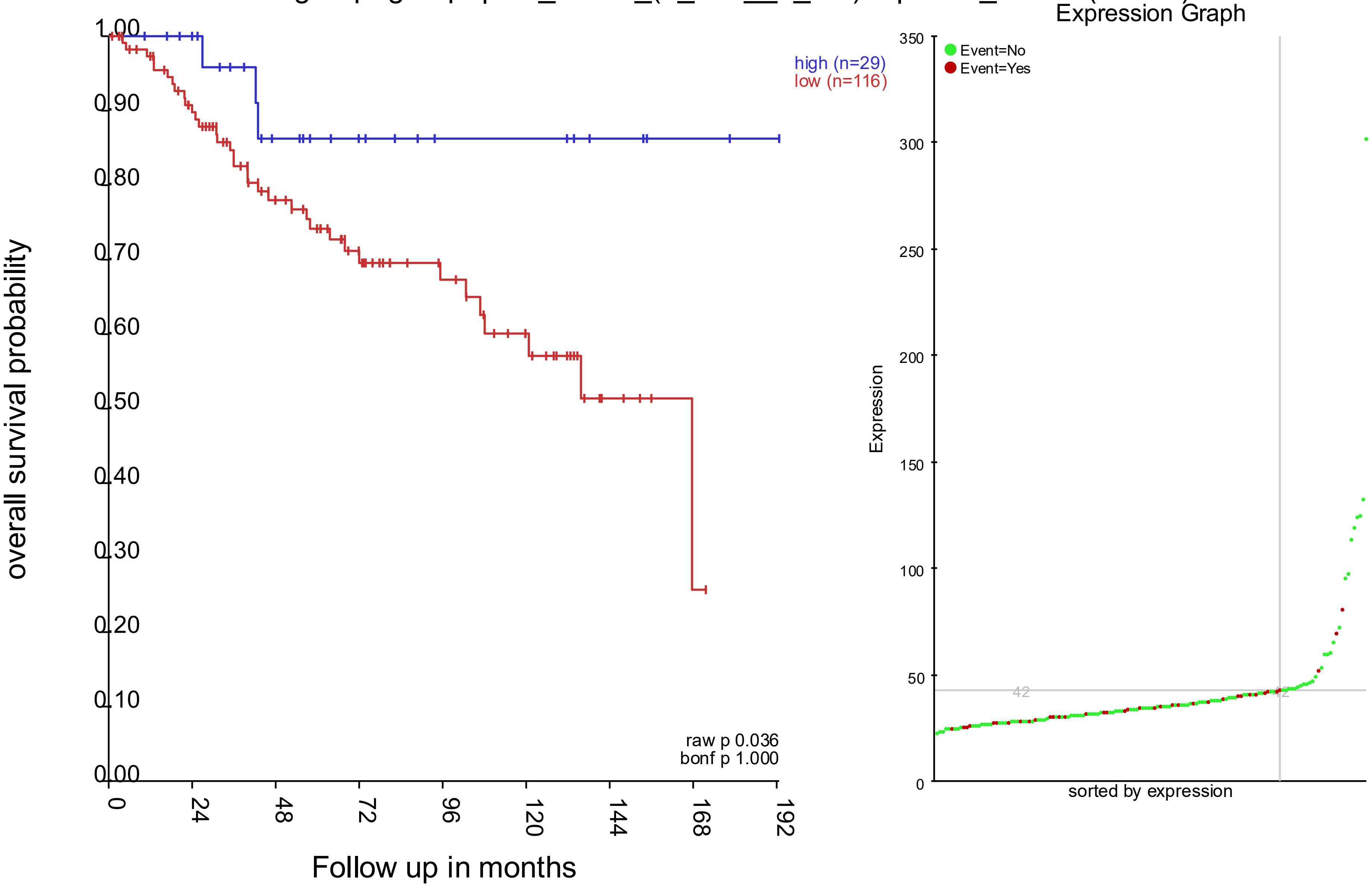

GROUP4 M1

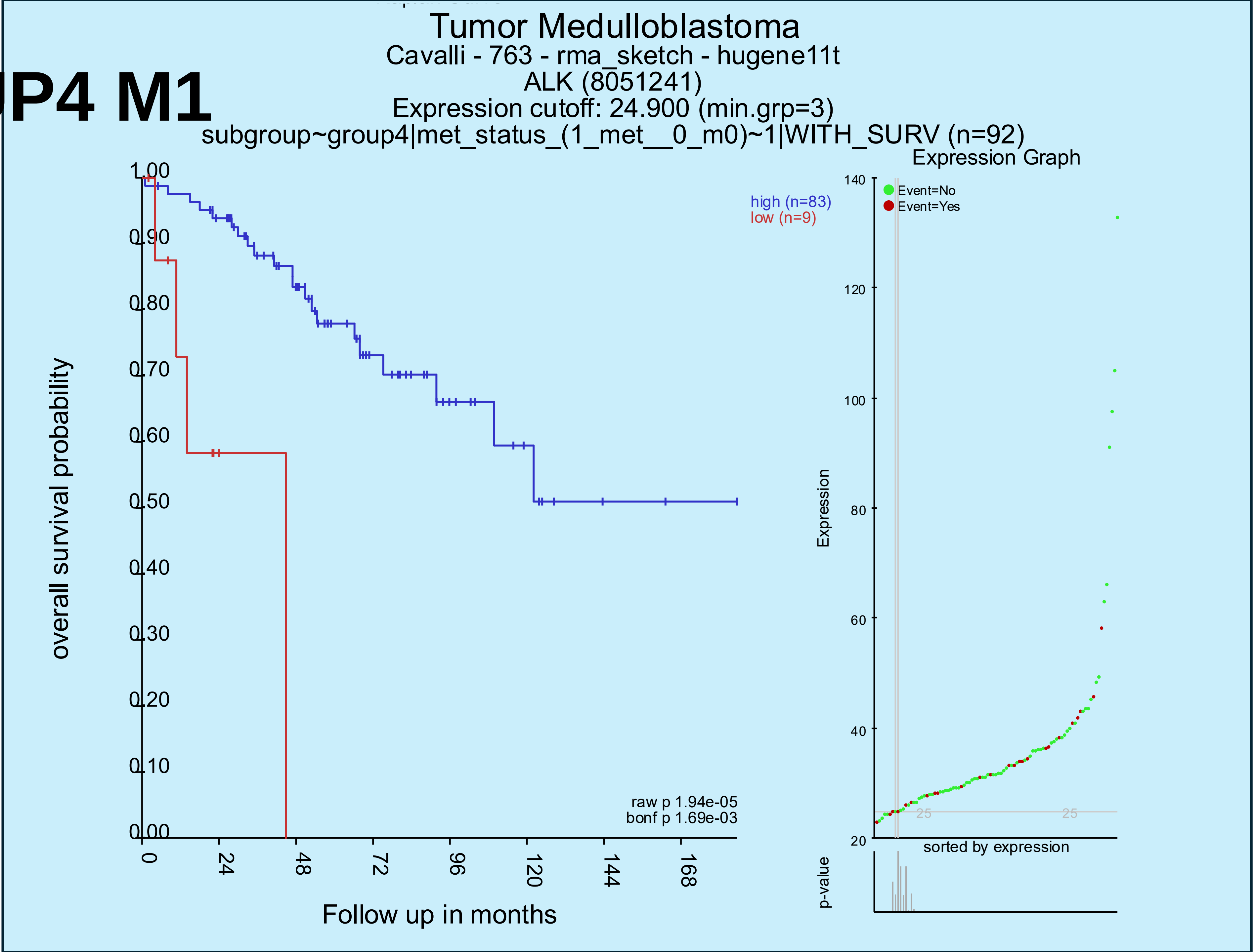

Tumor Medulloblastoma
Cavalli - 763 - rma_sketch - hugene11t
ALK (8051241)
Expression cutoff: 24.900 (min.grp=3)
subgroup~group4|met_status_(1_met__0_m0)~1|WITH_SURV (n=92)
overall survival probability
1.00
0.90
0.80
0.70
0.60
0.50
0.40
0.30
0.20
0.10
0.00
high (n=83)
low (n=9)
raw p 1.94e-05
bonf p 1.69e-03
0
24
48
72
96
120
144
168
Follow up in months
Expression Graph
Event=No
Event=Yes
Expression
140
120
100
80
60
40
20
25
25
sorted by expression
p-value

GROUP3 M0

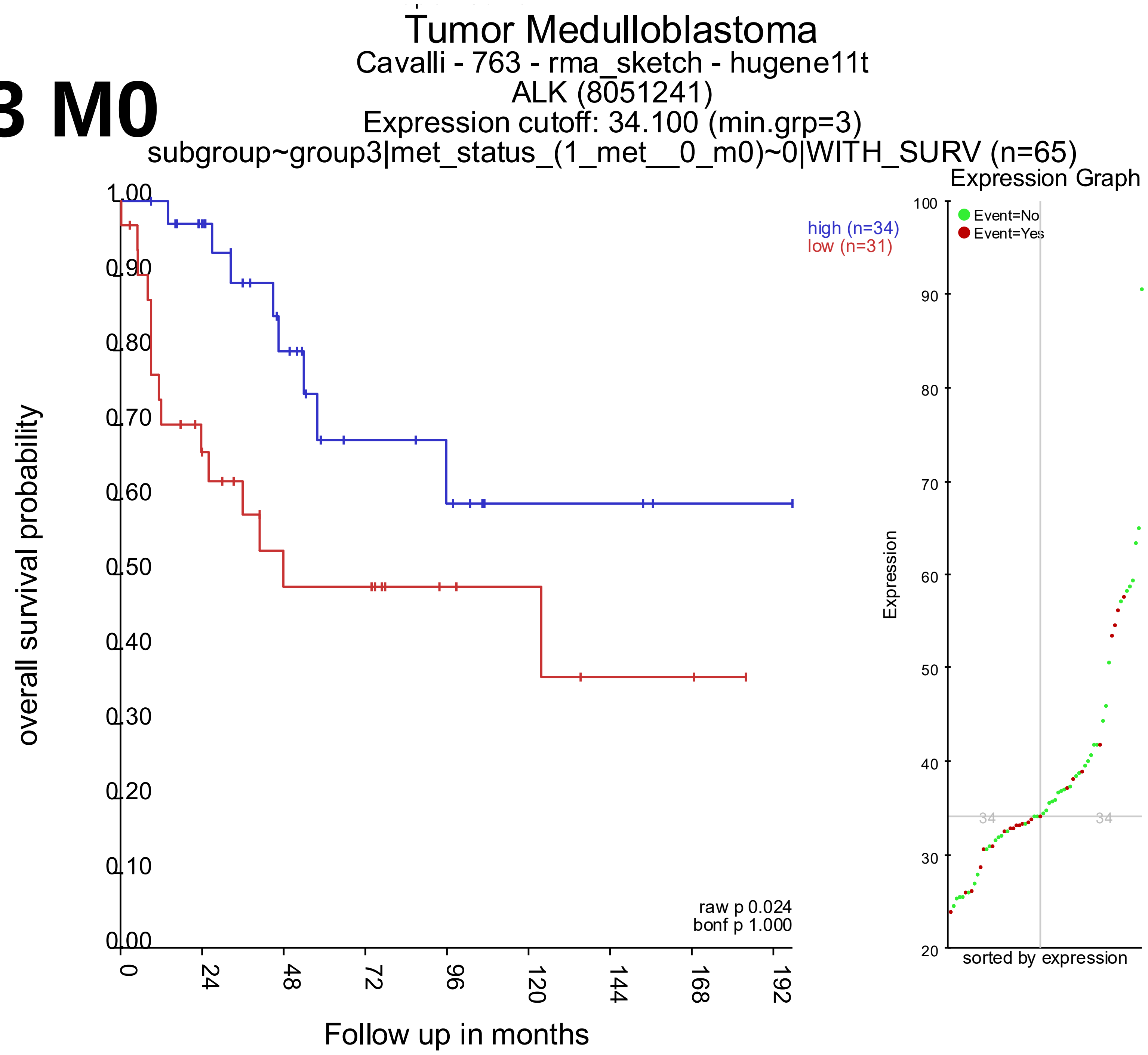
Tumor Medulloblastoma
Cavalli - 763 - rma_sketch - hugene11t
ALK (8051241)
Expression cutoff: 34.100 (min.grp=3)
subgroup~group3|met_status_(1_met__0_m0)~0|WITH_SURV (n=65)
overall survival probability
1.00
0.90
0.80
0.70
0.60
0.50
0.40
0.30
0.20
0.10
0.00
high (n=34)
low (n=31)
raw p 0.024
bonf p 1.000
0
24
48
72
96
120
144
168
192
Follow up in months
Expression Graph
Event=No
Event=Yes
Expression
100
90
80
70
60
50
40
30
20
34
34
sorted by expression

GROUP3 M1

Tumor Medulloblastoma
Cavalli - 763 - rma_sketch - hugene11t
ALK (8051241)
Expression cutoff: 31.200 (min.grp=3)
subgroup~group3|met_status_(1_met__0_m0)~1|WITH_SURV (n=41)

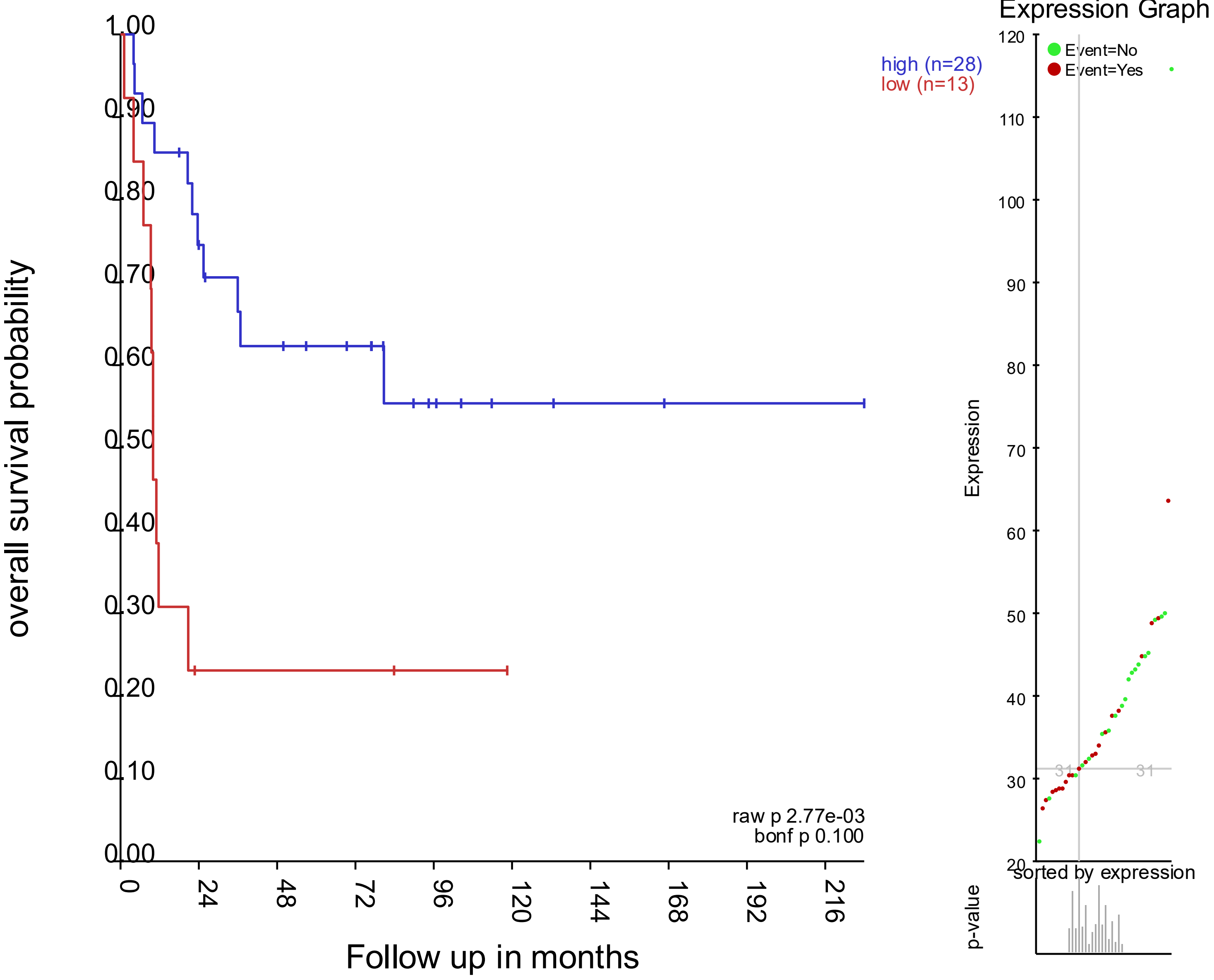

BCL2

WNT M0

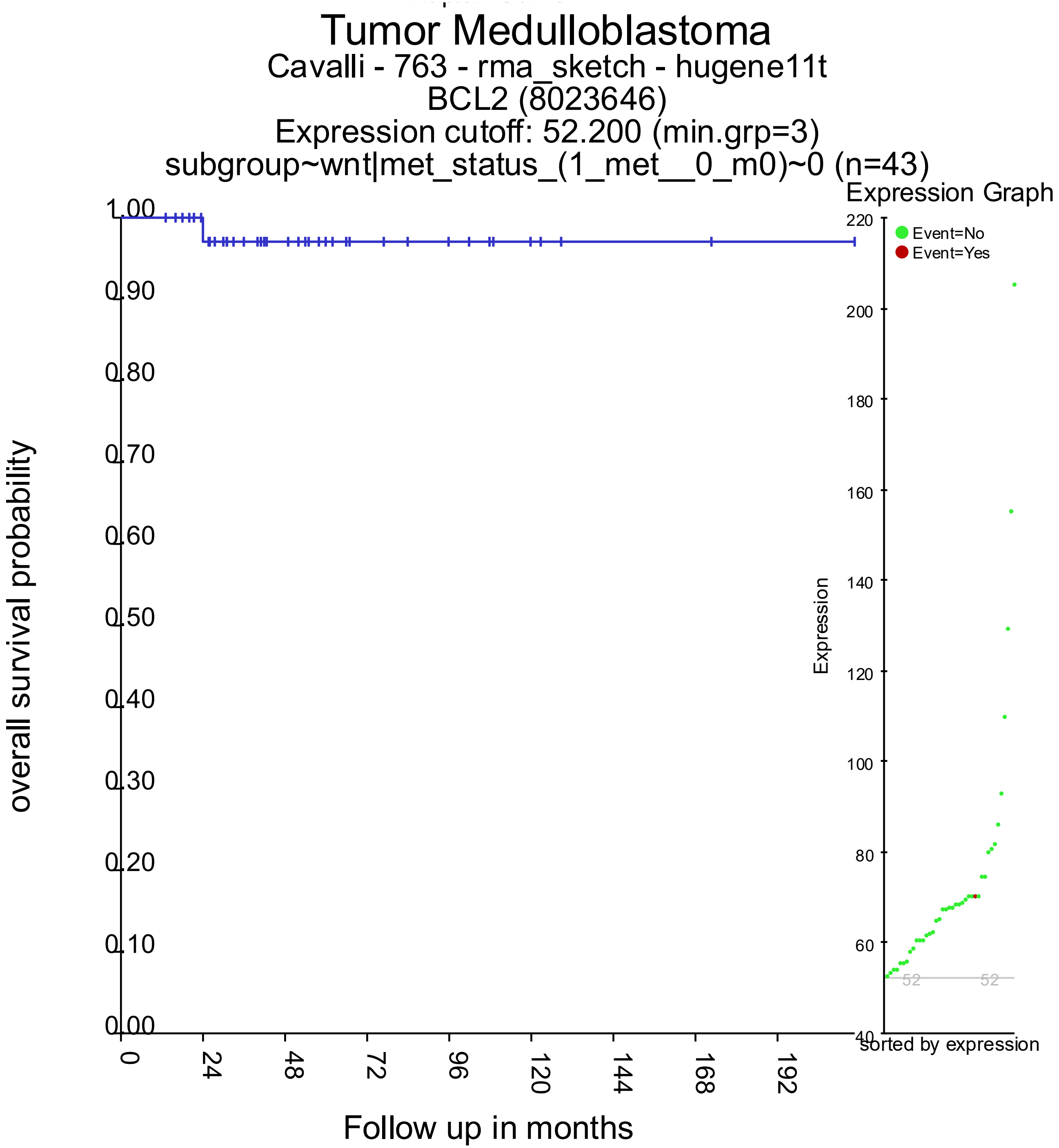

Tumor Medulloblastoma
Cavalli - 763 - rma_sketch - hugene11t
BCL2 (8023646)
Expression cutoff: 52.200 (min.grp=3)
subgroup~wnt|met_status_(1_met__0_m0)~0 (n=43)
overall survival probability
1.00
0.90
0.80
0.70
0.60
0.50
0.40
0.30
0.20
0.10
0.00
0
24
48
72
96
120
144
168
192
Follow up in months
Expression Graph
Event=No
Event=Yes
Expression
220
200
180
160
140
120
100
80
60
40
52
52
sorted by expression

WNT M1

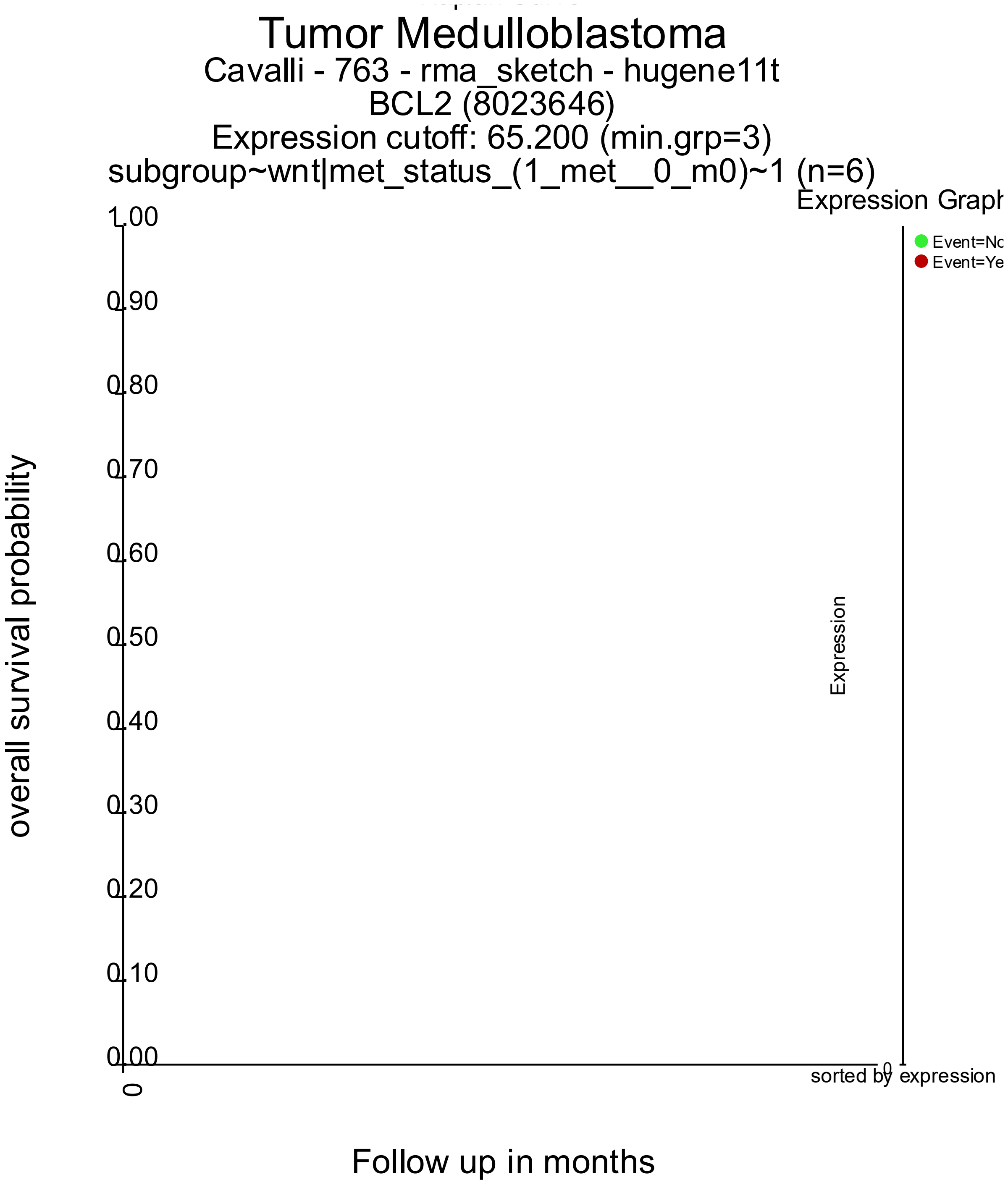

Tumor Medulloblastoma
Cavalli - 763 - rma_sketch - hugene11t
BCL2 (8023646)
Expression cutoff: 65.200 (min.grp=3)
subgroup~wnt|met_status_(1_met__0_m0)~1 (n=6)
overall survival probability
1.00
0.90
0.80
0.70
0.60
0.50
0.40
0.30
0.20
0.10
0.00
0
Follow up in months
Expression Graph
Event=No
Event=Yes
Expression
0
sorted by expression

SHH M0

Tumor Medulloblastoma
Cavalli - 763 - rma_sketch - hugene11t
BCL2 (8023646)
Expression cutoff: 166.800 (min.grp=3)
subgroup~shh|met_status_(1_met__0_m0)~0|WITH_SURV (n=124)

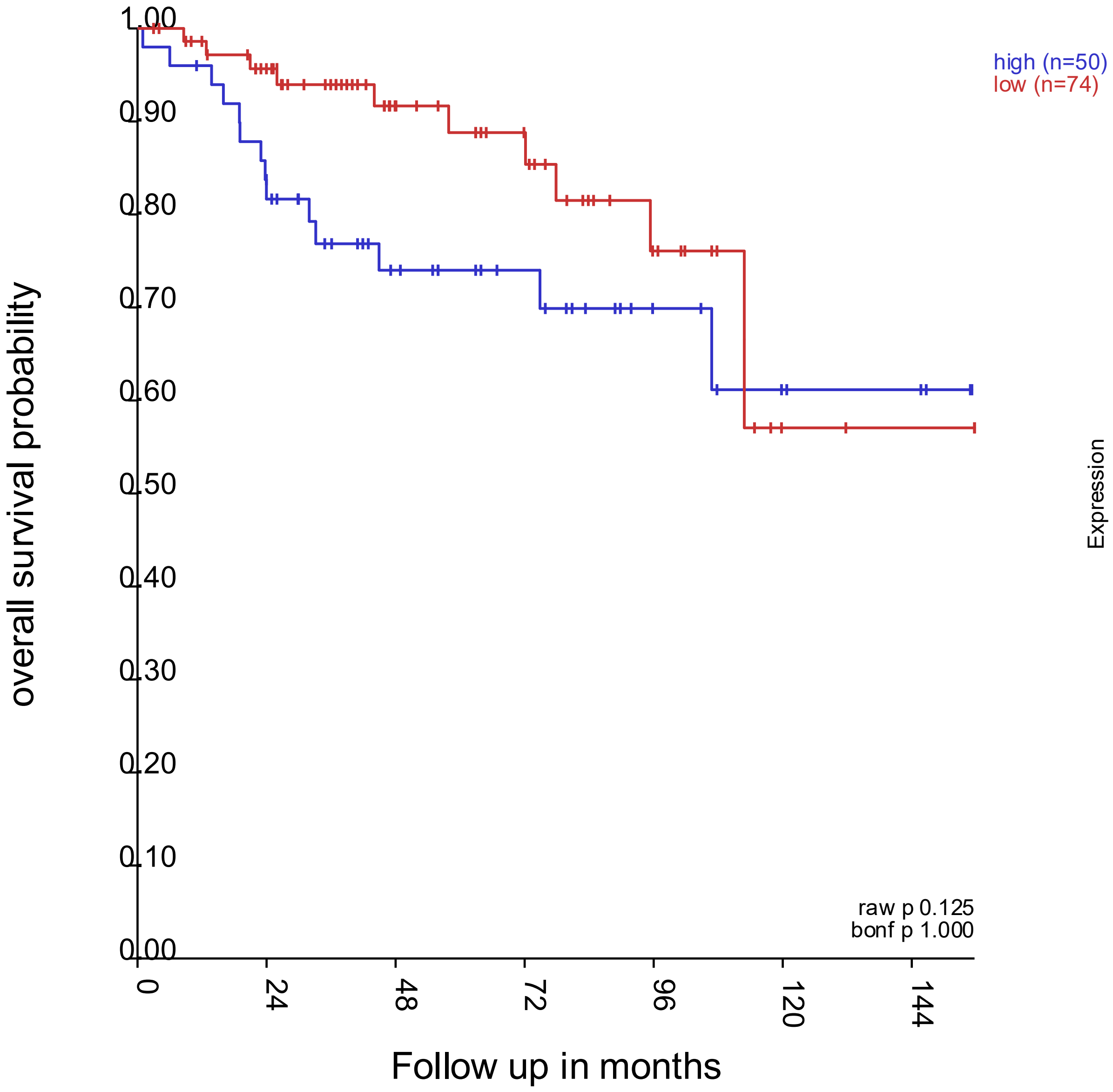

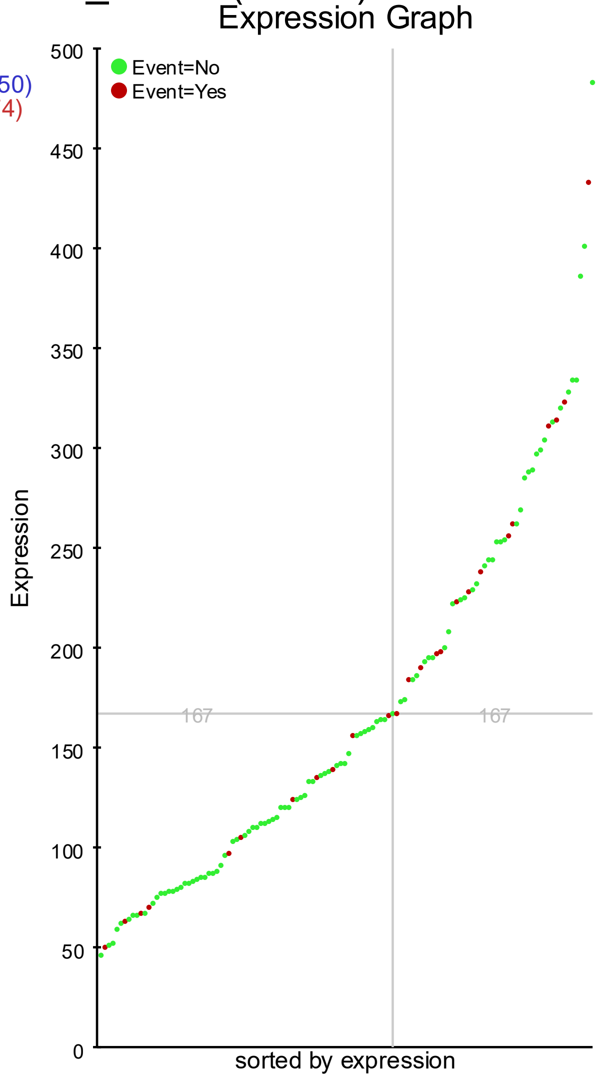

SHH M1

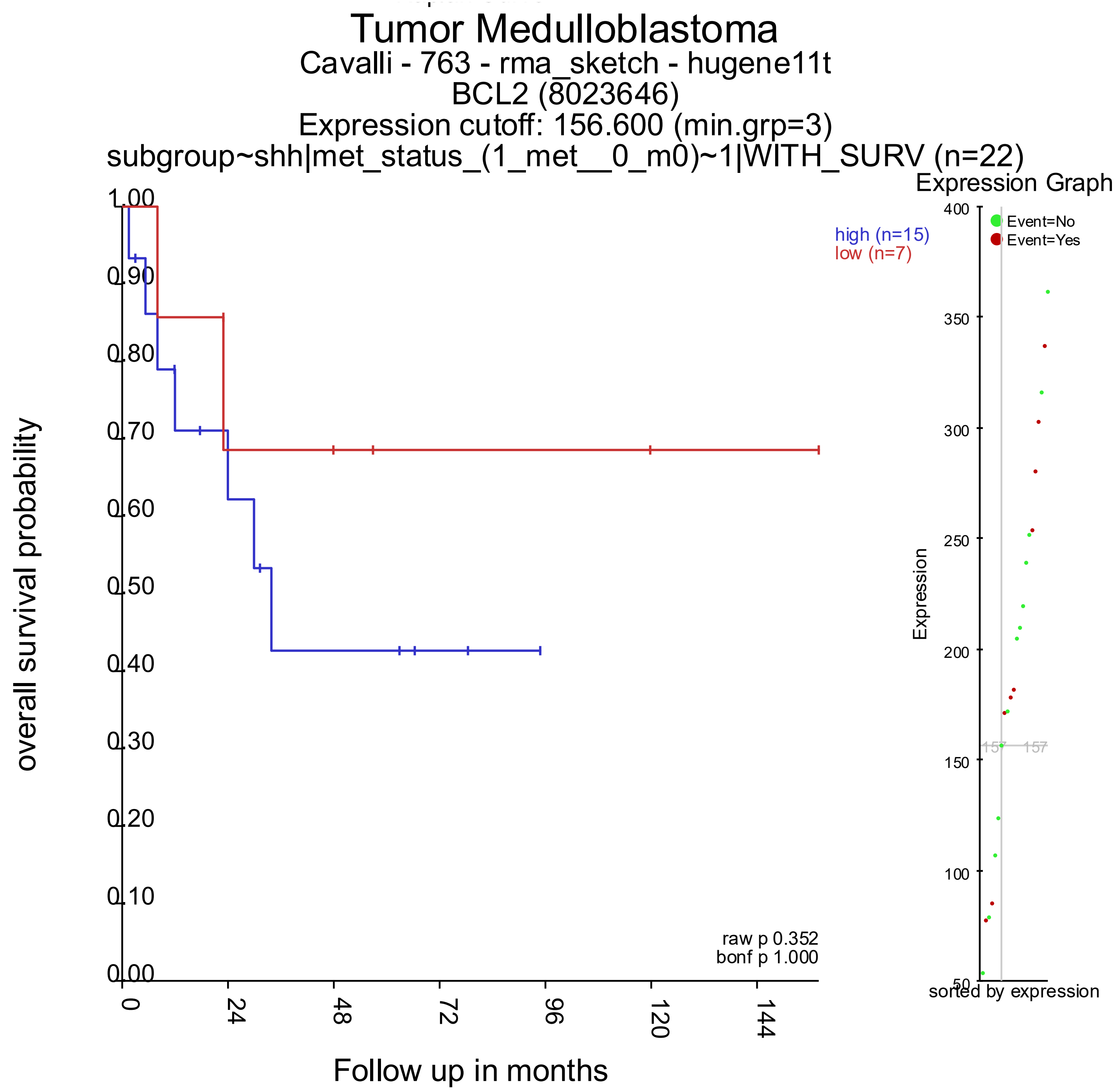

Tumor Medulloblastoma
Cavalli - 763 - rma_sketch - hugene11t
BCL2 (8023646)
Expression cutoff: 156.600 (min.grp=3)
subgroup~shh|met_status_(1_met__0_m0)~1|WITH_SURV (n=22)
high (n=15)
low (n=7)
overall survival probability
1.00
0.90
0.80
0.70
0.60
0.50
0.40
0.30
0.20
0.10
0.00
0
24
48
72
96
120
144
Follow up in months
raw p 0.352
bonf p 1.000
Expression Graph
Event=No
Event=Yes
Expression
400
350
300
250
200
150
100
50
157
157
sorted by expression

GROUP4 M0

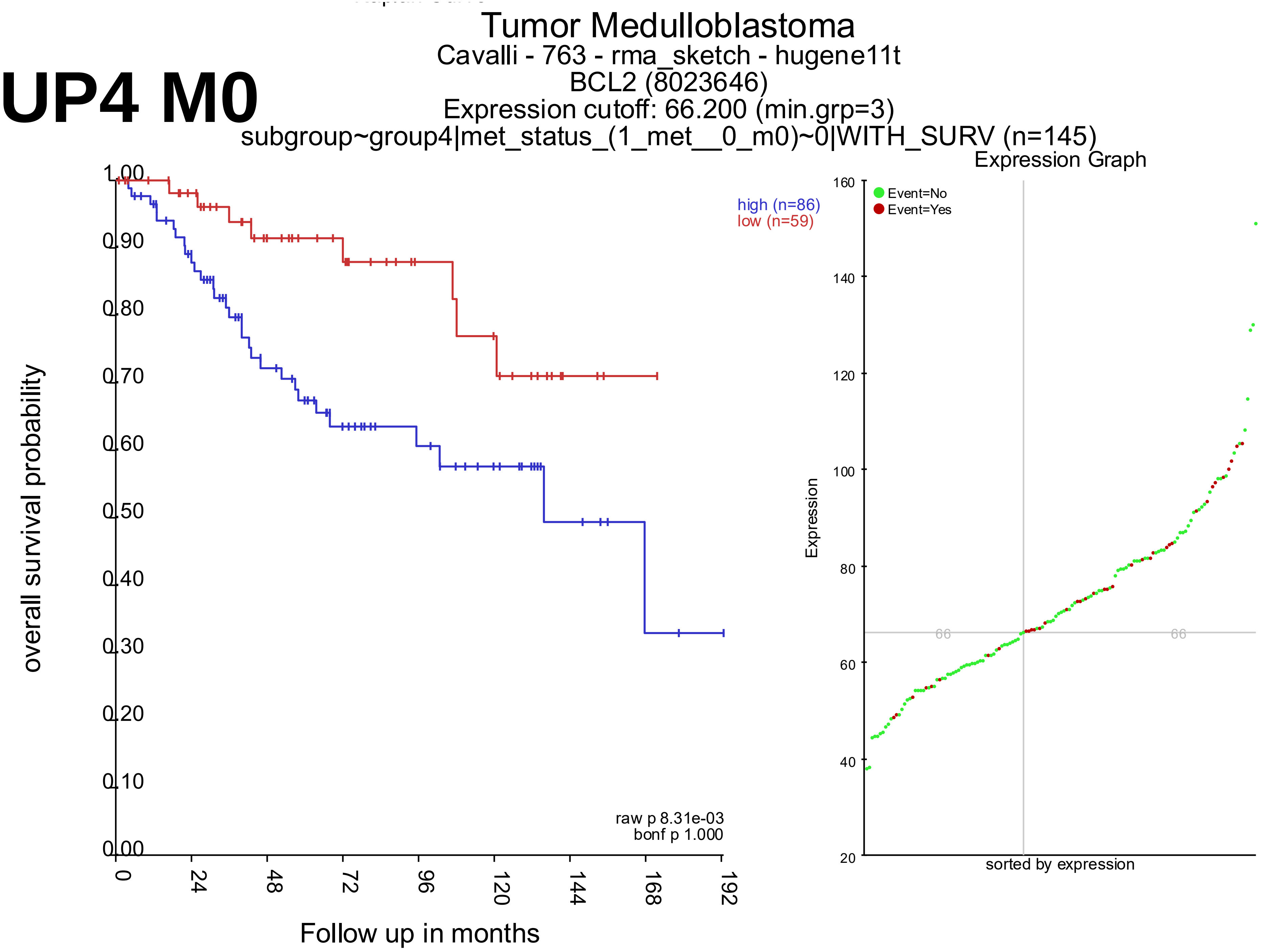

Tumor Medulloblastoma
Cavalli - 763 - rma_sketch - hugene11t
BCL2 (8023646)
Expression cutoff: 66.200 (min.grp=3)
subgroup~group4|met_status_(1_met__0_m0)~0|WITH_SURV (n=145)
Expression Graph
high (n=86)
low (n=59)
overall survival probability
1.00
0.90
0.80
0.70
0.60
0.50
0.40
0.30
0.20
0.10
0.00
raw p 8.31e-03
bonf p 1.000
0
24
48
72
96
120
144
168
192
Follow up in months
Event=No
Event=Yes
Expression
160
140
120
100
80
60
40
20
66
66
sorted by expression

GROUP4 M1

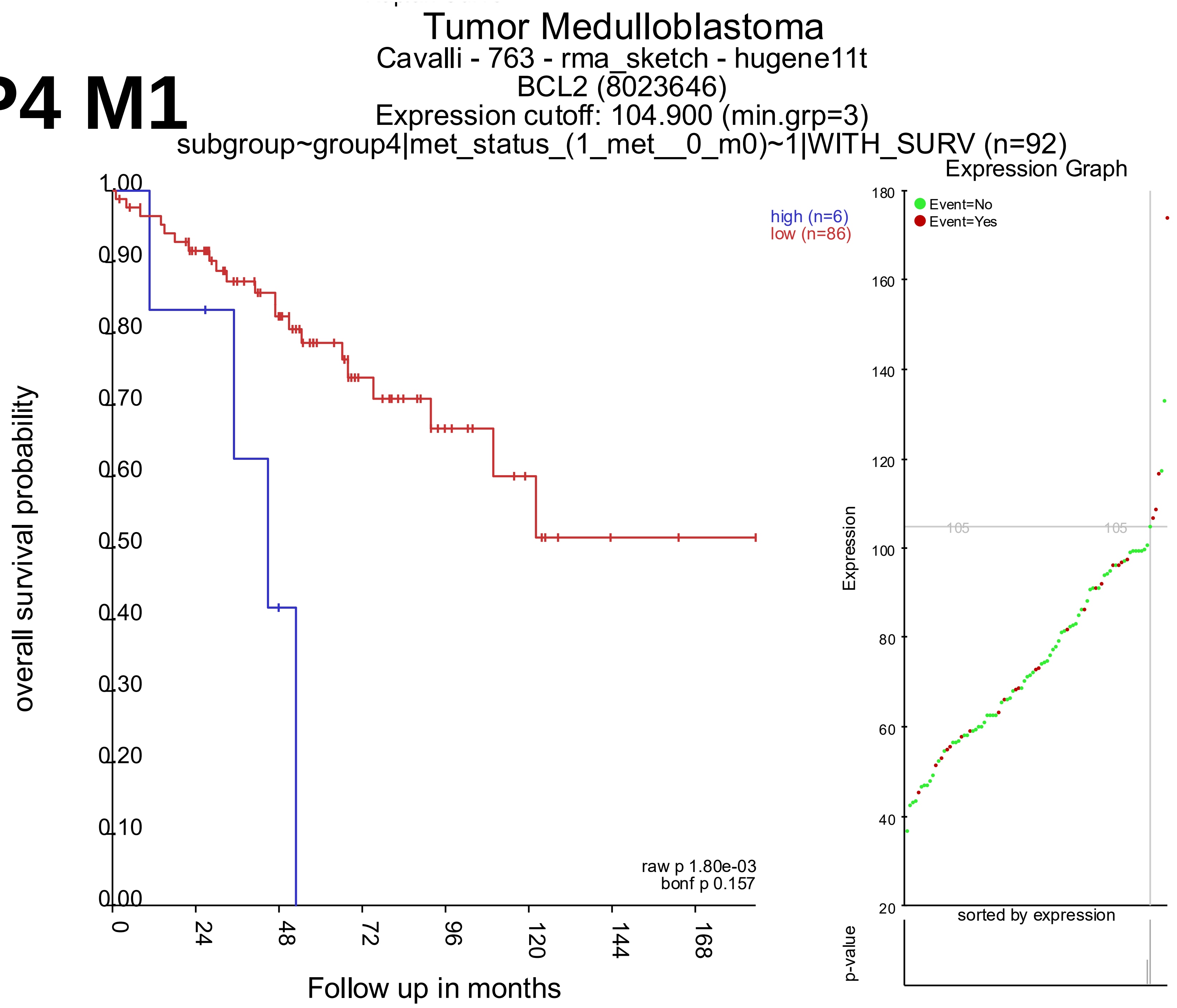
Tumor Medulloblastoma
Cavalli - 763 - rma_sketch - hugene11t
BCL2 (8023646)
Expression cutoff: 104.900 (min.grp=3)
subgroup~group4|met_status_(1_met__0_m0)~1|WITH_SURV (n=92)
overall survival probability
1.00
0.90
0.80
0.70
0.60
0.50
0.40
0.30
0.20
0.10
0.00
0
24
48
72
96
120
144
168
Follow up in months
high (n=6)
low (n=86)
raw p 1.80e-03
bonf p 0.157
Expression Graph
Event=No
Event=Yes
Expression
180
160
140
120
100
80
60
40
20
105
105
sorted by expression
p-value

GROUP3 M0

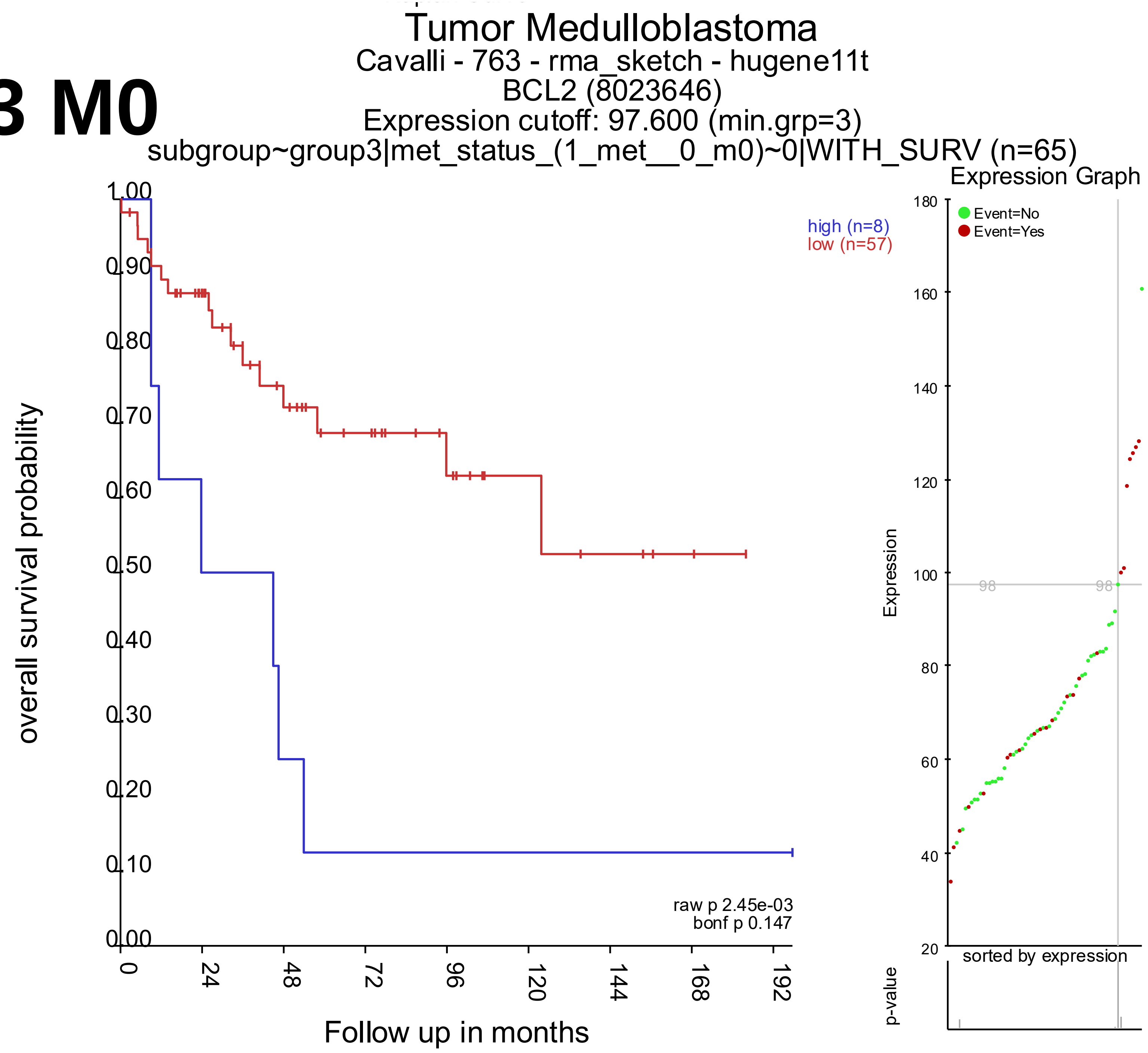

Tumor Medulloblastoma
Cavalli - 763 - rma_sketch - hugene11t
BCL2 (8023646)
Expression cutoff: 97.600 (min.grp=3)
subgroup~group3|met_status_(1_met__0_m0)~0|WITH_SURV (n=65)
overall survival probability
1.00
0.90
0.80
0.70
0.60
0.50
0.40
0.30
0.20
0.10
0.00
0
24
48
72
96
120
144
168
192
Follow up in months
high (n=8)
low (n=57)
raw p 2.45e-03
bonf p 0.147
Expression Graph
Event=No
Event=Yes
Expression
180
160
140
120
100
80
60
40
20
98
98
sorted by expression
p-value

GROUP3 M1

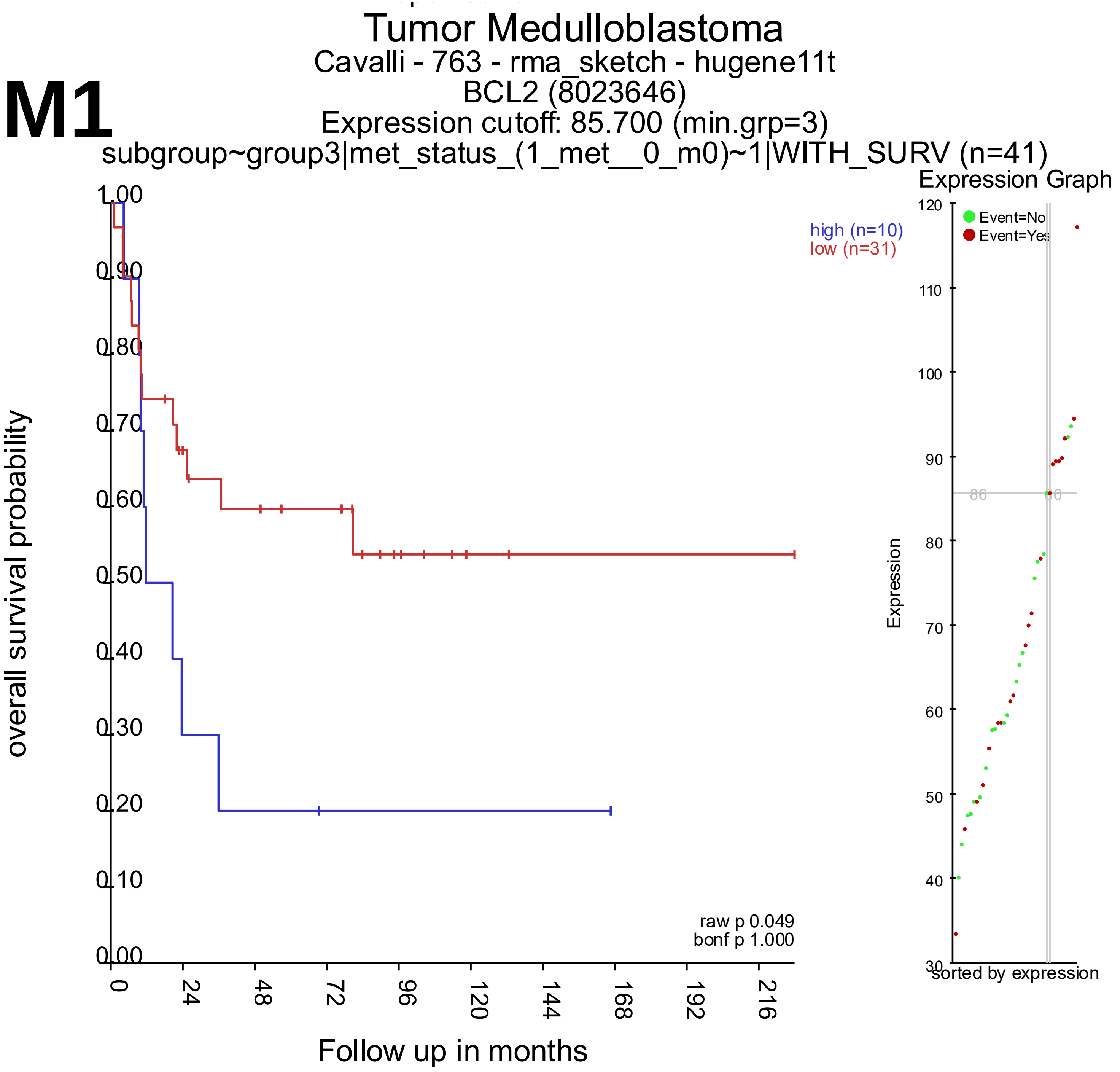
Tumor Medulloblastoma
Cavalli - 763 - rma_sketch - hugene11t
BCL2 (8023646)
Expression cutoff: 85.700 (min.grp=3)
subgroup~group3|met_status_(1_met__0_m0)~1|WITH_SURV (n=41)
overall survival probability
1.00
0.90
0.80
0.70
0.60
0.50
0.40
0.30
0.20
0.10
0.00
0
24
48
72
96
120
144
168
192
216
Follow up in months
high (n=10)
low (n=31)
raw p 0.049
bonf p 1.000
Expression Graph
Event=No
Event=Yes
Expression
120
110
100
90
80
70
60
50
40
30
86
sorted by expression

BCL2L1

WNT M0

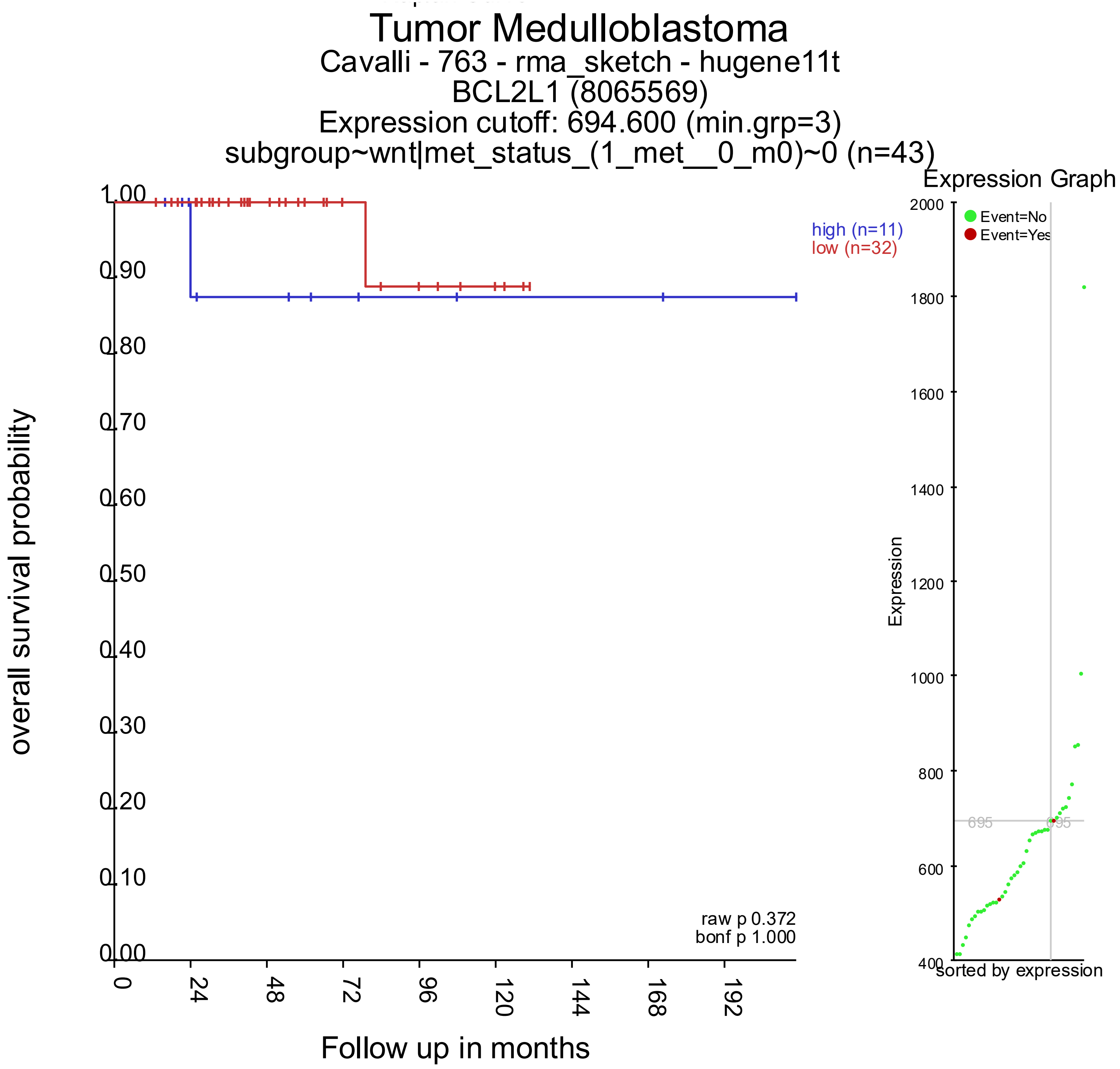

Tumor Medulloblastoma
Cavalli - 763 - rma_sketch - hugene11t
BCL2L1 (8065569)
Expression cutoff: 694.600 (min.grp=3)
subgroup~wnt|met_status_(1_met__0_m0)~0 (n=43)
overall survival probability
1.00
0.90
0.80
0.70
0.60
0.50
0.40
0.30
0.20
0.10
0.00
0
24
48
72
96
120
144
168
192
Follow up in months
high (n=11)
low (n=32)
raw p 0.372
bonf p 1.000
Expression Graph
Event=No
Event=Yes
Expression
2000
1800
1600
1400
1200
1000
800
600
400
695
sorted by expression

WNT M1

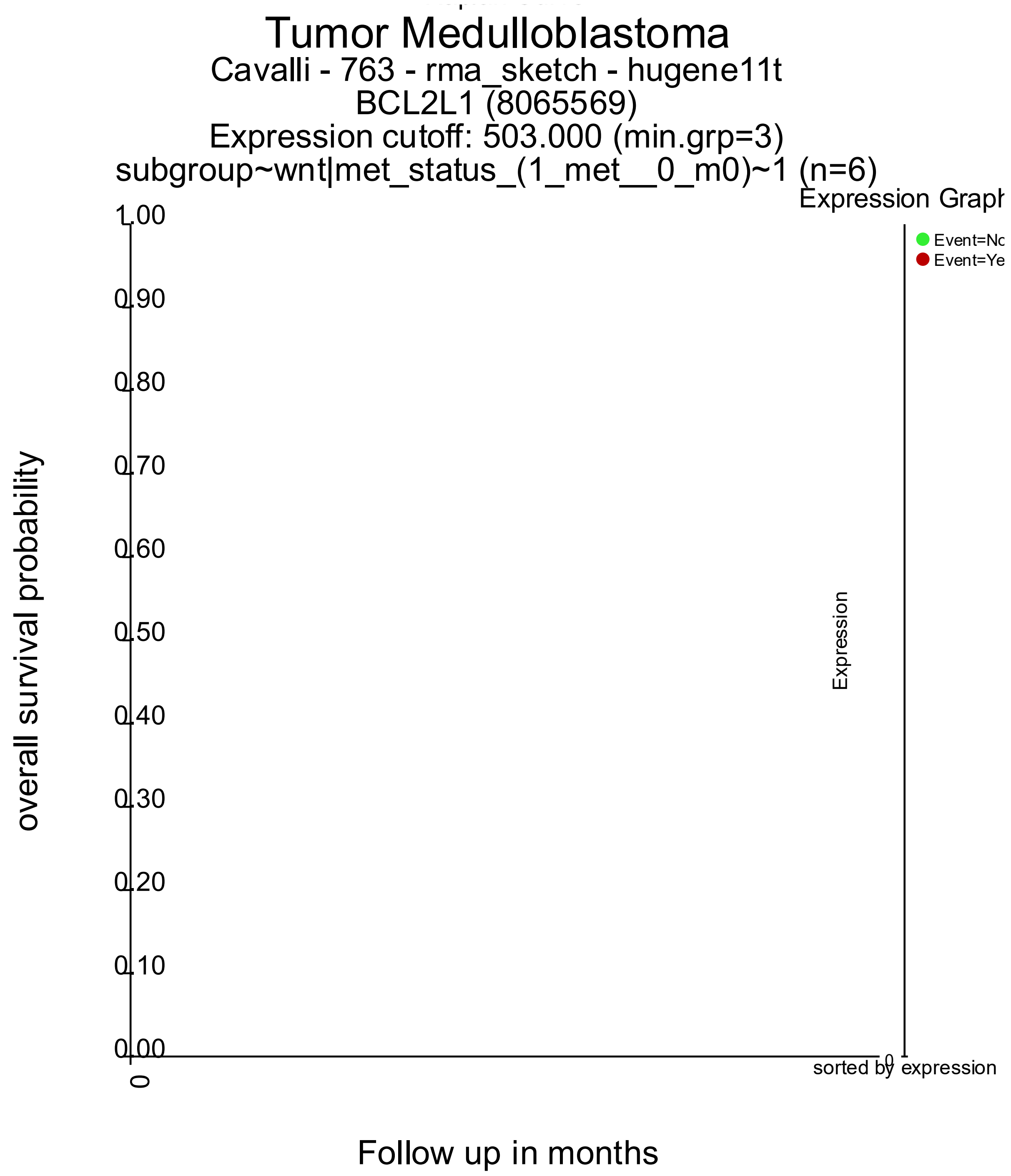

Tumor Medulloblastoma
Cavalli - 763 - rma_sketch - hugene11t
BCL2L1 (8065569)
Expression cutoff: 503.000 (min.grp=3)
subgroup~wnt|met_status_(1_met__0_m0)~1 (n=6)
Expression Graph
Event=No
Event=Ye
1.00
0.90
0.80
0.70
0.60
0.50
0.40
0.30
0.20
0.10
0.00
0
overall survival probability
Follow up in months
Expression
sorted by expression

SHH M0

Tumor Medulloblastoma
Cavalli - 763 - rma_sketch - hugene11t
BCL2L1 (8065569)
Expression cutoff: 388.900 (min.grp=3)
subgroup~shh|met_status_(1_met__0_m0)~0|WITH_SURV (n=124)

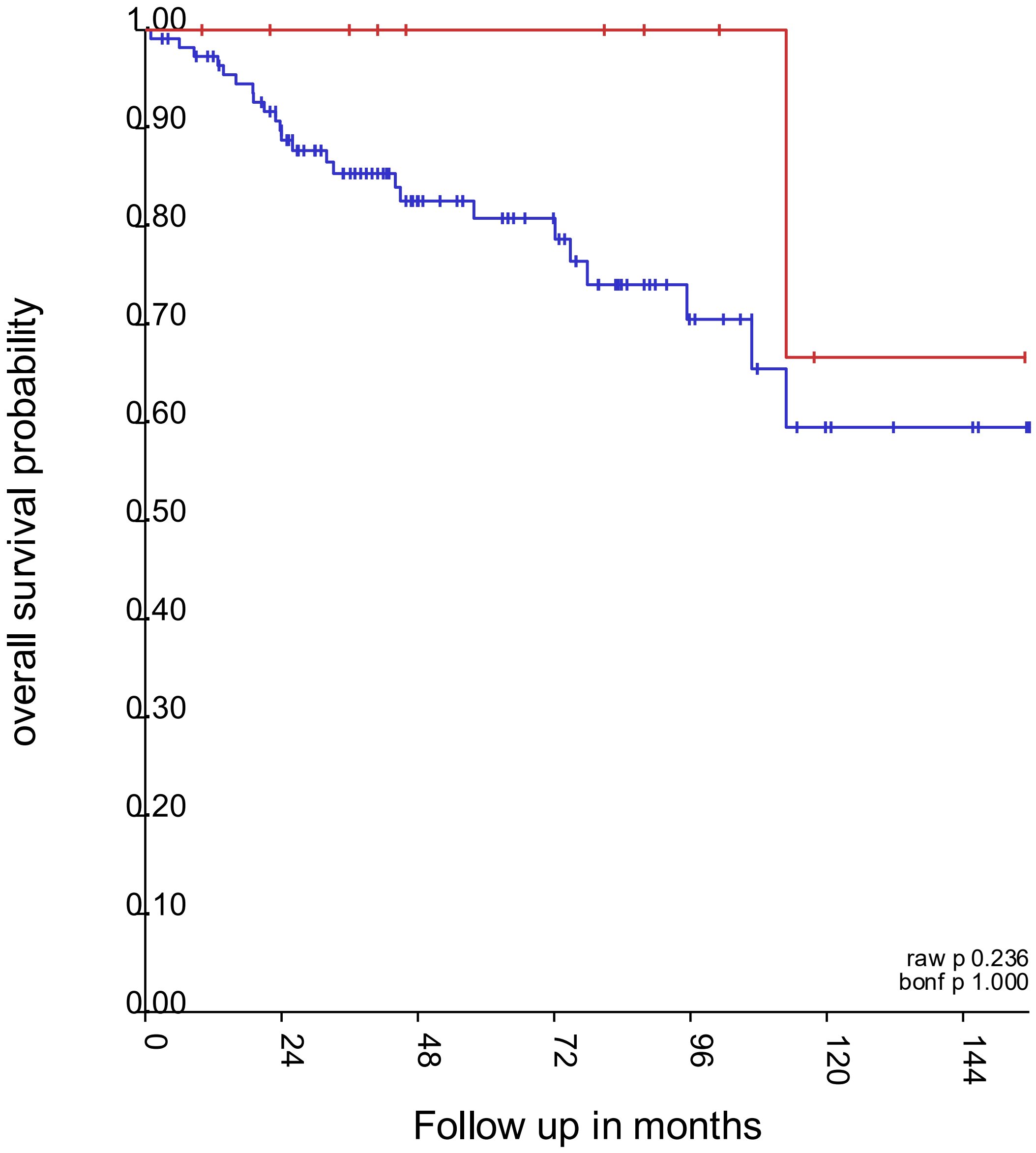

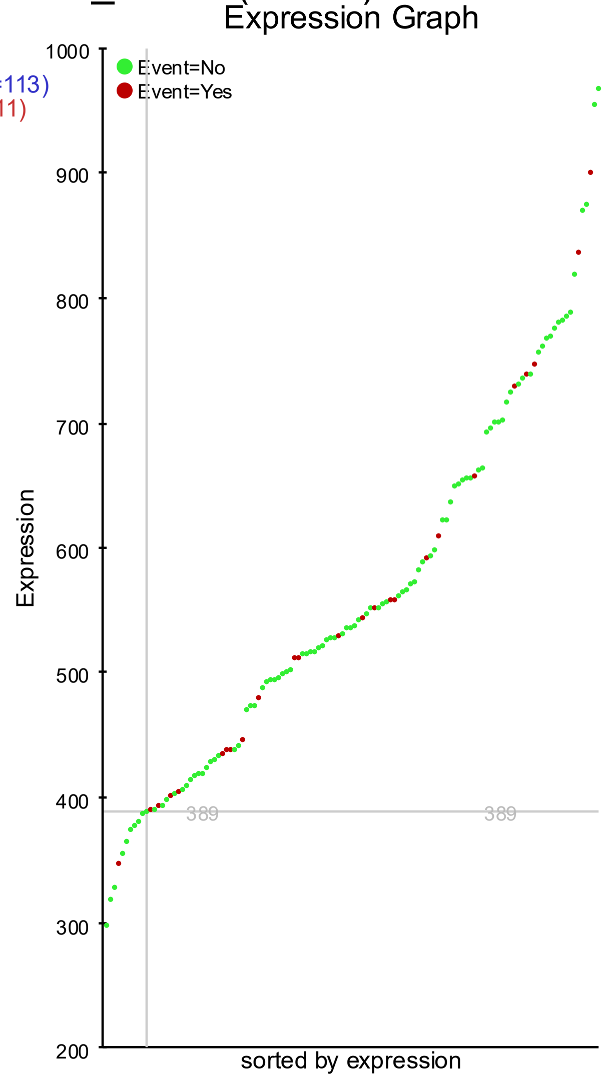

SHH M1

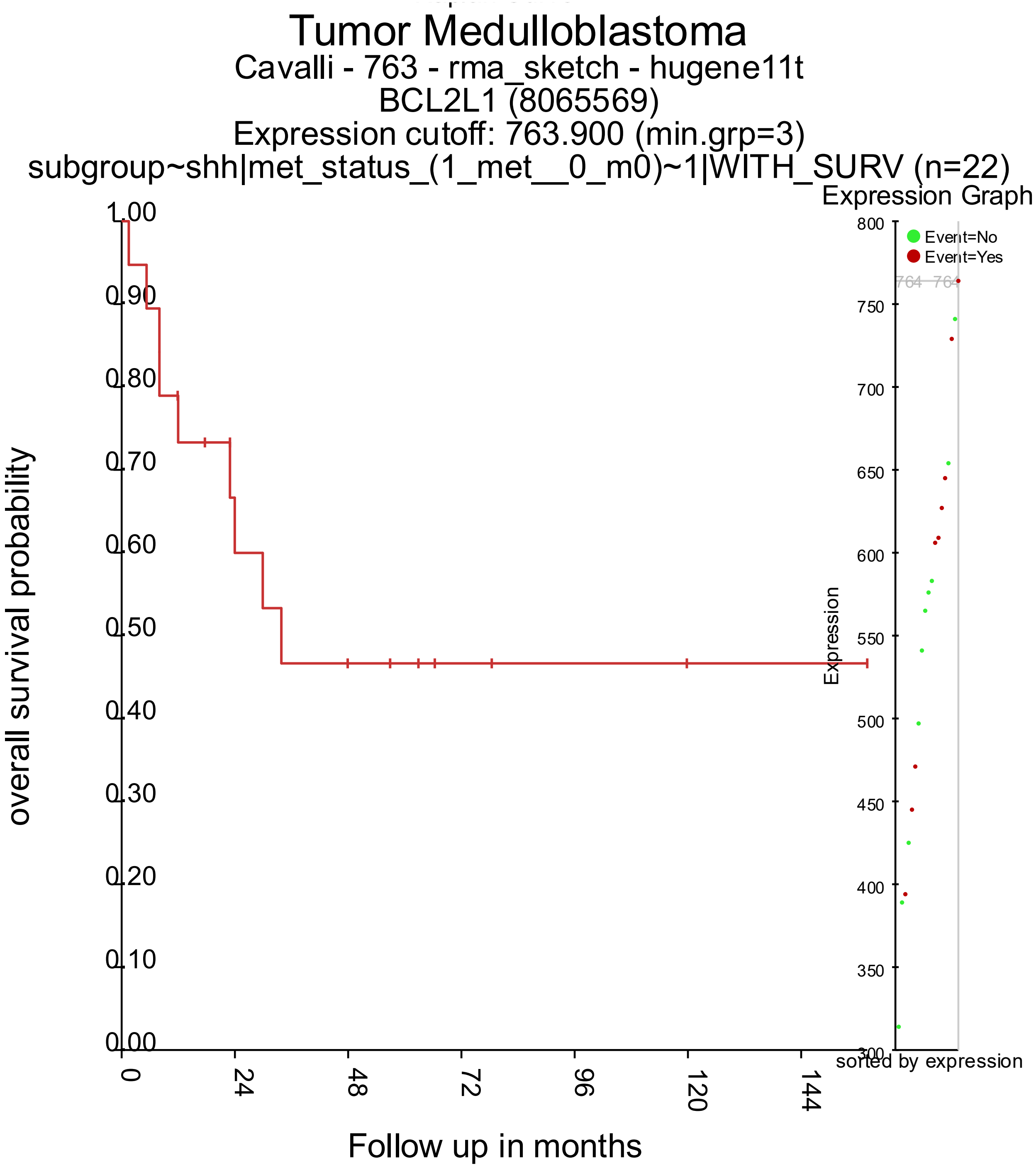

Tumor Medulloblastoma
Cavalli - 763 - rma_sketch - hugene11t
BCL2L1 (8065569)
Expression cutoff: 763.900 (min.grp=3)
subgroup~shh|met_status_(1_met__0_m0)~1|WITH_SURV (n=22)
overall survival probability
1.00
0.90
0.80
0.70
0.60
0.50
0.40
0.30
0.20
0.10
0.00
0
24
48
72
96
120
144
Follow up in months
Expression Graph
Event=No
Event=Yes
764
764
800
750
700
650
600
550
500
450
400
350
300
Expression
sorted by expression

GROUP4 M0

Tumor Medulloblastoma
Cavalli - 763 - rma_sketch - hugene11t
BCL2L1 (8065569)
Expression cutoff: 721.800 (min.grp=3)
subgroup~group4|met_status_(1_met__0_m0)~0|WITH_SURV (n=145)

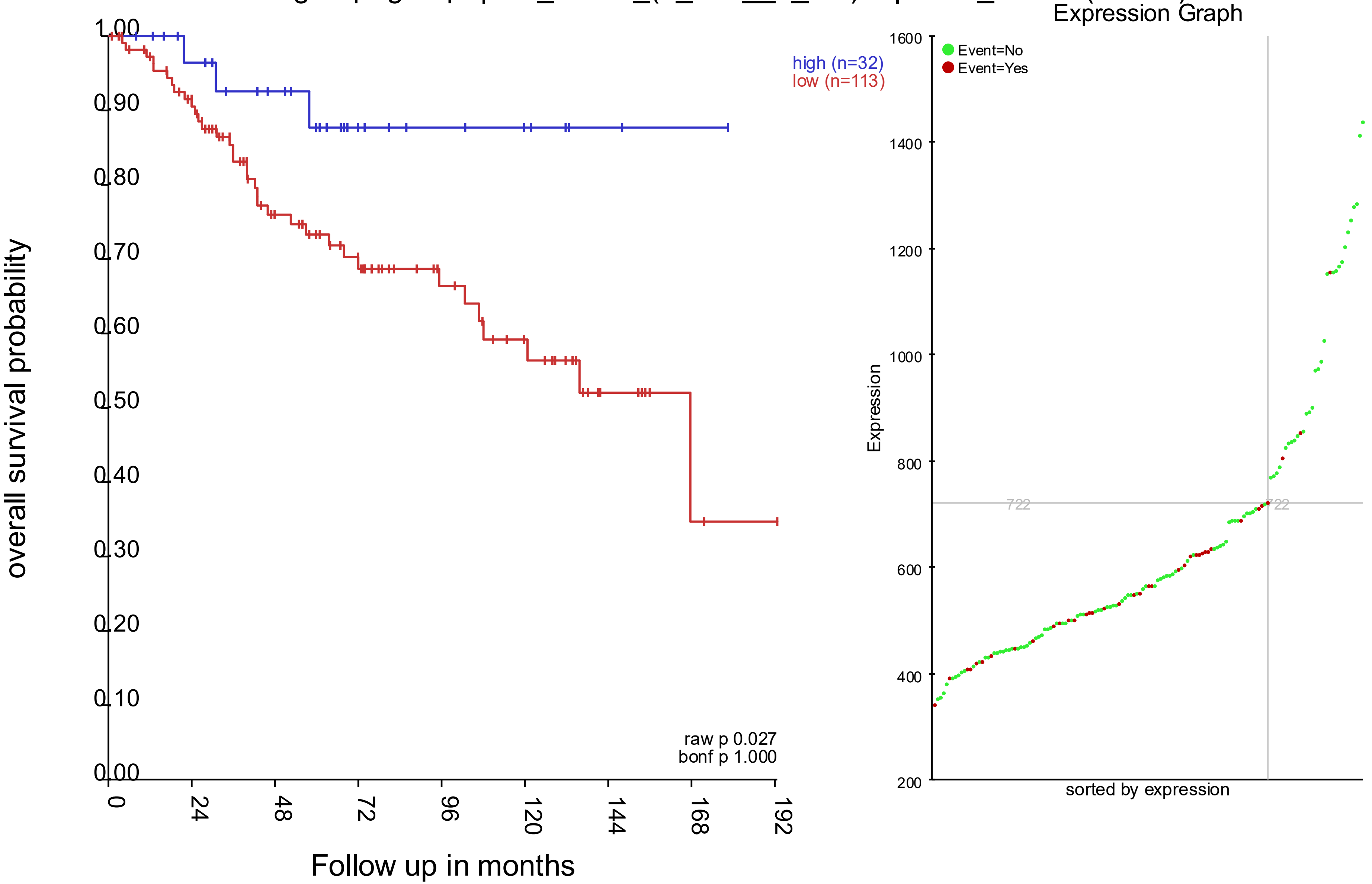

GROUP4 M1

Tumor Medulloblastoma
Cavalli - 763 - rma_sketch - hugene11t
BCL2L1 (8065569)
Expression cutoff: 461.100 (min.grp=3)
subgroup~group4|met_status_(1_met__0_m0)~1|WITH_SURV (n=92)

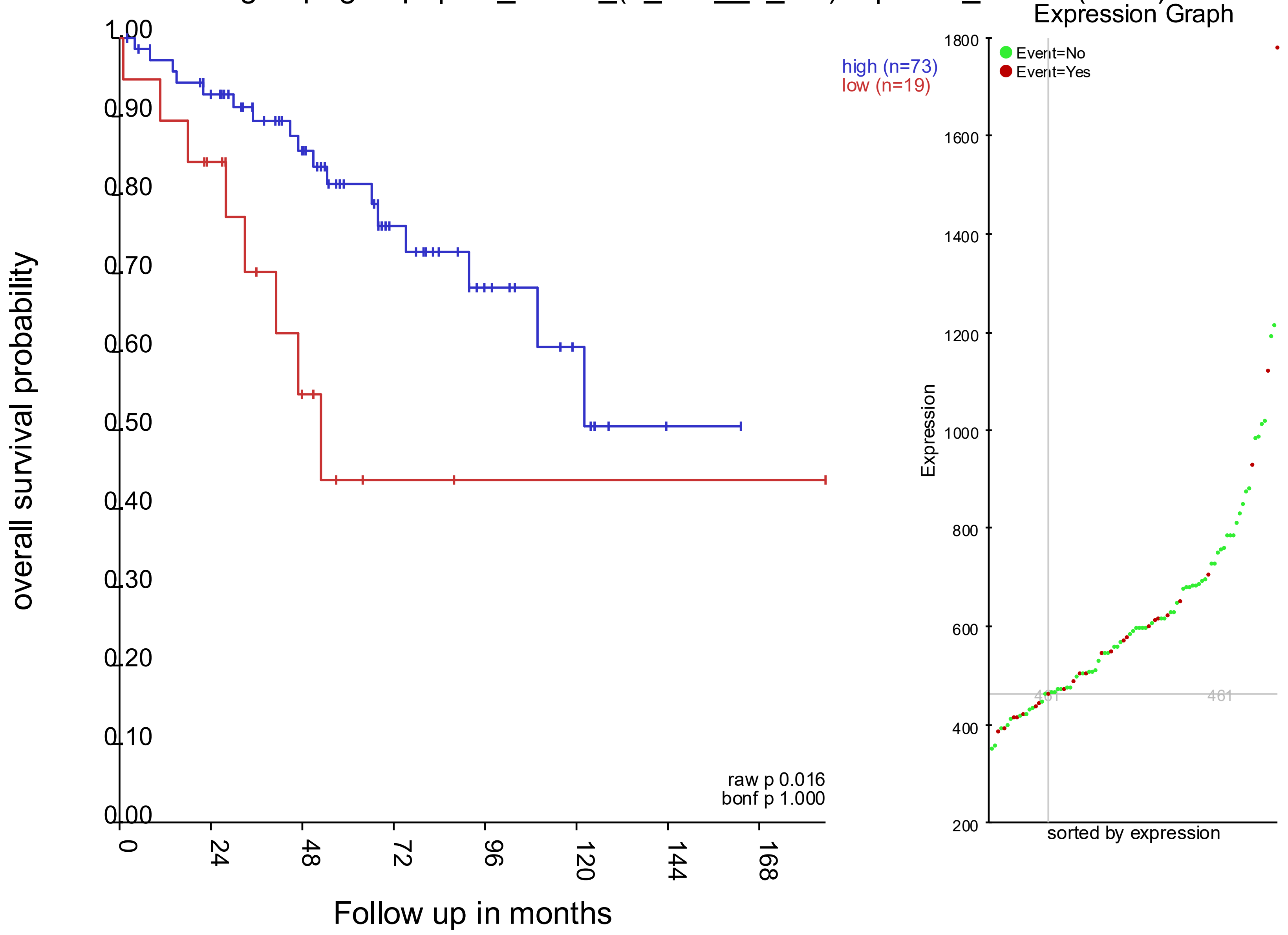

GROUP3 M0

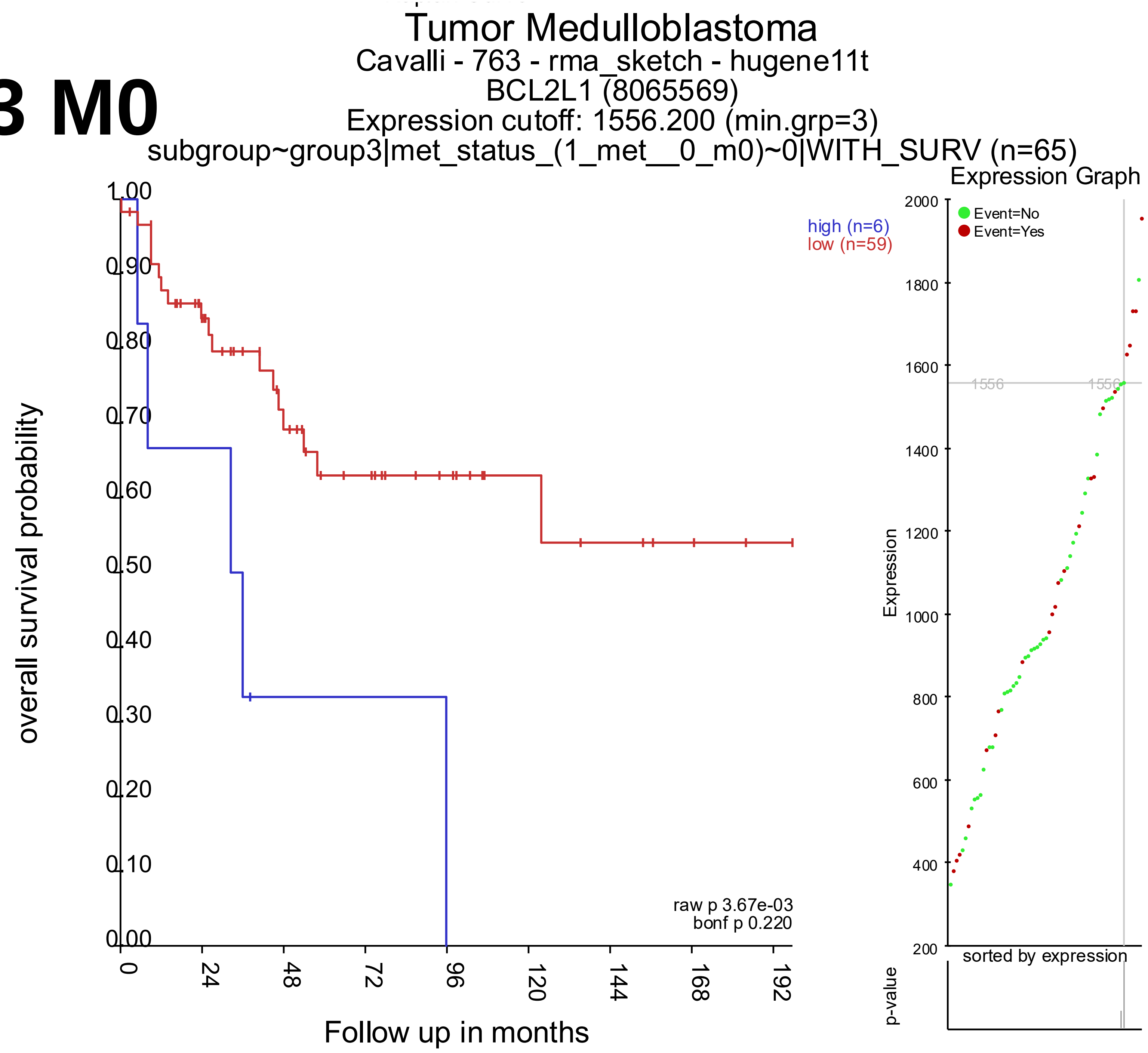

Tumor Medulloblastoma
Cavalli - 763 - rma_sketch - hugene11t
BCL2L1 (8065569)
Expression cutoff: 1556.200 (min.grp=3)
subgroup~group3|met_status_(1_met__0_m0)~0|WITH_SURV (n=65)
high (n=6)
low (n=59)
overall survival probability
1.00
0.90
0.80
0.70
0.60
0.50
0.40
0.30
0.20
0.10
0.00
0
24
48
72
96
120
144
168
192
Follow up in months
raw p 3.67e-03
bonf p 0.220
Expression Graph
Event=No
Event=Yes
Expression
2000
1800
1600
1400
1200
1000
800
600
400
200
1556
sorted by expression
p-value

GROUP3 M1

Tumor Medulloblastoma
Cavalli - 763 - rma_sketch - hugene11t
BCL2L1 (8065569)
Expression cutoff: 1585.800 (min.grp=3)
subgroup~group3|met_status_(1_met__0_m0)~1|WITH_SURV (n=41)

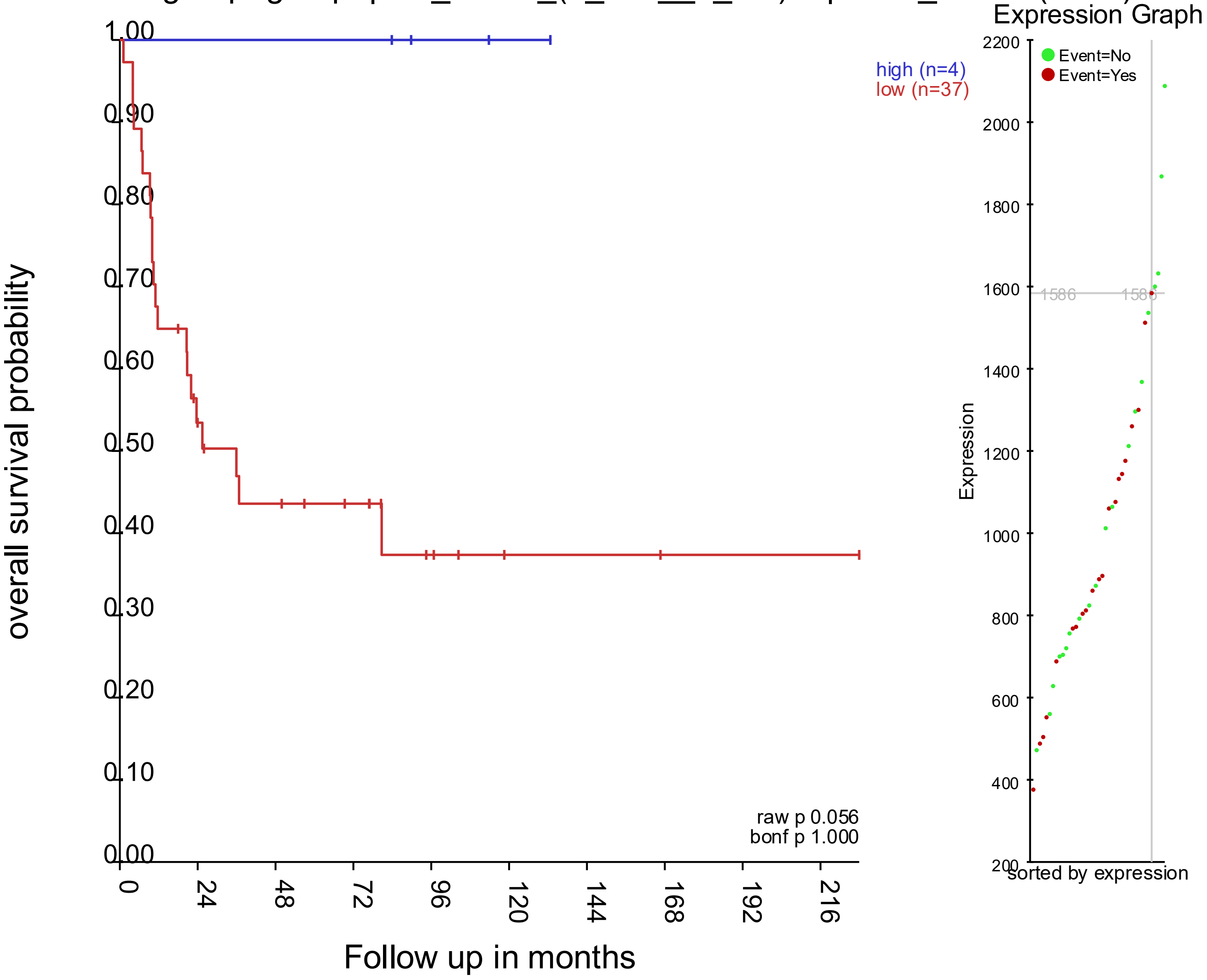

BCL2L2

WNT M0

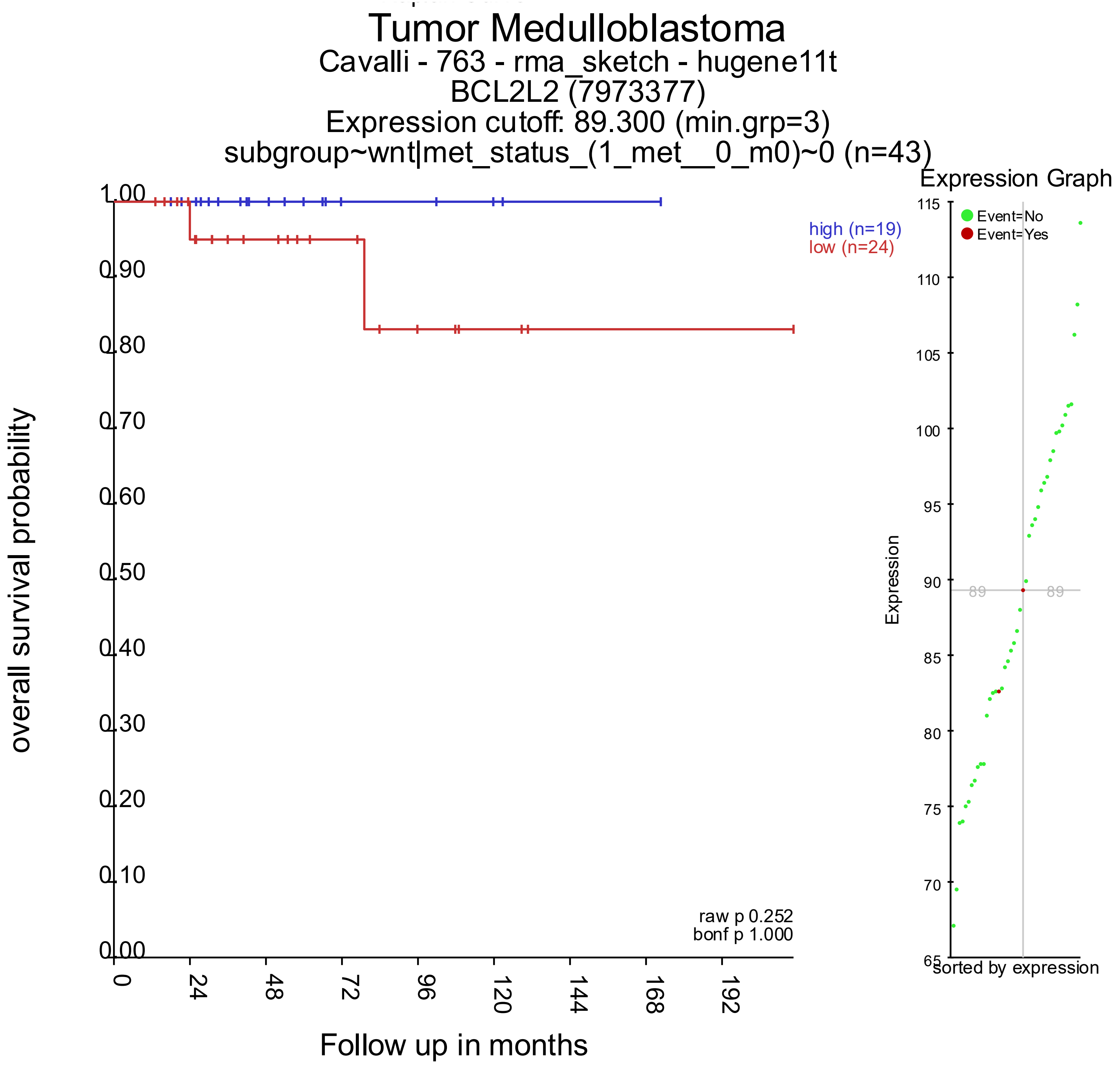

Tumor Medulloblastoma
Cavalli - 763 - rma_sketch - hugene11t
BCL2L2 (7973377)
Expression cutoff: 89.300 (min.grp=3)
subgroup~wnt|met_status_(1_met__0_m0)~0 (n=43)
high (n=19)
low (n=24)
overall survival probability
1.00
0.90
0.80
0.70
0.60
0.50
0.40
0.30
0.20
0.10
0.00
0
24
48
72
96
120
144
168
192
Follow up in months
raw p 0.252
bonf p 1.000
Expression Graph
Event=No
Event=Yes
Expression
115
110
105
100
95
90
85
80
75
70
65
89
89
sorted by expression

WNT M1

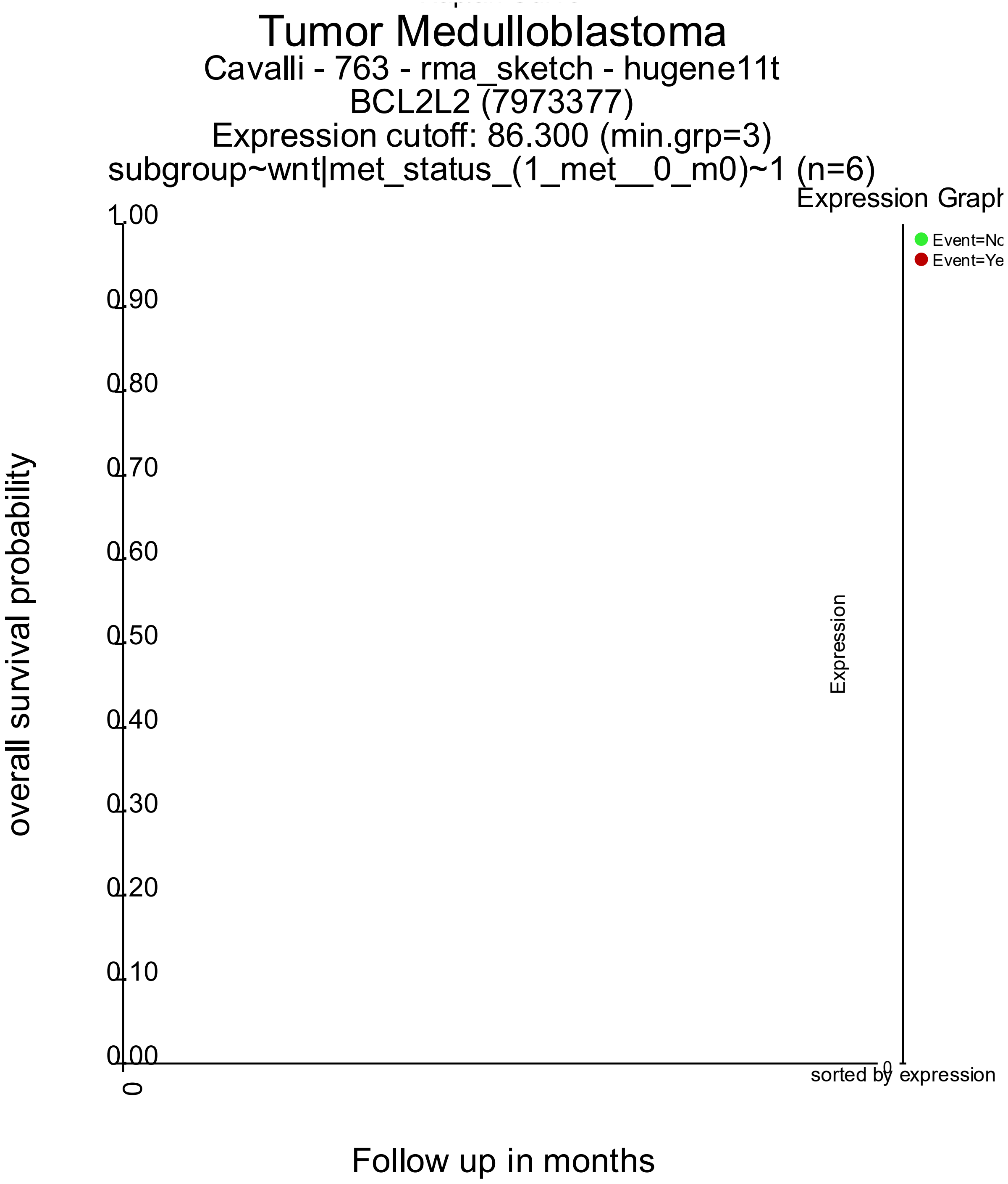

Tumor Medulloblastoma
Cavalli - 763 - rma_sketch - hugene11t
BCL2L2 (7973377)
Expression cutoff: 86.300 (min.grp=3)
subgroup~wnt|met_status_(1_met__0_m0)~1 (n=6)
Expression Graph
Event=N
Event=Ye
overall survival probability
1.00
0.90
0.80
0.70
0.60
0.50
0.40
0.30
0.20
0.10
0.00
0
Follow up in months
Expression
0
sorted by expression

SHH M0

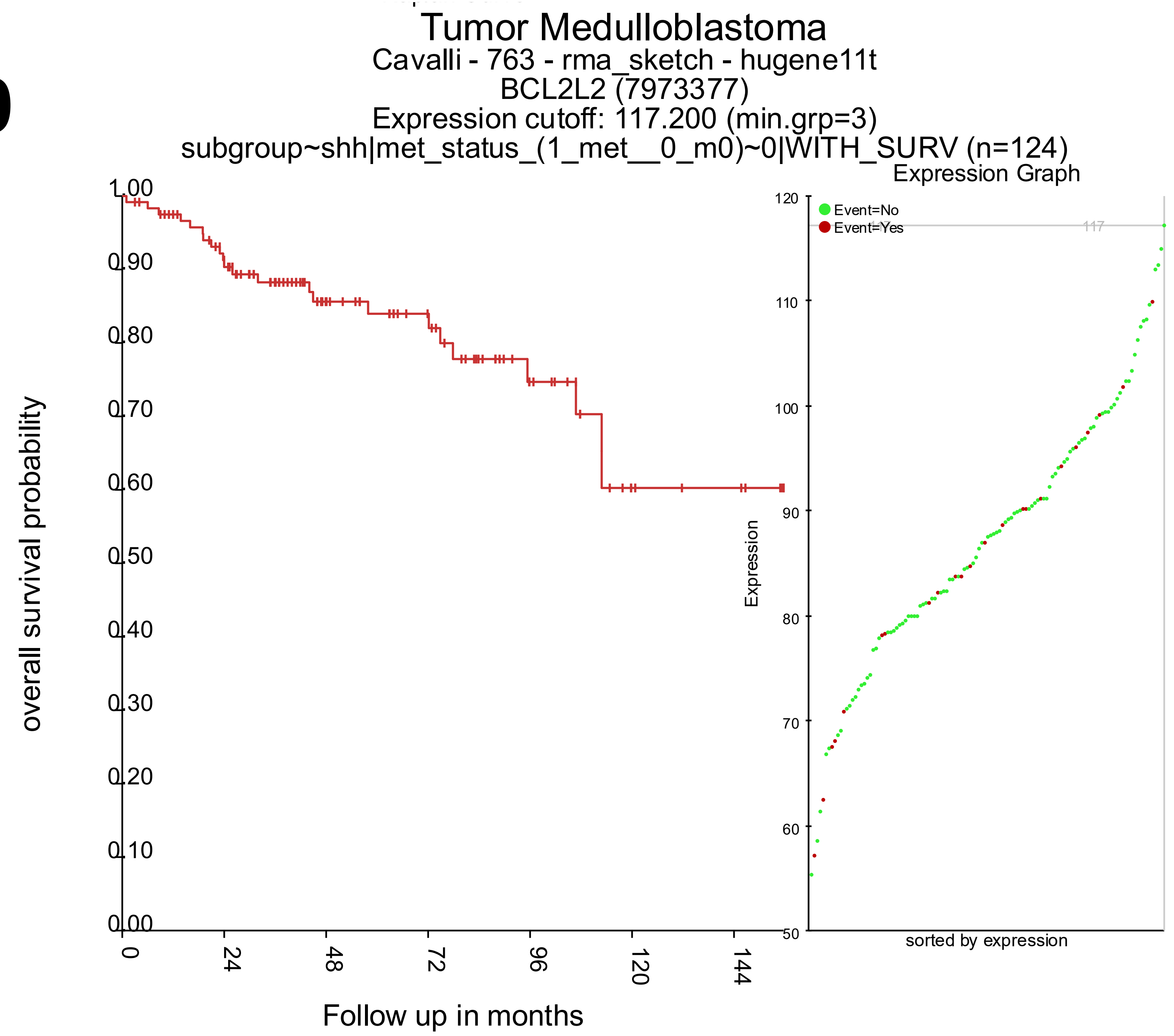

Tumor Medulloblastoma
Cavalli - 763 - rma_sketch - hugene11t
BCL2L2 (7973377)
Expression cutoff: 117.200 (min.grp=3)
subgroup~shh|met_status_(1_met__0_m0)~0|WITH_SURV (n=124)
overall survival probability
1.00
0.90
0.80
0.70
0.60
0.50
0.40
0.30
0.20
0.10
0.00
0
24
48
72
96
120
144
Follow up in months
Expression Graph
Event=No
Event=Yes
117
120
110
100
90
80
70
60
50
Expression
sorted by expression

SHH M1

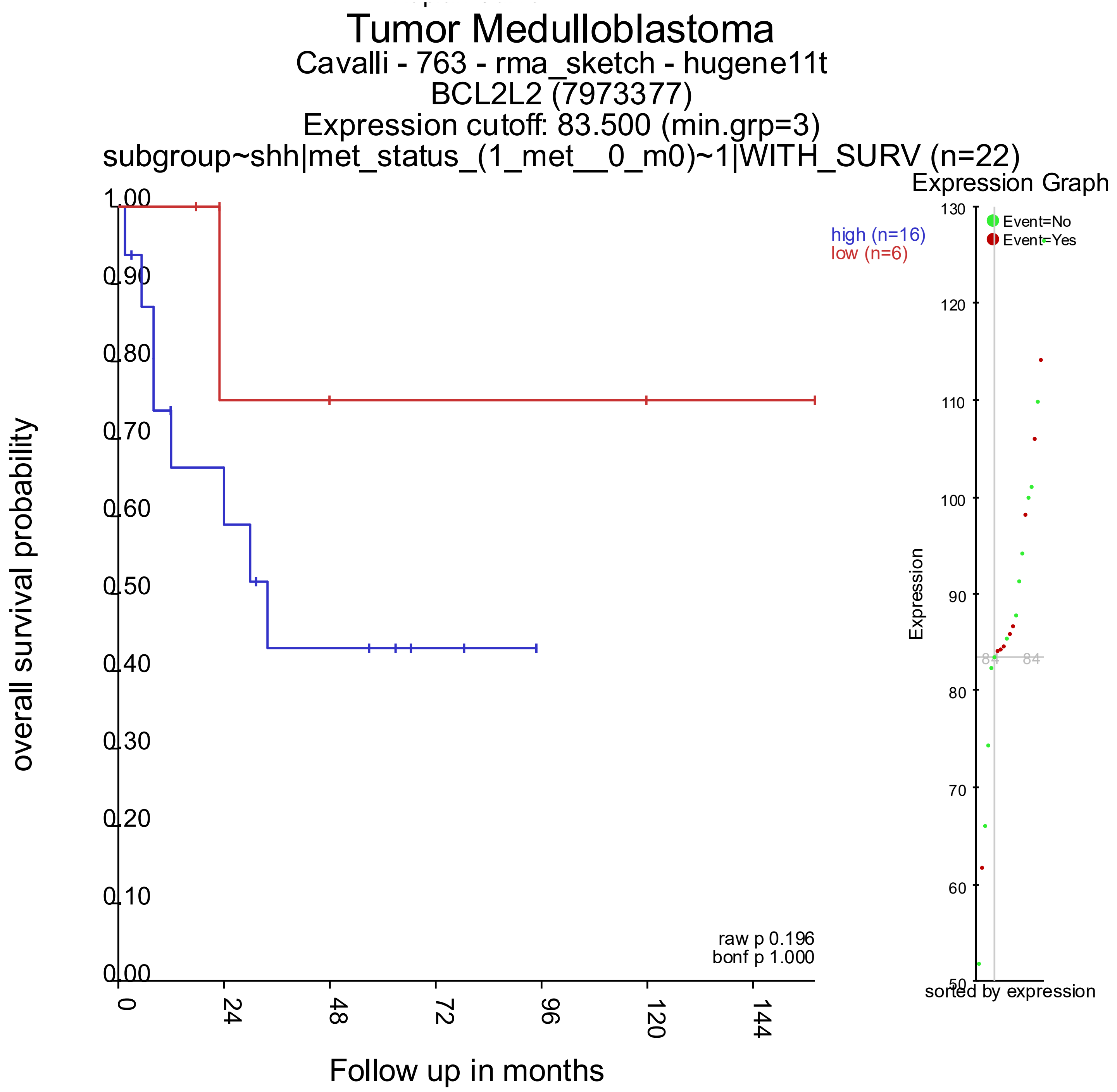

Tumor Medulloblastoma
Cavalli - 763 - rma_sketch - hugene11t
BCL2L2 (7973377)
Expression cutoff: 83.500 (min.grp=3)
subgroup~shh|met_status_(1_met__0_m0)~1|WITH_SURV (n=22)
overall survival probability
1.00
0.90
0.80
0.70
0.60
0.50
0.40
0.30
0.20
0.10
0.00
0
24
48
72
96
120
144
Follow up in months
high (n=16)
low (n=6)
raw p 0.196
bonf p 1.000
Expression Graph
Event=No
Event=Yes
Expression
130
120
110
100
90
80
70
60
50
84
sorted by expression

GROUP4 M0

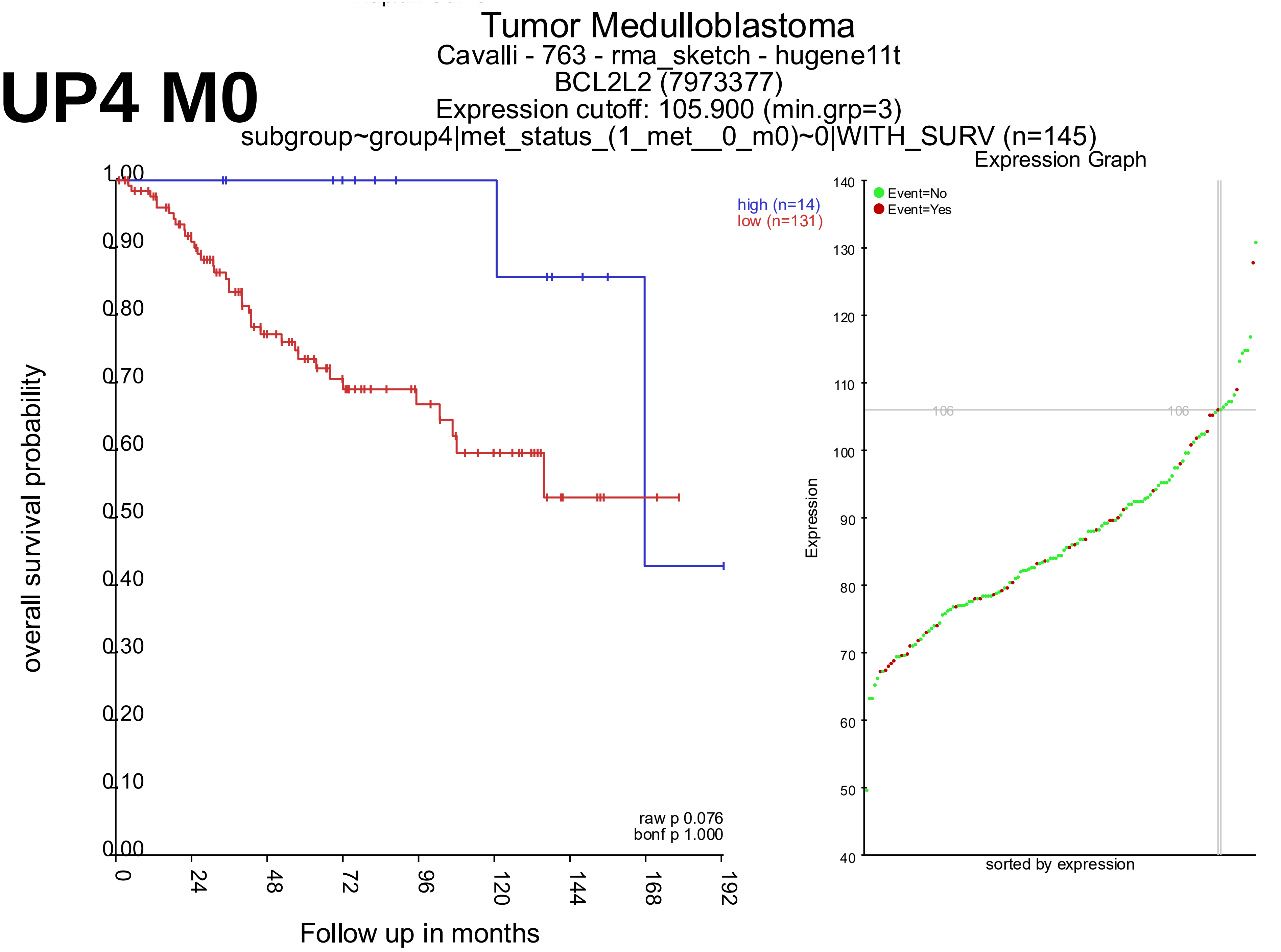

Tumor Medulloblastoma
Cavalli - 763 - rma_sketch - hugene11t
BCL2L2 (7973377)
Expression cutoff: 105.900 (min.grp=3)
subgroup~group4|met_status_(1_met__0_m0)~0|WITH_SURV (n=145)
overall survival probability
1.00
0.90
0.80
0.70
0.60
0.50
0.40
0.30
0.20
0.10
0.00
high (n=14)
low (n=131)
raw p 0.076
bonf p 1.000
0
24
48
72
96
120
144
168
192
Follow up in months
Expression Graph
Event=No
Event=Yes
140
130
120
110
100
90
80
70
60
50
40
Expression
106
106
sorted by expression

GROUP4 M1

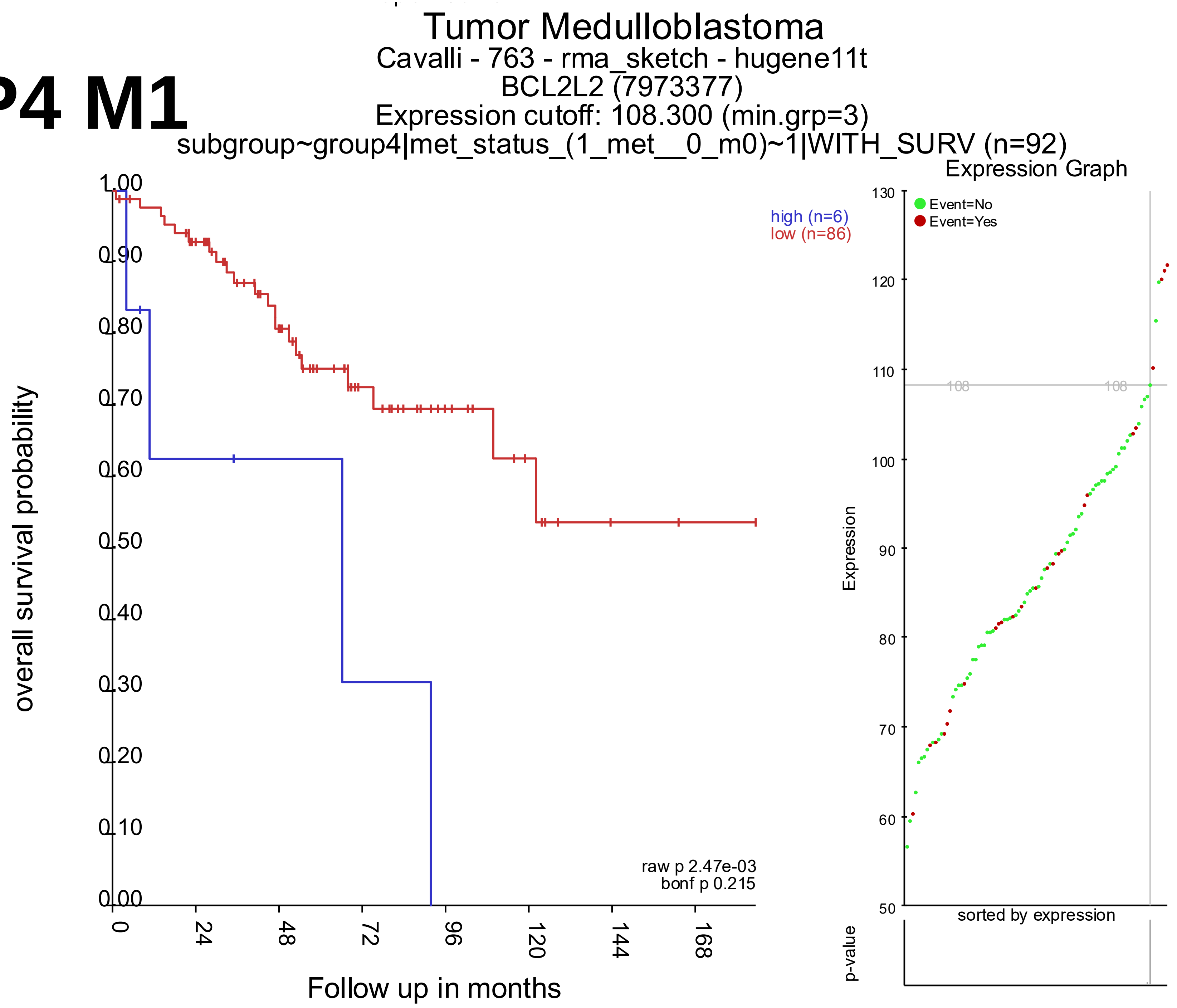

Tumor Medulloblastoma
Cavalli - 763 - rma_sketch - hugene11t
BCL2L2 (7973377)
Expression cutoff: 108.300 (min.grp=3)
subgroup~group4|met_status_(1_met__0_m0)~1|WITH_SURV (n=92)
overall survival probability
1.00
0.90
0.80
0.70
0.60
0.50
0.40
0.30
0.20
0.10
0.00
0
24
48
72
96
120
144
168
Follow up in months
high (n=6)
low (n=86)
raw p 2.47e-03
bonf p 0.215
Expression Graph
Event=No
Event=Yes
Expression
130
120
110
100
90
80
70
60
50
108
108
sorted by expression
p-value

GROUP3 M0

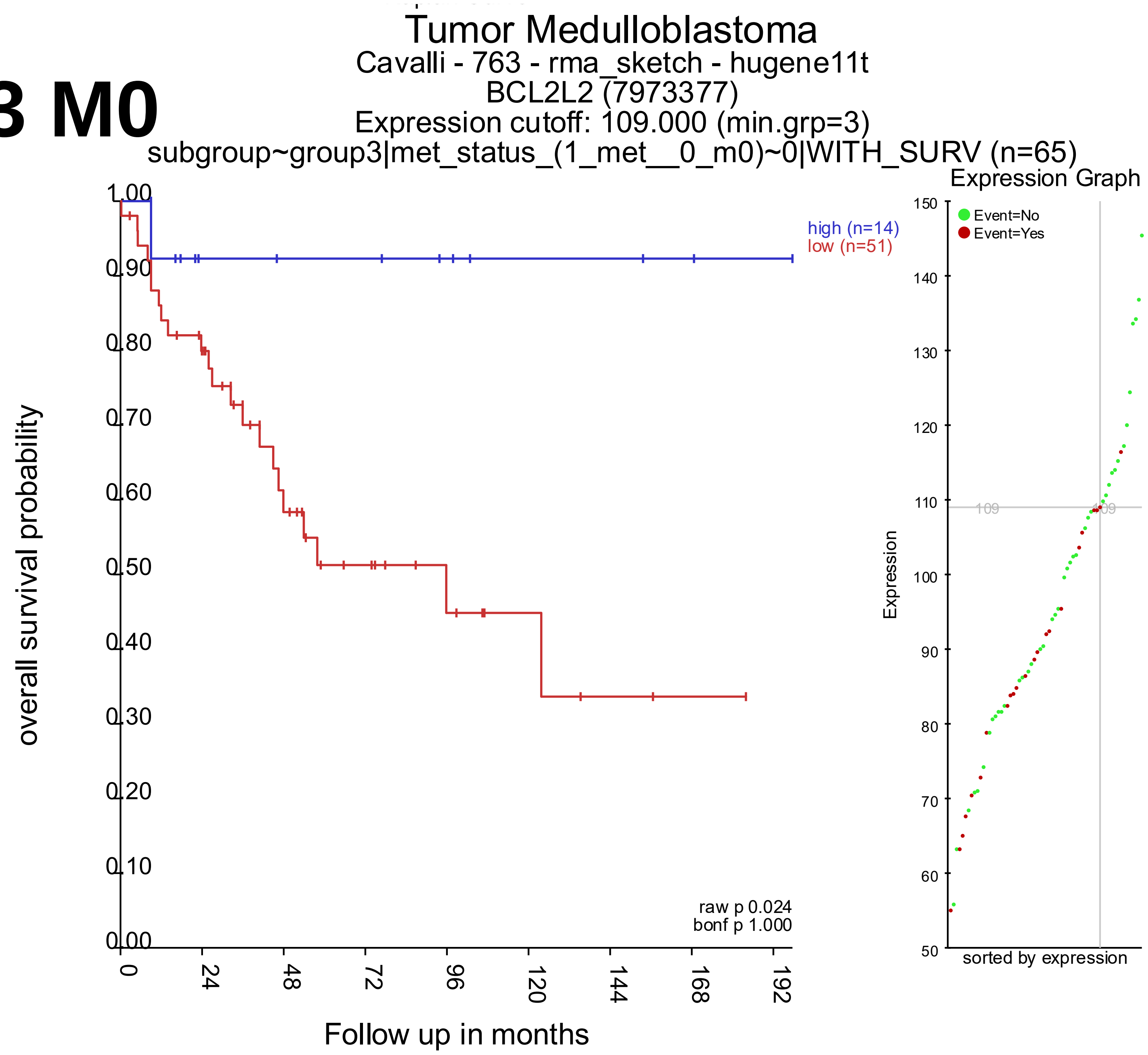
Tumor Medulloblastoma
Cavalli - 763 - rma_sketch - hugene11t
BCL2L2 (7973377)
Expression cutoff: 109.000 (min.grp=3)
subgroup~group3|met_status_(1_met__0_m0)~0|WITH_SURV (n=65)
overall survival probability
1.00
0.90
0.80
0.70
0.60
0.50
0.40
0.30
0.20
0.10
0.00
0
24
48
72
96
120
144
168
192
Follow up in months
high (n=14)
low (n=51)
raw p 0.024
bonf p 1.000
Expression Graph
Event=No
Event=Yes
Expression
150
140
130
120
110
100
90
80
70
60
50
109
sorted by expression

GROUP3 M1

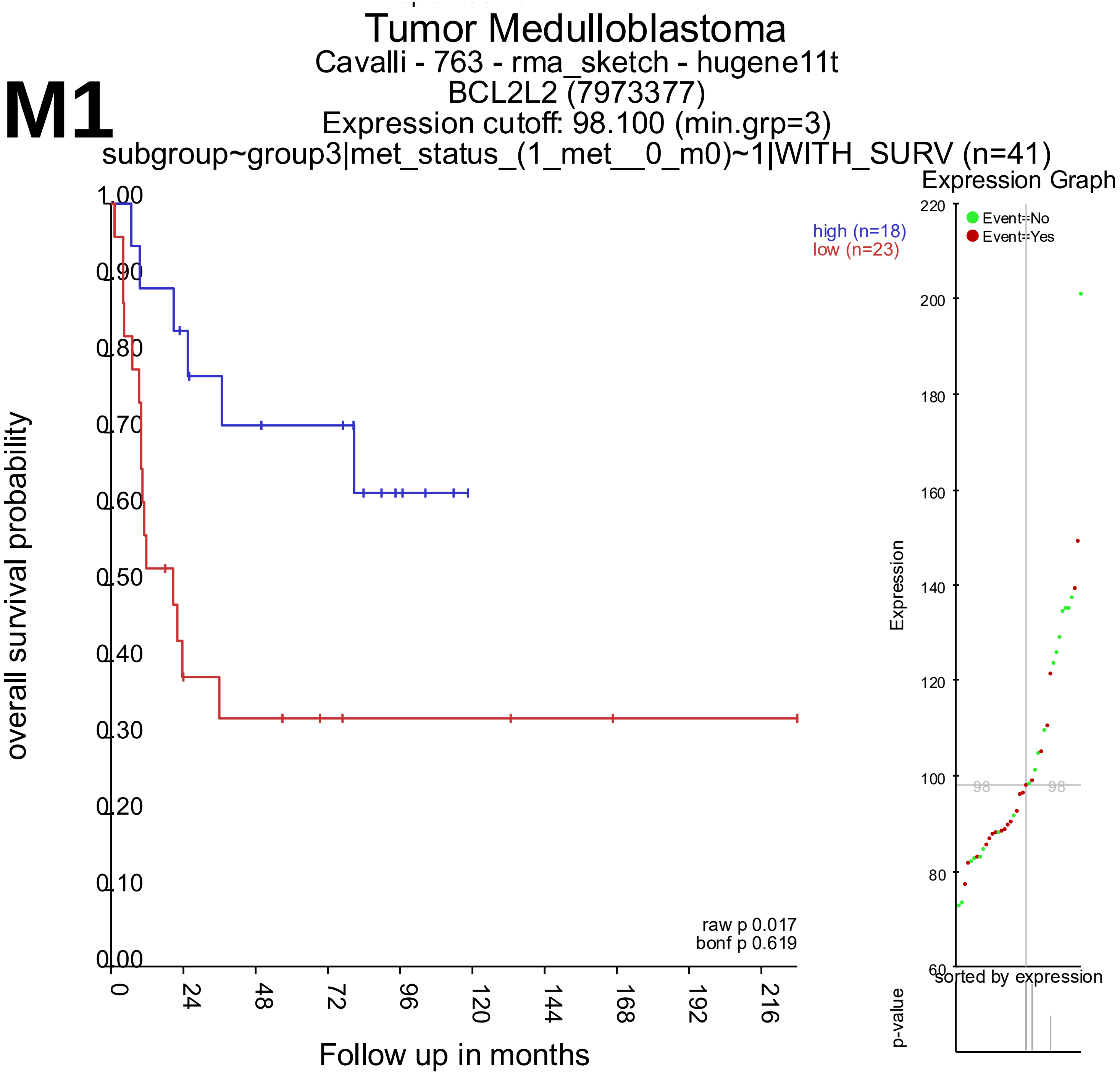
Tumor Medulloblastoma
Cavalli - 763 - rma_sketch - hugene11t
BCL2L2 (7973377)
Expression cutoff: 98.100 (min.grp=3)
subgroup~group3|met_status_(1_met__0_m0)~1|WITH_SURV (n=41)
high (n=18)
low (n=23)
overall survival probability
1.00
0.90
0.80
0.70
0.60
0.50
0.40
0.30
0.20
0.10
0.00
0
24
48
72
96
120
144
168
192
216
Follow up in months
raw p 0.017
bonf p 0.619
Expression Graph
Event=No
Event=Yes
Expression
220
200
180
160
140
120
100
80
60
98
98
sorted by expression
p-value

BTK

WNT M0

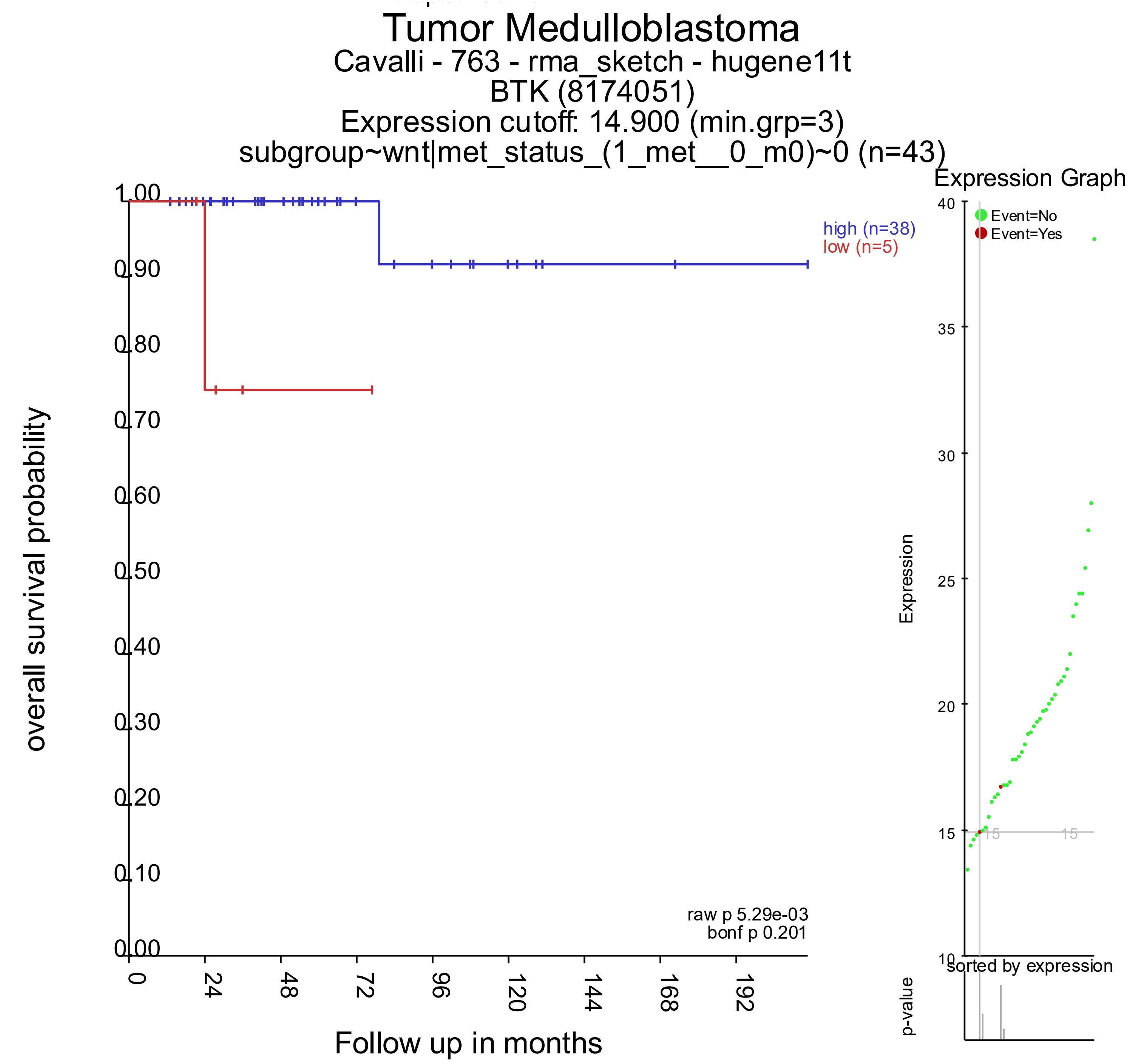
Tumor Medulloblastoma
Cavalli - 763 - rma_sketch - hugene11t
BTK (8174051)
Expression cutoff: 14.900 (min.grp=3)
subgroup~wnt|met_status_(1_met__0_m0)~0 (n=43)
overall survival probability
1.00
0.90
0.80
0.70
0.60
0.50
0.40
0.30
0.20
0.10
0.00
0
24
48
72
96
120
144
168
192
Follow up in months
high (n=38)
low (n=5)
raw p 5.29e-03
bonf p 0.201
Expression Graph
Event=No
Event=Yes
Expression
40
35
30
25
20
15
10
sorted by expression
p-value

WNT M1

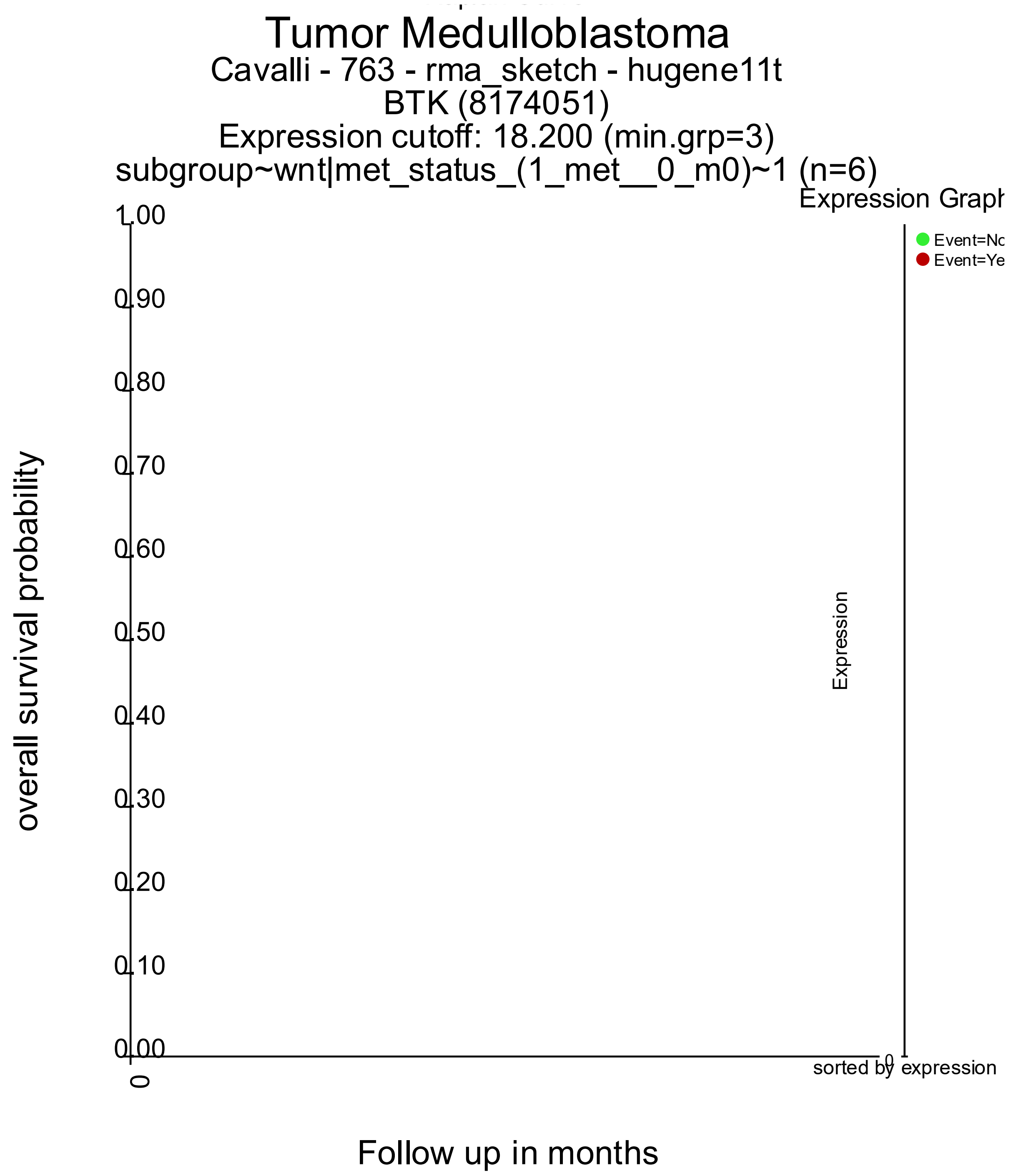

Tumor Medulloblastoma
Cavalli - 763 - rma_sketch - hugene11t
BTK (8174051)
Expression cutoff: 18.200 (min.grp=3)
subgroup~wnt|met_status_(1_met__0_m0)~1 (n=6)
Expression Graph
Event=No
Event=Yes
overall survival probability
1.00
0.90
0.80
0.70
0.60
0.50
0.40
0.30
0.20
0.10
0.00
0
Follow up in months
Expression
0
sorted by expression

SHH M0

Tumor Medulloblastoma
Cavalli - 763 - rma_sketch - hugene11t
BTK (8174051)
Expression cutoff: 27.700 (min.grp=3)
subgroup~shh|met_status_(1_met__0_m0)~0|WITH_SURV (n=124)

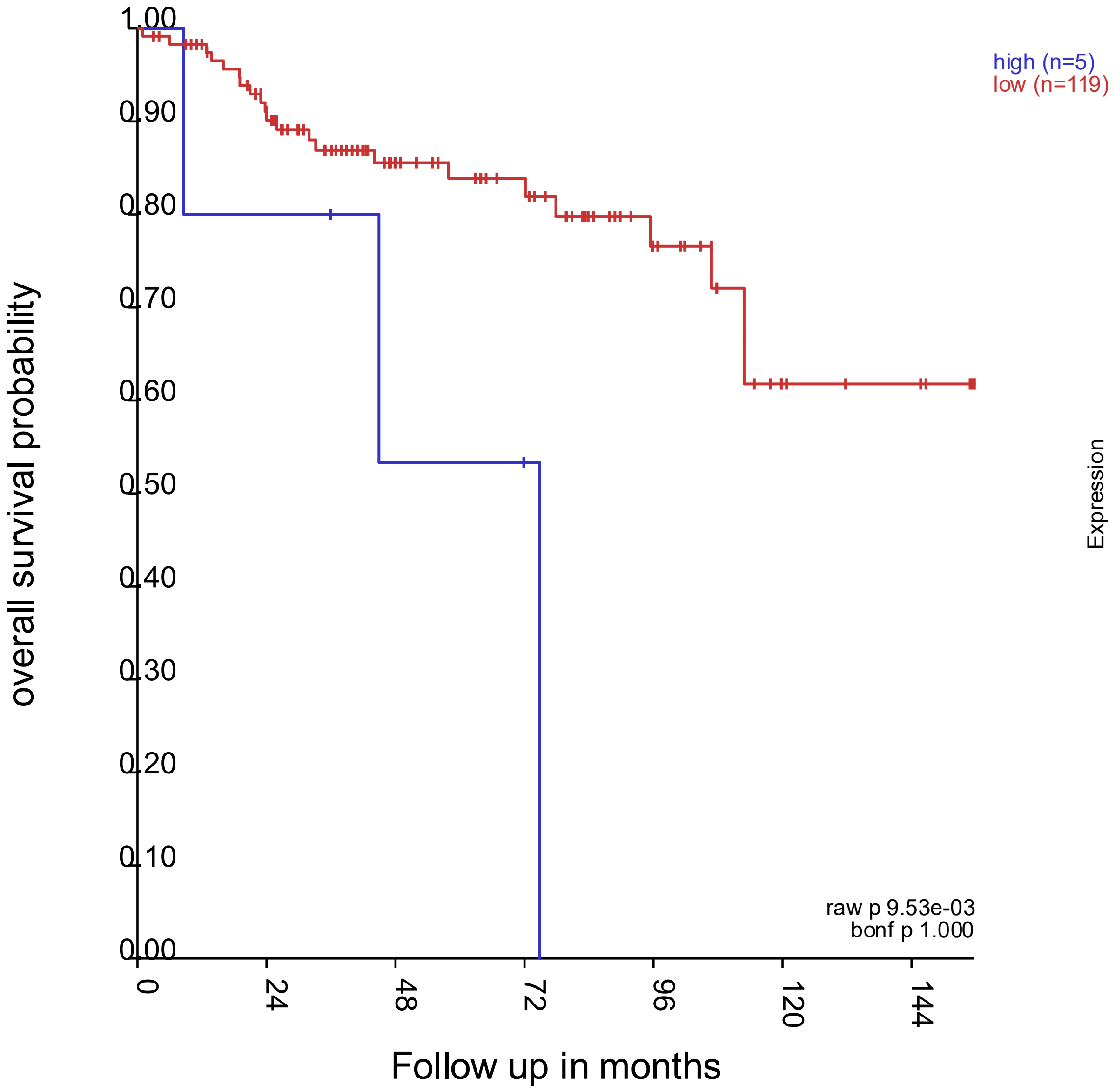

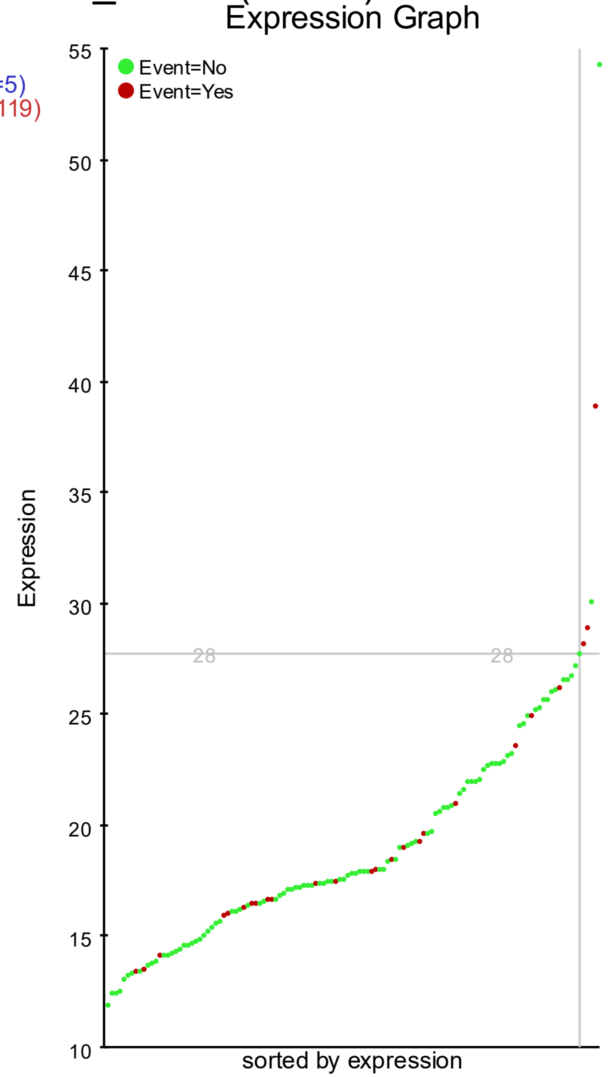

SHH M1

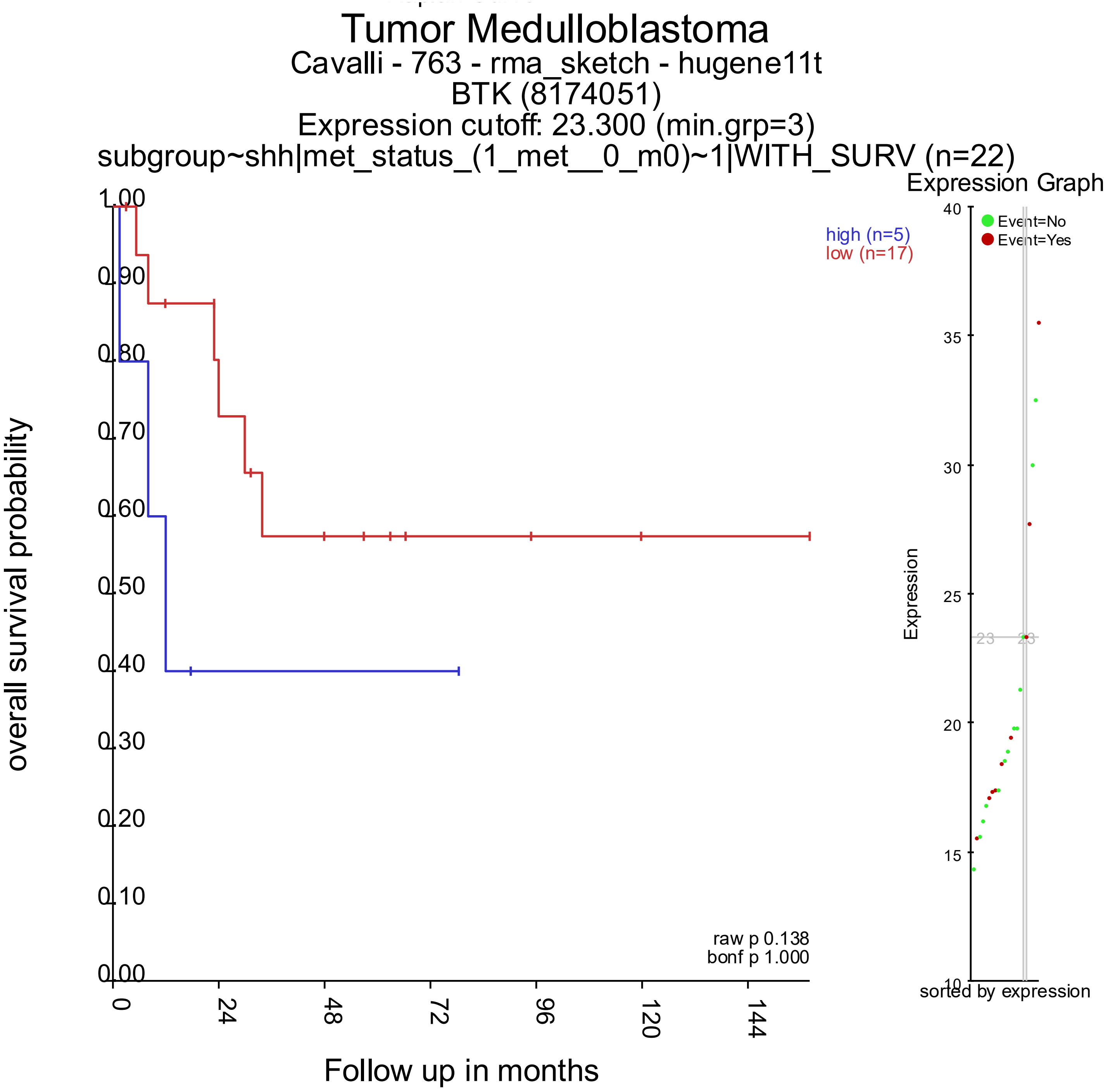

Tumor Medulloblastoma
Cavalli - 763 - rma_sketch - hugene11t
BTK (8174051)
Expression cutoff: 23.300 (min.grp=3)
subgroup~shh|met_status_(1_met__0_m0)~1|WITH_SURV (n=22)
overall survival probability
1.00
0.90
0.80
0.70
0.60
0.50
0.40
0.30
0.20
0.10
0.00
high (n=5)
low (n=17)
raw p 0.138
bonf p 1.000
0
24
48
72
96
120
144
Follow up in months
Expression Graph
Event=No
Event=Yes
Expression
40
35
30
25
20
15
10
23
sorted by expression

GROUP4 M0

Tumor Medulloblastoma
Cavalli - 763 - rma_sketch - hugene11t
BTK (8174051)
Expression cutoff: 27.700 (min.grp=3)
subgroup~group4|met_status_(1_met__0_m0)~0|WITH_SURV (n=145)

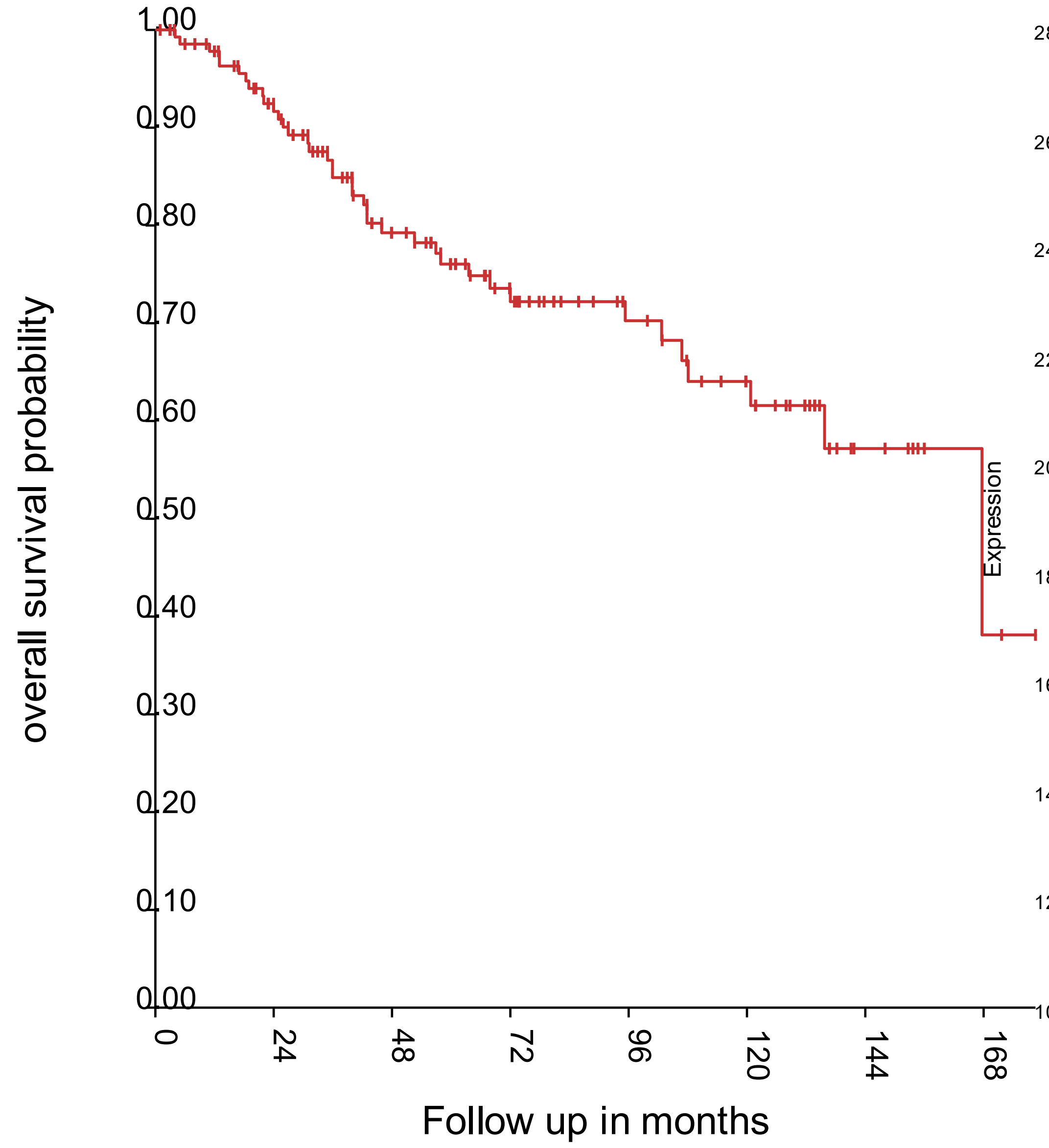

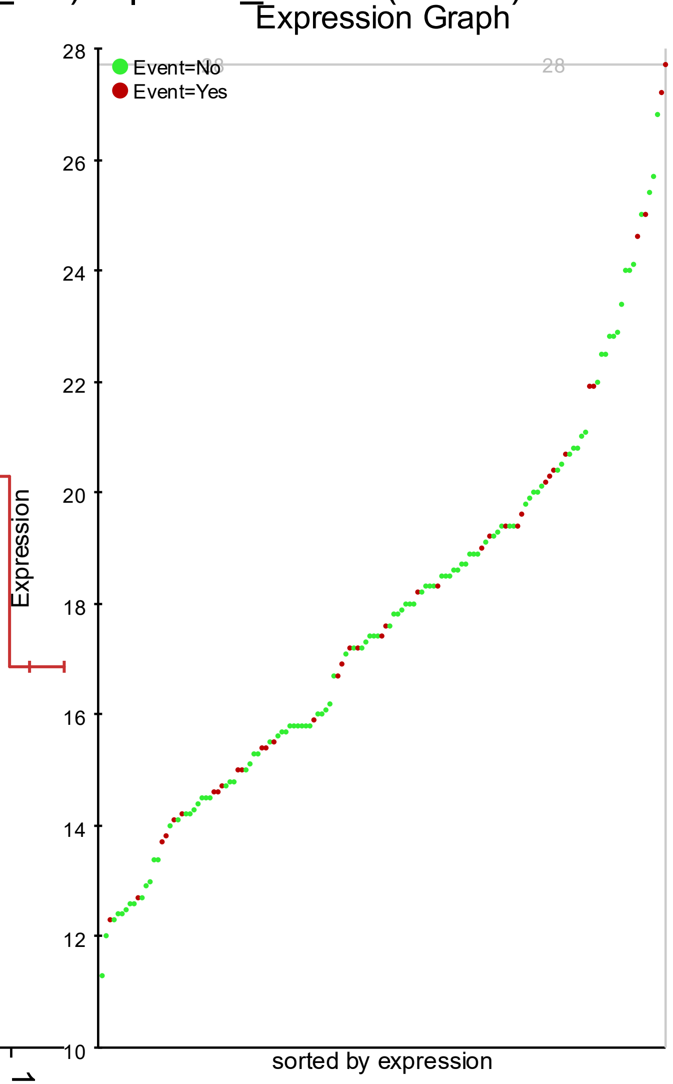

GROUP4 M1

Tumor Medulloblastoma
Cavalli - 763 - rma_sketch - hugene11t
BTK (8174051)
Expression cutoff: 12.700 (min.grp=3)
subgroup~group4|met_status_(1_met__0_m0)~1|WITH_SURV (n=92)

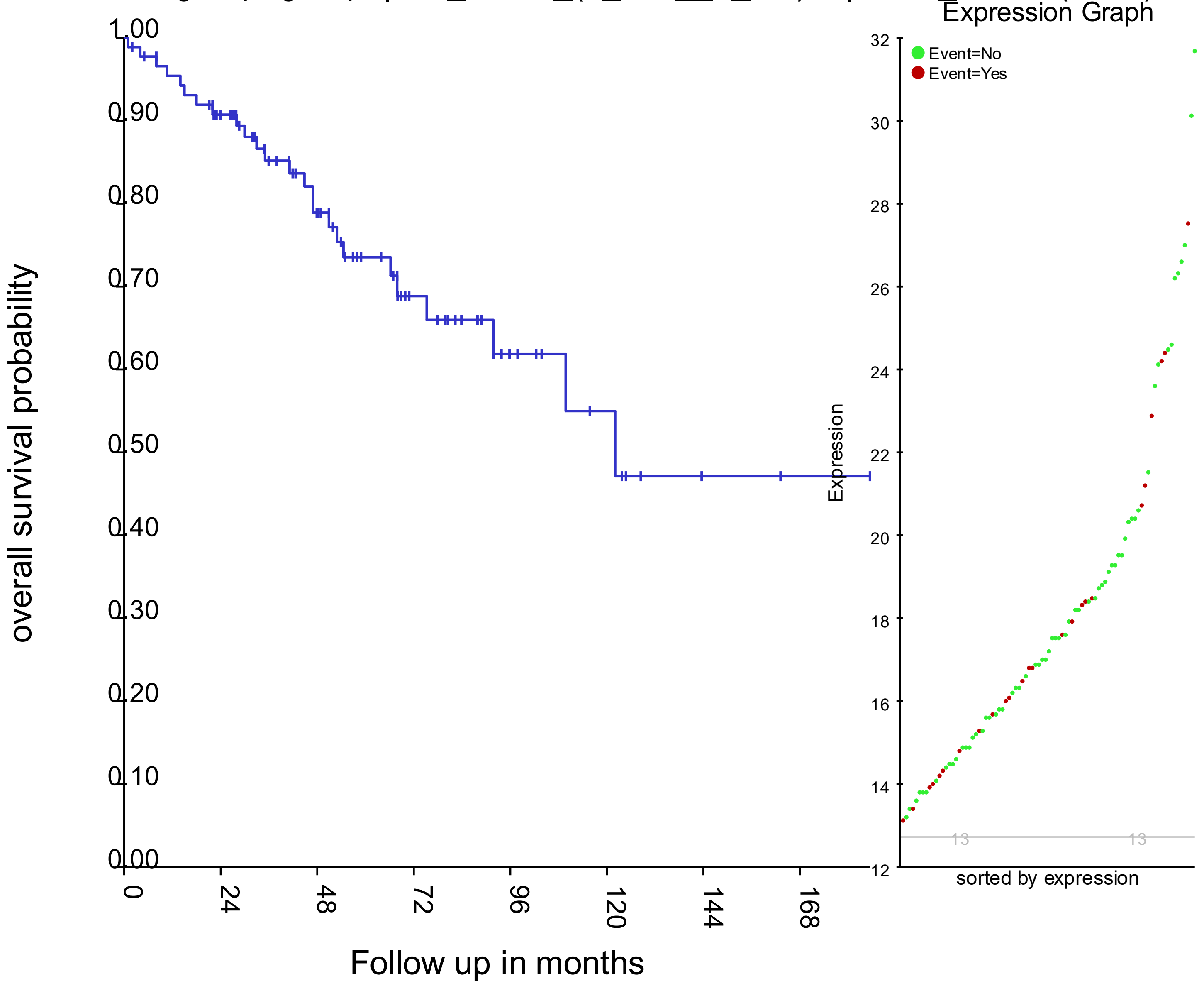

GROUP3 M0

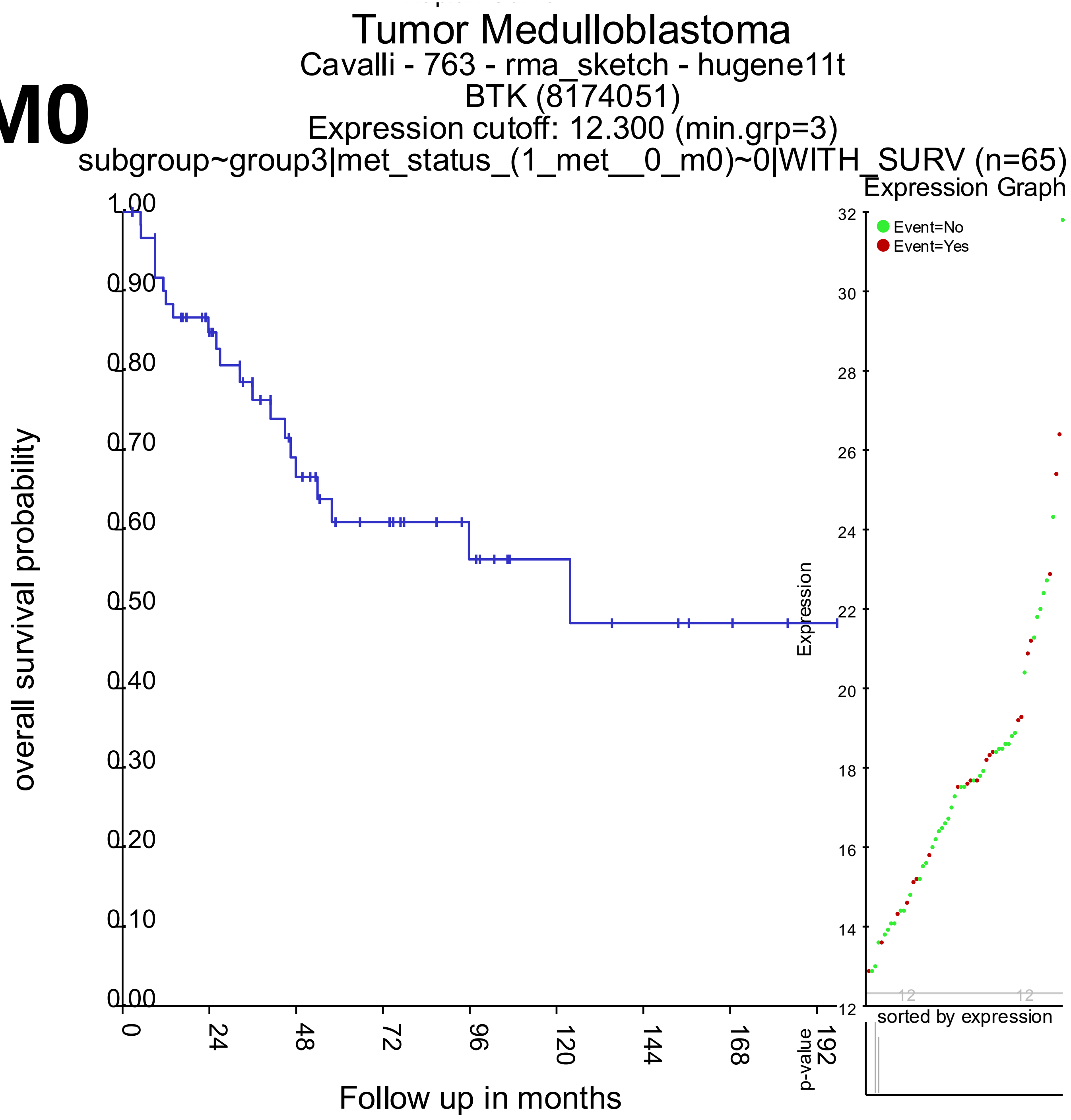

Tumor Medulloblastoma
Cavalli - 763 - rma_sketch - hugene11t
BTK (8174051)
Expression cutoff: 12.300 (min.grp=3)
subgroup~group3|met_status_(1_met__0_m0)~0|WITH_SURV (n=65)
overall survival probability
1.00
0.90
0.80
0.70
0.60
0.50
0.40
0.30
0.20
0.10
0.00
0
24
48
72
96
120
144
168
192
p-value
Follow up in months
Expression Graph
Event=No
Event=Yes
Expression
32
30
28
26
24
22
20
18
16
14
12
sorted by expression

GROUP3 M1

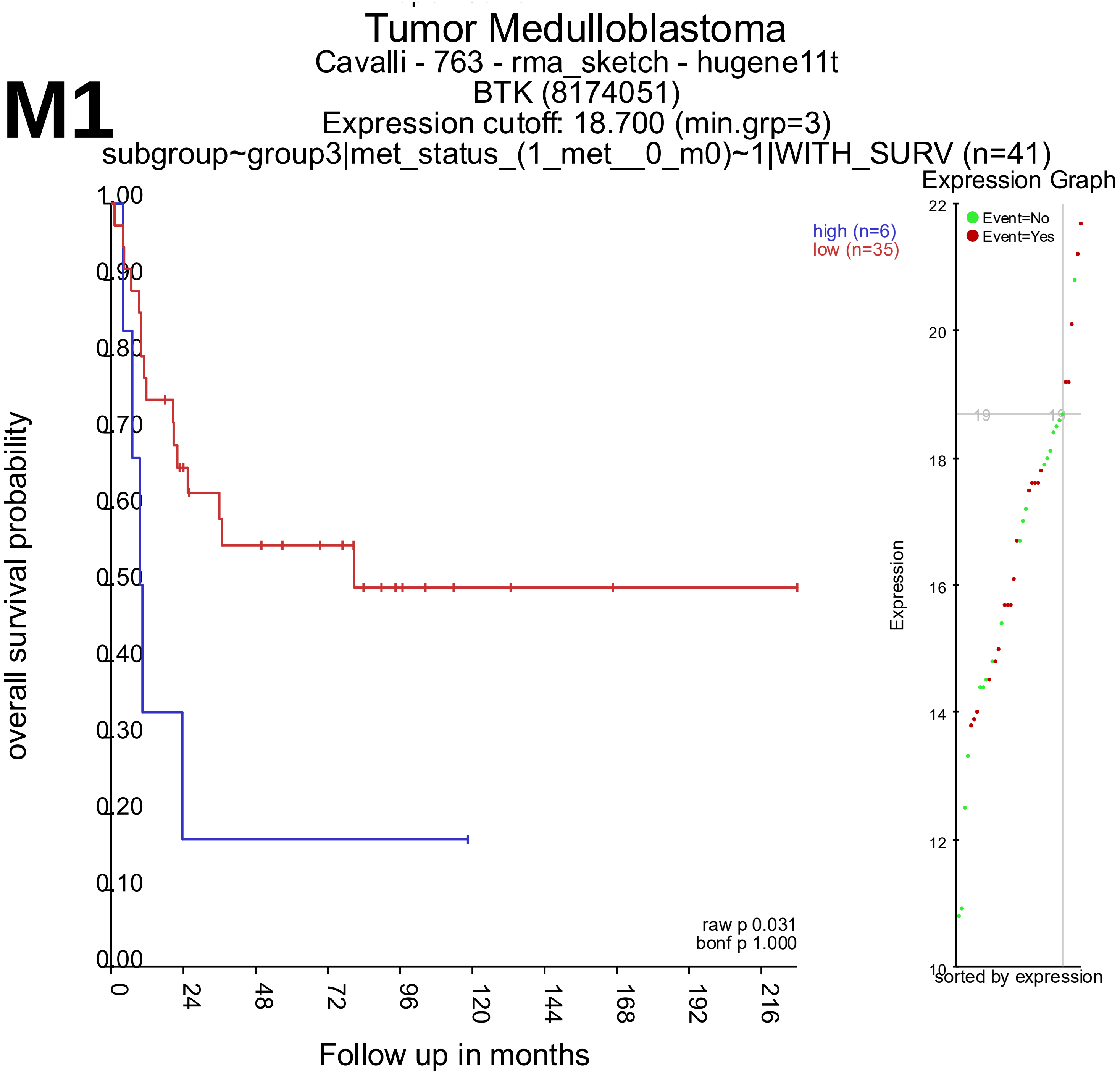

Tumor Medulloblastoma
Cavalli - 763 - rma_sketch - hugene11t
BTK (8174051)
Expression cutoff: 18.700 (min.grp=3)
subgroup~group3|met_status_(1_met__0_m0)~1|WITH_SURV (n=41)
overall survival probability
1.00
0.90
0.80
0.70
0.60
0.50
0.40
0.30
0.20
0.10
0.00
0
24
48
72
96
120
144
168
192
216
Follow up in months
high (n=6)
low (n=35)
raw p 0.031
bonf p 1.000
Expression Graph
Event=No
Event=Yes
Expression
22
20
18
16
14
12
10
19
19
sorted by expression

CD3D

WNT M0

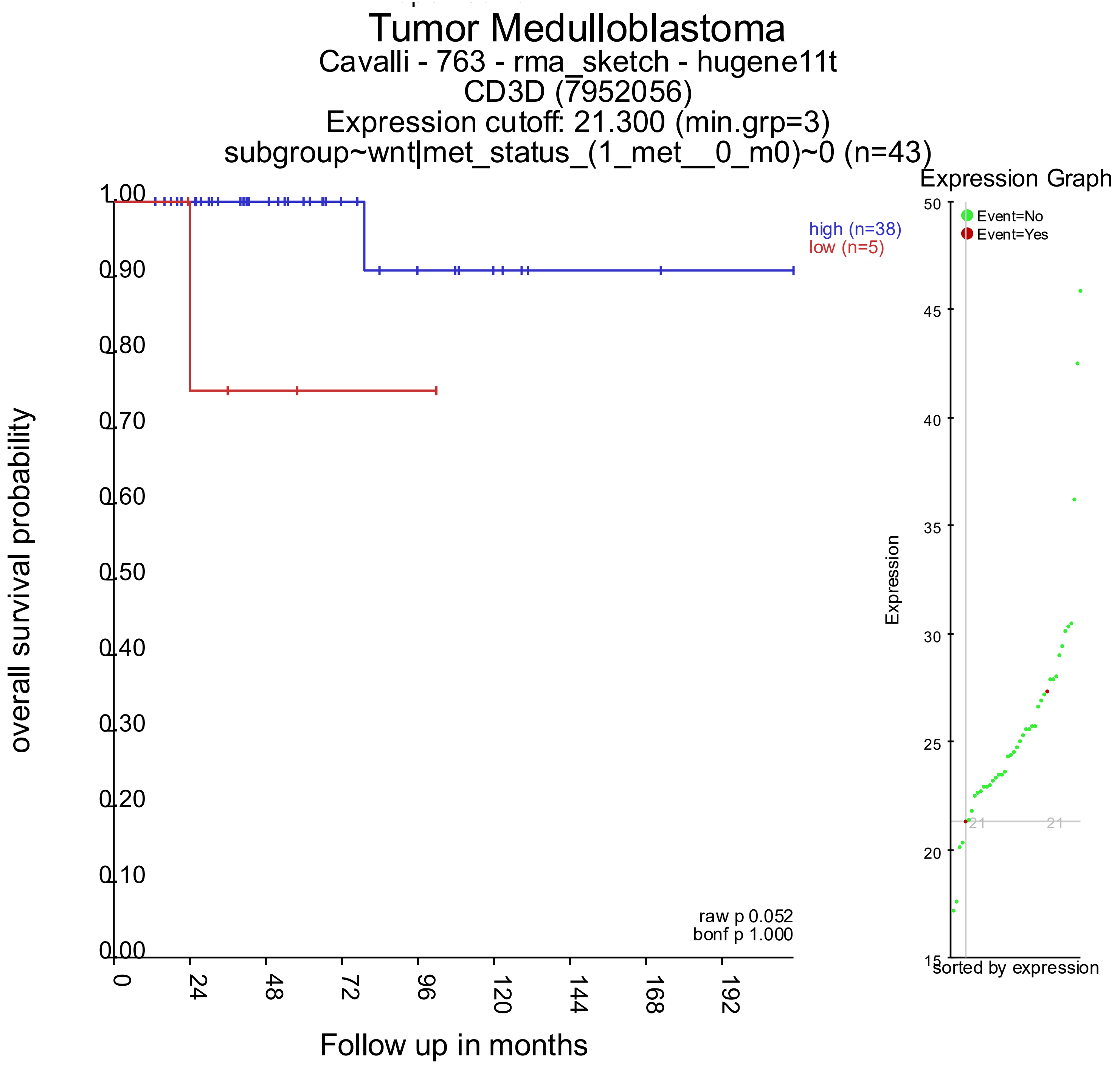

Tumor Medulloblastoma
Cavalli - 763 - rma_sketch - hugene11t
CD3D (7952056)
Expression cutoff: 21.300 (min.grp=3)
subgroup~wnt|met_status_(1_met__0_m0)~0 (n=43)
overall survival probability
1.00
0.90
0.80
0.70
0.60
0.50
0.40
0.30
0.20
0.10
0.00
high (n=38)
low (n=5)
raw p 0.052
bonf p 1.000
0
24
48
72
96
120
144
168
192
Follow up in months
Expression Graph
Event=No
Event=Yes
Expression
50
45
40
35
30
25
20
15
21
sorted by expression

WNT M1

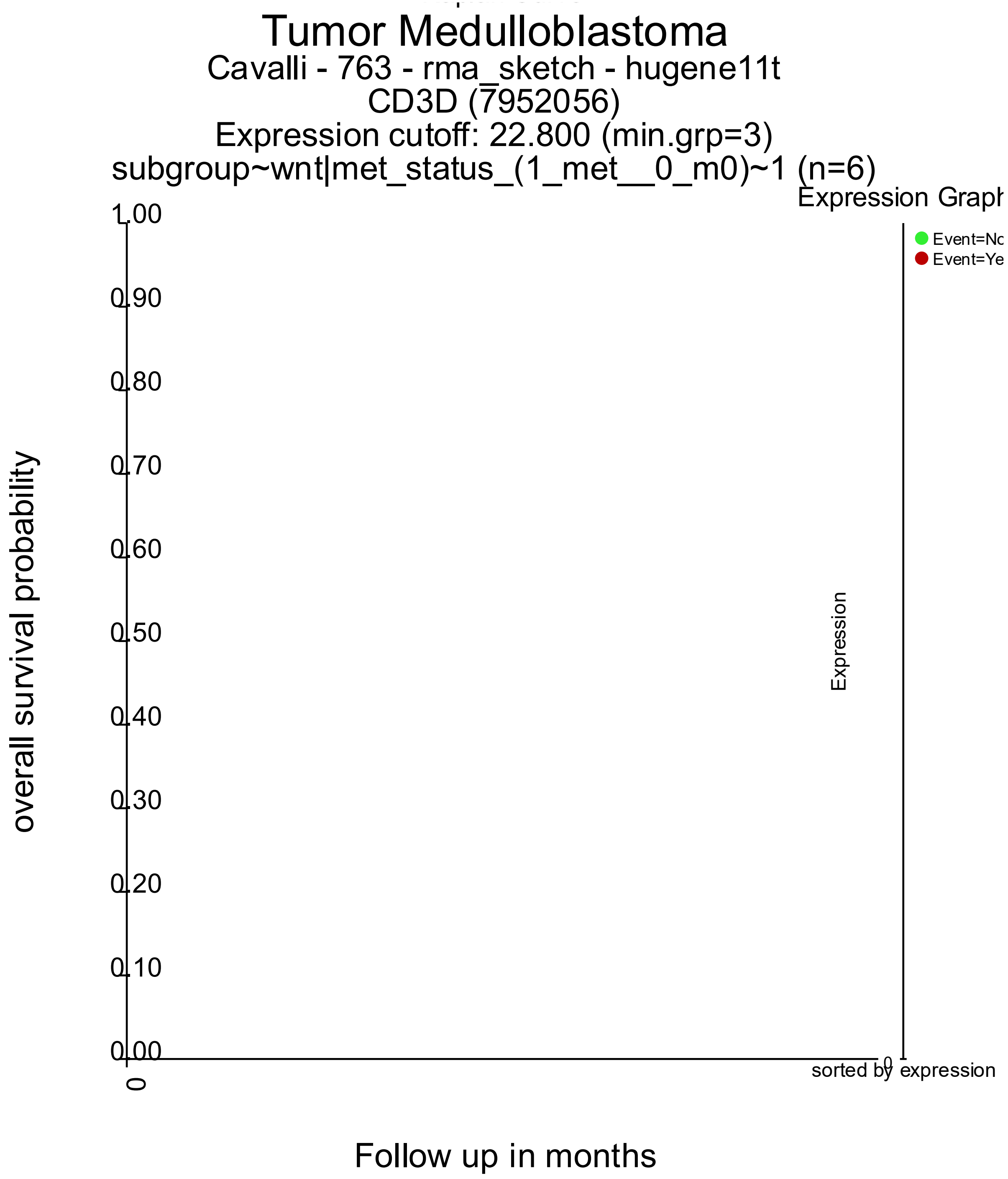

Tumor Medulloblastoma
Cavalli - 763 - rma_sketch - hugene11t
CD3D (7952056)
Expression cutoff: 22.800 (min.grp=3)
subgroup~wnt|met_status_(1_met__0_m0)~1 (n=6)
Expression Graph
Event=No
Event=Yes
1.00
0.90
0.80
0.70
0.60
0.50
0.40
0.30
0.20
0.10
0.00
0
overall survival probability
Expression
0
sorted by expression
Follow up in months

SHH M0

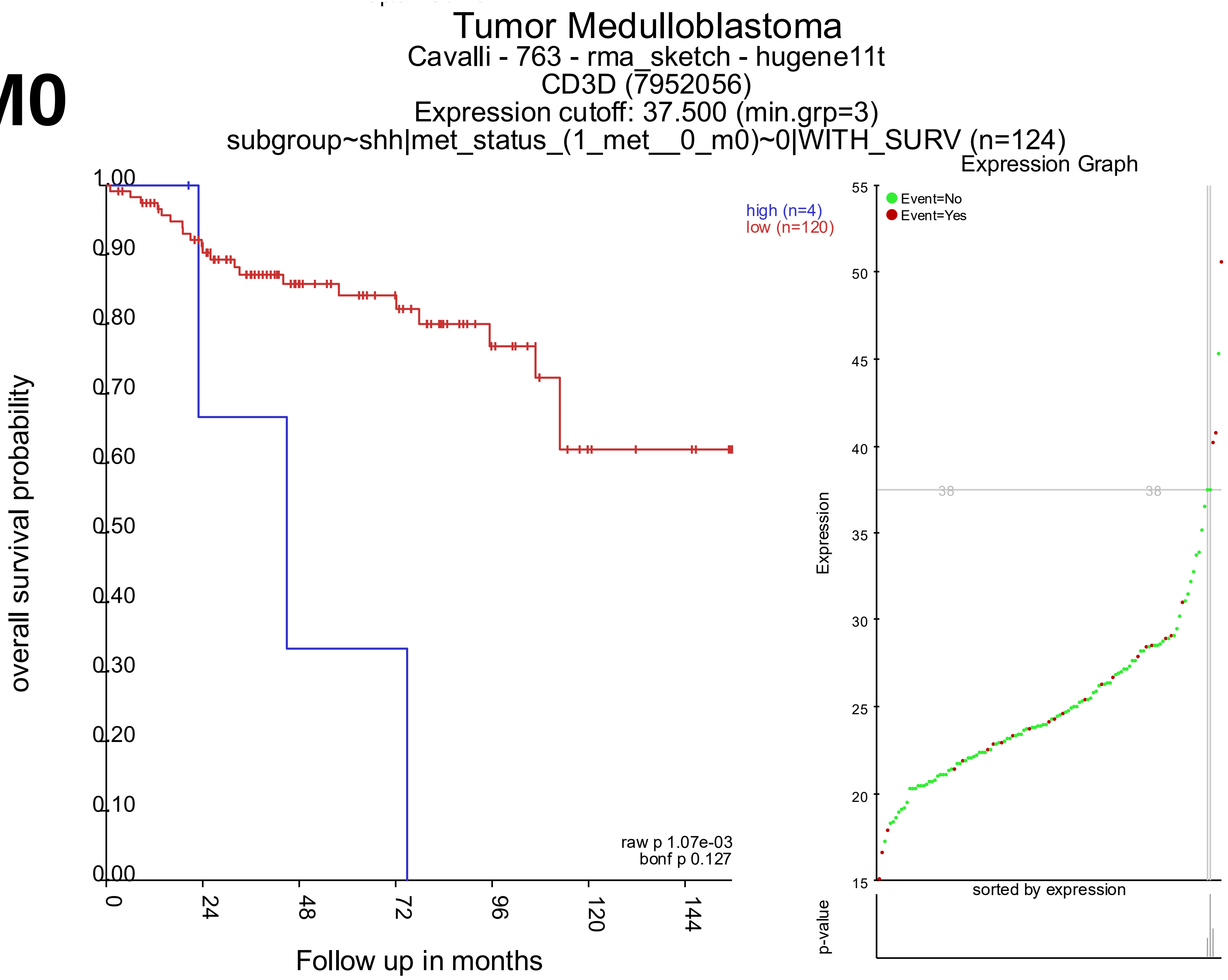

Tumor Medulloblastoma
Cavalli - 763 - rma_sketch - hugene11t
CD3D (7952056)
Expression cutoff: 37.500 (min.grp=3)
subgroup~shh|met_status_(1_met__0_m0)~0|WITH_SURV (n=124)
overall survival probability
1.00
0.90
0.80
0.70
0.60
0.50
0.40
0.30
0.20
0.10
0.00
0
24
48
72
96
120
144
Follow up in months
high (n=4)
low (n=120)
raw p 1.07e-03
bonf p 0.127
Expression Graph
Event=No
Event=Yes
Expression
55
50
45
40
35
30
25
20
15
38
38
sorted by expression
p-value

SHH M1

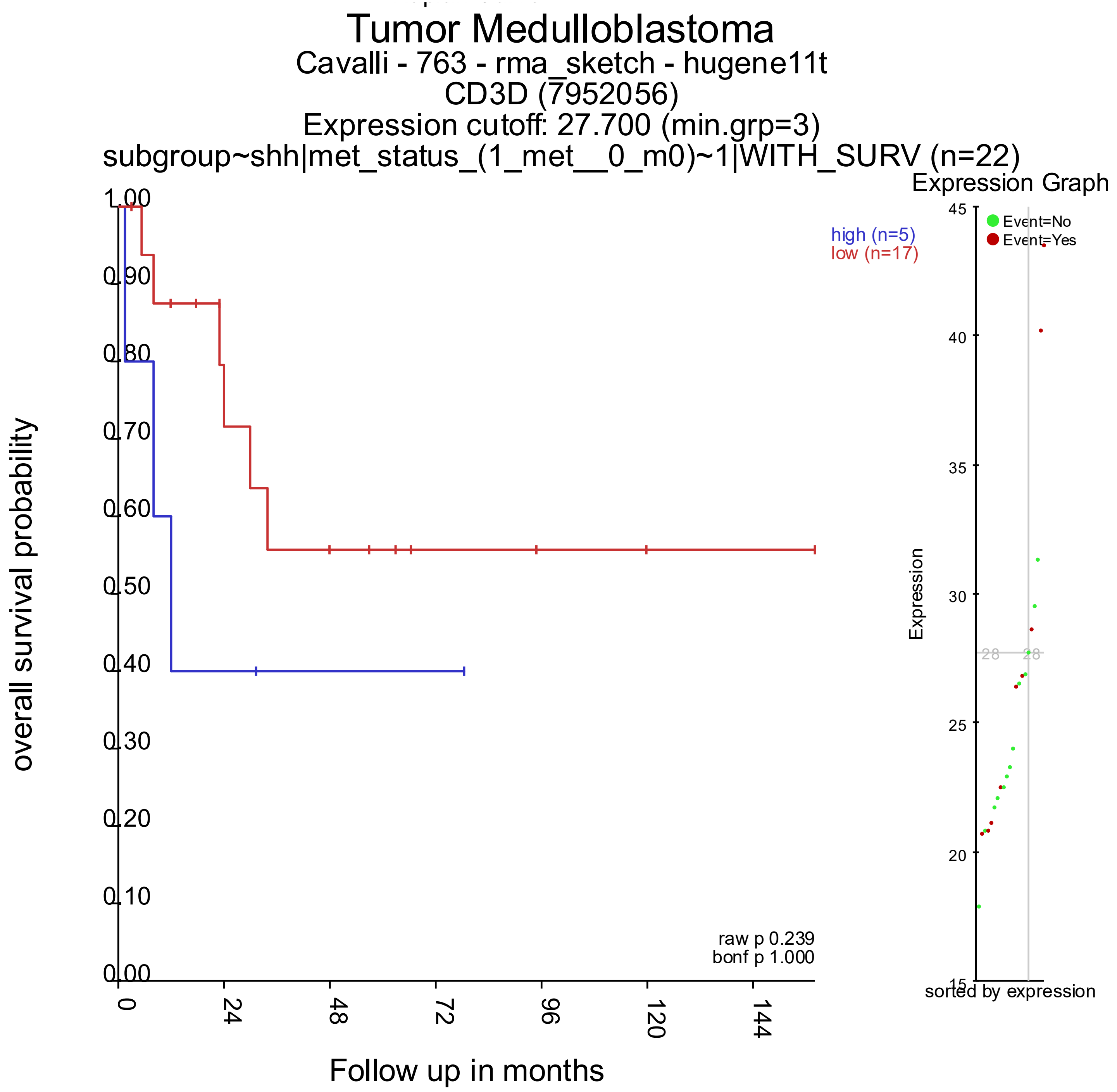

Tumor Medulloblastoma
Cavalli - 763 - rma_sketch - hugene11t
CD3D (7952056)
Expression cutoff: 27.700 (min.grp=3)
subgroup~shh|met_status_(1_met__0_m0)~1|WITH_SURV (n=22)
overall survival probability
1.00
0.90
0.80
0.70
0.60
0.50
0.40
0.30
0.20
0.10
0.00
0
24
48
72
96
120
144
Follow up in months
high (n=5)
low (n=17)
raw p 0.239
bonf p 1.000
Expression Graph
Event=No
Event=Yes
Expression
45
40
35
30
25
20
15
28
sorted by expression

GROUP4 M0

Tumor Medulloblastoma
Cavalli - 763 - rma_sketch - hugene11t
CD3D (7952056)
Expression cutoff: 15.500 (min.grp=3)
subgroup~group4|met_status_(1_met__0_m0)~0|WITH_SURV (n=145)

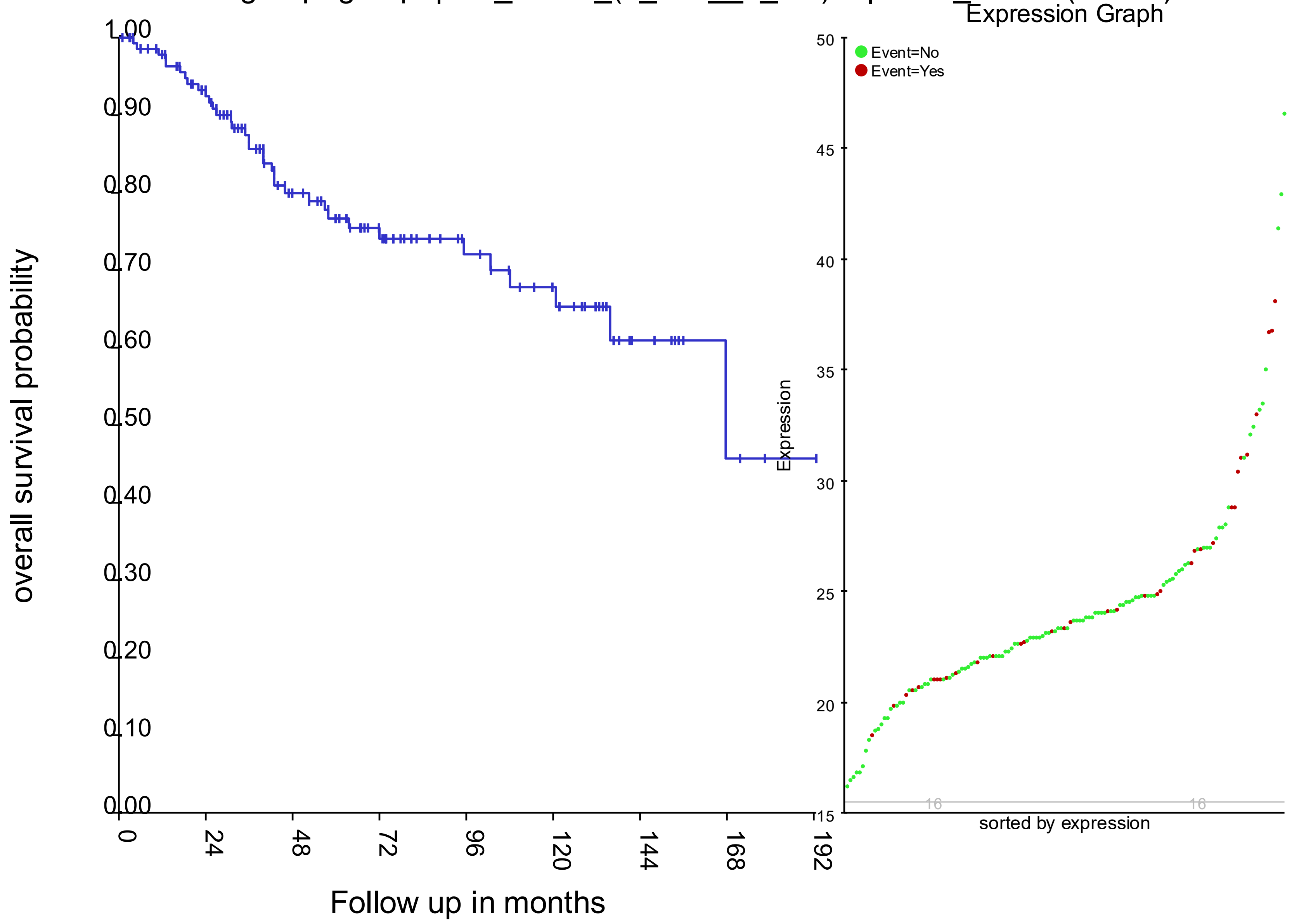

GROUP4 M1

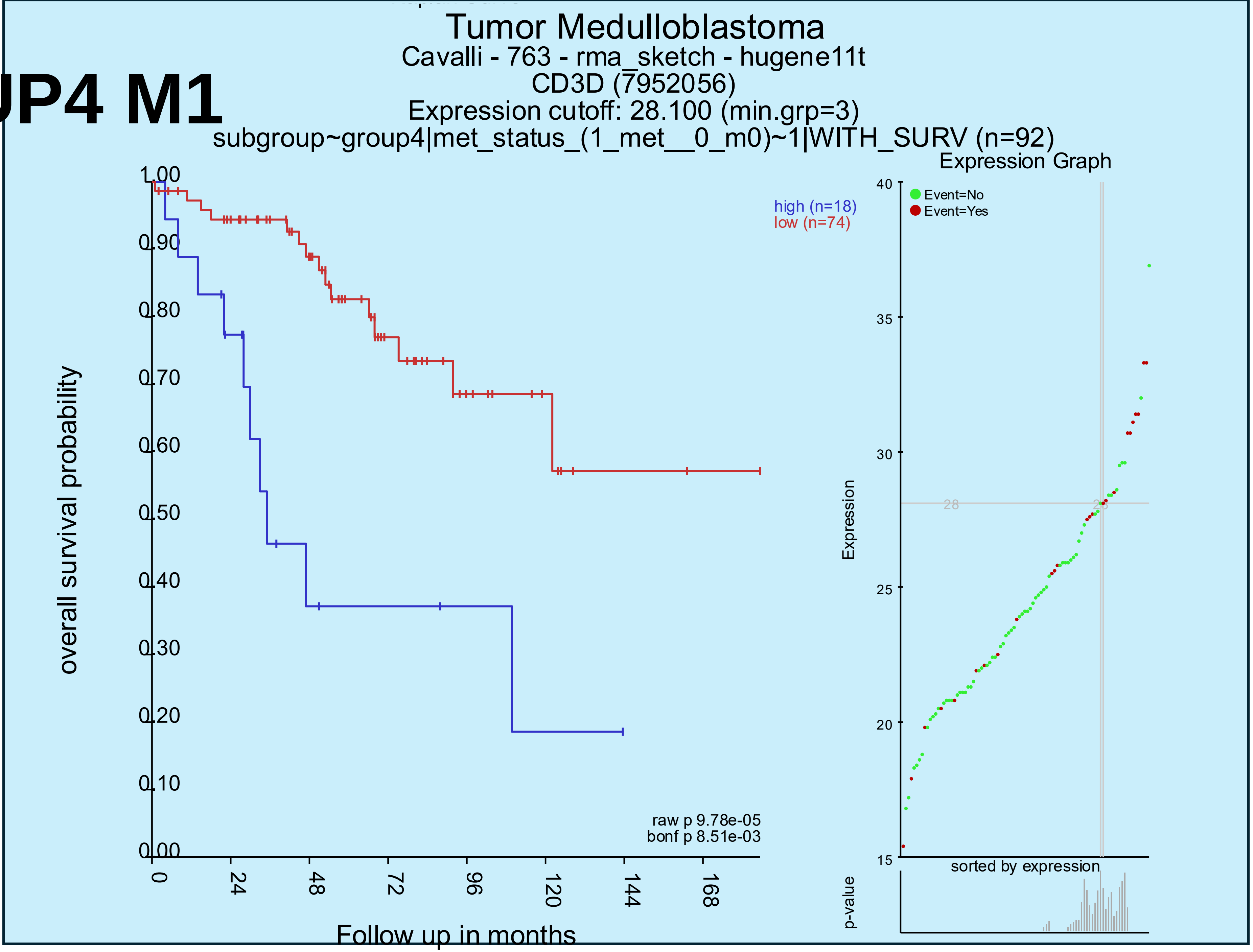
Tumor Medulloblastoma
Cavalli - 763 - rma_sketch - hugene11t
CD3D (7952056)
Expression cutoff: 28.100 (min.grp=3)
subgroup~group4|met_status_(1_met__0_m0)~1|WITH_SURV (n=92)
overall survival probability
1.00
0.90
0.80
0.70
0.60
0.50
0.40
0.30
0.20
0.10
0.00
high (n=18)
low (n=74)
raw p 9.78e-05
bonf p 8.51e-03
0
24
48
72
96
120
144
168
Follow up in months
Expression Graph
Event=No
Event=Yes
40
35
30
25
20
15
Expression
28
sorted by expression
p-value

GROUP3 M0

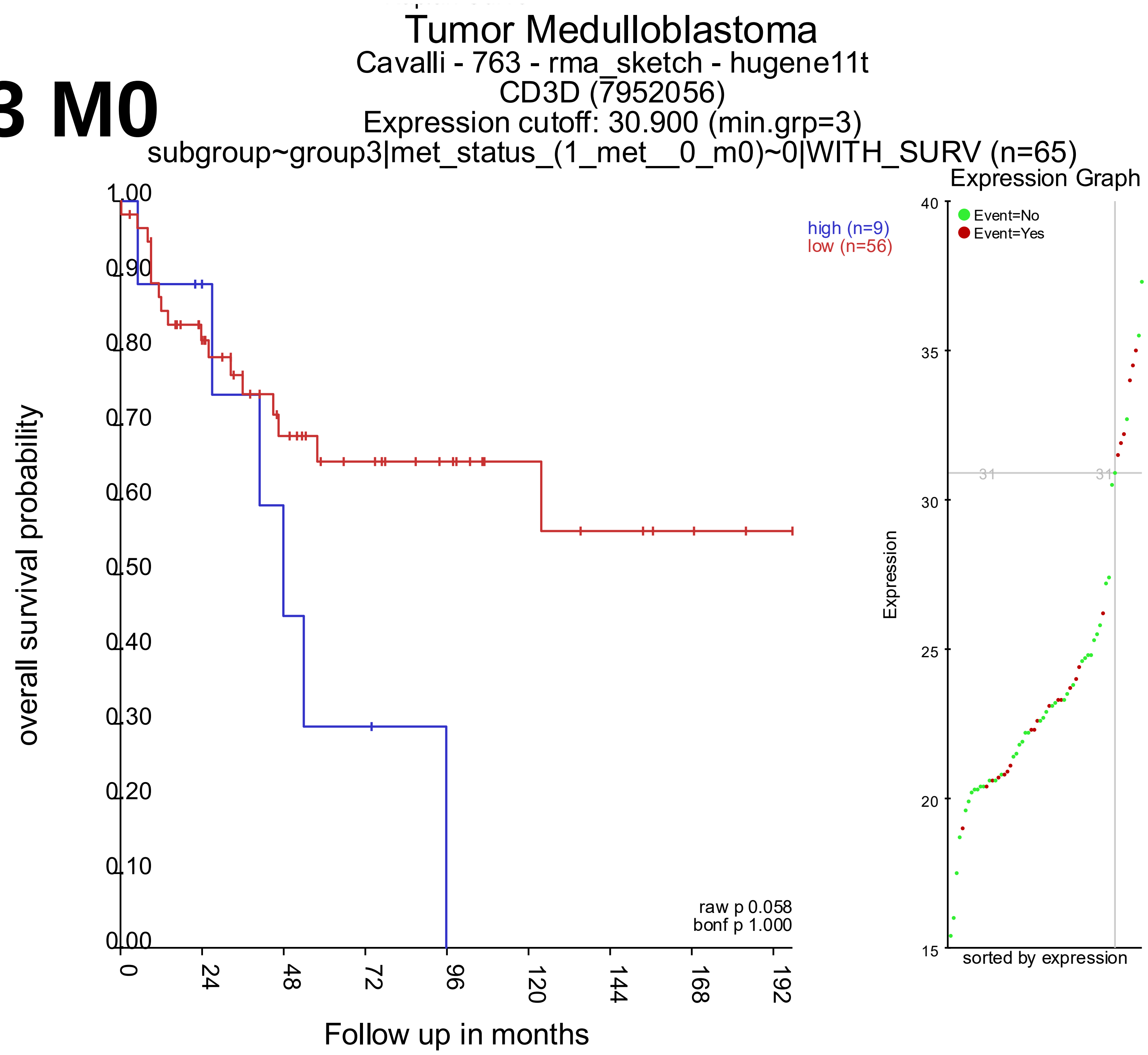

Tumor Medulloblastoma
Cavalli - 763 - rma_sketch - hugene11t
CD3D (7952056)
Expression cutoff: 30.900 (min.grp=3)
subgroup~group3|met_status_(1_met__0_m0)~0|WITH_SURV (n=65)
overall survival probability
1.00
0.90
0.80
0.70
0.60
0.50
0.40
0.30
0.20
0.10
0.00
high (n=9)
low (n=56)
raw p 0.058
bonf p 1.000
0
24
48
72
96
120
144
168
192
Follow up in months
Expression Graph
Event=No
Event=Yes
40
35
30
25
20
15
31
31
Expression
sorted by expression

GROUP3 M1

Tumor Medulloblastoma
Cavalli - 763 - rma_sketch - hugene11t
CD3D (7952056)
Expression cutoff: 19.300 (min.grp=3)
subgroup~group3|met_status_(1_met__0_m0)~1|WITH_SURV (n=41)

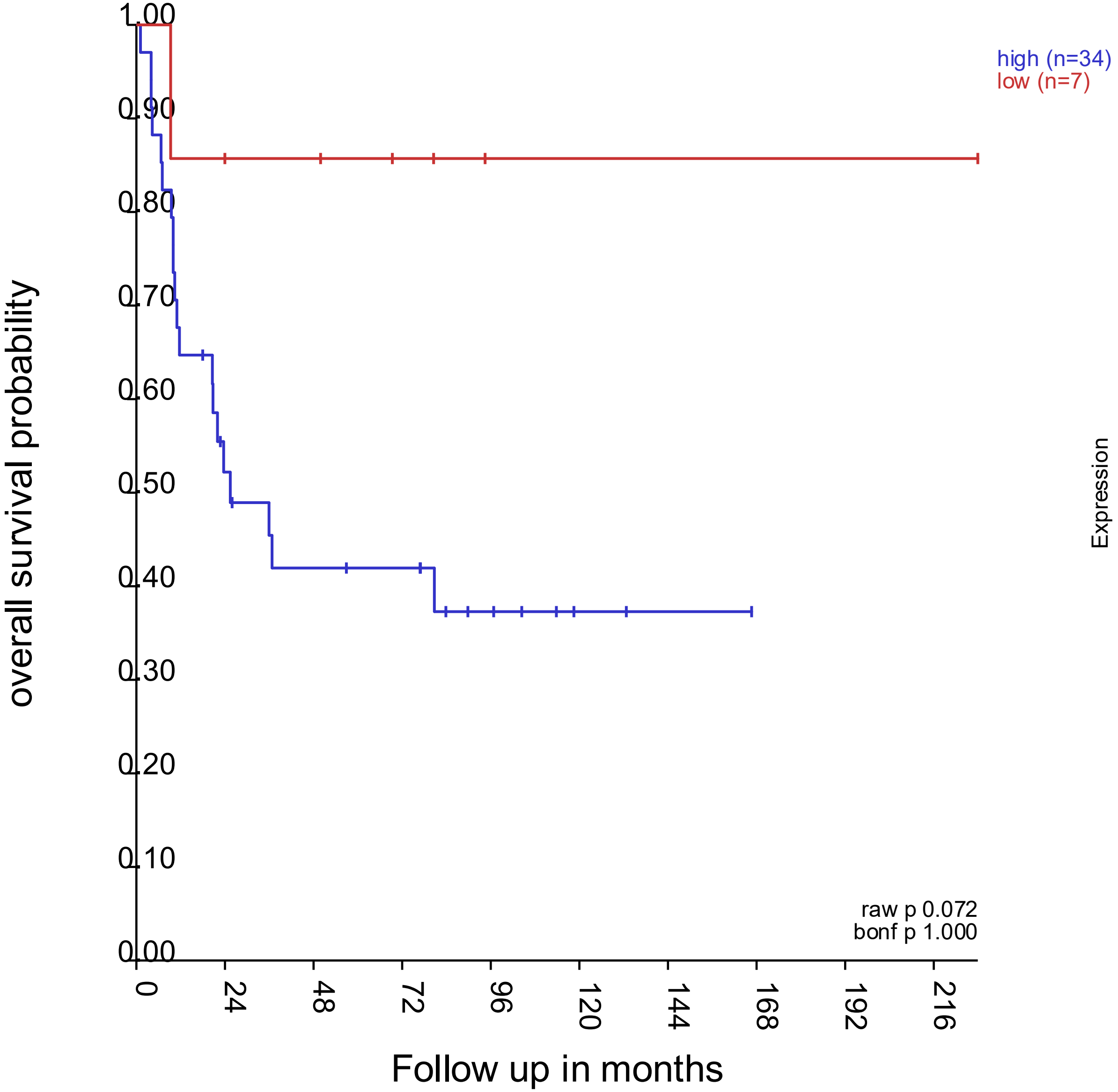

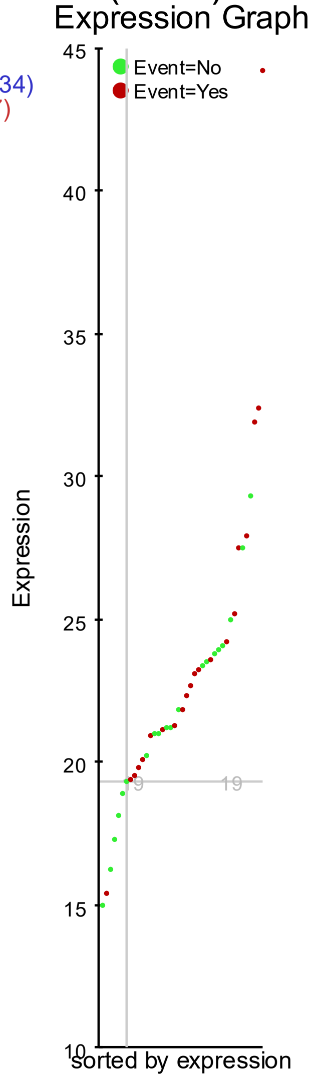

CD19

WNT M0

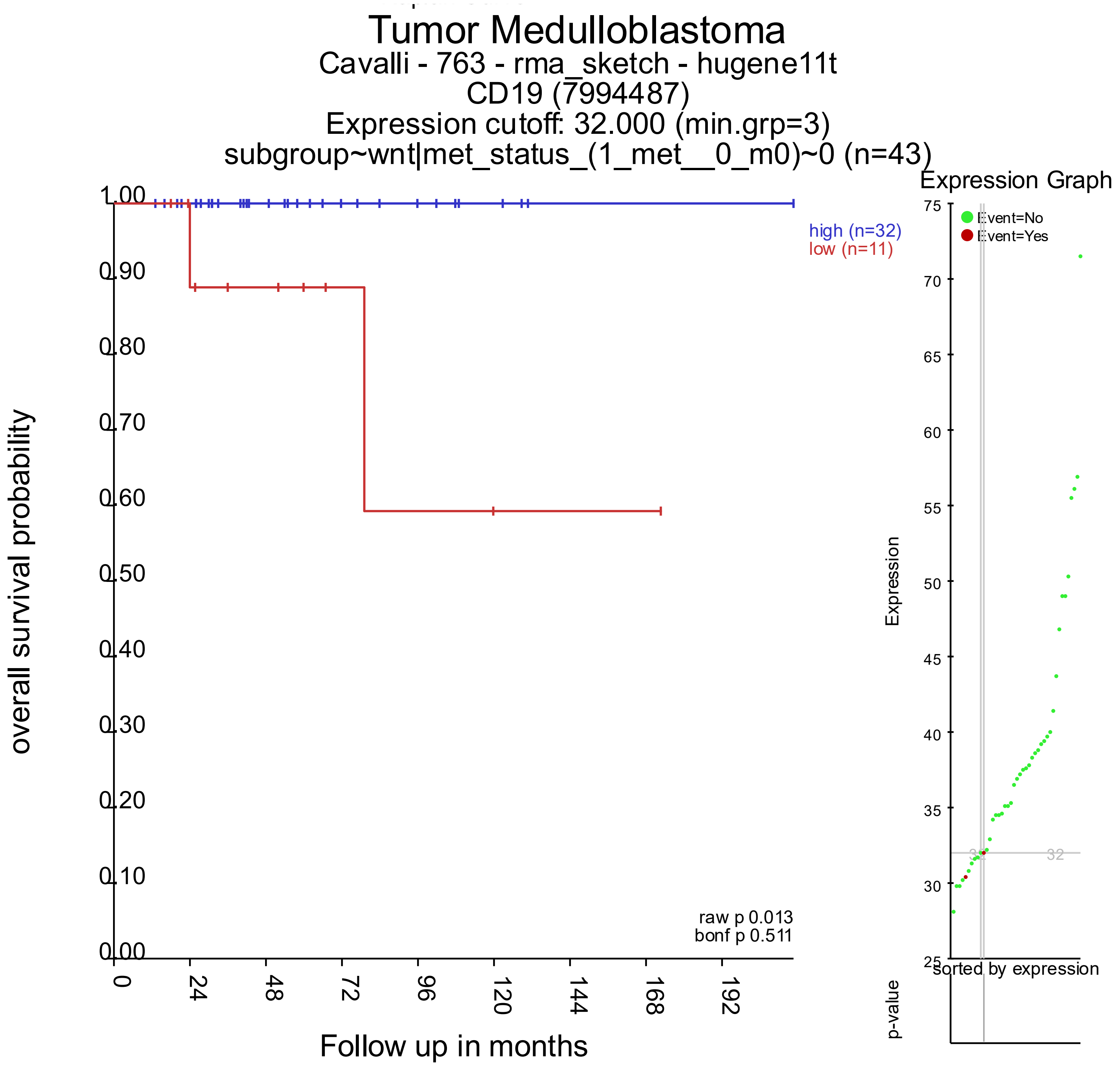

Tumor Medulloblastoma
Cavalli - 763 - rma_sketch - hugene11t
CD19 (7994487)
Expression cutoff: 32.000 (min.grp=3)
subgroup~wnt|met_status_(1_met__0_m0)~0 (n=43)
overall survival probability
1.00
0.90
0.80
0.70
0.60
0.50
0.40
0.30
0.20
0.10
0.00
0
24
48
72
96
120
144
168
192
Follow up in months
high (n=32)
low (n=11)
raw p 0.013
bonf p 0.511
Expression Graph
Event=No
Event=Yes
Expression
75
70
65
60
55
50
45
40
35
30
25
32
sorted by expression
p-value

WNT M1

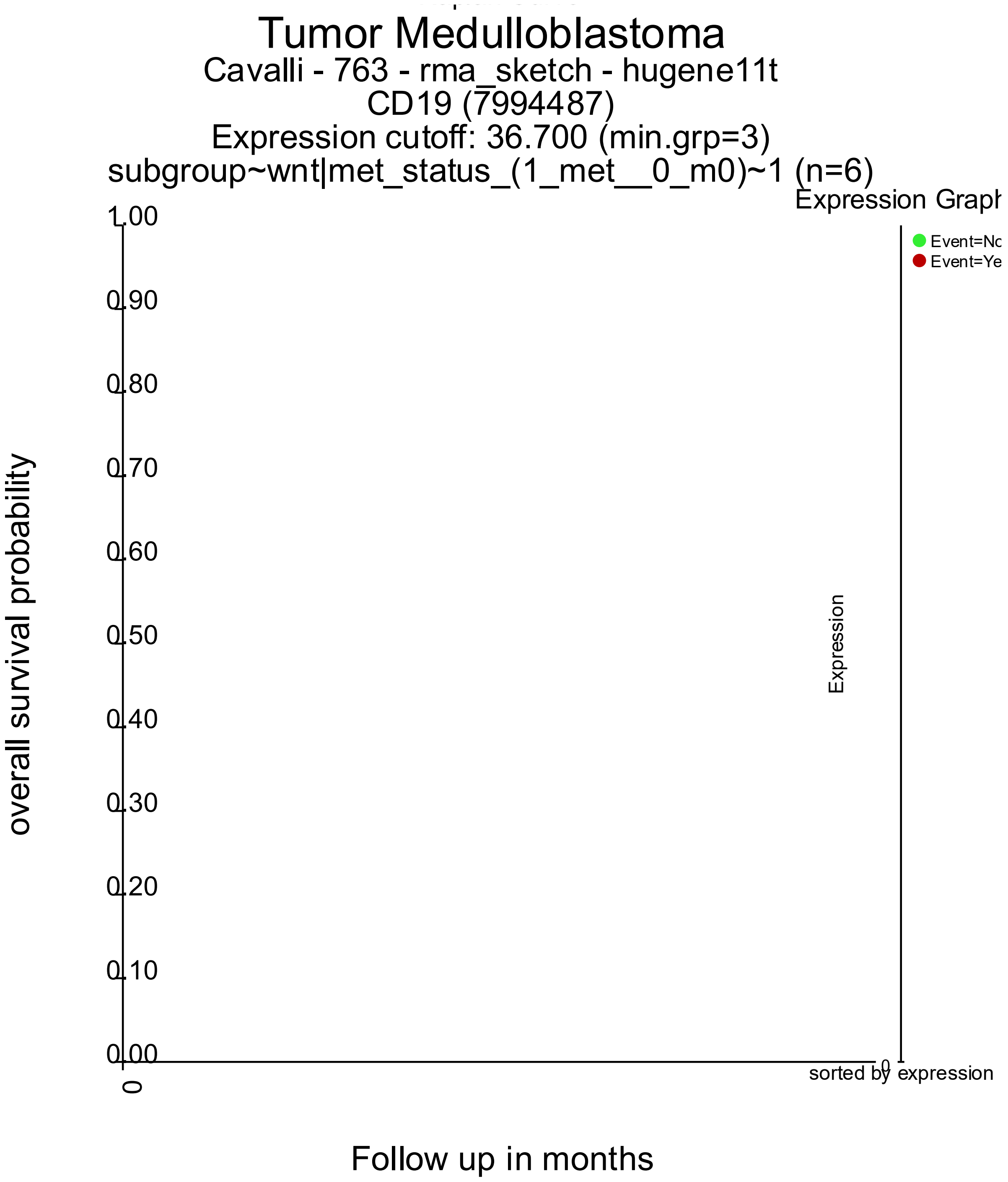
Tumor Medulloblastoma
Cavalli - 763 - rma_sketch - hugene11t
CD19 (7994487)
Expression cutoff: 36.700 (min.grp=3)
subgroup~wnt|met_status_(1_met__0_m0)~1 (n=6)
Expression Graph
Event=No
Event=Ye
1.00
0.90
0.80
0.70
0.60
0.50
0.40
0.30
0.20
0.10
0.00
overall survival probability
0
Follow up in months
Expression
0
sorted by expression

SHH M0

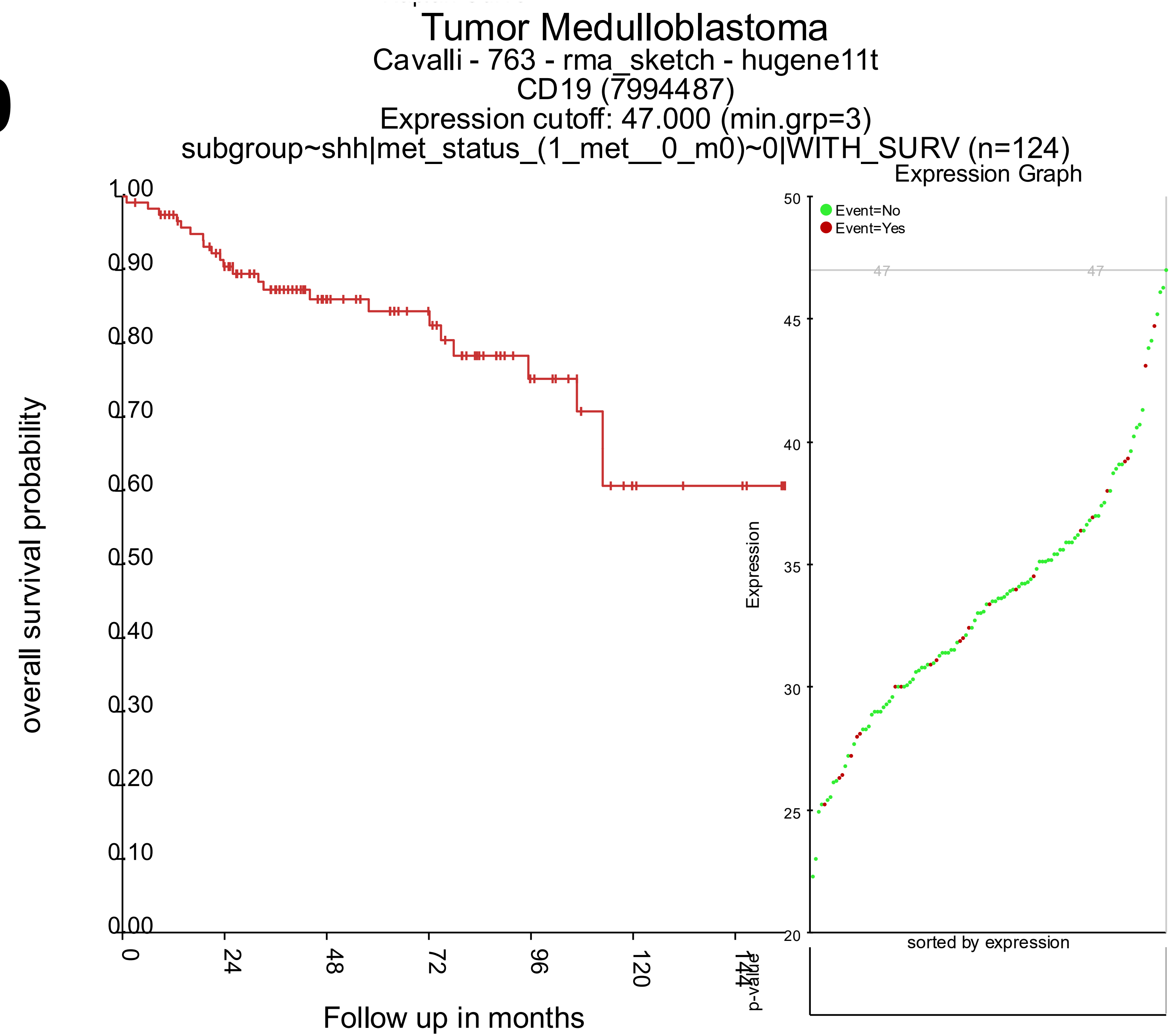
Tumor Medulloblastoma
Cavalli - 763 - rma_sketch - hugene11t
CD19 (7994487)
Expression cutoff: 47.000 (min.grp=3)
subgroup~shh|met_status_(1_met__0_m0)~0|WITH_SURV (n=124)
overall survival probability
1.00
0.90
0.80
0.70
0.60
0.50
0.40
0.30
0.20
0.10
0.00
0
24
48
72
96
120
144
Follow up in months
Expression Graph
Event=No
Event=Yes
47
47
Expression
50
45
40
35
30
25
20
sorted by expression

SHH M1

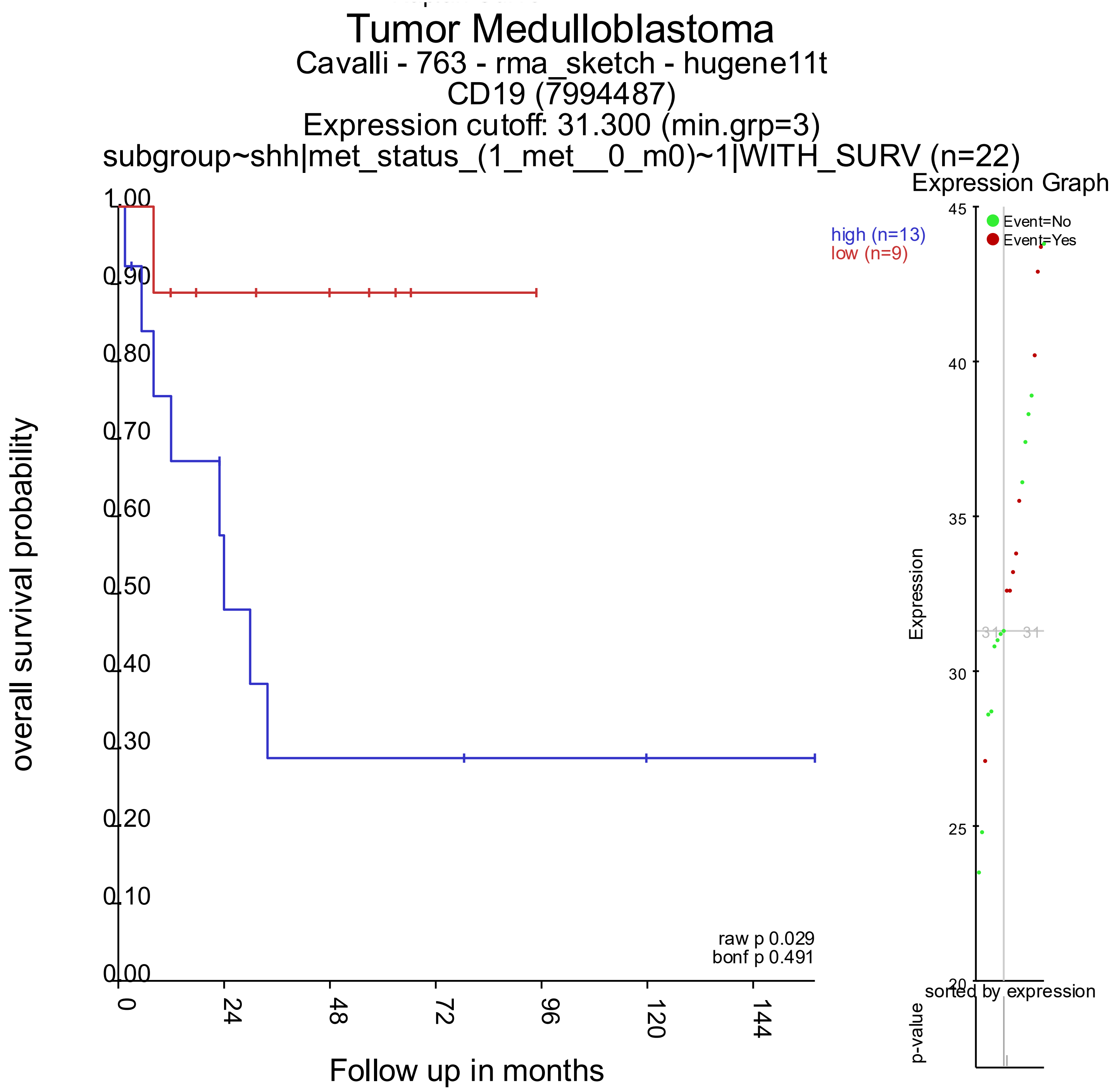

Tumor Medulloblastoma
Cavalli - 763 - rma_sketch - hugene11t
CD19 (7994487)
Expression cutoff: 31.300 (min.grp=3)
subgroup~shh|met_status_(1_met__0_m0)~1|WITH_SURV (n=22)
overall survival probability
1.00
0.90
0.80
0.70
0.60
0.50
0.40
0.30
0.20
0.10
0.00
0
24
48
72
96
120
144
Follow up in months
high (n=13)
low (n=9)
raw p 0.029
bonf p 0.491
Expression Graph
Event=No
Event=Yes
45
40
35
30
25
20
Expression
sorted by expression
p-value

GROUP4 M0

Tumor Medulloblastoma
Cavalli - 763 - rma_sketch - hugene11t
CD19 (7994487)
Expression cutoff: 26.200 (min.grp=3)
subgroup~group4|met_status_(1_met__0_m0)~0|WITH_SURV (n=145)

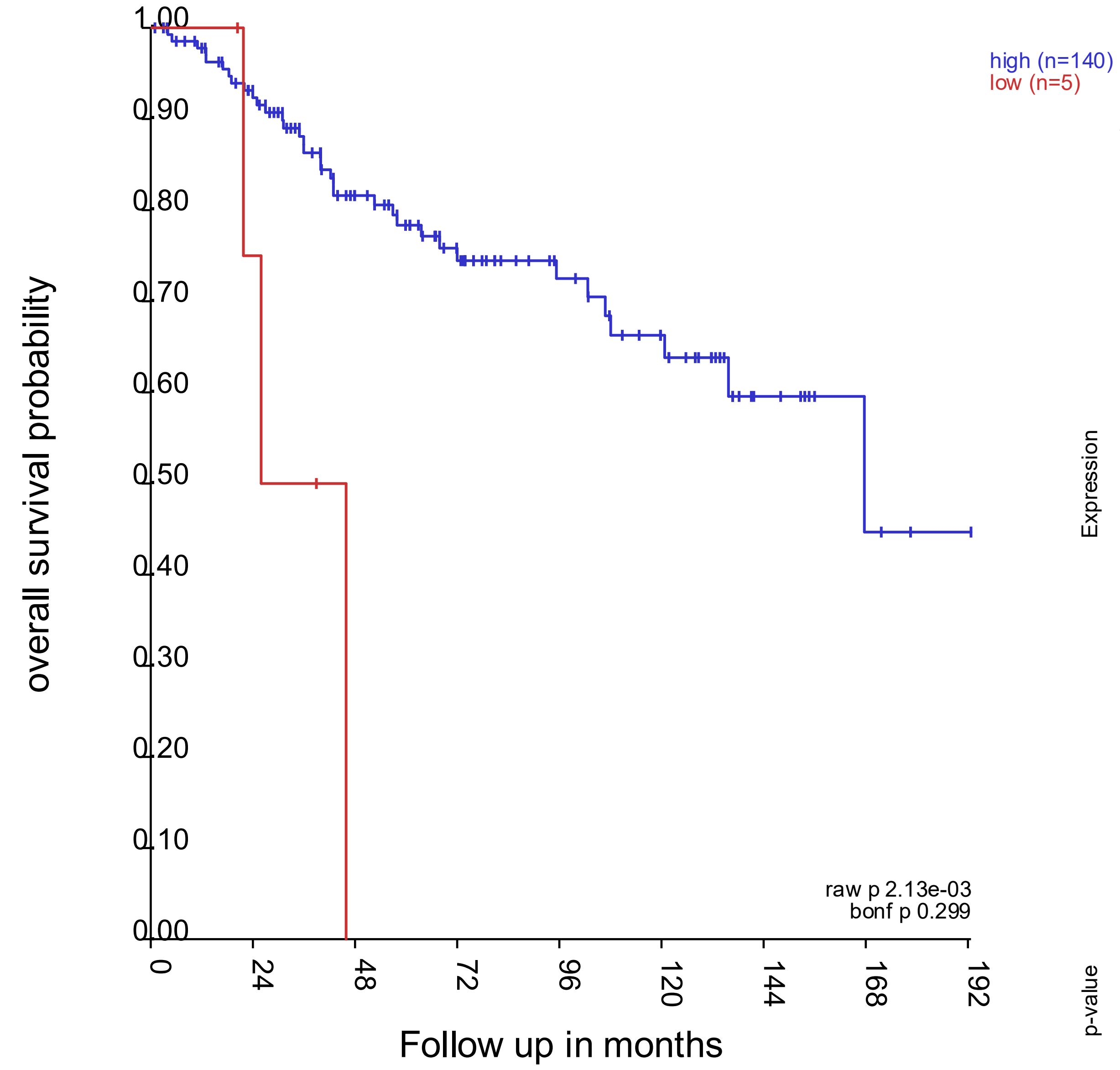

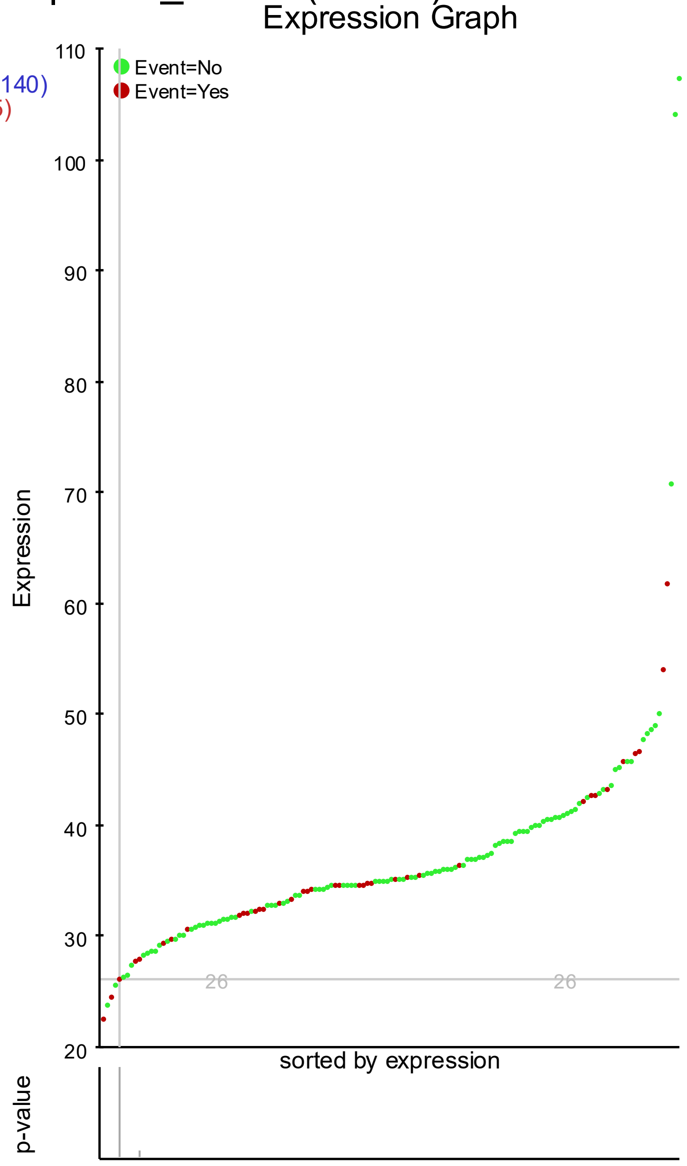

GROUP4 M1

Tumor Medulloblastoma
Cavalli - 763 - rma_sketch - hugene11t
CD19 (7994487)
Expression cutoff: 34.100 (min.grp=3)
subgroup~group4|met_status_(1_met__0_m0)~1|WITH_SURV (n=92)

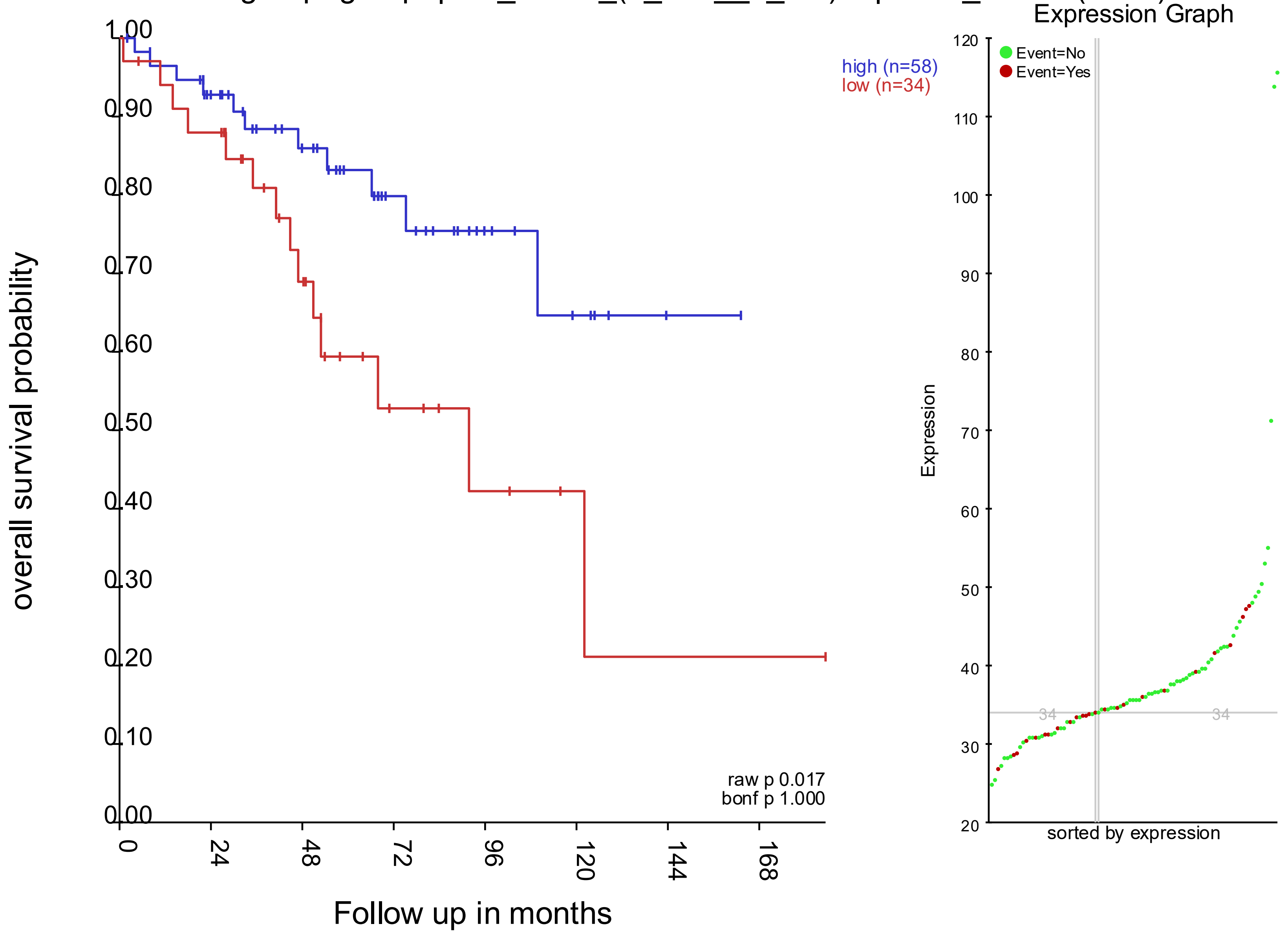

GROUP3 M0

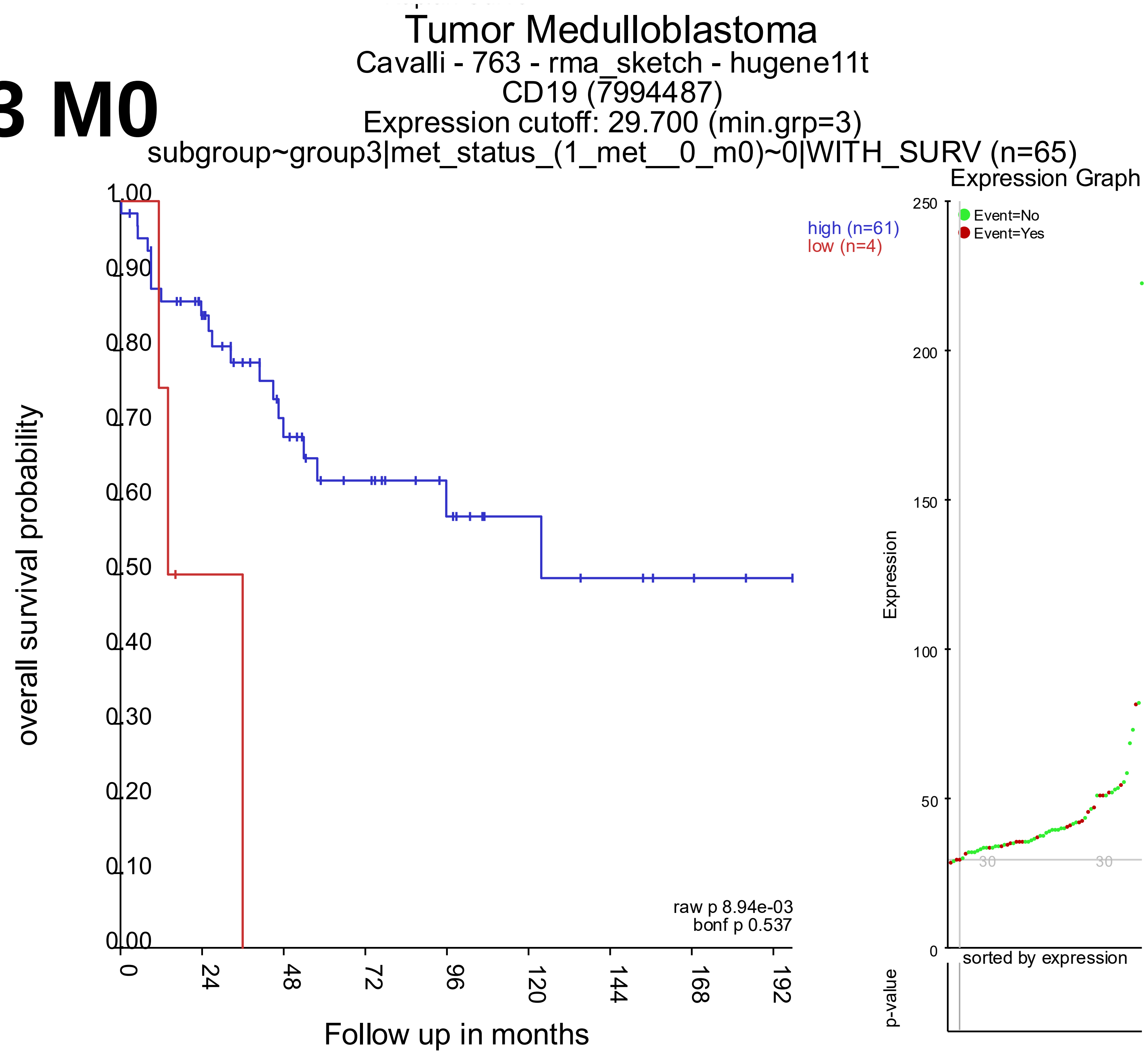

Tumor Medulloblastoma
Cavalli - 763 - rma_sketch - hugene11t
CD19 (7994487)
Expression cutoff: 29.700 (min.grp=3)
subgroup~group3|met_status_(1_met__0_m0)~0|WITH_SURV (n=65)
overall survival probability
1.00
0.90
0.80
0.70
0.60
0.50
0.40
0.30
0.20
0.10
0.00
high (n=61)
low (n=4)
raw p 8.94e-03
bonf p 0.537
0
24
48
72
96
120
144
168
192
Follow up in months
Expression Graph
Event=No
Event=Yes
250
200
150
100
50
0
Expression
30
30
sorted by expression
p-value

GROUP3 M1

Tumor Medulloblastoma
Cavalli - 763 - rma_sketch - hugene11t
CD19 (7994487)
Expression cutoff: 34.100 (min.grp=3)
subgroup~group3|met_status_(1_met__0_m0)~1|WITH_SURV (n=41)

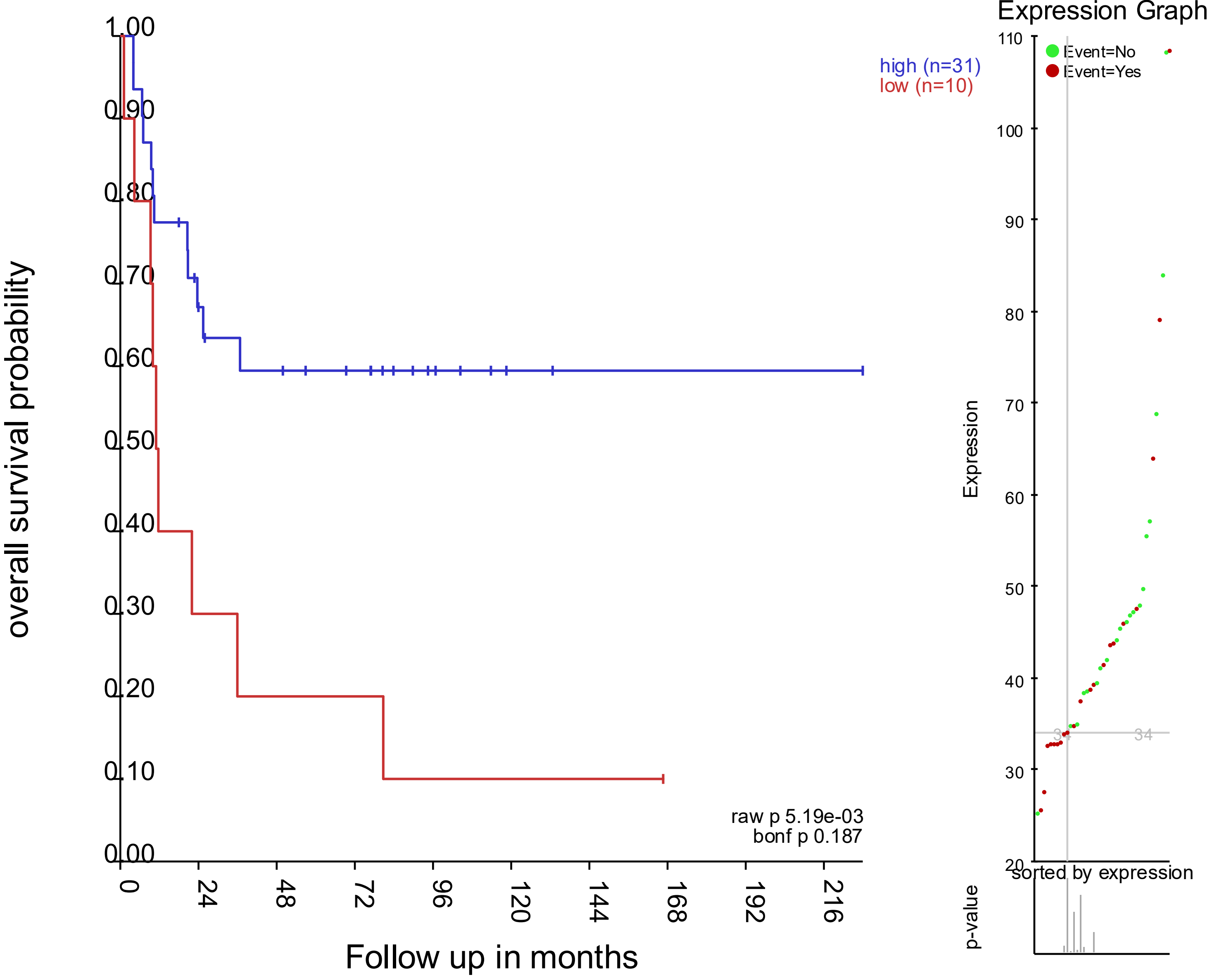

CD20

WNT M0

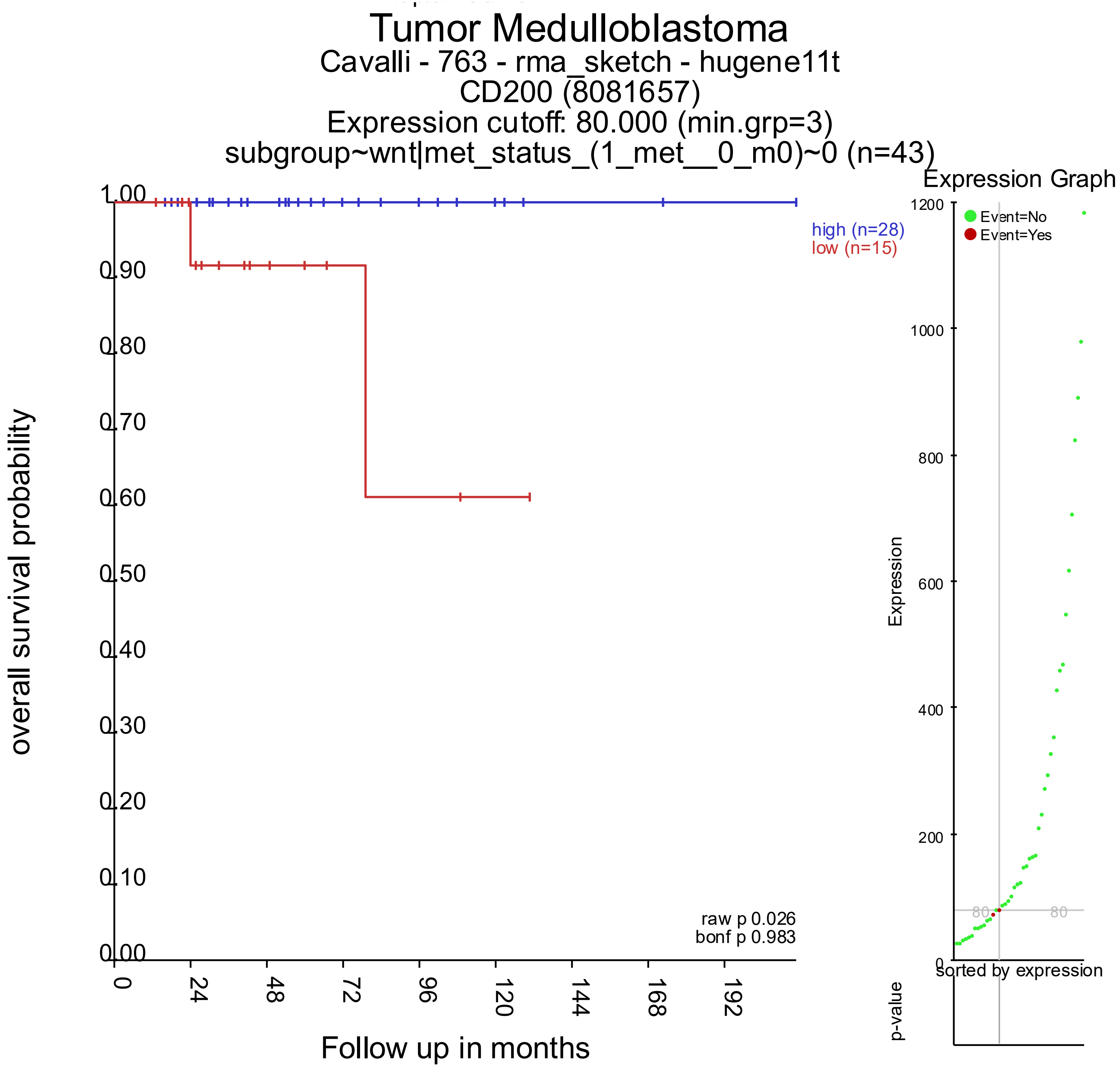
Tumor Medulloblastoma
Cavalli - 763 - rma_sketch - hugene11t
CD200 (8081657)
Expression cutoff: 80.000 (min.grp=3)
subgroup~wnt|met_status_(1_met__0_m0)~0 (n=43)
overall survival probability
1.00
0.90
0.80
0.70
0.60
0.50
0.40
0.30
0.20
0.10
0.00
0
24
48
72
96
120
144
168
192
Follow up in months
high (n=28)
low (n=15)
raw p 0.026
bonf p 0.983
Expression Graph
Event=No
Event=Yes
Expression
1200
1000
800
600
400
200
0
80
80
sorted by expression
p-value

WNT M1

Tumor Medulloblastoma
Cavalli - 763 - rma_sketch - hugene11t
CD200 (8081657)
Expression cutoff: 56.200 (min.grp=3)
subgroup~wnt|met_status_(1_met__0_m0)~1 (n=6)

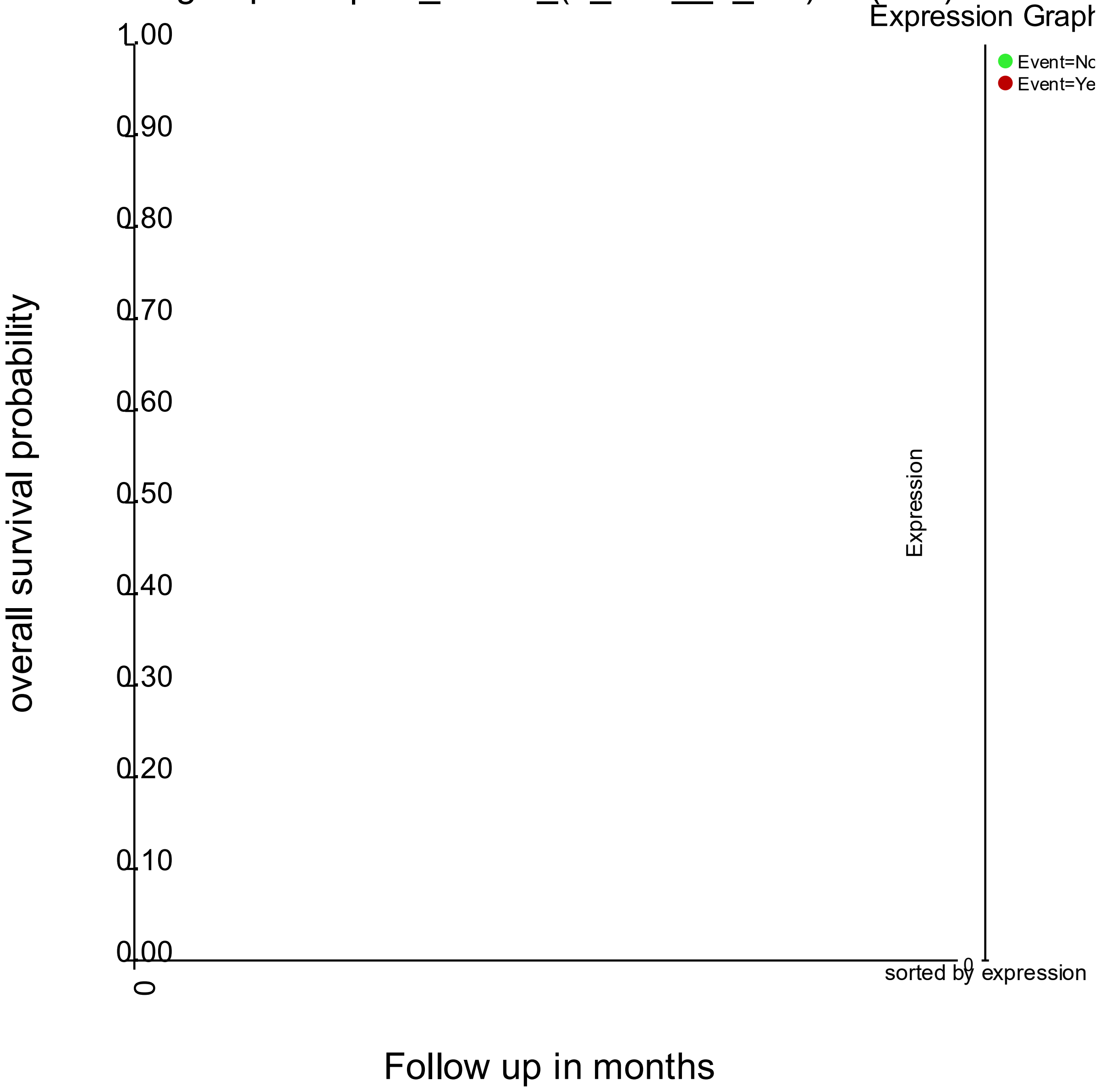

SHH M0

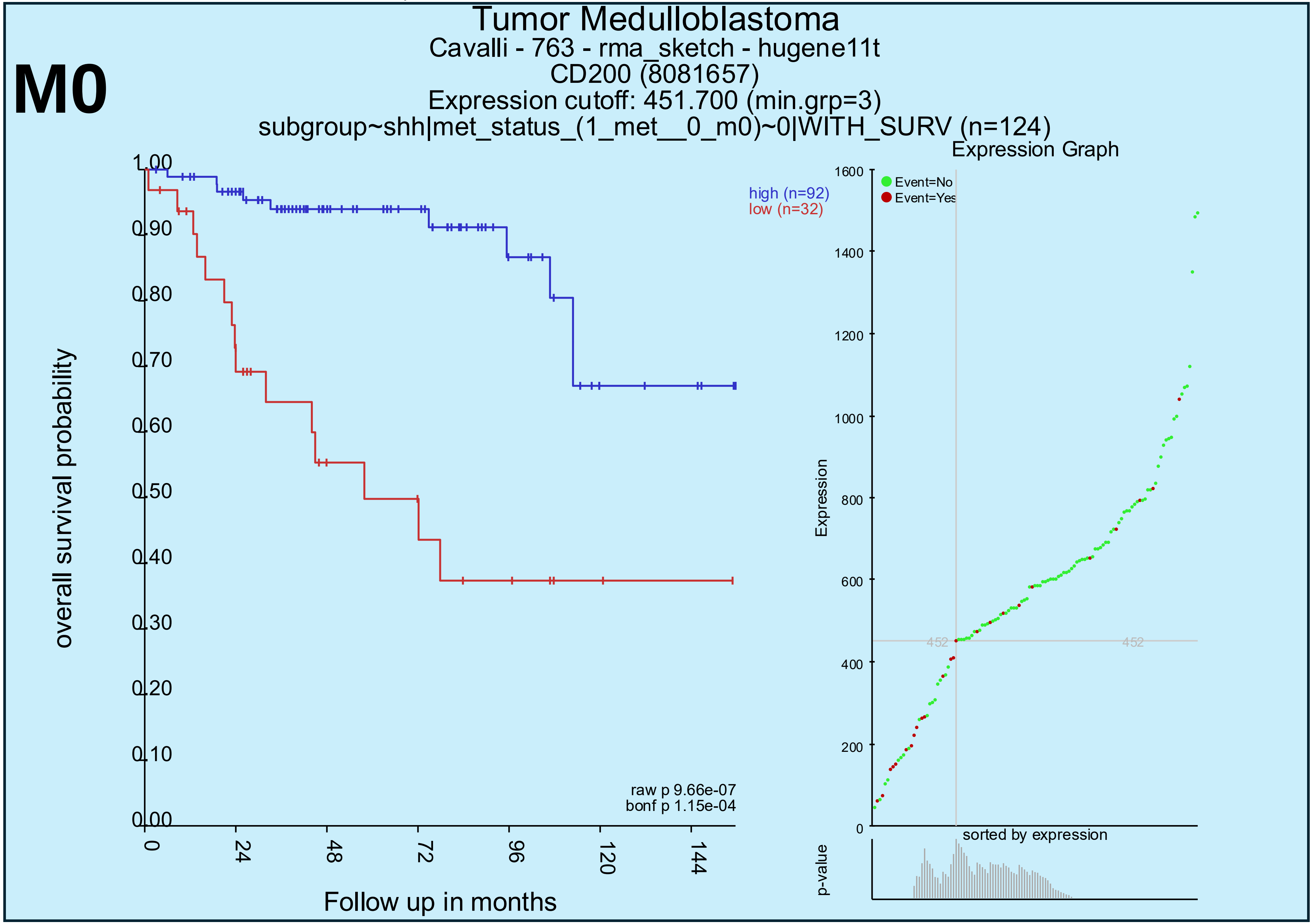
Tumor Medulloblastoma
Cavalli - 763 - rma_sketch - hugene11t
CD200 (8081657)
Expression cutoff: 451.700 (min.grp=3)
subgroup~shh|met_status_(1_met__0_m0)~0|WITH_SURV (n=124)
overall survival probability
1.00
0.90
0.80
0.70
0.60
0.50
0.40
0.30
0.20
0.10
0.00
0
24
48
72
96
120
144
Follow up in months
high (n=92)
low (n=32)
raw p 9.66e-07
bonf p 1.15e-04
Expression Graph
Event=No
Event=Yes
1600
1400
1200
1000
800
600
400
200
0
Expression
452
452
sorted by expression
p-value

SHH M1

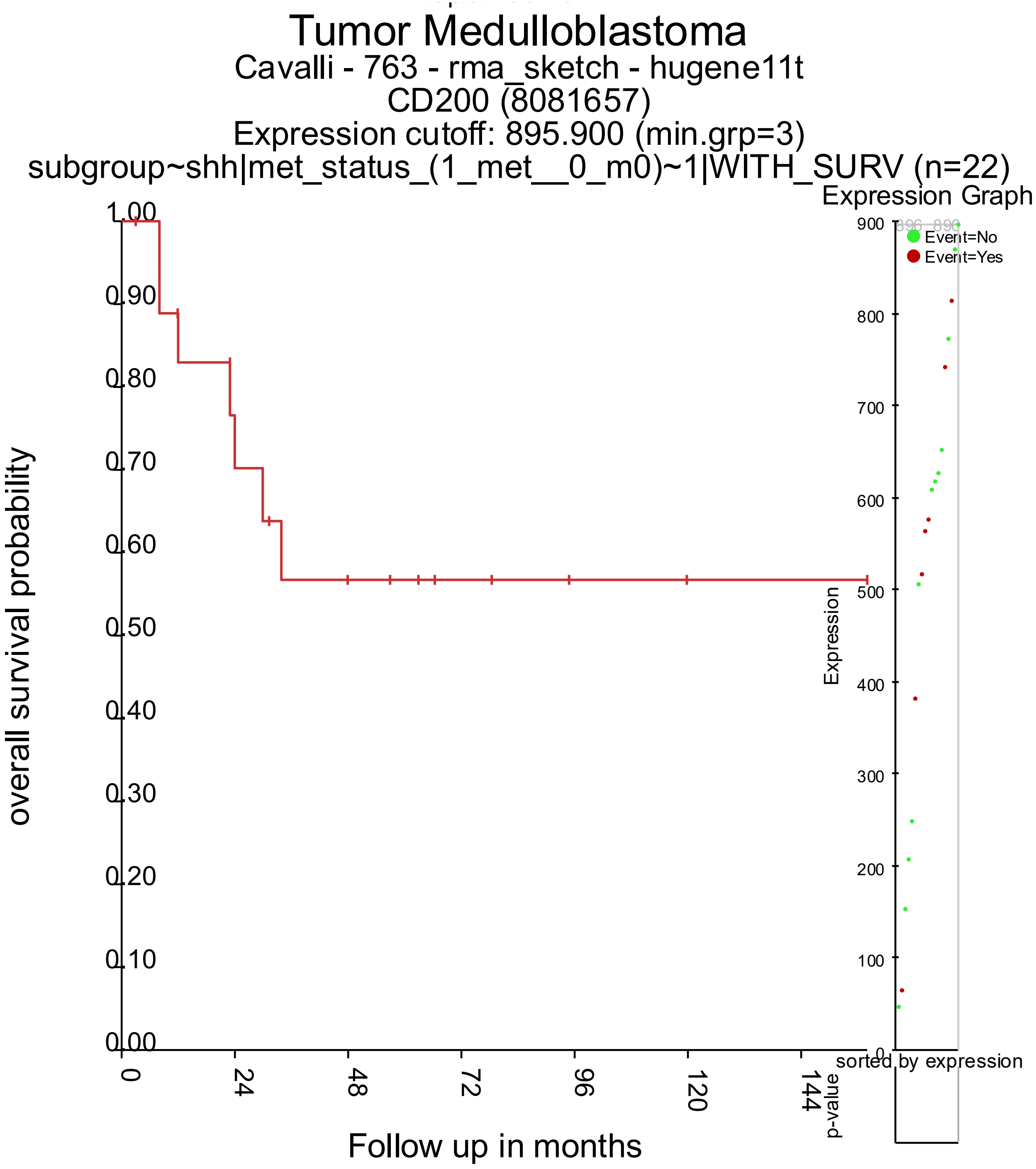
Tumor Medulloblastoma
Cavalli - 763 - rma_sketch - hugene11t
CD200 (8081657)
Expression cutoff: 895.900 (min.grp=3)
subgroup~shh|met_status_(1_met__0_m0)~1|WITH_SURV (n=22)
Expression Graph
overall survival probability
1.00
0.90
0.80
0.70
0.60
0.50
0.40
0.30
0.20
0.10
0.00
0
24
48
72
96
120
144
Follow up in months
p-value
Event=No
Event=Yes
Expression
900
800
700
600
500
400
300
200
100
0
sorted by expression

GROUP4 M0

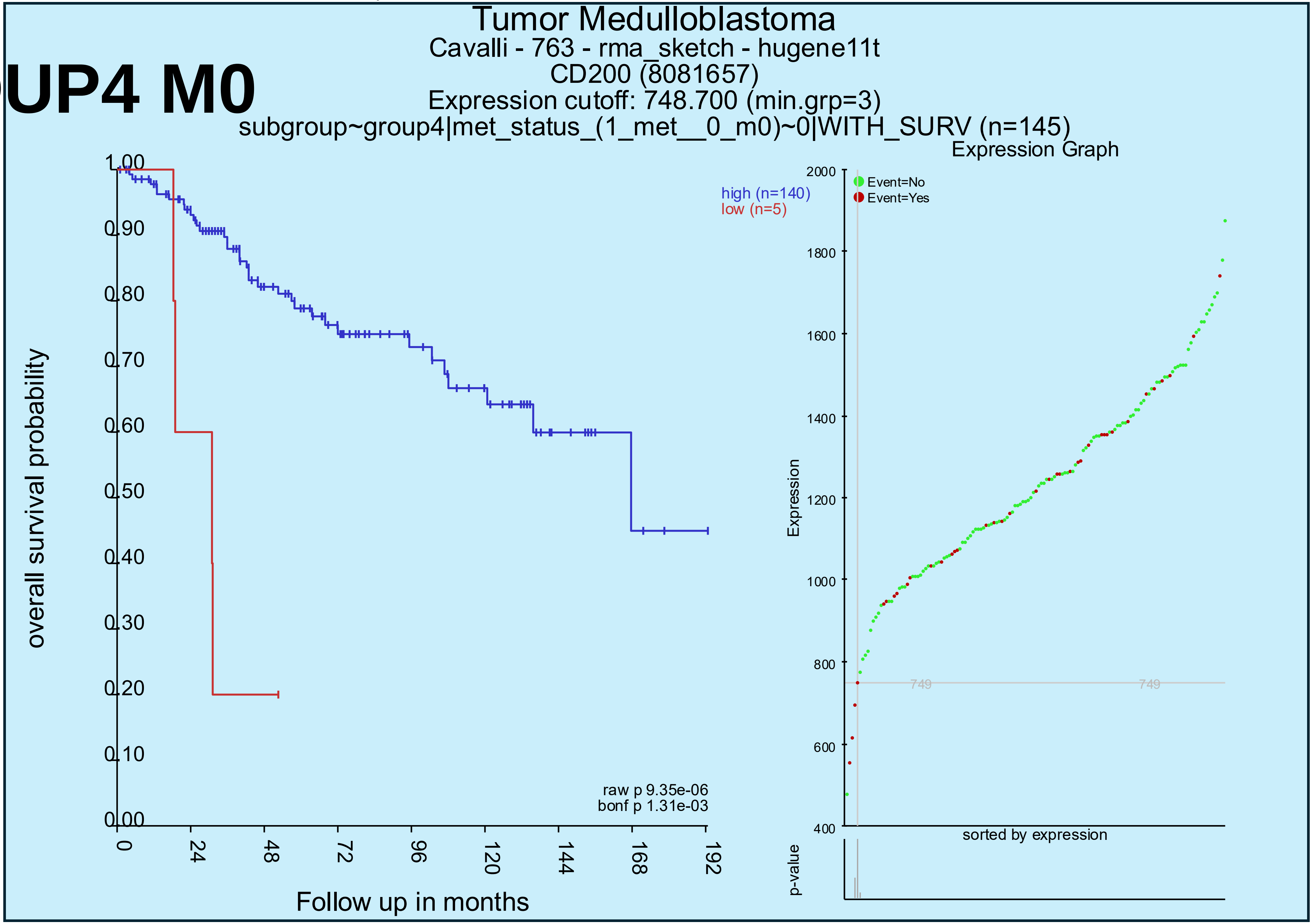

Tumor Medulloblastoma
Cavalli - 763 - rma_sketch - hugene11t
CD200 (8081657)
Expression cutoff: 748.700 (min.grp=3)
subgroup~group4|met_status_(1_met__0_m0)~0|WITH_SURV (n=145)
overall survival probability
1.00
0.90
0.80
0.70
0.60
0.50
0.40
0.30
0.20
0.10
0.00
0
24
48
72
96
120
144
168
192
Follow up in months
high (n=140)
low (n=5)
raw p 9.35e-06
bonf p 1.31e-03
Expression Graph
Event=No
Event=Yes
Expression
2000
1800
1600
1400
1200
1000
800
600
400
749
749
sorted by expression
p-value

GROUP4 M1

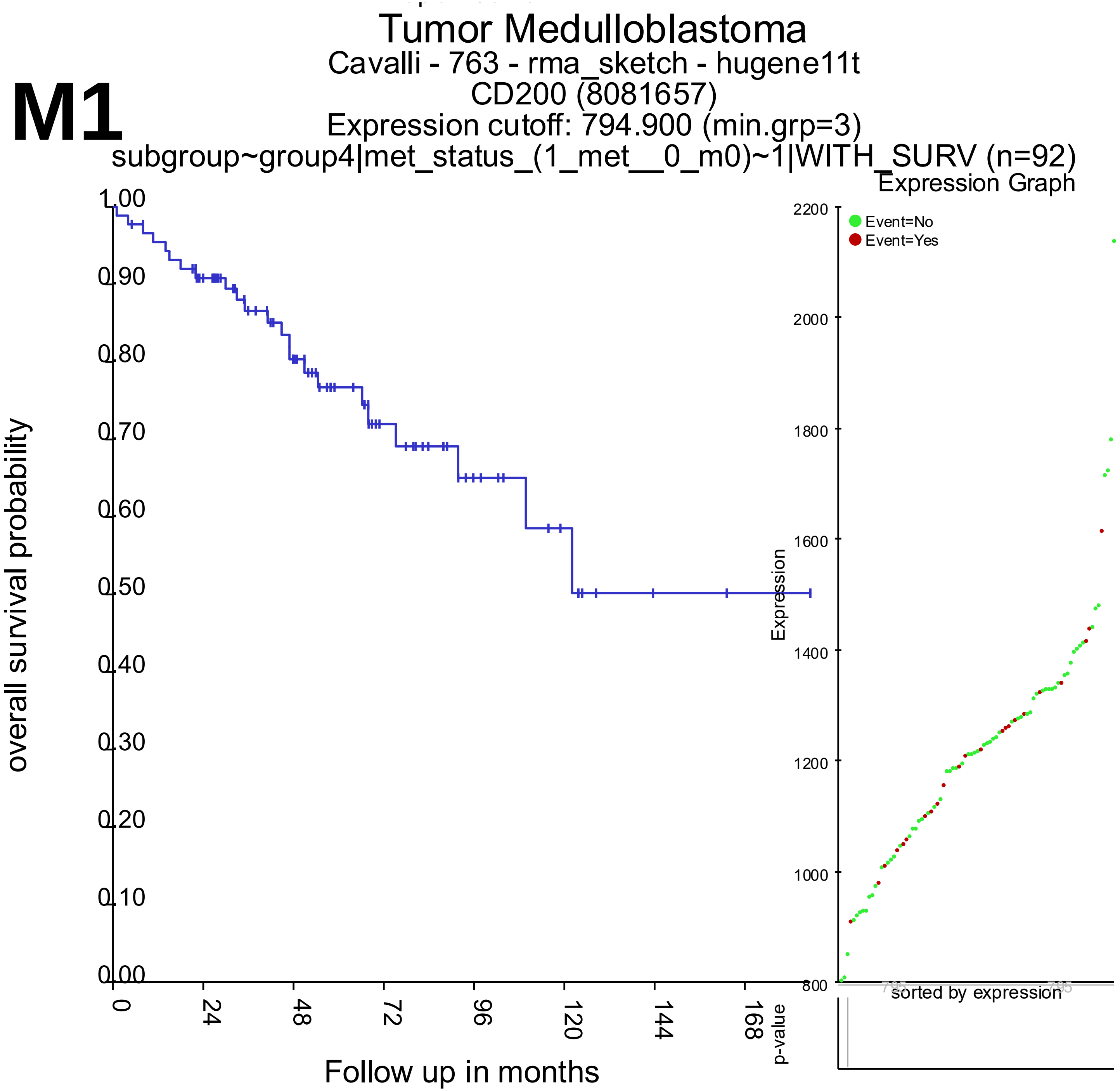

Tumor Medulloblastoma
Cavalli - 763 - rma_sketch - hugene11t
CD200 (8081657)
Expression cutoff: 794.900 (min.grp=3)
subgroup~group4|met_status_(1_met__0_m0)~1|WITH_SURV (n=92)
overall survival probability
1.00
0.90
0.80
0.70
0.60
0.50
0.40
0.30
0.20
0.10
0.00
0
24
48
72
96
120
144
168
Follow up in months
p-value
Expression Graph
Event=No
Event=Yes
Expression
2200
2000
1800
1600
1400
1200
1000
800
sorted by expression

GROUP3 M0

Tumor Medulloblastoma
Cavalli - 763 - rma_sketch - hugene11t
CD200 (8081657)
Expression cutoff: 121.000 (min.grp=3)
subgroup~group3|met_status_(1_met__0_m0)~0|WITH_SURV (n=65)

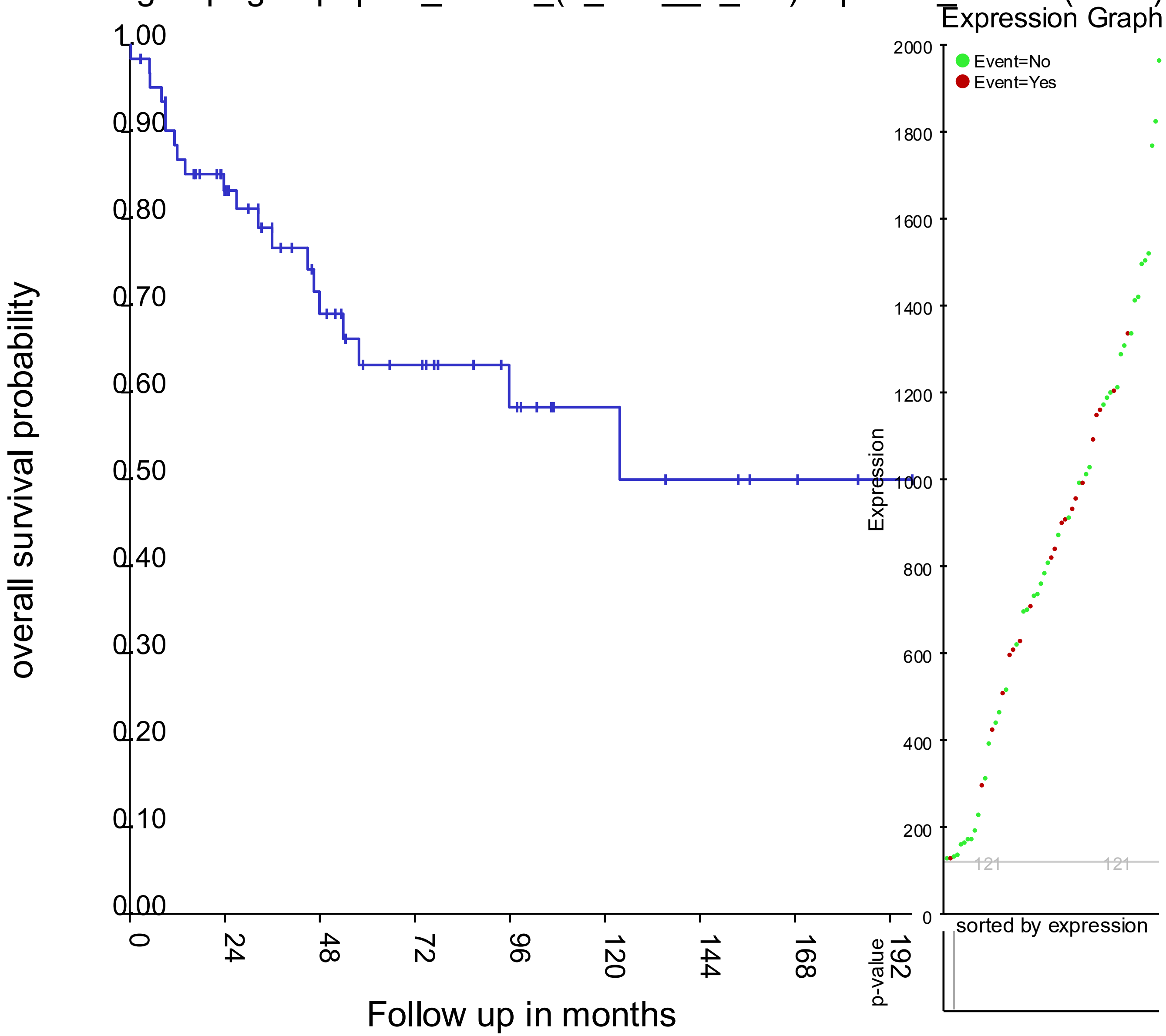

GROUP3 M1

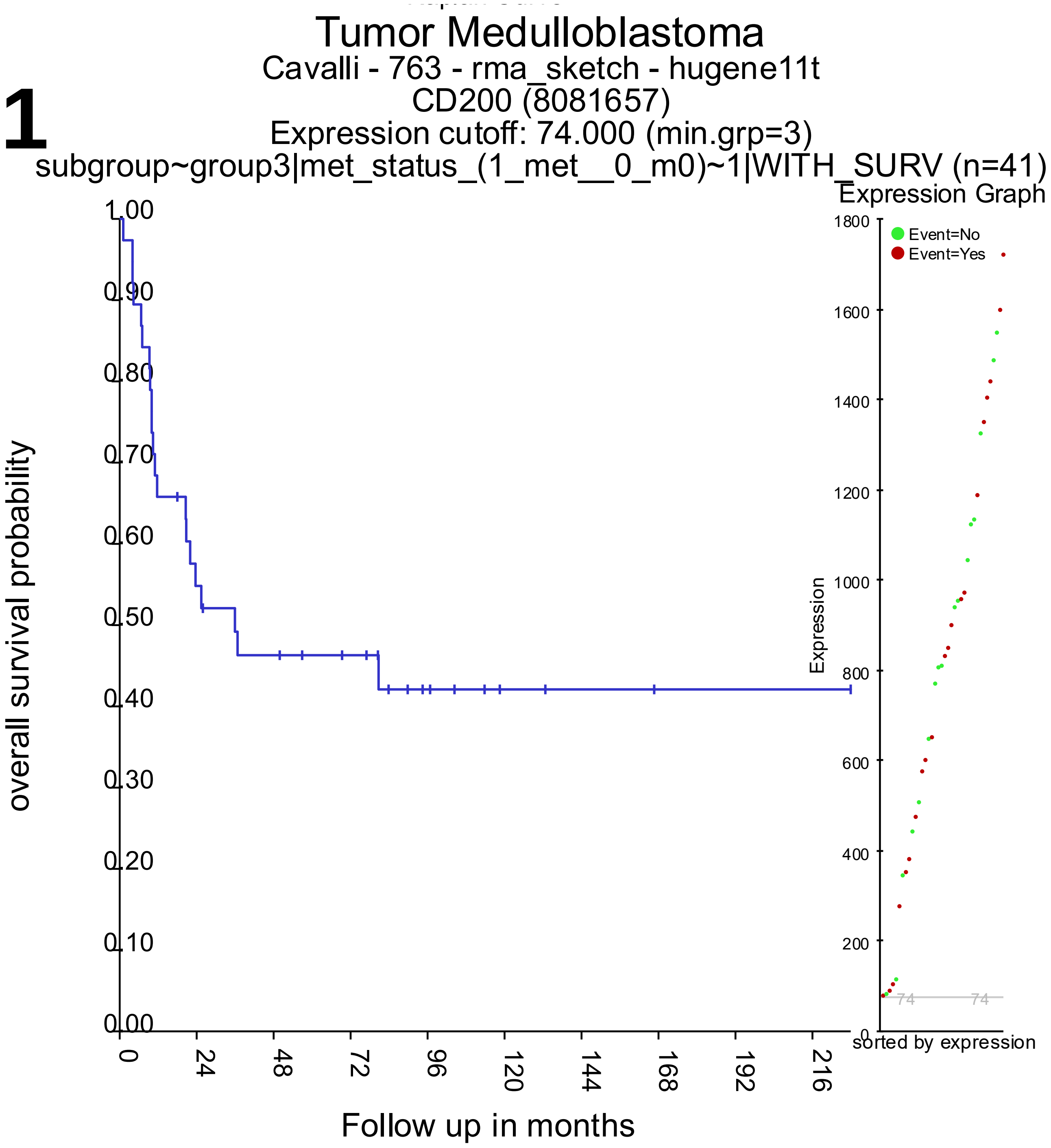
Tumor Medulloblastoma
Cavalli - 763 - rma_sketch - hugene11t
CD200 (8081657)
Expression cutoff: 74.000 (min.grp=3)
subgroup~group3|met_status_(1_met__0_m0)~1|WITH_SURV (n=41)
overall survival probability
1.00
0.90
0.80
0.70
0.60
0.50
0.40
0.30
0.20
0.10
0.00
0
24
48
72
96
120
144
168
192
216
Follow up in months
Expression Graph
Event=No
Event=Yes
Expression
1800
1600
1400
1200
1000
800
600
400
200
0
74
74
sorted by expression

CD25/ISG20

WNT M0

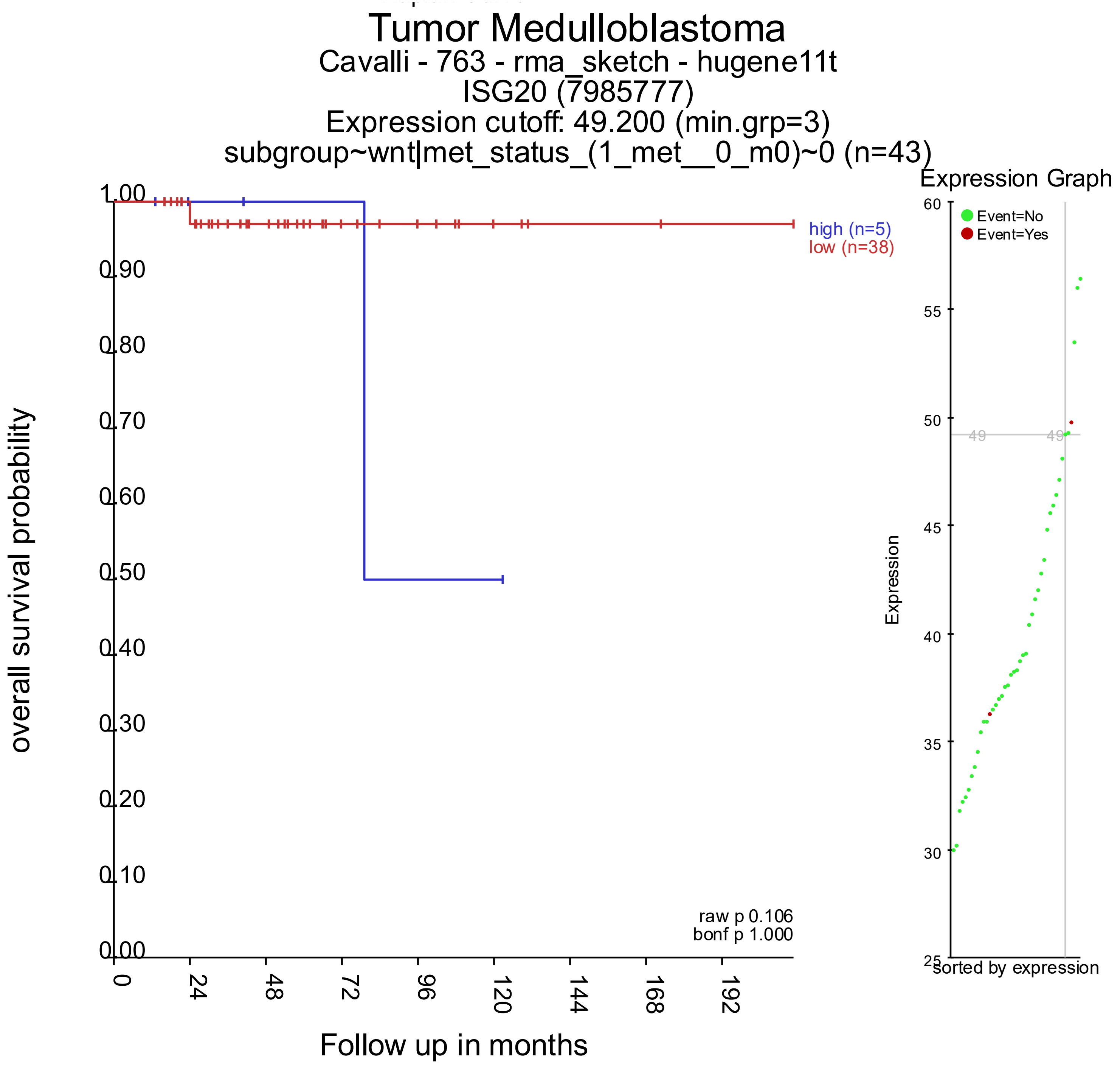

Tumor Medulloblastoma
Cavalli - 763 - rma_sketch - hugene11t
ISG20 (7985777)
Expression cutoff: 49.200 (min.grp=3)
subgroup~wnt|met_status_(1_met__0_m0)~0 (n=43)
overall survival probability
1.00
0.90
0.80
0.70
0.60
0.50
0.40
0.30
0.20
0.10
0.00
high (n=5)
low (n=38)
raw p 0.106
bonf p 1.000
0
24
48
72
96
120
144
168
192
Follow up in months
Expression Graph
Event=No
Event=Yes
60
55
50
45
40
35
30
25
Expression
49
49
sorted by expression

WNT M1

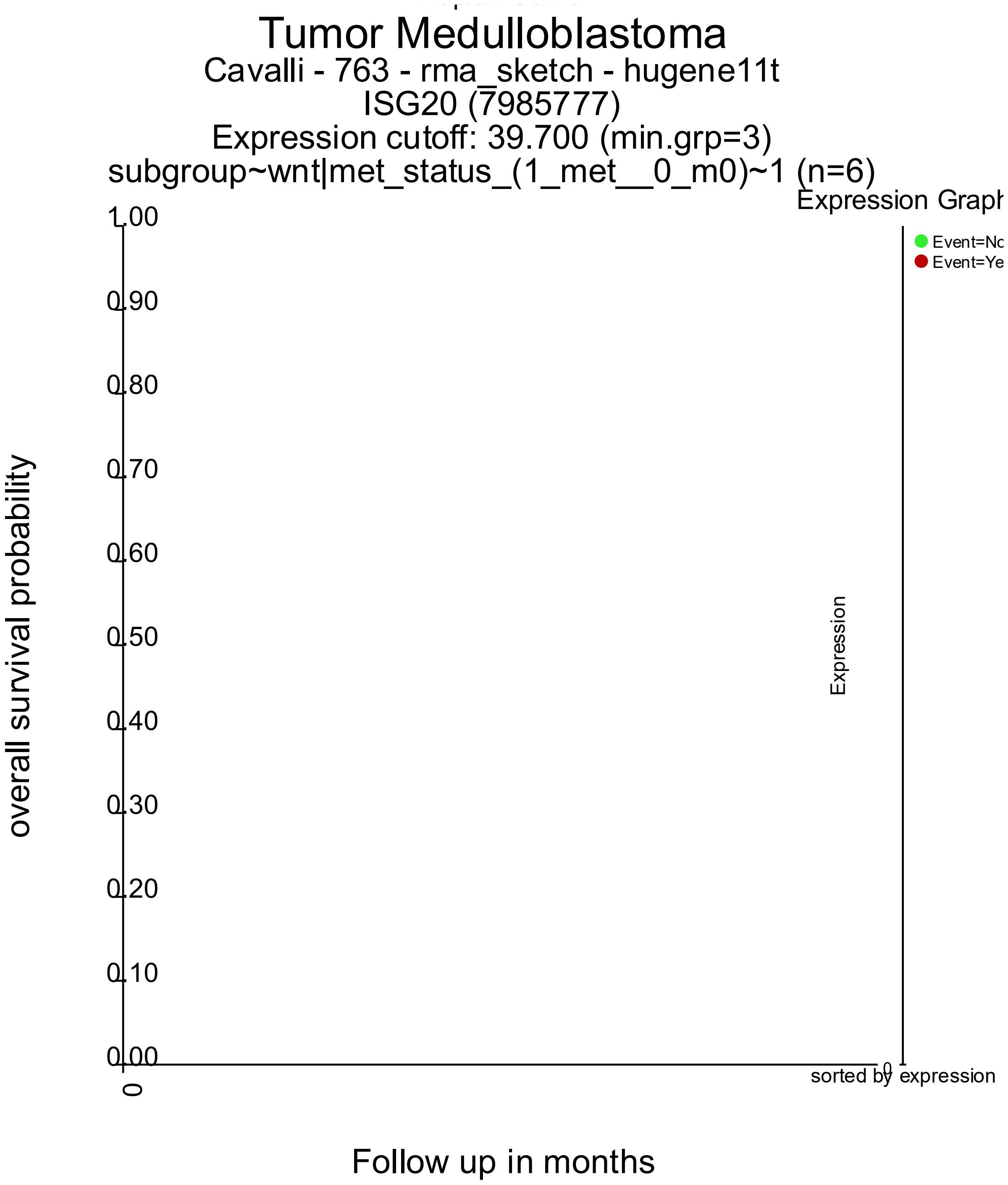

Tumor Medulloblastoma
Cavalli - 763 - rma_sketch - hugene11t
ISG20 (7985777)
Expression cutoff: 39.700 (min.grp=3)
subgroup~wnt|met_status_(1_met__0_m0)~1 (n=6)
Expression Graph
Event=No
Event=Ye
1.00
0.90
0.80
0.70
0.60
0.50
0.40
0.30
0.20
0.10
0.00
overall survival probability
0
Follow up in months
Expression
0
sorted by expression

SHH M0

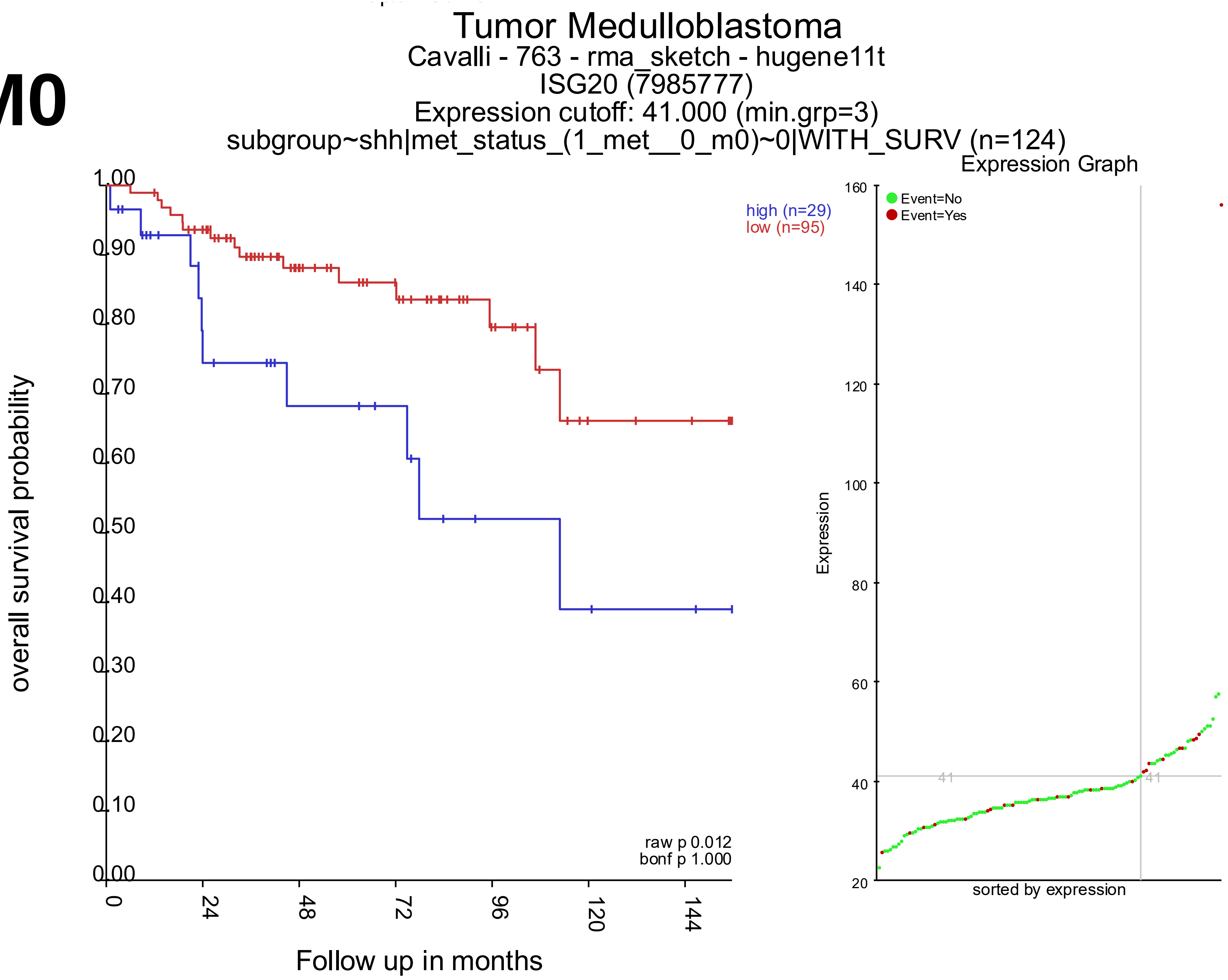
Tumor Medulloblastoma
Cavalli - 763 - rma_sketch - hugene11t
ISG20 (7985777)
Expression cutoff: 41.000 (min.grp=3)
subgroup~shh|met_status_(1_met__0_m0)~0|WITH_SURV (n=124)
overall survival probability
1.00
0.90
0.80
0.70
0.60
0.50
0.40
0.30
0.20
0.10
0.00
high (n=29)
low (n=95)
raw p 0.012
bonf p 1.000
0
24
48
72
96
120
144
Follow up in months
Expression Graph
Event=No
Event=Yes
Expression
160
140
120
100
80
60
40
20
41
sorted by expression

SHH M1

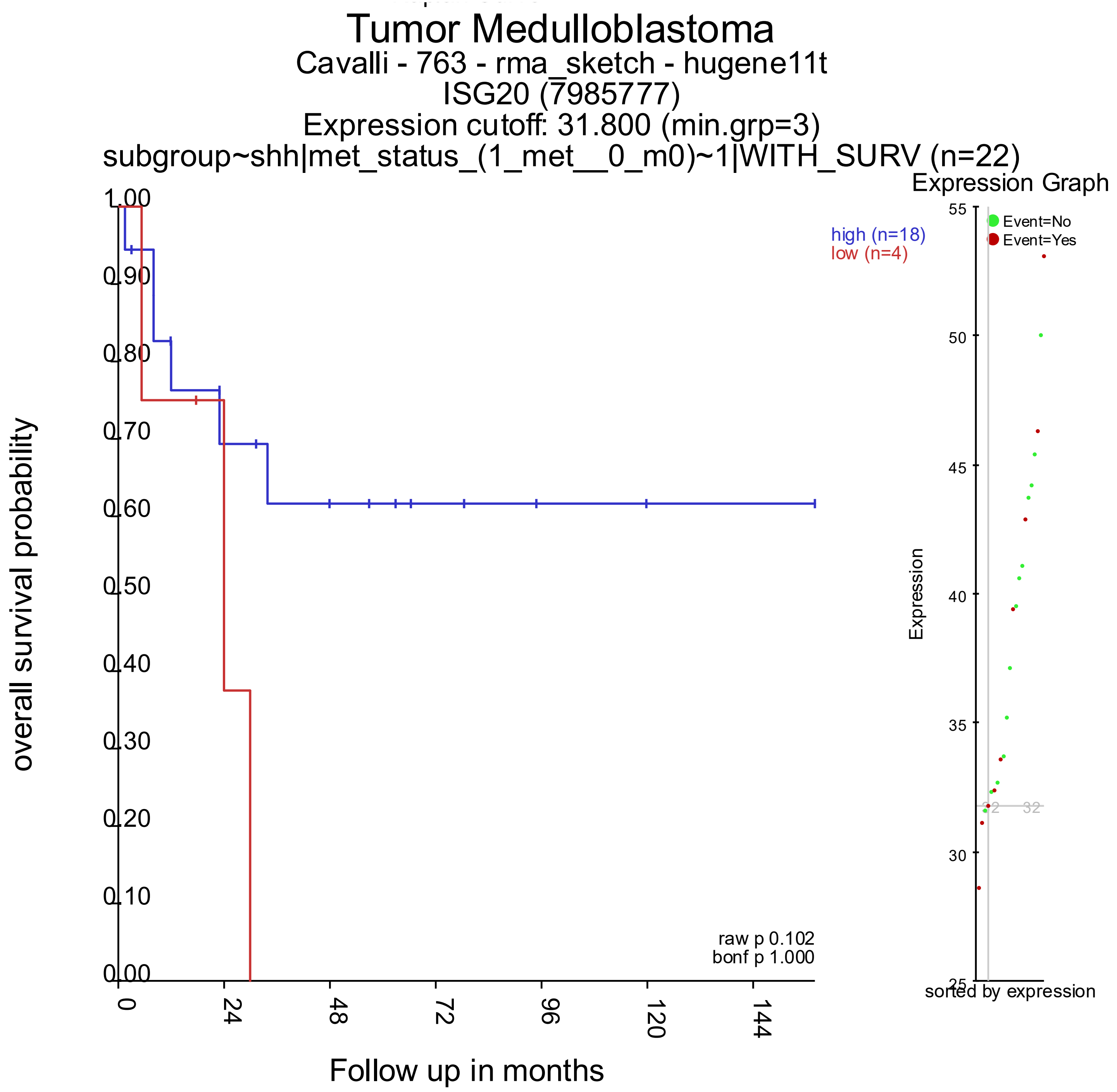

Tumor Medulloblastoma
Cavalli - 763 - rma_sketch - hugene11t
ISG20 (7985777)
Expression cutoff: 31.800 (min.grp=3)
subgroup~shh|met_status_(1_met__0_m0)~1|WITH_SURV (n=22)
overall survival probability
1.00
0.90
0.80
0.70
0.60
0.50
0.40
0.30
0.20
0.10
0.00
0
24
48
72
96
120
144
Follow up in months
high (n=18)
low (n=4)
raw p 0.102
bonf p 1.000
Expression Graph
Event=No
Event=Yes
Expression
55
50
45
40
35
30
25
32
sorted by expression

GROUP4 M0

Tumor Medulloblastoma
Cavalli - 763 - rma_sketch - hugene11t
ISG20 (7985777)
Expression cutoff: 47.400 (min.grp=3)
subgroup~group4|met_status_(1_met__0_m0)~0|WITH_SURV (n=145)

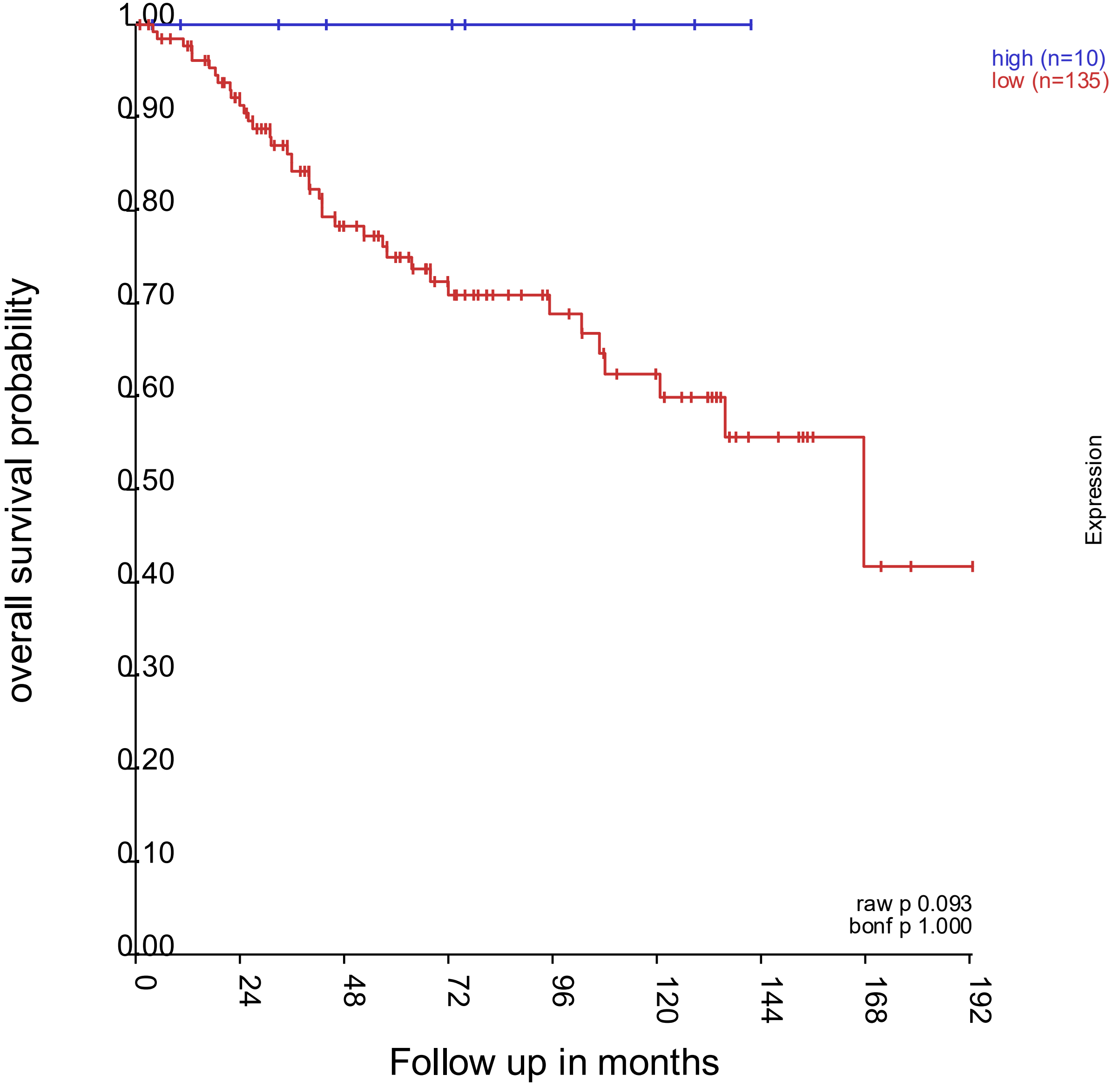

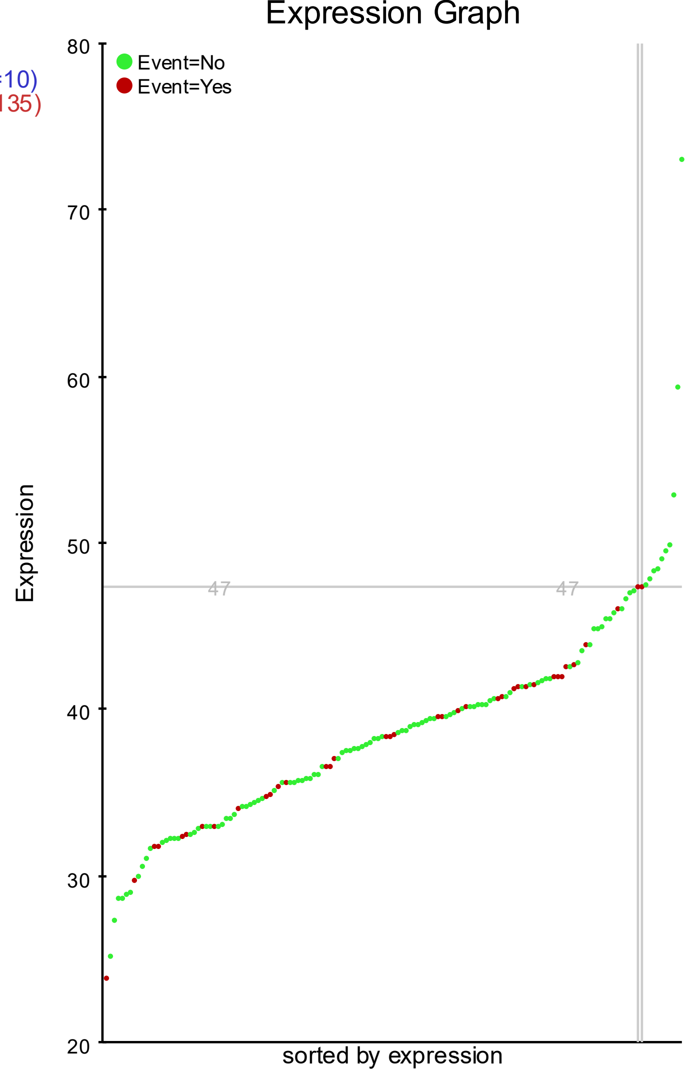

GROUP4 M1

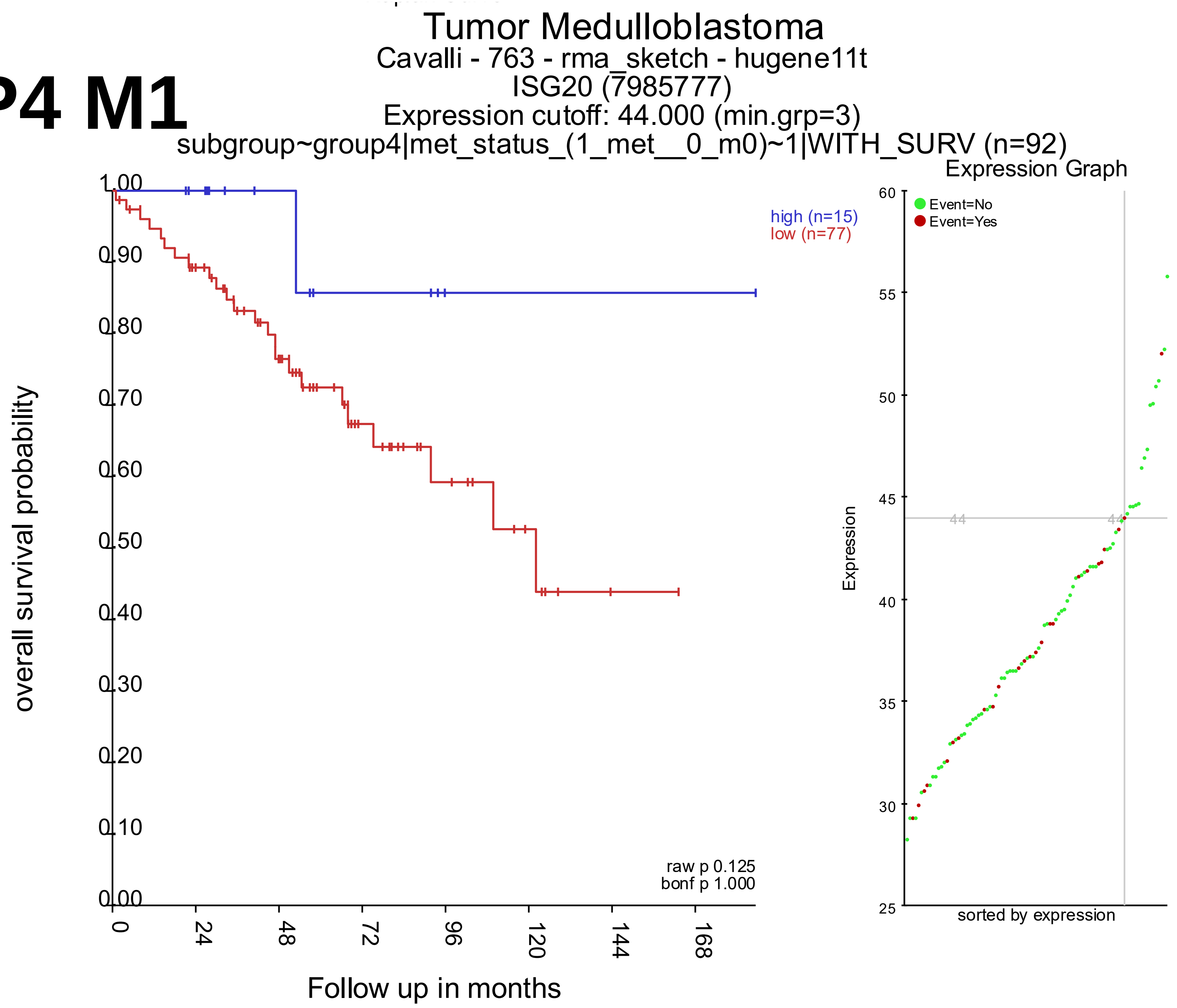
Tumor Medulloblastoma
Cavalli - 763 - rma_sketch - hugene11t
ISG20 (7985777)
Expression cutoff: 44.000 (min.grp=3)
subgroup~group4|met_status_(1_met__0_m0)~1|WITH_SURV (n=92)
overall survival probability
1.00
0.90
0.80
0.70
0.60
0.50
0.40
0.30
0.20
0.10
0.00
0
24
48
72
96
120
144
168
Follow up in months
high (n=15)
low (n=77)
raw p 0.125
bonf p 1.000
Expression Graph
Event=No
Event=Yes
Expression
60
55
50
45
40
35
30
25
44
sorted by expression

GROUP3 M0

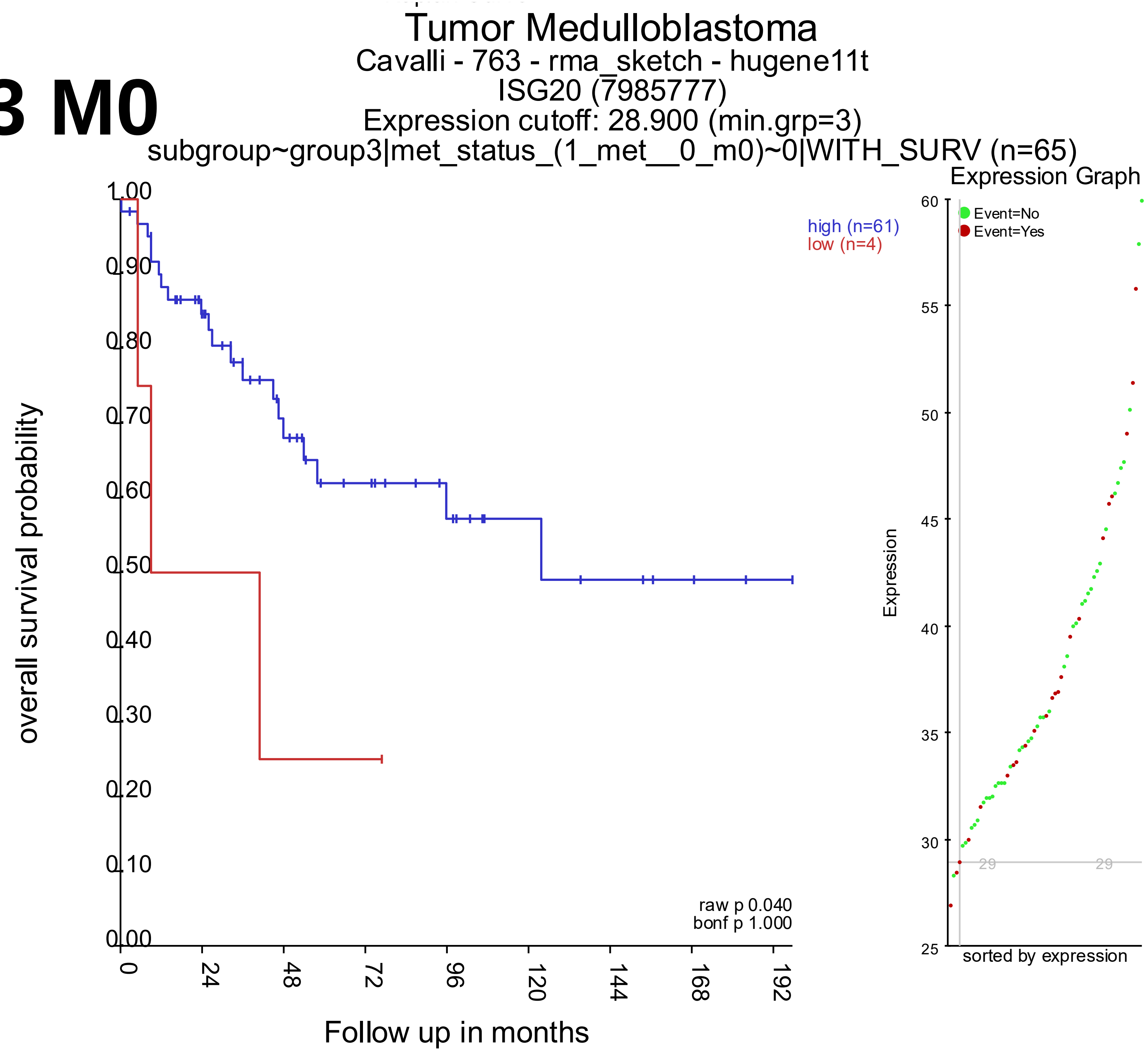

Tumor Medulloblastoma
Cavalli - 763 - rma_sketch - hugene11t
ISG20 (7985777)
Expression cutoff: 28.900 (min.grp=3)
subgroup~group3|met_status_(1_met__0_m0)~0|WITH_SURV (n=65)
overall survival probability
1.00
0.90
0.80
0.70
0.60
0.50
0.40
0.30
0.20
0.10
0.00
high (n=61)
low (n=4)
raw p 0.040
bonf p 1.000
0
24
48
72
96
120
144
168
192
Follow up in months
Expression Graph
Event=No
Event=Yes
Expression
60
55
50
45
40
35
30
25
29
29
sorted by expression

GROUP3 M1

Tumor Medulloblastoma
Cavalli - 763 - rma_sketch - hugene11t
ISG20 (7985777)
Expression cutoff: 27.200 (min.grp=3)
subgroup~group3|met_status_(1_met__0_m0)~1|WITH_SURV (n=41)

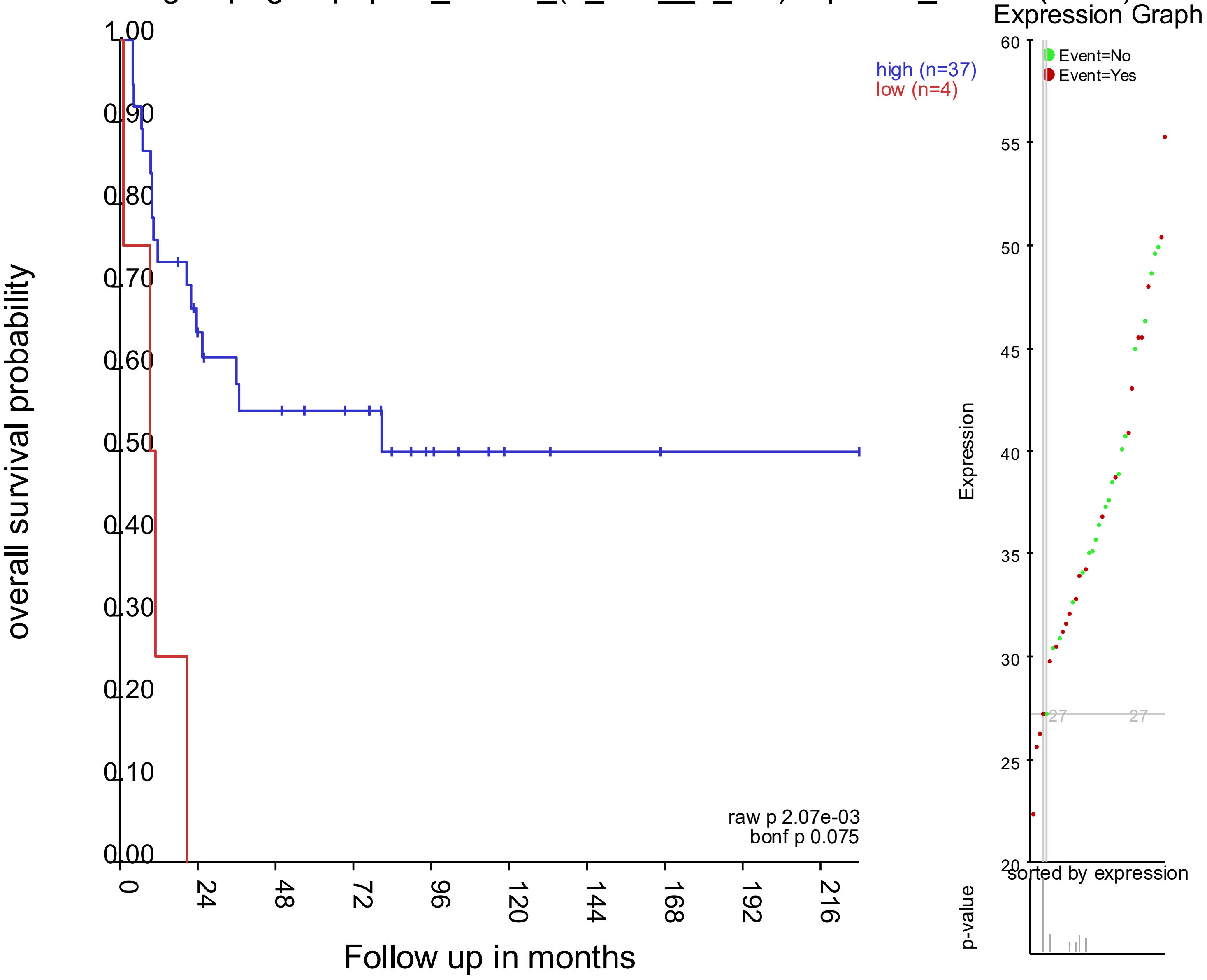

CD33

WNT M0

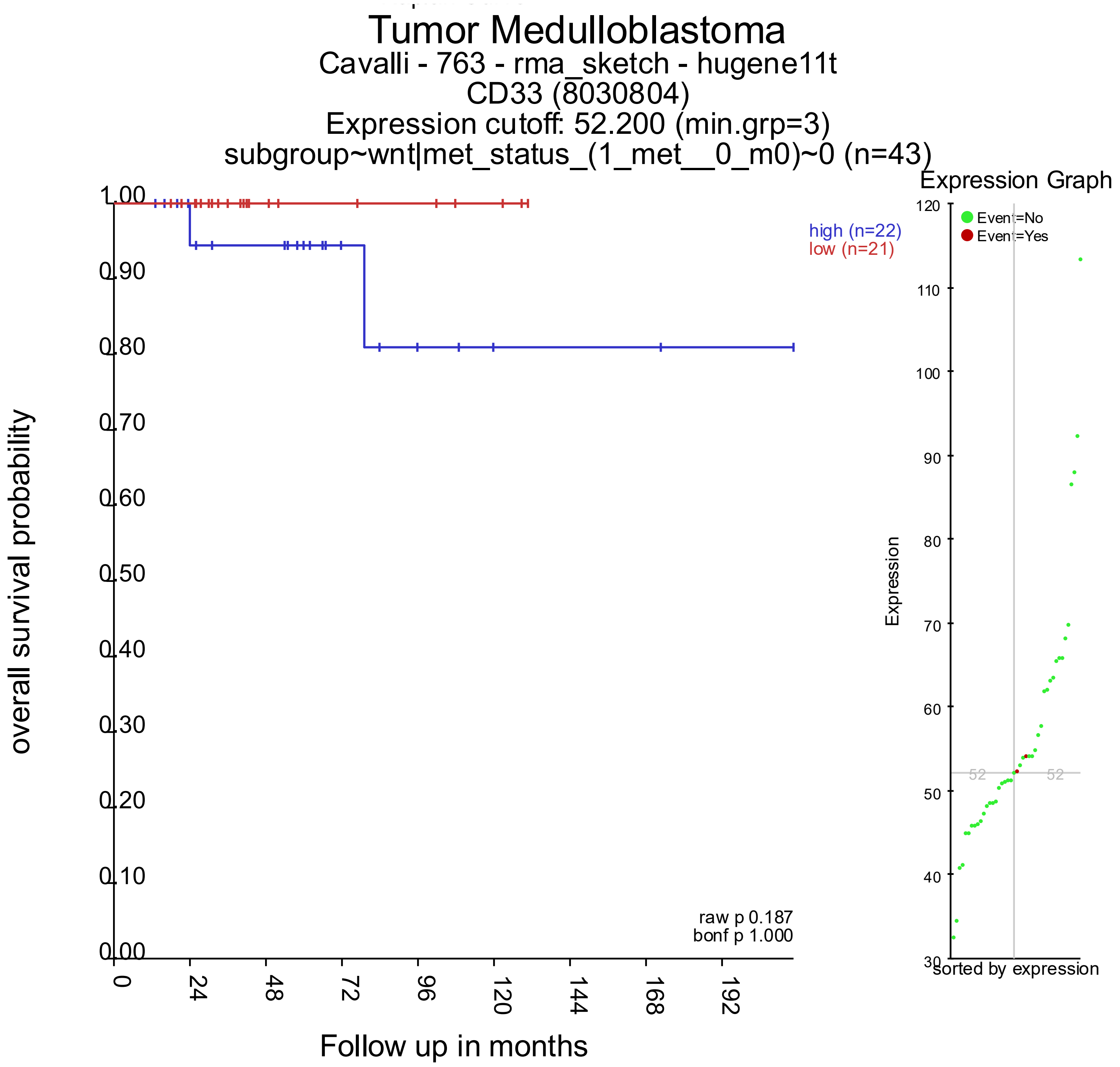

Tumor Medulloblastoma
Cavalli - 763 - rma_sketch - hugene11t
CD33 (8030804)
Expression cutoff: 52.200 (min.grp=3)
subgroup~wnt|met_status_(1_met__0_m0)~0 (n=43)
high (n=22)
low (n=21)
overall survival probability
1.00
0.90
0.80
0.70
0.60
0.50
0.40
0.30
0.20
0.10
0.00
raw p 0.187
bonf p 1.000
0
24
48
72
96
120
144
168
192
Follow up in months
Expression Graph
Event=No
Event=Yes
Expression
120
110
100
90
80
70
60
50
40
30
52
52
sorted by expression

WNT M1

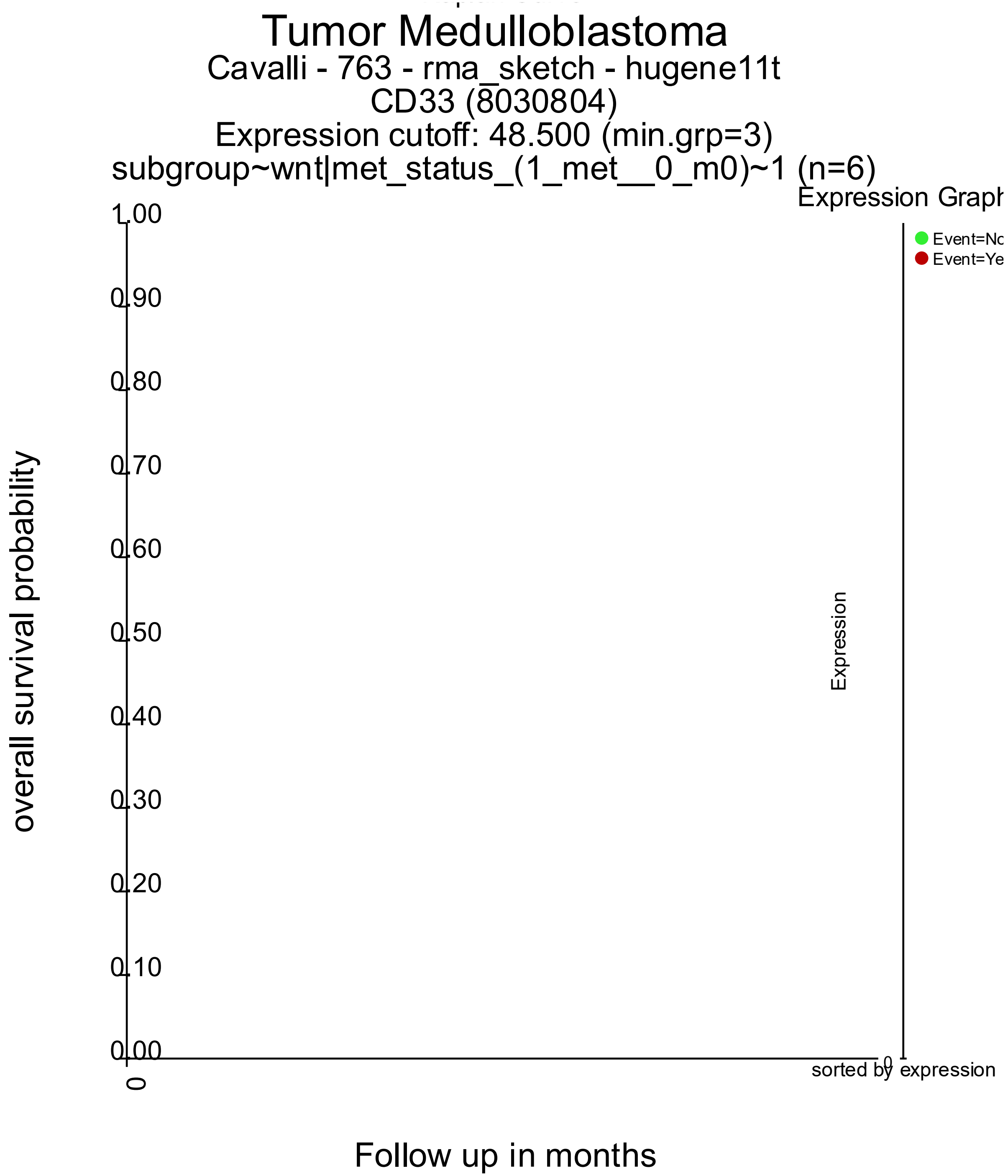
Tumor Medulloblastoma
Cavalli - 763 - rma_sketch - hugene11t
CD33 (8030804)
Expression cutoff: 48.500 (min.grp=3)
subgroup~wnt|met_status_(1_met__0_m0)~1 (n=6)
Expression Graph
Event=No
Event=Ye
1.00
0.90
0.80
0.70
0.60
0.50
0.40
0.30
0.20
0.10
0.00
overall survival probability
Expression
0
sorted by expression
0
Follow up in months

SHH M0

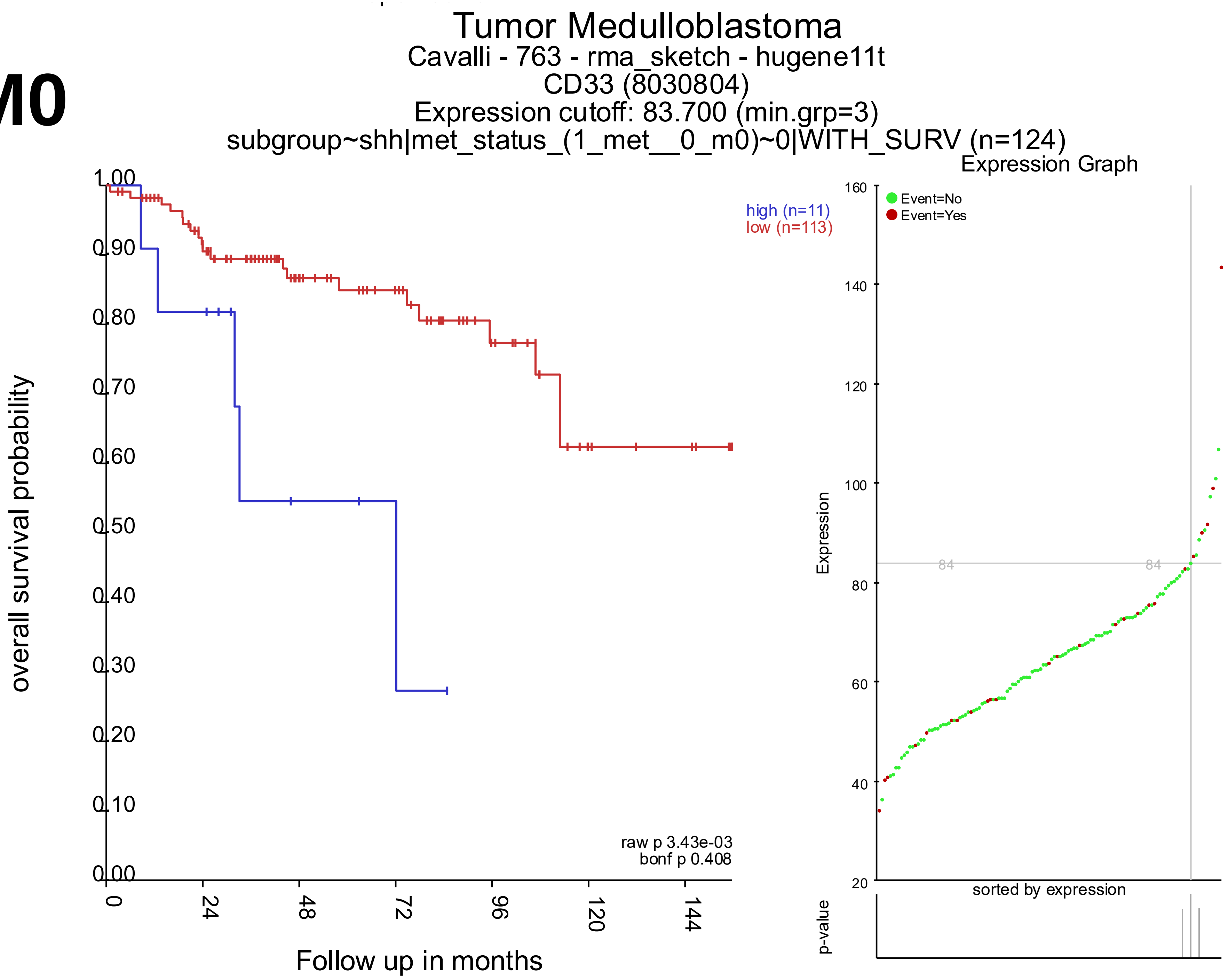

Tumor Medulloblastoma
Cavalli - 763 - rma_sketch - hugene11t
CD33 (8030804)
Expression cutoff: 83.700 (min.grp=3)
subgroup~shh|met_status_(1_met__0_m0)~0|WITH_SURV (n=124)
overall survival probability
1.00
0.90
0.80
0.70
0.60
0.50
0.40
0.30
0.20
0.10
0.00
0
24
48
72
96
120
144
Follow up in months
high (n=11)
low (n=113)
raw p 3.43e-03
bonf p 0.408
Expression Graph
Event=No
Event=Yes
Expression
160
140
120
100
80
60
40
20
84
84
sorted by expression
p-value

SHH M1

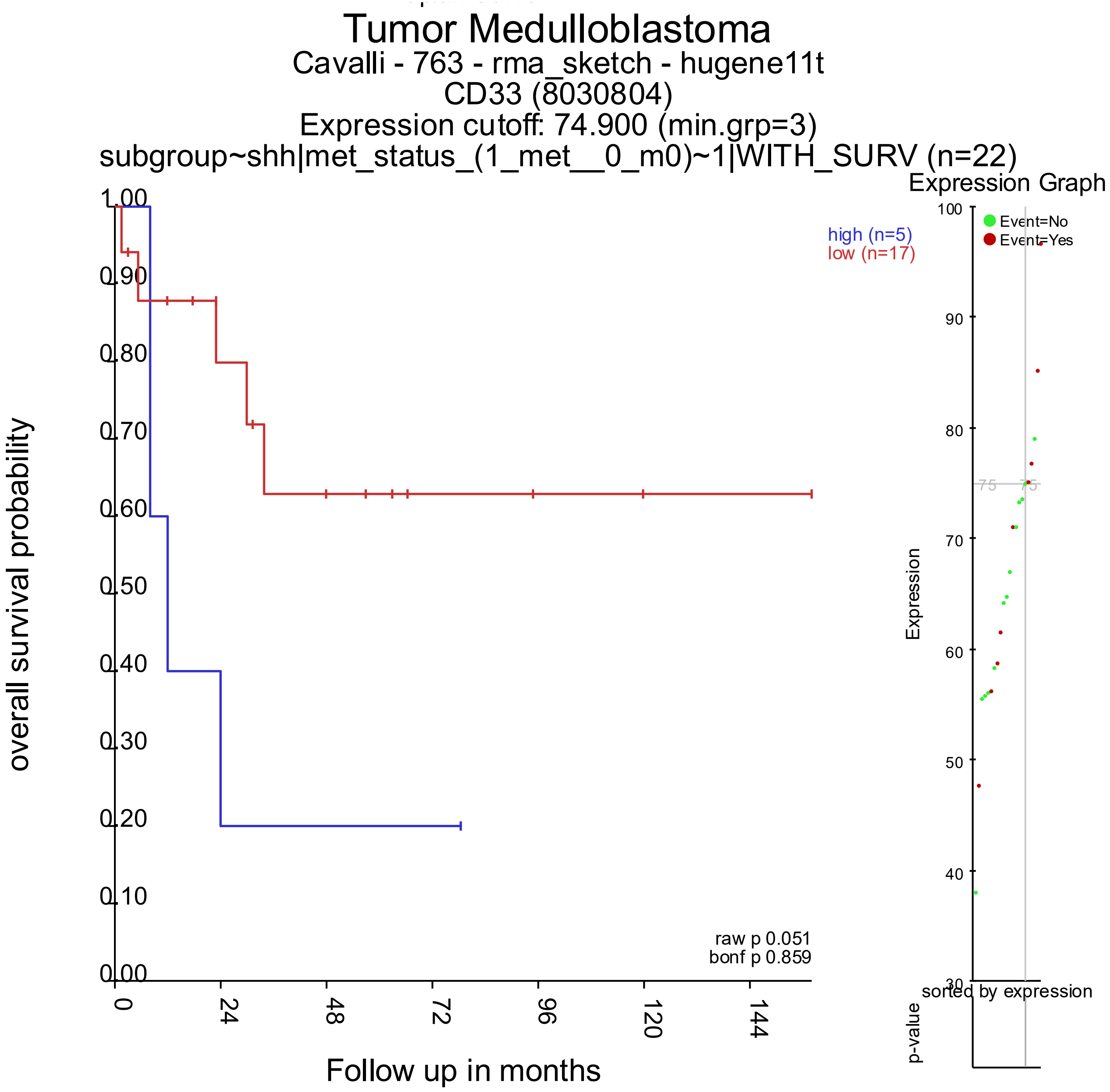

Tumor Medulloblastoma
Cavalli - 763 - rma_sketch - hugene11t
CD33 (8030804)
Expression cutoff: 74.900 (min.grp=3)
subgroup~shh|met_status_(1_met__0_m0)~1|WITH_SURV (n=22)
overall survival probability
1.00
0.90
0.80
0.70
0.60
0.50
0.40
0.30
0.20
0.10
0.00
0
24
48
72
96
120
144
Follow up in months
high (n=5)
low (n=17)
raw p 0.051
bonf p 0.859
Expression Graph
Event=No
Event=Yes
Expression
100
90
80
70
60
50
40
30
75
sorted by expression
p-value

GROUP4 M0

Tumor Medulloblastoma
Cavalli - 763 - rma_sketch - hugene11t
CD33 (8030804)
Expression cutoff: 43.400 (min.grp=3)
subgroup~group4|met_status_(1_met__0_m0)~0|WITH_SURV (n=145)

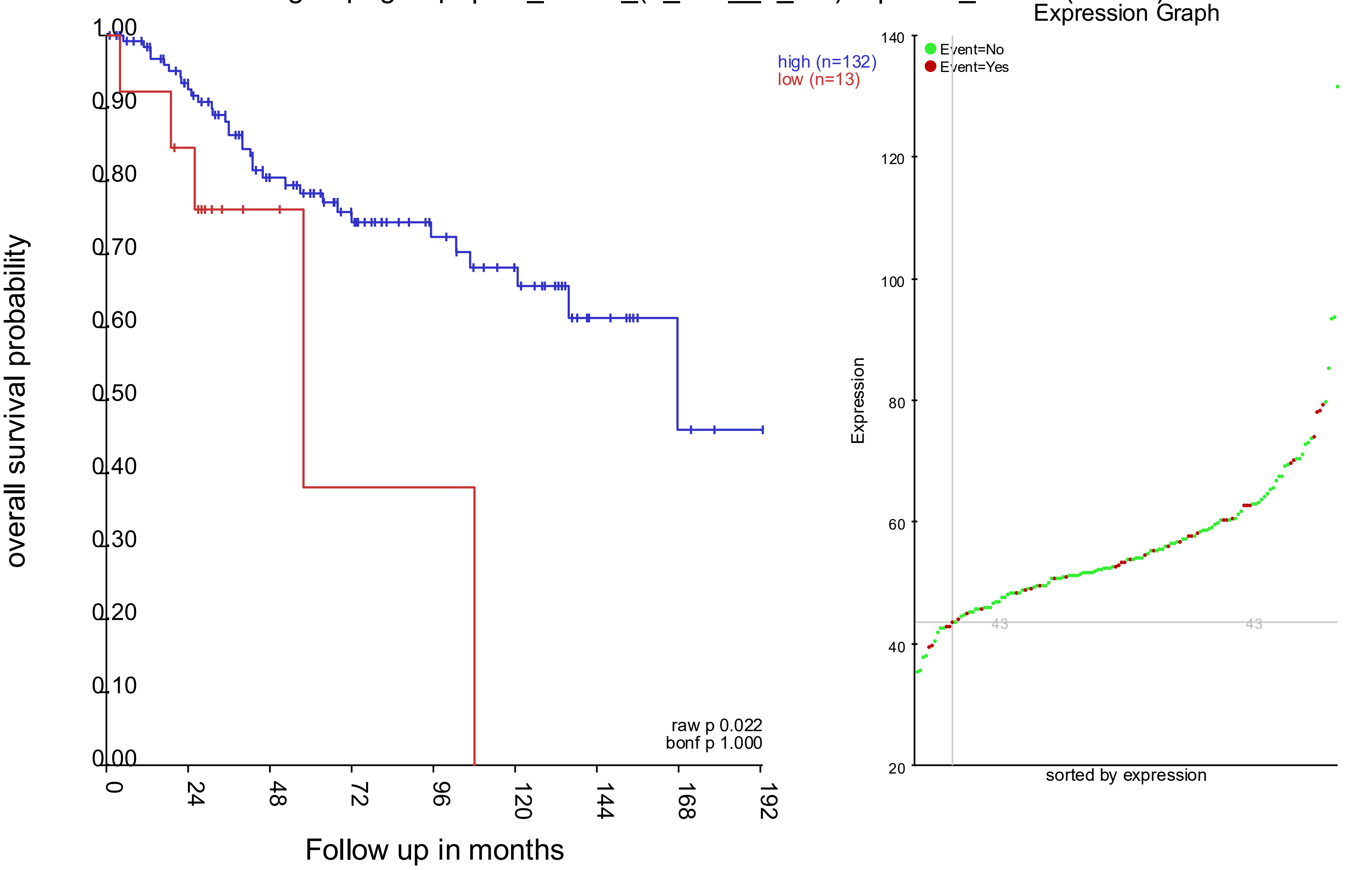

GROUP4 M1

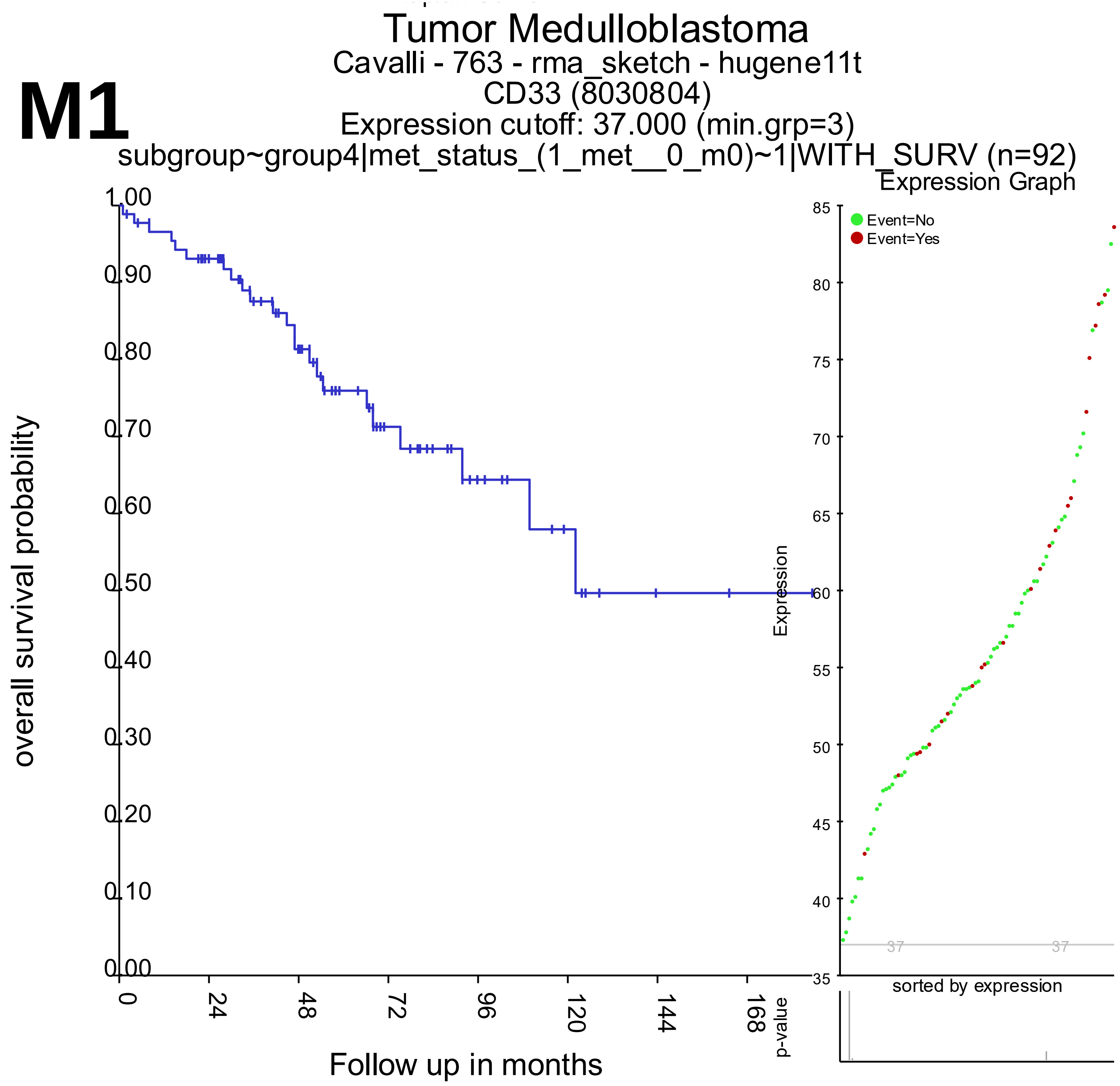

Tumor Medulloblastoma
Cavalli - 763 - rma_sketch - hugene11t
CD33 (8030804)
Expression cutoff: 37.000 (min.grp=3)
subgroup~group4|met_status_(1_met__0_m0)~1|WITH_SURV (n=92)
overall survival probability
1.00
0.90
0.80
0.70
0.60
0.50
0.40
0.30
0.20
0.10
0.00
0
24
48
72
96
120
144
168
Follow up in months
Expression Graph
Event=No
Event=Yes
Expression
85
80
75
70
65
60
55
50
45
40
35
37
sorted by expression
p-value

GROUP3 M0

Tumor Medulloblastoma

Cavalli - 763 - rma_sketch - hugene11t
CD33 (8030804)
Expression cutoff: 49.900 (min.grp=3)
subgroup~group3|met_status_(1_met__0_m0)~0|WITH_SURV (n=65)

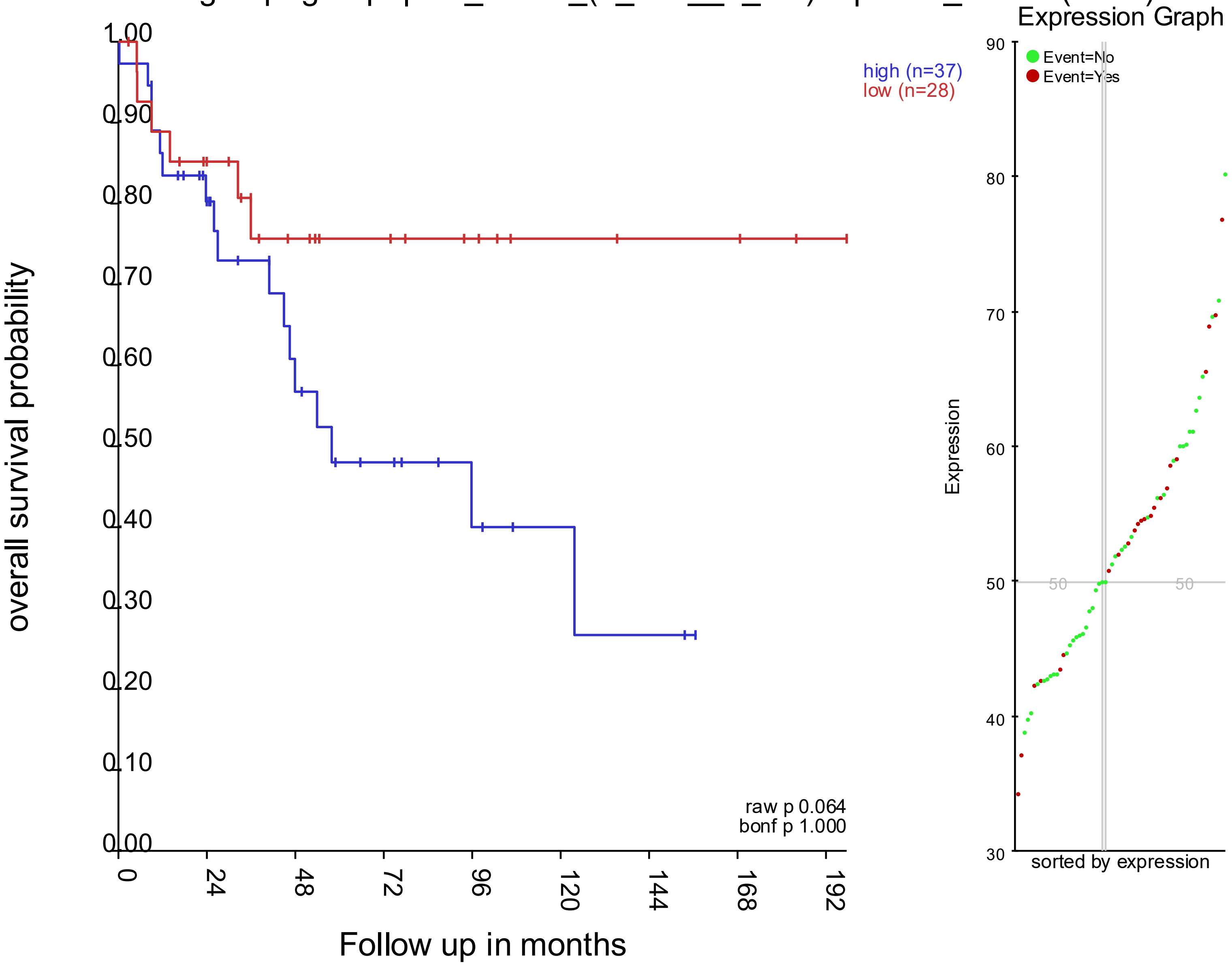

GROUP3 M1

Tumor Medulloblastoma
Cavalli - 763 - rma_sketch - hugene11t
CD33 (8030804)
Expression cutoff: 51.900 (min.grp=3)
subgroup~group3|met_status_(1_met__0_m0)~1|WITH_SURV (n=41)

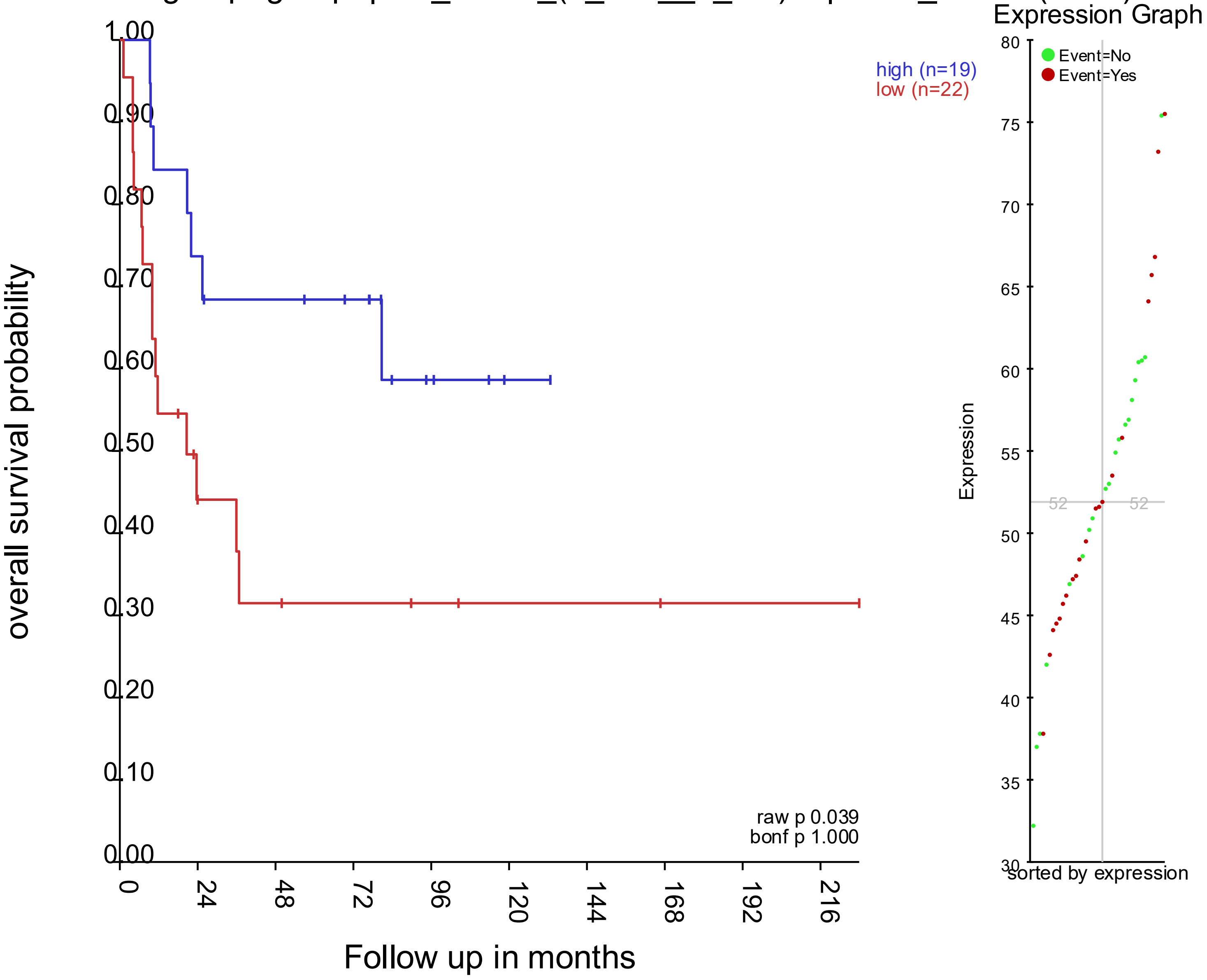

CD52

WNT M0

Tumor Medulloblastoma
Cavalli - 763 - rma_sketch - hugene11t
CD52 (7899160)
Expression cutoff: 57.500 (min.grp=3)
subgroup~wnt|met_status_(1_met__0_m0)~0 (n=43)

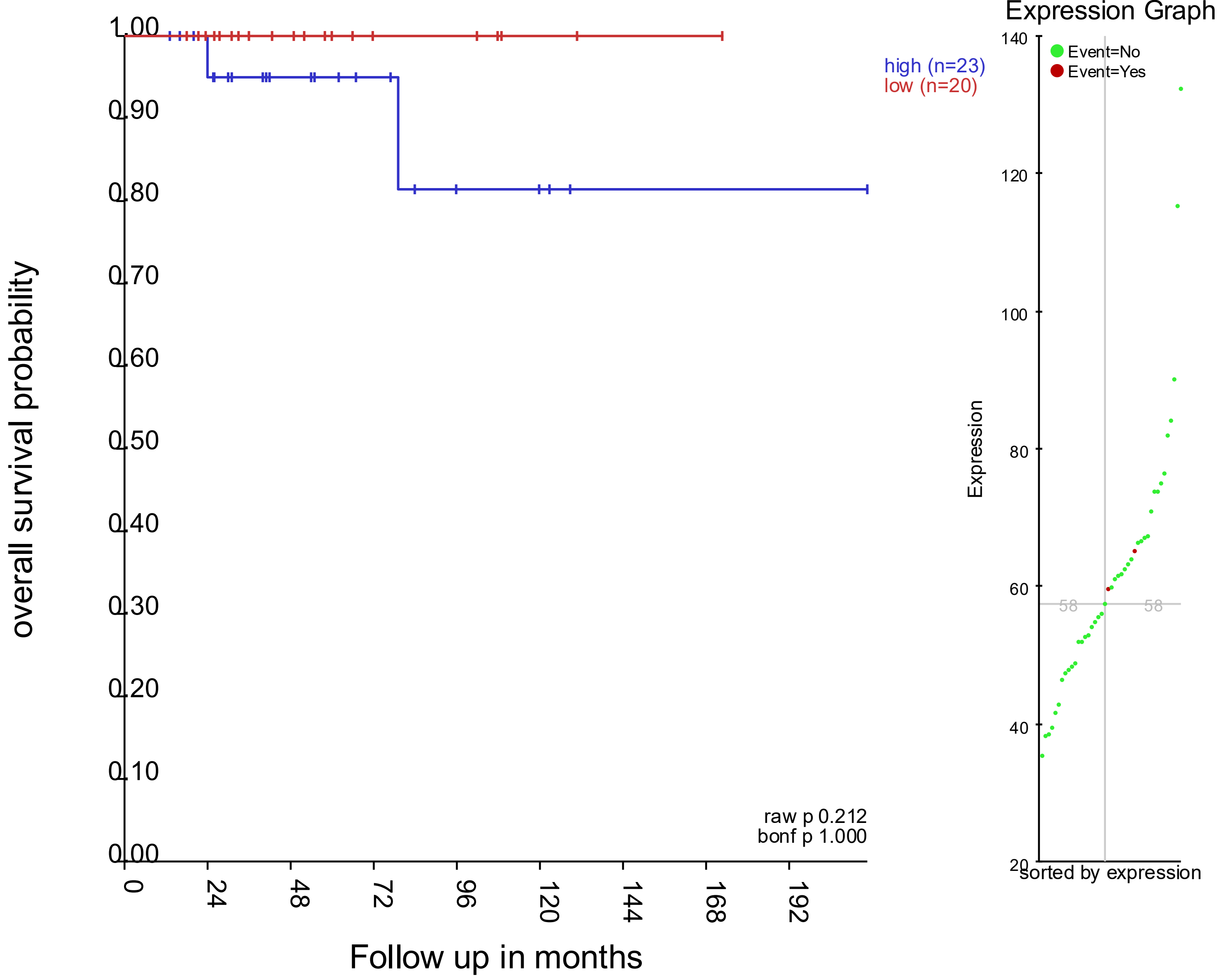

WNT M1

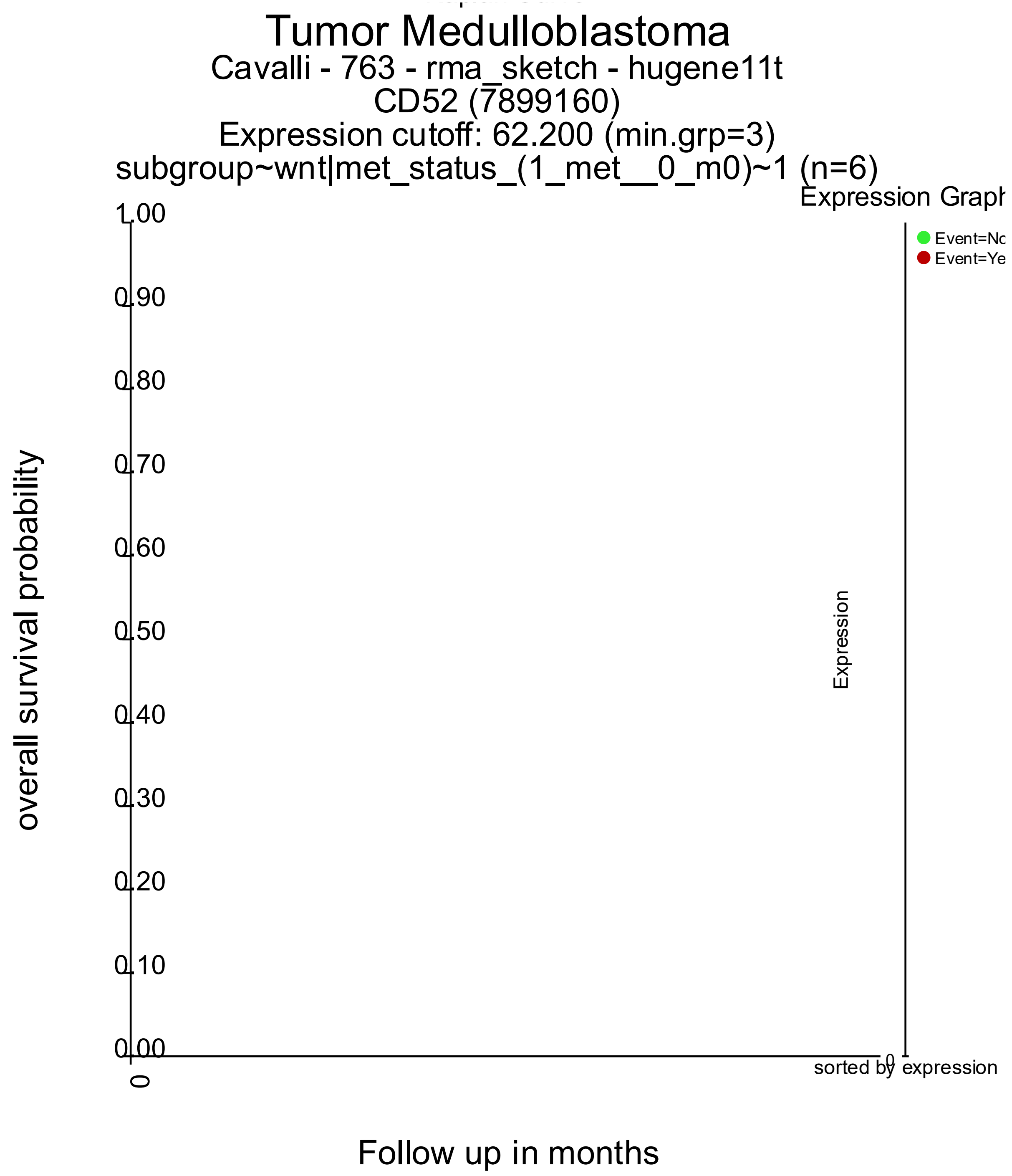
Tumor Medulloblastoma
Cavalli - 763 - rma_sketch - hugene11t
CD52 (7899160)
Expression cutoff: 62.200 (min.grp=3)
subgroup~wnt|met_status_(1_met__0_m0)~1 (n=6)
Expression Graph
Event=Nc
Event=Ye
overall survival probability
1.00
0.90
0.80
0.70
0.60
0.50
0.40
0.30
0.20
0.10
0.00
0
Follow up in months
Expression
0
sorted by expression

SHH M0

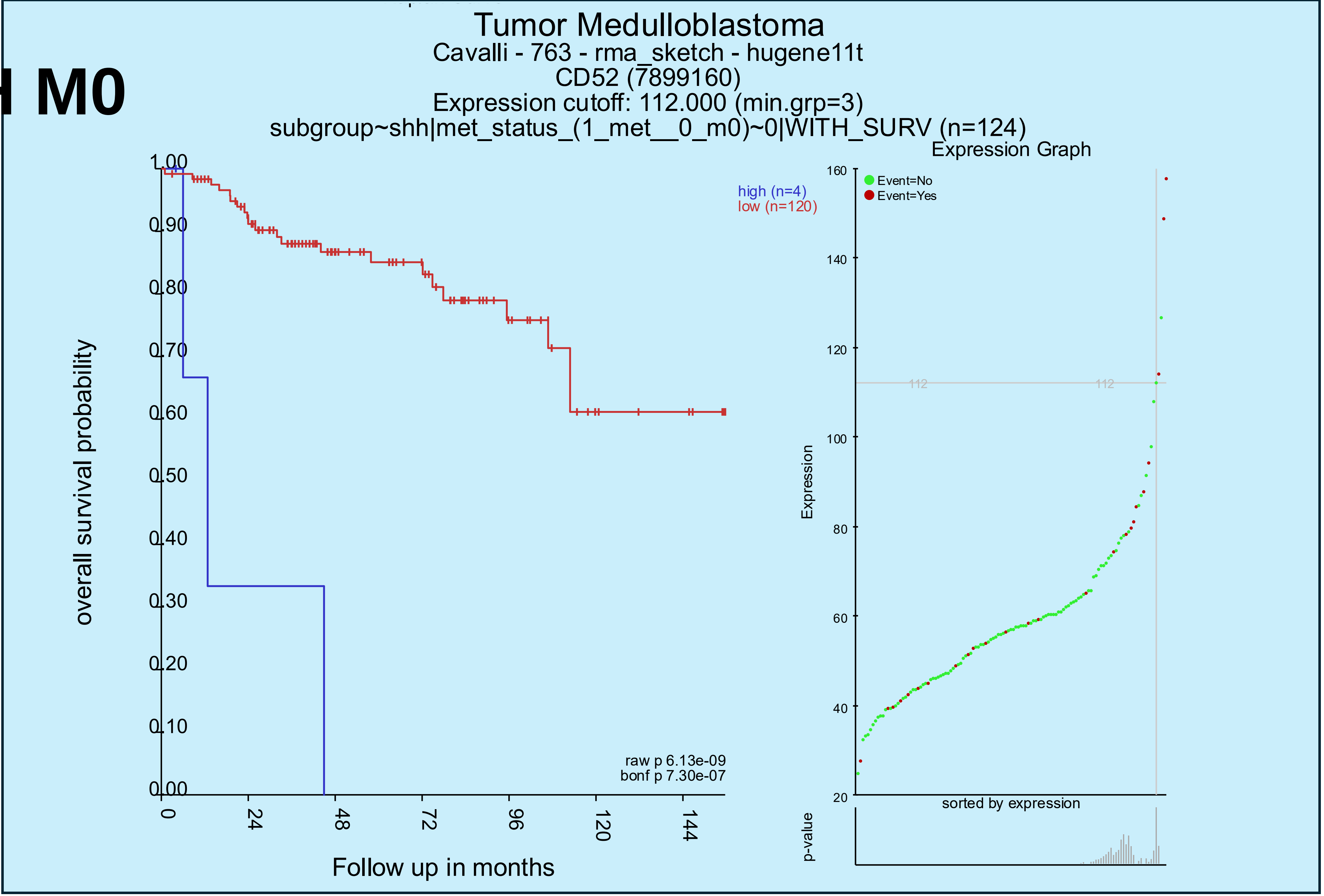

Tumor Medulloblastoma
Cavalli - 763 - rma_sketch - hugene11t
CD52 (7899160)
Expression cutoff: 112.000 (min.grp=3)
subgroup~shh|met_status_(1_met__0_m0)~0|WITH_SURV (n=124)
overall survival probability
1.00
0.90
0.80
0.70
0.60
0.50
0.40
0.30
0.20
0.10
0.00
0
24
48
72
96
120
144
Follow up in months
high (n=4)
low (n=120)
raw p 6.13e-09
bonf p 7.30e-07
Expression Graph
Event=No
Event=Yes
160
140
120
100
80
60
40
20
112
112
Expression
sorted by expression
p-value

SHH M1

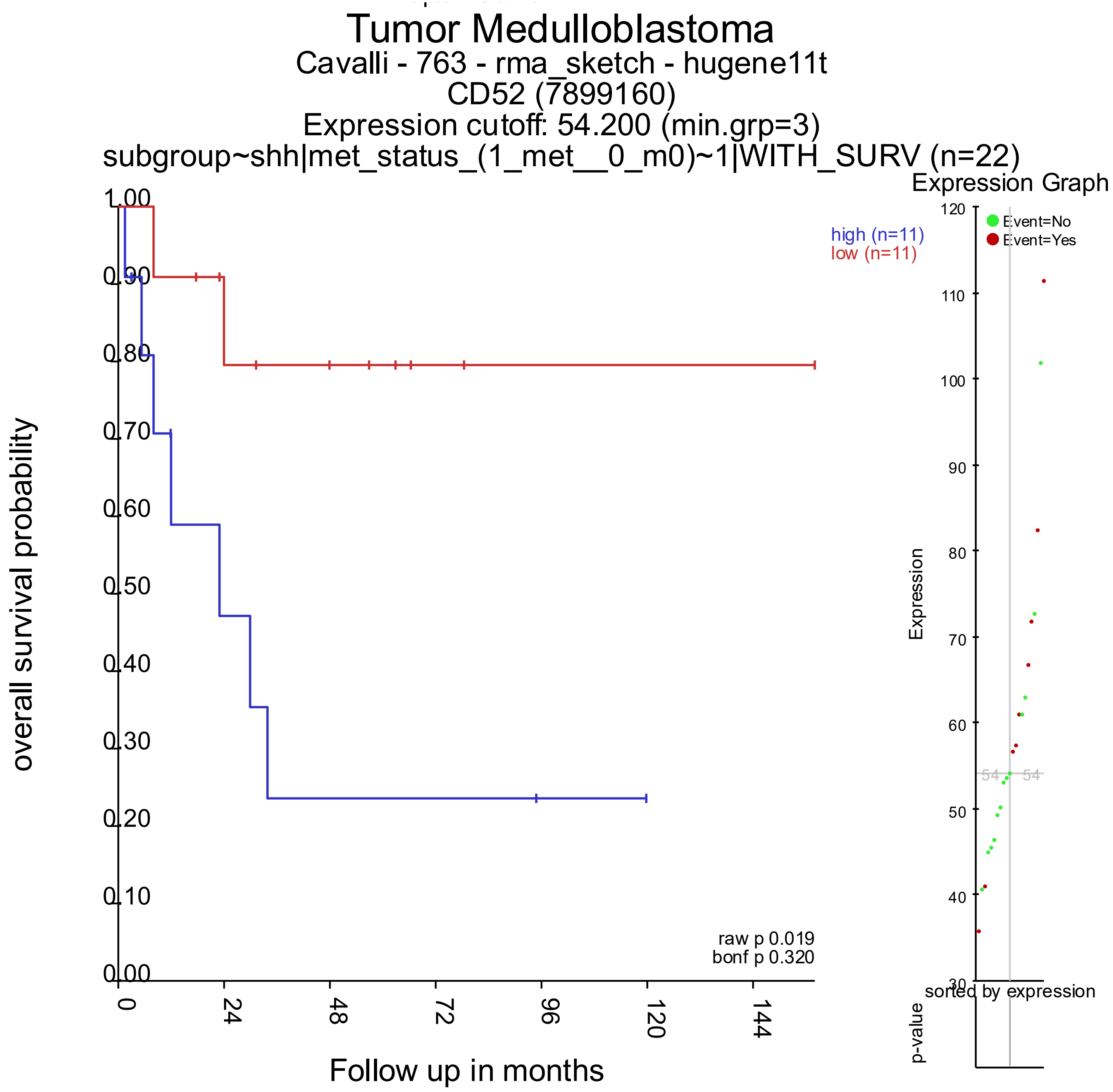
Tumor Medulloblastoma
Cavalli - 763 - rma_sketch - hugene11t
CD52 (7899160)
Expression cutoff: 54.200 (min.grp=3)
subgroup~shh|met_status_(1_met__0_m0)~1|WITH_SURV (n=22)
overall survival probability
1.00
0.90
0.80
0.70
0.60
0.50
0.40
0.30
0.20
0.10
0.00
0
24
48
72
96
120
144
Follow up in months
high (n=11)
low (n=11)
raw p 0.019
bonf p 0.320
Expression Graph
Event=No
Event=Yes
Expression
120
110
100
90
80
70
60
50
40
30
54
54
sorted by expression
p-value

GROUP4 M0

Tumor Medulloblastoma
Cavalli - 763 - rma_sketch - hugene11t
CD52 (7899160)
Expression cutoff: 59.400 (min.grp=3)
subgroup~group4|met_status_(1_met__0_m0)~0|WITH_SURV (n=145)

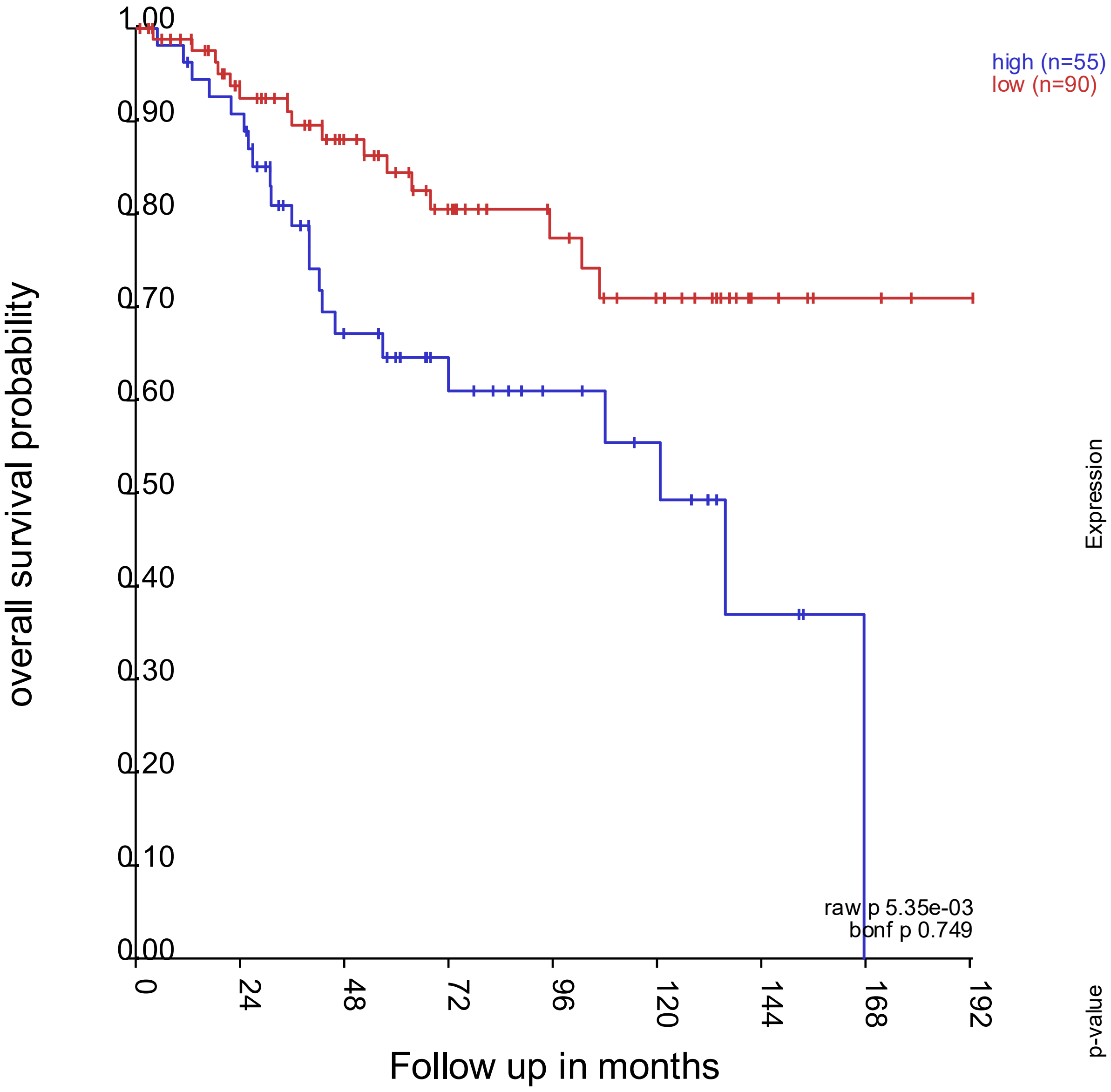

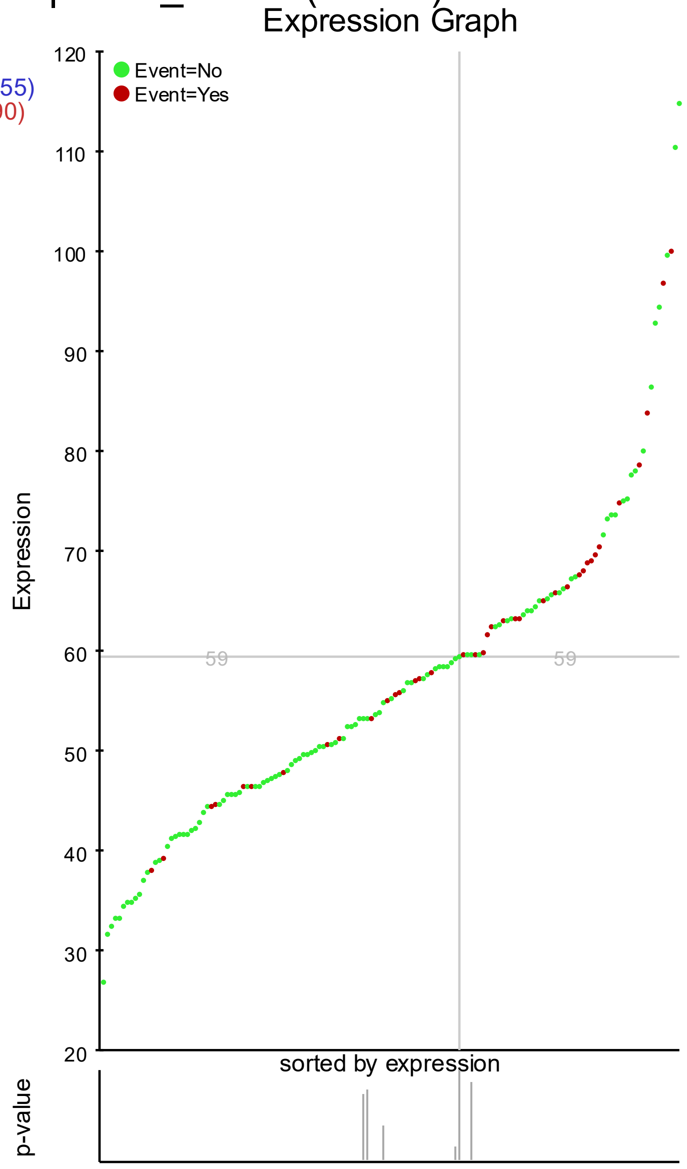

GROUP4 M1

Tumor Medulloblastoma
Cavalli - 763 - rma_sketch - hugene11t
CD52 (7899160)
Expression cutoff: 73.000 (min.grp=3)
subgroup~group4|met_status_(1_met__0_m0)~1|WITH_SURV (n=92)

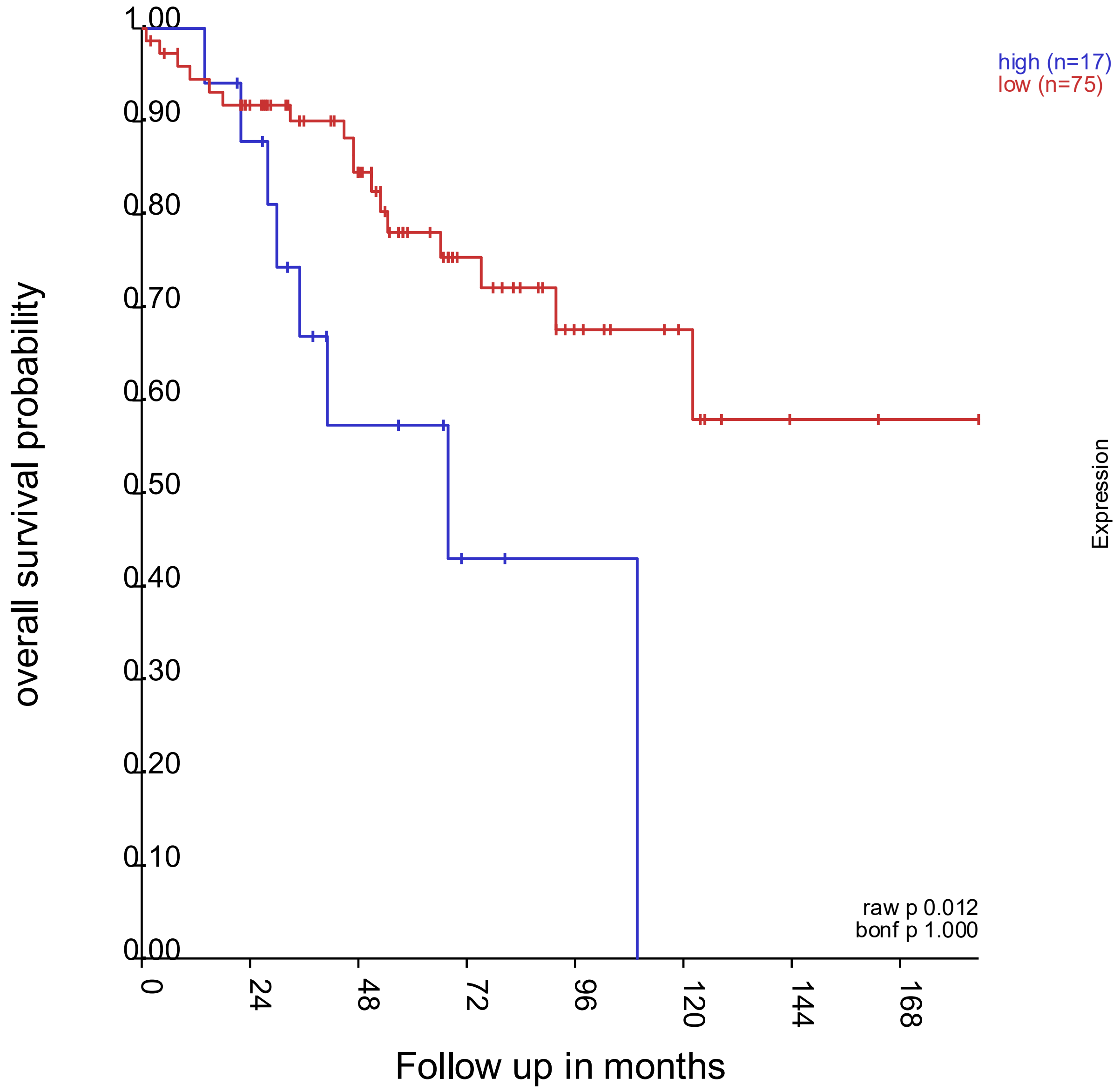

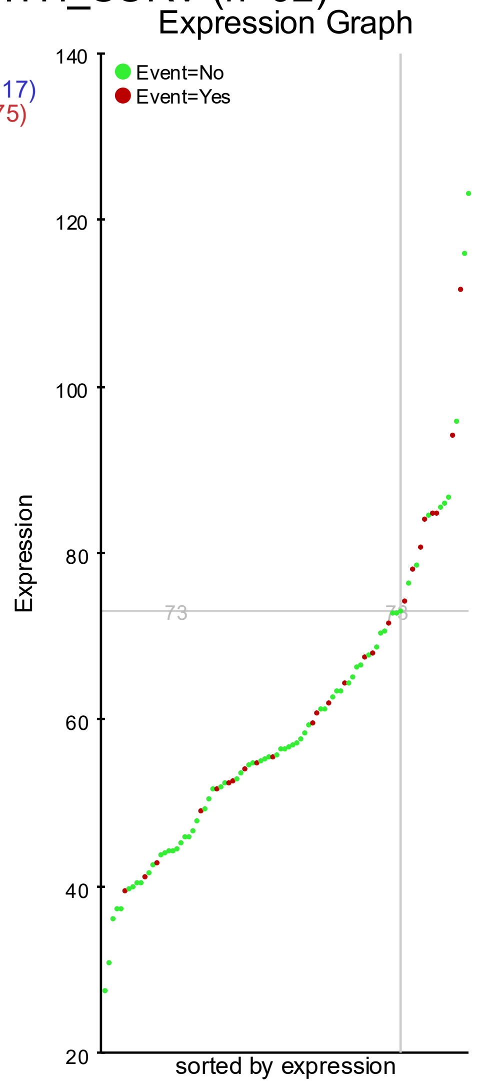

GROUP3 M0

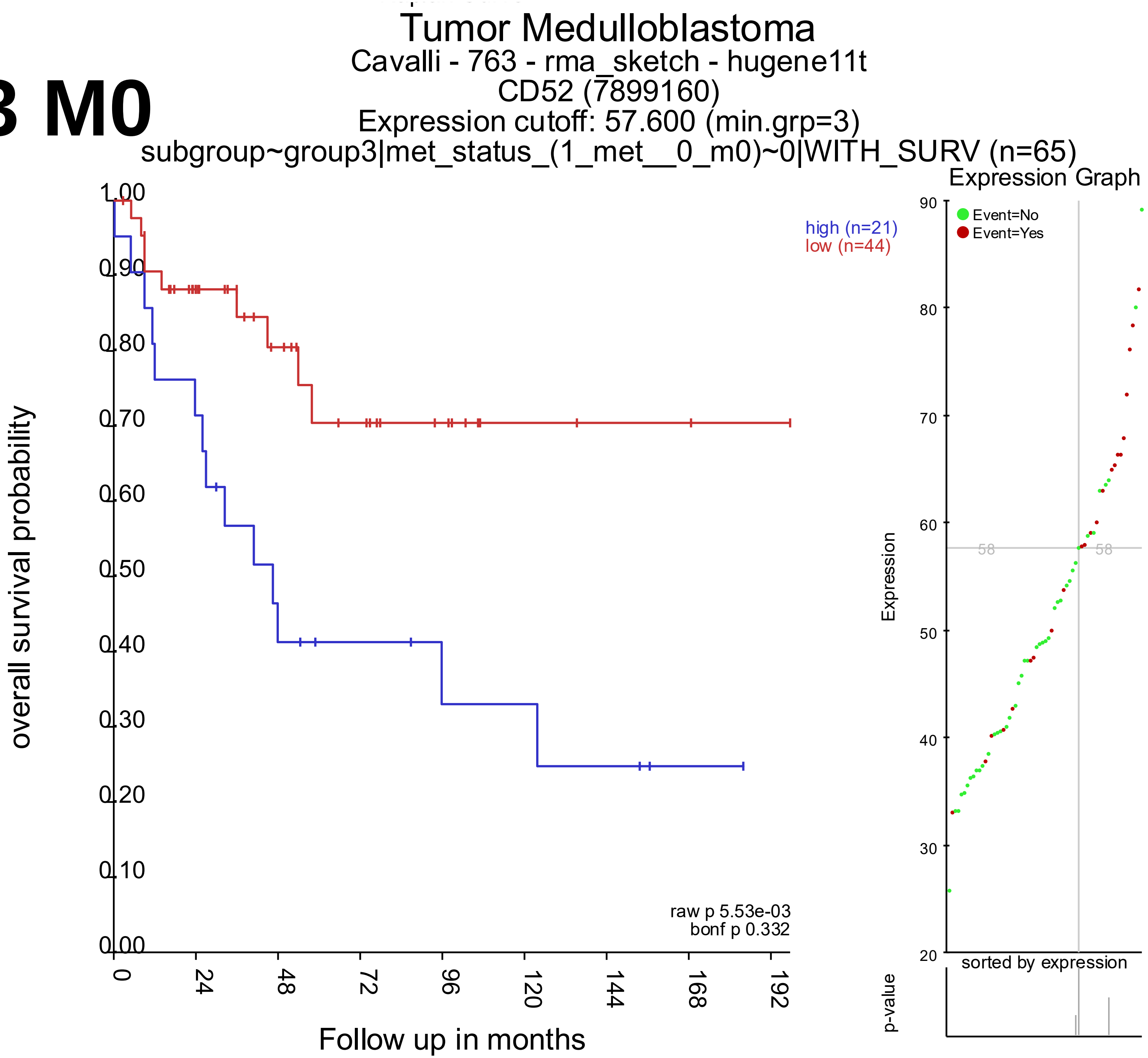

Tumor Medulloblastoma
Cavalli - 763 - rma_sketch - hugene11t
CD52 (7899160)
Expression cutoff: 57.600 (min.grp=3)
subgroup~group3|met_status_(1_met__0_m0)~0|WITH_SURV (n=65)
overall survival probability
1.00
0.90
0.80
0.70
0.60
0.50
0.40
0.30
0.20
0.10
0.00
0
24
48
72
96
120
144
168
192
Follow up in months
high (n=21)
low (n=44)
raw p 5.53e-03
bonf p 0.332
Expression Graph
Event=No
Event=Yes
90
80
70
60
50
40
30
20
Expression
58
58
sorted by expression
p-value

GROUP3 M1

Tumor Medulloblastoma
Cavalli - 763 - rma_sketch - hugene11t
CD52 (7899160)
Expression cutoff: 81.400 (min.grp=3)
subgroup~group3|met_status_(1_met__0_m0)~1|WITH_SURV (n=41)

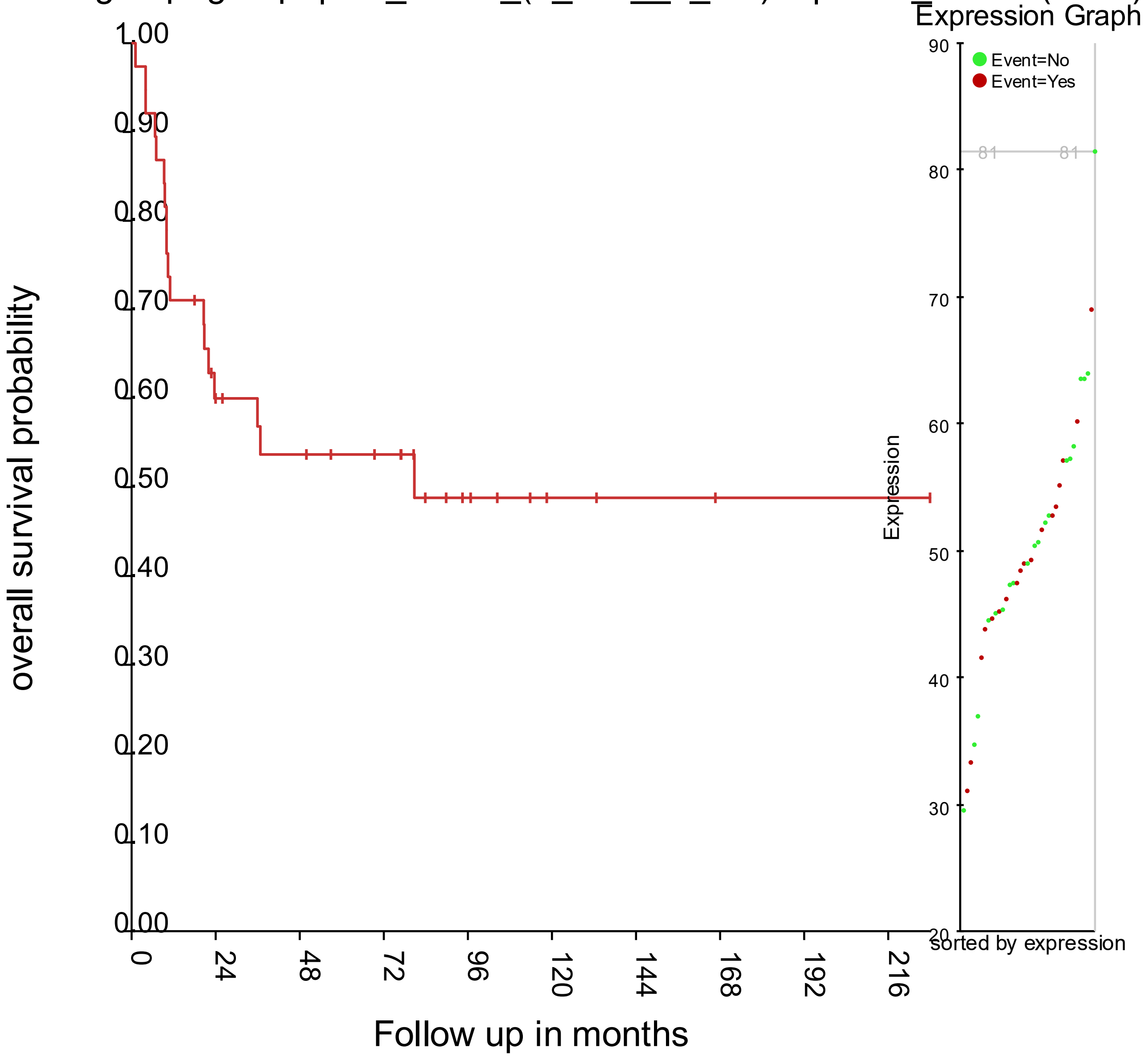

CDK4

WNT M0

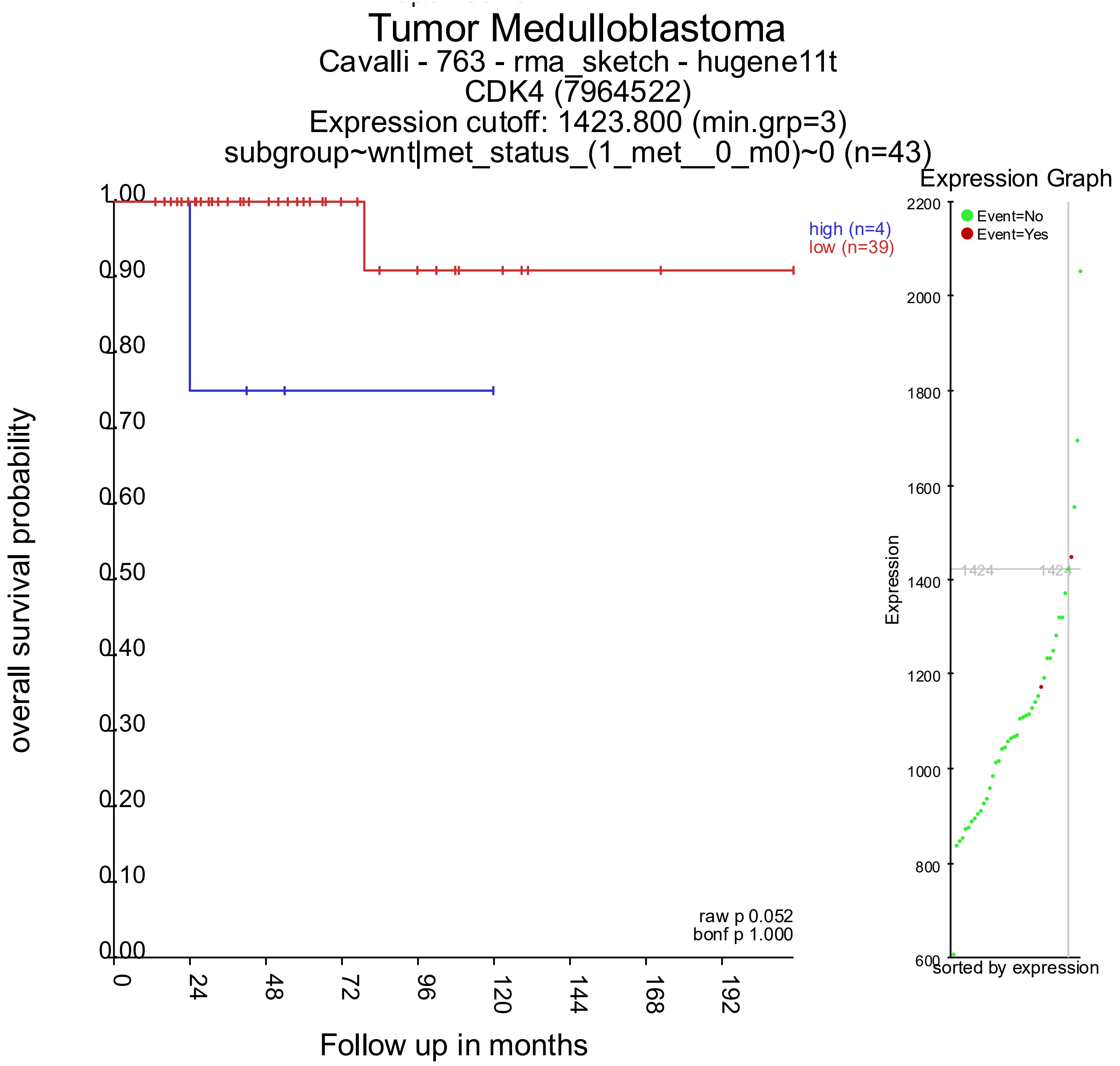

Tumor Medulloblastoma
Cavalli - 763 - rma_sketch - hugene11t
CDK4 (7964522)
Expression cutoff: 1423.800 (min.grp=3)
subgroup~wnt|met_status_(1_met__0_m0)~0 (n=43)
overall survival probability
1.00
0.90
0.80
0.70
0.60
0.50
0.40
0.30
0.20
0.10
0.00
0
24
48
72
96
120
144
168
192
Follow up in months
high (n=4)
low (n=39)
raw p 0.052
bonf p 1.000
Expression Graph
Event=No
Event=Yes
Expression
2200
2000
1800
1600
1400
1200
1000
800
600
1424
1424
sorted by expression

WNT M1

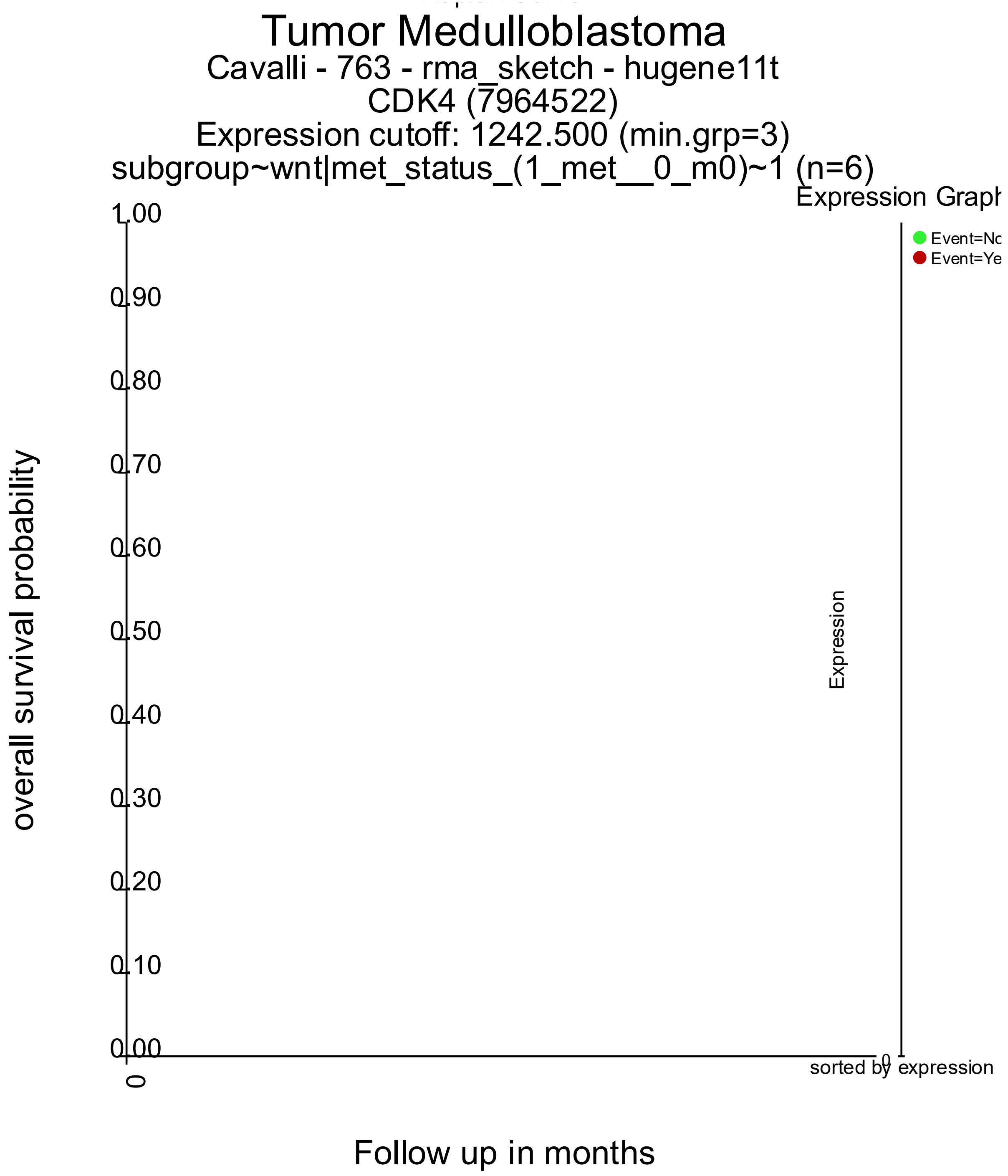
Tumor Medulloblastoma
Cavalli - 763 - rma_sketch - hugene11t
CDK4 (7964522)
Expression cutoff: 1242.500 (min.grp=3)
subgroup~wnt|met_status_(1_met__0_m0)~1 (n=6)
Expression Graph
Event=Nc
Event=Ye
overall survival probability
1.00
0.90
0.80
0.70
0.60
0.50
0.40
0.30
0.20
0.10
0.00
0
Follow up in months
Expression
0
sorted by expression

SHH M0

Tumor Medulloblastoma

Cavalli - 763 - rma_sketch - hugene11t
CDK4 (7964522)
Expression cutoff: 1232.800 (min.grp=3)
subgroup~shh|met_status_(1_met__0_m0)~0|WITH_SURV (n=124)

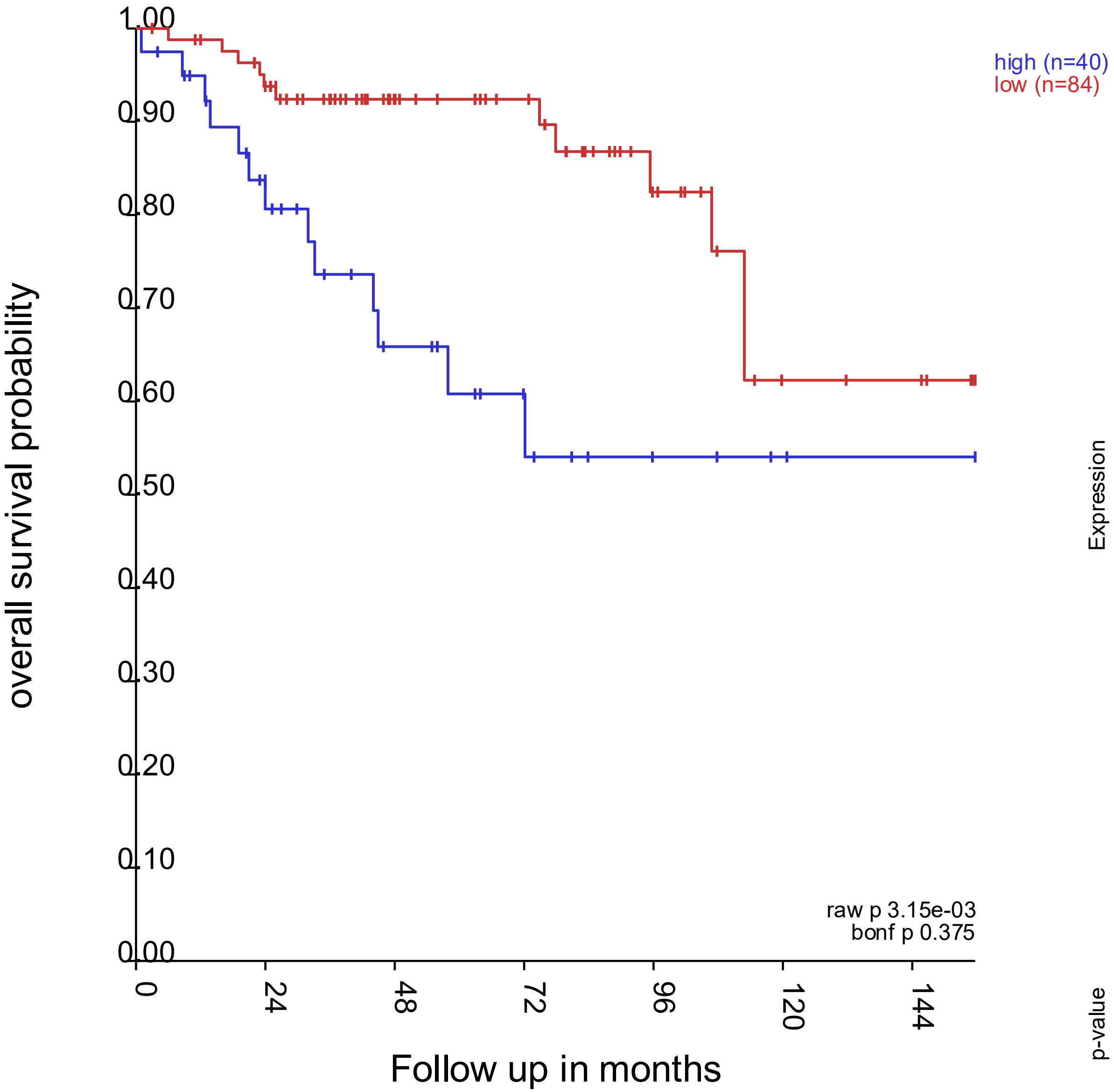

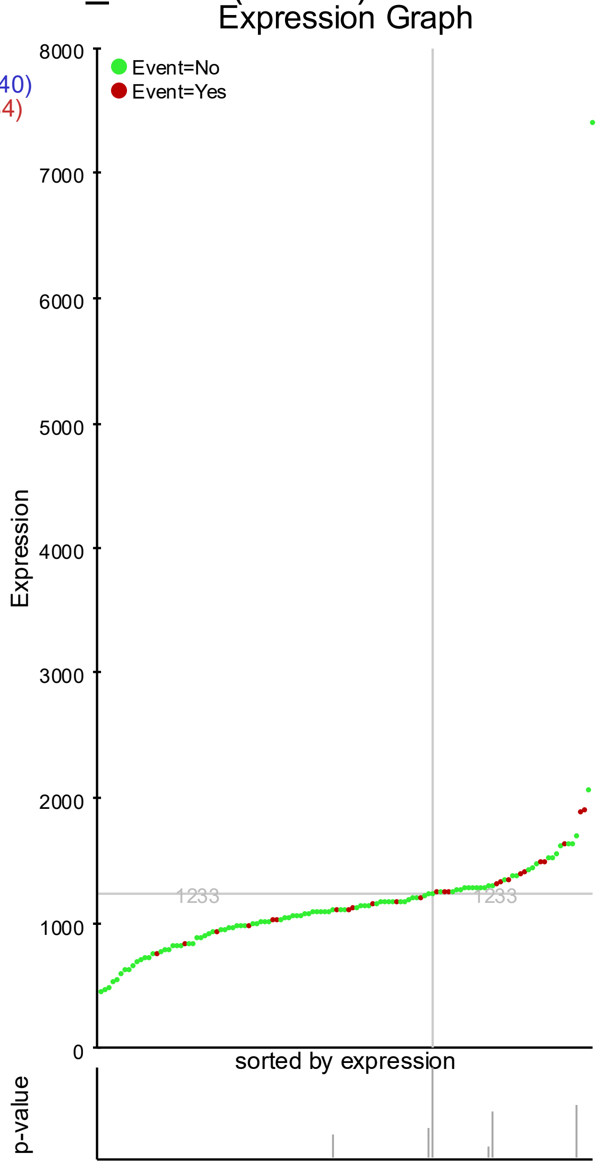

SHH M1

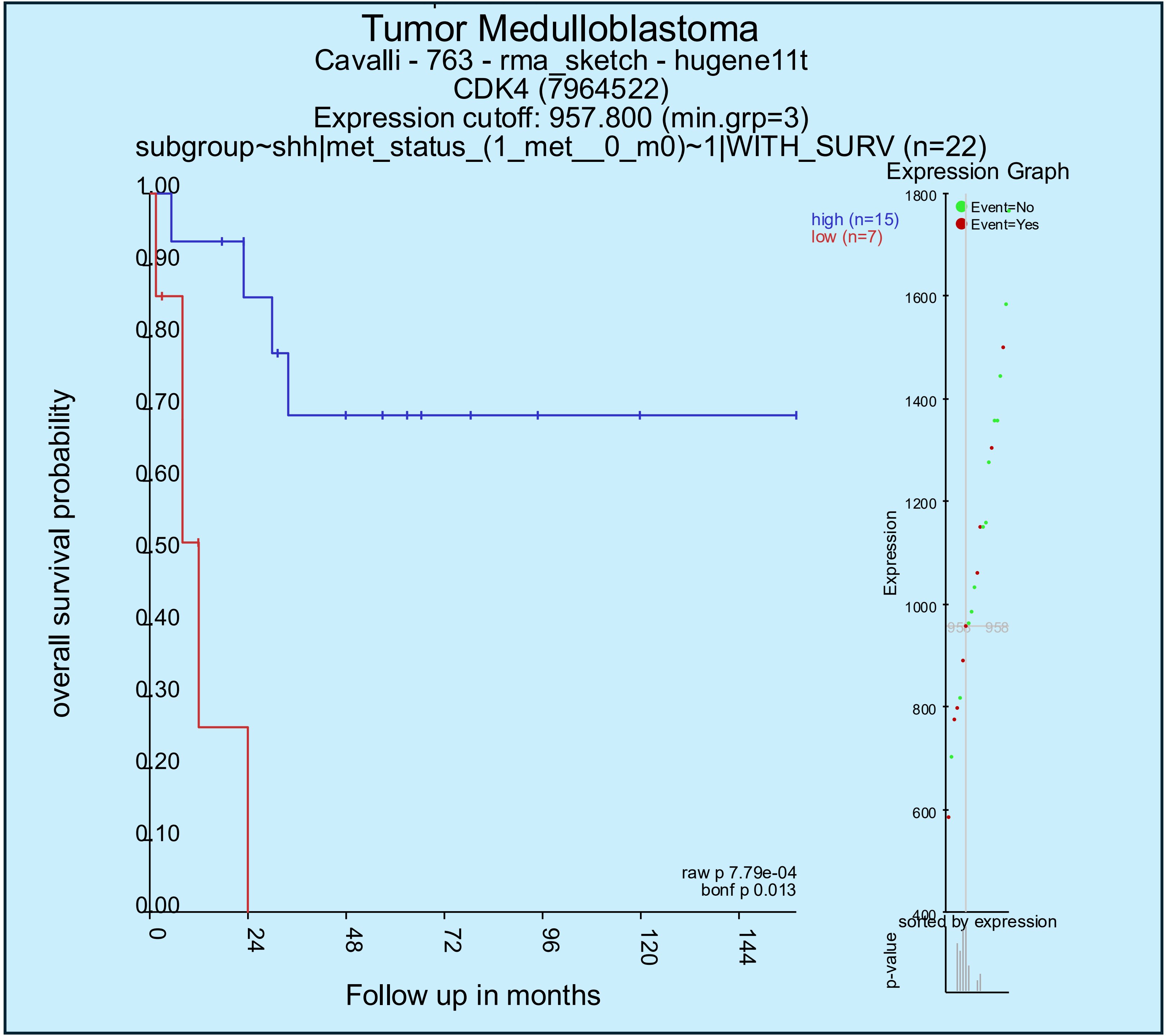
Tumor Medulloblastoma
Cavalli - 763 - rma_sketch - hugene11t
CDK4 (7964522)
Expression cutoff: 957.800 (min.grp=3)
subgroup~shh|met_status_(1_met__0_m0)~1|WITH_SURV (n=22)
Expression Graph
high (n=15)
low (n=7)
Event=No
Event=Yes
overall survival probability
1.00
0.90
0.80
0.70
0.60
0.50
0.40
0.30
0.20
0.10
0.00
raw p 7.79e-04
bonf p 0.013
0
24
48
72
96
120
144
Follow up in months
1800
1600
1400
1200
1000
800
600
400
Expression
958
sorted by expression
p-value

GROUP4 M0

Tumor Medulloblastoma
Cavalli - 763 - rma_sketch - hugene11t
CDK4 (7964522)
Expression cutoff: 1010.100 (min.grp=3)
subgroup~group4|met_status_(1_met__0_m0)~0|WITH_SURV (n=145)

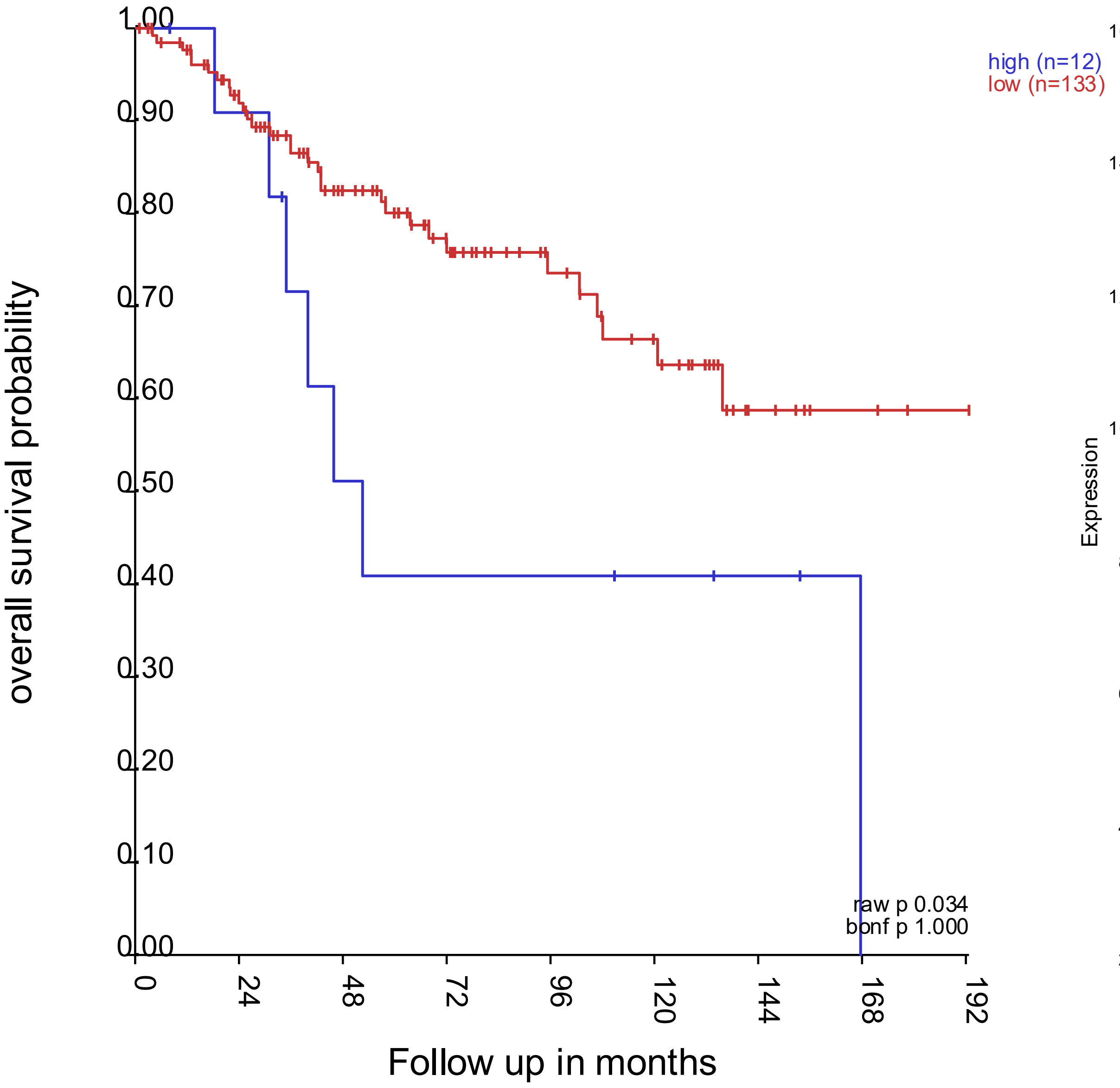

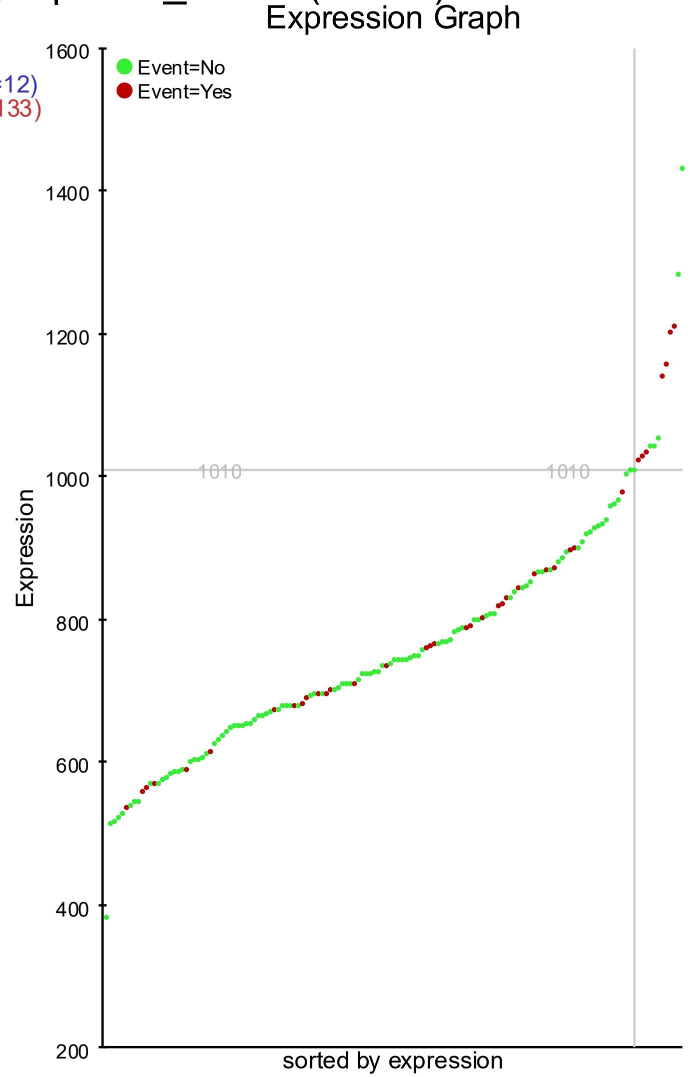

GROUP4 M1

Tumor Medulloblastoma
Cavalli - 763 - rma_sketch - hugene11t
CDK4 (7964522)
Expression cutoff: 879.500 (min.grp=3)
subgroup~group4|met_status_(1_met__0_m0)~1|WITH_SURV (n=92)

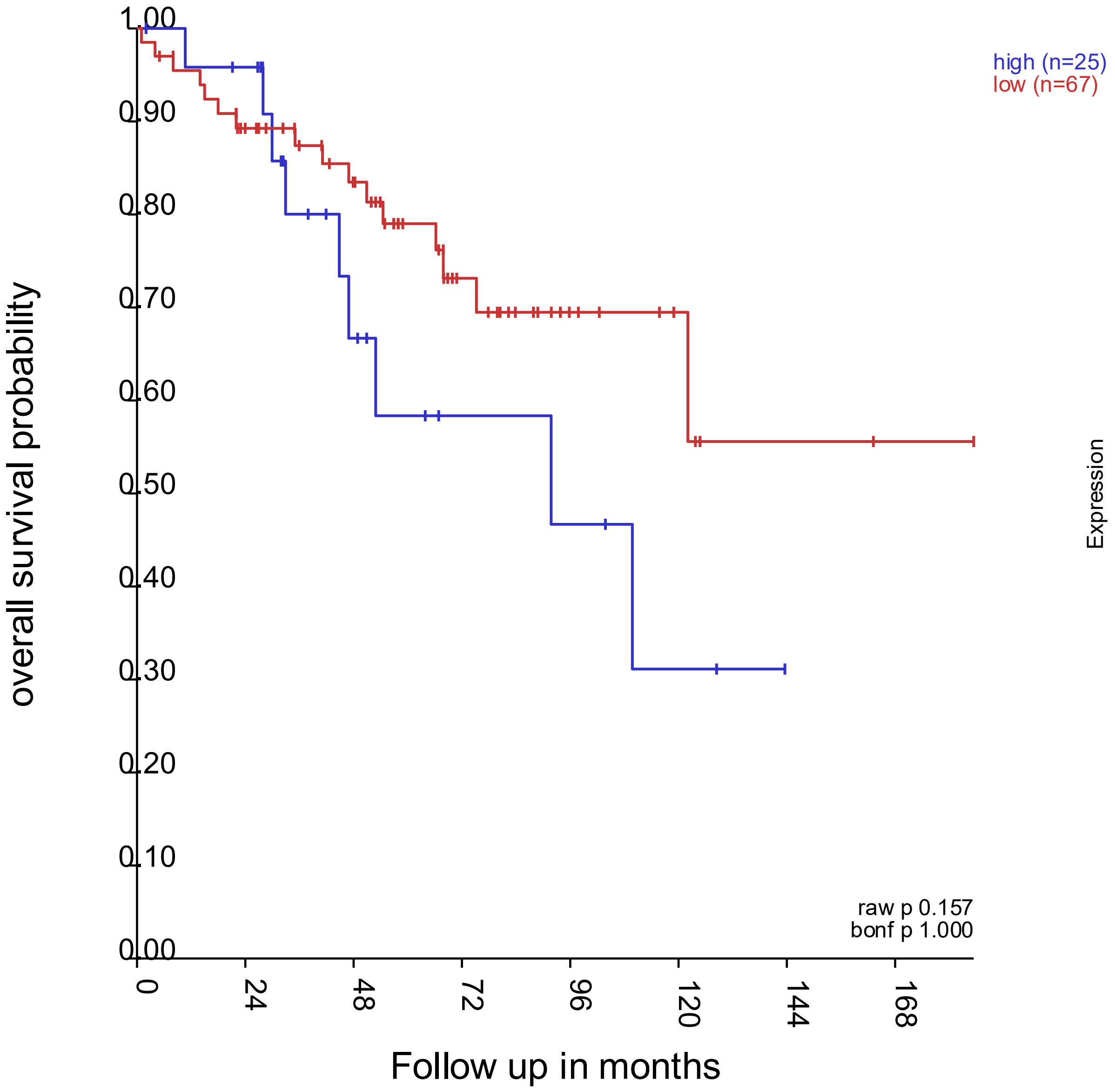

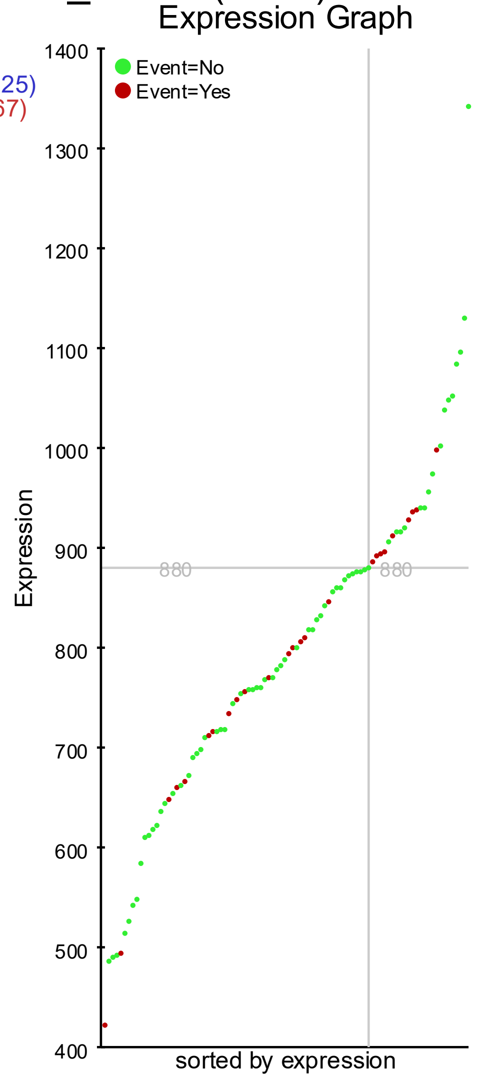

GROUP3 M0

Tumor Medulloblastoma
Cavalli - 763 - rma_sketch - hugene11t
CDK4 (7964522)
Expression cutoff: 943.400 (min.grp=3)
subgroup~group3|met_status_(1_met__0_m0)~0|WITH_SURV (n=65)

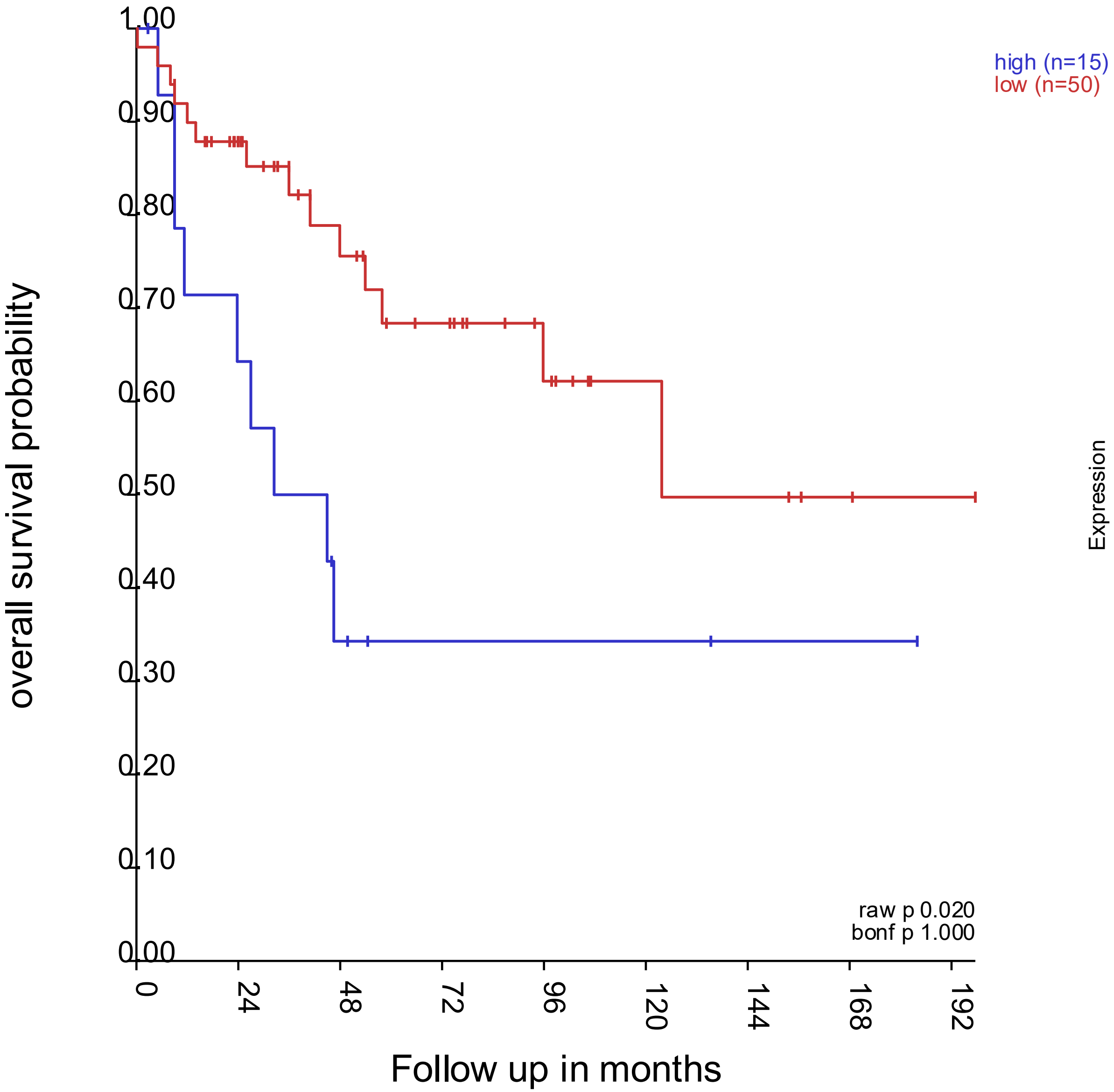

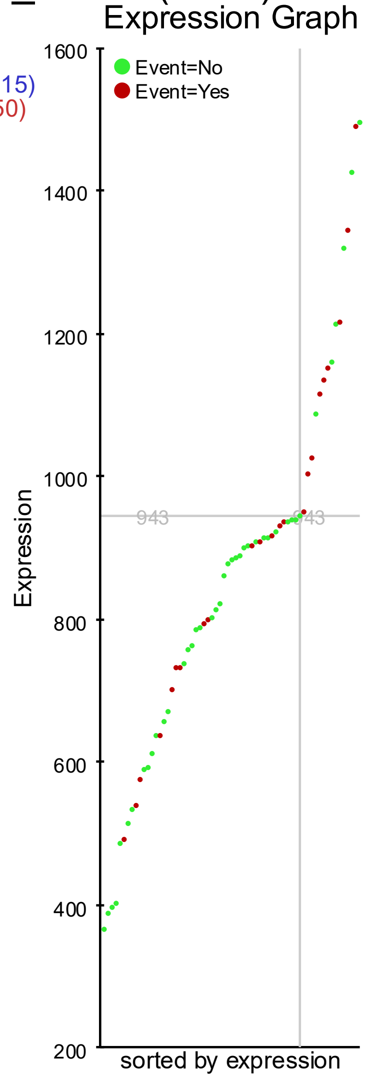

GROUP3 M1

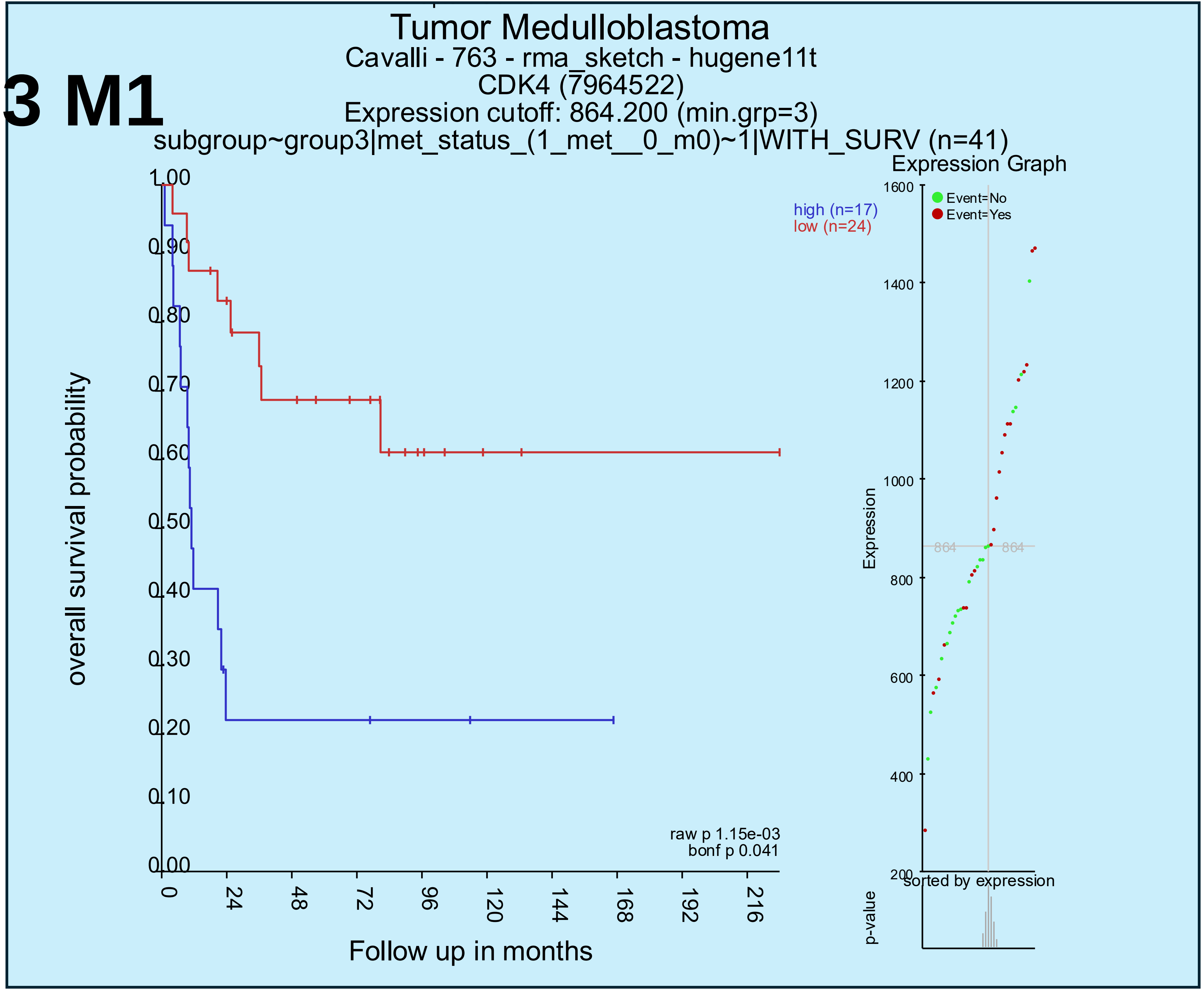

Tumor Medulloblastoma
Cavalli - 763 - rma_sketch - hugene11t
CDK4 (7964522)
Expression cutoff: 864.200 (min.grp=3)
subgroup~group3|met_status_(1_met__0_m0)~1|WITH_SURV (n=41)
overall survival probability
1.00
0.90
0.80
0.70
0.60
0.50
0.40
0.30
0.20
0.10
0.00
high (n=17)
low (n=24)
raw p 1.15e-03
bonf p 0.041
0
24
48
72
96
120
144
168
192
216
Follow up in months
Expression Graph
Event=No
Event=Yes
1600
1400
1200
1000
864
864
800
600
400
200
Expression
sorted by expression
p-value

CDK6

WNT M0

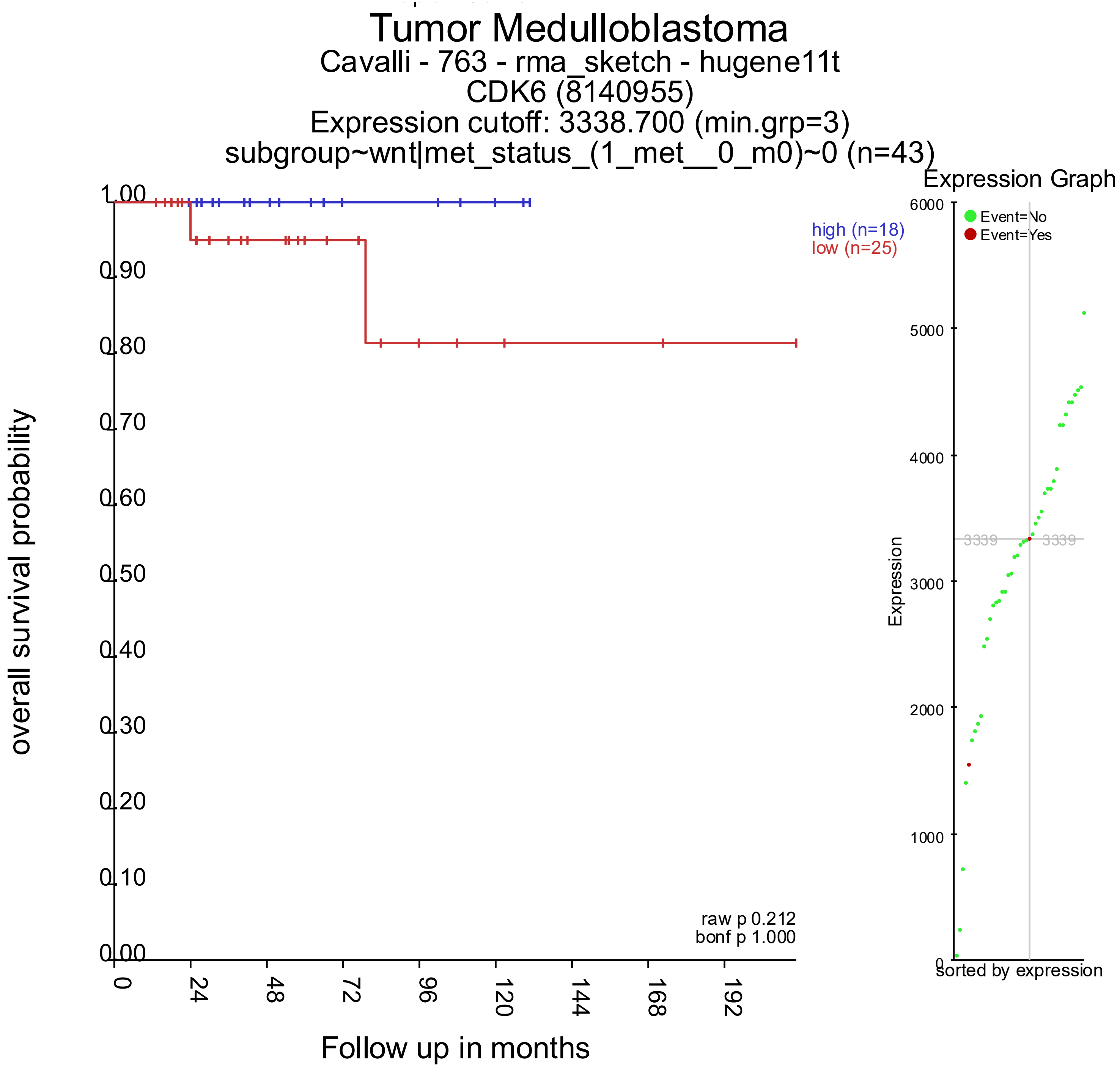
Tumor Medulloblastoma
Cavalli - 763 - rma_sketch - hugene11t
CDK6 (8140955)
Expression cutoff: 3338.700 (min.grp=3)
subgroup~wnt|met_status_(1_met__0_m0)~0 (n=43)
overall survival probability
1.00
0.90
0.80
0.70
0.60
0.50
0.40
0.30
0.20
0.10
0.00
0
24
48
72
96
120
144
168
192
Follow up in months
high (n=18)
low (n=25)
raw p 0.212
bonf p 1.000
Expression Graph
Event=No
Event=Yes
6000
5000
4000
3000
2000
1000
0
Expression
3339
3339
sorted by expression

WNT M1

Tumor Medulloblastoma
Cavalli - 763 - rma_sketch - hugene11t
CDK6 (8140955)
Expression cutoff: 3551.200 (min.grp=3)
subgroup~wnt|met_status_(1_met__0_m0)~1 (n=6)

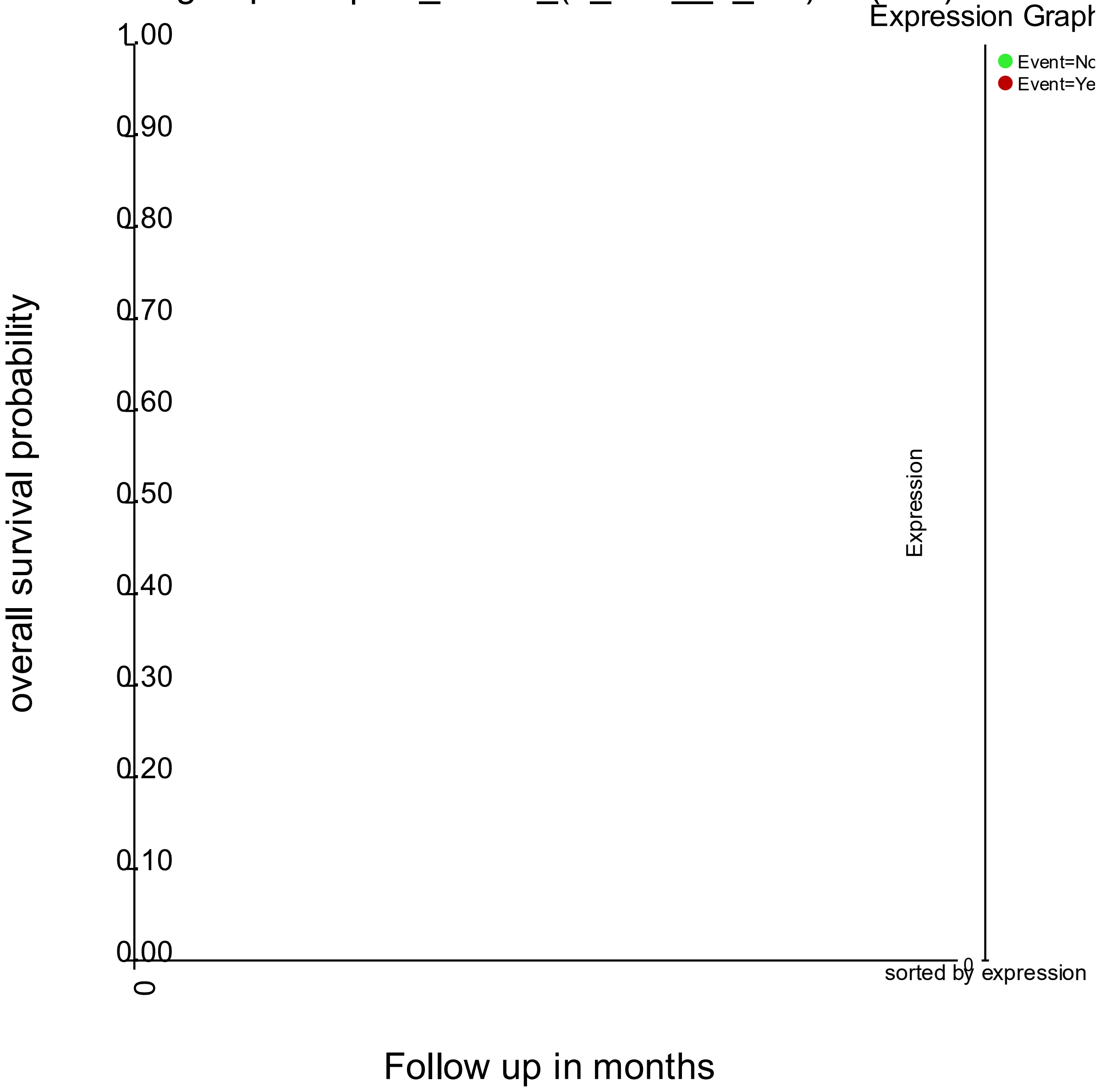

SHH M0

Tumor Medulloblastoma
Cavalli - 763 - rma_sketch - hugene11t
CDK6 (8140955)
Expression cutoff: 3324.600 (min.grp=3)
subgroup~shh|met_status_(1_met__0_m0)~0|WITH_SURV (n=124)

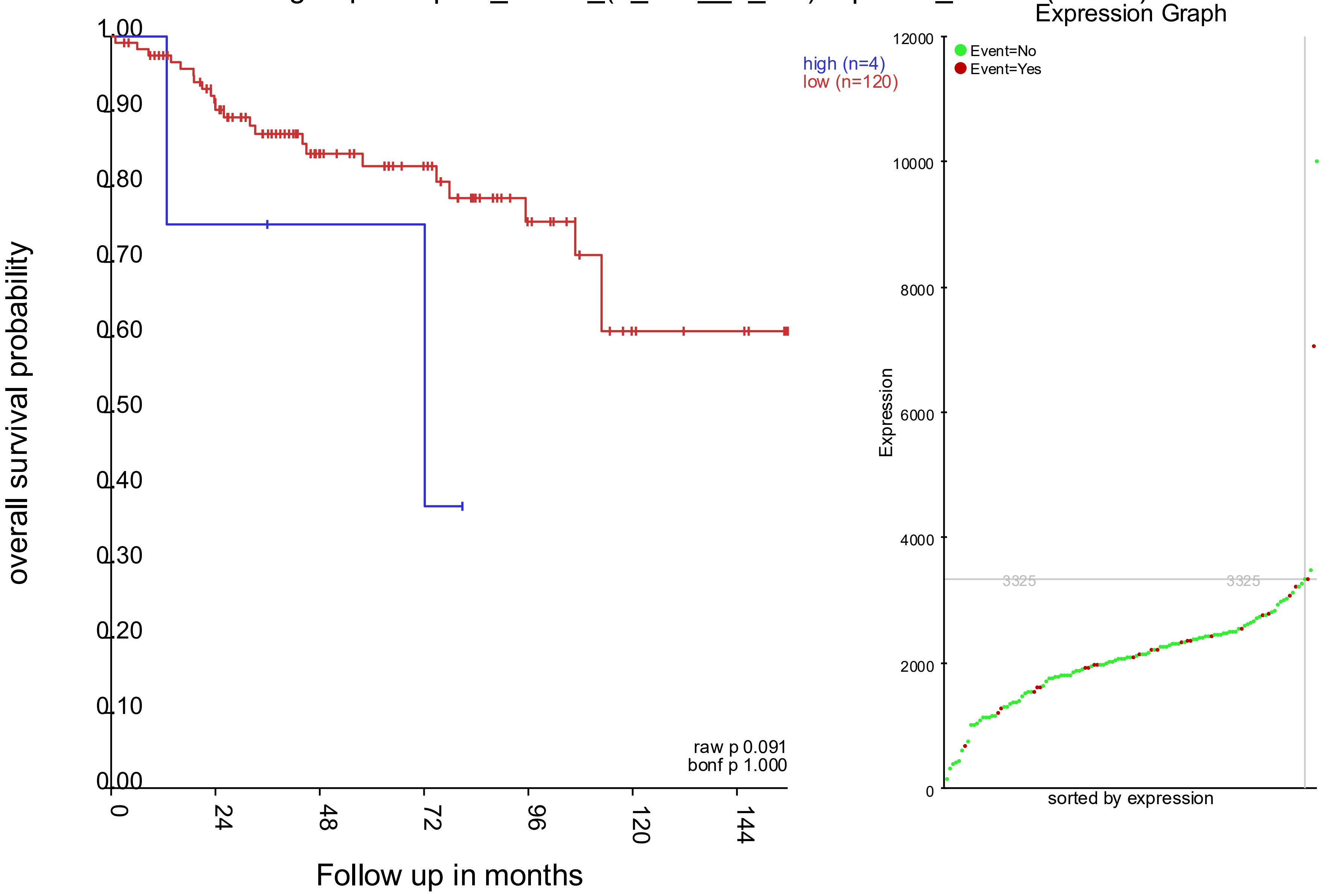

SHH M1

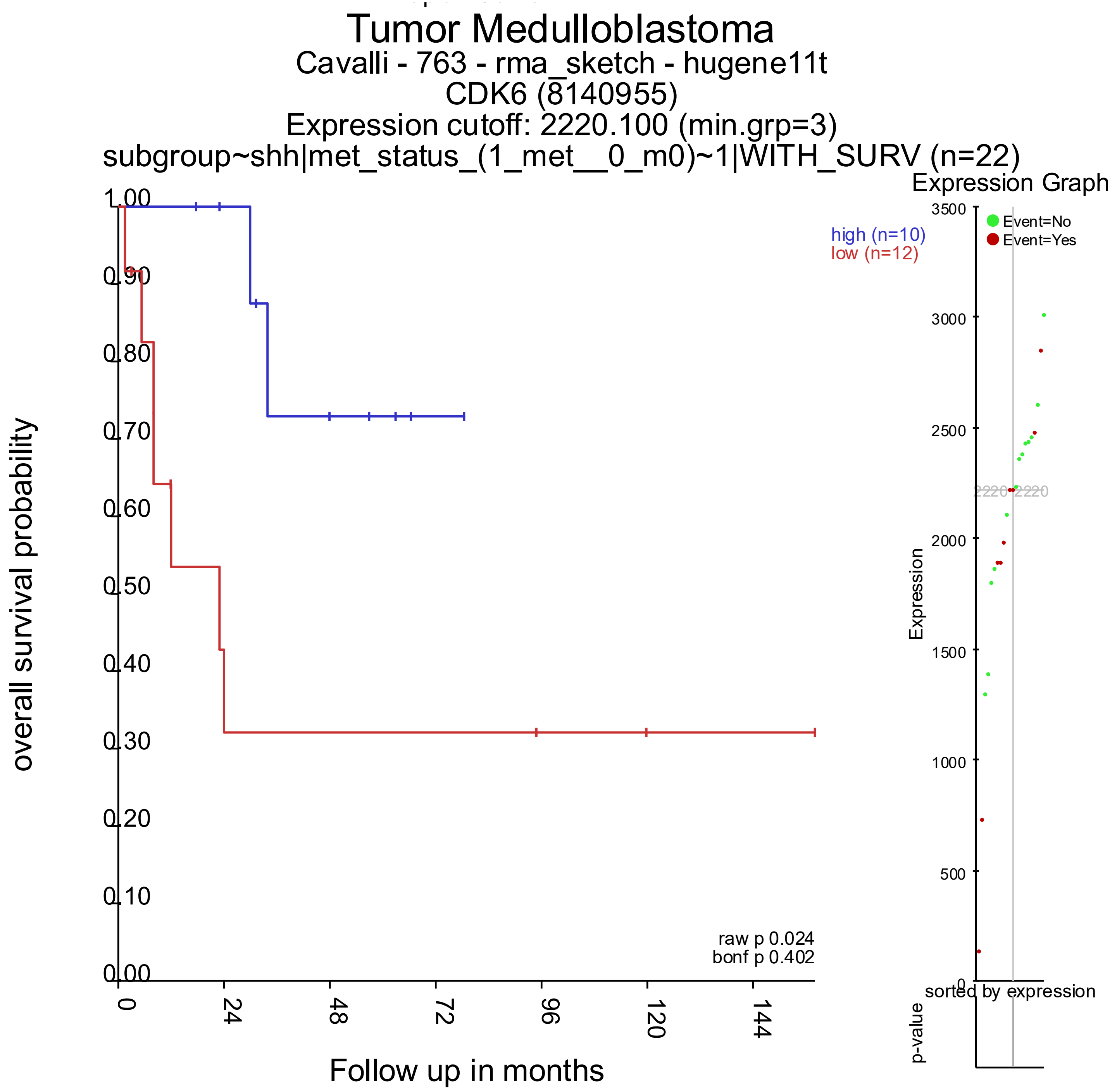
Tumor Medulloblastoma
Cavalli - 763 - rma_sketch - hugene11t
CDK6 (8140955)
Expression cutoff: 2220.100 (min.grp=3)
subgroup~shh|met_status_(1_met__0_m0)~1|WITH_SURV (n=22)
overall survival probability
1.00
0.90
0.80
0.70
0.60
0.50
0.40
0.30
0.20
0.10
0.00
0
24
48
72
96
120
144
Follow up in months
high (n=10)
low (n=12)
raw p 0.024
bonf p 0.402
Expression Graph
Event=No
Event=Yes
3500
3000
2500
2000
1500
1000
500
0
Expression
2220
2220
sorted by expression
p-value

GROUP4 M0

Tumor Medulloblastoma
Cavalli - 763 - rma_sketch - hugene11t
CDK6 (8140955)
Expression cutoff: 3204.900 (min.grp=3)
subgroup~group4|met_status_(1_met__0_m0)~0|WITH_SURV (n=145)

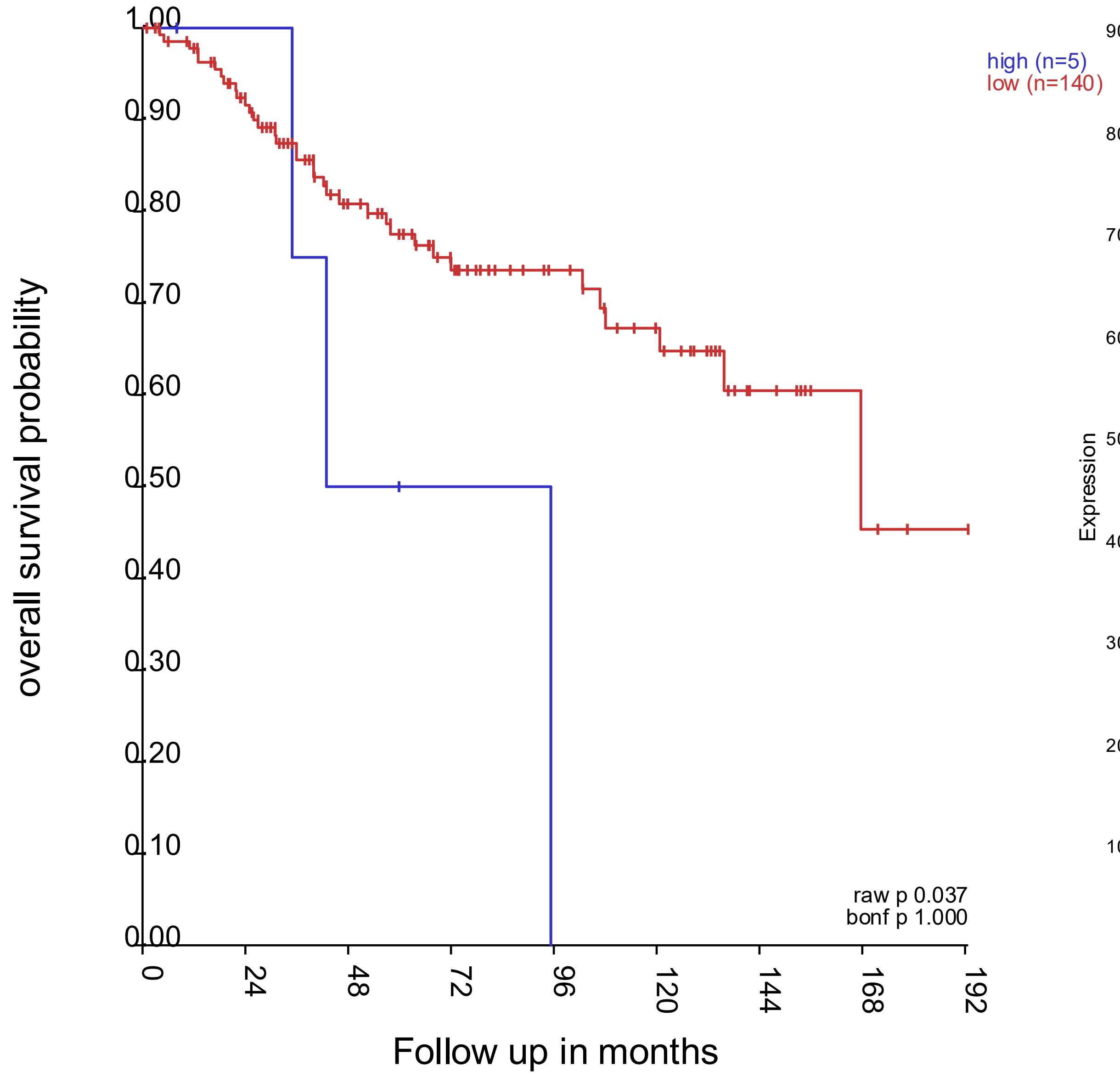

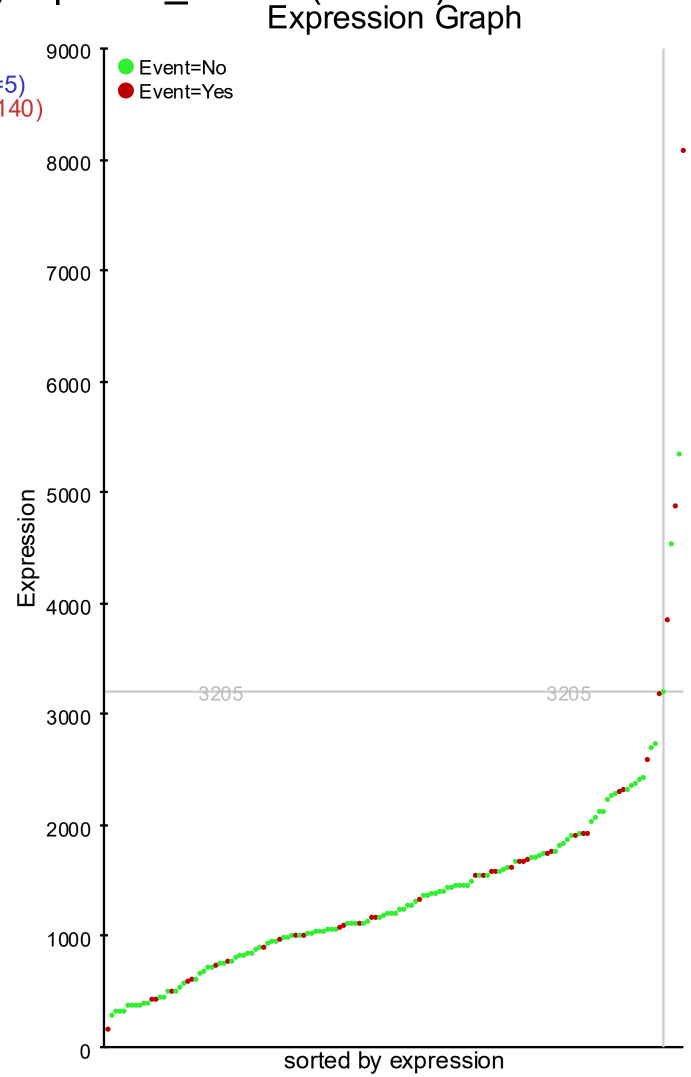

GROUP4 M1

Tumor Medulloblastoma
Cavalli - 763 - rma_sketch - hugene11t
CDK6 (8140955)
Expression cutoff: 3140.200 (min.grp=3)
subgroup~group4|met_status_(1_met__0_m0)~1|WITH_SURV (n=92)

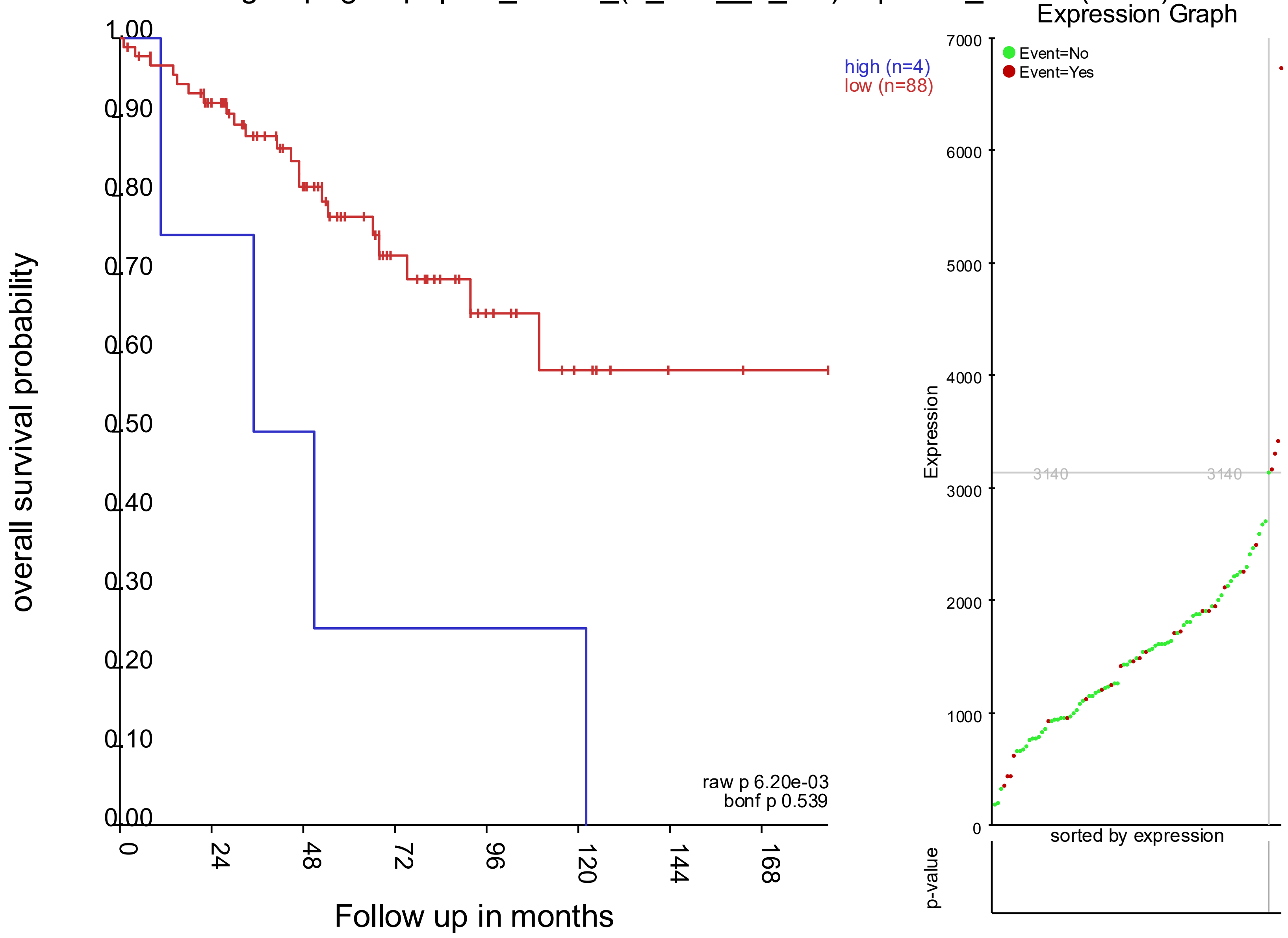

GROUP3 M0

Tumor Medulloblastoma
Cavalli - 763 - rma_sketch - hugene11t
CDK6 (8140955)
Expression cutoff: 1446.600 (min.grp=3)
subgroup~group3|met_status_(1_met__0_m0)~0|WITH_SURV (n=65)

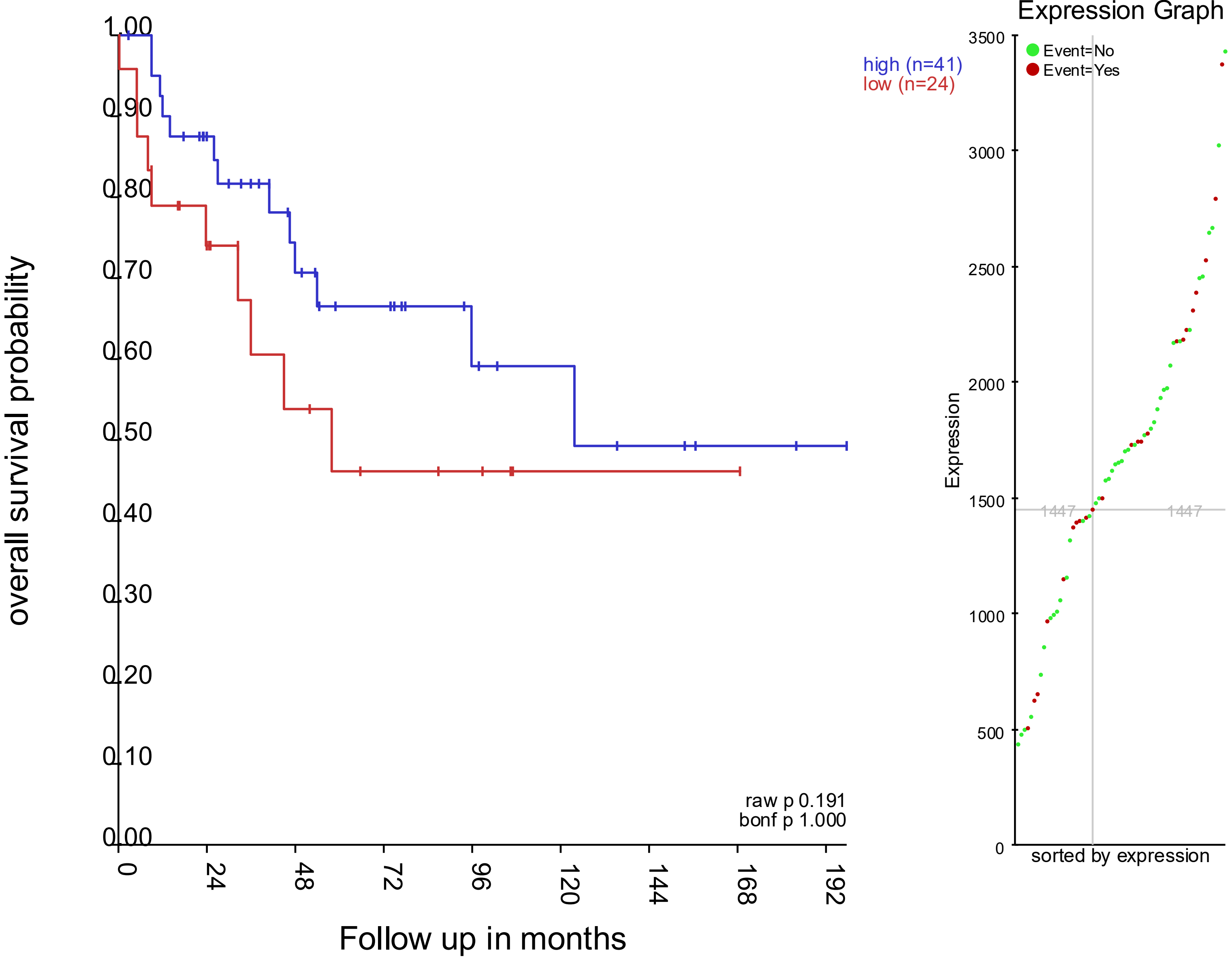

GROUP3 M1

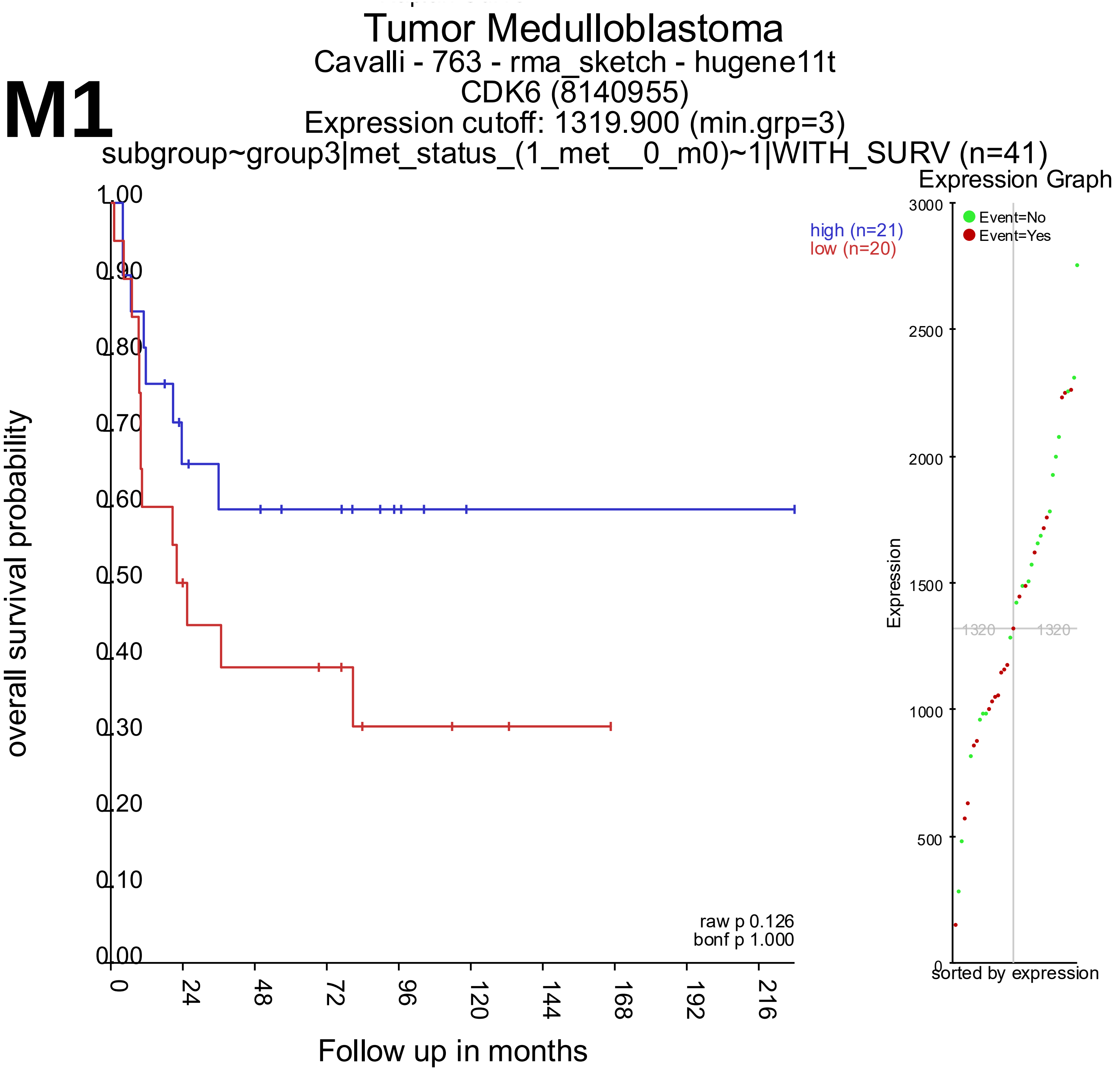

Tumor Medulloblastoma
Cavalli - 763 - rma_sketch - hugene11t
CDK6 (8140955)
Expression cutoff: 1319.900 (min.grp=3)
subgroup~group3|met_status_(1_met__0_m0)~1|WITH_SURV (n=41)
high (n=21)
low (n=20)
overall survival probability
Follow up in months
raw p 0.126
bonf p 1.000
Expression Graph
Event=No
Event=Yes
Expression
sorted by expression
1320

DDR2

WNT M0

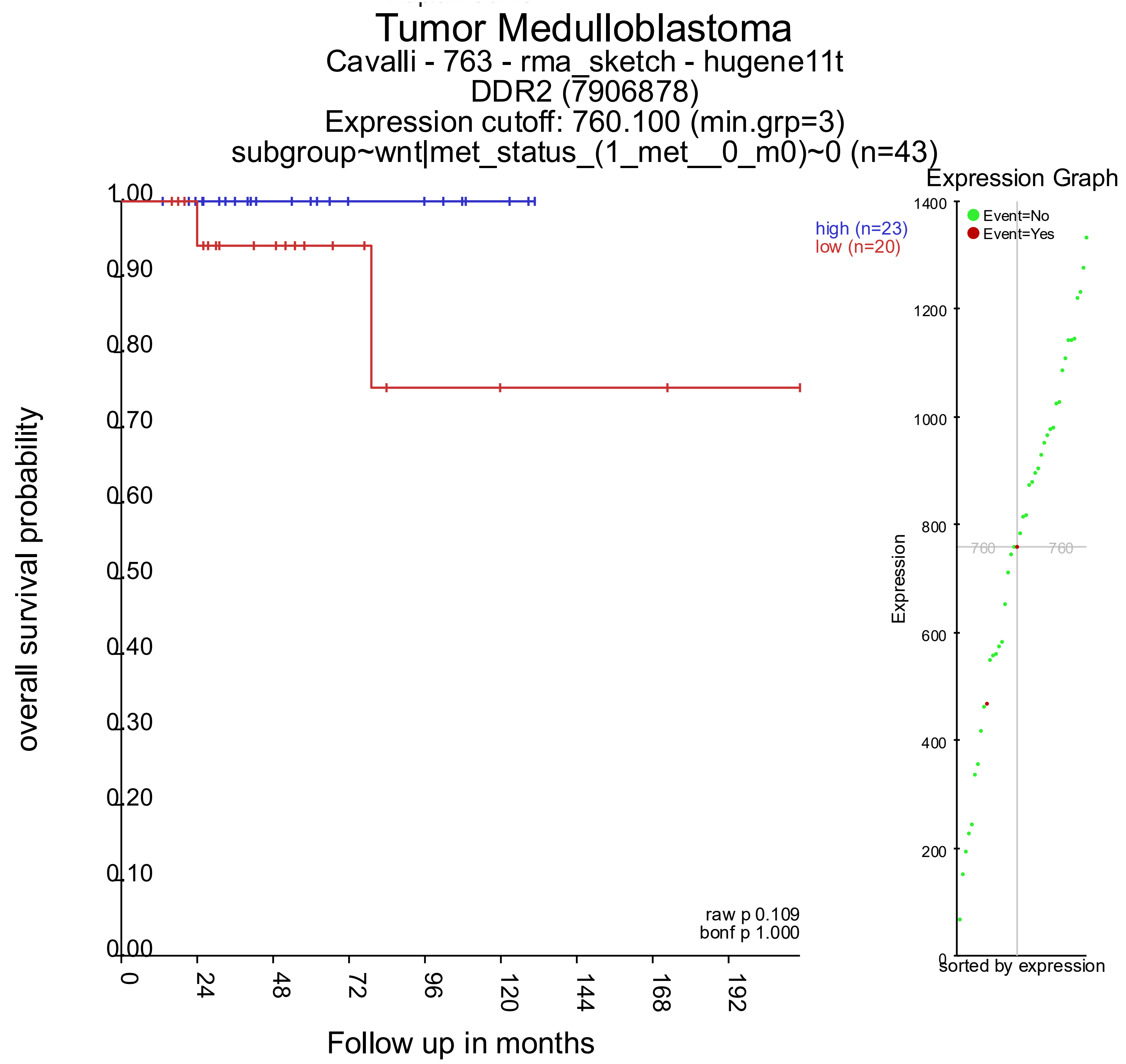

Tumor Medulloblastoma
Cavalli - 763 - rma_sketch - hugene11t
DDR2 (7906878)
Expression cutoff: 760.100 (min.grp=3)
subgroup~wnt|met_status_(1_met__0_m0)~0 (n=43)
high (n=23)
low (n=20)
overall survival probability
1.00
0.90
0.80
0.70
0.60
0.50
0.40
0.30
0.20
0.10
0.00
0
24
48
72
96
120
144
168
192
Follow up in months
raw p 0.109
bonf p 1.000
Expression Graph
Event=No
Event=Yes
Expression
1400
1200
1000
800
600
400
200
0
760
760
sorted by expression

WNT M1

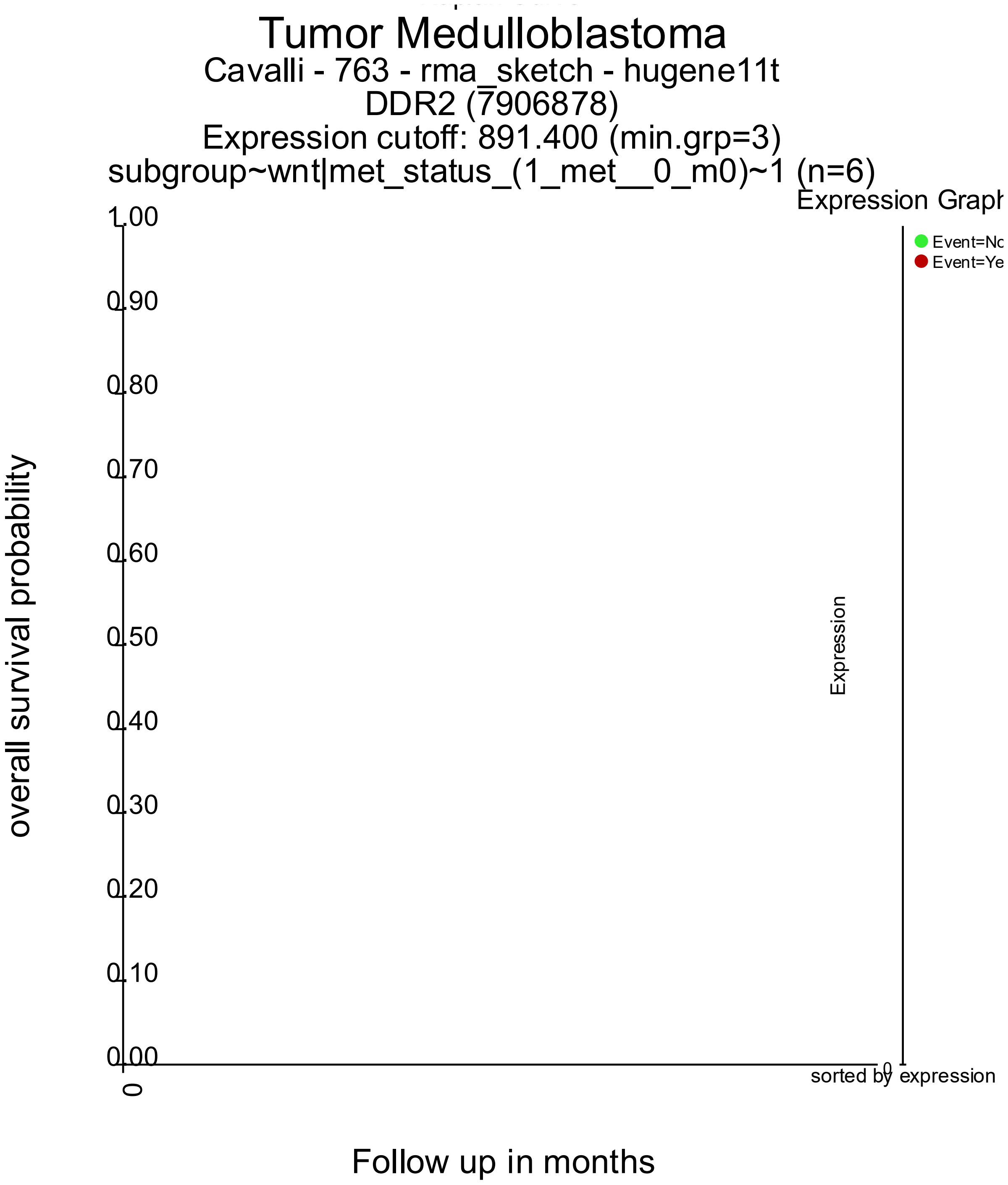

Tumor Medulloblastoma
Cavalli - 763 - rma_sketch - hugene11t
DDR2 (7906878)
Expression cutoff: 891.400 (min.grp=3)
subgroup~wnt|met_status_(1_met__0_m0)~1 (n=6)
Expression Graph
Event=No
Event=Ye
1.00
0.90
0.80
0.70
0.60
0.50
0.40
0.30
0.20
0.10
0.00
overall survival probability
0
Follow up in months
Expression
0
sorted by expression

SHH M0

Tumor Medulloblastoma
Cavalli - 763 - rma_sketch - hugene11t
DDR2 (7906878)
Expression cutoff: 82.000 (min.grp=3)
subgroup~shh|met_status_(1_met__0_m0)~0|WITH_SURV (n=124)

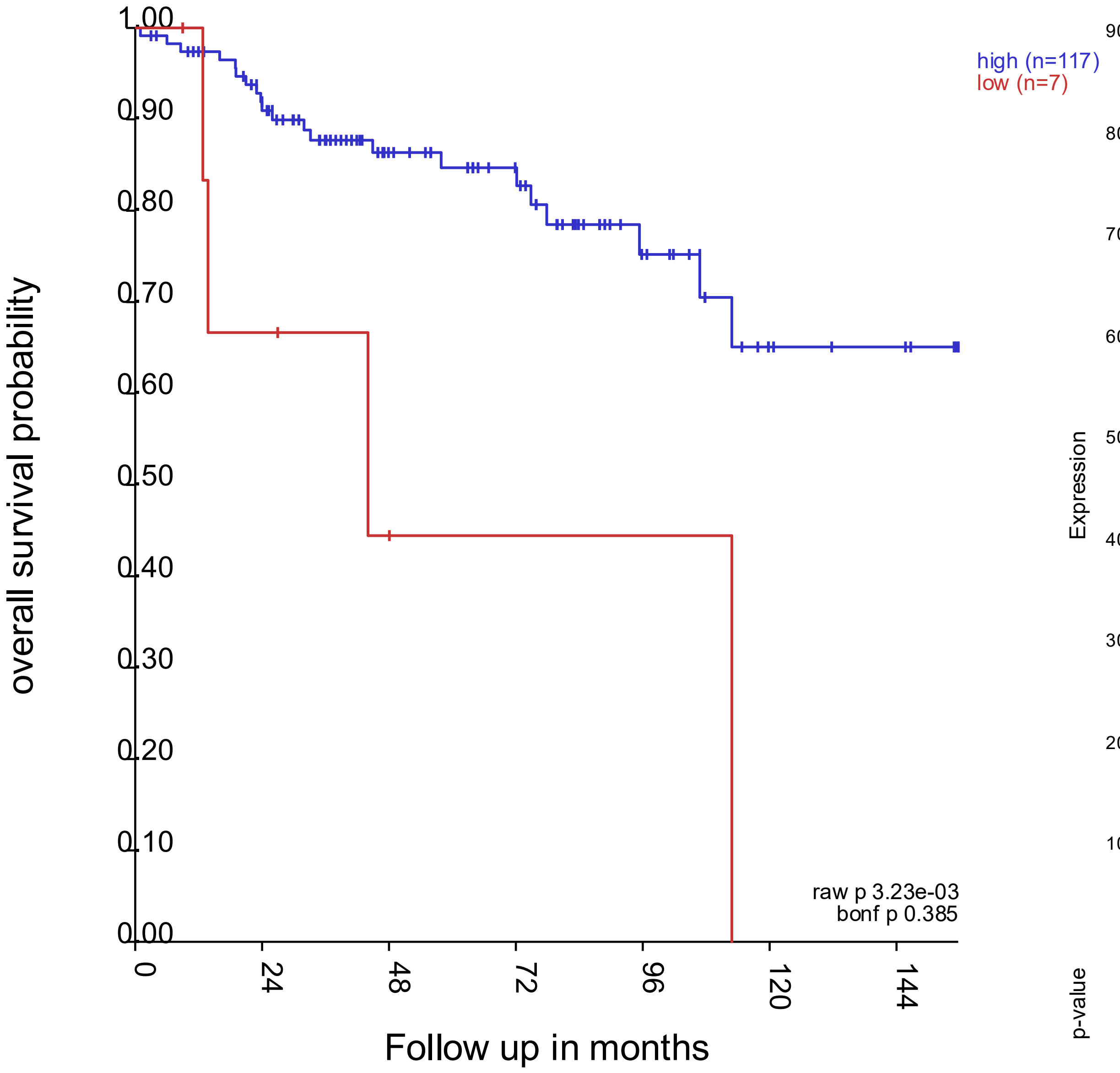

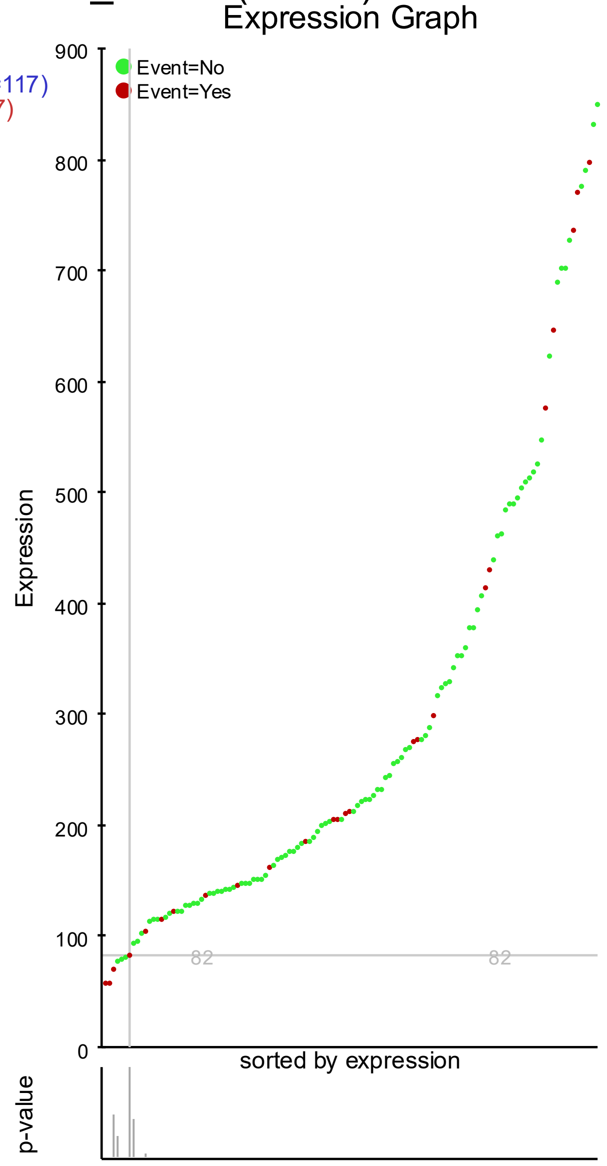

SHH M1

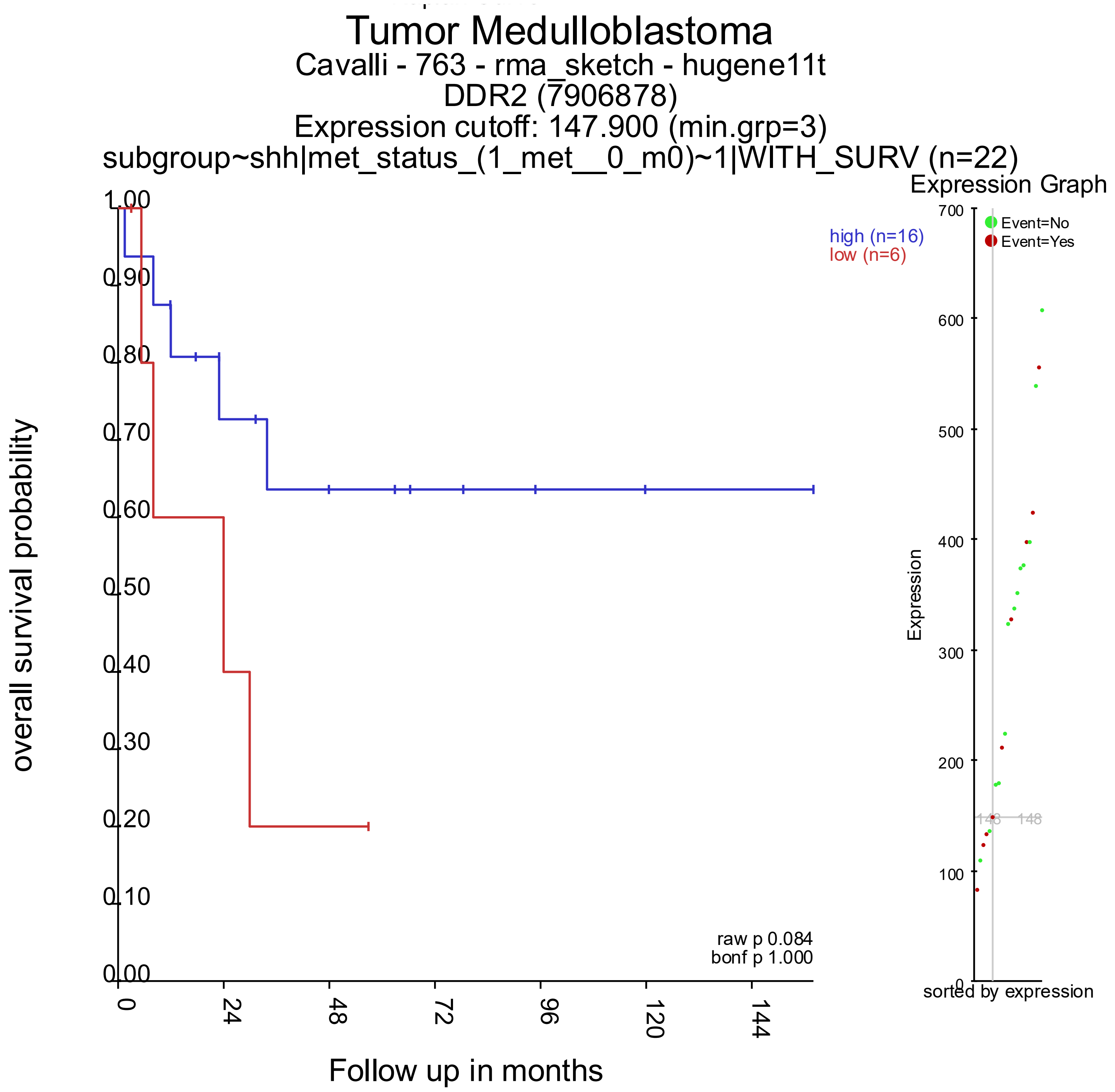
Tumor Medulloblastoma
Cavalli - 763 - rma_sketch - hugene11t
DDR2 (7906878)
Expression cutoff: 147.900 (min.grp=3)
subgroup~shh|met_status_(1_met__0_m0)~1|WITH_SURV (n=22)
high (n=16)
low (n=6)
overall survival probability
1.00
0.90
0.80
0.70
0.60
0.50
0.40
0.30
0.20
0.10
0.00
0
24
48
72
96
120
144
Follow up in months
raw p 0.084
bonf p 1.000
Expression Graph
Event=No
Event=Yes
Expression
700
600
500
400
300
200
100
0
148
148
sorted by expression

GROUP4 M0

Tumor Medulloblastoma
Cavalli - 763 - rma_sketch - hugene11t
DDR2 (7906878)
Expression cutoff: 67.700 (min.grp=3)
subgroup~group4|met_status_(1_met__0_m0)~0|WITH_SURV (n=145)

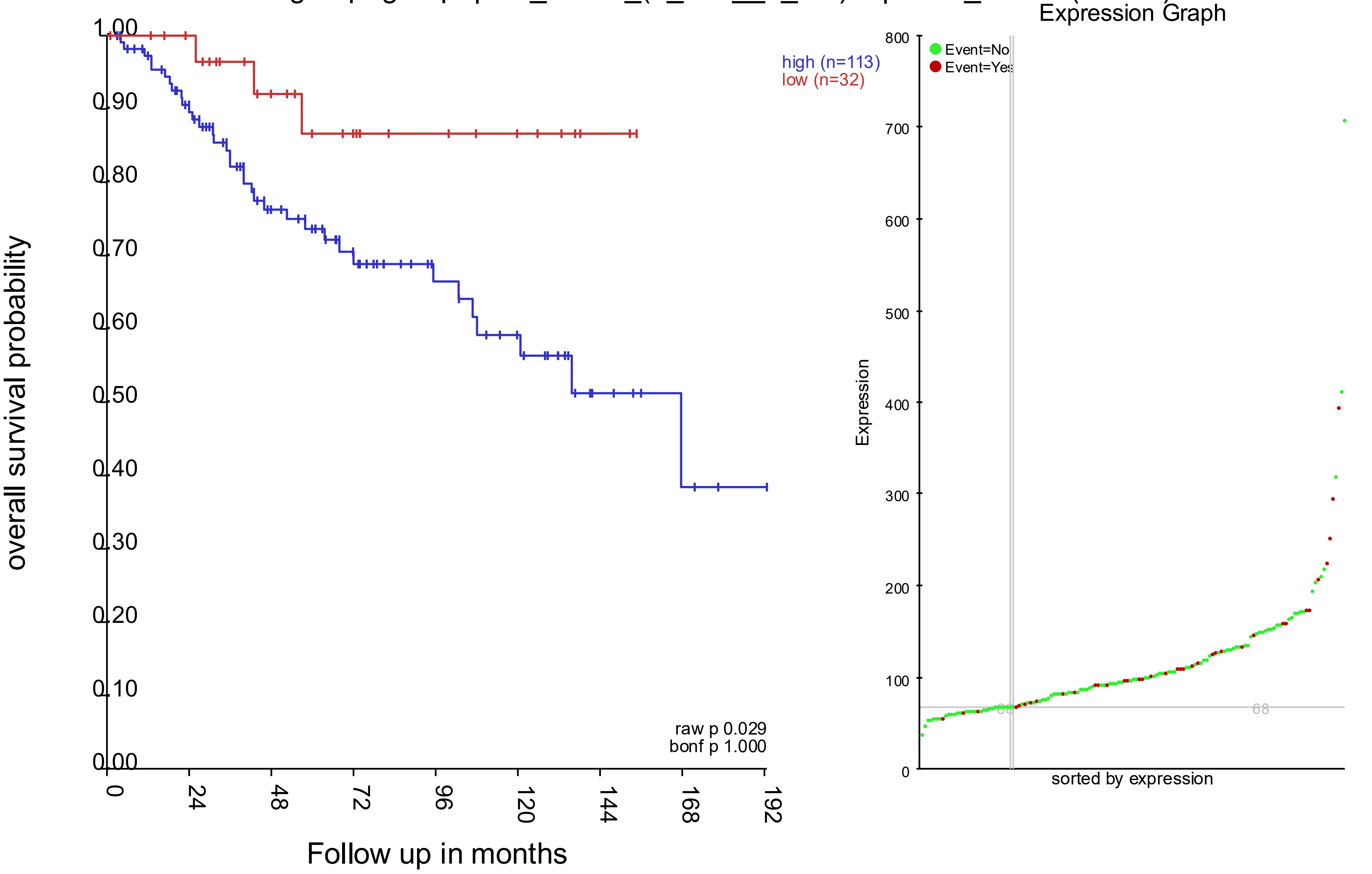

GROUP4 M1

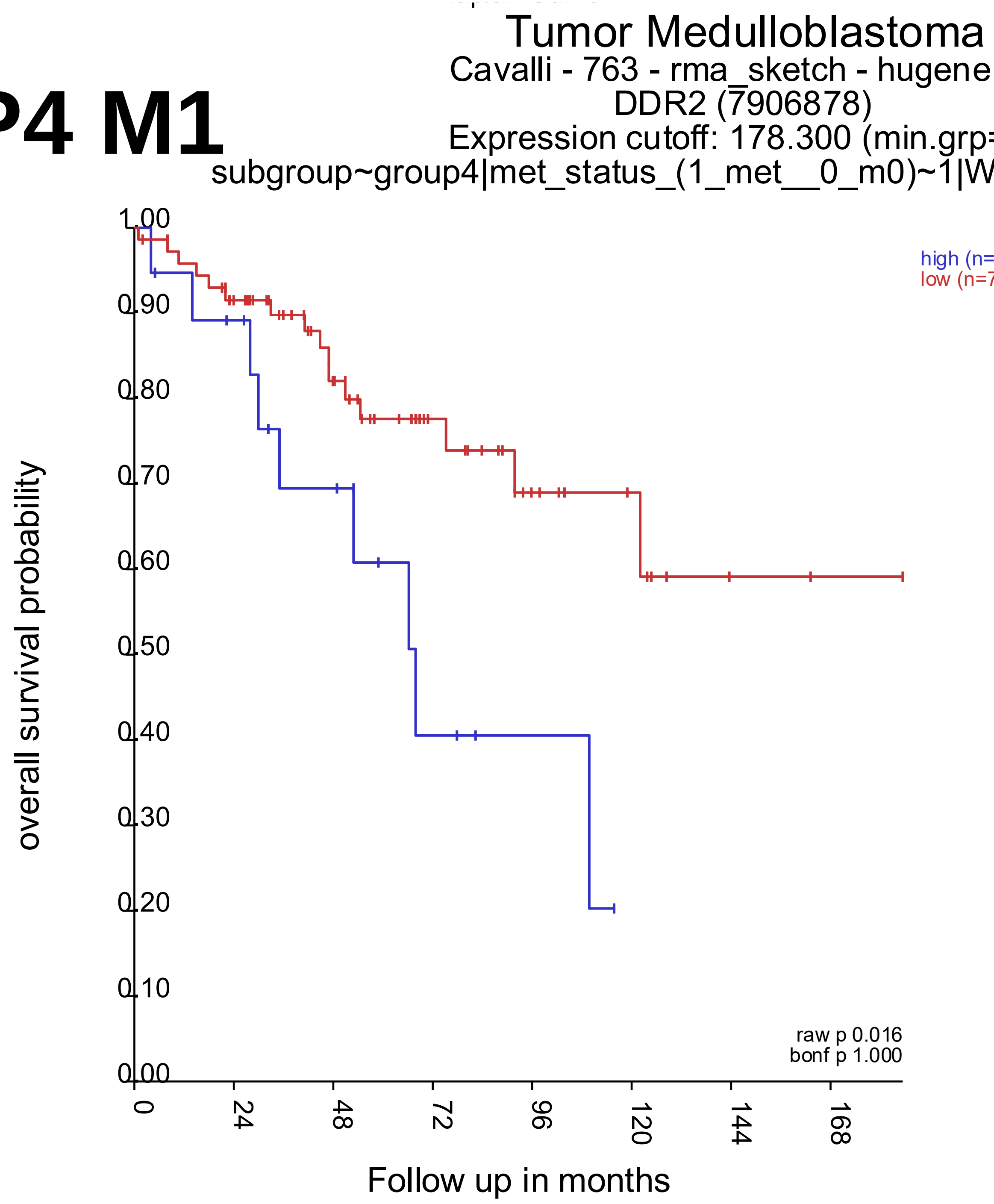
Tumor Medulloblastoma
Cavalli - 763 - rma_sketch - hugene11t
DDR2 (7906878)
Expression cutoff: 178.300 (min.grp=3)
subgroup~group4|met_status_(1_met__0_m0)~1|WITH_SURV (n=92)
overall survival probability
1.00
0.90
0.80
0.70
0.60
0.50
0.40
0.30
0.20
0.10
0.00
0
24
48
72
96
120
144
168
Follow up in months
high (n=19)
low (n=73)
raw p 0.016
bonf p 1.000

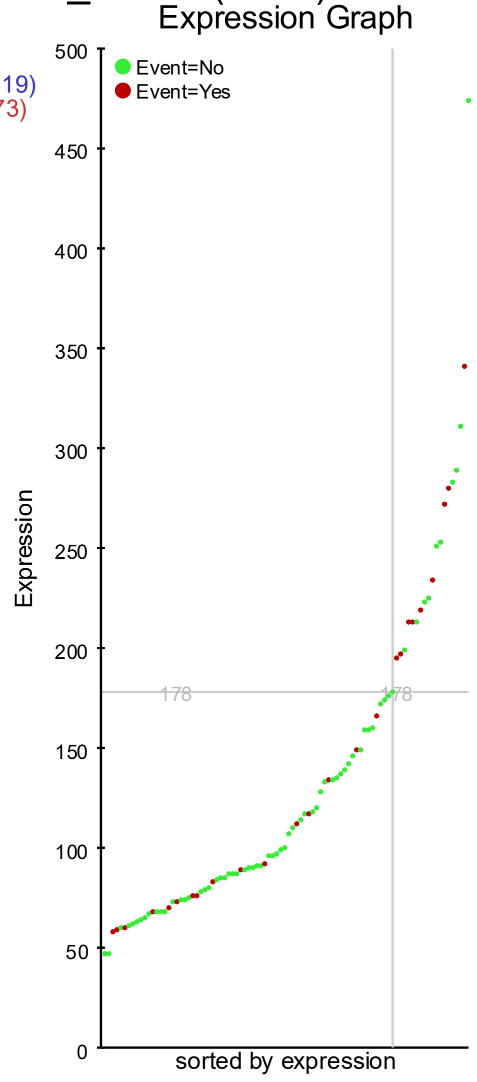
Expression Graph
Event=No
Event=Yes
Expression
500
450
400
350
300
250
200
150
100
50
0
178
178
sorted by expression

GROUP3 M0

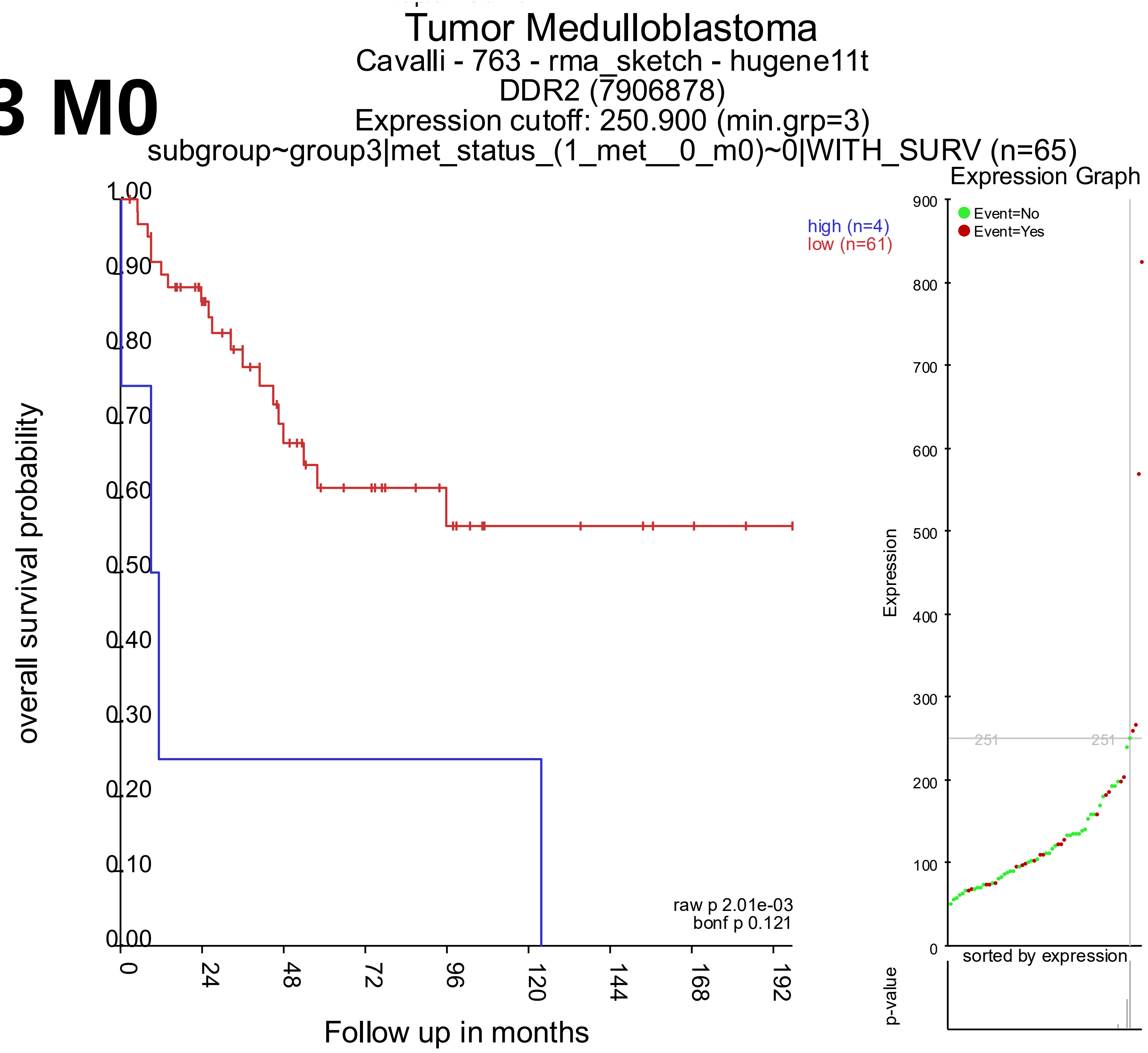

Tumor Medulloblastoma
Cavalli - 763 - rma_sketch - hugene11t
DDR2 (7906878)
Expression cutoff: 250.900 (min.grp=3)
subgroup~group3|met_status_(1_met__0_m0)~0|WITH_SURV (n=65)
overall survival probability
1.00
0.90
0.80
0.70
0.60
0.50
0.40
0.30
0.20
0.10
0.00
0
24
48
72
96
120
144
168
192
Follow up in months
high (n=4)
low (n=61)
raw p 2.01e-03
bonf p 0.121
Expression Graph
Event=No
Event=Yes
Expression
900
800
700
600
500
400
300
200
100
0
251
251
sorted by expression
p-value

GROUP3 M1

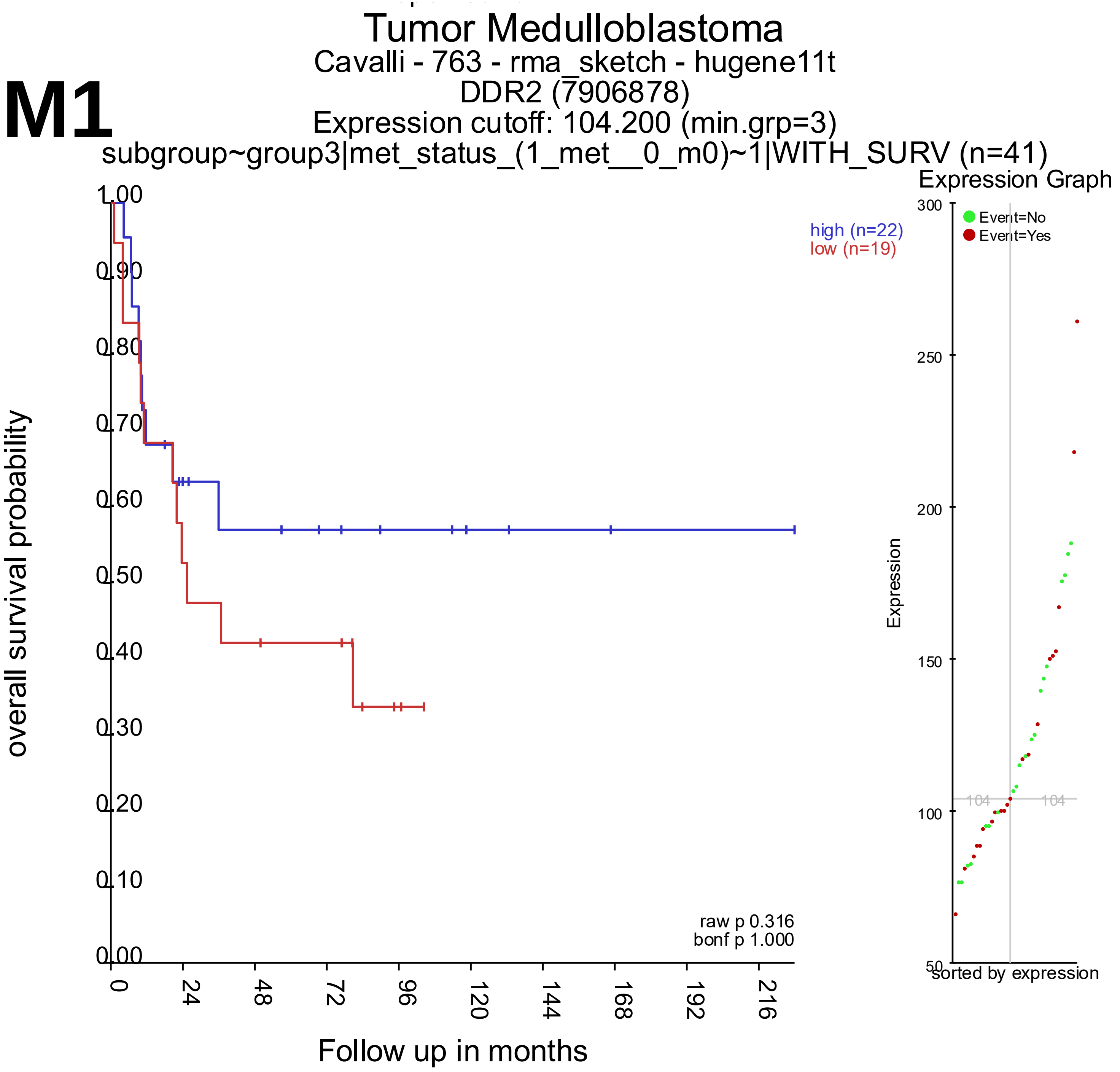

Tumor Medulloblastoma
Cavalli - 763 - rma_sketch - hugene11t
DDR2 (7906878)
Expression cutoff: 104.200 (min.grp=3)
subgroup~group3|met_status_(1_met__0_m0)~1|WITH_SURV (n=41)
overall survival probability
1.00
0.90
0.80
0.70
0.60
0.50
0.40
0.30
0.20
0.10
0.00
0
24
48
72
96
120
144
168
192
216
Follow up in months
high (n=22)
low (n=19)
raw p 0.316
bonf p 1.000
Expression Graph
Event=No
Event=Yes
300
250
200
150
100
50
Expression
104
104
sorted by expression

EPCAM

WNT M0

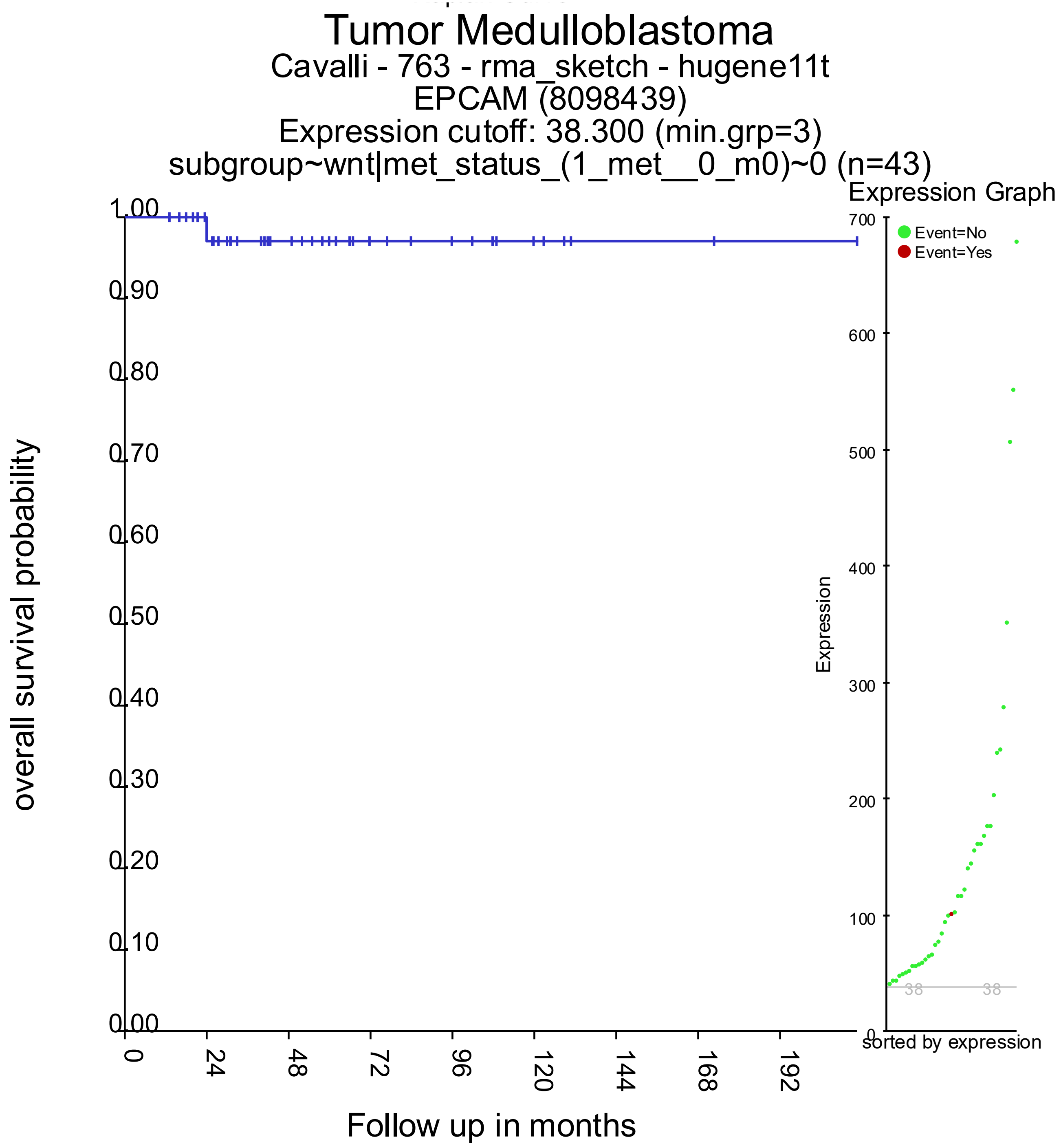
Tumor Medulloblastoma
Cavalli - 763 - rma_sketch - hugene11t
EPCAM (8098439)
Expression cutoff: 38.300 (min.grp=3)
subgroup~wnt|met_status_(1_met__0_m0)~0 (n=43)
overall survival probability
1.00
0.90
0.80
0.70
0.60
0.50
0.40
0.30
0.20
0.10
0.00
0
24
48
72
96
120
144
168
192
Follow up in months
Expression Graph
Event=No
Event=Yes
Expression
700
600
500
400
300
200
100
0
38
38
sorted by expression

WNT M1

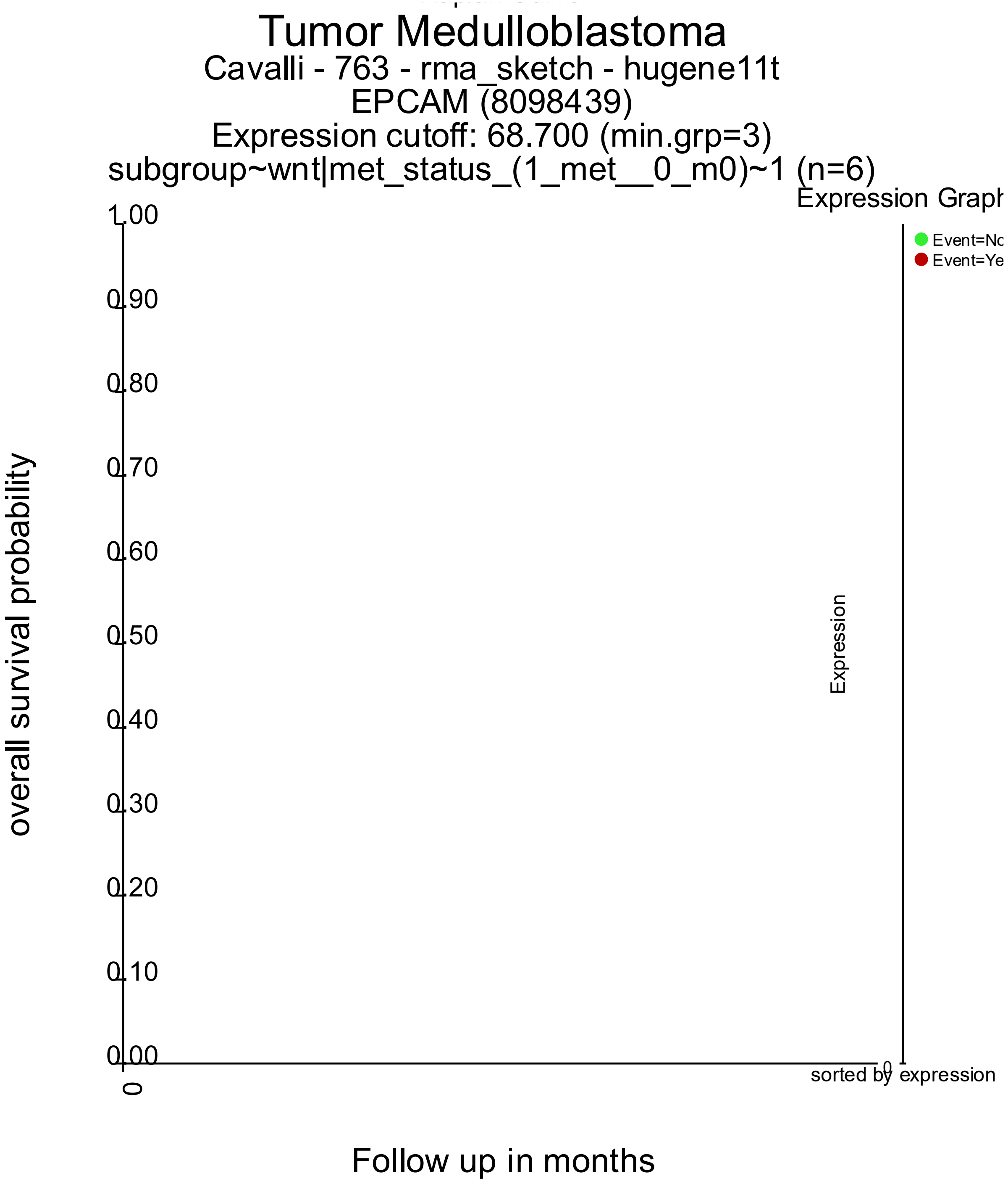
Tumor Medulloblastoma
Cavalli - 763 - rma_sketch - hugene11t
EPCAM (8098439)
Expression cutoff: 68.700 (min.grp=3)
subgroup~wnt|met_status_(1_met__0_m0)~1 (n=6)
Expression Graph
Event=No
Event=Yes
1.00
0.90
0.80
0.70
0.60
0.50
0.40
0.30
0.20
0.10
0.00
overall survival probability
0
Follow up in months
Expression
0
sorted by expression

SHH M0

Tumor Medulloblastoma
Cavalli - 763 - rma_sketch - hugene11t
EPCAM (8098439)
Expression cutoff: 40.100 (min.grp=3)
subgroup~shh|met_status_(1_met__0_m0)~0|WITH_SURV (n=124)

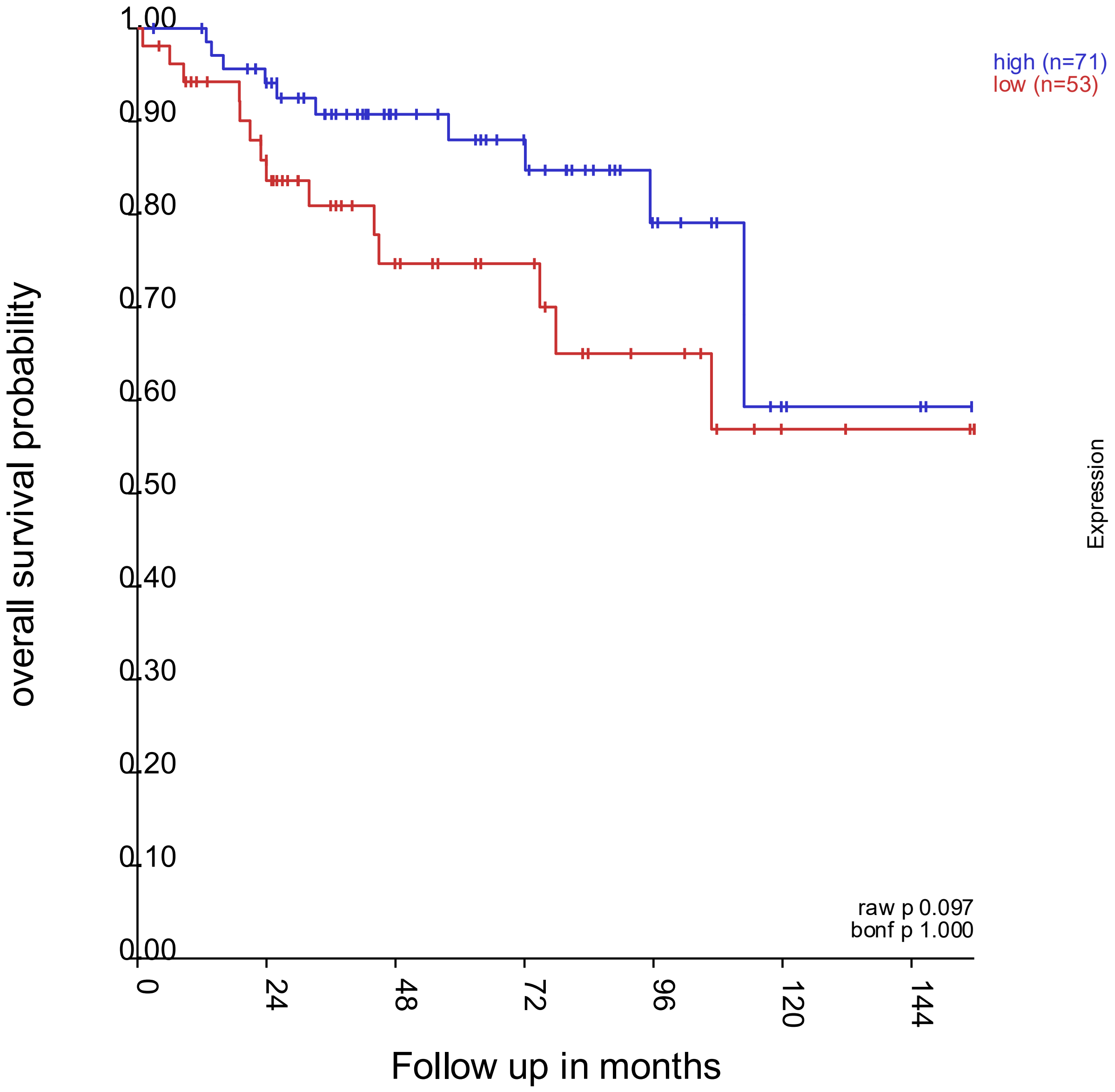

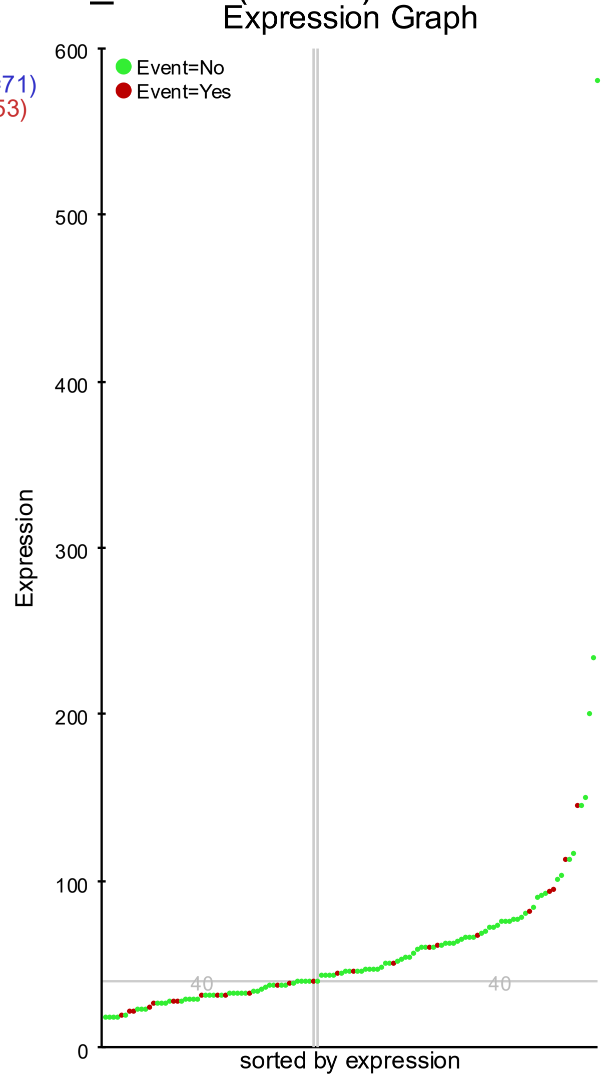

SHH M1

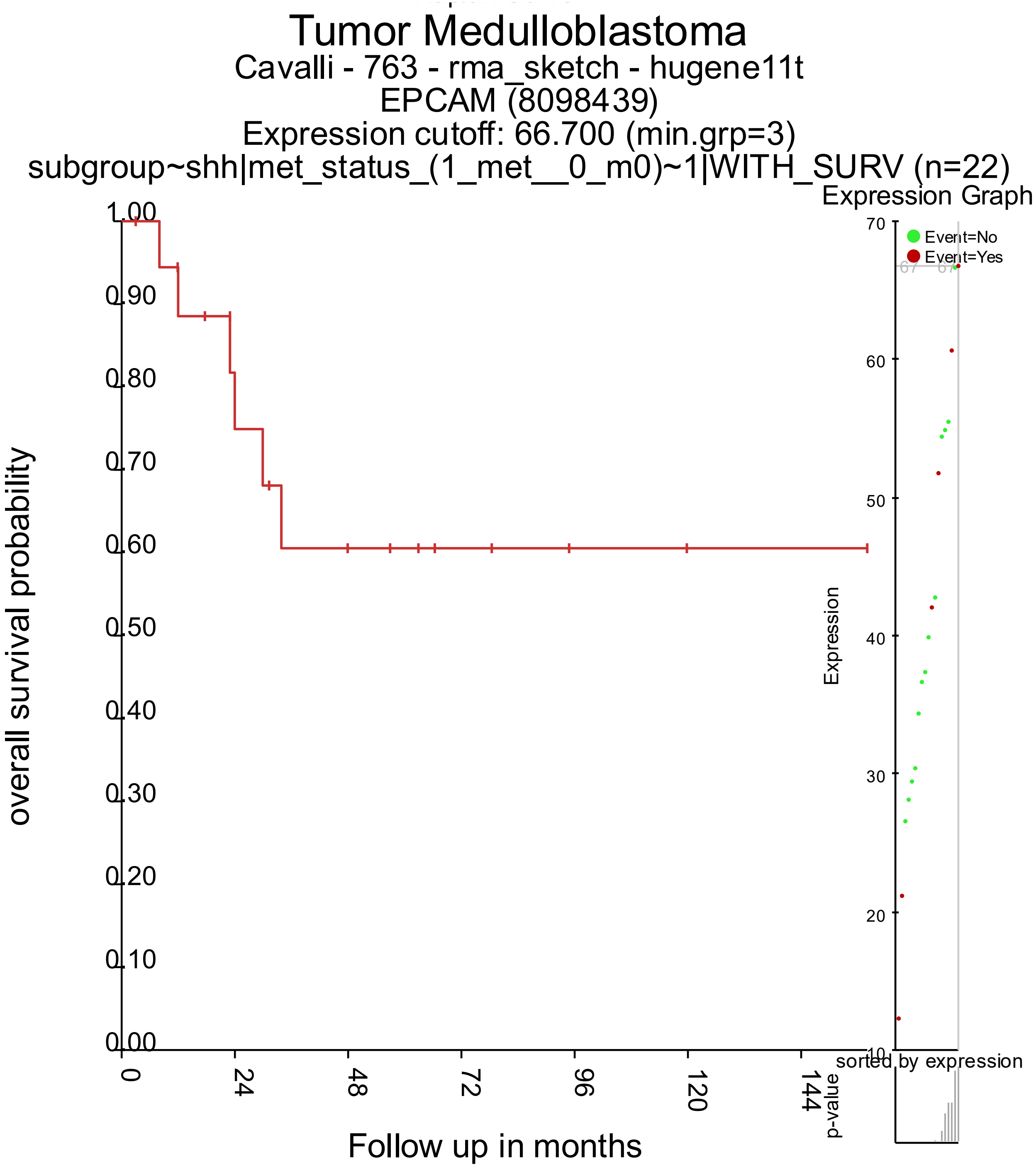
Tumor Medulloblastoma
Cavalli - 763 - rma_sketch - hugene11t
EPCAM (8098439)
Expression cutoff: 66.700 (min.grp=3)
subgroup~shh|met_status_(1_met__0_m0)~1|WITH_SURV (n=22)
overall survival probability
1.00
0.90
0.80
0.70
0.60
0.50
0.40
0.30
0.20
0.10
0.00
0
24
48
72
96
120
144
Follow up in months
Expression Graph
Event=No
Event=Yes
Expression
70
60
50
40
30
20
10
sorted by expression
p-value

GROUP4 M0

Tumor Medulloblastoma
Cavalli - 763 - rma_sketch - hugene11t
EPCAM (8098439)
Expression cutoff: 143.600 (min.grp=3)
subgroup~group4|met_status_(1_met__0_m0)~0|WITH_SURV (n=145)

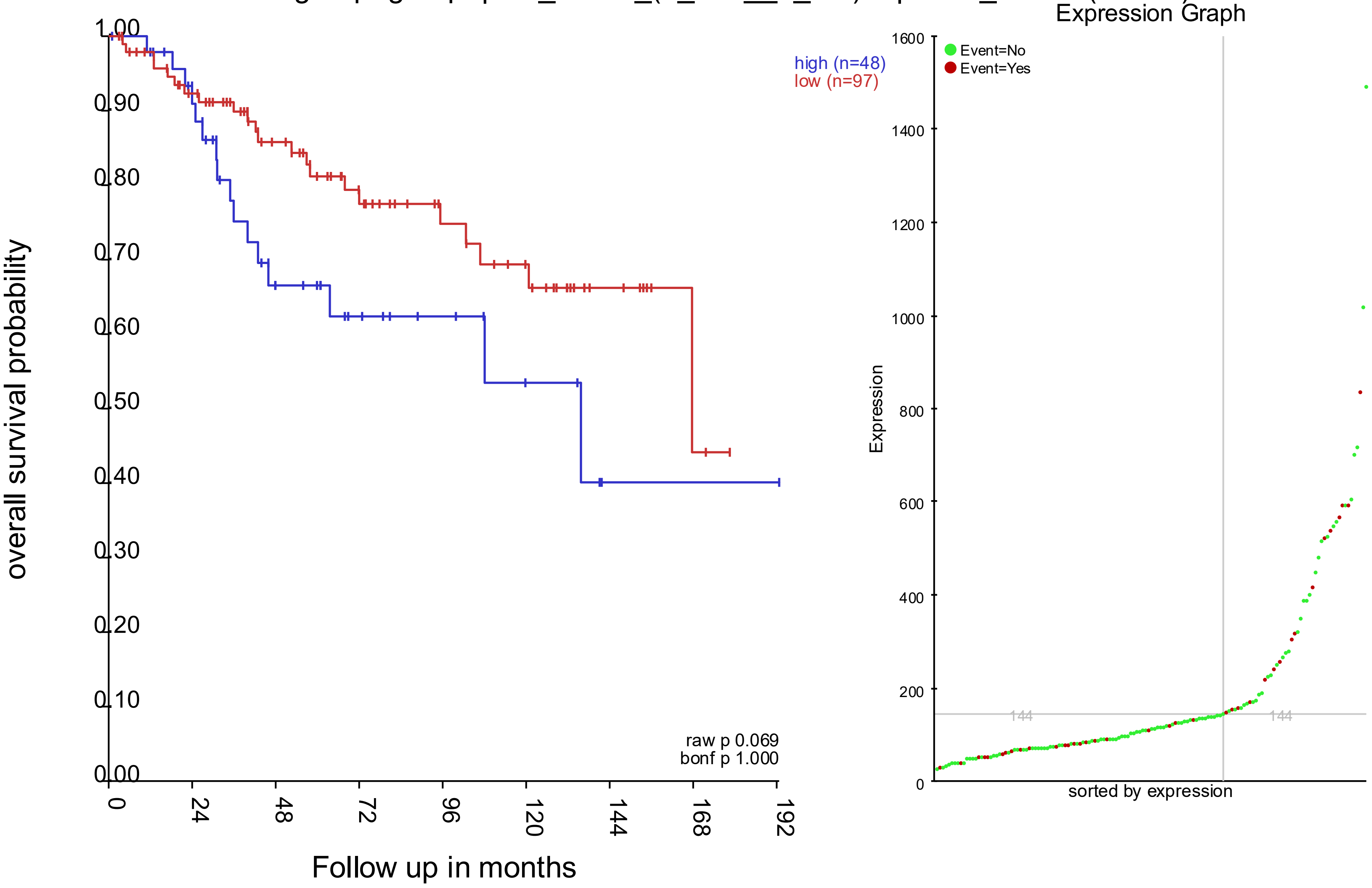

GROUP4 M1

Tumor Medulloblastoma
Cavalli - 763 - rma_sketch - hugene11t
EPCAM (8098439)
Expression cutoff: 39.800 (min.grp=3)
subgroup~group4|met_status_(1_met__0_m0)~1|WITH_SURV (n=92)

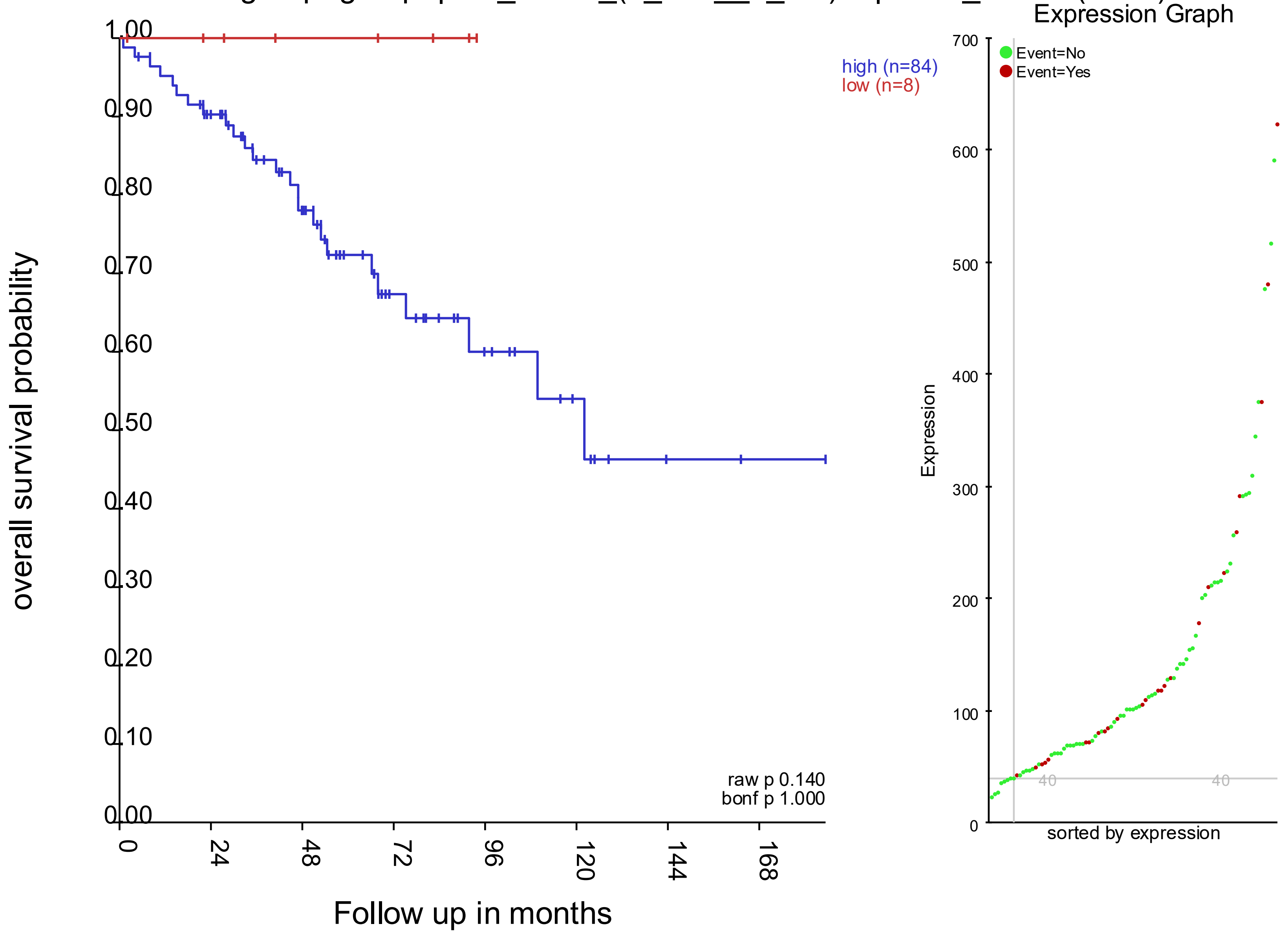

GROUP3 M0

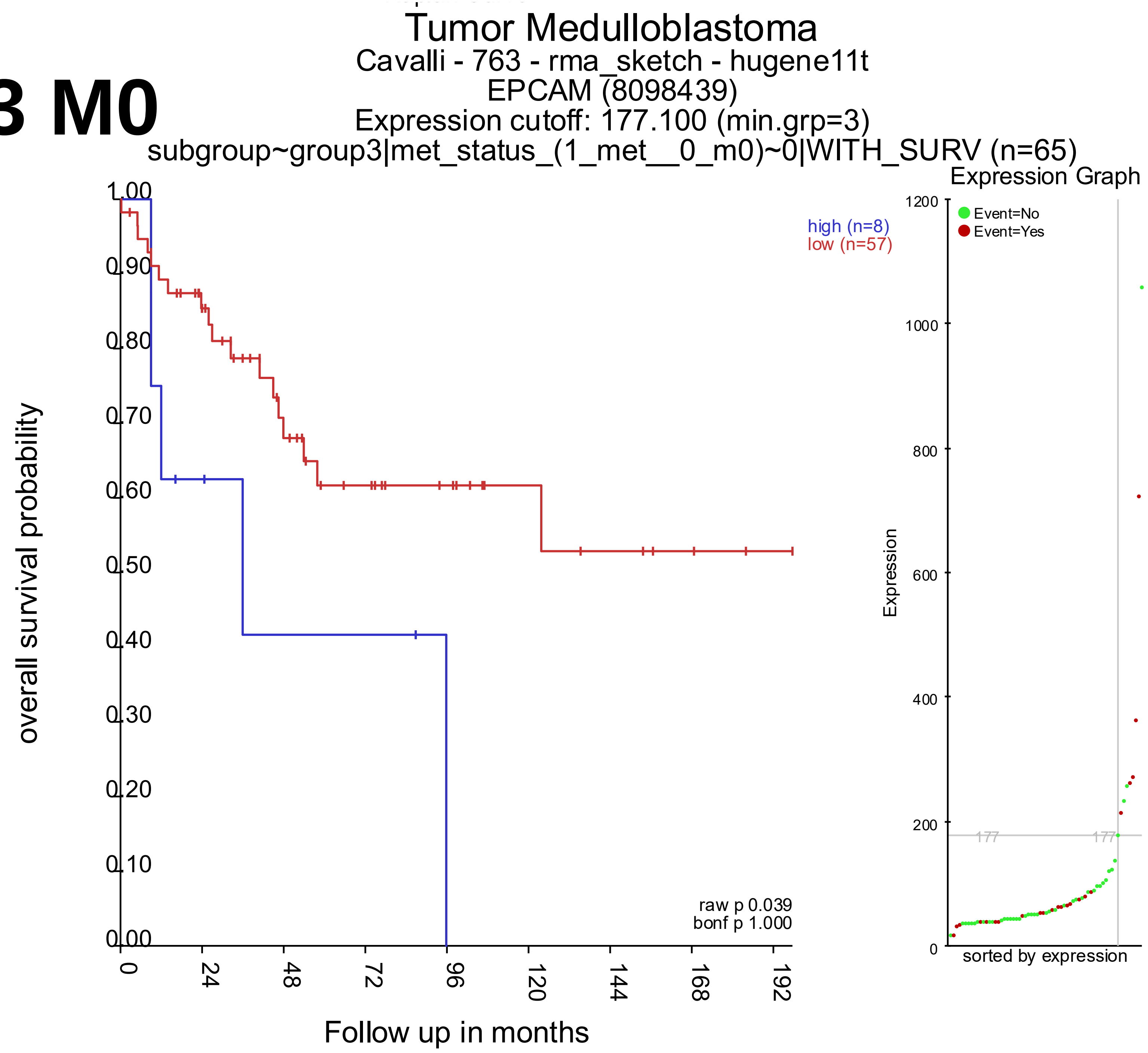

Tumor Medulloblastoma
Cavalli - 763 - rma_sketch - hugene11t
EPCAM (8098439)
Expression cutoff: 177.100 (min.grp=3)
subgroup~group3|met_status_(1_met__0_m0)~0|WITH_SURV (n=65)
overall survival probability
1.00
0.90
0.80
0.70
0.60
0.50
0.40
0.30
0.20
0.10
0.00
high (n=8)
low (n=57)
raw p 0.039
bonf p 1.000
0
24
48
72
96
120
144
168
192
Follow up in months
Expression Graph
Event=No
Event=Yes
Expression
1200
1000
800
600
400
200
0
177
sorted by expression

GROUP3 M1

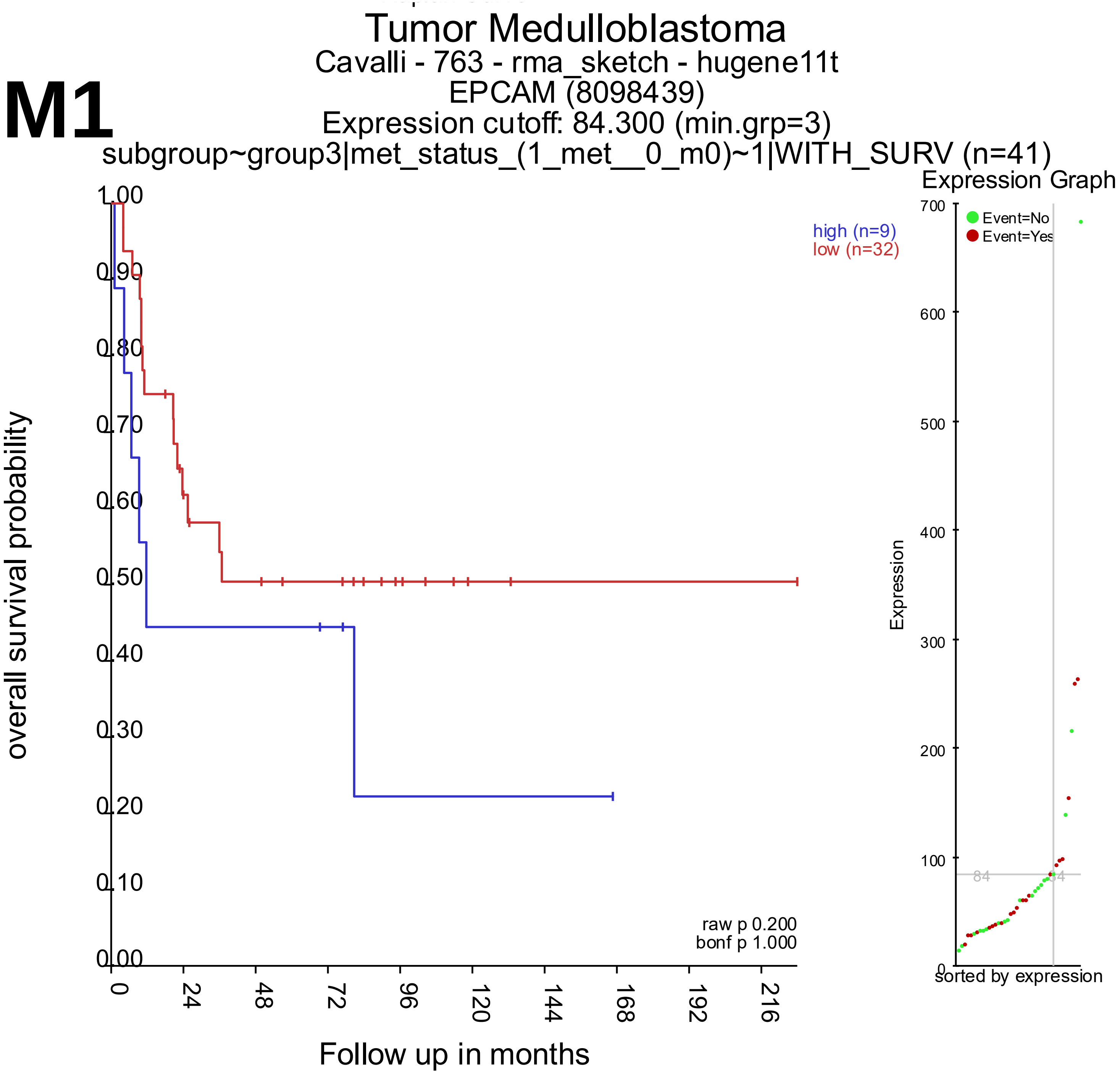

Tumor Medulloblastoma
Cavalli - 763 - rma_sketch - hugene11t
EPCAM (8098439)
Expression cutoff: 84.300 (min.grp=3)
subgroup~group3|met_status_(1_met__0_m0)~1|WITH_SURV (n=41)
overall survival probability
1.00
0.90
0.80
0.70
0.60
0.50
0.40
0.30
0.20
0.10
0.00
high (n=9)
low (n=32)
raw p 0.200
bonf p 1.000
0
24
48
72
96
120
144
168
192
216
Follow up in months
Expression Graph
Event=No
Event=Yes
Expression
700
600
500
400
300
200
100
0
84
sorted by expression

FGFR1

WNT M0

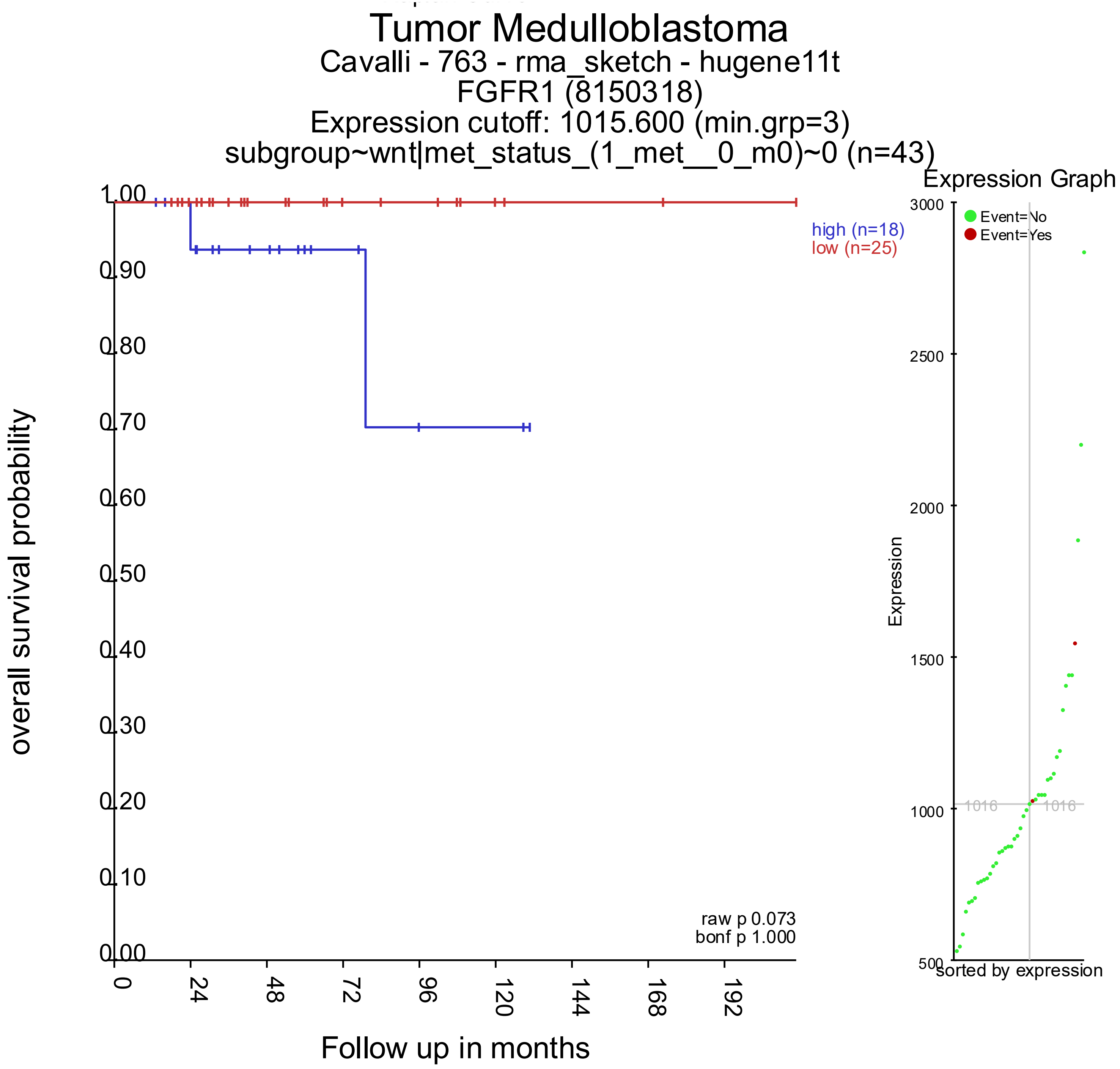

Tumor Medulloblastoma
Cavalli - 763 - rma_sketch - hugene11t
FGFR1 (8150318)
Expression cutoff: 1015.600 (min.grp=3)
subgroup~wnt|met_status_(1_met__0_m0)~0 (n=43)
high (n=18)
low (n=25)
overall survival probability
1.00
0.90
0.80
0.70
0.60
0.50
0.40
0.30
0.20
0.10
0.00
raw p 0.073
bonf p 1.000
0
24
48
72
96
120
144
168
192
Follow up in months
Expression Graph
Event=No
Event=Yes
Expression
3000
2500
2000
1500
1000
500
1016
1016
sorted by expression

WNT M1

Tumor Medulloblastoma
Cavalli - 763 - rma_sketch - hugene11t
FGFR1 (8150318)
Expression cutoff: 1263.100 (min.grp=3)
subgroup~wnt|met_status_(1_met__0_m0)~1 (n=6)

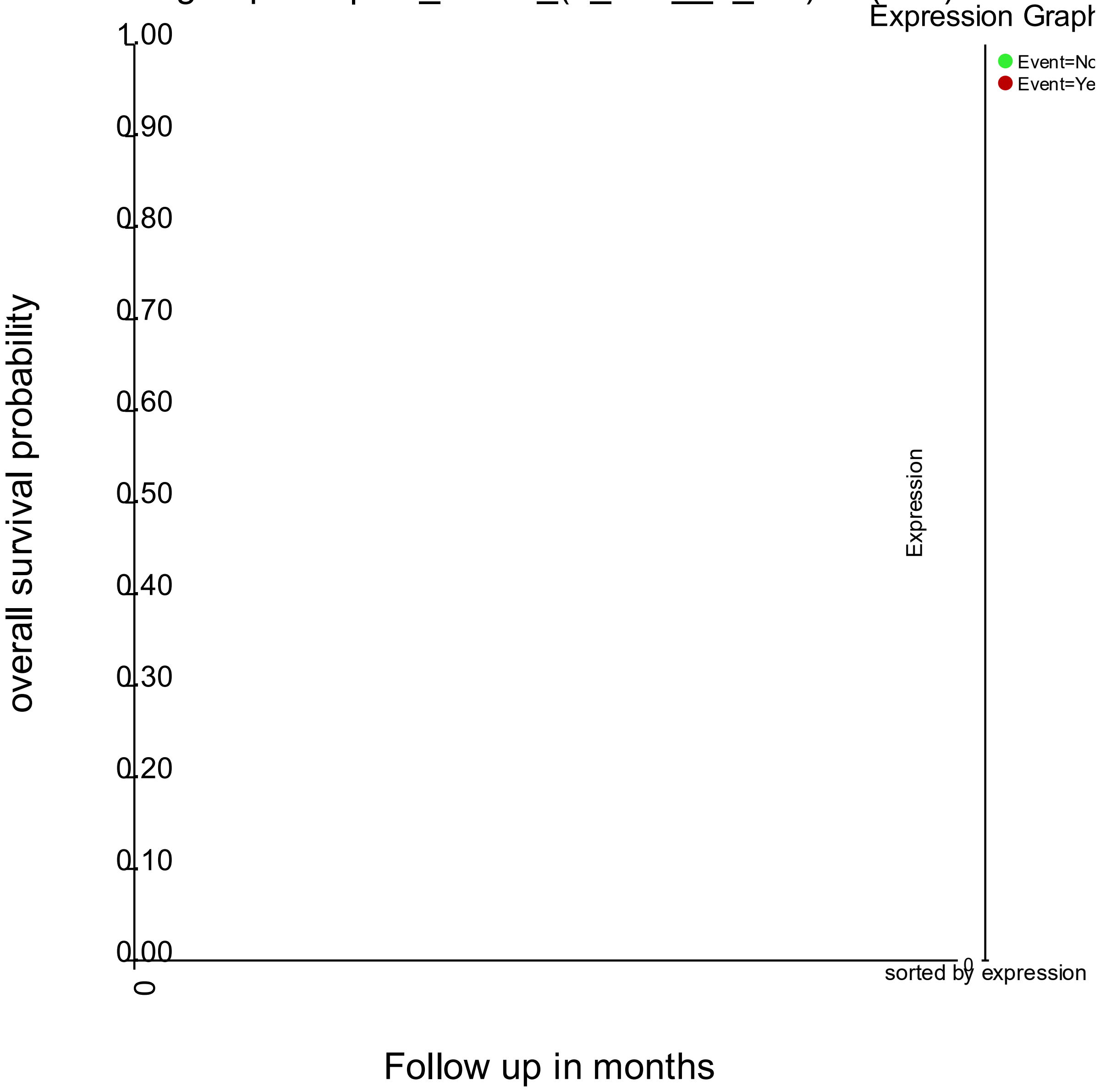

SHH M0

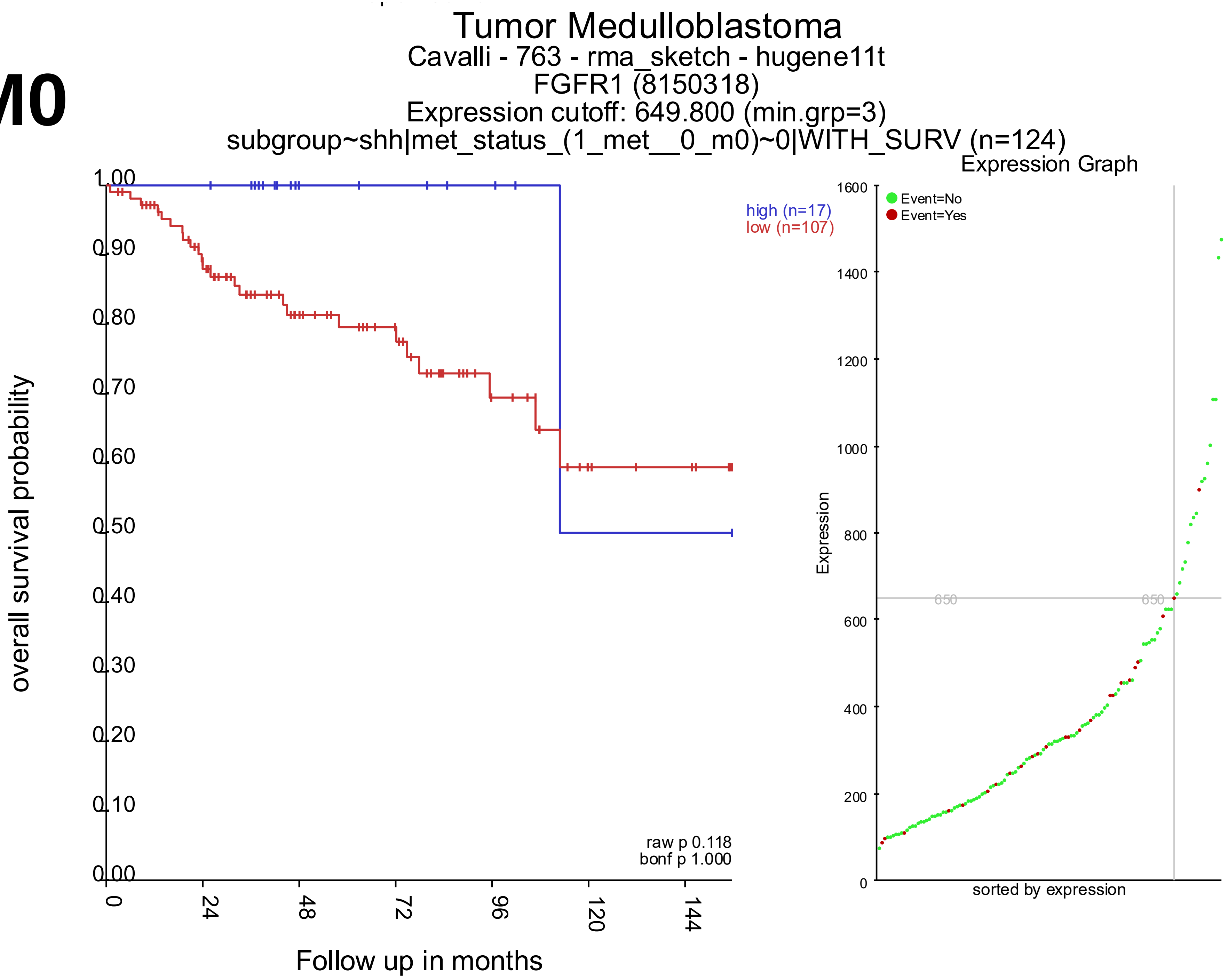
Tumor Medulloblastoma
Cavalli - 763 - rma_sketch - hugene11t
FGFR1 (8150318)
Expression cutoff: 649.800 (min.grp=3)
subgroup~shh|met_status_(1_met__0_m0)~0|WITH_SURV (n=124)
overall survival probability
1.00
0.90
0.80
0.70
0.60
0.50
0.40
0.30
0.20
0.10
0.00
0
24
48
72
96
120
144
Follow up in months
high (n=17)
low (n=107)
raw p 0.118
bonf p 1.000
Expression Graph
Event=No
Event=Yes
Expression
1600
1400
1200
1000
800
600
400
200
0
650
650
sorted by expression

SHH M1

Tumor Medulloblastoma
Cavalli - 763 - rma_sketch - hugene11t
FGFR1 (8150318)
Expression cutoff: 618.900 (min.grp=3)
subgroup~shh|met_status_(1_met__0_m0)~1|WITH_SURV (n=22)

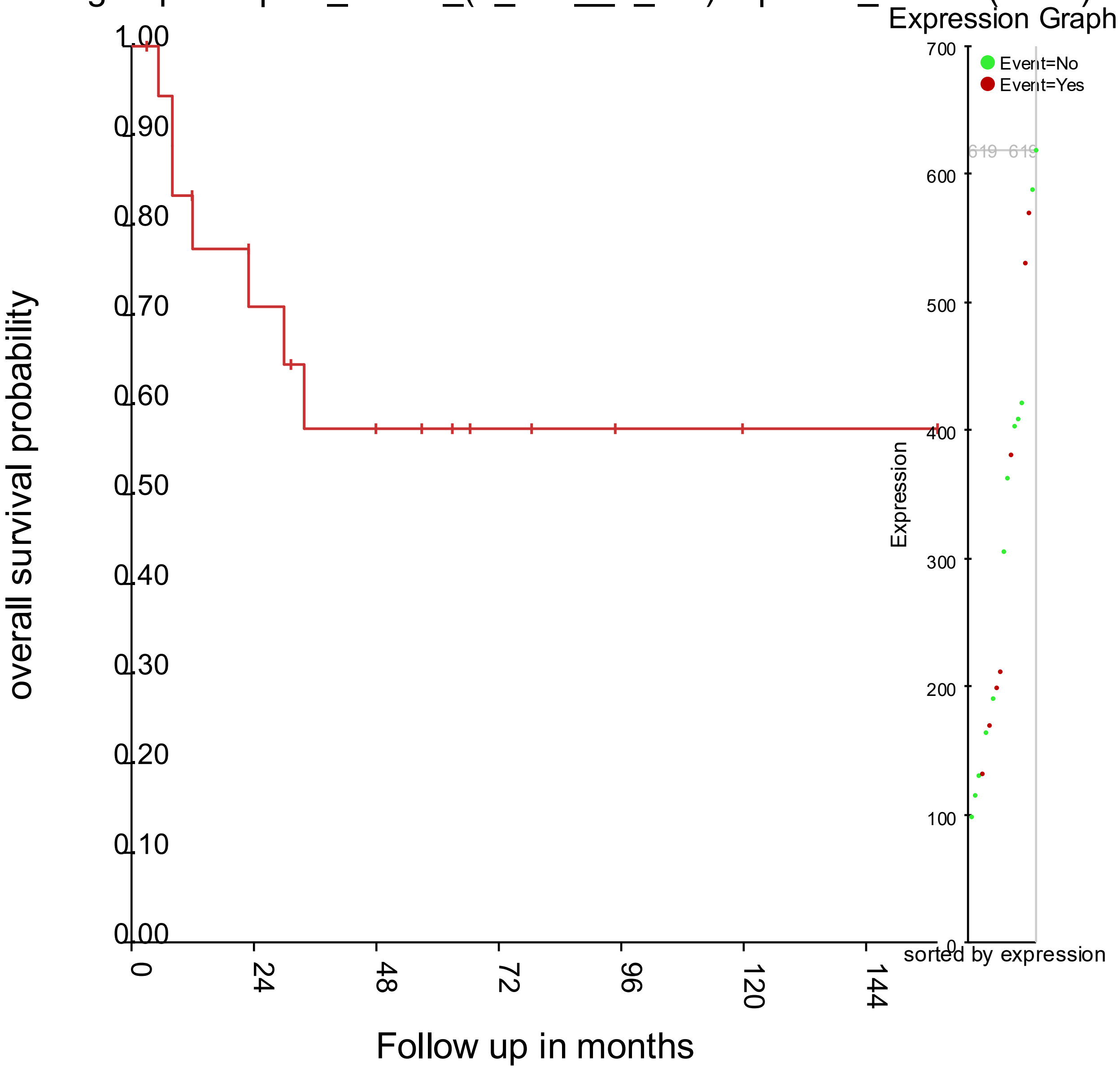

GROUP4 M0

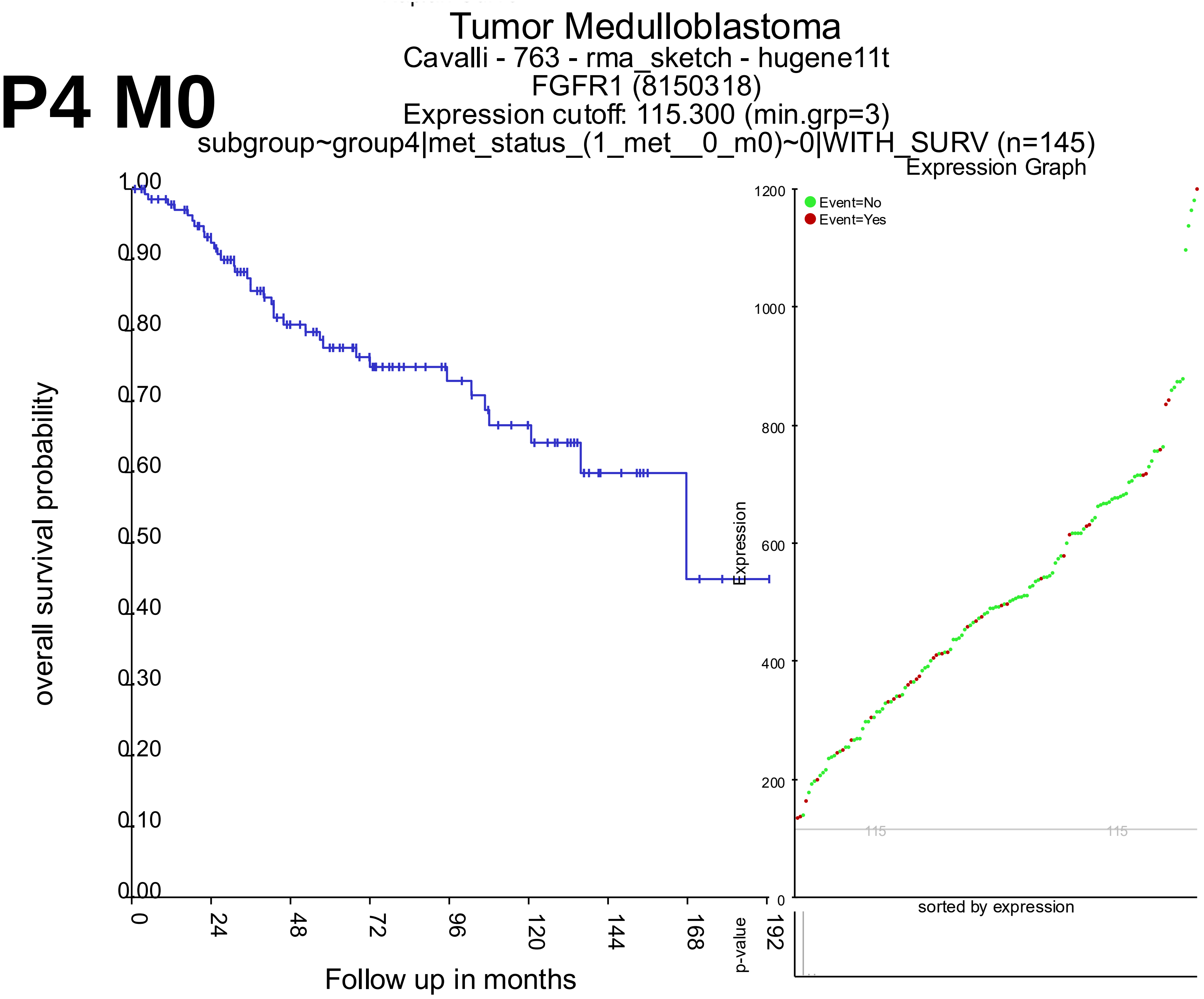

Tumor Medulloblastoma
Cavalli - 763 - rma_sketch - hugene11t
FGFR1 (8150318)
Expression cutoff: 115.300 (min.grp=3)
subgroup~group4|met_status_(1_met__0_m0)~0|WITH_SURV (n=145)
overall survival probability
1.00
0.90
0.80
0.70
0.60
0.50
0.40
0.30
0.20
0.10
0.00
0
24
48
72
96
120
144
168
192
Follow up in months
p-value
Expression Graph
Event=No
Event=Yes
Expression
1200
1000
800
600
400
200
0
115
115
sorted by expression

GROUP4 M1

Tumor Medulloblastoma
Cavalli - 763 - rma_sketch - hugene11t
FGFR1 (8150318)
Expression cutoff: 693.100 (min.grp=3)
subgroup~group4|met_status_(1_met__0_m0)~1|WITH_SURV (n=92)

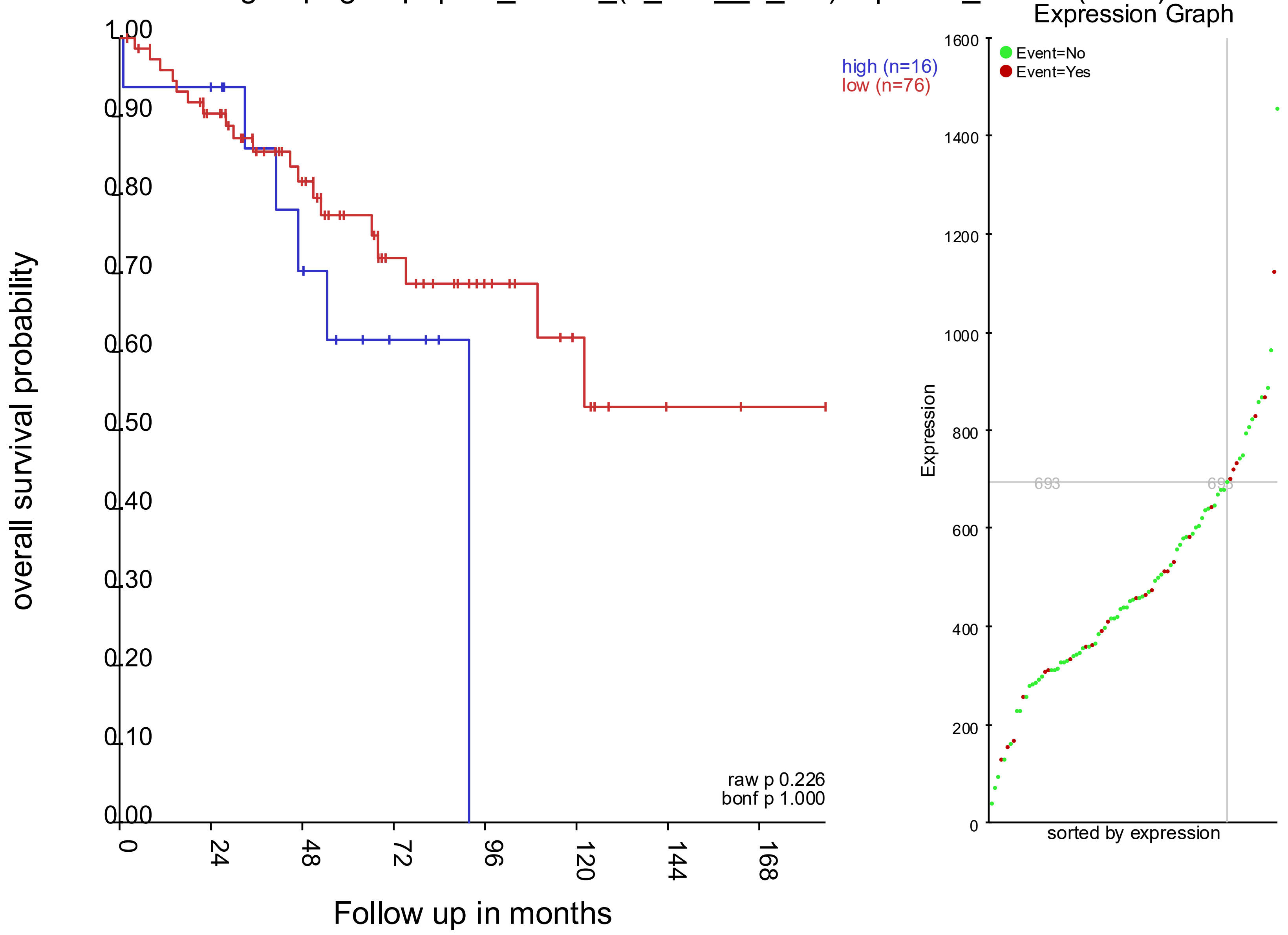

GROUP3 M0

Tumor Medulloblastoma
Cavalli - 763 - rma_sketch - hugene11t
FGFR1 (8150318)
Expression cutoff: 319.800 (min.grp=3)
subgroup~group3|met_status_(1_met__0_m0)~0|WITH_SURV (n=65)

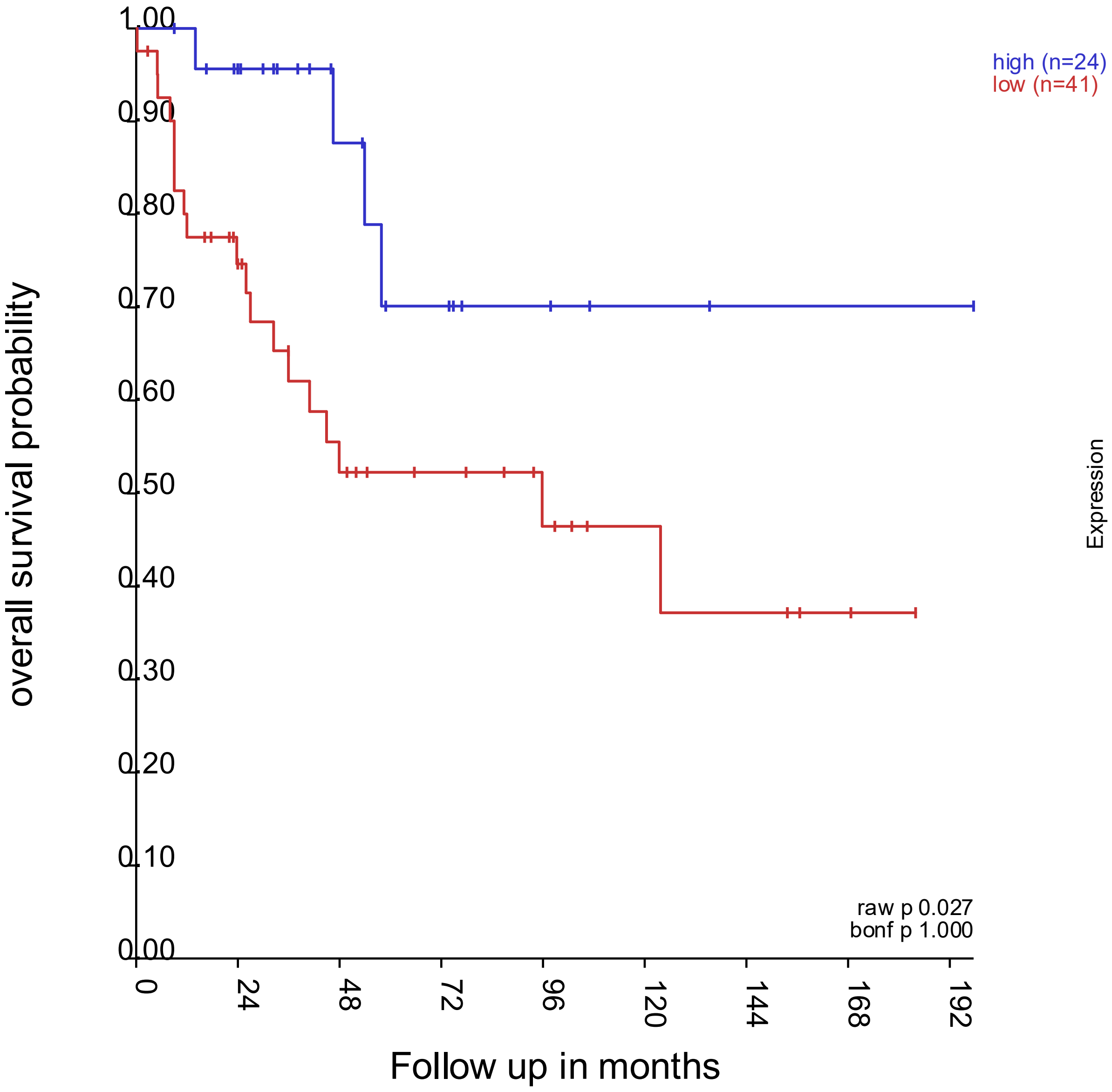

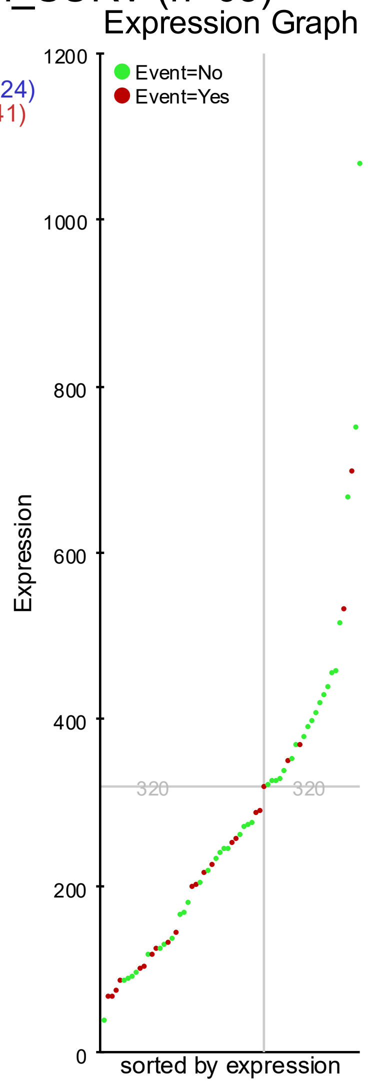

GROUP3 M1

Tumor Medulloblastoma
Cavalli - 763 - rma_sketch - hugene11t
FGFR1 (8150318)
Expression cutoff: 71.900 (min.grp=3)
subgroup~group3|met_status_(1_met__0_m0)~1|WITH_SURV (n=41)

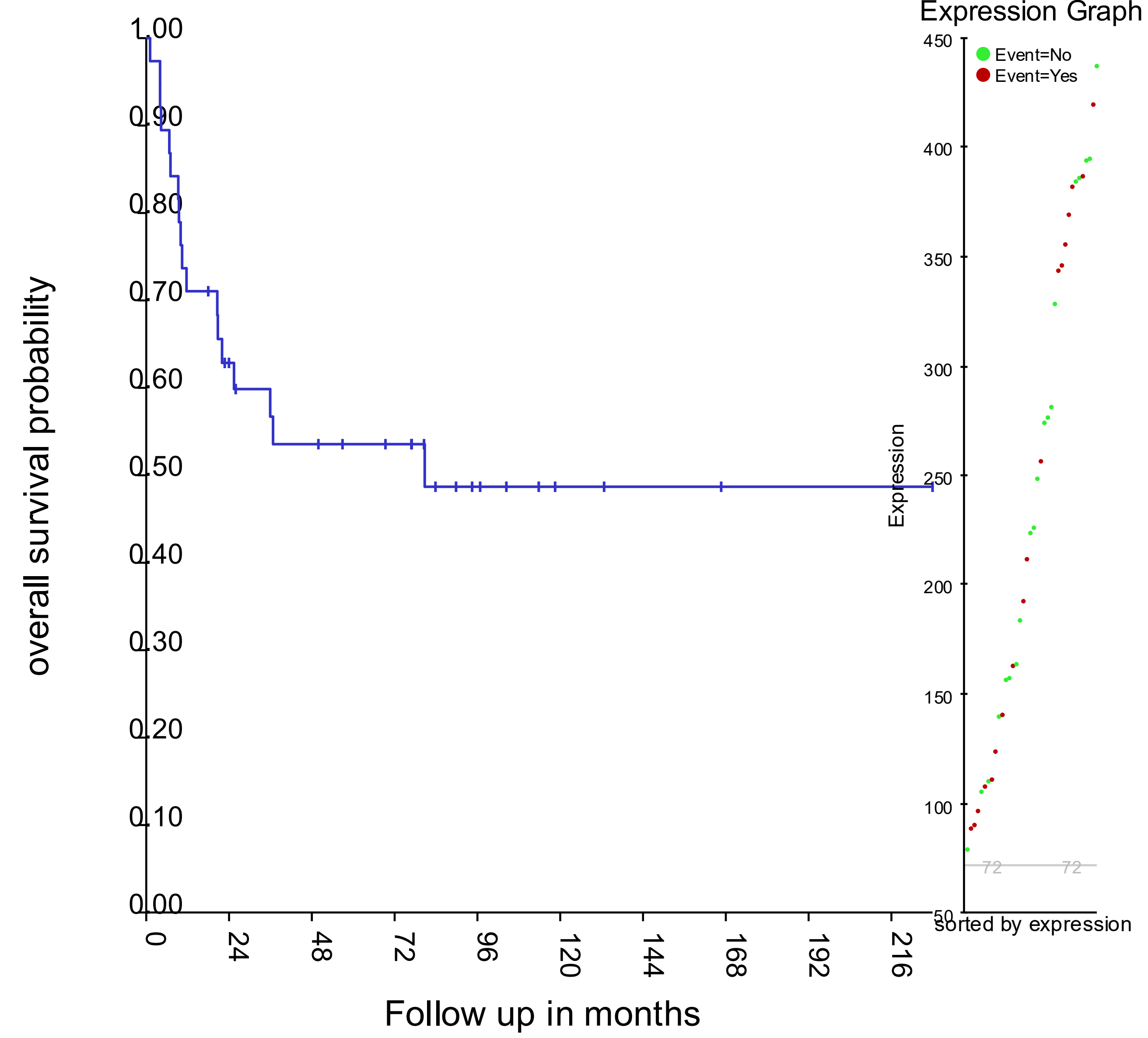

FGFR2

WNT M0

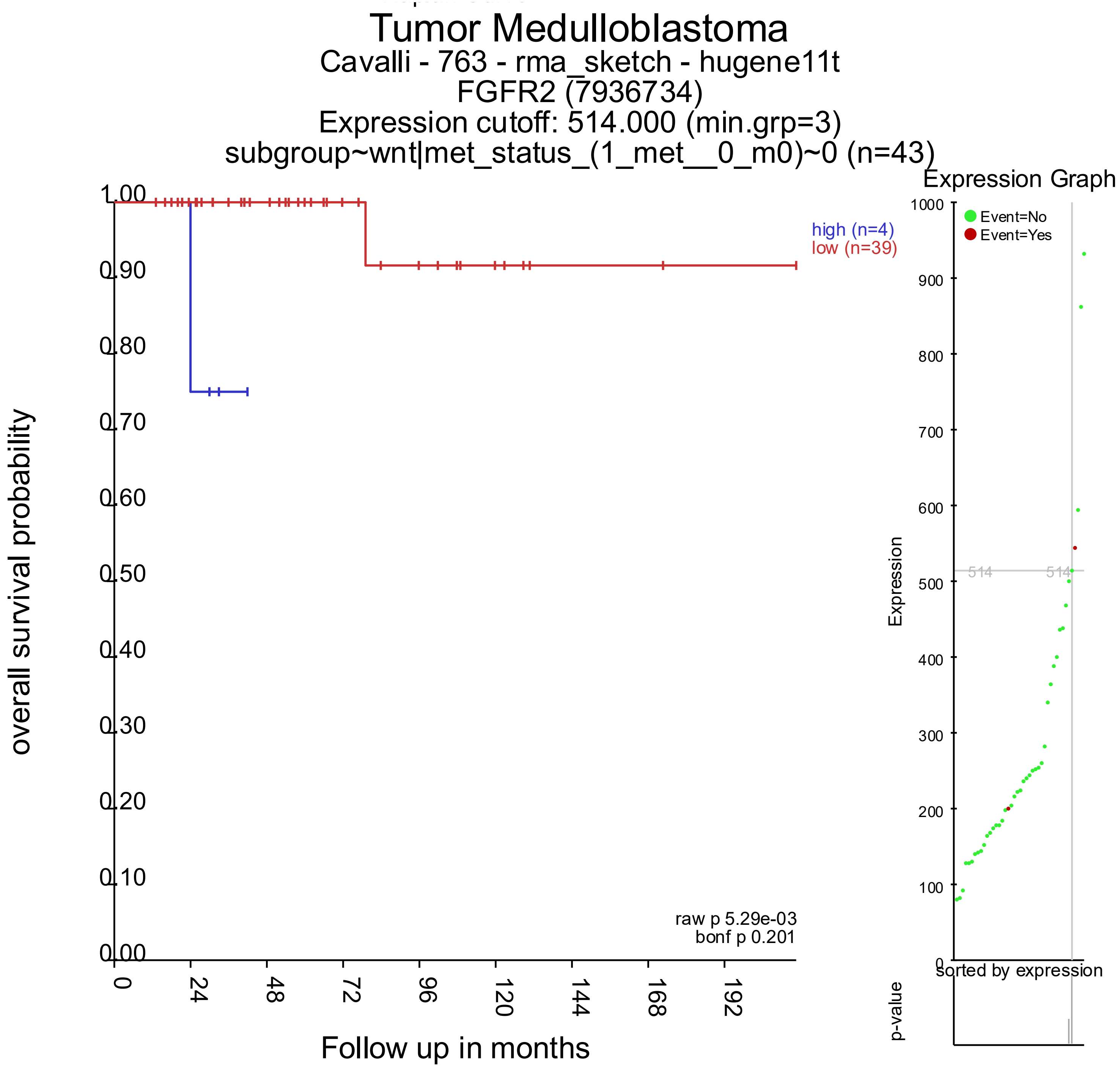
Tumor Medulloblastoma
Cavalli - 763 - rma_sketch - hugene11t
FGFR2 (7936734)
Expression cutoff: 514.000 (min.grp=3)
subgroup~wnt|met_status_(1_met__0_m0)~0 (n=43)
high (n=4)
low (n=39)
overall survival probability
1.00
0.90
0.80
0.70
0.60
0.50
0.40
0.30
0.20
0.10
0.00
0
24
48
72
96
120
144
168
192
Follow up in months
raw p 5.29e-03
bonf p 0.201
Expression Graph
Event=No
Event=Yes
Expression
1000
900
800
700
600
500
400
300
200
100
0
514
514
sorted by expression
p-value

WNT M1

Tumor Medulloblastoma
Cavalli - 763 - rma_sketch - hugene11t
FGFR2 (7936734)
Expression cutoff: 339.600 (min.grp=3)
subgroup~wnt|met_status_(1_met__0_m0)~1 (n=6)

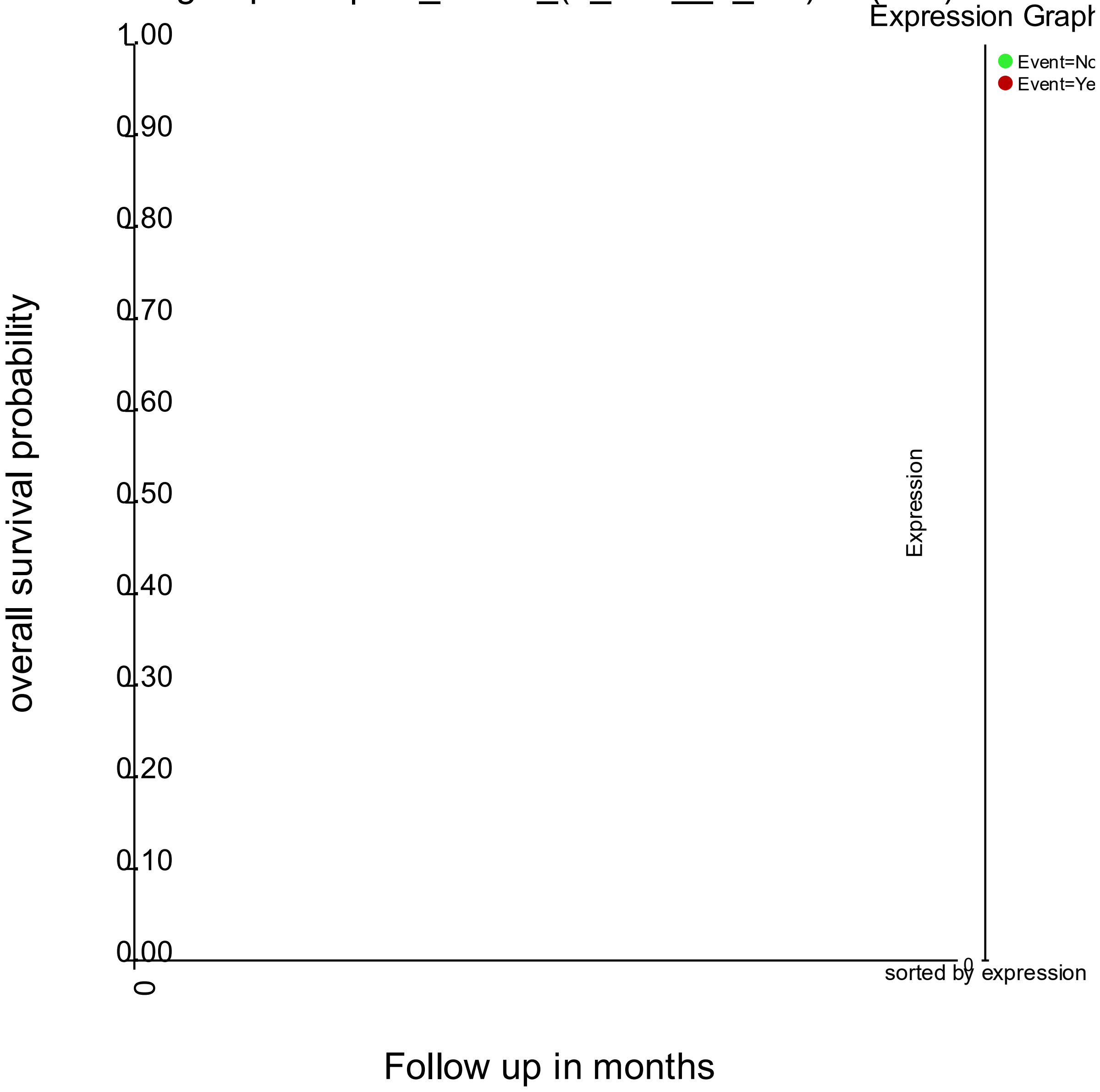

SHH M0

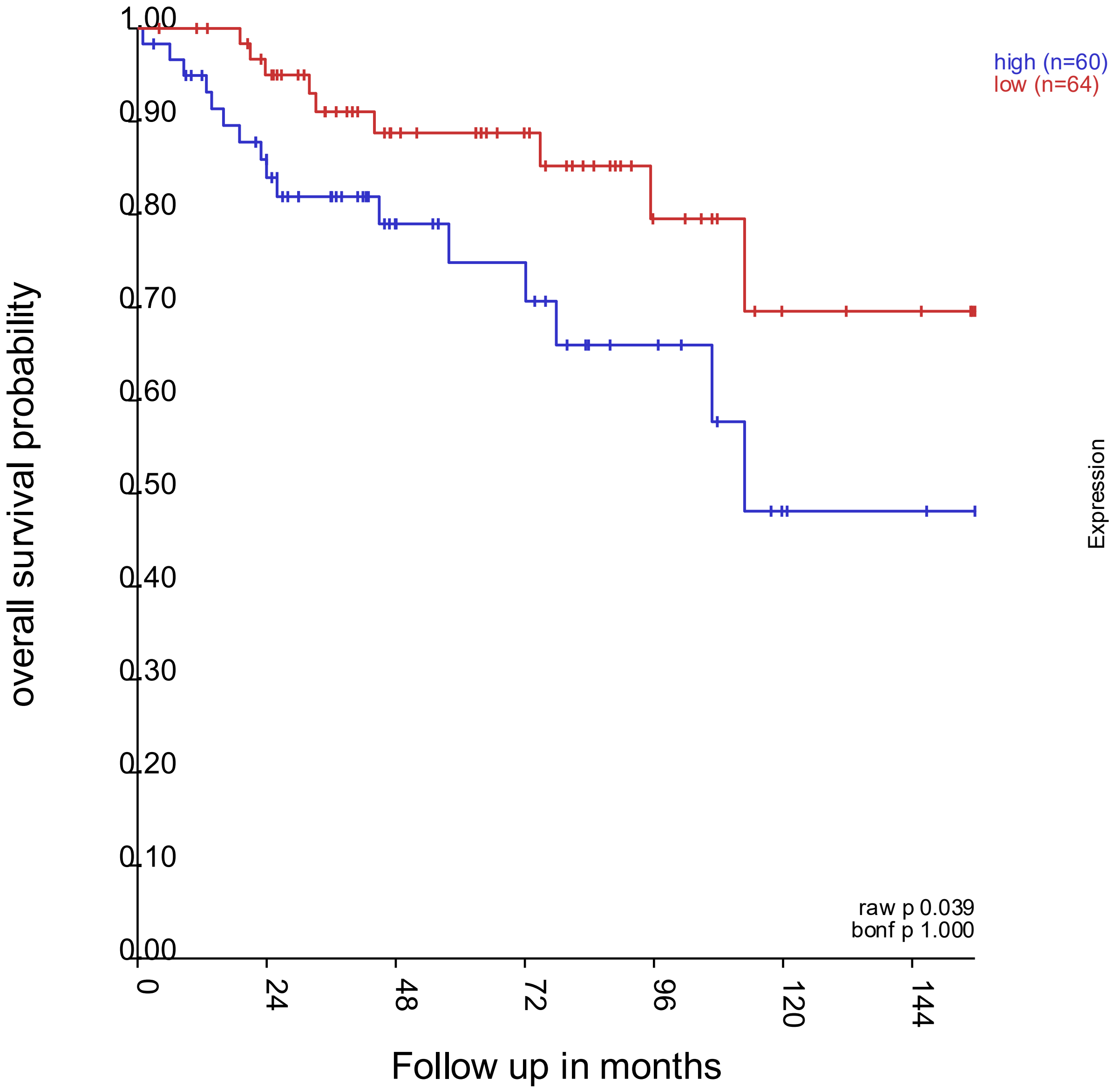

Tumor Medulloblastoma
Cavalli - 763 - rma_sketch - hugene11t
FGFR2 (7936734)
Expression cutoff: 108.800 (min.grp=3)
subgroup~shh|met_status_(1_met__0_m0)~0|WITH_SURV (n=124)
overall survival probability
1.00
0.90
0.80
0.70
0.60
0.50
0.40
0.30
0.20
0.10
0.00
high (n=60)
low (n=64)
raw p 0.039
bonf p 1.000
0
24
48
72
96
120
144
Follow up in months

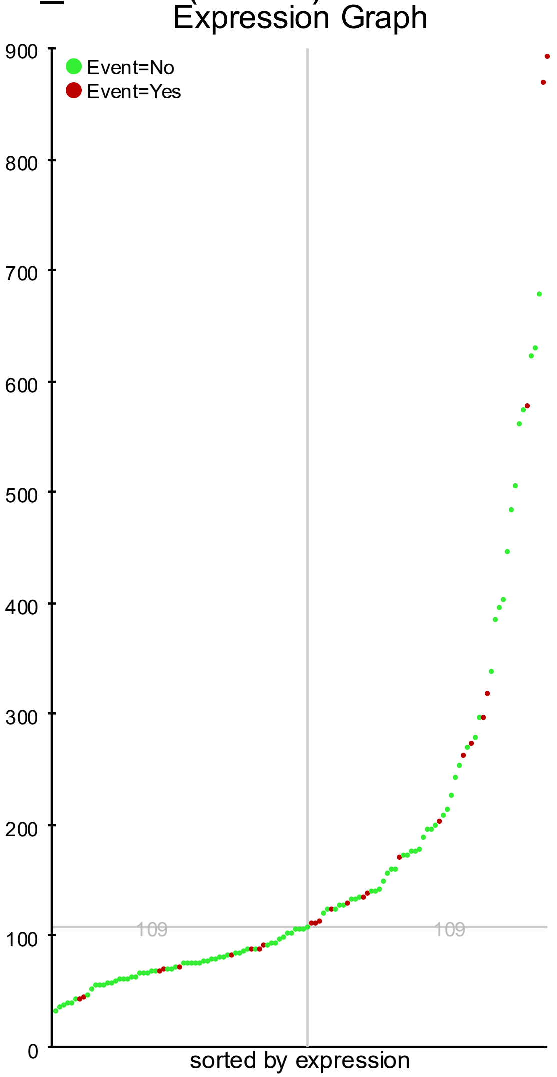

Expression Graph
Event=No
Event=Yes
Expression
900
800
700
600
500
400
300
200
100
0
109
109
sorted by expression

SHH M1

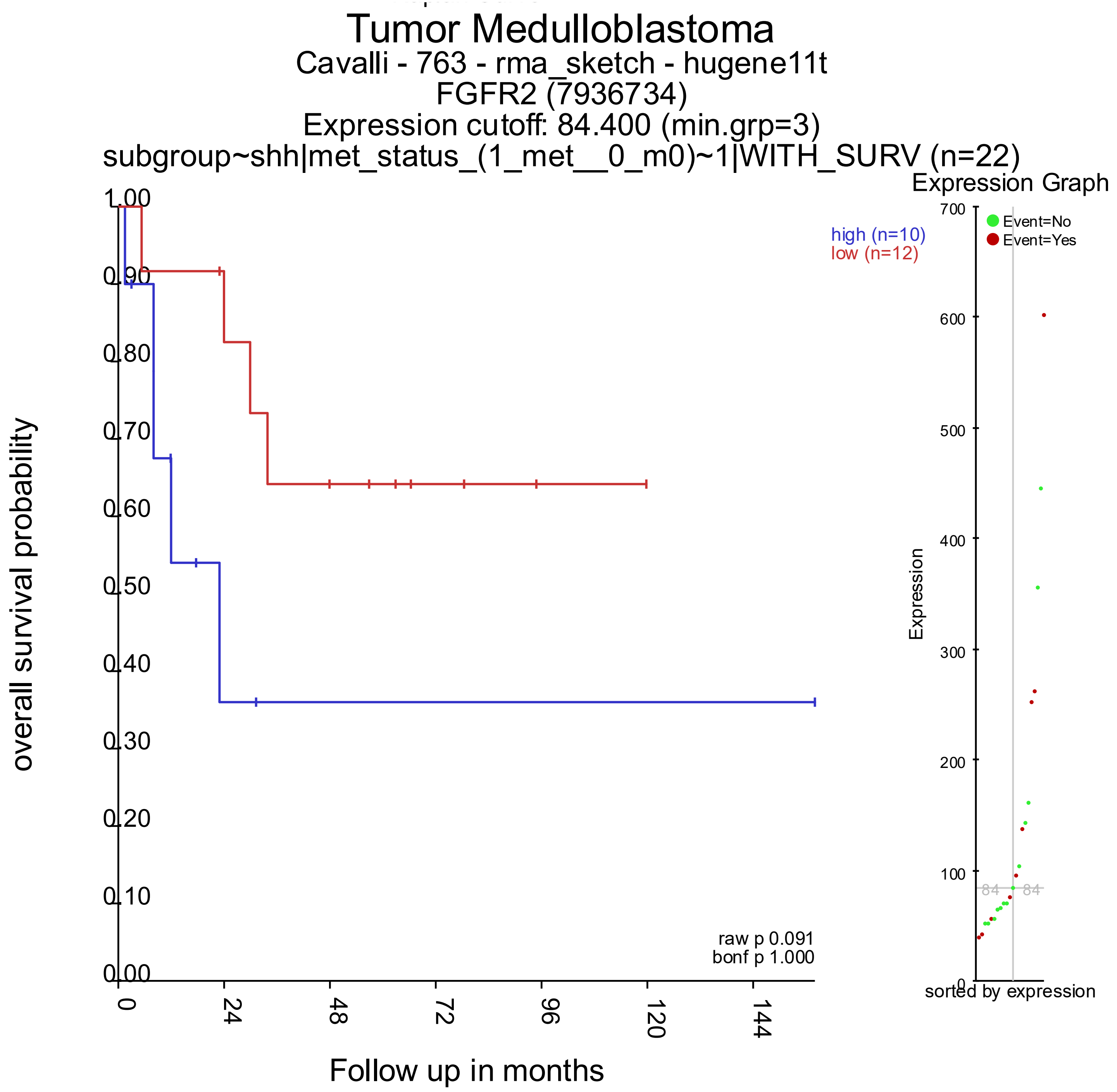

Tumor Medulloblastoma
Cavalli - 763 - rma_sketch - hugene11t
FGFR2 (7936734)
Expression cutoff: 84.400 (min.grp=3)
subgroup~shh|met_status_(1_met__0_m0)~1|WITH_SURV (n=22)
high (n=10)
low (n=12)
overall survival probability
1.00
0.90
0.80
0.70
0.60
0.50
0.40
0.30
0.20
0.10
0.00
0
24
48
72
96
120
144
Follow up in months
raw p 0.091
bonf p 1.000
Expression Graph
Event=No
Event=Yes
Expression
700
600
500
400
300
200
100
0
84
84
sorted by expression

GROUP4 M0

Tumor Medulloblastoma
Cavalli - 763 - rma_sketch - hugene11t
FGFR2 (7936734)
Expression cutoff: 35.000 (min.grp=3)
subgroup~group4|met_status_(1_met__0_m0)~0|WITH_SURV (n=145)

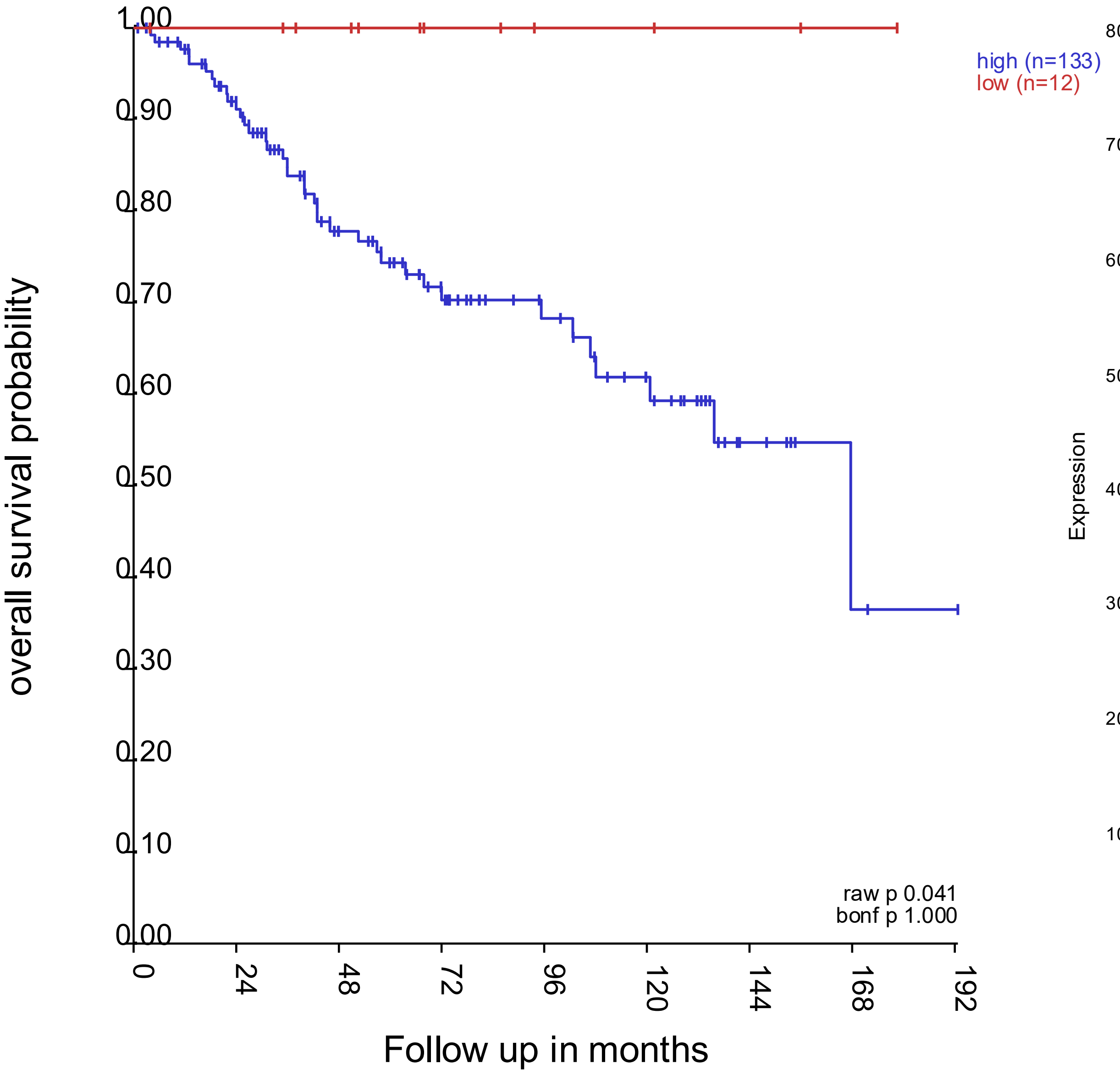

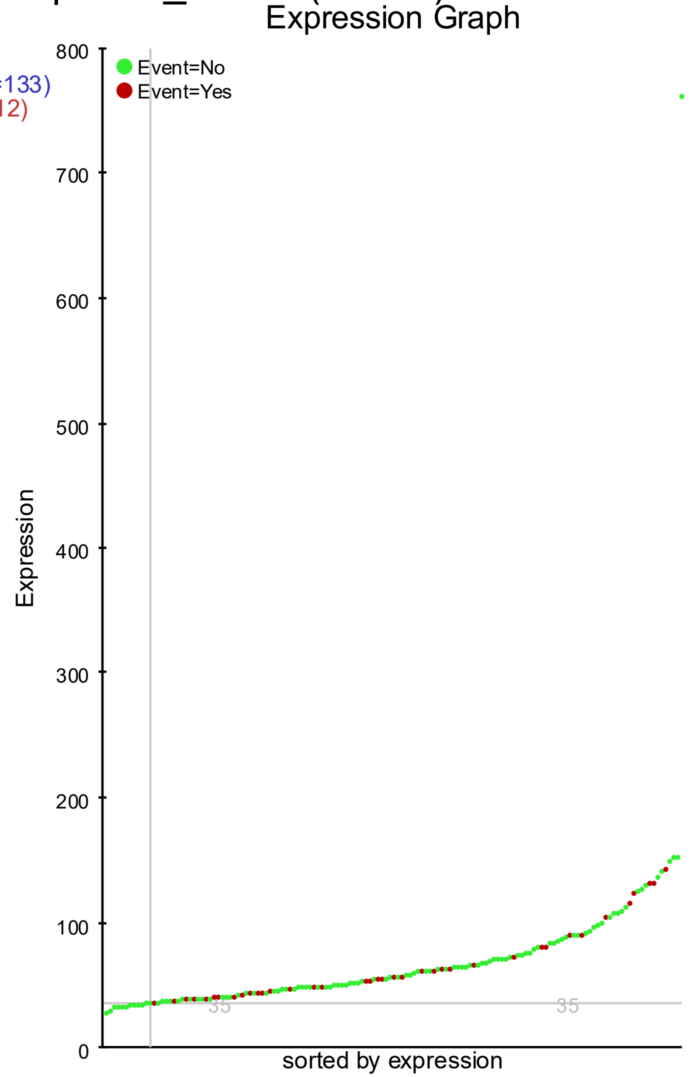

GROUP4 M1

Tumor Medulloblastoma
Cavalli - 763 - rma_sketch - hugene11t
FGFR2 (7936734)
Expression cutoff: 36.300 (min.grp=3)
subgroup~group4|met_status_(1_met__0_m0)~1|WITH_SURV (n=92)

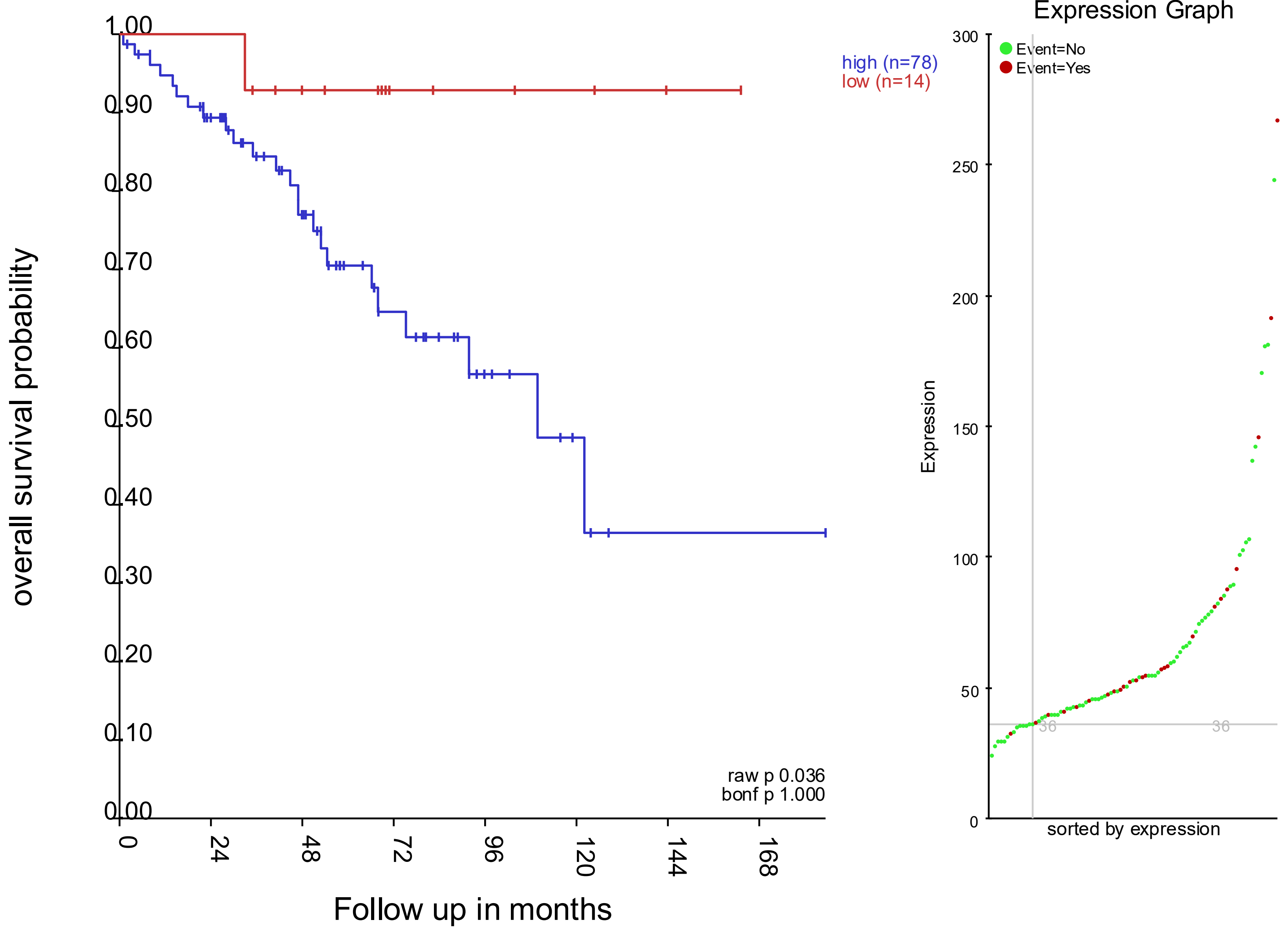

GROUP3 M0

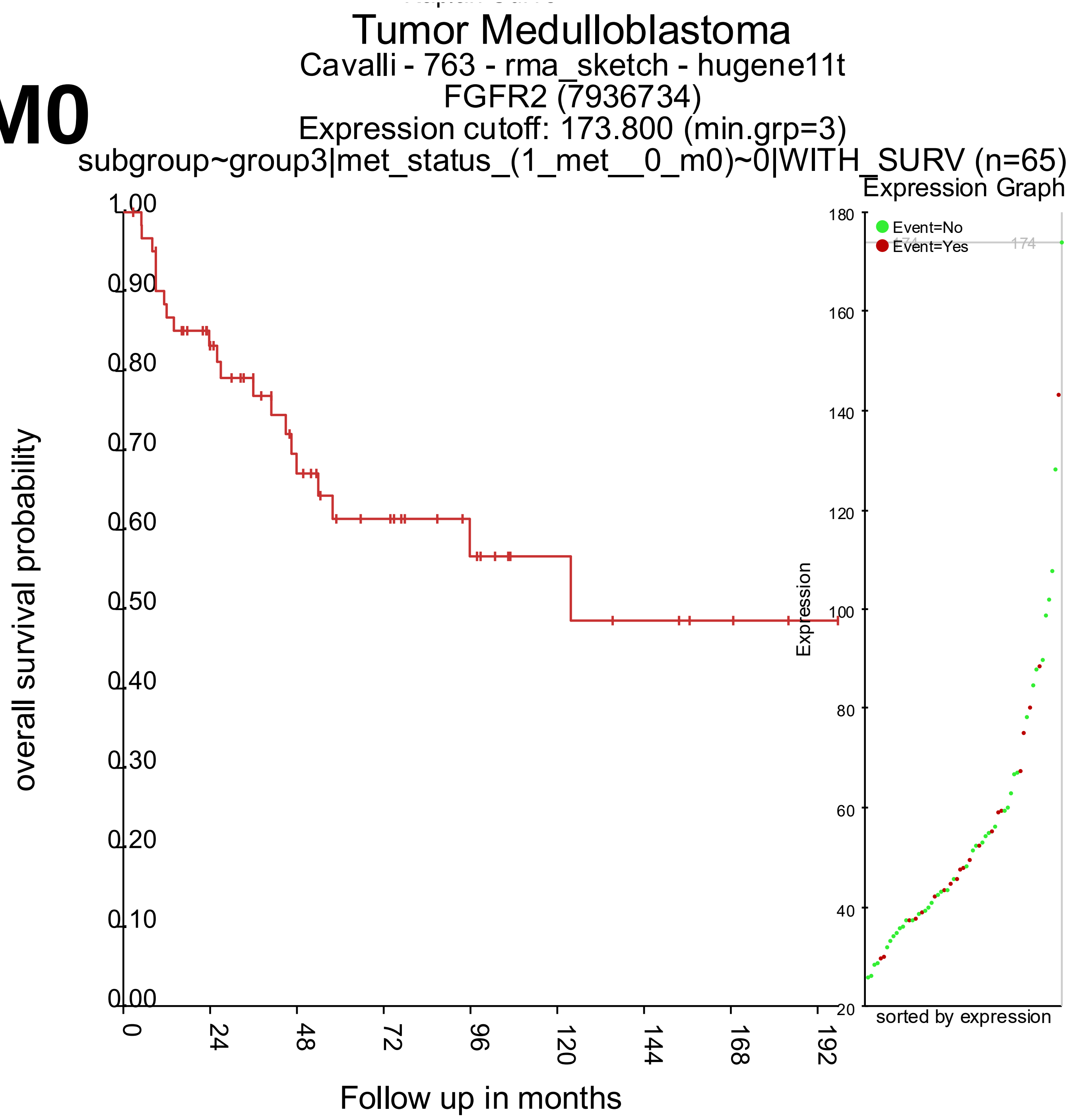

Tumor Medulloblastoma
Cavalli - 763 - rma_sketch - hugene11t
FGFR2 (7936734)
Expression cutoff: 173.800 (min.grp=3)
subgroup~group3|met_status_(1_met__0_m0)~0|WITH_SURV (n=65)
overall survival probability
1.00
0.90
0.80
0.70
0.60
0.50
0.40
0.30
0.20
0.10
0.00
0
24
48
72
96
120
144
168
192
Follow up in months
Expression Graph
Event=No
Event=Yes
174
180
160
140
120
100
80
60
40
20
Expression
sorted by expression

GROUP3 M1

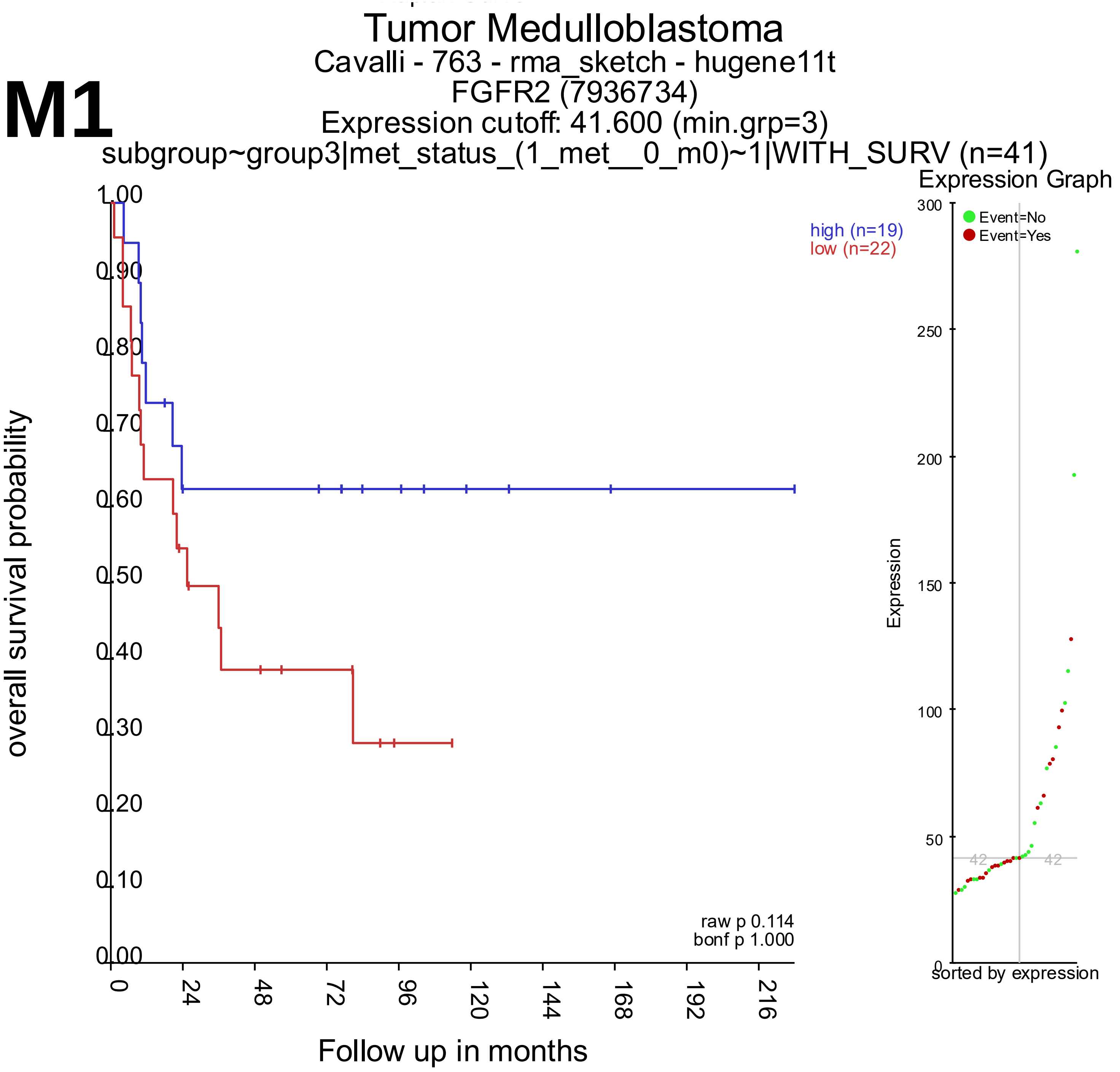

Tumor Medulloblastoma
Cavalli - 763 - rma_sketch - hugene11t
FGFR2 (7936734)
Expression cutoff: 41.600 (min.grp=3)
subgroup~group3|met_status_(1_met__0_m0)~1|WITH_SURV (n=41)
overall survival probability
1.00
0.90
0.80
0.70
0.60
0.50
0.40
0.30
0.20
0.10
0.00
high (n=19)
low (n=22)
raw p 0.114
bonf p 1.000
0
24
48
72
96
120
144
168
192
216
Follow up in months
Expression Graph
Event=No
Event=Yes
Expression
300
250
200
150
100
50
0
42
42
sorted by expression

FGFR3

WNT M0

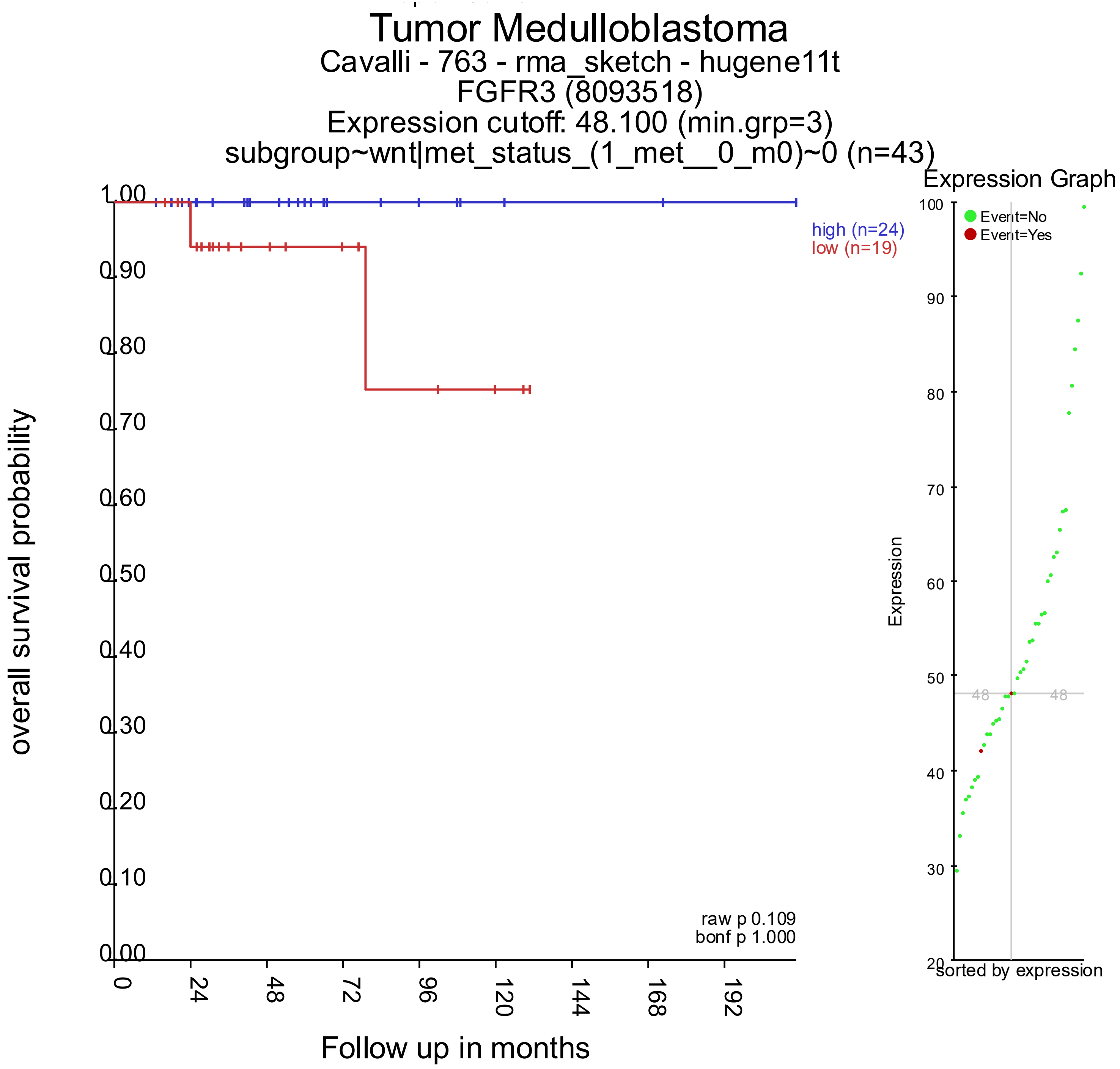

Tumor Medulloblastoma
Cavalli - 763 - rma_sketch - hugene11t
FGFR3 (8093518)
Expression cutoff: 48.100 (min.grp=3)
subgroup~wnt|met_status_(1_met__0_m0)~0 (n=43)
overall survival probability
1.00
0.90
0.80
0.70
0.60
0.50
0.40
0.30
0.20
0.10
0.00
high (n=24)
low (n=19)
raw p 0.109
bonf p 1.000
0
24
48
72
96
120
144
168
192
Follow up in months
Expression Graph
Event=No
Event=Yes
Expression
100
90
80
70
60
50
40
30
20
48
48
sorted by expression

WNT M1

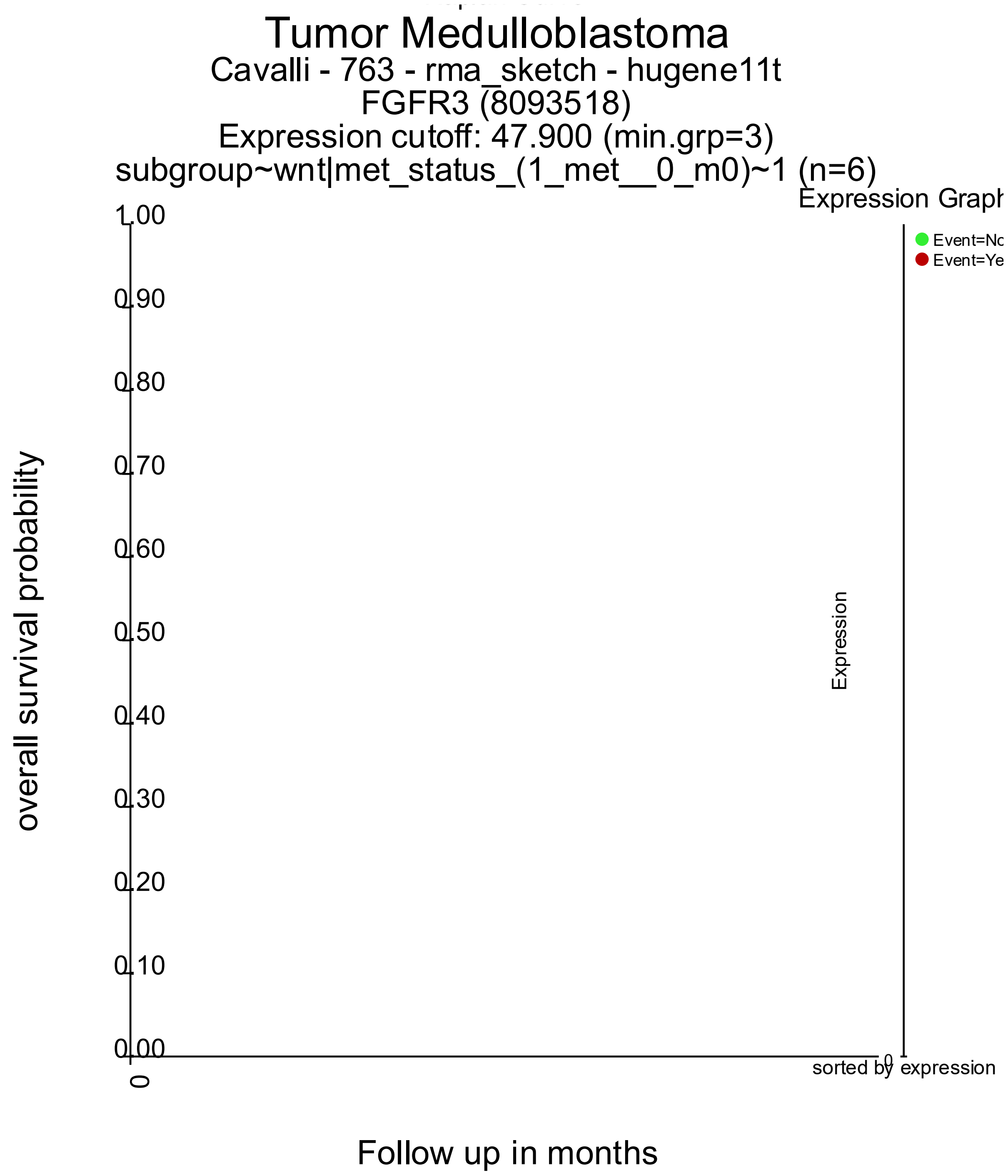

Tumor Medulloblastoma
Cavalli - 763 - rma_sketch - hugene11t
FGFR3 (8093518)
Expression cutoff: 47.900 (min.grp=3)
subgroup~wnt|met_status_(1_met__0_m0)~1 (n=6)
Expression Graph
Event=No
Event=Ye
1.00
0.90
0.80
0.70
0.60
0.50
0.40
0.30
0.20
0.10
0.00
0
overall survival probability
Follow up in months
Expression
0
sorted by expression

SHH M0

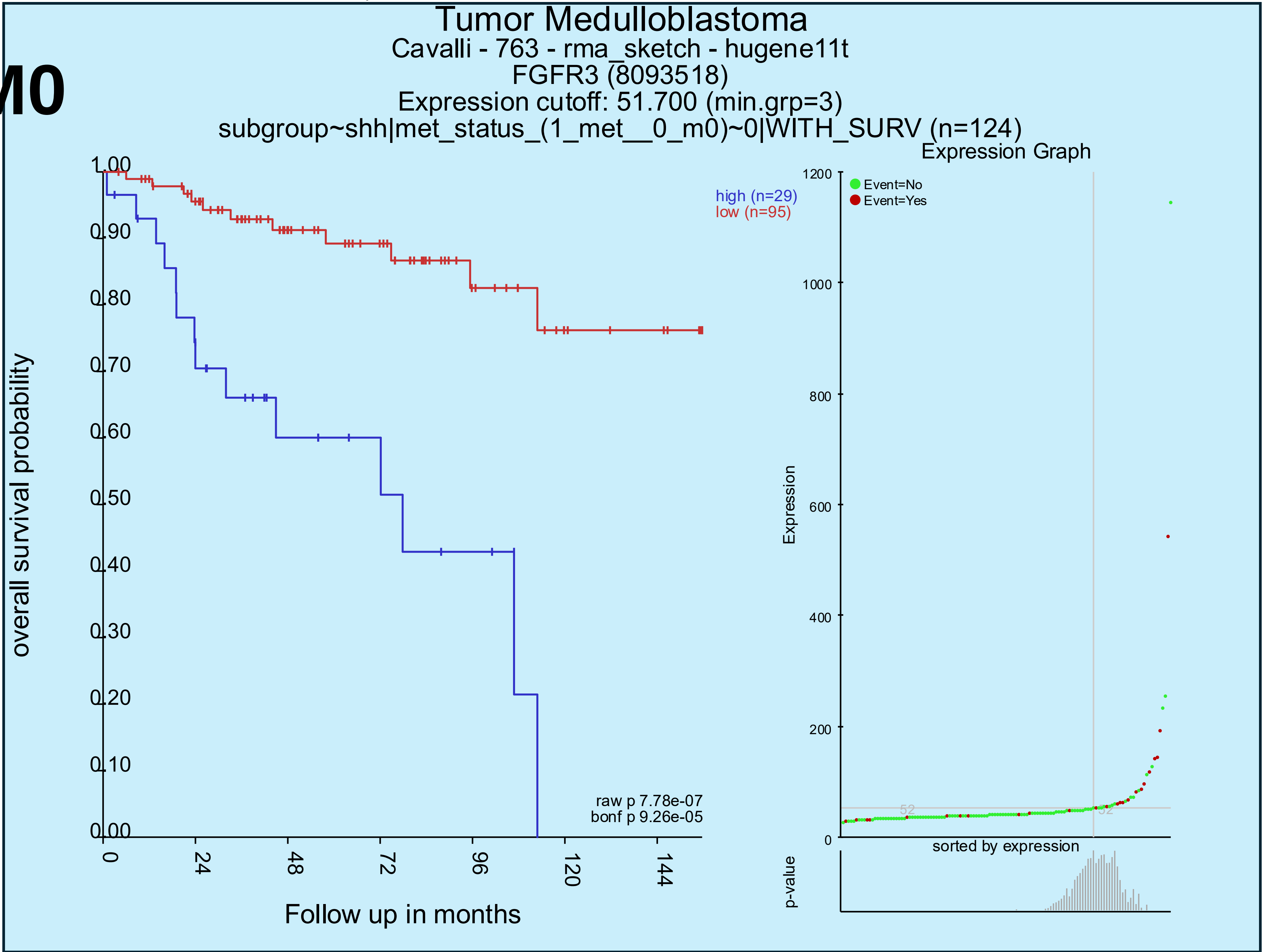

Tumor Medulloblastoma
Cavalli - 763 - rma_sketch - hugene11t
FGFR3 (8093518)
Expression cutoff: 51.700 (min.grp=3)
subgroup~shh|met_status_(1_met__0_m0)~0|WITH_SURV (n=124)
overall survival probability
1.00
0.90
0.80
0.70
0.60
0.50
0.40
0.30
0.20
0.10
0.00
0
24
48
72
96
120
144
Follow up in months
high (n=29)
low (n=95)
raw p 7.78e-07
bonf p 9.26e-05
Expression Graph
Event=No
Event=Yes
Expression
1200
1000
800
600
400
200
0
52
sorted by expression
p-value

SHH M1

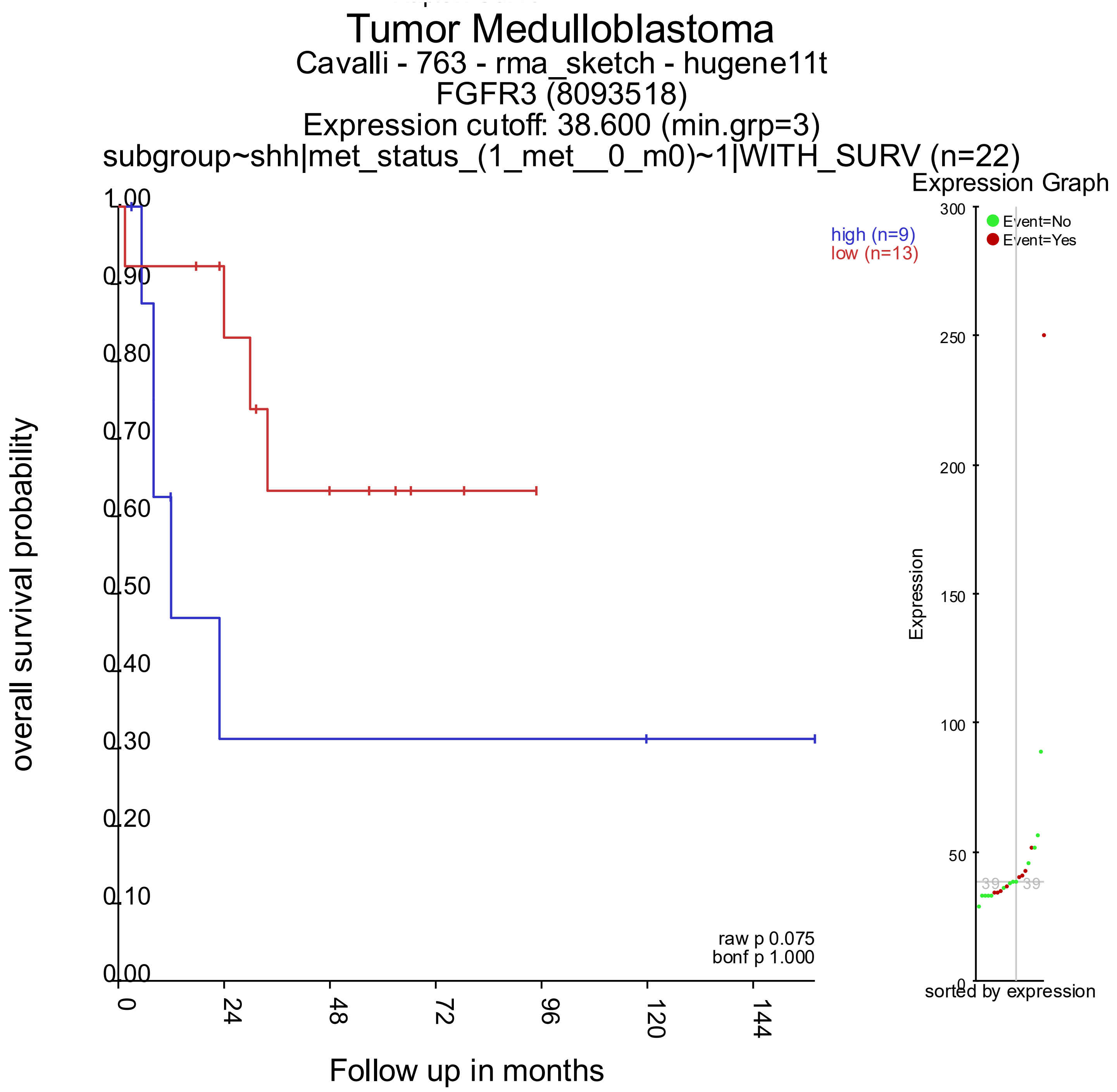

Tumor Medulloblastoma
Cavalli - 763 - rma_sketch - hugene11t
FGFR3 (8093518)
Expression cutoff: 38.600 (min.grp=3)
subgroup~shh|met_status_(1_met__0_m0)~1|WITH_SURV (n=22)
overall survival probability
1.00
0.90
0.80
0.70
0.60
0.50
0.40
0.30
0.20
0.10
0.00
0
24
48
72
96
120
144
Follow up in months
high (n=9)
low (n=13)
raw p 0.075
bonf p 1.000
Expression Graph
Event=No
Event=Yes
Expression
300
250
200
150
100
50
0
39
39
sorted by expression

GROUP4 M0

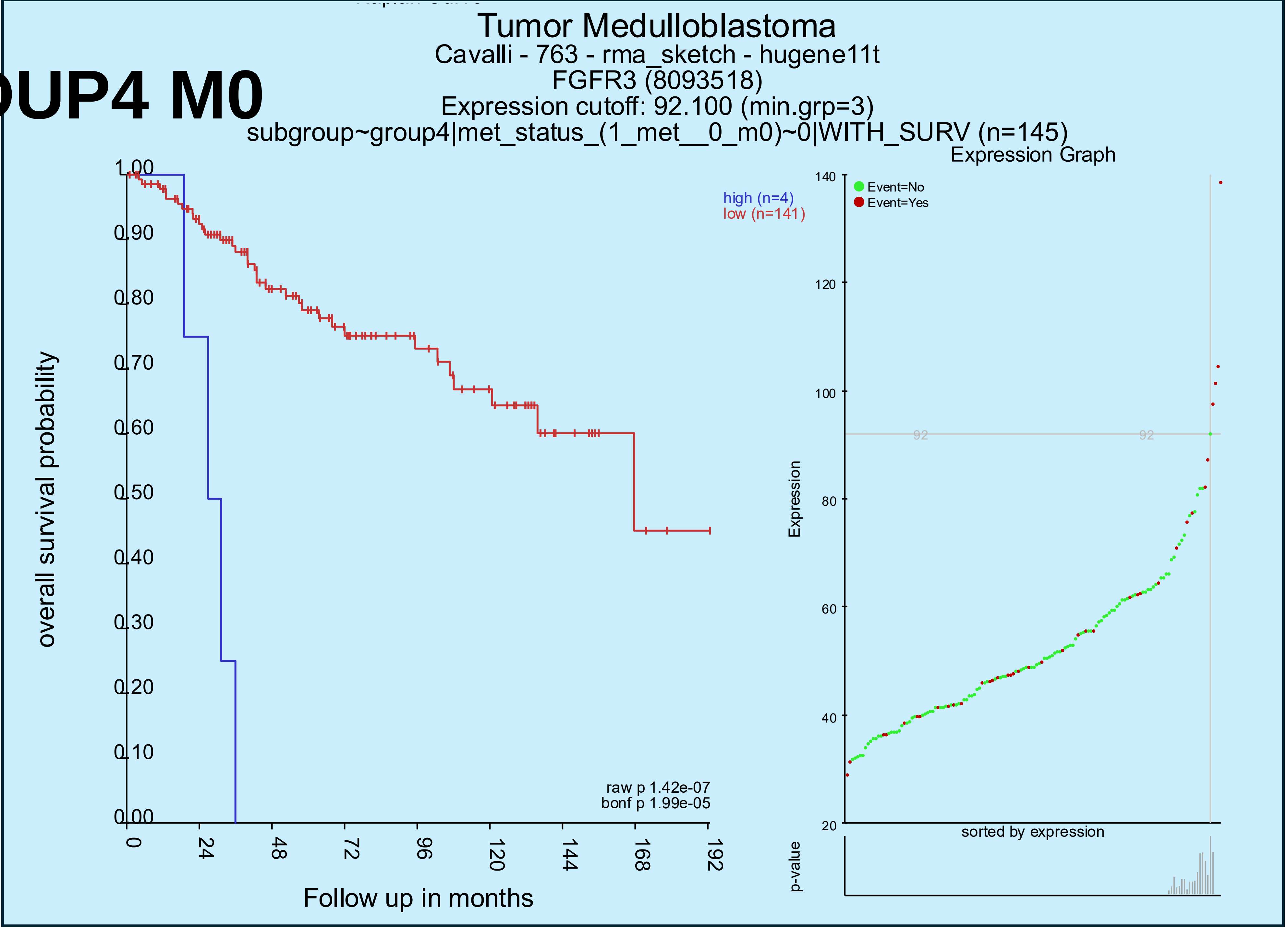

Tumor Medulloblastoma
Cavalli - 763 - rma_sketch - hugene11t
FGFR3 (8093518)
Expression cutoff: 92.100 (min.grp=3)
subgroup~group4|met_status_(1_met__0_m0)~0|WITH_SURV (n=145)
overall survival probability
1.00
0.90
0.80
0.70
0.60
0.50
0.40
0.30
0.20
0.10
0.00
0
24
48
72
96
120
144
168
192
Follow up in months
high (n=4)
low (n=141)
raw p 1.42e-07
bonf p 1.99e-05
Expression Graph
Event=No
Event=Yes
Expression
140
120
100
80
60
40
20
92
92
sorted by expression
p-value

GROUP4 M1

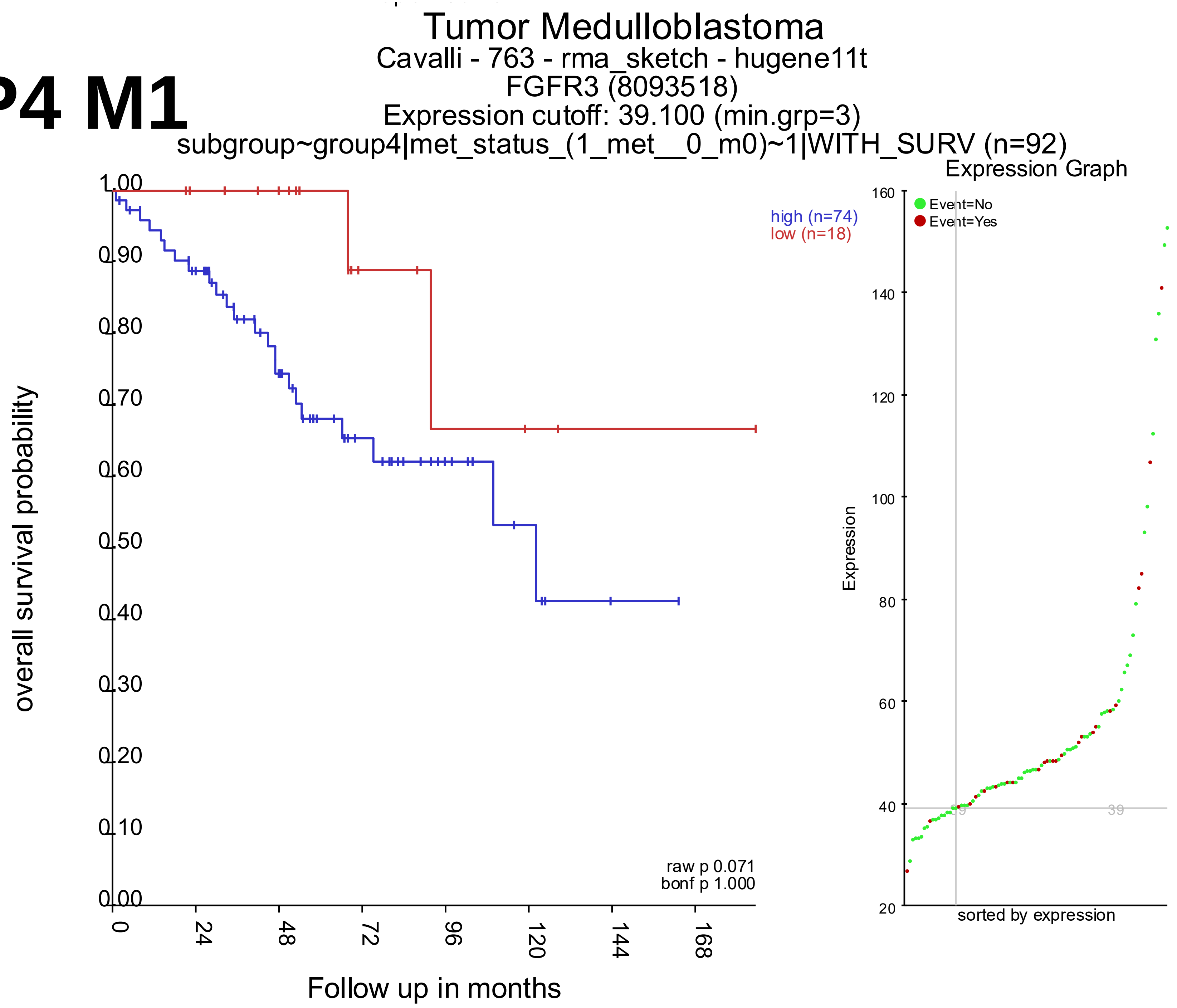

Tumor Medulloblastoma
Cavalli - 763 - rma_sketch - hugene11t
FGFR3 (8093518)
Expression cutoff: 39.100 (min.grp=3)
subgroup~group4|met_status_(1_met__0_m0)~1|WITH_SURV (n=92)
overall survival probability
1.00
0.90
0.80
0.70
0.60
0.50
0.40
0.30
0.20
0.10
0.00
0
24
48
72
96
120
144
168
Follow up in months
high (n=74)
low (n=18)
raw p 0.071
bonf p 1.000
Expression Graph
Event=No
Event=Yes
Expression
160
140
120
100
80
60
40
20
39
sorted by expression

GROUP3 M0

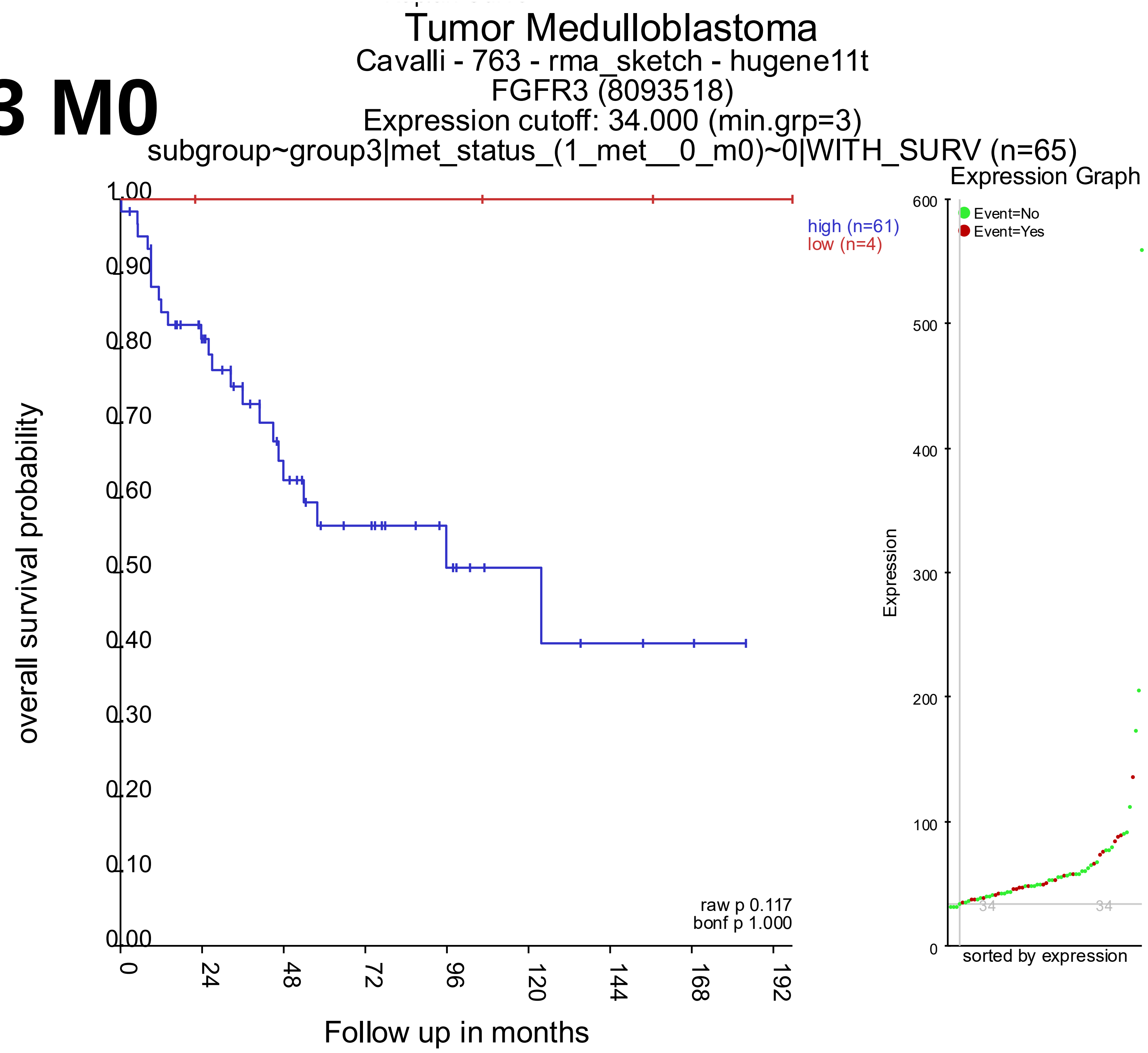

Tumor Medulloblastoma
Cavalli - 763 - rma_sketch - hugene11t
FGFR3 (8093518)
Expression cutoff: 34.000 (min.grp=3)
subgroup~group3|met_status_(1_met__0_m0)~0|WITH_SURV (n=65)
overall survival probability
1.00
0.90
0.80
0.70
0.60
0.50
0.40
0.30
0.20
0.10
0.00
high (n=61)
low (n=4)
raw p 0.117
bonf p 1.000
0
24
48
72
96
120
144
168
192
Follow up in months
Expression Graph
Event=No
Event=Yes
Expression
600
500
400
300
200
100
0
34
34
sorted by expression

GROUP3 M1

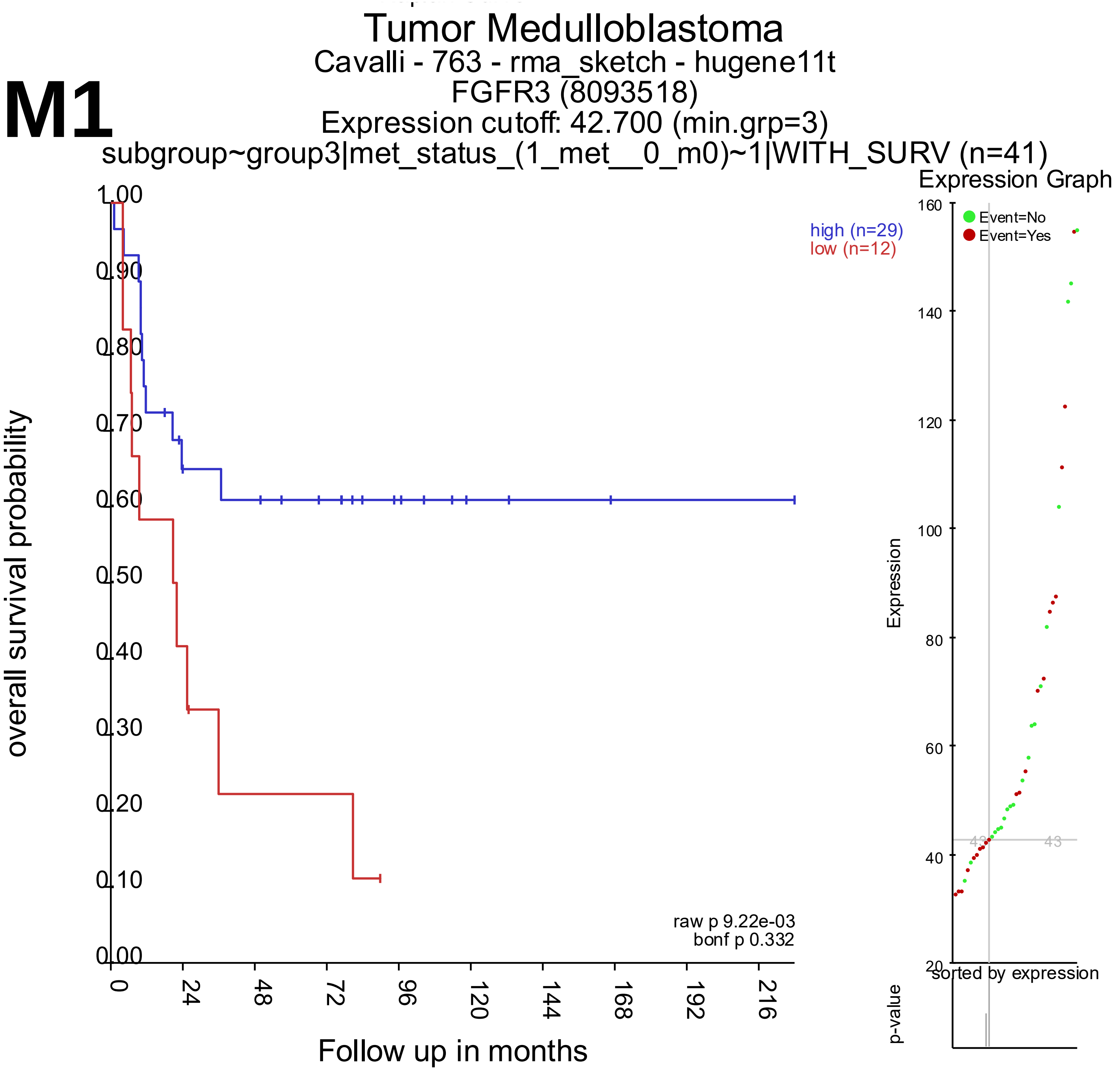

Tumor Medulloblastoma
Cavalli - 763 - rma_sketch - hugene11t
FGFR3 (8093518)
Expression cutoff: 42.700 (min.grp=3)
subgroup~group3|met_status_(1_met__0_m0)~1|WITH_SURV (n=41)
overall survival probability
1.00
0.90
0.80
0.70
0.60
0.50
0.40
0.30
0.20
0.10
0.00
0
24
48
72
96
120
144
168
192
216
Follow up in months
high (n=29)
low (n=12)
raw p 9.22e-03
bonf p 0.332
Expression Graph
Event=No
Event=Yes
Expression
160
140
120
100
80
60
40
20
43
sorted by expression
p-value

FLT3

WNT M0

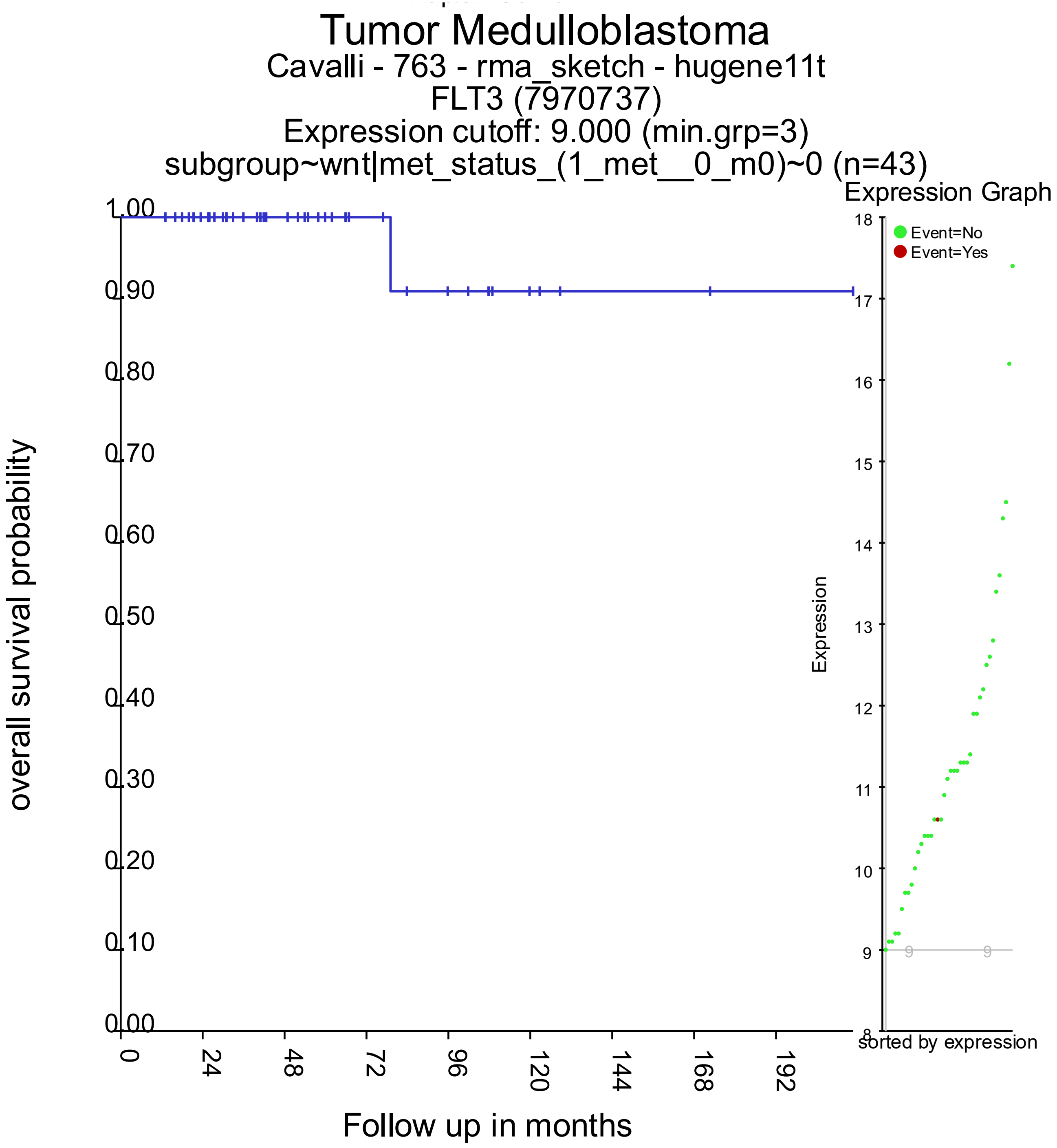
Tumor Medulloblastoma
Cavalli - 763 - rma_sketch - hugene11t
FLT3 (7970737)
Expression cutoff: 9.000 (min.grp=3)
subgroup~wnt|met_status_(1_met__0_m0)~0 (n=43)
overall survival probability
1.00
0.90
0.80
0.70
0.60
0.50
0.40
0.30
0.20
0.10
0.00
0
24
48
72
96
120
144
168
192
Follow up in months
Expression Graph
Event=No
Event=Yes
Expression
18
17
16
15
14
13
12
11
10
9
8
sorted by expression

WNT M1

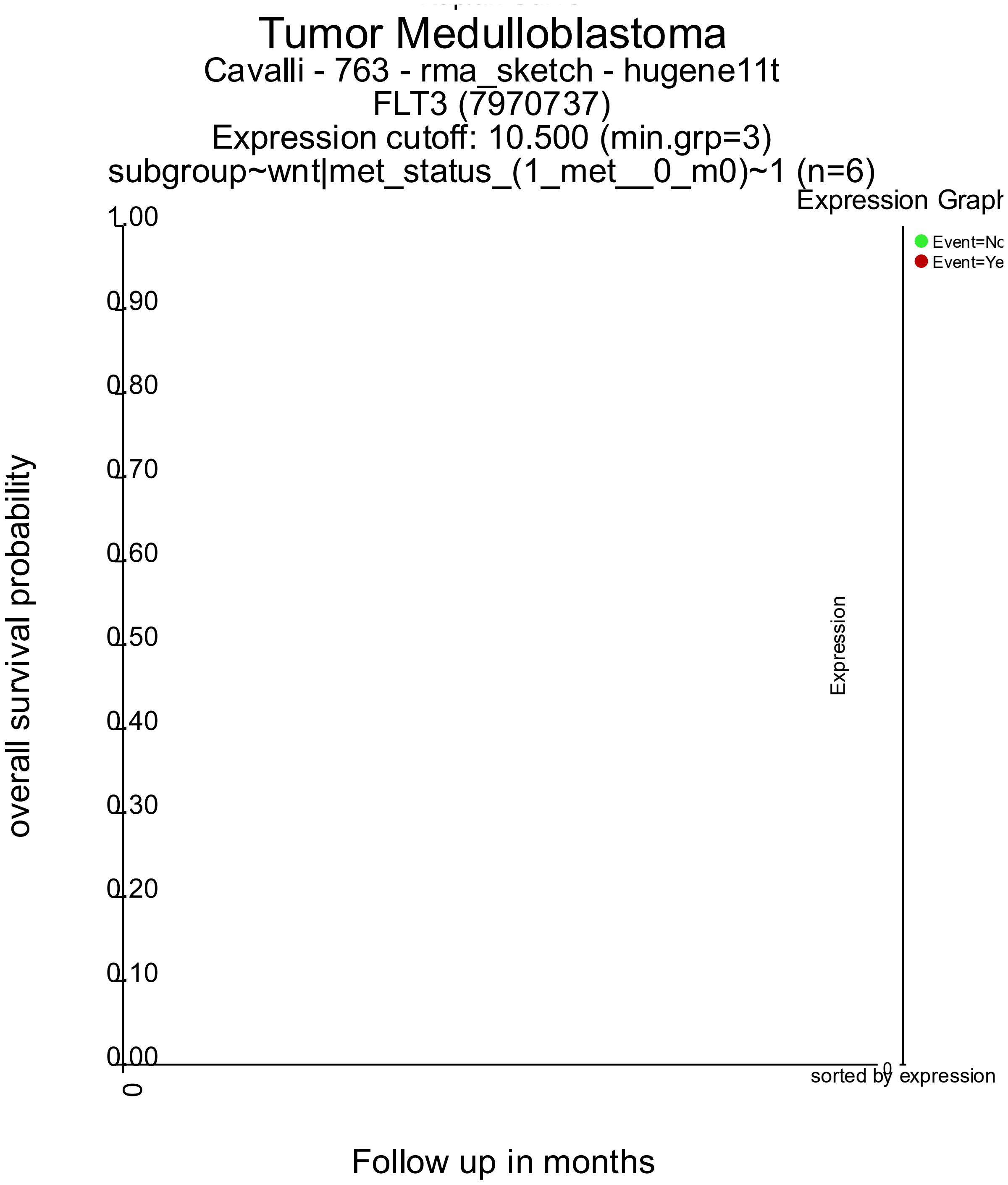

Tumor Medulloblastoma
Cavalli - 763 - rma_sketch - hugene11t
FLT3 (7970737)
Expression cutoff: 10.500 (min.grp=3)
subgroup~wnt|met_status_(1_met__0_m0)~1 (n=6)
Expression Graph
Event=No
Event=Yes
1.00
0.90
0.80
0.70
0.60
0.50
0.40
0.30
0.20
0.10
0.00
0
overall survival probability
Follow up in months
Expression
sorted by expression
0

SHH M0

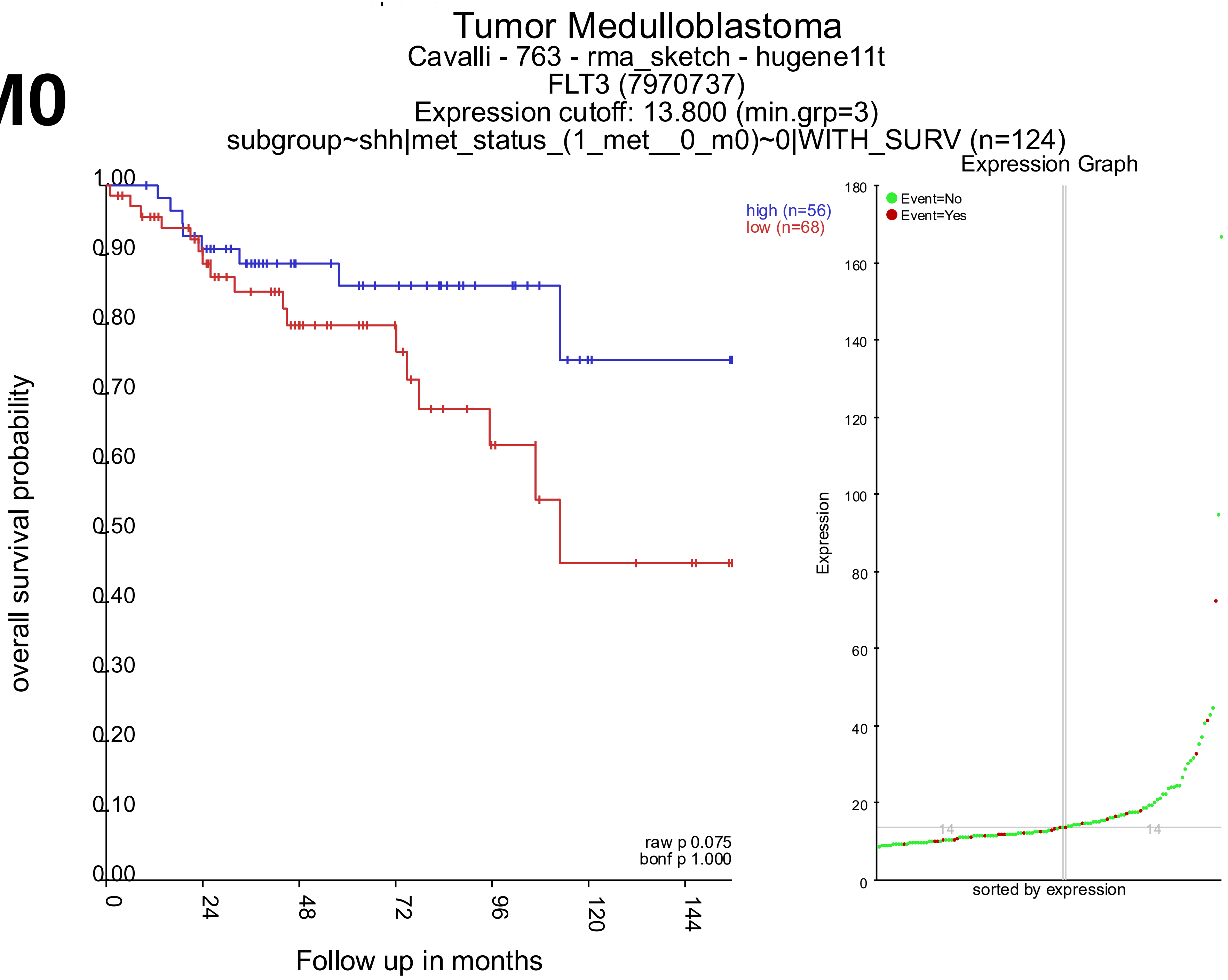

Tumor Medulloblastoma
Cavalli - 763 - rma_sketch - hugene11t
FLT3 (7970737)
Expression cutoff: 13.800 (min.grp=3)
subgroup~shh|met_status_(1_met__0_m0)~0|WITH_SURV (n=124)
overall survival probability
1.00
0.90
0.80
0.70
0.60
0.50
0.40
0.30
0.20
0.10
0.00
0
24
48
72
96
120
144
Follow up in months
high (n=56)
low (n=68)
raw p 0.075
bonf p 1.000
Expression Graph
Event=No
Event=Yes
Expression
180
160
140
120
100
80
60
40
20
0
14
sorted by expression

SHH M1

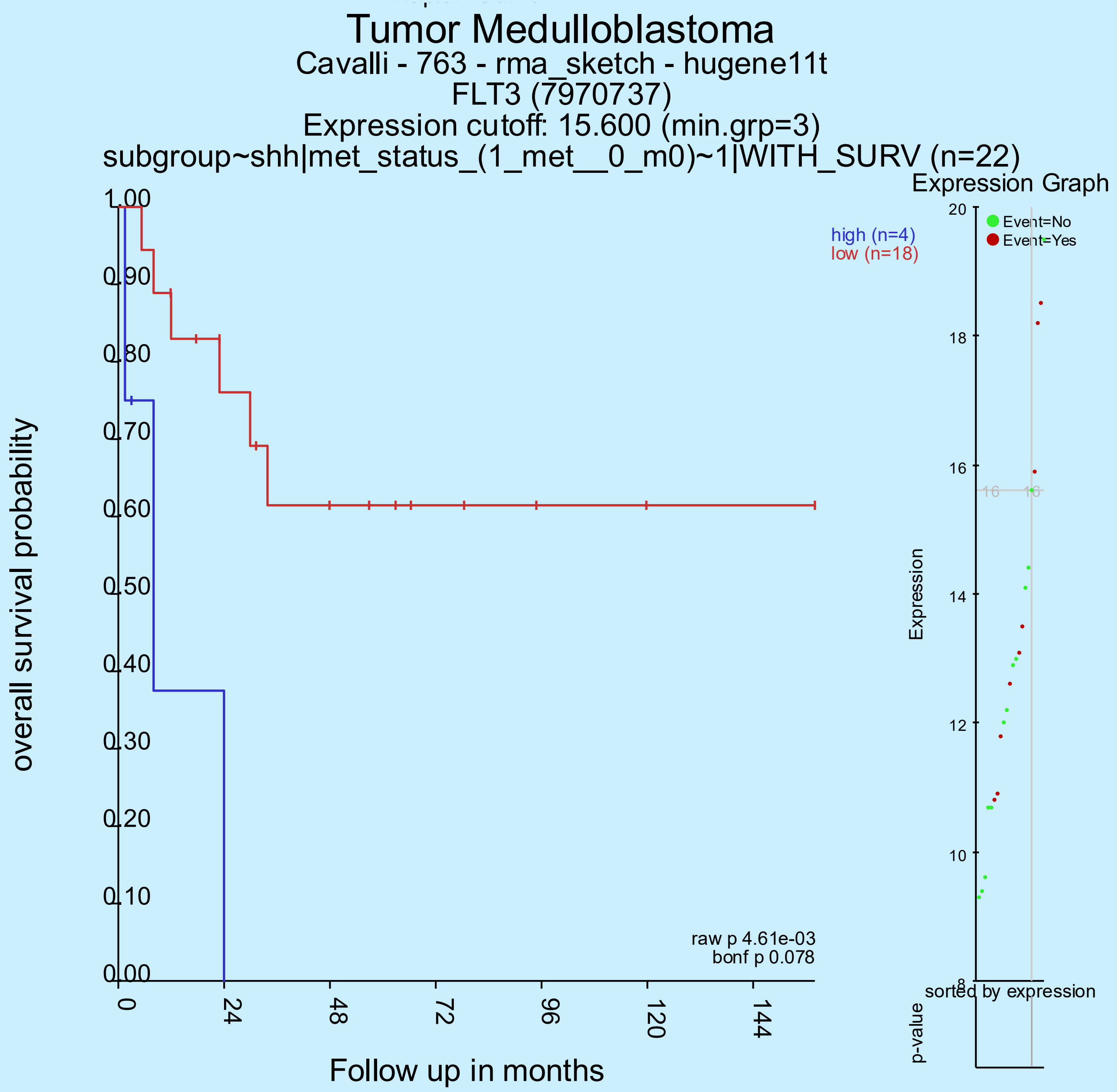
Tumor Medulloblastoma
Cavalli - 763 - rma_sketch - hugene11t
FLT3 (7970737)
Expression cutoff: 15.600 (min.grp=3)
subgroup~shh|met_status_(1_met__0_m0)~1|WITH_SURV (n=22)
overall survival probability
1.00
0.90
0.80
0.70
0.60
0.50
0.40
0.30
0.20
0.10
0.00
0
24
48
72
96
120
144
Follow up in months
high (n=4)
low (n=18)
raw p 4.61e-03
bonf p 0.078
Expression Graph
Event=No
Event=Yes
Expression
20
18
16
14
12
10
8
sorted by expression
p-value

GROUP4 M0

Tumor Medulloblastoma
Cavalli - 763 - rma_sketch - hugene11t
FLT3 (7970737)
Expression cutoff: 23.800 (min.grp=3)
subgroup~group4|met_status_(1_met__0_m0)~0|WITH_SURV (n=145)

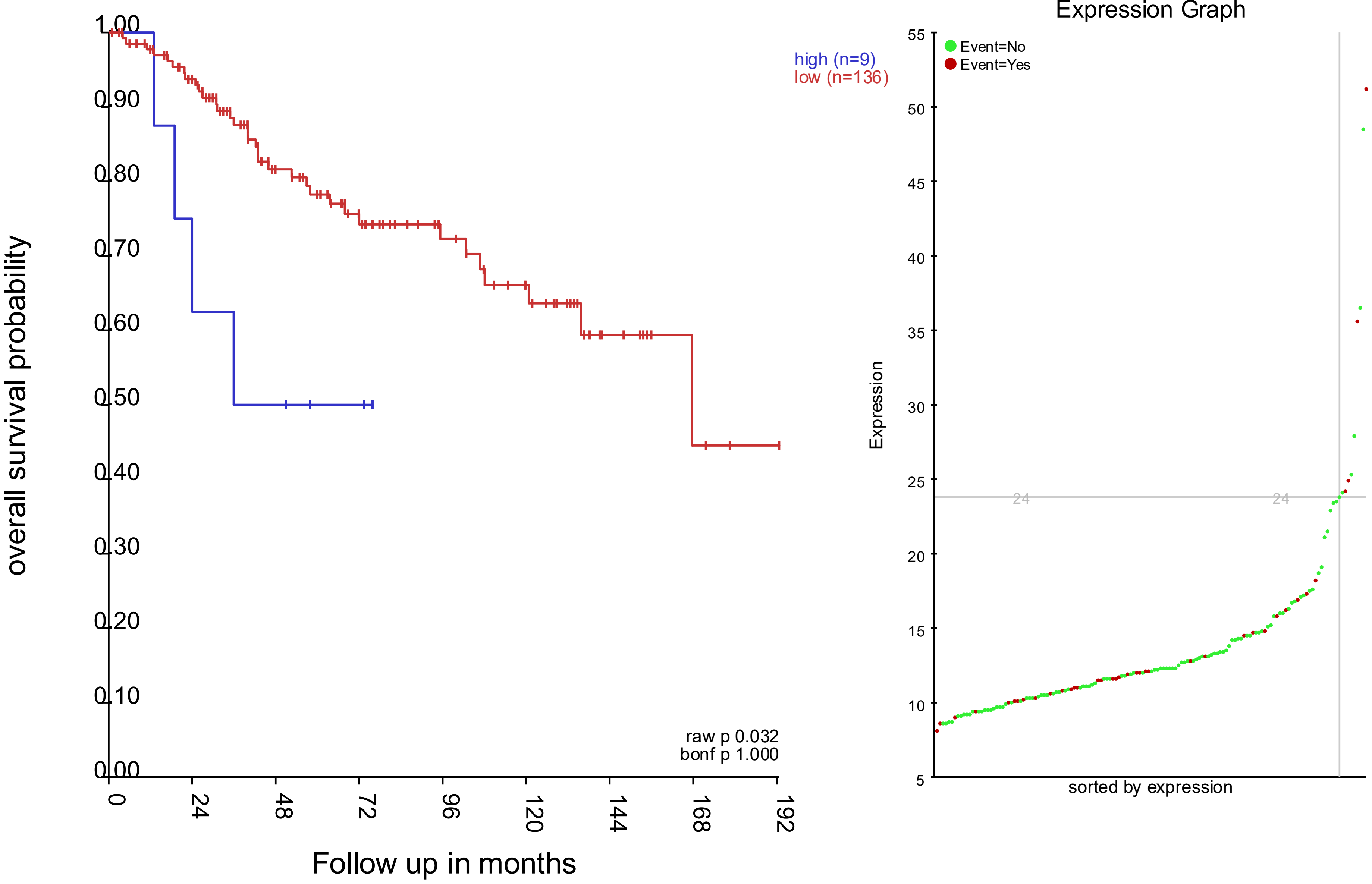

GROUP4 M1

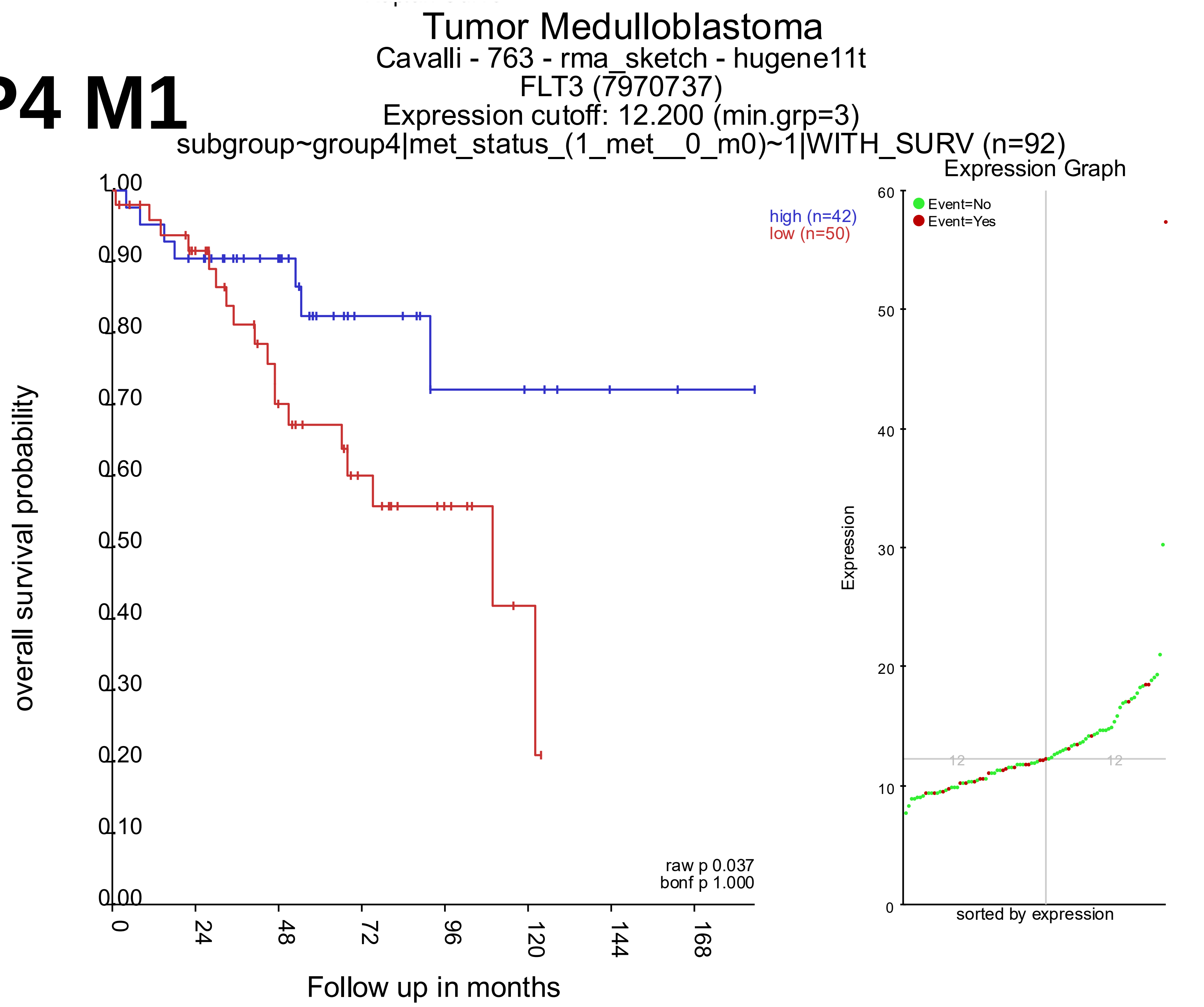

Tumor Medulloblastoma
Cavalli - 763 - rma_sketch - hugene11t
FLT3 (7970737)
Expression cutoff: 12.200 (min.grp=3)
subgroup~group4|met_status_(1_met__0_m0)~1|WITH_SURV (n=92)
overall survival probability
1.00
0.90
0.80
0.70
0.60
0.50
0.40
0.30
0.20
0.10
0.00
high (n=42)
low (n=50)
raw p 0.037
bonf p 1.000
0
24
48
72
96
120
144
168
Follow up in months
Expression Graph
Event=No
Event=Yes
Expression
60
50
40
30
20
10
0
12
12
sorted by expression

GROUP3 M0

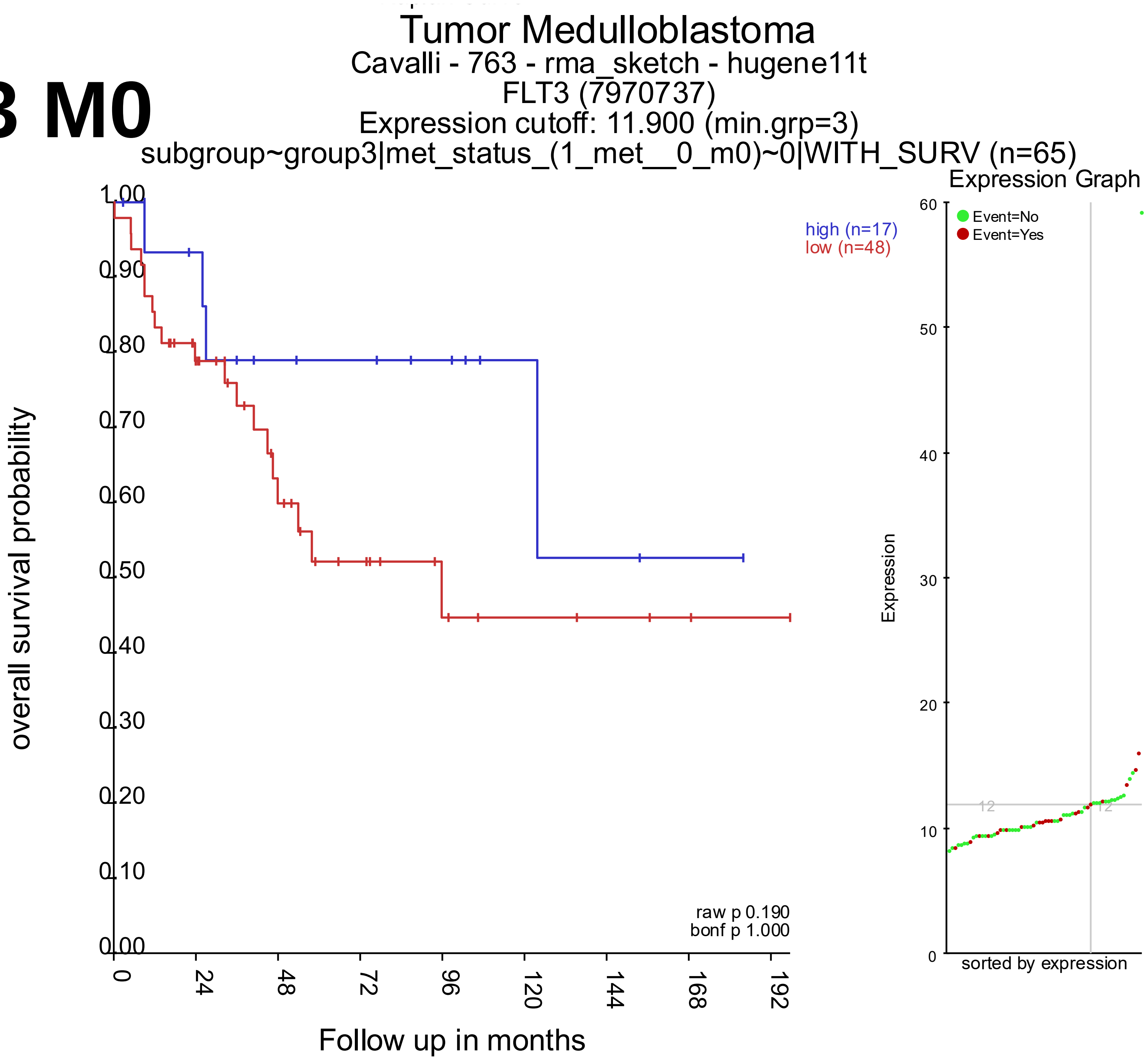

Tumor Medulloblastoma
Cavalli - 763 - rma_sketch - hugene11t
FLT3 (7970737)
Expression cutoff: 11.900 (min.grp=3)
subgroup~group3|met_status_(1_met__0_m0)~0|WITH_SURV (n=65)
overall survival probability
1.00
0.90
0.80
0.70
0.60
0.50
0.40
0.30
0.20
0.10
0.00
0
24
48
72
96
120
144
168
192
Follow up in months
high (n=17)
low (n=48)
raw p 0.190
bonf p 1.000
Expression Graph
Event=No
Event=Yes
Expression
60
50
40
30
20
10
0
sorted by expression

GROUP3 M1

Tumor Medulloblastoma
Cavalli - 763 - rma_sketch - hugene11t
FLT3 (7970737)
Expression cutoff: 9.300 (min.grp=3)
subgroup~group3|met_status_(1_met__0_m0)~1|WITH_SURV (n=41)

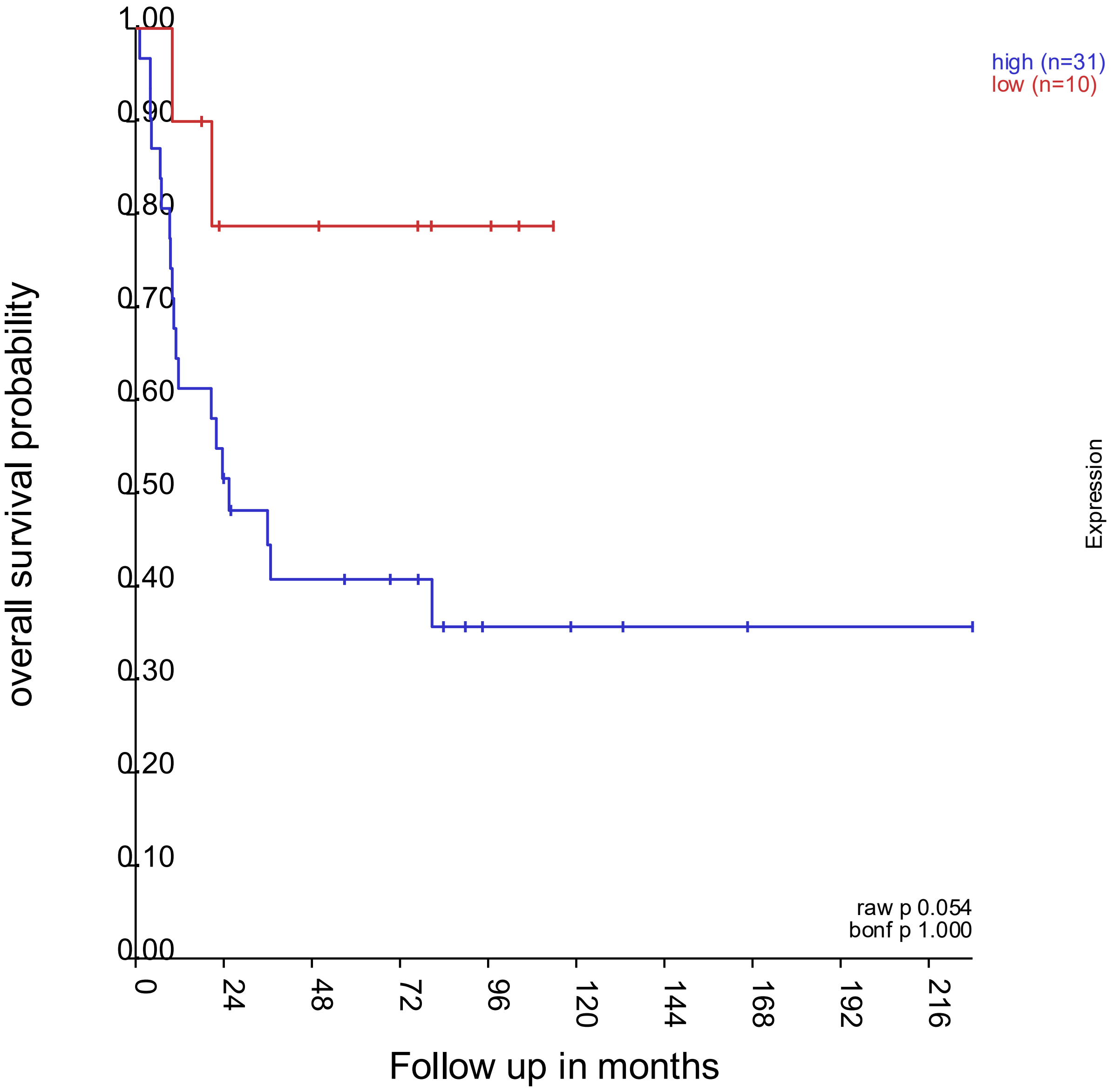

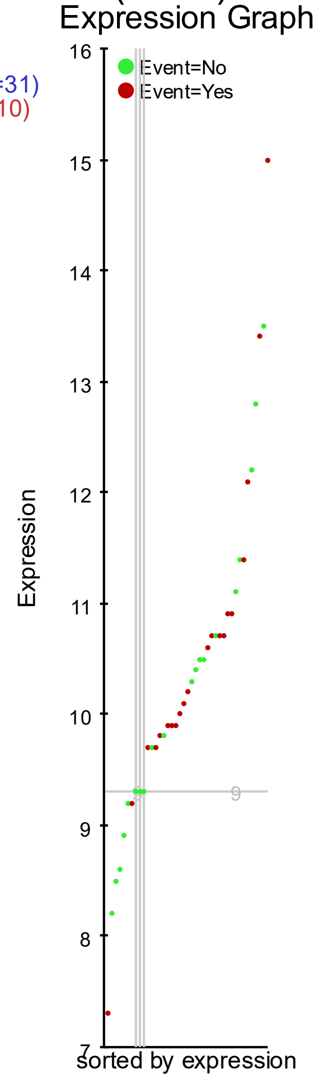

HER1/EGFR

WNT M0

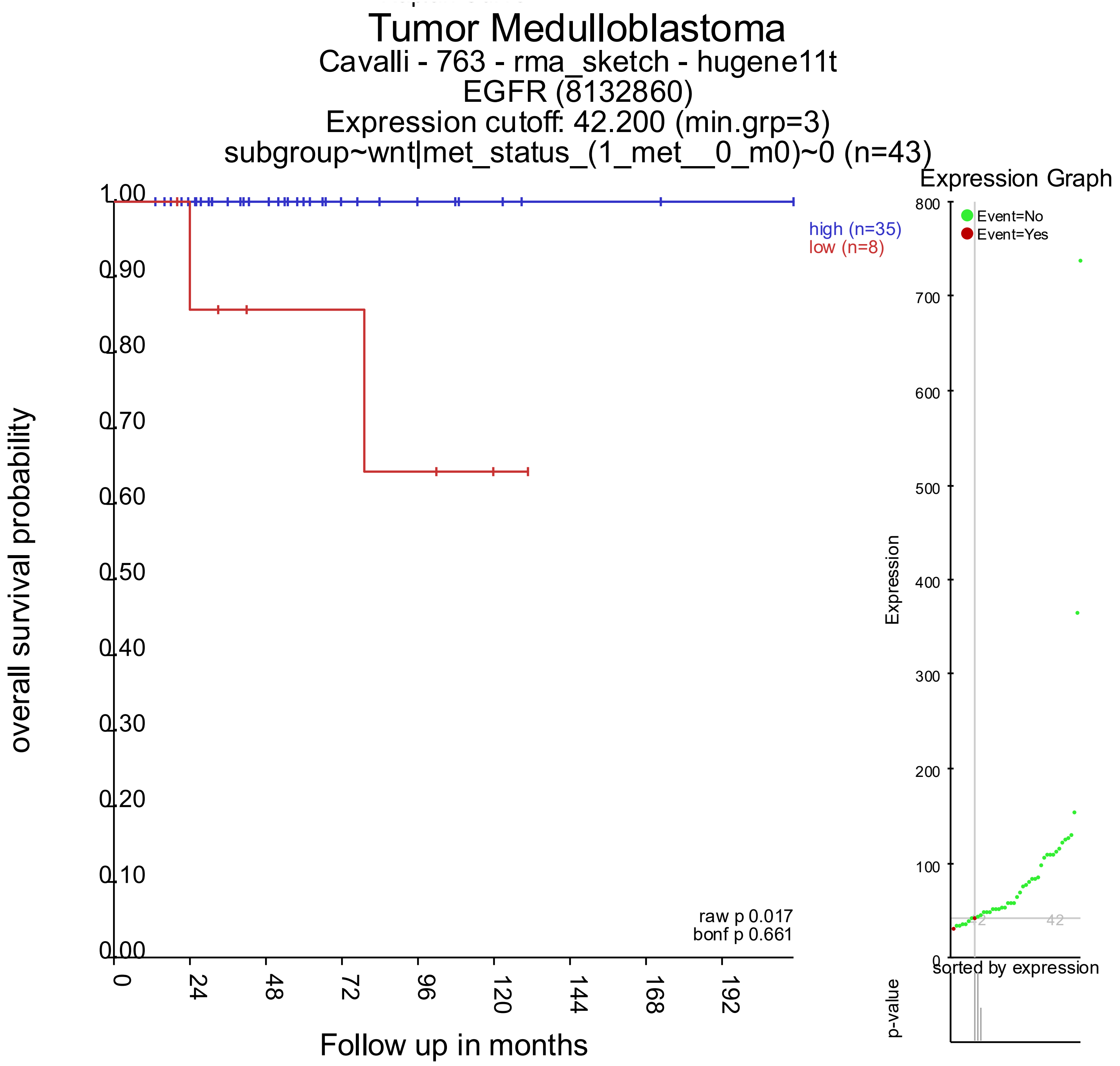
Tumor Medulloblastoma
Cavalli - 763 - rma_sketch - hugene11t
EGFR (8132860)
Expression cutoff: 42.200 (min.grp=3)
subgroup~wnt|met_status_(1_met__0_m0)~0 (n=43)
1.00
0.90
0.80
0.70
0.60
0.50
0.40
0.30
0.20
0.10
0.00
overall survival probability
high (n=35)
low (n=8)
raw p 0.017
bonf p 0.661
0
24
48
72
96
120
144
168
192
Follow up in months
Expression Graph
Event=No
Event=Yes
800
700
600
500
400
300
200
100
0
Expression
42
sorted by expression
p-value

WNT M1

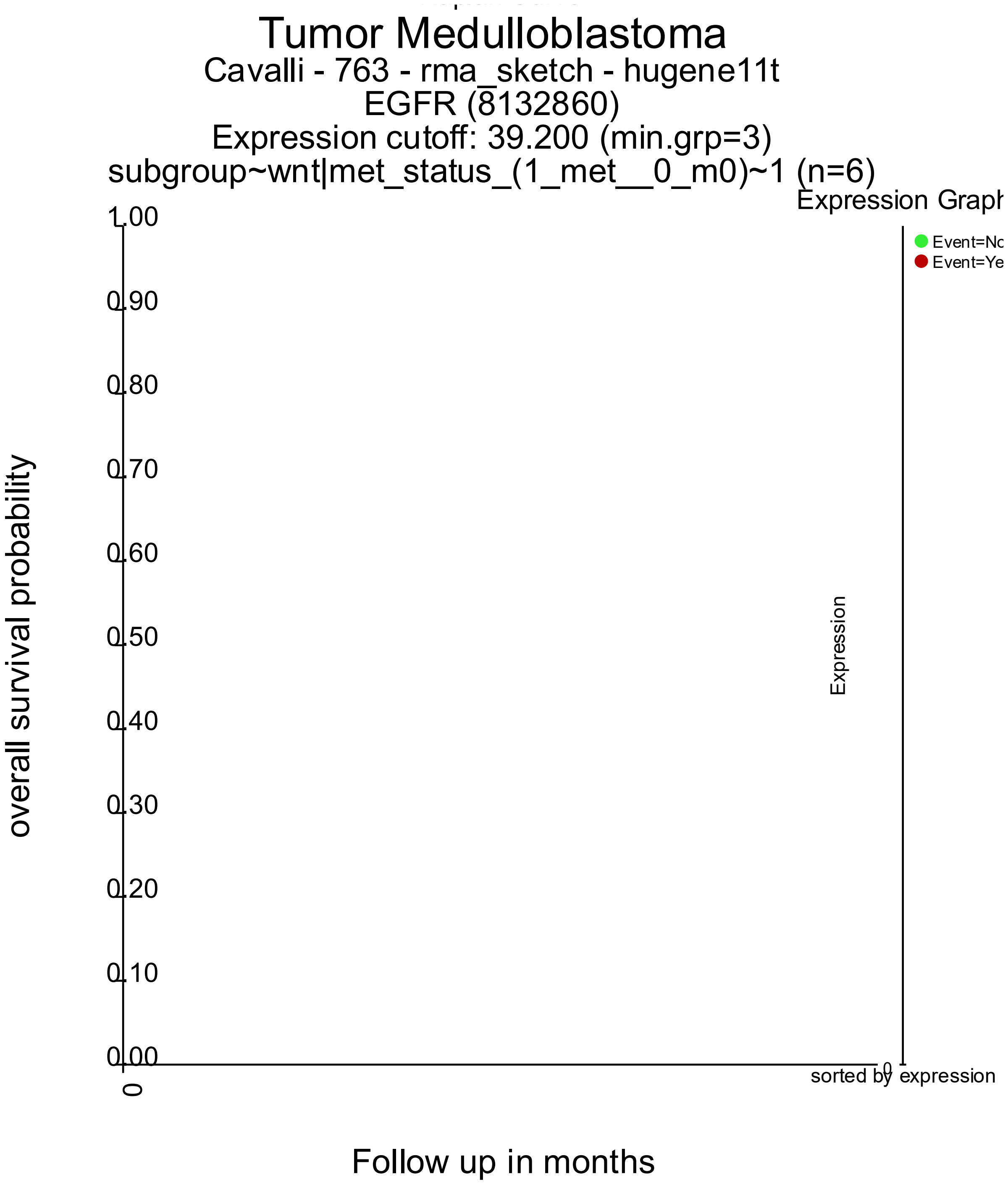

Tumor Medulloblastoma
Cavalli - 763 - rma_sketch - hugene11t
EGFR (8132860)
Expression cutoff: 39.200 (min.grp=3)
subgroup~wnt|met_status_(1_met__0_m0)~1 (n=6)
Expression Graph
Event=No
Event=Yes
overall survival probability
1.00
0.90
0.80
0.70
0.60
0.50
0.40
0.30
0.20
0.10
0.00
0
Follow up in months
Expression
0
sorted by expression

SHH M0

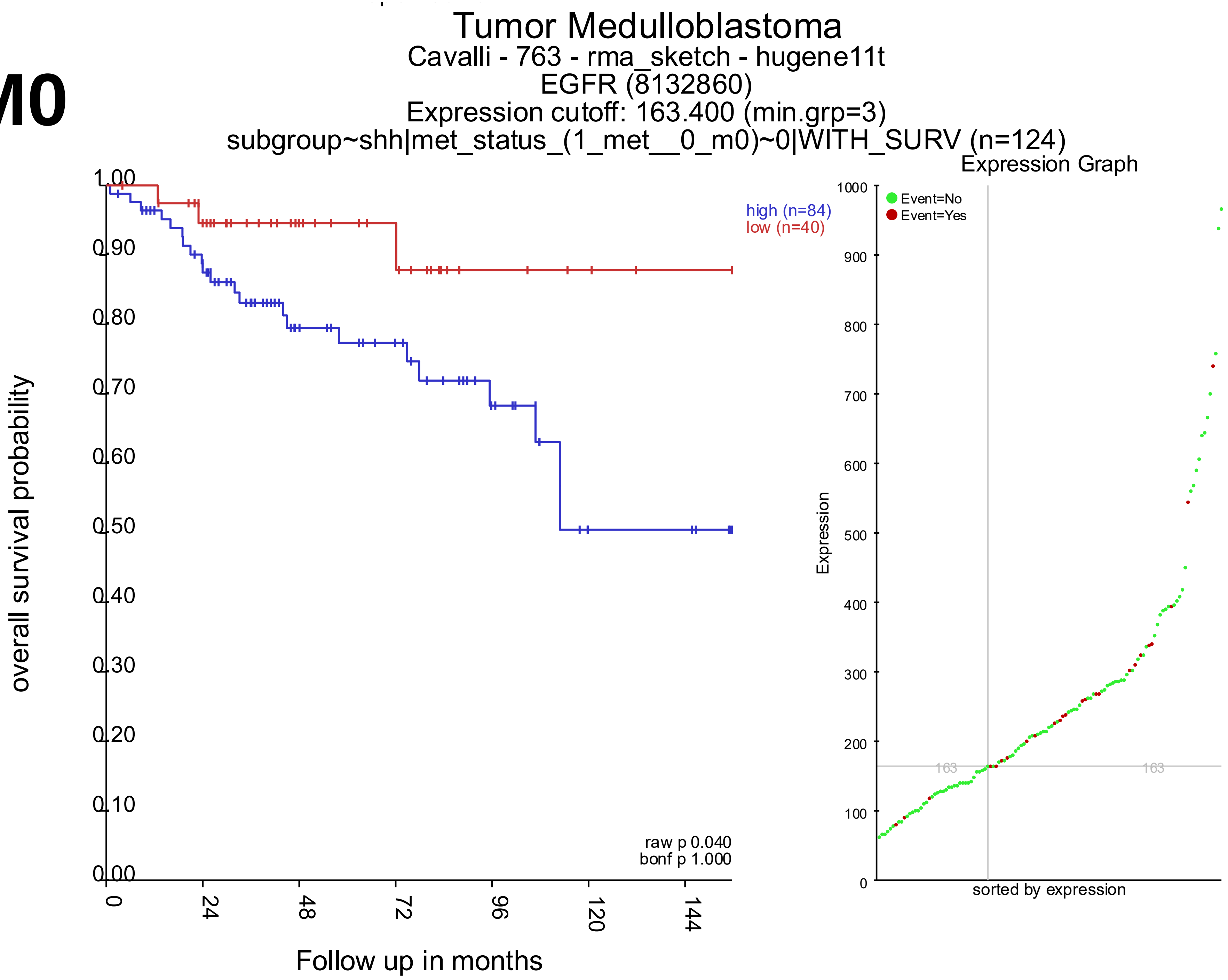
Tumor Medulloblastoma
Cavalli - 763 - rma_sketch - hugene11t
EGFR (8132860)
Expression cutoff: 163.400 (min.grp=3)
subgroup~shh|met_status_(1_met__0_m0)~0|WITH_SURV (n=124)
overall survival probability
1.00
0.90
0.80
0.70
0.60
0.50
0.40
0.30
0.20
0.10
0.00
0
24
48
72
96
120
144
Follow up in months
high (n=84)
low (n=40)
raw p 0.040
bonf p 1.000
Expression Graph
Event=No
Event=Yes
Expression
1000
900
800
700
600
500
400
300
200
100
0
163
163
sorted by expression

SHH M1

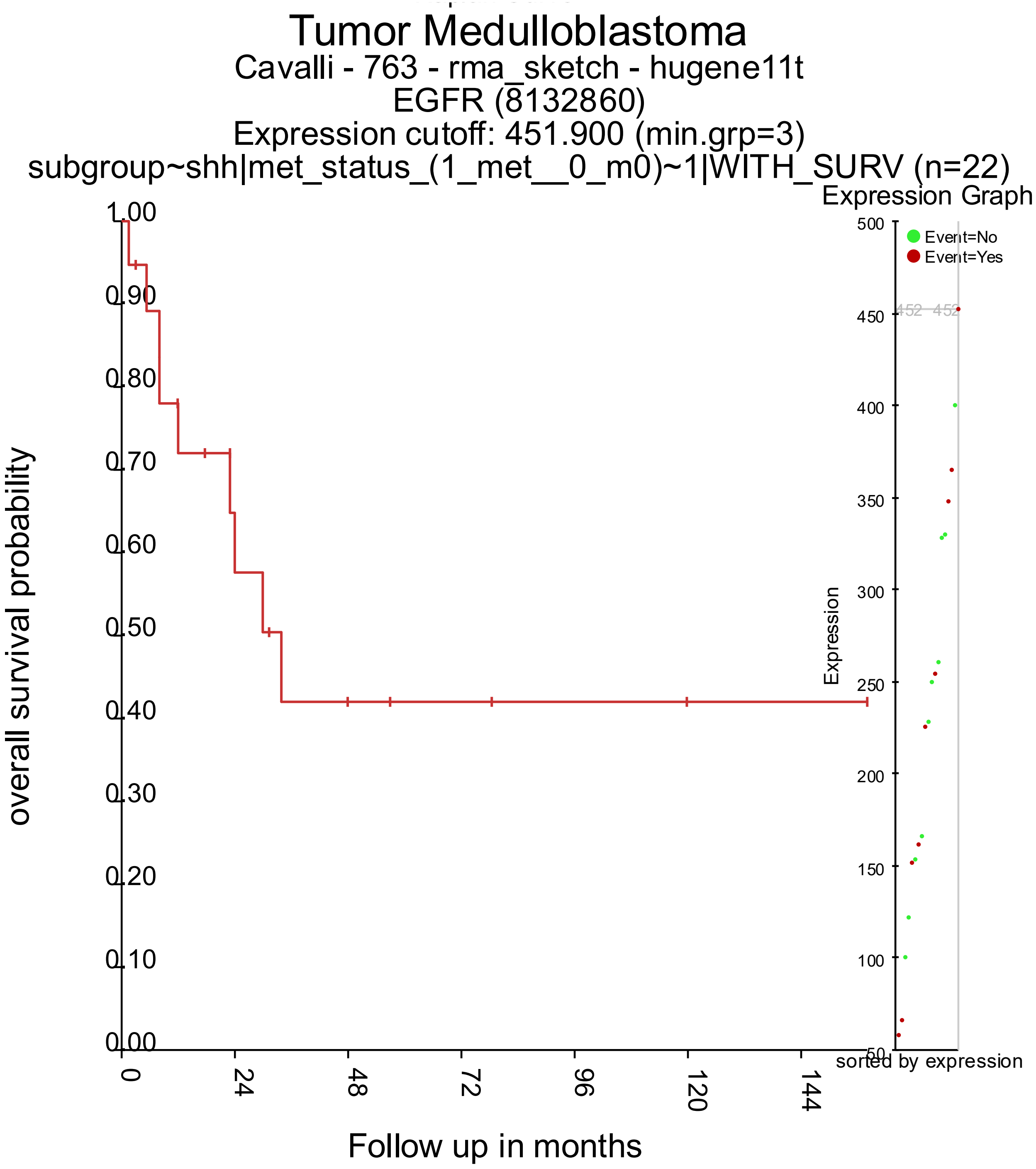

Tumor Medulloblastoma
Cavalli - 763 - rma_sketch - hugene11t
EGFR (8132860)
Expression cutoff: 451.900 (min.grp=3)
subgroup~shh|met_status_(1_met__0_m0)~1|WITH_SURV (n=22)
Expression Graph
overall survival probability
1.00
0.90
0.80
0.70
0.60
0.50
0.40
0.30
0.20
0.10
0.00
0
24
48
72
96
120
144
Follow up in months
Event=No
Event=Yes
Expression
500
450
400
350
300
250
200
150
100
50
452
452
sorted by expression

GROUP4 M0

Tumor Medulloblastoma
Cavalli - 763 - rma_sketch - hugene11t
EGFR (8132860)
Expression cutoff: 55.600 (min.grp=3)
subgroup~group4|met_status_(1_met__0_m0)~0|WITH_SURV (n=145)

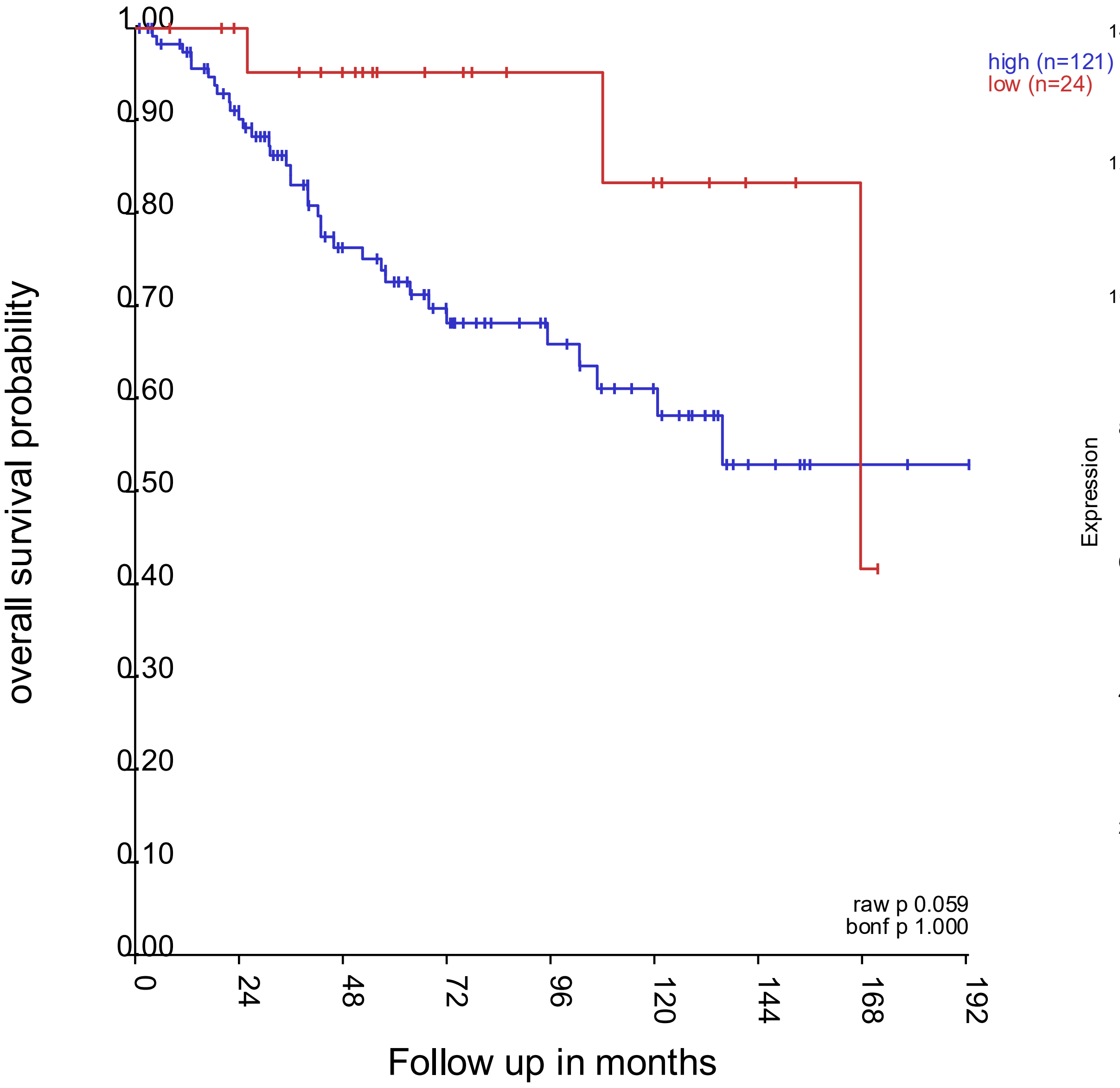

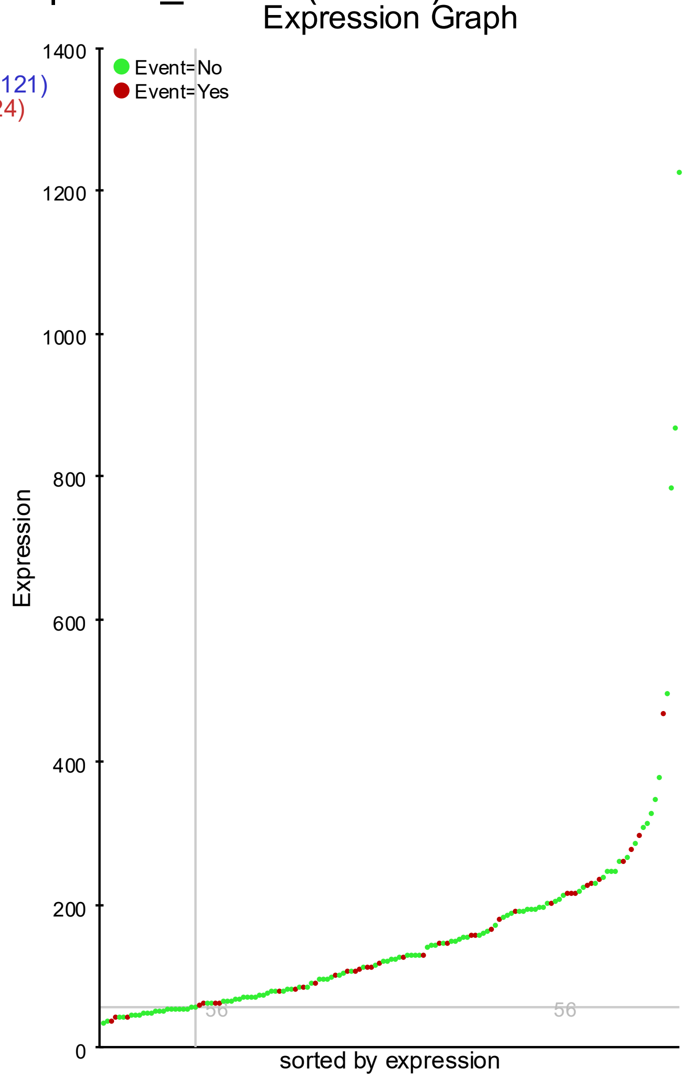

GROUP4 M1

Tumor Medulloblastoma
Cavalli - 763 - rma_sketch - hugene11t
EGFR (8132860)
Expression cutoff: 249.500 (min.grp=3)
subgroup~group4|met_status_(1_met__0_m0)~1|WITH_SURV (n=92)

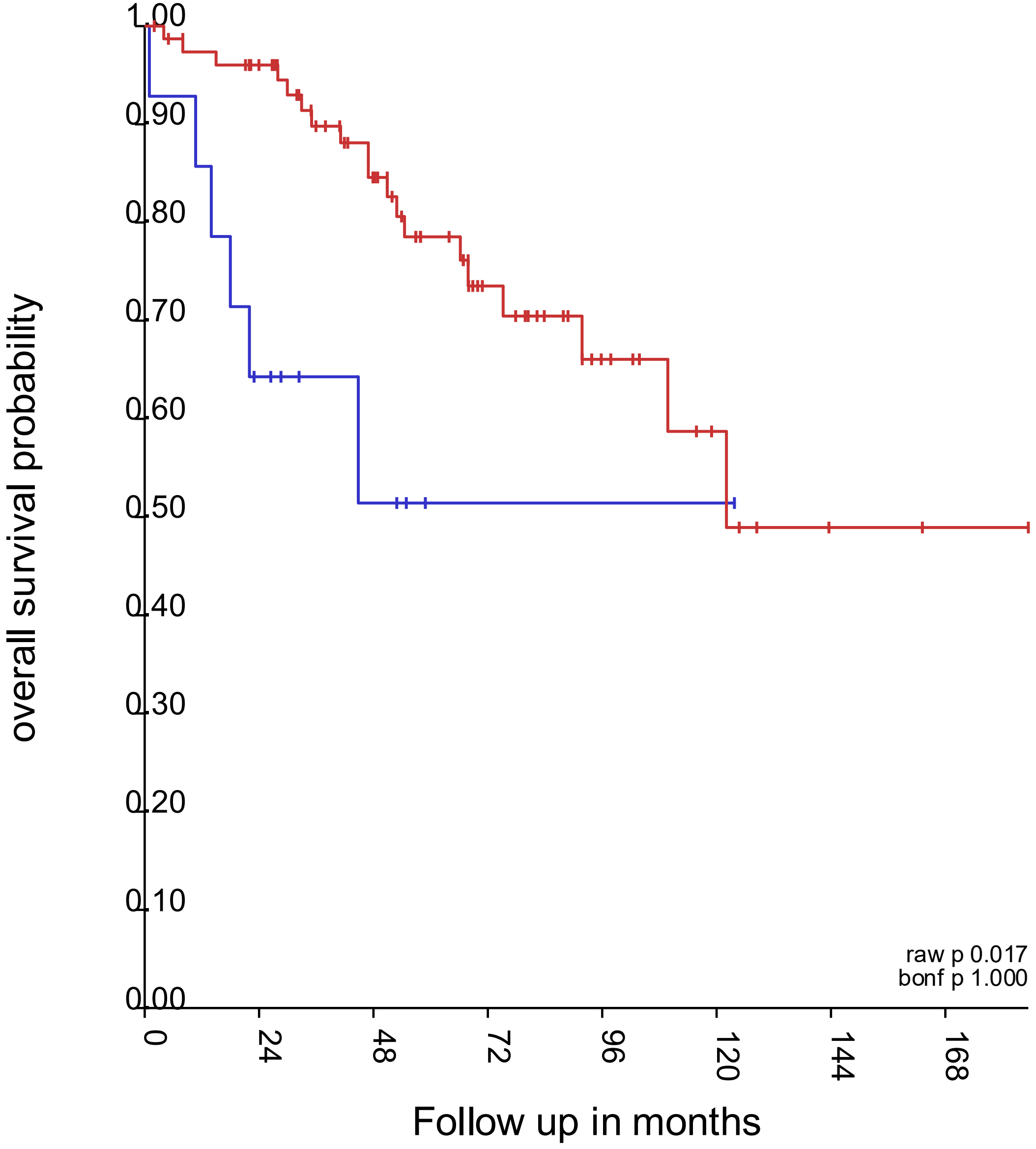

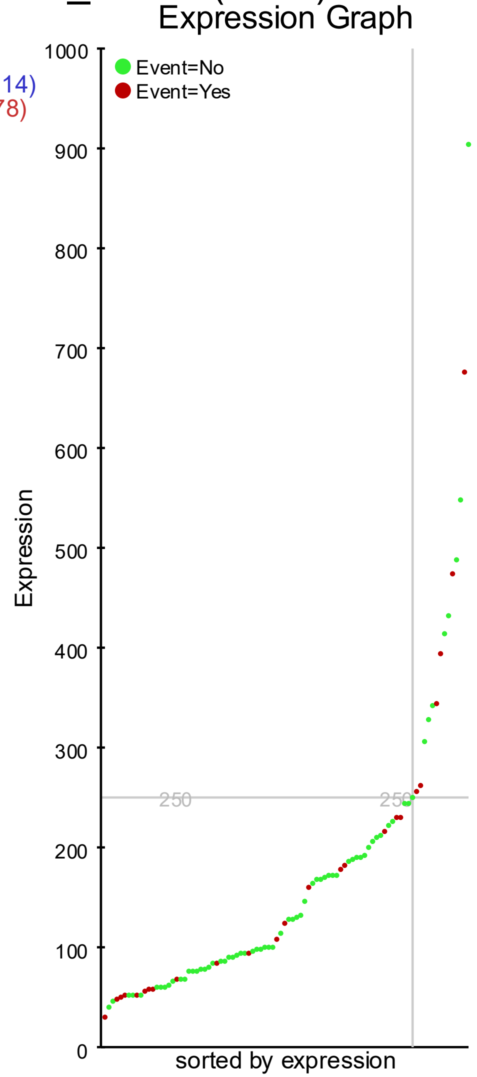

GROUP3 M0

Tumor Medulloblastoma
Cavalli - 763 - rma_sketch - hugene11t
EGFR (8132860)
Expression cutoff: 34.300 (min.grp=3)
subgroup~group3|met_status_(1_met__0_m0)~0|WITH_SURV (n=65)

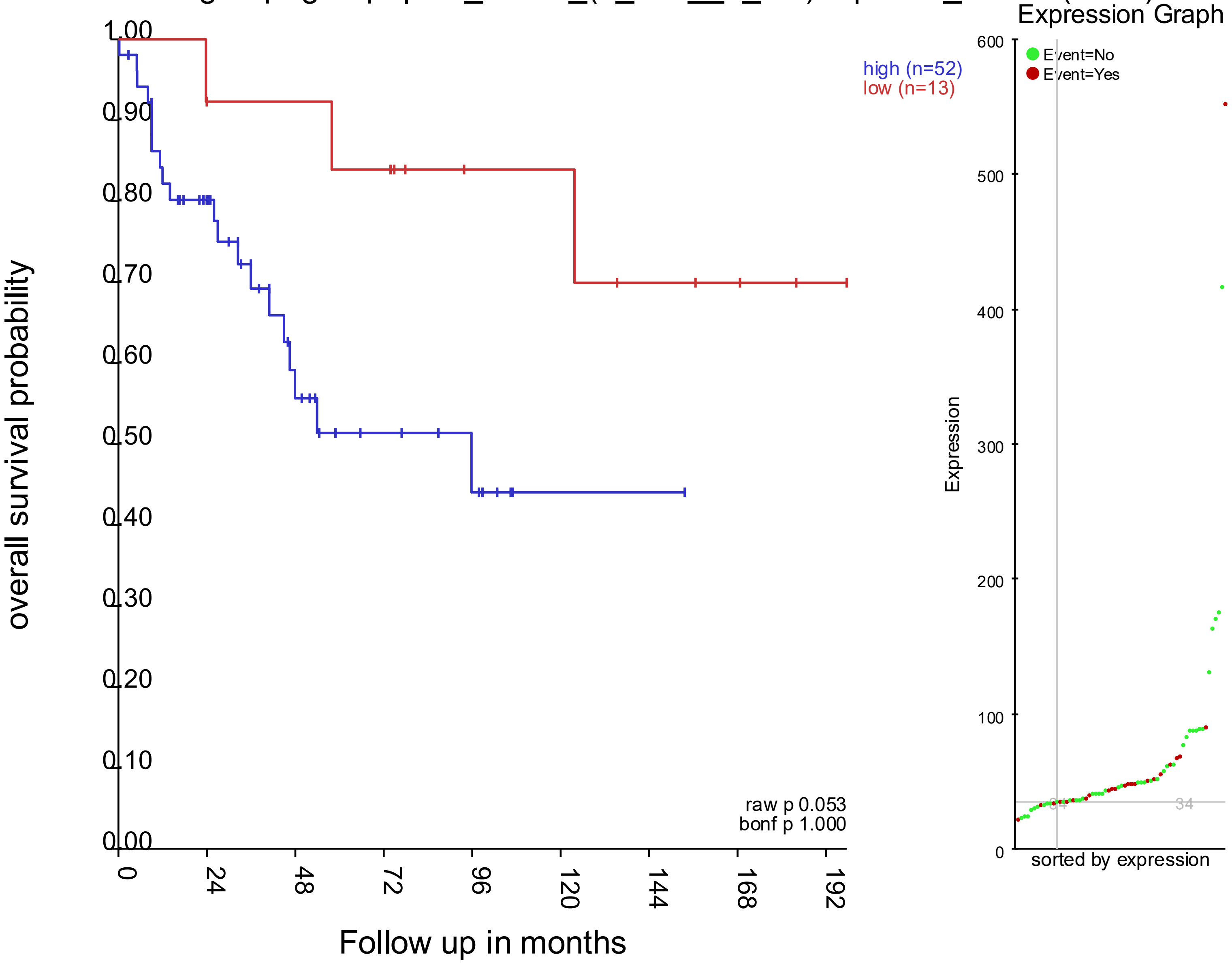

GROUP3 M1

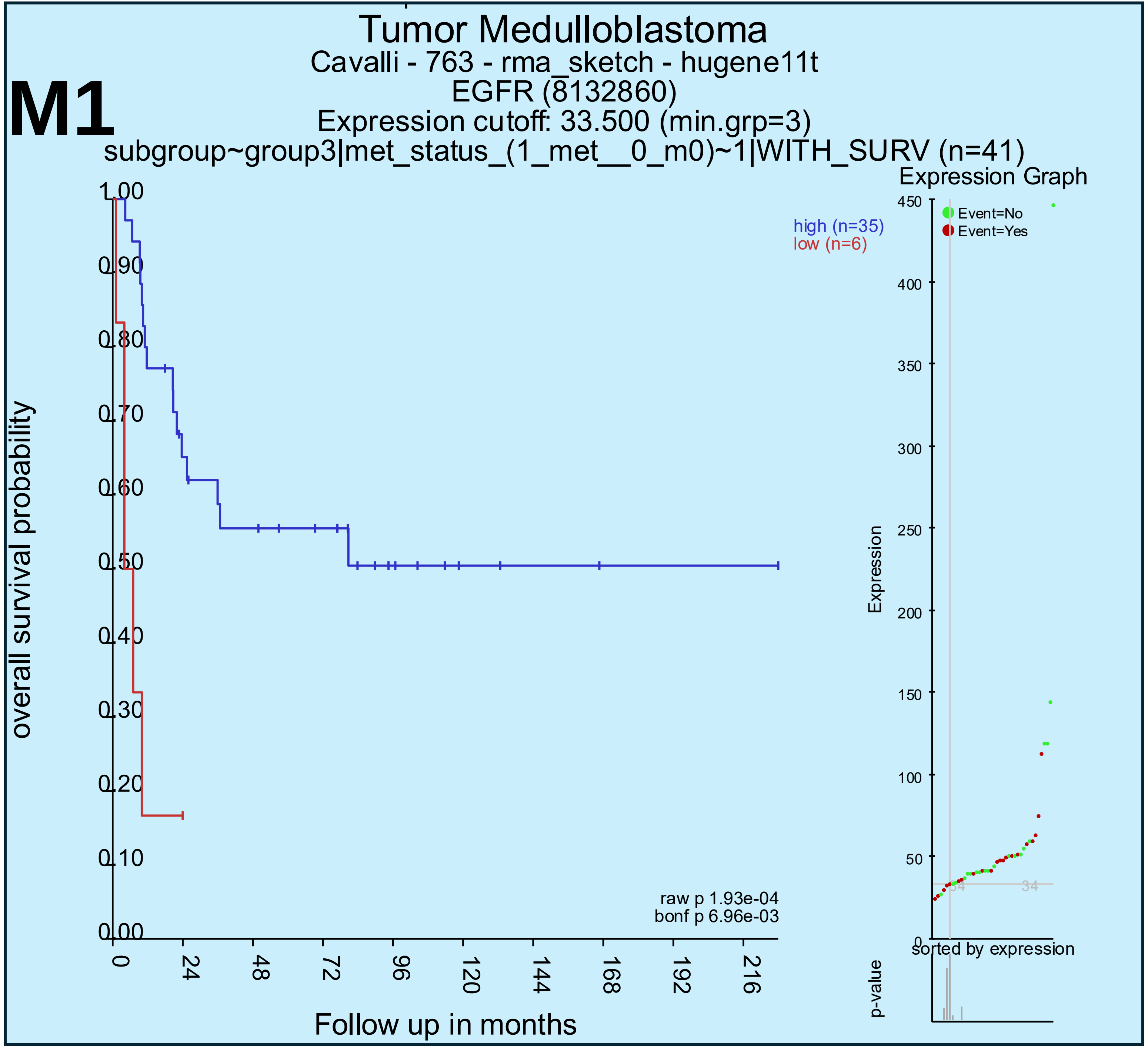

Tumor Medulloblastoma
Cavalli - 763 - rma_sketch - hugene11t
EGFR (8132860)
Expression cutoff: 33.500 (min.grp=3)
subgroup~group3|met_status_(1_met__0_m0)~1|WITH_SURV (n=41)
overall survival probability
1.00
0.90
0.80
0.70
0.60
0.50
0.40
0.30
0.20
0.10
0.00
high (n=35)
low (n=6)
raw p 1.93e-04
bonf p 6.96e-03
0
24
48
72
96
120
144
168
192
216
Follow up in months
Expression Graph
Event=No
Event=Yes
Expression
450
400
350
300
250
200
150
100
50
0
34
sorted by expression
p-value

HER2/ERBB2

WNT M0

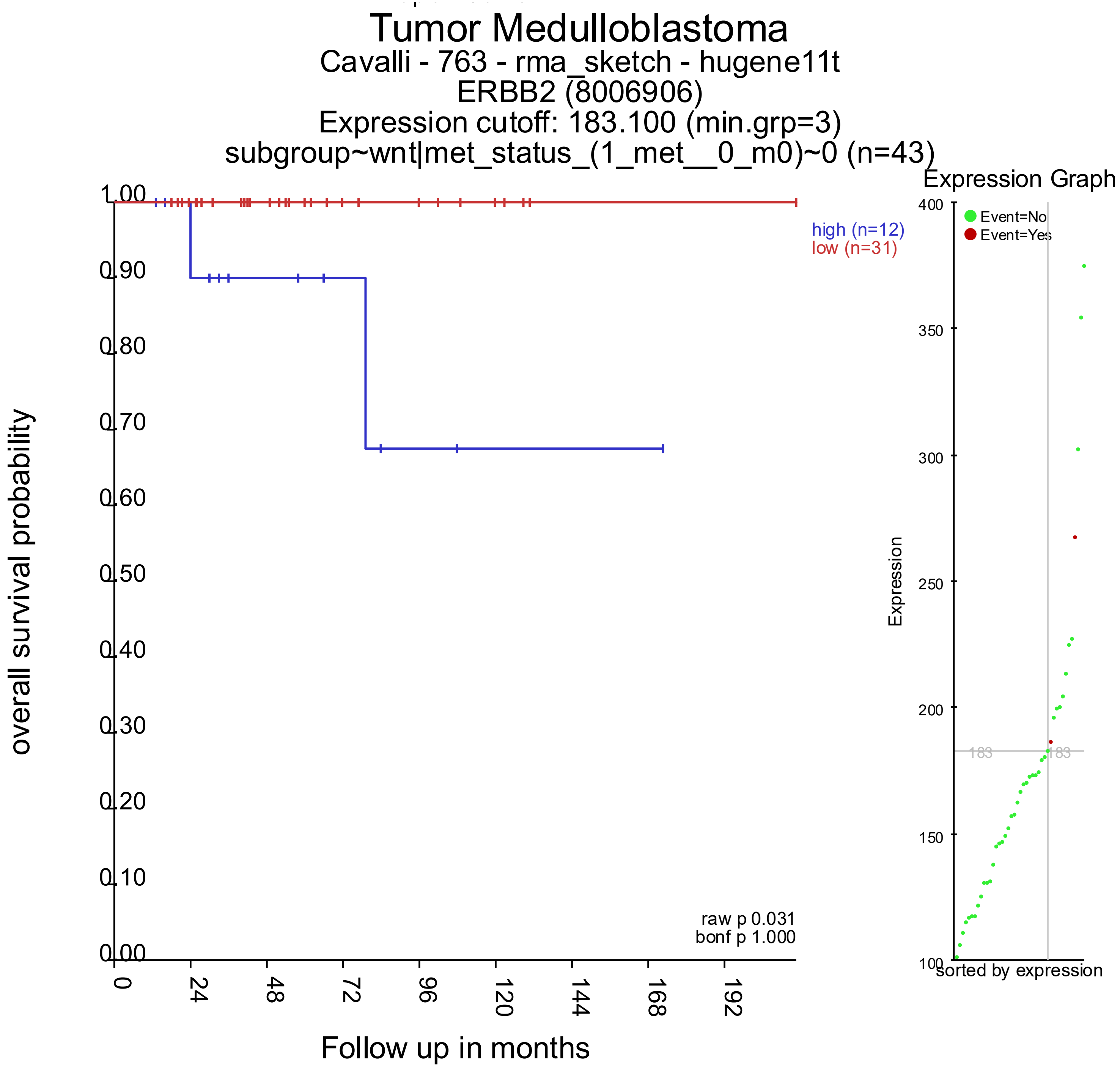

Tumor Medulloblastoma
Cavalli - 763 - rma_sketch - hugene11t
ERBB2 (8006906)
Expression cutoff: 183.100 (min.grp=3)
subgroup~wnt|met_status_(1_met__0_m0)~0 (n=43)
overall survival probability
1.00
0.90
0.80
0.70
0.60
0.50
0.40
0.30
0.20
0.10
0.00
0
24
48
72
96
120
144
168
192
Follow up in months
high (n=12)
low (n=31)
raw p 0.031
bonf p 1.000
Expression Graph
Event=No
Event=Yes
Expression
400
350
300
250
200
150
100
183
183
sorted by expression

WNT M1

Tumor Medulloblastoma
Cavalli - 763 - rma_sketch - hugene11t
ERBB2 (8006906)
Expression cutoff: 137.400 (min.grp=3)
subgroup~wnt|met_status_(1_met__0_m0)~1 (n=6)

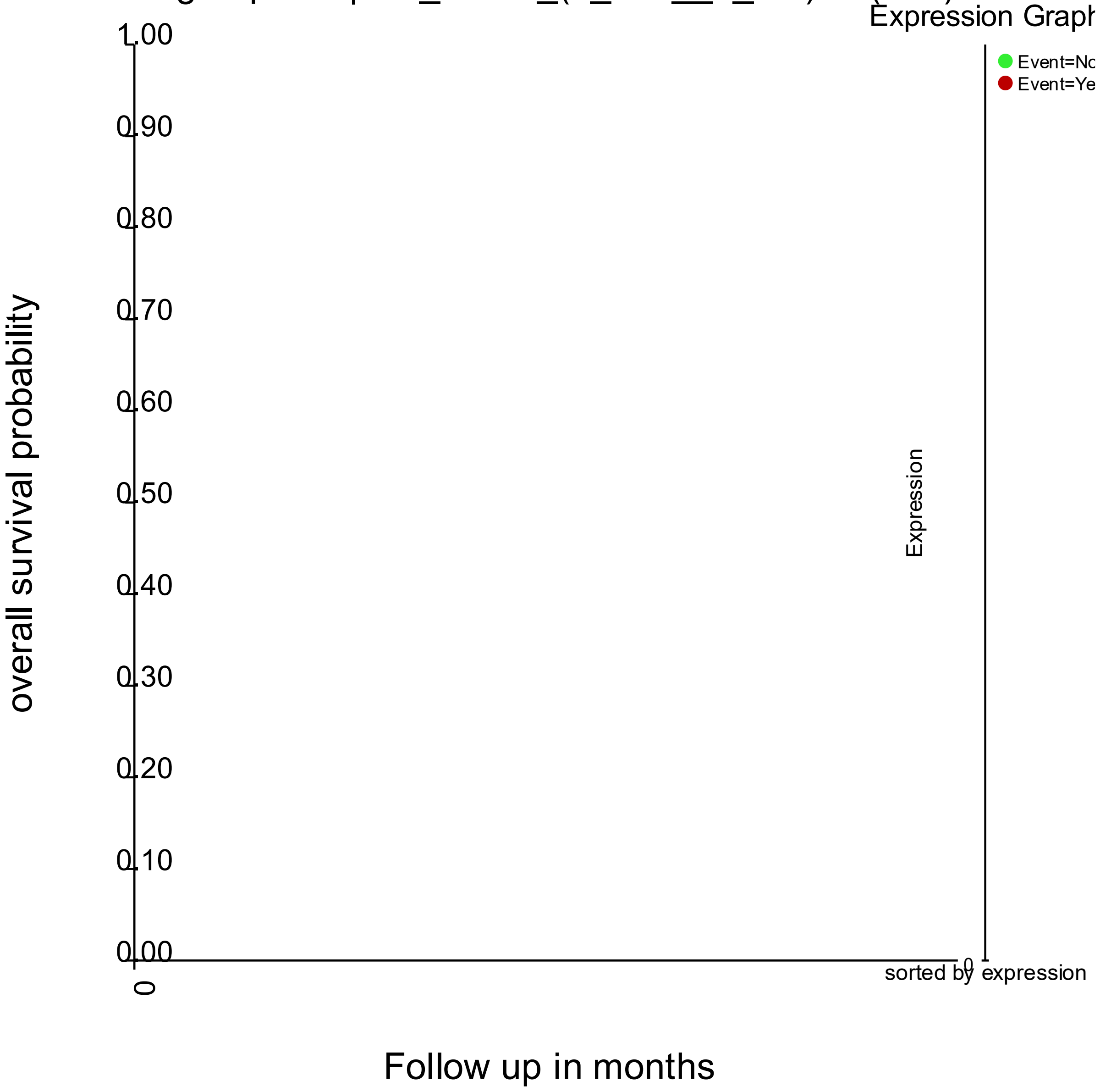

SHH M0

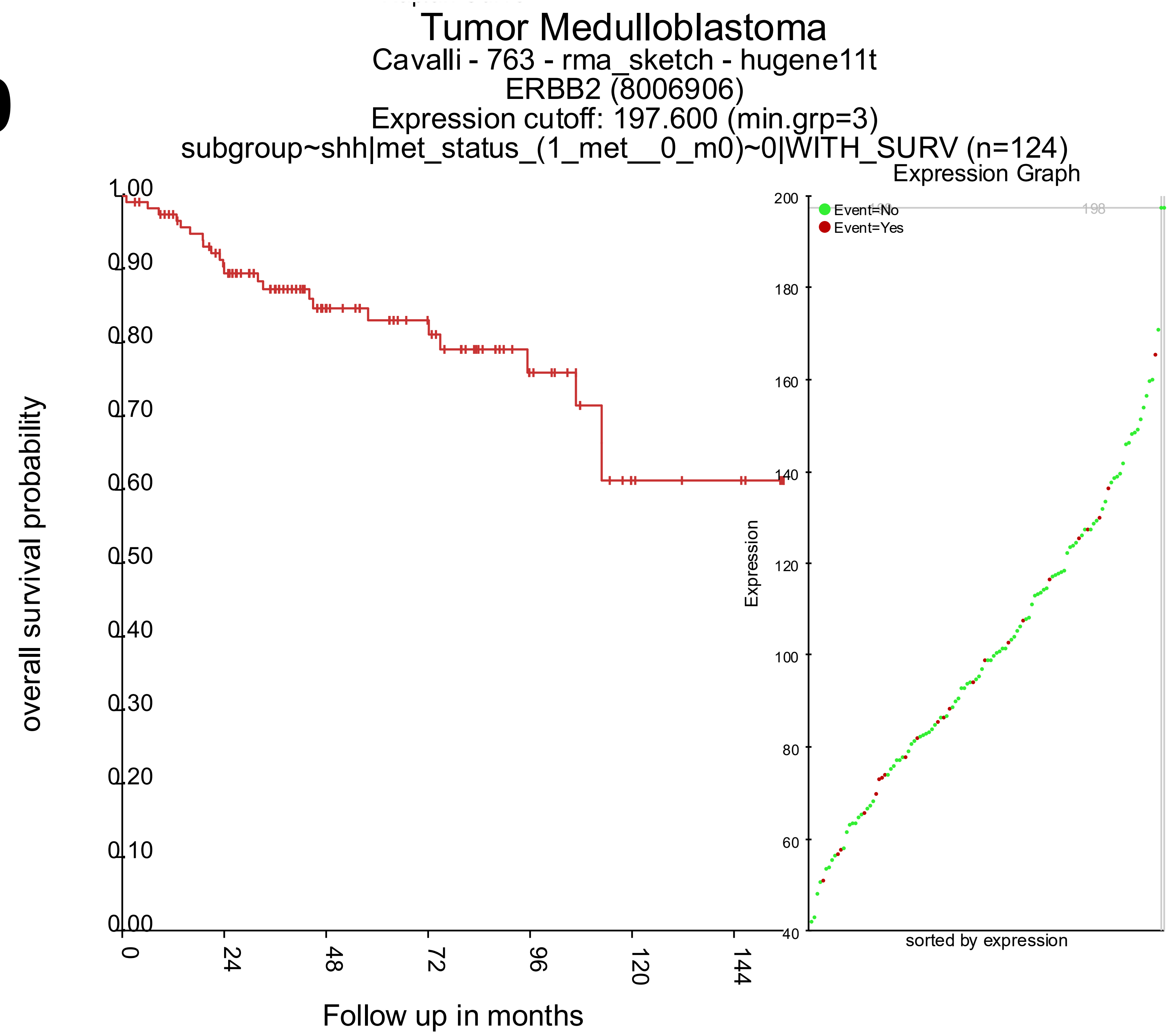

Tumor Medulloblastoma
Cavalli - 763 - rma_sketch - hugene11t
ERBB2 (8006906)
Expression cutoff: 197.600 (min.grp=3)
subgroup~shh|met_status_(1_met__0_m0)~0|WITH_SURV (n=124)
overall survival probability
1.00
0.90
0.80
0.70
0.60
0.50
0.40
0.30
0.20
0.10
0.00
0
24
48
72
96
120
144
Follow up in months
Expression Graph
Event=No
Event=Yes
198
200
180
160
140
120
100
80
60
40
Expression
sorted by expression

SHH M1

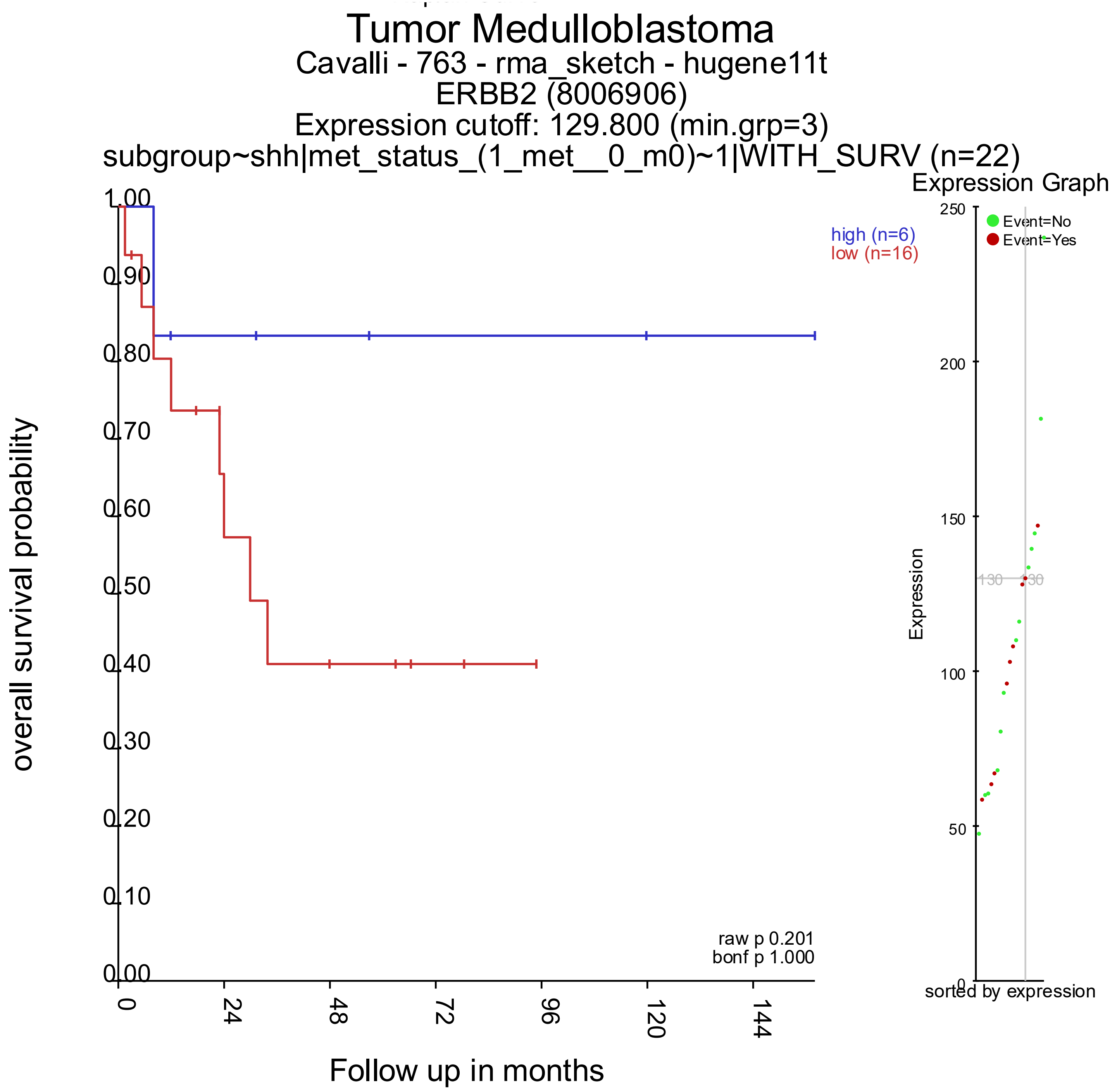

Tumor Medulloblastoma
Cavalli - 763 - rma_sketch - hugene11t
ERBB2 (8006906)
Expression cutoff: 129.800 (min.grp=3)
subgroup~shh|met_status_(1_met__0_m0)~1|WITH_SURV (n=22)
overall survival probability
1.00
0.90
0.80
0.70
0.60
0.50
0.40
0.30
0.20
0.10
0.00
high (n=6)
low (n=16)
raw p 0.201
bonf p 1.000
0
24
48
72
96
120
144
Follow up in months
Expression Graph
Event=No
Event=Yes
250
200
150
100
50
0
Expression
130
130
sorted by expression

GROUP4 M0

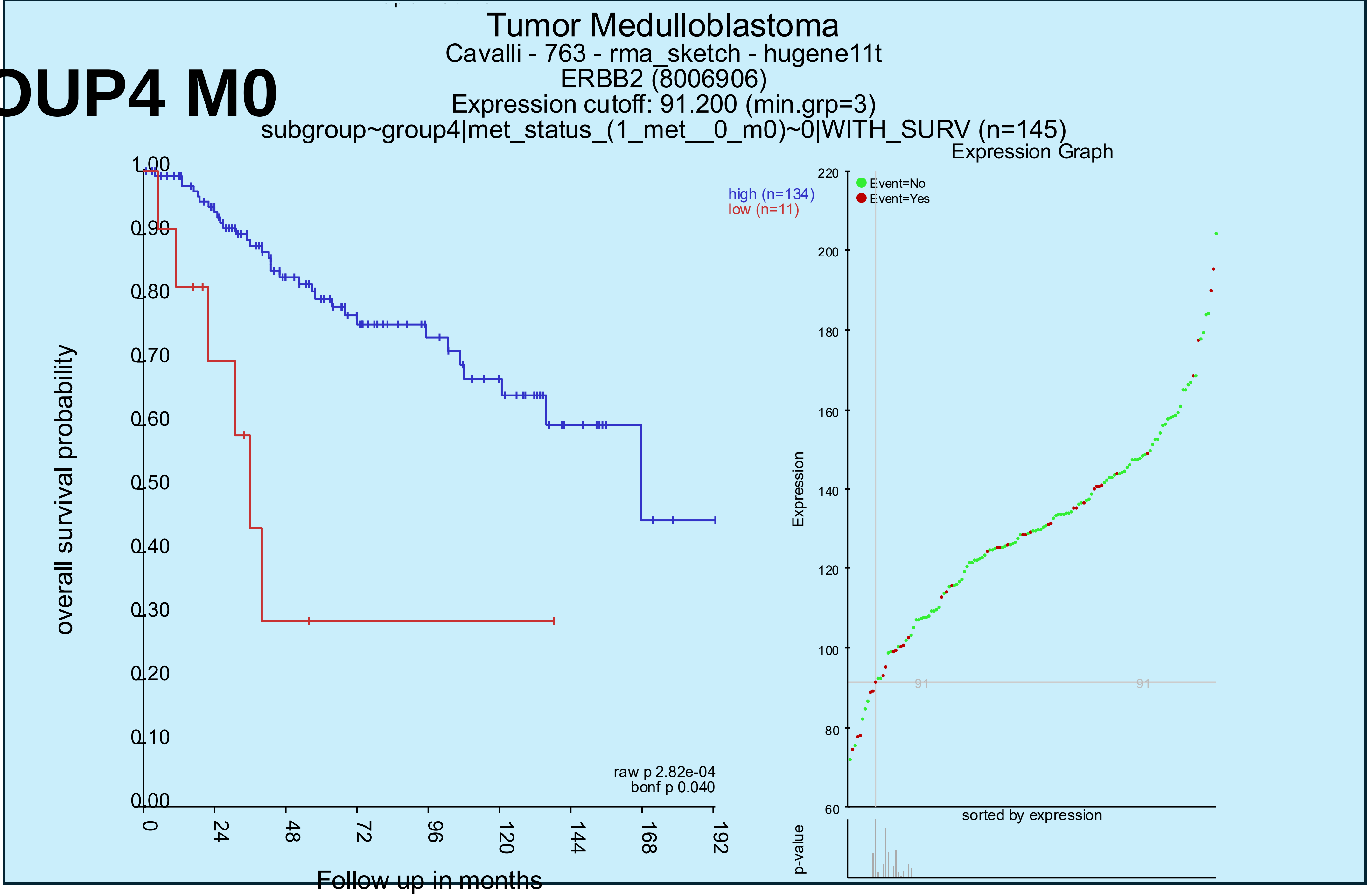
Tumor Medulloblastoma
Cavalli - 763 - rma_sketch - hugene11t
ERBB2 (8006906)
Expression cutoff: 91.200 (min.grp=3)
subgroup~group4|met_status_(1_met__0_m0)~0|WITH_SURV (n=145)
overall survival probability
1.00
0.90
0.80
0.70
0.60
0.50
0.40
0.30
0.20
0.10
0.00
0
24
48
72
96
120
144
168
192
Follow up in months
high (n=134)
low (n=11)
raw p 2.82e-04
bonf p 0.040
Expression Graph
Event=No
Event=Yes
Expression
220
200
180
160
140
120
100
80
60
91
91
sorted by expression
p-value

GROUP4 M1

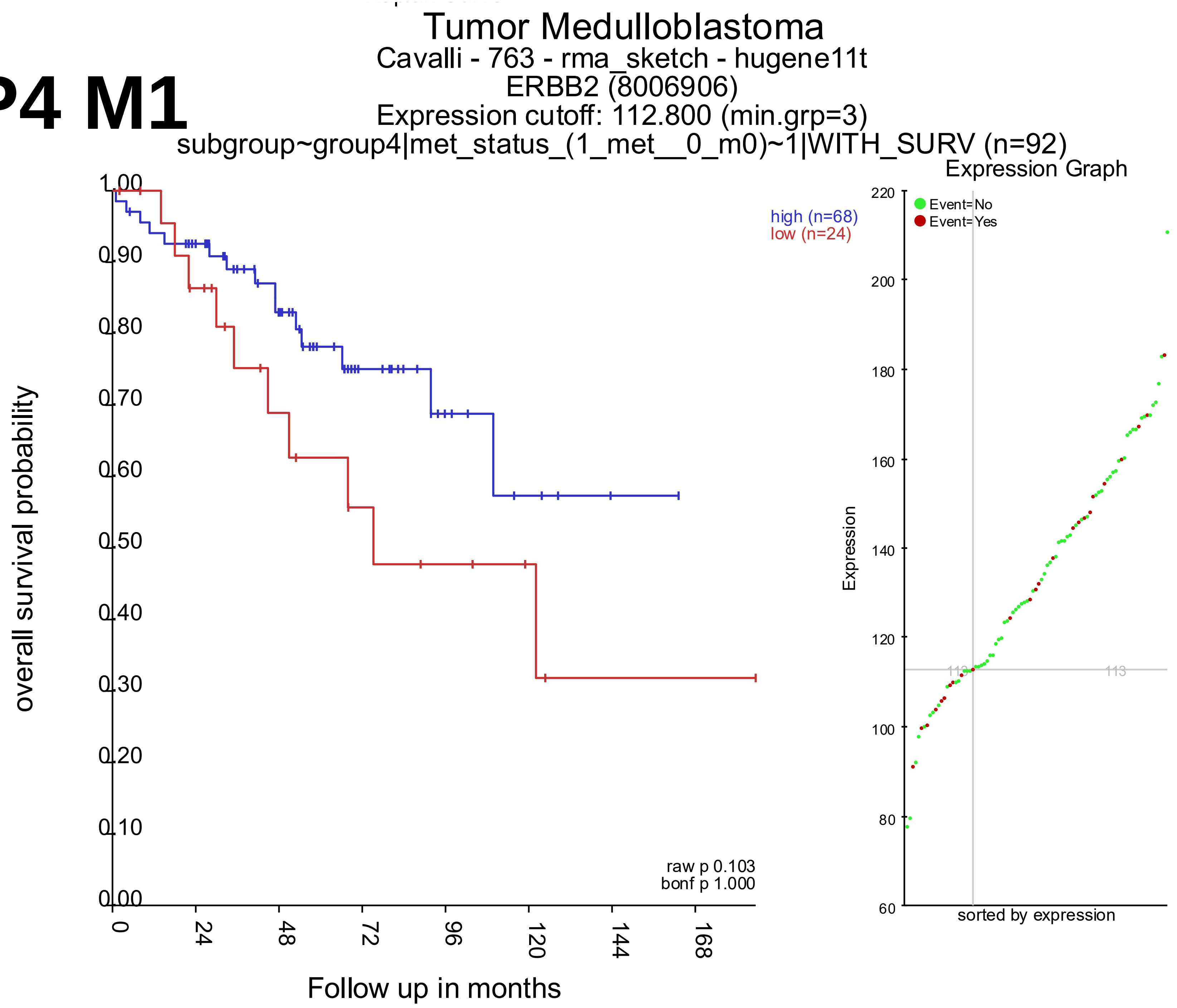

Tumor Medulloblastoma
Cavalli - 763 - rma_sketch - hugene11t
ERBB2 (8006906)
Expression cutoff: 112.800 (min.grp=3)
subgroup~group4|met_status_(1_met__0_m0)~1|WITH_SURV (n=92)
overall survival probability
1.00
0.90
0.80
0.70
0.60
0.50
0.40
0.30
0.20
0.10
0.00
0
24
48
72
96
120
144
168
Follow up in months
high (n=68)
low (n=24)
raw p 0.103
bonf p 1.000
Expression Graph
Event=No
Event=Yes
Expression
220
200
180
160
140
120
100
80
60
113
sorted by expression

GROUP3 M0

Tumor Medulloblastoma
Cavalli - 763 - rma_sketch - hugene11t
ERBB2 (8006906)
Expression cutoff: 159.000 (min.grp=3)
subgroup~group3|met_status_(1_met__0_m0)~0|WITH_SURV (n=65)

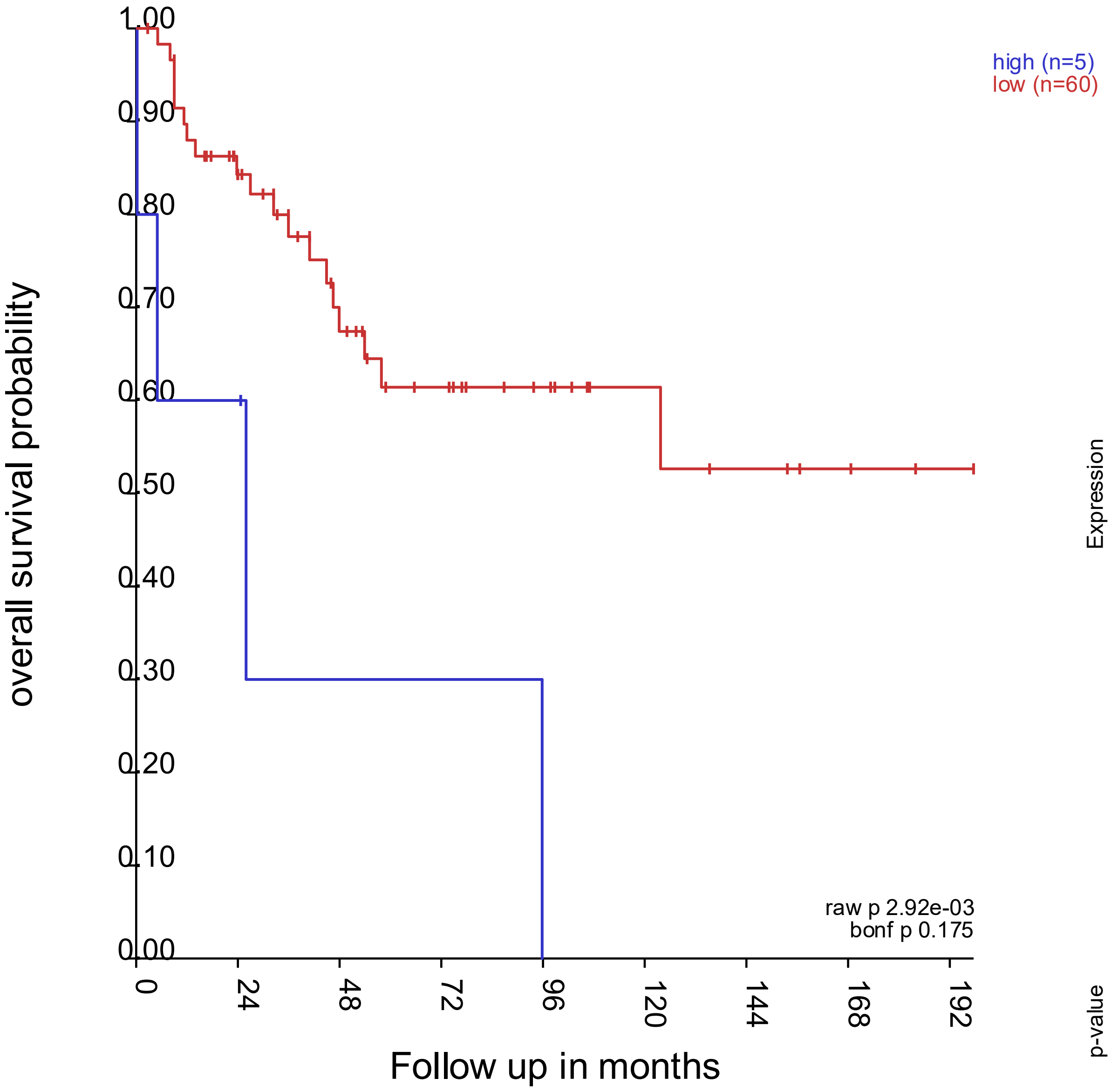

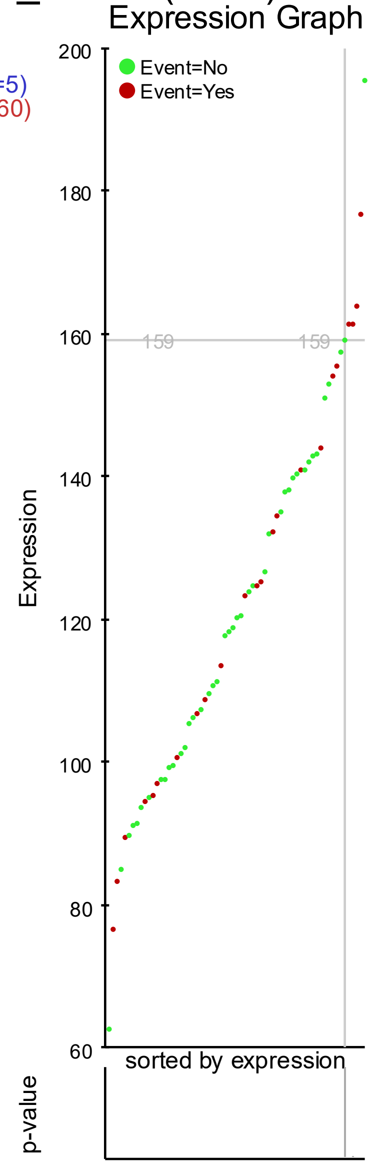

GROUP3 M1

Tumor Medulloblastoma
Cavalli - 763 - rma_sketch - hugene11t
ERBB2 (8006906)
Expression cutoff: 150.600 (min.grp=3)
subgroup~group3|met_status_(1_met__0_m0)~1|WITH_SURV (n=41)

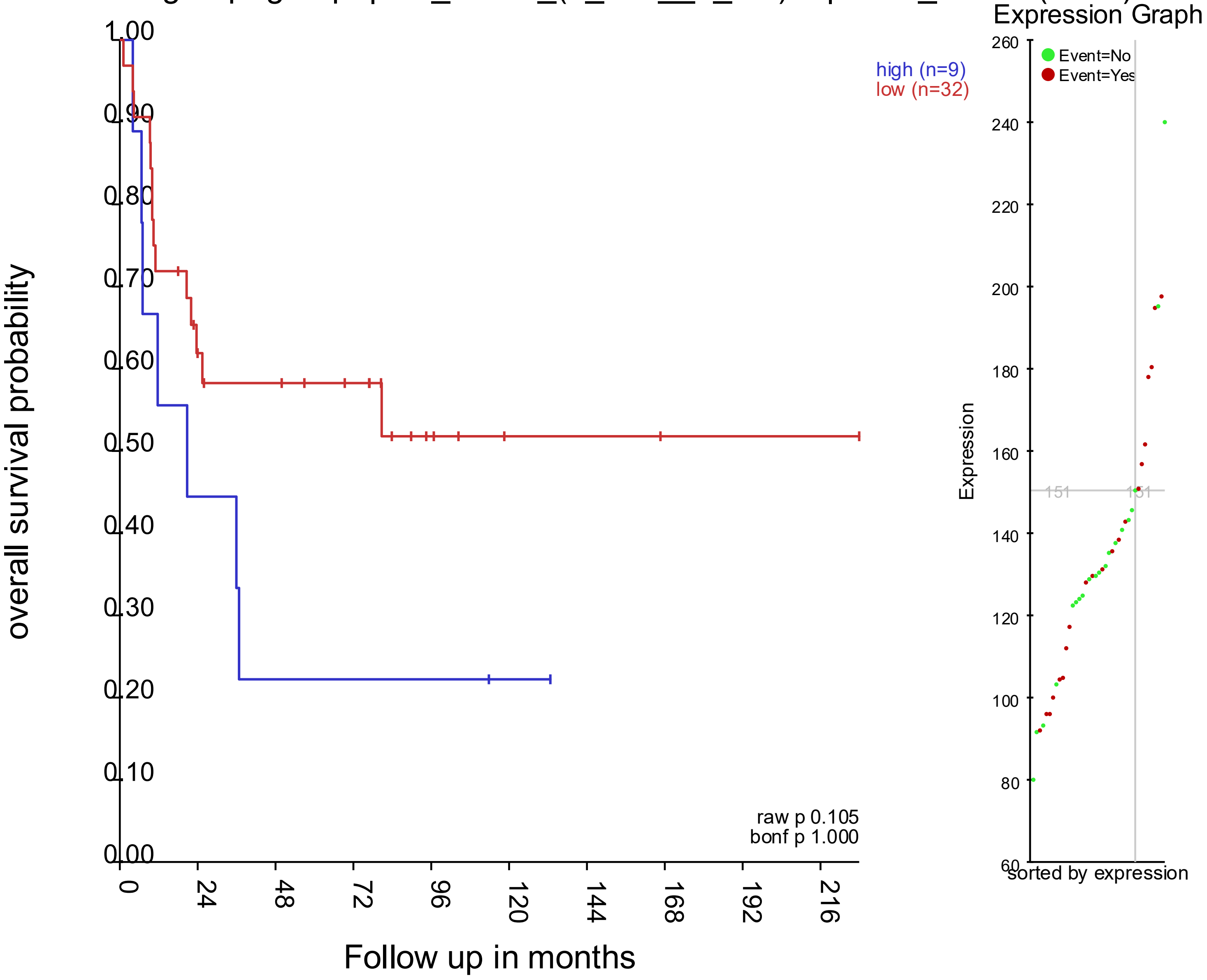

HER3/ERBB3

WNT M0

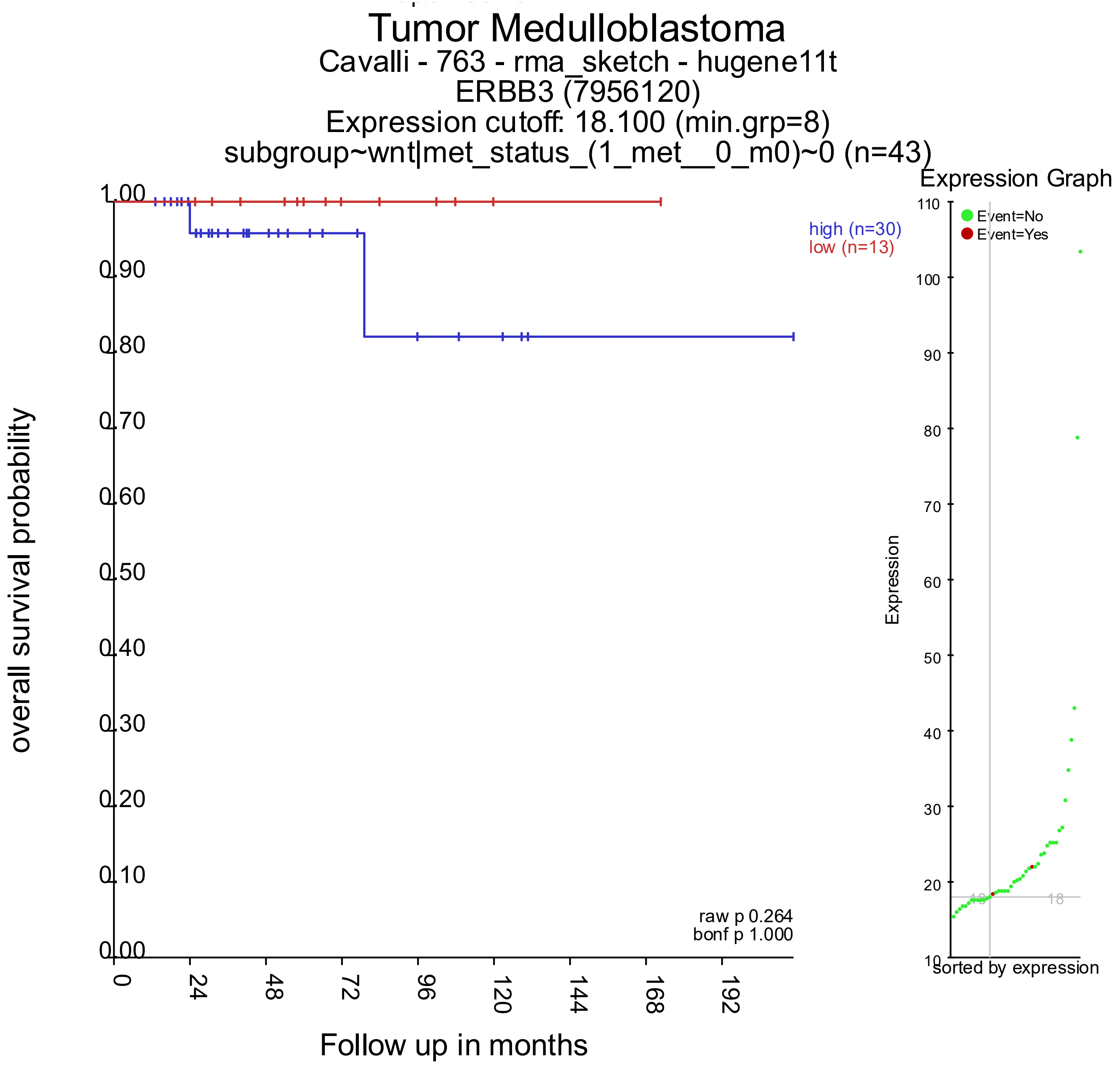

Tumor Medulloblastoma
Cavalli - 763 - rma_sketch - hugene11t
ERBB3 (7956120)
Expression cutoff: 18.100 (min.grp=8)
subgroup~wnt|met_status_(1_met__0_m0)~0 (n=43)
high (n=30)
low (n=13)
overall survival probability
1.00
0.90
0.80
0.70
0.60
0.50
0.40
0.30
0.20
0.10
0.00
raw p 0.264
bonf p 1.000
0
24
48
72
96
120
144
168
192
Follow up in months
Expression Graph
Event=No
Event=Yes
Expression
110
100
90
80
70
60
50
40
30
20
10
18
sorted by expression

WNT M1

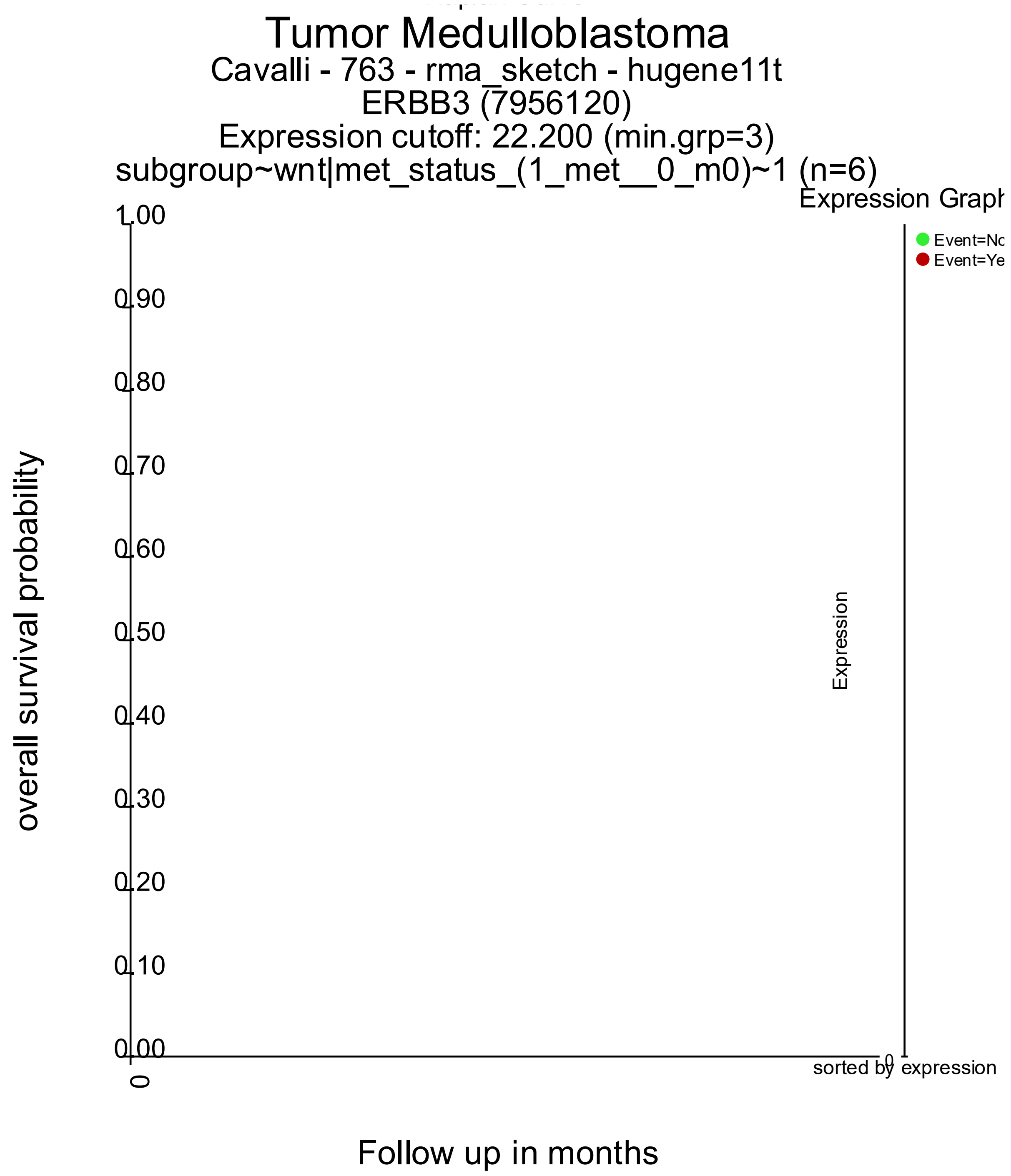
Tumor Medulloblastoma
Cavalli - 763 - rma_sketch - hugene11t
ERBB3 (7956120)
Expression cutoff: 22.200 (min.grp=3)
subgroup~wnt|met_status_(1_met__0_m0)~1 (n=6)
Expression Graph
Event=No
Event=Yes
1.00
0.90
0.80
0.70
0.60
0.50
0.40
0.30
0.20
0.10
0.00
0
overall survival probability
Follow up in months
Expression
sorted by expression
0

SHH M0

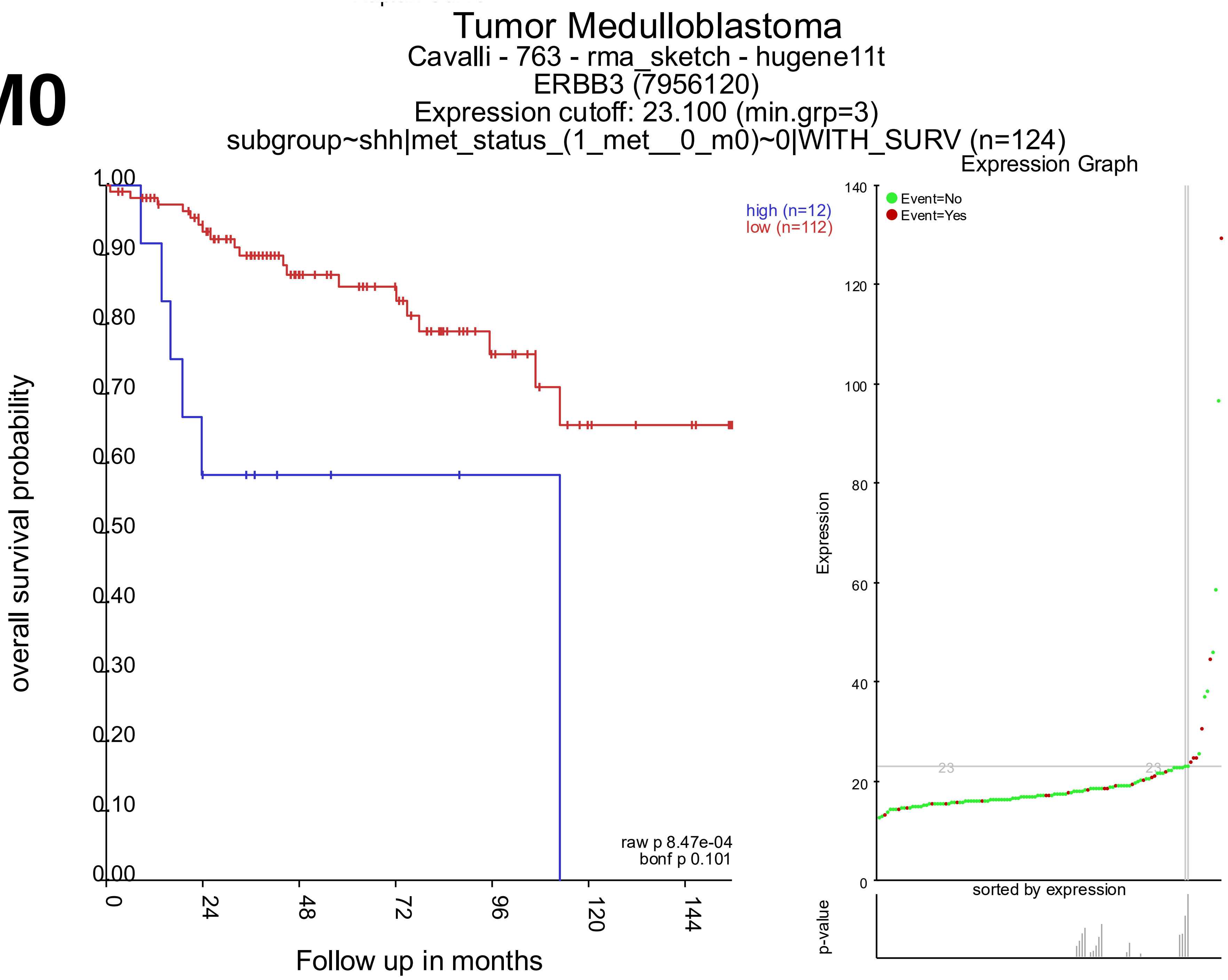

Tumor Medulloblastoma
Cavalli - 763 - rma_sketch - hugene11t
ERBB3 (7956120)
Expression cutoff: 23.100 (min.grp=3)
subgroup~shh|met_status_(1_met__0_m0)~0|WITH_SURV (n=124)
overall survival probability
1.00
0.90
0.80
0.70
0.60
0.50
0.40
0.30
0.20
0.10
0.00
high (n=12)
low (n=112)
raw p 8.47e-04
bonf p 0.101
0
24
48
72
96
120
144
Follow up in months
Expression Graph
Event=No
Event=Yes
Expression
140
120
100
80
60
40
20
0
23
sorted by expression
p-value

SHH M1

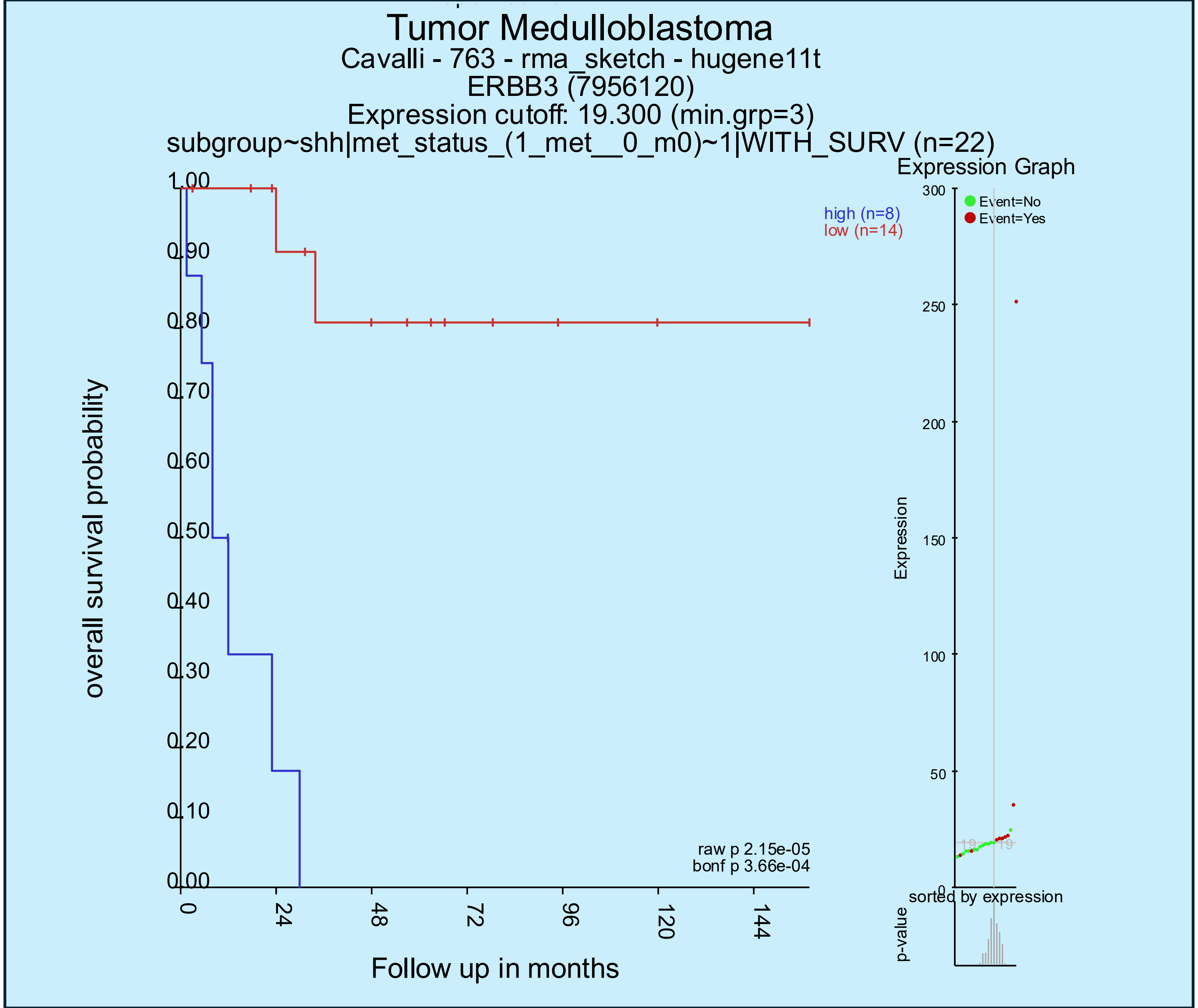

Tumor Medulloblastoma
Cavalli - 763 - rma_sketch - hugene11t
ERBB3 (7956120)
Expression cutoff: 19.300 (min.grp=3)
subgroup~shh|met_status_(1_met__0_m0)~1|WITH_SURV (n=22)
overall survival probability
1.00
0.90
0.80
0.70
0.60
0.50
0.40
0.30
0.20
0.10
0.00
0
24
48
72
96
120
144
Follow up in months
high (n=8)
low (n=14)
raw p 2.15e-05
bonf p 3.66e-04
Expression Graph
Event=No
Event=Yes
Expression
300
250
200
150
100
50
0
sorted by expression
p-value

GROUP4 M0

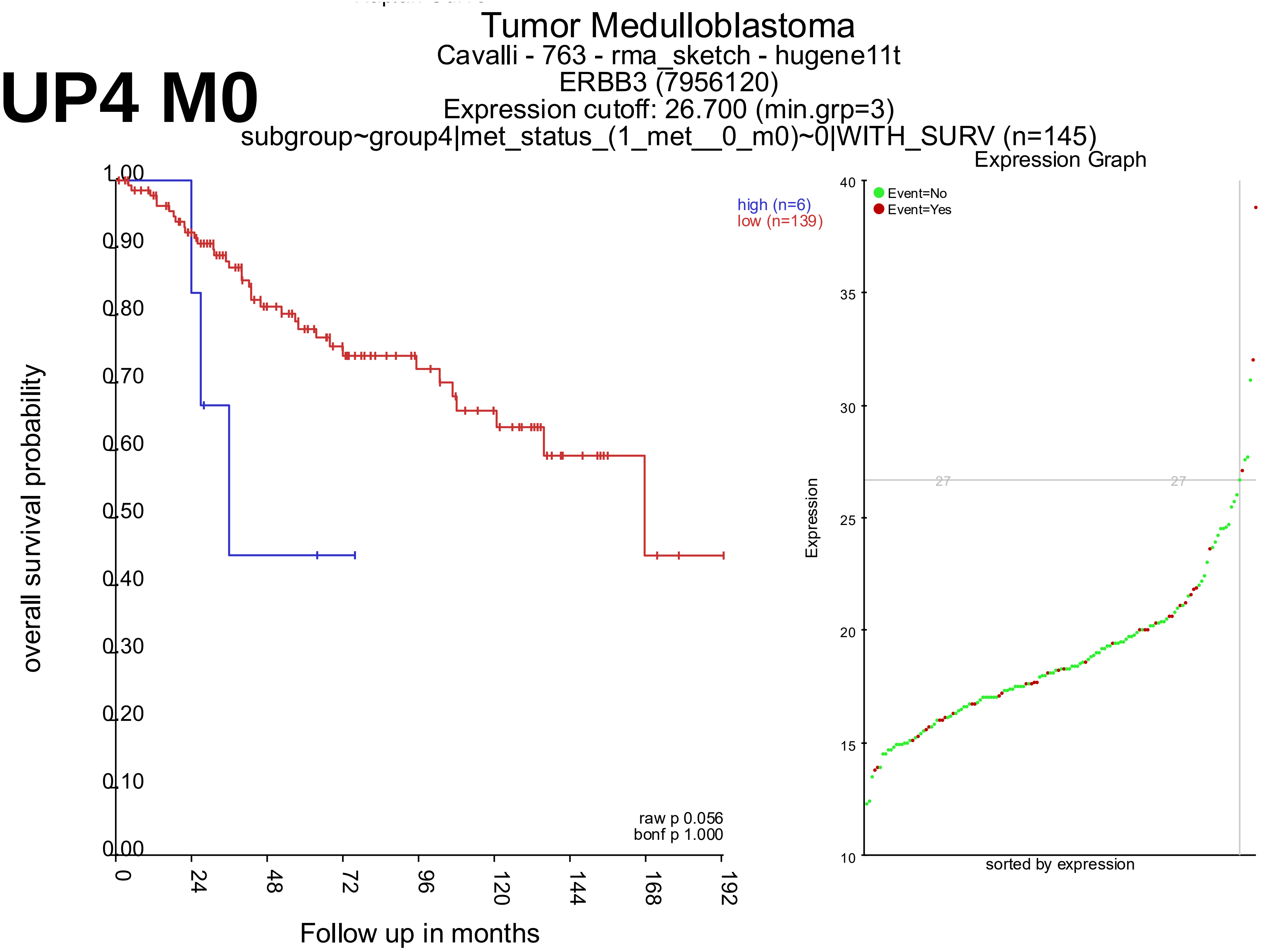

Tumor Medulloblastoma
Cavalli - 763 - rma_sketch - hugene11t
ERBB3 (7956120)
Expression cutoff: 26.700 (min.grp=3)
subgroup~group4|met_status_(1_met__0_m0)~0|WITH_SURV (n=145)
high (n=6)
low (n=139)
overall survival probability
Follow up in months
raw p 0.056
bonf p 1.000
Expression Graph
Event=No
Event=Yes
Expression
sorted by expression

GROUP4 M1

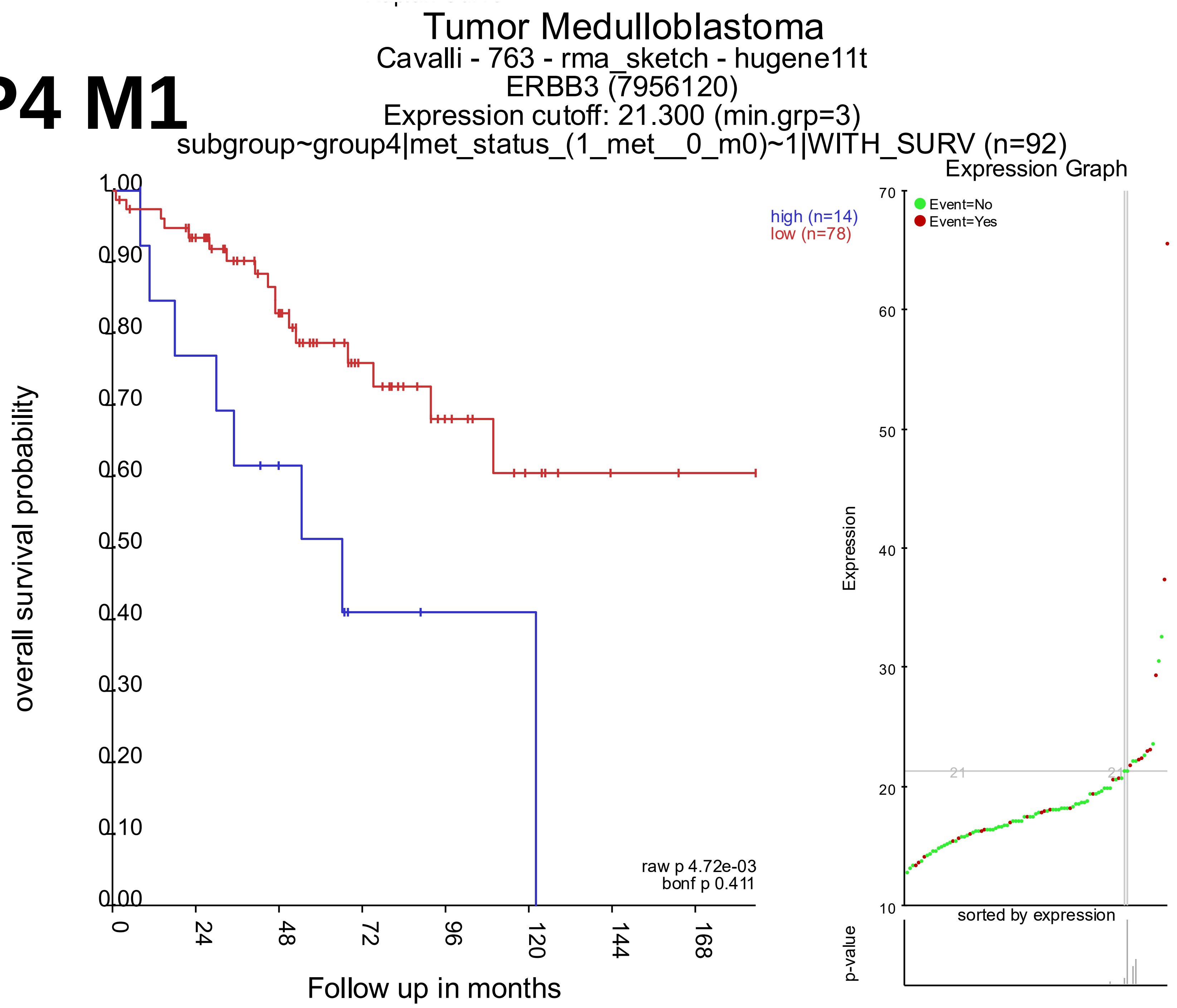

Tumor Medulloblastoma
Cavalli - 763 - rma_sketch - hugene11t
ERBB3 (7956120)
Expression cutoff: 21.300 (min.grp=3)
subgroup~group4|met_status_(1_met__0_m0)~1|WITH_SURV (n=92)
overall survival probability
1.00
0.90
0.80
0.70
0.60
0.50
0.40
0.30
0.20
0.10
0.00
0
24
48
72
96
120
144
168
Follow up in months
high (n=14)
low (n=78)
raw p 4.72e-03
bonf p 0.411
Expression Graph
Event=No
Event=Yes
Expression
70
60
50
40
30
20
10
21
sorted by expression
p-value

GROUP3 M0

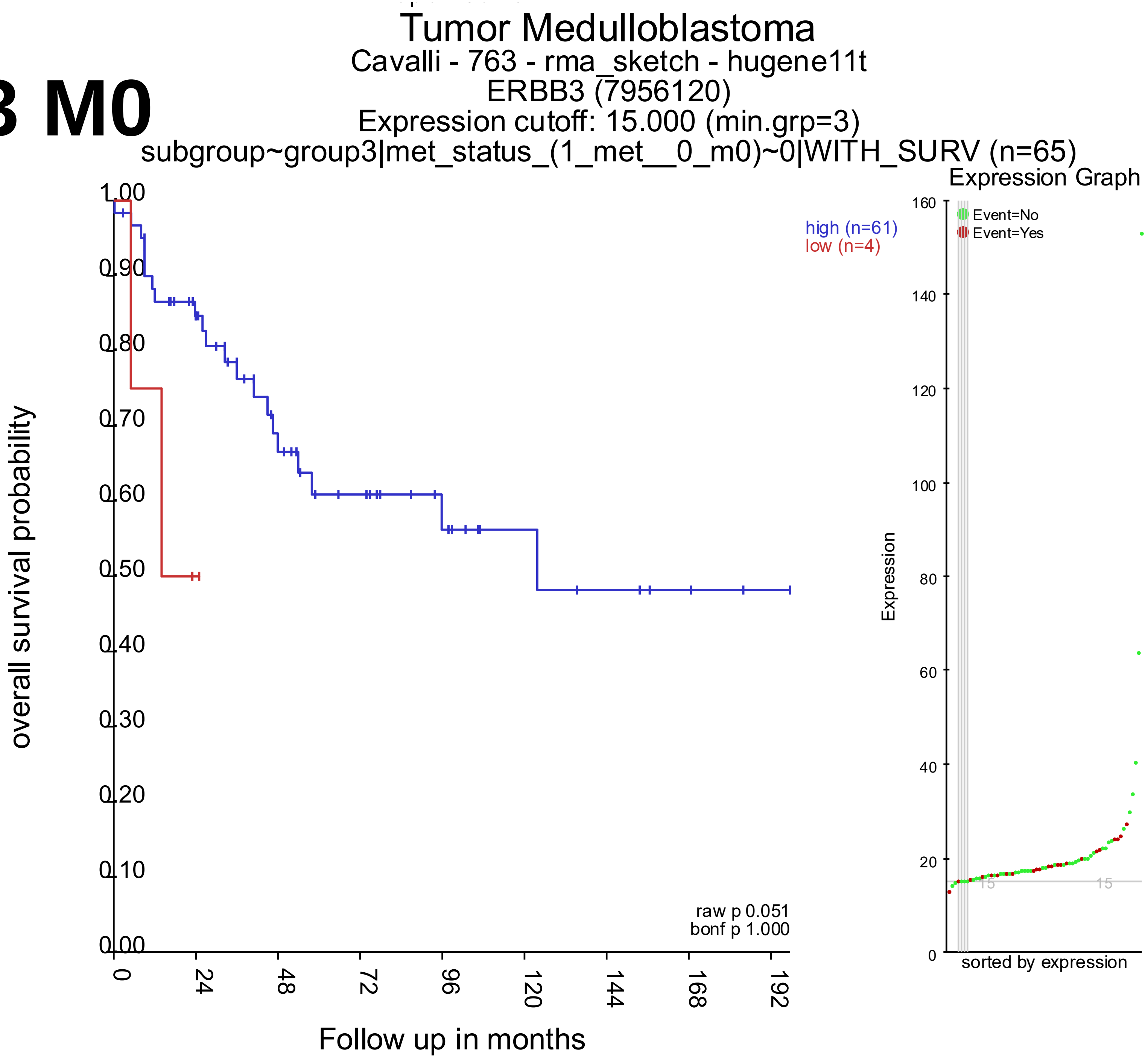

Tumor Medulloblastoma
Cavalli - 763 - rma_sketch - hugene11t
ERBB3 (7956120)
Expression cutoff: 15.000 (min.grp=3)
subgroup~group3|met_status_(1_met__0_m0)~0|WITH_SURV (n=65)
overall survival probability
1.00
0.90
0.80
0.70
0.60
0.50
0.40
0.30
0.20
0.10
0.00
high (n=61)
low (n=4)
raw p 0.051
bonf p 1.000
0
24
48
72
96
120
144
168
192
Follow up in months
Expression Graph
Event=No
Event=Yes
Expression
160
140
120
100
80
60
40
20
0
sorted by expression

GROUP3 M1

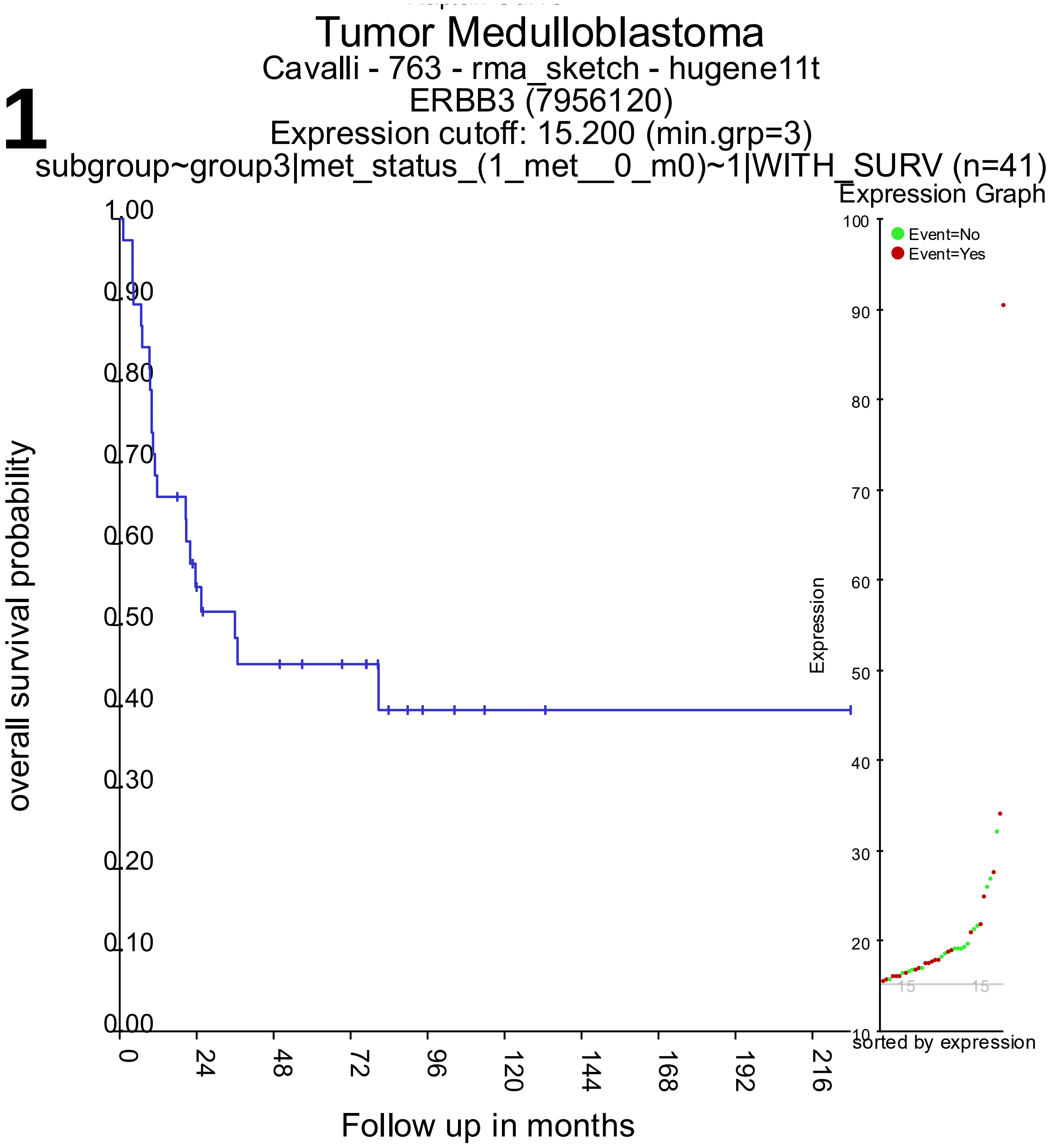

Tumor Medulloblastoma
Cavalli - 763 - rma_sketch - hugene11t
ERBB3 (7956120)
Expression cutoff: 15.200 (min.grp=3)
subgroup~group3|met_status_(1_met__0_m0)~1|WITH_SURV (n=41)
overall survival probability
1.00
0.90
0.80
0.70
0.60
0.50
0.40
0.30
0.20
0.10
0.00
0
24
48
72
96
120
144
168
192
216
Follow up in months
Expression Graph
Event=No
Event=Yes
Expression
100
90
80
70
60
50
40
30
20
10
15
sorted by expression

HER4/ERBB4

WNT M0

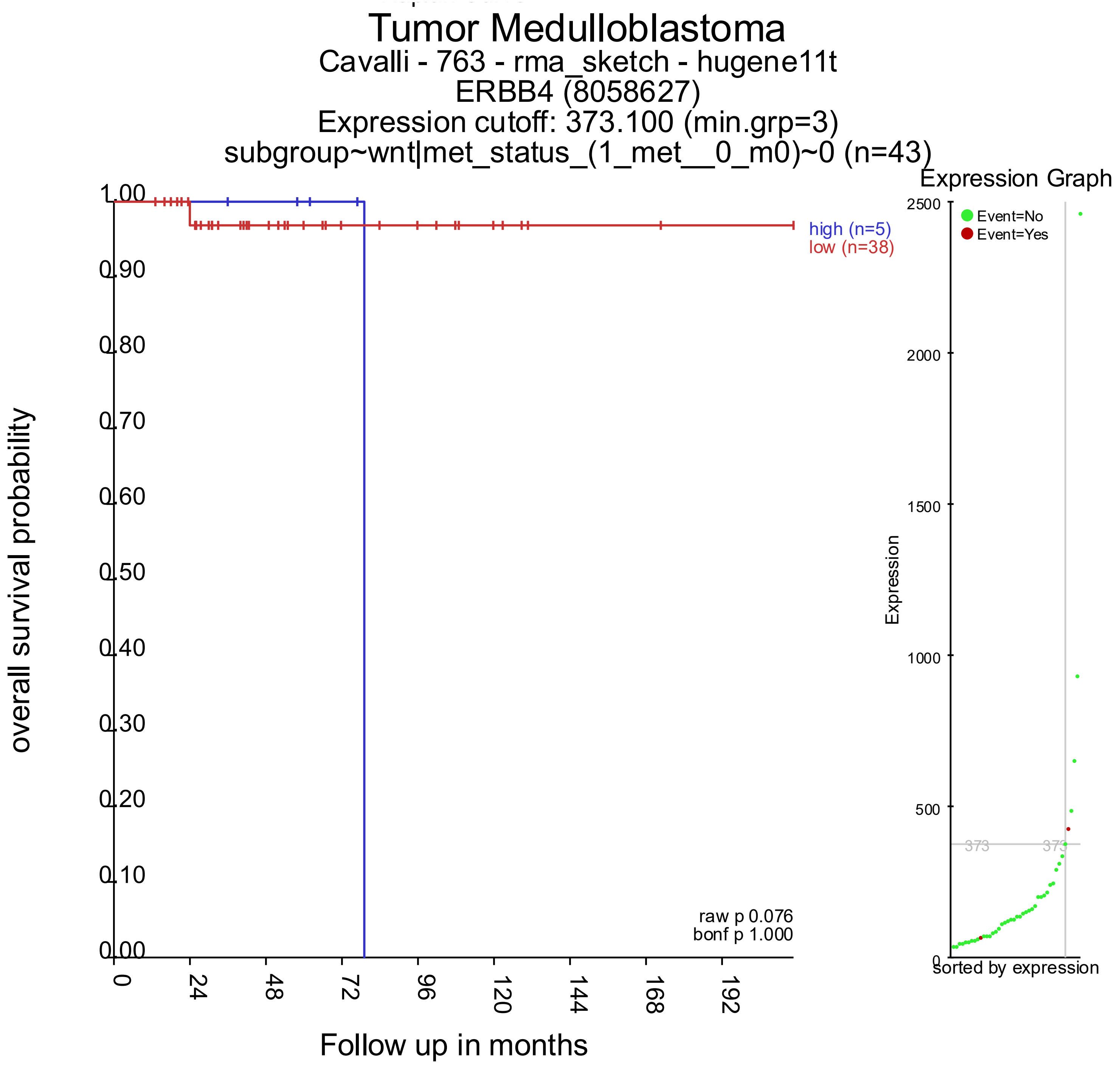

Tumor Medulloblastoma
Cavalli - 763 - rma_sketch - hugene11t
ERBB4 (8058627)
Expression cutoff: 373.100 (min.grp=3)
subgroup~wnt|met_status_(1_met__0_m0)~0 (n=43)
overall survival probability
1.00
0.90
0.80
0.70
0.60
0.50
0.40
0.30
0.20
0.10
0.00
0
24
48
72
96
120
144
168
192
Follow up in months
high (n=5)
low (n=38)
raw p 0.076
bonf p 1.000
Expression Graph
Event=No
Event=Yes
Expression
2500
2000
1500
1000
500
0
373
sorted by expression

WNT M1

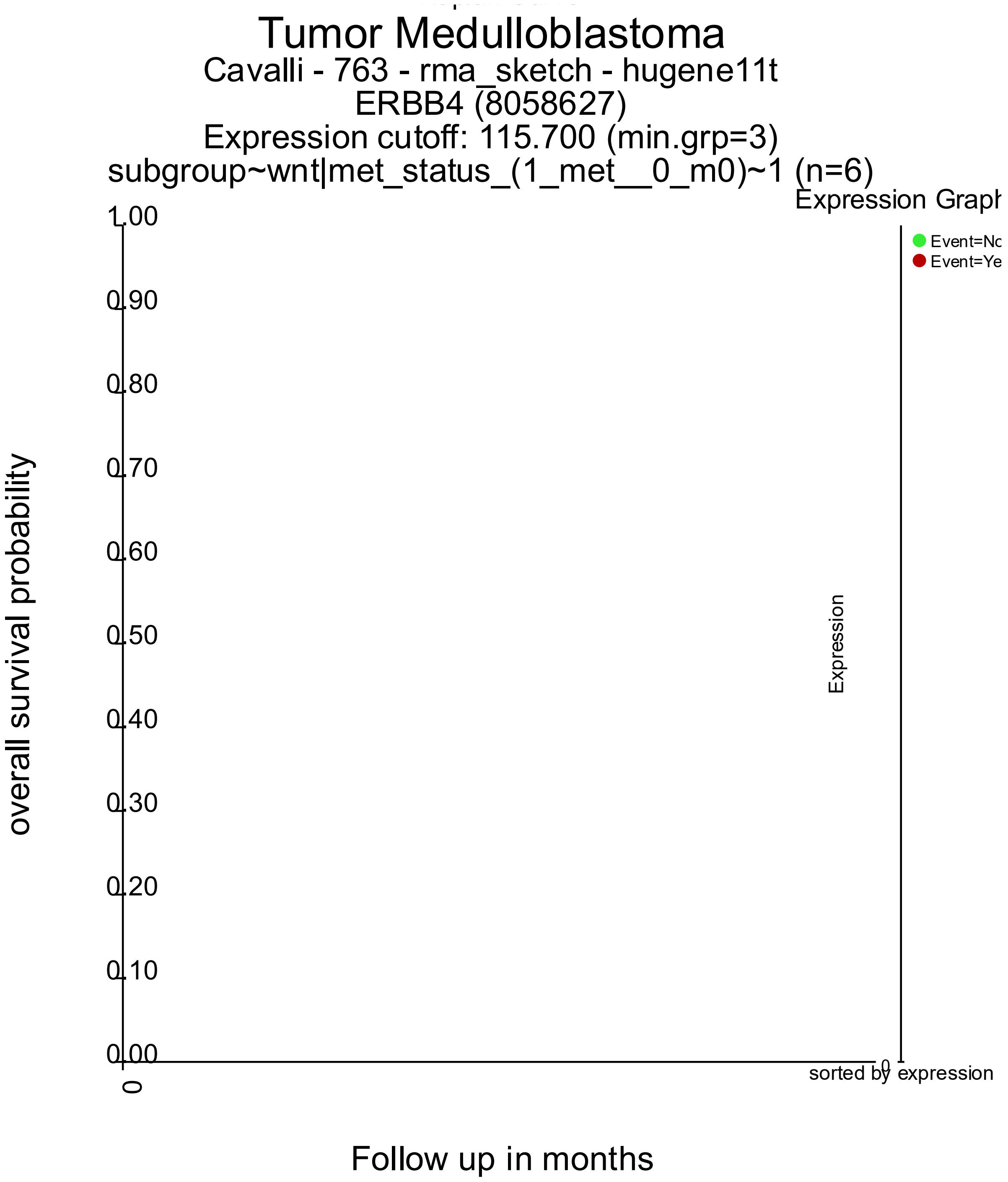

Tumor Medulloblastoma
Cavalli - 763 - rma_sketch - hugene11t
ERBB4 (8058627)
Expression cutoff: 115.700 (min.grp=3)
subgroup~wnt|met_status_(1_met__0_m0)~1 (n=6)
Expression Graph
Event=No
Event=Ye
1.00
0.90
0.80
0.70
0.60
0.50
0.40
0.30
0.20
0.10
0.00
0
overall survival probability
Follow up in months
Expression
sorted by expression
0

SHH M0

Tumor Medulloblastoma
Cavalli - 763 - rma_sketch - hugene11t
ERBB4 (8058627)
Expression cutoff: 232.300 (min.grp=3)
subgroup~shh|met_status_(1_met__0_m0)~0|WITH_SURV (n=124)

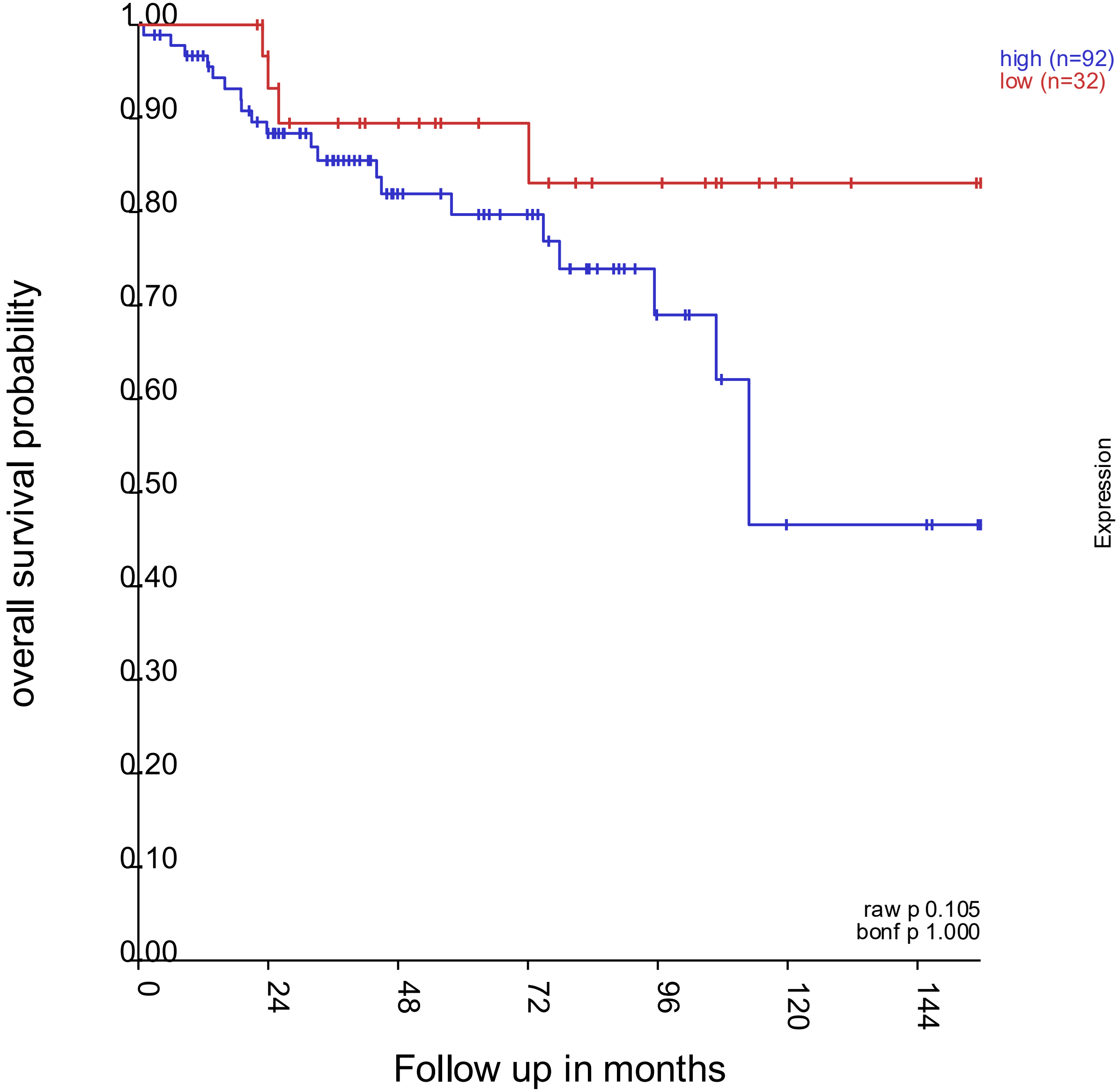

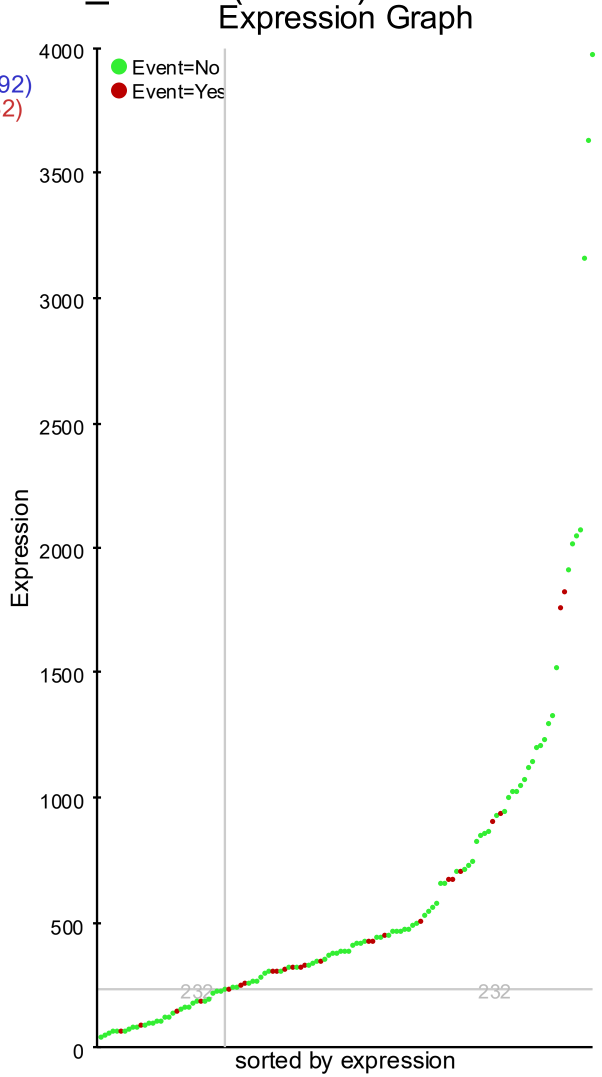

SHH M1

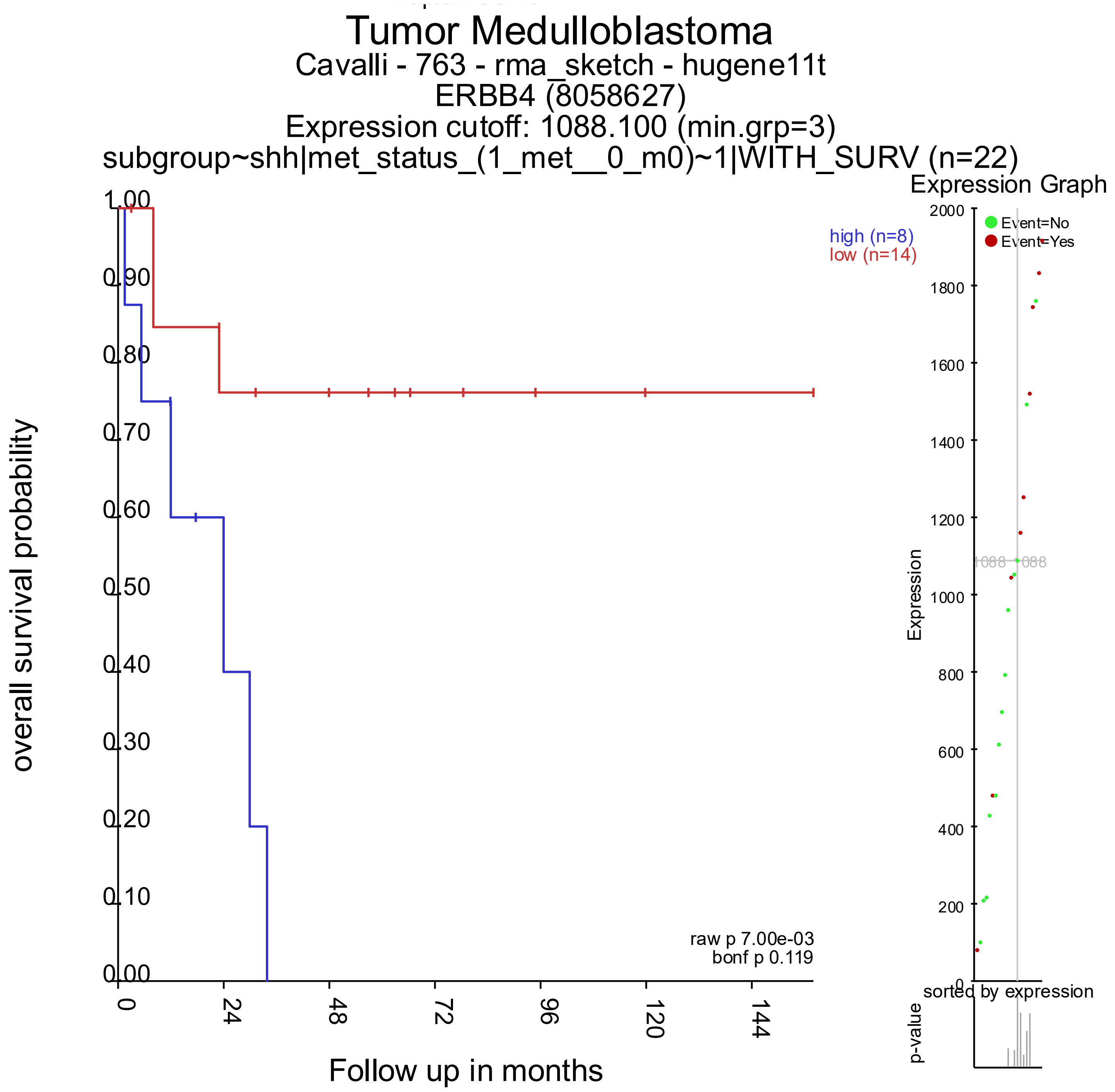

Tumor Medulloblastoma
Cavalli - 763 - rma_sketch - hugene11t
ERBB4 (8058627)
Expression cutoff: 1088.100 (min.grp=3)
subgroup~shh|met_status_(1_met__0_m0)~1|WITH_SURV (n=22)
overall survival probability
1.00
0.90
0.80
0.70
0.60
0.50
0.40
0.30
0.20
0.10
0.00
0
24
48
72
96
120
144
Follow up in months
high (n=8)
low (n=14)
raw p 7.00e-03
bonf p 0.119
Expression Graph
Event=No
Event=Yes
Expression
2000
1800
1600
1400
1200
1000
800
600
400
200
0
1088
sorted by expression
p-value

GROUP4 M0

Tumor Medulloblastoma
Cavalli - 763 - rma_sketch - hugene11t
ERBB4 (8058627)
Expression cutoff: 1741.600 (min.grp=3)
subgroup~group4|met_status_(1_met__0_m0)~0|WITH_SURV (n=145)

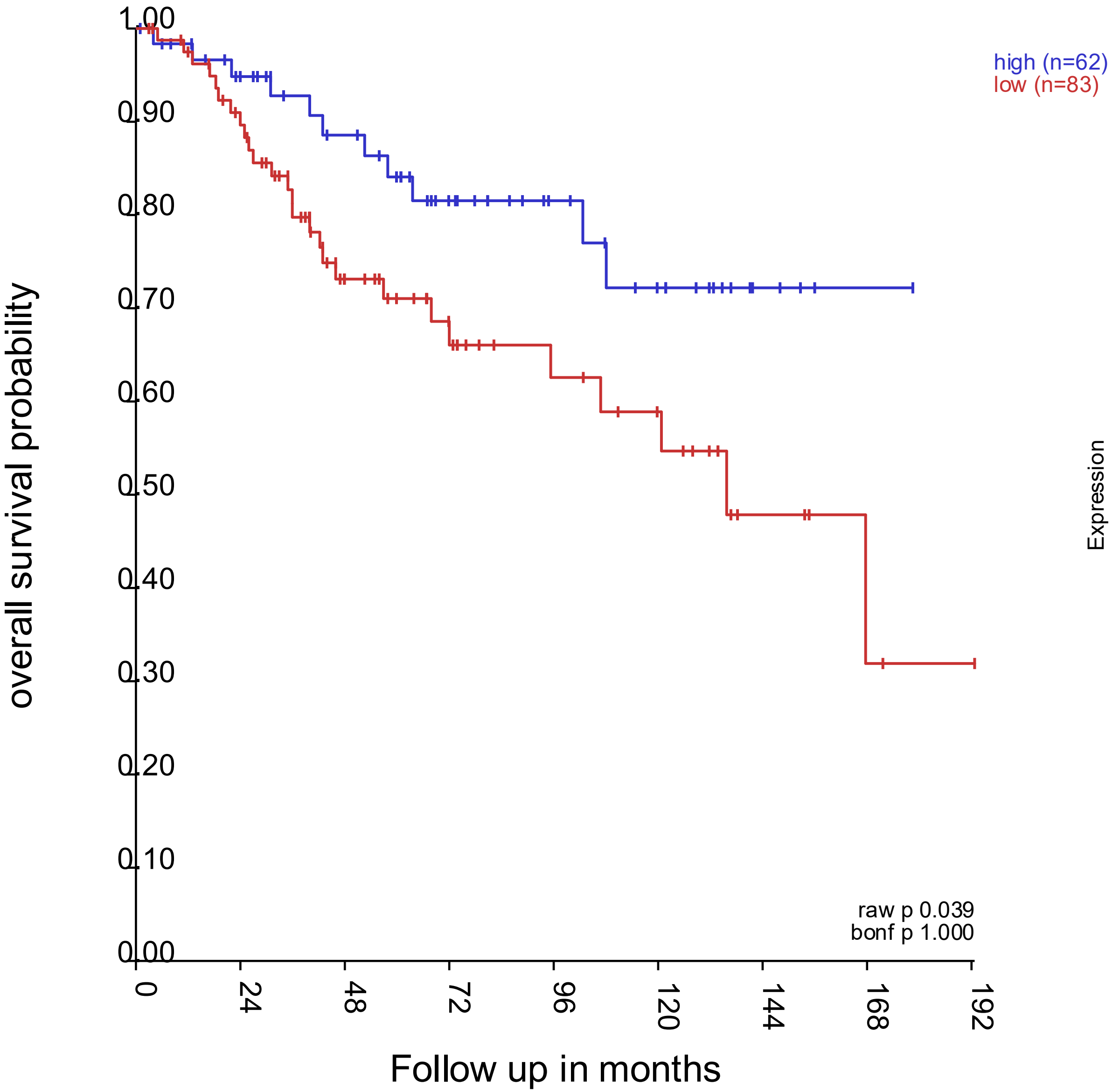

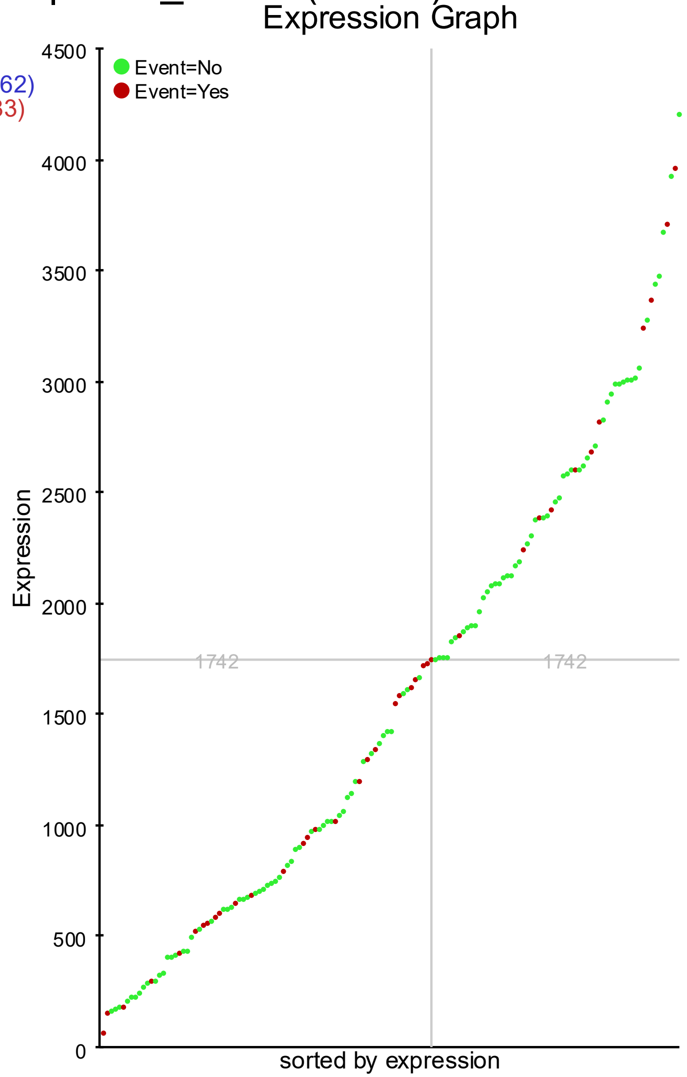

GROUP4 M1

Tumor Medulloblastoma
Cavalli - 763 - rma_sketch - hugene11t
ERBB4 (8058627)
Expression cutoff: 1196.000 (min.grp=3)
subgroup~group4|met_status_(1_met__0_m0)~1|WITH_SURV (n=92)

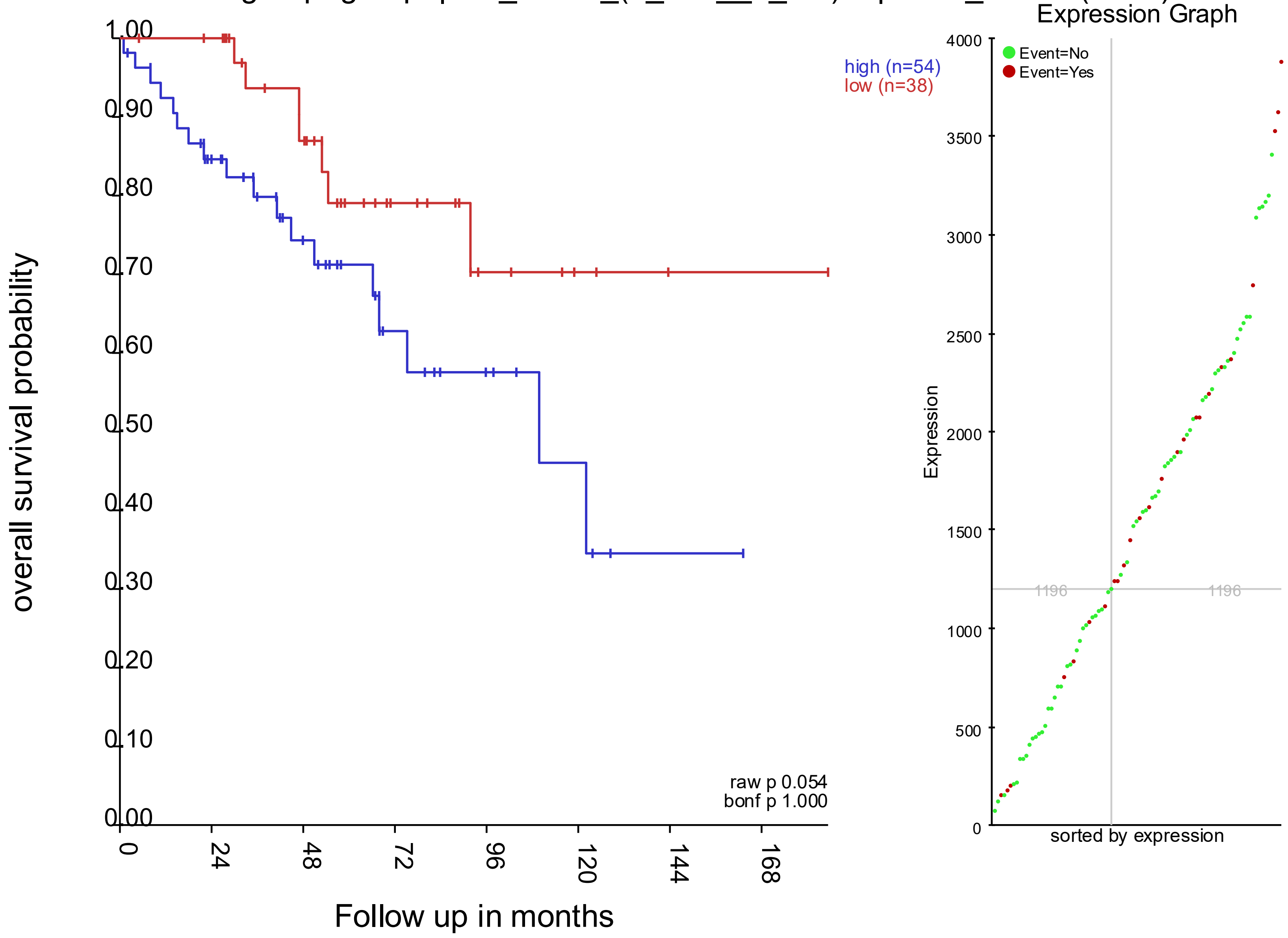

GROUP3 M0

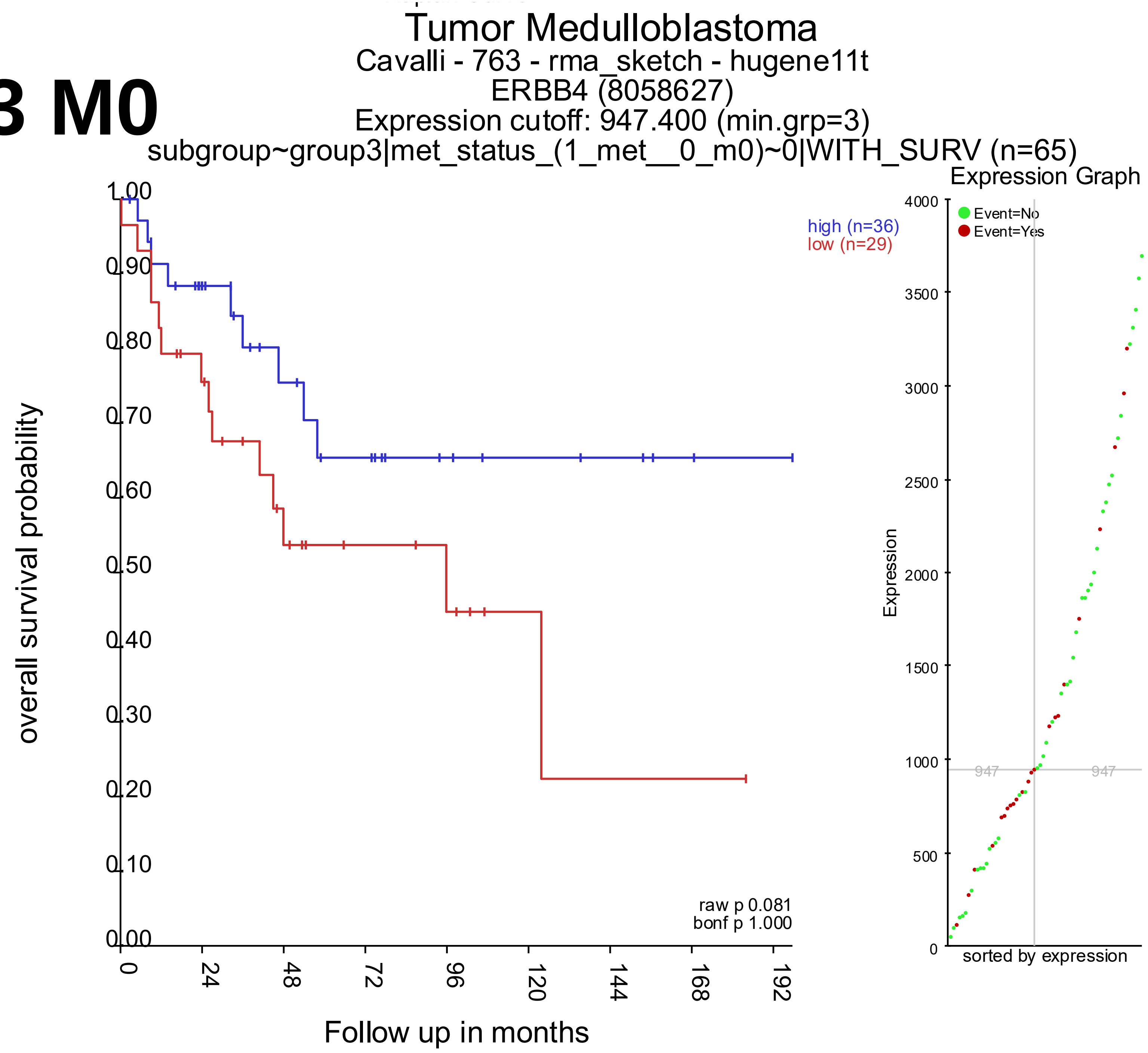

Tumor Medulloblastoma
Cavalli - 763 - rma_sketch - hugene11t
ERBB4 (8058627)
Expression cutoff: 947.400 (min.grp=3)
subgroup~group3|met_status_(1_met__0_m0)~0|WITH_SURV (n=65)
high (n=36)
low (n=29)
overall survival probability
1.00
0.90
0.80
0.70
0.60
0.50
0.40
0.30
0.20
0.10
0.00
0
24
48
72
96
120
144
168
192
Follow up in months
raw p 0.081
bonf p 1.000
Expression Graph
Event=No
Event=Yes
Expression
4000
3500
3000
2500
2000
1500
1000
500
0
947
947
sorted by expression

GROUP3 M1

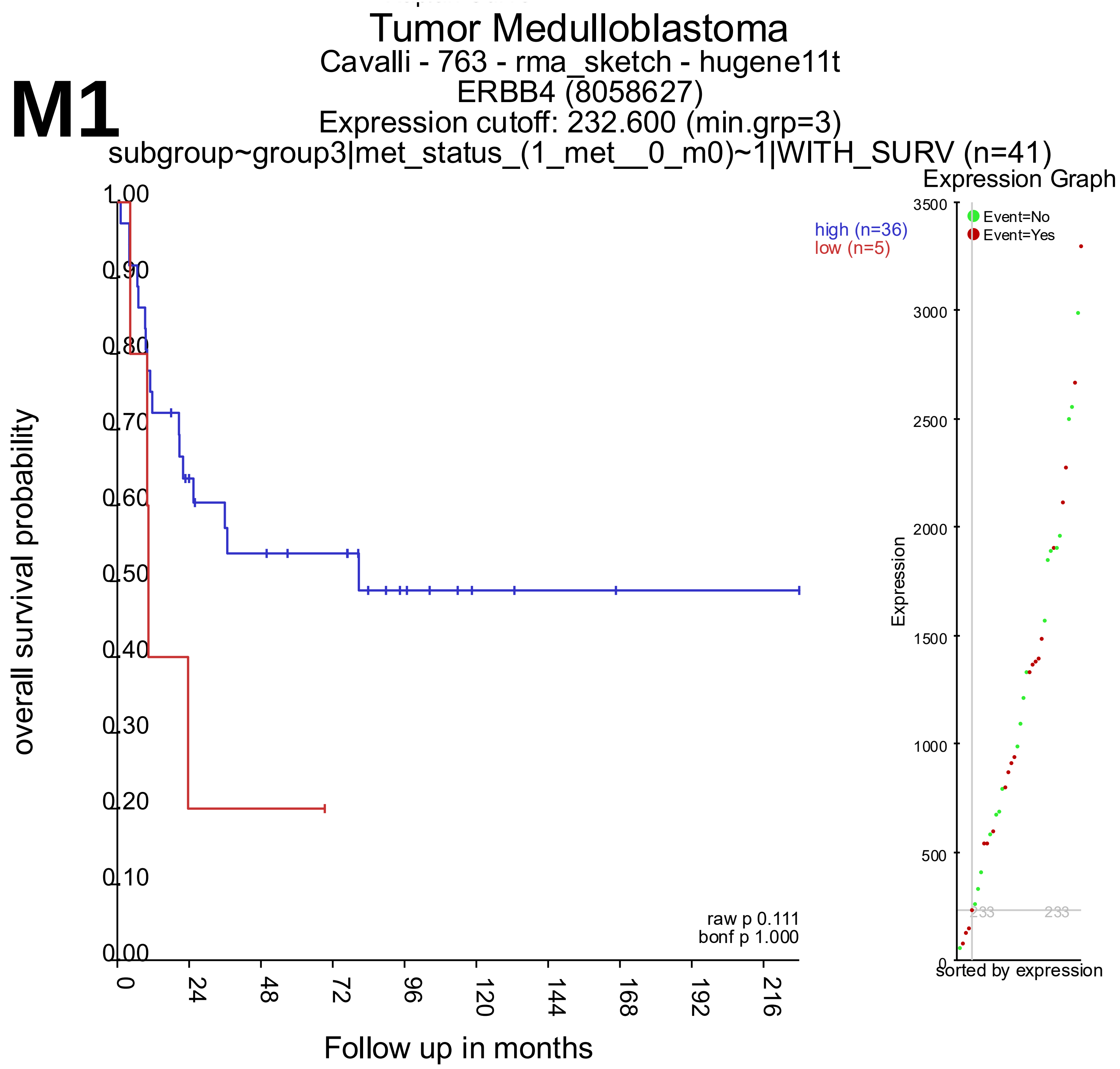

Tumor Medulloblastoma
Cavalli - 763 - rma_sketch - hugene11t
ERBB4 (8058627)
Expression cutoff: 232.600 (min.grp=3)
subgroup~group3|met_status_(1_met__0_m0)~1|WITH_SURV (n=41)
overall survival probability
1.00
0.90
0.80
0.70
0.60
0.50
0.40
0.30
0.20
0.10
0.00
0
24
48
72
96
120
144
168
192
216
Follow up in months
high (n=36)
low (n=5)
raw p 0.111
bonf p 1.000
Expression Graph
Event=No
Event=Yes
Expression
3500
3000
2500
2000
1500
1000
500
0
233
233
sorted by expression

PD1/PDCD1

WNT M0

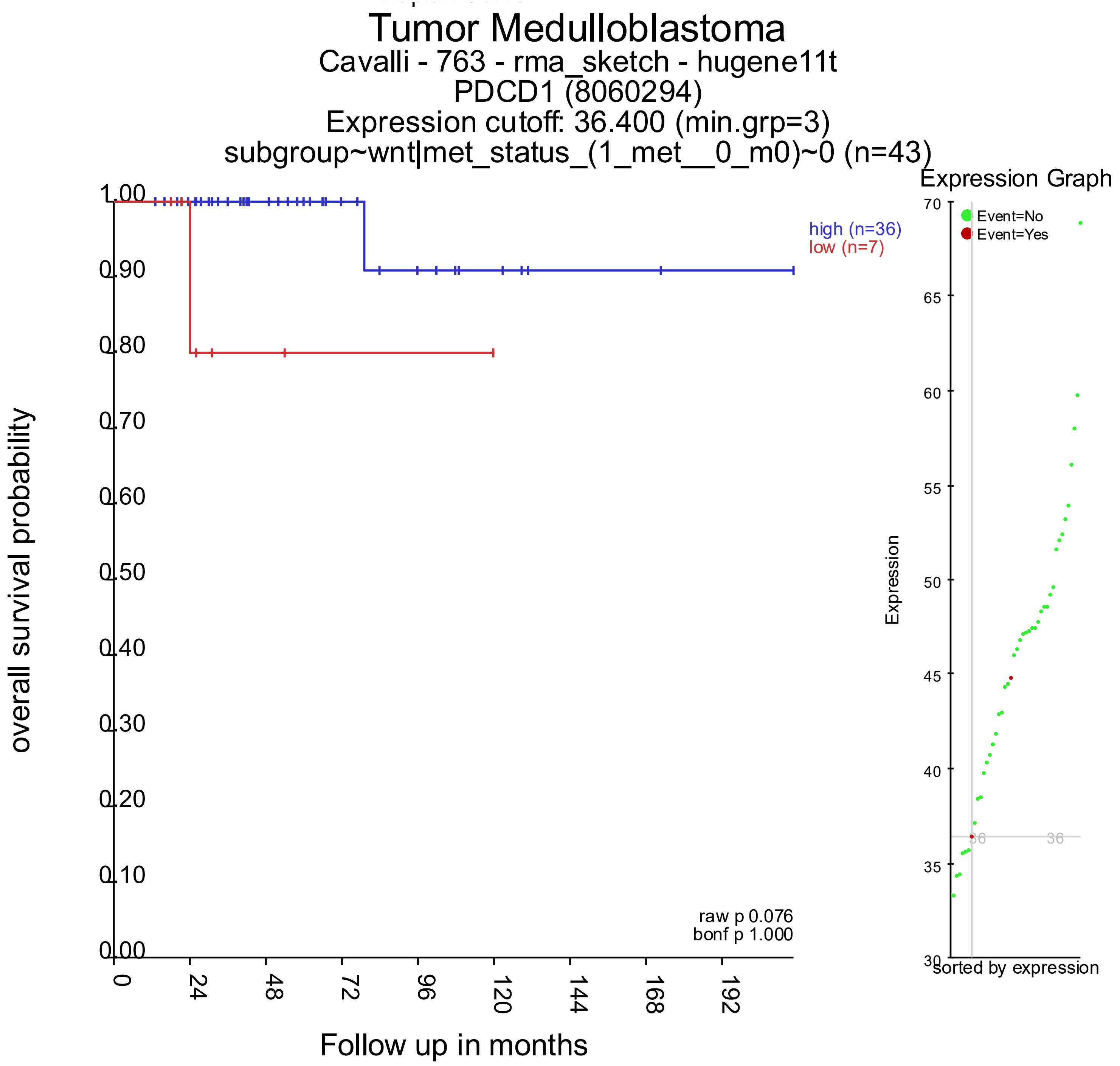

Tumor Medulloblastoma
Cavalli - 763 - rma_sketch - hugene11t
PDCD1 (8060294)
Expression cutoff: 36.400 (min.grp=3)
subgroup~wnt|met_status_(1_met__0_m0)~0 (n=43)
high (n=36)
low (n=7)
overall survival probability
1.00
0.90
0.80
0.70
0.60
0.50
0.40
0.30
0.20
0.10
0.00
0
24
48
72
96
120
144
168
192
Follow up in months
raw p 0.076
bonf p 1.000
Expression Graph
Event=No
Event=Yes
Expression
70
65
60
55
50
45
40
35
30
sorted by expression

WNT M1

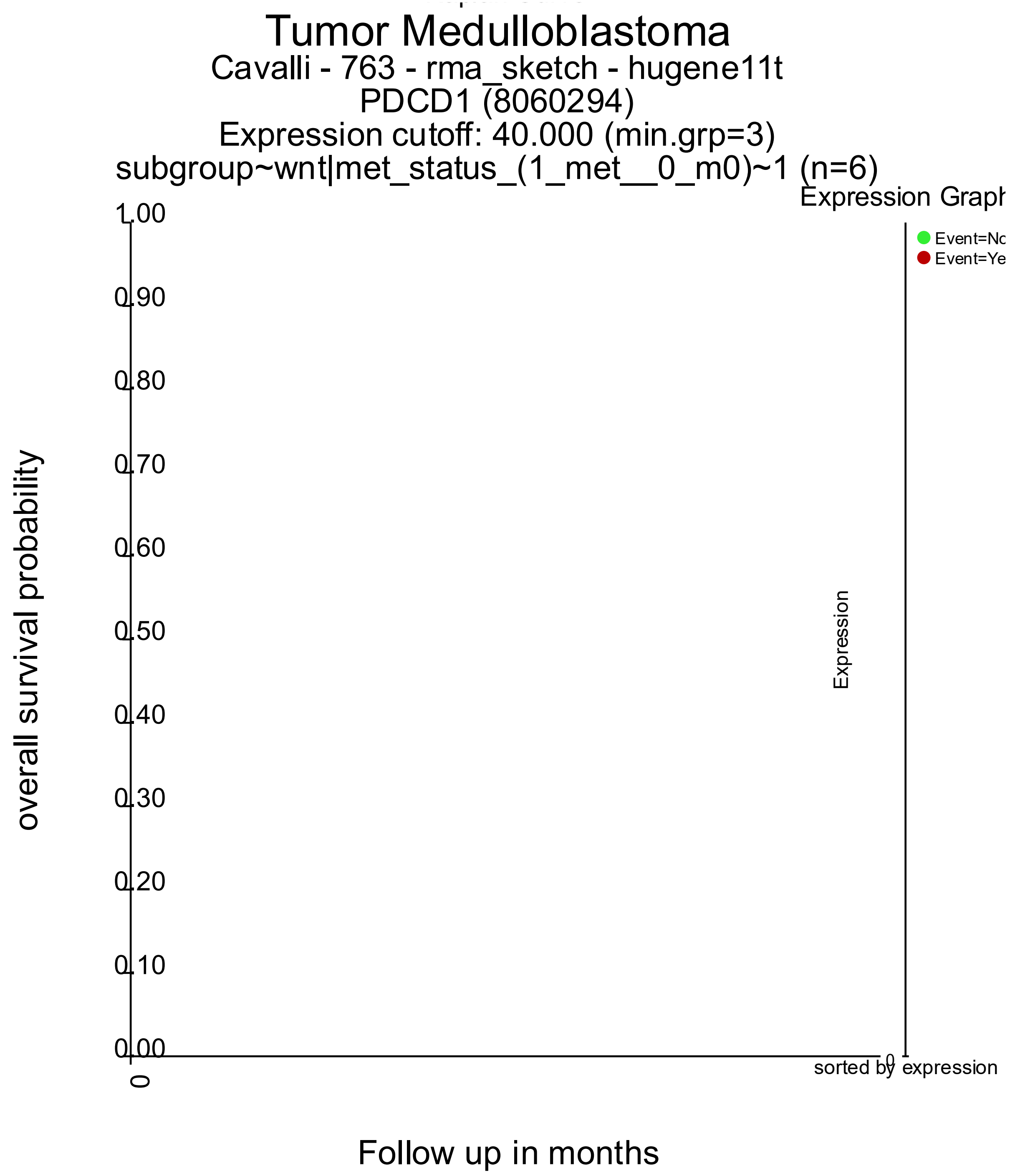

Tumor Medulloblastoma
Cavalli - 763 - rma_sketch - hugene11t
PDCD1 (8060294)
Expression cutoff: 40.000 (min.grp=3)
subgroup~wnt|met_status_(1_met__0_m0)~1 (n=6)
Expression Graph
Event=No
Event=Ye
overall survival probability
1.00
0.90
0.80
0.70
0.60
0.50
0.40
0.30
0.20
0.10
0.00
0
Follow up in months
Expression
0
sorted by expression

SHH M0

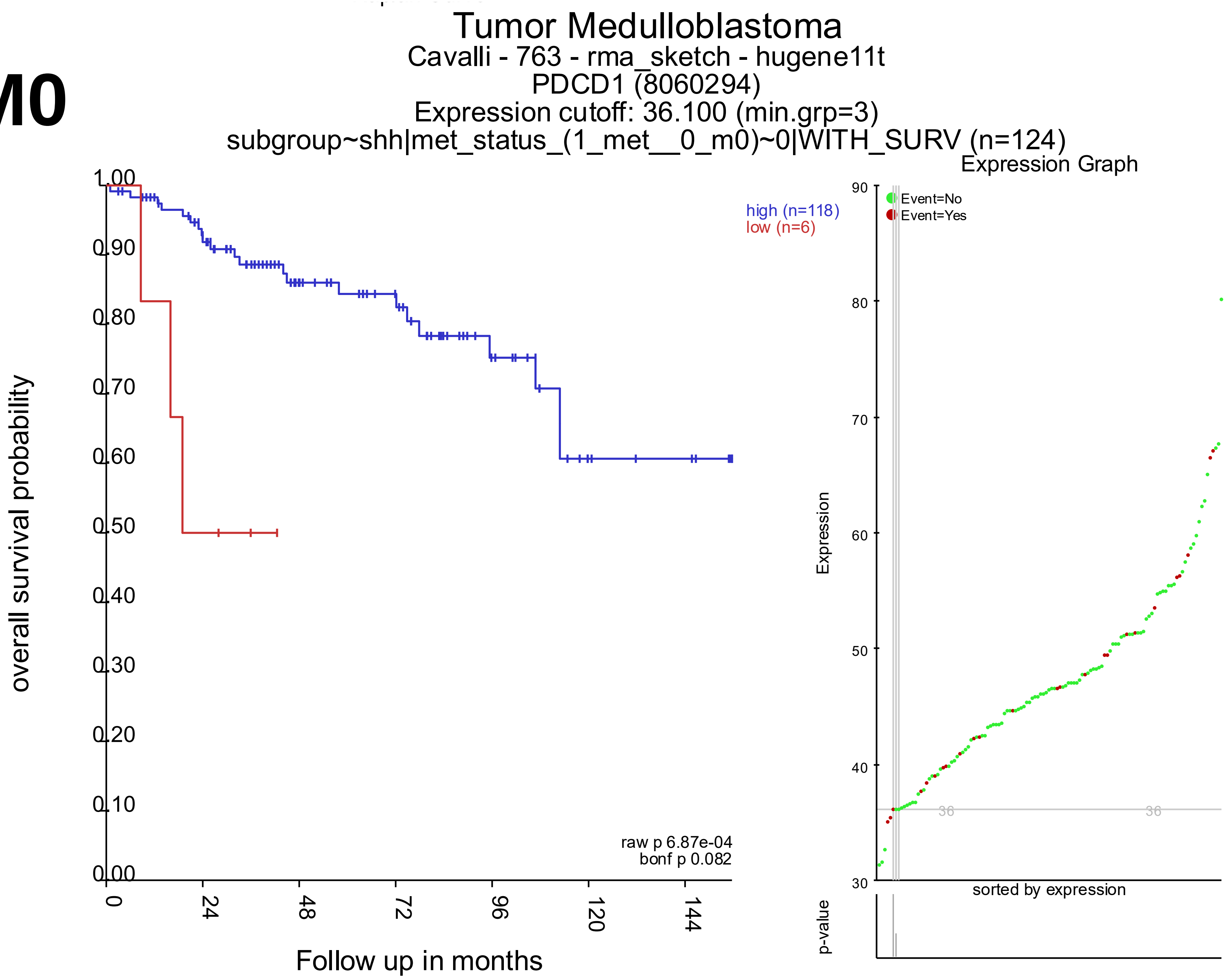

Tumor Medulloblastoma
Cavalli - 763 - rma_sketch - hugene11t
PDCD1 (8060294)
Expression cutoff: 36.100 (min.grp=3)
subgroup~shh|met_status_(1_met__0_m0)~0|WITH_SURV (n=124)
overall survival probability
1.00
0.90
0.80
0.70
0.60
0.50
0.40
0.30
0.20
0.10
0.00
0
24
48
72
96
120
144
Follow up in months
high (n=118)
low (n=6)
raw p 6.87e-04
bonf p 0.082
Expression Graph
Event=No
Event=Yes
Expression
90
80
70
60
50
40
30
36
36
sorted by expression
p-value

SHH M1

Tumor Medulloblastoma
Cavalli - 763 - rma_sketch - hugene11t
PDCD1 (8060294)
Expression cutoff: 39.100 (min.grp=3)
subgroup~shh|met_status_(1_met__0_m0)~1|WITH_SURV (n=22)

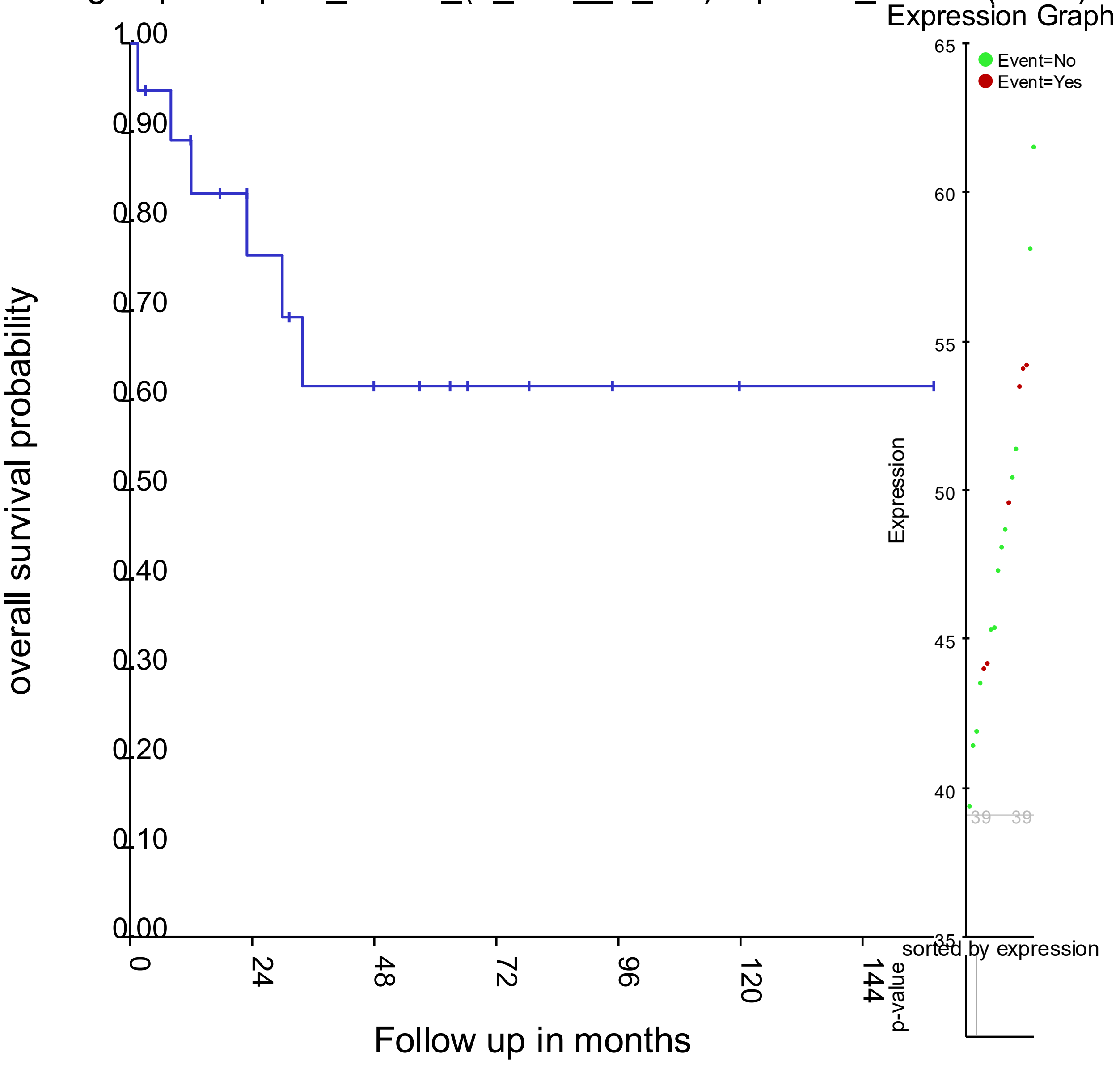

GROUP4 M0

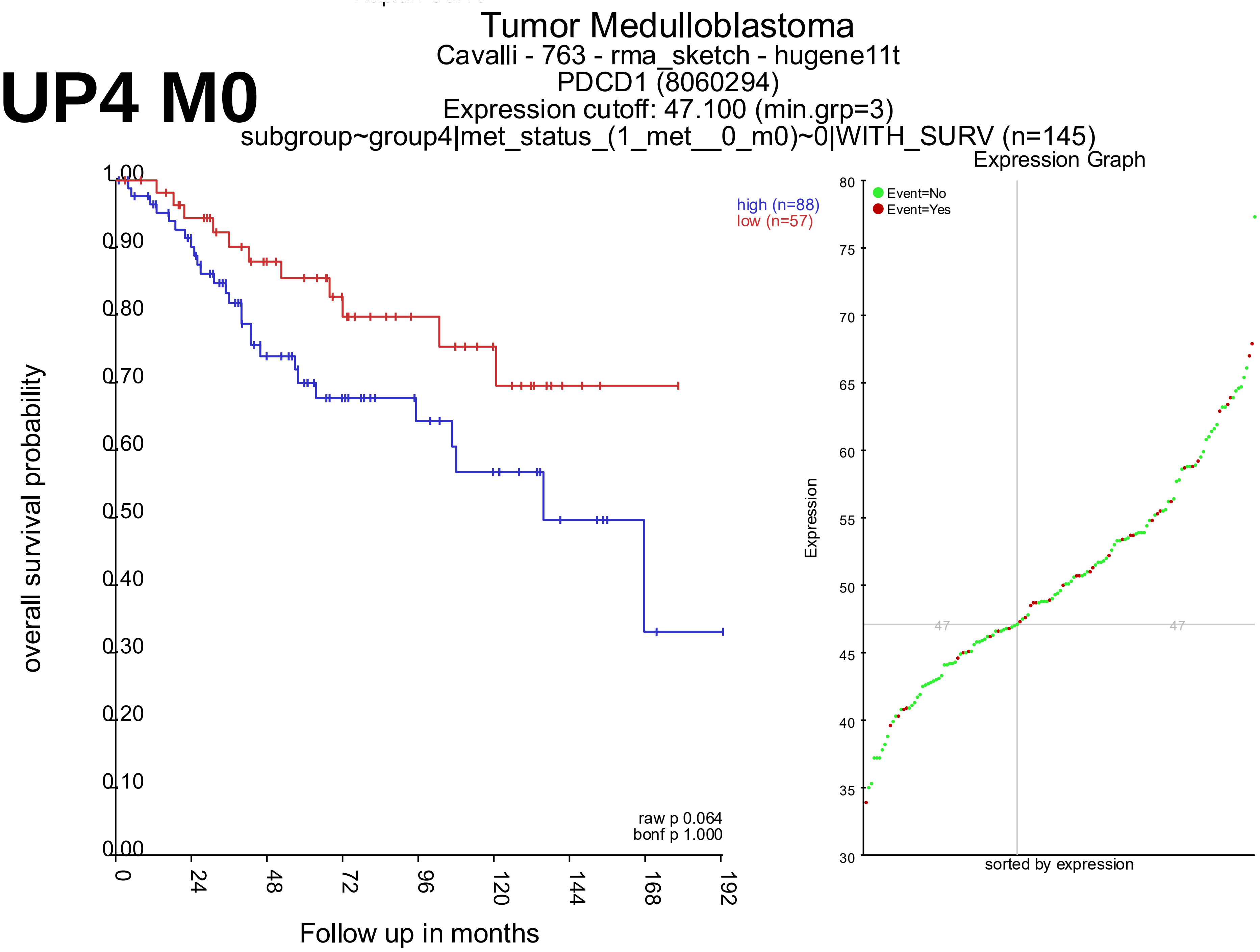
Tumor Medulloblastoma
Cavalli - 763 - rma_sketch - hugene11t
PDCD1 (8060294)
Expression cutoff: 47.100 (min.grp=3)
subgroup~group4|met_status_(1_met__0_m0)~0|WITH_SURV (n=145)
high (n=88)
low (n=57)
overall survival probability
1.00
0.90
0.80
0.70
0.60
0.50
0.40
0.30
0.20
0.10
0.00
0
24
48
72
96
120
144
168
192
Follow up in months
raw p 0.064
bonf p 1.000
Expression Graph
Event=No
Event=Yes
Expression
80
75
70
65
60
55
50
45
40
35
30
47
47
sorted by expression

GROUP4 M1

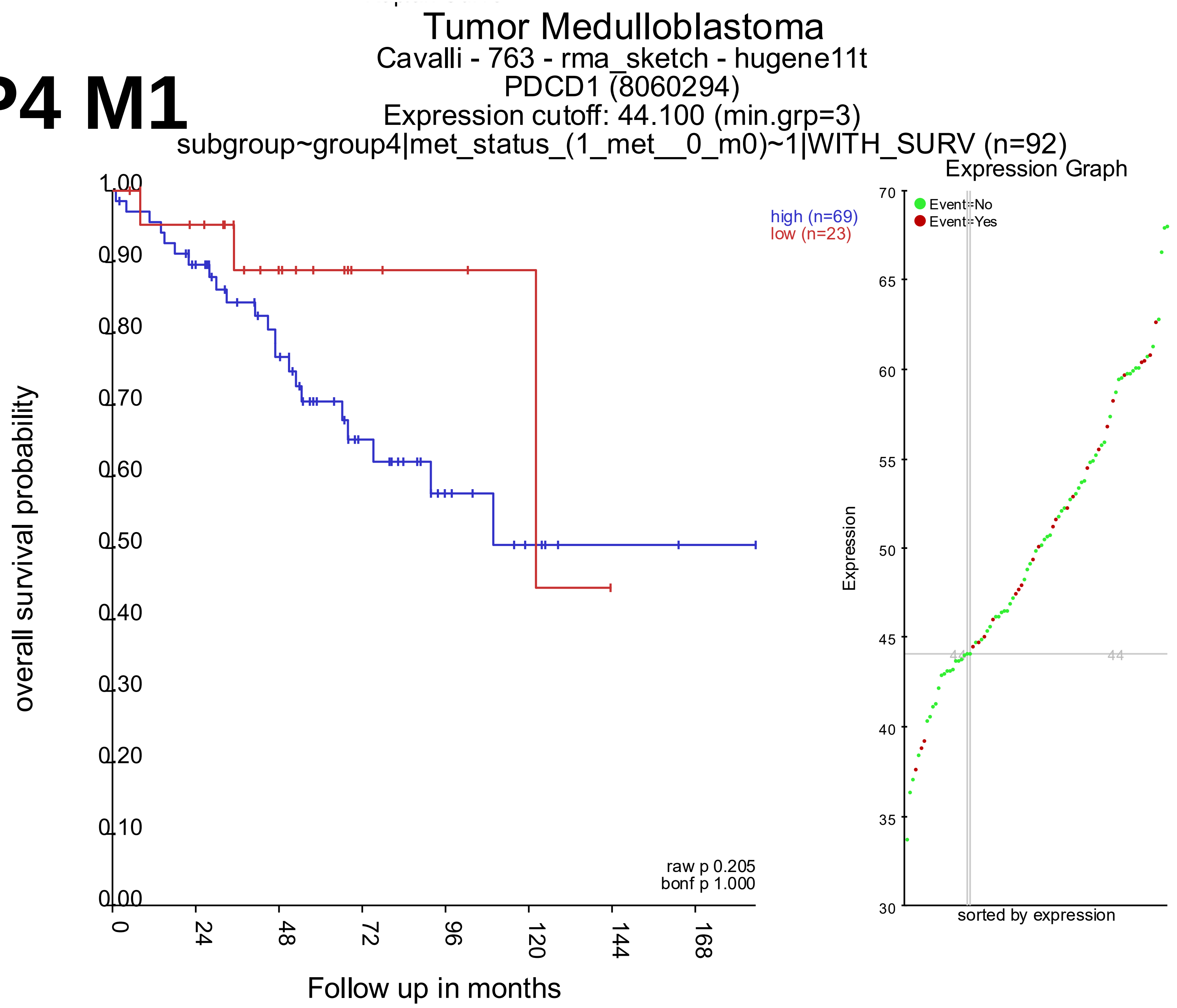

Tumor Medulloblastoma
Cavalli - 763 - rma_sketch - hugene11t
PDCD1 (8060294)
Expression cutoff: 44.100 (min.grp=3)
subgroup~group4|met_status_(1_met__0_m0)~1|WITH_SURV (n=92)
overall survival probability
1.00
0.90
0.80
0.70
0.60
0.50
0.40
0.30
0.20
0.10
0.00
0
24
48
72
96
120
144
168
Follow up in months
high (n=69)
low (n=23)
raw p 0.205
bonf p 1.000
Expression Graph
Event=No
Event=Yes
Expression
70
65
60
55
50
45
40
35
30
44
44
sorted by expression

GROUP3 M0

Tumor Medulloblastoma
Cavalli - 763 - rma_sketch - hugene11t
PDCD1 (8060294)
Expression cutoff: 42.600 (min.grp=3)
subgroup~group3|met_status_(1_met__0_m0)~0|WITH_SURV (n=65)

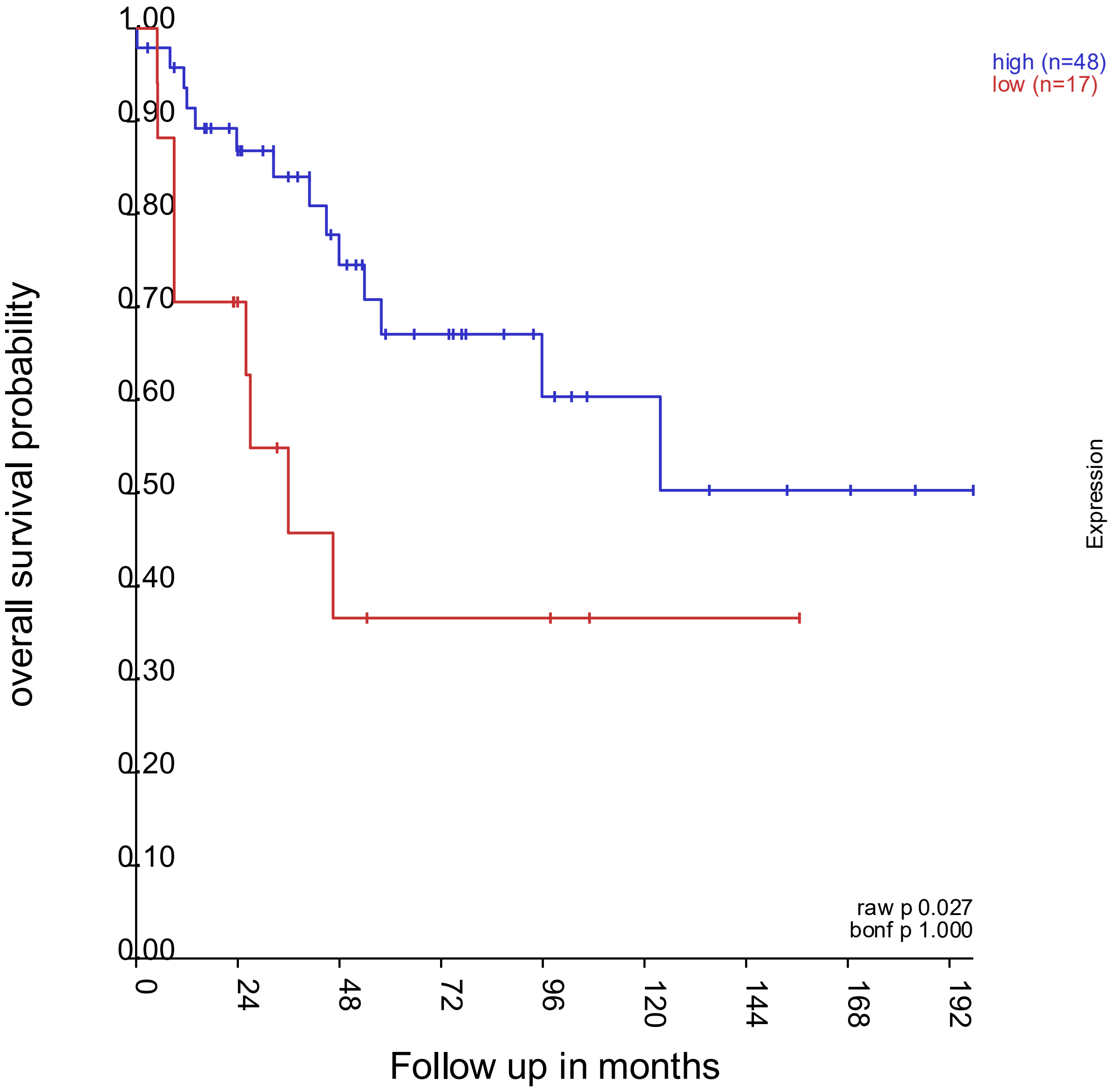

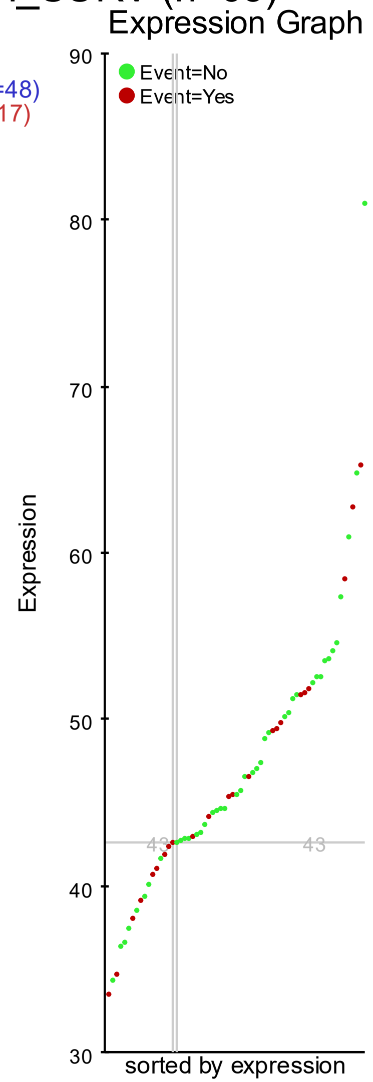

GROUP3 M1

Tumor Medulloblastoma
Cavalli - 763 - rma_sketch - hugene11t
PDCD1 (8060294)
Expression cutoff: 48.100 (min.grp=3)
subgroup~group3|met_status_(1_met__0_m0)~1|WITH_SURV (n=41)

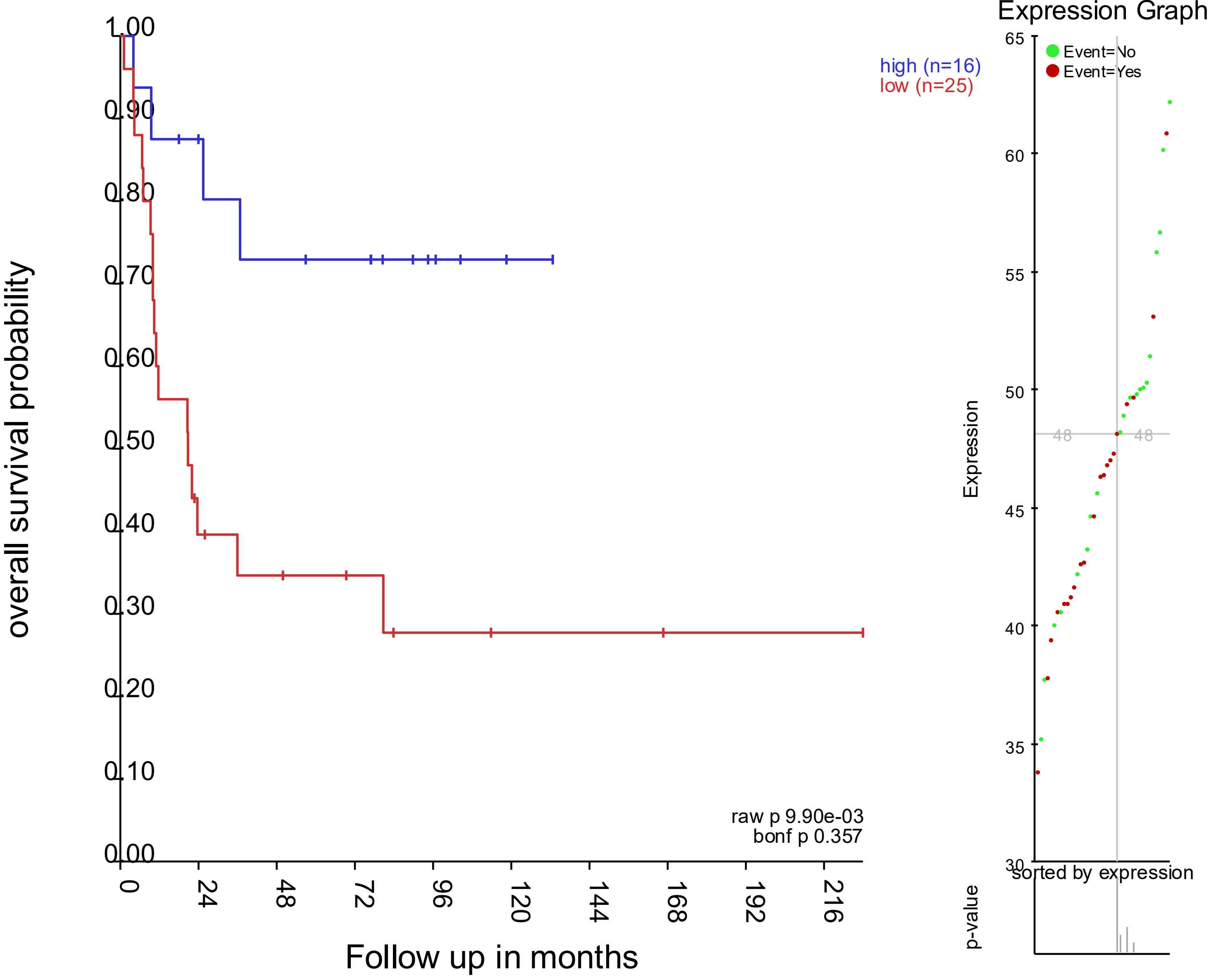

PD2/PDCD2

WNT M0

Tumor Medulloblastoma
Cavalli - 763 - rma_sketch - hugene11t
PDCD2 (8130962)
Expression cutoff: 194.600 (min.grp=3)
subgroup~wnt|met_status_(1_met__0_m0)~0 (n=43)

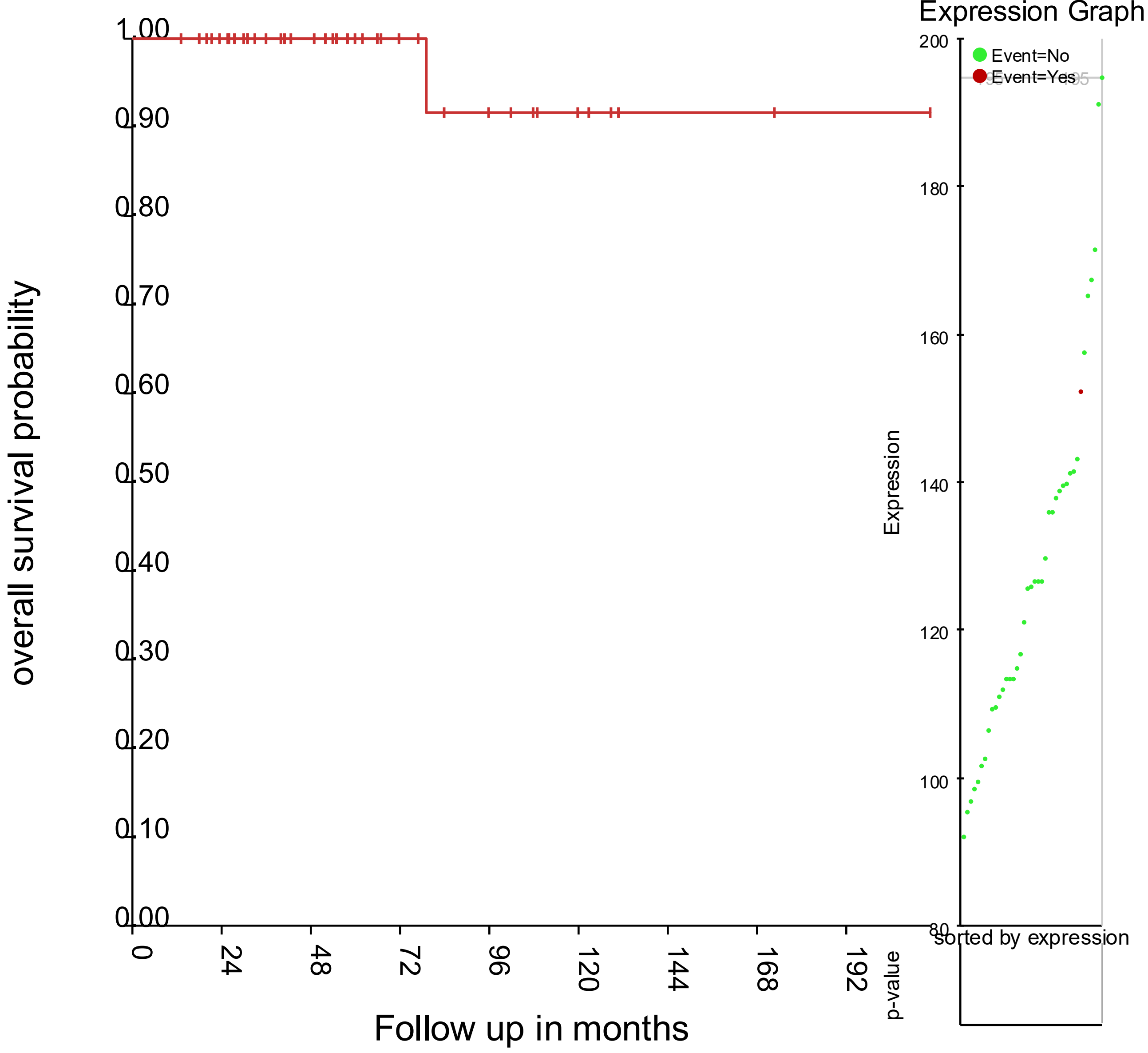

WNT M1

Tumor Medulloblastoma
Cavalli - 763 - rma_sketch - hugene11t
PDCD2 (8130962)
Expression cutoff: 124.600 (min.grp=3)
subgroup~wnt|met_status_(1_met__0_m0)~1 (n=6)

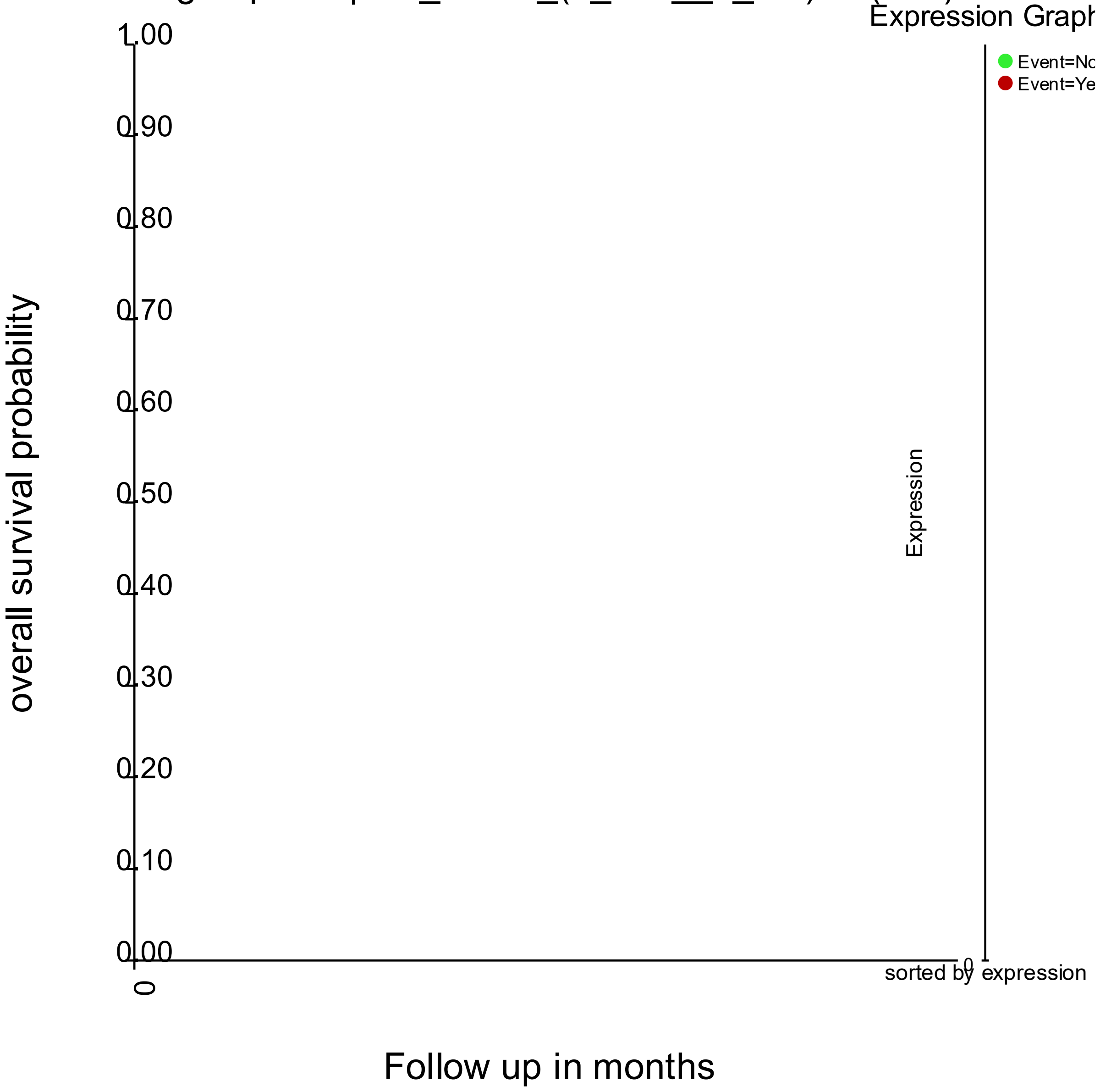

SHH M0

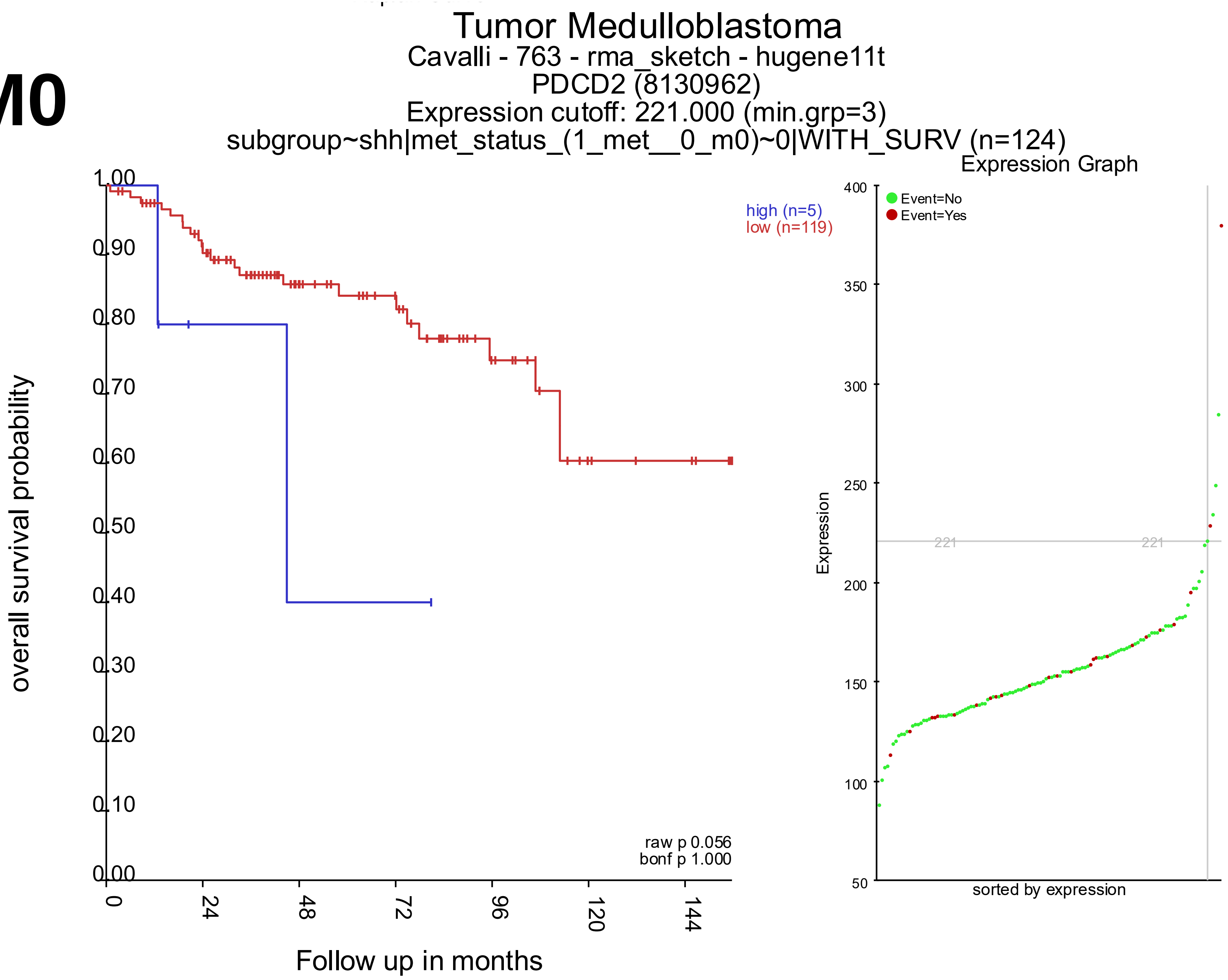
Tumor Medulloblastoma
Cavalli - 763 - rma_sketch - hugene11t
PDCD2 (8130962)
Expression cutoff: 221.000 (min.grp=3)
subgroup~shh|met_status_(1_met__0_m0)~0|WITH_SURV (n=124)
overall survival probability
1.00
0.90
0.80
0.70
0.60
0.50
0.40
0.30
0.20
0.10
0.00
0
24
48
72
96
120
144
Follow up in months
high (n=5)
low (n=119)
raw p 0.056
bonf p 1.000
Expression Graph
Event=No
Event=Yes
Expression
400
350
300
250
200
150
100
50
221
221
sorted by expression

SHH M1

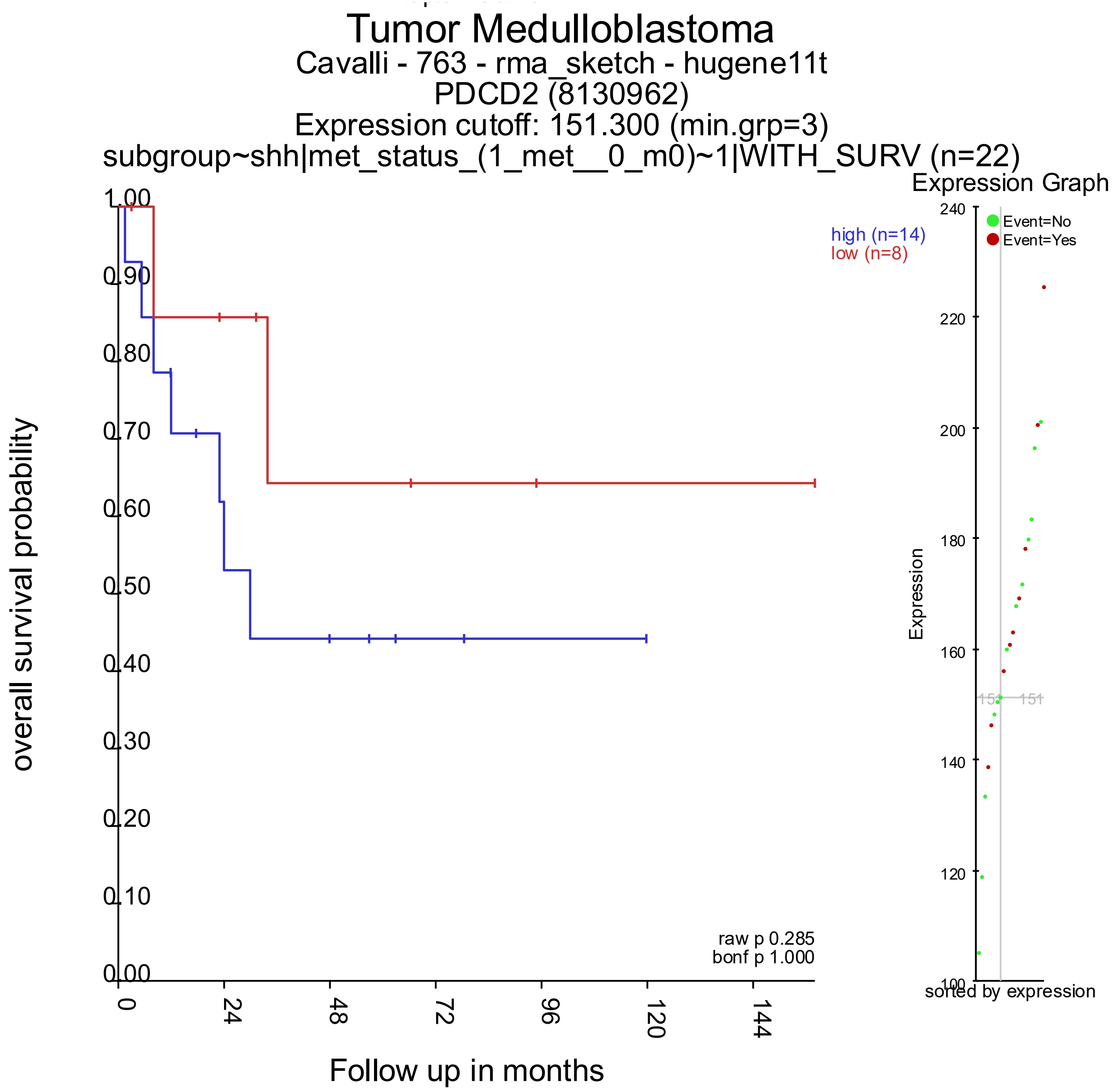
Tumor Medulloblastoma
Cavalli - 763 - rma_sketch - hugene11t
PDCD2 (8130962)
Expression cutoff: 151.300 (min.grp=3)
subgroup~shh|met_status_(1_met__0_m0)~1|WITH_SURV (n=22)
Expression Graph
high (n=14)
low (n=8)
Event=No
Event=Yes
overall survival probability
1.00
0.90
0.80
0.70
0.60
0.50
0.40
0.30
0.20
0.10
0.00
0
24
48
72
96
120
144
Follow up in months
raw p 0.285
bonf p 1.000
240
220
200
180
160
140
120
100
Expression
151
sorted by expression

GROUP4 M0

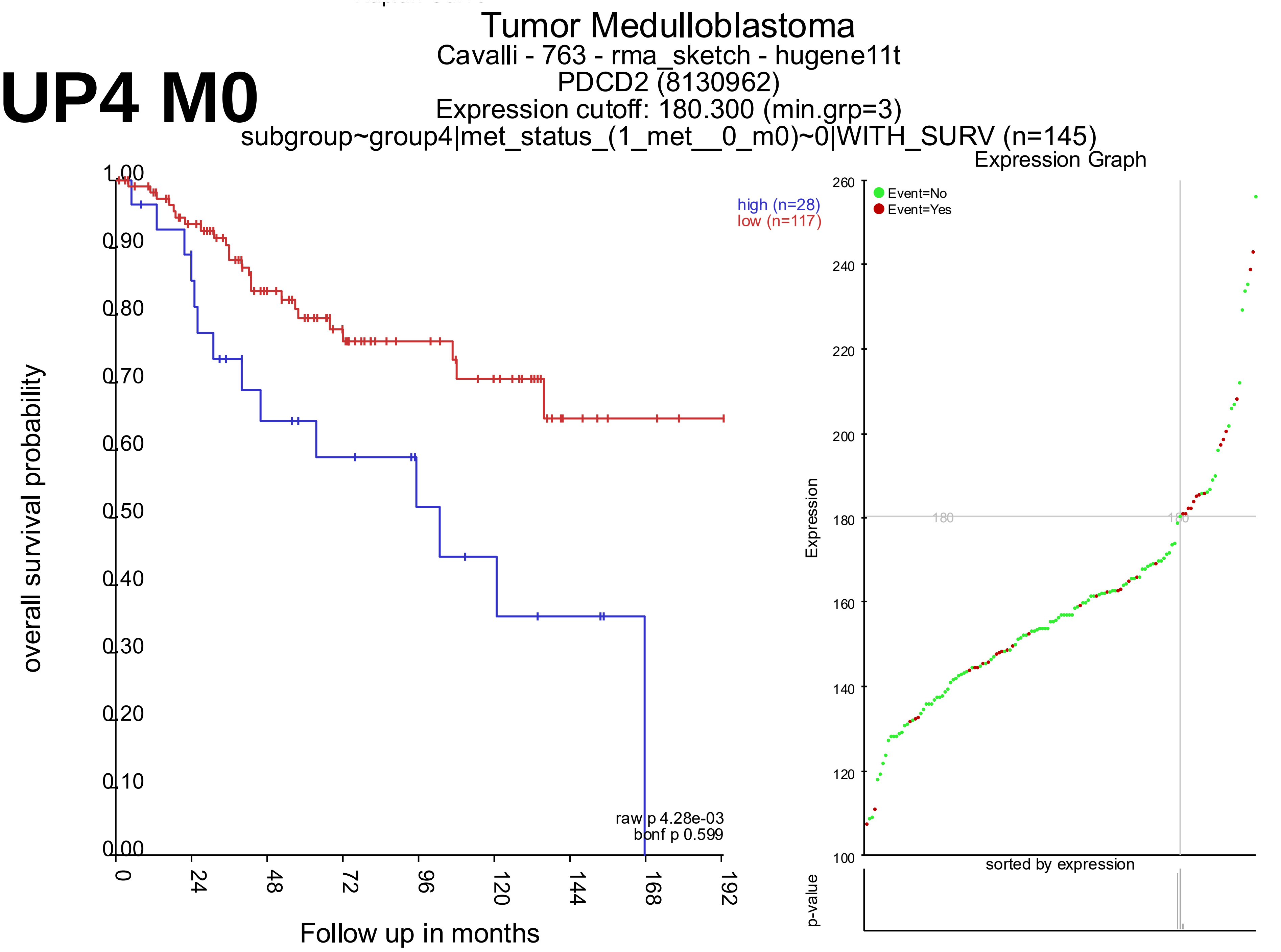

Tumor Medulloblastoma
Cavalli - 763 - rma_sketch - hugene11t
PDCD2 (8130962)
Expression cutoff: 180.300 (min.grp=3)
subgroup~group4|met_status_(1_met__0_m0)~0|WITH_SURV (n=145)
overall survival probability
1.00
0.90
0.80
0.70
0.60
0.50
0.40
0.30
0.20
0.10
0.00
high (n=28)
low (n=117)
raw p 4.28e-03
bonf p 0.599
0
24
48
72
96
120
144
168
192
Follow up in months
Expression Graph
Event=No
Event=Yes
Expression
260
240
220
200
180
160
140
120
100
sorted by expression
p-value

GROUP4 M1

Tumor Medulloblastoma
Cavalli - 763 - rma_sketch - hugene11t
PDCD2 (8130962)
Expression cutoff: 128.700 (min.grp=3)
subgroup~group4|met_status_(1_met__0_m0)~1|WITH_SURV (n=92)

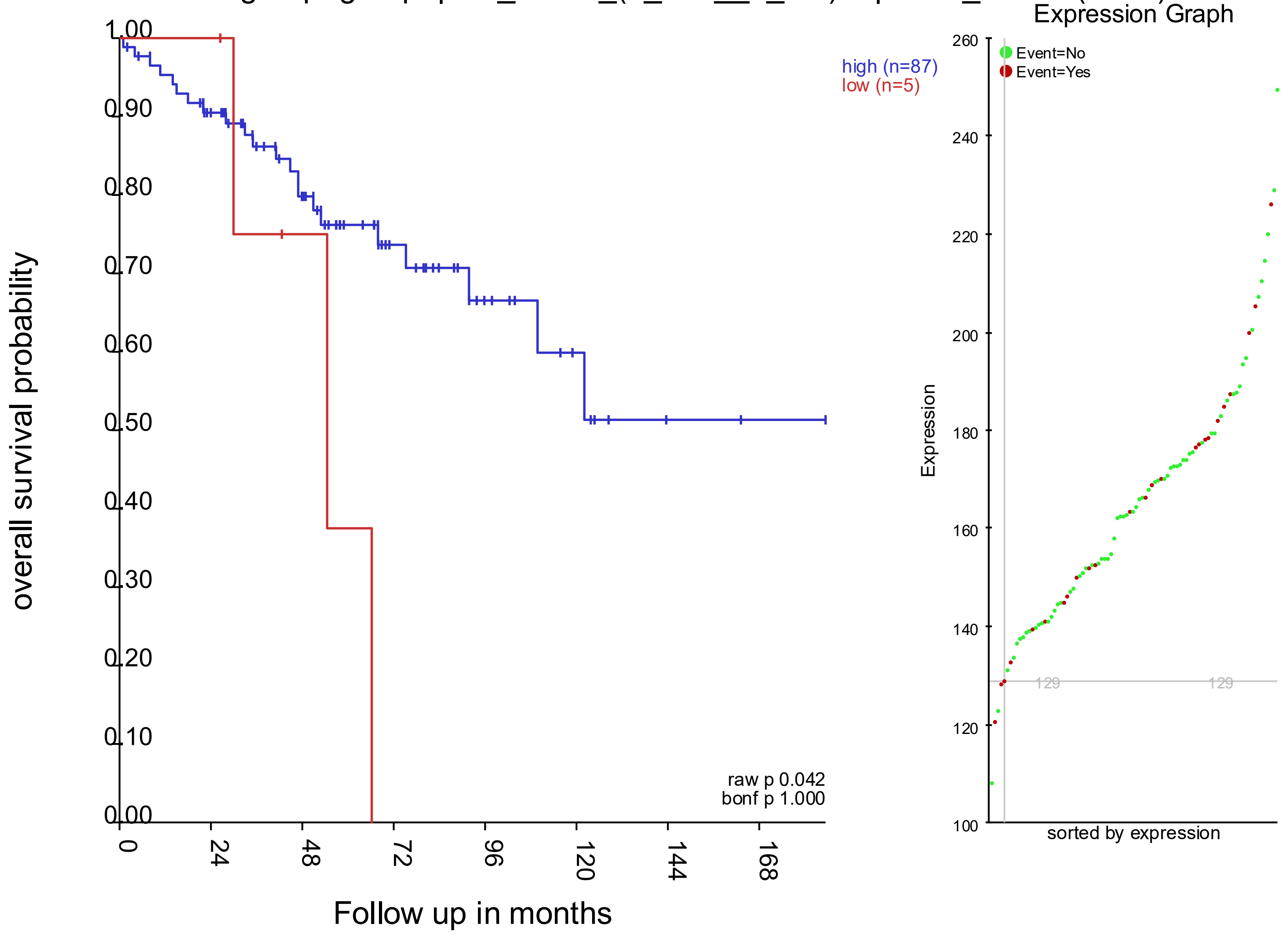

GROUP3 M0

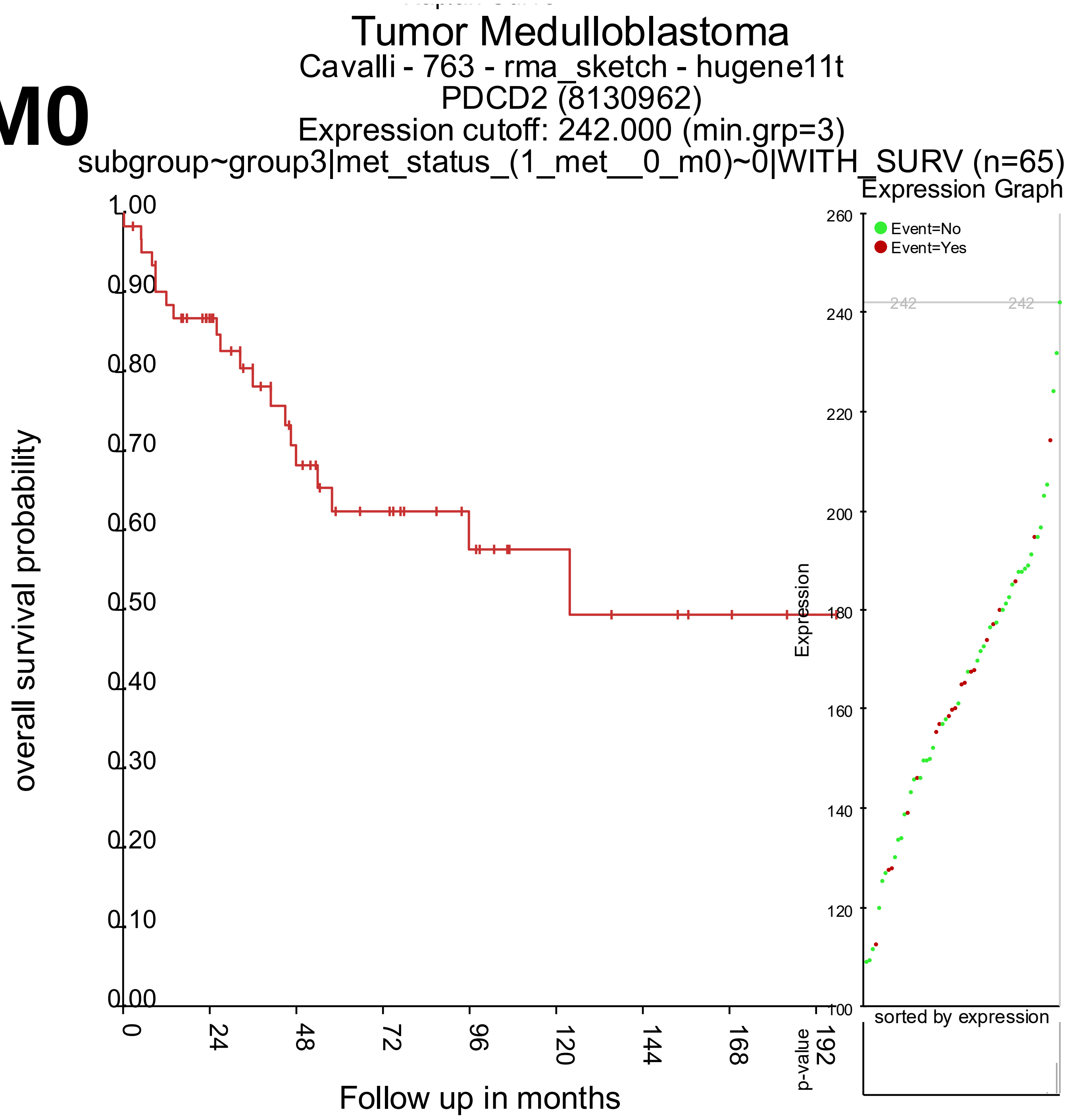
Tumor Medulloblastoma
Cavalli - 763 - rma_sketch - hugene11t
PDCD2 (8130962)
Expression cutoff: 242.000 (min.grp=3)
subgroup~group3|met_status_(1_met__0_m0)~0|WITH_SURV (n=65)
overall survival probability
1.00
0.90
0.80
0.70
0.60
0.50
0.40
0.30
0.20
0.10
0.00
0
24
48
72
96
120
144
168
192
Follow up in months
p-value
Expression Graph
Event=No
Event=Yes
242
242
260
240
220
200
180
160
140
120
100
Expression
sorted by expression

GROUP3 M1

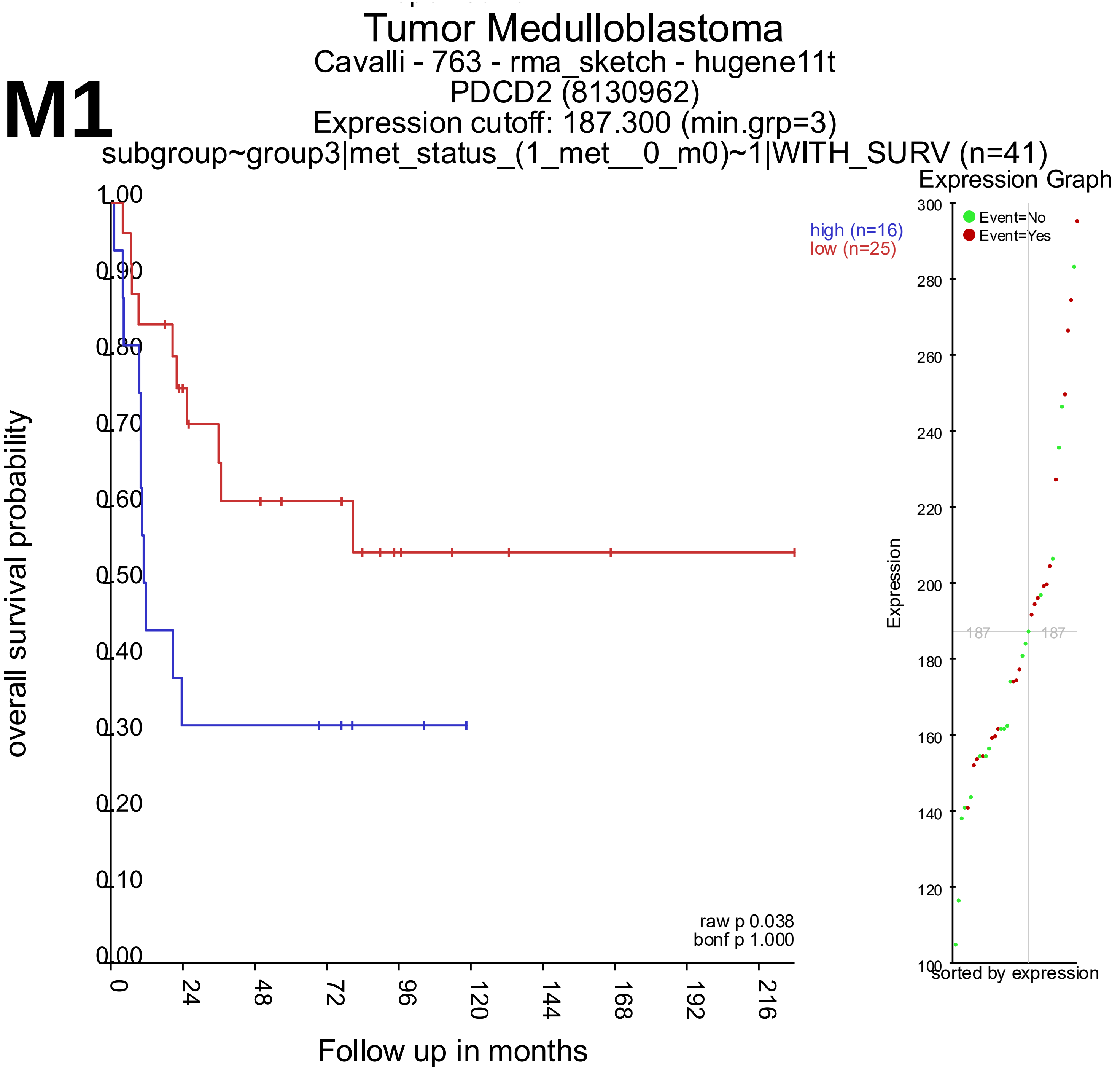

Tumor Medulloblastoma
Cavalli - 763 - rma_sketch - hugene11t
PDCD2 (8130962)
Expression cutoff: 187.300 (min.grp=3)
subgroup~group3|met_status_(1_met__0_m0)~1|WITH_SURV (n=41)
overall survival probability
1.00
0.90
0.80
0.70
0.60
0.50
0.40
0.30
0.20
0.10
0.00
0
24
48
72
96
120
144
168
192
216
Follow up in months
high (n=16)
low (n=25)
raw p 0.038
bonf p 1.000
Expression Graph
Event=No
Event=Yes
Expression
300
280
260
240
220
200
180
160
140
120
100
187
187
sorted by expression

PDL1/CD274

WNT M0

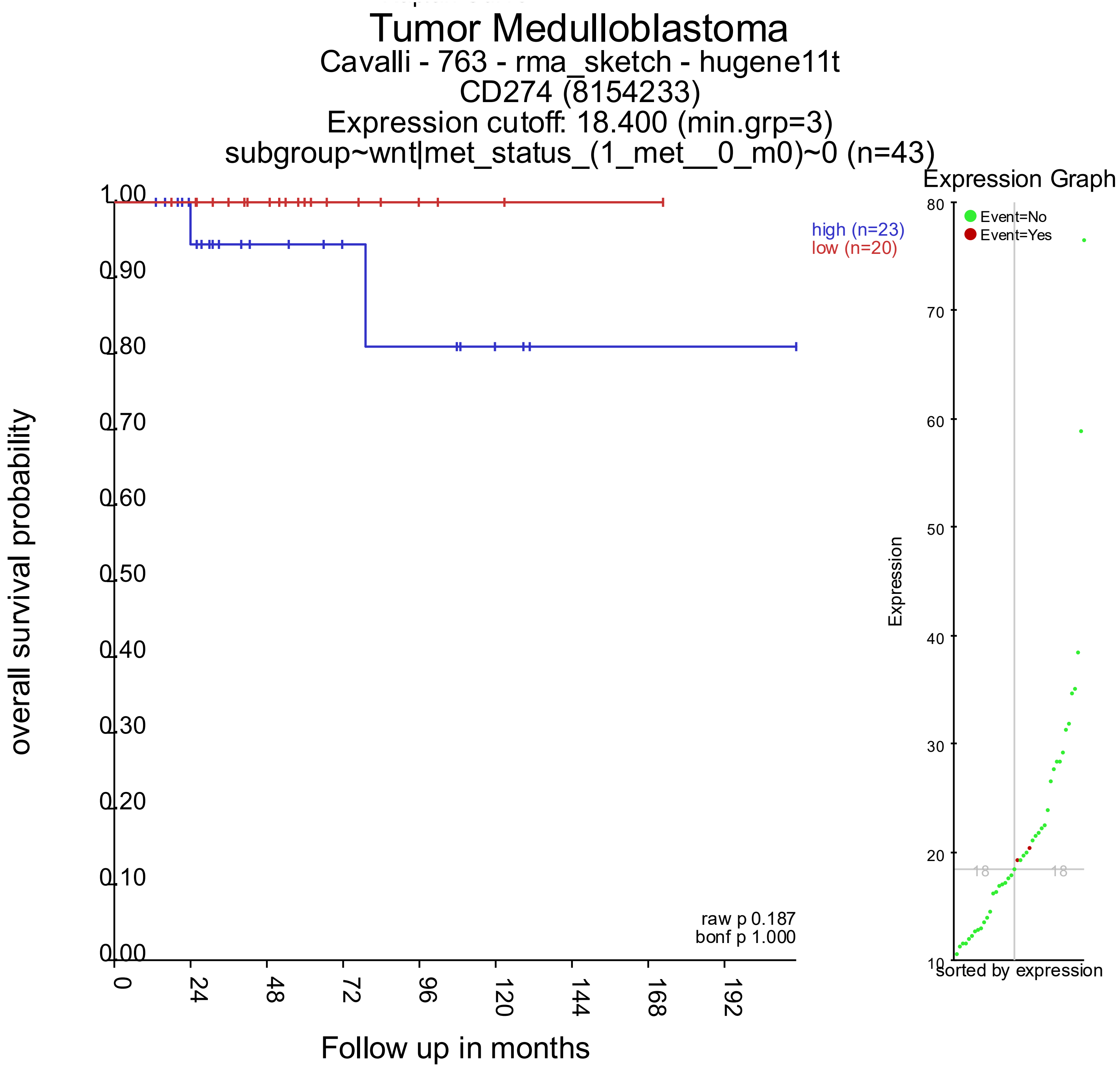

Tumor Medulloblastoma
Cavalli - 763 - rma_sketch - hugene11t
CD274 (8154233)
Expression cutoff: 18.400 (min.grp=3)
subgroup~wnt|met_status_(1_met__0_m0)~0 (n=43)
overall survival probability
1.00
0.90
0.80
0.70
0.60
0.50
0.40
0.30
0.20
0.10
0.00
0
24
48
72
96
120
144
168
192
Follow up in months
high (n=23)
low (n=20)
raw p 0.187
bonf p 1.000
Expression Graph
Event=No
Event=Yes
Expression
80
70
60
50
40
30
20
10
18
18
sorted by expression

WNT M1

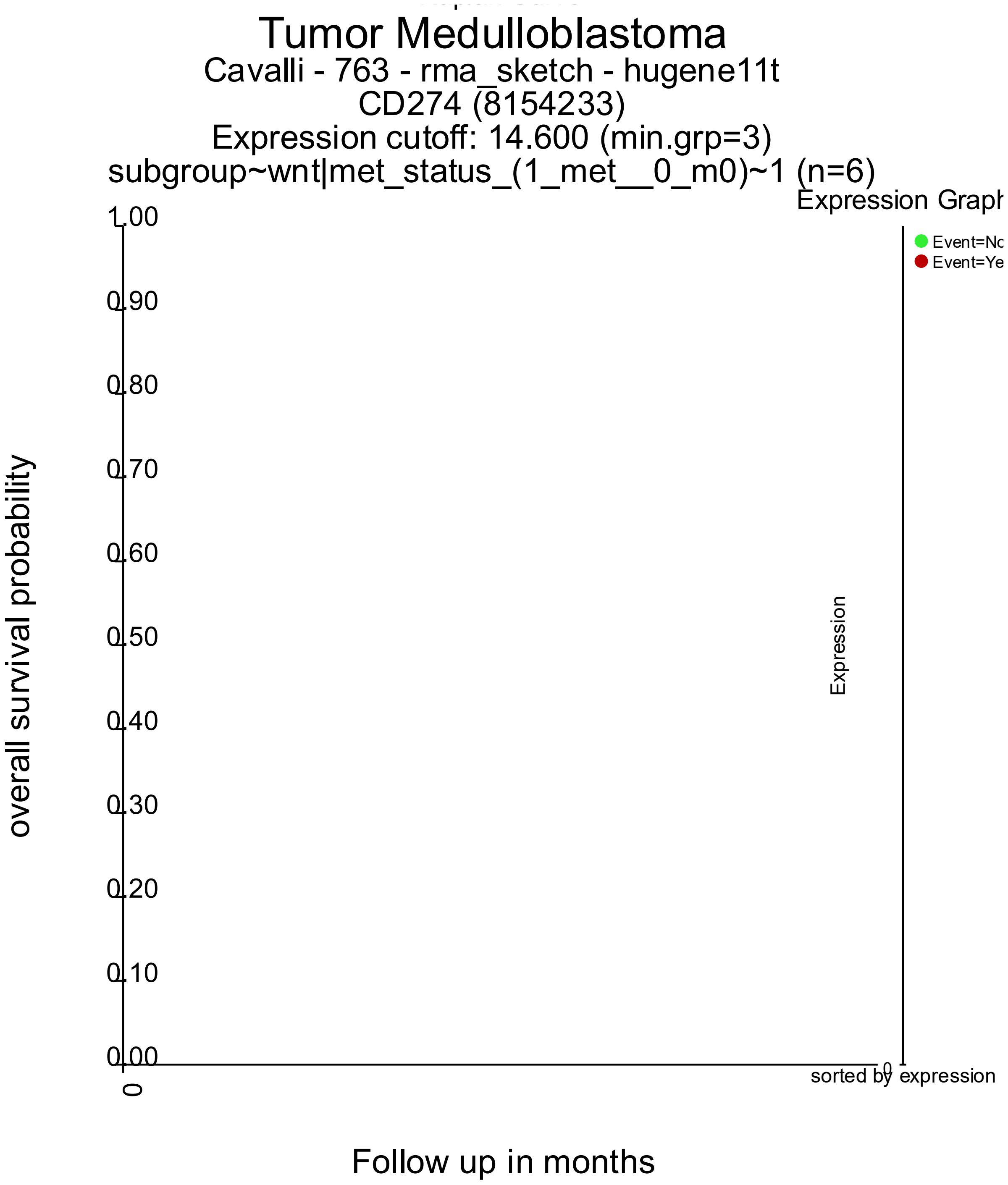

Tumor Medulloblastoma
Cavalli - 763 - rma_sketch - hugene11t
CD274 (8154233)
Expression cutoff: 14.600 (min.grp=3)
subgroup~wnt|met_status_(1_met__0_m0)~1 (n=6)
overall survival probability
1.00
0.90
0.80
0.70
0.60
0.50
0.40
0.30
0.20
0.10
0.00
0
Follow up in months
Expression Graph
Event=No
Event=Yes
Expression
0
sorted by expression

SHH M0

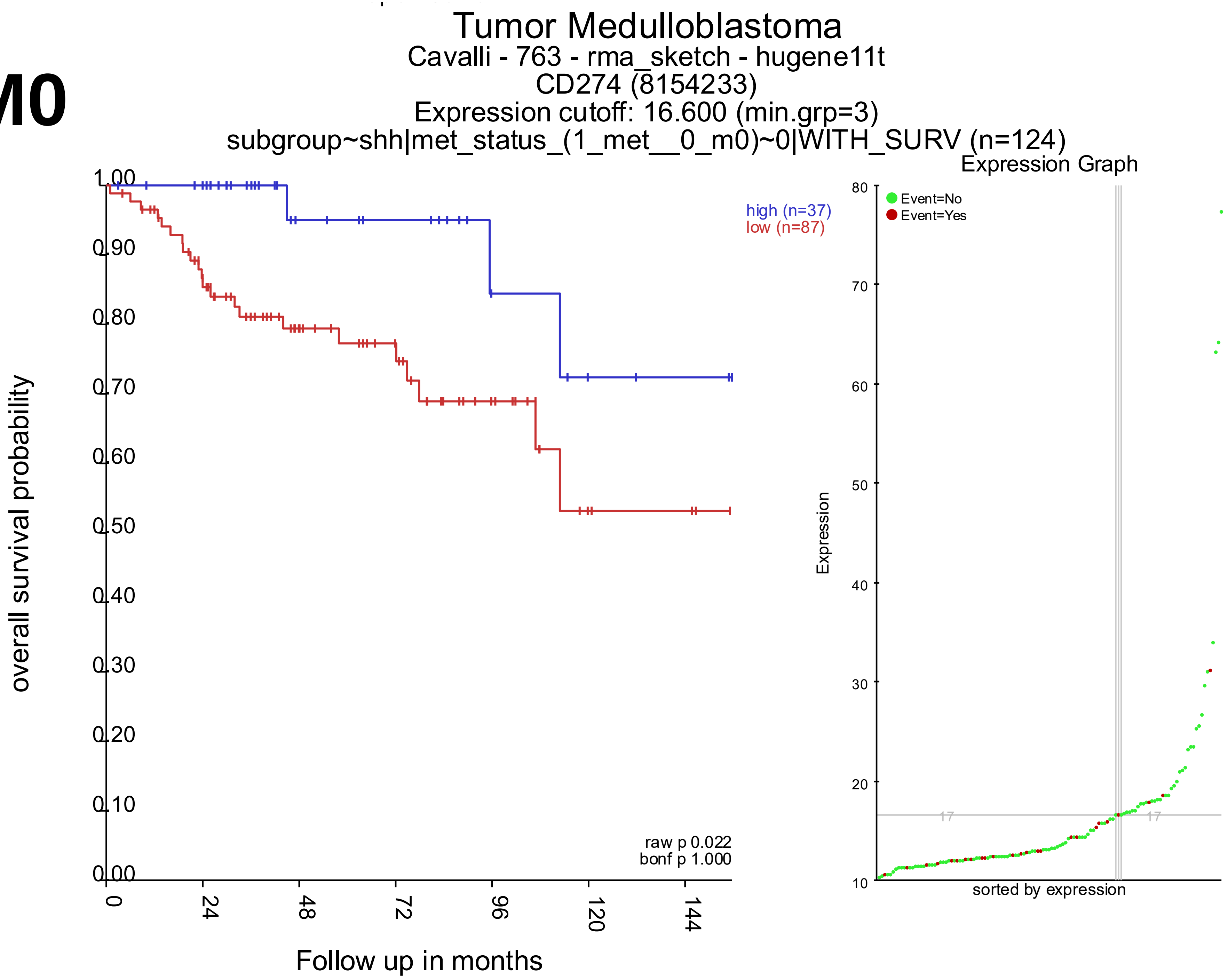

Tumor Medulloblastoma
Cavalli - 763 - rma_sketch - hugene11t
CD274 (8154233)
Expression cutoff: 16.600 (min.grp=3)
subgroup~shh|met_status_(1_met__0_m0)~0|WITH_SURV (n=124)
overall survival probability
1.00
0.90
0.80
0.70
0.60
0.50
0.40
0.30
0.20
0.10
0.00
0
24
48
72
96
120
144
Follow up in months
high (n=37)
low (n=87)
raw p 0.022
bonf p 1.000
Expression Graph
Event=No
Event=Yes
Expression
80
70
60
50
40
30
20
10
17
17
sorted by expression

SHH M1

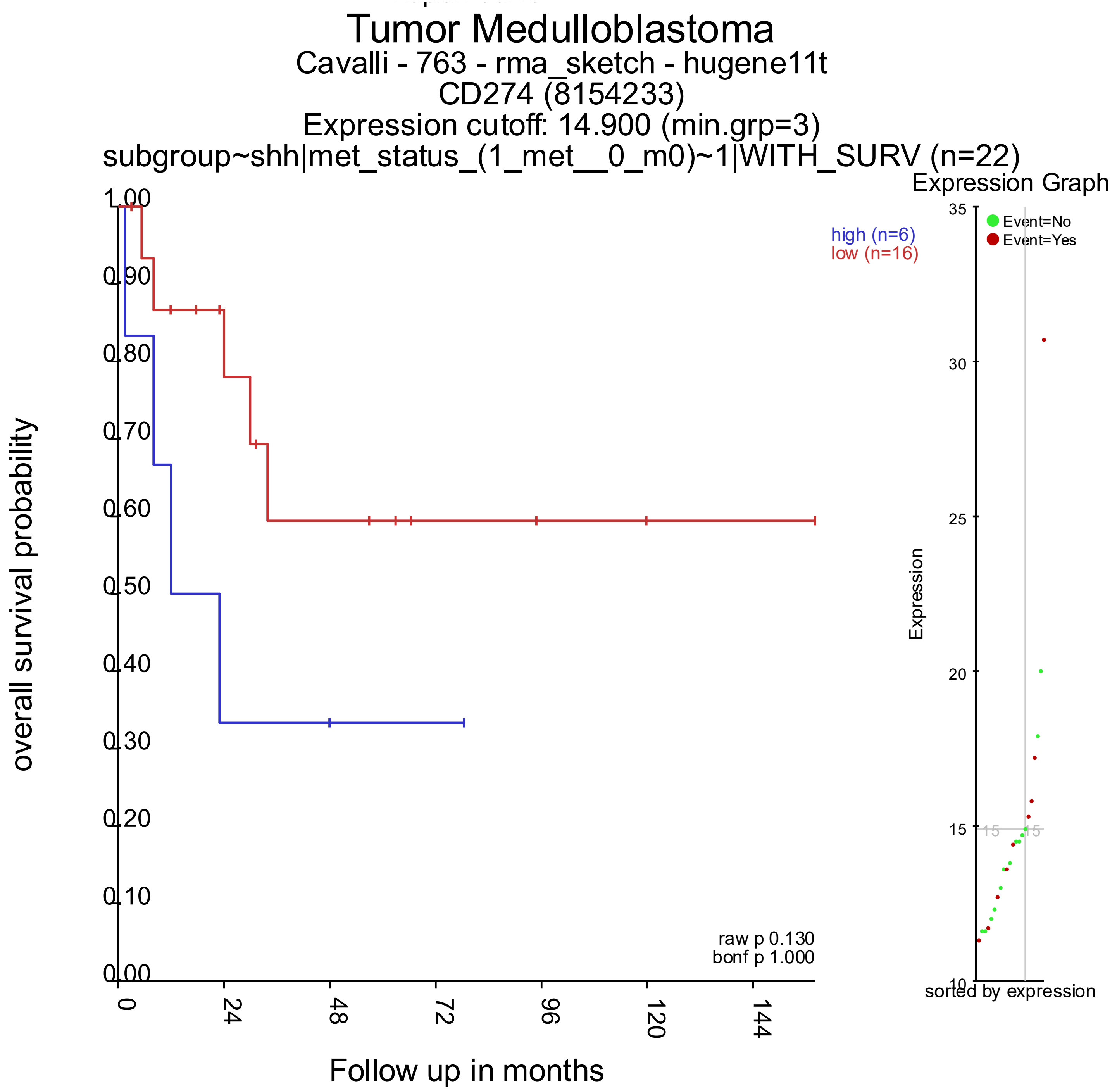

Tumor Medulloblastoma
Cavalli - 763 - rma_sketch - hugene11t
CD274 (8154233)
Expression cutoff: 14.900 (min.grp=3)
subgroup~shh|met_status_(1_met__0_m0)~1|WITH_SURV (n=22)
overall survival probability
1.00
0.90
0.80
0.70
0.60
0.50
0.40
0.30
0.20
0.10
0.00
0
24
48
72
96
120
144
Follow up in months
high (n=6)
low (n=16)
raw p 0.130
bonf p 1.000
Expression Graph
Event=No
Event=Yes
Expression
35
30
25
20
15
10
sorted by expression

GROUP4 M0

Tumor Medulloblastoma
Cavalli - 763 - rma_sketch - hugene11t
CD274 (8154233)
Expression cutoff: 12.000 (min.grp=3)
subgroup~group4|met_status_(1_met__0_m0)~0|WITH_SURV (n=145)

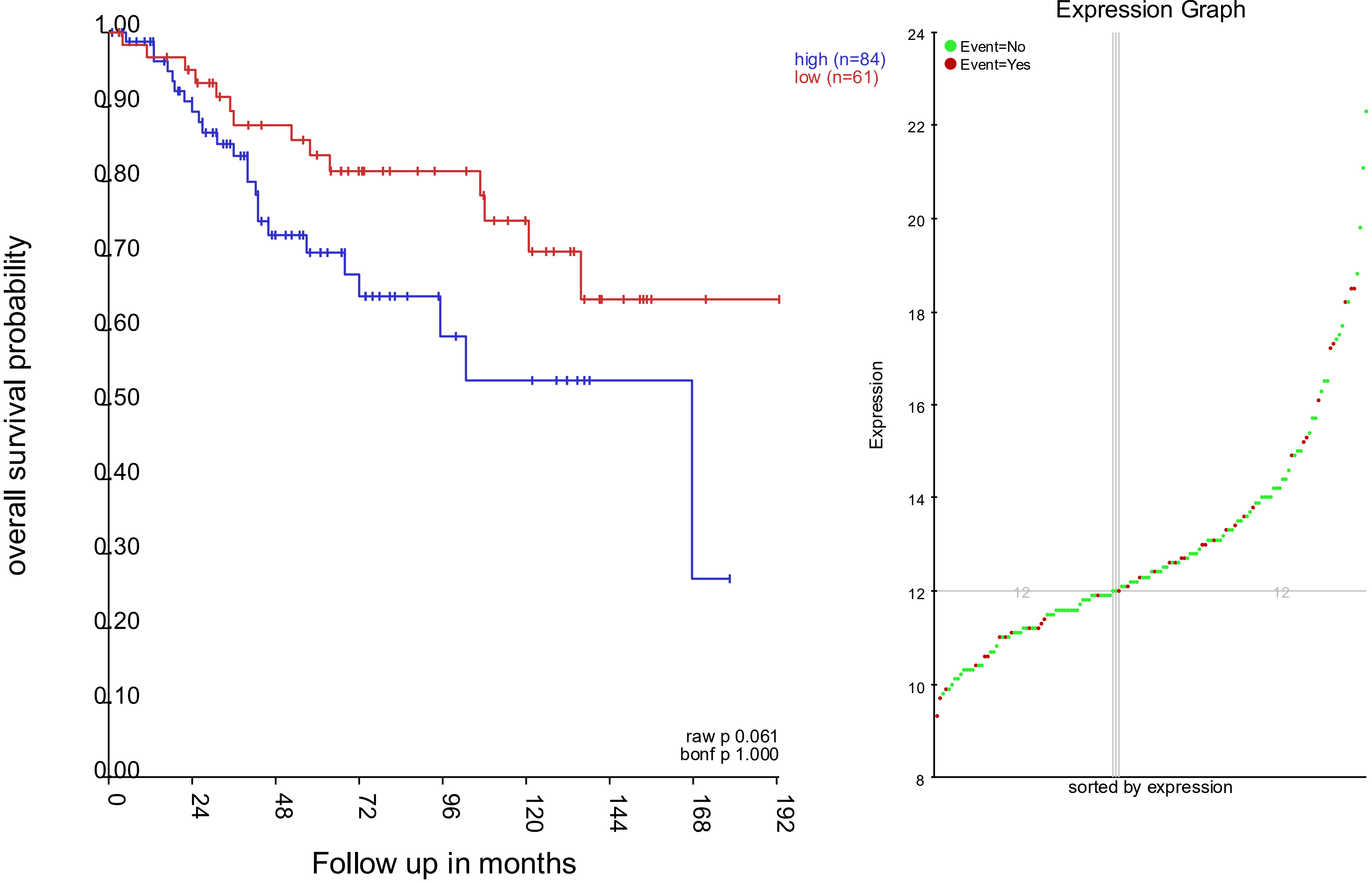

GROUP4 M1

Tumor Medulloblastoma
Cavalli - 763 - rma_sketch - hugene11t
CD274 (8154233)
Expression cutoff: 11.000 (min.grp=3)
subgroup~group4|met_status_(1_met__0_m0)~1|WITH_SURV (n=92)

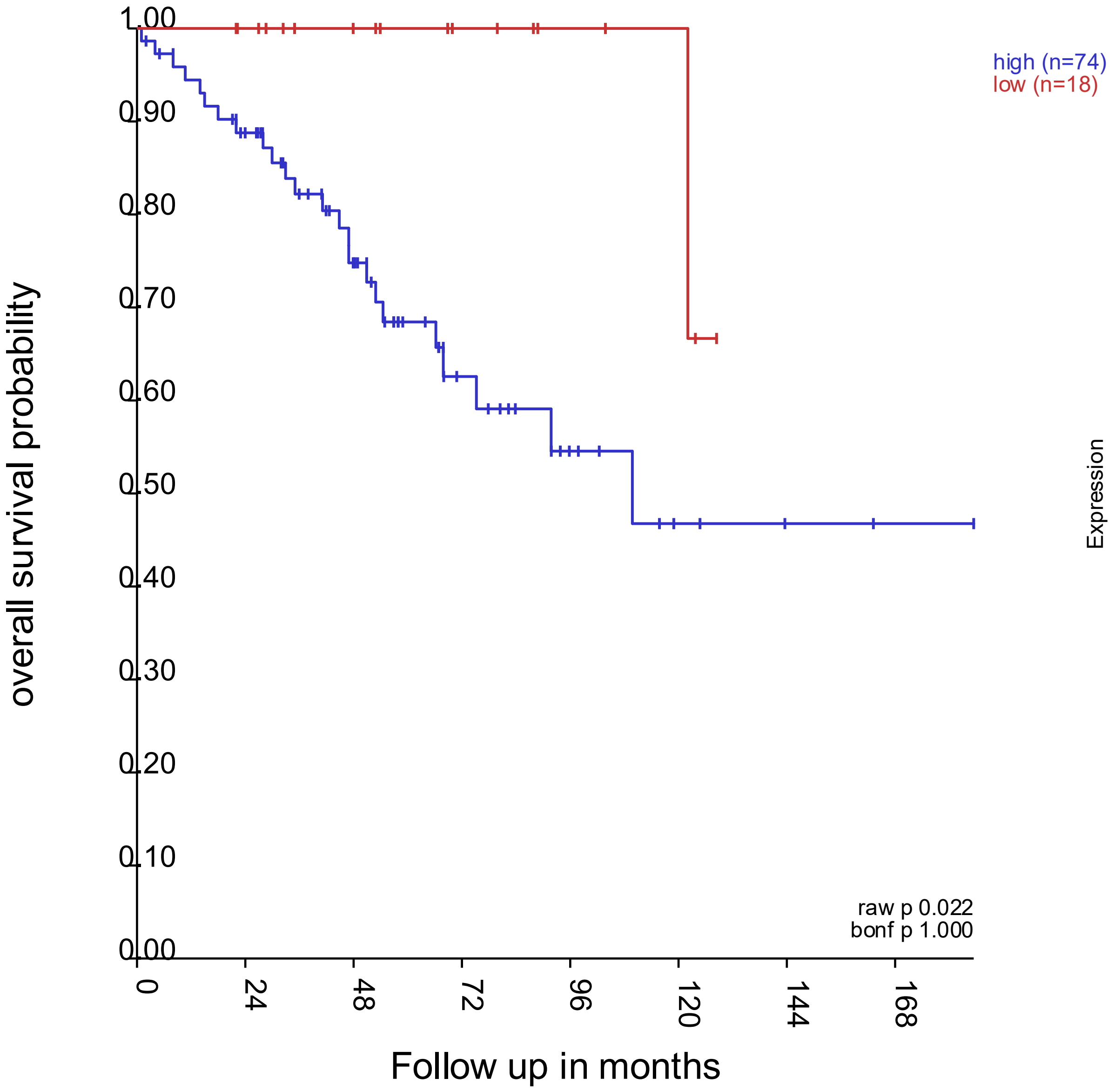

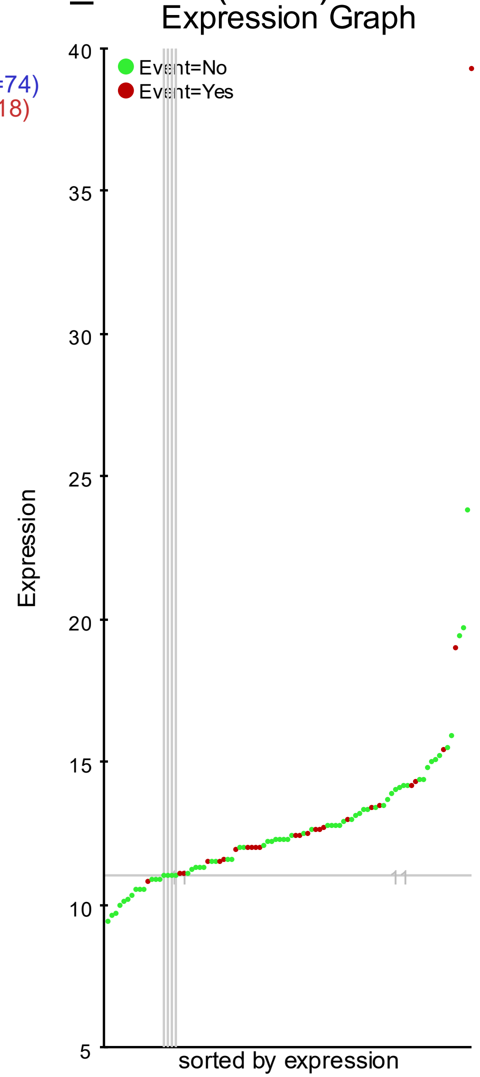

GROUP3 M0

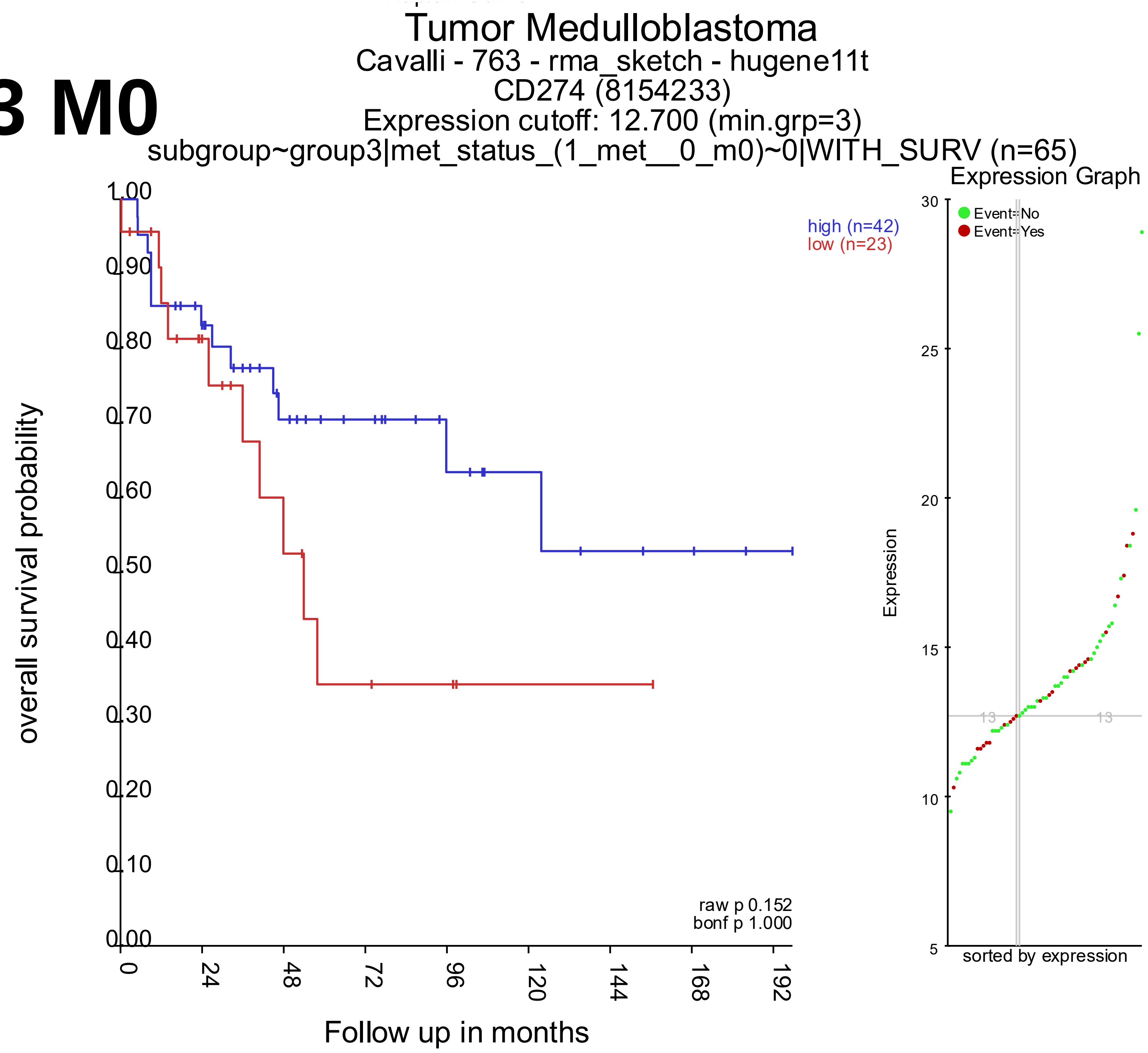

Tumor Medulloblastoma
Cavalli - 763 - rma_sketch - hugene11t
CD274 (8154233)
Expression cutoff: 12.700 (min.grp=3)
subgroup~group3|met_status_(1_met__0_m0)~0|WITH_SURV (n=65)
overall survival probability
1.00
0.90
0.80
0.70
0.60
0.50
0.40
0.30
0.20
0.10
0.00
0
24
48
72
96
120
144
168
192
Follow up in months
high (n=42)
low (n=23)
raw p 0.152
bonf p 1.000
Expression Graph
Event=No
Event=Yes
Expression
30
25
20
15
10
5
13
13
sorted by expression

GROUP3 M1

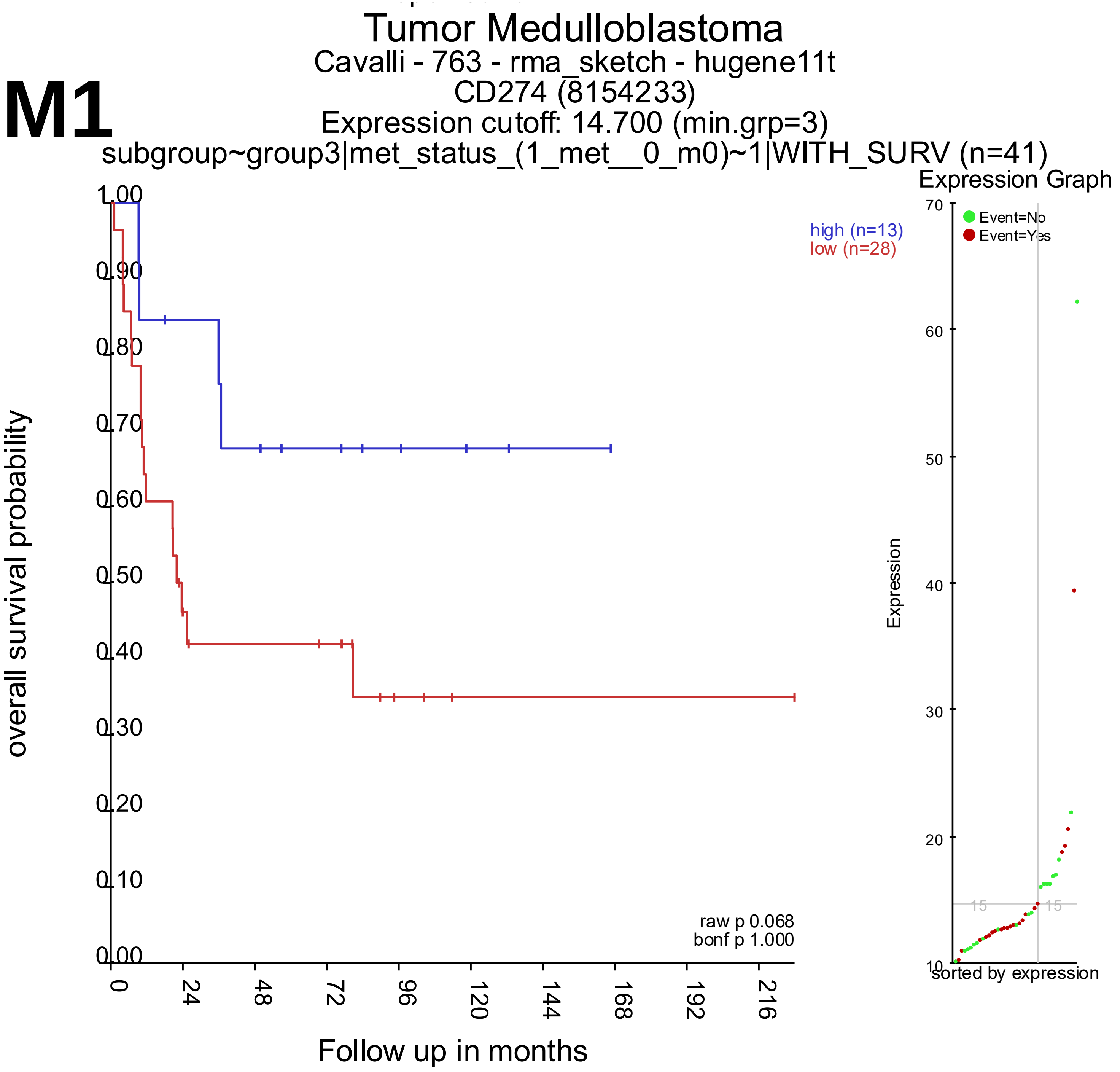

Tumor Medulloblastoma
Cavalli - 763 - rma_sketch - hugene11t
CD274 (8154233)
Expression cutoff: 14.700 (min.grp=3)
subgroup~group3|met_status_(1_met__0_m0)~1|WITH_SURV (n=41)
overall survival probability
1.00
0.90
0.80
0.70
0.60
0.50
0.40
0.30
0.20
0.10
0.00
high (n=13)
low (n=28)
raw p 0.068
bonf p 1.000
0
24
48
72
96
120
144
168
192
216
Follow up in months
Expression Graph
Event=No
Event=Yes
Expression
70
60
50
40
30
20
10
15
sorted by expression

PDL2/PDCD1LG2

WNT M0

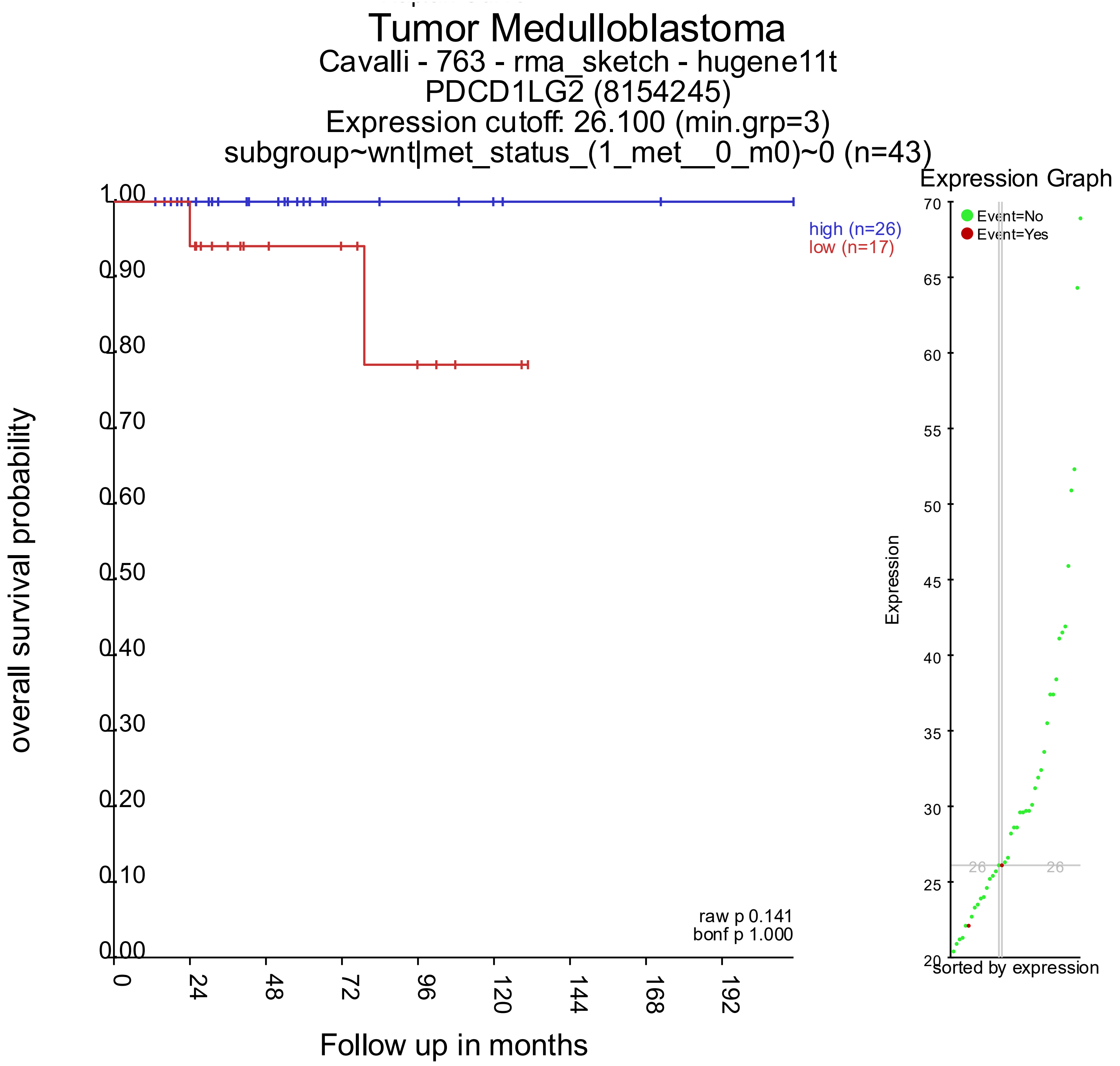
Tumor Medulloblastoma
Cavalli - 763 - rma_sketch - hugene11t
PDCD1LG2 (8154245)
Expression cutoff: 26.100 (min.grp=3)
subgroup~wnt|met_status_(1_met__0_m0)~0 (n=43)
high (n=26)
low (n=17)
overall survival probability
1.00
0.90
0.80
0.70
0.60
0.50
0.40
0.30
0.20
0.10
0.00
raw p 0.141
bonf p 1.000
0
24
48
72
96
120
144
168
192
Follow up in months
Expression Graph
Event=No
Event=Yes
Expression
70
65
60
55
50
45
40
35
30
25
20
26
26
sorted by expression

WNT M1

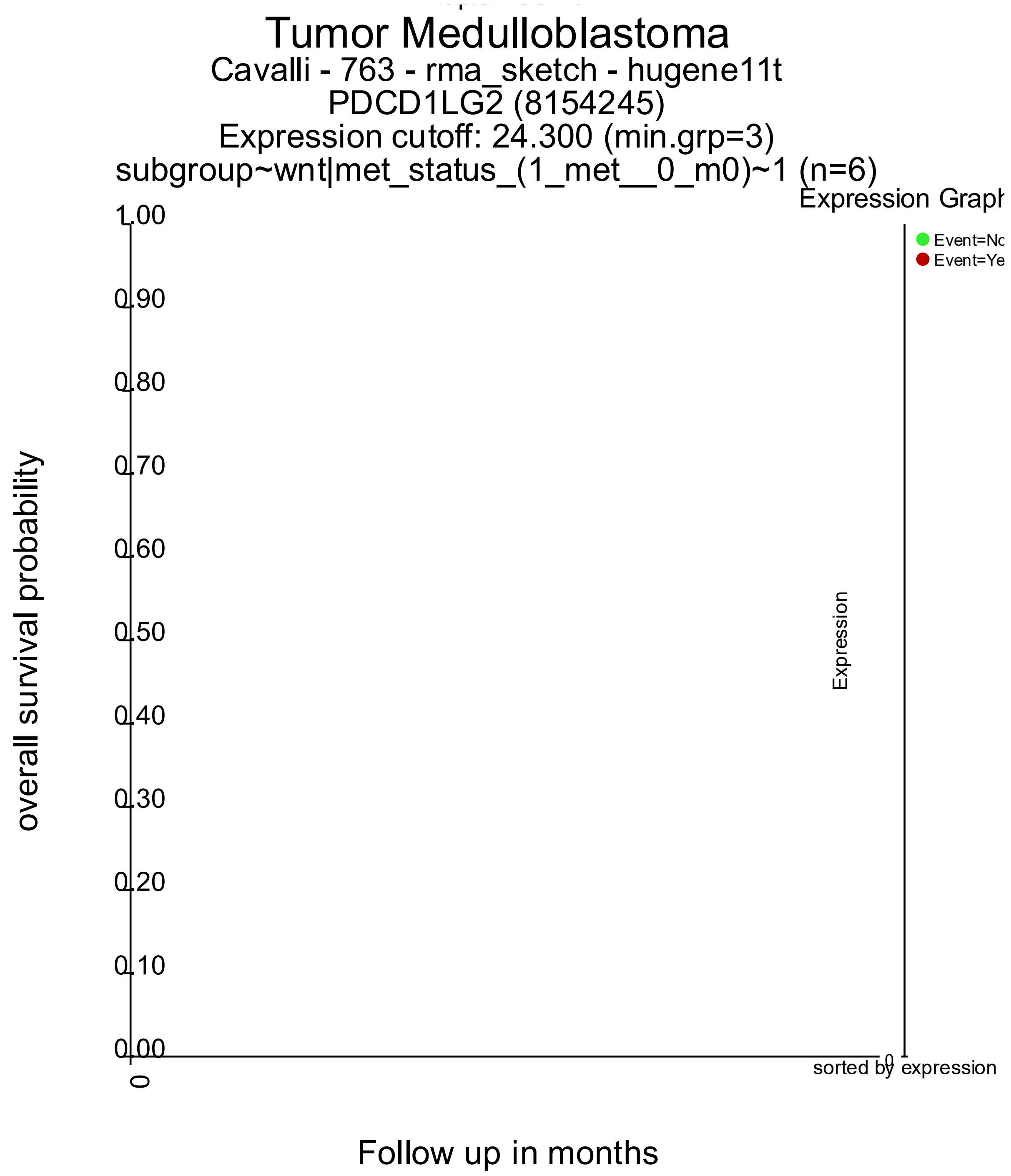

Tumor Medulloblastoma
Cavalli - 763 - rma_sketch - hugene11t
PDCD1LG2 (8154245)
Expression cutoff: 24.300 (min.grp=3)
subgroup~wnt|met_status_(1_met__0_m0)~1 (n=6)
Expression Graph
Event=No
Event=Yes
overall survival probability
1.00
0.90
0.80
0.70
0.60
0.50
0.40
0.30
0.20
0.10
0.00
0
Expression
0
sorted by expression
Follow up in months

SHH M0

Tumor Medulloblastoma
Cavalli - 763 - rma_sketch - hugene11t
PDCD1LG2 (8154245)
Expression cutoff: 19.400 (min.grp=3)
subgroup~shh|met_status_(1_met__0_m0)~0|WITH_SURV (n=124)

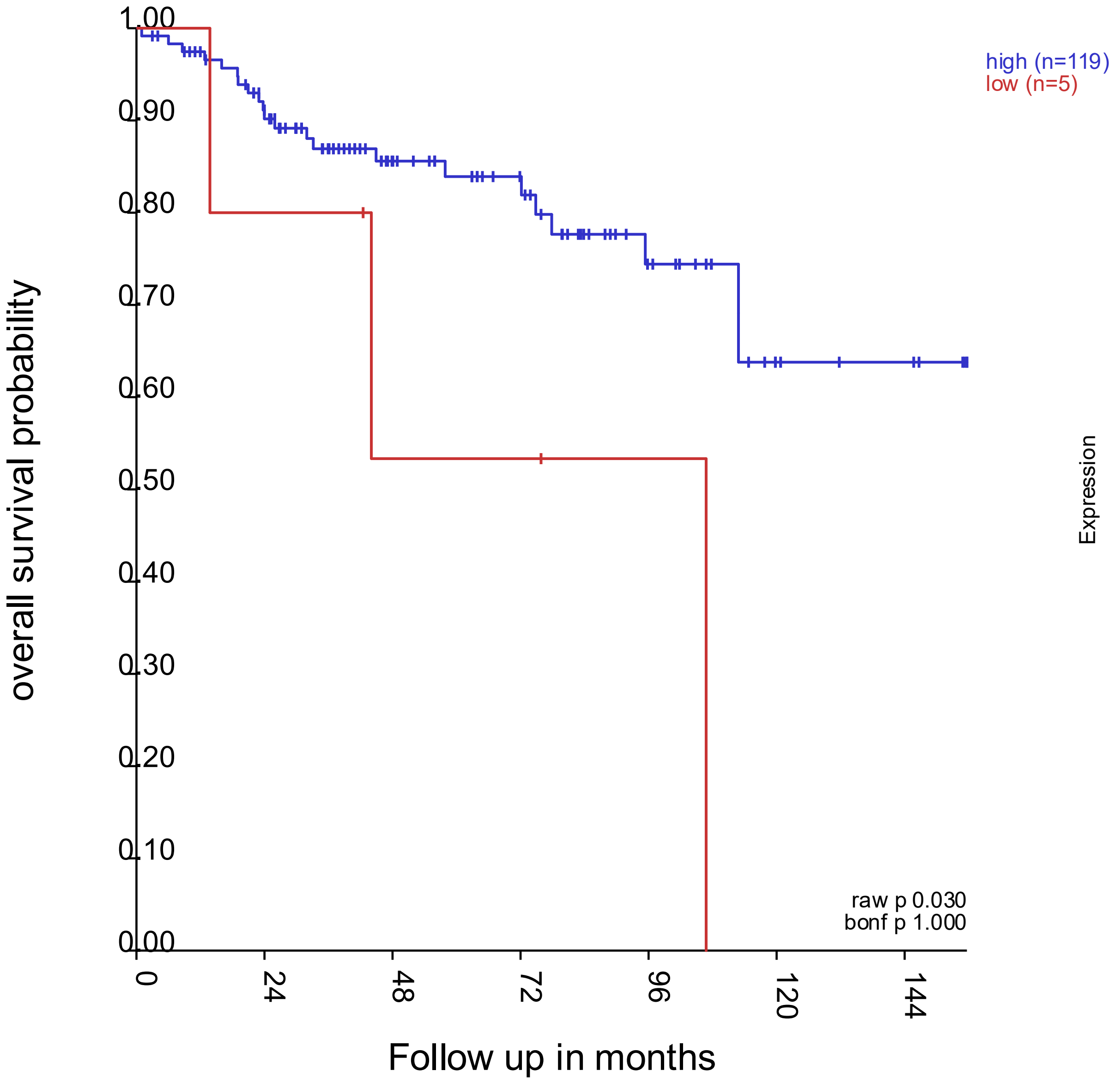

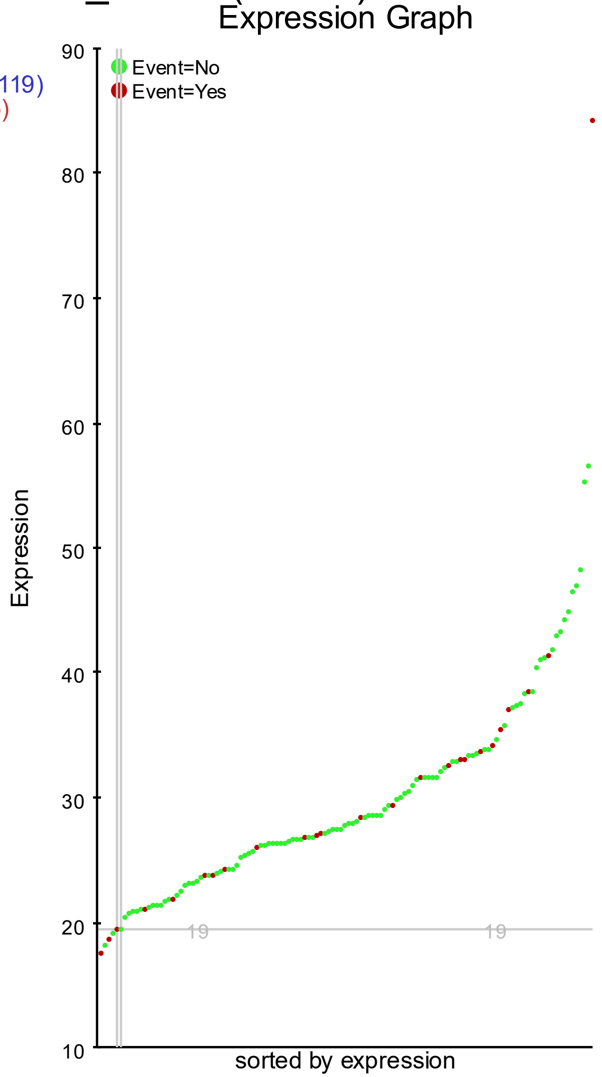

SHH M1

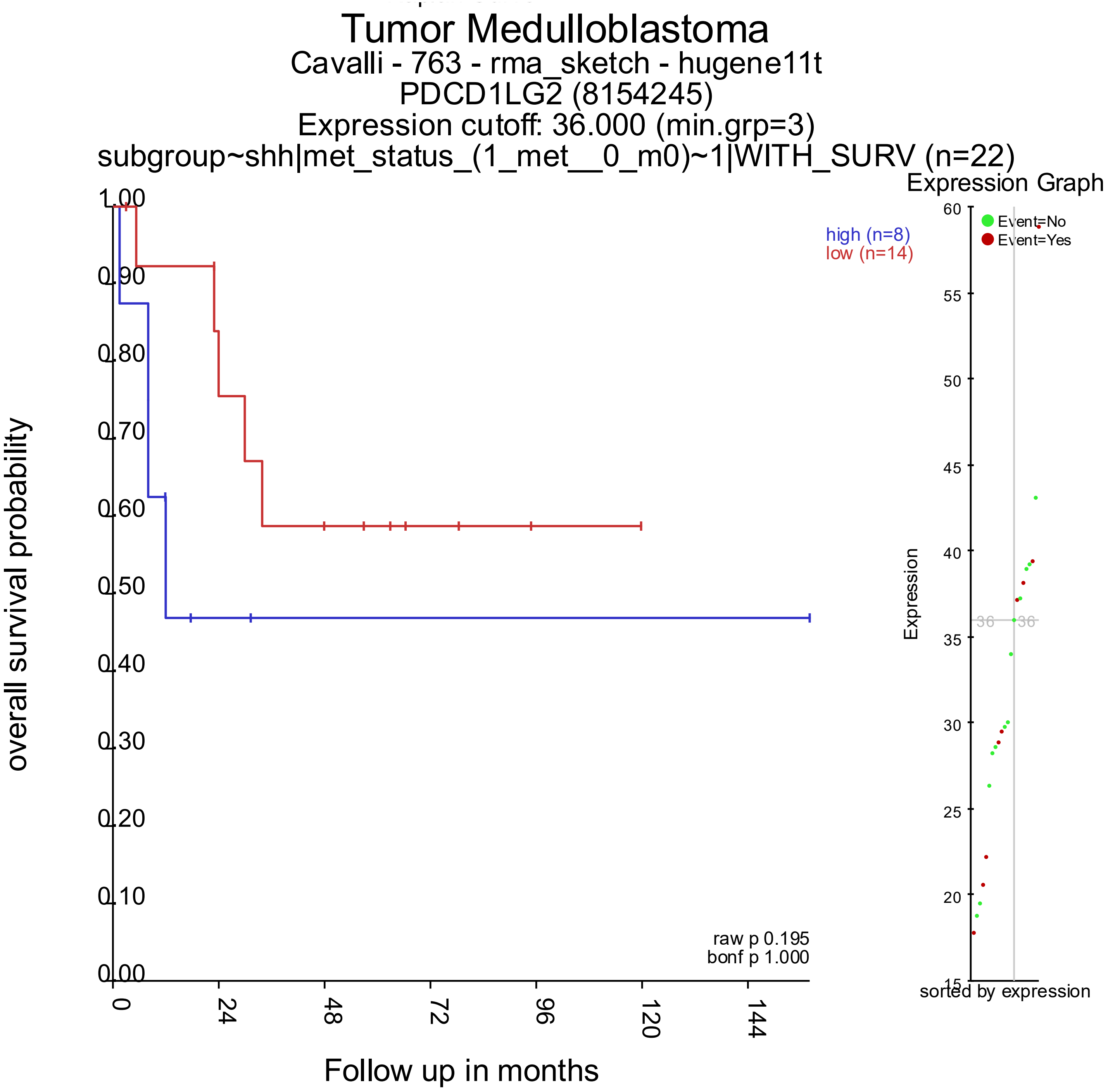

Tumor Medulloblastoma
Cavalli - 763 - rma_sketch - hugene11t
PDCD1LG2 (8154245)
Expression cutoff: 36.000 (min.grp=3)
subgroup~shh|met_status_(1_met__0_m0)~1|WITH_SURV (n=22)
overall survival probability
1.00
0.90
0.80
0.70
0.60
0.50
0.40
0.30
0.20
0.10
0.00
high (n=8)
low (n=14)
raw p 0.195
bonf p 1.000
0
24
48
72
96
120
144
Follow up in months
Expression Graph
Event=No
Event=Yes
Expression
60
55
50
45
40
35
30
25
20
15
36
sorted by expression

GROUP4 M0

Tumor Medulloblastoma
Cavalli - 763 - rma_sketch - hugene11t
PDCD1LG2 (8154245)
Expression cutoff: 30.800 (min.grp=3)
subgroup~group4|met_status_(1_met__0_m0)~0|WITH_SURV (n=145)

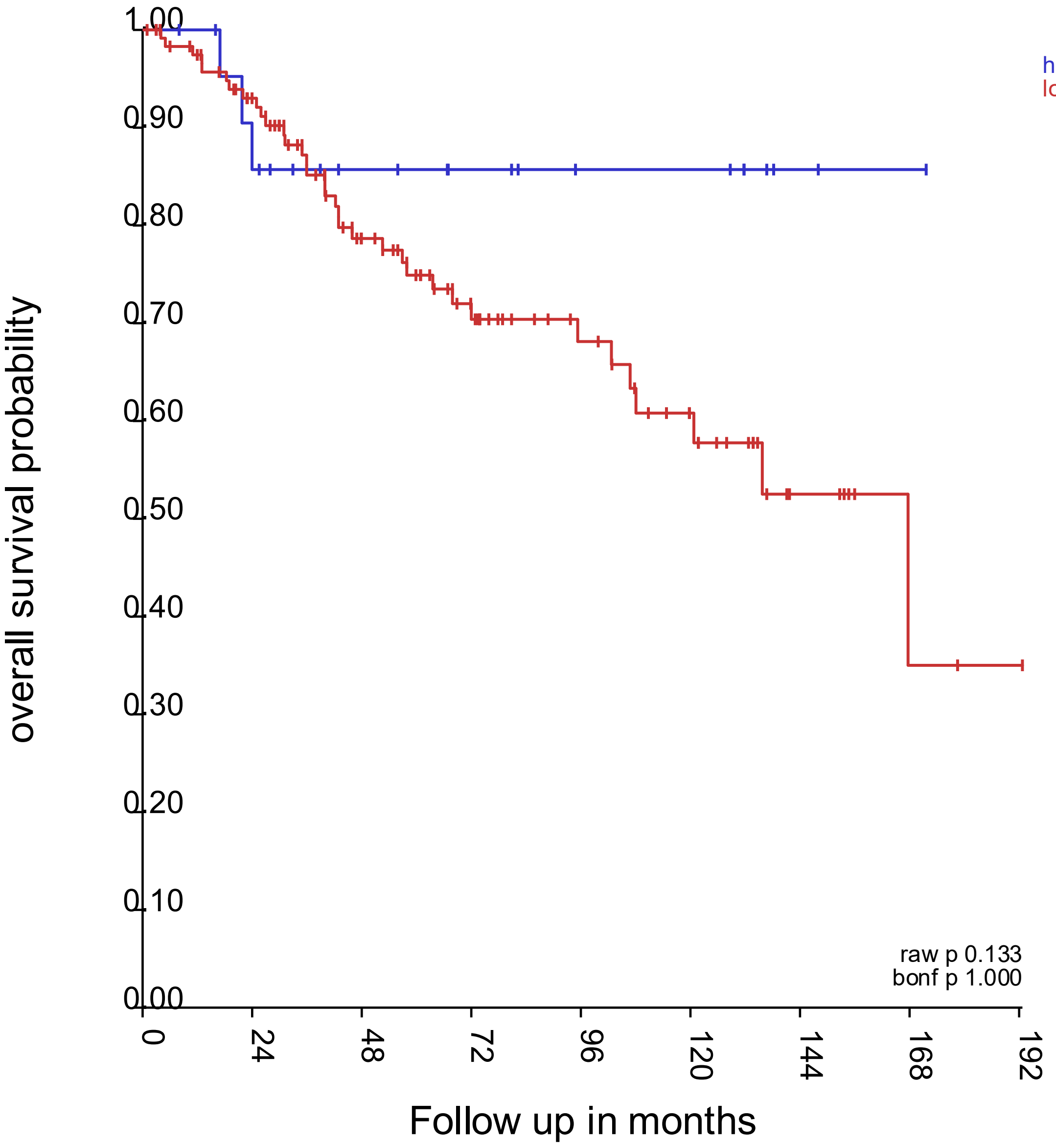

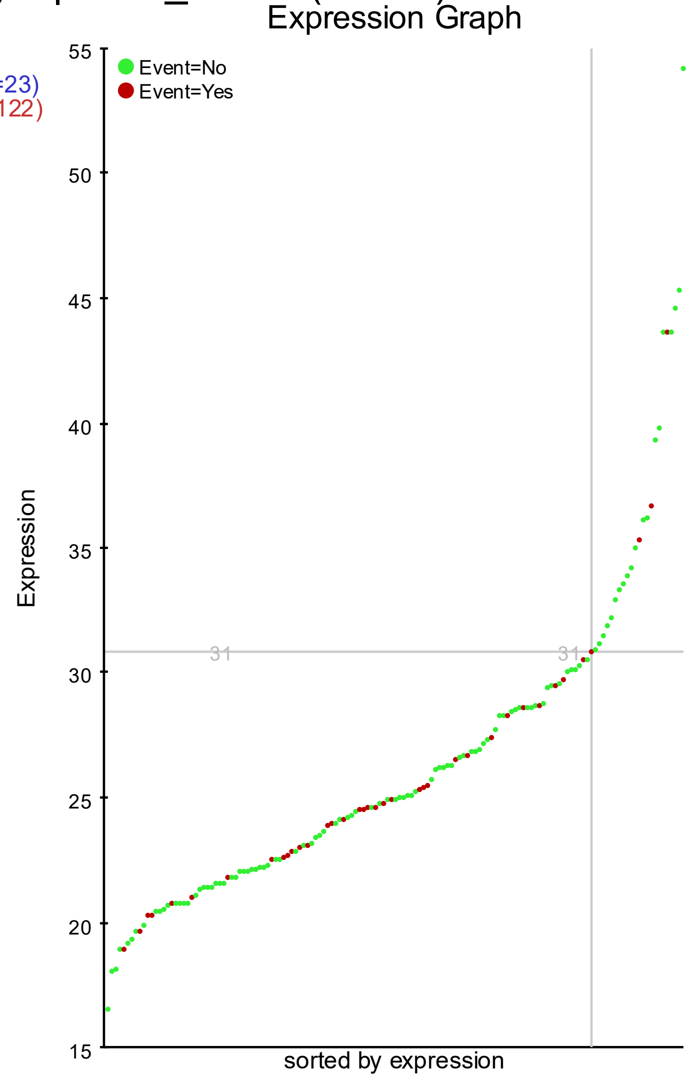

GROUP4 M1

Tumor Medulloblastoma
Cavalli - 763 - rma_sketch - hugene11t
PDCD1LG2 (8154245)
Expression cutoff: 21.600 (min.grp=3)
subgroup~group4|met_status_(1_met__0_m0)~1|WITH_SURV (n=92)

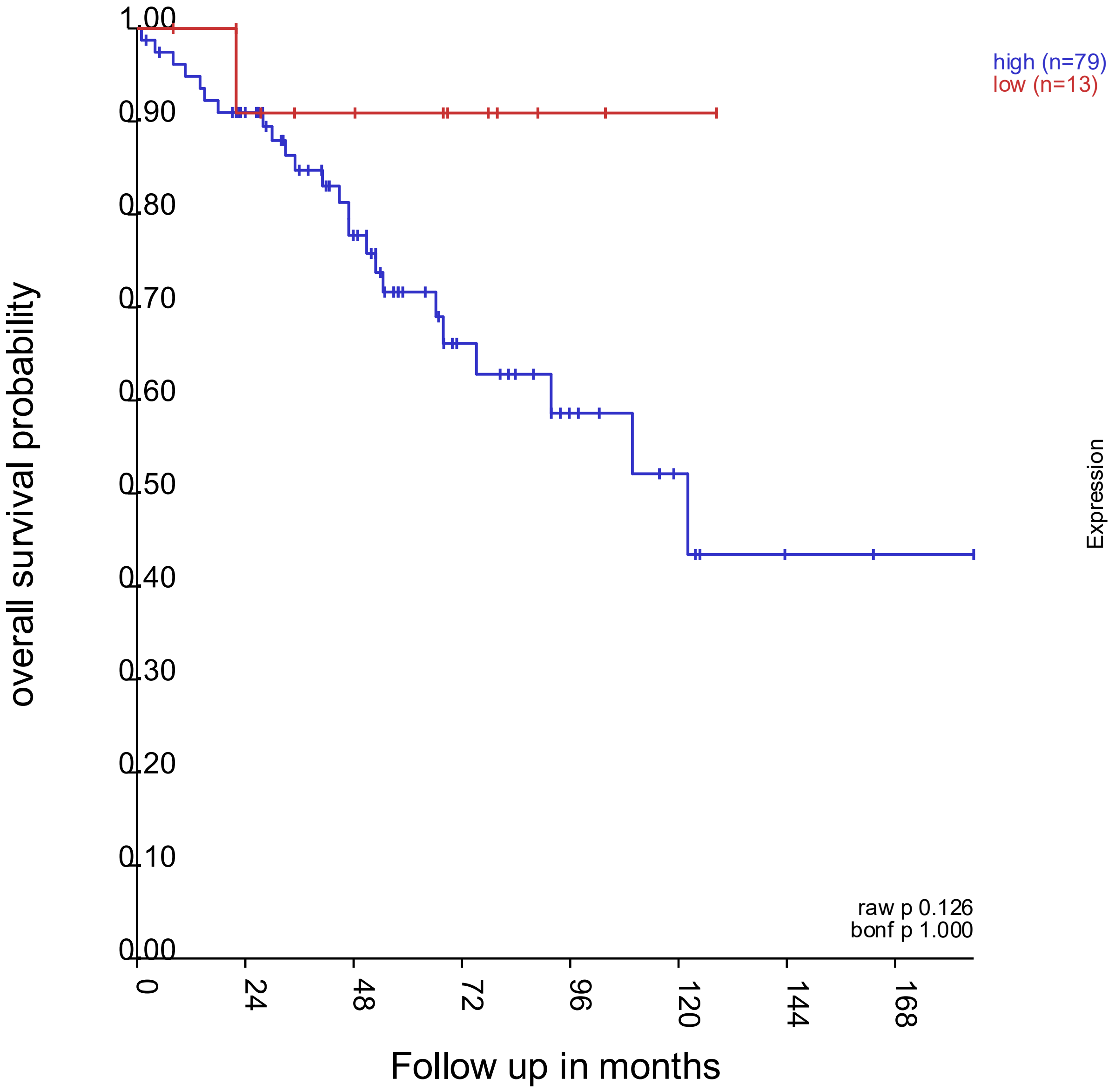

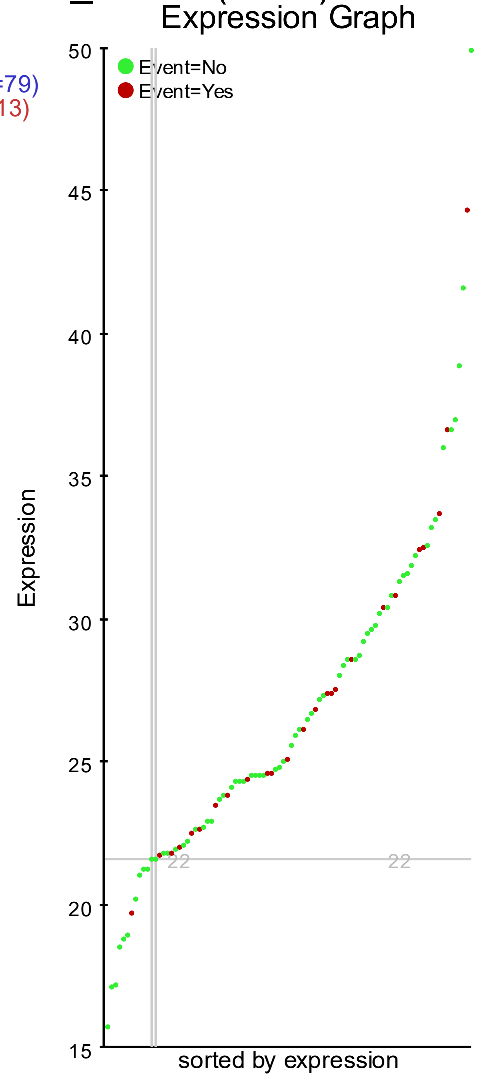

GROUP3 M0

Tumor Medulloblastoma
Cavalli - 763 - rma_sketch - hugene11t
PDCD1LG2 (8154245)
Expression cutoff: 28.600 (min.grp=3)
subgroup~group3|met_status_(1_met__0_m0)~0|WITH_SURV (n=65)

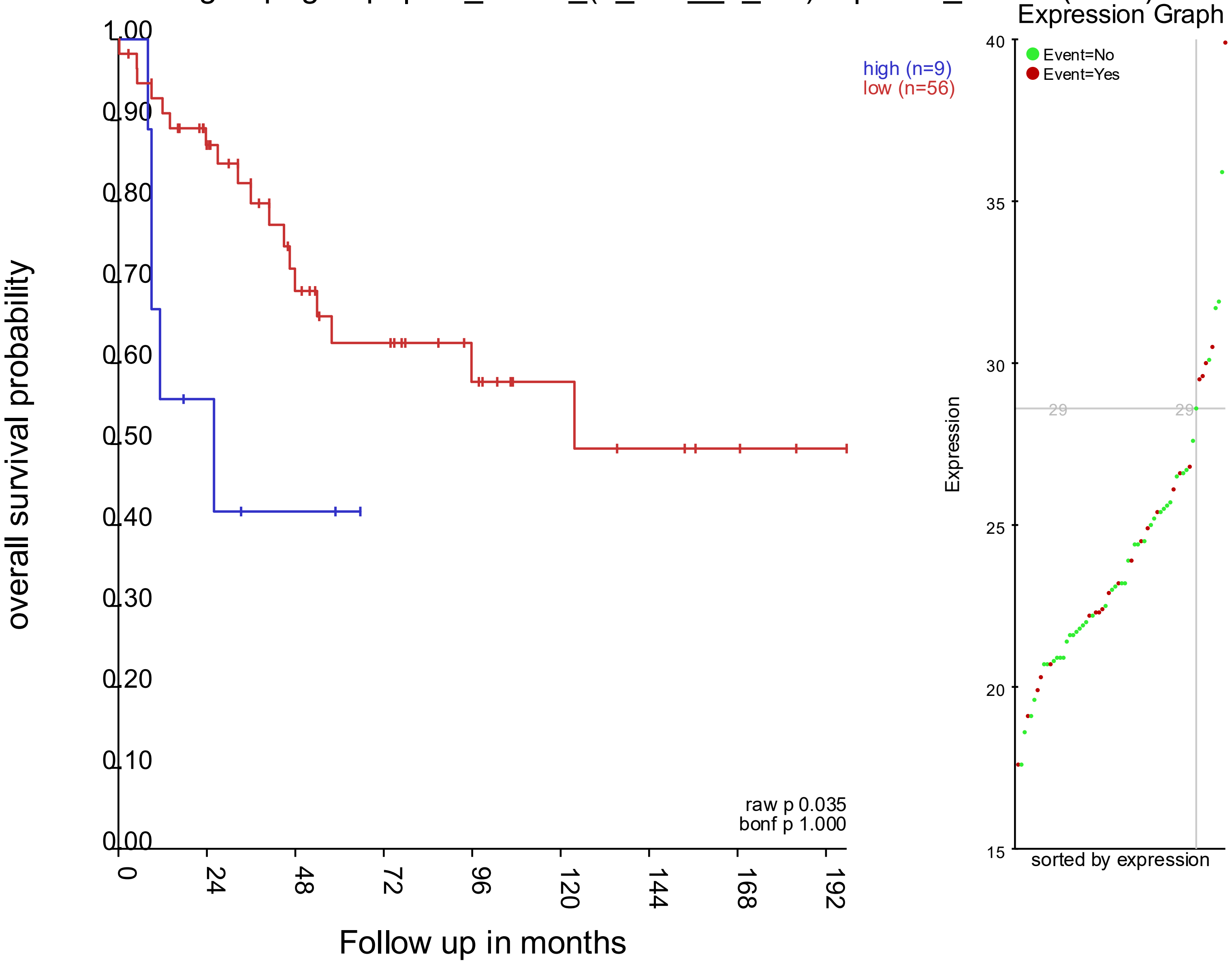

GROUP3 M1

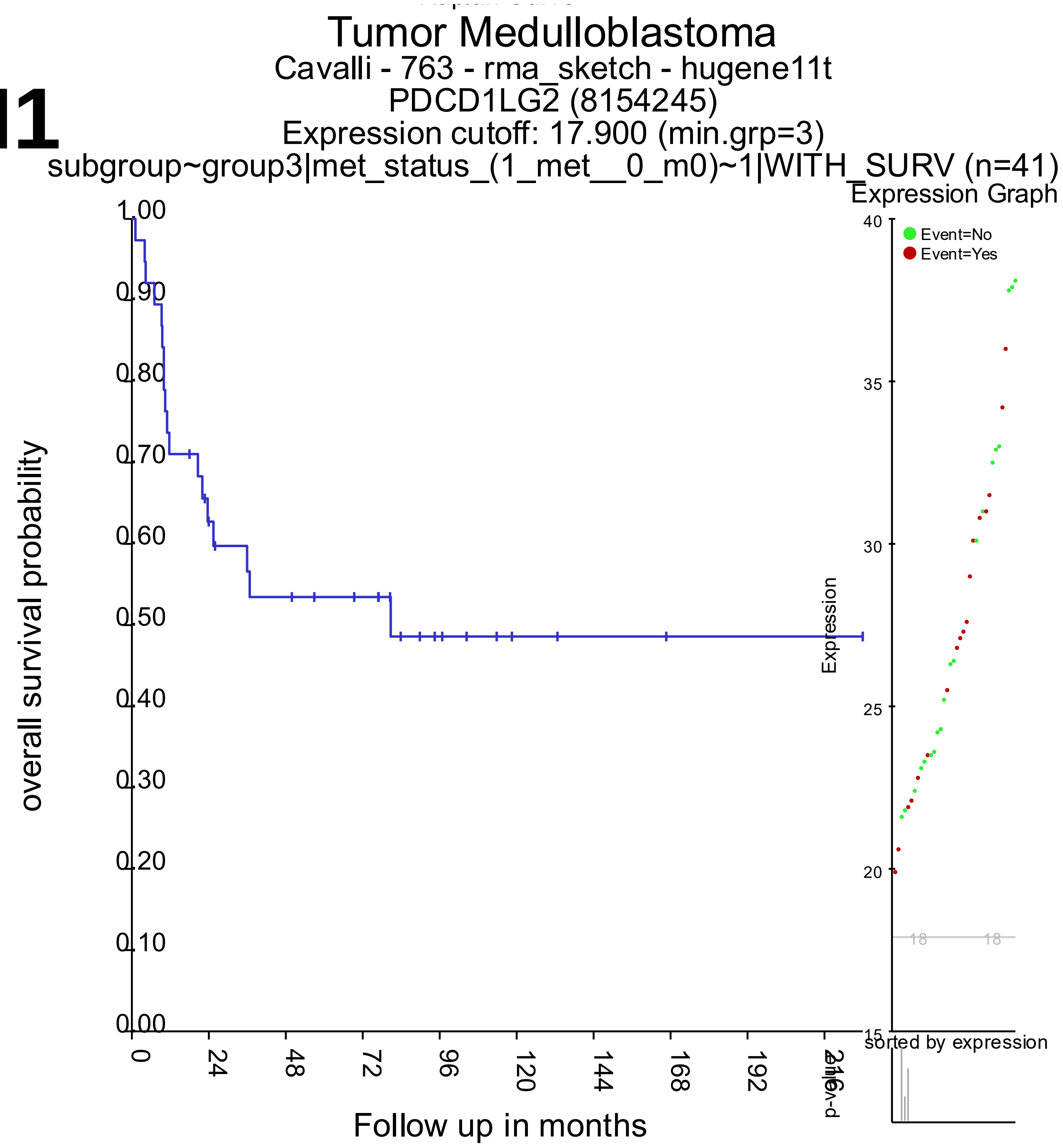

Tumor Medulloblastoma
Cavalli - 763 - rma_sketch - hugene11t
PDCD1LG2 (8154245)
Expression cutoff: 17.900 (min.grp=3)
subgroup~group3|met_status_(1_met__0_m0)~1|WITH_SURV (n=41)
overall survival probability
1.00
0.90
0.80
0.70
0.60
0.50
0.40
0.30
0.20
0.10
0.00
0
24
48
72
96
120
144
168
192
216
Follow up in months
Expression Graph
Event=No
Event=Yes
Expression
40
35
30
25
20
15
18
18
sorted by expression

CTLA4

WNT M0

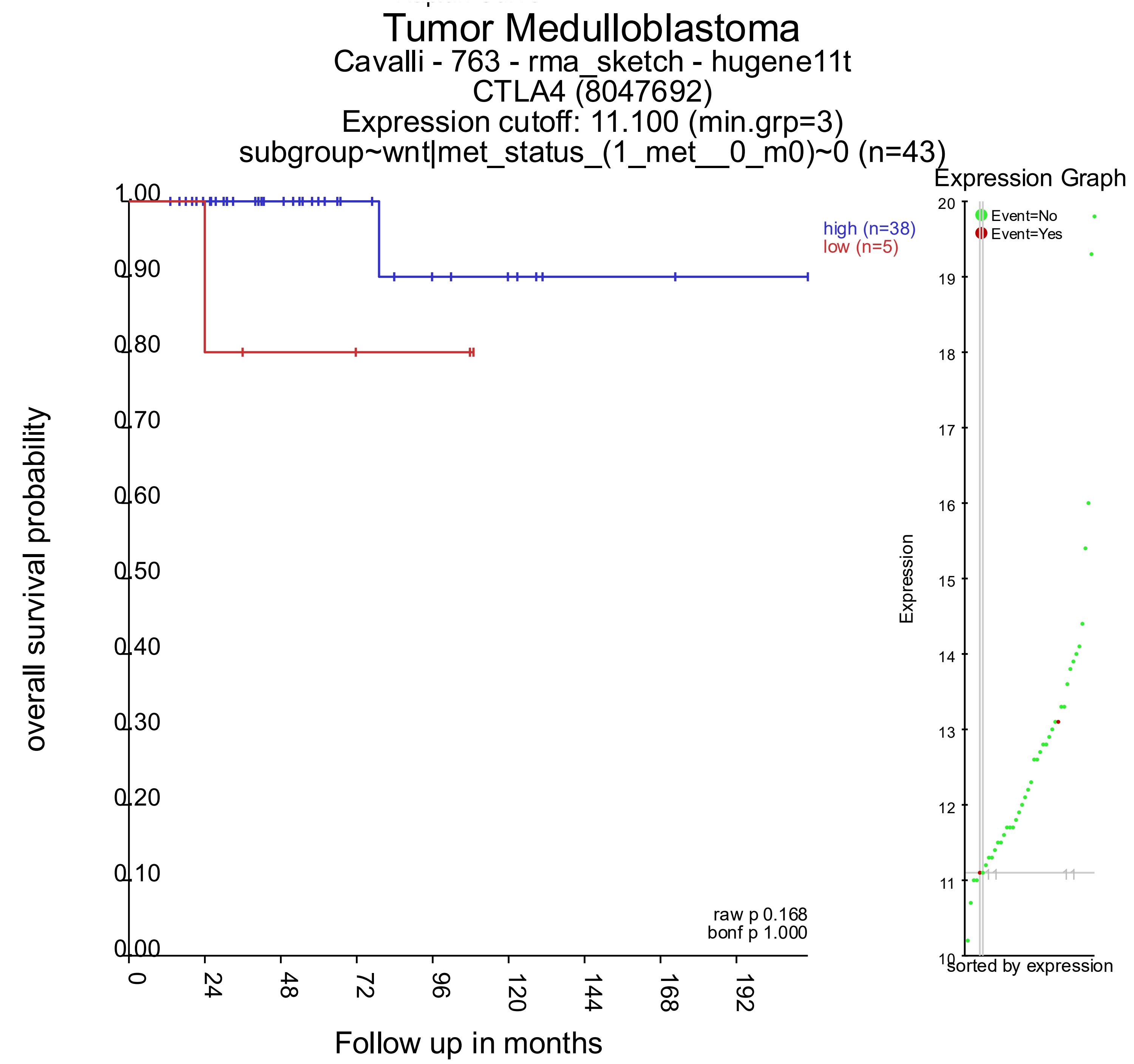

Tumor Medulloblastoma
Cavalli - 763 - rma_sketch - hugene11t
CTLA4 (8047692)
Expression cutoff: 11.100 (min.grp=3)
subgroup~wnt|met_status_(1_met__0_m0)~0 (n=43)
high (n=38)
low (n=5)
overall survival probability
1.00
0.90
0.80
0.70
0.60
0.50
0.40
0.30
0.20
0.10
0.00
raw p 0.168
bonf p 1.000
0
24
48
72
96
120
144
168
192
Follow up in months
Expression Graph
Event=No
Event=Yes
Expression
20
19
18
17
16
15
14
13
12
11
10
sorted by expression

WNT M1

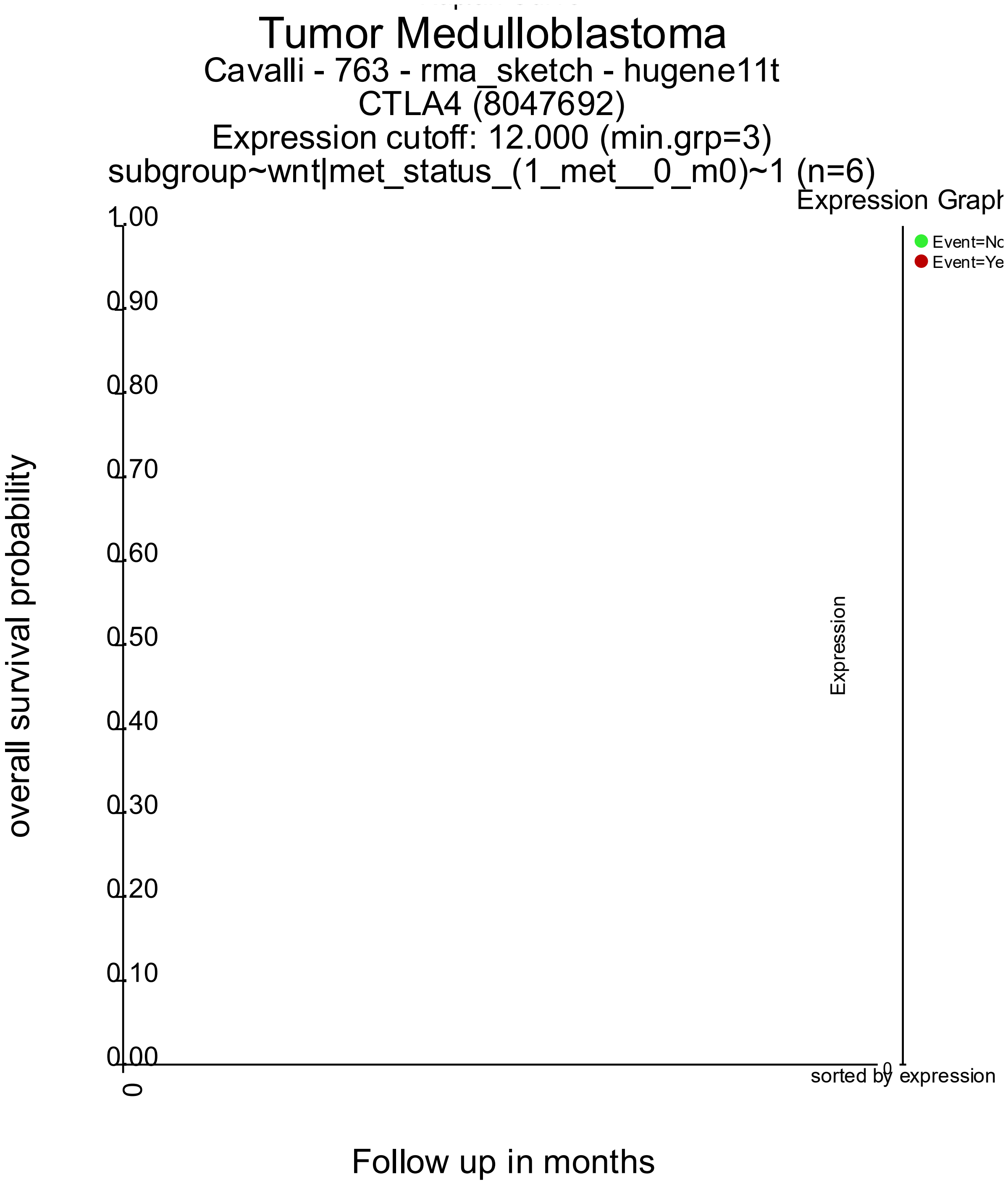

Tumor Medulloblastoma
Cavalli - 763 - rma_sketch - hugene11t
CTLA4 (8047692)
Expression cutoff: 12.000 (min.grp=3)
subgroup~wnt|met_status_(1_met__0_m0)~1 (n=6)
Expression Graph
Event=No
Event=Ye
overall survival probability
1.00
0.90
0.80
0.70
0.60
0.50
0.40
0.30
0.20
0.10
0.00
0
Follow up in months
Expression
0
sorted by expression

SHH M0

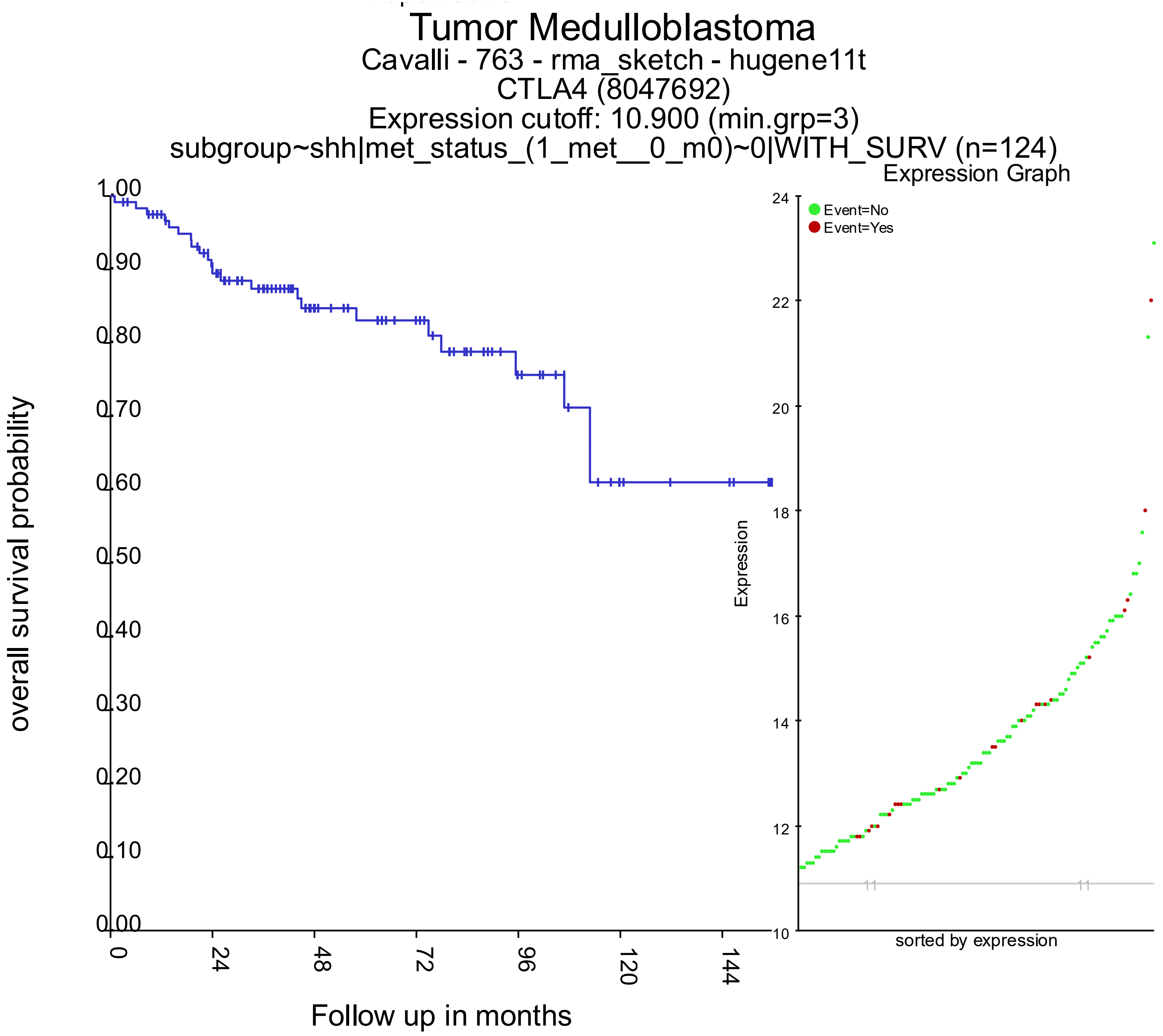

Tumor Medulloblastoma
Cavalli - 763 - rma_sketch - hugene11t
CTLA4 (8047692)
Expression cutoff: 10.900 (min.grp=3)
subgroup~shh|met_status_(1_met__0_m0)~0|WITH_SURV (n=124)
overall survival probability
1.00
0.90
0.80
0.70
0.60
0.50
0.40
0.30
0.20
0.10
0.00
0
24
48
72
96
120
144
Follow up in months
Expression Graph
Event=No
Event=Yes
Expression
24
22
20
18
16
14
12
10
sorted by expression

SHH M1

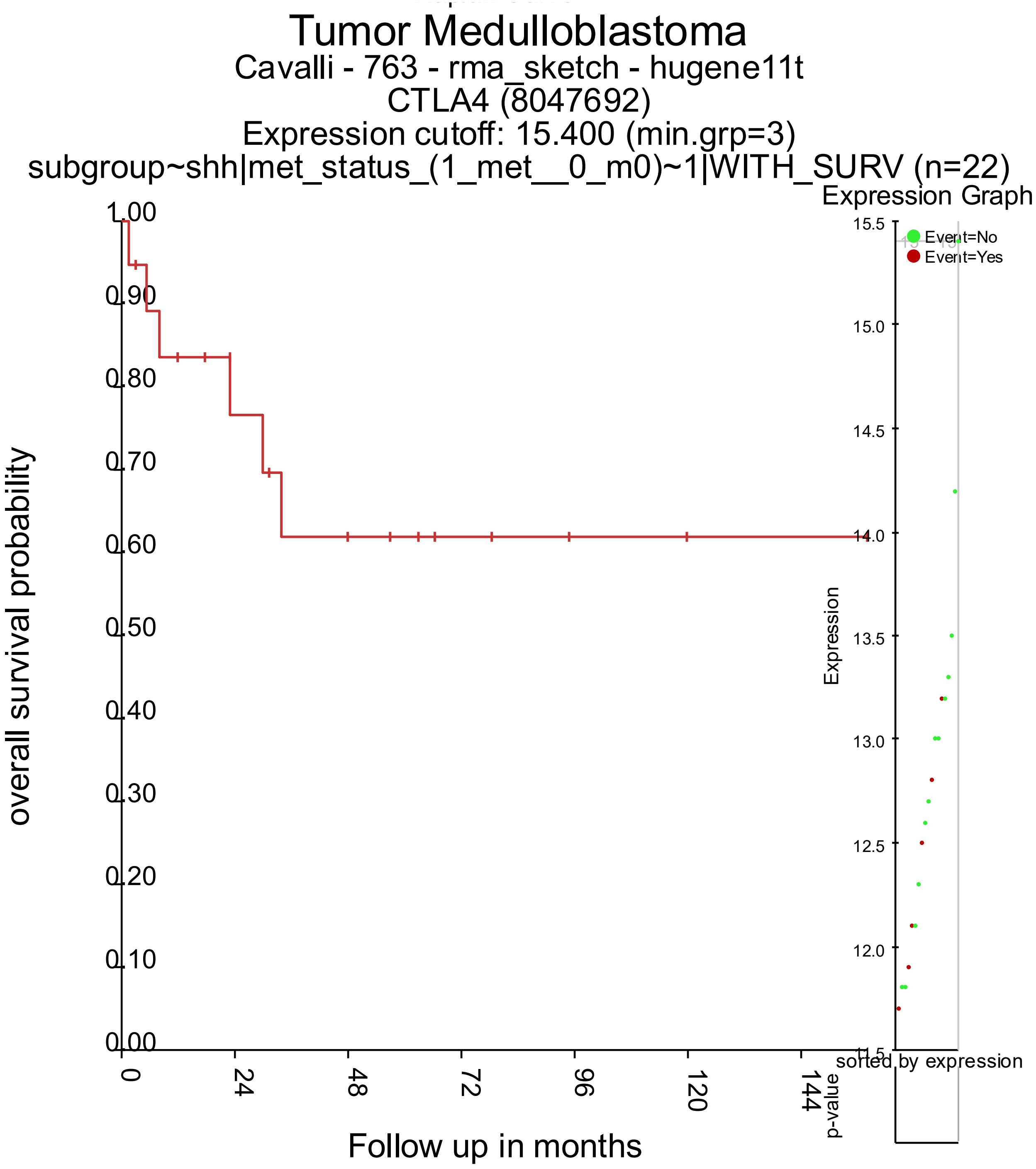

Tumor Medulloblastoma
Cavalli - 763 - rma_sketch - hugene11t
CTLA4 (8047692)
Expression cutoff: 15.400 (min.grp=3)
subgroup~shh|met_status_(1_met__0_m0)~1|WITH_SURV (n=22)
overall survival probability
1.00
0.90
0.80
0.70
0.60
0.50
0.40
0.30
0.20
0.10
0.00
0
24
48
72
96
120
144
Follow up in months
Expression Graph
Event=No
Event=Yes
Expression
15.5
15.0
14.5
14.0
13.5
13.0
12.5
12.0
11.5
sorted by expression
p-value

GROUP4 M0

Tumor Medulloblastoma
Cavalli - 763 - rma_sketch - hugene11t
CTLA4 (8047692)
Expression cutoff: 17.000 (min.grp=3)
subgroup~group4|met_status_(1_met__0_m0)~0|WITH_SURV (n=145)

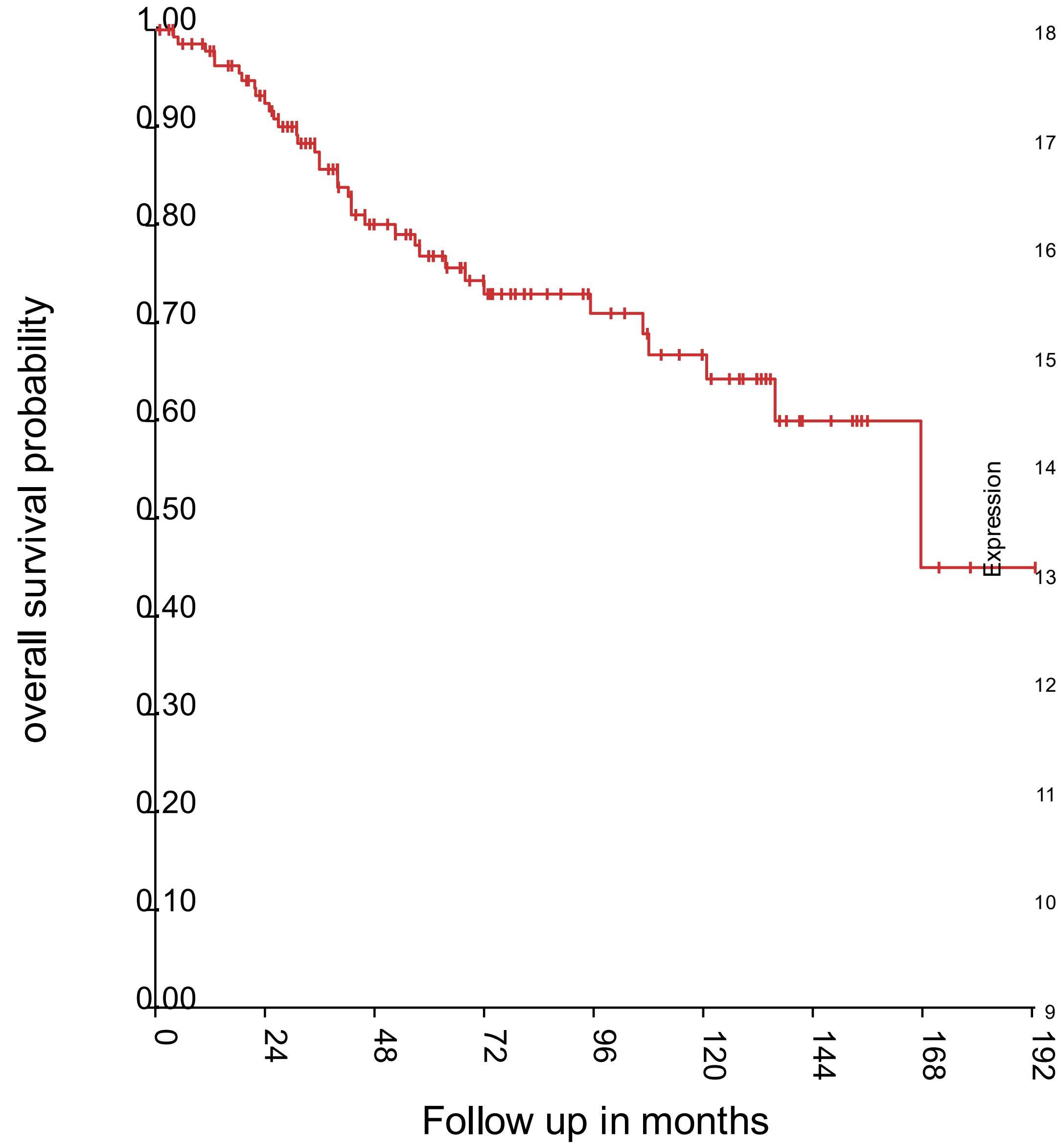

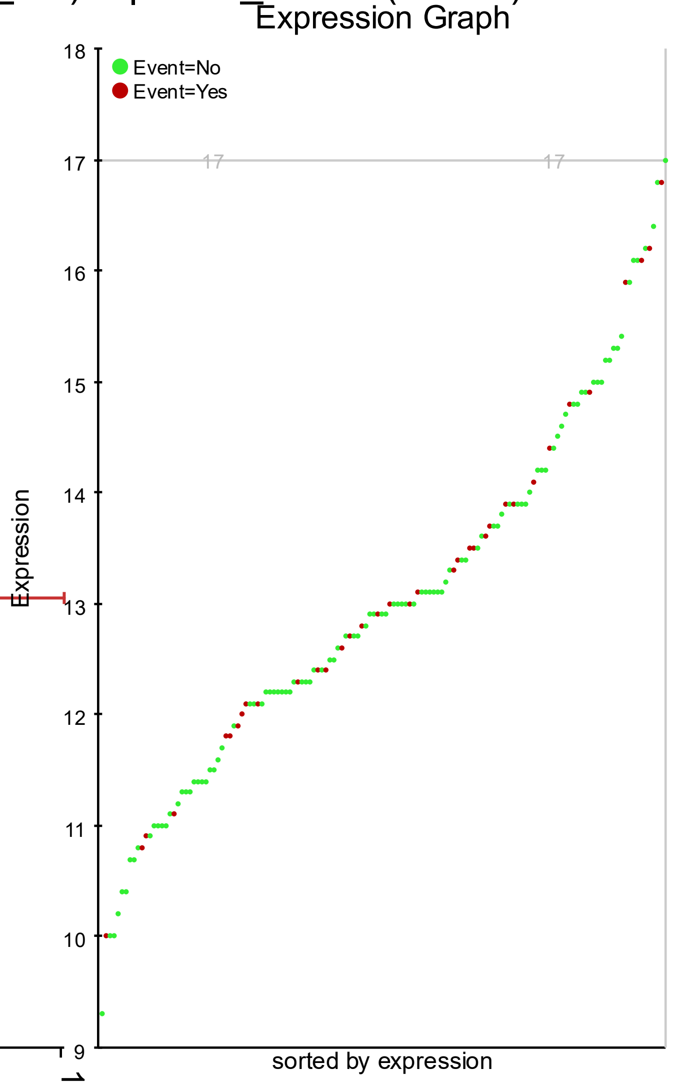

GROUP4 M1

Tumor Medulloblastoma
Cavalli - 763 - rma_sketch - hugene11t
CTLA4 (8047692)
Expression cutoff: 10.800 (min.grp=3)
subgroup~group4|met_status_(1_met__0_m0)~1|WITH_SURV (n=92)

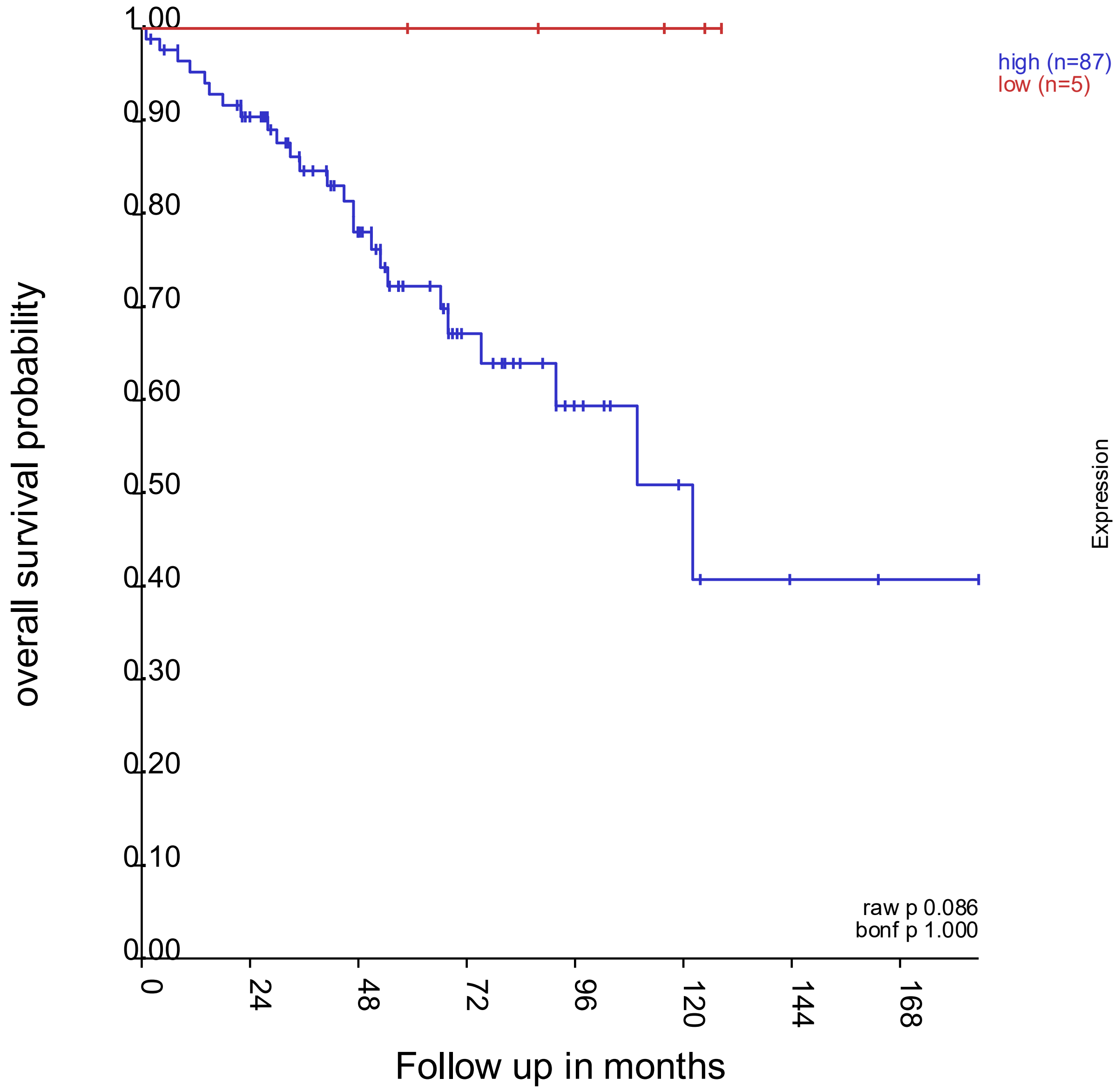

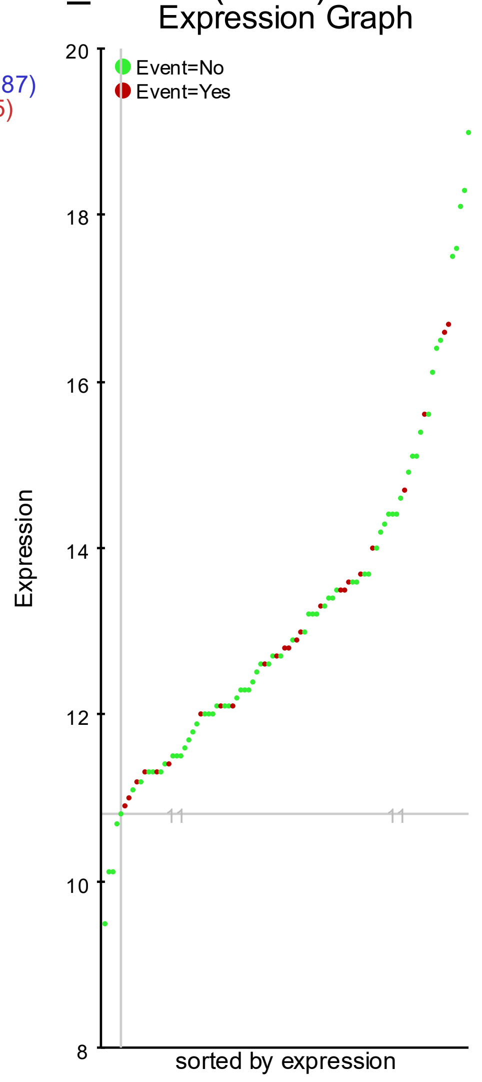

GROUP3 M0

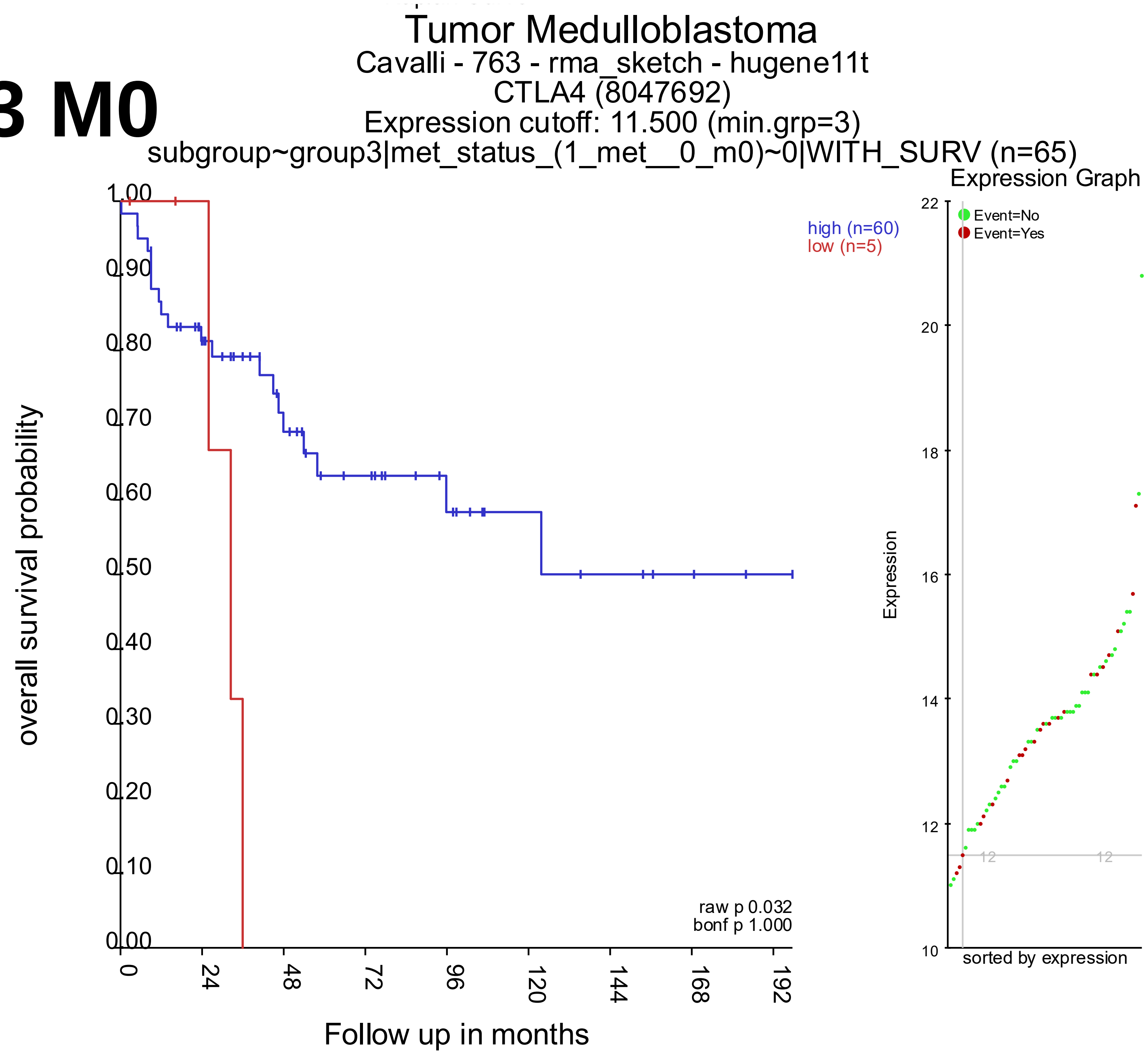

Tumor Medulloblastoma
Cavalli - 763 - rma_sketch - hugene11t
CTLA4 (8047692)
Expression cutoff: 11.500 (min.grp=3)
subgroup~group3|met_status_(1_met__0_m0)~0|WITH_SURV (n=65)
overall survival probability
1.00
0.90
0.80
0.70
0.60
0.50
0.40
0.30
0.20
0.10
0.00
high (n=60)
low (n=5)
raw p 0.032
bonf p 1.000
0
24
48
72
96
120
144
168
192
Follow up in months
Expression Graph
Event=No
Event=Yes
Expression
22
20
18
16
14
12
10
sorted by expression

GROUP3 M1

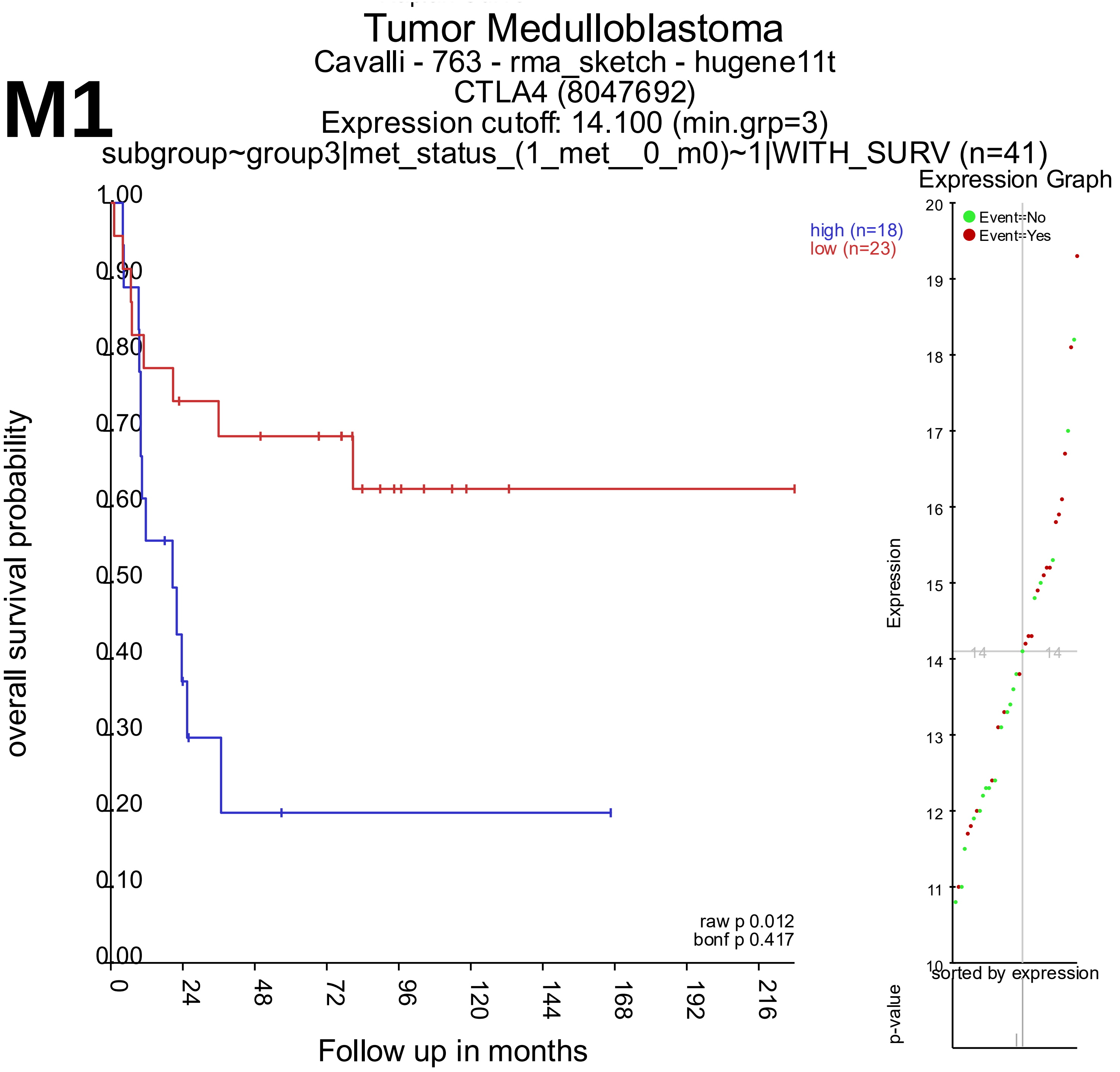
Tumor Medulloblastoma
Cavalli - 763 - rma_sketch - hugene11t
CTLA4 (8047692)
Expression cutoff: 14.100 (min.grp=3)
subgroup~group3|met_status_(1_met__0_m0)~1|WITH_SURV (n=41)
high (n=18)
low (n=23)
overall survival probability
Follow up in months
raw p 0.012
bonf p 0.417
Expression Graph
Event=No
Event=Yes
Expression
sorted by expression
p-value

HAVCR2/TIM3

WNT M0

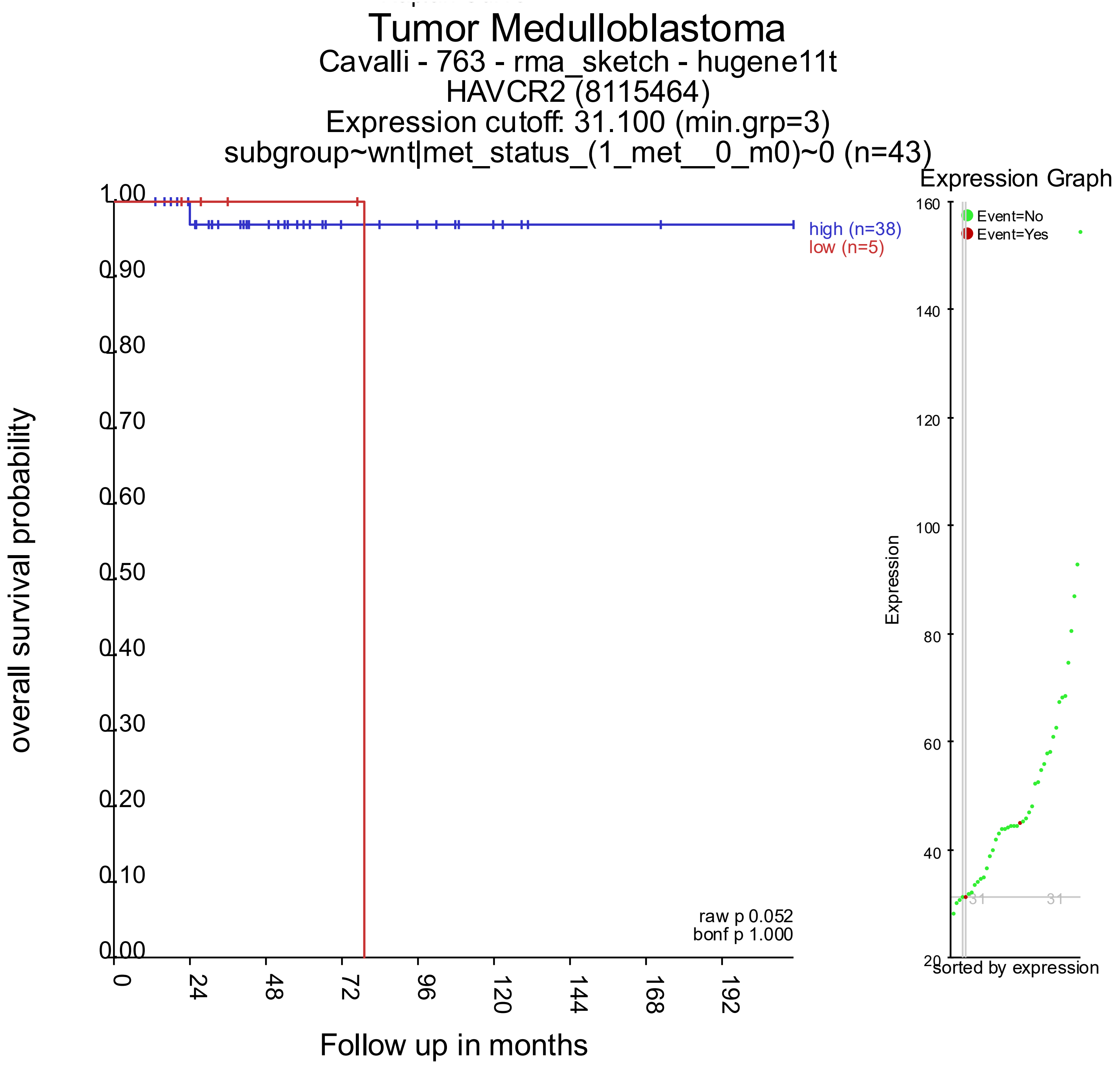

Tumor Medulloblastoma
Cavalli - 763 - rma_sketch - hugene11t
HAVCR2 (8115464)
Expression cutoff: 31.100 (min.grp=3)
subgroup~wnt|met_status_(1_met__0_m0)~0 (n=43)
overall survival probability
1.00
0.90
0.80
0.70
0.60
0.50
0.40
0.30
0.20
0.10
0.00
0
24
48
72
96
120
144
168
192
Follow up in months
high (n=38)
low (n=5)
raw p 0.052
bonf p 1.000
Expression Graph
Event=No
Event=Yes
Expression
160
140
120
100
80
60
40
20
31
31
sorted by expression

WNT M1

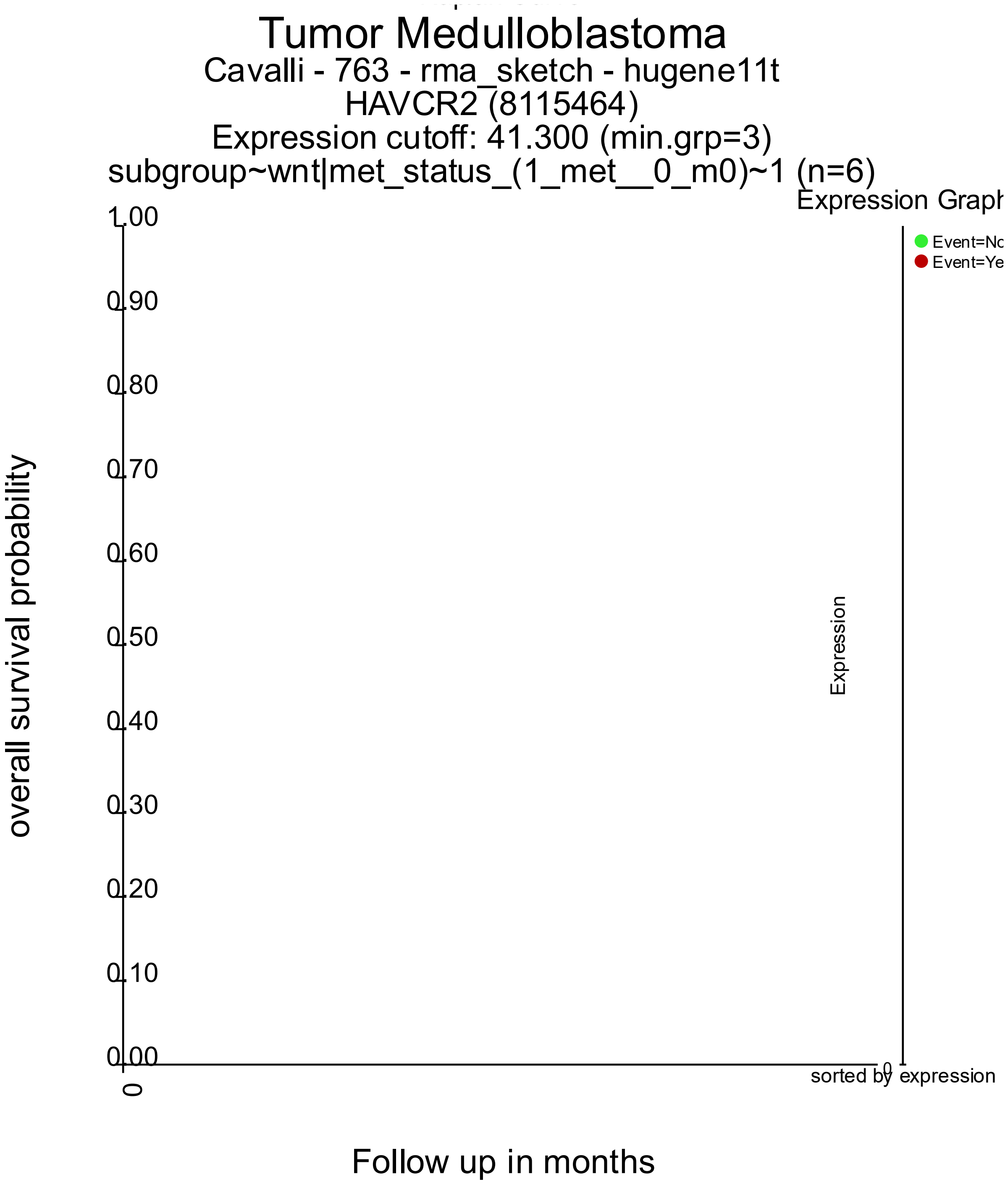

Tumor Medulloblastoma
Cavalli - 763 - rma_sketch - hugene11t
HAVCR2 (8115464)
Expression cutoff: 41.300 (min.grp=3)
subgroup~wnt|met_status_(1_met__0_m0)~1 (n=6)
Expression Graph
Event=No
Event=Ye
1.00
0.90
0.80
0.70
0.60
0.50
0.40
0.30
0.20
0.10
0.00
0
overall survival probability
Follow up in months
Expression
sorted by expression
0

SHH M0

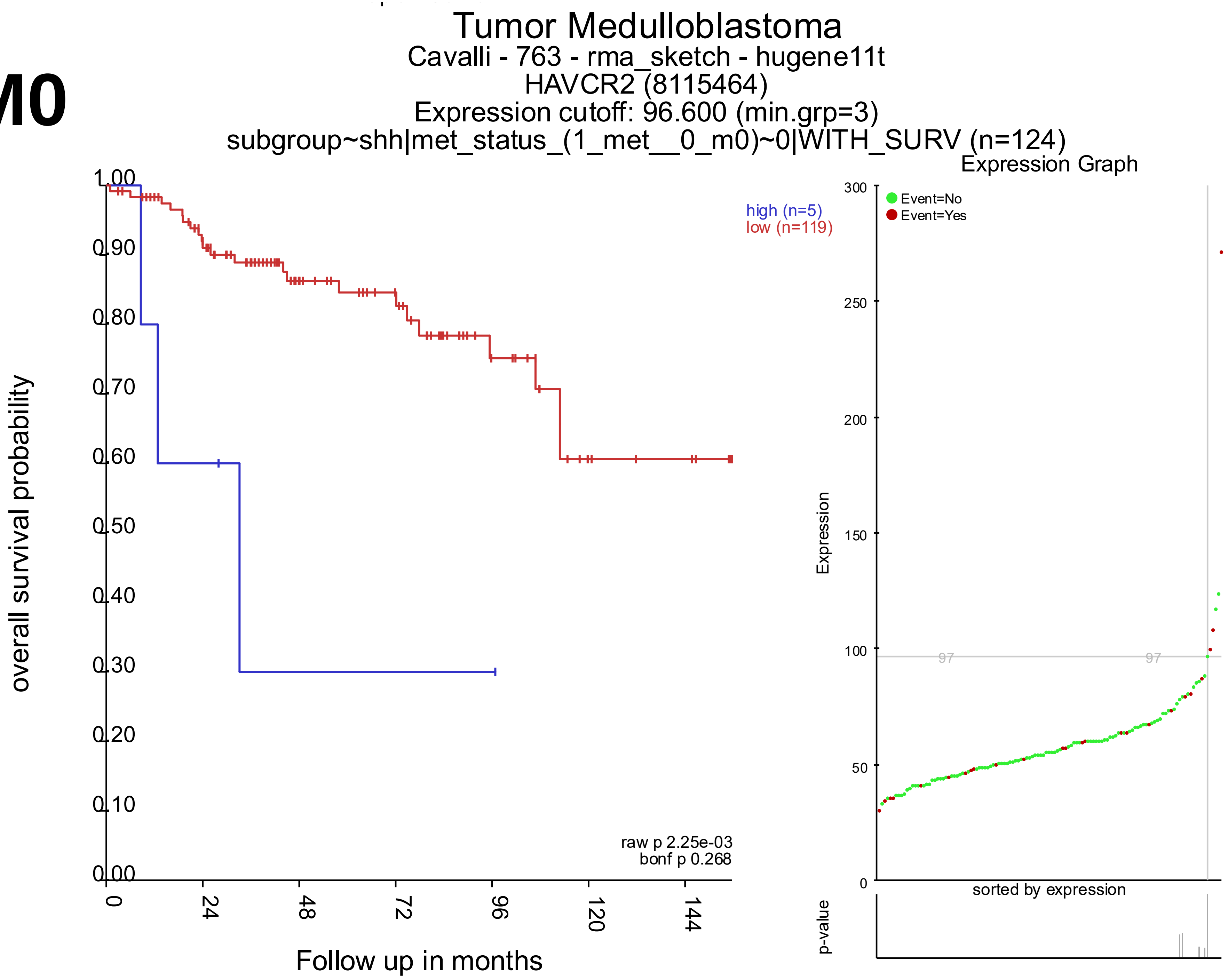

Tumor Medulloblastoma
Cavalli - 763 - rma_sketch - hugene11t
HAVCR2 (8115464)
Expression cutoff: 96.600 (min.grp=3)
subgroup~shh|met_status_(1_met__0_m0)~0|WITH_SURV (n=124)
overall survival probability
1.00
0.90
0.80
0.70
0.60
0.50
0.40
0.30
0.20
0.10
0.00
0
24
48
72
96
120
144
Follow up in months
high (n=5)
low (n=119)
raw p 2.25e-03
bonf p 0.268
Expression Graph
Event=No
Event=Yes
300
250
200
150
100
50
0
Expression
97
97
sorted by expression
p-value

SHH M1

Tumor Medulloblastoma
Cavalli - 763 - rma_sketch - hugene11t
HAVCR2 (8115464)
Expression cutoff: 97.100 (min.grp=3)
subgroup~shh|met_status_(1_met__0_m0)~1|WITH_SURV (n=22)

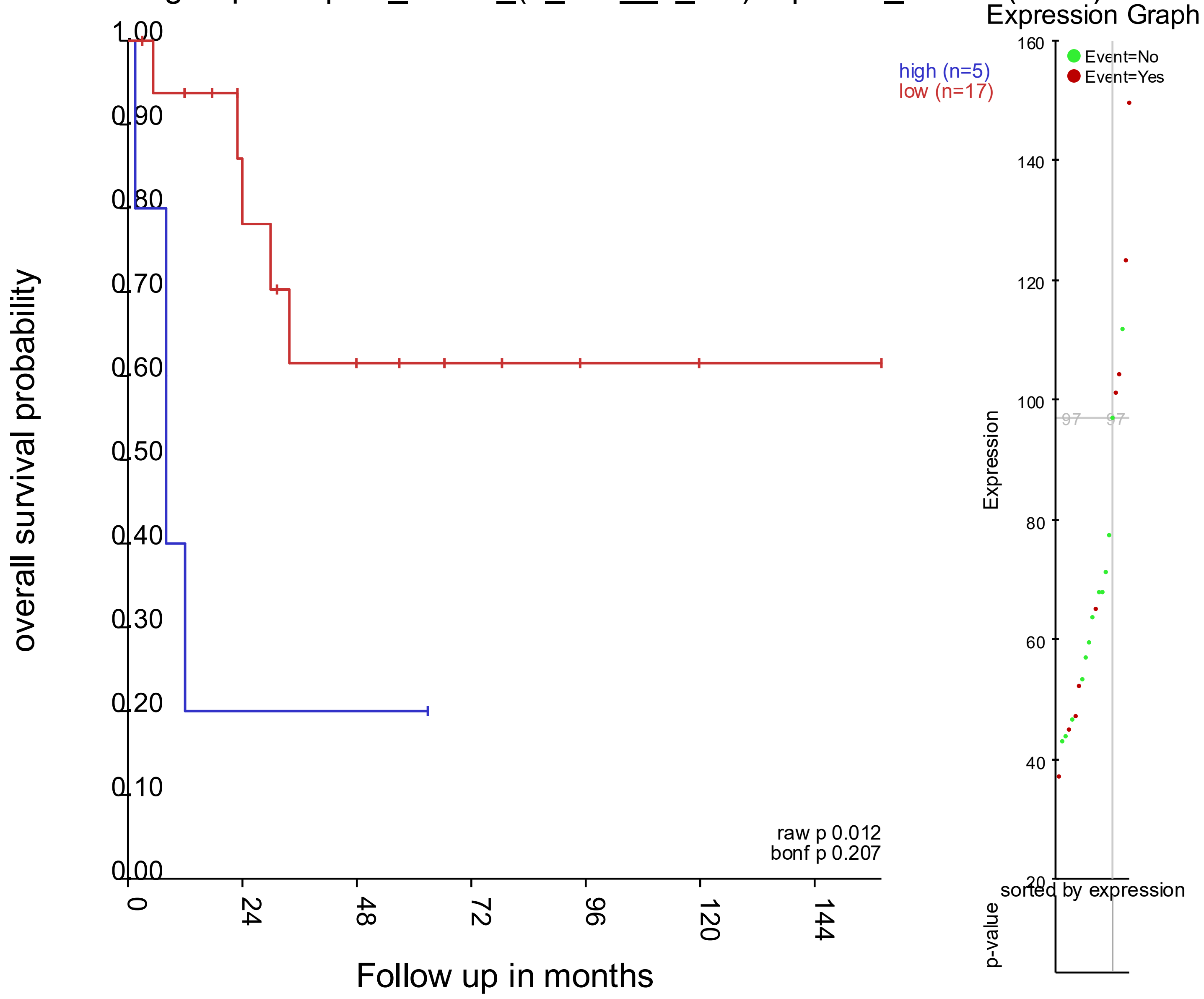

GROUP4 M0

Tumor Medulloblastoma
Cavalli - 763 - rma_sketch - hugene11t
HAVCR2 (8115464)
Expression cutoff: 59.700 (min.grp=3)
subgroup~group4|met_status_(1_met__0_m0)~0|WITH_SURV (n=145)

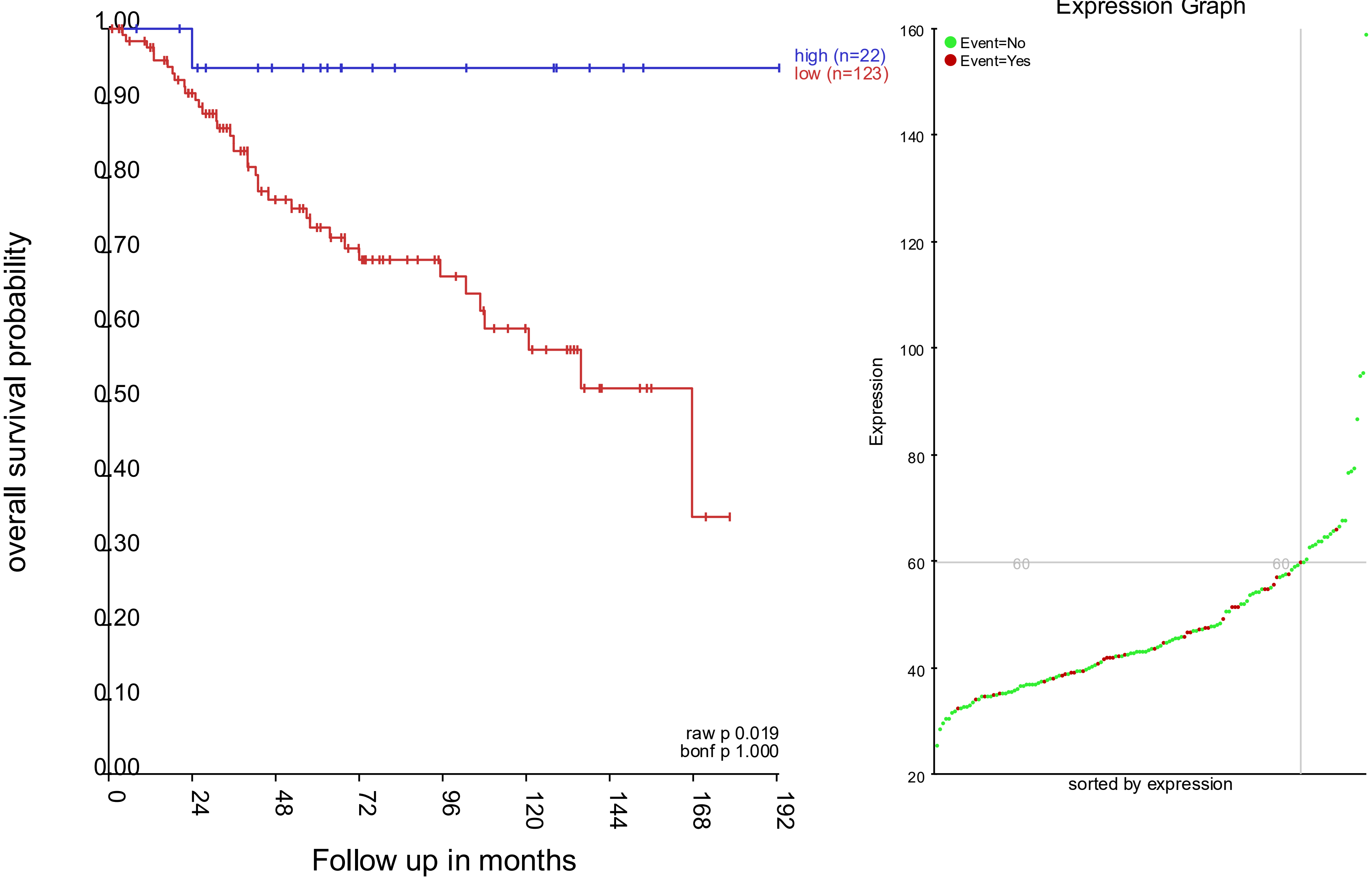

GROUP4 M1

Tumor Medulloblastoma
Cavalli - 763 - rma_sketch - hugene11t
HAVCR2 (8115464)
Expression cutoff: 30.900 (min.grp=3)
subgroup~group4|met_status_(1_met__0_m0)~1|WITH_SURV (n=92)

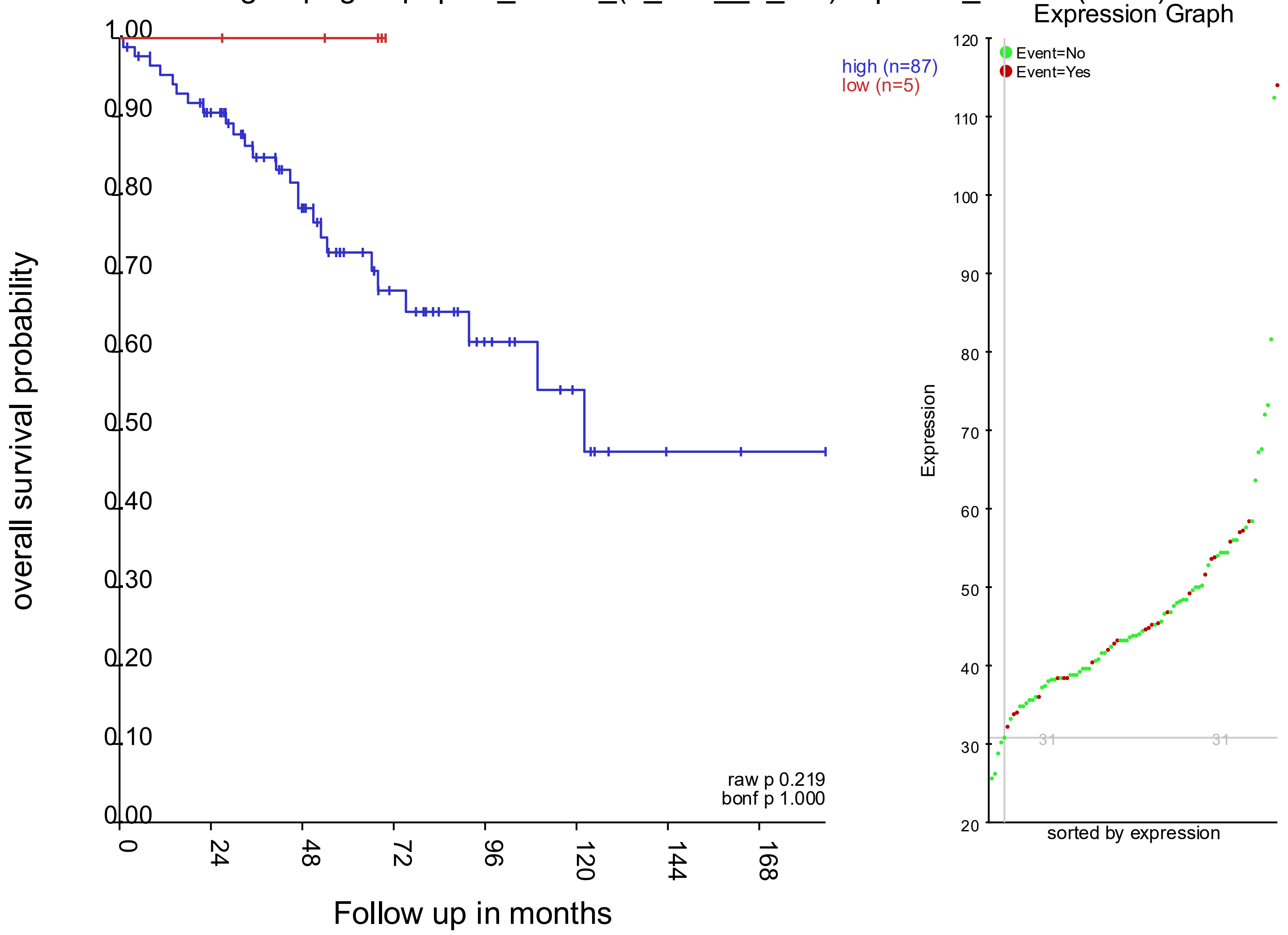

GROUP3 M0

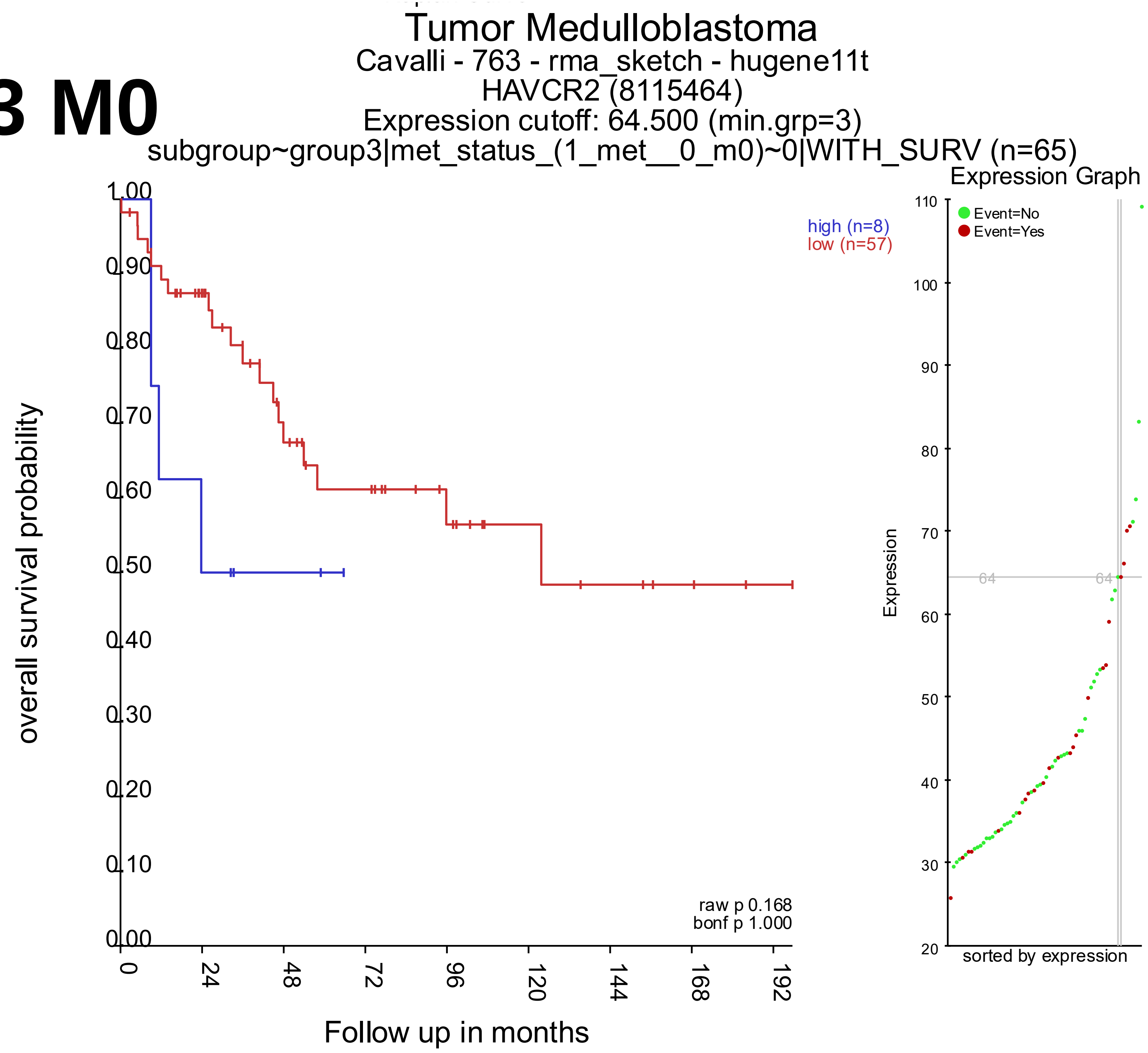

Tumor Medulloblastoma
Cavalli - 763 - rma_sketch - hugene11t
HAVCR2 (8115464)
Expression cutoff: 64.500 (min.grp=3)
subgroup~group3|met_status_(1_met__0_m0)~0|WITH_SURV (n=65)
overall survival probability
1.00
0.90
0.80
0.70
0.60
0.50
0.40
0.30
0.20
0.10
0.00
0
24
48
72
96
120
144
168
192
Follow up in months
high (n=8)
low (n=57)
raw p 0.168
bonf p 1.000
Expression Graph
Event=No
Event=Yes
Expression
110
100
90
80
70
60
50
40
30
20
64
64
sorted by expression

GROUP3 M1

Tumor Medulloblastoma
Cavalli - 763 - rma_sketch - hugene11t
HAVCR2 (8115464)
Expression cutoff: 39.800 (min.grp=3)
subgroup~group3|met_status_(1_met__0_m0)~1|WITH_SURV (n=41)

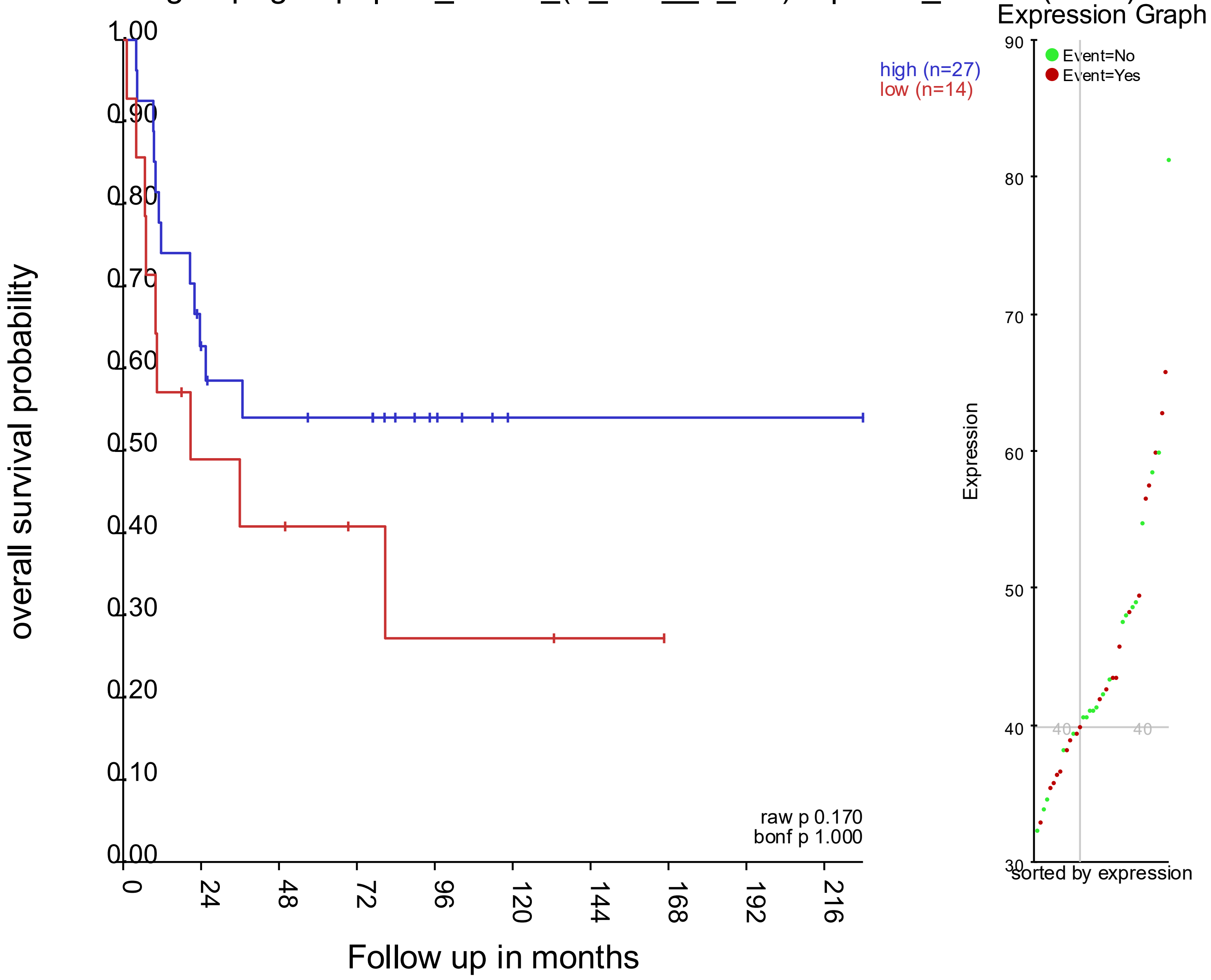

LAG3

WNT M0

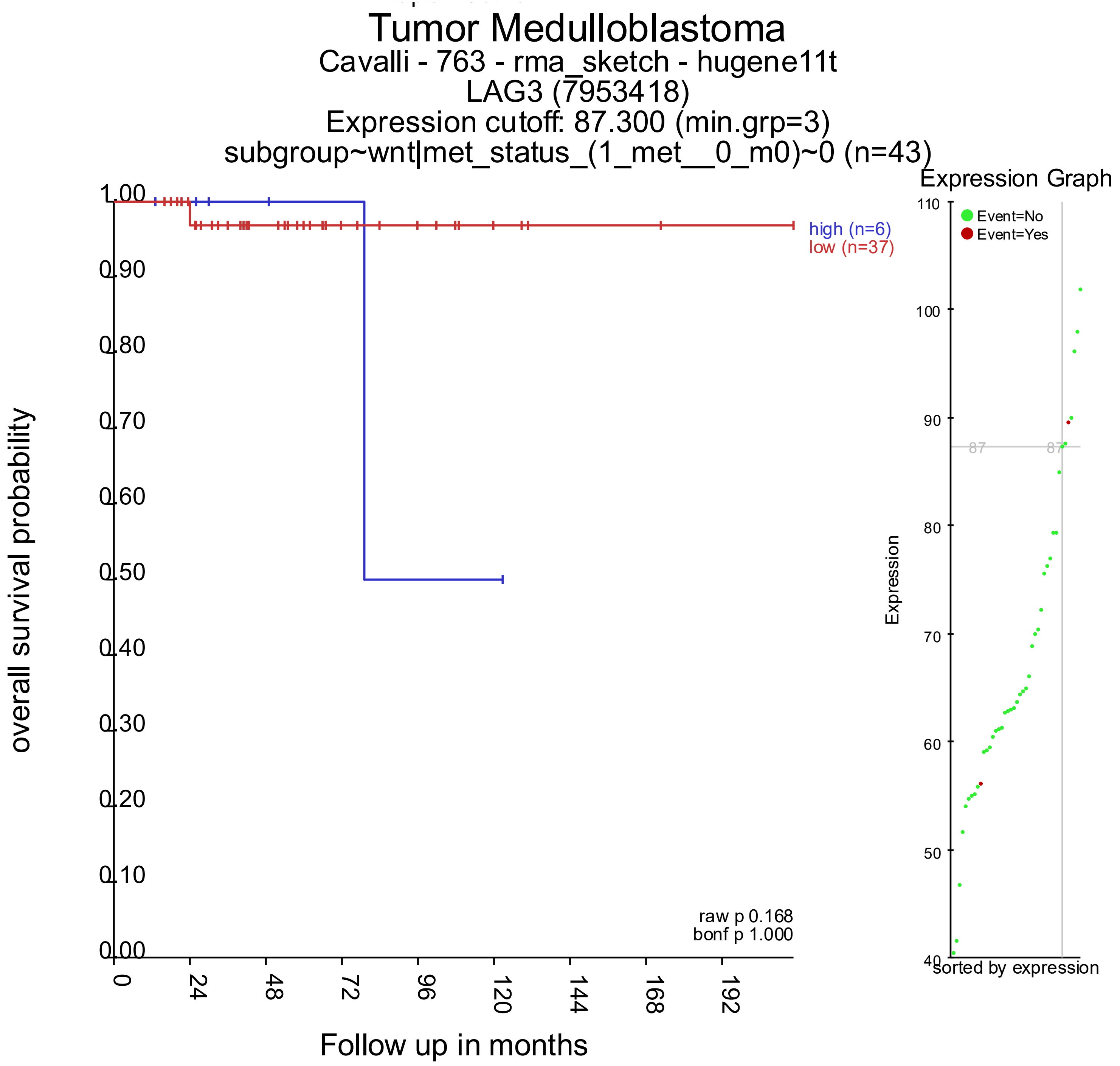

Tumor Medulloblastoma
Cavalli - 763 - rma_sketch - hugene11t
LAG3 (7953418)
Expression cutoff: 87.300 (min.grp=3)
subgroup~wnt|met_status_(1_met__0_m0)~0 (n=43)
overall survival probability
1.00
0.90
0.80
0.70
0.60
0.50
0.40
0.30
0.20
0.10
0.00
0
24
48
72
96
120
144
168
192
Follow up in months
high (n=6)
low (n=37)
raw p 0.168
bonf p 1.000
Expression Graph
Event=No
Event=Yes
Expression
110
100
90
80
70
60
50
40
87
87
sorted by expression

WNT M1

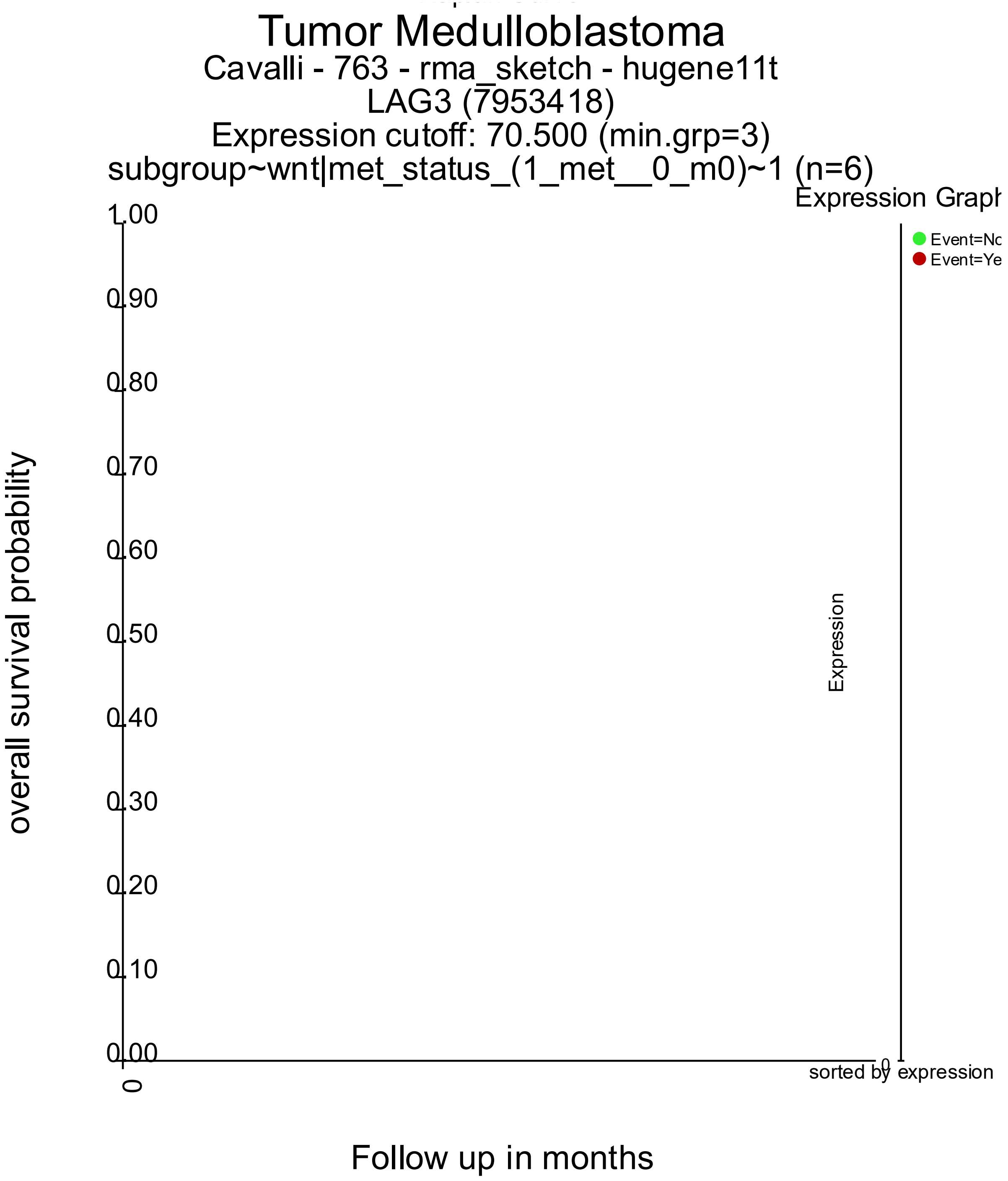

Tumor Medulloblastoma
Cavalli - 763 - rma_sketch - hugene11t
LAG3 (7953418)
Expression cutoff: 70.500 (min.grp=3)
subgroup~wnt|met_status_(1_met__0_m0)~1 (n=6)
overall survival probability
1.00
0.90
0.80
0.70
0.60
0.50
0.40
0.30
0.20
0.10
0.00
0
Follow up in months
Expression Graph
Event=No
Event=Ye
Expression
0
sorted by expression

SHH M0

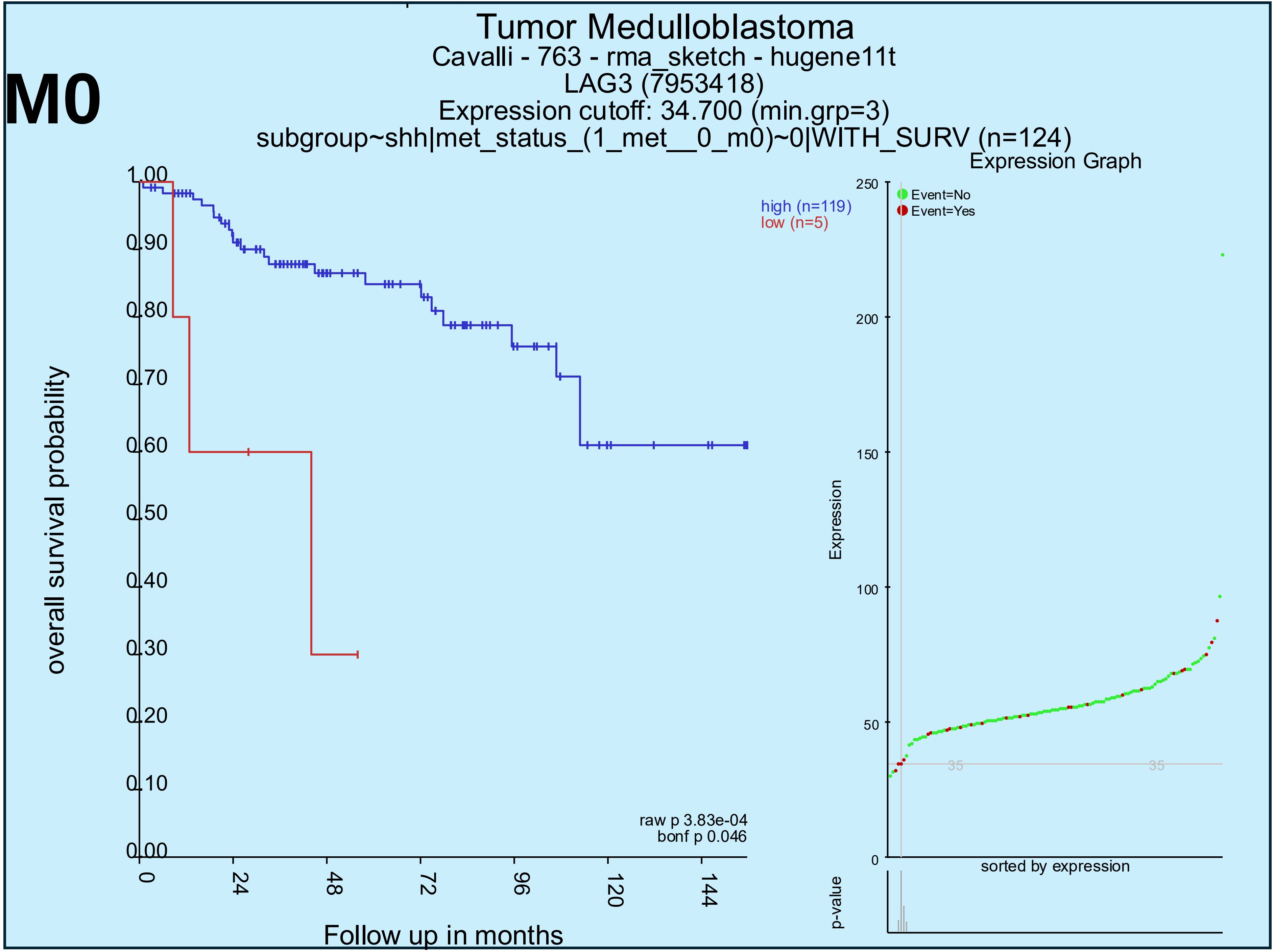

Tumor Medulloblastoma
Cavalli - 763 - rma_sketch - hugene11t
LAG3 (7953418)
Expression cutoff: 34.700 (min.grp=3)
subgroup~shh|met_status_(1_met__0_m0)~0|WITH_SURV (n=124)
Expression Graph
high (n=119)
low (n=5)
Event=No
Event=Yes
overall survival probability
1.00
0.90
0.80
0.70
0.60
0.50
0.40
0.30
0.20
0.10
0.00
raw p 3.83e-04
bonf p 0.046
0
24
48
72
96
120
144
Follow up in months
250
200
150
100
50
0
Expression
35
35
sorted by expression
p-value

SHH M1

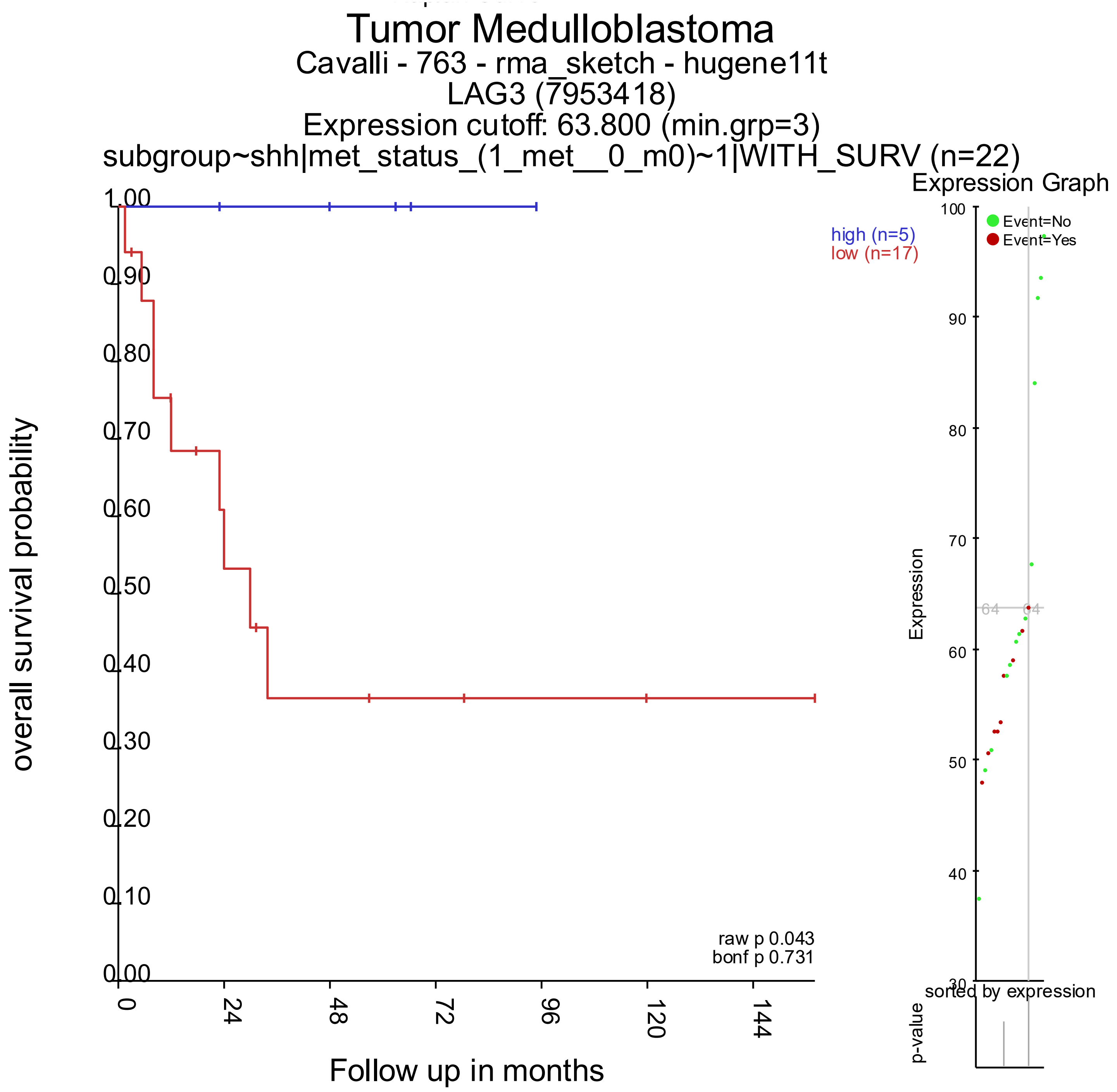

Tumor Medulloblastoma
Cavalli - 763 - rma_sketch - hugene11t
LAG3 (7953418)
Expression cutoff: 63.800 (min.grp=3)
subgroup~shh|met_status_(1_met__0_m0)~1|WITH_SURV (n=22)
overall survival probability
1.00
0.90
0.80
0.70
0.60
0.50
0.40
0.30
0.20
0.10
0.00
high (n=5)
low (n=17)
raw p 0.043
bonf p 0.731
0
24
48
72
96
120
144
Follow up in months
Expression Graph
Event=No
Event=Yes
Expression
100
90
80
70
64
60
50
40
30
sorted by expression
p-value

GROUP4 M0

Tumor Medulloblastoma
Cavalli - 763 - rma_sketch - hugene11t
LAG3 (7953418)
Expression cutoff: 46.300 (min.grp=3)
subgroup~group4|met_status_(1_met__0_m0)~0|WITH_SURV (n=145)

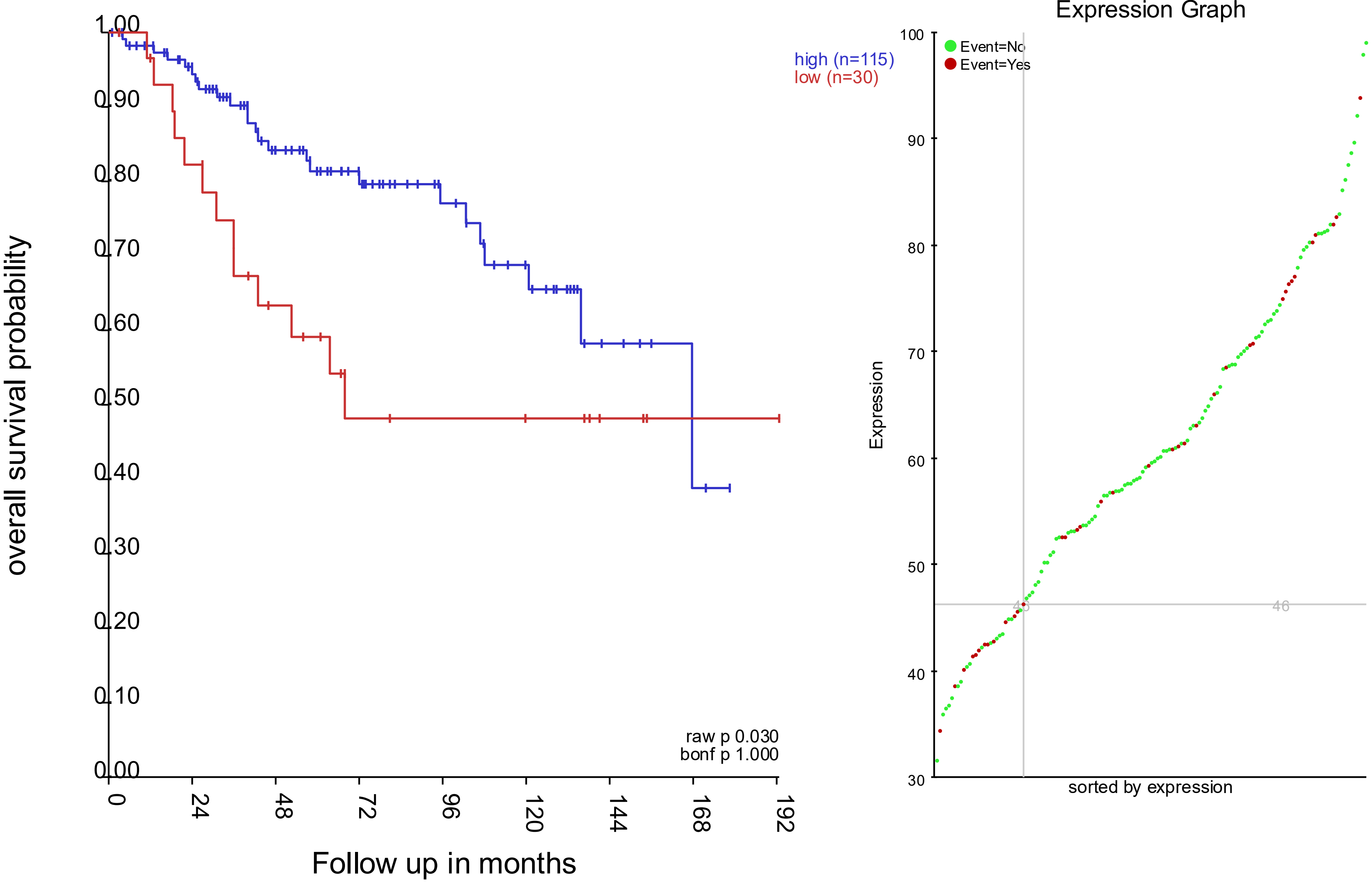

GROUP4 M1

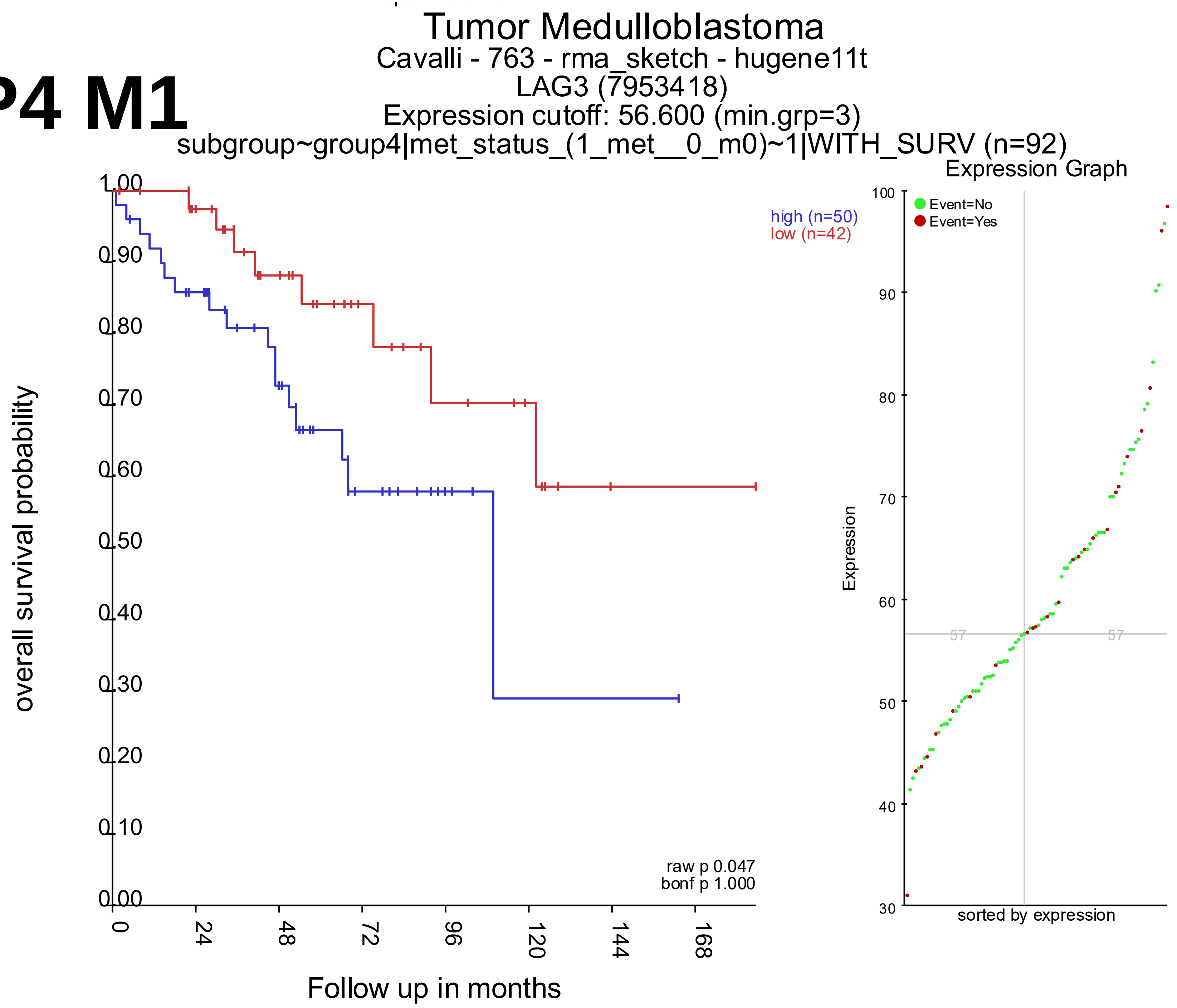

Tumor Medulloblastoma
Cavalli - 763 - rma_sketch - hugene11t
LAG3 (7953418)
Expression cutoff: 56.600 (min.grp=3)
subgroup~group4|met_status_(1_met__0_m0)~1|WITH_SURV (n=92)
overall survival probability
1.00
0.90
0.80
0.70
0.60
0.50
0.40
0.30
0.20
0.10
0.00
high (n=50)
low (n=42)
raw p 0.047
bonf p 1.000
0
24
48
72
96
120
144
168
Follow up in months
Expression Graph
Event=No
Event=Yes
Expression
100
90
80
70
60
50
40
30
57
57
sorted by expression

GROUP3 M0

Tumor Medulloblastoma
Cavalli - 763 - rma_sketch - hugene11t
LAG3 (7953418)
Expression cutoff: 38.100 (min.grp=3)
subgroup~group3|met_status_(1_met__0_m0)~0|WITH_SURV (n=65)

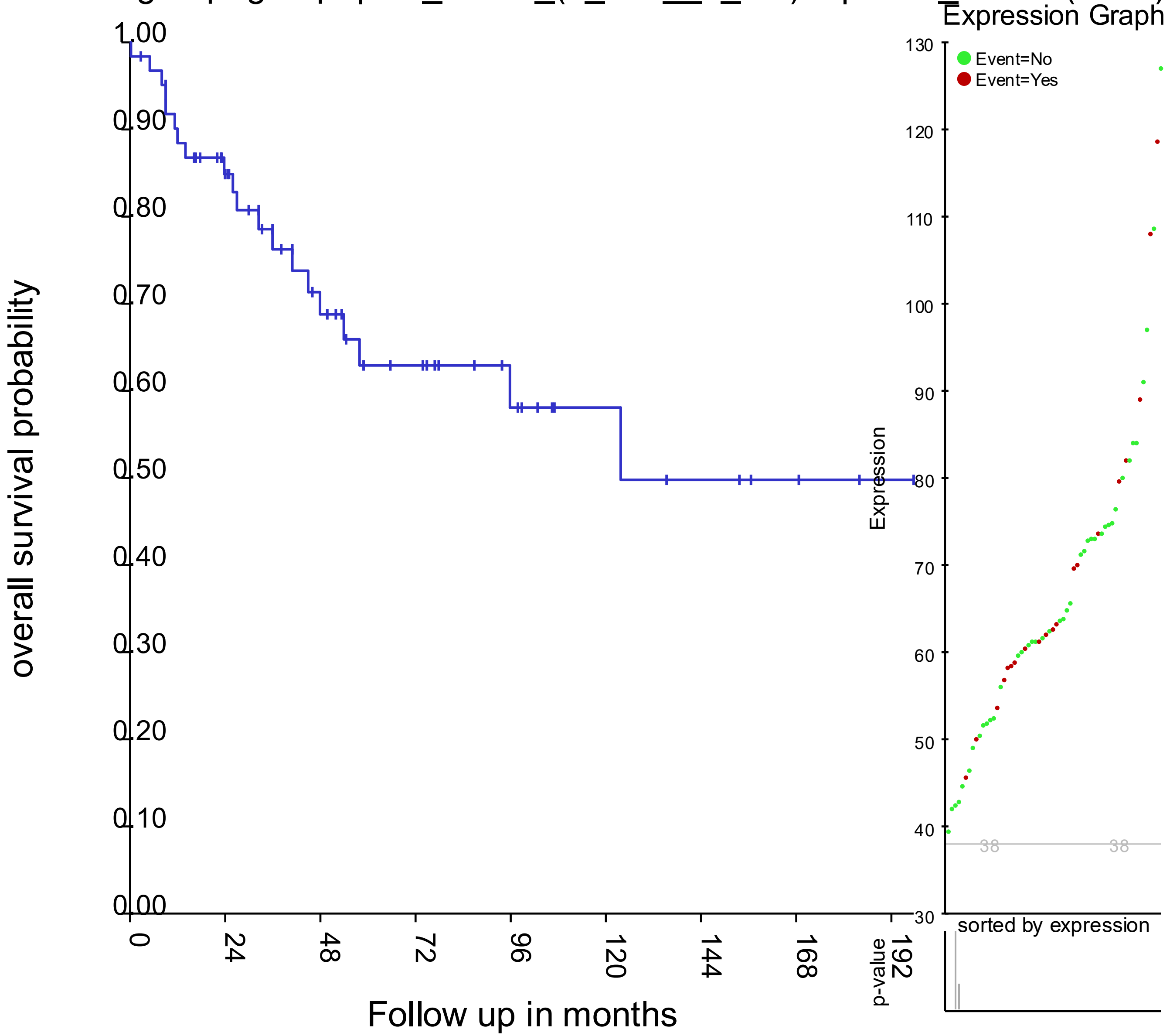

GROUP3 M1

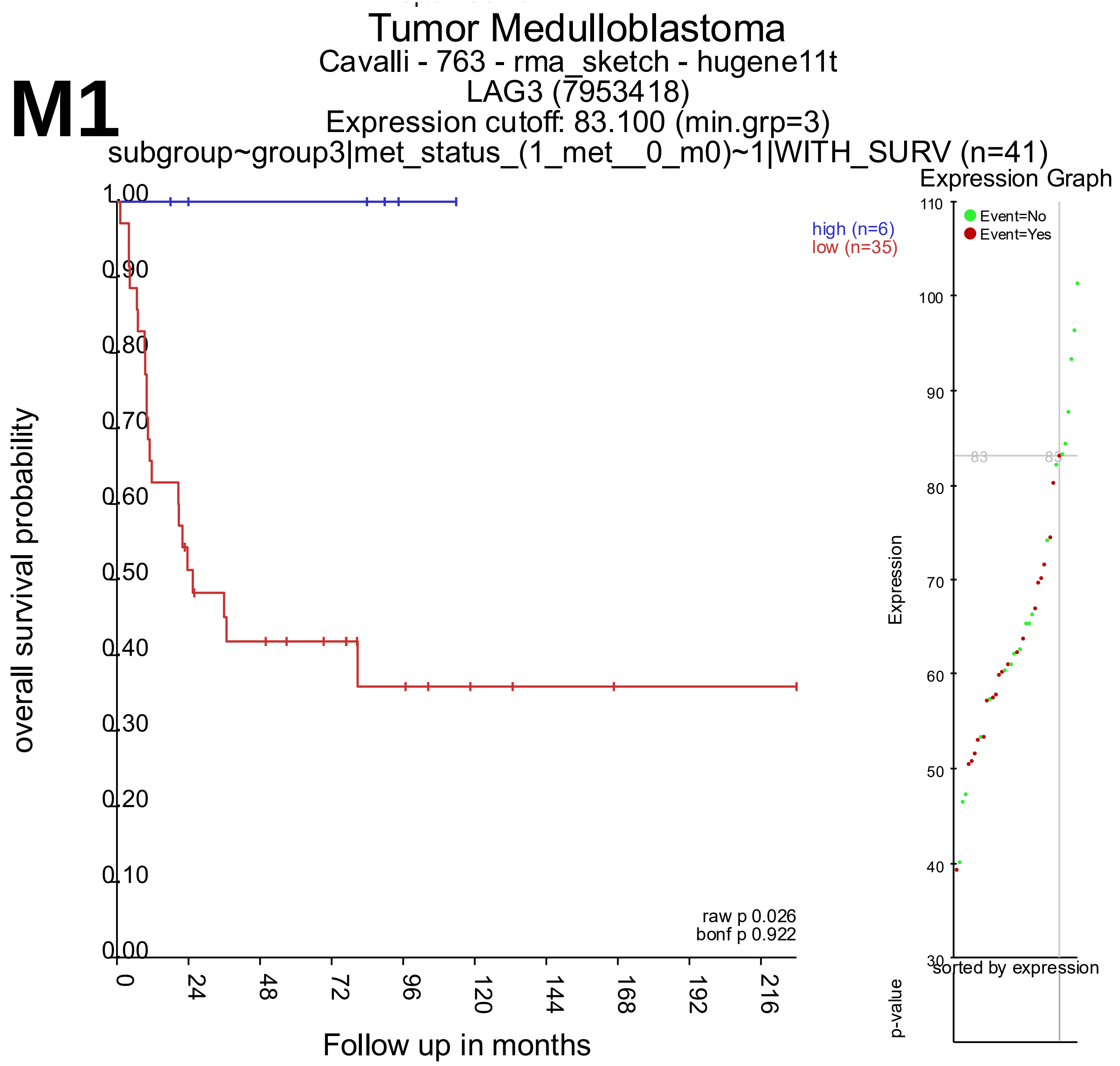

Tumor Medulloblastoma
Cavalli - 763 - rma_sketch - hugene11t
LAG3 (7953418)
Expression cutoff: 83.100 (min.grp=3)
subgroup~group3|met_status_(1_met__0_m0)~1|WITH_SURV (n=41)
overall survival probability
1.00
0.90
0.80
0.70
0.60
0.50
0.40
0.30
0.20
0.10
0.00
high (n=6)
low (n=35)
raw p 0.026
bonf p 0.922
0
24
48
72
96
120
144
168
192
216
Follow up in months
Expression Graph
Event=No
Event=Yes
110
100
90
80
70
60
50
40
30
Expression
83
sorted by expression
p-value

IDH1

WNT M0

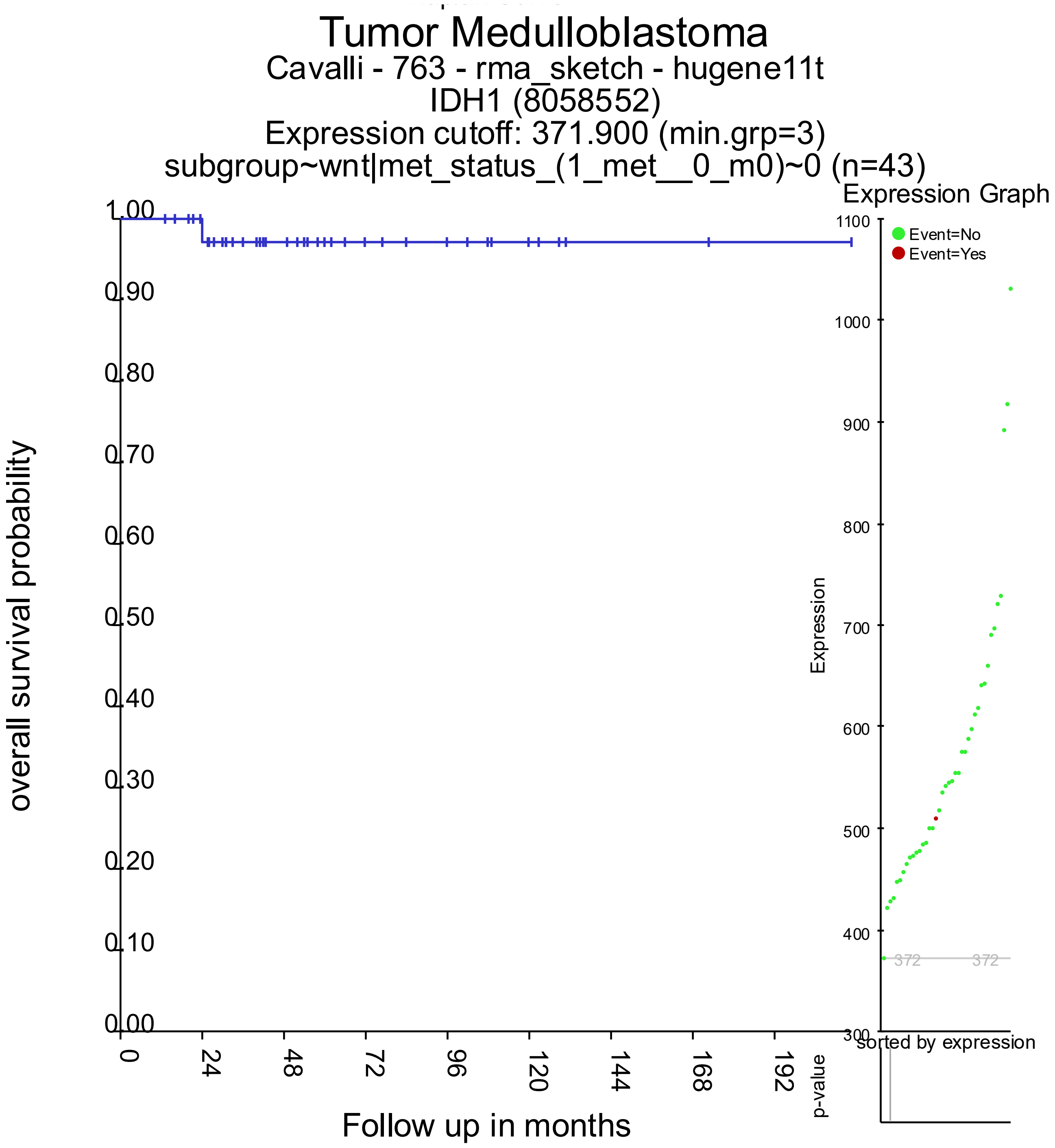

Tumor Medulloblastoma
Cavalli - 763 - rma_sketch - hugene11t
IDH1 (8058552)
Expression cutoff: 371.900 (min.grp=3)
subgroup~wnt|met_status_(1_met__0_m0)~0 (n=43)
overall survival probability
1.00
0.90
0.80
0.70
0.60
0.50
0.40
0.30
0.20
0.10
0.00
0
24
48
72
96
120
144
168
192
Follow up in months
p-value
Expression Graph
Event=No
Event=Yes
Expression
1100
1000
900
800
700
600
500
400
300
372
372
sorted by expression

WNT M1

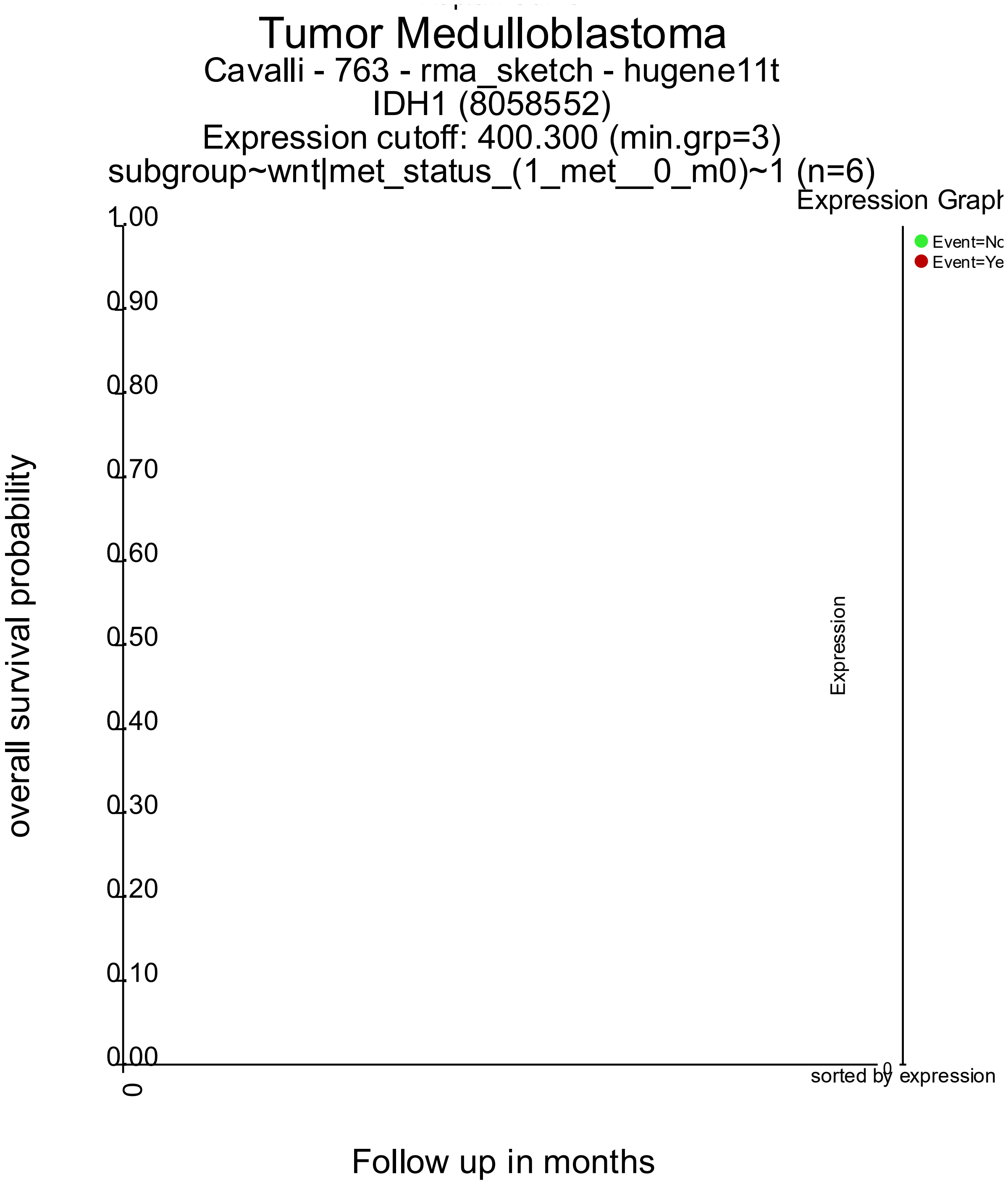

Tumor Medulloblastoma
Cavalli - 763 - rma_sketch - hugene11t
IDH1 (8058552)
Expression cutoff: 400.300 (min.grp=3)
subgroup~wnt|met_status_(1_met__0_m0)~1 (n=6)
Expression Graph
Event=No
Event=Yes
1.00
0.90
0.80
0.70
0.60
0.50
0.40
0.30
0.20
0.10
0.00
overall survival probability
0
Follow up in months
Expression
0
sorted by expression

SHH M0

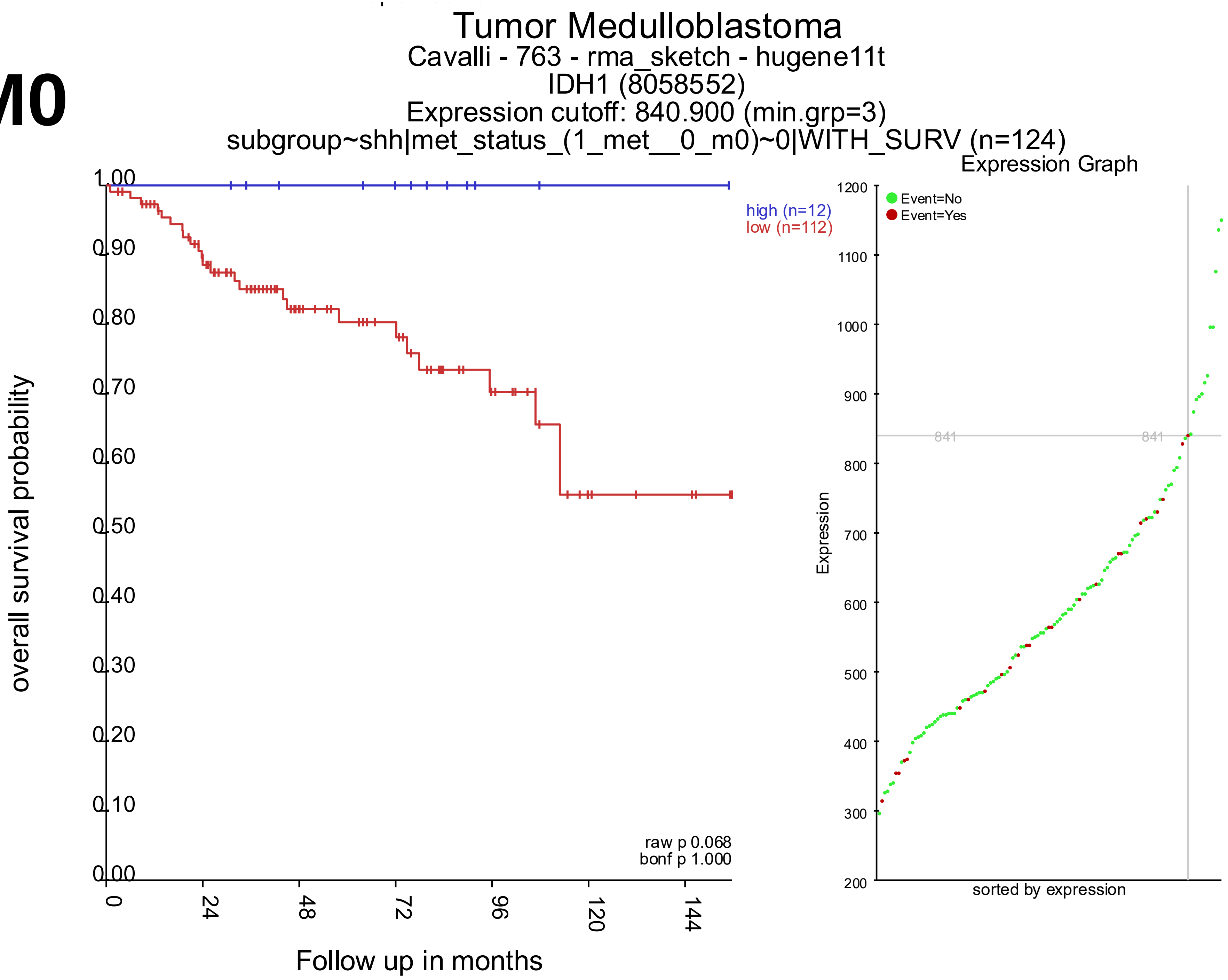

Tumor Medulloblastoma
Cavalli - 763 - rma_sketch - hugene11t
IDH1 (8058552)
Expression cutoff: 840.900 (min.grp=3)
subgroup~shh|met_status_(1_met__0_m0)~0|WITH_SURV (n=124)
overall survival probability
1.00
0.90
0.80
0.70
0.60
0.50
0.40
0.30
0.20
0.10
0.00
high (n=12)
low (n=112)
raw p 0.068
bonf p 1.000
0
24
48
72
96
120
144
Follow up in months
Expression Graph
Event=No
Event=Yes
1200
1100
1000
900
800
700
600
500
400
300
200
841
841
Expression
sorted by expression

SHH M1

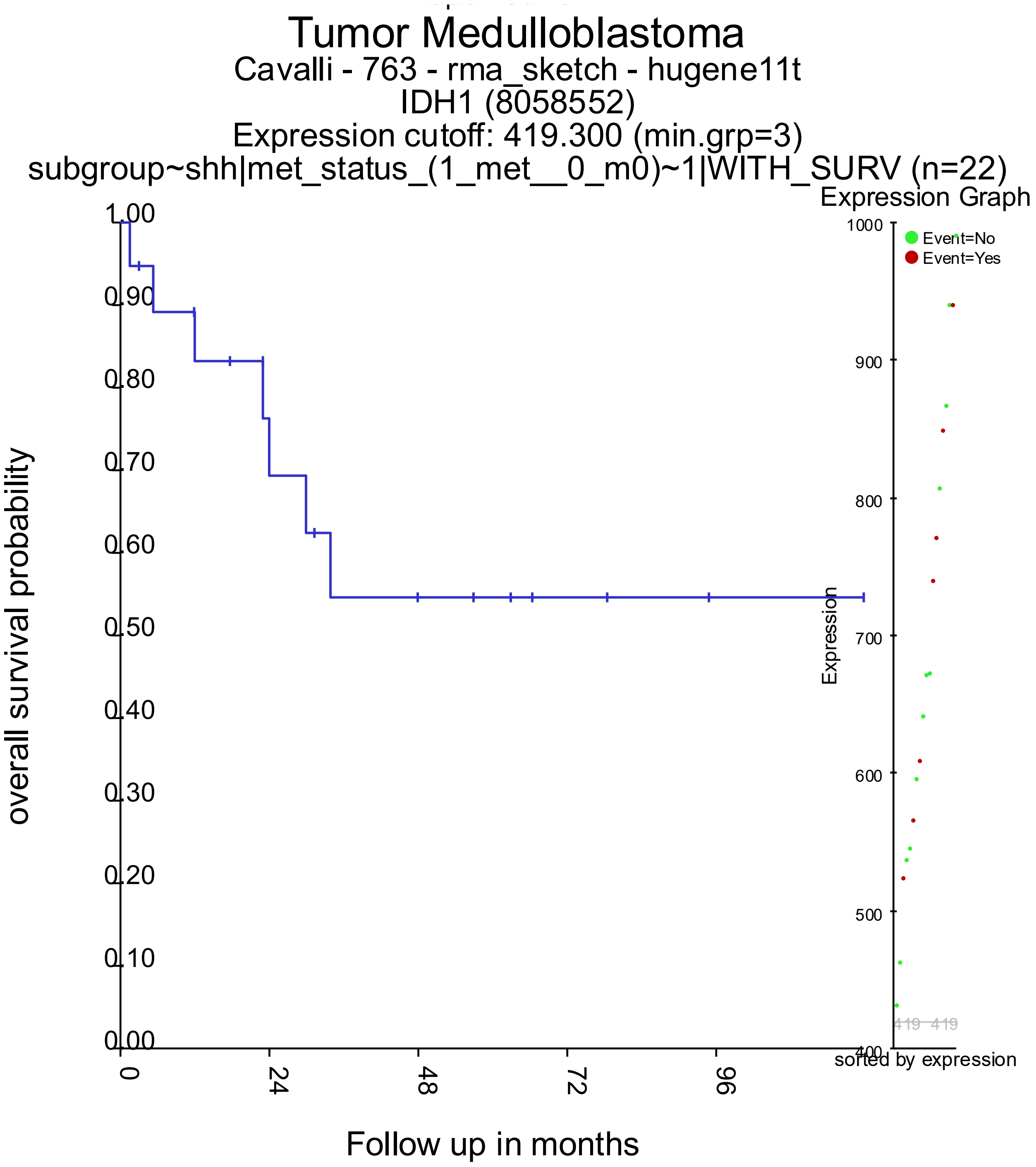

Tumor Medulloblastoma
Cavalli - 763 - rma_sketch - hugene11t
IDH1 (8058552)
Expression cutoff: 419.300 (min.grp=3)
subgroup~shh|met_status_(1_met__0_m0)~1|WITH_SURV (n=22)
overall survival probability
1.00
0.90
0.80
0.70
0.60
0.50
0.40
0.30
0.20
0.10
0.00
0
24
48
72
96
Follow up in months
Expression Graph
Event=No
Event=Yes
Expression
1000
900
800
700
600
500
400
419 419
sorted by expression

GROUP4 M0

Tumor Medulloblastoma
Cavalli - 763 - rma_sketch - hugene11t
IDH1 (8058552)
Expression cutoff: 249.200 (min.grp=3)
subgroup~group4|met_status_(1_met__0_m0)~0|WITH_SURV (n=145)

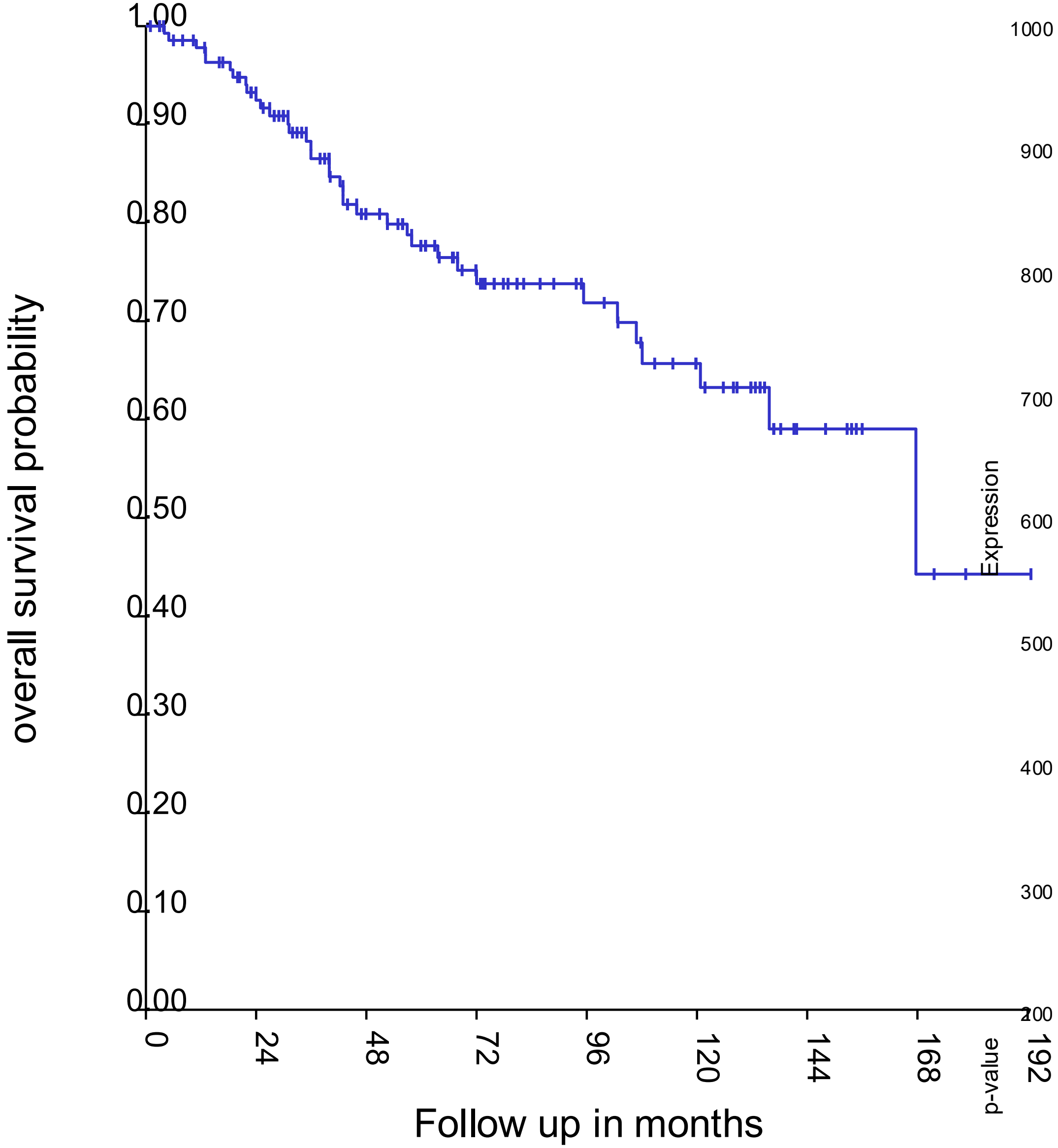

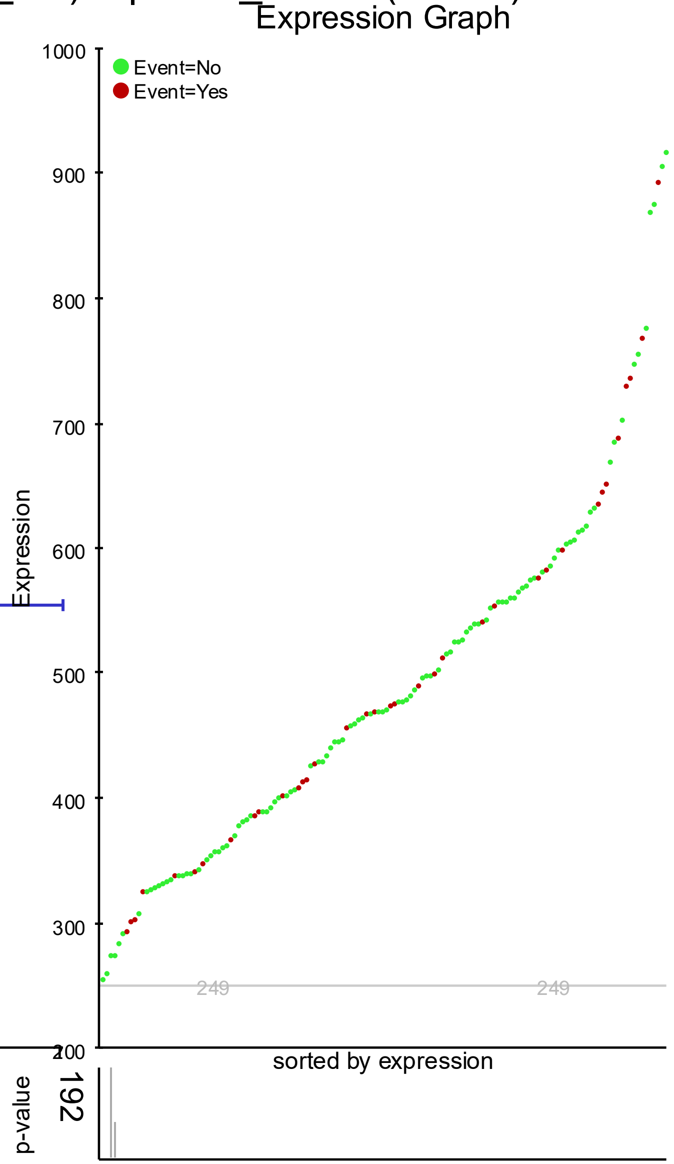

GROUP4 M1

Tumor Medulloblastoma
Cavalli - 763 - rma_sketch - hugene11t
IDH1 (8058552)
Expression cutoff: 325.300 (min.grp=3)
subgroup~group4|met_status_(1_met__0_m0)~1|WITH_SURV (n=92)

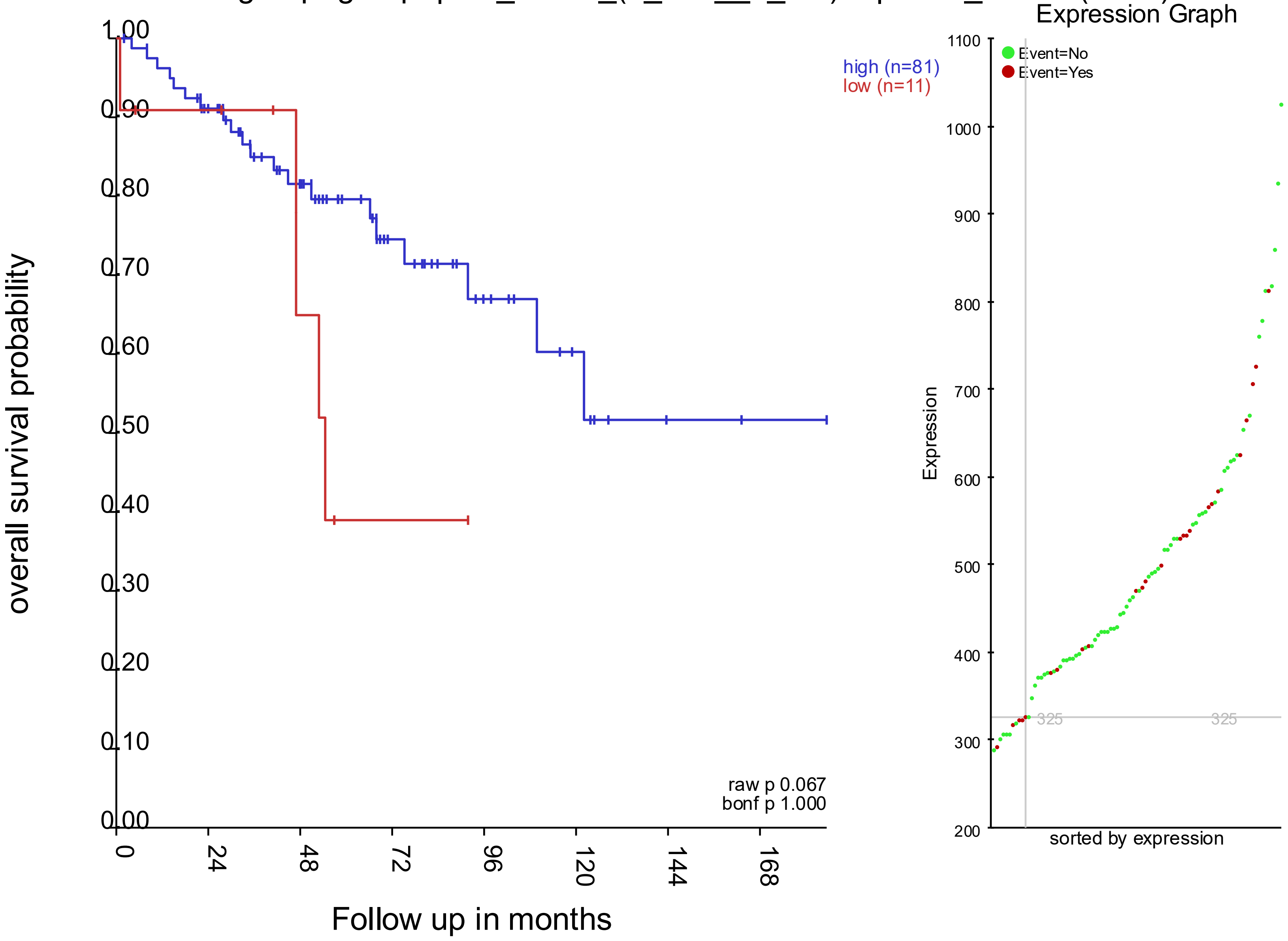

GROUP3 M0

Tumor Medulloblastoma
Cavalli - 763 - rma_sketch - hugene11t
IDH1 (8058552)
Expression cutoff: 542.400 (min.grp=3)
subgroup~group3|met_status_(1_met__0_m0)~0|WITH_SURV (n=65)

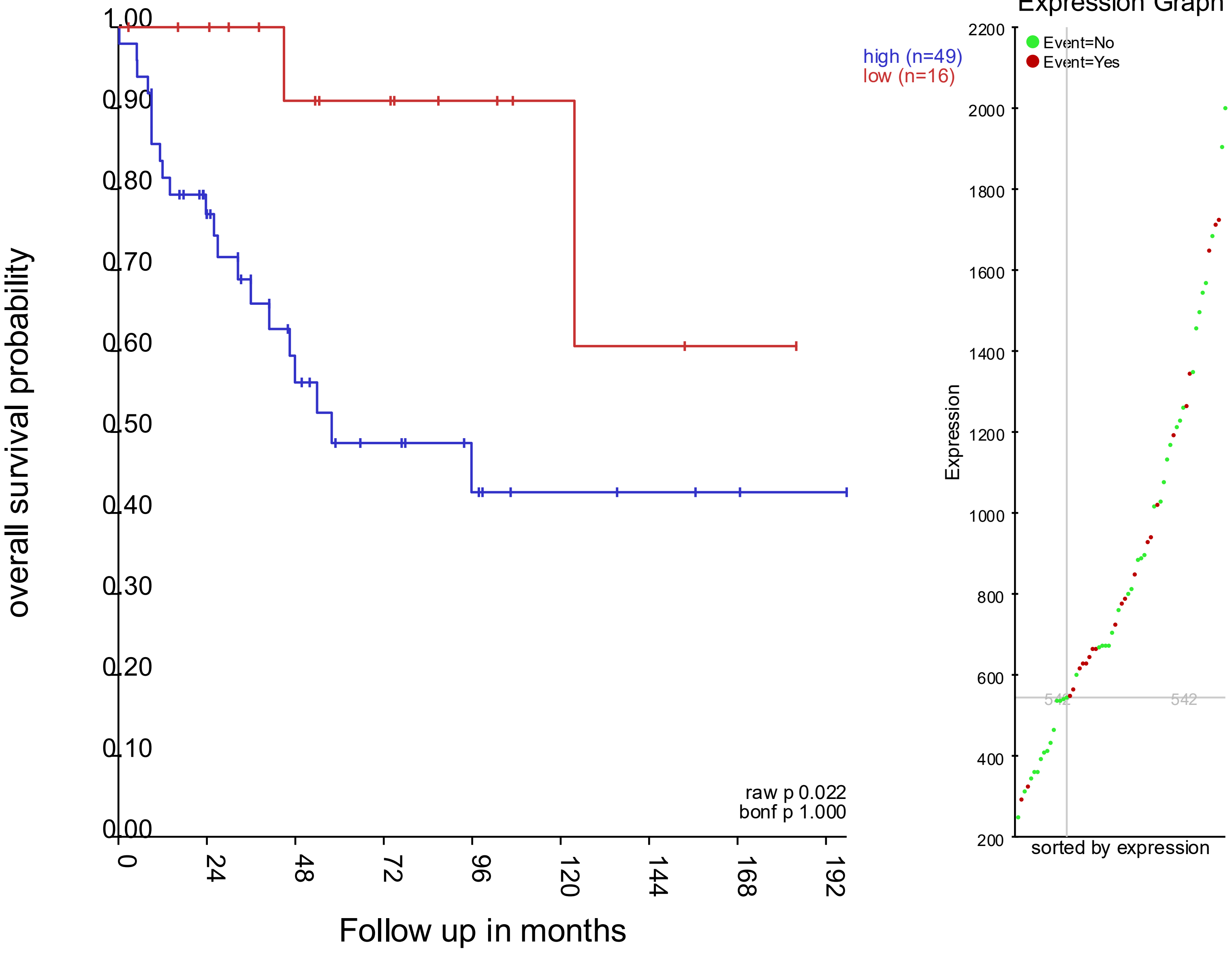

GROUP3 M1

Tumor Medulloblastoma
Cavalli - 763 - rma_sketch - hugene11t
IDH1 (8058552)
Expression cutoff: 722.200 (min.grp=3)
subgroup~group3|met_status_(1_met__0_m0)~1|WITH_SURV (n=41)

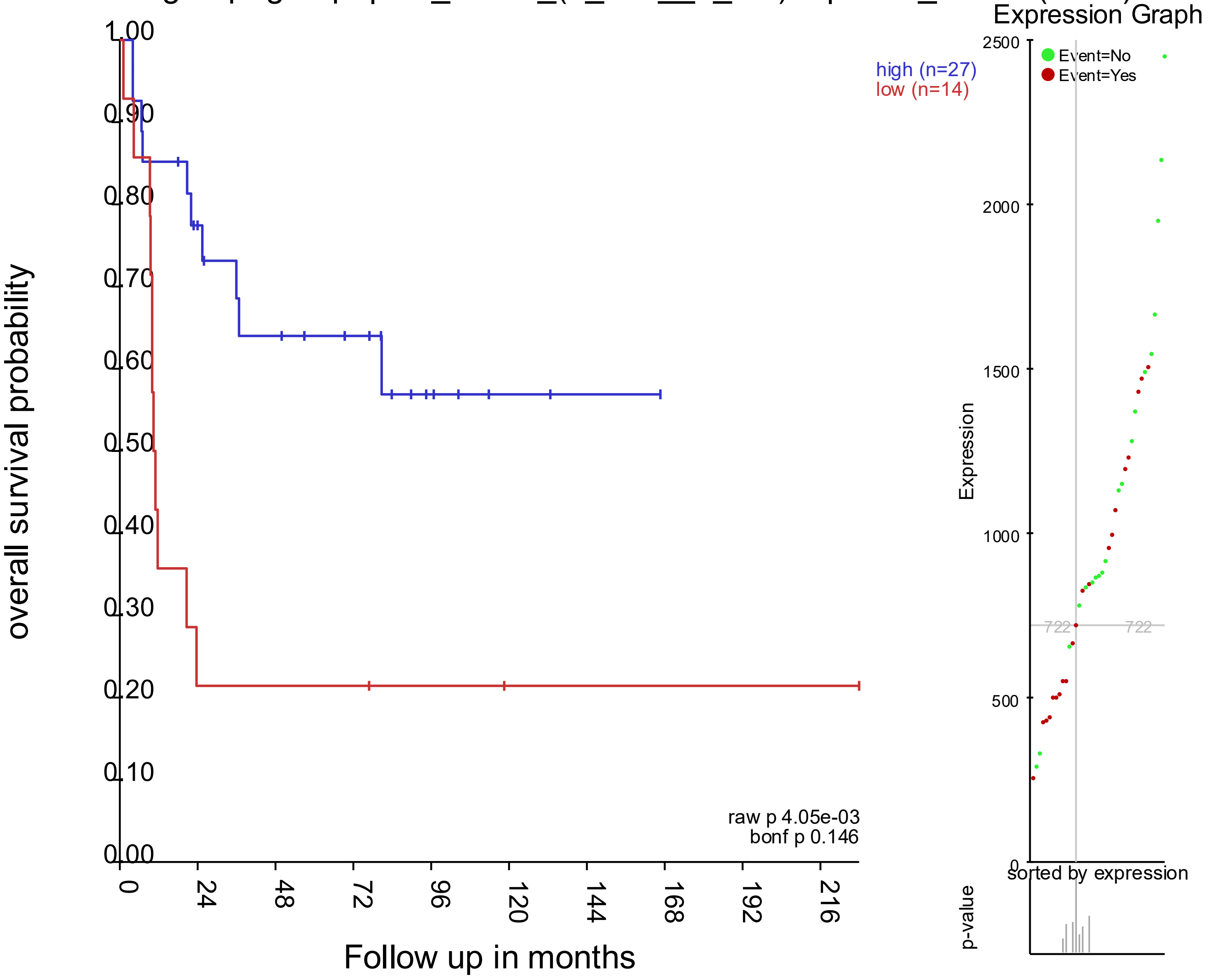

JAK1

WNT M0

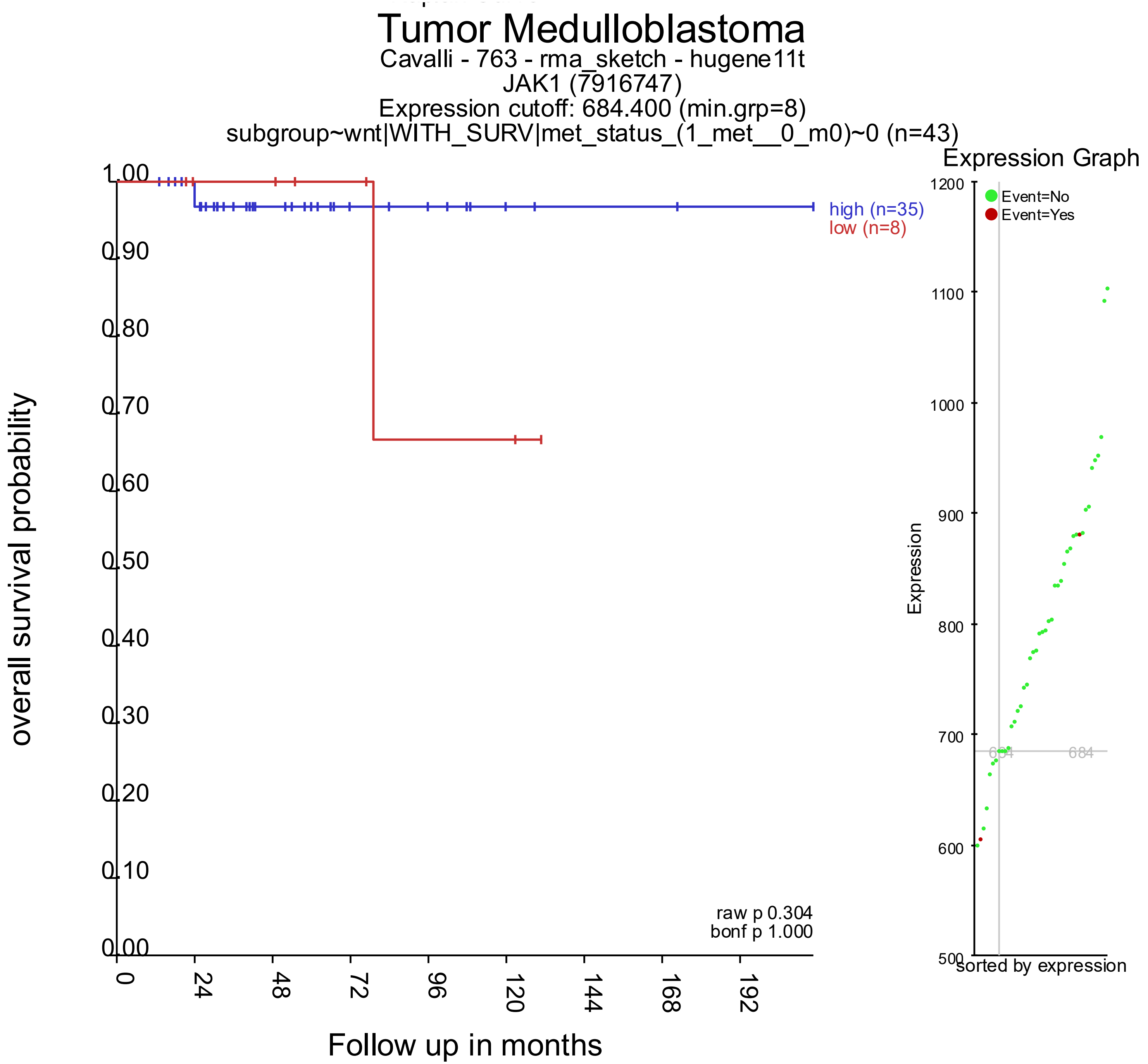

Tumor Medulloblastoma
Cavalli - 763 - rma_sketch - hugene11t
JAK1 (7916747)
Expression cutoff: 684.400 (min.grp=8)
subgroup~wnt|WITH_SURV|met_status_(1_met__0_m0)~0 (n=43)
overall survival probability
1.00
0.90
0.80
0.70
0.60
0.50
0.40
0.30
0.20
0.10
0.00
0
24
48
72
96
120
144
168
192
Follow up in months
high (n=35)
low (n=8)
raw p 0.304
bonf p 1.000
Expression Graph
Event=No
Event=Yes
Expression
1200
1100
1000
900
800
700
600
500
684
684
sorted by expression

WNT M1

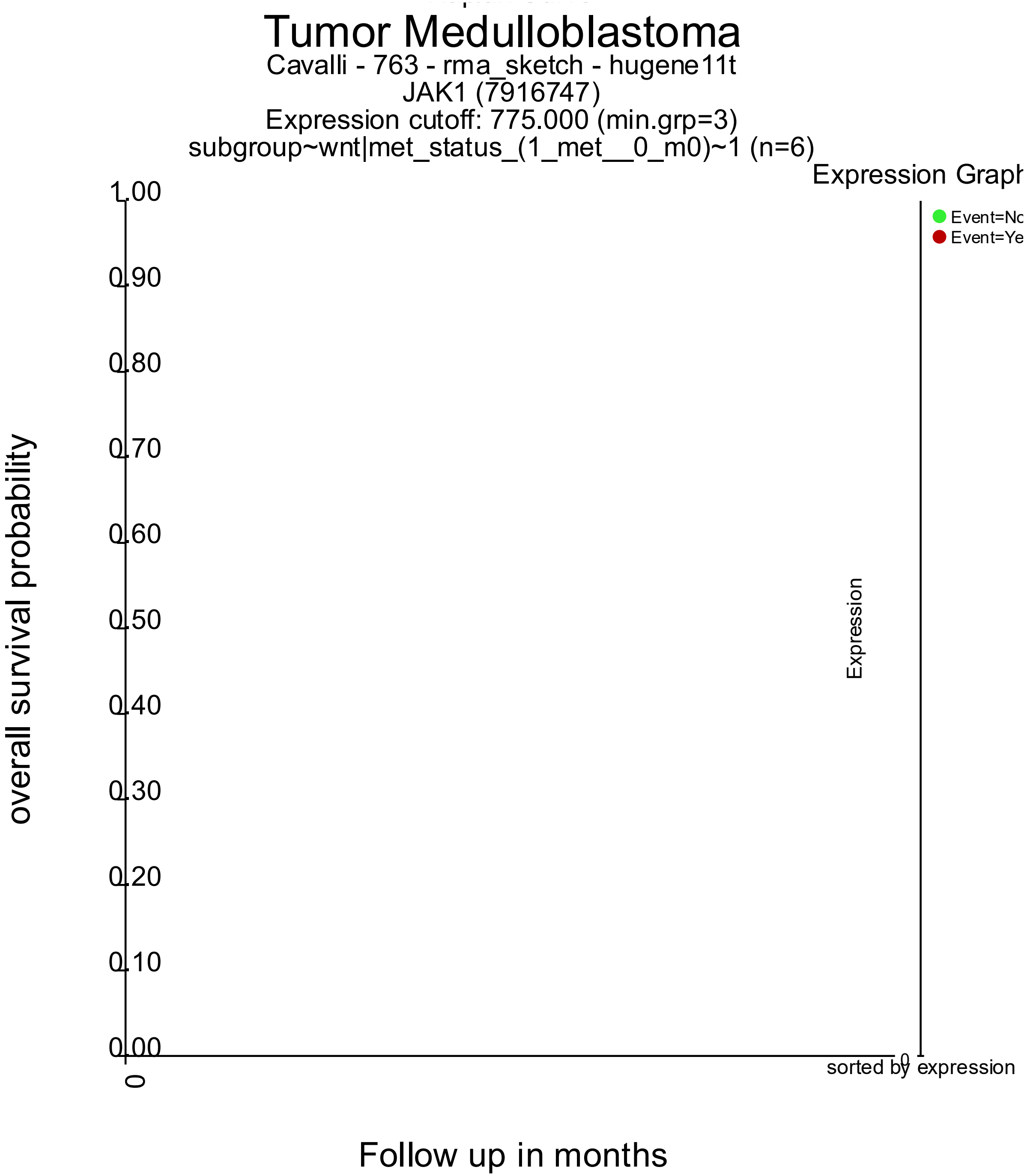

Tumor Medulloblastoma
Cavalli - 763 - rma_sketch - hugene11t
JAK1 (7916747)
Expression cutoff: 775.000 (min.grp=3)
subgroup~wnt|met_status_(1_met__0_m0)~1 (n=6)
Expression Graph
Event=No
Event=Yes
overall survival probability
1.00
0.90
0.80
0.70
0.60
0.50
0.40
0.30
0.20
0.10
0.00
0
Follow up in months
Expression
0
sorted by expression

SHH M0

Tumor Medulloblastoma
Cavalli - 763 - rma_sketch - hugene11t
JAK1 (7916747)
Expression cutoff: 597.400 (min.grp=8)
subgroup~shh|met_status_(1_met__0_m0)~0|WITH_SURV (n=124)

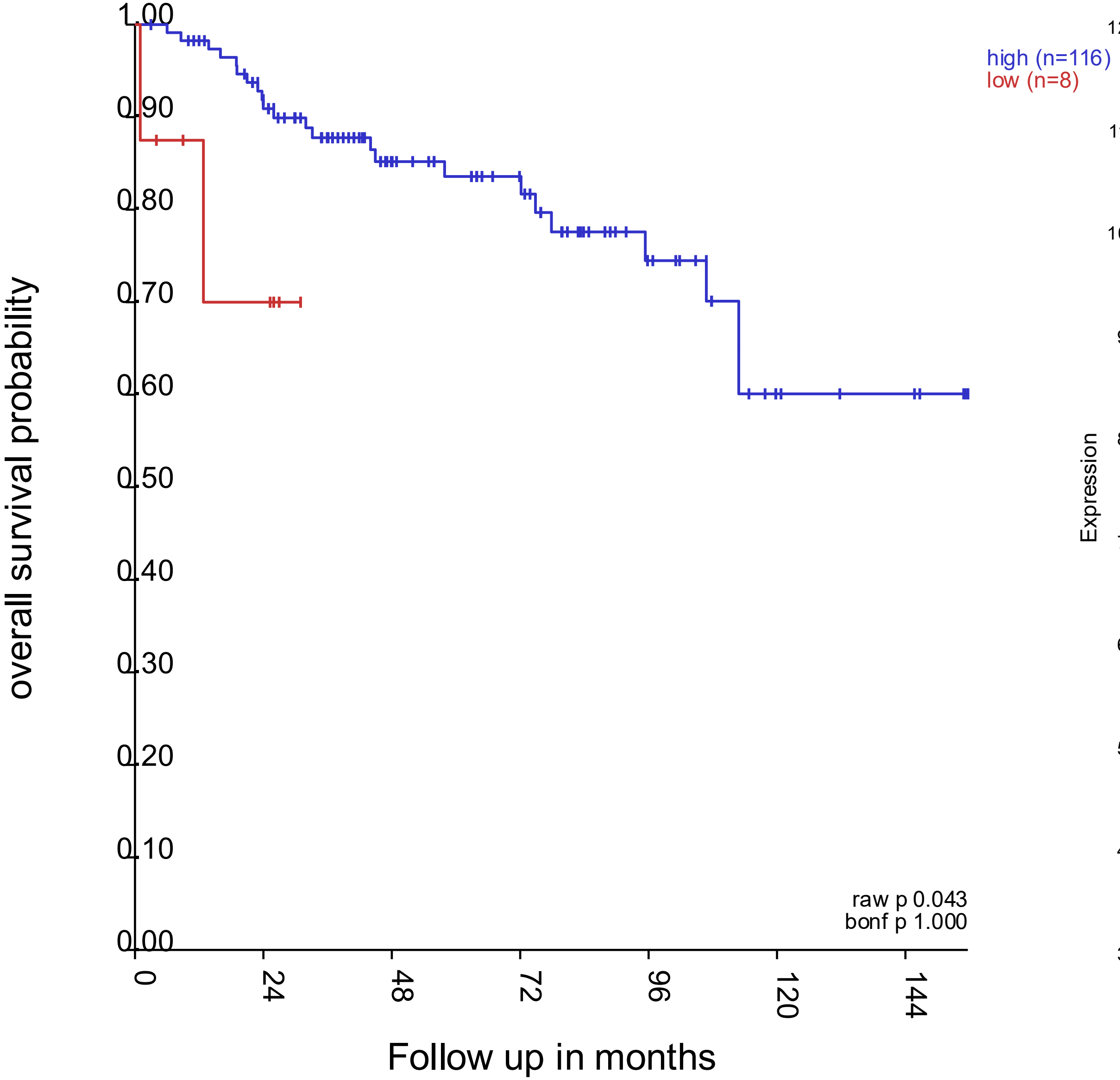

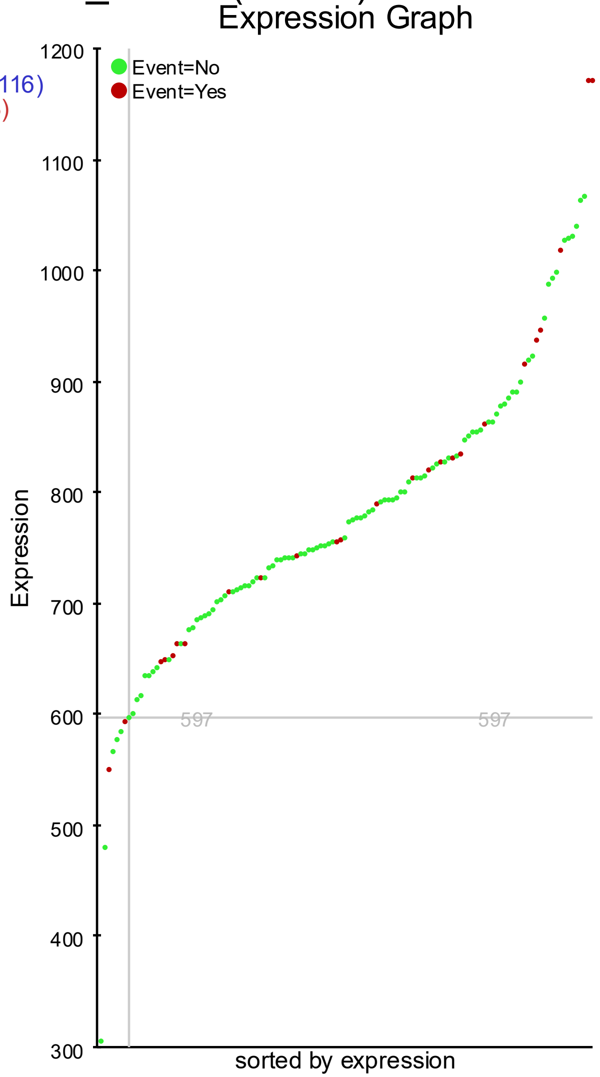

SHH M1

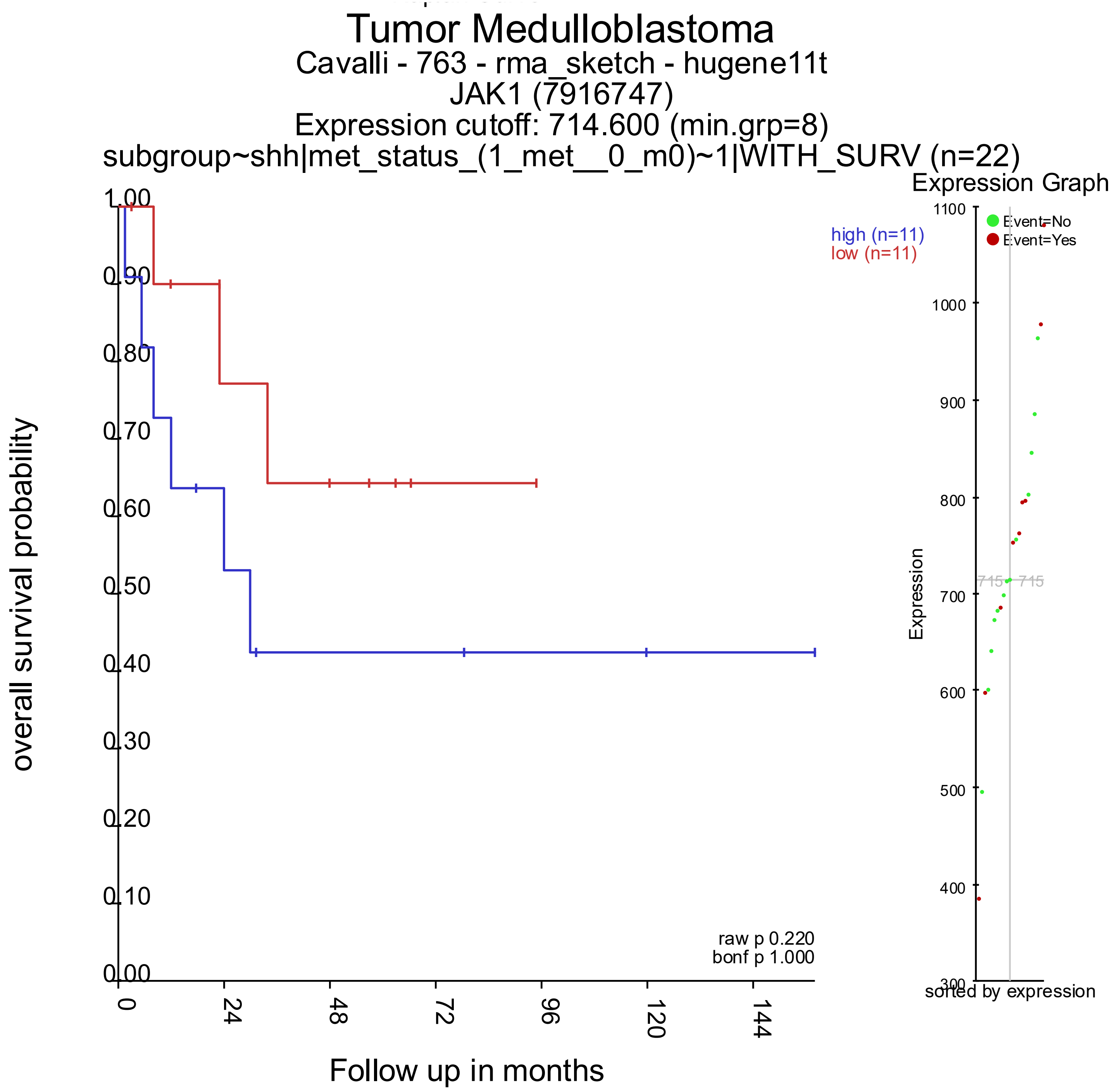

Tumor Medulloblastoma
Cavalli - 763 - rma_sketch - hugene11t
JAK1 (7916747)
Expression cutoff: 714.600 (min.grp=8)
subgroup~shh|met_status_(1_met__0_m0)~1|WITH_SURV (n=22)
overall survival probability
1.00
0.90
0.80
0.70
0.60
0.50
0.40
0.30
0.20
0.10
0.00
0
24
48
72
96
120
144
Follow up in months
high (n=11)
low (n=11)
raw p 0.220
bonf p 1.000
Expression Graph
Event=No
Event=Yes
Expression
1100
1000
900
800
700
600
500
400
300
715
715
sorted by expression

GROUP4 M0

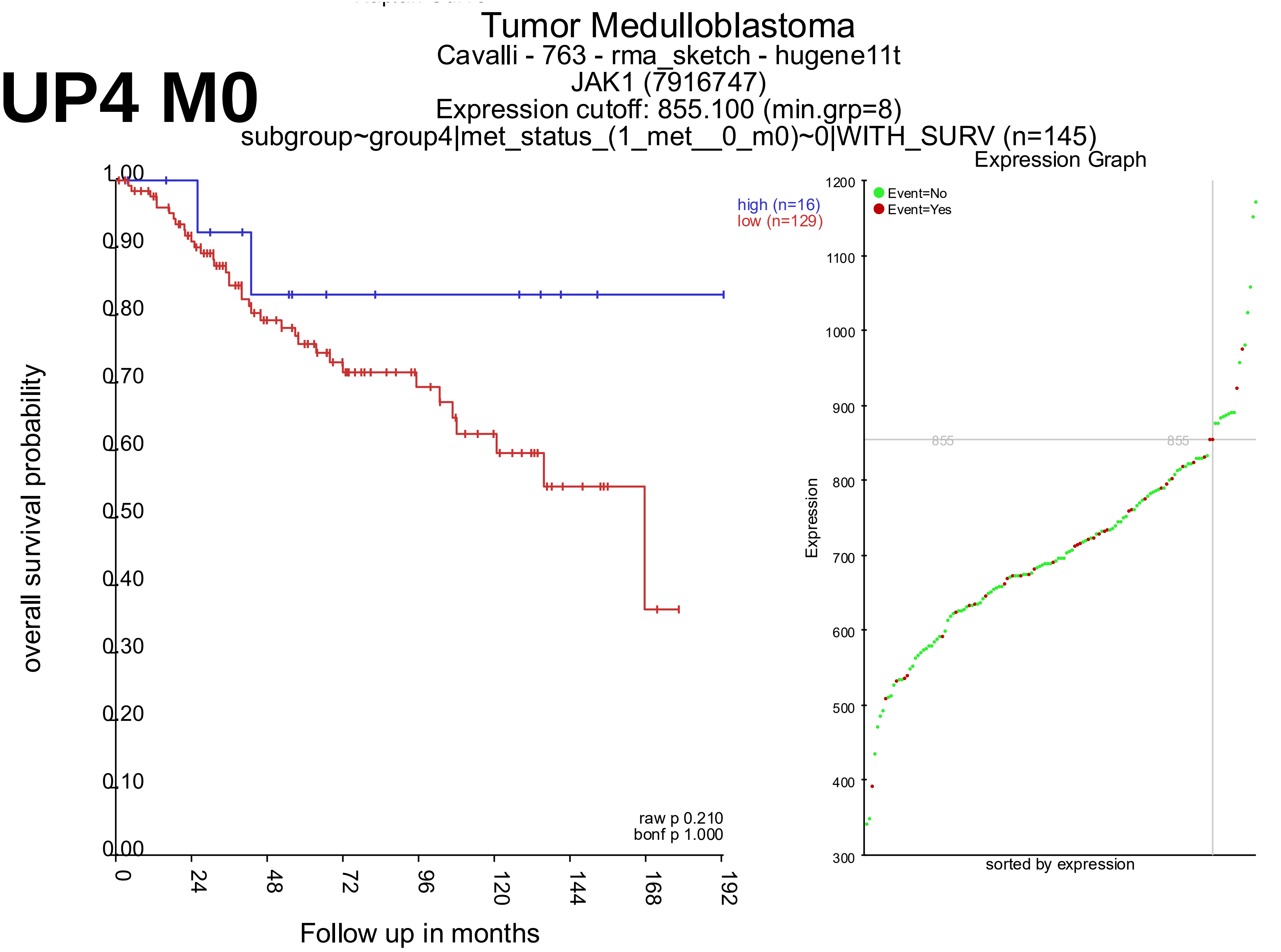

Tumor Medulloblastoma
Cavalli - 763 - rma_sketch - hugene11t
JAK1 (7916747)
Expression cutoff: 855.100 (min.grp=8)
subgroup~group4|met_status_(1_met__0_m0)~0|WITH_SURV (n=145)
overall survival probability
1.00
0.90
0.80
0.70
0.60
0.50
0.40
0.30
0.20
0.10
0.00
high (n=16)
low (n=129)
raw p 0.210
bonf p 1.000
0
24
48
72
96
120
144
168
192
Follow up in months
Expression Graph
Event=No
Event=Yes
Expression
1200
1100
1000
900
800
700
600
500
400
300
855
855
sorted by expression

GROUP4 M1

Tumor Medulloblastoma
Cavalli - 763 - rma_sketch - hugene11t
JAK1 (7916747)
Expression cutoff: 721.700 (min.grp=8)
subgroup~group4|met_status_(1_met__0_m0)~1|WITH_SURV (n=92)

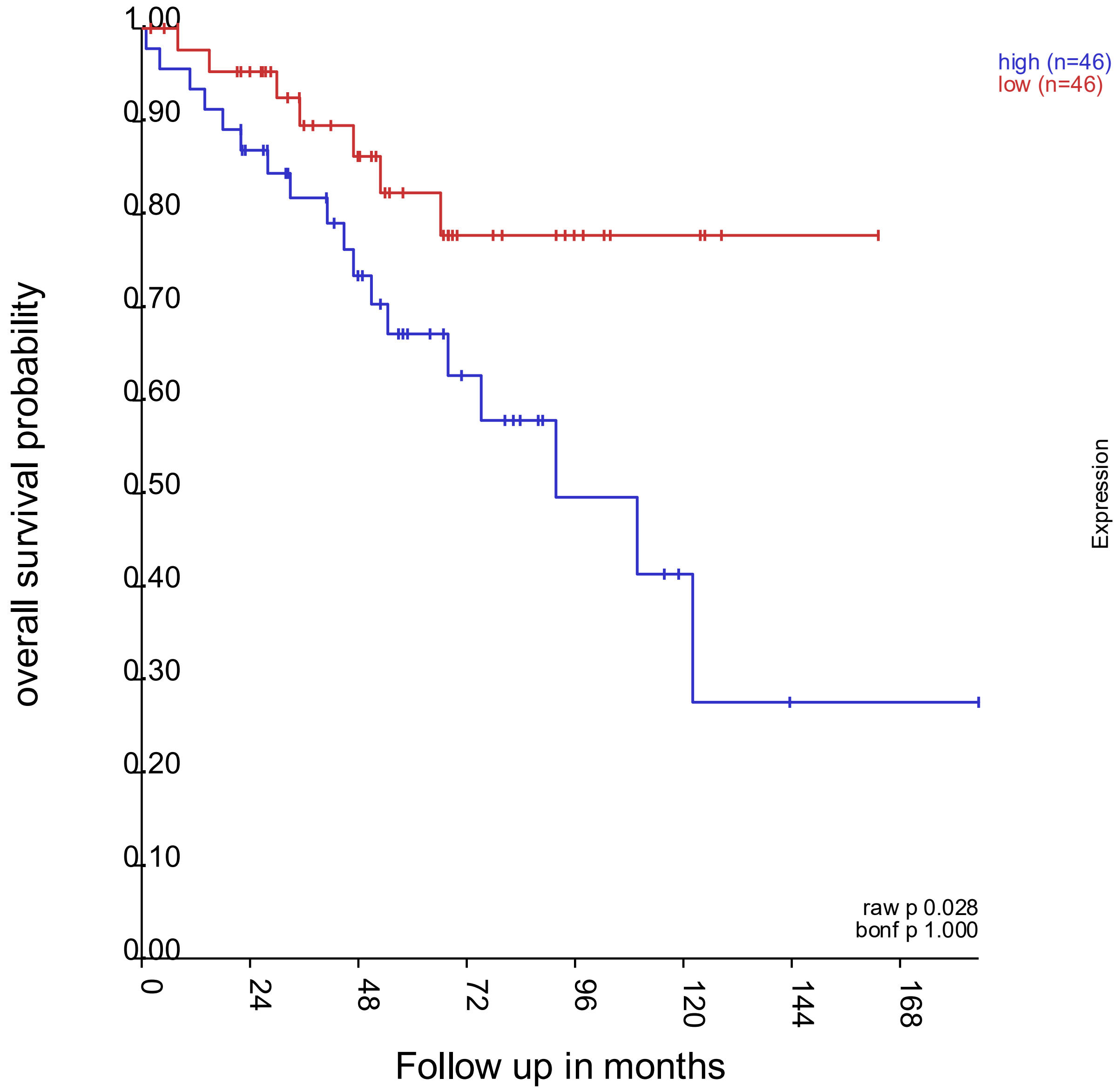

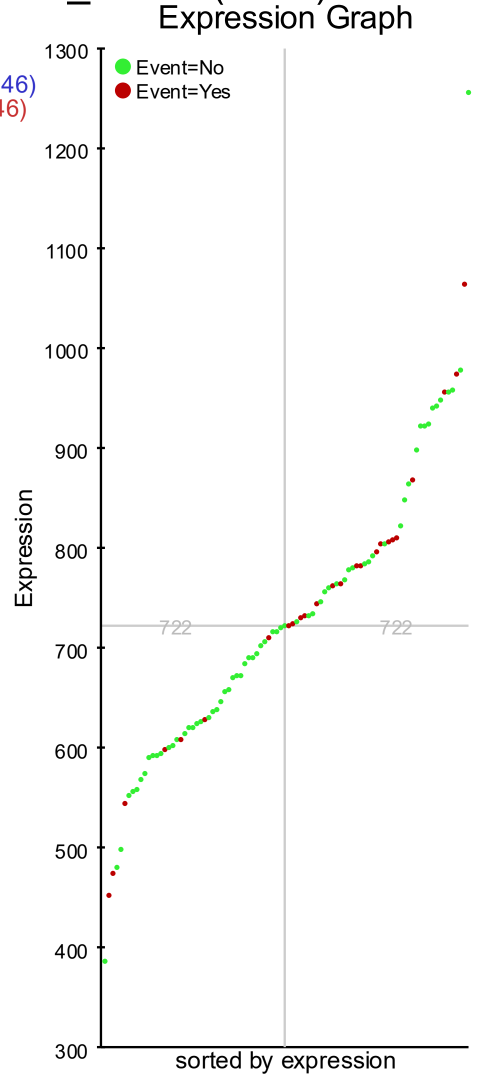

GROUP3 M0

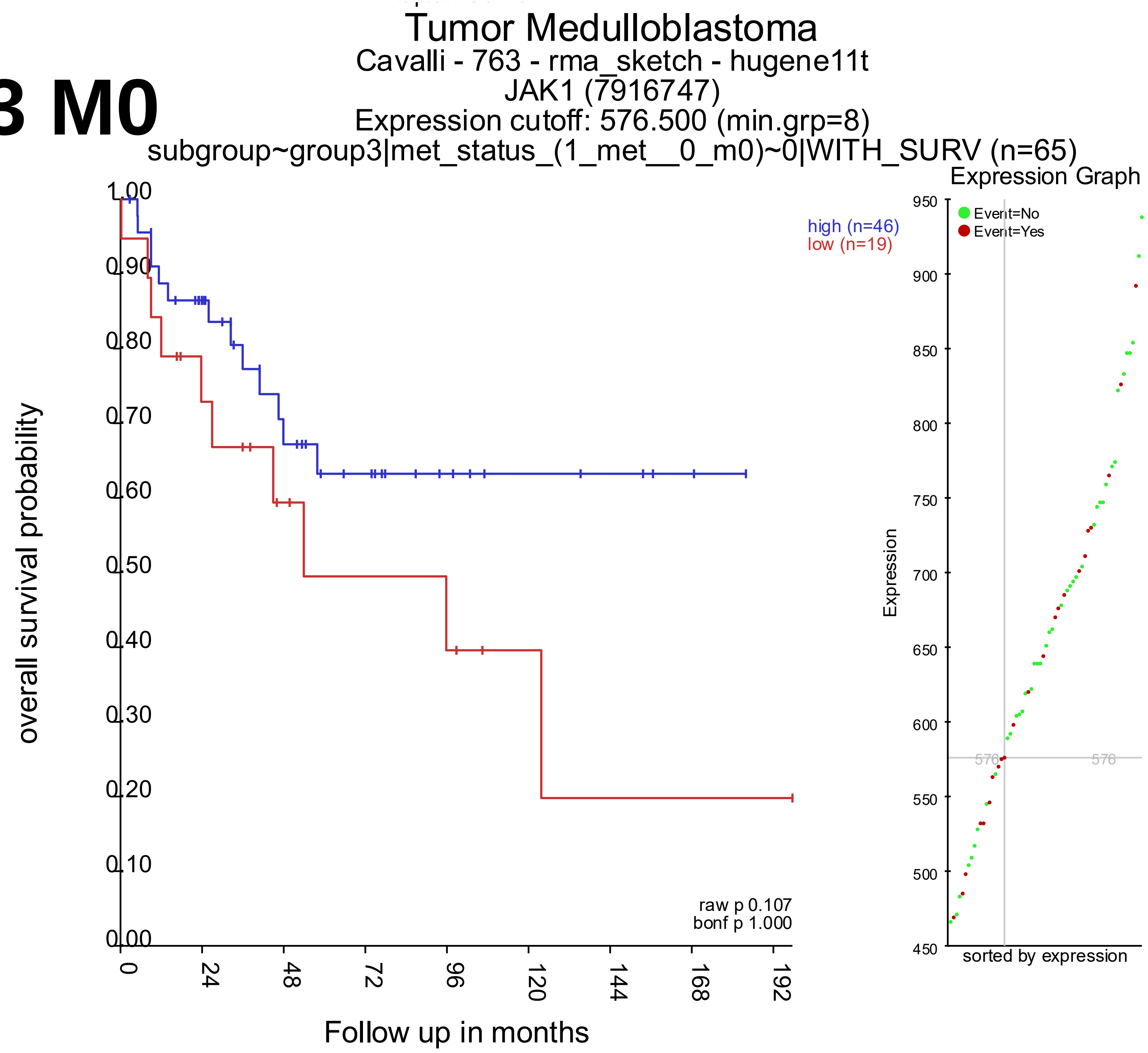

Tumor Medulloblastoma
Cavalli - 763 - rma_sketch - hugene11t
JAK1 (7916747)
Expression cutoff: 576.500 (min.grp=8)
subgroup~group3|met_status_(1_met__0_m0)~0|WITH_SURV (n=65)
overall survival probability
1.00
0.90
0.80
0.70
0.60
0.50
0.40
0.30
0.20
0.10
0.00
high (n=46)
low (n=19)
raw p 0.107
bonf p 1.000
0
24
48
72
96
120
144
168
192
Follow up in months
Expression Graph
Event=No
Event=Yes
950
900
850
800
750
700
650
600
550
500
450
Expression
576
576
sorted by expression

GROUP3 M1

Tumor Medulloblastoma
Cavalli - 763 - rma_sketch - hugene11t
JAK1 (7916747)
Expression cutoff: 612.800 (min.grp=8)
subgroup~group3|met_status_(1_met__0_m0)~1|WITH_SURV (n=41)

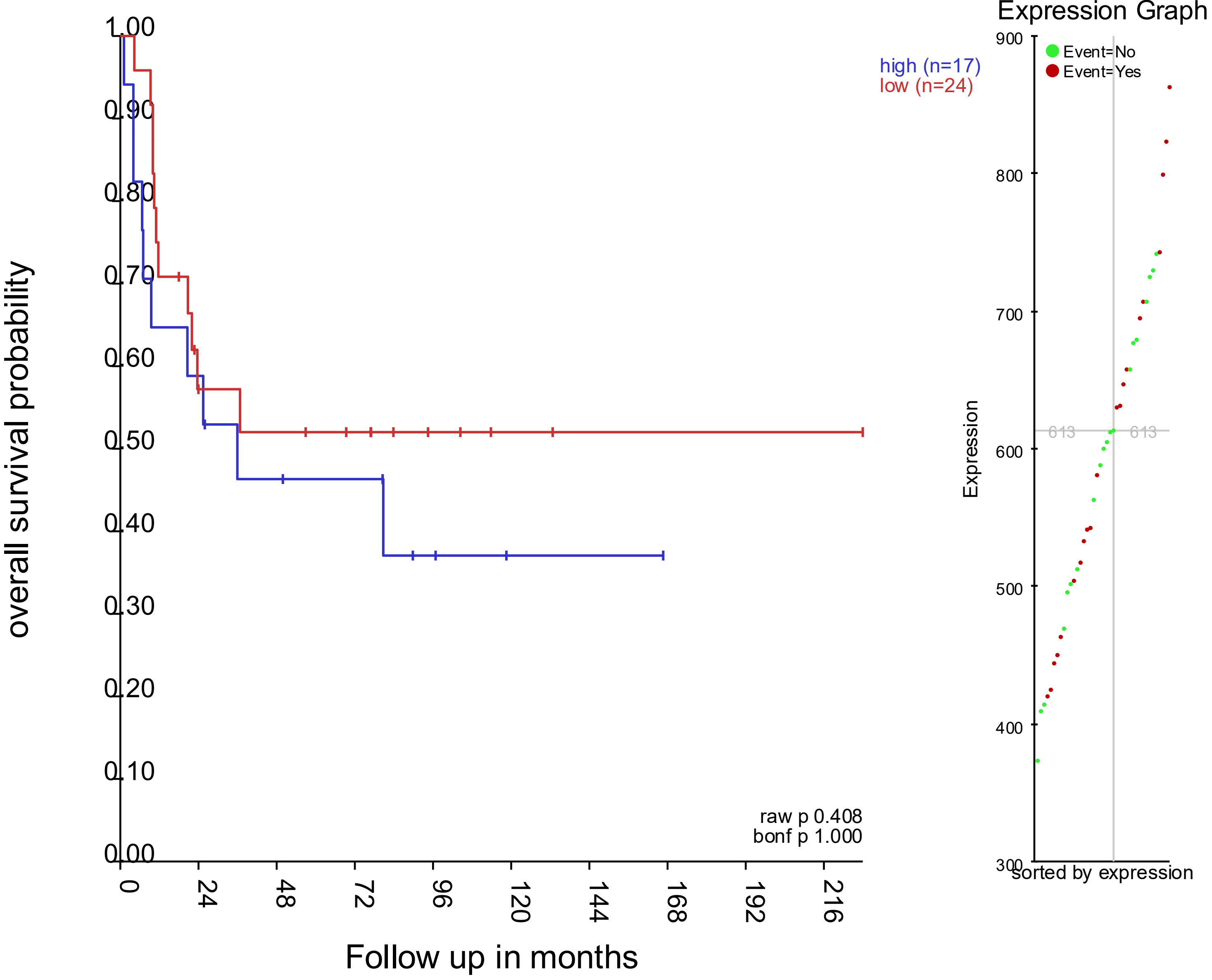

JAK2

WNT M0

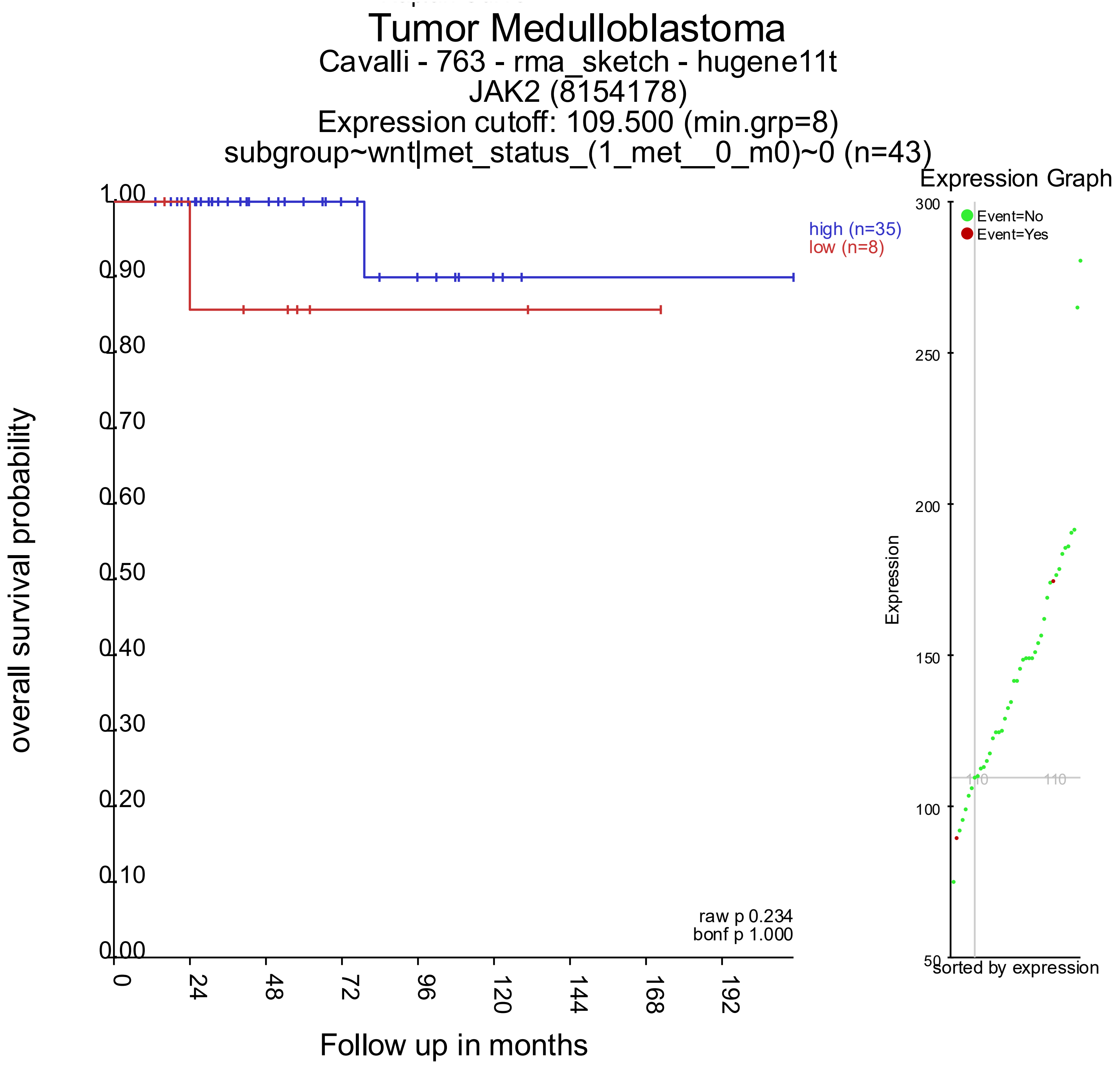

Tumor Medulloblastoma
Cavalli - 763 - rma_sketch - hugene11t
JAK2 (8154178)
Expression cutoff: 109.500 (min.grp=8)
subgroup~wnt|met_status_(1_met__0_m0)~0 (n=43)
overall survival probability
1.00
0.90
0.80
0.70
0.60
0.50
0.40
0.30
0.20
0.10
0.00
high (n=35)
low (n=8)
raw p 0.234
bonf p 1.000
0
24
48
72
96
120
144
168
192
Follow up in months
Expression Graph
Event=No
Event=Yes
Expression
300
250
200
150
100
50
110
110
sorted by expression

WNT M1

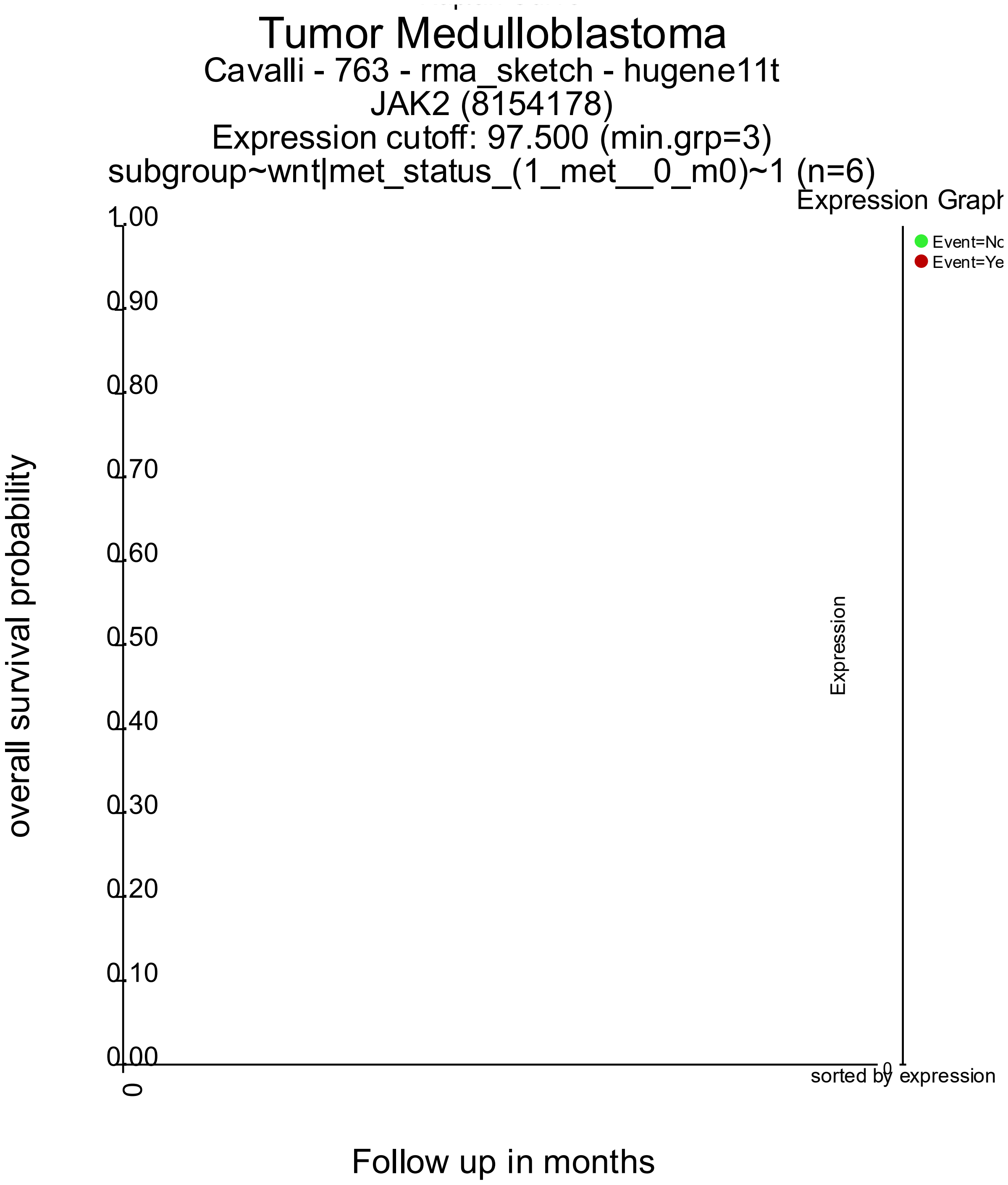

Tumor Medulloblastoma
Cavalli - 763 - rma_sketch - hugene11t
JAK2 (8154178)
Expression cutoff: 97.500 (min.grp=3)
subgroup~wnt|met_status_(1_met__0_m0)~1 (n=6)
Expression Graph
Event=No
Event=Yes
1.00
0.90
0.80
0.70
0.60
0.50
0.40
0.30
0.20
0.10
0.00
0
overall survival probability
Expression
sorted by expression
Follow up in months

SHH M0

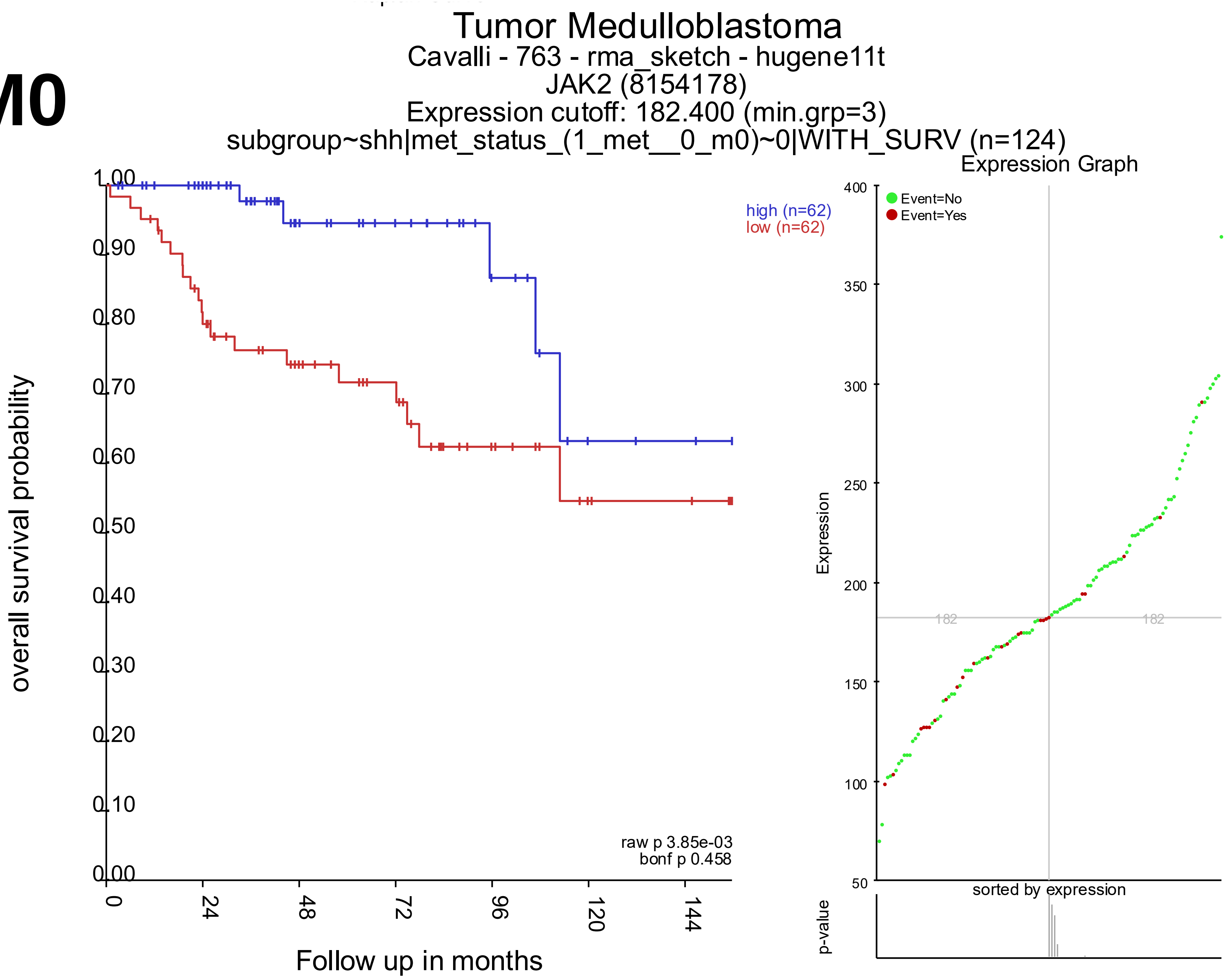

Tumor Medulloblastoma
Cavalli - 763 - rma_sketch - hugene11t
JAK2 (8154178)
Expression cutoff: 182.400 (min.grp=3)
subgroup~shh|met_status_(1_met__0_m0)~0|WITH_SURV (n=124)
overall survival probability
1.00
0.90
0.80
0.70
0.60
0.50
0.40
0.30
0.20
0.10
0.00
0
24
48
72
96
120
144
Follow up in months
high (n=62)
low (n=62)
raw p 3.85e-03
bonf p 0.458
Expression Graph
Event=No
Event=Yes
Expression
400
350
300
250
200
150
100
50
182
182
sorted by expression
p-value

SHH M1

Tumor Medulloblastoma
Cavalli - 763 - rma_sketch - hugene11t
JAK2 (8154178)
Expression cutoff: 142.400 (min.grp=3)
subgroup~shh|met_status_(1_met__0_m0)~1|WITH_SURV (n=22)

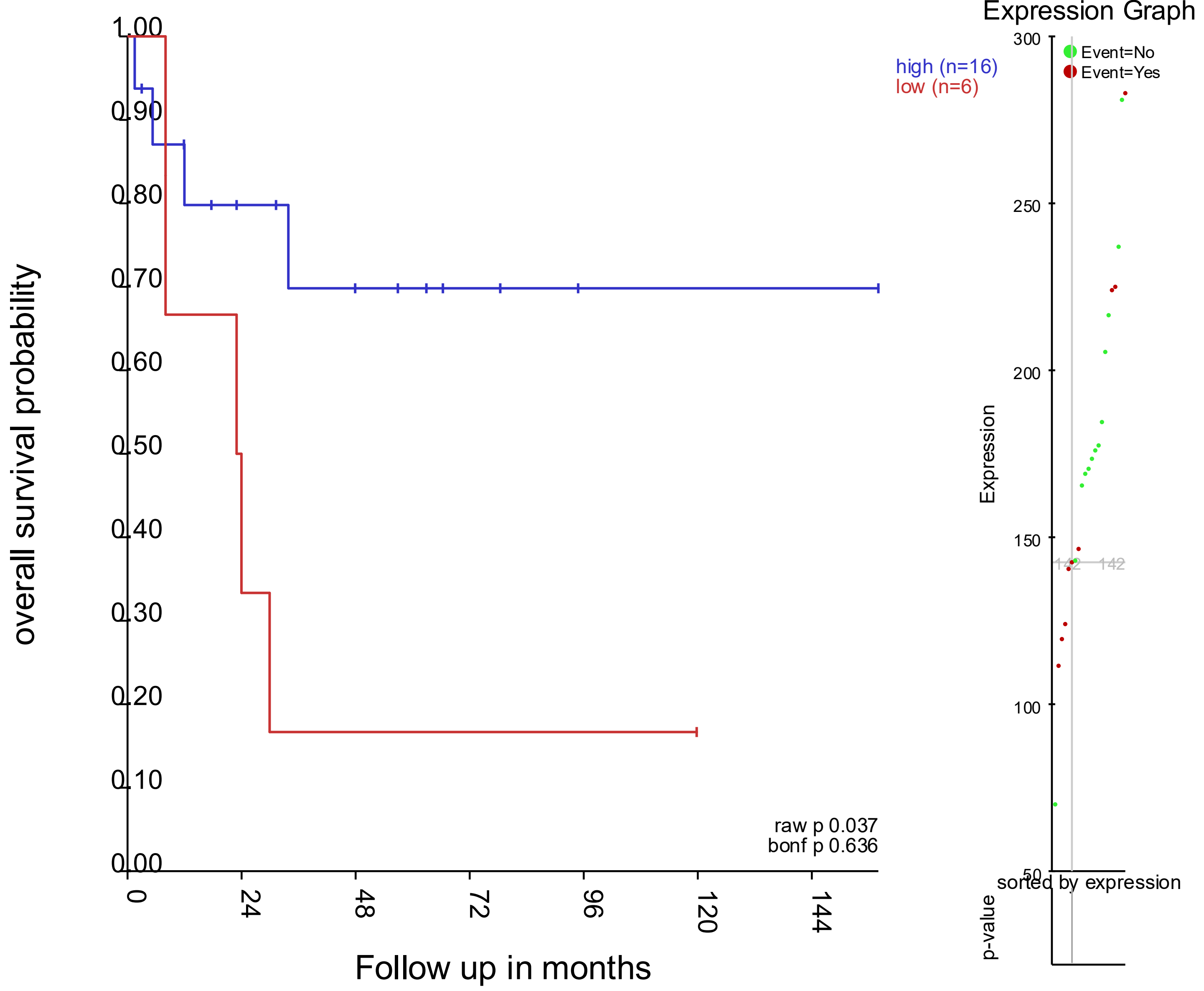

GROUP4 M0

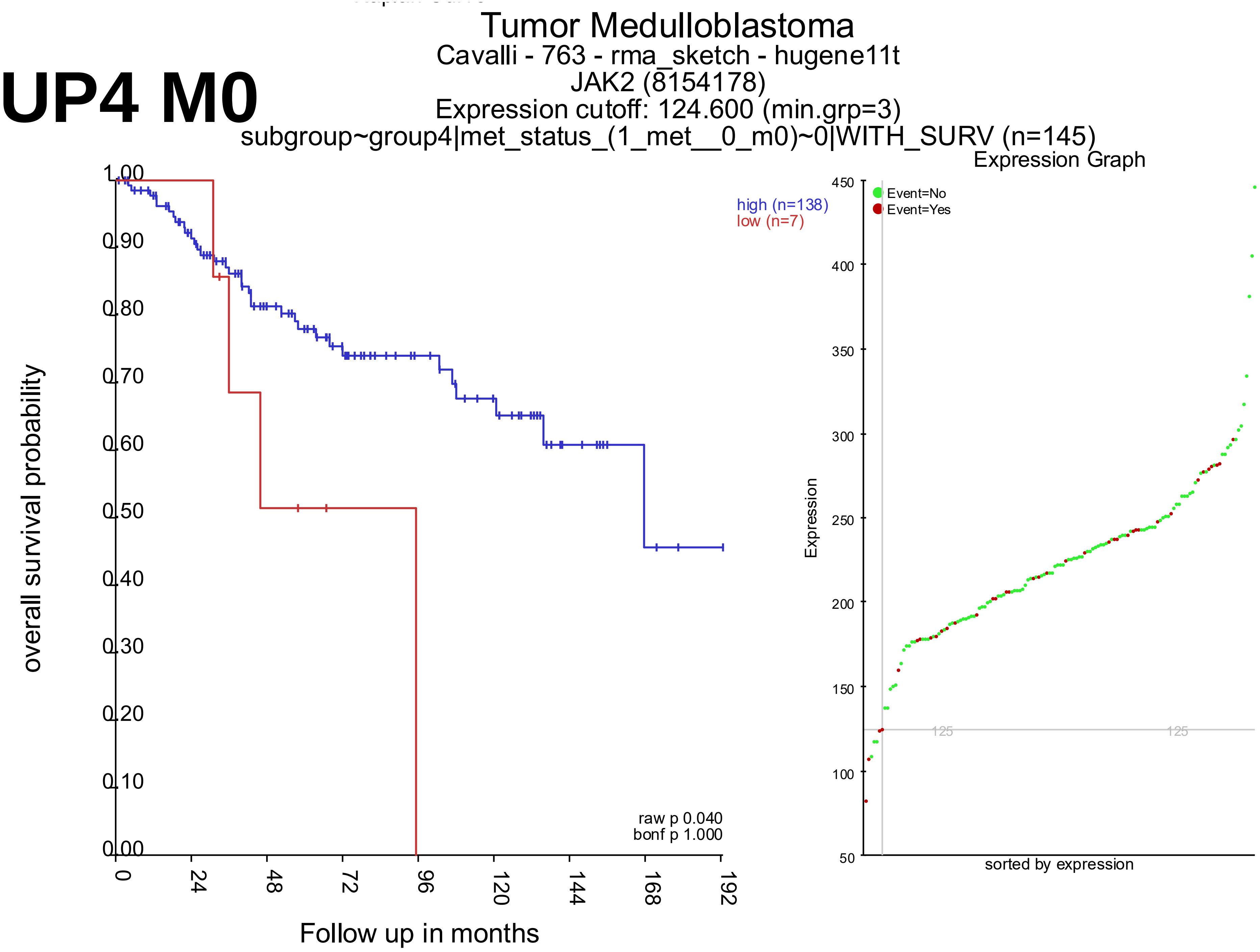

Tumor Medulloblastoma
Cavalli - 763 - rma_sketch - hugene11t
JAK2 (8154178)
Expression cutoff: 124.600 (min.grp=3)
subgroup~group4|met_status_(1_met__0_m0)~0|WITH_SURV (n=145)
high (n=138)
low (n=7)
overall survival probability
1.00
0.90
0.80
0.70
0.60
0.50
0.40
0.30
0.20
0.10
0.00
0
24
48
72
96
120
144
168
192
Follow up in months
raw p 0.040
bonf p 1.000
Expression Graph
Event=No
Event=Yes
Expression
450
400
350
300
250
200
150
100
50
125
125
sorted by expression

GROUP4 M1

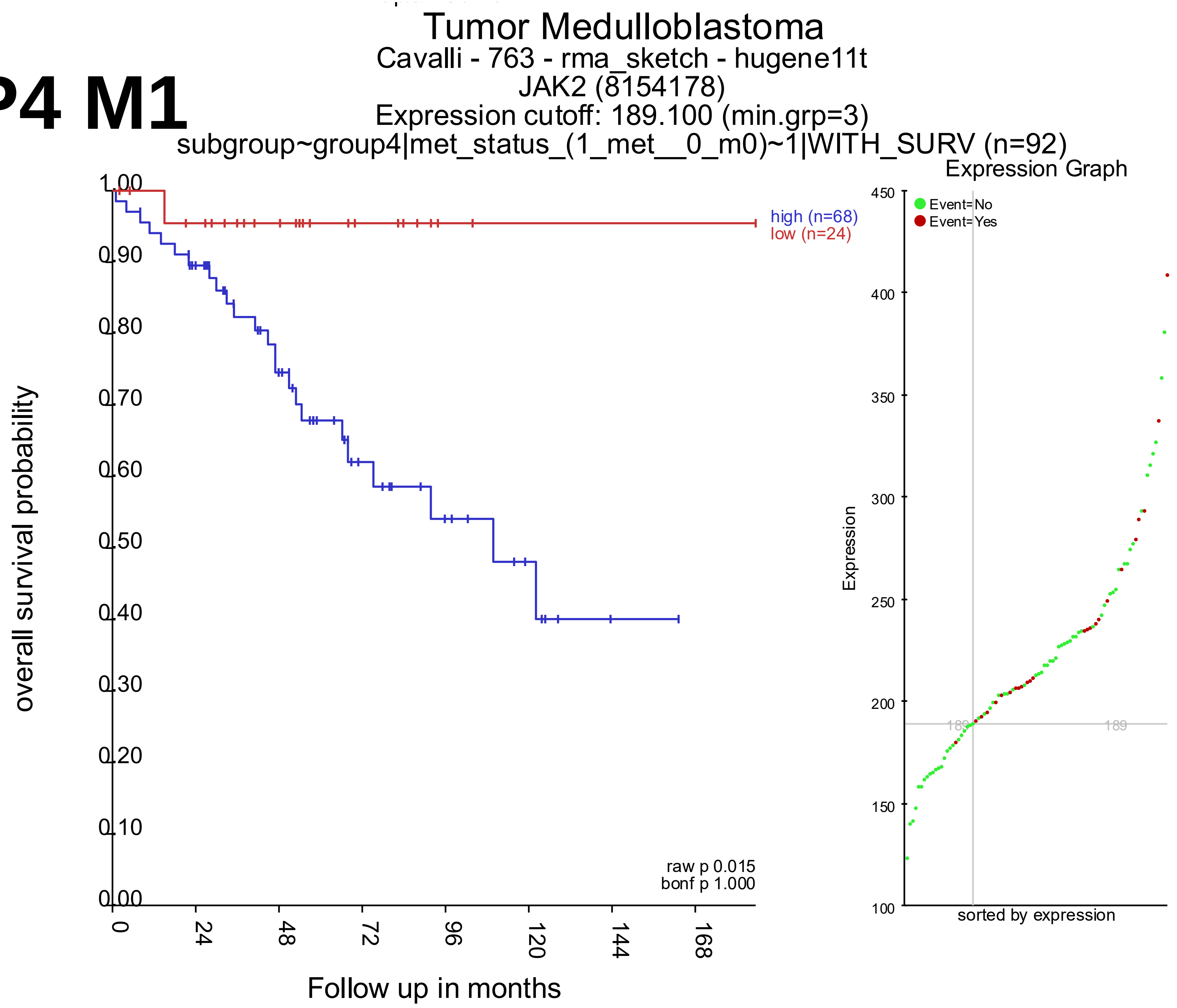
Tumor Medulloblastoma
Cavalli - 763 - rma_sketch - hugene11t
JAK2 (8154178)
Expression cutoff: 189.100 (min.grp=3)
subgroup~group4|met_status_(1_met__0_m0)~1|WITH_SURV (n=92)
overall survival probability
1.00
0.90
0.80
0.70
0.60
0.50
0.40
0.30
0.20
0.10
0.00
high (n=68)
low (n=24)
raw p 0.015
bonf p 1.000
0
24
48
72
96
120
144
168
Follow up in months
Expression Graph
Event=No
Event=Yes
Expression
450
400
350
300
250
200
150
100
189
189
sorted by expression

GROUP3 M0

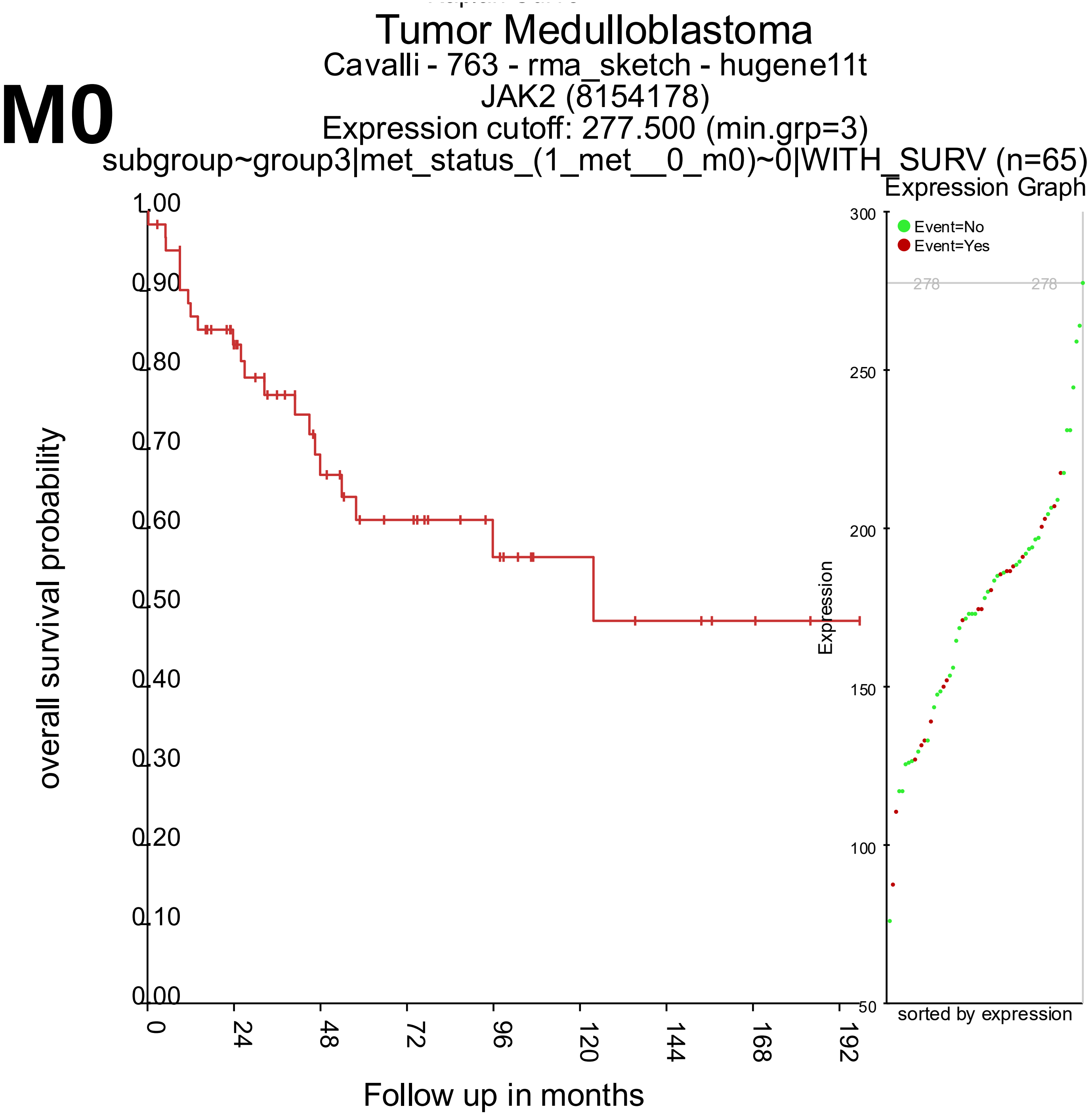

Tumor Medulloblastoma
Cavalli - 763 - rma_sketch - hugene11t
JAK2 (8154178)
Expression cutoff: 277.500 (min.grp=3)
subgroup~group3|met_status_(1_met__0_m0)~0|WITH_SURV (n=65)
overall survival probability
1.00
0.90
0.80
0.70
0.60
0.50
0.40
0.30
0.20
0.10
0.00
0
24
48
72
96
120
144
168
192
Follow up in months
Expression Graph
Event=No
Event=Yes
278
278
300
250
200
150
100
50
Expression
sorted by expression

GROUP3 M1

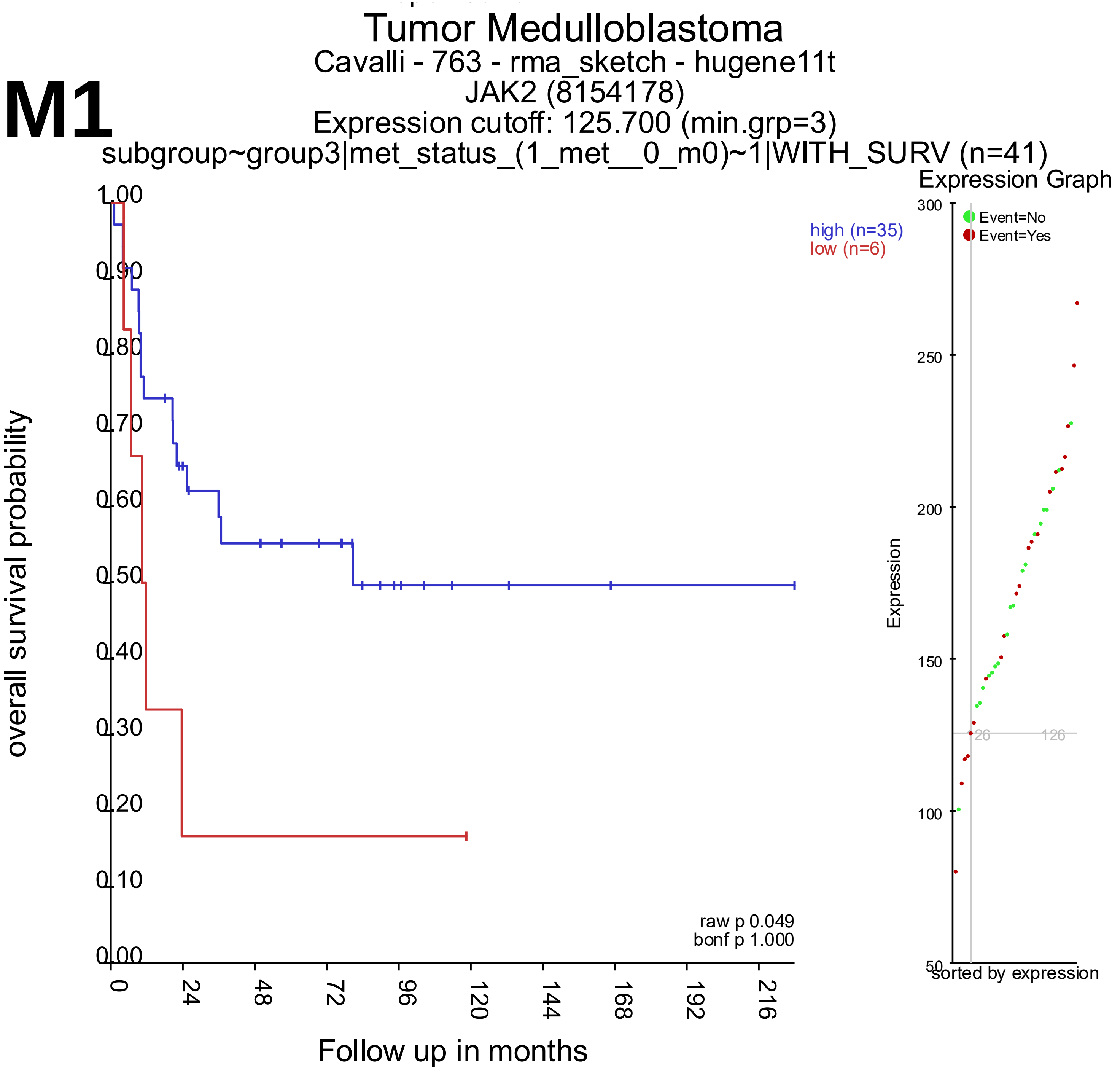

Tumor Medulloblastoma
Cavalli - 763 - rma_sketch - hugene11t
JAK2 (8154178)
Expression cutoff: 125.700 (min.grp=3)
subgroup~group3|met_status_(1_met__0_m0)~1|WITH_SURV (n=41)
overall survival probability
1.00
0.90
0.80
0.70
0.60
0.50
0.40
0.30
0.20
0.10
0.00
high (n=35)
low (n=6)
raw p 0.049
bonf p 1.000
0
24
48
72
96
120
144
168
192
216
Follow up in months
Expression Graph
Event=No
Event=Yes
Expression
300
250
200
150
100
50
26
126
sorted by expression

JAK3

WNT M0

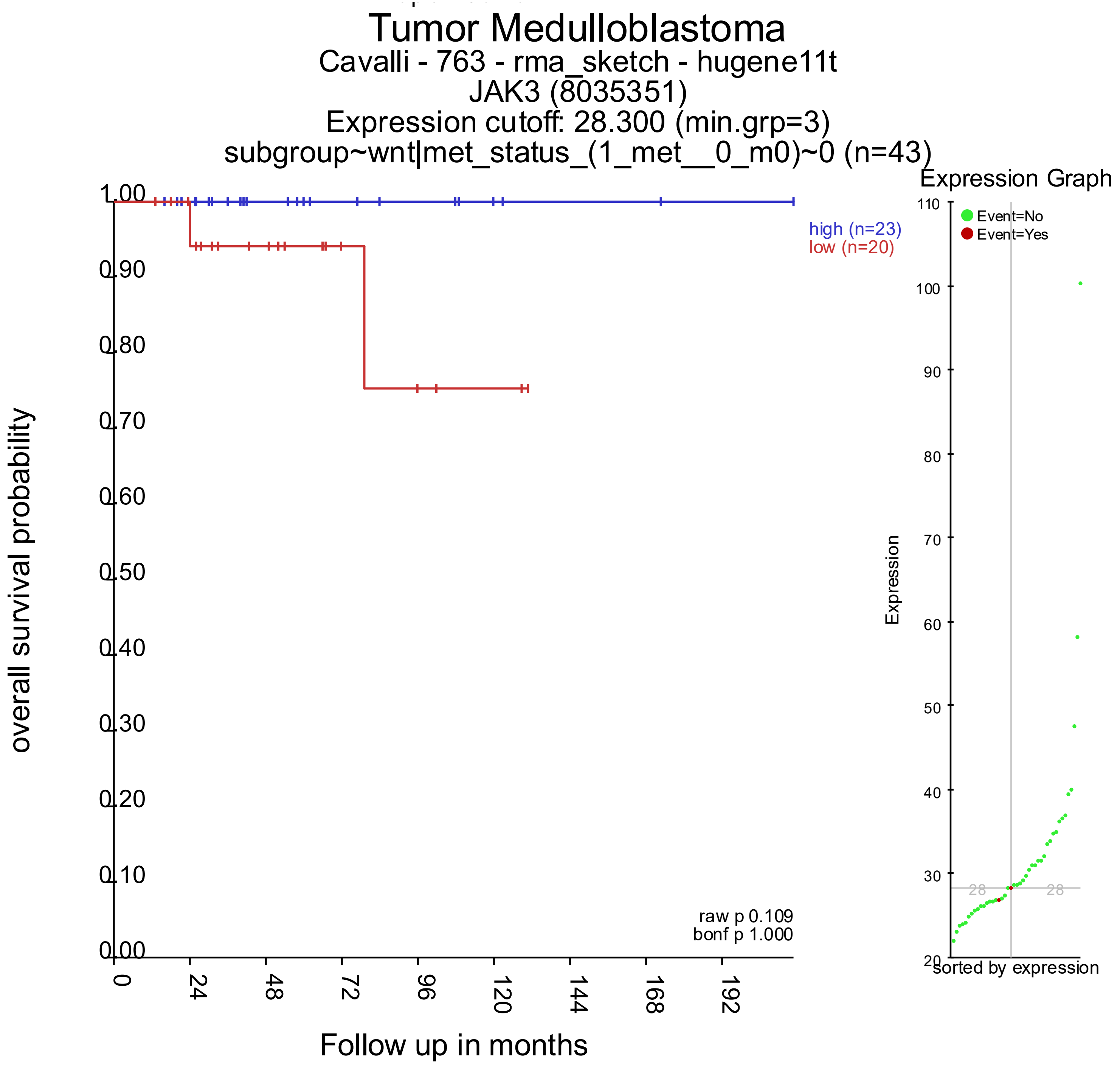

Tumor Medulloblastoma
Cavalli - 763 - rma_sketch - hugene11t
JAK3 (8035351)
Expression cutoff: 28.300 (min.grp=3)
subgroup~wnt|met_status_(1_met__0_m0)~0 (n=43)
overall survival probability
1.00
0.90
0.80
0.70
0.60
0.50
0.40
0.30
0.20
0.10
0.00
high (n=23)
low (n=20)
raw p 0.109
bonf p 1.000
0
24
48
72
96
120
144
168
192
Follow up in months
Expression Graph
Event=No
Event=Yes
Expression
110
100
90
80
70
60
50
40
30
20
28
28
sorted by expression

WNT M1

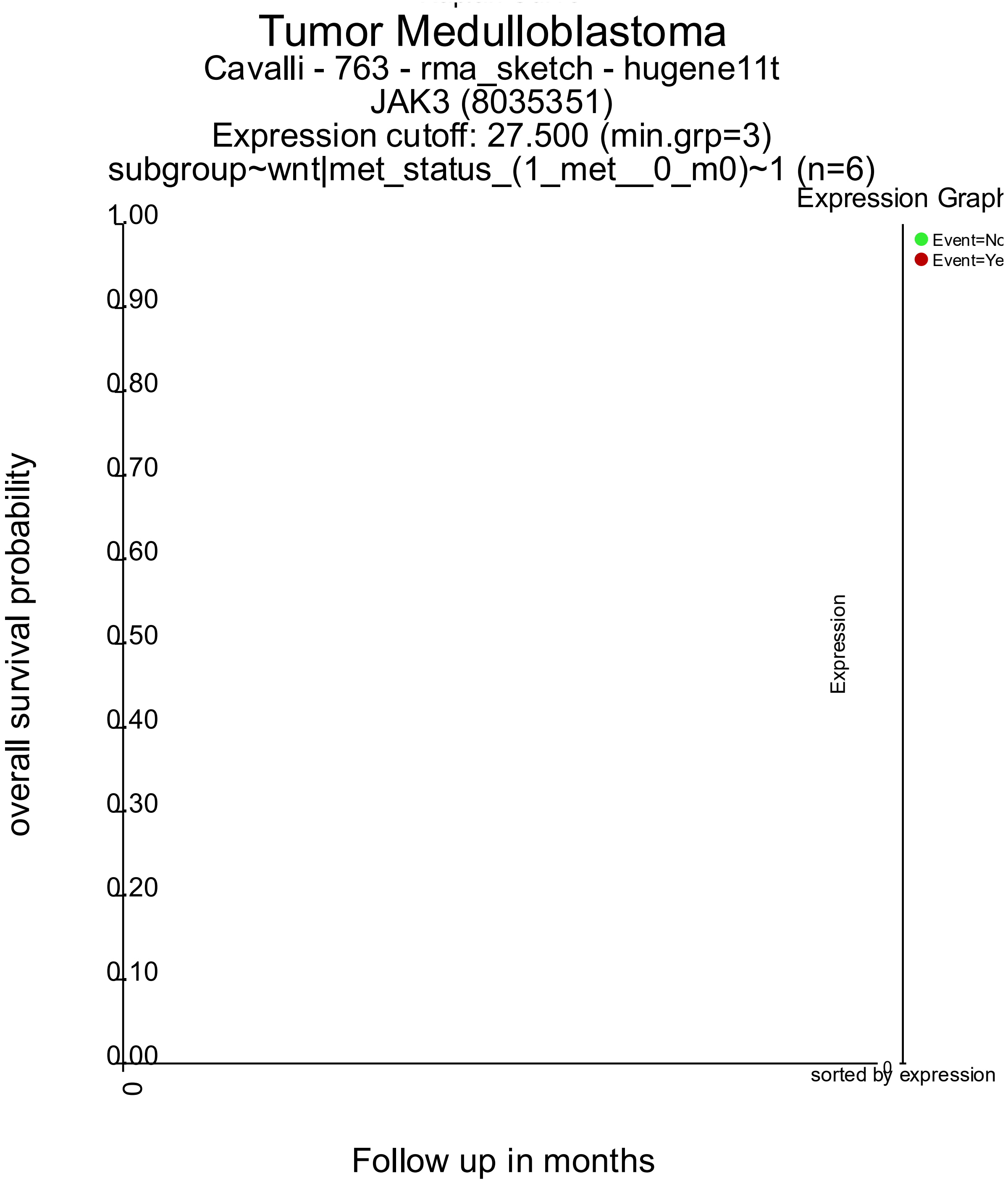
Tumor Medulloblastoma
Cavalli - 763 - rma_sketch - hugene11t
JAK3 (8035351)
Expression cutoff: 27.500 (min.grp=3)
subgroup~wnt|met_status_(1_met__0_m0)~1 (n=6)
Expression Graph
Event=No
Event=Yes
1.00
0.90
0.80
0.70
0.60
0.50
0.40
0.30
0.20
0.10
0.00
0
overall survival probability
Follow up in months
Expression
sorted by expression
0

SHH M0

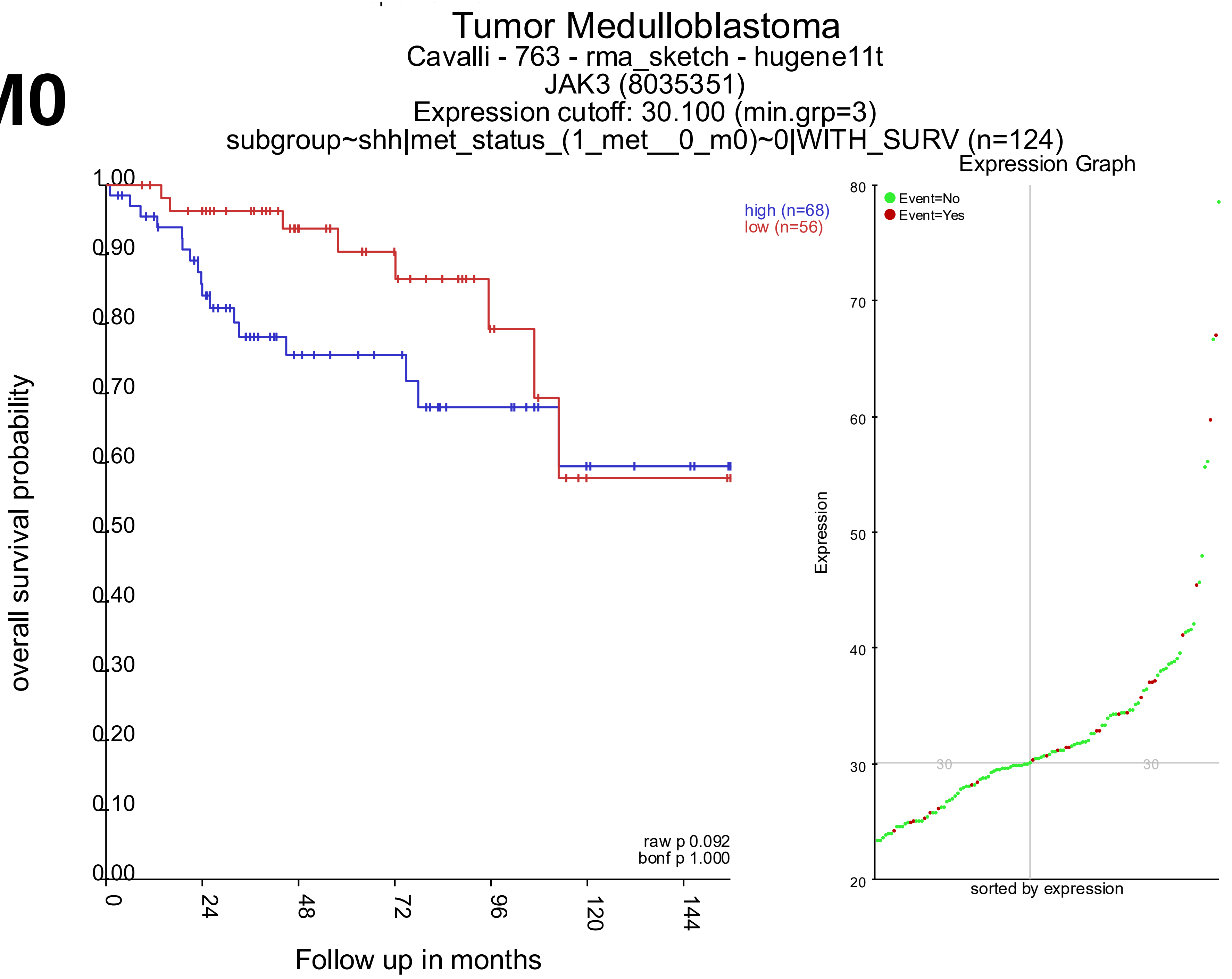

Tumor Medulloblastoma
Cavalli - 763 - rma_sketch - hugene11t
JAK3 (8035351)
Expression cutoff: 30.100 (min.grp=3)
subgroup~shh|met_status_(1_met__0_m0)~0|WITH_SURV (n=124)
overall survival probability
1.00
0.90
0.80
0.70
0.60
0.50
0.40
0.30
0.20
0.10
0.00
0
24
48
72
96
120
144
Follow up in months
high (n=68)
low (n=56)
raw p 0.092
bonf p 1.000
Expression Graph
Event=No
Event=Yes
Expression
80
70
60
50
40
30
20
sorted by expression

SHH M1

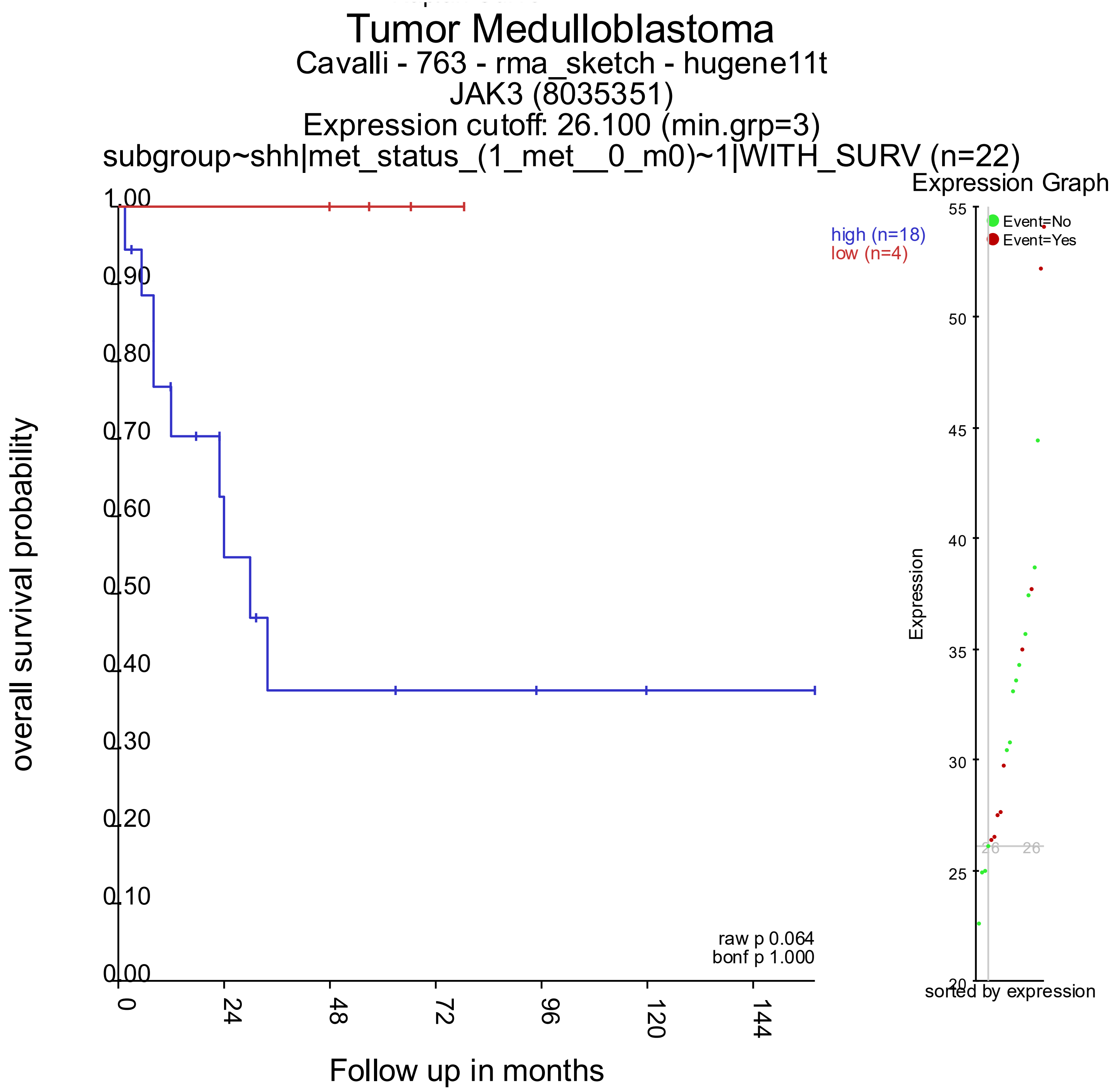
Tumor Medulloblastoma
Cavalli - 763 - rma_sketch - hugene11t
JAK3 (8035351)
Expression cutoff: 26.100 (min.grp=3)
subgroup~shh|met_status_(1_met__0_m0)~1|WITH_SURV (n=22)
overall survival probability
1.00
0.90
0.80
0.70
0.60
0.50
0.40
0.30
0.20
0.10
0.00
0
24
48
72
96
120
144
Follow up in months
high (n=18)
low (n=4)
raw p 0.064
bonf p 1.000
Expression Graph
Event=No
Event=Yes
Expression
55
50
45
40
35
30
25
20
26
26
sorted by expression

GROUP4 M0

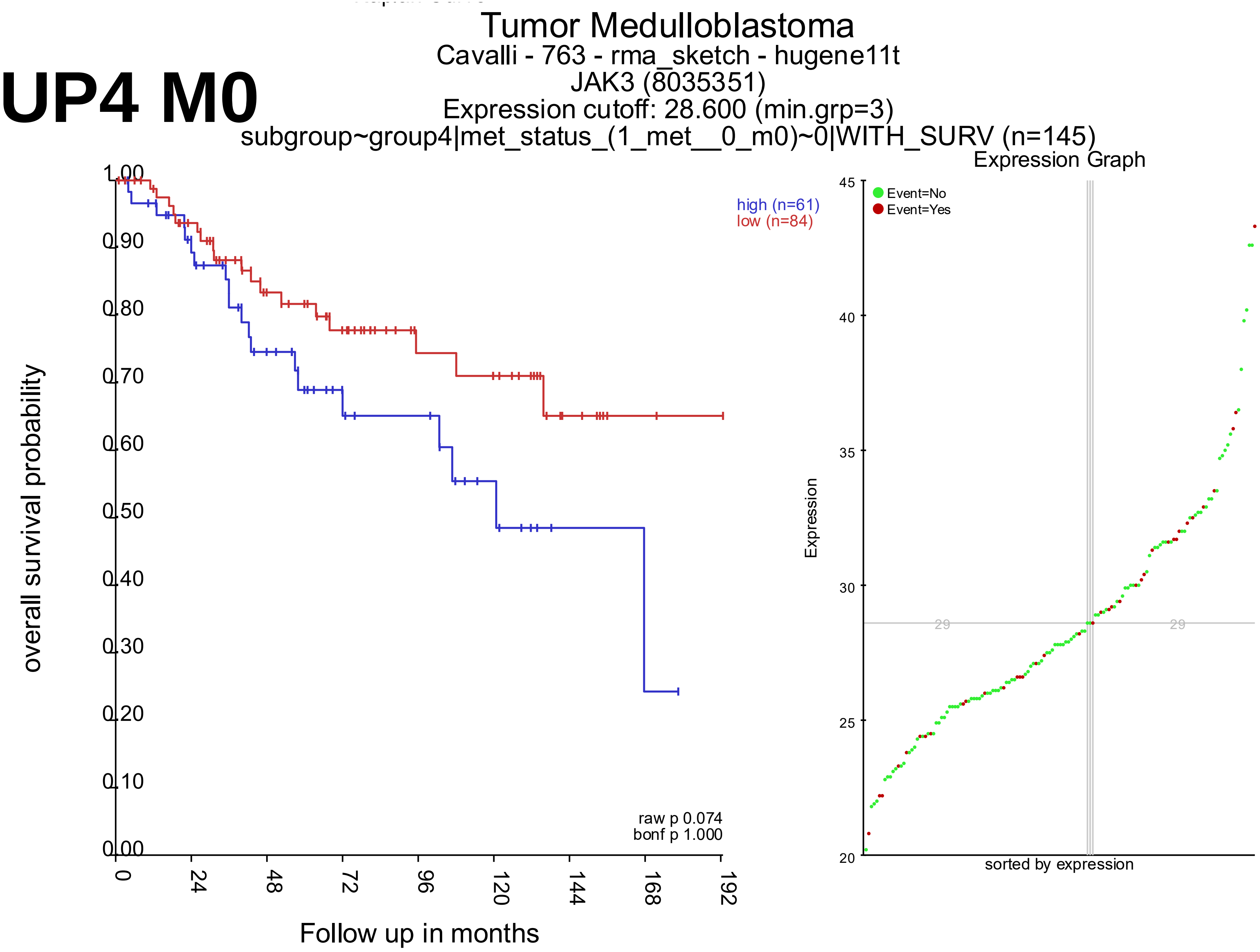
Tumor Medulloblastoma
Cavalli - 763 - rma_sketch - hugene11t
JAK3 (8035351)
Expression cutoff: 28.600 (min.grp=3)
subgroup~group4|met_status_(1_met__0_m0)~0|WITH_SURV (n=145)
high (n=61)
low (n=84)
overall survival probability
1.00
0.90
0.80
0.70
0.60
0.50
0.40
0.30
0.20
0.10
0.00
0
24
48
72
96
120
144
168
192
Follow up in months
raw p 0.074
bonf p 1.000
Expression Graph
Event=No
Event=Yes
Expression
45
40
35
30
25
20
29
29
sorted by expression

GROUP4 M1

Tumor Medulloblastoma
Cavalli - 763 - rma_sketch - hugene11t
JAK3 (8035351)
Expression cutoff: 39.600 (min.grp=3)
subgroup~group4|met_status_(1_met__0_m0)~1|WITH_SURV (n=92)

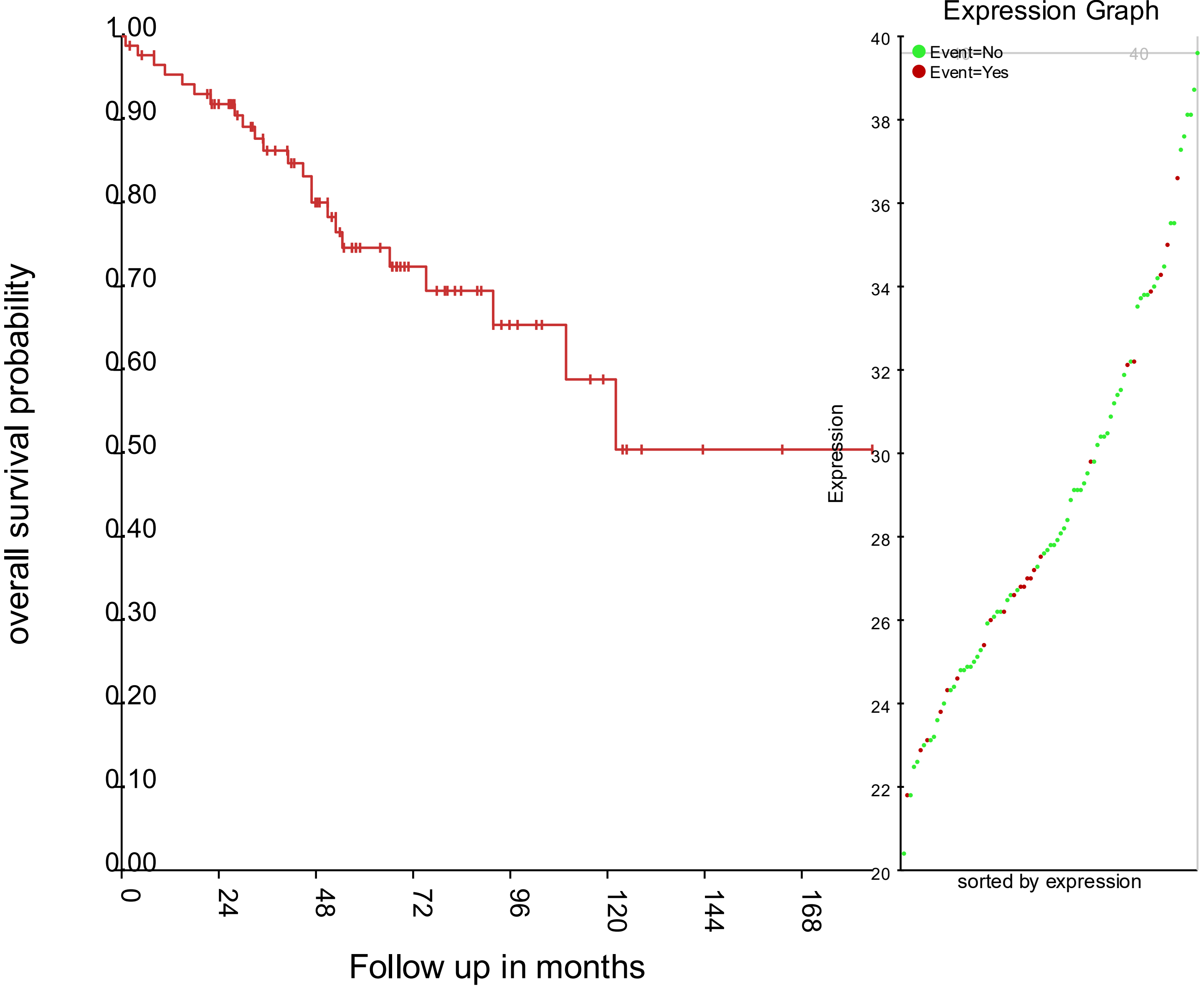

GROUP3 M0

Tumor Medulloblastoma
Cavalli - 763 - rma_sketch - hugene11t
JAK3 (8035351)
Expression cutoff: 22.300 (min.grp=3)
subgroup~group3|met_status_(1_met__0_m0)~0|WITH_SURV (n=65)

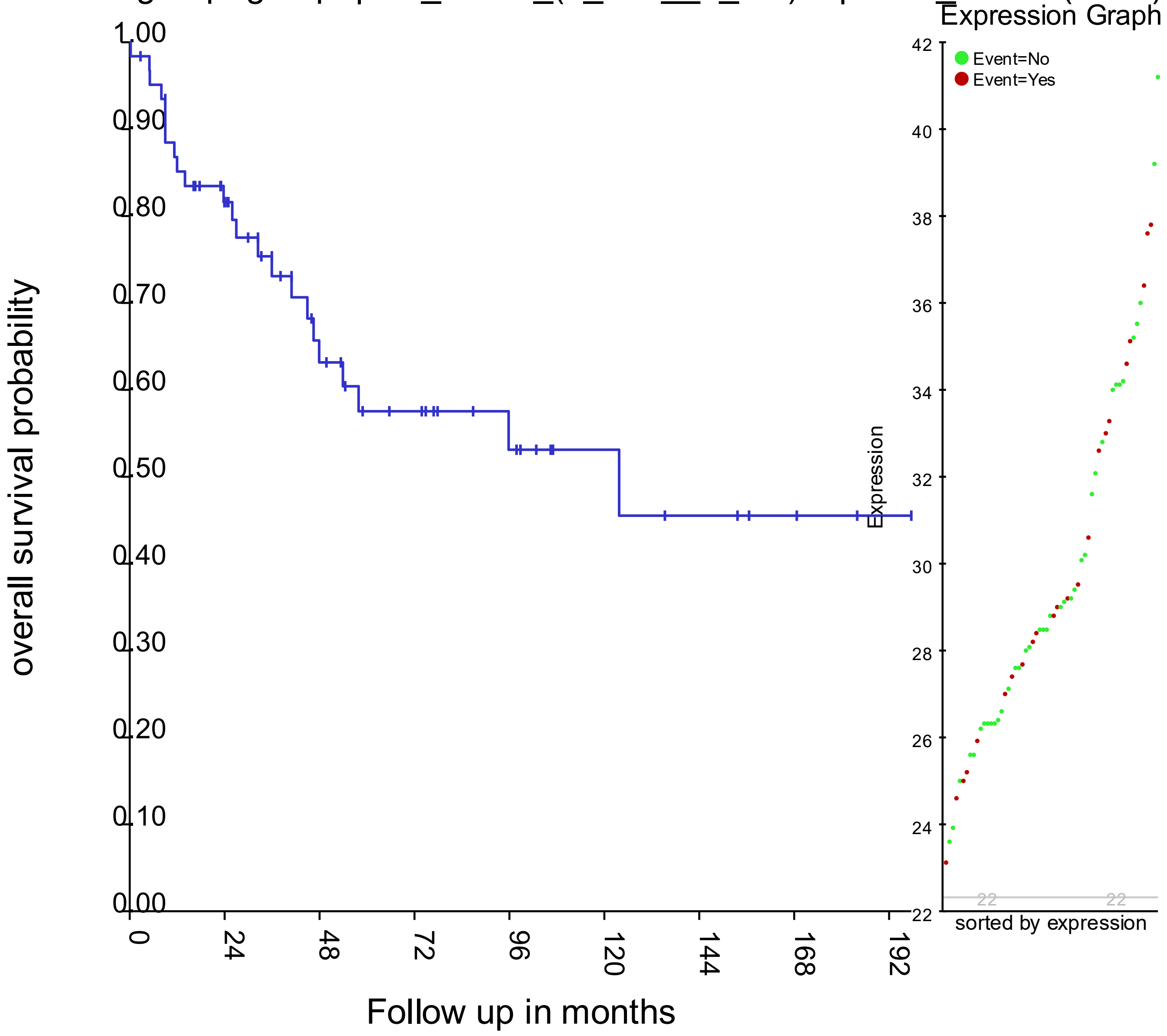

GROUP3 M1

Tumor Medulloblastoma
Cavalli - 763 - rma_sketch - hugene11t
JAK3 (8035351)
Expression cutoff: 31.200 (min.grp=3)
subgroup~group3|met_status_(1_met__0_m0)~1|WITH_SURV (n=41)

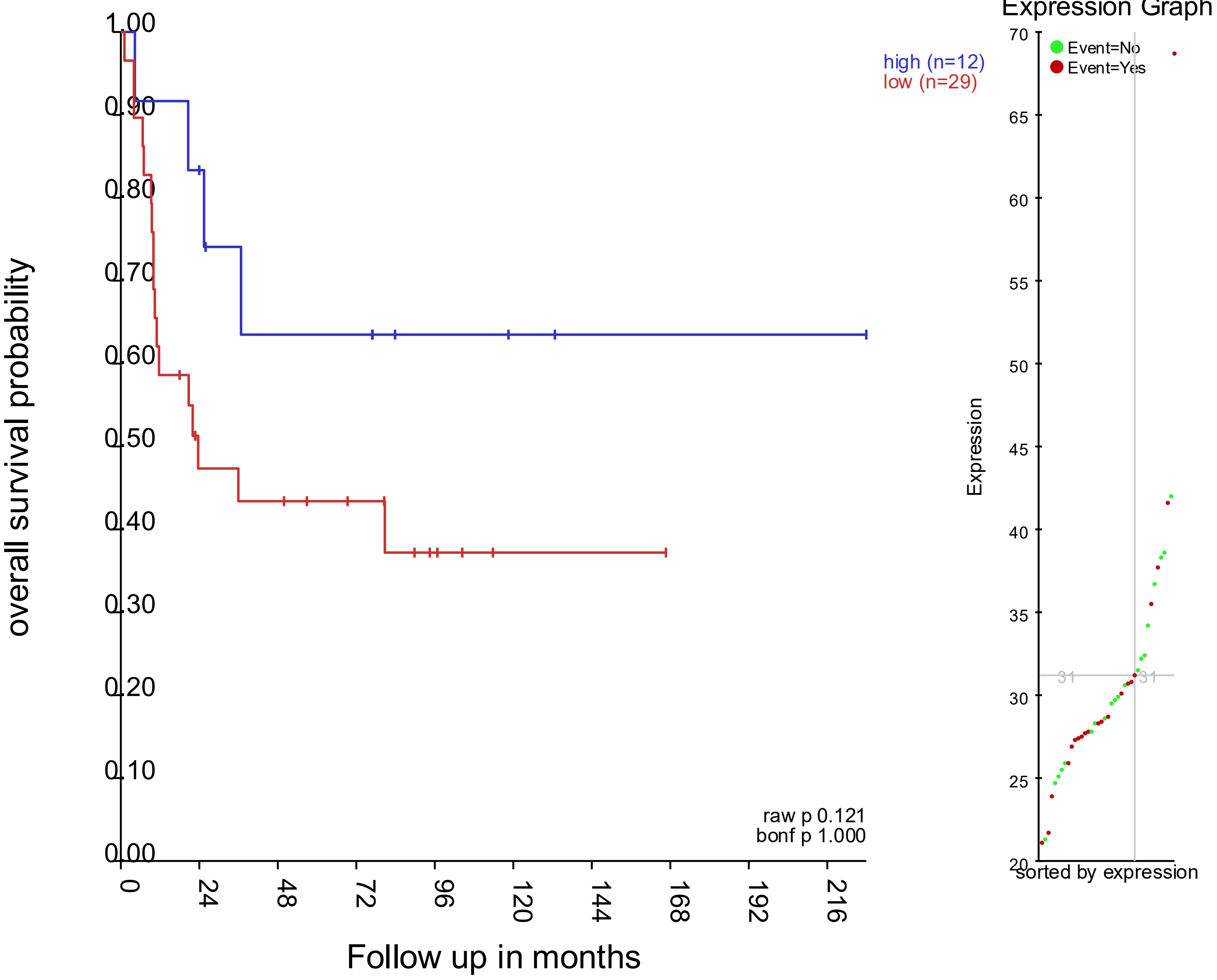

MAP2K2

WNT M0

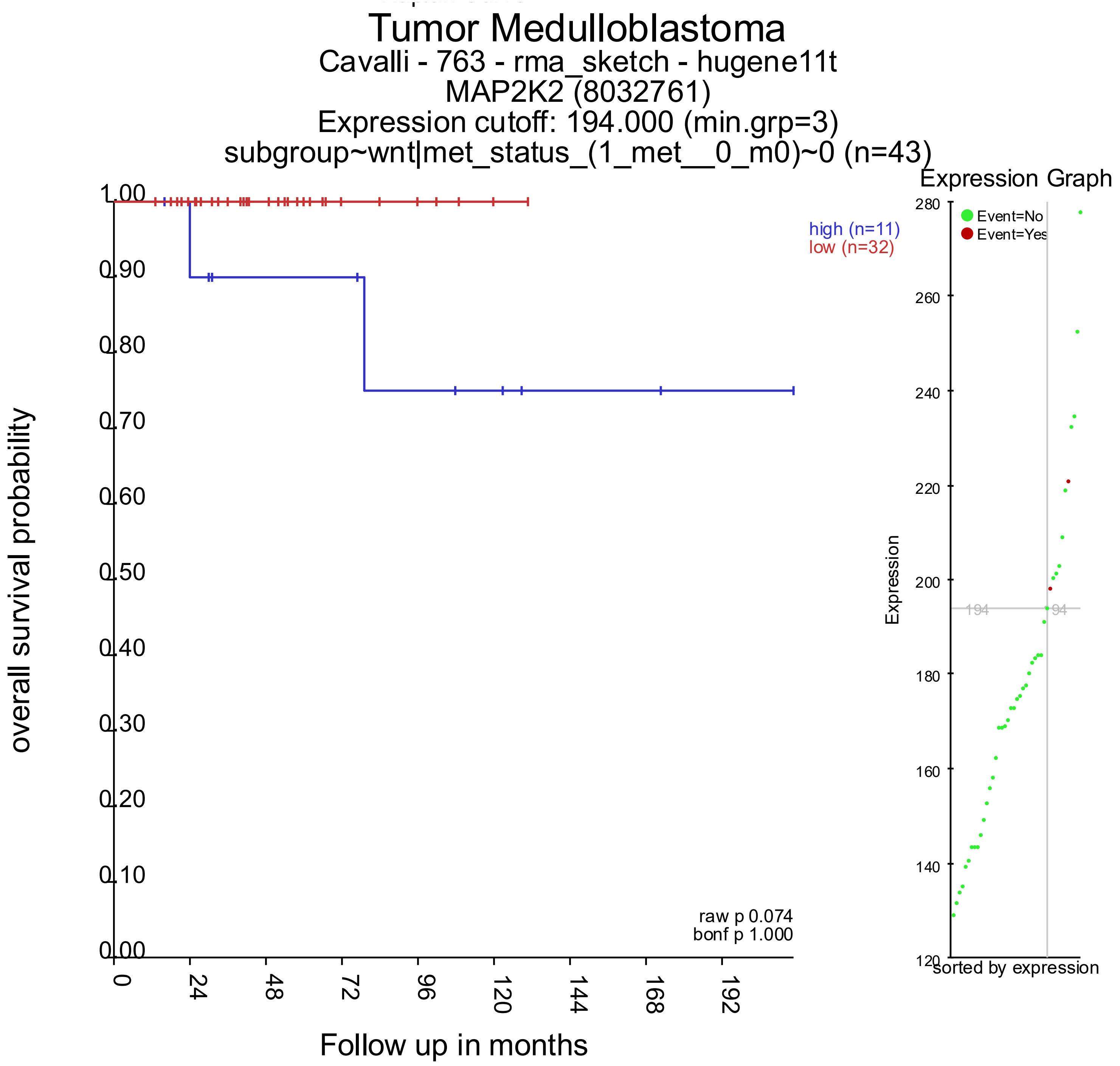
Tumor Medulloblastoma
Cavalli - 763 - rma_sketch - hugene11t
MAP2K2 (8032761)
Expression cutoff: 194.000 (min.grp=3)
subgroup~wnt|met_status_(1_met__0_m0)~0 (n=43)
high (n=11)
low (n=32)
overall survival probability
1.00
0.90
0.80
0.70
0.60
0.50
0.40
0.30
0.20
0.10
0.00
raw p 0.074
bonf p 1.000
0
24
48
72
96
120
144
168
192
Follow up in months
Expression Graph
Event=No
Event=Yes
Expression
280
260
240
220
200
180
160
140
120
194
sorted by expression

WNT M1

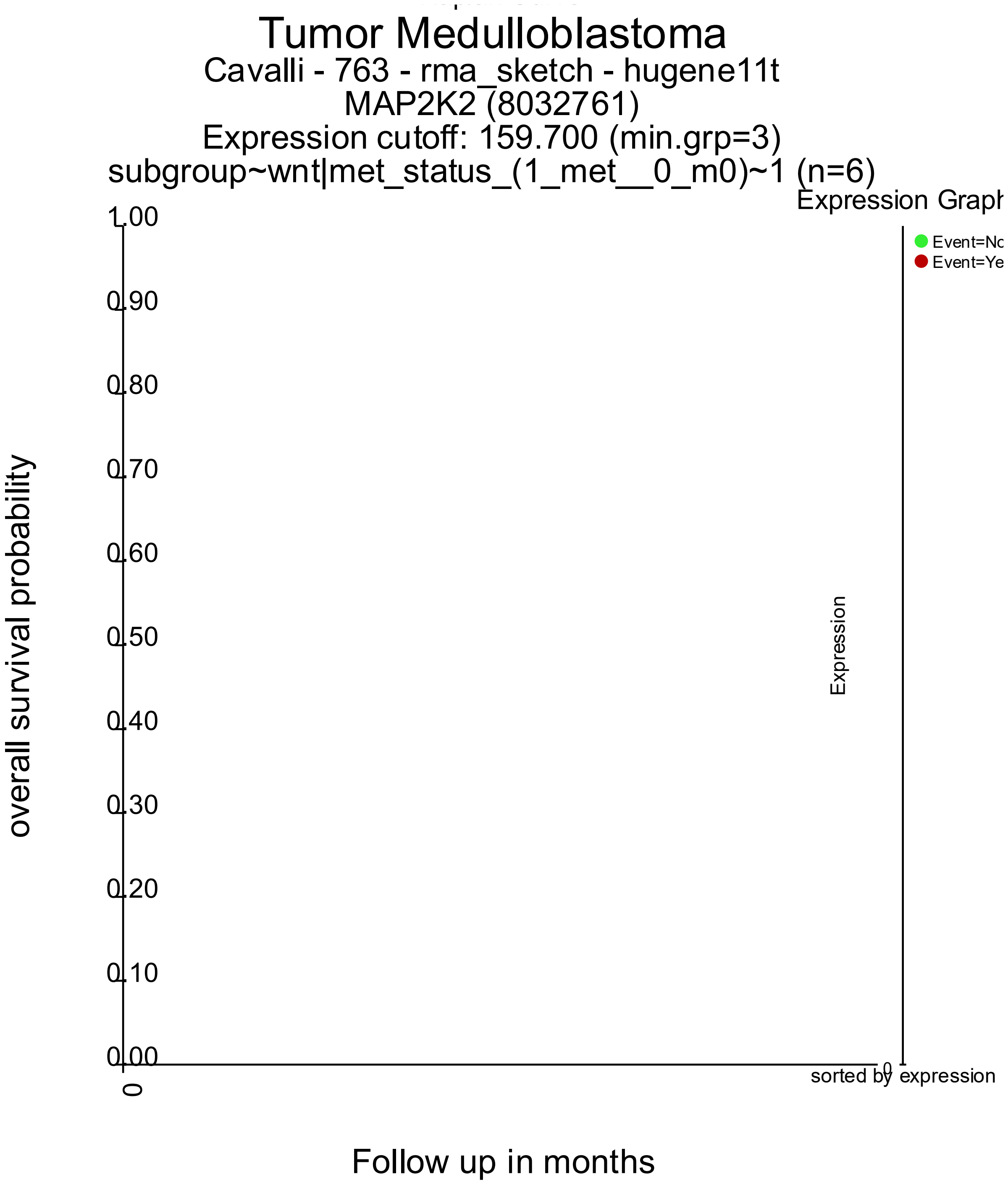

Tumor Medulloblastoma
Cavalli - 763 - rma_sketch - hugene11t
MAP2K2 (8032761)
Expression cutoff: 159.700 (min.grp=3)
subgroup~wnt|met_status_(1_met__0_m0)~1 (n=6)
Expression Graph
Event=No
Event=Ye
1.00
0.90
0.80
0.70
0.60
0.50
0.40
0.30
0.20
0.10
0.00
overall survival probability
0
Follow up in months
Expression
0
sorted by expression

SHH M0

Tumor Medulloblastoma
Cavalli - 763 - rma_sketch - hugene11t
MAP2K2 (8032761)
Expression cutoff: 175.200 (min.grp=3)
subgroup~shh|met_status_(1_met__0_m0)~0|WITH_SURV (n=124)

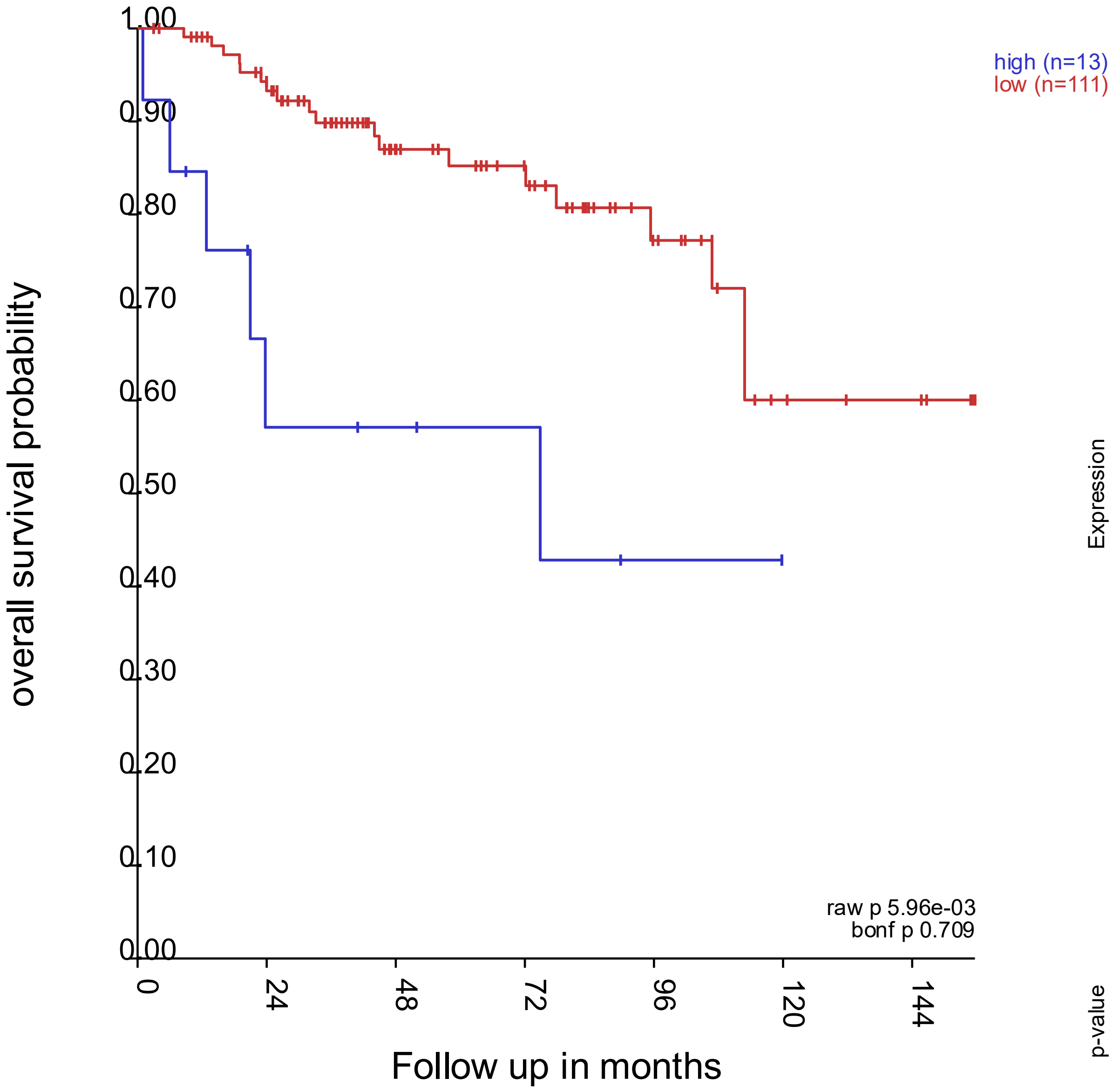

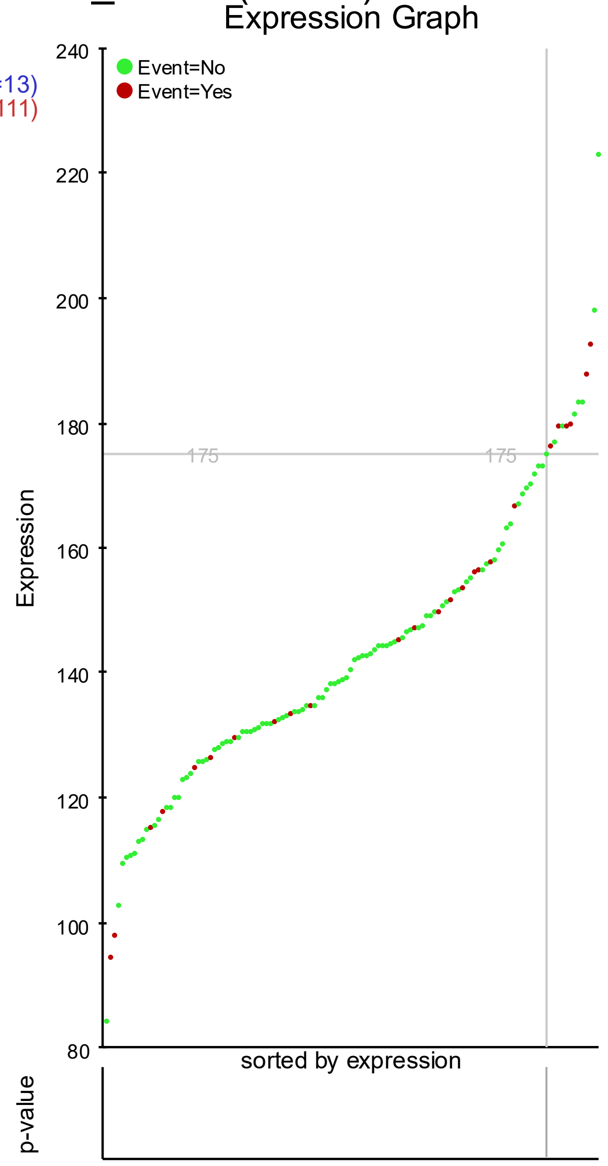

SHH M1

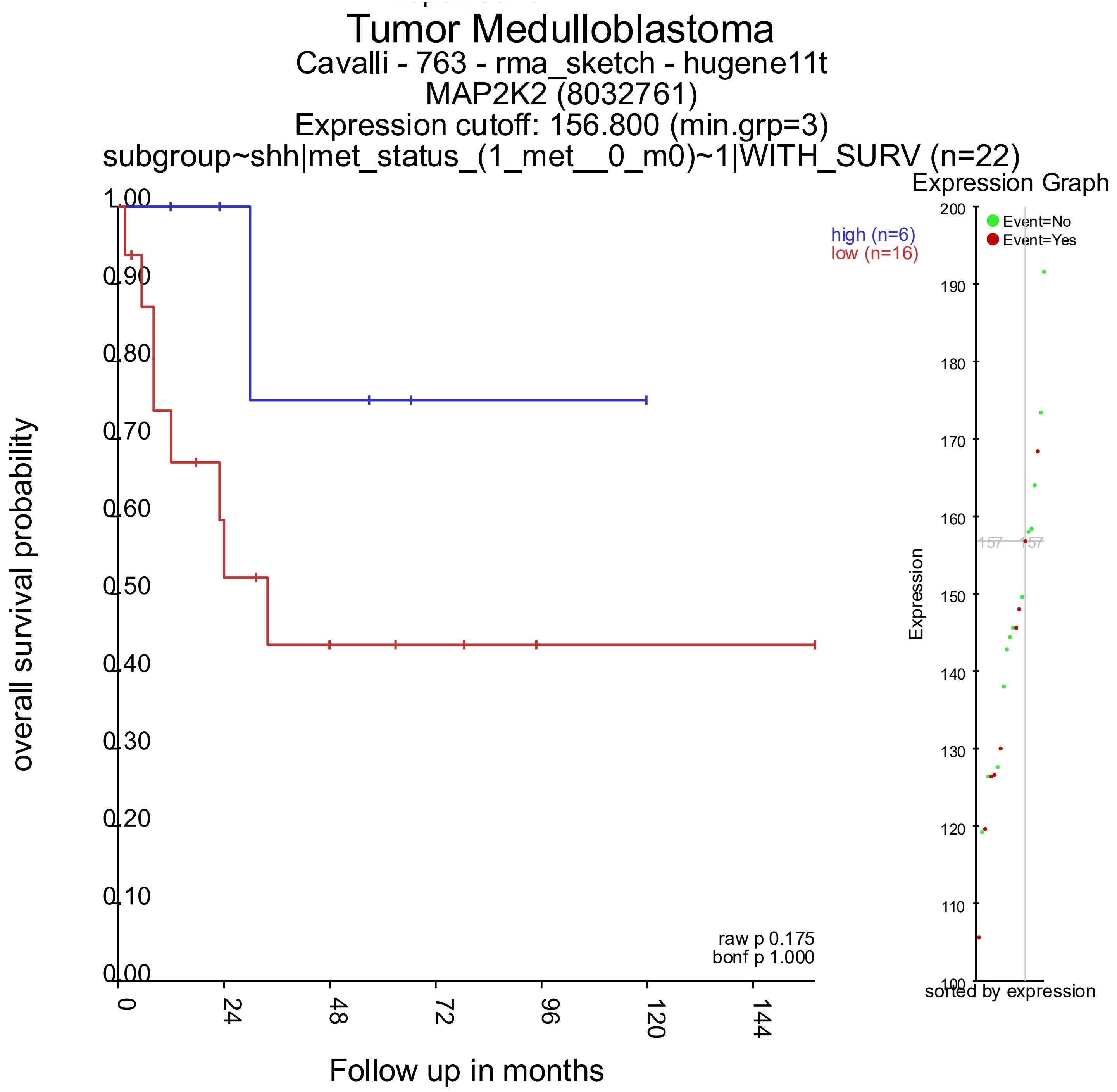

Tumor Medulloblastoma
Cavalli - 763 - rma_sketch - hugene11t
MAP2K2 (8032761)
Expression cutoff: 156.800 (min.grp=3)
subgroup~shh|met_status_(1_met__0_m0)~1|WITH_SURV (n=22)
overall survival probability
1.00
0.90
0.80
0.70
0.60
0.50
0.40
0.30
0.20
0.10
0.00
high (n=6)
low (n=16)
raw p 0.175
bonf p 1.000
0
24
48
72
96
120
144
Follow up in months
Expression Graph
Event=No
Event=Yes
200
190
180
170
160
150
140
130
120
110
100
157
Expression
sorted by expression

GROUP4 M0

Tumor Medulloblastoma
Cavalli - 763 - rma_sketch - hugene11t
MAP2K2 (8032761)
Expression cutoff: 86.100 (min.grp=3)
subgroup~group4|met_status_(1_met__0_m0)~0|WITH_SURV (n=145)

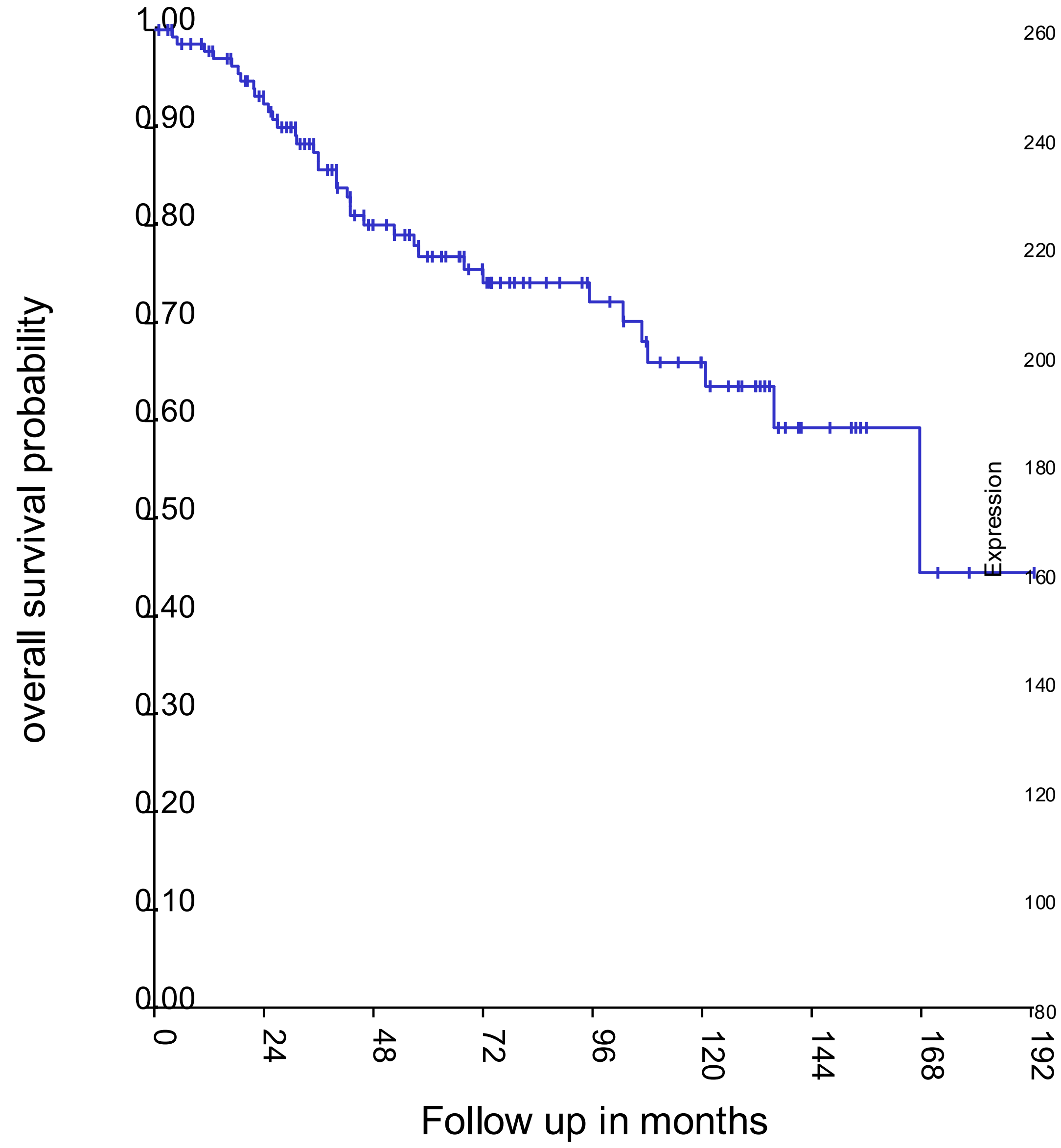

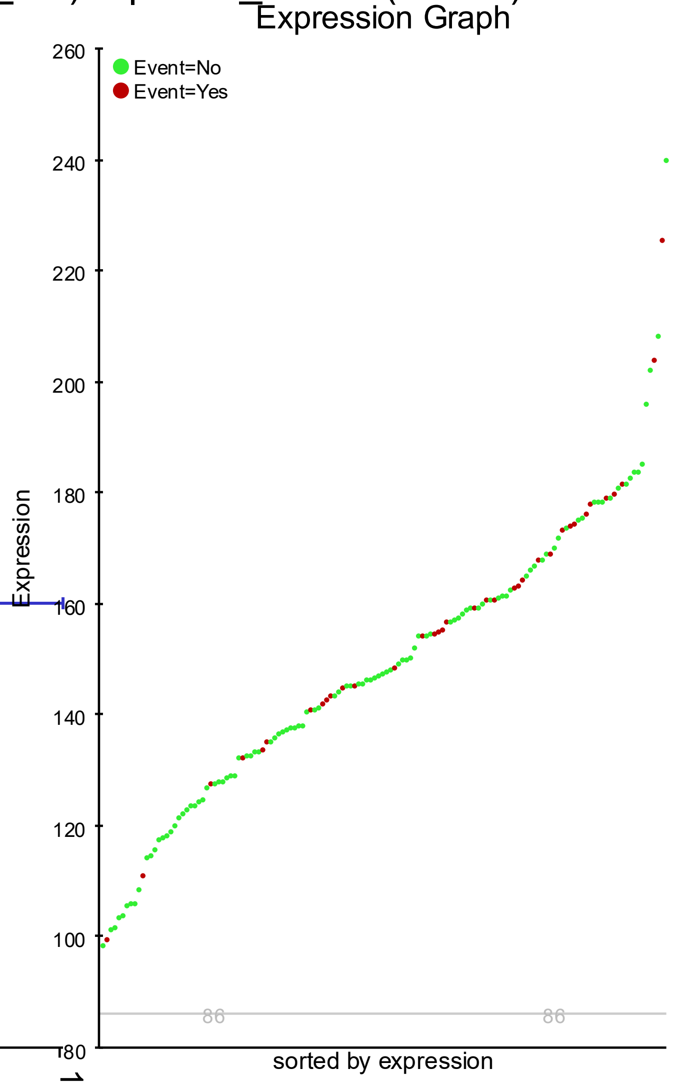

GROUP4 M1

Tumor Medulloblastoma
Cavalli - 763 - rma_sketch - hugene11t
MAP2K2 (8032761)
Expression cutoff: 130.000 (min.grp=3)
subgroup~group4|met_status_(1_met__0_m0)~1|WITH_SURV (n=92)

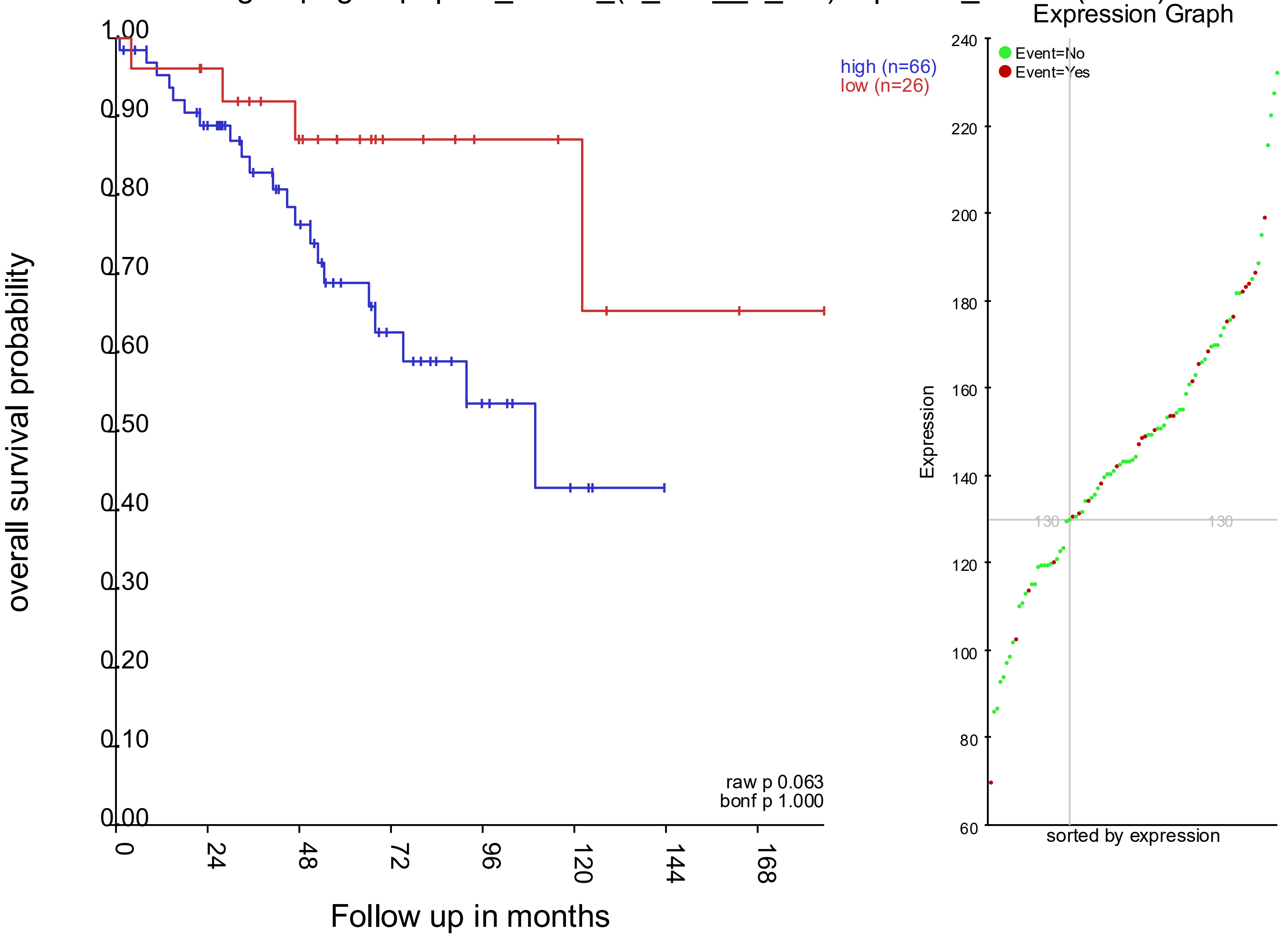

GROUP3 M0

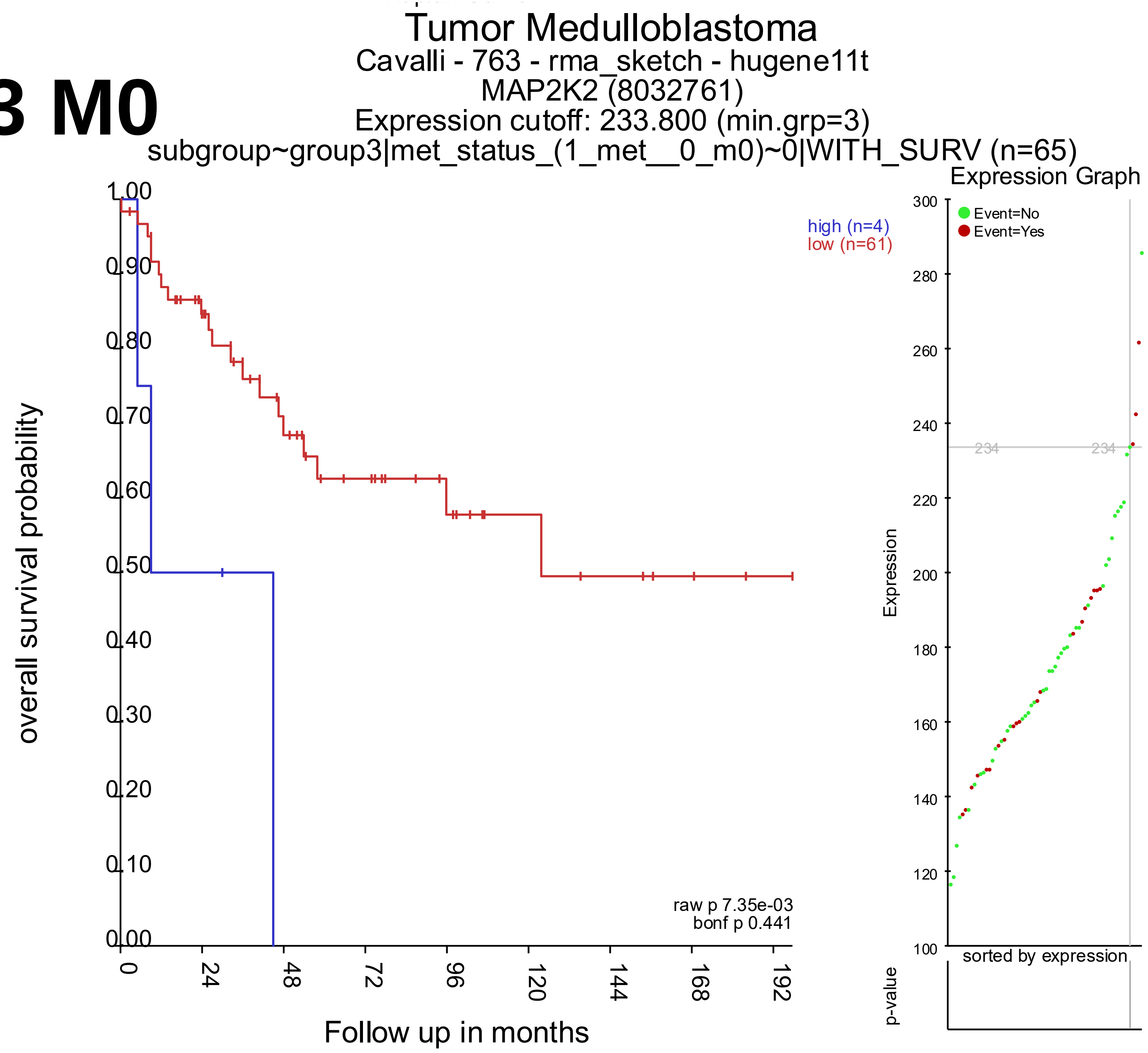

Tumor Medulloblastoma
Cavalli - 763 - rma_sketch - hugene11t
MAP2K2 (8032761)
Expression cutoff: 233.800 (min.grp=3)
subgroup~group3|met_status_(1_met__0_m0)~0|WITH_SURV (n=65)
high (n=4)
low (n=61)
overall survival probability
1.00
0.90
0.80
0.70
0.60
0.50
0.40
0.30
0.20
0.10
0.00
0
24
48
72
96
120
144
168
192
Follow up in months
raw p 7.35e-03
bonf p 0.441
Expression Graph
Event=No
Event=Yes
Expression
300
280
260
240
220
200
180
160
140
120
100
234
234
sorted by expression
p-value

GROUP3 M1

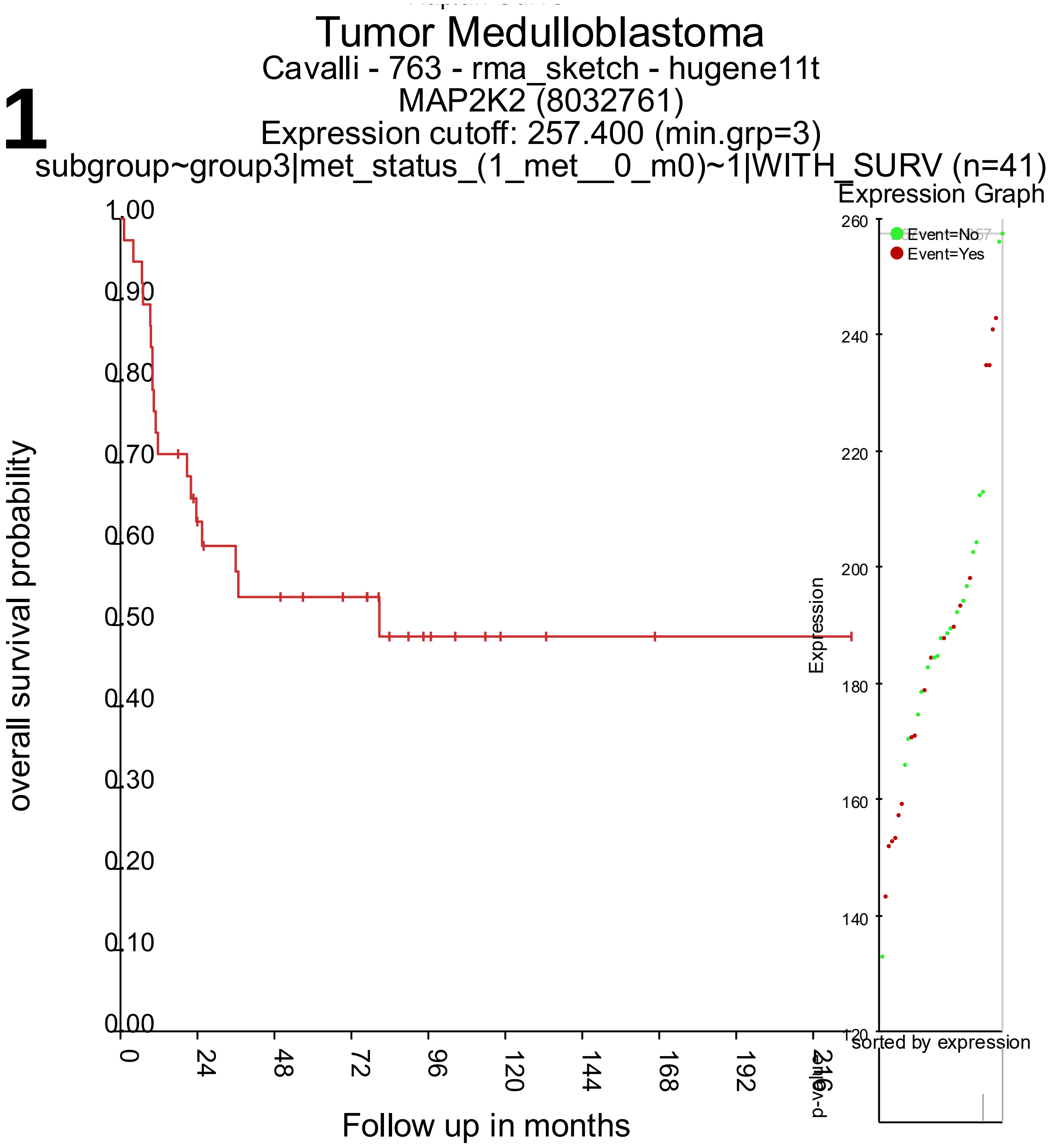

Tumor Medulloblastoma
Cavalli - 763 - rma_sketch - hugene11t
MAP2K2 (8032761)
Expression cutoff: 257.400 (min.grp=3)
subgroup~group3|met_status_(1_met__0_m0)~1|WITH_SURV (n=41)
overall survival probability
1.00
0.90
0.80
0.70
0.60
0.50
0.40
0.30
0.20
0.10
0.00
0
24
48
72
96
120
144
168
192
216
Follow up in months
Expression Graph
Event=No
Event=Yes
Expression
260
240
220
200
180
160
140
120
sorted by expression

MAPK1

WNT M0

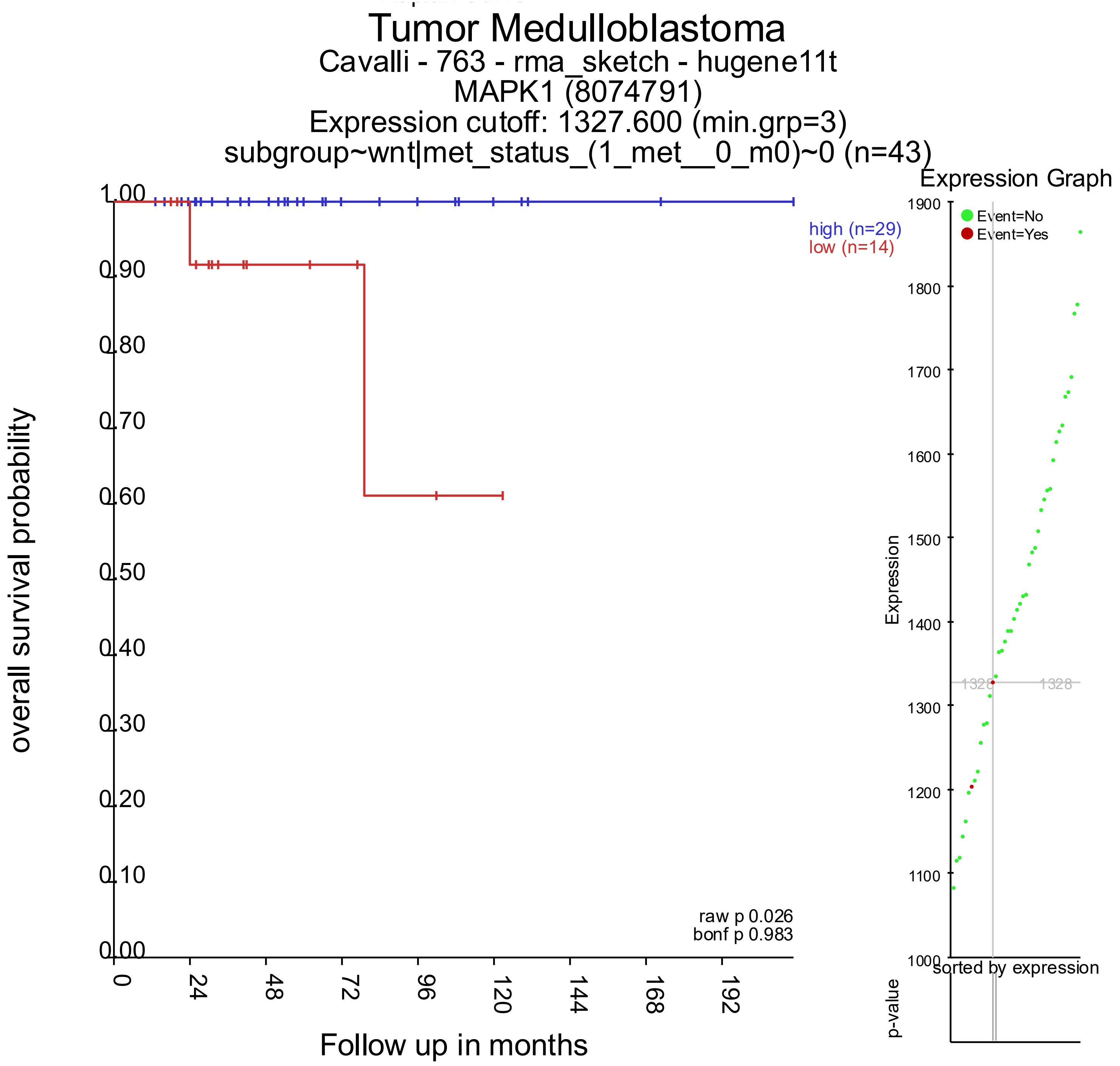

Tumor Medulloblastoma
Cavalli - 763 - rma_sketch - hugene11t
MAPK1 (8074791)
Expression cutoff: 1327.600 (min.grp=3)
subgroup~wnt|met_status_(1_met__0_m0)~0 (n=43)
overall survival probability
1.00
0.90
0.80
0.70
0.60
0.50
0.40
0.30
0.20
0.10
0.00
0
24
48
72
96
120
144
168
192
Follow up in months
high (n=29)
low (n=14)
raw p 0.026
bonf p 0.983
Expression Graph
Event=No
Event=Yes
Expression
1900
1800
1700
1600
1500
1400
1300
1200
1100
1000
1328
1328
sorted by expression
p-value

WNT M1

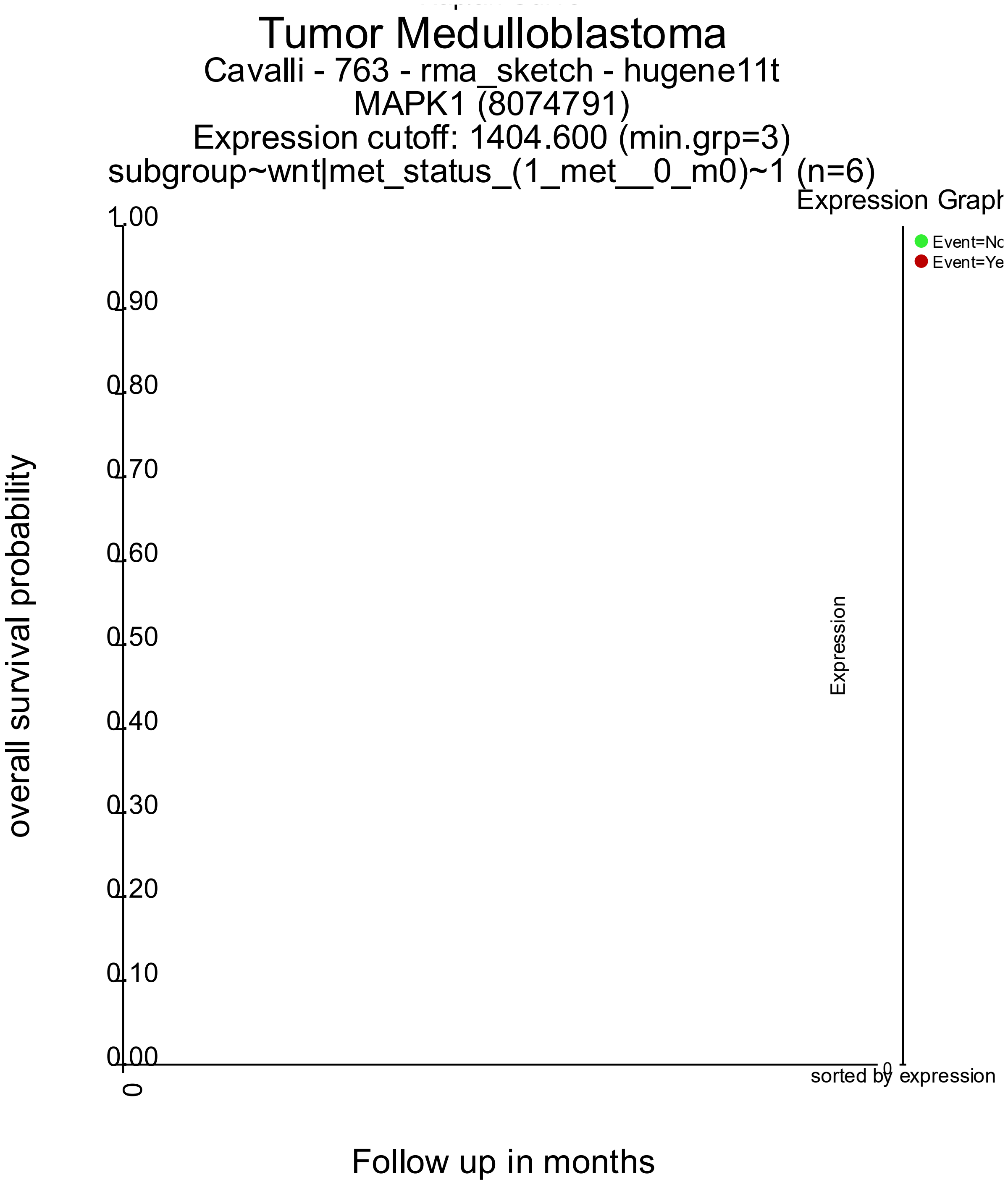

Tumor Medulloblastoma
Cavalli - 763 - rma_sketch - hugene11t
MAPK1 (8074791)
Expression cutoff: 1404.600 (min.grp=3)
subgroup~wnt|met_status_(1_met__0_m0)~1 (n=6)
Expression Graph
Event=No
Event=Ye
1.00
0.90
0.80
0.70
0.60
0.50
0.40
0.30
0.20
0.10
0.00
overall survival probability
0
Follow up in months
Expression
0
sorted by expression

SHH M0

Tumor Medulloblastoma
Cavalli - 763 - rma_sketch - hugene11t
MAPK1 (8074791)
Expression cutoff: 1064.300 (min.grp=3)
subgroup~shh|met_status_(1_met__0_m0)~0|WITH_SURV (n=124)

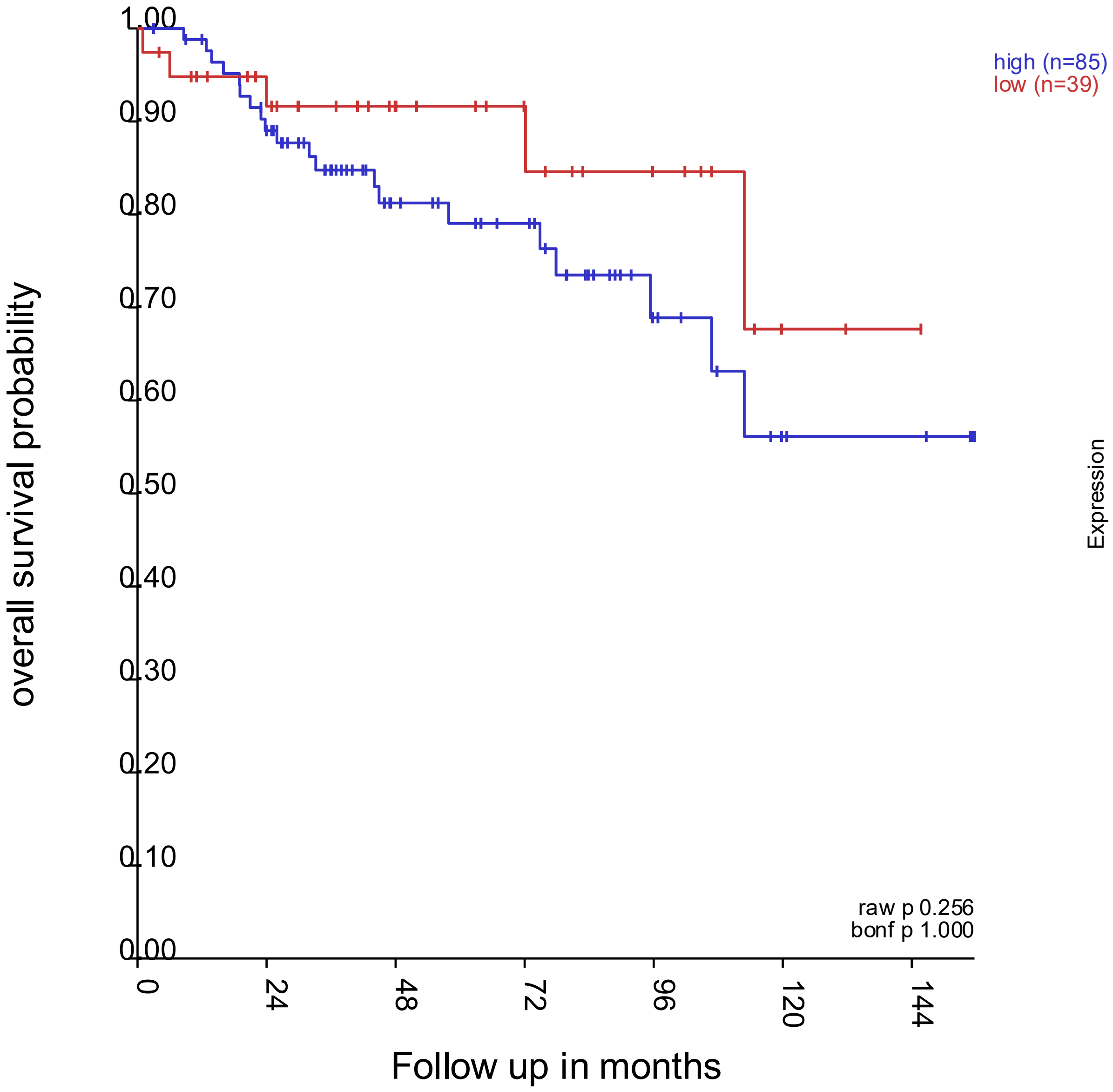

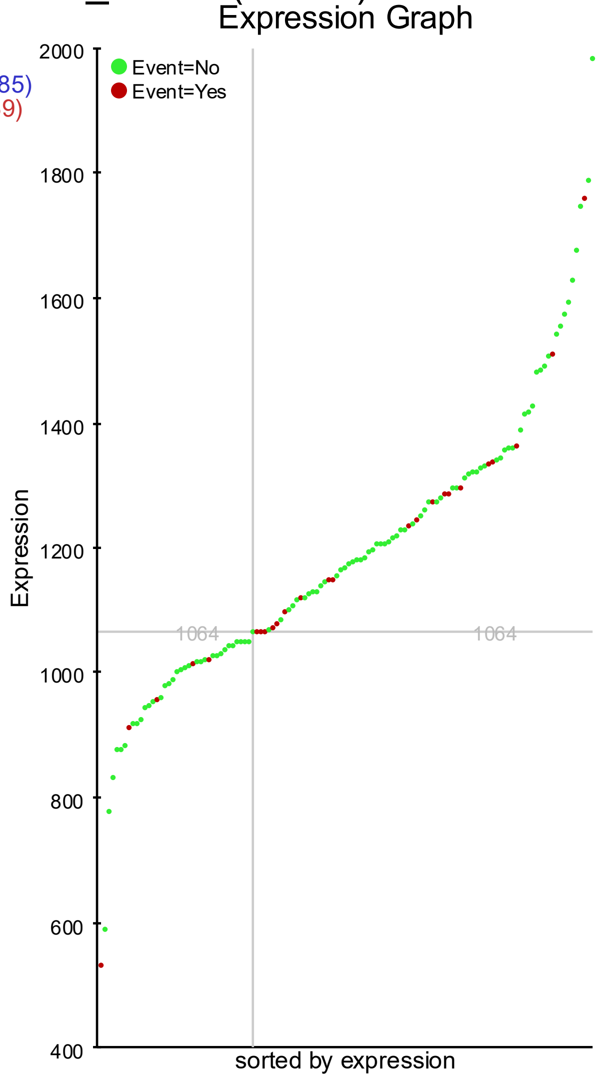

SHH M1

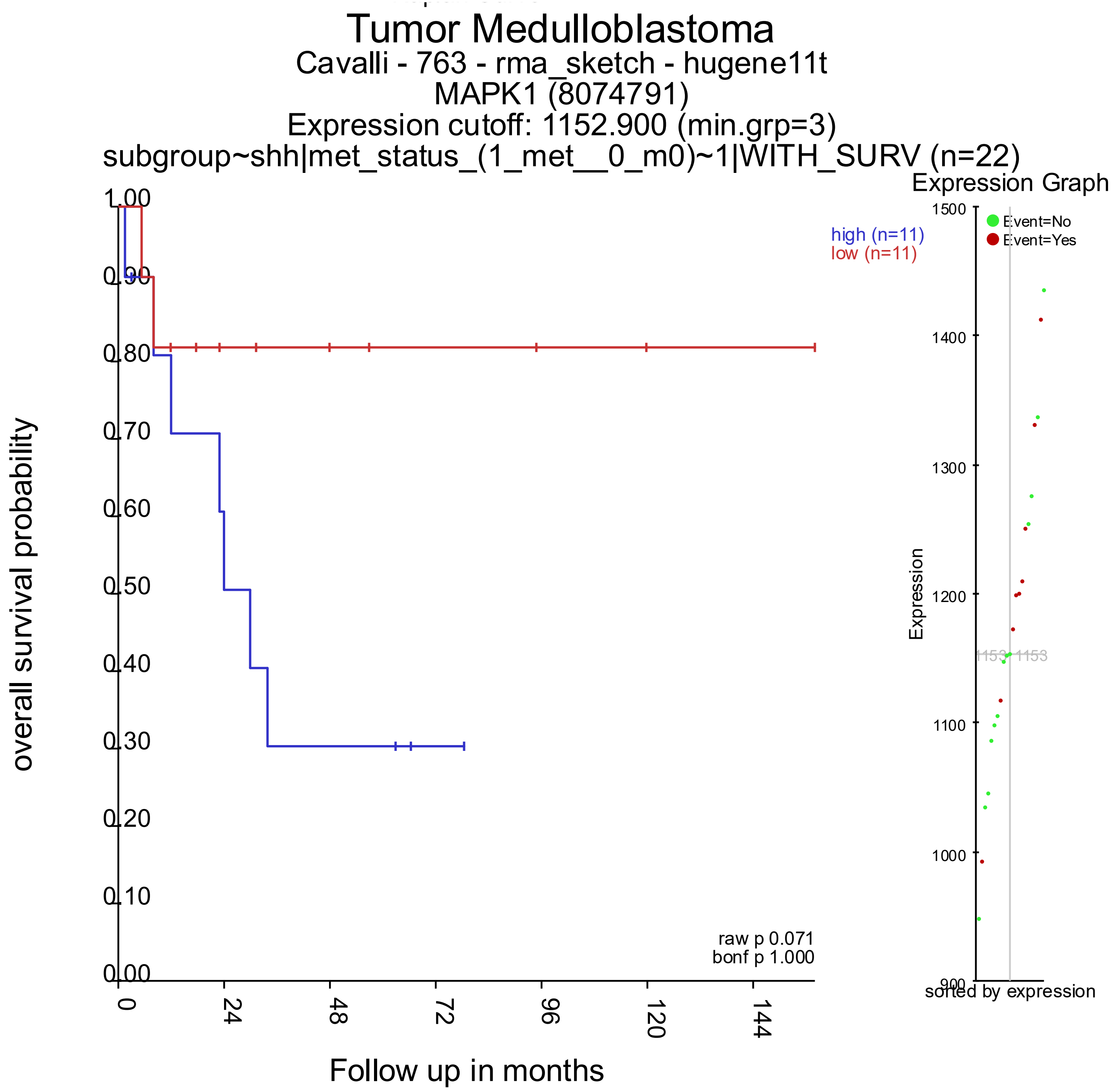
Tumor Medulloblastoma
Cavalli - 763 - rma_sketch - hugene11t
MAPK1 (8074791)
Expression cutoff: 1152.900 (min.grp=3)
subgroup~shh|met_status_(1_met__0_m0)~1|WITH_SURV (n=22)
high (n=11)
low (n=11)
overall survival probability
1.00
0.90
0.80
0.70
0.60
0.50
0.40
0.30
0.20
0.10
0.00
0
24
48
72
96
120
144
Follow up in months
raw p 0.071
bonf p 1.000
Expression Graph
Event=No
Event=Yes
1500
1400
1300
1200
1100
1000
900
Expression
1153
1153
sorted by expression

GROUP4 M0

Tumor Medulloblastoma
Cavalli - 763 - rma_sketch - hugene11t
MAPK1 (8074791)
Expression cutoff: 1329.500 (min.grp=3)
subgroup~group4|met_status_(1_met__0_m0)~0|WITH_SURV (n=145)

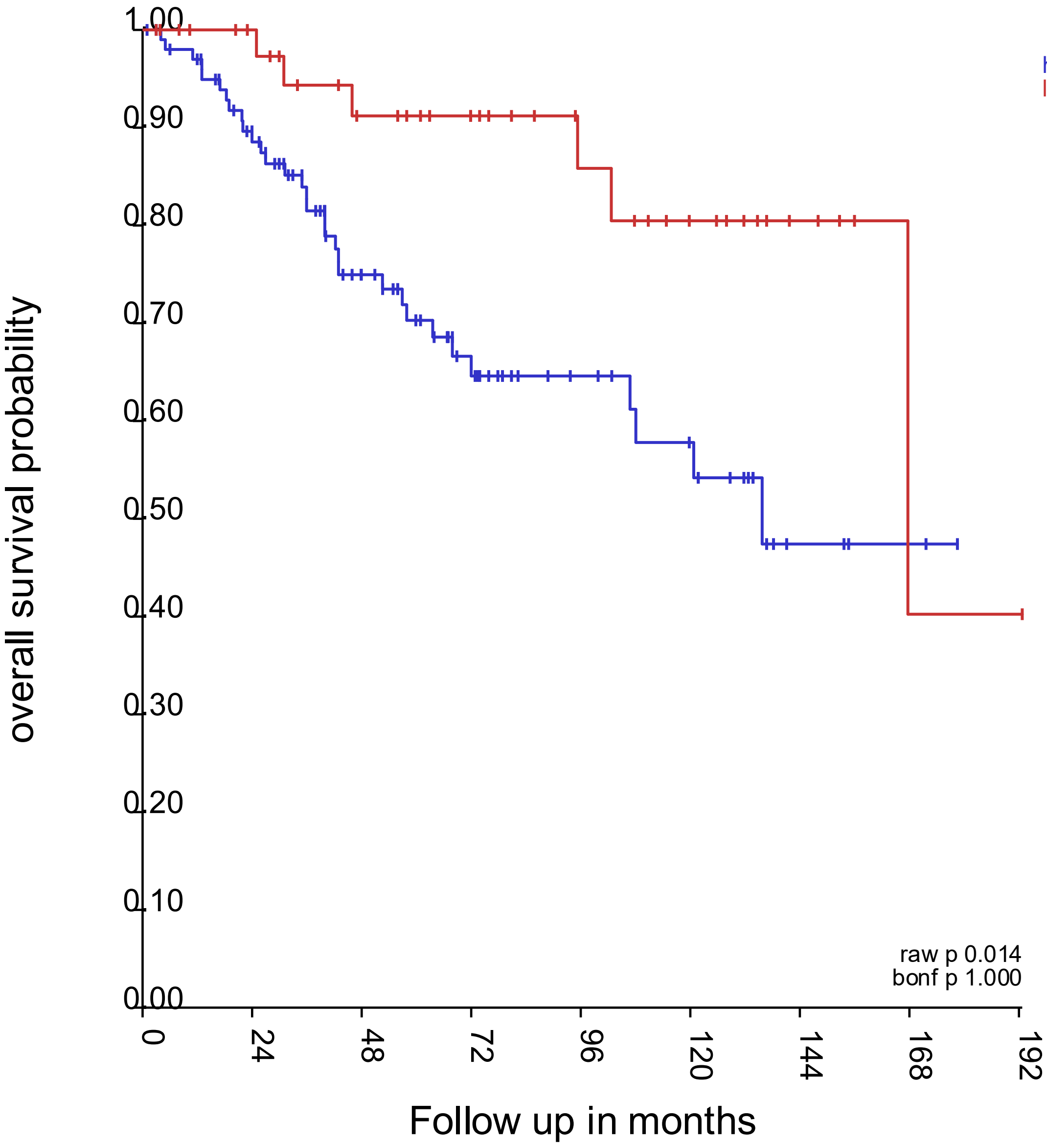

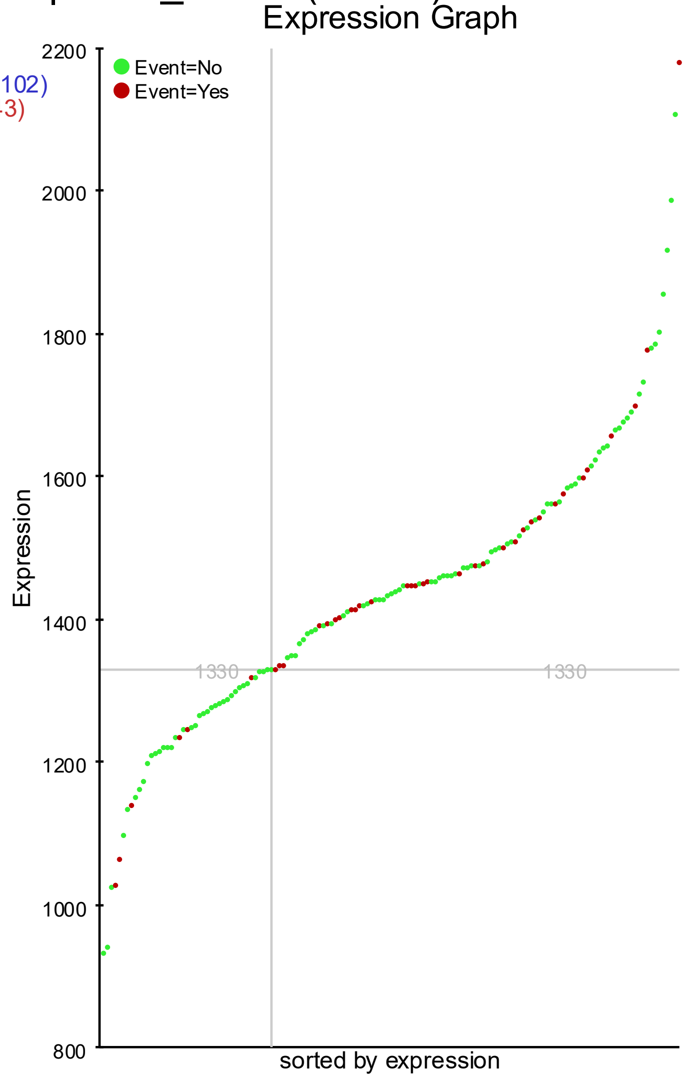

GROUP4 M1

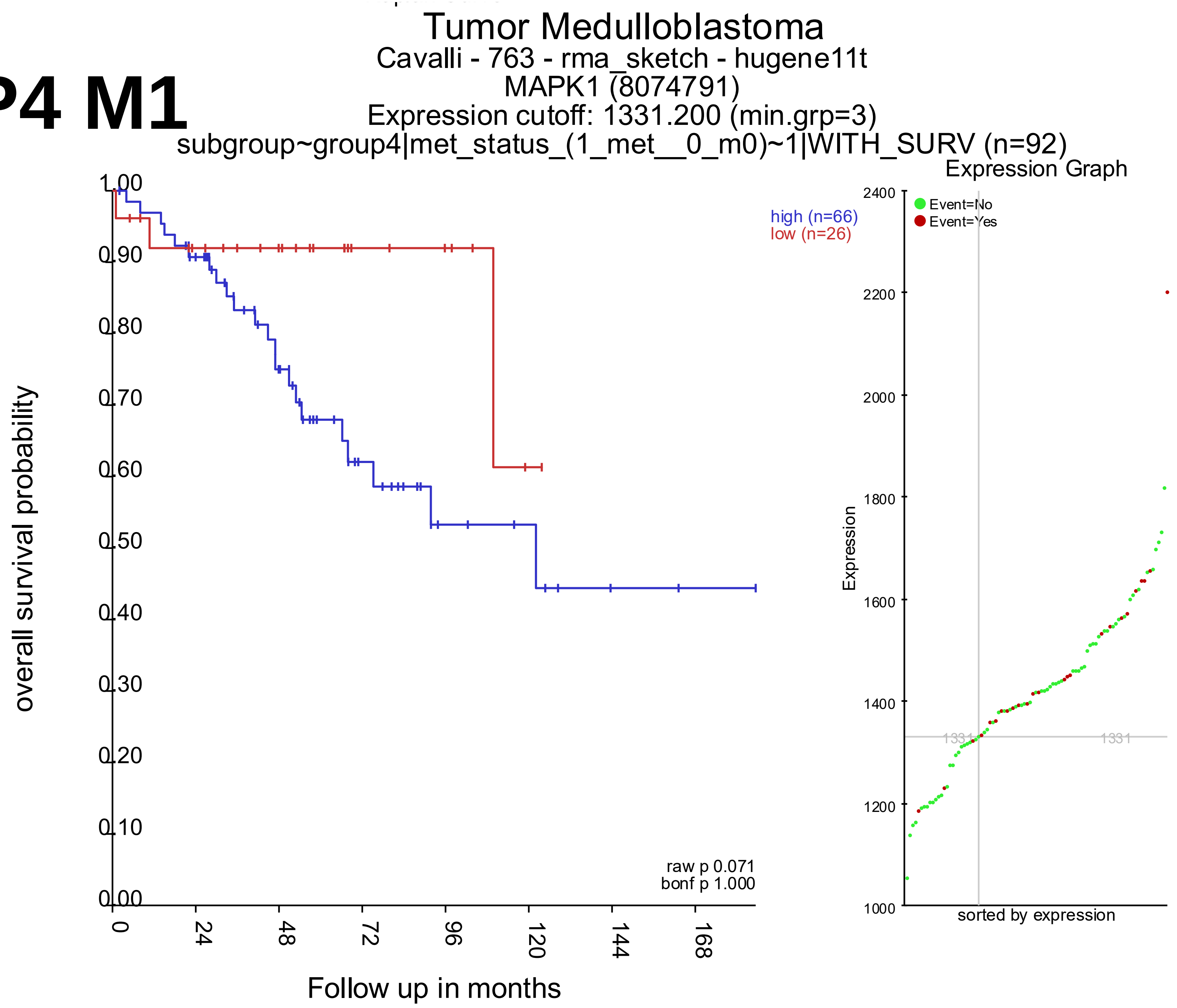

Tumor Medulloblastoma
Cavalli - 763 - rma_sketch - hugene11t
MAPK1 (8074791)
Expression cutoff: 1331.200 (min.grp=3)
subgroup~group4|met_status_(1_met__0_m0)~1|WITH_SURV (n=92)
overall survival probability
1.00
0.90
0.80
0.70
0.60
0.50
0.40
0.30
0.20
0.10
0.00
0
24
48
72
96
120
144
168
Follow up in months
high (n=66)
low (n=26)
raw p 0.071
bonf p 1.000
Expression Graph
Event=No
Event=Yes
Expression
2400
2200
2000
1800
1600
1400
1200
1000
1331
1331
sorted by expression

GROUP3 M0

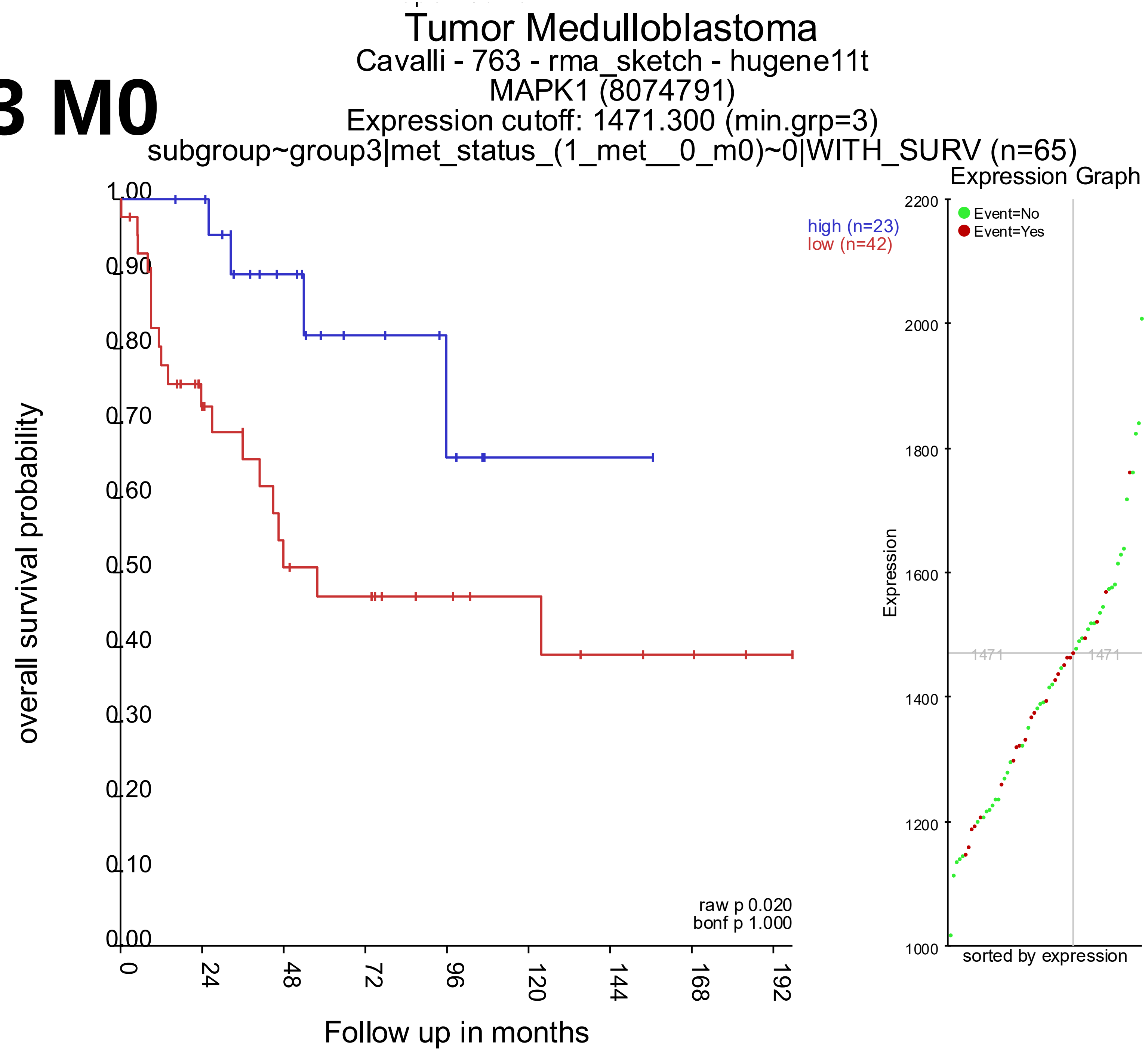

Tumor Medulloblastoma
Cavalli - 763 - rma_sketch - hugene11t
MAPK1 (8074791)
Expression cutoff: 1471.300 (min.grp=3)
subgroup~group3|met_status_(1_met__0_m0)~0|WITH_SURV (n=65)
high (n=23)
low (n=42)
overall survival probability
1.00
0.90
0.80
0.70
0.60
0.50
0.40
0.30
0.20
0.10
0.00
0
24
48
72
96
120
144
168
192
Follow up in months
raw p 0.020
bonf p 1.000
Expression Graph
Event=No
Event=Yes
Expression
2200
2000
1800
1600
1400
1200
1000
1471
sorted by expression

GROUP3 M1

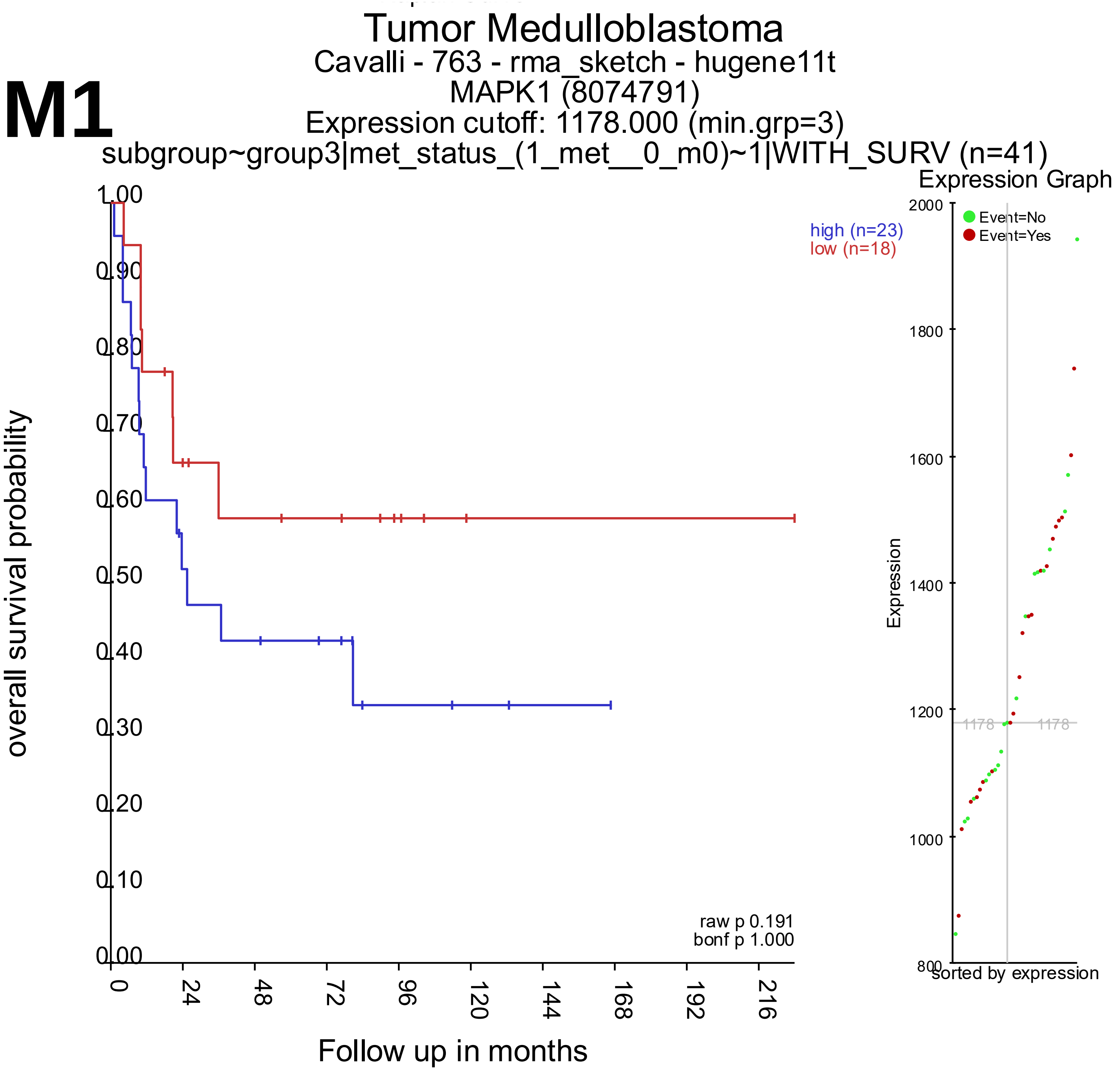

Tumor Medulloblastoma
Cavalli - 763 - rma_sketch - hugene11t
MAPK1 (8074791)
Expression cutoff: 1178.000 (min.grp=3)
subgroup~group3|met_status_(1_met__0_m0)~1|WITH_SURV (n=41)
overall survival probability
1.00
0.90
0.80
0.70
0.60
0.50
0.40
0.30
0.20
0.10
0.00
high (n=23)
low (n=18)
raw p 0.191
bonf p 1.000
0
24
48
72
96
120
144
168
192
216
Follow up in months
Expression Graph
Event=No
Event=Yes
2000
1800
1600
1400
1200
1000
800
Expression
1178
1178
sorted by expression

MAPK3

WNT M0

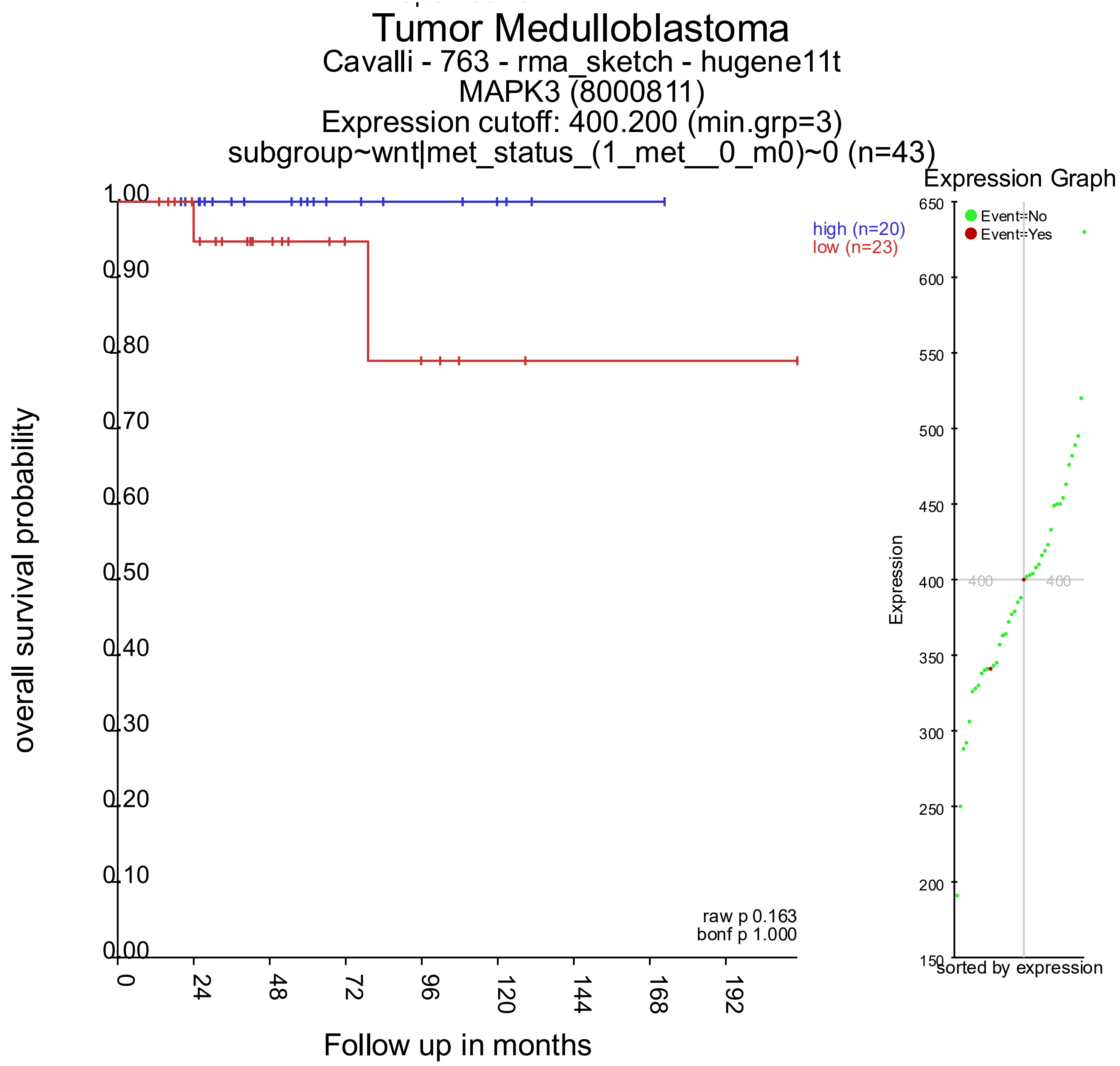

Tumor Medulloblastoma
Cavalli - 763 - rma_sketch - hugene11t
MAPK3 (8000811)
Expression cutoff: 400.200 (min.grp=3)
subgroup~wnt|met_status_(1_met__0_m0)~0 (n=43)
high (n=20)
low (n=23)
overall survival probability
1.00
0.90
0.80
0.70
0.60
0.50
0.40
0.30
0.20
0.10
0.00
raw p 0.163
bonf p 1.000
0
24
48
72
96
120
144
168
192
Follow up in months
Expression Graph
Event=No
Event=Yes
Expression
650
600
550
500
450
400
350
300
250
200
150
400
400
sorted by expression

WNT M1

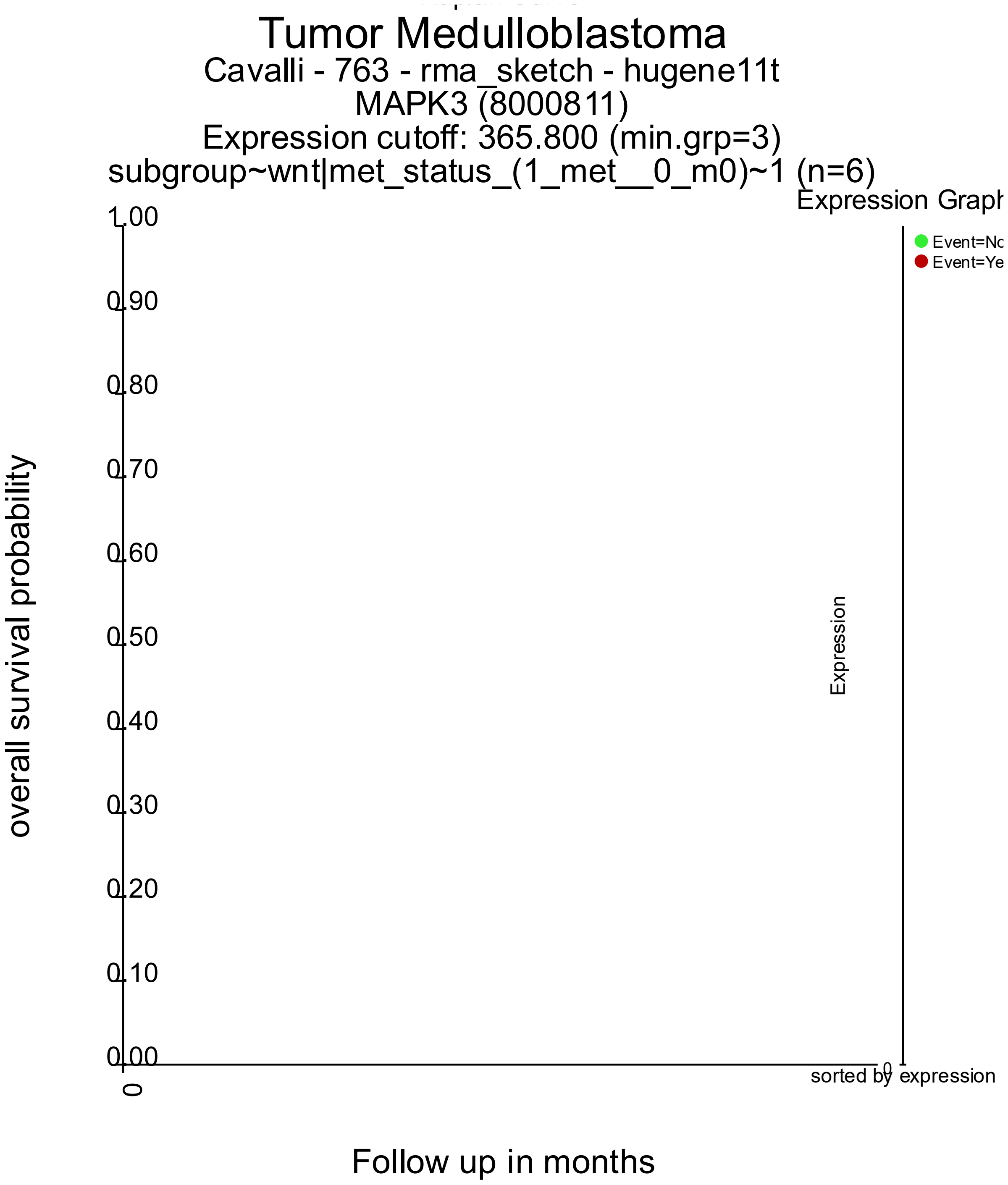

Tumor Medulloblastoma
Cavalli - 763 - rma_sketch - hugene11t
MAPK3 (8000811)
Expression cutoff: 365.800 (min.grp=3)
subgroup~wnt|met_status_(1_met__0_m0)~1 (n=6)
Expression Graph
Event=No
Event=Ye
1.00
0.90
0.80
0.70
0.60
0.50
0.40
0.30
0.20
0.10
0.00
0
overall survival probability
Follow up in months
Expression
0
sorted by expression

SHH M0

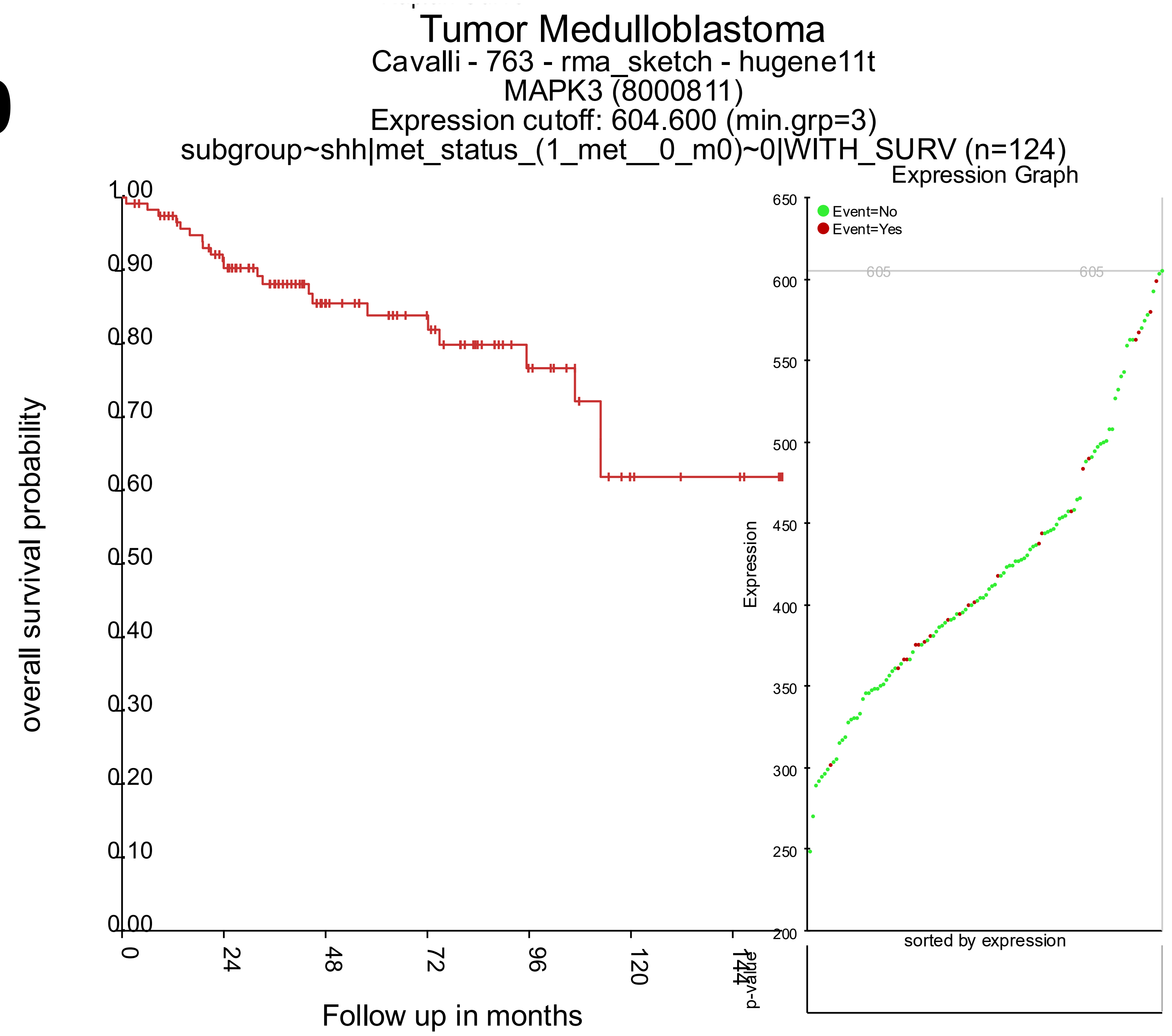

Tumor Medulloblastoma
Cavalli - 763 - rma_sketch - hugene11t
MAPK3 (8000811)
Expression cutoff: 604.600 (min.grp=3)
subgroup~shh|met_status_(1_met__0_m0)~0|WITH_SURV (n=124)
overall survival probability
1.00
0.90
0.80
0.70
0.60
0.50
0.40
0.30
0.20
0.10
0.00
0
24
48
72
96
120
144
Follow up in months
p-value
Expression Graph
Event=No
Event=Yes
605
605
Expression
650
600
550
500
450
400
350
300
250
200
sorted by expression

SHH M1

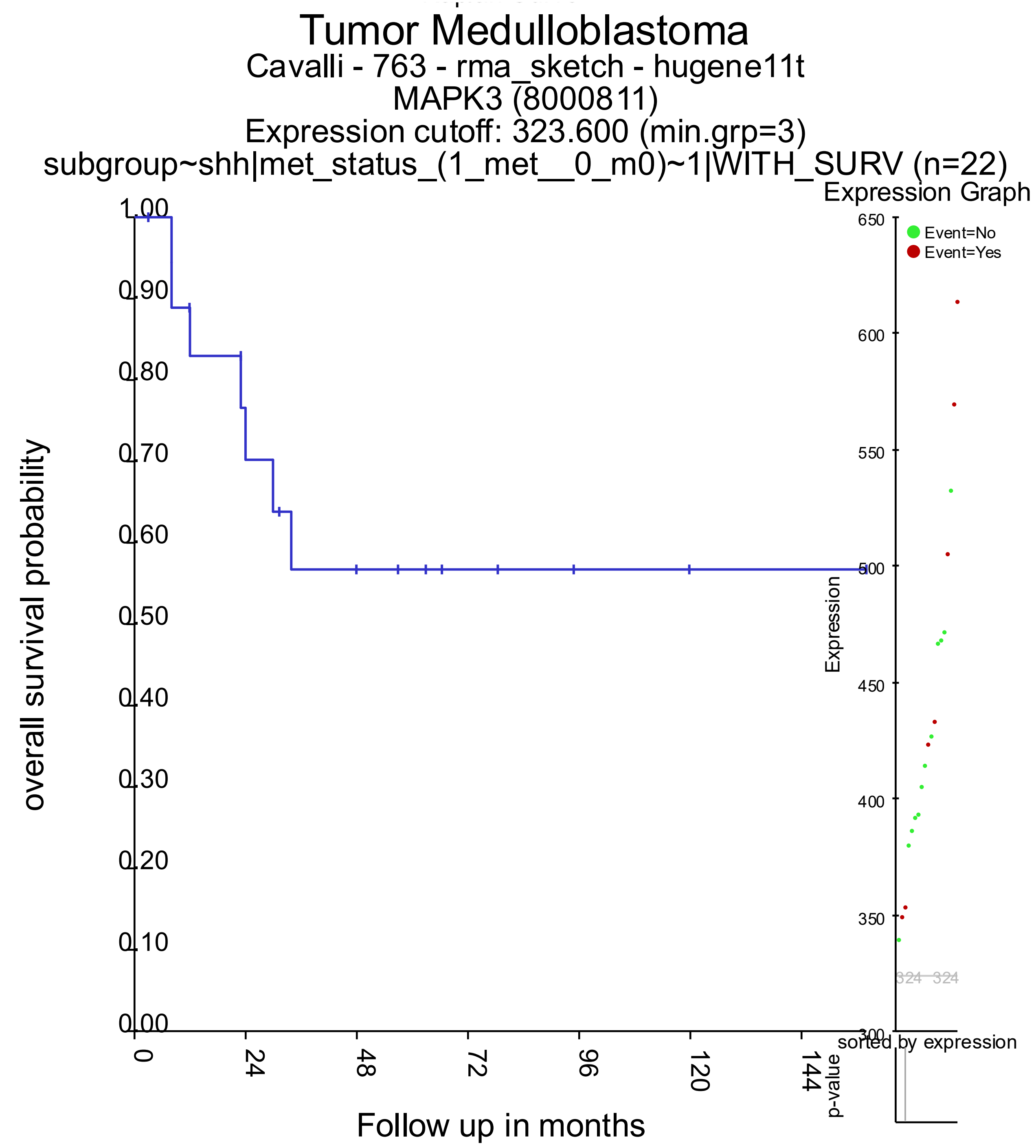

Tumor Medulloblastoma
Cavalli - 763 - rma_sketch - hugene11t
MAPK3 (8000811)
Expression cutoff: 323.600 (min.grp=3)
subgroup~shh|met_status_(1_met__0_m0)~1|WITH_SURV (n=22)
overall survival probability
1.00
0.90
0.80
0.70
0.60
0.50
0.40
0.30
0.20
0.10
0.00
0
24
48
72
96
120
144
Follow up in months
Expression Graph
Event=No
Event=Yes
650
600
550
500
450
400
350
300
Expression
324
324
sorted by expression
p-value

GROUP4 M0

Tumor Medulloblastoma
Cavalli - 763 - rma_sketch - hugene11t
MAPK3 (8000811)
Expression cutoff: 317.800 (min.grp=3)
subgroup~group4|met_status_(1_met__0_m0)~0|WITH_SURV (n=145)

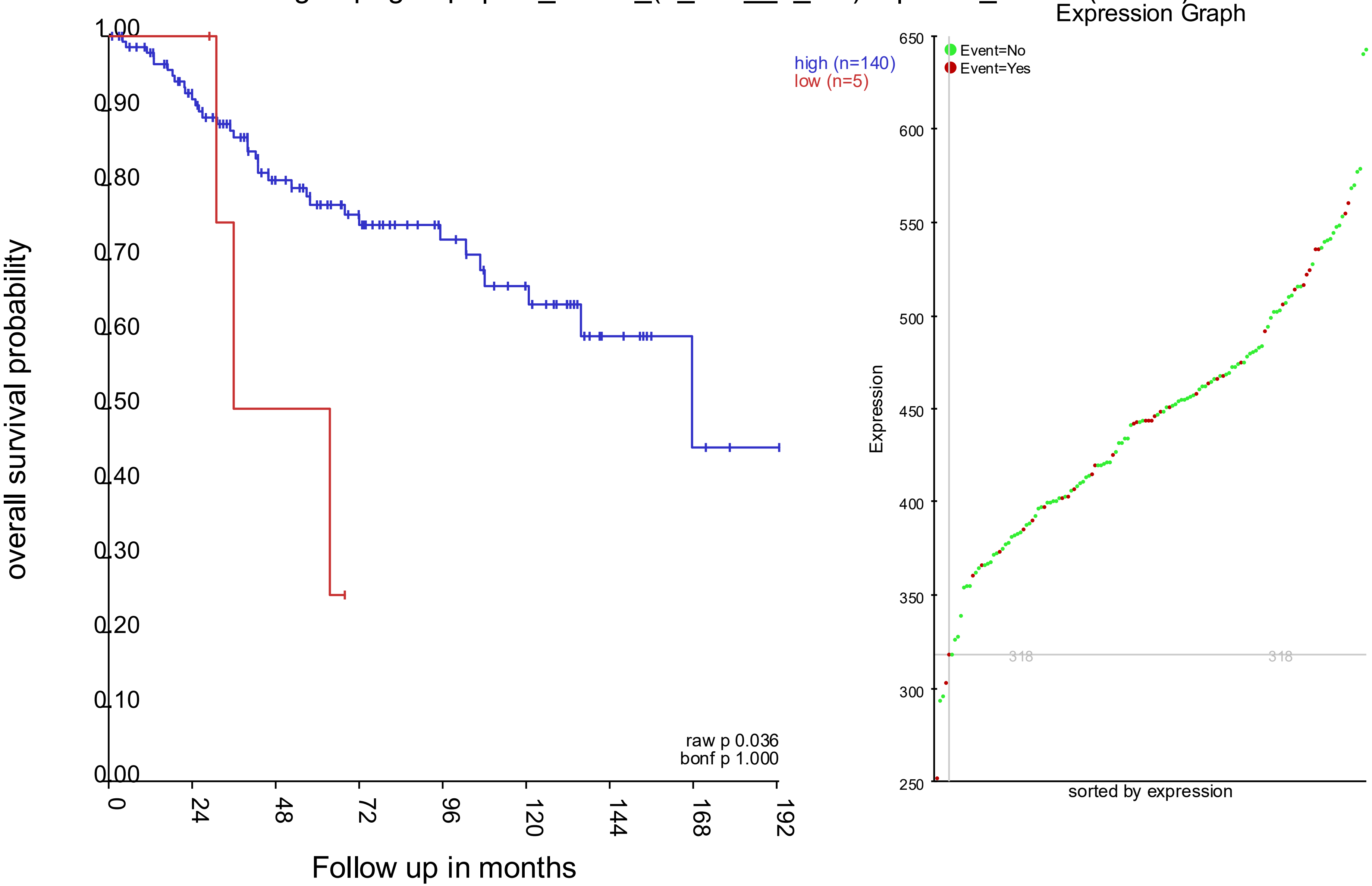

GROUP4 M1

Tumor Medulloblastoma
Cavalli - 763 - rma_sketch - hugene11t
MAPK3 (8000811)
Expression cutoff: 462.000 (min.grp=3)
subgroup~group4|met_status_(1_met__0_m0)~1|WITH_SURV (n=92)

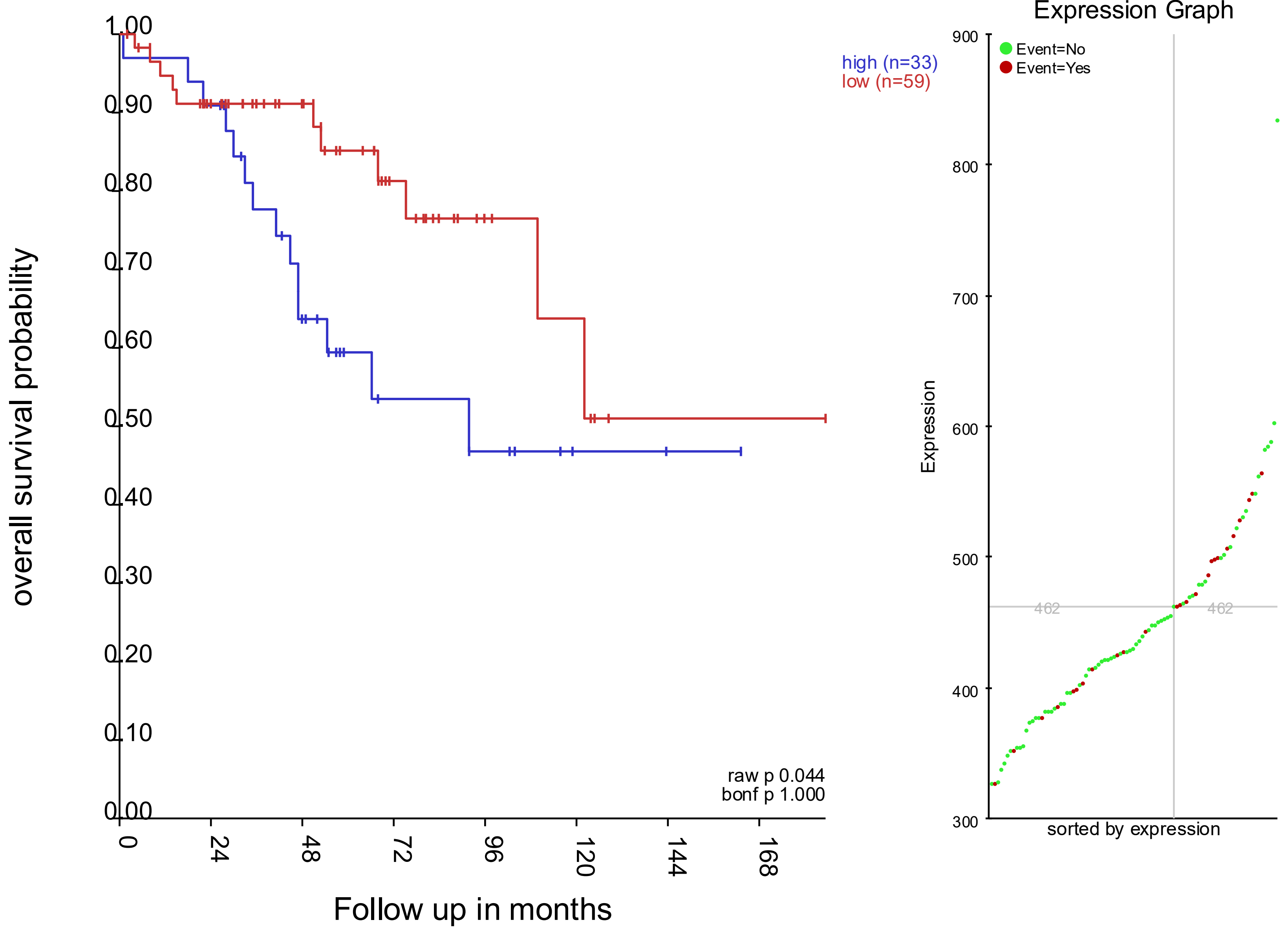

GROUP3 M0

Tumor Medulloblastoma
Cavalli - 763 - rma_sketch - hugene11t
MAPK3 (8000811)
Expression cutoff: 332.800 (min.grp=3)
subgroup~group3|met_status_(1_met__0_m0)~0|WITH_SURV (n=65)

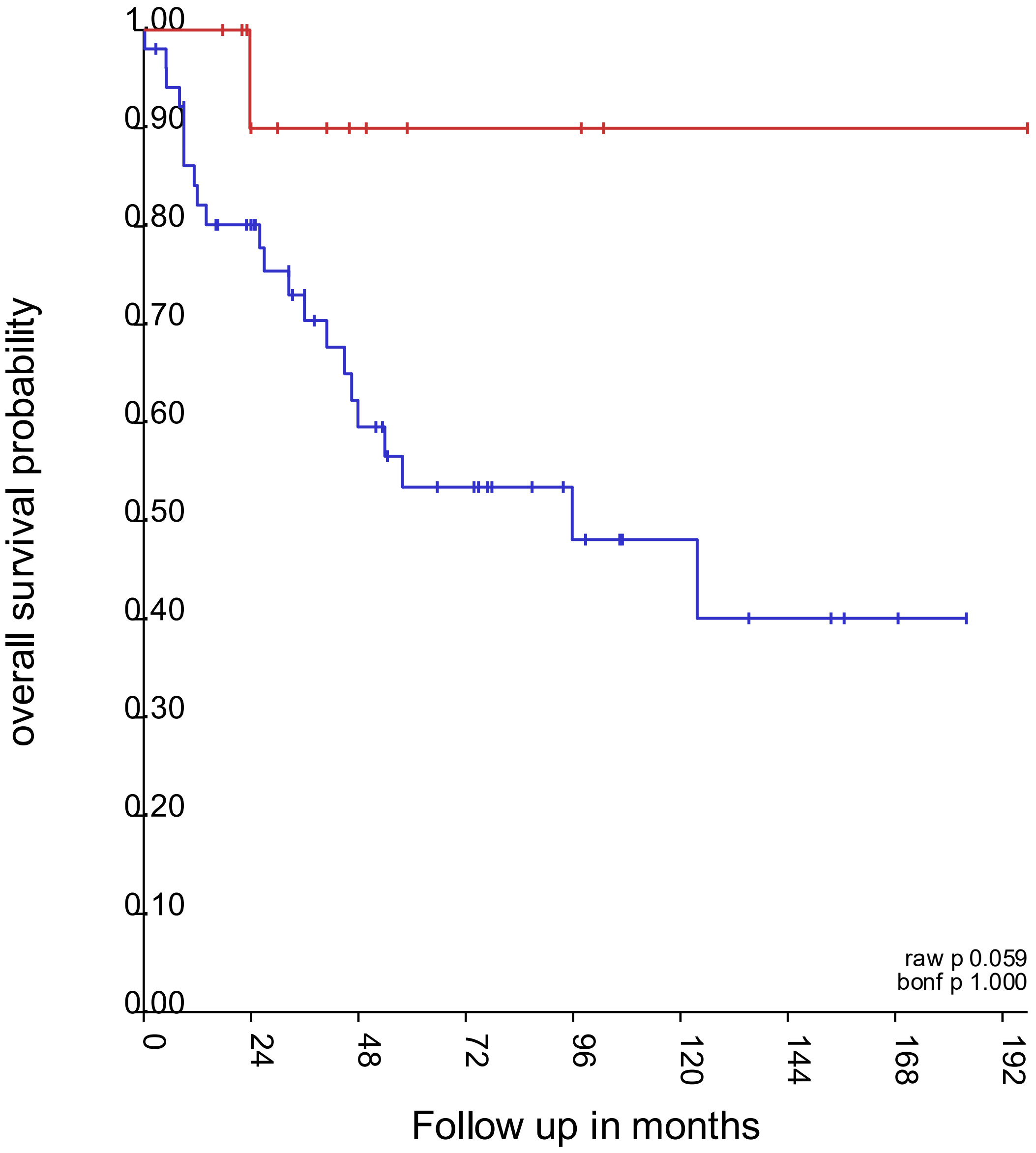

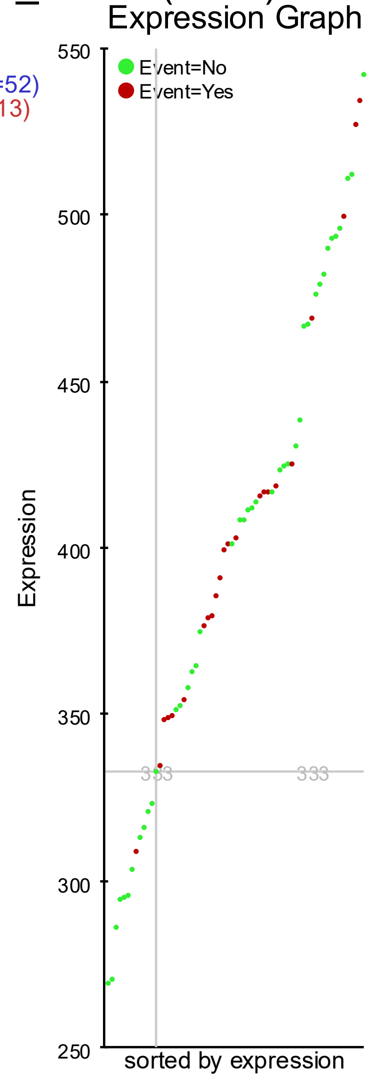

GROUP3 M1

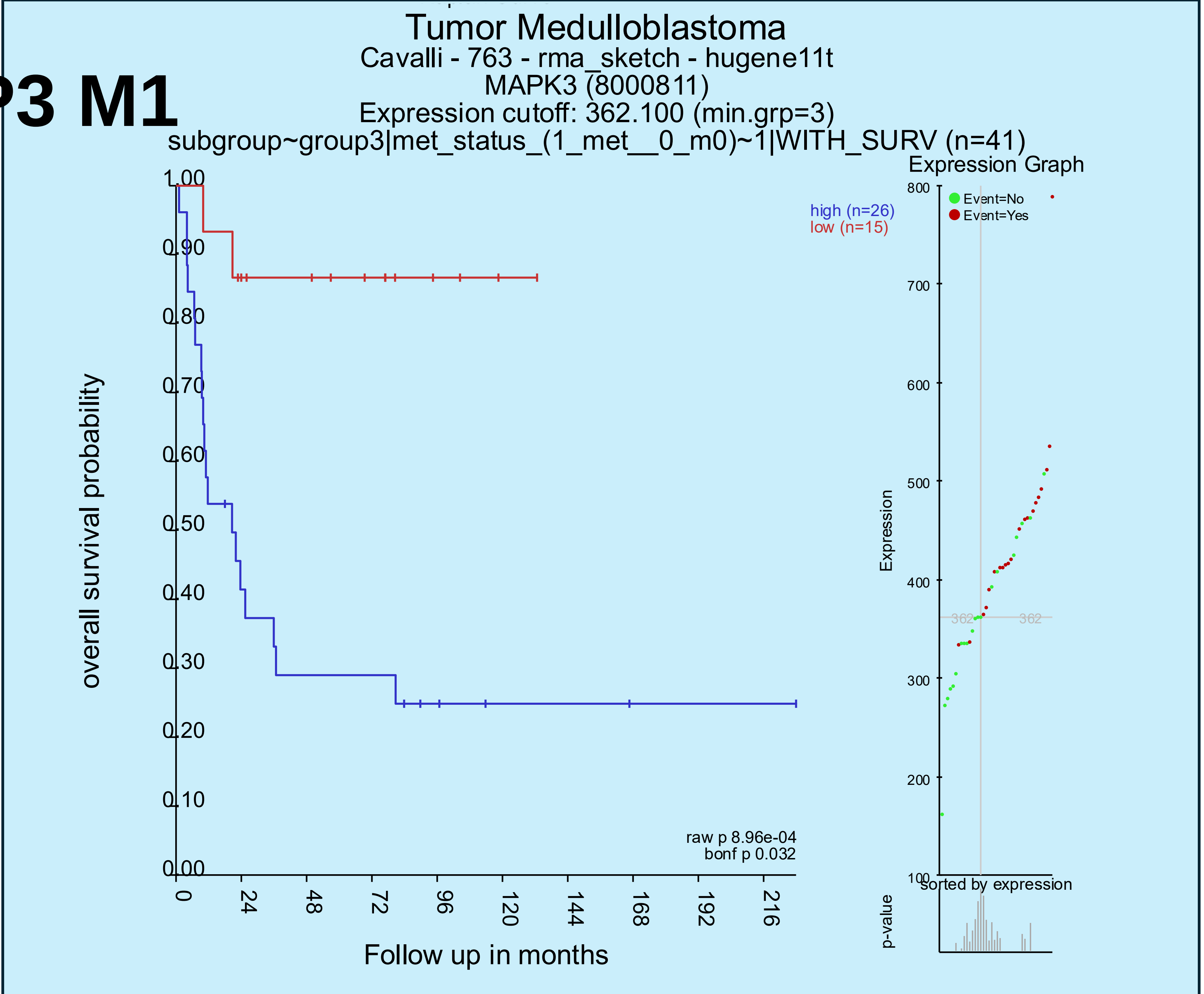
Tumor Medulloblastoma
Cavalli - 763 - rma_sketch - hugene11t
MAPK3 (8000811)
Expression cutoff: 362.100 (min.grp=3)
subgroup~group3|met_status_(1_met__0_m0)~1|WITH_SURV (n=41)
overall survival probability
1.00
0.90
0.80
0.70
0.60
0.50
0.40
0.30
0.20
0.10
0.00
high (n=26)
low (n=15)
raw p 8.96e-04
bonf p 0.032
0
24
48
72
96
120
144
168
192
216
Follow up in months
Expression Graph
Event=No
Event=Yes
Expression
800
700
600
500
400
300
200
100
362
362
sorted by expression
p-value

MET

WNT M0

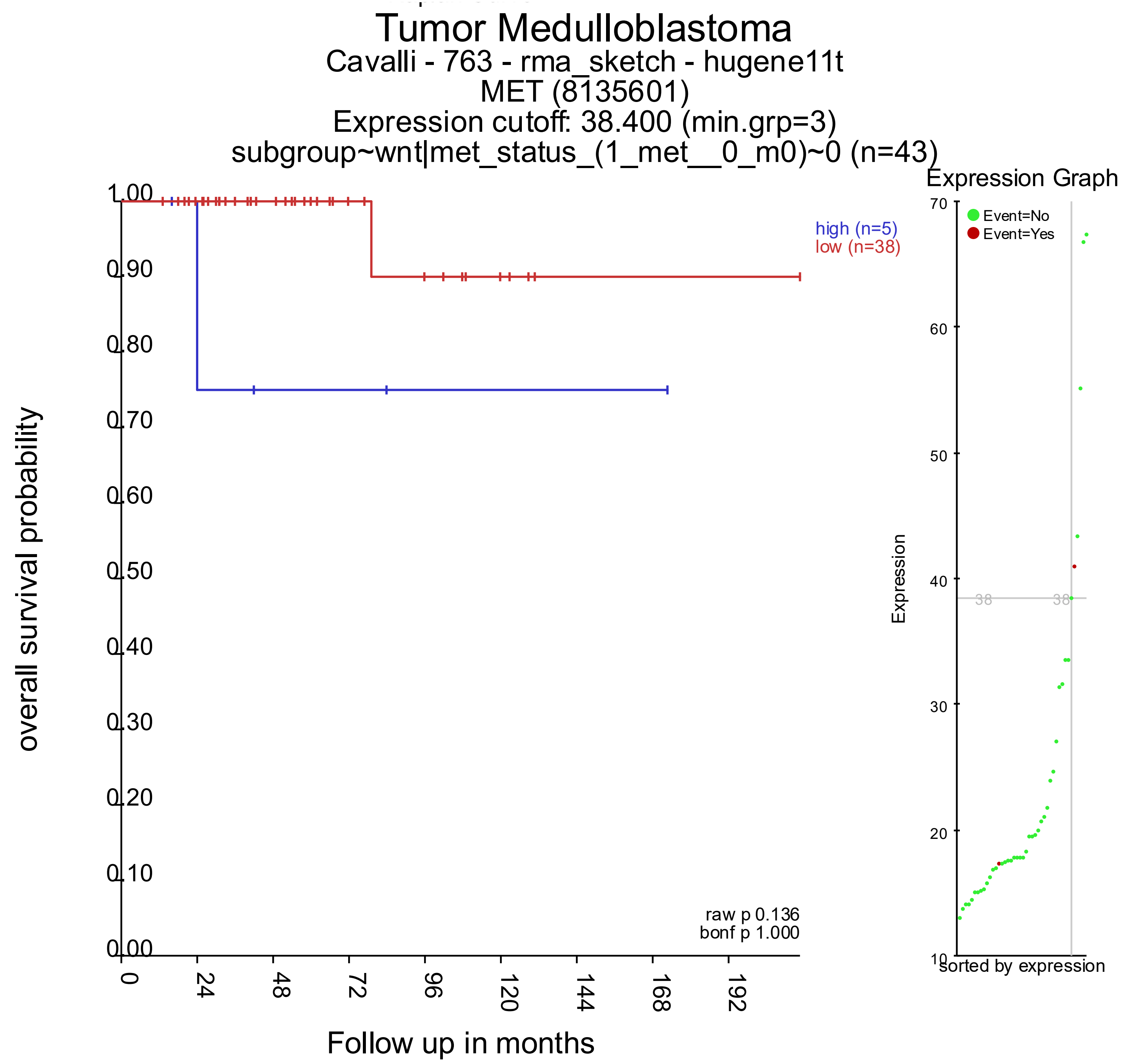

Tumor Medulloblastoma
Cavalli - 763 - rma_sketch - hugene11t
MET (8135601)
Expression cutoff: 38.400 (min.grp=3)
subgroup~wnt|met_status_(1_met__0_m0)~0 (n=43)
overall survival probability
1.00
0.90
0.80
0.70
0.60
0.50
0.40
0.30
0.20
0.10
0.00
0
24
48
72
96
120
144
168
192
Follow up in months
high (n=5)
low (n=38)
raw p 0.136
bonf p 1.000
Expression Graph
Event=No
Event=Yes
Expression
70
60
50
40
30
20
10
38
38
sorted by expression

WNT M1

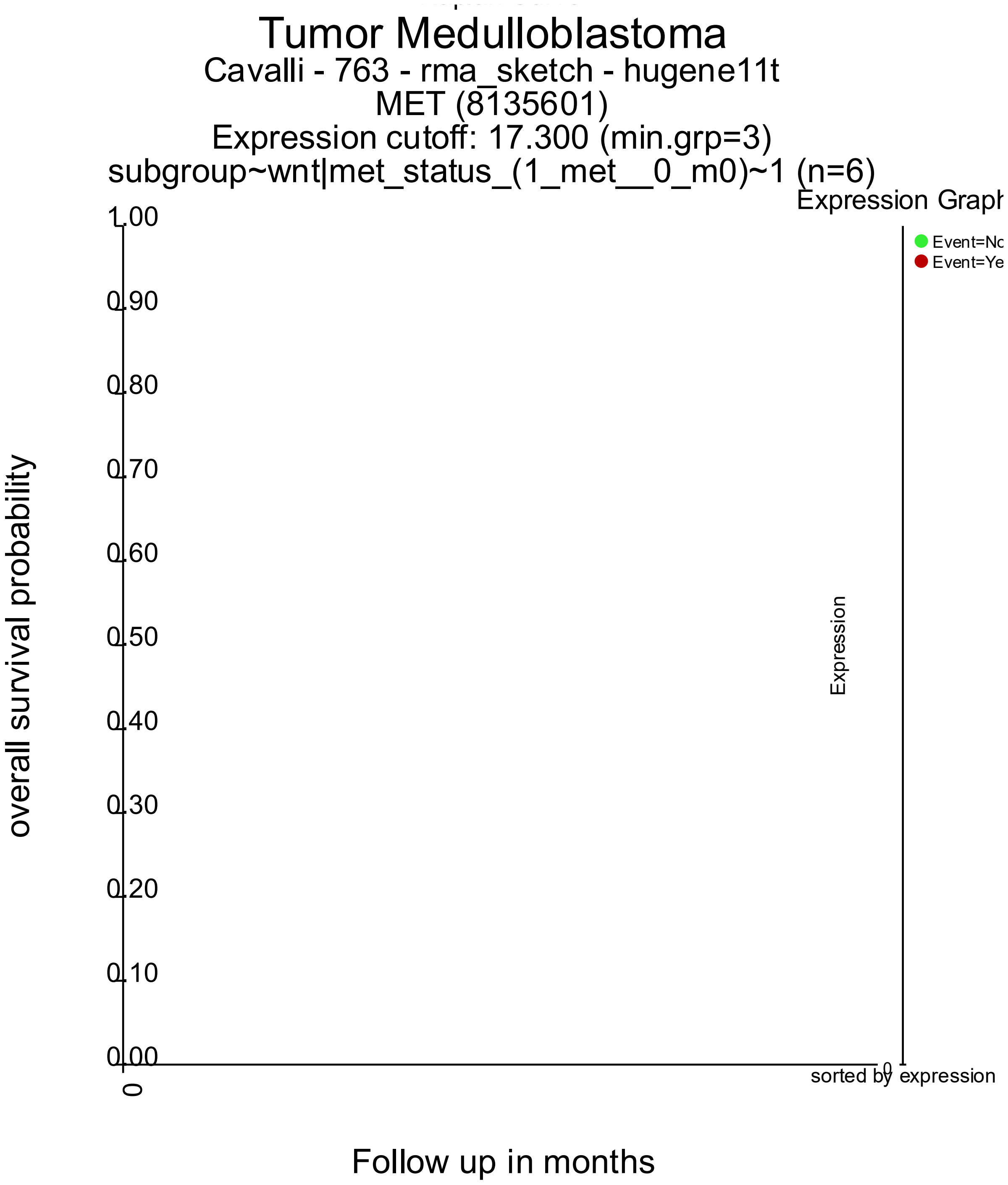

Tumor Medulloblastoma
Cavalli - 763 - rma_sketch - hugene11t
MET (8135601)
Expression cutoff: 17.300 (min.grp=3)
subgroup~wnt|met_status_(1_met__0_m0)~1 (n=6)
Expression Graph
Event=No
Event=Ye
overall survival probability
1.00
0.90
0.80
0.70
0.60
0.50
0.40
0.30
0.20
0.10
0.00
0
Follow up in months
Expression
0
sorted by expression

SHH M0

Tumor Medulloblastoma
Cavalli - 763 - rma_sketch - hugene11t
MET (8135601)
Expression cutoff: 358.800 (min.grp=3)
subgroup~shh|met_status_(1_met__0_m0)~0|WITH_SURV (n=124)

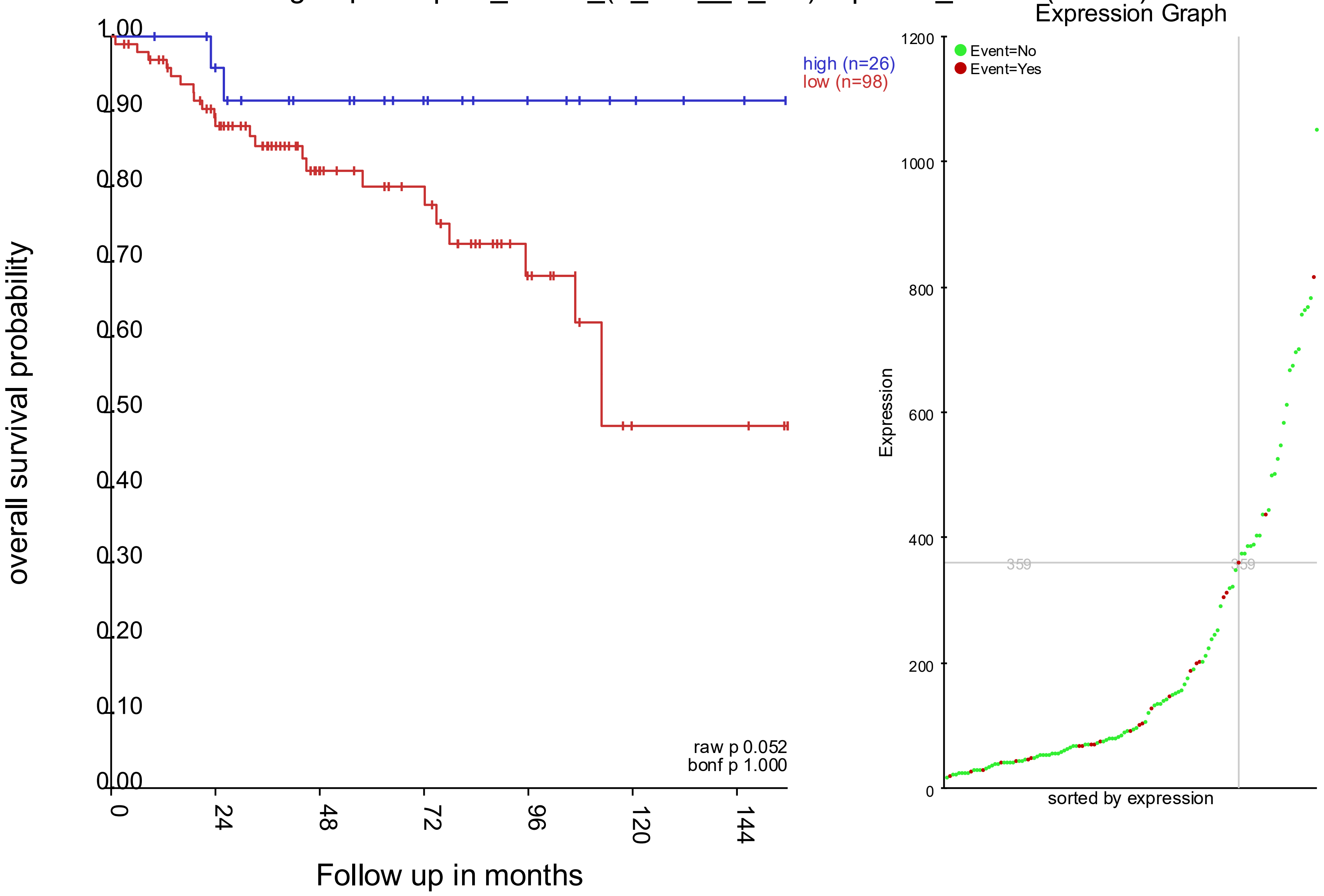

SHH M1

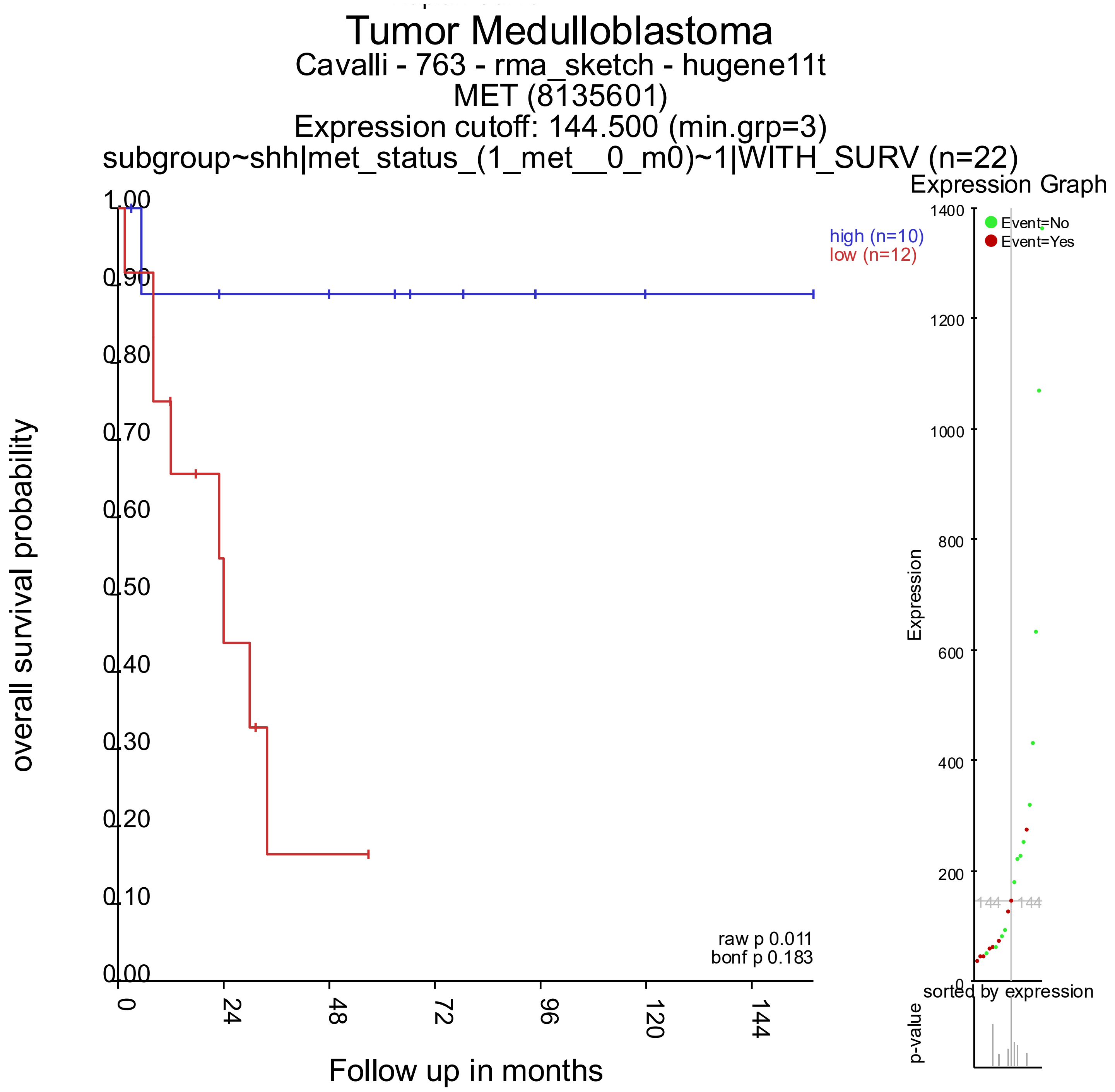

Tumor Medulloblastoma
Cavalli - 763 - rma_sketch - hugene11t
MET (8135601)
Expression cutoff: 144.500 (min.grp=3)
subgroup~shh|met_status_(1_met__0_m0)~1|WITH_SURV (n=22)
high (n=10)
low (n=12)
overall survival probability
1.00
0.90
0.80
0.70
0.60
0.50
0.40
0.30
0.20
0.10
0.00
0
24
48
72
96
120
144
Follow up in months
raw p 0.011
bonf p 0.183
Expression Graph
Event=No
Event=Yes
Expression
1400
1200
1000
800
600
400
200
0
144
sorted by expression
p-value

GROUP4 M0

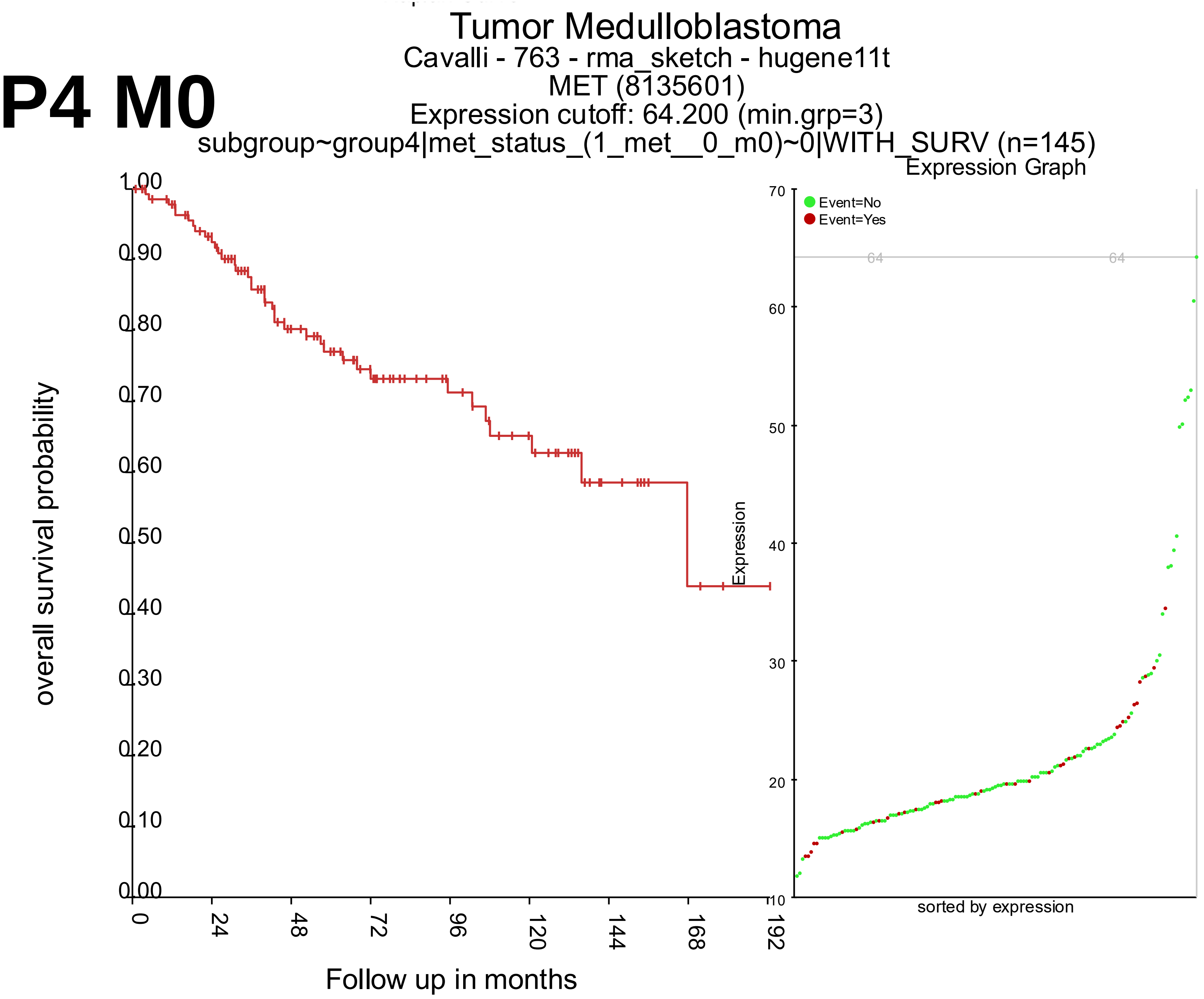
Tumor Medulloblastoma
Cavalli - 763 - rma_sketch - hugene11t
MET (8135601)
Expression cutoff: 64.200 (min.grp=3)
subgroup~group4|met_status_(1_met__0_m0)~0|WITH_SURV (n=145)
overall survival probability
1.00
0.90
0.80
0.70
0.60
0.50
0.40
0.30
0.20
0.10
0.00
0
24
48
72
96
120
144
168
192
Follow up in months
Expression Graph
Event=No
Event=Yes
70
60
50
40
30
20
10
64
64
Expression
sorted by expression

GROUP4 M1

Tumor Medulloblastoma
Cavalli - 763 - rma_sketch - hugene11t
MET (8135601)
Expression cutoff: 44.500 (min.grp=3)
subgroup~group4|met_status_(1_met__0_m0)~1|WITH_SURV (n=92)

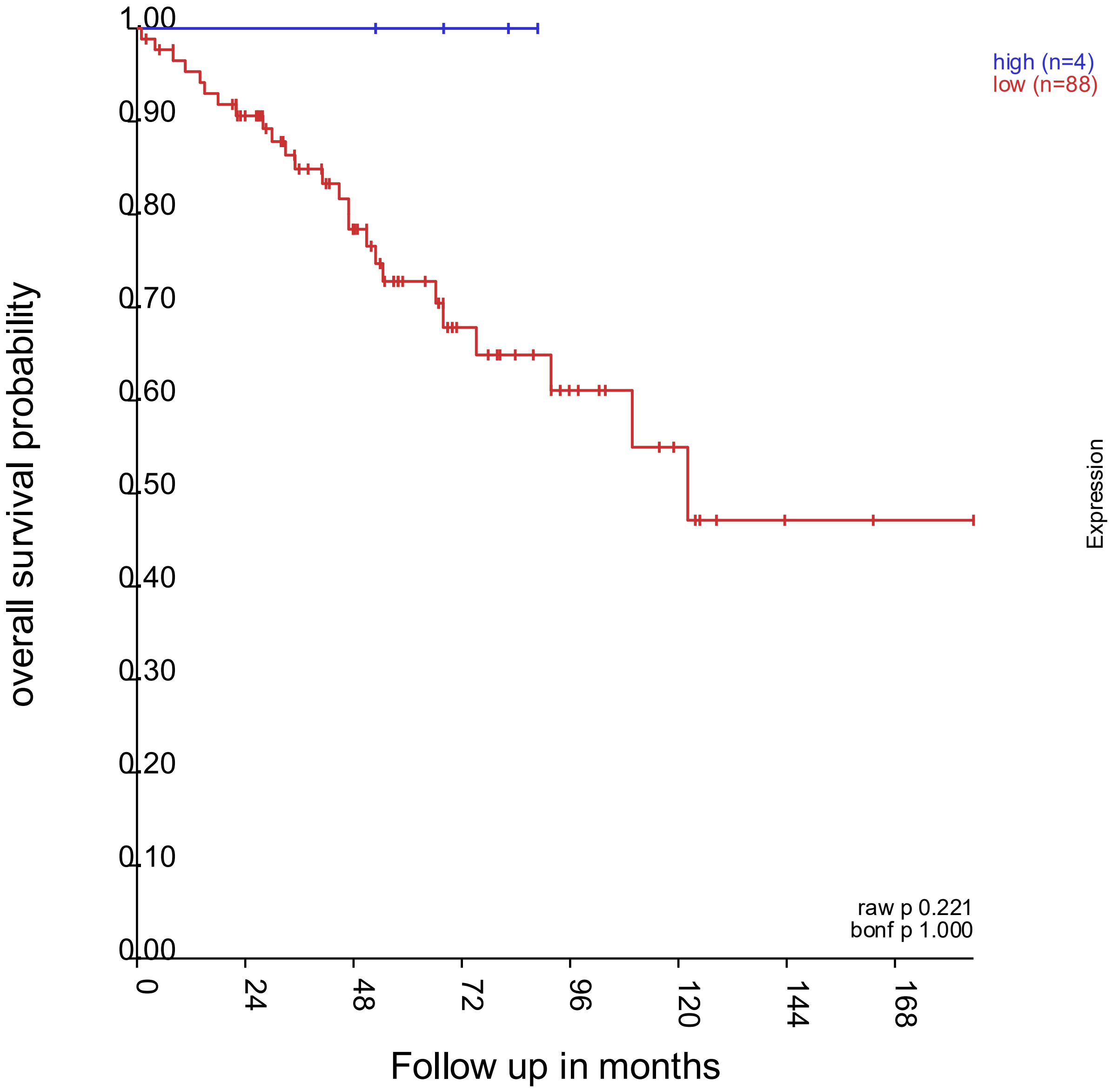

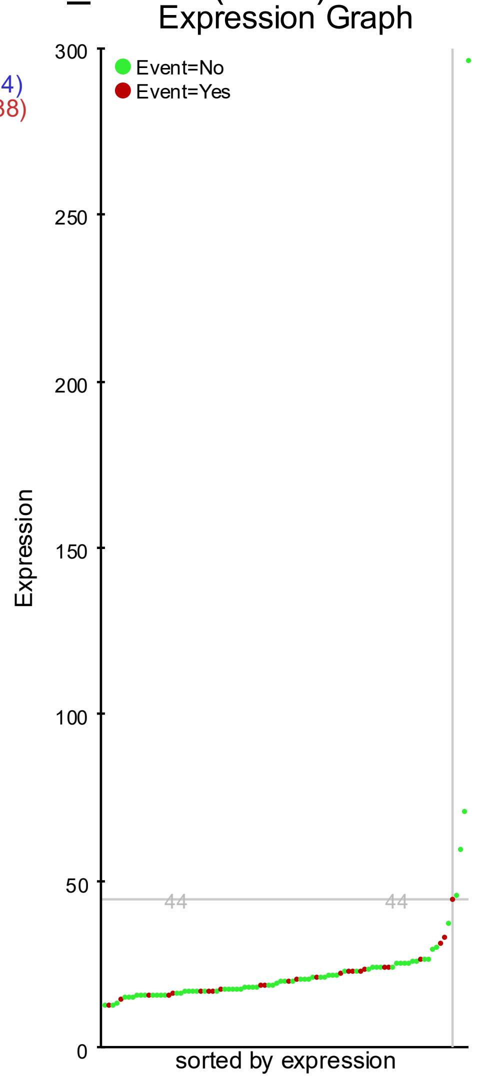

GROUP3 M0

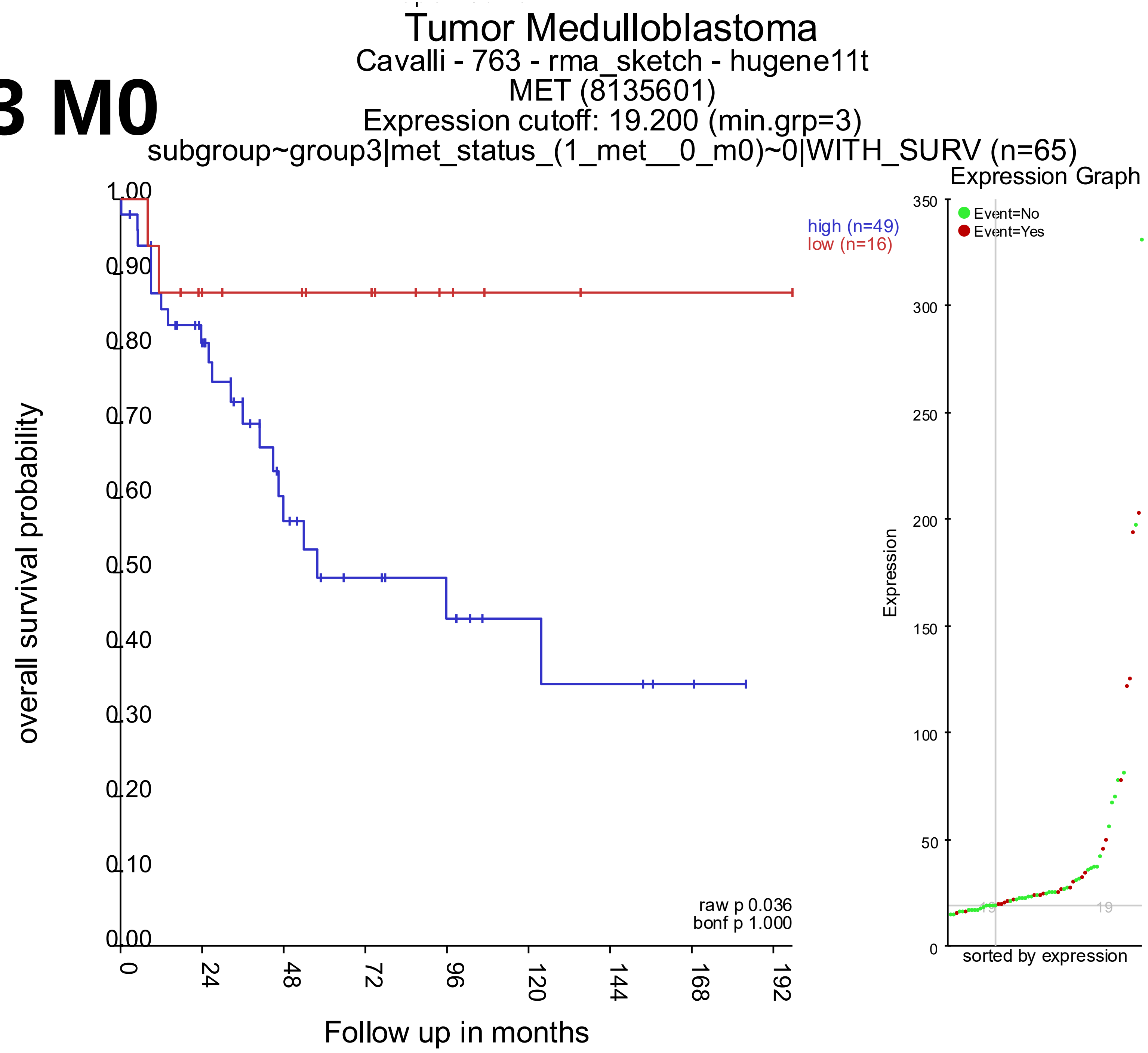

Tumor Medulloblastoma
Cavalli - 763 - rma_sketch - hugene11t
MET (8135601)
Expression cutoff: 19.200 (min.grp=3)
subgroup~group3|met_status_(1_met__0_m0)~0|WITH_SURV (n=65)
overall survival probability
1.00
0.90
0.80
0.70
0.60
0.50
0.40
0.30
0.20
0.10
0.00
0
24
48
72
96
120
144
168
192
Follow up in months
high (n=49)
low (n=16)
raw p 0.036
bonf p 1.000
Expression Graph
Event=No
Event=Yes
Expression
350
300
250
200
150
100
50
0
sorted by expression

GROUP3 M1

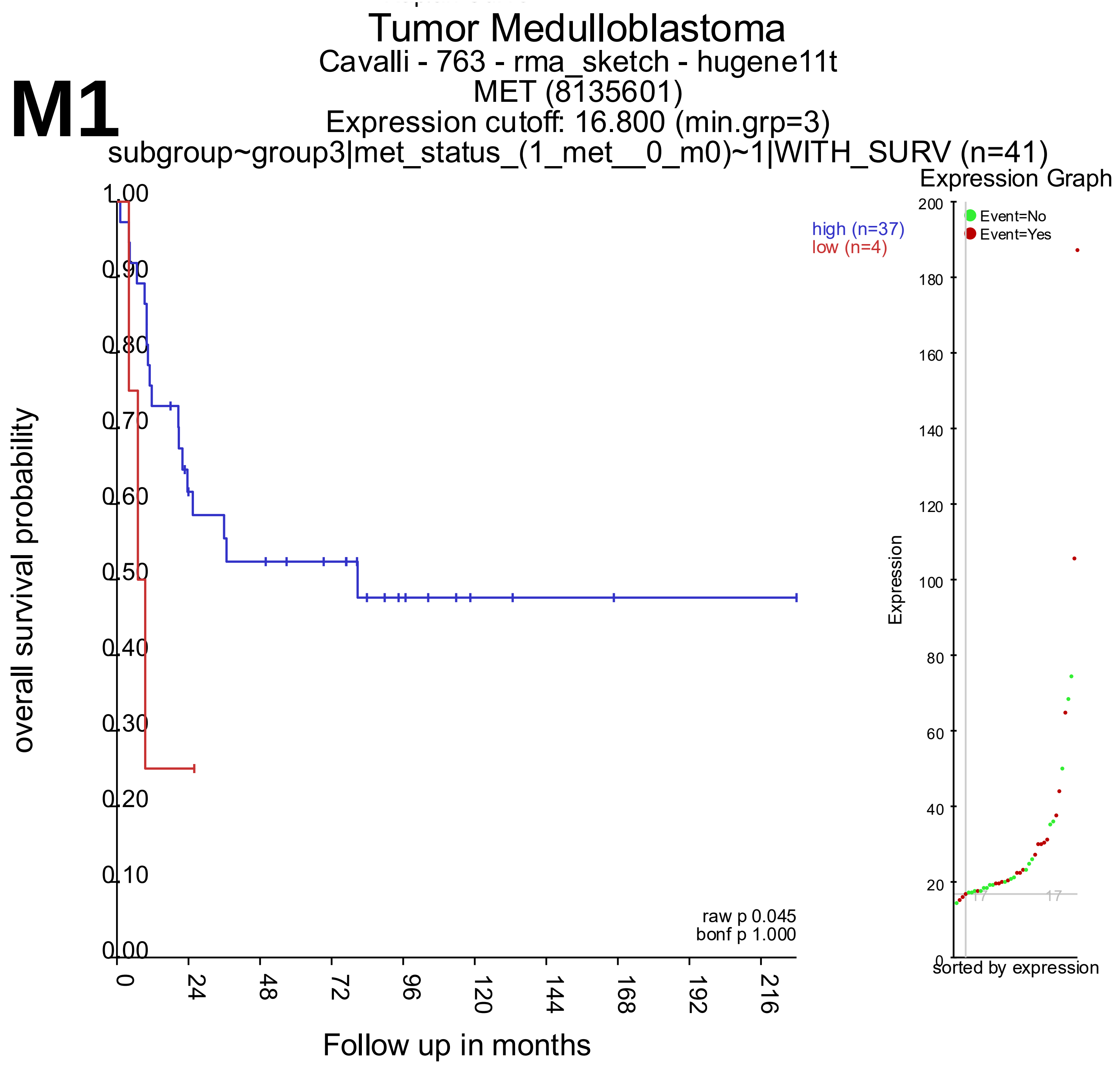

Tumor Medulloblastoma
Cavalli - 763 - rma_sketch - hugene11t
MET (8135601)
Expression cutoff: 16.800 (min.grp=3)
subgroup~group3|met_status_(1_met__0_m0)~1|WITH_SURV (n=41)
overall survival probability
1.00
0.90
0.80
0.70
0.60
0.50
0.40
0.30
0.20
0.10
0.00
high (n=37)
low (n=4)
raw p 0.045
bonf p 1.000
0
24
48
72
96
120
144
168
192
216
Follow up in months
Expression Graph
Event=No
Event=Yes
Expression
200
180
160
140
120
100
80
60
40
20
0
17
sorted by expression

MTOR

WNT M0

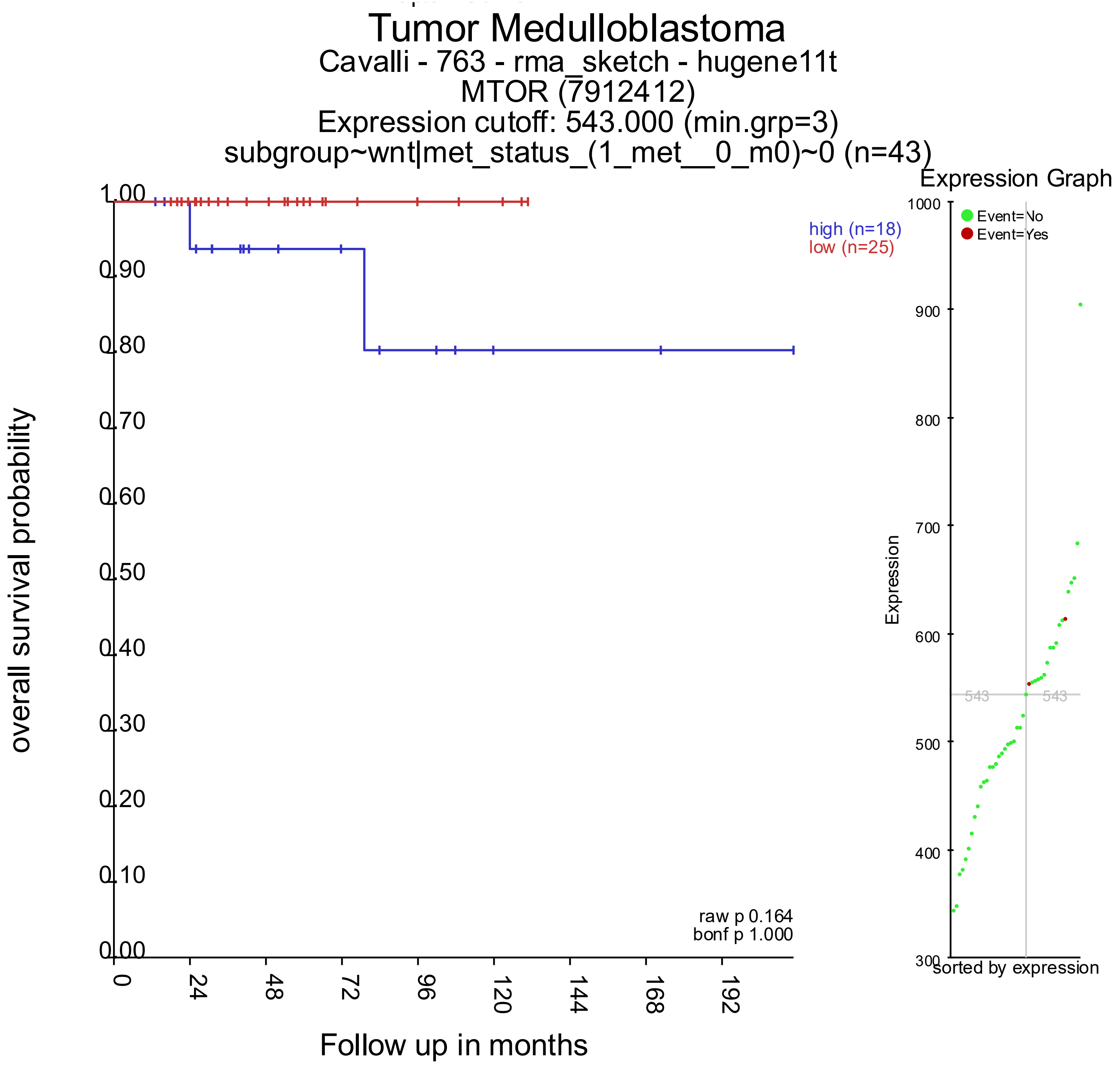

Tumor Medulloblastoma
Cavalli - 763 - rma_sketch - hugene11t
MTOR (7912412)
Expression cutoff: 543.000 (min.grp=3)
subgroup~wnt|met_status_(1_met__0_m0)~0 (n=43)
high (n=18)
low (n=25)
overall survival probability
1.00
0.90
0.80
0.70
0.60
0.50
0.40
0.30
0.20
0.10
0.00
0
24
48
72
96
120
144
168
192
Follow up in months
raw p 0.164
bonf p 1.000
Expression Graph
Event=No
Event=Yes
Expression
1000
900
800
700
600
500
400
300
543
543
sorted by expression

WNT M1

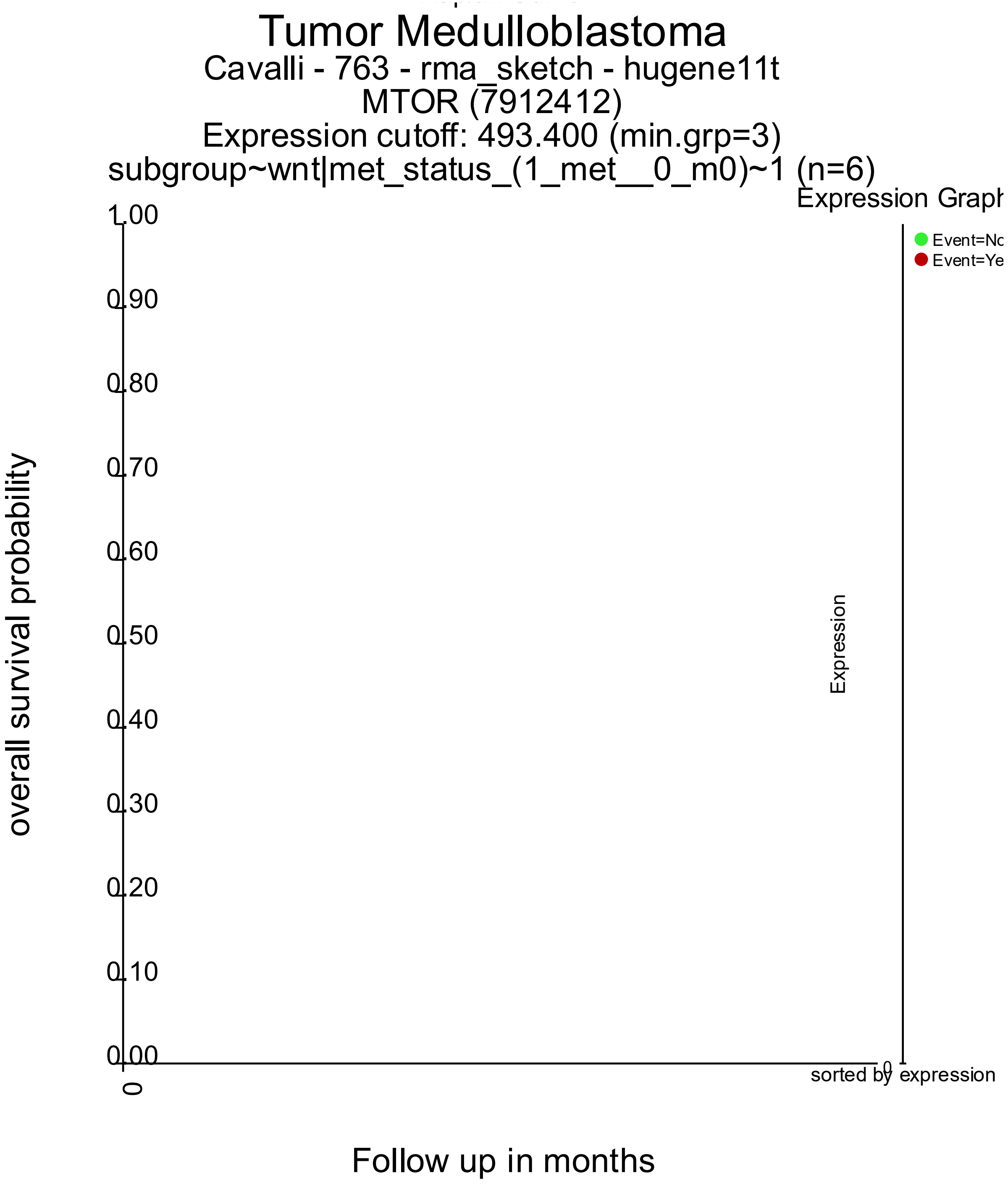

Tumor Medulloblastoma
Cavalli - 763 - rma_sketch - hugene11t
MTOR (7912412)
Expression cutoff: 493.400 (min.grp=3)
subgroup~wnt|met_status_(1_met__0_m0)~1 (n=6)
overall survival probability
1.00
0.90
0.80
0.70
0.60
0.50
0.40
0.30
0.20
0.10
0.00
0
Follow up in months
Expression Graph
Event=No
Event=Yes
Expression
0
sorted by expression

SHH M0

Tumor Medulloblastoma
Cavalli - 763 - rma_sketch - hugene11t
MTOR (7912412)
Expression cutoff: 523.800 (min.grp=3)
subgroup~shh|met_status_(1_met__0_m0)~0|WITH_SURV (n=124)

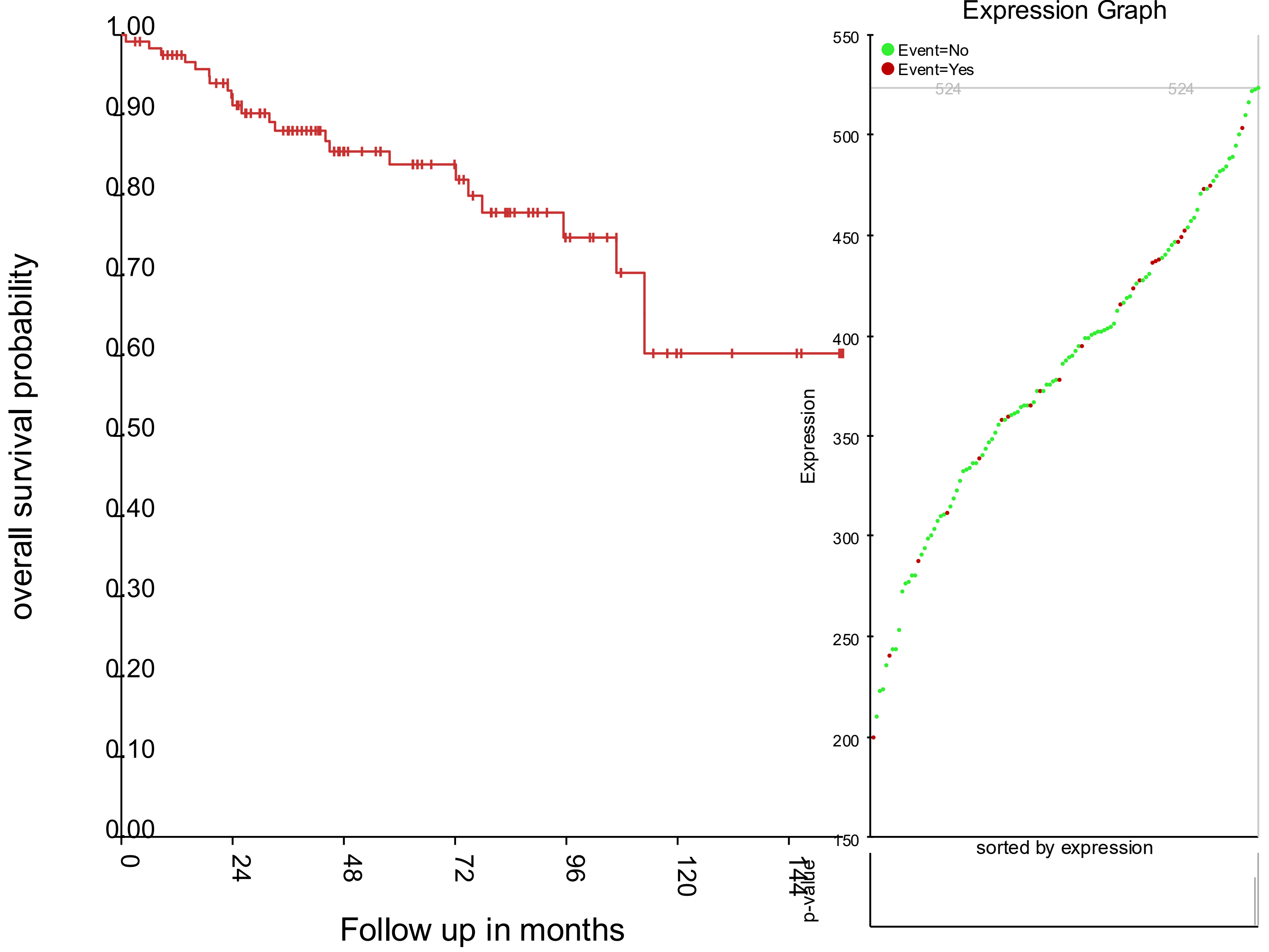

SHH M1

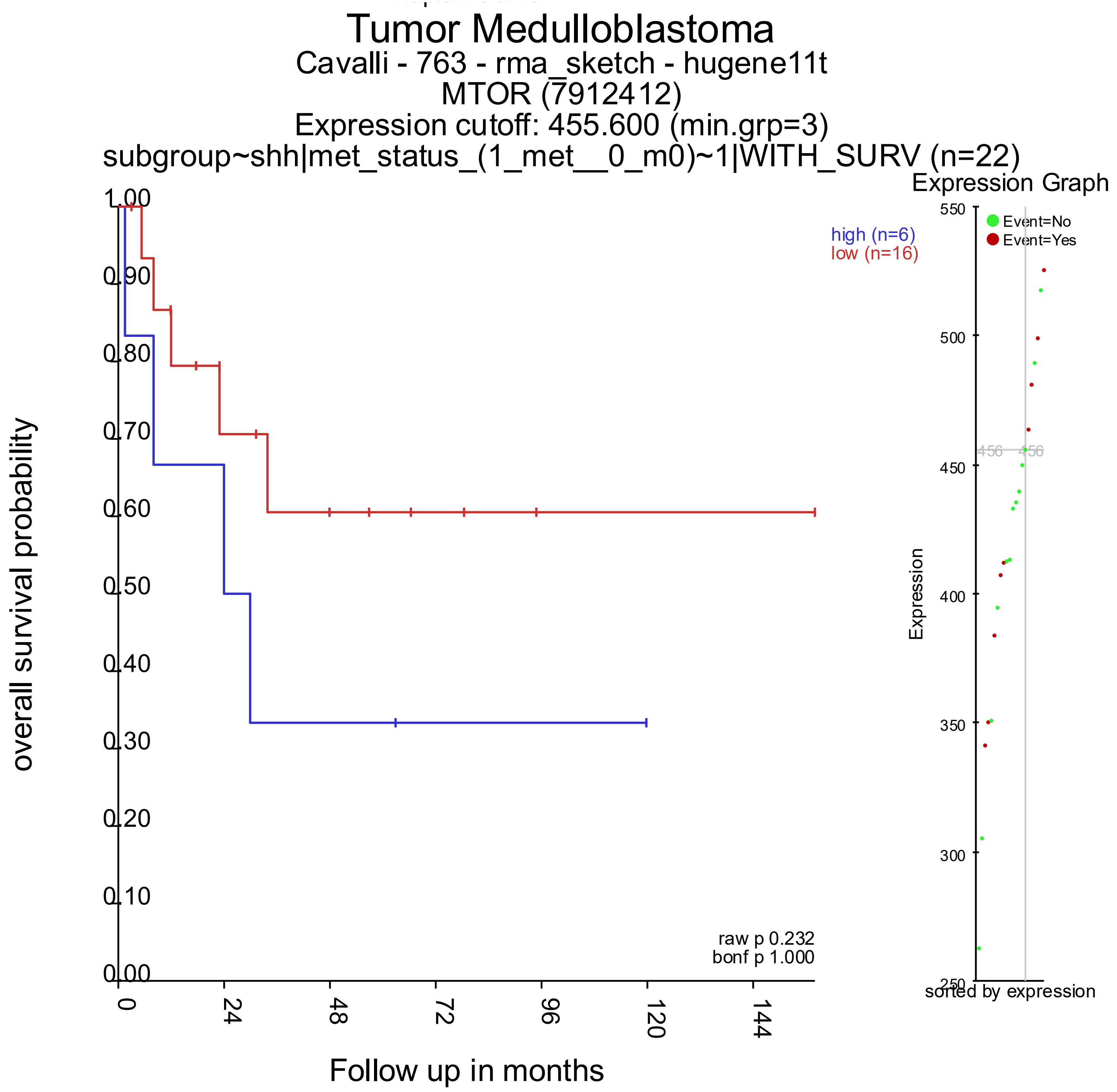

Tumor Medulloblastoma
Cavalli - 763 - rma_sketch - hugene11t
MTOR (7912412)
Expression cutoff: 455.600 (min.grp=3)
subgroup~shh|met_status_(1_met__0_m0)~1|WITH_SURV (n=22)
overall survival probability
1.00
0.90
0.80
0.70
0.60
0.50
0.40
0.30
0.20
0.10
0.00
high (n=6)
low (n=16)
raw p 0.232
bonf p 1.000
0
24
48
72
96
120
144
Follow up in months
Expression Graph
Event=No
Event=Yes
550
500
450
400
350
300
250
456
456
Expression
sorted by expression

GROUP4 M0

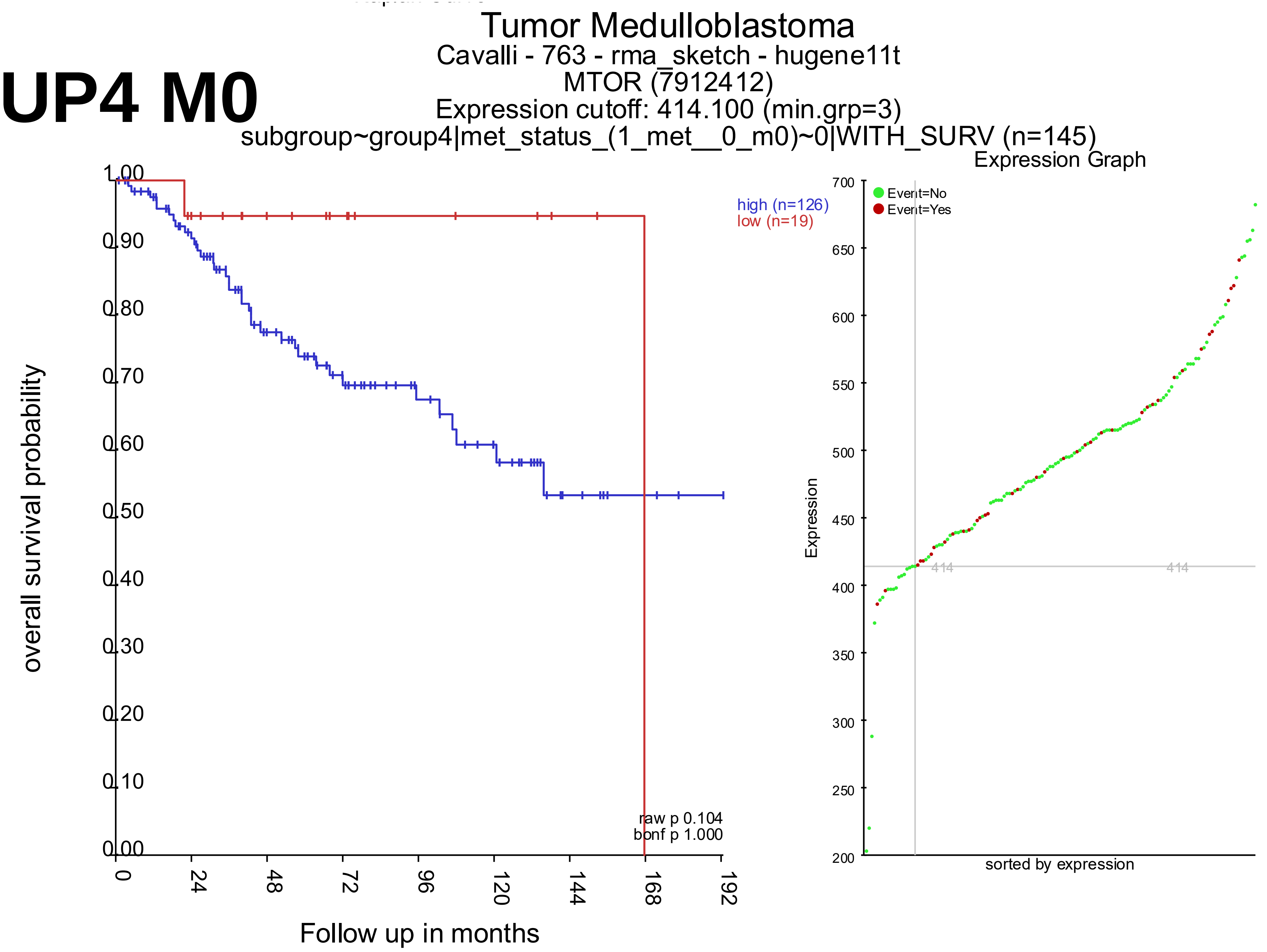
Tumor Medulloblastoma
Cavalli - 763 - rma_sketch - hugene11t
MTOR (7912412)
Expression cutoff: 414.100 (min.grp=3)
subgroup~group4|met_status_(1_met__0_m0)~0|WITH_SURV (n=145)
overall survival probability
1.00
0.90
0.80
0.70
0.60
0.50
0.40
0.30
0.20
0.10
0.00
0
24
48
72
96
120
144
168
192
Follow up in months
high (n=126)
low (n=19)
raw p 0.104
bonf p 1.000
Expression Graph
Event=No
Event=Yes
Expression
700
650
600
550
500
450
400
350
300
250
200
414
414
sorted by expression

GROUP4 M1

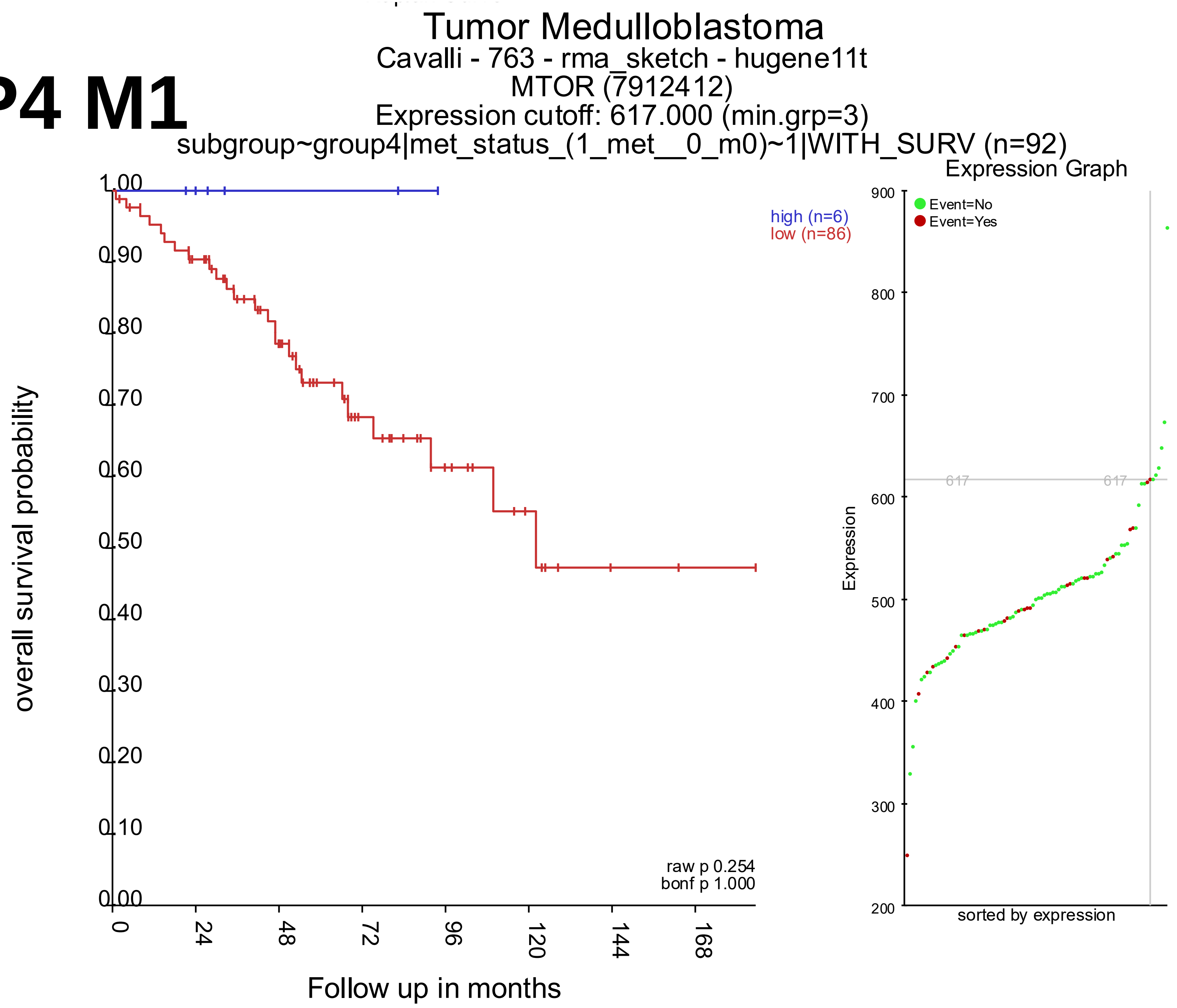

Tumor Medulloblastoma
Cavalli - 763 - rma_sketch - hugene11t
MTOR (7912412)
Expression cutoff: 617.000 (min.grp=3)
subgroup~group4|met_status_(1_met__0_m0)~1|WITH_SURV (n=92)
overall survival probability
1.00
0.90
0.80
0.70
0.60
0.50
0.40
0.30
0.20
0.10
0.00
high (n=6)
low (n=86)
raw p 0.254
bonf p 1.000
0
24
48
72
96
120
144
168
Follow up in months
Expression Graph
Event=No
Event=Yes
900
800
700
600
500
400
300
200
617
617
Expression
sorted by expression

GROUP3 M0

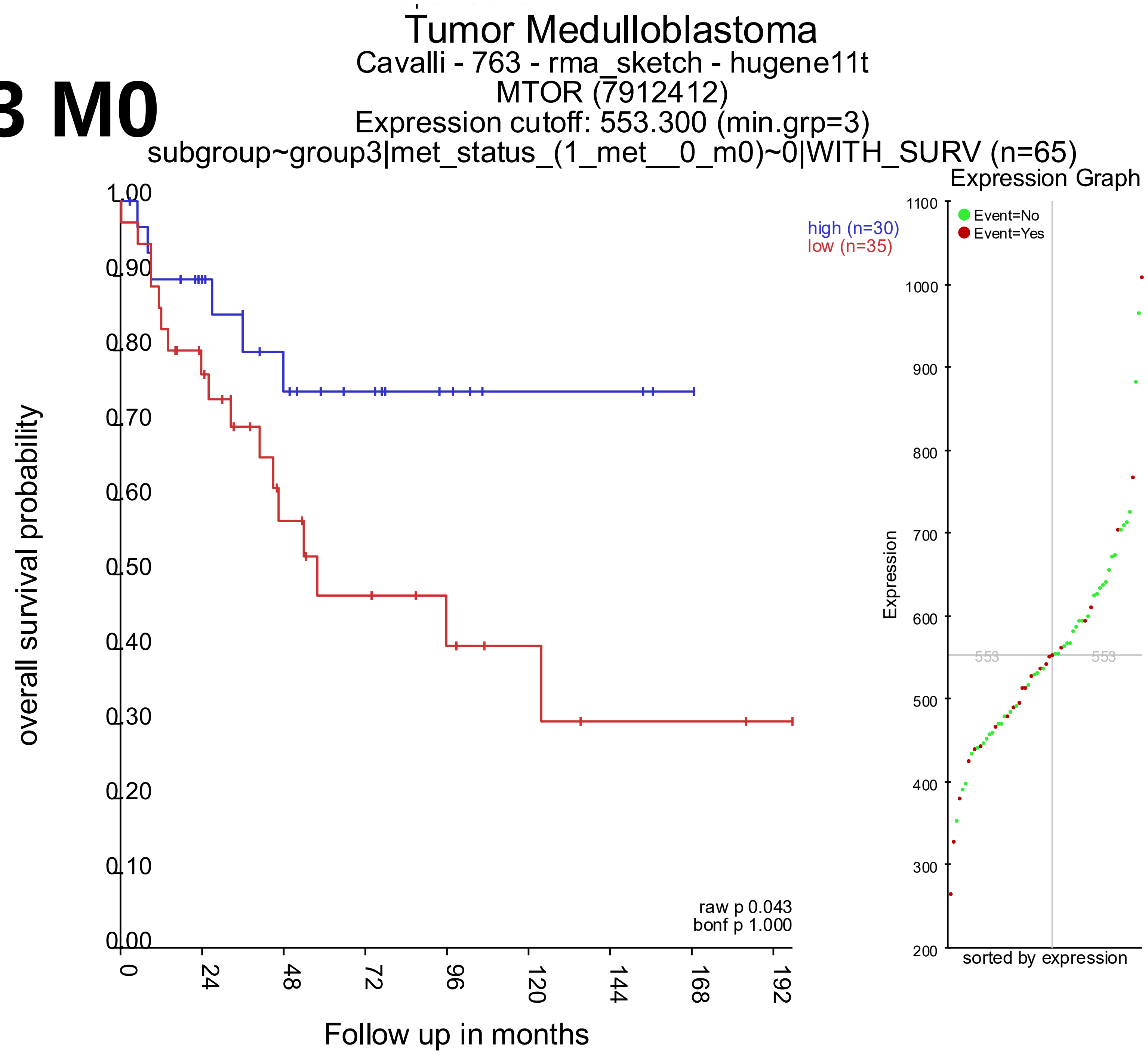

Tumor Medulloblastoma
Cavalli - 763 - rma_sketch - hugene11t
MTOR (7912412)
Expression cutoff: 553.300 (min.grp=3)
subgroup~group3|met_status_(1_met__0_m0)~0|WITH_SURV (n=65)
overall survival probability
1.00
0.90
0.80
0.70
0.60
0.50
0.40
0.30
0.20
0.10
0.00
0
24
48
72
96
120
144
168
192
Follow up in months
high (n=30)
low (n=35)
raw p 0.043
bonf p 1.000
Expression Graph
Event=No
Event=Yes
1100
1000
900
800
700
600
500
400
300
200
Expression
553
553
sorted by expression

GROUP3 M1

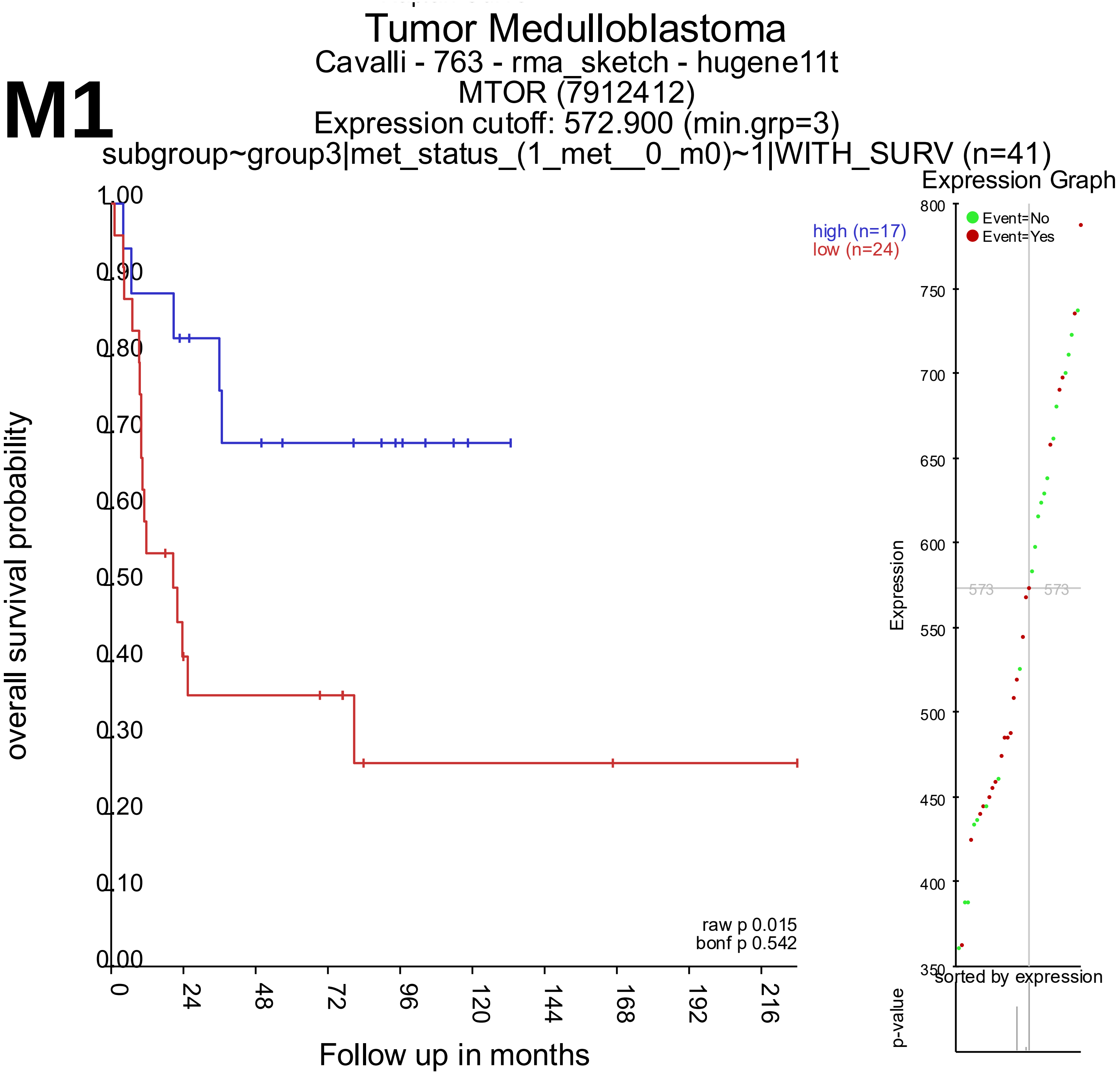

Tumor Medulloblastoma
Cavalli - 763 - rma_sketch - hugene11t
MTOR (7912412)
Expression cutoff: 572.900 (min.grp=3)
subgroup~group3|met_status_(1_met__0_m0)~1|WITH_SURV (n=41)
overall survival probability
1.00
0.90
0.80
0.70
0.60
0.50
0.40
0.30
0.20
0.10
0.00
0
24
48
72
96
120
144
168
192
216
Follow up in months
high (n=17)
low (n=24)
raw p 0.015
bonf p 0.542
Expression Graph
Event=No
Event=Yes
Expression
800
750
700
650
600
550
500
450
400
350
573
573
sorted by expression
p-value

NTRK1

WNT M0

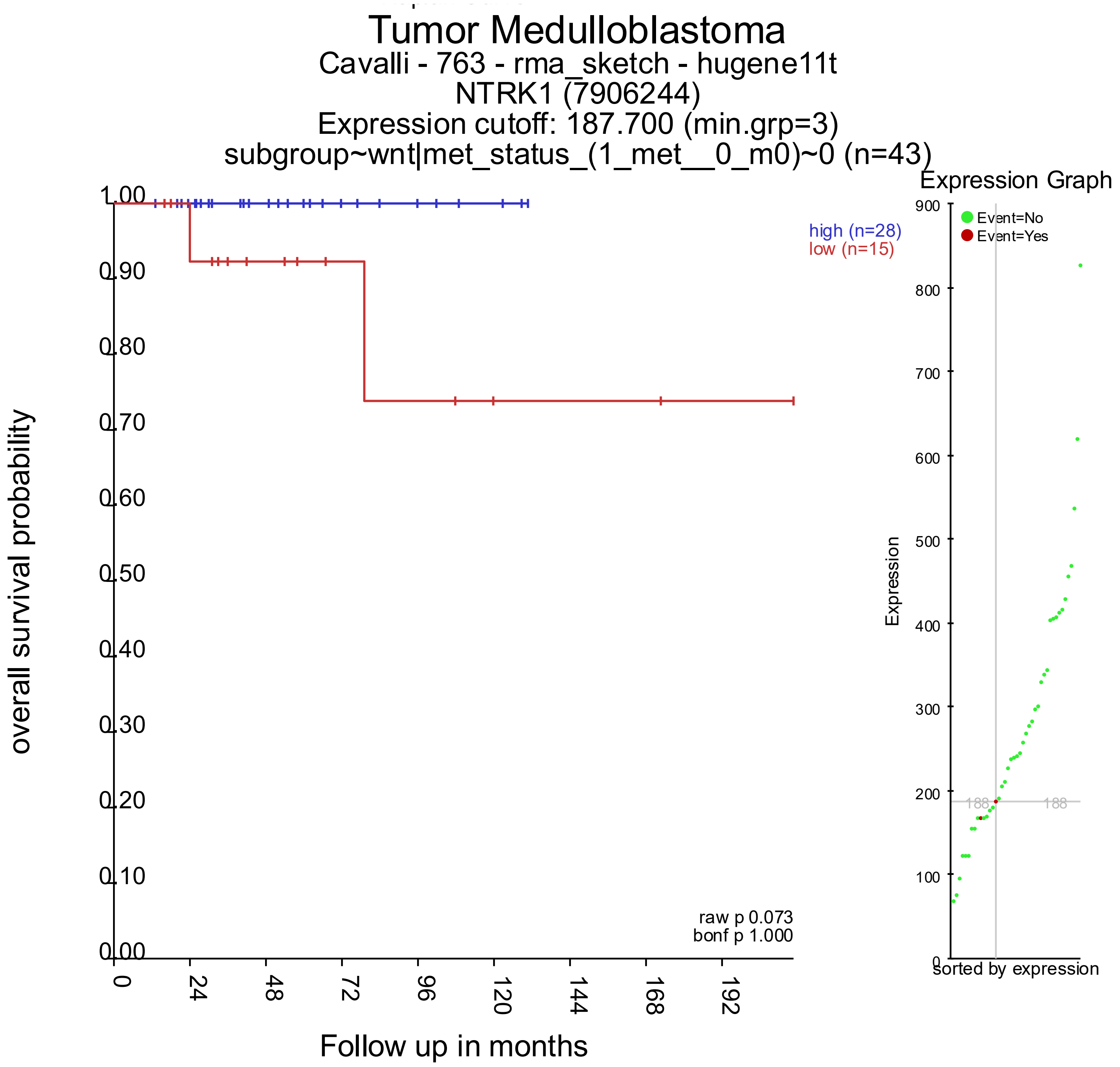
Tumor Medulloblastoma
Cavalli - 763 - rma_sketch - hugene11t
NTRK1 (7906244)
Expression cutoff: 187.700 (min.grp=3)
subgroup~wnt|met_status_(1_met__0_m0)~0 (n=43)
high (n=28)
low (n=15)
overall survival probability
1.00
0.90
0.80
0.70
0.60
0.50
0.40
0.30
0.20
0.10
0.00
0
24
48
72
96
120
144
168
192
Follow up in months
raw p 0.073
bonf p 1.000
Expression Graph
Event=No
Event=Yes
Expression
900
800
700
600
500
400
300
200
100
0
188
188
sorted by expression

WNT M1

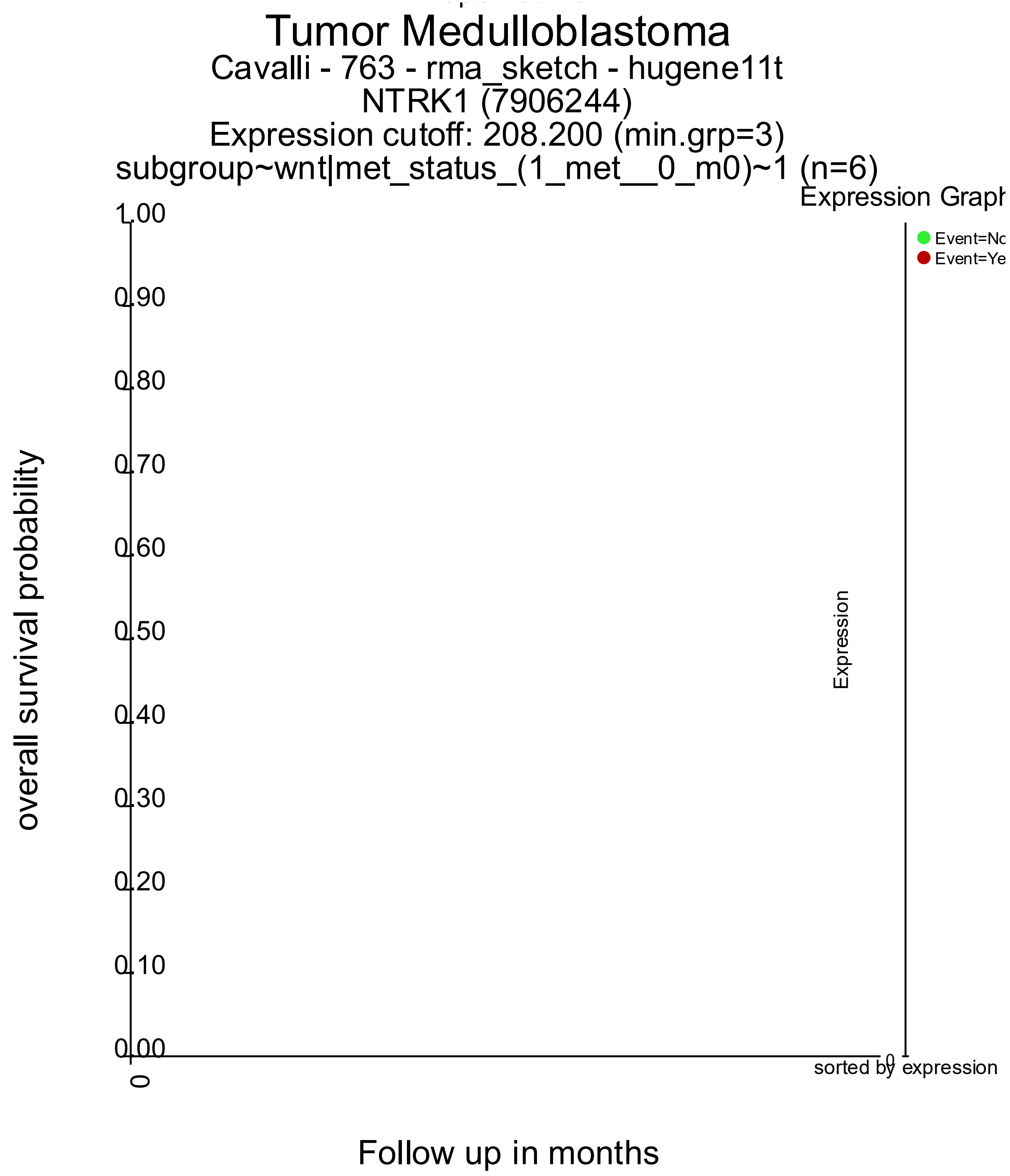

Tumor Medulloblastoma
Cavalli - 763 - rma_sketch - hugene11t
NTRK1 (7906244)
Expression cutoff: 208.200 (min.grp=3)
subgroup~wnt|met_status_(1_met__0_m0)~1 (n=6)
Expression Graph
Event=No
Event=Ye
overall survival probability
1.00
0.90
0.80
0.70
0.60
0.50
0.40
0.30
0.20
0.10
0.00
0
Expression
0
sorted by expression
Follow up in months

SHH M0

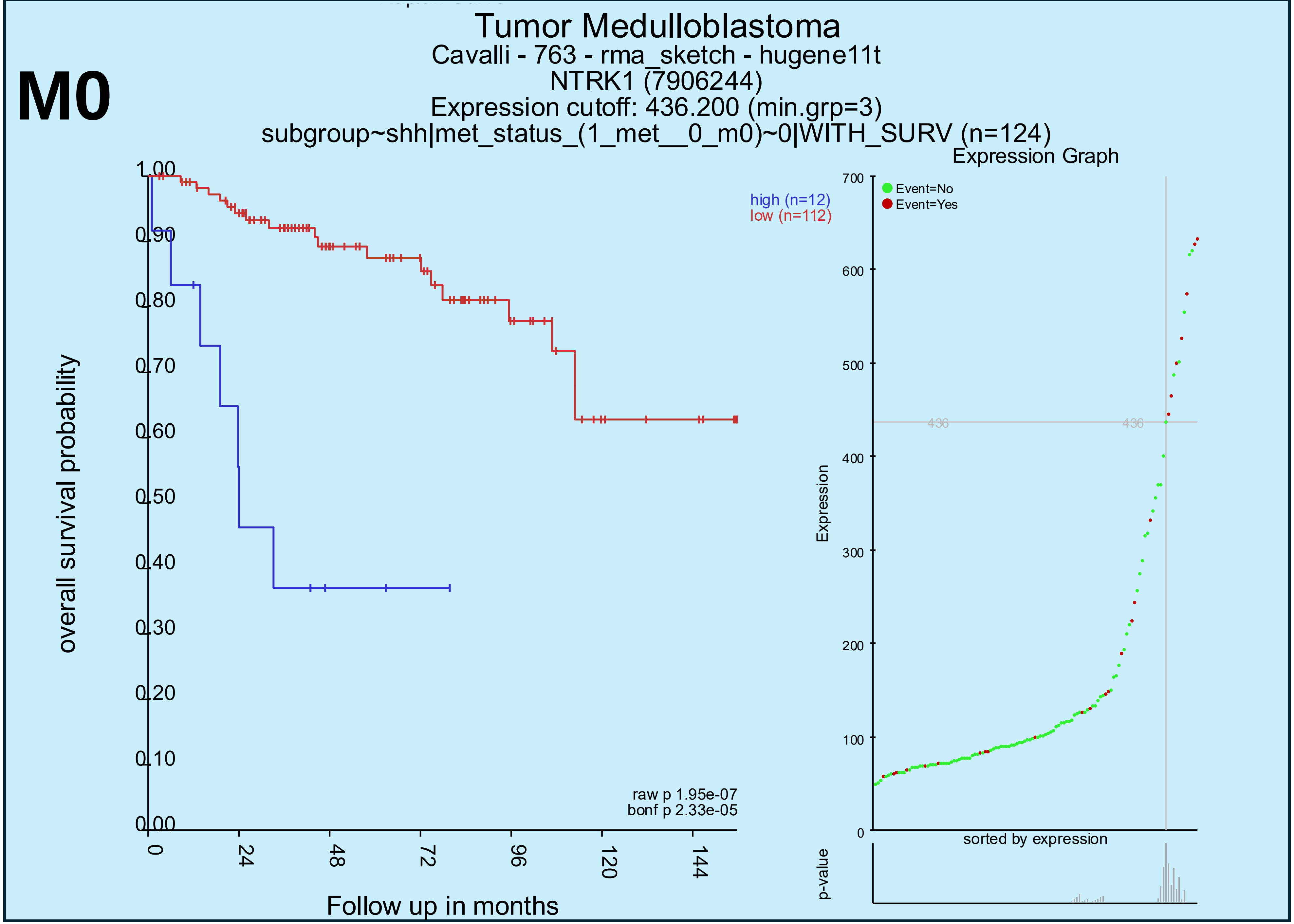

Tumor Medulloblastoma
Cavalli - 763 - rma_sketch - hugene11t
NTRK1 (7906244)
Expression cutoff: 436.200 (min.grp=3)
subgroup~shh|met_status_(1_met__0_m0)~0|WITH_SURV (n=124)
overall survival probability
1.00
0.90
0.80
0.70
0.60
0.50
0.40
0.30
0.20
0.10
0.00
0
24
48
72
96
120
144
Follow up in months
high (n=12)
low (n=112)
raw p 1.95e-07
bonf p 2.33e-05
Expression Graph
Event=No
Event=Yes
Expression
700
600
500
400
300
200
100
0
436
436
sorted by expression
p-value

SHH M1

Tumor Medulloblastoma
Cavalli - 763 - rma_sketch - hugene11t
NTRK1 (7906244)
Expression cutoff: 254.400 (min.grp=3)
subgroup~shh|met_status_(1_met__0_m0)~1|WITH_SURV (n=22)

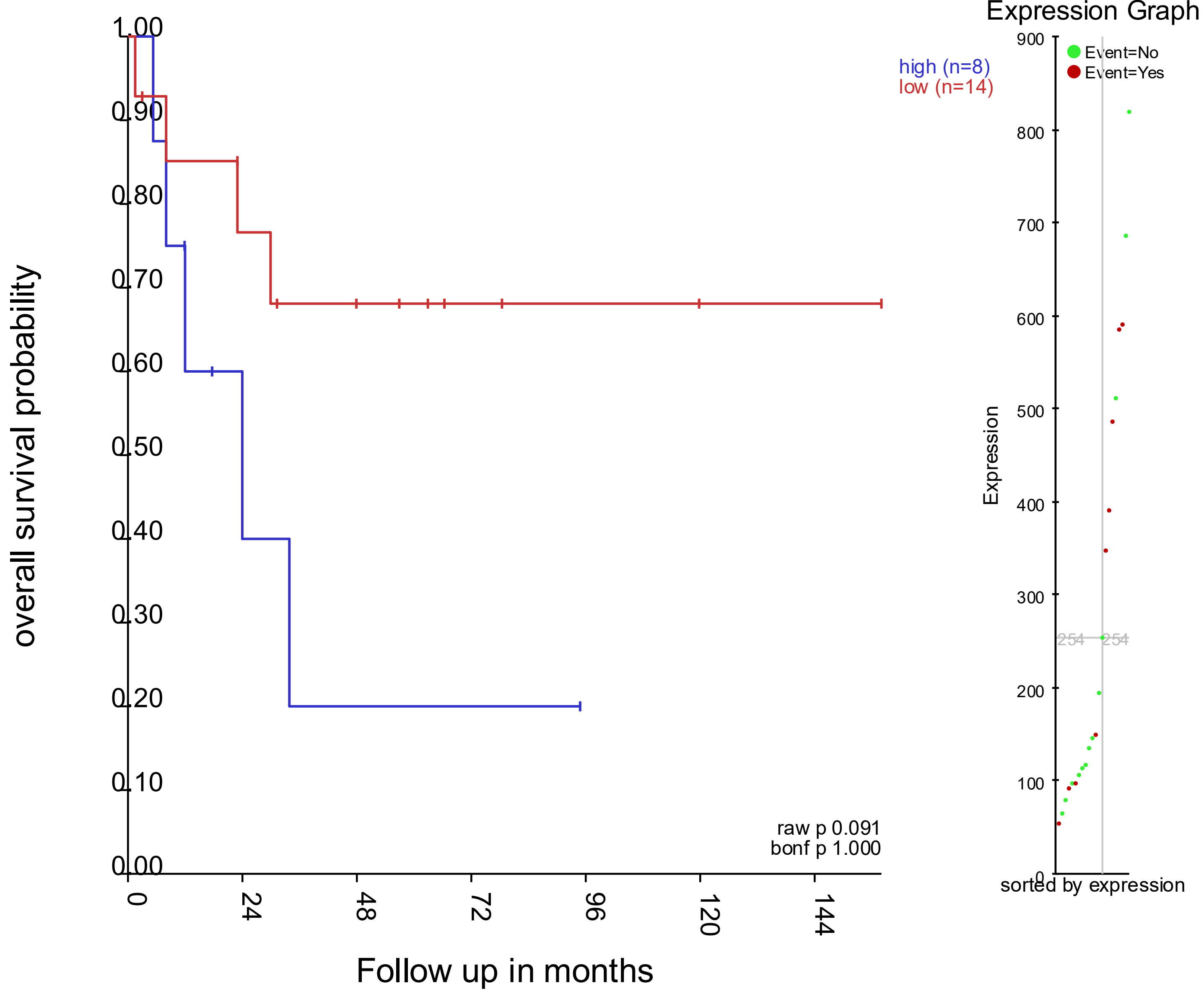

GROUP4 M0

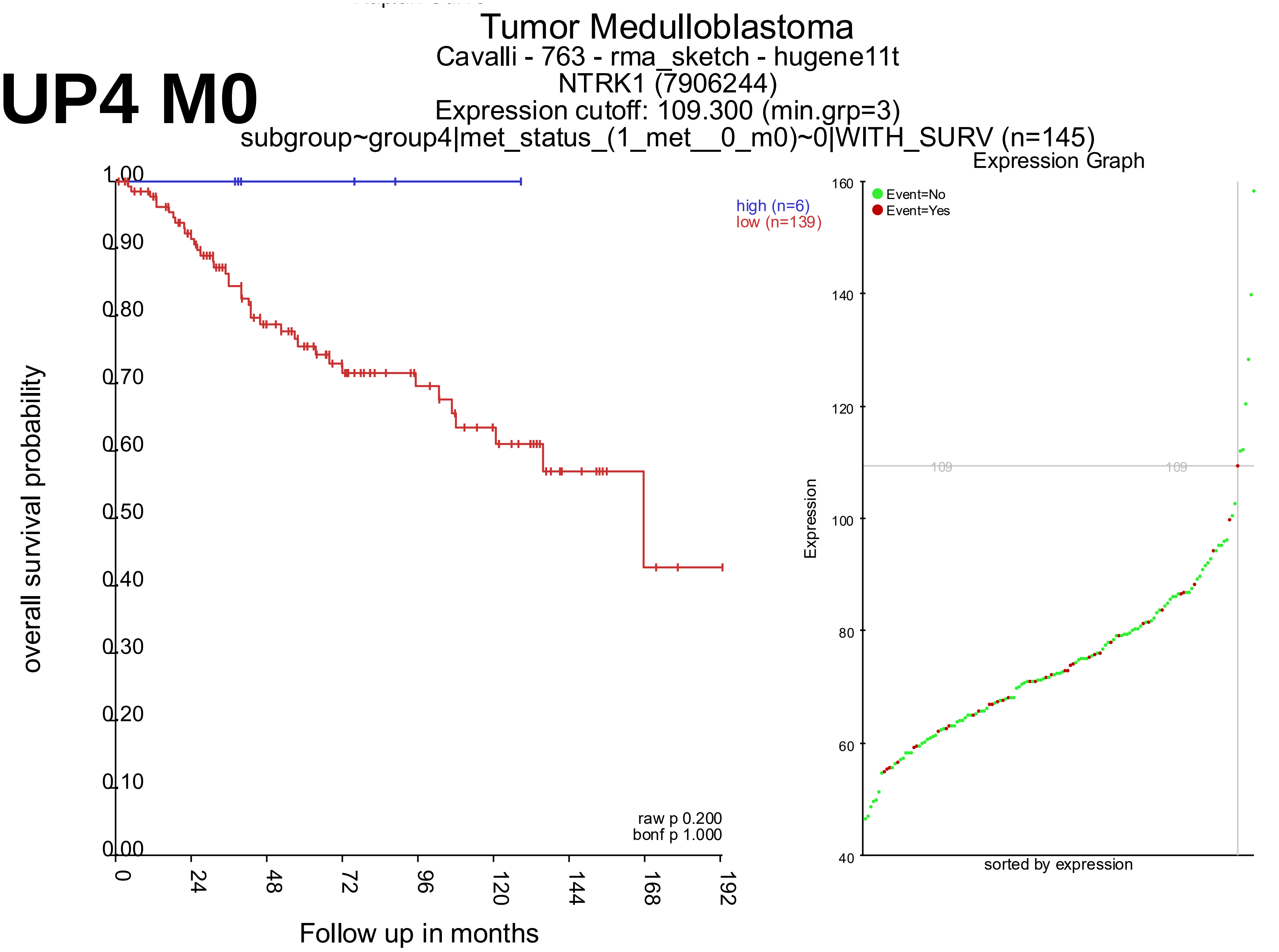

Tumor Medulloblastoma
Cavalli - 763 - rma_sketch - hugene11t
NTRK1 (7906244)
Expression cutoff: 109.300 (min.grp=3)
subgroup~group4|met_status_(1_met__0_m0)~0|WITH_SURV (n=145)
overall survival probability
1.00
0.90
0.80
0.70
0.60
0.50
0.40
0.30
0.20
0.10
0.00
high (n=6)
low (n=139)
raw p 0.200
bonf p 1.000
0
24
48
72
96
120
144
168
192
Follow up in months
Expression Graph
Event=No
Event=Yes
160
140
120
100
80
60
40
109
109
Expression
sorted by expression

GROUP4 M1

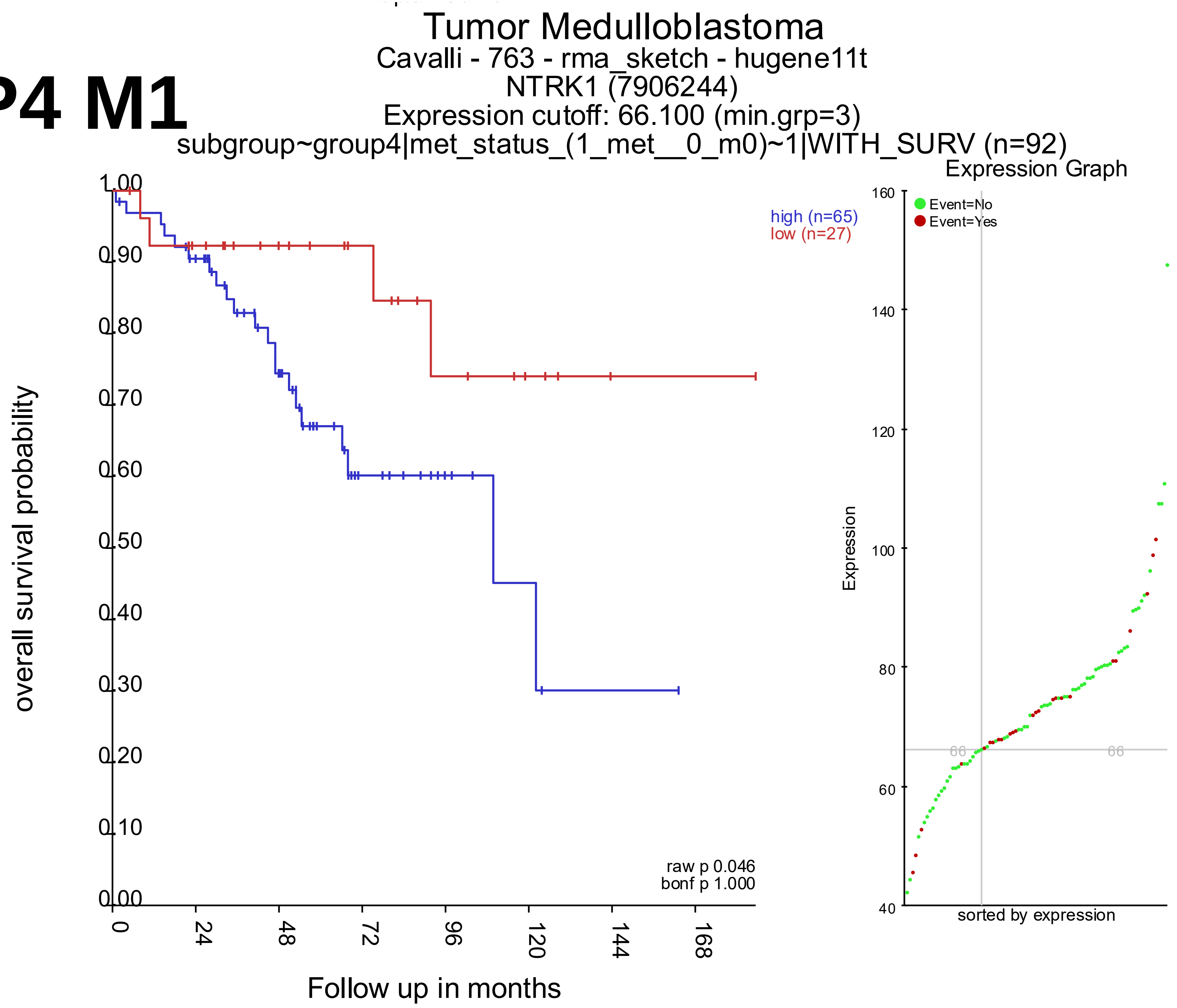

Tumor Medulloblastoma
Cavalli - 763 - rma_sketch - hugene11t
NTRK1 (7906244)
Expression cutoff: 66.100 (min.grp=3)
subgroup~group4|met_status_(1_met__0_m0)~1|WITH_SURV (n=92)
overall survival probability
1.00
0.90
0.80
0.70
0.60
0.50
0.40
0.30
0.20
0.10
0.00
0
24
48
72
96
120
144
168
Follow up in months
high (n=65)
low (n=27)
raw p 0.046
bonf p 1.000
Expression Graph
Event=No
Event=Yes
Expression
160
140
120
100
80
60
40
66
66
sorted by expression

GROUP3 M0

Tumor Medulloblastoma
Cavalli - 763 - rma_sketch - hugene11t
NTRK1 (7906244)
Expression cutoff: 75.600 (min.grp=3)
subgroup~group3|met_status_(1_met__0_m0)~0|WITH_SURV (n=65)

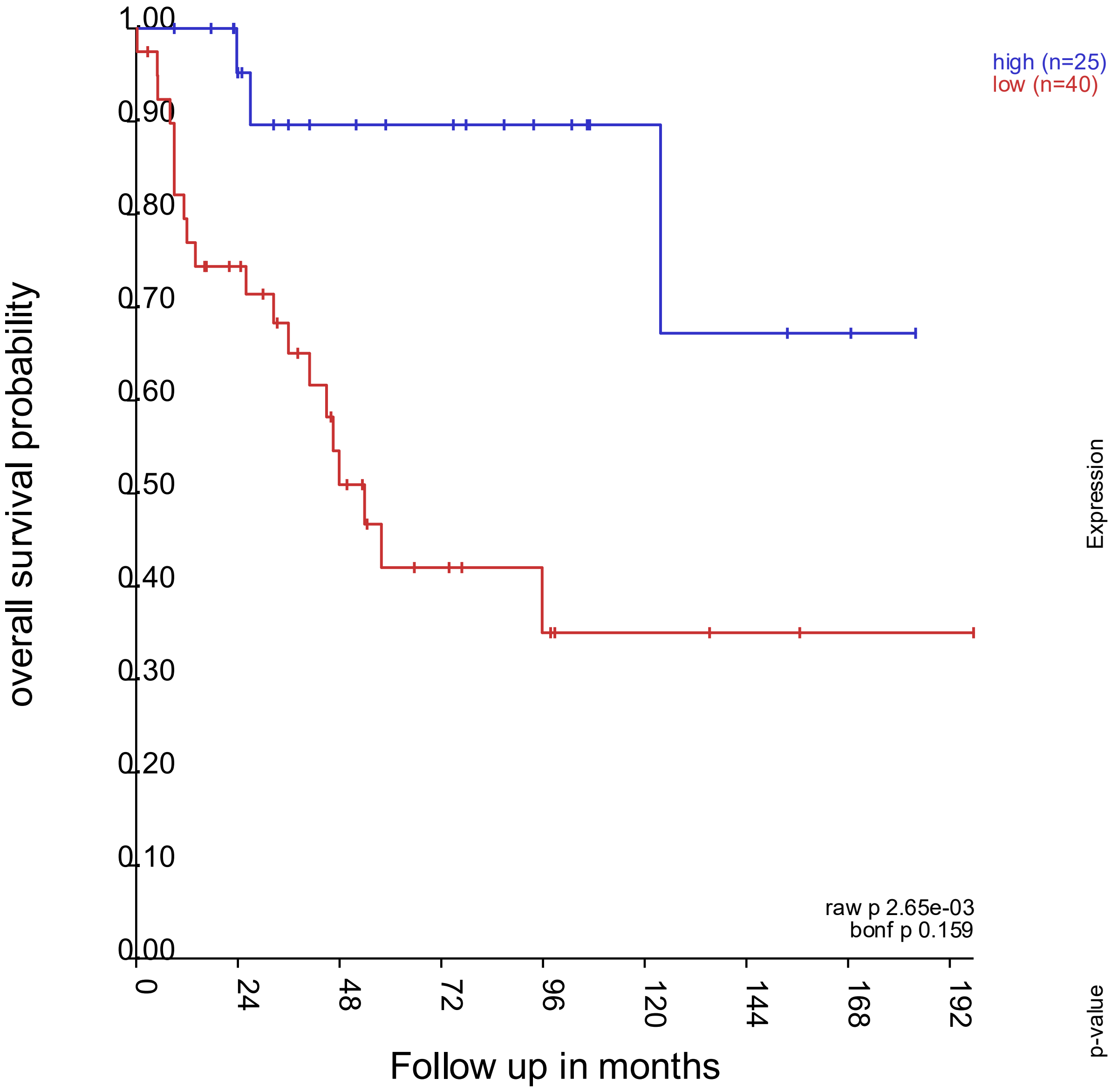

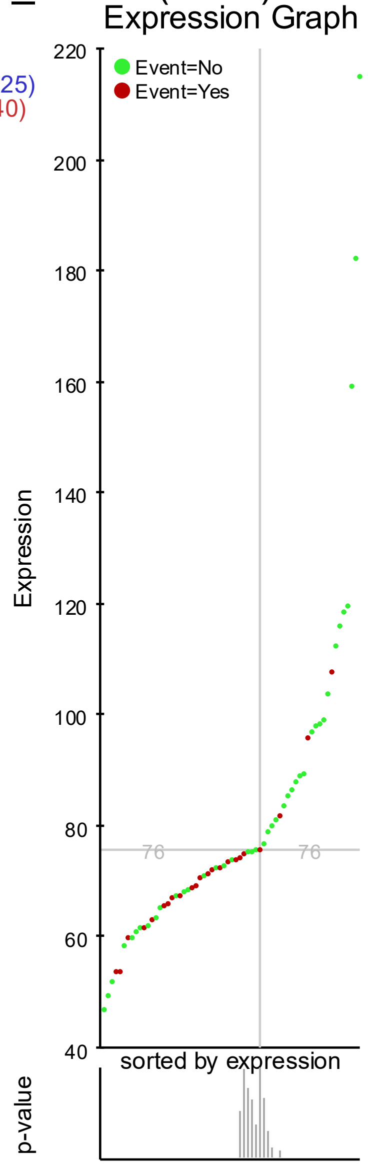

GROUP3 M1

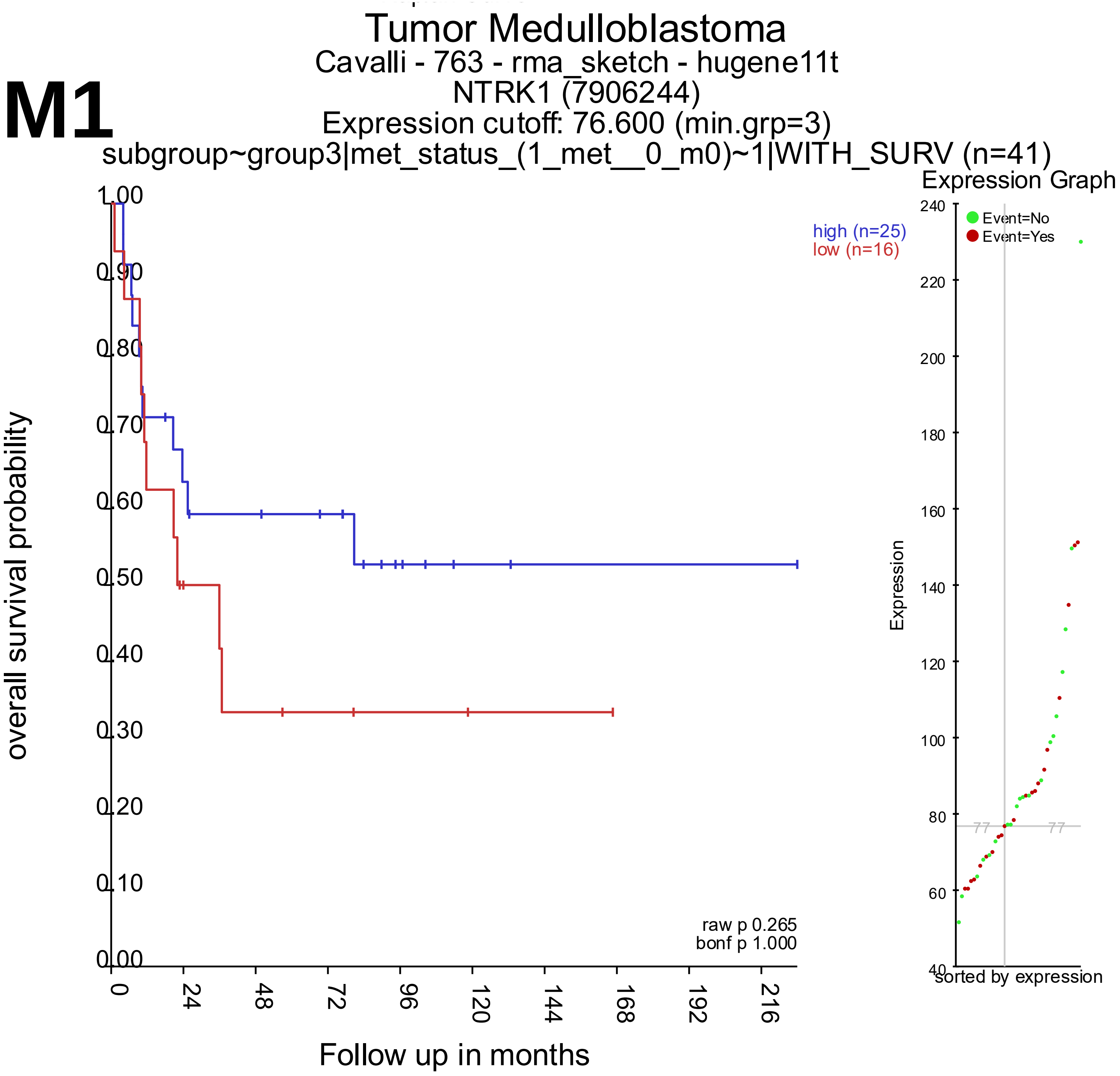
Tumor Medulloblastoma
Cavalli - 763 - rma_sketch - hugene11t
NTRK1 (7906244)
Expression cutoff: 76.600 (min.grp=3)
subgroup~group3|met_status_(1_met__0_m0)~1|WITH_SURV (n=41)
high (n=25)
low (n=16)
overall survival probability
1.00
0.90
0.80
0.70
0.60
0.50
0.40
0.30
0.20
0.10
0.00
0
24
48
72
96
120
144
168
192
216
Follow up in months
raw p 0.265
bonf p 1.000
Expression Graph
Event=No
Event=Yes
Expression
240
220
200
180
160
140
120
100
80
60
40
77
77
sorted by expression

NTRK2

WNT M0

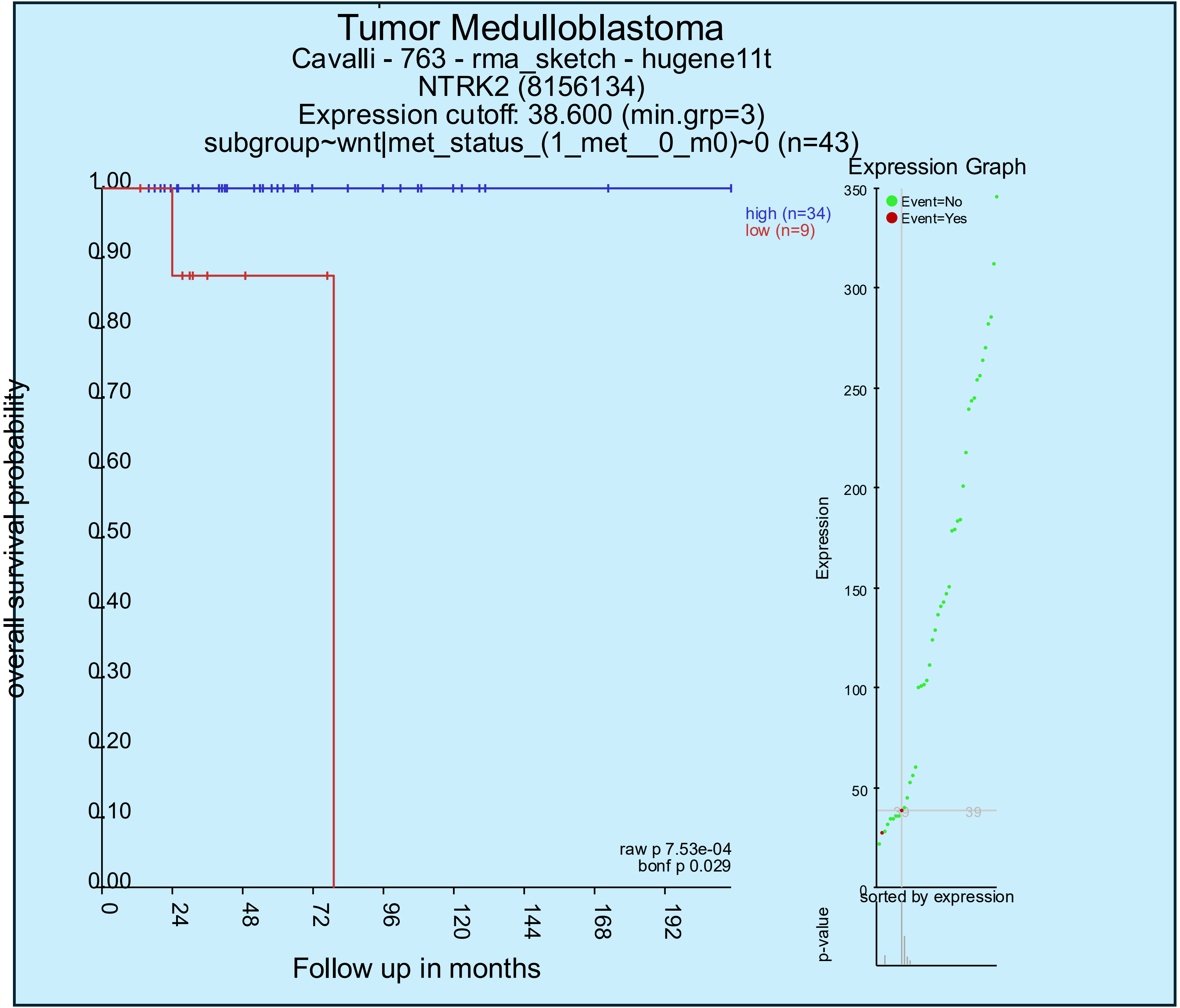

Tumor Medulloblastoma
Cavalli - 763 - rma_sketch - hugene11t
NTRK2 (8156134)
Expression cutoff: 38.600 (min.grp=3)
subgroup~wnt|met_status_(1_met__0_m0)~0 (n=43)
overall survival probability
1.00
0.90
0.80
0.70
0.60
0.50
0.40
0.30
0.20
0.10
0.00
0
24
48
72
96
120
144
168
192
Follow up in months
high (n=34)
low (n=9)
raw p 7.53e-04
bonf p 0.029
Expression Graph
Event=No
Event=Yes
Expression
350
300
250
200
150
100
50
0
39
sorted by expression
p-value

WNT M1

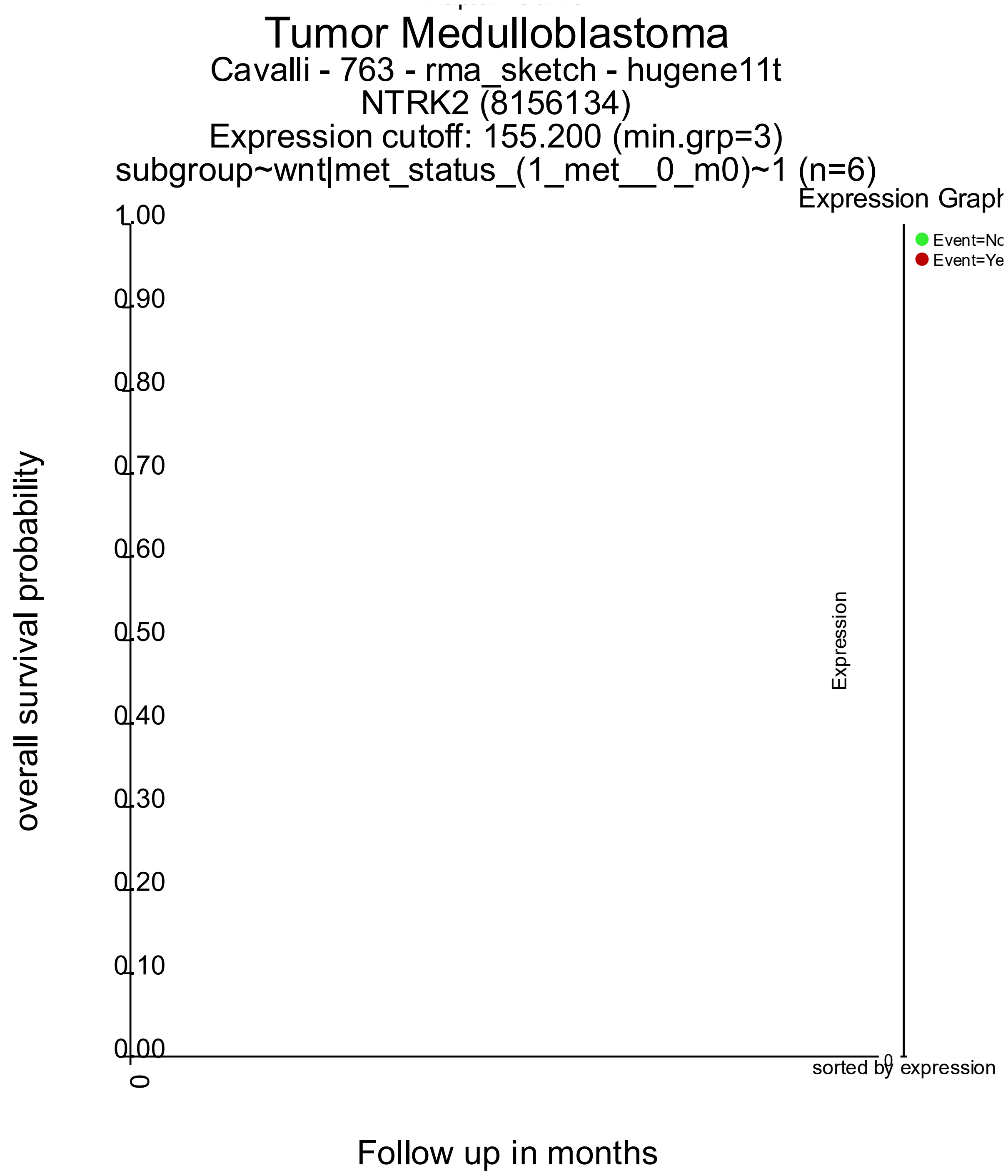
Tumor Medulloblastoma
Cavalli - 763 - rma_sketch - hugene11t
NTRK2 (8156134)
Expression cutoff: 155.200 (min.grp=3)
subgroup~wnt|met_status_(1_met__0_m0)~1 (n=6)
Expression Graph
Event=No
Event=Ye
overall survival probability
1.00
0.90
0.80
0.70
0.60
0.50
0.40
0.30
0.20
0.10
0.00
0
Follow up in months
Expression
0
sorted by expression

SHH M0

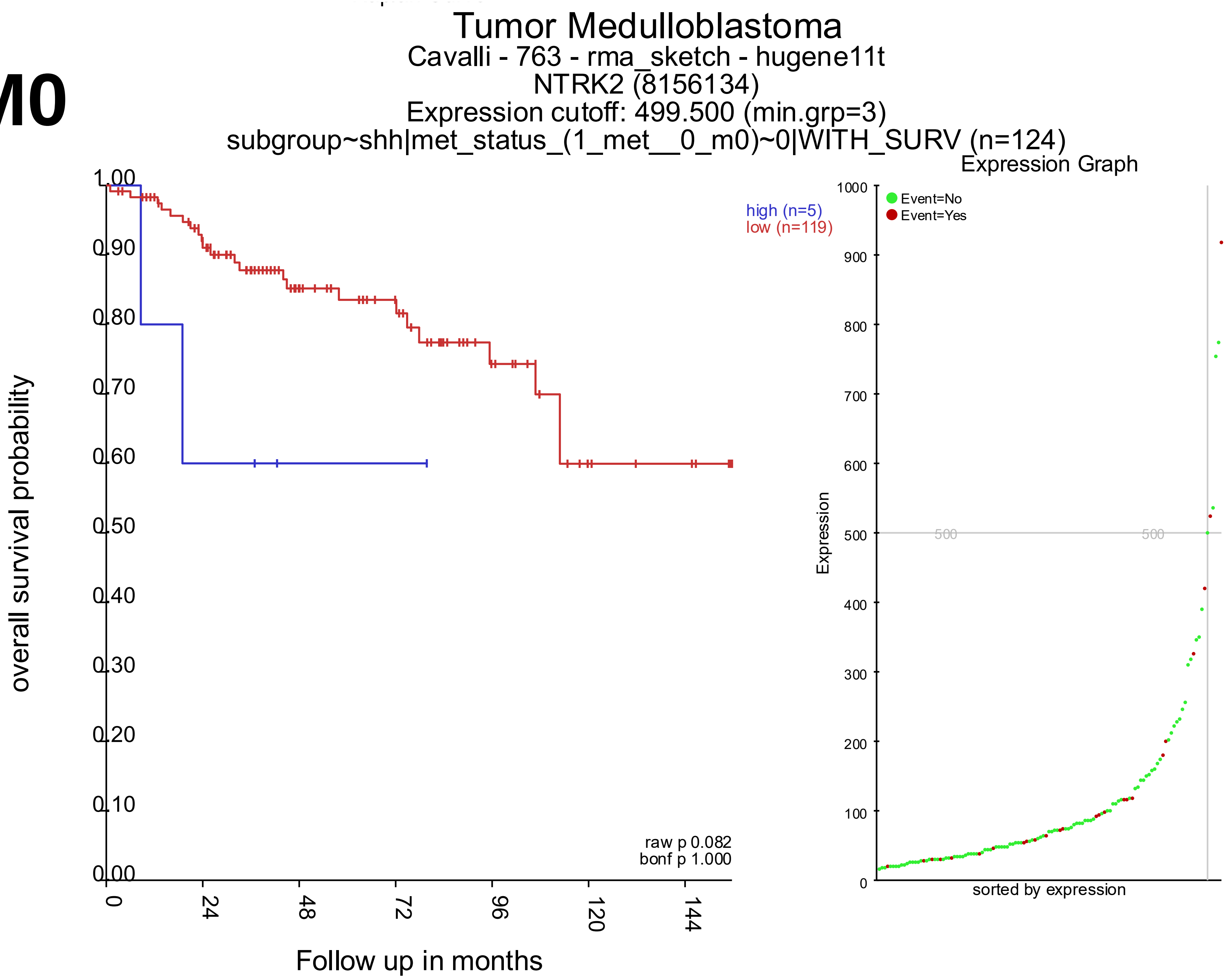

Tumor Medulloblastoma
Cavalli - 763 - rma_sketch - hugene11t
NTRK2 (8156134)
Expression cutoff: 499.500 (min.grp=3)
subgroup~shh|met_status_(1_met__0_m0)~0|WITH_SURV (n=124)
overall survival probability
1.00
0.90
0.80
0.70
0.60
0.50
0.40
0.30
0.20
0.10
0.00
high (n=5)
low (n=119)
raw p 0.082
bonf p 1.000
0
24
48
72
96
120
144
Follow up in months
Expression Graph
Event=No
Event=Yes
Expression
1000
900
800
700
600
500
400
300
200
100
0
500
500
sorted by expression

SHH M1

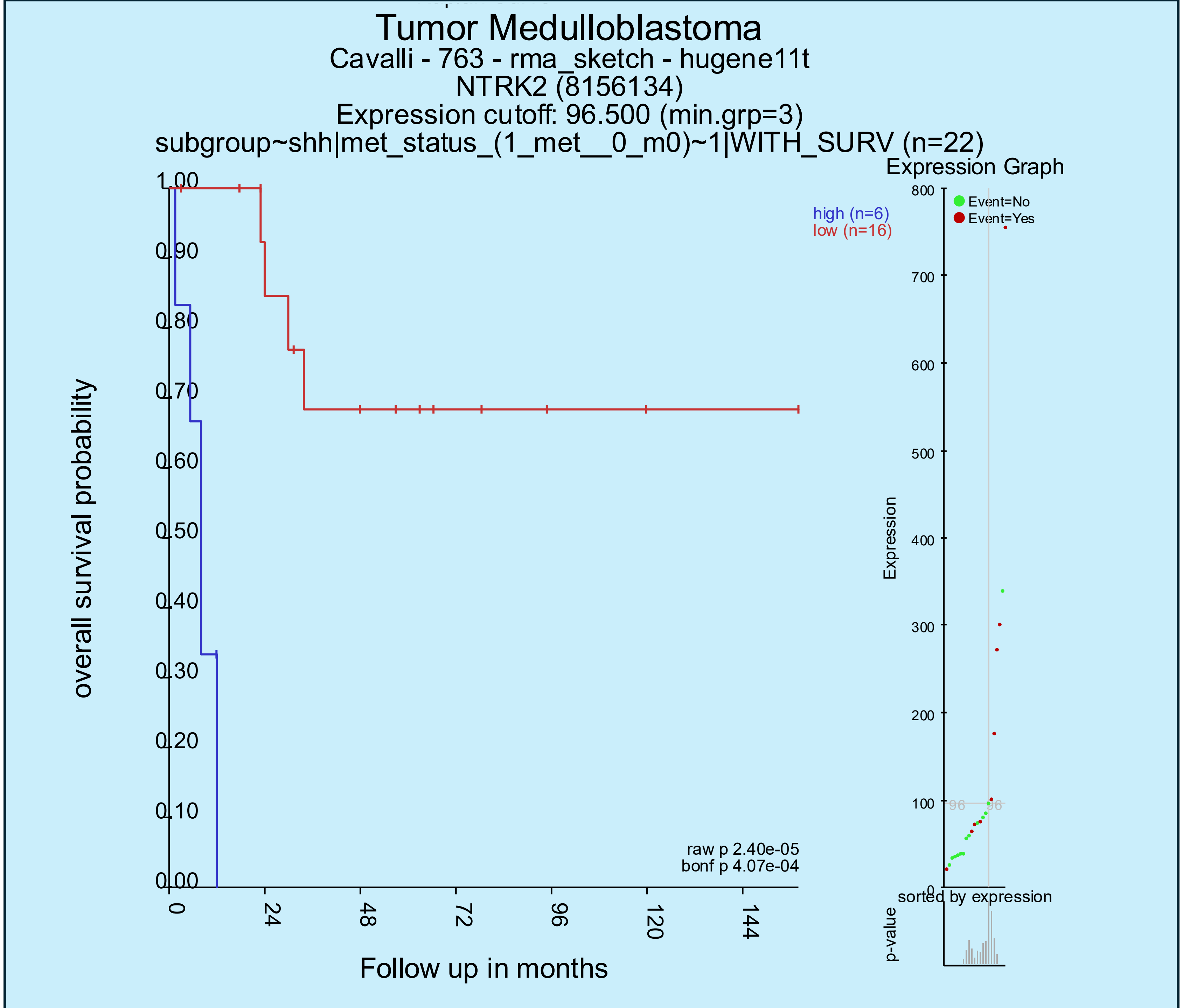
Tumor Medulloblastoma
Cavalli - 763 - rma_sketch - hugene11t
NTRK2 (8156134)
Expression cutoff: 96.500 (min.grp=3)
subgroup~shh|met_status_(1_met__0_m0)~1|WITH_SURV (n=22)
high (n=6)
low (n=16)
overall survival probability
Follow up in months
raw p 2.40e-05
bonf p 4.07e-04
Expression Graph
Event=No
Event=Yes
Expression
sorted by expression
p-value

GROUP4 M0

Tumor Medulloblastoma
Cavalli - 763 - rma_sketch - hugene11t
NTRK2 (8156134)
Expression cutoff: 209.300 (min.grp=3)
subgroup~group4|met_status_(1_met__0_m0)~0|WITH_SURV (n=145)

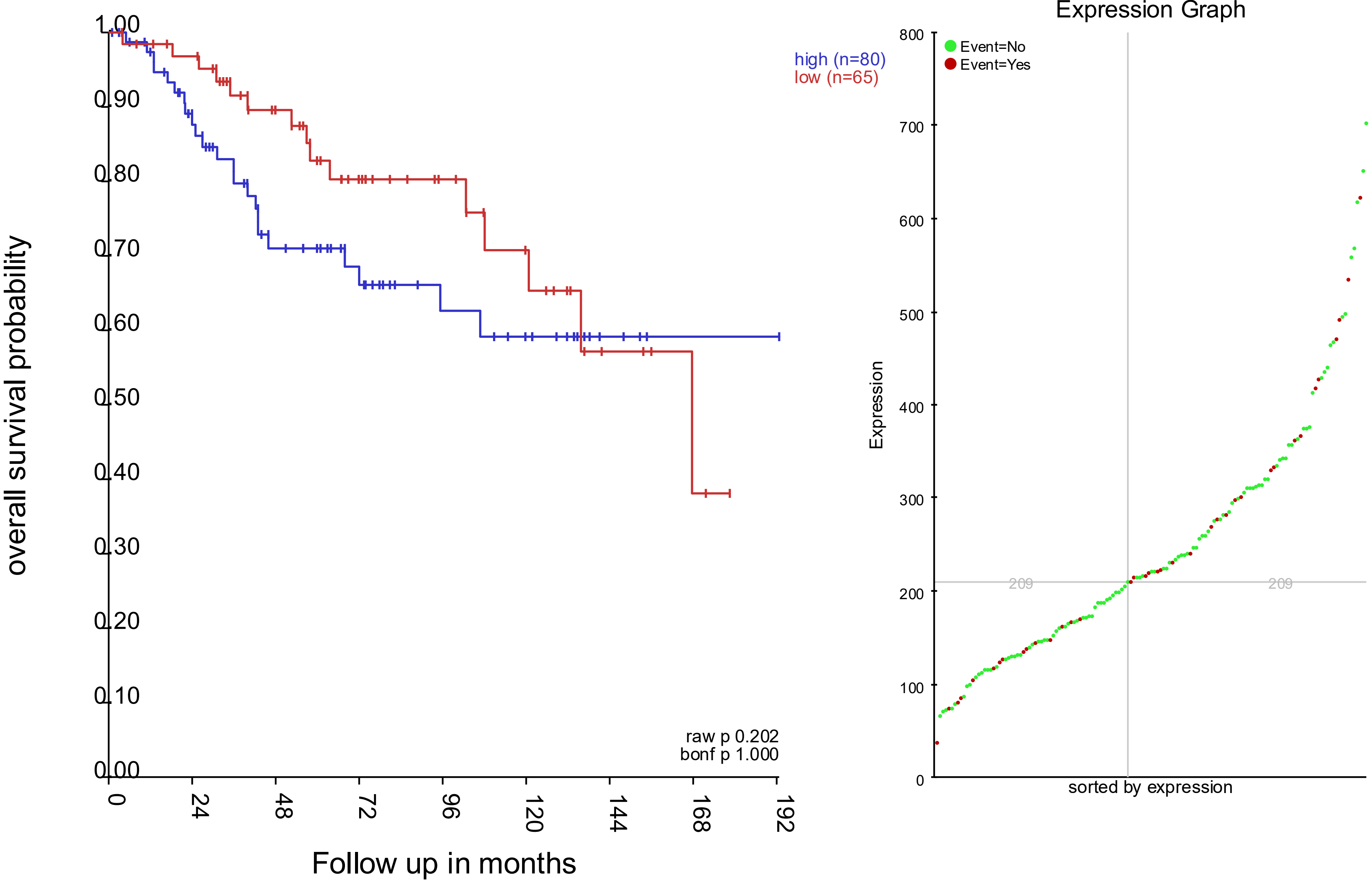

GROUP4 M1

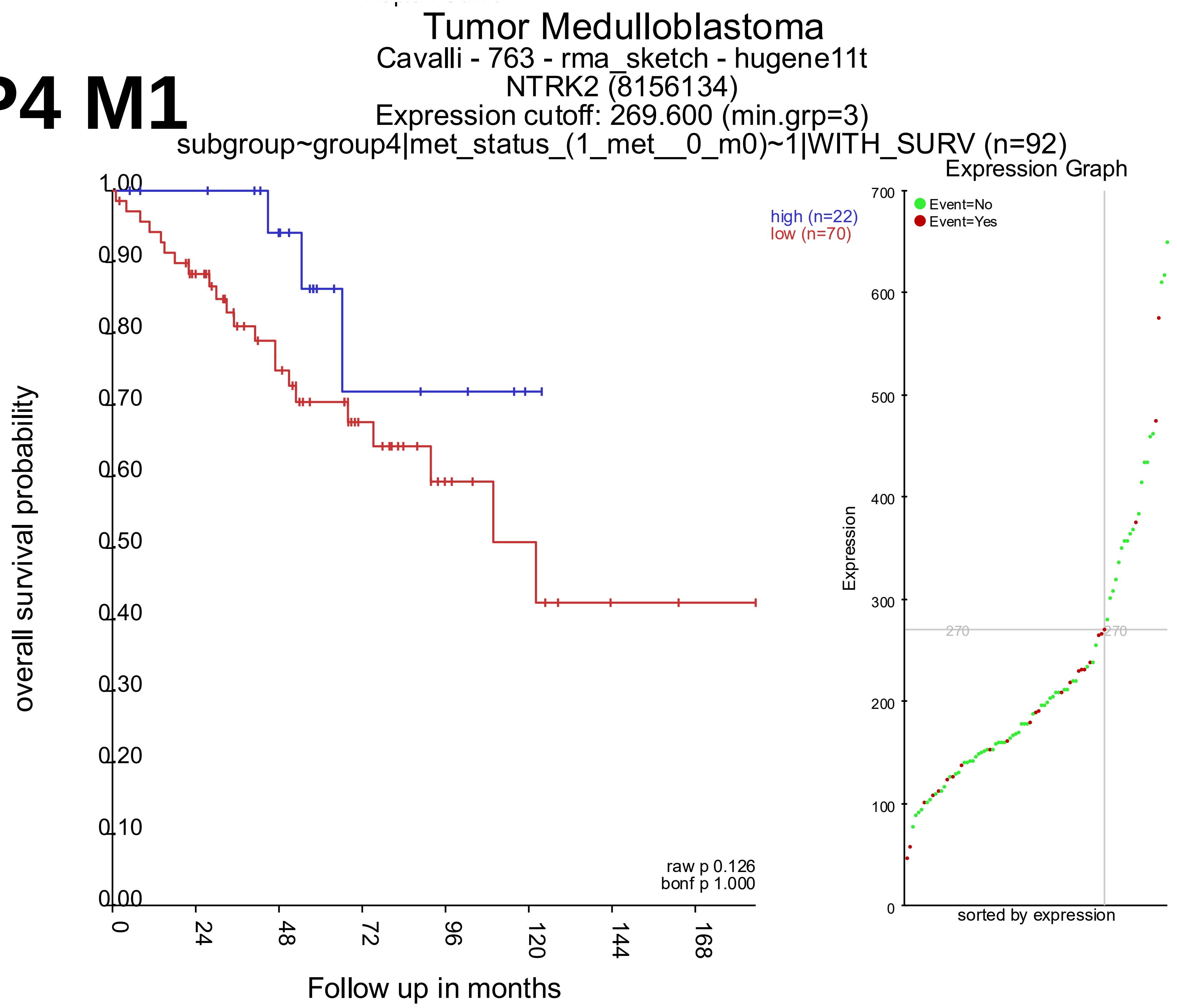

Tumor Medulloblastoma
Cavalli - 763 - rma_sketch - hugene11t
NTRK2 (8156134)
Expression cutoff: 269.600 (min.grp=3)
subgroup~group4|met_status_(1_met__0_m0)~1|WITH_SURV (n=92)
overall survival probability
1.00
0.90
0.80
0.70
0.60
0.50
0.40
0.30
0.20
0.10
0.00
high (n=22)
low (n=70)
raw p 0.126
bonf p 1.000
0
24
48
72
96
120
144
168
Follow up in months
Expression Graph
Event=No
Event=Yes
Expression
700
600
500
400
300
200
100
0
270
270
sorted by expression

GROUP3 M0

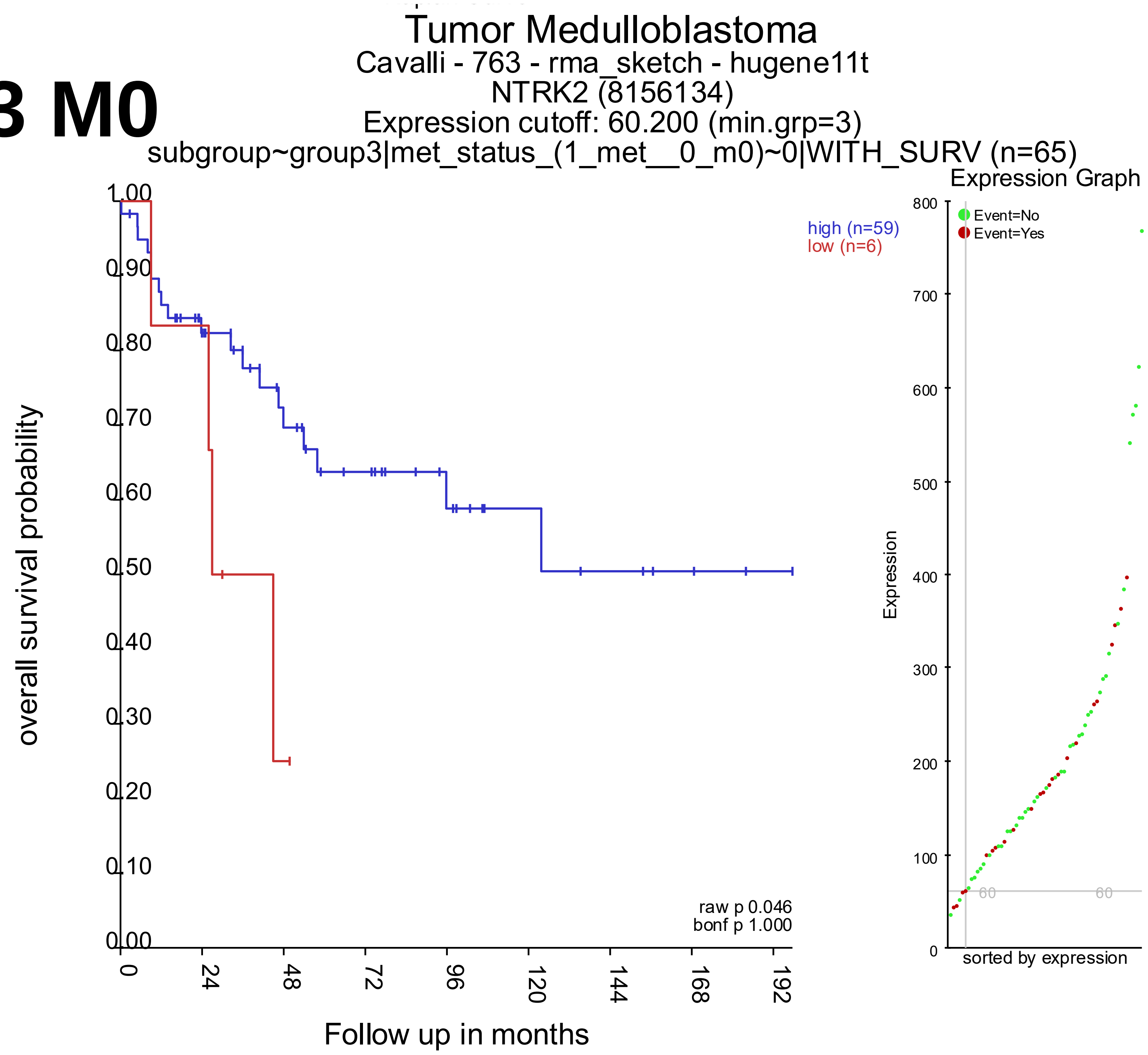

Tumor Medulloblastoma
Cavalli - 763 - rma_sketch - hugene11t
NTRK2 (8156134)
Expression cutoff: 60.200 (min.grp=3)
subgroup~group3|met_status_(1_met__0_m0)~0|WITH_SURV (n=65)
overall survival probability
1.00
0.90
0.80
0.70
0.60
0.50
0.40
0.30
0.20
0.10
0.00
0
24
48
72
96
120
144
168
192
Follow up in months
high (n=59)
low (n=6)
raw p 0.046
bonf p 1.000
Expression Graph
Event=No
Event=Yes
Expression
800
700
600
500
400
300
200
100
0
60
60
sorted by expression

GROUP3 M1

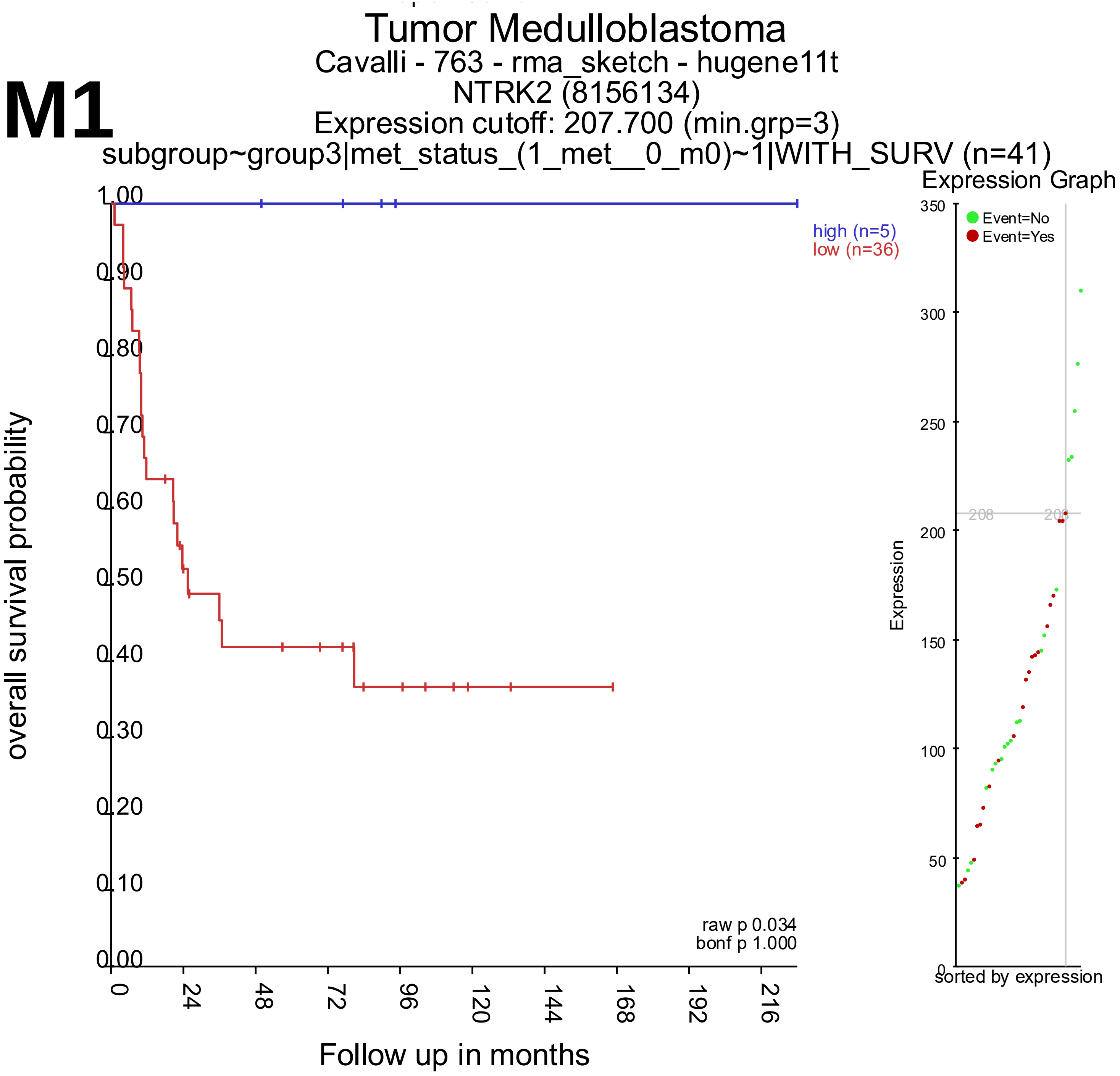

Tumor Medulloblastoma
Cavalli - 763 - rma_sketch - hugene11t
NTRK2 (8156134)
Expression cutoff: 207.700 (min.grp=3)
subgroup~group3|met_status_(1_met__0_m0)~1|WITH_SURV (n=41)
overall survival probability
1.00
0.90
0.80
0.70
0.60
0.50
0.40
0.30
0.20
0.10
0.00
high (n=5)
low (n=36)
raw p 0.034
bonf p 1.000
0
24
48
72
96
120
144
168
192
216
Follow up in months
Expression Graph
Event=No
Event=Yes
Expression
350
300
250
200
150
100
50
0
208
sorted by expression

NTRK3

WNT M0

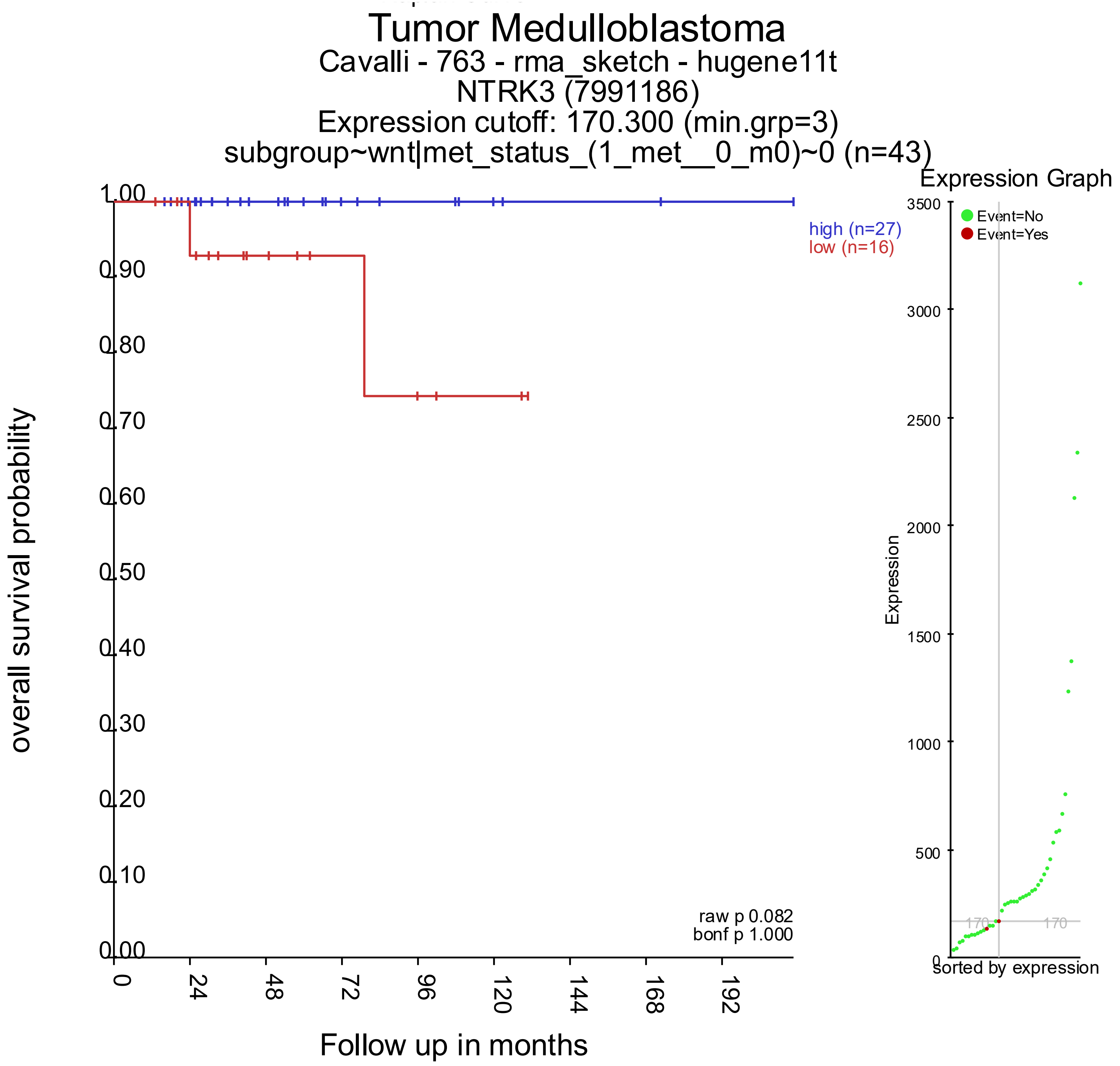
Tumor Medulloblastoma
Cavalli - 763 - rma_sketch - hugene11t
NTRK3 (7991186)
Expression cutoff: 170.300 (min.grp=3)
subgroup~wnt|met_status_(1_met__0_m0)~0 (n=43)
overall survival probability
1.00
0.90
0.80
0.70
0.60
0.50
0.40
0.30
0.20
0.10
0.00
0
24
48
72
96
120
144
168
192
Follow up in months
high (n=27)
low (n=16)
raw p 0.082
bonf p 1.000
Expression Graph
Event=No
Event=Yes
Expression
3500
3000
2500
2000
1500
1000
500
0
170
170
sorted by expression

WNT M1

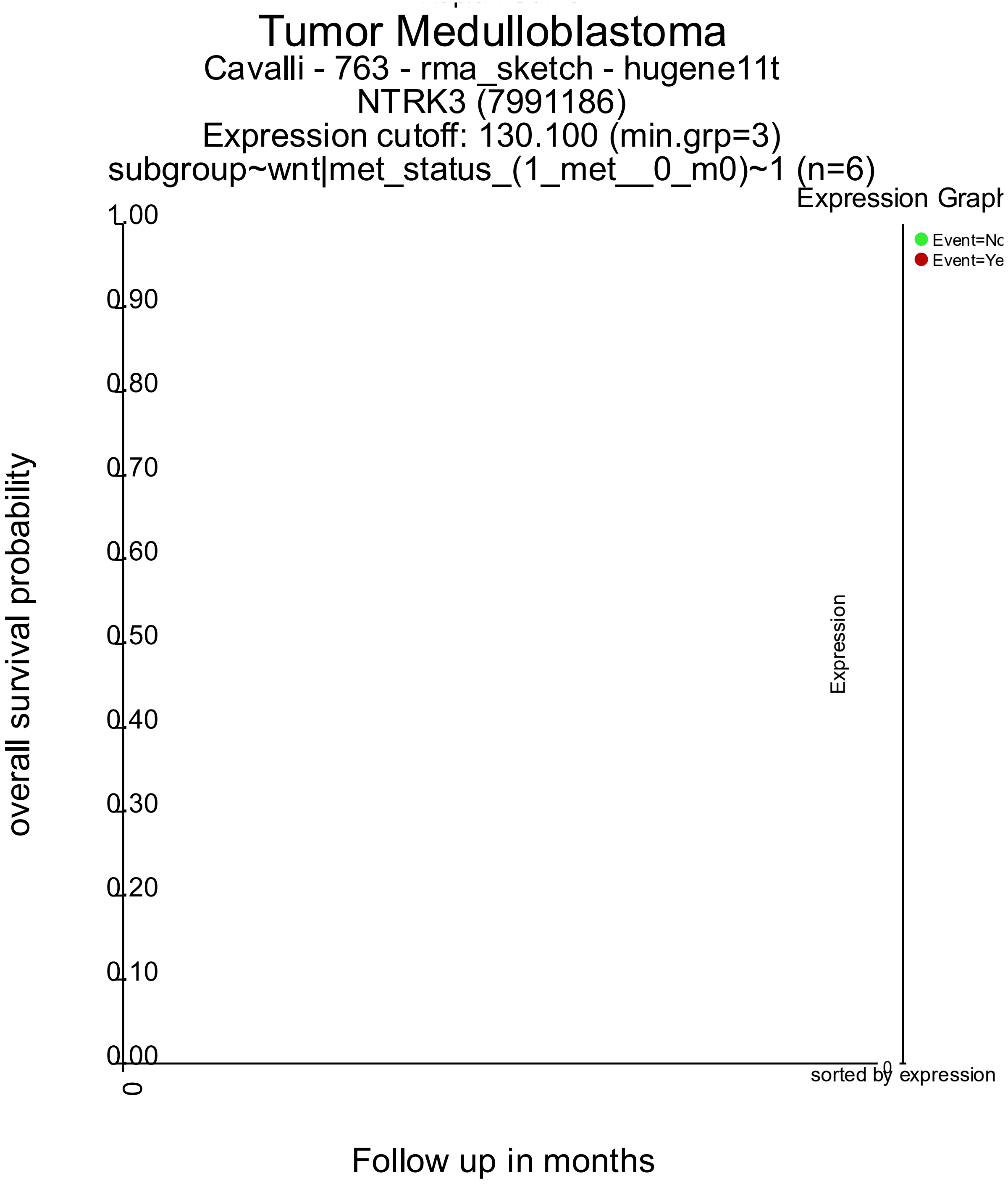

Tumor Medulloblastoma
Cavalli - 763 - rma_sketch - hugene11t
NTRK3 (7991186)
Expression cutoff: 130.100 (min.grp=3)
subgroup~wnt|met_status_(1_met__0_m0)~1 (n=6)
Expression Graph
Event=No
Event=Ye
1.00
0.90
0.80
0.70
0.60
0.50
0.40
0.30
0.20
0.10
0.00
0
overall survival probability
Follow up in months
Expression
0
sorted by expression

SHH M0

Tumor Medulloblastoma
Cavalli - 763 - rma_sketch - hugene11t
NTRK3 (7991186)
Expression cutoff: 954.500 (min.grp=3)
subgroup~shh|met_status_(1_met__0_m0)~0|WITH_SURV (n=124)

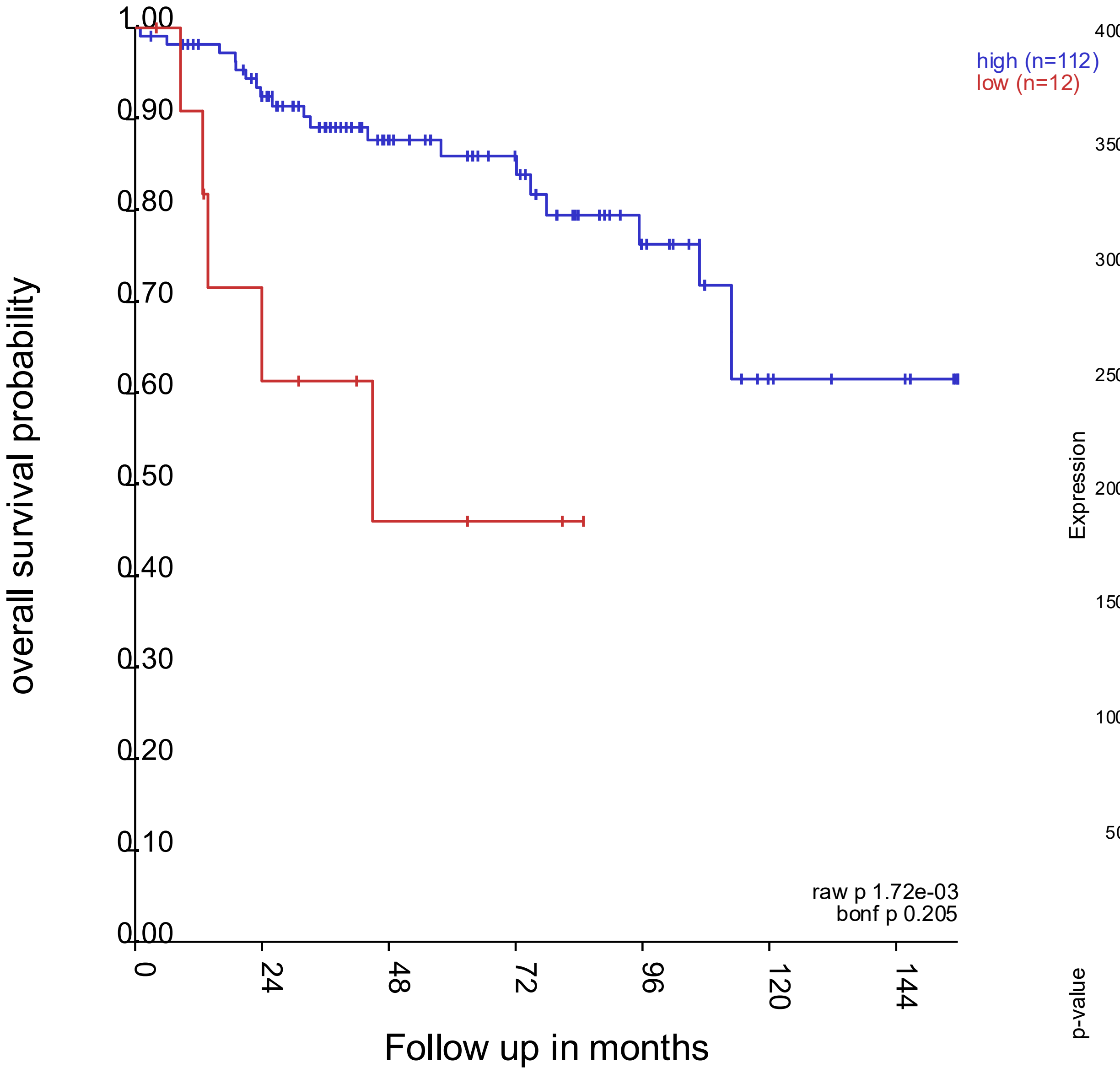

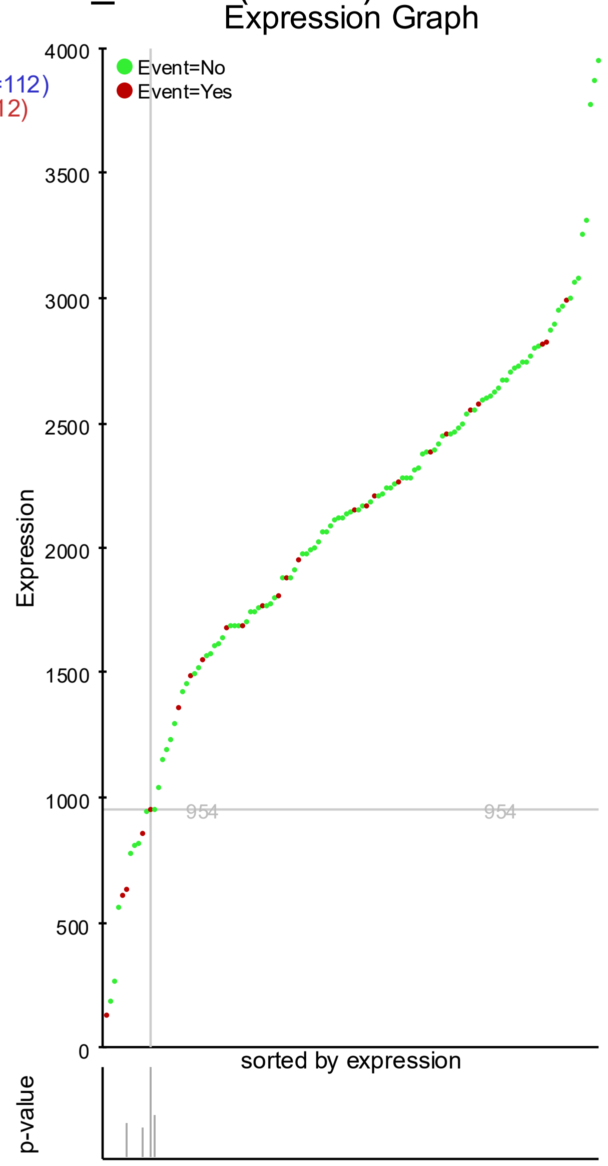

SHH M1

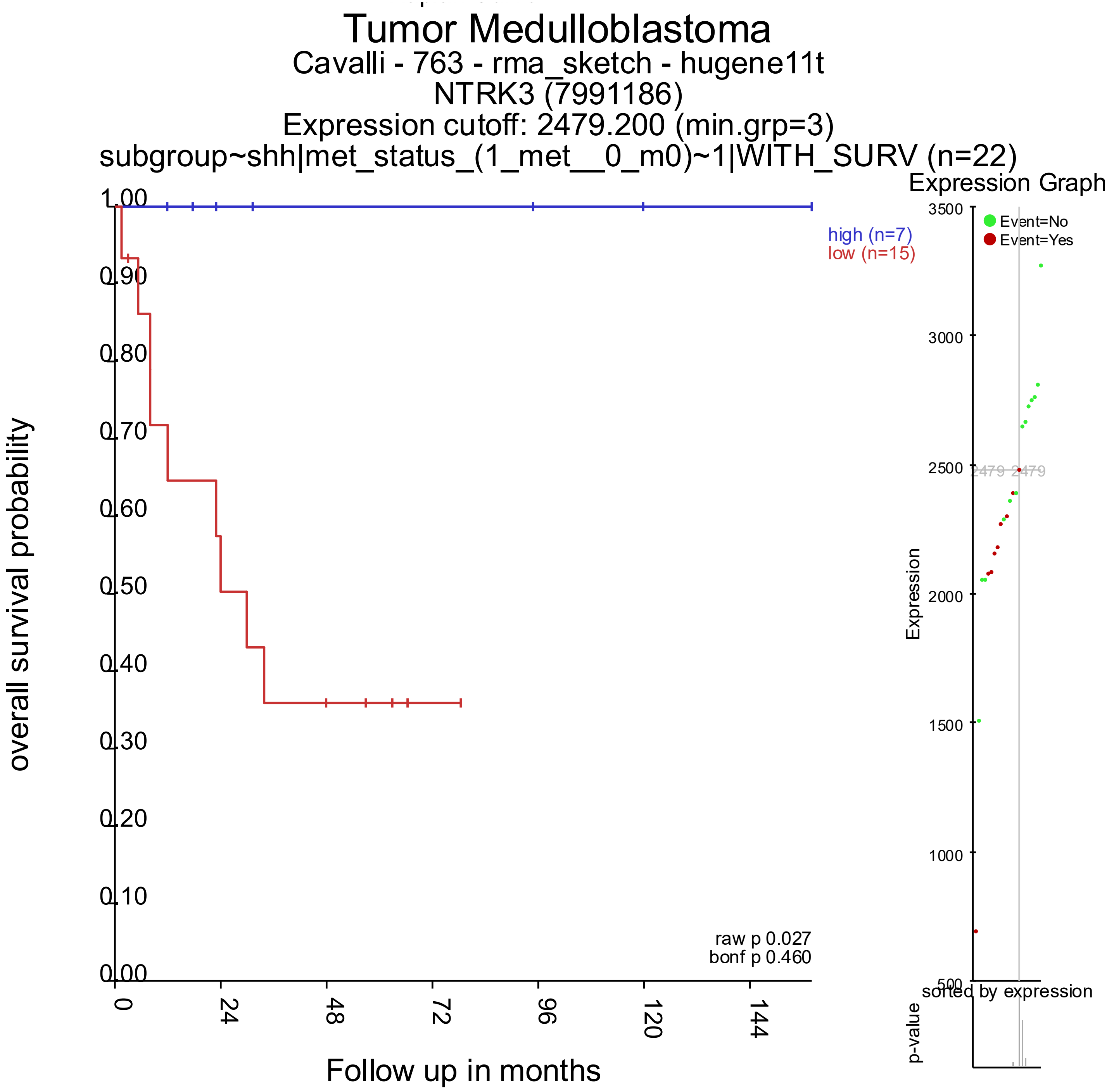
Tumor Medulloblastoma
Cavalli - 763 - rma_sketch - hugene11t
NTRK3 (7991186)
Expression cutoff: 2479.200 (min.grp=3)
subgroup~shh|met_status_(1_met__0_m0)~1|WITH_SURV (n=22)
high (n=7)
low (n=15)
overall survival probability
1.00
0.90
0.80
0.70
0.60
0.50
0.40
0.30
0.20
0.10
0.00
0
24
48
72
96
120
144
Follow up in months
raw p 0.027
bonf p 0.460
Expression Graph
Event=No
Event=Yes
3500
3000
2500
2000
1500
1000
500
2479
2479
Expression
sorted by expression
p-value

GROUP4 M0

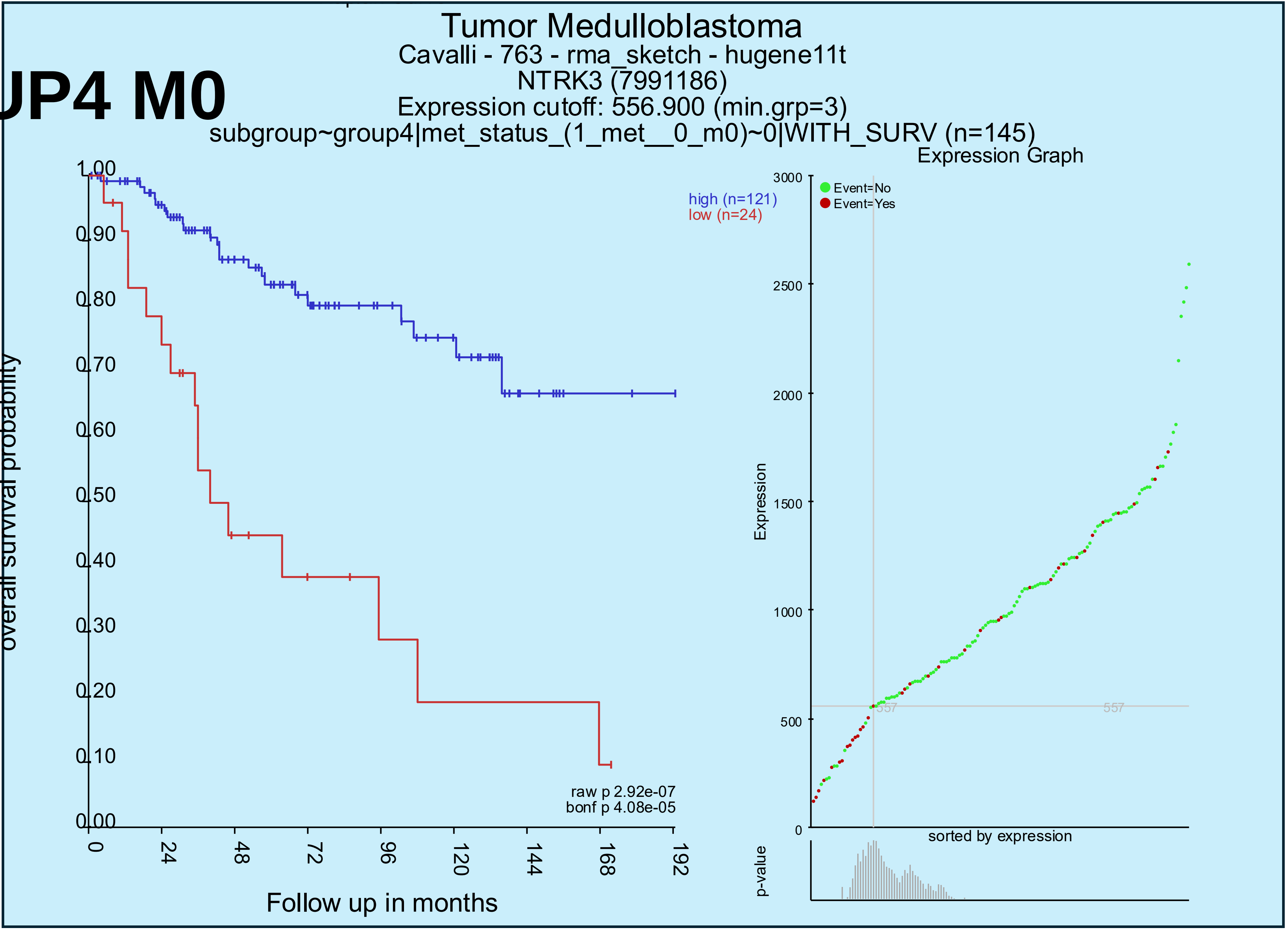
Tumor Medulloblastoma
Cavalli - 763 - rma_sketch - hugene11t
NTRK3 (7991186)
Expression cutoff: 556.900 (min.grp=3)
subgroup~group4|met_status_(1_met__0_m0)~0|WITH_SURV (n=145)
overall survival probability
1.00
0.90
0.80
0.70
0.60
0.50
0.40
0.30
0.20
0.10
0.00
high (n=121)
low (n=24)
raw p 2.92e-07
bonf p 4.08e-05
0
24
48
72
96
120
144
168
192
Follow up in months
Expression Graph
Event=No
Event=Yes
Expression
3000
2500
2000
1500
1000
500
0
557
557
sorted by expression
p-value

GROUP4 M1

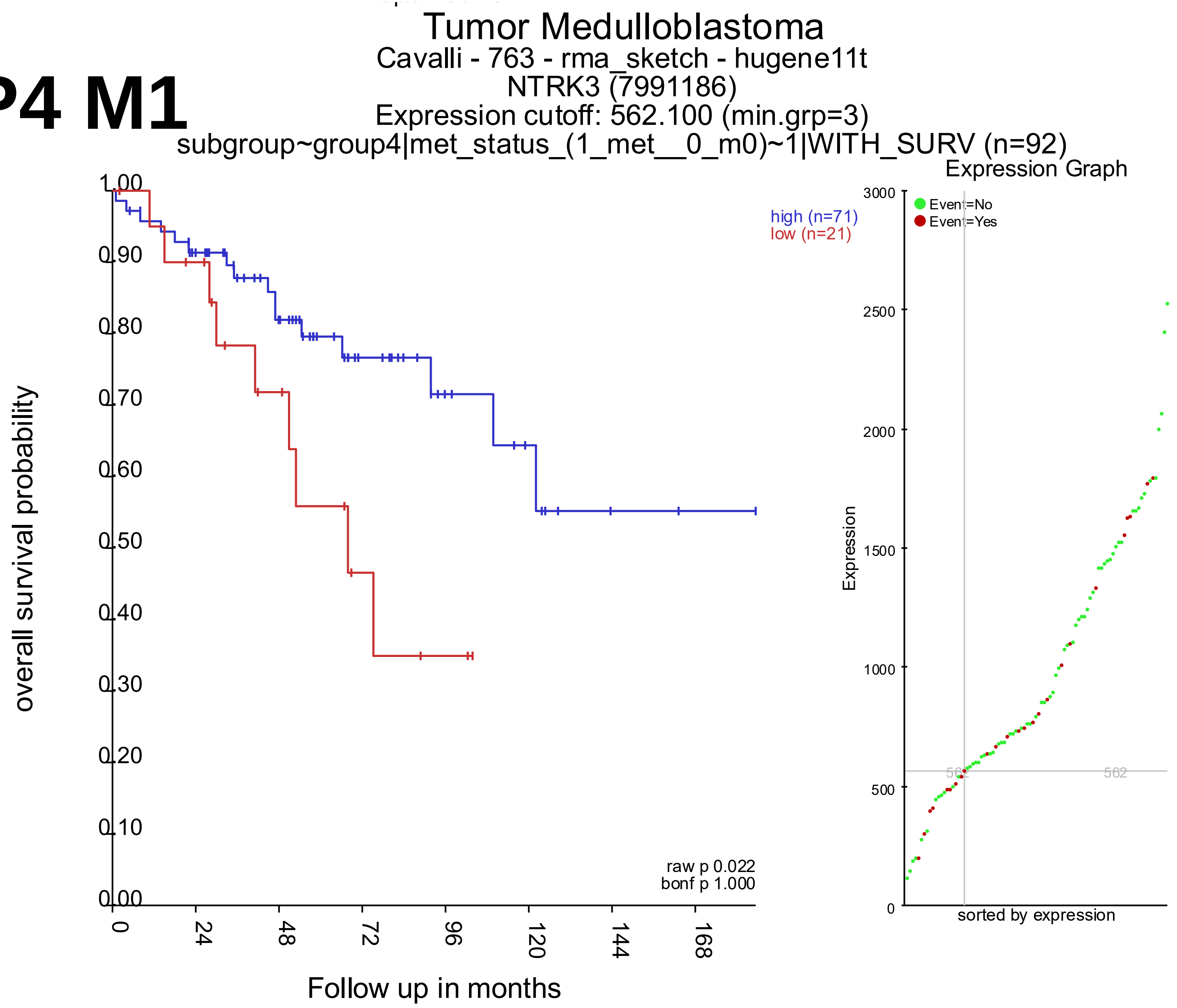

Tumor Medulloblastoma
Cavalli - 763 - rma_sketch - hugene11t
NTRK3 (7991186)
Expression cutoff: 562.100 (min.grp=3)
subgroup~group4|met_status_(1_met__0_m0)~1|WITH_SURV (n=92)
high (n=71)
low (n=21)
overall survival probability
1.00
0.90
0.80
0.70
0.60
0.50
0.40
0.30
0.20
0.10
0.00
raw p 0.022
bonf p 1.000
0
24
48
72
96
120
144
168
Follow up in months
Expression Graph
Event=No
Event=Yes
Expression
3000
2500
2000
1500
1000
500
0
562
sorted by expression

GROUP3 M0

Tumor Medulloblastoma
Cavalli - 763 - rma_sketch - hugene11t
NTRK3 (7991186)
Expression cutoff: 316.700 (min.grp=3)
subgroup~group3|met_status_(1_met__0_m0)~0|WITH_SURV (n=65)

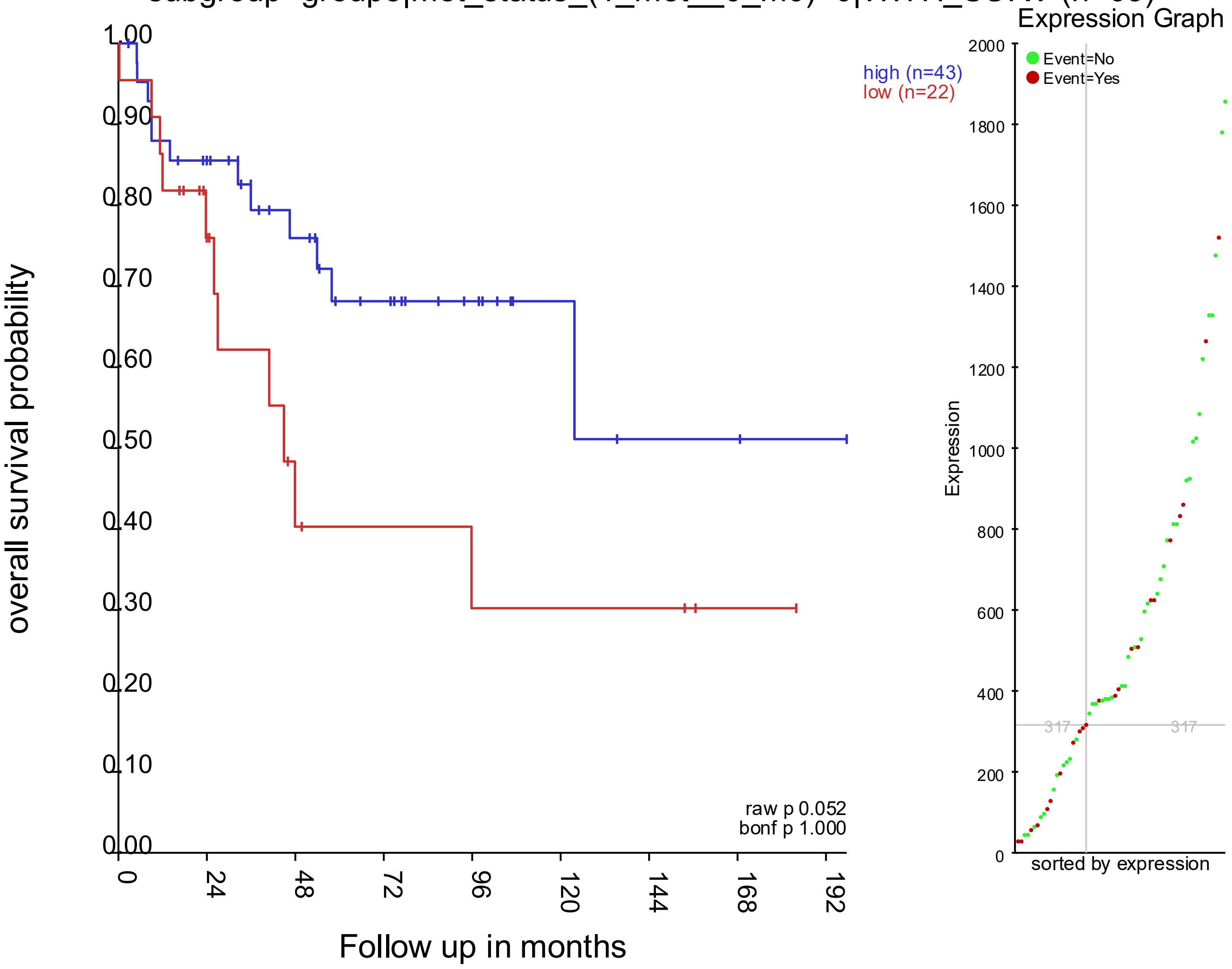

GROUP3 M1

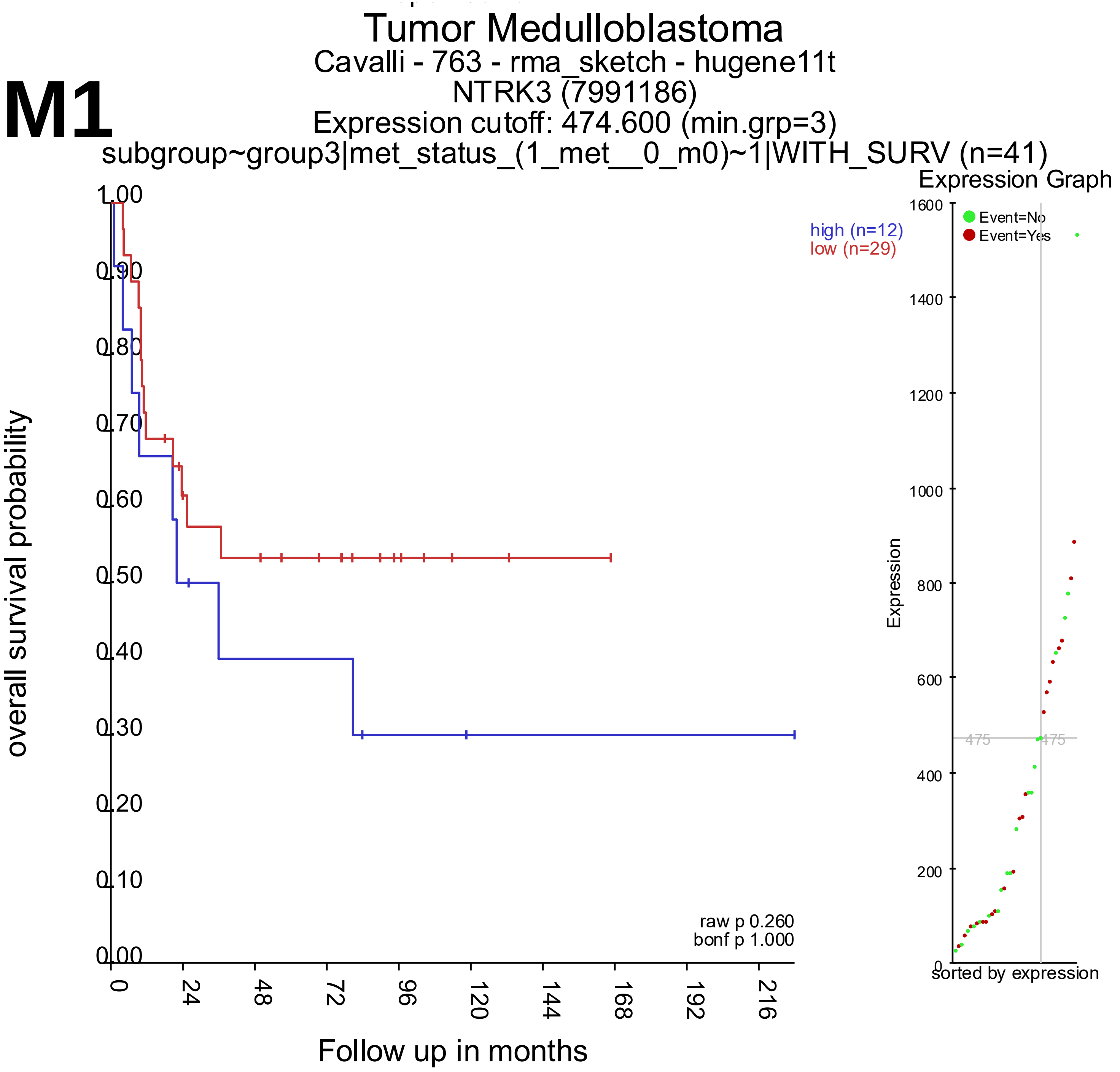
Tumor Medulloblastoma
Cavalli - 763 - rma_sketch - hugene11t
NTRK3 (7991186)
Expression cutoff: 474.600 (min.grp=3)
subgroup~group3|met_status_(1_met__0_m0)~1|WITH_SURV (n=41)
overall survival probability
1.00
0.90
0.80
0.70
0.60
0.50
0.40
0.30
0.20
0.10
0.00
0
24
48
72
96
120
144
168
192
216
Follow up in months
high (n=12)
low (n=29)
raw p 0.260
bonf p 1.000
Expression Graph
Event=No
Event=Yes
Expression
1600
1400
1200
1000
800
600
400
200
0
475
475
sorted by expression

PARP1

WNT M0

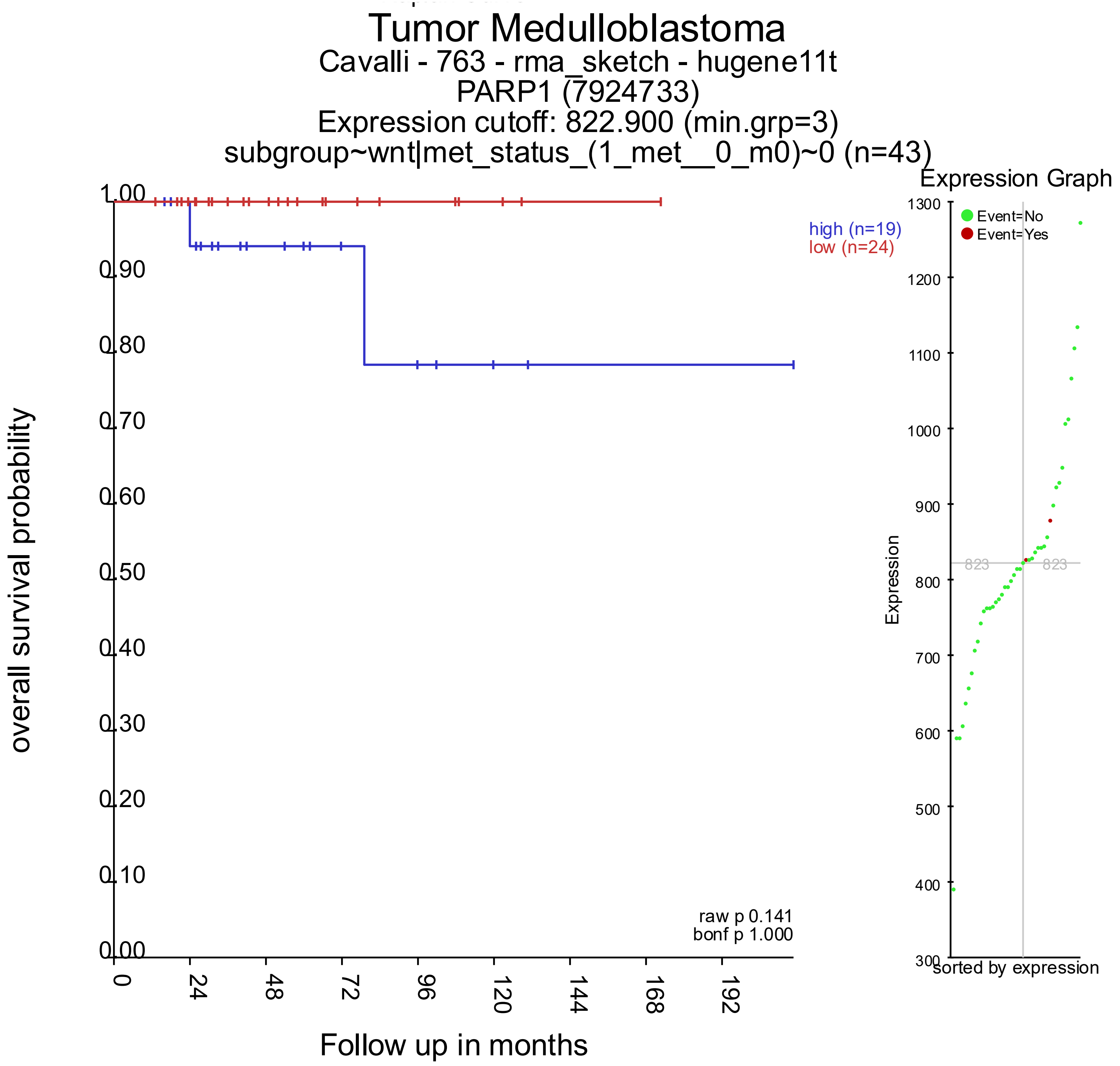

Tumor Medulloblastoma
Cavalli - 763 - rma_sketch - hugene11t
PARP1 (7924733)
Expression cutoff: 822.900 (min.grp=3)
subgroup~wnt|met_status_(1_met__0_m0)~0 (n=43)
high (n=19)
low (n=24)
overall survival probability
1.00
0.90
0.80
0.70
0.60
0.50
0.40
0.30
0.20
0.10
0.00
0
24
48
72
96
120
144
168
192
Follow up in months
raw p 0.141
bonf p 1.000
Expression Graph
Event=No
Event=Yes
Expression
1300
1200
1100
1000
900
800
700
600
500
400
300
823
823
sorted by expression

WNT M1

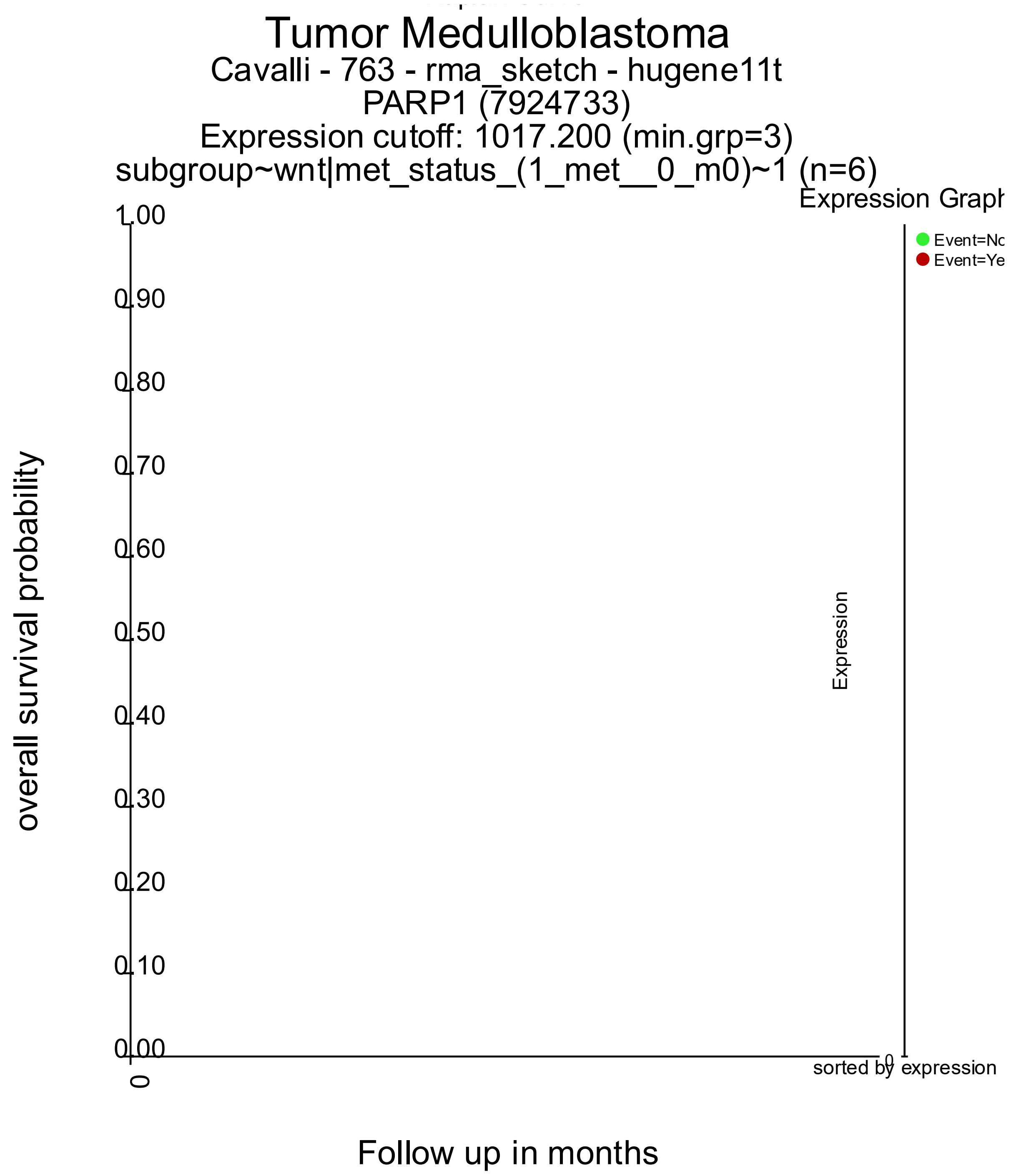

Tumor Medulloblastoma
Cavalli - 763 - rma_sketch - hugene11t
PARP1 (7924733)
Expression cutoff: 1017.200 (min.grp=3)
subgroup~wnt|met_status_(1_met__0_m0)~1 (n=6)
Expression Graph
Event=No
Event=Ye
1.00
0.90
0.80
0.70
0.60
0.50
0.40
0.30
0.20
0.10
0.00
overall survival probability
Expression
0
0
sorted by expression
Follow up in months

SHH M0

Tumor Medulloblastoma
Cavalli - 763 - rma_sketch - hugene11t
PARP1 (7924733)
Expression cutoff: 891.300 (min.grp=3)
subgroup~shh|met_status_(1_met__0_m0)~0|WITH_SURV (n=124)

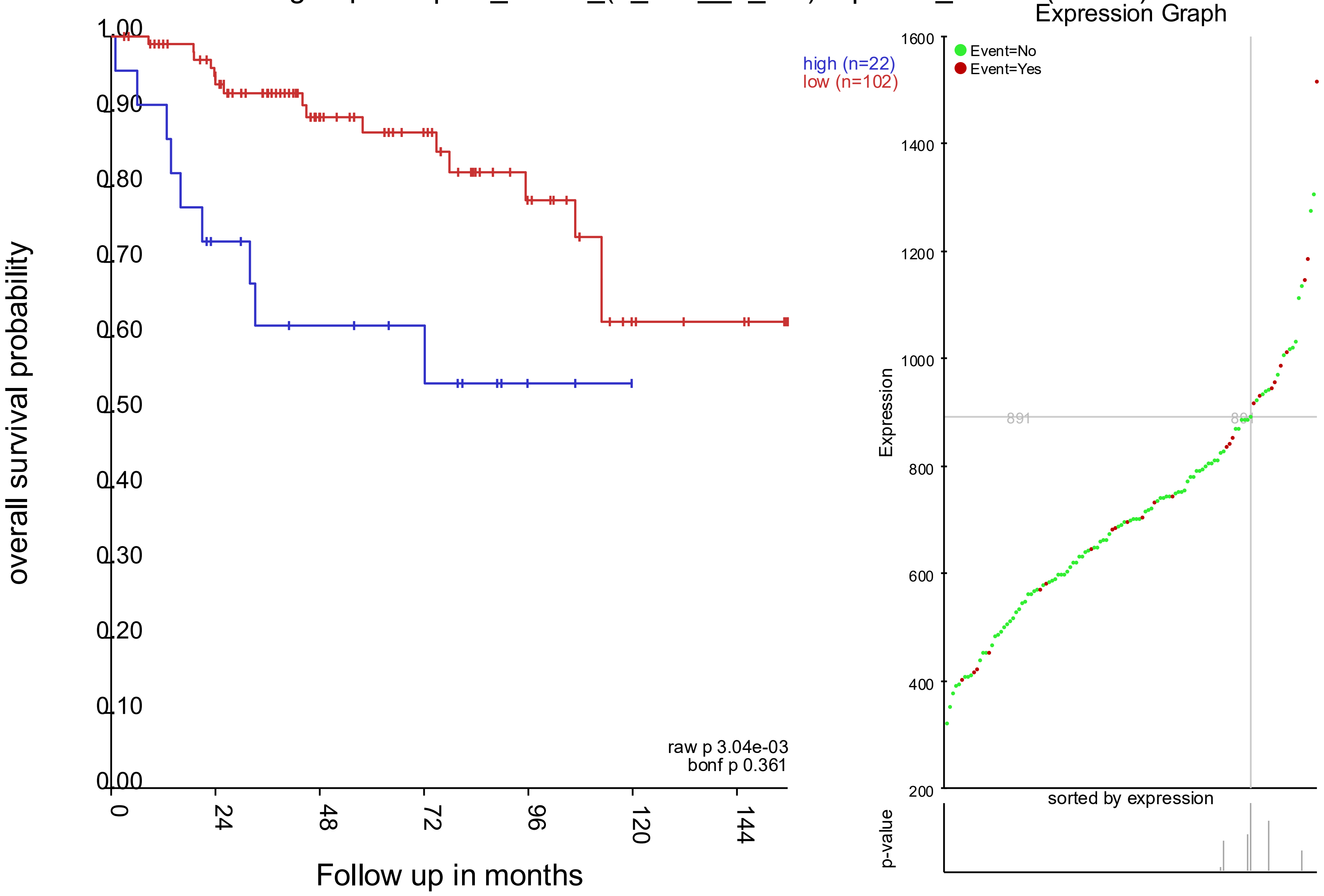

SHH M1

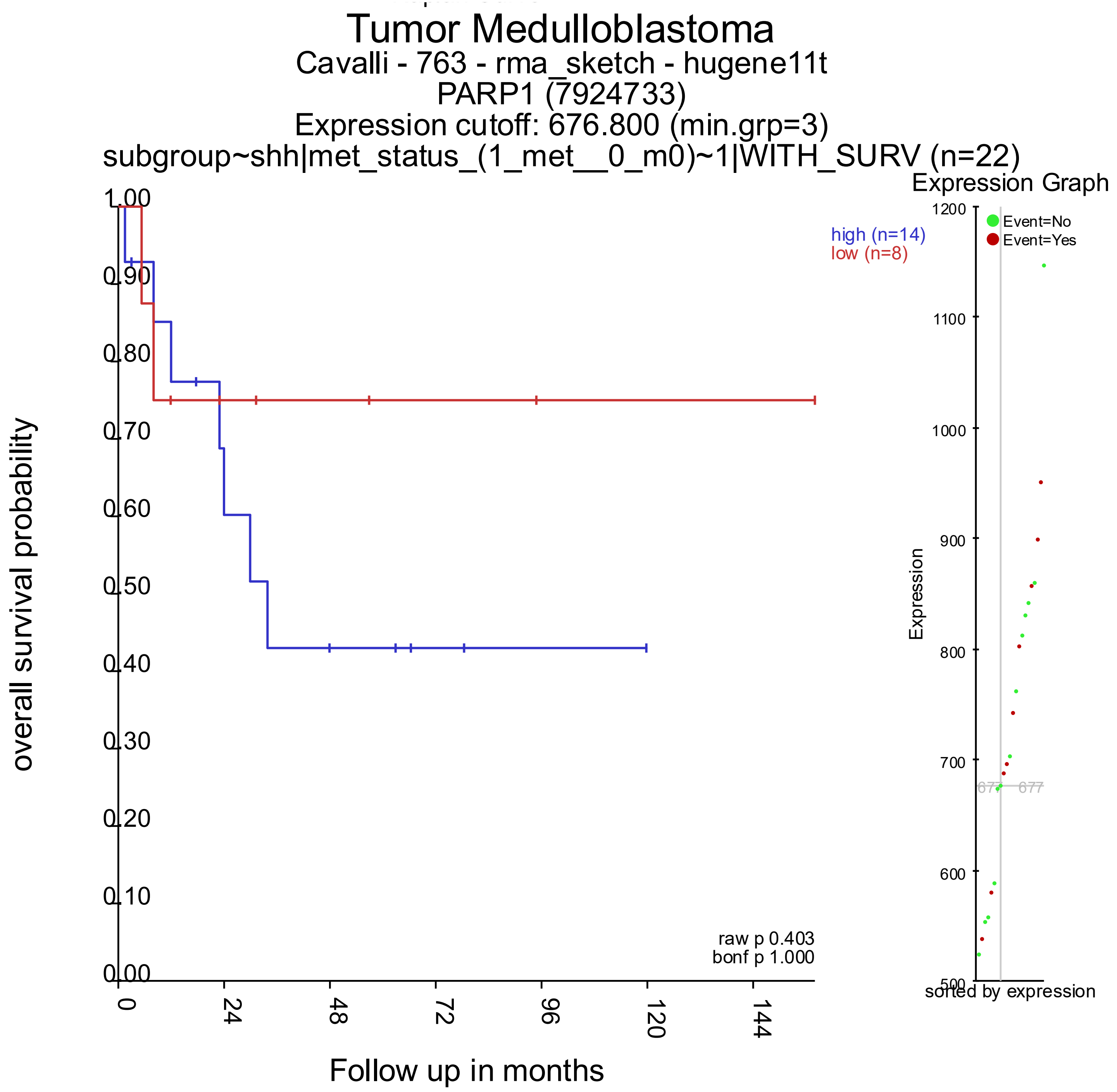

Tumor Medulloblastoma
Cavalli - 763 - rma_sketch - hugene11t
PARP1 (7924733)
Expression cutoff: 676.800 (min.grp=3)
subgroup~shh|met_status_(1_met__0_m0)~1|WITH_SURV (n=22)
overall survival probability
1.00
0.90
0.80
0.70
0.60
0.50
0.40
0.30
0.20
0.10
0.00
high (n=14)
low (n=8)
raw p 0.403
bonf p 1.000
0
24
48
72
96
120
144
Follow up in months
Expression Graph
Event=No
Event=Yes
Expression
1200
1100
1000
900
800
700
600
500
677
677
sorted by expression

GROUP4 M0

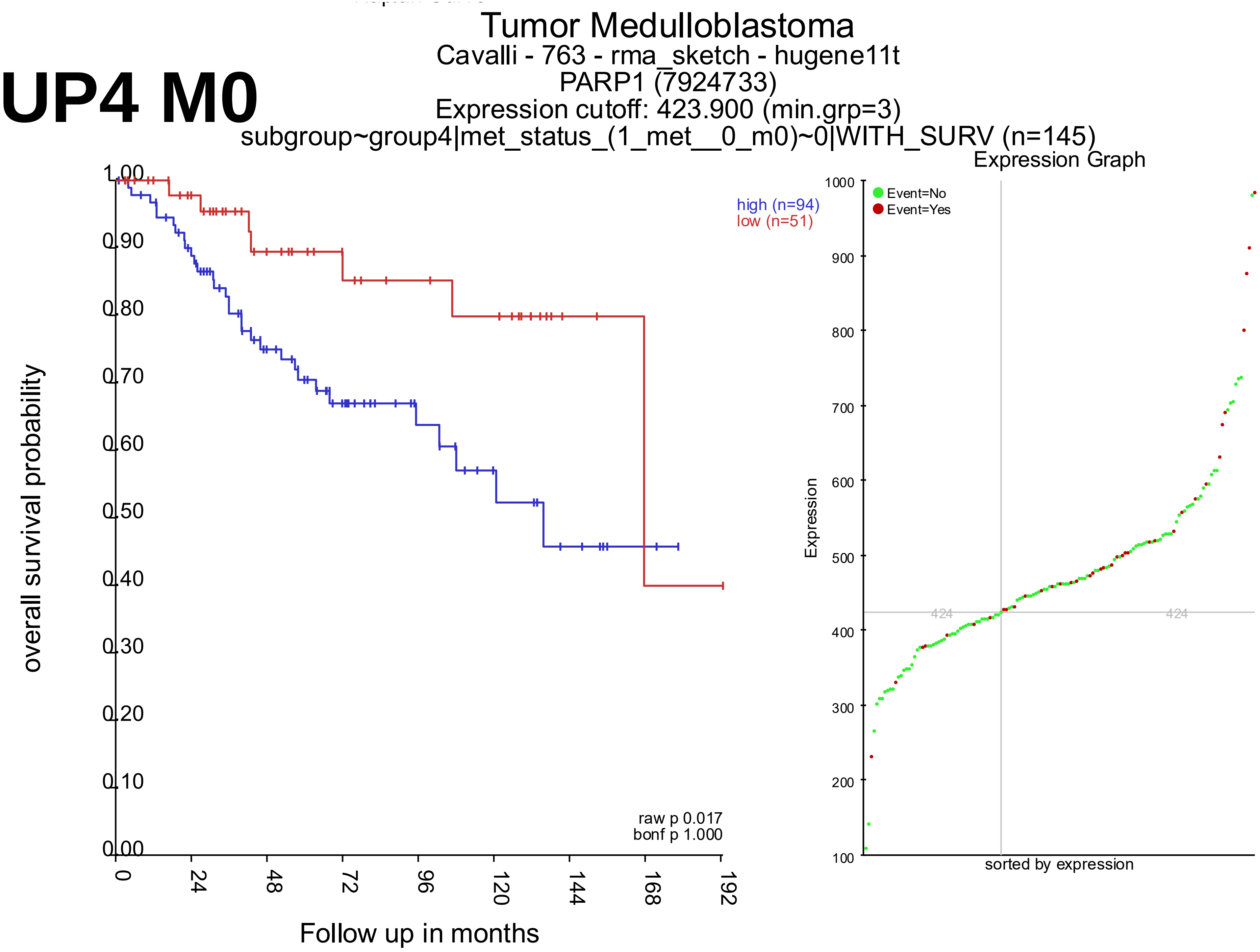

Tumor Medulloblastoma
Cavalli - 763 - rma_sketch - hugene11t
PARP1 (7924733)
Expression cutoff: 423.900 (min.grp=3)
subgroup~group4|met_status_(1_met__0_m0)~0|WITH_SURV (n=145)
high (n=94)
low (n=51)
overall survival probability
1.00
0.90
0.80
0.70
0.60
0.50
0.40
0.30
0.20
0.10
0.00
raw p 0.017
bonf p 1.000
0
24
48
72
96
120
144
168
192
Follow up in months
Expression Graph
Event=No
Event=Yes
Expression
1000
900
800
700
600
500
400
300
200
100
424
424
sorted by expression

GROUP4 M1

Tumor Medulloblastoma
Cavalli - 763 - rma_sketch - hugene11t
PARP1 (7924733)
Expression cutoff: 375.400 (min.grp=3)
subgroup~group4|met_status_(1_met__0_m0)~1|WITH_SURV (n=92)

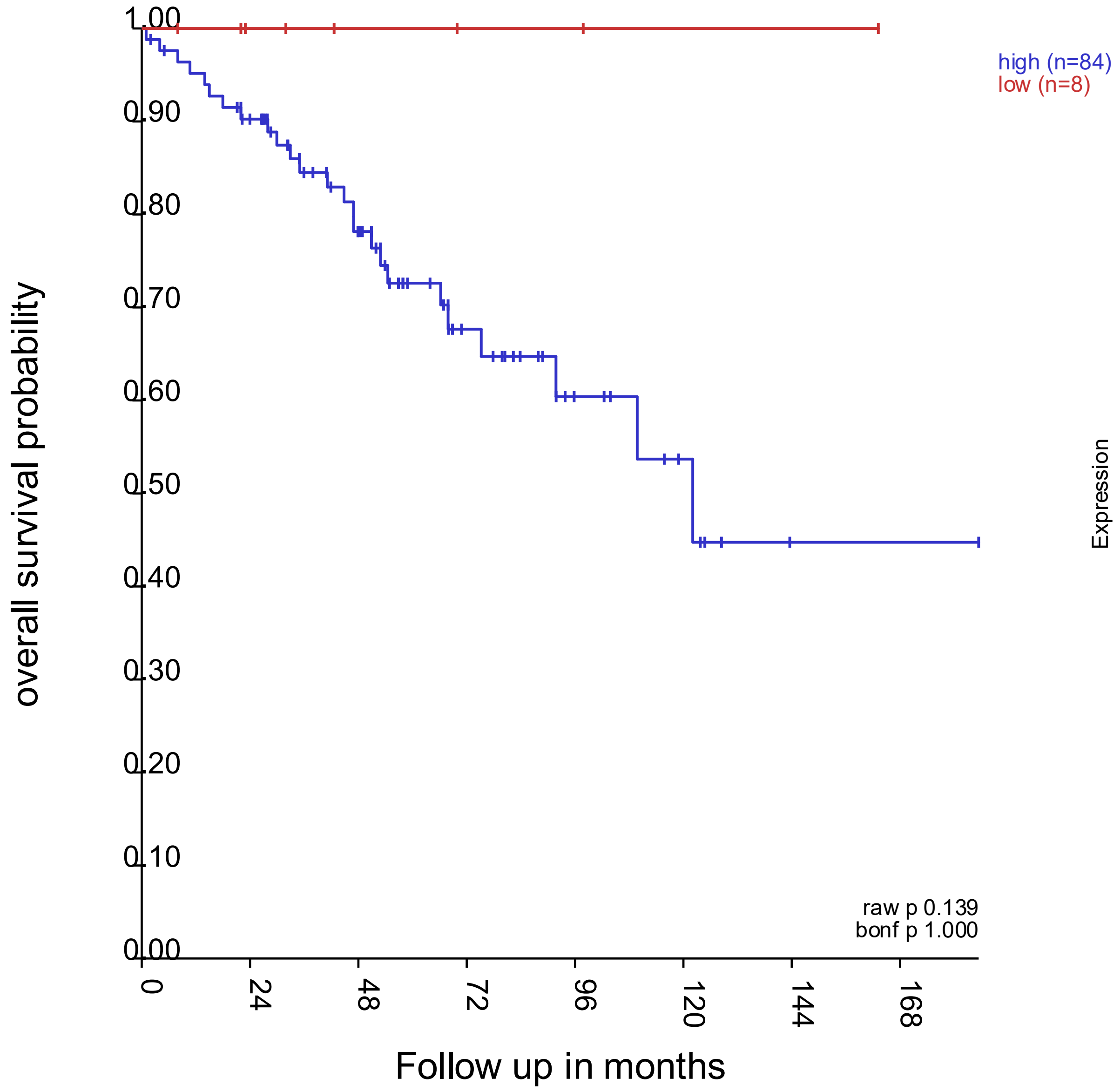

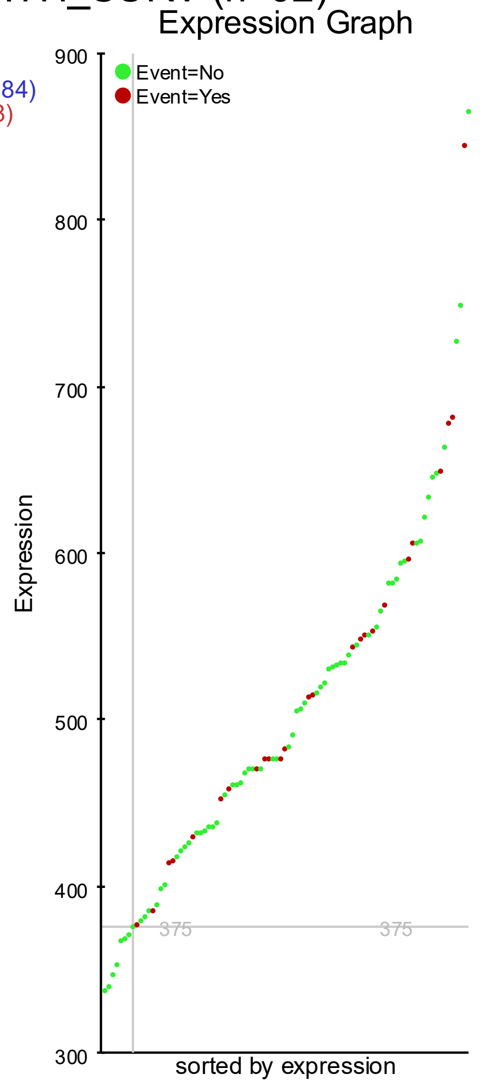

GROUP3 M0

Tumor Medulloblastoma
Cavalli - 763 - rma_sketch - hugene11t
PARP1 (7924733)
Expression cutoff: 861.900 (min.grp=3)
subgroup~group3|met_status_(1_met__0_m0)~0|WITH_SURV (n=65)

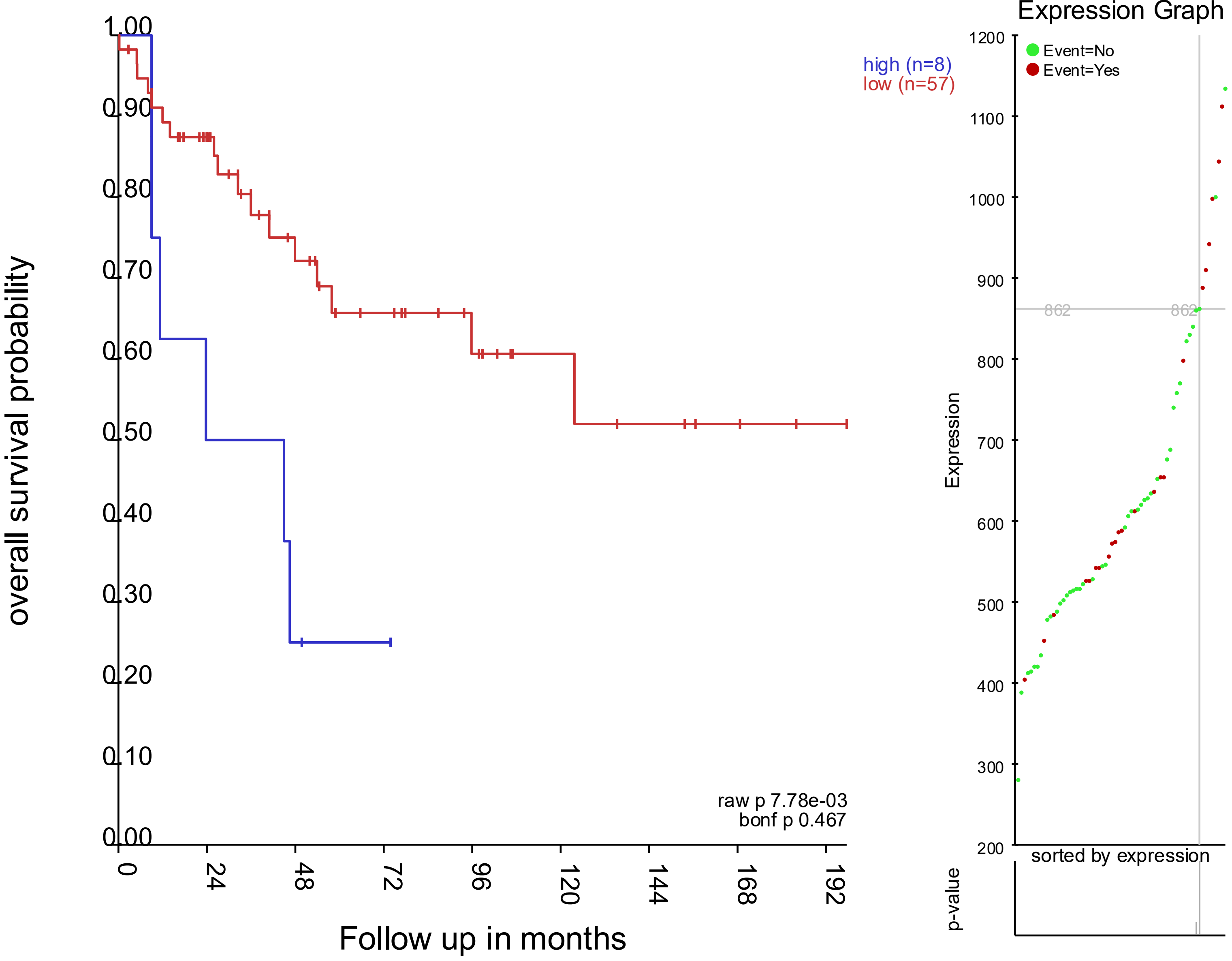

GROUP3 M1

Tumor Medulloblastoma
Cavalli - 763 - rma_sketch - hugene11t
PARP1 (7924733)
Expression cutoff: 475.400 (min.grp=3)
subgroup~group3|met_status_(1_met__0_m0)~1|WITH_SURV (n=41)

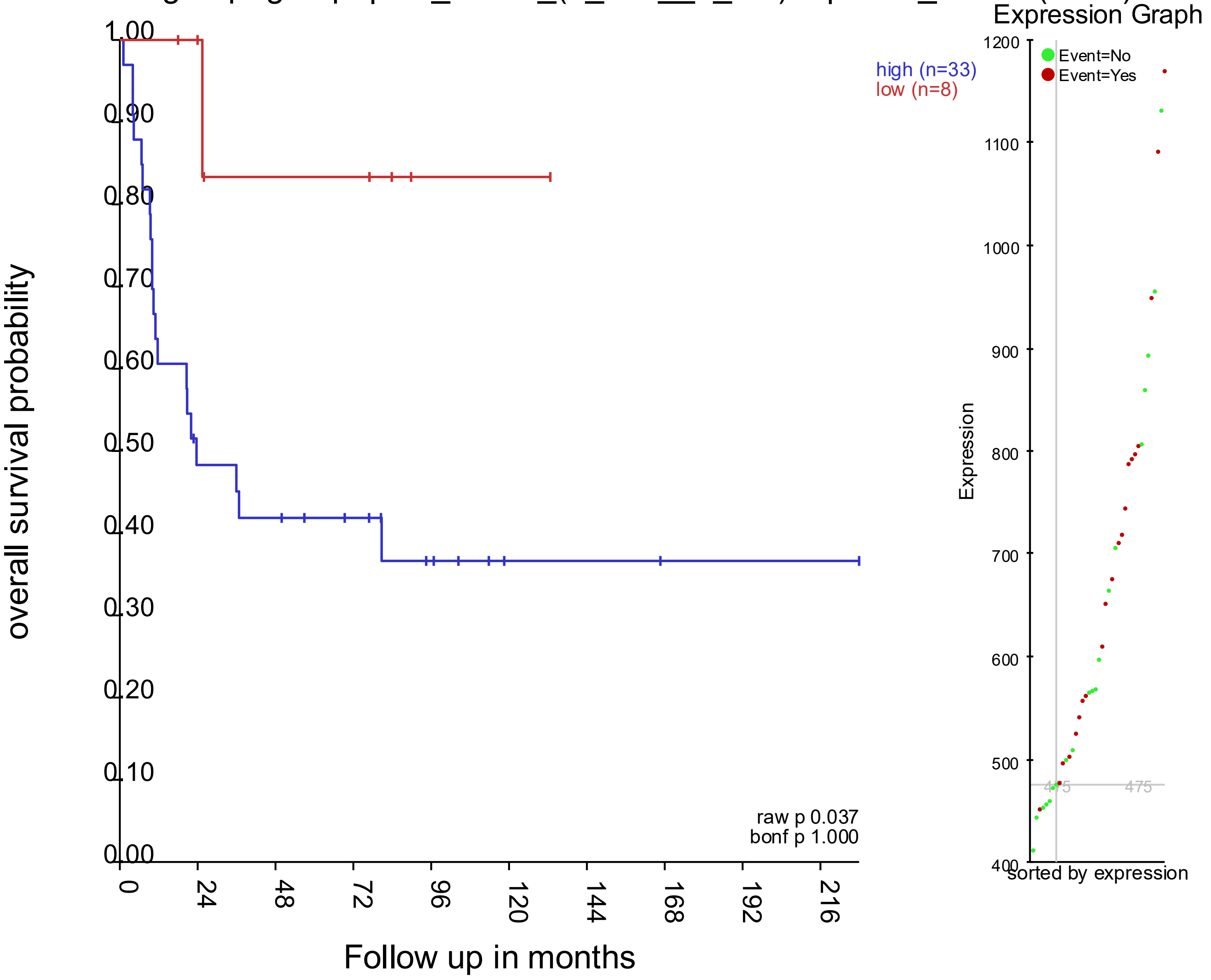

PDGFRA

WNT M0

Tumor Medulloblastoma
Cavalli - 763 - rma_sketch - hugene11t
PDGFRA (8095080)
Expression cutoff: 212.400 (min.grp=3)
subgroup~wnt|met_status_(1_met__0_m0)~0 (n=43)

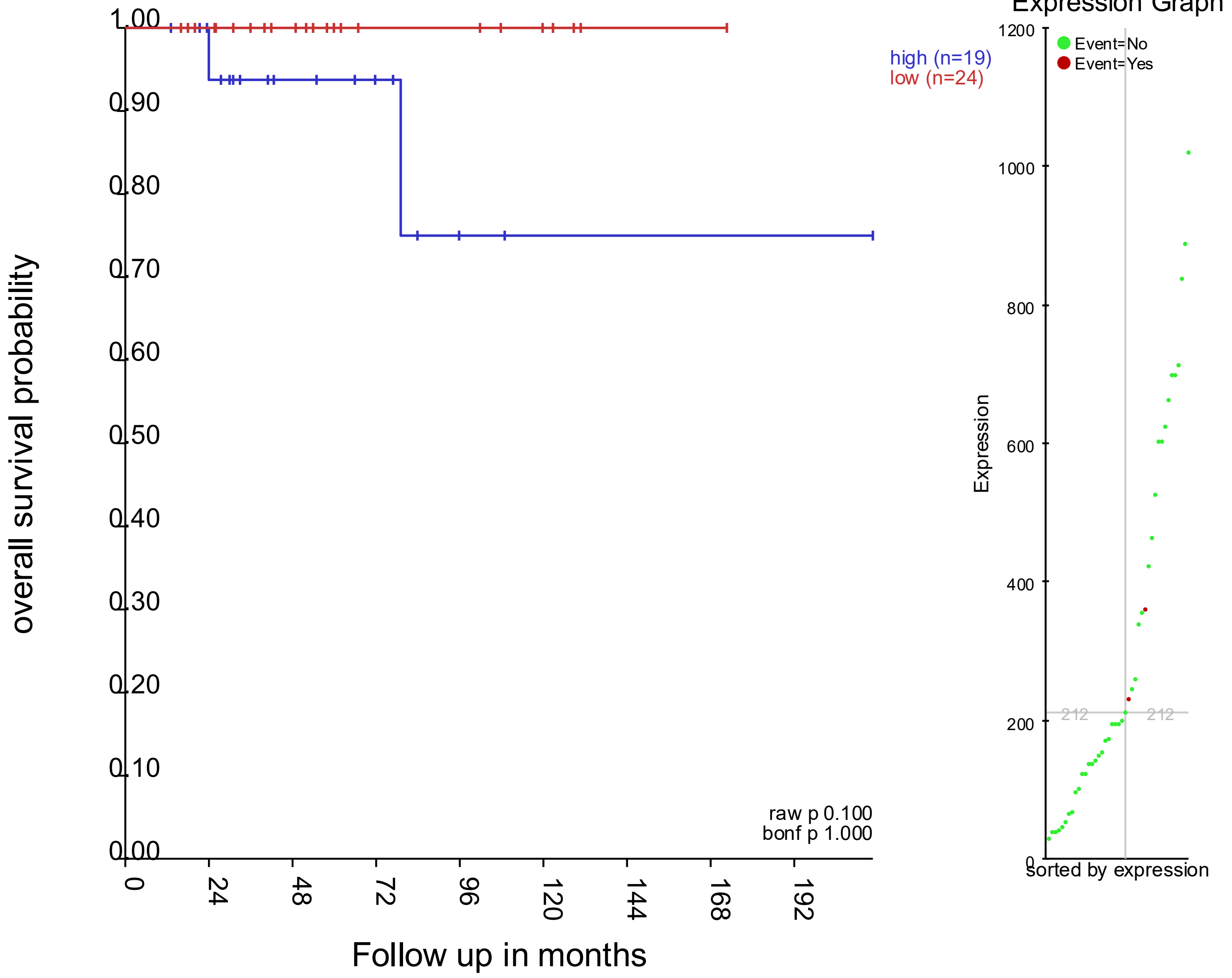

WNT M1

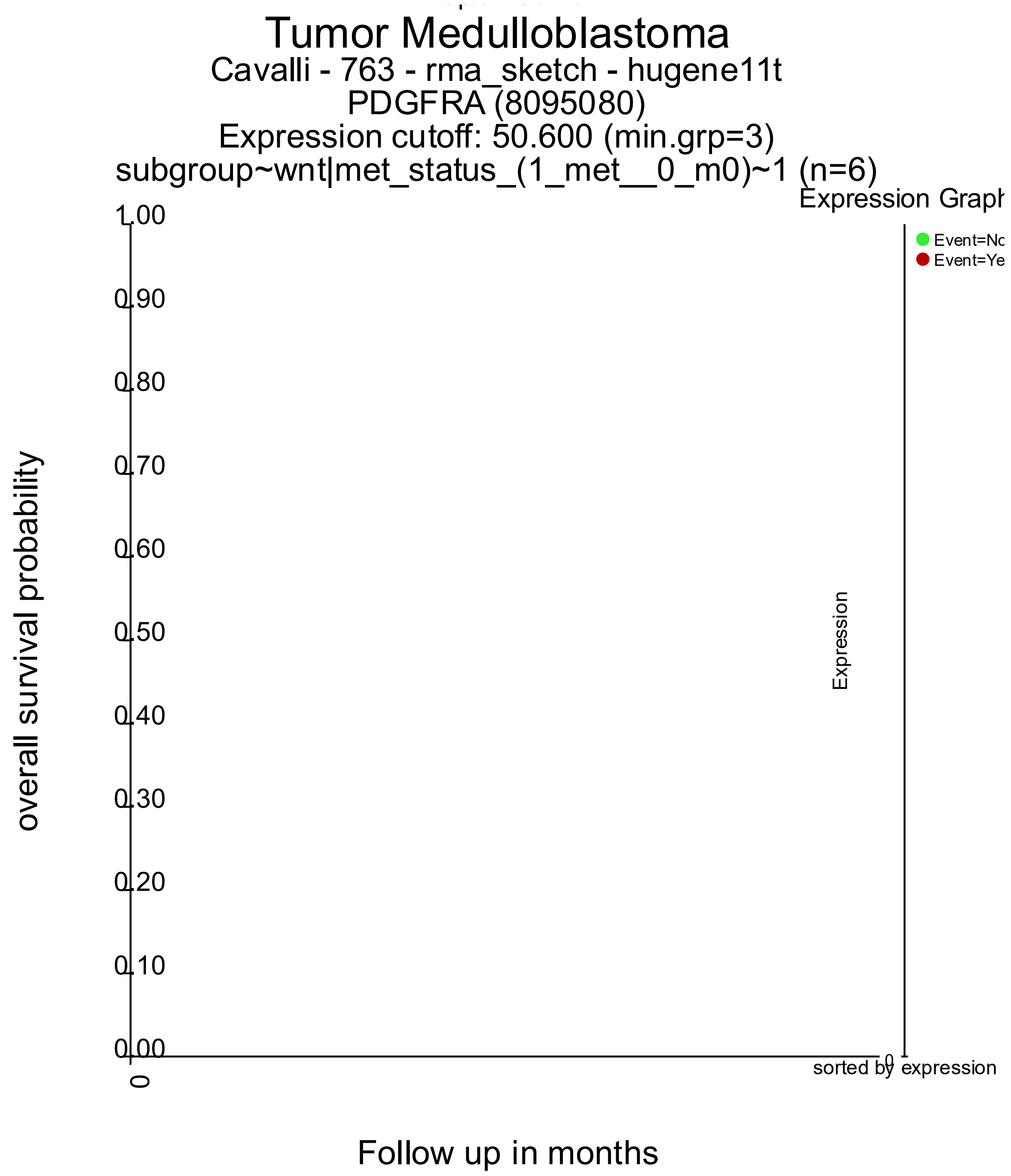

Tumor Medulloblastoma
Cavalli - 763 - rma_sketch - hugene11t
PDGFRA (8095080)
Expression cutoff: 50.600 (min.grp=3)
subgroup~wnt|met_status_(1_met__0_m0)~1 (n=6)
Expression Graph
Event=No
Event=Yes
overall survival probability
1.00
0.90
0.80
0.70
0.60
0.50
0.40
0.30
0.20
0.10
0.00
0
Follow up in months
Expression
0
sorted by expression

SHH M0

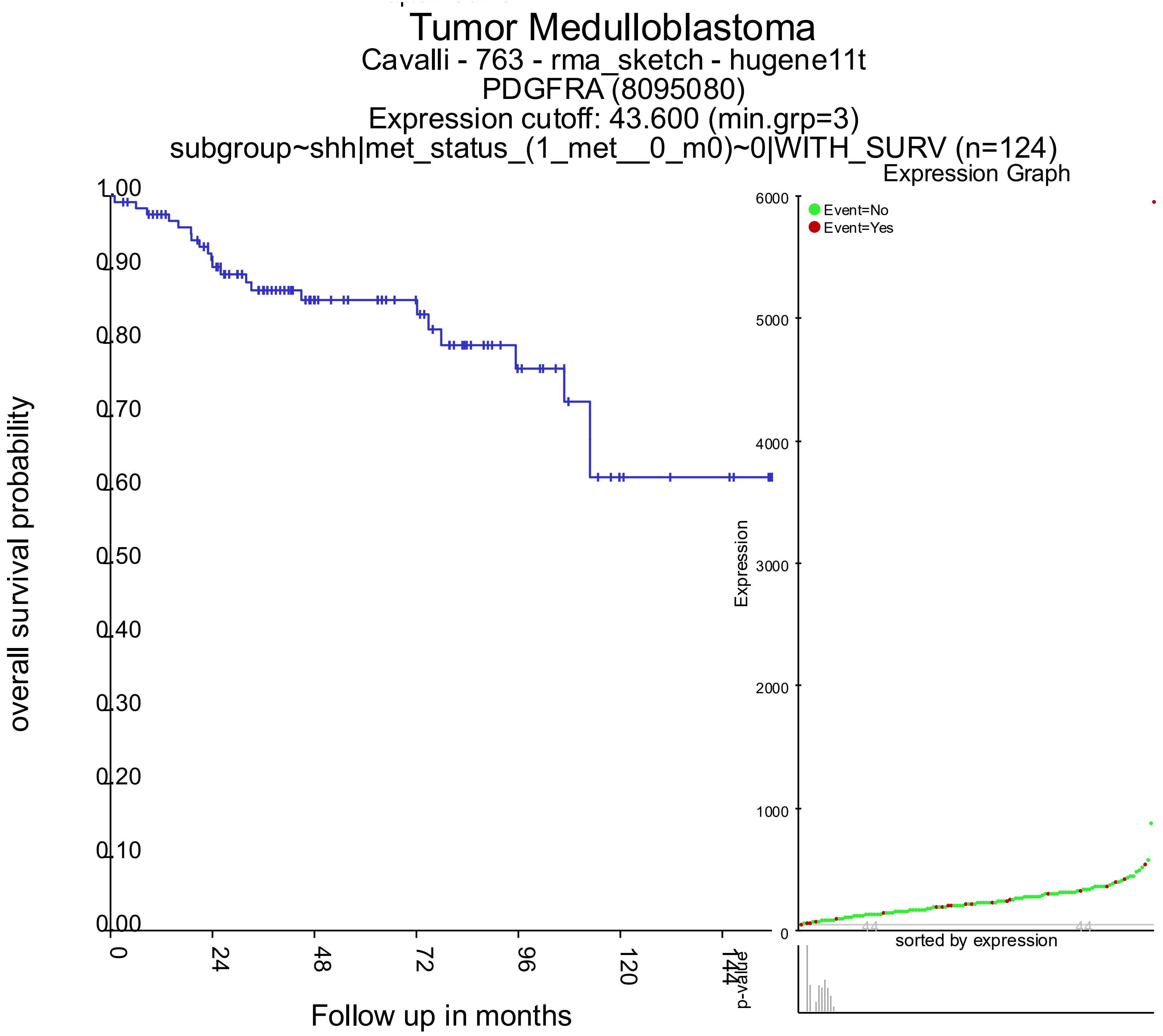

Tumor Medulloblastoma
Cavalli - 763 - rma_sketch - hugene11t
PDGFRA (8095080)
Expression cutoff: 43.600 (min.grp=3)
subgroup~shh|met_status_(1_met__0_m0)~0|WITH_SURV (n=124)
overall survival probability
1.00
0.90
0.80
0.70
0.60
0.50
0.40
0.30
0.20
0.10
0.00
0
24
48
72
96
120
144
Follow up in months
p-value
Expression Graph
Event=No
Event=Yes
Expression
6000
5000
4000
3000
2000
1000
0
sorted by expression

SHH M1

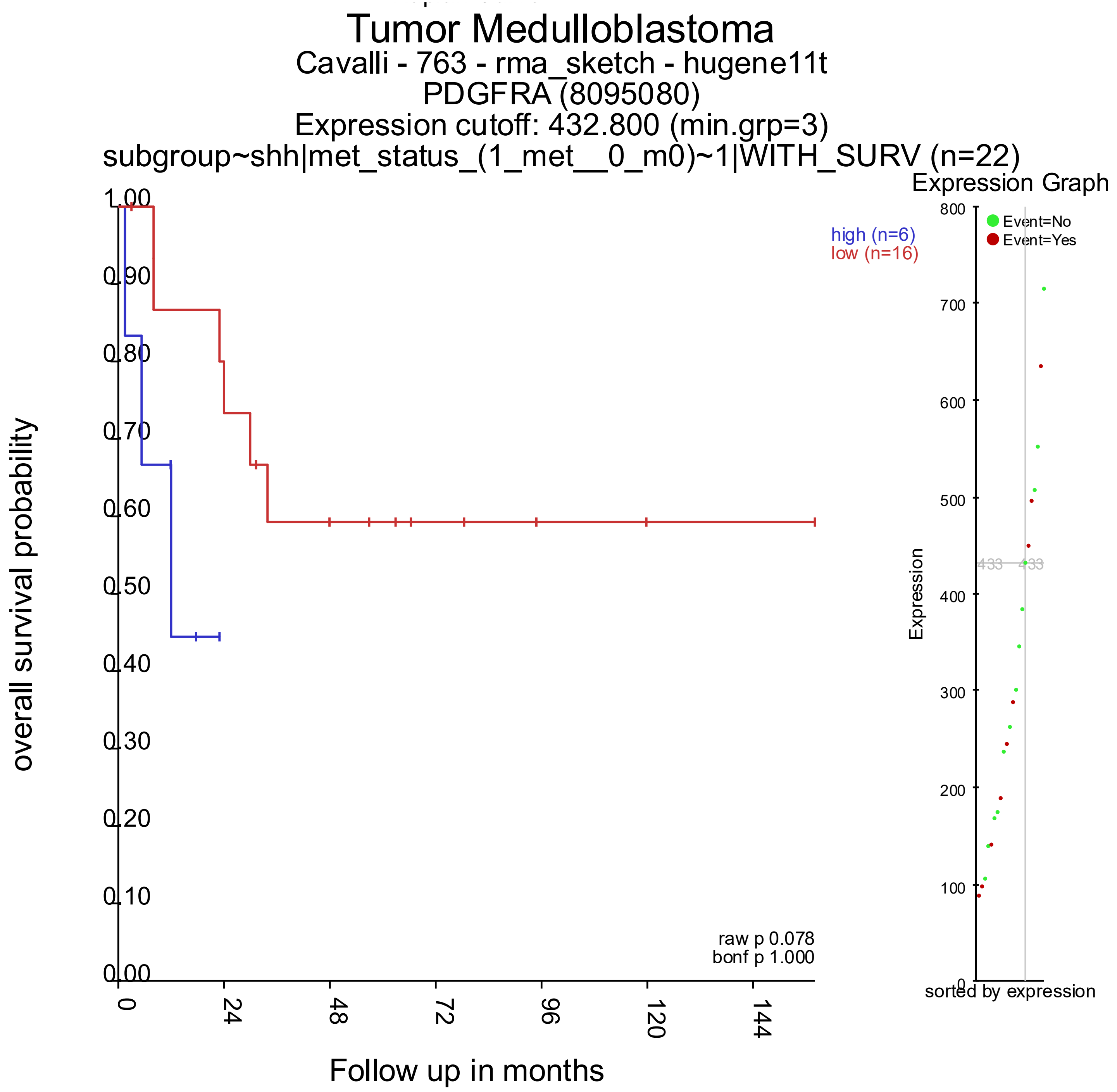
Tumor Medulloblastoma
Cavalli - 763 - rma_sketch - hugene11t
PDGFRA (8095080)
Expression cutoff: 432.800 (min.grp=3)
subgroup~shh|met_status_(1_met__0_m0)~1|WITH_SURV (n=22)
overall survival probability
high (n=6)
low (n=16)
raw p 0.078
bonf p 1.000
Follow up in months
Expression Graph
Event=No
Event=Yes
Expression
sorted by expression

GROUP4 M0

Tumor Medulloblastoma
Cavalli - 763 - rma_sketch - hugene11t
PDGFRA (8095080)
Expression cutoff: 32.600 (min.grp=3)
subgroup~group4|met_status_(1_met__0_m0)~0|WITH_SURV (n=145)

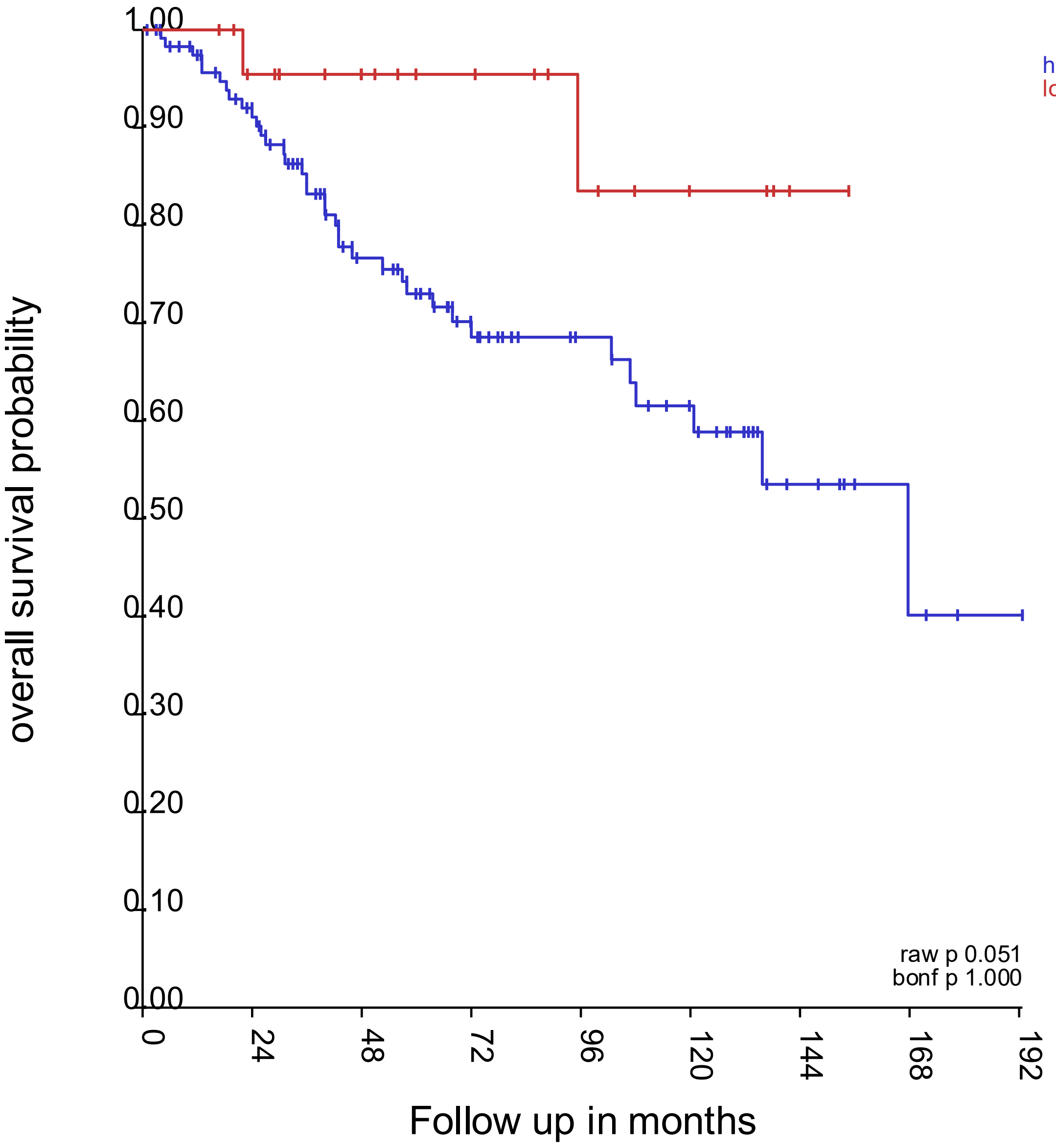

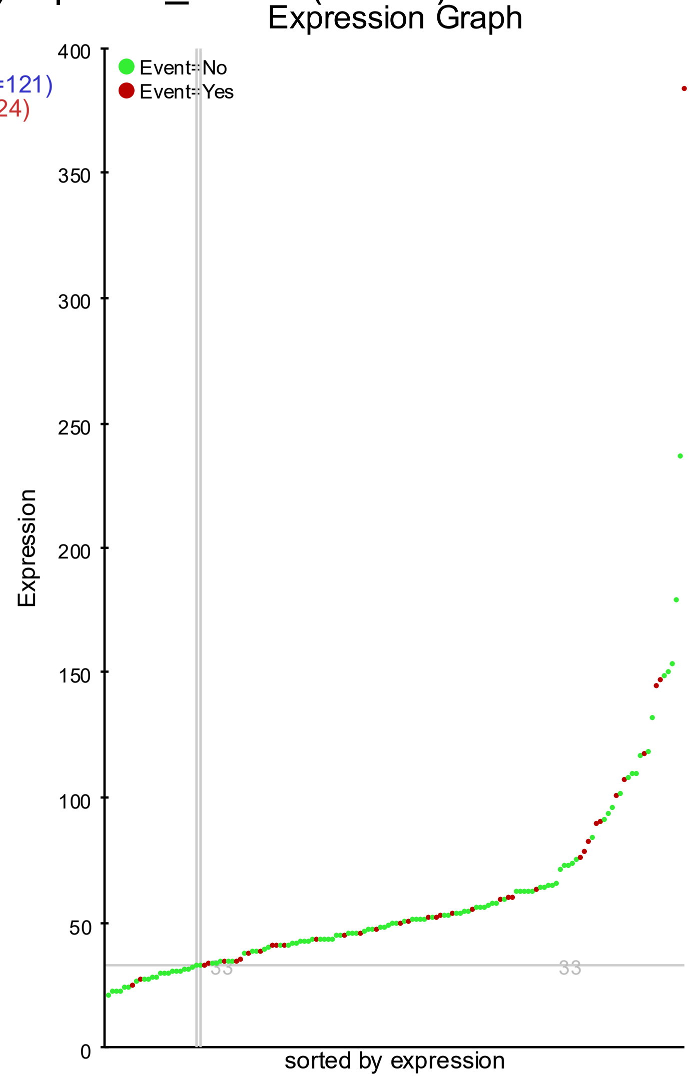

GROUP4 M1

Tumor Medulloblastoma
Cavalli - 763 - rma_sketch - hugene11t
PDGFRA (8095080)
Expression cutoff: 82.100 (min.grp=3)
subgroup~group4|met_status_(1_met__0_m0)~1|WITH_SURV (n=92)

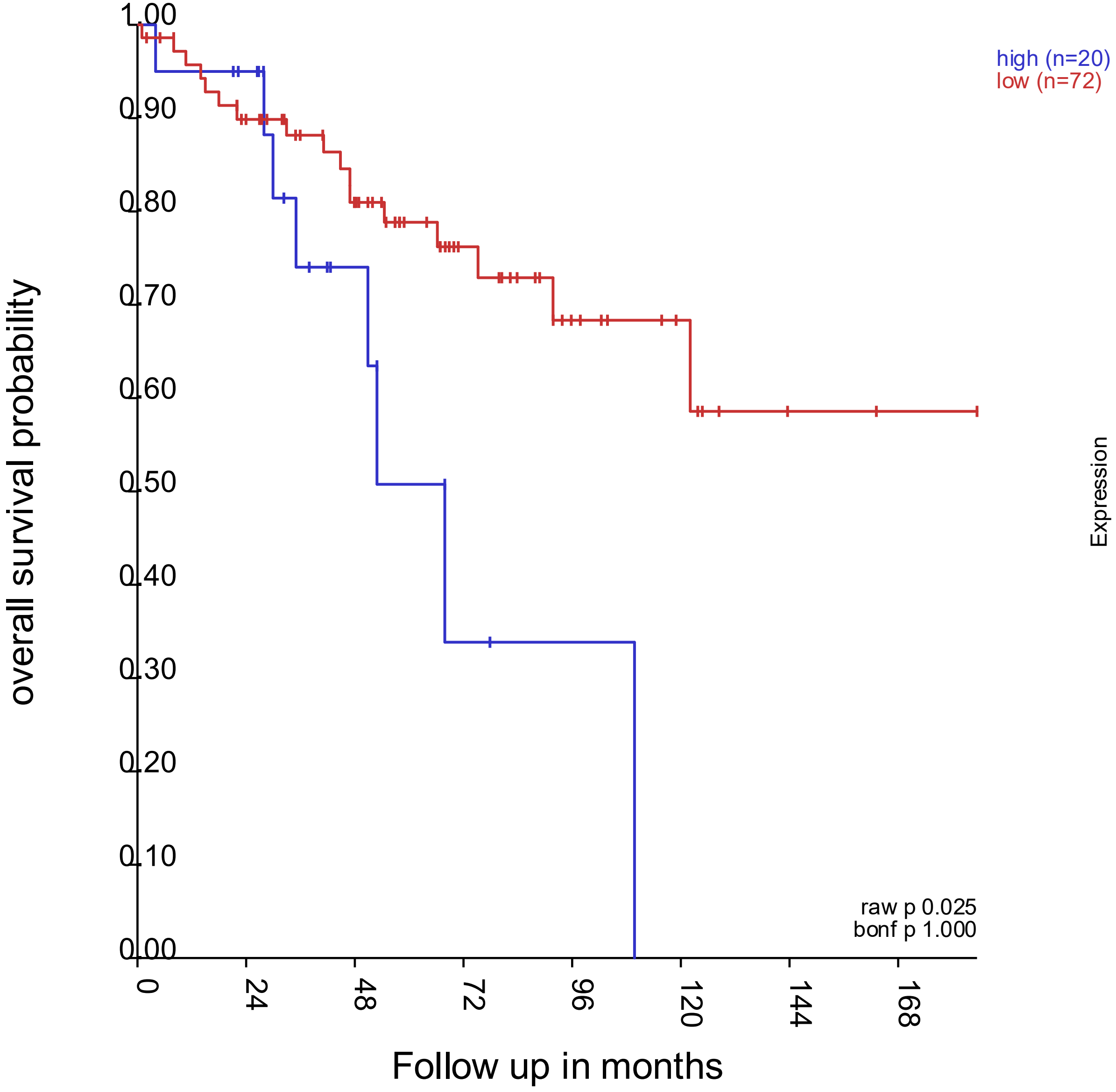

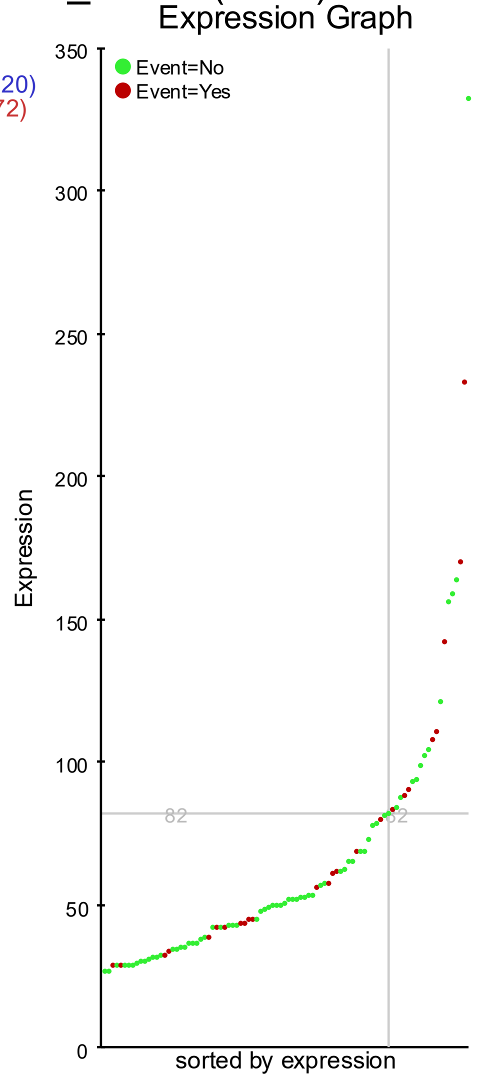

GROUP3 M0

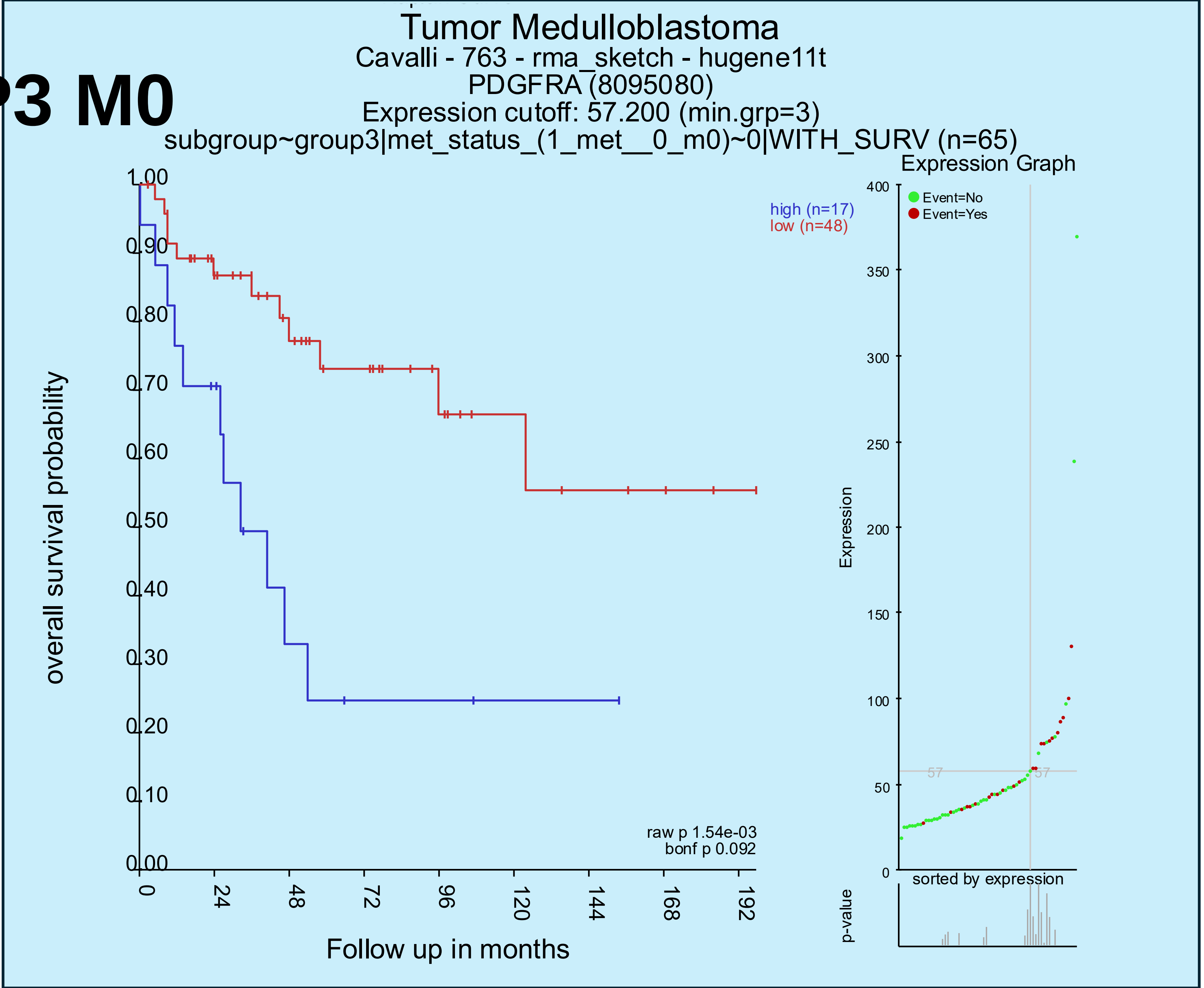
Tumor Medulloblastoma
Cavalli - 763 - rma_sketch - hugene11t
PDGFRA (8095080)
Expression cutoff: 57.200 (min.grp=3)
subgroup~group3|met_status_(1_met__0_m0)~0|WITH_SURV (n=65)
high (n=17)
low (n=48)
overall survival probability
Follow up in months
raw p 1.54e-03
bonf p 0.092
Expression Graph
Event=No
Event=Yes
Expression
sorted by expression
p-value

GROUP3 M1

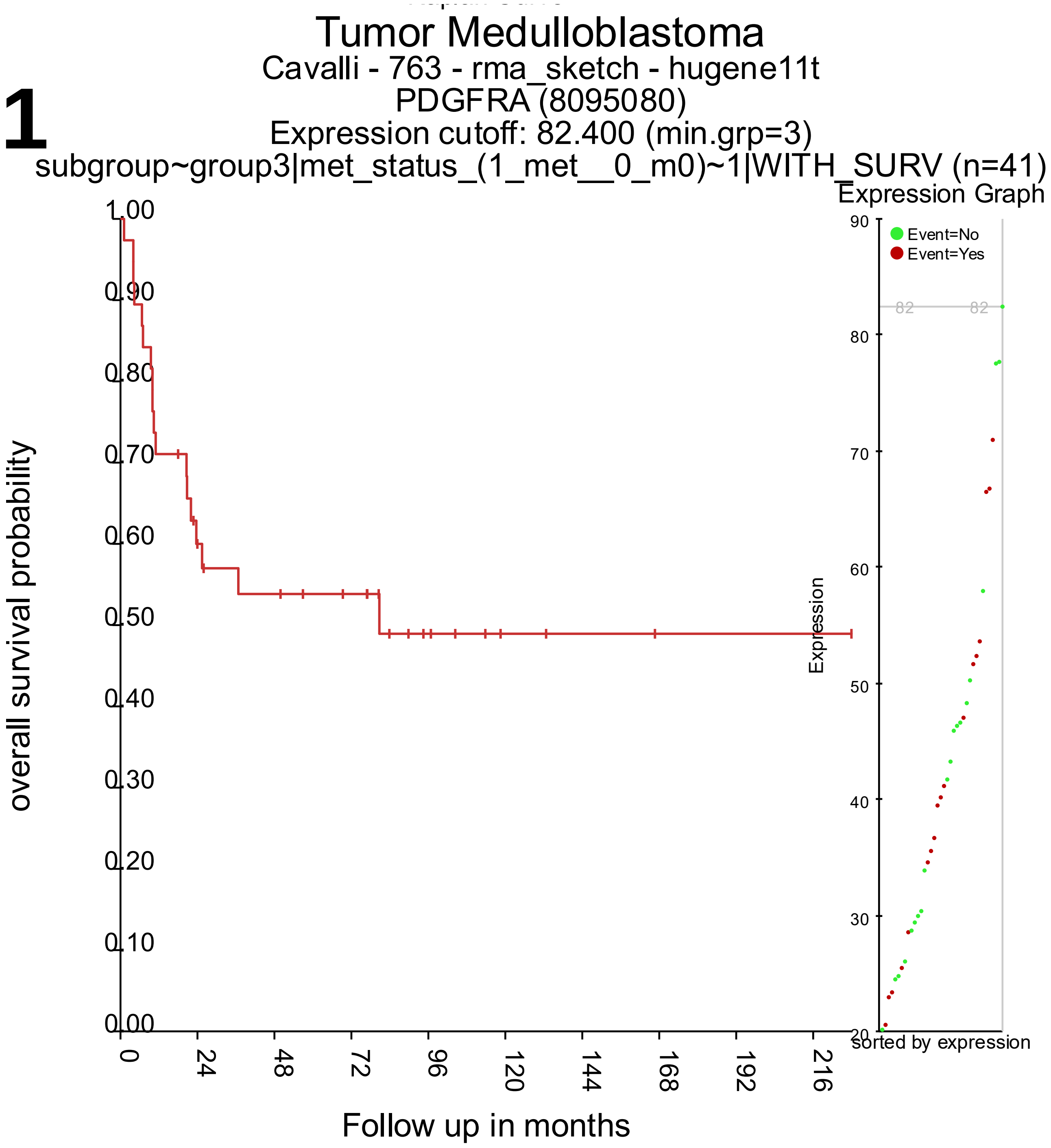

Tumor Medulloblastoma
Cavalli - 763 - rma_sketch - hugene11t
PDGFRA (8095080)
Expression cutoff: 82.400 (min.grp=3)
subgroup~group3|met_status_(1_met__0_m0)~1|WITH_SURV (n=41)
overall survival probability
1.00
0.90
0.80
0.70
0.60
0.50
0.40
0.30
0.20
0.10
0.00
0
24
48
72
96
120
144
168
192
216
Follow up in months
Expression Graph
Event=No
Event=Yes
Expression
90
80
70
60
50
40
30
20
82
82
sorted by expression

PDGFRB

WNT M0

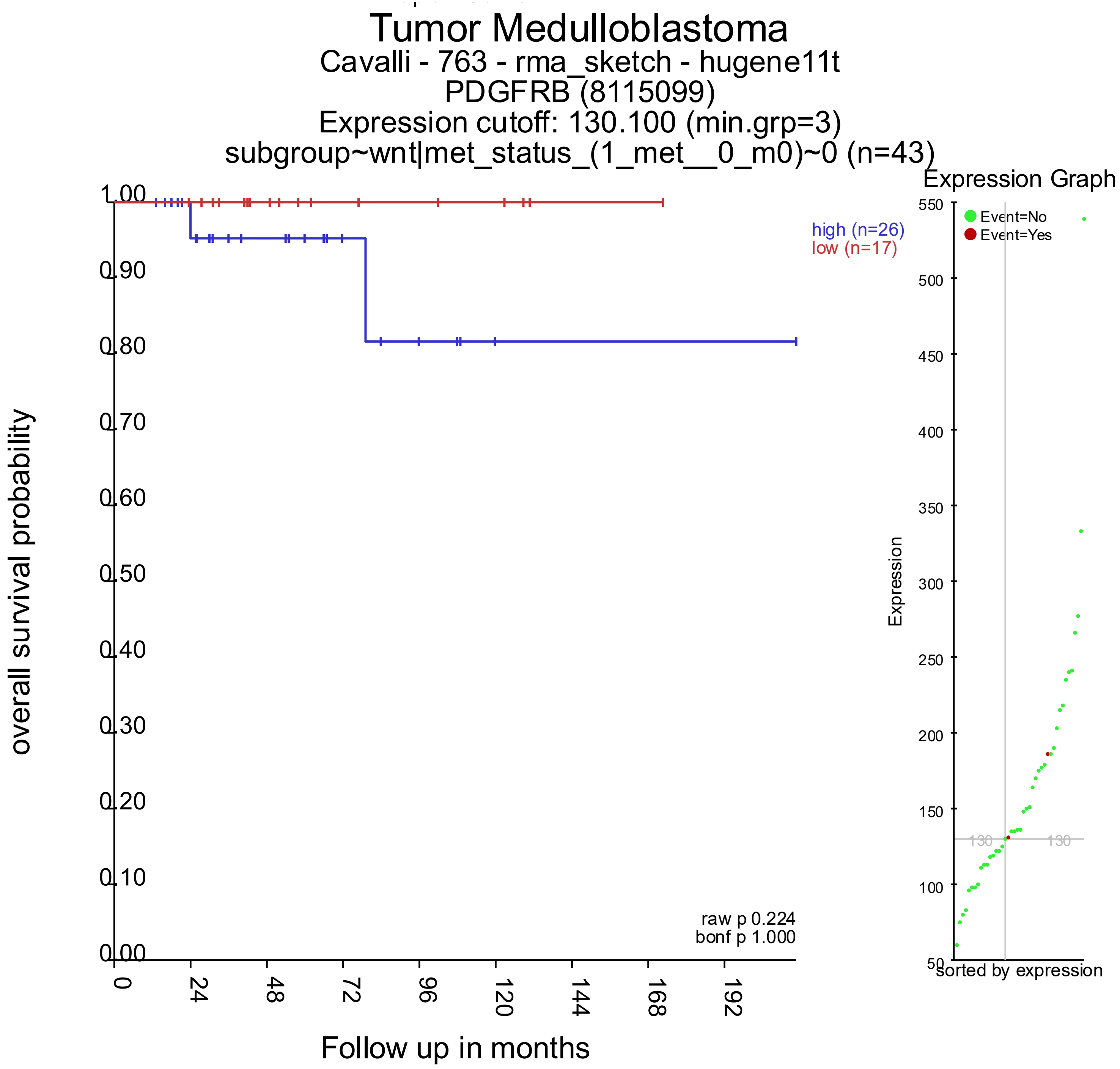
Tumor Medulloblastoma
Cavalli - 763 - rma_sketch - hugene11t
PDGFRB (8115099)
Expression cutoff: 130.100 (min.grp=3)
subgroup~wnt|met_status_(1_met__0_m0)~0 (n=43)
high (n=26)
low (n=17)
overall survival probability
1.00
0.90
0.80
0.70
0.60
0.50
0.40
0.30
0.20
0.10
0.00
0
24
48
72
96
120
144
168
192
Follow up in months
raw p 0.224
bonf p 1.000
Expression Graph
Event=No
Event=Yes
Expression
550
500
450
400
350
300
250
200
150
100
50
130
130
sorted by expression

WNT M1

Tumor Medulloblastoma
Cavalli - 763 - rma_sketch - hugene11t
PDGFRB (8115099)
Expression cutoff: 96.400 (min.grp=3)
subgroup~wnt|met_status_(1_met__0_m0)~1 (n=6)

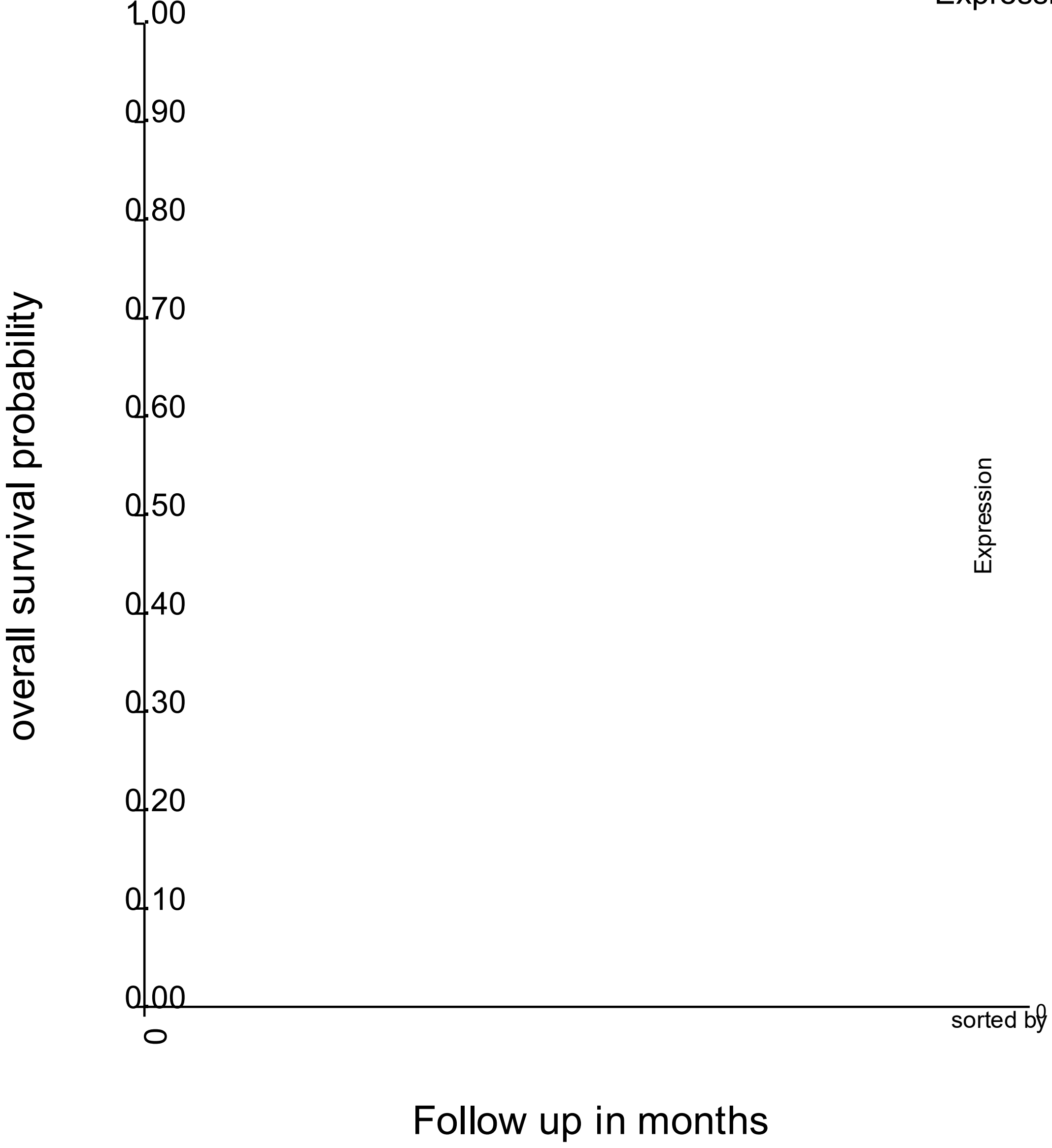

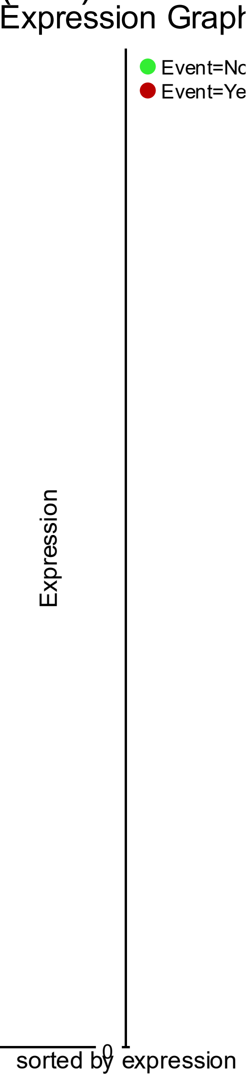

SHH M0

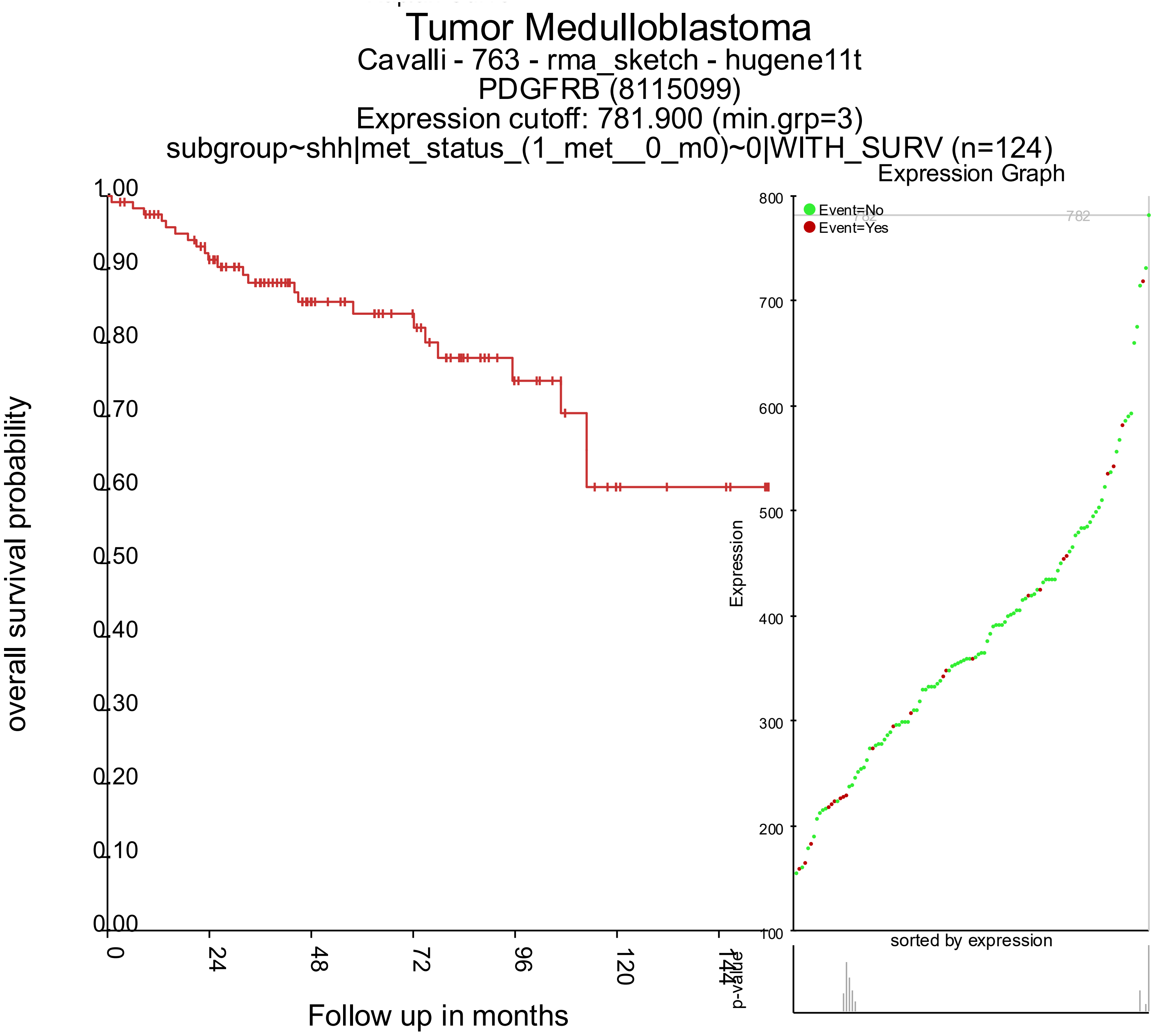

Tumor Medulloblastoma
Cavalli - 763 - rma_sketch - hugene11t
PDGFRB (8115099)
Expression cutoff: 781.900 (min.grp=3)
subgroup~shh|met_status_(1_met__0_m0)~0|WITH_SURV (n=124)
overall survival probability
1.00
0.90
0.80
0.70
0.60
0.50
0.40
0.30
0.20
0.10
0.00
0
24
48
72
96
120
144
Follow up in months
p-value
Expression Graph
Event=No
Event=Yes
782
Expression
800
700
600
500
400
300
200
100
sorted by expression

SHH M1

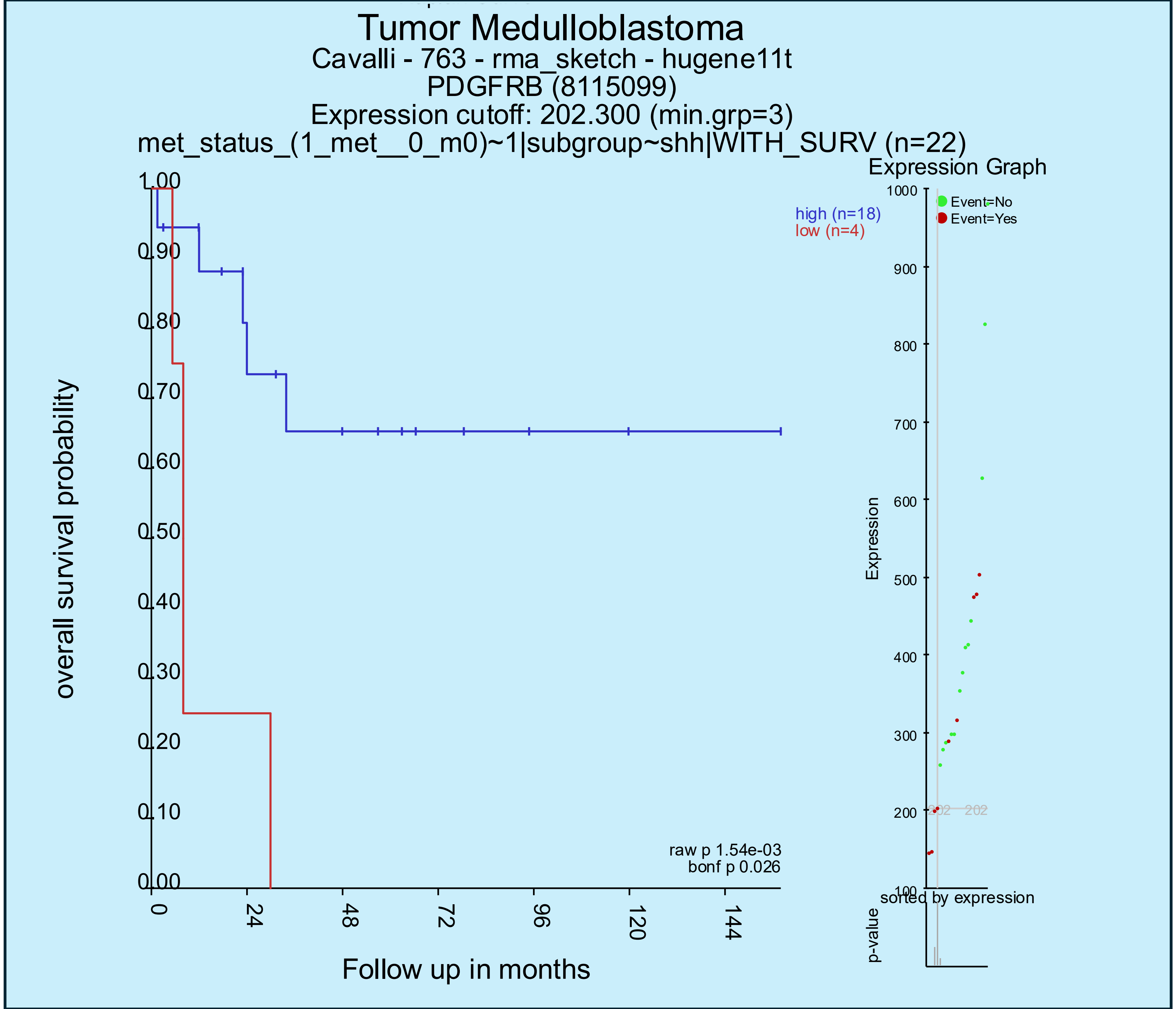

Tumor Medulloblastoma
Cavalli - 763 - rma_sketch - hugene11t
PDGFRB (8115099)
Expression cutoff: 202.300 (min.grp=3)
met_status_(1_met__0_m0)~1|subgroup~shh|WITH_SURV (n=22)
overall survival probability
1.00
0.90
0.80
0.70
0.60
0.50
0.40
0.30
0.20
0.10
0.00
high (n=18)
low (n=4)
raw p 1.54e-03
bonf p 0.026
0
24
48
72
96
120
144
Follow up in months
Expression Graph
Event=No
Event=Yes
1000
900
800
700
600
500
400
300
200
100
Expression
202
202
sorted by expression
p-value

GROUP4 M0

Tumor Medulloblastoma
Cavalli - 763 - rma_sketch - hugene11t
PDGFRB (8115099)
Expression cutoff: 174.000 (min.grp=3)
subgroup~group4|met_status_(1_met__0_m0)~0|WITH_SURV (n=145)

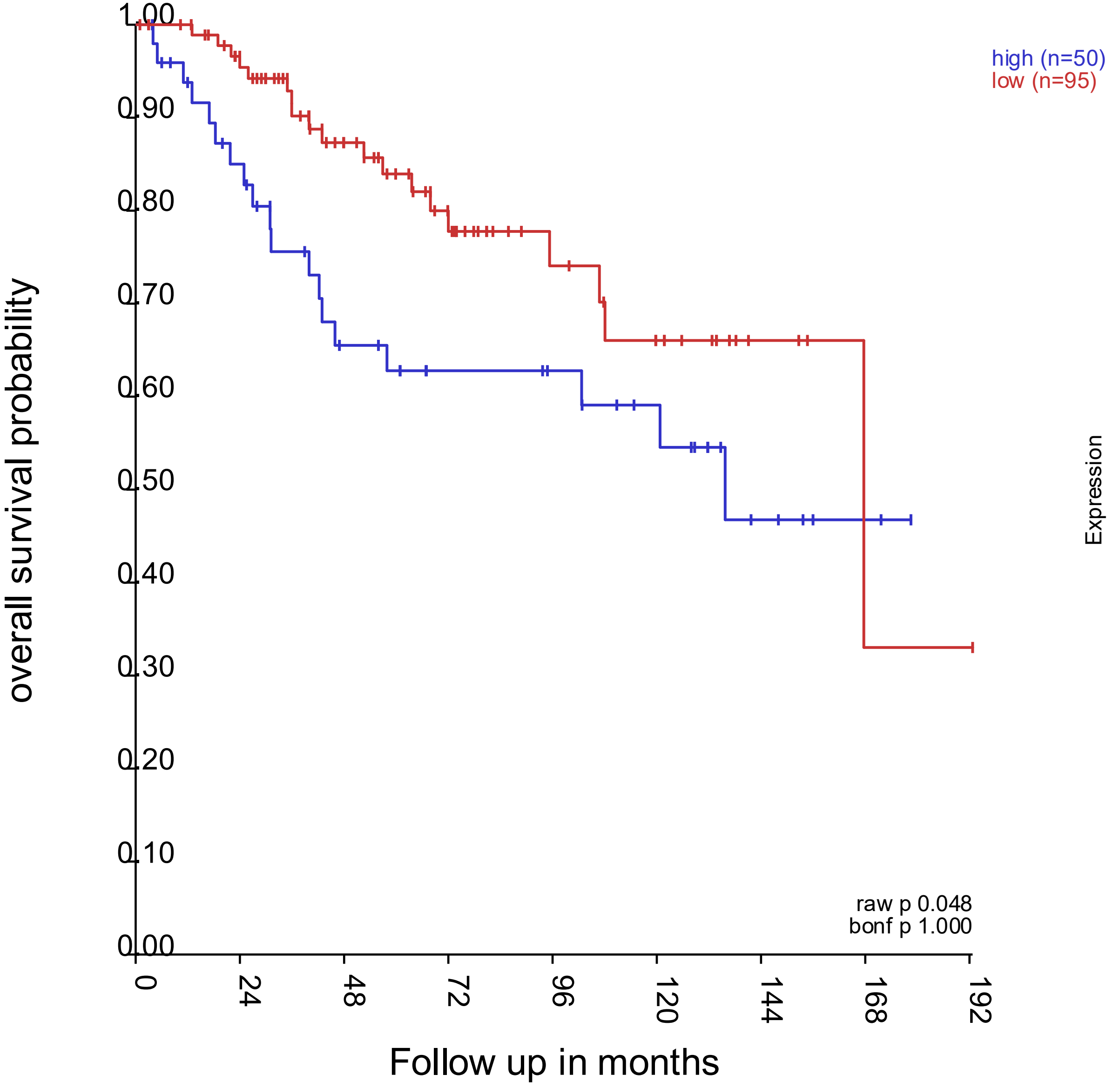

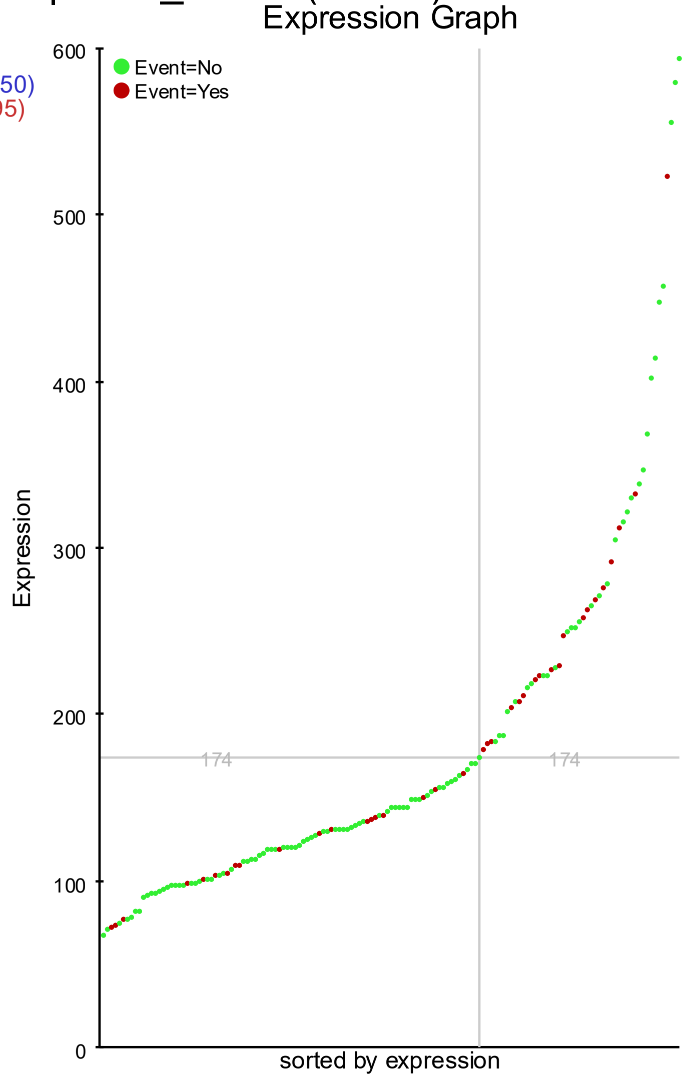

GROUP4 M1

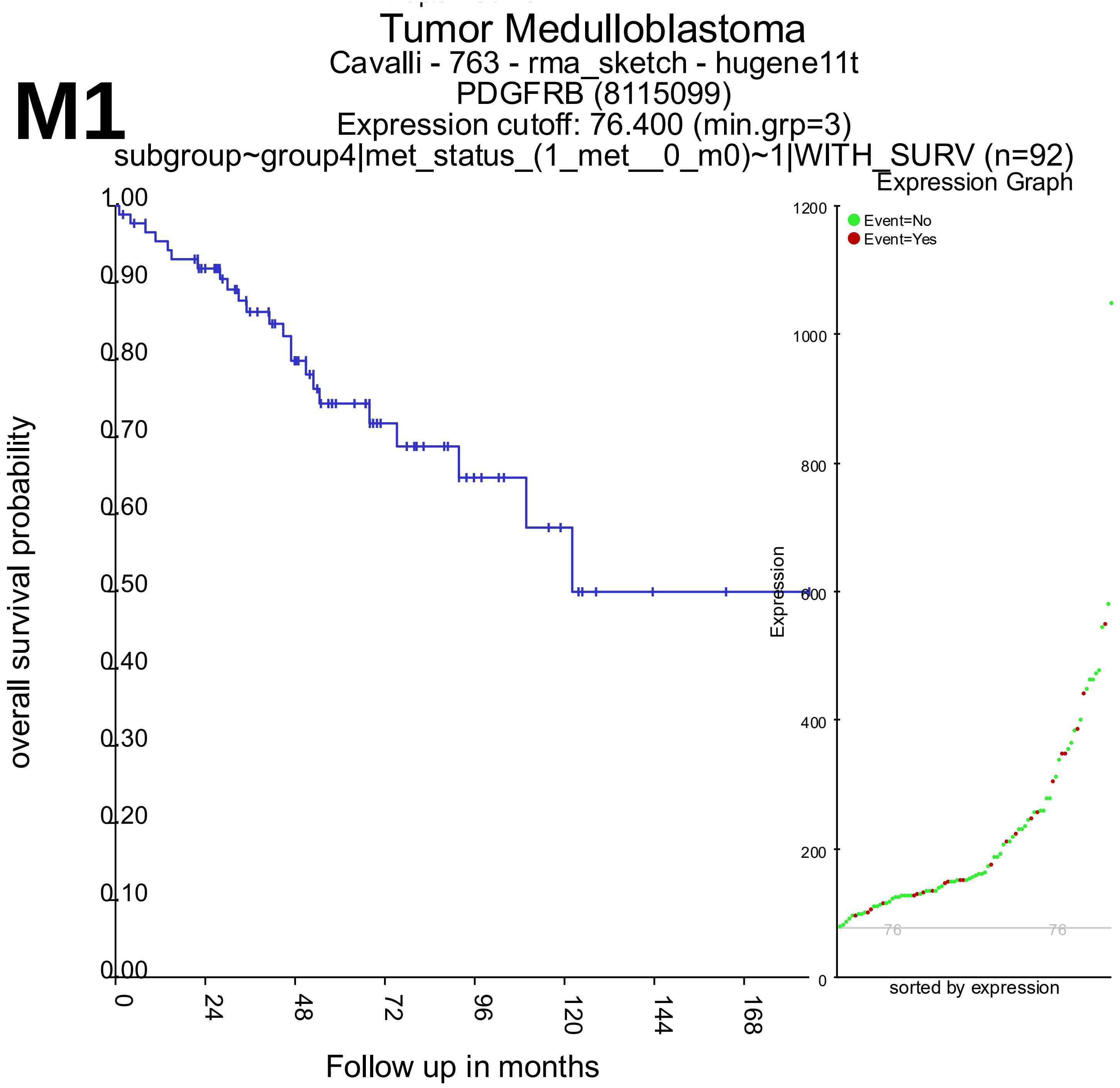
Tumor Medulloblastoma
Cavalli - 763 - rma_sketch - hugene11t
PDGFRB (8115099)
Expression cutoff: 76.400 (min.grp=3)
subgroup~group4|met_status_(1_met__0_m0)~1|WITH_SURV (n=92)
overall survival probability
1.00
0.90
0.80
0.70
0.60
0.50
0.40
0.30
0.20
0.10
0.00
0
24
48
72
96
120
144
168
Follow up in months
Expression Graph
Event=No
Event=Yes
Expression
1200
1000
800
600
400
200
0
76
76
sorted by expression

GROUP3 M0

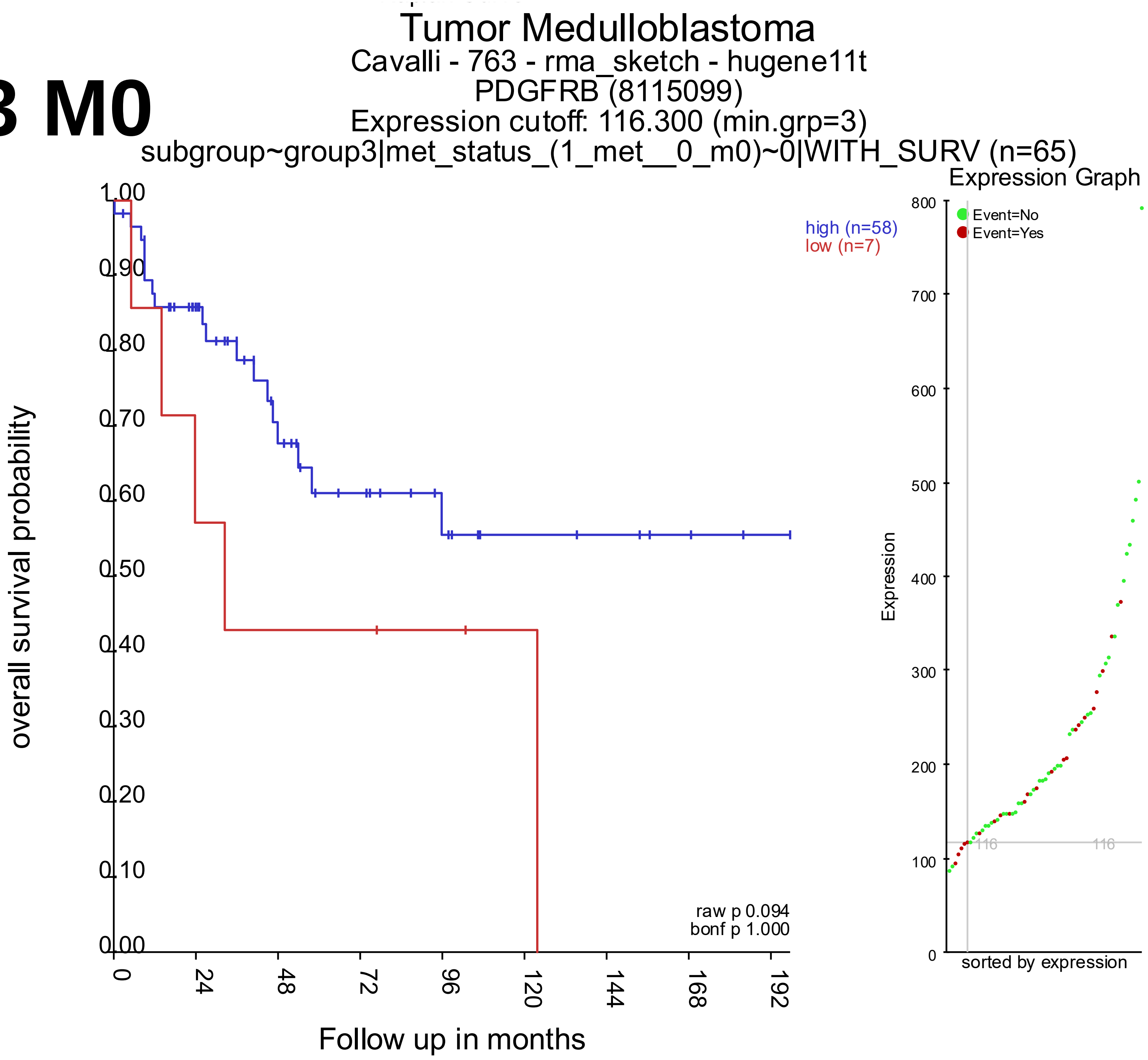

Tumor Medulloblastoma
Cavalli - 763 - rma_sketch - hugene11t
PDGFRB (8115099)
Expression cutoff: 116.300 (min.grp=3)
subgroup~group3|met_status_(1_met__0_m0)~0|WITH_SURV (n=65)
high (n=58)
low (n=7)
overall survival probability
1.00
0.90
0.80
0.70
0.60
0.50
0.40
0.30
0.20
0.10
0.00
0
24
48
72
96
120
144
168
192
Follow up in months
raw p 0.094
bonf p 1.000
Expression Graph
Event=No
Event=Yes
Expression
800
700
600
500
400
300
200
100
0
116
116
sorted by expression

GROUP3 M1

Tumor Medulloblastoma
Cavalli - 763 - rma_sketch - hugene11t
PDGFRB (8115099)
Expression cutoff: 126.100 (min.grp=3)
subgroup~group3|met_status_(1_met__0_m0)~1|WITH_SURV (n=41)

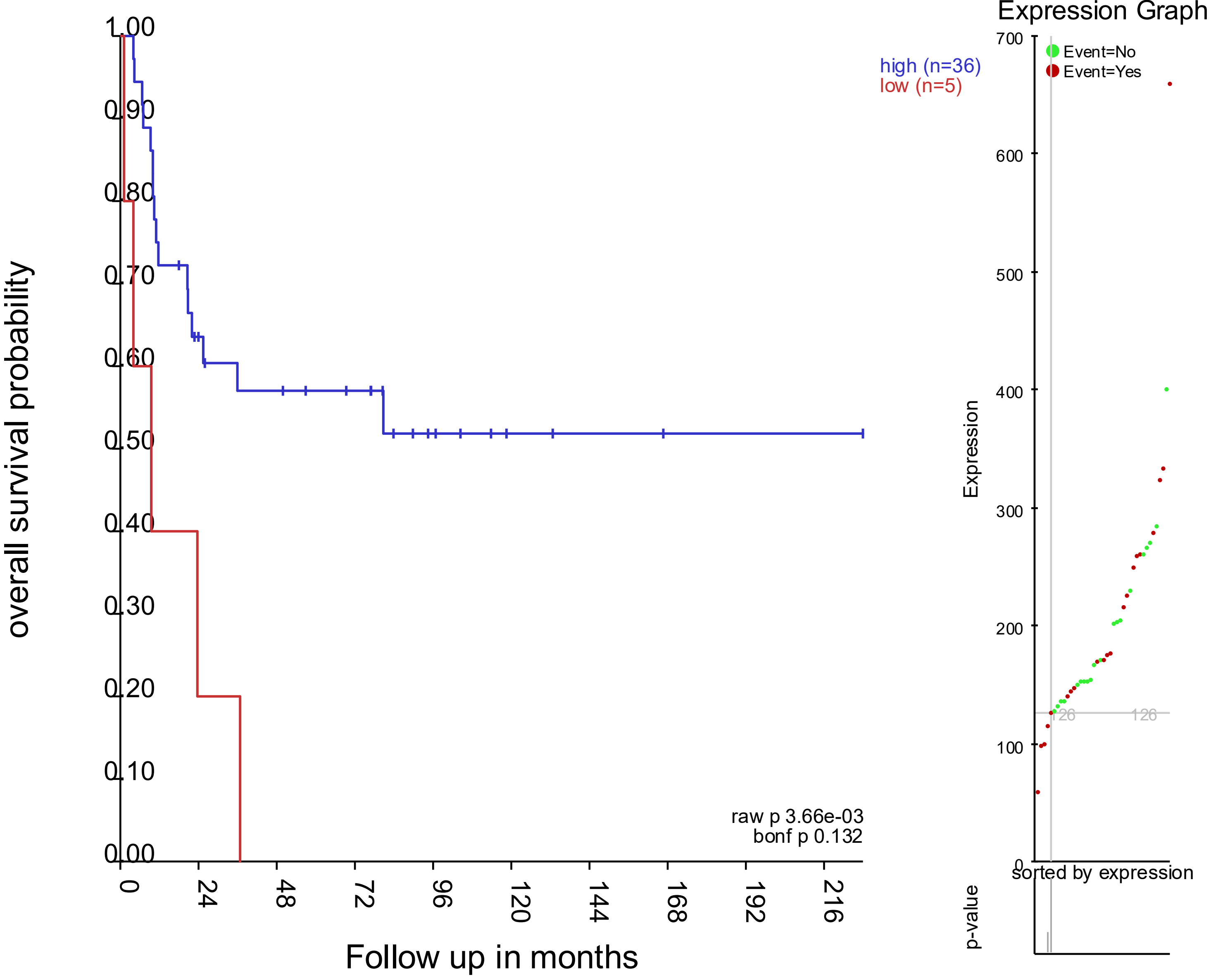

FIP1L1

WNT M0

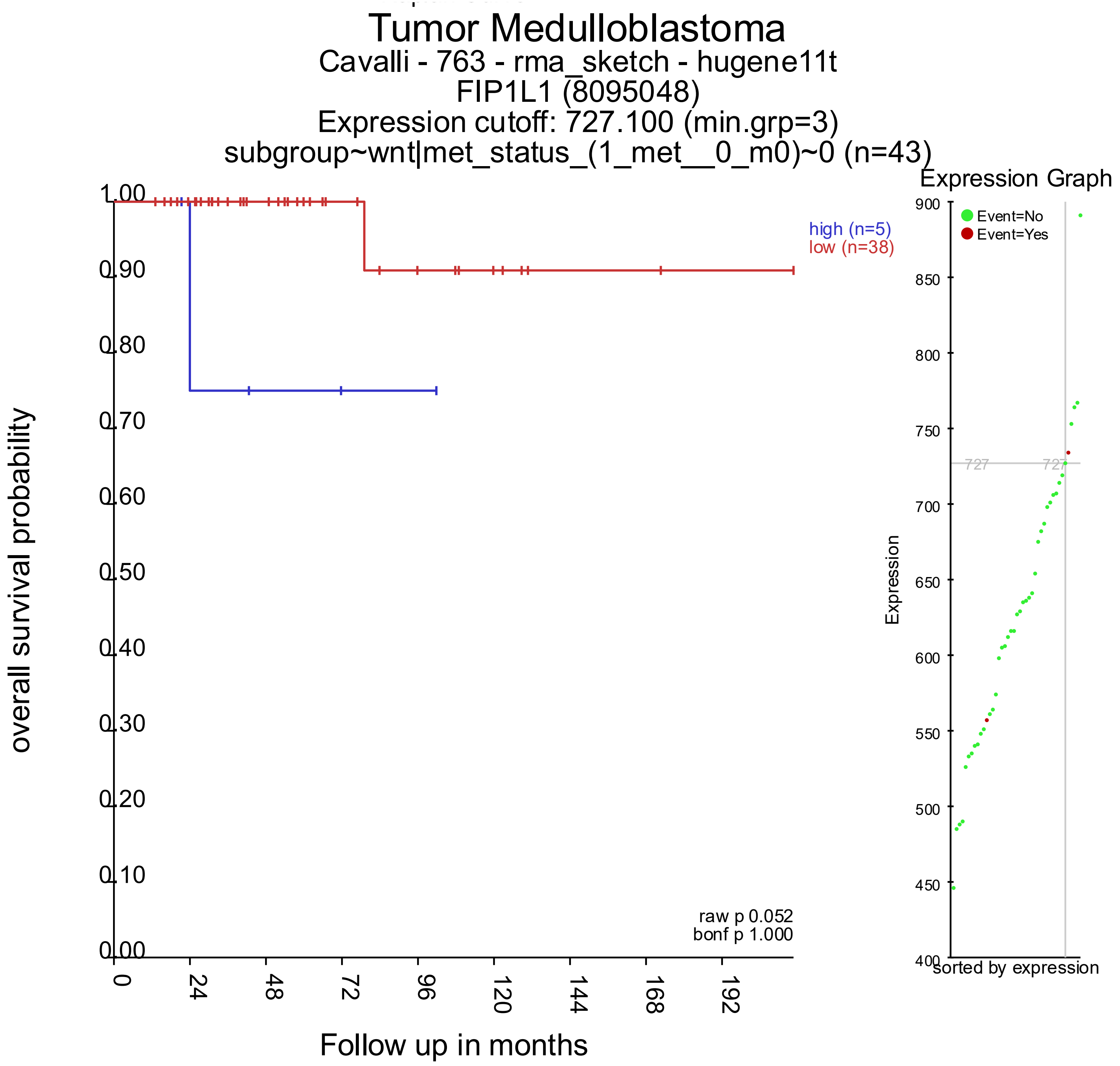

Tumor Medulloblastoma
Cavalli - 763 - rma_sketch - hugene11t
FIP1L1 (8095048)
Expression cutoff: 727.100 (min.grp=3)
subgroup~wnt|met_status_(1_met__0_m0)~0 (n=43)
overall survival probability
1.00
0.90
0.80
0.70
0.60
0.50
0.40
0.30
0.20
0.10
0.00
0
24
48
72
96
120
144
168
192
Follow up in months
high (n=5)
low (n=38)
raw p 0.052
bonf p 1.000
Expression Graph
Event=No
Event=Yes
Expression
900
850
800
750
727
727
700
650
600
550
500
450
400
sorted by expression

WNT M1

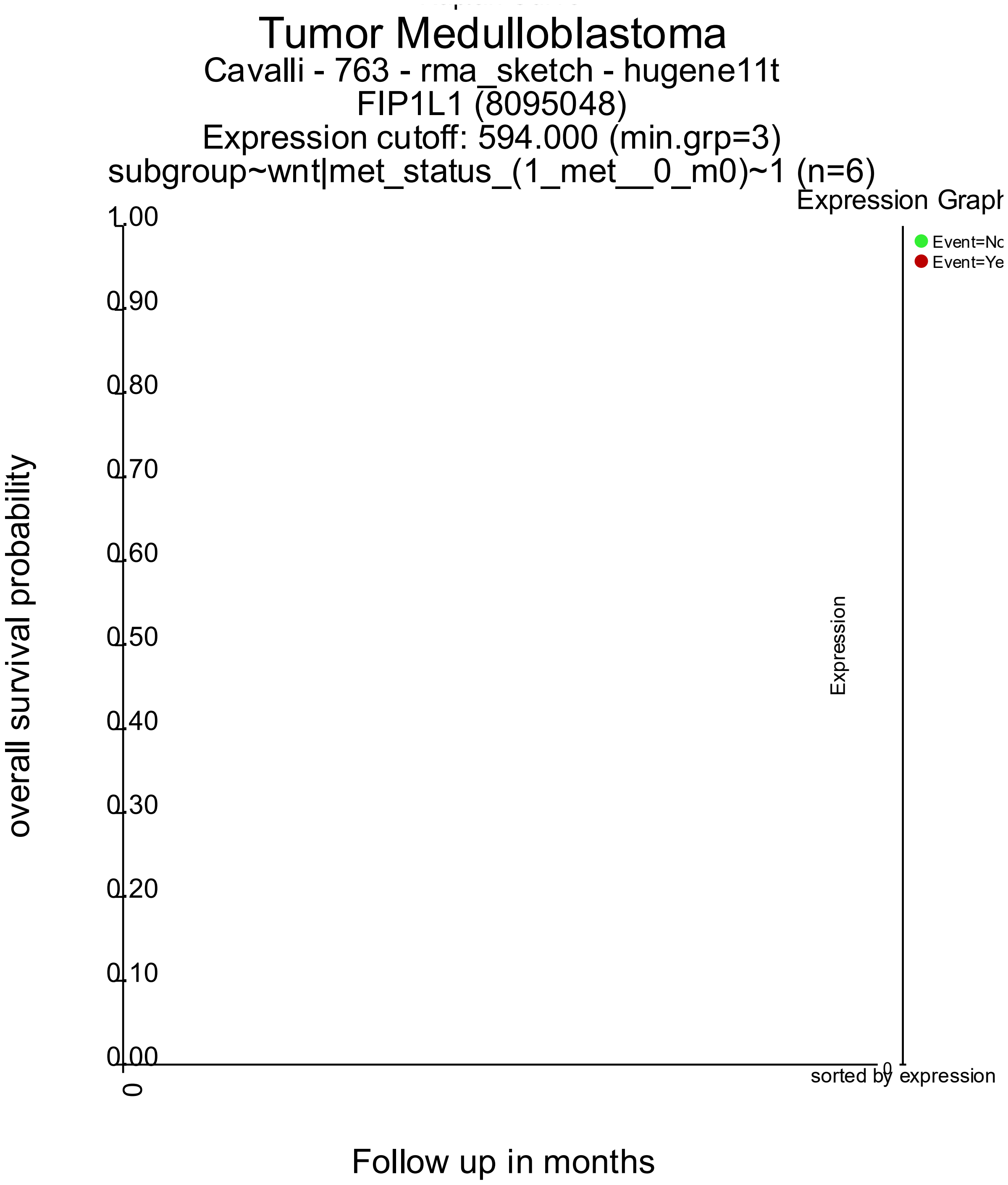

Tumor Medulloblastoma
Cavalli - 763 - rma_sketch - hugene11t
FIP1L1 (8095048)
Expression cutoff: 594.000 (min.grp=3)
subgroup~wnt|met_status_(1_met__0_m0)~1 (n=6)
Expression Graph
Event=No
Event=Ye
overall survival probability
1.00
0.90
0.80
0.70
0.60
0.50
0.40
0.30
0.20
0.10
0.00
0
Expression
0
sorted by expression
Follow up in months

SHH M0

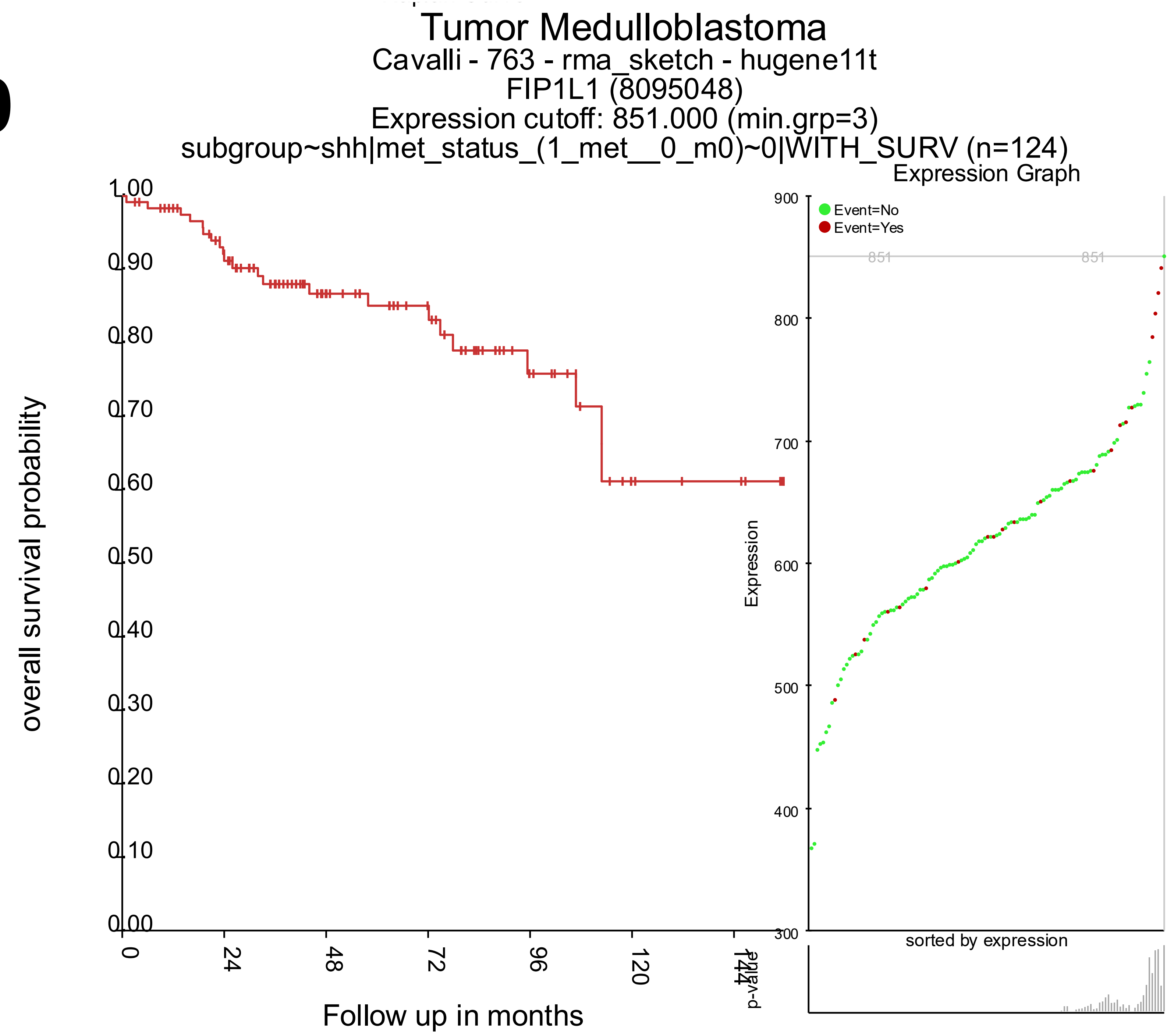

Tumor Medulloblastoma
Cavalli - 763 - rma_sketch - hugene11t
FIP1L1 (8095048)
Expression cutoff: 851.000 (min.grp=3)
subgroup~shh|met_status_(1_met__0_m0)~0|WITH_SURV (n=124)
overall survival probability
1.00
0.90
0.80
0.70
0.60
0.50
0.40
0.30
0.20
0.10
0.00
0
24
48
72
96
120
144
Follow up in months
Expression Graph
Event=No
Event=Yes
851
851
900
800
700
600
500
400
300
Expression
sorted by expression
p-value

SHH M1

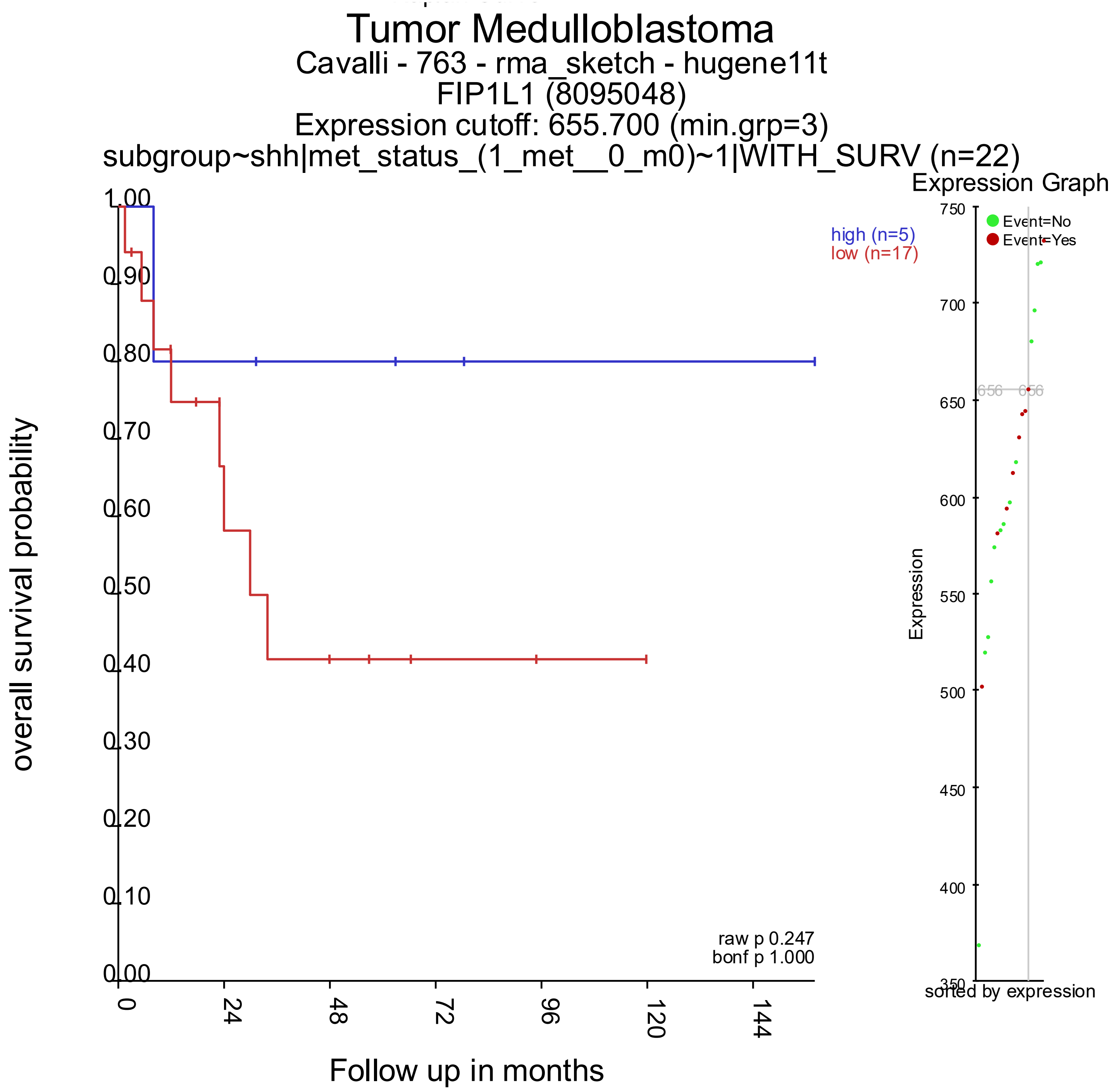
Tumor Medulloblastoma
Cavalli - 763 - rma_sketch - hugene11t
FIP1L1 (8095048)
Expression cutoff: 655.700 (min.grp=3)
subgroup~shh|met_status_(1_met__0_m0)~1|WITH_SURV (n=22)
overall survival probability
1.00
0.90
0.80
0.70
0.60
0.50
0.40
0.30
0.20
0.10
0.00
0
24
48
72
96
120
144
Follow up in months
high (n=5)
low (n=17)
raw p 0.247
bonf p 1.000
Expression Graph
Event=No
Event=Yes
Expression
750
700
650
600
550
500
450
400
350
656
656
sorted by expression

GROUP4 M0

Tumor Medulloblastoma
Cavalli - 763 - rma_sketch - hugene11t
FIP1L1 (8095048)
Expression cutoff: 887.900 (min.grp=3)
subgroup~group4|met_status_(1_met__0_m0)~0|WITH_SURV (n=145)

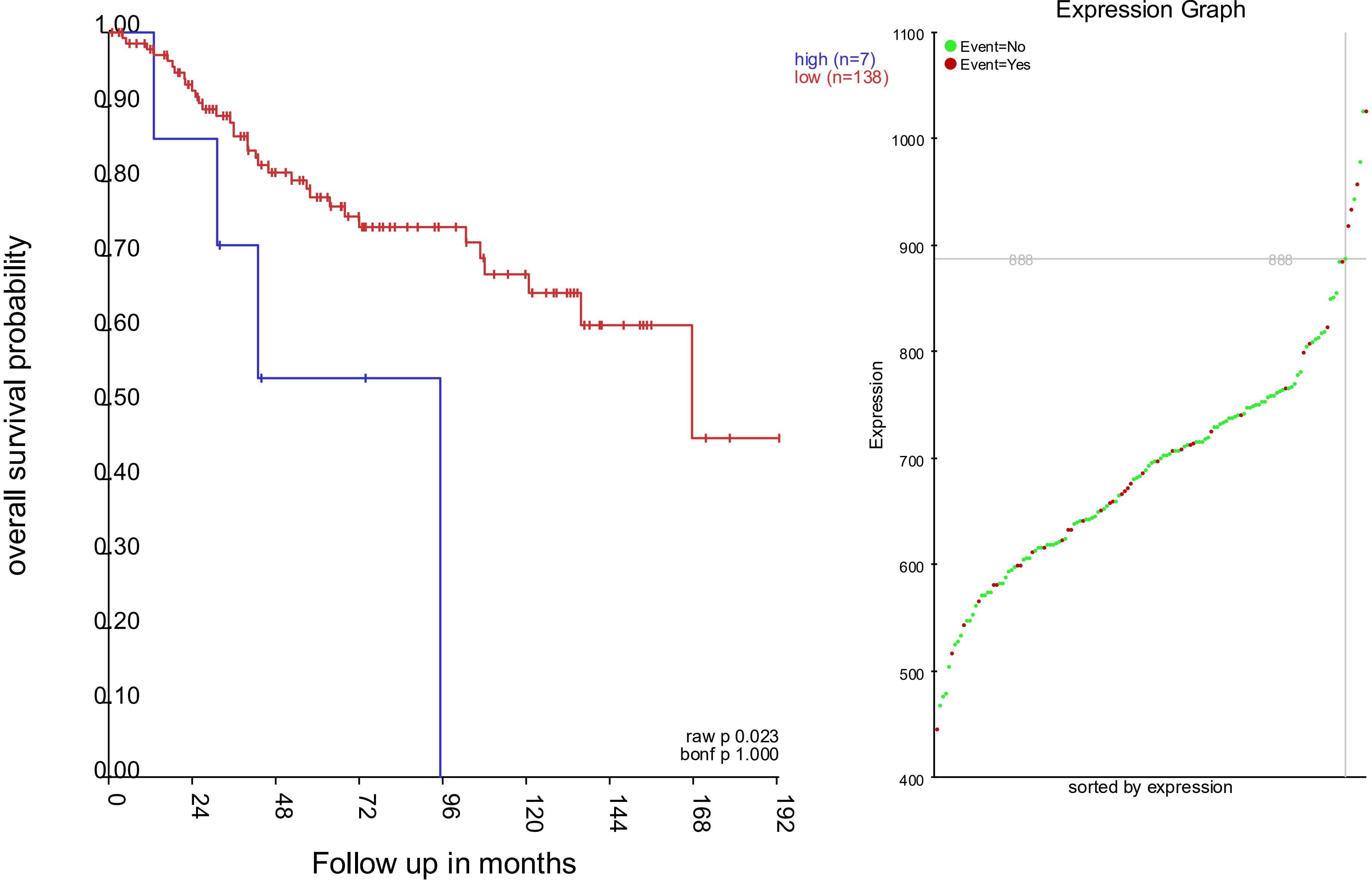

GROUP4 M1

Tumor Medulloblastoma
Cavalli - 763 - rma_sketch - hugene11t
FIP1L1 (8095048)
Expression cutoff: 847.600 (min.grp=3)
subgroup~group4|met_status_(1_met__0_m0)~1|WITH_SURV (n=92)

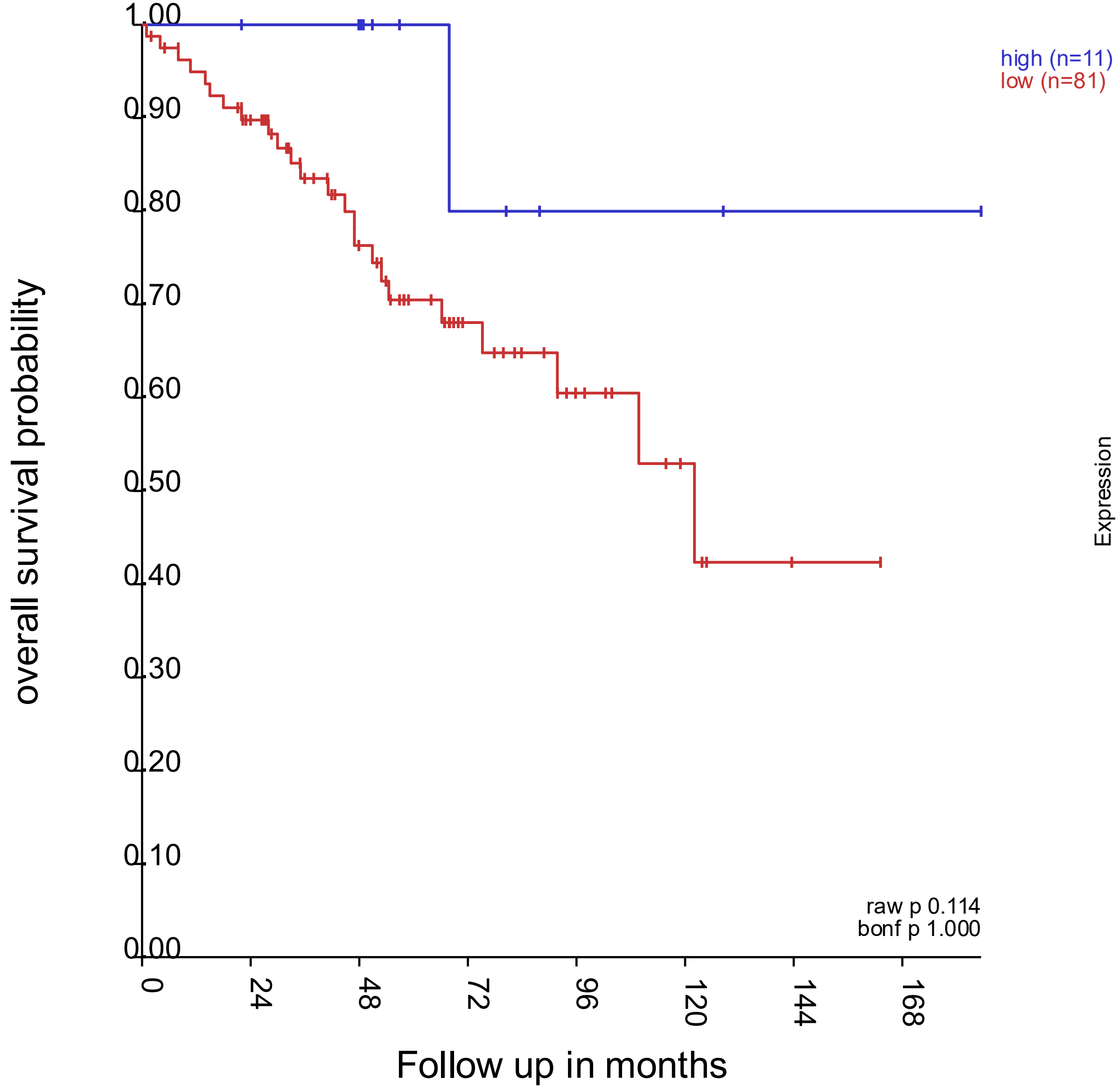

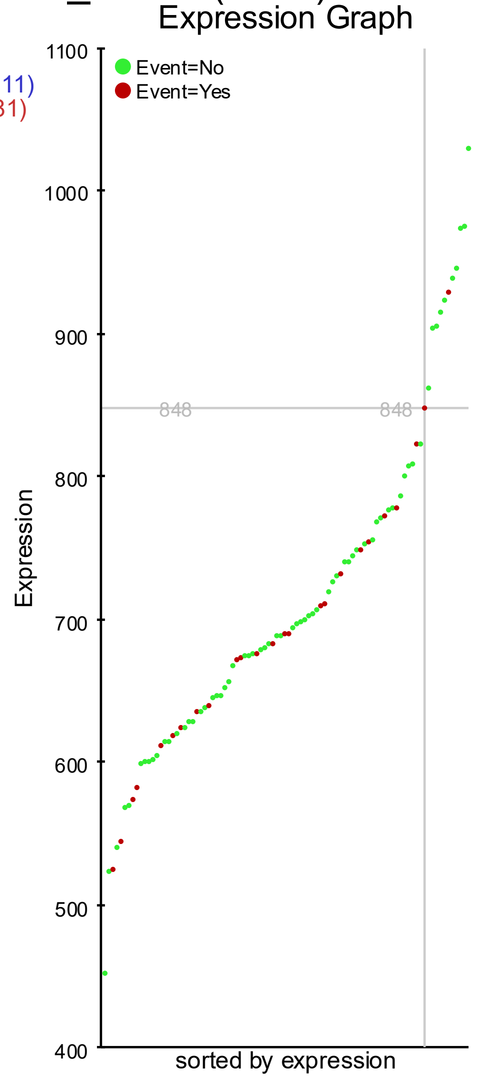

GROUP3 M0

Tumor Medulloblastoma
Cavalli - 763 - rma_sketch - hugene11t
FIP1L1 (8095048)
Expression cutoff: 579.000 (min.grp=3)
subgroup~group3|met_status_(1_met__0_m0)~0|WITH_SURV (n=65)

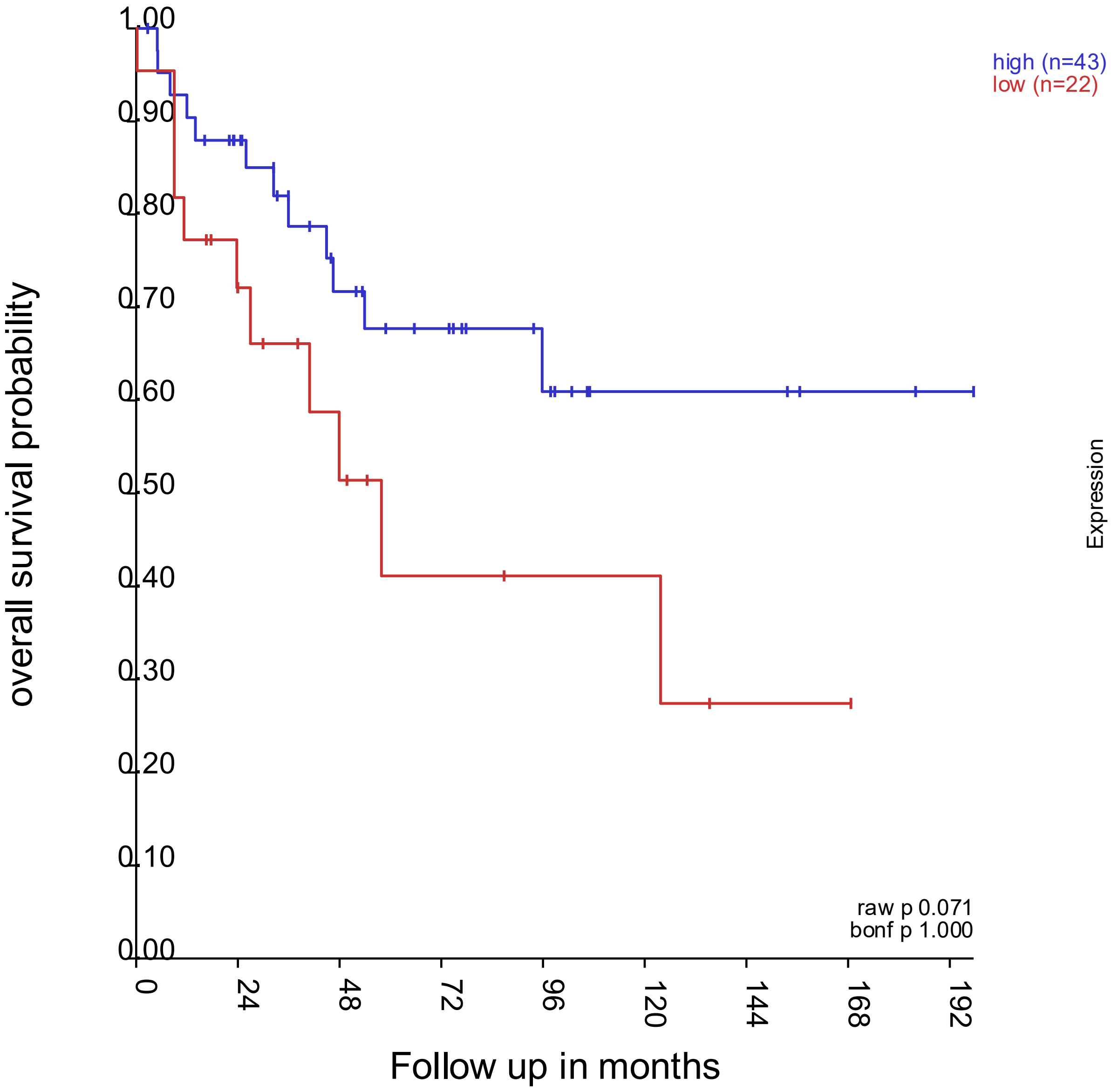

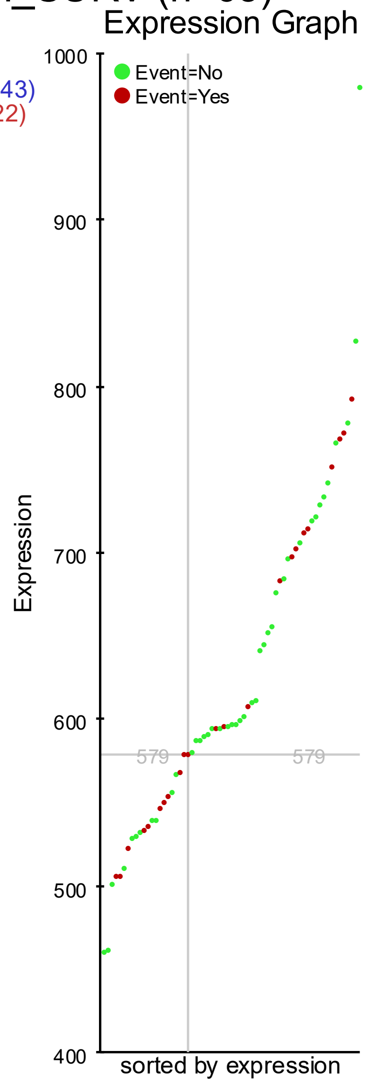

GROUP3 M1

Tumor Medulloblastoma
Cavalli - 763 - rma_sketch - hugene11t
FIP1L1 (8095048)
Expression cutoff: 791.300 (min.grp=3)
subgroup~group3|met_status_(1_met__0_m0)~1|WITH_SURV (n=41)

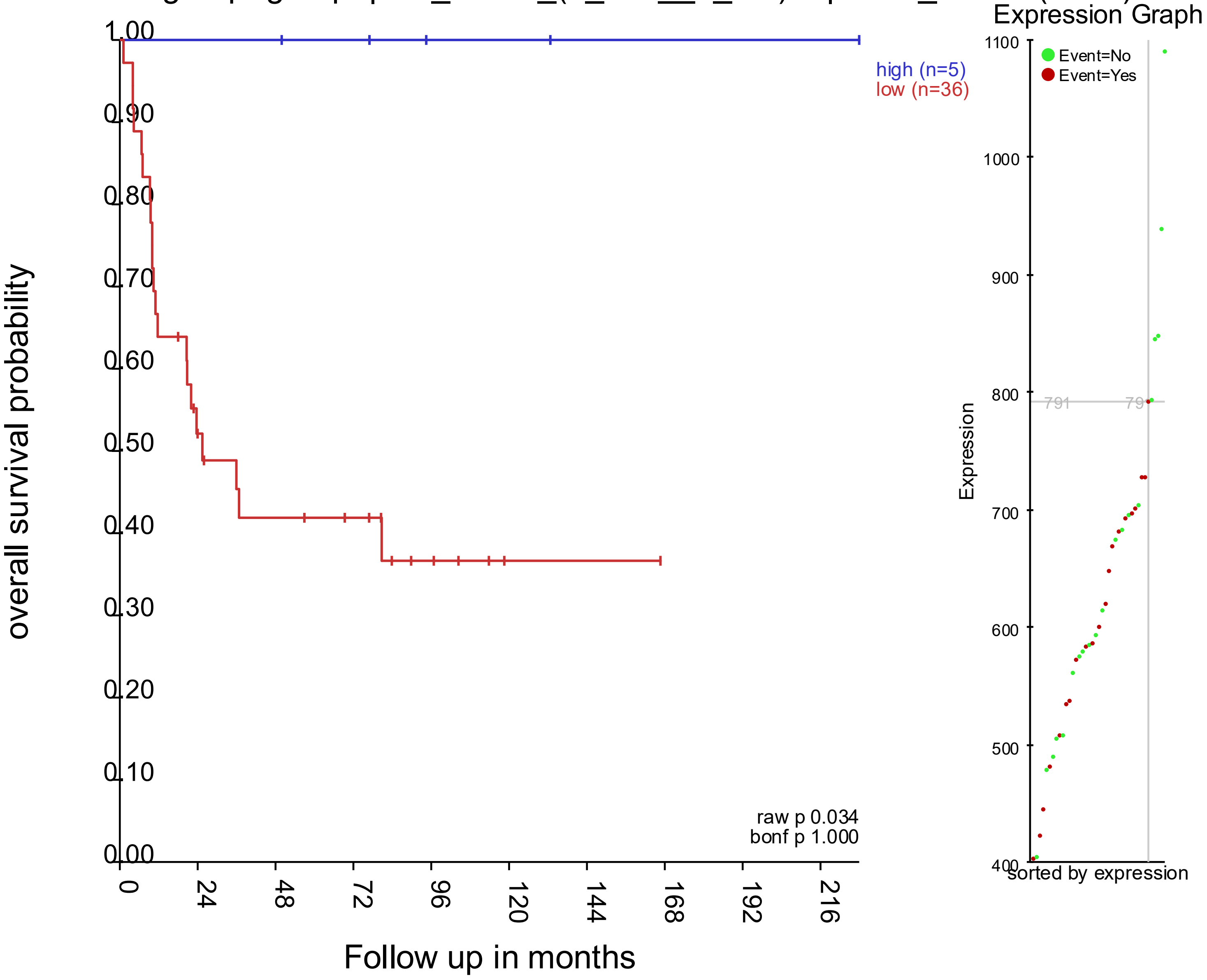

KIT

WNT M0

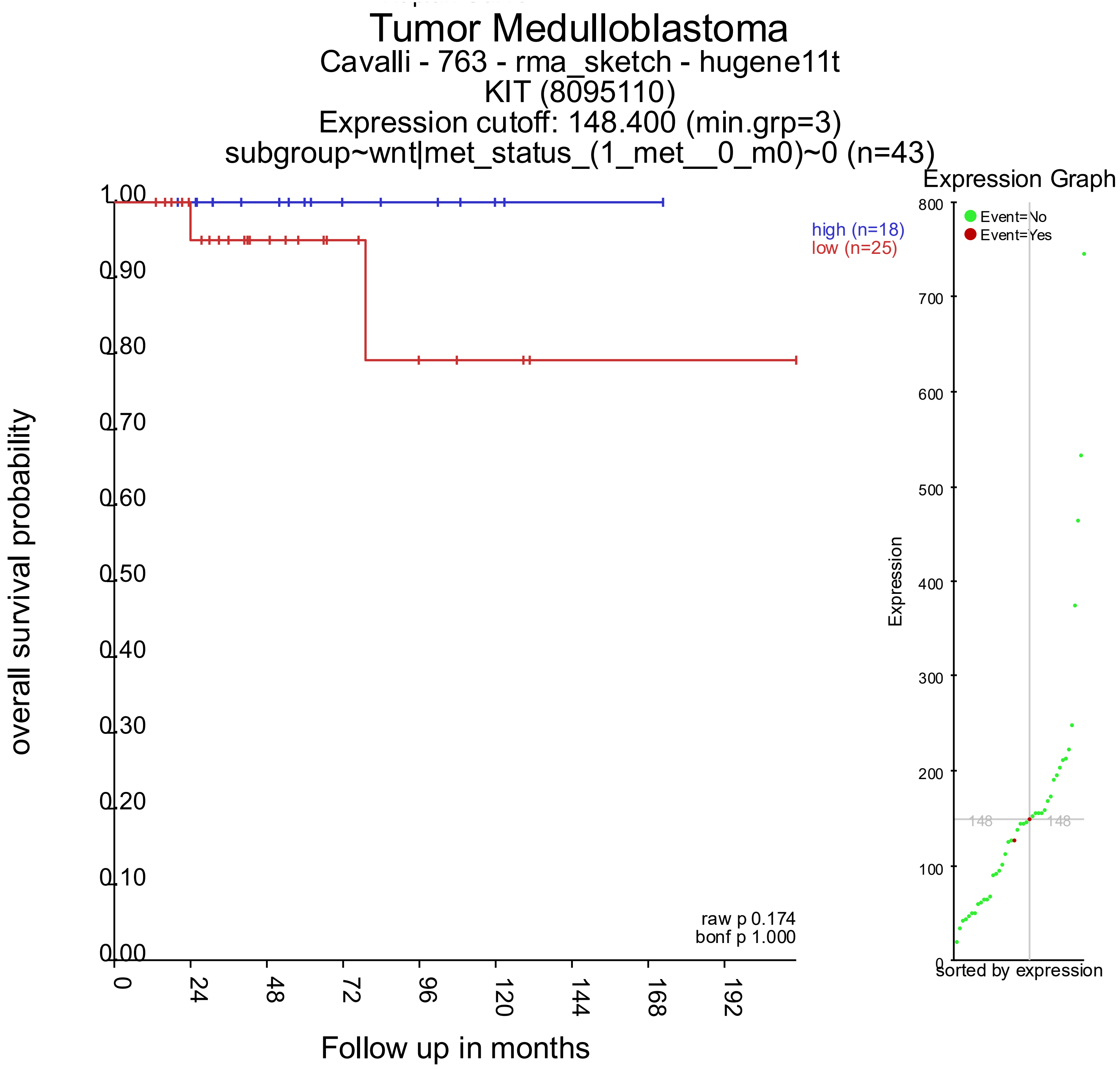

Tumor Medulloblastoma
Cavalli - 763 - rma_sketch - hugene11t
KIT (8095110)
Expression cutoff: 148.400 (min.grp=3)
subgroup~wnt|met_status_(1_met__0_m0)~0 (n=43)
overall survival probability
1.00
0.90
0.80
0.70
0.60
0.50
0.40
0.30
0.20
0.10
0.00
high (n=18)
low (n=25)
raw p 0.174
bonf p 1.000
0
24
48
72
96
120
144
168
192
Follow up in months
Expression Graph
Event=No
Event=Yes
Expression
800
700
600
500
400
300
200
100
0
148
148
sorted by expression

WNT M1

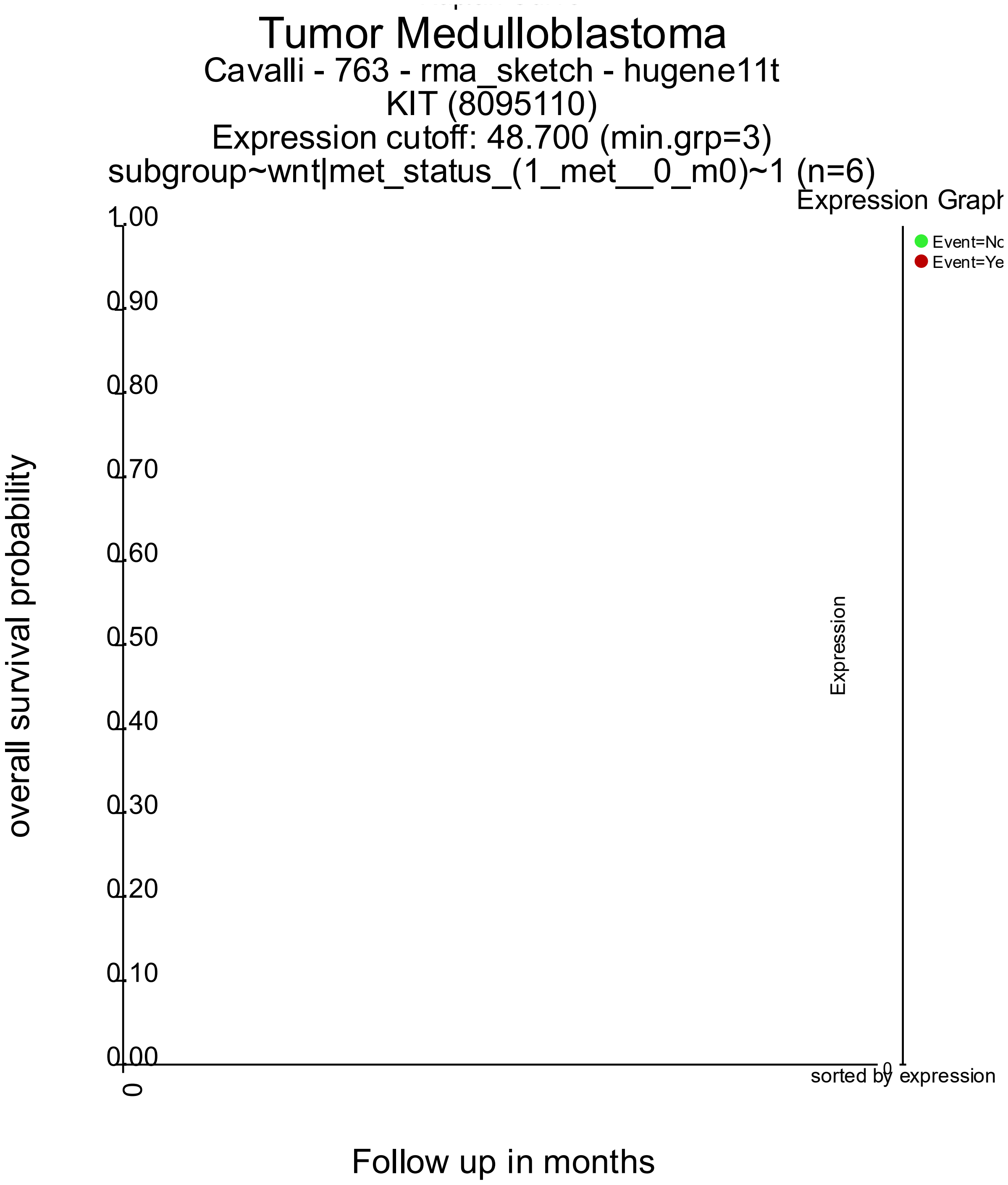

Tumor Medulloblastoma
Cavalli - 763 - rma_sketch - hugene11t
KIT (8095110)
Expression cutoff: 48.700 (min.grp=3)
subgroup~wnt|met_status_(1_met__0_m0)~1 (n=6)
Expression Graph
Event=No
Event=Yes
1.00
0.90
0.80
0.70
0.60
0.50
0.40
0.30
0.20
0.10
0.00
0
overall survival probability
Follow up in months
Expression
0
sorted by expression

SHH M0

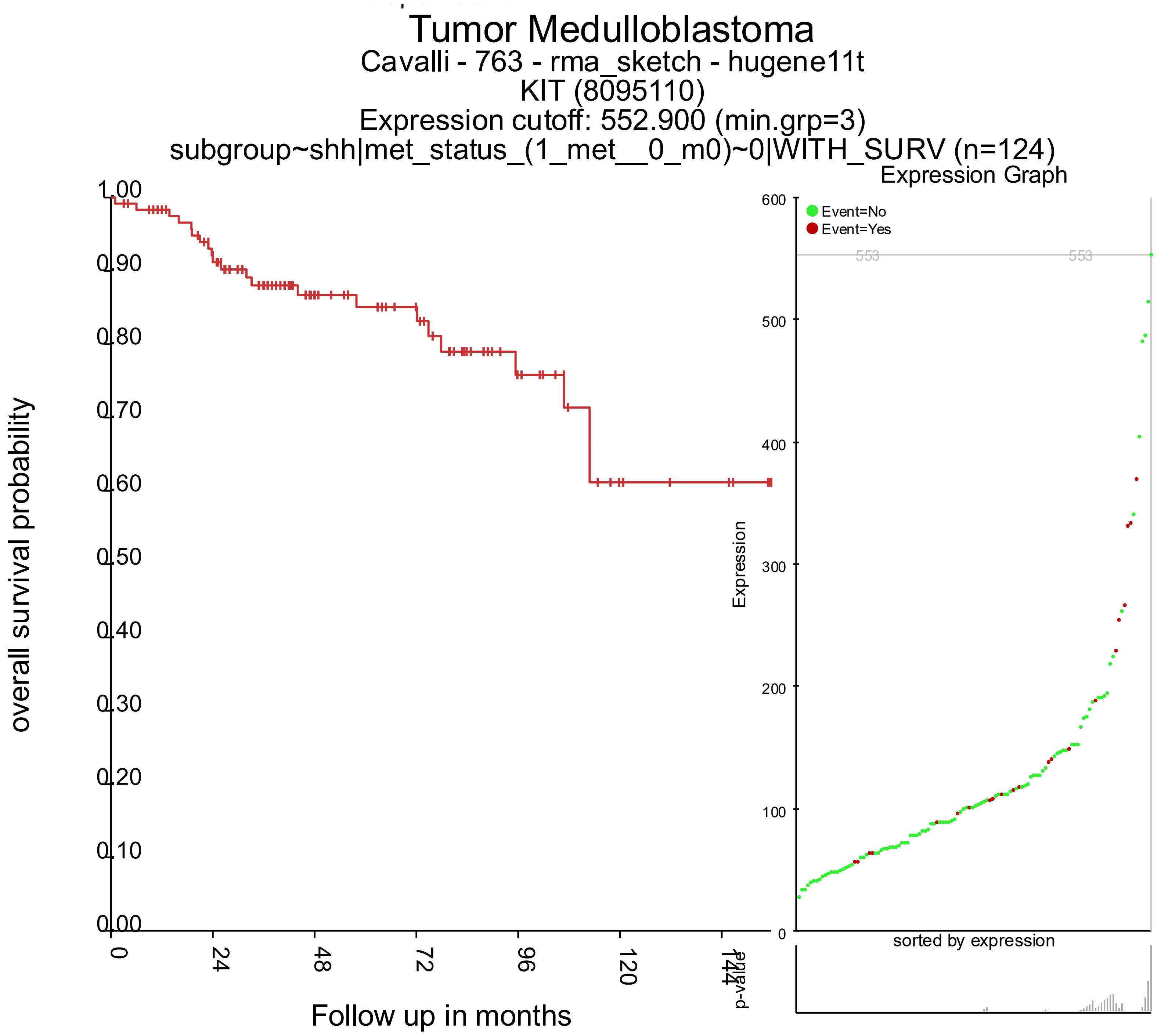

Tumor Medulloblastoma
Cavalli - 763 - rma_sketch - hugene11t
KIT (8095110)
Expression cutoff: 552.900 (min.grp=3)
subgroup~shh|met_status_(1_met__0_m0)~0|WITH_SURV (n=124)
overall survival probability
1.00
0.90
0.80
0.70
0.60
0.50
0.40
0.30
0.20
0.10
0.00
0
24
48
72
96
120
144
Follow up in months
p-value
Expression Graph
Event=No
Event=Yes
553
553
Expression
600
500
400
300
200
100
0
sorted by expression

SHH M1

Tumor Medulloblastoma
Cavalli - 763 - rma_sketch - hugene11t
KIT (8095110)
Expression cutoff: 294.000 (min.grp=3)
subgroup~shh|met_status_(1_met__0_m0)~1|WITH_SURV (n=22)

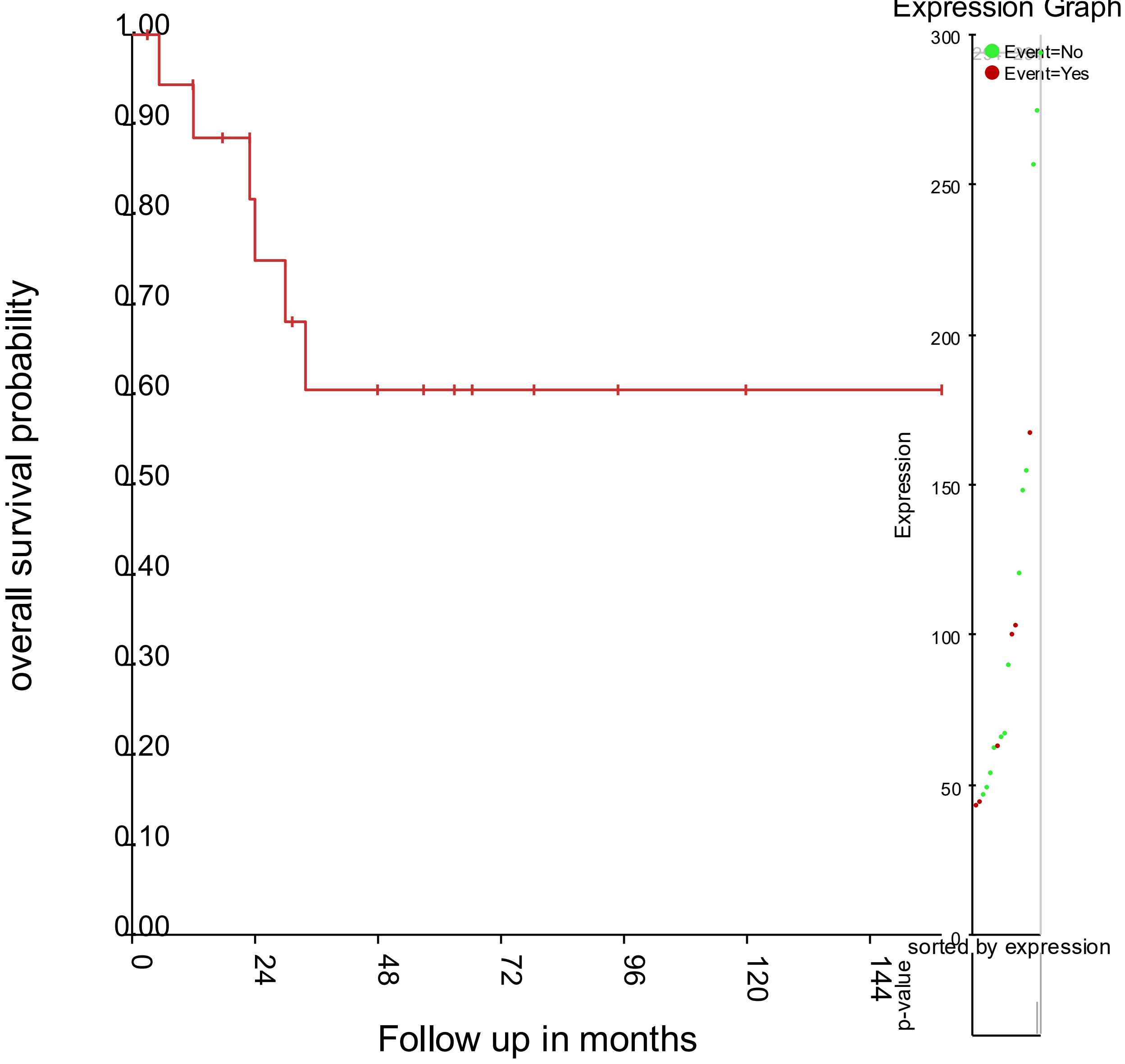

GROUP4 M0

Tumor Medulloblastoma
Cavalli - 763 - rma_sketch - hugene11t
KIT (8095110)
Expression cutoff: 153.400 (min.grp=3)
subgroup~group4|met_status_(1_met__0_m0)~0|WITH_SURV (n=145)

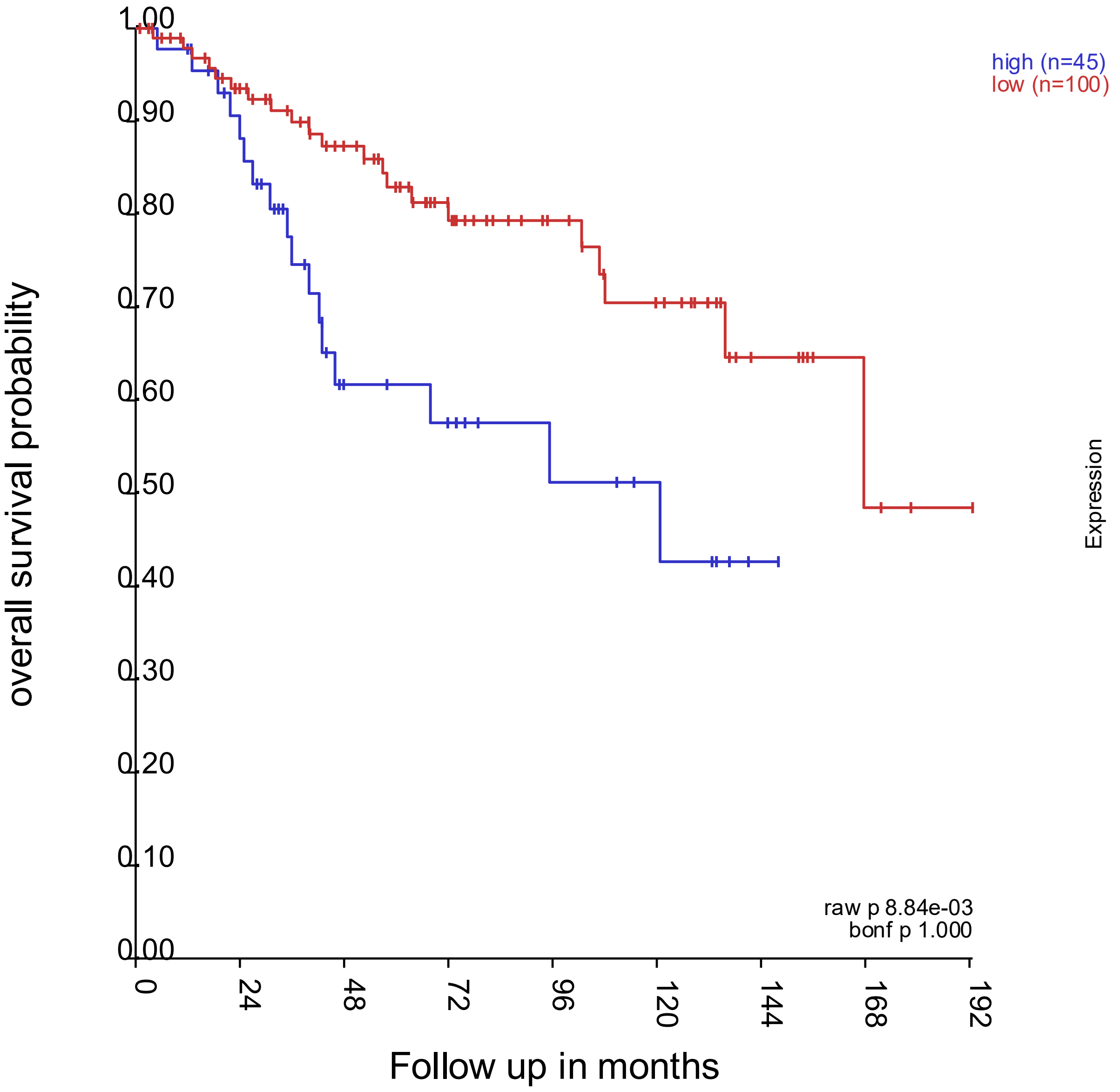

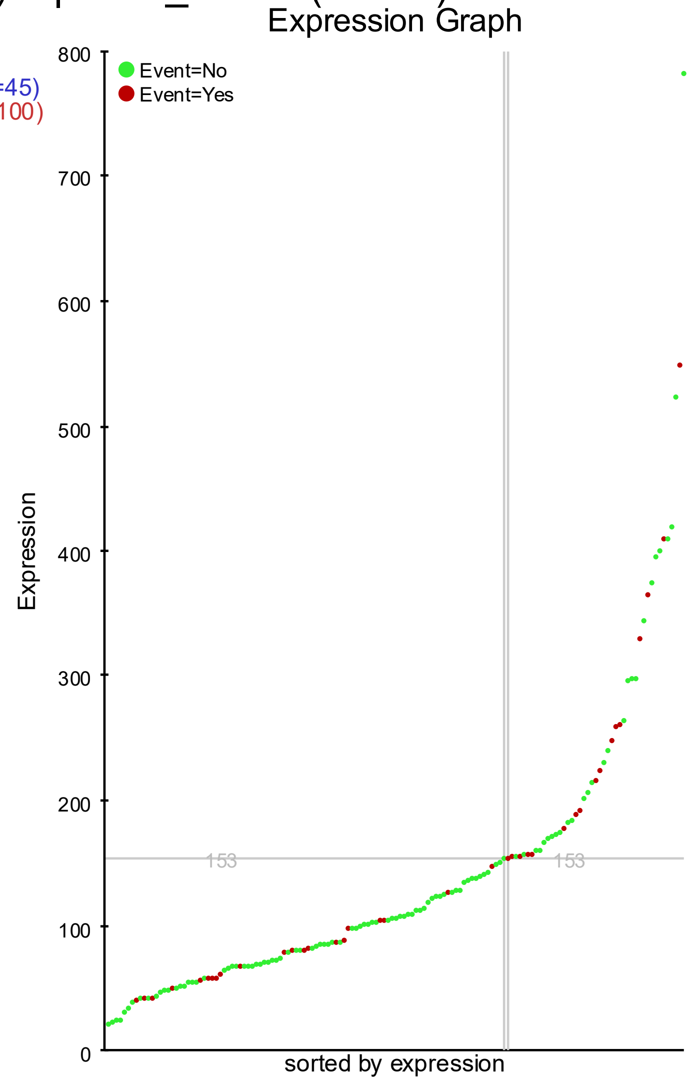

GROUP4 M1

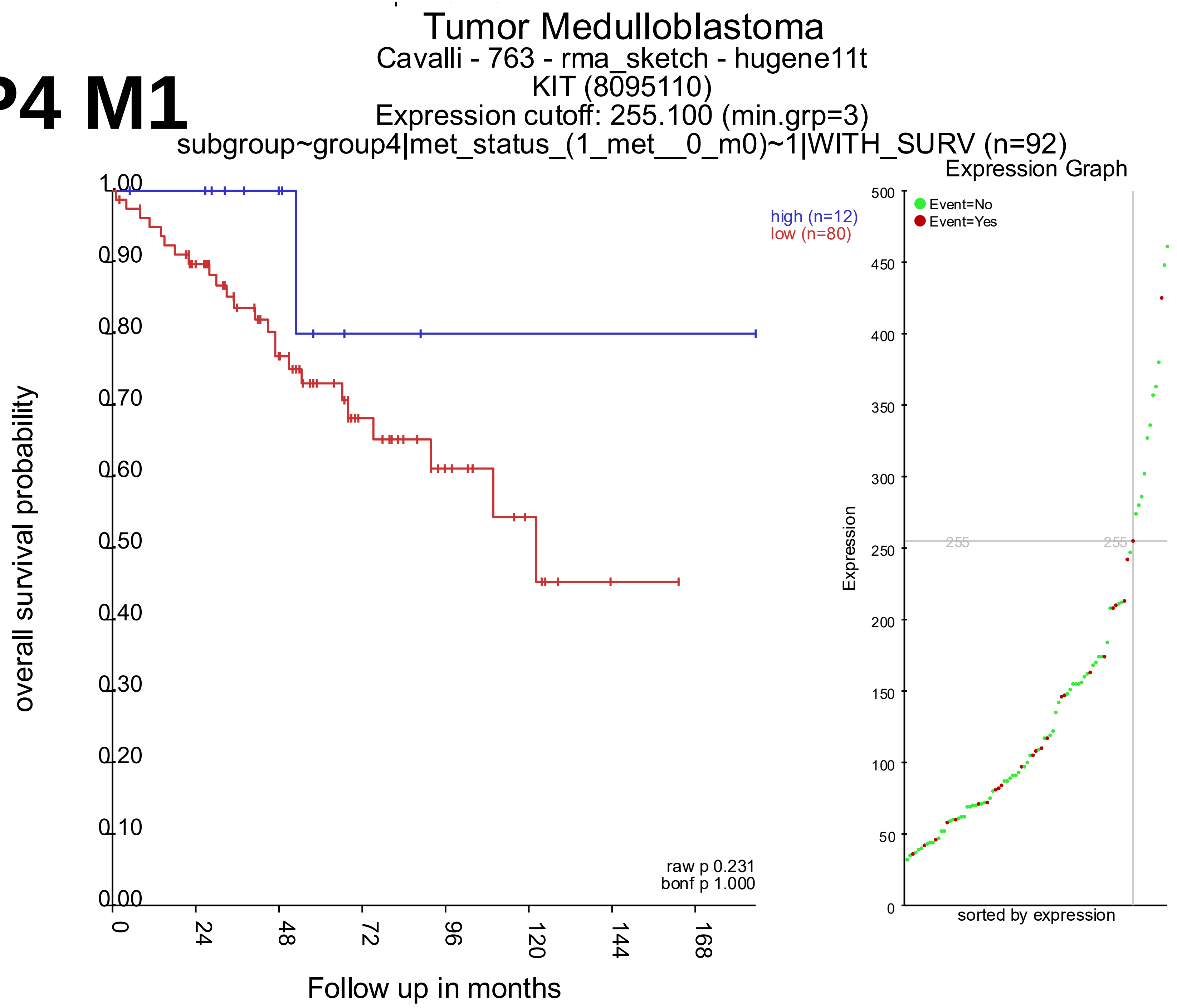

Tumor Medulloblastoma
Cavalli - 763 - rma_sketch - hugene11t
KIT (8095110)
Expression cutoff: 255.100 (min.grp=3)
subgroup~group4|met_status_(1_met__0_m0)~1|WITH_SURV (n=92)
overall survival probability
1.00
0.90
0.80
0.70
0.60
0.50
0.40
0.30
0.20
0.10
0.00
high (n=12)
low (n=80)
raw p 0.231
bonf p 1.000
0
24
48
72
96
120
144
168
Follow up in months
Expression Graph
Event=No
Event=Yes
Expression
500
450
400
350
300
250
200
150
100
50
0
255
255
sorted by expression

GROUP3 M0

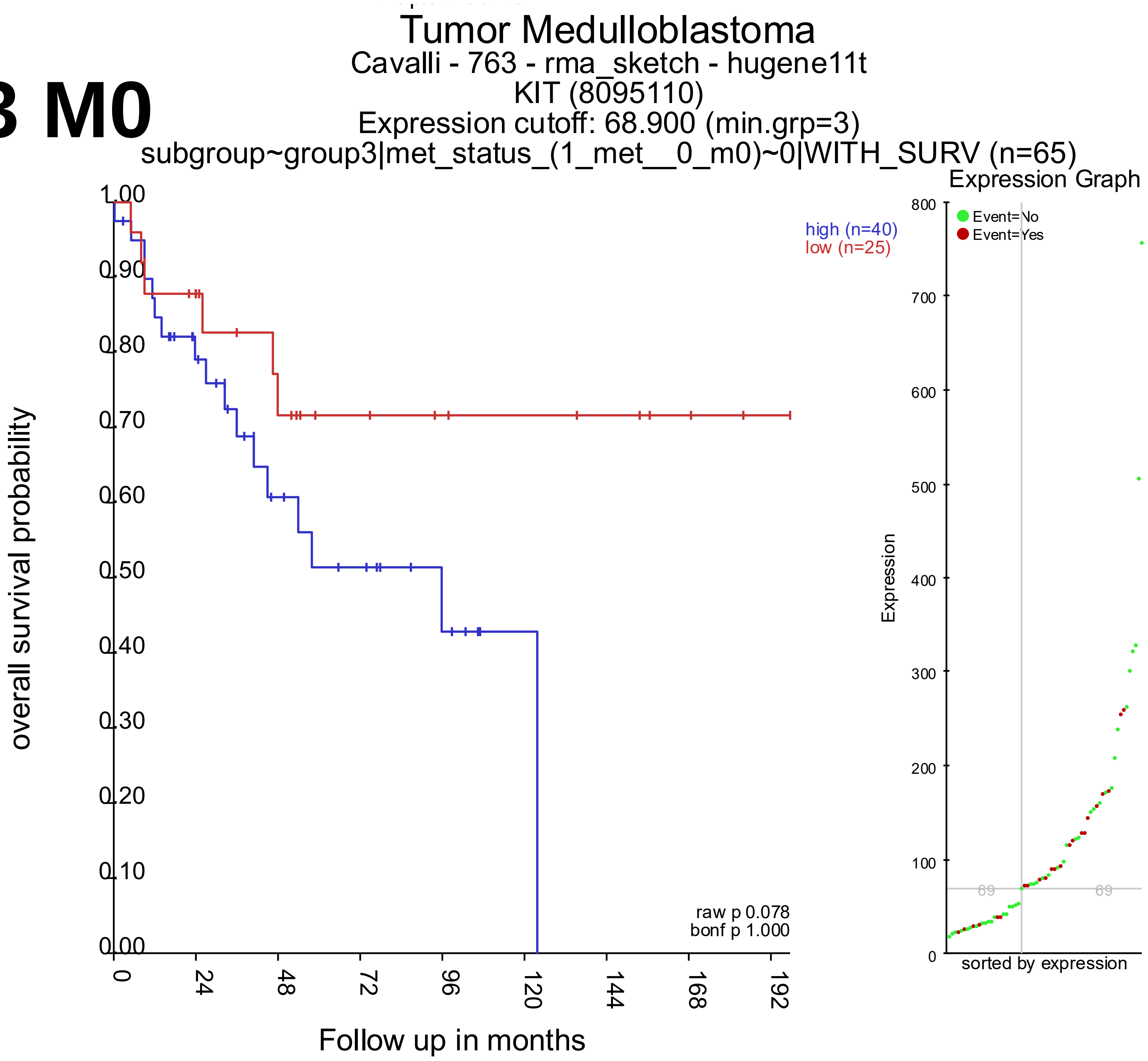

Tumor Medulloblastoma
Cavalli - 763 - rma_sketch - hugene11t
KIT (8095110)
Expression cutoff: 68.900 (min.grp=3)
subgroup~group3|met_status_(1_met__0_m0)~0|WITH_SURV (n=65)
high (n=40)
low (n=25)
overall survival probability
1.00
0.90
0.80
0.70
0.60
0.50
0.40
0.30
0.20
0.10
0.00
0
24
48
72
96
120
144
168
192
Follow up in months
raw p 0.078
bonf p 1.000
Expression Graph
Event=No
Event=Yes
Expression
800
700
600
500
400
300
200
100
0
69
69
sorted by expression

GROUP3 M1

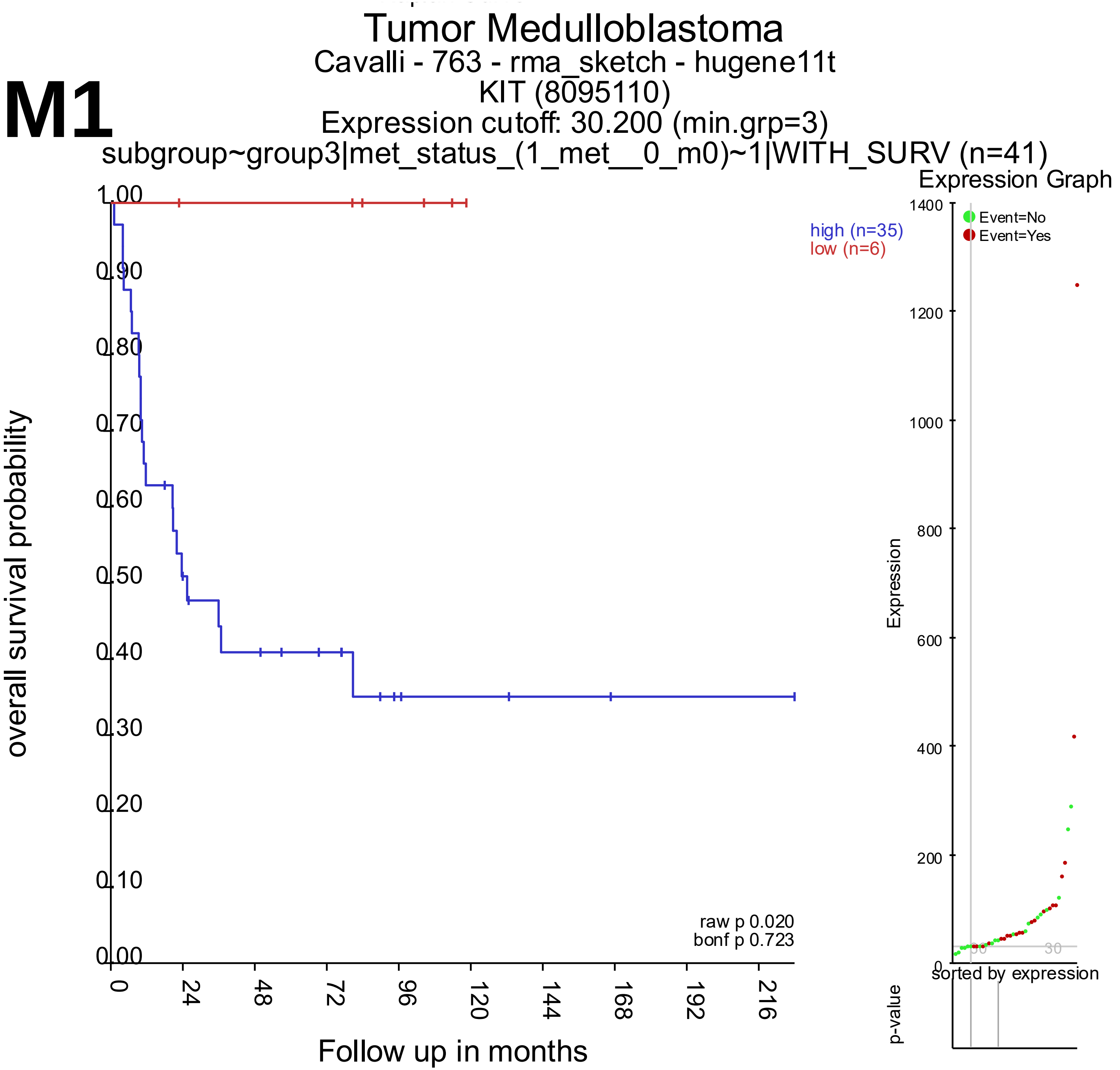
Tumor Medulloblastoma
Cavalli - 763 - rma_sketch - hugene11t
KIT (8095110)
Expression cutoff: 30.200 (min.grp=3)
subgroup~group3|met_status_(1_met__0_m0)~1|WITH_SURV (n=41)
high (n=35)
low (n=6)
overall survival probability
1.00
0.90
0.80
0.70
0.60
0.50
0.40
0.30
0.20
0.10
0.00
0
24
48
72
96
120
144
168
192
216
Follow up in months
raw p 0.020
bonf p 0.723
Expression Graph
Event=No
Event=Yes
Expression
1400
1200
1000
800
600
400
200
0
sorted by expression
p-value

PIK3CA

WNT M0

Tumor Medulloblastoma
Cavalli - 763 - rma_sketch - hugene11t
PIK3CA (8084016)
Expression cutoff: 261.800 (min.grp=3)
subgroup~wnt|met_status_(1_met__0_m0)~0 (n=43)

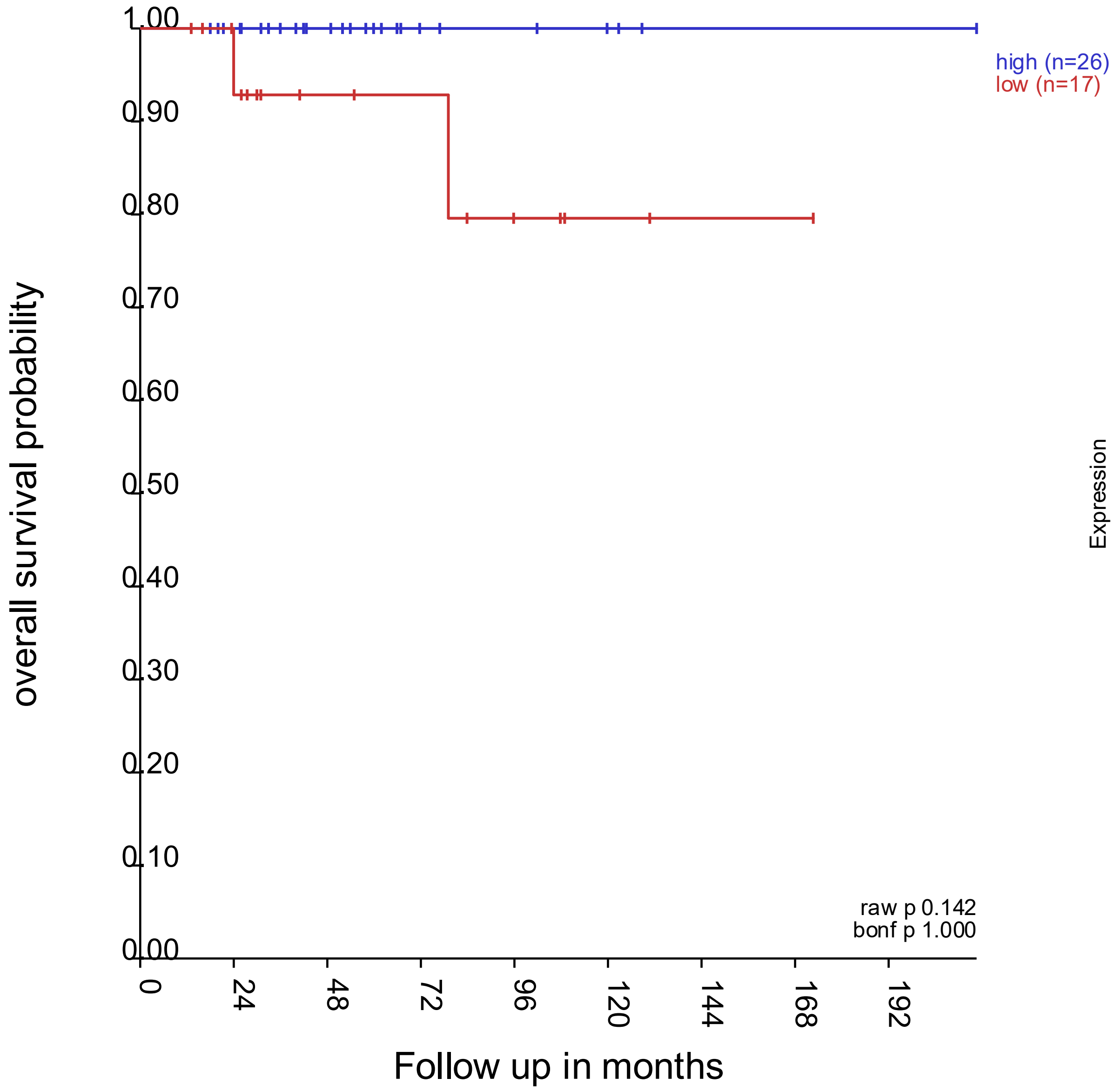

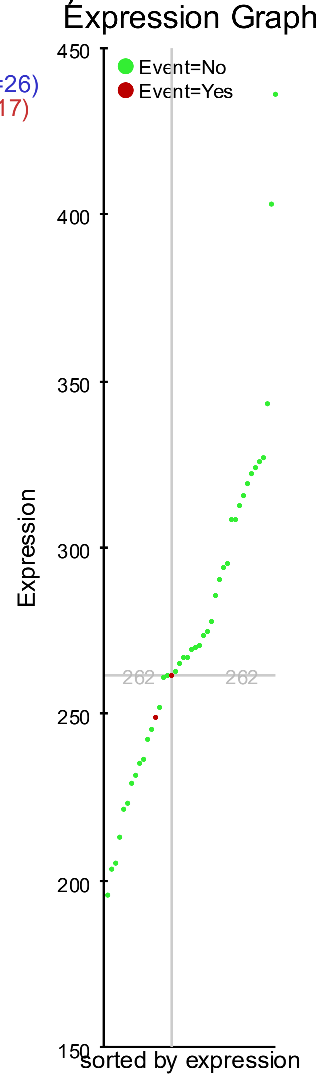

WNT M1

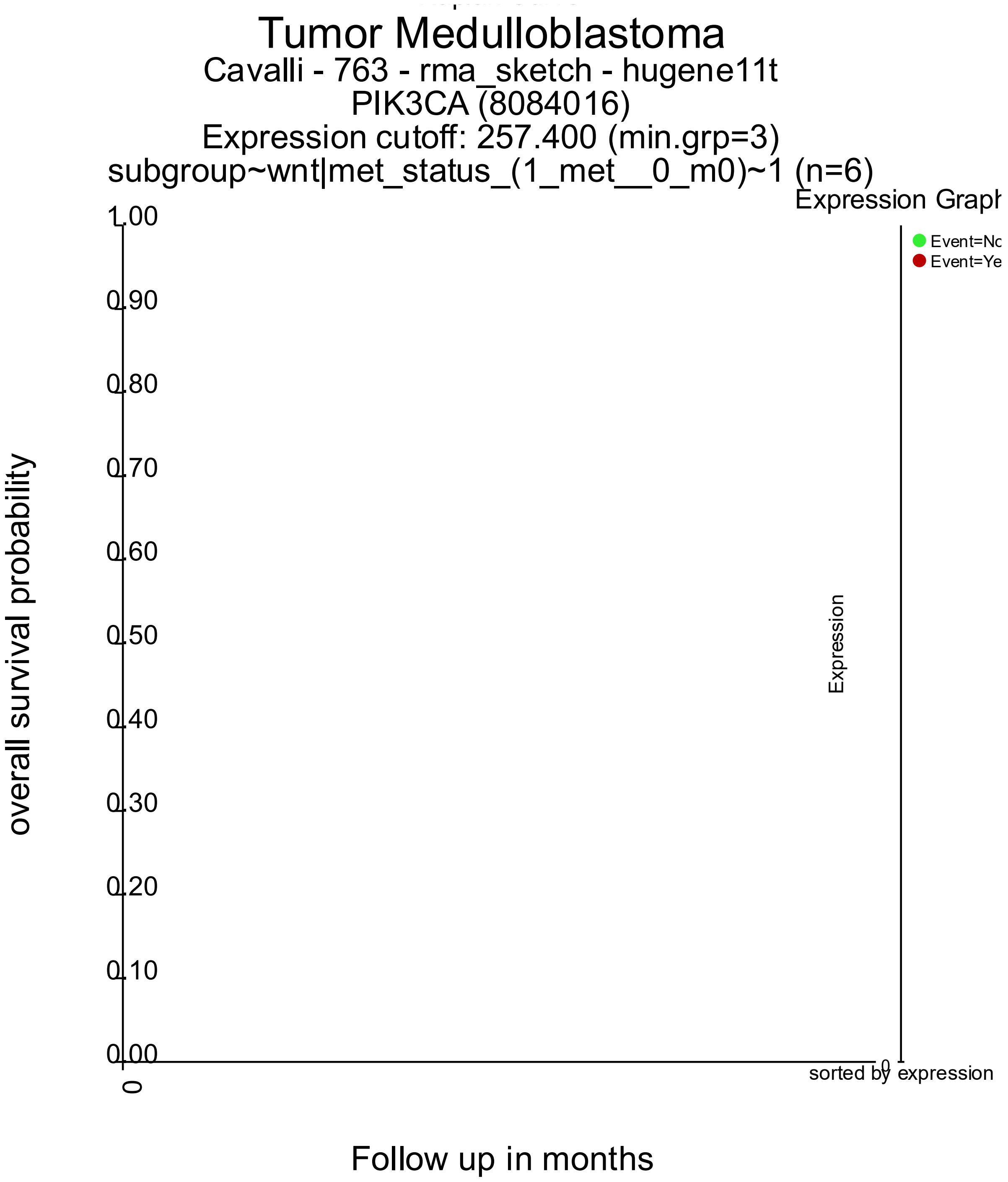

Tumor Medulloblastoma
Cavalli - 763 - rma_sketch - hugene11t
PIK3CA (8084016)
Expression cutoff: 257.400 (min.grp=3)
subgroup~wnt|met_status_(1_met__0_m0)~1 (n=6)
Expression Graph
Event=No
Event=Yes
overall survival probability
1.00
0.90
0.80
0.70
0.60
0.50
0.40
0.30
0.20
0.10
0.00
0
Expression
0
sorted by expression
Follow up in months

SHH M0

Tumor Medulloblastoma
Cavalli - 763 - rma_sketch - hugene11t
PIK3CA (8084016)
Expression cutoff: 373.900 (min.grp=3)
subgroup~shh|met_status_(1_met__0_m0)~0|WITH_SURV (n=124)

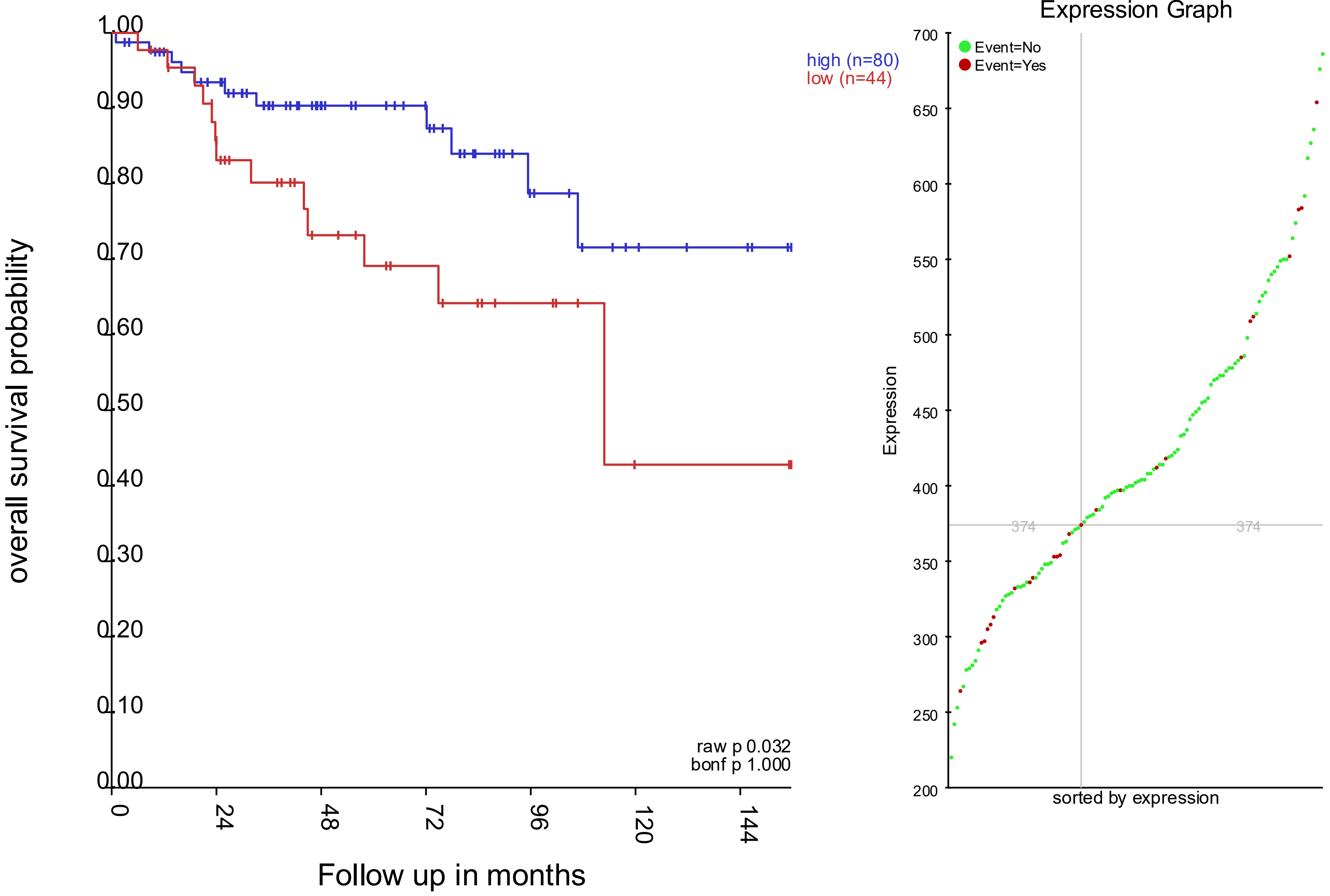

SHH M1

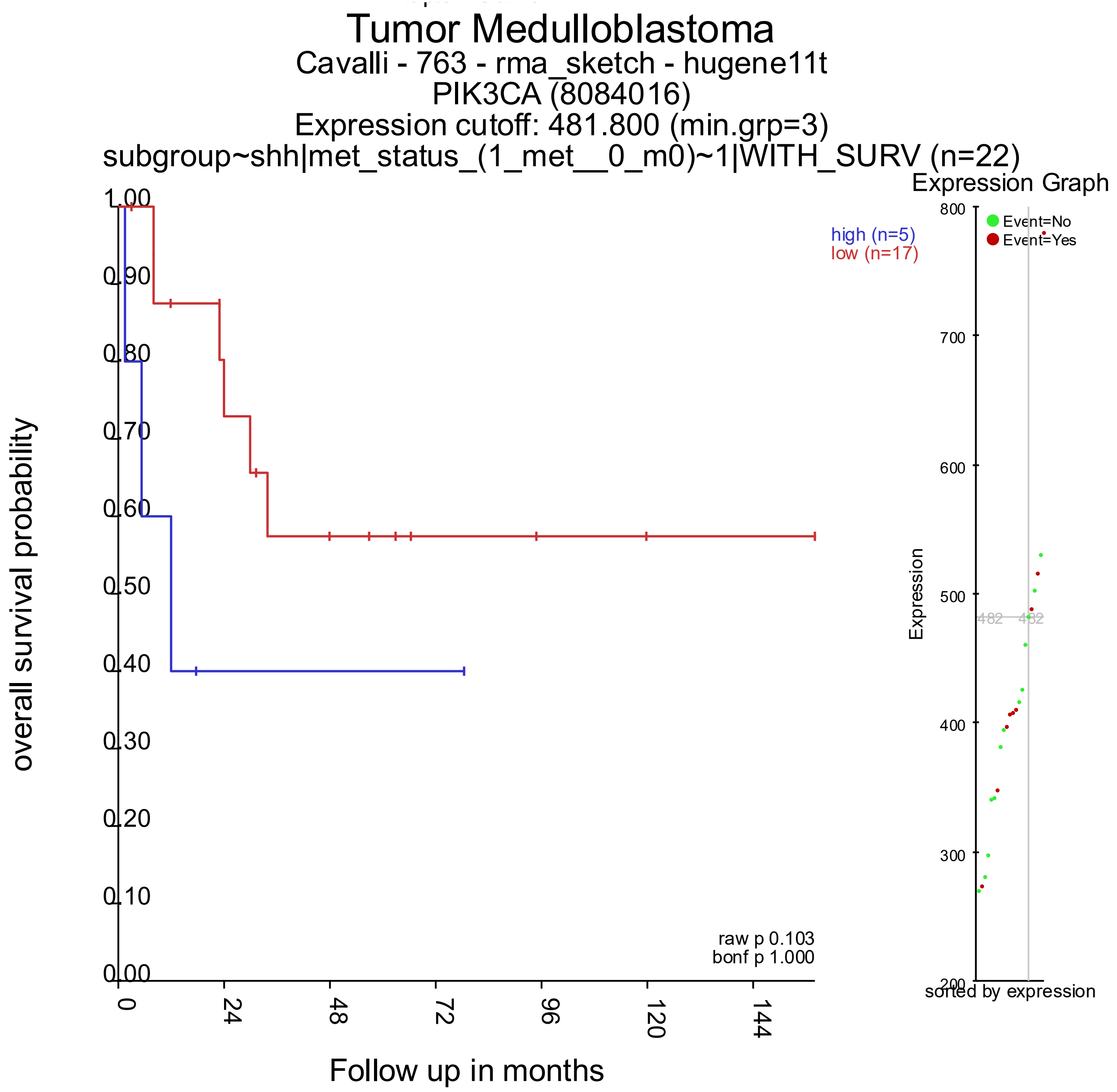

Tumor Medulloblastoma
Cavalli - 763 - rma_sketch - hugene11t
PIK3CA (8084016)
Expression cutoff: 481.800 (min.grp=3)
subgroup~shh|met_status_(1_met__0_m0)~1|WITH_SURV (n=22)
high (n=5)
low (n=17)
overall survival probability
1.00
0.90
0.80
0.70
0.60
0.50
0.40
0.30
0.20
0.10
0.00
raw p 0.103
bonf p 1.000
0
24
48
72
96
120
144
Follow up in months
Expression Graph
Event=No
Event=Yes
800
700
600
500
400
300
200
482
482
Expression
sorted by expression

GROUP4 M0

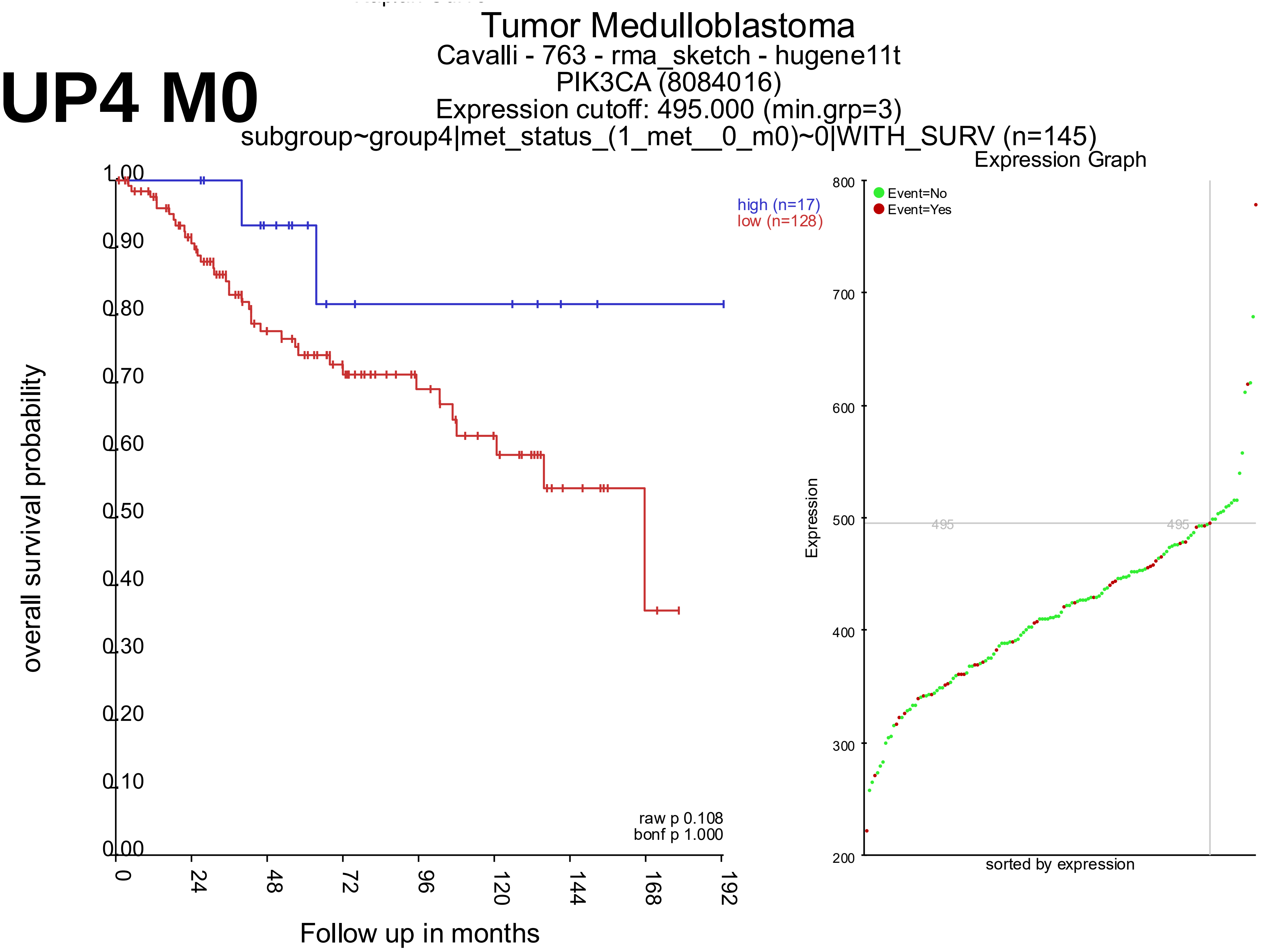

Tumor Medulloblastoma
Cavalli - 763 - rma_sketch - hugene11t
PIK3CA (8084016)
Expression cutoff: 495.000 (min.grp=3)
subgroup~group4|met_status_(1_met__0_m0)~0|WITH_SURV (n=145)
overall survival probability
1.00
0.90
0.80
0.70
0.60
0.50
0.40
0.30
0.20
0.10
0.00
high (n=17)
low (n=128)
raw p 0.108
bonf p 1.000
0
24
48
72
96
120
144
168
192
Follow up in months
Expression Graph
Event=No
Event=Yes
Expression
800
700
600
500
400
300
200
495
495
sorted by expression

GROUP4 M1

Tumor Medulloblastoma
Cavalli - 763 - rma_sketch - hugene11t
PIK3CA (8084016)
Expression cutoff: 539.400 (min.grp=3)
subgroup~group4|met_status_(1_met__0_m0)~1|WITH_SURV (n=92)

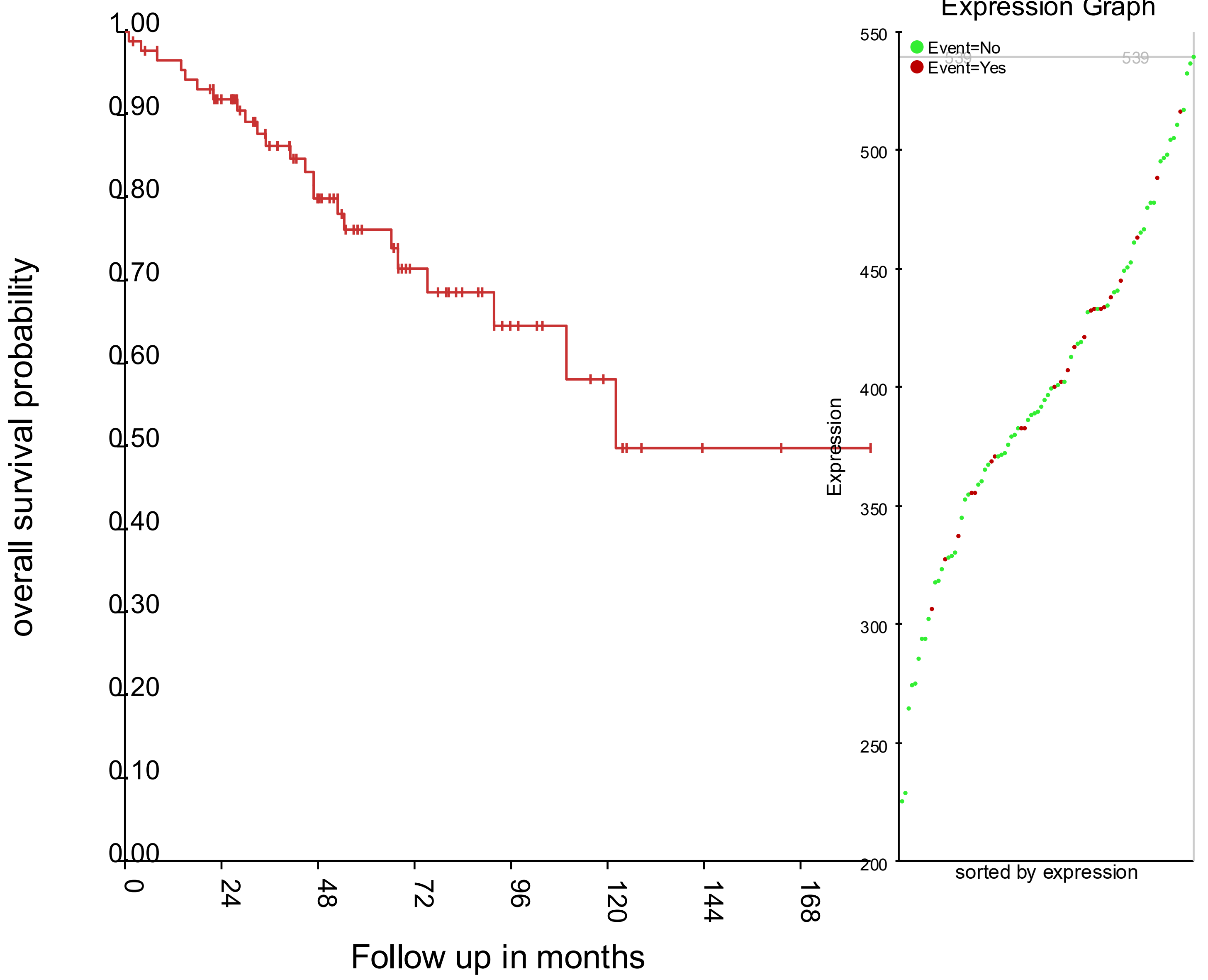

GROUP3 M0

Tumor Medulloblastoma
Cavalli - 763 - rma_sketch - hugene11t
PIK3CA (8084016)
Expression cutoff: 422.700 (min.grp=3)
subgroup~group3|met_status_(1_met__0_m0)~0|WITH_SURV (n=65)

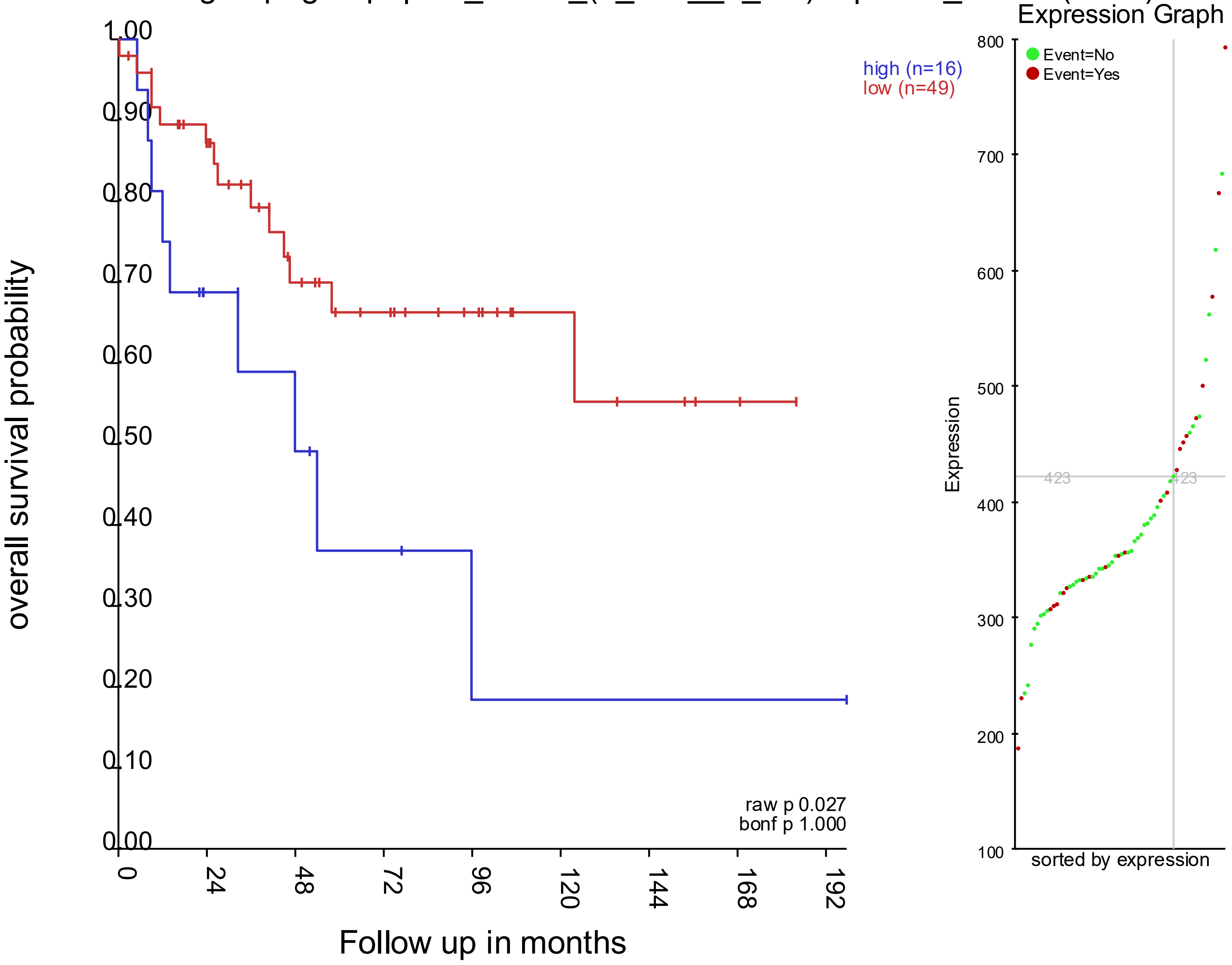

GROUP3 M1

Tumor Medulloblastoma
Cavalli - 763 - rma_sketch - hugene11t
PIK3CA (8084016)
Expression cutoff: 360.700 (min.grp=3)
subgroup~group3|met_status_(1_met__0_m0)~1|WITH_SURV (n=41)

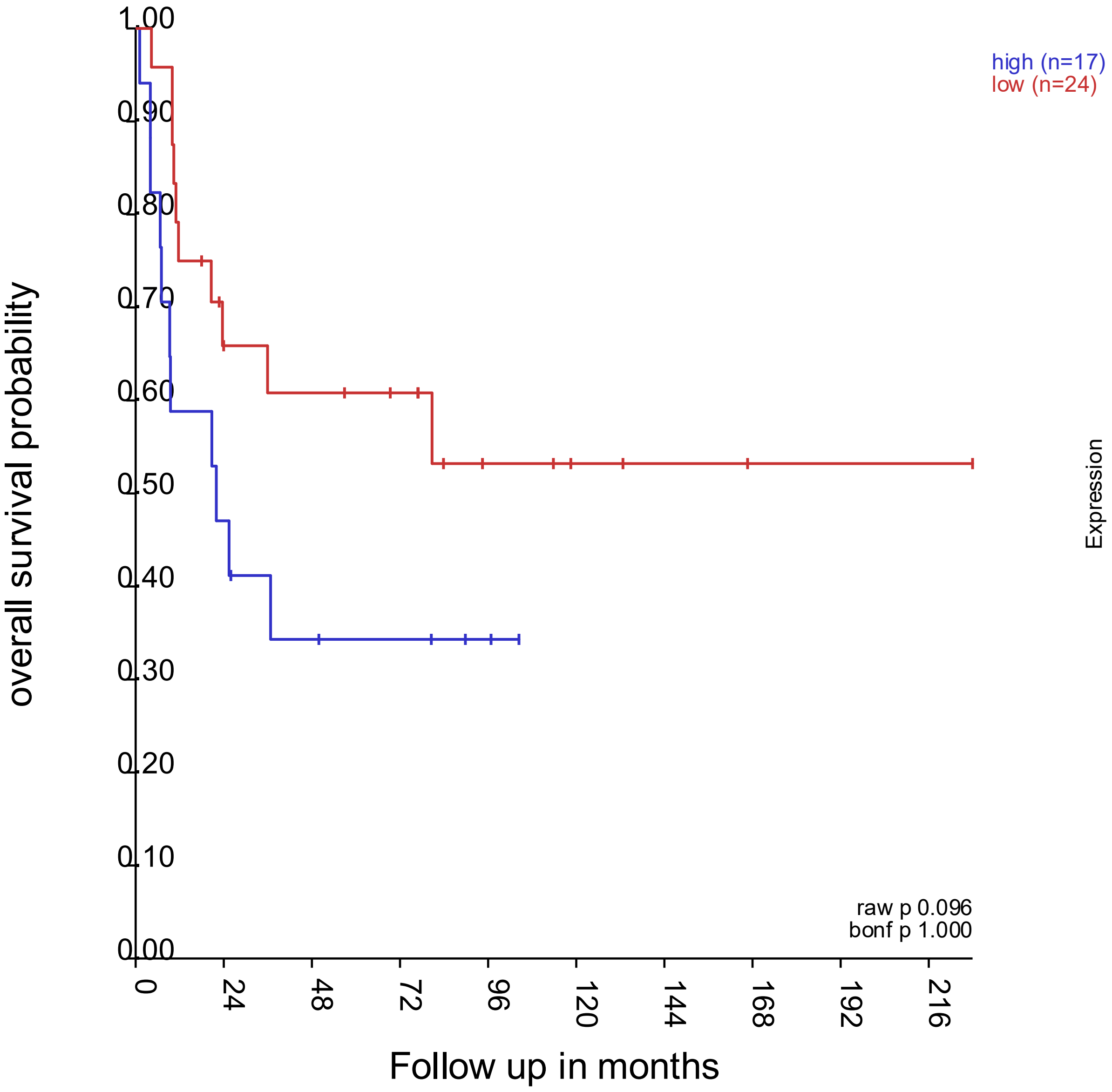

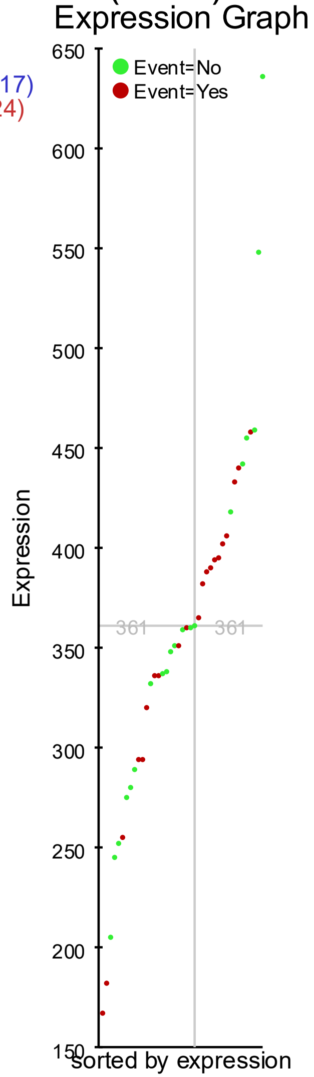

PIK3CB

WNT M0

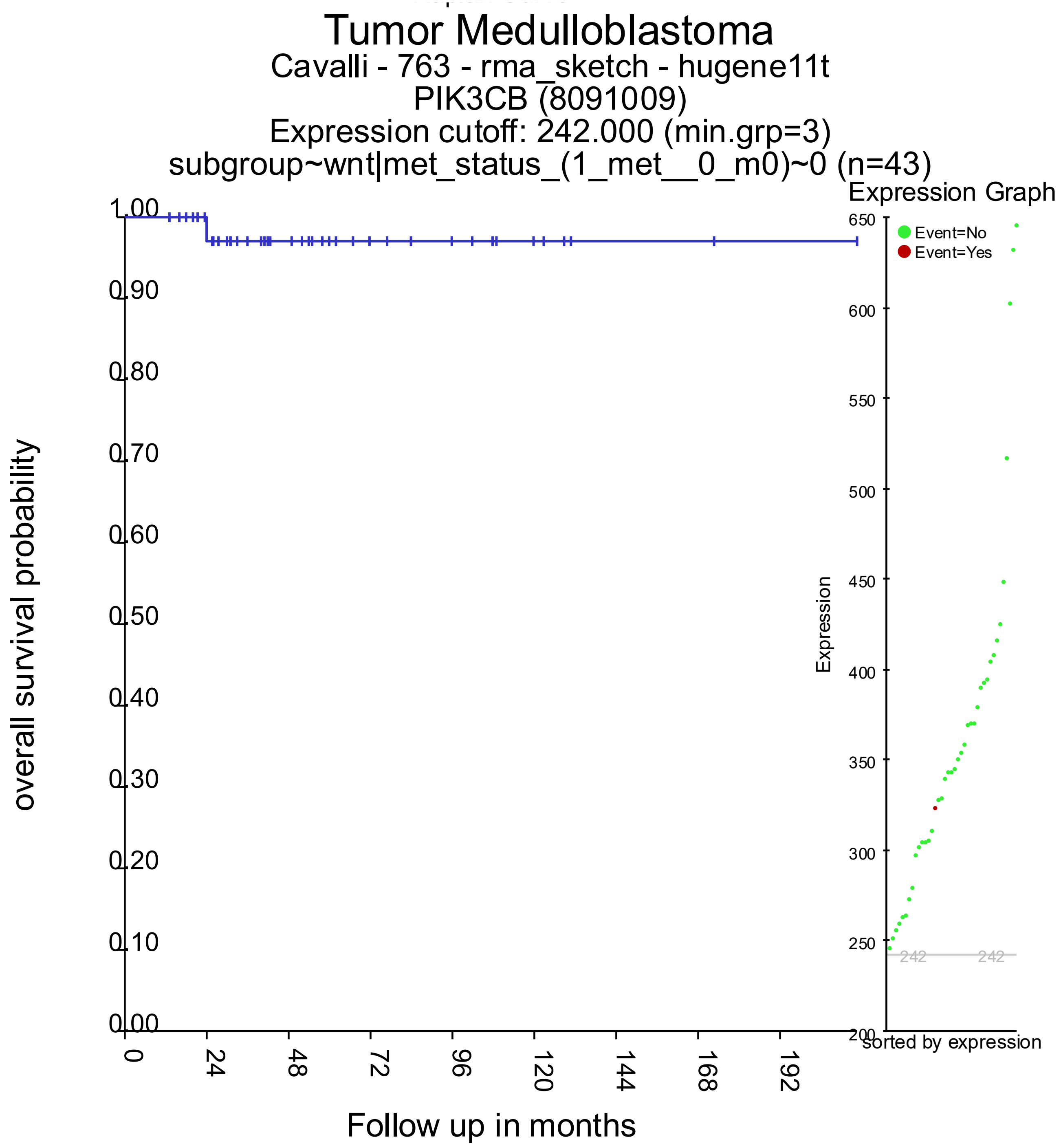
Tumor Medulloblastoma
Cavalli - 763 - rma_sketch - hugene11t
PIK3CB (8091009)
Expression cutoff: 242.000 (min.grp=3)
subgroup~wnt|met_status_(1_met__0_m0)~0 (n=43)
overall survival probability
1.00
0.90
0.80
0.70
0.60
0.50
0.40
0.30
0.20
0.10
0.00
0
24
48
72
96
120
144
168
192
Follow up in months
Expression Graph
Event=No
Event=Yes
650
600
550
500
450
400
350
300
250
200
Expression
242
242
sorted by expression

WNT M1

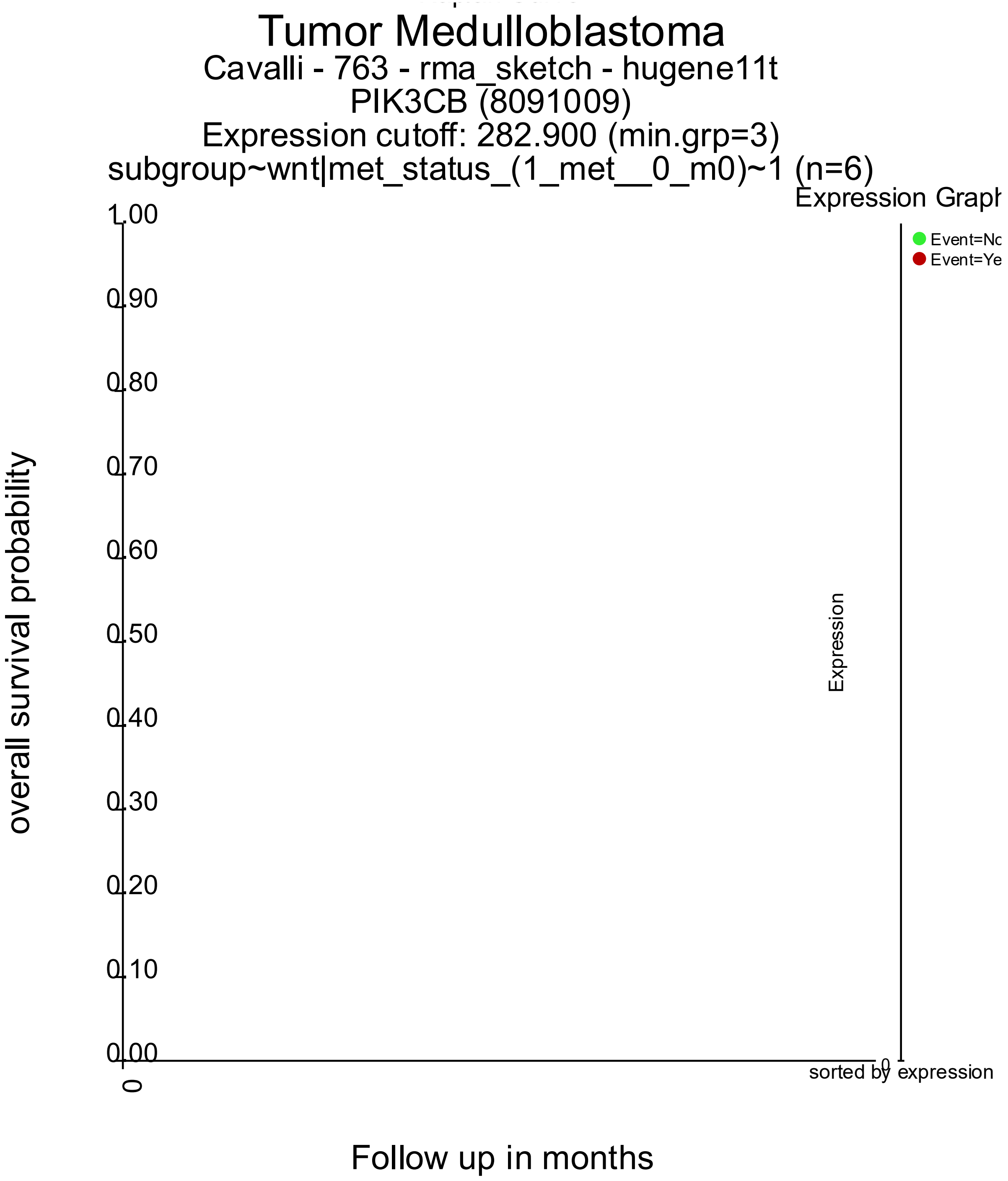

Tumor Medulloblastoma
Cavalli - 763 - rma_sketch - hugene11t
PIK3CB (8091009)
Expression cutoff: 282.900 (min.grp=3)
subgroup~wnt|met_status_(1_met__0_m0)~1 (n=6)
Expression Graph
Event=No
Event=Ye
overall survival probability
1.00
0.90
0.80
0.70
0.60
0.50
0.40
0.30
0.20
0.10
0.00
0
Expression
0
sorted by expression
Follow up in months

SHH M0

Tumor Medulloblastoma
Cavalli - 763 - rma_sketch - hugene11t
PIK3CB (8091009)
Expression cutoff: 439.200 (min.grp=3)
subgroup~shh|met_status_(1_met__0_m0)~0|WITH_SURV (n=124)

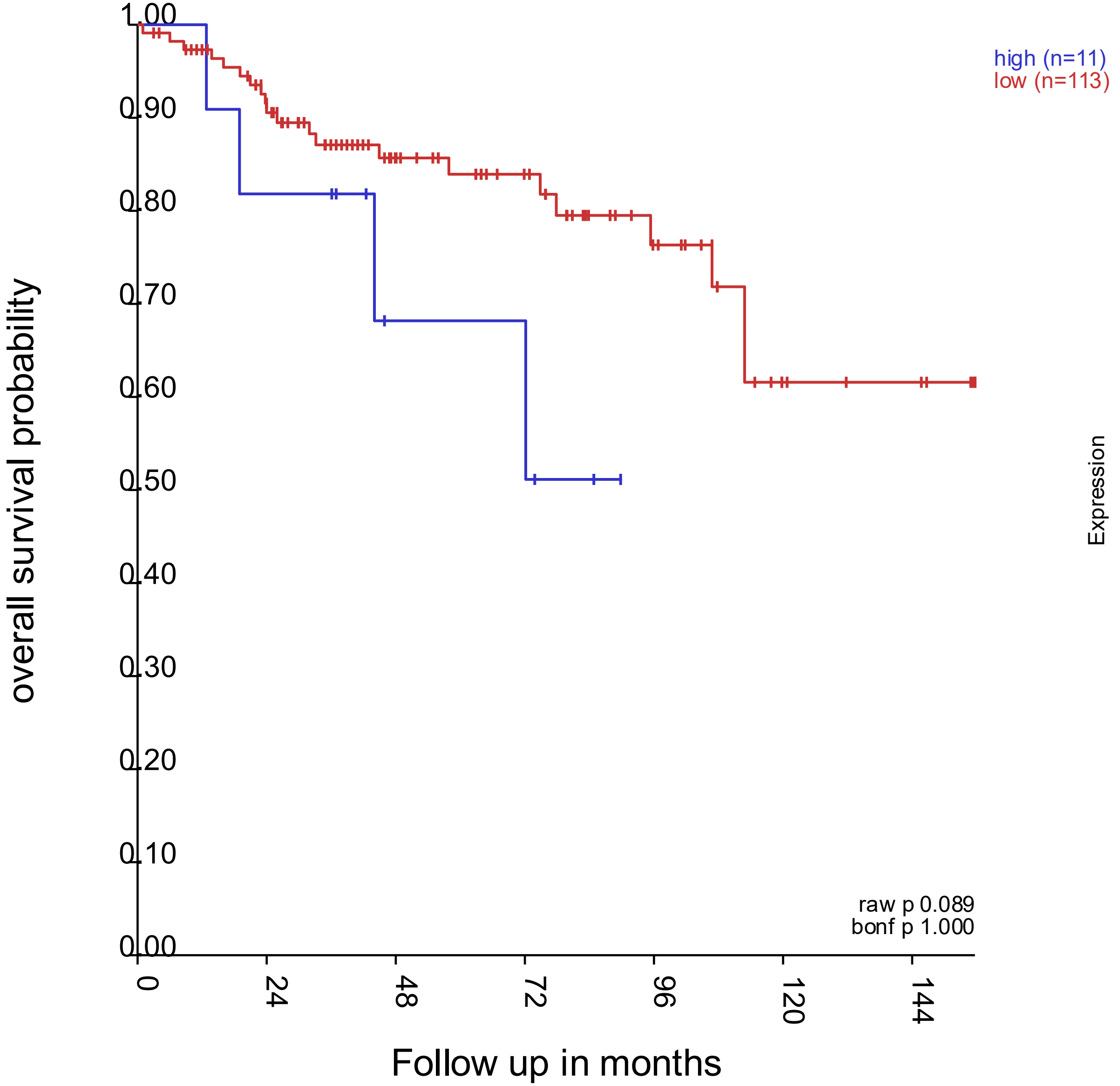

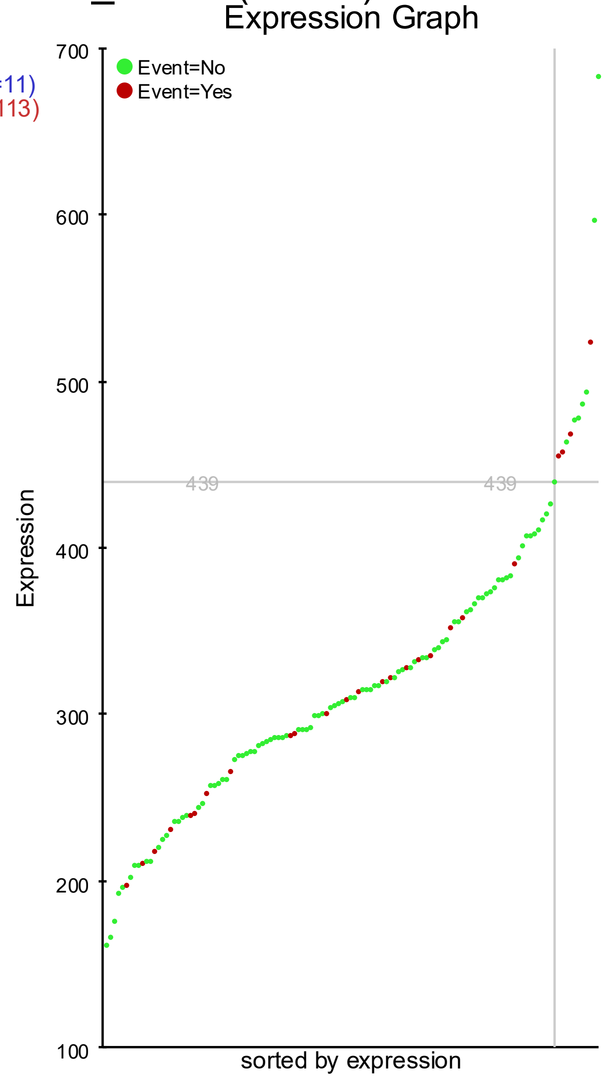

SHH M1

Tumor Medulloblastoma
Cavalli - 763 - rma_sketch - hugene11t
PIK3CB (8091009)
Expression cutoff: 231.600 (min.grp=3)
subgroup~shh|met_status_(1_met__0_m0)~1|WITH_SURV (n=22)

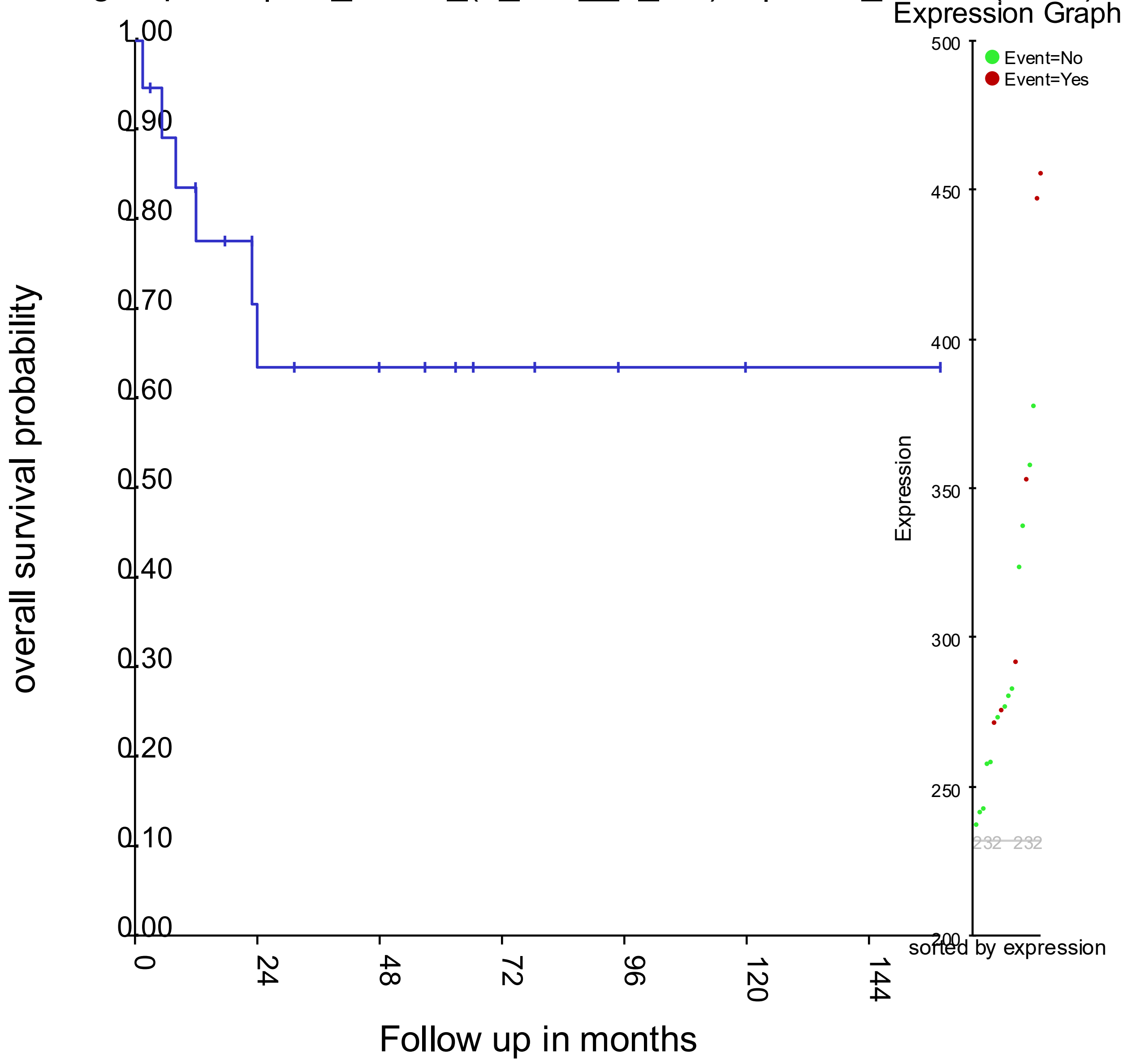

GROUP4 M0

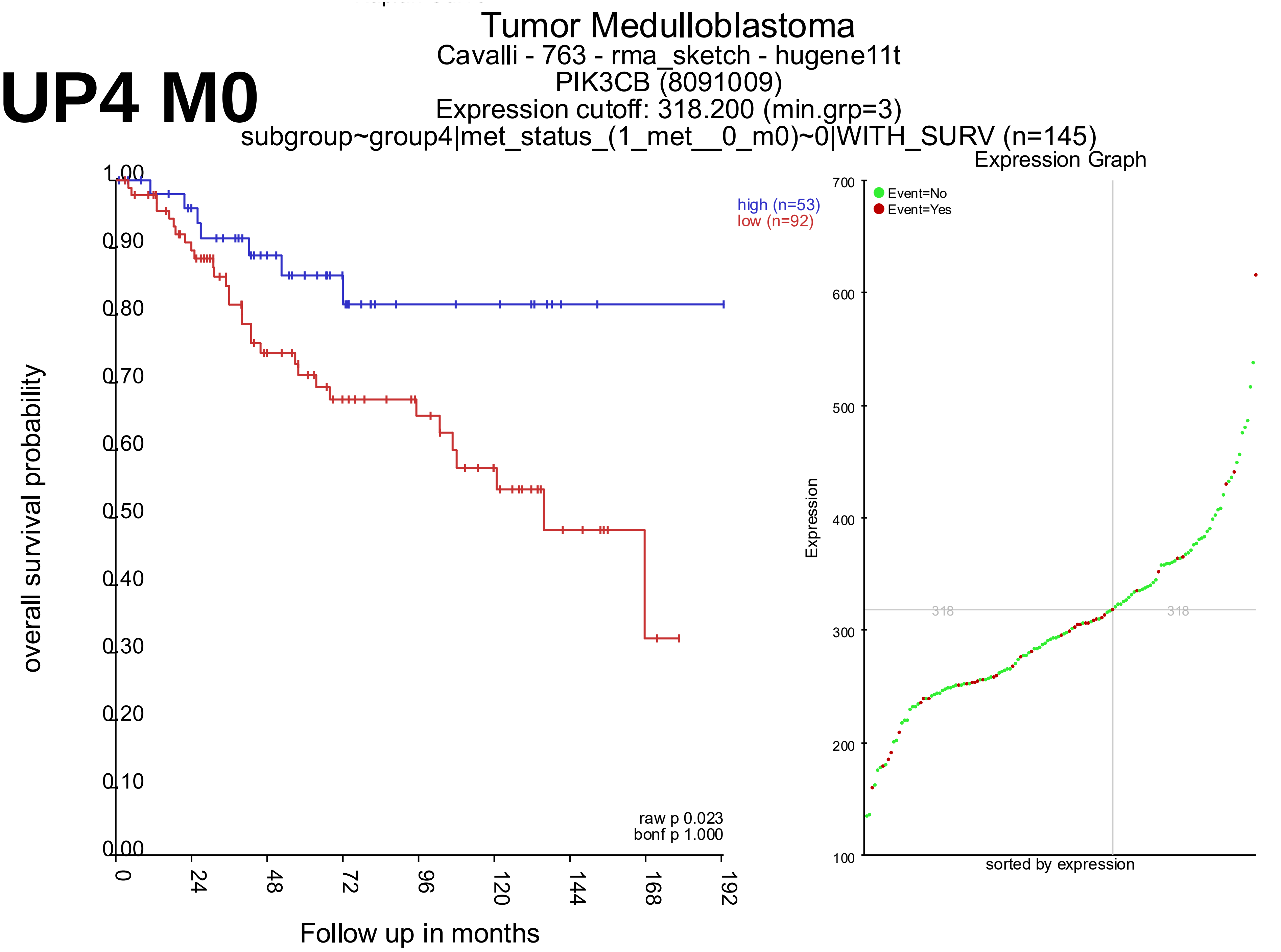

Tumor Medulloblastoma
Cavalli - 763 - rma_sketch - hugene11t
PIK3CB (8091009)
Expression cutoff: 318.200 (min.grp=3)
subgroup~group4|met_status_(1_met__0_m0)~0|WITH_SURV (n=145)
overall survival probability
1.00
0.90
0.80
0.70
0.60
0.50
0.40
0.30
0.20
0.10
0.00
high (n=53)
low (n=92)
raw p 0.023
bonf p 1.000
0
24
48
72
96
120
144
168
192
Follow up in months
Expression Graph
Event=No
Event=Yes
Expression
700
600
500
400
300
200
100
318
318
sorted by expression

GROUP4 M1

Tumor Medulloblastoma
Cavalli - 763 - rma_sketch - hugene11t
PIK3CB (8091009)
Expression cutoff: 270.400 (min.grp=3)
subgroup~group4|met_status_(1_met__0_m0)~1|WITH_SURV (n=92)

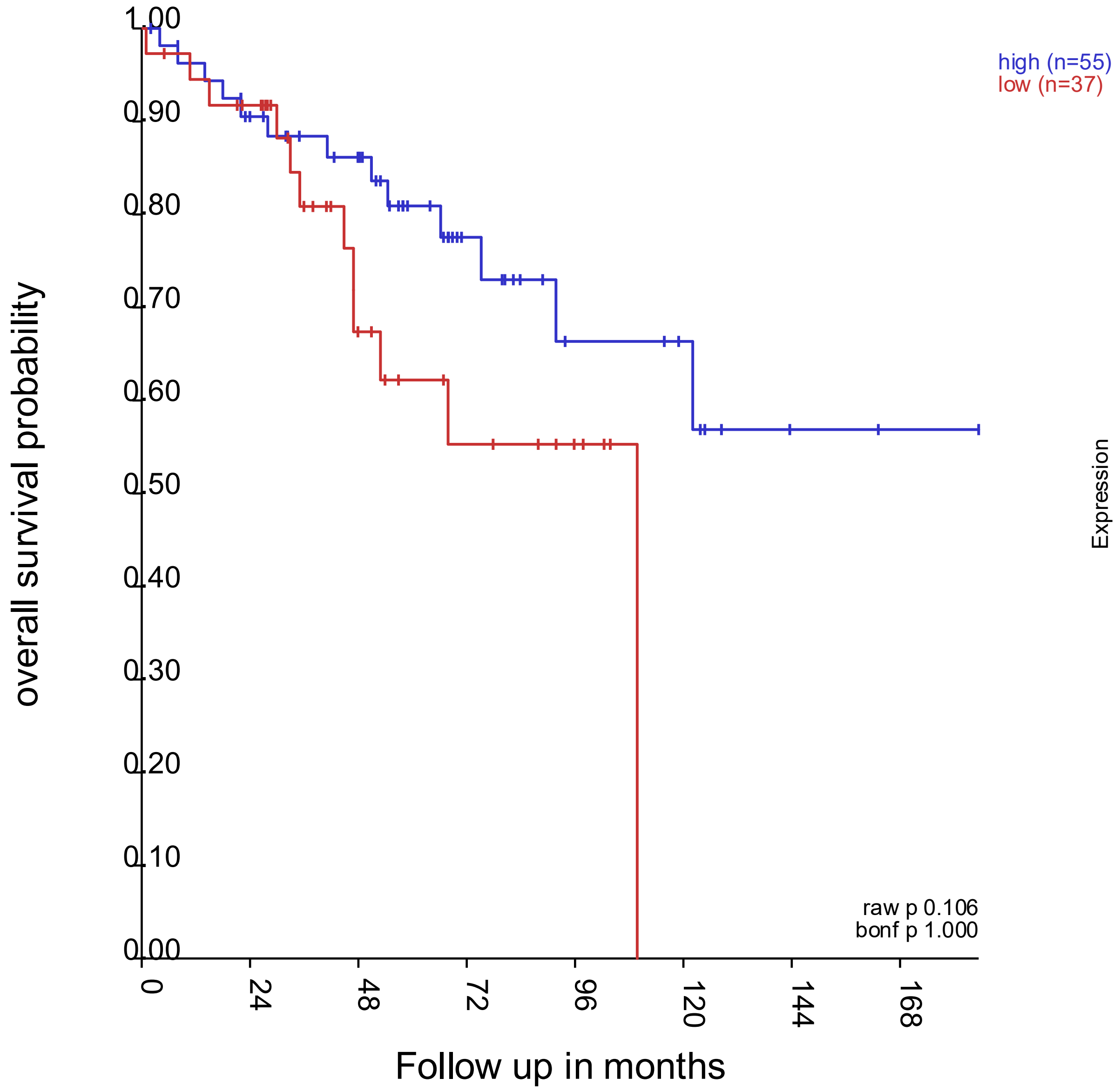

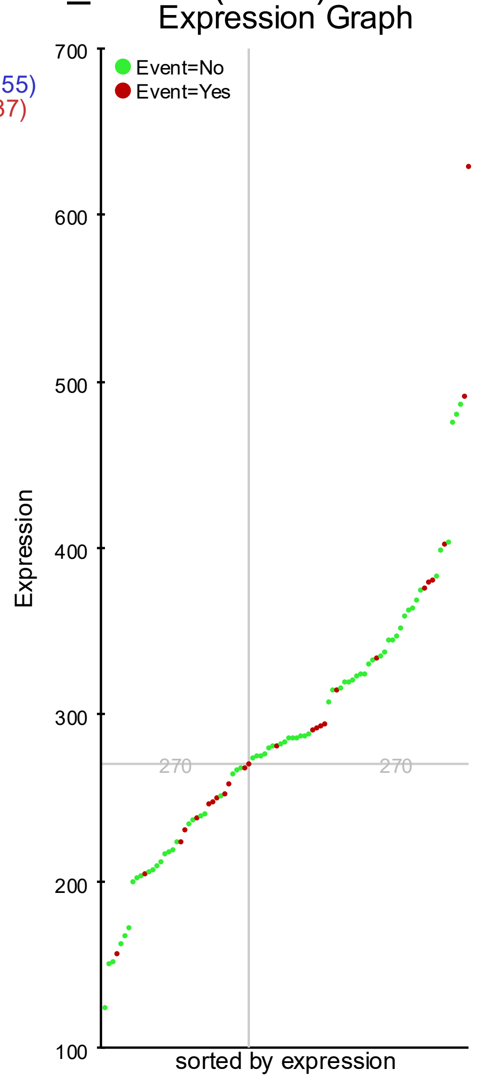

GROUP3 M0

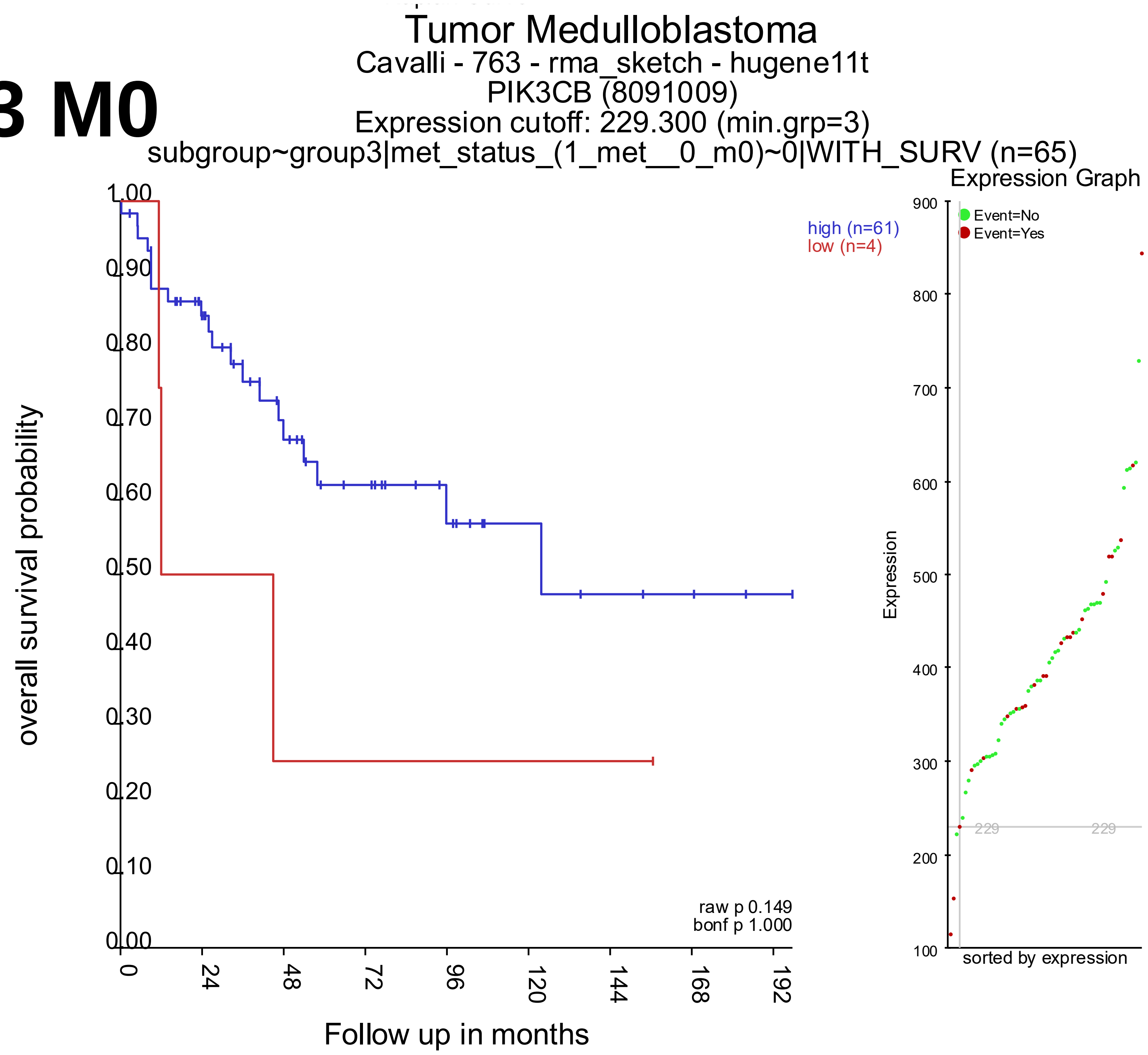

Tumor Medulloblastoma
Cavalli - 763 - rma_sketch - hugene11t
PIK3CB (8091009)
Expression cutoff: 229.300 (min.grp=3)
subgroup~group3|met_status_(1_met__0_m0)~0|WITH_SURV (n=65)
overall survival probability
1.00
0.90
0.80
0.70
0.60
0.50
0.40
0.30
0.20
0.10
0.00
0
24
48
72
96
120
144
168
192
Follow up in months
high (n=61)
low (n=4)
raw p 0.149
bonf p 1.000
Expression Graph
Event=No
Event=Yes
900
800
700
600
500
400
300
200
100
Expression
229
229
sorted by expression

GROUP3 M1

Tumor Medulloblastoma
Cavalli - 763 - rma_sketch - hugene11t
PIK3CB (8091009)
Expression cutoff: 377.200 (min.grp=3)
subgroup~group3|met_status_(1_met__0_m0)~1|WITH_SURV (n=41)

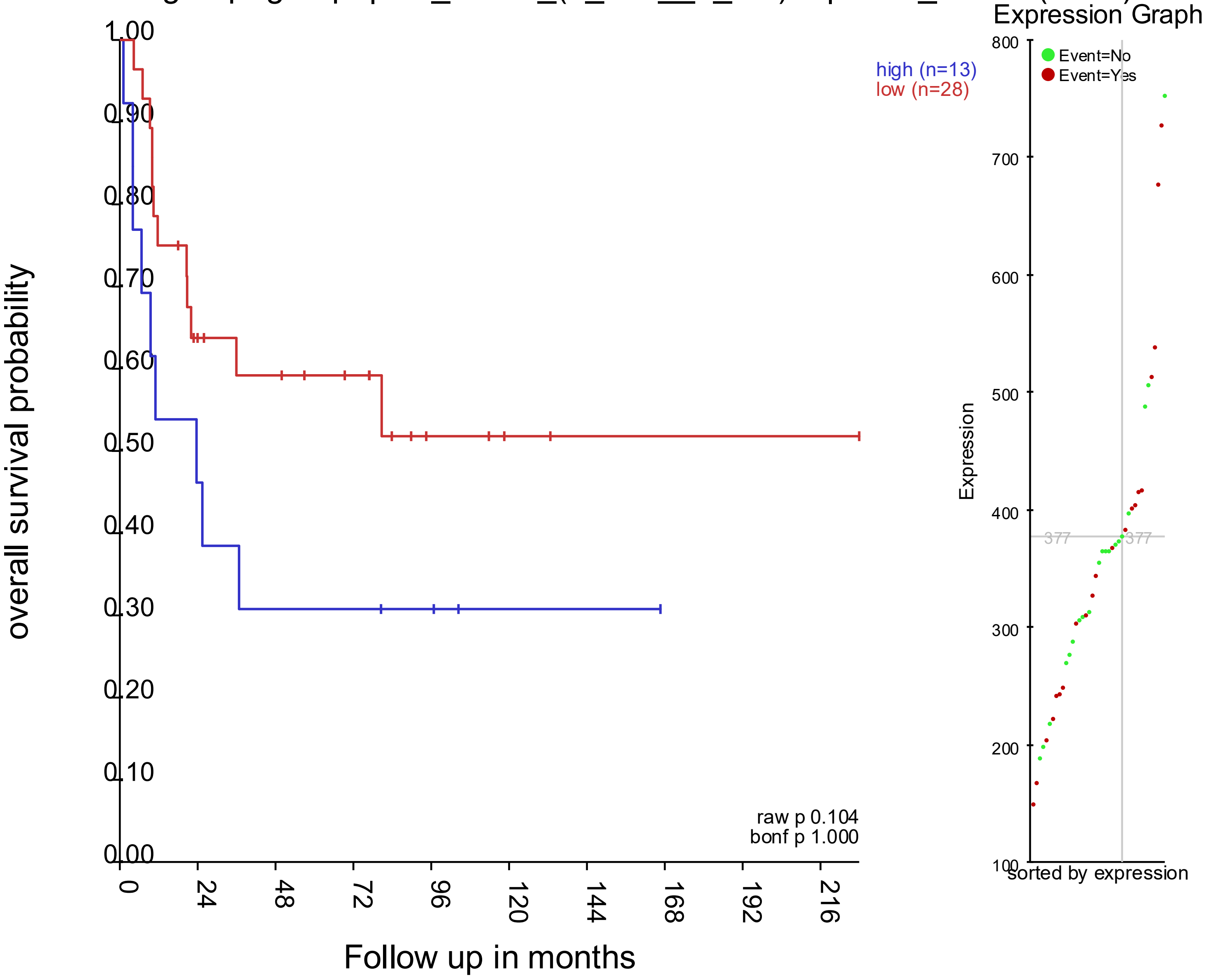

PIK3CD

WNT M0

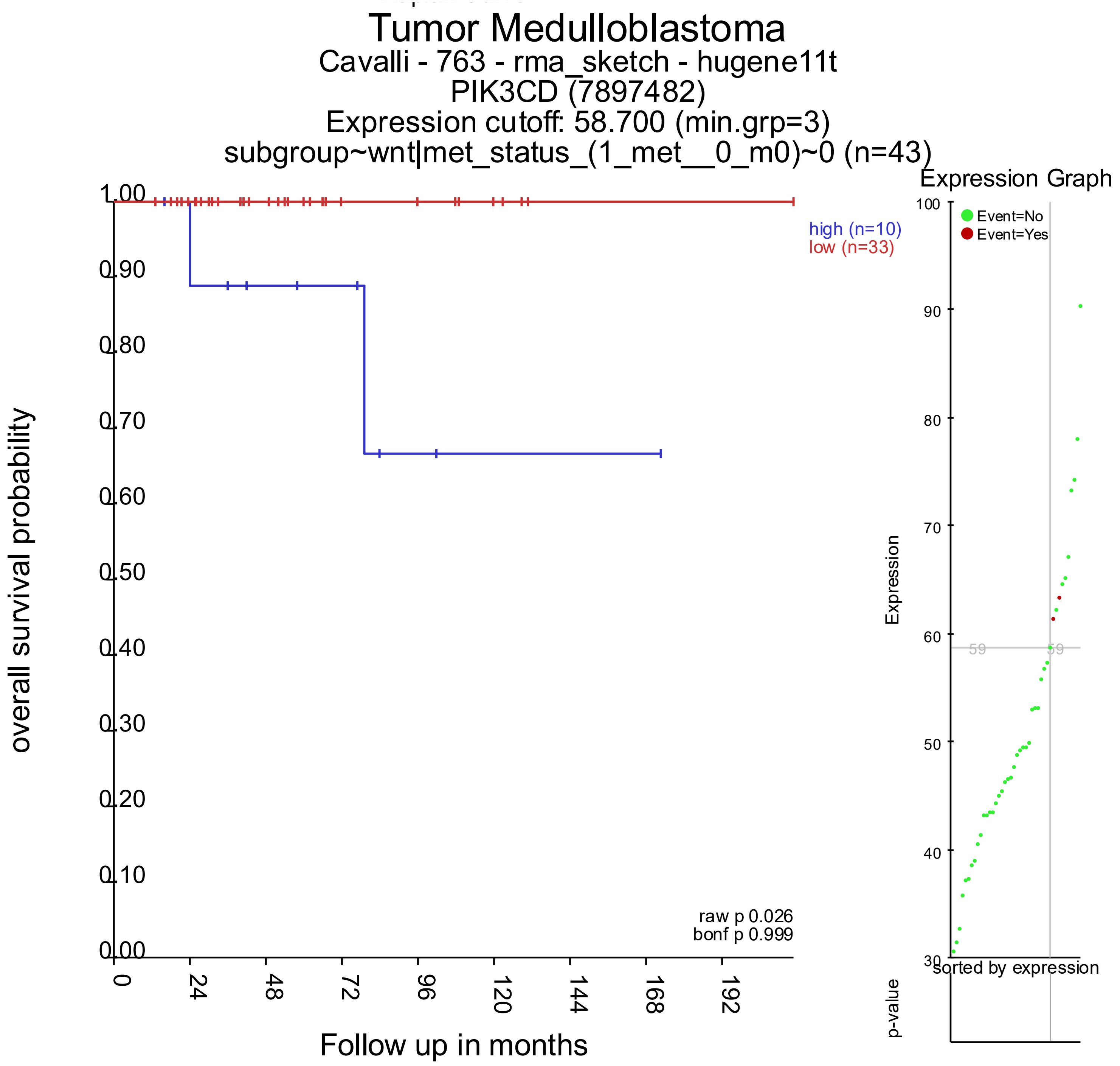
Tumor Medulloblastoma
Cavalli - 763 - rma_sketch - hugene11t
PIK3CD (7897482)
Expression cutoff: 58.700 (min.grp=3)
subgroup~wnt|met_status_(1_met__0_m0)~0 (n=43)
high (n=10)
low (n=33)
overall survival probability
1.00
0.90
0.80
0.70
0.60
0.50
0.40
0.30
0.20
0.10
0.00
0
24
48
72
96
120
144
168
192
Follow up in months
raw p 0.026
bonf p 0.999
Expression Graph
Event=No
Event=Yes
Expression
100
90
80
70
60
50
40
30
59
59
sorted by expression
p-value

WNT M1

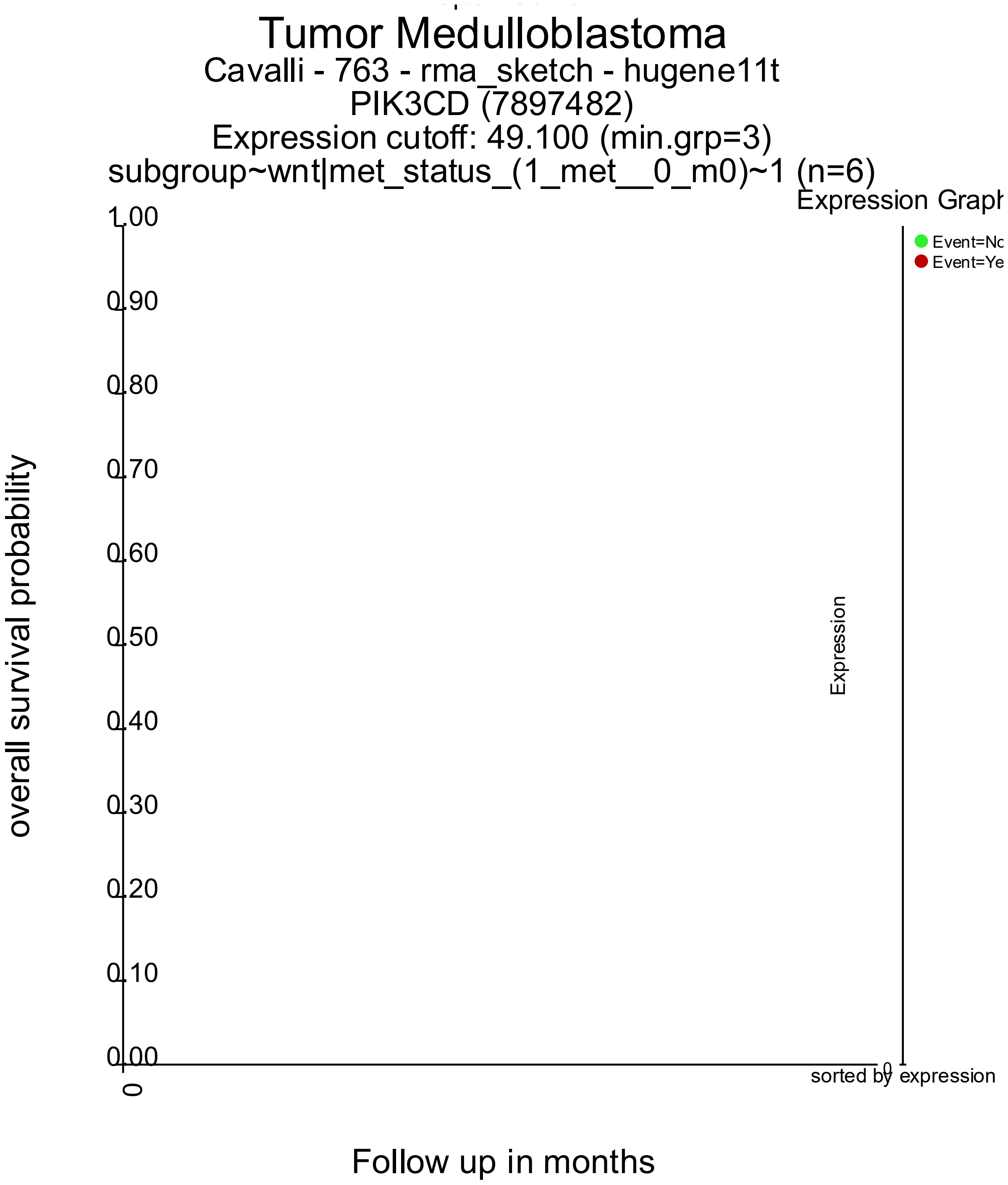

Tumor Medulloblastoma
Cavalli - 763 - rma_sketch - hugene11t
PIK3CD (7897482)
Expression cutoff: 49.100 (min.grp=3)
subgroup~wnt|met_status_(1_met__0_m0)~1 (n=6)
Expression Graph
Event=No
Event=Yes
overall survival probability
1.00
0.90
0.80
0.70
0.60
0.50
0.40
0.30
0.20
0.10
0.00
0
Expression
0
sorted by expression
Follow up in months

SHH M0

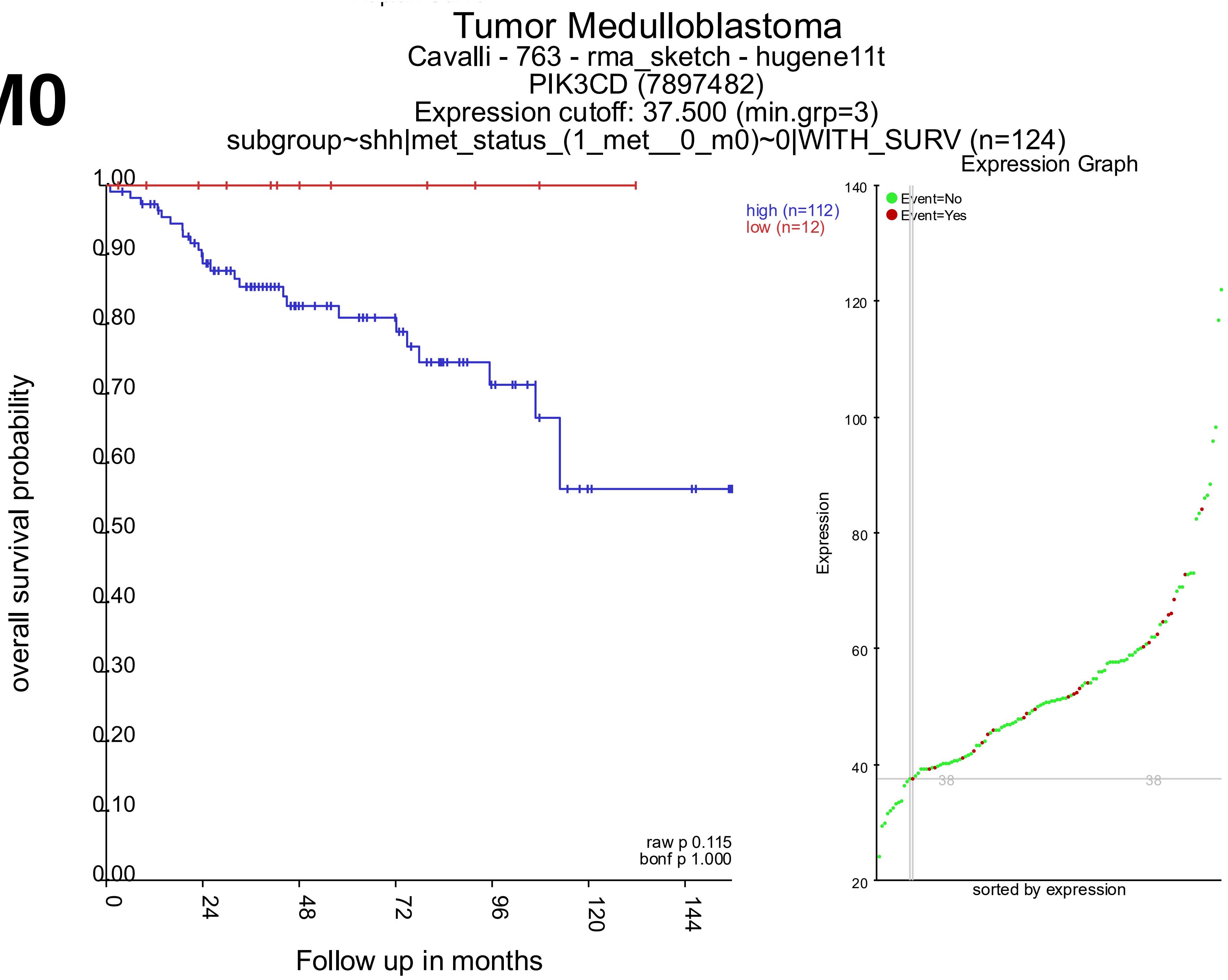

Tumor Medulloblastoma
Cavalli - 763 - rma_sketch - hugene11t
PIK3CD (7897482)
Expression cutoff: 37.500 (min.grp=3)
subgroup~shh|met_status_(1_met__0_m0)~0|WITH_SURV (n=124)
high (n=112)
low (n=12)
overall survival probability
1.00
0.90
0.80
0.70
0.60
0.50
0.40
0.30
0.20
0.10
0.00
0
24
48
72
96
120
144
Follow up in months
raw p 0.115
bonf p 1.000
Expression Graph
Event=No
Event=Yes
Expression
140
120
100
80
60
40
20
38
38
sorted by expression

SHH M1

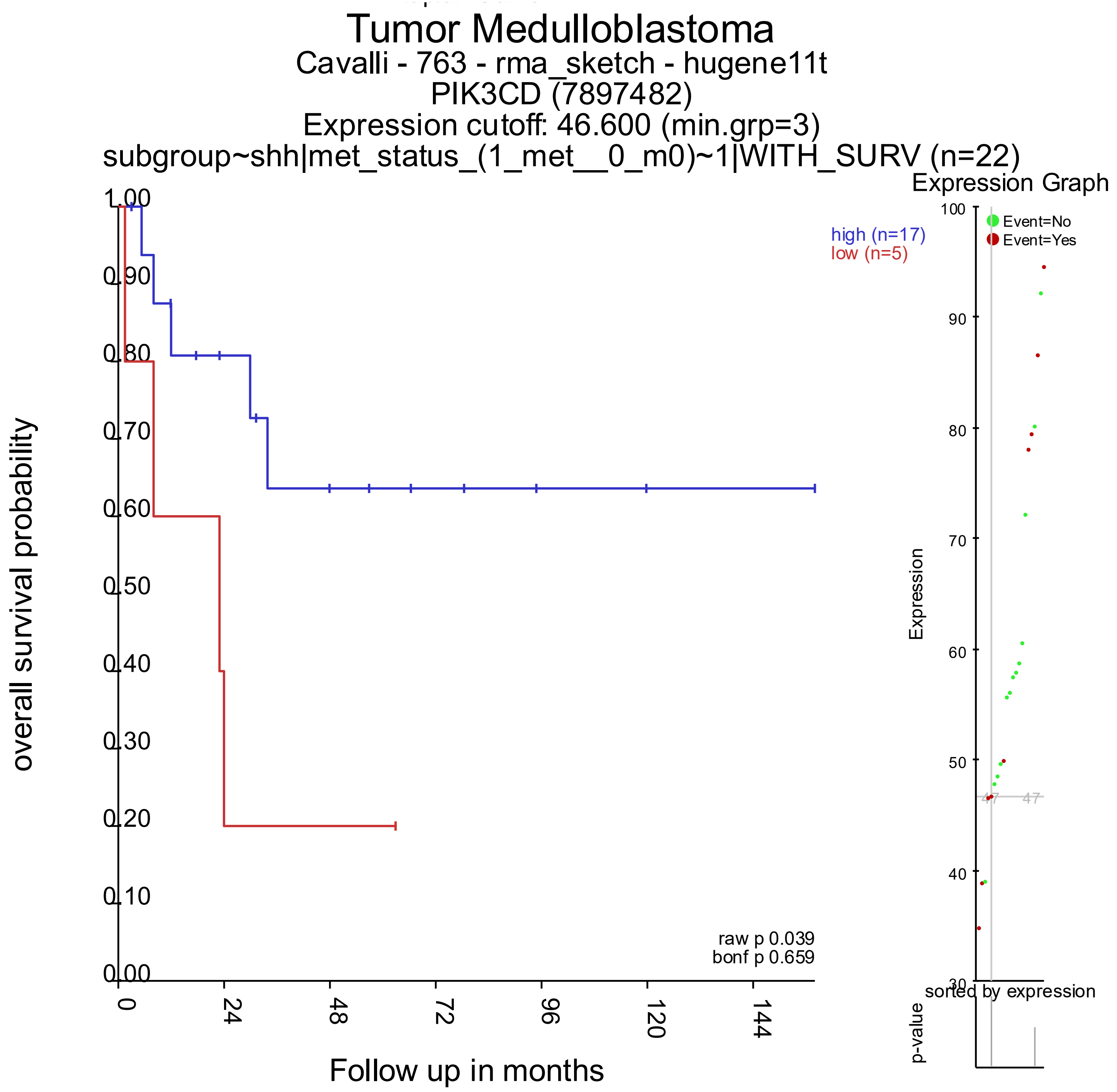

Tumor Medulloblastoma
Cavalli - 763 - rma_sketch - hugene11t
PIK3CD (7897482)
Expression cutoff: 46.600 (min.grp=3)
subgroup~shh|met_status_(1_met__0_m0)~1|WITH_SURV (n=22)
Expression Graph
high (n=17)
low (n=5)
Event=No
Event=Yes
overall survival probability
Follow up in months
Expression
sorted by expression
p-value
raw p 0.039
bonf p 0.659

GROUP4 M0

Tumor Medulloblastoma
Cavalli - 763 - rma_sketch - hugene11t
PIK3CD (7897482)
Expression cutoff: 48.600 (min.grp=3)
subgroup~group4|met_status_(1_met__0_m0)~0|WITH_SURV (n=145)

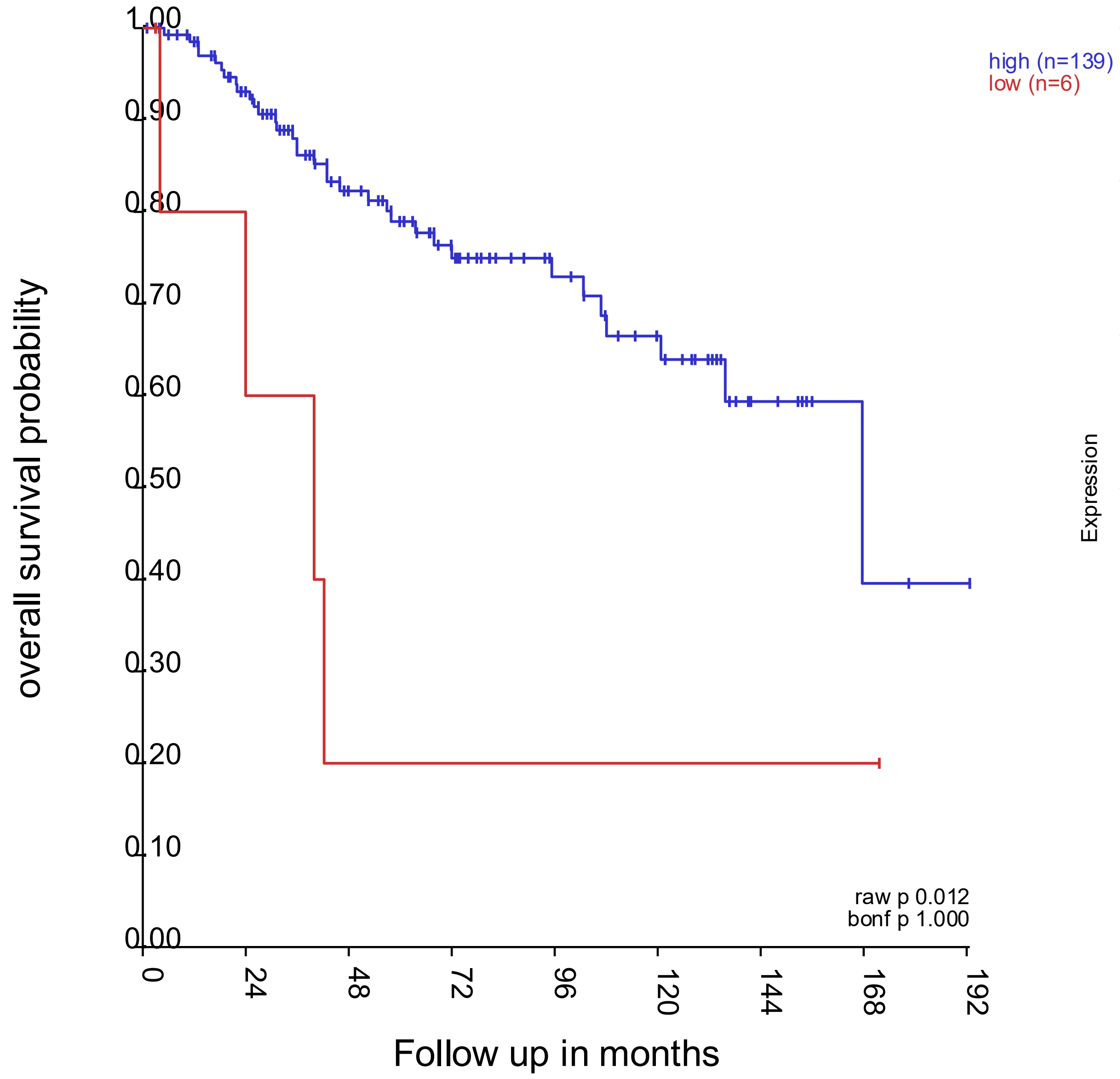

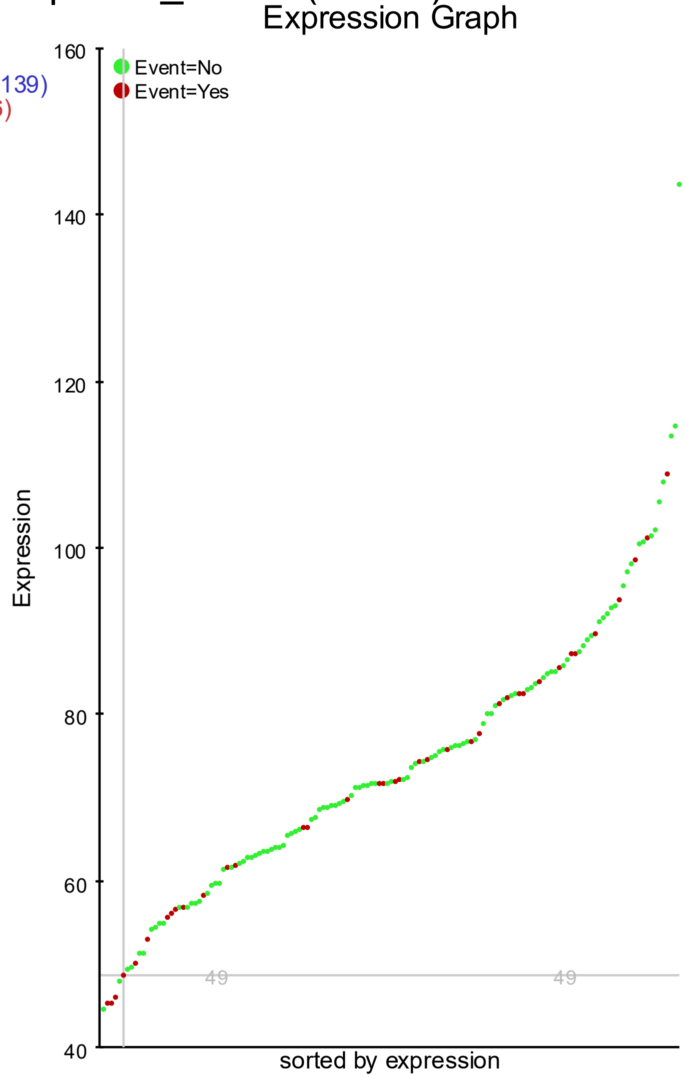

GROUP4 M1

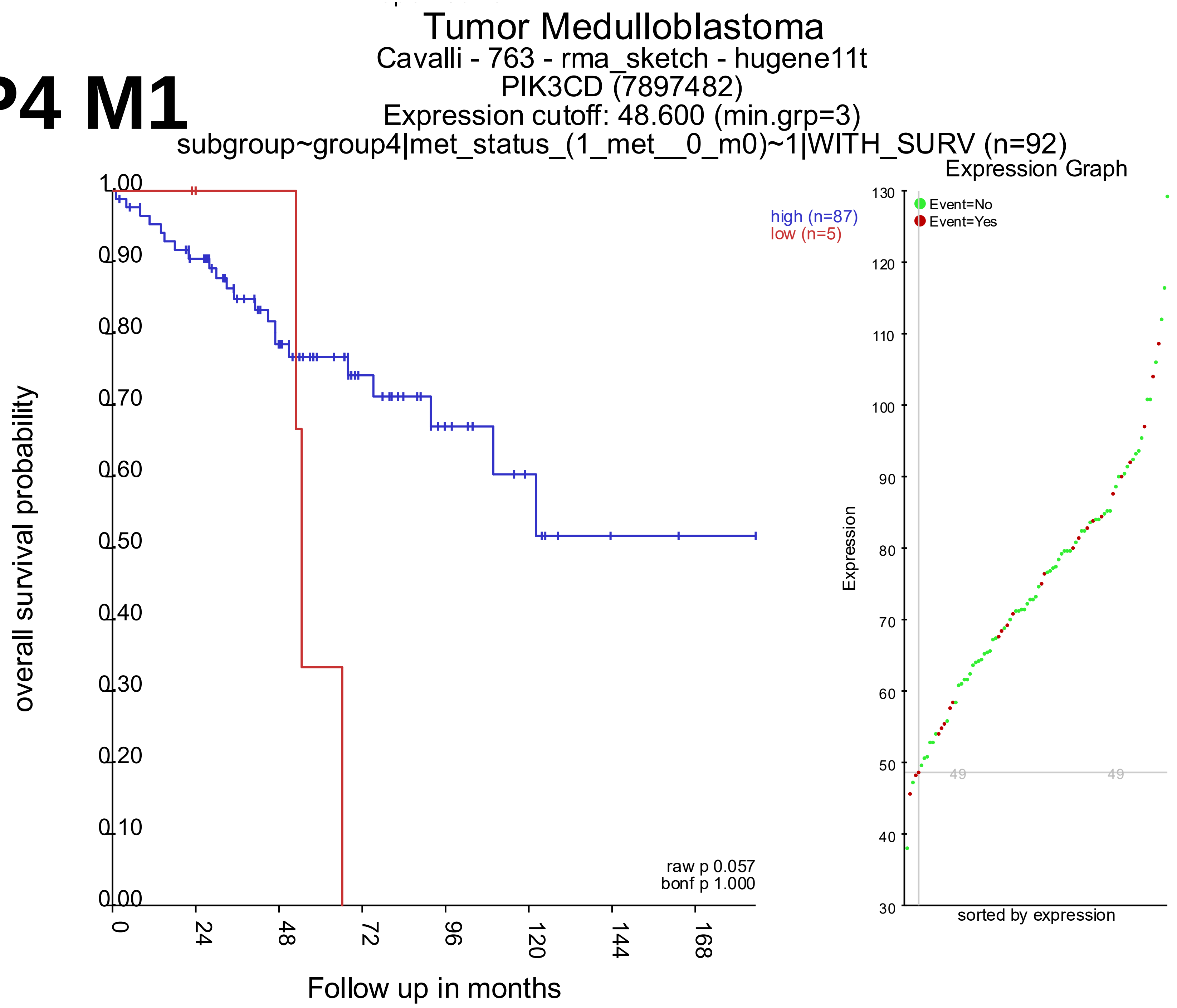

Tumor Medulloblastoma
Cavalli - 763 - rma_sketch - hugene11t
PIK3CD (7897482)
Expression cutoff: 48.600 (min.grp=3)
subgroup~group4|met_status_(1_met__0_m0)~1|WITH_SURV (n=92)
overall survival probability
1.00
0.90
0.80
0.70
0.60
0.50
0.40
0.30
0.20
0.10
0.00
0
24
48
72
96
120
144
168
Follow up in months
high (n=87)
low (n=5)
raw p 0.057
bonf p 1.000
Expression Graph
Event=No
Event=Yes
Expression
130
120
110
100
90
80
70
60
50
40
30
49
49
sorted by expression

GROUP3 M0

Tumor Medulloblastoma
Cavalli - 763 - rma_sketch - hugene11t
PIK3CD (7897482)
Expression cutoff: 38.500 (min.grp=3)
subgroup~group3|met_status_(1_met__0_m0)~0|WITH_SURV (n=65)

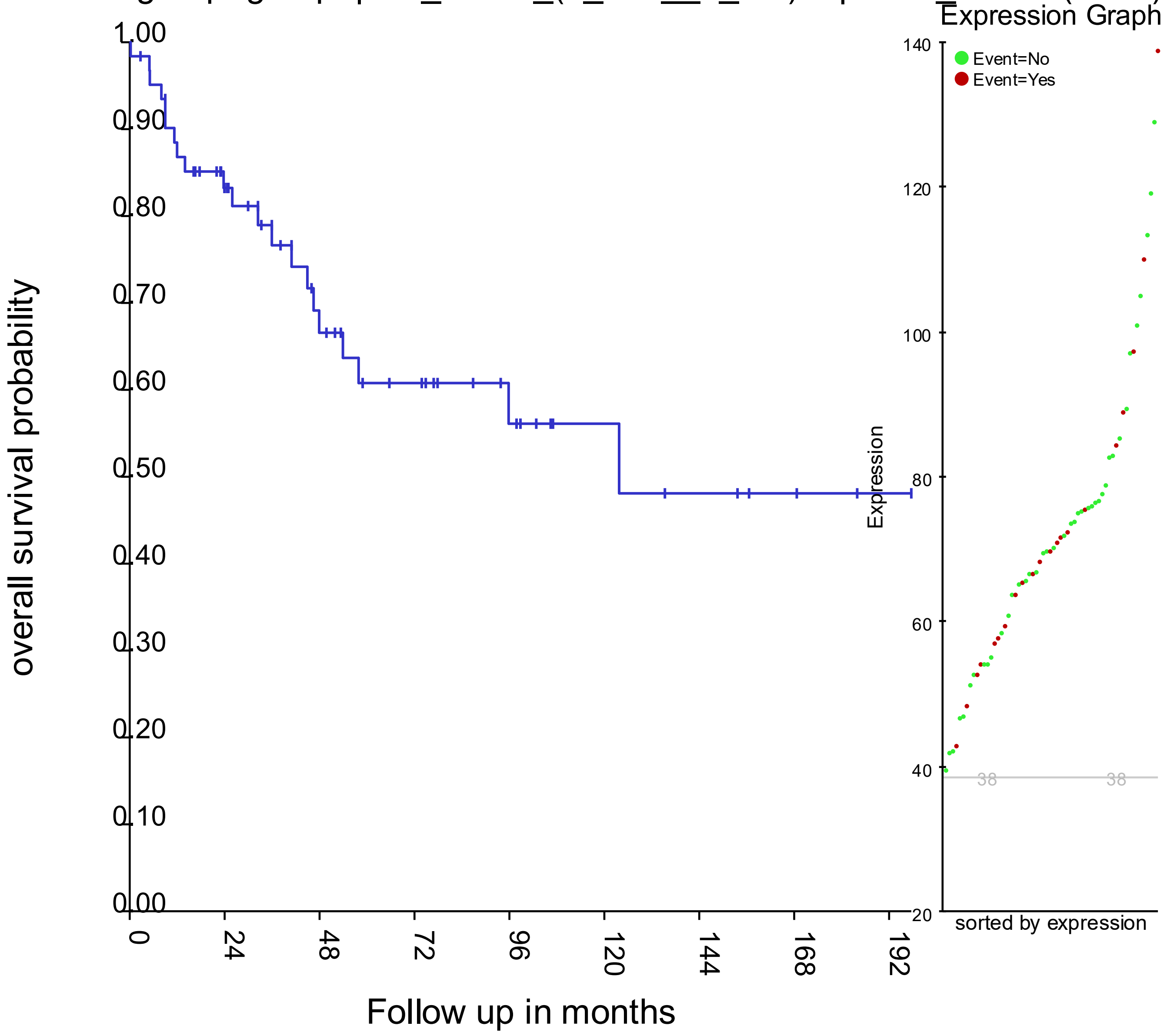

GROUP3 M1

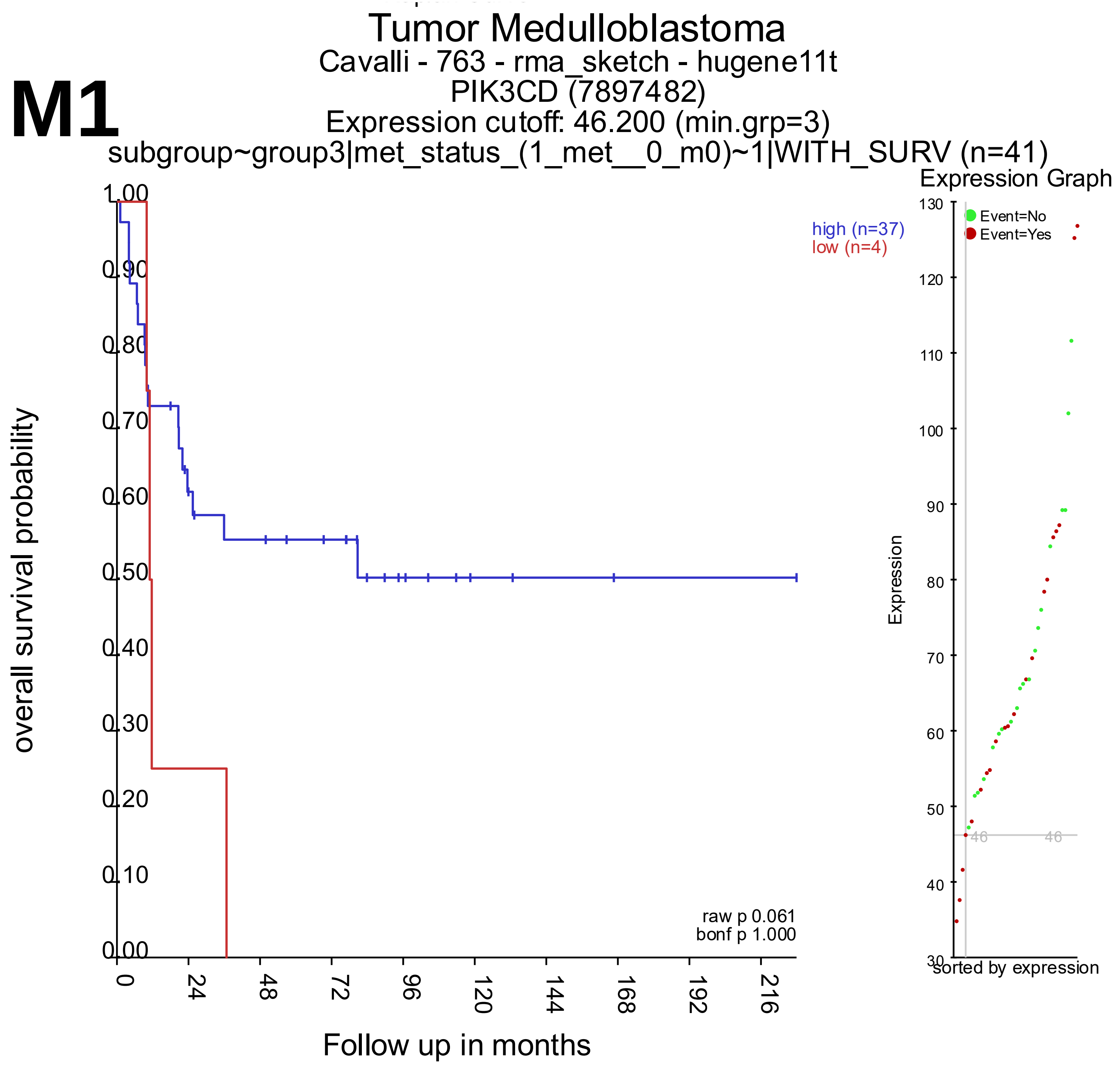

Tumor Medulloblastoma
Cavalli - 763 - rma_sketch - hugene11t
PIK3CD (7897482)
Expression cutoff: 46.200 (min.grp=3)
subgroup~group3|met_status_(1_met__0_m0)~1|WITH_SURV (n=41)
overall survival probability
1.00
0.90
0.80
0.70
0.60
0.50
0.40
0.30
0.20
0.10
0.00
0
24
48
72
96
120
144
168
192
216
Follow up in months
high (n=37)
low (n=4)
raw p 0.061
bonf p 1.000
Expression Graph
Event=No
Event=Yes
Expression
130
120
110
100
90
80
70
60
50
40
30
46
46
sorted by expression

PIK3CG

WNT M0

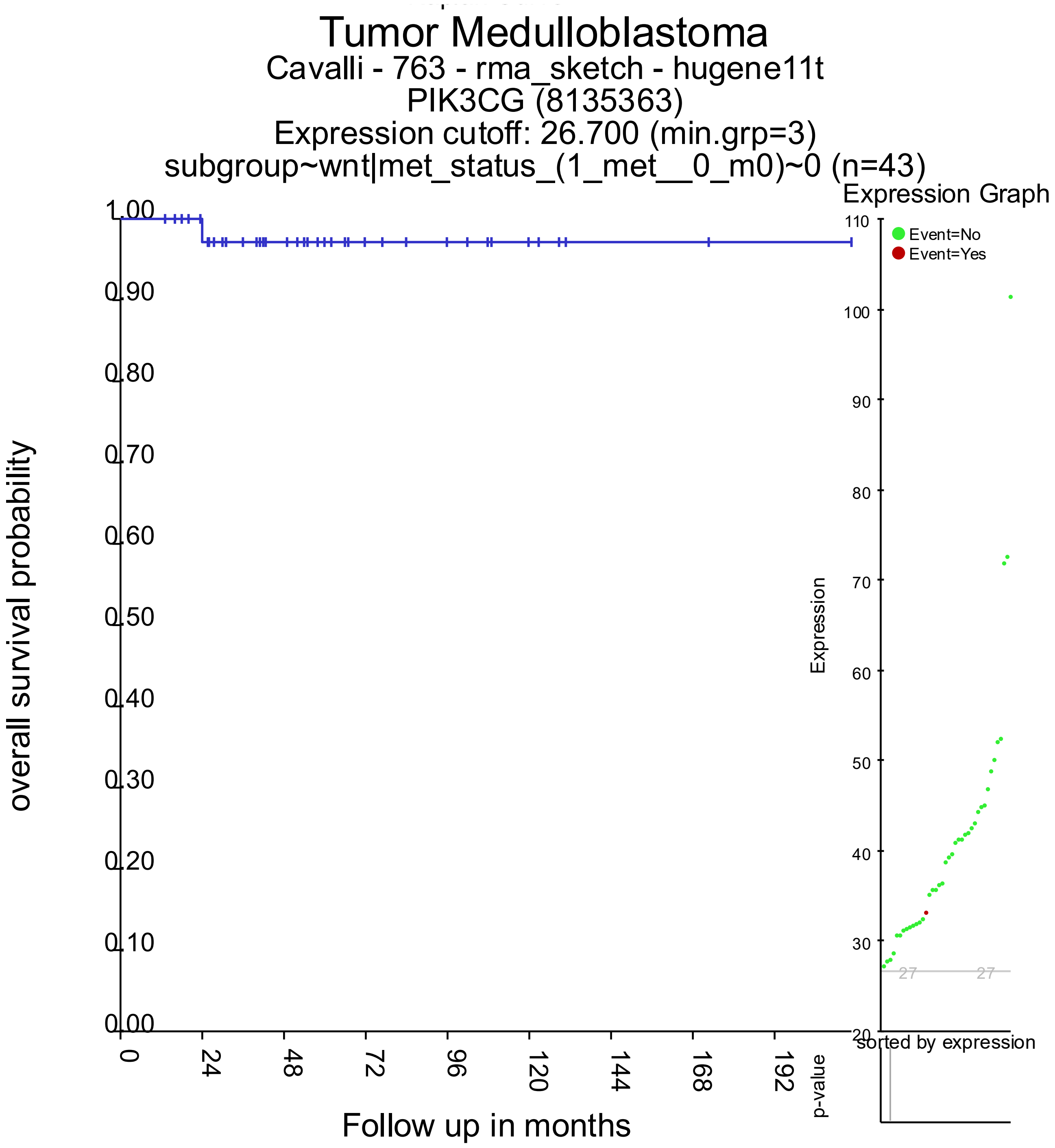
Tumor Medulloblastoma
Cavalli - 763 - rma_sketch - hugene11t
PIK3CG (8135363)
Expression cutoff: 26.700 (min.grp=3)
subgroup~wnt|met_status_(1_met__0_m0)~0 (n=43)
overall survival probability
1.00
0.90
0.80
0.70
0.60
0.50
0.40
0.30
0.20
0.10
0.00
0
24
48
72
96
120
144
168
192
Follow up in months
p-value
Expression Graph
Event=No
Event=Yes
Expression
110
100
90
80
70
60
50
40
30
20
27
27
sorted by expression

WNT M1

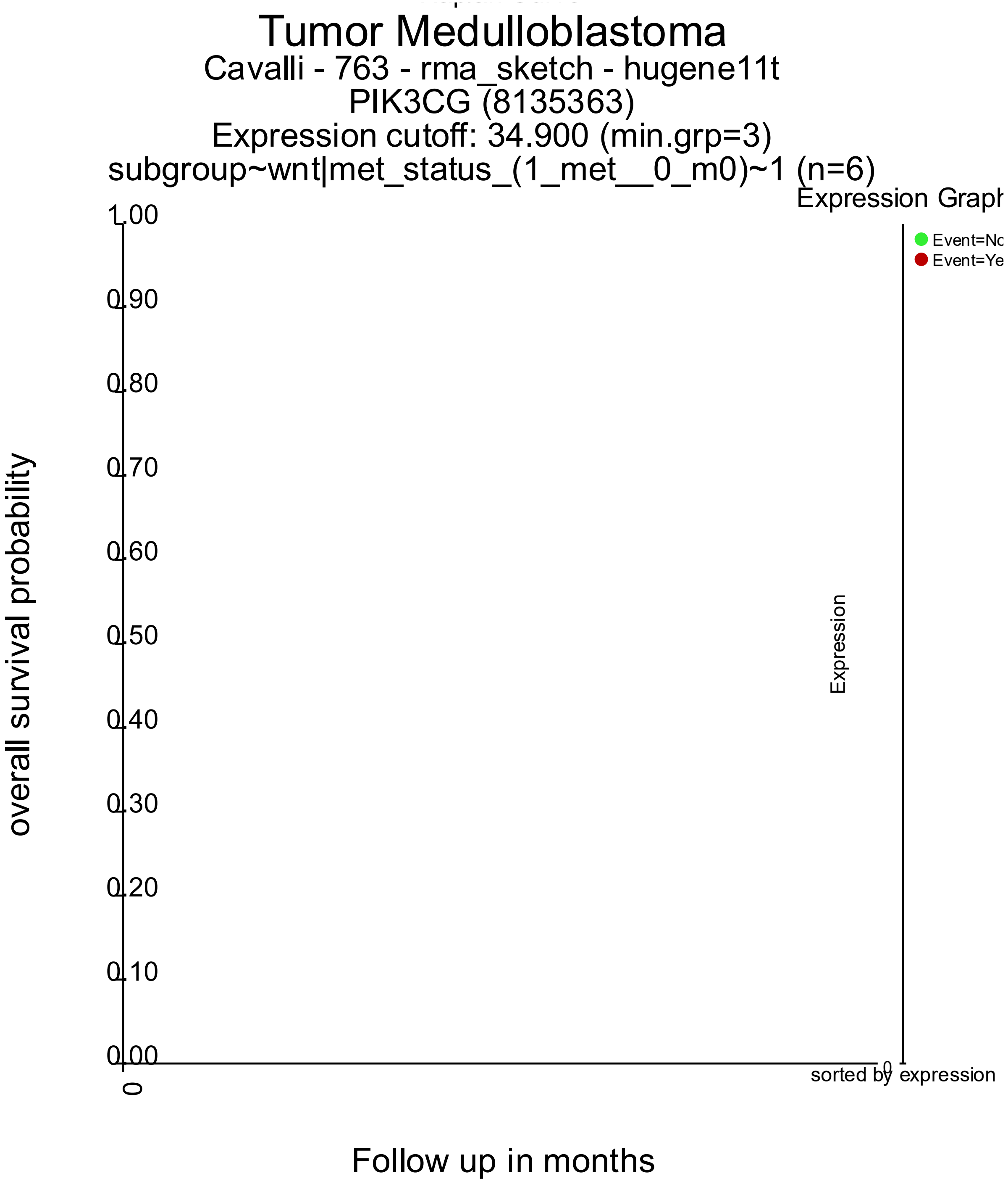

Tumor Medulloblastoma
Cavalli - 763 - rma_sketch - hugene11t
PIK3CG (8135363)
Expression cutoff: 34.900 (min.grp=3)
subgroup~wnt|met_status_(1_met__0_m0)~1 (n=6)
Expression Graph
Event=No
Event=Yes
overall survival probability
1.00
0.90
0.80
0.70
0.60
0.50
0.40
0.30
0.20
0.10
0.00
0
Follow up in months
Expression
0
sorted by expression

SHH M0

Tumor Medulloblastoma
Cavalli - 763 - rma_sketch - hugene11t
PIK3CG (8135363)
Expression cutoff: 81.000 (min.grp=3)
subgroup~shh|met_status_(1_met__0_m0)~0|WITH_SURV (n=124)

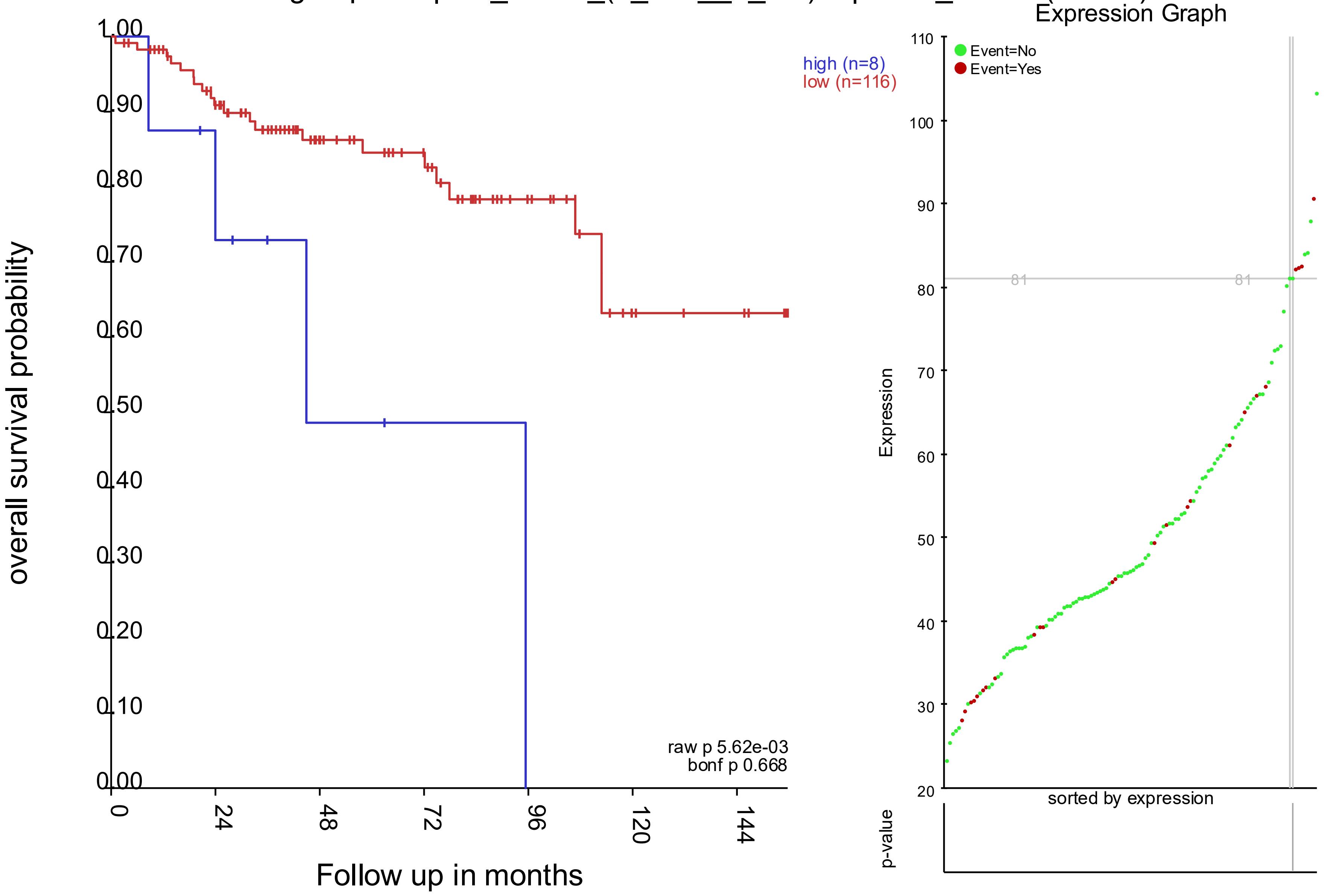

SHH M1

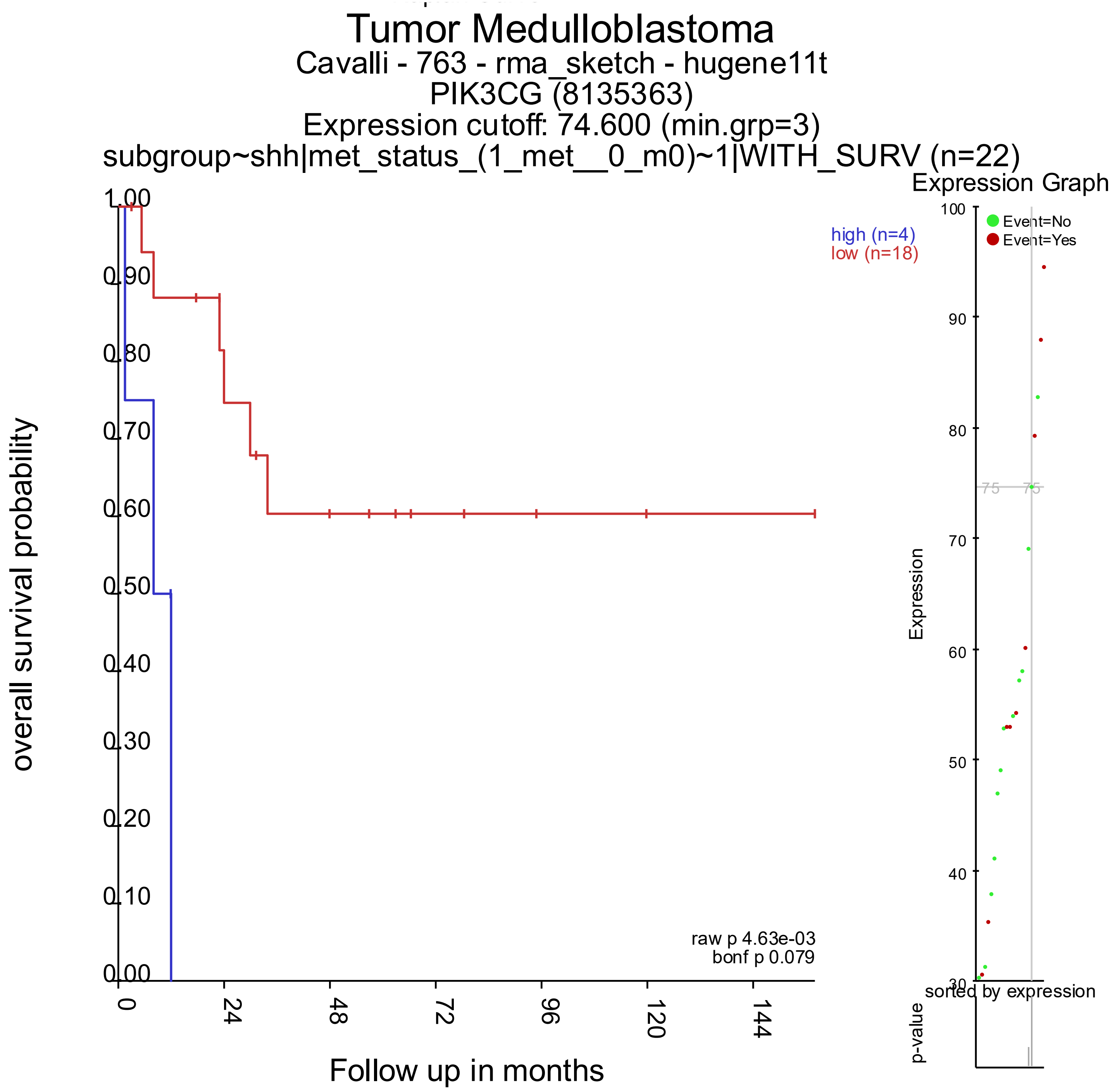

Tumor Medulloblastoma
Cavalli - 763 - rma_sketch - hugene11t
PIK3CG (8135363)
Expression cutoff: 74.600 (min.grp=3)
subgroup~shh|met_status_(1_met__0_m0)~1|WITH_SURV (n=22)
overall survival probability
1.00
0.90
0.80
0.70
0.60
0.50
0.40
0.30
0.20
0.10
0.00
high (n=4)
low (n=18)
raw p 4.63e-03
bonf p 0.079
0
24
48
72
96
120
144
Follow up in months
Expression Graph
Event=No
Event=Yes
100
90
80
70
60
50
40
30
Expression
sorted by expression
p-value

GROUP4 M0

Tumor Medulloblastoma
Cavalli - 763 - rma_sketch - hugene11t
PIK3CG (8135363)
Expression cutoff: 29.500 (min.grp=3)
subgroup~group4|met_status_(1_met__0_m0)~0|WITH_SURV (n=145)

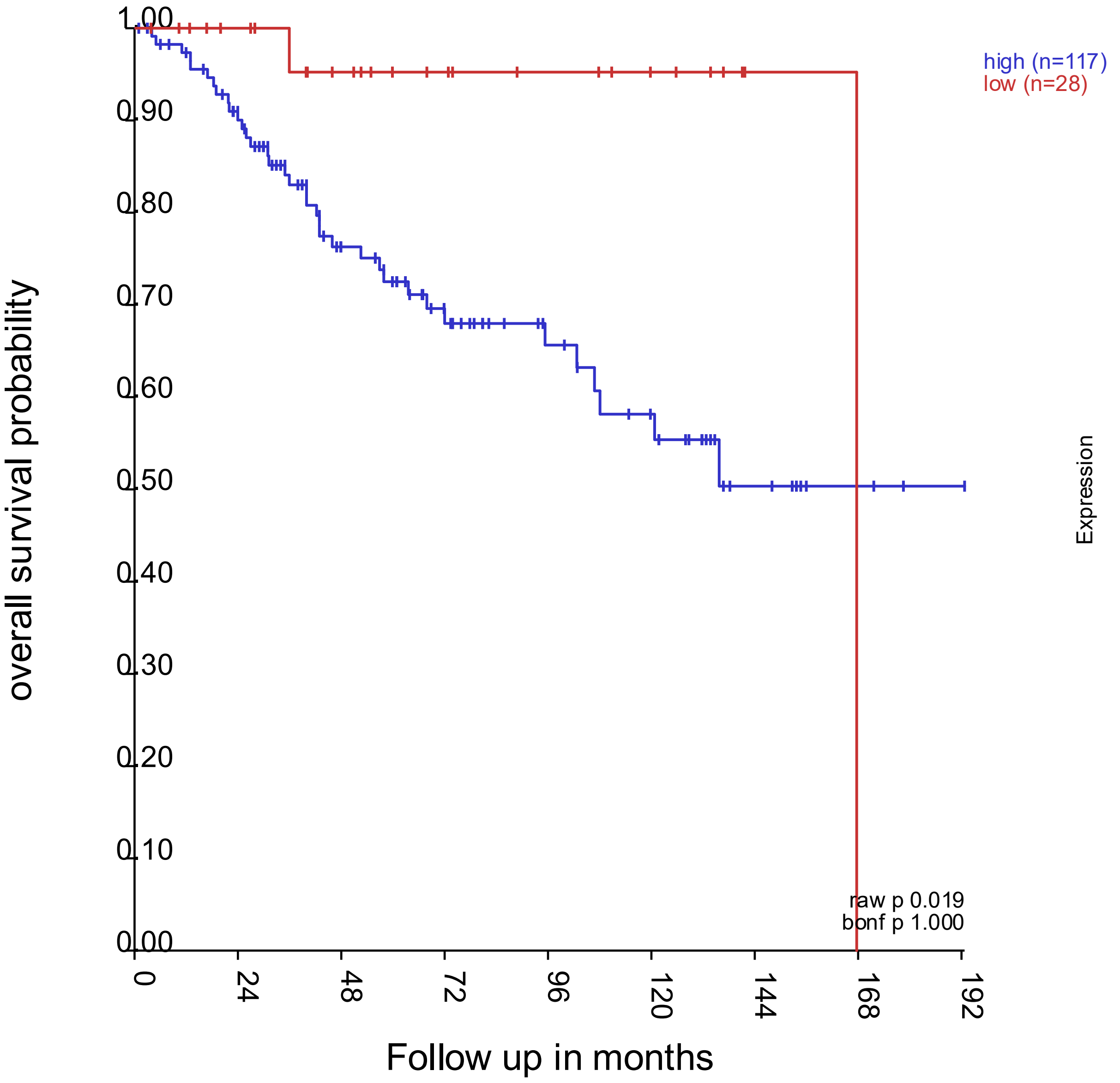

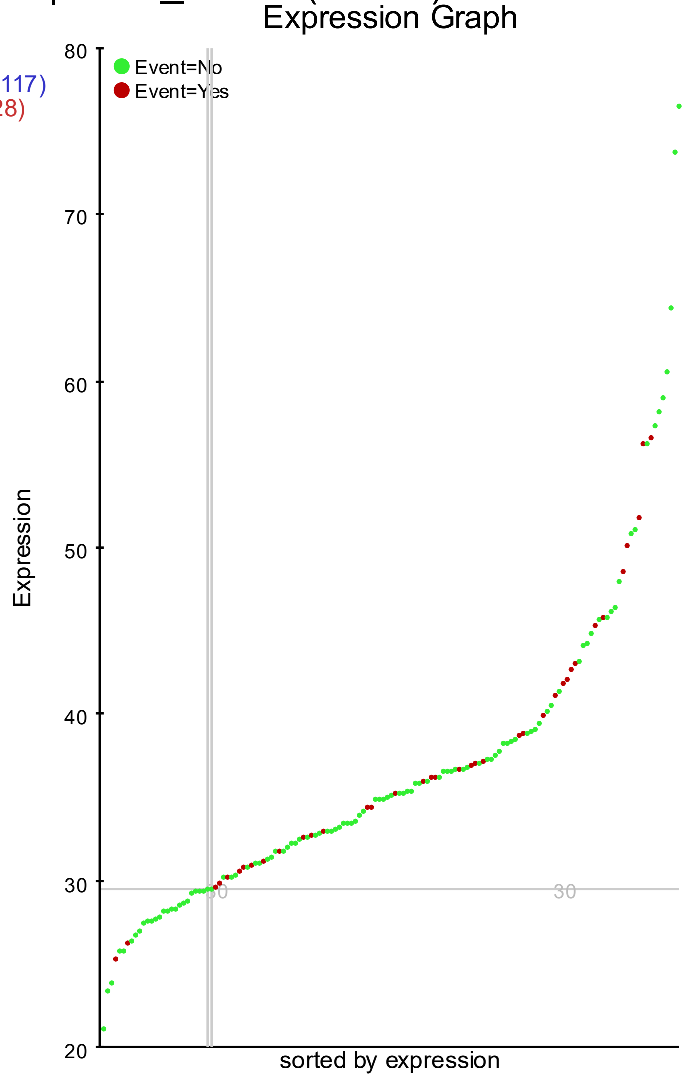

GROUP4 M1

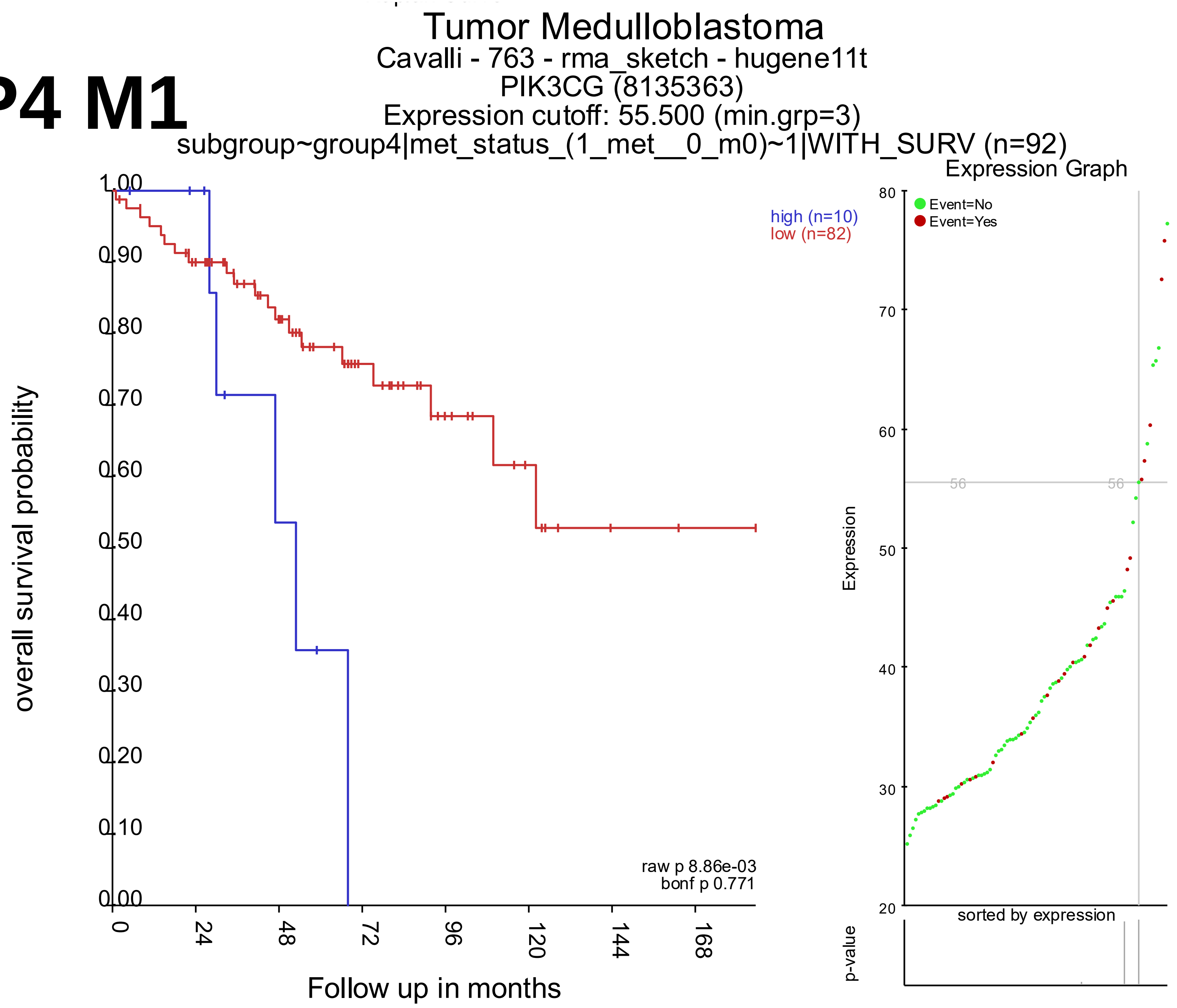

Tumor Medulloblastoma
Cavalli - 763 - rma_sketch - hugene11t
PIK3CG (8135363)
Expression cutoff: 55.500 (min.grp=3)
subgroup~group4|met_status_(1_met__0_m0)~1|WITH_SURV (n=92)
overall survival probability
1.00
0.90
0.80
0.70
0.60
0.50
0.40
0.30
0.20
0.10
0.00
high (n=10)
low (n=82)
raw p 8.86e-03
bonf p 0.771
0
24
48
72
96
120
144
168
Follow up in months
Expression Graph
Event=No
Event=Yes
Expression
80
70
60
50
40
30
20
56
56
sorted by expression
p-value

GROUP3 M0

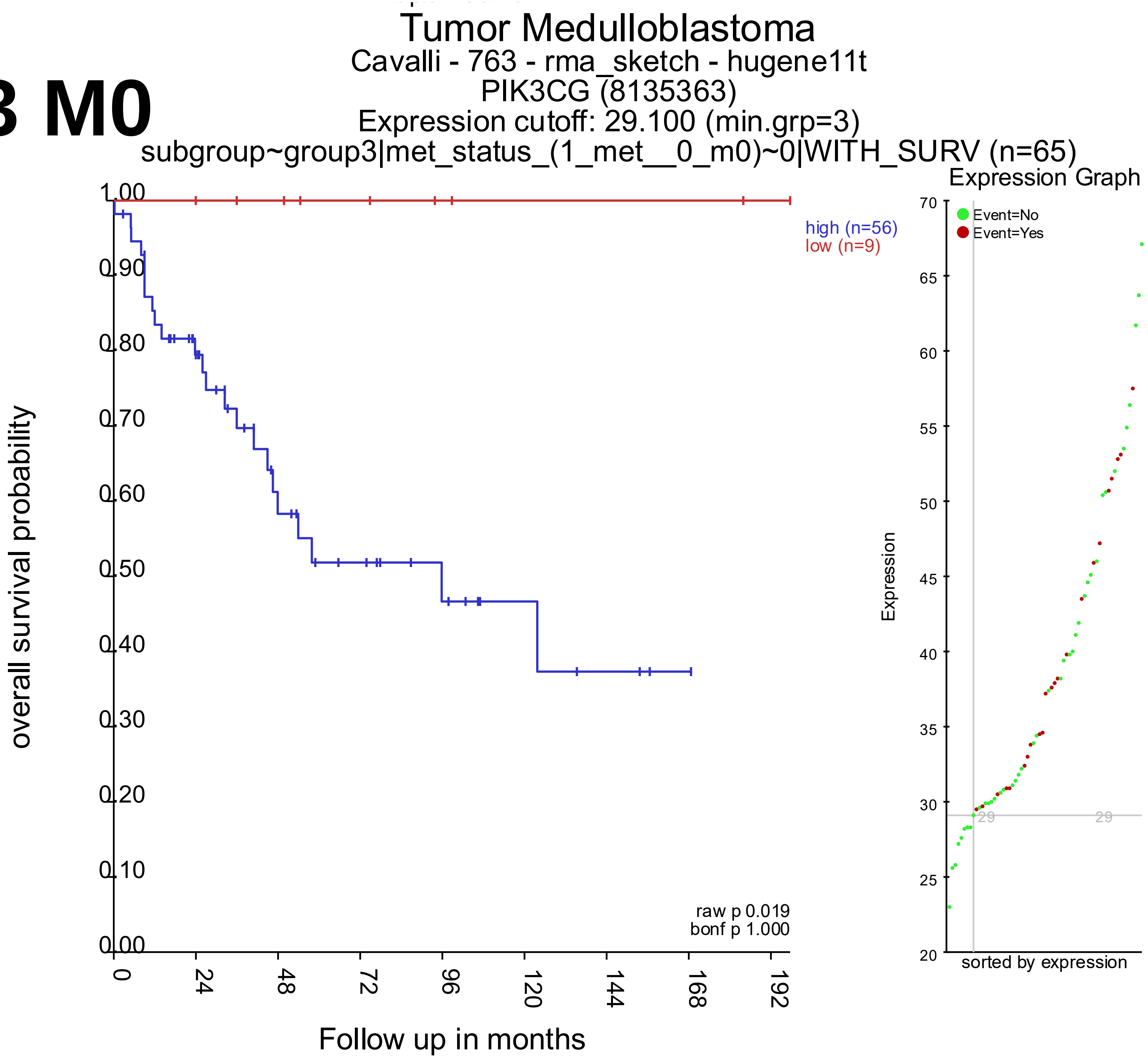

Tumor Medulloblastoma
Cavalli - 763 - rma_sketch - hugene11t
PIK3CG (8135363)
Expression cutoff: 29.100 (min.grp=3)
subgroup~group3|met_status_(1_met__0_m0)~0|WITH_SURV (n=65)
1.00
0.90
0.80
0.70
0.60
0.50
0.40
0.30
0.20
0.10
0.00
overall survival probability
high (n=56)
low (n=9)
raw p 0.019
bonf p 1.000
0
24
48
72
96
120
144
168
192
Follow up in months
Expression Graph
Event=No
Event=Yes
70
65
60
55
50
45
40
35
30
25
20
Expression
29
29
sorted by expression

GROUP3 M1

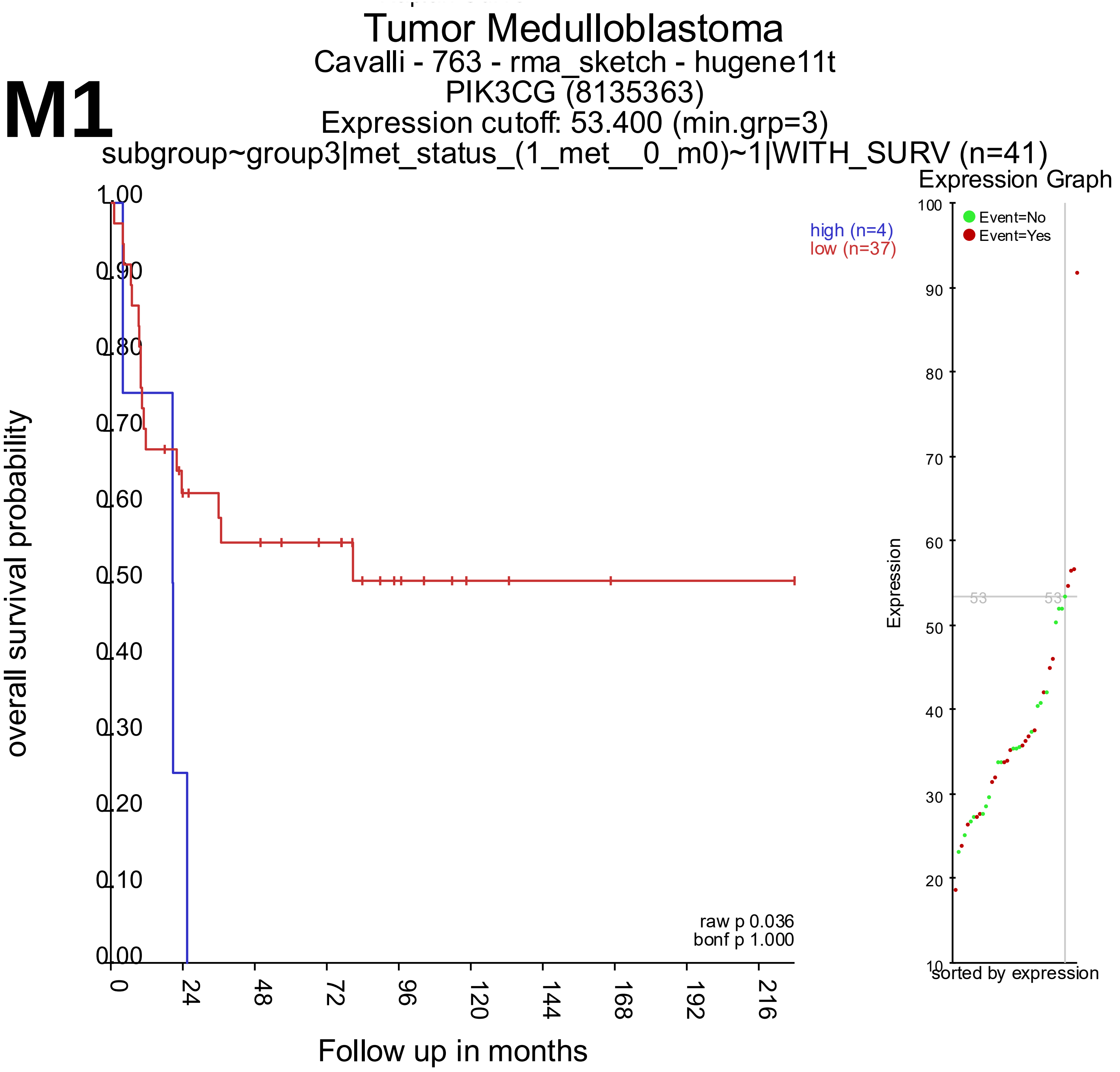

Tumor Medulloblastoma
Cavalli - 763 - rma_sketch - hugene11t
PIK3CG (8135363)
Expression cutoff: 53.400 (min.grp=3)
subgroup~group3|met_status_(1_met__0_m0)~1|WITH_SURV (n=41)
overall survival probability
1.00
0.90
0.80
0.70
0.60
0.50
0.40
0.30
0.20
0.10
0.00
0
24
48
72
96
120
144
168
192
216
Follow up in months
high (n=4)
low (n=37)
raw p 0.036
bonf p 1.000
Expression Graph
Event=No
Event=Yes
Expression
100
90
80
70
60
50
40
30
20
10
53
53
sorted by expression

PIK3C2A

WNT M0

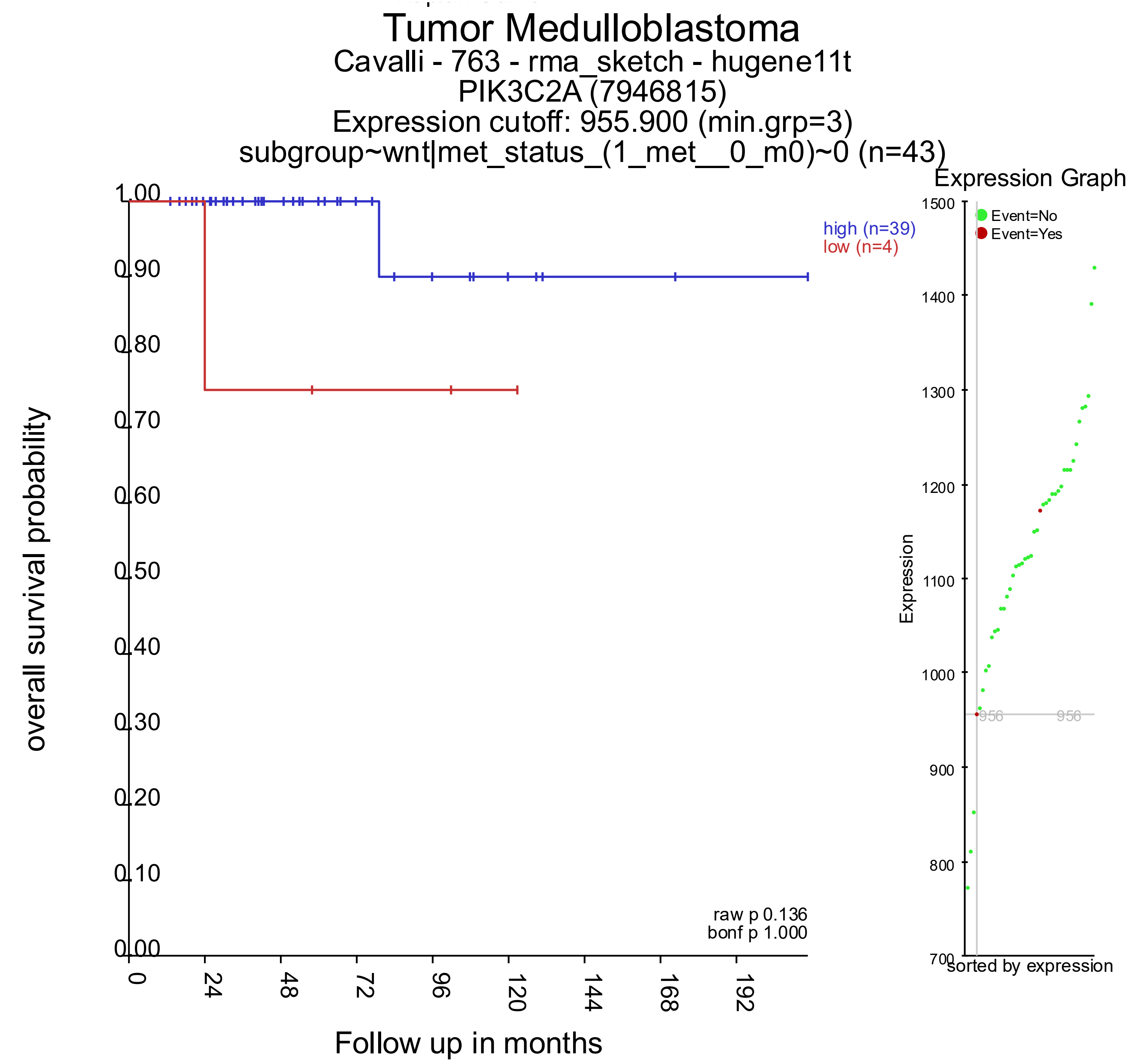
Tumor Medulloblastoma
Cavalli - 763 - rma_sketch - hugene11t
PIK3C2A (7946815)
Expression cutoff: 955.900 (min.grp=3)
subgroup~wnt|met_status_(1_met__0_m0)~0 (n=43)
high (n=39)
low (n=4)
overall survival probability
1.00
0.90
0.80
0.70
0.60
0.50
0.40
0.30
0.20
0.10
0.00
raw p 0.136
bonf p 1.000
0
24
48
72
96
120
144
168
192
Follow up in months
Expression Graph
Event=No
Event=Yes
Expression
1500
1400
1300
1200
1100
1000
900
800
700
956
956
sorted by expression

WNT M1

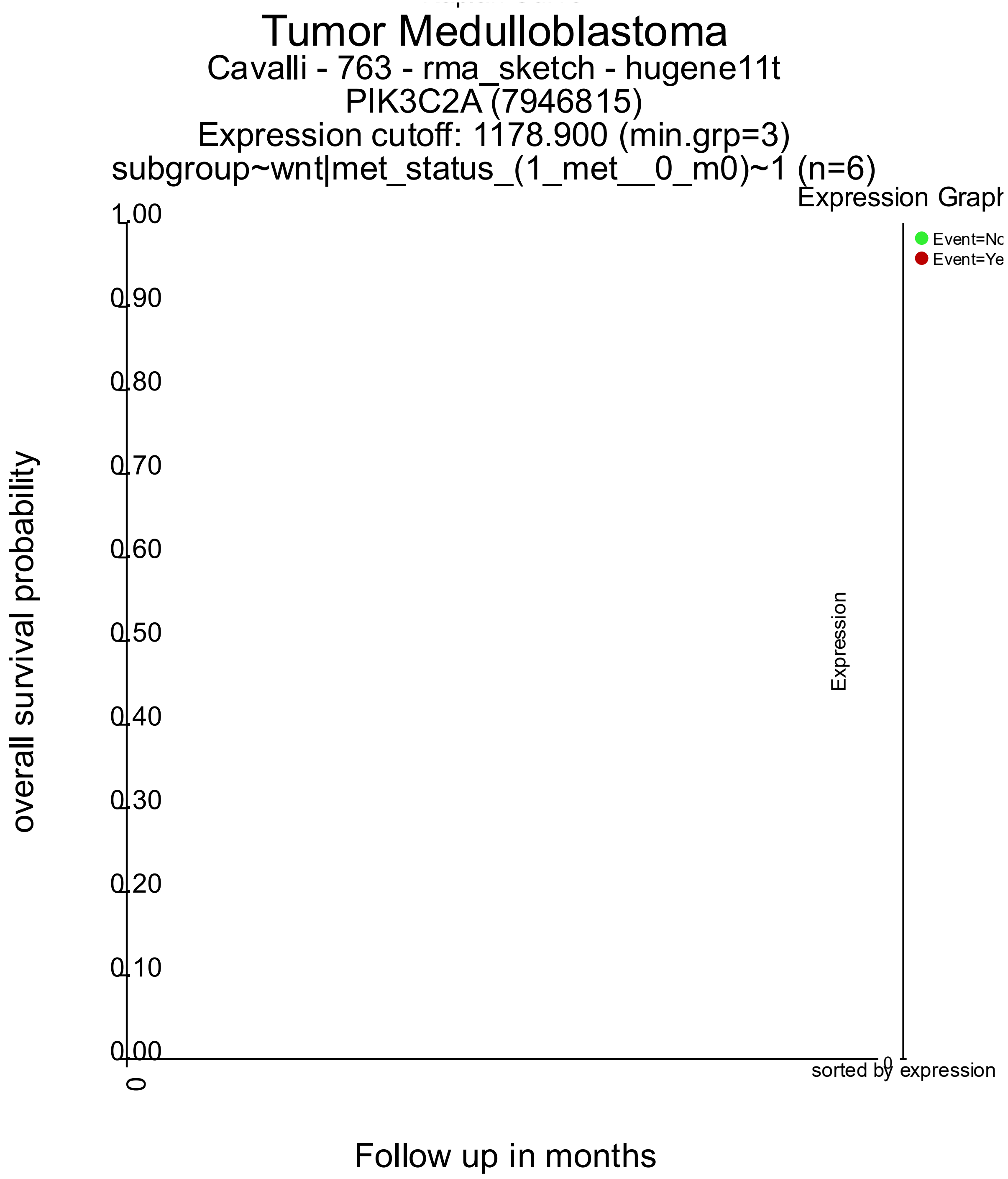
Tumor Medulloblastoma
Cavalli - 763 - rma_sketch - hugene11t
PIK3C2A (7946815)
Expression cutoff: 1178.900 (min.grp=3)
subgroup~wnt|met_status_(1_met__0_m0)~1 (n=6)
Expression Graph
Event=No
Event=Yes
1.00
0.90
0.80
0.70
0.60
0.50
0.40
0.30
0.20
0.10
0.00
overall survival probability
0
Follow up in months
Expression
0
sorted by expression

SHH M0

Tumor Medulloblastoma
Cavalli - 763 - rma_sketch - hugene11t
PIK3C2A (7946815)
Expression cutoff: 636.300 (min.grp=3)
subgroup~shh|met_status_(1_met__0_m0)~0|WITH_SURV (n=124)

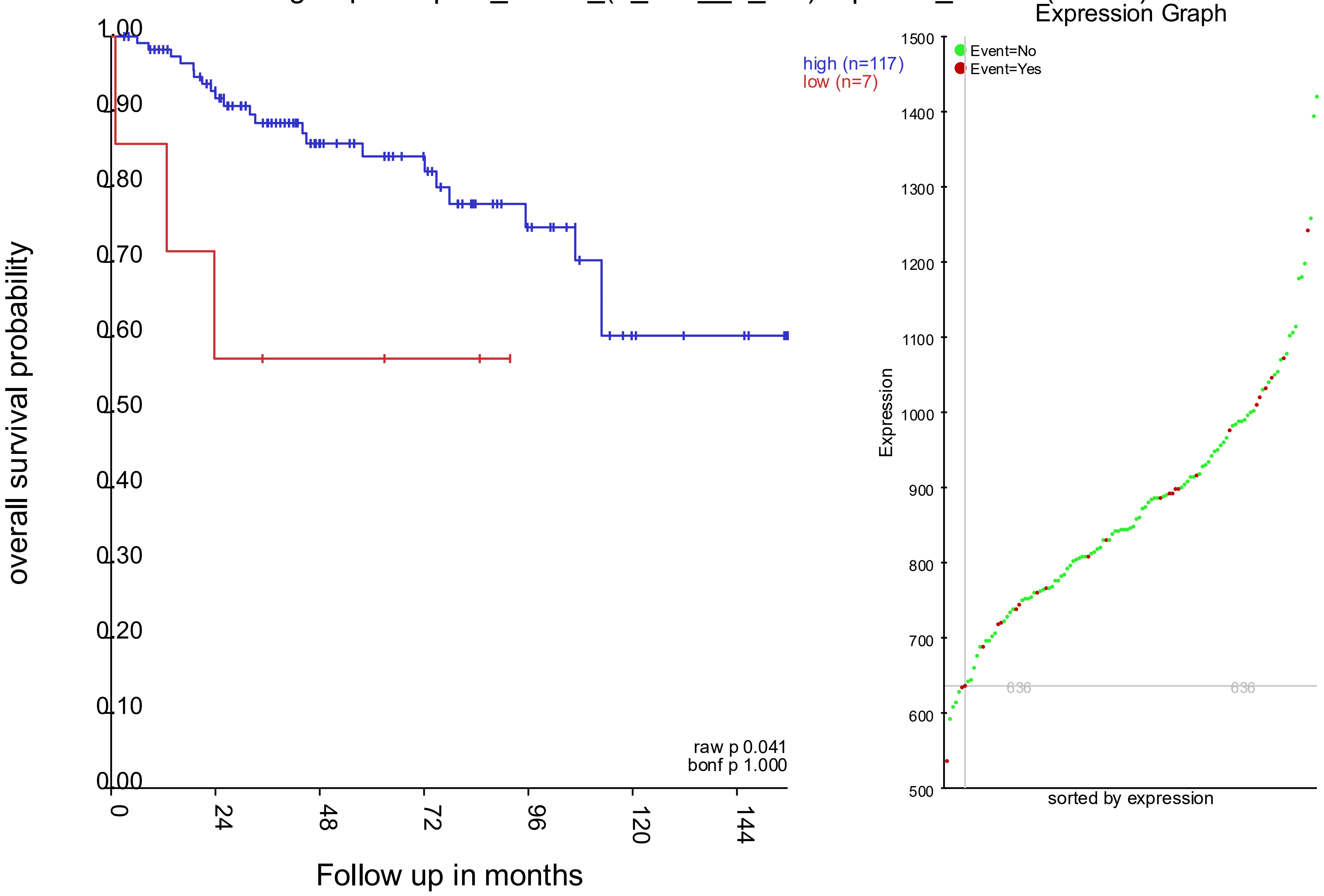

SHH M1

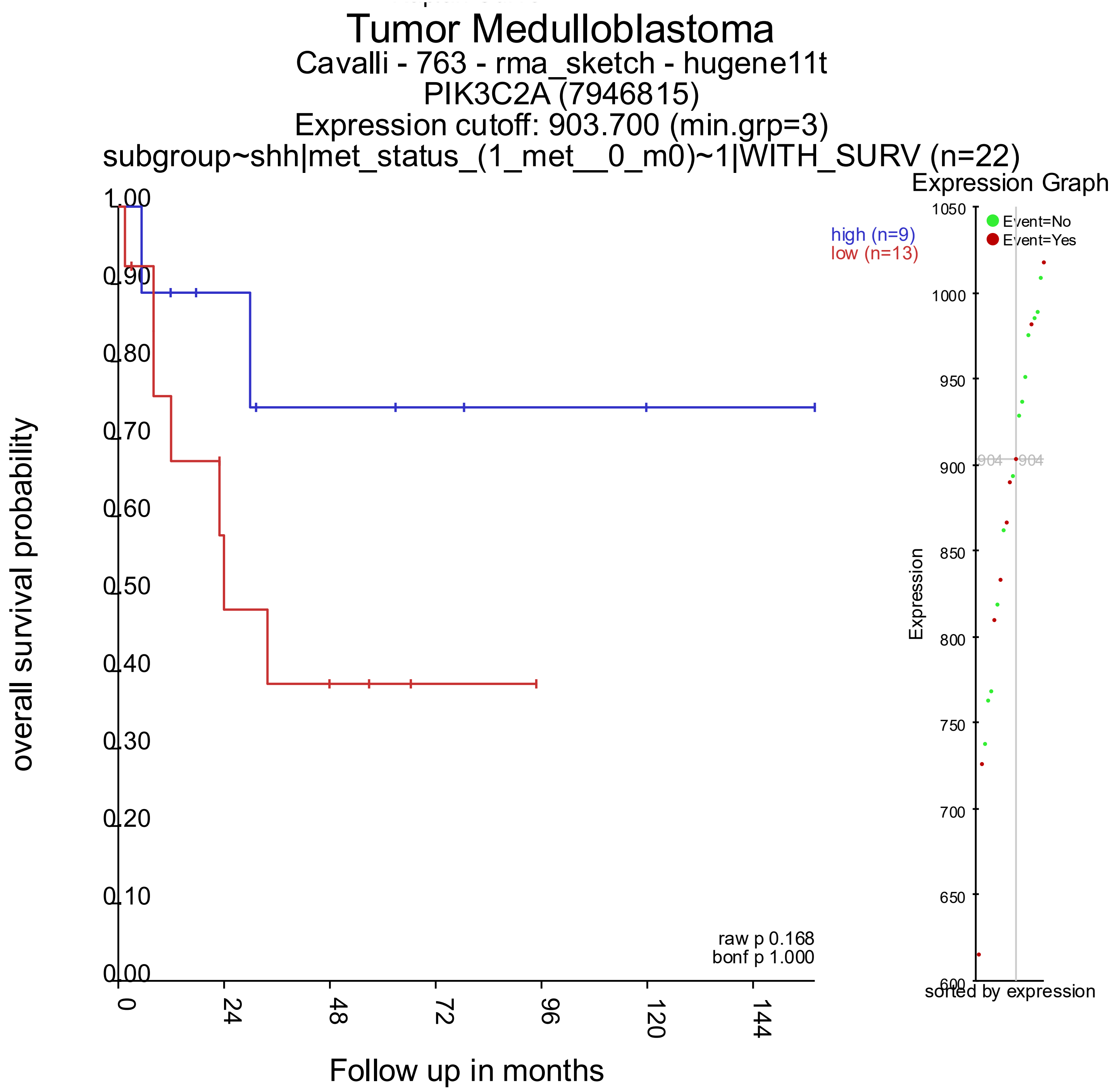

Tumor Medulloblastoma
Cavalli - 763 - rma_sketch - hugene11t
PIK3C2A (7946815)
Expression cutoff: 903.700 (min.grp=3)
subgroup~shh|met_status_(1_met__0_m0)~1|WITH_SURV (n=22)
overall survival probability
1.00
0.90
0.80
0.70
0.60
0.50
0.40
0.30
0.20
0.10
0.00
high (n=9)
low (n=13)
raw p 0.168
bonf p 1.000
0
24
48
72
96
120
144
Follow up in months
Expression Graph
Event=No
Event=Yes
Expression
1050
1000
950
900
850
800
750
700
650
600
904
904
sorted by expression

GROUP4 M0

Tumor Medulloblastoma
Cavalli - 763 - rma_sketch - hugene11t
PIK3C2A (7946815)
Expression cutoff: 980.600 (min.grp=3)
subgroup~group4|met_status_(1_met__0_m0)~0|WITH_SURV (n=145)

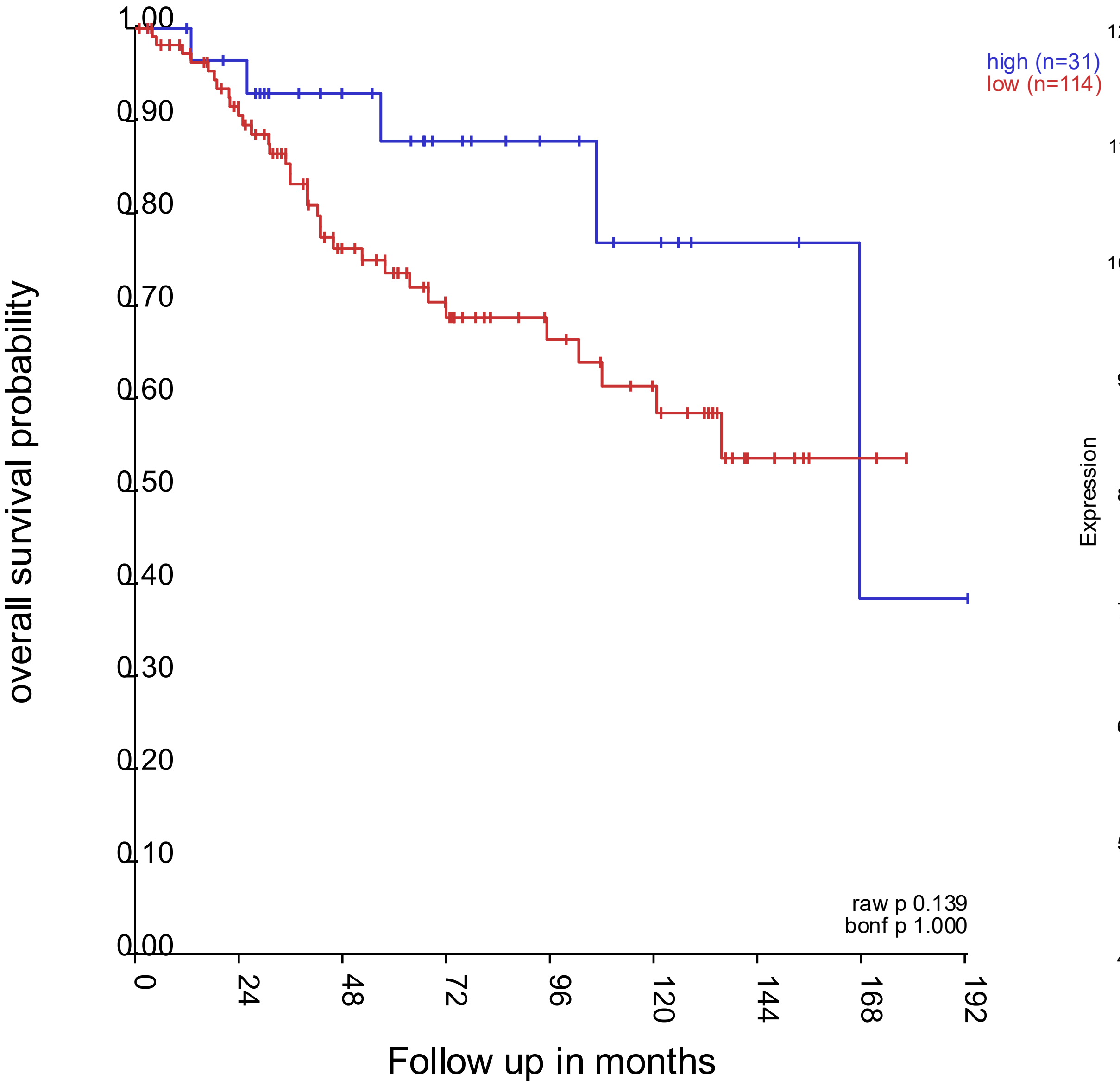

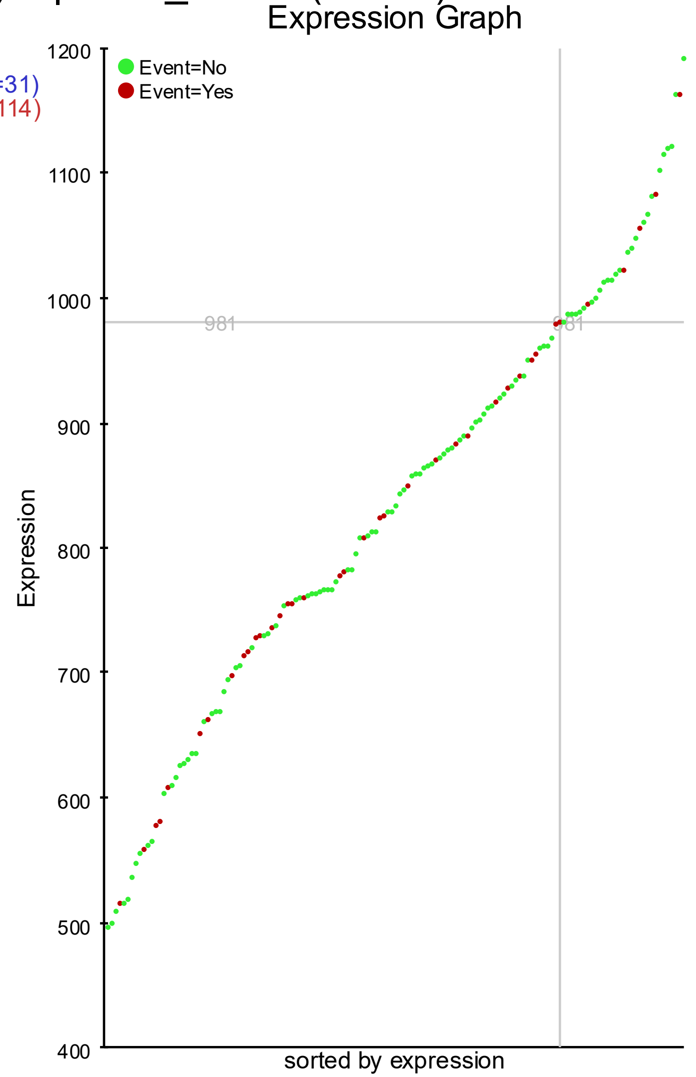

GROUP4 M1

Tumor Medulloblastoma
Cavalli - 763 - rma_sketch - hugene11t
PIK3C2A (7946815)
Expression cutoff: 879.900 (min.grp=3)
subgroup~group4|met_status_(1_met__0_m0)~1|WITH_SURV (n=92)

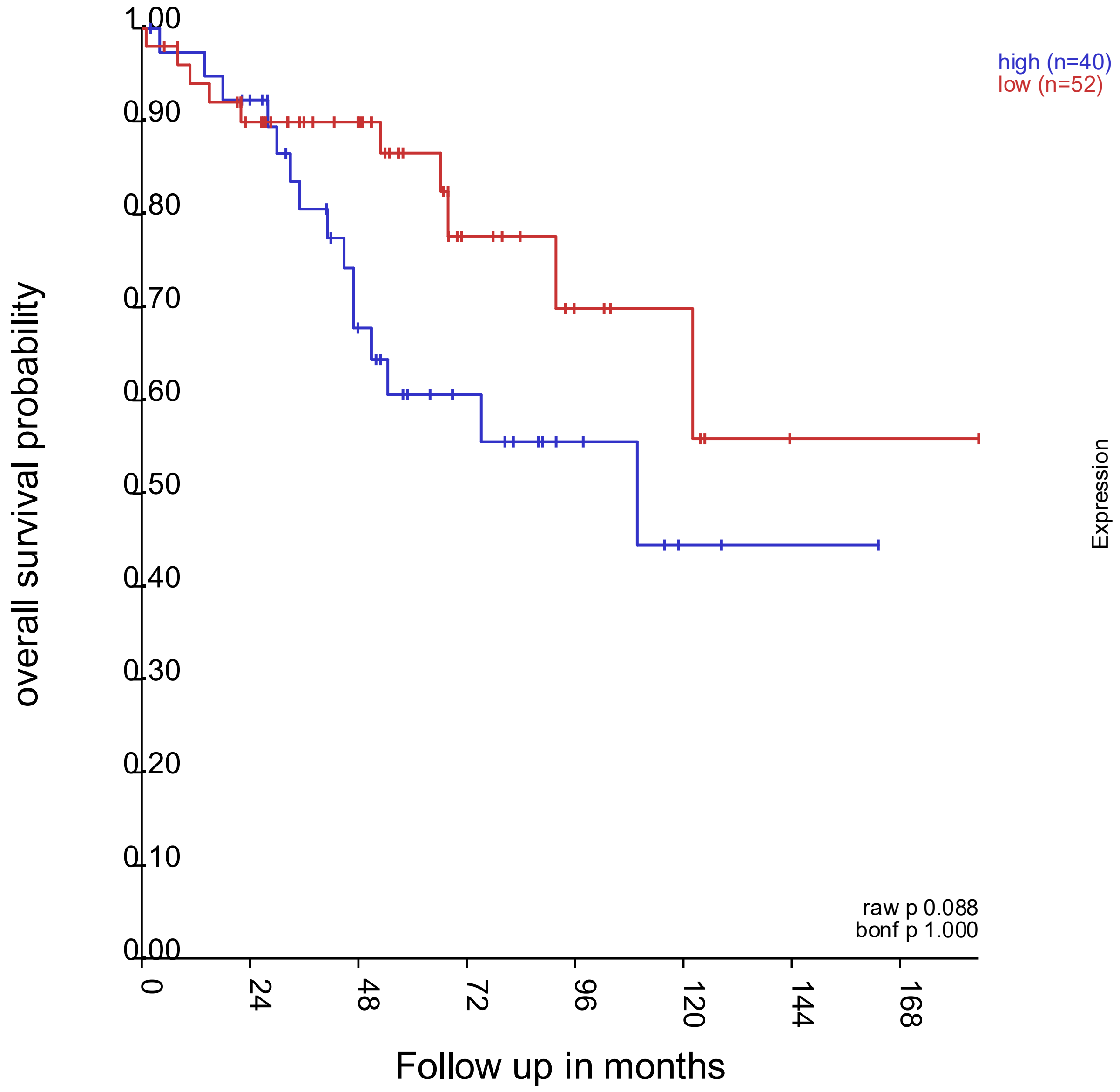

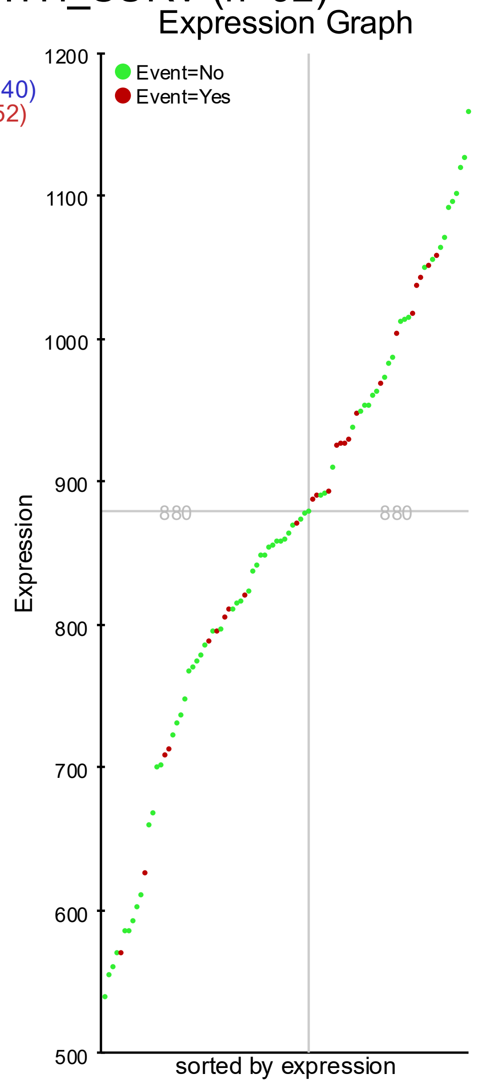

GROUP3 M0

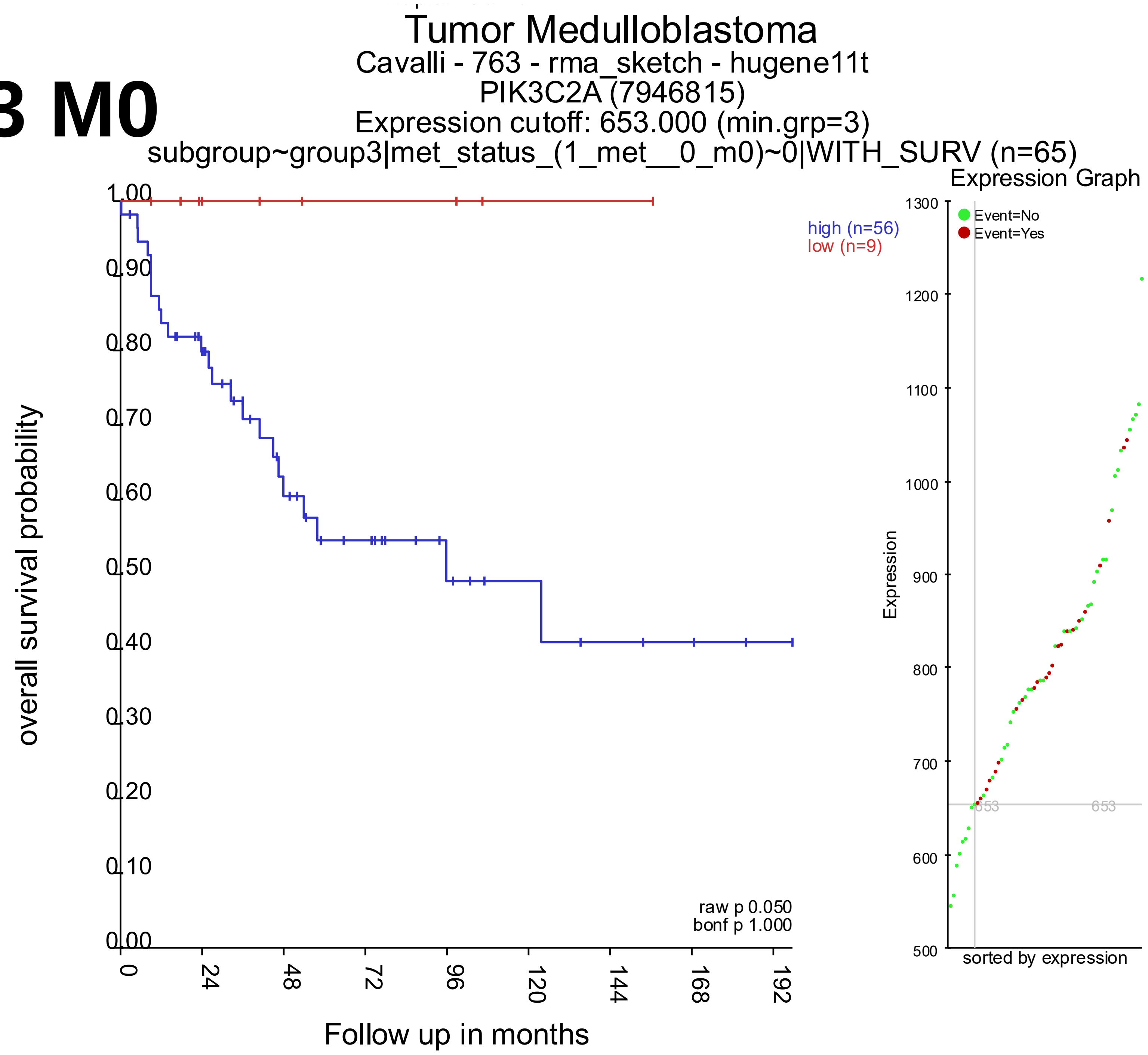

Tumor Medulloblastoma
Cavalli - 763 - rma_sketch - hugene11t
PIK3C2A (7946815)
Expression cutoff: 653.000 (min.grp=3)
subgroup~group3|met_status_(1_met__0_m0)~0|WITH_SURV (n=65)
overall survival probability
high (n=56)
low (n=9)
raw p 0.050
bonf p 1.000
Follow up in months
Expression Graph
Event=No
Event=Yes
Expression
sorted by expression

GROUP3 M1

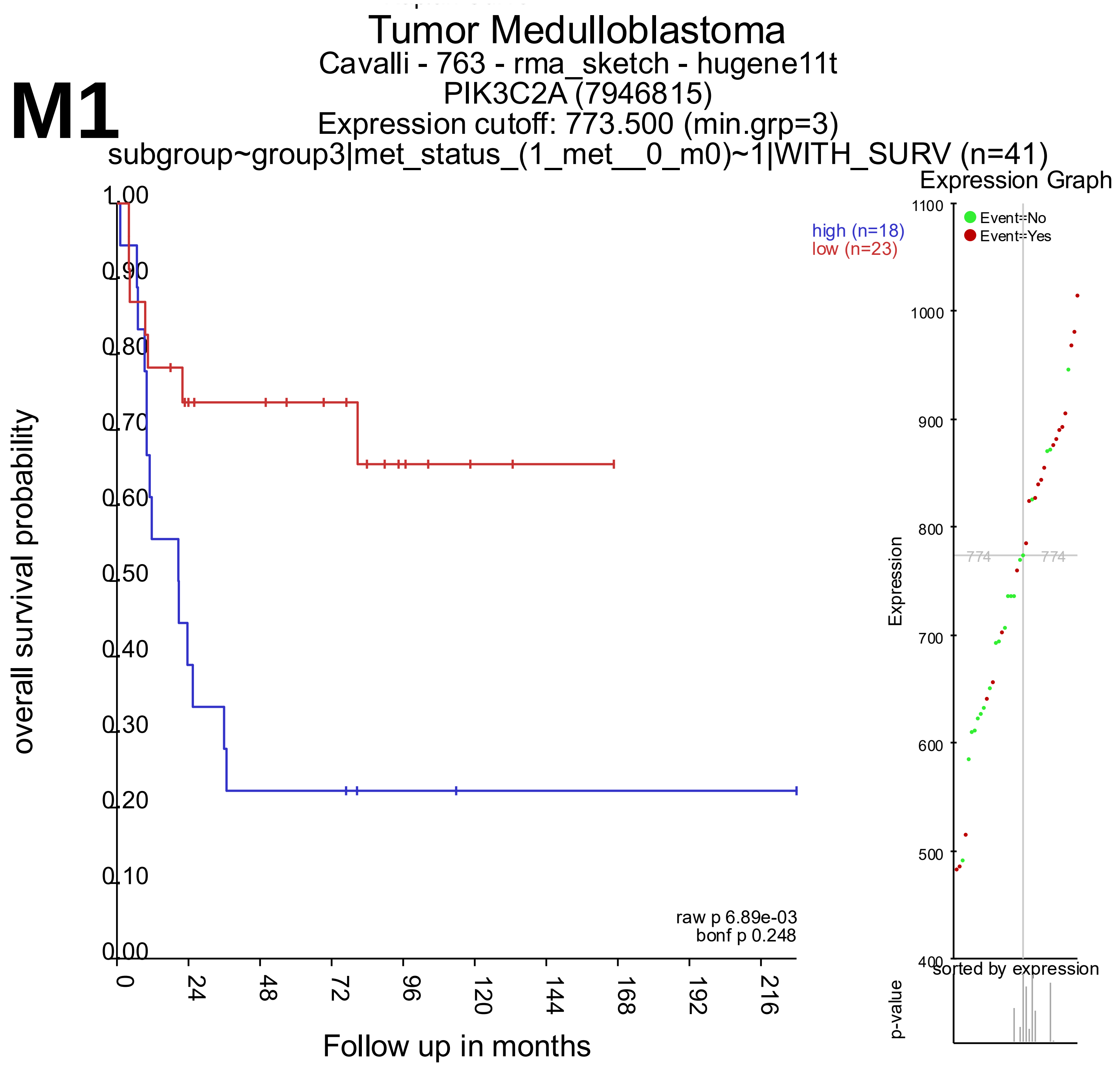

Tumor Medulloblastoma
Cavalli - 763 - rma_sketch - hugene11t
PIK3C2A (7946815)
Expression cutoff: 773.500 (min.grp=3)
subgroup~group3|met_status_(1_met__0_m0)~1|WITH_SURV (n=41)
overall survival probability
1.00
0.90
0.80
0.70
0.60
0.50
0.40
0.30
0.20
0.10
0.00
0
24
48
72
96
120
144
168
192
216
Follow up in months
high (n=18)
low (n=23)
raw p 6.89e-03
bonf p 0.248
Expression Graph
Event=No
Event=Yes
Expression
1100
1000
900
800
700
600
500
400
774
774
sorted by expression
p-value

PIK3C2B

WNT M0

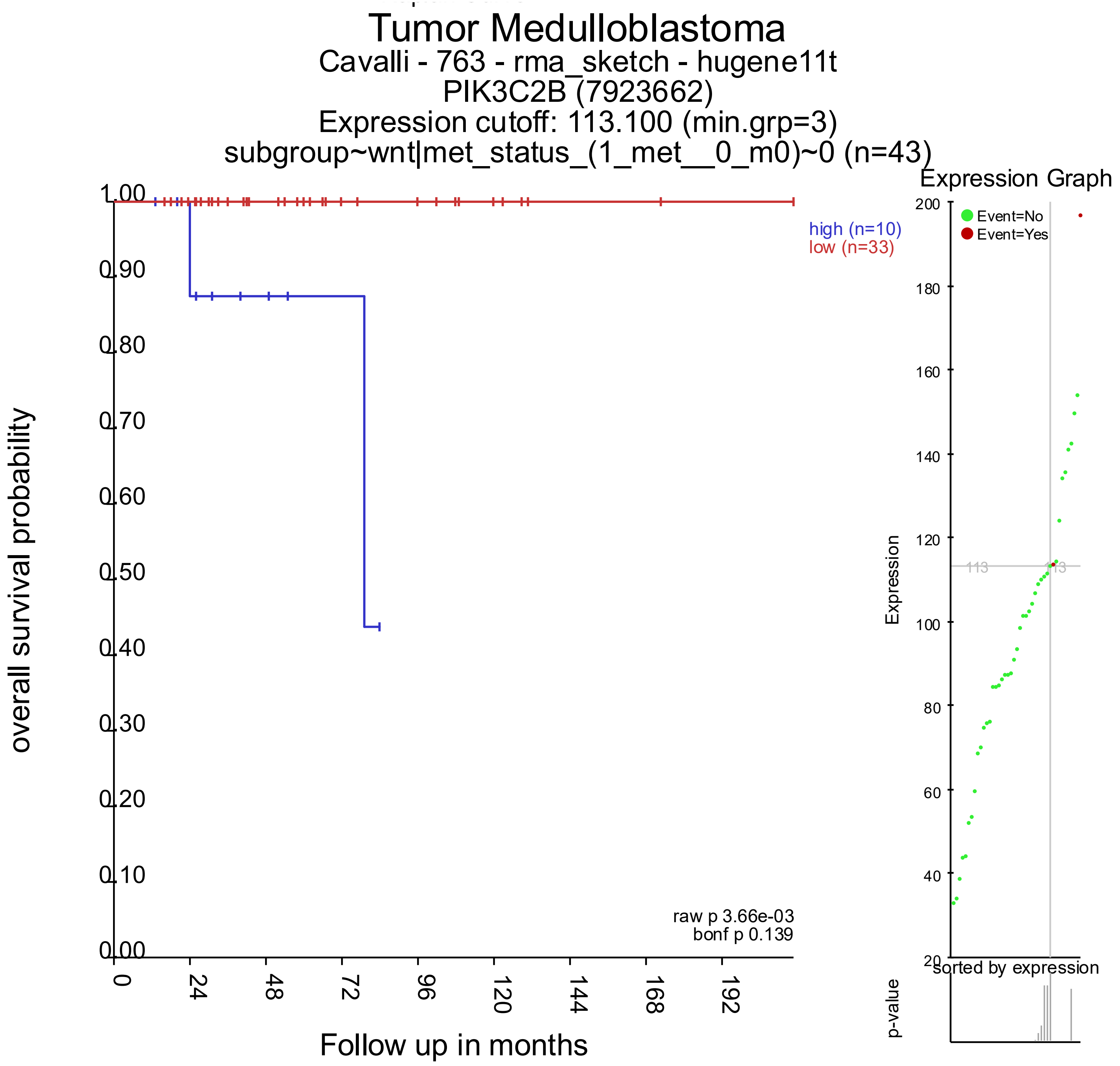

Tumor Medulloblastoma
Cavalli - 763 - rma_sketch - hugene11t
PIK3C2B (7923662)
Expression cutoff: 113.100 (min.grp=3)
subgroup~wnt|met_status_(1_met__0_m0)~0 (n=43)
overall survival probability
1.00
0.90
0.80
0.70
0.60
0.50
0.40
0.30
0.20
0.10
0.00
0
24
48
72
96
120
144
168
192
Follow up in months
high (n=10)
low (n=33)
raw p 3.66e-03
bonf p 0.139
Expression Graph
Event=No
Event=Yes
Expression
200
180
160
140
120
100
80
60
40
20
113
sorted by expression
p-value

WNT M1

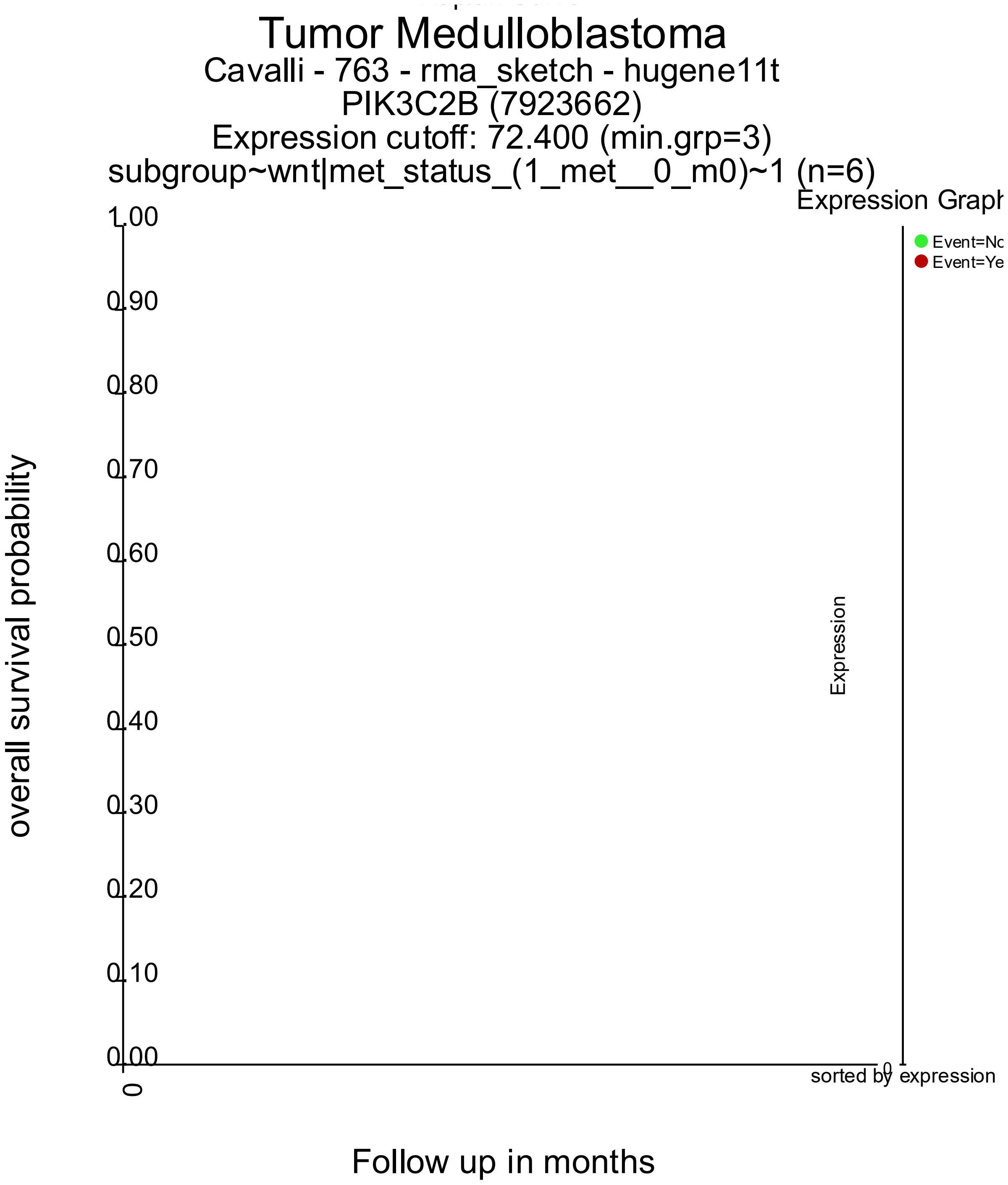

Tumor Medulloblastoma
Cavalli - 763 - rma_sketch - hugene11t
PIK3C2B (7923662)
Expression cutoff: 72.400 (min.grp=3)
subgroup~wnt|met_status_(1_met__0_m0)~1 (n=6)
Expression Graph
Event=No
Event=Yes
overall survival probability
1.00
0.90
0.80
0.70
0.60
0.50
0.40
0.30
0.20
0.10
0.00
0
Follow up in months
Expression
0
sorted by expression

SHH M0

Tumor Medulloblastoma
Cavalli - 763 - rma_sketch - hugene11t
PIK3C2B (7923662)
Expression cutoff: 175.000 (min.grp=3)
subgroup~shh|met_status_(1_met__0_m0)~0|WITH_SURV (n=124)

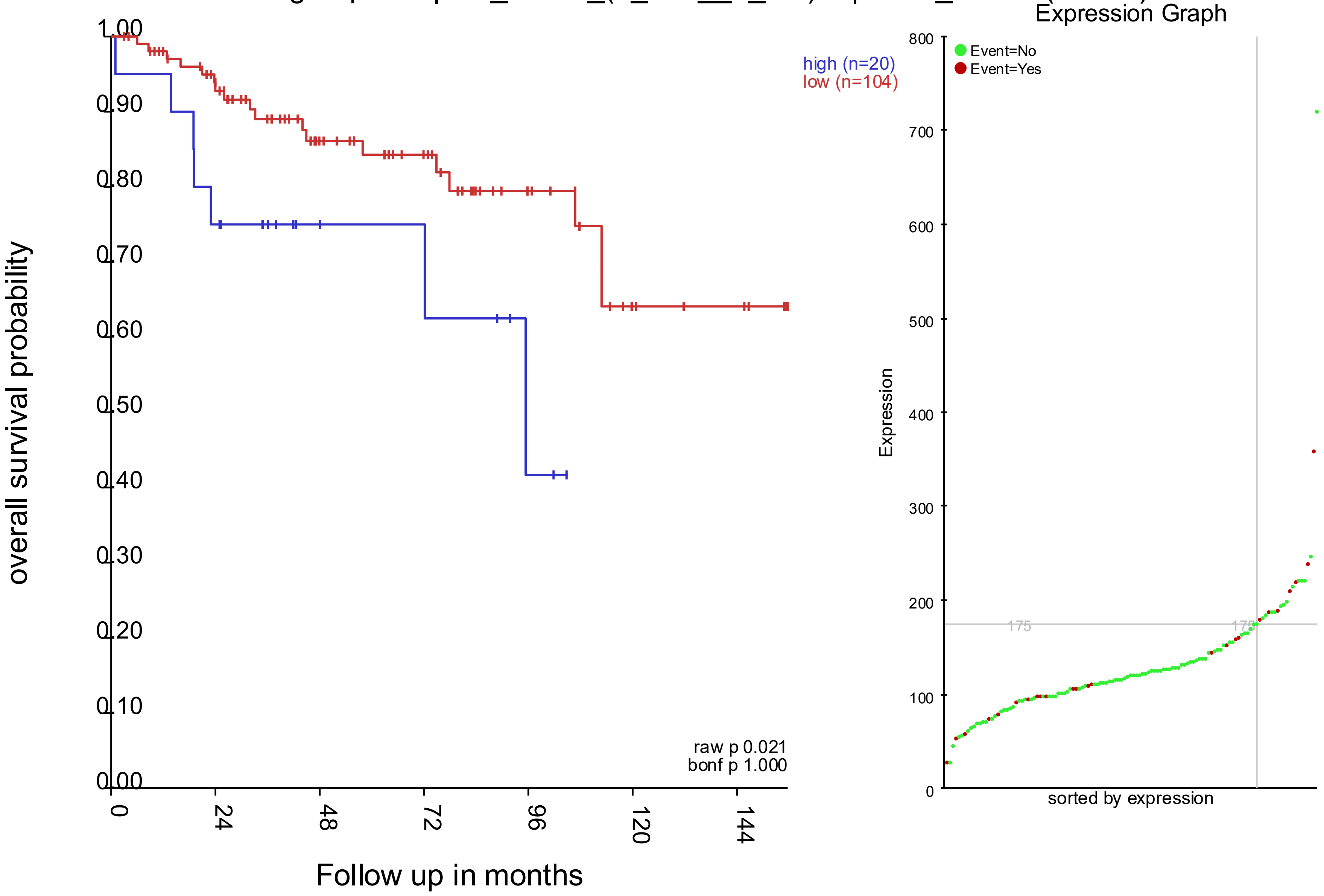

SHH M1

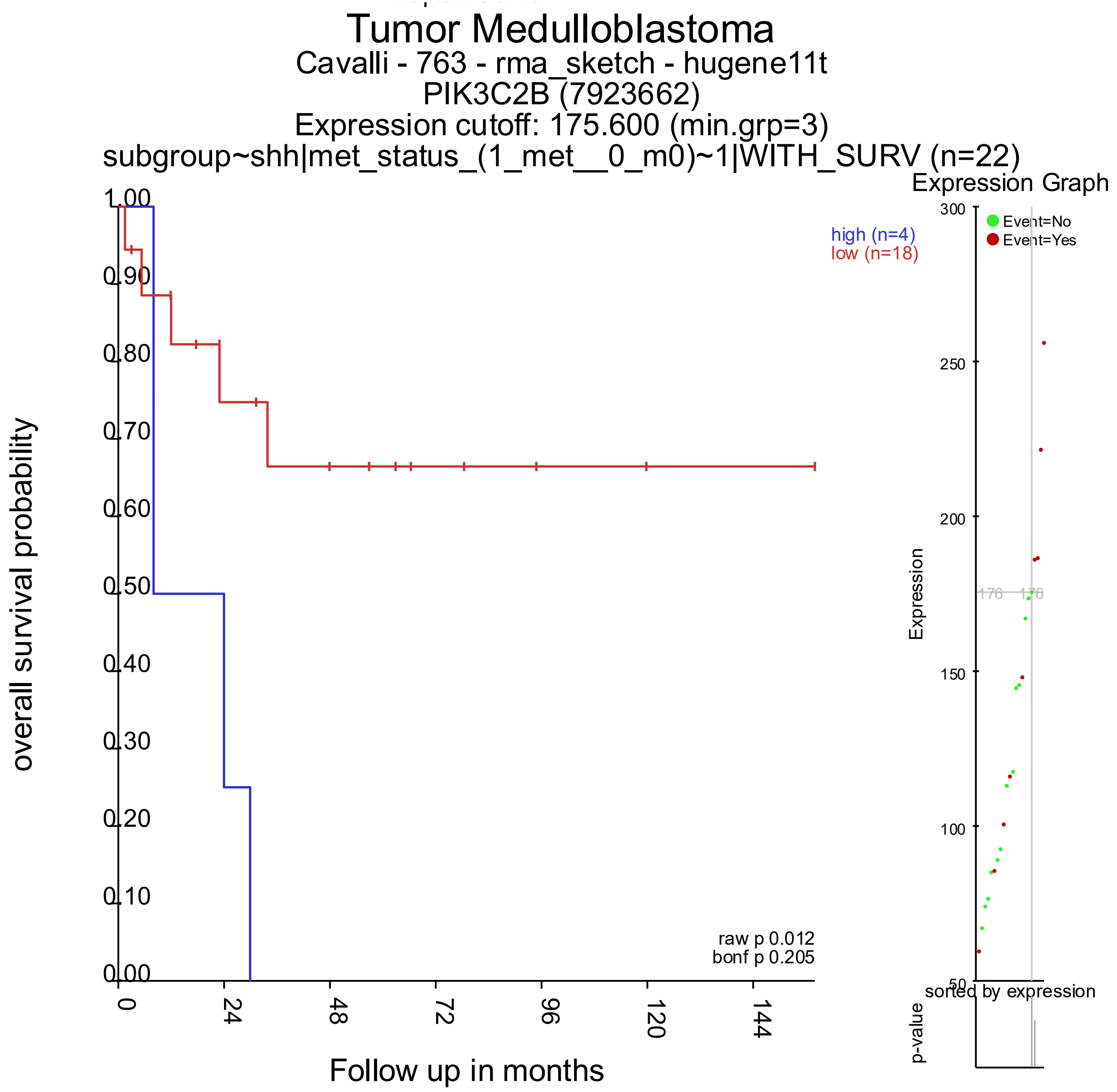

Tumor Medulloblastoma
Cavalli - 763 - rma_sketch - hugene11t
PIK3C2B (7923662)
Expression cutoff: 175.600 (min.grp=3)
subgroup~shh|met_status_(1_met__0_m0)~1|WITH_SURV (n=22)
overall survival probability
1.00
0.90
0.80
0.70
0.60
0.50
0.40
0.30
0.20
0.10
0.00
0
24
48
72
96
120
144
Follow up in months
high (n=4)
low (n=18)
raw p 0.012
bonf p 0.205
Expression Graph
Event=No
Event=Yes
Expression
300
250
200
150
100
50
176
176
sorted by expression
p-value

GROUP4 M0

Tumor Medulloblastoma
Cavalli - 763 - rma_sketch - hugene11t
PIK3C2B (7923662)
Expression cutoff: 138.000 (min.grp=3)
subgroup~group4|met_status_(1_met__0_m0)~0|WITH_SURV (n=145)

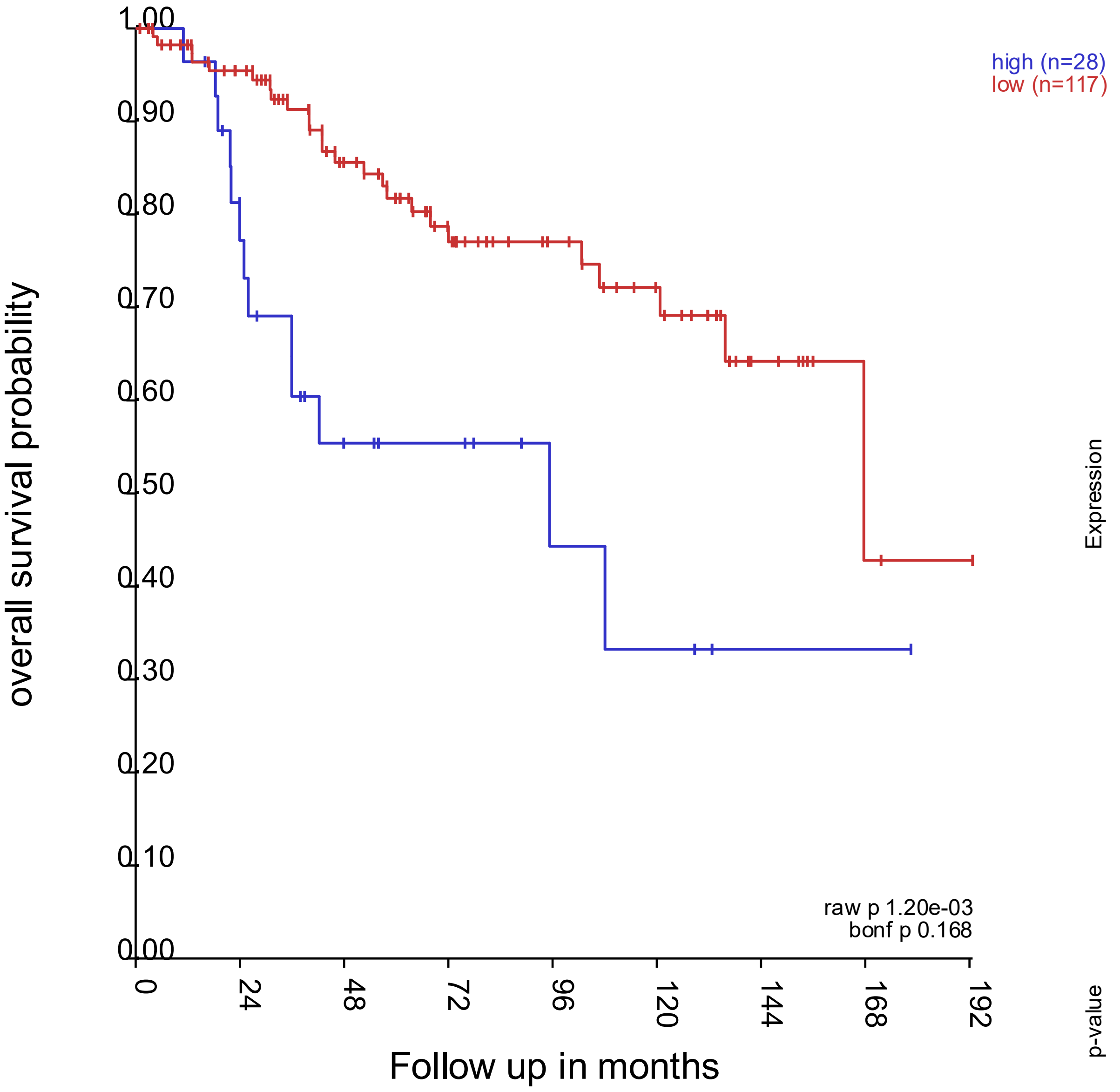

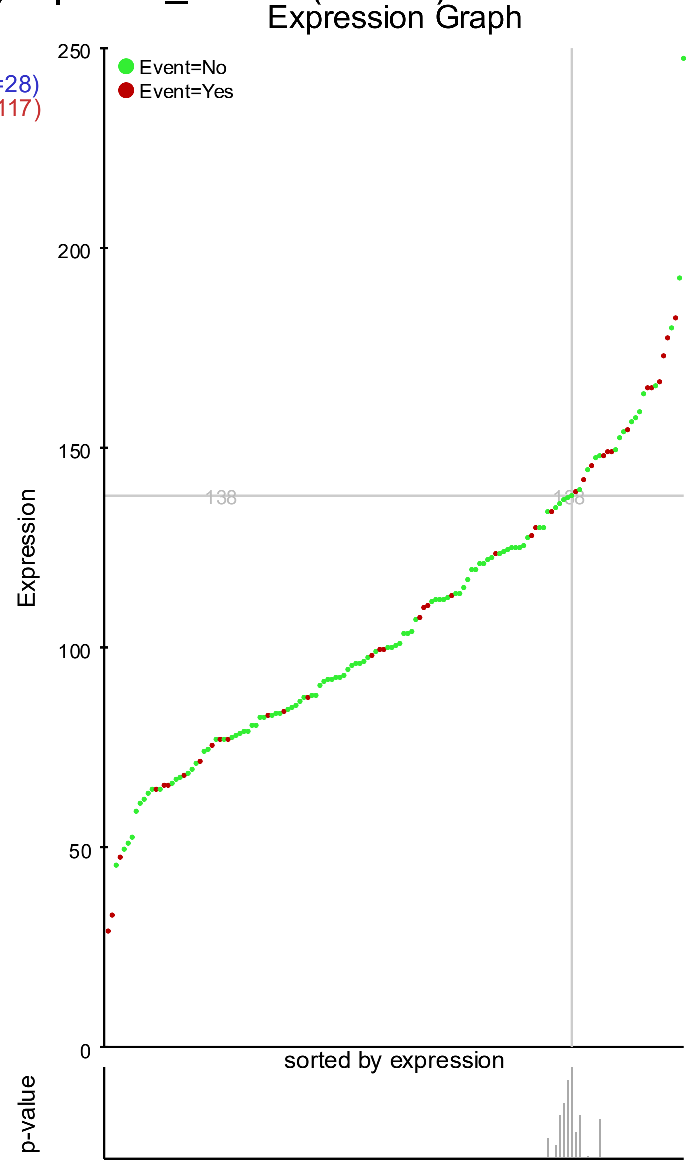

GROUP4 M1

Tumor Medulloblastoma
Cavalli - 763 - rma_sketch - hugene11t
PIK3C2B (7923662)
Expression cutoff: 83.700 (min.grp=3)
subgroup~group4|met_status_(1_met__0_m0)~1|WITH_SURV (n=92)

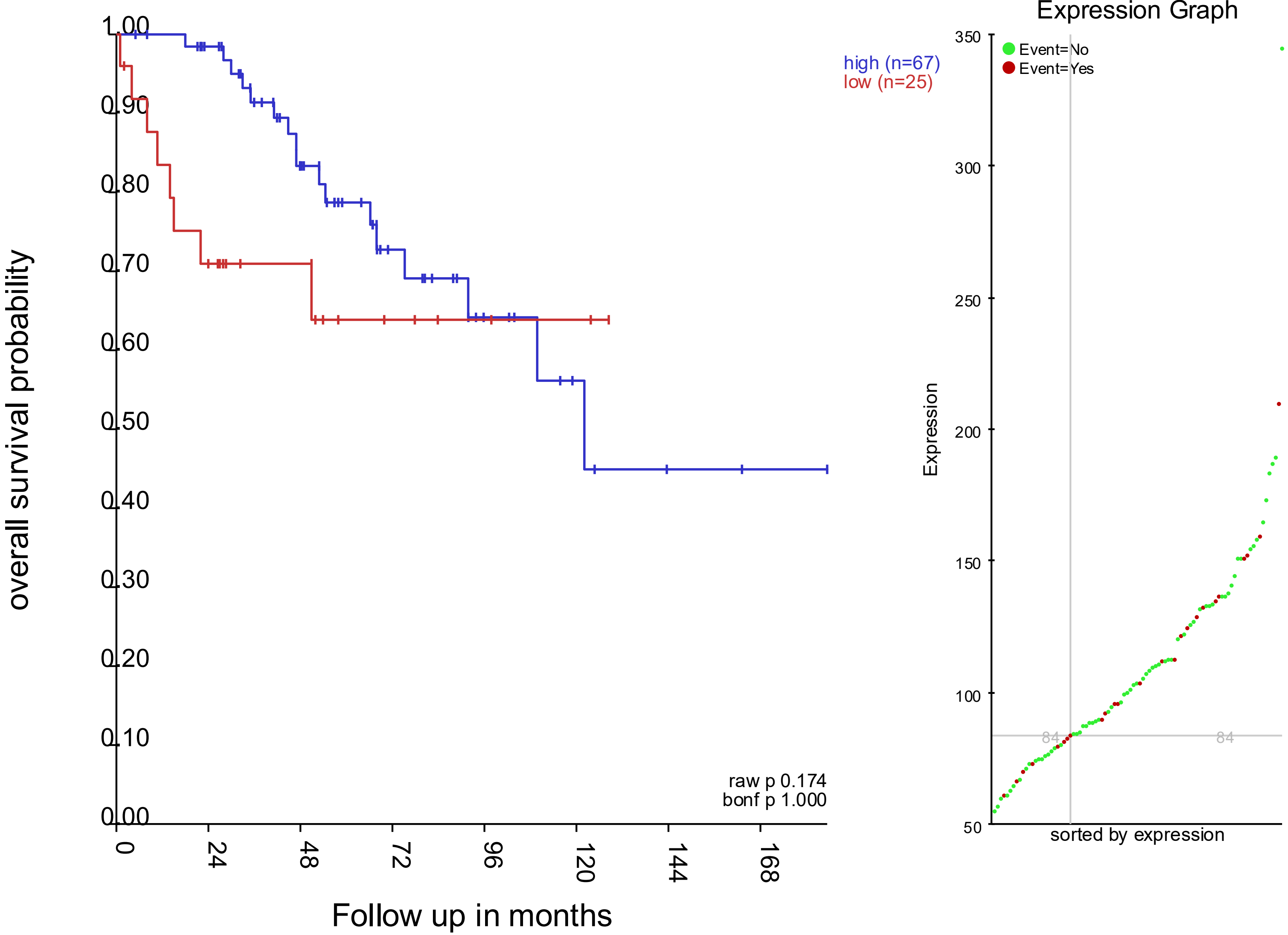

GROUP3 M0

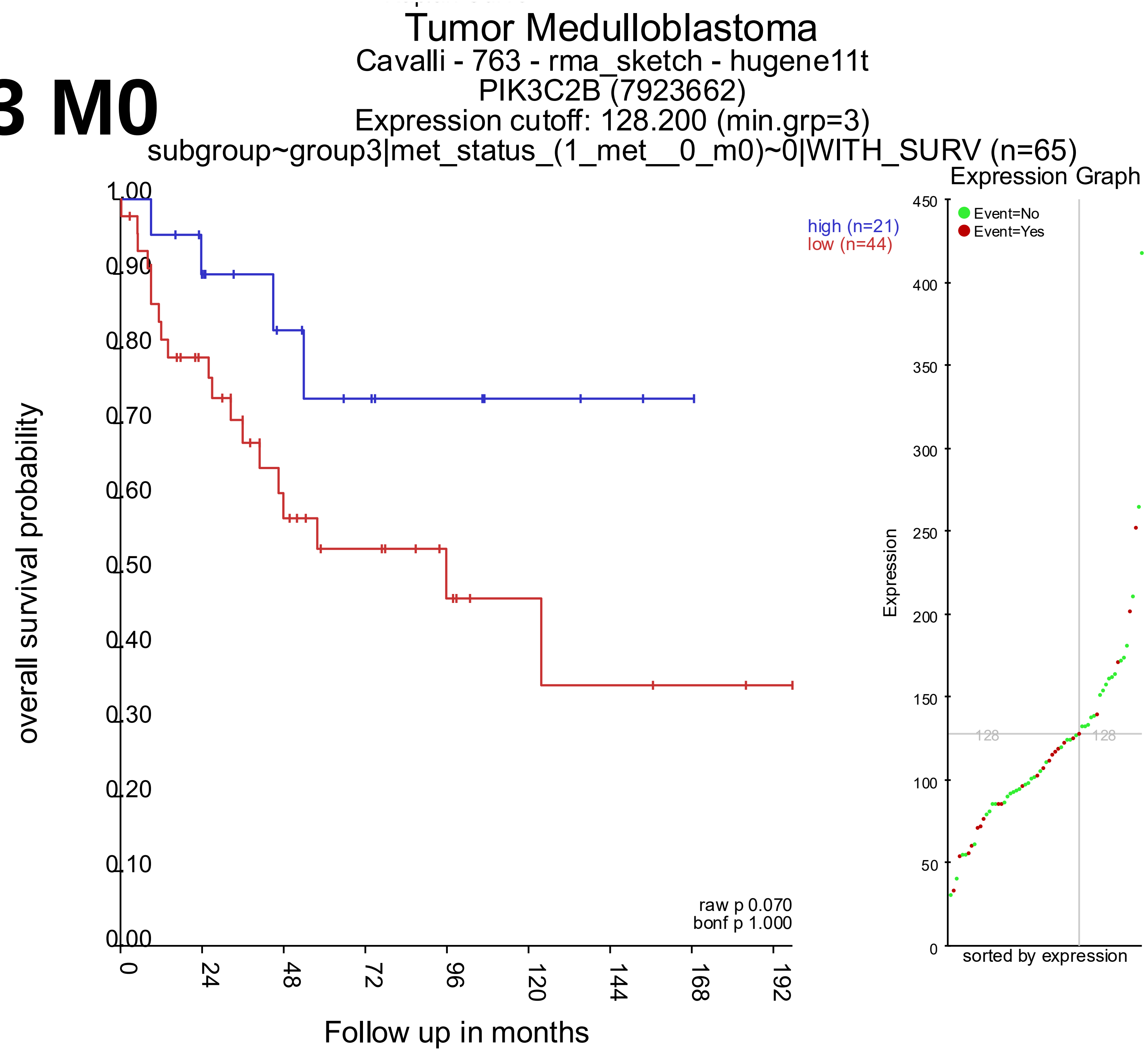

Tumor Medulloblastoma
Cavalli - 763 - rma_sketch - hugene11t
PIK3C2B (7923662)
Expression cutoff: 128.200 (min.grp=3)
subgroup~group3|met_status_(1_met__0_m0)~0|WITH_SURV (n=65)
overall survival probability
1.00
0.90
0.80
0.70
0.60
0.50
0.40
0.30
0.20
0.10
0.00
0
24
48
72
96
120
144
168
192
Follow up in months
high (n=21)
low (n=44)
raw p 0.070
bonf p 1.000
Expression Graph
Event=No
Event=Yes
Expression
450
400
350
300
250
200
150
100
50
0
128
128
sorted by expression

GROUP3 M1

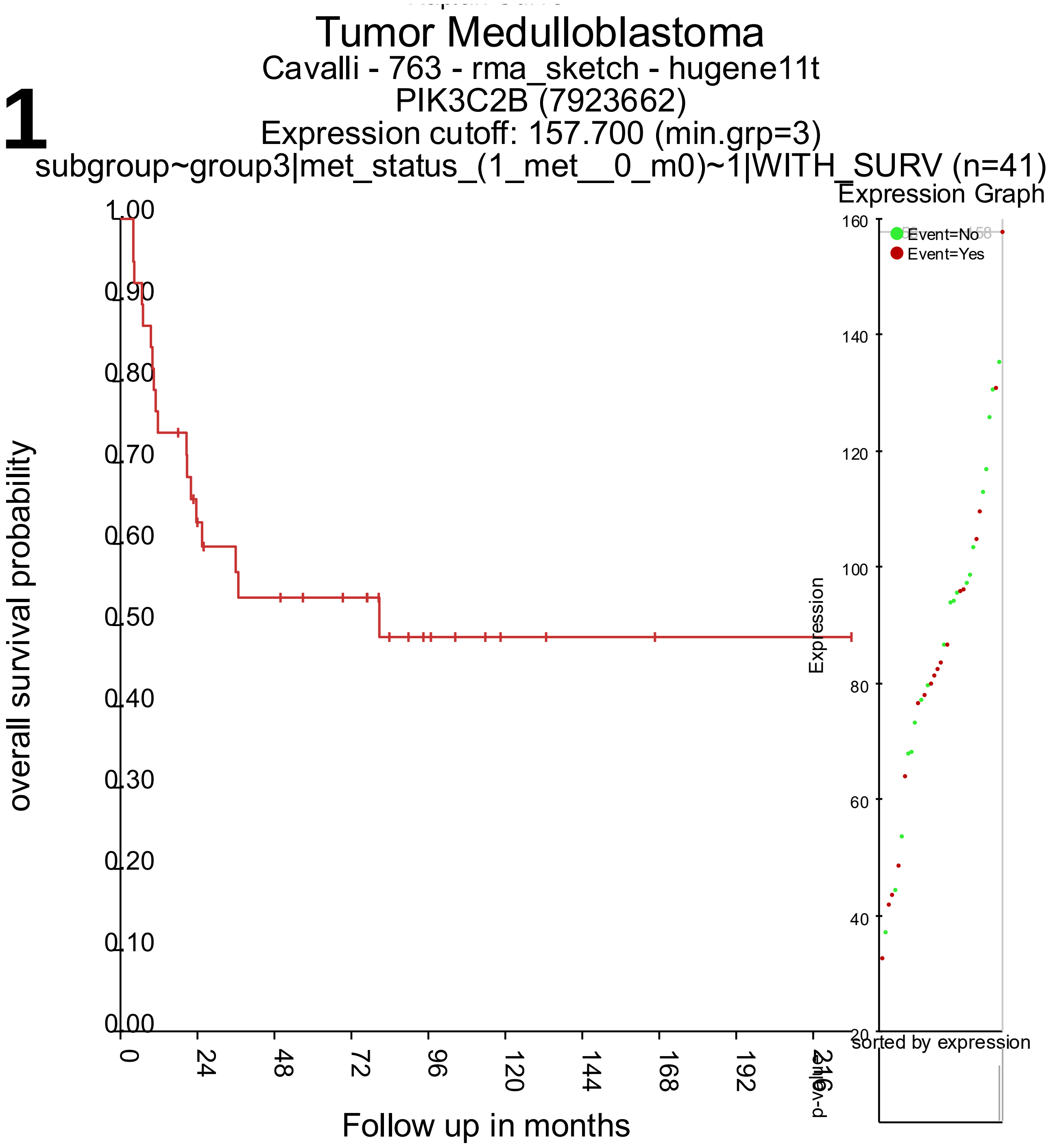

Tumor Medulloblastoma
Cavalli - 763 - rma_sketch - hugene11t
PIK3C2B (7923662)
Expression cutoff: 157.700 (min.grp=3)
subgroup~group3|met_status_(1_met__0_m0)~1|WITH_SURV (n=41)
overall survival probability
1.00
0.90
0.80
0.70
0.60
0.50
0.40
0.30
0.20
0.10
0.00
0
24
48
72
96
120
144
168
192
216
Follow up in months
Expression Graph
Event=No
Event=Yes
Expression
160
140
120
100
80
60
40
20
sorted by expression

PIK3C2G

WNT M0

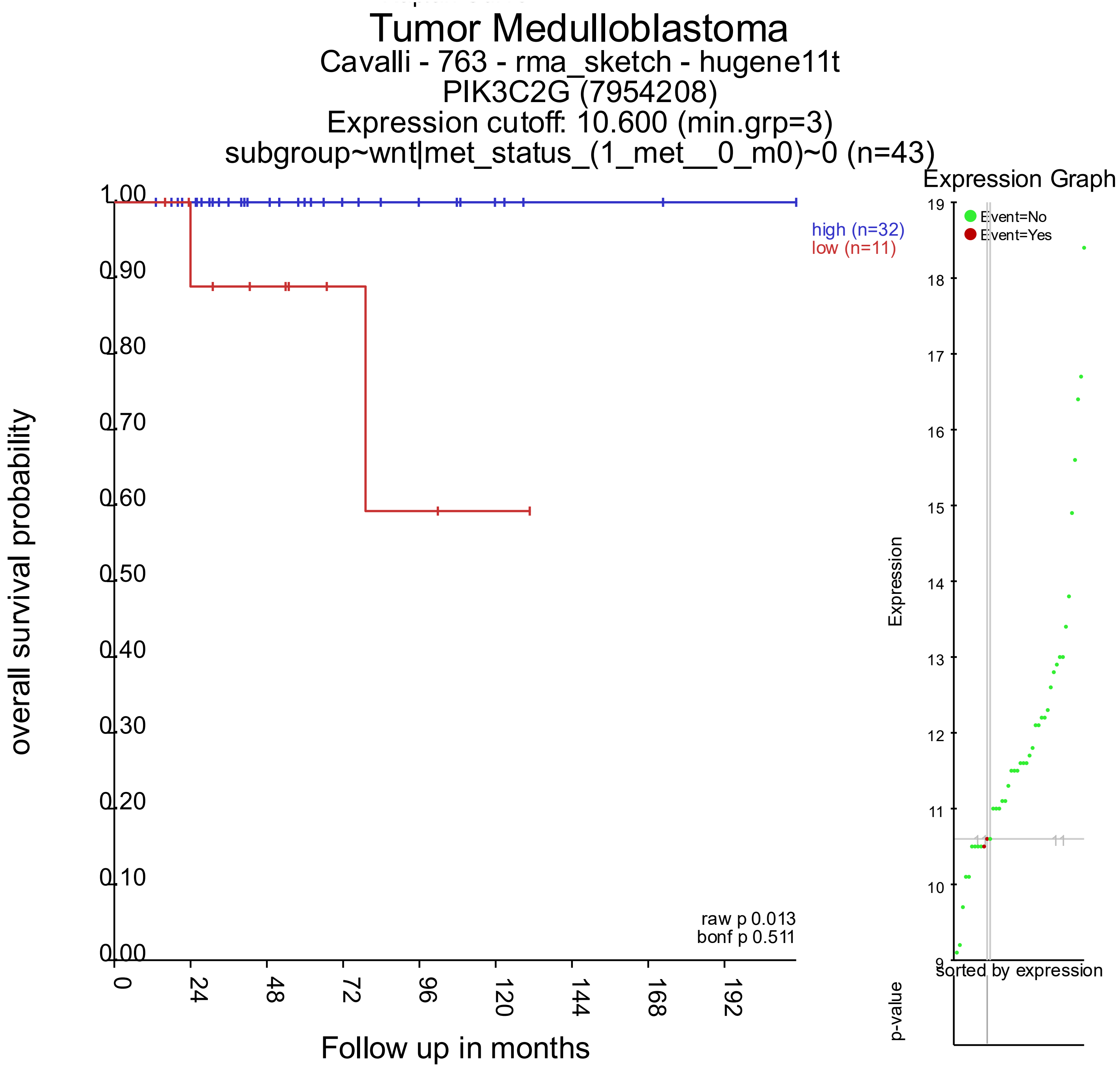

Tumor Medulloblastoma
Cavalli - 763 - rma_sketch - hugene11t
PIK3C2G (7954208)
Expression cutoff: 10.600 (min.grp=3)
subgroup~wnt|met_status_(1_met__0_m0)~0 (n=43)
overall survival probability
1.00
0.90
0.80
0.70
0.60
0.50
0.40
0.30
0.20
0.10
0.00
0
24
48
72
96
120
144
168
192
Follow up in months
high (n=32)
low (n=11)
raw p 0.013
bonf p 0.511
Expression Graph
Event=No
Event=Yes
Expression
19
18
17
16
15
14
13
12
11
10
9
sorted by expression
p-value

WNT M1

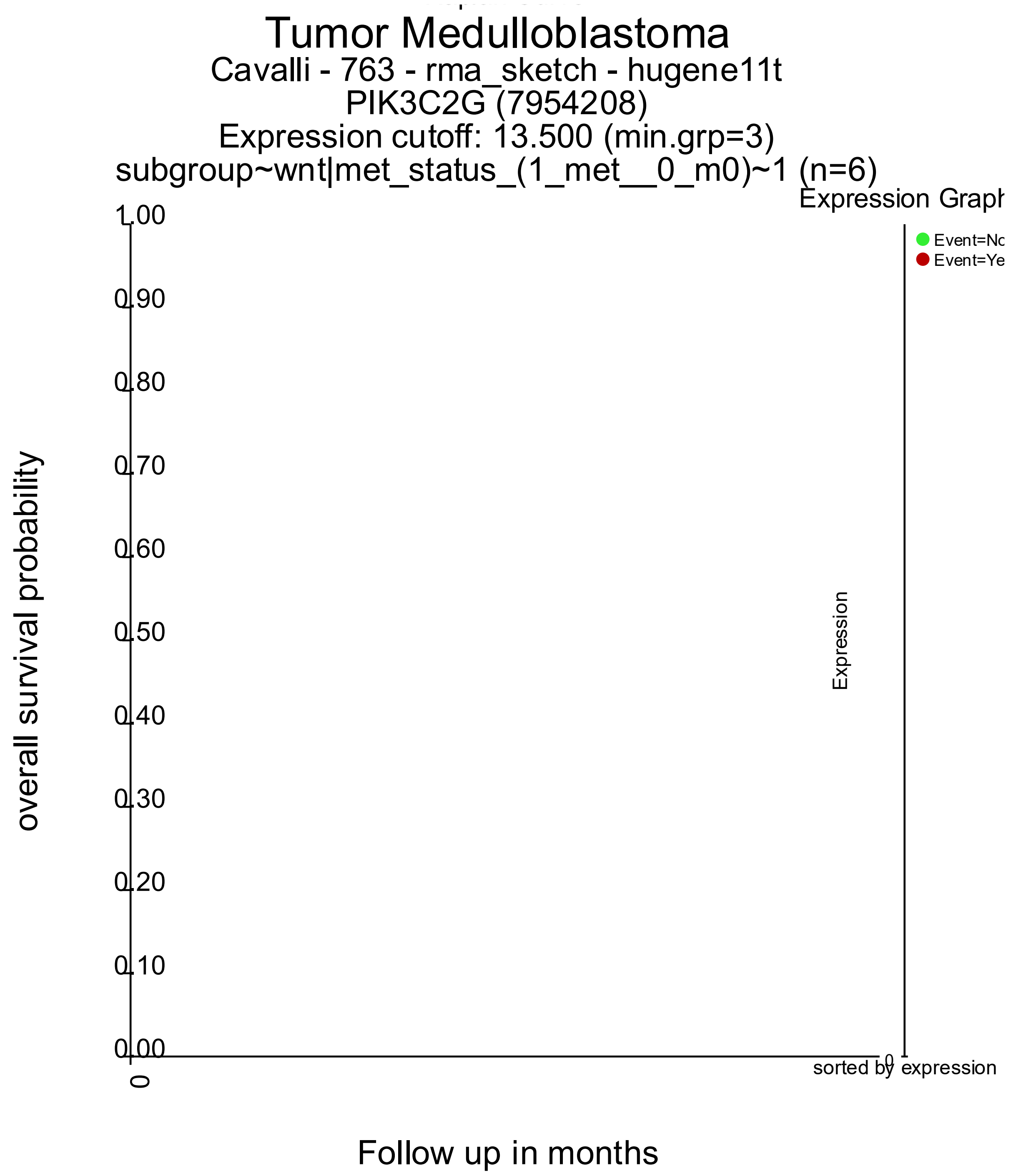
Tumor Medulloblastoma
Cavalli - 763 - rma_sketch - hugene11t
PIK3C2G (7954208)
Expression cutoff: 13.500 (min.grp=3)
subgroup~wnt|met_status_(1_met__0_m0)~1 (n=6)
Expression Graph
Event=No
Event=Yes
overall survival probability
1.00
0.90
0.80
0.70
0.60
0.50
0.40
0.30
0.20
0.10
0.00
0
Follow up in months
Expression
0
sorted by expression

SHH M0

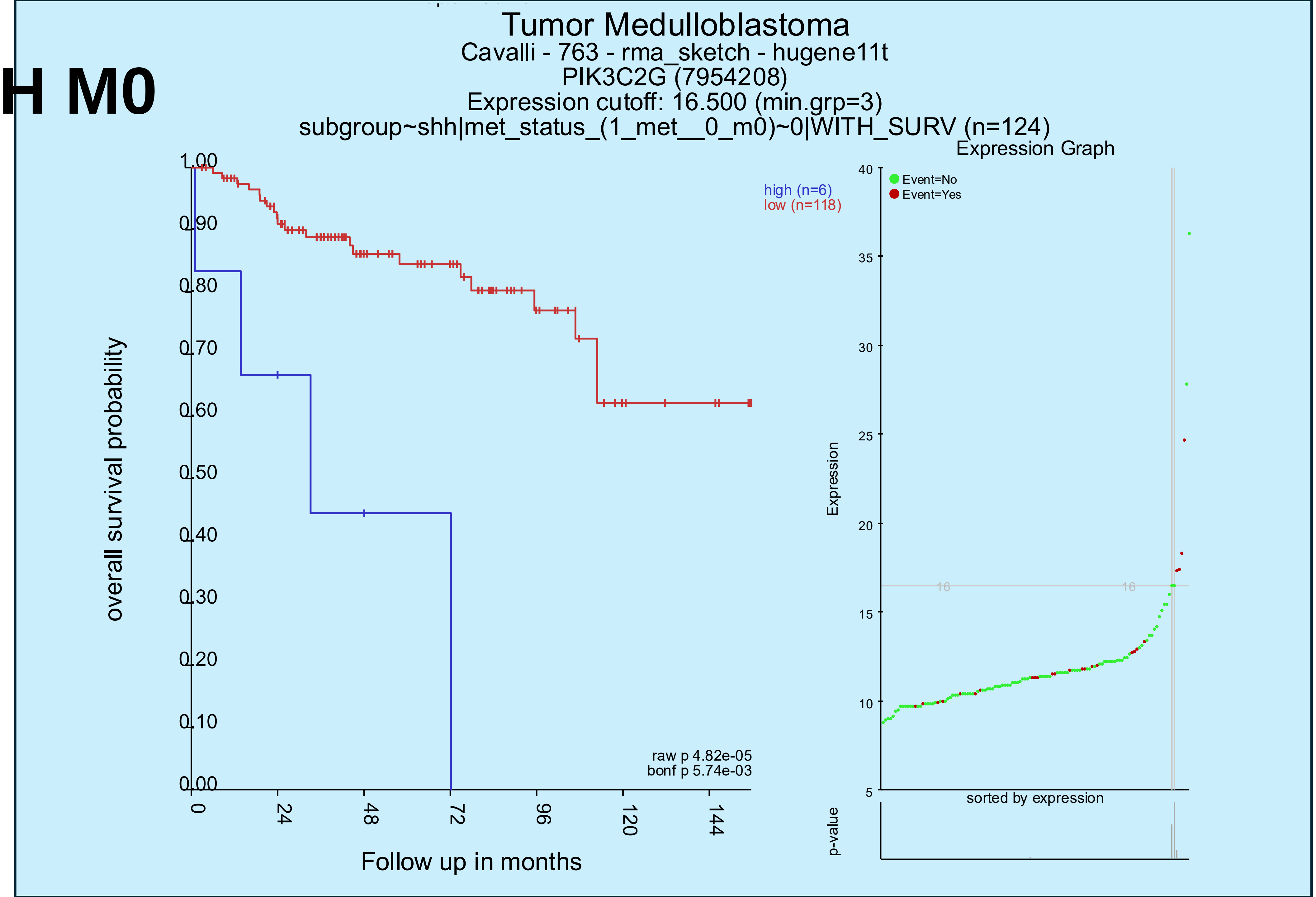

Tumor Medulloblastoma
Cavalli - 763 - rma_sketch - hugene11t
PIK3C2G (7954208)
Expression cutoff: 16.500 (min.grp=3)
subgroup~shh|met_status_(1_met__0_m0)~0|WITH_SURV (n=124)
overall survival probability
1.00
0.90
0.80
0.70
0.60
0.50
0.40
0.30
0.20
0.10
0.00
0
24
48
72
96
120
144
Follow up in months
high (n=6)
low (n=118)
raw p 4.82e-05
bonf p 5.74e-03
Expression Graph
Event=No
Event=Yes
Expression
40
35
30
25
20
15
10
5
16
16
sorted by expression
p-value

SHH M1

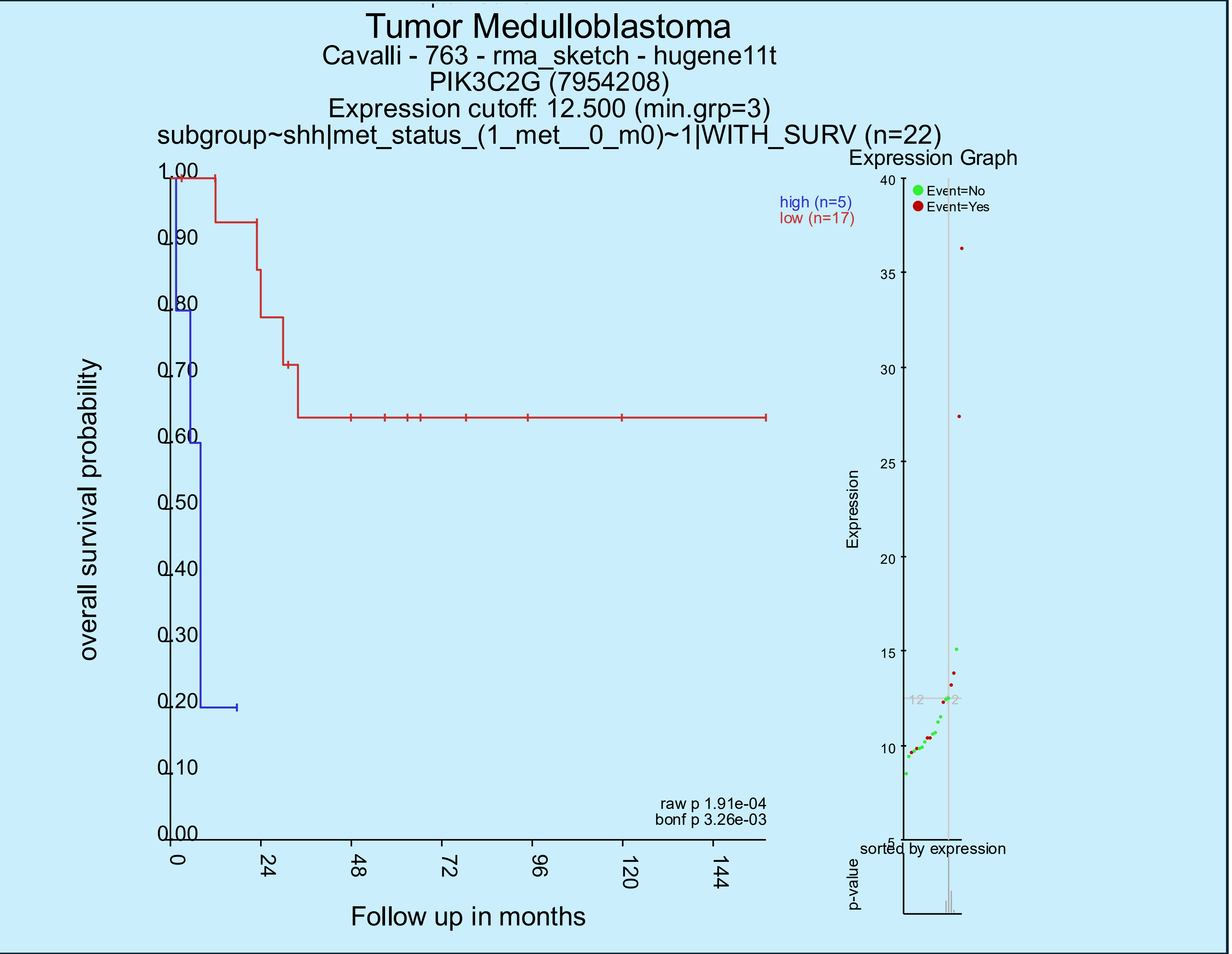
Tumor Medulloblastoma
Cavalli - 763 - rma_sketch - hugene11t
PIK3C2G (7954208)
Expression cutoff: 12.500 (min.grp=3)
subgroup~shh|met_status_(1_met__0_m0)~1|WITH_SURV (n=22)
overall survival probability
1.00
0.90
0.80
0.70
0.60
0.50
0.40
0.30
0.20
0.10
0.00
0
24
48
72
96
120
144
Follow up in months
high (n=5)
low (n=17)
raw p 1.91e-04
bonf p 3.26e-03
Expression Graph
Event=No
Event=Yes
Expression
40
35
30
25
20
15
10
5
sorted by expression
p-value

GROUP4 M0

Tumor Medulloblastoma
Cavalli - 763 - rma_sketch - hugene11t
PIK3C2G (7954208)
Expression cutoff: 10.000 (min.grp=3)
subgroup~group4|met_status_(1_met__0_m0)~0|WITH_SURV (n=145)

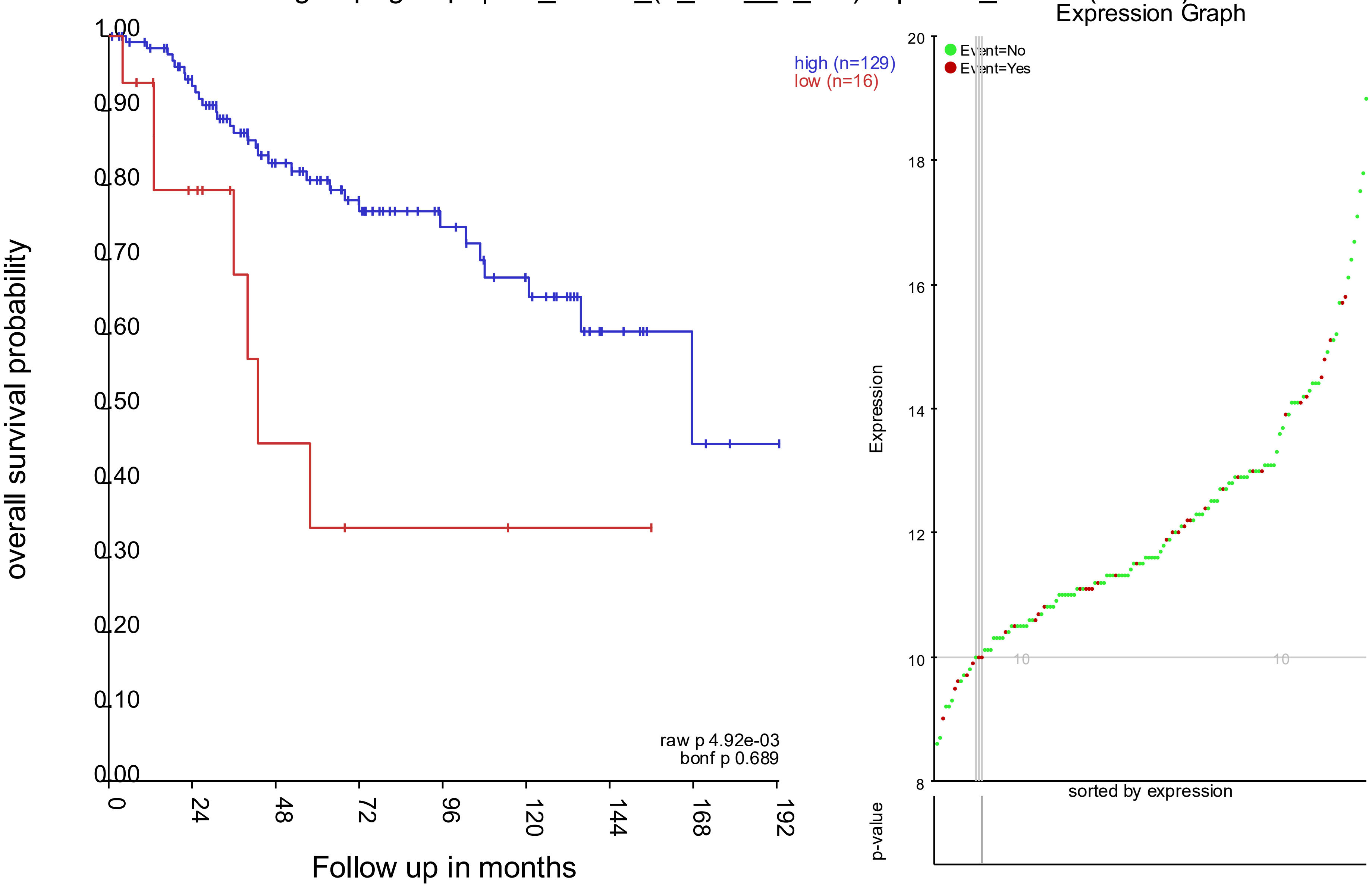

GROUP4 M1

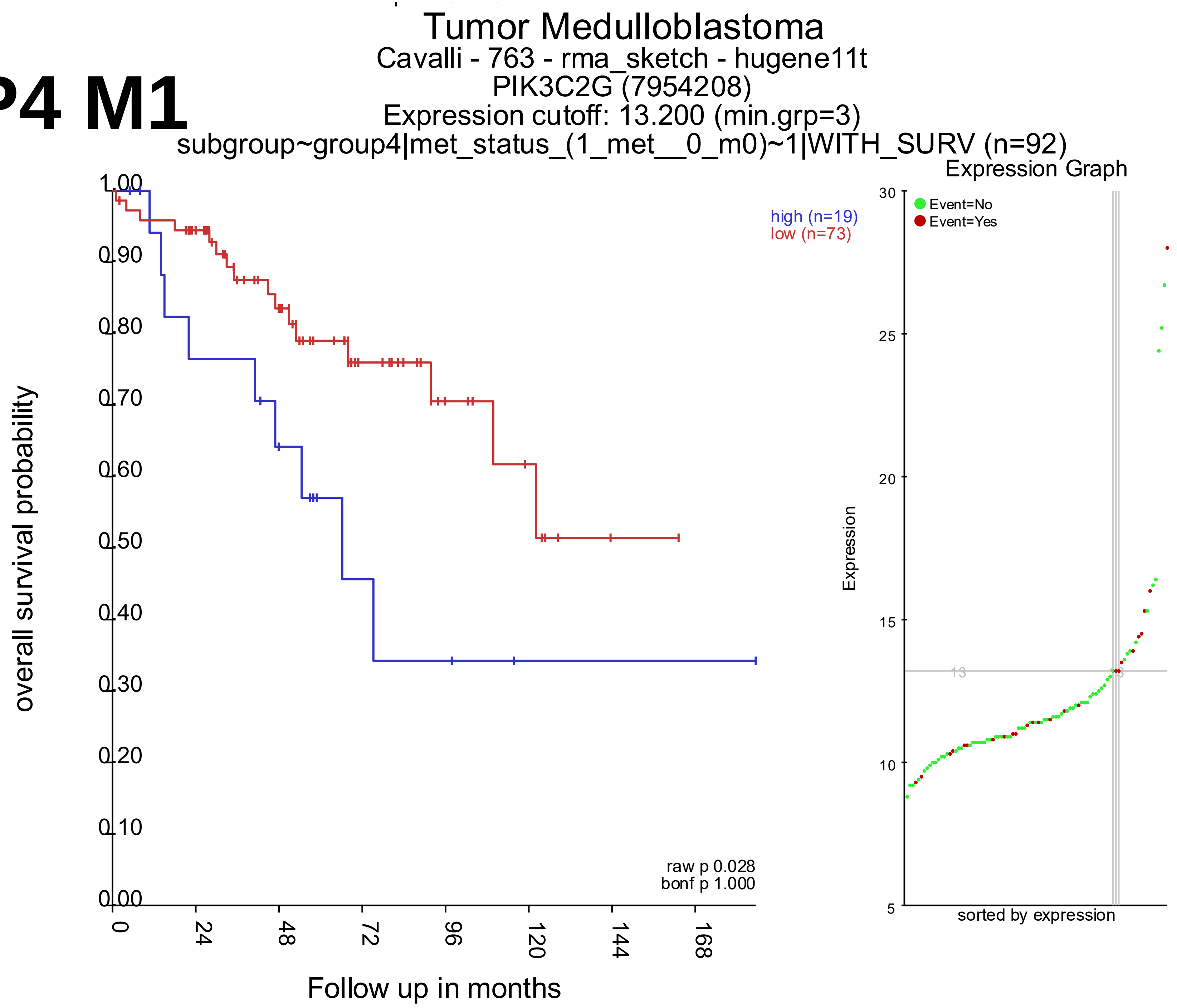

Tumor Medulloblastoma
Cavalli - 763 - rma_sketch - hugene11t
PIK3C2G (7954208)
Expression cutoff: 13.200 (min.grp=3)
subgroup~group4|met_status_(1_met__0_m0)~1|WITH_SURV (n=92)
overall survival probability
1.00
0.90
0.80
0.70
0.60
0.50
0.40
0.30
0.20
0.10
0.00
high (n=19)
low (n=73)
raw p 0.028
bonf p 1.000
0
24
48
72
96
120
144
168
Follow up in months
Expression Graph
Event=No
Event=Yes
30
25
20
15
10
5
Expression
13
sorted by expression

GROUP3 M0

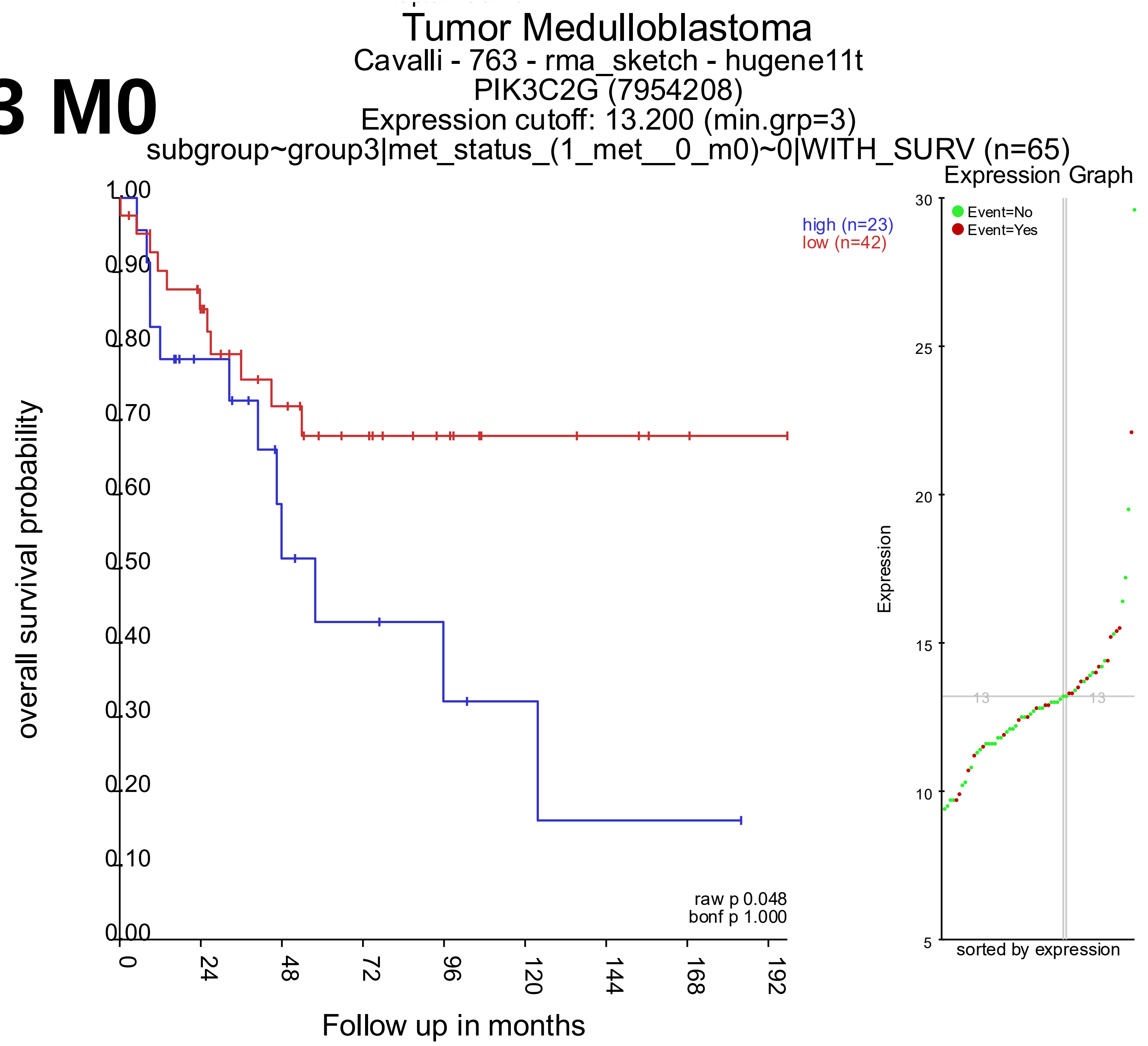

Tumor Medulloblastoma
Cavalli - 763 - rma_sketch - hugene11t
PIK3C2G (7954208)
Expression cutoff: 13.200 (min.grp=3)
subgroup~group3|met_status_(1_met__0_m0)~0|WITH_SURV (n=65)
high (n=23)
low (n=42)
overall survival probability
1.00
0.90
0.80
0.70
0.60
0.50
0.40
0.30
0.20
0.10
0.00
0
24
48
72
96
120
144
168
192
Follow up in months
raw p 0.048
bonf p 1.000
Expression Graph
Event=No
Event=Yes
Expression
30
25
20
15
10
5
13
sorted by expression

GROUP3 M1

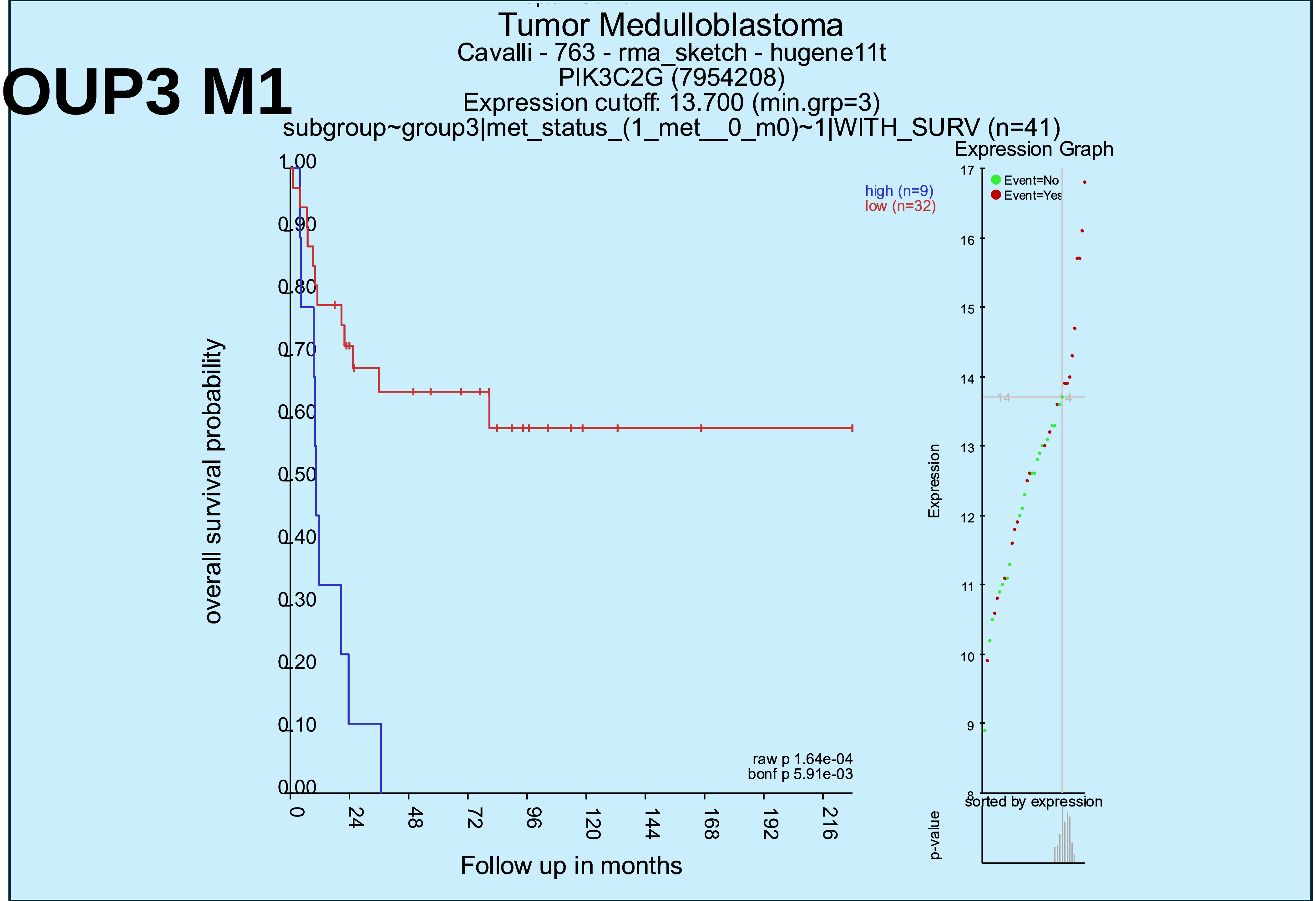

Tumor Medulloblastoma
Cavalli - 763 - rma_sketch - hugene11t
PIK3C2G (7954208)
Expression cutoff: 13.700 (min.grp=3)
subgroup~group3|met_status_(1_met__0_m0)~1|WITH_SURV (n=41)
overall survival probability
1.00
0.90
0.80
0.70
0.60
0.50
0.40
0.30
0.20
0.10
0.00
high (n=9)
low (n=32)
raw p 1.64e-04
bonf p 5.91e-03
0
24
48
72
96
120
144
168
192
216
Follow up in months
Expression Graph
Event=No
Event=Yes
Expression
17
16
15
14
13
12
11
10
9
8
sorted by expression
p-value

PIK3C3

WNT M0

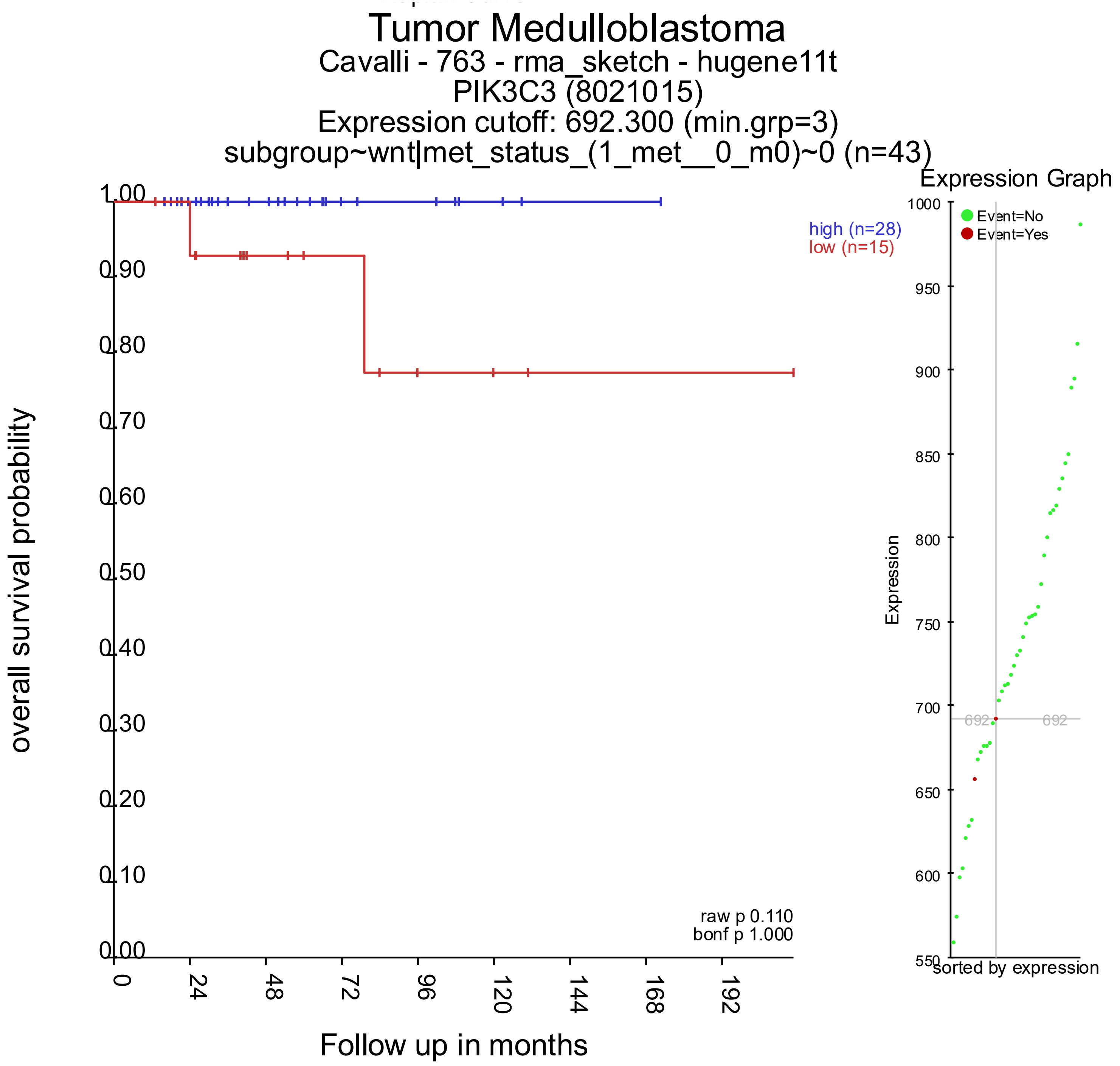

Tumor Medulloblastoma
Cavalli - 763 - rma_sketch - hugene11t
PIK3C3 (8021015)
Expression cutoff: 692.300 (min.grp=3)
subgroup~wnt|met_status_(1_met__0_m0)~0 (n=43)
overall survival probability
1.00
0.90
0.80
0.70
0.60
0.50
0.40
0.30
0.20
0.10
0.00
0
24
48
72
96
120
144
168
192
Follow up in months
high (n=28)
low (n=15)
raw p 0.110
bonf p 1.000
Expression Graph
Event=No
Event=Yes
Expression
1000
950
900
850
800
750
700
650
600
550
692
692
sorted by expression

WNT M1

Tumor Medulloblastoma
Cavalli - 763 - rma_sketch - hugene11t
PIK3C3 (8021015)
Expression cutoff: 727.000 (min.grp=3)
subgroup~wnt|met_status_(1_met__0_m0)~1 (n=6)

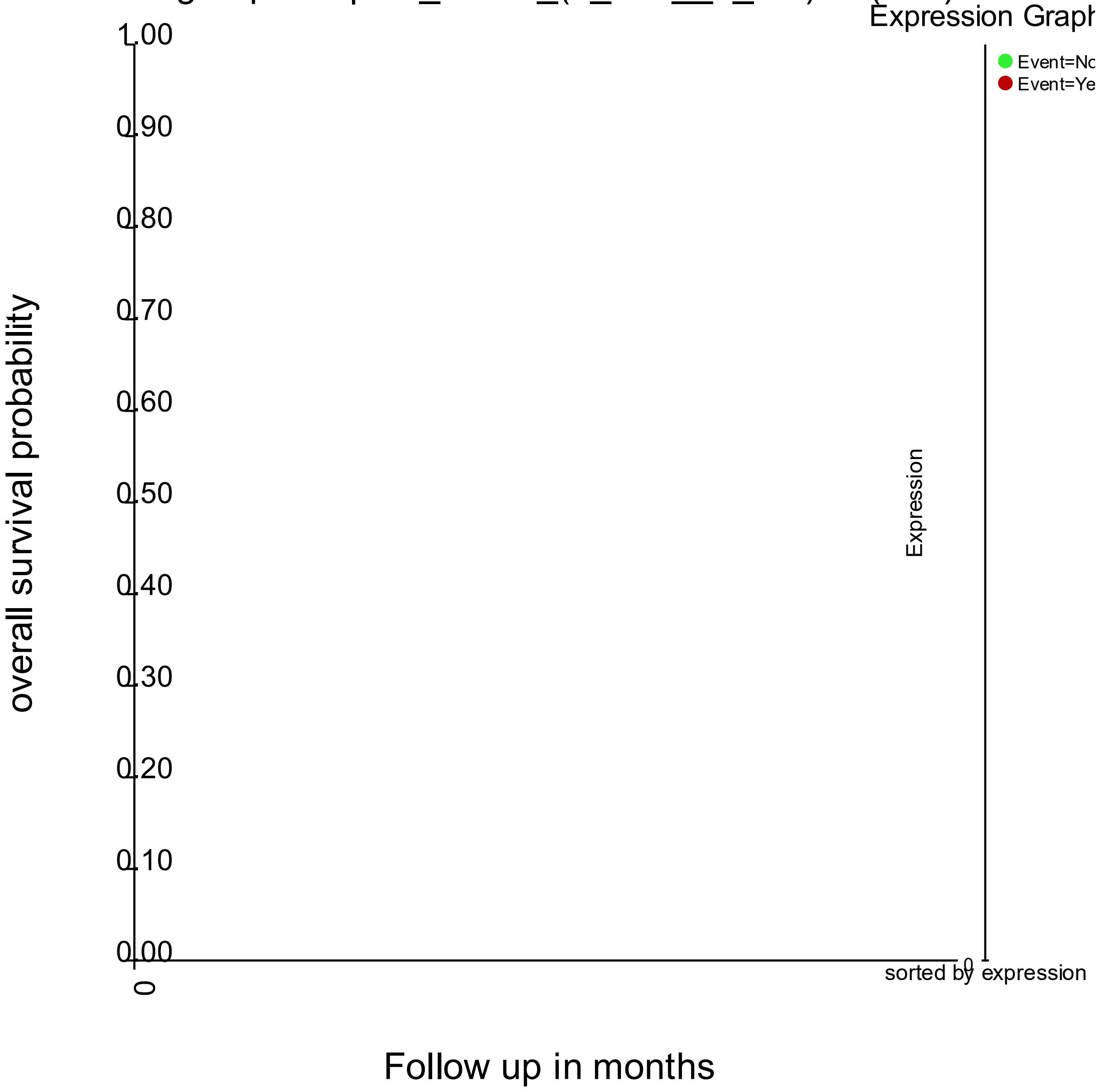

SHH M0

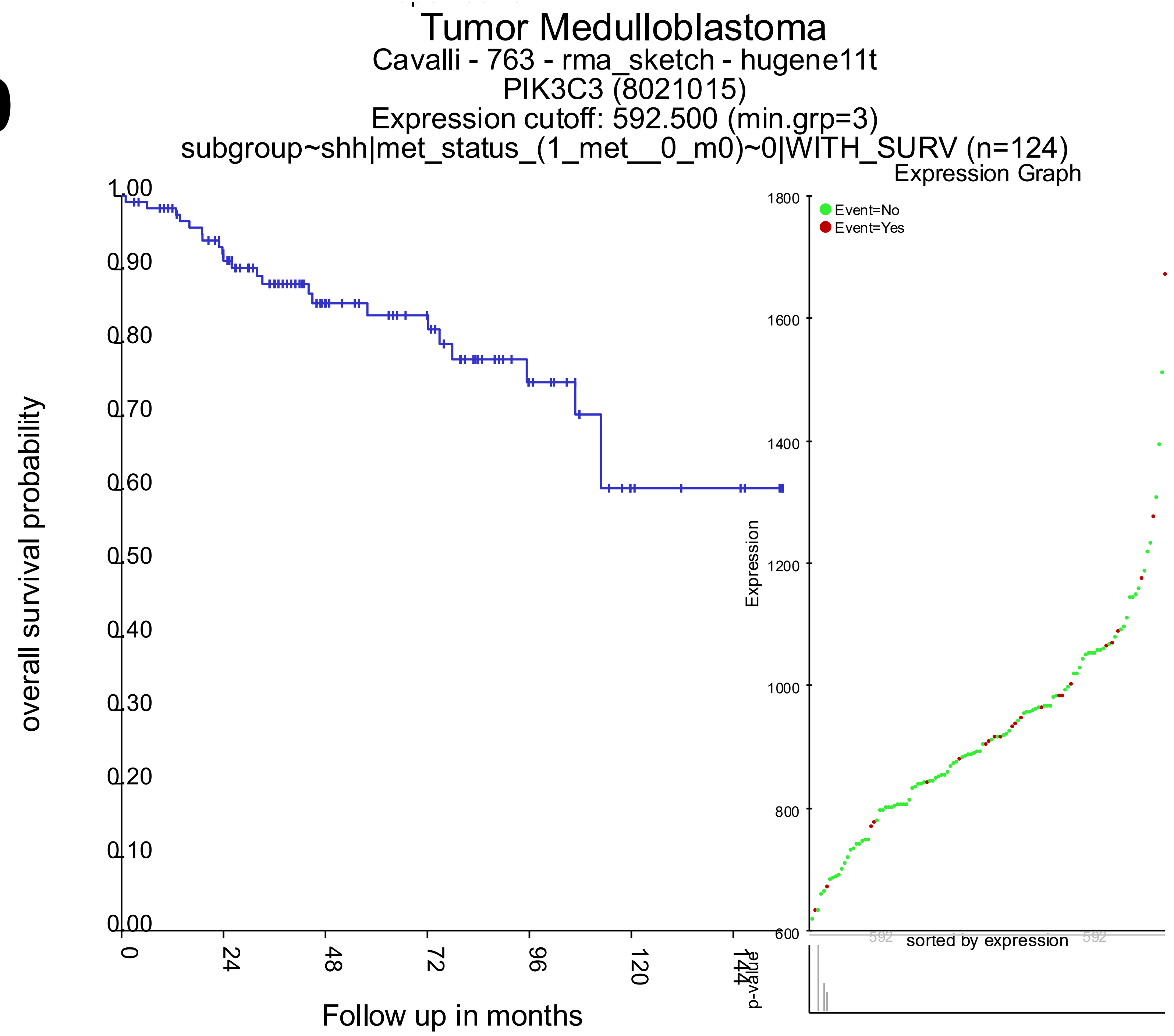

Tumor Medulloblastoma
Cavalli - 763 - rma_sketch - hugene11t
PIK3C3 (8021015)
Expression cutoff: 592.500 (min.grp=3)
subgroup~shh|met_status_(1_met__0_m0)~0|WITH_SURV (n=124)
overall survival probability
1.00
0.90
0.80
0.70
0.60
0.50
0.40
0.30
0.20
0.10
0.00
0
24
48
72
96
120
144
Follow up in months
Expression Graph
Event=No
Event=Yes
Expression
1800
1600
1400
1200
1000
800
600
592
sorted by expression
592
p-value

SHH M1

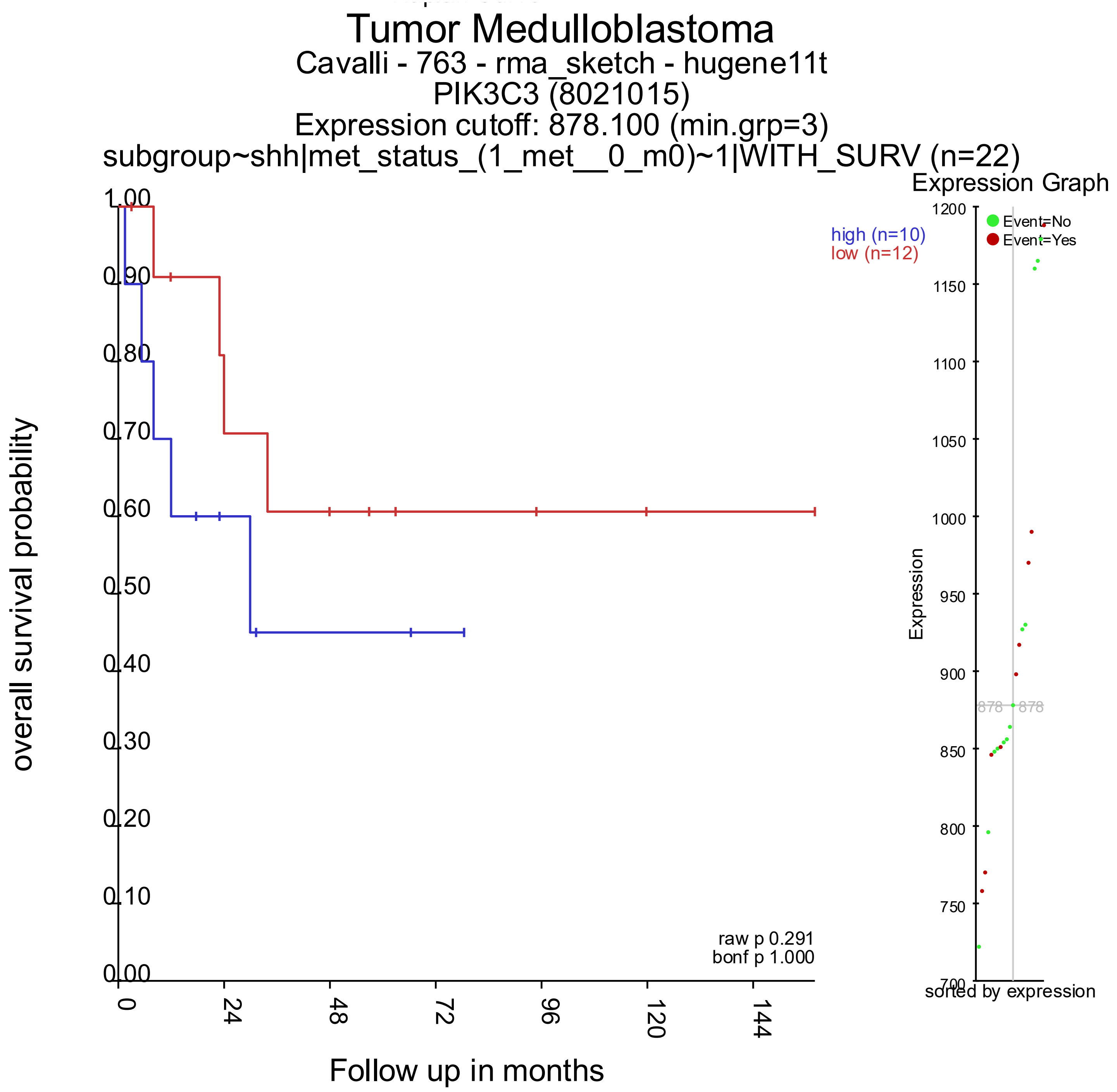
Tumor Medulloblastoma
Cavalli - 763 - rma_sketch - hugene11t
PIK3C3 (8021015)
Expression cutoff: 878.100 (min.grp=3)
subgroup~shh|met_status_(1_met__0_m0)~1|WITH_SURV (n=22)
overall survival probability
1.00
0.90
0.80
0.70
0.60
0.50
0.40
0.30
0.20
0.10
0.00
0
24
48
72
96
120
144
Follow up in months
high (n=10)
low (n=12)
raw p 0.291
bonf p 1.000
Expression Graph
Event=No
Event=Yes
Expression
1200
1150
1100
1050
1000
950
900
850
800
750
700
878
878
sorted by expression

GROUP4 M0

Tumor Medulloblastoma
Cavalli - 763 - rma_sketch - hugene11t
PIK3C3 (8021015)
Expression cutoff: 1085.700 (min.grp=3)
subgroup~group4|met_status_(1_met__0_m0)~0|WITH_SURV (n=145)

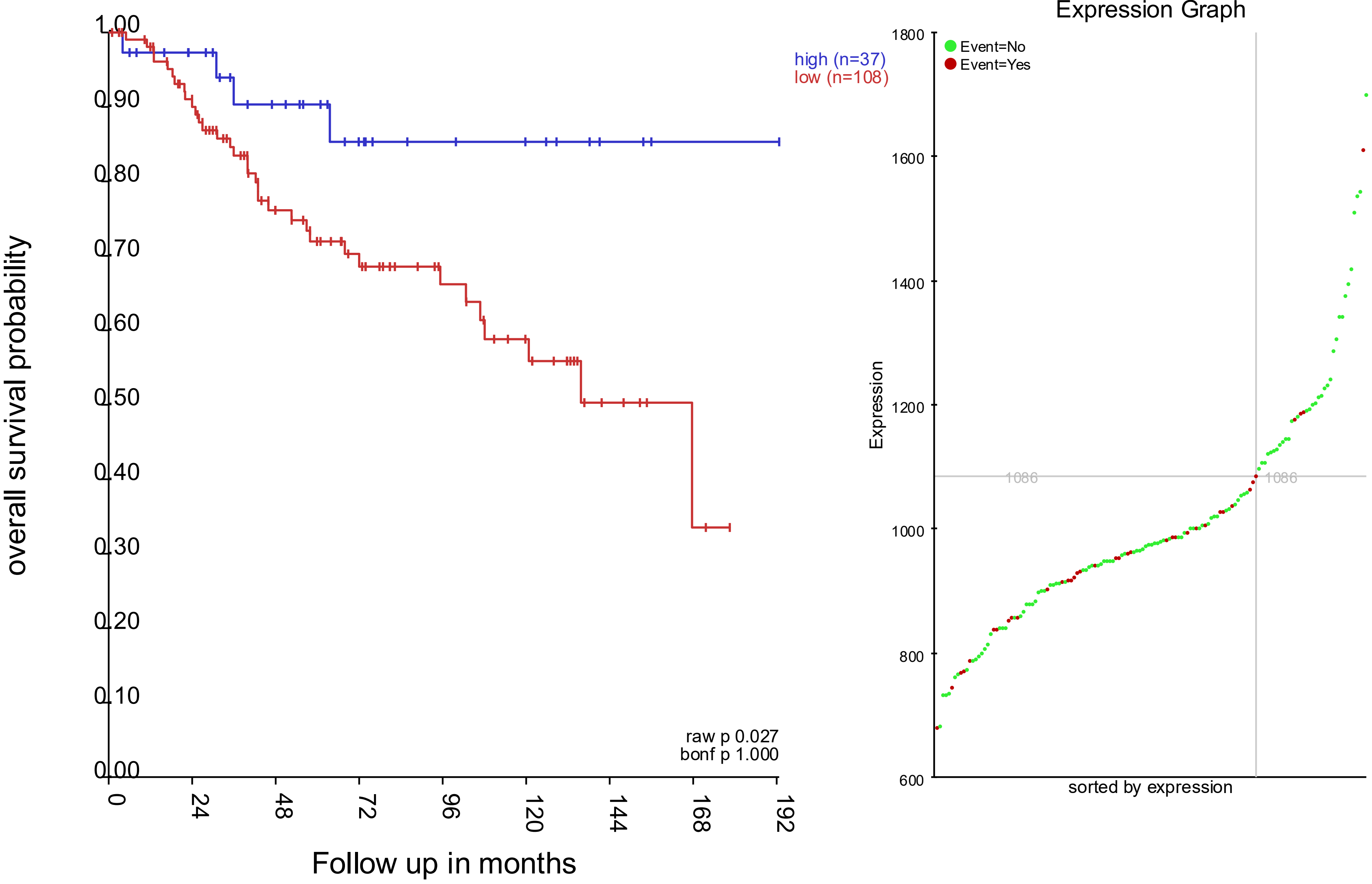

GROUP4 M1

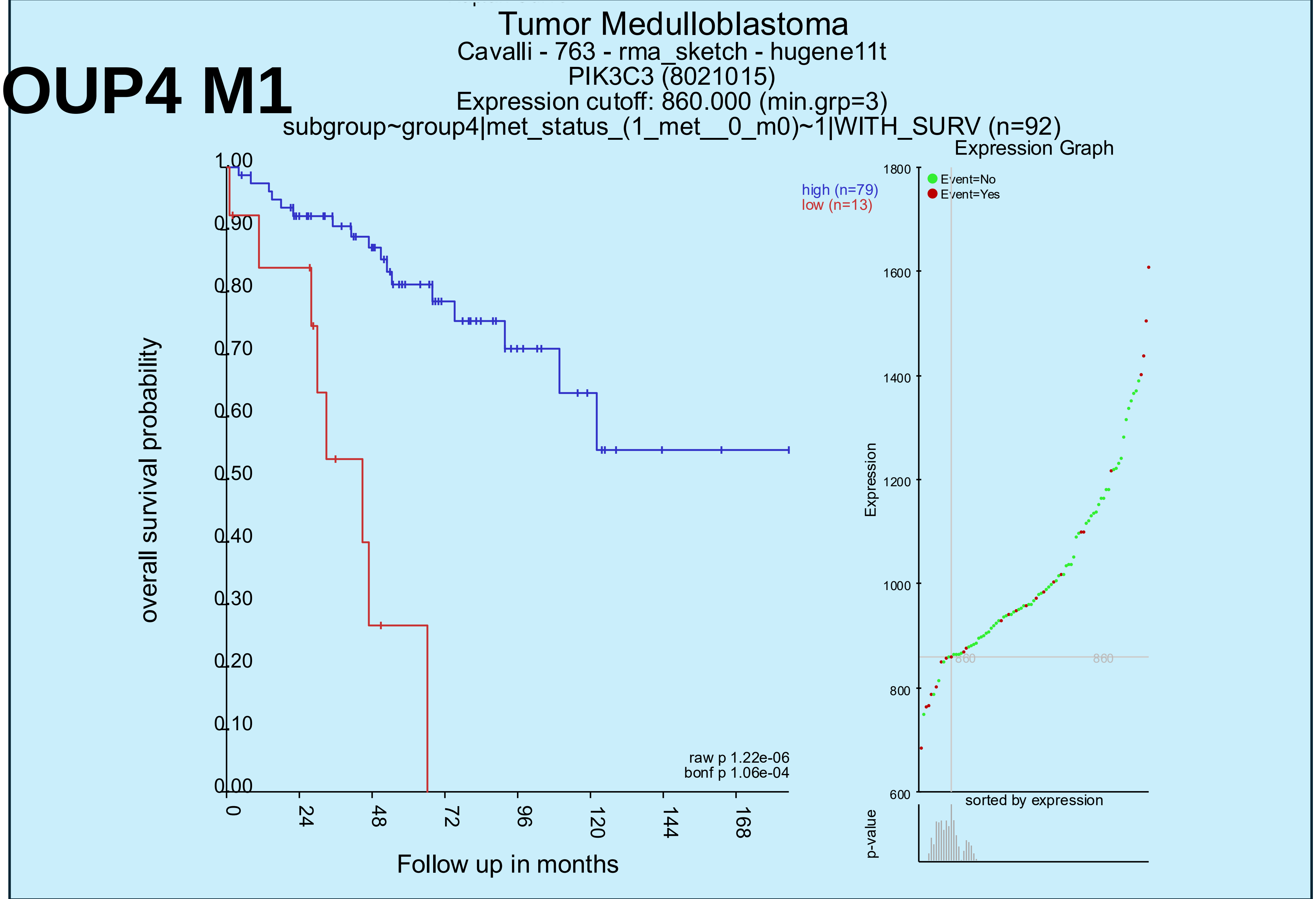

Tumor Medulloblastoma
Cavalli - 763 - rma_sketch - hugene11t
PIK3C3 (8021015)
Expression cutoff: 860.000 (min.grp=3)
subgroup~group4|met_status_(1_met__0_m0)~1|WITH_SURV (n=92)
overall survival probability
Follow up in months
high (n=79)
low (n=13)
raw p 1.22e-06
bonf p 1.06e-04
Expression Graph
Event=No
Event=Yes
Expression
sorted by expression
p-value

GROUP3 M0

Tumor Medulloblastoma
Cavalli - 763 - rma_sketch - hugene11t
PIK3C3 (8021015)
Expression cutoff: 1024.800 (min.grp=3)
subgroup~group3|met_status_(1_met__0_m0)~0|WITH_SURV (n=65)

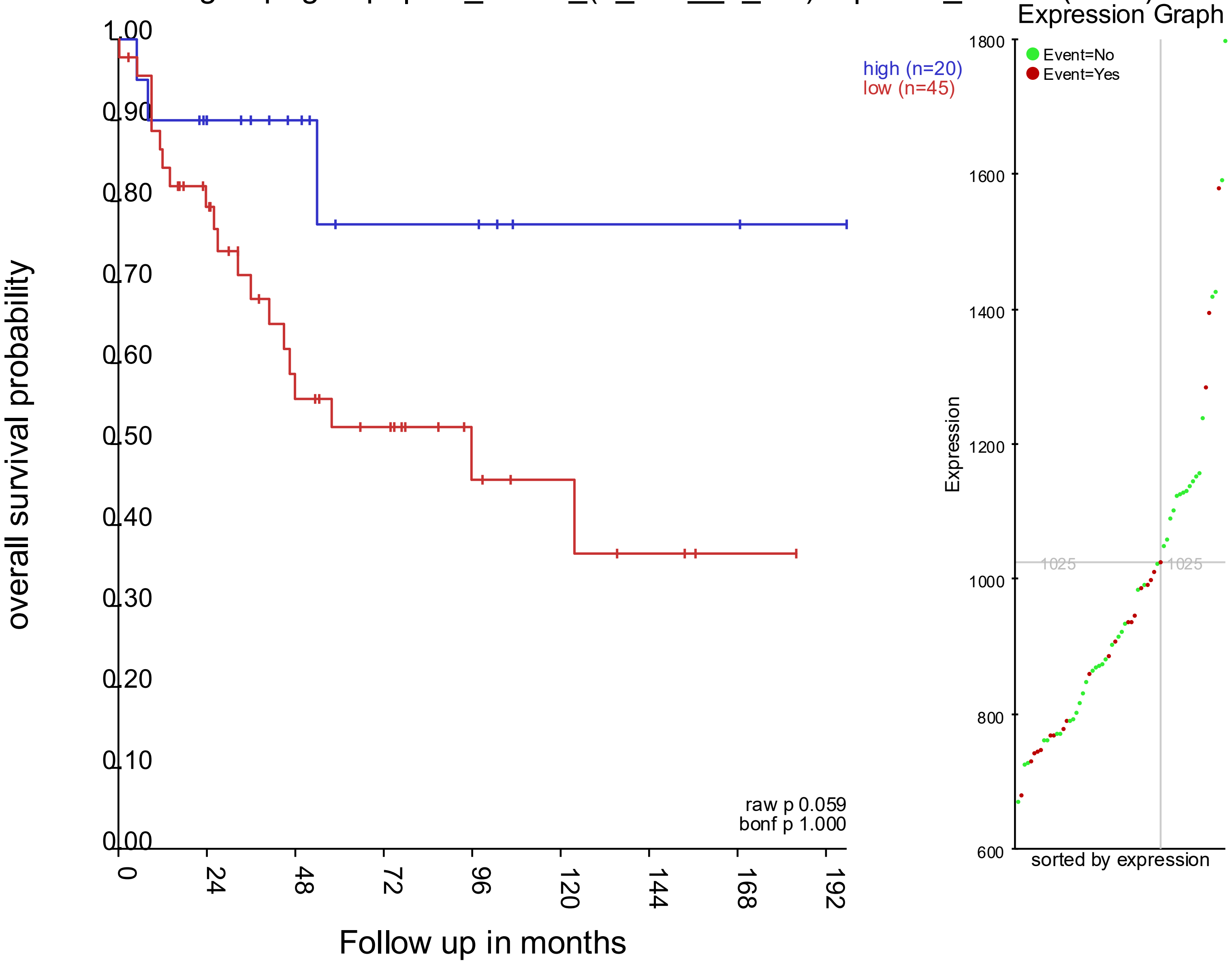

GROUP3 M1

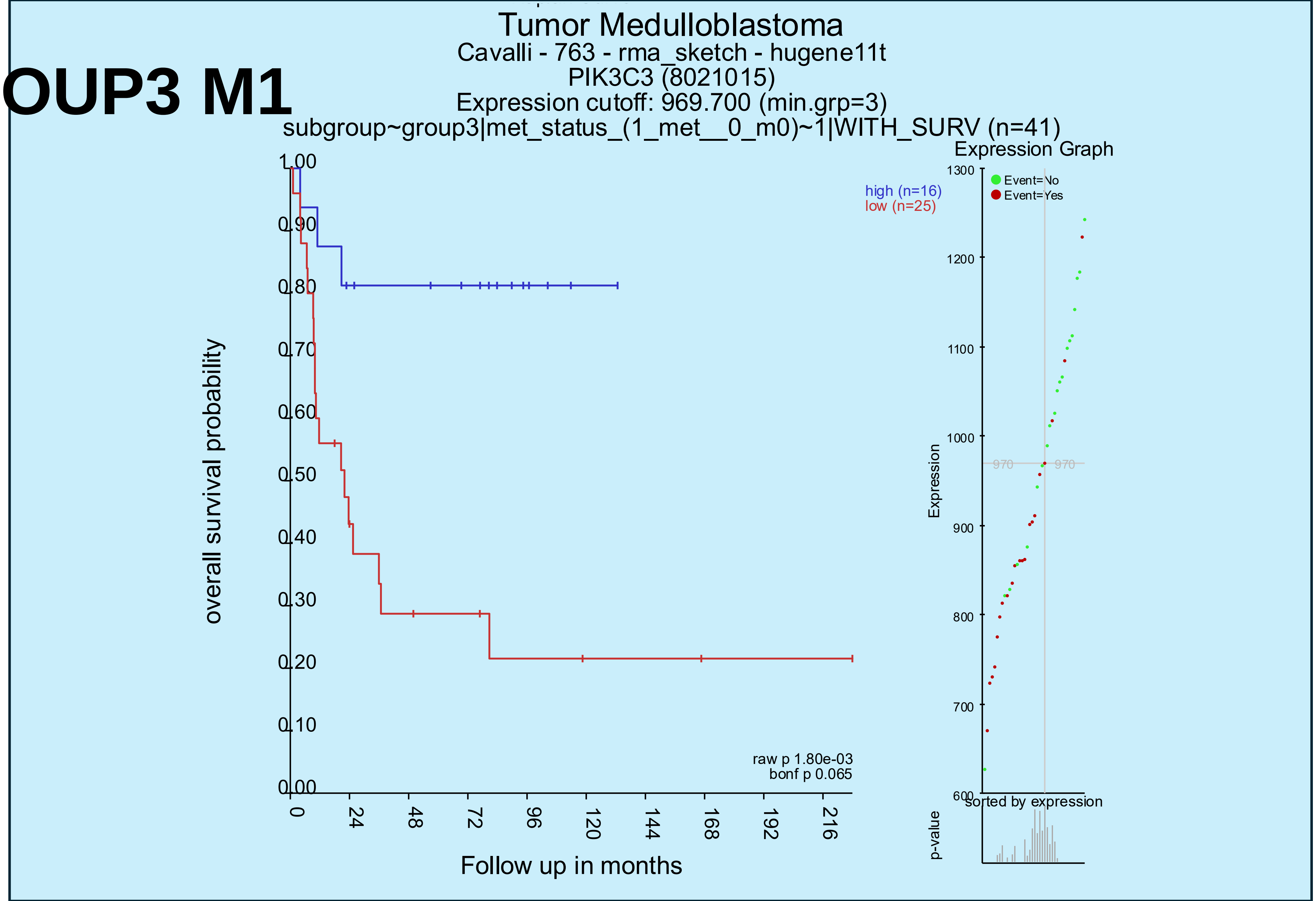
Tumor Medulloblastoma
Cavalli - 763 - rma_sketch - hugene11t
PIK3C3 (8021015)
Expression cutoff: 969.700 (min.grp=3)
subgroup~group3|met_status_(1_met__0_m0)~1|WITH_SURV (n=41)
overall survival probability
1.00
0.90
0.80
0.70
0.60
0.50
0.40
0.30
0.20
0.10
0.00
high (n=16)
low (n=25)
raw p 1.80e-03
bonf p 0.065
0
24
48
72
96
120
144
168
192
216
Follow up in months
Expression Graph
Event=No
Event=Yes
1300
1200
1100
1000
900
800
700
600
970
970
Expression
sorted by expression
p-value

ITPKC

WNT M0

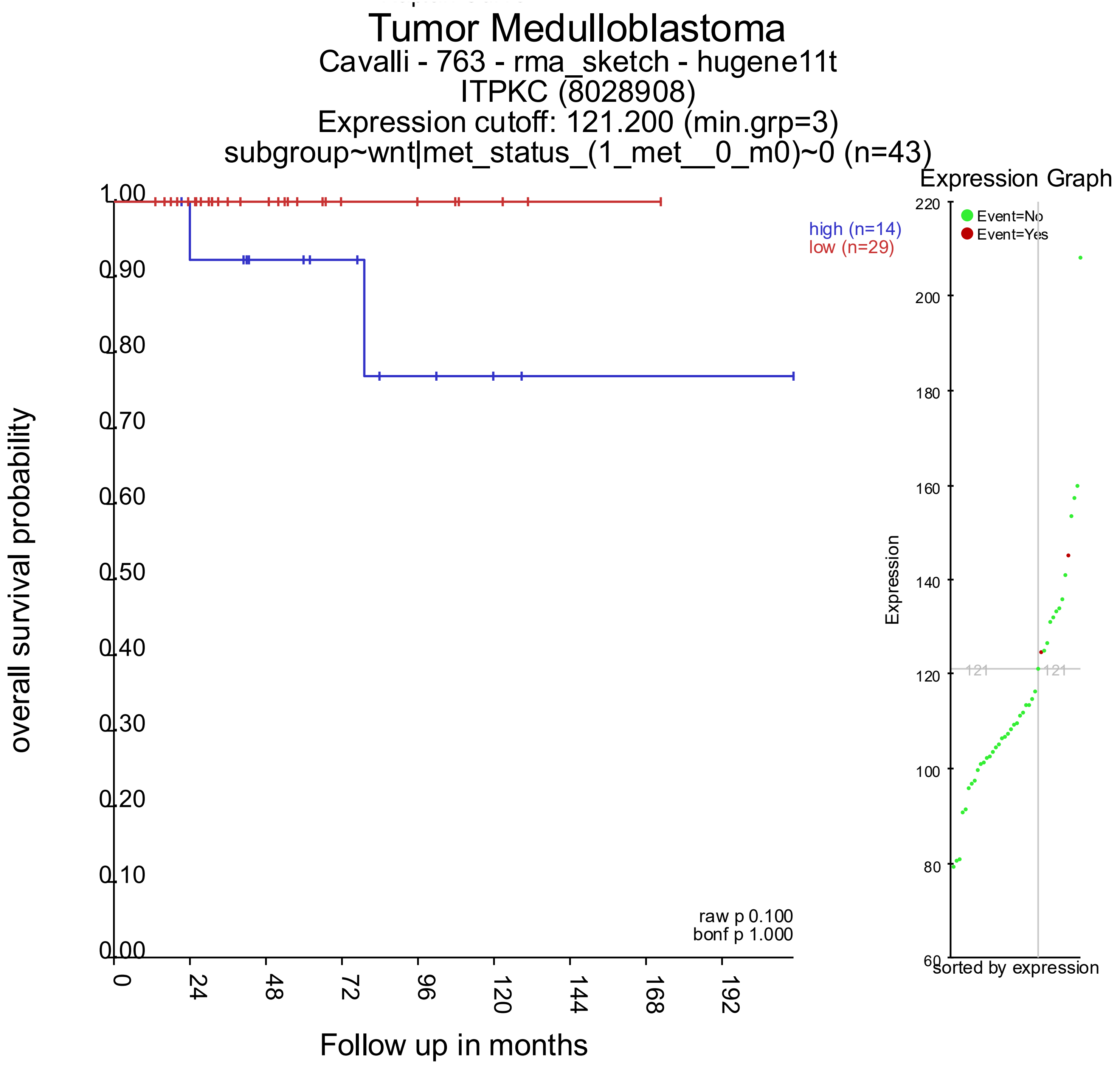

Tumor Medulloblastoma
Cavalli - 763 - rma_sketch - hugene11t
ITPKC (8028908)
Expression cutoff: 121.200 (min.grp=3)
subgroup~wnt|met_status_(1_met__0_m0)~0 (n=43)
high (n=14)
low (n=29)
overall survival probability
1.00
0.90
0.80
0.70
0.60
0.50
0.40
0.30
0.20
0.10
0.00
0
24
48
72
96
120
144
168
192
Follow up in months
raw p 0.100
bonf p 1.000
Expression Graph
Event=No
Event=Yes
Expression
220
200
180
160
140
120
100
80
60
121
121
sorted by expression

WNT M1

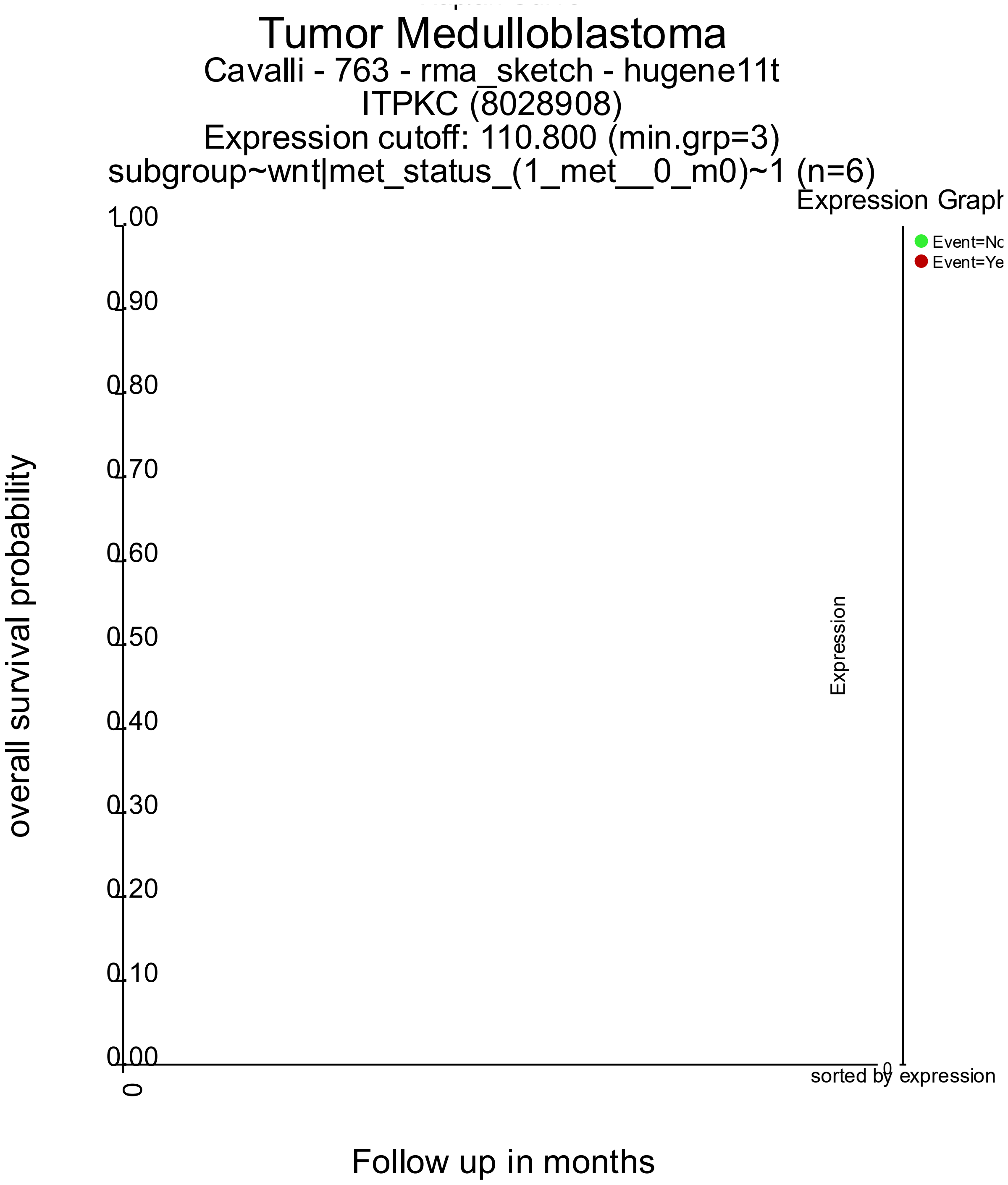

Tumor Medulloblastoma
Cavalli - 763 - rma_sketch - hugene11t
ITPKC (8028908)
Expression cutoff: 110.800 (min.grp=3)
subgroup~wnt|met_status_(1_met__0_m0)~1 (n=6)
Expression Graph
Event=No
Event=Ye
1.00
0.90
0.80
0.70
0.60
0.50
0.40
0.30
0.20
0.10
0.00
0
overall survival probability
Expression
0
sorted by expression
Follow up in months

SHH M0

Tumor Medulloblastoma
Cavalli - 763 - rma_sketch - hugene11t
ITPKC (8028908)
Expression cutoff: 130.700 (min.grp=3)
subgroup~shh|met_status_(1_met__0_m0)~0|WITH_SURV (n=124)

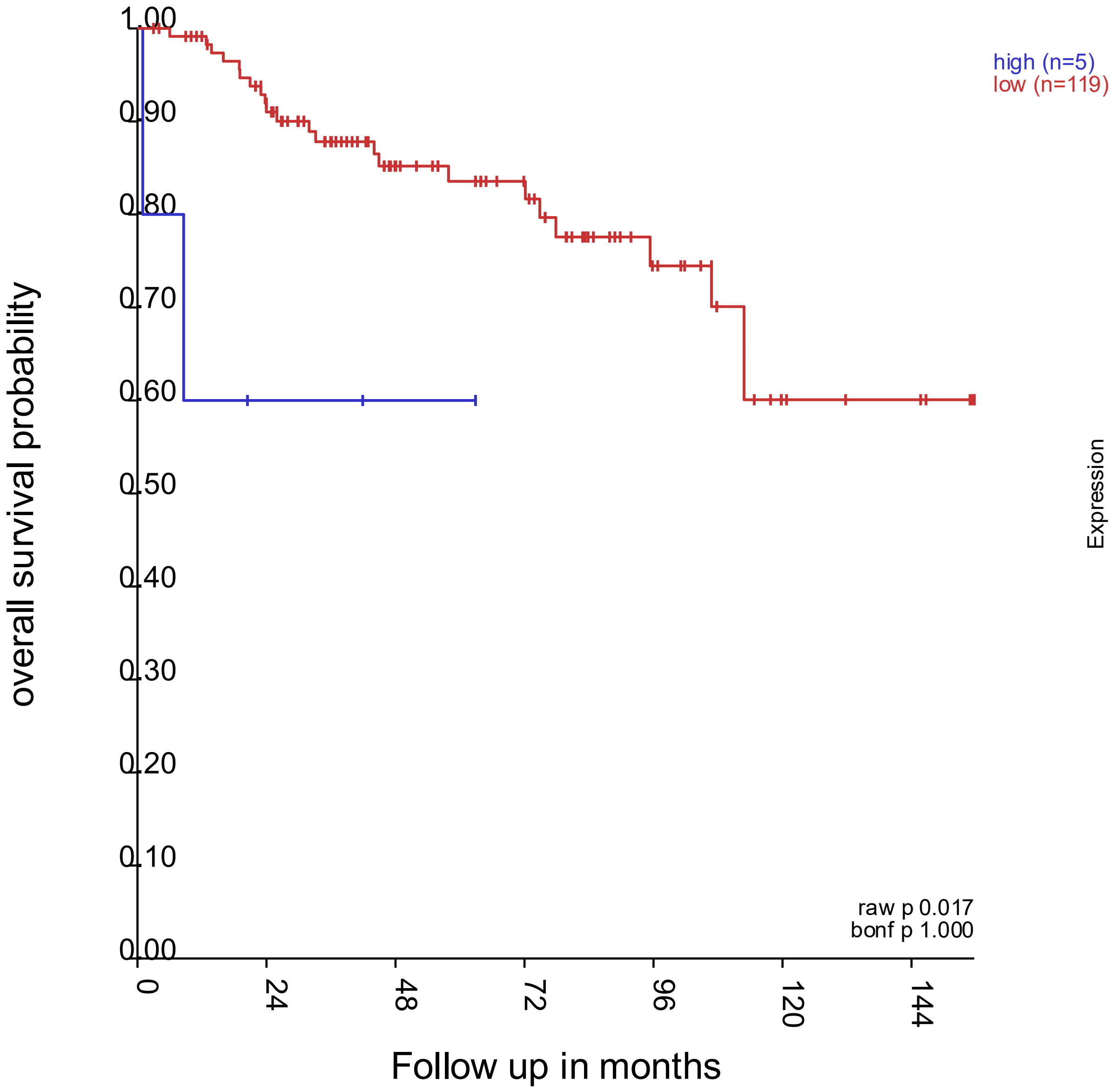

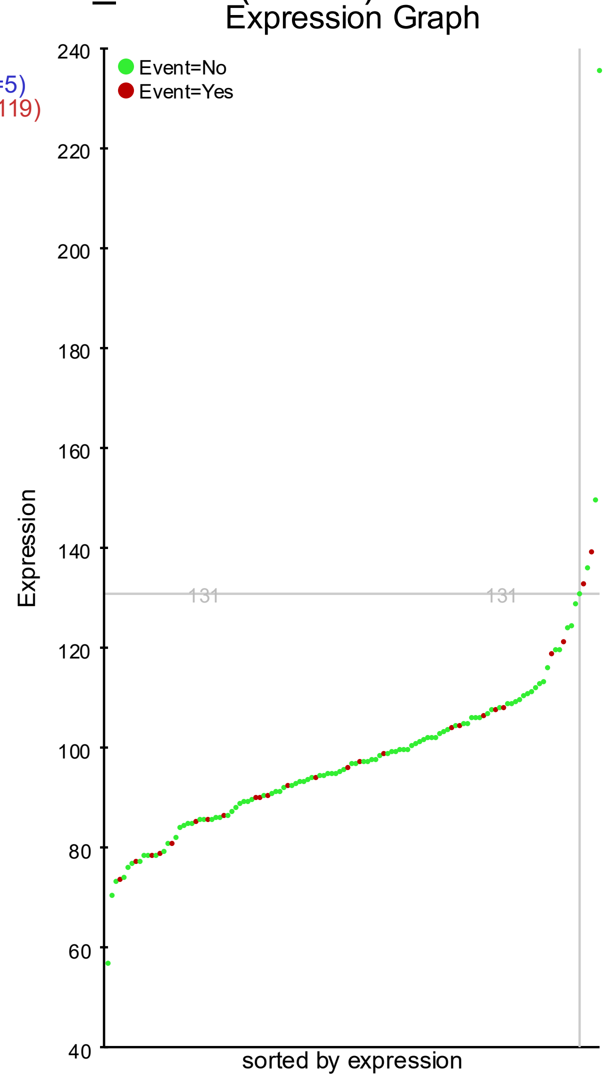

SHH M1

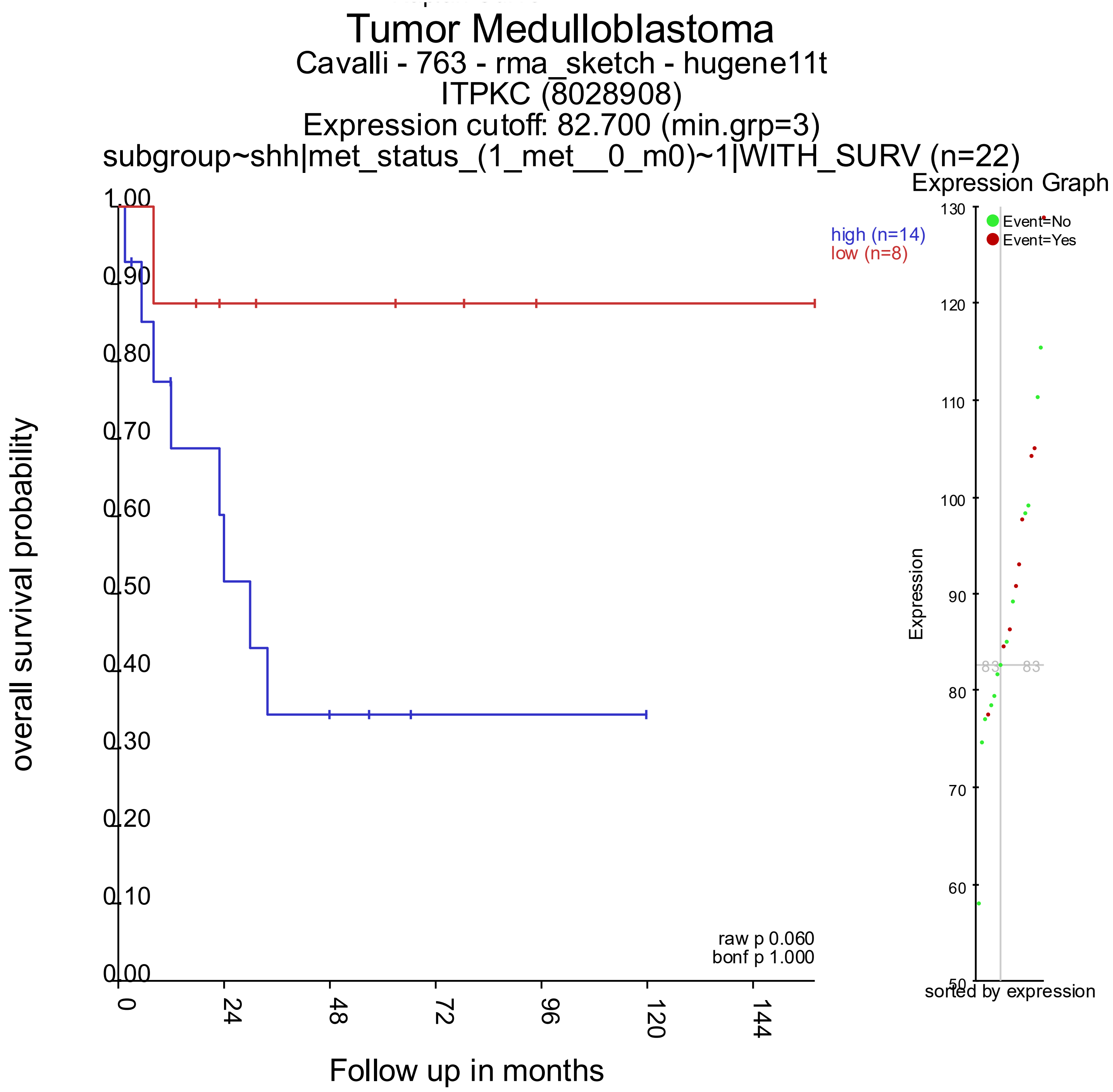

Tumor Medulloblastoma
Cavalli - 763 - rma_sketch - hugene11t
ITPKC (8028908)
Expression cutoff: 82.700 (min.grp=3)
subgroup~shh|met_status_(1_met__0_m0)~1|WITH_SURV (n=22)
overall survival probability
1.00
0.90
0.80
0.70
0.60
0.50
0.40
0.30
0.20
0.10
0.00
0
24
48
72
96
120
144
Follow up in months
high (n=14)
low (n=8)
raw p 0.060
bonf p 1.000
Expression Graph
Event=No
Event=Yes
130
120
110
100
90
80
70
60
50
Expression
83
83
sorted by expression

GROUP4 M0

Tumor Medulloblastoma
Cavalli - 763 - rma_sketch - hugene11t
ITPKC (8028908)
Expression cutoff: 128.400 (min.grp=3)
subgroup~group4|met_status_(1_met__0_m0)~0|WITH_SURV (n=145)

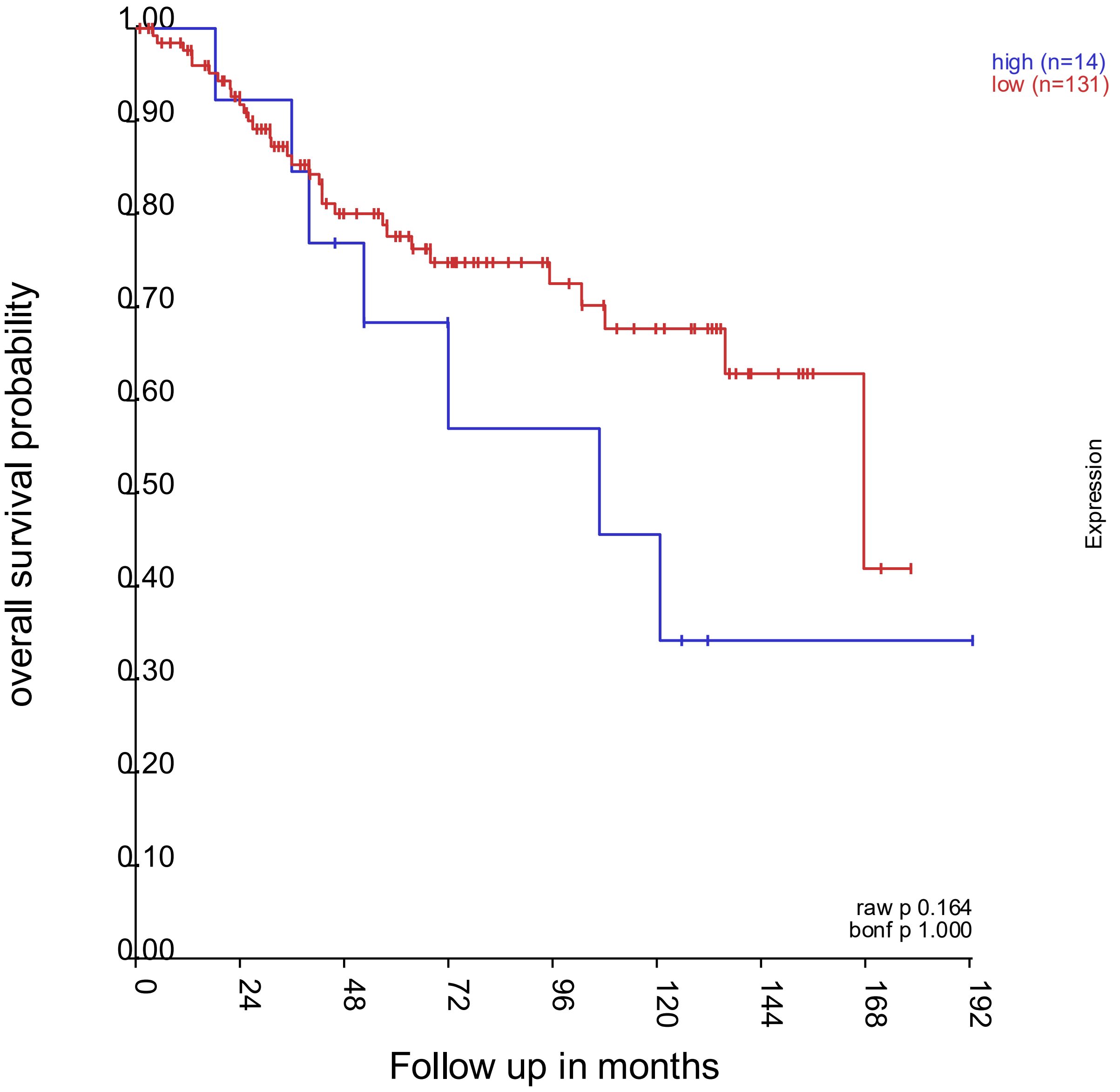

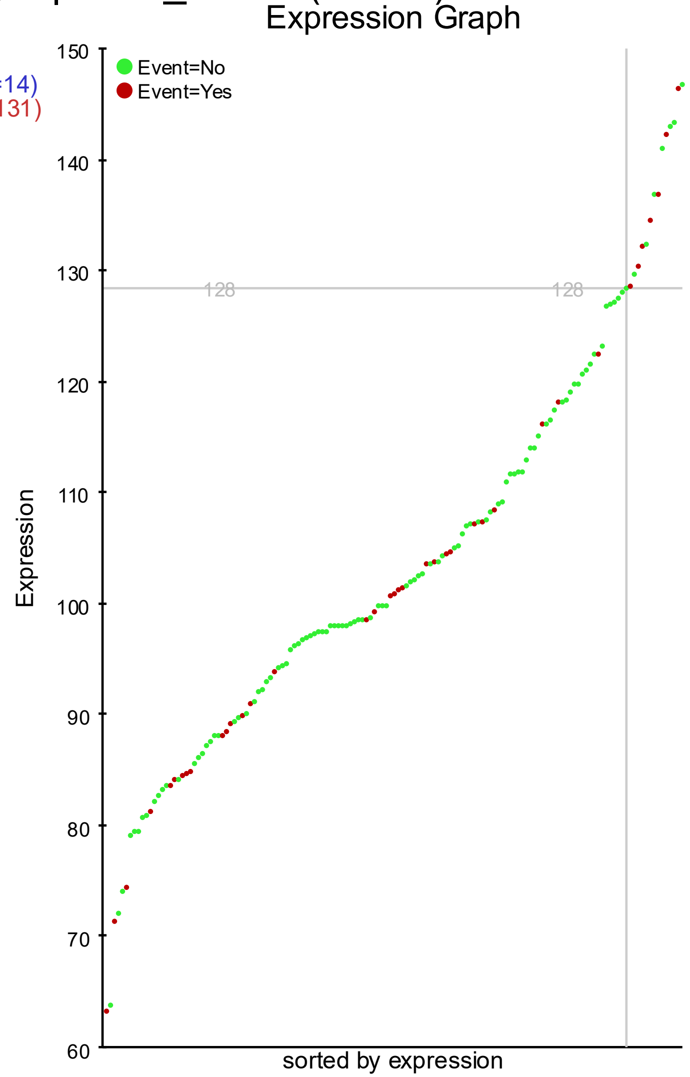

GROUP4 M1

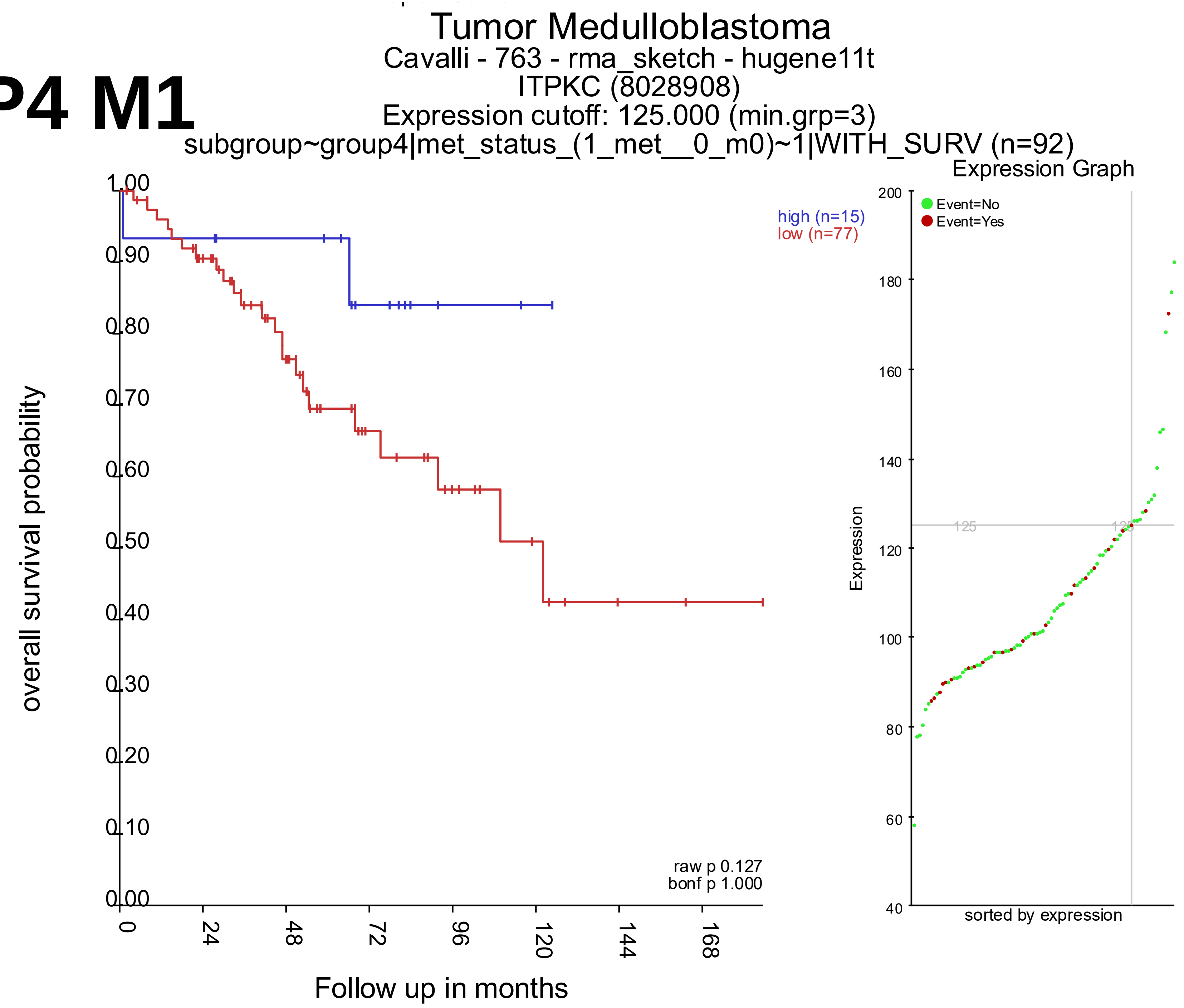

Tumor Medulloblastoma
Cavalli - 763 - rma_sketch - hugene11t
ITPKC (8028908)
Expression cutoff: 125.000 (min.grp=3)
subgroup~group4|met_status_(1_met__0_m0)~1|WITH_SURV (n=92)
overall survival probability
1.00
0.90
0.80
0.70
0.60
0.50
0.40
0.30
0.20
0.10
0.00
0
24
48
72
96
120
144
168
Follow up in months
high (n=15)
low (n=77)
raw p 0.127
bonf p 1.000
Expression Graph
Event=No
Event=Yes
Expression
200
180
160
140
120
100
80
60
40
125
sorted by expression

GROUP3 M0

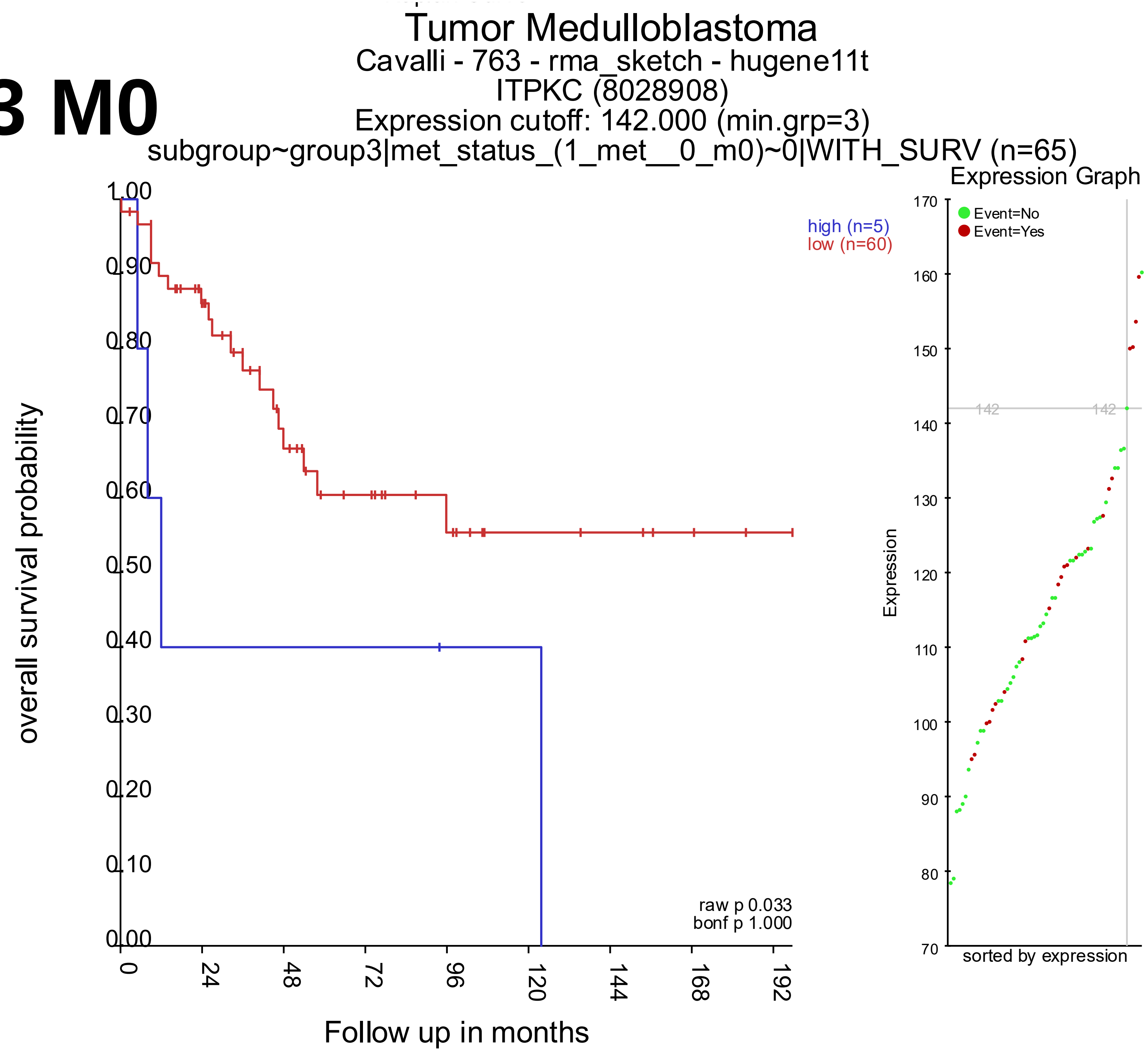

Tumor Medulloblastoma
Cavalli - 763 - rma_sketch - hugene11t
ITPKC (8028908)
Expression cutoff: 142.000 (min.grp=3)
subgroup~group3|met_status_(1_met__0_m0)~0|WITH_SURV (n=65)
overall survival probability
1.00
0.90
0.80
0.70
0.60
0.50
0.40
0.30
0.20
0.10
0.00
0
24
48
72
96
120
144
168
192
Follow up in months
high (n=5)
low (n=60)
raw p 0.033
bonf p 1.000
Expression Graph
Event=No
Event=Yes
Expression
170
160
150
140
130
120
110
100
90
80
70
142
142
sorted by expression

GROUP3 M1

Tumor Medulloblastoma
Cavalli - 763 - rma_sketch - hugene11t
ITPKC (8028908)
Expression cutoff: 125.800 (min.grp=3)
subgroup~group3|met_status_(1_met__0_m0)~1|WITH_SURV (n=41)

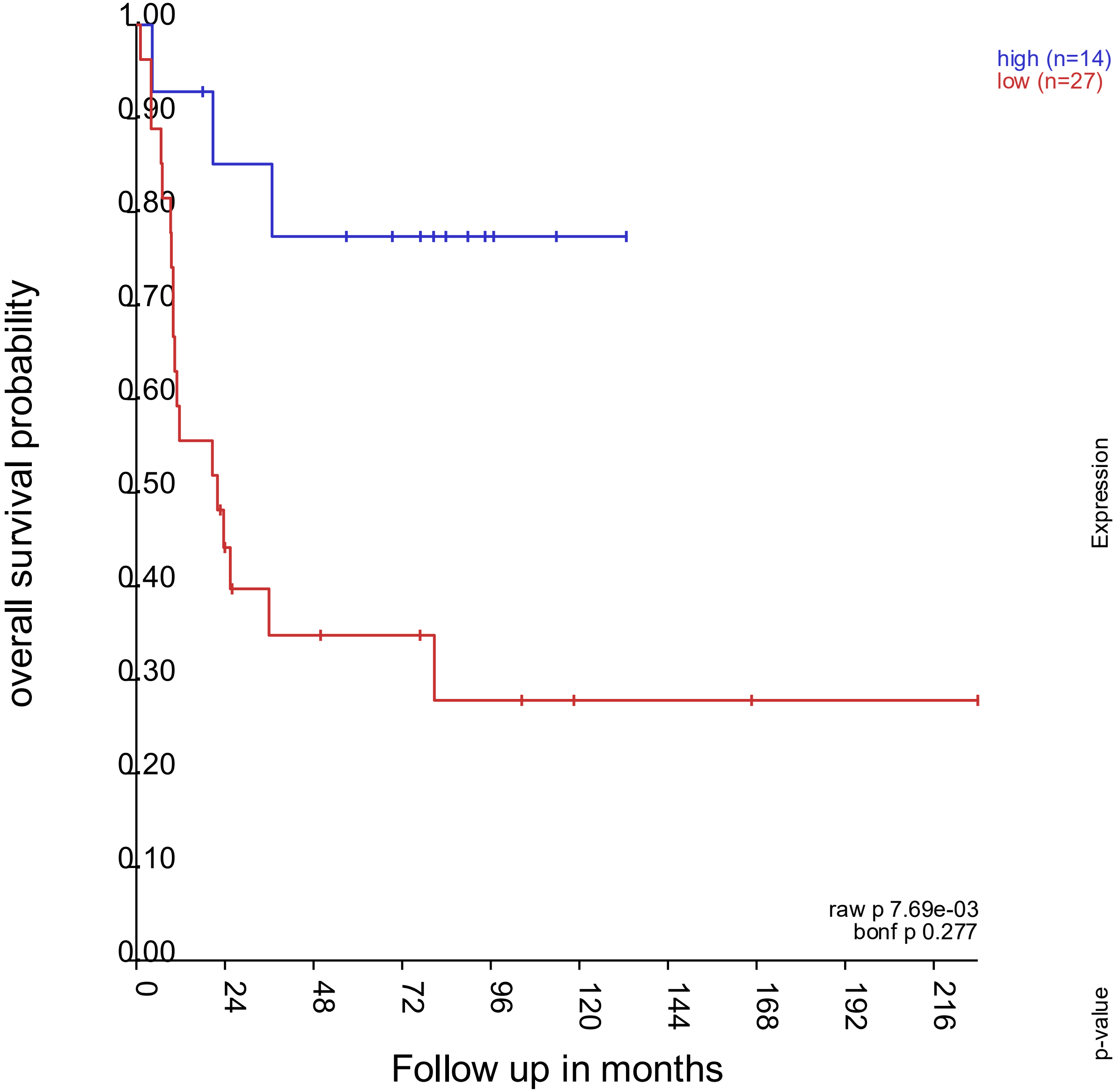

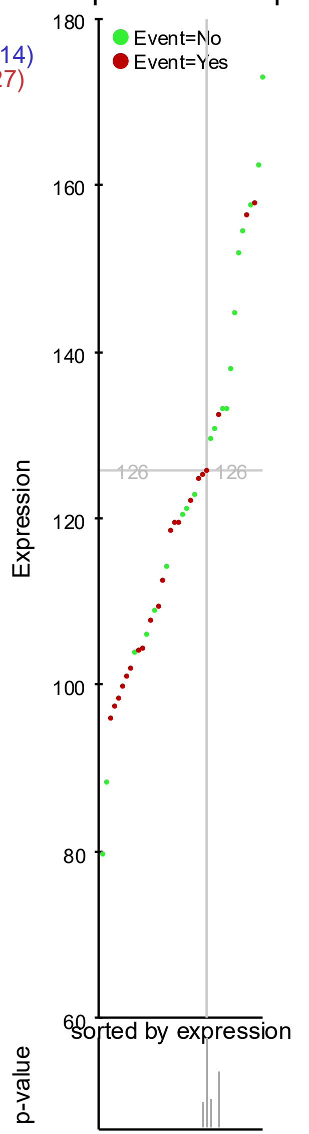

PRKCA

WNT M0

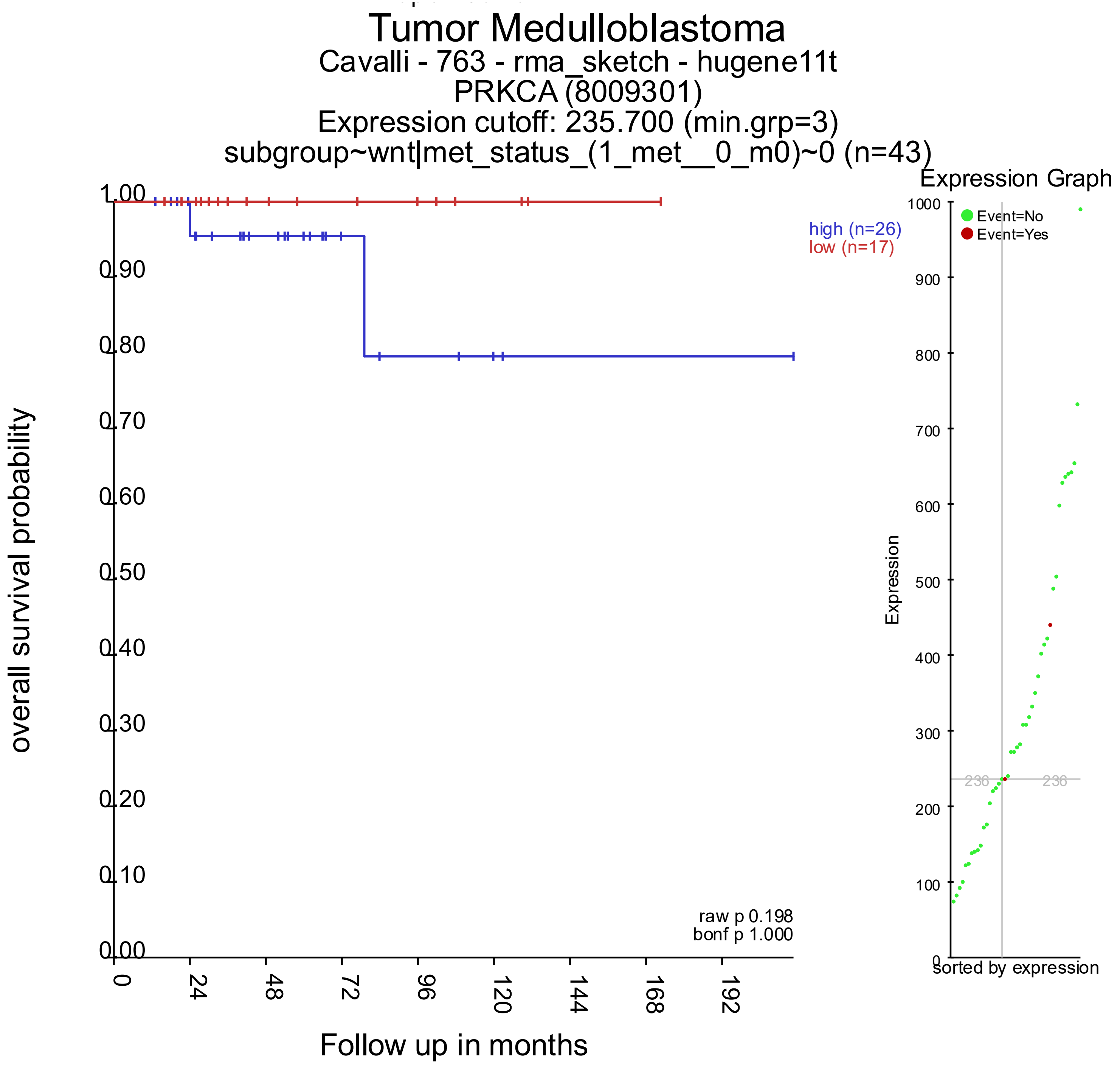

Tumor Medulloblastoma
Cavalli - 763 - rma_sketch - hugene11t
PRKCA (8009301)
Expression cutoff: 235.700 (min.grp=3)
subgroup~wnt|met_status_(1_met__0_m0)~0 (n=43)
high (n=26)
low (n=17)
overall survival probability
1.00
0.90
0.80
0.70
0.60
0.50
0.40
0.30
0.20
0.10
0.00
0
24
48
72
96
120
144
168
192
Follow up in months
raw p 0.198
bonf p 1.000
Expression Graph
Event=No
Event=Yes
Expression
1000
900
800
700
600
500
400
300
200
100
0
236
236
sorted by expression

WNT M1

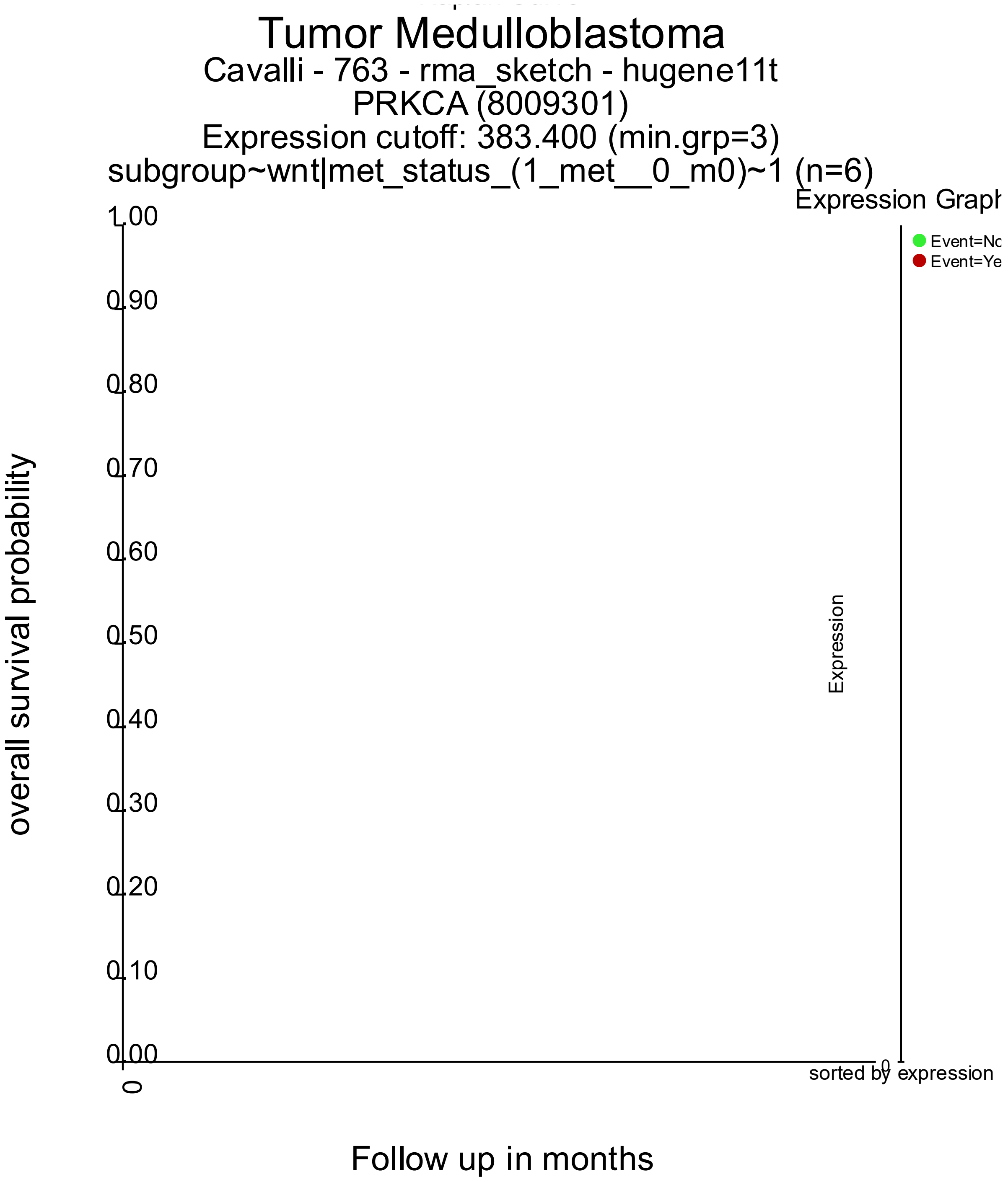
Tumor Medulloblastoma
Cavalli - 763 - rma_sketch - hugene11t
PRKCA (8009301)
Expression cutoff: 383.400 (min.grp=3)
subgroup~wnt|met_status_(1_met__0_m0)~1 (n=6)
Expression Graph
Event=No
Event=Ye
1.00
0.90
0.80
0.70
0.60
0.50
0.40
0.30
0.20
0.10
0.00
overall survival probability
0
Follow up in months
Expression
0
sorted by expression

SHH M0

Tumor Medulloblastoma
Cavalli - 763 - rma_sketch - hugene11t
PRKCA (8009301)
Expression cutoff: 704.500 (min.grp=3)
subgroup~shh|met_status_(1_met__0_m0)~0|WITH_SURV (n=124)

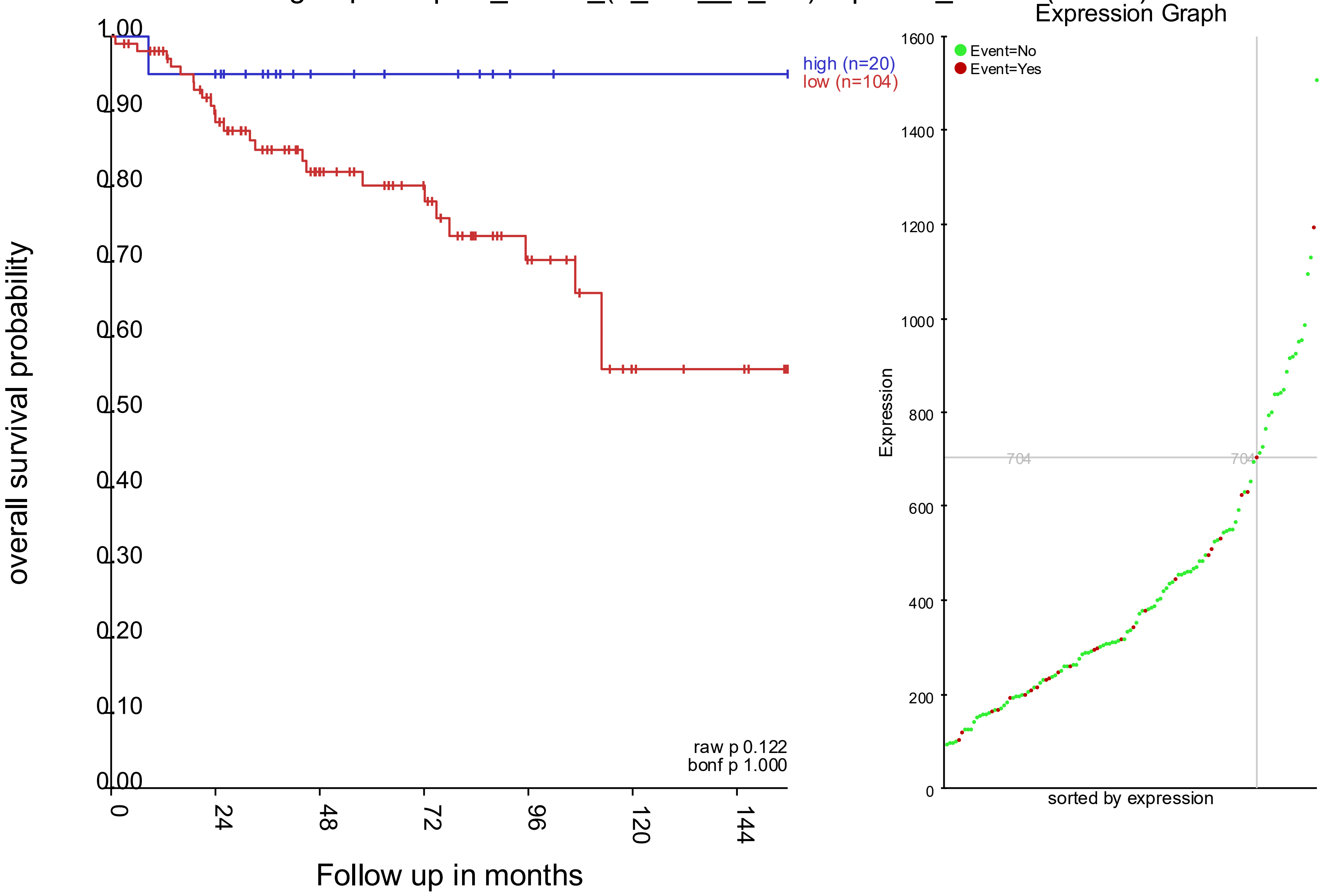

SHH M1

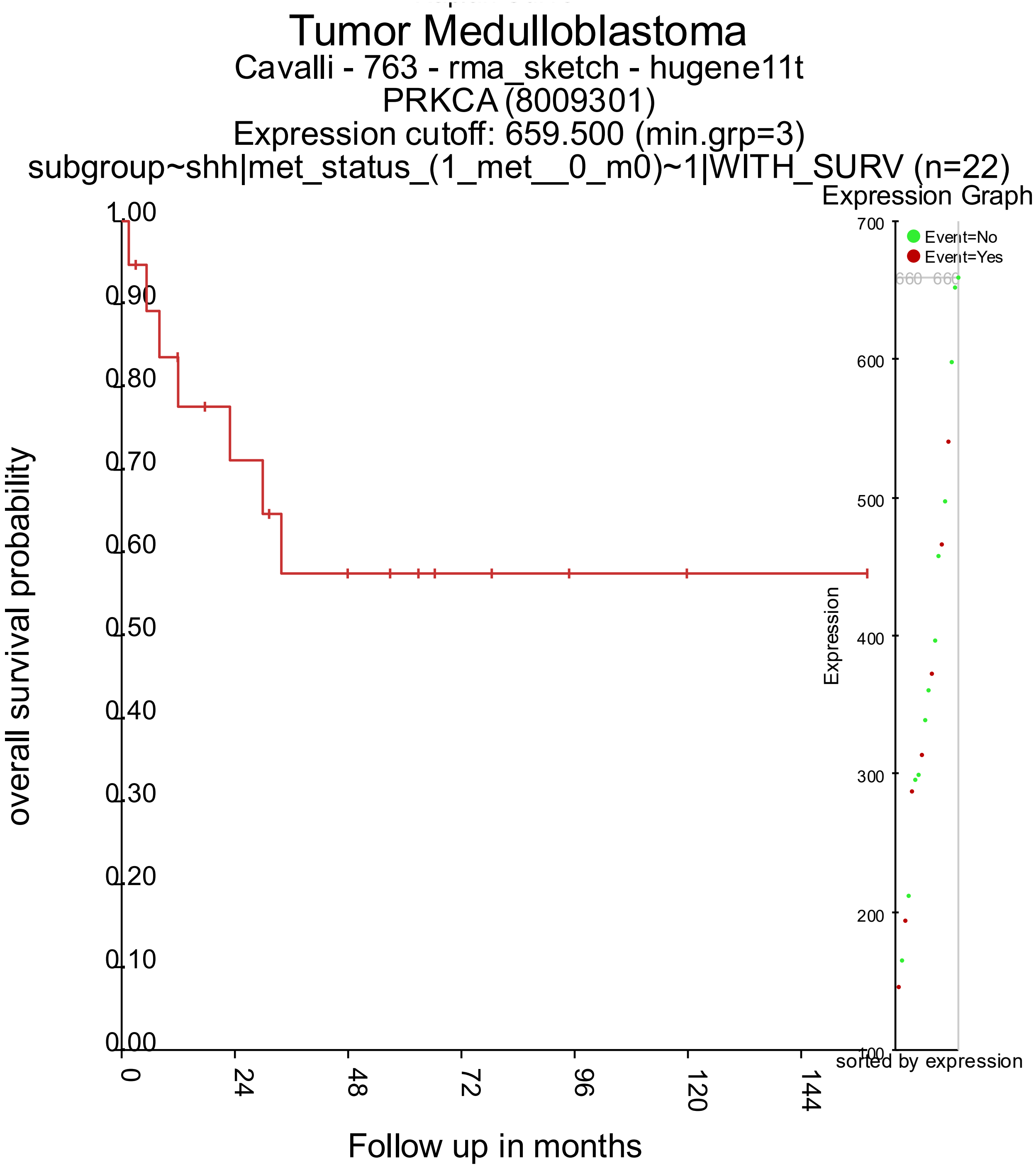

Tumor Medulloblastoma
Cavalli - 763 - rma_sketch - hugene11t
PRKCA (8009301)
Expression cutoff: 659.500 (min.grp=3)
subgroup~shh|met_status_(1_met__0_m0)~1|WITH_SURV (n=22)
overall survival probability
1.00
0.90
0.80
0.70
0.60
0.50
0.40
0.30
0.20
0.10
0.00
0
24
48
72
96
120
144
Follow up in months
Expression Graph
Event=No
Event=Yes
660
700
600
500
400
300
200
100
Expression
sorted by expression

GROUP4 M0

Tumor Medulloblastoma
Cavalli - 763 - rma_sketch - hugene11t
PRKCA (8009301)
Expression cutoff: 413.100 (min.grp=3)
subgroup~group4|met_status_(1_met__0_m0)~0|WITH_SURV (n=145)

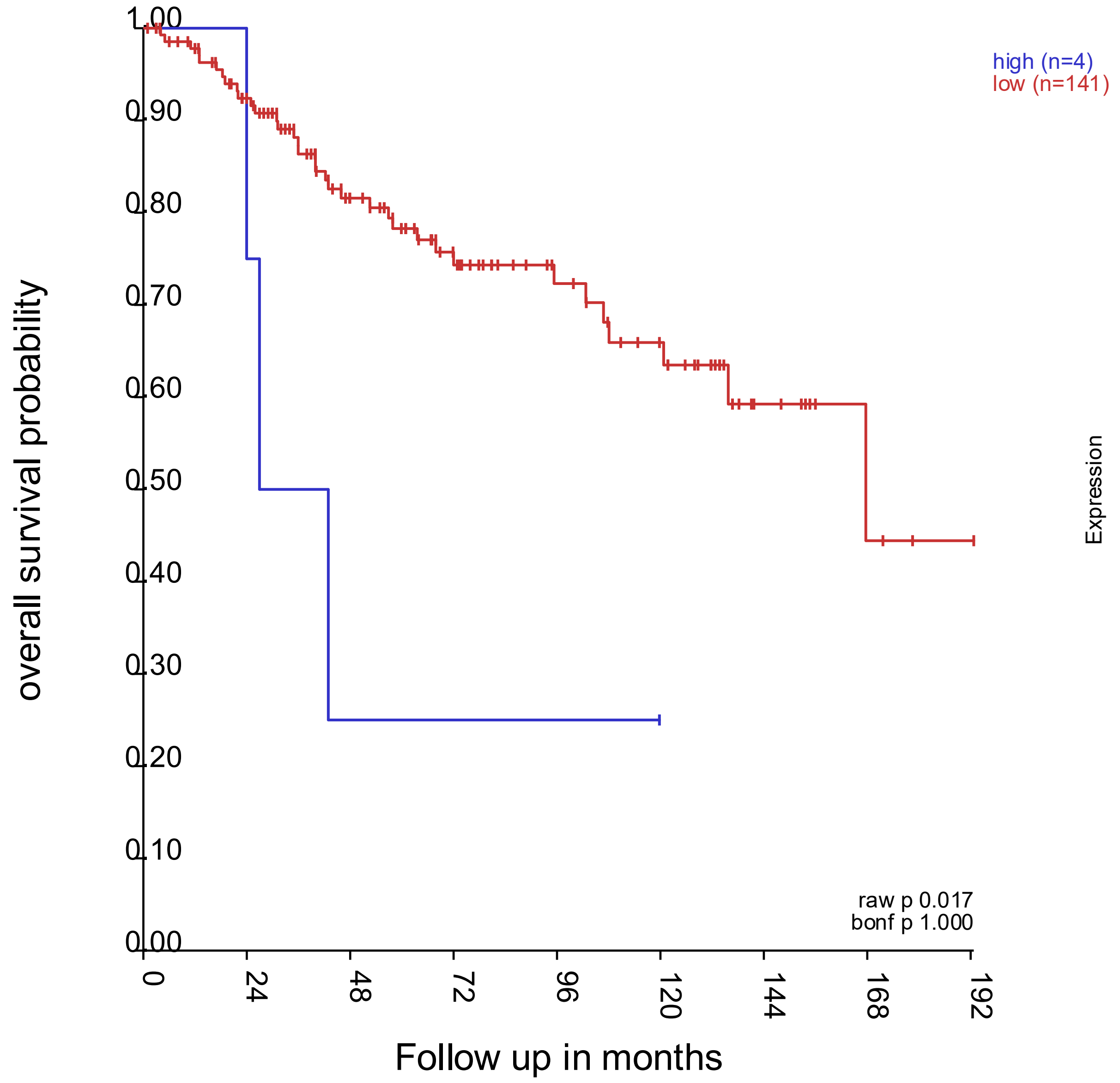

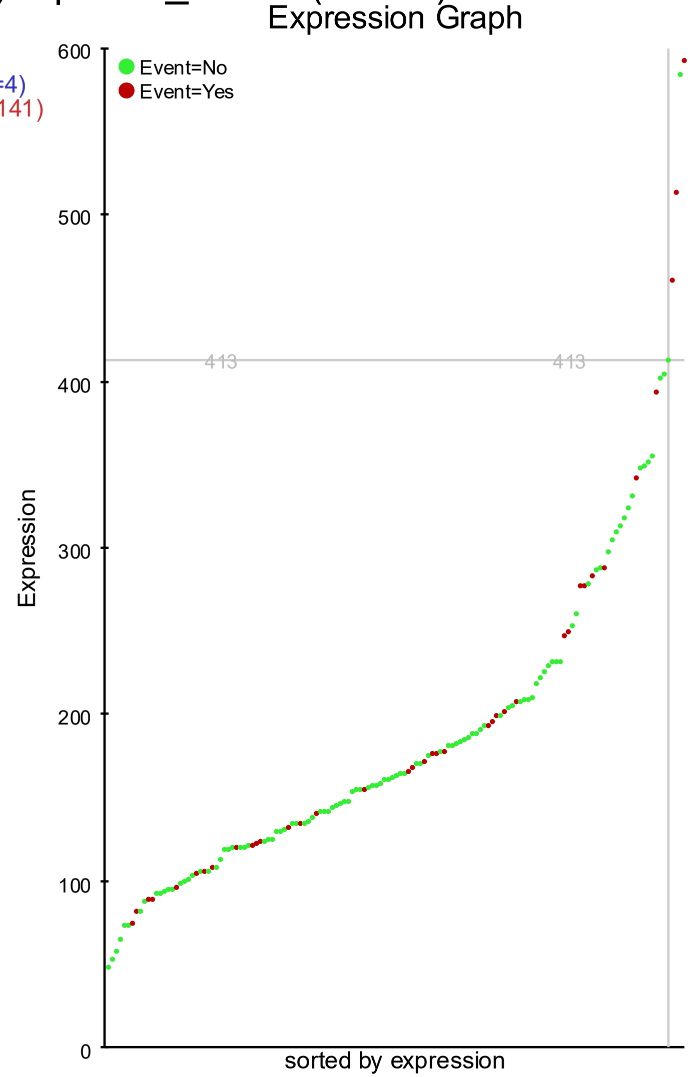

GROUP4 M1

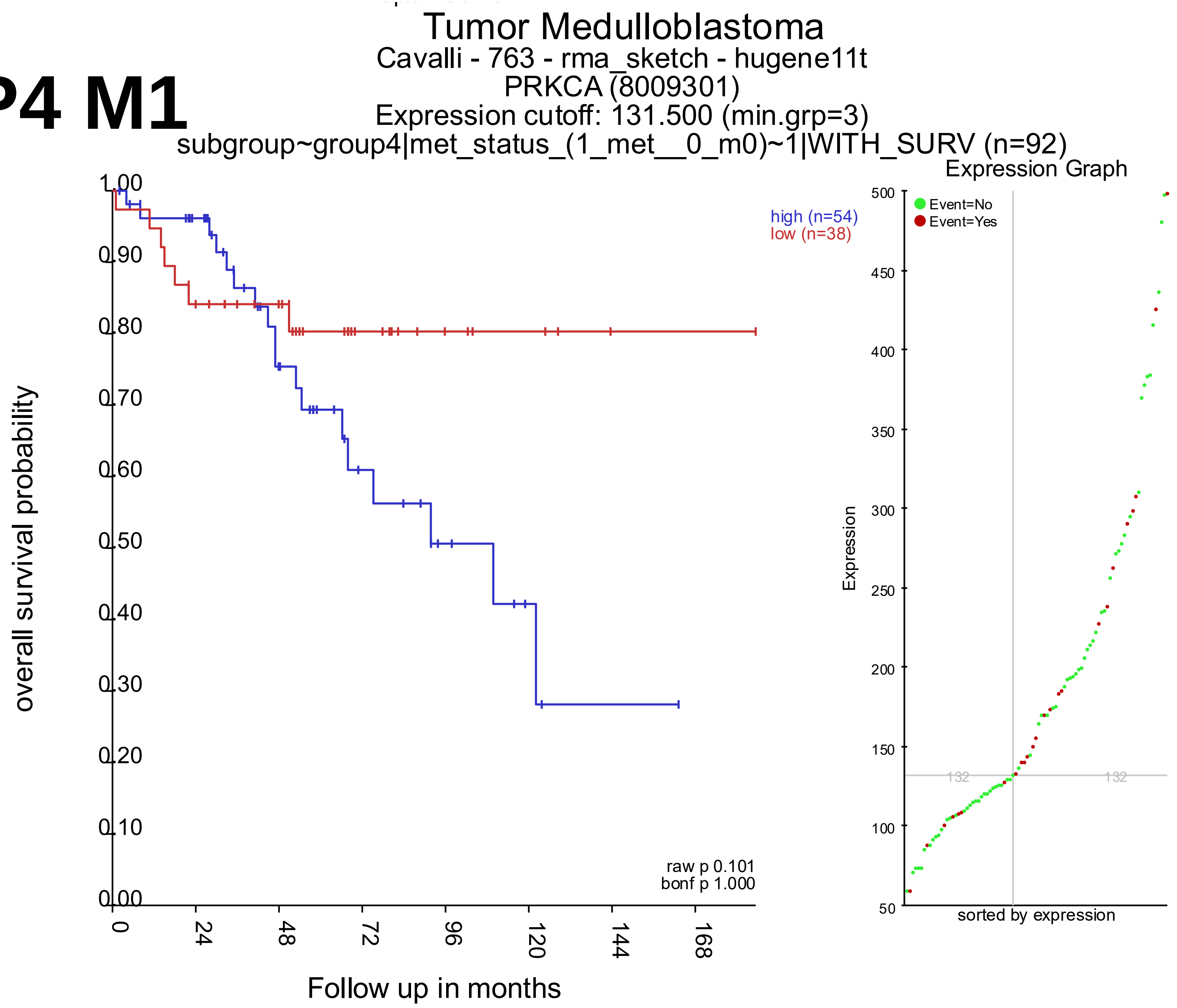

Tumor Medulloblastoma
Cavalli - 763 - rma_sketch - hugene11t
PRKCA (8009301)
Expression cutoff: 131.500 (min.grp=3)
subgroup~group4|met_status_(1_met__0_m0)~1|WITH_SURV (n=92)
overall survival probability
1.00
0.90
0.80
0.70
0.60
0.50
0.40
0.30
0.20
0.10
0.00
0
24
48
72
96
120
144
168
Follow up in months
high (n=54)
low (n=38)
raw p 0.101
bonf p 1.000
Expression Graph
Event=No
Event=Yes
Expression
500
450
400
350
300
250
200
150
100
50
132
132
sorted by expression

GROUP3 M0

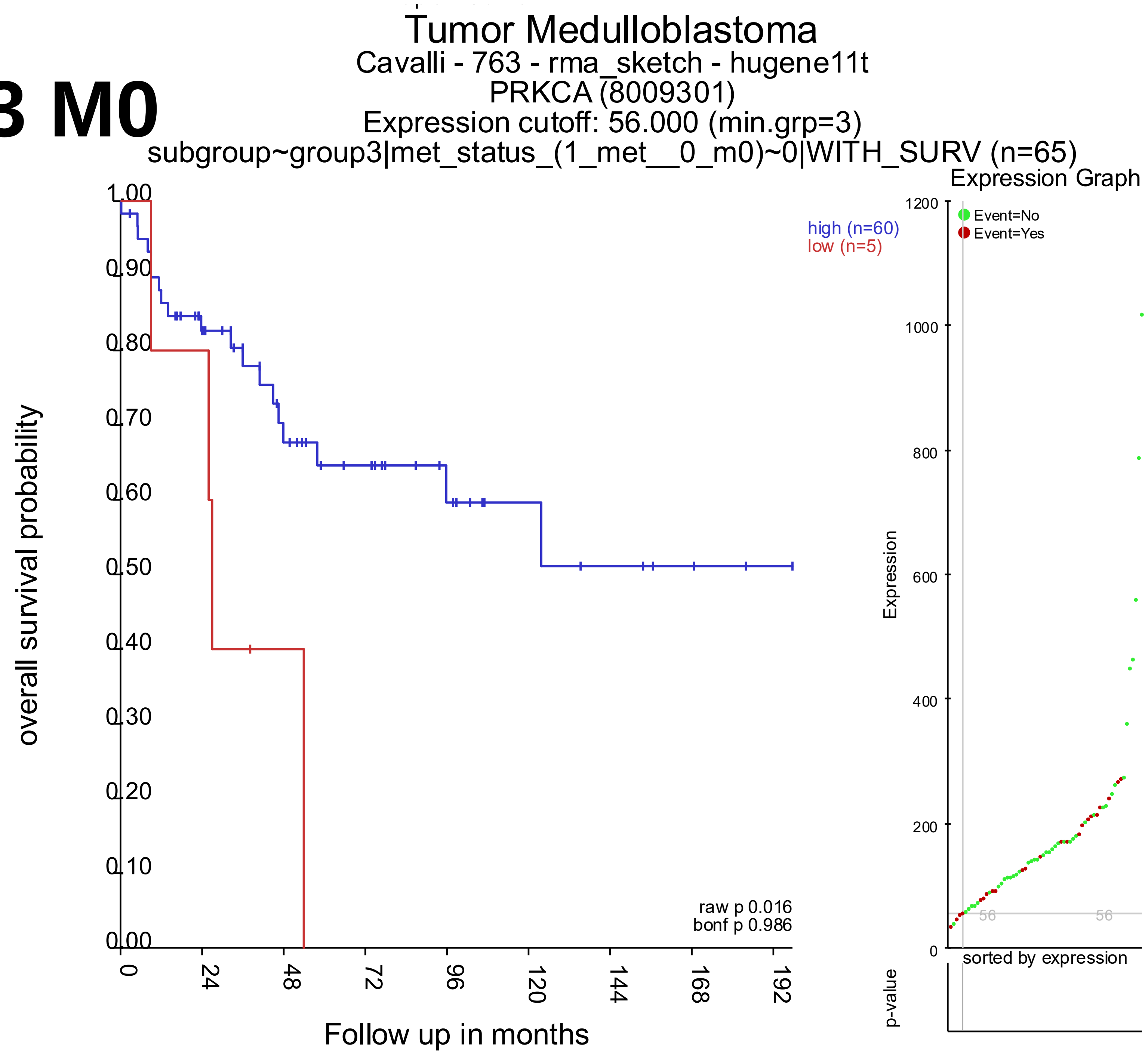

Tumor Medulloblastoma
Cavalli - 763 - rma_sketch - hugene11t
PRKCA (8009301)
Expression cutoff: 56.000 (min.grp=3)
subgroup~group3|met_status_(1_met__0_m0)~0|WITH_SURV (n=65)
overall survival probability
1.00
0.90
0.80
0.70
0.60
0.50
0.40
0.30
0.20
0.10
0.00
high (n=60)
low (n=5)
raw p 0.016
bonf p 0.986
0
24
48
72
96
120
144
168
192
Follow up in months
Expression Graph
Event=No
Event=Yes
Expression
1200
1000
800
600
400
200
0
56
56
sorted by expression
p-value

GROUP3 M1

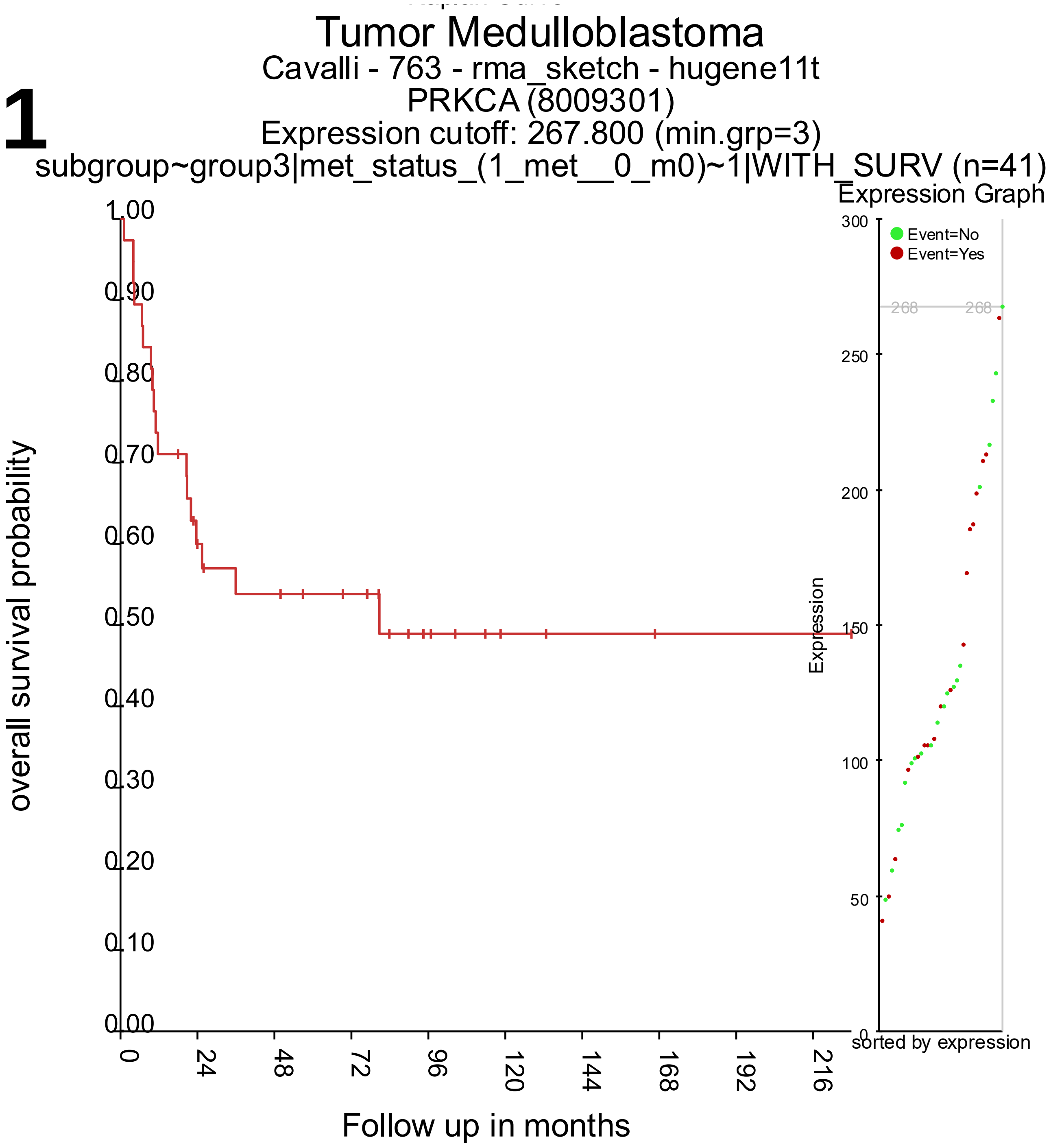

Tumor Medulloblastoma
Cavalli - 763 - rma_sketch - hugene11t
PRKCA (8009301)
Expression cutoff: 267.800 (min.grp=3)
subgroup~group3|met_status_(1_met__0_m0)~1|WITH_SURV (n=41)
overall survival probability
1.00
0.90
0.80
0.70
0.60
0.50
0.40
0.30
0.20
0.10
0.00
0
24
48
72
96
120
144
168
192
216
Follow up in months
Expression Graph
Event=No
Event=Yes
300
250
200
150
100
50
0
268
268
Expression
sorted by expression

PRKCB

WNT M0

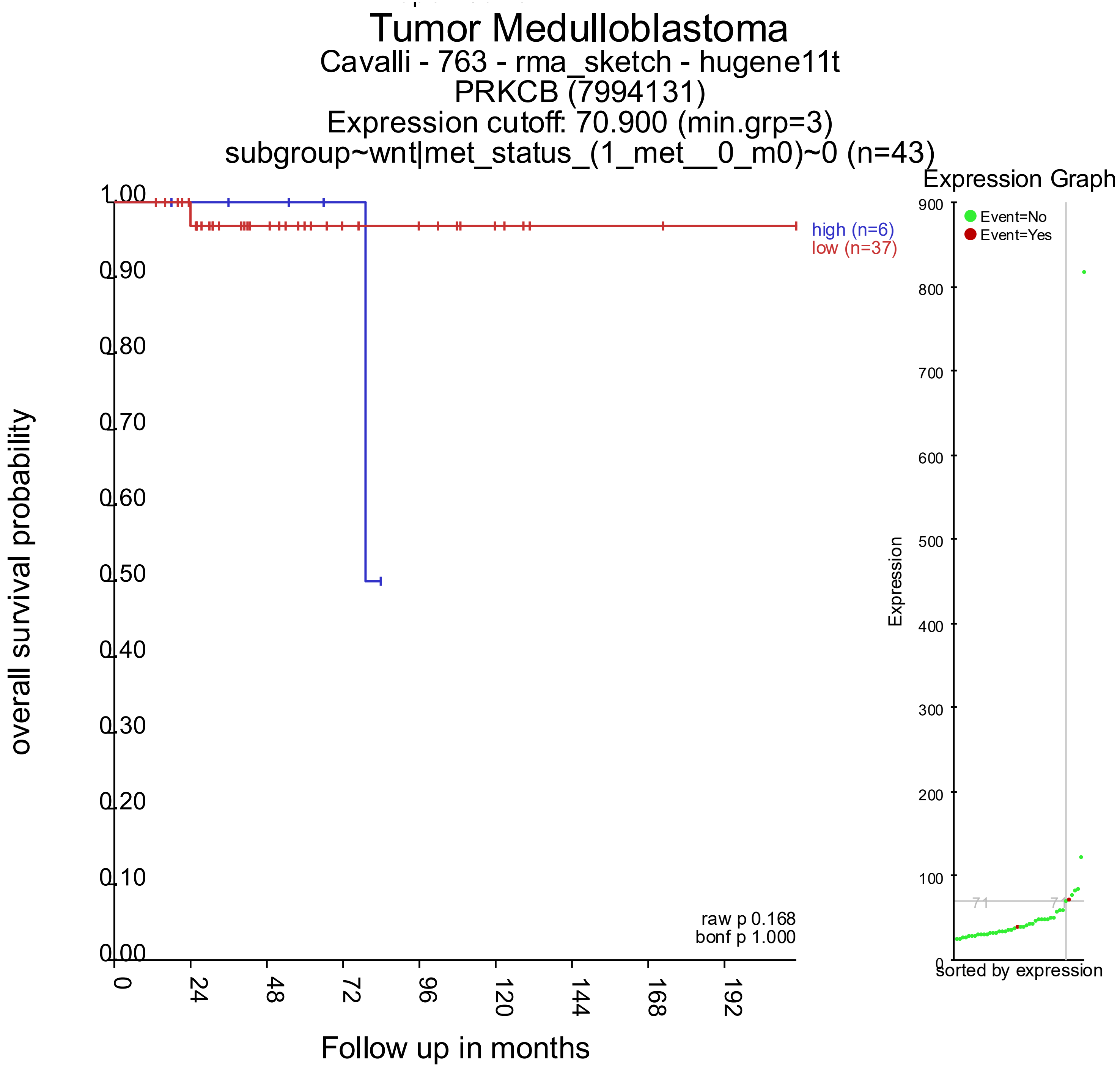

Tumor Medulloblastoma
Cavalli - 763 - rma_sketch - hugene11t
PRKCB (7994131)
Expression cutoff: 70.900 (min.grp=3)
subgroup~wnt|met_status_(1_met__0_m0)~0 (n=43)
overall survival probability
1.00
0.90
0.80
0.70
0.60
0.50
0.40
0.30
0.20
0.10
0.00
0
24
48
72
96
120
144
168
192
Follow up in months
high (n=6)
low (n=37)
raw p 0.168
bonf p 1.000
Expression Graph
Event=No
Event=Yes
Expression
900
800
700
600
500
400
300
200
100
0
sorted by expression

WNT M1

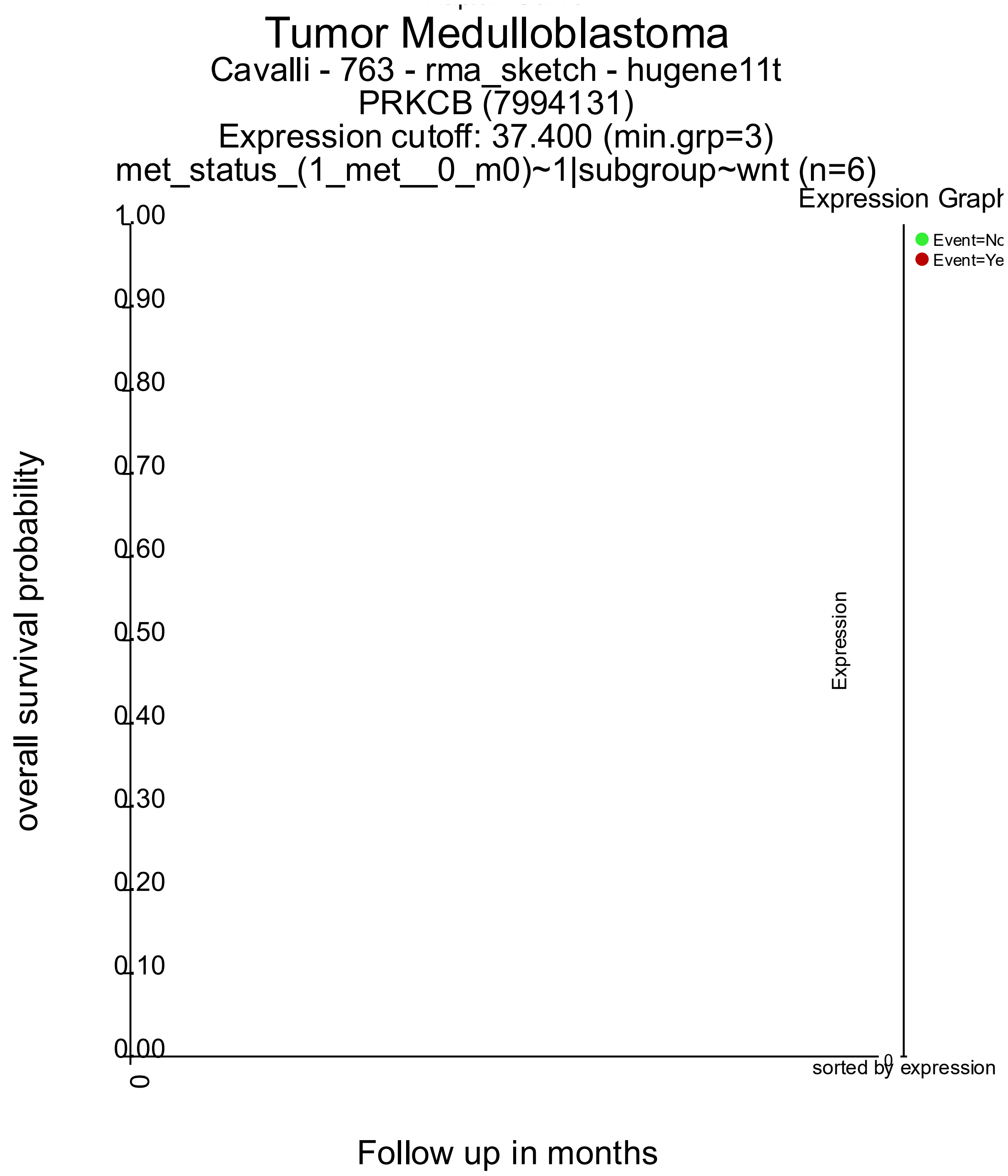

Tumor Medulloblastoma
Cavalli - 763 - rma_sketch - hugene11t
PRKCB (7994131)
Expression cutoff: 37.400 (min.grp=3)
met_status_(1_met__0_m0)~1|subgroup~wnt (n=6)
Expression Graph
Event=No
Event=Ye
overall survival probability
1.00
0.90
0.80
0.70
0.60
0.50
0.40
0.30
0.20
0.10
0.00
0
Follow up in months
Expression
0
sorted by expression

SHH M0

Tumor Medulloblastoma
Cavalli - 763 - rma_sketch - hugene11t
PRKCB (7994131)
Expression cutoff: 148.200 (min.grp=3)
subgroup~shh|met_status_(1_met__0_m0)~0|WITH_SURV (n=124)

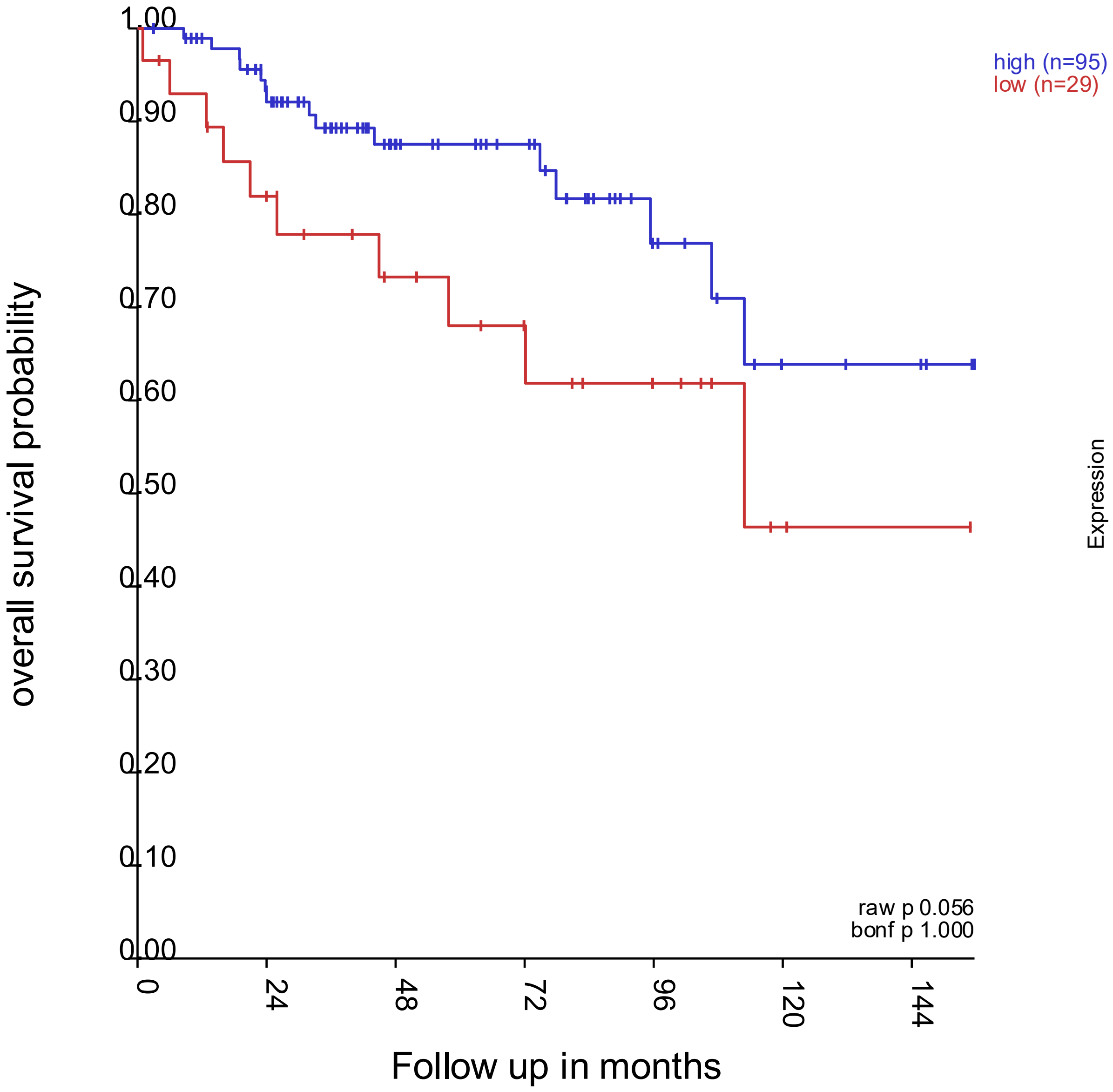

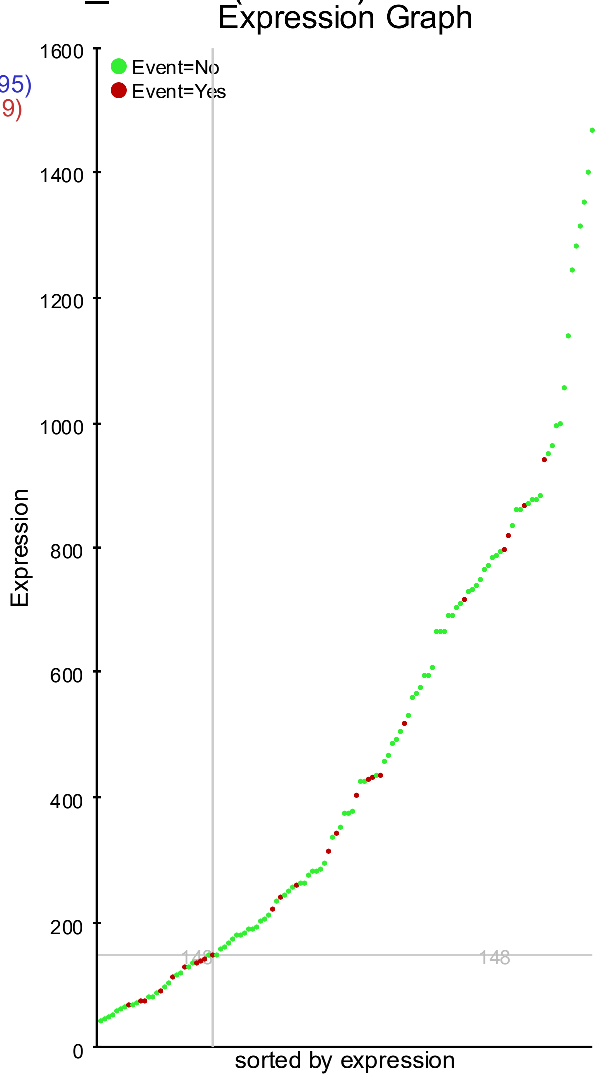

SHH M1

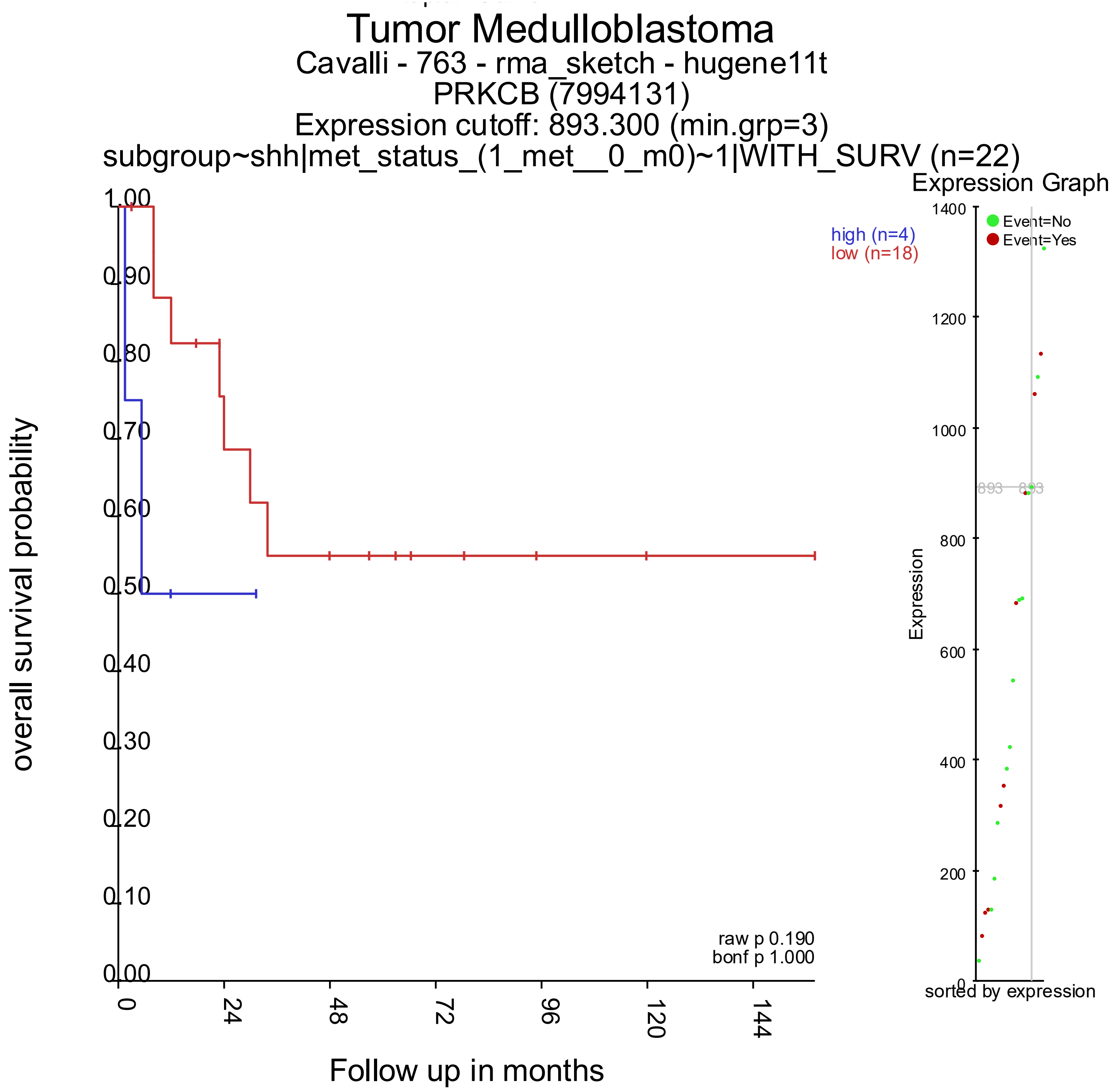

Tumor Medulloblastoma
Cavalli - 763 - rma_sketch - hugene11t
PRKCB (7994131)
Expression cutoff: 893.300 (min.grp=3)
subgroup~shh|met_status_(1_met__0_m0)~1|WITH_SURV (n=22)
overall survival probability
1.00
0.90
0.80
0.70
0.60
0.50
0.40
0.30
0.20
0.10
0.00
0
24
48
72
96
120
144
Follow up in months
high (n=4)
low (n=18)
raw p 0.190
bonf p 1.000
Expression Graph
Event=No
Event=Yes
Expression
1400
1200
1000
893
800
600
400
200
0
sorted by expression

GROUP4 M0

Tumor Medulloblastoma
Cavalli - 763 - rma_sketch - hugene11t
PRKCB (7994131)
Expression cutoff: 352.400 (min.grp=3)
subgroup~group4|met_status_(1_met__0_m0)~0|WITH_SURV (n=145)

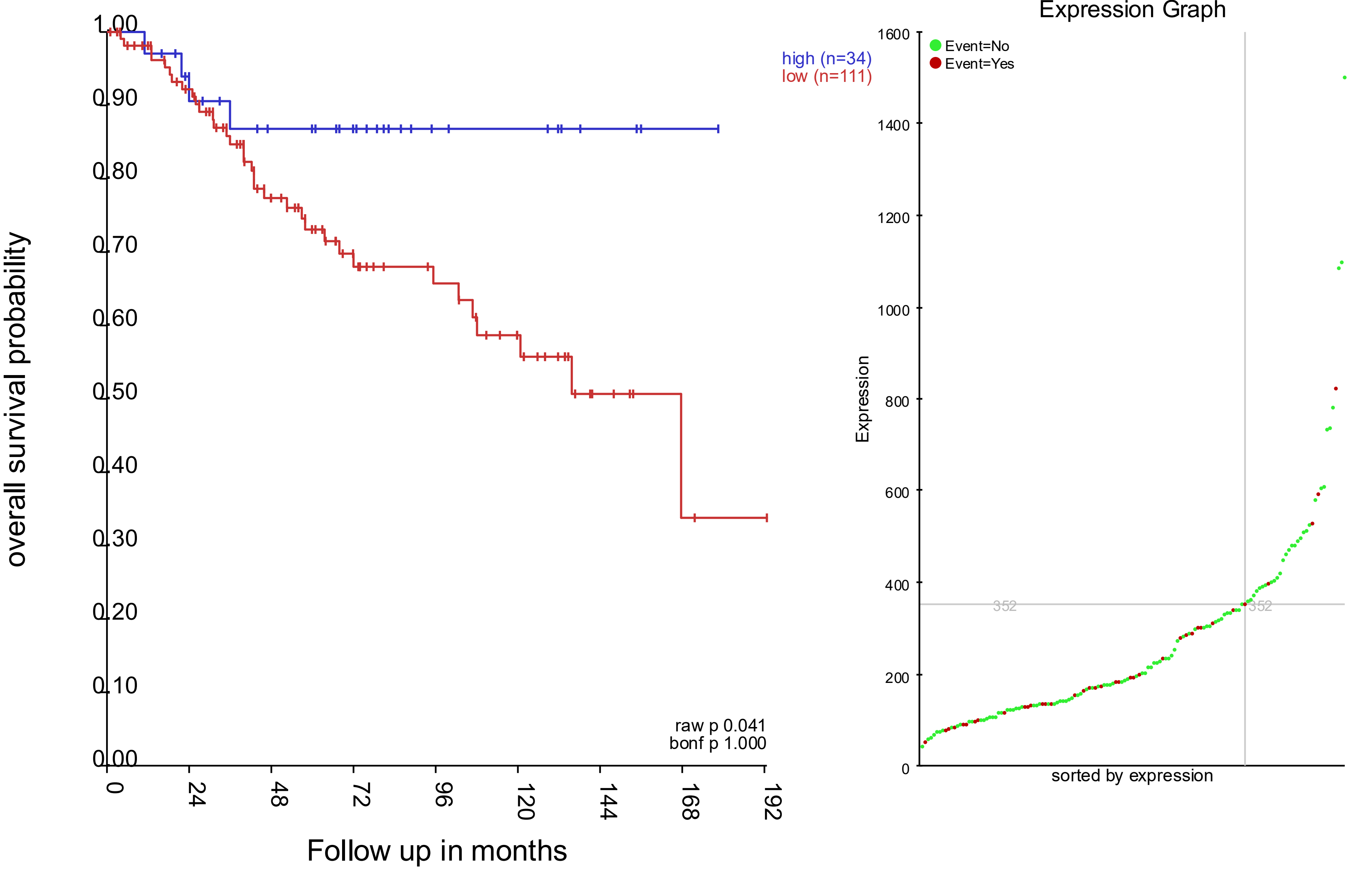

GROUP4 M1

Tumor Medulloblastoma

Cavalli - 763 - rma_sketch - hugene11t
PRKCB (7994131)
Expression cutoff: 95.200 (min.grp=3)
subgroup~group4|met_status_(1_met__0_m0)~1|WITH_SURV (n=92)

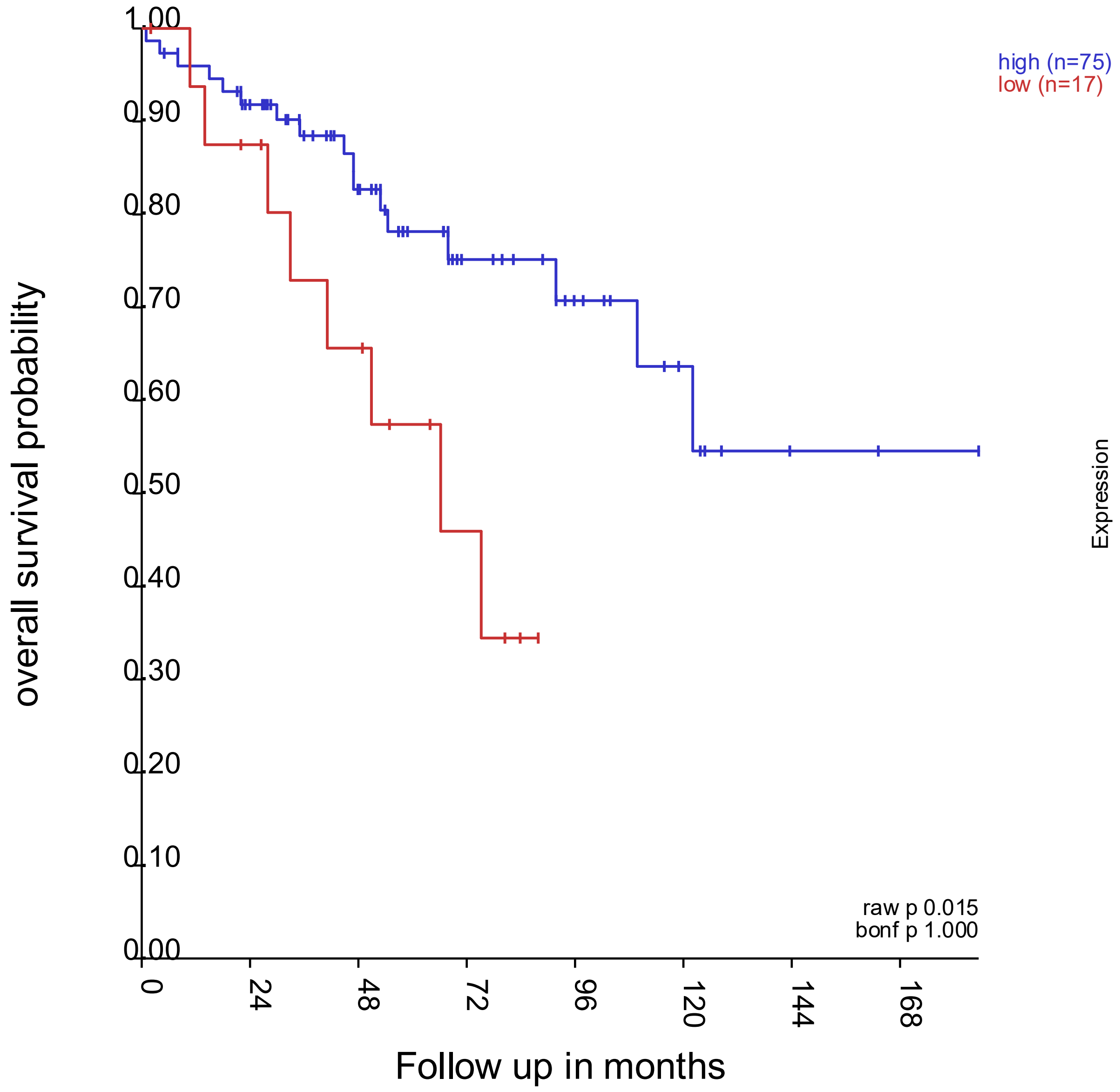

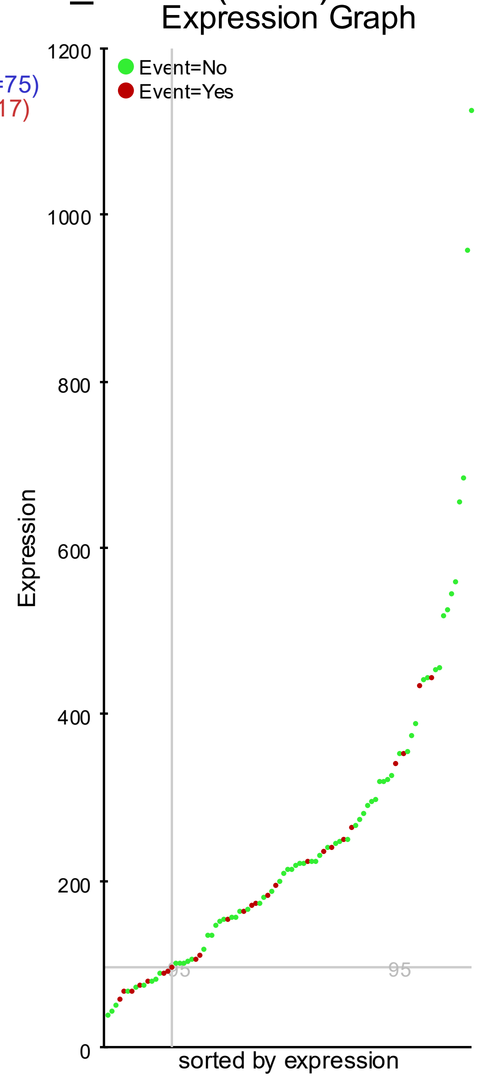

GROUP3 M0

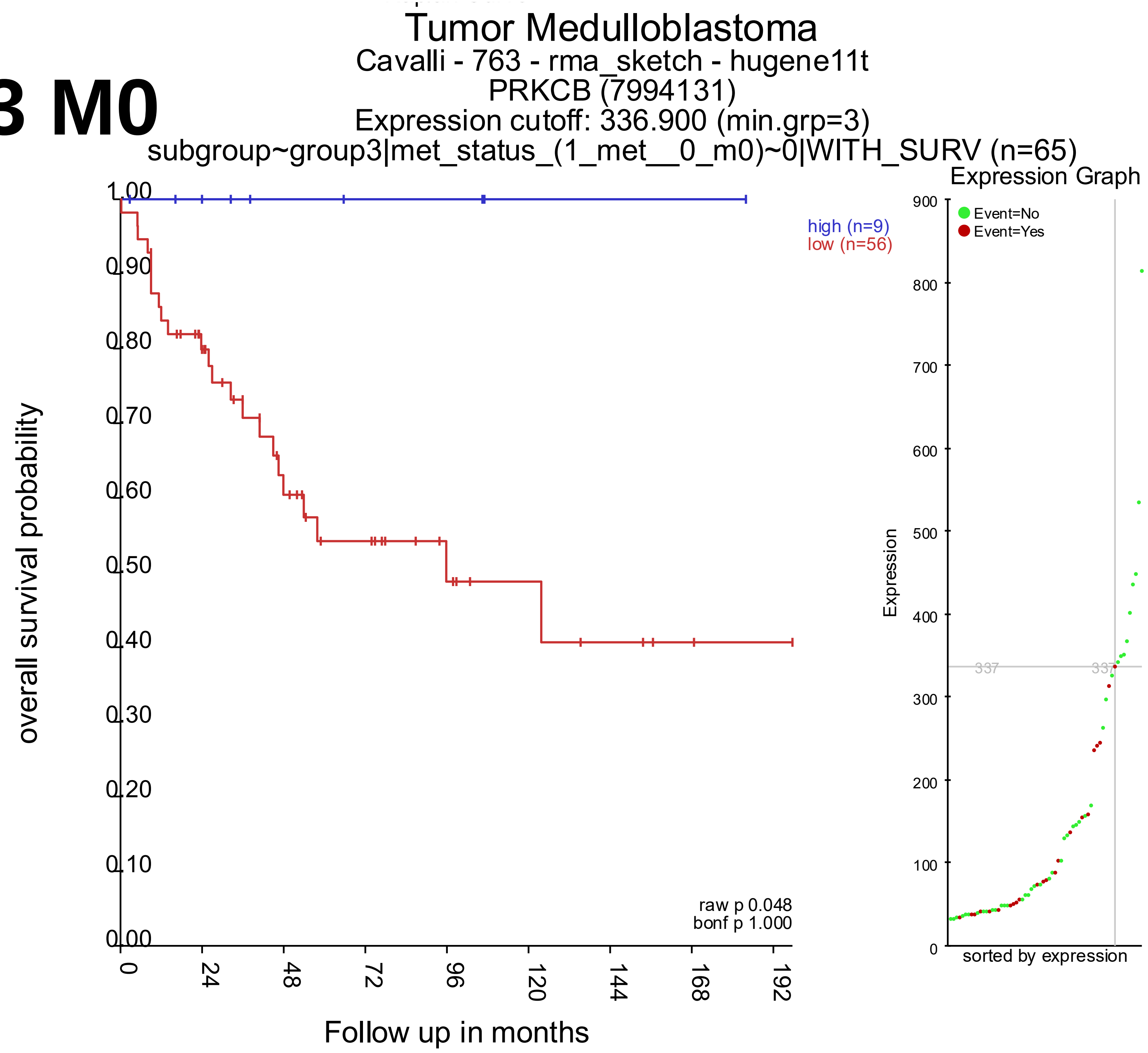
Tumor Medulloblastoma
Cavalli - 763 - rma_sketch - hugene11t
PRKCB (7994131)
Expression cutoff: 336.900 (min.grp=3)
subgroup~group3|met_status_(1_met__0_m0)~0|WITH_SURV (n=65)
overall survival probability
1.00
0.90
0.80
0.70
0.60
0.50
0.40
0.30
0.20
0.10
0.00
0
24
48
72
96
120
144
168
192
Follow up in months
high (n=9)
low (n=56)
raw p 0.048
bonf p 1.000
Expression Graph
Event=No
Event=Yes
Expression
900
800
700
600
500
400
300
200
100
0
337
337
sorted by expression

GROUP3 M1

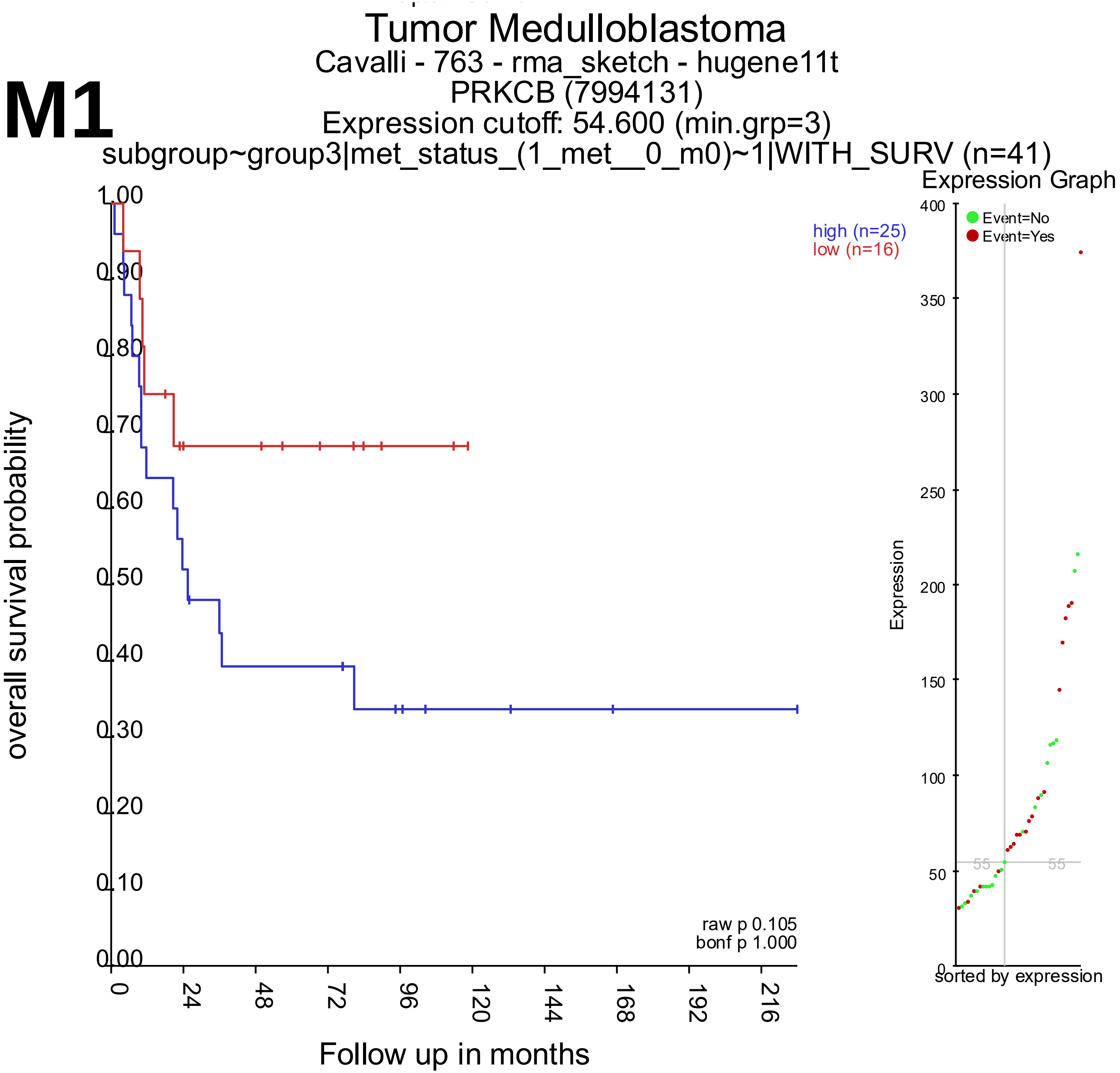

Tumor Medulloblastoma
Cavalli - 763 - rma_sketch - hugene11t
PRKCB (7994131)
Expression cutoff: 54.600 (min.grp=3)
subgroup~group3|met_status_(1_met__0_m0)~1|WITH_SURV (n=41)
overall survival probability
1.00
0.90
0.80
0.70
0.60
0.50
0.40
0.30
0.20
0.10
0.00
0
24
48
72
96
120
144
168
192
216
Follow up in months
high (n=25)
low (n=16)
raw p 0.105
bonf p 1.000
Expression Graph
Event=No
Event=Yes
Expression
400
350
300
250
200
150
100
50
0
55
55
sorted by expression

PRKCD

WNT M0

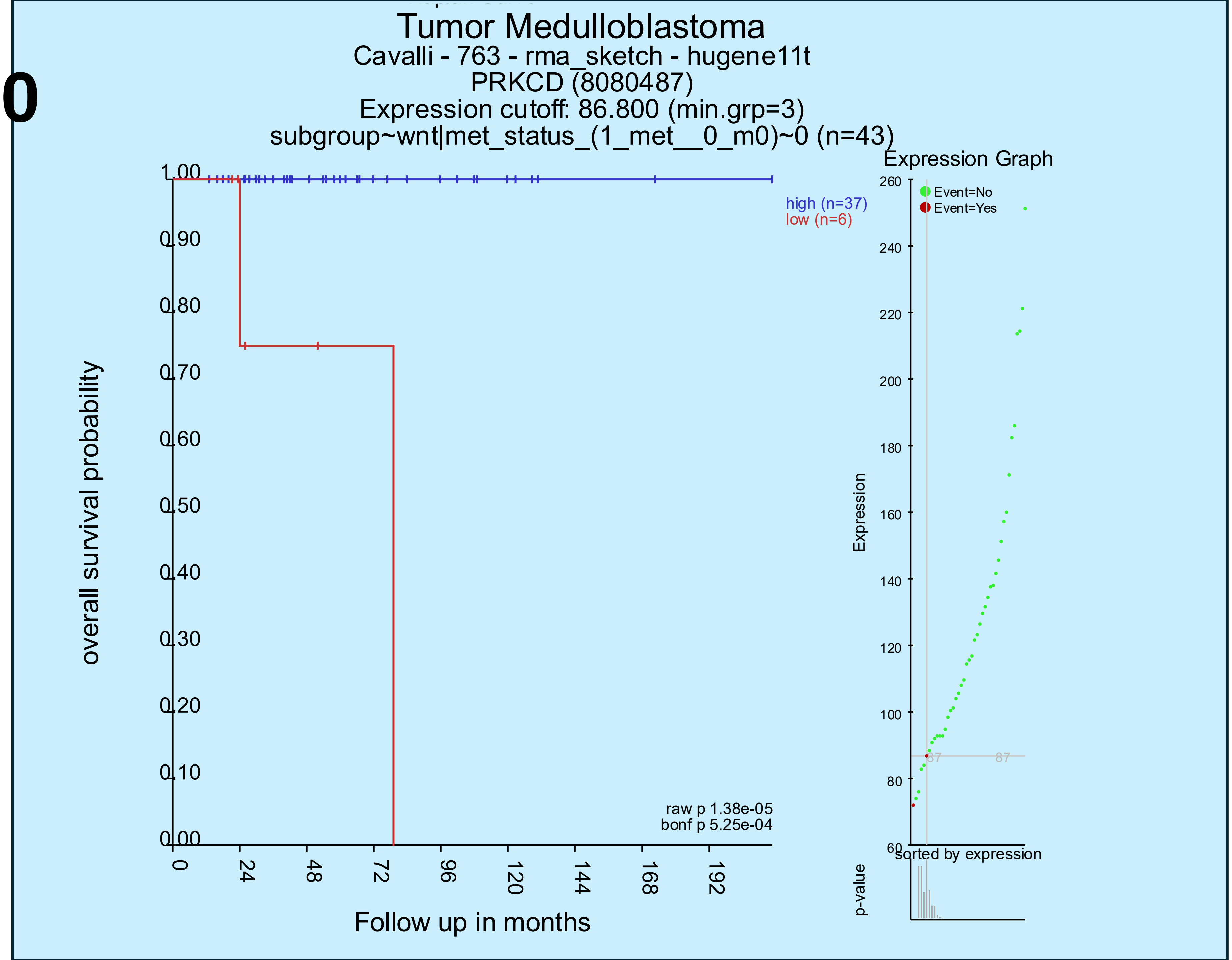

Tumor Medulloblastoma
Cavalli - 763 - rma_sketch - hugene11t
PRKCD (8080487)
Expression cutoff: 86.800 (min.grp=3)
subgroup~wnt|met_status_(1_met__0_m0)~0 (n=43)
overall survival probability
1.00
0.90
0.80
0.70
0.60
0.50
0.40
0.30
0.20
0.10
0.00
0
24
48
72
96
120
144
168
192
Follow up in months
high (n=37)
low (n=6)
raw p 1.38e-05
bonf p 5.25e-04
Expression Graph
Event=No
Event=Yes
Expression
260
240
220
200
180
160
140
120
100
80
60
sorted by expression
p-value

WNT M1

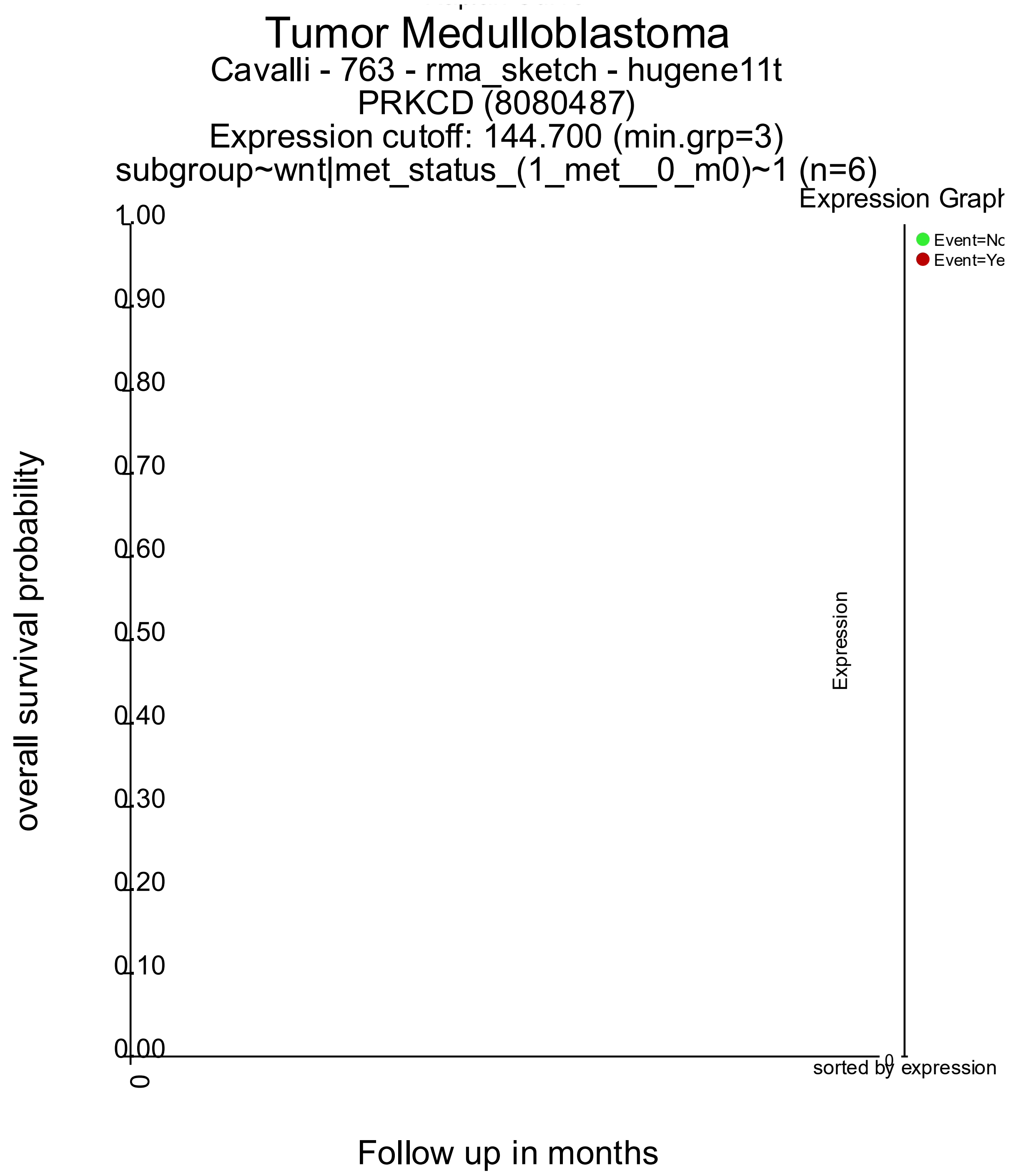

Tumor Medulloblastoma
Cavalli - 763 - rma_sketch - hugene11t
PRKCD (8080487)
Expression cutoff: 144.700 (min.grp=3)
subgroup~wnt|met_status_(1_met__0_m0)~1 (n=6)
Expression Graph
Event=No
Event=Yes
1.00
0.90
0.80
0.70
0.60
0.50
0.40
0.30
0.20
0.10
0.00
overall survival probability
0
Follow up in months
Expression
0
sorted by expression

SHH M0

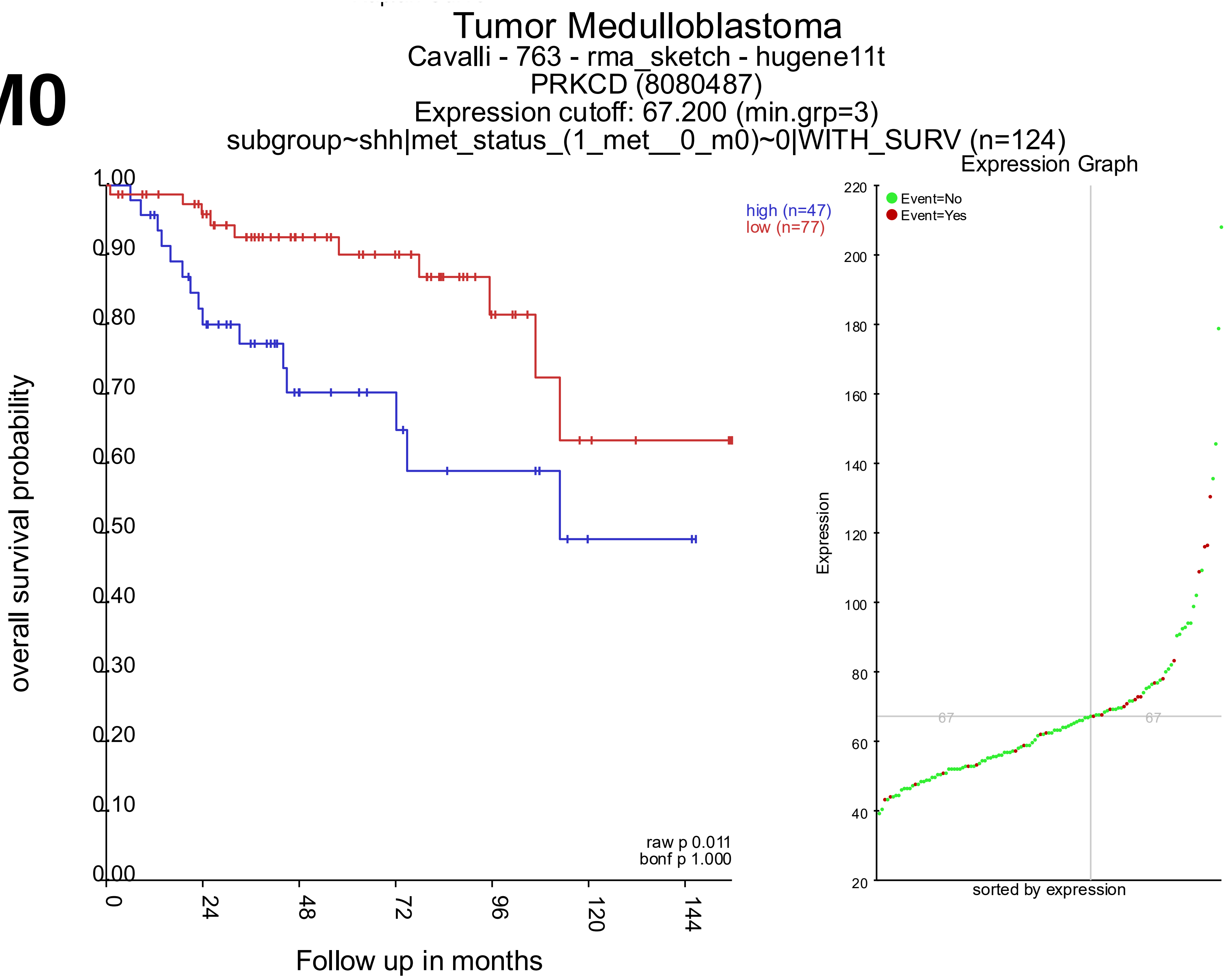

Tumor Medulloblastoma
Cavalli - 763 - rma_sketch - hugene11t
PRKCD (8080487)
Expression cutoff: 67.200 (min.grp=3)
subgroup~shh|met_status_(1_met__0_m0)~0|WITH_SURV (n=124)
overall survival probability
1.00
0.90
0.80
0.70
0.60
0.50
0.40
0.30
0.20
0.10
0.00
0
24
48
72
96
120
144
Follow up in months
high (n=47)
low (n=77)
raw p 0.011
bonf p 1.000
Expression Graph
Event=No
Event=Yes
Expression
220
200
180
160
140
120
100
80
60
40
20
67
67
sorted by expression

SHH M1

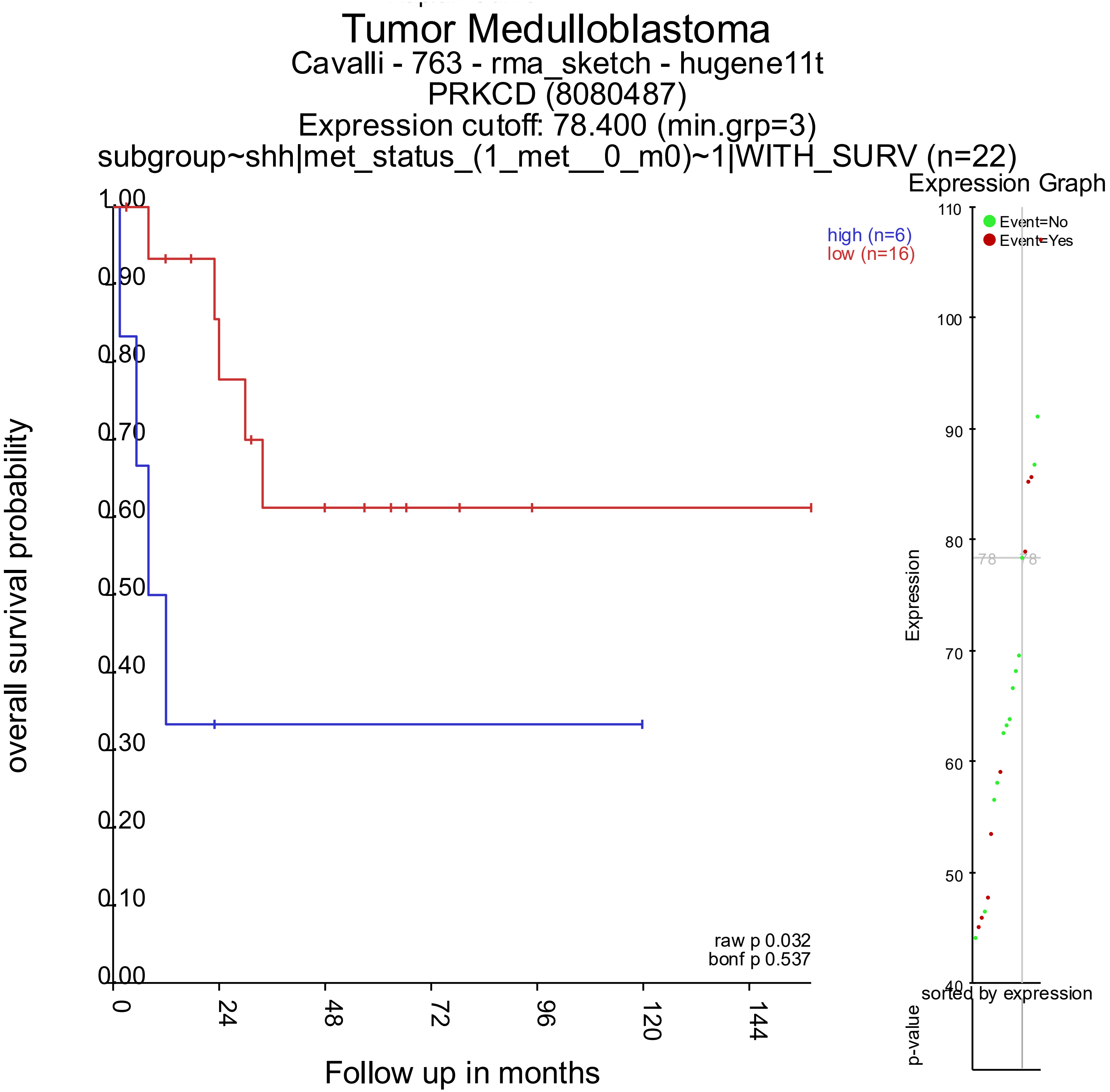

Tumor Medulloblastoma
Cavalli - 763 - rma_sketch - hugene11t
PRKCD (8080487)
Expression cutoff: 78.400 (min.grp=3)
subgroup~shh|met_status_(1_met__0_m0)~1|WITH_SURV (n=22)
high (n=6)
low (n=16)
overall survival probability
1.00
0.90
0.80
0.70
0.60
0.50
0.40
0.30
0.20
0.10
0.00
raw p 0.032
bonf p 0.537
0
24
48
72
96
120
144
Follow up in months
Expression Graph
Event=No
Event=Yes
110
100
90
80
70
60
50
40
78
Expression
sorted by expression
p-value

GROUP4 M0

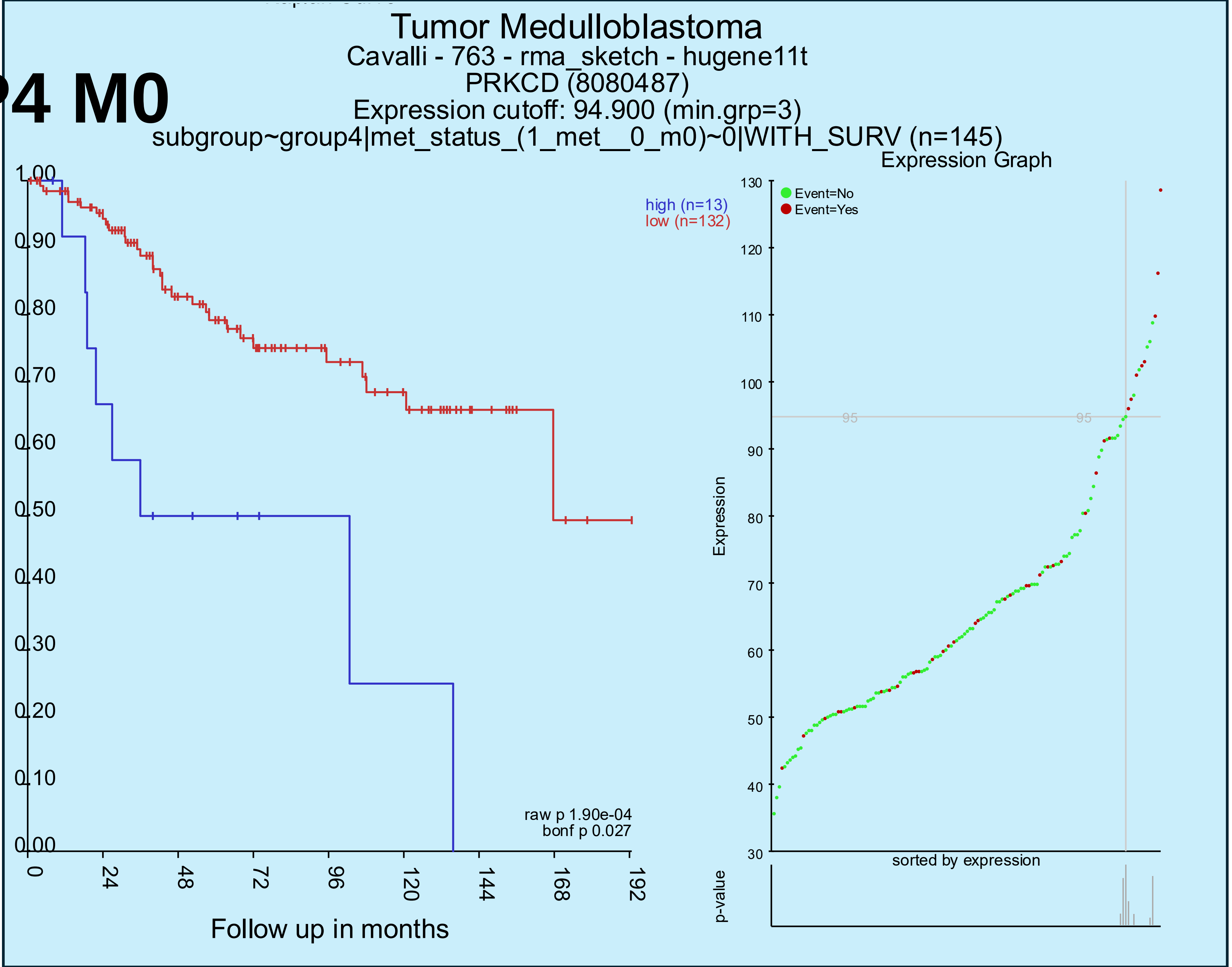

Tumor Medulloblastoma
Cavalli - 763 - rma_sketch - hugene11t
PRKCD (8080487)
Expression cutoff: 94.900 (min.grp=3)
subgroup~group4|met_status_(1_met__0_m0)~0|WITH_SURV (n=145)
overall survival probability
1.00
0.90
0.80
0.70
0.60
0.50
0.40
0.30
0.20
0.10
0.00
high (n=13)
low (n=132)
raw p 1.90e-04
bonf p 0.027
0
24
48
72
96
120
144
168
192
Follow up in months
Expression Graph
Event=No
Event=Yes
Expression
130
120
110
100
90
80
70
60
50
40
30
95
95
sorted by expression
p-value

GROUP4 M1

Tumor Medulloblastoma
Cavalli - 763 - rma_sketch - hugene11t
PRKCD (8080487)
Expression cutoff: 52.300 (min.grp=3)
subgroup~group4|met_status_(1_met__0_m0)~1|WITH_SURV (n=92)

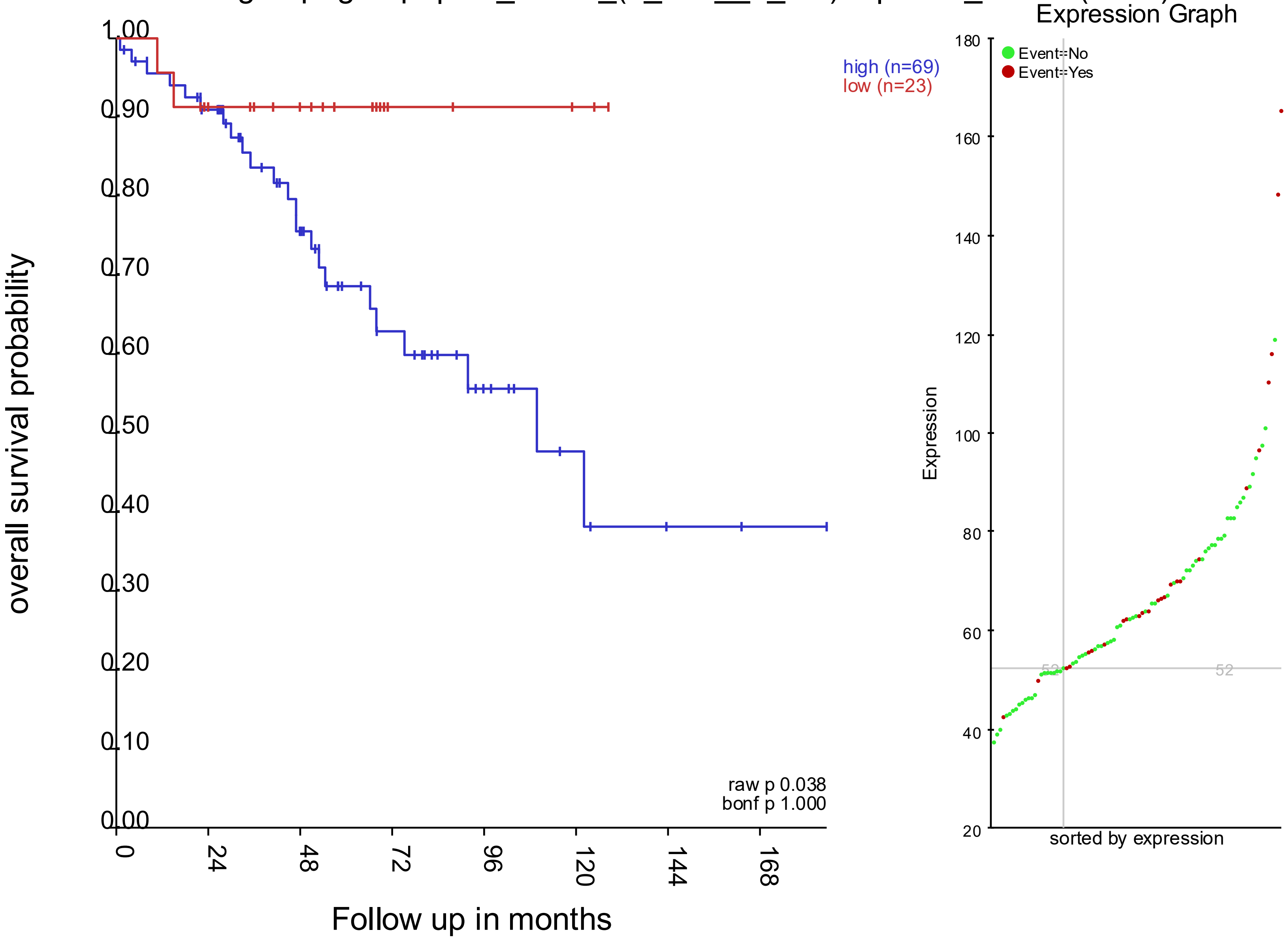

GROUP3 M0

Tumor Medulloblastoma
Cavalli - 763 - rma_sketch - hugene11t
PRKCD (8080487)
Expression cutoff: 103.300 (min.grp=3)
subgroup~group3|met_status_(1_met__0_m0)~0|WITH_SURV (n=65)

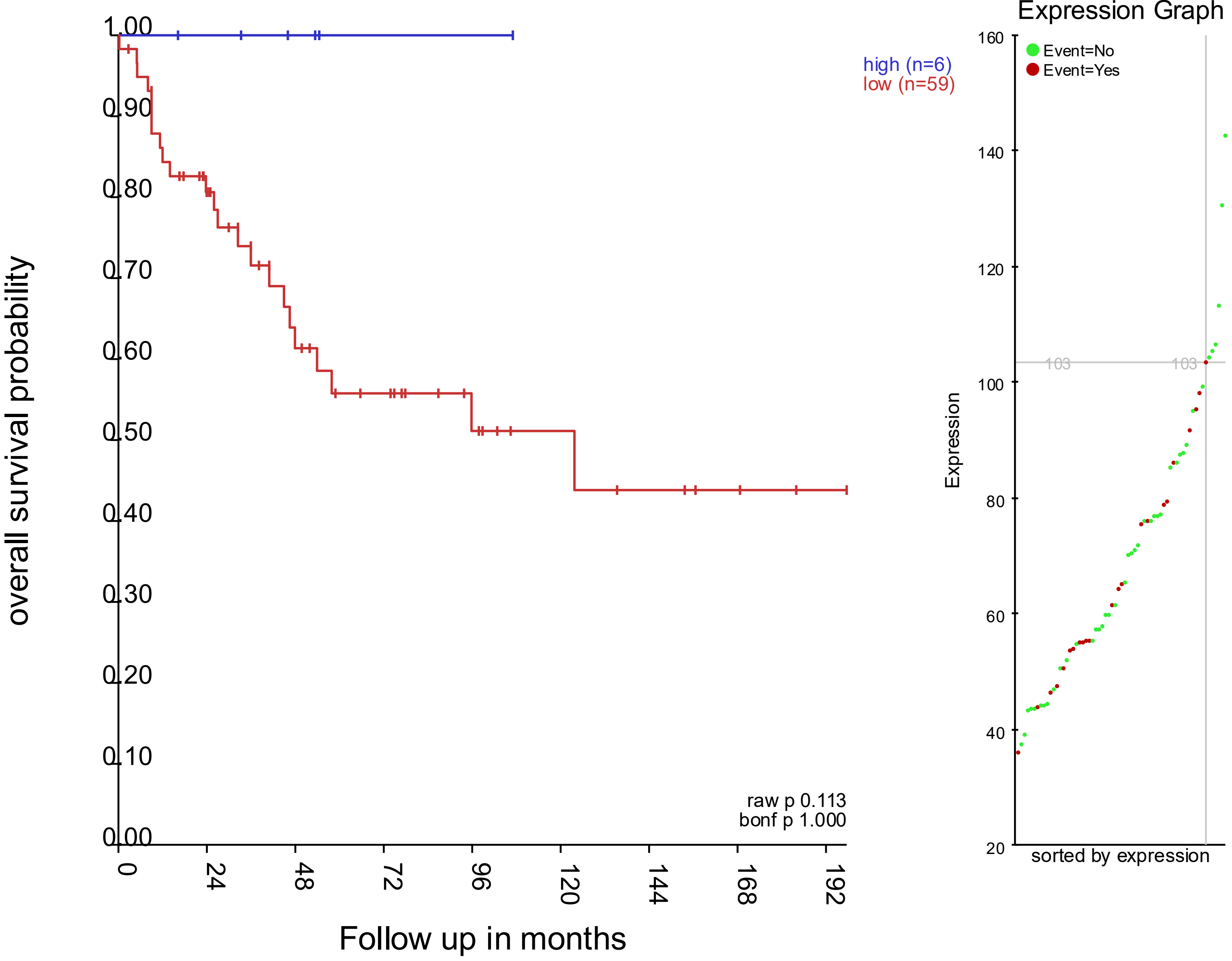

GROUP3 M1

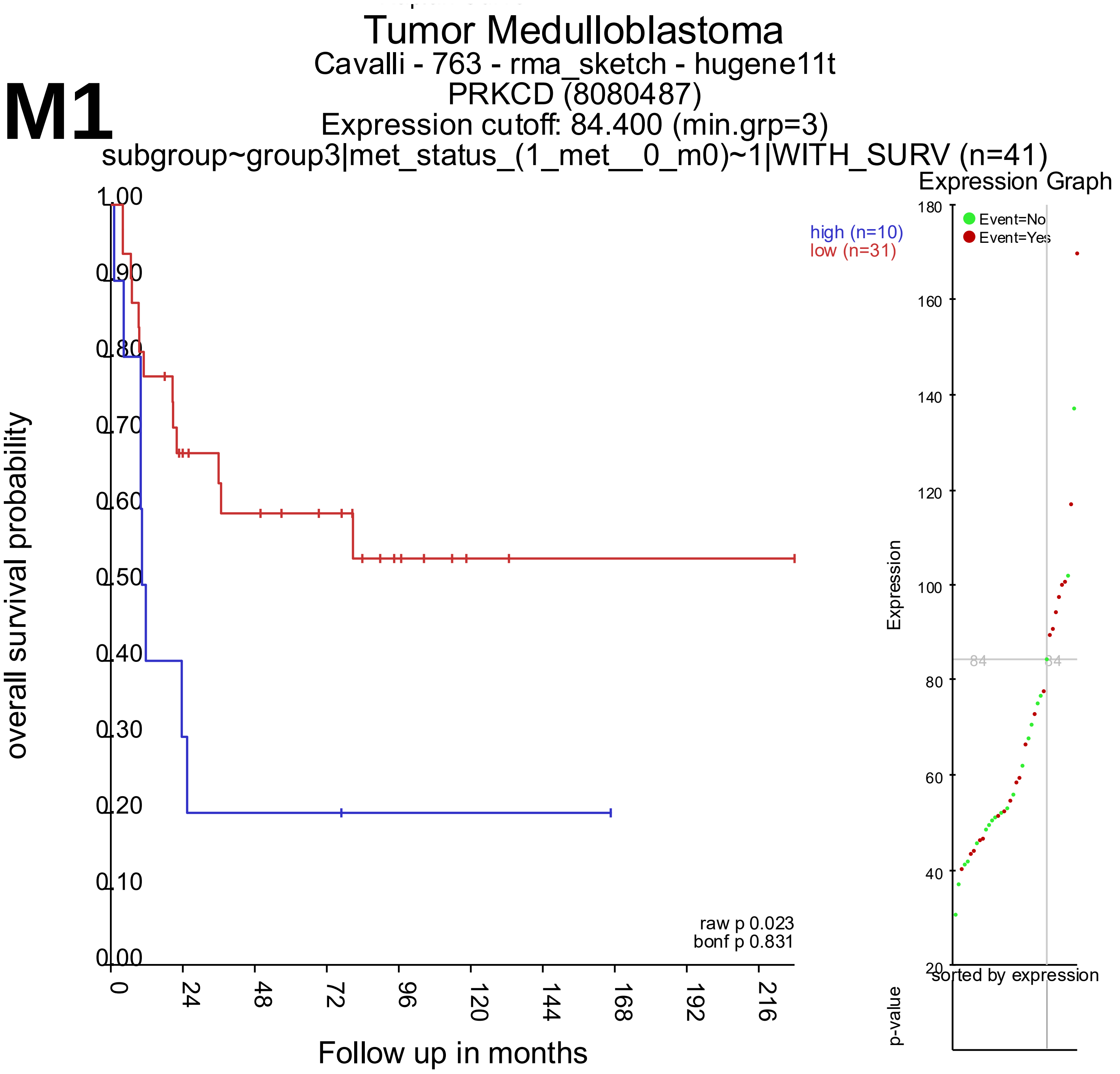

Tumor Medulloblastoma
Cavalli - 763 - rma_sketch - hugene11t
PRKCD (8080487)
Expression cutoff: 84.400 (min.grp=3)
subgroup~group3|met_status_(1_met__0_m0)~1|WITH_SURV (n=41)
high (n=10)
low (n=31)
overall survival probability
1.00
0.90
0.80
0.70
0.60
0.50
0.40
0.30
0.20
0.10
0.00
raw p 0.023
bonf p 0.831
0
24
48
72
96
120
144
168
192
216
Follow up in months
Expression Graph
Event=No
Event=Yes
Expression
180
160
140
120
100
80
60
40
20
84
sorted by expression
p-value

ARAF

WNT M0

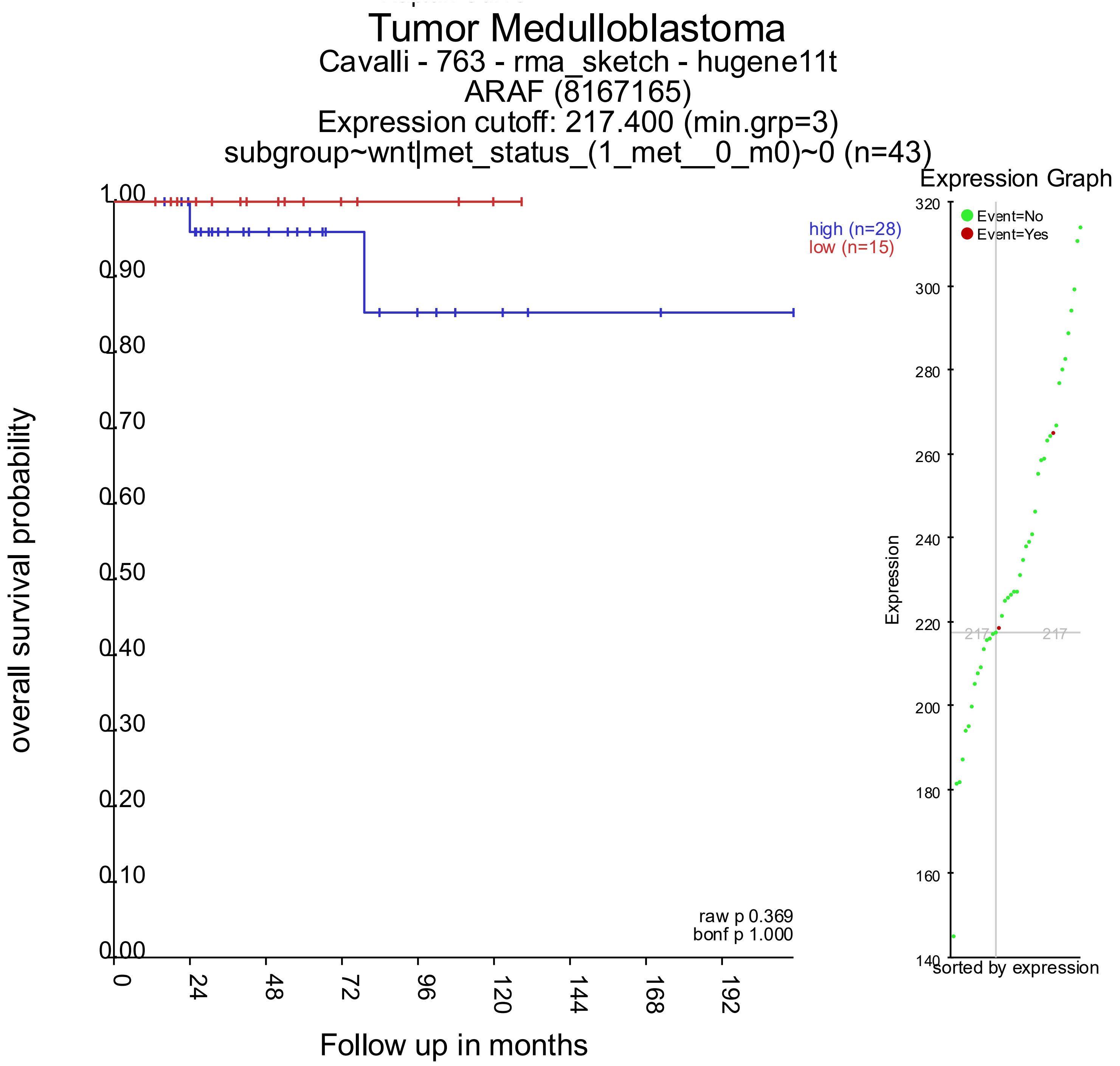

Tumor Medulloblastoma
Cavalli - 763 - rma_sketch - hugene11t
ARAF (8167165)
Expression cutoff: 217.400 (min.grp=3)
subgroup~wnt|met_status_(1_met__0_m0)~0 (n=43)
overall survival probability
1.00
0.90
0.80
0.70
0.60
0.50
0.40
0.30
0.20
0.10
0.00
0
24
48
72
96
120
144
168
192
Follow up in months
high (n=28)
low (n=15)
raw p 0.369
bonf p 1.000
Expression Graph
Event=No
Event=Yes
Expression
320
300
280
260
240
220
200
180
160
140
217
217
sorted by expression

WNT M1

Tumor Medulloblastoma
Cavalli - 763 - rma_sketch - hugene11t
ARAF (8167165)
Expression cutoff: 232.500 (min.grp=3)
subgroup~wnt|met_status_(1_met__0_m0)~1 (n=6)

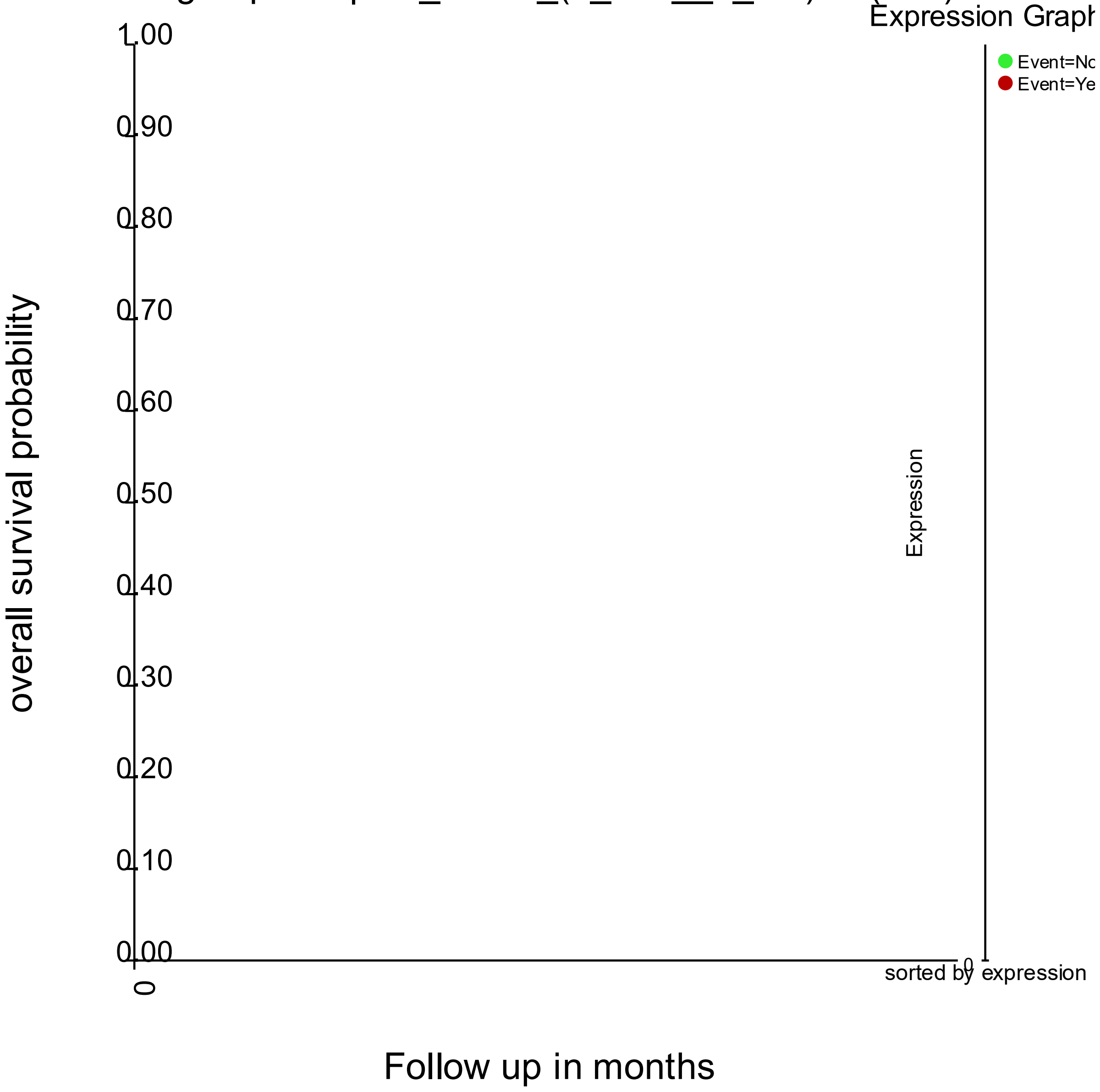

SHH M0

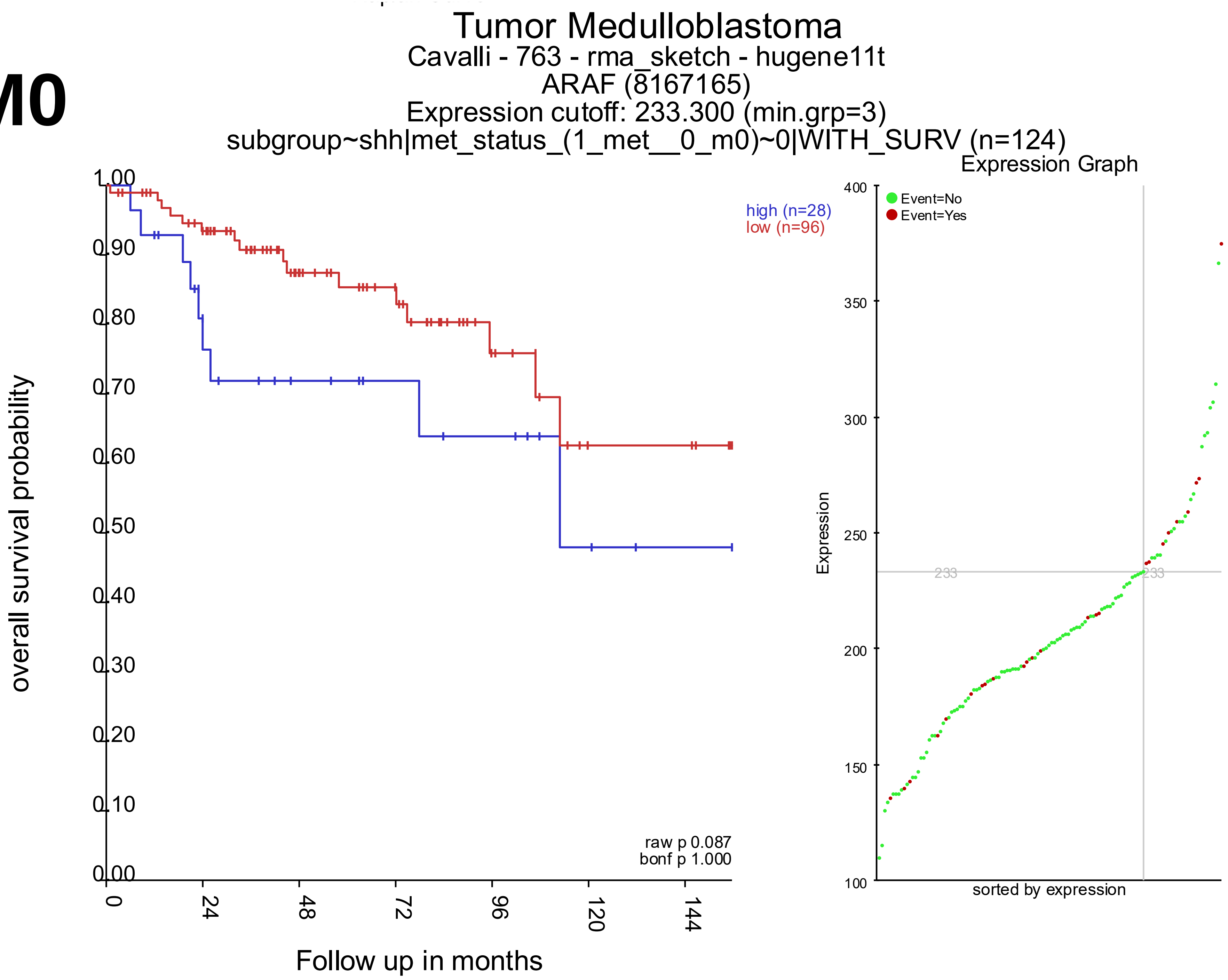

Tumor Medulloblastoma
Cavalli - 763 - rma_sketch - hugene11t
ARAF (8167165)
Expression cutoff: 233.300 (min.grp=3)
subgroup~shh|met_status_(1_met__0_m0)~0|WITH_SURV (n=124)
overall survival probability
1.00
0.90
0.80
0.70
0.60
0.50
0.40
0.30
0.20
0.10
0.00
high (n=28)
low (n=96)
raw p 0.087
bonf p 1.000
0
24
48
72
96
120
144
Follow up in months
Expression Graph
Event=No
Event=Yes
Expression
400
350
300
250
200
150
100
233
sorted by expression

SHH M1

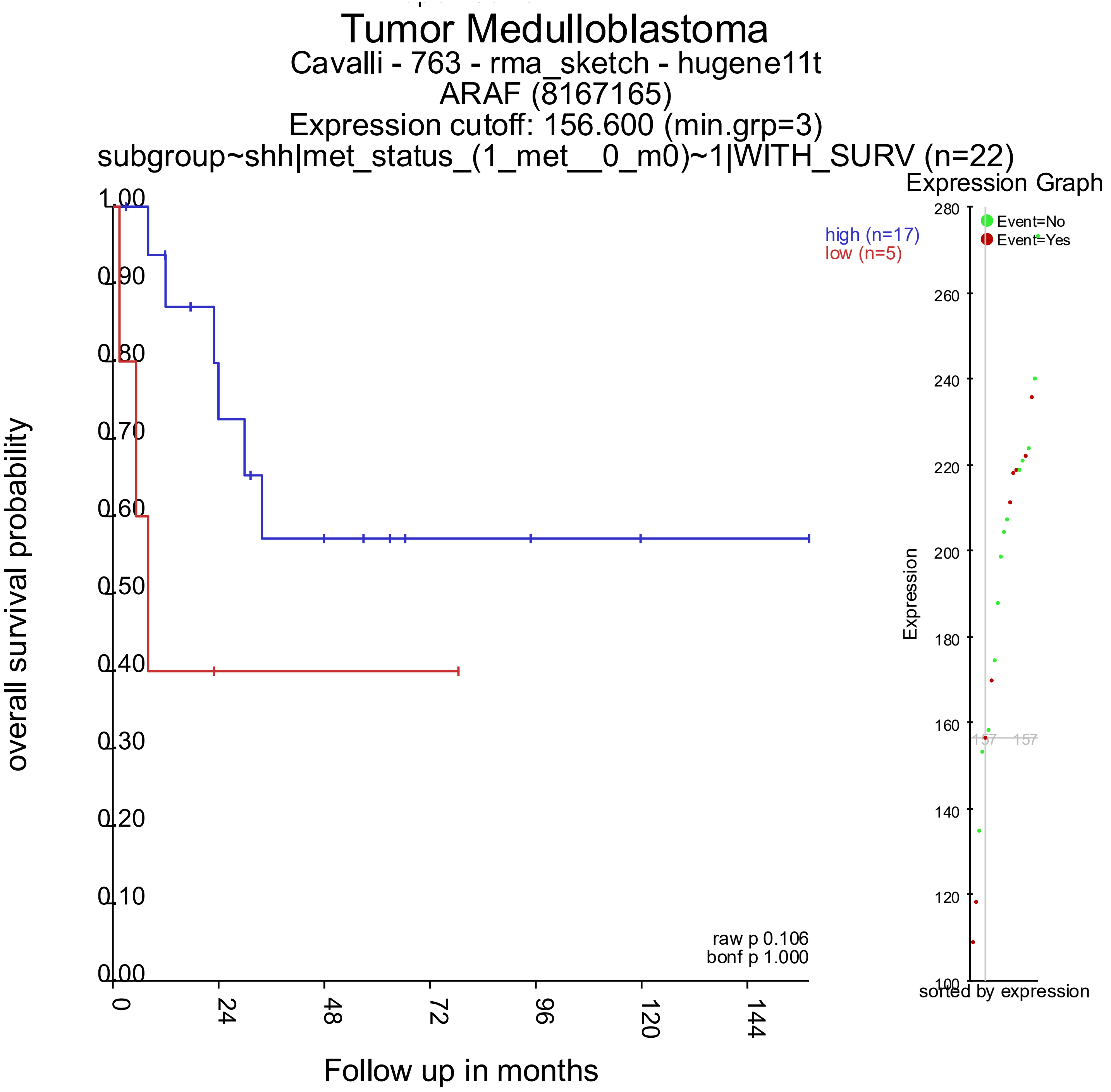

Tumor Medulloblastoma
Cavalli - 763 - rma_sketch - hugene11t
ARAF (8167165)
Expression cutoff: 156.600 (min.grp=3)
subgroup~shh|met_status_(1_met__0_m0)~1|WITH_SURV (n=22)
overall survival probability
1.00
0.90
0.80
0.70
0.60
0.50
0.40
0.30
0.20
0.10
0.00
high (n=17)
low (n=5)
raw p 0.106
bonf p 1.000
0
24
48
72
96
120
144
Follow up in months
Expression Graph
Event=No
Event=Yes
Expression
280
260
240
220
200
180
160
140
120
100
157
157
sorted by expression

GROUP4 M0

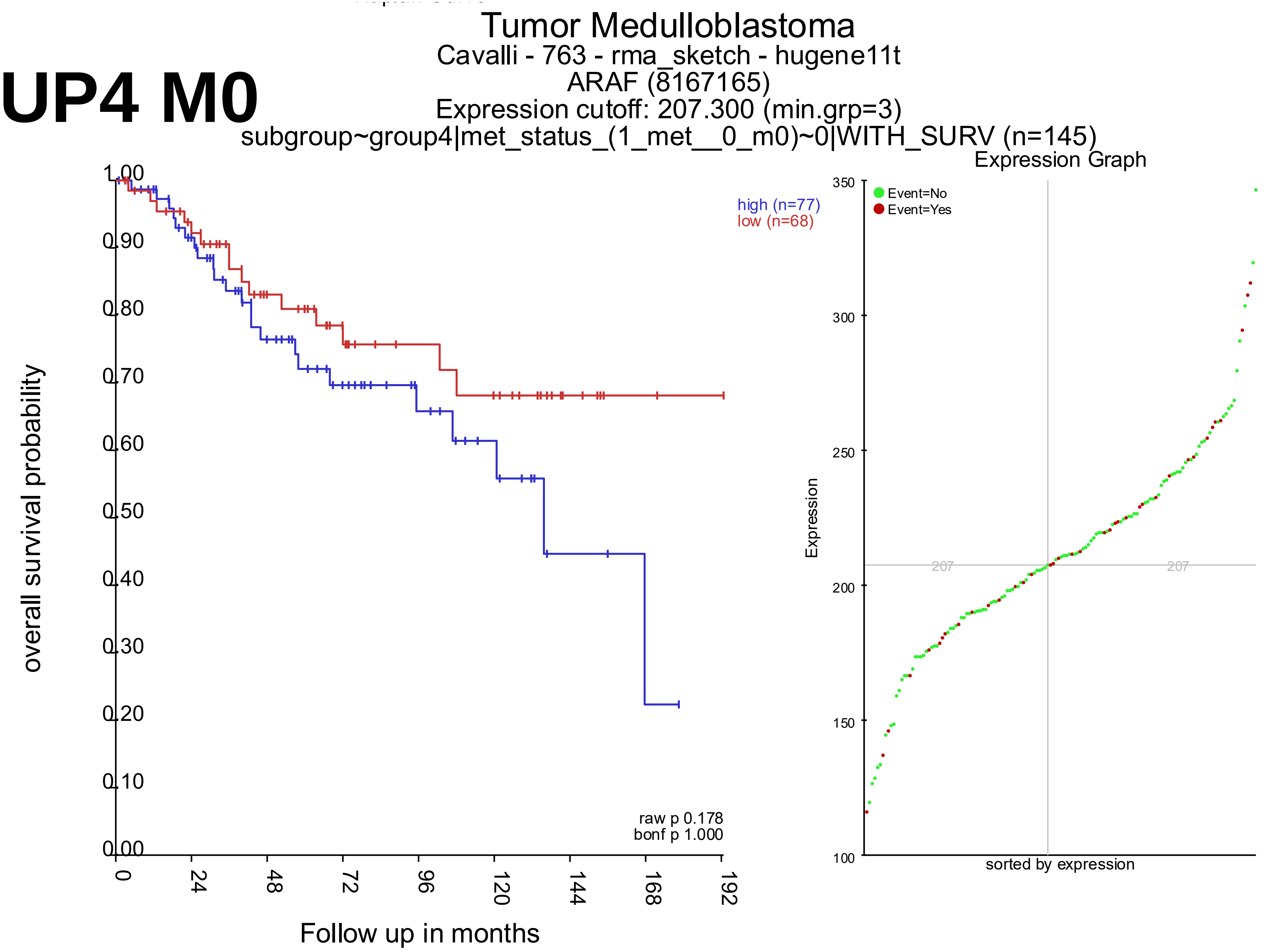

Tumor Medulloblastoma
Cavalli - 763 - rma_sketch - hugene11t
ARAF (8167165)
Expression cutoff: 207.300 (min.grp=3)
subgroup~group4|met_status_(1_met__0_m0)~0|WITH_SURV (n=145)
overall survival probability
1.00
0.90
0.80
0.70
0.60
0.50
0.40
0.30
0.20
0.10
0.00
high (n=77)
low (n=68)
raw p 0.178
bonf p 1.000
0
24
48
72
96
120
144
168
192
Follow up in months
Expression Graph
Event=No
Event=Yes
350
300
250
200
150
100
Expression
207
207
sorted by expression

GROUP4 M1

Tumor Medulloblastoma
Cavalli - 763 - rma_sketch - hugene11t
ARAF (8167165)
Expression cutoff: 184.000 (min.grp=3)
subgroup~group4|met_status_(1_met__0_m0)~1|WITH_SURV (n=92)

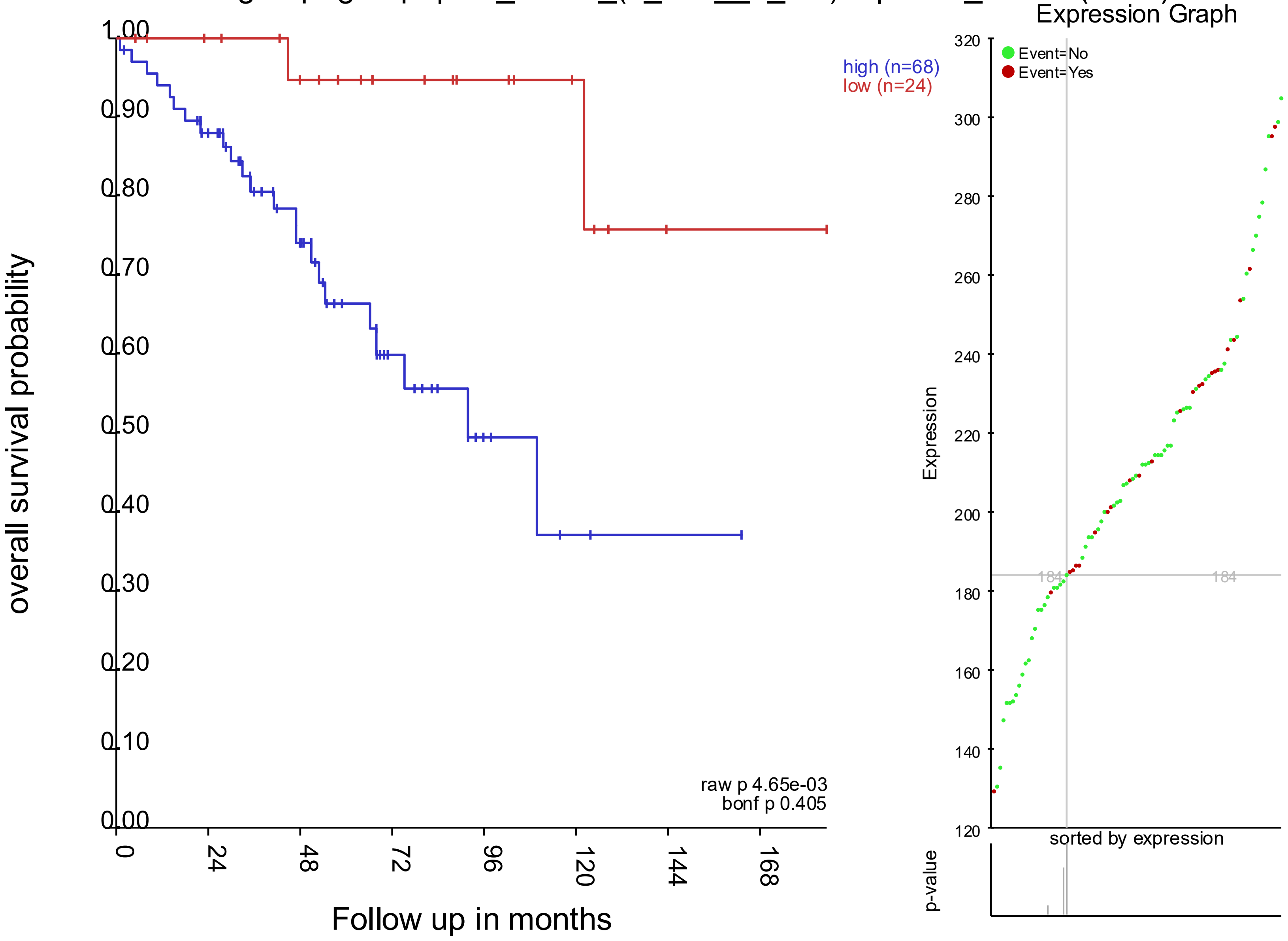

GROUP3 M0

Tumor Medulloblastoma
Cavalli - 763 - rma_sketch - hugene11t
ARAF (8167165)
Expression cutoff: 202.300 (min.grp=3)
subgroup~group3|met_status_(1_met__0_m0)~0|WITH_SURV (n=65)

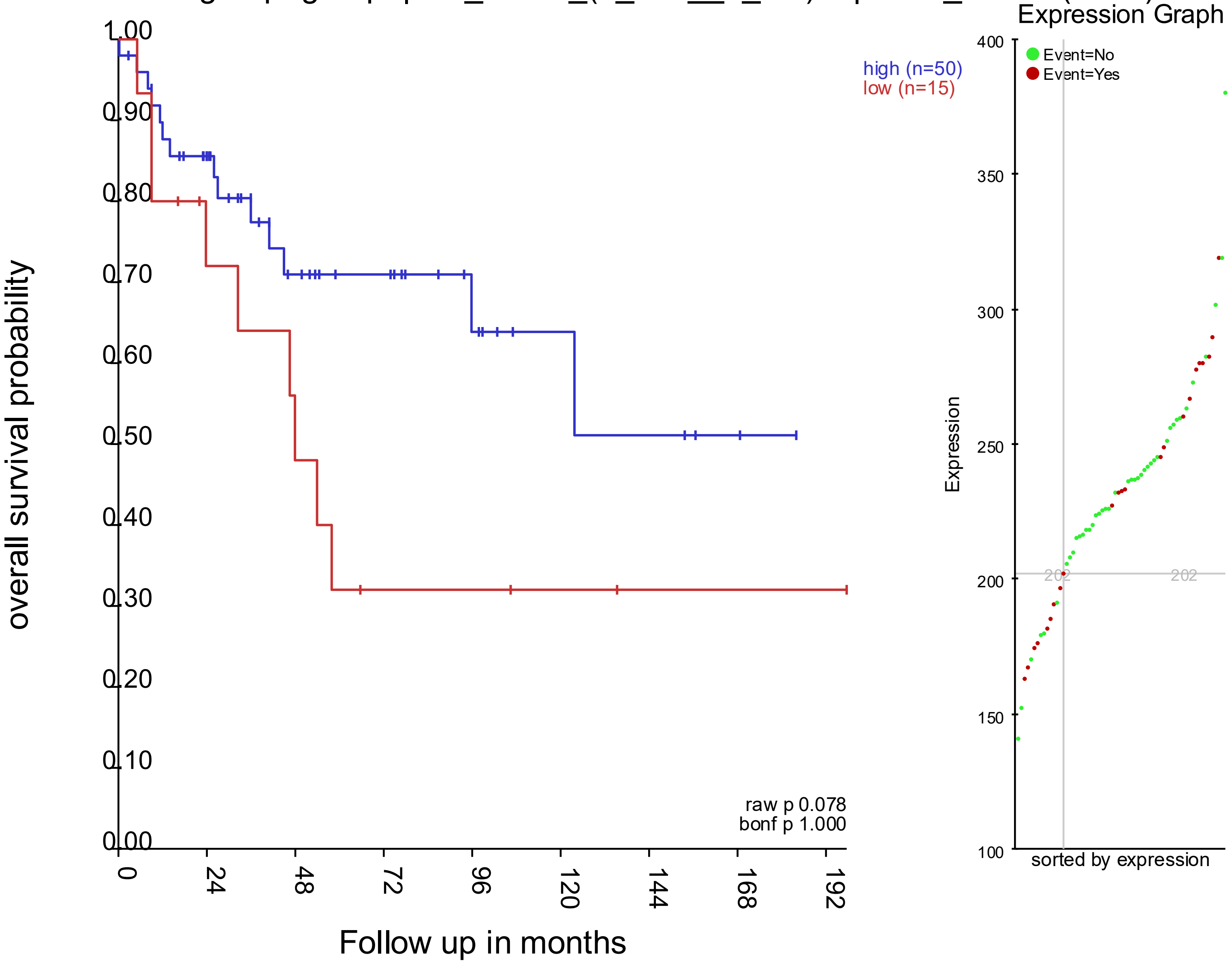

GROUP3 M1

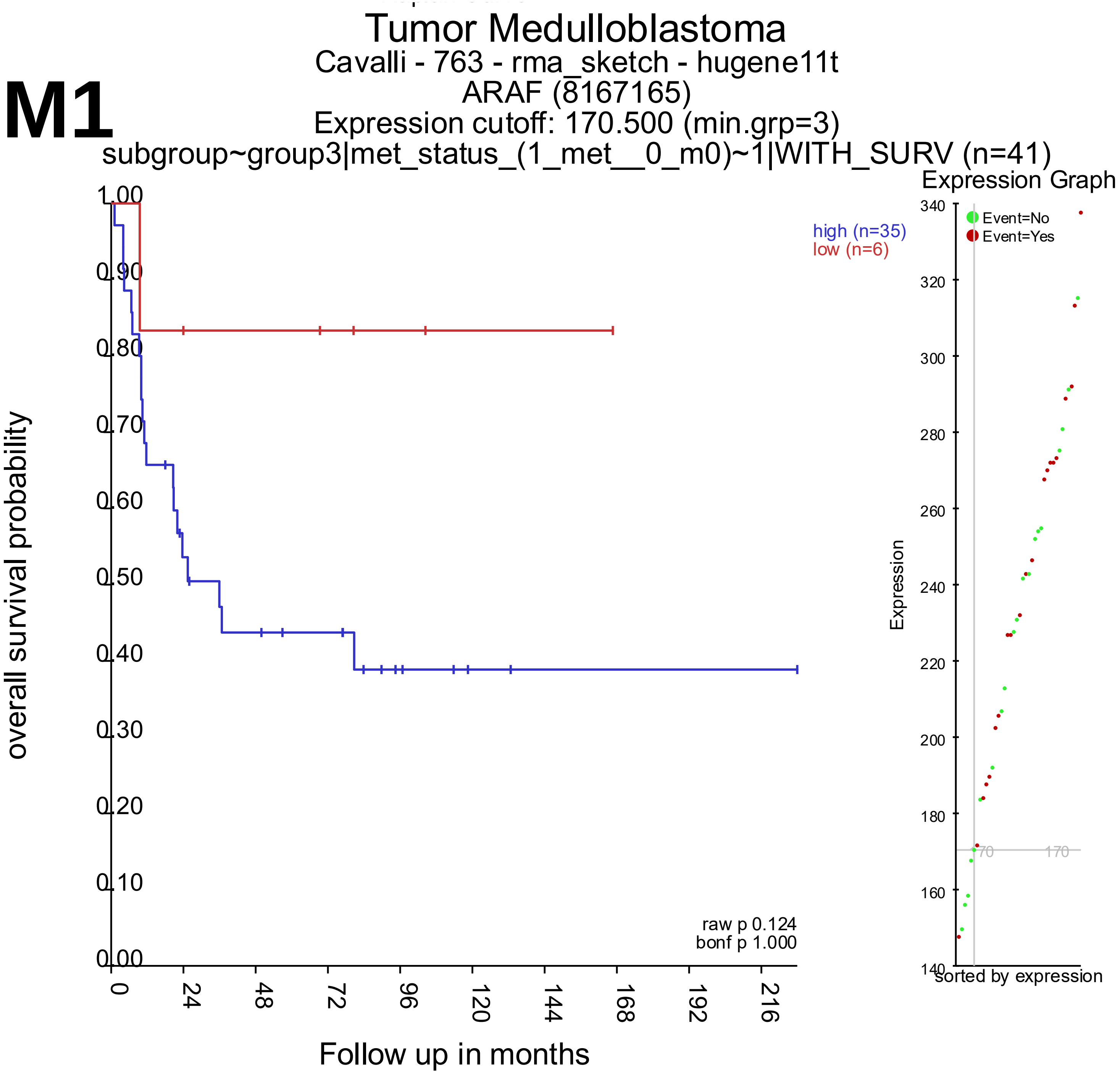

Tumor Medulloblastoma
Cavalli - 763 - rma_sketch - hugene11t
ARAF (8167165)
Expression cutoff: 170.500 (min.grp=3)
subgroup~group3|met_status_(1_met__0_m0)~1|WITH_SURV (n=41)
overall survival probability
1.00
0.90
0.80
0.70
0.60
0.50
0.40
0.30
0.20
0.10
0.00
0
24
48
72
96
120
144
168
192
216
Follow up in months
high (n=35)
low (n=6)
raw p 0.124
bonf p 1.000
Expression Graph
Event=No
Event=Yes
Expression
340
320
300
280
260
240
220
200
180
160
140
170
170
sorted by expression

BRAF

WNT M0

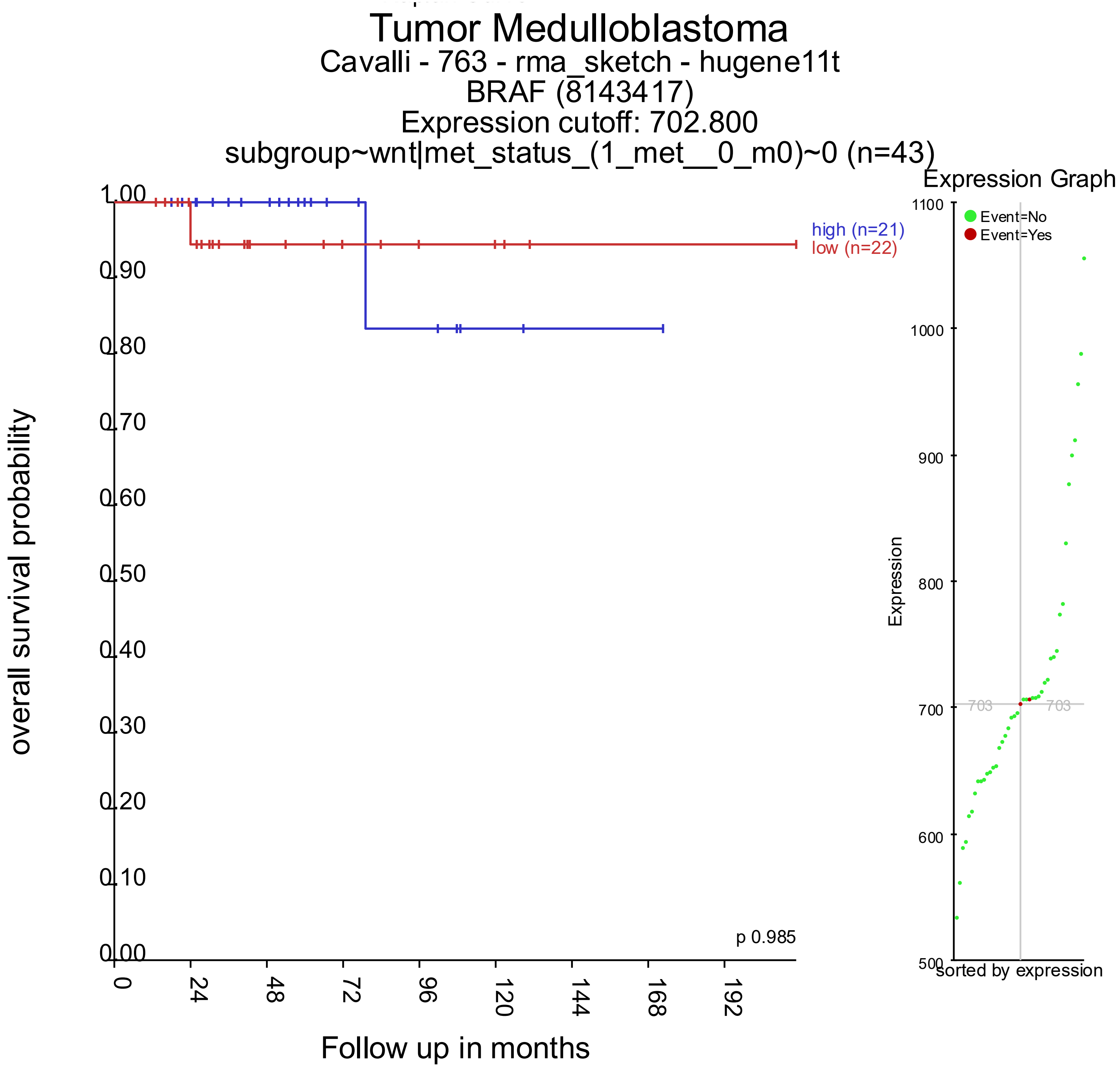

Tumor Medulloblastoma
Cavalli - 763 - rma_sketch - hugene11t
BRAF (8143417)
Expression cutoff: 702.800
subgroup~wnt|met_status_(1_met__0_m0)~0 (n=43)
overall survival probability
1.00
0.90
0.80
0.70
0.60
0.50
0.40
0.30
0.20
0.10
0.00
high (n=21)
low (n=22)
p 0.985
0
24
48
72
96
120
144
168
192
Follow up in months
Expression Graph
Event=No
Event=Yes
Expression
1100
1000
900
800
700
600
500
703
703
sorted by expression

WNT M1

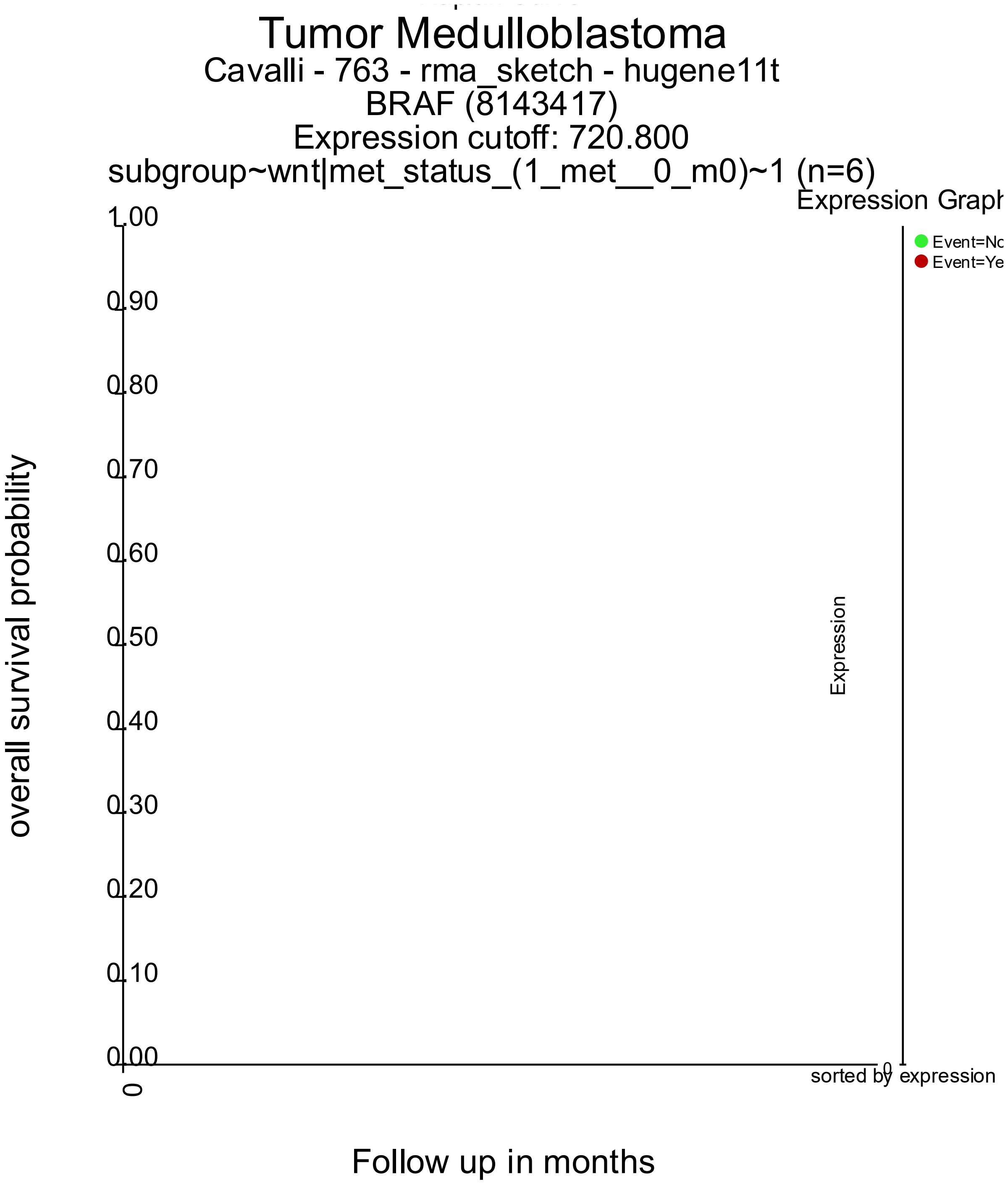

Tumor Medulloblastoma
Cavalli - 763 - rma_sketch - hugene11t
BRAF (8143417)
Expression cutoff: 720.800
subgroup~wnt|met_status_(1_met__0_m0)~1 (n=6)
Expression Graph
Event=No
Event=Yes
overall survival probability
1.00
0.90
0.80
0.70
0.60
0.50
0.40
0.30
0.20
0.10
0.00
0
Follow up in months
Expression
0
sorted by expression

SHH M0

Tumor Medulloblastoma
Cavalli - 763 - rma_sketch - hugene11t
BRAF (8143417)
Expression cutoff: 1204.200 (min.grp=3)
subgroup~shh|met_status_(1_met__0_m0)~0|WITH_SURV (n=124)

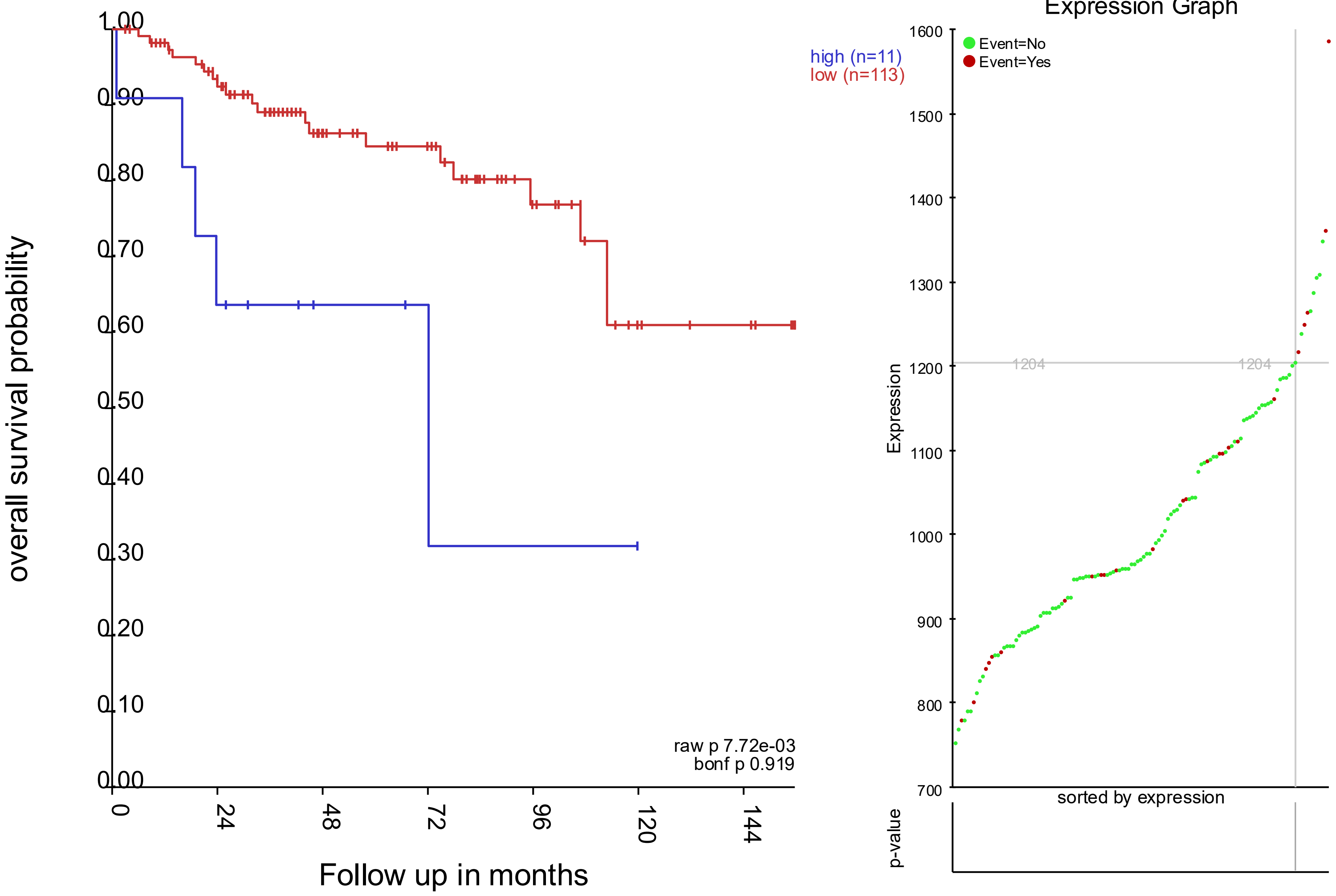

SHH M1

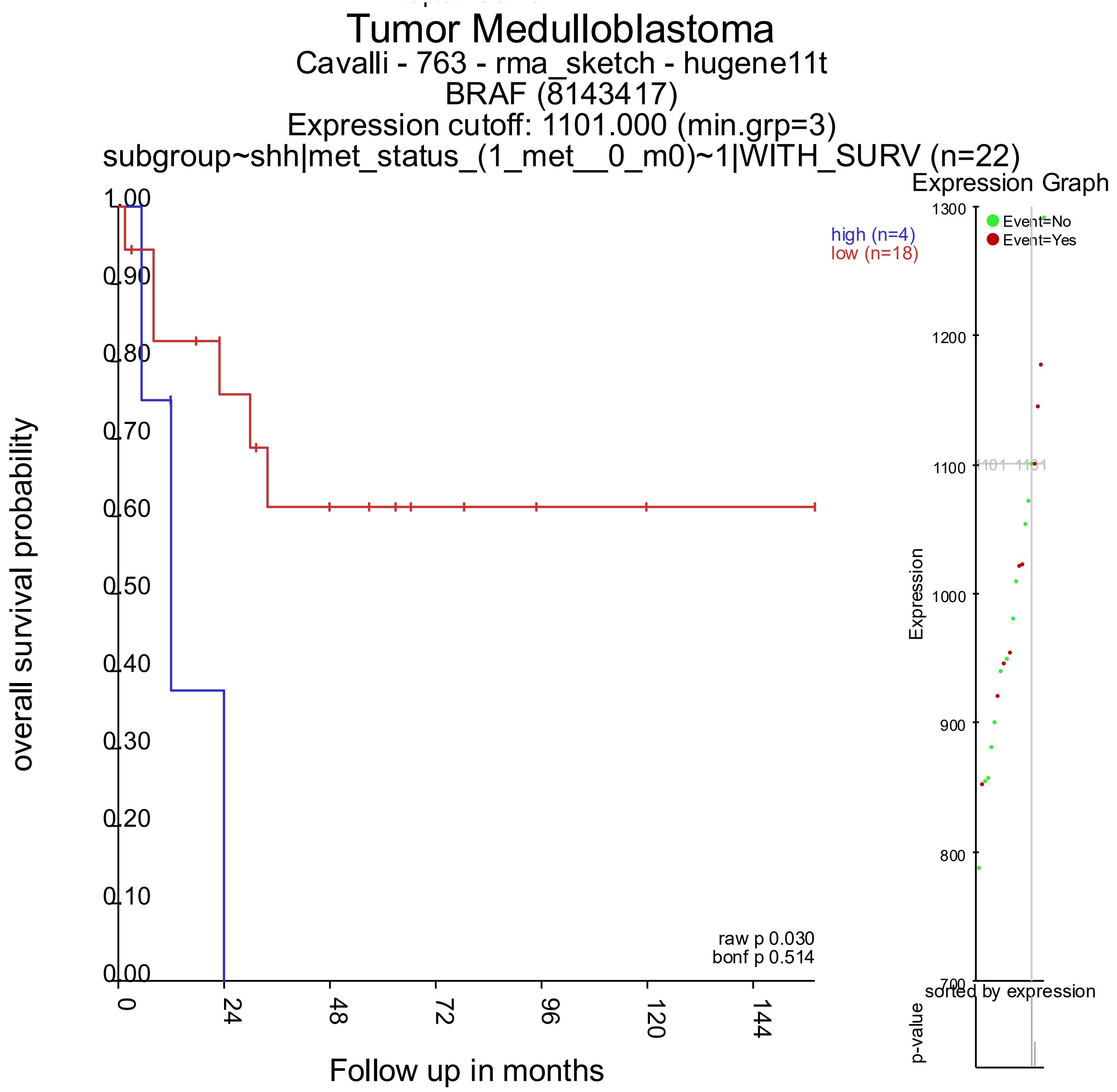

Tumor Medulloblastoma
Cavalli - 763 - rma_sketch - hugene11t
BRAF (8143417)
Expression cutoff: 1101.000 (min.grp=3)
subgroup~shh|met_status_(1_met__0_m0)~1|WITH_SURV (n=22)
high (n=4)
low (n=18)
overall survival probability
1.00
0.90
0.80
0.70
0.60
0.50
0.40
0.30
0.20
0.10
0.00
raw p 0.030
bonf p 0.514
0
24
48
72
96
120
144
Follow up in months
Expression Graph
Event=No
Event=Yes
1300
1200
1100
1000
900
800
700
Expression
sorted by expression
p-value

GROUP4 M0

Tumor Medulloblastoma
Cavalli - 763 - rma_sketch - hugene11t
BRAF (8143417)
Expression cutoff: 1757.800 (min.grp=3)
subgroup~group4|met_status_(1_met__0_m0)~0|WITH_SURV (n=145)

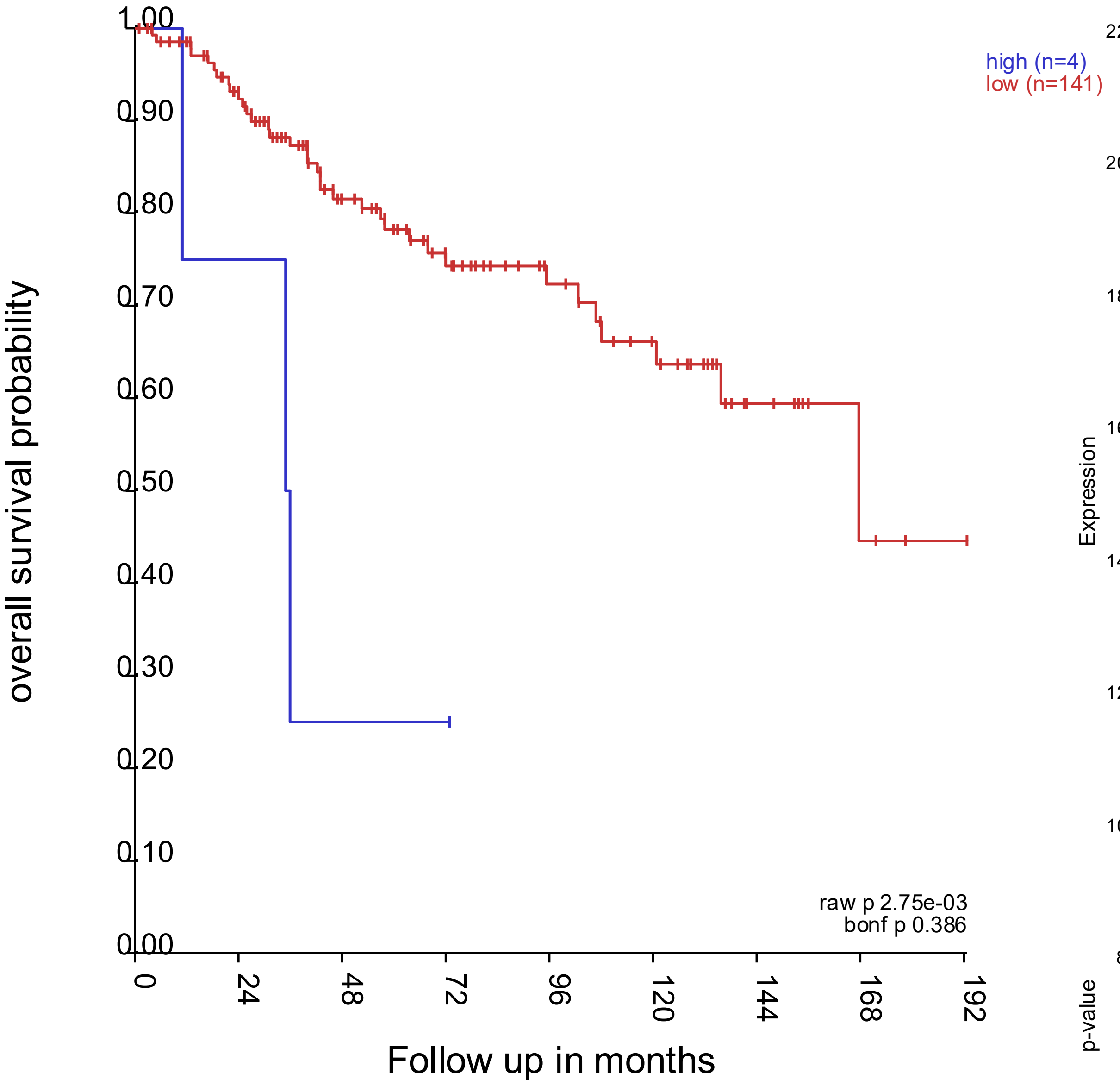

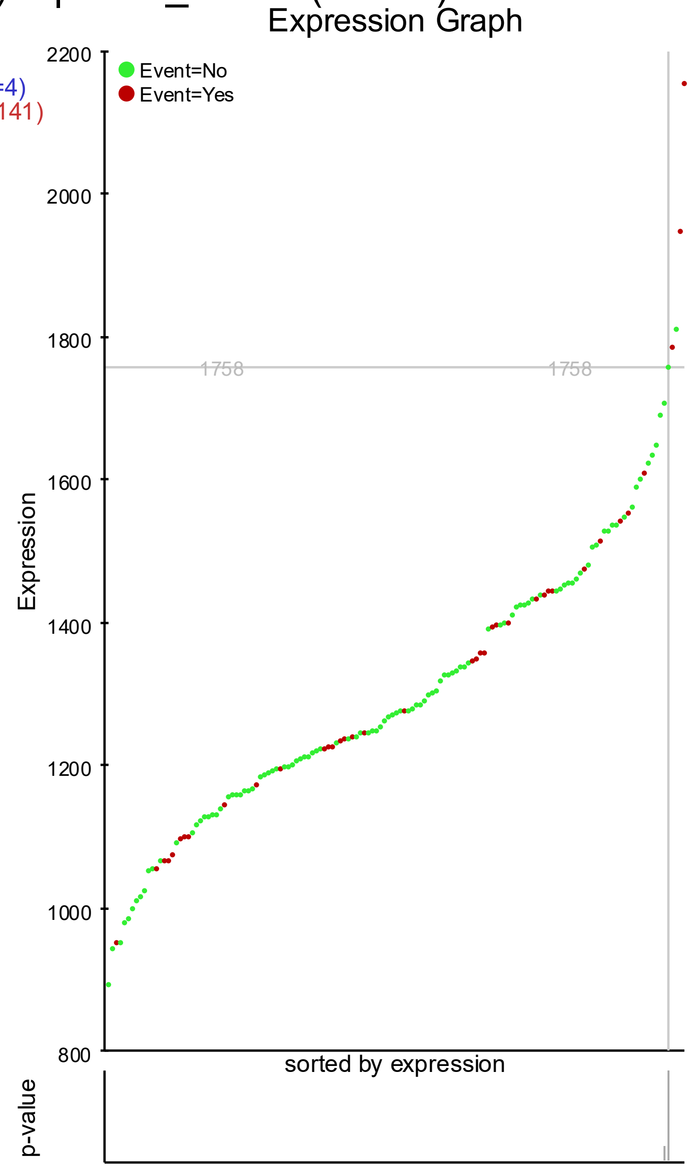

GROUP4 M1

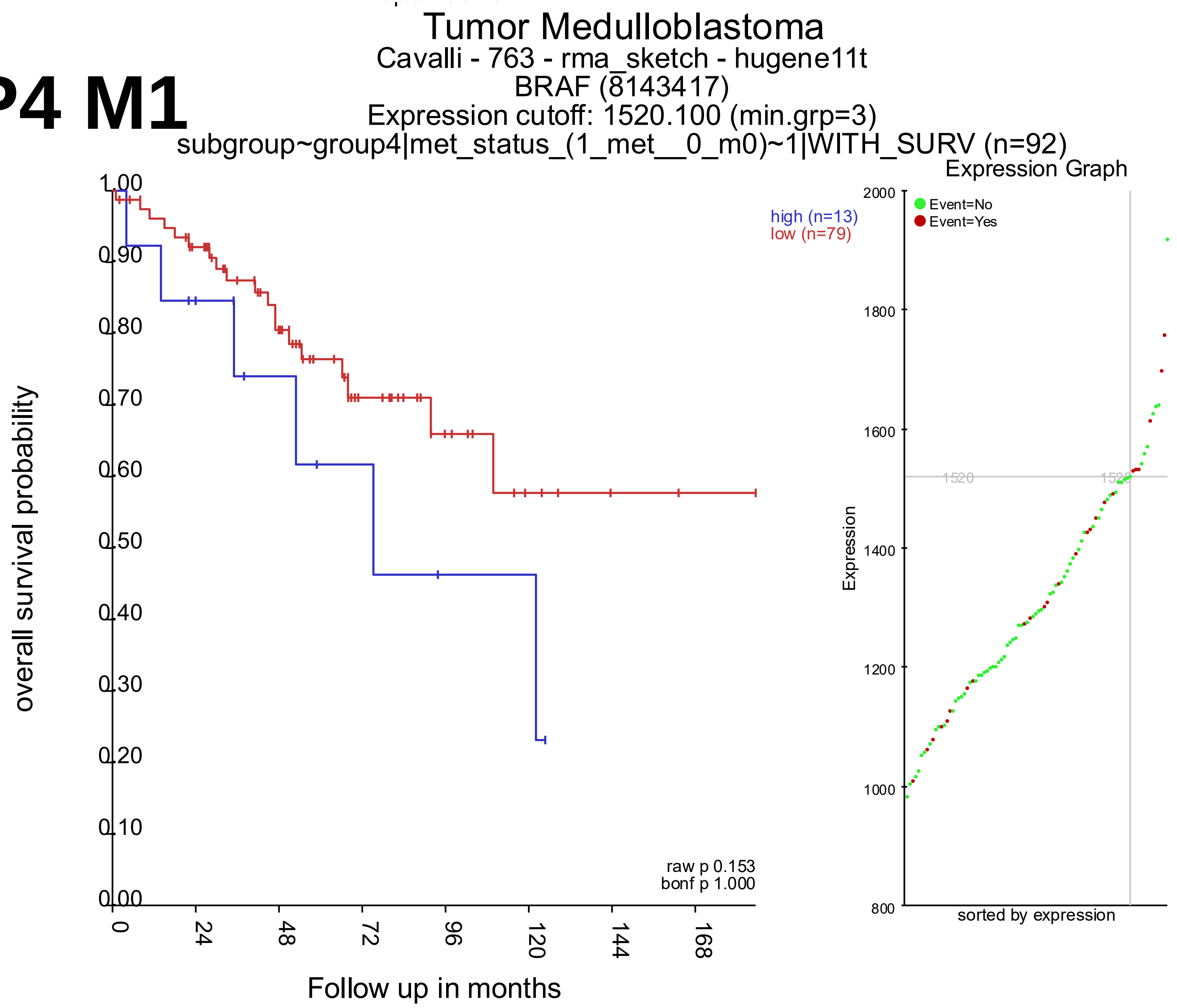
Tumor Medulloblastoma
Cavalli - 763 - rma_sketch - hugene11t
BRAF (8143417)
Expression cutoff: 1520.100 (min.grp=3)
subgroup~group4|met_status_(1_met__0_m0)~1|WITH_SURV (n=92)
overall survival probability
1.00
0.90
0.80
0.70
0.60
0.50
0.40
0.30
0.20
0.10
0.00
high (n=13)
low (n=79)
raw p 0.153
bonf p 1.000
0
24
48
72
96
120
144
168
Follow up in months
Expression Graph
Event=No
Event=Yes
Expression
2000
1800
1600
1400
1200
1000
800
1520
sorted by expression

GROUP3 M0

Tumor Medulloblastoma
Cavalli - 763 - rma_sketch - hugene11t
BRAF (8143417)
Expression cutoff: 887.400 (min.grp=3)
subgroup~group3|met_status_(1_met__0_m0)~0|WITH_SURV (n=65)

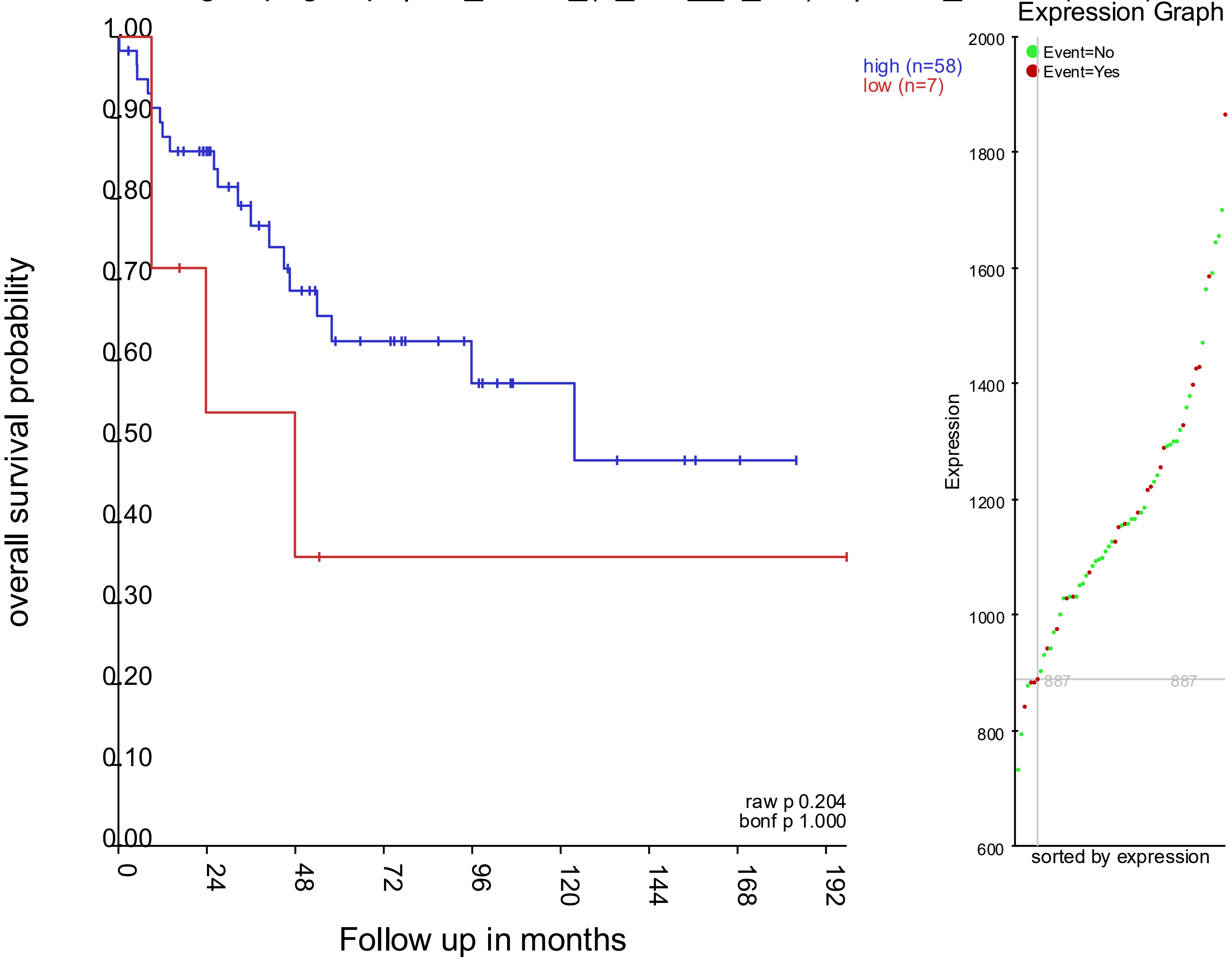

GROUP3 M1

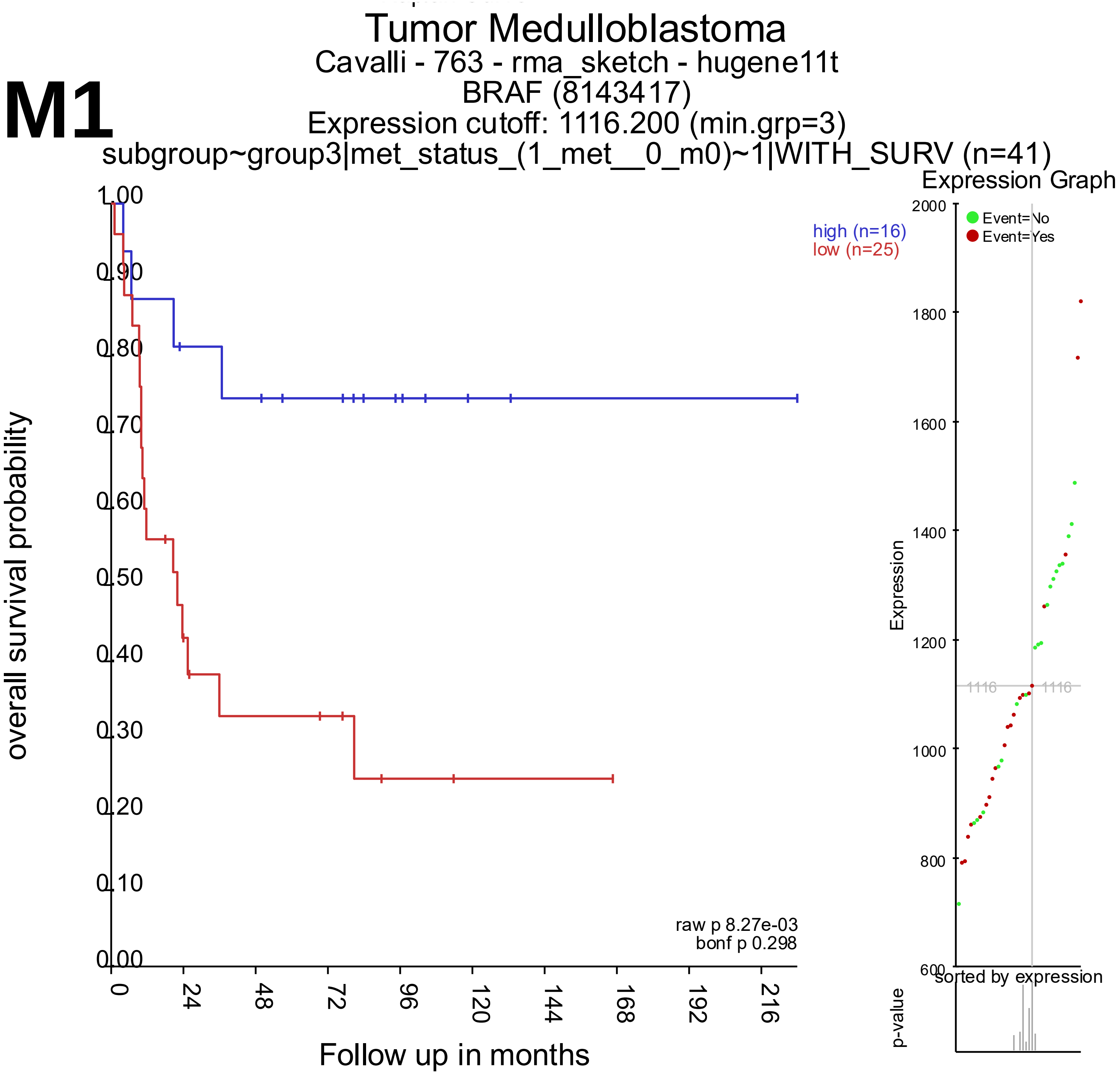

Tumor Medulloblastoma
Cavalli - 763 - rma_sketch - hugene11t
BRAF (8143417)
Expression cutoff: 1116.200 (min.grp=3)
subgroup~group3|met_status_(1_met__0_m0)~1|WITH_SURV (n=41)
high (n=16)
low (n=25)
overall survival probability
1.00
0.90
0.80
0.70
0.60
0.50
0.40
0.30
0.20
0.10
0.00
0
24
48
72
96
120
144
168
192
216
Follow up in months
raw p 8.27e-03
bonf p 0.298
Expression Graph
Event=No
Event=Yes
Expression
2000
1800
1600
1400
1200
1000
800
600
1116
sorted by expression
p-value

RAF1

WNT M0

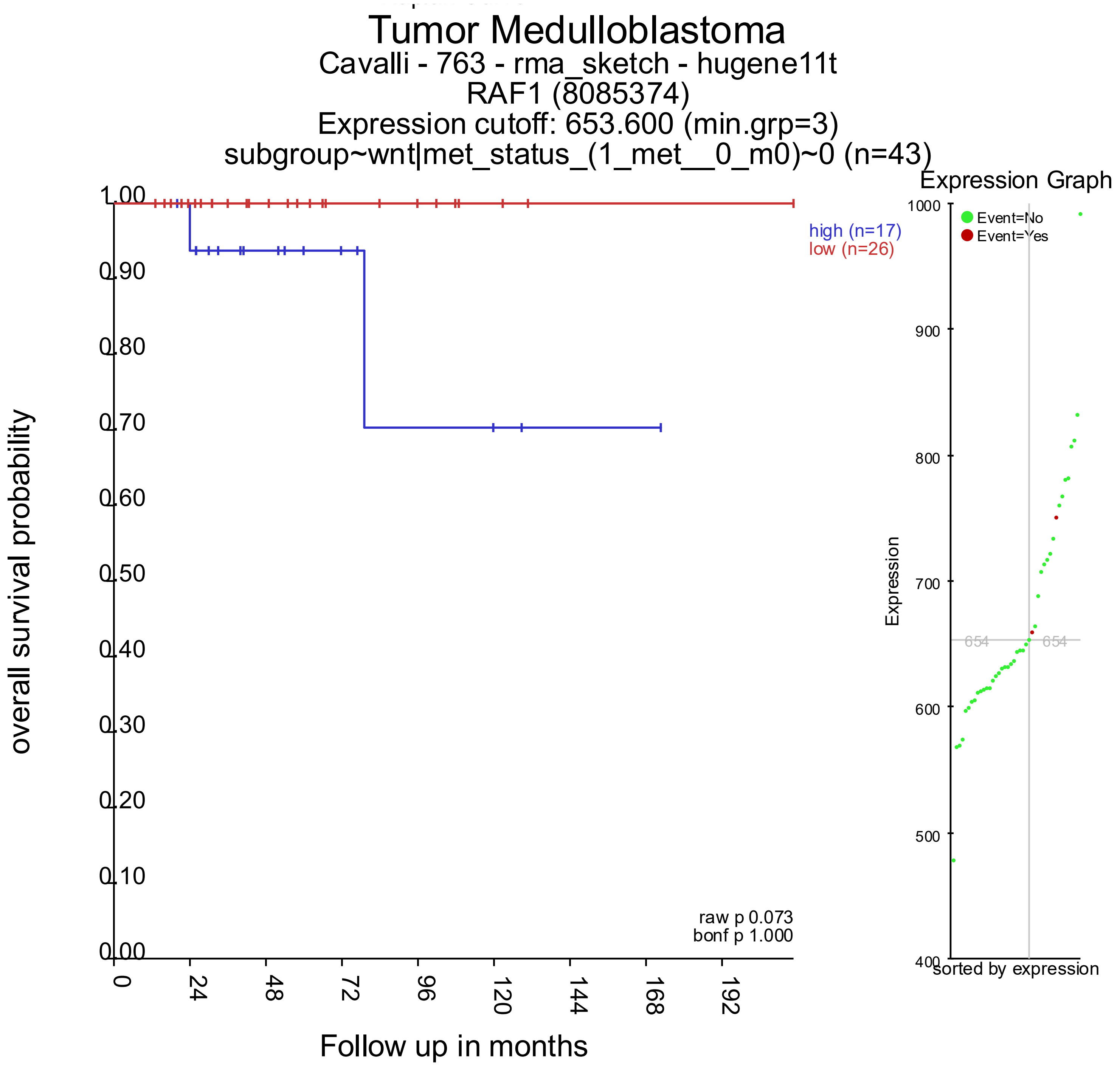
Tumor Medulloblastoma
Cavalli - 763 - rma_sketch - hugene11t
RAF1 (8085374)
Expression cutoff: 653.600 (min.grp=3)
subgroup~wnt|met_status_(1_met__0_m0)~0 (n=43)
high (n=17)
low (n=26)
overall survival probability
1.00
0.90
0.80
0.70
0.60
0.50
0.40
0.30
0.20
0.10
0.00
raw p 0.073
bonf p 1.000
0
24
48
72
96
120
144
168
192
Follow up in months
Expression Graph
Event=No
Event=Yes
Expression
1000
900
800
700
600
500
400
654
654
sorted by expression

WNT M1

Tumor Medulloblastoma
Cavalli - 763 - rma_sketch - hugene11t
RAF1 (8085374)
Expression cutoff: 675.400
subgroup~wnt|met_status_(1_met__0_m0)~1 (n=6)

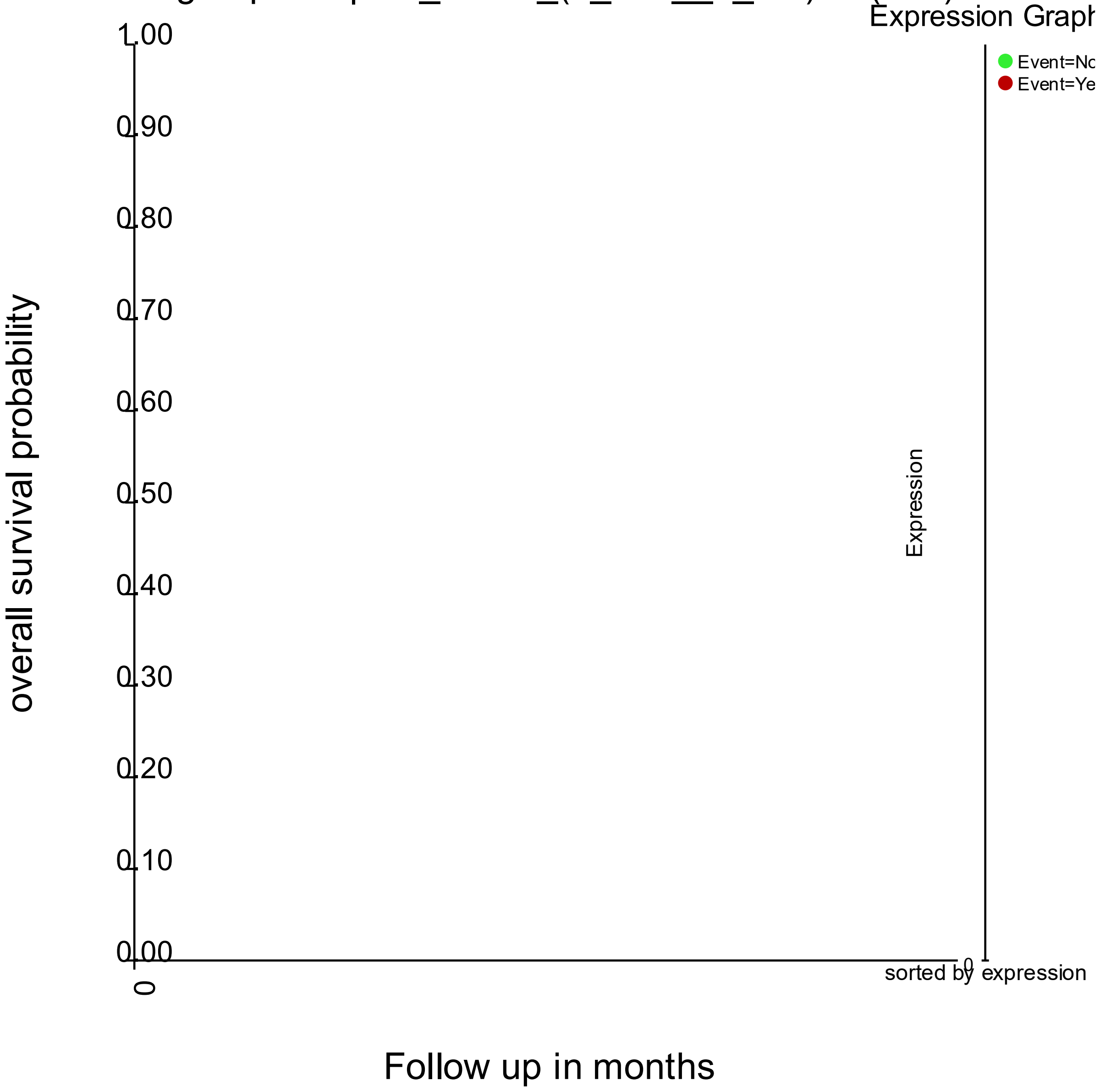

SHH M0

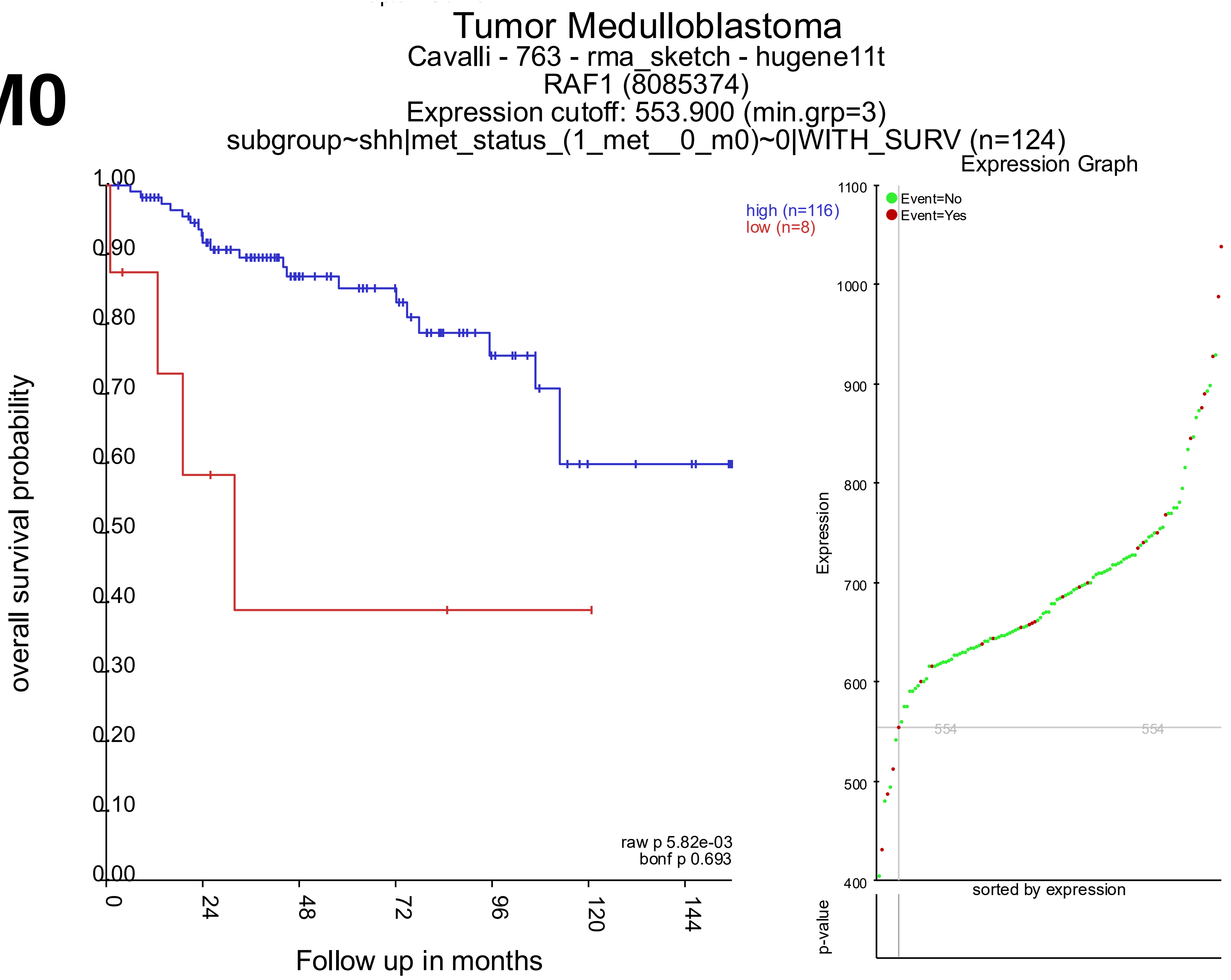
Tumor Medulloblastoma
Cavalli - 763 - rma_sketch - hugene11t
RAF1 (8085374)
Expression cutoff: 553.900 (min.grp=3)
subgroup~shh|met_status_(1_met__0_m0)~0|WITH_SURV (n=124)
overall survival probability
1.00
0.90
0.80
0.70
0.60
0.50
0.40
0.30
0.20
0.10
0.00
0
24
48
72
96
120
144
Follow up in months
high (n=116)
low (n=8)
raw p 5.82e-03
bonf p 0.693
Expression Graph
Event=No
Event=Yes
Expression
1100
1000
900
800
700
600
500
400
554
554
sorted by expression
p-value

SHH M1

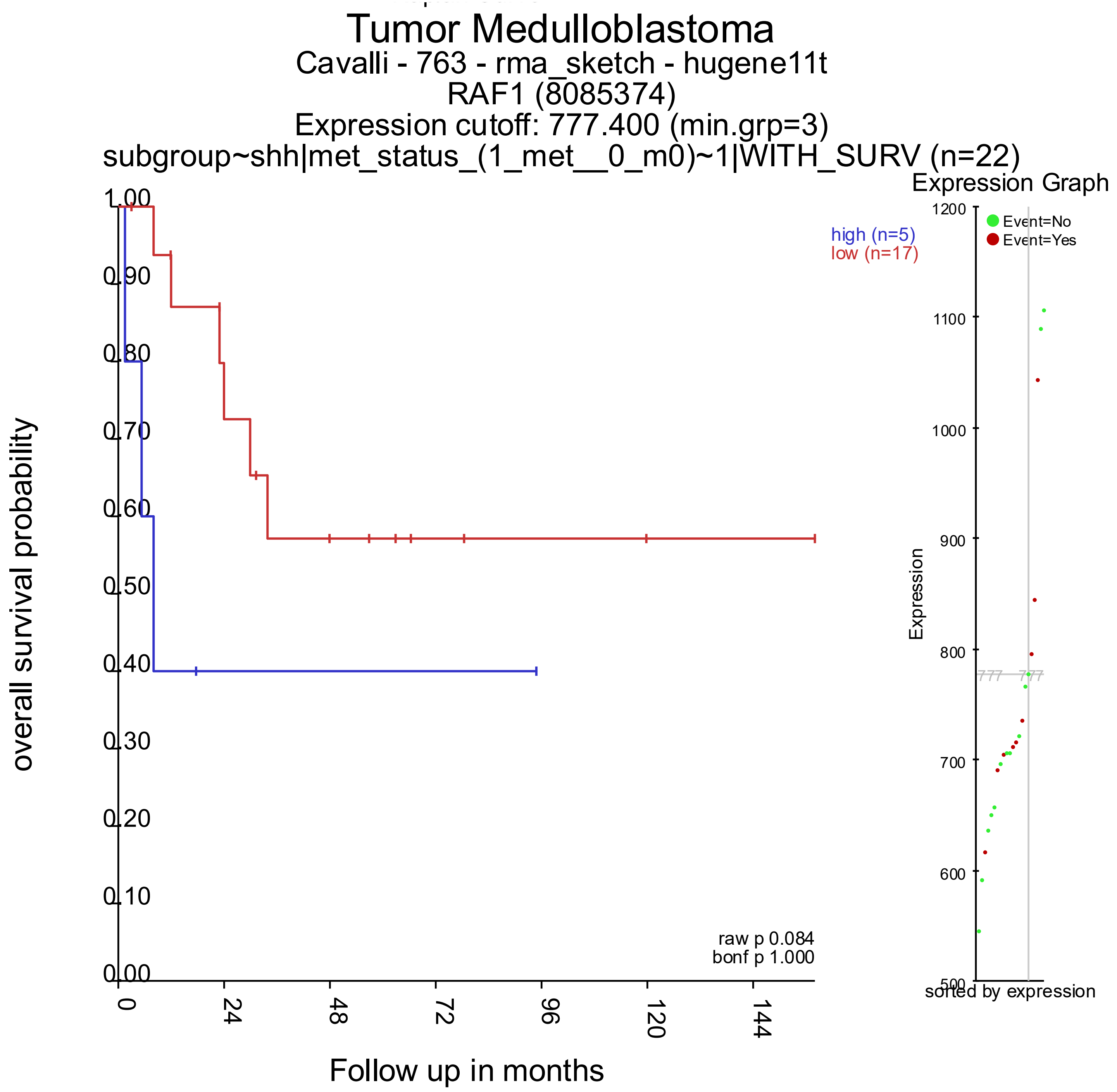

Tumor Medulloblastoma
Cavalli - 763 - rma_sketch - hugene11t
RAF1 (8085374)
Expression cutoff: 777.400 (min.grp=3)
subgroup~shh|met_status_(1_met__0_m0)~1|WITH_SURV (n=22)
overall survival probability
1.00
0.90
0.80
0.70
0.60
0.50
0.40
0.30
0.20
0.10
0.00
high (n=5)
low (n=17)
raw p 0.084
bonf p 1.000
0
24
48
72
96
120
144
Follow up in months
Expression Graph
Event=No
Event=Yes
Expression
1200
1100
1000
900
800
700
600
500
777
sorted by expression

GROUP4 M0

Tumor Medulloblastoma
Cavalli - 763 - rma_sketch - hugene11t
RAF1 (8085374)
Expression cutoff: 951.000 (min.grp=3)
subgroup~group4|met_status_(1_met__0_m0)~0|WITH_SURV (n=145)

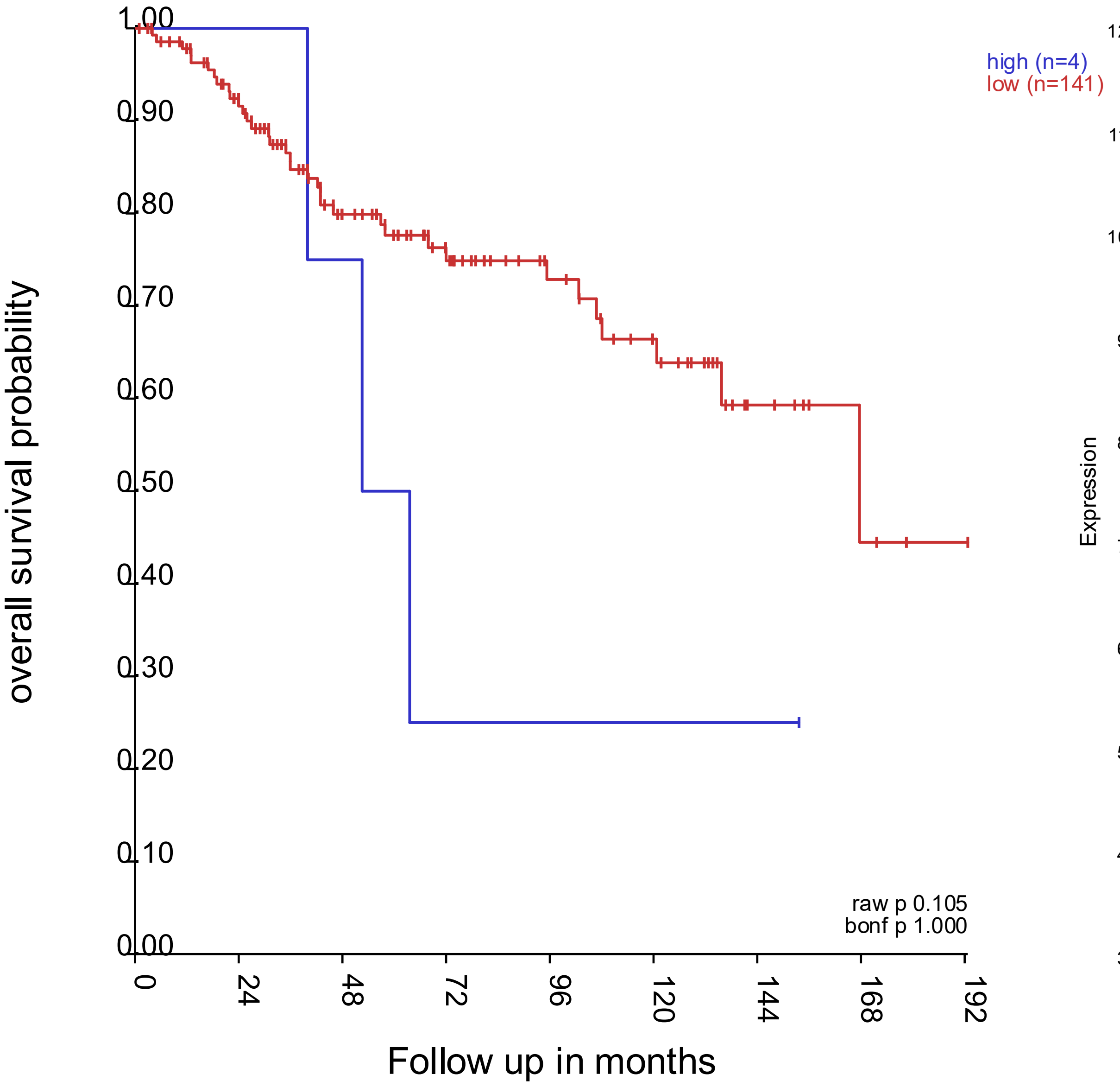

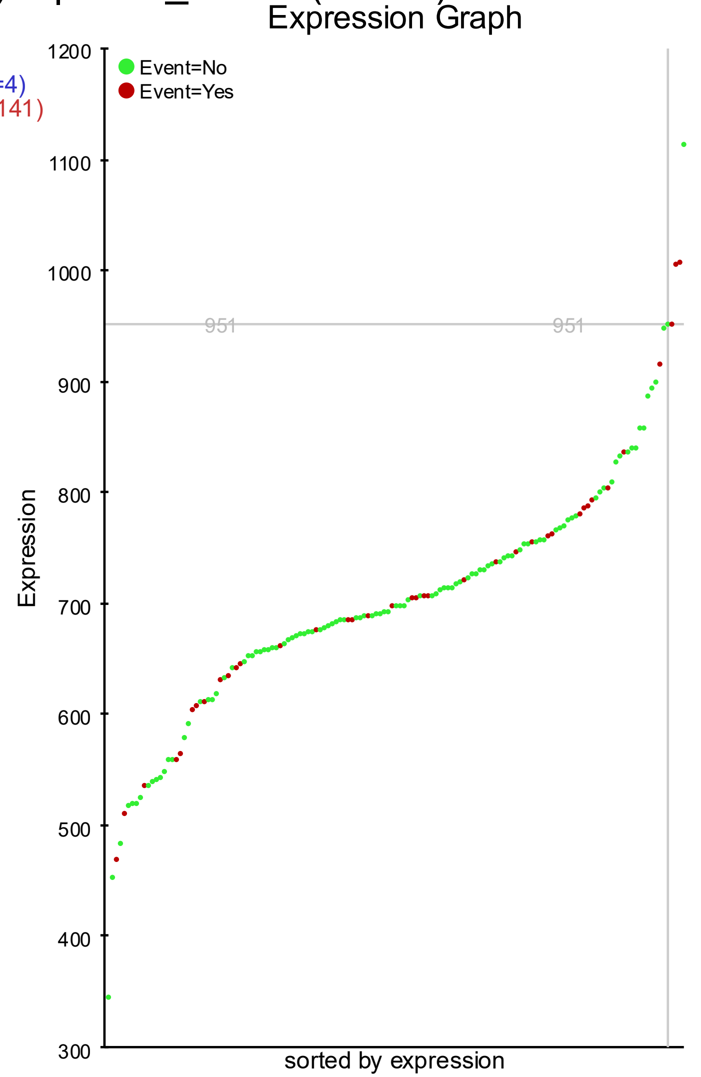

GROUP4 M1

Tumor Medulloblastoma
Cavalli - 763 - rma_sketch - hugene11t
RAF1 (8085374)
Expression cutoff: 756.200 (min.grp=3)
subgroup~group4|met_status_(1_met__0_m0)~1|WITH_SURV (n=92)

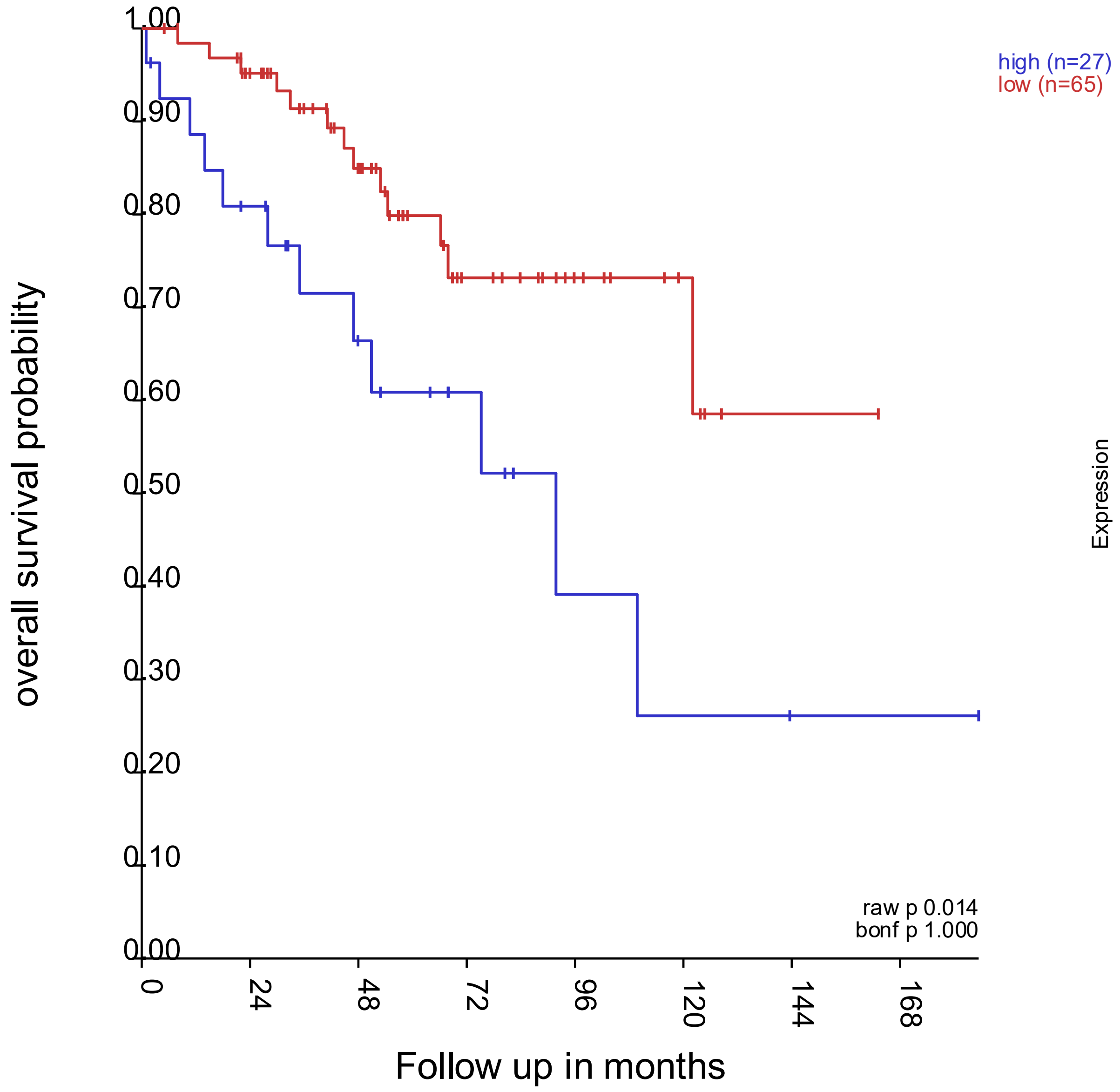

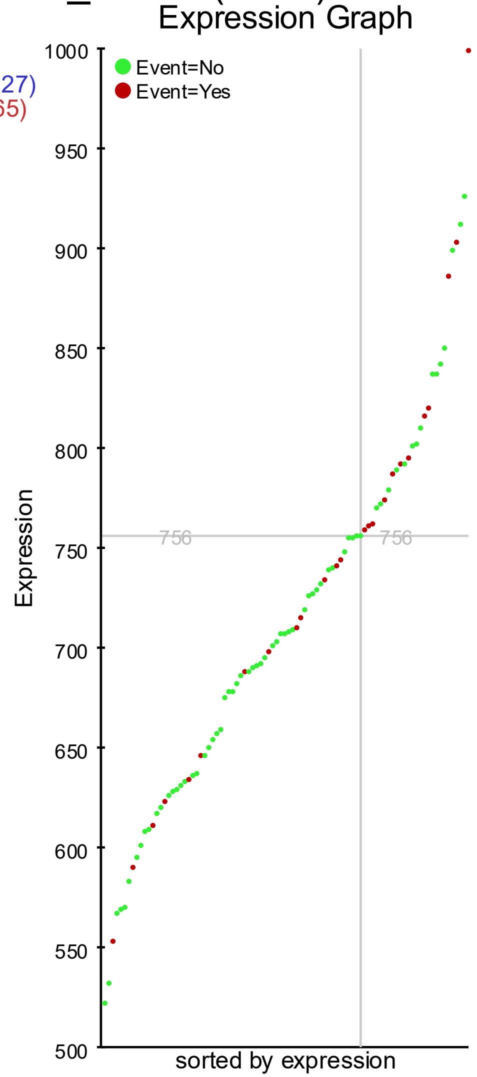

GROUP3 M0

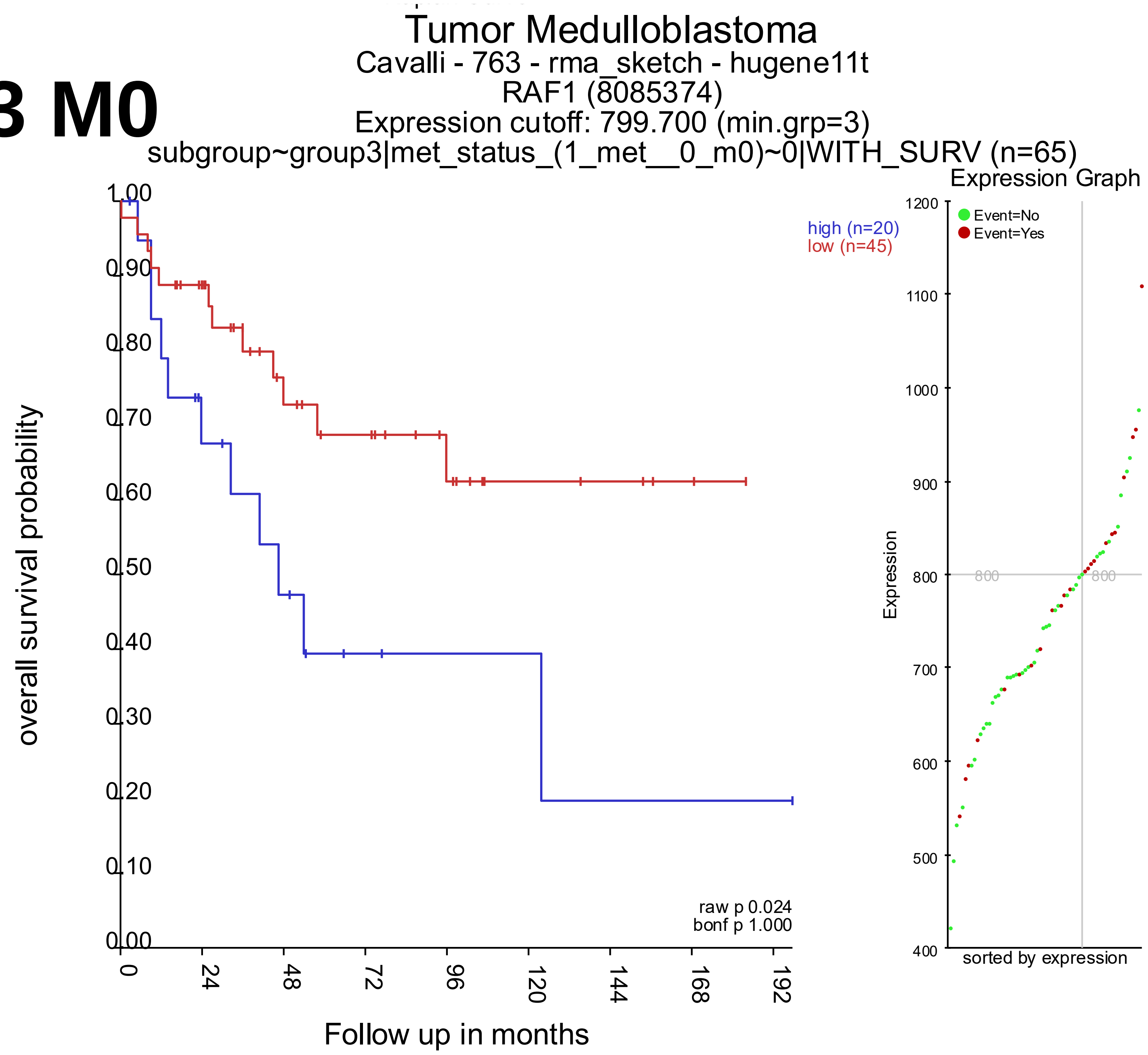

Tumor Medulloblastoma
Cavalli - 763 - rma_sketch - hugene11t
RAF1 (8085374)
Expression cutoff: 799.700 (min.grp=3)
subgroup~group3|met_status_(1_met__0_m0)~0|WITH_SURV (n=65)
high (n=20)
low (n=45)
overall survival probability
1.00
0.90
0.80
0.70
0.60
0.50
0.40
0.30
0.20
0.10
0.00
raw p 0.024
bonf p 1.000
0
24
48
72
96
120
144
168
192
Follow up in months
Expression Graph
Event=No
Event=Yes
Expression
1200
1100
1000
900
800
700
600
500
400
800
800
sorted by expression

GROUP3 M1

Tumor Medulloblastoma
Cavalli - 763 - rma_sketch - hugene11t
RAF1 (8085374)
Expression cutoff: 864.600 (min.grp=3)
subgroup~group3|met_status_(1_met__0_m0)~1|WITH_SURV (n=41)

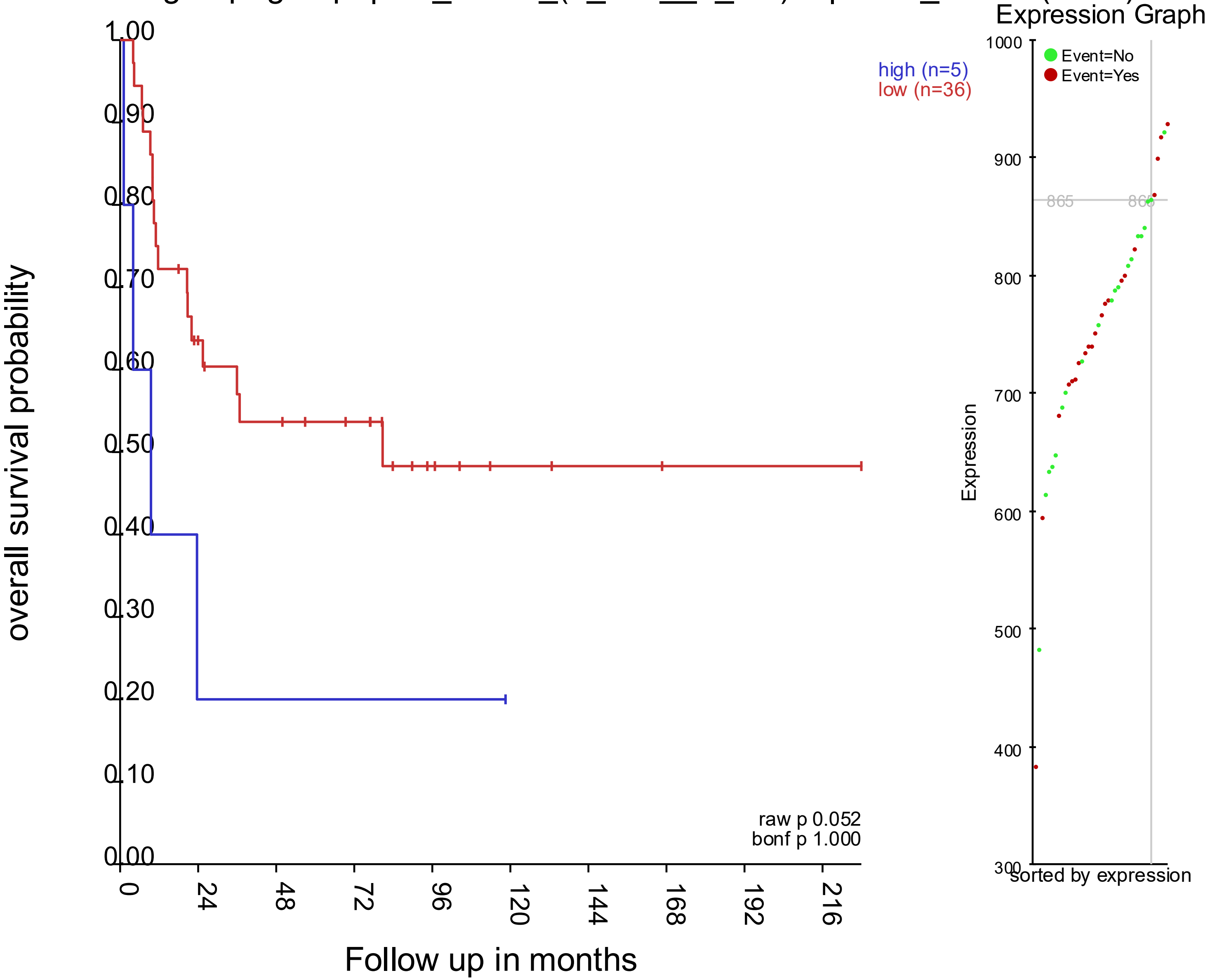

RET

WNT M0

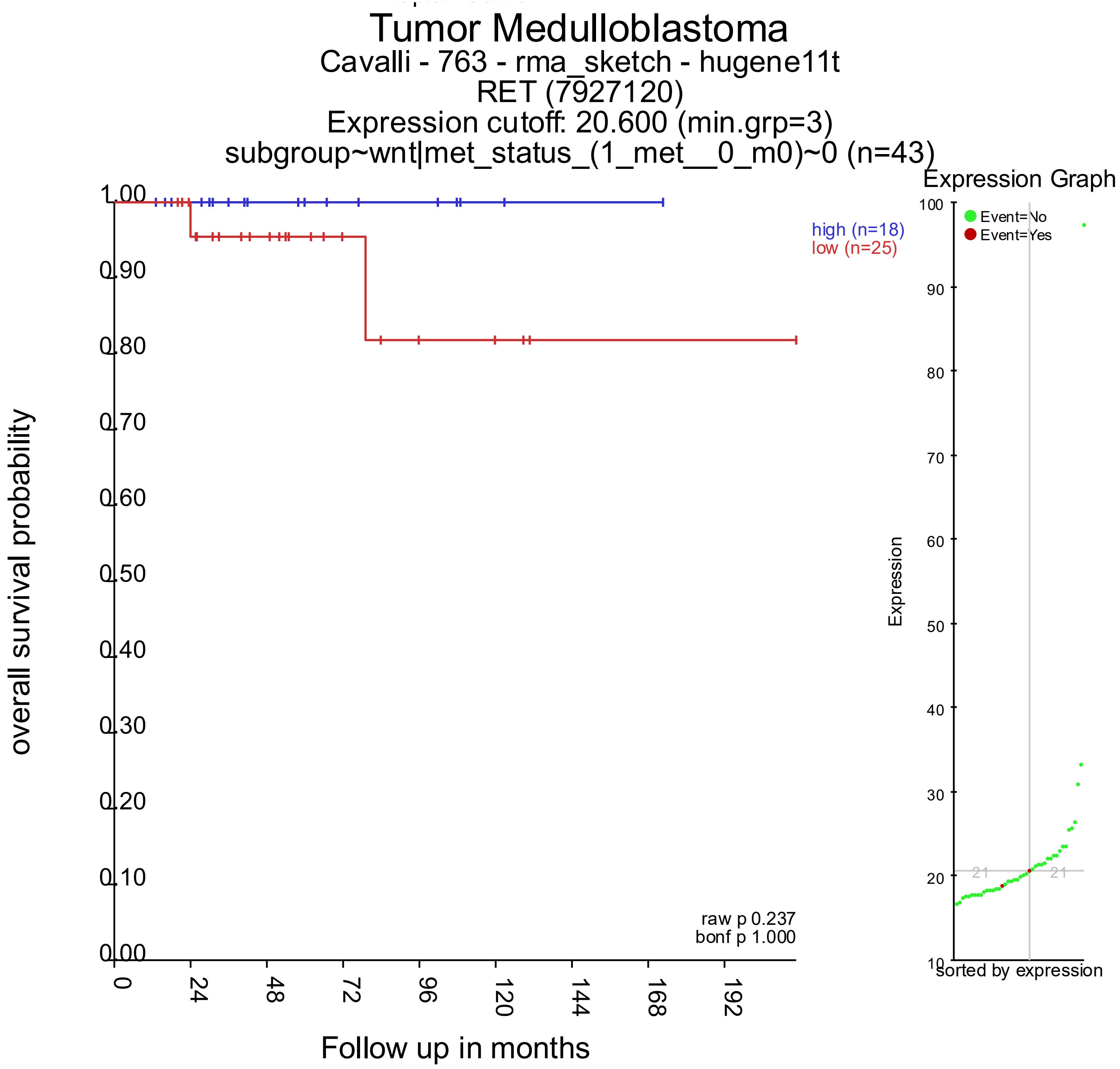

Tumor Medulloblastoma
Cavalli - 763 - rma_sketch - hugene11t
RET (7927120)
Expression cutoff: 20.600 (min.grp=3)
subgroup~wnt|met_status_(1_met__0_m0)~0 (n=43)
overall survival probability
1.00
0.90
0.80
0.70
0.60
0.50
0.40
0.30
0.20
0.10
0.00
0
24
48
72
96
120
144
168
192
Follow up in months
high (n=18)
low (n=25)
raw p 0.237
bonf p 1.000
Expression Graph
Event=No
Event=Yes
Expression
100
90
80
70
60
50
40
30
20
10
21
21
sorted by expression

WNT M1

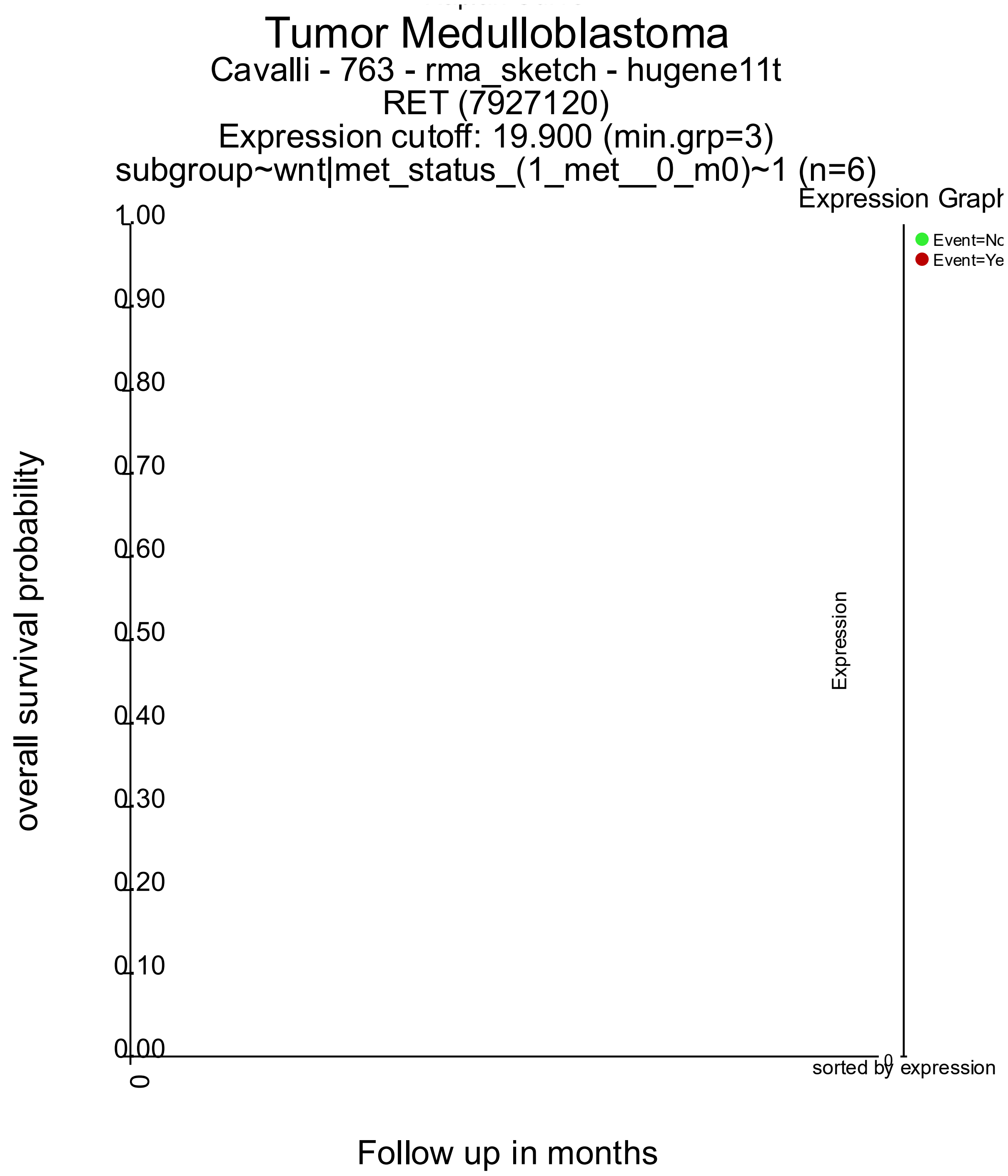

Tumor Medulloblastoma
Cavalli - 763 - rma_sketch - hugene11t
RET (7927120)
Expression cutoff: 19.900 (min.grp=3)
subgroup~wnt|met_status_(1_met__0_m0)~1 (n=6)
Expression Graph
Event=No
Event=Yes
1.00
0.90
0.80
0.70
0.60
0.50
0.40
0.30
0.20
0.10
0.00
overall survival probability
0
Follow up in months
Expression
0
sorted by expression

SHH M0

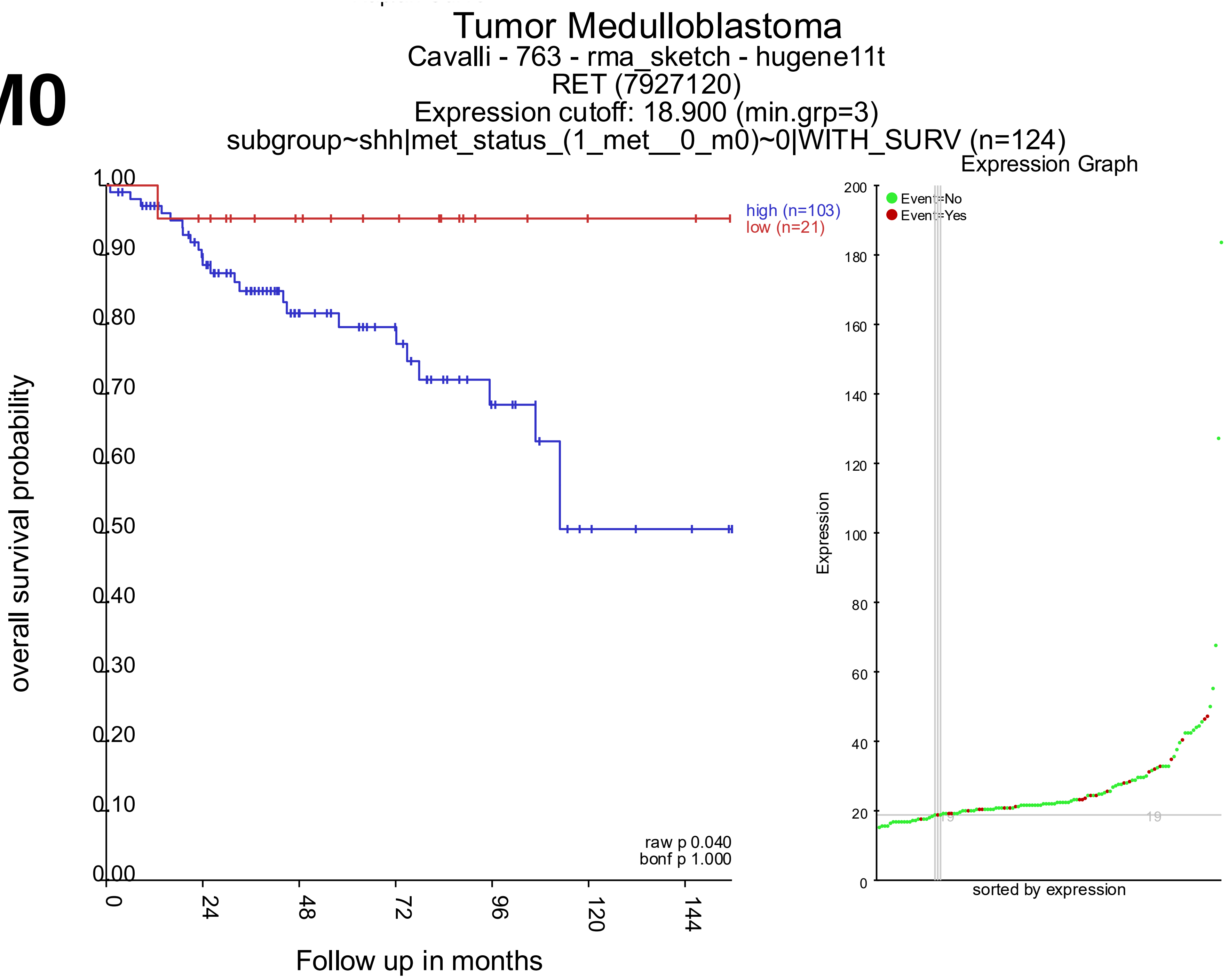
Tumor Medulloblastoma
Cavalli - 763 - rma_sketch - hugene11t
RET (7927120)
Expression cutoff: 18.900 (min.grp=3)
subgroup~shh|met_status_(1_met__0_m0)~0|WITH_SURV (n=124)
overall survival probability
1.00
0.90
0.80
0.70
0.60
0.50
0.40
0.30
0.20
0.10
0.00
high (n=103)
low (n=21)
raw p 0.040
bonf p 1.000
0
24
48
72
96
120
144
Follow up in months
Expression Graph
Event=No
Event=Yes
Expression
200
180
160
140
120
100
80
60
40
20
0
19
19
sorted by expression

SHH M1

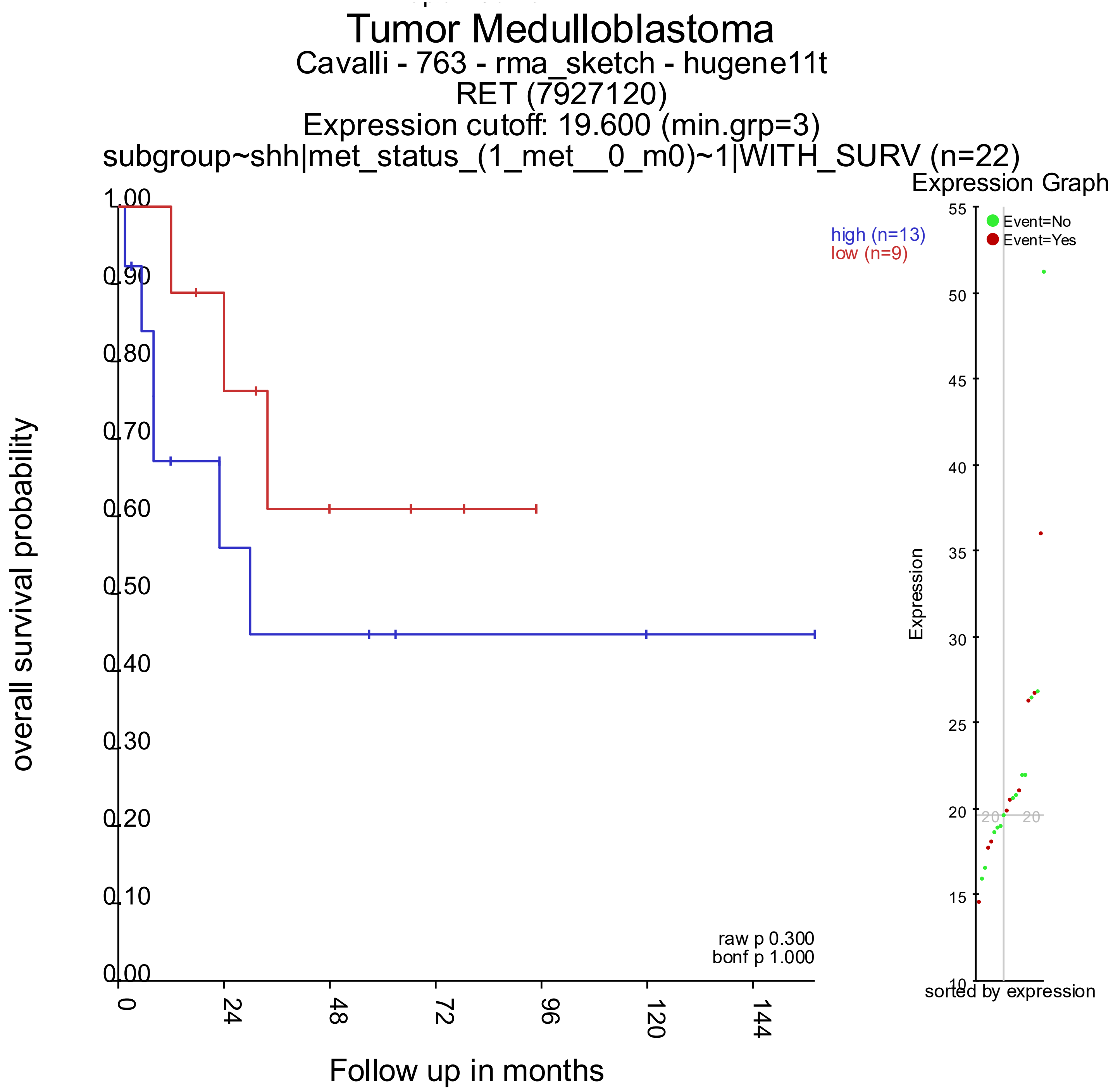

Tumor Medulloblastoma
Cavalli - 763 - rma_sketch - hugene11t
RET (7927120)
Expression cutoff: 19.600 (min.grp=3)
subgroup~shh|met_status_(1_met__0_m0)~1|WITH_SURV (n=22)
overall survival probability
Follow up in months
high (n=13)
low (n=9)
raw p 0.300
bonf p 1.000
Expression Graph
Event=No
Event=Yes
Expression
sorted by expression

GROUP4 M0

Tumor Medulloblastoma
Cavalli - 763 - rma_sketch - hugene11t
RET (7927120)
Expression cutoff: 21.100 (min.grp=3)
subgroup~group4|met_status_(1_met__0_m0)~0|WITH_SURV (n=145)

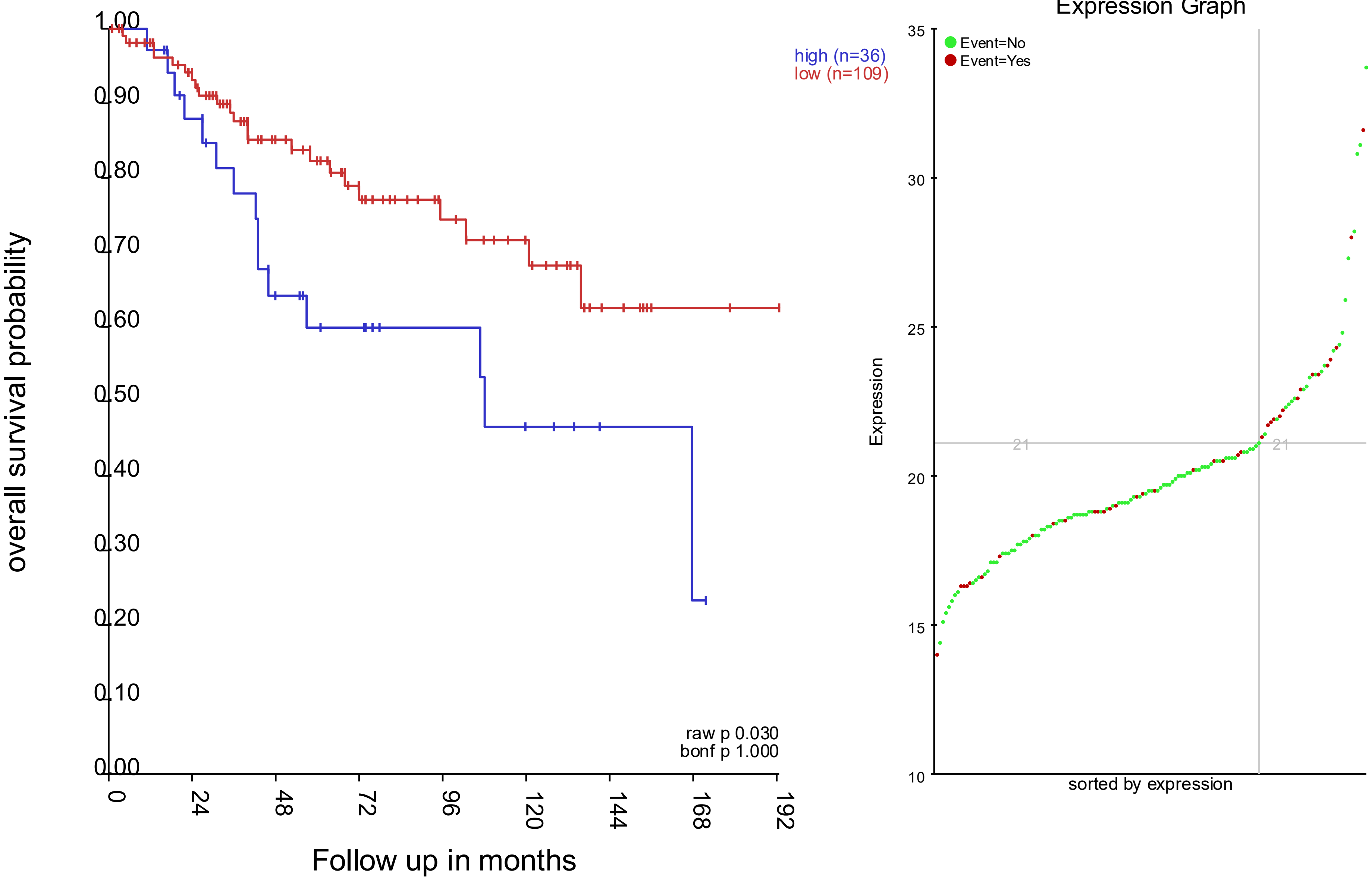

GROUP4 M1

Tumor Medulloblastoma
Cavalli - 763 - rma_sketch - hugene11t
RET (7927120)
Expression cutoff: 21.900 (min.grp=3)
subgroup~group4|met_status_(1_met__0_m0)~1|WITH_SURV (n=92)

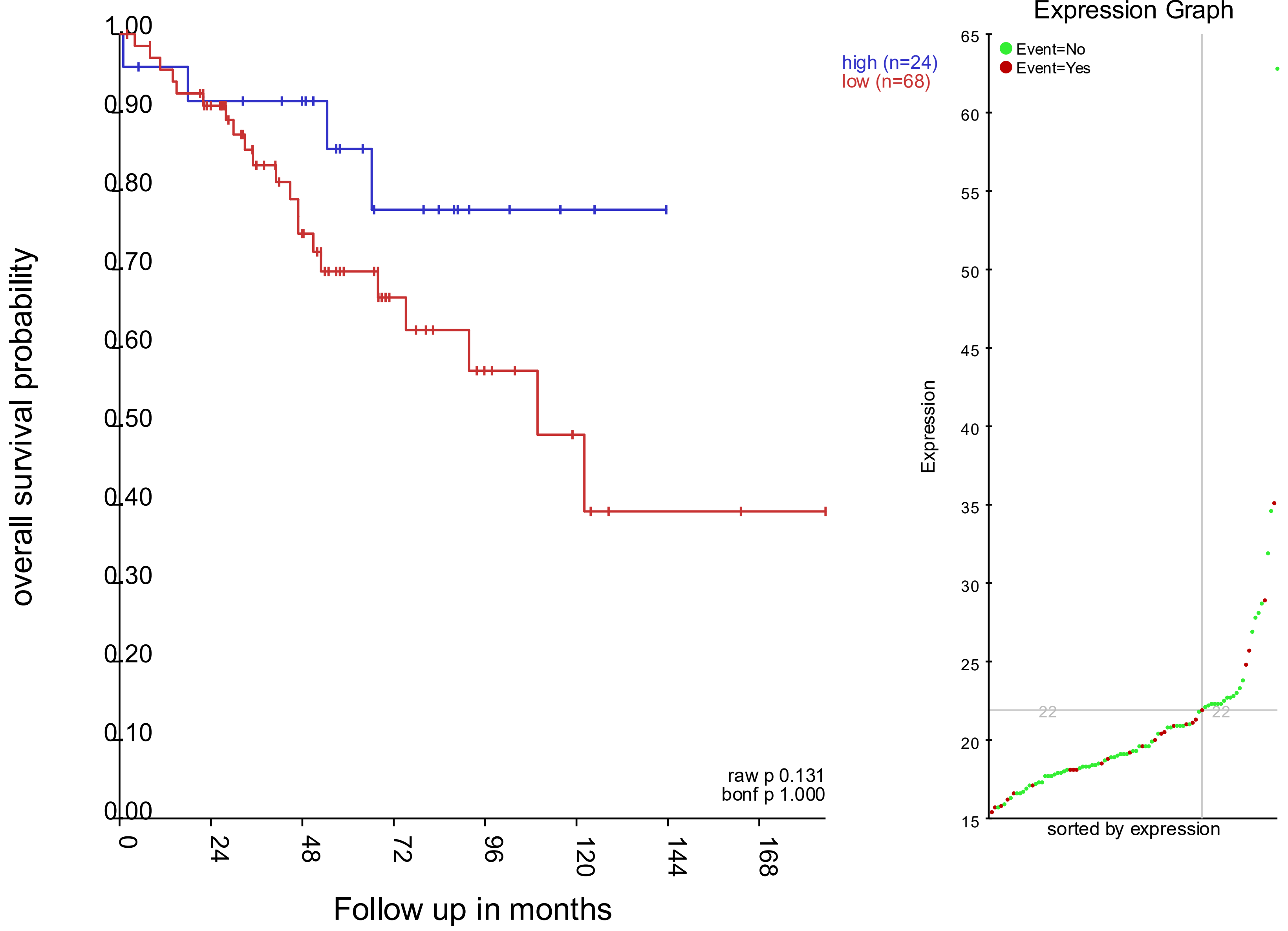

GROUP3 M0

Tumor Medulloblastoma
Cavalli - 763 - rma_sketch - hugene11t
RET (7927120)
Expression cutoff: 17.500 (min.grp=3)
subgroup~group3|met_status_(1_met__0_m0)~0|WITH_SURV (n=65)

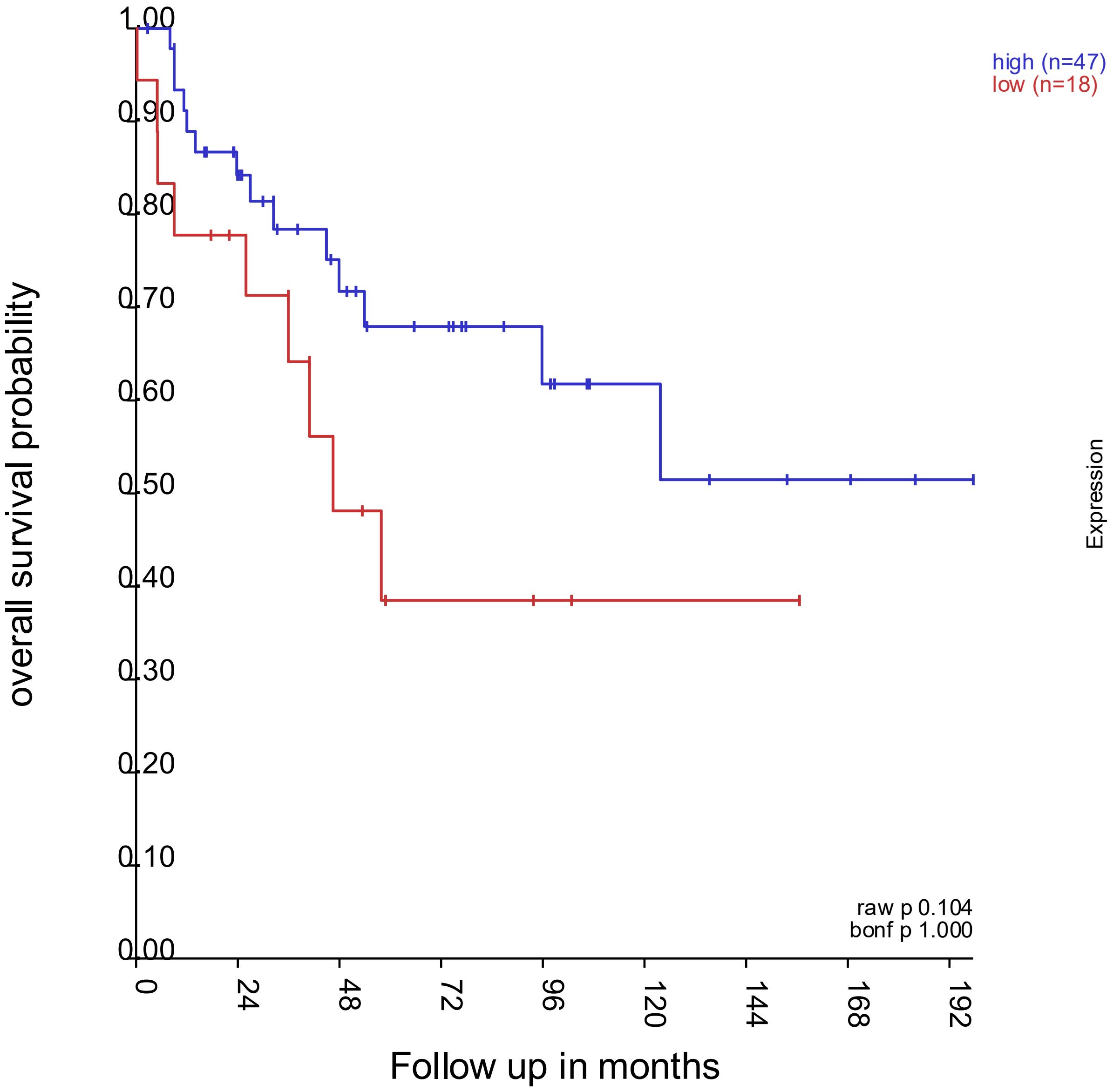

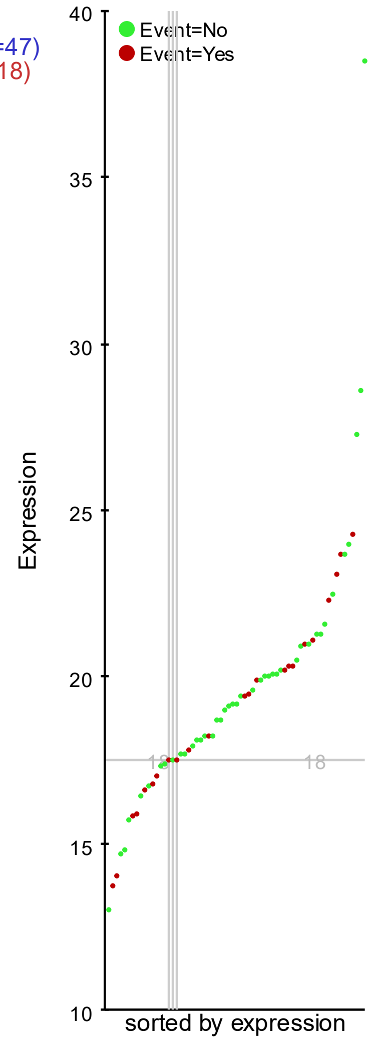

GROUP3 M1

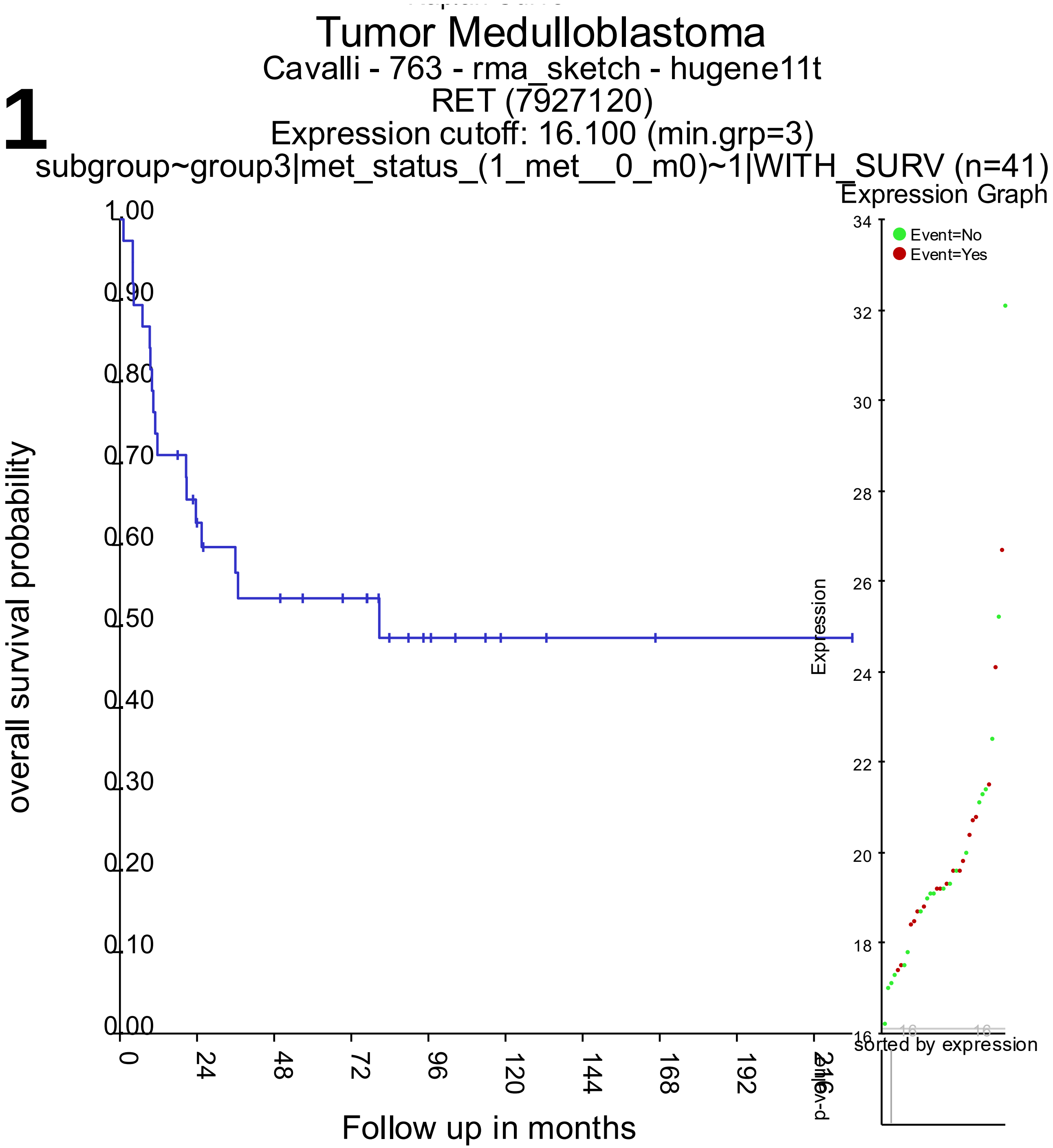
Tumor Medulloblastoma
Cavalli - 763 - rma_sketch - hugene11t
RET (7927120)
Expression cutoff: 16.100 (min.grp=3)
subgroup~group3|met_status_(1_met__0_m0)~1|WITH_SURV (n=41)
overall survival probability
1.00
0.90
0.80
0.70
0.60
0.50
0.40
0.30
0.20
0.10
0.00
0
24
48
72
96
120
144
168
192
216
Follow up in months
Expression Graph
Event=No
Event=Yes
Expression
34
32
30
28
26
24
22
20
18
16
sorted by expression

ROS1

WNT M0

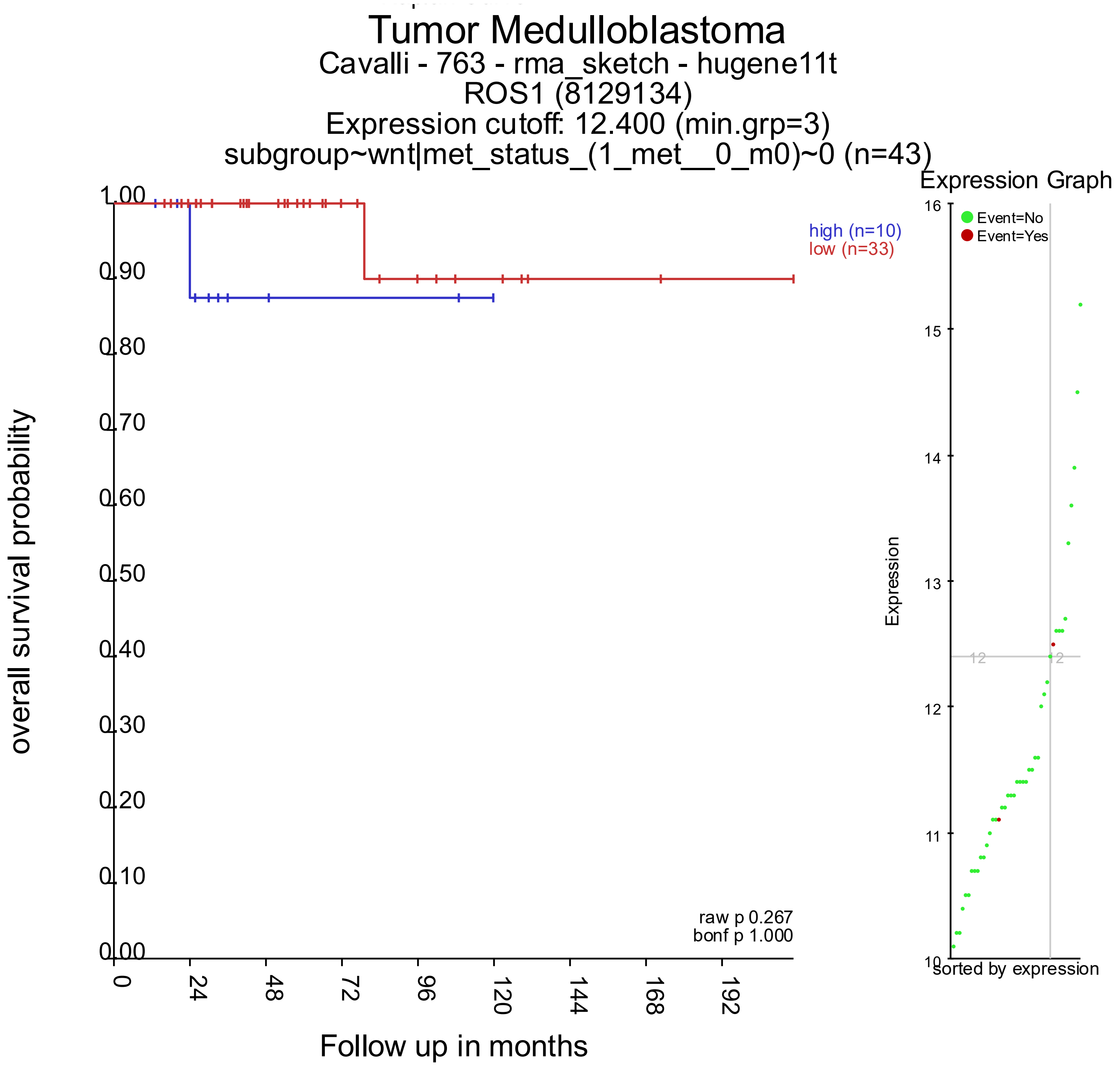

Tumor Medulloblastoma
Cavalli - 763 - rma_sketch - hugene11t
ROS1 (8129134)
Expression cutoff: 12.400 (min.grp=3)
subgroup~wnt|met_status_(1_met__0_m0)~0 (n=43)
overall survival probability
1.00
0.90
0.80
0.70
0.60
0.50
0.40
0.30
0.20
0.10
0.00
0
24
48
72
96
120
144
168
192
Follow up in months
high (n=10)
low (n=33)
raw p 0.267
bonf p 1.000
Expression Graph
Event=No
Event=Yes
Expression
16
15
14
13
12
11
10
sorted by expression

WNT M1

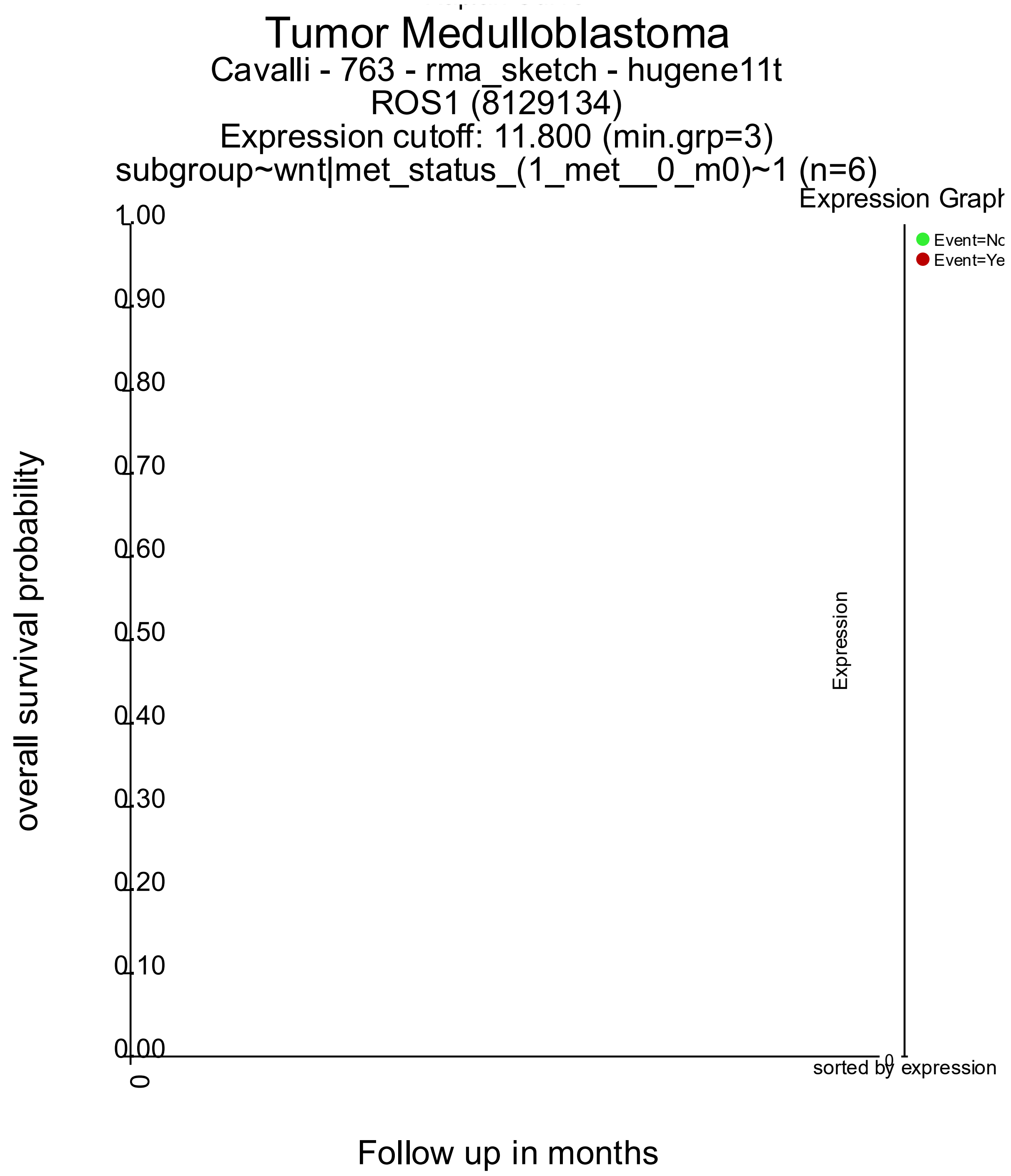

Tumor Medulloblastoma
Cavalli - 763 - rma_sketch - hugene11t
ROS1 (8129134)
Expression cutoff: 11.800 (min.grp=3)
subgroup~wnt|met_status_(1_met__0_m0)~1 (n=6)
Expression Graph
Event=No
Event=Yes
overall survival probability
1.00
0.90
0.80
0.70
0.60
0.50
0.40
0.30
0.20
0.10
0.00
0
Follow up in months
Expression
0
sorted by expression

SHH M0

Tumor Medulloblastoma
Cavalli - 763 - rma_sketch - hugene11t
ROS1 (8129134)
Expression cutoff: 10.200 (min.grp=3)
subgroup~shh|met_status_(1_met__0_m0)~0|WITH_SURV (n=124)

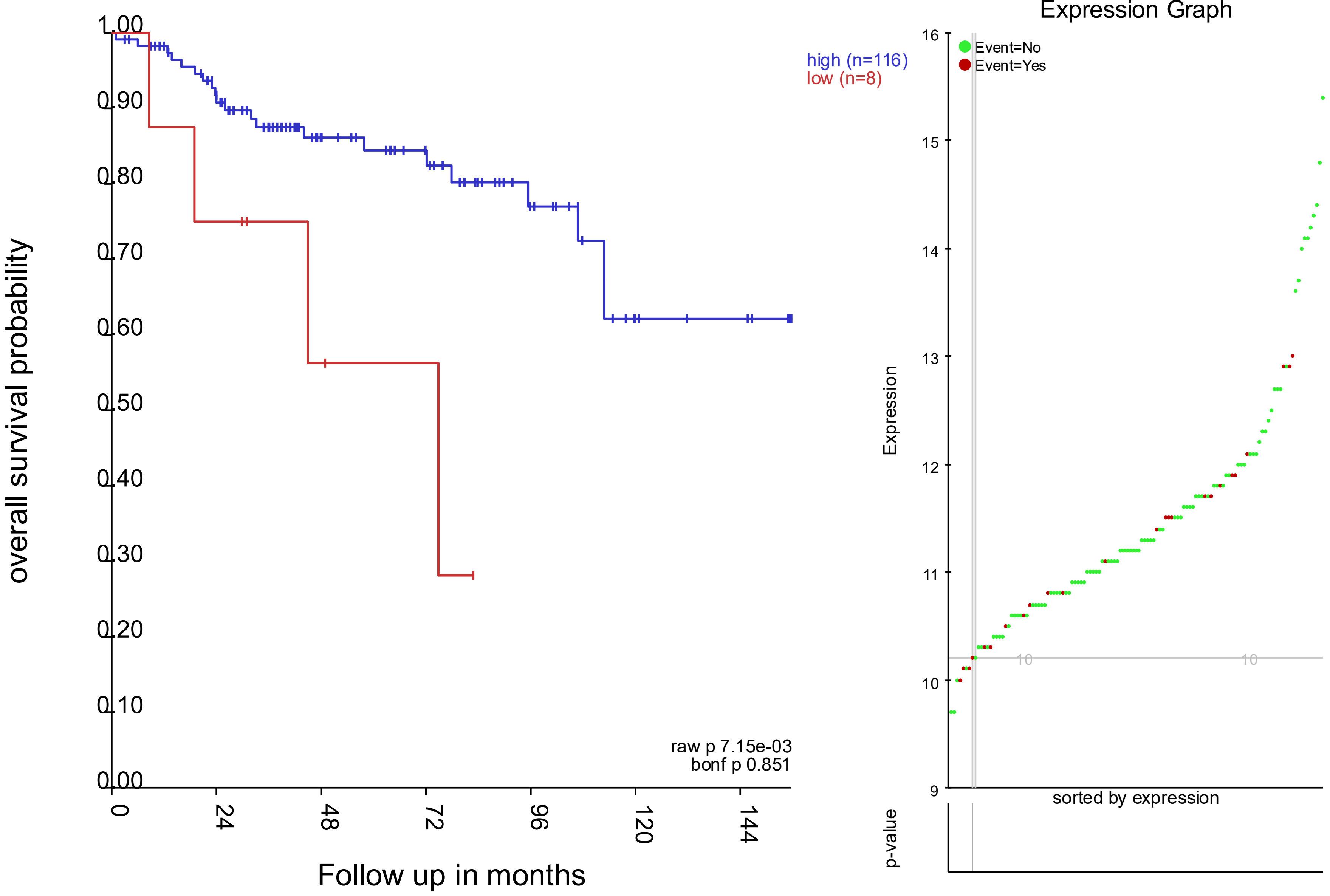

SHH M1

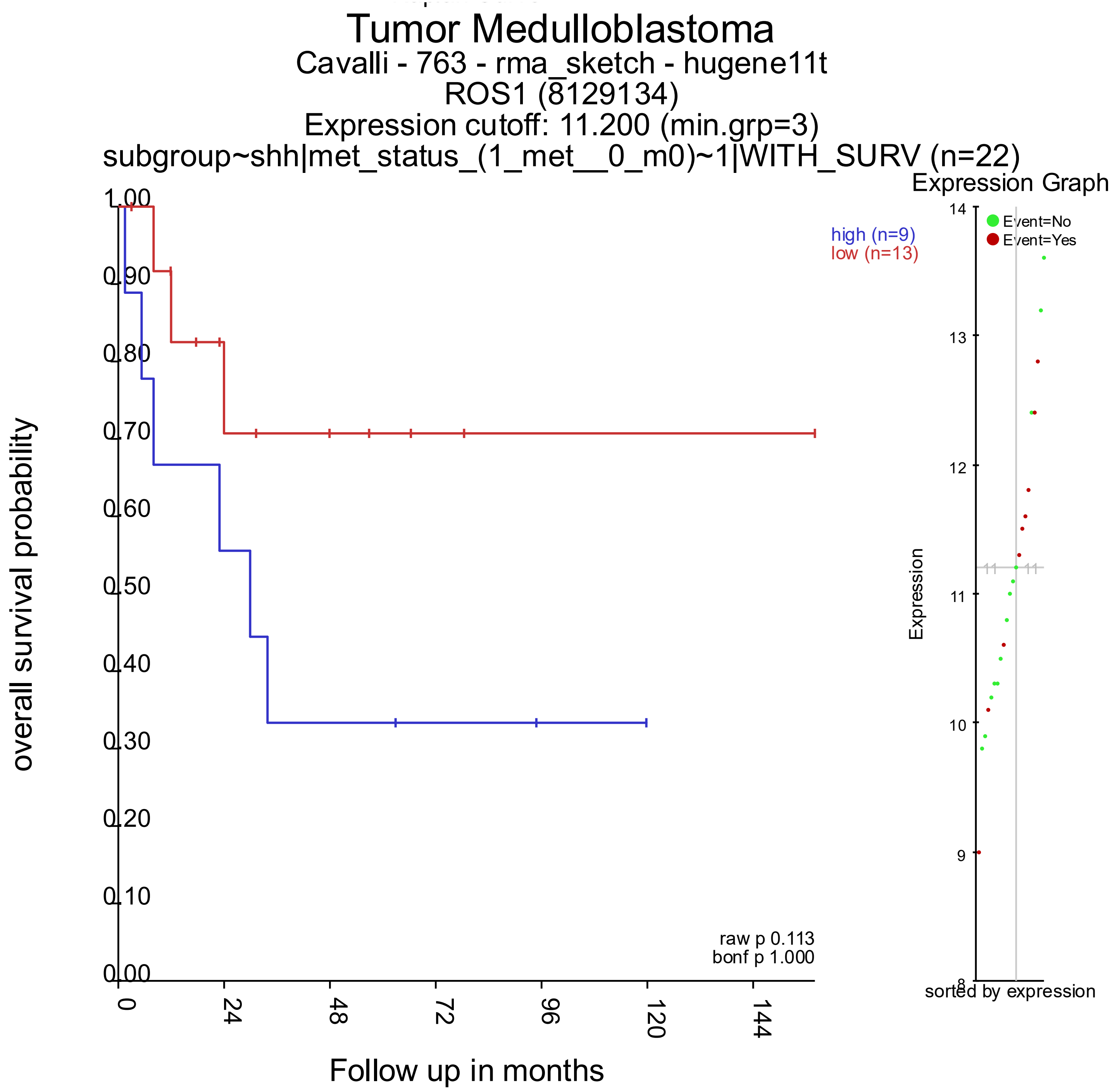
Tumor Medulloblastoma
Cavalli - 763 - rma_sketch - hugene11t
ROS1 (8129134)
Expression cutoff: 11.200 (min.grp=3)
subgroup~shh|met_status_(1_met__0_m0)~1|WITH_SURV (n=22)
high (n=9)
low (n=13)
overall survival probability
1.00
0.90
0.80
0.70
0.60
0.50
0.40
0.30
0.20
0.10
0.00
0
24
48
72
96
120
144
Follow up in months
raw p 0.113
bonf p 1.000
Expression Graph
Event=No
Event=Yes
Expression
14
13
12
11
10
9
8
sorted by expression

GROUP4 M0

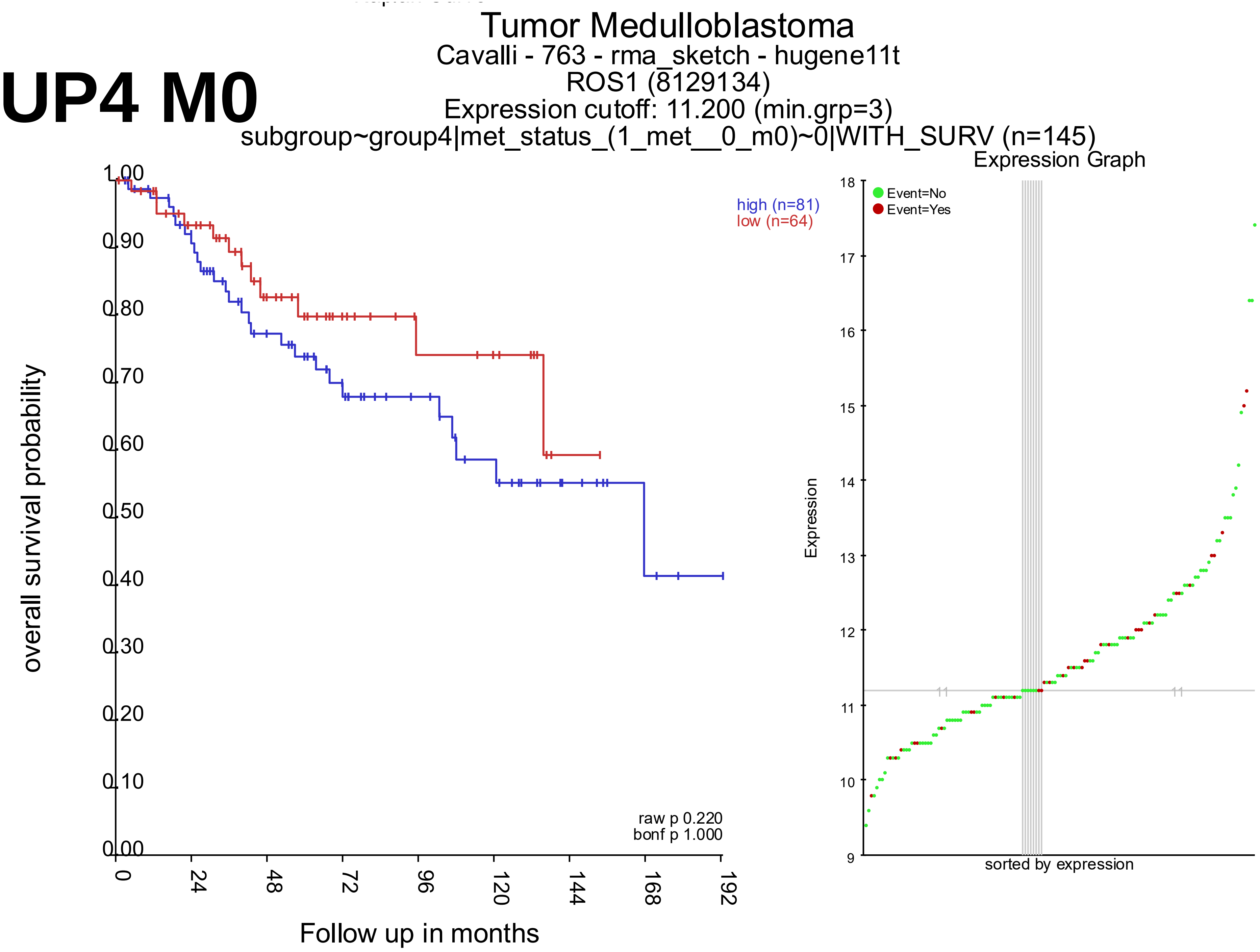
Tumor Medulloblastoma
Cavalli - 763 - rma_sketch - hugene11t
ROS1 (8129134)
Expression cutoff: 11.200 (min.grp=3)
subgroup~group4|met_status_(1_met__0_m0)~0|WITH_SURV (n=145)
overall survival probability
1.00
0.90
0.80
0.70
0.60
0.50
0.40
0.30
0.20
0.10
0.00
0
24
48
72
96
120
144
168
192
Follow up in months
high (n=81)
low (n=64)
raw p 0.220
bonf p 1.000
Expression Graph
Event=No
Event=Yes
Expression
18
17
16
15
14
13
12
11
10
9
sorted by expression

GROUP4 M1

Tumor Medulloblastoma
Cavalli - 763 - rma_sketch - hugene11t
ROS1 (8129134)
Expression cutoff: 11.000 (min.grp=3)
subgroup~group4|met_status_(1_met__0_m0)~1|WITH_SURV (n=92)

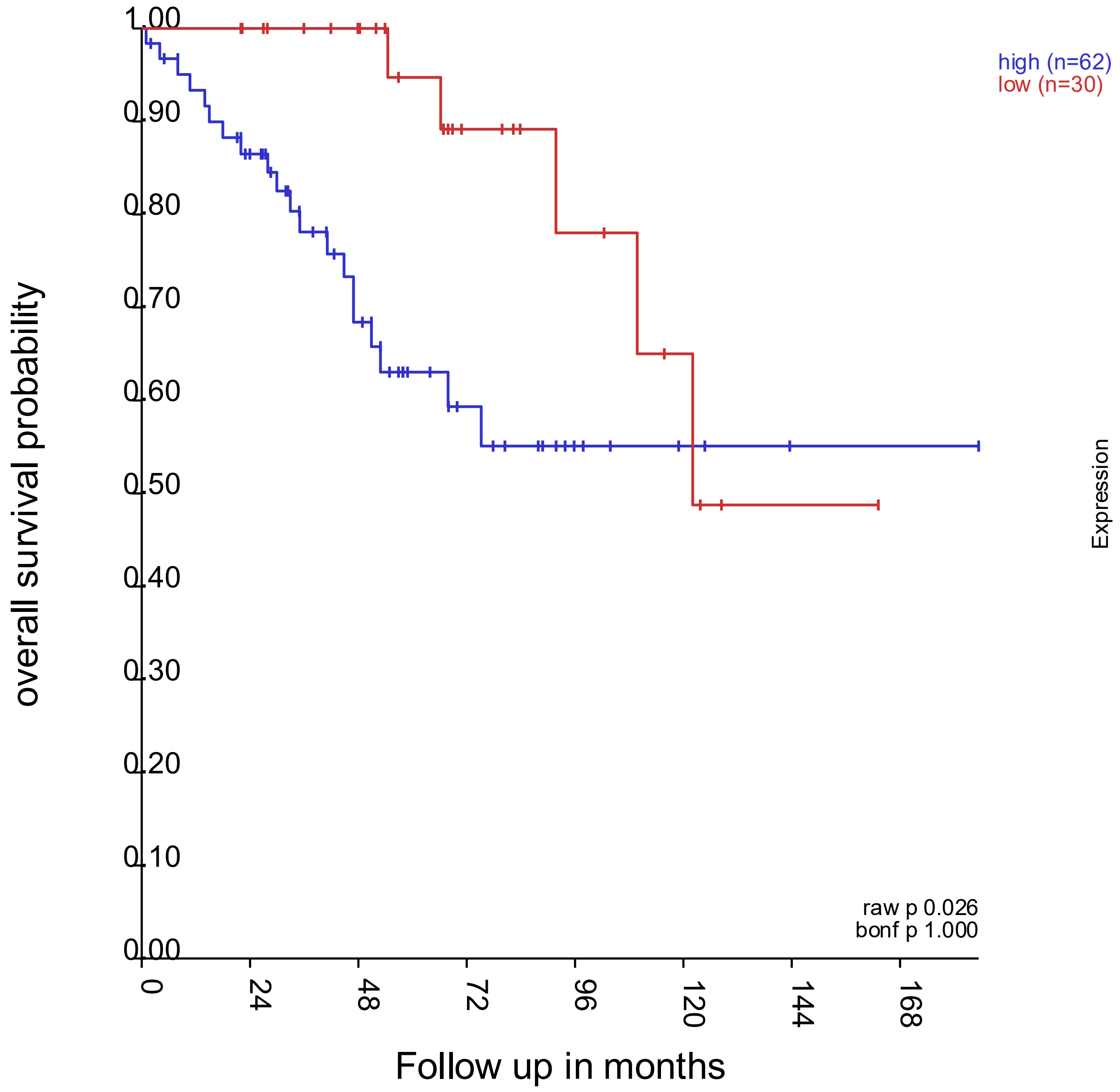

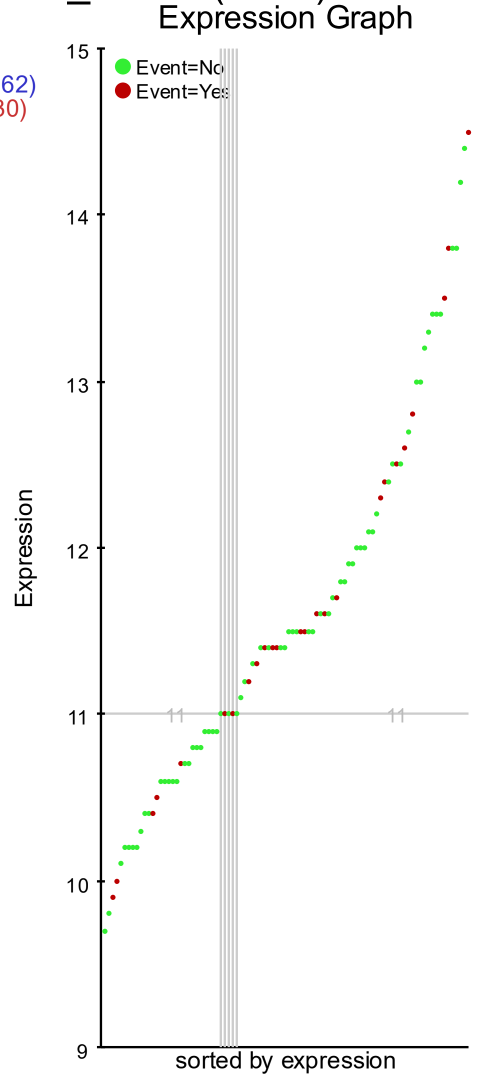

GROUP3 M0

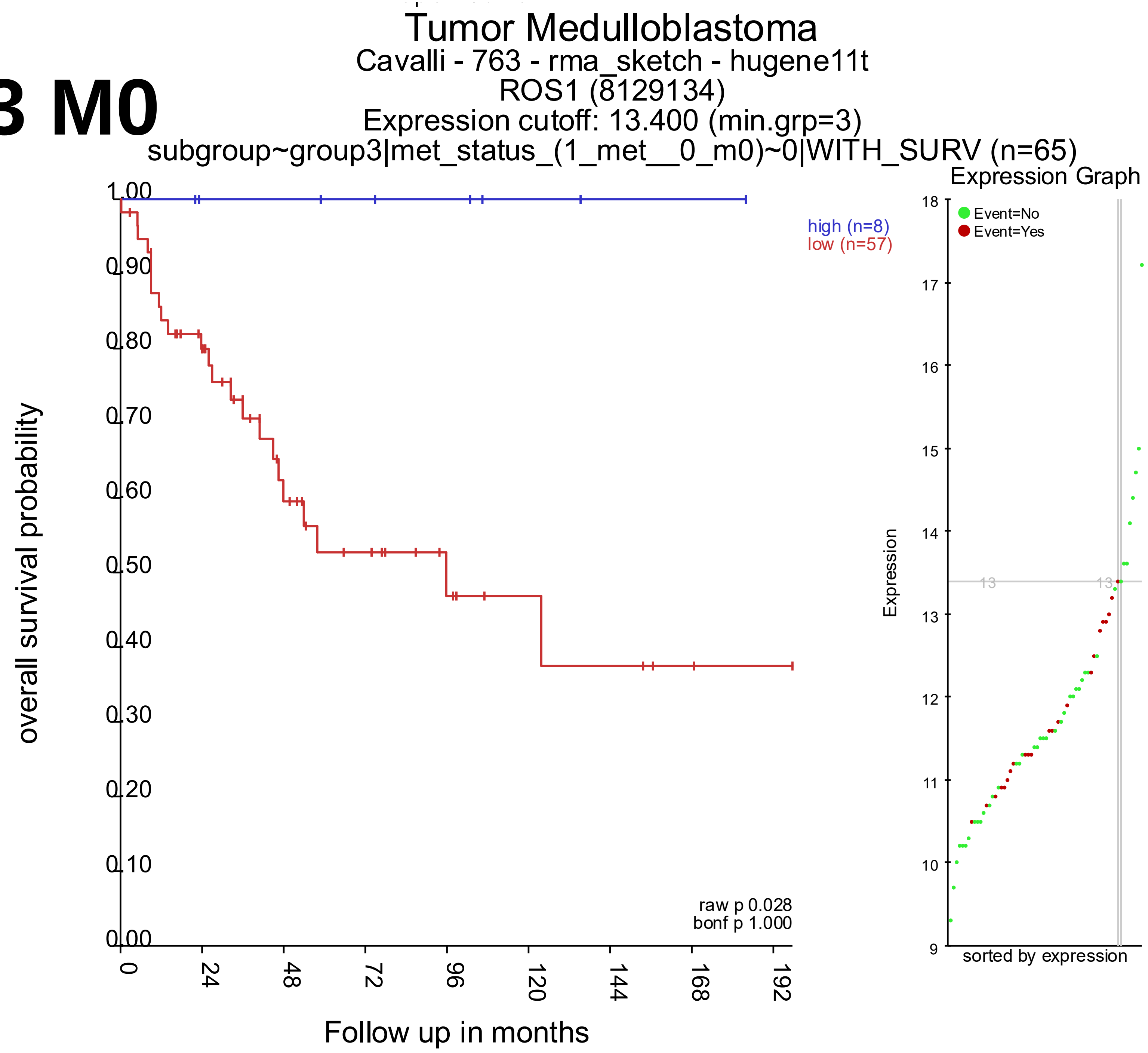

Tumor Medulloblastoma
Cavalli - 763 - rma_sketch - hugene11t
ROS1 (8129134)
Expression cutoff: 13.400 (min.grp=3)
subgroup~group3|met_status_(1_met__0_m0)~0|WITH_SURV (n=65)
overall survival probability
1.00
0.90
0.80
0.70
0.60
0.50
0.40
0.30
0.20
0.10
0.00
0
24
48
72
96
120
144
168
192
Follow up in months
high (n=8)
low (n=57)
raw p 0.028
bonf p 1.000
Expression Graph
Event=No
Event=Yes
Expression
18
17
16
15
14
13
12
11
10
9
sorted by expression

GROUP3 M1

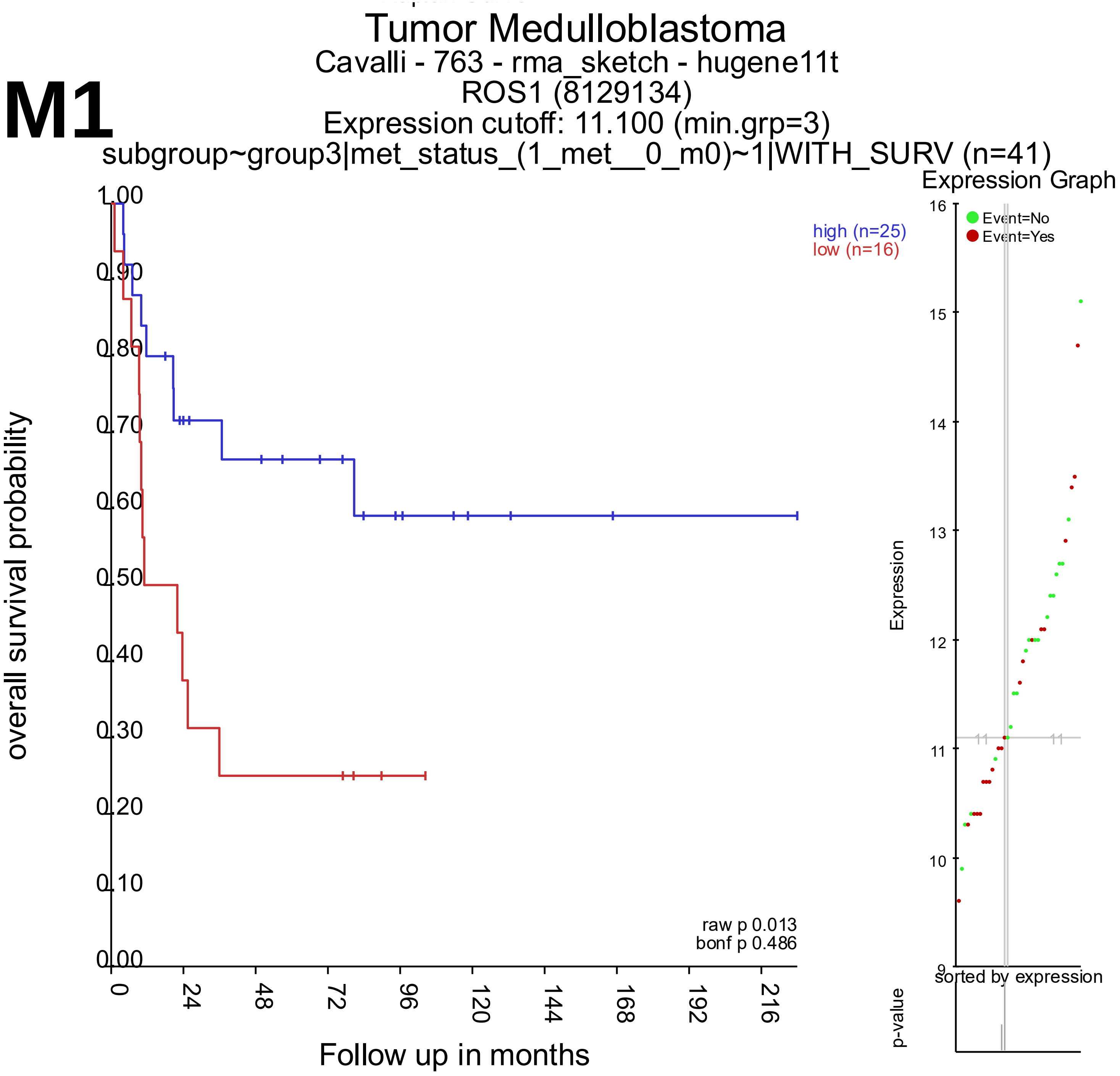

Tumor Medulloblastoma
Cavalli - 763 - rma_sketch - hugene11t
ROS1 (8129134)
Expression cutoff: 11.100 (min.grp=3)
subgroup~group3|met_status_(1_met__0_m0)~1|WITH_SURV (n=41)
high (n=25)
low (n=16)
overall survival probability
1.00
0.90
0.80
0.70
0.60
0.50
0.40
0.30
0.20
0.10
0.00
0
24
48
72
96
120
144
168
192
216
Follow up in months
raw p 0.013
bonf p 0.486
Expression Graph
Event=No
Event=Yes
Expression
16
15
14
13
12
11
10
9
sorted by expression
p-value

SMO

WNT M0

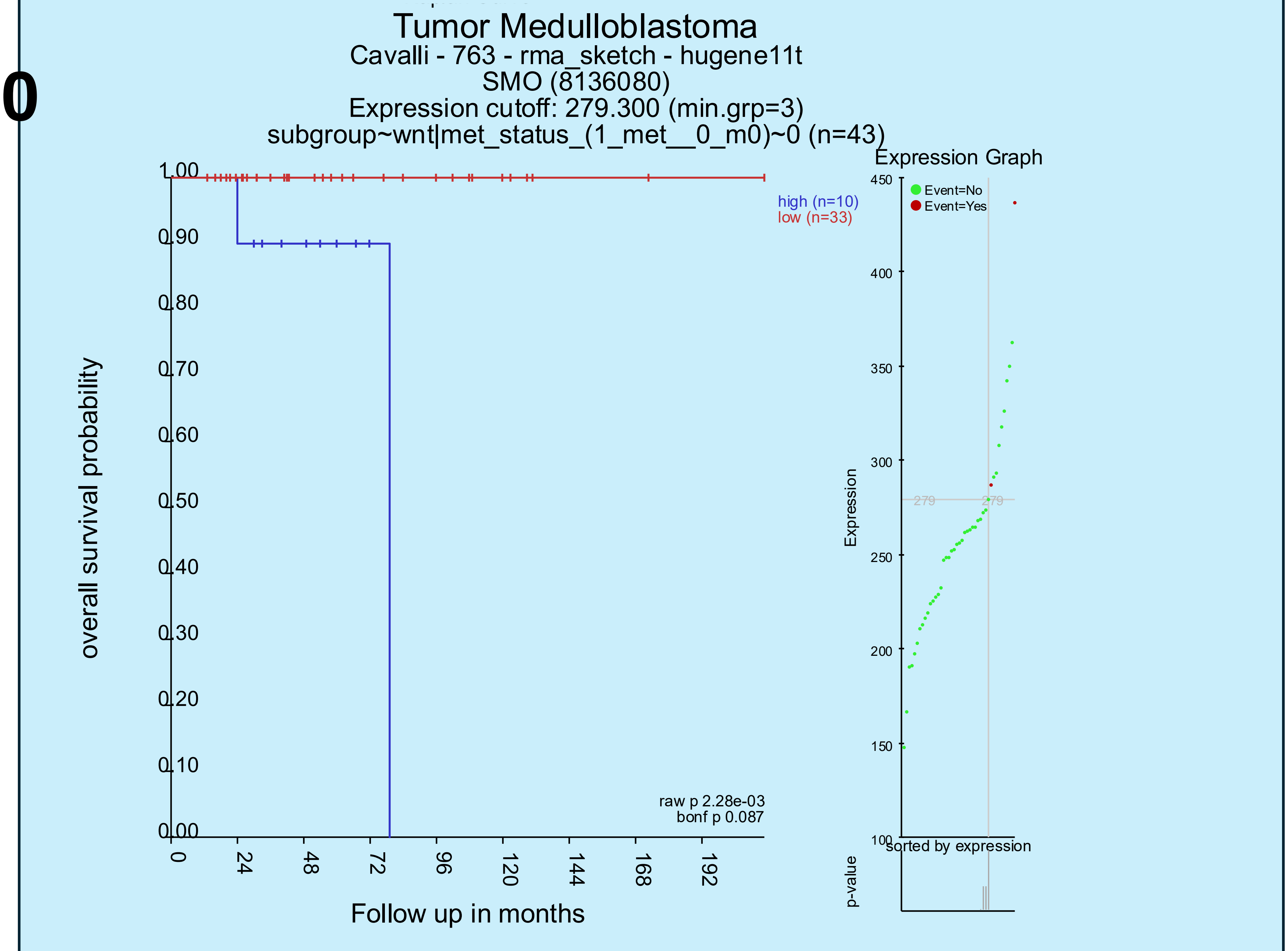

Tumor Medulloblastoma
Cavalli - 763 - rma_sketch - hugene11t
SMO (8136080)
Expression cutoff: 279.300 (min.grp=3)
subgroup~wnt|met_status_(1_met__0_m0)~0 (n=43)
overall survival probability
1.00
0.90
0.80
0.70
0.60
0.50
0.40
0.30
0.20
0.10
0.00
high (n=10)
low (n=33)
raw p 2.28e-03
bonf p 0.087
0
24
48
72
96
120
144
168
192
Follow up in months
Expression Graph
Event=No
Event=Yes
450
400
350
300
250
200
150
100
Expression
279
sorted by expression
p-value

WNT M1

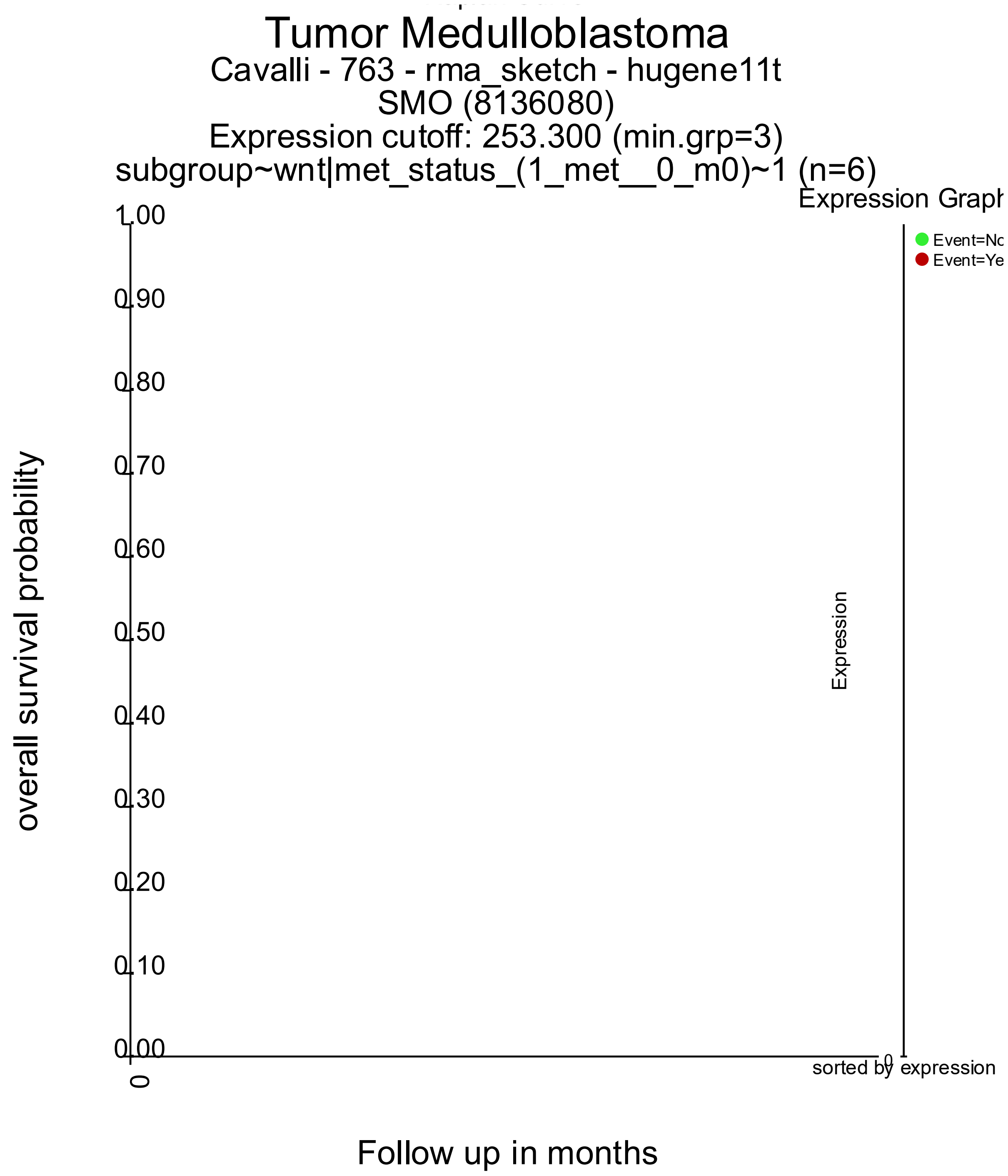

Tumor Medulloblastoma
Cavalli - 763 - rma_sketch - hugene11t
SMO (8136080)
Expression cutoff: 253.300 (min.grp=3)
subgroup~wnt|met_status_(1_met__0_m0)~1 (n=6)
Expression Graph
Event=No
Event=Ye
1.00
0.90
0.80
0.70
0.60
0.50
0.40
0.30
0.20
0.10
0.00
overall survival probability
0
Follow up in months
Expression
0
sorted by expression

SHH M0

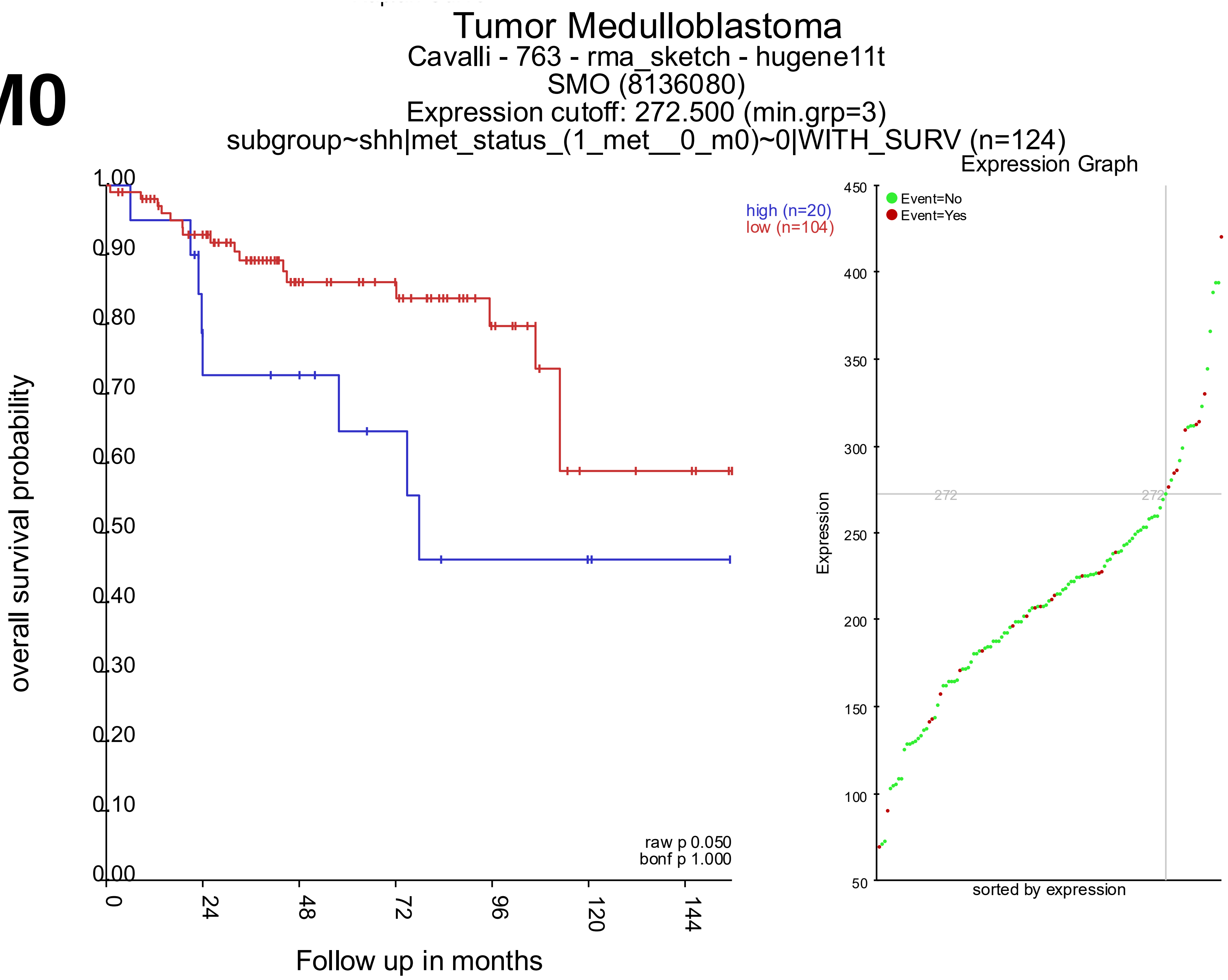

Tumor Medulloblastoma
Cavalli - 763 - rma_sketch - hugene11t
SMO (8136080)
Expression cutoff: 272.500 (min.grp=3)
subgroup~shh|met_status_(1_met__0_m0)~0|WITH_SURV (n=124)
overall survival probability
1.00
0.90
0.80
0.70
0.60
0.50
0.40
0.30
0.20
0.10
0.00
0
24
48
72
96
120
144
Follow up in months
high (n=20)
low (n=104)
raw p 0.050
bonf p 1.000
Expression Graph
Event=No
Event=Yes
Expression
450
400
350
300
250
200
150
100
50
272
272
sorted by expression

SHH M1

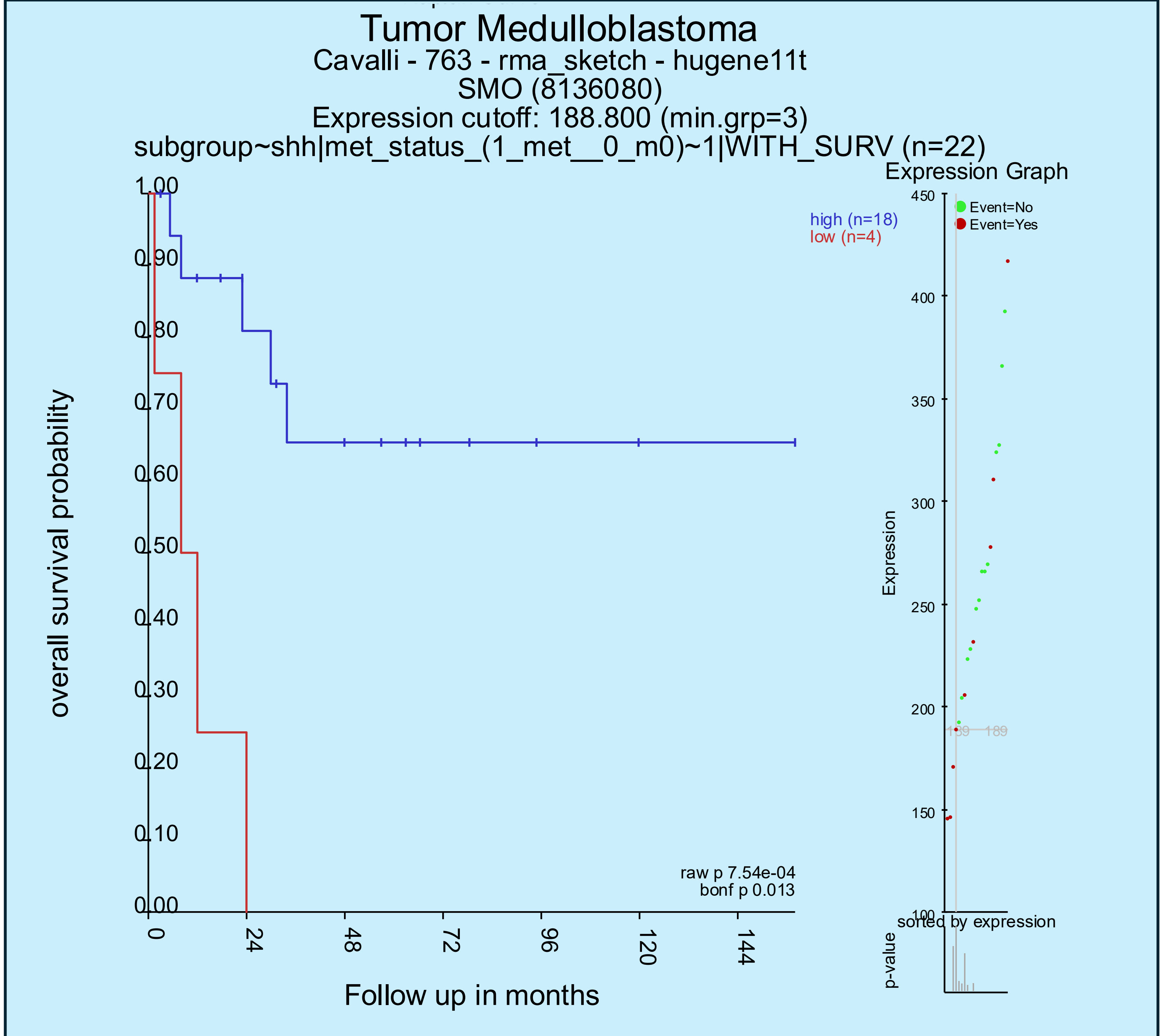

Tumor Medulloblastoma
Cavalli - 763 - rma_sketch - hugene11t
SMO (8136080)
Expression cutoff: 188.800 (min.grp=3)
subgroup~shh|met_status_(1_met__0_m0)~1|WITH_SURV (n=22)
overall survival probability
1.00
0.90
0.80
0.70
0.60
0.50
0.40
0.30
0.20
0.10
0.00
0
24
48
72
96
120
144
Follow up in months
high (n=18)
low (n=4)
raw p 7.54e-04
bonf p 0.013
Expression Graph
Event=No
Event=Yes
Expression
450
400
350
300
250
200
150
100
189
189
sorted by expression
p-value

GROUP4 M0

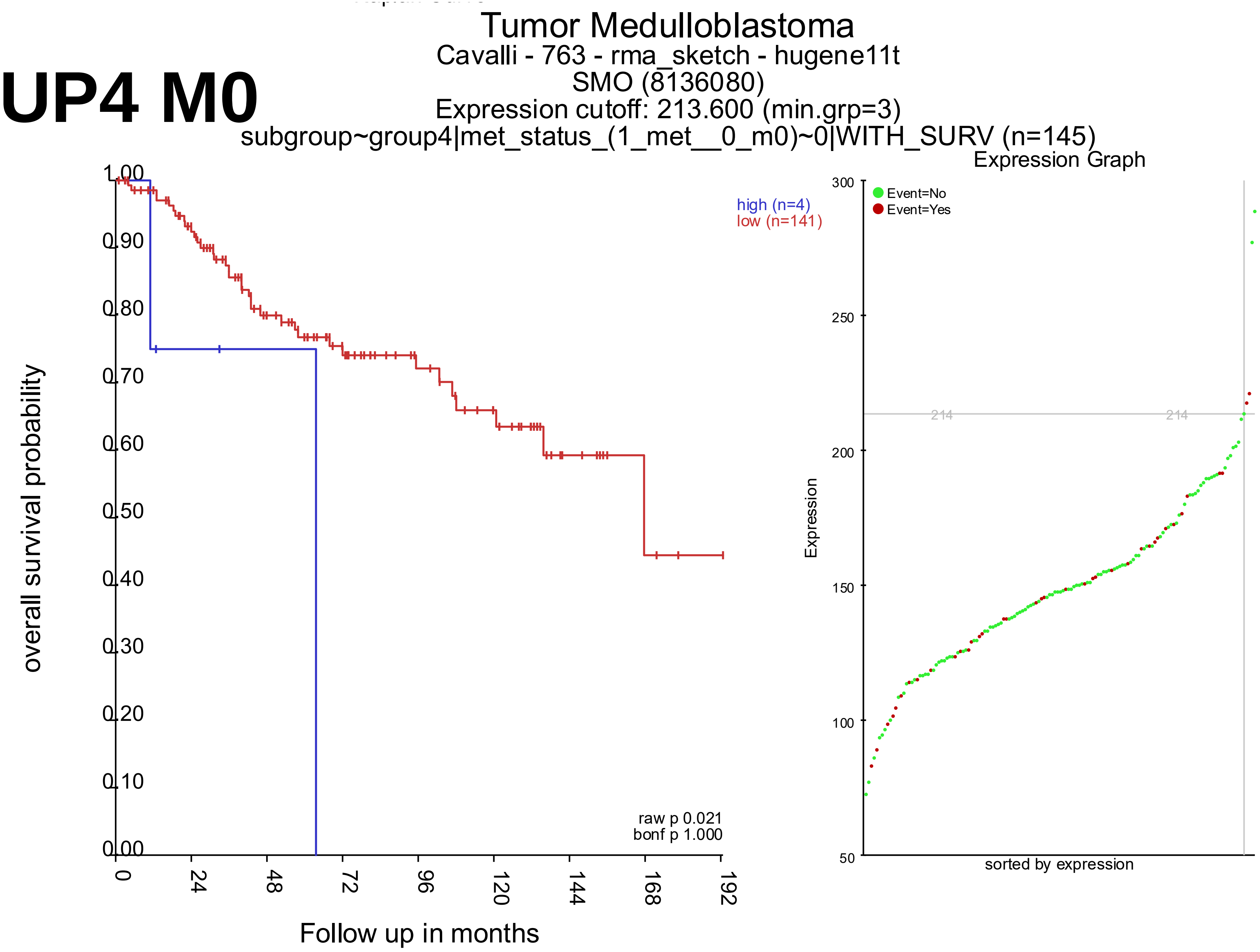

Tumor Medulloblastoma
Cavalli - 763 - rma_sketch - hugene11t
SMO (8136080)
Expression cutoff: 213.600 (min.grp=3)
subgroup~group4|met_status_(1_met__0_m0)~0|WITH_SURV (n=145)
overall survival probability
1.00
0.90
0.80
0.70
0.60
0.50
0.40
0.30
0.20
0.10
0.00
high (n=4)
low (n=141)
raw p 0.021
bonf p 1.000
0
24
48
72
96
120
144
168
192
Follow up in months
Expression Graph
Event=No
Event=Yes
300
250
200
150
100
50
214
214
Expression
sorted by expression

GROUP4 M1

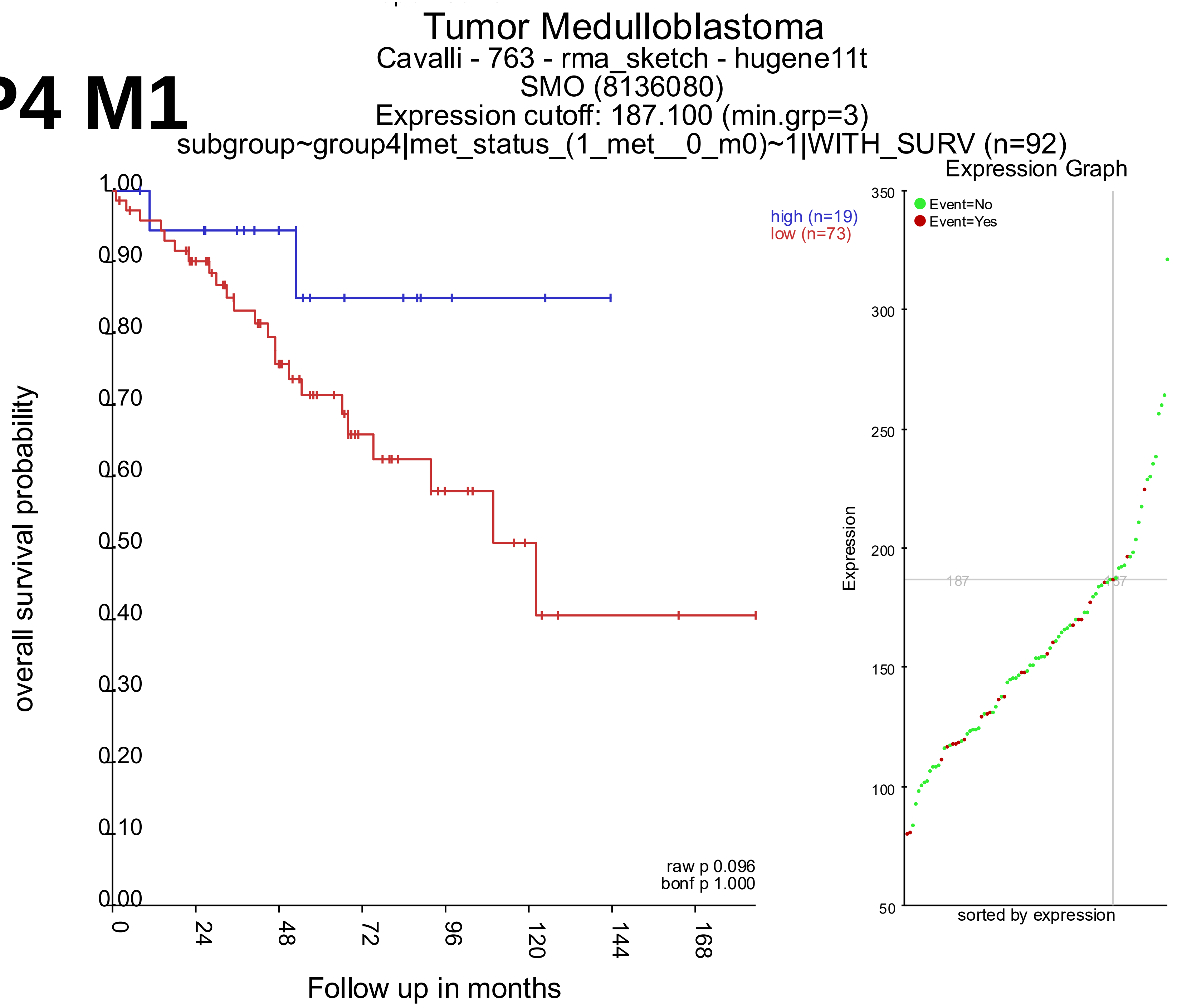

Tumor Medulloblastoma
Cavalli - 763 - rma_sketch - hugene11t
SMO (8136080)
Expression cutoff: 187.100 (min.grp=3)
subgroup~group4|met_status_(1_met__0_m0)~1|WITH_SURV (n=92)
overall survival probability
1.00
0.90
0.80
0.70
0.60
0.50
0.40
0.30
0.20
0.10
0.00
0
24
48
72
96
120
144
168
Follow up in months
high (n=19)
low (n=73)
raw p 0.096
bonf p 1.000
Expression Graph
Event=No
Event=Yes
Expression
350
300
250
200
150
100
50
187
sorted by expression

GROUP3 M0

Tumor Medulloblastoma
Cavalli - 763 - rma_sketch - hugene11t
SMO (8136080)
Expression cutoff: 124.200 (min.grp=3)
subgroup~group3|met_status_(1_met__0_m0)~0|WITH_SURV (n=65)

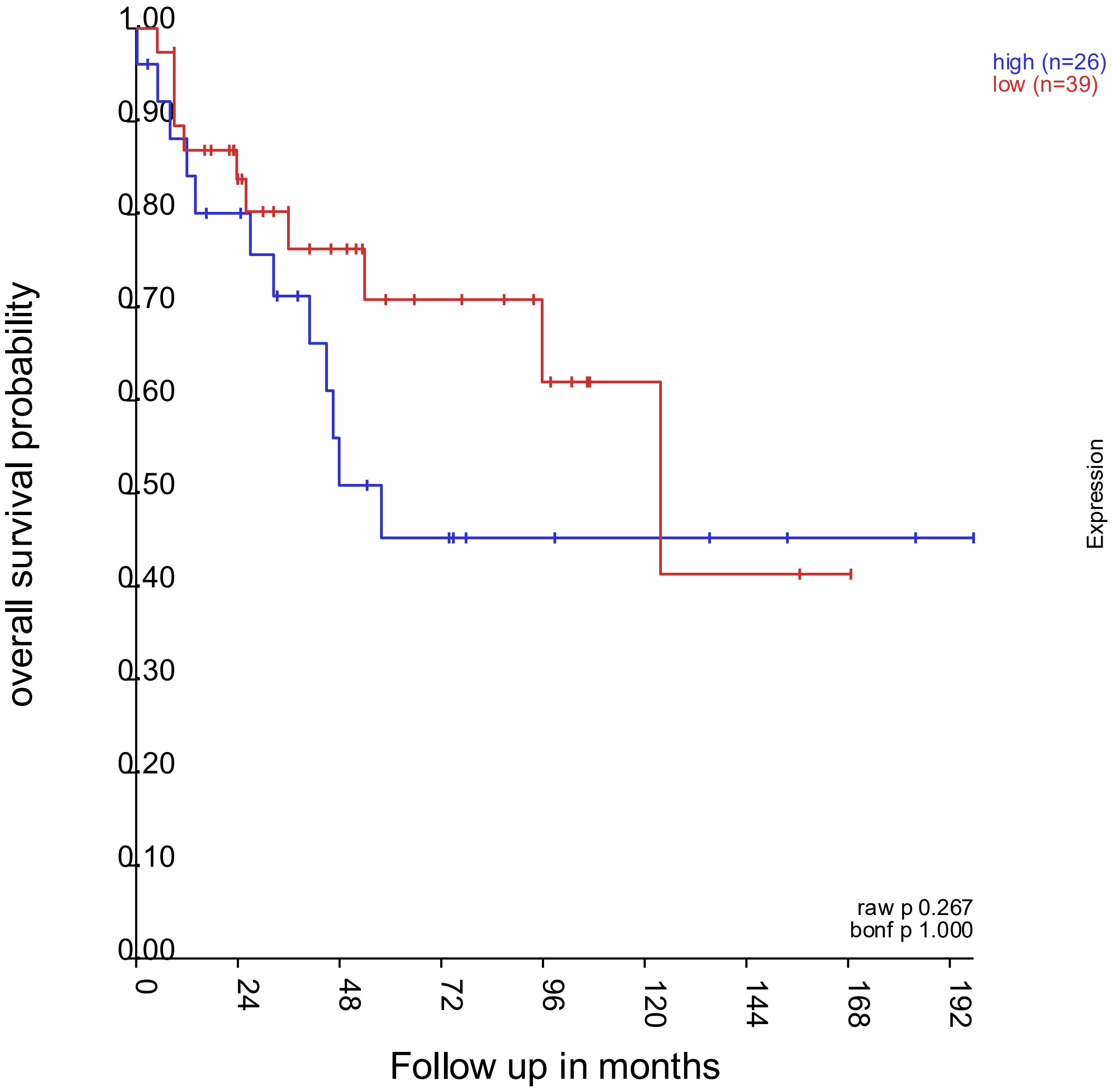

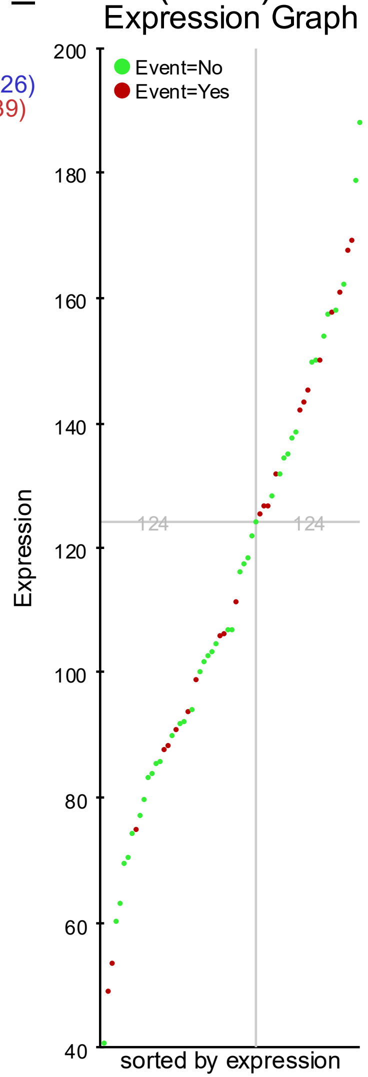

GROUP3 M1

Tumor Medulloblastoma
Cavalli - 763 - rma_sketch - hugene11t
SMO (8136080)
Expression cutoff: 151.200 (min.grp=3)
subgroup~group3|met_status_(1_met__0_m0)~1|WITH_SURV (n=41)

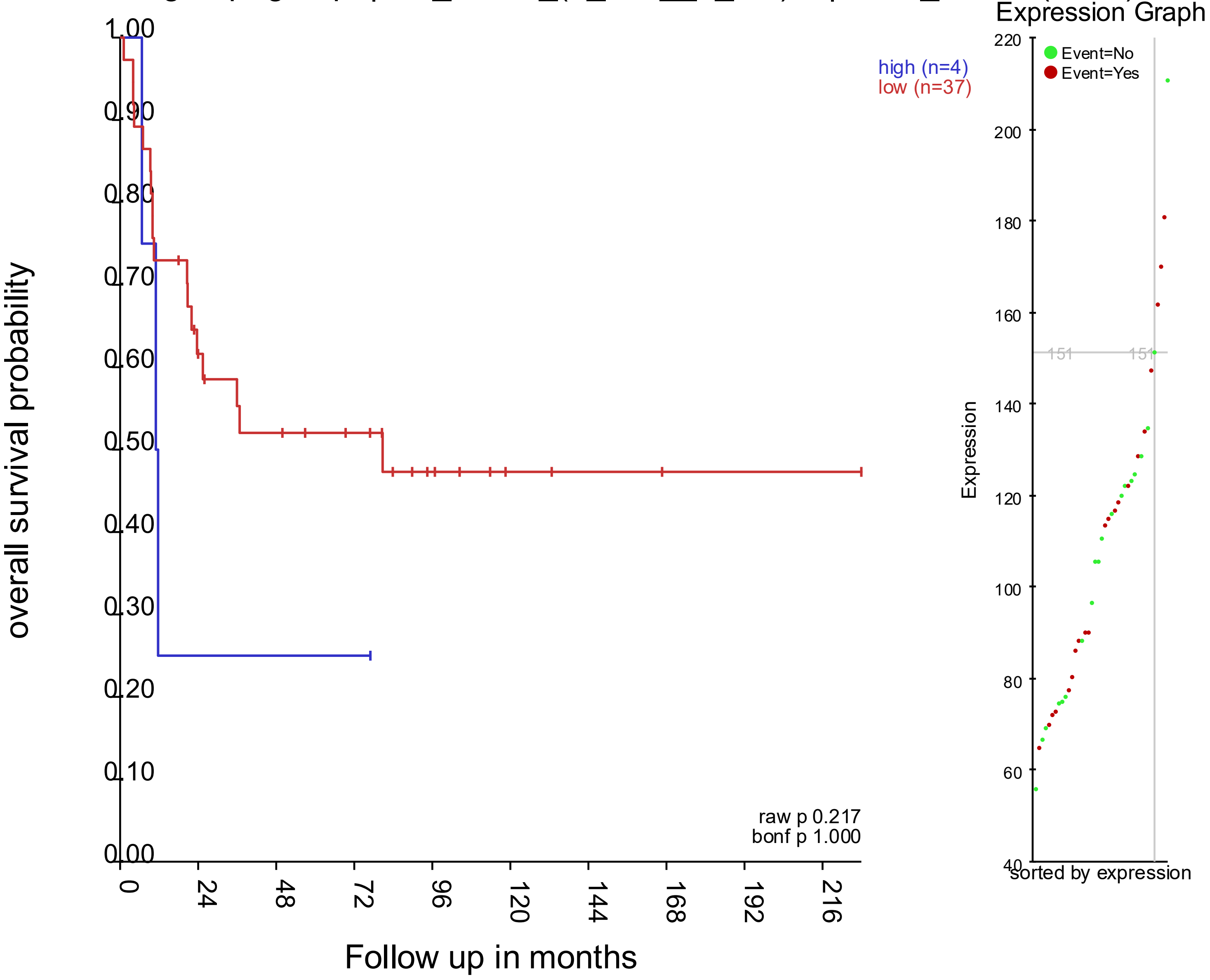

ABL1

WNT M0

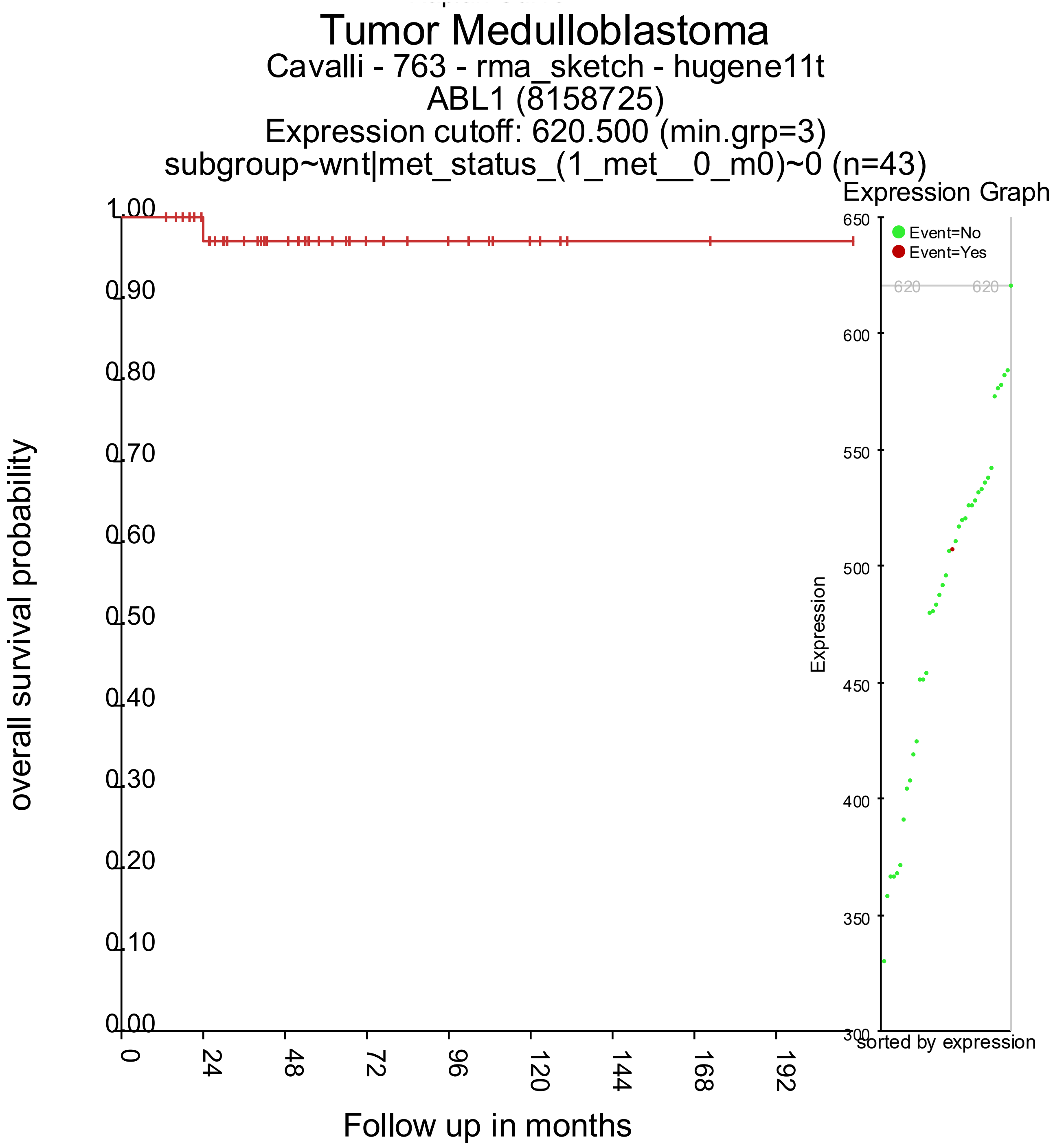

Tumor Medulloblastoma
Cavalli - 763 - rma_sketch - hugene11t
ABL1 (8158725)
Expression cutoff: 620.500 (min.grp=3)
subgroup~wnt|met_status_(1_met__0_m0)~0 (n=43)
overall survival probability
1.00
0.90
0.80
0.70
0.60
0.50
0.40
0.30
0.20
0.10
0.00
0
24
48
72
96
120
144
168
192
Follow up in months
Expression Graph
Event=No
Event=Yes
620
620
650
600
550
500
450
400
350
300
Expression
sorted by expression

WNT M1

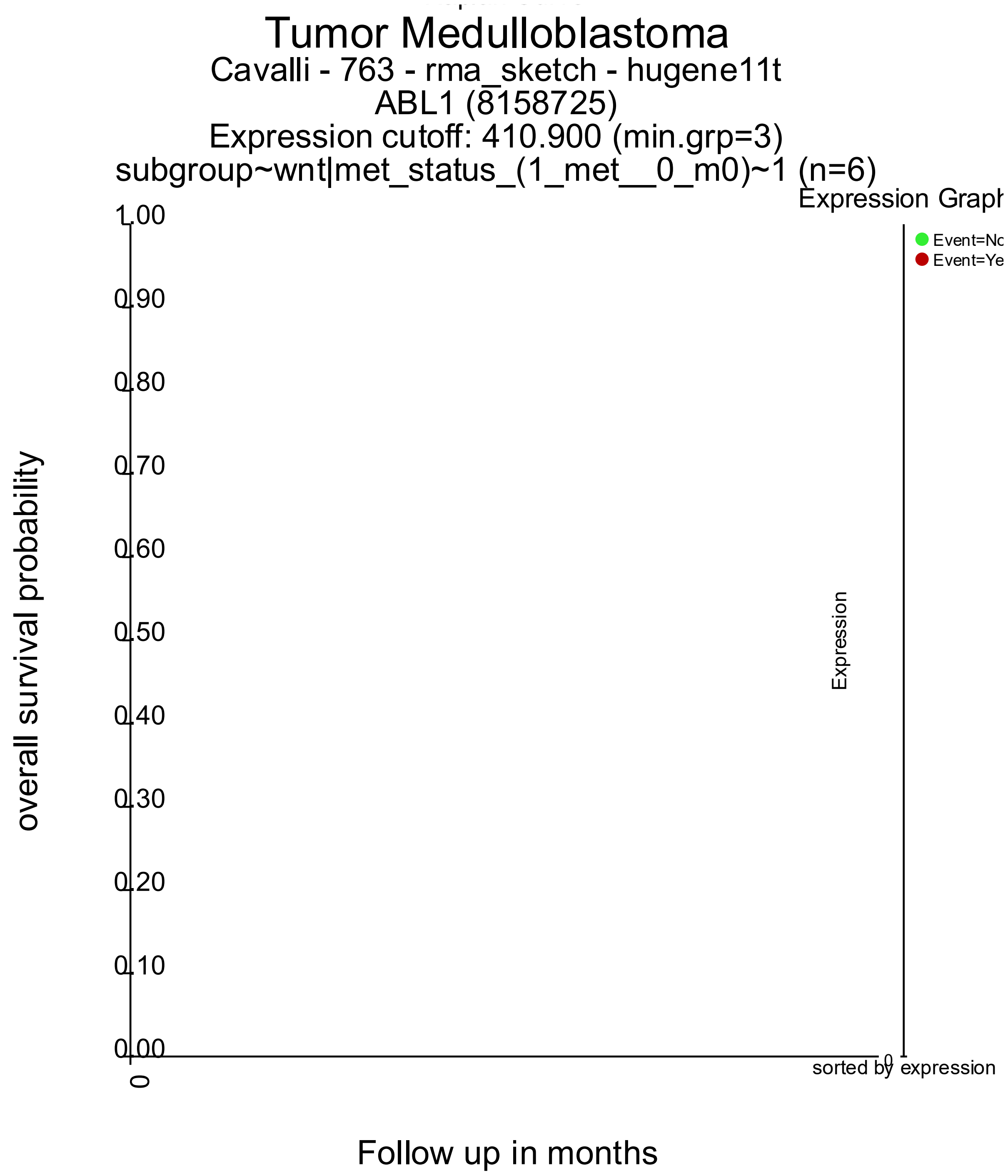

Tumor Medulloblastoma
Cavalli - 763 - rma_sketch - hugene11t
ABL1 (8158725)
Expression cutoff: 410.900 (min.grp=3)
subgroup~wnt|met_status_(1_met__0_m0)~1 (n=6)
Expression Graph
Event=No
Event=Yes
1.00
0.90
0.80
0.70
0.60
0.50
0.40
0.30
0.20
0.10
0.00
0
overall survival probability
Follow up in months
Expression
sorted by expression
0

SHH M0

Tumor Medulloblastoma
Cavalli - 763 - rma_sketch - hugene11t
ABL1 (8158725)
Expression cutoff: 333.100 (min.grp=3)
subgroup~shh|met_status_(1_met__0_m0)~0|WITH_SURV (n=124)

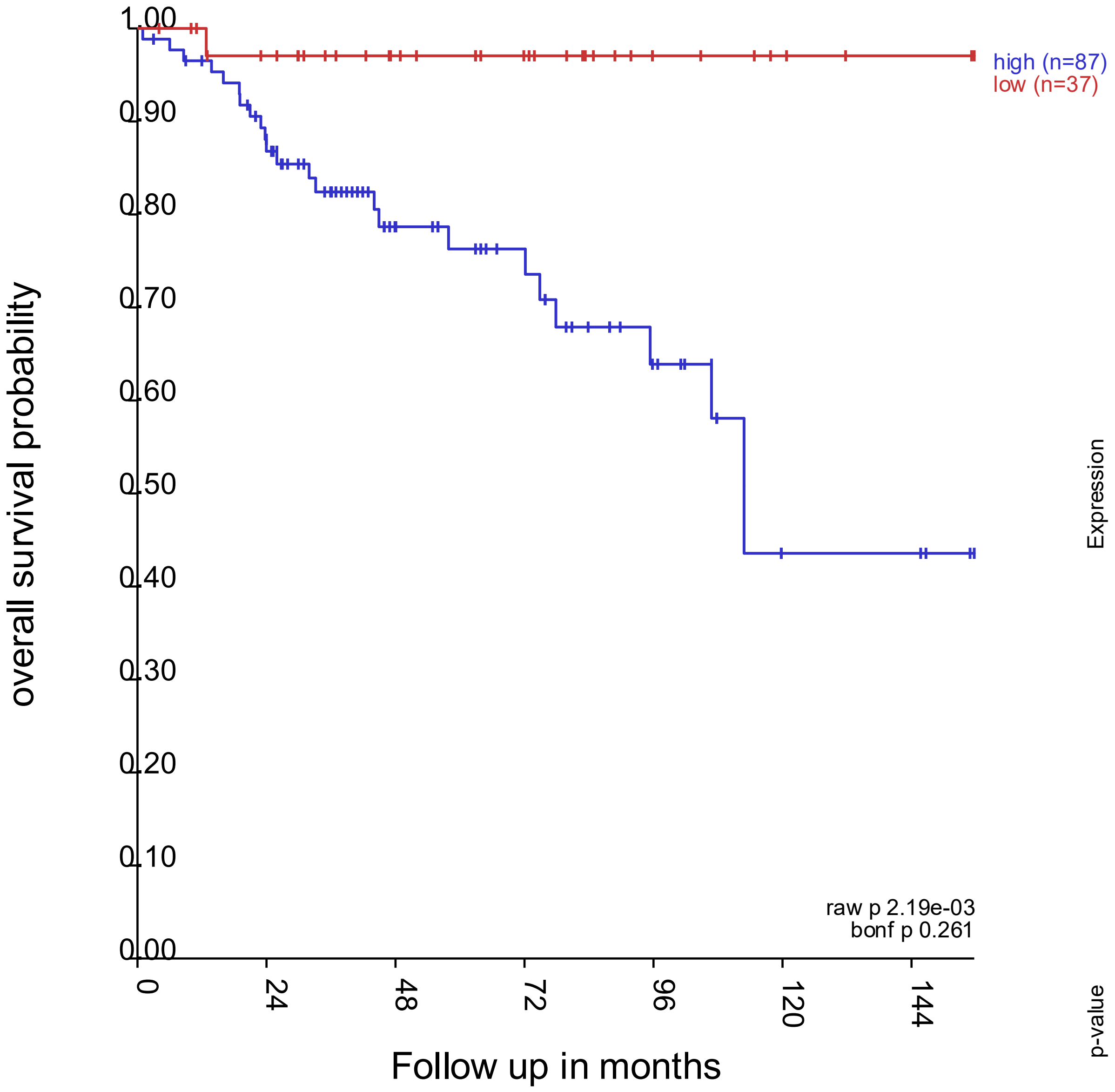

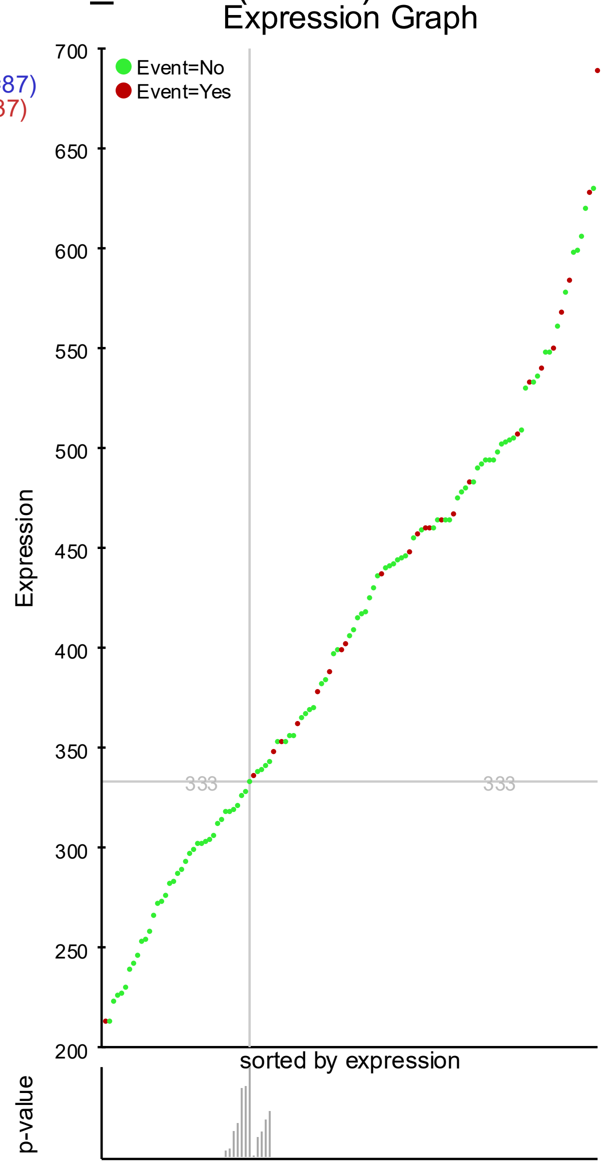

SHH M1

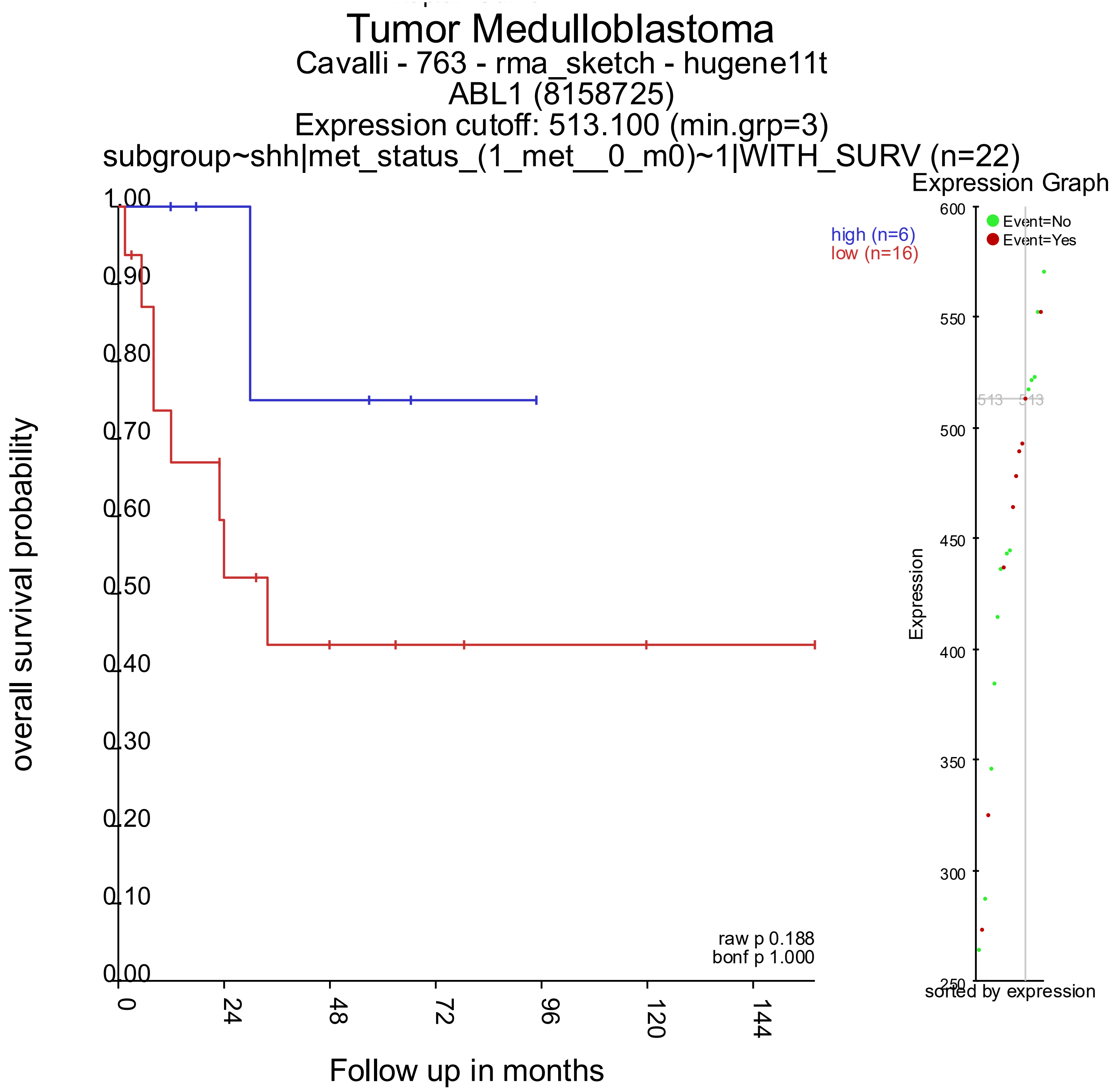

Tumor Medulloblastoma
Cavalli - 763 - rma_sketch - hugene11t
ABL1 (8158725)
Expression cutoff: 513.100 (min.grp=3)
subgroup~shh|met_status_(1_met__0_m0)~1|WITH_SURV (n=22)
overall survival probability
1.00
0.90
0.80
0.70
0.60
0.50
0.40
0.30
0.20
0.10
0.00
0
24
48
72
96
120
144
Follow up in months
high (n=6)
low (n=16)
raw p 0.188
bonf p 1.000
Expression Graph
Event=No
Event=Yes
Expression
600
550
500
450
400
350
300
250
513
513
sorted by expression

GROUP4 M0

Tumor Medulloblastoma
Cavalli - 763 - rma_sketch - hugene11t
ABL1 (8158725)
Expression cutoff: 969.700 (min.grp=3)
subgroup~group4|met_status_(1_met__0_m0)~0|WITH_SURV (n=145)

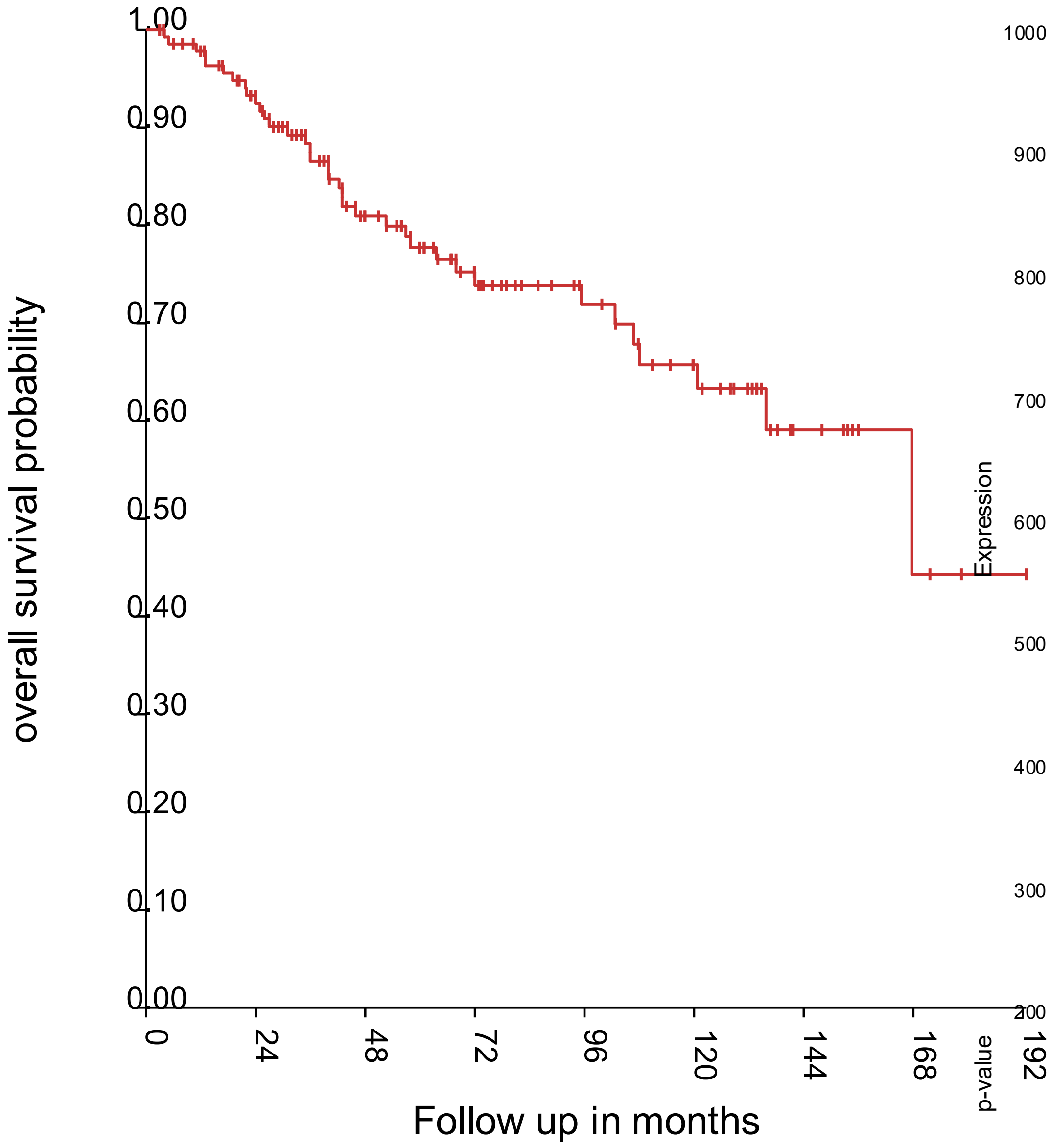

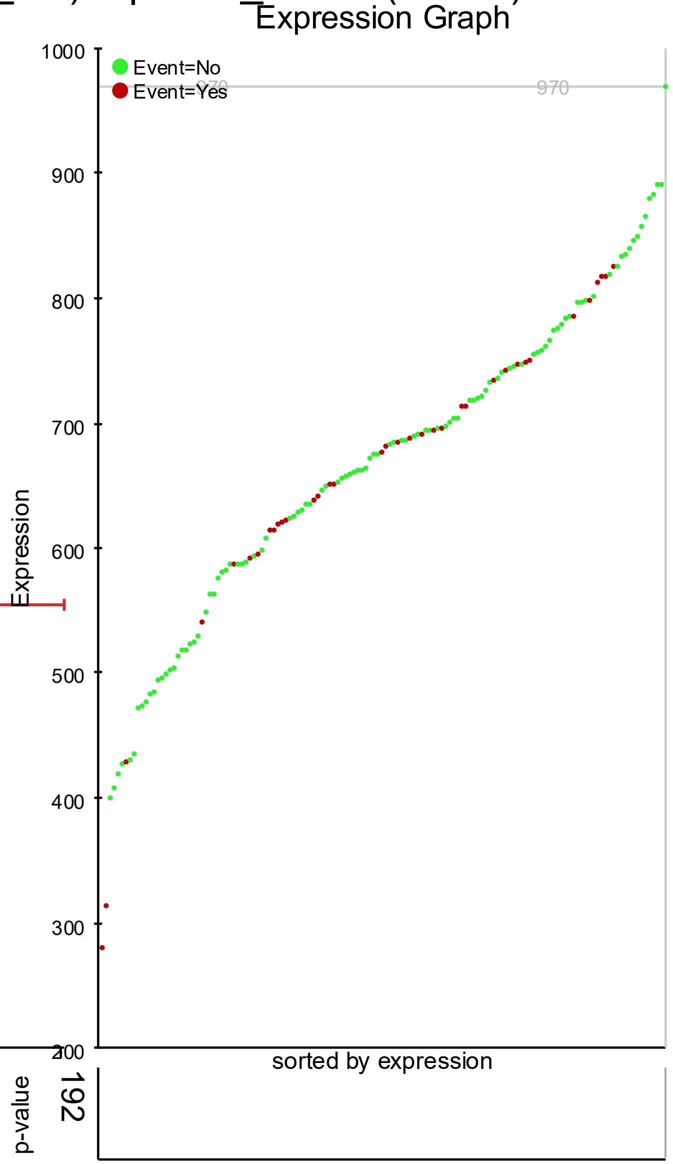

GROUP4 M1

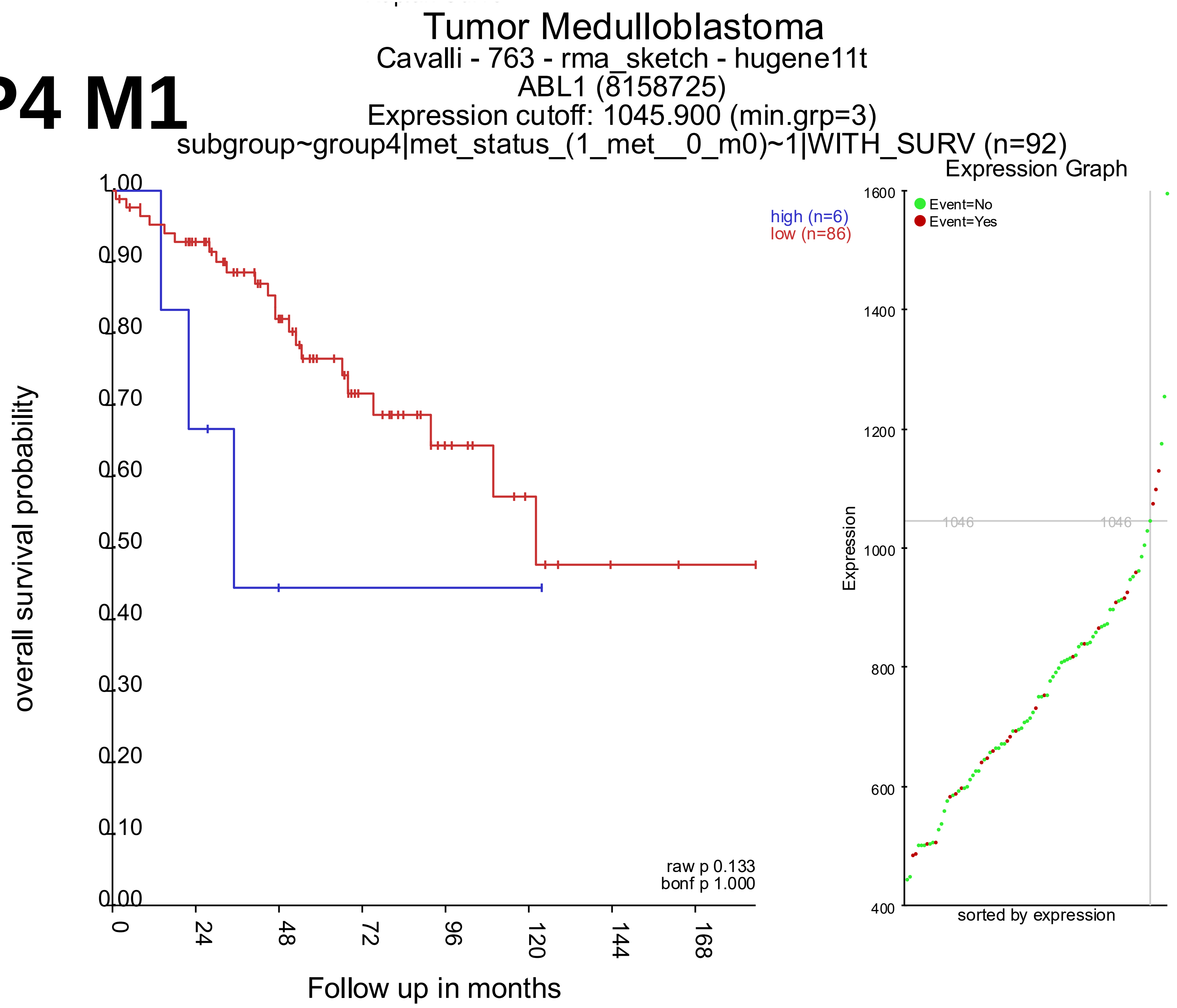

Tumor Medulloblastoma
Cavalli - 763 - rma_sketch - hugene11t
ABL1 (8158725)
Expression cutoff: 1045.900 (min.grp=3)
subgroup~group4|met_status_(1_met__0_m0)~1|WITH_SURV (n=92)
overall survival probability
1.00
0.90
0.80
0.70
0.60
0.50
0.40
0.30
0.20
0.10
0.00
0
24
48
72
96
120
144
168
Follow up in months
high (n=6)
low (n=86)
raw p 0.133
bonf p 1.000
Expression Graph
Event=No
Event=Yes
Expression
1600
1400
1200
1000
800
600
400
1046
1046
sorted by expression

GROUP3 M0

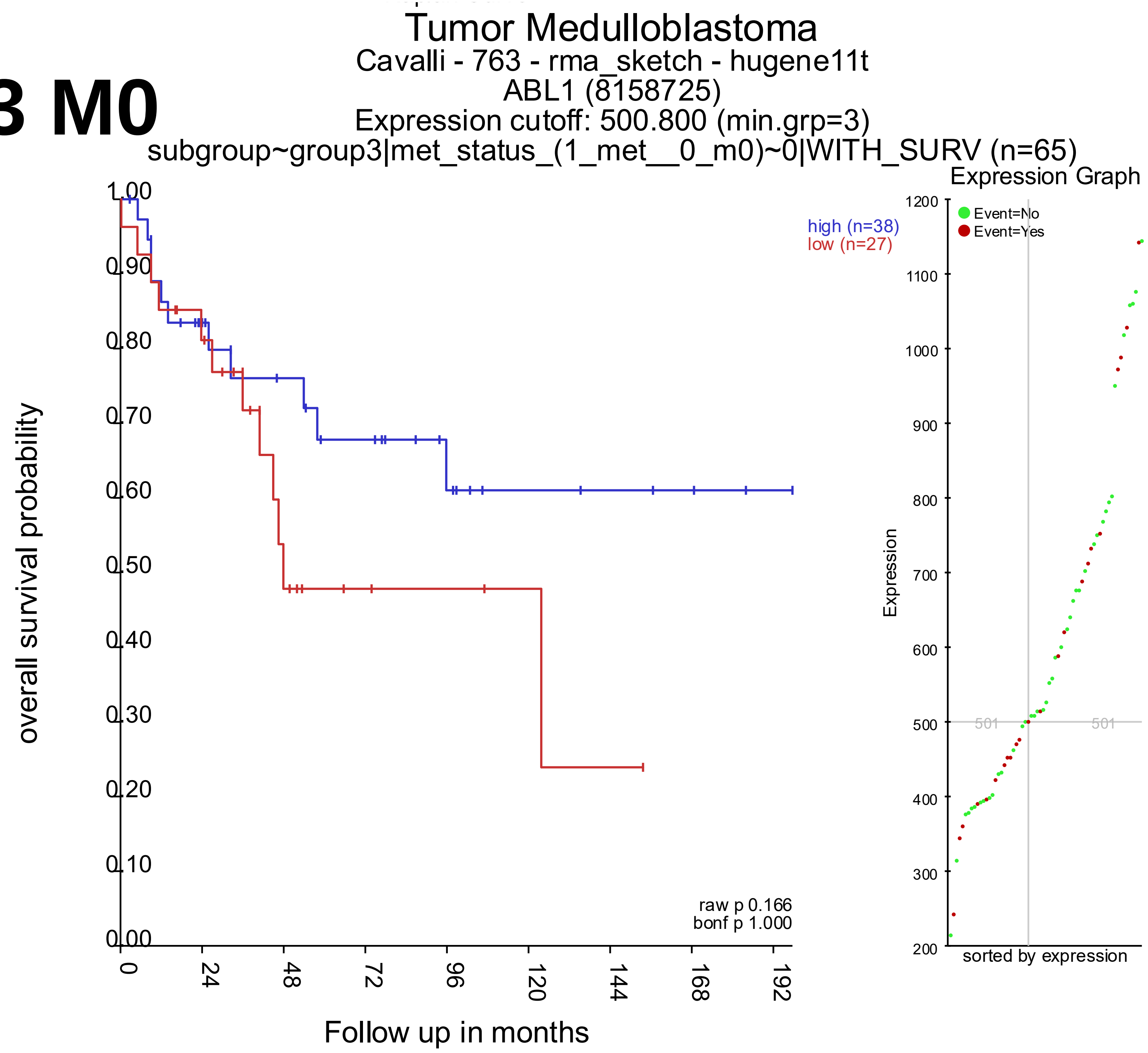

Tumor Medulloblastoma
Cavalli - 763 - rma_sketch - hugene11t
ABL1 (8158725)
Expression cutoff: 500.800 (min.grp=3)
subgroup~group3|met_status_(1_met__0_m0)~0|WITH_SURV (n=65)
high (n=38)
low (n=27)
overall survival probability
1.00
0.90
0.80
0.70
0.60
0.50
0.40
0.30
0.20
0.10
0.00
0
24
48
72
96
120
144
168
192
Follow up in months
raw p 0.166
bonf p 1.000
Expression Graph
Event=No
Event=Yes
Expression
1200
1100
1000
900
800
700
600
500
400
300
200
501
501
sorted by expression

GROUP3 M1

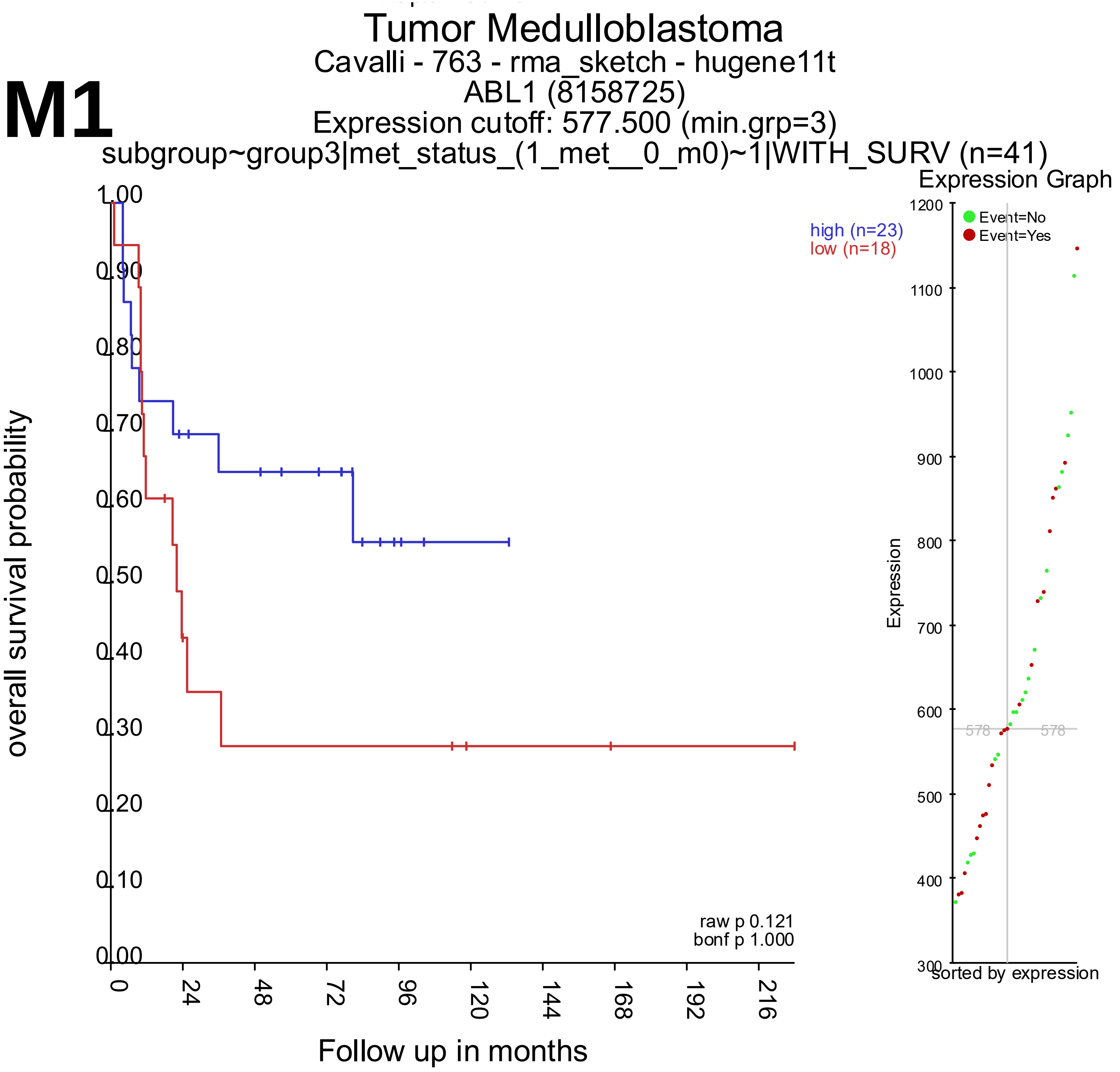

Tumor Medulloblastoma
Cavalli - 763 - rma_sketch - hugene11t
ABL1 (8158725)
Expression cutoff: 577.500 (min.grp=3)
subgroup~group3|met_status_(1_met__0_m0)~1|WITH_SURV (n=41)
overall survival probability
1.00
0.90
0.80
0.70
0.60
0.50
0.40
0.30
0.20
0.10
0.00
high (n=23)
low (n=18)
raw p 0.121
bonf p 1.000
0
24
48
72
96
120
144
168
192
216
Follow up in months
Expression Graph
Event=No
Event=Yes
Expression
1200
1100
1000
900
800
700
600
500
400
300
578
578
sorted by expression

SRC

WNT M0

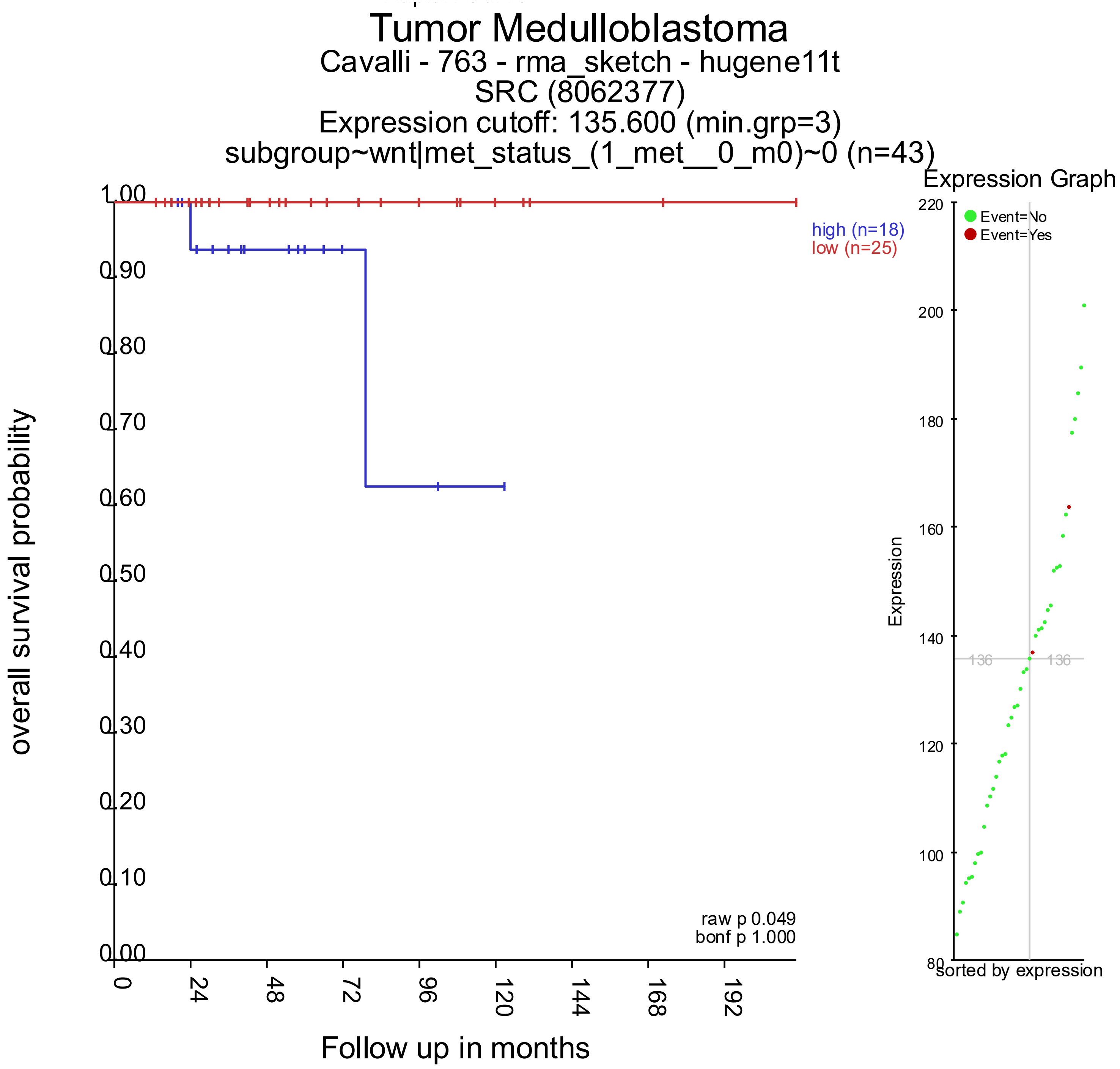
Tumor Medulloblastoma
Cavalli - 763 - rma_sketch - hugene11t
SRC (8062377)
Expression cutoff: 135.600 (min.grp=3)
subgroup~wnt|met_status_(1_met__0_m0)~0 (n=43)
high (n=18)
low (n=25)
overall survival probability
1.00
0.90
0.80
0.70
0.60
0.50
0.40
0.30
0.20
0.10
0.00
0
24
48
72
96
120
144
168
192
Follow up in months
raw p 0.049
bonf p 1.000
Expression Graph
Event=No
Event=Yes
Expression
220
200
180
160
140
120
100
80
136
sorted by expression

WNT M1

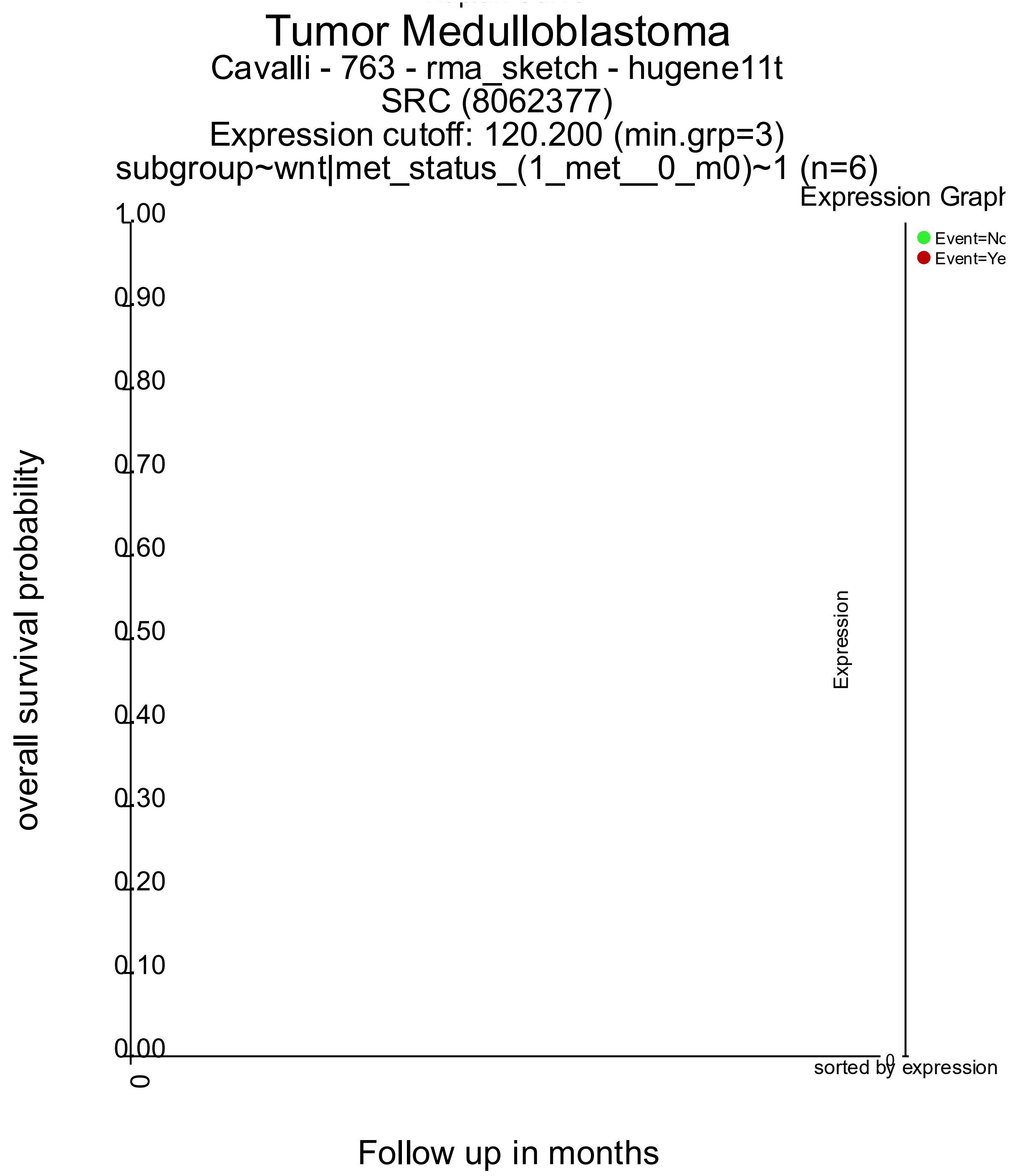

Tumor Medulloblastoma
Cavalli - 763 - rma_sketch - hugene11t
SRC (8062377)
Expression cutoff: 120.200 (min.grp=3)
subgroup~wnt|met_status_(1_met__0_m0)~1 (n=6)
Expression Graph
Event=No
Event=Yes
overall survival probability
1.00
0.90
0.80
0.70
0.60
0.50
0.40
0.30
0.20
0.10
0.00
0
Follow up in months
Expression
0
sorted by expression

SHH M0

Tumor Medulloblastoma
Cavalli - 763 - rma_sketch - hugene11t
SRC (8062377)
Expression cutoff: 94.000 (min.grp=3)
subgroup~shh|met_status_(1_met__0_m0)~0|WITH_SURV (n=124)

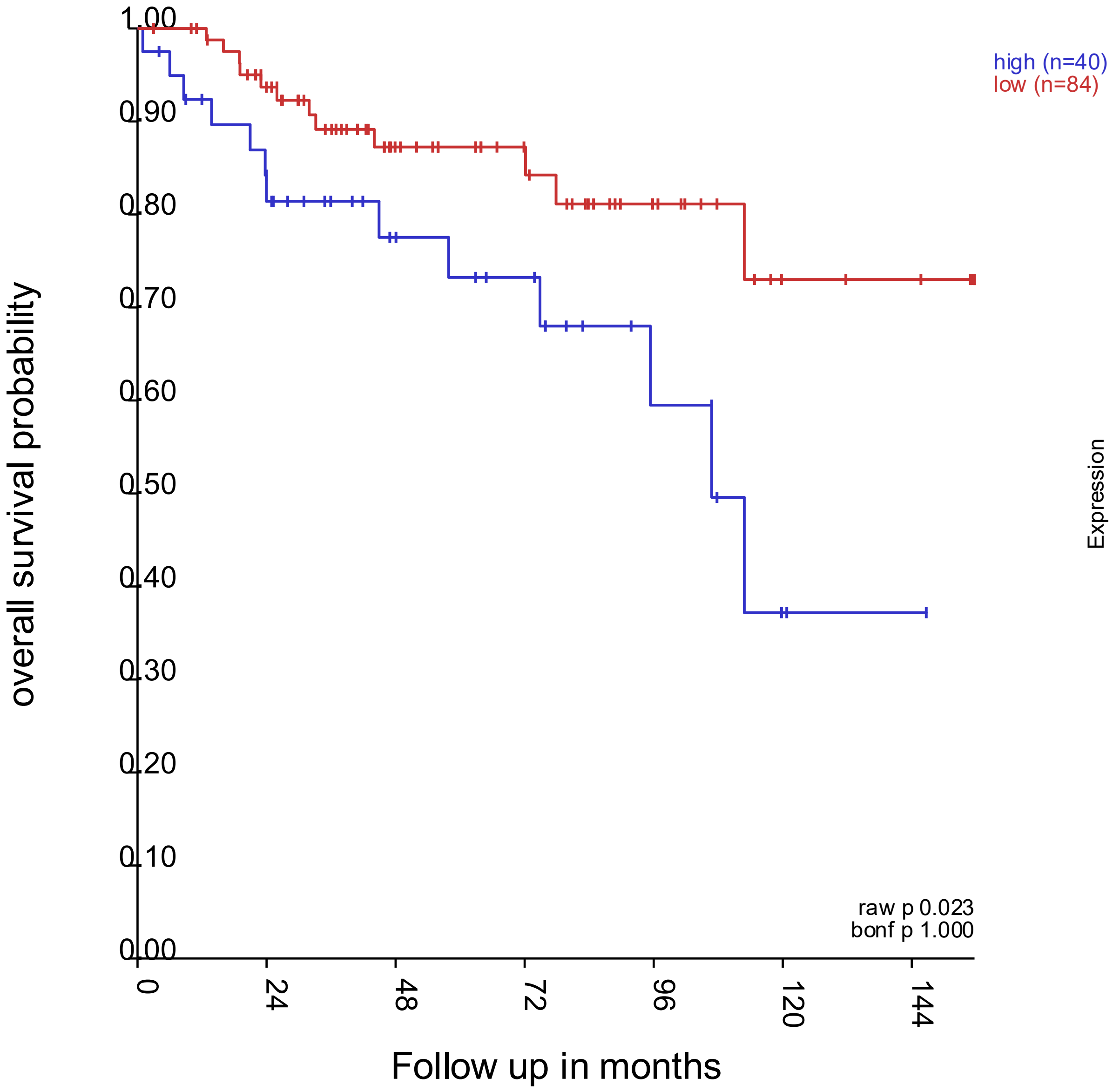

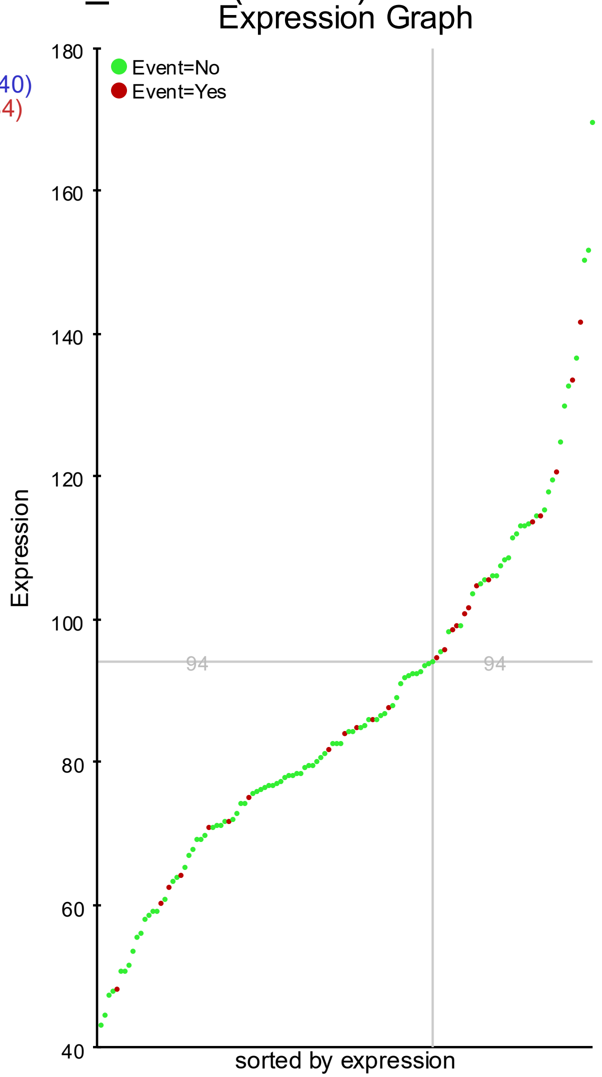

SHH M1

Tumor Medulloblastoma
Cavalli - 763 - rma_sketch - hugene11t
SRC (8062377)
Expression cutoff: 75.500 (min.grp=3)
subgroup~shh|met_status_(1_met__0_m0)~1|WITH_SURV (n=22)

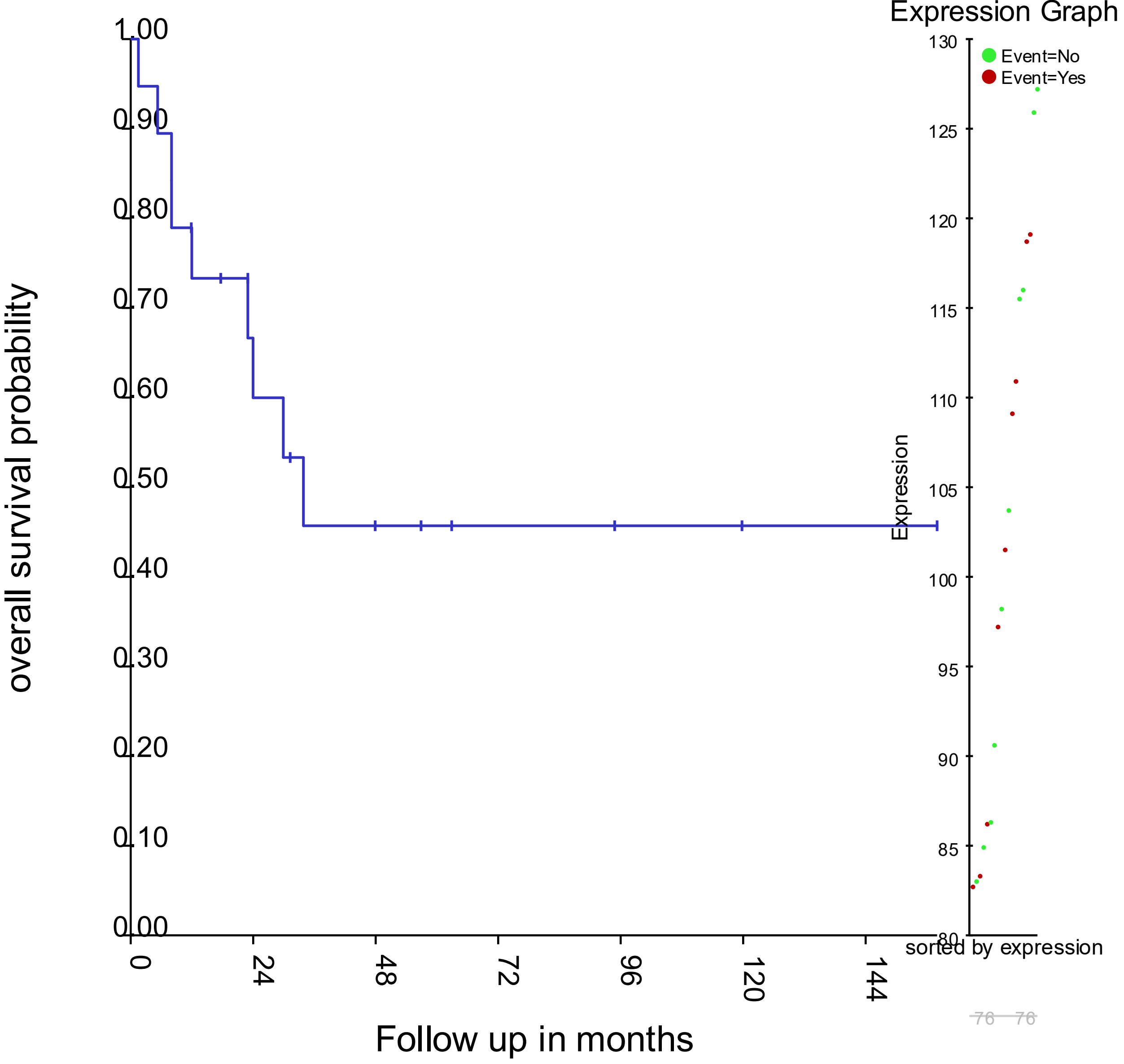

GROUP4 M0

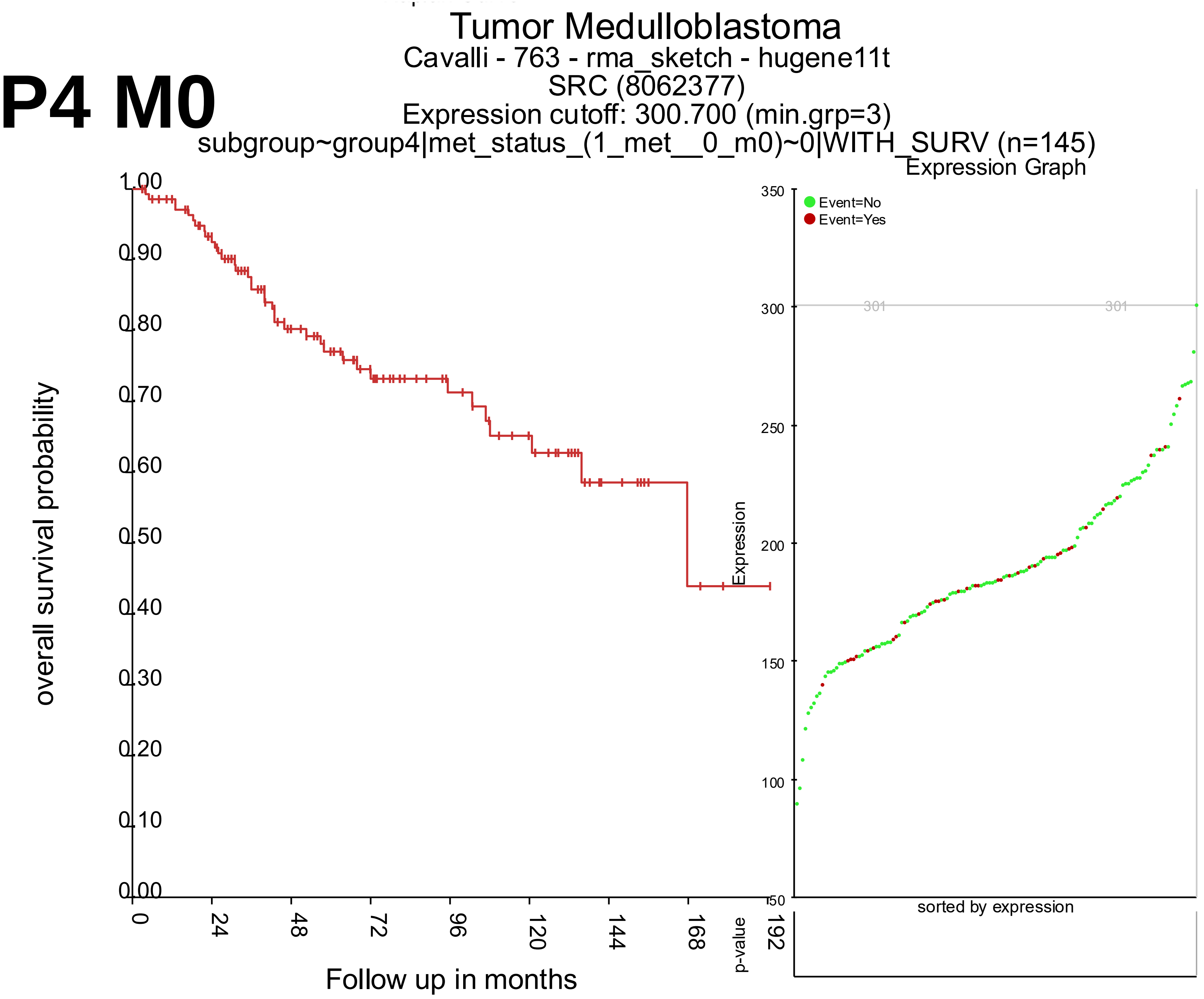

Tumor Medulloblastoma
Cavalli - 763 - rma_sketch - hugene11t
SRC (8062377)
Expression cutoff: 300.700 (min.grp=3)
subgroup~group4|met_status_(1_met__0_m0)~0|WITH_SURV (n=145)
overall survival probability
1.00
0.90
0.80
0.70
0.60
0.50
0.40
0.30
0.20
0.10
0.00
0
24
48
72
96
120
144
168
192
Follow up in months
p-value
Expression Graph
Event=No
Event=Yes
350
300
250
200
150
100
50
301
301
Expression
sorted by expression

GROUP4 M1

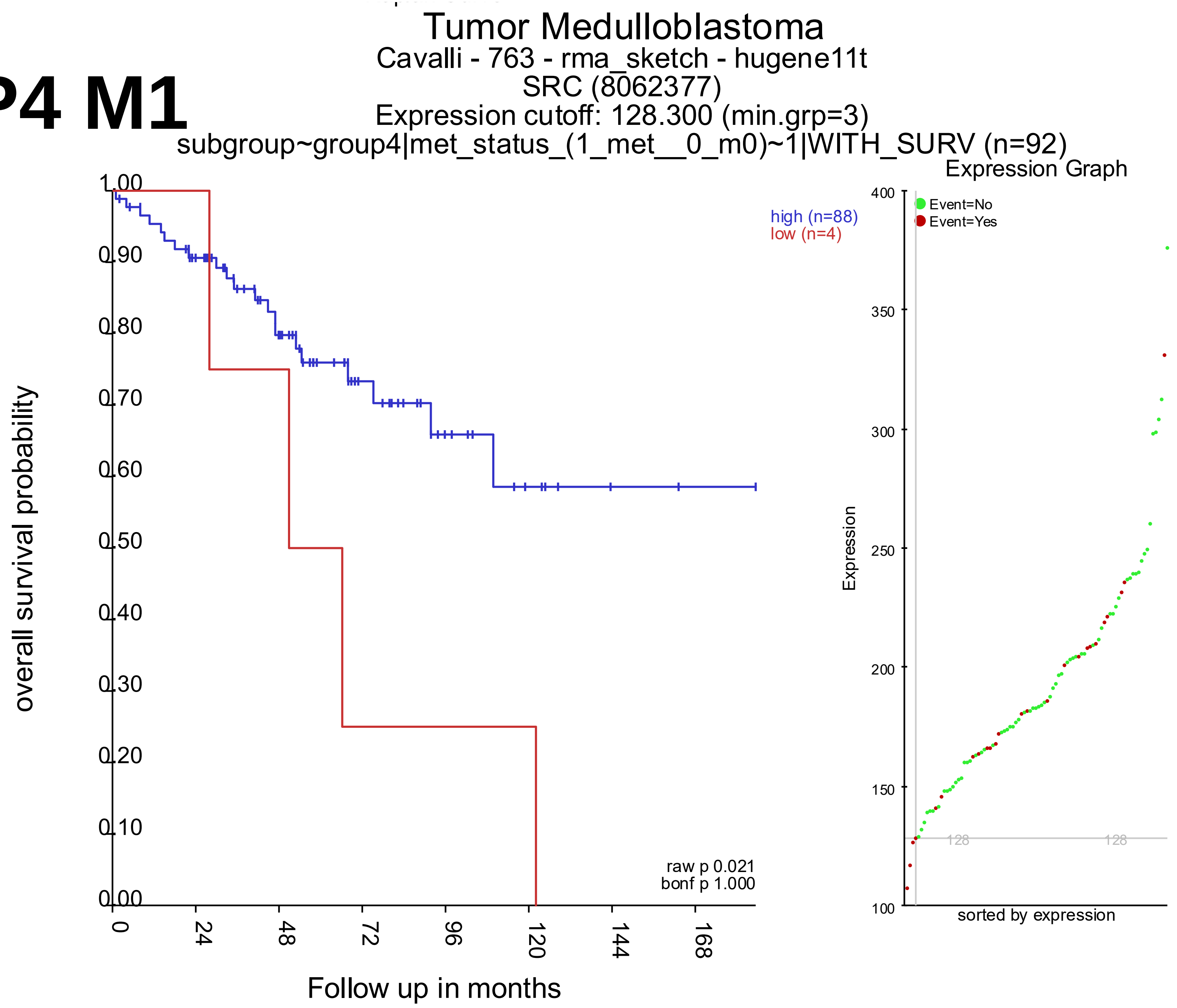

Tumor Medulloblastoma
Cavalli - 763 - rma_sketch - hugene11t
SRC (8062377)
Expression cutoff: 128.300 (min.grp=3)
subgroup~group4|met_status_(1_met__0_m0)~1|WITH_SURV (n=92)
overall survival probability
1.00
0.90
0.80
0.70
0.60
0.50
0.40
0.30
0.20
0.10
0.00
0
24
48
72
96
120
144
168
Follow up in months
high (n=88)
low (n=4)
raw p 0.021
bonf p 1.000
Expression Graph
Event=No
Event=Yes
Expression
400
350
300
250
200
150
100
128
128
sorted by expression

GROUP3 M0

Tumor Medulloblastoma
Cavalli - 763 - rma_sketch - hugene11t
SRC (8062377)
Expression cutoff: 114.800 (min.grp=3)
subgroup~group3|met_status_(1_met__0_m0)~0|WITH_SURV (n=65)

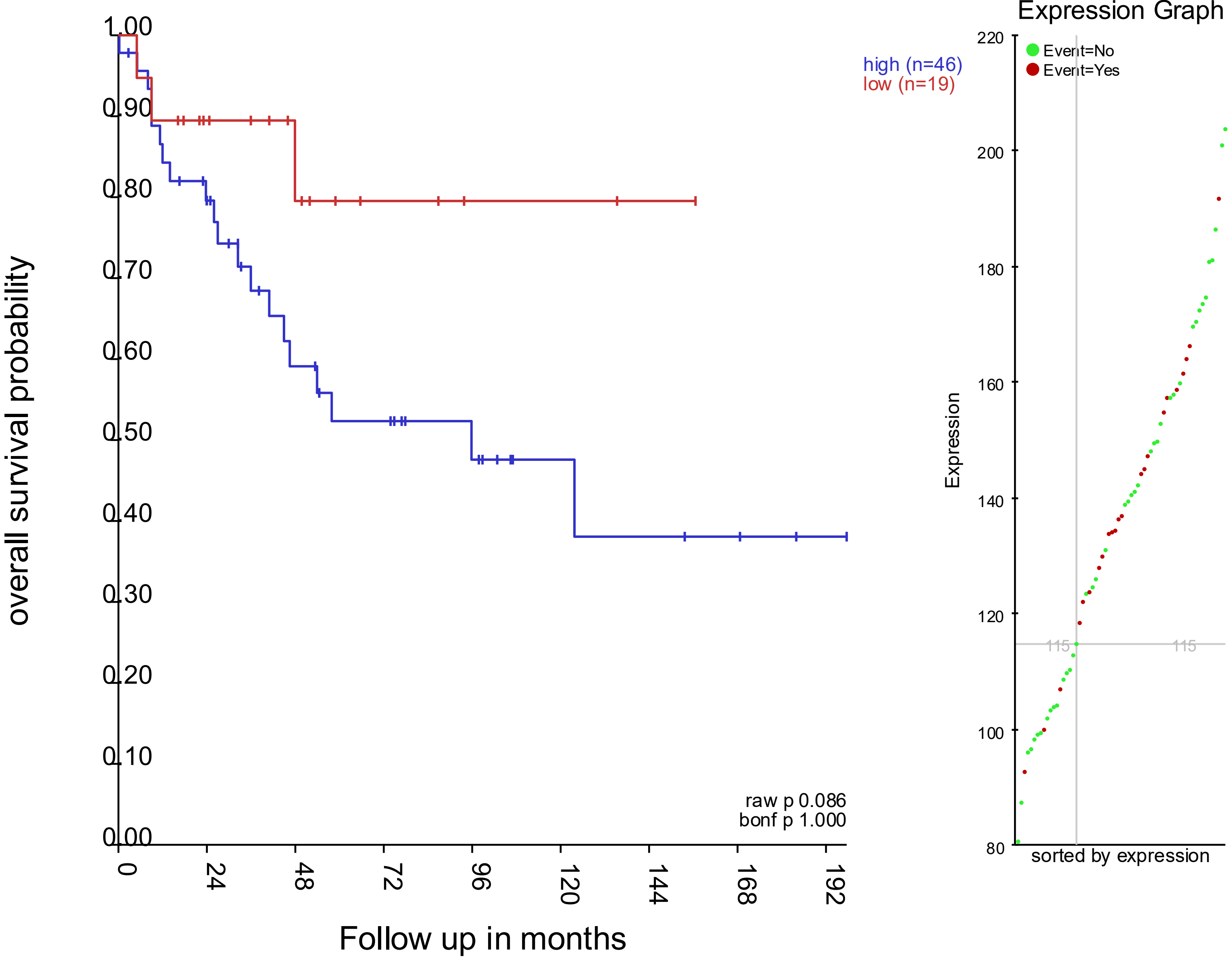

GROUP3 M1

Tumor Medulloblastoma
Cavalli - 763 - rma_sketch - hugene11t
SRC (8062377)
Expression cutoff: 127.000 (min.grp=3)
met_status_(1_met__0_m0)~1|subgroup~group3|WITH_SURV (n=41)

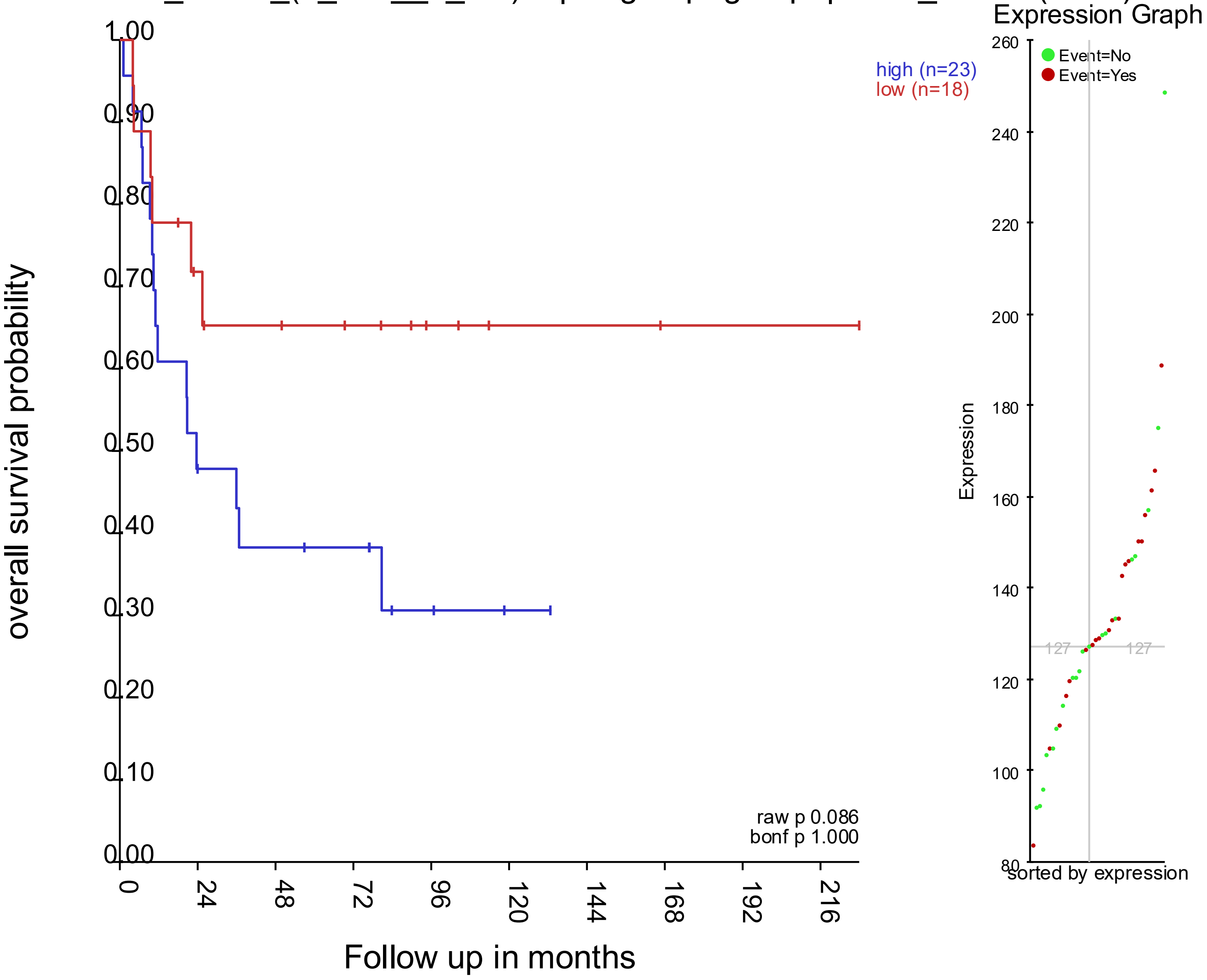

Fyn

WNT M0

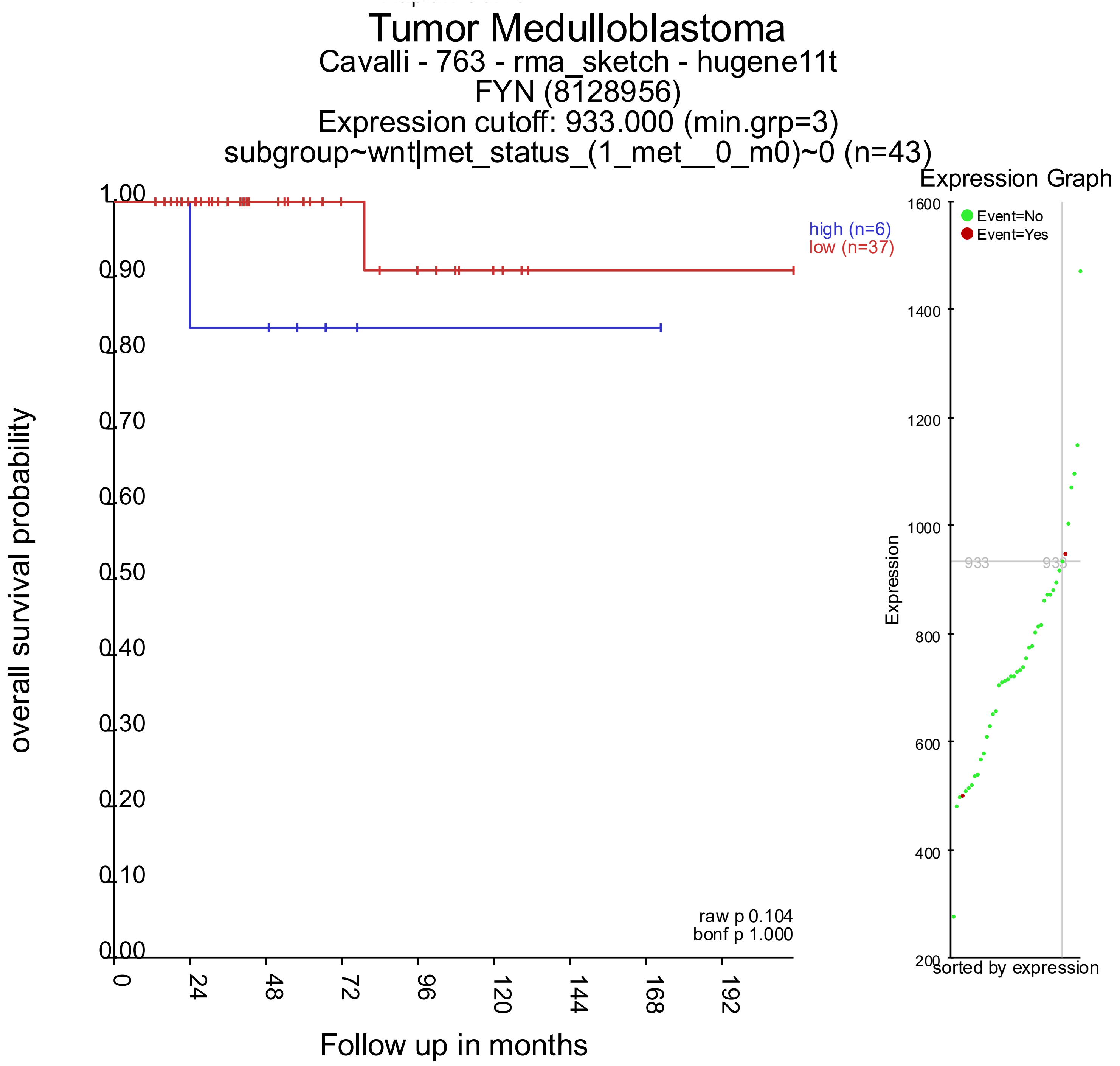

Tumor Medulloblastoma
Cavalli - 763 - rma_sketch - hugene11t
FYN (8128956)
Expression cutoff: 933.000 (min.grp=3)
subgroup~wnt|met_status_(1_met__0_m0)~0 (n=43)
overall survival probability
1.00
0.90
0.80
0.70
0.60
0.50
0.40
0.30
0.20
0.10
0.00
0
24
48
72
96
120
144
168
192
Follow up in months
high (n=6)
low (n=37)
raw p 0.104
bonf p 1.000
Expression Graph
Event=No
Event=Yes
Expression
1600
1400
1200
1000
800
600
400
200
933
933
sorted by expression

WNT M1

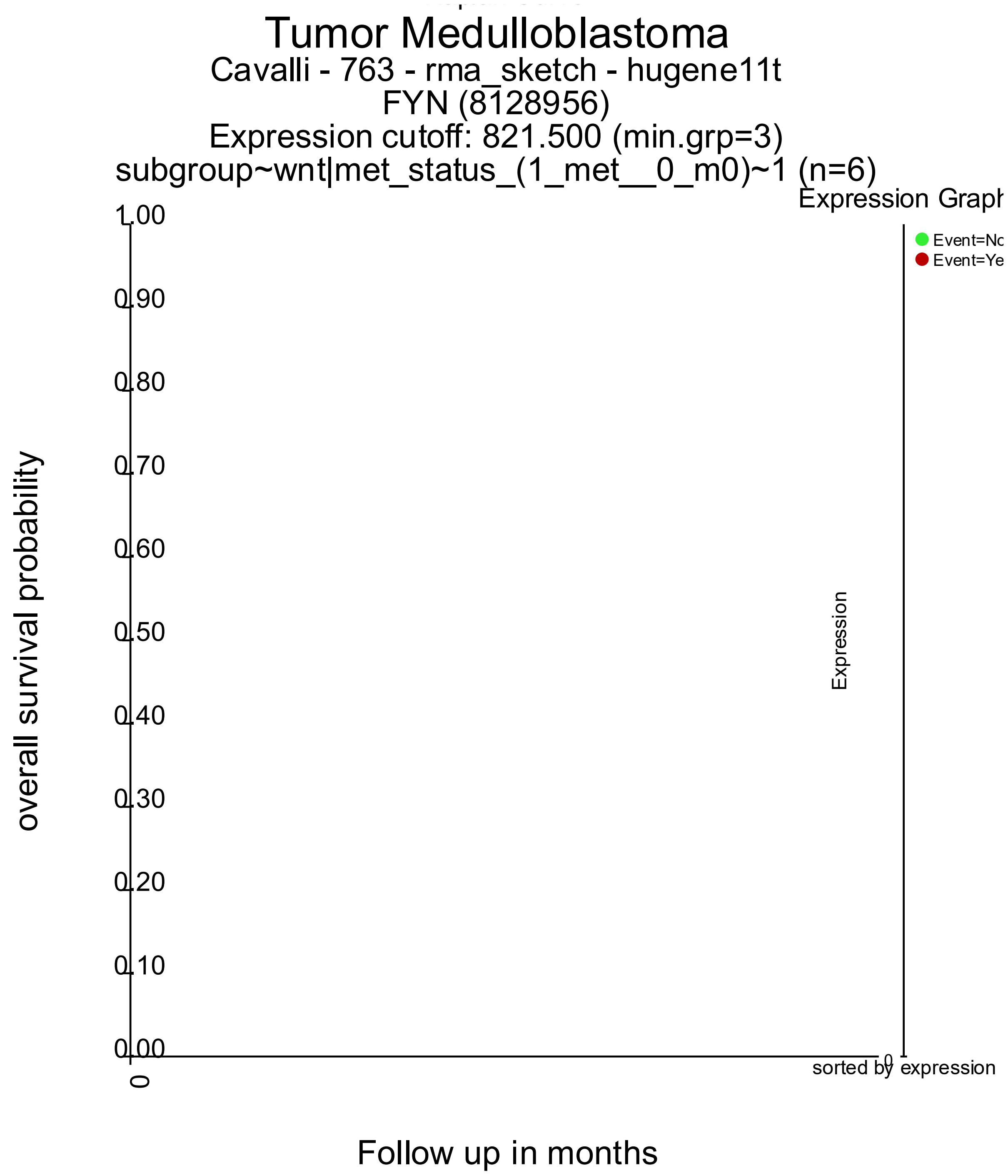

Tumor Medulloblastoma
Cavalli - 763 - rma_sketch - hugene11t
FYN (8128956)
Expression cutoff: 821.500 (min.grp=3)
subgroup~wnt|met_status_(1_met__0_m0)~1 (n=6)
Expression Graph
Event=No
Event=Yes
1.00
0.90
0.80
0.70
0.60
0.50
0.40
0.30
0.20
0.10
0.00
overall survival probability
0
Follow up in months
Expression
0
sorted by expression

SHH M0

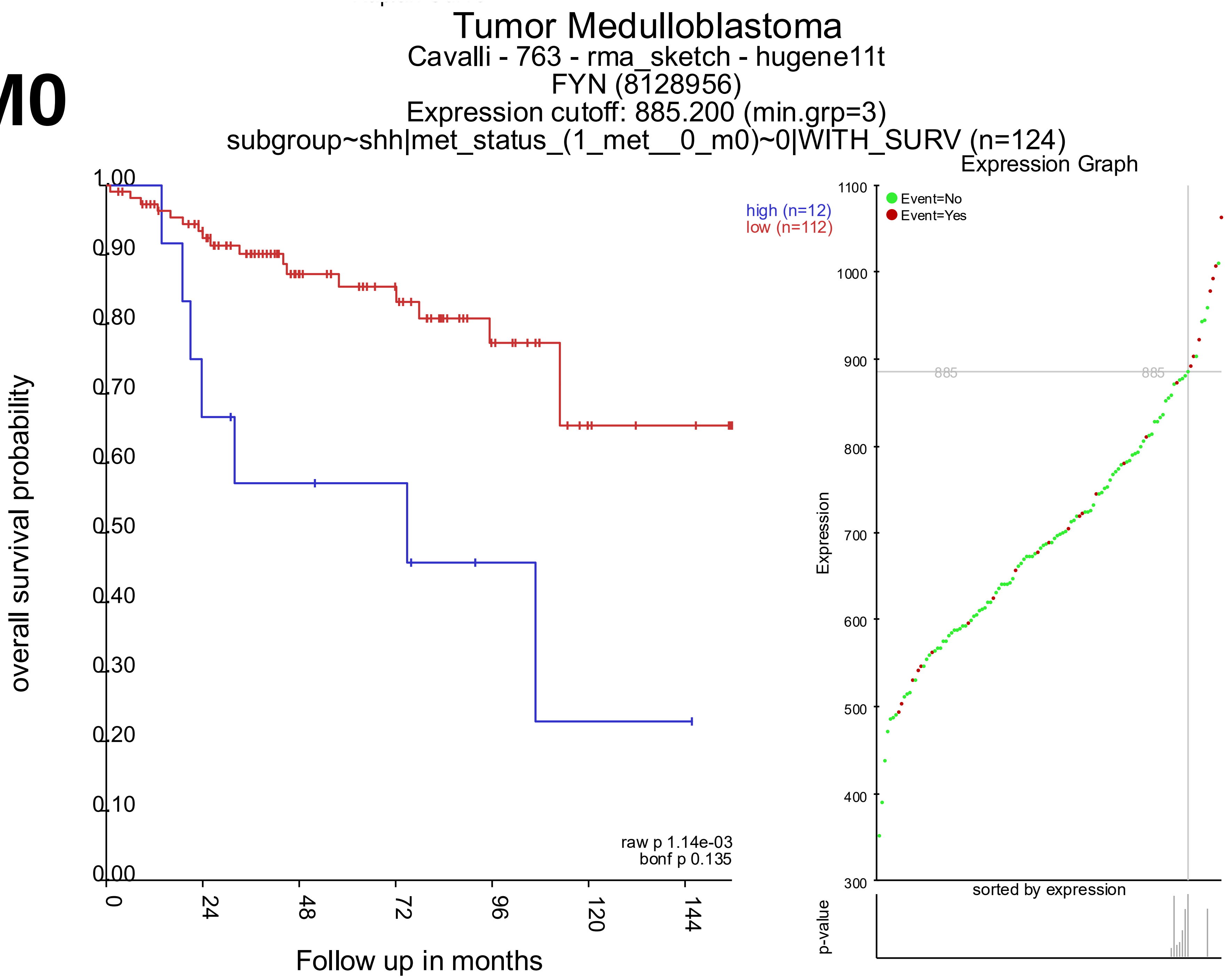
Tumor Medulloblastoma
Cavalli - 763 - rma_sketch - hugene11t
FYN (8128956)
Expression cutoff: 885.200 (min.grp=3)
subgroup~shh|met_status_(1_met__0_m0)~0|WITH_SURV (n=124)
overall survival probability
1.00
0.90
0.80
0.70
0.60
0.50
0.40
0.30
0.20
0.10
0.00
0
24
48
72
96
120
144
Follow up in months
high (n=12)
low (n=112)
raw p 1.14e-03
bonf p 0.135
Expression Graph
Event=No
Event=Yes
Expression
1100
1000
900
800
700
600
500
400
300
885
885
sorted by expression
p-value

SHH M1

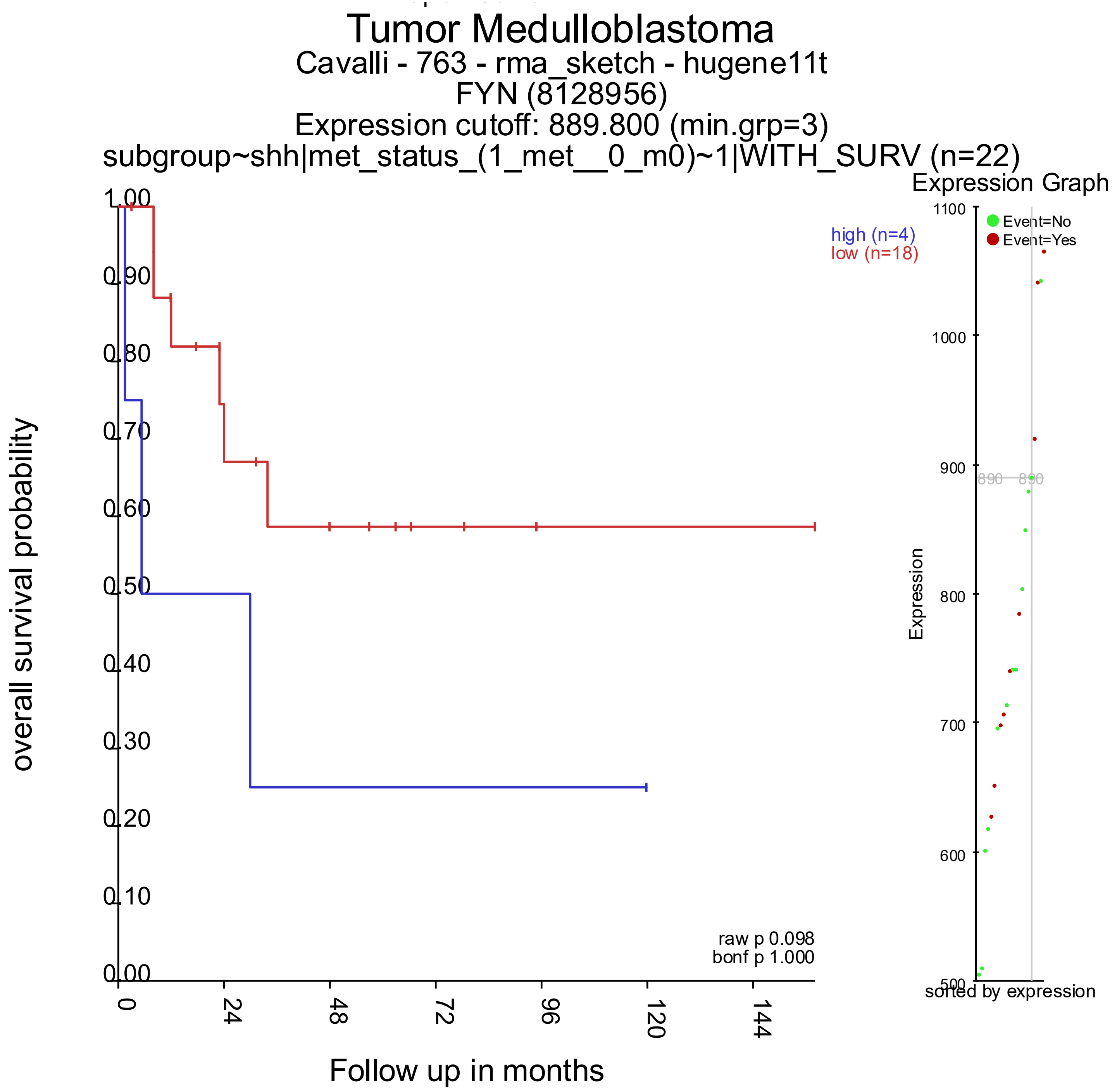

Tumor Medulloblastoma
Cavalli - 763 - rma_sketch - hugene11t
FYN (8128956)
Expression cutoff: 889.800 (min.grp=3)
subgroup~shh|met_status_(1_met__0_m0)~1|WITH_SURV (n=22)
overall survival probability
1.00
0.90
0.80
0.70
0.60
0.50
0.40
0.30
0.20
0.10
0.00
0
24
48
72
96
120
144
Follow up in months
high (n=4)
low (n=18)
raw p 0.098
bonf p 1.000
Expression Graph
Event=No
Event=Yes
Expression
1100
1000
900
800
700
600
500
890
sorted by expression

GROUP4 M0

Tumor Medulloblastoma
Cavalli - 763 - rma_sketch - hugene11t
FYN (8128956)
Expression cutoff: 677.800 (min.grp=3)
subgroup~group4|met_status_(1_met__0_m0)~0|WITH_SURV (n=145)

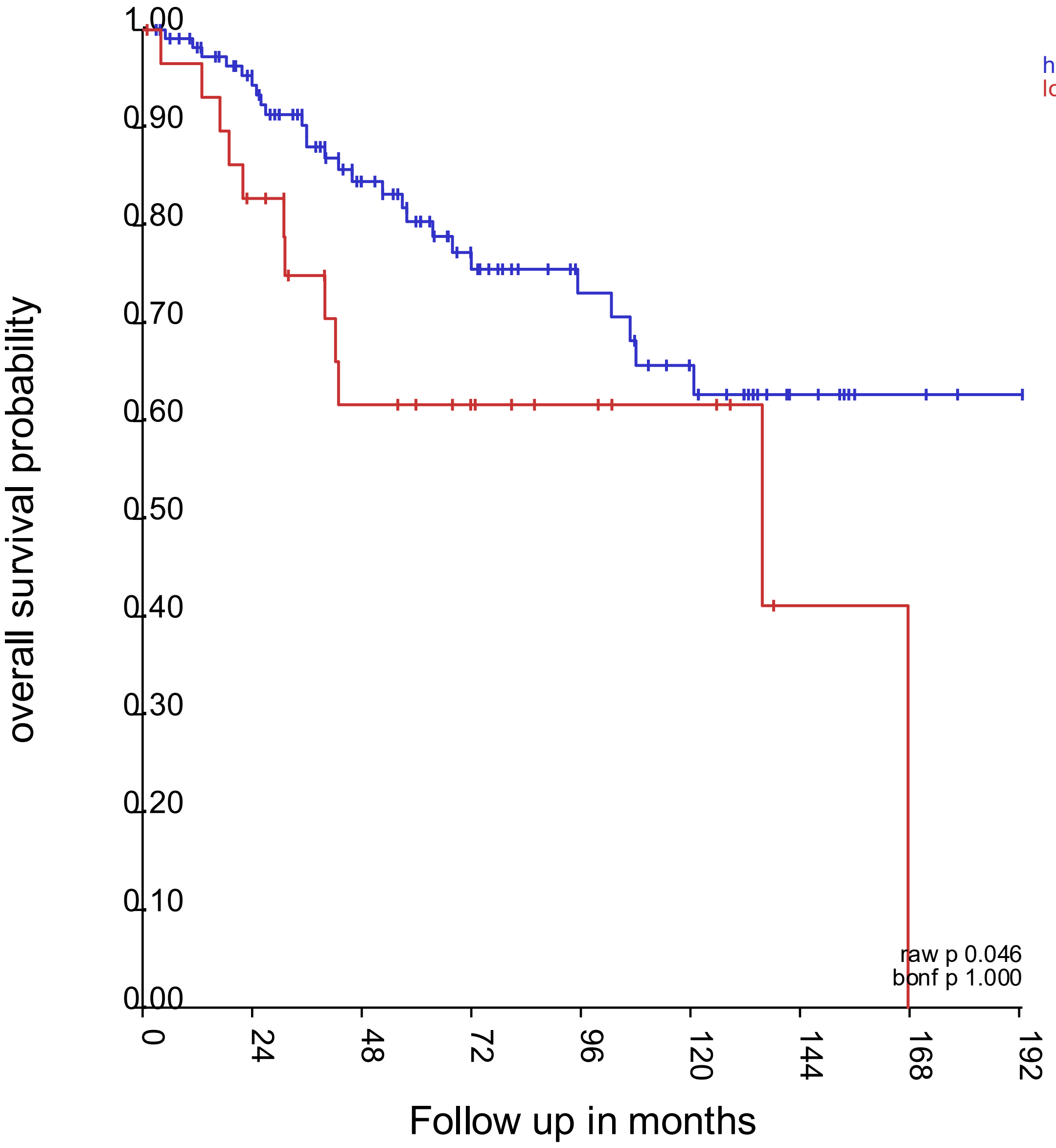

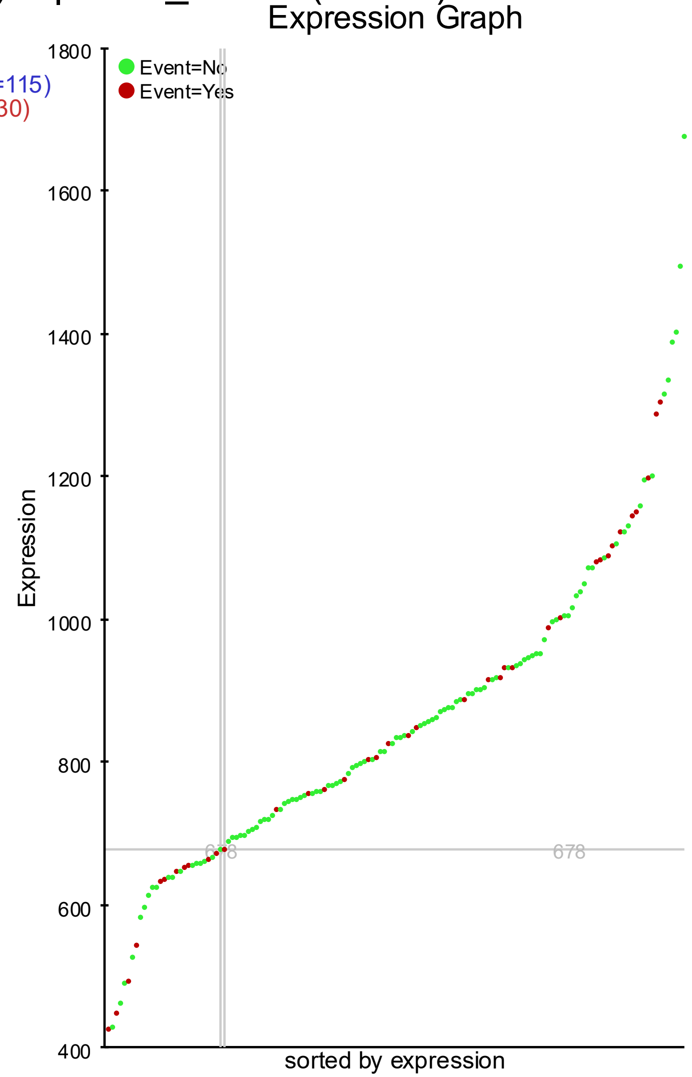

GROUP4 M1

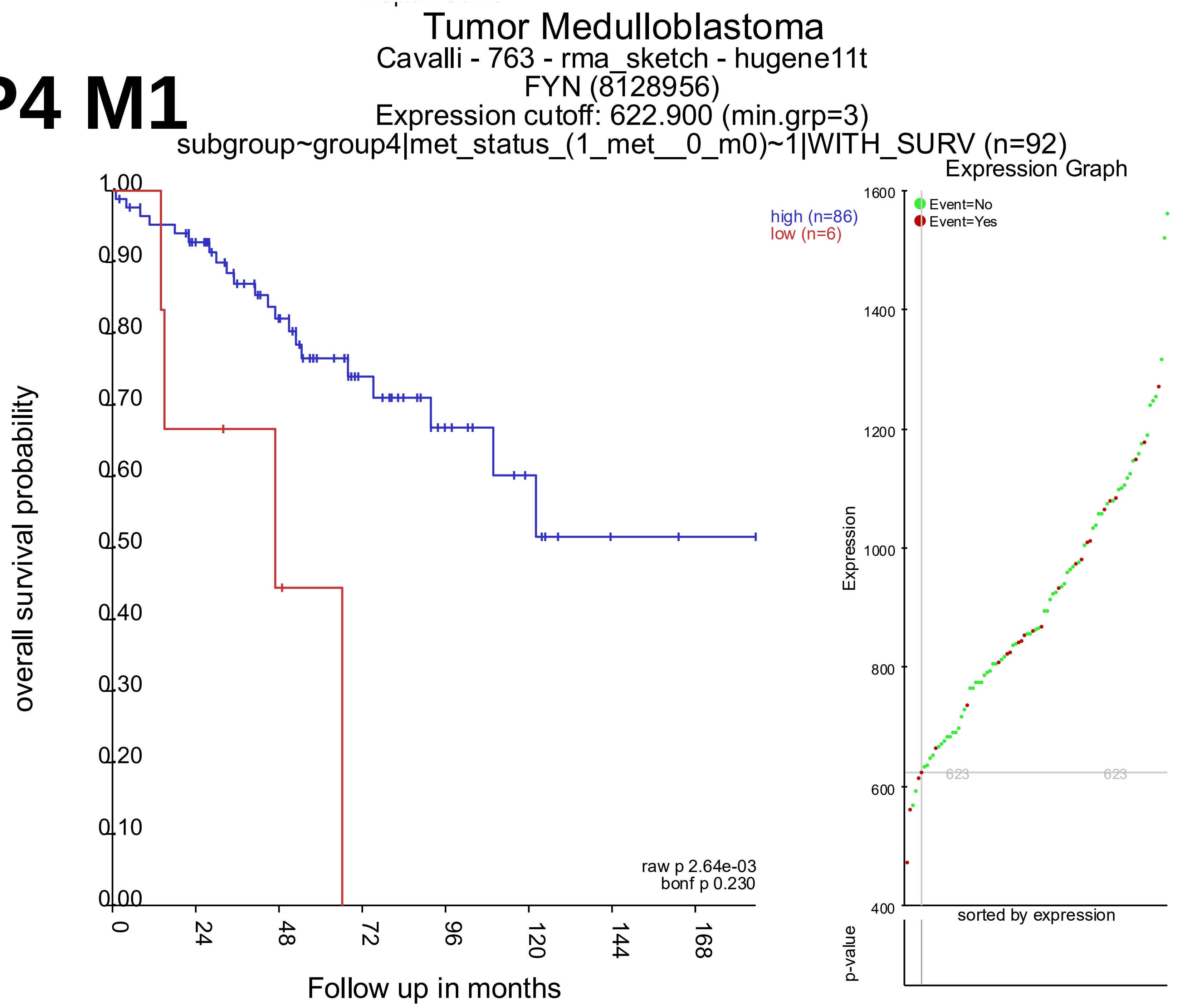

Tumor Medulloblastoma
Cavalli - 763 - rma_sketch - hugene11t
FYN (8128956)
Expression cutoff: 622.900 (min.grp=3)
subgroup~group4|met_status_(1_met__0_m0)~1|WITH_SURV (n=92)
overall survival probability
1.00
0.90
0.80
0.70
0.60
0.50
0.40
0.30
0.20
0.10
0.00
high (n=86)
low (n=6)
raw p 2.64e-03
bonf p 0.230
0
24
48
72
96
120
144
168
Follow up in months
Expression Graph
Event=No
Event=Yes
Expression
1600
1400
1200
1000
800
600
400
623
623
sorted by expression
p-value

GROUP3 M0

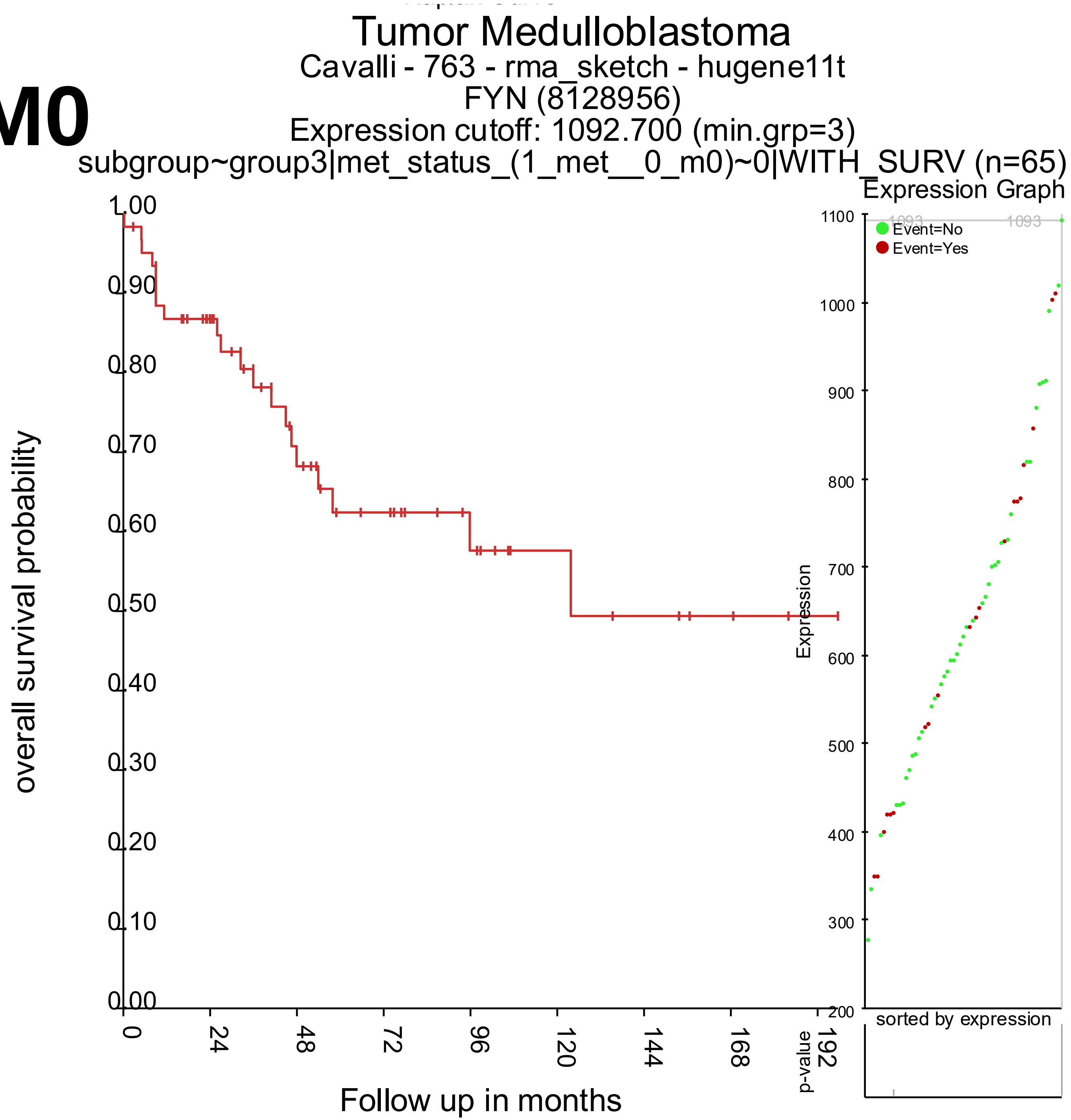

Tumor Medulloblastoma
Cavalli - 763 - rma_sketch - hugene11t
FYN (8128956)
Expression cutoff: 1092.700 (min.grp=3)
subgroup~group3|met_status_(1_met__0_m0)~0|WITH_SURV (n=65)
overall survival probability
1.00
0.90
0.80
0.70
0.60
0.50
0.40
0.30
0.20
0.10
0.00
0
24
48
72
96
120
144
168
192
Follow up in months
Expression Graph
Event=No
Event=Yes
1093
1100
1000
900
800
700
600
500
400
300
200
Expression
sorted by expression
p-value

GROUP3 M1

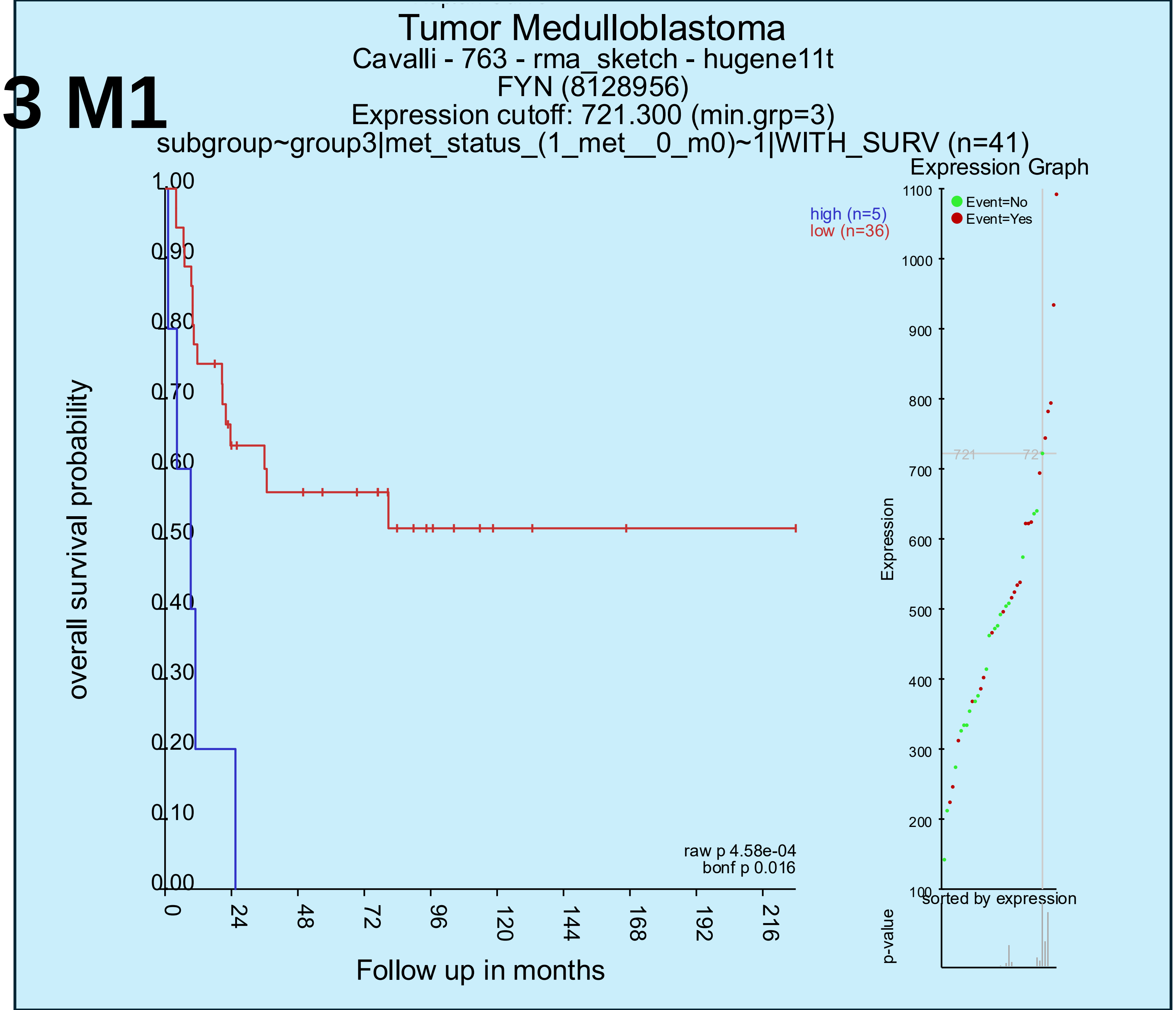
Tumor Medulloblastoma
Cavalli - 763 - rma_sketch - hugene11t
FYN (8128956)
Expression cutoff: 721.300 (min.grp=3)
subgroup~group3|met_status_(1_met__0_m0)~1|WITH_SURV (n=41)
overall survival probability
1.00
0.90
0.80
0.70
0.60
0.50
0.40
0.30
0.20
0.10
0.00
0
24
48
72
96
120
144
168
192
216
Follow up in months
high (n=5)
low (n=36)
raw p 4.58e-04
bonf p 0.016
Expression Graph
Event=No
Event=Yes
1100
1000
900
800
700
600
500
400
300
200
100
Expression
721
sorted by expression
p-value

YES

WNT M0

Tumor Medulloblastoma
Cavalli - 763 - rma_sketch - hugene11t
YES1 (8021984)
Expression cutoff: 259.200 (min.grp=3)
subgroup~wnt|met_status_(1_met__0_m0)~0 (n=43)

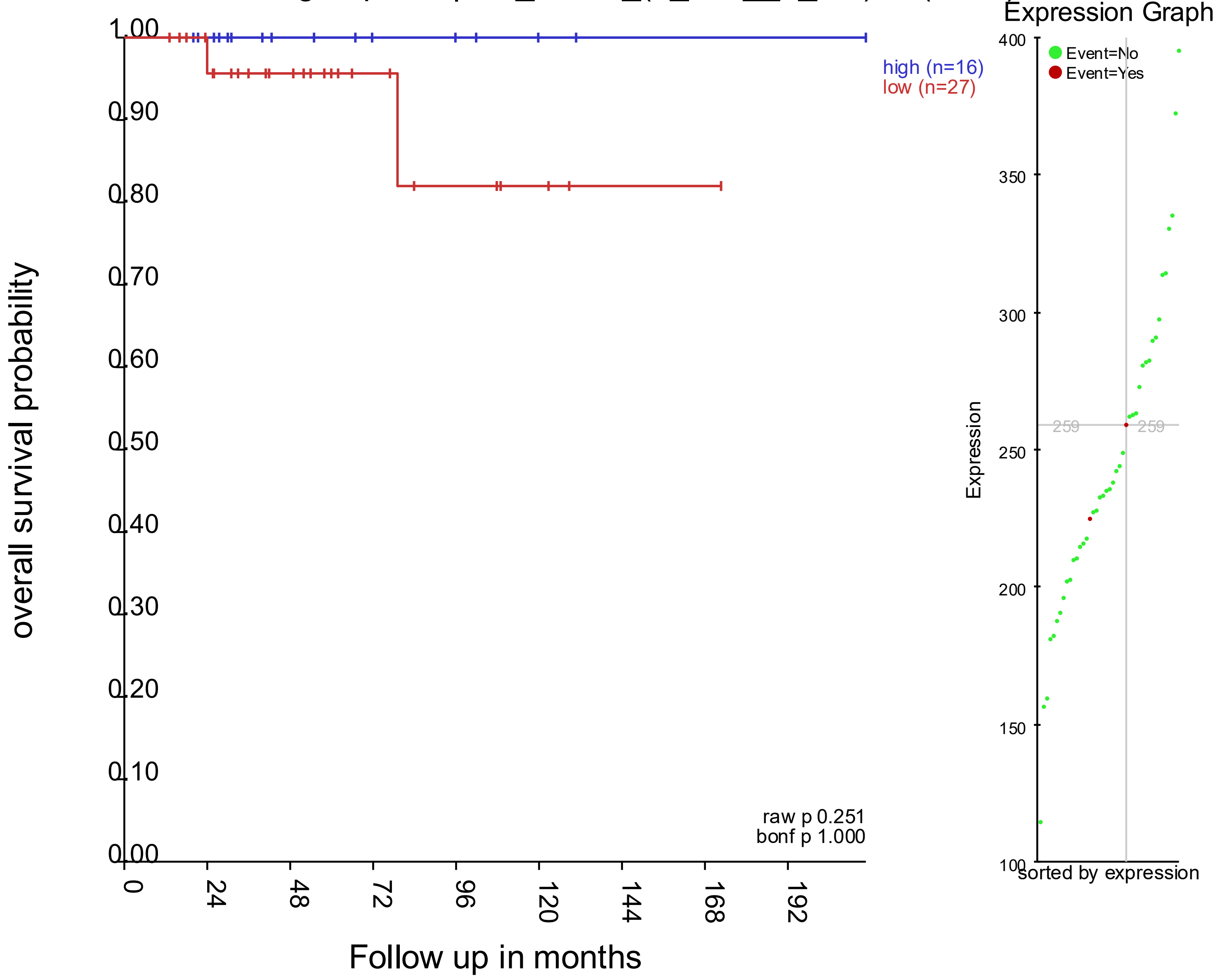

WNT M1

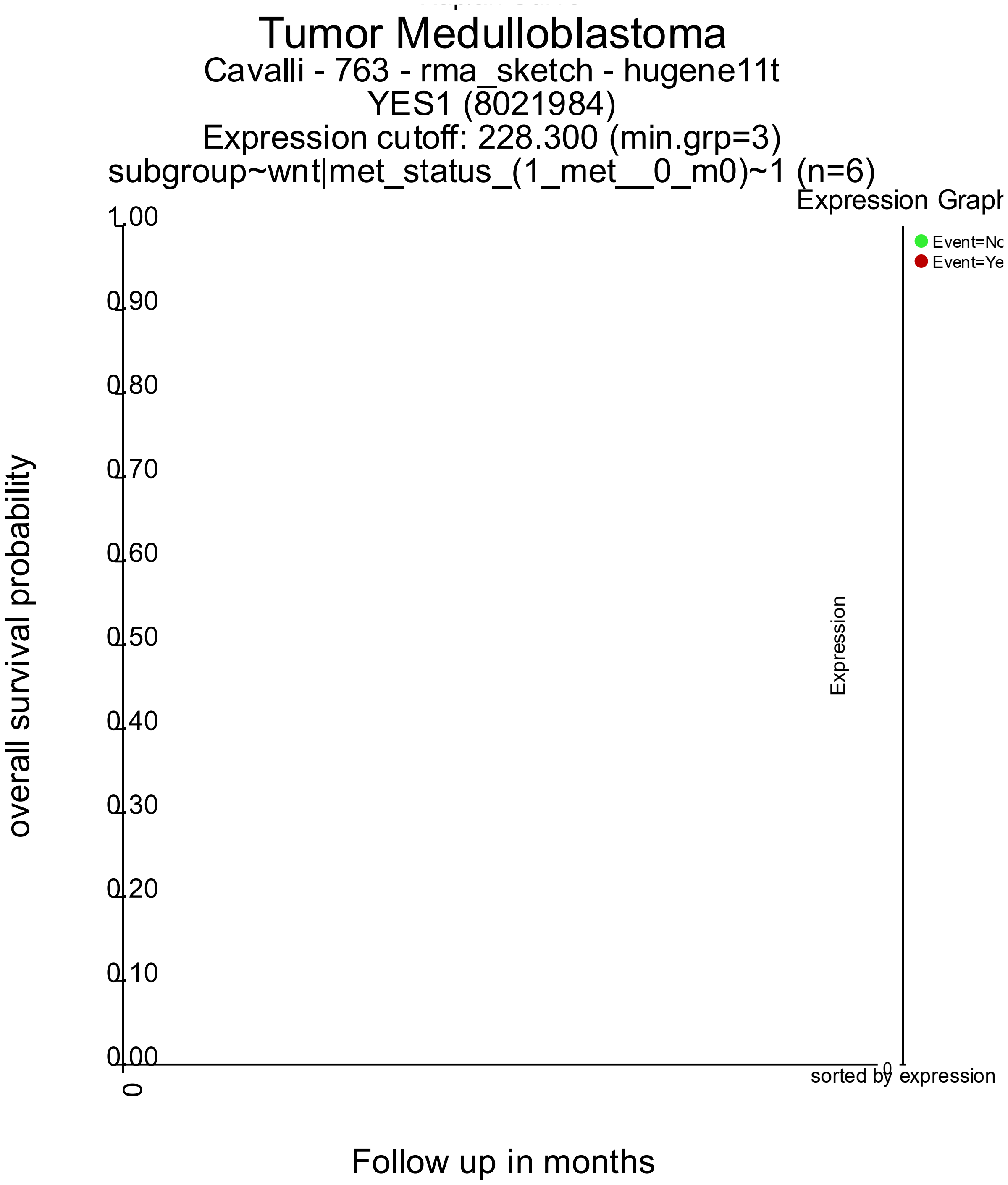

Tumor Medulloblastoma
Cavalli - 763 - rma_sketch - hugene11t
YES1 (8021984)
Expression cutoff: 228.300 (min.grp=3)
subgroup~wnt|met_status_(1_met__0_m0)~1 (n=6)
Expression Graph
Event=No
Event=Yes
1.00
0.90
0.80
0.70
0.60
0.50
0.40
0.30
0.20
0.10
0.00
overall survival probability
0
Follow up in months
Expression
0
sorted by expression

SHH M0

Tumor Medulloblastoma
Cavalli - 763 - rma_sketch - hugene11t
YES1 (8021984)
Expression cutoff: 196.600 (min.grp=3)
subgroup~shh|met_status_(1_met__0_m0)~0|WITH_SURV (n=124)

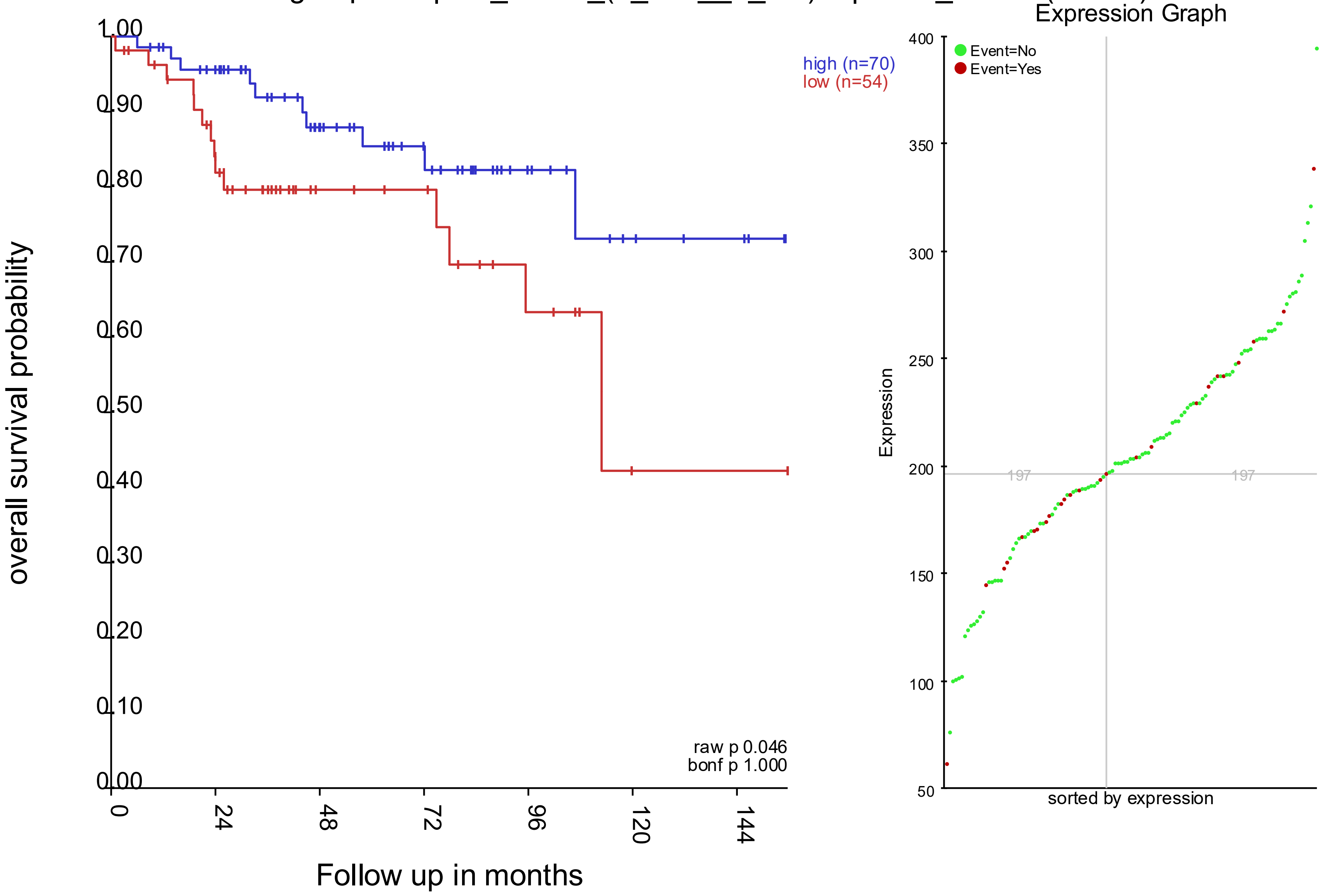

SHH M1

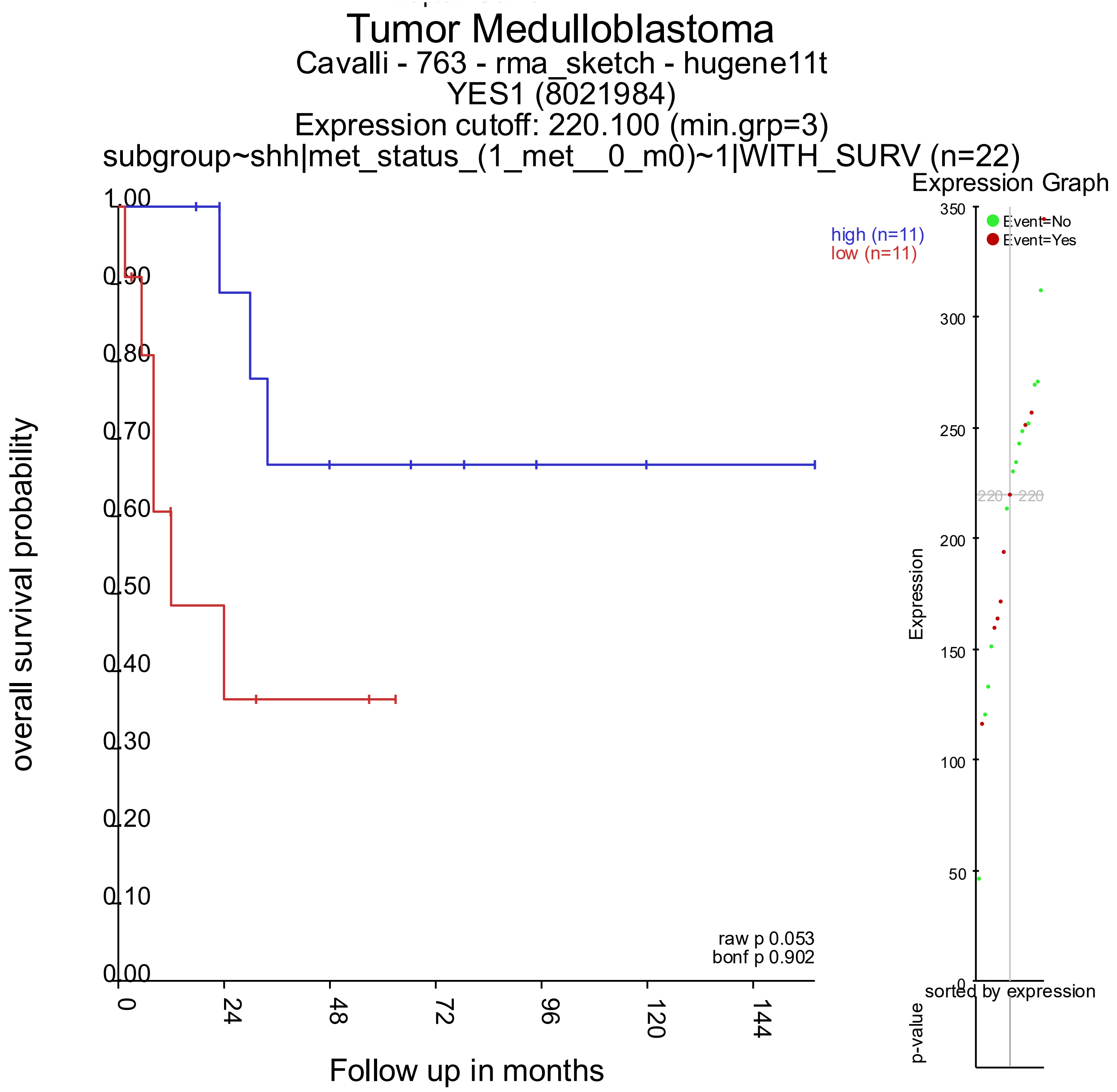

Tumor Medulloblastoma
Cavalli - 763 - rma_sketch - hugene11t
YES1 (8021984)
Expression cutoff: 220.100 (min.grp=3)
subgroup~shh|met_status_(1_met__0_m0)~1|WITH_SURV (n=22)
overall survival probability
1.00
0.90
0.80
0.70
0.60
0.50
0.40
0.30
0.20
0.10
0.00
high (n=11)
low (n=11)
raw p 0.053
bonf p 0.902
0
24
48
72
96
120
144
Follow up in months
Expression Graph
Event=No
Event=Yes
Expression
350
300
250
200
150
100
50
0
220
220
sorted by expression
p-value

GROUP4 M0

Tumor Medulloblastoma
Cavalli - 763 - rma_sketch - hugene11t
YES1 (8021984)
Expression cutoff: 131.300 (min.grp=3)
subgroup~group4|met_status_(1_met__0_m0)~0|WITH_SURV (n=145)

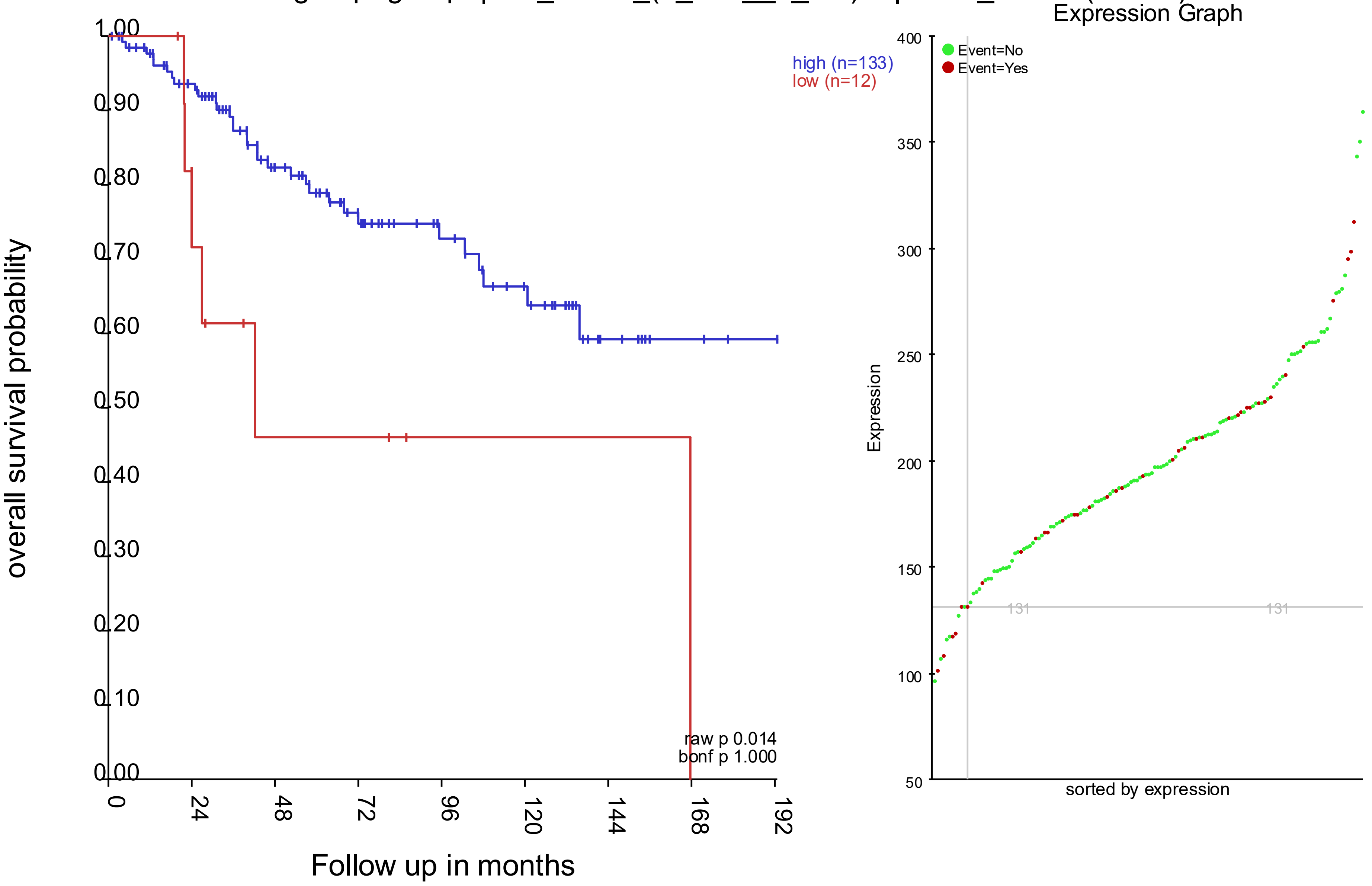

GROUP4 M1

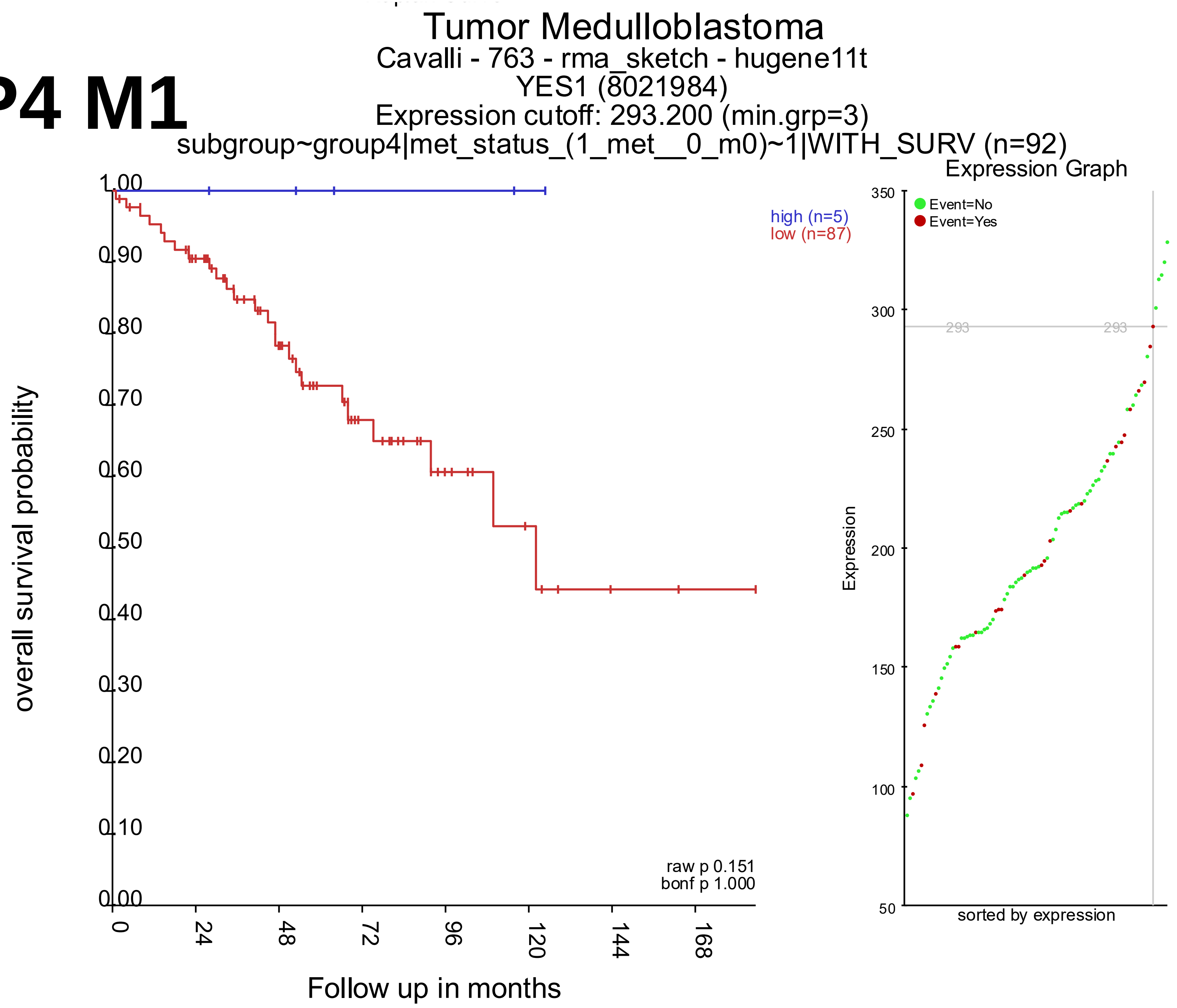

Tumor Medulloblastoma
Cavalli - 763 - rma_sketch - hugene11t
YES1 (8021984)
Expression cutoff: 293.200 (min.grp=3)
subgroup~group4|met_status_(1_met__0_m0)~1|WITH_SURV (n=92)
overall survival probability
1.00
0.90
0.80
0.70
0.60
0.50
0.40
0.30
0.20
0.10
0.00
0
24
48
72
96
120
144
168
Follow up in months
high (n=5)
low (n=87)
raw p 0.151
bonf p 1.000
Expression Graph
Event=No
Event=Yes
Expression
350
300
250
200
150
100
50
293
293
sorted by expression

GROUP3 M0

Tumor Medulloblastoma
Cavalli - 763 - rma_sketch - hugene11t
YES1 (8021984)
Expression cutoff: 154.800 (min.grp=3)
subgroup~group3|met_status_(1_met__0_m0)~0|WITH_SURV (n=65)

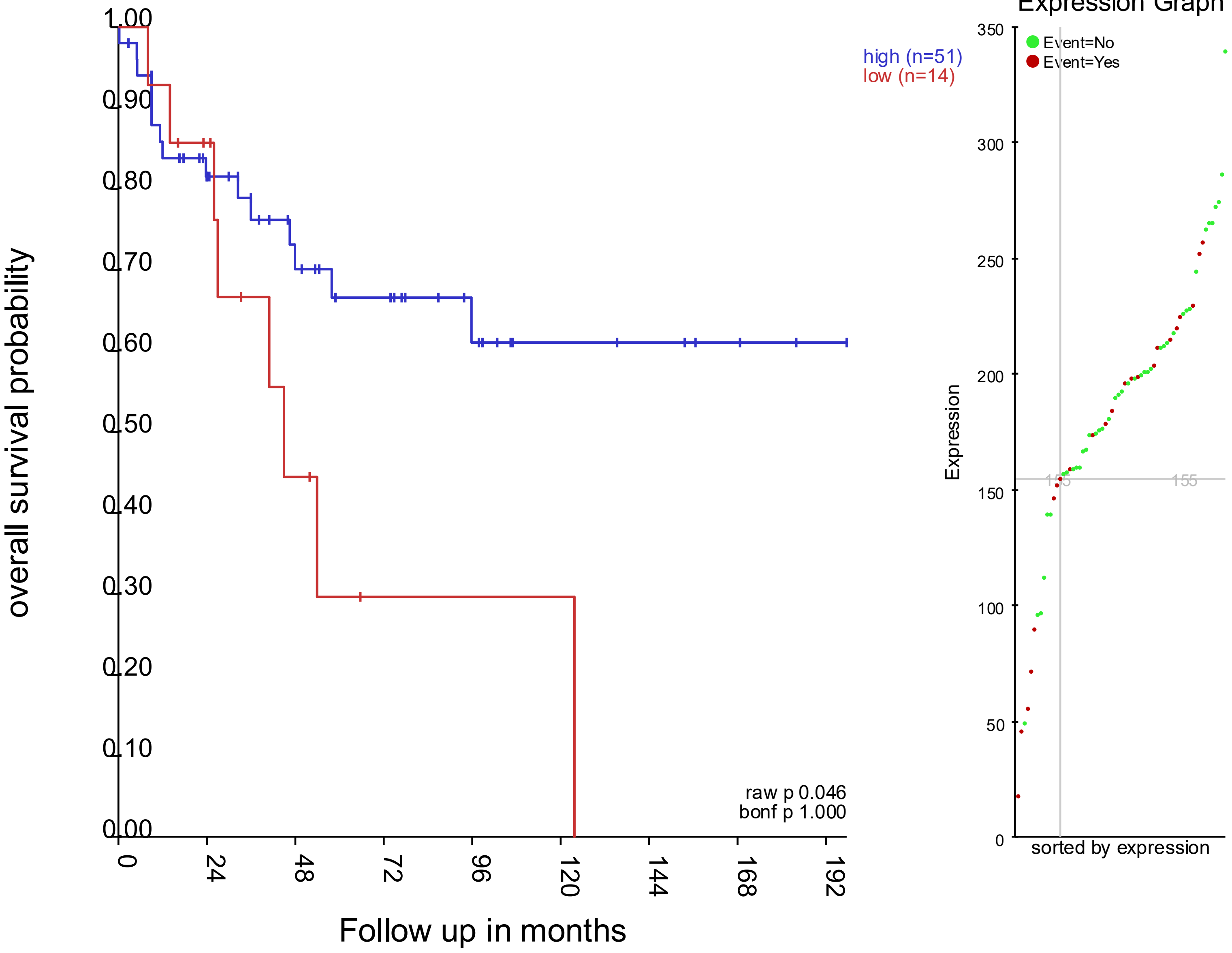

GROUP3 M1

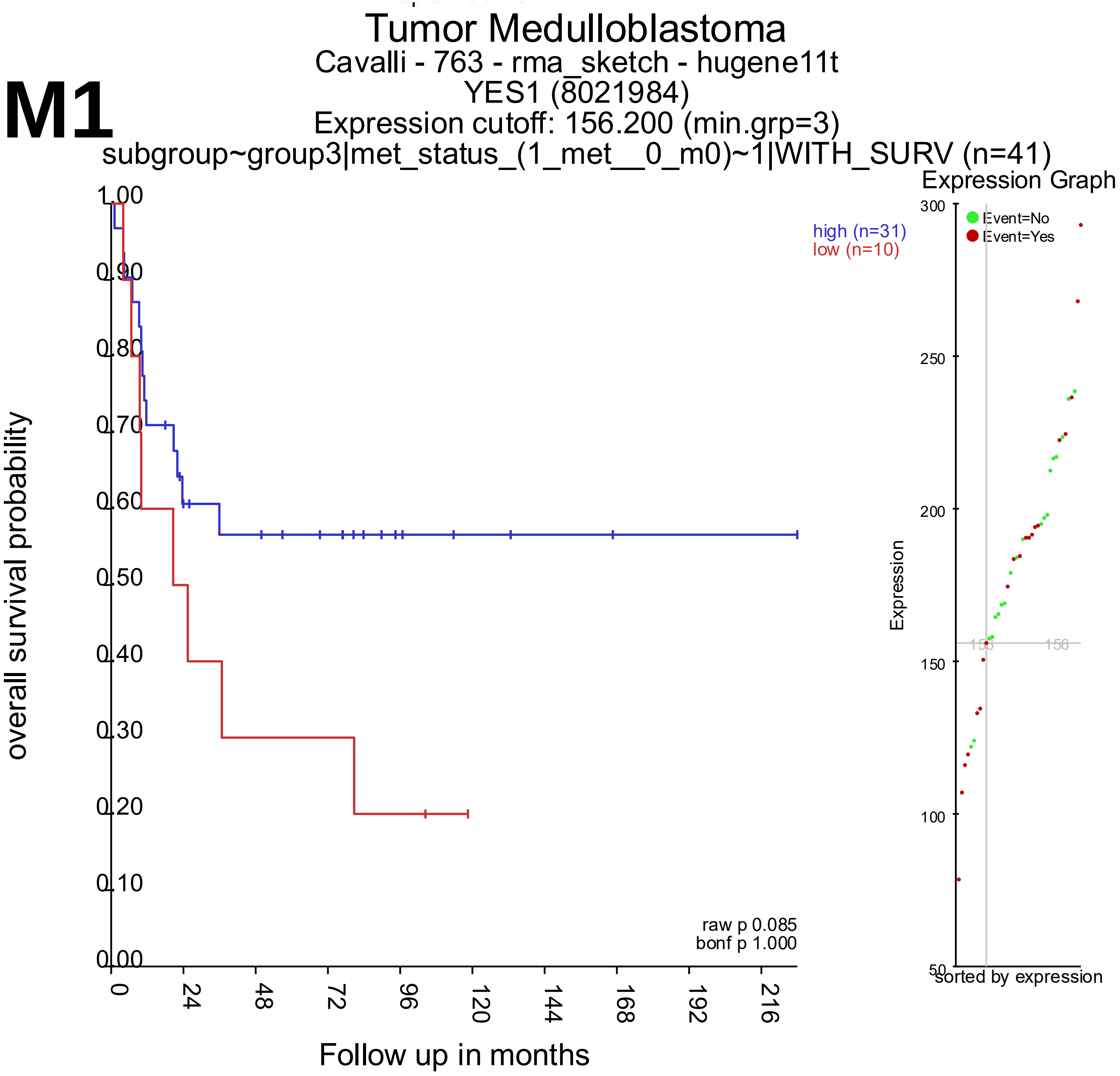
Tumor Medulloblastoma
Cavalli - 763 - rma_sketch - hugene11t
YES1 (8021984)
Expression cutoff: 156.200 (min.grp=3)
subgroup~group3|met_status_(1_met__0_m0)~1|WITH_SURV (n=41)
overall survival probability
1.00
0.90
0.80
0.70
0.60
0.50
0.40
0.30
0.20
0.10
0.00
0
24
48
72
96
120
144
168
192
216
Follow up in months
high (n=31)
low (n=10)
raw p 0.085
bonf p 1.000
Expression Graph
Event=No
Event=Yes
Expression
300
250
200
150
100
50
156
sorted by expression

LYN

WNT M0

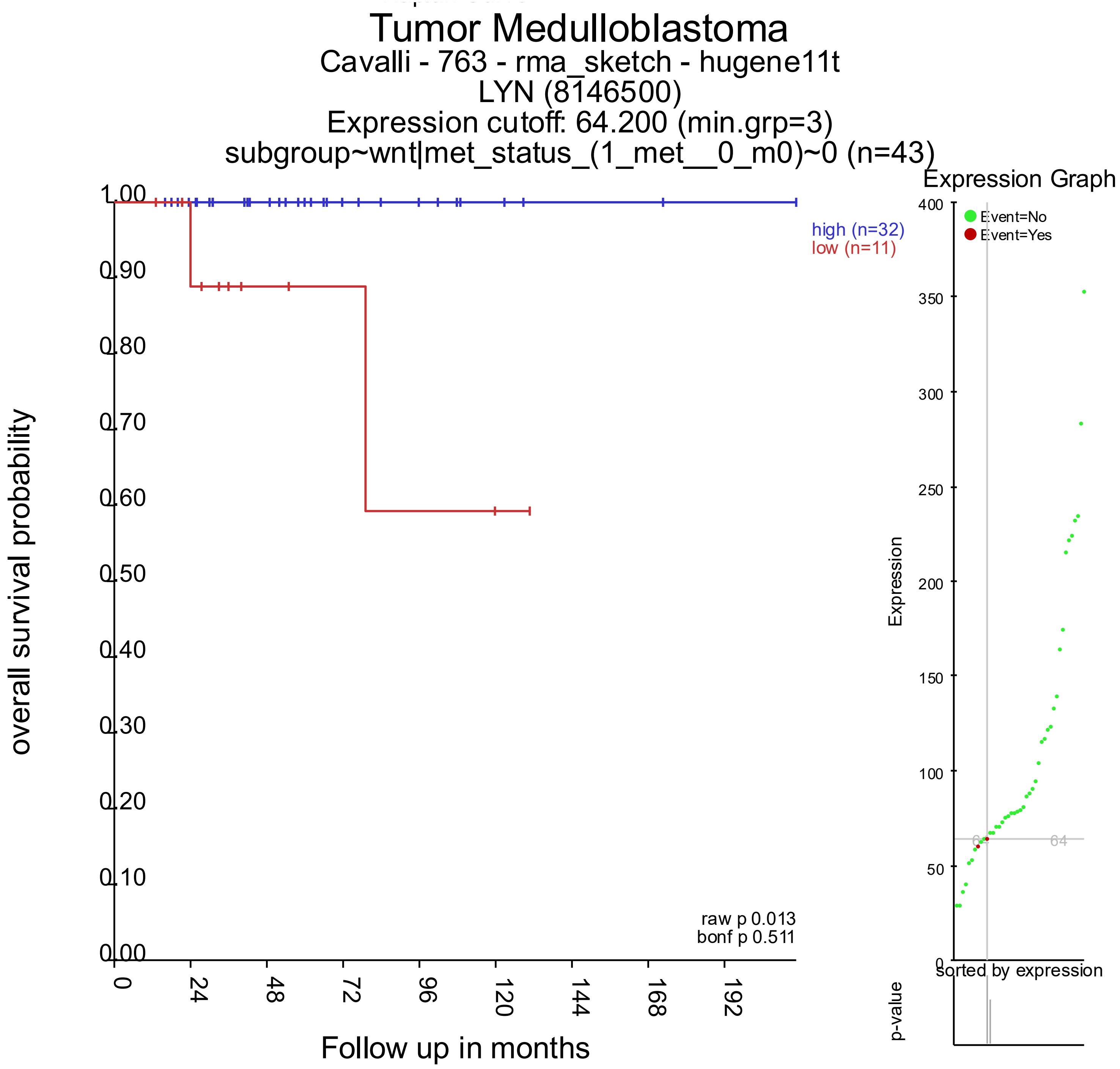

Tumor Medulloblastoma
Cavalli - 763 - rma_sketch - hugene11t
LYN (8146500)
Expression cutoff: 64.200 (min.grp=3)
subgroup~wnt|met_status_(1_met__0_m0)~0 (n=43)
overall survival probability
1.00
0.90
0.80
0.70
0.60
0.50
0.40
0.30
0.20
0.10
0.00
high (n=32)
low (n=11)
raw p 0.013
bonf p 0.511
0
24
48
72
96
120
144
168
192
Follow up in months
Expression Graph
Event=No
Event=Yes
400
350
300
250
200
150
100
50
0
Expression
64
sorted by expression
p-value

WNT M1

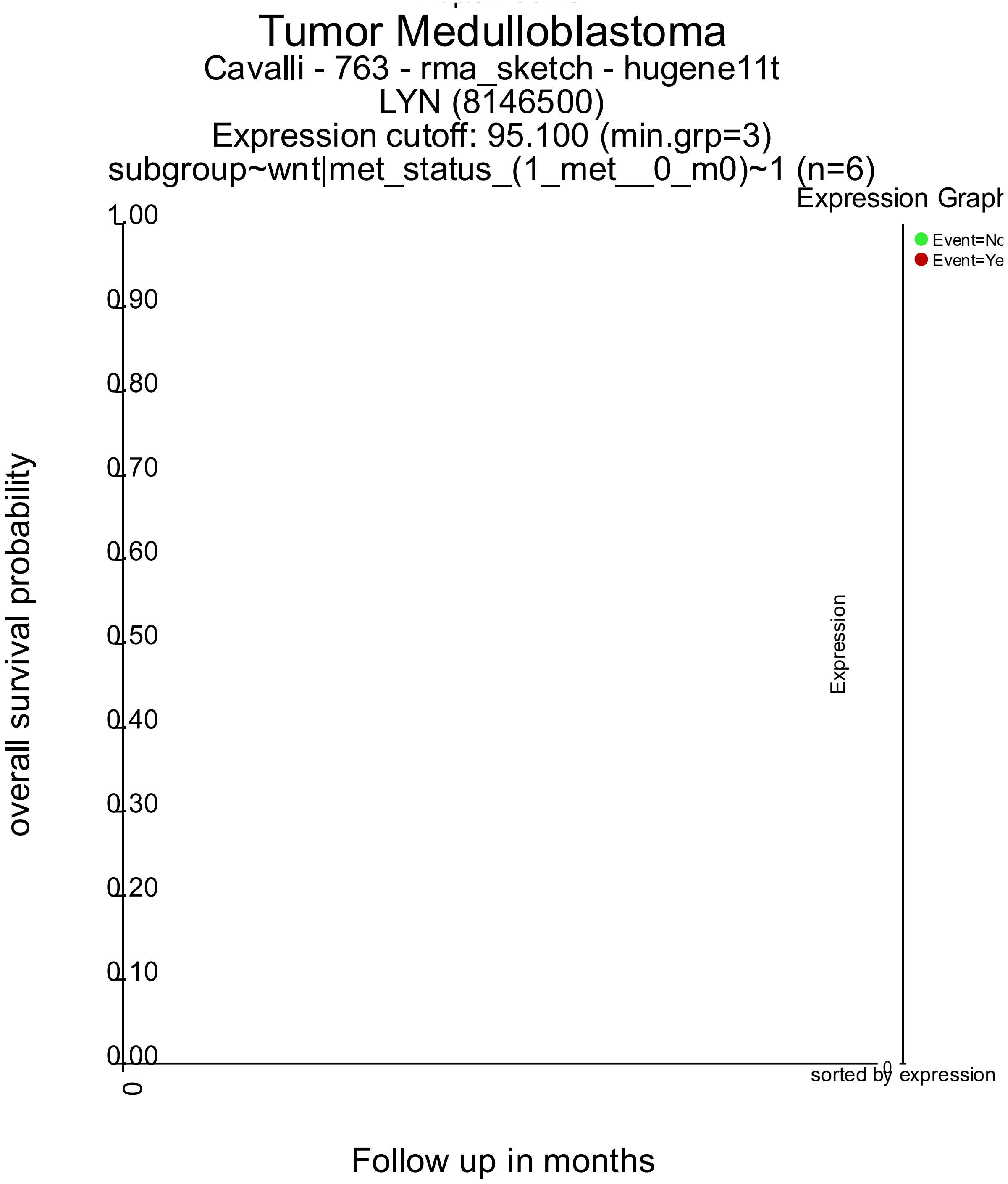

Tumor Medulloblastoma
Cavalli - 763 - rma_sketch - hugene11t
LYN (8146500)
Expression cutoff: 95.100 (min.grp=3)
subgroup~wnt|met_status_(1_met__0_m0)~1 (n=6)
Expression Graph
Event=No
Event=Yes
1.00
0.90
0.80
0.70
0.60
0.50
0.40
0.30
0.20
0.10
0.00
0
overall survival probability
Follow up in months
Expression
sorted by expression
0

SHH M0

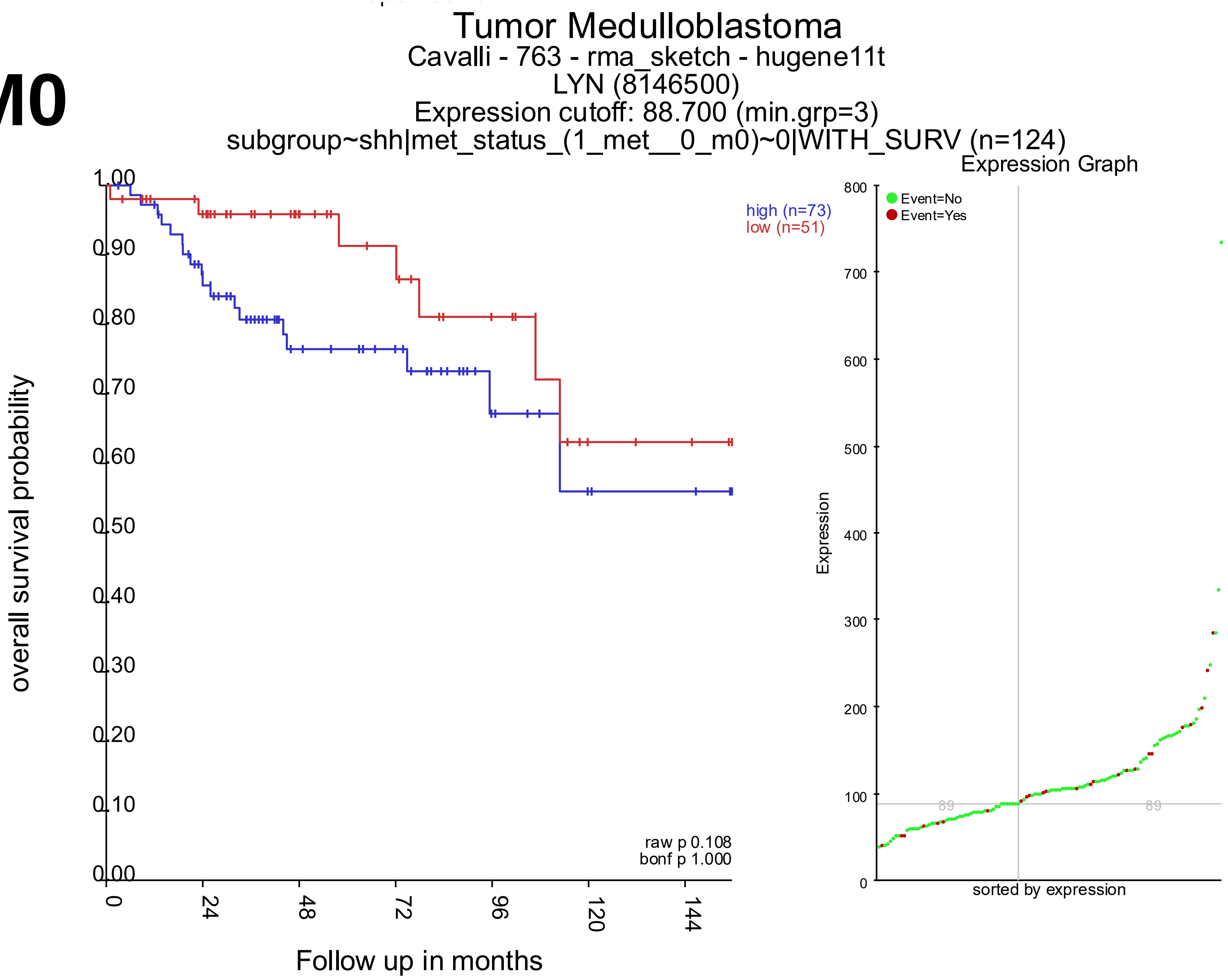
Tumor Medulloblastoma
Cavalli - 763 - rma_sketch - hugene11t
LYN (8146500)
Expression cutoff: 88.700 (min.grp=3)
subgroup~shh|met_status_(1_met__0_m0)~0|WITH_SURV (n=124)
overall survival probability
1.00
0.90
0.80
0.70
0.60
0.50
0.40
0.30
0.20
0.10
0.00
0
24
48
72
96
120
144
Follow up in months
high (n=73)
low (n=51)
raw p 0.108
bonf p 1.000
Expression Graph
Event=No
Event=Yes
Expression
800
700
600
500
400
300
200
100
0
89
89
sorted by expression

SHH M1

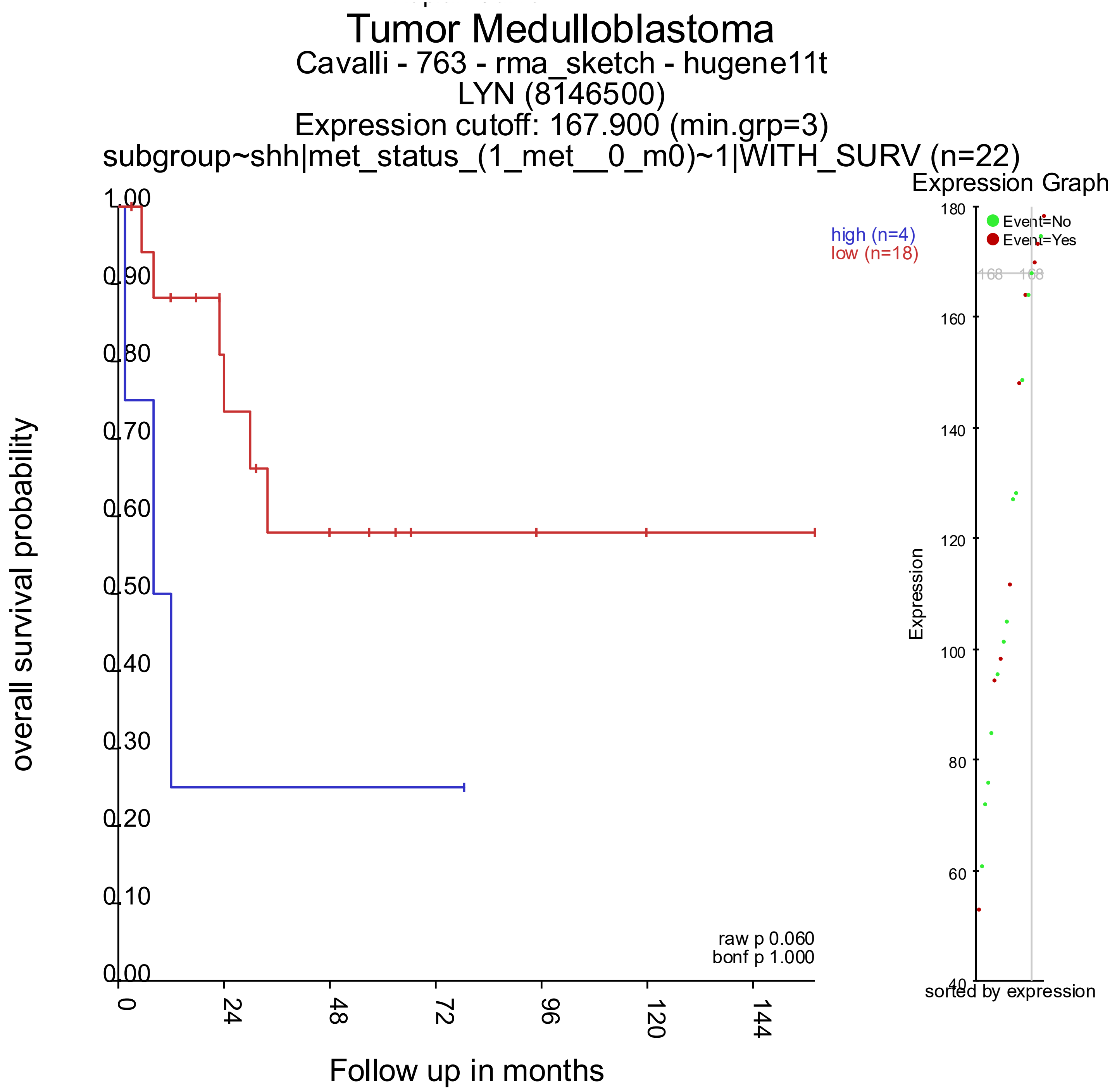

Tumor Medulloblastoma
Cavalli - 763 - rma_sketch - hugene11t
LYN (8146500)
Expression cutoff: 167.900 (min.grp=3)
subgroup~shh|met_status_(1_met__0_m0)~1|WITH_SURV (n=22)
overall survival probability
1.00
0.90
0.80
0.70
0.60
0.50
0.40
0.30
0.20
0.10
0.00
0
24
48
72
96
120
144
Follow up in months
high (n=4)
low (n=18)
raw p 0.060
bonf p 1.000
Expression Graph
Event=No
Event=Yes
168
Expression
180
160
140
120
100
80
60
40
sorted by expression

GROUP4 M0

Tumor Medulloblastoma
Cavalli - 763 - rma_sketch - hugene11t
LYN (8146500)
Expression cutoff: 211.800 (min.grp=3)
subgroup~group4|met_status_(1_met__0_m0)~0|WITH_SURV (n=145)

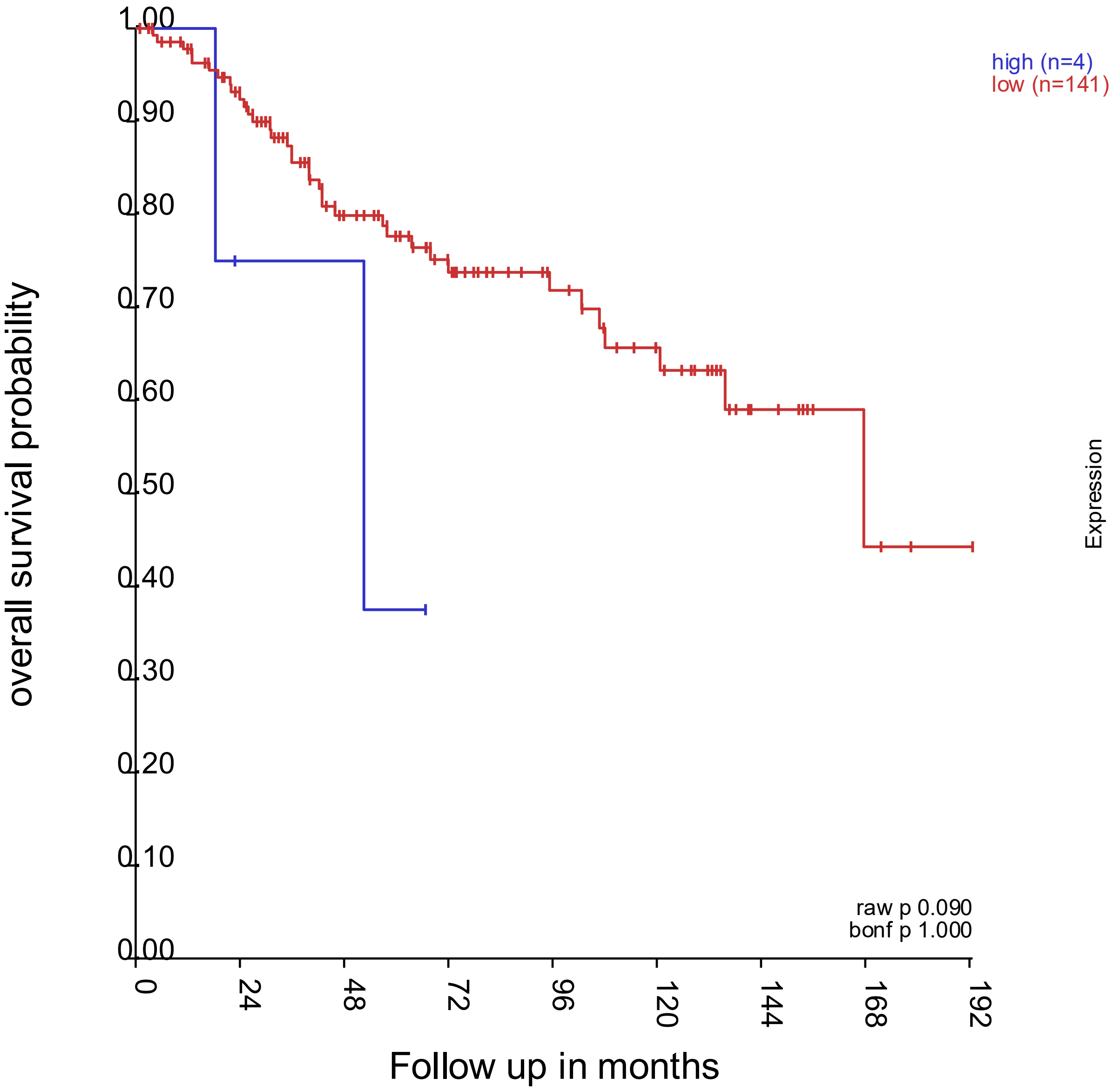

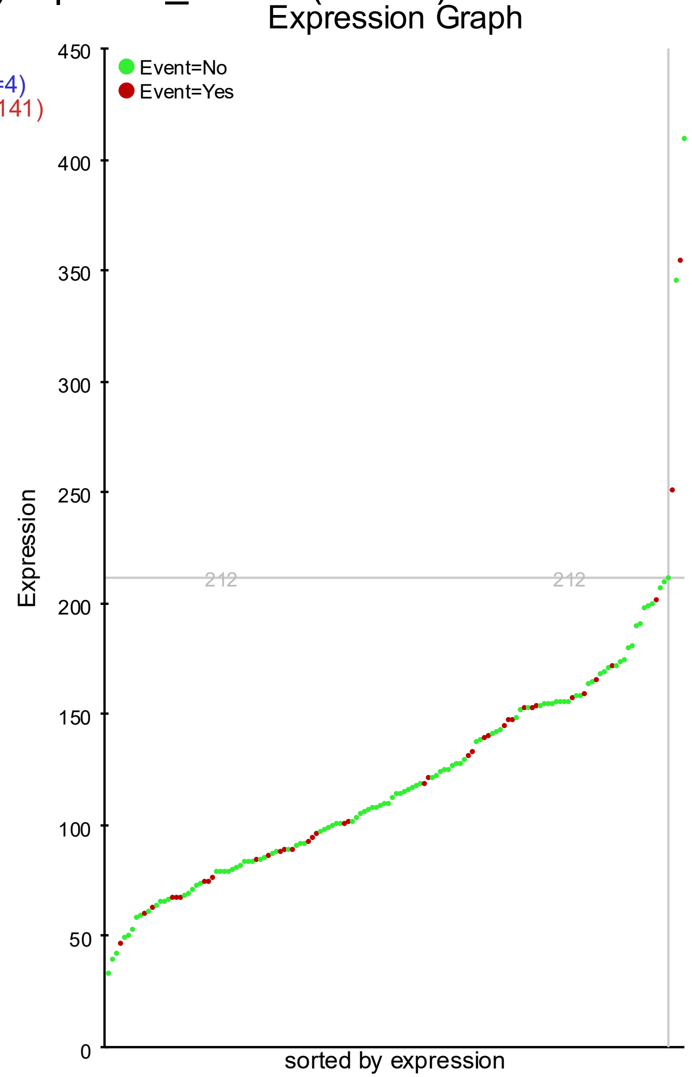

GROUP4 M1

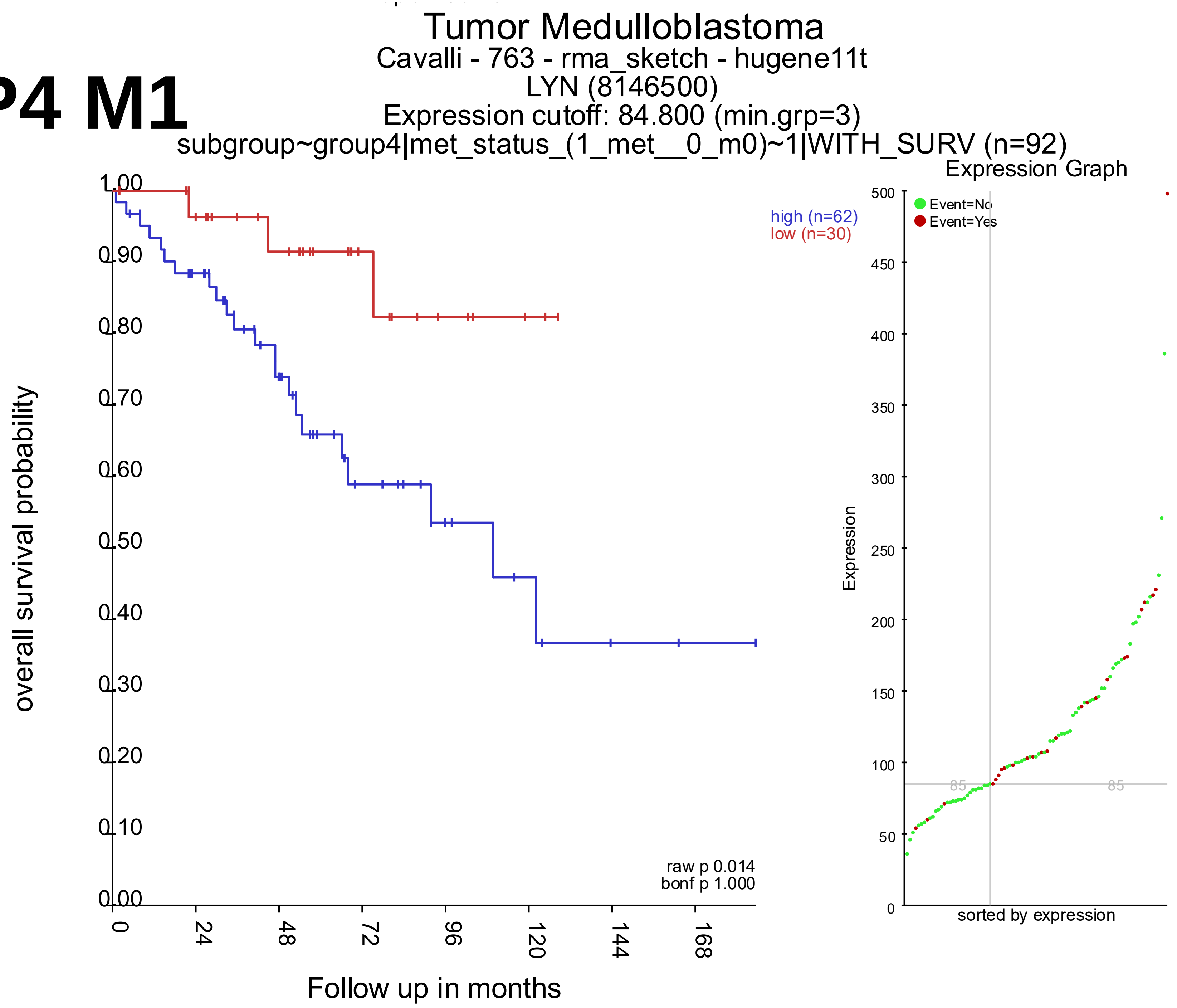

Tumor Medulloblastoma
Cavalli - 763 - rma_sketch - hugene11t
LYN (8146500)
Expression cutoff: 84.800 (min.grp=3)
subgroup~group4|met_status_(1_met__0_m0)~1|WITH_SURV (n=92)
overall survival probability
1.00
0.90
0.80
0.70
0.60
0.50
0.40
0.30
0.20
0.10
0.00
0
24
48
72
96
120
144
168
Follow up in months
high (n=62)
low (n=30)
raw p 0.014
bonf p 1.000
Expression Graph
Event=No
Event=Yes
Expression
500
450
400
350
300
250
200
150
100
50
0
85
85
sorted by expression

GROUP3 M0

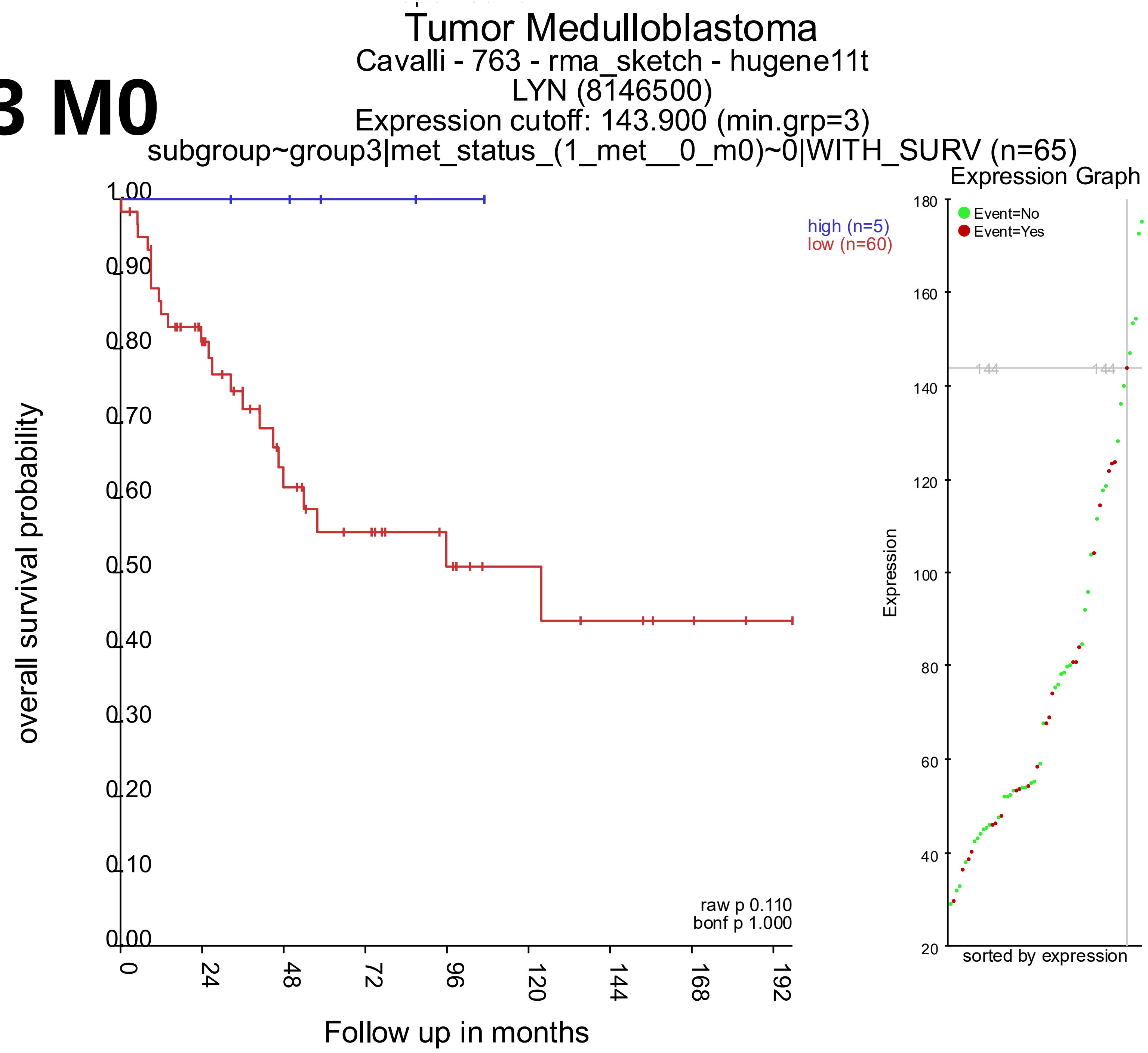

Tumor Medulloblastoma
Cavalli - 763 - rma_sketch - hugene11t
LYN (8146500)
Expression cutoff: 143.900 (min.grp=3)
subgroup~group3|met_status_(1_met__0_m0)~0|WITH_SURV (n=65)
overall survival probability
1.00
0.90
0.80
0.70
0.60
0.50
0.40
0.30
0.20
0.10
0.00
high (n=5)
low (n=60)
raw p 0.110
bonf p 1.000
0
24
48
72
96
120
144
168
192
Follow up in months
Expression Graph
Event=No
Event=Yes
Expression
180
160
144
144
140
120
100
80
60
40
20
sorted by expression

GROUP3 M1

Tumor Medulloblastoma
Cavalli - 763 - rma_sketch - hugene11t
LYN (8146500)
Expression cutoff: 48.700 (min.grp=3)
subgroup~group3|met_status_(1_met__0_m0)~1|WITH_SURV (n=41)

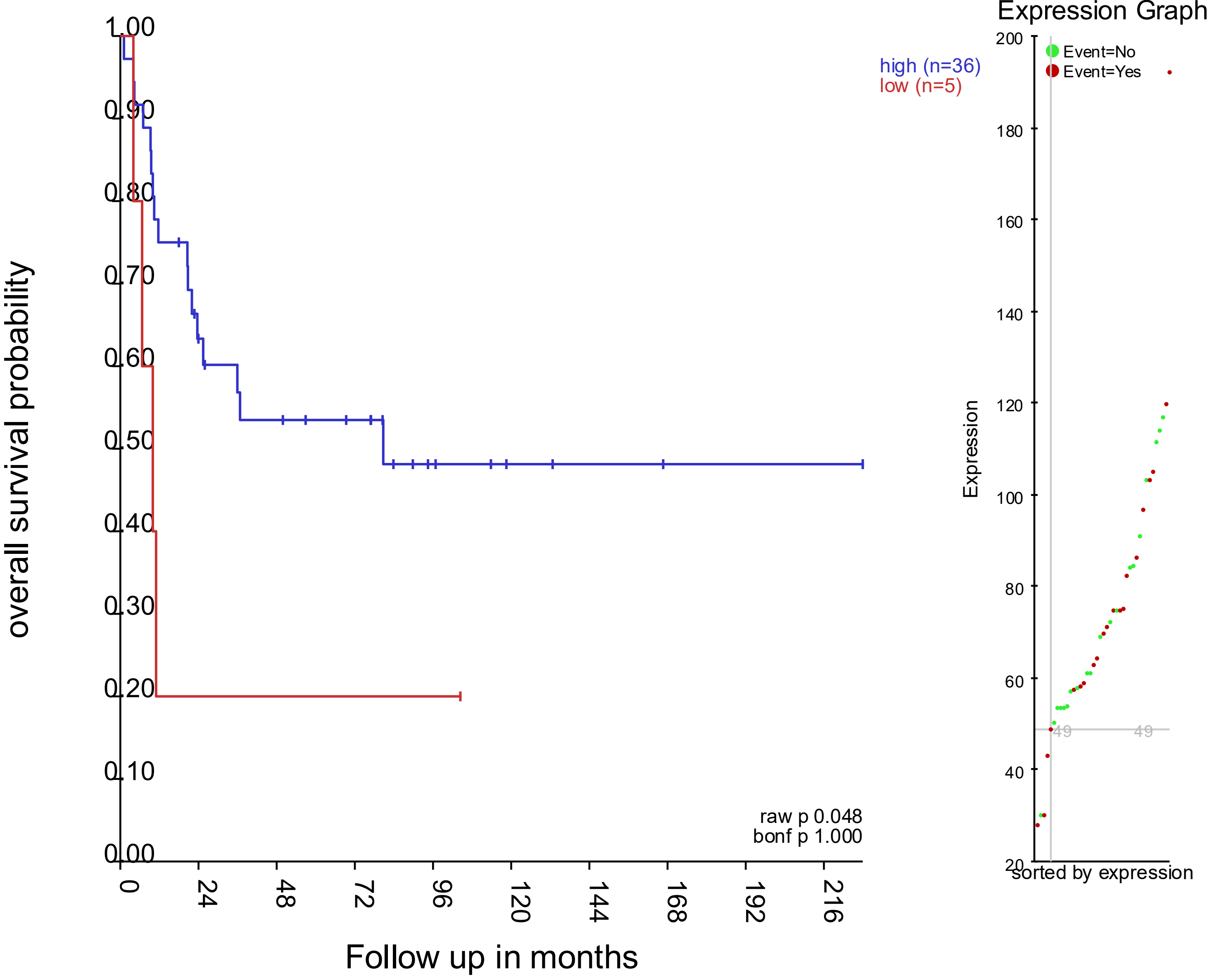

FLT1

WNT M0

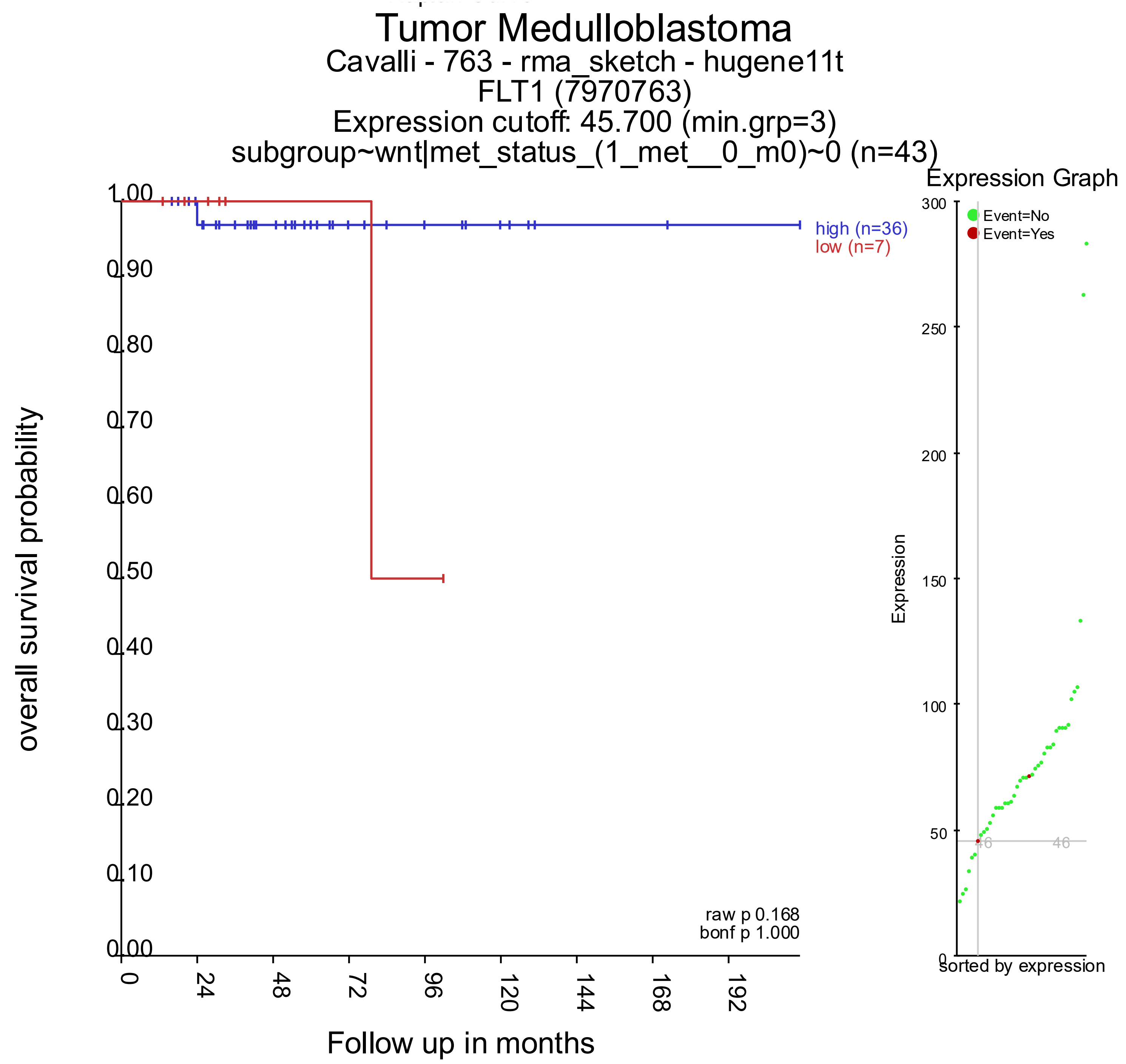

Tumor Medulloblastoma
Cavalli - 763 - rma_sketch - hugene11t
FLT1 (7970763)
Expression cutoff: 45.700 (min.grp=3)
subgroup~wnt|met_status_(1_met__0_m0)~0 (n=43)
overall survival probability
1.00
0.90
0.80
0.70
0.60
0.50
0.40
0.30
0.20
0.10
0.00
high (n=36)
low (n=7)
raw p 0.168
bonf p 1.000
0
24
48
72
96
120
144
168
192
Follow up in months
Expression Graph
Event=No
Event=Yes
Expression
300
250
200
150
100
50
0
46
sorted by expression

WNT M1

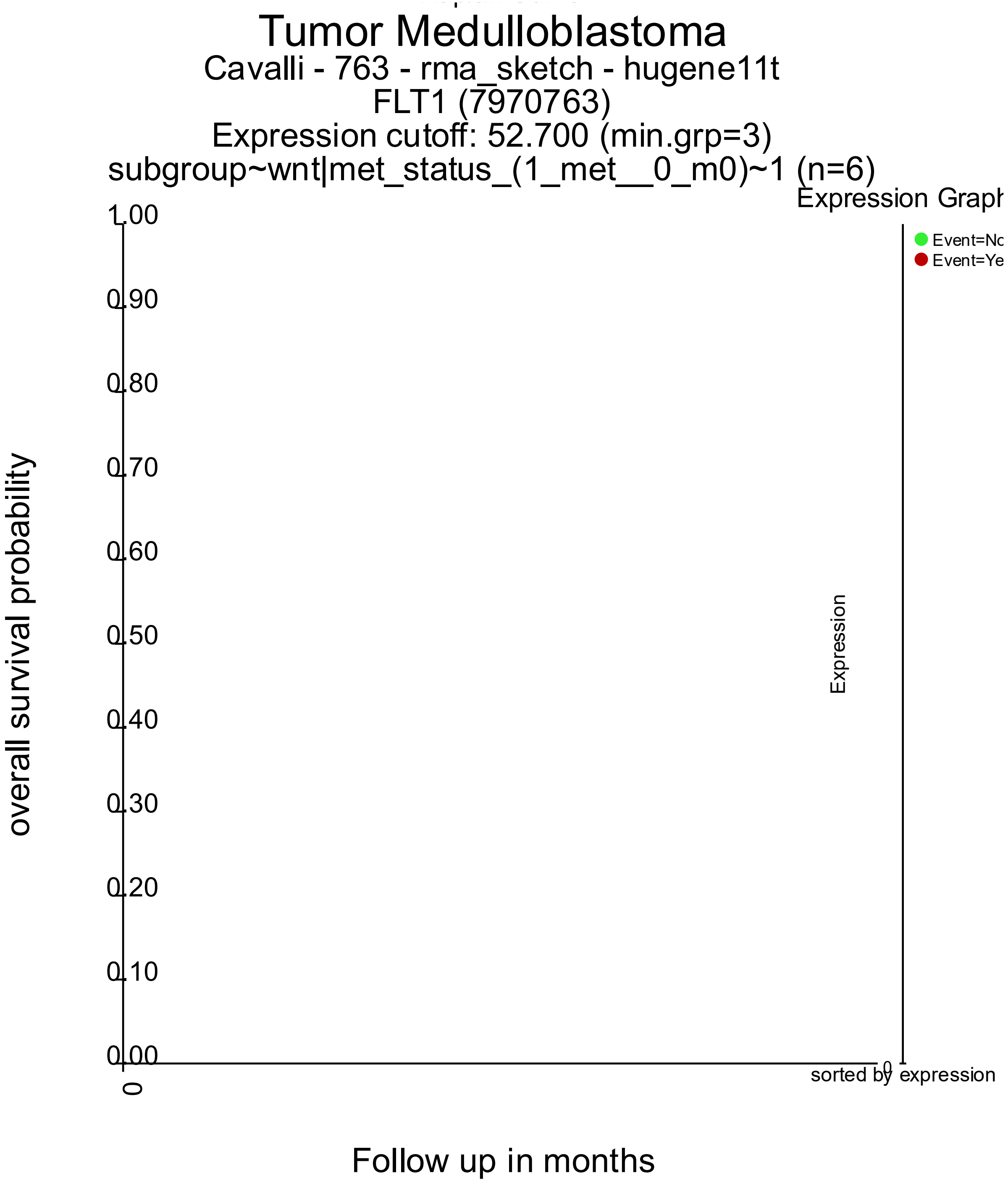
Tumor Medulloblastoma
Cavalli - 763 - rma_sketch - hugene11t
FLT1 (7970763)
Expression cutoff: 52.700 (min.grp=3)
subgroup~wnt|met_status_(1_met__0_m0)~1 (n=6)
Expression Graph
Event=No
Event=Yes
overall survival probability
1.00
0.90
0.80
0.70
0.60
0.50
0.40
0.30
0.20
0.10
0.00
0
Expression
0
sorted by expression
Follow up in months

SHH M0

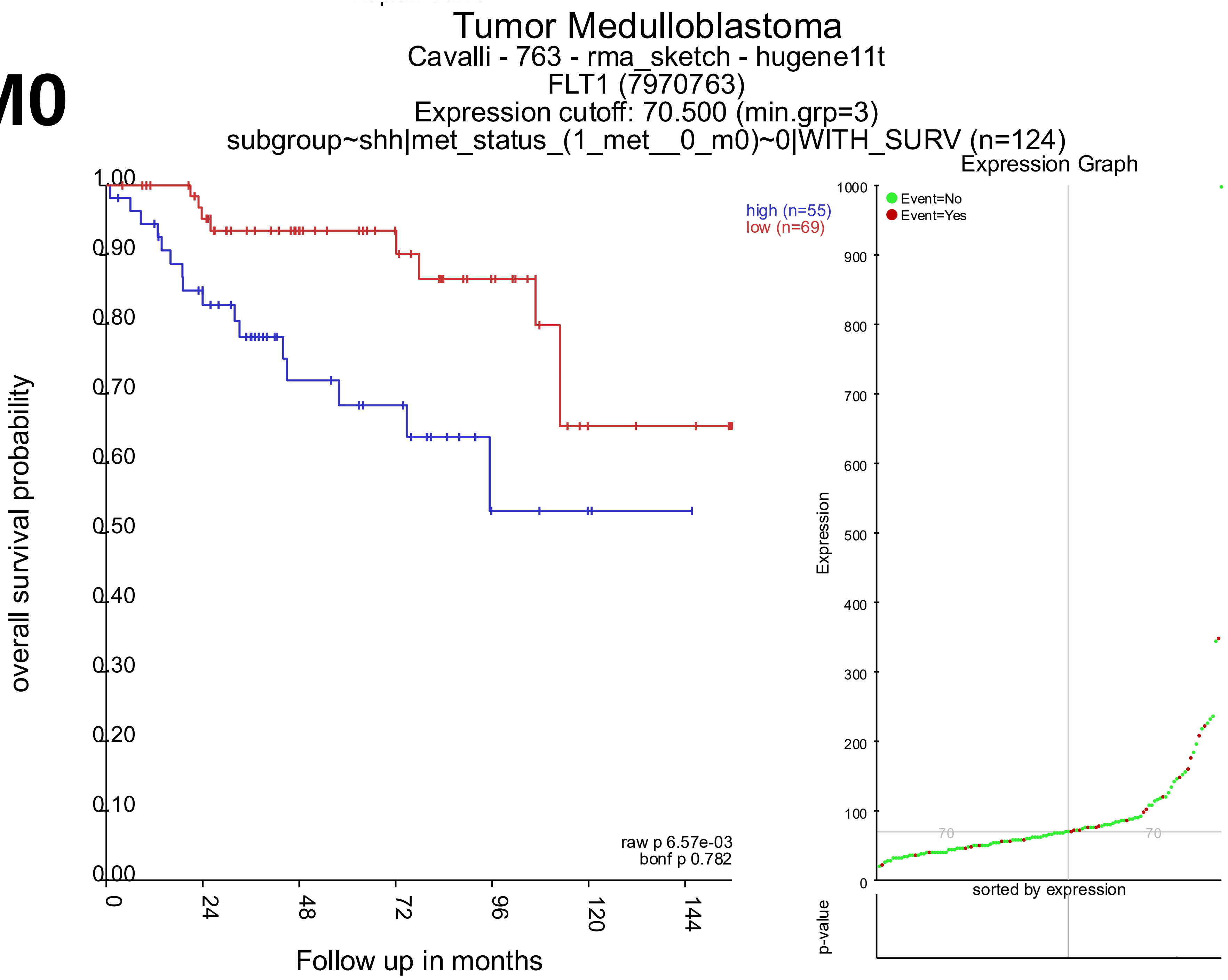
Tumor Medulloblastoma
Cavalli - 763 - rma_sketch - hugene11t
FLT1 (7970763)
Expression cutoff: 70.500 (min.grp=3)
subgroup~shh|met_status_(1_met__0_m0)~0|WITH_SURV (n=124)
overall survival probability
1.00
0.90
0.80
0.70
0.60
0.50
0.40
0.30
0.20
0.10
0.00
0
24
48
72
96
120
144
Follow up in months
high (n=55)
low (n=69)
raw p 6.57e-03
bonf p 0.782
Expression Graph
Event=No
Event=Yes
Expression
1000
900
800
700
600
500
400
300
200
100
0
70
70
sorted by expression
p-value

SHH M1

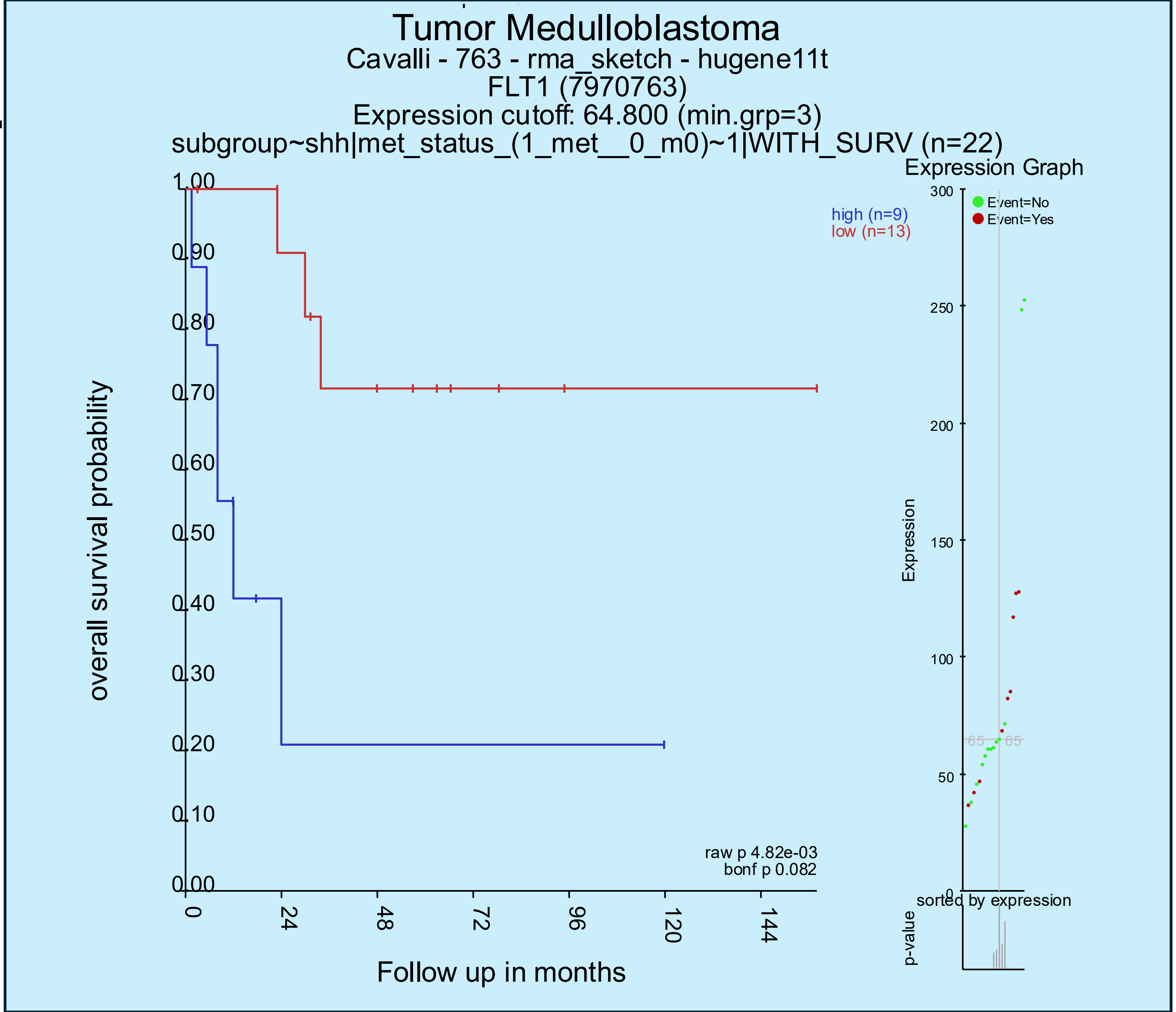

Tumor Medulloblastoma
Cavalli - 763 - rma_sketch - hugene11t
FLT1 (7970763)
Expression cutoff: 64.800 (min.grp=3)
subgroup~shh|met_status_(1_met__0_m0)~1|WITH_SURV (n=22)
overall survival probability
1.00
0.90
0.80
0.70
0.60
0.50
0.40
0.30
0.20
0.10
0.00
high (n=9)
low (n=13)
raw p 4.82e-03
bonf p 0.082
0
24
48
72
96
120
144
Follow up in months
Expression Graph
Event=No
Event=Yes
Expression
300
250
200
150
100
50
0
65
65
sorted by expression
p-value

GROUP4 M0

Tumor Medulloblastoma
Cavalli - 763 - rma_sketch - hugene11t
FLT1 (7970763)
Expression cutoff: 78.000 (min.grp=3)
subgroup~group4|met_status_(1_met__0_m0)~0|WITH_SURV (n=145)

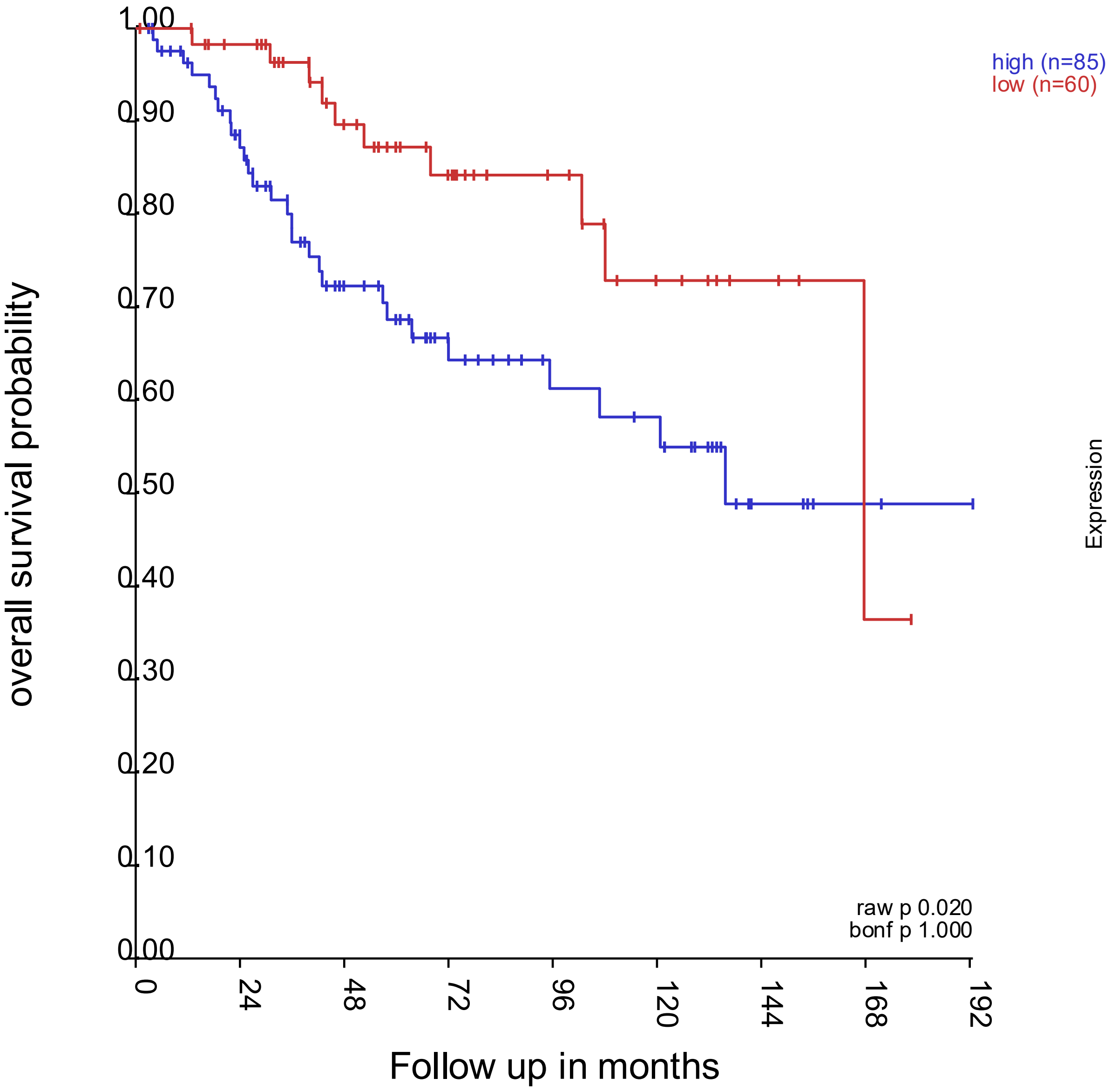

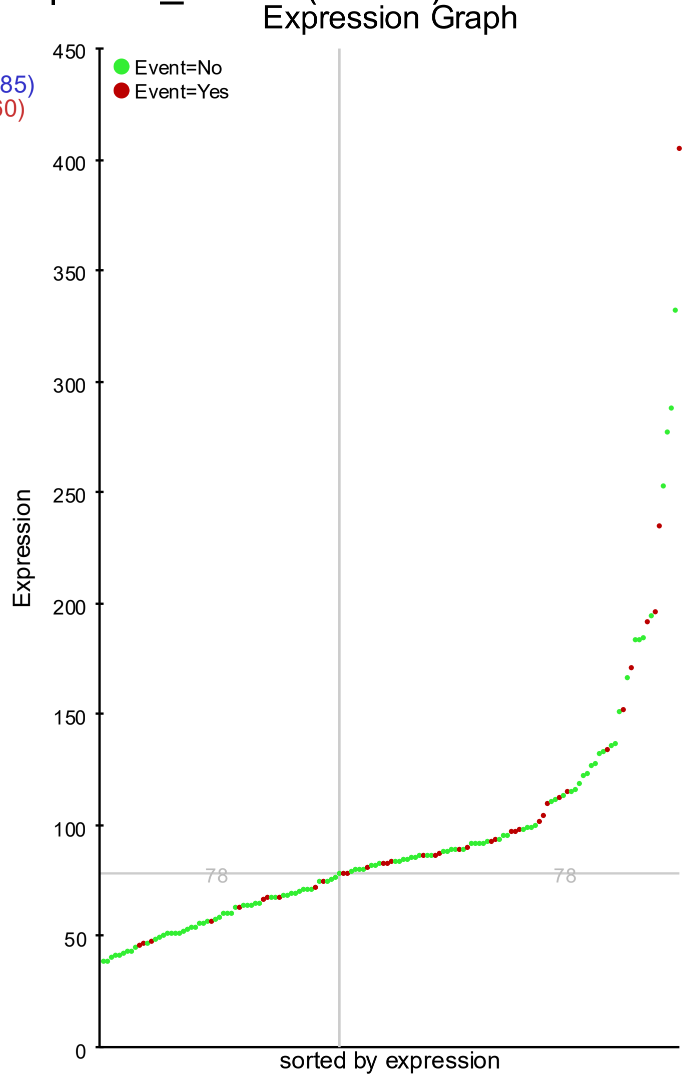

GROUP4 M1

Tumor Medulloblastoma
Cavalli - 763 - rma_sketch - hugene11t
FLT1 (7970763)
Expression cutoff: 61.300 (min.grp=3)
subgroup~group4|met_status_(1_met__0_m0)~1|WITH_SURV (n=92)

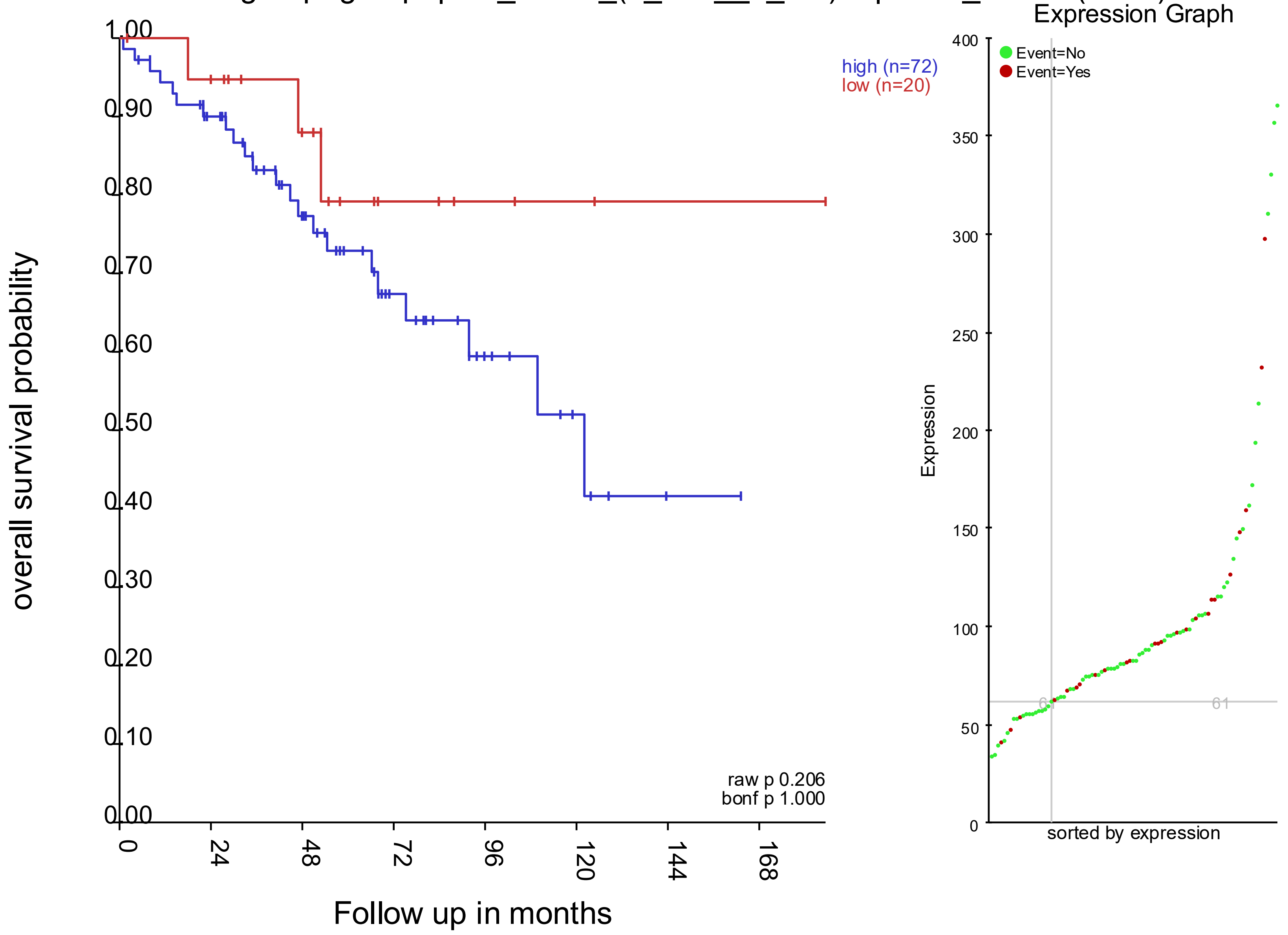

GROUP3 M0

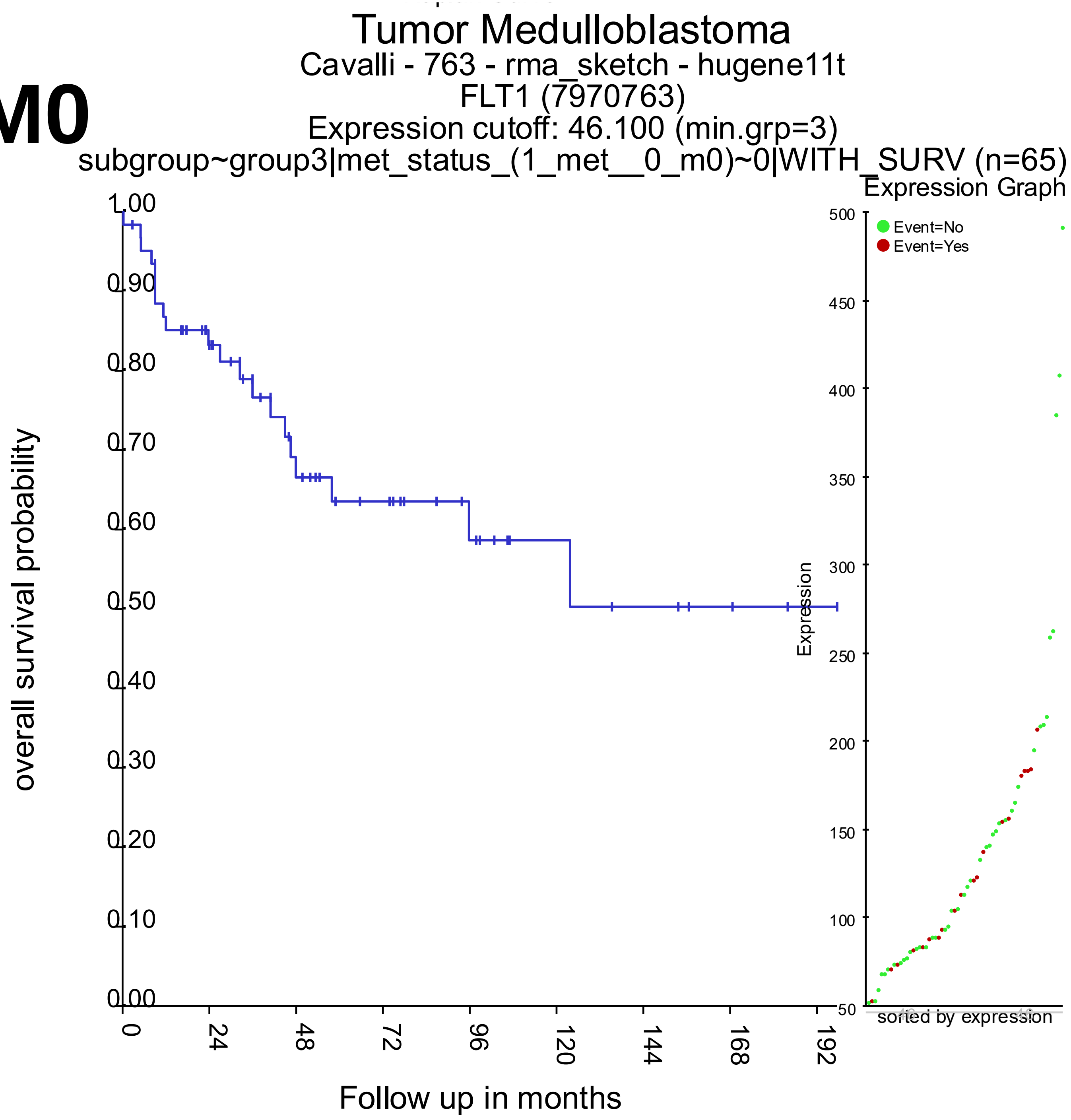

Tumor Medulloblastoma
Cavalli - 763 - rma_sketch - hugene11t
FLT1 (7970763)
Expression cutoff: 46.100 (min.grp=3)
subgroup~group3|met_status_(1_met__0_m0)~0|WITH_SURV (n=65)
overall survival probability
1.00
0.90
0.80
0.70
0.60
0.50
0.40
0.30
0.20
0.10
0.00
0
24
48
72
96
120
144
168
192
Follow up in months
Expression Graph
Event=No
Event=Yes
Expression
500
450
400
350
300
250
200
150
100
50
sorted by expression

GROUP3 M1

Tumor Medulloblastoma
Cavalli - 763 - rma_sketch - hugene11t
FLT1 (7970763)
Expression cutoff: 56.500 (min.grp=3)
subgroup~group3|met_status_(1_met__0_m0)~1|WITH_SURV (n=41)

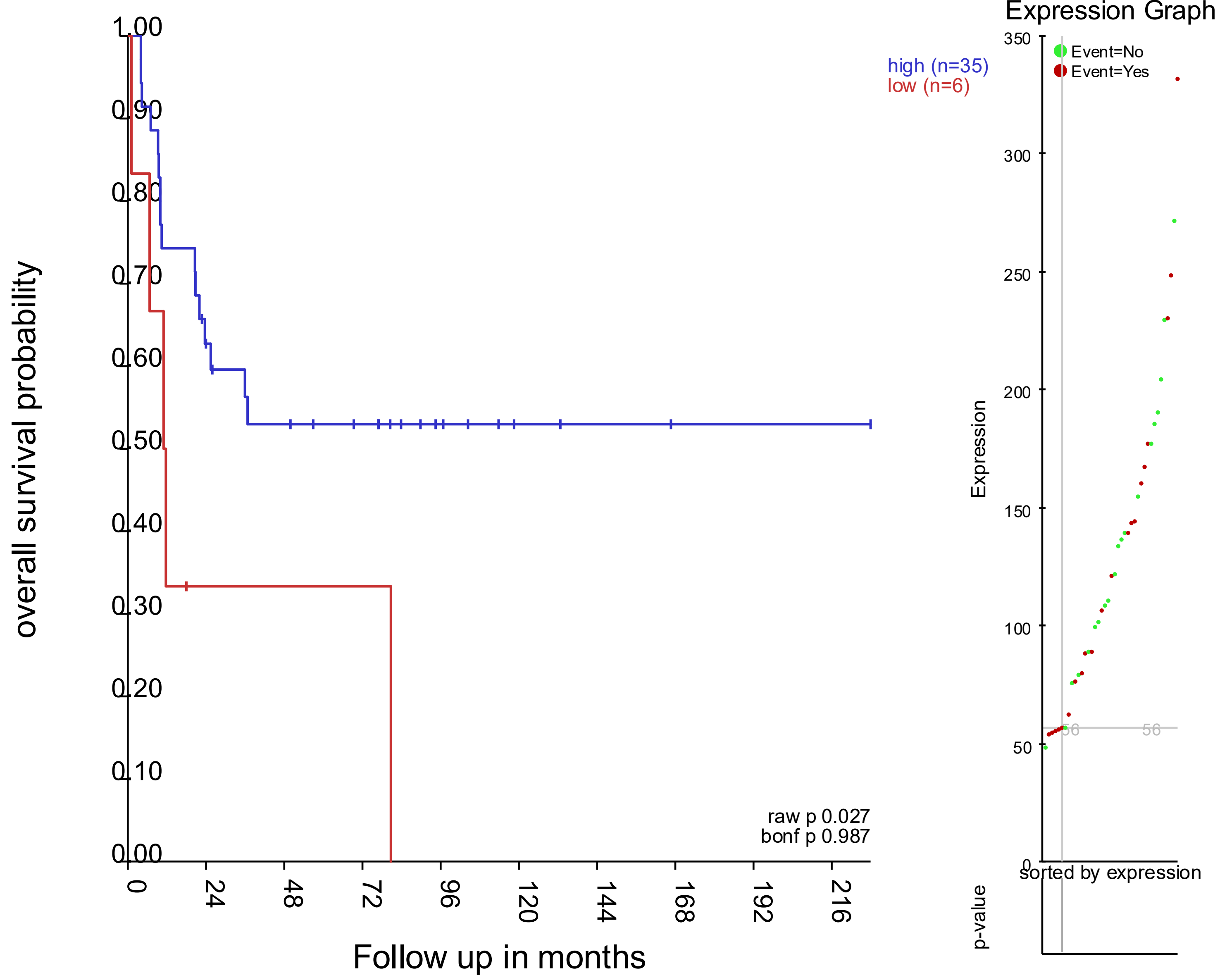

KDR

WNT M0

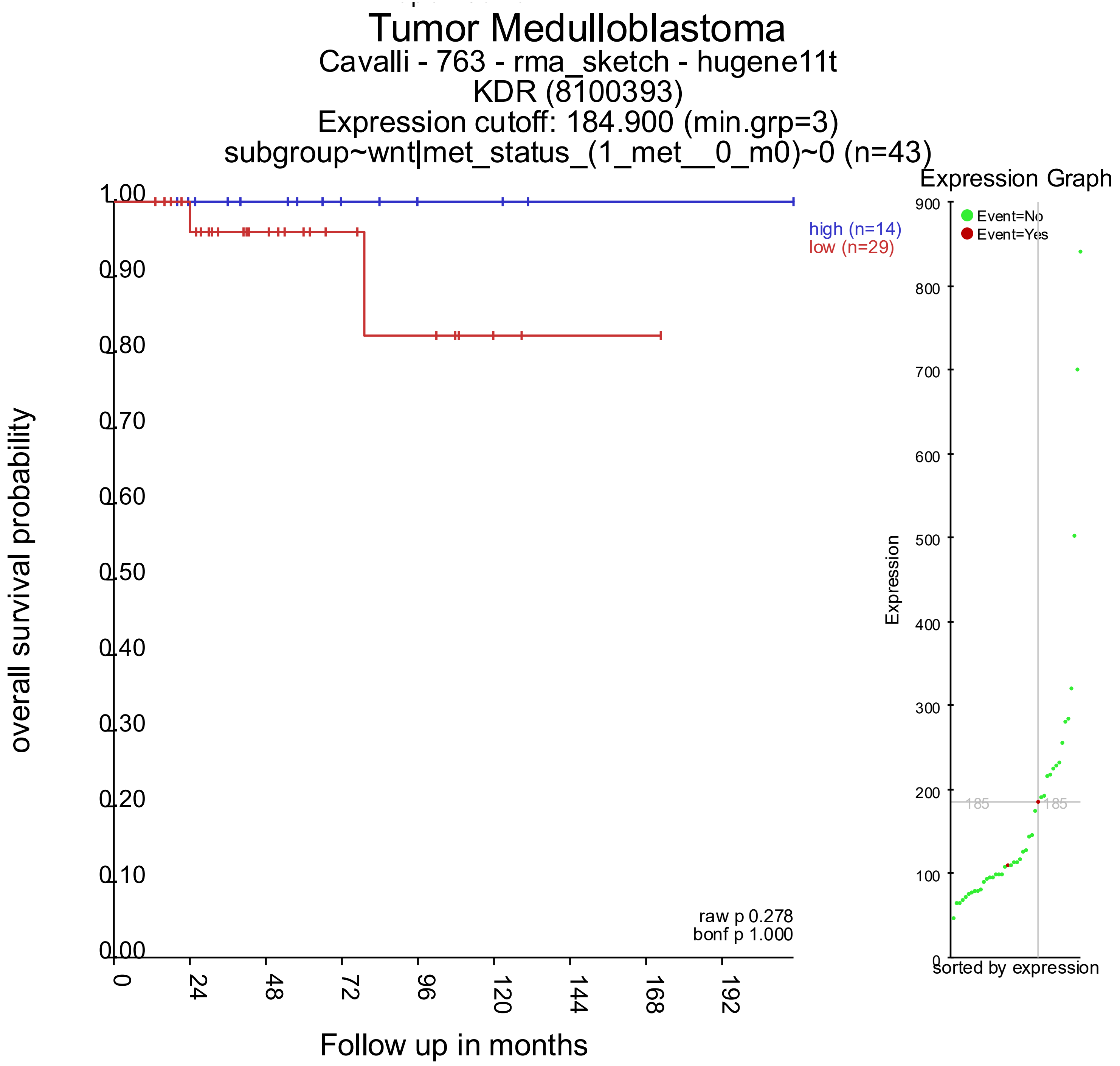

Tumor Medulloblastoma
Cavalli - 763 - rma_sketch - hugene11t
KDR (8100393)
Expression cutoff: 184.900 (min.grp=3)
subgroup~wnt|met_status_(1_met__0_m0)~0 (n=43)
overall survival probability
1.00
0.90
0.80
0.70
0.60
0.50
0.40
0.30
0.20
0.10
0.00
high (n=14)
low (n=29)
raw p 0.278
bonf p 1.000
0
24
48
72
96
120
144
168
192
Follow up in months
Expression Graph
Event=No
Event=Yes
Expression
900
800
700
600
500
400
300
200
100
0
185
185
sorted by expression

WNT M1

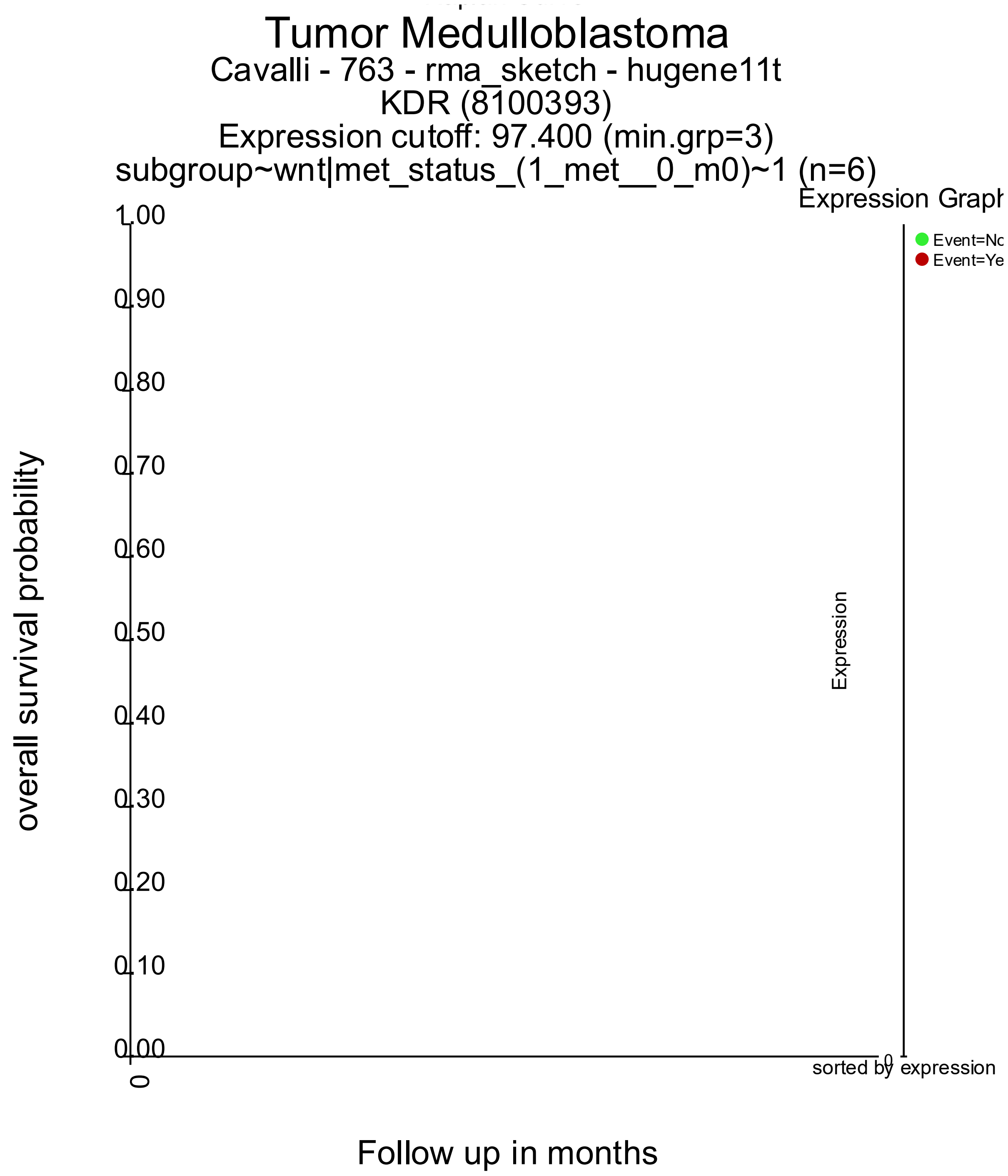
Tumor Medulloblastoma
Cavalli - 763 - rma_sketch - hugene11t
KDR (8100393)
Expression cutoff: 97.400 (min.grp=3)
subgroup~wnt|met_status_(1_met__0_m0)~1 (n=6)
Expression Graph
Event=No
Event=Yes
1.00
0.90
0.80
0.70
0.60
0.50
0.40
0.30
0.20
0.10
0.00
overall survival probability
0
Follow up in months
Expression
0
sorted by expression

SHH M0

Tumor Medulloblastoma
Cavalli - 763 - rma_sketch - hugene11t
KDR (8100393)
Expression cutoff: 128.900 (min.grp=3)
subgroup~shh|met_status_(1_met__0_m0)~0|WITH_SURV (n=124)

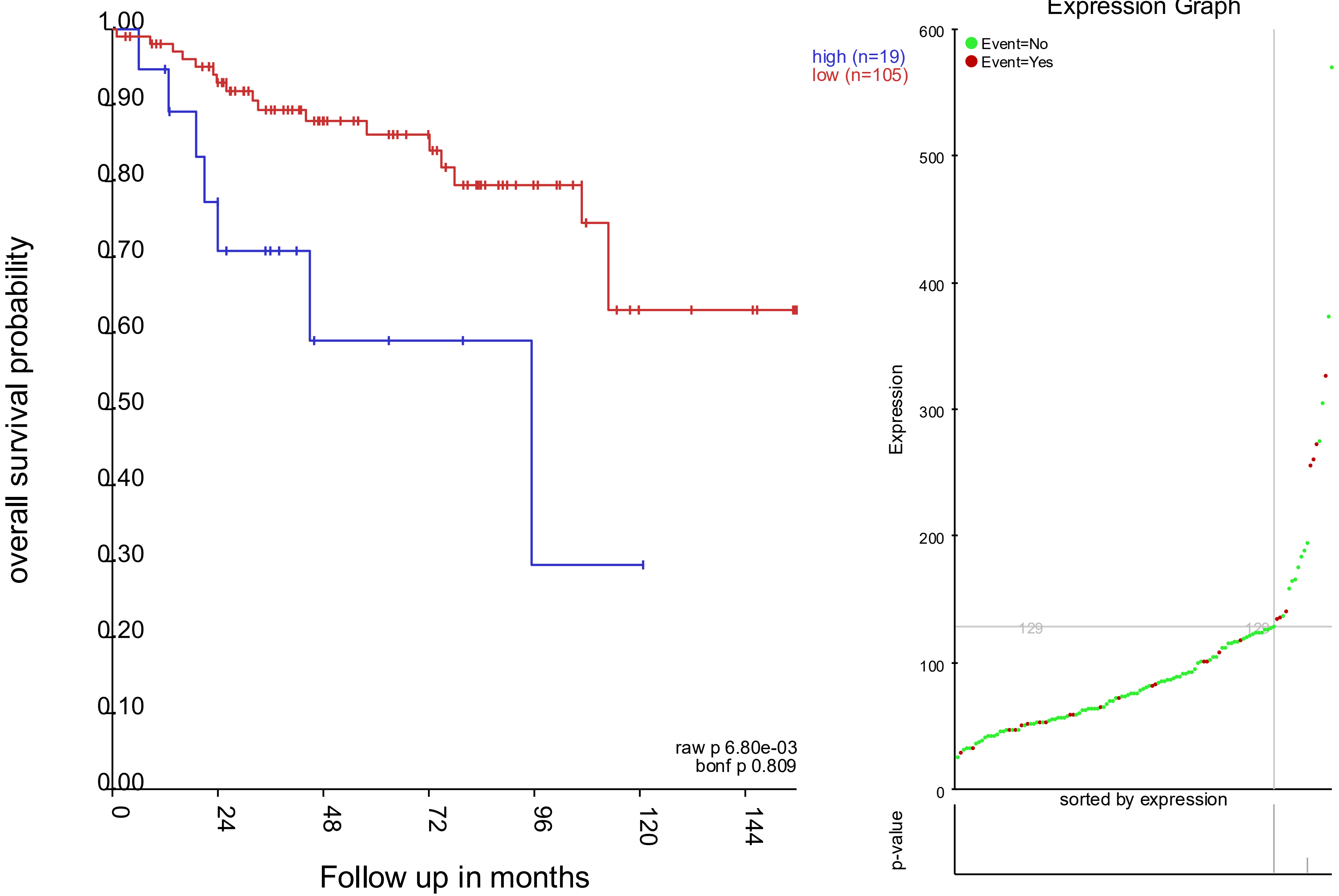

SHH M1

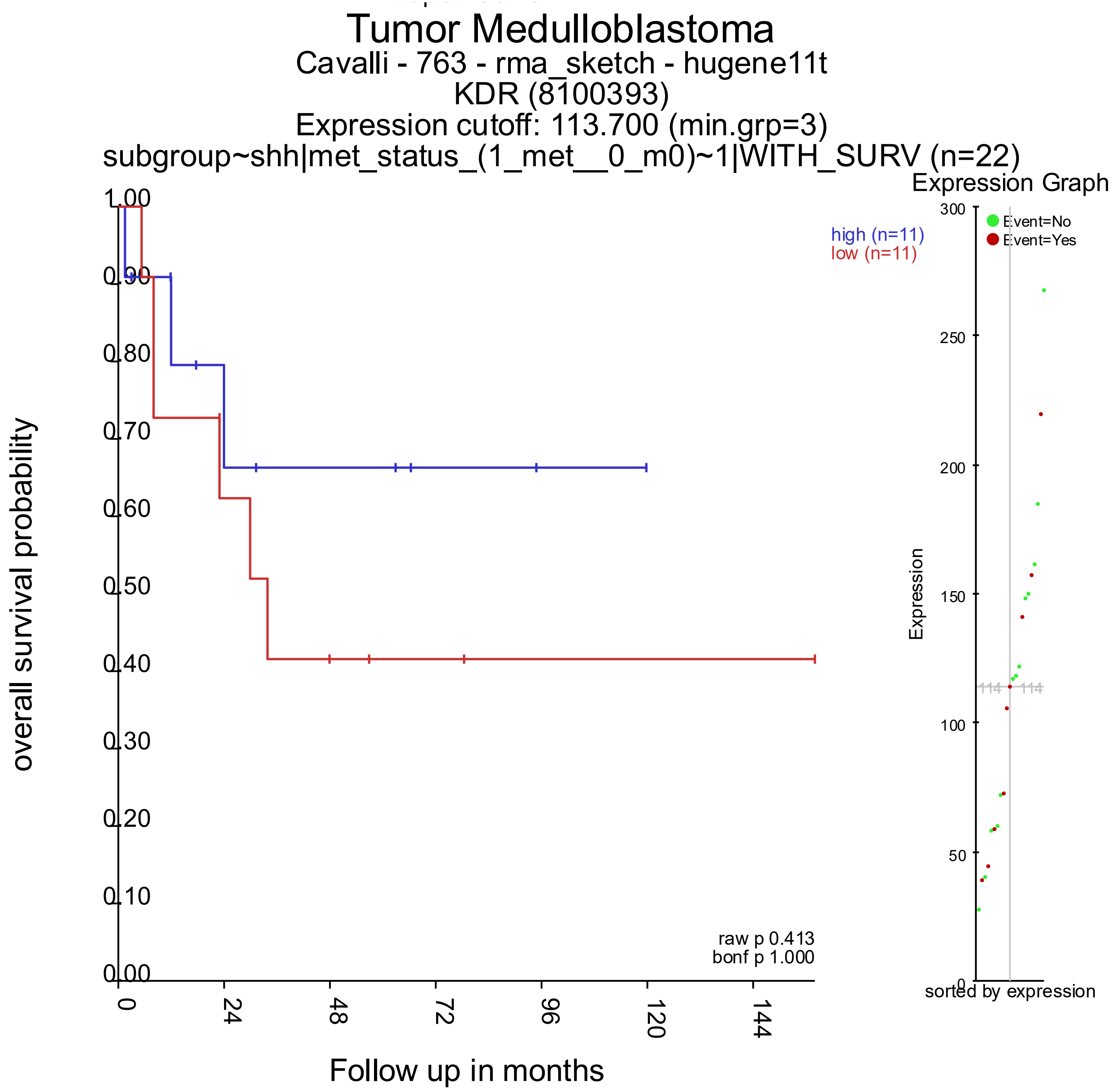

Tumor Medulloblastoma
Cavalli - 763 - rma_sketch - hugene11t
KDR (8100393)
Expression cutoff: 113.700 (min.grp=3)
subgroup~shh|met_status_(1_met__0_m0)~1|WITH_SURV (n=22)
overall survival probability
1.00
0.90
0.80
0.70
0.60
0.50
0.40
0.30
0.20
0.10
0.00
0
24
48
72
96
120
144
Follow up in months
high (n=11)
low (n=11)
raw p 0.413
bonf p 1.000
Expression Graph
Event=No
Event=Yes
Expression
300
250
200
150
100
50
0
114
114
sorted by expression

GROUP4 M0

Tumor Medulloblastoma
Cavalli - 763 - rma_sketch - hugene11t
KDR (8100393)
Expression cutoff: 52.300 (min.grp=3)
subgroup~group4|met_status_(1_met__0_m0)~0|WITH_SURV (n=145)

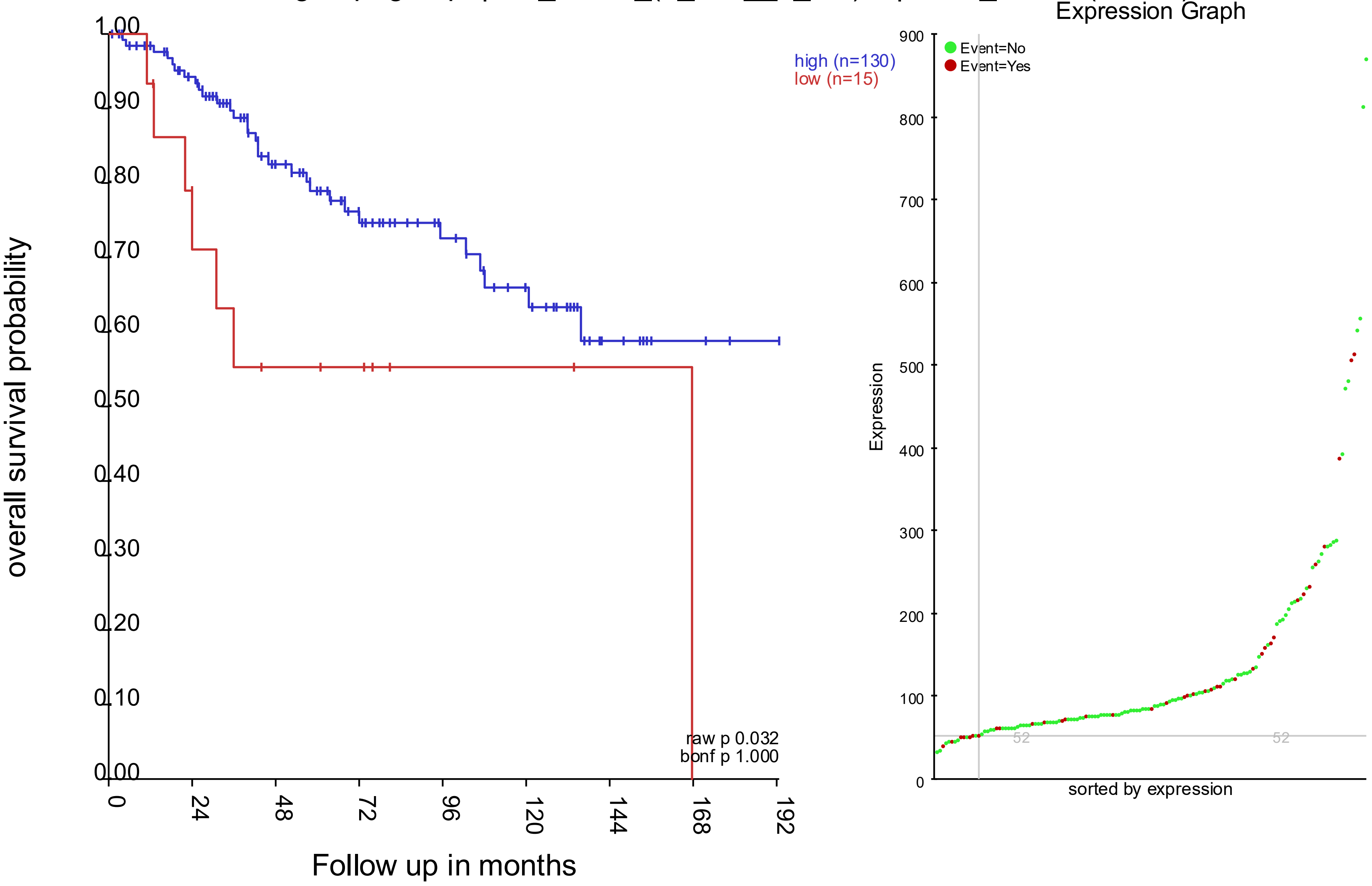

GROUP4 M1

Tumor Medulloblastoma
Cavalli - 763 - rma_sketch - hugene11t
KDR (8100393)
Expression cutoff: 82.400 (min.grp=3)
subgroup~group4|met_status_(1_met__0_m0)~1|WITH_SURV (n=92)

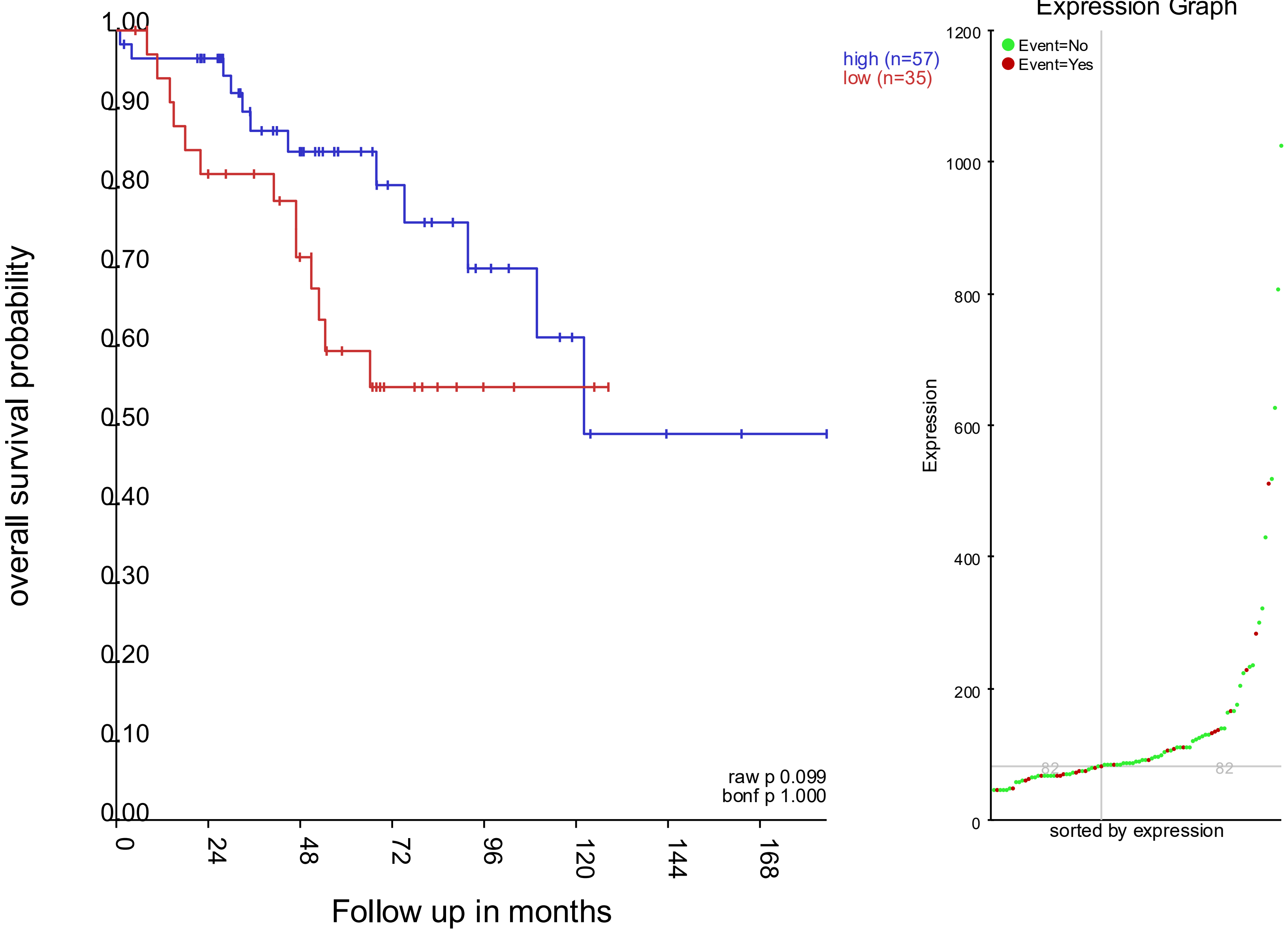

GROUP3 M0

Tumor Medulloblastoma
Cavalli - 763 - rma_sketch - hugene11t
KDR (8100393)
Expression cutoff: 53.700 (min.grp=3)
subgroup~group3|met_status_(1_met__0_m0)~0|WITH_SURV (n=65)

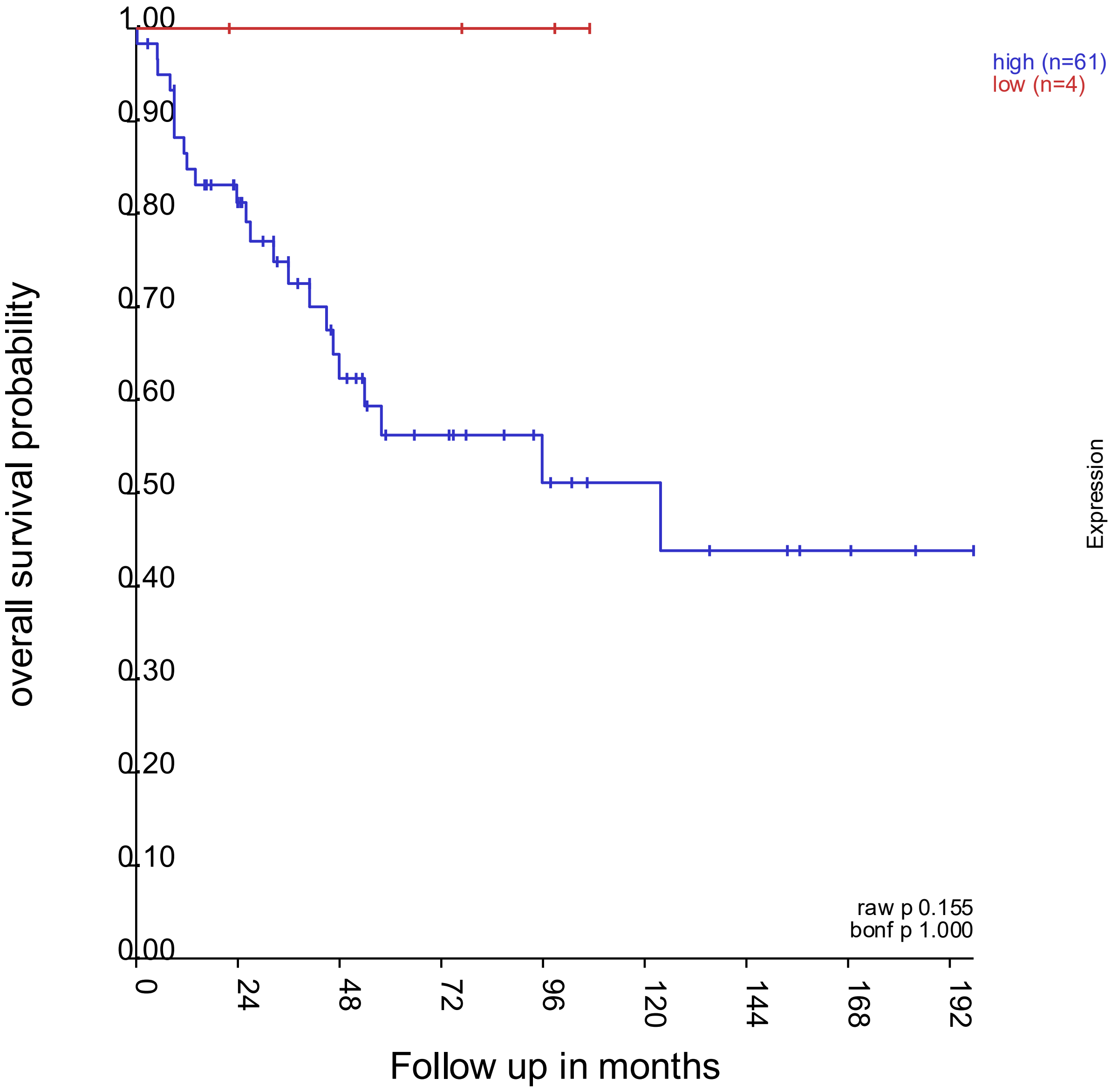

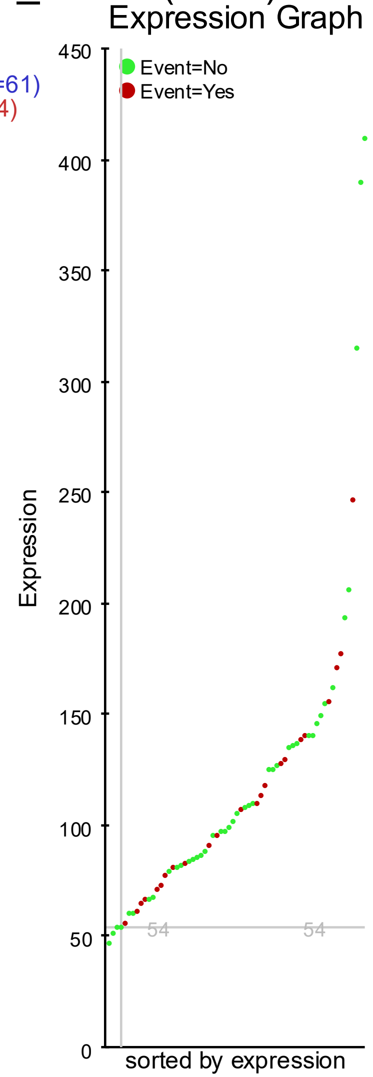

GROUP3 M1

Tumor Medulloblastoma
Cavalli - 763 - rma_sketch - hugene11t
KDR (8100393)
Expression cutoff: 213.500 (min.grp=3)
subgroup~group3|met_status_(1_met__0_m0)~1|WITH_SURV (n=41)

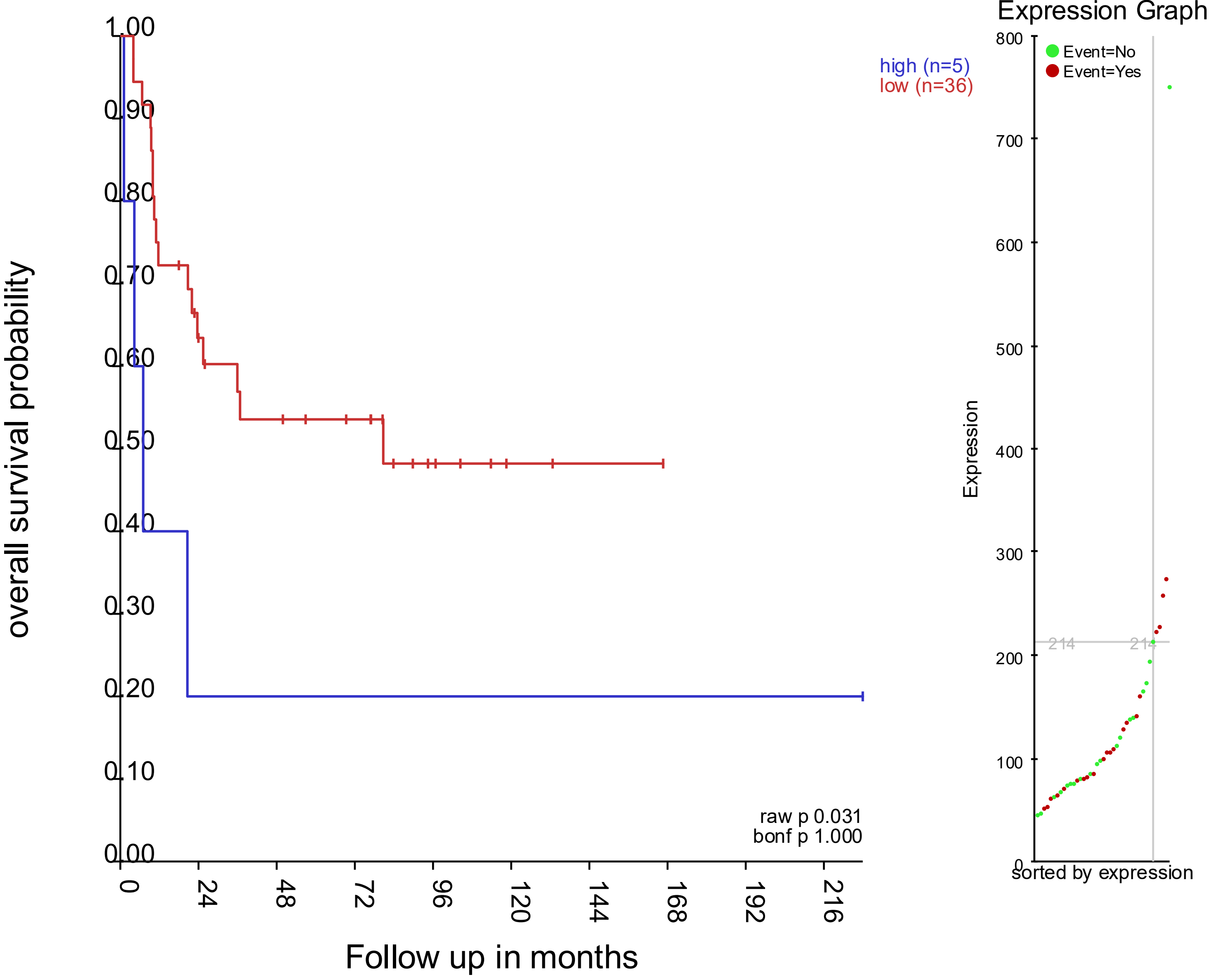

FLT4

WNT M0

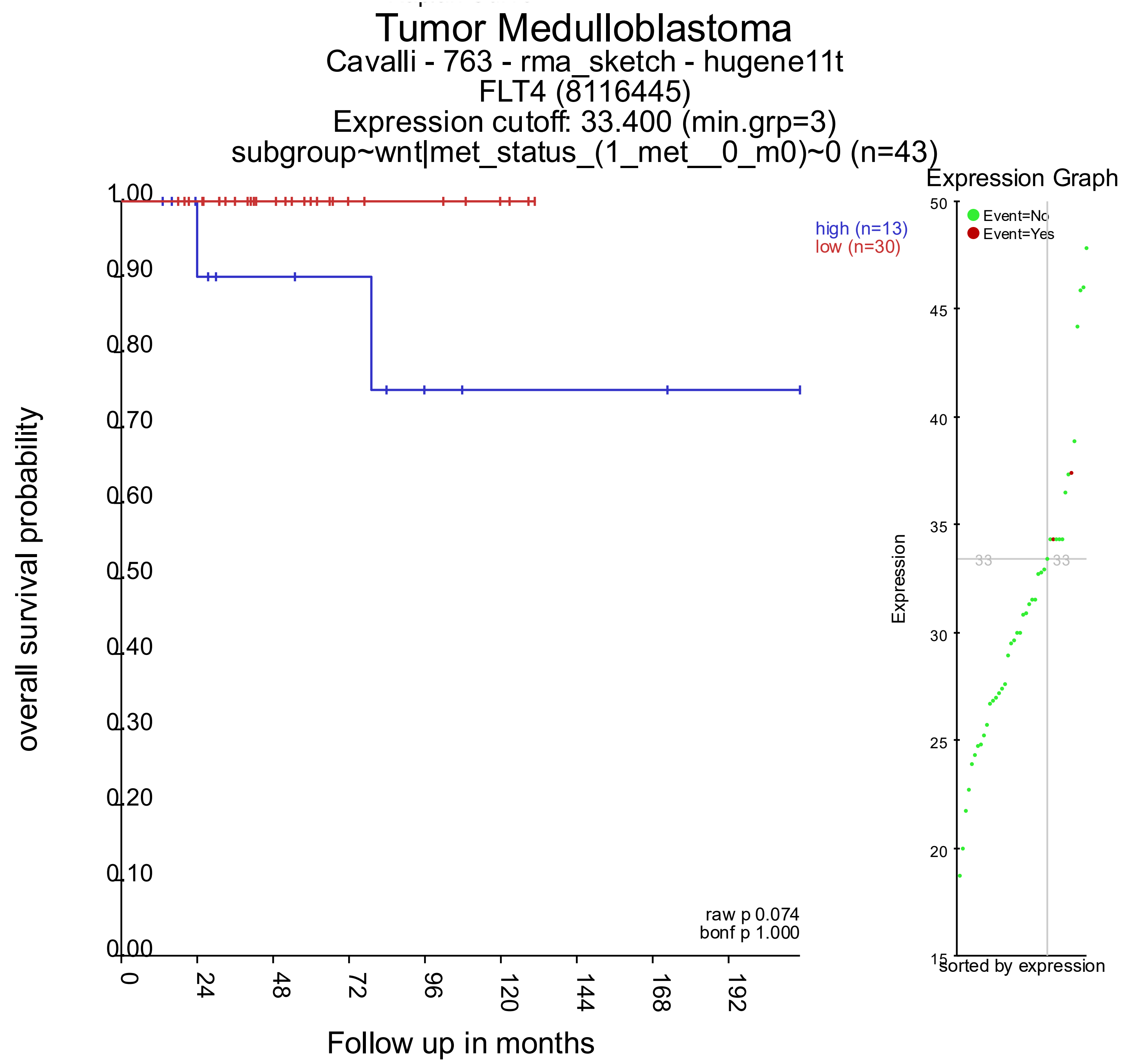

Tumor Medulloblastoma
Cavalli - 763 - rma_sketch - hugene11t
FLT4 (8116445)
Expression cutoff: 33.400 (min.grp=3)
subgroup~wnt|met_status_(1_met__0_m0)~0 (n=43)
overall survival probability
1.00
0.90
0.80
0.70
0.60
0.50
0.40
0.30
0.20
0.10
0.00
0
24
48
72
96
120
144
168
192
Follow up in months
high (n=13)
low (n=30)
raw p 0.074
bonf p 1.000
Expression Graph
Event=No
Event=Yes
Expression
50
45
40
35
30
25
20
15
33
33
sorted by expression

WNT M1

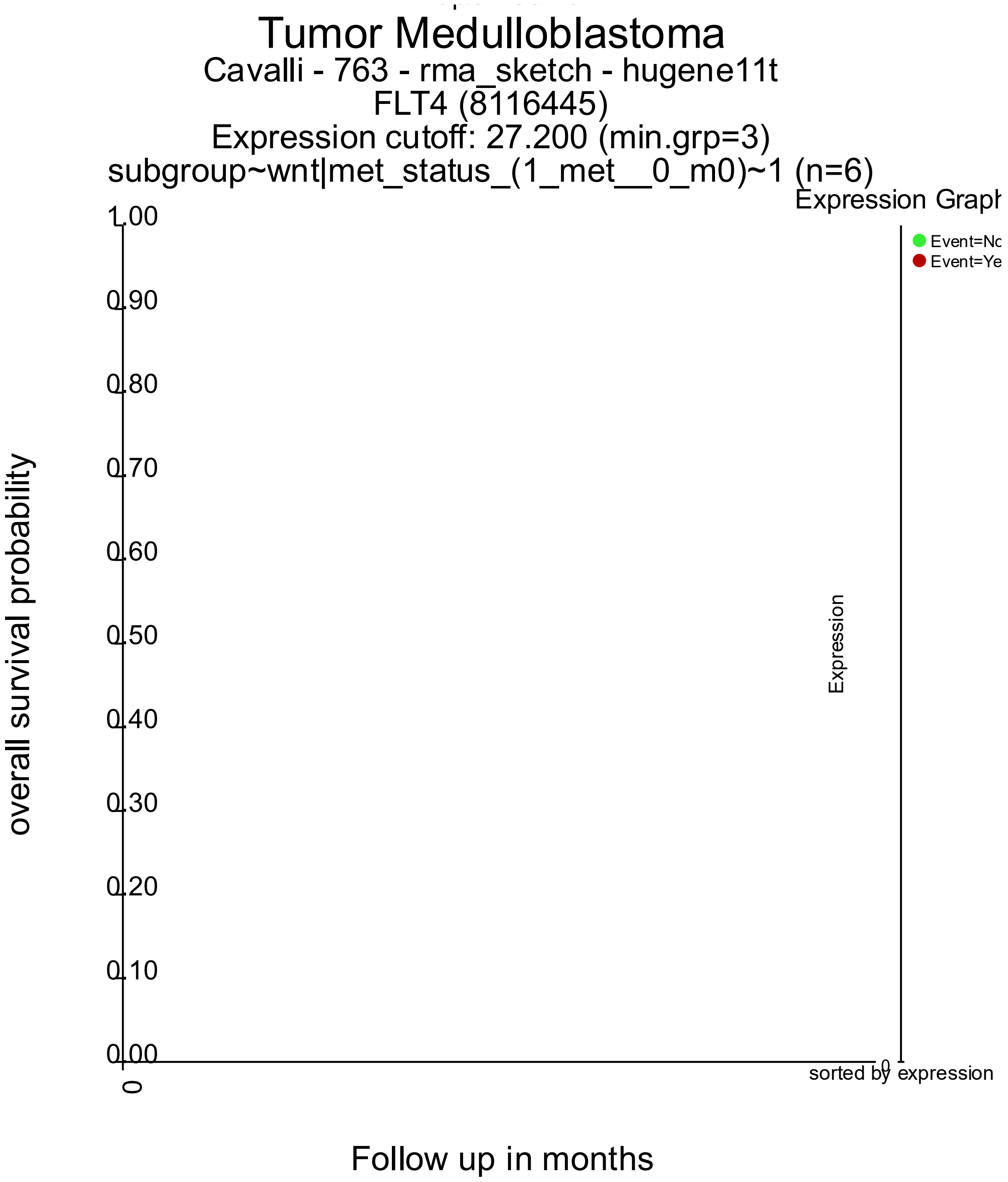
Tumor Medulloblastoma
Cavalli - 763 - rma_sketch - hugene11t
FLT4 (8116445)
Expression cutoff: 27.200 (min.grp=3)
subgroup~wnt|met_status_(1_met__0_m0)~1 (n=6)
Expression Graph
Event=No
Event=Ye
overall survival probability
1.00
0.90
0.80
0.70
0.60
0.50
0.40
0.30
0.20
0.10
0.00
0
Follow up in months
Expression
0
sorted by expression

SHH M0

Tumor Medulloblastoma
Cavalli - 763 - rma_sketch - hugene11t
FLT4 (8116445)
Expression cutoff: 34.600 (min.grp=3)
subgroup~shh|met_status_(1_met__0_m0)~0|WITH_SURV (n=124)

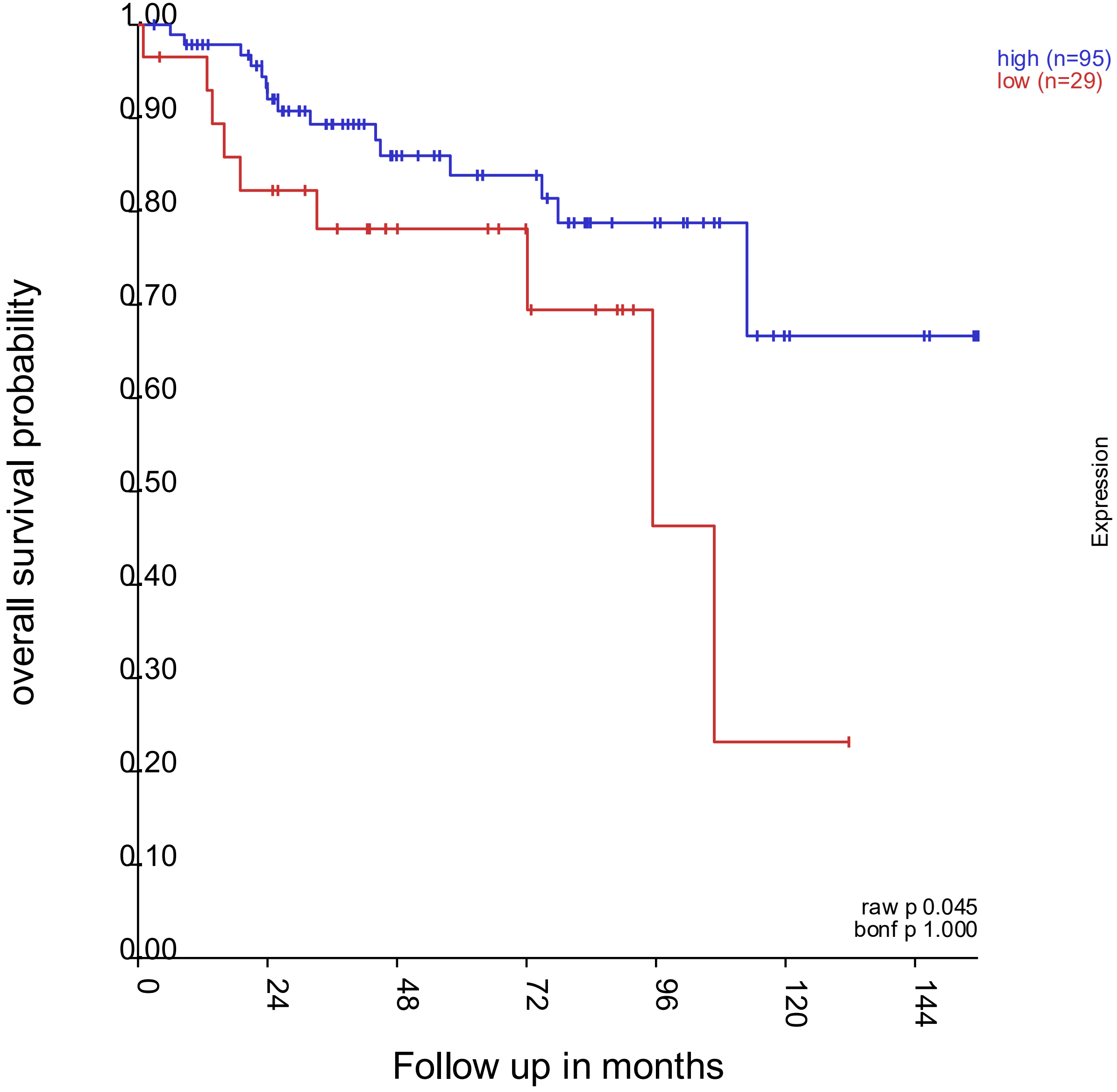

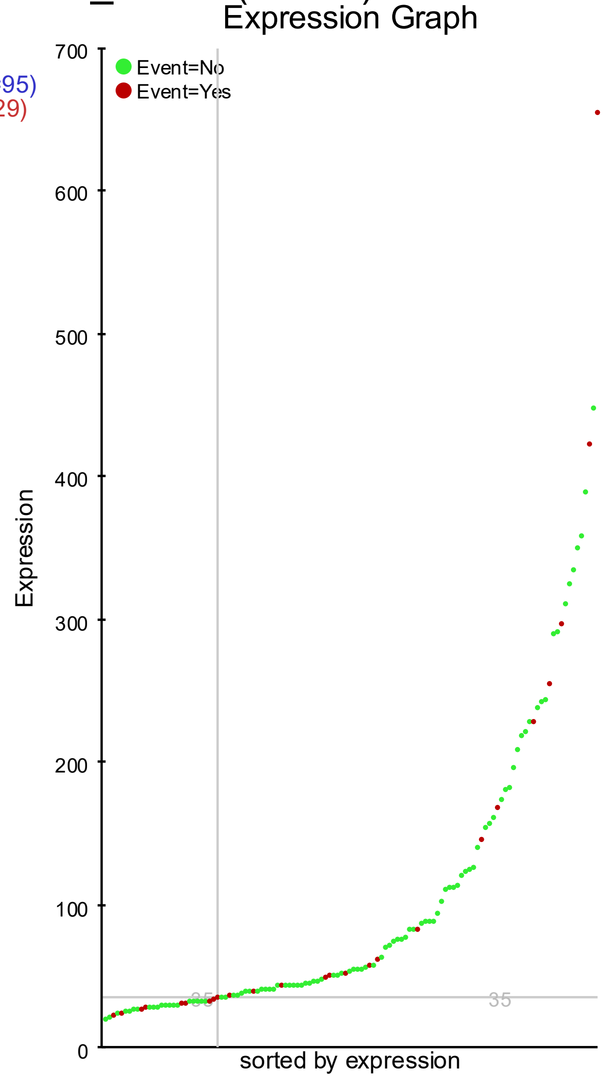

SHH M1

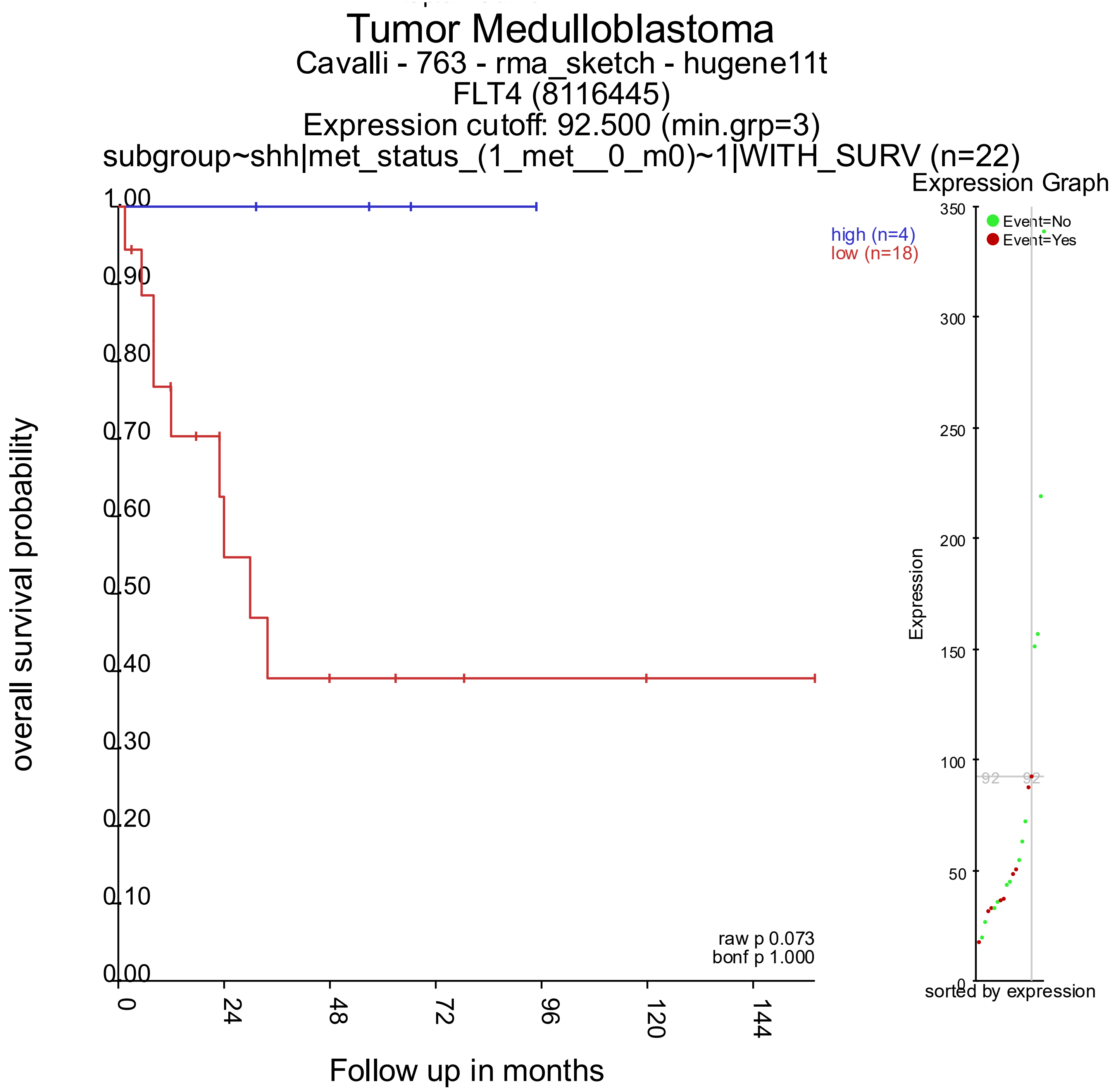
Tumor Medulloblastoma
Cavalli - 763 - rma_sketch - hugene11t
FLT4 (8116445)
Expression cutoff: 92.500 (min.grp=3)
subgroup~shh|met_status_(1_met__0_m0)~1|WITH_SURV (n=22)
overall survival probability
Follow up in months
high (n=4)
low (n=18)
raw p 0.073
bonf p 1.000
Expression Graph
Event=No
Event=Yes
Expression
sorted by expression

GROUP4 M0

Tumor Medulloblastoma
Cavalli - 763 - rma_sketch - hugene11t
FLT4 (8116445)
Expression cutoff: 37.800 (min.grp=3)
subgroup~group4|met_status_(1_met__0_m0)~0|WITH_SURV (n=145)

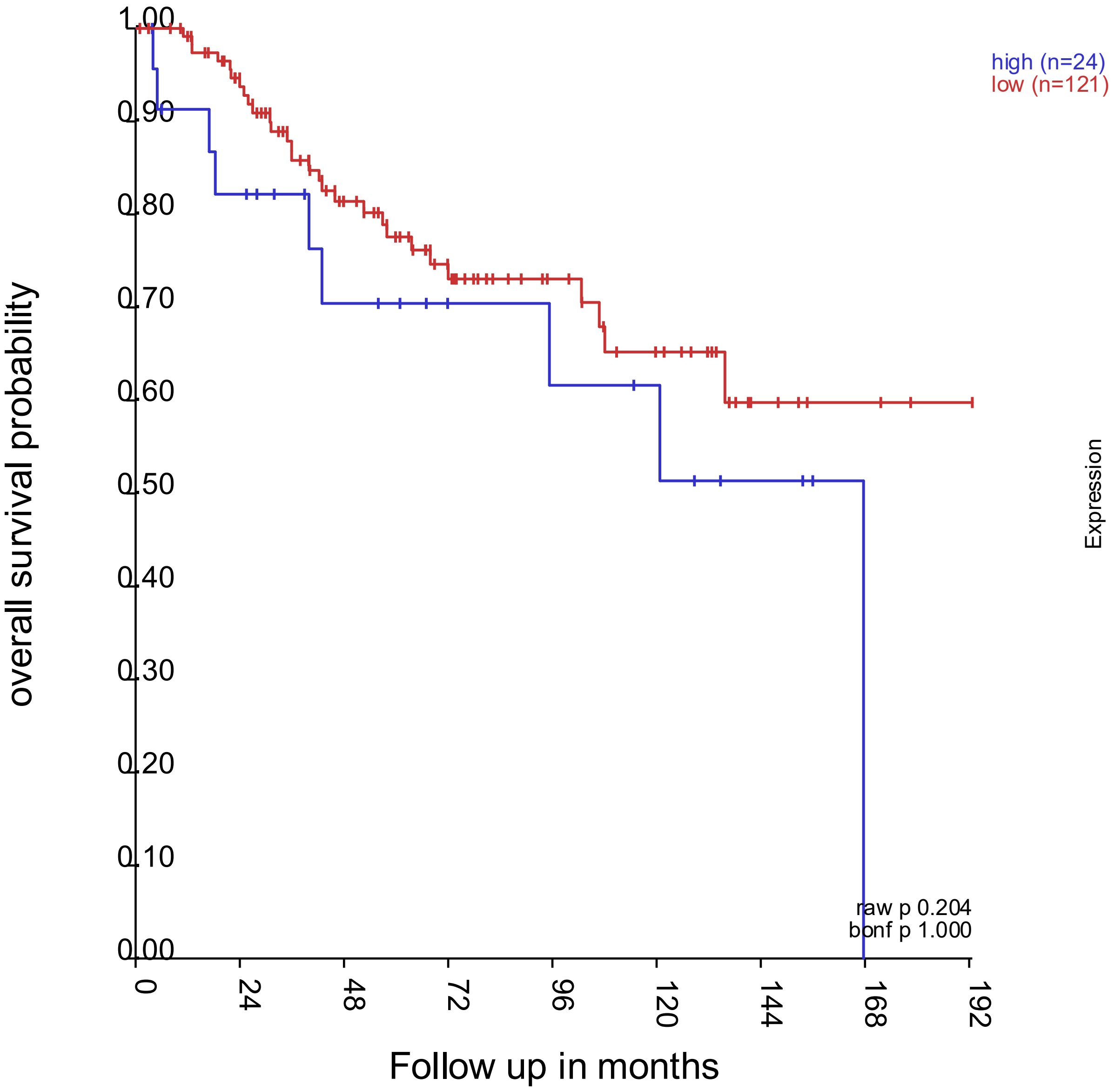

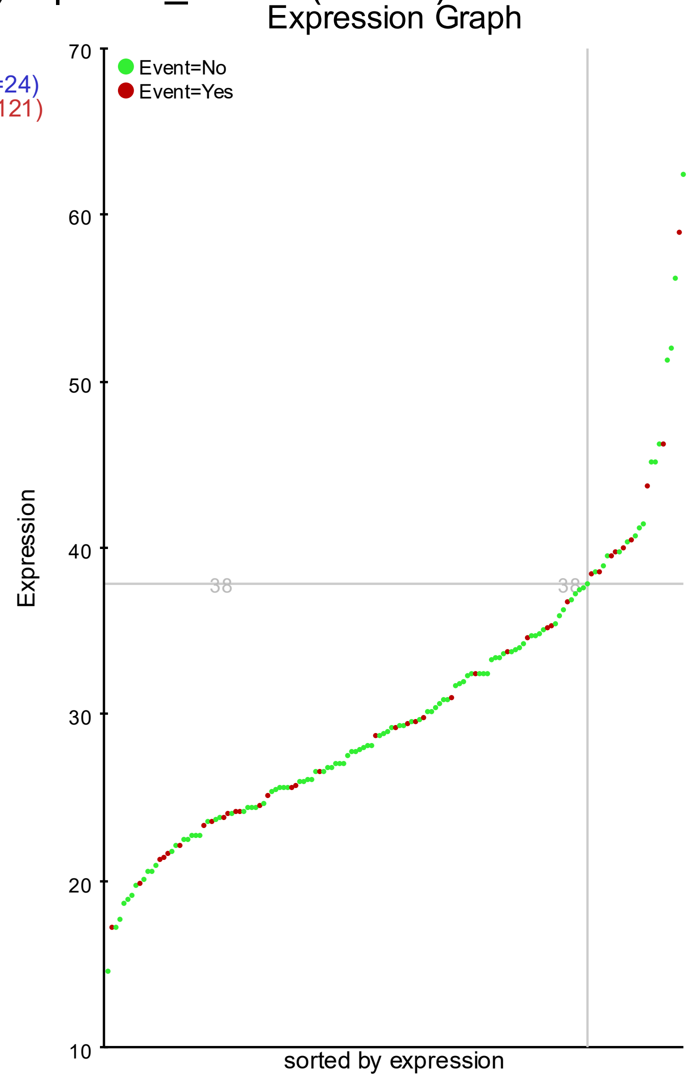

GROUP4 M1

Tumor Medulloblastoma
Cavalli - 763 - rma_sketch - hugene11t
FLT4 (8116445)
Expression cutoff: 36.000 (min.grp=3)
subgroup~group4|met_status_(1_met__0_m0)~1|WITH_SURV (n=92)

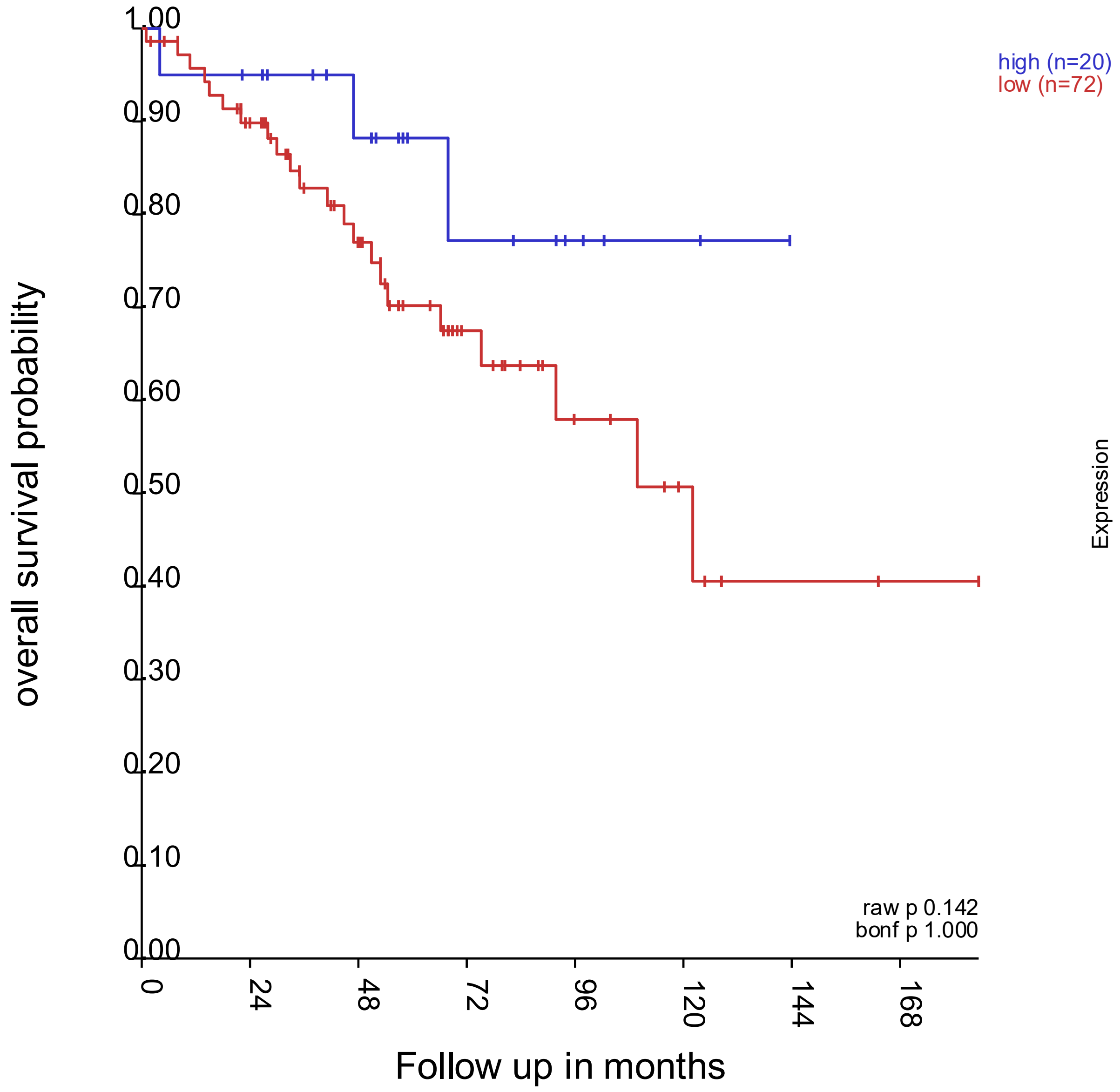

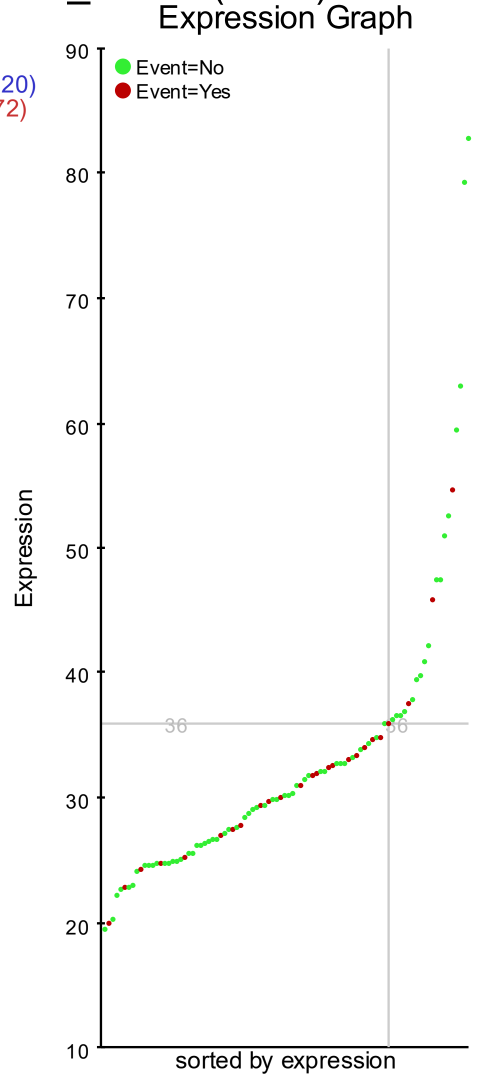

GROUP3 M0

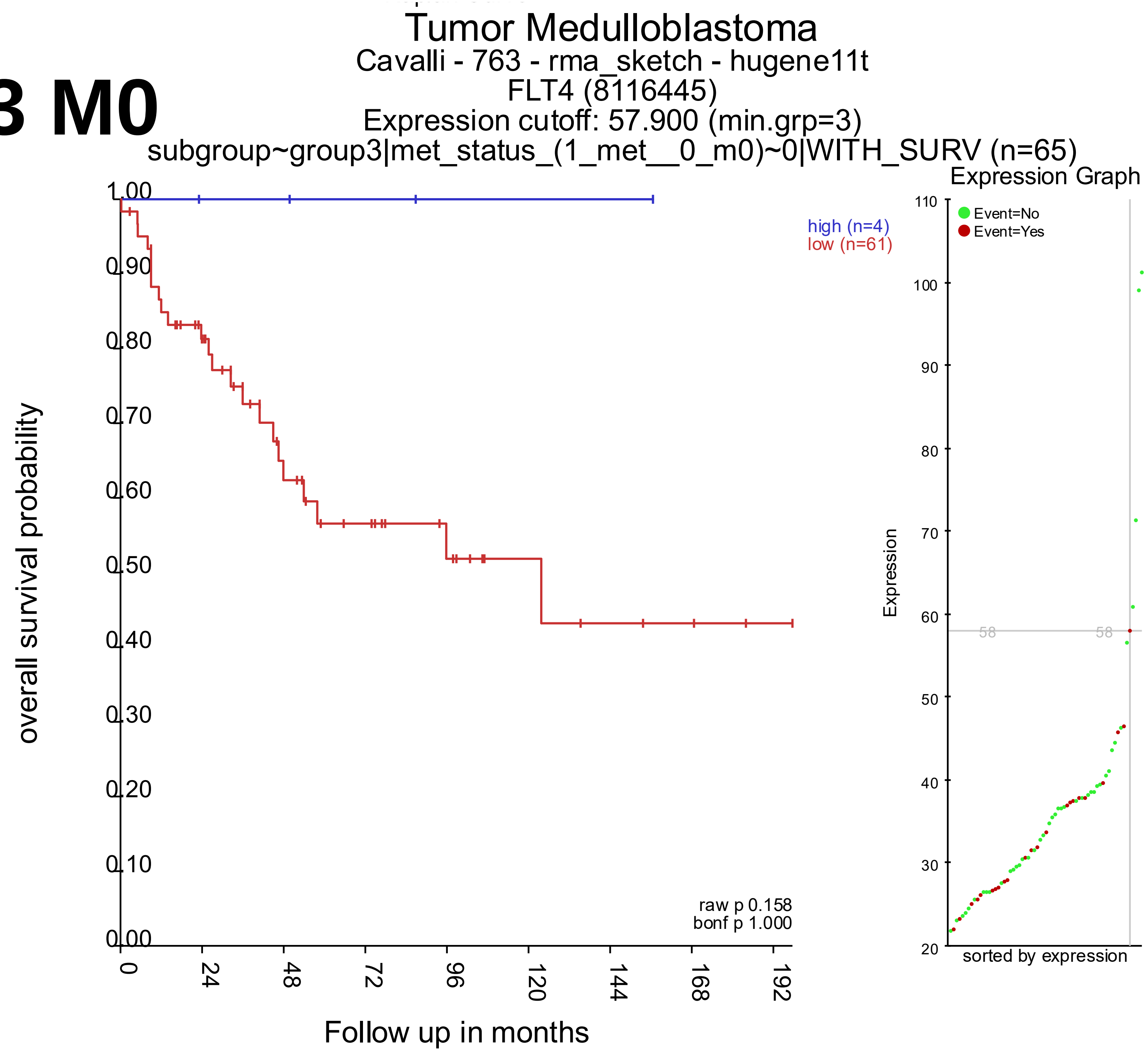

Tumor Medulloblastoma
Cavalli - 763 - rma_sketch - hugene11t
FLT4 (8116445)
Expression cutoff: 57.900 (min.grp=3)
subgroup~group3|met_status_(1_met__0_m0)~0|WITH_SURV (n=65)
high (n=4)
low (n=61)
overall survival probability
1.00
0.90
0.80
0.70
0.60
0.50
0.40
0.30
0.20
0.10
0.00
0
24
48
72
96
120
144
168
192
Follow up in months
raw p 0.158
bonf p 1.000
Expression Graph
Event=No
Event=Yes
Expression
110
100
90
80
70
60
50
40
30
20
58
58
sorted by expression

GROUP3 M1

Tumor Medulloblastoma
Cavalli - 763 - rma_sketch - hugene11t
FLT4 (8116445)
Expression cutoff: 31.500 (min.grp=3)
subgroup~group3|met_status_(1_met__0_m0)~1|WITH_SURV (n=41)

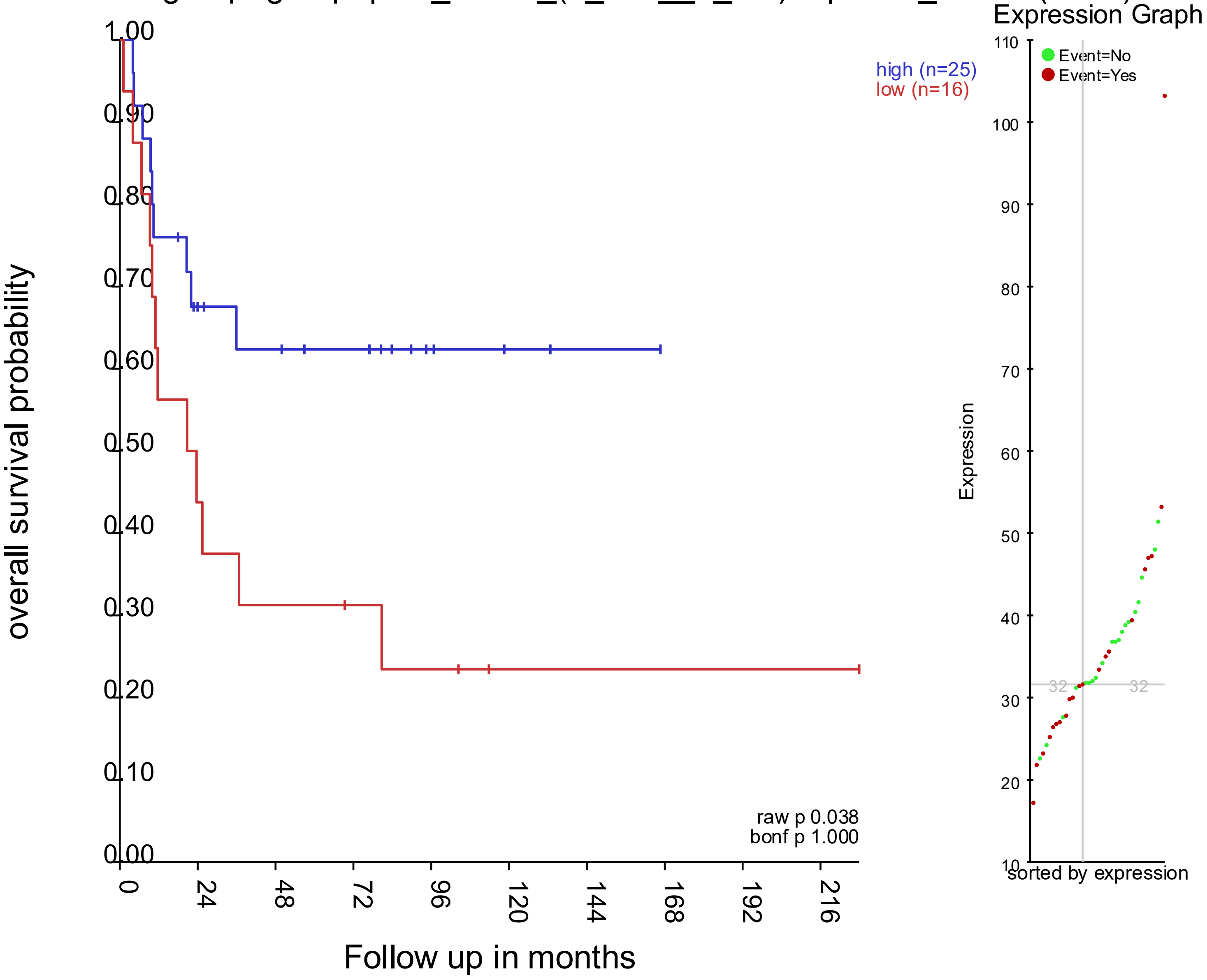

Supplement: Supplementary file 1 [file cancers-17-03659-s001.zip › Supplementary Figure S2.pdf]
