# Supplementary material for: Exploring Novel Applications: Repositioning Clinically Approved Therapies for Medulloblastoma Treatment
Source: Cancers (Basel). 2025 Nov 14;17(22):3659. doi: 10.3390/cancers17223659 (PMC12650705; doi:10.3390/cancers17223659)
Supplement: Supplementary file 1 [file cancers-17-03659-s001.zip › Supplementary Tables S1-S4.pdf]

Type of the Paper (Article.)

Title: Exploring Novel Applications: Repositioning Clinically Approved Therapies for Medulloblastoma Treatment

Arthur Karaulic [1], and Gilles Pagès [1,*]

[1] University Côte d'Azur, Institute for research on cancer and ageing of Nice (IRCAN°, UMR CNRS 7284/U INSERM 1081
* Correspondence: gpages@unice.fr; Tel.: (0033 4 89153479)

Supplementary Materials: The following supporting information can be downloaded at: https://www.mdpi.com/article/doi/s1, Figure S1: title; Table S1: title; Video S1: title.

Legends to supplementary figures

Supplementary Figure 1: Kaplan-Meier analysis of the relationship between targetable genes and survival in different medulloblastoma subgroups. The Kaplan-Meier method was used to generate survival curves, and analyses of censored data were performed using Cox proportional hazards models. Raw and Bonferroni-corrected p-values are indicated for statistical significance.

Supplementary Figure 2: Kaplan-Meier analysis of the relationship between targetable genes and survival in different medulloblastoma subgroups considering the non-metastatic (M0) and metastatic (M1) patients. The Kaplan-Meier method was used to generate survival curves, and analyses of censored data were performed using Cox proportional hazards models. Raw and Bonferroni-corrected p-values are indicated for statistical significance.

Academic Editor: Firstname Lastname

Received: date
Revised: date
Accepted: date
Published: date

Citation: To be added by editorial staff during production.

Copyright: © 2025 by the authors. Submitted for possible open access publication under the terms and conditions of the Creative Commons Attribution (CC BY) license (https://creativecommons.org/licenses/by/4.0/).

	ALK	BCL2	BCL2L1	BCL2L2	BTK	CD3D	CD19	CD20	CD25	CD33	CD52	CDK		DDR2	EPCAM	FGFR			FLT3
	ALK	BCL2	BCL2L1	BCL2L2	BTK	CD3D	CD19	CD20	CD25/ISG20	CD33	CD52	CDK4	CDK6	DDR2	EPCAM	FGFR1	FGFR2	FGFR3	FLT3
WNT	NS	NS	NS	NS	3e-3/NS	NS	NS	0.02/NS	NS	NS	NS	0.057/NS	NS	NS	NS	0.06/NS	NS	NS	0.04/NS
SHH	1e-2/NS	0.03/NS	NS	NS	0.01/NS	2e-2/NS	0.046/NS	1.4e-3/NS	0.01/NS	2e-3/NS	**2.8e-4/0.04**	9e-3/NS	0.07/NS	1.4e-3/NS	NS	NS	0.03/NS	**9e-5/0.01**	0.04/NS
GR4	0.02/NS	0.02/NS	1e-2/NS	NS	NS	**9e-5/0.02**	4.3e-4/NS	**3e-4/0.07**	NS	0.015/NS	5.5e-4/NS	0.01/NS	2e-3/NS	3e-3/NS	NS	9e-4/NS	5e-3/NS	7e-4/NS	7e-3/NS
GR3	1.5e-3/NS	**5e-4/0.05**	NS	3e-3/NS	0.04/NS	0.03/NS	0.05/NS	NS	6.7e-4/NS	NS	0.01/NS	**2e-4/0.02**	0.03/NS	0.04/NS	0.015/NS	0.03/NS	NS	NS	0.035/NS

	HER				Immune check points							IDH1	JAK			MAPK		
	EGFR/HER1	ERBB2/HER2	ERBB3/HER3	ERBB4/HER4	PD1	PD2	PDL1	PDL2	CTLA4	TIM3	LAG3	IDH1	JAK1	JAK2	JAK3	MAP2K2	MAPK1	MAPK3
WNT	5e-3/NS	0.05/NS	NS	NS	NS	NS	NS	NS	NS	0.08/NS	NS	0.07/NS	NS	0.095/NS	NS	NS	0.03/NS	NS
SHH	NS	0.04/NS	**2e-5/3.5e-3**	0.02/NS	0.03/NS	NS	9e-3/NS	0.02/NS	0.07/NS	**3e-5/5e-3**	NS	NS	4e-3/0.6	5e-3/0.8	8e-3/NS	0.04/NS	NS	1.2e-3/NS
GR4	NS	0.01/NS	0.03/NS	0.01/NS	NS	0.02/NS	3e-3/NS	NS	0.02/NS	0.06/NS	NS	0.05/NS	0.07/NS	NS	0.06/NS	0.05/NS	4e-3/NS	0.05/NS
GR3	0.02/NS	0.01/NS	NS	0.02/NS	6e-3/NS	**8e-4/0.08**	NS	0.03/NS	7e-3/NS	0.03/NS	NS	0.08/NS	0.05/NS	NS	NS	**5e-4/0.05**	0.04/NS	6e-3/NS

	MET	MTOR	NTRK			PARP1	PDGFR				PIK3G							
	MET	MTOR	NTRK1	NTRK2	NTRK3	PARP1	PDGFRA	PDGFRB	FIP1L1	KIT	PIK3CA	PIK3CB	PIK3CD	PIK3G	PIK3C2A	PIK3C2B	PIK3C2G	PIK3C3
WNT	NS	NS	NS	4e-4/NS	0.04/NS	NS	NS	NS	0.04/NS	NS	NS	0.04/NS	0.04/NS	0.02/NS	0.06/NS	0.01/NS	6e-3/NS	NS
SHH	0.03/NS	NS	**1e-7/1.5e-5**	0.01/NS	5e-5/9e-3	5e-3/NS	**7e-5/0.01**	5e-8/8e-6	**3.5e-7/5.5e-5**	**9e-5/0.01**	8.9e-3/NS	0.02/NS	0.06/NS	4e-4/0.06	0.04/NS	3e-3/NS	**5e-8/8e-6**	5e-3/NS
GR4	0.08/NS	NS	0.07/NS	NS	**8e-7/2e-4**	0.02/NS	0.06/NS	0.02/NS	0.04/NS	0.08/NS	0.03/NS	0.02/NS	0.01/NS	3e-4/0.07	0.095/NS	0.04/NS	0.05/NS	2e-3/NS
GR3	0.02/NS	0.01/NS	0.02/NS	NS	0.03/NS	0.01/NS	0.01/NS	0.02/NS	0.07/NS	0.04/NS	0.057/NS	0.05/NS	0.08/NS	0.05/NS	0.03/NS	NS	1.4e-3/NS	1.6e-3/NS

	PKC				RAF			RET	ROS1	SMO	SRC					VEGFR		
	ITPKC	PRKCA	PRKCB	PRKCD	ARAF	BRAF	RAF1	RET	ROS1	SMO	ABL1	SRC	FYN	YES	LYN	FLT1	KDR	FLT4
WNT	NS	NS	NS	**2e-6/1.2e-4**	NS	NS	NS	NS	NS	0.02/NS	0.06/NS	0.035/NS	NS	NS	7e-3/NS	NS	NS	NS
SHH	NS	0.014/NS	0.04/NS	1.6e-3/NS	NS	0.03/NS	8e-4/NS	NS	7e-3/NS	0.05/NS	3e-3/NS	4e-3/NS	**2e-5/3e-3**	6.5e-3/NS	NS	6e-3/NS	5e-3/NS	0.02/NS
GR4	NS	0.03/NS	1e-2/NS	**1e-4/0.024**	0.07/NS	1e-2/NS	0.02/NS	0.07/NS	NS	0.03/NS	0.02/NS	NS	0.05/NS	0.04/NS	0.02/NS	5e-3/NS	0.04/NS	NS
GR3	0.03/NS	0.04/NS	0.07/NS	NS	0.01/NS	0.01/NS	0.02/NS	0.05/NS	0.01/NS	NS	NS	0.04/NS	6e-3/NS	0.01/NS	NS	0.04/NS	NS	0.08/NS

Table S1: Association Between Targeted Genes and Patient Survival Across Medulloblastoma Subgroups. This table illustrates the relationship between genes targeted by the therapies listed in Table 1 and overall survival (OS) across various genetic subgroups of medulloblastoma. The analyzed genes or gene families are displayed alongside their respective P-values, calculated using the R2 platform with the optimal cutoff method. Two P-values are provided for each gene: the first represents the raw significance, while the second reflects the Bonferroni-corrected significance. Genes are visually categorized based on their prognostic association using background colors: **White background**: Genes linked to shorter OS; **Black background**: Genes linked to longer OS; **Dark grey background with enlarged text**: Genes associated with a poor prognosis, indicated by both raw and Bonferroni-corrected P-values < 0.05; **Light grey background**: Non-significant (NS) genes with no clear survival impact. This classification provides an intuitive visual summary of the survival impact of specific genes across medulloblastoma subgroups, facilitating a better understanding of their prognostic relevance.

	ALK	BCL2	BCL2L1	BCL2L2	BTK	CD3D	CD19	CD20	CD25	CD33	CD52	CDK		DDR2	EPCAM	FGFR			FLT3
	ALK	BCL2	BCL2L1	BCL2L2	BTK	CD3D	CD19	CD20	CD25	CD33	CD52	CDK4	CDK6	DDR2	EPCAM	FGFR1	FGFR2	FGFR3	FLT3
WNT M0	NS	NS	NS	NS	5.3e-3/NS	0.05/NS	0.01/NS	0.02/NS	NS	NS	NS	0.05/NS	NS	NS	NS	NS	5.9e-3/NS	NS	NS
WNT M1	NS	NS	NS	NS	NS	NS	NS	NS	NS	NS	NS	NS	NS	NS	NS	NS	NS	NS	NS
SHH M0	NS	NS	NS	NS	1e-2/NS	1e-3/NS	NS	1e-6/1e-4	0.01/NS	3e-3/NS	6e-9/7e-7	3e-3/NS	NS	3e-3/NS	NS	NS	0.04/NS	7.8e-7/9e-5	NS
SHH M1	NS	NS	NS	NS	NS	NS	0.03/NS	NS	NS	0.05/NS	0.02/NS	7.8e-4/0.01	0.02/NS	NS	NS	NS	NS	0.075/NS	2e-3/0.08
GR4 M0	0.04/NS	2e-3/NS	0.03/NS	0.076/NS	NS	NS	2e-3/NS	9e-6/1e-3	NS	0.02/NS	5e-3/NS	0.03/NS	0.04/NS	0.03/NS	NS	NS	0.04/NS	1.4e-7/2e-5	0.04/NS
GR4 M1	2e-4/1.7e-3	8e-3/NS	0.02/NS	2.5e-3/NS	NS	1e-4/8.5e-3	0.02/NS	NS	NS	NS	0.02/NS	NS	6e-3/NS	0.02/NS	NS	NS	0.04/NS	0.071/NS	0.03/NS
GR3 M0	0.02/NS	2.5e-3/NS	3.7e-3/NS	0.02/NS	NS	0.06/NS	9e-3/NS	NS	0.04/NS	0.06/NS	5.5e-3/NS	0.02/NS	NS	2e-3/NS	0.04/NS	0.02/NS	NS	NS	NS
GR3 M1	3e-3/NS	0.05/NS	0.056/NS	0.02/NS	0.03/NS	0.07/NS	5e-3/NS	NS	2e-3/0.075	0.04/NS	NS	1e-3/0.04	NS	NS	NS	NS	NS	9e-3/NS	0.05/NS

	HER				Immune checkpoints							IDH1	JAK			MAPK		
	EGFR/HER1	ERBB2/HER2	ERBB3/HER3	ERBB4/HER4	PD1	PD2	PDL1	PDL2	CTLA4	TIM3	LAG3	IDH1	JAK1	JAK2	JAK3	MAP2K2	MAPK1	MAPK3
WNT M0	0.02/NS	0.02/NS	NS	NS	NS	NS	NS	NS	NS	0.05/NS	NS	NS	NS	NS	NS	NS	0.03/NS	NS
WNT M1	NS	NS	NS	NS	NS	NS	NS	NS	NS	NS	NS	NS	NS	NS	NS	NS	NS	NS
SHH M0	0.04/NS	NS	2e-5/4e-4	NS	7e-4/0.08	0.056/NS	0.02/NS	0.03/NS	NS	2e-3/NS	4e-4/0.046	0.07/NS	0.04/NS	3.9e-3/NS	NS	6e-3/NS	NS	NS
SHH M1	0.05/NS	NS	8.5e-4/NS	7e-3/NS	NS	NS	NS	NS	NS	0.01/NS	0.04/NS	NS	NS	0.04/NS	NS	NS	NS	NS
GR4 M0	0.06/NS	2.8e-4/0.04	0.056/NS	0.04/NS	0.064/NS	4e-3/NS	0.06/NS	NS	NS	0.02/NS	0.03/NS	NS	NS	0.04/NS	0.074/NS	NS	0.01/NS	0.04/NS
GR4 M1	0.02/NS	NS	4.7e-3/NS	0.05/NS	NS	0.04/NS	0.02/NS	NS	NS	NS	0.047/NS	NS	0.03/NS	0.015/NS	NS	NS	NS	0.04/NS
GR3 M0	0.05/NS	2.9e-3/NS	0.05/NS	NS	0.02/NS	NS	0.07/NS	0.035/NS	0.03/NS	NS	NS	0.02/NS	NS	NS	NS	7.5e-3/NS	0.02/NS	0.059/NS
GR3 M1	2e-4/7e-3	NS	NS	NS	1e-2/NS	0.04/NS	NS	NS	0.01/NS	NS	0.03/NS	4.5e-3/NS	NS	0.05/NS	NS	NS	NS	9e-4/0.03

	MET	MTOR	NTRK			PARP1	PDGFR				PIK3G							
	MET	MTOR	NTRK1	NTRK2	NTRK3	PARP1	PDGFRA	PDGFRB	FIP1L1	KIT	PIK3CA	PIK3CB	PIK3CD	PIK3G	PIK3C2A	PIK3C2B	PIK3C2G	PIK3C3
WNT M0	NS	NS	NS	7.5e-4/0.03	NS	NS	NS	NS	0.05/NS	NS	NS	NS	0.03NS	NS	NS	3.6e-3/NS	0.01/NS	NS
WNT M1	NS	NS	NS	NS	NS	NS	NS	NS	NS	NS	NS	NS	NS	NS	NS	NS	NS	NS
SHH M0	0.05/NS	NS	2e-7/2e-5	NS	1.7e-3/NS	3e-3/NS	NS	NS	NS	NS	0.03/NS	NS	NS	5.7e-3/NS	0.04/NS	0.03/NS	4.8e-5/5.7e-3	NS
SHH M1	0.01/NS	NS	NS	2e-5/4e-4	0.03/NS	NS	NS	1.5e-3/0.03	NS	NS	NS	NS	0.04/NS	4.6e-3/NS	NS	0.01/NS	2e-4/3.3e-3	NS
GR4 M0	NS	NS	NS	NS	3e-7/4e-5	0.02/NS	0.035/NS	0.05/NS	0.02/NS	8.8e-3/NS	NS	0.02/NS	0.01/NS	0.03/NS	NS	1.2e-3/NS	5e-3/NS	0.03/NS
GR4 M1	NS	NS	0.046/NS	NS	0.02/NS	NS	0.025/NS	NS	NS	NS	NS	NS	0.06/NS	9e-3/NS	NS	NS	0.03/NS	1.2e-6/1e-4
GR3 M0	0.04/NS	0.04/NS	3e-3/NS	0.046/NS	0.05/NS	7.8e-3/NS	1.5e-3/0.09	0.09/NS	0.07/NS	0.08/NS	0.03/NS	NS	NS	0.02/NS	0.05/NS	NS	0.05/NS	0.06/NS
GR3 M1	0.045/NS	0.015/NS	NS	0.03/NS	NS	0.04/NS	NS	1.5e-3/0.03	0.03/NS	0.02/NS	NS	NS	NS	0.04/NS	7e-3/NS	NS	1.6e-4/6e-3	1.8e-3/0.07

	PKC				RAF			RET	ROS1	SMO	SRC					VEGFR		
	ITPKC	PRKCA	PRKCB	PRKCD	ARAF	BRAF	RAF1	RET	ROS1	SMO	ABL1	SRC	FYN	YES	LYN	FLT1	KDR	FLT4
WNT M0	NS	NS	NS	1.4e-5/5e-4	NS	NS	NS	NS	NS	2.3e-3/0.087	NS	0.05NS	NS	NS	0.01/NS	NS	NS	NS
WNT M1	NS	NS	NS	NS	NS	NS	NS	NS	NS	NS	NS	NS	NS	NS	NS	NS	NS	NS
SHH M0	0.06/NS	NS	0.056/NS	0.01/NS	NS	7.7e-3/NS	5.8e-3/0.03	0.04/NS	7e-3/NS	0.05/NS	2e-3/NS	0.02NS	1.4e-3/NS	0.05/NS	NS	6.8e-3/NS	6.6e-3/NS	0.045/NS
SHH M1	0.02/NS	NS	NS	0.03/NS	NS	0.03/NS	NS	NS	NS	7.5e-4/0.01	NS	NS	0.098/NS	0.05/NS	NS	4.8e-3/0.08	NS	0.07/NS
GR4 M0	NS	0.02/NS	0.015/NS	1.9e-4/0.03	NS	2.8e-3/NS	NS	0.03/NS	NS	0.02/NS	NS	NS	0.014/NS	0.02/NS	NS	0.03/NS	0.03/NS	NS
GR4 M1	NS	NS	0.04/NS	0.04/NS	4.7e-3/NS	NS	0.014/NS	NS	0.03/NS	NS	NS	0.02/NS	2.6e-3/NS	NS	0.014/NS	NS	NS	NS
GR3 M0	0.03/NS	0.02/NS	0.05/NS	NS	NS	NS	0.02/NS	NS	0.03/NS	NS	NS	NS	NS	0.05/NS	NS	NS	NS	NS
GR3 M1	7.7e-3/NS	NS	NS	0.02/NS	NS	1.5e-3/0.03	0.05/NS	NS	0.01/NS	NS	NS	NS	4.6e-4/0.02	0.085/NS	0.05/NS	0.03/NS	0.03/NS	0.04/NS

Table S2: Association Between Targeted Genes and Patient Survival Across Medulloblastoma Subgroups Considering Metastatic Status (M0, M1). This table explores the association of genes targeted by the therapies listed in Table 1 with overall survival (OS) in medulloblastoma patients, stratified by metastatic status (M0: non-metastatic, M1: metastatic). The analyzed genes or gene families are presented alongside their respective P-values, calculated using the R2 platform based on the optimal cutoff approach. Two P-values are reported for each gene: the raw significance value and the Bonferroni-corrected significance value. Genes are visually categorized using background colors to reflect their prognostic impact: **White background**: Genes associated with shorter OS; **Black background**: Genes associated with longer OS; **Dark grey background** with enlarged text: Genes associated with a poor prognosis, indicated by both raw and Bonferroni-corrected P-values < 0.05; **Light grey background**: Non-significant (NS) genes with no discernible impact on OS. This visual classification highlights the survival implications of specific genes across medulloblastoma subgroups while considering the patients' metastatic status, providing valuable insights into prognostic and therapeutic considerations.

Cohort Name	Mutation (Number of case)	Phenotype	Treatment
**PCGP Medulloblastoma Nature 2012 37 patients**			
CTNNB1 (beta Cat) 2 patients 5%.	Mutations S33F (1), G34R (1)	Likely Oncogenic	Elraglusib, Tideglusib
PI3KCA 1 patient 2.7 %	Mutation Q546K (1)	Oncogenic	Capivasertib
PTCH1 1 patient 2.7 %	Mutation S861Mfs*2 (1)	Likely oncogenic truncating mutation	Sonidegig Vismodegib
**Medulloblastoma Broad Nature 2012 92 Samples**			
CTNNB1 (beta Cat) 6 Patients 6.5 %	Mutation D32G (1), Mutation S33F (1), S33Y (1) G34R (2), S37P (1)	Likely Oncogenic	
PTCH1 5 Patients 6.5 %	Mutation X406 Splice (1), I1055Sfs*3 (1), Q242Vfs*9 (1) Q889Afs*7 (1), n V439Qfs*59 (1)	Likely oncogenic truncating mutations Likely oncogenic truncating mutation	Sonidegig Vismodegib Sonidegig Vismodegib
FGFR1 2 Patients 2.2 %	Mutation N577K (1), K687E (1)	Likely Oncogenic	Erdafitinib, Fexagratinib
SMO 1 Patient 1.1 %	Mutation L412F (1)	Likely Oncogenic	Resistance to Vismodegib
PTEN 1 Patient 1.1 %	Mutation G165E (1)	Oncogenic	Capivasertib, GSK2636771, AZD8186
PIK3CA 1 Patient 1.1 %	Mutation H1047L (1)	Oncogenic	Alpelisib Capivasertib RLY-2608
SUFU 1 Patient 1.1 %	Mutation X386_Splice (1)	Likely Oncogenic	Resistance to Vismodegib
**Medulloblastoma DKFZ Nature 2017 491 Samples**			
PTCH1 25 Patients 5.1 %	Mutation C92* (1), W129* (1), Q177* (1), E374* (1), F583* (1) R602* (1), C618* (1), Q839* (1), C1093* (1), E1183* (1) Mutation V169Pfs*82 (1), Q242Vfs*9 (1), C226Ffs*2 (1) Mutation D301Ifs*23 (1), X406_splice (1), L448Nfs*9 (1) L450Pfs*5 (1), A451Pfs*5 (1), C462Wfs*27 (1) F495Nfs*50 (1), X750_splice (1), Y797Sfs*24 (1) L819Tfs*10 (1), I1055Sfs*3 (1), X1150_splice (1)	Likely oncogenic truncating mutations	Sonidegig Vismodegib
CTNNB1 (beta Cat) 25 Patients 5.1 %	Mutation D32Y (2), S33C (2), S33F (6), S33Y (4), G34E (1) G34R (4), G34V (1), S37C (1), S37F (1), S37P (1) S37Y (1), T41A (1)	Likely Oncogenic	Elraglusib, Tideglusib
ERBB4 1 Patient 0.2 %	Fusion ERBB4-LCLAT1 (1)	Likely Oncogenic	Lapatinib Dacomitinib
SMO1 11 Patients 2.2 %	Mutation L412F (8), W535L (3)	Likely Oncogenic	Resistance to Vismodegib
SUFU 11 Patients 2.2 %	Mutation I291* (1), E437* (1), P12Rfs*90 (1), A25Gfs*23 (1) Y147Vfs*21 (1), F149Sfs*20 (1), T190Nfs*25, T261Gfs*8 (1) P341Rfs*20 (1), X386_splice (1), SUFU-CYP17A1-AS1 Fusion (1)	Likely Oncogenic	Resistance to Vismodegib
IDH1 1 Patient 0.2 %	Mutation R132C	Oncogenic	Ivosidenib Vorasidenib
PTEN 9 Patients 1.83 %	lutation W111R (1), G132V (1), G165E (1), D324Y (1), M134del (1 T319Ifs*2 (1), K322Rfs*23 (1), PTEN-NR2F1-AS1 Fusion (1) Mutation PTEN-THAP9 Fusion (1)	Oncogenic	Capivasertib GSK2636771 AZD8186
PIK3R1 3 Patients 0.61 %	X582_splice (1), Y452_N453ins* (1), D68Gfs*38 (1)	Likely Oncogenic	Capivasertib
**Medulloblastoma ICGC Nature 2012 125 Samples**			
CTNNB1 (beta Cat) 15 Patients 12 %	Mutation D32A (2), D32Y (3), S33C (4), S33F (2) G34R (2), S37F (1), S37Y (1)	Likely Oncogenic	Elraglusib, Tideglusib
PTCH1 6 Patients 4.8 %	Mutation E374* (1), Y1009* (1), F495Efs*50 (1) D773Rfs*16 (1), Y804Cfs*3 (1), L819Tfs*10 (1)	Likely oncogenic truncating mutations	Sonidegig Vismodegib
PIK3CA 2 Patients 1.6 %	Mutation H1047L (1), C420R (1)	Oncogenic	Alpelisib Capivasertib RLY-2608
NRAS 1 Patient 0.8 %	Mutation G13V	Oncogenic	Binimetinib Cobimetinib Trametinib
CDKN2A 1 Patient 0.8 %	Mutation D84N	Likely oncogenic truncating mutation	Palbociclib Ribociclib Abemaciclib
SMO 8 Patient 6.4 %	Mutation L412F	Likely Oncogenic	Resistance to Vismodegib
PTEN 1 Patient 0.8 %	Mutation H93Y	Oncogenic	Capivasertib GSK2636771 AZD8186
PIK3R1 1 Patient 0.8 %	Mutation X582_splice	Likely Oncogenic	Capivasertib
ATM 1 Patient 0.8 %	Mutation Y1915*	Likely Oncogenic	Olaparib Talazoparib + Enzalutamide
FGFR2 1 Patient 0.8 %	Mutation K659E	Likely Oncogenic	Erdafitinib RLY-4008 AZD4547
**Medulloblastoma PCGP Nature 2012 37 Samples**			
CTNNB1 (beta Cat) 4 Patients 10.8 %	Mutation D32G (1), S33F (1), S33Y (1), G34R (1)	Likely Oncogenic	Elraglusib, Tideglusib
PIK3CA 1 Patient 2.7 %	Mutation Q546K	Oncogenic	Capivasertib + Fulvestrant RLY-2608
SUFU 2 Patients 5.4 %	Mutation T261Gfs*8 (1), V148Sfs*30 (1)	Likely Oncogenic	Resistance to Vismodegib
**Medulloblastoma Sickkids Nature 2016 46 Samples**			
PTCH1 4 Patients 8.7 %	Mutation E675* (1), V442Gfs*54 (1), S444Afs*11 (1), Y452Lfs*4 (1)	Likely oncogenic truncating mutation	Sonidegig Vismodegib
PTEN 2 Patients 4.3 %	Mutation A126T (1), G132V (1)	Oncogenic	Capivasertib GSK2636771 AZD8186
CTNNB1 (beta Cat) 2 Patients 4.3 %	Mutation D32V (1), G34R (1)	Likely Oncogenic	Elraglusib, Tideglusib
SMO 1 Patient 2.2 %	Mutation L412F	Likely Oncogenic	Resistance to Vismodegib

Table S3: Alignment of Targeted Therapies with Specific Gene Mutations. Alignment of targeted therapies with specific mutations that are not addressable by conventional therapies. It includes the names of patient cohorts from the TCGA, the specific genes and mutations identified, their associated expected phenotypes, and the names of treatments tailored to these mutations.

TREATMENT	Clinical application				Research (in vitro/vivo)					
	Used in MB	Reference	Other brain tumor	Reference	Used in MB	Reference	Other brain tumor	Reference	Cross the BBB	Reference(s)
**Abemaciclib**	No		No		No		Glioblastoma	PMID: 38999983	Yes (low)	PMID: 38999983
**Acalabrutinib**	No		Leptomeninges/CNS	PMID: 35732356	No		Glioblastoma<br>Neuroblastoma (+in vivo)	PMID: 34577576<br>PMID: 32705581		
**Afatinib**	No				No					
**Alemtuzumab**	No		CD4+ / Tcells in brain<br>Tcells / CNS	PMID: 36362494<br>PMID: 32802528	No		No		Yes	PMID: 36362494<br>PMID: 33728194
**Alpelisib**	No		No		Yes (+in vivo)	PMID: 31492956	Neuroblastoma (+in vivo)<br>Glioblastoma	PMID: 33491755<br>PMID: 33318517<br>PMID: 27120806		
**Asciminib**	No		No		Yes (+in vivo)	PMID: 37781087	No			
**Atezolizumab**	No		Glioblastoma	PMID: 30073642	No		No			
**Avapritinib**	No		No		No		Myxoid glioneuronal tumors	PMID: 38532028	Yes	PMID: 38167404
**Avelumab**	No				No					
**Axitinib**	Yes	PMID: 38778441			Yes	PMID: 35008234<br>PMID: 34234256<br>PMID: 29377550<br>PMID: 31035676			Yes	PMID: 35008234<br>PMID: 31035676
**Baricitinib**	No				No					
**Basiliximab**	No				No					
**Blinatumomab**	No				No					
**Cetuximab**	Yes	PMID: 23426003<br>PMID: 24092425								
**Cobimetinib**	No				No					
**Dacomitinib**	No				Yes (+in vivo)	PMID: 29574250	lioblastoma & Pineoblastoma (+in viv	PMID: 29574250		
**Durvalumab**	No				No					
**Erlotinib**	Yes	PMID: 29778738	Glioblastoma	PMID: 32550606						
**Gefitinib**	No				Yes<br>Yes (+in vivo)	PMID: 21726539<br>PMID: 19033425<br>PMID: 18829483	Glioblastoma<br>Glioma	PMID: 21726539<br>PMID: 18829483		
**Gemtuzumab**	No				No					
**Gilteritinib**	No				No					
**Idelalisib**	No				No					
**Imatinib**	No				Yes	PMID: 37987713<br>PMID: 19417143	Glioblastoma<br>Myxoid glioneuronal tumors	PMID: 37987713<br>PMID: 38532028		
**Ipilimumab**	Yes	PMID: 36808285	Glioblastoma	PMID: 36808285						
**Nilotinib**	No				Yes	PMID: 31539380				
**Nivolumab**	Yes	PMID: 36808285<br>PMID: 30681550	Glioblastoma	PMID: 36808285<br>PMID: 30681550						
**Olaparib**	No				Yes<br>Yes (+in vivo)<br>Yes (+in vivo)<br>Yes	PMID: 37783879<br>PMID: 35001340<br>PMID: 34508175<br>PMID: 22184287	Neuroblastoma (+in vivo)<br>Glioblastoma<br>High grade glioma and Ependymoma	PMID: 34508175<br>PMID: 35001340<br>PMID: 22184287		
**Palbociclib**	No				Yes (+in vivo)<br>Yes (+in vivo)<br>Yes<br>Yes (+in vivo)	PMID: 39605517<br>PMID: 37333134<br>PMID: 37127652<br>PMID: 35080986				
**Pembrolizumab**	No				No					
**Pemigatinib**	No				No					
**Pertuzumab**	No				No					
**Ribociclib**	No				Bioinformatic study<br>Yes (+in vivo)<br>Yes (+in vivo)	PMID: 37783879<br>PMID: 36318650<br>PMID: 35709750				
**Rucaparib**	No				Yes	PMID: 20978505				
**Sabatolimab**	No				No					
**Selumetinib**	No				Yes (+in vivo)<br>Yes (+in vivo)	PMID: 35835937<br>PMID: 29930101				
**Sonidegib**	Yes	PMID: 31362788<br>PMID: 28605510<br>PMID: 24523439<br>PMID: 35316996<br>PMID: 35053576<br>PMID: 31835472<br>PMID: 34409296			Yes<br><br>Yes (+in vivo)	PMID: 36688010<br>PMID: 31835472<br>PMID: 35475274				
**Sunitinib**	No				Yes (+in vivo)<br>Yes<br>Yes	PMID: 35008234<br>PMID: 20524040<br>PMID: 20053726			Yes	PMID: 35008234<br>PMID: 20524040
**Talazoparib**	No				Yes (+in vivo)<br>Yes (+in vivo)	PMID: 37910601<br>PMID: 25263539			No	PMID: 37910601
**Tofacitinib**	No				Yes	PMID: 30332965				
**Trametinib**	No				Yes	PMID: 37726268				
**Trastuzumab**	No				No					
**Venetoclax**	No				Yes<br>Yes (+in vivo)<br>Yes	PMID: 35056150<br>PMID: 32855528<br>PMID: 26394044				

Table S4: Overview of treatments that have been approved for clinical use or previously described in the literature. Potentially repositionable treatments were extensively reviewed to evaluate their relevance for medulloblastoma or other brain tumors. The table also indicates whether each treatment can cross the blood-brain barrier (BBB) and provides the corresponding PubMed ID (PMID) for reference.
