# Supplementary figures and images for: Exploring Novel Applications: Repositioning Clinically Approved Therapies for Medulloblastoma Treatment
Source: Cancers (Basel). 2025 Nov 14;17(22):3659. doi: 10.3390/cancers17223659 (PMC12650705; doi:10.3390/cancers17223659)

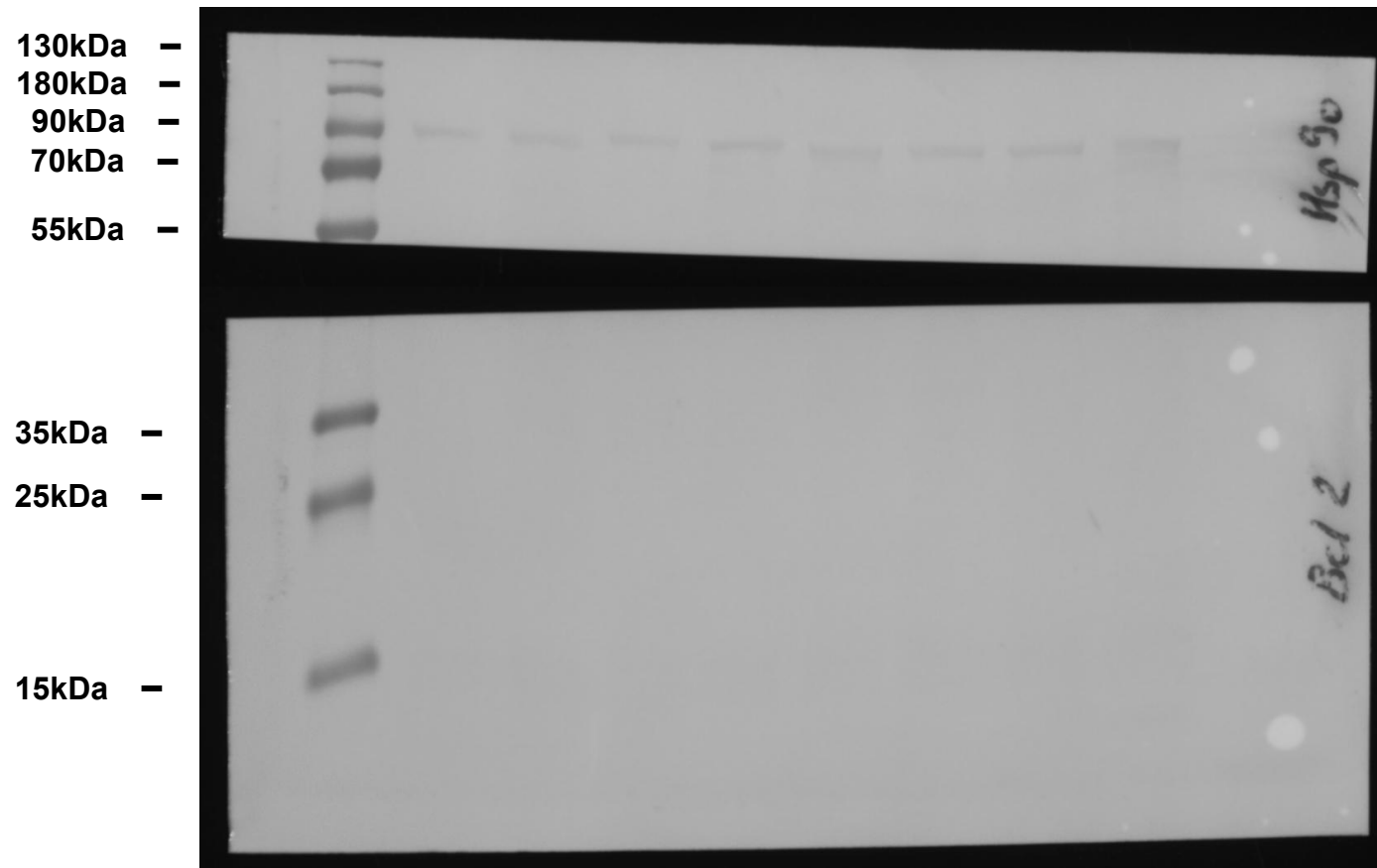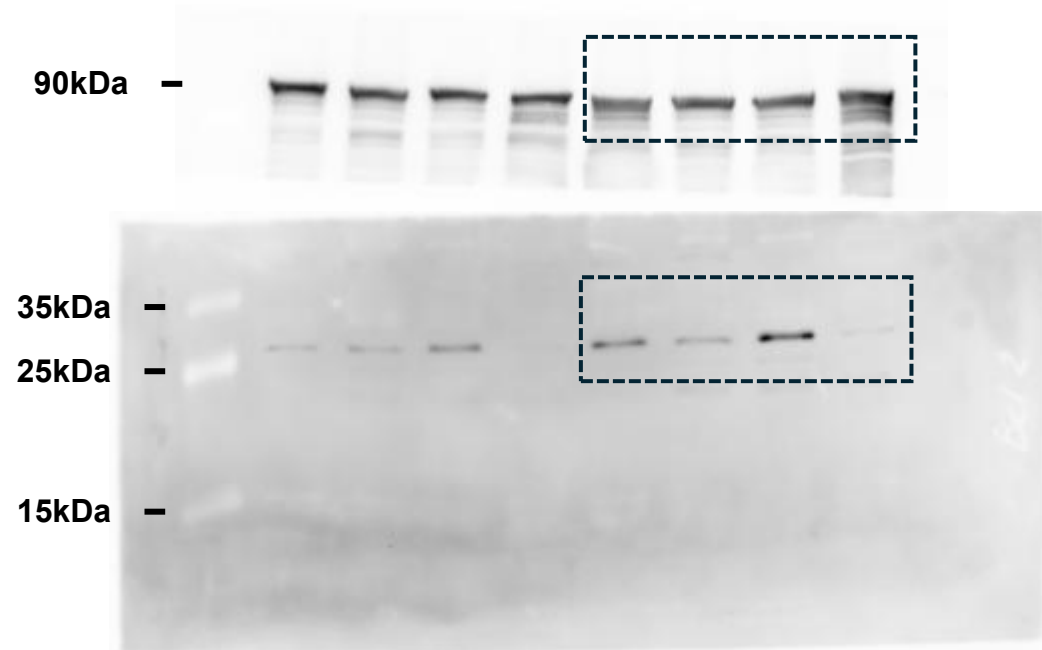

Supplement: Supplementary file 1 [file cancers-17-03659-s001.zip › Supplementary File S1/wb BCL2 data brut DEF.pdf]

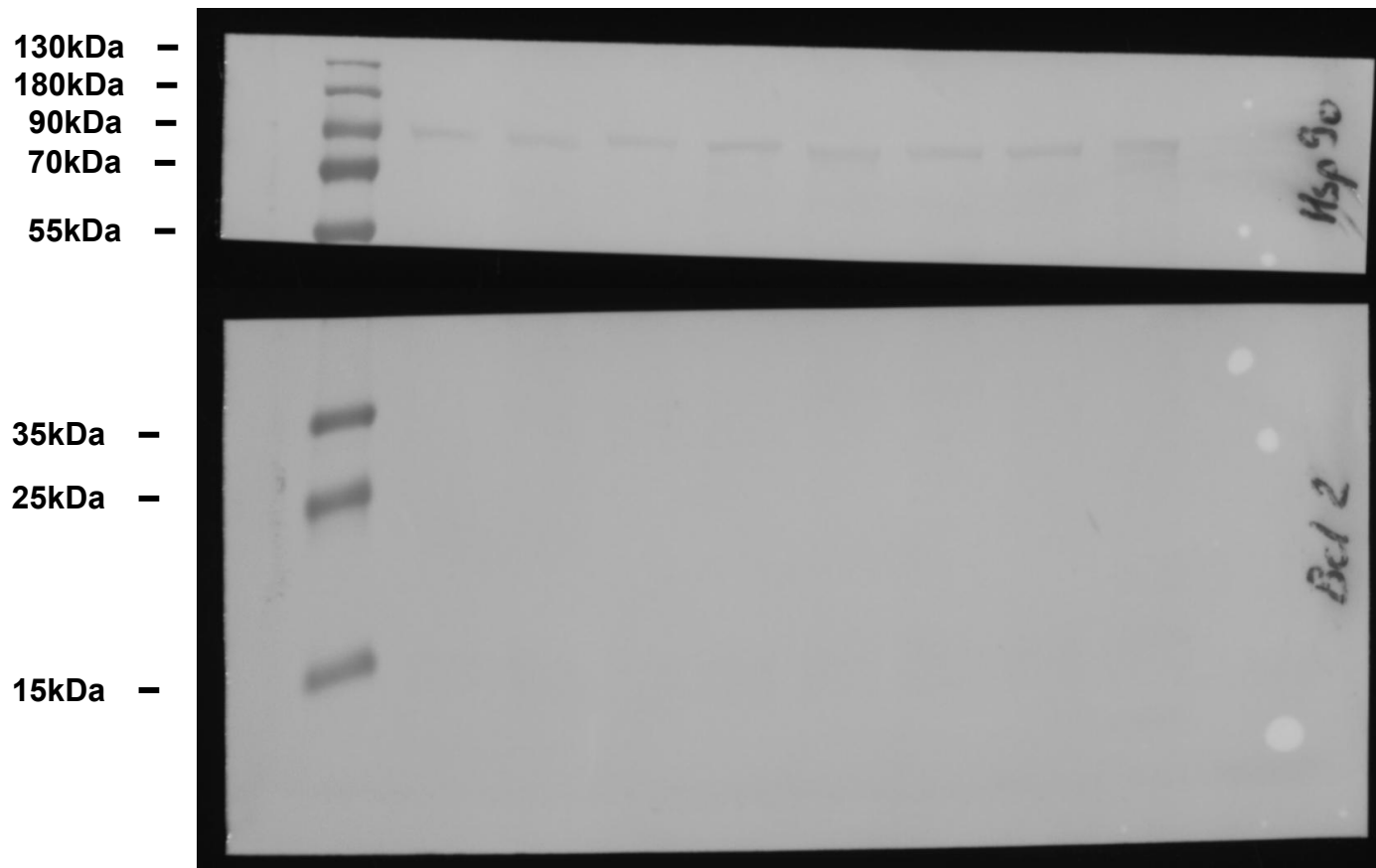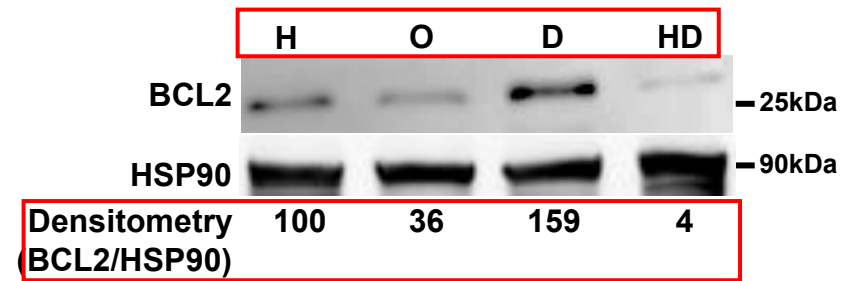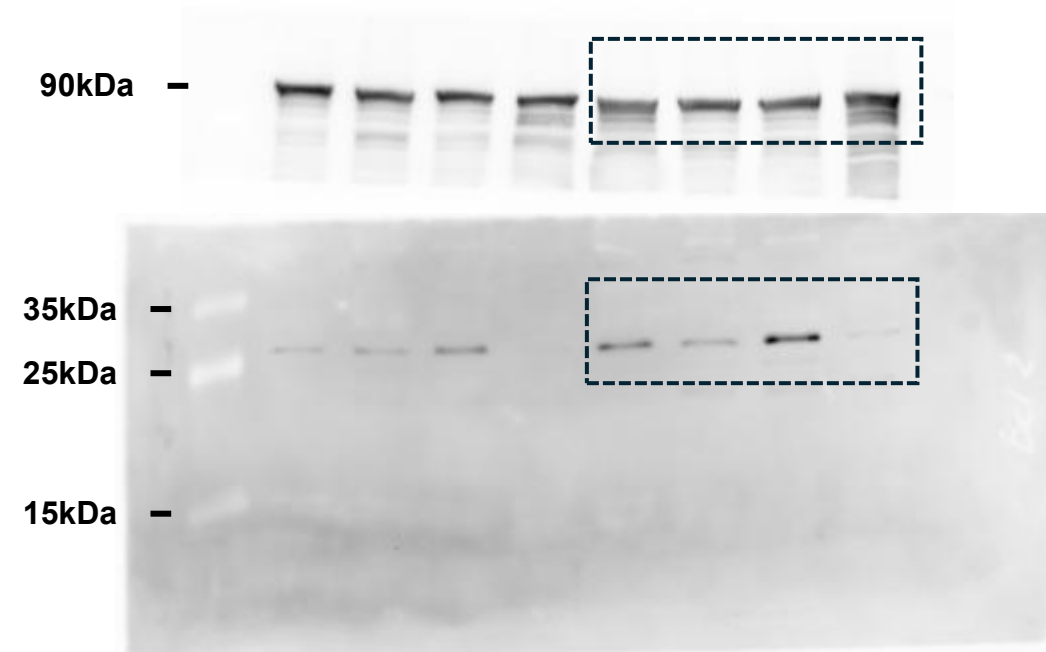

Supplement: Supplementary file 1 [file cancers-17-03659-s001.zip › Supplementary File S1/wb BCL2 data brut DEFDEF.pdf]
